# Personalized neoantigen viro-immunotherapy platform for triple-negative breast cancer

**DOI:** 10.1136/jitc-2023-007336

**Published:** 2023-08-16

**Authors:** Renato Brito Baleeiro, Peng Liu, Louisa S Chard Dunmall, Carmela Di Gioia, Ai Nagano, Lauren Cutmore, Jun Wang, Claude Chelala, Lydon Wainaina Nyambura, Peter Walden, Nicholas Lemoine, Yaohe Wang

**Affiliations:** 1 Centre for Cancer Biomarkers and Biotherapeutics, Queen Mary University of London, London, UK; 2 Department of Dermatology, Venerology and Allergology, Charité Universitätsmedizin Berlin, Berlin, Germany; 3 Zhengzhou University, Zhengzhou, Henan, China

**Keywords:** Breast Neoplasms, Immunotherapy, Immunogenicity, Vaccine, Oncolytic Virotherapy

## Abstract

**Background:**

Triple-negative breast cancer (TNBC) corresponds to approximately 20% of all breast tumors, with a high propensity for metastasis and a poor prognosis. Because TNBC displays a high mutational load compared with other breast cancer types, a neoantigen-based immunotherapy strategy could be effective. One major bottleneck in the development of a neoantigen-based vaccine for TNBC is the selection of the best targets, that is, tumor-specific neoantigens which are presented at the surface of tumor cells and capable of eliciting robust immune responses. In this study, we aimed to set up a platform for identification and delivery of immunogenic neoantigens in a vaccine regimen for TNBC using oncolytic vaccinia virus (VV).

**Methods:**

We used bioinformatic tools and cell-based assays to identify immunogenic neoantigens in TNBC patients’ samples, human and murine cell lines. Immunogenicity of the neoantigens was tested in vitro (human) and ex vivo (murine) in T-cell assays. To assess the efficacy of our regimen, we used a preclinical model of TNBC where we treated tumor-bearing mice with neoantigens together with oncolytic VV and evaluated the effect on induction of neoantigen-specific CD8+T cells, tumor growth and survival.

**Results:**

We successfully identified immunogenic neoantigens and generated neoantigen-specific CD8+T cells capable of recognizing a human TNBC cell line expressing the mutated gene. Using a preclinical model of TNBC, we showed that our tumor-specific oncolytic VV was able to change the tumor microenvironment, attracting and maintaining mature cross-presenting CD8α+dendritic cells and effector T-cells. Moreover, when delivered in a prime/boost regimen together with oncolytic VV, long peptides encompassing neoantigens were able to induce neoantigen-specific CD8+T cells, slow tumor growth and increase survival.

**Conclusions:**

Our study provides a promising approach for the development of neoantigen-based immunotherapies for TNBC. By identifying immunogenic neoantigens and developing a delivery system through tumor-specific oncolytic VV, we have demonstrated that neoantigen-based vaccines could be effective in inducing neoantigen-specific CD8+T cells response with significant impact on tumor growth. Further studies are needed to determine the safety and efficacy of this approach in clinical trials.

**WHAT IS ALREADY KNOWN ON THIS TOPIC**

- Triple-negative breast cancer (TNBC) has a poor prognosis and few treatment options and unlike other breast cancer types, TNBC has a high mutational load and is often infiltrated by T lymphocytes. However, it is unclear whether TNBC can generate neoantigens that can be targeted by immunotherapeutic strategies.

**WHAT THIS STUDY ADDS**

- This study shows that TNBC can generate immunogenic neoantigens that can be recognized by cytotoxic T lymphocytes. Moreover, it presents a platform for the identification and delivery of immunogenic neoantigens in a personalized vaccine where neoantigens are given together with a tumor-specific oncolytic vaccinia virus.

**HOW THIS STUDY MIGHT AFFECT RESEARCH, PRACTICE OR POLICY**

- This study offers a platform for the identification of the best antigen targets and a delivery system to administer those antigens to stimulate an immune response against such targets. This approach has the potential to improve the outcome of patients with TNBC.

## Introduction

Triple-negative breast cancer (TNBC) is a subset of breast cancer characterized by the absence of estrogen receptor (ER), progesterone receptor (PR), and HER-2 expression. The disease represents approximately 20% of newly diagnosed breast tumors.^1 2^ TNBC carries the most dismal prognosis of breast cancers, with the aggressive and heterogeneous nature of the disease increasing the likelihood of disease dissemination and recurrence post-treatment.^3 4^ Therefore, new therapeutic regimens are urgently needed.

Cancer immunotherapy is currently one of the most promising and rapidly advancing cancer treatment modalities.^5–8^ The promise of immunotherapy is particularly evident in the recent success of antibodies targeting cytotoxic T-lymphocyte-associated protein 4 (CTLA-4), programmed death 1 (PD1) or PD-L1 (programmed death ligand-1) immune checkpoints in promoting antitumor immune responses and extending survival in a number of malignancies.^8^ Early phase clinical trials for TNBC patients have demonstrated durable single agent responses to PD1 and PD-L1 checkpoint inhibitors,^9 10^ but these responses have been low, despite TNBCs having been characterized as extremely favorable candidates for immunotherapeutic intervention, in part due to overexpression of PD-L1^11 12^ and the increased frequency of tumor-infiltrating lymphocytes compared with other breast cancer subtypes.^13^

Cancer vaccination has emerged as the most effective way to promote antitumor immune responses.^14^ Historically, cancer vaccines have targeted tumor-associated antigens (TAAs), which are self-antigens expressed in normal tissues, but which are ectopically expressed or overexpressed in cancer cells. Most clinical trials targeting TAAs have failed to demonstrate durable benefit compared with standard-of-care treatment,^15^ as treatments are limited by off-tumor toxicity and pre-existing immune tolerance to self-antigens. In contrast, cancer vaccines targeting neoantigens have the advantage of targeting tumor-exclusive mutated sequences that have not been subjected to central immune tolerance, increasing the potential for immune recognition without off-target effects.^16^

Recently, advances in precision oncology via the advent of high-throughput, low-cost sequencing and bioinformatics platforms has enabled genomic sequencing from patient biopsy samples and surrounding normal tissues. This platform is enabling rapid identification of neoantigens, tumor-specific antigens resulting from somatic DNA alterations, expressed by individual patients that are potentially more clinically viable vaccine targets.^17^ Although whole-exome and mRNA sequencing can rapidly identify potential neoantigens in individual tumors, defining the neoantigens that can be generated by the tumor cell MHC-I processing machinery, can bind to the individual’s HLA and be presented to the cognate CD8+T cell in an immunogenic fashion, therefore, clinically relevant, remains a challenge.

Selection of epitopes for cancer immunotherapy is a complex multistep process. Epitopes are often selected by using online programs such as NetMHC that can predict the binding affinity of mutant peptides for the patients’ HLA.^18^ Besides being able to bind to HLA, a good vaccine candidate must also be immunogenic, that is, be capable of eliciting an immune response. This capacity is mainly dependent on the existence and frequency of clones capable of recognising that epitope in the individual’s T-cell repertoire. The most common way to determine immunogenicity of cancer peptides is by testing patients’ T-cells reactivity against the candidate neoantigens in T-cell assays. However, it has been shown that despite the large number of predicted epitopes, neoantigen-specific T-cell reactivity in cancer patients is generally limited to just a few mutant epitopes.^19–21^ It has recently been reported that melanoma patients vaccinated with neoantigen-loaded dendritic cells (DCs) increased dramatically the number of neoantigen-specific T-cell clones, compared with the number prior to vaccination.^22^ These data indicate that an effector T-cell pool targeting many tumor-expressed neoantigens in patients with cancer may be undetected because of poor priming or due to tolerisation of the tumor-specific T-cells. To circumvent the need for the use of blood from immunocompromised patients, T-cell repertoires from HLA-matched healthy blood donors could be applied for identification of neoantigens. Evidence that such a screening with donor T-cell repertoires is possible was shown in two recent studies in which a much higher frequency of neoantigens was shown to be immunogenic than was anticipated from analyzing melanoma patient’s autologous ex vivo T-cell responses.^23 24^ To date, the vast majority of studies analyzing T-cell-based immune response in cancer have by and large focused on melanoma. However, for the development and clinical use of effective immunotherapy for epithelial cancers, such as TNBC, the establishment of approaches to delve into T-cell-mediated tumor recognition in this tumor type is of paramount importance.

Once immunogenic neoantigens are identified, the next challenge for a cancer vaccine is how to deliver these antigens effectively. Usually, selected antigens are delivered together with adjuvants to induce a proper immune response.^24–26^ Use of replicating oncolytic viruses (OV) to deliver antigens into the tumor could offer a tremendous advantage over common adjuvants, as when administered directly into the tumor they can double as both adjuvants and oncolytic agents, resulting in an enhanced overall antitumor effect. We have recently developed a new generation of tumor-targeted replicating vaccinia virus (VV) (VVLΔTKΔN1L) with deletion of the thymidine kinase and N1L genes that enhance safety (selectively targeting tumors with deregulated Ras-EGFR pathways). This mutant virus resulted in development of higher tumor-specific immunity in vivo compared with N1L-intact VV.^27–29^

Here, we report a platform that combines the identification and the delivery of neoantigens in an effective vaccine regimen for TNBC. The most promising neoantigen candidates were identified and validated through exome/RNA sequencing, proteome analysis, prediction algorithms and cell-binding assays. This was followed by the delivery of the neoantigens in a prime/boost regimen by mixing peptides with a tumor-selective oncolytic VV. This platform demonstrated significant efficacy regarding the induction of specific antitumoral responses and antitumor efficacy using a murine TNBC model.

## Results

### Selection of HLA-binding neoantigens

To ascertain whether breast cancer can be targeted by neoantigen immunotherapy, we established patient-derived xenografts from four TNBC patients, named SW1360, SW2163, SW2183 and SW2388 and additionally assessed the human TNBC cell line MDA-MB-231. Whole exomes from patients’ tumor and normal tissue were sequenced and transcriptome data were used to confirm the expression of the mutated genes. For the human TNBC cell line MDA-MB-231, exome sequencing data available on Catalog Of Somatic Mutations In Cancer (COSMIC) (https://cancer.sanger.ac.uk/cosmic) was used. Using the NetMHC 4.0 algorithm,^30^ a total of 95 candidate neoantigens were predicted in silico to bind to patients’ HLA-A alleles, with a range of 5–57 neoantigens predicted per patient (online supplemental table 1). Of the 95 mutant transcripts, 66 were found in proteome analysis (online supplemental tables 1 and 2), which indicates that not all transcripts are translated into proteins, although the possibility that the proteins were present in too low amounts to be detected by mass spectrometry cannot be ruled out. From the MDA-MB-231 cell line, a total of 27 candidate neoantigens were predicted in silico to bind to HLA-A*02:01 (online supplemental table 3). Peptides corresponding to the 66 candidate neoantigens from the TNBC patients and 27 from the MDA-MB-231 line were synthesized and tested for their binding capacity to the corresponding patient HLA class I allele using T2 cells (HLA-A*02:01), T2 cells transfected with HLA-A*03:01 or a lymphoblastoid cell line homozygous for HLA-A*24:02 (BRIP). Of the 66 patient-derived peptides tested, 44 (66.6%) bound to the predicted HLA-A allele (online supplemental figure S1A–D and table 4). While the in silico prediction corresponded to the actual binding capacity of the patient-derived peptides in ~66% of the cases, there were some discrepancies. For instance, peptide #4 from patient SW2388 had a predicted affinity of 225 nM to HLA-A*03:01, but failed to bind to HLA in the cell-binding assay. In contrast, peptide #8 from the same patient with a predicted affinity of 1332 nM, way above the 500 nM threshold that predicts binding, turned out to bind effectively. For the MDA-MB-231 line, 21 out of 27 epitopes were confirmed to bind to HLA-A*02:01 in cell-binding assays (online supplemental figure S1E and table 5). Altogether, these results highlight the importance of the use of binding assays in order to select the epitopes that can physically bind to the patients’ MHC.

10.1136/jitc-2023-007336.supp1 Supplementary data

### Immunogenicity of TNBC-derived neoantigens

**Figure 1:**
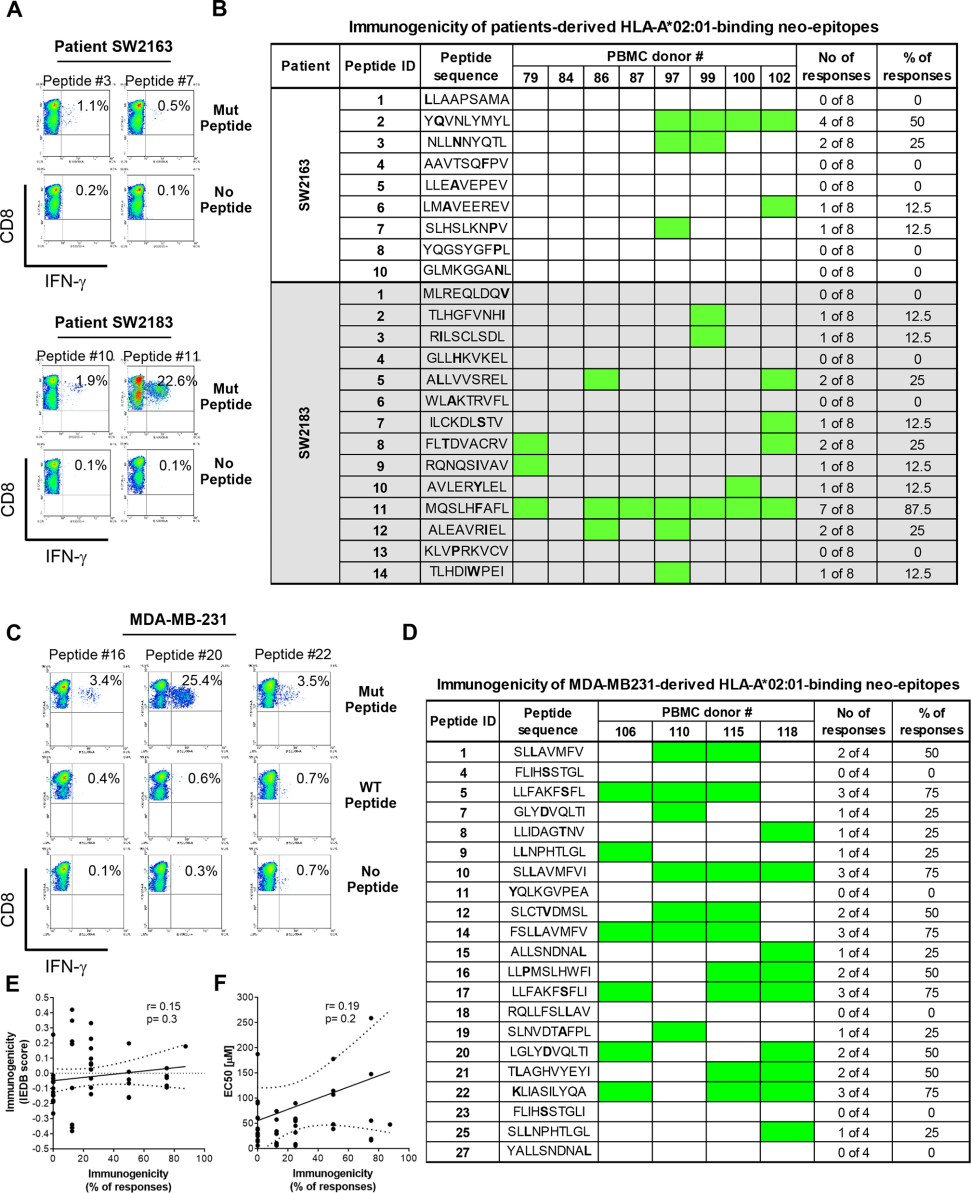
TNBC-derived neoantigens are immunogenic. TNBC peptides were examined for their ability to promote immune responses in immunogenicity T-cell assays using healthy blood samples from nine donors positive for HLA-A*02:01. CD8+T cells isolated from healthy donor blood were stimulated three times with autologous DC pulsed with each of the HLA-A*02:01-binding peptides. Seven days after the last round of stimulation, CD8+T cells were challenged with T2 cells pulsed with the mutant, wild-type peptide (for MDA-MB-231 cells) or left without peptide. Intracellular IFN-γ was measured by FACS. The dot plots in (A, C) depict mutant peptides from TNBC patients and MDA-MB-231 cells, respectively, that elicited strong CD8+T cell responses from one representative donor and in (B, D) is shown the response of all donors analyzed; green represents the responses. (E) Correlation between the immunogenicity according to the IEDB score and T-cell assays; (F) Correlation between binding capacity of the peptides as per the EC50 values from cell-based binding assays and immunogenicity, expressed as the percentage of donors responding to each individual peptide. Responders in (B, D) were determined when percentage of IFN-γ-positive within the CD8+ population in the peptide-stimulated group was twice or higher compared with the non-stimulated T-cells. DCs, dendritic cells; IEDB, Immune Epitope DataBase; PBMC, peripheral blood mononuclear cell; TNBC, triple-negative breast cancer.

To evaluate the immunogenicity of the candidate neoantigens in vitro, we developed a T-cell assay in which in vitro differentiated mature DCs from healthy HLA-A-matched donors were pulsed with individual epitopes and used to stimulate autologous CD8+T cells in 3-weekly stimulation rounds. Seven days after the third stimulation, CD8+T cells were challenged overnight with epitope-pulsed APCs (T2 cells or autologous monocytes) and intracellular IFN-γ production used as a measure of T-cell response. Of the 44 patient-derived epitopes that were confirmed to effectively bind their HLA alleles, only 14 were capable of eliciting CD8+T cell responses (figure 1A,B, online supplemental figure S2 and table 1). Interestingly, not all immunogenic epitopes elicited response in all donors. The most immunogenic peptide was peptide #11 from patient SW2183, which triggered responses in seven out of eight donors tested (figure 1B). Sixteen out of the 21 MDA-MB-231-derived epitopes tested induced CD8+T cell responses and, as we had observed with the patients, not all donors responded to all neoantigen peptides (figure 1C,D, online supplemental figure S3 and table 1). Importantly, in most cases the neoantigen-specific T-cells did not recognize the wild-type peptide. However, CD8+T cells specific for neoantigens #1, #5 and #17 cross-reacted with their wild-type counterparts in 1/2, 3/3 and 2/3 of the responding donors, respectively (online supplemental table 6). It is worth noting that all the epitopes that were immunogenic in our T-cell assays were predicted to be poorly or non-immunogenic by the Immune Epitope DataBase (IEDB) immunogenicity prediction algorithm (figure 1E).

**Figure 2:**
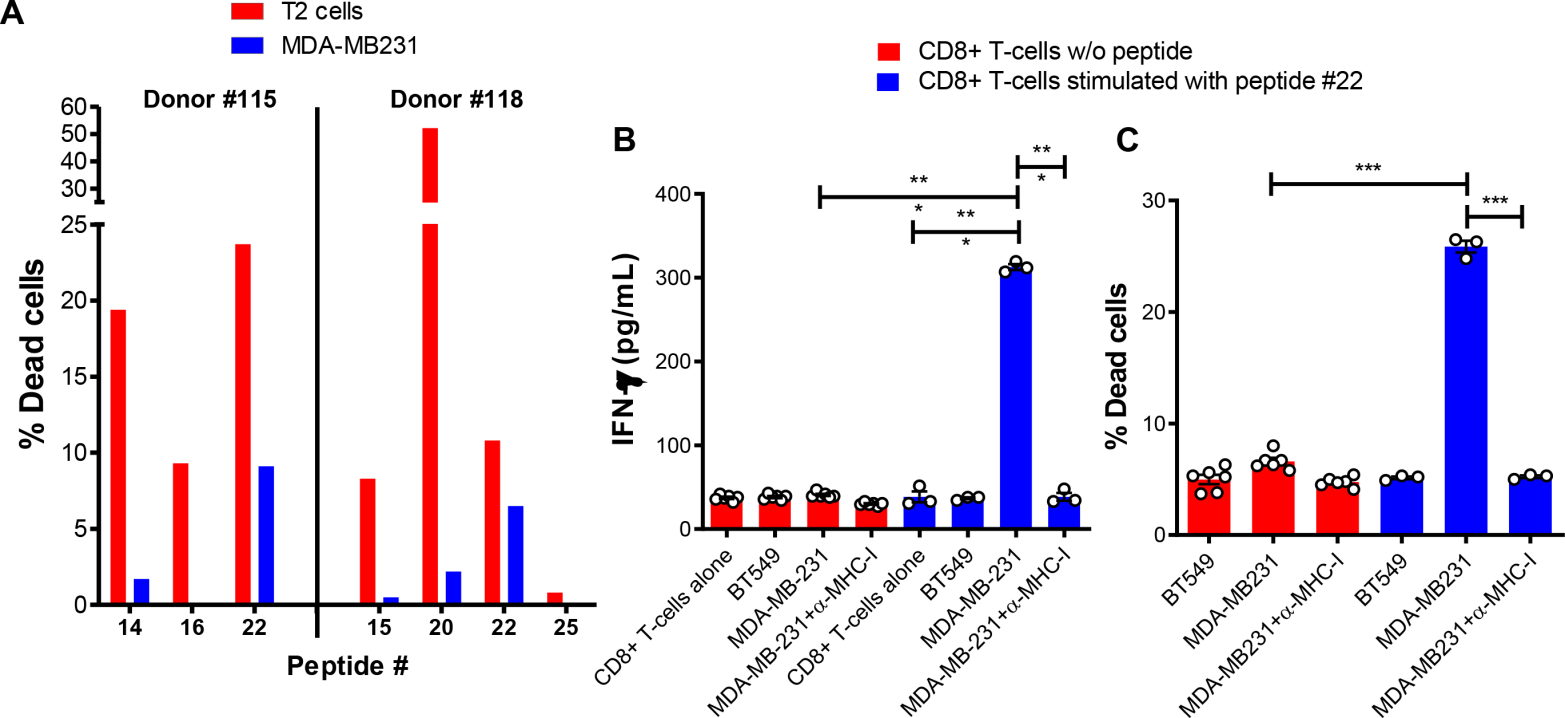
Validation of candidate MDA-MB-231-derived neoantigens. (A) For determination of cytotoxic activity of neoantigen-stimulated CD8+T cells, the bulk of CD8+T cells from donors 115 and 118 stimulated with the indicated neoantigen peptides were incubated overnight with carboxyfluorescein succinimidyl ester (CFSE)-labeled T2 cells pulsed with the neoantigen (red) or CFSE-labeled MDA-MB-231 cells (blue). Cell viability of target T-cells in these co-cultures was determined by staining with EthD-1 followed by flow cytometry analysis. Target T-cells gated on the CFSE-positive population that were positive for EthD-1 were considered dead cells. (B, C) CD8+T cells from a HLA-A*02:01-positive donor were stimulated as in (A) with peptide #22 and incubated overnight with peptide #22-negative/HLA-A*02:01-positive BT549 or peptide #22-positive/HLA-A*02:01-positive MDA-MB-231 cells with or without blocking anti-MHC-I antibodies. (B) Supernatants were collected and assayed for IFN-γ by ELISA and (B) cytotoxicity was determined as in (A). The data were analyzed by one-way ANOVA*p<0.05; **p<0.01; ***p<0.001. Graph shows mean±SEM. ANOVA, analysis of variance.

**Figure 3:**
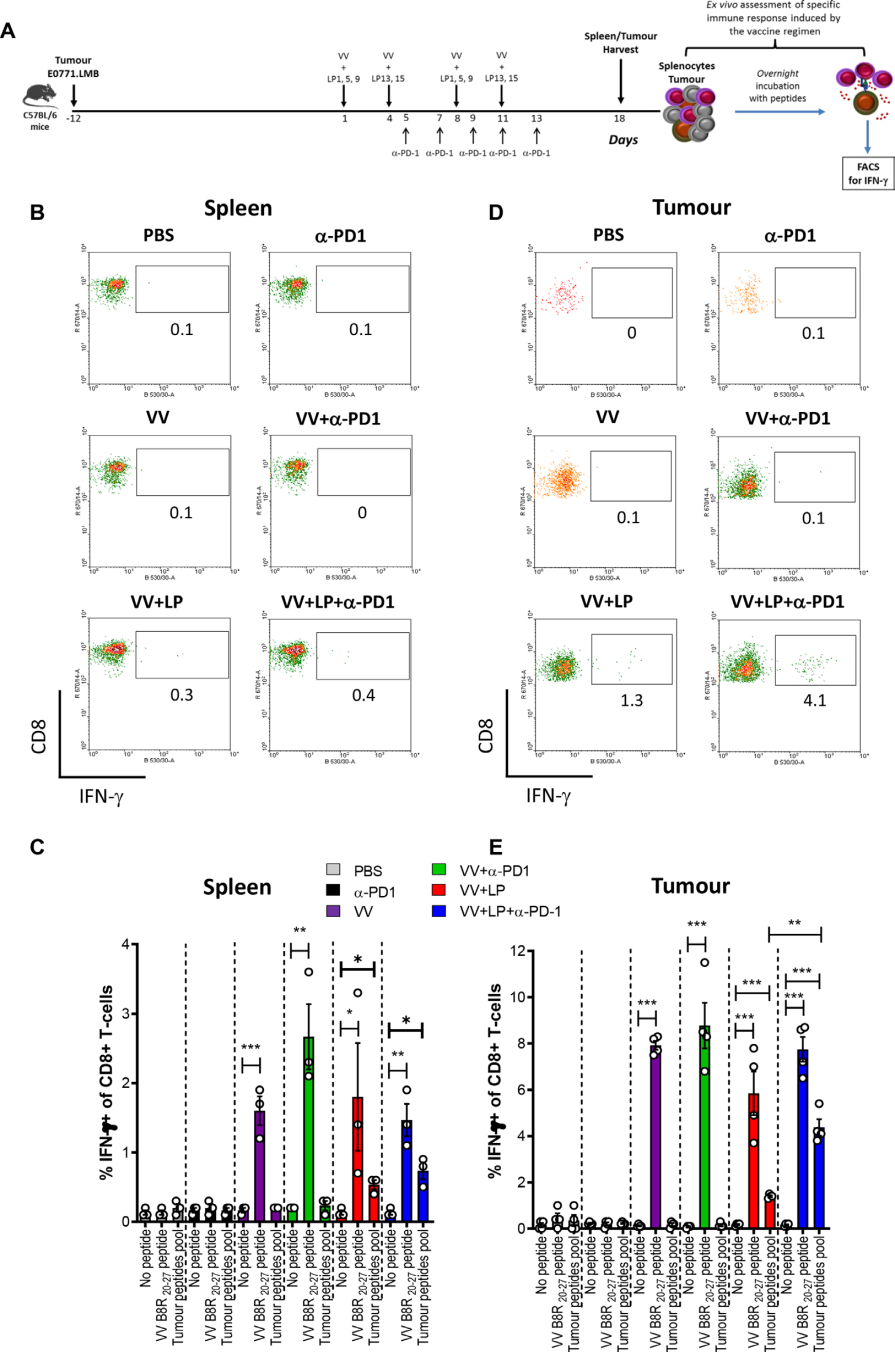
Induction of neoepitope specific CD8+T cells in E0771.LMB-bearing mice. Experimental setup of vaccination of E0771.LMB-bearing C57BL/6 mice with 31-mer long peptides admixed with VV is depicted in A. Seven days after the booster injection, spleens and tumors were harvested, processed and single cell suspensions were incubated ex vivo overnight with a pool of minimal short neoepitope peptides, VV B8R^20–27^ peptide or left with no peptide. Intracellular IFN-γ was measured by FACS and results are shown as the percentage of IFN-γ-positive cells gated on CD8+T cells. (B, D) Depict representative dot plots of the CD8+T cells response from spleen and tumor, respectively; and the graphs in C and E show the means with SEM of the percentage of IFN-γ-positive cells of CD8+T cells in spleen (C) and tumor (E) from all experiments combined. The data shown in (C, E) were analyzed by one-way ANOVA. *p<0.05; **p<0.01; ***p<0.001. ANOVA, analysis of variance; SEM, SE of the mean; VV, vaccinia virus.

**Table 1:** Summary of the TNBC neoantigen candidates

Patient/cell line	HLA allele	Predicted binder*	Actual binder†	Immunogenic‡	Displayed by the tumor§
SW1360	HLA-A*24:02	4	2	0	n.d
SW2163	HLA-A*02:01	10	9	4	n.d
	HLA-A*24:02	9	4	0	n.d
SW2183	HLA-A*02:01	14	14	10	n.d
	HLA-A*24:02	20	8	0	n.d
SW2388	HLA-A*03:01	9	7	0	n.d
MDA-MB-231	HLA-A*02:01	27	21	16	1

*As determined by NetMHC 4.0 program.

†Binding assay evaluated using cell-based binding assays.

‡Reactivity as determined by intracellular IFN-γ in CD8+T cells by FACS.

§Reactivity as determined by intracellular IFN-γ in CD8+T cells by FACS and CTL assay.

n.d, not determined; TNBC, triple-negative breast cancer.

The first requirement for any peptide to trigger a T-cell response is its capacity to bind to HLA, but the affinity to which it binds does not seem to be a good predictor of immunogenicity, as we found no correlation between the binding capacity of the peptides to the HLA and immunogenicity in our assays (figure 1F). In conclusion, of the 93 (66 from the 4 TNBC patients and 27 from MDA-MB-231 cell line) peptides predicted by bioinformatics tools, only 30 hold the potential to translate into an effective CD8+T-cell-based immune response (table 1). These results highlight the need for screening of neoepitope candidates in cell-based assays, which may additionally inform required improvements of the current binding and immunogenicity prediction algorithms.

### TNBC cells expressing neoantigens can be recognized and killed by CD8+ T-cells

We next sought to determine whether the immunogenic neoantigen peptides were being actively presented by the tumor cells. As the tumor samples from the TNBC patients were limited, the human TNBC cell line MDA-MB-231 was used. Immunoaffinity purification of the HLA-A2 molecules from the MDA-MB-231 cells was performed, followed by chromatography and tandem mass spectrometric analysis of HLA-A2 peptides. The mass spectrometry spectra were analyzed using Sequit software tool and queried against the human proteome dataset (Uniprot) and against the list of neoantigens identified by exome sequencing. Mass spectrometry measurement detected 974 HLA-A*02:01-binding peptides (online supplemental file 1 and figure S4), but none of these were neoantigens. If neoantigen peptides are displayed on the cell surface on HLA-A*02:01, they are probably below the threshold for detection by mass spectrometry. Another way to identify neoantigens presented by the tumor cells is to assess whether neoantigen-speciﬁc CD8+T cells can recognize the tumor. On stimulation with the neoantigen peptide #22, T-cells recognized and killed MDA-MB-231 tumor cells (figure 2A), thus confirming that the peptide #22 is generated by the MHC class I processing machinery of the tumor cell for presentation on the cell surface. In contrast, CD8+T cells stimulated with other peptides, including the immunogenic neoantigen peptides #14, #15, #16 and #20 did not recognize MDA-MB-231 tumor cells. Furthermore, incubation of neoantigen peptide #22-stimulated CD8+T cells with MDA-MB-231 in the presence of anti-MHC-I antibodies or with BT549 (HLA-A*02:01-positive/peptide #22-negative) resulted in negligible IFN-γ production (figure 2B) and poor killing activity (figure 2C), showing that the recognition of the tumor cells by the neoantigen peptide #22-stimulated CD8+T cells is HLA-restricted and antigen-specific. Control CD8+T cells cultured the same way, but without peptides produced low amounts of IFN-γ, and displayed poor cytotoxic activity against MDA-MB-231 cells, which further confirms that the peptide-specific T-cells were recognizing the tumor cells in a specific manner. Taken together, these data indicate that neoantigen peptides are most likely presented on the surface of tumor cells in levels under the threshold for detection by mass spectrometry, yet in sufficient amounts for recognition by the specific CD8+T cells.

### Neoantigens are immunogenic in vivo

Given that human TNBC can generate immunogenic neoantigen peptides that are presented on the tumor cell surface to be targeted by cytotoxic CD8+T cells, we set out to test a neoantigen-based vaccine in a mouse model of TNBC. Whole-exome sequencing of the E0771.LMB murine TNBC cell line revealed 1768 non-synonymous mutations (online supplemental file 2) with 63 neoantigens predicted to bind to H2-Db or H2-Kb (online supplemental table 7). The binding capacity of the neoantigen candidates was assessed by stabilization assay using TAP-deficient RMA-S cell line expressing the murine MHC-I alleles H-2Db and H-2Kb. Based on binding, we selected 30 of these 63 epitopes for assessment of immunogenicity (online supplemental figure S5 and table 8). To evaluate the immunogenicity of these 30 neoepitopes, peptides were synthesized as 31-mer long peptides (LP), each LP composed of one H2-Db and one H2-Kb minimal epitope (online supplemental table 9) and used together with adjuvants to immunize C57BL/6 mice. LP were used because we had observed that they are superior to short peptides in triggering peptide-specific CD8+T cell responses (online supplemental figure S6). Following immunization with the neoantigen candidates, the splenocytes were challenged ex vivo with the minimal mutated epitopes or their wild-type counterparts and intracellular IFN-γ was used as a measure of CD8+T cell response. Nine out of 30 peptides elicited CD8+T cell responses against the mutated, but not the wild-type peptide (online supplemental figure S7). Remarkably, as we observed in the human TNBC patients and MDA-MB-231 cell line, the best MHC-I-binders were not always the most immunogenic neoantigen peptides, confirming that MHC-I-binding affinity may not be a good predictor of immunogenicity.

10.1136/jitc-2023-007336.supp2 Supplementary data

### VV+LP prime/boost vaccination combined with anti-PD-1 enhances neoepitope specific CD8+ T-cell infiltration into the tumor

Next we sought to determine the best way to deliver neoantigens for induction of neoantigen-specific CD8+T cells in a therapeutic setting. The promotion of immunogenic cell death by OVs, whose hallmark is the exposure of calreticulin to the cell surface, is key to the uptake of the dying tumor cells by DCs and subsequent induction of T-cells against tumor antigens.^31^ We found that our previously reported oncolytic VV (VVLΔTKΔN1L) was very effective in promoting exposure of calreticulin to the cell surface in a panel of human and murine TNBC cell lines (online supplemental figure S8) and to efficiently kill the mouse TNBC E0771.LMB cell line (online supplemental figure S9).

There are reports that OVs coated with poly-K-modified peptides via electrostatic interactions can induce peptide-specific CD8 T-cells.^32 33^ Examination of the delivery of peptides by oncolytic VV using electrostatic interactions between poly-K modified peptide and the virus versus delivery by simple mixing of unmodified peptides with VV demonstrated no advantage of using a poly-K peptide delivery process over use of an unmodified peptide delivery process in the induction of peptide-specific CD8+T cells (online supplemental figure S10). Therefore, we used unmodified LP mixed with oncolytic VV in all immunization experiments.

E0771.LMB subcutaneous tumors were established in female C57BL/6 mice and the animals received intratumoral injections of VV+LP on days 1, 4, 8 and 11 (figure 3A). We used the LP1, 5, 9, 13 and 15, which were shown to be immunogenic in the previous experiment (online supplemental figure S7). Because TNBC is reported to respond to anti-PD1 therapy,^9 10^ some groups received anti-PD-1 on days 5, 7, 9, 11 and 13. Control groups received only PBS, VV alone or anti-PD-1 alone. Seven days after the last virus injection, mice were culled, had their spleen and tumors harvested for assessment of antitumor immune response. Neoepitope-specific CD8+T cells were detected in the spleen (figure 3B,C) and tumor (figure 3D,E) in groups treated with VV+LP. Interestingly, although addition of anti-PD-1 did not increase the frequency of neoepitope-specific CD8+T cells in the spleen, it induced a more robust infiltration of the specific T-cells into the tumor (figure 3B–E).

### VV+LP+α-PD-1 treatment promotes an immune-stimulatory tumor microenvironment

**Figure 4:**
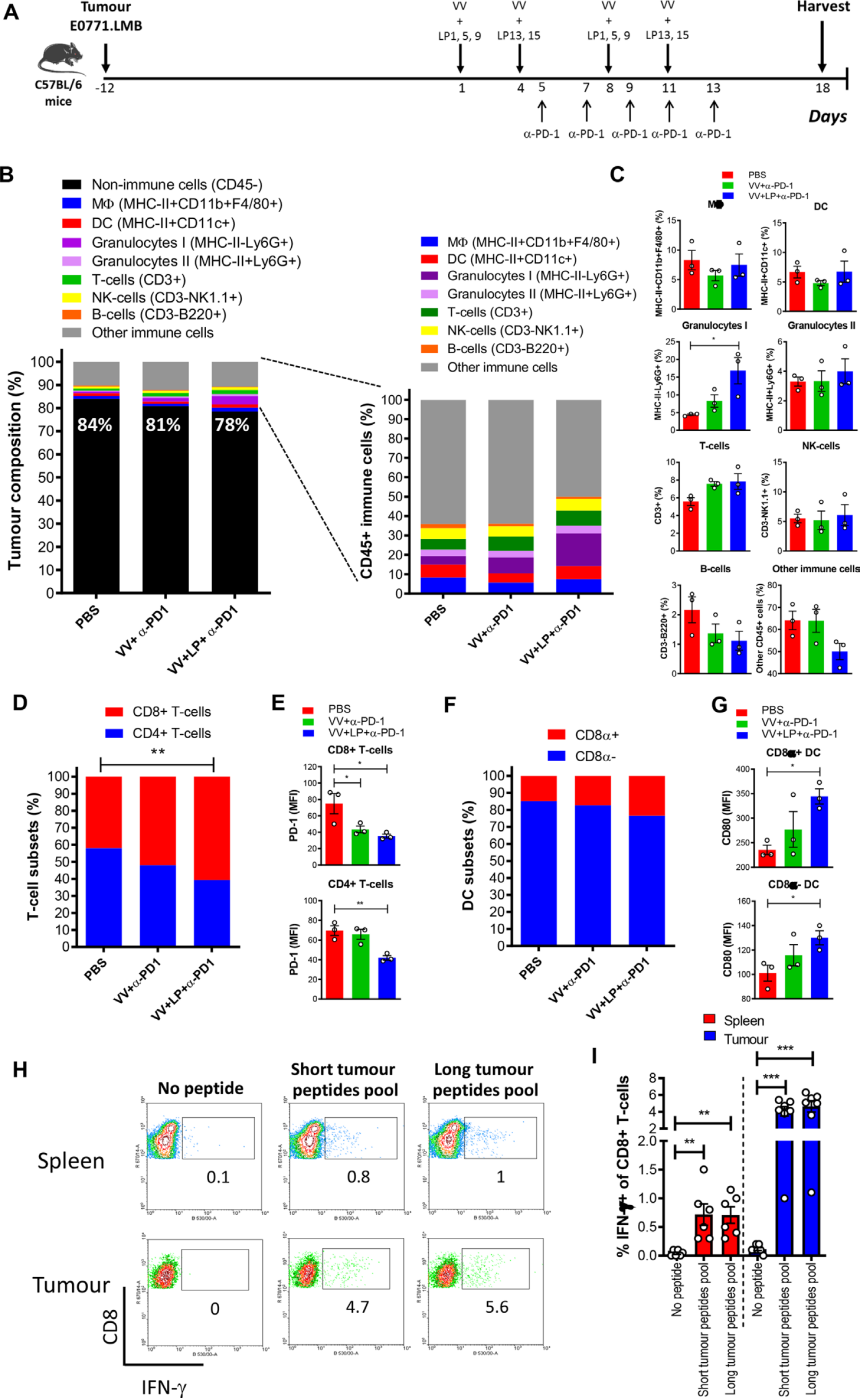
Immune cell infiltration in the TME. (A) Treatment of E0771.LMB-bearing C57BL/6 mice with 31-mer long peptides admixed with VV. Seven days after the booster injection, tumors were harvested, processed and single cell suspensions were analyzed by FACS. (B) Percentage of immune and non-immune cells; (C) Percentage of immune cells subsets in the tumor; (D) Ratio between CD4+ and CD8+ T cells and (E) expression of PD-1 on CD4+ (E, upper graph) and CD8+ (E, bottom graph) T-cells; (F) Ratio between cross-presenting and conventional DC subsets and level of expression of the maturation marker CD80 on the cross-presenting CD8α+ (G, upper graph) and conventional CD8α- DC subset (G, bottom graph). Single cell suspensions of spleens and tumors were incubated overnight with a pool of short minimal neoepitopes or long peptides encompassing these neoepitopes and activation was measured by detecting IFN-γ in CD8+T cells by FACS. The dot plots in (H) depict CD8+T cell responses from one representative mouse and in (I) is shown the response of all mice analyzed. The data shown in (C, D, E, G, I) were analyzed by one-way ANOVA. *p<0.05; **p<0.01; ***p<0.001. ANOVA, analysis of variance; DC, dendritic cell; TME, tumor microenvironment; VV, analysis of variance.

We then sought to determine the effect of VV+LP vaccination in combination with anti-PD-1 on immune cell infiltration into the E0771.LMB tumor microenvironment (TME). E0771.LMB tumors were harvested from mice following vaccine plus anti-PD-1 treatment 7 days after last VV injection, and the immune composition was profiled by flow cytometry (figure 4A and online supplemental figure S11). Tumors from mice receiving VV+LP+α-PD-1 contained a higher proportion of immune cells overall (figure 4B). Significantly more granulocyte infiltration was found in tumors from mice treated with VV+LP+α-PD-1 compared with PBS or VV+α-PD-1 alone (figure 4C). Treatment with VV+LP+α-PD-1 favored infiltration of CD8+T cells over the CD4+subset (figure 4D). Both CD4+ and CD8+ T cells from the VV+LP+α-PD-1 group displayed reduced expression of PD-1 on the cell surface (figure 4E). As reported by Bu *et al,* 2022, the clone RMP1-14 used in vivo does not block the binding of the clone 29F.1A12, used to stain the cells for flow cytometry.^34^ Therefore, it is likely that the lower expression of PD-1 by the tumor-infiltrating T-cells in the animals treated with LP+VV+a-PD-1 is due to the change in the TME caused by the treatment regimen as opposed to any potential block of the target by the treatment antibody. The cross-presenting CD8α+subset of DC was found in larger proportion in tumors from mice treated with VV+LP+α-PD-1, compared with the groups that received PBS or only VV+α-PD-1 (figure 4F). Importantly, the two DC subsets from the VV+LP+α-PD-1 group exhibited a more mature phenotype, observed by their higher levels of the costimulatory molecule CD80 (figure 4G). Incubation of the tumor digestion product with LP, resulted in response of the epitope specific CD8+T cells (figure 4H,I), indicating cross-presentation activity within the tumor, as LP cannot be presented by MHC-I molecules without prior uptake and intracellular processing by the specialized cross-presenting DC (online supplemental figure S12). These findings are in line with the results showing presence of the cross-presenting CD8α+DC infiltrating these tumors. Overall, these data show that VV are not only an effective antigen delivery system, but more importantly, they can alter the TME by promoting recruitment of the neoepitope-specific CD8+T cells into the tumor.

### Evaluation of VV+LP+α-PD-1 in a mouse model of TNBC

**Figure 5:**
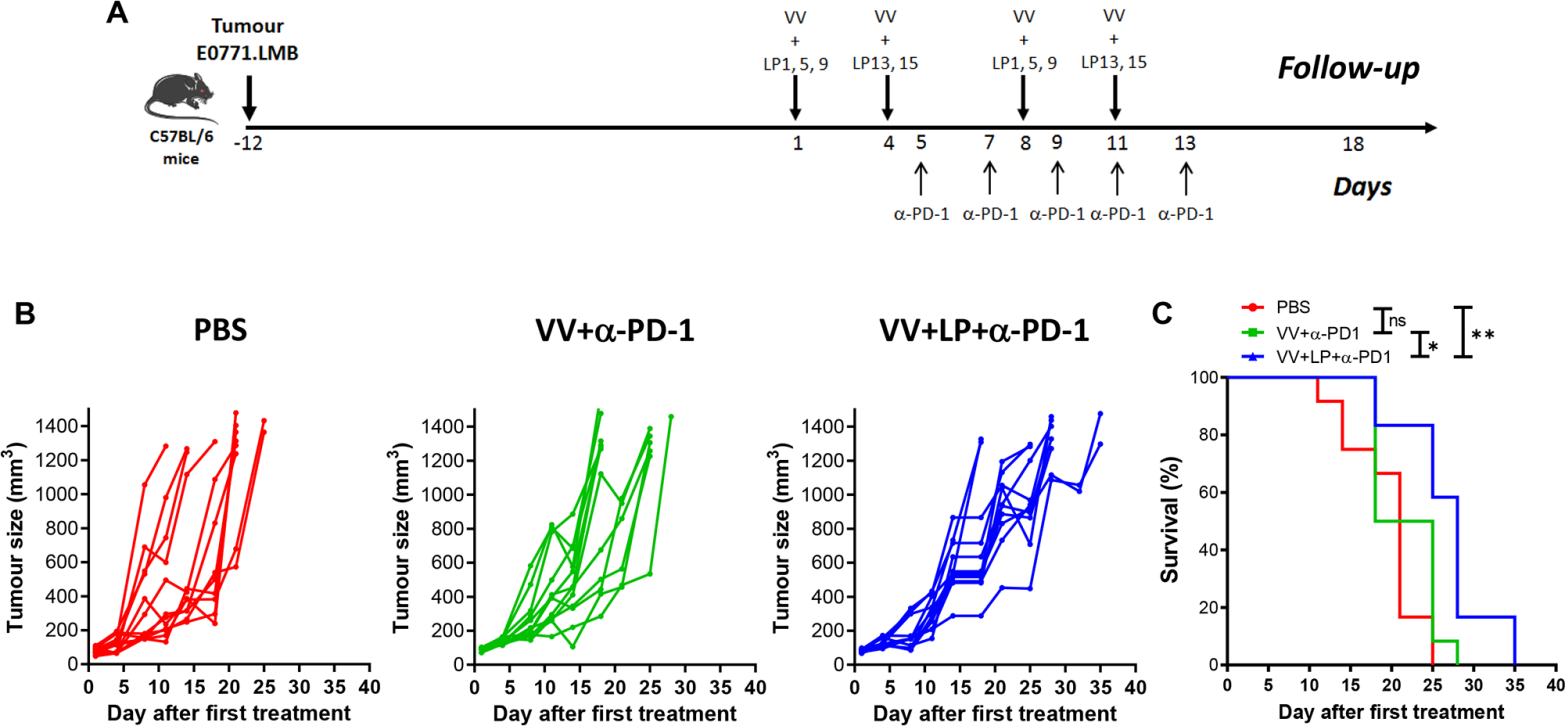
In vivo VV+LP+α-PD-1 treatment delays tumor growth and increases survival. (A) Therapeutic regimen. (B) Evolution of tumor volume (MM^3^) after treatment as a function of time (days). (C) Kaplan-Meier survival analysis with log rank (Mantel-Cox) tests were used to assess survival. (n=12/group). *p<0.05; **p<0.01; n.s non-significant. VV, vaccinia virus.

To determine whether VV+LP+α-PD-1 could affect tumors in vivo, we assessed the treatment regimen in E0771.LMB-bearing mice (figure 5A). Even in this more challenging therapeutic setting, tumor-bearing mice treated with VV+LP+α-PD-1 showed slower tumor growth and longer survival compared with untreated control or VV+α-PD-1 alone groups (figure 5B,C). Although the survival was significantly longer in the group receiving VV+LP+α-PD-1 (p<0.05, Kaplan-Meier survival analysis with log rank Mantel-Cox tests), the effect size was modest (figure 5C). The fact that the use of LP impacted the tumor growth and survival strongly indicates presentation of the neoantigens by the tumor, despite our failure to detect them by mass spectrometry (online supplemental file 1 and figure S13).

### Assessment of the TME of treated mice at the endpoint

**Figure 6:**
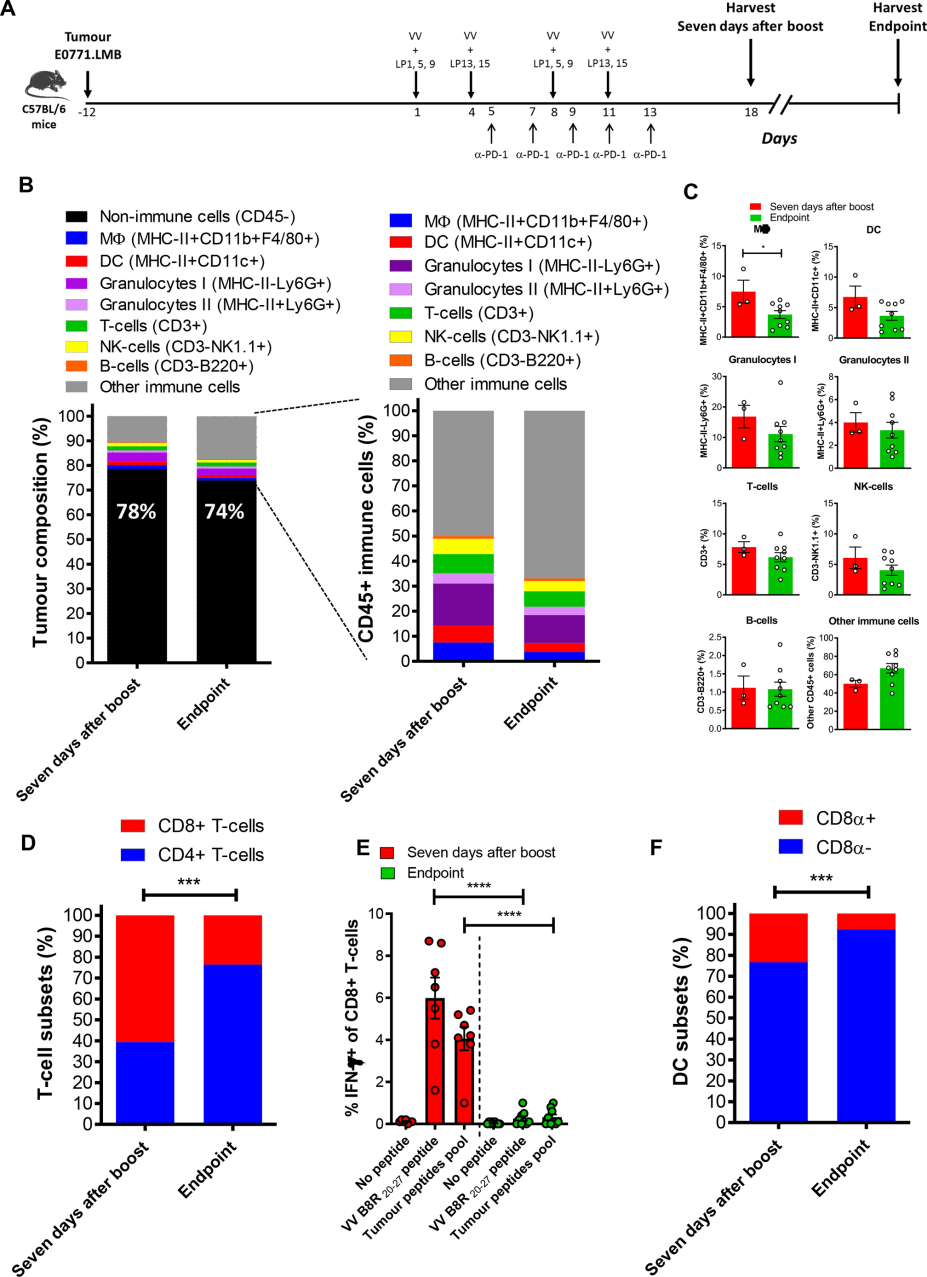
Immune cell infiltration changes in the TME. (A) Treatment of E0771.LMB-bearing C57BL/6 mice with 31-mer long peptides admixed with VV. Seven days after the booster injection or at the endpoint, tumors were harvested, processed and single cell suspensions were analyzed by FACS. (B) Percentage of immune and non-immune cells; (C) Percentage of immune cells subsets in the tumor; (D) Ratio between CD4+ and CD8+ T cells; (E) Single cell suspensions of tumors were incubated overnight with VV B8R 20–27 peptide or a pool of short minimal neoepitopes and activation was measured by detecting IFN-γ in CD8+T cells by FACS; (F) Ratio between cross-presenting CD8α+ and conventional CD8α- DC subsets. The data shown in (C–F) were analyzed by one-way ANOVA. *p<0.05, ***p<0.001, ****p<0.0001. ANOVA, analysis of variance; DC, dendritic cell; VV, vaccinia virus.

Although the mice treated with VV+LP+α-PD-1 had a slower tumor growth and longer survival compared with the other groups, they eventually succumbed to the tumor. We set out to investigate the composition of the tumor of the mice at their endpoint in comparison to that observed 7 days after boost (figure 6A) to see whether there was a change in the immune infiltrates that could explain the loss of tumor control in these mice. Despite a slight increase in the immune infiltrate at the endpoint (figure 6B), there was a trend toward lower frequency of granulocytes and DC (figure 6C) and a statistically significant reduced proportion of macrophages (figure 6C). Strikingly, the proportion of CD4+ in relation to CD8+T cells was increased, with a higher frequency of CD4+T cells at the endpoint, as opposed to a higher frequency of CD8+T cells 7 days after the booster injection (figure 6D). Moreover, both virus-specific and neoepitope-specific CD8+T cells were dramatically reduced in the tumors at the endpoint (figure 6E). Cross-presenting CD8α+DC was also significantly decreased at the endpoint (figure 6F). Altogether, these data indicate that despite treatment, the TME is eventually shifted to a state that favors tumor growth.

### Evaluation of an extended treatment schedule with VV+LP+α-PD-1 on tumor growth and survival in a TNBC model

**Figure 7:**
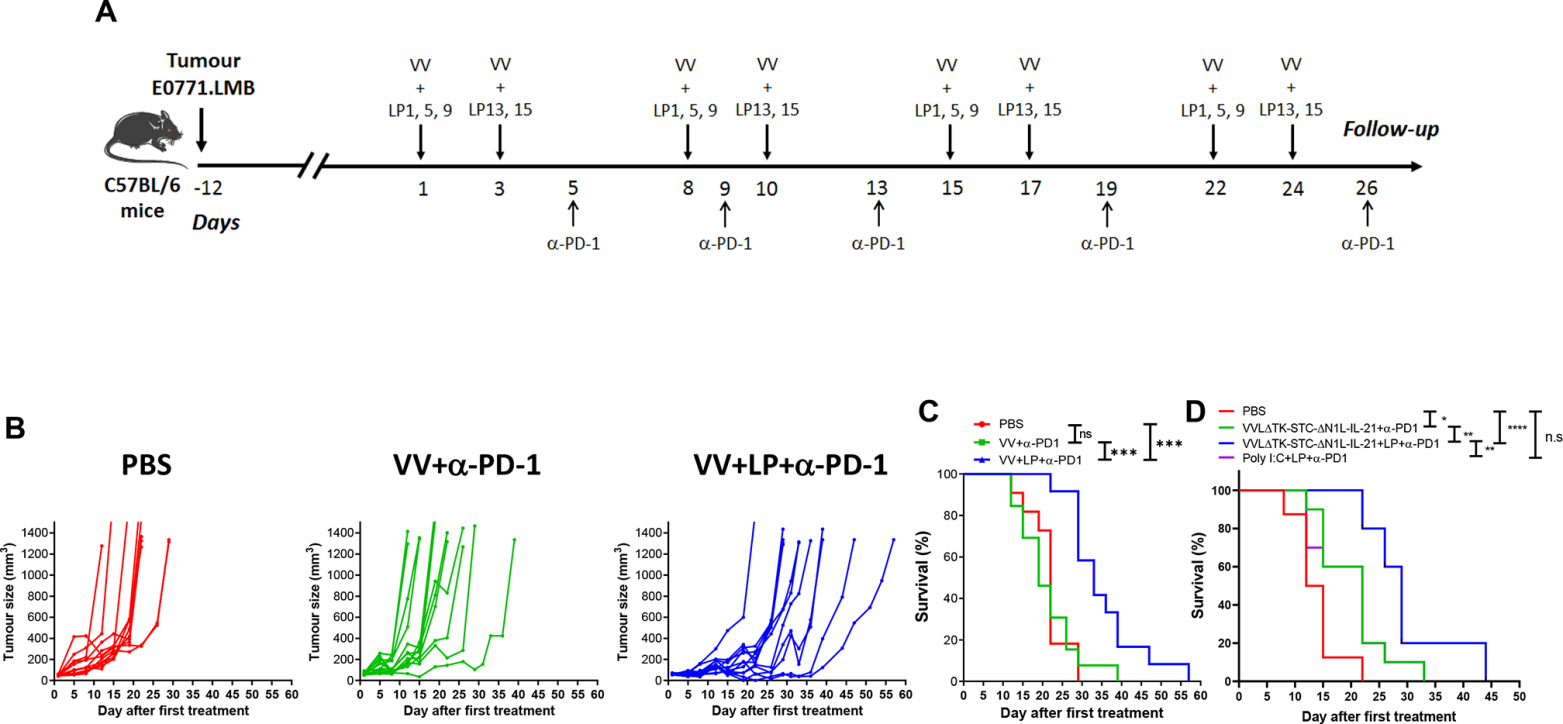
Extended in vivo VV+LP+α-PD-1 treatment delays tumor growth and increases survival. (A) Therapeutic regimen. (B) Evolution of tumor volume (mm^3^) after treatment as a function of time (days). (C) Kaplan-Meier survival analysis with log rank (Mantel-Cox) tests were used to assess survival. (n=11/13/group). *p<0.05; **p<0.01; ***p<0.001; n.s, not significant. (D) VV armed with IL-21 (VVLΔTK-STC-ΔN1L-IL-21) is more efficacious than Poly I:C in increasing survival when administered in conjunction with neoantigen peptides and α-PD-1 antibodies. Kaplan-Meier survival analysis with log rank (Mantel-Cox) tests were used to assess survival. (n=8/10 mice per group). *p<0.05, **p<0.01, ****p<0.0001; n.s not significant. VV, vaccinia virus.

We reasoned that a treatment over a longer period of time would sustain the immune response to the tumor and improve survival. Tumor-bearing mice were treated with VV+LP+α-PD-1 in a schedule of 8 injections of VV+LP over 24 days (figure 7A), as opposed to the previous treatment with four injections over a 13-day period (figure 6A). As previously, tumor-bearing mice treated with VV+LP+α-PD-1 exhibited slower tumor growth and longer survival compared with untreated control or VV+α-PD-1 alone groups (figure 7B,C). However, this extended treatment schedule resulted in a greater effect size and a longer overall survival time. Using neoantigen peptides with a VV armed with IL-21 did not improve survival of the mice, but it resulted in better outcomes compared with the use of neoantigens plus Poly I:C, an adjuvant commonly used in cancer vaccines (figure 7D).

## Discussion

Despite the success of vaccines for prevention and control of infectious diseases, there are no effective vaccines against cancer.^35^ Among the reasons for the limited efficacy of cancer vaccines is that while current vaccines were designed to induce neutralizing antibodies-based immunity, for defense against cancer, cytotoxic T-cell-based responses are crucial. Furthermore, current vaccines are prophylactic and development of therapeutic vaccines is a more difficult task, requiring the induction of vigorous and sustained T-cell immune responses.^35 36^ Another factor that can potentially limit the efficacy of cancer vaccines is the difficulty in determining specific and immunogenic tumor antigens.^37^ In addition, to attack tumor cells effectively, tumor-specific T-cells mobilized by the vaccine must avoid negative regulatory signals in the TME that dampen their activation or induce tolerance programs such as anergy or exhaustion.^38^ We sought to develop a therapeutic vaccine platform that fulfills the above requirements by combining bioinformatics tools and cell-based assays for selection of cancer neoantigens and further exploring the potential of oncolytic VV to deliver these neoantigens in an immunogenic fashion.

Neoantigens (or neoepitopes) found in tumors are unique peptides presented on MHC molecules for recognition by T-cells and represent promising targets for cancer immunotherapy.^35 37^ In this study, we assessed the feasibility of harnessing neoantigens for a personalized immunotherapy for TNBC. Although the antitumor potential of neoantigen-specific T-cells has been demonstrated in several studies for a number of malignancies,^24 39^ to our knowledge, this is the first report of a neoantigen-based immunotherapy combined with oncolytic virotherapy in TNBC.

One of the greatest challenges in harnessing the neoantigenic repertoire of cancers is the selection and validation of suitable targets from a large pool of predicted neoepitopes. Although computational algorithms to predict binding affinity of peptide and MHC-I are usually employed to narrow down the large number of candidate neoepitopes, their accuracy to predict stability of the peptide–MHC-I complexes and immunogenicity are limited. Using cell-based assays, we observed that only about 70% of the human and the murine predicted epitopes can in fact bind to their corresponding MHC class I alleles. Furthermore, not all binders were capable of eliciting a CD8+T-cell-based response, regardless of their binding capacity. These observations contradict previous works that show a correlation between affinity of the peptide for the MHC and immunogenicity.^22 40^ The fact that some epitopes can bind to MHC molecules with high affinity without inducing an immune response could be explained by the absence of T-cell clones capable of recognizing these epitopes. On the other hand, epitopes that despite binding to the MHC with low affinity are capable of inducing a strong CD8+T cell response indicate the presence of T-cell clones capable of recognizing those MHC-I/epitope complexes. Our results thus suggest that immunogenicity is primarily determined by the T-cell repertoire for the different MHC-I/epitope complexes, rather than affinity.

Immunogenicity of peptides can be predicted through computational algorithms (eg, IEDB; http://tools.iedb.org/immunogenicity/) designed to predict the immunogenicity of peptides based on their properties, such as amino acids in position 4–6 and presence of aromatic side chains.^41^ Intriguingly, by using the IEDB tool, we failed to find a correlation between predicted immunogenicity and true immunogenicity of our neoepitopes. Balachandran *et al* have proposed peptide foreigness or non-selfness as a better predictor of immunogenicity of neoepitopes in patients with pancreatic cancer.^42^ While several parameters could be integrated for the selection of the best targets for a vaccine, it is possible (and likely) that many tumors will not yield such high quality neoepitopes, as advanced cancers seem to undergo immune editing, thereby getting rid of the most immunogenic epitopes.^43^ Thus, despite improvements of the algorithms for identification of MHC-binding and immunogenic peptides, determining the actual immunogenicity of those peptides without validation through cell-based assays still remains a challenge. These results highlight the need for the use of T-cell-based immunogenicity assays in the selection of the best epitopes for a vaccine, as not all binders will induce a T-cell response. This is key to a successful vaccine, as if too many epitopes for the same MHC allele are used in the vaccine, there is a high likelihood that they will compete for processing and binding to the MHC, which may cause some of the most immunogenic peptides to be excluded from presentation, and thereby impairing T-cell induction against the relevant epitopes.

One major concern about any targeted immunotherapeutic approach is specificity. To prevent the induction of neoepitope-specific CD8+T cells with capacity to recognize their non-mutated counterparts, we selected peptides containing mutations that are exposed to the T-cell receptor or peptides with mutations that create new anchor residues that increase binding affinity for their MHC class I molecules.^40 44^ In most cases the neoantigen-specific T-cells did not recognize the wild-type peptide. However, CD8+T cells specific for the MDA-MB-231-derived neoantigens #1, #5 and #17 cross-reacted with their wild-type counterparts in some of the responding donors. The mutated residue present in the neoantigen #1, #5 and #17 are located in the inner region of the peptides, which are supposed to interact with the TCR.^40 44^ Therefore, it was expected that the CD8+T cell clones that react to the mutant peptide would be distinct from those reactive to the corresponding wild-type. The possibility that the clones raised against the mutant peptide can recognize its wild-type counterpart should be taken into account in the selection of neoantigens for a vaccine to avoid immune reactions against non-altered epitopes present in non-malignant cells.

Another challenge for the selection of the best targets for a vaccine is to identify which of the immunogenic epitopes are generated by the MHC class I processing machinery and displayed by the tumor cells for CTL recognition. The identification of peptides presented by tumor cells is usually addressed by mass spectrometry through the analysis of peptides eluted from MHC molecules. Although mass spectrometry measurement detected nearly 1000 HLA-A*02:01-binding peptides from the human MDA-MB-231 and around 1300 H2-Db/Kb-binding peptides from the murine E0771.LMB TNBC cell line, none of these were neoepitopes. However, CD8+T cells generated against the MDA-MB-231-derived peptide 22 recognized and killed MDA-MB-231 cells in a specific manner. Additionally, mice bearing E0771.LMB tumor vaccinated with a selection of immunogenic neoepitopes showed delayed tumor growth and longer survival. These results strongly indicate the presentation of those neoepitopes by the tumor cells on MHC class I molecules, despite being presented at levels below the threshold for detection by mass spectrometry. Although effector CD8+T cells need only a few molecules to recognize and kill the target tumor cells,^45^ administration of OV into the tumor induces inflammation which can result in upregulation of MHC-I molecules in tumor cells. As a consequence, it is likely that the presentation of neoepitopes will be increased.

To be effective, a cancer vaccine needs to induce vigorous and sustained T-cell-based immune responses.^35 36^ To achieve this, delivery of the vaccine to address and co-ordinately activate DCs and CD8+cytotoxic T-cells is essential. Replication-competent viral vectors such as VV have become attractive choices as antigen delivery systems.^46^ Traditionally tumor antigen sequences are introduced into the viral vector for delivery.^47^ However, personalized treatments would necessitate genetic manipulation of viruses for each patient, a time-consuming process that requires re-evaluation of each virus produced. To address this constraint, we administered oncolytic VV in a mixture with neoantigen peptides in the form of 31-mer LP and observed a vigorous neoantigen-specific CD8+T cell response in tumor-bearing mice. Delivery of peptides with OV have been shown before, where several virus vectors were coated with poly-K-engineered peptides via an electrostatic interaction.^32 33^ In a side-by-side comparison, we show that a mixture of viruses and peptides is as efficient as viruses coated with engineered poly-K peptides in inducing epitope-specific immunity. Among the advantages of peptide-based vaccines is that they are easy to synthesize and have been well tolerated and proven safe in clinical trials.^35^ Furthermore, it is important to consider that LP, as we used here, are more efficient at eliciting CD8+T cells by a variety of reasons. First, for the priming of naïve CD8+T cells, professional APCs such as DC must take up antigens, process them and present peptides on MHC-I to the peptide-specific CD8+T cells. When short minimal epitopes are used, they can bind directly to all cells expressing MHC-I molecules, leaving too little for the professional APCs. LP will not bind to MHC-I molecules, resulting in more antigens for the professional APCs to take up and process for presentation. Second, in the form of LP, the minimal epitope may be more protected from the cleavage by proteases in the tissue, leading to a longer half-life of the antigen. Third, some long epitopes may contain epitopes for CD4+T cells, which may in turn enhance the activation of the CD8+T cells.^48^ Although all three mechanisms could contribute to the higher efficiency of LP, it is not entirely known which one is the most important. The fact that oncolytic VV plus neoantigen peptides can elicit peptide-specific CD8+T cells strongly indicates that neoantigen delivery together with OV could be an effective ‘off-the-shelf’ approach to stimulate antitumor immunity in TNBC patients.

After induction of antitumor specific CD8+T cells, it is crucial to ensure a prompt and selective homing of activated neoantigen-specific T-cells to tumor tissues where they are required. We reasoned that neoantigen peptides administered intratumorally together with oncolytic VV could induce local inflammation, thereby recruiting into the TME the adaptive immune cells elicited by the vaccine. In fact, the treatment with virus enhanced the recruitment of granulocytes and mature DC subsets into the TME. Moreover, oncolytic VV achieved efficient antigen delivery and promoted infiltration of activated neoantigen-specific CD8+T cells. We show that replicating oncolytic VV vectors mixed with neoepitopes from E0771.LMB cells is an effective regimen for induction of peptide-specific CD8+T cells. Additionally, by adding anti-PD-1 to our regimen, we observed a significantly higher frequency of neoepitope-specific CD8+T cells within the tumor. Moreover, both CD4+ and CD8+ within the tumor displayed lower expression of PD-1, a marker of exhausted T-cells. The value of combination therapy was clearly shown in a recent neoantigen vaccine trial for melanoma, in which the clinical benefit of the vaccination was significantly improved with administration of antibodies against immune checkpoint molecules.^35^ Although this regimen slowed the tumor growth and increased survival, the tumor eventually escaped control resulting in the demise of all treated mice. At the endpoint, the tumors were marked by a dramatic change in some immune cell populations, in particular, a significant decrease in CD8+T cells and cross-presenting CD8α+DCs. Furthermore, negligible amounts of VV and neoantigen-specific CD8+T cells were found in the tumors at that stage. The reduction in cell types thought to be key to an anti-tumor immune response could be behind the tumor growth that led to the demise of the mice. Extending the treatment led to increased survival of the mice treated with VV+LP+α-PD-1, indicating that to sustain an effective immune response to keep the tumor under control, the treatment should be continued for a longer period of time. Interestingly, Li *et al* observed tumor control in mice vaccinated with a DNA-based vaccine encoding multiple neoepitopes in conjunction with anti-PD-1 in E0771 and 4T1 models.^49^ An important difference between that study and ours is that they used a less aggressive E0771 cell line, whereas in our study, we used a parental line, E0771.LMB, which is more aggressive. That could explain why they achieved tumor growth control with their regimen, while in our study, the tumor growth resumed after treatment cessation. Moreover, they started the treatment before tumor injection, meaning that by the time the tumor was injected, there were tumor-specific CD8+T cells already primed.

We believe that in a clinical setting, where PD-1/PDL-1 blockade is already used as part of standard of care,^9 10^ a neoantigen-based vaccine could improve patients’ outcomes significantly. The vaccine would induce activation of neoantigen-specific T-cells. The use of OV can not only provide the dangers signals, key to the induction of an effective adaptive immune response, but also change the TME favoring the infiltration of the tumor-specific T-cells mobilized by the vaccine, as we show here. The anti-PD-1/PDL1 would ensure those T-cells can perform their job at eliminating the tumor cells without becoming exhausted. Furthermore, as we show here and in line with findings reported by Roy *et al*,^50^ using OV plus peptides improve the overall antitumor effect compared with poly I:C. The current mRNA vaccines provide antigen plus adjuvant, as the mRNA can engage innate immunity receptors in a similar way as viral RNA does. However, mRNA cannot kill tumor cells as OV do. mRNA vaccines are likely similar to peptide plus poly I:C vaccines, in which it provides antigen plus adjuvant in one package, but not cytotoxic activity. Two landmark studies show similar results when using mRNA^51^ and LP plus poly I:C^24^ to treat melanoma patients. In that context, we believe that OV plus neoantigens would be more effective than the current mRNA and poly I:C plus neoantigen peptides vaccines.

One limitation of the oncolytic viroimmunotherapy described here was in vivo efficacy. Although our vaccination regimen generated neoantigen-specific CD8+T cells, altered the TME and improved survival of the mice, it did not cause complete tumor regression. It is possible that the regimen can be optimized by using OV in combination with other anticancer agents to target other elements of the TME, such as adenosine and MDSC. Alternatively, we could administer the virus plus peptides systemically, as reported by Roy *et al*.^50^ The advantage of systemic administration of OV is that they can reach small metastatic lesions. Furthermore, when used together with tumor antigens, they can elicit a vigorous CD8+T-cell-based response.^50^

In summary, we used sequencing data from TNBC samples and bioinformatics tools to predict the best neoepitope candidates for a personalized neoantigen vaccine. Each predicted neoantigen was evaluated using peptide-binding assays, T-cell cultures that measure the ability of CD8+T cells to recognize candidate neoantigens, and a preclinical model in which we measured antitumor immunity. Our results demonstrate that TNBC-derived neoantigens can be recognized by the immune system, and that immunization of tumor-bearing mice with neoepitopes induced neoepitope-specific CD8+T cells, slowed tumor growth and increased survival of mice. We conclude that sequencing and epitope-prediction strategies coupled to cell-based assays can identify and prioritize candidate neoantigens for immune targeting in TNBC. Although our platform was tested in TNBC, we believe that the approach can be extended to other malignancies.

## Material and methods

### Tumor specimens

Frozen breast cancer tissues from four TNBC patients collected at cancer resection were obtained from the Barts Cancer Institute Breast Tissue Bank and Barts Health National Health Service (NHS) Trust, London, UK as described previously^52^ (East of England - Cambridge Central Research Ethics Committee Protocol Number 15-EE-0192), with written informed consent obtained from each patient. The clinical characteristics of the TNBC patients are described elsewhere.^52^

### Exome and RNA sequencing of TNBC tissue samples

RNA sequencing was performed on tumor samples from 4 TNBC patients and surrounding non-tumor tissue 10 cm away from the lesion was taken as a control for somatic mutations. RNA-seq reads were aligned to human genome hg38 using Hisat2. Count files were generated by mapping reads using HTseq Pair comparison between tumor and surrounding tissue to identify somatic mutation variants using VarScan2. Mutations were selected if there was greater than ×10 coverage across tumor and surrounding tissue, and if the VAF (the number of variant amino acid changes observed out of the total number of samples) in surround tissue was less than 1% and greater than 15% in tumor. Mutations also had to be present on both the forward and reverse DNA strand. The remaining mutations were annotated with ANNOVAR. Variants present in 1000 Genomes and ESP 6500 exomes with MAF>1% were also removed. The mutations were annotated against RefSeq annotation to extract exonic variants. The allelic HLA pair was estimated from fastq files of RNA-seq using ATHLATE software^53^ and was used to select epitopes that bind to the specific HLA pairing from that patient.

### Proteomics analysis of TNBC tissue samples

Proteomics data for each TNBC patient were also obtained. Galaxy integrated-omics online software was used to search for neoepitopes predicted by RNAseq in the proteomics data, as described elsewhere.^52^

### Exome sequencing data of the MDA-MB-231 cell line

Exome sequencing data of the human MDA-MB-231 cell line was obtained online on the COSMIC. Identification of HLA-A*02:01 neoantigen candidates was performed with SNPnexus software (http://snp-nexus.org),^54^ which employs NetMHCpan^55^ to report the predicted peptide-MHC (pMHC) binding affinity of each mutated and wild-type peptide pair in nanoMolar (nM) units and rank percentages.

### Neoantigen binding affinity prediction

Candidate neoantigens were created from mutated peptide sequences using R statistical software. Nine amino acid LP were created for each non-synonymous mutation that binds to each patient’s HLA-A allele. For each neoepitope the mutant amino acid could occupy any of the positions. The MHC binding affinity for each specific HLA allele was calculated using NetMHC V.4.0 Server.^30^ Peptides were selected based on predicted affinity and location of the mutant amino acid within the peptide. When the mutated amino acid was located in anchor positions (ie, 1, 2 or C-terminus), binding affinity less than 1000 nM for mutant and above 1000 nM for wild-type counterpart for HLA-A*02:01; HLA-A*24:02 below 2500 nM; and HLA-A*03:01 below 1500 nM. In cases where a mutant and its wild-type counterpart peptide had similar predicted binding affinities below the threshold, the mutant peptide was selected when the mutated amino acid was not located in anchor positions, that is, position 3, 4, 5, 6, 7 or 8.

### Identification of neoantigens from the murine TNBC E0771.LMB cell line

For the identification of potential neoantigens from the somatic missense mutations detected from whole-exome sequencing analysis, exome sequencing data were used to compile a list of expressed somatic missense mutations. Amino-acid substitutions corresponding to each of the coding missense mutations were translated into a 21-mer amino acid FASTA sequence, with 10 amino acids flanking the mutated amino acid on each side. These 21-mer amino-acid sequences were evaluated through the MHC class I peptide-binding algorithm NetMHC 4.0^30^ to identify high-affinity 8mer, 9mer and 10mer neoepitopes predicted to bind with high affinity to the C57BL/6 mouse MHC-I alleles H2-D_b_ and H2-K_b_. Neoantigens falling within the 1% percentile rank were selected for validation in cell-based binding assays.

### Cell lines and viruses

The human TNBC cell lines BT549, CAL-120, HCC1143, HCC1937, MDA-MB-231, MDA-MB436, MDA-MB453 and MDA-MB468, and murine 4T1 were obtained from the American Type Culture Collection (ATCC) and cultured in DMEM containing 10% FBS. The murine TNBC cell line E0771.LMB was kindly provided by Professor Kairbaan Hodivala-Dilke from the Barts Cancer Institute and cultured in RPMI1640 containing 10% FBS and 10 mM HEPES. CV1 (African monkey kidney) cells and JH293 cells were obtained from the ATCC and maintained in DMEM containing 5% FBS. All cell lines were authenticated through short tandem repeat profiling (Public Health England). To prevent phenotypic drift, after 50 passages for each cell line, a new vial was thawed. All cell lines were routinely tested for mycoplasma contamination and only used when mycoplasma-free. Construction and production of adenovirus triple-deleted Ad-TD and VV Lister strain VVLΔTKΔN1L and VVLΔTK-STC-ΔN1L-IL-21, were previously described.^26 56 57^

### HLA-typing

The HLA-A alleles of the human TNBC cell lines and all peripheral blood mononuclear cells (PBMCs) donors used for peptide-stimulation assays were determined by VH Bio Ltd (Gateshead, UK) using HLA typing with sequence-specific oligonucleotide primed PCR.

### Coating of peptides onto Ad and VV

The coupling of 31-mer OVA peptide onto AdV and VV was performed as described elsewhere.^32 33^ Briefly, the electrostatic binding potential for 31-mer OVA peptide to Ad-TD and VVLΔTKΔN1L were determined by mixing viruses and peptides at a ratio of a 1:100, 1:200 or 1:500 (µg viral protein: µg peptide) for 30 min at pH 7.4. All measurements were performed at 25°C with a Zetasizer Nano ZS (Malvern). After coupling, the viruses/peptide complexes were used to immunize the mice.

### Cell surface exposure of calreticulin

Cells were incubated with VVLΔTKΔN1L (MOI: 1), left without virus (negative control) or with 2 µM mitoxantrone (positive control). Cells were harvested at different time points and stained with Alexa fluor 647-labeled anti-calreticulin antibody (Abcam, UK). Data analysis was performed on BD FACSDIVA software and WinMDI softwares.

### Cell-based binding assays

Binding of human HLA-A*02:01 and HLA-A*03:01 and murine H2-D_b_ and H2-K_b_ peptides was assessed in stabilization assays using TAP-deficient lymphoblastoid T2 and RMA-S cells, respectively. For the assessment of HLA-A*24:02 peptides, a cell-based competitive binding assay with lymphoblastoid BRIP cell line (European Collection of Authenticated Cell Cultures, UK) was employed. T2 and T2 cells transfected with HLA-A*03:01 were kindly provided by Dr. Louise Boyle from University of Cambridge and by Professor Peter Cresswell from Yale University, respectively. All cell lines were grown and kept in culture in RPMI medium with 10% FCS at 37°C with 5% CO_2_. For the stabilization assays, the cells were washed and incubated in serum-free RPMI medium for 4 hours at 37°C with peptide concentrations ranging from 100 µM (in eight serial dilution steps). The peptide-loaded stable MHC class I on the cell surface was measured by flow cytometry by staining the cells with fluorochrome-labeled monoclonal antibodies to HLA-A*02:01 (clone BB7.2; BD Biosciences), HLA-A*03:01 (clone GAP.A3), H2-D_b_ (clone 28-14-8) and H2-K_b_ (clone AF6-88.5.5.3) (all from Fisher Scientific/eBioscience). The mean fluorescence intensity (MFI) was taken as measure for the peptide stabilizing effect, thereby implicating peptide binding. EC50 values were calculated as the peptide concentration required for half-maximal MFI. For the cell-based competitive binding assay, lymphoblastoid BRIP cells, homozygous for HLA-A*24:02 were incubated in serum-free RPMI medium for 4 hours at 37°C with peptide concentrations ranging from 100 µM (in eight serial dilution steps) in the presence of 500 nM of FITC-labeled reference peptide RYLKK(FITC)QQLL.^58^ The relative binding of the unlabeled competitor peptides were expressed as inhibitory concentration (IC50), that is, the concentration of competitor peptide required to inhibit 50% of binding of the FITC-labeled reference peptide. All measurements were performed with a FACSCalibur flow cytometer (BD Bioscience, Heidelberg, Germany). Data analysis was done with WinMDi 2.9 (Purdue University, USA), and EC50/IC50 calculation with Graphpad Software Prism V.7.03 for Windows (GraphPad Software, La Jolla, California, USA).

### In vitro generation of human DCs

DCs were generated in vitro from magnetic-sorted CD14+cells (Miltenyi Biotec, Bergisch Gladbach, Germany) from PBMCs of healthy donors. Briefly, CD14+cells were cultured in 175 cm^2^ cell culture flasks in RPMI 1640 GlutaMax culture medium (Invitrogen, Carlsbad, California, USA) with 10% heat-inactivated fetal bovine serum (Gibco), 1% pen/strep (Thermo Scientific), 1% sodium pyruvate (Thermo Scientific) and 1% non-essential amino acids (Thermo Scientific). For differentiation of CD14+cells into immature DCs (iDCs), on days 0 and 4 the cultures were supplemented with 50 ng/mL recombinant human GM-CSF and 50 ng/mL IL-4 (BioLegend, San Diego, California, USA). The cultures were maintained at 37°C in humidized atmosphere with 5% CO_2_. For DCs maturation, on day 5 of culture, LPS (Sigma-Aldrich, Germany) at 100 ng/mL was added to the iDCs and the culture continued. The cells were harvested on day 7, loaded with peptides and used in co-cultures with autologous CD8+T cells.

### In vitro T-cell assays

In vitro studies to evaluate the immunogenicity of candidate TNBC-patients and MDA-MB-231-derived neoantigens were performed using HLA-matched healthy donors’ autologous PBMCs. Briefly, mature DCs were pulsed with individual neoantigens at 10 µg/mL and co-cultured with autologous magnetic-sorted CD8+T cells (Miltenyi Biotec, Bergisch Gladbach, Germany) in RPMI with 5% AB human serum, 10 units/mL penicillin–streptomycin, 2 mmol/L L-glutamine, 1% nonessential amino acid, IL-6 (1000 U/mL) and IL-12 (5 ng/mL). IL-2 (30 U/mL) and IL-15 (5 ng/mL) were added every 2 days. CD8+T cells underwent another 2 rounds of stimulation with peptide-pulsed mature DCs on days 7 and 14. Seven days after the third stimulation, CD8+T cells were challenged by overnight incubation with peptide-loaded T2 cells or autologous monocytes. As a control, CD8+T cells were incubated with T2 or autologous monocytes without peptide. Reactivity of the T-cells was determined by intracellular IFN-γ staining measured by flow cytometry.

### Cytotoxicity assay

For the cytotoxicity assays, the bulk of neoepitope-reactive CD8+T cells were incubated with carboxyfluorescein succinimidyl ester (CFSE)-labeled target cells, which were either peptide-loaded T2 cells, BT549 or MDA-MB-231 cell line. After overnight incubation the cells were stained with Ethidium Homodimer 1 (Invitrogen) to determine cell death. Target cells were gated on the CFSE-positive population and cell death was determined by the percentage of cells positive for Ethidium Homodimer 1.

### ELISA for IFN-γ

The supernatants of co-cultures of tumor and CD8+T cells were collected after overnight incubation and stored at −80°C. IFN-γ levels from the co-cultures were measured by ELISA Max Standard kit (BioLegend) in 96-well microtiter plates according to the manufacturer’s instructions. Results are expressed as pg/mL.

### Flow cytometry

For cell surface labeling, cell suspensions were incubated with antibodies for 30 min at room temperature. For intracellular staining, cells were fixed and permeabilized using Leucoperm kit (Bio-Rad) in accordance with the manufacturer’s guidelines. Cell viability was analyzed by Ethidium homodimer 1 staining using LIVE/DEAD Viability/Cytotoxicity Kit, for mammalian cells according to the manufacturer’s instructions (Invitrogen). Flow cytometry was done using a FACSCalibur (Becton Dickinson, Heidelberg, Germany) or BD LSRII (Becton Dickinson) flow cytometer. The data were processed with the CellQuest software (Becton Dickinson, Heidelberg, Germany) and analyzed with the WinMDi V.2.9 (Purdue University, USA).

### Isolation, purification and LC-MS/MS analysis of MHC-presented peptides from human and murine TNBC cell lines

MHC molecules were isolated as previously described.^59 60^ In brief, 4×10^9^ MDA-MB-231 and 2×10^9^ E0771.LMB shock frozen cells were lysed respectively in 0.3% CHAPS, 0.2% NP-40, 145 mM NaCl, 1 mM EDTA, 1 mM Pefabloc, 20 mM Tris-HCl buffer at pH 7.4 and ultracentrifuged for 1 hour at 100,000 × g. MDA-MB-231 cell lysates were treated with antibody of irrelevant specificity for preclearing and supernatants were subsequently purified using the monoclonal anti-HLA-A2-specific antibody BB7.2 coupled to CNBr-activated sepharose (Amersham Biosciences, Uppsala, Sweden). Cell lysates from E0771.LMB were precleared by incubation with antibodies of irrelevant specificities and H2Kb and H2Db molecules were purified using the monoclonal anti-H2Kb and H2Db antibody 28-8-6S coupled to CNBr-activated sepharose. The anti-human and anti-mouse MHC columns with MHC-peptide complexes were washed successively with 20 mM Tris, 145 mM NaCl pH 7.4 (TBS), 0.3% CHAPS in TBS, TBS, 0.3% ß-octylglycoside in TBS, TBS and finally ultrapure water. MHC-peptide complexes were eluted using 0.7% TFA in ultrapure water. MHC-peptide fractionates were obtained using an acetonitrile gradient of 5%–90% of solvent B (90% acetonitrile, 0.1% TFA in ultrapure water) in solvent A (0.1% TFA in ultrapure water) with a reverse phase column μRPC C2/C18, SC2.1/10 on a Smart HPLC system (Amersham Biosciences, Freiburg, Germany), respectively. The peptide fractionates were analyzed by reverse phase liquid chromatography (Ultimate 3000 RSLCnano, ThermoFisher Scientific) coupled on-line with Q Exactive Plus mass spectrometer (ThermoFisher Scientific). Peptide fractionates were injected onto a C18 precolumn at 30 µL/min (2% acetonitrile, 0.1% TFA) for 4 min. Subsequently, peptides were separated at a flow rate of 300 nl/min onto a 75 µm×25 cm PepMap nano-HPLC column with a gradient of 3%–30% of 80% acetonitrile and 0.1%FA acid in ultrapure water over 90 min. Eluted peptides were nanospray-ionized and fragmented based on the 10 most intense precursor ions signals, with a 20 s dynamic exclusion time to avoid repeated fragmentation. MS and MS/MS spectra were processed and peptides were identified against Swissprot human protein sequence database version 56.3 (20,418 reviewed non-redundant protein sequences) and Swissprot mouse protein sequence database version 56.3 (17,016 reviewed non-redundant protein sequences) using MASCOT server (V.2.4). Precursor and fragment mass tolerances of 5ppm and 0.02 Da were used respectively, oxidation of methionine was allowed as a possible modification. Peptide-spectrum matches were validated using a statistical evaluation −10logP, where logP is the logarithm to the base 10 of P (p<0.05) and FDR of <5%. De novo sequencing using Sequit software^61^ and manual inspection were used to further validate the identified peptides. Peptides source proteins were annotated and classified according to subcellular locations and biological functions using Uniprot.^62^ Binding motifs for the peptides were visualized using sequence logos.^63 64^

### Animal experiments

All mouse studies were carried out under the terms of the Home Office Project Licence PPL 70/6030 and PP9448177 and subject to Queen Mary University of London ethical review, according to the guidelines for the welfare and use of animals in cancer research. To test the immunogenicity of OVA peptide when delivered as a short 8-mer minimal epitope or as 31-mer LP, on days 0 and 14 C57BL/6 mice were injected subcutaneously in the right flank with 100 µg of the peptide together with 50 µg of Poly I:C (Invivogen). One week after the second injection the mice were culled, had their spleen harvested and used in ex vivo stimulation experiments. To determine the efficacy of the delivery of 31-mer OVA along with OV, on days 0 and 14, female C57BL/6 mice were injected with 31mer OVA alone or together with Ad (day 0) and VV (day 14). Control groups were injected with OVA peptide alone or Ad/VV without OVA peptide. One week after the second injection, mice were culled, their spleen removed and used in ex vivo stimulation assays to determine the efficacy of the immunization. To test the immunogenicity of the E0771.LMB-derived neoantigens, female C57BL/6 mice were injected subcutaneously on the right flank with three LP each containing one epitope for H2-D_b_ and one for H2-K_b_. Peptides were injected in conjunction with the following adjuvants: anti-mouse CD40 antibody (5 µg; BioLegend), CpG (10 µg; Invivogen), IFN-γ (100 ng; BioLegend), MPLA (10 µg; Invivogen) and poly I:C (50 µg; Invivogen). To determine the efficacy of the delivery of E0771.LMB-derived neoepitopes in conjunction with oncolytic VV on the tumor growth, female C57BL/6 mice were injected with 5×10^5^ E0771.LMB cells in the right flank. When the tumor became palpable, each mouse received an injection of two or three 31-mer LP together with VV (1×10^8^ pfu) on day 1 and 2 (priming), 8 and 11 (boost). Control groups were injected with PBS or virus alone. Some groups received anti-PD-1 (200 µg/mouse) antibodies via intraperitoneal injections on days 5, 7, 9, 11 and 13. In all experiments, tumors were measured twice a week until the tumor size reached the maximum allowed, and mice were then culled. Animals were excluded from the experiments only if tumors did not form or if health concerns were reported. For all animal experiments, mice were randomly grouped and the measurements for tumor size were carried out blind for the groups.

### Spleen and tumor processing

Spleens were harvested from mice, combined with cell culture medium (RPMI medium, 10% FBS, 1% penicillin–streptomycin, and 1% sodium pyruvate), and single cell suspensions were prepared by mashing the spleen against a 70 µm cell strainer. Cells were resuspended in red blood cell lysis buffer (Sigma-Aldrich), washed in PBS, and the pellet was resuspended in cell culture medium and used for peptide stimulation. Tumor samples were minced with a scalpel and incubated with 2 mg/mL collagenase, 0.1 mg/mL hyaluronidase and 0.1 mg/mL DNase I for 2 hours at 37°C. Cell suspensions were filtered using a 70 µm cell strainer, resuspended in culture medium (RPMI medium, 10% FBS, 1% penicillin–streptomycin, and 1% sodium pyruvate) and used for flow cytometry and peptide stimulation.

### Ex vivo peptide stimulation of splenocytes and tumor cell digestion product

Splenocytes or cell suspension from tumor digestion (2×10^6^) were plated into each well of a U-bottom 96-well plate and restimulated with 10 µg/mL of the indicated peptides. When a pool of peptides was used, each peptide in the pool was at 5 µg/mL. Restimulated splenocytes were incubated at 37°C and 5% CO_2_ for 48 hours and the supernatant was collected and IFN-γ measured by ELISA. Alternatively, splenocytes or tumor cell suspensions were incubated overnight in the presence of 5 µg/mL brefeldin A (BFA; Sigma-Aldrich), and intracellular IFN-γ measured by flow cytometry.

### Statistical analysis

Statistical analysis was carried out using the Graphpad Software Prism V.5.01 or V.7.03 for Windows (GraphPad Software). The results were presented as mean±SE of the mean. Differences between groups were analyzed using one-way analysis of variance test. Survival data were represented in a Kaplan-Meier plot and log rank analysis was used to determine if differences between groups were significant. Differences with a p<0.05 were considered significant.

## Data Availability

All data relevant to the study are included in the article or uploaded as online supplemental information.
