# Supplementary material for: Personalized neoantigen viro-immunotherapy platform for triple-negative breast cancer
Source: J Immunother Cancer. 2023 Aug 16;11(8):e007336. doi: 10.1136/jitc-2023-007336 (PMC10432671; doi:10.1136/jitc-2023-007336)
Supplement: Supplementary data [file jitc-2023-007336supp001.pdf]

Supplementary Table 1 - Characteristics of neo-antigens predicted to bind to the patients' HLA alleles. In green and red are the peptides found and not found in the proteome analysis, respectively. Of the 95 neo-antigens predicted to bind to the HLA-A, 66 were found in the proteome and selected for binding assays.

Patient	HLA Allele	Mutant Peptide		Wild Type Peptide		Gene	Mut protein expressed?
		Amino Acid Sequence	Aff (nM) NetMHC	Amino Acid Sequence	Aff (nM) NetMHC		
SW1360	HLA-A*24:02	C**W**NVEQARF	368.8	C**R**NVEQARF	17195	PTAR1	[green]
		EYC**G**TLMSL	1353.7	EYC**S**TLMSL	914.2	NCF1	[red]
		RVSQE**S**FVF	164.6	RVSQE**P**FVF	180.6	ZNF587	[green]
		T**Y**FLVCFIP	524.1	T**D**FLVCFIP	26166.5	ABCA6	[green]
		RVAVILNE**F**	1569.4	RVAVILNE**S**	37184.7	CBWD5	[green]
SW2163	HLA-A*02:01	**L**LAAPSAMA	385.2	**P**LAAPSAMA	12488.9	CREB3L1	[green]
		Y**Q**VNLYMYL	14.1	Y**E**VNLYMYL	2869.9	SCN4A	[green]
		NLL**N**NYQTL	223.9	NLL**K**NYQTL	508	FAM111B	[green]
		AAVTSQ**F**PV	384.5	AAVTSQ**S**PV	7360.8	C18orf8	[green]
		KL**Y**GGKKKV	621.4	KL**H**GGKKKV	3850.5	STAB2	[red]
		LLE**A**VEPEV	33.3	LLE**V**VEPEV	89.4	NBPF12	[green]
		LM**A**VEEREV	822.7	LM**E**VEEREV	5820.1	NBPF10	[green]
		SLHSLKN**P**V	64.7	SLHSLKN**R**V	471	TCF12	[green]
		YQGSYGF**P**L	15.2	YQGSYGF**R**L	97.9	TP53	[green]
		LLFLPL**L**PV	12.6	LLFLPL**F**PV	4.9	ZDHHC11	[red]
		FLPL**L**PVQV	20.3	FLPL**F**PVQV	14.7	ZDHHC11	[red]
		L**L**PVQVQTL	692.8	L**F**PVQVQTL	17817.6	ZDHHC11	[red]
		SQ**F**PVPCKL	544.1	SQ**S**PVPCKL	2884.3	C18orf8	[green]
		GLMKGGA**N**L	64.7	GLMKGGA**D**L	263.8	APMAP	[green]
SW2163	HLA-A*24:02	KT**S**ICYLSF	486.1	KT**N**ICYLSF	1250.3	DENND6A	[green]
		K**Y**ISTSLPV	240.3	K**S**ISTSLPV	17263.1	PLS1	[green]
		IWEQLAS**R**F	94.8	IWEQLAS**G**F	404.5	CLASP2	[green]
		**I**KPEVIFKI	2001.5	**T**KPEVIFKI	3144.8	ZNF248	[green]
		QY**Q**VNLYMY	2019.5	QY**E**VNLYMY	7252.8	SCN4A	[green]
		IKSMAQ**Y**LI	2264.2	IKSMAQ**H**LI	5389.6	DOCK9	[green]
		**F**FNKGGSVF	598.7	**L**FNKGGSVF	677.5	ADGRV1	[green]
		**L**RNPGTWTF	1194.2	**P**RNPGTWTF	4060.6	VSIG10L	[green]
		LMKGGA**N**LF	821.1	LMKGGA**D**LF	1989	APMAP	[green]
SW2183	HLA-A*02:01	MLREQLDQ**V**	113	MLREQLDQ**A**	1235.4	CROCC	[green]
		SLYTV**L**PEG	382	SLYTV**P**PEG	1489.8	IFT140	[red]
		TLHGFVNH**I**	139.2	TLHGFVNH**T**	1369.4	ANO2	[green]
		R**I**LSCLSDL	478.5	R**S**LSCLSDL	7013.8	PPP1R3D	[green]
		GLL**H**KVKEL	254.9	GLL**R**KVKEL	648.8	SH2D3C	[green]
		NLTEVP**A**DL	497.1	NLTEVP**T**DL	654.5	TPBG	[red]
		A**L**LVVSREL	286.9	A**V**LVVSREL	3392.1	NLRP2	[green]
		A**L**AASSSSA	347.6	A**S**AASSSSA	22794	KMT2A	[red]
		WL**A**KTRVFL	188.5	WL**E**KTRVFL	1943.3	FLAD1	[green]
		**A**LTSQSAEI	283.2	**T**LTSQSAEI	553.8	TPCN1	[red]
		ILCKDL**S**TV	55.5	ILCKDL**P**TV	14.9	CFAP69	[green]
		GTQ**H**LCFCV	623.3	GTQ**Q**LCFCV	977	SLC7A6	[red]
		FL**T**DVACRV	5.2	FL**I**DVACRV	3.3	S1PR3	[green]
		RMAP**M**LPSV	5	RMAP**V**LPSV	4.1	MRPL35	[red]
		RQNQS**I**VAV	652.4	RQNQS**T**VAV	1222.1	ARNTL2	[green]
		SAVNY**I**TQV	554.5	SAVNY**M**TQV	967.1	CRISPLD2	[red]
		LLNFTG**F**AV	44.5	LLNFTG**C**AV	286.1	CHURC1-FNTB	[red]
		YIVALP**V**AV	15.1	YIVALP**L**AV	14.4	DNAJC22	[red]
		LLGSSSSP**A**	573.3	LLGSSSSP**T**	3047.2	TPCN1	[red]
		AVLER**Y**LEL	145.7	AVLER**D**LEL	244.9	MYO15B	[green]
		LI**I**MLEAAL	547.6	LI**T**MLEAAL	1985.3	CLTC	[red]
		MQSLH**F**AFL	224.1	MQSLH**S**AFL	254.4	URB2	[green]
		ALEAVR**I**EL	710	ALEAVR**S**EL	2100.3	JMJD7	[green]
		YLT**S**LMTPV	2.8	YLT**C**LMTPV	2.9	ATP10A	[red]
		KLV**P**RKVCV	286.4	KLV**A**RKVCV	298.5	PPIP5K1	[green]
		AMQE**M**VDAL	48.7	AMQE**T**VDAL	87.4	KIF21A	[red]
		TLHDI**W**PEI	15	TLHDI**G**PEI	48.6	CACNA1D	[green]
		EL**F**TKLHPA	198.4	EL**L**TKLHPA	391.6	GPR68	[red]
SW2183	HLA-A*24:02	SFLLNFTG**F**	213.4	SFLLNFTG**C**	17287.2	CHURC1-FNTB	[red]
		L**F**TKLHPAF	490.7	L**L**TKLHPAF	5145.8	GPR68	[green]
		S**Y**YMPQLFW	67.4	S**N**YMPQLFW	9466.6	PLCB1	[green]
		YRMV**V**YHNW	471.6	YRMV**L**YHNW	517	PDE11A	[green]
		SS**Y**YMPQLF	339.3	SS**N**YMPQLF	960.5	PLCB1	[red]
		EMQSLH**F**AF	435.2	EMQSLH**S**AF	3592.3	URB2	[green]
		R**Y**LELSLRP	196.2	R**D**LELSLRP	25291.4	MYO15B	[red]
		RFFPLV**L**DL	402.3	RFFPLV**P**DL	564.7	N6AMT1	[green]
		KYSDV**I**KVL	439.6	KYSDV**M**KVL	581.4	ZFYVE16	[green]
		DYPGQG**F**SF	100.1	DYPGQG**S**SF	642.3	ZMIZ2	[green]
		RMAP**M**LPSV	2007.8	RMAP**V**LPSV	3245.9	MRPL35	[green]
		**S**YEEITSPW	512.9	**P**YEEITSPW	1555.9	KIAA1586	[red]
		RTGESS**F**PF	711.2	RTGESS**L**PF	1782	ABCC9	[green]
		SVM**K**KNNLF	629.6	SVM**E**KNNLF	1015.7	SLC24A1	[green]
		**Y**YMPQLFWN	788.9	**N**YMPQLFWN	2957.5	PLCB1	[green]
		SF**P**YESVPW	703.3	SF**A**YESVPW	882.5	ZMIZ2	[green]
		MIFA**A**VVLF	1021.2	MIFA**V**VVLF	1687.3	CCR4	[green]
		IFA**A**VVLFL	815.4	IFA**V**VVLFL	1513.2	CCR4	[green]
		TVMLPD**Y**SF	1324.2	TVMLPD**H**SF	3805.1	NBEAL1	[red]
		D**Y**SFCDSLW	898.3	D**H**SFCDSLW	18479.2	NBEAL1	[red]
		FYLT**S**LMTP	1103.1	FYLT**C**LMTP	1293.3	ATP10A	[green]
		RLA**T**EPFPF	195.7	RLA**A**EPFPF	178.4	TCAF2	[red]
		QYAAAS**S**AW	372.6	QYAAAS**P**AW	312.1	ARID1B	[green]
		VNSGIY**W**VL	1041.7	VNSGIY**R**VL	9800.5	HSPA14	[red]
		IWL**A**KTRVF	54.3	IWL**E**KTRVF	63.7	FLAD1	[red]
		VCAHTAII**F**	2296	VCAHTAII**Y**	30529.6	WDR66	[green]
		HTAII**F**NVF	1198.5	HTAII**Y**NVF	786.1	WDR66	[green]
		RFTL**S**VPGF	636.3	RFTL**P**VPGF	714.9	NFATC2	[green]
		VYSFL**C**KTI	55	VYSFL**Y**KTI	22.1	AP1M2	[green]
SW2388	HLA-A*03:01	RMLPSGLS**R**	48.1	RMLPSGLS**W**	2994.6	ATG2B	[green]
		RQGLLQ**M**QK	251.7	RQGLLQ**K**QK	2473	CCDC66	[green]
		KA**L**PRAGVK	79.5	KA**F**PRAGVK	31	ZNF385D	[green]
		VLALA**V**HFK	36.5	VLALA**A**HFK	27.8	DIXDC1	[red]
		HQRIHTG**G**K	225.3	HQRIHTG**E**K	404.7	ZNF84	[green]
		RIHTG**G**KPY	1008.5	RIHTG**E**KPY	2186.1	ZNF84	[green]
		QSKVAAWL**K**	1141.3	QSKVAAWL**Q**	34034.3	OSBPL6	[green]
		RLEM**S**CLSK	200.8	RLEM**C**CLSK	214.5	ZCWPW2	[green]
		CIVYL**S**LSR	1332	CIVYL**P**LSR	1751.5	SEMA6C	[green]
		HLI**H**HRMLH	137.3	HLI**R**HRMLH	155.2	ZNF746	[green]

Supplementary Table 2 – Characteristics of TNBC patient's-derived neo-antigens predicted to bind to the patients' HLA alleles.

Patient	HLA Allele	Peptide ID	Mutated Peptide			Wild Type Peptide			Gene	Amino Acid Substitution
			Amino Acid Sequence	Aff (nM) NetMHC	Rank (%) NetMHC	Amino Acid Sequence	Aff (nM) NetMHC	Rank (%) NetMHC		
SW1360	HLA-A*24:02	1	C**W**NVEQARF	368.8	0.6	C**R**NVEQARF	17195	11	PTAR1	p.R382W
		2	RVSQE**S**FVF	164.6	0.3	RVSQE**P**FVF	180.6	0.4	ZNF587	p.P169S
		3	T**Y**FLVCFIP	524.1	0.8	T**D**FLVCFIP	26166.5	19	ABCA6	p.D1196Y
		4	RVAVILNE**F**	1569.4	1.5	RVAVILNE**S**	37184.7	38	CBWD5	p.S78F
SW2163	HLA-A*02:01	1	**L**LAAPSAMA	385.2	2.5	**P**LAAPSAMA	12488.9	18	CREB3L1	p.P140L
		2	Y**Q**VNLYMYL	14.1	0.175	Y**E**VNLYMYL	2869.9	7	SCN4A	p.E1266Q
		3	NLL**N**NYQTL	223.9	1.7	NLL**K**NYQTL	508	3	FAM111B	p.K389N
		4	AAVTSQ**F**PV	384.5	2.5	AAVTSQ**S**PV	7360.8	12	C18orf8	p.S329F
		5	LLE**A**VEPEV	33.3	0.5	LLE**V**VEPEV	89.4	1	NBPF12	p.V1047A
		6	LM**A**VEEREV	822.7	4	LM**E**VEEREV	5820.1	11	NBPF10	p.E3722A
		7	SLHSLKN**P**V	64.7	0.8	SLHSLKN**R**V	471	3	TCF12	p.R399P
		8	YQGSYGF**P**L	15.2	0.2	YQGSYGF**R**L	97.9	1	TP53	p.R71P
		9	SQ**F**PVPCKL	544.1	3	SQ**S**PVPCKL	2884.3	7	C18orf8	p.S329F
		10	GLMKGGA**N**L	64.7	0.8	GLMKGGA**D**L	263.8	1.9	APMAP	p.D295N
	HLA-A*24:02	1	KT**S**ICYLSF	486.1	0.7	KT**N**ICYLSF	1250.3	1.3	DENND6A	p.N95S
		2	K**Y**ISTSLPV	240.3	0.5	K**S**ISTSLPV	17263.1	11	PLS1	p.S546Y
		3	IWEQLAS**R**F	94.8	0.175	IWEQLAS**G**F	404.5	0.6	CLASP2	p.G135R
		4	**I**KPEVIFKI	2001.5	1.8	**T**KPEVIFKI	3144.8	2.5	ZNF248	p.T52I
		5	QY**Q**VNLYMY	2019.5	1.8	QY**E**VNLYMY	7252.8	5	SCN4A	p.E1266Q
		6	IKSMAQ**Y**LI	2264.2	2	IKSMAQ**H**LI	5389.6	4	DOCK9	p.H973Y
		7	**F**FNKGGSVF	598.7	0.8	**L**FNKGGSVF	677.5	0.9	ADGRV1	p.L3412F
		8	**L**RNPGTWTF	1194.2	1.3	**P**RNPGTWTF	4060.6	3	VSIG10L	p.P739L
		9	LMKGGA**N**LF	821.1	1	LMKGGA**D**LF	1989	1.8	APMAP	p.D295N
SW2183	HLA-A*02:01	1	MLREQLDQ**V**	113	1.1	MLREQLDQ**A**	1235.4	4.5	CROCC	p.A246V
		2	TLHGFVNH**I**	139.2	1.3	TLHGFVNH**T**	1369.4	5	ANO2	p.T847I
		3	R**I**LSCLSDL	478.5	3	R**S**LSCLSDL	7013.8	12	PPP1R3D	p.S23I
		4	GLL**H**KVKEL	254.9	1.9	GLL**R**KVKEL	648.8	3.5	SH2D3C	p.R576H
		5	A**L**LVVSREL	286.9	2	A**V**LVVSREL	3392.1	8	NLRP2	p.V840L
		6	WL**A**KTRVFL	188.5	1.6	WL**E**KTRVFL	1943.3	6	FLAD1	p.E25A
		7	ILCKDL**S**TV	55.5	0.7	ILCKDL**P**TV	14.9	0.2	CFAP69	p.P364S
		8	FL**T**DVACRV	5.2	0.04	FL**I**DVACRV	3.3	0.01	S1PR3	p.I265T
		9	RQNQS**I**VAV	652.4	3.5	RQNQS**T**VAV	1222.1	4.5	ARNTL2	p.T581I
		10	AVLER**Y**LEL	145.7	1.4	AVLER**D**LEL	244.9	1.8	MYO15B	p.D693Y
		11	MQSLH**F**AFL	224.1	1.7	MQSLH**S**AFL	254.4	1.9	URB2	p.S809F
		12	ALEAVR**I**EL	710	3.5	ALEAVR**S**EL	2100.3	6	JMJD7	p.S11I
		13	KLV**P**RKVCV	286.4	2	KLV**A**RKVCV	298.5	2	PPIP5K1	p.A305P
		14	TLHDI**W**PEI	15	0.2	TLHDI**G**PEI	48.6	0.6	CACNA1D	p.G1671W
	HLA-A*24:02	1	L**F**TKLHPAF	490.7	0.7	L**L**TKLHPAF	5145.8	3.5	GPR68	p.L341F
		2	S**Y**YMPQLFW	67.4	0.125	S**N**YMPQLFW	9466.6	6	PLCB1	p.N608Y
		3	YRMV**V**YHNW	471.6	0.7	YRMV**L**YHNW	517	0.8	PDE11A	p.L412V
		4	EMQSLH**F**AF	435.2	0.7	EMQSLH**S**AF	3592.3	3	URB2	p.S809F
		5	RFFPLV**L**DL	402.3	0.6	RFFPLV**P**DL	564.7	0.8	N6AMT1	p.P160L
		6	KYSDV**I**KVL	439.6	0.7	KYSDV**M**KVL	581.4	0.8	ZFYVE16	p.M1267I
		7	DYPGQG**F**SF	100.1	0.2	DYPGQG**S**SF	642.3	0.9	ZMIZ2	p.S749F
		8	RMAP**M**LPSV	2007.8	1.8	RMAP**V**LPSV	3245.9	2.5	MRPL35	p.V82M
		9	RTGESS**F**PF	711.2	0.9	RTGESS**L**PF	1782	1.7	ABCC9	p.L626F
		10	SVM**K**KNNLF	629.6	0.9	SVM**E**KNNLF	1015.7	1.2	SLC24A1	p.E277K
		11	**Y**YMPQLFWN	788.9	1	**N**YMPQLFWN	2957.5	2.5	PLCB1	p.N608Y
		12	SF**P**YESVPW	703.3	0.9	SF**A**YESVPW	882.5	1.1	ZMIZ2	p.A22P
		13	MIFA**A**VVLF	1021.2	1.2	MIFA**V**VVLF	1687.3	1.6	CCR4	p.V247A
		14	IFA**A**VVLFL	815.4	1	IFA**V**VVLFL	1513.2	1.5	CCR4	p.V247A
		15	FYLT**S**LMTP	1103.1	1.2	FYLT**C**LMTP	1293.3	1.4	ATP10A	p.C1278S
		16	QYAAAS**S**AW	372.6	0.6	QYAAAS**P**AW	312.1	0.5	ARID1B	p.P488S
		17	VCAHTAII**F**	2296	2	VCAHTAII**Y**	30529.6	24	WDR66	p.Y285F
		18	HTAII**F**NVF	1198.5	1.3	HTAII**Y**NVF	786.1	1	WDR66	p.Y285F
		19	RFTL**S**VPGF	636.3	0.9	RFTL**P**VPGF	714.9	0.9	NFATC2	p.P154S
		20	VYSFL**C**KTI	55	0.1	VYSFL**Y**KTI	22.1	0.03	AP1M2	p.Y85C
SW2388	HLA-A*03:01	1	RMLPSGLS**R**	48.1	0.25	RMLPSGLS**W**	2994.6	4	ATG2B	p.W1211R
		2	RQGLLQ**M**QK	251.7	0.9	RQGLLQ**K**QK	2473	3.5	CCDC66	p.K925M
		3	KA**L**PRAGVK	79.5	0.4	KA**F**PRAGVK	31	0.15	ZNF385D	p.F244L
		4	HQRIHTG**G**K	225.3	0.8	HQRIHTG**E**K	404.7	1.2	ZNF84	p.E708G
		5	RIHTG**G**KPY	1008.5	2	RIHTG**E**KPY	2186.1	3.5	ZNF84	p.E708G
		6	QSKVAAWL**K**	1141.3	2.5	QSKVAAWL**Q**	34034.3	55	OSBPL6	p.Q253K
		7	RLEM**S**CLSK	200.8	0.8	RLEM**C**CLSK	214.5	0.8	ZCWPW2	p.C197S
		8	CIVYL**S**LSR	1332	2.5	CIVYL**P**LSR	1751.5	3	SEMA6C	p.P515S
		9	HLI**H**HRMLH	137.3	0.6	HLI**R**HRMLH	155.2	0.6	ZNF746	p.R527H

Supplementary Table 3 – Characteristics of MDA-MB231-derived neo-antigens predicted to bind to HLA-A*02:01.

Peptide ID	Mutated Peptide			Wild Type Peptide			Gene	Amino Acid Substitution
	Amino Acid Sequence	Aff (nM) NetMHC	Rank (%) NetMHC	Amino Acid Sequence	Aff (nM) NetMHC	Rank (%) NetMHC		
1	SL**L**AVMFV	40.2	0.6	SL**V**AVMFV	142.2	1.3	OR2Z1	p.V31L
2	LLFSL**L**AV	57.1	0.7	LLFSL**V**AV	70.4	0.8	OR2Z1	p.V31L
3	YI**V**ILWAV	106.1	1.1	YI**L**ILWAV	34.1	0.5	OR51D1	p.L233V
4	FLIH**S**STGL	8	0.08	FLIH**P**STGL	14.6	0.2	CDHR1	p.P523S
5	LLFAKF**S**FL	12.6	0.15	LLFAKF**F**FL	4.9	0.03	OR2M2	p.F327S
6	QLLFSL**L**AV	28.5	0.4	QLLFSL**V**AV	29.4	0.4	OR2Z1	p.V31L
7	GLY**D**VQLTI	9.5	0.125	GLY**A**VQLTI	23.3	0.4	TSPO2	p.A84D
8	LLIDAG**T**NV	14.5	0.2	LLIDAG**A**NV	13.7	0.175	FANK1	p.A269T
9	L**L**NPHTLGL	41.9	0.6	L**R**NPHTLGL	23737.8	33	ZNF703	p.R535L
10	SL**L**AVMFVI	11.6	0.15	SL**V**AVMFVI	32.6	0.5	OR2Z1	p.V31L
11	**Y**QLKGVPEA	25.5	0.4	**H**QLKGVPEA	1033.3	4	PROM2	p.H334Y
12	SLCT**V**DMSL	71.5	0.8	SLCT**A**DMSL	130.6	1.3	CFLAR	p.A408V
13	LLFSL**L**AVM	62.8	0.8	LLFSL**V**AVM	83.9	0.9	OR2Z1	p.V31L
14	FSL**L**AVMFV	117.7	1.2	FSL**V**AVMFV	110.9	1.1	OR2Z1	p.V31L
15	ALLSNDNA**L**	139.4	1.3	ALLSNDNA**F**	7376.9	12	IDUA	p.F352L
16	LL**P**MSLHWFI	7.9	0.08	LL**L**MSLHWFI	7.8	0.08	CNIH4	p.L63P
17	LLFAKF**S**FLI	20.8	0.3	LLFAKF**F**FLI	19	0.25	OR2M2	p.F327S
18	RQLLFSL**L**AV	50.7	0.7	RQLLFSL**V**AV	57.3	0.7	OR2Z1	p.V31L
19	SLNVDT**A**FPL	33.8	0.5	SLNVDT**P**FPL	26.6	0.4	CRTC3	p.P578A
20	LGLY**D**VQLTI	38.7	0.5	LGLY**A**VQLTI	78.5	0.9	TSPO2	p.A84D
21	T**L**AGHVYEYI	25.2	0.4	T**P**AGHVYEYI	19199.5	26	SLITRK5	p.P744L
22	**K**LIASILYQA	26.3	0.4	**E**LIASILYQA	698.2	3.5	DPY19L4	p.E142K
23	FLIH**S**STGLI	74.1	0.9	FLIH**P**STGLI	112.3	1.1	CDHR1	p.P523S
24	FSL**L**AVMFVI	15.4	0.2	FSL**V**AVMFVI	35.4	0.5	OR2Z1	p.V31L
25	SL**L**NPHTLGL	61.5	0.8	SL**R**NPHTLGL	450.6	3	ZNF703	p.R535L
26	L**L**AVMFVIGL	114.8	1.1	L**V**AVMFVIGL	1164	4.5	OR2Z1	p.V31L
27	YALLSNDNA**L**	150.9	1.4	YALLSNDNA**F**	5850.1	11	IDUA	p.F352L

Brito Baleeiro R, *et al. J Immunother Cancer* 2023; 11:e007336. doi: 10.1136/jitc-2023-007336

Supplementary Table 4 – Predicted vs actual binding capacity of Patients' neo-antigen candidates.

Patient	HLA Allele	Peptide ID	Amino Acid Sequence	Aff (nM) NetMHC	Rank (%) NetMHC	*EC/IC50 (µM) Binding Assays
SW1360	HLA-A*24:02	1	CWNVEQARF	368.8	0.6	1.925
		2	RVSQESFVF	164.6	0.3	n.d
		3	TYFLVCFIP	524.1	0.8	n.d
		4	RVAVILNEF	1569.4	1.5	2.53
		CMV-pp65 341-349	***QYDPVAALF***	***104.98***	***0.2***	***0.7074***
SW2163	HLA-A*02:01	1	LLAAPSAMA	385.2	2.5	37.12
		2	YQVNLYMYL	14.1	0.175	974.1
		3	NLLNNYQTL	223.9	1.7	89.76
		4	AAVTSQFPV	384.5	2.5	27.71
		5	LLEAVEPEV	33.3	0.5	89.82
		6	LMAVEEREV	822.7	4	34.11
		7	SLHSLKNPV	64.7	0.8	74.01
		8	YQGSYGFPL	15.2	0.2	187.2
		9	SQFPVPCKL	544.1	3	n.d
		10	GLMKGGANL	64.7	0.8	16.99
		Flu-MP 58-66	***GILGFVFTL***	***15.71***	***0.2***	***7.048***
	HLA-A*24:02	1	KTSICYLSF	486.1	0.7	3649
		2	KYISTSLPV	240.3	0.5	7.135
		3	IWEQLASRF	94.8	0.175	6.405
		4	IKPEVIFKI	2001.5	1.8	n.d
		5	QYQVNLYMY	2019.5	1.8	1527
		6	IKSMAQYLI	2264.2	2	n.d
		7	FFNKGGSVF	598.7	0.8	13.26
		8	LRNPGTWTF	1194.2	1.3	n.d
		9	LMKGGANLF	821.1	1	26.72
		CMV-pp65 341-349	***QYDPVAALF***	***104.98***	***0.2***	***0.7074***
SW2183	HLA-A*02:01	1	MLREQLDQV	113	1.1	13.87
		2	TLHGFVNHI	139.2	1.3	10.65
		3	RILSCLSDL	478.5	3	29.32
		4	GLLHKVKEL	254.9	1.9	30.14
		5	ALLVVSREL	286.9	2	5.522
		6	WLAKTRVFL	188.5	1.6	5.871
		7	ILCKDLSTV	55.5	0.7	9.327
		8	FLTDVACRV	5.2	0.04	5.38
		9	RQNQSIVAV	652.4	3.5	57.38
		10	AVLERYLEL	145.7	1.4	5.962
		11	MQSLHFAFL	224.1	1.7	47.66
		12	ALEAVRIEL	710	3.5	5.843
		13	KLVPRKVCV	286.4	2	386.5
		14	TLHDIWPEI	15	0.2	9.314
		Flu-MP 58-66	***GILGFVFTL***	***15.71***	***0.2***	***2.826***
	HLA-A*24:02	1	LFTKLHPAF	490.7	0.7	19.6
		2	SYYMPQLFW	67.4	0.125	27.77
		3	YRMVVYHNW	471.6	0.7	n.d
		4	EMQSLHFAF	435.2	0.7	4.845
		5	RFFPLVLDL	402.3	0.6	1445
		6	KYSDVIKVL	439.6	0.7	1.376
		7	DYPGQGFSF	100.1	0.2	5.841
		8	RMAPMLPSV	2007.8	1.8	n.d
		9	RTGESSFPF	711.2	0.9	227.7
		10	SVMKKNNLF	629.6	0.9	339.9
		11	YYMPQLFWN	788.9	1	15637
		12	SFPYESVPW	703.3	0.9	4174
		13	MIFAAVLF	1021.2	1.2	12049
		14	IFAAVVLFL	815.4	1	415.2
		15	FYLTSLMTP	1103.1	1.2	n.d
		16	QYAAASSAW	372.6	0.6	51.99
		17	VCAHTAIIF	2296	2	3587
		18	HTAIIFNVF	1198.5	1.3	141.1
		19	RFTLSVPGF	636.3	0.9	1.597
		20	VYSFLCKTI	55	0.1	0.6069
		CMV-pp65 341-349	***QYDPVAALF***	***104.98***	***0.2***	***0.5824***
SW2388	HLA-A*03:01	1	RMLPSGLSR	48.1	0.25	51.74
		2	RQGLLQMQK	251.7	0.9	229
		3	KALPRAGVK	79.5	0.4	84.91
		4	HQRIHTGGK	225.3	0.8	n.d
		5	RIHTGGKPY	1008.5	2	61.96
		6	QSKVAAWLK	1141.3	2.5	n.d
		7	RLEMSCLSK	200.8	0.8	285.4
		8	CIVYLSLSR	1332	2.5	39.86
		9	HLIHHRMLH	137.3	0.6	144.3
		Flu-NP 265-273	***ILRGSVAHK***	***19.94***	***0.08***	***9.46***

*EC50 values obtained from stabilization assays were used for HLA-A*02:01 and HLA-A*03:01 peptides and IC50 values from competitive binding assay were used for HLA-A*24:02 peptides.

n.d: not determined.

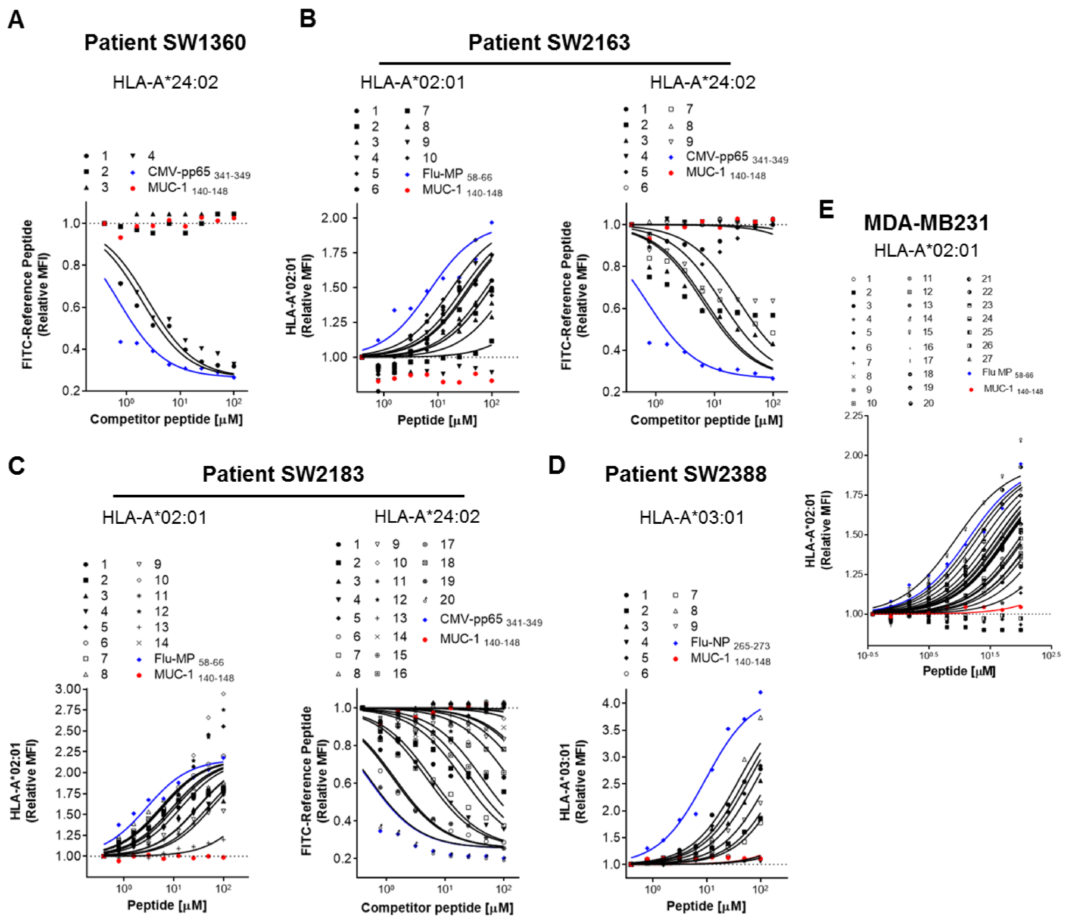

Figure S1 – Screening of neo-antigen candidates for a personalized vaccine for TNBC. Neo-antigen peptide candidates were identified through whole exome and mRNA sequencing plus proteome analysis from tumour samples of four TNBC patients and whole exome from one TNBC cell line. Potential binders for the HLA allele expressed by each patient/cell line were selected using NetMHC 4.0 program. The neo-antigen peptides with predicted high affinity were assessed for their capacity to bind to the patients' HLA in cell-based binding assays. HLA-A*02:01 and HLA-A*03:01-binding peptides were tested in MHC-I stabilization assays using TAP-deficient T2 cell lines expressing HLA-A*02:01 or HLA-A*03:01; HLA-A*24:02 peptides were tested in a competitive MHC-I-binding assay using a lymphoblastoid cell line (BRIP) homozygous for HLA-A*24:02. In (**A**) is shown results for patient SW1360; in (**B**) for SW2163; in (**C**) for SW2183; in (**D**) for SW2388; and in (**E**) for MDA-MB231 cells. The binding of each peptide was analysed three times and the mean of all of the experiments performed is shown. MUC-1 $_{140\text{-}148}$ peptide was used as negative control in all experiments; as positive control, Flu MP $_{58\text{-}66}$ and Flu-NP $_{265\text{-}273}$ peptides were used for HLA-A*02:01 and HLA-A*03:01 respectively; CMV pp65 $_{341\text{-}349}$ peptide was used as positive control for HLA-A*24:02

Supplementary Table 5 – Predicted vs actual binding capacity of MDA-MB231-derived neo-antigen candidates.

Peptide ID	Amino Acid Sequence	Aff (nM) NetMHC	Rank (%) NetMHC	EC50 (μM) Binding Assays
1	SL**L**AVMFV	40.2	0.6	177.8
2	LLFSL**L**AV	57.1	0.7	n.d
3	YI**V**ILWAV	106.1	1.1	n.d
4	FLIH**S**STGL	8	0.08	60.2
5	LLFAKF**S**FL	12.6	0.15	258.5
6	QLLFSL**L**AV	28.5	0.4	n.d
7	GLY**D**VQLTI	9.5	0.125	31.72
8	LLIDAG**T**NV	14.5	0.2	59.32
9	L**L**NPHTLGL	41.9	0.6	57.08
10	SL**L**AVMFVI	11.6	0.15	147.5
11	**Y**QLKGVPEA	25.5	0.4	93.29
12	SLCT**V**DMSL	71.5	0.8	114.4
13	LLFSL**L**AVM	62.8	0.8	478.5
14	FSL**L**AVMFV	117.7	1.2	55.32
15	ALLSNDNA**L**	139.4	1.3	8.692
16	LL**P**MSLHWFI	7.9	0.08	38.82
17	LLFAKF**S**FLI	20.8	0.3	19.15
18	RQLLFSL**L**AV	50.7	0.7	24.61
19	SLNVDT**A**FPL	33.8	0.5	45.06
20	LGLY**D**VQLTI	38.7	0.5	106.9
21	T**L**AGHVYEYI	25.2	0.4	48.08
22	**K**LIASILYQA	26.3	0.4	16.22
23	FLIH**S**STGLI	74.1	0.9	39.57
24	FSL**L**AVMFVI	15.4	0.2	152
25	SL**L**NPHTLGL	61.5	0.8	54.46
26	L**L**AVMFVIGL	114.8	1.1	n.d
27	YALLSNDNA**L**	150.9	1.4	61.05
Flu-MP 58-66	***GILGFVFTL***	***15.71***	***0.2***	***13.84***

n.d: not determined.

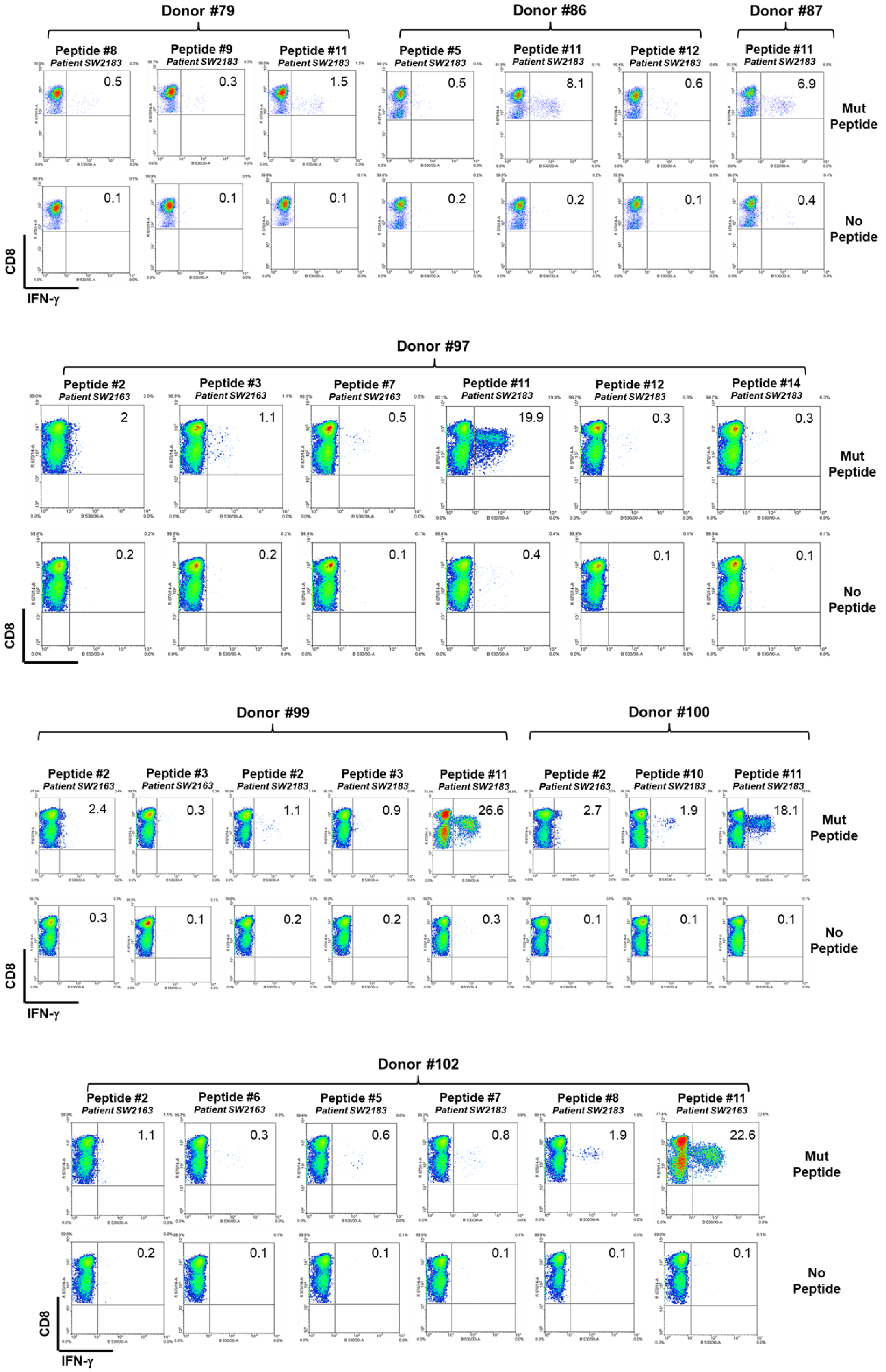

Figure S2 – TNBC-derived neo-antigens are immunogenic. TNBC peptides were examined for their ability to promote immune responses in immunogenicity T-cell assays using healthy blood samples from 9 donors positive for HLA-A*02:01. CD8+ T-cells isolated from healthy donor blood

were stimulated three times with autologous DC pulsed with each of the HLA-A*02:01-binding peptides. Seven days after the last round of stimulation, CD8+ T-cells were challenged with T2 cells pulsed with the mutant or left without peptide. Intracellular IFN-γ was measured by FACS. The dot plots depict mutant peptides from TNBC patients that elicited CD8+ T-cell responses from all responding donors analysed.

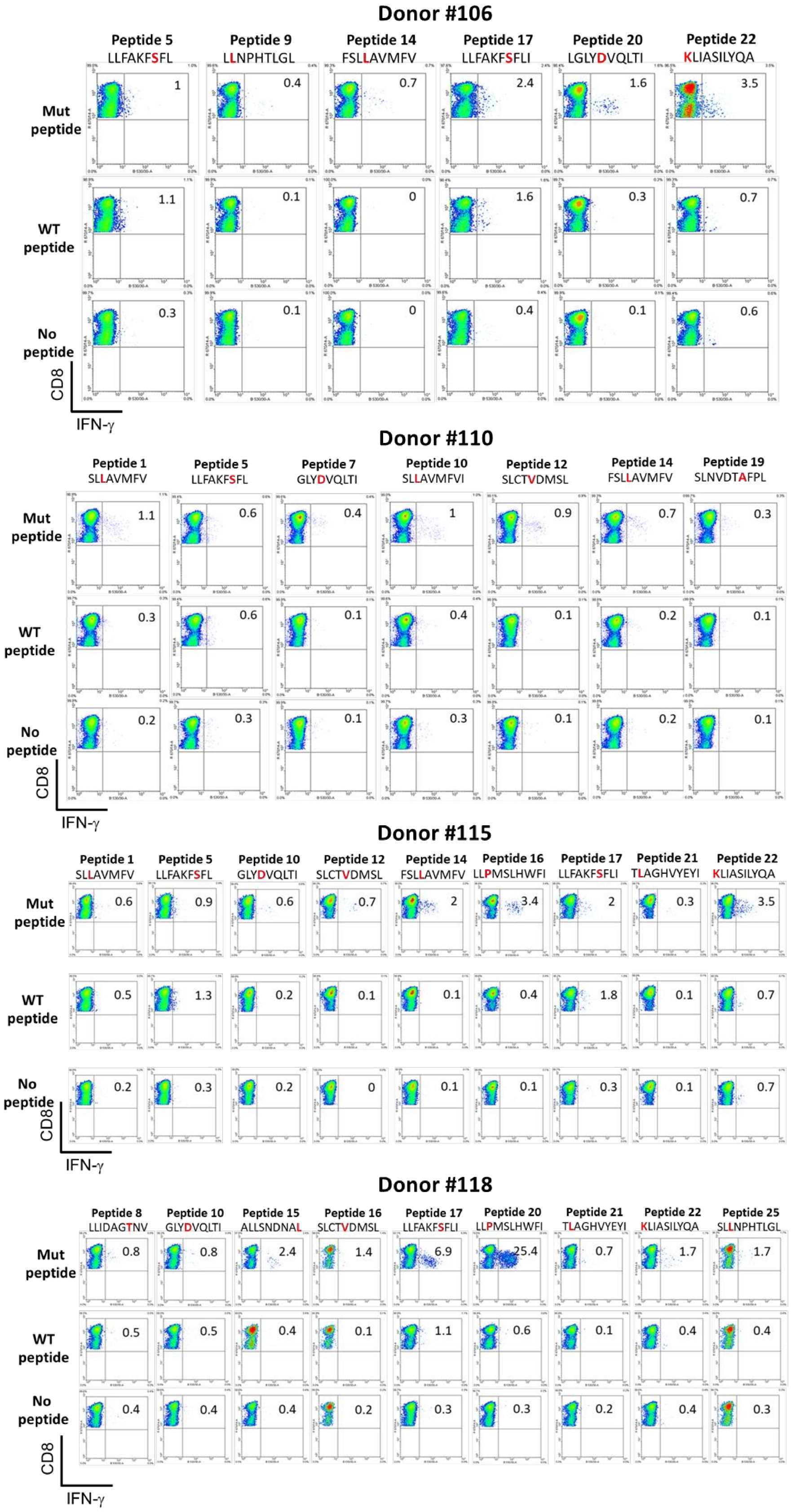

Figure S3 – TNBC-derived neo-antigens are immunogenic. TNBC peptides were examined for their ability to promote immune responses in immunogenicity T-cell assays using healthy blood samples from 4 donors positive for HLA-A*02:01. CD8+ T-cells isolated from healthy donor blood

Brito Baleeiro R, *et al. J Immunother Cancer* 2023; 11:e007336. doi: 10.1136/jitc-2023-007336

were stimulated three times with autologous DC pulsed with each of the HLA-A*02:01-binding peptides. Seven days after the last round of stimulation, CD8+ T-cells were challenged with T2 cells pulsed with the mutant, wild type peptide or left without peptide. Intracellular IFN-γ was measured by FACS. The dot plots depict mutant peptides from MDA-MB-231 cells that elicited strong CD8+ T-cell responses the responding donors analysed.

Supplementary Table 6 – Immunogenicity of MDA-MB231-derived neo-antigens. Green and yellow represent responses against the mutated and wild type peptide, respectively.

Peptide	Status	PBMC Donor			
		#106	#110	#115	#118
1	Mut				
	WT				
4	Mut				
	WT				
5	Mut				
	WT				
7	Mut				
	WT				
8	Mut				
	WT				
9	Mut				
	WT				
10	Mut				
	WT				
11	Mut				
	WT				
12	Mut				
	WT				
14	Mut				
	WT				
15	Mut				
	WT				
16	Mut				
	WT				
17	Mut				
	WT				
18	Mut				
	WT				
19	Mut				
	WT				
20	Mut				
	WT				
21	Mut				
	WT				
22	Mut				
	WT				
23	Mut				
	WT				
25	Mut				
	WT				
27	Mut				
	WT				

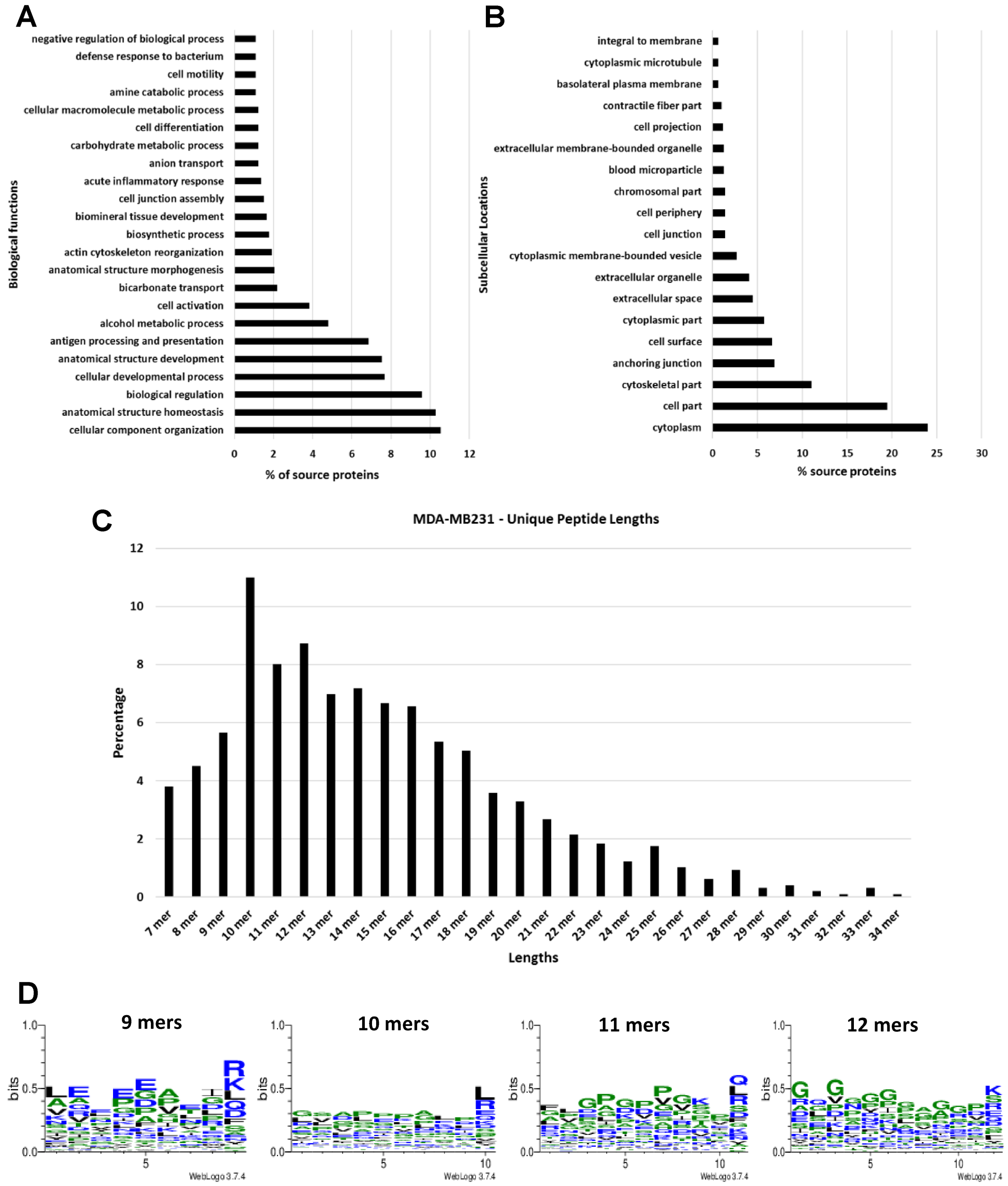

Figure S4 – Characterisation of peptides eluted from HLA-A2 molecules of MDA-MB231 cells. Biological functions (**A**) and subcellular locations (**B**) of the source proteins of the HLA-A2–bound peptides from MDA-MB231 cells were assigned using the Human Protein Reference Database. The amino acid lengths of the HLA-A2-eluted peptides are shown in (**C**). Binding motifs for HLA-A2 molecules are shown as logo plots of residue frequency at each position of HLA-A2 peptides according to their length (**D**).

Supplementary Table 7 – Characteristics of E0771.LMB-derived neo-antigens predicted to bind to the murine MHC-I alleles H2-Db and H2-Kb.

Brito Baleeiro R, *et al. J Immunother Cancer* 2023; 11:e007336. doi: 10.1136/jitc-2023-007336

Peptide ID	MHC Allele	Mutated Peptide			Wild Type Peptide			Gene	Amino Acid Substitution
		Amino Acid Sequence	Aff (nM) NetMHC	Rank (%) NetMHC	Amino Acid Sequence	Aff (nM) NetMHC	Rank (%) NetMHC		
1	**Db**	FGLINVT**P**NM	7.79	0.01	FGLINVT**L**NM	29.87	0.02	Dhrs9	L146P
2	Kb	SSIYFLY**R**L	7.76	0.02	SSIYFLY**L**L	12.27	0.04	Slc9a4	L134R
3	Kb	SIYFLY**R**L	10.69	0.03	SIYFLY**L**L	23.62	0.07	Slc9a4	L134R
4	**Db**	FPAL**G**NCCTI	53.2	0.04	FPAL**V**NCCTI	8.45	0.01	Dnah7b	V2537G
5	**Db**	LGLENI**C**WL	75.72	0.05	LGLENI**Y**WL	43.85	0.04	Angptl2	Y355C
6	**Db**	FTFRN**N**VCM	89.67	0.06	FTFRN**H**VCM	50.14	0.04	Dyrk3	H282N
7	Kb	VAFV**R**FARS	24.24	0.07	VAFV**K**FARS	33.93	0.1	Rbm45	K72R
8	**Db**	SQLNN**F**LDQL	195.36	0.1	SQLNN**L**LDQL	205.65	0.12	Lamc1	L1518F
9	**Db**	FGLINVT**P**N	181.36	0.1	FGLINVT**L**N	2246.46	0.7	Dhrs9	L146P
10	Kb	TIP**Q**YFCDL	42.63	0.12	TIP**H**YFCDL	66.08	0.2	Olfr340	H176Q
11	Kb	VVYLMR**S**I	37.05	0.12	VVYLMR**W**I	287.26	0.7	Fastkd1	W447S
12	**Db**	I**A**MVSYIHI	228.76	0.12	I**V**MVSYIHI	3320.59	0.9	Olfr1107	V214A
13	**Db**	LQLN**N**QLFV	288.94	0.15	LQLN**S**QLFV	8494.87	2.5	Cntnap5a	S912N
14	Kb	T**G**ISFSSV	45.43	0.15	T**D**ISFSSV	4068.58	6	Olfr345	D70G
15	Kb	VASVFY**S**IV	48.77	0.15	VASVFY**T**IV	80.76	0.25	Olfr1099	T279S
16	Kb	**V**SNVFYKIV	55.16	0.17	**D**SNVFYKIV	3132.17	26	Pkhd1	D2250V
17	Kb	DSSIYFLY**R**L	54.05	0.17	DSSIYFLY**L**L	115.36	0.3	Slc9a4	L134R
18	**Db**	VLVLVNAVY**V**	384.31	0.17	VLVLVNAVY**F**	1797.55	0.6	Serpinb12	F211V
19	**Db**	LG**V**YNHSVL	333.34	0.17	LG**E**YNHSVL	10377.83	3	Ttc17	E310V
20	**Db**	KV**V**SNVFYKI	413.33	0.2	KV**D**SNVFYKI	6402.63	1.7	Pkhd1	D2250V
21	Kb	SSIYFLY**R**LP	68.87	0.2	SSIYFLY**L**LP	145.19	0.4	Slc9a4	L134R
22	Kb	WVYDHAK**L**	63.04	0.2	WVYDHAK**W**	3447.63	5	Nrp2	W664L
23	**Db**	FAPK**T**LPPL	406.61	0.2	FAPK**N**LPPL	8.19	0.01	Itih5	N288T
24	Kb	VT**P**NMLPL	66.93	0.2	VT**L**NMLPL	37.76	0.12	Dhrs9	L146P
25	Kb	**V**SNVFYKI	75.94	0.25	**D**SNVFYKI	8890.77	12	Pkhd1	D2250V
26	Kb	TVF**P**FIDM	84.98	0.25	TVF**S**FIDM	50.67	0.15	Dnah7a	S875P
27	Kb	TVF**P**FIDMNL	75.99	0.25	TVF**S**FIDMNL	47.38	0.15	Dnah7a	S875P
28	Kb	VSFSTEA**P**YL	84.36	0.25	VSFSTEA**S**YL	120.29	0.4	Cntnap5a	S800P
29	Kb	LMYLRF**P**AT	88.99	0.25	LMYLRF**S**AT	57.37	0.17	Olfr424	S262P
30	Kb	VAFV**R**FAR	79.68	0.25	VAFV**K**FAR	129.09	0.4	Rbm45	K72R
31	Kb	R**T**FLRSAL	80.05	0.25	R**K**FLRSAL	1991.65	3.5	Nckap1	K344T
32	Kb	GTL**P**FMYL	89.38	0.25	GTL**L**FMYL	47.16	0.15	Olfr1008	L256P
33	Kb	MAYD**S**YVAI	77.21	0.25	MAYD**R**YVAI	27.08	0.08	Olfr1100	R122S
34	**Db**	FII**A**MVSYI	545.06	0.25	FII**V**MVSYI	283.99	0.15	Olfr1107	V214A
35	**Db**	RLQLN**N**QLFV	789.71	0.3	RLQLN**S**QLFV	12174.44	3.5	Cntnap5a	S912N
36	Kb	LMYLRF**P**ATF	107.02	0.3	LMYLRF**S**ATF	81.67	0.25	Olfr424	S262P
37	**Db**	WLGLENI**C**WL	774	0.3	WLGLENI**Y**WL	510.2	0.25	Angptl2	Y355C
38	**Db**	INVT**P**NMLPL	663.43	0.3	INVT**L**NMLPL	663.43	0.3	Dhrs9	L146P
39	**Db**	YM**Y**IIRKSYI	1127.71	0.4	YM**N**IIRKSYI	3140.56	0.9	Dnah7c	N2766Y
40	**Db**	SFTFRN**N**VCM	952.51	0.4	SFTFRN**H**VCM	506.43	0.25	Dyrk3	H282N
41	Kb	LESFTFRN**N**V	142.75	0.4	LESFTFRN**H**V	216.88	0.6	Dyrk3	H282N
42	**Db**	SSILA**M**LAI	964.66	0.4	SSILA**L**LAI	1351.94	0.5	Adora1	L98M
43	**Db**	YGFIAV**T**VI	1167.44	0.4	YGFIAV**A**VI	2676.8	0.8	Olfr368	A228T
44	Kb	SS**L**LSVGNL	120.66	0.4	SS**V**LSVGNL	266.6	0.7	Scn2a	V791L
45	Kb	YVAI**Y**NPL	126.86	0.4	YVAI**C**NPL	4443.65	6	Olfr1009	C127Y
46	Kb	ASMAYD**S**YV	157	0.4	ASMAYD**R**YV	282.68	0.7	Olfr1100	R122S
47	**Db**	I**A**MVSYIHII	1225.41	0.4	I**V**MVSYIHII	7668.72	2	Olfr1107	V214A
48	**Db**	LCL**Q**NKTHV	1116.03	0.4	LCL**R**NKTHV	1970.56	0.6	Pla2g4e	R176Q
49	**Db**	RAIDR**R**DYYL	1321.58	0.5	RAIDR**G**DYYL	6188.22	1.6	Pcmtd1	G39R
50	**Db**	SFIDMN**M**EPF	1387.37	0.5	SFIDMN**L**EPF	1780.46	0.6	Dnah7b	L925M
51	**Db**	FIDMN**M**EPFL	1489.23	0.5	FIDMN**L**EPFL	1428.76	0.5	Dnah7b	L925M
52	**Db**	IAL**P**TNFQVT	1699.06	0.5	IAL**L**TNFQVT	1712.88	0.6	Cps1	L1464P
53	**Db**	SVLC**H**DNYL	1466.43	0.5	SVLC**Q**DNYL	4683.37	1.2	Cfh	Q837H
54	Kb	QSMEEF**Q**SL	177.93	0.5	QSMEEF**R**SL	191.38	0.5	Ints7	R679Q
55	Kb	RTYRC**G**HPL	183.75	0.5	RTYRC**A**HPL	72.22	0.2	Lrrc8a	A311G
56	Kb	MTEQFSS**L**	203.77	0.5	MTEQFSS**V**	1409.12	2.5	Scn2a	V791L
57	Kb	VFYGTL**P**FM	180.53	0.5	VFYGTL**L**FM	343.04	0.8	Olfr1008	L256P
58	**Db**	NAIAHTANT**I**	1696.56	0.5	NAIAHTANT**F**	13466.14	4	Olfr1047	F164I
59	Kb	INCGYPNN**L**	274.07	0.7	INCGYPNN**F**	1362.26	2.5	Mrc1	F920L
60	Kb	LSPIH**Y**SSA	302.4	0.7	LSPIH**D**SSA	12193.02	16	Mastl	D366Y
61	Kb	**V**AGTYAWM	302.64	0.7	**A**AGTYAWM	1870.04	3	Map3k9	A302V
62	Kb	V**A**KTEFQLL	449.55	1	V**V**KTEFQLL	475.34	1	Med23	V1242A
63	Kb	K**P**FEMFREL	685.17	1.4	K**R**FEMFREL	467.16	1	Trp53	R331P

Brito Baleeiro R, *et al. J Immunother Cancer* 2023; 11:e007336. doi: 10.1136/jitc-2023-007336

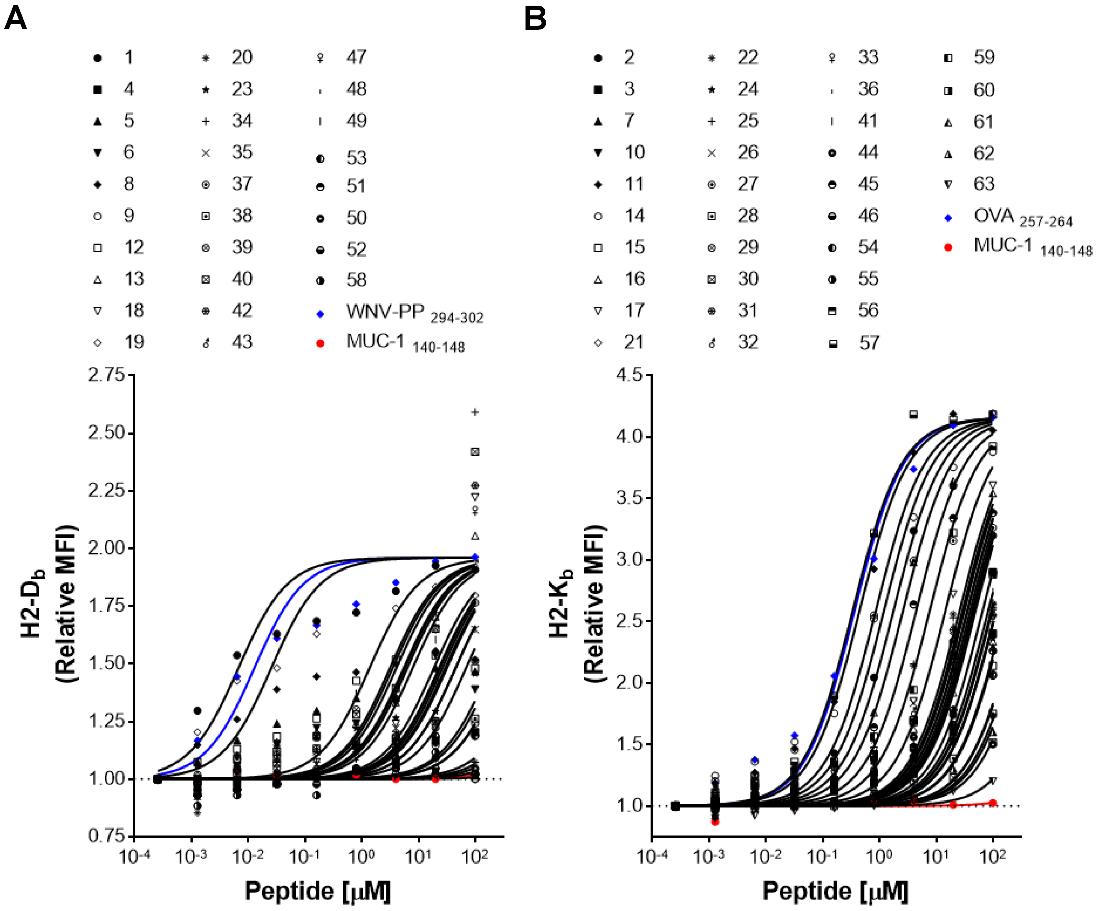

Figure S5 – Screening of neo-antigen candidates for a personalised vaccine for murine TNBC. Neo-antigen peptide candidates were identified through whole exome from the murine TNBC cell line E0771.LMB. Potential binders for the mouse MHC-I alleles were selected using NetMHC 4.0 program. The neo-antigen peptides with predicted high affinity were assessed for their capacity to bind to the MHC-I in cell-based binding assays. H2-Db- and H2-Kb-binding peptides were tested in MHC-I stabilization assays using TAP-deficient RMA-S cell line expressing H2-Db (**a**) and H2-Kb (**b**). The binding of each peptide was analysed three times and the mean of all of the experiments performed is shown. MUC-1 $_{140\text{-}148}$ peptide was used as negative control in all experiments; as positive control, West Nile Virus poly protein (WNV-PP) $_{294\text{-}302}$ and ovalbumin (OVA) $_{257\text{-}264}$ peptides were used for H2-Db and H2-Kb, respectively.

Supplementary Table 8 – Predicted vs actual binding capacity of E0771.LMB-derived neo-antigen candidates.

Brito Baleeiro R, *et al. J Immunother Cancer* 2023; 11:e007336. doi: 10.1136/jitc-2023-007336

Peptide ID	MHC Allele	Amino Acid Sequence	Aff (nM) NetMHC	Rank (%) NetMHC	EC50 (μM) Binding Assays
1	**Db**	FGLINVT**P**NM	7.79	0.01	0.006684
2	Kb	SSIYFLY**R**L	7.76	0.02	1.755
3	Kb	SIYFLY**R**L	10.69	0.03	111.4
4	**Db**	FPAL**G**NCCTI	53.2	0.04	297.1
5	**Db**	LGLENI**C**WL	75.72	0.05	22.87
6	**Db**	FTFRN**N**VCM	89.67	0.06	26.23
7	Kb	VAFV**R**FARS	24.24	0.07	112
8	**Db**	SQLNN**F**LDQL	195.36	0.1	1.367
9	**Db**	FGLINVT**P**N	181.36	0.1	211.5
10	Kb	TIP**Q**YFCDL	42.63	0.12	277.7
11	Kb	VVYLMR**S**I	37.05	0.12	0.442
12	**Db**	I**A**MVSYIHI	228.76	0.12	3.484
13	**Db**	LQLN**N**QLFV	288.94	0.15	5.469
14	Kb	T**G**ISFSSV	45.43	0.15	0.8549
15	Kb	VASVFY**S**IV	48.77	0.15	96.07
16	Kb	**V**SNVFYKIV	55.16	0.17	92.46
17	Kb	DSSIYFLY**R**L	54.05	0.17	14.42
18	**Db**	VLVLVNAVY**V**	384.31	0.17	7.852
19	**Db**	LG**V**YNHSVL	333.34	0.17	0.02539
20	**Db**	KV**V**SNVFYKI	413.33	0.2	932.5
21	Kb	SSIYFLY**R**LP	68.87	0.2	30.92
22	Kb	WVYDHAK**L**	63.04	0.2	25.58
23	**Db**	FAPK**T**LPPL	406.61	0.2	68.45
24	Kb	VT**P**NMLPL	66.93	0.2	81.61
25	Kb	**V**SNVFYKI	75.94	0.25	28.62
26	Kb	TVF**P**FIDM	84.98	0.25	43.77
27	Kb	TVF**P**FIDMNL	75.99	0.25	1.189
28	Kb	VSFSTEA**P**YL	84.36	0.25	285.1
29	Kb	LMYLRF**P**AT	88.99	0.25	165
30	Kb	VAFV**R**FAR	79.68	0.25	478.3
31	Kb	R**T**FLRSAL	80.05	0.25	45.61
32	Kb	GTL**P**FMYL	89.38	0.25	42.76
33	Kb	MAYD**S**YVAI	77.21	0.25	65.15
34	**Db**	FII**A**MVSYI	545.06	0.25	6.312
35	**Db**	RLQLN**N**QLFV	789.71	0.3	44.78
36	Kb	LMYLRF**P**ATF	107.02	0.3	34.85
37	**Db**	WLGLENI**C**WL	774	0.3	44.81
38	**Db**	INVT**P**NMLPL	663.43	0.3	181.5
39	**Db**	YM**Y**IIRKSYI	1127.71	0.4	29.44
40	**Db**	SFTFRN**N**VCM	952.51	0.4	4.053
41	Kb	LESFTFRN**N**V	142.75	0.4	30.44
42	**Db**	SSILA**M**LAI	964.66	0.4	18.57
43	**Db**	YGFIAV**T**VI	1167.44	0.4	31.51
44	Kb	SS**L**LSVGNL	120.66	0.4	345.4
45	Kb	YVAI**Y**NPL	126.86	0.4	4.414
46	Kb	ASMAYD**S**YV	157	0.4	48.97
47	**Db**	I**A**MVSYIHII	1225.41	0.4	23.78
48	**Db**	LCL**Q**NKTHV	1116.03	0.4	5.74
49	**Db**	RAIDR**R**DYYL	1321.58	0.5	3.137
50	**Db**	SFIDMN**M**EPF	1387.37	0.5	1349
51	**Db**	FIDMN**M**EPFL	1489.23	0.5	14031
52	**Db**	IAL**P**TNFQVT	1699.06	0.5	2920
53	**Db**	SVLC**H**DNYL	1466.43	0.5	301.9
54	Kb	QSMEEF**Q**SL	177.93	0.5	157.5
55	Kb	RTYRC**G**HPL	183.75	0.5	114
56	Kb	MTEQFSS**L**	203.77	0.5	35.52
57	Kb	VFYGTL**P**FM	180.53	0.5	0.3351
58	**Db**	NAIAHTANT**I**	1696.56	0.5	1842
59	Kb	INCGYPNN**L**	274.07	0.7	7.617
60	Kb	LSPIH**Y**SSA	302.4	0.7	39.4
61	Kb	**V**AGTYAWM	302.64	0.7	2.578
62	Kb	V**A**KTEFQLL	449.55	1	386.1
63	Kb	K**P**FEMFREL	685.17	1.4	1335
WNV-PP 294-302	***Db***	***LGMSNRDFL***	***7.95***	***0.01***	***0.01277***
OVA 257-264	***Kb***	***SIINFEKL***	***19.37***	***0.06***	***0.3609***

Supplementary Table 9 – Long peptides (LP) used to immunize C57BL/6 mice. Each mouse group received three LP. Each LP encompasses one H2-Db and one H2-Kb epitope, which appears underlined in the long peptide sequence. Mutated residues appear in bold.

Group	Treatment	Long Peptide Sequence	Epitopes Composition
1	LP1	NLFGLINVTPNMLPLVMDSSIYFLYRLPPIV	**#1 Db**-FGLINVT**P**NM
			**#2 Kb**-SSIYFLY**R**L
	LP2	EYWLGLENICWLTNQGNDLVVYLMRSIQSDL	**#5 Db**-LGLENI**C**WL
			**#11 Kb**-VVYLMR**S**I
	LP3	MLESFTFRNNVCMAFEHLAFTGISFSSVTAP	**#6 Db**-FTFRN**N**VCM
			**#14 Kb**-T**G**ISFSSV
2	LP4	SLLSQLNNFLDQLGQLMDSSIYFLYRLPPIV	**#8 Db**-SQLNN**F**LDQL
			**#17 Kb**-DSSIYFLY**R**L
	LP5	NLFGLINVTPNMLPLVCGWVYDHAKLLRSTW	**#9 Db**-FGLINVT**P**N
			**#22 Kb**-WVYDHAK**L**
	LP6	FTFIIAMVSYIHIIGLINVTPNMLPLVKKA	**#12 Db**-I**A**MVSYIHI
			**#24 Kb**-VT**P**NMLPL
3	LP7	GHFRLQLNNQLFVGGTWGLKVVSNVFYKIVG	***#13 Db***-LQLN**N**QLFV
			#25 Kb-**V**SNVFYKI
	LP8	YAMLGVYNHSVLCYDSDDSTVFPFIDMNLEP	***#19 Db***-LG**V**YNHSVL
			#26 Kb-TVF**P**FIDM
	LP9	VHYFAPKTLPPLPKNDDSTVFPFIDMNLEPF	***#23 Db***-FAPK**T**LPPL
			#27 Kb-TVF**P**FIDMNL
4	LP10	IFTFIIAMVSYIHIIHMLESFTFRNNVCMA	***#34 Db***-FII**A**MVSYI
			#41 Kb-LESFTFRN**N**V
	LP11	GHFRLQLNNQLFVGGTYDRYVAIYNPLLYSV	***#35 Db***-RLQLN**N**QLFV
			#45 Kb-YVAI**Y**NPL
	LP12	IPPAYMYIIRKSYIPNVVVVFYGTLPFMYLQ	***#39 Db***-YM**Y**IIRKSYI
			#57 Kb-VFYGTL**P**FM
5	LP13	MLESFTFRNNVCMAFEWNDINCGYPNNLICQ	***#40 Db***-SFTFRN**N**VCM
			#59 Kb-INCGYPNN**L**
	LP14	DLSKLCLQNKTHVKFPELALSPIHYSSAIPA	***#48 Db***-LCL**Q**NKTHV
			#60 Kb-LSPIH**Y**SSA
	LP15	AFRAIDRRDYYLEGYTKMSVAGTYAWMAPE	***#49 Db***-RAIDR**R**DYYL
			#61 Kb-**V**AGTYAWM

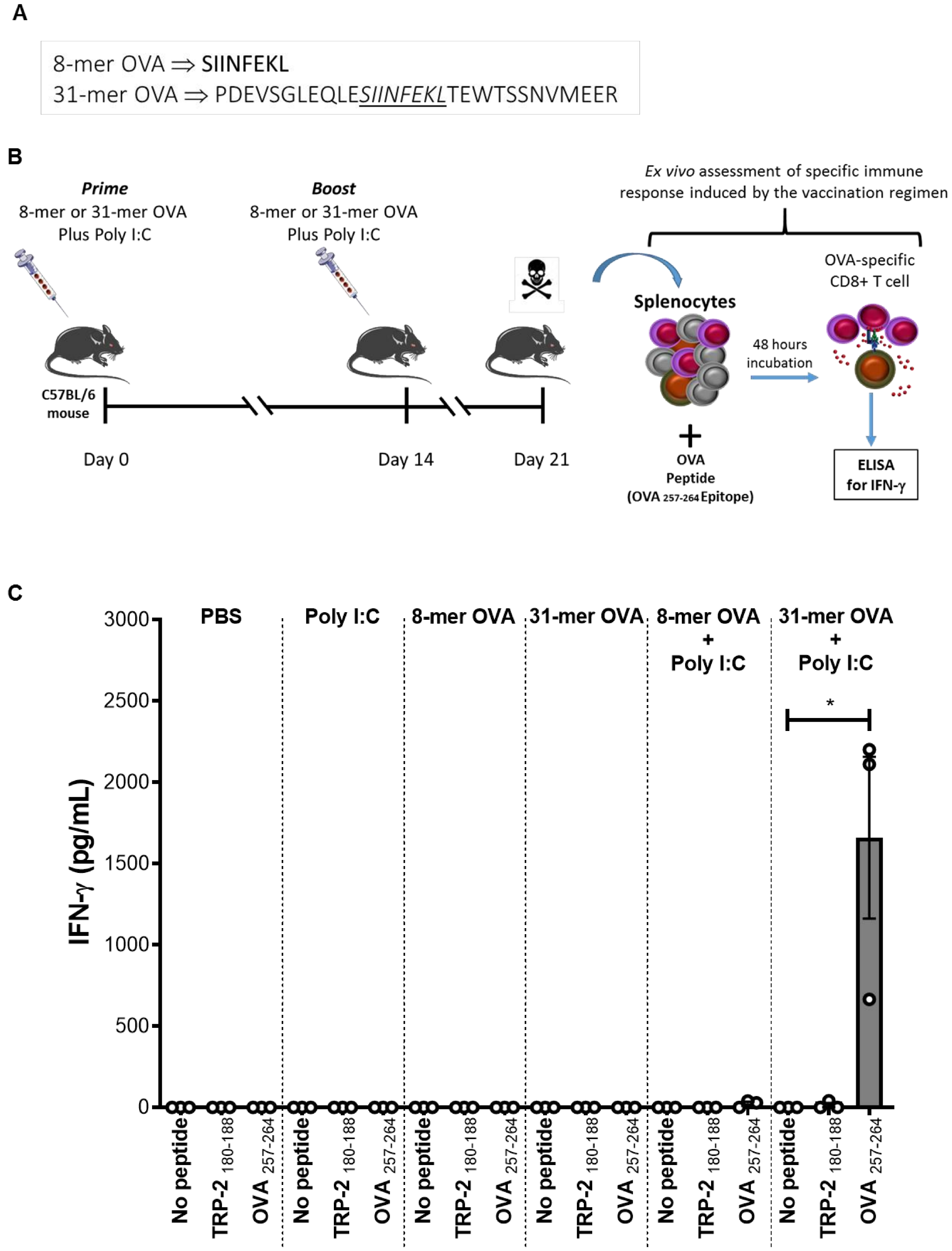

Figure S6 – Antigens administered as long peptides induce more vigorous epitope-specific CD8+ T-cells than the short minimal epitope. To determine the optimal peptide length to achieve immunisation, C57BL/6 mice were immunised at days 1 and 14 with either 8-mer or 31-mer OVA peptide administered either alone or together with Poly I:C (**A** and **B**). One week following the booster injection, spleens were harvested, processed and splenocytes were incubated *ex vivo* for 48h with 8-mer OVA peptide, Tyrosinase-related protein-2 (TRP-2) peptide or left with no peptide. Production of IFN-γ in the supernatant was measured by ELISA (**C**). The data were analysed by one-way ANOVA. *P< 0.05.

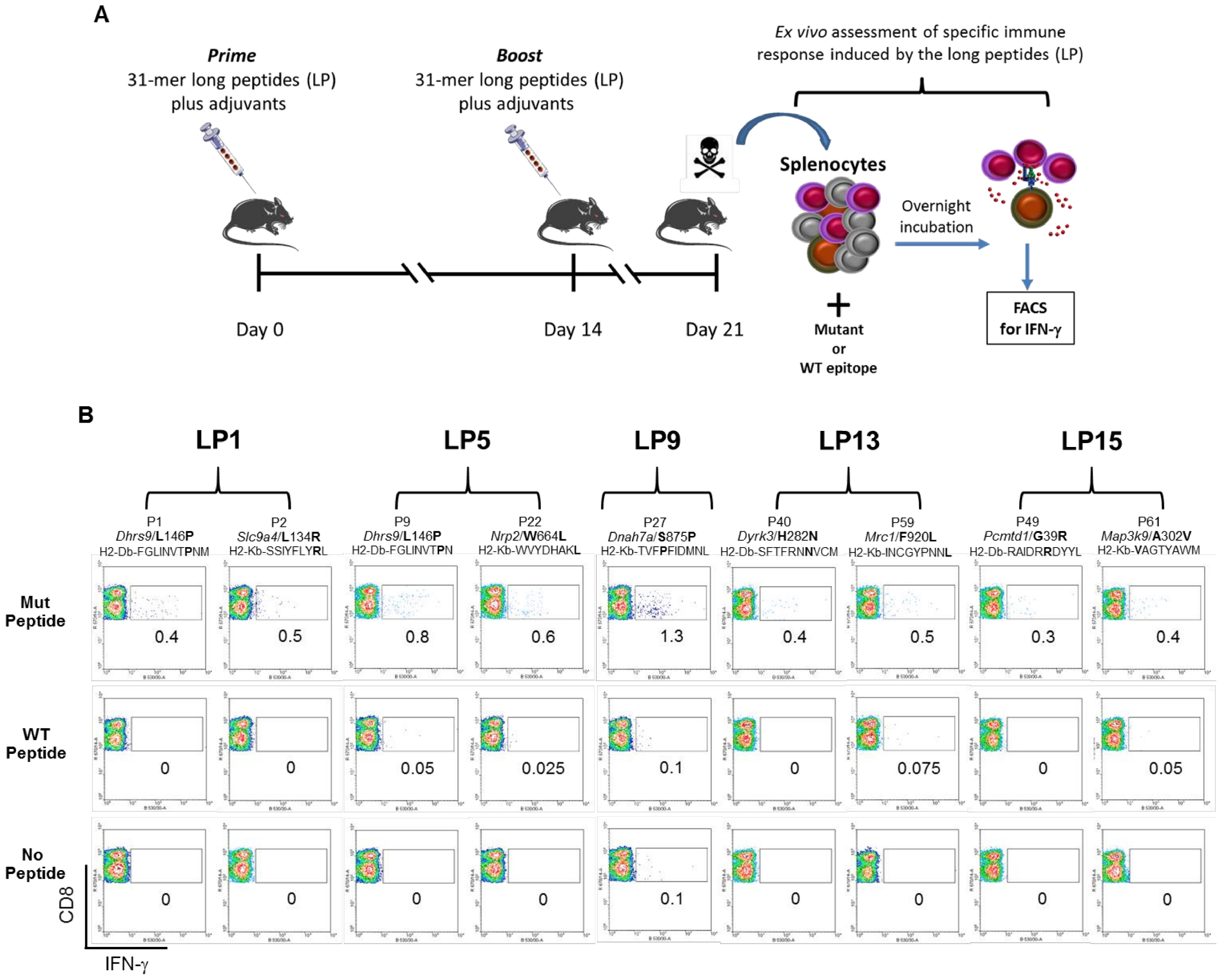

Figure S7 – Immunogenicity in vivo of E0771.LMB-derived neo-antigens. (**A**) To determine which of the neo-antigen candidates were capable of triggering in vivo CD8+ T-cell response, C57BL/6 mice were immunised at days 1 and 14 with either 31-mer long peptides encompassing minimal neo-antigen candidates together with adjuvants. One week following the booster injection, spleens were harvested, processed and splenocytes were incubated *ex vivo* for overnight with the minimal mutant epitopes, their wild type counterparts or left without peptide. Intracellular IFN-γ was measured by FACS and results are shown as the percentage of IFN-γ-positive cells gated on CD8+ T-cells (**B**). The dot pots show responses against the immunogenic peptides.

.

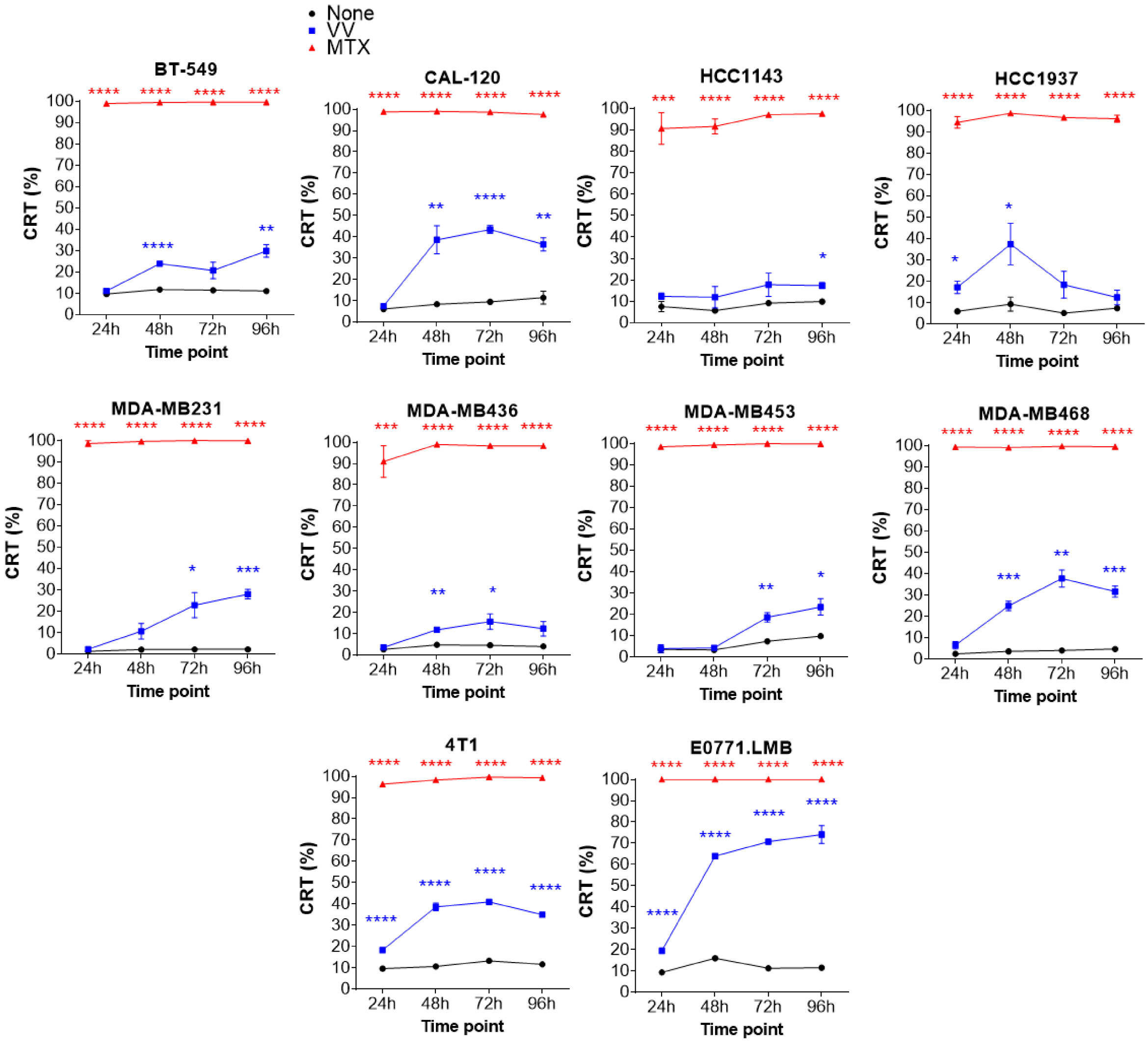

Figure S8 – Expression of calreticulin (CRT) on human and murine TNBC cell lines. The indicated human and murine TNBC cell lines wereincubated for 24h, 48h, 72h or 96h in the presence of 1 MOI of Vaccinia virus (VV) and cell-surface CRT expression was determined by flow cytometry. Negative controls consisted of cells incubated without virus (None) and as positive control, cells were incubated with mitoxantrone (MTX) for the indicated time period. The graphs show the means of 3 independent experiments and standard error of the mean. Data were analysed by two-tailed paired t test. *P< 0.05; **p< 0.01; ***p< 0.001; ****p<0.0001.

Brito Baleeiro R, *et al. J Immunother Cancer* 2023; 11:e007336. doi: 10.1136/jitc-2023-007336

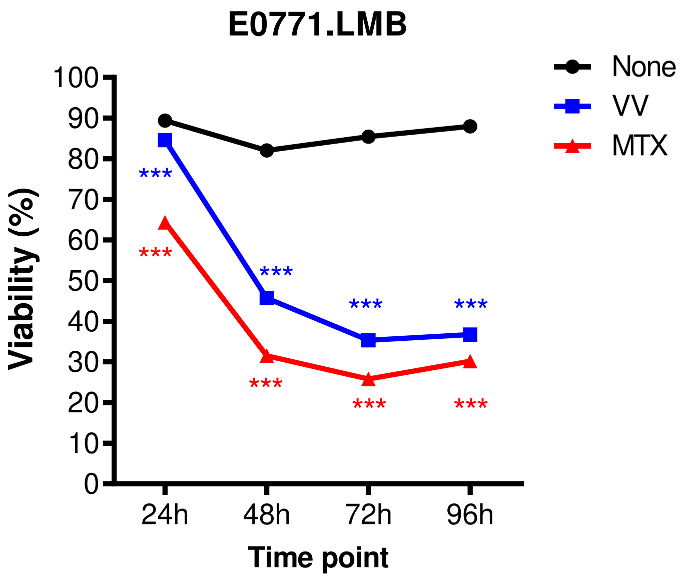

Figure S9 – Effect of VV on the viability of the murine TNBC cell line E0771.LMB. E0771.LMB cells were incubated for 24h, 48h, 72h or 96h in the presence of 1 MOI of Vaccinia virus (VV) and viability was determined by incorporation of EthD-1 by flow cytometry. Negative control consisted of cells incubated without virus (None) and as positive control, cells were incubated with mitoxantrone (MTX) for the indicated time period. The graphs show the means of 3 independent experiments and standard error of the mean. Data were analysed by two-tailed paired t test. ***p< 0.001.

Brito Baleeiro R, *et al. J Immunother Cancer* 2023; 11:e007336. doi: 10.1136/jitc-2023-007336

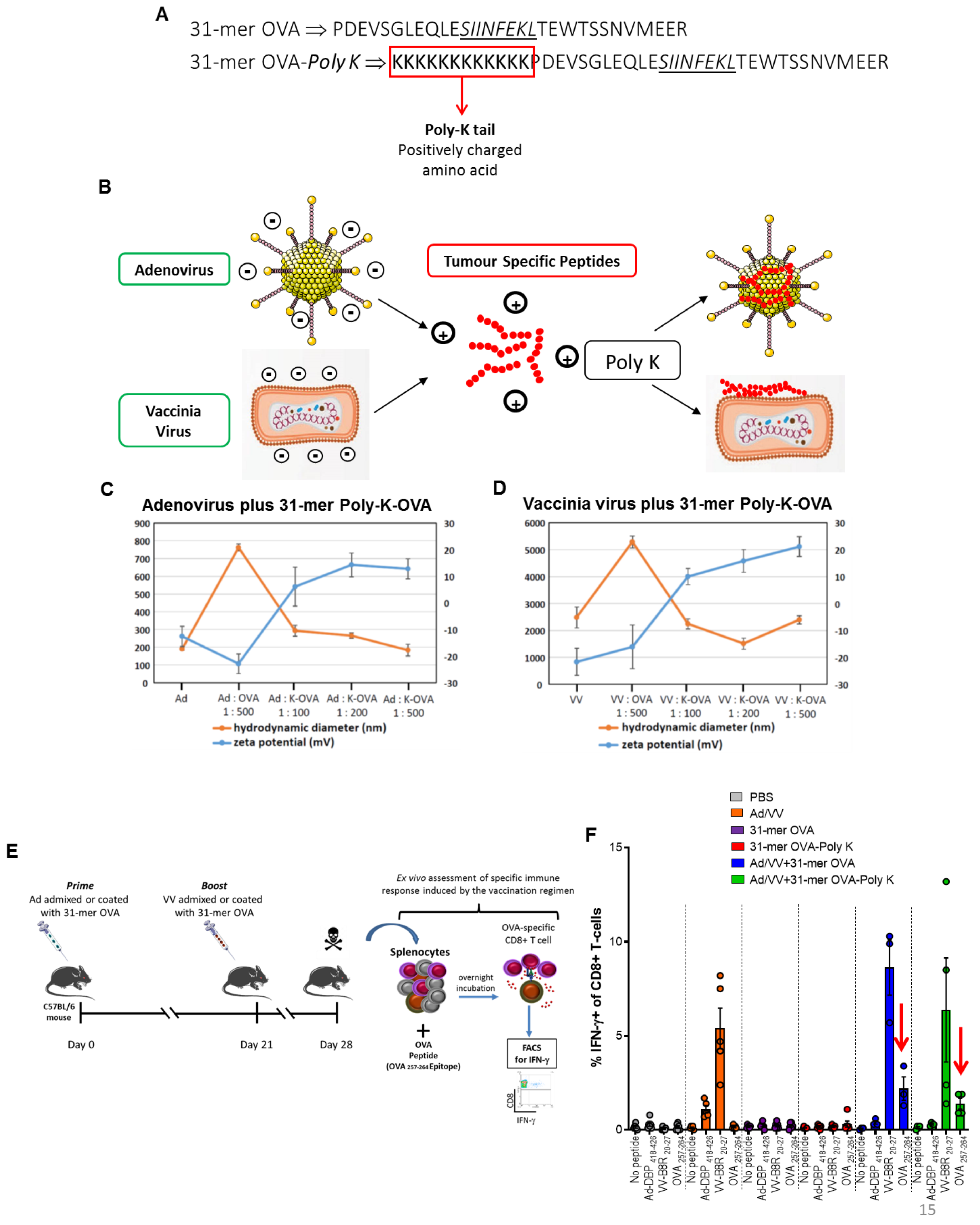

Figure S10 - Oncolytic viruses can be used as delivery systems for induction of antigen-specific CD8+ T-cell response *in vivo* and represent effective peptide delivery platforms. (**A**) 31-mer OVA peptides used in the experiments. (**B**) Illustration of the coupling of peptides onto the surface of adenovirus and vaccinia virus vectors for cancer viro-immunotherapy. The electrostatic binding potential for 31-mer OVA peptide to Ad-TD (**C**) and VVLΔTKΔN1L (**D**) were determined by mixing viruses and peptides at a ratio of a 1:100, 1:200 or 1:500 (µg viral protein: µg peptide) for 30 min at pH 7.4. All measurements were performed at 25°C with a Zetasizer Nano ZS (Malvern). **(C)** Without a poly-K modification, heavy aggregation of viral particles was observed, as indicated by an increase in the size of the Ad-TD/OVA complexes (762±19 nm). The lowest Ad-TD: peptide (poly-K-OVA) ratio (1:100) was able to increase the charge of the viral particles from -12.8±7.3mV to +6.1±3.7mV. Above 1:100, the net charge reached a plateau-like kinetic as we measured zeta potentials of +6.1±3.7 mV, +14.3±4.4 mV and +12.8±3.7 mV for the 1:100, 1:200

and 1:500 ratios, respectively. However, only at a ratio of 1:500 to represent the normal diameter of Ad-TD particles. (**D**) As for Ad, without a poly-K modification, heavy aggregation of VV was observed, as indicated by an increase in the size of the V.V/OVA complexes (5287±223nm). The lowest VV: peptide (poly-K-OVA) ratio (1:100) was able to increase the charge of the viral particles from -21.1±3.6 mV to +10.0±2.9 mV. Above 1:100, the net charge increased in a dose-dependent manner, as we measured zeta potentials of +10.0±2.9 mV, +15.8±4.2 mV and +21.1±3.6 mV for the 1:100, 1:200 and 1:500 ratios, respectively. However, only at a ratio of 1:500 the hydrodynamic diameter of the complex decreased (reaching~2400 nm), which represents the normal diameter of VV particles. These data demonstrate that both Ad-TD and VVLΔTKΔN1L are able to form a stable electrostatic interaction at a ratio of 1:500 with poly-K-antigens and as such are suitable delivery vectors for peptides. (**E**) C57BL/6 mice were immunised at days 1 and 21 with 31-mer OVA peptide admixed with Ad or VV or (poly-K) 31-mer OVA peptide coated on the capsid of Adenovirus (Ad) or Vaccinia virus (VV). Viruses or peptides alone were used as controls. One week following the booster injection, spleens were harvested, processed and splenocytes were incubated *ex vivo* overnight with OVA peptide, Tyrosinase-related protein-2 (TRP-2) peptide, Ad-DBP peptide, VV B8R peptide or left with no peptide. (**F**) Intracellular IFN-γ was measured by FACS and results are shown as the percentage of IFN-γ-positive cells gated on CD8+ T cells.

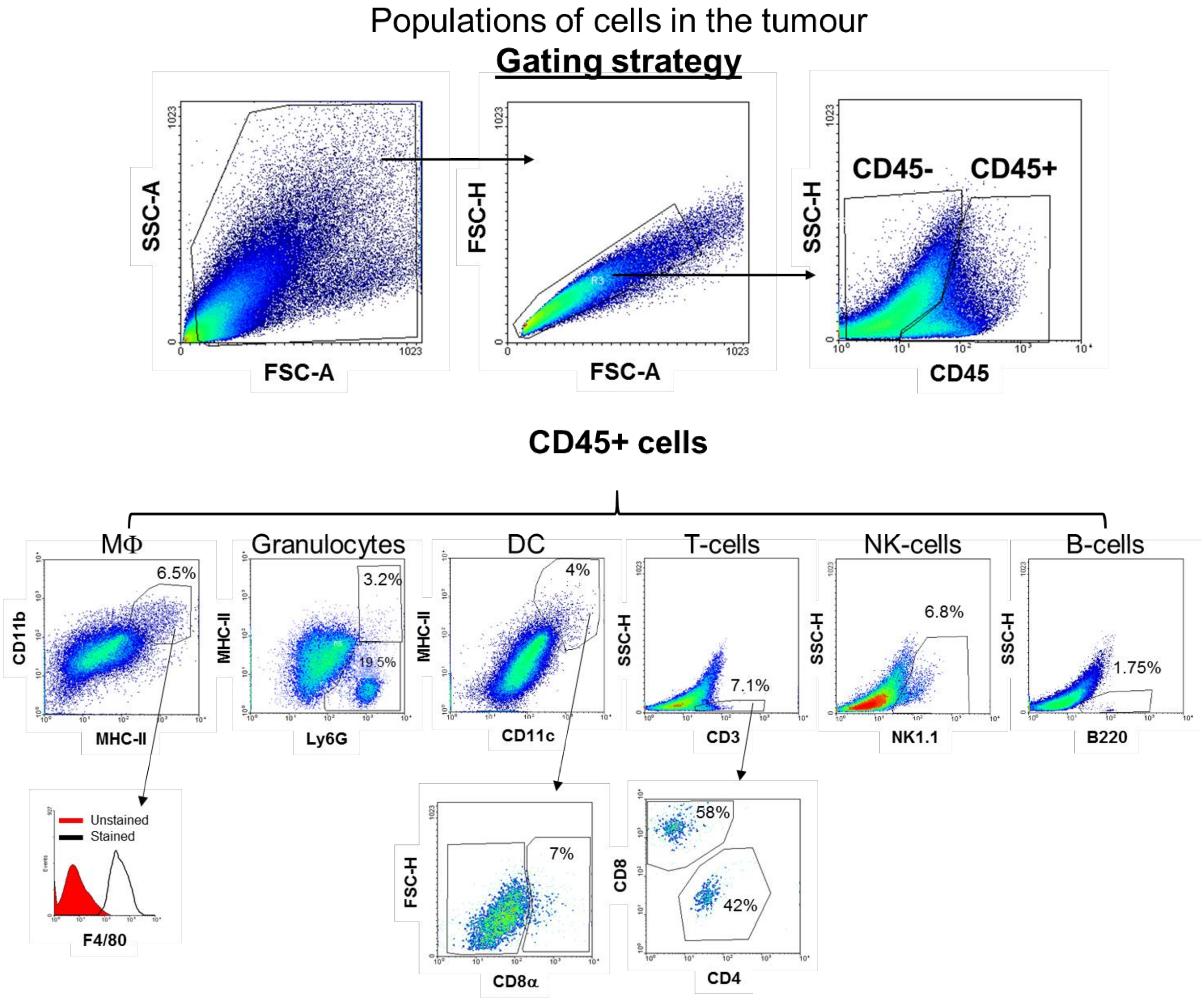

Figure S11 – Gating strategy for the analysis of the tumour-infiltrating immune cell types.

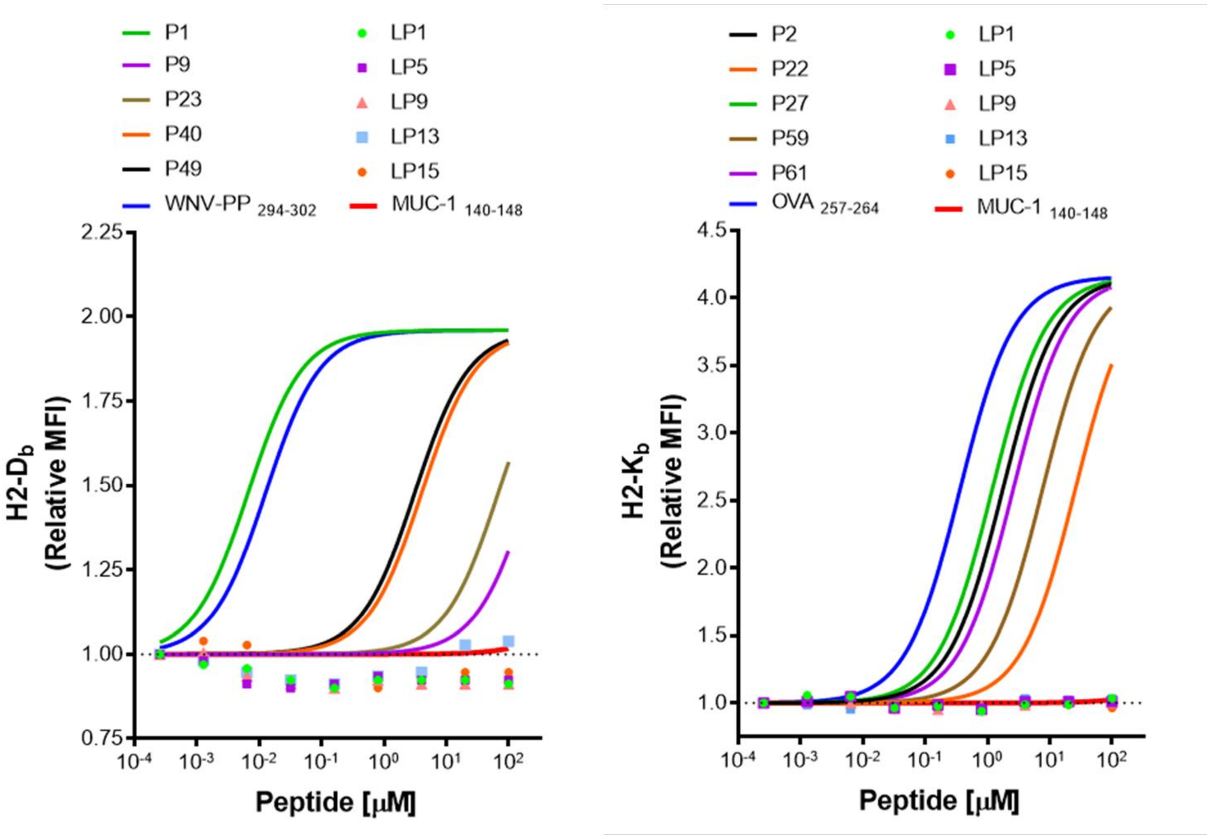

Figure S12 – Testing the binding of long peptides to H2-Db and H2-Kb. Long peptides (LP) used for immunisation as well as their short minimal epitopes were assessed for their capacity to bind to the MHC-I in cell-based binding assays. H2-Db- and H2-Kb-binding peptides were tested in MHC-I stabilization assays using TAP-deficient RMA-S cell line expressing H2-Db (**A**) and H2-Kb (**B**). The binding of each peptide was analysed three times and the mean of all of the experiments performed is shown. MUC-1 $_{140\text{-}148}$ peptide was used as negative control in all experiments; as positive control, West Nile Virus poly protein (WNV-PP) $_{294\text{-}302}$ and ovalbumin (OVA) $_{257\text{-}264}$ peptides were used for H2-Db and H2-Kb, respectively.

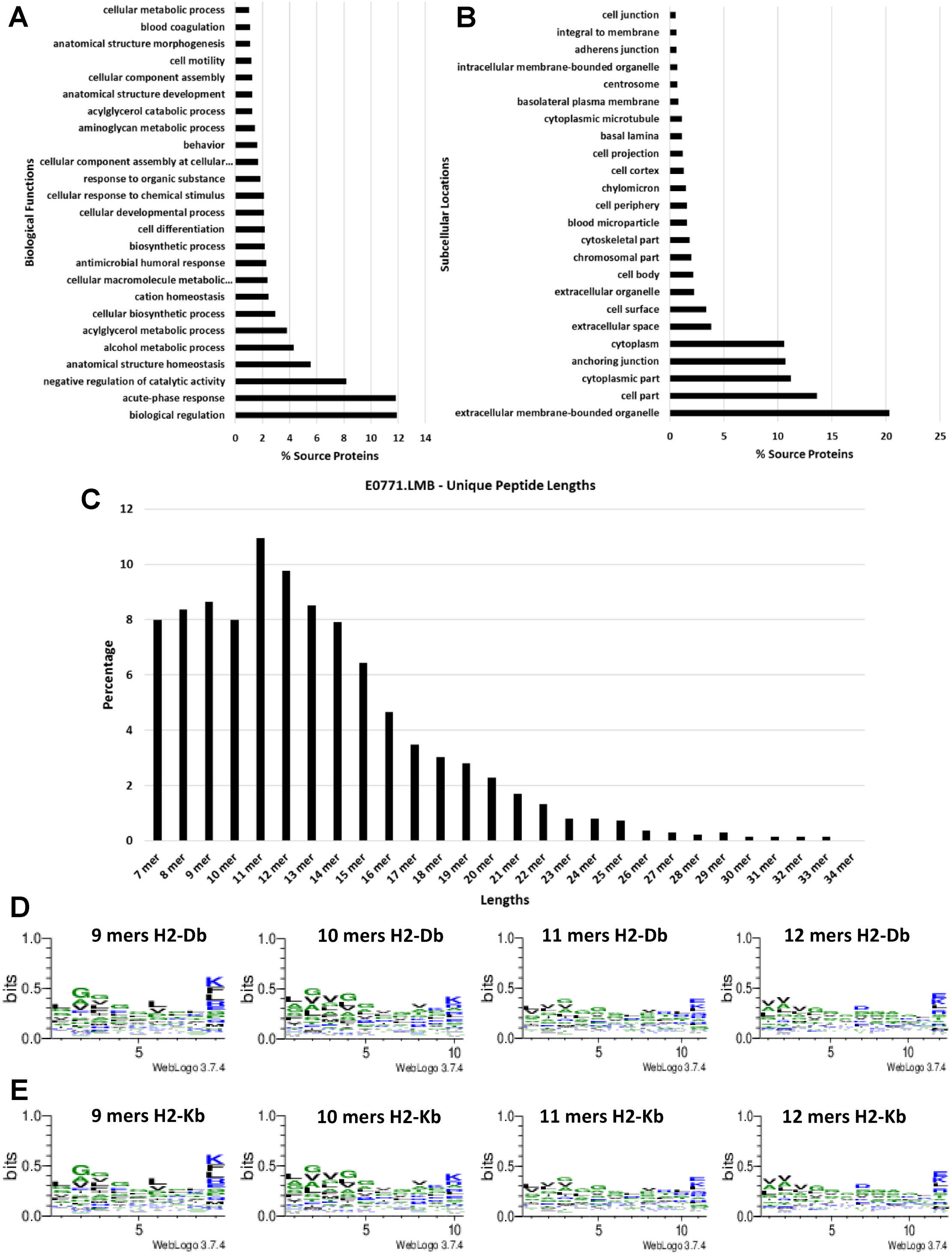

Figure S13 – Characterisation of peptides eluted from mouse H2-Db and H2-Kb molecules of E0771.LMB cells. Biological functions (**A**) and subcellular locations (**B**) of the source proteins of the MHC-I–bound peptides from E0771.LMB cells were assigned using the Mouse Protein Reference Database. The amino acid lengths of the MHC-I-eluted peptides are shown in (**C**). Binding motifs for MHC-I molecules are shown as logo plots of residue frequency at each position of H2-Db (**D**) and H2-Kb (**E**) peptides according to their length.
