# Supplementary material for: Personalized neoantigen viro-immunotherapy platform for triple-negative breast cancer
Source: J Immunother Cancer. 2023 Aug 16;11(8):e007336. doi: 10.1136/jitc-2023-007336 (PMC10432671; doi:10.1136/jitc-2023-007336)
Supplement: Supplementary data [file jitc-2023-007336supp002.pdf]

ESGO 2019 Book

Plenary Session 1: Best oral communications - Cervical Cancer

SUCCOR study. An international European cohort observational study comparing minimally invasive surgery versus open abdominal radical hysterectomy in patients with stage IB1 (FIGO 2009,< 4 cm) cervical cancer operated in 2013-2014

L. Chiva[1], V. Zanagnolo[2], A. Kucukmetin[3], G. Chakalova[4], F. Raspagliesi[5], F. Narducci[6], T. Toptas[7], M. Meydanli[8], A. Fagotti[9], D. Cibula[10], D. Wydra[11], R. Póka[12], R. Jach[13], M. Tavares[14], K. Tamussino[15], D. Haidopoulos[16], J. Ponce[17], I. Berlev[18], F. Roldán[19], S. Domingo[20], I. Zapardiel[21], E. Goncalves[22], M. Malzoni[23], O. Arencibia[24], K. Kukk[25], H. Haller[26], G. Vorgias[27], F. Ghezzi[28], F. Guyon[29], S. Herrero[30], J. Haesen[31], J.G. Feron[32], J. Minguez[33], E. Chacon[33], D. Vazquez[34], T. Castellanos[34], J. Arevalo[35], N. Martin Calvo[36], J.L. Alcazar[33], et al on behalf of Succor Study Group
[1]Clinica Universidad de Navarra, Madrid, Spain, [2]European Institute of Oncology, Milano, Italy, [3]Northern Gynaecological Oncology Centre, Gateshead, United Kingdom, [4]University Hospital of Oncology, Sofia, Bulgaria, [5]Istituto Nazionale Tumori - Milan, Milan, Italy, [6]Centre Oscar Lambret, Lille, France, [7]Saglik Bilimleri University Antalya Research and Training Hospital, Antalya, [8]Zekai Tahir Burak Women's Health Training Hospital, Ankara, Turkey, [9]Fondazione Policlinico A. Gemelli, Rome, Italy, [10]General Faculty Hospital, Prague, Czech Republic, [11]MU of Gdansk, Gdansk, Poland, [12]Gynecologic Oncology, University of Debrecen Faculty of Medicine, Debrecen, Hungary, [13]Jagiellonian University Medical College, Krakow, Poland, [14]Instituto Português de Oncologia, Porto, Portugal, [15]Medical University of Graz, Graz, Austria, [16]Alexandra Hospital, Athens, Greece, [17]Hospital del Belvitge, Barcelona, Spain, [18]N.N.Petrov National Medical Research Center of Oncology, Saint-Petersburg, Russian Federation, [19]Hospital Clínico Universitario Lozano Blesa, Zaragoza, [20]University Hospital La Fe, Valencia, [21]La Paz University Hospital, Madrid, Spain, [22]Chua-HDF, Faro, Portugal, [23]Advanced Endoscopic Gynecologic Surgery, Avellino, Italy, [24]Hospital Universitario Materno Infantil de Canarias, Las Palmas de Gran Canaria, Spain, [25]Gynecology, North Estonia Medical Foundation, Tallinn, Estonia, [26]Clinical Hospital Center Rijeka, Rijeka, Croatia, [27]Metaxa Memorial Cancer Hospital, Piraeus, Greece, [28]University of Insubria, Varese, Italy, [29]Institut Bergonié, Bordeaux, France, [30]Hospital Puerta de Hierro, Madrid, Spain, [31]University Hospitals Leuven, Leuven, Belgium, [32]Institut Curie, Paris, France, [33]Clinica Universidad de Navarra, Pamplona, [34]Clinica Universidad de Navarra, MADRID, [35]Internal Medicine, Hospital Principe de Asturias, [36]Preventive Medicine and Public Health, Clinica Universidad de Navarra, Madrid, Spain

Introduction / Background: Minimally invasive surgery (MIS) was adopted as an alternative to laparotomy for radical hysterectomy in patients with early-stage cervical cancer before obtaining solid evidence regarding its effect on survival. We studied what was the outcome of patients that underwent Radical hysterectomy for stage IB1 cervical cancer , depending on the different approaches.
Methodology: We performed a cohort study involving women who underwent radical hysterectomy for stage IB1 cervical cancer during the 2013-2014 period in 89 centers belonging to 23 European countries . The study included patients with a histologic subtype of squamous-cell carcinoma, adenocarcinoma, or adenosquamous carcinoma. Either preoperative pelvic MRI or vaginal ultrasound indicating tumor diameter < 4 cm and no parametrial invasion was mandatory. The primary outcome was the rate of disease-free survival at 4.5 years between MIS vs. open surgery. Patients with history of previous conization were also analyzed separately. A propensity matching score was applied to balance all the relevant variables that were found significant to modify the rate of relapse. Inverse probability weighting-adjusted disease-free survival evaluated the impact of the use of uterine manipulator in MIS.
Results: 624 patients were registered of whom 582 fulfilled all the inclusion criteria.
With a median follow up of 58 months, patients that underwent open surgery showed a DFS at 4,5y of 93% vs. 82 % in the group of MIS (p= 0,023, HR 3.48; 95% CI: 1.17-9.48). The use of manipulator was associated with a worse DFS in the MIS group (HR 2.38; 95% CI: 1.32-4.29). Overall survival at 4.5y was significantly lower (96% vs.88%) in the group of MIS (p=0,016). Patients with previous conization did not show differences between both approaches.
Conclusion: The risk of relapse and death in the group of MIS was significantly higher. The use of manipulator worsened the outcome among MIS patients.

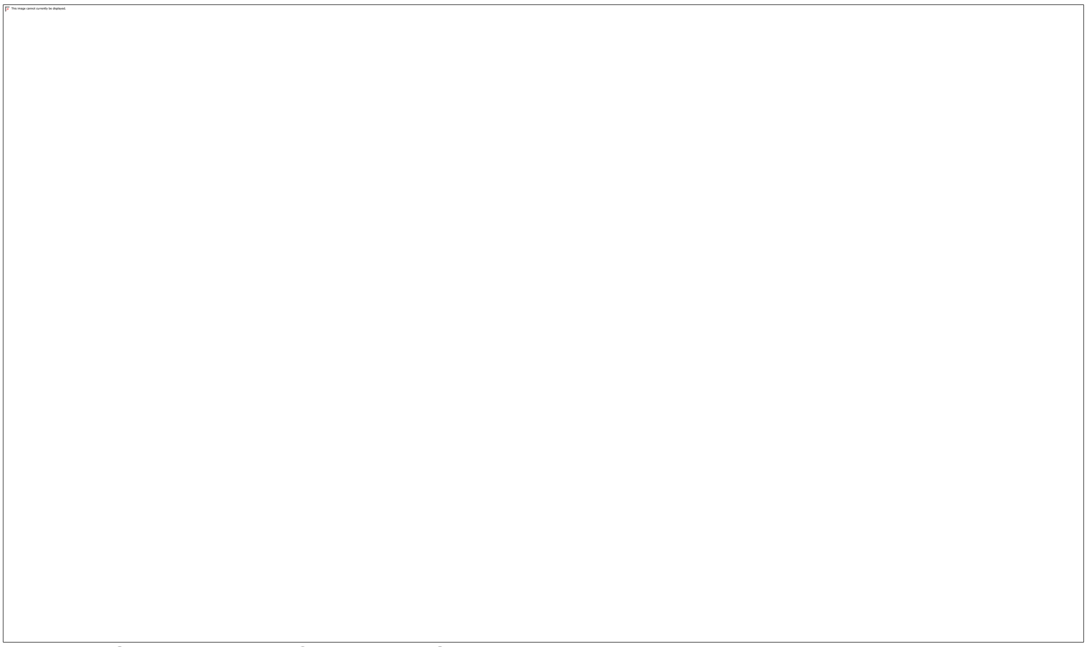

[Fig 1. DFS in both groups Open and MIS after Propensity matching score]

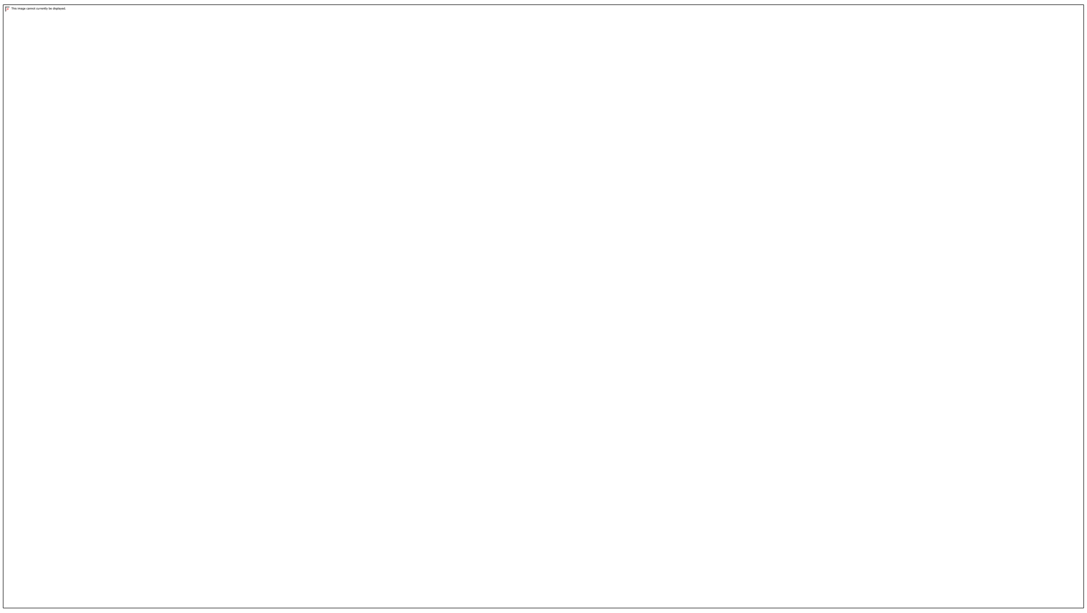

[Fig2. Impact of the use of manipulator in the DFS of the MIS approach]

VARIABLES	OPEN	MIS
Mean BMI (kg/m2)	26	24

Mean original tumor size by clinical evaluation	20.8 mm	17.6 mm
Mean MRI largest diameter	20.6 mm	15.9 mm
Previous cone biopsy	27.7%	47%
Mean tumor largest diameter in Pathology Report	22.2 mm	16.5 mm
% of positive pelvic nodes	12.7 %	9.6 %
Adjuvant therapy after surgery	48 %	37 %
% of Relapse	9.3 %	13.2 %
% of Death from any cause	4.3 %	7.1 %

[TABLE 1. Characteristics of both groups BEFORE the Propensity Matching Score]

VARIABLES	OPEN	MIS
Mean BMI (kg/m2)	26	26
Mean original tumor size by clinical evaluation	23 mm	21 mm
Mean MRI largest diameter	24 mM	22 mm
Previous cone biopsy	0 %	0 %
Mean tumor largest diameter in Pathology Report	23 mm	22 mm
% of positive pelvic nodes	7 %	7 %
Adjuvant therapy after surgery	50 %	50 %
% of Relapse	11 %	22 %
% of Death from any cause	6.4 %	10.8 %

[TABLE2 Characteristics of both groups AFTER the Propensity Matching Score]

Disclosure: Nothing to disclose

Frozen section examination of sentinel lymph nodes can be used as a decisional tool in the surgical management of early cervical cancer

A. Rychlik[1], M.A. Angeles[2], F. Migliorelli[3], E. Mery[4], S. Croce[5], F. Guyon[1], G. Ferron[2], A. Martinez[2], D. Querleu[1]

[1]Department of Surgery, Institut Bergonié, Bordeaux, [2]Department of Surgery, Institut Claudius Regard, IUCT Oncopole, Toulouse, France, [3]Department of Obstetrics and Gynecology, Geneva University Hospitals, Geneva, Switzerland, [4]Department of Pathology, Institut Claudius Regard, IUCT Oncopole, Toulouse, [5]Department of Pathology, Institut Bergonié, Bordeaux, France

Introduction / Background: The sentinel node procedure has been found to be accurate in detecting lymph node metastasis in early stage cervical cancer. The objective of this study is to evaluate the accuracy of frozen sections in the assessment of the sentinel node, with the objective to correctly orientate the intraoperative decision.

Methodology: 176 patients with early stage cervical cancer undergoing sentinel lymph node (SLN) dissection with frozen section examination from two French Comprehensive Cancer Centers were

included. A combined technique using isosulfan blue dye and technetium-99 was used in the majority of patients.
Results: A total of 506 SLN were analyzed by frozen sections. Twenty-eight lymph nodes (5.5%) were positive at the time of definitive pathologic examination including 16 macrometastases, 9 micrometastases and 3 lymph nodes with isolated tumor cells. Three macrometastases, 2 micrometastases and 3 lymph nodes with isolated tumor cells were missed on frozen sections. Bilateral mapping was detected in 153 (86.7%) out of 176 patients. Nineteen of these patients (12.4%) had SLN involvement including 3 patients with micrometastases and 3 patients with isolated tumor cells. Macrometastatic disease was identified at definitive pathology exam in 3 patients with negative frozen section analysis.Considering macrometastases only as positive lymph nodes, sensitivity of the frozen section was 76.9% (95% confidence interval 49.7-91.8) and negative predictive value was 97.9% (95% confidence interval 94.0-99.3). Including micrometastasis, sensitivity was 81.2% (95% confidence interval 57.0 - 93.4) and negative predictive value remained 97.9% (95% confidence interval 93.9 - 99.3).
Conclusion: With a prevalence of positive lymph nodes on the order of 10%, the negative predictive value of frozen section examination of sentinel lymph nodes is 98%, with an inferior limit of the confidence interval superior to 94% in our series. Diagnostic accuracy remained acceptable even if micrometastases were included. Frozen section examination can be incorporated in the intraoperative decision algorithm.
Disclosure: Nothing to disclose

Surgical algorithm for sentinel lymph nodes detection in early-stage cervical cancer
V. Balaya[1], B. Guani[2], L. Magaud[3], H. Bonsang-Kitzis[1], M. Deloménie[1], H.-T. Nguyen-Xuan[1], M. Koual[1], R. Montero macias[1], A. Bresset[1], C. Ngo[1], A.-S. Bats[1], P. Mathevet[2], F. Lécuru[1], SENTICOL
[1]*Gynecologic and Breast Oncologic Surgery Department, Georges Pompidou European Hospital, Paris, France,* [2]*Gynecologic Department, University Hospital of Vaud, Lausanne, Switzerland,* [3]*Pôle IMER, Hospices Civils de Lyon, Lyon, France*

Introduction / Background: The aim of this study was to describe and assess a surgical algorithm for sentinel lymph nodes (SLN) detection in early-stage cervical cancer to improve lymph node staging.
Methodology: Two French prospective multicentric database on SLN biopsy for cervical cancer (SENTICOL I and II) were analyzed. SLN detection was performed with a combined labeling technique (Patent blue and radioactive tracer). Patients who underwent both SLN biopsy and pelvic lymphadenectomy (PLND) were included. SLN and non-SLN were recorded according to the Marnitz classification. The surgical algorithm was retrospectively applied and consisted in exploration the following area:
(1) external iliac and interiliac,
(2) the common iliac,
(3) parametrial and presacral and
(4) paraaortic area.
In case of no SLN detected in a hemi-pelvis, a full PLND was performed.
Results: Between January 2005 and July 2012, 305 patients from 23 centers fulfilled the inclusion criteria. Median SLN detected was 3 and median total LN harvested was 17. At least one SLN was found in 92.8% of patients while bilateral detection rate was 74.6%. At final pathologic examination, 61 patients (20%) had LN metastases. Among the cases that at least one SLN detected, SLN diagnosed 52 of 57 patients with LN involvement (Sensitivity = 91.2% and Negative predictive value = 97.8%). By applying the algorithm, all patients with LN metastasis were identified. In case of bilateral SLN detection, no false negative was found and bilateral negative SLN predicted accurately the absence of LN metastases. In these cases, 79.8% of PLND would be avoided.
Conclusion: This multicentric evaluation validates the use of a surgical SLN algorithm in early-stage cervical cancer. Full lymphadenectomy could be omitted in case of bilateral negative SLN. This algorithm permits to identify all patients with nodal spread and may imply a decrease of unjustified PLND.

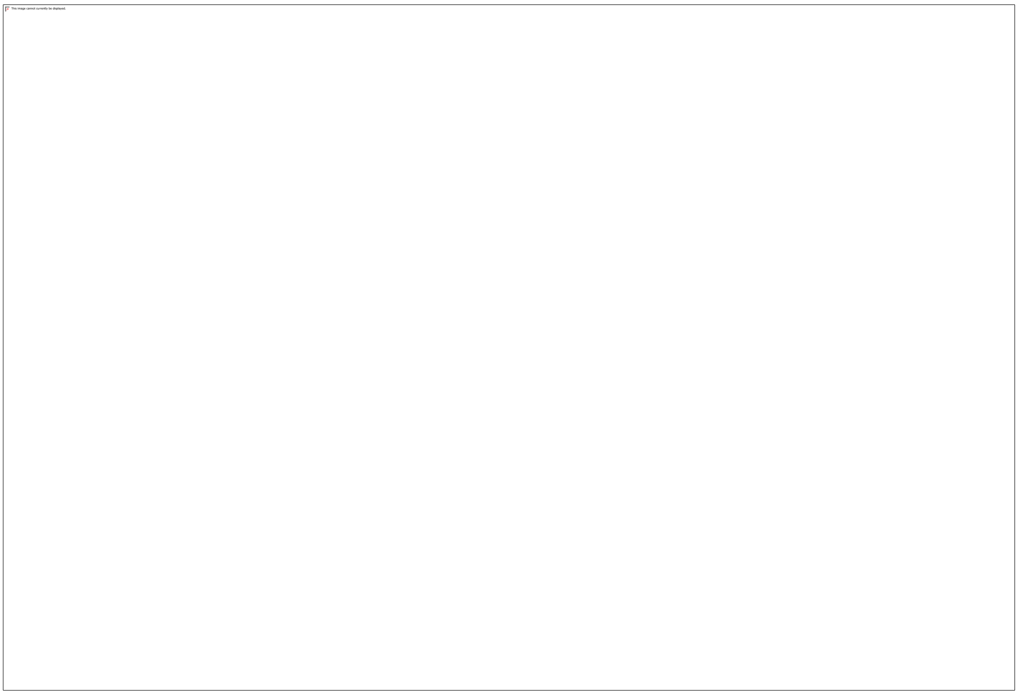

[Surgical algorithm for SLN mapping]

Disclosure: Nothing to disclose

SENTIX - Sentinel lymph node in patients with cervical cancer: time to voiding recovery after surgery (CEEGOG-CX01; ENGOT-CX2; NCT02494063)

I. Zapardiel[1], R. Kocian[2], C. Köhler[3], J. Klat[4], A. Germanova[2], A. Jacob[3], S. Bajsova[4], G. Böhmer[3], L. Lay[5], A. Torne[6], P. Havelka[7], B. Kipp[8], G. Szewczyk[9], R. Toth[10], J. Staringer[11], F.J. De Santiago[12], P.J. Coronado[13], R. Poka[14], R. Laky[15], M. Luyckx[16], M. Fastrez[17], L. Dusek[18], A. Hernandez[1], D. Cibula[2]
[1]Gynecologic Oncology Unit, La Paz University Hospital, Madrid, Spain, [2]Gynecologic Oncology Center, Department of Obstetrics and Gynecology, First Faculty of Medicine, Charles University and General University Hospital, CEEGOG, Prague, Czech Republic, [3]Department of Special Operative and Oncologic Gynaecology, Asklepios-Clinic Hamburg, Hamburg, Germany, [4]Department of Obstetrics and Gynecology, University Hospital Ostrava, Ostrava Poruba, Czech Republic, [5]Departament of Gynecology, Institute of Oncology Angel H Roffo University of Buenos Aires, Buenos Aires, Argentina, [6]Unit of Gynecological Oncology, Institute Clinic of Gynecology, Obstetrics and Neonatology (ICGON), Hospital Clinic of Barcelona, Barcelona, Spain, [7]Department of Obstetrics and Gynaecology, Bata Hospital Zlin, CEEGOG, Zlin, Czech Republic, [8]Department of Obstetrics and Gynecology, Cantonal Hospital of Lucerne, Lucerne, Switzerland, [9]Department of Obstetrics and Gynecology, Institute of Mother and Child, Warsaw, Poland, [10]Oncology Institute of East Slovakia, Košice, Slovakia, [11]Department of Gynecology and Obstetrics, Hospital Español de Buenos Aires, Buenos Aires, Argentina, [12]Gynecology, MD Anderson Cancer Center, [13]Departamento de Obstetricia y Ginecología, Hospital Clínico San Carlos, Madrid, Spain, [14]Institute of Obstetrics and Gynaecology, Faculty of Medicine, University of Debrecen, Debrecen, Hungary, [15]Gynecology, Medical University of Graz, Graz, Austria, [16]Department of Gynecology, Université catholique de Louvain, Cliniques Universitaires St Luc, [17]Department of Obstetrics and Gynaecology, St Pierre University Hospital, Université Libre de Bruxelles, Brussels, Belgium, [18]Institute of Biostatistics and Analyses, Faculty of Medicine, Masaryk University, Brno, Czech Republic

Introduction / Background: Despite the use of nerve-sparing technique, bladder dysfunction remains the leading morbidity associated with parametrectomy (radical hysterectomy or trachelectomy). Our aim was to analyze the time to postoperative voiding recovery and the factors influencing it in the SENTIX trial cohort, in whom different types of surgical approach and parametrectomy was used.
Methodology: The SENTIX trial is a prospective cohort international study on sentinel lymph node biopsy without pelvic lymphadenectomy in patients with early-stage cervical cancer (≤4 cm or ≤2cm in fertility sparing). It entails 46 sites from 18 countries. Ad hoc analysis of data from the SENTIX trial was performed to assess factors influencing voiding recovery, which was defined as the number of days from surgery to bladder catheter or epicystostomy removal with post-voiding urine residuum < 50 mL.
Results: The characteristics of the cohort of 372 patients are shown in Table 1. Median (range) voiding recovery time was 3 (0-21) days; it was < 7 days in 288 (78.5%) patients and < 1 month in 357 (97.3%). Among 10 (2.7%) patients with recovery >1 month, only 1 case (80 days) was not associated with any severe intraoperative complication (6 cases) or adjuvant radiotherapy (3 cases). Tumor size >2 cm, open surgery, and a more extensive type of parametrectomy significantly influenced voiding recovery (Figure 1). In multivariate analysis, only previous pregnancy and type of parametrectomy were significant (Table 2).
Conclusion: Voiding recovery time is directly related to the extent of parametrectomy. It is, however, temporary and almost all patients recover within 1 month, even after a more radical procedure (C2). The higher risk associated with laparotomy reflects a tendency to be more radical with open surgery.

[Figure 1: Kaplan - Meier curve for time to voiding recovery according to type of parametrectomy]

Predictors		OR (95% CI)	p
Type of parametrectomy	B	5.248 (1.683; 16.366)	0.004
	C1	4.401 (1.458; 13.285)	0.009
	C2	6.190 (1.939; 19.761)	0.002
	not done	reference	
Previous pregnancy	no	0.270 (0.092; 0.790)	0.017

	yes	reference	

[Table 2: Predictors of voiding recovery > 7 days: multivariate analysis]

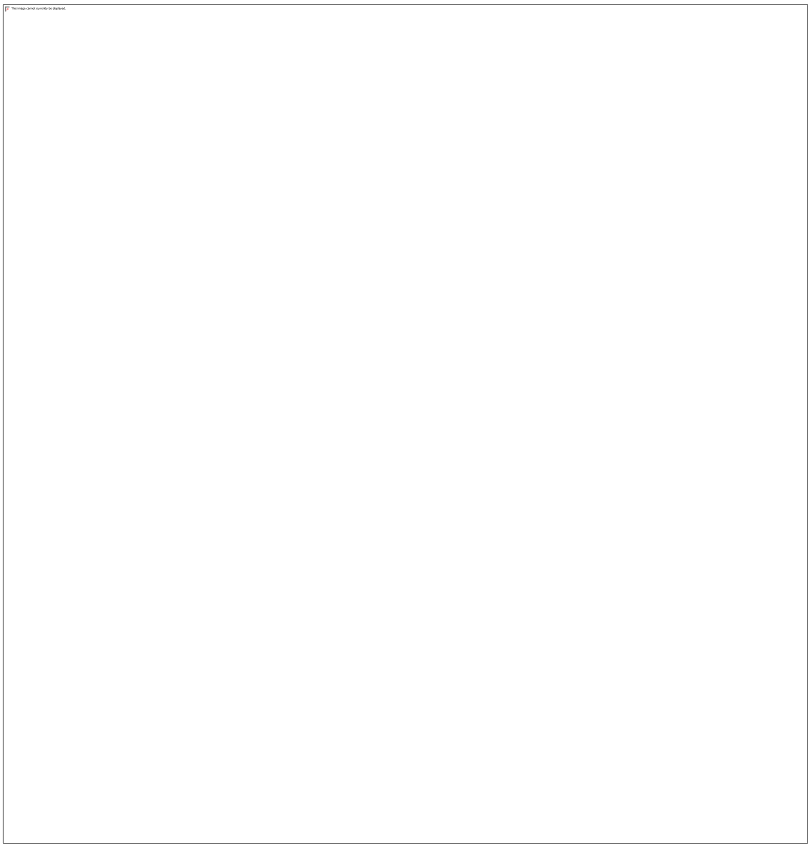

[Table 1: Main characteristics of patients (N = 372)]

Disclosure: This work was supported by a grant from the Czech Research Council (No 16-31643A). There are no conflicts of interest

Best Oral Communications / Late-Breaking Abstracts 2 - Endometrial Cancer

Conservative treatment of atypical endometrial hyperplasia and early endometrial cancer: a prospective single institution study of 418 patients

O. Novikova[1], E. Novikova[1], V. Nosov[2], A. Shevchuk[1], C. Avasova[1]
[1]*Moscow Herzen Oncology Institute, Branch of the National Medical Research Radiological Centre of Ministriy of Health of Russian Federation,* [2]*European Medical Center, Moscow, Russian Federation*

Introduction / Background: High-dose progestins have been the mainstay of conservative treatment of atypical endometrial hyperplasia (AEH) and early EC since Bokhman's first publication in 1985 until today. One of the treatment options is levonorgestrel-releasing intrauterine device (LNG IUD) but data in regard to its effectiveness is limited.
Methodology: Total of 418 patients aged 19-46 years receiving hormonal therapy (HT) (228 (55%)

with AEH and 190 (45%) with early EC) were prospectively analyzed from 2009 till 2019. Treatment variants for AEH included LNG IUD (n=169), LNG IUD + gonadotropin-releasing hormone (GnRH) agonist (n=20), high-dose oral medroxyprogesteron acetate (MPA) (n=39). EC treatment options were LNG IUD + GnRH agonist (n=139), LG IUD + GnRH + MPA (n=139), MPA (n=27).
Results: HT duration was 6-9 months. Complete response (CR) was observed in 96% of AEH and 88% of EC patients. Post-treatment follow-up for 332 patients lasted for a median of 26 months (from 3 to 149 months). 64% of patients attempted conception, 38% of them via ART. 40% of women in this group gave birth. The rate of recurrence was 23.6% in AEH group and 31.2% in EC group. 65% and 55% of patients respectively were disease-free at 5 years (p=0,021). Possible recurrence risk factors were analyzed: age at diagnosis, body mass index, parity, menstrual history, initial diagnosis, tumor grade, early myometrial invasion, use of ART, birth after HT, LNG IUD maintenance use. Regular menstruations prior to treatment, birth or LNG IUD maintenance use after treatment were associated with better DFS in univariate analysis. Only two latter factors kept significance in multivariate analysis: HR=0,215; 95% CI 0,092-0,506; p<0,001 and HR=0,262; 95% CI 0,119-0,575; p=0,001, respectively.
Conclusion: Hormonal therapy of EC is highly effective but carries high risk of recurrence. Birth or maintenance LNG IUD use after treatment were associated with decreased rates of recurrence.
Disclosure: Nothing to disclose

Lympho-vascular space invasion is strongly associated with lymph node metastases and decreased survival in endometrioid endometrial cancer: a Swedish Gynecologic Cancer Group (SweGCG) study

K. Stålberg[1], M. Bjurberg[2], C. Borgfeldt[3], J. Carlson[4], P. Dahm Kähler[5], A. Flöter-Rådestad[6], K. Hellman[4], E. Hjerpe[4], E. Holmberg[7], P. Kjølhede[8], J. Marcickiewicz[9], P. Rosenberg[10], B. Tholander[11], E. Åvall-Lundqvist[12], T. Högberg[13]
[1]Department of Women's and Children' Health, Uppsala University, Uppsala, [2]Department of Clinical Sciences, Lunds University, [3]Department of Obstetrics and Gynecology, Lund University, Lund, [4]Department for Oncology and Pathology, Karolinska Institutet, Stockholm, [5]Department of Obstetrics and Gynecology, Sahlgrenska Academy at University of Gothenburg, Gothenburg, [6]Department of Obstetrics and Gynecology, Karolinska Institutet, Stockholm, [7]Regional Cancer Center Western, Sahlgrenska University Hospital, Gothenburg, [8]Department of Children and Women's Health, Linköpings University, Linköping, [9]Department of Obstetrics and Gynecology, Hallands Hospital, Varberg, [10]Linköping University, Department of Clinical Oncology and Department of Clinical and Experimental Medicine, Linköping, [11]Department of Immunology, Genetics and Pathology, Uppsala University, Uppsala, [12]Department of Clinical Oncology and Department of Clinical and Experimental Medicine, Linköpings University, Linköping, [13]Department of Cancer Epidemiology, Lund University, Lund, Sweden

Introduction / Background: The aim of this study was to evaluate the impact of lymphovascular space invasion (LVSI) on the risk of lymph node metastases and survival in endometrioid endometrial adenocarcinoma.
Methodology: This is a retrospective cohort study based on prospectively recorded data. Patients with endometrioid adenocarcinoma registered in the Swedish Quality Registry for Gynecologic Cancer 2010-2017 with FIGO stages I-III and verified nodal status were identified (n=1587). LVSI together with DNA ploidy, FIGO grade, myometrial invasion and age, were included in multivariable regression analyses with lymph node metastases as the dependent variable. Associations between the risk factors and overall and relative survival were included in multivariable models. Estimates of risk ratios (RR), hazard ratios (HR), excess mortality rate ratios (EMR), and 95% confidence intervals (95% CI) were calculated.
Results: The presence of LVSI presented the strongest association with lymph node metastases (RR=5.46, CI 3.69-8.07, p< 0.001) followed by deep myometrial invasion (RR=1.64, CI 1.13-2.37, p=0.008). In the multivariable survival analyses, LVSI (EMR=7.69, CI 2.03-29.10, p=0.003) and non-diploidy (EMR=3.23, CI 1.25-8.41, p=0.016) were associated with decreased relative survival. In sub-analyses including only patients with complete para-aortic and pelvic lymphadenectomy and negative lymph nodes (n=404), only LVSI (HR=2.50, CI 1.05-5.98, p=0.039) was associated with a worsened

overall survival.
Conclusion: This large nationwide study identified LVSI as the strongest independent risk factor for lymph node metastases and decreased survival in patients with endometrioid adenocarcinomas. Moreover, decreased overall survival was also seen in patients with LVSI-positive tumors and negative lymph nodes, indicating that hematogenous dissemination might also be important.
Disclosure: Nothing to disclose

Development and validation of an endometrial carcinoma preoperative Bayesian network using molecular and clinical biomarkers (ENDORISK): an ENITEC collaboration study

C. Reijnen[1,2], E. Gogou[3], L. van der Putten[1], N. Visser[4], K. van de Vijver[5], M. Santacana[6], J. Bulten[4], E. Colas[7], A. Gil-Moreno[8], A. Reques[9], G. Mancebo[10], C. Krakstad[11], J. Trovik[12], I. Haldorsen[13], H. Engerud[11], J. Huvila[14], M. Koskas[15], V. Weinberger[16], L. Minar[16], E. Jandakova[17], A. van der Wurff[18], X. Matias-Guiu[6], F. Amant[19], H. Küsters-Vandevelde[20], J. Ramjith[21], L. Massuger[1], M. Snijders[2], P. Lucas[3], J. Pijnenborg[1]
[1]Obstetrics and Gynaecology, Radboud UMC Nijmegen, [2]Obstetrics and Gynaecology, Canisius-Wilhelmina Hospital, [3]Department of Computing Sciences, Radboud University, [4]Pathology, Radboud UMC Nijmegen, Nijmegen, The Netherlands, [5]Pathology, Ghent University Hospital, Ghent, Belgium, [6]Department of Pathology and Molecular Genetics and Research Laboratory, Hospital Universitari Arnau de Vilanova, Lleida, [7]Biomedical Research Group in Gynecology, Vall Hebron Institute of Research, Universitat Autònoma de Barcelona, [8]Gynecological Department, [9]Pathology Department, Vall Hebron University Hospital, [10]Obstetrics and Gynecology, Hospital del Mar, Barcelona, Spain, [11]Centre for Cancer Biomarkers, Department of Clinical Science, University of Bergen, [12]Obstetrics and Gynecology, [13]Mohn Medical Imaging and Visualization Centre, Department of Radiology, Haukeland University Hospital, Bergen, Norway, [14]Pathology, University of Turku, Turku, Finland, [15]Obstetrics and Gynecology Department, Bichat-Claude Bernard Hospital, Paris, France, [16]Gynecology and Obstetrics, Faculty of Medicine, [17]Institute of Pathology, Faculty of Medicine, Masaryk University, Brno, Czech Republic, [18]Pathology, Elisabeth-TweeSteden Hospital, Nijmegen, [19]Gynaecologic Oncology, Center for Gynaecologic Oncology Amsterdam, Location Antoni van Leeuwenhoek, Amsterdam, [20]Pathology, Canisius-Wilhelmina Hospital, [21]Radboud University, Department for Health Evidence, Nijmegen, The Netherlands

Introduction / Background: The presence of pelvic and/or para-aortic lymph node metastasis (LNM) is one of the most important prognostic factors for poor outcome in endometrial carcinoma (EC). Current risk stratification for lymphadenectomy is mainly based on preoperative tumor grade, results in over- and undertreated of approximately 25% and 15% of the patients. Use of preoperative prediction models allow a personalized risk estimation and contribute to shared decision making, balancing risks and clinical benefit in tailored treatment. The aim of this study is to develop a Bayesian network (BN), based on easily-accessible clinical, histopathological and molecular biomarkers, for the prediction of lymph node metastasis and outcome in endometrial carcinoma patients. Second, the calibration and discrimination this network will be tested by means of external validation.
Methodology: This network was constructed within the European Network for Individualized Treatment of Endometrial Cancer (ENITEC), using a cohort including 809 patients treated for EC. The network was based both on expert knowledge of EC progression and learned from data of the construction cohort. Variables used to construct to BN included: preoperative tumor grade; immunohistochemical profile including estrogen receptor-, progesterone receptor-, p53- and L1CAM-expression; cancer antigen 125 serum levels, thrombocyte count, imaging results and cervical cytology. Internal cross-validation and external validation was performed using two independent validation cohorts comprising 431 and 400 patients.
Results: A Bayesian network was constructed to predict the presence of lymph node metastasis and 1-, 3- and 5-year disease-specific survival (**Figure 1**). Internal cross-validation showed good discrimination (area under the receiver operator characteristic curve 0.86) and was calibrated well with respect to the prediction of lymph node metastasis. External validation will be completed soon.
Conclusion: We have developed and externally validated a Bayesian network predicting lymph node metastasis in endometrial carcinoma using preoperative markers with high diagnostic accuracy.

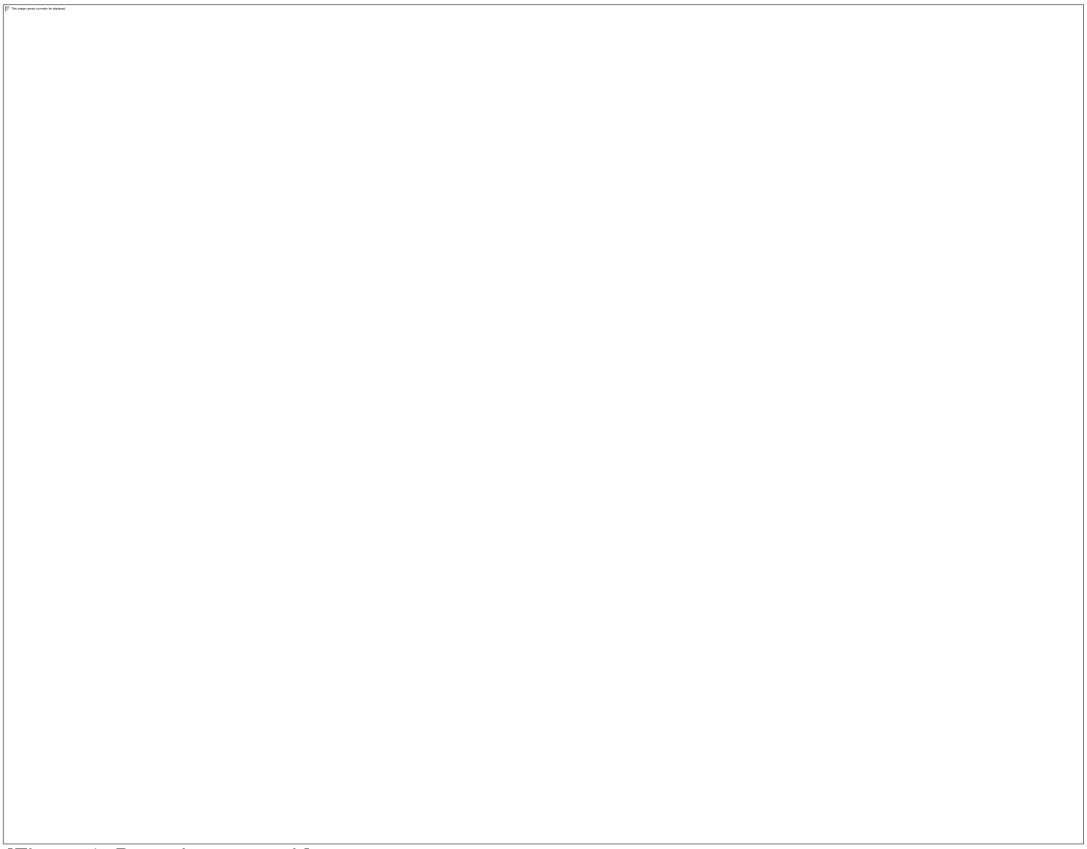

[Figure 1. Bayesian network]

Disclosure: Nothing to disclose

Prognostic relevance of the molecular classification in high-risk endometrial cancer: analysis of the PORTEC-3 trial

A. Leon-Castillo[1], S.M. de Boer[2], M.E. Powell[3], L.R. Mileshkin[4], H.J. Mackay[5], A. Leary[6], H.W. Nijman[7], N. Singh[8], P. Pollock[9], P. Bessette[10], C. Haie-Meder[11], V.T.H.B.M. Smit[1], R.J. Edmondson[12], H. Putter[13], H.C. Kitchener[12], E.J. Crosbie[12], M. de Bruyn[7], R.A. Nout[2], N. Horeweg[2], T. Bosse[1], C.L. Creutzberg[2]

[1]Department of Pathology, [2]Department of Radiation Oncology, Leiden University Medical Center, Leiden, The Netherlands, [3]Department of Clinical Oncology, Barts Health NHS Trust, London, United Kingdom, [4]Division of Cancer Medicine, Peter MacCallum Cancer Centre, Melbourne, VIC, Australia, [5]Division of Medical Oncology and Hematology, Sunnybrook Odette Cancer Centre, Toronto, ON, Canada, [6]Department of Medical Oncology, Gustave Roussy, Villejuif, France, [7]Department of Gynecology, University Medical Center Groningen, University of Groningen, Groningen, The Netherlands, [8]Department of Pathology, Barts Health NHS Trust, London, United Kingdom, [9]Institute of Health and Biomedical Innovation, Queensland University of Technology, Brisbane, QLD, Australia, [10]CCTG, Department of Obstetrics and Gynaecology, University of Sherbrooke, Quebec, QC, Canada, [11]Department of Radiotherapy, Gustave Roussy, Villejuif, France, [12]Institute of Cancer Sciences, University of Manchester, St Mary's Hospital, Manchester, United Kingdom, [13]Department of

Biostatistics, Leiden University Medical Center, Leiden, The Netherlands

Introduction / Background: The TCGA molecular classification of endometrial cancer (EC) has proven prognostic impact for patients with intermediate-risk EC. The randomised PORTEC-3 trial investigated the benefit of combined adjuvant chemotherapy and radiotherapy (CTRT) compared with RT alone for women with high-risk EC (HREC). We evaluated the prognostic significance of the molecular classification in HREC using tissues from consenting PORTEC-3 trial participants.
Methodology: 423 paraffin-embedded tissue samples (64% of 660 participants) were collected. Through targeted DNA-sequencing for pathogenic *POLE*-exonuclease domain mutations (EDM) and immunohistochemistry for p53 and mismatch repair (MMR) proteins, the HREC were classified as *POLE*-ultramutated (*POLE*mut), p53 mutant staining (p53abn), MMR-deficient (MMRd) or no specific molecular profile (NSMP). The Kaplan-Meier method, log-rank test and Cox's proportional hazard model were used for analysis.
Results: Molecular analysis was successful in 410 HREC (97% of the 423), identifying 4 molecular subgroups: p53abn (n=92, 22%), *POLE*mut (n=52, 13%), MMRd (n=137, 33%) and NSMP (n=129, 32%). Five-year recurrence-free survival (RFS) for patients with *POLE*mut, p53abn, MMRd and NSMP EC was 98%, 50%, 74% and 76%, respectively (p< 0.0001), and overall survival was 98%, 55%, 81% and 88% (p< 0.0001). Multivariable analysis showed that p53abn was the strongest prognostic factor for decreased survival, while pathogenic *POLE* EDM was the strongest favourable factor (table 1). Patients with p53abn HREC had significant benefit of combined adjuvant chemotherapy and radiotherapy (5-year RFS with CTRT 61% versus 37% for RT, log-rank p=0.015).
Conclusion: The molecular classification provides better risk stratification than histopathology alone. Patients with *POLE*mut HREC have excellent clinical outcome, suggesting these should be classified as low-risk, independent of other pathologic variables. P53abn EC is the strongest predictor of poor clinical outcome, and these patients had significant benefit from added chemotherapy. Molecular characteristics should be incorporated in clinical diagnostics and decision making and future trials should address molecular subgroup-based treatments.

[Table 1. Multivariable analysis of molecular subgroups and clinicopathological features (n=410)]

Disclosure: AL reports receiving advisory board fees from AstraZeneca, Tesaro, Clovis, MSD,

Grisdstone, Seattle Genetics, Gamamabs, and Biocad, and travel support paid to her institution from Roche and AstraZeneca. HN reports is the founder of SME Vicinivax and has collaborated with Aduro, TRON & Merck.

Best Oral Communications / Late-Breaking Abstracts 3 - Ovarian Cancer

Niraparib therapy in patients with newly diagnosed advanced ovarian cancer after chemotherapy: PRIMA/ENGOT-OV26/GOG-3012 study

A. González-Martín[1], B. Pothuri[2], I. Vergote[3], R.D. Christensen[4], W. Graybill[5], M.R. Mirza[6], C. McCormick[7], D. Lorusso[8], P. Hoskins[9], G. Freyer[10], K. Baumann[11], K. Jardon[12], A. Redondo[13], R.G. Moore[14], C. Vulsteke[15], R.E. O'Cearbhaill[16], B. Lund[17], F. Backes[18], P. Barretina-Ginesta[19], A.F. Haggerty[20], M.J. Rubio-Pérez[21], M.S. Shahin[22], G. Mangili[23], W.H. Bradley[24], I. Bruchim[25], K. Sun[26], I. Malinowska[26], Y. Li[26], D. Gupta[26], B.J. Monk[27]

[1]Grupo Español de Investigación en Cáncer de Ovario (GEICO) and Medical Oncology Department, Clínica Universidad de Navarra, Madrid, Spain, [2]Gynecologic Oncology Group (GOG) and Department of Obstetrics/Gynecology, Perlmutter Cancer Center, NYU Langone Health, New York, NY, United States, [3]Belgium and Luxembourg Gynaecological Oncology Group (BGOG) and Department of Gynaecology & Obstetrics, Division of Gynaecological Oncology, University Hospitals Leuven, Leuven Cancer Institute, Leuven, Belgium, [4]Nordic Society of Gynaecological Oncology (NSGO) and Research Unit of General Practice, Institute of Public Health, University of Southern Denmark, Odense, Denmark, [5]GOG and Gynecologic Oncology, Medical University of South Carolina, Charleston, SC, United States, [6]NSGO and Rigshospitalet-Copenhagen University Hospital, Copenhagen, Denmark, [7]GOG and Legacy Medical Group Gynecologic Oncology, Portland, OR, United States, [8]Multicentre Italian Trials in Ovarian Cancer and Gynecologic Malignancies (MITO) and Fondazione IRCCS National Cancer Institute of Milan, Milan, Italy, [9]US Oncology Research (USOR) and Department of Medical Oncology, BC Cancer - Vancouver, Vancouver, BC, Canada, [10]Groupe d'Investigateurs Nationaux pour l'Etude des Cancers Ovariens (GINECO) and Service d'Oncologie Médicale, Centre Hospitalier Lyon-Sud, Lyon, France, [11]Arbeitsgemeinschaft Gynäkologische Onkologie (AGO) and Department of Gynecology and Obstetrics, Klinikum der Stadt Ludwigshafen, Ludwigshafen, Germany, [12]GOG and Department of Obstetrics and Gynecology, McGill University, Department of Oncology, McGill University Health Centre, Division of Gynecologic Oncology, Montreal, QC, Canada, [13]GEICO and Hospital Universitario La Paz-IdiPAZ, Madrid, Spain, [14]USOR and Division of Gynecologic Oncology, Wilmot Cancer Institute, Department of Obstetrics and Gynecology, University of Rochester, Rochester, NY, United States, [15]BGOG and Department of Medical Oncology and Hematology, AZ Maria Middelares, Gent, and Department of Molecular Imaging, Pathology, Radiotherapy & Oncology, Center for Oncological Research, Antwerp University, Antwerp, Belgium, [16]GOG and Department of Medicine, Memorial Sloan Kettering Cancer Center, Department of Medicine, Weill Cornell Medical College, New York, NY, United States, [17]NSGO and Department of Oncology, Aalborg University, Aalborg, Denmark, [18]Division of Gynecologic Oncology, Ohio State University, Columbus, OH, United States, [19]GEICO and Medical Oncology Department, Catalan Institute of Oncology (ICO) - Hospital Universitari Dr Josep Trueta, and Department of Medical Sciences, Medical School University of Girona, Girona, Spain, [20]GOG and Division of Gynecologic Oncology, University of Pennsylvania, Philadelphia, PA, United States, [21]GEICO and Hospital Universitario Reina Sofía, Cordoba, Spain, [22]GOG and Hanjani Institute for Gynecologic Oncology, Asplundh Cancer Pavilion, Abington Jefferson Hospital, Sidney Kimmel Medical College of Thomas Jefferson University, Willow Grove, PA, United States, [23]MITO and Department of Obstetrics and Gynaecology, San Raffaele Scientific Institute, Milan, Italy, [24]GOG and Department of Obstetrics and Gynecology, Medical College of Wisconsin, Milwaukee, WI, United States, [25]Israeli Society of Gynecologic Oncology (ISGO) and Department of Gynecology and Gynecologic Oncology, Hillel Yaffe Medical Center, Technion Israel Institute of Technology, Haifa, Israel, [26]TESARO: A GSK Company, Waltham, MA, [27]Arizona Oncology (US Oncology Network), University of Arizona College of Medicine, Creighton University School of Medicine, Phoenix, AZ, United States

Introduction / Background: Niraparib improves progression-free survival (PFS) in patients (pts) with newly diagnosed advanced ovarian cancer after 1st-line (1L) platinum-based chemotherapy (CT). We report the efficacy of niraparib in pts by biomarker status.

Methodology: This double-blind, placebo (PBO)-controlled, phase 3 study randomized 733 pts with newly diagnosed advanced ovarian, primary peritoneal, or fallopian tube cancer with a complete or partial response (CR or PR) to 1L platinum-based CT. Stratification factors were best response to the 1L CT (CR/PR), receipt of neoadjuvant CT (yes/no), and homologous recombination status (deficient/proficient/not determined). Pts received niraparib or PBO once daily. The primary endpoint of PFS assessed by blinded independent central review was analyzed using a stratified Cox proportional hazards model and hierarchically tested in homologous recombination deficient pts, then the overall population.
Results: Of 733 randomized pts (niraparib, 487; PBO, 246), 373 (51%) were homologous recombination deficient (niraparib, 247; PBO, 126) and 249 (34%) were homologous recombination proficient (niraparib, 169; PBO, 80). Overall, 35% had stage IV disease, 67% received NACT, and 31% had a PR to 1L CT. Niraparib-treated pts in all the biomarkers groups had a statistically significant and clinically meaningful benefit in PFS (Table). The most common grade ≥3 adverse events were anemia (31%), thrombocytopenia (29%), and neutropenia (13%).
Conclusion: Niraparib improved PFS as evidenced by reduction in the risk of recurrence or death due to any cause in the overall population of advanced ovarian cancer. No new safety signals were identified.

Disease characteristics	Hazard ratio (95% CI)	P Value
Overall	0.62 (0.502-0.755)	<0.0001
Homologous recombination deficient	0.43 (0.310-0.588)	<0.0001
BRCAmut	0.40 (0.265-0.618)	<0.0001
BRCAwt	0.50 (0.305-0.831)	0.0064
Homologous recombination proficient	0.68 (0.492-0.944)	0.0203

[CI=confidence interval; mut=mutated; wt=wild type]

Disclosure: AMG: Consulting: AstraZeneca, TESARO, Roche, Pharmamar, Clovis, Merck, Genmab, ImmunoGen, Oncoinvent AS BP, RDC: Advisory: TESARO IV: Personal: Advaxis, Eisai, MSD Belgium, Roche NV, Genmab, Roche, Pharmamar, Millennium Pharmaceuticals, Clovis, Astrazeneca NV, Tesaro, Immunogen, Sotio. Grants: Amgen, Stichting tegen Kanker, Roche. Contracted Research: Oncoinvent AS, Genmab WG: Consulting: TESARO MM: Leadership/Other ownership: Karyopharm Therapeutics, Sera Prognostics. Personal Fees: Roche, AstraZeneca, Clovis, Pfizer, TESARO, Genmab, BioCad, Sotio, Geneos Therapeutics, Merck, Oncology Venture, Seattle Genetics, Sera Prognostics, Takeda, Zailab. Grants: AstraZeneca, Clovis, Pfizer, TESARO, Boehringer Ingelheim CCM, PH, KHB, KJ, CGAV, BL, AFH, MJR-P, WHB, IB: none DL: Personal: AstraZeneca, Clovis, Genmab, Immunogen, Pharma Mar SA, Amgen, Merck. Grants: Pharma Mar SA, Merck GF: Personal: TESARO, AstraZeneca, Clovis, Roche, Bristol-Meyers Squibb, MSD, Pfizer, Novartis. Grants: AstraZeneca, Roche AR: Research funding/Advisory role: Pharmamar, Roche, Eisai,AstraZeneca, TESARO RGM: Research: Angle PLC. Consulting: Fujirebio Diagnostics REO: Advisory: Clovis, TESARO, GlaxoSmithKline FB: Advisory: TESARO, Clovis, Agenus, Merck, Eisai. Grant: Clovis, Merck, Eisai, Immunogen. Lecture: CEC Oncology MPBG: Lecture/Advisory board: TESARO, AstraZeneca, Roche, Clovis, Pharmamar, MSD. MSS: Personal: TESARO, Merck, Astra Zeneca, Clovis, Pacira Pharamceuticals. Grant: TESARO GM: Personal: AstraZeneca, TESARO. Non-Financial Support: TESARO, Roche. BJM: Speaker bureau, Grant: TESARO KS, IM, YL, DG: TESARO employee

Phase III PAOLA-1/ENGOT-ov25 trial: olaparib plus bevacizumab as maintenance therapy in patients with newly diagnosed, advanced ovarian cancer treated with platinum-based

chemotherapy plus bevacizumab

P. Harter[1], M.A. Mouret-Reynier[2], S. Pignata[3], C. Cropet[4], A. González-Martin[5], G. Bogner[6], K. Fujiwara[7], I. Vergote[8], N. Colombo[9], J. Mäenpää[10], A. Floquet[11], A. El-Balat[12], D. Lorusso[13], E.M. Guerra Alia[14], M. Fabbro[15], B. Schmalfeldt[16], A.C. Hardy Blessard[17], I. Runnebaum[18], E. Pujade-Lauraine[19], I. Ray-Coquard[20]

[1]Kliniken Essen Mitte, Essen and AGO, Essen, Germany, [2]Centre Jean Perrin, Clermont and Gineco, Clermont-Ferrand, France, [3]Istituto Nazionale Tumori, Naples and IRCCS, Mito, Naples, Italy, [4]Centre Léon Bérard and Gineco, Lyon, France, [5]MD Anderson Cancer Center Madrid and GEICO, Madrid, Spain, [6]Paracelsus Medical University Salzburg and Ago Au, Salzburg, Austria, [7]Saitama Medical University and Gotic, Saitama, Japan, [8]University Hospital Leuven, Leuven Cancer Institute, Leuven and BGOG, Leuven, Belgium, [9]European Institute of Oncology, Milan and Mango, Milan, Italy, [10]Tampere University and University Hospital, Tampere and NSGO, Tampere, Finland, [11]Institut Bergonié, Bordeaux and Gineco, Bordeaux, France, [12]Klinikum der Johann Wolfgang Goethe-Universität, Frankfurt and Ago, Germany, [13]Fondazione IRCCS Istituto Nazionale Tumori, Milan and Mito, Italy, [14]Hospital Universitario Ramón y Cajal, Madrid and Geico, Spain, [15]ICM Val d'Aurelle, Montpellier and Gineco, Montpellier, France, [16]Universitätsklinikum Hamburg-Eppendorf, Hamburg and AGO, Hamburg, Germany, [17]Centre CARIO - HPCA, Plérin sur mer and Gineco, Plérin, France, [18]Universitätsklinikum Jena, Jena and AGO, Jena, Germany, [19]ARCAGY Research, Paris and Gineco, Paris, [20]Centre Léon Bérard, Lyon and Gineco, Lyon, France

Introduction / Background: In the Phase III SOLO1 first-line trial, maintenance therapy with the PARP inhibitor olaparib provided a substantial progression-free survival (PFS) benefit to advanced ovarian cancer (OC) patients who have a *BRCA1/2* mutation (*BRCA*m) and are in complete or partial response to first-line chemotherapy (Moore K *et al. N Engl J Med* 2018). VEGF inhibitor may increase PARP sensitivity, particularly in some OC patients with *BRCA* wild type (Liu JF *et al*. *Lancet Oncol* 2014). PAOLA-1/ENGOT-ov25 (NCT02477644) is the first Phase III trial to evaluate the efficacy and safety of a PARP inhibitor with bevacizumab as first-line maintenance therapy, and in patients regardless of *BRCA*m status.

Methodology: PAOLA-1 is a randomized, double-blind, international Phase III trial. Eligible patients had newly diagnosed, advanced (FIGO stage IIIA-IV) high-grade serous or endometrioid ovarian, fallopian tube or peritoneal cancer. Patients had no evidence of disease or were in partial response following first-line platinum-based chemotherapy plus bevacizumab. Bevacizumab was to be given for up to 15 months, including the initial combination with chemotherapy. 806 patients were randomized (2:1) in maintenance therapy to olaparib (300 mg bid) or placebo plus bevacizumab (15 mg/kg, d1, q3w), stratified by first-line treatment outcome and tumour *BRCA*m status. The primary endpoint is investigator-assessed PFS (modified RECIST v1.1). The 458 PFS events required for the primary analysis to show significance at the two-sided 5% level with an estimated >80% power have been reached in March 2019. Database lock was in July and analyses are ongoing.

Results: The LBA will report: investigator-assessed PFS, PFS by *BRCA*m/HRD status, health- related quality of life, and safety and tolerability results.

Conclusion: PAOLA-1 will demonstrate whether addition of olaparib to bevacizumab maintenance therapy following first-line platinum-based chemotherapy plus bevacizumab provides a clinically meaningful benefit to OC patients.

Disclosure: P Harter declares honoraria from AstraZeneca, Roche, Sotio, Tesaro, Stryker, ASCO, Zai Lab and MSD; and consultancy/advisory board fees from AstraZeneca, Roche, Tesaro, Lilly, Clovis, Immunogen and MSD/Merck. Marie Ange Mouret-Reynier declares no conflicts of interest. S Pignata declares honoraria from Roche, AstraZeneca, MSD, Pfizer, Incyte, Novartis, PharmaMar, Clovis and Tesaro; and travel expenses from Roche, AstraZeneca and MSD. Claire Cropet declares no conflicts of interest. A Gonzalez-Martin declares consulting/advisory board fees from Roche, AstraZeneca, Tesaro, Clovis, Pfizer, ImmunoGen, PharmaMar, MSD, Genmad and Novartis; and speaker bureau/expert testimony fees from Roche, AstraZeneca, MSD Tesaro and PharmaMar. Gerhard Bogner declares consulting/advisory board fees from AstraZeneca Tesaro and Roche; and travel expenses from GSK, Roche, Pharmamar, AstraZeneca and Tesaro. K Fujiwara declares honoraria from AstraZeneca, Chugai Roche, Zeria, Taiho, Nihon Kayaku, Kyowahakko Kirin, Janssen and Daiichi Sankyo; consulting/advisory board fees from Pfizer, MSD and Eisai; research funding from AstraZeneca, MSD, Chugai Roche, Eisai and Kaken; and travel expenses from Pfizer. I Vergote declares consulting/advisory board fees from Advaxis, BIOCAD, Eisai, MSD, Roche, Genmab, Roche, PharmaMar, Millenium Pharmaceuticals, Clovis, AstraZeneca, Tesaro, Oncoinvent, ImmunoGen and Sotio; contracted research with Oncoinvent and Genmab; research funding from Amgen, Roche and Stichting tegen Kanker; and Takeda Oncology, PharmaMar, Genmab, Roche, AstraZeneca, Tesaro, Clovis and Immunogen. N Colombo declares honoraria from Roche, AstraZeneca, PharmaMar, Tesaro, Clovis, Pfizer, MSD, BIOCAD and Takeda; consulting/advisory board fees from Roche,

AstraZeneca, PharmaMar, Tesaro, Clovis, Pfizer, MSD, BIOCAD, Takeda; and travel expenses from PharmaMar, Tesaro and Roche. J Mäenpää declares honoraria from Roche, AstraZeneca and Tesaro; consultancy/advisory board fees from AstraZeneca, Tesaro, Clovis and MSD; and travel expenses from Roche. Anne Floquet declares consultancy/advisory board fees from GSK, AstraZeneca and Clovis; and travel expenses from Roche, GSK and AstraZeneca. Ahmed El-Balat declares honoraria from AstraZeneca, Roche, Pharmamar, MSD, Tesaro, Clovis; and consultancy/advisory board fees from AstraZeneca and Roche. D Lorusso declares honoraria from Merck; consultancy/advisory board fees from Merck, AstraZeneca, Tesaro, Clovis, Immunogen, PharmaMar and Roche; research funding from Clovis, PharmaMar, Merck and Roche; and travel expenses from Tesaro, Roche and PharmaMar. EM Guerra Alia declares consultancy/advisory board fees from Roche, Clovis and AstraZeneca; and travel expenses from Roche and Baxter. Michel Fabro declares honoraria from Tesaro and AstraZeneca; and consultancy/advisory board fees from Tesaro and AstraZeneca. Barbara Schmalfeldt declares honoraria from Roche, AstraZeneca, Tesaro, Clovis, Ethicon, MSD; consultancy/advisory fees from Roche, AstraZeneca, Tesaro, Clovis, Ethicon, MDS; speaker bureau/expert testimony fees from Roche, AstraZeneca, Tesaro, Clovis, Ethicon, MDS; research grant funding from Roche, AstraZeneca, Tesaro, Clovis, Ethicon, MDS and travel expenses from Roche, AstraZeneca, Tesaro, Clovis, Ethicon, MDS. Anne Claire Hardy Blessard declares honoraria from AstraZeneca, Lilly, Novartis, Pfizer, Roche and Tesaro. Ingo Runnebaum declares honoraria from AstraZeneca. I Ray-Coquard declares honoraria from AstraZeneca, Clovis, Tesaro and PharmaMar; consulting/advisory board fees from AstraZeneca, Roche, Clovis, Tesaro, Genmab, PharmaMar, MSD and Pfizer; research funding from MSD; and travel expenses from AstraZeneca and Roche; consulting/advisory board fees from Roche; and travel expenses from Roche, MSD, Tesaro and AstraZeneca. E Pujade-Lauraine declares honoraria from AstraZeneca and Tesaro; consulting/advisory board fees from AstraZeneca, Roche, Clovis, Tesaro, Genmab, Incyte, MSD and Pfizer; research funding from AstraZeneca, Roche and Tesaro; and travel expenses from AstraZeneca, Roche and Tesaro.

TP53 mutations in cell-free DNA as early markers of therapeutic response in platinum-resistant relapsed ovarian cancer (PROC): a prospective translational analysis of the phase II GANNET53 clinical trial

A. Vanderstichele[1], N. Concin[2], P. Busschaert[1], I. Braicu[3], P. Combe[4], I. Ray-Coquard[5], F. Joly[6], P. Harter[7], P. Wimberger[8], F. Selle[4], A. Ignatov[9], B. Schmalfeldt[10], E. Van Nieuwenhuysen[1], S. Darb-Esfahani[3], A. Zeimet[2], S. Mahner[10,11], E. Pujade-Lauraine[4], C. Marth[2], R. Berger[2], J. Sehouli[3], U. Moll[12], R. Zeillinger[13], D. Lambrechts[14,15], I. Vergote[1]

[1]Division of Gynaecological Oncology, University Hospitals Leuven, and Department of Oncology, Leuven Cancer Institute, KU Leuven, Leuven, European Union, Belgium, [2]Medical University Innsbruck, Innsbruck, Austria, [3]Charite Medical University of Berlin, Berlin, Germany, [4]Assistance Publique des Hôpitaux de Paris (AP-HP), Paris, [5]Centre Anticancereux Léon Bérard, Lyon, [6]Centre de Lutte Contre le Cancer, Francois Baclesse, Caen, France, [7]Klinik Essen-Mitte, Essen, [8]Technische Universität Dresden University Hospital, Dresden, [9]Otto-von-Guericke-Universität Magdeburg, Magdeburg, [10]Universitätsklinikum Hamburg-Eppendorf, Hamburg, [11]Ludwig Maximilian University of Munich, Munich, Germany, [12]Department of Pathology, Renaissance School of Medicine, Stony Brook University, Stony Brook, NY, United States, [13]Molecular Oncology Group, Medical University of Vienna, Vienna, Austria, [14]Laboratory for Translational Genetics, Department of Oncology, [15]Center for Cancer Biology, VIB, KU Leuven, Leuven, Belgium

Introduction / Background: Detecting tumor-specific genetic alterations in cell-free DNA (cfDNA) obtained from cancer patients allows for a quantification of the tumoral fraction, i.e. the circulating tumor DNA (ctDNA). Previous studies in metastatic cancer patients showed that early changes of this fraction during therapy are indicative of therapeutic response. We tested this hypothesis on cfDNA samples collected in the GANNET53 clinical trial (FP7, funded by the European Commission, grant no 602602).

Methodology: Patients with high-grade serous/endometrioid and/or undifferentiated PROC were recruited for treatment with weekly paclitaxel with or without Hsp90-inhibitor ganetespib. Archival

biopsy samples were used for tumor TP53 genotyping. CfDNA was prospectively collected prior to treatment at baseline (cycle 1 day 1, C1D1), 24 hours later (cycle 1 day 2, C1D2) and after 4 and 8 weeks, at day 1 of cycle 2 (C2D1) and 3 (C3D1) respectively. Targeted TP53 resequencing was performed on cfDNA and allelic frequencies of the known TP53 variant (TP53 VAF) were quantified and correlated with clinical outcome.
Results: For 125 of the 133 randomized patients, at least 1 C1D1 cfDNA sample was available. For 119 tumor samples, TP53 genotyping was successful and identified deleterious TP53 mutations in 106 patients (89.1%). The median ctDNA level was 1.82% (IQR:0.17-8.34%) at C1D1 which decreased significantly after 4-8 weeks of therapy. Overall, ctDNA was detectable in 64.6% (64/99) of baseline samples. Baseline CA125 did not differ between cases with and without detectable ctDNA at C1D1. Detection of ctDNA at C1D1 (HR 2.3; 95%CI:1.4-3.9), C1D2 (HR 2.2; 95%CI:1.3-3.9) and C2D1 (HR 2.8; 95%CI:1.6-4.9) predicted a worse overall survival. A subgroup of patients for whom TP53 ctDNA was undetectable at C2D1 or C3D1 (14/64) had a high overall response rate of 64.2%.
Conclusion: Quantification of TP53 mutations in cfDNA of PROC patients has prognostic value at baseline. Favorable early changes during treatment may predict therapeutic response.
Disclosure: The presenting author, A.Vanderstichele, has no conflict of interest.

Ovarian cancer detection combining an innovative catheter for uterine and tubal lavage with ultra-sensitive TP53 sequencing

P. Speiser[1], E. Maritschnegg[1], F. Heitz[2,3], N. Pecha[4], J. Bouda[5], F. Trillsch[6], C. Grimm[7], A. Vanderstichele[8], C. Agreiter[9], P. Harter[2], E. Obermayr[9], I. Vergote[8], R. Zeillinger[10], J.J. Salk[11], R.A. Risques[12], AGO Austria
[1]Medical University of Vienna, Vienna, Austria, [2]Department of Gynaecology and Gynaecologic Oncology, Kliniken Essen Mitte, Essen, [3]Department for Gynecology and Gynecologic Oncology, Charité Campus Virchow-Klinikum, Berlin, Germany, [4]Department of Urology, Municipal Hospital Rudolfstiftung, Vienna, Austria, [5]Department of Gynecology and Obstetrics, Faculty of Medicine and University Hospital in Pilsen, Charles University in Prague, Pilsen, Czech Republic, [6]DepartmentofObstetricsandGynaecology, University Hospital, LMU Munich, Munich, Germany, [7]Department of Gynaecology and Gynaecologic Oncology, Medical University Vienna, Vienna, Austria, [8]Department of Gynecologic Oncology, Leuven Cancer Institute, University Hospitals Leuven ,KU, Leuven, Belgium, [9]Department of Gynaecology and Gynaecologic Oncology, [10]Molecular Oncology Group, Medical University of Vienna, Vienna, Austria, [11]TwinStrand Biosciences, [12]Department of Pathology, University of Washington, Seattle, WA, United States

Introduction / Background: Poor survival of high-grade serous pelvic cancer (HGSC) is caused by a lack of effective screening measures. Uterine and tubal lavage (UtL) is the sampling technique for HGSC detection with the highest sensitivity ever reported. We combined UtL with the highest accuracy sequencing technology for *TP53* mutations, Duplex Sequencing (DS) in order to develop a test potentially feasible for HGSC screening.
Methodology: In study I, the clinical feasibility of UtL using a new catheter was examined in 93 patients undergoing surgery for suspected HGSC. In study II, pain at UtL under local anesthesia was evaluated in 22 healthy women. In Study III, uterine lavages from 10 HGSC patients and 11 controls were subjected to DS to identify *TP53* mutations.
Results: In study I, UtL was performed successfully in 92 (98.9%) of 93 cases by 16 gynecologists. In 84.8% of patients no cervical dilatation was required. For benign conditions, the need for dilatation was associated with menopause status (OR, 4.929; 95% CI, 1.439-16.884; P = 0.016).
In study II, pain during UtL was rated with a median VAS score of 1.6. During 4 weeks after UtL, no serious complications occurred. UtL took 6.5 minutes on average. The amount of extracted DNA was above the lower limit for sensitive, deep-sequencing mutation analysis in all cases.
In study III, in 80% of all HGSC tumor-derived *TP53* mutations in UtL samples were detected by DS. However, we detected low frequency *TP53* mutations in nearly all samples tested. Mutations increased with age and shared the selection traits of clonal *TP53* mutations commonly found in human tumors. However, tumor-derived *TP53* mutations were present at frequencies at least 10-fold higher than background mutations.

Conclusion: The UtL concept fulfils all prerequisites to be used in a potential screening setting. Interpretation of DS results needs to consider *TP53* biologic background mutations.
Disclosure: Philipp Harter COI: Honoraria: Astra Zeneca, Roche, Sotio, Tesaro, Stryker, ASCO, Zai Lab, MSD; Advisory Board: Astra Zeneca, Roche, Tesaro, Lilly, Clovis, Immunogen, MSD/Merck; Research funding (Inst): Astra Zeneca, Roche, GSK, Boehringer Ingelheim, Medac, DFG, European Union, DKH, Tesaro, Genmab Rosana Risques COI: RAR shares equity in NanoString Technologies Inc. and is the principal investigator on an NIH SBIR subcontract research agreement with TwinStrand Biosciences Inc. Florian Heitz COI: Honoraria: AstraZeneca, Roche, Tesaro, Clovis; Advisory Board: Astra Zeneca, Roche, Tesaro, Clovis Christoph Grimm COI: Consultant: AstraZeneca, Celgene, MSD, PharmaMar, Roche, Tesaro, Vifor Pharma Speaker: Amgen, AstraZeneca, MSD, PharmaMar, Roche, Tesaro Direct research funding: Meda Pharma, Roche Diagnostics Ignace Vergote COI: CONSULTING = ADVISORY BOARD: Advaxis, Inc., Eisai Inc., MSD Belgium, Roche NV, Genmab, F. Hoffmann-La Roche Ltd, Pharmamar, Millennium Pharmaceuticals, Clovis Oncology Inc., AstraZeneca NV, Tesaro, Oncoinvent AS, Immunogen Inc, Sotio CONTRACTED RESEARCH (via KULeuven): Oncoinvent AS, Genmab GRANT = CORPORATE SPONSORED RESEARCH: Amgen, Roche, Stichting tegen Kanker Accomodations, travel Fabian Trillsch COI:Consultant: AstraZeneca, Roche, Tesaro Speaker / Travel Expenses: AstraZeneca, Medac, PharmaMar, Roche, Tesaro Paul Speisr: Share Holder OVARTEC GmbH

Best Oral / Late-Breaking Abstracts 4 - Miscellaneous

Results from neoadjuvant chemotherapy followed by surgery compared to chemoradiation for Stage IB2-IIB cervical cancer: EORTC55994

S. Greggi[1], G. Kenter[2], I. Vergote[3], D. Katsaros[4], J. Kobierski[5], L.F.A.G. Massuger[6], P.A. van Doorn[7], F. Landoni[8], J. van de Velden[9], N. Reed[10], C. Coens[11], I. van Luijk[12], P. Ottevanger[6], N. Colombo[13], A. Casado Herraez[14]
[1]Gynecologic Oncology Surgery, Istituto Nazionale Tumori IRCCS 'Fondazione G. Pascale', Naples, Italy, [2]Center Gynaecologic Oncology Amsterdam, Amsterdam, The Netherlands, [3]University Hospitals, Leuven, Belgium, [4]AOU Citta della Salute, PO S. Anna and University of Torino, Torino, Italy, [5]Medical University of Gdansk, Gdansk, Poland, [6]Radboud University Medical Center, Nijmegen, [7]Erasmus University Medical Center Rotterdam, Rotterdam, The Netherlands, [8]Milano Bicocca University, Ospedale San Gerardo, Monza, Italy, [9]Amsterdam University Medical Center, Amsterdam, The Netherlands, [10]Gartnavel General Hospital, Glasgow, United Kingdom, [11]European Organization on Research and Treatment of Cancer EORTC, Brussels, Belgium, [12]MC Haaglanden, The Hague, The Netherlands, [13]University of Milan Bicocca and European Institute of Oncology, Milan, Italy, [14]University Hospital San Carlos, Madrid, Spain

Introduction / Background: Within EORTC-GCCG we conducted a randomized multinational multicenter trial in order to compare the value of neoadjuvant chemotherapy followed by radical surgery with standard concomitant chemoradiation in Stage IB2-IIB cervical carcinoma. As the trial (55994) is approaching completion of its follow-up, preliminary results are presented here.
Methodology: Between May 2002 and June 2014 a total of 620 patients with FIGO stage Ib2-IIb were randomized between neoadjuvant chemotherapy followed by surgery (NACTS, arm1, N=311) with standard concomitant chemoradiotherapy (CCRT, arm2, N=309) . In arm1, radical hysterectomy was required within 6 weeks after completion of cisplatin-based chemotherapy with a cumulative minimum of 225mg/m2, in arm2, radiation consisted of 45-50Gy plus boost concurrent with weekly cisplatin chemotherapy (40mg/m2 per week). Primary endpoint was 5-yr overall survival (OS).
Results: Median follow-up time was 8.2 years (95%CI =7.8yrs-8.6yrs) and similar between both arms. A total of 191 deaths (31%) occurred. Age, stage and histological cell type were balanced in both arms. Protocol treatment was completed in 459 (74%) patients (71% for NACTS; 82% for CCRT). In arm1 238 (76%) patients underwent surgery. Main reasons for not having surgery as per protocol, were toxicity (25/74, 34%), progressive disease (18/74, 24%) and insufficient response to NACT (12/74, 16%). Additional radiotherapy was given to 113 patients (36.3%) in arm1; additional surgery performed in 9 patients (2.9%) in arm2. Short term severe adverse events (≥G3) occurred more frequently in arm1 than in arm2 (35% vs 21%, p < 0.001). The 5 year OS was 72% in arm1 and 76% in arm2 (not statistically significant, difference =4.0% (95%CI: -4%-12%); HR 0.87, 95%CI: 0.65-0.15,

p=0.332).
Conclusion: These preliminary results revealed no difference in 5-year OS between NACTS and CCRT, indicating that quality of life and long term toxicity across prognostic factors are important to decide on optimal treatment.
Disclosure: Nothing to disclose

Dose-dense neoadjuvant chemotherapy followed by sentinel node mapping and laparoscopic pelvic lymphadenectomy and simple trachelectomy in cervical cancer: update results

H. Robova[1], L. Rob[1], M.J. Halaska[1], M. Hruda[1], T. Pichlik[1], J. Drozenova[2], P. Skapa[3]
[1]Obstetrics and Gynecology, [2]Pathology, Charles University, 3rd Medical Facculty, [3]Pathology and Molecular Biology, Charles University, 2nd Medical Facculty, Prague, Czech Republic

Introduction / Background: Fertility-sparing surgery is safe only if tumor doesn't exceeded 2 cm in the biggest diameter. When the tumor bigger, surgery must be more radical (abdominal trachelectomy type C2), but pregnancy results aren't promising. Neoadjuvant chemotherapy (NAC) followed by simple trachelectomy could be option.
Methodology: Women with squamous cell, adeno and adenosquamous cancers IB2 and IB3 infiltrated less than two third of cervical stroma, were included to prospective study. They received 3 cycles of NAC in ten-days interval (cisplatin 75mg/m^2, ifosfamide 2g/m^2 (max. 3g) in squamous cancers, cisplatin 75mg/m^2, doxorubicin 35mg/m^2 in adeno and adenosquamous cancers). Women underwent sentinel lymph node mapping and laparoscopic pelvic lymphadenectomy. When lymph nodes were negative, simple trachelectomy were performed after one week.
Results: Forty women were included in to study (28 IB3 and 12 IB3). Fertility was saved in 29 women (72.5%), five (17.2%) of them recurred (4local and 1 distant) and tree (10.3%) patient died (10.3%). Three women lost fertility after treatment of recurrence; definitively fertility was saved in 26 women. Twenty women want be pregnant until now and 18 (90.0%) became pregnant. Fifteen women delivered 18 babies (1 in 24 weeks, 4 in 28-34 weeks, 3 in 34-36 weeks, 10 in terms). One woman miscarried in first trimester, one in second trimester and one is now pregnant.
Conclusion: Oncological results in NAC followed by simple trachelectomy in cervical cancers bigger than 2cm are acceptable (mortality rate 10.3%) and pregnancy results are excellent (pregnancy rate 90.0%), but still it is experimental protocol for full instructed women.
Work was supported Charles University research program PROGRES Q 28 (Oncology).
Disclosure: Nothing to disclose

Randomised trial of unselected BRCA testing in Ashkenazi Jews: Long term outcomes and factors affecting uptake of testing

R. Manchanda[1,2,3], M. Burnell[3], F. Gaba[1,2], R. Desai[3], J. Wardle[3], S. Gessler[3], L. Side[4], S. Sanderson[3], K. Loggenberg[4], A. Brady[5], H. Dorkins[6], Y. Wallis[7], C. Chapman[7], C. Jacobs[8,9], R. Legood[10], U. Beller[11], I. Tomlinson[12], U. Menon[3], I. Jacobs[13]
[1]Barts Cancer Institute, [2]Barts Health NHS Trust, [3]University College London, [4]North East Thames Regional Genetics Unit, Great Ormond Street Hospital, [5]North West Thames Regional Genetics Service, Northwick Park Hospital, London, [6]University of Oxford, Oxford, [7]Birmingham Women's and Children's NHS Foundation Trust, Birmingham, [8]Dept Clinical Genetics, Guy's Hospital, London, United Kingdom, [9]University of Technology Sydney, Sydney, NSW, Australia, [10]London School of Hygiene and Tropical Medicine, London, United Kingdom, [11]Shaare Zedek Medical Center, Jerusalem, Israel, [12]University of Birmingham, Birmingham, United Kingdom, [13]University of New South Wales,

Sydney, NSW, Australia

Introduction / Background: Unselected population-based *BRCA*-testing enables application of genomics on a population-scale to maximise primary-prevention for breast-&-ovarian cancer. We present factors affecting uptake and long-term follow-up results of the GCaPPS-trial comparing population-based and Family-History (FH)/Clinical-criteria based *BRCA*-testing.
Methodology: Design: Randomised-Controlled-Trial (ISRCTN73338115) GCaPPS, with two-arms: (a)Population-Screening (PS); (b)FH/Clinical-criteria based testing.
Setting: North-London Ashkenazi-Jewish(AJ) population
Population-based RCT (1:1). Participants recruited through self-referral, from North-London AJ-population.
Inclusion-criteria: AJ women/men >18years. Exclusion-criteria: prior *BRCA*-testing or first-degree-relatives of *BRCA*-carriers.
Interventions: Pre-test counselling for BRCA-testing through recruitment-clinics (clusters). Genetic-testing for Jewish *BRCA* founder-mutations: 185delAG(c.68_69delAG), 5382insC(c.5266dupC) and 6174delT(c.5946delT), for (a)all participants in PS-arm; (b)those fulfilling FH/clinical-criteria in FH-arm. Validated questionnaires (HADS/MICRA/HAI/SF12) analysed psychological well-being/quality-of-life outcomes at baseline, 1-year, 2-years and 3-years follow-up. Linear mixed-models and appropriate contrast-tests analysed the impact of *BRCA*-testing on psychological and quality-of-life outcomes over 3-years. Socio-demographic/family-history/knowledge/psychological well-being data along-with benefits/risks/cultural-influences (18-item-questionnaire measuring 'attitude') were collected. Logistic-regression models evaluated factors affecting uptake/interest/intention-to undergo *BRCA*-testing.
Results: 1034 (women=691/men=343) participants randomized to PS (n=530) or FH (n=504) arms. There was a statistically significant decrease in anxiety(p=0.046) and total anxiety-&-depression scores(p=0.0.012) in the PS-arm compared to FH-arm over 3years. No significant difference was observed between FH/PS arms for depression, health-anxiety, distress, uncertainty, quality-of-life or experience scores associated with BRCA-testing.
18/30(60%) *BRCA*-carriers identified did not fulfil *BRCA*-testing clinical-criteria. The total BRCA-prevalence= 2.9%(CI:1.97%,4.12%) (BRCA1=1.55%(CI:0.89%,2.5%); BRCA2=1.35%(CI:0.74%,2.26%)). The BRCA-testing uptake was 88%. Being married/cohabiting had four-fold higher-odds for *BRCA*-testing uptake(p=0.009). Reduced uncertainty/reassurance were the most important factors affecting decision-making. Increased importance/concern towards risks/limitations (confidentiality/insurance/emotional-impact/inability to prevent cancer/marriage-ability/ethnic-focus/stigmatization) were significantly associated with lower-odds of undergoing *BRCA*-testing, discriminating between acceptors and decliners.
Conclusion: Population-based AJ *BRCA*-testing has high acceptability, doesn't adversely affect long-term psychological well-being or quality-of-life, decreases anxiety and could identify up-to 150% additional *BRCA*-carriers. Pre-test counselling increases awareness of disadvantages/limitations of *BRCA*-testing, influencing final cost-benefit perception and decision-making on undergoing testing.
Disclosure: IJ and UM have a financial interest in Abcodia, Ltd., a company formed to develop academic and commercial development of biomarkers for screening and risk prediction. IJ is a member of the board of Abcodia Ltd, a Director of Women's Health Specialists Ltd and received consultancy from Beckton Dickinson. RM declares research funding from The Eve Appeal and Cancer Research UK into population testing and from Barts & the London Charity and Rose Trees Trust outside this work, an honorarium for grant review from Israel National Institute for Health Policy Research and honorarium for advisory board membership from Astrazeneca/MSD. RM is supported by an NHS Innovation Accelerator (NIA) Fellowship for population testing. The other authors declare no conflict of interest.

Radiotherapy instead of inguinofemoral lymphadenectomy in vulvar cancer patients with a metastatic sentinel node: results of GROINSS-V II

M.H.M. Oonk[1], B. Slomovitz[2], P. Baldwin[3], H. van Doorn[4], J. van der Velden[5], J. de Hullu[6], B. Slangen[7], K. Gaarenstroom[8], I. Vergote[9], M. Brannstrom[10], E. van Dorst[11], W. van Driel[12], R. Hermans[13], D. Nunns[14], M. Widschwendter[15], D. Nugent[16], C. Holland[17], P. DiSilvestro[18], A. Sharma[19],

R. Mannel[20], D. Boll[13], A. Covens[21], D. Cibula[22], D. Provencher[23], D. Luesley[24], P. Ellis[25], T. Duncan[5], M. Tjiong[26], D. Cruickshank[27], P. Kjolhede[28], C. Levenback[29], J. Bouda[30], K. Kieser[31], I. Runnebaum[32], C. Palle[33], N. Spirtos[34], D. O'Malley[35], M. Leitao[36], M. Geller[37], K. Tamussino[38], K. Dhar[39], D. Tobias[40], C. Borgfeldt[41], T. Myers[42], J. Lea[43], J. Bailey[44], P. Persson[45], B. Monk[46], C. Creutzberg[8], A. van der Zee[47], GROINSS-V II Study Group
[1]University Medical Center Groningen, Groningen, The Netherlands, [2]University of Miami, Miami, FL, United States, [3]Cambridge University Hospitals NHS Foundation Trust, Addenbrooke's Hospital, Cambridge, United Kingdom, [4]Erasmus MC Cancer Institute, University Medical Center Rotterdam, Rotterdam, [5]Amsterdam University Medical Centers, University of Amsterdam, Amsterdam, [6]Radboud University Medical Center, Nijmegen, [7]Maastricht University Medical Center, Maastricht, [8]Leiden University Medical Center, Leiden, The Netherlands, [9]University Hospital Leuven, Leuven, Belgium, [10]Sahlgrenska Academy at University of Gothenburg, Gothenburg, Sweden, [11]UMC Utrecht Cancer Center, Utrecht, [12]CGOA, Location The Netherlands Cancer Institute, Amsterdam, [13]Catharina Hospital, Eindhoven, The Netherlands, [14]Nottingham University Hospitals NHS Trust, Nottingham, [15]University Collega London, Institute for Women's Health, London, [16]Leeds Teaching Hospitals NHS Trust, Leeds, [17]Saint Mary's Hospital, Manchester University NHS Trust, Manchester, United Kingdom, [18]Rhode Island Hospital, Providence, RI, United States, [19]University Hospital Llandough, Llandough, United Kingdom, [20]Stephenson Cancer Center, University of Oklahoma, Oklahoma, OK, United States, [21]University of Toronto, Odette Cancer Center, Sunnybrook Health Sciences Centre, Toronto, ON, Canada, [22]First Faculty of Medicine and General University Hospital, Charles University, Prague, Czech Republic, [23]University of Montreal, Cancer Institute Montreal CHUM, Montreal, QC, Canada, [24]Birmingham Women's and Children's NHS Foundation Trust, Birmingham, [25]Royal Surrey County Hospital NHS Trust, Guildford, United Kingdom, [26]Amsterdam UMC, Location VUmc, Amsterdam, The Netherlands, [27]James Cook University Hospital, Middlesbrough, United Kingdom, [28]Linkoping University, Linköping, Sweden, [29]MD Anderson Cancer Center, Houston, TX, United States, [30]Medical Faculty Hospital Pilsen, Charles University, Pilsen, Czech Republic, [31]Dalhousie University, Halifax, NS, Canada, [32]Jena University Hospital, Friedrich Schiller University, Jena, Germany, [33]Herlev and Gentofte Hospital, Copenhagen University Hospital, Copenhagen, Denmark, [34]Women's Cancer Center of Nevada, Las Vegas, NV, [35]Ohio State University Comprehensive Cancer Center, Columbus, OH, [36]Memorial Sloan Kettering Cancer Center, New York City, NY, [37]University of Minnesota, Minneapolis, MN, United States, [38]Medical University Graz, Graz, Austria, [39]Singleton Hospital, Swansea, United Kingdom, [40]Morristown Medical Center, Morristown, NJ, United States, [41]Skane University Hospital, Lund University, Lund, Sweden, [42]Baystate Medical Center, Springfield, MA, [43]University of Texas Southwestern Medical Center, Dallas, TX, United States, [44]St Michaels Hospital, Bristol, United Kingdom, [45]Uppsala University, Uppsala, Sweden, [46]St. Joseph's Hospital and Medical Center, Phoenix, AZ, United States, [47]University Medical Center Groningen, University of Groningen, Groningen, The Netherlands

Introduction / Background: Introduction: GROINSS-V II investigated whether radiotherapy is a safe alternative for inguinofemoral lymphadenectomy (IFL) in vulvar cancer patients with a metastatic sentinel node (SN).
Methodology: Methods: In GROINSS-V II, a prospective multicentre phase II trial, patients were included with early-stage squamous cell carcinoma (SCC) of the vulva (diameter < 4cm) without suspicious lymph nodes at imaging, who had primary surgery with SN-procedure. In case of a metastatic SN (metastasis of any size), radiotherapy was given to the groin(s) (50Gy). In case of a negative SN, patients were followed-up for ≥2 years. Stopping rules were defined for both groups to monitor groin recurrence rate.
Results: Results: From December 2005 until October 2016, 1552 eligible patients were registered. SN-metastasis occurred in 324/1552 (21%) patients. After 54 months of inclusion the stopping-rule for SN-positive patients was activated; interim analysis showed an increased risk for groin recurrence in case of SN-metastasis >2mm and/or extranodal extension (ENE). After amendment of the protocol only patients with SN-micrometastasis ≤2mm received radiotherapy, while those with SN metastasis >2mm underwent IFL (with radiotherapy if >1 metastasis or ENE). Final analysis after ≥2 years of follow-up revealed six isolated groin recurrences in 157 patients with a SN-micrometastasis (3.8%). Four could not be considered radiotherapy failures: two developed recurrence in the contralateral (SN-negative) groin, two refused radiotherapy. Twenty-eight patients did not get radiotherapy (2 recurrences;7.1%). Among 129 patients who received radiotherapy to the groin(s) only two isolated groin recurrences were diagnosed (1.6%: 95%CI:0-3.8%). The combination of radiotherapy with SN was associated with minimal toxicity: 5/118(4.2%) grade 3 toxicity, no grade 4 or 5 toxicity. In 38/1222 SN-negative patients (3.1%: 95%CI:2.1-4.1%) isolated groin recurrences were diagnosed with clear protocol violations in 6 patients: incomplete treatment of the groin (n=3); primary tumor >4cm (n=1); not all SNs visualized on the lymphoscintigram removed (n=2). After exclusion of these protocol

violations an isolated groin recurrence rate of 2.6% (95%CI:1.7-3.5%) was observed.
Conclusion: Conclusion: Radiotherapy to the groins is a safe alternative for IFL in patients with SN metastasis ≤2mm, with minimal toxicity. We further established the safety of omitting IFL in patients with SCC of the vulva < 4cm and a negative SN. For patients with SN metastasis >2mm, radiotherapy with a total dose of 50Gy was no safe alternative for IFL; dose escalation and/or chemoradiation should be investigated in these patients.
Disclosure: Funded by Dutch Cancer Society

Oral Communication 1 - Gynaecological Cancer

Should we offer multi-gene testing to all patients with breast cancer: a cost-effectiveness analysis

L. Sun[1,2], A. Brentnall[3], S. Patel[1,2], D. Buist[4], E. Bowles[4], D.G. Evans[5], D. Eccles[6], J. Hopper[7], S. Li[7], S. Duffy[3], J. Cuzick[3], I. dos-Santos-Silva[8], Z. Sadique[1], L. Yang[9], R. Legood[1], R. Manchanda[2,10,11]
[1]Department of Health Services Research and Policy, London School of Hygiene and Tropical Medicine, [2]Barts Cancer Institute, Queen Mary University of London, [3]Wolfson Institute of Preventive Medicine, Queen Mary University of London, London, United Kingdom, [4]Kaiser Permanente Washington Health Research Institute, Seattle, WA, United States, [5]Manchester Academic Health Science Centre (MAHSC), Manchester Universities Foundation Trust, St. Mary's Hospital, The University of Manchester, Manchester, [6]Cancer Sciences Academic Unit, Faculty of Medicine and Cancer Sciences, University of Southampton, Southampton, United Kingdom, [7]Centre for Epidemiology & Biostatistics, Melbourne School of Population & Global Health, Faculty of Medicine, Dentistry & Health Sciences, University of Melbourne, Melbourne, VIC, Australia, [8]Department of Non-communicable Disease Epidemiology, London School of Hygiene and Tropical Medicine, London, United Kingdom, [9]School of Public Health, Peking University, Beijing, China, [10]Barts Health NHS Trust, Royal London Hospital, [11]MRC Clinical Trials Unit at UCL, Institute of Clinical Trials & Methodology, Faculty of Population Health Sciences, University College London, London, United Kingdom

Introduction / Background: Currently breast cancer (BC) patients are offered genetic-testing only if they have a ≥10% risk of being a BRCA carrier based on family history and clinical criteria. However, this approach misses a large proportion (~50%) of overall mutation carriers as they fall below this 10% threshold. Mutation identification enables primary prevention for ovarian cancer (OC) in BC-patients and BC-&-OC in unaffected relatives. We estimate incremental lifetime-effects, costs, cost-effectiveness and population impact of multigene-testing all BC patients compared to current practice of family-history/clinical-criteria based genetic (*BRCA*)-testing.
Methodology: Cost-effectiveness microsimulation modelling study comparing lifetime costs-&-effects of *BRCA1/BRCA2/PALB2* (multigene) testing all unselected BC-cases (Strategy-A) with family-history/clinical-criteria based *BRCA1/BRCA2*-testing (Strategy-B) in both UK and US populations. Data obtained from 11,836 population-based BC-patients (regardless of family-history) recruited to four large research studies in the UK (Predicting-Risk-of-Breast-Cancer-at-Screening (PROCAS: 1389 out of 57,000 women) & Prospective-Outcomes-in-Sporadic-versus-Hereditary-breast-cancer (POSH: 2885) studies); US (Kaiser-Permanente Washington Breast-Cancer-Surveillance-Consortium (BCSC) registry: 5892 out of 132,139 women) and Australia (Population-based BC-cases of the Australian-Breast-Cancer-Family-Study (ABCFS: 1670 women)). The main outcome measure was the incremental cost per quality-adjusted life-year (QALY) gained with a 3.5% annual discount. Parameter uncertainty was explored using one-way and probabilistic sensitivity analyses.
Results: Compared with current clinical-criteria/family-history-based *BRCA*-testing, (*BRCA1/BRCA2/PALB2*) multigene-testing for all BC-patients would cost £10,470/QALY (UK) or $58,702/QALY (US) gained, well below UK/NICE and US cost-effectiveness thresholds of £30,000/QALY & $100,000/QALY. Probabilistic sensitivity-analysis shows unselected multigene-testing remains cost-effective for 98% UK/ 77% US health-system simulations. One year's unselected panel-genetic testing can prevent 1,776 BC/OC-cases and 557 deaths in the UK; and 8,258 BC/OC-cases and 2,143 deaths in the US. Correspondingly, 7 UK/32 US excess heart-disease deaths occur annually.
Conclusion: Unselected panel genetic-testing for all BC patients compared to current clinical-criteria

restricted testing is extremely cost-effective. We recommend changing the current policy to expand genetic testing to all BC patients.

Disclosure: RM declares research funding from The Eve Appeal and Cancer Research UK into population testing and from Barts & the London Charity and Rosetree Charity outside this work, as well as an honorarium for grant review from Israel National Institute for Health Policy Research and honorarium for advisory board meeting for MSD and Astrazeneca. DGE declares travel grants paid by Astrazeneca. The other authors declare no conflict of interest.

Maintenance olaparib after platinum-based chemotherapy in patients with newly diagnosed advanced ovarian cancer and a BRCA mutation: efficacy by the timing of surgery and residual tumour status following upfront or interval cytoreductive surgery in the Phase III SOLO1 trial

K. Moore[1], N. Colombo[2], G. Scambia[3], B.-G. Kim[4], A. Oaknin[5], M. Friedlander[6], A. Lisyanskaya[7], A. Floquet[8,9], A. Leary[9,10], G.S. Sonke[11], C. Gourley[12], S. Banerjee[13], A. Oza[14], A. González-Martín[15], C. Aghajanian[16], W. Bradley[17], C. Mathews[18], J. Liu[19], E.S. Lowe[20], R. Bloomfield[21], P. DiSilvestro[18]
[1]Stephenson Cancer Center at the University of Oklahoma, Oklahoma City, OK, United States, [2]University of Milan-Bicocca and IEO European Institute of Oncology IRCCS, Milan, [3]Fondazione Policlinico Universitario A Gemelli IRCCS, Università Cattolica, Rome, Italy, [4]Samsung Medical Center, Sungkyunkwan University School of Medicine, Seoul, Republic of Korea, [5]Vall d'Hebron University Hospital, Vall d'Hebron Institute of Oncology (VHIO), Barcelona, Spain, [6]University of New South Wales Clinical School, Prince of Wales Hospital, Randwick, NSW, Australia, [7]St Petersburg City Oncology Dispensary, St Petersburg, Russian Federation, [8]Institut Bergonié, Comprehensive Cancer Centre, Bordeaux, [9]Groupe d'Investigateurs Nationaux pour l'Etude des Cancers Ovariens, Paris, [10]Gustave-Roussy Cancer Campus, Villejuif, France, [11]The Netherlands Cancer Institute, Amsterdam, The Netherlands, [12]Cancer Research UK Edinburgh Centre, Institute of Genetics and Molecular Medicine, University of Edinburgh, Edinburgh, [13]The Royal Marsden NHS Foundation Trust and Institute of Cancer Research, London, United Kingdom, [14]Princess Margaret Cancer Centre, Toronto, ON, Canada, [15]Clínica Universidad de Navarra, Madrid, Spain, [16]Memorial Sloan Kettering Cancer Center, New York, NY, [17]Froedtert and the Medical College of Wisconsin, Milwaukee, WI, [18]Women & Infants Hospital, Providence, RI, [19]Dana-Farber Cancer Institute, Boston, MA, [20]AstraZeneca, Gaithersburg, MD, United States, [21]AstraZeneca, Cambridge, United Kingdom

Introduction / Background: In SOLO1 (NCT01844986; GOG-3004), maintenance olaparib significantly improved progression-free survival (PFS) vs placebo (HR 0.30; 95% CI 0.23-0.41; *P*< 0.0001; Moore *et al. NEJM* 2018) in patients with newly diagnosed advanced ovarian cancer (OC) and a BRCA mutation (BRCAm) who were in clinical complete response (CR) or partial response (PR) following platinum-based chemotherapy. This analysis evaluates olaparib efficacy in SOLO1 by timing of surgery and presence of residual tumour following surgery.

Methodology: SOLO1 recruited patients who had undergone upfront or interval cytoreductive surgery and had no residual or residual macroscopic disease. Patients were stratified by platinum response (clinical CR or PR) and received olaparib tablets 300 mg twice daily or placebo. PFS was assessed by investigators using modified RECIST v1.1.

Results: 260 patients were randomized to olaparib and 131 to placebo; one patient did not receive placebo. Median follow-up was 41 months in both arms. 63% and 35% of patients underwent upfront and interval surgery, respectively; 76% and 21% had no residual and residual macroscopic disease, respectively, after surgery regardless of timing; and 44% of SOLO1 patients had stage III disease, underwent upfront surgery and had no residual disease following surgery. PFS was significantly improved with olaparib, regardless of the timing of surgery or residual disease status after surgery (Table). PFS was also significantly improved in the subgroup of patients with stage III disease who underwent upfront surgery and had no residual disease following surgery.

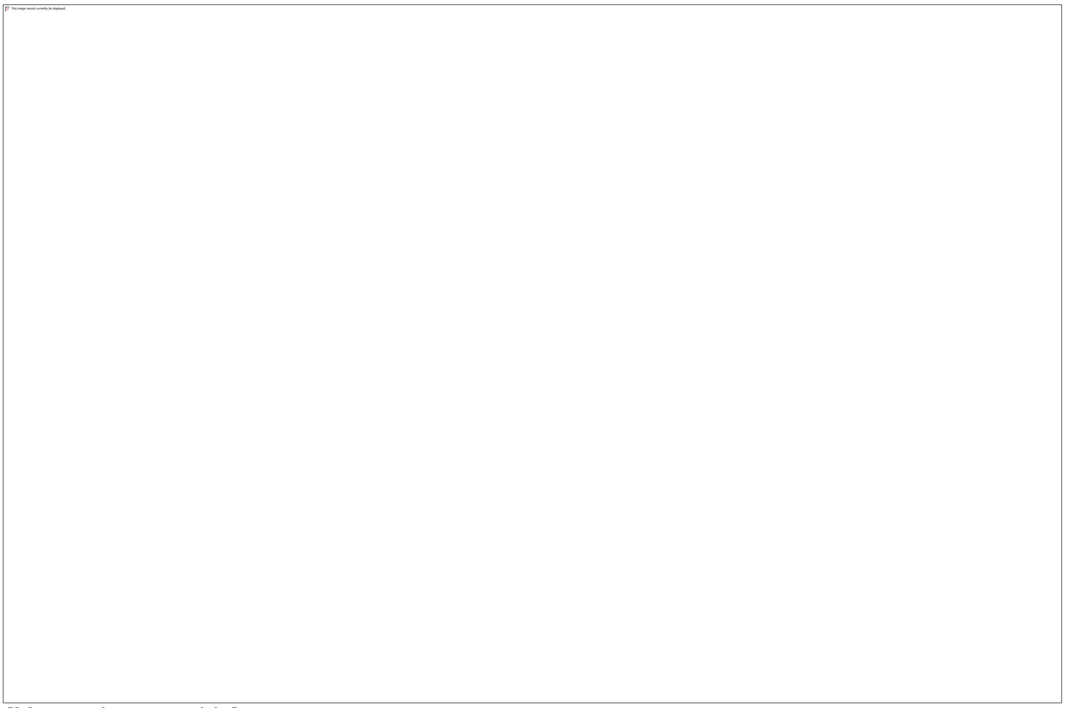

[Moore abstract table]

Conclusion: Maintenance olaparib improved outcomes compared with placebo in patients with newly diagnosed advanced OC and a BRCAm, regardless of the timing of surgery or residual disease status after surgery. Olaparib improved PFS in stage III patients who had undergone upfront surgery and had no residual disease, a population at high risk of progression, but typically excluded from first-line OC trials.

Disclosure: KM: Ad board: AZ, Advaxis, Clovis, Tesaro, Genentech/Roche, Immunogen, VBL Therapeutics, Merck, Janssen, Aravive, Pfizer, Eisai, Samumed, Oncomed. NC: Honoraria/reimbursement, speaker role/advisory board fees: AZ, Roche, Tesaro, PharmaMar; advisory board fees: Clovis, Pfizer. GS: None. B-GK: None. AO: Consulting fees: Roche, AZ, PharmaMar, Clovis, Tesaro, Immunogen, Genmab. MF: Honoraria, speaker role/advisory boards: AZ; advisory board fees: MSD, Lilly, Takeda; non-comp consulting: AbbVie. AL: None. AF: Advisory board fees: AZ, Clovis; congress support: Roche, PharmaMar; advisory board fees/congress support: Tesaro. AL: Honoraria/reimbursement, advisory board fees: AZ; advisory board fees: Clovis, Biocad; consulting fees: Seattle Genetics. GS: Research funding: AZ, Merck, Novartis, Roche. CG: Honoraria/reimbursement, consulting fees, grants: AZ, Tesaro, Nucana; honoraria/reimbursement, consulting fees: Roche, Clovis, Foundation One, MSD, Sierra Oncology; grants: Novartis, Aprea. SB: Grants, honoraria/reimbursement: AZ; honoraria/reimbursement: Tesaro, Clovis, Merck, PharmaMar, Roche, Seattle Genetics. AO: Steering committee role (non-comp): AZ, Clovis, Tesaro. AG-M: Speaker role/consulting fees: AZ, Tesaro, PharmaMar, Roche; consulting fees: Clovis, Inmunogen, Genmab, Pfizer, MSD. CA: Honoraria/reimbursement: Tesaro, ImmunoGen, Clovis. WB: None. CM: None. JL: Advisory board fees: AZ, Clovis, Mersana, Tesaro. ESL: Shareholder, employee: AZ. RB: Shareholder, employee: AZ. PD: Consulting fees: AZ, Tesaro.

Long-term safety assessment of niraparib in patients with recurrent ovarian cancer: results from the ENGOT-OV16/NOVA trial

M.R. Mirza[1], A. Dørum[2], B. Benigno[3], S. Mahner[4], P. Bessette[5], I.M. Bover Barcelo[6], D. Berton[7], J.

Ledermann[8], B.J. Rimel[9], J. Herrstedt[10], S. Lau[11], U. Canzler[12], I. Palacio Vázquez[13], E. Kalbacher[14], J. Buscema[15], D. Lorusso[16], P. Debruyne[17], I. Bruchim[18], W. Guo[19], D. Gupta[19], F.A. de Jong[19], U.A. Matulonis[20]
[1]Nordic Society of Gynaecological Oncology (NGSO) and Rigshospitalet, Copenhagen University Hospital, Copenhagen, Denmark, [2]Nordic Society of Gynaecological Oncology (NSGO), Radiumhospitalet, Oslo, Norway, [3]Northside Hospital, Atlanta, GA, United States, [4]Arbeitsgemeinschaft Gynäkologische Onkologie (AGO), University Hospital, Ludwig-Maximilians University of Munich, Munich, Germany, [5]University of Sherbrooke, Sherbrooke, QC, Canada, [6]Hospital Son Llàtzer, Palma, Spain, [7]Groupe d'Investigateurs Nationaux pour l'Étude des Cancers Ovariens (GINECO), Institut de Cancérologie de l'Ouest (ICO) Centre René Gauducheau, Saint-Herblain, France, [8]National Cancer Research Institute (NCRI), University College London, London, United Kingdom, [9]Cedars-Sinai Medical Center, West Hollywood, CA, United States, [10]University of Copenhagen, Copenhagen, Denmark, [11]McGill University, Montreal, QC, Canada, [12]TU Dresden, Dresden, Germany, [13]Hospital Universitario Central de Asturias, Asturias, Spain, [14]CHRU Besançon, Hôpital Jean Minjoz, Besançon, France, [15]Arizona Oncology Associates, Tucson, AZ, United States, [16]Multicentre Italian Trials in Ovarian Cancer/Mario Negri Gynecologic Oncology (MITO/MaNGO), Fondazione IRCCS National Cancer Institute, Milan, Italy, [17]Belgian Gynaecological Oncology Group (BGOG), Kortrijk Cancer Centre, AZ Groeninge, Kortrijk, Belgium, [18]Israeli Society of Gynecologic Oncology (ISGO), Hillel Yaffe Medical Center, Hadera, and Technion-Israel Institute of Technology, Haifa, Israel, [19]TESARO: A GSK Company, Waltham, [20]Dana-Farber Cancer Institute, Boston, MA, United States

Introduction / Background: Niraparib is a poly(ADP-ribose) polymerase inhibitor (PARPi) approved in the United States and Europe for maintenance treatment of patients with recurrent ovarian, fallopian tube, or primary peritoneal cancer (ROC) following complete or partial response to platinum-based chemotherapy. Here, we present long-term safety data of niraparib, reporting the incidence of treatment-emergent adverse events (TEAEs) among patients in the ENGOT-OV16/NOVA trial.
Methodology: ENGOT-OV16/NOVA enrolled 553 patients with ROC who received 2-3 prior lines of platinum-based chemotherapy. Patients were randomised 2:1 to receive niraparib (300 mg once daily) or placebo in independent germline *BRCA*-mutated (g*BRCA*mut) or non-g*BRCA*mut cohorts. We report TEAE incidence monthly for the first 6 months and for months 7-12 combined. After month 12, we report TEAEs to the safety data cutoff of September 2017.
Results: Overall, 367 patients received niraparib 300 mg once daily. Dose reductions due to TEAEs were highest in month 1 (34%). In month 5, only 7% of patients required dose reductions. The incidence of any-grade and grade ≥3 haematologic and symptomatic TEAEs decreased each month in the first 6 months and remained low thereafter. Incidence of grade ≥3 thrombocytopenia was highest in month 1 (28%) and decreased to 10% and 5% at months 2 and 3, respectively. Treatment discontinuations due to TEAEs were < 5% in each month and time interval measured.
Conclusion: The incidence of haematologic and symptomatic TEAEs decreased quickly over the first 3 months and continued to decrease over the duration of the trial. Dose reductions and grade ≥3 thrombocytopenia events were highest in month 1 and decreased rapidly thereafter. Treatment discontinuations due to TEAEs were < 5% for all months and time intervals measured. These data demonstrate the importance of appropriate dose selection and support the safe, long-term use of niraparib for maintenance treatment in patients with ROC.

[Any Grade and Grade ≥3 TEAEs]

[TEAE Summary]

Disclosure: AD, IMBB, DB-R, JH, EK, JB, PD, IB, SL: Nothing to disclose WG, SH, FdJ: Employee of Tesaro MRM: Consulting for Clovis, AstraZeneca, Tesaro BB: Honoraria from AstraZeneca, Insys, Funding from Tesaro SM: Grant and Personal Fees from Tesaro, AstraZeneca, Medac, MSD, PharmaMar, Roche, Teva. Personal Fees from Clovis, Sensor Kinesis PB: Site payment for clinical trial conduct from Tesaro JL: Ad Boards and PI for trials with AstraZeneca, Clovis, Pfizer. Ad Boards for Roche. Clinical Trials with MSD/Merck. BJR: Consulting with AstraZeneca, Tesaro, Genentech/Roche. Honoraria from Genentech UC: Honoraria and fees for consulting and Ad boards from Roche, AstraZeneca IPV: Travel and Accomodations from PharmaMar, Roche. Expert Testimony

for AstraZeneca. Research Funding from Novartis, Tesaro DL: Ad Board for Tesaro, Clovis, AstraZeneca. Research Support from Clovis. Study Conduction support from Tesaro and PharmaMar UAM: Consulting for Merck KGaA, Clovis, Geneos, Eli Lilly, 2x Oncology

Child development at 6 years after maternal cancer diagnosis and treatment during pregnancy

C. Maggen[1,2], K. Van Calsteren[3,4], E. Cardonick[5], R.G. Shmakov[6], M.M. Gziri[7], A. Cabrera Garcia[8], R. Fruscio[9], C.A.R. Lok[10], M.J. Halaska[11], I.A. Boere[12], P. Zola[13], P.B. Ottevanger[14], C.J.M. de Groot[15], G. Scarfone[16], M. Fumagalli[17], R.C. Painter[18], J. de Haan[15], F. Amant[1,19,20]

[1]University of Leuven, University Hospitals Leuven, Antoni van Leeuwenhoek - Netherlands Cancer Institute, KU Leuven, [2]Department of Obstetrics and Gynaecology, University Hospitals Leuven, [3]Department of Obstetrics, University Hospitals Leuven, UZ Leuven, [4]Department of Development and Regeneration, KU Leuven - University of Leuven, Leuven, Belgium, [5]Department of Obstetrics and Gynaecology, Cooper University Health Care, Camden, NJ, United States, [6]Federal State Budget Institution 'Research Centre for Obstetrics, Gynaecology and Perinatology', Ministry of Healthcare of the Russian Federation, Moscow, Russian Federation, [7]KU Leuven - University of Leuven, University Hospitals Leuven | Antoni van Leeuwenhoek - Netherlands Cancer Institute, Cliniques Universitaires St Luc, Leuven, Belgium, [8]Hospital Regional de Alta Especialidad de Ixtapaluca (HRAEI), Mexico, Mexico, [9]Clinic of Obstetrics and Gynecology, University of Milan Bicocca, Milan, Italy, [10]Centre for Gynaecological Oncology Amsterdam (CGOA), Antoni van Leeuwenhoek Hospital, Amsterdam, The Netherlands, [11]Faculty Hospital Kralovske Vinohrady, Charles University, 3rd Medical Facculty, Prague, Czech Republic, [12]Medical Oncology, Erasmus MC, Rotterdam, The Netherlands, [13]Department of Surgical Sciences, University of Turin, Turin, Italy, [14]Department of Medical Oncology, Radboud UMC Nijmegen, Nijmegen, [15]Department of Obstetrics and Gynaecology, Amsterdam UMC, Location VU University Medical Center, Amsterdam, The Netherlands, [16]Gynaecological Oncology Unit, Fondazione IRCCS Cà Granda Ospedale Maggiore Policlinico, Università degli Studi di Milano, Milan, Italy, [17]Neonatal Intensive Care Unit, Fondazione IRCCS Cà Granda Ospedale Maggiore Policlinico, Università degli Studi di Milano, Milan, [18]Department of Obstetrics and Gynaecology, Amsterdam UMC, Location AMC, Amsterdam, The Netherlands, [19]Department of Gynaecological Oncology, UZ Leuven, Leuven, Belgium, [20]Centre of Gynaecological Oncology (CGOA), Amsterdam UMC, Location AMC, VUmc, NKI, Amsterdam, The Netherlands

Introduction / Background: The International Network of Cancer, Infertility and Pregnancy was launched in order to register women of reproductive age with a cancer diagnosis. Over the years, the project has expanded with currently 2653 cases registered by 114 centres and an annual registration rate of 150 patients. The expected rising numbers of cancer diagnosis during pregnancy as a result of an increased age at first childbirth and the possibility of early cancer detection by the non-invasive prenatal testing calls for an ongoing evaluation of clinical practice. Moreover, women might become pregnant while exposed to new target therapies that are being introduced into oncological practice.
Methodology: The INCIP database consists of a secured on-line registration tool. Oncological, obstetric and neonatal data are registered by members. Annual scientific meetings give updates on the ongoing research projects.
Results: Most patients were registered in Belgium, the Netherlands, Italy and USA and one third of participating centres are non-European. Currently 2059 patients with a cancer diagnosis or treatment during pregnancy are registered, 395 women that received fertility preservation and 199 patients with a postnatal cancer diagnosis (Figure 1). Breast cancer, lymphoma and cervical cancer are the most frequent registered cancer types and the majority of patients (67%) received antenatal cancer treatment (Figure 2). Most women delivered a live born baby (88%), however 47% delivered preterm and 80% of preterm deliveries were medically induced. One-fifth of neonates (21%) were small for gestational age. Congenital malformations were reported in 3% of live births.
Conclusion: Cancer occurring in women endangers obstetrical and neonatal outcome and potentially future fertility. The INCIP registry is open for further collection of data since for such a relatively rare situation, only a large scale project will provide better insights on maternal and foetal risks assessment, which is essential for optimal patient counselling and care.

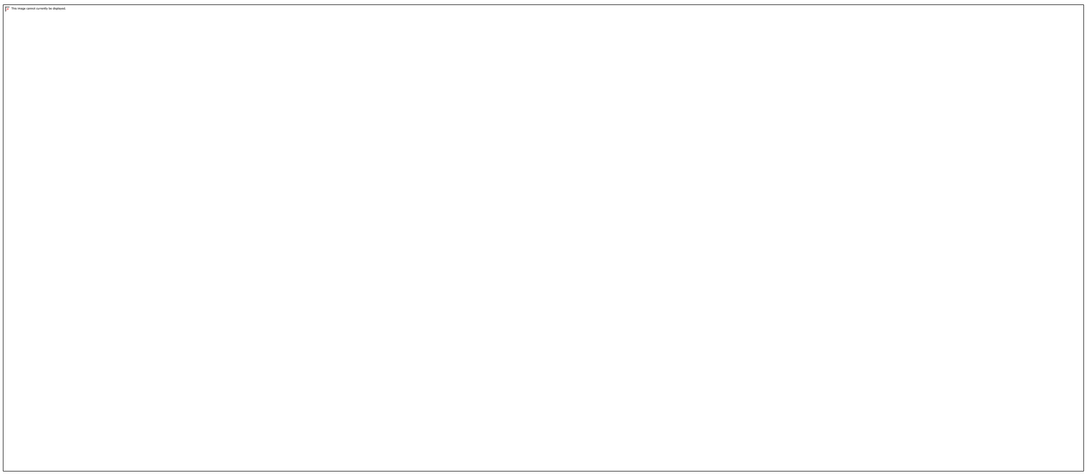

[Figure 1: Registered cases by INCIP]

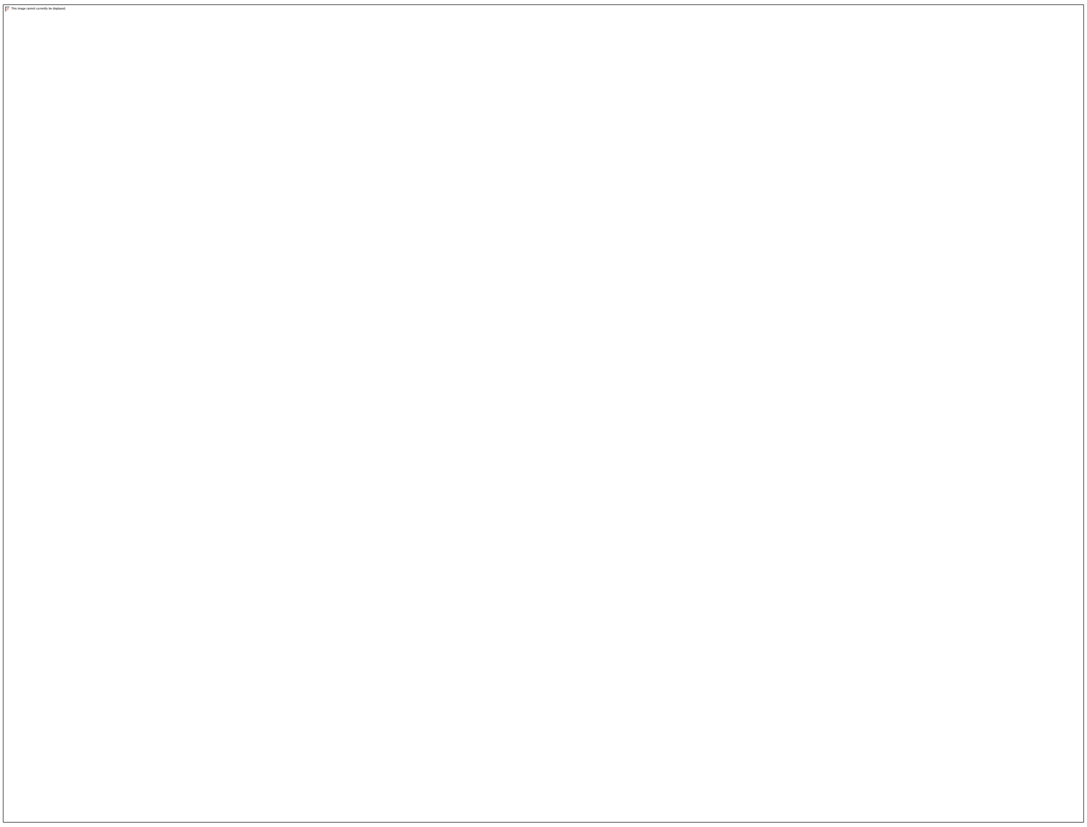

[Figure 2: Management of cancer during pregnancy according to trimester of diagnosis]

Disclosure: The INCIP network would not be able to operate without the ongoing support of ESGO. Furthermore the project is supported by the Research Foundation—Flanders (FWO) in Belgium (grant no G070514N) and the European Union's Horizon 2020 research and innovation program under grant agreement No 647047. There are no conflicting interests to declare.

When to stop futile treatment towards end of life in gynaecological cancer patients: a population-based study in Oslo county, Norway

K. Lindemann[1,2], H. Liland[1], B. Kloppen[1], M. Sylten Engh[1], M. Turzer[3], T. Paulsen[1]
[1]Department of Gynaecological Cancer, Oslo University Hospital, [2]University of Oslo, Oslo, [3]Department of Oncology, Hospital Østfold Kalnes, Kalnes, Norway

Introduction / Background: End of life (EOL) care for patients with gynaecologic malignancies often includes futile chemotherapy and invasive procedures. At the same time symptom burden is high and integration of palliative care is crucial. We aimed to assess care in the last weeks of life in gynaecological cancer patients.
Methodology: This retrospective study included all gynecological cancer patients who died between Jan 1st 2015 and Dec 31st, 2017 in Oslo County, Norway. The modified Glasgow Prognostic score (mGPS) was calculated at start of last line of chemotherapy. Descriptive statistics were used. Survival was estimated with the Kaplan-Meier method. P-values of < 0.05 were considered statistically significant.
Results: We included 215 patients, of those 53% with ovarian-, 30% with endometrial-, 14% with cervical cancer and 3% with other gynecological cancers. 15% had >3 lines of treatment prior to death. 163 patients died of their cancer and were analyzed for EOL care. In the last 30 days of life, 15% of the patients received chemotherapy, 67% were admitted to hospital and 35% had a surgical intervention (i.e. drainage of pleural effusions/ascites, surgery for bowel obstruction). In 62% a palliative care team was involved, in half of them in the last 30 days only. There was less use of chemotherapy towards EOL when a palliative care team was involved earlier (within 60 days before death) (6.8% vs 21.3%, p< 0.019). Patients with a mGPS of 2 had significantly shorter survival from start of last chemotherapy compared to patients with a score ≤1 (median 2.5 vs 6.3 months, p< 001).
Conclusion: Too few patients received multidisciplinary care including palliative care towards EOL. Gynaecological cancer patients may benefit from early integration of palliative care to avoid futile treatment towards EOL. The modified GPS seems to be a helpful tool when deciding on palliative chemotherapy.
Disclosure: Nothing to disclose

Oral Communication 2 - Cervical Cancer

Diagnostic performance of ultrasound in the assessment of parametrial involvement before surgery in cervical cancer patients (diagnostic accuracy study)

D. Fischerova[1], T. Ballaschova[1], L. Dostalek[1], F. Frühauf[1], M. Zikan[2], J. Slama[1], R. Kocian[1], A. Germanova[1], K. Němejcova[3], J. Jarkovsky[4], D. Cibula[1]
[1]Gynecologic Oncology Centre, Department of Obstetrics and Gynecology, First Faculty of Medicine and General University Hospital, Charles University, [2]Department of Obstetrics and Gynecology, Bulovka Hospital, First Faculty of Medicine, Charles University, [3]Department of Pathology, First Faculty of Medicine and General University Hospital, Charles University in Prague, [4]Institute of Health Information and Statistics of the Czech Republic, Prague, Czech Republic

Introduction / Background: To evaluate the performance of 2-dimensional (2D) ultrasound in preoperative assessment of parametria involvement in patients with cervical cancer planned for primary surgical treatment. Histopathological findings from radical hysterectomy/trachelectomy/parametrectomy were used as a reference standard.
Methodology: Patients with biopsy-proven cervical cancer evaluated on ultrasound and planned for primary surgery 01/2004 and 12/2017 were prospectivelly enrolled. The predefined protocol for ultrasound assessment included the presence of tumor, maximum tumor size, tumor-free distance (TFD), the parametrial involvement status, grade of parametrial infiltration and lymph node status.
Results: Altogether, 722 patients were included, out of them 77 patients (11%) were excluded for tumor duplicity, absence of index test or reference standard. Data of 645 patients were analysed, 555

early stage cases (T1[except T1b3], T2a1, FIGO 2018) and 90 locally advanced stage (T1b3 and higher [except T2a1], FIGO 2018). The median age was 42 years and BMI was 24.5. Sixty-two patients (10%) underwent neoadjuvant chemotherapy (NACT). The maximum tumor size was 19.5 mm.

Age	45.4 (13.4) / 41.8 (28.1; 70.9)
BMI	25.6 (5.1) / 24.5 (18.8; 35.8)
Date of surgery	19-APR-10 / 27-MAY-10 (16-SEP-04; 21-APR-16)
Pathologic maximum tumor size (mm)	91.9 (1736.1) / 19.5 (0.4; 55)
Histotype 1, 2, 3, 4	498 (77.9%), 112 (17.5%), 19 (3%), 10 (1.6%)
Grade 1, 2, 3	64 (12.5%), 245 (48%), 201 (39.4%)
LVSI	208 (32.2%)

[Tabulka 1 Study cohort characteristics (mean (standard deviation) / median (5; 95th percentile), histot.1-squamoc., 2- adenoca, 3-adenosquam, 4-other)]

The histologically confirmed prevalence of infiltrated parametria was 3.9% (25/645), with 12 cases correctly identified by the ultrasound. Ultrasound did not detect 11 cases (1.7%) with microscopic spread (< 2 mm) and 2 cases (0.3%) with macroscopic infiltration (2.75 and 10.0 mm). The accuracy of ultrasound in parametrial detection reached 97% with diagnostic odds ratio 62.7.
Conclusion: Ultrasound is very good method for preoperative assessment of parametrial involvement in cervical cancer with a minimal risk of failure to detect infiltration > 2 mm.
Disclosure: This work was supported by Charles University in Prague (UNCE 204065 and PROGRES Q28/LF1) and by a grant from the Czech Research Council (No 16-31643A). None of the authors declare any conflict of interest.

European perspective on surgical approach in early cervical cancer after LACC trial. An international ESGO survey

L. Chiva[1,2], E. Chacon[3], I. Carriles[3], D. Vazquez[1], M. Platero[1], T. Castellanos[1], J. Minguez[3], A. Gonzalez-Martin[4], J. Espinos[4], L. Sanchez[4], J.M. Aramendia[5], I. Espinosa[6], M.A. Idoate[7], M. Cambeiro[8], R. Martinez Monje[9], F. Calvo[8], J.L. Alcazar[3]
[1]Obstetrics and Gynecology, Clinica Universidad de Navarra, Madrid, [2]Obstetrics and Gynecology, Universidad de Navarra, [3]Obstetrics and Gynecology, Clinica Universidad de Navarra, Pamplona, [4]Medical Oncology, Clinica Universidad de Navarra, Madrid, [5]Medical Oncology, Clinica Universidad de Navarra, Pamplona, [6]Pathology, Clinica Universidad de Navarra, Madrid, [7]Pathology, Clinica Universidad de Navarra, Pamplona, [8]Radiation Therapy Oncology, Clinica Universidad de Navarra, Madrid, [9]Radiation Therapy Oncology, Clinica Universidad de Navarra, Pamplona, Spain

Introduction / Background: This study aims to understand what was the impact of the LACC trial (Laparoscopy in cervical cancer) among the members of ESGO. We have explored members´ understanding of the results and changes in the clinical practice.
Methodology: Methods: A questionnaire addressing multiple issues related to the LACC trial and surgical management of early cervical cancer was designed and sent to ESGO members before and after the publication of the LACC trial in the NEJM. The survey was entirely confidentially and could be completed in a short period of time. Responses from 394 and 326 members from 33 European countries were obtained. The respondents were divided into two groups depending on whether or not they belong to European countries.
Results: Characteristics and perceptions of the responders are shown in Tables 1 and 2. 97 % of responders knew the results of the trial and 92 % had read the manuscript. 83 % did not expect the results . 56 % of members confirmed that have changed their practice after the publication of the trial. Before the trial, only 29 % operated cervical cancer by laparotomy and after the publication 63 % does. Fig1-2. In cervical cancer patients who have undergone conization with clear margins 73 %

would offer MIS to the patient while only 49 % would if the specimen margins was positive. Members consider that uterine manipulator an incorrect tumor handling occasioned the poorer result of MIS. 9 out 10 of members are willing to discuss the LACC trial with their patients and 80% consider unethical avoiding this discussion. 85 % think that ESGO should promote a new randomized clinical trial and meanwhile 65 % would be interested to participate in a observational study.
Conclusion: Among ESGO members, the publication of LACC trial has objectively modified the surgical approach in early cervical cancer.

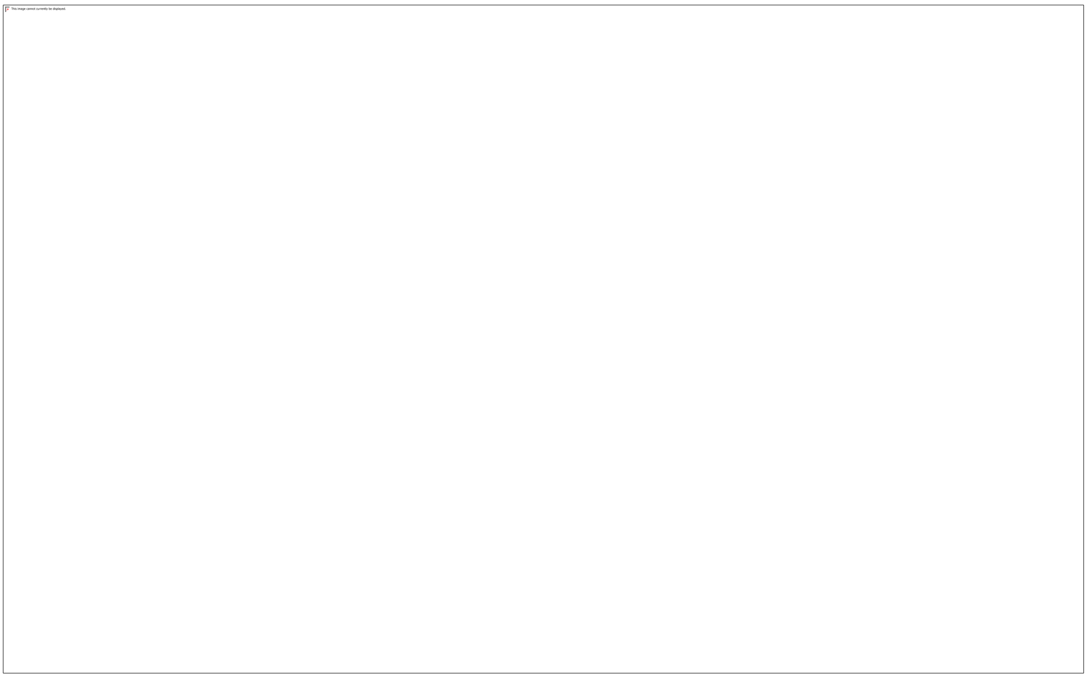

[Fig.1 Surgical approach among ESGO members before LACC trial]

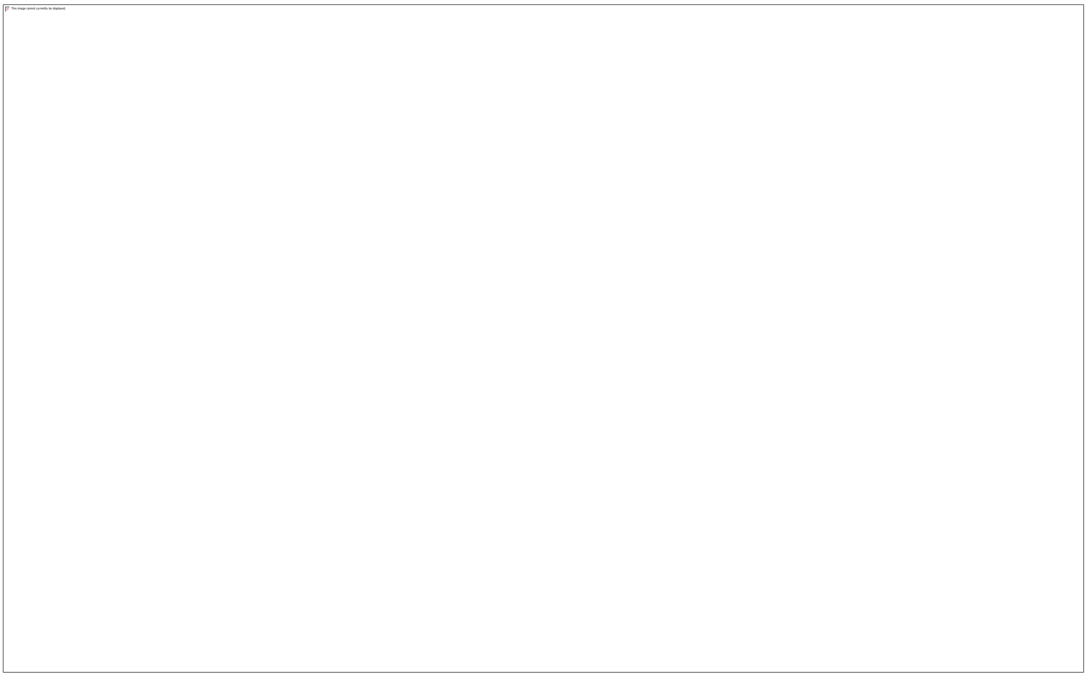

[Fig.2 Surgical approach among ESGO members after LACC trial]

Number of paritcipants	394
Working in ESGO zone	76 %
Age (y) Mean	49.5 (31-76)
Gender	M: 74%, F: 26%
Years of practice after residency program	16.3 (1-43)
Academic public hospital	69,8 %
Accredited fellowship	72.1 %
Regularly operate patients with cervical cancer	94 %
Surgical procedures for early cervical cancer per institution and year	25

[Table1 Responders characteristics]

I feel comfortable operating a radical Hysterectomy by	Laparotomy:90%	Laparoscopy:58%	Robotics:19%	Vaginally:5%
I feel comfortable operating a radical Trachelectomy by	Laparotomy:62%	Laparoscopy:27%	Robotics:11%	Vaginally:26%
I would offer a MIS approach in stage (FIGO 2019)	IA1with LVSI: 62%	IA2:63%	IB1<2 cm:63%	any IB1:10%
I would recommend MIS to candidates for radical trachelectomy	Yes:34%	No:36%	Maybe:24%	I am lost:5%
I would carry out as first step the SNLB by MIS, independently of the eventual surgical approach (MIS or open) of the Radical Histerectomy	Yes:61%	No:34%		

[Table 2. ESGO members perceptions after LACC trial]

Disclosure: Nothing to disclose

The role of robotic assisted laparoscopy in early stage cervical cancer, the Danish experience

T.H. Schnack[1], P.T. Jensen[2], S.F. Bjørn[3], L. Frøding[3], Z. Ketabi[3], K. Jochumsen[4], K. Fuglsang[2], J. Dinesen[2], C.H. Soegaard[3], E. Soegaard-Andersen[5], M.M. Jensen[5], L.H. Oester[6], A.A. Knudsen[5], C. Høgdall[3]

[1]Gynecologic and Obstetrics, Copenhagen University Hospital, Rigshospitalet, Copenhagen, [2]Gynecology, Aarhus University Hospital, Aarhus, [3]Gynecology, Copenhagen University Hospital, Rigshospitalet, Copenhagen, [4]Gynecology, Odense University Hospital, Odense, [5]Gynecology, Aalborg University Hospital, Aalborg, [6]Gynecology, Herlev University Hospital, Herlev, Denmark

Introduction / Background: Since the introduction of robotic assisted laparoscopy it has been the preferred surgical approach for early stage cervical cancer (ECC) in the Nordic countries. Lately, a randomized trial comparing open access vs. minimal invasive surgery has questioned the oncological safety of robotic surgery in ECC. The aim of the present study was to evaluate the safety of robotic surgery in the treatment of ECC in Denmark.
Methodology: Clinical and follow-up data on all consecutive patients with ECC stage IA2-IB1 who underwent radical hysterectomy in the period January 1st 2005 - June 30th 2017 were derived from the Danish Gynecologic Cancer Database (DGCD). Data was validated with other registers and patient files. Descriptive statistics with univariate and multivariate analyses were used.
Results: 1125 ECC patients were included; 530 who underwent a radical hysterectomy before the introduction of robotic-assisted laparoscopy (period 1) and 595 who underwent surgery after the introduction of robotic-assisted laparoscopy (period 2). No significant differences in the rate of recurrence, recurrence location, or use of chemo-radiation between the two cohorts was observed. Furthermore, no significant differences in the five-year cancer specific survival between cases diagnosed before and after the introduction of robotic-assisted laparoscopy was found - five year cancer specific survivals of 94.1% and 95.9% (p-value = 0.10) in period 1 and period 2, respectively.
Conclusion: Based on national and prospectively collected data our cohort study found no indication that the oncologic safety such as risk of recurrence and survival has been compromised by the introduction of the robotic platform. However, use of robotic surgery for early cervical cancer will be followed closely throug our national cancer database (DGCD) in the future.
Disclosure: Nothing to disclose

Bevacizumab combined with first-line carboplatin and paclitaxel for metastatic/recurrent/persistent cervical cancer: primary results from the global single-arm phase II CECILIA study

N. Colombo[1], L. Dreosti[2], M. McCormack[3], A. Nogueira-Rodrigues[4], G. Scambia[5], A. Roszak[6], M. Donica[7], B. Ulker[7], A. González-Martín[8], A. Redondo[9]
[1]European Institute of Oncology and University of Milan-Bicocca, Milan, Italy, [2]University of Pretoria, Pretoria, South Africa, [3]University College London Hospitals, London, United Kingdom, [4]Federal University of Minas Gerais Brazil and Brazilian Group of Gynecologic Oncology, Belo Horizonte, Brazil, [5]Policlinico Universitario 'Agostino Gemelli', Università Cattolica del Sacro Cuore, Rome, Italy, [6]Wielkopolskie Centrum Onkologii im. Marii Skłodowskiej-Curie, Poznań, Poland, [7]F Hoffmann-La Roche Ltd, Basel, Switzerland, [8]Clínica Universidad de Navarra, [9]Hospital Universitario La Paz, Madrid, Spain

Introduction / Background: In the phase III GOG240 trial, combining bevacizumab with paclitaxel and cisplatin/topotecan for advanced cervical cancer significantly improved overall and progression-free survival. The single-arm phase II CECILIA study (NCT02467907) evaluated bevacizumab with a more widely used chemotherapy backbone.
Methodology: The primary objective was to determine the safety of bevacizumab/carboplatin/paclitaxel for advanced cervical cancer, defined by the frequency and severity of gastrointestinal (GI) perforation/fistula, GI-vaginal fistula and genitourinary (GU) fistula. Eligible patients had metastatic/recurrent/persistent cervical cancer not amenable to curative surgery and/or radiotherapy. Patients with ongoing bladder/rectal involvement, prior cobalt radiotherapy, history of fistula/GI perforation, or bowel resection ≤6 weeks or chemoradiation ≤3 months before starting bevacizumab were excluded. Patients received bevacizumab 15mg/kg, paclitaxel 175mg/m^2 and carboplatin AUC5 q3w until disease progression, unacceptable toxicity or consent withdrawal. If any drug was stopped for toxicity, the remaining drug(s) could be continued alone.
Results: Between July 2015 and December 2016, 150 patients began treatment. Baseline

characteristics at study entry were: 20%/53%/27% with persistent/recurrent/de novo metastatic disease; 71%/58%/59% with prior radiotherapy/chemoradiation/platinum. Median treatment duration was 6.7 (range < 1-39) months for bevacizumab and 3.9 (range 0-14.6) months for chemotherapy; 57% of patients received single-agent bevacizumab after discontinuing chemotherapy. Table 1 summarises primary endpoint results. In 17 patients with fistula/perforation events, median time to onset was 3.8 (range 0.3-12.4) months. Grade 3/4 adverse events occurred in 73% of patients, most commonly neutropenia/neutrophil count decreased (25%), anaemia (19%) and hypertension (14%). Grade 5 adverse events occurred in 3%. Figure 1 shows progression-free and overall survival. The objective response rate was 61% (95% CI 52-69%), including complete responses in 14% (9-21%).
Conclusion: Bevacizumab can be combined with carboplatin/paclitaxel chemotherapy in the cervical cancer population treated in CECILIA. The fistula/GI perforation incidence is in line with GOG240. Efficacy results are encouraging.

[Table 1. Summary of primary endpoint results]

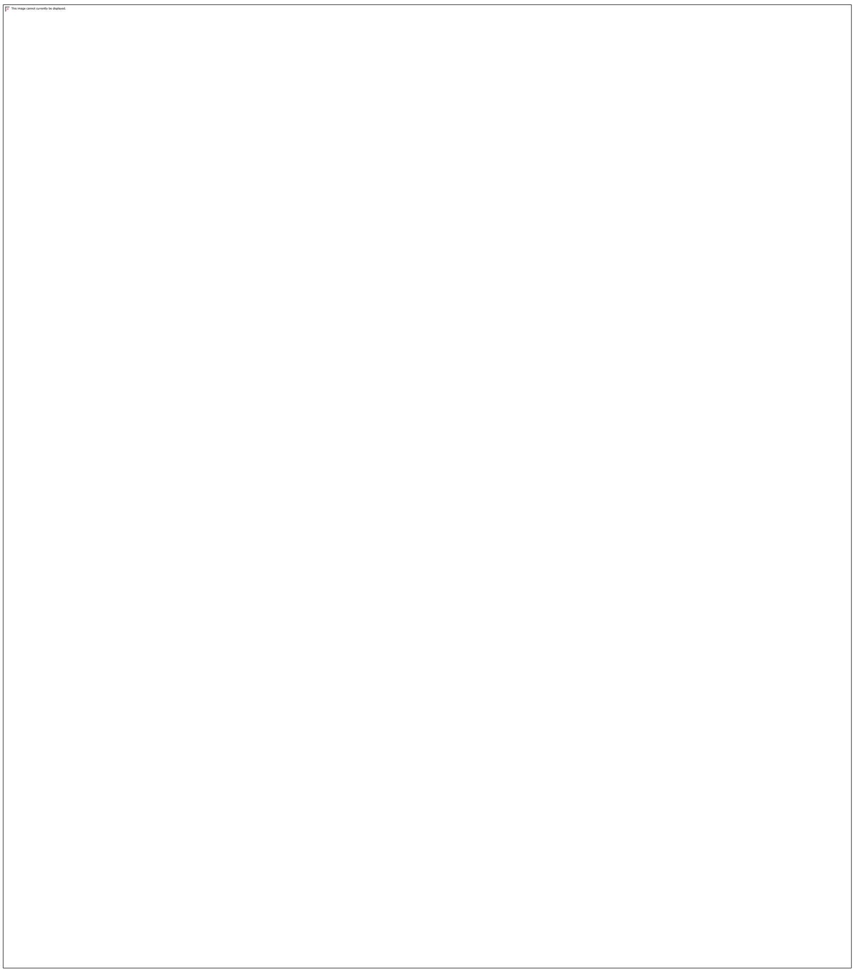

[Figure 1. Progression-free and overall survival]

Disclosure: Nicoletta Colombo: advisor/consultant for Roche, PharmaMar, AstraZeneca, Tesaro, Clovis, Pfizer, MSD, BIOCAD, Takeda, Lilly; travel/accommodation expenses from Roche, PharmaMar, Tesaro. Lydia Dreosti: honoraria for advisory/consultancy roles from Eli Lilly, MSD; travel/accommodation expenses from Janssen, Merck. Mary McCormack: honoraria from AstraZeneca, Roche. Angélica Nogueira-Rodrigues: honoraria for advisory boards/consultancy from Roche, AstraZeneca, MSD; research funding to institution from Roche, MSD. Giovanni Scambia, Andrzej Roszak: no conflict of interest. Margarita Donica: employee of F Hoffmann-La Roche; shares in Novartis. Bulent Ulker: contracted to work on behalf of F Hoffmann-La Roche. Antonio González-Martín: advisory boards for Roche, AstraZeneca, Tesaro, Clovis, Pfizer, ImmunoGen, PharmaMar, MSD, Genmad; speaker bureau for Roche, PharmaMar, Tesaro, AstraZeneca; research funding to institution from Roche, Tesaro. Andrés Redondo: honoraria from AstraZeneca, Roche Farma, Tesaro, Clovis, Pharmamar, Lilly, Eisai; advisory boards for AstraZeneca, Roche Farma, Tesaro, Clovis, Pharmamar, Lilly; travel/accommodation from AstraZeneca, Tesaro, PharmaMar, Roche Farma; research funding to institution from Eisai, PharmaMar, Roche Farma.

Patient-reported sexual frequency and vaginal functioning in patients with locally advanced

cervical cancer following definitive radiochemotherapy and image-guided adaptive brachytherapy (EMBRACE study)

K. Kirchheiner[1], I.M. Jürgenliemk-Schulz[2], C. Haie-Meder[3], J.C. Lindegaard[4], A. Sturdza[1], U. Mahantshetty[5], B. Segedin[6], K. Bruheim[7], B. Rai[8], R. Cooper[9], E. van der Steen-Banasik[10], E. Wiebe[11], M. Sundset[12], E. van Limbergen[13], E. Villafranca[14], H. Westerveld[15], L.T. Tan[16], K. Tanderup[4], R. Pötter[1], R.A. Nout[17], EMBRACE Collaborative Group

[1]Department of Radiotherapy, General Hospital Vienna, Medical University Vienna, Vienna, Austria, [2]Department of Radiation Oncology, University Medical Centre Utrecht - Utrecht Cancer Centre, Utrecht, The Netherlands, [3]Department of Radiotherapy, Gustave-Roussy Cancer Campus, Villejuif, France, [4]Department of Oncology, Aarhus University Hospital, Aarhus, Denmark, [5]Department of Radiation Oncology, Tata Memorial Hospital, Mumbai, India, [6]Department of Radiotherapy, Institute of Oncology Ljubljana, Ljubljana, Slovenia, [7]Department of Oncology, The Norwegian Radium Hospital, Oslo University Hospital, Oslo, Norway, [8]Department of Radiotherapy and Oncology, Postgraduate Institute of Medical Education and Research, Chandigarh, India, [9]Leeds Cancer Centre, St James's University Hospital, Leeds, United Kingdom, [10]Department of Radiotherapy, Radiotherapiegroep Arnhem, Arnhem, The Netherlands, [11]Department of Oncology, Cross Cancer Institute and University of Alberta, Edmonton, AB, Canada, [12]Clinic of Oncology and Women's Clinic, St. Olavs Hospital, Trondheim, Norway, [13]Department of Radiation Oncology, UZ Leuven, Leuven, Belgium, [14]Department of Radiation Oncology, Hospital of Navarra, Pamplona, Spain, [15]Department of Radiation Oncology, University of Amsterdam, Amsterdam UMC, Location Academic Medical Center, Amsterdam, The Netherlands, [16]Oncology Centre, Cambridge University Hospitals NHS Foundation Trust, Addenbrooke's Hospital, Cambridge, United Kingdom, [17]Department of Radiation Oncology, Leiden University Medical Center, Leiden, The Netherlands

Introduction / Background: To evaluate sexual frequency and vaginal functioning in patients treated with combined external beam radiotherapy ± chemotherapy and image-guided adaptive brachytherapy in the prospective, observational, multi-center EMBRACE study (www.embracestudy.dk).

Methodology: Patient-reported sexual outcomes were prospectively assessed according to EORTC-QLQ-CX24 questionnaires at baseline (available for 1027 patients) and follow-ups: 1st year every 3M (months), 2nd/3rd year every 6M and yearly thereafter (available for 1044 patients with no evidence of disease). Sexual activity was analysed longitudinally, and defined as frequent if reported (to some degree) in ≥50% of follow-up and as occasional if reported in < 50%. Prevalence rates over time were displayed. Associations between dyspareunia and vaginal functioning were evaluated with Spearman´s rank correlations, pooling observations over all follow-ups (N=2670).

Results: After the diagnosis of cancer, 230/1027 (22.4%) of patients reported some degree of sexual activity. During follow-up (median 36M), 433/1044 (41.5%) patients reported no sexual activity, 128 (12.3%) occasional and 483 (46.3%) frequent sexual activity. Prevalence rates (figure 1A-F) revealed a slow increase of sexual activity during the 1st year of follow-up, reaching a plateau up to 5 years. Vaginal functioning problems during intercourse showed a substantial increase after treatment and remained either elevated or showed signs of slight improvement over time. Sexual enjoyment was impaired, with 37-48% of patients reporting to have “not at all”/“a little” sexual enjoyment over time. Patient-reported vaginal dryness, shortening and tightening were significantly correlated with dyspareunia to r=0.407, 0.539, 0.552, respectively (figure 2A-C).

Conclusion: More than half of locally advanced cervical cancer patients are sexually active and the majority of those report frequent sexual activity. Treatment-induced vaginal changes (dryness, shortening and tightening) are associated with dyspareunia. Sexual enjoyment is compromised in almost half of sexually active patients. This underlines the importance of efforts directed towards prevention of vaginal morbidity and sexual rehabilitation after treatment.

This image cannot currently be displayed.

[Figure 1 A-F]

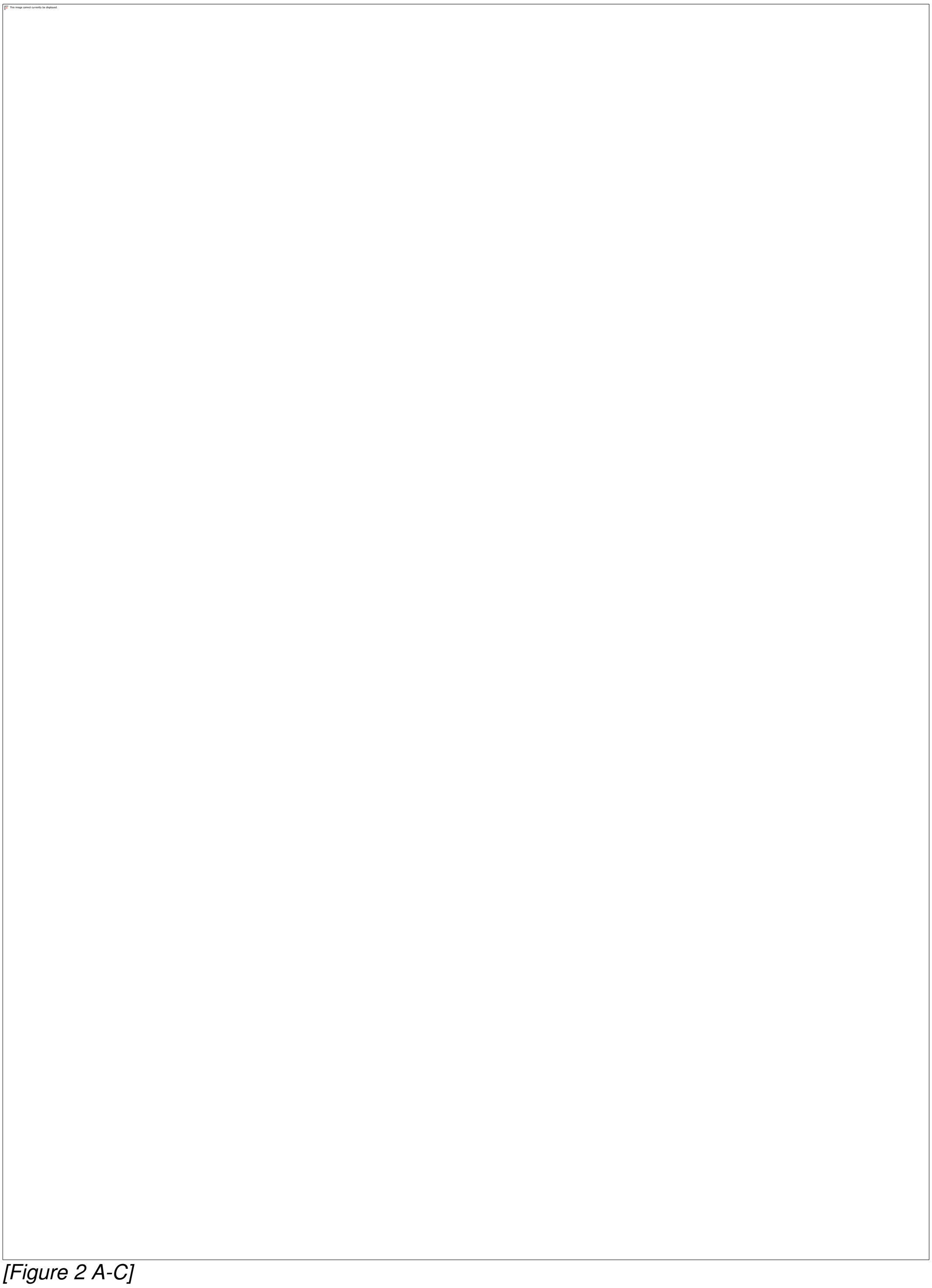

[Figure 2 A-C]

Disclosure: The findings of this abstract were submitted to ESTRO (European Society for Radiotherapy & Oncology, accepted for oral presentation in April 2019, Milano, Italy) and ASTRO (American Society for Radiation Oncology, accepted for oral presentation in September 2019, Chicago, USA). All authors declare no conflict of interest. The EMBRACE study was sponsored by the Medical University of Vienna. Unrestricted research funding was provided by Nucletron, an Elekta company and Varian Medical Systems. Financial support by the Austrian Federal Ministry of Science,

Research and Economy, and the Austrian Foundation for Research, Technology, and Development, the Danish Cancer Society and CIRRO - The Lundbeck Foundation Center for Investigational Research in Radiation Oncology is gratefully acknowledged.

Oral Communication 3 - Ovarian Cancer

Whole genome sequencing of ovarian granulosa cell tumours show heterogeneity, genomic instability and tumour evolution

J.F. Roze[1], G.M. Monroe[1], J.W. Groeneweg[1], E. Stelloo[2], S.T. Paijens[3], H.W. Nijman[3], H.S. van Meurs[4], L.R.C.W. van Lonkhuijzen[4], J.M.J. Piek[5], C.A.R. Lok[6], G.N. Jonges[7], R.P. Zweemer[1]
[1]Department of Gynaecological Oncology, UMC Utrecht Cancer Center, [2]Center for Molecular Medicine, University Medical Center Utrecht, Utrecht University, Utrecht, [3]Department of Obstetrics and Gynaecology, University Medical Center Groningen, Groningen, [4]Department of Gynecological Oncology, Centre for Gynaecological Oncology, Amsterdam UMC, Location AMC, Amsterdam, [5]Department of Obstetrics and Gynaecology, Catharina Hospital, Eindhoven, [6]Department of Gynaecological Oncology, Centre for Gynaecological Oncology, The Netherlands Cancer Institute, Antoni van Leeuwenhoek Hospital, Amsterdam, [7]Department of Pathology, University Medical Center Utrecht, Utrecht University, Utrecht, The Netherlands

Introduction / Background: Adult granulosa cell tumours (aGCTs) are thought to arise from the stromal cells of the ovary and are molecularly characterized by a specific *FOXL2* c.402C>G mutation. Cytoreductive surgery remains the mainstay of treatment of both primary and recurrent disease, because the effect of chemotherapy is limited. Unravelling this tumour on a genomic level could possibly identify targets for systemic therapy. However, most genomic studies on ovarian cancer do not focus on aGCTs. We investigated the genomic landscape and tumour stability of aGCTs, a crucial step towards targeted treatment.
Methodology: We performed whole genome sequencing (WGS; 30-90X coverage) on 31 fresh frozen aGCT samples (5 primary aGCTs and 26 recurrences) to detect single nucleotide variants and copy number variants. From 23 samples matched germline DNA was available, enabling somatic variation identification. We compared multiple tumour samples taken from the same patient at different locations and time points. The cohort will be further extended to identify novel (targetable) cohort mutations and affected pathways.
Results: Cohort analysis detected the FOXL2 mutation in 90% (28/31 samples) and a TERT promoter mutation in 68% (C228T in 14/31 and C250T in 7/31) of samples. Copy number analyses showed polyploidy in 70% of the matched samples (16/23) and confirmed previous reported gains in chromosome 14 and losses in 22. Intra-patient comparisons showed 40-72% unique somatic variants with 1562-1650 overlapping somatic variants detected in all samples. Somatic variant analysis in the matched samples showed 1431 to 8102 (median 4103) single nucleotide variants and 462 to 2963 (median 2065) insertions/deletions.
Conclusion: The view of aGCTs as monogenetic and genetically stable is no longer supported. The large number of unique somatic mutations in intra-patient comparisons reveals a high degree of tumour heterogeneity. Future targeted treatment should focus on consistent genomic changes shared both between and within patients.

[Figure 1]

Disclosure: No competing interests. Applicable funding sources: Granulosafonds Philine van Esch.

Effect of Progression-Free Interval (PFI) following penultimate platinum-based regimen on the efficacy of rucaparib maintenance treatment in patients with platinum-sensitive, recurrent ovarian carcinoma: an analysis from the Phase 3 study ARIEL3

A.R. Clamp[1], A.M. Oza[2], D. Lorusso[3], C. Aghajanian[4], A. Oaknin[5], A. Dean[6], N. Colombo[7], J.I. Weberpals[8], G. Scambia[3], A. Leary[9], R.W. Holloway[10], M. Amenedo Gancedo[11], P.C. Fong[12], J.C. Goh[13,14], D.M. O'Malley[15], D.K. Armstrong[16], S. Banerjee[17], J. García-Donas[18], E.M. Swisher[19], T. Cameron[20], L. Maloney[21], S. Goble[22], R.L. Coleman[23], J.A. Ledermann[24]
[1]Department of Medical Oncology, The Christie NHS Foundation Trust and University of Manchester, Manchester, United Kingdom, [2]Division of Medical Oncology and Hematology, Princess Margaret Cancer Centre, University Health Network, Toronto, ON, Canada, [3]Gynecologic Oncology Unit, Fondazione Policlinico Universitario A. Gemelli IRCCS, Rome, Italy, [4]Gynecologic Medical Oncology, Memorial Sloan Kettering Cancer Center, New York, NY, United States, [5]Medical Oncology Department, Vall d'Hebron University Hospital, Vall d'Hebron Institute of Oncology (VHIO), Barcelona, Spain, [6]Department of Oncology, St John of God Subiaco Hospital, Subiaco, WA, Australia, [7]Gynecologic Cancer Program, European Institute of Oncology and University of Milan-Bicocca, Milan, Italy, [8]Division of Gynecologic Oncology, Ottawa Hospital Research Institute, Ottawa, ON, Canada, [9]Gynecological Unit, Gustave Roussy Cancer Center, INSERM U 981, and Groupe d'Investigateurs Nationaux pour l'Etude des Cancers Ovariens (GINECO), Villejuif, France, [10]Department of Gynecologic Oncology, Florida Hospital Cancer Institute, Orlando, FL, United States, [11]Medical Oncology Department, Oncology Center of Galicia, La Coruña, Spain, [12]Medical Oncology Department, Auckland City Hospital, Grafton, Auckland, New Zealand, [13]Department of Oncology, Cancer Care Services, Royal Brisbane and Women's Hospital, Herston, [14]University of Queensland, St Lucia, QLD, Australia, [15]Clinical Research Gynecologic Oncology, The Ohio State University, James Cancer Center, Columbus, OH, [16]Department of Medical Oncology, Johns Hopkins University

Medical Center, Baltimore, MD, United States, [17]Medical Oncology Service, HM Hospitales - Centro Integral Oncológico Hospital de Madrid Clara Campal, Madrid, Spain, [18]Gynaecology Unit, The Royal Marsden NHS Foundation Trust, London, United Kingdom, [19]Division of Gynecologic Oncology, University of Washington, Seattle, WA, United States, [20]Clinical Science, Clovis Oncology UK Ltd., Cambridge, United Kingdom, [21]Clinical Development, [22]Biostatistics, Clovis Oncology, Inc., Boulder, CO, [23]Department of Gynecologic Oncology and Reproductive Medicine, The University of Texas MD Anderson Cancer Center, Houston, TX, United States, [24]Department of Oncology, UCL Cancer Institute, University College London and UCL Hospitals, London, United Kingdom

Introduction / Background: In ARIEL3, rucaparib maintenance treatment significantly improved progression-free survival (PFS) vs placebo in all patient populations, regardless of biomarker status (Coleman et al. *Lancet*. 2017;390:1949-61). This subgroup analysis examined the effect of the stratification factor PFI following penultimate platinum-based chemotherapy (also a prognostic factor in ovarian cancer) on primary and secondary endpoints of investigator-assessed and blinded independent central review (BICR)-assessed PFS in ARIEL3.
Methodology: Patients were randomised 2:1 to oral rucaparib (600 mg BID) or placebo. Analysis was based on the randomisation stratification factor of PFI following penultimate platinum-based regimen: 6-12 months or >12 months. PFS was assessed in 3 predefined cohorts: *BRCA* mutant; *BRCA* mutant + *BRCA* wild type/high loss of heterozygosity (LOH high); and intent-to-treat (ITT) population. Safety was assessed in all patients who received ≥1 dose of rucaparib.
Results: Visit cutoff dates for efficacy and safety were 15 April 2017 and 31 December 2017, respectively. For all predefined cohorts, investigator and BICR assessments showed a significant PFS improvement with rucaparib vs placebo in both PFI subgroups (Figure).

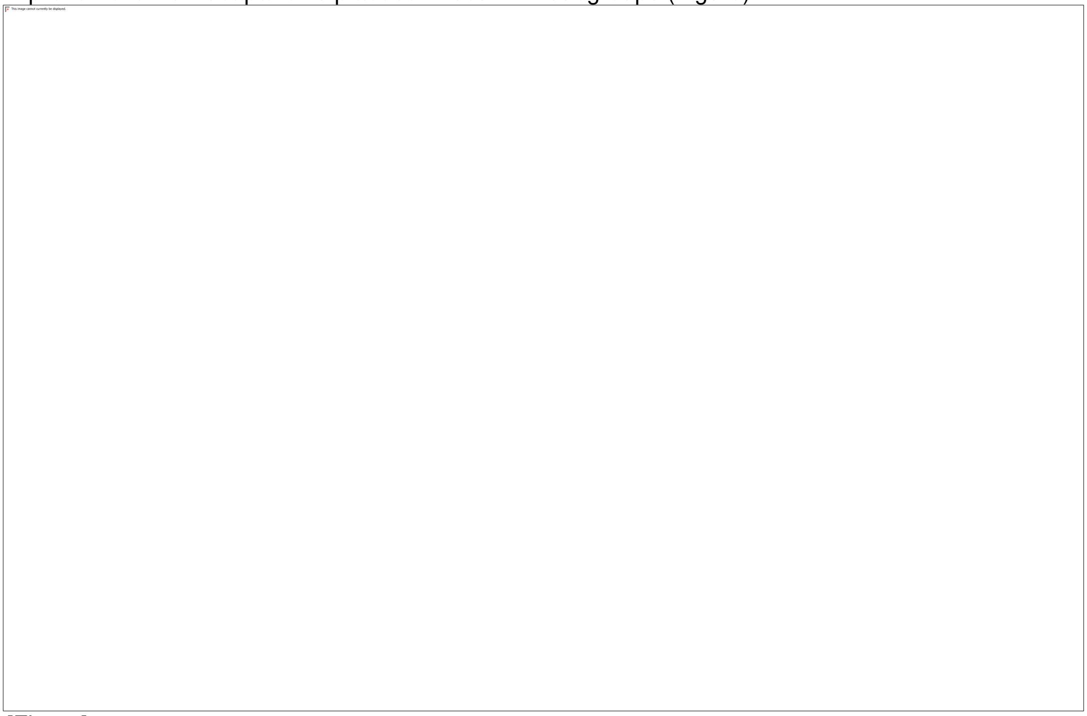

[Figure]

As expected, patients receiving placebo with a PFI 6-12 months had a shorter median PFS than those with a PFI >12 months. The treatment by PFI subgroup interaction was not significant, indicating that the treatment benefit was similar in both PFI subgroups. Safety data in the PFI subgroups were consistent with the overall study population, as previously reported.
Conclusion: In ARIEL3, rucaparib maintenance treatment significantly improved PFS vs placebo in all cohorts, irrespective of PFI subgroup. The magnitude of treatment effect was similar for both PFI subgroups.
Disclosure: ARC: AstraZeneca, Roche, Clovis AMO: Clovis, Amgen, Immunovaccine, Verastem, AstraZeneca, WebRx DL: Clovis, AstraZeneca, ImmunoGen, Merck, PharmaMar, Roche, Takeda, Tesaro CA: Clovis, Mateon, Bayer, Cerulean, Tesaro, VentiRx AO: Clovis, AstraZeneca, ImmunoGen, Genmab/Seattle Genetics, PharmaMar, Roche, Tesaro AD: Precision Oncology Australia, Shire

Pharmaceuticals, Specialised Therapeutics Australia NC: Clovis, Advaxis, AstraZeneca, BIOCAD, MSD, Pfizer, PharmaMar, Roche, Takeda, Tesaro JIW: AbbVie, AstraZeneca GS: Clovis, AstraZeneca, PharmaMar, Roche, Tesaro AL: Clovis, Pfizer, PharmaMar, GamaMabs, Merus, AstraZeneca RWH: Clovis, AstraZeneca, Tesaro MAG: Clovis, AstraZeneca, PharmaMar, Roche PCF: Clovis, AstraZeneca JCG: AstraZeneca, BMS, Janssen, Ipsen, MSD, Astellas DMO'M: Clovis, AstraZeneca, Gynecologic Oncology Group, Janssen, Myriad, Tesaro, Amgen, ImmunoGen, AbbVie, Ambry, Health Analytics, Agenus, Ajinomoto, Array BioPharma, BMS, ERGOMED Clinical Research, Exelixis, Genentech, GSK, INC Research, inVentiv Health Clinical, Ludwig Institute for Cancer Research, Novartis, PRA International, Regeneron, Serono, Stemcentrx, TRACON DKA: Morphotek, Clovis, Advaxis, AstraZeneca, Pfizer, Syndax, Tesaro SB: Clovis, AstraZeneca, ImmunoGen, GamaMabs, Merck Serono, PharmaMar, Roche, Seattle Genetics, Tesaro JG-D: AstraZeneca, Clovis, Genentech/Roche, Janssen EMS: nothing to disclose TC, LM, SG: Clovis RLC: Clovis, AbbVie, AstraZeneca, Esperance, Janssen, Merck, Millennium, OncoMed, Roche/Genentech, Bayer, GamaMabs, Genmab, Gradalis, Pfizer, Tesaro JAL: Clovis, AstraZeneca, Pfizer, Artios Pharma, Cristal Therapeutics, MSD, Regeneron, Roche, Seattle Genetics, Tesaro

Patient-centred outcomes in the Phase 3 study ARIEL3 of rucaparib maintenance treatment in patients with platinum-sensitive, recurrent ovarian carcinoma: post hoc exploratory analyses by BRCA mutation status and patient age

N. Colombo[1], A.M. Oza[2], D. Lorusso[3], C. Aghajanian[4], A. Oaknin[5], A. Dean[6], J.I. Weberpals[7], A.R. Clamp[8], G. Scambia[3], A. Leary[9], R.W. Holloway[10], M. Amenedo Gancedo[11], P.C. Fong[12], J.C. Goh[13,14], D.M. O'Malley[15], D.K. Armstrong[16], S. Banerjee[17], J. García-Donas[18], E.M. Swisher[19], J. Meunier[20], T. Cameron[21], L. Maloney[22], S. Goble[23], J. Bedel[24], R.L. Coleman[25], J.A. Ledermann[26]
[1]Gynecologic Cancer Program, European Institute of Oncology and University of Milan-Bicocca, Milan, Italy, [2]Division of Medical Oncology and Hematology, Princess Margaret Cancer Centre, University Health Network, Toronto, ON, Canada, [3]Gynecologic Oncology Unit, Fondazione Policlinico Universitario A. Gemelli IRCCS, Rome, Italy, [4]Gynecologic Medical Oncology, Memorial Sloan Kettering Cancer Center, New York, NY, United States, [5]Medical Oncology Department, Vall d'Hebron University Hospital, Vall d'Hebron Institute of Oncology (VHIO), Barcelona, Spain, [6]Department of Oncology, St John of God Subiaco Hospital, Subiaco, WA, Australia, [7]Division of Gynecologic Oncology, Ottawa Hospital Research Institute, Ottawa, ON, Canada, [8]Department of Medical Oncology, The Christie NHS Foundation Trust and University of Manchester, Manchester, United Kingdom, [9]Gynecological Unit, Gustave Roussy Cancer Center, INSERM U 981, and Groupe d'Investigateurs Nationaux pour l'Etude des Cancers Ovariens (GINECO), Villejuif, France, [10]Department of Gynecologic Oncology, Florida Hospital Cancer Institute, Orlando, FL, United States, [11]Medical Oncology Department, Oncology Center of Galicia, La Coruña, Spain, [12]Medical Oncology Department, Auckland City Hospital, Grafton, Auckland, New Zealand, [13]Department of Oncology, Cancer Care Services, Royal Brisbane and Women's Hospital, Herston, [14]University of Queensland, St Lucia, QLD, Australia, [15]Clinical Research Gynecologic Oncology, The Ohio State University, James Cancer Center, Columbus, OH, [16]Department of Medical Oncology, Johns Hopkins University Medical Center, Baltimore, MD, United States, [17]Medical Oncology Service, HM Hospitales—Centro Integral Oncológico Hospital de Madrid Clara Campal, Madrid, Spain, [18]Gynaecology Unit, The Royal Marsden NHS Foundation Trust, London, United Kingdom, [19]Division of Gynecologic Oncology, University of Washington, Seattle, WA, United States, [20]Modus Outcomes, Lyon, France, [21]Clinical Science, Clovis Oncology UK Ltd., Cambridge, United Kingdom, [22]Clinical Development, [23]Biostatistics, Clovis Oncology, Inc., Boulder, CO, United States, [24]Pricing & Market Access - Europe, Clovis Oncology Switzerland GmbH, Zurich, Switzerland, [25]Department of Gynecologic Oncology and Reproductive Medicine, The University of Texas MD Anderson Cancer Center, Houston, TX, United States, [26]Department of Oncology, UCL Cancer Institute, University College London and UCL Hospitals, London, United Kingdom

Introduction / Background: Maintenance therapy for recurrent ovarian cancer is intended to extend progression-free survival (PFS) without compromising patient quality of life; therefore, the clinical benefits of prolonged PFS should be evaluated in the context of toxicities that may compromise

patients' wellbeing. In ARIEL3 (CO-338-014; NCT01968213), rucaparib significantly improved PFS vs placebo in all predefined patient cohorts regardless of biomarker status (Coleman et al. *Lancet*. 2017;390:1949-61) or age (Ledermann et al. Presented at SGO 2019; abst 4). This post hoc exploratory analysis examined quality-adjusted PFS (QA-PFS) and quality-adjusted time without symptoms or toxicity (Q-TWiST) in patients from ARIEL3, including the subgroup of patients with a *BRCA* mutation and subgroups based on patient age.

Methodology: Patients were randomised 2:1 to oral rucaparib (600 mg BID) or placebo. QA-PFS and Q-TWiST were analysed in patients with a *BRCA* mutation (germline, somatic, or origin unknown), the ITT population (ie, all randomised patients), and subgroups of the ITT population defined by patient age at baseline (< 65, 65-74, or ≥75 years). Q-TWiST was performed using 2 sets of treatment-emergent adverse events (TEAEs): all grade ≥3 TEAEs and grade ≥2 TEAEs of nausea, vomiting, fatigue, and asthenia only.

Results: The visit cutoff date for these analyses was 15 April 2017. QA-PFS, Q-TWiST considering grade ≥3 TEAEs, and Q-TWiST considering select grade ≥2 TEAEs were significantly longer with rucaparib than placebo in patients with a *BRCA* mutation and in the ITT population (Table). Across all age subgroups, QA-PFS and Q-TWiST (both analyses) were significantly longer with rucaparib than placebo (Table).

[Table]

Conclusion: In the ITT population, *BRCA*-mutant subgroup, and age subgroups analysed, the quality-adjusted analyses, which incorporated patient-centred perspectives, confirmed the benefit of rucaparib vs placebo.

			Rucaparib vs placebo					
			QA-PFS[a]		Q-TWiST[b] (grade ≥3 TEAEs)		Q-TWiST[b] (select grade ≥2 TEAEs[c])	
**Cohort**	**Rucaparib, n**	**Placebo, n**	**Mean, mo**	**Mean difference (95% CI)**	**Mean, mo**	**Expects mean difference (95% CI)**	**Mean, mo**	**Expected mean difference (95% CI)**
BRCA	130	66	15.28 vs	9.37 (6.65-	16.42 vs	9.73 (7.10-	16.24 vs	9.56 (6.99-

mutant			5.92	11.85)	6.70	11.94)	6.68	11.81)
ITT population	375	189	12.02 vs 5.74	6.28 (4.85-7.47)	13.32 vs 6.44	6.88 (7.71-8.23)	13.16 vs 6.40	6.77 (5.64-8.14)
Subgroup analysis by patient age (ITT population)								
<65 years	237	117	12.70 vs 6.02	6.68 (4.95-8.54)	13.81 vs 6.54	7.27 (5.49-8.96)	13.70 vs 6.53	7.17 (5.47-8.88)
65-74 years	113	64	10.73 vs 5.36	5.37 (2.84-7.34)	12.20 vs 6.20	6.00 (3.28-8.08)	11.99 vs 6.14	5.85 (3.16-7.88)
≥75 years	25	8	9.51 vs 4.77	4.74 (1.93-7.19)	11.75 vs 6.06	5.69 (1.02-8.50)	11.58 vs 6.05	5.53 (0.90-8.32)
Visit cutoff 15 April 2017.<br>[a]QA-PFS was calculated as PFS function × EQ-5D index score function.<br>[b]Q-TWiST was calculated as (μTOX × TOX) + TWiST.<br>[c]Nausea, vomiting, fatigue, and asthenia.<br>CI, confidence interval; TOX, mean duration with AEs; TWiST, mean duration without AEs or symptoms of progression; μTOX, standarised mean EQ-5D index score.								

[Table]

Disclosure: NC: Clovis, Advaxis, AstraZeneca, BIOCAD, MSD, Pfizer, PharmaMar, Roche, Takeda, Tesaro AMO: Clovis, Amgen, Immunovaccine, Verastem, AstraZeneca, WebRx DL: Clovis, AstraZeneca, ImmunoGen, Merck, PharmaMar, Roche, Takeda, Tesaro CA: Clovis, Mateon, Bayer, Cerulean, Tesaro, VentiRx AO: Clovis, AstraZeneca, ImmunoGen, Genmab/Seattle Genetics, PharmaMar, Roche, Tesaro AD: Precision Oncology Australia, Shire, Specialised Therapeutics Australia JIW: AbbVie, AstraZeneca ARC: AstraZeneca, Roche, Clovis GS: Clovis, AstraZeneca, PharmaMar, Roche, Tesaro AL: Clovis, Pfizer, PharmaMar, GamaMabs, Merus, AstraZeneca RWH: Clovis, AstraZeneca, Tesaro MAG: Clovis, AstraZeneca, PharmaMar, Roche PCF: Clovis, AstraZeneca JCG: AstraZeneca, BMS, Janssen, Ipsen, MSD, Astellas DMO'M: Clovis, AstraZeneca, Gynecologic Oncology Group, Janssen, Myriad, Tesaro, Amgen, ImmunoGen, AbbVie, Ambry, Health Analytics, Agenus, Ajinomoto, Array, BMS, ERGOMED Clinical Research, Exelixis, Genentech, GSK, INC Research, inVentiv Health Clinical, Ludwig Institute for Cancer Research, Novartis, PRA International, Regeneron, Serono, Stemcentrx, TRACON DKA: Morphotek, Clovis, Advaxis, AstraZeneca, Pfizer, Syndax, Tesaro SB: Clovis, AstraZeneca, ImmunoGen, GamaMabs, Merck Serono, PharmaMar, Roche, Seattle Genetics, Tesaro JG-D: AstraZeneca, Clovis, Genentech/Roche, Janssen EMS: nothing to disclose JM: Modus Outcomes TC, LM, SG, JB: Clovis RLC: Clovis, AbbVie, AstraZeneca, Esperance, Janssen, Merck, Millennium, OncoMed, Roche/Genentech, Bayer, GamaMabs, Genmab, Gradalis, Millennium, Pfizer, Tesaro JAL: Clovis, AstraZeneca, Pfizer, Artios Pharma, Cristal Therapeutics, Merck/MSD, Regeneron, Roche, Seattle Genetics, Tesaro

Dilution of molecular-pathologic gene signatures (MPG) by parameter unrelated to tumor biology (UTB) may prevent prediction of the resection status after debulking surgery in patients with advanced ovarian cancer

F. Heitz[1,2], S. Kommoss[2,3], R. Tourani[4], A. Grandelis[5], L. Uppendahl[5], C. Aliferis[4], A. Burges[2,6], C. Wang[7], U. Canzler[2,8], J. Wang[4], A. Belau[2,9], S. Prader[1], L. Hanker[2,10], S. Ma[4], B. Ataseven[1,6], F. Hilpert[2,11], S. Schneider[1], J. Sehouli[12], R. Kimmig[2,13], C. Kurzeder[14,15], B. Schmalfeldt[2,16,17], I. Braicu[12], P. Harter[1,2], S.C. Dowdy[7], B.J.N. Winterhoff[5], J. Pfisterer[2,18], A. du Bois[1,2]
[1]Klinik für Gynäkologie und Gynäkologische Onkologie, Kliniken Essen-Mitte, Essen, [2]AGO Study Group, Wiesbaden, [3]Department of Women's Health, Tuebingen University Hospital, Tuebingen, Germany, [4]Institute for Health Informatics (IHI), Academic Health Center, University of Minnesota, Minneapolis, [5]Department of Gynecology, Obstetrics and Women's Health, Division of Gynecologic Oncology, University of Minnesota, Minnesota, MN, United States, [6]Department of Gynecology, University Hospital Munich-Großhadern, Munich, Germany, [7]Division of Gynecologic Surgery,

Department of Obstetrics and Gynecology, Mayo Clinic Hospital, Rochester, MN, United States, [8]Department of Gynecology and Obstetrics, Technische Universität Dresden, Dresden, [9]Klinik und Poliklinik für Frauenheilkunde und Geburtshilfe, Ernst Moritz Arndt Universität Greifswald, Greifswald, [10]Klinik für Frauenheilkunde und Geburtshilfe, University of Schleswig-Holstein, Lübeck, [11]Department for Tumortherapy, Krankenhaus Jerusalem Hamburg, Hamburg, [12]Department of Gynecology, Charité - Universitätsmedizin Berlin, Humboldt-Universität zu Berlin, Berlin Institute of Health, Berlin, [13]Department of Gynecology and Obstetrics, University of Duisburg-Essen, Essen, Germany, [14]Department for Gynecology, Universitätsspital Basel, Basel, Switzerland, [15]Department of Obstrics and Gynecology, Universtity of Ulm, Ulm, [16]Zentrum für Department of Gynecology, University Medical Center Hamburg-Eppendorf, Hamburg, [17]Department of Gynecology and Obstetrics, Technical University of Munich, Munich, [18]Gynecologic Oncology Center, Kiel, Germany

Introduction / Background: Predicting surgical outcome could improve individualizing treatment strategies for pts with AOC. Several earlier papers have proposed that debulking signatures using RNA expression analyses might help in this regard.
Methodology: FFPE tumor tissue of FIGO stage IIIC/IV pts of AGO-OVAR 11 were used to generate whole-exome data. Previously identified molecular signatures (MS), including TCGA molecular subtype and three other published MPG, were tested. We used state-of the art biostatistical approaches for analyses. A theoretical model using data of a tertiary gynecologic cancer center was implemented to evaluate the impact of predictive factors for RD not directly related to tumor biology on the performance of a debulking signature model that predicts RD status (RD0 and RD< 1).
Results: Of the 266 pts that met inclusion criteria, 104 (39.1%) underwent complete resection. Previously reported MPG did not predict RD in this cohort. Best Area under the Curve (AUC) was generated using LR: 0.53 ± 0.04. Similarly, TCGA molecular subtypes (AUC=0.56 ± 0.04), an independent de novo signature (AUC=0.51 ± 0.04), and the total gene expression data set using all 21,000 genes (AUC=0.55 ± 0.03) were not able to predict RD status. We identified reasons unrelated to tumor biology that serve as potential limiting factors in the ability to use MS for predicting RD. Even in a centre with a complete resection rate higher than 65% in all comers, a debulking signature which perfectly predicts RD (100%) would have a predictive performance of only AUC 0.83, due to UTB.
Conclusion: Previously identified MPG cannot be generalized. Our theoretical model showed that factors unrelated to tumor biology limit the ability to identify a molecular debulking signature for RD. UTB may be the main obstacle to predict surgical outcome in an all comer Population.
Disclosure: FH: Advisory board: Roche, Tesaro, Honoraria: AstraZeneca, Roche, Tesaro, Clovis, travel/accommodation expenses: PharmaMar; Tesaro; SK: none ; RT: none; AG: none; LU: none; CA: none; AB:none; CW: none; UC: Advisory board/honoraria: Roche, Astra Zeneca; JW:none; AB: none; SP: none; LH:none; SM: none; BA: Advisory board: Roche, Tesaro, Amgen; honoraria: Celgene, Clovis, Astra Zeneca; travel/accommodation expenses: PharmaMar; FH: Honoraria: Roche, Tesaro, Clovis, Astra Zeneca, PharmaMar travel/ accommodations/ expenses: Astra Zeneca, Pharmamar; SS: none; JS:; RK: Advisory Role: Medtronic, Roche, Teva; Honoraria: AstraZeneca, Intuitive Surgical, Prostrakan, RIEMSER; travel/ accommodations/ expenses: Cambridge Medical Robotics; CK: none; BS: none; IB: Honoraria: Roche Pharma, CLOVIS, Tesaro, AstraZeneca, Seattle Genetics, Amgen; PH: Advisory Board: Astra Zeneca, Roche, Tesaro, Lilly, Clovis, Immunogen, MSD/Merck; Honoraria: Astra Zeneca, Roche, Sotio, Tesaro, Stryker, ASCO, Zai Lab, MSD; Research funding (Inst): Astra Zeneca, Roche, GSK, Boehringer Ingelheim, Medac, DFG, European Union, DKH, Tesaro, Genmab; SD: none; BW: none; JP: Advisory board: Clovis, Roche, AstraZeneca, Tesaro; Honoraria: Roche, AstraZeneca, Tesaro, Amgen, Clovis, MSD; travel/ accommodations/ expenses: Roche, Tesaro; AdB: advisory board: Roche, Astra Zeneca, Tesaro, Clovis, Pfizer, Biocad, Genmab, Seattle Genetics, MSD; Honoraria: Roche, Astra Zeneca, Tesaro, Clovis, Pfizer, Biocad, Genmab, Seattle Genetics, MSD

Risk factors for high-grade serous carcinoma in *BRCA1/2* mutation carriers undergoing risk-reducing salpingo-oophorectomy - a preliminary report from the nationwide Dutch HEBON study

J. Brouwer[1], H. Hollema[1], M. Rookus[2], M. van Beurden[3], H.H. van Boven[3], J. Bulten[4], H.C. van

Doorn[5], E.B.L. van Dorst[6], K.N. Gaarenstroom[7], J. De Hullu[4], B. Slangen[8], G.H. De Bock[1], M.J.E. Mourits[1]
[1]University Medical Center Groningen, Groningen, [2]Netherlands Cancer Institute, [3]Antoni van Leeuwenhoek Hospital, Amsterdam, [4]Radboud University Medical Center, Nijmegen, [5]Erasmus University Medical Center, Rotterdam, [6]University Medical Center Utrecht, Utrecht, [7]Leiden University Medical Center, Leiden, [8]Maastricht University Medical Center, Maastricht, The Netherlands

Introduction / Background: *BRCA1/2* mutation carriers are advised to undergo timely risk-reducing salpingo-oophorectomy (RRSO) to prevent high-grade serous carcinoma (HGSC). However, some women are diagnosed with serous tubal intra-epithelial carcinoma (STIC) or HGSC at time of RRSO while others develop HGSC after RRSO. We studied occurrence and risk factors for STIC and HGSC in a prospective nationwide cohort of *BRCA1/2* mutation carriers.
Methodology: In the prospective HEBON cohort, asymptomatic women with a pathogenic *BRCA1* or *BRCA2* mutation who underwent RRSO between 1995 and 2014 were linked to the national pathology database and followed until 2018. Clinical and histopathological data were retrieved for multivariate logistic and Cox regression analyses. STIC and HGSC cases are currently being revised and another 732 women with RRSOs until 2018 are additionally being included.
Results: Among 1,253 *BRCA1* and 758 *BRCA2* carriers, 1,539 (77%) had their RRSO before 2010 and 1,218 (61%) of fallopian tubes were totally embedded. Four women were diagnosed with STIC and 28 with HGSC at time of RRSO (1.4%). Twenty HGSCs (71%) had coexisting STIC. STIC and HGSC were associated with age at time of RRSO (OR=1.07 per year), *BRCA1* mutation (OR=3.0), total embedding (TE) of fallopian tubes (OR=8.9) and duration of oral contraceptive use (OR=0.9 per year). Among the 1,979 women with histological normal RRSO, 14 developed HGSC within 1.8 and 10.0 years after RRSO (0,9%). Age at time of RRSO (HR=1.06 per year) and *BRCA1* mutation (HR=8.8) increased HGSC risk whereas TE of fallopian tubes (HR=0.21) reduced HGSC risk after RRSO.
Conclusion: Incidence of STIC and HGSC is low and is associated with several clinical and histopathological factors. These data suggest that most HGSCs that develop after RRSO originate from occult STIC or tubal HGSC in RRSOs that were inadequately analyzed due to prior lack of knowledge of the importance of the fallopian tube.
Disclosure: Nothing to disclose

Oral Communication 4 - Gynaecological Cancer

Incidence and clinical presentation of lymphoedema in legs after endometrial cancer surgery - The LASEC (Lymphoedema After Surgery of Endometrial Cancer) trial

<u>M. Wedin</u>[1], K. Stålberg[2], J. Marcickiewicz[3], E. Ahlner[4], Å. Åkesson[5], P. Rosenberg[4], P. Kjølhede[6]
[1]Obstetrics and Gynecology, Linköping University Hospital, Linköping, [2]Women and Children´s Health, Uppsala University, Uppsala, [3]Varberg Central Hospital, Varberg, [4]Oncology, Linköping University Hospital, Linköping, [5]Obstetrics and Gynecology, Sahlgrenska Academy at University of Gothenburg, Gothenburg, [6]Obstetrics and Gynecology, Linköping University, Linköping, Linköping, Sweden

Introduction / Background: Development of lymphoedema after treatment of endometrial cancer (EC), is still an unexplored area. The aim of this study was to determine the incidence and clinical presentation of lymphoedema of the legs after surgery for EC including pelvic and para-aortic lymphadenectomy one year postoperatively.
Methodology: An observational multicentre study conducted in 14 centres in Sweden recruited 262 women. 130 women with high-risk early-stage EC underwent surgery including pelvic and para-aortic lymphadenectomy and 132 with low-risk EC had surgery without lymphadenectomy. Systematic leg circumference measurements enabling calculation of leg volumes were performed on four occasions; preoperatively, 4-6 weeks, 6 months, and 1 year postoperatively. Clinical evaluation of lymphoedema by a lymph therapist was conducted on the same occasion. A 10% increase in volume from baseline was categorised as lymphoedema.
Results: The incidence of lymphoedema increased from 1.8% at the 4-6 weeks assessment to 15% one year postoperatively when lymphadenectomy had been performed. The corresponding figures in the non-lymphadenectomy group were 1.7% to 9.3%. There was no significant difference in incidence

on any occasion between the lymphadenectomy and non-lymphadenectomy group (Table 1). In women with more than 10% increase in leg volume, 60.7% was objectively clinically graded as normal by lymph therapists. The distribution of the occurrence of lymphoedema differed significantly between those who had less than 10% increase in leg volume and those who had ≥ 10% (Table 2).
Conclusion: The incidence of lymphoedema in legs caused by lymphadenectomy after surgery for EC seemed to be low when assessed by leg volume measurements. Clinically grading of lymphoedema seemed to be underestimated by lymph therapists especially in those who had ≥ 10% increase in leg volume. This raise concerns about the methods of determining and reporting lymphoedema.

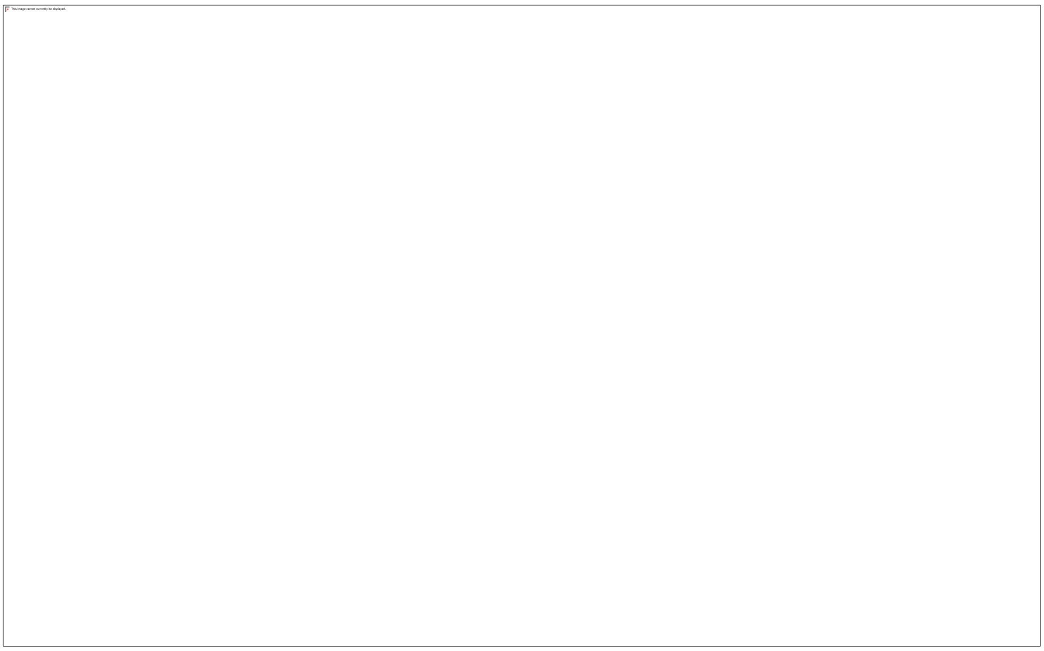

[Table 1 and 2]

Disclosure: Nothing to disclose

Enhanced recovery after surgery in advanced ovarian cancer: a prospective randomized trial

J.L. Sánchez-Iglesias[1], M. Carbonell Socias[1], A. Pérez Benavente[1], N.R. Gómez-Hidalgo[1], S. Manrique Muñoz[2], M. García Gorriz[2], R. Burgos Peláez[3], H. Segurola Gurrutxaga[3], P. Gutierrez Barceló[4], C. Pérez Barragan[4], M. Pamies Serrano[4], S. Serrano Castro[4], A.A. Scaillet Hopuberechts[4], A.M. Álvarez López[4], D. Monroy Parada[5], Y. Cossio Gil[5], A. Gil-Moreno[1]
[1]Gynecology Oncology Department, [2]Anesthesia Department, [3]Endocrinology and Nutrition Department, [4]Oncology Nurse Department, [5]Preventive Medicine Department, Vall d´Hebron University Hospital, Barcelona, Spain

Introduction / Background: Enhanced recovery after surgery program (ERAS) had demonstrated to reduce hospitalizations without increasing the rate of complications. However, information on how to apply the program to advanced ovarian cancer patients is still limited. We postulate that the ERAS program improve the postoperative recovery, decreasing the length of stay at hospital, the readmissions and medical cost-related.
Methodology: We designed the PROFAST trial (ClinicalTrials: NCT02172638) a prospective randomized trial. Patients diagnosed with ovarian carcinoma based on clinical or radiological suspicion and ovarian recurrences from 2014 to 2018 at Vall D'Hebron Hospital, were randomized to an

specifically designed ERAS protocol or to a conventional management (CM). Reduction in the median length of stay was considered as a primary outcome. The incidence of perioperative complications and readmissions rated up to 28 days after surgery and medical cost-related were considered as secondary outcomes.
Results: 99 patients were included, 50 patients were randomized to the ERAS group, and 49 to the CM group. Demographic and clinical characteristics, stage at diagnoses and surgical procedures were similar in both groups with no statistically significant difference (p >0.005). The adherence to the ERAS program was > 90% for all required items except for the needed of abdominal drainage. Median length of stay at hospital was 9 days vs 7 days, respectively (p< 0.05). Readmission rates were highly reduced in theERAS group. There were not statistically significant difference in complication rates. The ERAS reduced the medical costs up to 13330 euros.
Conclusion: In the PROFAST trial, ERAS appears to be a safe and effective in reducing the median length of stay at hospital, readmission rates and medical- costs in advanced ovarian cancer surgery.

	Conventional (n=49)	ERAS (n=50)	Total (n=99)	p-Value
Age	59	58	58	0.869
Readmissions	10 (20%)	3 (6%)	13 (13%)	0.033
Major Complications	8(16%)	9(18%)	17(17%)	0.825
Anastomotic Leak	1(2%)	2(4%)	3(3%)	1
Mortality	2(4%)	2(4%)	3(61%)	0.7
Reoperation	5(10%)	4(8%)	9(9%)	0.74
Median Length of Stay (days)	9	7	8	0.009
Medical Costs	276.800	248.500	525.300	Under evaluation

[Table 1. Complications of surgery related]

Disclosure: Nothing to disclose

Trial of intraoperative cell salvage versus transfusion in ovarian cancer (TIC TOC): a multi-centre randomised controlled feasibility study

K. Galaal[1], J. Vickery[2], A. Lopes[3], C. Pritchard[4], A. Barton[5], J. Wingham[3], E.M. Elsa Marques[6], J. Palmer[4], C. Ralph[7], J. Fauld[4], P. Ewings[5], TIC TOC trial study group
[1]Royal Cornwall Hospital NHS Trust | Gynaecology, Royal Cornwall Hospital, Truro, [2]Peninsula Clinical Trials Unit, Plymouth University, Plymouth, [3]Exeter University, Exeter, [4]Research, Development and Innovation, Royal Cornwall Hospitals NHS Trust, Truro, [5]NIHR Research Design Service (South West), Plymouth, [6]Bristol Medical School, University of Bristol, Bristol, [7]Department of Anaesthesia, Royal Cornwall Hospitals NHS Trust, Truro, United Kingdom

Introduction / Background: Ovarian cancer is the leading cause of death from gynaecological cancer, with more than 7000 new cases registered in the UK in 2014. Standard treatment includes surgical resection of all macroscopic tumour, followed by chemotherapy. Surgery can be extensive and associated with substantial blood loss which is conventionally replaced with a donor blood transfusion. While often necessary and lifesaving, the use of donor blood is associated with increased risks of complications and adverse surgical outcomes. Intraoperative cell salvage (ICS) is a blood conservation strategy in which red cells collected from blood lost during surgery are returned to the patient thus minimising the use of donor blood. The aim of this feasibility study was to assess whether ICS is a safe alternative to blood transfusion and if a larger, fully powered trial can successfully be planned and delivered.
Methodology: This is a randomised, controlled multi-centre feasibility trial conducted in four UK acute

NHS hospitals over 12 months. Adult women requiring primary or interval surgery for suspected ovarian cancer were eligible for the trial if CT scan evidence supported FIGO stage III/IV ovarian or primary peritoneal cancer. Participants were randomised 1:1 ratio to receive either intra-operative cell salvage (ICS) reinfusion or donor blood transfusion intra-operatively,with stratification by study site.
Results: 173 women were approached, and 59 took part. In the ICS group, 16 (62%) of the 26 participants received ICS reinfusion. In the donor blood group, 14 of the 29 (48%) received donor blood. Twenty-two (40%) participants (9 ICS, 13 control) experienced infections, mainly wound, urinary tract or chest. Six participants died (2 ICS, 4 control) were all due to disease progression.
Conclusion: We have demonstrated that ICS is safe and acceptable alternative to blood transfusion in ovarian cancer surgery. Further larger randomised trial is needed to determine the long term outcomes.
Disclosure: Nothing to disclose

What is life like after pelvic exenteration? The longitudinal assessment of quality of life after pelvic exenteration for recurrent / persistent gynaecological cancer

A. Fernandes[1], S. Bryan[1], D. Kolomainen[2], R. Phipps[3], K. Power[4], A. Thompson[5], D. Barton[1]
[1]Gynaecology, The Royal Marsden NHS Foundation Trust, [2]Gynaecology, Southend University Hospital NHS Foundation Trust, [3]The Royal Marsden NHS Foundation Trust, [4]Plastic Surgery, [5]Urology, The Royal Marsden NHS Foundation Trust, London, United Kingdom

Introduction / Background: Pelvic exenteration (PE) can result in considerable acute and long-term morbidity. Routine longitudinal assessment of quality of life (QoL) of women with recurrent/persistent gynaecological cancer undergoing/had undergone PE, was implemented in 2012 to evaluate trends in QoL prospectively.
Methodology: The European Organisation for Research and Treatment of Cancer quality of life questionnaire (EORTC QLQ-C30) was completed by women before and/or after PE attending out-patient consultations between 2012 and 2018. Data were collected at various time-points from baseline to up to12 years post-surgery. Descriptive analysis, frequencies and independent chi-square tests were performed.
Results: Forty-two women underwent PE with curative intent, with a mean age of 58 years (SD 10.8) were included, 40 had received pelvic radiotherapy, and 60% had cervical or endometrial cancer. A total PE was performed in 52% (n=22) of women, anterior PE in 28% (N=12) and posterior PE in 20% (n=8). In 31% of total PE cases a double-barrelled stoma was formed.
QoL at baseline was poor, further decreased at 1-month post-surgery, showed a steady improvement after 6 months, which continued long-term (>5 years) both in Global Health status and all functioning scores. With the exception of diarrhoea and insomnia, all symptoms significantly improved 1-month post-surgery. Pain scores halved from baseline to 1-2 years (43.5 to 20). In women with a total PE, those with a double-barrelled stoma had better emotional, social and cognitive functioning compared to those who had a colostomy and urostomy(e.g. $x^2(8, 68) = 17.58$ $p < 0.05$).
Conclusion: PE can improve the QoL of women with recurrent/persistent gynaecological cancer who are symptomatic. In cases of total PE, the double-barrelled stoma have better outcomes compared to two stomas. These findings will better inform women for whom PE is offered as a treatment option. They highlight the importance of continued interdisciplinary collaboration from the planning of surgery to long-term follow-up.

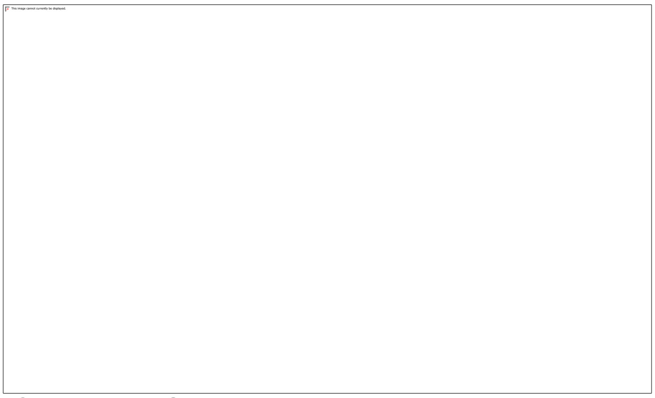

[Global Health Status]

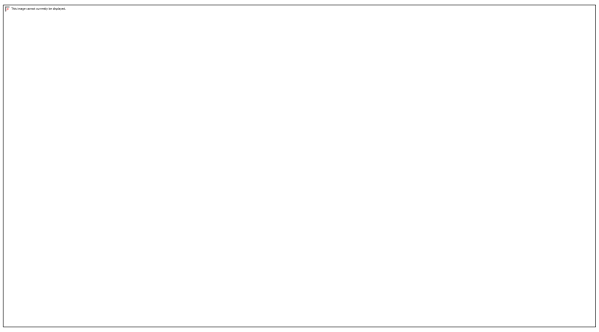

[Functional Scores]

Disclosure: Nothing to disclose

On the value of a prognostic tumour score in locally advanced cervical cancer

J.C. Lindegaard, P. Petric, A.M. Lindegaard, K. Tanderup, L.U. Fokdal
Department of Oncology, Aarhus University Hospital, Aarhus N, Denmark

Introduction / Background: The integral results of clinical examination and magnetic resonance imaging (MRI) in patients with locally advanced cervical cancer (LACC) may provide prognostic information that cannot readily be placed in current staging systems such as proximal versus distal parametrial invasion, unilateral versus bilateral involvement or organ infiltration on MRI. The aim was to investigate the performance of a simple but wide-ranging tumour score for reporting and prognostication.
Methodology: 400 consecutive patients with LACC treated 2005-2018 with external beam radiotherapy +/- concomitant cisplatin and image guided adaptive brachytherapy were analysed. The diagnostic work-up included clinical examination, positron emission tomography-computed tomography (PET/CT) and MRI. International Federation of Gynecology and Obstetrics (FIGO) 2009 stage distribution was IB-IIA 9%, IIB 61% and III-IV 30%. The degree of involvement of 8 anatomical locations (cervix, left parametrium, right parametrium, vagina, bladder, ureters, rectum and uterine corpus) was scored according to a ranked ordinal scale with 0-3 points. The total sum of points constituted the T-score.
Results: The median T-score was 6 (range 0-20). The likelihood of receiving full course concomitant

chemotherapy decreased significantly with T-score (Figure 1), with only about 50% of the patients being able to receive ≥5 courses when T-score= 8. Prediction of the use of interstitial needles for brachytherapy increased sharply with 45% and 90% of patients with T-score of 6 and 12 requiring needles, respectively. Based on the frequency distribution of the T-score, 4 equally sized risk groups were formed: 0-4, 5-6, 7-9 and >9 (Figure 2). The T-score was highly significant in both univariate and multivariate analysis and outperformed FIGO stage for both survival and local control.
Conclusion: The T-score may predict the ability of patients to undergo concomitant chemotherapy and the need for interstitial brachytherapy. In addition, the T-score is a powerful full prognostic factor for survival and local control.

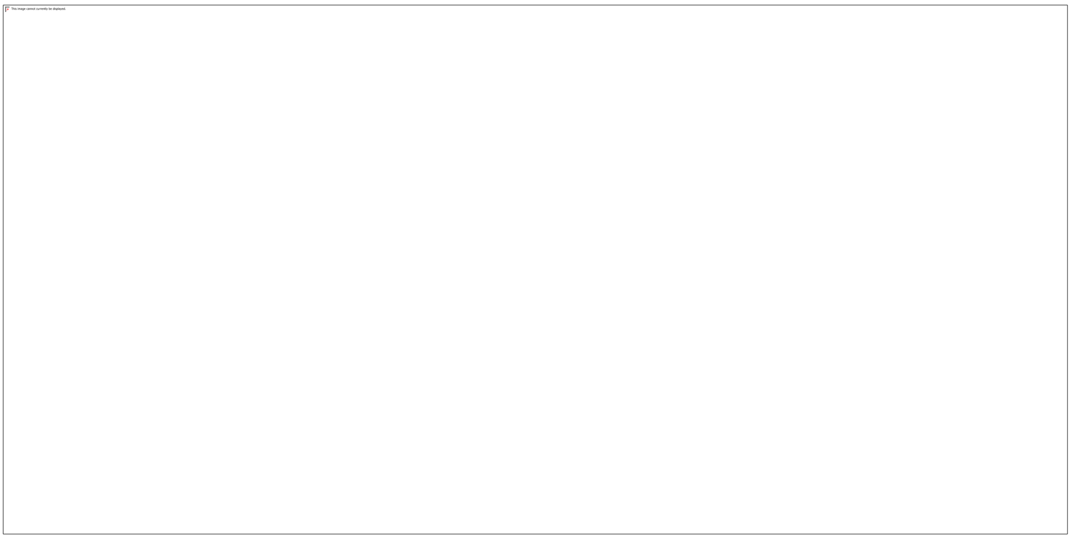

[Figure 1: Logit curves for concomitant chemotherapy (left) and interstitial brachytherapy (right).]

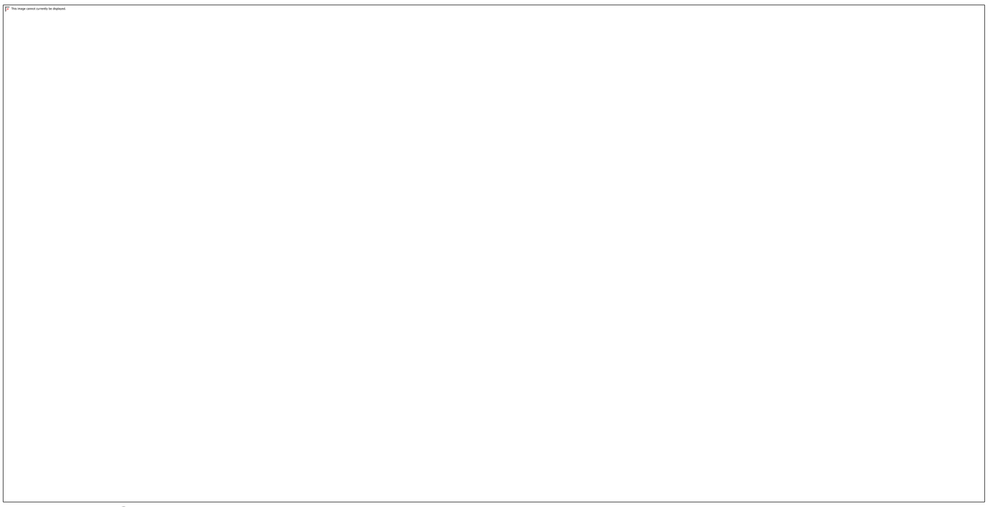

[Figure 2: Overall survival according to T-score for all patients (left) and for stage IIB (right).]

Disclosure: Nothing to disclose

Young Investigators Oral Presentations (ENYGO Session)

Impact of different adjuvant treatment strategies on survival in stage III endometrial cancer: a population-based study

W.J. van Weelden[1], C. Reijnen[1,2], F. Eggink[3], N. de Lange[3], H. Nijman[4], D. Boll[5], M. van der Aa[6], J. Pijnenborg[1,7]
[1]Obstetrics & Gynecology, Radboud University Medical Center, [2]Obstetrics & Gynecology, Canisius-Wilhelmina Hospital, Nijmegen, [3]Obstetrics & Gynecology, University Medical Center Groningen, [4]Obstetrics & Gynecology, University Medical Center Groningen, University of Groningen, Groningen, [5]Obstetrics & Gynecology, Catharina Cancer Institute, Catharina Hospital, Eindhoven, [6]Research, Netherlands Comprehensive Cancer Organisation (IKNL), Utrecht, [7]Radboud Institute for Health Sciences, Nijmegen, The Netherlands

Introduction / Background: Endometrial cancer (EC) is the most common gynecological cancer in the Western world. For those with advanced stage EC outcome is significantly worse despite adjuvant treatment. Different adjuvant treatment strategies for these patients have been compared in the GOG-258 and PORTEC-3 studies. Since EC patients are mainly elderly women with substantial comorbidity, these patients are often underrepresented in clinical trials. The aim of this study is to evaluate outcome in stage III EC patients, according different applied treatment strategies in a population based study.
Methodology: All patients treated for FIGO stage III EC in the period 2005-2015 were extracted from the Netherlands Cancer Registry (NCR). Patients without surgery, and those with residual tumor after surgery were excluded from analyses. The 5-year overall survival was compared for different adjuvant treatment strategies: observation, vaginal brachytherapy (VBT), external beam radiation (EBRT), chemotherapy (CT), and chemoradiation (CRT).
Results: A total of 997 EC patients were eligible for analysis. The mean age at diagnosis was 67, and most patients (68%) were diagnosed with endometrioid histology. Adjuvant treatment was applied in 83%, and consisted of EBRT/VBT (53%), CT (12%), and CRT (15%). Patients >70 years more often received no adjuvant treatment, 23% compared to 13% (< 70). Chemoradiation was more frequent applied for serous (24%) and carcinosarcoma (28%), compared to endometrioid (11%) and clear cell (9%) EC. The 5-year overall survival was significantly better in patients that CRT compared to EBRT, 66% (53-76%) and 58% (49-61%) respectively. This survival benefit for patients treated with adjuvant CRT remained significant in multivariable Cox regression analysis (HR 0.68, 0.49-0.94 95% CI).
Conclusion: This population based study demonstrates the benefit of chemoradiation in FIGO III EC , with a significant better 5-year overall survival compared to external beam radiation.

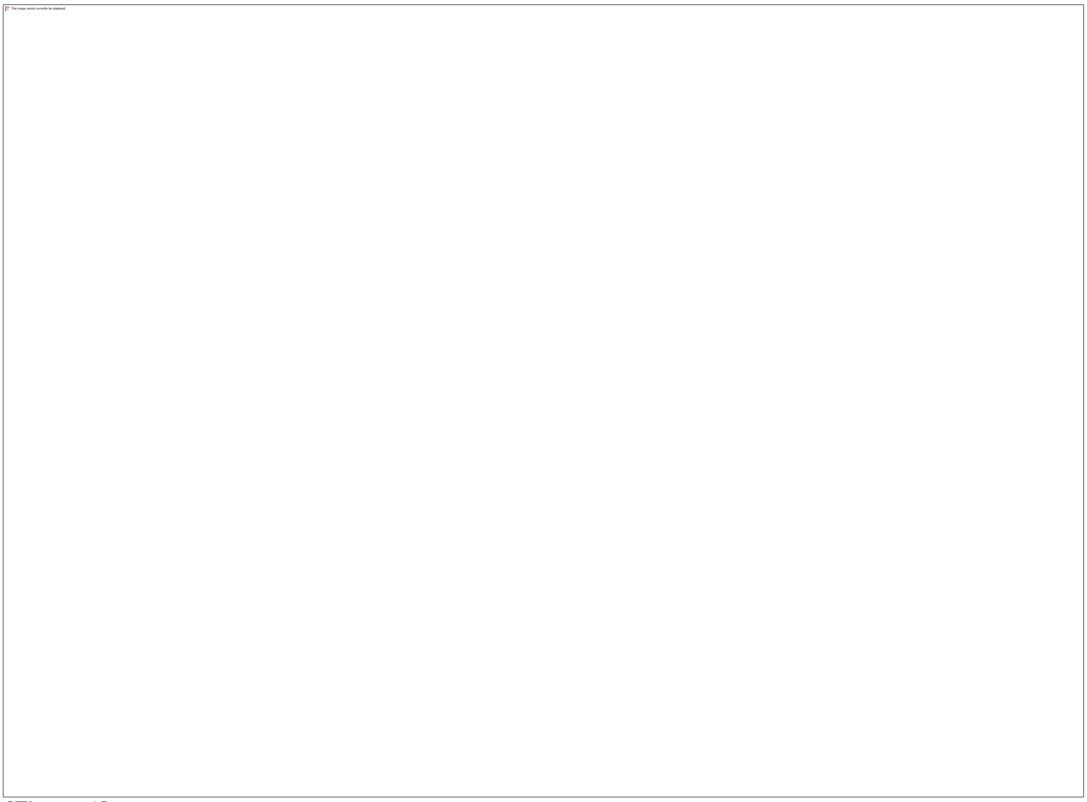

[Figure 1]

Disclosure: Nothing to disclose

Vascular endothelial growth factor receptor 2 (VEGFR2) correlates with long-term survival in patients with advanced high-grade serous ovarian cancer (HGSOC). A study from the tumor bank ovarian cancer (TOC) consortium

J. Guan[1], S. Darb-Esfahani[1], R. Richter[1], E. Taube[1], I. Ruscito[1], S. Mahner[2], L. Woelber[3], K. Prieske[3], N. Concin[4], I. Vergote[5], E. Van Nieuwenhuysen[5], P. Achimas-Cadariu[6], J. Glajzer[1], H. Woopen[1], M. Stanske[1], H. Kulbe[1], C. Denkert[1], J. Sehouli[1], E. Braicu[1]
[1]Charite Medical University of Berlin, Berlin, [2]University Hospital, Ludwig-Maximilians-University Munich, Munich, [3]University-Medical-Center Hamburg-Eppendorf, Hamburg, Germany, [4]Department of Gynecology, Medical University of Innsbruck, Innsbruck, Austria, [5]University Hospital Leuven, Leuven, Belgium, [6]University of Medicine and Pharmacy Iuliu Hatieganu Cluj-Napoca, Cluj-Napoca, Romania

Introduction / Background: The impact of angiogenesis on long-term survival of high-grade serous ovarian cancer (HGSOC) patients remains unclear. This study investigated whether angiogenic markers correlated with 5-year progression-free survival (PFS) ina large cohort ofmatched advanced HGSOC tissue samples.
Methodology: Tumor samples from 124 primary HGSOC patients were retrospectively collected within the Tumor Bank Ovarian Cancer (www.toc-network.de). All patients were in advanced stages (FIGOstage III-IV). No patient had received anti-angiogenesis therapy. The cohort contains 62 long-term survivors and 62 controls matched by age and post-surgical tumor residuals. Long-term survivors were defined as patients with no relapse within 5 years after the end of first-line chemotherapy.

Controls were patients who suffered from first relapse within 6 to 36 monthsafter primary treatment. Samples were assessed for immunohistochemical expression of vascular endothelial growth factor (VEGF) A and VEGF receptor 2 (VEGFR2). Expression profiles of VEGFA and VEGFR2 were compared between the two groups.
Results: Significant correlation betweenVEGFA and VEGFR2 expression was observed (p< 0.0001, Spearman coefficient 0.347).VEGFR2 high expression ($VEGFR2^{high}$) was found more frequently in long-term survivors (77.4%, 48/62) than in controls (51.6%, 30/62, p=0.001), independent of FIGO stage and VEGFA expression in multivariate analysis (p=0.005). Also, $VEGFR2^{high}$was found the most frequently in women with PFS ≥ 10 years (p=0.001) among all 124 patients. However, no significant association was detected between VEGFA expression and 5-year PFS (p=0.075).
Conclusion: VEGFR2 overexpression significantly correlated with long-term PFS in HGSOC patients, independent of age, FIGO stage, tumor residual and VEGFA expression.
Disclosure: Dr. Elena Ioana reports grants, personal fees and other from Roche Pharma, personal fees from Clovis, personal fees and other from TesaroBio, personal fees and other from AstraZeneca, personal fees from Amgen, personal fees from Incyte, grants from Molecular Health, outside the submitted work; Dr. Sven Mahner reports research support, advisory board, honoraria and travel ex-penses from AstraZeneca, Bayer, Boehringer Ingelheim, Clovis, Eisai, Glax-oSmithKline, Jenapharm, Janssen-Cilag, Medac, MSD, Novartis, PharmaMar, Roche, Sensor Kinesis, Teva, Tesaro; Dr. Linn Woelber reports grants from medac oncology, during the conduct of the study; grants, personal fees and non-financial support from medac oncology, person-al fees and non-financial support from Tesaro, personal fees from Roche, Pharma-mar, Tewa, Astra Zeneca, Jenapharm and Janssen-Cilag, outside the submitted work; Dr. Carsten Denkert is the cofounder and shareholder of Sividon Diagnostics and in advisory board of Teva, Roche, AstraZeneca, Celgene, Pfizer, Novartis, Daiichi, and MSD. Other authors have no conflict of interest.

Comparison of survival outcomes in optimally or maximally cytoreducted stage IIIC ovarian high-grade serous carcinoma: women with only peritoneal tumor burden versus women with both peritoneal and lymphogenous dissemination

I. Selcuk[1], M.M. Meydanli[1], I. Yalcin[1], K. Gungorduk[2], S. Akgol[3], H. Celik[4], A. Ayhan[5]
[1]*Gynecologic Oncology, Zekai Tahir Burak Women's Health Training and Research Hospital, Faculty of Medicine, University of Health Sciences, Ankara,* [2]*Gynecologic Oncology, Mugla Sitki Kocman University Training and Research Hospital, Mugla,* [3]*Gynecologic Oncology, Kanuni Sultan Suleyman Teaching and Research Hospital, University of Health Sciences, Faculty of Medicine, Istanbul,* [4]*Gynecologic Oncology, Baskent University Faculty of Medicine, Adana,* [5]*Gynecologic Oncology, Baskent University Faculty of Medicine, Ankara, Turkey*

Introduction / Background: The aim of this study was to analyze the survival outcomes of stage IIIC ovarian high-grade serous carcinoma (HGSC) patients with both peritoneal and lymphatic dissemination (IP(+)/RP(+)) who had undergone maximal or optimal cytoreduction followed by intravenous carboplatin/paclitaxel chemotherapy to those women with stage IIIC ovarian HGSC who have only peritoneal involvement (IP(+)/RP(-)) treated similarly.
Methodology: We performed aretrospective, multicenter study by participation of five gynecologic cancer centers. At first, stage IIIC ovarian HGSC patients were classified asoptimally or maximally debulked cohorts. Then in each cohort, patients were divided into two groups:
1) The IP(+)/RP(-) group included women with transcoelomic spread outside the pelvis with no nodal disease,
2) The IP(+)/RP(+) group included patients with transcoelomic dissemination outside the pelvis in addition to positive nodal status.
Survival outcomes were compared between the two groups in each cohort.
Results: A total of 405 ovarian HGSC patients were analyzed. In the optimally debulked cohort (n=257), the median progression-free survival (PFS) and overall survival (OS) for the IP(+)/RP(-) group (n=69) was 24 and 57 months, respectively compared to 21 and 58 months in the IP(+)/RP(+) group (n=188) (p=0.78, and p= 0.40; respectively). In the maximally debulked cohort (n=148), the median PFS and OS for the IP(+)/RP(-) group (n=55) was 35 and 63 months, respectively compared

to 25 and 51 months in the IP(+)/RP(+) group (n=93) (p=0.49, and p= 0.31; respectively).
Conclusion: Our findings indicated no survival difference between the IP(+)/RP(-) and the IP(+)/RP(+) groups after maximal or optimal cytoreduction.
Disclosure: Nothing to disclose

Detection of mRNA of CDKN2A, MKi67 and TOP2A in liquid-based cytology as biomarker of high grade lesions of uterine cervix

N. Carreras Diéguez[1], C. Martí[1], A. Rodríguiez-Trujillo[1], E. Barnadas[2], A. Torné[1], J. Ordi[3], M. del Pino[1]
[1]Gynecology and Obstetrics, Hospital Clinic Barcelona, [2]IDIBAPS, Hospital Clínic de Barcelona, [3]Pathology Department, Hospital Clinic Barcelona, Barcelona, Spain

Introduction / Background: It has been suggested that the evaluation of the expression of host-cell genes involved in cervical carcinogenesis induced by high-risk human papillomavirus (HR-HPV) may be useful to identify patients harbouring high-grade squamous intraepithelial lesions (HSIL). We aimed to evaluate the performance of mRNA detection of three biomarkers (CDKN2A, MKi67, TOP2A) in liquid-based cytology (LBC) to identify HSIL.
Methodology: Observational prospective study including 250 patients referred to colposcopy. Patients underwent Pap-test, HR-HPV testing, colposcopy, and at least one biopsy. CDKN2A, MKi67 and TOP2A mRNA was detected in LBC specimens by reverse transcription and quantitative polymerase chain reaction.
Among HR-HPV positive patients, 49 showed negative pathological findings, 38 harboured low-grade squamous intraepithelial lesions (LSIL), 105 harboured HSIL, and 17 showed discordant results between cytology (HSIL) and biopsy (LSIL). Thirty-five patients were HR-HPV negative and presented negative biopsies (control group).
mRNA expression was quantified using *ΔCycle threshold* and compared between groups. We performed ROC analysis and computed sensitivity and specificity of mRNA expression for HSIL detection.
Results: Two hundred and forty-three (97.2%) LBC samples yielded adequate material for mRNA determination.
We found higher expression of the three biomarkers in HSIL group compared to control group ($p < 0.05$). TOP2A expression was higher in HSIL group compared with women with positive HR-HPV testing and negative biopsy ($p < 0.05$). Patients with discordant results between cytology and biopsy, showed higher expression of all biomarkers compared with the control group ($p < 0.05$).
Table 1 shows area under the curve (AUC), sensitivity and specificity of the biomarkers for HSIL detection, at the optimal cut-off point according to ROC curve.

	AUC (95% CI)	Cut-off point (ΔCycle threshold)	Sensitivity (95% CI)	Specificity (95% CI)
CDKN2A	0.72 (0.61 - 0.83)	-1.04	59.8% (48.9 - 69.7)	80.0% (62.7 - 90.5)
MKi67	0.74 (0.64 - 0.84)	3.43	64.5% (54.4 - 73.5)	76.7% (59.1- 88.2)
TOP2A	0.72 (0.62 - 0.81)	3.55	72.0% (62.2 - 80.1)	66.7% (48.8 - 80.8)

[Table 1. Area under the curve (AUC), sensitivity and specificity of CKN2A, MKi67 and TOP2A for HSIL detection.]

Conclusion: Detection of mRNA of CKN2A, MKi67 and TOP2A in LBC samples showed higher sensitivity than Pap-test to identify women with HSIL. Further studies are needed to confirm that host-cell mRNA detection might have a role in secondary prevention of cervical cancer.
Disclosure: Nothing to disclose

The role of the vaginal microbiota in the regression of untreated CIN2 lesions

A. Mitra[1], D. Macintyre[1], G. Ntritsos[2], A. Smith[3], K. Tsilidis[1,2], J. Marchesi[1,3], P. Bennett[1], A.-B. Moscicki[4], M. Kyrgiou[1]
[1]*Imperial College London, London, United Kingdom,* [2]*University of Ioannina, Ioannina, Greece,* [3]*Cardiff University, Cardiff,* [4]*University of California Los Angeles, Los Angeles, United Kingdom*

Introduction / Background: Emerging evidence suggests associations between the vaginal microbiota (VMB) composition, HPV infection, and cervical intra-epithelial neoplasia (CIN), however, causal inference remains uncertain. The use of longitudinal cohorts can enable temporal assessment of the relationship between VMB and the natural history of CIN. We aimed to assess whether VMB composition associates with rates of progression, persistence or clearance in a cohort of young women with histologically confirmed untreated CIN2 lesions.
Methodology: 16S rRNA was extracted and sequenced from serial collected liquid based cytology samples of young women with histologically-proven CIN2 that was managed conservatively in Northern California, USA over a 24-month period.
Results: Of the 87 women in the cohort, 48.3% had regressed by 12 months (42/87), 72.4% (63/87) by 24 months. High-diversity, *Lactobacillus* spp. depleted, VMB composition at time of CIN2 diagnosis was associated with higher rates of disease persistence at 12 months, compared to those with *Lactobacillus* spp. dominance at baseline. Relative abundances of taxa including *Megasphaera* spp. (p=0.0004), *Prevotella timonensis* (p=0.015) and *Gardnerella vaginalis* (p=0.036) were higher in women with persistent disease at 12 months compared to those who regressed. A similar trend was seen by 24-month follow-up, but was not statistically significant. The rate of CIN2 regression was slower in women with *Lactobacillus* spp. depletion at baseline.
Conclusion: Depletion of *Lactobacillus* spp. and presence of specific anaerobic taxa are associated with CIN2 persistence and slower regression. Further research on the impact of VMB on the natural history of CIN may help identify VMB compositions and specific taxa associated with increased risk of progression and thus offer rational targets for the development of new prevention and treatment strategies.
Disclosure: Nothing to disclose

Application of precision medicine: can molecular risk stratification provide new management options for low stage endometrioid ovarian carcinoma?

P. Krämer[1], A. Talhouk[2], T. Bosse[3], F. Heitz[4,5], S. Prader[4], N. Singh[6], F. Kommoss[7], B. Krämer[1], A. Hartkopf[1], S. Brucker[1], J. McAlpine[2,8], M. Koebel[9], M. Anglesio[2,10], S. Kommoss[1]
[1]*Department of Women's Health, Tuebingen University Hospital, Tuebingen, Germany,* [2]*Department of Obstetrics and Gynecology, University of British Columbia, Vancouver, BC, Canada,* [3]*Department of Pathology, Leiden University Medical Centre, Leiden, The Netherlands,* [4]*Department of Gynecology and Gynecologic Oncology, Kliniken Essen Mitte, Essen,* [5]*Department of Gynecology and Gynecologic Oncology, Charité Campus Virchow-Klinikum, Berlin, Germany,* [6]*Department of Pathology, Barts Health NHS Trust, London, United Kingdom,* [7]*Institute of Pathology, Heidelberg University Hospital, Heidelberg, Germany,* [8]*Department of Molecular Oncology, BC Cancer Research Centre, Vancouver, BC,* [9]*Department of Pathology and Laboratory Medicine, University of Calgary and Calgary Laboratory Services, Calgary, AB,* [10]*Department of Pathology and Laboratory Medicine, University of British Columbia, Vancouver, BC, Canada*

Introduction / Background: Endometrioid ovarian carcinoma (ENOC) is generally associated with a more favorable prognosis compared to other ovarian carcinoma histotypes. Nonetheless, patients are still treated according to a "one size fits all" approach. While tumor staging offers some stratification,

the development of personalized treatment concepts remain elusive. Our group has recently shown that the Proactive Molecular Risk Classifier for Endometrial Cancer (ProMisE), also provides additional prognostic information in it's ovarian endometrioid counterpart. The aim of this study was to investigate ProMisE in a large cohort of low stage (FIGO I-II) ovarian endometrioid carcinoma.
Methodology: After establishment of prognostic groups in >500 ENOC we examined a subset of n=379 FIGO stage I-II cases. Cases were aligned into low risk POLE mutant (POLE); moderate risk mismatch repair deficient (MMRd); high-risk p53 abnormal (p53abn); and a final moderate risk category lacking these biomarkers (p53wt). Kaplan-Meier survival analysis was performed.
Results: 4% of cases were POLE, 15% MMRd, 73% p53wt and 8% p53abn. Groups showed distinct progression-free and overall survival (p < 0.01). 5-year PFS and OS was 74% in p53abn, 89% in MMRd, 93% in p53wt, and 100% in POLE cases.
Conclusion: ProMisE risk classification provides additional prognostic information in a large cohort of low stage ENOC. In the context of low-stage ENOC, and especially younger women wishing to preserve fertility, the introduction of ProMisE-stratified treatment algorithms may serve not only to improve patient care overall but to reduce chemotherapy burden and highlight cases where fertility-sparing surgeries pose little or no risk.
Disclosure: Nothing to disclose

The efficacy of an algorithm using sentinel lymph node biopsy and frozen section in the avoidance of a combined treatment in early-stage cervical cancer management

L. Dostalek[1], J. Slama[1], D. Fischerova[1], R. Kocian[1], A. Germanova[1], F. Fruhauf[1], K. Nemejcova[2], P. Dundr[2], J. Jarkovsky[3], D. Cibula[1]
[1]Gynecologic Oncology Center, Department of Obstetrics and Gynecology, First Faculty of Medicine, Charles University in Prague and General University Hospital in Prague, [2]Department of Pathology, First Faculty of Medicine, Charles University in Prague and General University Hospital in Prague, [3]Institute of Health Information and Statistics of the Czech Republic, Prague, Czech Republic

Introduction / Background: The need for a combined treatment composed of radical surgery followed by adjuvant radiotherapy can be decreased by abandoning radical surgery in patients with intraoperatively detected lymph node (LN) involvement. The aim of the study was to analyze efficacy of the algorithm using intraoperative pathological assessment of sentinel LN (SLN).
Methodology: Cervical cancer patients were included into the study who had stage T1a - T2b squamous, adeno- or adenosquamous carcinoma, were referred for primary surgical treatment, and had at least one SLN detected and submitted for frozen section evaluation. In the case of intraoperatively detected SLN involvement, parametrectomy (radical hysterectomy or trachelectomy) was abandoned and the patient was referred for primary chemoradiation. Radical surgery was completed in patients with intraoperatively negative SLNs. Indications for postoperative adjuvant radiotherapy included LN involvement from final pathology, parametrial involvement, or positive resection margins.
Results: The trial included 309 patients (Table 1). LN positivity was intraoperatively detected in 18 (6%) patients in whom radical hysterectomy was abandoned. Adjuvant radiotherapy after completed radical surgery was given to 29 (9%) cases, including 20 cases with macrometastases (MAC; 8) or micrometastases (MIC; 12) reported from the final histology, 8 cases with positive parametria (all ≤ 3mm), and 1 case with a positive vaginal resection margin (Figure 1).
Conclusion: Out of 47 (15%) patients with high-risk prognostic risk factors (LN, parametria or surgical margin involvement), in only 19 (38%) of them was a combined treatment successfully avoided. This was due to the low sensitivity of frozen section in the detection of MIC and low accuracy of clinical staging in the detection of microscopic parametrial involvement.

	All patients	pN1 (incl. macromet. and micromet.)	p (chi square, Kruskall Wallis)	pN0 (incl. isolated tumor cells)

			From frozen section	From final histology		
N		309	18	20		271
Age		44.4 ± 12.7	47.9 ± 12.1	45.7 ± 12.4	0.90	44.0 ± 12.7
Sentinel lymph node detection	bilateral	266 (86%)	14 (78%)	18 (90%)	0.48	234 (86%)
Stage	T1a - T1b1	239 (77%)	8 (44%)	13 (65%)	0.39	218 (80%)
	T1b2 - T2b	70 (23%)	10 (56%)	7 (35%)	0.47	53 (20%)
Pathologic largest tumor diameter	[mm]	21.6 ± 14.2	15.7 ± 14.2	31.5 ± 16.3	0.07	21.0 ± 13.8
Lymphovascular space invasion	yes	108 (35%)	6 (33%)	16 (80%)	0.03	86 (32%)
Size of nodal macrometastasis	[mm]	7.9 ± 6.7	10.9 ± 6.0	5.7 ± 6.5	0.14	

[Table 1. Demographic data]

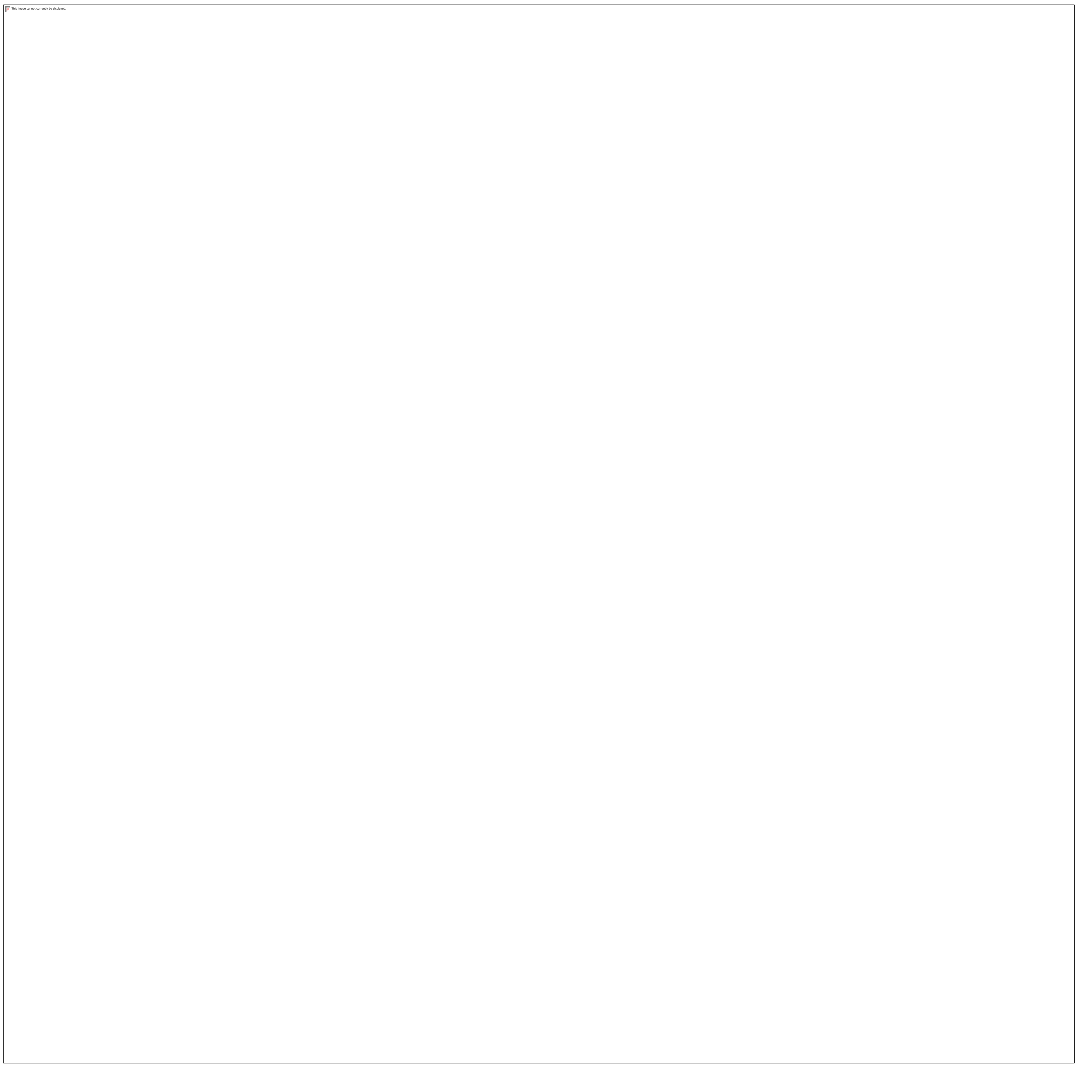

[Figure 1. Flowchart of patients with early stage cervical cancer (2005 - 2015)]

Disclosure: Nothing to disclose

^{99m}Tc-NC+ICG versus ^{99m}Tc-NC+MB technique for sentinel lymph node biopsy in early-stage endometrial cancer

V. Bebia[1], C. Forcada-Guasch[1], D. Villasboas-Rosciolesi[2], S. Franco-Camps[1], A. Pérez-Benavente[1], A. Gil-Moreno[1], S. Cabrera-Díaz[1]
[1]Department of Gynecology - Gynecologic Oncology Unit, Vall d'Hebron University Hospital,
[2]Department of Nuclear Medicine, Vall d´Hebron University Hospital, Barcelona, Spain

Introduction / Background: Several techniques for sentinel lymph node biopsy (SLNB) in early-stage endometrial cancer have been previously described. However, little or no evidence is available regarding combined indocyanine green plus ^{99m}Tc-albumin nanocolloid (^{99m}Tc-NC+ICG). Combining ^{99m}Tc-NC+ICG may decrease the incidence of "empty lymph node packets" and restrict ICG high diffusion and background signal, diminishing total SLNs per patient without compromising its accuracy.Our aim was to compare two different approaches to SLNB in early-stage endometrial cancer: combining methylene blue plus ^{99m}Tc-NC (^{99m}Tc-NC+MB) *versus* ^{99m}Tc-NC+ICG.
Methodology: Consecutive patients with preoperative diagnosis of early-stage endometrial carcinoma in a single centre were offered to be included in our trial from February 2017 until March 2019. Patients were assigned to ^{99m}Tc-NC+MB or ^{99m}Tc-NC+ICG group depending on availability of special ICG-detector cameras.
Results: A total of 71 patients were included for analysis, 67.6% (n=48) in ^{99m}Tc-NC+MB group and 32.4% (n=23) in ^{99m}Tc-NC+ICG group. Most patients presented early stage, grade 2, low risk endometrioid carcinoma. A minimally invasive approach was largely preferred (95.8%). Both groups were comparable regarding demographic and clinical variables, except for a bigger proportion of patients undergoing robotic surgery in the ^{99m}Tc-NC+ICG group (78.3% *vs* 35.4%, p=0.002).
Global detection rate (DR) was 94.4% (CI: 86.2-98.4), and was not statistically different among groups (^{99m}Tc-NC+MB: 93.8% *vs* ^{99m}Tc-NC+ICG: 95.7%). A better optimal (bilateral) mapping rate was observed among ^{99m}Tc-NC+ICG patients (69.5% *vs* 41.7%, p=0.028), with a statistically significant increase in the median of SLN analyzed per patient (3 *vs* 2, p=0.040).
A total of 5 (7.5%) patients had positive lymph nodes in definitive lymphadenectomy histology, all of whom had positive SLNs. Then, no false-negative SLN were observed in this cohort, achieving a sensitivity rate of 100%.
Conclusion: ^{99m}Tc-NC+ICG is a feasible, oncologically safe technique for SLNB in early-stage endometrial cancer patients, appearing to be superior in terms of bilateral mapping to ^{99m}Tc-NC+MB.

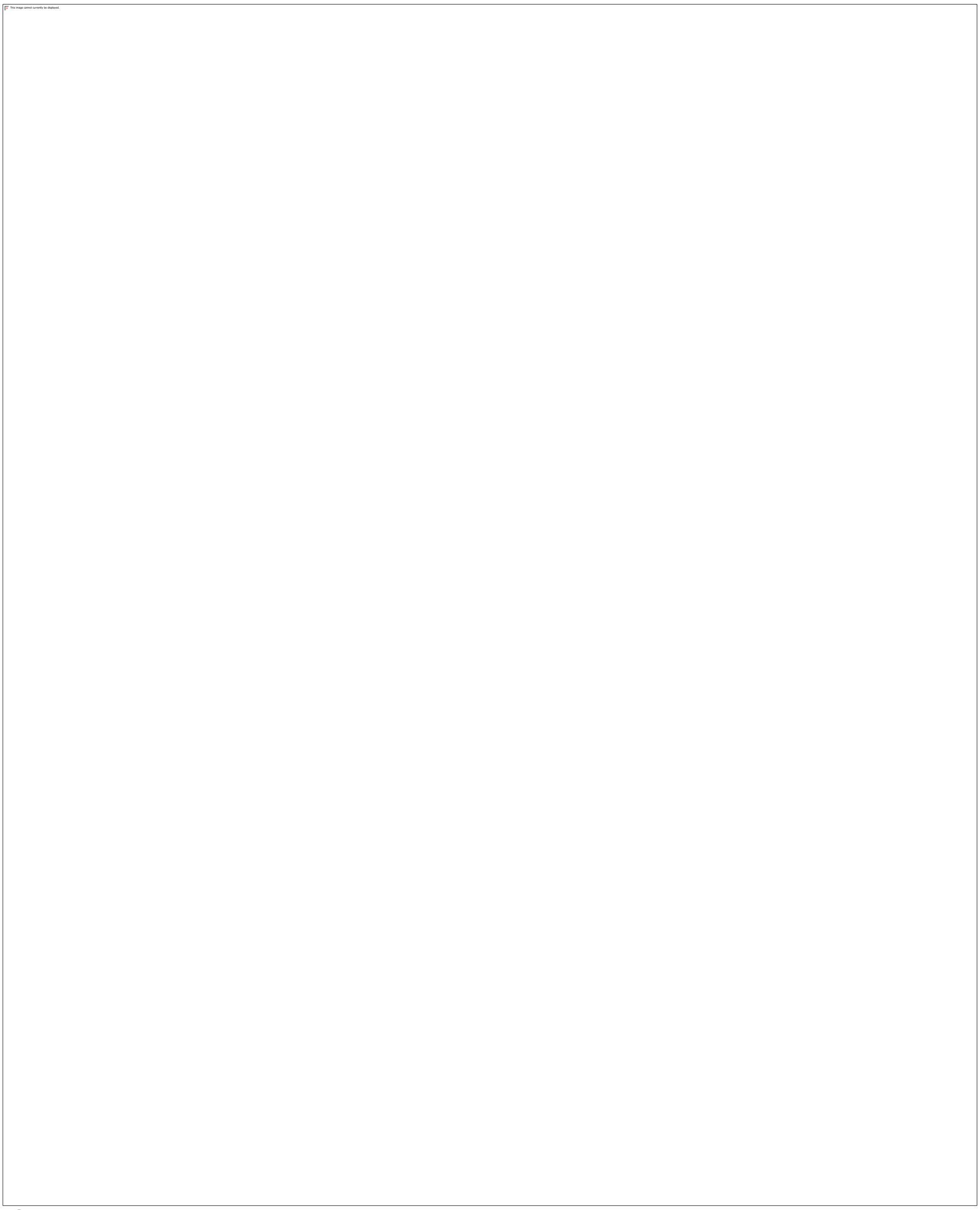

[SLNs anatomic distribution]

	General (n=71)	99mTc-NC+MB (n=48)	99mTc-NC+ICG (n=23)	p-value
**Age (years) [mean (CI)]**	62.4 (59.6-65.2)	63.2 (59.7-66.6)	60.9 (56.0-65.8)	0.455
**Hx: Endometrioid**	63 (88.7%)	42 (87.5%)	21 (91.3%)	
**Hx: Serous carcinoma**	6 (8.5%)	4 (8.3%)	2 (8.7%)	

**Hx: Carcinosarcoma**	2 (2.8%)	2 (4.2%)	0 (0%)	1.000
**St: Stage I**	53 (74.6%)	34 (70.8%)	19 (82.6%)	
**St: Stage II**	10 (14.1%)	7 (14.6%)	3 (13.0%)	
**St: Stage III**	8 (11.2%)	5 (10.5%)	2 (2.8%)	0.696
**Route: Laparotomy**	3 (4.2%)	3 (6.3%)	0 (0%)	
**Route: Minimally invasive**	68 (95.8%)	45 (93.7%)	23 (100.0%)	0.002

[Clinical variables]

	General (n=71)	99mTc-NC+MB (n=48)	99mTc-NC+ICG (n=23)	p-value
**Drainage: Absence**	4 (5.6%)	3 (6.3%)	1 (4.4%)	
**Drainage: Unilateral pelvic**	25 (35.2%)	19 (39.6%)	6 (26.1%)	
**Drainage: Unilateral pelvic + aortic**	3 (4.2%)	3 (6.3%)	0 (0%)	
**Drainage: Bilateral pelvic +/- aortic**	35 (49.3%)	19 (39.6%)	16 (69.6%)	
**Drainage: Aortic**	4 (5.6%)	4 (8.3%)	0 (0%)	0.082
**Detection rate (%, CI)**	94.4% (86.2-98.4%)	93.8 (82.8-92.7)	95.7 (78.1-99.9)	0.745
**Optimal mapping (%, CI)**	50.7% (38.6-62.8)	41.7 (27.6-56.8)	69.6 (47.1-86.8)	0.028
**SLNB, total nodes (SN per patient) [median (IQR)]**	2 (1-3)	2 (1-3)	3 (2-4)	0.040
**Positive SLNB**	5 (7.5%)	5 (11.1%)	0 (0%)	0.163

[Sentinel lymph node biopsy variables]

Disclosure: Nothing to disclose

Validation of an ERAS protocol in gynecological surgery: interim analysis of an italian randomized controlled trial

F. Ferrari[1], S. Forte[2], N. Sbalzer[3], M. Mauri[2], V. Zizioli[1], E. Sartori[2], F. Odicino[2]
[1]Department of Obstetrics and Gynecology, Spedali Civili Brescia, [2]Department of Obstetrics and Gynecology, University of Brescia, [3]Department of Anesthesia, Spedali Civili Brescia, Brescia, Italy

Introduction / Background: Enhanced Recovery After Surgery (ERAS) is a new emerging standard of perioperative care for gynecological surgery. The aim of the study is to compare outcomes of conventional perioperative care with those of an ERAS protocol in women undergoing major gynecological surgery.
Methodology: In this trial (clinicaltrials.gov NCT03347409), involving patients affected by benign

gynecological pathology, endometrial or ovarian cancer, we randomly assigned patients to undergo conventional perioperative care (Standard) or ERAS protocol. The primary outcome was the length of hospitalization (LOH) in days with noninferiority claimed if the lower boundary of the two-sided 95% confidence interval of the between-group difference (Standard versus ERAS) was equal or greater than 1.5 days. Secondary outcomes include comparison of postoperative pain, vomiting and nausea, anesthesiological and surgical complications up to thirty days after surgery and the time-to-event in hours for bowel movements, flatus, drink, hunger, eating and walking and quality of recovery using a validated questionnaire. We further evaluated compliance to ERAS protocol.
Results: We enrolled 138 patients and of them 70 (51%) underwent Standard protocol while 68 (49%) were assigned to ERAS. The two groups were similar with respect to age, BMI, Charlson-comorbidity-index, anesthesiological risk, smoking habits, surgical access and complexity of surgical procedures. Fifty-two patients (38%) underwent hysterectomy for benign surgery, 44 (32%) for endometrial cancer and 42 (30%) for ovarian cancer. LOH was 3.5 days shorter in ERAS protocol (CI95% 2.1-4.2; p = 0.00). Subgroup analysis showed a greater advantage in patients underwent laparotomic surgery with a LOH 4.1 days shorter (CI95% 3.6-4.7; p = 0.00), while for laparoscopic surgery LOH was 2.1 days shorter (CI95% 1.6-3.2; p = 0.00). Secondary outcomes and update of the aforementioned data should be assessed yet.
Conclusion: Application of ERAS protocol in gynecological surgery translated in a shorter LOH, regardless surgical access and pathology.
Disclosure: Nothing to disclose

Comparaison of laparoscopic and abdominal radical hysterectomy in cervical cancer : a French multicentric study

F. Zaccarini, Y. Dabi, C. Touboul, FRANCOGYN
Department of Obstetrics and Gynecology, Hôpital Intercommunal de Créteil, Créteil, France

Introduction / Background: Radical hysterectomy (RH) is recommended as a standard treatment for early stage cervical cancer (FIGO IA1 to IIA2). There is no guideline to recommend either laparoscopy or abdominal laparotomy for treating these patients. Laparoscopic surgery has been increasingly used in this indication since it is associated with less morbidity. However, a recent randomized trial showed that patients treated with minimally invasive surgery experienced higher recurrence rate and a lower overall survival rate. As this is the first and only trial showing such results, we aimed to evaluate oncological and surgical outcomes of these patients treated by minimal invasive surgery or laparotomy for an early stage cervical cancer using a large french multicentric database.
Methodology: Data of 1447 patients with cervical cancer treated between 1996 and 2016 were extracted from maintained databases of 10 French University hospitals. Patients treated with RH for an early stage cervical cancer were selected for further analysis.
A propensity score-matched comparison (1:3) was developed through multivariable logistic regression model including age, stage, histological type, tumor size, body mass index and adjuvant therapy as covariates. The primary outcome was overall survival. Secondary outcomes included surgical parameters such as the operating time, blood loss, complication rate and oncological data such as disease free survival and site of recurrence.
Results: After performing propensity score matching, 34 patients treated with abdominal surgery were matched with 101 patients treated by minimal invasive surgery. The groups were similar for the main parameters influencing survival in early stage cervical cancer i.e. age, FIGO stage, tumor size and adjuvant therapy received.
The laparoscopic group displayed a poorer overall survival (median OS 32,26 vs 56,88 months ; p< 0,05).
Conclusion: In this multicenter study, minimal invasive surgery was associated with lower rates of overall survival among patients treated with RH for an early stage cervical cancer.
Disclosure: Nothing to disclose

ePoster Talks: cervical cancer

EP1213

Comparison of survival between abdominal versus laparoscopic radical hysterectomy in cervical cancer: 10 year experience in a single UK cancer centre

N. Gomes, A. Dasgupta, D. Blake, A. Fisher, N. Ratnavelu, A. Kucukmetin
Queen Elizabeth Hospital, Northern Gynaecologic Oncology Centre, Gateshead, United Kingdom

Introduction / Background: Minimal access approaches to radical hysterectomy for cervical cancer have become increasingly popular in the last decade and have been adopted broadly. Laparoscopic approach for cervical cancer trial (LACC) concluded more recurrence and higher mortality in minimal access group. These findings are supported by subsequent epidemiological data.To demonstrate that patients' care had not been compromised, we compared the outcomes of TARH (Abdominal radical hysterectomy) and TLRH (Laparoscopic radical hysterectomy) in our hospital in the past 10 years.
Methodology: We performed a retrospective cohort study involving women who underwent radical hysterectomy for stage 1A2 - 2A cervical cancer during the January 2008 to December 2018 period at Northern Gynaecological Oncology Centre. All TARH was performed between 2008 to 2010. Overall and progression free survival were compared in the groups and compared with clinical variables using Cox proportional hazards regression and log rank test.
Results: A total of 239 patients were included in our study.69 patients underwent TARH and 170 had TLRH. Both groups were comparable in baseline characteristics.1A2=1%, 1B1= 81%, 1B2= 15%, 2A= 3%. With a median follow up time of 52 months there were no significant differences in mortality 13% vs 5% (hazard ratio 1.01; 95% confidence interval [CI], 0.36 to 2.82; P=0.98 by the log-rank test) or recurrence rate 15.9 % and 7% (Hazard Ratio 1.46,95% CI:0.59-3.59, p=0.41) in TARH and TLRH group respectively. Although mortality was not associated with route of surgery, it was associated with tumour size, and the presence of lymphovascular space invasion(p=0.007). Blood loss, transfusion requirements and post-operative complications were significantly lower in TLRH arm (p=0.03).
Conclusion: Neither mortality nor recurrence was associated with route of surgery. There was a tendency of greater recurrence in the open radical hysterectomy arm, but this was not statistically significant.
Disclosure: Nothing to disclose

EP1232

The next generation sequencing of cancer-related genes in small cell neuroendocrine carcinoma of the cervix

X. Pei, H. Yang, L. Xiang, W. Jiang, S. Ye, Y. Wu
Fudan University Shanghai Cancer Center, Shanghai, China

Introduction / Background: Small cell neuroendocrine carcinoma of the cervix (SCNEC) is a lethal malignancy and little treatment progress has been made for decades. We sought to identify whether SCNEC harbor mutations and potential targets for therapeutic interventions.
Methodology: Primary tumor tissue and blood samples were obtained from 51 patients with SCNEC treated at Fudan University Shanghai Cancer Center (FUSCC). The next generation sequencing was carried out to detect mutations of 520 cancer-related genes, including the entire exon regions of 312 genes and the hotspot mutation regions of 208 genes.
Results: Of the 51 detected patients, 98% (50/51) harbor genetic alterations. The most prominent mutations were mutations in PIK3CA (12%), TP53(12%), followed by KRAS(10%), KTM2D(10%), ATM(8%), PRKDC(8%), PTEN(8%). SCNEC had a high incidence of copy number variants with a rate of 60.8%. IRS2 and MYC was the gene most frequently amplified (14% of the samples respectively), with frequent amplification in CDK8(10%), IL7R (10%), RICTOR(10%). 68.6% of the samples were demonstrated with variants of potential clinical significance (Tier II) and most of which could be therapeutically intervened.

Conclusion: We identified several significantly genetic alterations in SCNEC, including previously reported PIK3CA, TP53, KRAS, MYC and newly found KTM2D, ATM, PRKDC, IRS2, CDK8, IL7R,RICTOR.The gene profile of SCENC provided here will serve as a reference to further advance the understanding and precision treatment of SCNEC.

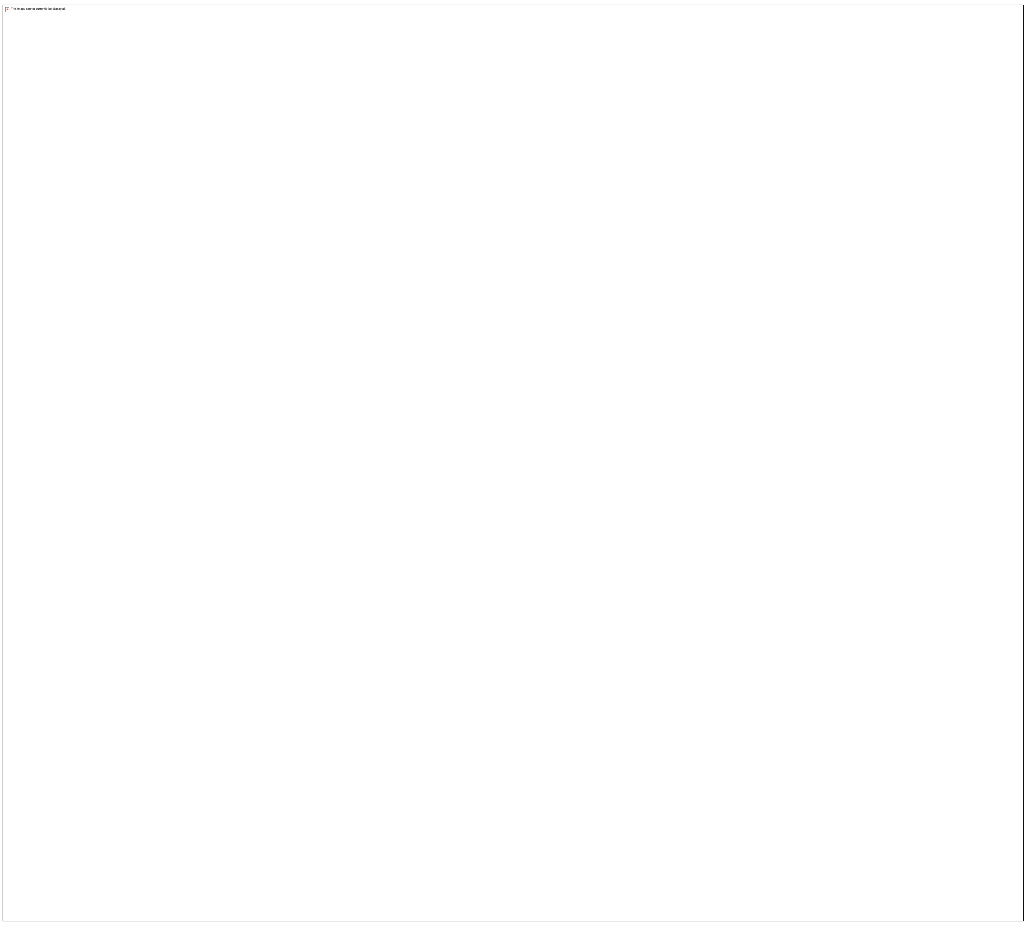

[The Genomic Landscape of SCNEC]

Disclosure: Nothing to disclose

EP1215

Postoperative radiotherapy does not benefit stage IB-IIA cervical squamous cell carcinoma with intermediate risk according to Sedlis criteria

L. Cao, H. Wen, Z. Feng, X. Han, X. Wu
Gynecologic Oncology, Fudan University Shanghai Cancer Center, Shanghai, China

Introduction / Background: Postoperative radiotherapy was proved to reduce recurrence of intermediate-risk stage IB cervical cancer by GOG 92 trial initiated in 1988.This study was aimed to demonstrate whether postoperative radiotherapy (w/o concurrent chemotherapy) influences the outcomes of early-stage cervical squamous cell carcinoma with intermediate risk in the current background.
Methodology: Patients with stage IB1-IIA2 (FIGO 2009) cervical squamous cell carcinoma who

underwent radical hysterectomy and pelvic lymph node dissection at our institution between March 2006 and February 2014 were reviewed. Only patients with no positive lymph nodes, surgical margin or parametrium but with intermediate-risk factors according to Sedlis criteria were included. Patients who underwent observation (Obs.), radiotherapy alone (RT) and radiotherapy with concurrent chemotherapy (CCRT) postoperatively were compared for recurrence-free survival (RFS), disease-specific survival (DSS), and site of first relapse (pelvic *vs* distant *vs* combined pelvic and distant).
Results: 94 patients in Obs. group, 279 patients in RT group and 488 patients in CCRT group were included. Five-year RFS were 84.8%, 84.5% and 89.9% for Obs., RT and CCRT group, respectively (p=0.22).Corresponding five-year DSS were 90.7%, 88.0% and 91.5%, respectively (p=0.21). Adjuvant radiotherapy (w/o concurrent chemotherapy) was not an independent prognostic factor for RFS and DSS compared with no further treatment (all p>0.05). There was no significant difference in the distribution of site of first relapse among three groups (p>0.05). Among entities of Sedlis criteria, patients with combination of positive lymph vascular space invasion (LVSI) and deep 1/3 stromal invasion had much poorer RFS (p=0.02) and DSS (p=0.004) compared with those with combination of negative LVSI, middle or deep 1/3 stromal invasion and tumor diameter ≥ 4cm.
Conclusion: Postoperative radiotherapy (w/o concurrent chemotherapy) doesn't benefit patients with intermediate risk according to Sedlis criteria. The prognosis of patients meeting Sedlis criteria is not even balanced.
Disclosure: Nothing to disclose

EP1216

What are the real-world outcomes of locally advanced cervical cancer patients who receive neo-adjuvant chemotherapy?

K. Baillie[1], C. Crearie[1], J. Laskey[1], M. Bennie[2,3], R. Harrand[1], A. Sadozye[1], A. Kerr[1], N. Reed[1], K. Graham[1]
[1]Beatson West of Scotland Cancer Centre, NHS Greater Glasgow and Clyde, [2]Strathclyde Institute for Pharmacy and Biomedical Sciences, University of Strathclyde, Glasgow, [3]Public Health and Intelligence Strategic Business Unit, NHS National Services Scotland, Edinburgh, United Kingdom

Introduction / Background: Concurrent chemoradiotherapy (CCRT) is the standard of care for locally advanced cervical cancer. However, a significant number of women develop local and/or systemic relapse. Limited studies suggest that neoadjuvant chemotherapy (NACT) may improve efficacy, with 3 year overall survival (OS) of 67% in a recent Phase II study (McCormack M, et al; 2013). In the West of Scotland Cancer Network (WoSCAN), 3-weekly NACT has been used for over a decade in high-risk women. A previous local study in 2012 demonstrated 55% of NACT patients proceeded to CCRT. This study aimed to identify the proportion of patients proceeding to definitive CCRT following NACT in the real-world setting and evaluate survival.
Methodology: Data were collected retrospectively from electronic clinical records of WoSCAN patients who commenced NACT between January 2012 and December 2016. Statistical analysis was performed using R®. OS was estimated using Kaplan-Meier method and Cox's proportional-hazards models used to estimate unadjusted hazard ratios (HR).
Results: 126 patients were identified; median age was 45 years (range 22-75). The majority had at least FIGO Stage II disease (91.3%) and squamous pathology (85.7%). Para-aortic nodes were present in 10.4% and 4.8% had Stage IVB disease (encompassed within a radical field). NACT regimens consisted primarily of Cisplatin/ Paclitaxel (62.7%) or Carboplatin/ Paclitaxel (34.9%); a median of 3 cycles were administered. 85.7% proceeded to CCRT, 11.1% received radiotherapy (RT) only, and 3.2% progressed or defaulted. 3 year OS was 74.2% (95% CI 66.4-82.9). Treatment with RT only, significantly increased the death hazard (Unadjusted HR 2.35 (95% CI 1.15-4.79)).
Conclusion: Almost 90% of patients proceeded to CCRT. Survival compares favourably with recent Phase II data illustrating that NACT followed by CCRT is a feasible approach for locally advanced cervical cancer, especially in women with bulky disease. Preliminary data are encouraging, but results of Phase III INTERLACE trial are still awaited.
Disclosure: This study was funded by the Scottish Government. The authors declare no competing interests.

EP1217

Surgical staging prior to chemoradiation is beneficial for survival in patients with high stage cervical cancer

G. Kenter[1], M. Popovic[2], S. Alran[3], V. Fourchotte[3], S. Dureau[3], A. Mandic[2], H. Zijlmans[1], P. Hillemans[4], F. Lecuru[5], C. Ngo[5], P. Wimberger[6], R. Rouzier[3], M. Kamal[3], S. Scholl[3], RAIDs Consortium

[1]Center Gynaecologic Oncoloy Amsterdam, Amsterdam UMC, Location AMC, VUmc, NKI, Amsterdam, The Netherlands, [2]Operative Oncology, Institute of Oncology, Voyvodina, Serbia, [3]Drug Development and Innovation, Institut Curie PSL Research University, Paris, France, [4]Hannover Medical School, Hannover, Germany, [5]Service de Chirurgie Cancerologique, Universitee de Paris Descartes Hospital Europeain George Pompidou, Paris, France, [6]Gynaecology and Obstertrics, Universitatsklinikum Carl Gustav Carus, Dresden, Germany

Introduction / Background: The RAIDs consortium (http://www.raids-fp7.eu/) conducted a prospective study [BioRAIDs (NCT02428842)] with the objectives to stratify patient populations as well as to identify clinical and molecular patterns associated with poor outcome.. Debate is ongoing about the benefit of surgical staging prior to chemoradiation. Here we report on the value of diagnostic pelvic and para-aortal lymphadenectomy (LA) for patients scheduled for primary chemoradiation.
Methodology: Between2013-2017, the RAIDs Network collected a clinical dataset involving 419 participant patients from 18 centers in seven EU countries, of whom 377 patients are evaluable. Treatment decisions had been based on guidelines defined in the clinical protocol as detailed in Ngo et al, 2015 and were also based on the policy of the center. Systematic MRI imaging was mandatory. LA as well as PET imaging was recommended. From 262 patients in stage IIB-IV who were scheduled for primary chemoradiation 126underwent LA.
Results: Patients who underwent surgical LA followed by chemoradiation showed better PFS at 24 months compared to those who did not. This difference was statistically significant (p-value of univariate : p = 0.008) . If we adjust with the FIGO, the effect of staging LA remains.
Conclusion: Although these data were collected retrospectively and might be biased we found that staging LA improved PFS also after adjusting for stage. These data are in line with literature and advocate for a randomized trial to proof the value of staging lymphadenectomy prior to chemoradiation for patients with high stage cervical cancer.

multivariate	hr	1c95% (hr)	pvalue
lymphadenectomy			
no LA/surgery plus LA	1		
staging LA	0.52	0.32;0.83	0.006
figo 2018			
1b-2a	1		
2b-3a	1.42	0.51;3.90	0.50
3b-4	2.7	1.08;6.75	0.03

[LA according to FIGO stage]

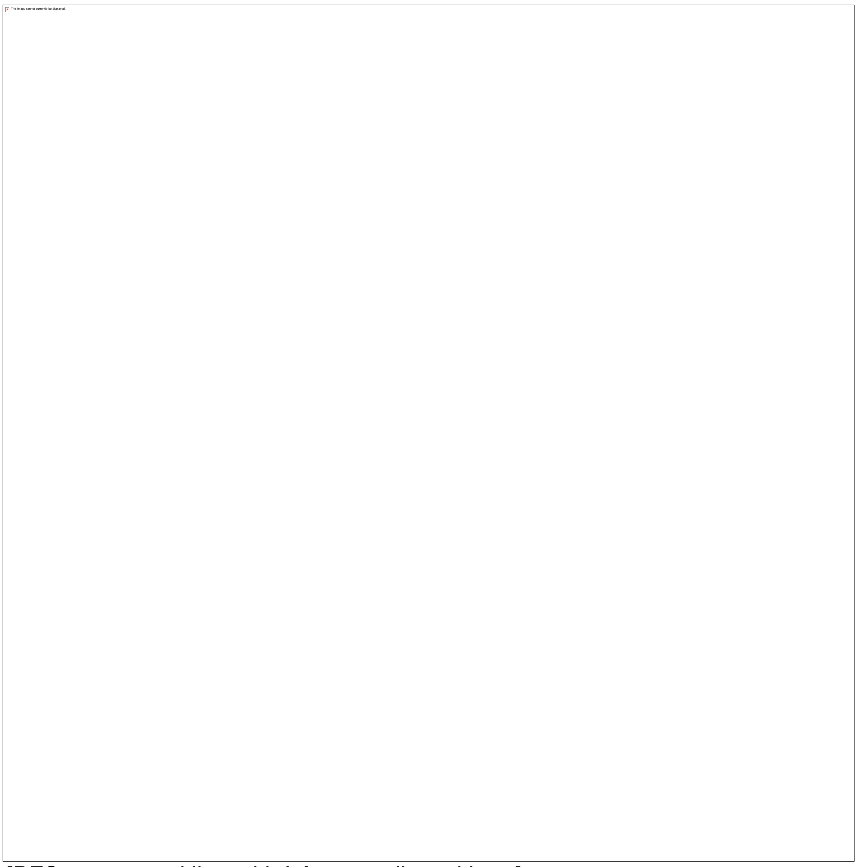

[PFS curves: red line with LA, green line without]

Disclosure: This project has received funding from the European Union's Seventh Program for research, technological development and demonstration under grant agreement No 30481

EP1218

Outcome in 186 patients with cervical cancer stage IB1 treated with radical hysterectomy without adjuvant radiochemotherapy, unless presenting with metastatic lymph nodes

J. Haesen[1], R. Salihi[1], A.-S. Van Rompuy[2], E. Van Nieuwenhuysen[1], M. Christiaens[3], T. Van Gorp[1], S. Han[1], P. Neven[1], I. Vergote[1]

[1]Division of Gynaecological Oncology, Department of Gynaecology and Obstetrics, [2]Department of Pathology, [3]Department of Radiotherapy-Oncology, University Hospitals Leuven, Leuven, Belgium

Introduction / Background: Lymphovascular space invasion (LVSI), deep stromal invasion (DSI) and large tumor size have been identified as predictors for intermediate risk for recurrence. Adjuvant radiotherapy (RT) has been advocated in these patients (Sedlis Gynecol Oncol 1999,73:177). However, it is still controversial whether adjuvant radiochemotherapy (RCT) improves overall survival (OS).

Methodology: All consecutive patients (1997-2017) with cervical cancer FIGO (2009) stage IB1 (≤ 4cm) treated in our department were included. Intermediate risk for recurrence was defined as presence of two of the following factors in patients with negative lymph nodes: LVSI, DSI (>10mm) and large tumor size (>2cm).

Results: Two hundred-and-one patients were included into the study, of which 186 patients

underwent primary surgery and 15 patients received a fertility sparing treatment (table 1). Mean time of follow-up was 11 years (IQR 6-15). Postoperatively, 24 patients received adjuvant therapy due to presence of positive lymph nodes, positive section margins or vaginal intra-epithelial neoplasia: RCT (n=16), RT (n=7) or chemotherapy (n=1). None of the patients with intermediate risk factors according underwent adjuvant RT/RCT. Disease recurrence was observed in 23 patients (11%). The recurrences were local (n=14), distant (n=2) or both local and distant (n=7). Ten patients died of disease. The 5-year OS was 93 % and the 5-year progression-free-survival (PFS) was 89%. Cox proportional hazards models showed that intermediate risk factors did not significantly influence OS or PFS.
Conclusion: Recurrence rate was rather low (11%), despite the fact that only 12% of the patients with cervix cancer stage IB1 received postoperative radio(chemo)therapy. Intermediate risk factors such as LVSI, DSI (>10mm) or tumor size > 2 cm did not predict recurrence nor survival. Future studies are needed to elucidate whether adjuvant radiotherapy is needed in the current era with better survival than in the nineties when the GOG99 (Sedlis et al.) was performed.

Follow - up period (years; IQR)	11.18 (5.94 - 15.38)
Age (IQR)	42 (36-51)
BMI (IQR)	23.1 (21.3-26.3)
Type of surgery (%)	
Cone excision	15 (8)
Total hysterectomy	25 (12)
Radical hysterectomy	161 (80)
Pelvic lymphadenectomy (%)	189 (94)
Number of pelvic nodes resected (IQR)	26 (21-35)

[Table 1. Patient characteristics in Cervical cancer IB1 (FIGO 2009)]

Histology (%)	
Adenocarcinoma	44 (22)
Squamous	135 (67)
Other	22 (11)
Median size tumor clinically (mm; IQR)	25 (20-30)
Size tumor on imaging (mm; IQR)	22 (15-28)
Size tumor pathology report (mm; IQR)	15 (10-25)
Positive lymph nodes (%)	18 (9)
Positive resection margin (%)	2 (1)

[Table 2. Tumor characteristics]

Disclosure: Nothing to disclose

EP1219

Added value of para-aortic surgical staging compared to F18 FDG PET/CT on the external beam radiation field of patients with locally advanced cervical cancer: an ONCO-GF study

M. De Cuypere[1], P. Lovinfosse[2], F. Goffin[1], S. Schoenen[1], R. Rovira[3], J. Duch[4], M. Fastrez[5], G.

Gebhart[6], M. Luyckx[7], J.-L. Squifflet[7], G. Charaf[8], K. Crener[9], F. Buxant[10], D. Bucella[11], M. Jouret[12], C. Gennigens[13], ONCO-GF: Gynaecological Oncology Working Group from the GGOLFB (Groupement des Gynécologues Obstétriciens de Langue Française de Belgique)
[1]Department of Gynaecological Oncology, University of Liège, [2]Department of Nuclear Medicine, University of Liège, Liège, Belgium, [3]Department of Gynaecological Oncology and Laparoscopy, Hospital de la Santa Creu I Sant Pau of Barcelona, [4]Department of Nuclear Medicine, Hospital de la Santa Creu i Sant Pau of Barcelona, Barcelona, Spain, [5]Department of Gynaecology, St Pierre University Hospital, Free University of Brussels, [6]Department of Nuclear Medicine, Institut Jules Bordet, Free University of Brussels, [7]Department of Gynaecology, Saint Luc Academic Hospital, Catholic University of Louvain, Brussels, Belgium, [8]Department of Obstetrics and Gynaecology, University Hospital Ambroise Paré of Mons, Lille, France, [9]Department of Obstetrics and Gynaecology, University Hospital Ambroise Paré of Mons, Mons, [10]Department of Obstetrics and Gynaecology, Iris South Hospital, [11]Department of Nuclear Medicine, Iris South Hospital, Brussels, [12]Department of Obstetrics and Gynaecology, Hospital of Picard Wallonia, Tournai, [13]Department of Medical Oncology, University of Liège, Liège, Belgium

Introduction / Background: Extended field chemoradiation is recommended for patients with locally advanced cervical cancer (LACC) and para-aortic lymph node (PALN) metastases. The radiation planning may be based on the positron emission tomography-computed tomography (PET/CT) while others recommend to rely on a surgical staging. We report the rate of patients for whom the radiation field defined on a PET/CT basis was modified by the histological PALN status.
Methodology: Between March 2010 and December 2016, 168 consecutive patients with LACC underwent a pre-therapeutic PET/CT and PALN dissection. The data were reviewed retrospectively. The diagnostic performance of the PET/CT for definition of PALN status was calculated. We determined the percentage of patients for whom PALN dissection altered the external beam radiation treatment (EBRT) field defined on the PET/CT basis.
Results: Of the 151 patients with negative PALNs on PET/CT, 26 had histological PALN metastasis. Of the 17 patients with positive PALNs on PET/CT, 9 were negative on histology. Seven of these 9 false positive cases were located in the common iliac region. Sensitivity, specificity, positive and negative predictive value of PET/CT were 23.5, 93.3, 47.1 and 82.8% respectively. In total, 35 out of 168 patients underwent EBRT-field adaptation (pelvic vs extended field). The rate of radiation field modification was particularly high (27,7%) in the subgroup of patients with metastatic pelvic lymph nodes (PLNs) on PET/CT.
Conclusion: Para-aortic surgical staging contributes significantly to individualize the radiation treatment of patients with LACC, particularly for those with positive PLN at PET/CT. Indication of surgical staging deserves particular attention when the PET/CT suggests positive lymph nodes in the common iliac region.
Disclosure: Nothing to disclose

EP1220

Comparison of laparoscopic versus open radical hysterectomy in early cervical cancer after completing learning curve and reducing intraperitoneal tumor exposure

J.-Y. Park, D.-Y. Kim, D.-S. Suh, J.-H. Kim, Y.-M. Kim, Y.-T. Kim, J.-H. Nam
Obstetrics and Gynecology, University of Ulsan College of Medicine, Asan Medical Center, Seoul, Republic of Korea

Introduction / Background: The main limitation of LACC trial is that the surgeon validation criteria for laparoscopic radical hysterectomy was not enough to include surgeons who completed learning curve for laparoscopic radical hysterectomy. So, the comparison between open and laparoscopic surgery was unfair. In addition, there was no safety measure to reduce intraperitoneal tumor exposure during laparoscopic radical hysterectomy. The aim of this study was to compare the survival outcomes between laparoscopic versus open radical hysterectomy in early cervical cancer after completing learning curve and reducing intraperitoneal tumor exposure.
Methodology: This was a retrospective study including patients with stage IA2 - IIA2 cervical cancer who underwent laparoscopic or open radical hysterectomy. All surgeons completed the learning curve

for laparoscopic radical hysterectomy. To reduce the intraperitoneal tumor exposure during laparoscopic radical hysterectomy, all broken tumor tissues were washed out before colpotomy, and colpotomy and stump repair has been performed transvaginally. The survival outcomes were compared between laparoscopic and open radical hysterectomy.
Results: During the study period, 859 and 1388 patients underwent open and laparoscopic radical hysterectomy, respectively. The 5-year DFS and OS did not differ between laparoscopic and open radical hysterectomy. The survival outcomes did not differ between laparoscopic and open radical hysterectomy both in patients with tumor < 4 cm and with tumor > 4 cm. The use of laparscopic surgery increased gradually and has become the main surgical approach during the study period. Each year's survival rate was not compromised despite the increased use of laparoscopic radical hysterectomy.
Conclusion: The survival outcome of laparoscopic radical hysterectomy is comparable to open radical hysterectomy after completing learning curve and reducing intraperitoneal tumor exposure during surgery.
Disclosure: Nothing to disclose

EP1233

Wide variation in self-management skills in gynaecological cancer survivors from the Netherlands, Denmark and Norway - first results from the InCHARGE study

B.H. de Rooij[1,2], M. Skorstad[3], M. Moustgaard Jeppesen[4,5], S. Holm Bergholdt[6,7], N.P.M. Ezendam[1,2], P.T. Jensen[6,7], I. Vistad[3], L.V. van de Poll-Franse[1,2,8]
[1]Netherlands Comprehensive Cancer Organisation (IKNL), Utrecht, [2]Medical and Clinical Psychology, Tilburg University, Tilburg, The Netherlands, [3]Department of Obstetrics and Gynaecology, Sørlandet Hospital, Kristiansand, Norway, [4]Department of Gynecology and Obstetrics, Lillebaelt Hospital, Kolding, [5]OPEN, Odense Patient Data Explorative Network, Odense University Hospital, Odense, [6]Department of Gynecology and Obstetrics, Aarhus University Hospital and Faculty of Health, Institute for Clinical Medicine, Aarhus University, Aarhus, [7]Faculty of Health Sciences, Odense University Hospital, Odense, Denmark, [8]Division of Psychosocial Research and Epidemiology, Netherlands Cancer Institute, Amsterdam, The Netherlands

Introduction / Background: With improved survivorship and limited resources for follow-up care, patients are expected to manage their disease well beyond active treatment. Little is known about the factors that empower patients to thrive after gynaecological cancer. In three Northern European countries, we aimed to 1) identify and characterize subgroups of gynaecological cancer survivors based on self-management skills, and 2) assess what socio-demographic and clinical factors are associated with these subgroups.
Methodology: In the InCHARGE study *(International Collaboration of Healthcare professionals And Researchers for Gynaecological cancer survivors' Empowerment)*, patients with ovarian, endometrial and cervical cancers participated in a self-administered survey at 1-7 years after initial diagnosis. Questionnaires included sociodemographic and clinical variables, and assessed self-management in 7 domains. Subgroups with similar scores on self-management domains were identified and characterized using latent class cluster analysis, and factors associated with the subgroups were assessed using multiple logistic regression analysis.
Results: Out of 1,387 participants, cluster analysis identified 4 subgroups of survivors: 1) high self-management on all domains (51%), 2) activity-focused self-management (19%), 3) relationship-focused self-management (15%), and 4) low self-management on all domains (16%) (**Figure 1**). Compared to cluster 1, survivors in cluster 2 more often had ovarian cancer, were more often younger and Danish and had more comorbidity; survivors in cluster 3 had lower cancer stages and less often underwent chemotherapy; and survivors in cluster 4 were more often cervical cancer patients with higher cancer stages, more often underwent chemotherapy or radiotherapy and were younger, lower-educated and had more comorbidity.
Conclusion: The substantial variation in self-management skills among gynaecological cancer survivors suggests that subgroups of survivors need additional support for long-term management of their cancer. Younger and lower-educated cervical cancer patients with higher cancer stages may particularly require support with regard to skills and techniques to cope with their symptoms.

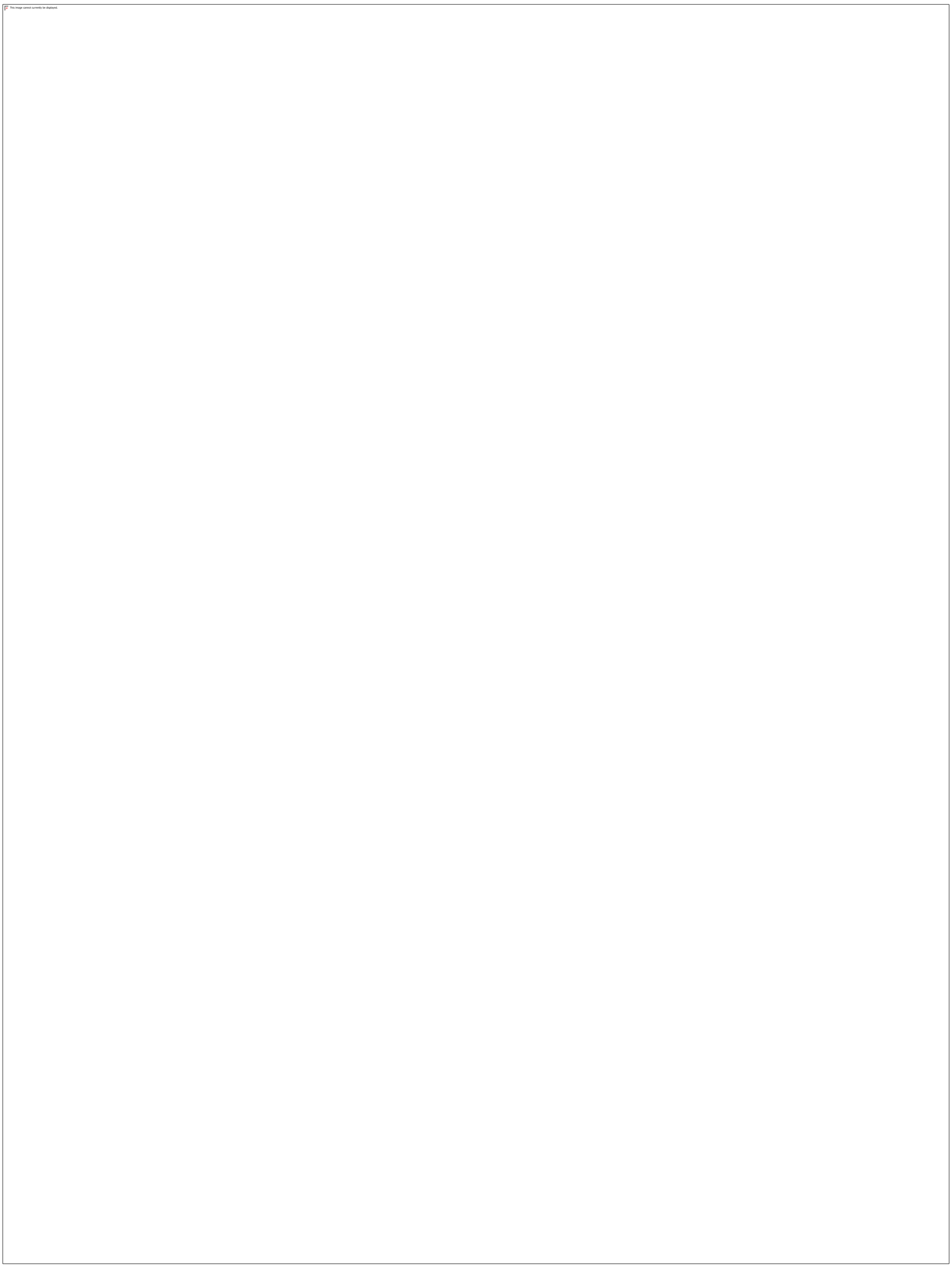

[Figure 1: Spider plot of self-management scores by self-management cluster]

Disclosure: Funding for this study was provided by the Norwegian Symptom Management Network (NORSMAN).

P09

FIGO 2018 stage IB2 (≥2 to < 4 cm) cervical cancer treated with neoadjuvant chemotherapy followed by fertility sparing surgery (CoNteSSa)/Neoadjuvant chemotherapy and conservative surgery in cervical cancer to preserve fertility (NeoCon-F)

M. Plante[1], N. van Trommel[2], S. Lheureux[3], A. Oza[3], L. Wang[3], K. Sikorska[4], S. Ferguson[5], K. Han[6], F. Amant[7,8,9]

[1]Division of Gynecologic Oncology, Laval University - Centre Hospitalier Universitaire de Québec - L'Hôtel-Dieu de Québec, Quebec, QC, Canada, [2]Gynecologic Oncology, Netherlands Cancer Institute/Antoni van Leeuwenhoek Hospital, Amsterdam, The Netherlands, [3]Drug Development Program, Princess Margaret Consortium, Toronto, ON, Canada, [4]Department of Biostatistics, The Netherlands Cancer Institute/Antoni van Leeuwenhoek Hospital, Amsterdam, The Netherlands, [5]Department of Obstetrics and Gynecology, [6]Radiation Medicine Program - Department of Radiation Oncology, Princess Margaret Cancer Center, Toronto, ON, Canada, [7]Gynecologic Oncology, Antoni van Leeuwenhoek Hospital - The Netherlands Cancer Institute, [8]Gynaecologic Oncology, Department Woman & Child, Amsterdam UMC, University of Amsterdam, Amsterdam, The Netherlands, [9]Gynecologic Oncology, KU Leuven - University of Leuven, University Hospitals Leuven, Leuven, Belgium

Introduction / Background: There is limited data regarding the optimal management of premenopausal women with cervical lesions measuring ≥2 to < 4 cm who desire to preserve fertility.
Methodology:
Study hypothesis: Neoadjuvant chemotherapy (NACT) will be effective in reducing the size of the tumor and will enable fertility-sparing surgery (FSS) without compromising oncologic outcome.
Primary objective: To evaluate the feasibility of preserving fertility.
Secondary objectives: To evaluate response rate to NACT, surgical complications and rate of FSS, overall survival up to 3 years for patients who completed NACT and FSS.
Trial design: Premenopausal women diagnosed with stage IB2, ≥2 to < 4 cm cervical cancer who wish to preserve fertility will receive 3 cycles of platinum/paclitaxel chemotherapy. Patients with complete/partial response (residual lesion < 2 cm) will undergo FSS. Patients will be followed for 3 years to monitor outcome. Patients with suboptimal response (residual lesion ≥2 cm) will receive definitive radical hysterectomy and/or chemoradiation.
Major eligibility criteria: Patients must have histologically confirmed invasive cervical cancer, ≥2 to < 4 cm lesion by clinical evaluation and MRI, negative nodes and premenopausal (≤40 years old). Following 3 cycles of NACT, patients must achieve a complete/partial response. Exclusion criteria include high-risk histology, tumor extension to uterine corpus/isthmus (as per MRI) and suboptimal response/progression following NACT.
Results:
Primary endpoints: Assess the rate of functional uterus defined as successful FSS and no adjuvant therapy.
Exploratory objectives: Evaluate patients reported outcome and quality of life, ovarian function and rate of pregnancy during follow-up period (3 years). Translational research part is included.
Conclusion: This is a Phase II, prospective international trial. 90 evaluable patients will be needed to complete the study. A stopping rule will be activated if the recurrence rate at 2 years exceeds 10%. Activation is planned for the summer 2019.
Disclosure: Nothing to disclose

ePoster Talks: Ovarian + Endometria

EP1222

Is conservative surgery appropriate in ovarian granulosa cell tumors with tumor rupture?

S. Gouy, G. Miailhe, H. Gauthier, P. Pautier, A. Maulard, C. Genestie, P. Morice, A. Leary
Gustave Roussy, Villejuif, France

Introduction / Background: To evaluate oncological and fertility outcomes after conservative surgery for ovarian granulosa cell tumors (GCT) associated with tumor rupture.
Methodology: Retrospective study of patients (pts) < 40 years, referred to our center with a GCT, who were treated with conservative surgery (contralateral ovarian and uterine preservation) and stages IC 1 and 2 according to the 2014 FIGO classification.
Results: Sixteen patients were identified (adult GCT 11 pts, juvenile GCT 5 pts); median age was 27 [19-40] years. Nine patients had stage IC1 (54%) and 7 (44%) stage IC2. Eight (50 %) patients received adjuvant chemotherapy with bleomycin, etoposide and cisplatin (PEB). With a median follow up of 9.2 [0.7-31] years, the relapse rate was high at 68% (11/16) with a median delay of 4.5 [0.6-19.6] years. The locations of relapse were pure peritoneal (n=8), contralateral ovary + peritoneal (n=2), contralateral ovary + peritoneal+ liver metastasis (N=1). Amongst these 16 patients, 11 were in remission, 3 were alive with disease and 2 died due to disease (in 2 juvenile types). Of the 11 patients with an adult GCT, 5 received adjuvant chemotherapy and 8 relapsed. Two (40%) relapsed in the adjuvant chemotherapy group whilst all of the 6 (100%) without adjuvant chemotherapy relapsed. Four patients had a pregnancy.
Conclusion: For adult GCT, conservative surgery followed by adjuvant chemotherapy with PEB as an alternative after complete surgical staging for ICs who desire fertility preservation. Completion surgery should be done after childbearing or after an age which remains to be defined (potentially at 40 years old) in order to reduce the risk of relapse. For juvenile GCT in young adults we are unable to recommend the same strategy due to the lack of data.
Disclosure: Nothing to disclose

EP1223

Fertility-sparing surgery in patients with borderline ovarian tumors and reproductive outcomes

P. Helmut[1,2], P. Harter[1], B. Ataseven[1,3], F. Heitz[1,2], S. Prader[1], S. Schneider[1], S. Heikaus[4], A. Traut[1], A. du Bois[1]
[1]Gynecology Oncology, Kliniken Essen Mitte, Essen, [2]Department of Gynecology with Center for Oncological Surgery, Charité - Universitätsmedizin Berlin, Corporate Member of Freie Universität Berlin, Humboldt-Universität zu Berlin, and Berlin Institute of Health, Berlin, [3]Department of Obstetrics and Gynecology, University Hospital, LMU, Munich, [4]Center for Pathology, Kliniken Essen Mitte, Essen, Germany

Introduction / Background: Borderline ovarian tumours (BOTs) are recognized as a unique entity of ovarian tumours that do not exert infiltrative destructive growth or stromal invasion. BOTs occur often in young patients and fertility sparing surgery (FSS) is an important issue. With this study we aimed to evaluate relapses and fertility of patients after FSS.
Methodology: Patients with BOT were included from our prospective registry database between 2000-2018. An external histopathological review was conducted in all cases. FSS was performed after individual discussion, a complete staging was defined according to FIGO, without lymphadenectomy and with a waiver for preservation of uterus and one ovary in case of FSS. Prognostic factors for Recurrence-Rates (RR) and data on fertility were studied.
Results: Among 352 Patients 80.2% had FIGO-Stage I disease and 63.9% had a serous BOT. 95 patients underwent FSS. After a median follow-up of 63 months 19 patients (5.4%) relapsed; in 5 cases a malignant transformation was reported (1.4%). After FSS 13 patients relapsed, all as BOT. The all-over recurrence-rate (RR) was 1.4% in FIGO-Stage I and 25.5 % in FIGO III-IV (HR20; 95%-CI 6.6-62; p=< .001). The corresponding RRs for Radical Surgery (RS) in stage I were 0.5%, in stage II-IV 9.8% and for FSS in stage I 3.9% and 55.6% in stage II-IV. In multivariate analysis stage II-IV (HR=22; 95%-CI: 7.2-77; p=< .001) and FSS (HR=11; 95%-CI: 2.9-44; p=.003) remained significant risk factors for recurrent disease. 36 patients (37.9%) had pregnancy desire. The cumulative incidence of first pregnancy was 77.7%. 28 patients experienced at least one term pregnancy, in total 36 Life-births were reported.

Conclusion: FSS in stage I is a safe procedure. In other stages FSS has to be discussed individually. An external second opinion regarding pathology is mandatory, this might explain partially our low rate of invasive relapses. Life-birth-rates after FSS are high.
Disclosure: H. Plett: nothing to disclose. P.Harter: Honoraria: Astra Zeneca, Roche, Sotio, Tesaro, Stryker, ASCO, Zai Lab, MSD; Advisory Board: Astra Zeneca, Roche, Tesaro, Lilly, Clovis, Immunogen, MSD/Merck; Research funding (Inst): Astra Zeneca, Roche, GSK, Boehringer Ingelheim, Medac, DFG, European Union, DKH, Tesaro, Genmab F. Heitz: Honoraria: AstraZeneca, Roche, Tesaro, Clovis; Advisory Board: Astra Zeneca, Roche, Tesaro, Clovis A. du Bois: personal fees from Roche, personal fees from Astra Zeneca, personal fees from Tesaro, personal fees from Clovis, personal fees from Pfizer, personal fees from Genmab, personal fees from Pharmar, personal fees from Biocad, outside the submitted work; B. Ataseven: Roche Advisory board, lecture, travel/accommodation expenses Tesaro Advisory board, travel/accommodation expenses Amgen Advisory board Celgene lecture PharmaMar travel/accommodation expenses Clovis lecture Astra Zeneca lecture S. Schneider: Roche: lecture Tesaro: Advisory board,lecture, travel/accommodation expenses Clovis: lecture Astra: Zeneca lecture A. Traut, S. Prader, S. Heikaus: nothing to disclose

EP1224

Predictors and clinical outcome of pancreatic fistula in patients receiving splenectomy for advanced or recurrent ovarian cancer: a large multicentric experience

G. Sozzi[1,2], C. Cicero[1], A. Fagotti[3], M. Petrillo[4], S. Domingo[5], V. Lago[5], R. Berretta[6], G. Paci[7], V. Vargiu[3], G. Scambia[3], V. Chiantera[1,2]
[1]Department of Gynecologic Oncology, ARNAS Civico Hospital, [2]University of Palermo, Palermo, [3]Department of Women's and Children's Health, Catholic University of the Sacred Heart, Roma, [4]Department of Obstetrics and Gynecology, University of Sassari, Sassari, Italy, [5]Department of Gynecologic Oncology, University Hospital La Fe, Valencia, Spain, [6]Department of Obstetrics and Gynecology, University of Parma, Parma, [7]Department of Gynecologic Oncology, University of Palermo, Palermo, Italy

Introduction / Background: The aim of the surgical treatment of advanced ovarian cancer is to completely remove all macroscopic disease.To achieve optimal cytoreduction, the use of extensive upper abdominal surgery has become widely accepted.Although the disease doesn't usually invade the splenic parenchyma, the capsule may commonly be infiltrated. These metastases may be difficult to be dissected from the pancreatic tail and a partial pancreatic resection en bloc with spleen may be required. The objective of this study is to evaluate predictors and clinical outcome of pancreatic fistula in patients receiving splenectomy for advanced or recurrent ovarian cancer.
Methodology: Data of women receiving splenectomy during cytoreductive surgery for advanced or recurrent ovarian cancer from December 2012 to May 2018 were retrospectively retrieved from the oncological databases of five institutions. Criteria's used to identify pancreatic leaks were measurement of lipase and amylase level on peri-pancreatic drainage fluid, imaging and abdominal pain referable to pancreatitis.Surgical and post-operative data were analyzed.
Results: Overall 260 patients were included in the study.Pancreatic resection was performed in 45 (17.6%) women, among them 23 patients received only pancreatic capsule resection and 22 received distal pancreatic tail resection. Intraperitoneal hypertermic chemotherapy (HIPEC) was performed in 28 (10.8%) cases. Pancreatic fistula was detected in 32 (12.3%) cases. At multivariable analysis pancreatic resection (OR: 2.064; p: 0.040), in particular pancreatic tail resection (OR:3.055; p: 0.003) and HIPEC administration (OR: 1.910; p: 0.057) were identified as independent predictors of pancreatic fistula.
Conclusion: Twelve percent of patients who received splenectomy during cytoreductive surgery for ovarian cancer developed a pancreatic leak.Distal pancreatectomy and HIPEC administration have been identified as independent predictors of pancreatic fistula in this subset of patients. No significant delay in initiation of adjuvant chemotherapy was observed in women with leakage.

	Univariate Analysis	Multivariate Analysis

Variable	Odds Ratio	p-value	Odds Ratio	p-value
Age (as continuous variable)	0.145	0.83	-	-
BMI (as continuous variable)	0.127	0.28	-	-
Hemoglobin levels (as continuous variable)	0.655	0.42	-	-
Albumin levels(<2.4 vs >2.5)	1.134	0.30	-	-
Pancreatic resection (No/Yes)	4.769	0.03	2.064	0.04
Type of pancreatic resection(No/Capsule/Tail)	13.074	0.001	3.055	0.003
HIPEC administration (No/Yes)	4.733	0.03	1.910	0.05
Operatory time (as continuous variable)	1.097	0.30	-	-

[Univariate and multivariate analysis of predictors of pancreatic fistula in the overall study population]

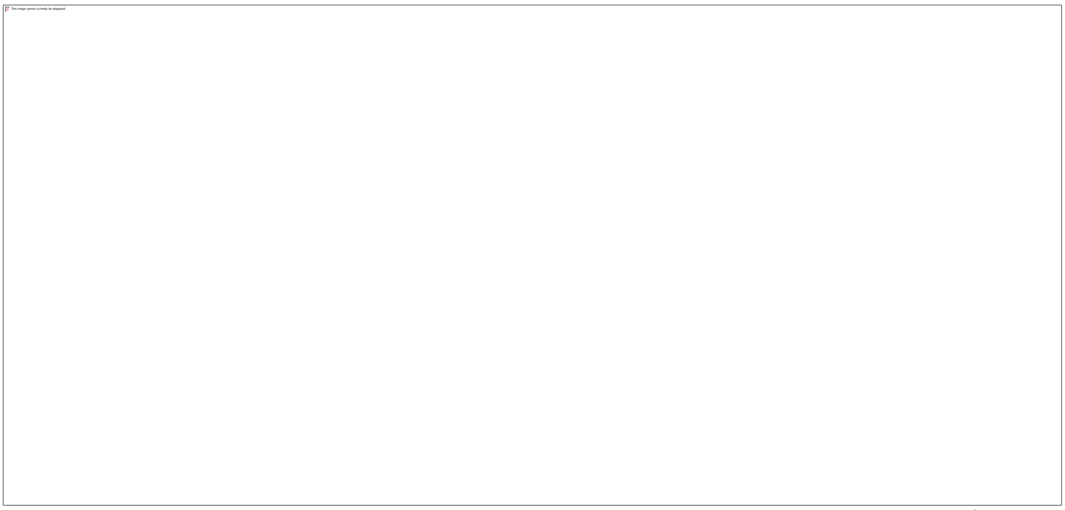

[Differences in term of time to chemotherapy according to pancreatic resection (A) or HIPEC (B)]

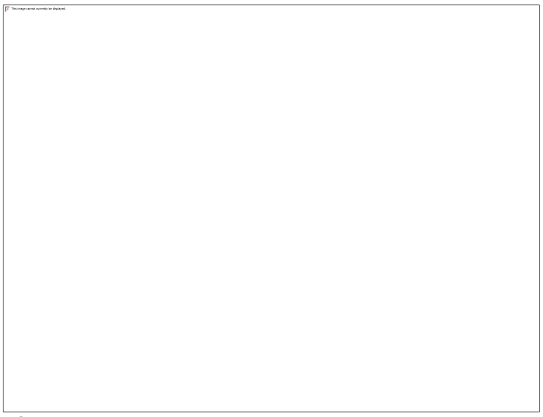

[Spleen resected en bloc with omentum, parietal and diaphragmatic peritoneum, uterus and rectum]

Disclosure: Nothing to disclose

EP1225

How the conformity of surgical practice with the national guidelines improved the quality of management of ovarian Granulosa Cell Tumors (GCT)? A TMRG and GINECO group study

C. Lenck[1], N. Chopin[1], S. Gouy[2], H. Bonsang Kitzis[3], C. Martinez Gomez[4], N. Radosevic-Robin[5], S. Martin[6], C. Lefeuvre-Plesse[7], E. Lambaudies[8], E. Leblanc[9], F. Guyon[10], J.-M. Classe[11], R. Ramanah[12], F. Beurrier[1], M. Angeles-Fite[4], C. Pomel[13], F. Joly[14], T. De La Motte Rouge[7], M. Provansal[15], A. Lesoin[16], A. Floquet[17], D. Berton-Rigaud[18], E. Kalbacher[19], P. Meus[1], F. Selle[20], I. Treilleux[21], F. Lecuru[3], P. Pautier[22], I. Ray-Coquard[23,24]

[1]Surgery, Centre Anticancereux Léon Bérard, Lyon, [2]Institut Gustave Roussy, Villejuif, [3]Surgery, Assistance Publique des Hôpitaux de Paris (AP-HP) Hôpital Georges Pompidou, Paris, [4]Surgery, Institut Universitaire du Cancer, Toulouse, [5]Pathology, Centre Jean Perrin, Clermont-Ferrand, [6]Surgery, Centre Francois Baclesse, Caen, [7]Medical Oncology, Centre Eugene Marquis, Rennes, [8]Surgery, Institut Paoli Calmettes, Marseille, [9]Centre Oscar Lambret, Lille, [10]Surgery, Institut Bergonié, Bordeaux, [11]Surgery, Institut de Cancérologie de l'Ouest, Nantes, [12]Surgery, CHRU de Besancon, Hôpital Jean Minjoz, Besancon, [13]Surgery, Centre Jean Perrin, Clermont-Ferrand, [14]Medical Oncology, Centre Francois Baclesse, Caen, [15]Medical Oncology, Institut Paoli Calmettes, Marseille, [16]Medical Oncology, Centre Oscar Lambret, Lille, [17]Medical Oncology, Institut Bergonié, Bordeaux, [18]Medical Oncology, Institut de Cancérologie de l'Ouest (site René Gauducheau), Nantes, [19]Medical Oncology, CHRU de Besancon, Hôpital Jean Minjoz, Besancon, [20]Medical Oncology, Groupe Hospitalier Diaconesses Croix Saint Simon, Hôpital de La Croix Saint Simon, Paris, [21]Pathology, Centre Anticancereux Léon Bérard, Lyon, [22]Medical Oncology, Institut Gustave Roussy, Villejuif, [23]Medical Oncology, Centre Anticancereux Léon Bérard, [24]University Claude Bernard Lyon 1, Lyon, France

Introduction / Background: Surgery is the cornerstone of ovarian Granulosa Cell Tumor (GCT). Complete resection with adequate staging is the gold standard in 1st line setting.
The aim of this study is to assess the impact of an appropriate surgery according to the guidelines.
Methodology: This is a nationwide five-year retrospective study, including 463 patients (of the 639 included patients) with a definitive diagnosis of GCT between 2011 and 2016. Medical and surgical practices were analyzed for conformity. Conformity to guidelines was defined by a conservative (unilateral salpingo-oophorectomy) or radical surgery (hysteretomy and bilateral salpingo-oophorectomy) including surgical staging (omentectomy, peritoneal biopsy and peritoneal cytology) according to the FIGO classification.
Results: Median age at diagnosis was 49 years old (range 10-89). The median size of tumor was 94 mm (range 5-400). Radical surgery was performed in 240 patients (51,8%), while a fertility-sparing surgery was performed in 98 cases (21,2%). A complete surgical staging was performed in only 76 cases (16,4%). 289 (62.4%) patients received an evaluation of the endometrium (hysterectomy or endometrial curettage).
Surgery was fully compliant with the guidelines in 65 patients (14%), partially compliant in 213 patients (46%), non-compliant in 137 patients (29,6%) and not assessable in 48 cases (10,4%) respectively. A statistically significant improvement in the surgical management was observed among patients treated after 2012 compared to those treated before 2012. This Improvement included: endometrial evaluation (p=0,026), tumour rupture rate during surgery (p=0,010) and the global compliance of the surgery (p< 0,001). Survival data will be reported.
Conclusion: Staging surgery provides a better assessment and an appropriate treatment of this rare tumors. According to this study, the implementation of a national network and guidelines dedicated to rare gynaecologic tumors seems to significantly improve the surgical management of the patients with GCT.

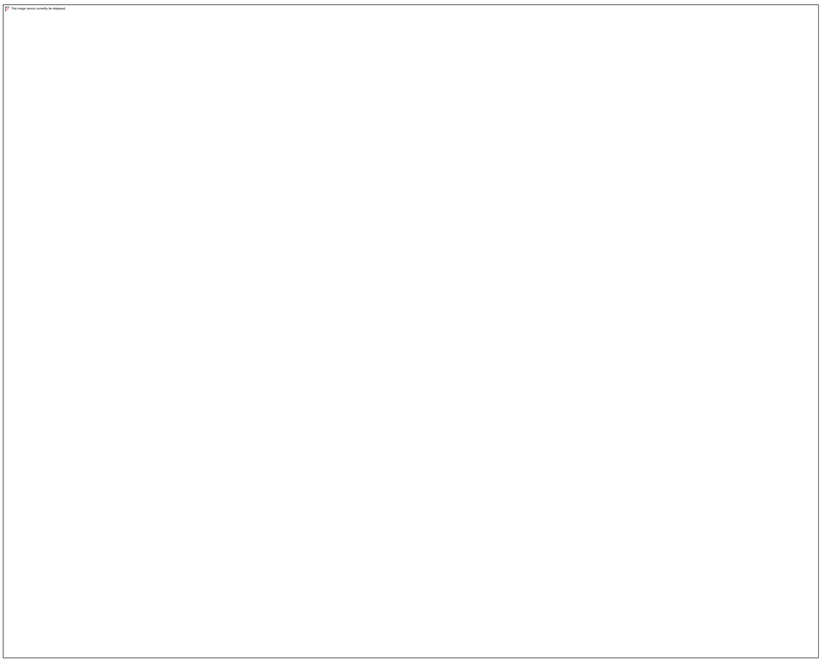

[Progression Free Survival compared to the surgical conformity]

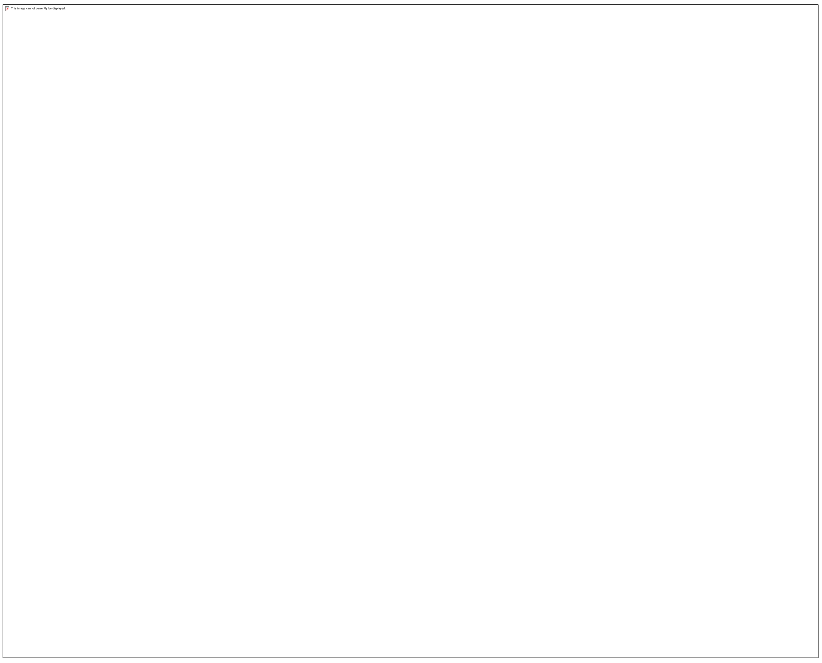

[Overall Survival compared to the surgical conformity]

Disclosure: Nothing to disclose

EP1226

Molecular and phenotypic characterisation of intra-tumoural heterogeneity in high grade serous ovarian cancer

P. Cunnea[1], E. Curry[1], K. Nixon[1], K. Thol[1], R. Wulandari[1], D. Bowtell[2], I. McNeish[1], C. Fotopoulou[1]
[1]Department of Surgery & Cancer, Imperial College London, London, United Kingdom, [2]Peter MacCallum Cancer Centre, Melbourne, VIC, Australia

Introduction / Background: High-grade serous ovarian cancer (HGSOC) is typified by high degrees of genomic instability and heterogeneity, with the majority of patients eventually acquiring resistance to platinum chemotherapy. Matching the best treatment options to patients remains challenging due to diverse platinum resistance mechanisms and limited effective predictive biomarkers. This study aims to understand the extent of intra-tumoural heterogeneity (ITH) in advanced stage HGSOC, and how this changes over time at relapse, and define the link between ITH at molecular and phenotypic levels.
Methodology: High tumour-load HGSOC patients (n=53) undergoing radical upfront-debulking underwent a tumour mapping of their tumour dissemination patterns. Tumour biopsies were collected (range 4-15, median=9), placed in short-term cultures, treated with cisplatin and apoptosis/viability assayed. DNA was extracted (5 tumours per patient) and Illumina Human OmniExpress genotyping performed. Allele-specific copy number (CN) was quantified using ASCAT. The sum of differences in gene-level CN estimates was used to define genomic dissimilarity of deposits. When relapsed, patients had paired biopsies.
Results: Tumour deposits varied extensively to each other at the copy-number level, possibly reflecting divergence from different tumour sub-clones. Different degrees of heterogeneity were observed in different patients, which appeared to correlate with progression-free survival and variance in apoptosis induction across tumours in the patient series. Patients who developed platinum-resistant disease tended to be more polyclonal. Higher *CCNE1* CN associated with *in-vitro* chemoresistance (p=0.019, r=-0.170), and poorer patient outcome (p=0.041). However, extensive variations in *CCNE1* CN were observed across multiple tumours in the same patients.
Conclusion: Vast heterogeneity is observed at the phenotypic and genomic level in HGSOC patients with direct correlation with later development of platinum-resistant disease. Extensive variations in *CCNE1* CN across multiple intra-abdominal samples within the same patient, suggests that single site biopsies cannot accurately represent overall disseminated HGSOC biology and has implications for overinterpretation of studies relating to outcome and platinum-resistance.
Disclosure: Professor Christina Fotopoulou COI: advisory boards and honoraria from Roche, Tesaro, Sequana, Olympus, Astra Zeneca

EP1227

Nutritional interventions to improve clinical outcomes in ovarian cancer: a systematic review of randomized controlled trials

C. Marchetti[1], M.C. Mele[2], E. Rinninella[2], M. Cintoni[2], P. Raoul[2], G. Scaletta[3], A. Gasbarrini[2], G. Scambia[3], A. Fagotti[3]
[1]Fondazione Policlinico Agostino Gemelli, IRCCS, Dipartimento Scienze della Salute della Donna, del Bambino e di Sanità Pubblica, UOC di Ginecologia Oncologica, Roma, [2]Fondazione Policlinico A. Gemelli, IRCCS, [3]Fondazione Policlinico Agostino Gemelli, IRCCS, Dipartimento Scienze della Salute della Donna, del Bambino e di Sanità Pubblica, UOC di Ginecologia Oncologica, Rome, Italy

Introduction / Background: Among all gynaecological neoplasms, ovarian cancer has the highest rate of disease-related malnutrition, representing an important risk factor of postoperative mortality and morbidity. Hence, the importance of finding effective nutritional interventions is crucial to improveovarian cancer patient's well-being and survival.
Methodology: This systematic review of randomized controlled trials (RCTs) aims at assessing the effects of nutritional interventions on clinical outcomes such as overall survival, progression-free survival, length of hospital stay (LOS), complications following surgery and/or chemotherapy in ovarian cancer patients.
Methods: Three electronic bibliographic databases — MEDLINE, Web of Science and Cochrane Central Register of Controlled Trials - were used to conduct a systematic literature search based on

fixed inclusion and exclusion criteria, until December 2018.
Results: A total of 14 studies were identified. Several early postoperative feeding interventions studies (n=8) were retrieved mainly demonstrating a reduction in LOS and an ameliorated intestinal recovery after surgery. Moreover, innovative nutritional approaches such as chewing gum intervention (n=1), coffee consumption (n=1), ketogenic diet intervention (n=2) or fruit and vegetable juice concentrate supplementation diet (n=1) and short-term fasting (n=1) have been shown as valid and well-tolerated nutritional strategies improving postoperative care.
Conclusion: despite an acceptable number of prospective trials, there is still a lack of homogeneous and robust endpoints. In particular, there is an urgent need ofRCTs evaluating overall survival and progression-free survival during ovarian oncology treatments. Further high-quality studies are warranted, especially prospective study and large RCTs, with more homogeneous types of intervention and clinical outcomes, including a more specific sampling of ovarian cancer women, to identify appropriate and effective nutritional strategies for this cancer at high risk of malnutrition.
Disclosure: Nothing to disclose

EP1228

LIBUSE trial - Algorithm for cervical cancer screening in the Czech Republic with usage of HPV DNA testing with HPV 16/18 genotyping and p16/Ki-67 dual-stained cytology

J. Slama[1], V. Dvorak[2], M. Trnkova[3], A. Skrivanek[4], O. Novackova[5], P. Ovesna[5]
[1]Department of Gynecology and Obstetrics, 1st Faculty of Medicine, Charles University and General University Hospital, Prague, [2]Center of Primary Care, Brno, [3]Department of Pathology, Aesculab, Prague, [4]Center of Primary Care, Olomouc, [5]Institute of Biostatistics, Masaryk University, Brno, Czech Republic

Introduction / Background: The incidence of cervical cancer in the Czech Republic remains stable over more than 30 years irrespective of existing national screening based on annual collecting of Pap smears. The aim of our prospective trial was to evaluate the role of HPV DNA testing with 16/18 genotyping and triage with p16/Ki-67 immunocytochemistry.
Methodology: Women between 30 and 60 years who had in 12 collaborating centres regular annual Pap smear were co-tested for HPV DNA with selective 16/18 genotyping (Cobas 4800, Roche). All HPV 16/18 positive cases and/or those with severe abnormality in cytology were directly refered for colposcopy; HPV non-16/18 positive cases and LSILs were triaged using p16/Ki-67 dual-stained cytology (CINtec Plus, Roche) and positive cases were refered for colposcopy while negative cases were further followed.
Results: Altogether 2407 patiens were eligible for analysis after first round of screening. Mean age of subjects was 43 years. Pap smears showed 8 cases with severe and 105 cases with mild abnormalities. There were 7.4 % (180/2418) patients with HPV positivity, from which 50 had HPV 16 and/or 18. Biopsy confirmed 32 high-grade squamous and 2 glandular lesions, all of them were HPV positive. Triage using p16/Ki-67 was positive in 22.5 % cases (29/129).
Conclusion: Screening based on HPV testing with selective 16/18 genotyping and p16/Ki-67 triage found during the first round four times more high-grade lesions including glandular lesions than standard screening based on Pap smears.
Disclosure: Nothing to disclose

EP1229

Preventing Ovarian Cancer through early Excision of Tubes and late Ovarian Removal (PROTECTOR) Study

F. Gaba[1,2], D. Chandrasekaran[1,2], U. Menon[3], G. Evans[4], N. Singh[2], G. McCluggage[5], N. Wilkinson[6],

R. Ganesan[7], G. Rowlands[8], G. Bryson[9], H. Hanson[10], E. Saridogan[6], M. Burnell[3], R. Legood[11], R. Manchanda[1,2]
[1]Barts Cancer Institute, [2]Barts Health NHS Trust, [3]University College London, London, [4]University of Manchester, Manchester, [5]Belfast Health & Social Care Trust, Belfast, [6]University College Hospitals NHS Foundation Trust, London, [7]Birmingham Women's and Children's NHS Foundation Trust, Birmingham, [8]University Hospital of Wales, Cardiff, [9]Queen Elizabeth University Hospital, Glasgow, [10]St George's University Hospitals NHS Foundation Trust, [11]London School of Hygiene and Tropical Medicine, London, United Kingdom

Introduction / Background: Aims - To evaluate impact on sexual function, endocrine function, quality-of-life (QoL), health and well-being and determine cost-effectiveness of risk-reducing early-salpingectomy and delayed oophorectomy (RRESDO), as a two-step ovarian cancer (OC) prevention strategy in pre-menopausal women at increased risk of OC.
Methodology: Design - Multi-centre, observational cohort controlled trial with three arms: RRESDO; risk-reducing salpingo-oophorectomy (RRSO); controls (no surgery).
Inclusion-criteria: Pre-menopausal women; ≥30-years; at increased risk of OC (mutation carriers or on basis of a strong family-history; completed their family (for surgical arms).
Exclusion-criteria: Post-menopausal women; previous bilateral salpingectomy or bilateral oophorectomy; pregnancy; previous tubal/ovarian/peritoneal malignancy; < 12 months post cancer treatment; clinical suspicion of tubal/ovarian cancer at baseline.
Recruitment: Through NHS cancer genetics/high-risk familial cancer clinics/general gynaecology clinics/gynaecological oncology clinics/GP surgeries/clinical referrals/supporting charities/self-referral.
Primary-outcome: Sexual function.
Secondary-outcomes: Endocrine function/menopause; regret/satisfaction; surgical morbidity; QoL/psychological health; number of intraepithelial carcinomas/invasive cancers; utility scores for early-salpingectomy; cost-effectiveness; health and well-being.
Consenting individuals for the surgical arms undergo an ultrasound, CA125, FSH and complete questionnaires collecting information on medical history, family-history, QoL, sexual function, cancer worry, psychological well-being and satisfaction/regret. All women undergoing surgery have cytological and histological assessment using a SEE-FIMM protocol, with centralised pathology review. Post-surgery FSH levels are tested and follow-up questionnaires sent at 3 months and annually. Controls have FSH levels checked and complete questionnaires at baseline and annually for three years. Participants from all three arms are invited to take part in semi-structured in-depth interviews.
Results: PROTECTOR is open to recruitment across several centres throughout the UK.
Conclusion: With growing evidence implicating the role of the fallopian tubes in ovarian carcinogenesis and the detrimental sequelae of surgical menopause in pre-menopausal women following RRSO, PROTECTOR is essential to investigate RRESDO as an alternative risk-reducing strategy in women who do not wish to undergo oophorectomy.
Disclosure: RM declares research funding from Barts and The London Charity and Roseetrees Trust for the PROTECTOR Study and is Chief Investigator. RM declares research funding from The Eve Appeal and Cancer Research UK outside this work, as well as an honorarium for grant review from Israel National Institute for Health Policy Research. RM is supported by an NHS Innovation Accelerator (NIA) Fellowship for population testing. FG is an investigator and the study coordinator for the PROTECTOR study. UM has a financial interest in Abcodia Ltd., a company formed to develop academic and commercial development of biomarkers for screening and risk prediction. NS, GM, NW, RG, GR, GB are members of the PROTECTOR central pathology review committee. GE, ES, HH, UM are members of the PROTECTOR trial management committee. MB, RL have nothing to declare.

EP1230

Development of a pragmatic assay to assess homologous recombination competency in endometrial cancer

C.J.H. Kramer[1], L.M. van Wijk[2], M.M. de Jonge[1], V. Serra[3], A. Llop-Guevara[3], C.D. de Kroon[4], M.P.G. Vreeswijk[2], T. Bosse[1]
[1]Department of Pathology, [2]Department of Human Genetics, Leiden University Medical Center, Leiden, The Netherlands, [3]Experimental Therapeutics Group, Vall d'Hebron University Hospital, Vall

d'Hebron Institute of Oncology (VHIO), Barcelona, Spain, [4]Department of Gynecology, Leiden University Medical Center, Leiden, The Netherlands

Introduction / Background: Homologous recombination deficiency (HRD) is frequent in non-endometrioid endometrial cancer (EC), justifying clinical trials testing PARP-inhibitors. To facilitate this development a pragmatic test to asses HR competency is required. Accumulation of RAD51 protein at sites of DNA double strand breaks, has been put forward as a reliable functional test for HR competency. Here we aimed to test the performance of this pragmatic assay on formalin-fixed paraffin-embedded (FFPE) tumour tissue.
Methodology: We used a previously described, prospective collected series of 21 EC that underwent primary resection with known HR status based on genomic analyses (next-generation sequencing for HR-core genes and array comparative genomic hybridization/SNP array) as our training cohort. To assure the presence of endogenous DNA damage, a gamma-H2AX staining was performed. Co-immunofluorescence (IF) was performed for both Geminin (marker staining cells in G2/S phase) and RAD51. RAD51 score was calculated as the fraction of Geminin positive cells that showed RAD51 foci (≥ 2) in the nucleus. Final RAD51 scores were correlated to known HR-status.
Results: All 21 EC showed positivity for gamma-H2AX staining assuring presence of endogenous DNA damage in routine diagnostic FFPE tumour tissue. Among all cases, the RAD51scores varied form 0-72%. The range of RAD51 scores in the five HRD EC was 0-4%, whereas the 16 HRP EC tested showed RAD51 scores ranging from 16-72% ($P < 0.001$).
Conclusion: This RAD51 assay can successfully be performed on diagnostic FFPE material and reliably determined HR competency in 100% of our EC cases using cut-off RAD51 score of 10%. We will present the performance of this cut-off on our test-set of HR-annotated EC. This RAD51 assay is a promising cheap, pragmatic assay that can be used in the clinic to select patients that may benefit from platinum compounds and PARP-inhibitor therapy.
Disclosure: Nothing to disclose

EP1231

Impact of population genetic testing and ovarian cancer risk stratification on the emotional well-being and health of unselected women in a general population

F. Gaba[1,2], X. Liu[1], D. Chandrasekaran[1,2], J. Kalsi[3], A. Antoniou[4], L. Side[5], U. Menon[3], I. Jacobs[6], D. Marks[7], R. Manchanda[1,2]
[1]Barts Cancer Institute, [2]Department of Gynaecological Oncology, St Bartholomew's Hospital, [3]University College London, London, [4]University of Cambridge, Cambridge, [5]University Hospital Southampton NHS Foundation Trust, Southampton, United Kingdom, [6]University of New South Wales, Sydney, NSW, Australia, [7]London School of Hygiene and Tropical Medicine, London, United Kingdom

Introduction / Background: Algorithms for predicting ovarian-cancer (OC) risk, have been developed and validated in the 'Predicting-Risk-of-Ovarian-Malignancy-Improved-Screening and Early-detection' (PROMISE) programme. This enables population stratification for OC-risk prediction, screening and prevention. We present the results of a qualitative-study exploring the range of attitudes, experiences and impact on emotional wellbeing, lifestyle and health following unselected panel-genetic-testing (PGT) and OC-risk stratification in unselected women ≥18-years ascertained through primary care networks in the PROMISE Feasibility-Study (ISRCTN 54246466). This is the first qualitative-study in unselected general-population women undergoing PGT. Qualitative data enables care to be provided according to insights of users by gaining a better understanding of, and reasons for choices made.
Methodology: In-depth semi-structured 1:1 interviews were conducted using a pre-developed topic-guide (development informed by literature review and expert consultation) until informational saturation reached. Wording and sequencing of questions were left open with probes used to elicit additional information. All interviews were audio recorded and transcribed verbatim. Questions were fine-tuned during a pilot-interview. Transcripts were analysed using an inductive theoretical framework and data managed using NVIVO-v12.
Results: Informational saturation was reached following ten interviews. Eight interconnected themes were identified: health behavioural choices; interest; counselling; decision making; facilitators/barriers determining acceptability; effect of results on health/wellbeing; results communication; satisfaction.

Overall satisfaction with PGT/OC risk stratification was high and no interviewees expressed regret. The most important facilitators were ease of testing, learning about children's risk, access and ease of surgical prevention. Barriers included change in family dynamics, insurance, stigmatization, having personality traits associated with stress/worry.
Conclusion: Population-based genetic-testing for OC-risk prediction in general-population women is associated with high acceptability and satisfaction. The facilitators and barriers observed are largely similar to those reported with genetic-testing seen in high-risk cancer clinics and unselected testing in the Jewish population.
Disclosure: RM declares research funding from Cancer Research UK and The Eve Appeal for the PROMISE Feasibility Study and is Chief Investigator. RM declares research funding from Barts & the London Charity and Rosetrees Trust outside this work, as well as an honorarium for grant review from Israel National Institute for Health Policy Research and honorarium for advisory board membership from Astrazeneca/MSD. RM is supported by an NHS Innovation Accelerator (NIA) Fellowship for population testing. IJ and UM have a financial interest in Abcodia, Ltd., a company formed to develop academic and commercial development of biomarkers for screening and risk prediction. IJ is a member of the board of Abcodia Ltd, a Director of Women's Health Specialists Ltd and received consultancy from Beckton Dickinson. FG is an investigator and the study coordinator for the PROMISE Feasibility study. XL, DC, DM, LS, JK, AA have nothing to declare.

Poster exhibition Day 1

P01

Molecular investigation of the DNA methylation status of one four-gene panel in Greek patients from breast FNAC samples

E. Vavoulidis[1], N. Kougioumtsidou[1], M. Nasioutziki[1], E. Mareti[1], G.C. Pratilas[1], A. Liberis[1], S. Petousis[1], E. Chasioti[1], A. Daniilidis[1], A. Papanikolaou[1], L. Zepiridis[2], K. Dinas[1]
[1]2nd Obstetrics & Gynaecology, [2]1rst Obstetrics & Gynaecology, Aristotle University of Thessaloniki, Thessaloniki, Greece

Introduction / Background: Alterations in DNA methylation pattern are common in various cancers including, breast tumors. Aberrant methylation of normally unmethylated CpG-rich areas which are in/near the promoter region, has been associated with transcriptional inactivation of various genes in breast cancer patients, with most studies focusing on tissue samples. In this study, we determined the frequency of aberrant promoter methylation of the *APC*, *CDH13*, *HIN-1* & *CYCLIND2* genes through molecular analysis of cytological material obtained with fine needle aspiration (FNAC).
Methodology: Our study retrospectively analyzed 103 women who visited our Breast Pathology Clinic. FNAC was collected in Cytolyt (Hologic). DNA was extracted from breast cells with Quick-gDNA MiniPrep kit (Zymo) and was subjected to complete bisulfite conversion with EZ DNA Methylation-Gold Kit (Zymo). A methylation specific PCR was carried out with primers designed for amplification of either the methylated or the unmethylated condition for each gene. The final PCR-products were separated in 2% agarose gels with electrophoresis.
Results: DNA methylation was observed for all 4 genes in over 75% of the C4 and C5 cytologically-classified samples (n=66). In the "gray zone" of C3 cytologically-classified samples (n=11), the methylation frequencies were 72.7% for *CND2* & *APC* and 63.7% for *CDH13* & *HIN1*. In comparison, the vast majority of C1 and C2 cytologically-classified samples were unmethylated for all 4 genes with methylation frequencies ranging from 0% to 25%.
Conclusion: It seems that in patients with C3 to C5 cytological diagnosis, high methylation frequencies were observed for all candidate genes in contrast with the low frequencies in patients negative for neoplasia or with benign breast lesions. A multiplex genetic panel for detection of DNA methylation, combined with cytological diagnosis and other clinical/lifestyle parameters, during a non-operative FNA could provide important information for clinicians pre-operatively leading to a more accurate detection and staging of breast tumors.
Disclosure: Nothing to disclose

P02

Is it still necessary to perform sentinel lymph node biopsy in breast cancer patients with negative axillary ultrasound in post-Z0011 era? - retrospective analysis

L. Dostalek[1], P. Saskova[1], P. Steyerova[2], D. Pavlista[1]
[1]*Gynecologic Oncology Center, Department of Obstetrics and Gynecology, First Faculty of Medicine, Charles University in Prague and General University Hospital in Prague,* [2]*Depeartment of Radiology, First Faculty of Medicine, Charles University in Prague and General University Hospital in Prague, Prague, Czech Republic*

Introduction / Background: According to the results of ACOSOG Z0011 trial, there is no need to complete axillary dissection in patients after breast conserving surgery and one or two positive sentinel lymph nodes (SLN).
Because axillary ultrasound has high sensitivity and specificity in detection of macrometastases in axilla, chance of missing more than two macrometastases is supposed to be low.
Aim of this trial was to assess the number patients with negative axillary ultrasound who had more than two infiltrated SLNs in definitive histology (and did not fulfill the inclusion criteria for Z0011 trial) and the number of patients in whom axillary macometastases would be missed if SLN biopsy (and axillary dissection) was not performed.
Methodology: Breast cancer patients with tumors up to 5cm and no metastases on axillary ultrasound treated in our institution in 2016 were included into the retrospective analysis.
Macrometastsis in SLN was an indication for axillary dissection.
Number of patients with more than two macrometastases in SLNs and total number of patients with nodal macrometastases were evaluated.
Results: There were 87 patients enrolled into the trial - 11 of them had macrometastases in SLNs: 10 patients had one and 1 had three macrometastases in SLNs.
All these 11 patients were subject to axillary dissection - in one patient there were 4 more macrometastases in other axillary lymph nodes.
Conclusion: 1 patient (1%) did not fulfill the inclusion criteria of Z0011 trial and thus SLN biopsy would change the management. If SLN biopsy wasn't performed, macrometastases in 11(13%) patients would be missed. Of these, one had 3 and one had 5 macrometastases.
Nowadays it is not clear if these missed axillary nodal macrometastases are of any impact on oncological outcome. Future prospective trials will bring more evidence on this topic.
Disclosure: Nothing to disclose

P03

Management of ovarian cysts in premenopausal women with tamoxifen

A. Goeminne[1], C. Maggen[1], D. Timmerman[1], P. Neven[2]
[1]*Obstetrics and Gynaecology,* [2]*Multidisciplinary Breast Centre, KU Leuven - University of Leuven, Leuven, Belgium*

Introduction / Background: In this retrospective study, we investigated the ovarian function of 69 premenopausal patients with hormone-receptor positive, human epidermal growth factor receptor 2 (HER2) negative breast cancer receiving tamoxifen with a focus on the management and survival of patients with ovarian cyst formation.
Methodology: 69 premenopausal women treated with tamoxifen for early primary HER2- negative breast cancer - referred to the gynaecology ultrasound department of the university hospitals of Leuven between 2003 and 2018 - were selected. Patients that received chemotherapy, adjuvant radiotherapy or ovarian suppression were excluded. Oncological data, gynaecological ultrasound reports, details on menstruation pattern during tamoxifen and hormonal blood tests were obtained from patient records.
Results: In total, 31 patients developed a cyst during tamoxifen treatment. Median age at diagnosis of breast cancer was 47 years (37-53) and younger patients were more likely to develop ovarian cysts

(p< 0.002). Median interval between initiation of tamoxifen and detection of ovarian cysts was 10 months (1-59). Median size of ovarian cysts was 47 mm (30-96). Most patients (20/31) were followed conservatively, with spontaneous regression of the cysts. In 4/31 patients regression occurred after interruption of tamoxifen or additional ovarian suppression. Surgery was performed in 7/31 patients for large symptomatic cysts or torsion. Bleeding pattern changed in 55 out of 68 of the patients (one patient was excluded because of hysterectomy), most frequently oligomenorrhea or amenorrhea. 13/49 available records of the patients had high oestradiol levels (>400 pg/ml) while treated with tamoxifen. Survival of all patients was excellent; with a median follow-up of 73 months no events occurred.
Conclusion: We reported that the administration of tamoxifen in premenopausal women is associated with ovarian cyst formation and high estradiol levels. Ovarian cysts mostly can be managed conservatively. In this series, the impact of tamoxifen on ovarian function does not appear to affect survival.
Disclosure: Nothing to disclose

P04

Serum concentration of selected molecules involved in angiogenesis in different breast cancer subtypes

D. Balalis[1], S. Kokkali[2], N. Koufopoulos[3], P. Provatopoulou[4], E. Kalogera[4], G. Bletsa[4], I. Misitzis[5], D. Dimas[5], N. Arkadopoulos[6], C. Spiliopoulou[7], D. Vlachodimitropoulos[7]
[1]Surgery Department, [2]First Medical Oncology, Saint-Savvas Anticancer Hospital, [3]2nd Pathology Department, Attikon University Hospital, Medical School of Athens, [4]Research Center, Hellenic Anticancer Institute, [5]Breast Cancer Surgical Department, Saint-Savvas Anticancer Hospital, [6]Surgery Department, Attikon University Hospital, [7]Department of Forensic Medicine and Toxicology, Faculty of Medicine, National and Kapodistrian University of Athens, Athens, Greece

Introduction / Background: Angiogenesis is a hallmark of BC. Heparin-binding-EGF-like (HB-EGF) belongs to epidermal growing factors (EGF) family and binds to EGFR. In combination to platelet-derived growing factor (PDGF) it induces tumor growth. In triple negative breast cancer (TNBC) HB-EGF is associated with aggressiveness. VEGF co-receptor neurolipin-1 is also involved in VEGF pathway.
Methodology: We measured serum levels of VEGF, HB-EGF, PDGF and neuropilin-1 in patients diagnosed with early BC in Saint-Savvas Hospital and healthy donors (HD), using ELISA. All individuals signed an informed consent.
Results: Sixty patients with early BC and 20 HD were included. Serum concentrations of all biomarkers were higher in patients versus HD (table 1). The four biomarkers were analyzed across BC subtypes (table 2). VEGF, HB-EGF and PDGF were lower in HER2-overexpressing versus non-HER2 over-expressing subtypes. In contrast, neuropilin-1 was higher in HER2-overexpressing subtype. Patients with TNBC presented the highest level of VEGF and HB-EGF.
Conclusion: Concentration of angiogenic molecules seem to differ across BC subtypes. TNBC patients have high levels of growing factors and lower levels of neuropilin-1. Angiogenesis seems to play an important role in TNBC.

	Patients	Healthy donors
VEGF (pg/ml)	315.0 (33.7-1011.0)	307.8 (52.7-723.0)
HB-EGF (pg/ml)	148.8 (56.8-276.2)	137.1 (41.8-293.0)
PDGF (pg/ml)	960.4 (459.4-1923.0)	958.2 (367.9-1354.0)
NEUROPILIN-1 (ng/ml)	241.7 (114.8-395.4)	237.7 (158.7-320.4)

[Serum concentration of the 4 biomarkers in breast cancer patients and healthy donors. Concentrations are expressed in mean value and range.]

	Luminal A (N=20)	Luminal B (N=20)	Triple negative (N=11)	Her2-overexpressing (N=9)
VEGF (pg/ml)	250.2 (63.2-651.8)	378.1 (58.71-1011.0)	424.1 (274.4-891.3)	135.8 (33.7-659.2)
HB-EGF (pg/ml)	154.2 (68.1-276.2)	151.6 (56.8-274.0)	179.8 (91.9-255.4)	127.7 (99.4-179.1)
PDGF (pg/ml)	1042.0 (645.9-1840.0)	947.9 (528.7-1923.0)	1033.0 (459.4-1544.0)	891.2 (563.2-1320.0)
NEUROPILIN-1 (ng/ml)	224.1 (128.6-370.2)	242.7 (174.0-376.5)	223.0 (114.8-321.9)	269.4 (158.9-395.4)

[Serum concentration of the 4 biomarkers in breast cancer patients across different histotypes. Concentrations are expressed in mean value and range.]

Disclosure: Nothing to disclose

P05

Cardiac safety of the trastuzumab biosimilar ABP 980 in women with HER2-positive early breast cancer in the LILAC study

H.-C. Kolberg[1], M. Colleoni[2], G. Savva Demetriou[3], P. Santi[4], H. Tesch[5], Y. Fujiwara[6], Z. Tomasevic[7], V. Hanes[8]
[1]Marienhospital Bottropg GmbH, Klinik für Gynäkologie und Geburtshilfe, Bottrop, Germany, [2]European Institute of Oncology, Milan, Italy, [3]Wits Oncology Wits Donald Gordon Medical Centre, Parktown, South Africa, [4]Centro de Estudos de Hematologia e Oncologia, São Paolo, Brazil, [5]Haemato-Oncology Practice, Bethanien Hospital, Frankfurt am Main, Germany, [6]National Cancer Center Hospital, Tokyo, Japan, [7]Institute for Oncology and Radiology of Serbia, Daily Chemotherapy Hospital, Belgrade, Serbia, [8]Amgen Inc., Thousand Oaks, CA, United States

Introduction / Background: Although well-tolerated, trastuzumab can lead to severe heart failure in 2-4% of patients. In the phase 3 LILAC trial, trastuzumab biosimilar ABP 980 demonstrated similar efficacy, safety, and immunogenicity to trastuzumab reference product (RP) in women with HER2-positive early breast cancer. Here we report on cardiac safety of ABP 980 versus RP.
Methodology: In the LILAC trial, all 725 patients randomly assigned to receive ABP 980 (n=364) or trastuzumab RP (n=361) were assessed for adverse events (AEs) every 3 weeks (Q3W) and for cardiac safety by left ventricular ejection fraction (LVEF) 2D echocardiogram every 3 months. LVEF decline was defined as the value decrease from study baseline by ≥10 percentage points and to < 50%. After breast surgery, patients received investigational product (IP) Q3W for up to 1 year; ABP 980-treated patients continued ABP 980, and RP-treated patients either continued RP (n=190) or switched to ABP 980 (RP/ABP 980; n=171).
Results: IP disposition was well balanced between treatment groups. Over the entire trial, 22 (3.1%) patients had LVEF decline by ≥10 percentage points from baseline and to < 50%; no meaningful between-group differences were observed (ABP 980:10/359 [2.8%], RP: 6/184 [3.3%], RP/ABP 980: 6/171 [3.5%]). The incidence of cardiac AEs was low and comparable between treatment groups. One grade 3 cardiac failure event was reported in the RP/ABP 980 arm; another in the RP arm was coincident with LVEF decline. During the adjuvant phase, no patient discontinued IP due to cardiac failure.
Conclusion: These pre-specified analyses confirm the tolerability of ABP 980 and demonstrate clinical similarity of ABP 980 and RP with respect to cardiac safety. The incidence of LVEF decline was consistent with the known cardiac safety profile of the RP. No new cardiac safety signals were observed whether patients were on ABP 980 or switched from RP to ABP 980.

Disclosure: H-C Kolberg received consulting fees from Novartis, GSK, Pfizer, Carl Zeiss Meditec, Genomic Health, LIV Pharma, SurgVision, Roche, TEVA, Theraclion, Amgen Inc, Janssen; honoraria from AstraZeneca, Amgen, Theraclion, ZEISS, Novartis, Pfizer, Roche, Genomic Health, SurgVision; contracted Research with Amgen Inc.; and holds stocks in Theraclion and Phaon Scientific. M. Colleoni received honoraria from Novartis; consulting fees from Pierre Fabre, Pfizer, OBI Pharma, Puma Biotechnology, Celldex, AstraZeneca; and contracted Research with Amgen Inc. G. Demetriou received consulting fees from AstraZeneca and Profmed; received honoraria from Roche/Genentech; and honoraria for Speakers' bureau from Merck Serono. P. Santi declares no conflict of interest. H. Tesch received consulting fees as advisor and Research funding from Amgen. Y. Fujiwara received consulting fees from Bristol-Myers Squibb Japan and AstraZeneca KK; and honoraria from AstraZeneca KK, Daiichi Sankyo, and SRL Diagnostics. Z. Tomasevic received honoraria and consulting fees from Roche, Pfizer, and Novartis; honoraria for Speakers' Bureau from AstraZeneca, Roche, and Pfizer; and contracted Research with Amgen Inc. V. Hanes is an employee of Amgen and holds stock in Amgen.

P06

Low dose metronomic chemotherapy as an efficient treatment option in metastatic breast cancer - results of an exploratory case-control study

S. Krajnak, C. Schnatz, K. Almstedt, W. Brenner, A.-S. Heimes, S. Nezi-Cahn, R. Schwab, A. Hasenburg, M. Schmidt, M.J. Battista
Department of Gynaecology and Obstetrics, University Medical Centre Mainz, Mainz, Germany

Introduction / Background: There is a growing importance of low-dose metronomic chemotherapy (LDMC) in metastatic breast cancer (MBC). In this retrospective case-control-analysis we compared the efficacy of LDMC and conventional chemotherapy in MBC.
Methodology: Each LDMC patient receiving oral cyclophosphamide (CTX) (50 mg daily) and methotrexate (MTX) (2.5 mg every other day) was matched with two patients who received conventional chemotherapy. Age, number of chemotherapy lines and metastatic lesions as well as hormone receptor (HR) status were considered as matching criteria. Primary endpoint was disease control rate greater than 24 weeks (DCR). Secondary endpoints were DCR in subgroups (age at begin of therapy, number of chemotherapy lines, number of different metastatic lesions and HR status), progression-free survival (PFS) and duration of response (DoR).
Results: A total of 40 cases and 80 controls entered the study. 30% patients with LDMC and 23% patients with conventional chemotherapy showed DCR (p=0.380). Among younger patients DCR was 40% in LDMC vs. 25% in the control group (p=0.249). In addition, DCR was achieved in 33% vs. 26% patients with ≤ 2 chemotherapy lines (p=0.568) and in 36% vs. 18% patients with ≤ 2 different metastatic lesions (p=0.096), respectively. In the triple negative group 30% LDMC vs. 5% control patients showed DCR (p=0.095). The median PFS was 12.5 weeks in both groups (p=0.218). The median DoR was 31 weeks in LDMC and 21 weeks in the control group (p=0.383).
Conclusion: In this retrospective case-control study we demonstrated a similar efficacy of LDMC compared to conventional chemotherapy in the treatment of MBC. Moreover, no significant differences were found in the subgroups studied. Therefore, the concept of LDMC may also be a treatment option in both younger and non-heavily pre-treated MBC patients who do not need rapid remission.
Disclosure: Nothing to disclose

P07

Feasibility of preserving intercostobrachial nerve and the medial pectoral nerve during radical mastectomy and breast conservative surgery

G. Psaras[1], H. Dudarovska[1], S. Voloshin[1], O. Kalinina[1], M. Diasamidze[1], T. Marharynt[1], O. Kichyk[1], V. Savran[2], O. Logutov[1], V. Saienko[1], M. Doroshenko[1], Y. Kovalova[1]
[1]Surgical, Mariupol Cancer Center, Mariupol, [2]Internal Deseases #2, Lviv Medical Institute, Lviv, Ukraine

Introduction / Background: Preserving intercostobrachial nerve (IBN) and the medial pectoral nerve (MPN) during radical mastectomy (RM) and breast conservative surgery (BCS) improves the quality of life of patients (pts) with breast cancer. The aim of the study was to assess the feasibility of preserving IBN and MPN during RM and BCS.
Methodology: The analyses were done on a retrospective consecutive series of 164 pts with breast cancer treated by RM and BCS in the Mariupol Cancer Center from January 2017 to December 2018. Of these, 119 pts underwent RM, and 45 - BCS.
Results: Postoperative mortality was not observed. Morbidity was 7.6±2.4% after RM and 8.9±4.2% after BCS. After RM and BCS, it was possible to save IBN and MPN in 34.7±3.7% pts, after RM - in 27,7±4.1% pts, after BCS - in 53,3±7.4% pts. It was possible to save one of the nerves (or IBN or MPN) after RM in 1,7±1.2% pts, after BCS - in 20,0±6.0% pts. In BCS, axillary lymph node dissection from a single access was performed in 23 pts, and from a separate access in 22 pts. When using a single access, the preservation of IBN and MPN was possible in 65.2±9.9% of pts, from a separate - in 40.9±10.5%. The duration of BCS was 158.7 ± 13.2 min. The duration of BCS with save IBN and MPN was 163.4 ± 14.5 min, while BCS without nerve preservation - 145.8 ± 29.6 min. The duration of RM with save IBN and MPN was 133.5 ± 10.5 min, while the RM without nerve preservation - 101.1 ± 10.0 min.
Conclusion: During RM and BCS, it is possible to save IBN and MPN in one third of pts.
Disclosure: Nothing to disclose

P08

Treatment outcomes of locally advanced cervical cancer (LACC) after concurrent chemoradiation followed by high-dose-rate brachytherapy (HDR): a sixteen year single institution experience

M. Agulto-Mercadal, G. Amparo
Section of Gynecologic Oncology and Trophoblastic Disease, Jose R. Reyes Memorial Medical Center, Manila, Philippines

Introduction / Background: A retrospective study of patients with LACC, Stage IB2-IVA,treated with concurrent chemoradiation followed by HDR brachytherapy at a single tertiary institution was conducted. This paper assessed the outcomes, prognostic factors, overall disease-free survival (DFS) and overall survival (OS) of cervical cancer after such treatment.
Methodology: Patients treated with concurrent chemoradiation followed by HDR brachytherapy for cervical cancer from January 2001 to December 2016 were analysed. Treatment consisted of pelvic external beam radiation therapy with concurrent chemotherapy followed by HDR brachytherapy. DFS, OS and prognostic variables were assessed.
Results: A total of 313 cervical cancer patients treated with concurrent chemoradiation followed by HDR brachytherapy was included. Mean age was 50.4±9.7 years, mostly presenting with Stage III disease (67.73%) and squamous cell histology (77.64%). Recurrent disease was seen in 21.09% of patients at a median of 29.32 months during median follow-up of 34.09 months (range: 3-180 months). Most common sites of recurrence were pelvic (12.78%), osseous (2.24%) and nodal (2.24%). DFS at 5 and 10 years were 60% and 53.61%, respectively. OS at 5 and 10 years were at 91.68% and 74.78%, respectively. Tumor size 3 months post-brachytherapy (HR 1.646 (1.164-2.329), p-value: 0.005), and overall treatment time (HR 1.022 (1.007-1.038), p-value: 0.004) were significant prognostic factors for recurrence while age ≤ 40 years (HR 3.497 (1.189-10.282), p-value: 0.023), presence of recurrence (HR 39.906 (5.043-315.785), p-value: 0.000), and tumor size 3 months post-brachytherapy (HR 2.497 (1.271-4.906), p-value: 0.008) were important prognostic factors for survival.
Conclusion: This study showed that concurrent chemoradiation followed by HDR brachytherapy for locally advanced cervical cancer has a 5-year DFS and OS of 60% and 91.68%, respectively. Tumor size 3 months post-brachytherapy and overall treatment time were significant prognostic factors for

recurrence, while age ≤ 40 years, presence of recurrence and tumor size 3 months post-brachytherapy were important prognostic factors for survival.
Disclosure: Nothing to disclose

P11

Immediate colposcopy findings among women with high risk HPV other than HVPV16 / 18 and normal cytology

S. Aydın[1], H.N. Kayahan[1], D.S. Arıcı[2]
[1]*Obstetric and Gynecology,* [2]*Pathology, Bezmialem Vakif University, İstanbul, Turkey*

Introduction / Background: Persistent infection with 1 of 14 high-risk genotypes Human Papilloma Virus (HPV) genotypes is the crucitial for the development of cervical cancer. high grade cervical cancer precursors. These HPV genotypes can be named as high risk HPV types (HR-HPV). The reassuring management of women with cytology negative, HR-HPV positive is important especially after the widespread use of HPV testing either as a cotest or primary screening. The aim of our study was to compare the colposcopic biopsy results of women with HPV 16/18 with other HR-HPV genotypes and determine positive predictive values (PPV) for CIN2+ of other HR HPV genotypes.
Methodology: We prospectively had included the women with negative cytology and positive HR-HPV test other than HPV 16 /18. Control group was composed of women with negative cytology positive test result for either HPV 16 or HPV 18. Women with HR-HPV positive, cytology negative referred to immediate colposcopy.
Results: Totaly 185 colposcopies were performed for HRHPV test positive, of these 97 had HRHPV positive test other than HPV 16/18. The prevalance of CIN1 and CIN2 is significantly higher in HPV16/18 group than pooled other HRHPV group (34.1% vs 17.5%, p=0.01 for CIN 1+; 14.8% vs 5.2%, p=0.03 for CIN 2+). The prevalance of CIN3 was almost three fold in women with HPV 16/18 (9.1% vs 3.1%). PPV for CIN 2+ was 16.4 (9.1-27.3) for HPV16, 11.7 (2-37.7) for HPV18, 20 (3.5-55.7) for HPV 31, 11.1 (0.6-49.3) for HPV 51, 12.5 (0.6-53.3) for HPV 58 and 59.
Conclusion: Although HPV 16 preserve high PPV among basal cytology negative population, especially HPV 33, 51, 58, 59 and 18 had similiar predictive values. Full genotyping or genotyping of HR-HPV could help reassuring traiage of women with negative cytology.

	HPV 16-18 (n=88)	O-HRHPV (n=97)	p value
CIN 1+ (n, %)	30 (34.1)	17 (17.5)	0.01
CIN 2+ (n, %)	13 (14.8)	5 (5.2)	0.03
CIN 3 (n, %)	8 (9.1)	3 (3.1)	0.08

[Table 1: Immediate colposcopic biopsy diagnosis among HPV16/18 and O-HRHPV groups. HPV16/18 includes any type of infection with HPV16+ or HPV18+; OHRH]

	CIN 2+ PPV (95% CI)	CIN 3 PPV (95% CI)
HPV 16/18	14.7 (8.4-24.3)	9.1 (4.2-17.6)
HPV 16	16.4 (9.1-27.3)	10.1 (5.1-20.9)
HPV 18	11.7 (2-37.7)	-
O-HRHPV	5.1 (1.9-12.1)	3.1 (0.8-9)
HRHPV	9.7 (6-15)	5.9 (3.1-10.6)
HPV 31	20 (3.5-55.7)	-
HPV 51	11.1 (0.6-49.3)	11.1 (0.6-49.3)

HPV 58	12.5 (0.6-53.3)	-
HPV 59	12.5 (0.6-53.3)	12.5 (0.6-53.3)

[Table 2 Diagnostic value of HRHPV for screening high level cervical lesions in cytology negative population.]

Disclosure: Nothing to disclose

P12

Risk factors for failure of bilateral sentinel lymph node mapping in early-stage cervical cancer

A. Bresset[1], V. Balaya[1], B. Guani[2], L. Magaud[3], H. Bonsang-Kitzis[1], M. Deloménie[1], H.-T. Nguyen-Xuan[1], M. Koual[1], R. Montero Macias[1], C. Ngo[1], A.-S. Bats[1], P. Mathevet[2], F. Lécuru[1], SENTICOL
[1]Gynecologic and Breast Oncologic Surgery Department, Georges Pompidou European Hospital, Paris, France, [2]Gynecologic Department, University Hospital of Vaud, Lausanne, Switzerland, [3]Pôle IMER, Hospices Civils de Lyon, Lyon, France

Introduction / Background: The objective of this study was to determine clinical, tumor and surgical factors associated with sucessful bilateral sentinel lymph node mapping (SBM) in early-stage cervical cancer.
Methodology: We performed an ancillary work on the data of two prospective trials on SLN biopsy (SENTICOL I & II). Patients having lymph node dissection for FIGO IA-IIA cervical cancer were included between 2005 and 2012 from 25 French oncologic centers. Sentinel lymph node (SLN) was detected by a combined labeling technique (blue and isotopic).
Results: 405 patients were included for analysis: SLNs were identified on at least one side of the pelvis in 381 patients (94.1%) and bilaterally in 326 patients (80.5%). No SLNs were found in 24 patients (6%). The mean age was 43 years [22-85 years]. Most patients (88.1%) had IB1 clinical FIGO stage. The majority of patients (71%) had squamous cells carcinoma. Surgeries were mainly performed by mini-invasive approach (368 patients - 90.9%). By multivariate analysis, SBM was associated with inclusion during the period 2009-2012 (ORa= 2.24, 95%CI = [1.09 - 4.61], p =0.03) compared to the period 2005-2007. Failure of bilateral SLN mapping was associated with Age ≥ 70 years (ORa= 0.02, 95%CI= [0.002 - 0.29], p = 0.003), BMI > 30 kg/m2 (ORa= 0.33, 95%CI= [0.15 - 0.75], p = 0.01) and tumor size ≥ 20 mm (ORa= 0.41, 95%CI= [0.19 - 0.87], p = 0.02)
Conclusion: A better experience of SLN biopsy technique improved SBM rate in early-stage cervical cancer. Age higher than 70 years, obesity and tumor larger than 20 mm decreased siignificantly SBM.
Disclosure: Nothing to disclose

P13

Role of one-step nucleic acid amplification (OSNA) to detect sentinel lymph node micro-metastasis in early-stage cervical cancer

N. Bizzarri[1], L. Pedone Anchora[1], G.F. Zannoni[2], A. Santoro[2], M. Valente[2], V. Gallotta[1], C. Conte[1], E. Teodorico[1], G.L.M. Di Fiore[1], V. Ghirardi[1], A. Fagotti[1], G. Ferrandina[1], G. Scambia[1]
[1]Gynecologic Oncology Division, [2]Pathology Department, Policlinico Universitario Agostino Gemelli I.R.C.C.S. Universita Cattolica Di Roma, Rome, Italy

Introduction / Background: Evidence in literature supports the accuracy of sentinel lymph node (SLN) in early-stage cervical cancer. SLN is usually analyzed through ultra-staging examination, which cannot be performed intraoperatively as it is too cumbersome and time-consuming. One-step nucleic

acid amplification (OSNA) is a rapid assay able to detect cytokeratin (CK)19-mRNA in SLN and it can be used for intra-operative detection of macro-metastasis, micro-metastasis and isolated tumor cells (ITC). It consists of a short homogenization step followed by amplification of CK19-mRNA directly from the lysate. We aim to evaluate if OSNA could be used to detect micro-metastasis in patients with early cervical cancer.

Methodology: Consecutive patients who underwent surgery for FIGO stage IA1 LVSI+ to IB1 between 01/2015-02/2019, and had SLN biopsy+/-pelvic lymphadenectomy (PLN), were included. SLN was detected with cervical injection of indocyanine-green (1.25mg/ml) and sent intra-operatively for OSNA. OSNA result was considered: negative: < 160 CK19-copies/µL; ITC 160-249 CK19-copies/µL; micro-metastasis 250-5000 CK19-copies/µL; macro-metastasis >5000 CK19-copies/µL. The first 12 OSNA cases had immunohistochemistry staining for CK19 in the primary tumour.

Results: 109 patients underwent SLN biopsy: 59 (54.1%) were analyzed with H&E, 29 (26.6%) with OSNA and 21 (19.3%) with ultra-staging. 12/12 (100%) primary tumors were CK19-positive. Patients' characteristics are demonstrated in Table 1. In the OSNA group, 5 (17.2%) patients had unilateral and 24 (82.8%) bilateral mapping. OSNA detected micro-metastasis in 7/29 (24.1%) patients. In 5/7 (71.4%) cases, systematic bilateral PLN was performed and in all these patients, SLN was the only positive node. When PLN was performed, no false negative in bilateral detection and 1/5 (20%) false negative with unilateral detection was found.

Conclusion: This is one of the first series investigating the application of SLN CK19-OSNA in early-cervical cancer. OSNA is able to detect micro-metastasis in SLN. However, further cases are necessary to define the positive predictive value of this technique.

[Table 1]

Disclosure: Nothing to disclose

P14

Long-term survival outcomes after inframesenteric versus infrarenal extraperitoneal para-aortic lymphadenectomy for the pretherapeutic staging of locally advanced cervical cancer

A. Boscher[1], E. Tresch-Bruneel[2], A. Cordoba[3], A. Lemaire[4], M. Ben Haj-Amor[5], A. Chevalier[6], F. Narducci[1], E. Leblanc[1]
[1]Oncogynecologic Surgery, [2]Department of Statistics, [3]Department of Radiotherapy, [4]Pathology Unit, [5]Department of Medical Imaging, [6]Department of Oncocology, Centre Oscar Lambret, Lille, France

Introduction / Background: The surgical pretherapeutic staging of para-aortic (PA) lymph nodes in locally advanced cervical cancers (LACC) was historically limited to the left renal vein cranially. Given the low rate of isolated skip metastasis above the IMA, the potential surgical morbidity and the absence of evidence regarding the survival benefits of the supramesenteric dissection, a study was conducted to compare inframesenteric (IM-PALND) and infrarenal (IR-PALND) PA lymphadenectomy for patients with LACC. The long-term survival outcomes of these patients are presented hereunder.
Methodology: A prospective cohort of IM-PALND was compared with a retrospective series of patients with IR-PALND in a single referral center, from 2011 to 2015. All patients had a negative pretherapeutic imaging (MRI and PET/CT) at the PA level. The therapeutic sequence included an extraperitoneal laparoscopic PALND, concomitant chemoradiation and uterovaginal brachytherapy, followed or not by surgery, for primary or recurrent LACC.
Results: 119 patients were included: 55 in the IM-PALND group, and 64 in the IR-PALND group. The populations were comparable in terms of comorbidities, histology, and FIGO stage. Nodal PA status were similar for both groups. The median follow-up was 65 months. At 5 years, overall survival (58.6% versus 69.8%, p=0.49), recurrence-free survival (57.1% versus 55.3%, p=0.90), distant metastasis-free survival (56.9% versus 58.5%, p=0.69) and paraaortic recurrence-free survival (58.5% versus 66.5%, p=0.50) were not significantly different between IM-PALND and IR-PALND, respectively.
Conclusion: This is the first report on comparative long-term survival outcomes between IM-PALND and IR-PALND for the pretherapeutic staging of LACC. Long-term survival outcomes were not significantly different between IM-PALND and IR-PALND.
Disclosure: Nothing to disclose

P15

Cervical intraepithelial neoplasia and cervical cancer: a genome wide association study (GWAS) of the UK BioBank Cohort

S. Bowden[1], I. Kalliala[1], M. Wielscher[2], B. Bodinier[2], J. Flanagan[1], M. Chadeau-Hyam[2], M.-R. Jarvelin[2], M. Kyrgiou[1]
[1]Surgery and Cancer, [2]Biostatistics and Epidemiology, Imperial College London, London, United Kingdom

Introduction / Background: Persistent infection with high-risk human papillomavirus (HPV) is causally associated with cervical cancer. However, only ~1% of women with HPV infection progress to cervical neoplasia (CIN). It is estimated that heritability may explain 25-30% of total variation in liability for cervical cancer. Common genetic variants have been detected in HLA (Human Leukocyte Antigen) regions responsible for the immune response, but this is not well understood. We aimed to conduct the largest genome-wide association study (GWAS) of cervical cancer to date, using UK Biobank data to identify genetic polymorphisms associated with CIN and cervical cancer.
Methodology: Using phenotypic data available from linked UK cancer registries, hospital ICD-10 records, operative procedures and participant interviews we identified 6378 women with CIN3/cervical cancer and 198,441 controls. With UK Biobank genotyping calls (Affymetrix UK Biobank Axiom® array) we conducted genome wide analyses, adjusting for population structure and potential confounders. As a stringent correction for multiple testing, we considered a pre-test significance level of 10^{-8} to declare single nucleotide polymorphisms (SNPs).
Results: In the first UK Biobank iteration we have identified potential SNPs (p< 5x10E-8) associated

with CIN3/cervical cancer, with a large number of significant loci residing within Chromosomes 2 and 6 (Fig1). Independent loci in the Major Histocompatibility Complex (MHC) region at 6p21.3 were associated with CIN3/cervical cancer, including loci adjacent to the MHC class 1 polypeptide-related sequence A gene (MICA) and HLA-DRB1 (Fig2), which replicates previously reported associations from published GWAS.

Conclusion: We observed genetic variants significantly associated with CIN3/cervical cancer in both cohorts. Loci within the MHC may affect susceptibility to development of CIN3/cervical cancer through altered immune responses. We will next undertake fine-mapping within the UK Biobank cohort, to further classify any novel causal variants that may explain the estimated genetic susceptibility to cervical cancer.

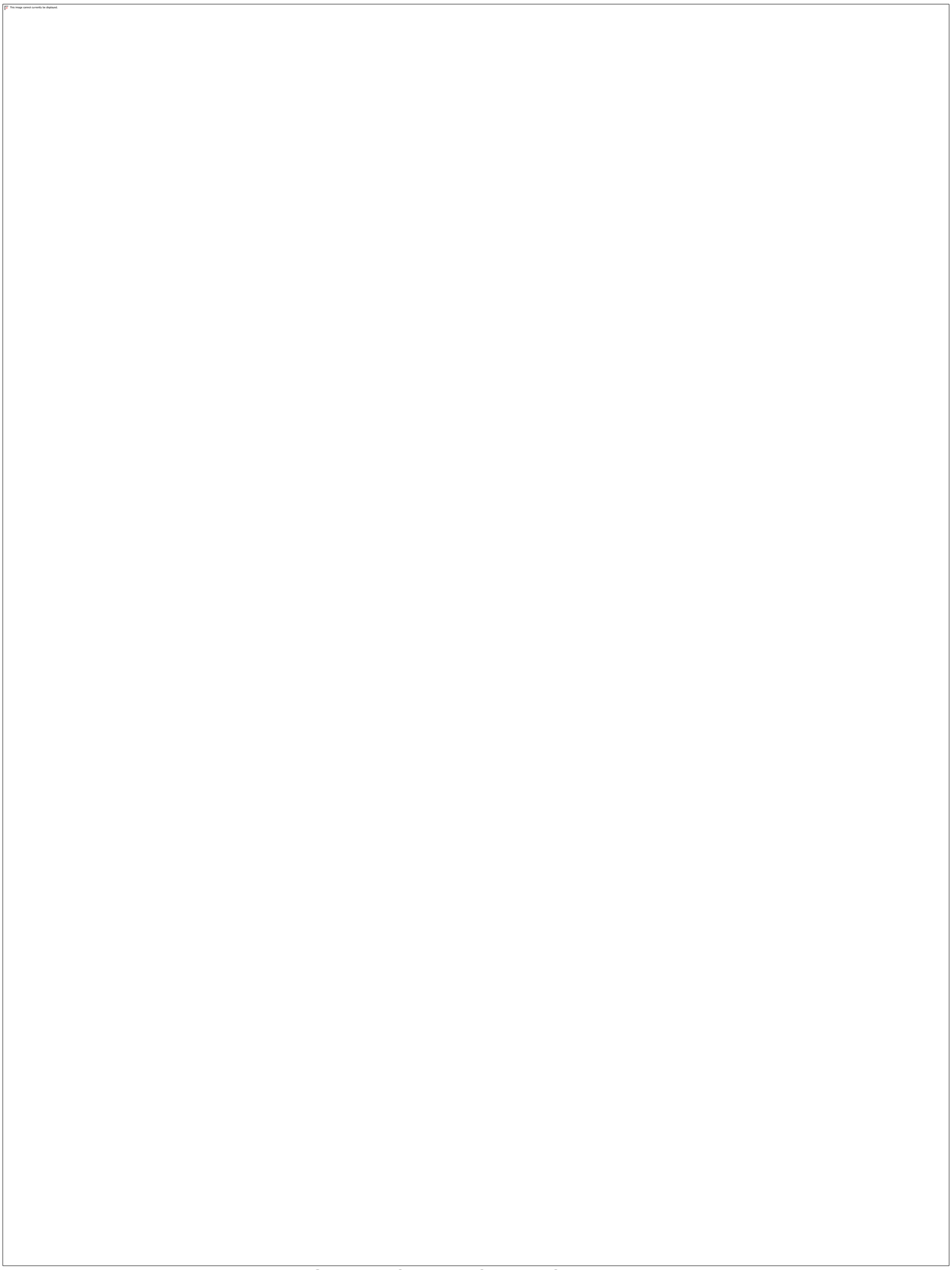

[Manhattan plot of genome wide SNPs in Cervical Cancer/CIN3 in UK Biobank cohort]

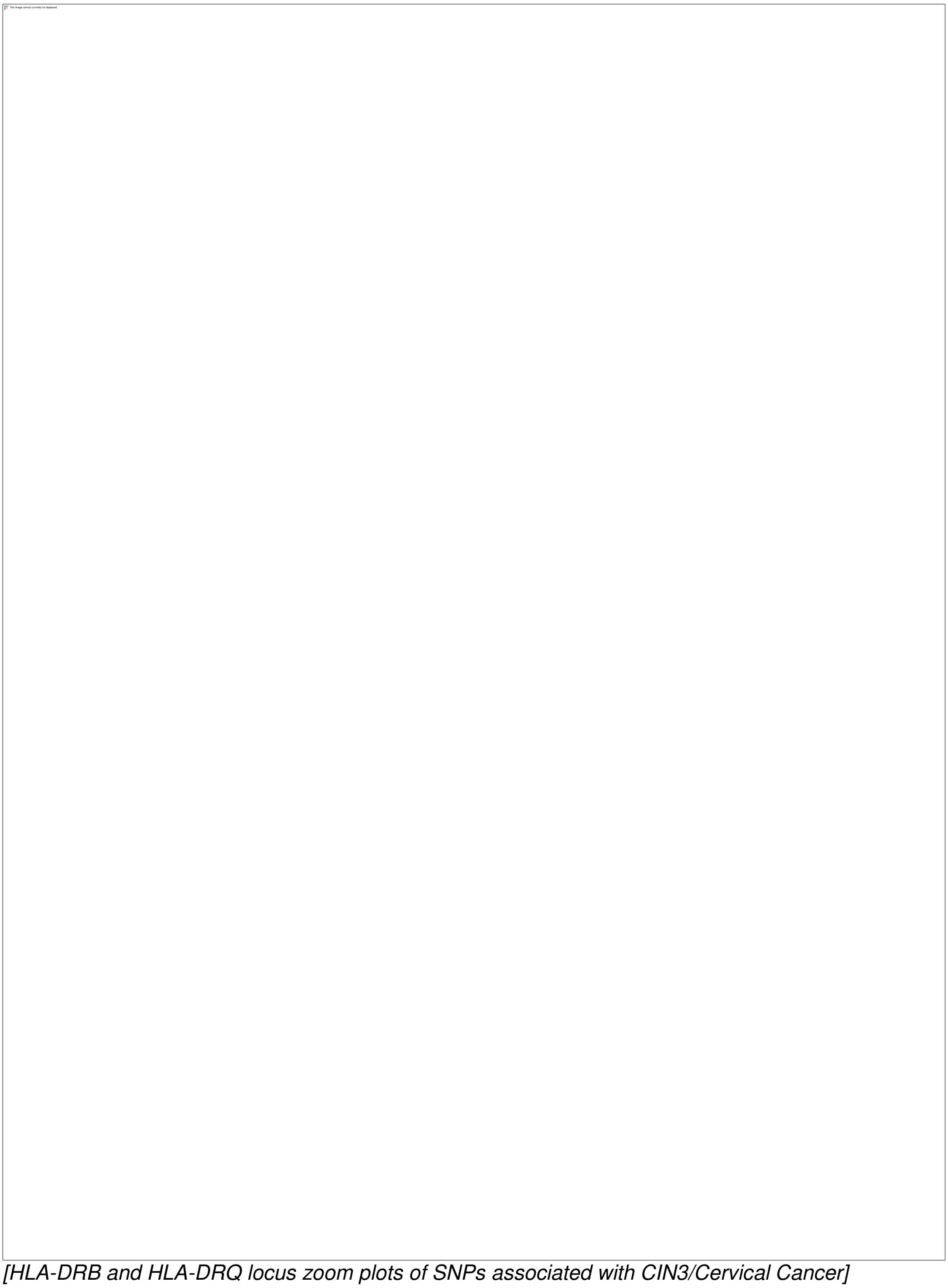

[HLA-DRB and HLA-DRQ locus zoom plots of SNPs associated with CIN3/Cervical Cancer]

Disclosure: Funding provided by an NIHR Academic Clinical Fellowship (SL) and a Wellcome Trust Clinician Scientist Fellowship (SL). No conflicts of interest to declare.

P16

Factors influencing recurrence in patients undergoing laparoscopic treatment for early stage cervical cancer

J. Casarin[1], G. Bogani[2], C. Pinelli[1], A. Papadia[3], M.D. Mueller[3], F. Raspagliesi[2], F. Ghezzi[1]
[1]Obstetrics and Gynecology, University of Insubria, Varese, [2]Gynaecology Oncology, National Cancer Institute, Milan, Italy, [3]Gynaecology Oncology, University Hospital of Bern, Bern, Switzerland

Introduction / Background: Following the results of LACC trial, laparoscopic treatment for early stage cervical cancer has become matter of debate. In the present investigation we aimed to evaluate predictors of recurrence after laparoscopic surgery for early stage cervical cancer (CC)
Methodology: A multi-centre retrospective study was conducted among patients who underwent laparoscopic radical surgery for FIGO 2009 stage IA (positive LVSI) - IB1 at three referral gynecologic oncology centers, between January 2006 and June 2018. All patients had radical hysterectomy (B-C1) with or without lymph node dissection according with tumour characteristics. Surgical and oncological outcomes were analysed
Results: Among 186 patients who met the inclusion criteria, 16 (8,6%) experienced recurrence (6 vault, 9 pelvis, 5 abdominal, 3 distant, 7 multiple), and 9 (4.8%) died of disease over the follow-up period (median follow-up 38.0 months). Surgical-related complications did not influence survival outcomes. All the recurrences occurred in stage Ib1 disease, which has been found to be the main predictor of recurrence (p=0.02), while no association between positive lymph node and relapse was detected. Patients who had preoperative conization (93, 50%) had a significant lower rate of recurrence compared to those who had biopsy (93, 50%) (1/93, 1,1% vs. 15/93: 16,1% p=0.0003). After stratification by FIGO stage, patients with Ib1 tumor undergoing conization had significant favourable DFS compared to those who had only biopsy (p=0.012)
Conclusion: Preoperative conization might play a crucial role in patients undergoing laparoscopic treatment for early stage CC. Further studies are warranted to strength our finding.

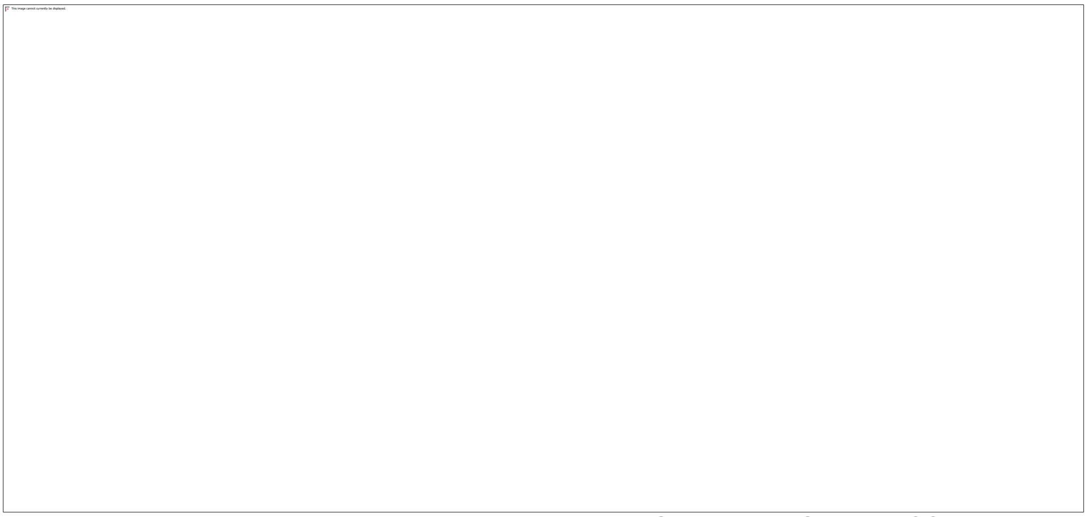

[Analysis of consecutive patients laparoscopically treated for Stage Ia1 (LVSI+) - Ib1 CC]

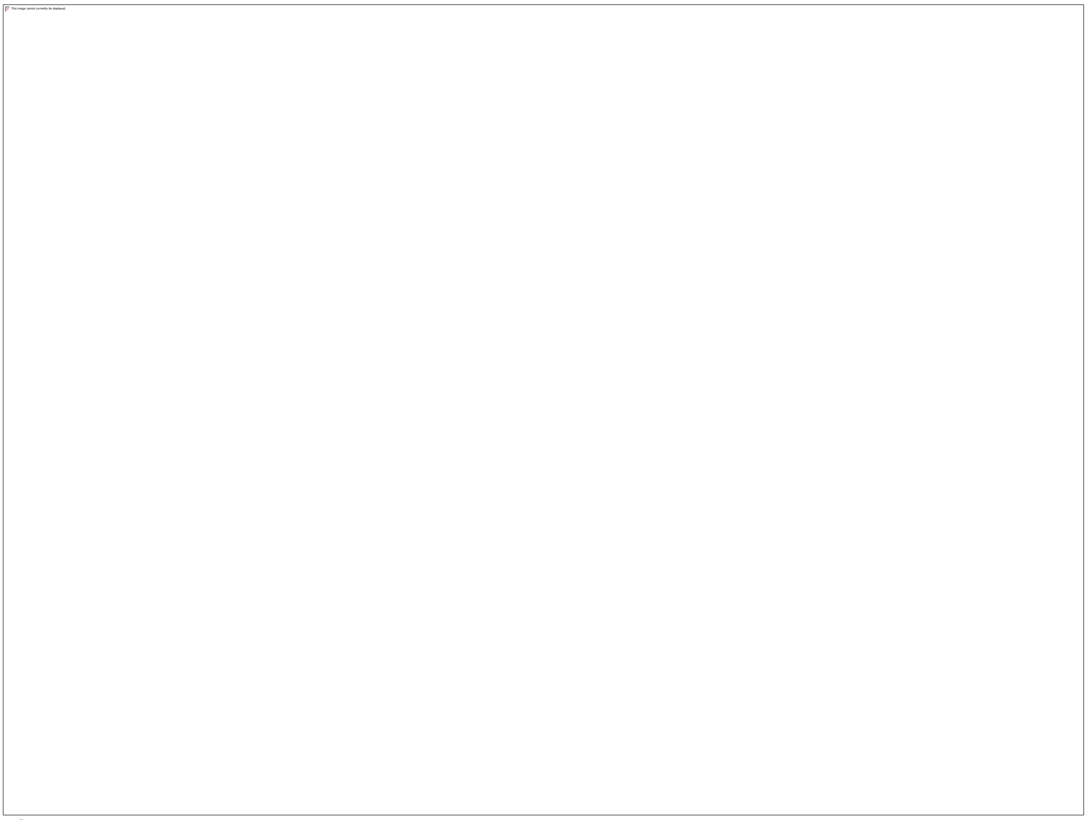

[Survival outcomes and pattern of recurrence of the study population]

Disclosure: Nothing to disclose

P17

The clinicopathological characteristics and prognosis of cervical stump carcinoma: a retrospective analysis

J. Cheng, X. Wu, H. Wen
Fudan University Shanghai Cancer Center, Shanghai, China

Introduction / Background: Carcinoma of cervical stump rarely happens to patients who underwent supracervical hysterectomy (SCH) previously. It is difficult to be treated with brachytherapy because of the pelvic anatomic modification, so most patients are treated with surgery. This study aimed to evaluate clinicopathological characteristics and prognosis of cervical stump carcinoma compared to matched controls with ordinary cervical carcinoma.
Methodology: In this retrospective analysis, 96 consecutive cervical stump carcinoma cases who received radical surgery in Fudan University Shanghai Cancer Center between Apr 2006 and Feb 2014. Three controls to each case were randomly selected from ordinary cervical carcinoma using propensity score matching analysis (matched to FIGO stage, age, diagnostic year, ECOG performance status, menopause status, childbirth status and histologic type). The clinicopathological characteristics and follow-up data were analyzed and compared between controls and cases.
Results: The average age of cervical stump carcinoma was 50.7 years old and pathological examination showed 86.5 % of squamous cell carcinoma, 10.4% of adenocarcinoma and 3.1% of adenosquamous carcinoma. There were 63.5% cases in FIGO stage I, 35.4% cases in stage II and 1% cases in stage IIIa. The significant prognostic prognostic factors included FIGO stage, tumor size

and lymph node metastasis. Compared to controls, less patients with cervical stump carcinoma had stromal invasion >1/2 (p=0. 001) and received adjuvant therapy(p=0.009). Otherwise, there was no statistical difference between cervical stump carcinoma and ordinary cervical carcinoma in 5-years OS (84.6% vs 85.5%, p=0.750) and RFS (85.2% vs 81.4%, p=0.389).
Conclusion: There are no statistical difference in survival between Cervical stump carcinoma and ordinary cervical carcinoma.

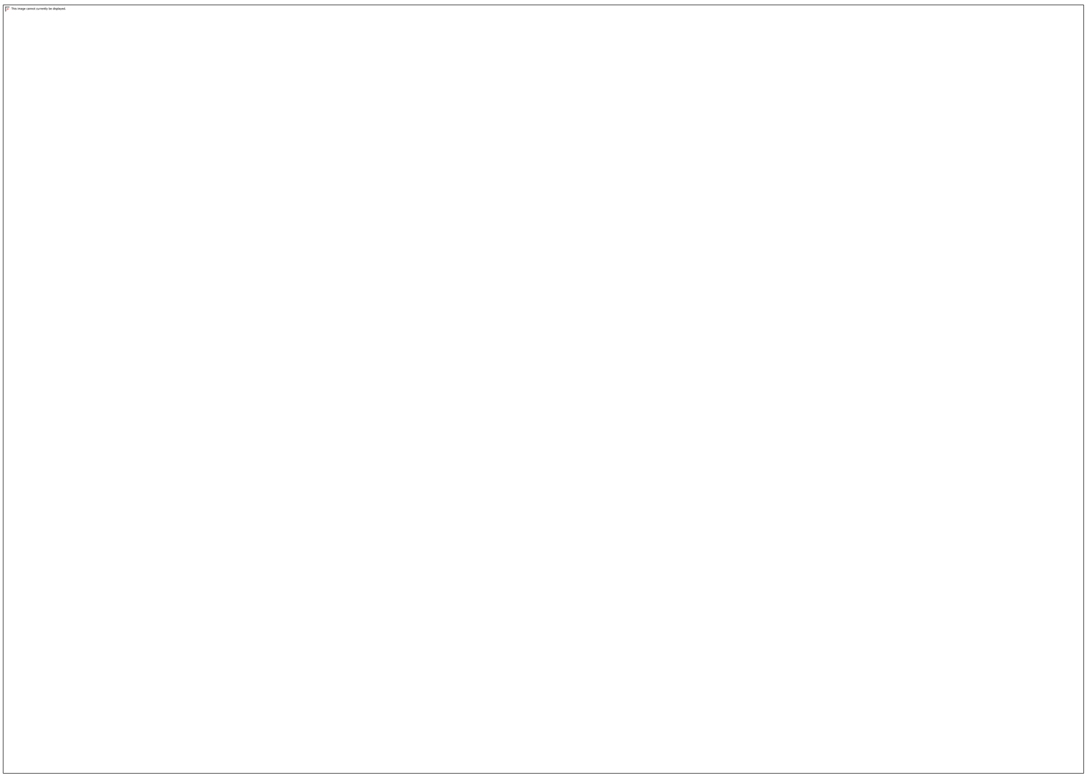

[Figure1. OS and RFS of cervical stump carcinoma compared to matched controls.]

Disclosure: Nothing to disclose

P18
Tumor free distance is the best predictive marker in patients with early-stage cervical cancer treated by primary surgery

D. Cibula[1], R. Kocián[1], L. Dostálek[1], D. Fischerová[1], A. Germanová[1], F. Frühauf[1], P. Dundr[2], K. Němejcová[2], J. Jarkovský[3], A. Burgetová[4], J. Sláma[1]
[1]Gynecologic Oncology Center, Department of Obstetrics and Gynecology, First Faculty of Medicine, Charles University in Prague and General University Hospital in Prague, [2]Department of Pathology, First Faculty of Medicine, Charles University in Prague and General University Hospital in Prague, [3]Institute of Health Information and Statistics of the Czech Republic, [4]Department of radiology, First Faculty of Medicine, Charles University in Prague and General University Hospital in Prague, Prague, Czech Republic

Introduction / Background: Main limitation of the majority of previous studies on prognostic markers lied in an insufficient standardisation of both clinical management and the method of assessment of individual parameters.

Methodology: All consecutive patients with early-stage cervical cancer treated by primary surgery in a single centre between 01/2007 and 12/2016 were eligible if they were assessed by standardized protocols for preoperative imaging and pathology. Fifteen prognostic parameters were evaluated, including age, 11 tumour-related (stage; largest tumour size; tumour size binarized; depth of stromal invasion; minimal tumour free distance (TFD); TFD binarized; lymphovascular space invasion (LVSI); tumour type; grade; parametrial invasion) and 3 lymph node (LN) status related ones (number of positive LNs; LN involvement; type of metastasis in LN).
Results: Data from 378 consecutive patients were analysed. Table 1 shows characteristics of the whole group (Cohort A) and LN negative patients (Cohort B). All parameters were associated with a risk of recurrence (RR), except for age and grade, in Cohort A, but only 4 remained significant in Cohort B (tumor type, grade, minimal TFD, TFD binarized). The best predictive model for Cohort A entailed a combination of TFD≤3.5 mm and LN positivity, which discriminated a subgroup of 42 patients with RR 36% versus 6.5% in the rest of the cohort (Figure1). In Cohort B a combination of TFD≤3.5 mm and adenosquamous tumour type discriminated a small group of 9 patients (RR 33% versus 6% (Figure 2).
Conclusion: TFD surpassed all other traditional tumor-related markers in the assessment of the recurrence risk in both cohorts. Predictive models combining TFD with LN status (whole cohort) or histological type (LN negative cases) can easily be used in daily practice and can identify the smallest possible group of patients with the highest risk of recurrence.

BMJ Publishing Group Limited (BMJ) disclaims all liability and responsibility arising from any reliance placed on this supplemental material which has been supplied by the author(s)

This image cannot currently be displayed.

[Table 1: Summary of demographic and clinical parameters in Cohort A (all cases) and Cohort B (LN neg]

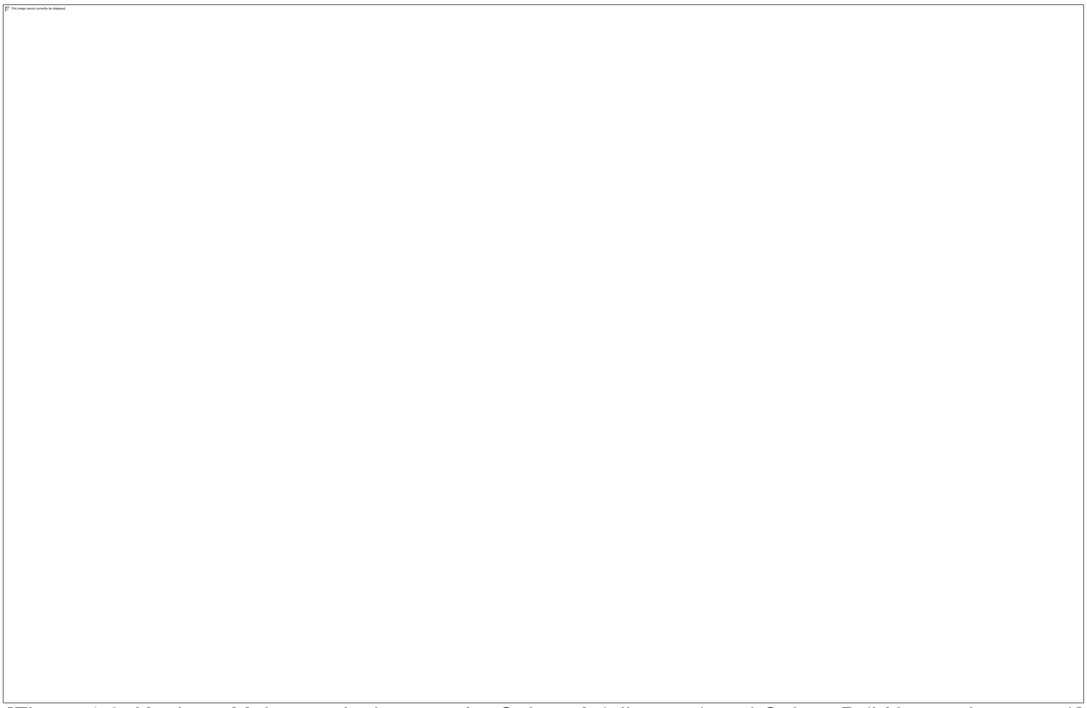

[Figure 1,2: Kaplan - Meier survival curves for Cohort A (all cases) and Cohort B (LN negative cases)]

Disclosure: Disclosures: None of the authors declare a conflict of interest. **Acknowledgements:** This work was supported by Charles University in Prague (UNCE 204065 and PROGRES Q28/LF1), by the project of Ministry of Health of the Czech Republic (MZ CR - RVO VFN64165) and by a grant from the Czech Research Council (No 16-31643A).

P19

Villoglandular adenocarcinoma of the uterine cervix: support for less invasive management

A. Dietl[1], K. Aumann[2]
[1]Obstetrics and Gynecology, University of Erlangen, Erlangen, [2]Medical Centre - University of Freiburg, Institute for Surgical Pathology, Freiburg, Germany

Introduction / Background: Villoglandular adenocarcinoma (VGA) of the uterine cervix is a rare tumour and has been classified as a variant of a cervical adenocarcinoma with good prognosis, but radical therapy still remains the standard treatment. Less invasive management could be an option.
Methodology: Case series and case reports were identified by searching PubMed, and from references in relevant articles from 1989 (the year VGA was first described) to 2018. We used the search terms: “villoglandular”, “cervical cancer”, “adenocarcinoma”. The extracted data on tumour characteristics were: stage Ia-Ib1, lnos, LVSI status, specific surgical approach, pure VGA). Three patients were excluded because of a complex histology (VGA combined with underlying adenocarcinoma or squamous cell carcinoma).
Results: fter exclusion for repeated publications, we identified 54 reports, involving 320 patients, in which VGA was analysed. 91 patients were excluded because of stage Ib2, II, III, unknown (48), no follow up data (n=40), and no pure VGA (n=3). A total of 229 patients was evaluated, 45 (20 %) were treated by a “conservative management” (conisation, simple hysterectomy, trachelectomy) and 184 (80 %) by an “invasive management” (radical hysterectomy with or without chemoradiationtherapy). Recurrences have been found in the “conservative group” in one case (2,2%, ROD: 44 months) and in the “invasive group” in 10 cases (5,4%), respectively (ROD: 12, 17, 22, 24, 34, 42, 42, 44, 61, ?(NED):

153 months). Four patients (2, 2%) died after “invasive therapy” (DOD: “a few months later”, 42 months, “fifth year”, 79 months).
Conclusion: The excellent prognosis of pure VGA and the young age of these patients are justifiable that this tumour may be managed by a less radical procedure. The histological diagnosis of VGA is a challenge and pre-treatment should not be based only on a simple punch biopsy but a conisation with widely tumour-free margins.
Disclosure: Nothing to disclose

P20

Lymphocytes count at diagnosis is a potential survival predictor in patients treated by chemoradiation in locally advanced cervical cancer

C. Gennigens[1], F. Kridelka[2], G. Jerusalem[1], L. Seidel[3], F. Goffin[2], A. Barbeaux[4], A. Kakkos[2], J. Hermesse[5], B. Sautois[1], E. Gonne[1], M. De Cuypere[2]
[1]Medical Oncology, [2]Obstetrics and Gynecology, [3]Biostatistics, University of Liège, Liège, [4]Medical Oncology, CHR East Belgium, Verviers, [5]Radiotherapy, University of Liège, Liège, Belgium

Introduction / Background: Cancer-mediated inflammation and immunosuppression together with concomitant chemoradiation (CCRT) affects the hematological profile of patients treated for locally advanced cervical cancer (LACC). We investigated the predictive value of a low hemoglobin (Hb) and lymphocytes counts on the outcome of patients receiving CCRT for advanced cervical neoplasia.
Methodology: Between January 2010 and May 2017, 134 consecutive patients with LACC (FIGO 2009, stages IB2-IVA), received CCRT at the CHU of Liège (Belgium). The data were reviewed retrospectively. Detailed hematological parameters, at initiation (week1) and during CCRT (week >=2) were collected. Cox univariate analysis measured the impact of Hb and lymphocytes variations on patients recurrence-free-survival (RFS) and cancer-specific-survival (CSS).
Results: Median follow-up was 37.5 months. Twenty five patients developed recurrence of whom 20 died from the disease. The mCSS is 69.7 months and mRFS is 36.7 months.
Hemoglobin level at diagnosis (p = 0.67) (HR = 1.046-IC = 0.850-1.288) or during treatment (p = 0.96) (HR = 0.989-IC = 0.640-1.528) does not affect survival. With a p value of 0,077 and a HR of 0.417 (IC = 0.158-1.100) absolute lymphocyte count at week1 demonstrates a borderline significance on CSS. We also demonstrate the same trend with percentage of lymphocytes with a p = 0.060 and a HR of 0.952-IC = 0.906-1.001.
Conclusion: Lymphocytes count at initiation of CCRT could be an attractive prognostic factor for patients treated for LACC. In the era of immunotherapy, it could be considered as a potential biomarker for selecting patients benefiting from a combination of radiotherapy and immunotherapy (checkpoint inhibitors).
Disclosure: Nothing to disclose

P21

Role of the mode of delivery on the rate of regression of cytologic abnormalities during pregnancy

M. Hernandez Ontoria, R. Bermejo Martínez, F. Martínez Hernández, A. Villalba Gutierrez, C. Almansa, E. Lorenzo Hernando, L. Muñoz Hernando, V. Bravo Violeta
Gynecology and Obstetrics, Hospital 12 Octubre, Madrid, Spain

Introduction / Background: The physiologic change of the cervix during pregnancy and delivery may theoretically have positive influences on spontaneous regression of abnormal cervical cytology. The aim of the study is to determine the correlation between the role of delivery and the clearance of

cervical cytologic abnormalities.
Methodology: Unicentric retrospective study of 116 patients with cytologic abnormalities detected during pregnancy followed at Hospital 12 de Octubre (Madrid, Spain) between 2009 and 2018.
Results: The mean age at diagnosis was 29.7 years (15-52). 19.8% patients were smokers, 8 had a history of sexually transmitted infection (STI) and 5 were immunocompromised. 4.3% had been vaccinated against HPV before diagnosis. The most common cervical cytologic abnormality was ASC-US in 35.3%, followed by LSIL in 34.5% and HSIL in 23.3%. High risk HPV types were detected in 53 patients. 85.3% of the patients underwent a colposcopy. In 29 cases a biopsy was performed: LSIL-CIN1 in 17.2%, HSIL-CIN2 in 13.8% and HSIL-CIN3 in 51.7% was found. 81.1% were delivered vaginally while the others by caesarean section. 90 patients underwent 6-weeks postpartum re-evaluation: clearance of HPV was 18.3% and the rate of regression of cytologic abnormalities was 44.4%. No significant difference was found between the two groups (vaginal delivery: 76 patients and caesarean section: 14 patients) according to age, race, body mass index, smoking, state of immunosuppression, history of STI or parity. Normalization of smears was shown in 45.3% women who delivered vaginally and in 40.0% women who underwent caesarean section (p=0.70).
Conclusion: The role of the mode of delivery in the rate of regression of citologic abnormalities is debated. Some reports found higher rate of regression of cervical dysplasia in association with vaginal delivery compared to caesarean section, as ours does, while others showed no relationship.
Disclosure: The results from this study are hypothesis generating. The higher rate of regression of cervical dysplasia in patients that delivered vaginally is in agreement with the idea that the cervical trauma generated by mechanical dilatation in labour produces an inflammatory reaction that could explain this regression phenomenon. Large population prospective studies are needed to confirm the hypothetical role of the vaginal delivery in the clearance of cervical cytologic abnormalities.

P22

An exploratory safety and immunogenicity study of human papillomavirus (HPV16+) immunotherapy VB10.16 in women with high grade cervical intraepithelial neoplasia (HSIL; CIN 2/3)

P. Hillemanns[1], K.U. Petry[2], G. Böhmer[3], M. Jentschke[4], L. Wölber[5], I. Skjørestad[6], A. Frederiksen[6], M. Axelsen[6]
[1]Klinik für Frauenheilkunde und Geburtshilfe, Medizinische Hochschule Hannover, Hannover, [2]Department of Obstetrics and Gynecology, Clinic of the City of Wolfsburg, Wolfsburg, [3]IZD Institut für Zytologie und Dysplasie, [4]Medizinische Hochschule Hannover, Hannover, [5]University Medical Center Hamburg-Eppendorf, Hamburg, Germany, [6]Vaccibody A/S, Oslo, Norway

Introduction / Background: This was an exploratory, multicenter study of VB10.16 immunotherapy in patients with HPV16+ CIN 2/3.
Methodology: VB10.16 is a naked DNA plasmid vaccine. It encodes for a recombinant protein consisting of 3 modules: mutation-inactivated HPV16 E7/E6 protein linked to human chemokine MIP-1α via a dimerization module derived from human IgG3. The unique mode of action is that it directly targets APCs, then HPV16+ infected tissue and mediates cell killing.
This study with 34 CIN 2/3 patients was divided into a Dosing Phase (two vaccination schedules, 3 injections) and an Expansion Phase (4 vaccinations) to assess safety/tolerability immunogenicity and preliminary signs of efficacy with VB10.16. Patients were followed for 24 weeks by colposcopy, cytology, HPV16+ testing and histology. Immunological testing were IFN-γ ELISpot, IHC, PD-L1. The median age was 29.0 years, 70.6% had CIN 2, 29.4% had CIN 3, all were HPV16 positive (one patient withdrew as she was HPV16 negative), and 44% positive for other HPV types at inclusion.
Results: Treatment with VB10.16 was well tolerated, and mostly injection site related adverse events were reported. No patients experienced SAEs, or discontinued the study vaccine. The highest grade AEs were Grade 3 in 3 patients. IFN-γ ELISpot data from the Dosing Phase identified the superior dose regimen and suggested a further enhancement with adding a fourth dose. All patients in the Expansion Phase elicited a HPV16-specific T-cell response where 94% showed an increased response after vaccination. Signs of efficacy (CIN regression, HPV16+ clearance, lesion size reduction) correlating with immune responses could be observed. In patients without signs of efficacy, an upregulation of PD-L1 was observed which could have inhibited the T-cells response induced by

the vaccine.
Conclusion: VB10.16 immunotherapy in patients with HPV16+ CIN 2/3 showed promising results. A study for cervical cancer in combination with a checkpoint inhibitor is in Progress.
Disclosure: Study was supported by VACCIBODY A.S., Oslo, Norway. Agnete Fredriksen and Mads Axelsen are employees and warrant holders of Vaccibody. Dr. Woelber reports personal fees from Medac Oncology, Tesaro, Roche, Pharmamar, GSK, Jenapharm, Merck, grants from Greiner, outside the submitted work.

P23

Using 3-dimensional tumour mapping to study patterns of relapse after pelvic exenteration for gynaecological malignancy

G. Imseeh[1,2], D.P.J. Barton[1], D. Kolomainen[1], S. Bryan[1], A. Fernandes[1], J. Ash[1], A. Sohaib[1], N.M. de Souza[2], A. Taylor[1,2]
[1]Department of Gynaecological Oncology, The Royal Marsden NHS Foundation Trust, [2]Department of Imaging and Radiotherapy, The Institute of Cancer Research, London, United Kingdom

Introduction / Background: Pelvic exenteration (PE) can provide long-term survival for patients with recurrent gynaecological malignancies, but there is a high risk of relapse. To develop evidence-based strategies for post-operative treatment, this study aimed to identify the anatomical regions at highest risk of recurrence and establish the relationship between pre- and post-PE relapse sites by comparing the anatomical overlap in their volumes.
Methodology: All patients who underwent PE for gynaecological malignancy between 2006-2017 at the Royal Marsden Hospital were identified. Clinical information, surgical reports, histopathology and imaging data were retrospectively analysed. DICOM data for tumour recurrences were transferred to the Eclipse radiotherapy planning system. Three-dimensional tumour volumes (3DTV) were generated to create cumulative anatomical maps of recurrence on a reference dataset. For patients with locoregional recurrence (LRR), pre-exenteration (pre_3DTV) and relapse (rel_3DTV) tumour volumes were then outlined on co-registered images. Volumes of overlap were calculated to assess relationship to margins (figure 1).

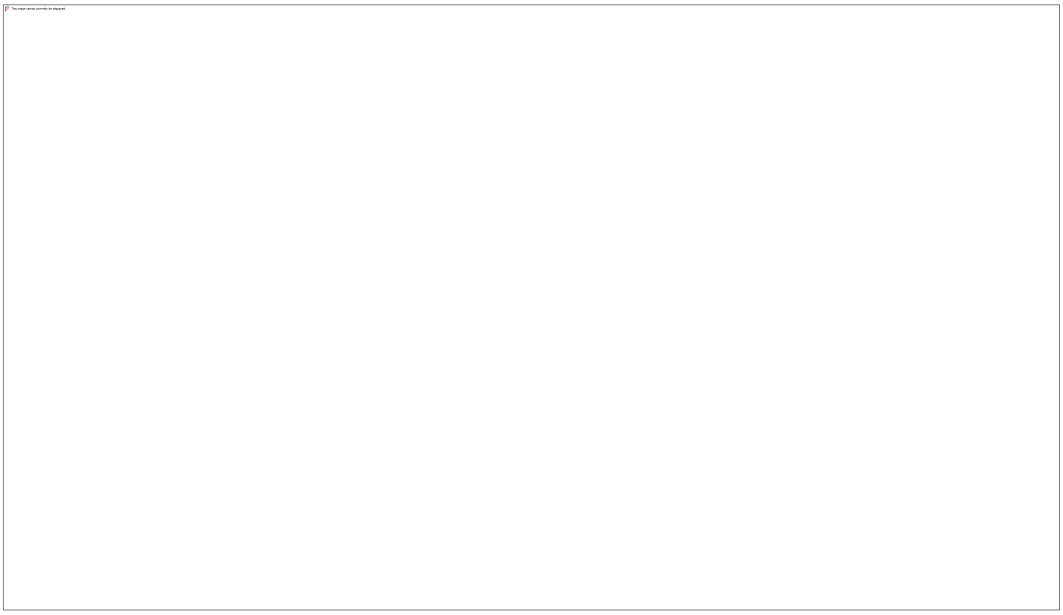

[Figure 1]

Results: 58 patients were evaluated with median age 56 years (27-83). Cervical cancer (43.1%) and squamous histology (56.9%) were the commonest presentations. At median follow up of 59.4 months, 32 patients (55%) had recurrence of which 26 (81%) had LRR. Cumulative anatomical mapping showed 42% LRR occurred centrally, 27% pelvic side-wall (PSW), 23% anterior, 8% posterior and 19% inguinal. After anterior PE, LRR is predominantly central (50%), posterior PE predominantly PSW (50%) and after total PE anterior (40%). 3DTVs were outlined for 21 patients, 15 with close/positive margins. Mean pre_3DTV was 61.2cm^3 (1.4-474.8) and mean rel_3DTV was 70.4cm^3 (1.3-436.6). With 5mm isotropic expansion of pre_3DTV, there was overlap in 65% cases. Mean overlap volume was 14.3cm^3 (0-138.5).
Conclusion: Loco-regional relapse is the dominant pattern of recurrence following exenteration. There is frequently an anatomical relationship between pre- and post-PE relapsed tumour volumes. This suggests additional targeted local therapy such as re-irradiation may improve outcomes
Disclosure: Nothing to disclose

P24

The role of neoadjuvant chemotherapy in patients with high-grade neuroendocrine carcinoma of the uterine cervix

J.-H. Kim[1], S.-H. Shim[2], S.-H. Kim[3], S.-W. Lee[3], J.-Y. Park[3], D.-Y. Kim[3], D.-S. Suh[3], J.-H. Kim[3], Y.-M. Kim[3], Y.-T. Kim[3], J.-H. Nam[3]
[1]Obstetrics and Gynecology, Asan Medical Center, University of Ulsan College of Medicine, [2]Konkuk University Medical Center, [3]Asan Medical Center, University of Ulsan College of Medicine, Seoul, Republic of Korea

Introduction / Background: To evaluate the feasibility and efficacy of neoadjuvant chemotherapy in the management of patients with high-grade neuroendocrine(HGNE) carcinoma of the uterine cervix using a matched case-control study.
Methodology: We performed a single-center, retrospective study of 58 surgically treated HGNE cervical cancer patients. Of these patients, 13 patients (20.90%) received neoadjuvant chemotherapy before surgery. For a 1-to-2 matched case-control study, the rest 45 patients who underwent initially radical hysterectomy were selected as a control group.
Results: Between neoadjuvant chemotherapy group and initial radical surgery group, stage(P=0.027), pelvic exam (size>4cm versus ≤4cm, P=0.021) and operation type (open radical hysterectomy versus laparoscopic radical hysterectomy, P=0.005) were statistically different variables. We matched these three variables to the control group. The overall survival rate was not different (66.67 % of neoadjuvant chemotherapy group vs. 60.00% of matched control group, P=1.00). The recurrence rate was not different (77.78 % of neoadjuvant chemotherapy group vs. 60.00% of matched control group, P=0.575). The estimated blood loss during operation(P=0.04). and intraoperative complication (P=0.026) was lower in neoadjuvant chemotherapy group than matched control group.
Conclusion: Neoadjuvant chemotherapy followed by surgery was feasible and safe in the treatment of HGNE carcinoma of the uterine cervix.
Disclosure: Nothing to disclose

P25

Prognostic factors and impact of the minimally invasive surgery in early stage neuroendocrine carcinoma of cervix

D.-Y. Kim[1], J.-H. Kim[1], S.-H. Shim[2], S.-W. Lee[1], J.-Y. Park[1], D.-S. Suh[1], J.-H. Kim[1], Y.-M. Kim[1], Y.-T. Kim[1], J.-H. Nam[1]
[1]Asan Medical Center, University of Ulsan College of Medicine, [2]Konkuk University Medical Center,

Seoul, Republic of Korea

Introduction / Background: We investigate prognostic factors and impact of the minimally invasive surgery (MIS) in surgically treated early-stage high-grade (HG) neuroendocrine cervical carcinoma (NECC).
Methodology: Between 1993 and 2017, 47 patients with FIGO (2009) stage IA-IIA HG NECC were initially treated with radical hysterectomy. Clinicopathological variables of the patients were retrospectively reviewed from electronic medical records. Cox proportional hazards regression was used to identify potential prognostic factors. According to surgical approach, survival curves were compared using the Kaplan-Meier method with log rank test.
Results: The median follow-up period was 28.2 months (range, 3.83-202.5). Stage IB1 disease was the most common (70.2%). Twenty-nine patients (61.7%) underwent RH via MIS. The overall survival (OS) rate was 63.8.0%, and the disease-free survival (DFS) rate was 38.3%. Lymph-node (LN) metastasis was an independent significant risk factor for OS (HR, 2.594; 95% CI, 1.152-5.842; P=0.021) and DFS (HR, 3.236; 95% CI, 1.188-8.815; P=0.022). The Kaplan-Meier survival curves revealed that there was no significant difference in OS and DFS between the laparotomy group and MIS group (50% vs. 72.4% log-rank p value=0.196, 38.9% vs. 37.9% p= 0.975). Moreover, the type of surgical approach was not a risk factor for OS (HR, 0.467; 95% CI, 0.714-1.257; P=0.132) and DFS (HR, 1.048; 95% CI, 0.494-2.224; P=0.093).
Conclusion: LN metastasis was a poor prognostic factor of survival outcomes in initially surgically treated early-stage HG NECC. There was no difference in survival between the MIS and laparotomy approached.
Disclosure: Nothing to disclose

P26

Micrometastasis in sentinel lymph nodes is a significant negative prognostic marker in early-stage cervical cancer

R. Kocian[1], K. Němejcová[2], J. Sláma[1], P. Dundr[2], D. Fischerová[1], A. Germanová[1], F. Frühauf[1], L. Dostálek[1], J. Jarkovský[3], D. Cibula[1]
[1]Department of Obstetrics and Gynecology, Gynecologic Oncology Center, First Faculty of Medicine, Charles University and General University Hospital, [2]Institute of Pathology, First Faculty of Medicine, Charles University and General University Hospital, [3]Institute of Biostatistics and Analyses, Faculty of Medicine, Masaryk University, Brno, Prague, Czech Republic

Introduction / Background: Since the risk of recurrence is low in early-stage cervical cancer, any assessment of prognostic importance of micrometastases (MIC) requires a large cohort. The aim of the study was to retrospectively analyze a large group of patients treated at a single site where sentinel lymph node (SLN) has been used in the management of cervical cancer since 2004 and which has an intensive protocol for SLN pathological ultrastaging.
Methodology: Patients with stage T1a - T2b cervical cancer treated by primary surgery with curative intent, who had common tumor types, at least unilateral SLN detection, and who were treated between 01/2007 - 12/2016 were included in the study. Radical surgery was abandoned if metastasis in SLN was detected by frozen section. All SLN were processed by an intensive protocol for SLN ultrastaging (entire SLN processed, 2 sections (H&E / cytokeratin) in 150 µm intervals from each block).
Results: The analysis included data from 234 patients (Table 1). Adjuvant treatment was given to 79% of patients with macrometastasis (MAC), 77% with MIC, and 11% with isolated tumor cells (ITC). Within the median follow-up of 67 months 29 recurrences occurred. The recurrence rate of patients with negative LN, with MAC, MIC or ITC reached 9%, 29%, 35% and 22% respectively. Table 2 shows parameters significant for the risk of recurrence. Kaplan-Meier curves for PFS were significantly different for cases with MAC and MIC in comparison to LN-negative cases (Figure 1).
Conclusion: The presence of MIC in SLN was associated with significantly decreased PFS, similar to MAC. There was a similar trend for ITC, but it did not reach significance due to low numbers. Patients with MIC and MAC should be managed identically and SLN ultrastaging should become an integral part of the management of patients with early-stage cervical cancer.

[Figure 1: Kaplan - Meier model for progression free survival according to the type of LN involvement]

[Table2: Significant parameters for the risk of recurrence/death from univariate analysis.]

Age (years), BMI		41.8 (26.3; 68.1), 24.3 (18.5; 36.3)
Stage pT (pathology)	1a1, 1a2, 1b1, 1b2, 2a1	9 (3.8%), 7 (3.0%), 159 (67.9%), 33 (14.1%), 25 (10.7%)
Tumor type	Adenocarcinoma, Adenosquamous, Squamous	51 (21.8%), 7 (3.0%), 170 (72.6%)
Grade	1, 2, 3, missing	21 (9.0%), 96 (41.0%), 97 (41.5%), 20 (8.5%)
LVSI, FST, Number of LN per patient	lymphovascular space invasion, fertility sparing treatment	100 (42.7%), 29 (12.4.%), 36.0 (4.0;59.5)
Type of parametrectomy	B, C1, C2, simple hyst/trachel.	13 (5.6%), 110 (47.1%), 94 (38.2%), 25 (10.6%)
Type of positivity	MAC, MIC, ITC, Negative	14 (6.0%), 17 (7.3.%), 9 (3.8%), 194 (82.9%)
Adjuvant treatment		36 (15.4%)
Follow up length (months)		67.6 (8.65;127.86)
Recurrence		29 (12.4%)

[Table 1: Characteristics of the group.]

Disclosure: Acknowledgements This work was supported by Charles University in Prague (UNCE 204065 and PROGRES Q28/LF1) and by a grant from the Czech Research Council (No 16-31643A). None of the authors declare any conflict of interest.

P27

Conservative management of stage ia cervical cancer: outcomes following loop excision as a fertility sparing policy with critical emphasis on margin status

P. Korompelis, S. Rundle, M. Adishesh, R. O'Donnell, R. Naik
QE Hospital, NGOC, Gateshead, United Kingdom

Introduction / Background: Controversies in stage IA cervical cancer include 1) need for radical treatment in IA2 disease, 2) lymphadenectomy in LVSI positive cases, 3) reflex hysterectomy as 1ry treatment or delayed hysterectomy after fertility completion. These controversies are highlighted by a trend towards conservative treatment consisting solely of loop excision.
We present the largest series of a conservative treatment policy in stage IA cervical cancer.
Methodology: A cross-referenced analysis utilising national and organisational databases was performed ensuring capture of all stage IA cervical cancers within a defined geographical area of the North of England over a 10yr period (2006-16), population 1.6million women. The study objectives were to determine 1) regional incidence, 2) recurrence rate invasive disease, 3) recurrence rate CIN, and 4) reflex and delayed hysterectomy rates.
Results: ***Diagnosis:*** 247 stage 1A cases were identified, 232(94%) 1A1 and 15(6%) IA2 (incidence 15/100,000 women). 89% squamous histology, 5% LVSI positive, 4 occult cases at hysterectomy, remaining 243 loop diagnoses, 22% complete excision of invasive disease & CIN at first loop (11% incomplete excision invasive, 67% incomplete excision CIN).
Treatment: 20% underwent one loop, 53% two loops and 1 patient 3 loops; 27% had hysterectomy. 7% underwent bilateral pelvic lymphadenectomy (0/17 positive nodes).
Outcomes: Recurrence rate for invasive disease was 2/247(0.8%) with no deaths (median follow-up 46mths), CIN recurrence was 8/247(3%). Delayed hysterectomy rate was 13/177(7%). Both invasive recurrences were in stage IA1, LVSI negative cases. Case1: hysterectomy with CIN3 at the vaginal

margin. Case2: loop treatment with CIN3 at the ectocervical margin. The difference in recurrence rates following hysterectomy (1.4%) or loop excision (0.6%) was non-significant (p=0.85).
Conclusion: Recurrent invasive disease following a conservative treatment policy is < 1%. Irrespective of loop excision/hysterectomy treatment, margin status defines the risk of recurrence, clear margins=0% (0/230), CIN incompletely excised=12% (2/17), CIN3 incompletely excised=33% (2/6)[p< 0.05].
Disclosure: Nothing to disclose

P28

Immune checkpoint expression patterns in tumor-infiltrating lymphocytes from preclinical cervical carcinoma specimens

O. Kurmyshkina[1], T. Volkova[1], P. Kovchur[2]
[1]*Petrozavodsk State University, Institute of High-Tech Biomedicine,* [2]*Petrozavodsk State University, Institute of Medicine, Petrozavodsk, Russian Federation*

Introduction / Background: Achieving positive outcomes of using immunotherapeutic approaches in cancer treatment implies multifaceted targeting of functionally different mechanisms of immune response, including mechanisms governed by molecules of immune checkpoint system. Functional roles of immune checkpoints in different immune cell subsets of cancer patients are not comprehensively characterized; the existing data are sometimes controversial. Studying the immune checkpoint expression patterns in initial stages of invasive tumor growth and progression, in comparison with non-invasive neoplastic lesions, may help better understand their involvement in regulating tumor-immunity interactions. Little or virtually no data are available for very early (preclinical) cervical cancer.
Methodology: Fresh cervical epithelial tissue specimens (along with peripheral blood) were collected from women with preclinical early invasive and non-invasive squamous carcinoma of the cervix. Cell suspensions obtained by gentle enzymatic dissociation were subjected to multicolor flow cytometry. Co- expression of PD-1/CD279, PD-L1/CD274, TIM-3/CD366, and LAG-3/CD223 markers was evaluated in major populations of tumor-infiltrating CD4 and CD8 T lymphocytes, including their regulatory subset (Treg), and compared to circulating T cells.
Results: PD-1/-L1, TIM-3 and LAG-3 were analyzed in total CD4/CD8 T cells, CD4/8CD25(+/-) T subsets, and CD4/8CD25(+/high)CD127(-/low) Treg. Tumor-infiltrating (TILs) and peripheral blood (PBLs) lymphocytes, either CD4 or CD8, were shown to have different expression levels of immune checkpoint markers with significantly higher levels found on TILs. Early invasive and non-invasive carcinoma samples could also be stratified according to the frequencies of TILs. TIM-3 and LAG-3 were strongly co-expressed with PD-1/-L1 on TILs. The prevalence of CD25(+/high)CD127(-/low) cells among CD4 (CD8) TILs was significantly higher than among PBLs with the highest difference observed for CD8 subset, and the majority of these cells co-expressed PD-1 with TIM-3/LAG-3. All analyzed TILs subsets were found to have exclusively high levels of PD-L1.
Conclusion: The findings indicate deep involvement of immune checkpoint mechanisms in initiation of invasive growth of cervical carcinoma.
Disclosure: The work is supported by the Russian Science Foundation (grant No.17-15-01024).

P29

Predictive value of p16/Ki-67 dual-stained cytology for the progression of cervical dysplasia

Y.K. Lee[1,2], S.R. Hong[3]
[1]*Gynecologic oncology, Sheik Khalifha Specialty Hospital, Ras Al Khaimah, United Arab Emirates,* [2]*Seoul National University Hospital,* [3]*Cheil General Hospital & Women's Healthcare Center, Seoul,*

Republic of Korea

Introduction / Background: Dual immunocytochemistry for p16/Ki-67 has been reported as a useful biomarker to identify cells of underlying high-grade cervical intraepithelial neoplasia in the Papanicolaou (Pap) cytology. Regression rates for atypical squamous cells of undetermined significance (ASCUS) or low-grade squamous intraepithelial lesion (LSIL) range widely, from 30% to 80%. The objective of this study was to investigate the predictive value of p16/Ki-67 dual-stained cytology for the progression of disease.
Methodology: Cytology p16/Ki-67 dual-staining test was performed on 691 liquid-based residual samples from a cohort of women with ASC-US/ LSIL and co-testing human papillomavirus (HPV) positive. Among them, 250 women were followed up for at least a year. Study end points were HSIL detection in 1 and 2 year's follow-up.
Results: Positivity of p16/Ki-67 dual stained cytology was well correlated to progression of disease compared with positivity of HPV 16/18. During 1 year follow-up, 23 of 250 women experienced progression of disease into HSIL. For positivity of p16/Ki-67 dual stained cytology, Sensitivity (60.9%) for the detection of HSIL or specificity (81.5%) for normal or low grade cytology was higher than those of HPV 16/18 tests (13.0% and 89.0%, respectively) (p< 0.001). During 2 years follow-up, 11 of 190 women experienced progression of disease into HSIL. For positivity of p16/Ki-67 dual stained cytology, Sensitivity (45.5%) for the detection of HSIL or specificity (82.1%) for normal or low grade cytology was higher than those of HPV 16/18 tests (18.2% and 89.9%, respectively) (p=0.025).
Conclusion: p16/Ki-67 dual stained cytology could provide both high sensitivity and specificity for the prediction of HSIL in Pap cytology in the future. Positive p16/Ki-67 dual-stained cytology in low grade cytology was highly associated with the progression of disease in 1 or 2 years follow-up. Therefore, in cases of positive dual-stained cells morphologically showing benign atypical features, further follow-up would be necessary.
Disclosure: Nothing to disclose

P30

FIGO stage 1B1 cervical cancer - an evaluation of a treatment strategy based on pelvic MRI and pelvic examination at Oslo University Hospital, Norway

K. Lindemann[1,2], G.B. Kristensen[1,3], K. Skogsfjord[1], B. Eyjolfsdottir[1], K. Bruheim[4]
[1]Department of Gynaecological Cancer, Oslo University Hospital, [2]University of Oslo, [3]Institute for Cancer Genetics and Informatics, [4]Department of Oncology, Oslo University Hospital, Oslo, Norway

Introduction / Background: In early stage cervical cancer (CC), ESMO/ESTRO guidelines recommend the avoidance of combining radical surgery and radiotherapy due to the higher morbidity. Surgery is the preferred treatment for selected patients to avoid late-effects from chemoradiotherapy (CRT). Still, there are considerable differences in the workup and treatment in Europe. Pelvic MRI and clinical examination is part of the mandatory work-up for treatment selection at Oslo University Hospital (OUH). We evaluated this strategy in FIGO (2009) IB1 patients.
Methodology: Patients with stage IB1 treated at OUH between 2005 and 2015 were identified from our institutional database. Patients with fertility-sparing surgery or neuroendocrine tumors were excluded. End of follow-up was Febr 1st 2019. Descriptive statistics were used. Survival was calculated with the Kaplan-Meier method.
Results: 340 patients were identified, 63% with squamous carcinoma and 34% with adenocarcinoma. Mean age was 46 years. The median follow-up time was 6.0 years. 19 patients (6%) were treated with neoadjuvant chemotherapy. 293 patients (86%) underwent surgery, of which 141 (48%) with minimally invasive surgery (MIS). 32 patients (11%) received postoperative (CRT) due to either deep stromal infiltration (n=8), metastatic lymph nodes (n=11), parametrial infiltration (n=5), positive margins (n=1) or a combination of several risk factors (n=5). 47 patients (14%) were treated with radical CRT. 40 patients (12%) developed recurrence, (13%) after surgery and 6% after CRT. 21 patients developed pelvic recurrence, 6 distant and 13 both local and distant. Most of the both local and distant recurrences (12 of 13) occurred in patients who had undergone MIS. There was only one pelvic failure after radical CRT. 3- and 5-year survival was 96% and 93%, respectively.
Conclusion: Selection of patients based on MRI resulted in few patients who needed postoperative

CRT and yielded excellent survival. However, the surgical technique warrants careful evaluation to reduce relapse rates.
Disclosure: Nothing to disclose

P31

Good interobserver agreement for mri-based assessment of tumor size≥4 cm, parametrial invasion and lymph node metastasis in uterine cervical cancer

N. Lura, Bergen Research Group for Gynecological Cancer
Radiology, Haukeland University Hospital, Bergen, Norway

Introduction / Background: Pelvic magnetic resonance imaging (MRI)-findings indicating tumor size ≥4 cm, parametrial invasion and pelvic/paraaortic lymph node metastases are presently incorporated in the newly revised cervical cancer FIGO staging (2018) system and guide choice of treatment. Good interobserver agreement between radiologists for these staging parameters is however, critical if these are to guide therapy. This study aimed to evaluate the interobserver agreement for these staging parameters based on MRI in a large cervical cancer patient cohort.
Methodology: A total of 421 patients with histologically confirmed uterine cervical cancer who had pelvic MRI at primary diagnostic work-up were included. Their MRI scans were read independently by two radiologists (with 4 and 10 yrs. of MRI reading experience, respectively) blinded for clinical information/FIGO stage. MRI findings indicating tumor size ≥4 cm, parametrial invasion and/or pelvic/paraaortic lymph node metastases (when >10 mm in short-axis diameter) were recorded. Interobserver agreement for these staging parameters were assessed using exact Fleiss kappa (κ). Agreement beyond chance was interpreted as poor (κ≤0.20), fair (κ=0.21-0.40), moderate (κ=0.41-0.60), good (κ=0.61-0.80) or very good (κ=0.81-1.00). The prognostic value of the MRI based staging parameters was assessed using Kaplan-Meier and log-rank test.
Results: Interobserver agreement between the two radiologists for the evaluation of tumor size ≥4 cm was *very good* with a κ value of 0.83. For the evaluation of parametrial invasion and lymph node metastases, the agreement was *good* with κ values of 0.74 and 0.73, respectively. Presence of these imaging findings were also significantly associated with poor prognosis (Figure).
Conclusion: The interobserver reproducibility for the MRI-based staging parameters: tumor size ≥4 cm, parametrial invasion and pelvic/paraaortic lymph node metastases are *very good* and *good*, supporting the robustness of these imaging parameters for safe incorporation into the 2018 FIGO staging system.

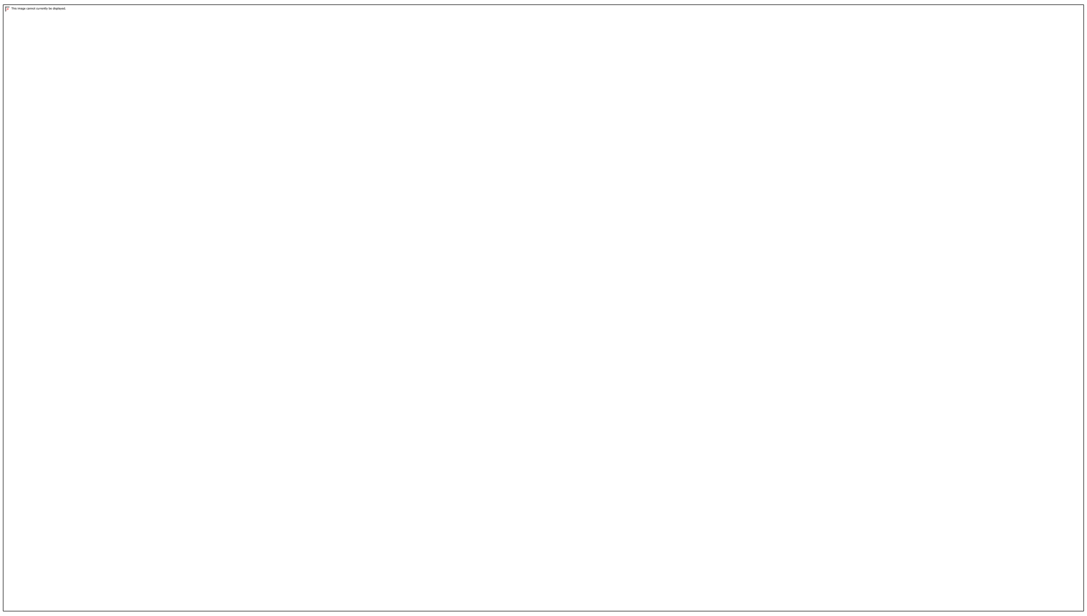

[survival curves]

Disclosure: Nothing to disclose

P32

Neoadjuvant chemoimmunotherapy increases the overall five-year survival of patients with locally advanced cervical cancer

A. Menshenina, T. Moiseenko, E. Frantsiyants, E. Verenikina, N. Ushakova, M. Adamyan, T. Chalabova, G. Selezneva, N. Maksimova, A. Pustovalova
Rostov Research Institute of Oncology, Rostov-on-Don, Russian Federation

Introduction / Background: Our aim was to assess the effect of various modifications of neoadjuvant chemotherapy (NACT) on the overall five-year survival (OS) of patients with locally advanced cervical cancer (LACC) with the inclusion of therapeutic plasmapheresis (PA) and nonspecific immunotherapy in the treatment programme.
Methodology: As the first stage of treatment of 51 patients with LACC of T2a-2bN0-1M0 stages, NACT courses were performed according to the scheme: cisplatin and bleocin.
The first group consisted of the patients (n = 31) who underwent a session of PA, a day after which they started a course of NACT. A course of nonspecific immunotherapy with Allokin-alpha was performed in parallel with the course of NAPCT
The second group consisted of 20 patients who received standard NACT scheme: cisplatin, bleomycin.
Results: Among the studied groups, the maximum OS was observed in the patients of the second group who received PA, courses on NAPCT and immunomodulator Allokin-alpha.
In this group, OS was 83.5±4.6%; which is much higher than the rates of OS in the group of patients treated according to the standard method (50.6±6.6%).
The differences are statistically significant, p=0.0462 (Log-Rank test).
Conclusion: Carrying out NACT in combination with PA and nonspecific immunotherapy in the treatment of patients with LACC is pathogenetically justified, since it allows to heighten the effect of cytostatics on the tumor, has an immunomodulatory effect on the body of patients and significantly increases the overall 5-year survival of patients.
Disclosure: Nothing to disclose

P33

Up-regulation of FOXP3 T regulatory lymphocytes in patients with high-grade squamous intraepithelial lesions correlated with HPV infection

A. Mitildzans[1,2], S. Isajevs[3,4], D. Rezeberga[2,5]
[1]Oncogynecology, Riga East University Hospital, [2]Gynecology and Obstetrics, Riga Stradins University, [3]Pathology, Riga East University Hospital, [4]Pathology, University of Latvia, [5]Gynecology, Riga East University Hospital, Riga, Latvia

Introduction / Background: Regulatory (FOXP3+) T cells (Tregs) comprise a subpopulation of CD4+ T cells that suppress autoreactive immune cells, thereby protecting organs and tissues from autoimmunity. Novel therapeutic strategies for cervical cancer and squamous intraepithelial lesions (SIL) focus on immune-modulatory and cancer vaccination approaches.
The aim of this study was to analyze the role of T regulatory cells (Tregs), CD4 and CD8 T lymphocytes and HPV status in low and high grade squamous intraepithelial lesions (LSIL and HSIL).
Methodology: 62 patients were enrolled in the study in Riga East University Hospital. Each patient had undergone a biopsy or electroexcision of the cervix. The Immunostaining for CD4, CD8 and FOXP3 was performed on tissue samples of normal (n=10) and LSIL (n=32) and HSIL (20) lesions. The *Aptima* HPV assay was used to assess HPV infection.
Results: Obtained results showed that patients the numbers of CD4 T-lymphocytes did not differed between the patients with LSIL and HSIL. However, patients with HSIL had significant CD8 T-lymphocytes upregulation compared to patients with LSIL (16± 4 vs. 8 ± 2 cells/mm^2, p = 0.001). In addition patients with HSIL with concomitant epithelial koilocytosis demonstrated increased numbers of FOXP3 positive T-lymphocytes compared to patients with LSIL (12± 6 vs. 6 ± 2 cells/mm^2, p = 0.02). The HPV was detected in 92% of HSIL and 82% of LSIL patients. The numbers of FOXP3 T lymphocytes correlated with HPV status (Rho=+ 0.82; p< 0.0001).
Conclusion: Upregulation of T regulatory lymphocytes in patients with HSIL suggested the pivotal role of Tregs for counteracting the host immune response for the progression from LSIL to HSIL, which correlated with HPV infection. Prime targets for new immune-based non-invasive therapies for the HSIL treatment could be beneficial.
Disclosure: Nothing to disclose

P34

Cervical intraepithelial neoplasia is associated with an altered vaginal microbiome and innate immune disruption

A. Mitra[1], D. Macintyre[1], Y. Lee[1], A. Smith[2], J. Marchesi[1,2], D. Lyons[3], S. Stock[4], P. Bennett[1], M. Kyrgiou[1]
[1]Imperial College London, London, [2]Cardiff University, Cardiff, [3]Imperial College NHS Healthcare Trust, London, [4]Edinburgh University, Edinburgh, United Kingdom

Introduction / Background: The innate immune system is a key factor in the fate of an HPV infection and a proinflammatory environment has been shown to facilitate HPV persistence and cervical carcinogenesis. Antimicrobial peptides (AMPs) have innate antibacterial and antiviral properties, as well as a role in tissue remodelling but have not been studied in cervical intraepithelial neoplasia (CIN). Bacterial vaginosis is known to be associated with HPV infection and CIN, therefore the vaginal microbiota (VMB) may also influence the innate immune response and subsequent disease state.
We aimed to investigate changes in VMB composition and expression of Human Beta Defensin-1 (hBD-1), Secretory Leucocyte Protease Inhibitor (SLPI) and inflammatory cytokines according to

disease status.
Methodology: Population: Non-pregnant, premenopausal women attending the colposcopy clinic in London, UK with cytological +/- histological disease classification.
Analysis: Vaginal swab samples were used for 16s rRNA bacterial sequencing to assess VMB composition and for enzyme-linked immunosorbent assay (ELISA) to determine hBD-1, SLPI, IL-1b and IL-8 levels.
Results: A total of 232 women were included and classified into three groups; 34 normal, 81 low-grade CIN, and 117 high-grade CIN. Lower levels of *Lactobacillus* spp. with increased bacterial species diversity, were seen with increasing disease severity. A bacterial vaginosis-like VMB was observed twice as frequently in women with high-grade disease (28%) compared to controls (14%). Higher levels of pro-inflammatory cytokines; IL-1b and IL-8 and AMPs; hBD-1 and SLPI increase with increasing disease severity.
Conclusion: *Lactobacillus* spp. are thought to protect the female reproductive tract from pathogens, including viruses and the lower levels observed with increasing disease severity, along with higher levels of pro-inflammatory cytokines may play a role in disease outcomes. AMPs are known to be overexpressed in several cancers, and therefore higher levels may have a deleterious effect due their ability to facilitate tissue remodeling that is required for carcinogenesis.
Disclosure: Nothing to disclose

P35

Effectiveness of folate receptor-mediated detection (FRD) staining solution for cervical cancer screening

Y. Dai[1], L. Wan[1], D. Moos[2], D. Li[3]
[1]Guangren Hospital, School of Medicine, Xi'an Jiaotong University, Jilin, China, [2]Universidad Autonoma de Guadalajara, Guadalajara, Mexico, [3]Second Hospital of Jilin University, Jilin, China

Introduction / Background: The detection of HPV infection and Cytology are currently recognized for cervical cancer screening， in many regions there are conditions that make it difficult to establish or maintain high-quality, high-coverage cytology based screening programs. Folate Receptor-mediated Diagnosis（FRD） has been proposed as a reliable method to screen cervical intraepithelial neoplasia and cancer. This study aims to investigate the clinical significance of FRD by comparing the accuracy of FRD with human papillomavirus (HPV) testing and Thinprep cytologic test (TCT).
Methodology: From December 2018 to March 2019, patients in the gynecology clinic of the Second Hospital of Jilin University were screened with FRD, TCT, and HPV examinations during the same visit. If any of the three test results were positive, colposcopy was performed with cervical biopsy being the gold standard for pathological diagnosis.
Results: The sensitivity of FRD, TCT, and HPV in the diagnosis of cervical intraepithelial neoplasia were 72.22%, 72.22%, and 83.33% respectively. The specificity of FRD, TCT, and HPV in diagnosis was 65.07%, 60.31%, and 25.39% respectively. The accuracy of FRD, TCT, and HPV in diagnosis was 66.67%, 62.96% and 38.27%. The positive predictive value (PPV) of FRD, TCT, and HPV in diagnosis was 37.14%, 34.21% and 38.27% respectively, while the negative predictive value (NPV) was 89.13%, 88.37% and 84.21% respectively.
Conclusion: Overall, FRD provided high values of sensitivity, specificity and accuracy. While ensuring high detection rate, FRD has its own unique advantages in detection speed (results in less than 60 seconds), economic cost, and patient compliance. FRD can be a very beneficial and useful tool in the primary screening of cervical intraepithelial neoplasia.
Disclosure: Nothing to disclose

P36

SENTIX - a prospective cohort international study on sentinel lymph node biopsy without pelvic lymph node dissection in cervical cancer patients: central quality control of SLN pathological assessment (CEEGOG-CX01; ENGOT-CX2; NCT02494063)

K. Němejcová[1], R. Kocian[2], C. Köhler[3], J. Klat[4], P. Dundr[1], A. Germanova[5], A. Plaikner[3], S. Bajsova[4], P. Mallmann[3], M. Ostojich[6], A. Berjón[7], A. Torne[8], F. Raspagliesi[9], M. Pakiz[10], P. Kascak[11], M. Redecha[12], L. Snyman[13], M. Misiek[14], C. Zorrero[15], S. Tingulstad[16], A. Vinnytska[17], I. Vergote[18], M. Michal[19], J. Jarkovsky[20], D. Cibula[5], Central and Eastern European Gynecologic Oncology Group Belgium and Luxembourg Gynaecological Oncology Group

[1]Institute of Pathology, First Faculty of Medicine, Charles University and General University Hospital in Prague, Prague, [2]Gynecologic Oncology Center, Department of Obstetrics and Gynecology, First Faculty of Medicine, Charles University and General University Hospital in Prague, CEEGOG, Pragu, Czech Republic, [3]Department of Special Operative and Oncologic Gynaecology, Asklepios-Clinic Hamburg, Hamburg, Germany, [4]Department of Obstetrics and Gynecology, University Hospital Ostrava, CEEGOG*, Ostrava, [5]Gynecologic Oncology Center, Department of Obstetrics and Gynecology, First Faculty of Medicine, Charles University and General University Hospital in Prague, CEEGOG*, Prague, Czech Republic, [6]Department of Gynecology,Institute of Oncology Angel H Roffo University of Buenos Aires, Buenos Aires, Argentina, [7]Department of Pathology, Hospital Universitario La Paz, Madrid, [8]Unit of Gynecological Oncology, Institute Clinic of Gynecology, Obstetrics and Neonatology (ICGON), Hospital Clinic of Barcelona, Barcelona, Spain, [9]Fondazione IRCCS Istituto Nazionale dei Tumori di Milano, Milano, Italy, [10]University Clinic for Gynaecology and Perinatology, University Medical Centre Maribor, CEEGOG*, Maribor, Slovenia, [11]Department of Obstetrics and Gynecology, Faculty Hospital Trencin, CEEGOG*, Trencin, [12]Department of Gynaecology and Obstetrics, University Hospital, Comenius University, CEEGOG*, Bratislava, Slovakia, [13]Gynaecologic Oncology Unit, Department of Obstetrics and Gynaecology, University of Pretoria, Pretoria, South Africa, [14]Department of Gynecologic Oncology, Holycross Cancer Center, CEEGOG*, Kielce, Poland, [15]Gynecology Department, Instituto Valenciano de Oncología (IVO), Valencia, Spain, [16]Department of Gynaecology, St Olav's Hospital, Trondheim, Norway, [17]LISOD - Israeli Oncological Hospital, CEEGOG*, Plyuty, Ukraine, [18]Department of Gynecology and Obstetrics, University Hospital Leuven, BGOG#, Leuven, Belgium, [19]Department of Obstetrics and Gynaecology, Hospital České Budějovice, JSC., CEEGOG*, České Budějovice, [20]Institute of Biostatistics and Analyses, Faculty of Medicine, Masaryk University, Brno, Czech Republic*

Introduction / Background: The quality of pathological assessment is of pivotal importance in the management of cervical cancer patients if pelvic lymph node dissection (PLND) is to be replaced by sentinel lymph node (SLN) biopsy only.

Methodology: The aim was to report the results of the quality control of pathological SLN assessment in the group of 300 patients treated per protocol. All specimens and full pathology reports from at least two patients per site were reviewed centrally in Prague. SLN ultrastaging protocol included a complete processing of all SLN tissue in slices of 2 mm thickness, 2 sections in 150 µm from each block until no tissue left, one stained with H&E and second examined immunohistochemically. The findings were classified as: Minor (minor protocol deviations), Major (additional sections completed at central pathology), or Critical (deviations could not have been fixed at central pathology). In the case of Major or Critical findings, sites were requested to submit all samples from all additional patients for the second-round assessment.

Results: Samples from 77 cases from 36 sites with at least 1 patient treated per protocol were submitted for the central reading. Minor findings were identified in 27% of cases, Major in 30%, and Critical in 4%. Samples from 18 patients were submitted for the second control round; 9 were without deviations 7 had Minor, 2 Major, and 1 Critical findings. One site was prematurely closed due to repeated Critical findings. The most frequent deviations included incompletely processed SLN (36/95) and a lower number of immunohistochemical staining (31/95).

Conclusion: Major deviations from the SLN protocol were identified in 33% of sites and 31% of samples. These deviations can impact detection of small metastases and, consequently, the safety of the SLN concept. Central pathology reading allowed for substantial improvement of SLN pathological assessment during the trial.

Disclosure: Acknowledgements: This work was supported by a grant from the Czech Research Council (No 16-31643A). None of the authors declare a conflict of interest.

P37

Phase 1 evaluation of bintrafusp alfa (M7824), a bifunctional fusion protein targeting TGF-β and PD-L1, in cervical cancer

S. Allan[1], F. Braiteh[2], E. Calvo Aller[3], A. Cervantes[4], W. Edenfield[5], T. Li[6], M. Rasschaert[7], T.-W. Park-Simon[8], F. Longo[9], L. Paz-Ares[10], A. Spira[11,12], J. Strauss[13], L.S. Ojalvo[14], C. Helwig[15], I. Dussault[16], J.L. Gulley[17]
[1]Tasman Oncology Research, Ltd, Southport, QLD, Australia, [2]Comprehensive Cancer Centers of Nevada, Las Vegas, NV, United States, [3]START Madrid - CIOCC, Centro Integral Oncológico Clara Campal, Hospital Madrid Norte Sanchinarro, Madrid, [4]Biomedical Research Institute INCLIVA, University of Valencia, Valencia, Spain, [5]Greenville Hospital System University Medical Center (ITOR), Greenville, SC, [6]University of California Davis Comprehensive Cancer Center, Sacramento, CA, United States, [7]Universitair Ziekenhuis Antwerpen, Edegem, Belgium, [8]Medizinische Hochschule Hannover, Hannover, Germany, [9]Hospital Universitario Ramón y Cajal, Instituto Ramón y Cajal de Investigación Sanitaria (IRICYS), CIBERONC, [10]Hospital Universitario 12 de Octubre, Madrid, Spain, [11]Virginia Cancer Specialists, Fairfax, VA, [12]US Oncology Research, The Woodlands, TX, [13]Laboratory of Tumor Immunology and Biology, Center for Cancer Research, National Cancer Institute, National Institutes of Health, Bethesda, MD, [14]Immuno-Oncology, EMD Serono, Inc., Billerica, MA, United States, [15]Merck Healthcare KGaA, Darmstadt, Germany, [16]EMD Serono, Inc., Billerica, MA, [17]Genitourinary Malignancies Branch, Center for Cancer Research, National Cancer Institute, National Institutes of Health, Bethesda, MD, United States

Introduction / Background: Globally, cervical cancer is one of the most common and lethal gynaecological cancers. Advanced/metastatic disease is typically treated with chemotherapy, often with poor objective response rates (ORRs) and durations of response (DORs) in pretreated patients; ORRs with anti-PD-1 therapies range from 12%-26%. Bintrafusp alfa* (M7824) is an innovative first-in-class bifunctional fusion protein composed of the extracellular domain of the TGF-βRII receptor (a TGF-β “trap”) fused to a human IgG1 mAb blocking PD-L1. We report safety and efficacy in patients with cervical cancer treated with bintrafusp alfa in dose-escalation and expansion phases of an ongoing phase 1 trial (NCT02517398).
Methodology: Patients with advanced/metastatic disease received bintrafusp alfa 0.3-30 mg/kg (dose-escalation, n=10) or 1200 mg (expansion, n=15) Q2W until PD, unacceptable toxicity, or withdrawal. Treatment past PD was allowed if adverse events (AEs) were tolerable and no new treatment was indicated. Primary endpoints were safety (dose-escalation) and BOR (expansion).
Results: As of 9 January 2019, 25 patients were treated for a median duration of 9.6 (range, 2.0-72.0) weeks. Six patients had confirmed responses per RECIST 1.1 (ORR, 24.0%); 5/6 responses were ongoing at data cutoff (median DOR not reached; range, 2.3+-24.9+ months). One additional patient had a delayed PR that was ongoing for 8.7 months at data cutoff, with 73.3% disease shrinkage after initial PD (total clinical response rate, 28.0%). Responses occurred irrespective of PD-L1 protein expression: 1 patient with PD-L1-negative disease had a response. Grade 3 treatment-related AEs (TRAEs) occurred in 6 patients (24.0%); 1 patient experienced a grade 4 TRAE (4.0%; hypokalaemia). Six patients discontinued treatment due to TRAEs. No treatment-related deaths occurred.
Conclusion: Bintrafusp alfa has a manageable safety profile and promising clinical activity with durable responses. Further investigation of bintrafusp alfa in cervical and other HPV-associated cancers is ongoing (NCT03427411). Updated results will be presented.

*Proposed INN

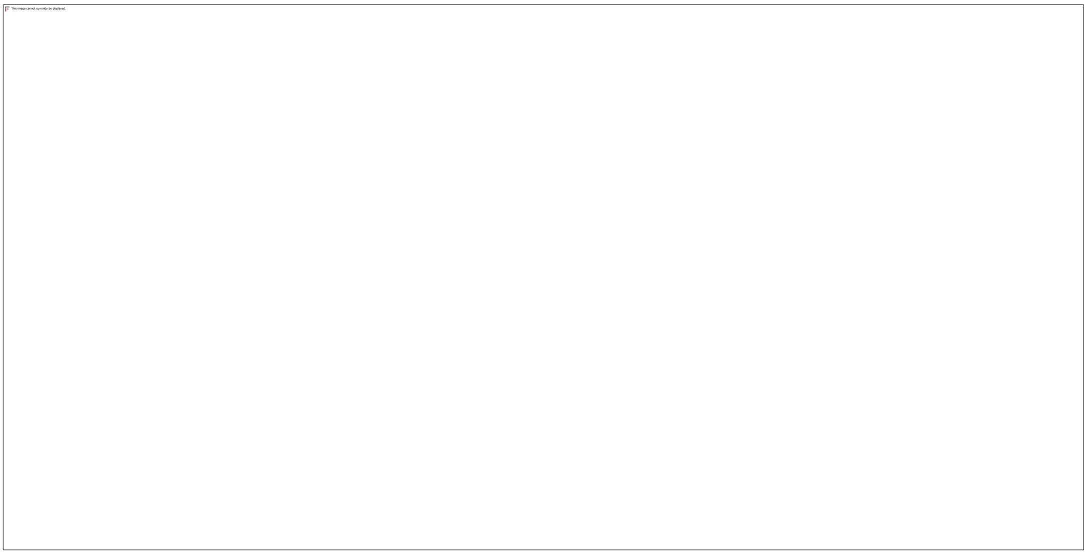

[Response rates of bintrafusp alfa in cervical cancer]

Disclosure: TL discloses honoraria from Foundation Medicine, Takeda, and PUMA, and receives grant/research support from Foundation Medicine, Pfizer, AstraZeneca, Hengrui, and Eureka. JS discloses inventorship on a National Institutes of Health patent related to the work. LSO and ID disclose employment with EMD Serono, Inc. CH discloses employment with Merck Healthcare KGaA. JLG discloses that National Cancer Institute has a cooperative research and development agreement with EMD Serono, Inc. and discloses inventorship on a National Institutes of Health patent related to the work. All remaining authors have nothing to disclose. Funding for this trial was provided in part by the Center for Cancer Research, National Cancer Institute, and National Institutes of Health Clinical Center. This trial was sponsored by Merck Healthcare KGaA, Darmstadt, Germany, and is part of an alliance between Merck Healthcare KGaA and GlaxoSmithKline.

P38

The use of rapid evaporative ionisation mass spectrometry (REIMS) as a real-time bedside test in cervical cancer screening

M. Paraskevaidi, S. Cameron, E. Whelan, M. Tzafetas, A. Mitra, S. Lever, A. Semertzidou, Z. Takats, M. Kyrgiou
Imperial College London, London, United Kingdom

Introduction / Background: Cervical cancer is the 4th most common cancer among women while its incidence is expected to rise by 43% in the UK by 2035 (Cancer Research UK, 2019). The microscopic examination of cervical cells currently carried out to screen asymptomatic women is prone to human error and can lead to high numbers of false-positives and false-negatives. Primary HPV DNA testing has been shown to be more accurate in screening and therefore is projected to replace cytology in the UK by the end of 2019 (Rebolj *et al.*, 2019). Rapid evaporative ionization mass spectrometry (REIMS) is an innovative technique that allows interrogation of biological samples without any need for laborious sample preparation.
Our main objective is to establish whether REIMS can be employed for the detection and grading of pre-invasive cervical changes. We also seek to assess the ability of REIMS to distinguish women with high-risk HPV (hrHPV) infection from women without infection.
Methodology: Cell pellet from liquid-based cytology (LBC) samples has been analysed with REIMS. During REIMS, laser energy is directed to the sample of interest and rapid heating results in a vapour containing gas phase ions. The generated ions are introduced into a spectrometer and a mass

spectrum with molecular information is produced. Samples from more than 400 women, with recorded hrHPV genotype and cytological / histological results, have been analysed. The derived mass spectra are used to differentiate between women with precancerous changes as well as those being hrHPV positive and negative.
Results: Our preliminary results show that REIMS can predict the presence and grade of disease with higher accuracy than current cytology. The diagnostic accuracy of REIMS was also comparable to the gold-standard HPV genotyping.
Conclusion: Using a near-real-time, bedside technique such as REIMS would reduce cost, repeated visits and prolonged waiting times, benefitting both clinicians and patients.
Disclosure: Nothing to disclose

P39

ITIC2 trial - topical imiquimod versus conization to treat cervical intraepithelial neoplasia: randomised controlled, non-inferiority trial

S. Polterauer[1], O. Reich[2], A. Widschwendter[3], J. Ganhoer-Schimboeck[4], G. Bogner[5], R. Berger[6], R. Horvat[7], A. Gleiss[8], M. Fraunschiel[8], S. Aberle[9], A. Reinthaller[1], C. Grimm[1], AGO Austria
[1]Department of Obstetrics and Gynecology, Medical University of Vienna, Vienna, [2]Department of Obstetrics and Gynecology, Medical University Graz, Graz, [3]Department of Obstetrics and Gynecology, Medical University Innsbruck, Innsbruck, [4]Department of Obstetrics and Gynecology, Ordensklinikum Linz und Konventhospital Barmherzige Brüder, Linz, [5]Department of Obstetrics and Gynecology, Universitätsklinik Salzburg Paracelsus Medzinische Universität PMU, Salzburg, [6]AGO Austria, Innsbruck, [7]Department for Pathology, [8]Center of Medical Statistics, Informatics and Intelligent Systems, Section for Medical Information Management and Imaging, Medical University of Vienna, [9]Center for Virology, Medical University Vienna, Vienna, Austria

Introduction / Background: In a recently published randomized, placebo-controlled phase II trial, we showed that topical imiquimod (IMQ) therapy is an efficacious and feasible treatment for selected patients with high-grade squamous intraepithelial lesion (HSIL). Aim of the present study was to investigate the non-inferiority of a 16-week topical IMQ therapy compared to large loop excision of the transformation zone (LLETZ) in patients diagnosed with cervical intraepithelial neoplasia (CIN) 2/3 (HSIL).
Methodology: Phase III randomized, controlled, multicenter, open, non-inferiority trial performed by Austrian Gynecologic Oncology group (AGO-Austria). Patients with histologically proven CIN 2 (older than 30 years) and CIN3 and satisfactory colposcopy were randomized (1:1) to topical IMQ treatment or LLETZ. Successful treatment was defined as negative HPV high-risk test (Roche cobas) result 6 months after start of the treatment. Secondary endpoints were long-term success of treatment and histological outcome.
Results: In total 93 patients were randomized and received the allocated treatment. The trial was stopped early due to poor accrual. CIN 2 and 3 at inclusion was present in 17 and 76 patients. In the IMQ group negative HPV test at 6 months after treatment start was observed in 66.7% of patients compared to 69.1% in LLETZ group (ITT analysis, rate difference 2.4%, 95% CI: -16.9 to -21.0). Negative HPV test at 12 months after treatment start was observed in 56.9% (IMQ) compared to 52.4% (LLETZ) of patients. In the IMQ group histologic regression 6 months after treatment was observed in 72.7% of patients. Complete resection was observed in 84.4% in the LLETZ group.
Conclusion: IMQ and LLETZ treatment seem to result in similar rates of negative HPV test results. We observed major difficulties in recruiting participants in the present academic RCT.
Disclosure: The present academic trial was partly funded by the Anniversary Fund of the Oesterreichische Nationalbank (OeNB). Stephan Polterauer, Alexander Reinthaller and Christoph Grimm have received honoraria for consultation from AstraZeneca, Celgene, MSD, PharmaMar, Roche,Roche Diagnostics, Meda Pharma, Tesaro, Vifor Pharma, outside the submitted work. Andreas Gleiss, Melanie Fraunschiel, Regina Berger, Gerhard Bogner, Olaf Reich, Gerda Trutnovsky, Stephan Aberle, Reinhard Horvat, Julia Ganhoer-Schimboeck, and Andreas Widschwendter have nothing to disclose.

P40

Operative prognostic scores of pelvic exenterations for gynecological cancer: when should we do the surgery?

L. Sabiani, G. Houvenaeghel, S. Knight, G. Atrous, C. Jauffret, E. Lambaudie
Institute Paoli-Calmettes, Marseille, France

Introduction / Background: The use of pelvic exenteration (PE) for locally advanced gynecological cancer or local recurrence is sometimes the only possible treatment. Because of the impact of this major surgery, our objective was to identify prognostic factors in order to establish an operative prognostic score.
Methodology: Patients were characterized by age, type of PE (anterior, posterior, total, atypical, preservation of pelvic floor and perineal excision, extended or not to other structures), indication, curative or palliative surgery and prior radiation therapy.
Results: Over 30 years, 277 patients were included. Overall survival at 3 and 5 years was 36.8% and 32.3%. The rate of non-curative resection was significantly correlated with posterior PE (OR: 2.29), tumors developed in lateral pelvic (OR: 2.91), PE requiring enlargement (OR: 2.61) and lack of radiotherapy or < 45 Gy (OR: 1.73). In multivariate analysis, factors impacting significantly on survival were: total PE (HR: 2.04, p < 0.0001), extended PE (HR: 1.56, p = 0.017) and lack of radiotherapy or < 45 Gy (HR: 1.40; p = 0.033).Based on these factors, we created 4 differents groups in order to establish a pre and post-operative prognostic score. Overall 5-year survival for each group was respectively 48.7%, 29.0%, 28.7% and 14.8% (p < 0.0001). A preoperative high score was a strong negative predictive predictor on overall survival (HR = 3.01, p < 0.0001).
Conclusion: These scores could help to predict the overall survival and to decide when it would be necessary to perform this surgery.

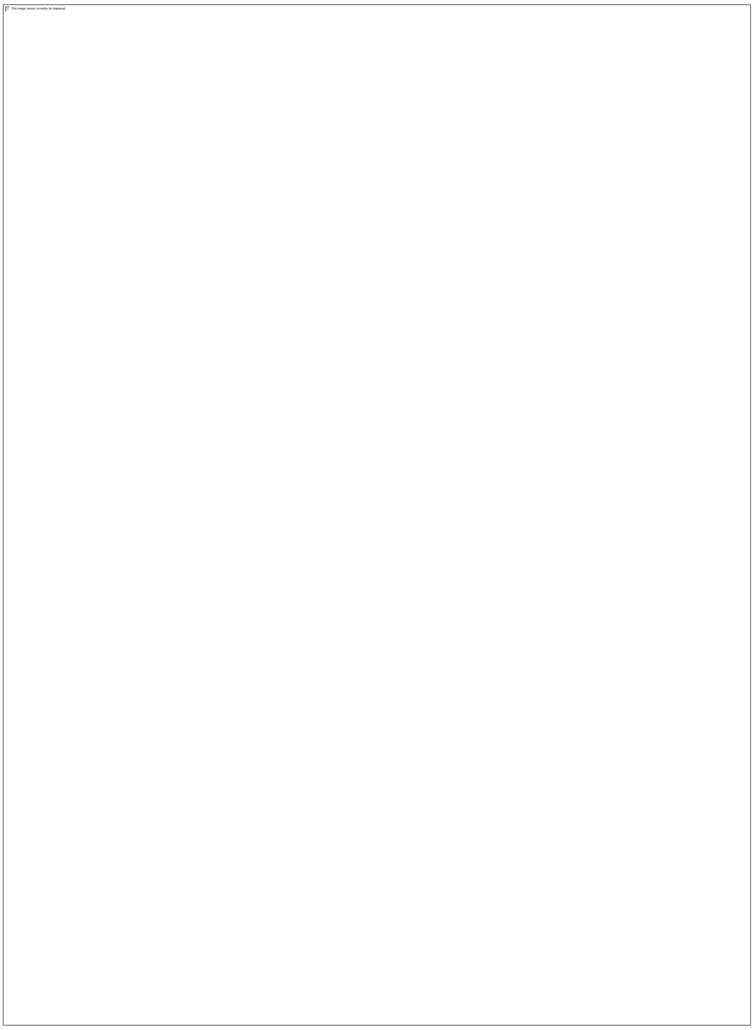

[Figure 1 . How to calculate the Pre Operative Prognostic Predictive Score]

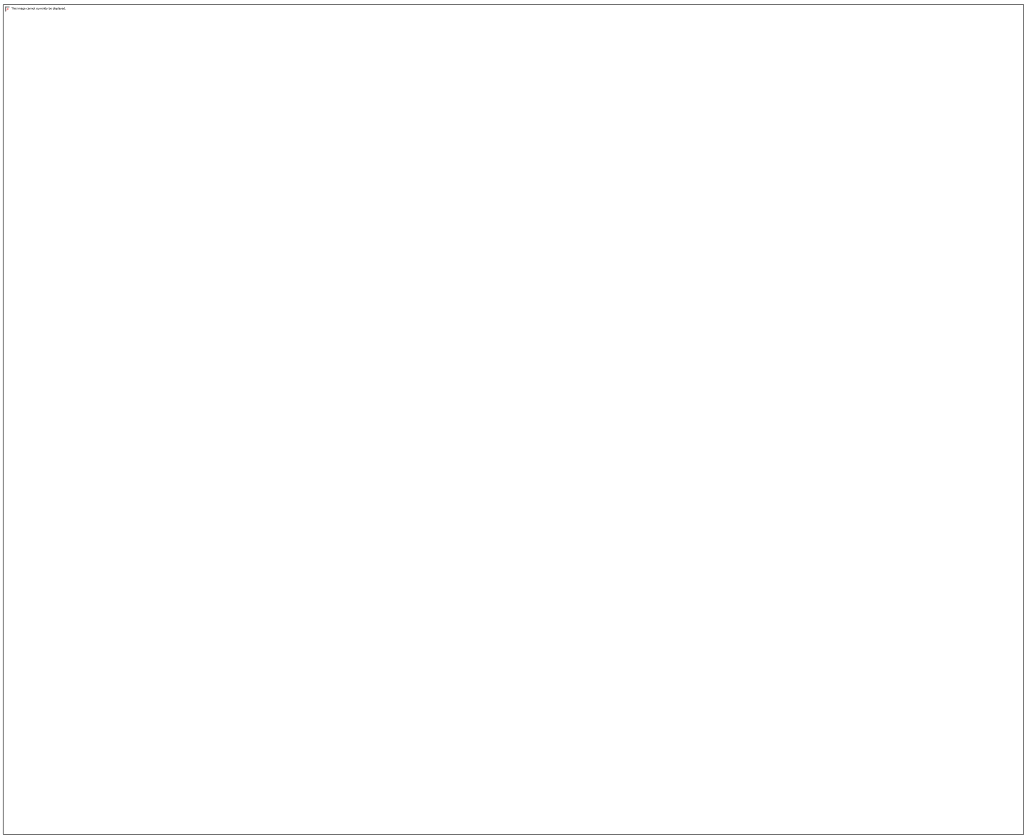

[Figure 2. Overall survival curves according to Pre Operative and Post Operative Prognostic Score]

Disclosure: Nothing to disclose

P41

HPV vaccination and recurrent disease after conization: a meta-analysis

V. Paspalj[1], R. Schwameis[1], S. Pils[1], R. Ristl[2], E. Joura[1]
[1]Department of General Gynecology and Gynecologic Oncology, Medical University Vienna, [2]Section for Medical Statistics, Center for Medical Statistics, Informatics, and Intelligent Systems, Medical University of Vienna, Vienna, Austria

Introduction / Background: Women with treatment of HSIL of the cervix have an increased risk for subsequent HPV related diseases. Developed for primary prevention, the HPV vaccines have demonstrated disease reduction in females and males after treatment of HPV related disease. We investigated the influence of HPV vaccination before or after conization on the incidence of recurrent HSIL of the cervix.
Methodology: A PubMed search provided five appropriate studies addressing the incidence of recurrent HSIL (CIN 2+) after conization in vaccinated or unvaccinated women. Three studies were post-hoc analyses of large prospective randomized trials. One study was retrospective and another study was prospective but not randomized. Data of 3202 women with conization were included into this meta-analysis.
Results: In the HPV vaccinated cohorts, the risk for recurrent disease was reduced by two thirds (RR 0.34 [0.21, 0.56]). Four of five studies demonstrated recurrent disease reduction of >= 65%, the only negative study was underpowered to address this issue.

Conclusion: HPV vaccination before or after conization appears to have a significant effect on recurrent disease. In addition, a reduction of subsequent vulvovaginal disease after treatment has been demonstrated. A randomized controlled trial has already been started in the Netherlands. A cost-effectiveness analysis is missing to this date.
Disclosure: VP: non to declare RS: travel expenses: Amgen, Roche, Pharma Mar (not related to this work) SP: lecture fee: MSD (not related to this work) RR: none to declare EJ: Research grant: MSD Advisory board fee and lecture fee: MSD, Roche

P42

ICG sentinel lymph node mapping in early cervical cancer (FRIENDS Study)

P. Stanciu[1], T. Ind[1], M. Kaur[1], K. Vroobel[2], A. Attygalle[2], M. Nobbenhuis[1]
[1]Gynaecological Oncology Surgery Department, [2]Pathology Department, The Royal Marsden NHS Foundation Trust, London, United Kingdom

Introduction / Background: The purpose of this study is to assess the detection rate of sentinel lymph nodes (SLNs) using Indocyanine Green (ICG) during robotic surgery for early stage cervical cancer.
Methodology: This is a prospective, observational study that included women undergoing robotic surgery for early stage (FIGO ≤IB1) cervical cancer at Royal Marsden Hospital in London. From March 2015 until October 2018, 71 consecutive women were included and had SLN dissection using ICG. Thereafter, completion of surgery consisting of either trachelectomy, or radical hysterectomy was performed followed by pelvic lymphadenectomy if indicated according to hospital guidelines. No ultra-staging was applied.
Results: A mean of four SLNs (SD 2.8) per patient were identified. SLNs were detected most frequently in common iliac, external iliac and obturator chains. Unilateral and bilateral SLN detection rates were 100% and 95.8%, respectively. Positive SLNs were detected in eight patients (11.3%). No additional positive nodes were found in the full lymphadenectomy samples. The sensitivity and negative predictive value of SLN dissection were both 100%.
Conclusion: Florescent Robotic Indocyanine Endoscopic Node Detection Study (FRIENDS) has demonstrated that the use of ICG in robotic SLN dissection for early stage cervical cancer is feasible. In this setting, SLN dissection has the potential to replace a full lymphadenectomy and reduce morbidity. Replacement of ultra-staging with perioperative frozen section analysis of SLN to determine the extent of lymph node dissection might be justified.
Disclosure: Nothing to disclose

P43

Dose received by ovaries during preoperative uterovaginal brachytherapy

R. Trigui[1], S. Yahiaoui[1], S. Zarraa[1], R. Abidi[1], K. Mahjoubi[1], A. Belaid[1], C. Nasr[1], I. Saidani[2], A. Abessi[2], K. Aouinti[2], I. Jlassi[2], M. Besbes[2], I. Dkhil[3], S. Sghaier[3], R. Aouini[3], I. Megdiche[3], A. Zidi[3]
[1]Radiotherapy, [2]Medical Physics, [3]Radiology, Salah Azaiez, Tunis, Tunisia

Introduction / Background: Preoperative uterovaginal brachytherapy at dose of 60 Gy is a therapeutic option for patients with localized cervical cancer. In our study, we will evaluate the dose received by the ovaries of these patients.
Methodology: This is a prospective study of eighteen premenopausal patients with cervical cancer treated at the Salah Azaiez Institute between December 2018 and March 2019. These patients received uterovaginal brachytherapy. the minimal and mean doses received by left and right ovaries were evaluated. We also evaluated the doses received by a volume formed by the ovaries to which

predictive margins for their mobility were added.
This volume is defined by a margin of 3.8 cm in longitudinal and 3.3cm in anteroposterior and 5.8 cm in lateral for the right ovary.
It is defined by a margin of 5.3 cm in longitudinal and 7.8 cm in anteroposterior and 1.7 cm in lateral for the left ovary
Results: The age of the patients was between 25 and 45 years old. For the right ovary, the average of minimal dose was 9.67Gy (3.12-33.6) . The average of mean dose was 15.87 Gy (6.12-54). For the predictive volume of right ovarian mobility,the average of minimal dose was 3.83 GY (1.36-15.28) and the average of mean dose received by this volume was 19.99GY (6.64-66.8). For the left ovary, the average of minimal dose was 9.62Gy (5.2-26). The average of mean dose was 15.75Gy (4.8-55.6). For the predictive volume of left ovarian mobility, the average of minimal dose was 4.14 GY (2.8-13.48) and the average of mean dose was 17.07 GY (5.34-77.6).
Conclusion: Uterovaginal Brachytherapy may cause early menopause by delivering more than the tolerance dose to the ovaries. Ovarian transposition should be offered to premenopausal patients.
Disclosure: Nothing to disclose

P44

Oncological and reproductive outcomes after fertility-sparing surgery in women with Cervical Cancer: a systematic review and meta-analysis

M. Tzafetas, A. Mitra, S. Lever, I. Kalliala, D. MacIntyre, S. Ghaem-Maghami, M. Kyrgiou
Imperial College London, London, United Kingdom

Introduction / Background: Several approaches to fertility-sparing surgery in women with cervical cancer exist. Differences in radicality of parametrial excision are thought to exist according to surgical technique. Minimal access approaches may offer benefits in terms of enhanced patient recovery and nerve-sparing, however comparative oncological safety compared to an open approach is highly debated, especially in tumour volume >2cm. Pregnancy outcomes may also vary according to approach. This is the first systematic review and meta-analysis to compare the oncological and reproductive outcomes of simple or radical trachelectomy surgeries.
Methodology: We searched relevant studies in MEDLINE, PUBMED and CENTRAL from inception to September 2018. Studies were eligible if they investigated oncological and/or reproductive outcomes following any radical trachelectomy surgical approach. Data were extracted in duplicate and additionally requested from authors where necessary. The primary outcome was cervical cancer recurrence rate after treatment. Secondary outcomes were margin involvement rate, residual tumour rate, conception rate and pregnancy outcomes (including 1stand 2ndtrimester miscarriage rates, delivery rate, preterm labour rate (< 37 weeks)). Random effects models were applied in STATA IC v15 to determine pooled estimates and corresponding heterogeneity. Sensitivity analyses were performed to analyse subgroups and where significant heterogeneity was detected.
Results: We identified 59 eligible studies including over 2150 women. Recurrence rate varied by surgical approach: simple vaginal trachelectomy (SVT) 2% (95%CI 1-3%, I^2=NA), vaginal radical trachelectomy (VRT) 3% (95%CI 2-4%, I^2=0%) abdominal radical trachelectomy (ART) 1% (95%CI 0-2%, I^2=35%), laparoscopic radical trachelectomy (LRT) 2% (95%CI 0-6%, I^2=31%), radical robotic trachelectomy (RRT) 0% (95%CI 0-4%, I^2=0%). Involved margins rate was lowest for LRT (7%, 95%CI 0-3%, I^2=0%) and highest for RRT (7%, 95%CI 0-25%, I^2=50.8%). Conception rate was highest for VRT (62%, 95%CI 47-75%, I^2=84%) and lowest for ART (34%, 95%CI 25-44%, I^2=55%).
Conclusion: Balancing the oncological and fertility outcomes for fertility preservation techniques is challenging but achievable.
Disclosure: Nothing to disclose

P45

Pathology review of stage IA2 microinvasive and small diameter stage IB1 cervical cancer

B. Vranes
Gynecology and Obstetrics, University Clinical Center Zvezdara, Belgrade, Serbia

Introduction / Background: FIGO definition from 2009, limits stage IA2 to 5x7 mm, and stage IB1 to a gross tumor diameter of 4 cm. Our study reviews the pathology in 32 cases of stage IA2 and 205 cases of small diameter stage IB1 cervical cancer
Methodology: Pathology review was done including the „third dimension" measure in stage IA2 not exceeding 7 mm and maximal measure in small diameter IB1 not exceeding 20 mm. Therefore exclusion criteria further limited these two groups of patients. The main focus was to emphasize the risk factors contributing to tumor spread.
the disease beyond cervix and defining the spread pathways.
Results: Metastatic pelvic nodes were found in 3.1% of patients in stage IA2, and 6,3% in small stage IB1. LVSI was detected in 9,1% in stage IA2, and in 32.2% in small stage IB1 cervical cancer. There were no cases of parametrial involvement in stage IA2, but 12 cases in small stage IB1; 9 in patients with positive and only 3 with negative pelvic nodes. Two of these patients were LVSI positive, and only one LVSI negative with superficial invasion. The risk of parametrial involvement in small IB1 tumors was 0.5% with superficialinvasion and no LVSI. The single most important predicting factor for nodal metastases in both groups was LVSI, 33% of patients in stage IA2, and 84.6% in small stage IB1 had nodes positive. The depth of invasion showed importance only in small diameter IB1 tumors. Tumor volume had no significant impact limited with maximal measures applied.
Conclusion: The review shows no risk for parametrial tumor spread in stage IA2 but very low risk in small diameter IB1 tumors. Tumors belonging to different FIGO stages, ie IA2 and IB1 with diameter less than 20 mm seem to be acting biologically closer compared to the two substages of their own stage.
Disclosure: Nothing to disclose

P46

Predictors of short-term surgical complications after radical hysterectomy for early-stage cervical cancer

H. Wenzel[1], T. van Gorp[2,3], R. Bekkers[4], C. de Kroon[5], L. van Lonkhuijzen[6], L. Massuger[7], H. Nijman[8], R. Smolders[9], N. van Trommel[10], R. Yigit[8], R. Zweemer[11], R. Kruitwagen[2], M. van der Aa[1]
[1]Department of Research, Netherlands Comprehensive Cancer Organisation (IKNL), Utrecht, [2]Department of Obstetrics and Gynaecology and GROW - School for Oncology and Developmental Biology, Maastricht University Medical Centre+, Maastricht, The Netherlands, [3]Department of Gynaecology and Obstetrics, Division of Gynaecologic Oncology, University Hospitals Leuven, Leuven Cancer Institute, Leuven, Belgium, [4]Department of Obstetrics and Gynaecology, Catharina Hospital, Eindhoven, [5]Department of Gynaecology, Leiden University Medical Centre, Leiden, [6]Department of Gynaecologic Oncology, Amsterdam University Medical Centre - Centre for Gynaecological Oncology Amsterdam, Amsterdam, [7]Department of Obstetrics and Gynaecology, Radboud University Medical Centre, Nijmegen, [8]Department of Obstetrics and Gynaecology, University Medical Centre Groningen, Groningen, [9]Department of Gynaecological Oncology, Erasmus Medical Centre - Cancer Centre, Rotterdam, [10]Department of Gynaecologic Oncology, Netherlands Cancer Institute - Antoni van Leeuwenhoek, Amsterdam, [11]Department of Gynaecological Oncology, University Medical Centre Utrecht - Utrecht Cancer Centre, Utrecht, The Netherlands

Introduction / Background: Recently published data have renewed the debate on surgical approach (open vs. laparoscopic) in radical hysterectomy and its effect on survival and recurrence. In addition, an estimation of risk factors of surgical complications would be beneficial in the management of these patients. Therefore, we aimed to evaluate possible predictors of short-term surgical complications after radical hysterectomy for early-stage cervical cancer.

Methodology: Patients diagnosed with cervical cancer FIGO (2009) stage IB1 and IIA1 between January 2015 and December 2017 who underwent radical hysterectomy with pelvic lymphadenectomy in one of the nine specialized centres in the Netherlands, were identified from the Netherlands Cancer Registry. Patients were excluded if primary treatment consisted of simple hysterectomy (i.e. without parametrial dissection) or radical trachelectomy. Complications and type of complications, developing within 30 days after surgery, were registered. Multivariable logistic regression analysis was used to identify predictors for surgical complications.
Results: Of the 472 patients, 166 (35%) developed surgical complications. Predominant FIGO stage was IB1 (97%), mean age was 45 ± 12 years, mean body mass index was 26 ± 5 kg/m^2. Most patients were treated with open surgery (58%). The most frequent complications were urinary retention with catheterisation in 73 patients (15%) and excessive perioperative blood loss > 1000 mL (EBL) in 50 patients (11%) (Table 1). Open surgery, chronic pulmonary disease, vascular disease and medical centre emerged as independent predictors of the occurrence of complications (Table 2). BMI was found as negative predictor for urinary retention. Open surgery and BMI were found to be independent predictors for EBL.
Conclusion: We conclude that open surgery, chronic pulmonary disease, vascular disease and BMI negatively affect the occurrence of short-term surgical complications. We believe these findings should also be taken into consideration, together with data on survival and recurrence, in the choice of surgical approach.

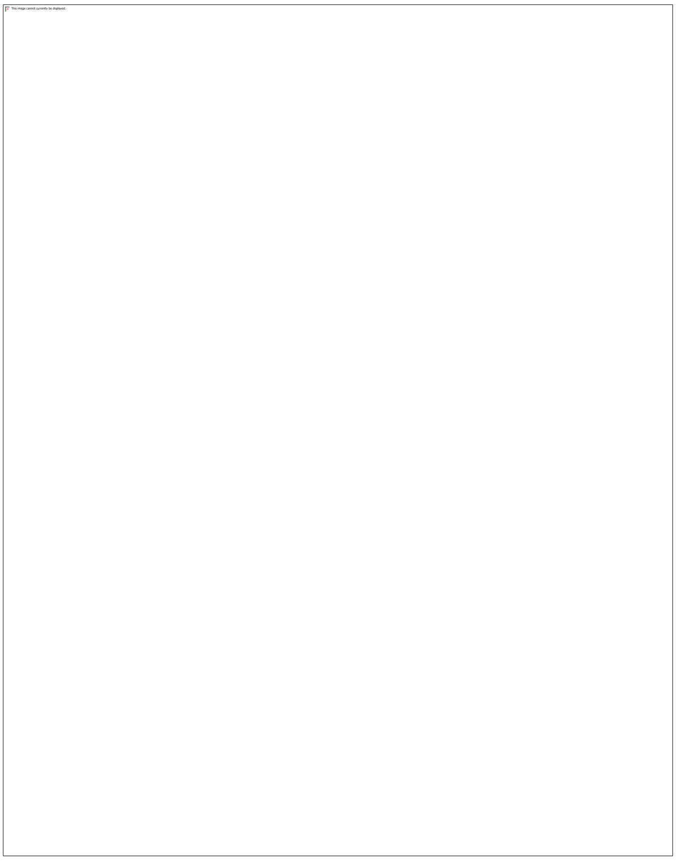

[ ]

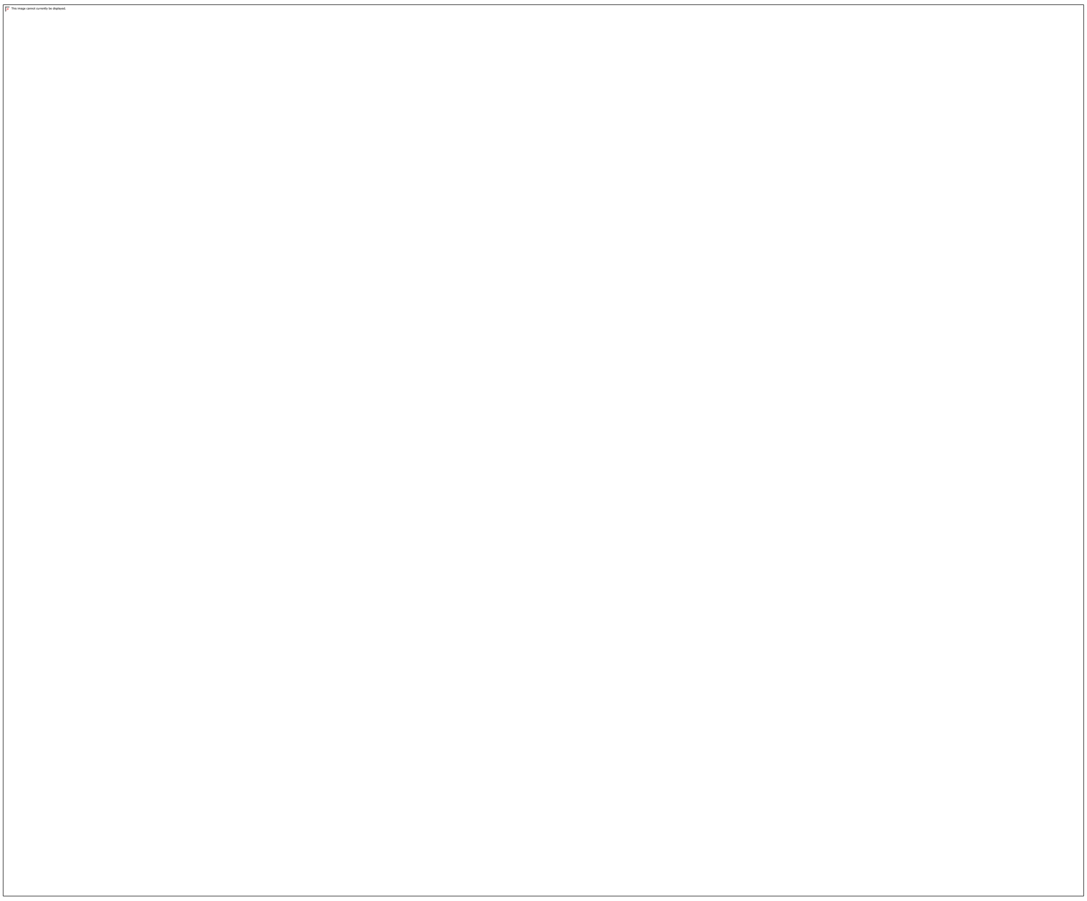

[ ]

Disclosure: None of the authors received financial support for the research and/or authorship of this article. Hans Wenzel - Nothing to disclose; Toon van Gorp - Nothing to disclose; Ruud Bekkers - Nothing to disclose; Cor de Kroon - Nothing to disclose; Luc van Lonkhuijzen - Nothing to disclose; Leon Massuger - Nothing to disclose; Hans Nijman - Grant Dutch Cancer Society; Stock owner SME Vicinivax; Grant Aduro; Ramon Smolders - Nothing to disclose; Nienke van Trommel - Nothing to disclose; Refika Yigit - Nothing to disclose; Ronald Zweemer - Proctor Intuitive Surgical; Roy Kruitwagen - Nothing to disclose; Maaike van der Aa - Nothing to disclose;

P47

Guidline adherence and survival in locally advanced cervical cancer in the Netherlands between 2009 - 2016

H. Wenzel[1], R. Bekkers[2], V. Lemmens[1,3], H. Nijman[4], M. van der Aa[1]
[1]Department of Research, Netherlands Comprehensive Cancer Organisation (IKNL), Utrecht, [2]Department of Obstetrics and Gynaecology, Catharina Hospital, Eindhoven, [3]Department of Public Health, Erasmus University Medical Centre, Rotterdam, [4]Department of Obstetrics and Gynaecology, University Medical Centre Groningen, Groningen, The Netherlands

Introduction / Background: In the Netherlands, approximately one third of the cervical cancer patients is diagnosed with locally advanced cervical cancer (LACC). In the past decades, chemoradiation (CRT) has replaced radiotherapy (RT) as preferred treatment. Alternatively,

radiotherapy with hyperthermia and radical hysterectomy ± neoadjuvant chemotherapy (RH) are applied in LACC treatment as well. We aimed to evaluate trends in guideline adherence and survival since the introduction of CRT as standard of treatment for LACC in the national guidelines of 2004.
Methodology: In this retrospective study, patients diagnosed with cervical cancer (FIGO stages IB2, IIA2-IVA 2009) between 2005-2016, were identified from the Netherlands Cancer Registry. Patients were classified guideline-adherent when receiving primary treatment according to the guideline. Trends, from 2005-2007 to 2014-2016, were analysed with the Cochran-Armitage trend test and 5-year relative survival (RS) was calculated. Multivariable Poisson regression was applied. Subanalyses for FIGO IB2 (only stage for which all treatment modalities apply) and IIB (most prevalent stage) were conducted.
Results: 2915 patients (mean age 57 ± 17 years) were diagnosed with LACC - mainly with FIGO IIB (47%). Guideline adherence was observed in 73% of all patients, with higher RS compared to non-adherent patients (see Table 1). Over time, increases were seen in guideline adherence and 5-year RS (see Figure 1) and CRT was administered more often (73% - 86%). Of the 532 IB2 patients, guideline adherence was observed in 86%, CRT was the most common treatment (58%) and those treated with RH (*n*=173) showed highest survival (82%). Of the 1371 IIB patients, guideline adherence was observed in 79%, CRT was the most common treatment (86%) and those treated with RH (*n*=43) showed highest survival (84%).
Conclusion: Guideline adherence is associated with higher survival. This may partly be explained by confounding by indication. Expectedly, survival rates improved with the replacement of RT by CRT.

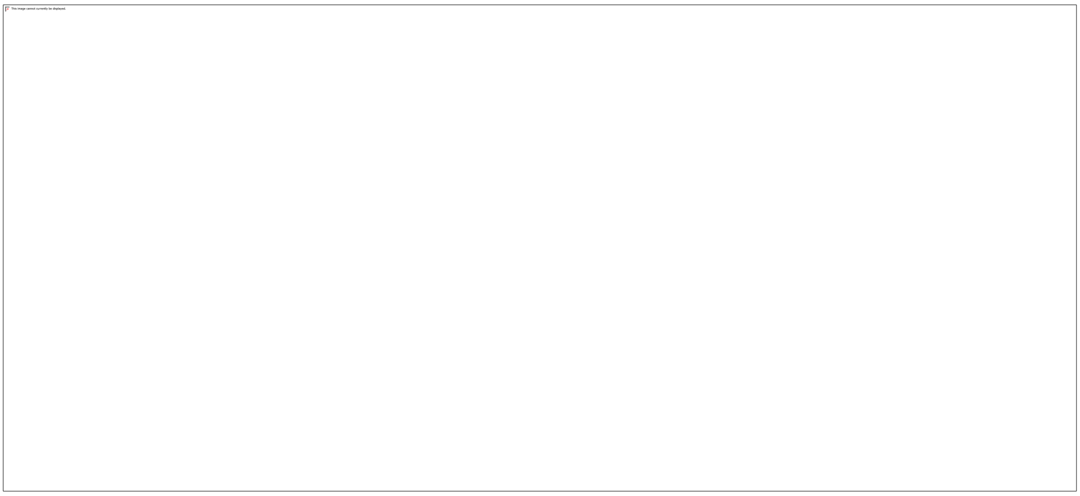

[ ]

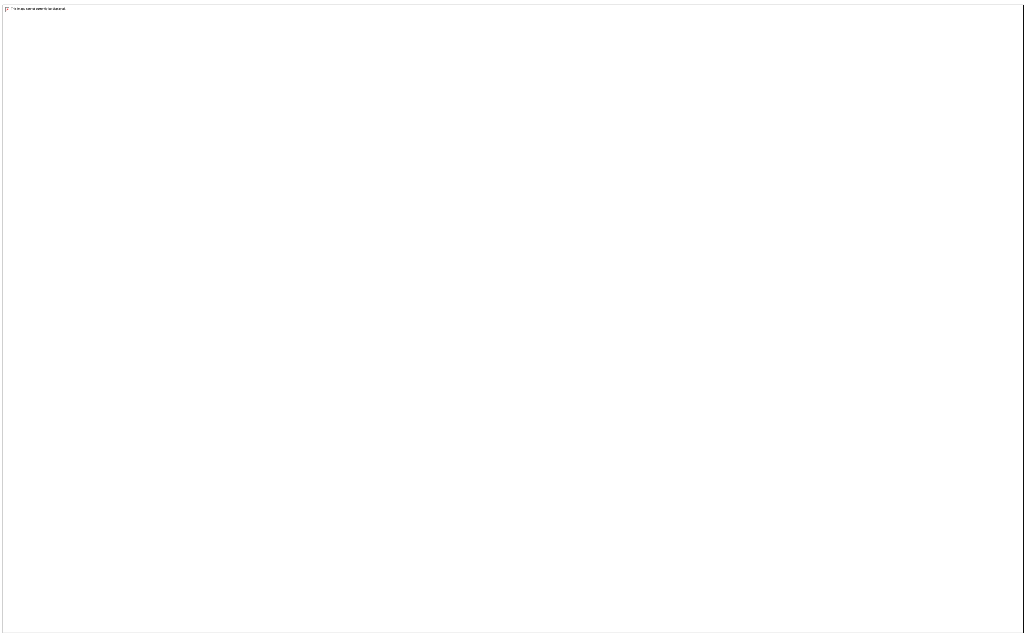

[Figure 1. Trends in guideline adherence and survival]

Disclosure: None of the authors received financial support for the research and/or authorship of this article. Hans Wenzel - Nothing to disclose; Ruud Bekkers - Research funds from Roche Diagnostics; Hans Nijman - Grants from Dutch Cancer Society; Collaborations with Aduro, DCprime, MSD, TRON; Founder of ViciniVax; Valery Lemmens - Unrestricted research grants and educational grants from Roche; Maaike van der Aa - Nothing to disclose

P48

Obstetric outcomes after abdominal radical trachelectomy (ART) for early-stage cervical cancers: a retrospective analysis of 341 cases in China

X. Wu[1,2], X. Li[1,2]
[1]Gynaecologic Oncology, Fudan University Shanghai Cancer Center, [2]Oncology, Shanghai Medical College, Shanghai, China

Introduction / Background: To report the obstetric outcomes of young patients undergoing abdominal radical trachelectomy (ART) for the treatment of early-stage cervical cancers in China.
Methodology: We retrospectively reviewed 341 patients with stage IA-IB1 cervical cancer who underwent ART between April 2004 and September 2017 in China.
Results: Of the 341 patients underwent ART, 325 had their fertilities preserved. 200 women (61.5%) did not plan to get pregnant after ART, and 85.0% of them were due to childbearing before surgery (55.0%) or unmarried (30.0%). Among 125 women attempted to conceive, 23 women achieved a total of 26 pregnancies. Seventy-four patients had infertility problems, 35 conceived with assisted reproductive technologies, and 13 of them succeeded. Cervical stenosis (22, 25.9%) and fallopian tube obstruction (18, 21.2%) were the most frequently cause of fertility treatment after surgery. Among patients who conceived, 3 patients had first trimester miscarriage and 5 patient had second trimester miscarriage. Two woman elected to have pregnancy termination and 1 were currently pregnant. A total of 15 pregnancies reached the third trimester, and 80% of them ended more than 36 weeks of gestation. Among 13 cases of miscarriages and preterm birth, 7 occurred in patients without cerclage placed. In 10 cases of full-time birth, 8 patients had cerclage placed.
Conclusion: The majority of patients did not attempt to conceive or experienced infertility after ART in

China. Assisted reproductive technology should be encouraged to improve the fertility rate. Cerclage is effective in the prevention of miscarriage and pre-term labor.
Disclosure: Nothing to disclose

P49

LncRNA-mRNA co-expression involved in high risk HPV-related cervical cancer cells

L. Yang, X. Zeng, M. Xi
West China Second University Hospital | Gynecology, Sichuan University, Chengdu, China

Introduction / Background: Long non-coding RNAs are emerging to be novel regulators in gene expression. In current study, lncRNAs microarray and lncRNA-mRNA co-expression analysis were performed to explore the alternation and function of lncRNAs in cervical cancer cells. Our study might help to understand the interplay between lncRNAs and coding genes anticipated in oncogenic HPV proliferation, and lncRNAs may be the missing links of well-known oncogenic and tumor suppressor networks.
Methodology: The high-throughput analysis was performed to detect the global lncRNAs and mRNAs expression changes between oncogenic HPV-positive cervical cancer cells (SiHa and HeLa) and oncogenic HPV-negative cervical cancer cells (C33-A). Additionally, we constructed a co-expression network of lncRNAs and coding gene transcripts to predict and validate the potential biological function of differentially expressed lncRNAs. Western blot、PCR and transwell test were performed to validate the biological function of ENST00000420168, ENST00000564977 and TCONS_00010232 in cervical cancer cells.
Results: With the help of mRNA-lncRNA co-expression network, we found that ENST00000503812 was significantly correlated with RAD51B and IL-28A expression in HPV-16 positive cervical cancer cells (SiHa), while ENST00000420168, ENST00000564977 and TCONS_00010232 had significant correlation with FOXQ1 and CASP3 expression in HPV-18 positive cervical cancer cells (HeLa). Up-regulation of ENST00000503812 inhibited RAD51B and IL-28A expression and result in deficiency of DNA repair pathway and immune responses in HPV-16 positive cervical cancer cells. Up-regulation of ENST00000420168, ENST00000564977 and down-regulation of TCONS_00010232 stimulated FOXQ1 expression and suppressed CASP3 expression in HPV-18 positive cervical cancer cell, which leads to HPV-induced proliferation and deficiency in apoptosis.
Conclusion: Our study demonstrated that integration of oncogenic HPV DNA into host genome could alter the expression profiles of lncRNAs in cervical cancer cells. Co-expression network revealed that certain lncRNAs can lead to differentially expression of their target coding genes including several crucial regulators of DNA repairing, cell cycle, proliferation and apoptosis in HPV-positive cervical cancer cells.

This image cannot currently be displayed.

[Fig_1]

This image cannot currently be displayed.

[Fig_2]

Disclosure: Nothing to disclose

P50

Up-regulated circPOLD1 promotes tumorigenesis in cervical cancer

L. Zhao, Y. Zhang, W. Lv, X. Cheng, X. Xie
Zhejiang University, Hang Zhou, China

Introduction / Background: The role of circular RNAs (circRNAs) still need to be explored in cancers. We aimed to investigate differentially expressed circRNAs in onconegic progress of cervical cancer, and focus on one of them to verify its effects on the development in cervical cancer.
Methodology: High-throughput sequencing technique was performed to identify circRNAs expression profiles in cervical tissues of normal, High Squamous Intraepithelial Lesion(HSIL), and squamous-cell carcinoma (SCC)(each n=6). The results were validated by quantitative real-time PCR (qPCR) in 69 samples. The effect of circRNAs on proliferation, clonal formation and apoptosis were observed in cervical cancer cells by circRNA knockdown. RNA pulldown assay and RNA Binding Protein Immunoprecipitation Assay (RIP) was used to verify the combination between circRNA and its potential RNA binding protein, based on the bioinformatics prediction program.
Results: Totally 798 differentially expressed circRNAs with more than 2-fold change were identified(P< 0.05), of those, 2 down-regulated and 6 up-regulated ones were validated by qPCR. CircPOLD1 was selected to further investigate the function. Functional assays indicated that of circPOLD1 knockdown inhibited cellular proliferation and induced apoptosis in cervical cancer cells. The bioinformatics prediction program predicted several RNA binding proteins including Y-box binding protein-1 (YB1), an oncogene that has been widely researched, that could have bind with circPOLD1. The circPOLD1 directly bound to YBX1 and inhibited its function. Same as circPOLD1, knockdown of YB1 could inhibite cellular proliferation and apoptosis in cervical cancer cells.
Conclusion: CircPOLD1 may play an oncogenic role on cervical cancer tumorigenesis by targeting YBX1.
Disclosure: Nothing to disclose

P51

The use of human papillomavirus DNA methylation in cervical intraepithelial neoplasia: a systematic review and meta-analysis

S. Bowden[1], I. Kalliala[1], A. Veroniki[1], M. Arbyn[2], A. Mitra[1], K. Lathouras[1], L. Mirabello[3], M. Chadeau-Hyam[4], E. Paraskevaidis[5], J. Flanagan[1], M. Kyrgiou[1]
[1]Surgery and Cancer, Imperial College London, London, United Kingdom, [2]Unit Cancer Epidemiology, Belgian Cancer Centre, Brussels, Belgium, [3]NCI/NIH, Bethesda, WA, United States, [4]Biostatistics and Epidemiology, Imperial College London, London, United Kingdom, [5]University of Ioannina, Ioannina, Greece

Introduction / Background: Methylation of viral DNA has been proposed as a novel biomarker for triage of HPV positive women at screening.This systematic review and meta-analysis aims to assess how methylation levels change with disease severity and to determine its diagnostic test accuracy in detecting high-grade cervical intra-epithelial neoplasia (CIN) in HPV positive women.
Methodology: We performed searches in MEDLINE, EMBASE and CENTRAL from inception to September 2018. Studies were eligible if they explored HPV methylation levels in HPV positive women. Data were extracted in duplicate and requested from authors where necessary. Random-effects models and a bivariate mixed-effects binary regression modelwere applied to determine pooled effect estimates.
Results: 43 studies with 8775 high-risk HPV positive women were eligible. The pooled estimates for positive methylation rate in HPV16 L1 gene were higher for ≥CIN2/HSIL (72·7% (47·8-92·2)) vs ≤CIN1/LSIL(44·4% (16·0-74·1)). The pooled difference in mean methylation level was significantly higher in ≥CIN2/HSIL vs ≤CIN1/LSILfor the HPV16 L1 gene (11·3% (6·5-16·1)). The pooled odds ratio of HPV16 methylation in the L1 gene was 6·57 (3·49-12·39) for ≥CIN2/HSIL vs. ≤CIN1/LSIL. HPV16 L1/L2 genes performed best in predicting CIN2 or worse (pooled sensitivity 77% (63-87), specificity

64% (55-71), area under the curve (AUC) 0·73 (0·69-0·76)) (Fig1). HPV16 L1/L2 methylation improved triage of HPV16 positive women (Fig2).
Conclusion: Higher HPV methylation is associated with increased disease severity, whilst HPV16 L1/L2 genes demonstrated high diagnostic accuracy to detect high-grade CIN in HPV16 positive women. The direct clinical use of this marker in triage is limited by the need of a multi-genotype assay. Next-generation multiplex sequencing assays containing all HPV types are under development and have the potential to allow rapid, automated and low-cost methylation testing.

BMJ Publishing Group Limited (BMJ) disclaims all liability and responsibility arising from any reliance placed on this supplemental material which has been supplied by the author(s)

This image cannot currently be displayed.

[Diagnostic Accuracy of HPV16 L1/L2 in predicting CIN2/HSIL or worse]

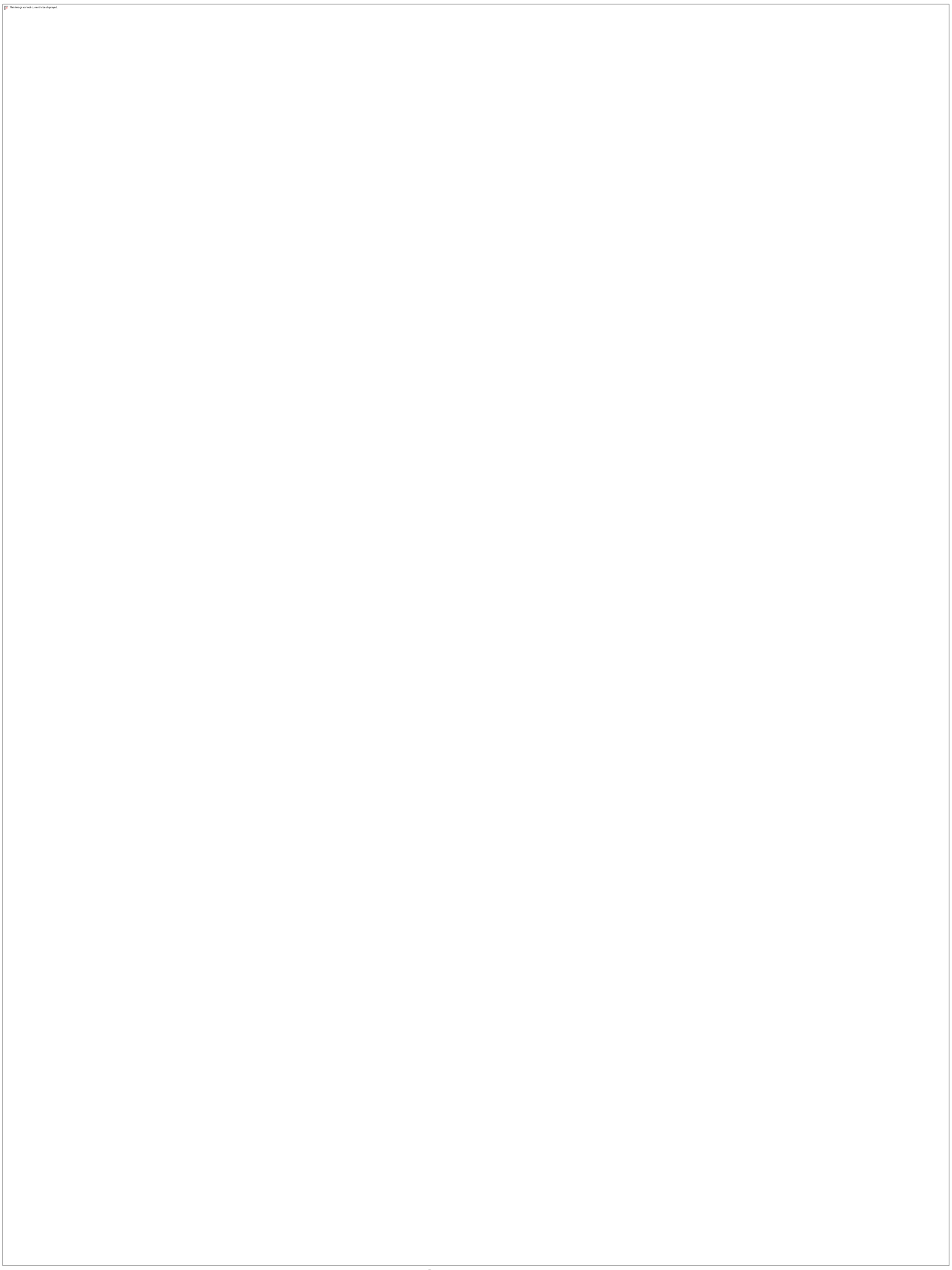

[Pre- and post methylation test probability of CIN2/3 in HPV16 positive women]

Disclosure: Funded by NIHR, Genesis Research Trust, Imperial Healthcare Charity, European Unions Horizon 2020, Wellcome Trust PhD Fellowship. No conflicts of interest

P52

Diagnostic workup in women with a first episode of postmenopausal bleeding: a multicentre prospective observational study

J.A. van der Zande[1], L.A. van Werkhoven[1], M.C. Breijer[2], M.Z. Dorman[3], H.C. van Doorn[1], EIN Study Group South West Holland
[1]Gynaecologic Oncology, Erasmus MC Cancer Institute, University Medical Centre Rotterdam, Rotterdam, [2]Obstetrics and Gynaecology, Spaarne Gasthuis, Haarlem, [3]Obstetrics and Gynaecology, Bravis Hospital, Roosendaal, The Netherlands

Introduction / Background: The Dutch diagnostic guideline for women with a first episode of postmenopausal bleeding (PMB) was updated in 2015, and aims at detecting (pre)malignancies and uterine polyps (Figure 1). The main aim of this study was to evaluate the adherence to the guideline. Secondly, if recurrent bleeding and detection of (pre)malignancies in the first year of follow up relates to guideline-adherence or violation.
Methodology: We performed a multicentre, prospective cohort study in four hospitals in The Netherlands including women undergoing an aspiration endometrial biopsy for a first episode of PMB after providing written informed consent. Women with a benign or insufficient sample were assigned to the diagnostic workup group (DWG) when the workup was according to the guideline. If not, women were assigned to the protocol violation group (PVG). Statistical analysis was performed with SPSS24. Central study approval was obtained at the ErasmusMC, (MEC 2015-740), the study was registered on www.trialregister.nl (NL7608).
Results: From 01.2016 till 02.2019 we enrolled 341 women, of which 78% had an increased or not measurable endometrial thickness and a benign or insufficient endometrial sample. The guideline was subsequently followed in 52%, revealing a (pre)malignancy in 6% of these women. In the DWG, 8.7% experienced recurrent bleeding within the first year, not attributable to a (pre)malignancy (Figure 2). In the PVG, recurrent bleeding occurred in 15.6% (RR 0.60; 95% CI 0.29-1.21), with detection of a (pre)malignancy in three cases (RR 0.27; CI 95% 0.02-4.88) (Table 1).
Conclusion: In women with a first episode of postmenopausal bleeding, an increased or not measurable endometrial thickness, and a benign or insufficient endometrial sample the guideline was followed in 52%. In women with guideline violation more recurrent bleeding and (pre)malignancies were seen in the first year of follow-up, although significance was not reached.

	Diagnostic workup in accordance with protocol (n=11)	Violation diagnostic workup protocol (n=19)	Relative risk	95% CI
Benign	8 (72,7%)	10 (52,6%)	1,22	0,59-2,53
Benign polyp(s)	3 (27,3%)	6 (31,6%)	0,89	0,26-3,03
(Pre) malignant	0	3 (15,8%)	0,27	0,02-4,88

[Table 1. Histopathology results for women with recurrent bleeding during 1st year follow up]

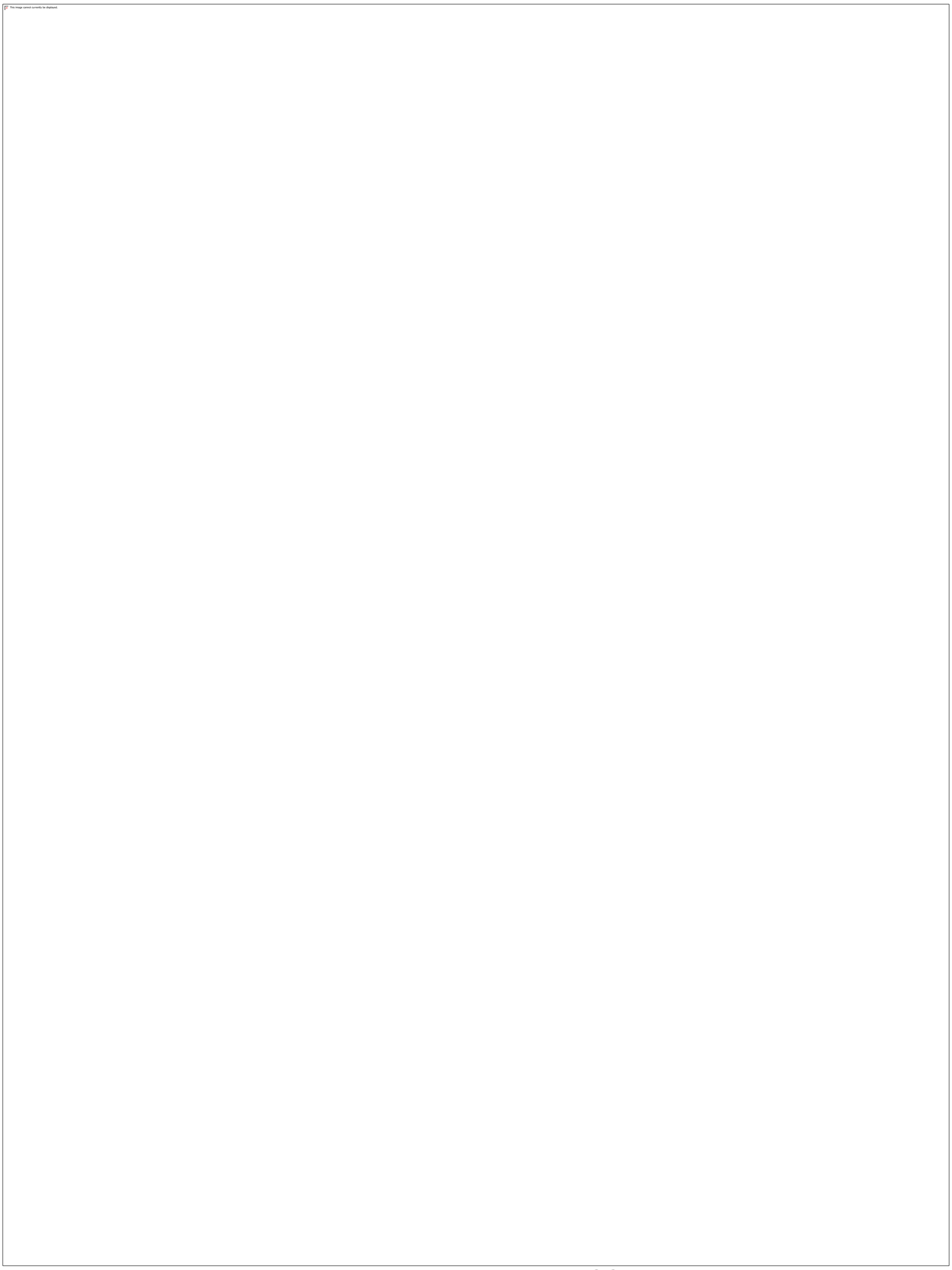

[Figure 1, Algorithm first episode postmenopausal bleeding NVOG guideline Postmenoposal bleeding 2015]

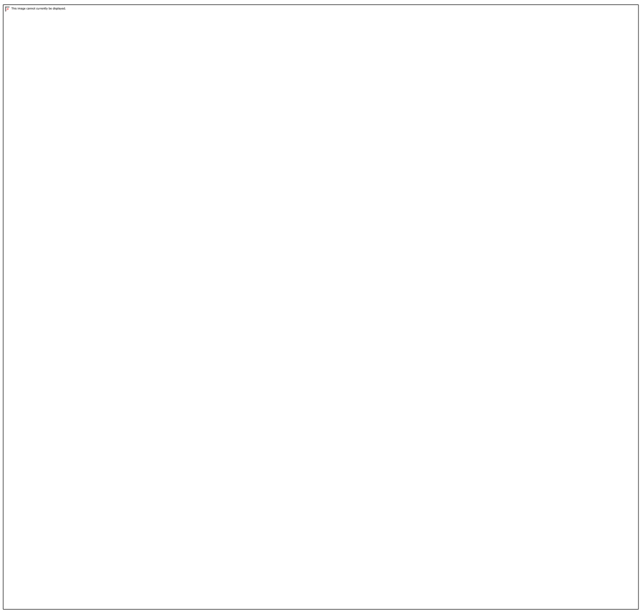

[Figure 2, Kaplan-Meier curves for risk of recurrent bleeding after diagnostic workup]

Disclosure: Nothing to disclose

P53

Endometrial Intraepithelial Neoplasia (EIN) in minimally invasive endometrial biopsies, preliminary results of a prospective observational study

L.A. van Werkhoven[1], J.A. van der Zande[1], M.Z. Dorman[2], K. Schelfout[3], P.C. Ewing-Graham[4], H.C. van Doorn[1], EIN Study Group South West Holland
[1]Gynaecologic Oncology, ErasmusMC Cancer Institute, University Medical Centre Rotterdam, Rotterdam, [2]Obstetrics and Gynaecology, Bravis Hospital, Roosendaal, [3]Pathology, Bravis Hospital, Bergen op Zoom, [4]Pathology, Erasmus MC Cancer Institute, University Medical Centre Rotterdam, Rotterdam, The Netherlands

Introduction / Background: To examine the accuracy of the diagnosis endometrial intraepithelial neoplasia (EIN) in an office endometrium sample (OES).
Methodology: Consecutive patients aged ≥40 years with any indication for OES by aspiration curettage (OES-A) or outpatient hysteroscopy (OES-H) were asked for written consent. Pathology was assessed according to the EIN system and the WHO94 classification. The pathology of any further, more extensive, specimen obtained within 12 months of OES, was regarded as the final pathological diagnosis. Statistical analysis was performed with SPSS24. Central study approval was obtained at the ErasmusMC, (MEC 2015-740), the study was registered on www.trialregister.nl (NL7608).
Results: From 01-2016 to 02-2019, 343 patients were included, as summarized in Table 1. OES-A was the first sampling for 340 patients. Of these, 43 samples were non-diagnostic (12,4%). Benign, EIN or malignancy was the diagnosis in 258 (76,1%), 6 (1,8%) and 33 (9,7%) cases respectively (Figure 1). Final pathological diagnosis showed 290 benign cases (85,5%), 3 of EIN (0,9%) and 46 malignancies (13,6%). The accuracy of diagnosing EIN in an OES-A was 85,5%, with sensitivity of 46,2% and specificity of 100%. Subsequently, 66 OES-H samples were obtained, the accuracy of diagnosing EIN in an OES-H was 91,3%. The incidence of EIN in any OES was 2,5%. In eight out of ten patients a malignancy was ultimately diagnosed. Analyses of the EIN cases show that, despite not all five EIN histopathological criteria were met, the diagnosis EIN was set.
Conclusion: EIN was diagnosed in specimen obtained through OES, although the diagnosis EIN was

made despite not all histological criteria were being met. The EIN and WHO94 systems showed a considerable overlap. Given the low incidence of EIN, our study cohort will be extended to further define the place of the EIN system in an office sampling setting.

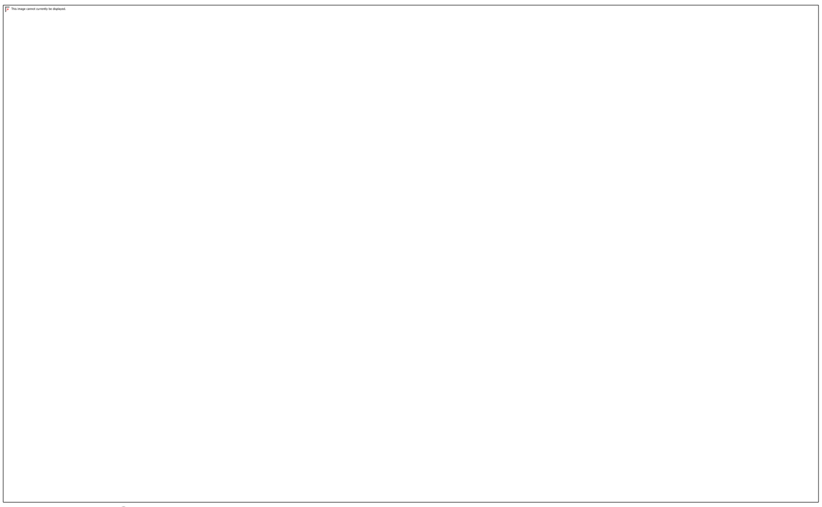

[Figure 1, Outcome of office endometrium sample vs. final diagnosis, including 12 months follow up]

Number of patients	343
Age (years), mean (±SD)	59,5 ± 10,6
Pre menopausal	97 (28,8%)
Indication: pre menopausal menstrual disorder	87 (25,4%)
Indication: postmenopausal bleeding	228 (66,5%)
Indication: bleeding under hormonal replacement therapy	8 (2,3%)
Indication: abnormal cervix cytology in population screening	2 (0,6%)
Indication: otherwise	29 (8,5%)

[Table 1. Patient characteristics]

Disclosure: Nothing to disclose

P54

Comparison of clinical and ultrasound characteristics in low and high grade serous cancer

D. Fischerova[1], B. Cornelli[2], B. Crnobrnja[3], F. Frühauf[1], R. Kocian[1], D. Indrova[1], K. Němejcova[4], I. Dostalkova[5], D. Cibula[1]

[1]Gynecologic Oncology Centre, Department of Obstetrics and Gynecology, First Faculty of Medicine and General University Hospital, Charles University, Prague, Czech Republic, [2]Department of Obstetrics and Gynecology, University of Brescia, Brescia, Italy, [3]Gynecological Oncology Center, Department of Gynecology and Breast Oncology, University Medical Centre Maribor, Maribor, Slovenia, [4]Department of Pathology, First Faculty of Medicine and General University Hospital, Charles University, Prague, [5]Faculty of Science, University of South Bohemia, Ceske Budejovice,

Czech Republic

Introduction / Background: This is the first study up to date aiming to evaluate different ultrasound features of low and high grade serous epithelial ovarian cancer (LGSC, HGSC). In the histopathology, LGSC contains psammoma bodies more often than HGSC. The formation of psammoma bodies is due to collagen production by neoplastic cells with subsequent calcification.
Methodology: This was a retrospective study comprising two cohorts (LGSC and HGSC) of 53 cases each (17 early and 36 advanced stage). Clinical information, ultrasound assessment of primary lesion using IOTA terms and additional ultrasound features of primary tumor and carcinomatosis (hyperechogenic spots, sheet/nodal type and echogenicity of carcinomatosis etc.) were compared.
Results: Women with LGSC were younger (mean age 49.0 vs 60.4 years), less often postmenopausal (60 vs 90%), presenting with lower level of CA125 (mean 380 vs 1059 U/mL) and rarer ascites (15 vs 36%). LGSC in comparison to HGSC were often presenting as unilocular-solid lesions (31 vs 2%) with more papillary projections (52 vs 31%), with hyperechogenic appearance of solid tumor components (55 vs 17%) and hyperechogenic peritoneal spread (100 vs 13%) with multiple hyperechogenic spots (74 vs 7%) and shadowing (46 vs 4%) corresponding to the presence of calcified psammoma bodies. In contrast, HGSC were frequently solid (62.5 vs 29%), richly perfused tumors (87 vs 67%) presenting with hypoechogenic peritoneal spread (77 vs 0.0%). All differences were statistically significant.
Conclusion: LGSC and its peritoneal spread usually manifest as hyperechogenic (almost white) while HGSC are typically hypoechogenic. The significant difference in tumor and its carcinomatosis appearance contributes to the ultrasound-based tumor-specific preoperative diagnosis and individualized planning of care.

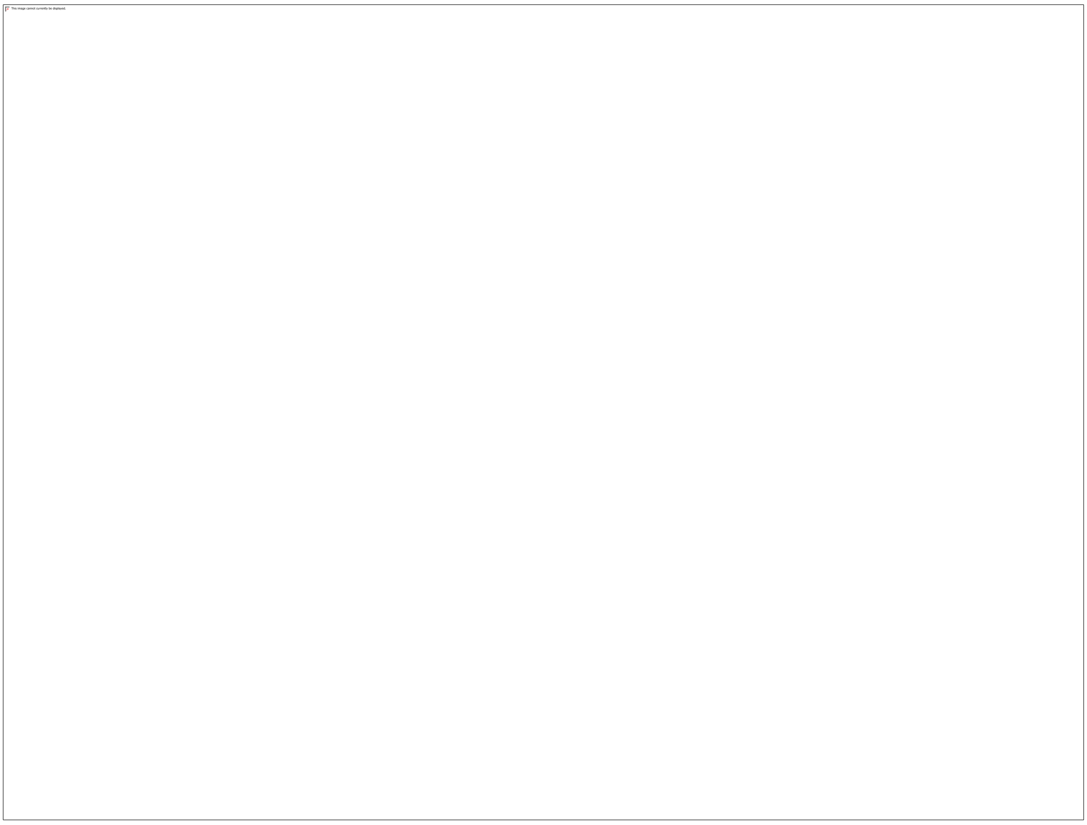

[Figure 1. Ultrasound characteristics of LGSC and HGSC (Solid component of ovarian tumor)]*

Disclosure: This work was supported by Charles University in Prague (UNCE 204065 and PROGRES Q28/LF1) and by a grant from the Czech Research Council (AZV NV19-03-00552).

P55

Triage by methylation marker analysis versus colposcopy biopsy in women who test HPV-positive or abnormal LBC results on cervical samples to triage cervical cancer and HSIL for further treatment

Y. Gu, G. Zhou, J. Ding, K. Hua
Department of Gynecology, Obstetrics and Gynecology Hospital of Fudan University, Shanghai, China

Introduction / Background: While colposcopy screening was successful in screening cervical cance, it has limitations like subjectivity, interobserver variability, modest sensitivity. The study aims to evaluate the efficacy of colposcopy and CareMe Pluse methylation for triage cervical cancer and HSIL for future treatment.
Methodology: In the prospective observational multi-center study, 4070 patients with HPV-positive or abnormal LBC results underwent colposcopy-examination to identify the grade of cervical lesion, meanwhile the cervical scrapes were tested for DNA methylations. 607 women diagnosed as ≥HSIL via colposcopy-biopsy and then underwent LEEP (Loop Eelectrosurgical Excision Procedure). Depending on the pathology after LEEP as golden standard, the predictive performance of methylation analysis and colposcopy-biopsy were compared. The sensitivity and specificity of several other methylation tests were analyzed to determine the optimal methylation strategy.
Results: The predictive performance of CareMe-plus methylation was evaluated by comparing the sensitivity and specificity of methylation tests and colposcopy biopsy in patients who underwent LEEP (n=607). Depending on the pathology after LEEP as golden standard, the predictive performance of CareMe-plus methylation was significantly greater than colposcopy-biopsy for cervical cancer, CareMe-plus methylation analysis revealed a significant discrimination of women with cervical cancer from those with ≤HSIL (P=0.022). ROC curve analysis showed an AUC of 0.869. The predictive performance of CareMe-plus methylation was significantly greater than colposcopy-biopsy for HSIL+, CareMe-plus methylation analysis revealed a significant discrimination of women with HSIL+ from those with ≤LSIL (P=0.026). The CareMe-plus methylation analysis was superior to other alternative methylations to triage patients for further treatment. When compared the predictive performance of several methylation tests for cervical cancer, CareMe and CareMe-plus is greater than EPB41L3 and QIsure.
Conclusion: CareMe-plus methylation assay may be applicable to identify HPV-positive or abnormal LBC women with a high-risk of high grade lesion or cervical cancer. It may triage patients for accurate further treatment when combining with colposcopy biopsy.

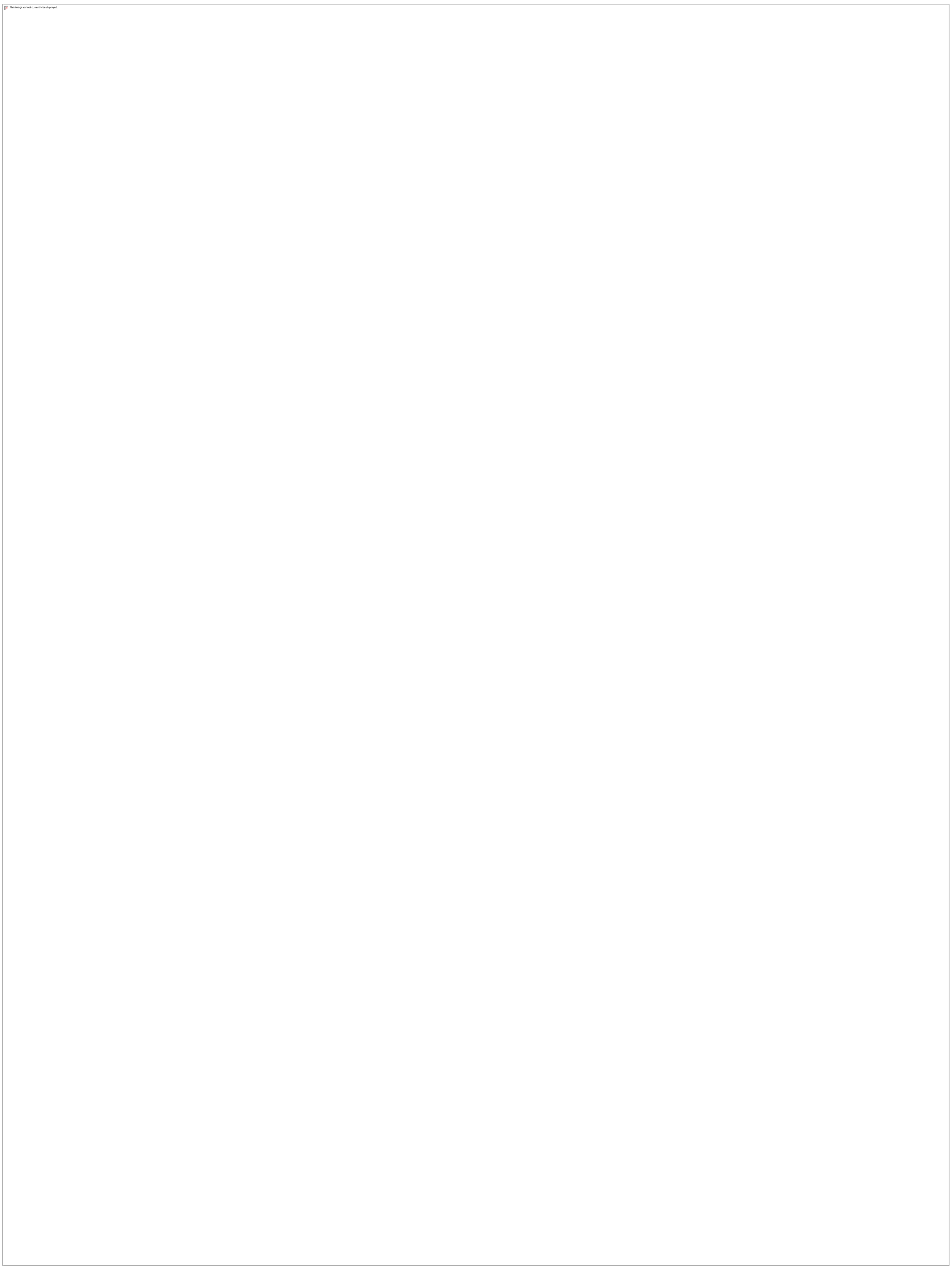

[Recruitment and follow-up flow chart.]

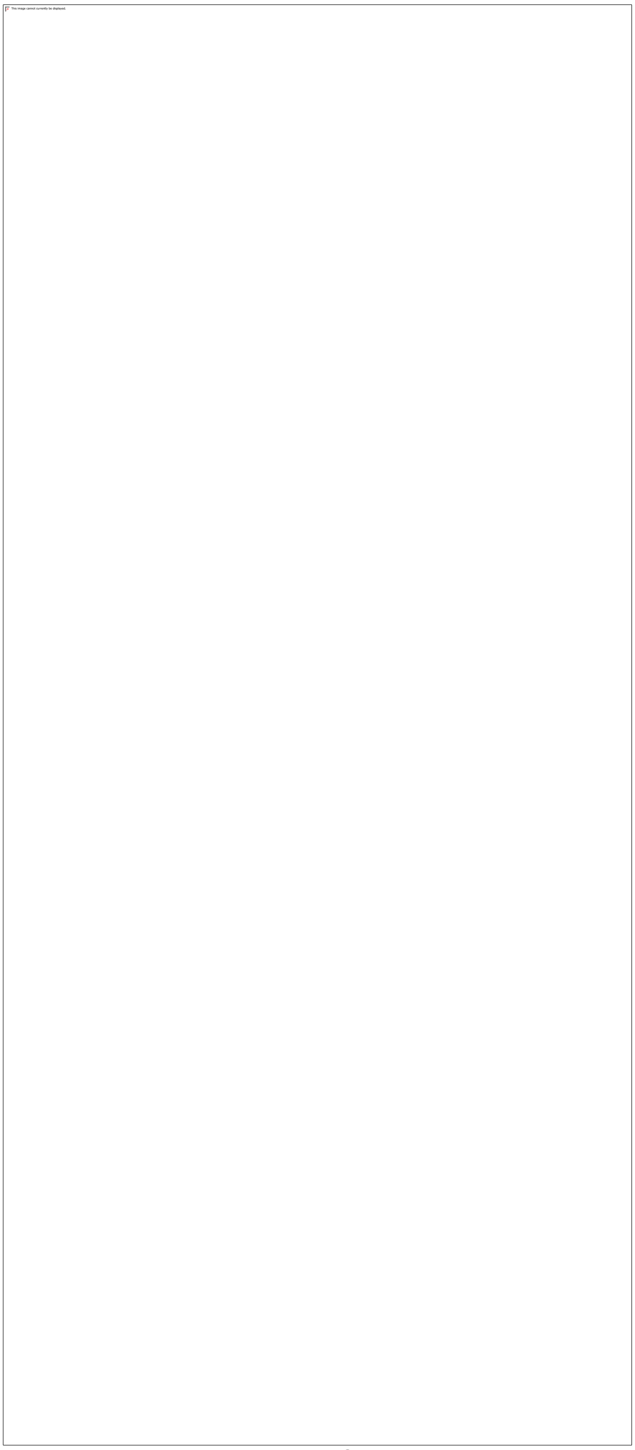

[The predictive performance of CareMe-plus methylation to triage cervical cancer and HSIL]

Disclosure: Nothing to disclose

P56

Comparison of the first 650 and the latest 524 laparoscopic transperitoneal pelvic/paraaortic lymphadenectomies in a series of 2535 patients treated for different gynaecological cancers

A. Jacob[1], A. Plaikner[1], P. Mallmann[2], G. Di Favero[3], R. Tozzi[4], P. Martus[5], S. Marnitz[6], C. Köhler[1]
[1]Department of Advanced Operative and Oncologic Gynecology, Asklepios Klinik Altona, Hamburg, [2]Department of Gynecology, University of Cologne, Medical Faculty, Cologne, [3]Helios Mariahilf Klinik Hamburg, Hamburg, Germany, [4]Department of Gynaecologic Oncology, University of Oxford, Oxford, United Kingdom, [5]Institute for Clinical Epidemiology and Applied Biostatistics, University of Tuebingen, Tuebingen, [6]Department of Radiation Oncology, University of Cologne, Medical Faculty, Cologne, Germany

Introduction / Background: Lymphadenectomy is an integral part of operative staging and treatment of gynaecological malignancies. Since its introduction over 25 years ago, laparoscopic lymphadenectomy has proven to be feasible, safe and oncologically adequate in comparison to open surgery while morbidity is lower and hospital stay is considerably shorter.
Methodology: In this retrospective study we analysed our prospectively collected data of laparoscopic pelvic and/or paraaortic lymphadenectomies performed between 1994 and 2018 by one team of gynaecological oncology surgeons. Data was analysed with regards to operation time, intra- and postoperative complications, number of harvested lymph nodes and influence of BMI. Previously published data of the first 650 patients treated after introduction of the method between 1994 and 2003 (period 1) was compared with the data of the latest 524 patients treated between 2014 and 2018 (period 2).
Results: Indications for pelvic and/ or paraaortic lymphadenectomies in the 2535 patients were cervical cancer, endometrial cancer, vulvar cancer with positive groin nodes, early ovarian cancer, vaginal cancer, uterine carcinosarcoma and others in a number of 1893, 361, 62, 99, 28, 13 and 79 patients, respectively. The learning curve for the two procedures was already completed during the first 650 cases. Since 1999 a steady and oncologically adequate number of lymph nodes were harvested in both areas independent of patients' BMI. OR-time for paraaortic lymph node dissection was shorter in period 2 (69 vs 100min). In contrast to period 1 (2.9%), there were no intraoperative complications in period 2, whereas postoperative complications are similarly frequent (5.8 vs 7.6%).
Conclusion: In this large cohort of patients who underwent laparoscopic lymphadenectomy the oncological equivalency and minimal morbidity of this approach was once again demonstrated. With growing experience intraoperative complication rates can be minimised. Teaching and integration of new team members does not impair surgical results when performed under supervision.
Disclosure: Nothing to disclose

P57

Value of circulating tumor DNA in the differentiation between malignant and benign ovarian tumors

P. Lof[1], E.A. Sistermans[2], L. Wessels[3], F. Amant[1,4], D. van den Broek[5], C.H. Mom[4], M. Hemelaar[6], W.M. van Baal[7], M. Verbruggen[8], F. Rosier - van Dunné[9], B. Hermsen[10], H.M. Horlings[11], C.A.R. Lok[1]
[1]Gynecology, Netherlands Cancer Institute - Antoni van Leeuwenhoek, [2]Genome Diagnostics, Amsterdam UMC, Location VU University Medical Center, [3]Molecular Carcinogenesis, Netherlands Cancer Institute - Antoni van Leeuwenhoek, [4]Gynecologic Oncology, Amsterdam UMC, Location Amsterdam Medical Center and University of Amsterdam, [5]Laboratory Medicine, Netherlands Cancer Institute - Antoni van Leeuwenhoek, Amsterdam, [6]Gynecology, Dijklander Hospital, Hoorn, [7]Gynecology, Flevo Hospital, Almere, [8]Gynecology, Zaans Medical Center, Zaandam, [9]Gynecology, Tergooi, Blaricum / Hilversum, [10]Gynecology, Onze Lieve Vrouwe Gasthuis, [11]Pathology, Netherlands Cancer Institute - Antoni van Leeuwenhoek, Amsterdam, The Netherlands

Introduction / Background: In the Netherlands, yearly 7600 women are diagnosed with an ovarian tumor. Only five percent of these tumors is malignant. It is challenging to identify all malignant tumors

preoperatively. An accurate pre-operative diagnosis is important, because patients with ovarian cancer should undergo surgical staging in an oncology center performed by a gynecologic oncologist. Tissue biopsies could confirm or exclude malignancy preoperatively, but may cause spreading of tumor cells in case of malignancy. 'Liquid' biopsies overcome the important limitations of current tests as it is minimally invasive and holds the promise of increasing sensitivity and specificity in detecting ovarian cancer. The aim of this study is to find accurate molecular biomarkers in liquid biopsies to identify malignant tumors in patients with an ovarian tumor.
Methodology: In this prospective case-control study, we collected liquid biopsies from patients with an ovarian tumor (n=64). We analyzed copy number aberrations in circulating tumor DNA (ctDNA) with low-coverage whole genome sequencing (0.5x). Sequencing data were analyzed using WISECONDOR (WIthin-SamplE COpy Number aberration DetectOR).
Results: We included 32 patients with a benign ovarian tumor and 32 patients with a clinically low-stage malignant ovarian tumor. Nine patients showed a highly abnormal genetic profile. They were all diagnosed with ovarian cancer (specificity 100%). The rest of the patients had a normal to slightly abnormal genetic profile, including 23 patients with a malignant tumor (sensitivity 28%), of which five turned out to have advanced ovarian cancer after surgery
Conclusion: Low-coverage whole genome sequencing of ctDNA to identify early stage ovarian cancer has an extremely high specificity. However, the sensitivity is low, which makes it not implementable as a single test in clinical practice yet. Further training of the algorithm and development of specific software for ovarian tumor analysis could improve the performance. Also combination with other biomarkers or ultrasound models should be investigated.

		Malignant	Benign	Total	
Genetic tumor profile	Highly abnormal	9	0	9	PPV = 100%
	Normal or slightly abnormal	23	32	55	NPV = 42%
	Total	32	32	64	
		Sensitivity = 28%	Specificity = 100%		

[Performance of circulating tumor DNA in identifying ovarian cancer, PPV = positive predictive value, NPV = negative predictive value]

Disclosure: Nothing to disclose

P58

Differential molecular diagnosis of uterine leiomyomas and leiomyosarcomas using DNA and RNA sequencing

A. Mas[1], R. Alonso[1], P. Escorcia[1], J. Jimenez-Almazán[2], J. Monleón[3], B. Montero[3], V. Lago[3], C. Simón[1,2,4]
[1]Igenomix Foundation, [2]Igenomix S.L, [3]Institute of Health Research, La Fe Hospital, [4]Department of Pediatrics, Obstetrics and Gynecology, University of Valencia, Valencia, Spain

Introduction / Background: Nowadays, the absence of standardized criteria to identify and differentiate uterine leiomyomas (LM) and leiomyosarcomas (LMS) prior to surgery, cause a significant stress in the patient, leading to unnecessary invasive procedures and additional costs to the National Health System. As consequence, the development of an accurate and non-invasive differential diagnostic methods in patients with surgical indication is needed to avoid the potential dissemination of hidden LMS from morcellation. We aim to identify differential genetic targets in LMS vs LM using Next Generation Sequencing to advance our knowledge in their differential diagnosis.
Methodology: A total of 13 LM and 13 LMS from formalin-fixed paraffin-embedded samples were

collected, processed and targeted sequenced for DNA and RNA coding regions for 170 solid tumours associated-genes. DNA sequencing data were assessed to identify copy number variations (CNVs), single nucleotide variants (SNVs) and small insertions/deletions(indels). Through the RNA analysis we got the gene expression profile, splice variants and gene fusions, being validated by immunohistochemistry and fluorescence in situ hybridization.
Results: Tumor mutation burden was higher in LMS vs LM specimens. In terms of CNVs, 20 genes in LMS vs 6 genes in LM were affected by deletions. Gains were also more frequently identified in LMS compared to LM (19 genes vs 3 genes). Regarding SNVs and indels, we identified 105 of 159 genes in LMS samples, while mutations in 82 genes were distinguished in LM samples. Specific transcriptomic profile was also observed for 11 genes of 55 in LMS samples, while 8.5% of initially diagnosed LMS showed high confidence gene fusions.
Conclusion: This study represents a scientific-technological innovation that allows, through an integrated genomic and transcriptomic analysis, the discovery of novel genes and pathways that potentially differentiate LM vs LMS, providing a new insight for the molecular diagnosis that may shift the current therapeutic focus.
Disclosure: Drs. Jimenez-Almazán and Simón are employed by Igenomix SL. The remaining authors report no conflict of interest.

P59

Patients' and gynaecologists' views on sentinel lymph node procedure in low and intermediate risk endometrial cancer management: a vignette study

J.W. Aarts[1], L. Burg[2], J. Kasius[3], H. Groenewoud[4], A. Kraayenbrink[2], P. Stalmeier[4], P. Zusterzeel[1]
[1]Radboud University Medical Centre, Nijmegen, [2]Rijnstate Hospital, Arnhem, [3]Amsterdam UMC, Location AMC, Amsterdam, [4]Radboudumcm Health Evidence, Nijmegen, The Netherlands

Introduction / Background: Over 200 articles about the sentinel lymph node procedure (SNP) in endometrial cancer (EC) have been published in the last decade, but none assesses patients' and gynaecologists' views on this innovative technique. In this vignette study we established which factors are important for both patients and gynaecologists when considering SNP in EC.
Methodology: Based on literature and patients' and gynaecologists' interviews, a list of attributes regarding SNP was composed: 1)risk of complications of SNP, 2)chance of finding a metastasis, 3)survival gain, 4)risk of complications after radiotherapy, 5)additional operation time and 6)hospital of surgery (travel time). A questionnaire with eighteen hypothetical scenarios was developed, in which the levels of each attribute varied. Patients previously treated for low or intermediate risk EC and gynaecologists with interest in oncology were invited to participate. In each scenario they were asked how strongly they would prefer SNP on a scale from 1 to 7. The strength of preference for each scenario was analyzed using a mixed model.
Results: Both patients (41/108) and gynaecologists (42/126) had a preference for SNP. On a scale from 1-7 the mean overall preference for patients was 4.29 (95% CI 3.72-4.85) and 4.39 (95% CI 3.99-4.78) for gynaecologists. Patients' preferences increased from 3.4 in case of no survival gain to 4.9 in case of three years survival gain (P=0.000), and decreased when travel time increased to over 60 minutes (-0.4, P=0.024) or with an increased risk of complications after adjuvant radiotherapy (-0.6, P=0.002). The chance of finding a metastasis was not important to patients. For gynaecologists all attributes except travel time were significantly important.
Conclusion: This study is the first to describe several characteristics being important for patients and gynaecologists when considering SNP in low and intermediate risk EC management. This study can guide counselling of patients when considering SNP.
Disclosure: Nothing to disclose

P60

Correlation of ganglionar affection predictive factors in endometrial cancer: a prospective cohort study

I. Aizpuru, M. Mancisidor, M. Gorostidi, R. Ruiz, I. Ruiz, A. Lekuona
Donostia Hospital, San Sebastian, Spain

Introduction / Background: Lymph node involvement is the most important prognostic factor in endometrial cancer. Identifying predictive factors of involvement prior to surgery would help us to plan the surgery and even avoid lymphadenectomy in low risk patients.
The aim of this study is to analyze the pre- and post-surgery correlation of these predictive factors and their association with lymph node involvement.
Methodology: Prospective cohort study including 207 patients with endometrial cancer at Donostia University Hospital (June 2014 - December 2018). Inclusion criteria was indication for primary surgery. Pre-surgery variables to study were histology (Endometroid subtype defined as type I and the rest as type II) and tumor grade (G1, G2 and G3) determined in preoperative endometrial biopsy, size and myometrial infiltration (MI) < or ≥ 50% by MRI. Then we analyzed the previous ones and lymphovascular space involvement (LVSI) in the definitive surgical piece.
Results: The correlation was very high for the size (rho 0.98) (Figure 1), high for the IM (rho 0.66) and the G (rho 0.66). The last one, became very high eliminating the G2 (rho 0.85). For histology, the correlation was moderate (rho 0.54).
All factors were associated with lymph node involvement (Table 2). Best predictive model was constructed by a multivariate regression combining the LVSI (OR 2.4 CI 95% 1.0 - 5.6), ≥50% myometrial infiltration (OR 6.0 CI 95% 2.5-14.3) and type II histology (OR 3.5 CI 95% 1.2 - 9.9).
Finally, we assessed the diagnostic output of tumor size for lymph node involvement using the ROC curve and its area under the curve was 0.68. (Figure 2)

VARIABLES	VALUES
Age (mean ± SD, years)	63.1 ± 10.6
BMI (mean ± SD, kg/m2)	28.6 ± 5.7
Tumor size (mean ± SD, mm)	32.6 ±14.8
Tumor size n(%) < 35mm n (%) > 35mm n (%)	108 (53.2) 82 (56.8)
Histological type n (%) Complex hyperplasia with atypia Type I: Endometroid Type II Serous Clear cells Carcinosarcoma Mixed	1 (0.5) 176 (85.0) 16 (7.7) 5 (2.4) 4 (1.9) 5 (2.4)
Stage n (%) I II III IV	163 (84.5) 5 (2.6) 21 (10.9) 4 (2.1)
Miometrial infiltration n (%) < 50% ≥ 50%	145 (70.0) 62 (30.0)
Tumoral grade n (%) G1 G2 G3	101 (48.8) 58 (28.0) 48 (23.2)
LVSI n (%)	52 (25.1)

[Table 1. Clinical and pathological characteristics of patients]

VARIABLES	Odds Ratio	CI 95%	p
Histology (type II)	3.3	1.3-8.1	0.012
Tumoral grade (G3)	2.4	1.5-3.4	<0.001
Miometrial infiltration (≥ 50%)	5.9	2.8-11.5	<0.001
Tumor size (> 35mm)	2.6	1.2-5.5	<0.001
LVSI	4.1	2.0-8.7	<0.001

[Table 2. Predictors of lymph node involvement (univariate regression)]

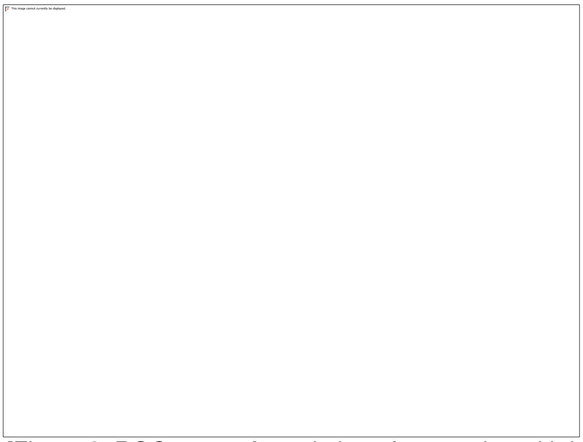

[Figure 2. ROC curve. Association of tumor size with lymph node involvement.]

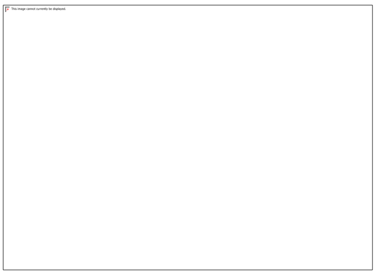

[Figure 1. Positive correlation between tumor size by MRI and in the definitive histology of the surg]

Conclusion: Studied predictive factors have good pre- and post-surgery correlation. All are associated with lymph node involvement. The best model to predict lymph node involvement is the one that includes MI, histology and LVSI.
Disclosure: Nothing to disclose

P61

Sentinel node mapping using indocyanine green for uterine malignancies: three-year experience with the da Vinci XI system

M. Anttila, M.-L. Eloranta, K. Louhiranta, H. Kärkkäinen, H. Sallinen, K. Hämäläinen
Kuopio University Hospital, Kuopio, Finland

Introduction / Background: Lymphadenectomy (LND) is essential when evaluating the stage and need for adjuvant treatment in endometrial cancer. However, its therapeutic value is still open. In cervical cancer, the role of LND in treatment selection is much more established. The aim of this study was to evaluate indocyanine green (ICG) and near infrared (NIR) fluorescence mapping used consecutively both in endometrial and cervical cancer.
Methodology: This is a cohort study of patients with clinical stage I endometrial (n=178) and cervical (n=28) cancer undergoing robotic surgery (da Vinci Xi, Intuitive Surgery, Sunnyvale, CA, USA) in Kuopio University Hospital between February 2016 and 2019. ICG tracer (Verdye, 1.25 mg/ml) was injected in the cervix at the 3 and 9 o'clock positions submucosal and stromally.
Results: SLN was detected bilaterally in 84% of the patients, and at least unilaterally in 86.4%. On pathologic evaluation, sentinel node included lymph node tissue in 98% of the cases. In 29 (14%) patients, SLN harbored metastasis. In endometrial and cervical cancer, the metastasis was observed in 13.3% and 18.5% of the cases, respectively. In those patients (n=124) having intermediate or high-

risk disease and fit for completion lymphadenectomy, non-SLN harbored metastasis in eight patients (8%) although SLN did not. Accordingly, the false negative rate was 25%. The SLN was the only metastatic node in twelve patients (12%). Sensitivity to detect node-positive disease was 78.4%(95% CI 61.8-90.2%) and a negative predictive value was 91.8% (95% CI 85.9-95.4%).
Conclusion: SLN detection using ICG proved to be feasible and accurate. The results correlate with previous single institution studies and meta-analyses.
Disclosure: Nothing to disclose

P62

Management of endometrial cancer in elderly patients identifying patients who may benefit from a curative treatment

V. Benito[1], A. Lubrano[1], M. Andújar[1], M. Mori[2], M. Federico[2]
[1]University Hospital of Canary Islands, [2]University Hospital Dr. Negrin, Las Palmas GC, Spain

Introduction / Background: Endometrial cancer is the most common pelvic gynecological cancer in European countries and its frequency increases with age. Given the gradual increase in life expectancy in European countries, it is expected that this malignancy will be diagnosed more frequently in women of advanced ages. The goal of this study was to evaluate the demographic characteristics, pathology, treatment, prognostic factors and survival rates in elderly patients with EC and to compare their results with those of younger ones, in order to define the specific characteristics of this malignancy in this population.
Methodology: Analysis of all EC patients managed at the University Hospital of the Canary Islands between 1990-2016. Survival curves were calculated by using the Kaplan-Meier method and compared with the log-rank test. Logistic regression analysis was used to assess the independent effect of different variables on CSS. Statistical significance was considered for $p< 0.05$.
Results: The study included 1799 EC patients; 170 of them (9.4%) were 80 years old or older. Elderly patients received less surgery (68.2% vs. 92.4%), lymphadenectomy (10.3% vs. 26.2%) and adjuvant treatment (37.1% vs. 51.2%) than younger ones, and presented higher probability of receiving palliative treatment (27.6% vs. 4%). Endometrioid tumors were more frequently diagnosed in younger patients (78.8% vs. 62.9%), while type 2-EC was more frequently diagnosed in elderly ones (37.1% vs. 21.2%).
CSS in older patients was significantly poorer than in younger ones, with a mean of 61.4 months (95%CI 51.7-71.1) versus 226 months (95%CI 218.9-233.1), respectively. In a multivariate analysis: age, FIGO stage, histology, tumor differentiation and adjuvant treatment were independently associated with survival.
Conclusion: Although EC is more aggressive in older patients, they are less likely to receive optimal treatment, which negatively affects their survival. Specific guidelines for the management of this population, including a comprehensive geriatric assessment, should be developed to improve their prognosis.
Disclosure: Nothing to disclose

P63

Targeted therapies in uterine sarcomas and carcinosarcomas- the summary of ENITEC (European Network for Individualised Treatment of Endometrial Cancer) collaboration project

M. Bobiński[1], K. Okła[1], P. Garcia Sanz[2], G. Moreno Bueno[2], A. Wawruszak[3], M. Dmoszyńska-Graniczka[3], W. Bednarek[1], J. Kotarski[1], ENITEC (European Network for Individualised Treatment of Endometrial Cancer)
[1]I Chair and Department of Gynaecological Oncology and Gynaecology, Medical University of Lublin,

Lublin, Poland, [2]MD Anderson Cancer Center, Madrid, Spain, [3]Department of Biochemistry and Molecular Biology, Medical University of Lublin, Lublin, Poland

Introduction / Background: The results of systemic therapy among the most of patients suffering from uterine sarcomas and carcinosarcomas remains unsatisfactory. Strong need of further research on multi-drug regimens is widely postulated. To investigate this problem the collaboration in ENITEC group was raised.
Methodology: Two uterine sarcomas (MES-SA, ESS-1), two carcinosarcoma (SK-UT-1, SKUT-1B) cell lines and control line HSF (Human Skin Fibroblasts) were cultured. Cells were incubated in presence of gemcitabine, mTOR inhibitors (rapamycin, MLN0128) and natural compound (fucoidan). Cell viability was assessed with MTT test. The combinations of investigated drugs activities were assessed using isobolography. Additionally, apoptosis and cell cycle were assessed.
Results: Gemcitabine decreases cell viability among all tested cell lines with strongest effect in SK-UT-1, SK-UT-1B, and ESS-1. Rapamycin affects cell viability in ESS-1 and MES-SA but not carcinosarcoma and HSF cell lines. MLN0128 influenced cell viability among all tested lines but in ESS-1 and MES-SA in significantly lower concentration comparing to the others. Fucoidan significantly decreases cell viability in SK-UT-1, SK-UT-1B, and ESS-1 cell lines, but not in MES-SA. Fucoidan was not substantially affecting proliferation among normal cells. The isobolographic analysis revealed synergistic effect of following combinations: gemcitabine+fucoidan and gemcitabine+MLN0128 in SK-UT-1B. Antagonism of gemcitabine+MLN0128 was noticed in MES-SA cell line. The effects of other combinations of tested drugs were additive.
Conclusion:
1. Fucoidan showed high activity against carcinosarcoma cell lines- due to its similarity to endometrial cancer further experiments with this substance are justified.
2. MES-SA and ESS-1 lines are significantly more sensitive to mTORi comparing to others cell lines.
3. Antagonistic effect between gemcitabine and MLN0128 in MES-SA might be avoided in designing future clinical trials- it's mechanism should be clarified.
4. Synergism observed between gemcitabine and novel agents (fucoidan and MLN0128) in carcinosarcoma cell line might be used in planning future therapies.
Disclosure: Nothing to disclose

P64

Examination of genes encoding telomerase associated proteins suggests a prognostic relevance for NHP2 and NOP10 in endometrial cancer

L. Button[1], R. Alnafakh[1], J. Drury[2], S.B. DeCruze[2], G. Saretzki[3], M. Adishesh[4], D. Hapangama[5]
[1]University of Liverpool, Liverpool, [2]University of Liverpool, Crown St, Liverpool, [3]University of Newcastle, Newcastle, [4]Gynaecological Oncology, Liverpool Women's Hospital, [5]Department of Women's & Children's Health, University of Liverpool, Liverpool, United Kingdom

Introduction / Background: Risk of recurrence of endometrial cancer (EC) after surgical treatment is 13% and recurrent disease carries a poor prognosis. Research into prognostic indicators is essential to improve EC management and patient outcome. Cancer cell immortality is dependent on telomerase, but the role of telomerase-associated proteins is poorly understood. Protein encoding telomerase-associated genes such as *NHP2* and *NOP10* in the endometrium has not yet been described.
Aims:
1. To examine the prognostic association of genes encoding telomerase-associated proteins in EC in The Cancer Genome Atlas (TCGA) dataset.
2. To characterise expression of the gene products with prognostic relevance in an independent sample set.
Methodology: In silico study interrogated TCGA dataset; NHP2 and NOP10 were selected for further study in 20 healthy control and 19 EC samples. Proteins were detected using Western blotting; NHP2 co-expression with dyskerin (with known alteration in EC) was visualised using immunofluorescence and NHP2 immunohistochemical staining was semi-quantified using a quickscore.
Results: Alterations in NHP2 and NOP10 RNA expression levels were associated with poor prognosis in EC; the respective proteins were detected for the first time in non-malignant premenopausal and

postmenopausal endometrium as well as in EC in an independent patient cohort. Immunofluorescence demonstrates NHP2 co-expression with dyskerin in healthy and malignant endometrium, with an apparent loss of NHP2 and dyskerin in EC samples. A statistically significant decrease in NHP2 Quickscore was observed in EC relative to premenopausal (p=0.0008) and postmenopausal (p=0.01) samples.
Conclusion: Our findings suggest a prognostic role for two telomerase-associated proteins NHP2 and NOP10 in EC. Further work is warranted to examine the functional role these proteins play in normal endometrial cellular proliferation as well as in EC.
Disclosure: Nothing to disclose

P65

Adjuvant management of operated uterine sarcomas: a single institution experience

A. Cordoba, J. Prades, L. Basson, Y.-M. Robin, S. Taieb, F. Narducci, D. Hudry, L. Bresson, A. Chevalier, F. Le Tinier, X. Mirabel, E. Lartigau, N. Penel, E. Leblanc
Centre Oscar Lambret, Lille, France

Introduction / Background: Describe the adjuvant management of high grade Uterine Sarcoma (US) and highlight prognostic factors for overall survival (OS) and pelvic-free survival (PFS).
Methodology: Between 01/2000 and 01/2015, 91 patients undergoing surgery were presented at the Multidisciplinary Team Meeting of our institution. The type of surgery, the anatomopathological features, adjuvant treatments, dates and sites of recurrence were collected. The prognostic value of the various factors was evaluated with the multivariate Cox model.
Results: A total of 50 women were identified including 43 (86%) leiomyosarcomas and 7 (14%) high grade sarcoma. 18 (36%) patients received adjuvant pelvic radiotherapy (AR) and 6 (12%%) received adjuvant systemic therapy. The median follow-up time was 63 months. 39 (78%) patients had a recurrence: 22 (58%) only metastatic recurrence, 2 (5%) isolated pelvic recurrence and 15 (38%) pelvic and metastatic recurrence. AR (p=0.005, HR=0,15) was associated with survival without pelvic recurrence in univariate analysis; age > 55 years (p=0.015, HR=2.37) and AR (p=0.013, HR=0.41) were associated with metastatic free survival in multivariate analysis.
Conclusion: According to the results of our series, there is a benefit of radiotherapy after surgery in terms of local control of US. It is necessary to identify the subgroup of patients who will benefit from an AR in order to provide them with more optimal care.
Disclosure: The authors declare that they have no potential conflict of interest relevant to this article.

P66

Single-site versus multiport robotic hysterectomy in obese patients with endometrial cancer: a multicentre retrospective evaluation of surgical and oncological outcomes

E. Vizza[1], L. Mereu[2], S. Bogliolo[3], V. Cela[4], B. Gardella[5], C. Certelli[1], A. Pellegrini[2], G. Posar[4], S. Tateo[2], A. Gadducci[4], A. Spinillo[3], G. Corrado[6]
[1]Regina Elena National Cancer Institute, Rome, [2]Santa Chiara Hospital, Trento, [3]European Institute of Oncology, Milan, [4]University of Pisa, Pisa, [5]IRCCS - Policlinico San Matteo and University, Pavia, [6]Policlinico Universitario Agostino Gemelli I.R.C.C.S. Universita Cattolica Di Roma, Roma, Italy

Introduction / Background: The majority of women who undergo radical hysterectomy for endometrial cancer (EC) are obese and they may benefit from robotic surgery. The aim of this study is to compare robotic single-site hysterectomies (RSSH) and robotic multiport hysterectomies (RMPH) for EC in obese patients.
Methodology: This study compares RSSH with RMPH in obese patients with EC and FIGO stage I or

II. Data were collected from July 2010 to October 2018 in four Italian institutes: Regina Elena National Cancer Institute of Rome, Fondazione Policlinico San Matteo and University of Pavia, Santa Chiara Hospital of Trento and University of Pisa. According to their BMI, patients were divided in 3 classes: A from 30 to 34.9 kg/m^2 , B from 35 to 39.9 kg/m^2 and C 40 kg/m^2 or higher.
Results: We included 225 patients: 76 in the RSSH group divided, respectively, in 53, 18 and 5 in the A, B and C classes; 149 in the RMPH group divided, respectively, in 76, 37 and 36 in the A, B and C classes. In the RSSH group, the median operation time (OT) and blood loss (BL) were, respectively, 139min and 50ml in the A class, 142.5min and 50ml in the B class and 165min and 100ml in the class C. In the RMPH group, the median OT and the median BL were, respectively, 195min and 100ml in the class A, 170min and 50ml in the class B and 122.5min and 75ml in the class C. The conversion rate increased in the 3 classes, especially in the RSSH group (in the A, B and C classes, respectively, 1.8%, 5.5% and 60%).
Conclusion: Technical difficulties in single-site surgery increase with the patients' BMI and when the BMI is 40 kg/m^2 or higher this technique is most of the times unfeasible.
Disclosure: Nothing to disclose

P67

Cost effectiveness of robotic versus open surgery hysterectomy and pelvic lymphadenectomy for endometrial cancer. The medico-economic study of a private hospital in Athens, Greece during the last decade

E. Karatrasoglou[1], V. Hatzirafail[1], G. Thomou[1], A. Paulaki[2], A. Mpakas[3]
[1]Department of Gynecologic Surgery, Euroclinic Hospital, [2]Department of Pathological Anatomy, IASO Women's Hospital, [3]Department of Anaesthesiology, Euroclinic Hospital, Athens, Greece

Introduction / Background: The purpose of our study is to compare the costs of conventional open-surgery versus the robotic-assisted laparoscopy in the treatment of endometrial cancer.
Methodology: 108 patients with endometrial cancer who were operated at the department of Gynaecologic Surgery of "Euroclinic Hospital", Athens, Greece from 2010 to 2019, were randomised for this study. All women underwent hysterectomy, bilateral salpingo-oophorectomy (BSOE) and pelvic lymphadenectomy for endometrial carcinoma according to evident-based guidelines.50 patients were operated with the use of robotic system da Vinci S & Si while 58 patients were operated via laparotomy. In the hospital perspective the costs were defined by examining the medical pathway for each type of intervention Patients' BMI was similar for the two groups. We analysed the stage and grade of disease, the comorbidity factors, the number of lymph nodes removed and the percentage of complications (such as blood loss) and readmissions.
We defined the overall medical care cost by adding the cost of surgery, the post-operative hospital stay and/or hospitalisation within one month after surgery.
Results: For endometrial cancer, the surgical procedure cost for robotic-assisted laparoscopy was €5.950 versus €4.950 for conventional laparotomy. Considering the overall medical care, the average cost of the in-patient treatment was €7.467 (median €6.302) for the robotic group (with average hospital stay of 3,12 days), compared to €8.086 (median €7.024) for the open-surgery group (with average hospital stay of 5,34 days). p value for the cost of surgeries is calculated to 0,37 while p value for the average stay is 0,00001.
Conclusion: The cost of a robotic hysterectomy-BSOE-pelvic lymphadenectomy exceeds the cost of the open-surgery for endometrial cancer due to the higher cost of instruments and equipment. However, when considering overall medical care, conventional surgery for endometrial cancer is found 1,08 times more expensive than robotic-assisted surgery.

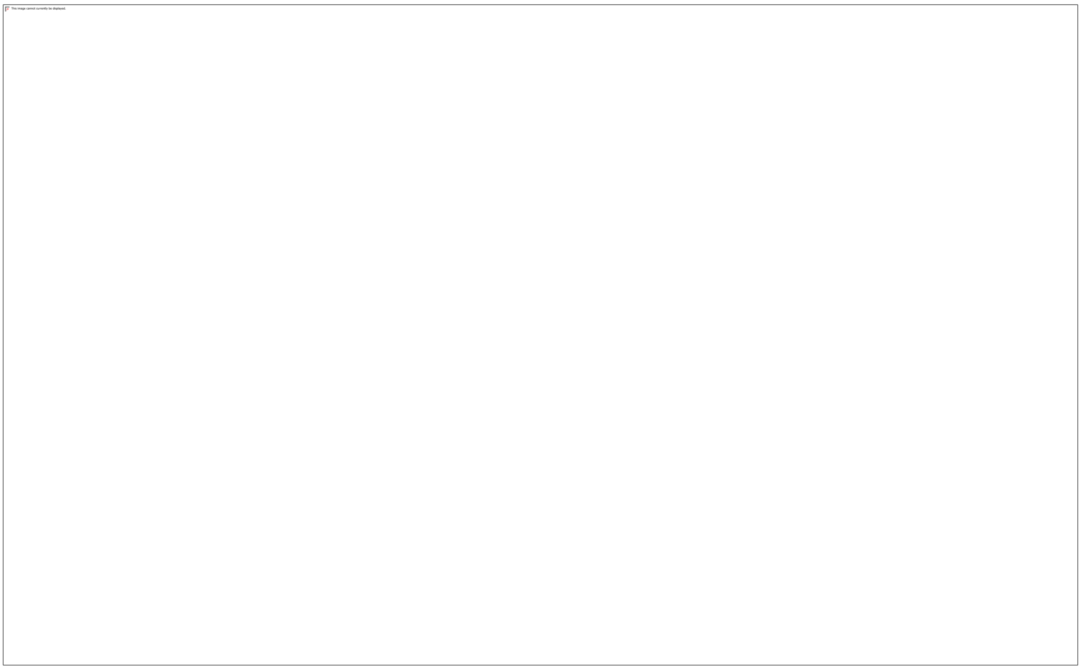

[Medical overall cost (robotic vs laparotomy)]

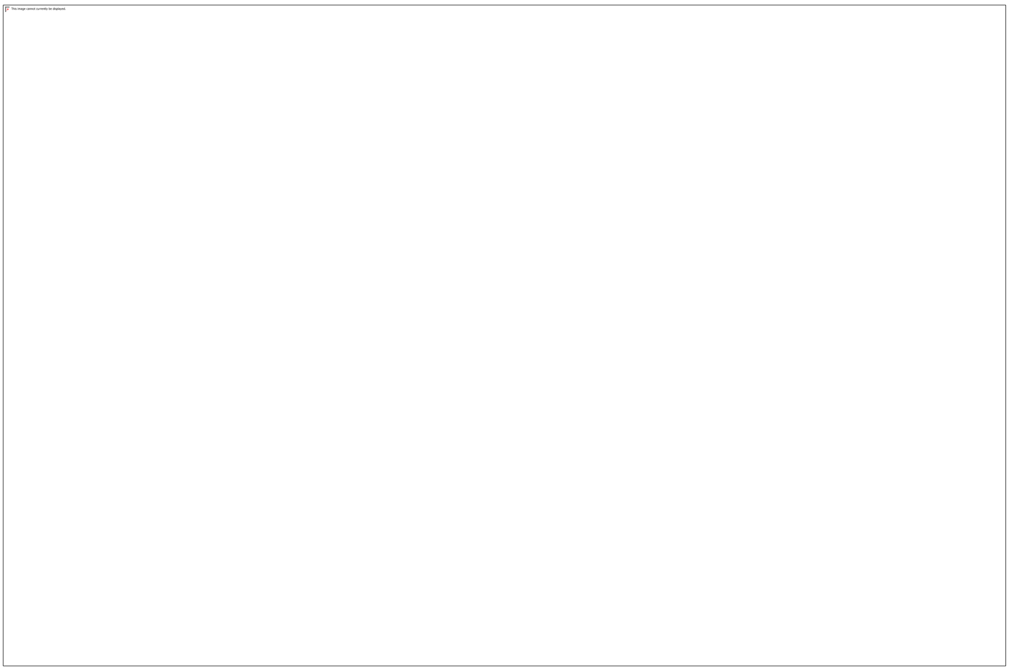

[Number of inpatient hysterectomies (robotic vs laparotomy)]

Disclosure: Nothing to disclose

P68

Diagnostic accuracy of endometrial biopsy in endometrial carcinoma grading, correlated to the amount of tissue

A.M.C. Hulsman[1], C. Reijnen[1], J. Bulten[1], H.V.N. Kusters[2], K. van de Vijver[3], M. Santacana[4], E. Colas[5], G. Mancebo[6], A. Reques[7], A. Gil-Moreno[5,7], J. Trovik[8], C. Krakstad[8,9], J. Huvila[10], I.S. Haldorsen[8,9], H.R. Engerud[8,9], M. Koskas[11], V. Weinberger[12], L. Minar[12], E. Jandakova[12], X. Matias-Guiu[4], F. Armant[13,14], L.F.A.G. Massuger[1], M.P.L.M. Snijders[2], J.M.A. Pijnenborg[1], ENITEC-consortium
[1]Radboud University Medical Centre, [2]Canisius-Wilhelmina Hospital, Nijmegen, The Netherlands, [3]Ghent University Hospital, Cancer Research Institute Ghent (CRIG), Ghent, Belgium, [4]Hospital Universitari Arnau de Vilanova, Lleida, [5]Vall Hebron Institute of Research, [6]Hospital del Mar, [7]Vall Hebron University Hospital, Barcelona, Spain, [8]Haukeland University Hospital, [9]University of Bergen, Bergen, Norway, [10]University of Turku, Turku, Finland, [11]Bichat-Claude Bernard Hospital, Paris, France, [12]Masaryk University, Brno, Czech Republic, [13]KU Leuven, Leuven, Belgium, [14]Centre for Gynaecological Oncology Amsterdam, Amsterdam University Medical Center, Amsterdam, The Netherlands

Introduction / Background: The diagnostic accuracy of endometrial biopsy has been related to the amount of tissue. In endometrial cancer (EC) there is only moderate agreement on tumor grade between preoperative endometrial sampling and final diagnosis with the lowest agreement for grade 2 carcinoma. Since the amount of preoperative endometrial tumor tissue has not yet been related to the degree of discordance, we aim to determine this relation with regard to final tumor type and grade.
Methodology: Within the European Network for Individualized Treatment of Endometrial Cancer (ENITEC), 582 preoperative endometrial biopsy samples of EC patients were retrospectively collected and classified into low-grade (grade 1-2) and high-grade carcinoma (grade 3). The endometrial tissue surface was digitally calculated using ImageJ software and the correlation between the amount of tissue and concordance of diagnosis was calculated.
Results: Agreement on tumor grade between preoperative endometrial sampling and final diagnosis was 62.5%. Up- or downgrading was found in respectively 27.1% and 10.3%. Clinically relevant up- or downgrading was found in respectively 7.8% and 27% of the cases. Premalignant endometrial tissue contained less endometrium compared to malignant endometrium (4.4 vs. 19.3 mm^2,p=0.03). Serous EC contained less endometrial tissue compared with grade 1-3 endometrioid EC (13.0 vs. 19.1 mm^2,p=0.017). The median endometrial tissue surfaces of grade 1-3 did not significantly differ from each other (18.7 vs. 19.8 vs. 24.1 mm^2). Samples with a discordant diagnosis consisted of significantly more endometrial tissue compared to the concordant group (p=0.018).
Conclusion: Based on postoperative diagnosis, there is no difference in the amount of endometrial tissue between the different endometrial grades. However, there is a tendency to underestimate a sample with less and overestimate a sample with more endometrial tissue. Awareness is especially needed, in serous EC with in general less tissue.
Disclosure: Nothing to disclose

P69

Surgical treatment of recurrent endometrial cancer

N. Kado, N. Takahashi, M. Sugiyama, A. Mochizuki, Y. Kasamatsu, M. Abe, M. Takekuma, Y. Hirashima
Shizuoka Cancer Center, Nagaizumicho, Japan

Introduction / Background: Standard therapy for recurrent endometrial cancer is chemotherapy. There is less conclusive evidence about surgical treatment of recurrent endometrial cancer. We investigated outcomes after surgical treatment for recurrent endometrial cancer at our hospital and verified its effectiveness.
Methodology: We reviewed the medical records retrospectively for patients who underwent surgical

treatment for primary recurrent endometrial cancer between September 2002 and March 2016. We evaluated clinicopathological characteristics, time to recurrence, age at the time of surgical treatment for recurrence, the site of recurrence, residual tumor and postoperative therapy of recurrent surgery, progression-free survival (PFS) and overall survival (OS).
Results: 18 patients were evaluated. The median follow-up was 28.9 months (12.7-134.2). Median age was 65.5 (48-78) years old. The site of recurrence were lung 7, peritoneal dissemination 7, and lymph nodes 5. 14 of 18 patients had underwent macroscopic complete tumor resection. Median PFS was 18.1 months (0.9-134.2) and median OS was 119.1 months (31.1-189.2).Prognostic factors for surgical treatment of PFS were lung metastases (HR 0.18 [95% CI: 0.042-0.77], P < 0.05), age at the time of surgical treatment for recurrence (66 years or older) (HR 0.25 [95% CI: 0.07-0.95] , P < 0.05) ,and those of OS were residual tumor (HR 10.6 [95% CI: 1.69-66.9], P < 0.05), time to recurrence(758 days or more) (HR 0.12 [95% CI: 0.02-0.80], P < 0.05).
Conclusion: Surgical treatment of recurrent endometrial cancer may be effective for patients who had longer time to recurrence and underwent macroscopic complete tumor resection.
Disclosure: Nothing to disclose

P70

Description of the surgical management of patients with uterine corpus cancers in Belgium: results from a multicentric prospective observational study (EFFECT)

A. Kakkos[1], A. De Geyndt[2], G. Silversmit[2], G. Bouche[3], E. de Jonge[4], G. Jacomen[5], H. Denys[6], E. Van Limbergen[7], B. Vandermeersch[8], J. Kerger[9], D. Vander Steichel[10], M. Baldewijns[11], E. Lauwers[12], N. Van Damme[2], F. Goffin[1], F. Amant[7]
[1]Gynecologic Oncology, CHU de Liège, Liège, [2]Belgian Cancer Registry, [3]Anticancer Fund, Brussels, [4]Gynecology-Obstetrics, Ziekenhuis Oost-Limburg, Genk, [5]Anatomopathology, AZ Sint-Maarten, Duffel, [6]Medical Oncology, University Hospital Ghent, Gent, [7]Gynecologic Oncology, University Hospital Leuven, Leuven, [8]CHIREC Cancer Institute, [9]Medical Oncology, Jules Bordet Institute, [10]Fondation contre le Cancer, Brussels, [11]Anatomopathology, University Hospital Leuven, Leuven, [12]Kom Op Tegen Kanker, Brussels, Belgium

Introduction / Background: Uterine corpus cancer is the most frequent pelvic gynecological cancer in Belgium, however the adherence to the management guidelines is widely heterogeneous. In order to assess the quality of management, the EFFECT (EFFectiveness of Endometrial Cancer Treatment) project was initiated by the Anticancer Fund. Here we report the results regarding the surgical management of the patients included in EFFECT.
Methodology: Patients with uterine malignant tumors diagnosed between 2012 and 2016 were registered prospectively and on a voluntary basis in an online secured database hosted by the Belgian Cancer Registry (EFFECT study). Data on pathologic assessment, preoperative management, surgery, adjuvant treatment and follow-up were collected. We present the demographic characteristics, pathological assessment and surgical management of the whole study population.
Results: Overall, 4037 new cases were registered by 59 participating centers. The mean number of patients treated by each center was 65, 14 centers registered more than 100 patients during the whole period. The median patients' age was 69 years (range, 22-98 years). 95,5% of operated malignant tumors were carcinomas, 4,5% were sarcomas. 78% of the patients with a carcinoma and 75% of the sarcomas were FIGO stage I. 56% of operated patients were treated by minimally invasive surgery (laparoscopy or robotic-assisted laparoscopy), 36% by laparotomy and 7% by exclusive vaginal surgery. 44% of the operated patients had surgical lymph node staging (72% pelvic lymphadenectomy, 2% para-aortic lymphadenectomy, 22% pelvic and para-aortic lymphadenectomy). The median number of pelvic nodes resected was 17 (0-73) and for para-aortic nodes 11 (0-84).
Conclusion: Only half of the patients with clinical stage I were operated by minimally invasive surgery, 7% of patients were operated by exclusive vaginal surgery inappropriate for fit patients. Further analysis will assess the Quality of Care and hopefully permit to improve surgical and oncologic outcomes by the feedback provided to the different centers.
Disclosure: Nothing to disclose

P71

Does mismatch repair (MMR) deficiency have prognostic significance in low-risk endometrioid endometrial cancers?

S.R. Kim[1], A. Pina[2], A. Albert[3], J. McAlpine[4], R. Wolber[5], B. Gilks[5], M. Carey[4], J. Kwon[4]
[1]Gynecologic Oncology, University of British Columbia, Vancouver, BC, [2]Division of Gynecology Oncology, Universite de Montreal, Montreal, QC, [3]Women's Health Research Institute, [4]Division of Gynecologic Oncology, [5]Department of Pathology and Laboratory Medicine, University of British Columbia, Vancouver, BC, Canada

Introduction / Background: Mismatch repair deficiency (MMRd) is observed in 25-30% of all endometrial cancers. This can be detected by the absence of MMR protein staining on immunohistochemistry (IHC). Only 10% of women with MMRd have Lynch syndrome, but MMRd may still have prognostic significance. The objective of this study was to compare clinical outcomes between MMR deficient and proficient low-risk endometrioid endometrial cancers (stage IA, grade 1/2).
Methodology: This was a retrospective population-based cohort study of all low-risk endometrial cancers from Vancouver Coastal Health authority region from 2011 to 2016 that were assessed for MMR deficiency. Primary outcome measures were recurrence rates expressed per person-years (py), progression free survival (PFS) and overall survival (OS) calculated using Kaplan-Meier method and log-rank tests. Cox proportional hazards model estimated the association between MMRd and recurrence/death after adjustment for covariates.
Results: There were 477 low-risk patients, including 132 MMRd (27.7%) and 345 MMRp (proficient) patients. Women with MMRd tumors had higher recurrence rates (3.53p100py vs 1.21p100py) and worse PFS (p=0.0086) compared to women with MMRp tumors. After adjustment for age, LVSI status, adjuvant therapy, and post-operative grade, MMRd status remained associated with a higher risk of recurrence (HR 2.99, 95% CI 1.27-7.04). There was no significant difference in OS between MMR groups (HR 1.38, 95% CI 0.57-3.33).
Conclusion: In low-risk stage IA grade 1 or 2 endometrioid endometrial cancers, MMR deficiency is associated with a higher recurrence rate than in MMR proficient cases, after adjustment for covariates, implying that MMR deficiency reflects a different biology in endometrial cancer.
Disclosure: Nothing to disclose

P72

Choice of treatment for endometrioid EC $T_{1a}N_0M_0$ G_1 - G_2 and obesity III-IV

N. Kucheryna[1], S. Kartashov[1], I. Muryzina[2], V. Lazurenko[3], K. Oleshko[1], S.T. Bui[1], M. Kartashova[2]
[1]Obstetrics, Gynaecology and Gynaecologic Oncology, Kharkov Medical Academy of Postgraduate Education, [2]Obstetrics and Gynaecology N1, [3]Obstetrics and Gynaecology N2, Kharkov National Medical University, Kharkov, Ukraine

Introduction / Background: According to Bulletin of National Cancer Registry of Ukraine vol. 20, 2017 - 2018 endometrial cancer (EC) is the third most commonly occurring cancer in women in Ukraine, it constituted 9.4% (age-standardised rate is 21.4 per 100,000), but 5-year survival rate is lower than in high-income countries. This study aimed to find the best approach for treatment of endometrioid EC in women with obesity III-IV degree to balance appropriate extent of surgery and risk of intra- and postoperative complications.
Methodology: The study involved patients with endometrioid EC T1aN0M0 G1 - G2 and obesity III-IV: 107 of them underwent total abdominal hysterectomy + bilateral salpingo-oophoreectomy (TAH+BSO), other 23 patients were treated by laparoscopically assisted vaginal hysterectomy (LAVH). The criteria for accrual had included CT, MRI and then intraoperative evidence allowing to omit lymphadenectomy (selective lymphadenectomy paradigm).

Results: Women of both subsets matched by age (respectively 60.9 and 62.4 years). TAH+BSO time constituted 154 min, blood loss was 320 ml, intraoperative complications occurred in 8 cases (7.5%), postoperative - 2 (8.7%), duration of stay in hospital reached 16.9 days. LAVH time was slightly shorter (135 min), blood loss was significantly reduced (240 ml) as well as the rate of intra- and postoperative complications: respectively 2 (8.7%) and 2 (8.7%), time of hospital stay was 2.7 days. Both groups matched by tumour grading: TAH+BSO - G1 in 80 pts (74.8%), G2 - 27 (25.2%); LAVT - 17 (73.9%) and 6 (26.1%) respectively. Throughout 4.5 years of follow-up there were 5 recurrences (4.67%) in TAH+BSO group and 1 recurrence in LAVT group (4.35%).
Conclusion: LAVH as a treatment for endometrioid EC T1aN0M0 G1 - G2 and obesity III-IV with preoperative and intraoperative evidence against lymphadenectomy is safe and gains benefits, namely: reduction of procedure's time, blood loss, intra- and postoperative complications´ rate and duration of hospital stay
Disclosure: Nothing to disclose

P73

Is intraoperative frozen section needed to assess myometrial infiltration in endometrial cancer (EC)? A multicentre study in Oncological Network of Piemonte and Valle d'Aosta

C. Baima Poma[1], I. Cotrino[2], M. Ribotta[3], L. Fuso[4], A. Ferrero[4], L.L. Mariani[4], C. Macchi[1], M.E. Laudani[1], D. Lerda[1], E. Potenza[1], P. Zola[1]
[1]Surgical Science, University of Turin, [2]Gynecology and obstetrics, A.O.U Città della Salute e della Scienza, [3]Pathological anatomy, Città della Salute e della Scienza, [4]Gynecology and Obstetrics, AO Ordine Mauriziano, Turin, Italy

Introduction / Background: In developed countries, endometrial cancer is the fourth most common cancer in women. ESGO guidelines propose appropriate strategies based on risk factors. Lymphadenectomy is a staging procedure and even if it does not seem to have a therapeutic role, it is crucial for tailoring adjuvant therapy. The aim of the study is to verify the reliability of expert transvaginal sonographer (TVS) and frozen section (FS) to assess myometrial infiltration (< 50% vs ≥50%) to limit systematic lymphadenectomy where not indicated.
Methodology: To verify the accuracy of expert TVS and intraoperative frozen section to evaluate myometrial infiltration, 364 consecutive patients affected by EC were prospectively followed in 3 centres of the Oncological Network of Piemonte and Valle d'Aosta from 01/2016 to 10/2018. Definitive pathological report sensitivity, specificity, PPV, NPV, LR + LR- of TVUS and FS are obtained. The results were evaluated overall and by centre.
Results: TVUS by expert ultra-sonographer has obtained a diagnostic accuracy with sensitivity 76% specificity 73% PPV 66% NPP 82%. The variability observed between hospitals: centre A sensitivity 76% specificity 86% PPV 85% NPP 82%; centre B sensitivity 60% specificity 75% PPV 59% NPP 76%; centre C sensitivity 95% specificity 58% PPV 58% NPP 95%. The overall FS examination has the following values: sensitivity 86% specificity 97% PPV 95% NPP 92%. Stratifying by centres: A sensitivity 91% specificity 96% PPV 95% NPP 93%; B sensitivity 87% specificity 97% PPV 93% NPP 95%; C sensitivity 85% specificity 100% PPV 100% NPP 91%. Patients who add FS to TVUS had a reclassification improvement of 7.5%.
Conclusion: FS remains the most reliable method in the “real world” to assess myometrial infiltration and to direct the staging procedures in endometrial cancer. The variability of FS among centres belonging to an oncological network is lower than what observed for TVUS.
Disclosure: Prof. Paolo Zola has received grants from Astrazeneca, Roche, Pharmamar and Tesaro.

P74

Endometrial cancer: can the iKnife diagnose endometrial cancer?

D. Marcus, A. Savage, J. Balog, H. Kudo, J. Abda, R. Dina, Z. Takats, S. Ghaem-Maghami
Cancer and Surgery, Imperial College London, London, United Kingdom

Introduction / Background: Endometrial cancer is the most common gynaecological cancer in the western world with 88'000 new cases diagnosed per year in the European Union. Diagnosis of endometrial cancer is confirmed by histological examination of endometrial curetting's or outpatient endometrial biopsies e.g. pipelle; but can take up to two weeks. To-date there is no Point-Of-Care (POC) diagnosis available. The intelligent surgical knife ('iKnife') analyses tissue real-time but has never been used to diagnose endometrial cancer.
This study aims to establish whether the iKnife could distinguish between normal and malignant endometrial pipelle biopsy samples based on differences in their lipidomic profiles.
Methodology: Research pipelle biopsy samples were obtained for women needing biopsies for clinical reasons (samples concurrently sent to conventional histopathology).
A Waters G2-XS Xevo Q-Tof mass spectrometer (MS) was used in this study in conjunction with a modified handheld diathermy (collectively coined the 'iKnife').
The resultant surgical aerosol containing ionic species produced during diathermy was then analysed with this technology; producing spectra that are background subtracted, lock mass corrected and in the phospholipid range. Principal component analysis (PCA) and linear discriminant analysis (LDA) were then performed to find the variance in spectral signatures. A leave one patient out cross validation was used to obtain diagnostic accuracy.
Results: 134 pipelle biopsy samples (73 normal and 61 malignant) were obtained. Each tissue sample was processed as described; producing an individual spectrum per burn.
The iKnife differentiated between normal and malignant endometrial tissues on the basis of differences in their unique phospholipid spectral signatures. Cross validation revealed a diagnostic accuracy of 80%.
Conclusion: This pilot study is the first to use the iKnife as a tool to differentiate between normal and malignant endometrial pipelle samples. These results are encouraging and suggest that the iKnife could be used as an adjunct in clinic to provide a POC diagnosis.
Disclosure: Nothing to disclose

P75

Long-term results of endometrium cancer Ib G3 stage treatment

S. Mavrichev, A. Shushkevich
Oncogynecology, N.N. Alexandrov Cancer Center of Belarus, Minsk, Belarus

Introduction / Background: The article analyzes treatment outcomes in 370 patients with stage Ia G3 of endometrium cancer
Methodology: We analyzed retrospective data between 2006 and 2010 years. The study include women with endometrium cancer (EC) stage Ia G3. There were´t morphological evidence of myometrial invasion - 55 patients, whereas 315 women had stage Ia G3 EC with myometrial invasion.We used Kaplan-Meier curves to evaluate the 5 years overall survival, cancer-specific and relapse-free survival among patients with and without myometrial invasion. We compare results of treatment between group of patients (151) who underwent surgical treatment (ST) with radiation therapy (RT) and group of patients (191) who underwent ST with RT and adjuvant chemotherapy.(OS)
Results: Among patients without myometrial invasion the 5-year overall, cancer specific and relapse-free survival rates were 79.7 ± 5.5; 86.6 ± 4.7 and 84.6 ± 5.0 % respectively, whereas patients with myometrial invasion 75.6 ± 2.4; 81.3 ± 2.2. and 78.0 ± 2.4 % respectively. In both groups chemotherapy did´t improve survival. Among various combined treatment, the best results were achieved in regimen that included adjuvant external RT on the pelvic area, but onlu in group who did´t undergo pelvic lymphnodes dissection
Conclusion: Chemotherapy does not improve treatment outcomes for patients with EC stage Ia G3., although the majority of relapses manifested as distant metastasis. Combination of ST with RT remains one of the best results. We do´t find any statistic difference between two groups with/without myometrial invasion: overall survival - p=0,526; cancer specific and relapse-free survival rates -

p=0,236 and p=0,154 respectively
Disclosure: Nothing to disclose

P76

Prospective investigation of pre-diagnostic urinary Bisphenol A and phthalates in relation to endometrial cancer risk in the Multiethnic Cohort (MEC) Study

D. Sarink[1], L. Le Marchand[1], I. Cheng[2], A.H. Wu[3], A.A. Franke[1], L.R. Wilkens[1], K. White[1], H. Yu[1], M.A. Merritt[1]
[1]*University of Hawaii Cancer Center, Honolulu, HI,* [2]*University of California, San Francisco, San Francisco,* [3]*Keck School of Medicine, University of Southern California, Los Angeles, CA, United States*

Introduction / Background: In the Multiethnic Cohort (MEC) Study based in Hawaii and Los Angeles, the age-adjusted incidence of endometrial cancer (per 100,000, ages 60-90) is highest in Native Hawaiians (112.0) vs. 68.9 in white women. Since endometrial cancer develops under conditions of high circulating estrogen levels, we hypothesized that exposure to estrogenically active BPA and phthalates will increase endometrial cancer risk and these associations may be most relevant for Native Hawaiian women who have high endometrial cancer incidence rates.
Methodology: We initiated a nested case-control study to measure urinary BPA and phthalates from the MEC. We identified 146 postmenopausal endometrial cancer cases and controls matched 1:1 on race/ethnicity, area, birth year, fasting hours, urine type, date/time of collection and postmenopausal hormone use. Conditional logistic regression will be used to calculate odds ratios and 95% confidence intervals for the association between BPA or phthalate concentrations with endometrial cancer risk accounting for the matching criteria, creatinine concentration and endometrial cancer risk factors.
Results: There were differences in endometrial cancer risk factors between the cases and controls; for example, cases were more likely to never use oral contraceptives (46% of cases vs. 39% of controls) and cases had a higher body mass index (32% of cases vs. 19% of controls with BMI ≥30 kg/m2). There were no differences in the frequency of cases and controls who were parous or by education level. We are currently analyzing the associations with urinary BPA and phthalate metabolite concentrations and will present our findings at the meeting.
Conclusion: To our knowledge this is the first study to examine the role of BPA and phthalates in relation to endometrial cancer risk using prospective samples and to explore whether risk associations differ across racial/ethnic groups. This work will highlight new avenues for further research that aim to correct observed endometrial cancer disparities.
Disclosure: Nothing to disclose

P77

L1CAM is an independent predictor of poor survival of endometrial cancer undergoing full lymphadenectomy and adjuvant chemotherapy

A. Oku[1,2], H. Asano[1,2], K. C Hatanaka[3,4], R. Matsuoka[5], T. Kato[2], Y. Konno[2], T. Mitamura[2], K. Ihira[2], A. Nozaki[2], M. Takeda[2], N. Kobayashi[2], M. Kudo[2], Y. Matsuno[6], Y. Hatanaka[4], H. Watari[1,2]
[1]*Deprtment of Obstetrics and Gynecology, Hokkaido University Graduate School of Medicine,* [2]*Department of Gynecology,* [3]*Clinical Research and Medical Innovation Center,* [4]*Research Division of Genome Companion Diagnostics, Hokkaido University Hospital, Sapporo,* [5]*Department of Pathology, International University of Health and Welfare, Mita Hospital, Tokyo,* [6]*Department of Surgical Pathology, Hokkaido University Hospital, Sapporo, Japan*

Introduction / Background: L1 cell adhesion molecule (L1CAM), a transmembrane protein of the

immunoglobulin family that has been implicated in promoting cancer cell proliferation, invasion, migration, and metastasis, has been established as an important predictor of poor survival of early-stage endometrial cancer patients. We aimed to investigate whether L1CAM remains significant predictor of poor survival of the patients with endometrial cancer undergoing extensive surgical staging and adjuvant chemotherapy in this retrospective analysis.
Methodology: We prepared tissue microarray (TMA) from surgical tissue specimens of 185 endometrial cancer patients who underwent full lymphadenectomy combined with adjuvant chemotherapy for patients at risk for recurrence, and evaluated expression of L1CAM by immunohistochemistry (IHC). L1CAM positivity was correlated with clinicopathological factors and its prognostic significance was investigated by multivariate analysis using Cox regression model.
Results: Among 169 cases with over three years of follow-up period (median 95 months, 5-186 months), 53 cases (31.4 %) showed positivity of L1CAM. L1CAM positivity was significantly correlated with non-endometrioid histology (p< 0.0001), carcinosarcoma (p=0.0008), positive cytology (p=0.005), and p53 positivity (p< 0.0001). L1CAM positive patients showed higher recurrent rate(15/53 (28.30%) vs 16/116 (13.79%), p=0.0277) and frequency of distant failure (11/14 (78.57%) vs 5/16 (31.25%), p=0.0361) than L1CAM negative patients. L1CAM positivity was a significant predictor of poor survival among advanced-stage patients (5-year OS; 49.0% vs 87.8%, p=0.0008, PFS; 35.7% vs 75.6%, p=0.0026), but not early-stage patients (5-year OS; 100% vs 98.7%, p=0.3456, PFS; 83.2% vs 93.3% p=0.2112). Multivariate analysis revealed that surgical stage and L1CAM positivity were independent predictors of poor survival. OS can be stratified into three groups by the combination of stage and L1CAM positivity.
Conclusion: L1CAM is an independent predictor of poor survival of Japanese patients with advanced-stage endometrial cancer undergoing full lymphadenectomy and adjuvant chemotherapy. New treatment strategy should be developed for L1CAM positive patients with endometrial cancer.
Disclosure: Nothing to disclose

P78

Robot-assisted management of endometrial cancer in Belgium: an analysis of 615 patients by the Belgian and Luxembourg Gynaecological Oncology Group

S. Ongaro[1], C. Ver Eecke[2], F. Peeters[3], K. Traen[4], P. Van Trappen[5], A. Kakkos[1], A. Laenen[2], E. Despierre[4], E. Van Nieuwenhuysen[2], T. Van Gorp[2], F. Kridelka[1], I. Vergote[2], F. Goffin[1]
[1]Department of Obstetrics and Gynaecology, Centre Hospitalier Université de Liège, Site Notre Dame des Bruyères et Centre Hospitalier Régional Liège, Université de Liège, Liège, [2]Division of Gynaecological Oncology, Department of Obstetrics and Gynaecology, Leuven Cancer Institute, Catholic University of Leuven, Leuven, [3]Department of Obstetrics and Gynaecology, General Hospital Klina, Brasschaat, [4]Department of Obstetrics and Gynaecology, Onze-Lieve-Vrouwziekenhuis, Aalst, [5]Department of Obstetrics and Gynaecology, General Hospital Sint-Jan, Bruges, Belgium

Introduction / Background: The aim of this prospective study was to report perioperative outcomes, disease-free and overall survival of robot-assisted management of patients with endometrial cancer, treated in 5 centers in Belgium.
Methodology: Patients undergoing surgery between August 2007 and December 2018 (n=615) were prospectively collected.
Results: Median age was 68 years (range, 31-92), median BMI 28 kg/m^2 (range, 17-61). Five-hundred-ninety-nine patients underwent robot-assisted hysterectomy and bilateral salpingo-oophorectomy. In addition, full pelvic lymphadenectomy was performed in 151 (25%), and pelvic sentinel nodes biopsies only in 181 patients (29%). Sixteen patients were referred for robotic node staging after hysterectomy performed elsewhere. Histological types were endometrioid (n=512, 84%), serous (n=43, 7%), clear cell (n=8, 1%), mucinous (n=3, 0.5%), squamous (n=2, 0.3 %) and carcinosarcoma (n=20, 3%). FIGO stage was: IA (61%), IB (24%), II (4%), IIIA (2%), IIIB (0,3%), IIIC (8%), IV (1%). Metastatic pelvic nodes were found in 43 (7%), positive para-aortic nodes in 3 (0,4%), pelvic and para-aortic nodal metastases in 7 patients (1,2%). Median number of removed pelvic and para-aortic nodes was 15 (range, 0-64) and 10 (range 1-47), respectively. Median estimated blood loss was 50 mL (range 0 - 1000), median skin-to-skin time 150 minutes (range 55-480) and conversion rate 0.8% (n=5). Intra-operative complications occurred in 5% (n = 30). At least one postoperative

complication occurred in 15 % (n = 89). Median follow-up was 27 months (range, 1-131). Recurrence occurred in 63 patients (11%) and 38 patients (6%) died of endometrial cancer. The 2- and 5-year recurrence-free survival rates were 89% and 84%, respectively. The 2- and 5-year overall survival rates were 92 % and 83 %, respectively.
Conclusion: This study confirms the feasibility and safety of robot-assisted management of endometrial cancer with a low complication, conversion and recurrence rate.
Disclosure: Nothing to disclose

P79

Hysterectomy-corrected mortality rates of corpus uteri cancer in Denmark during 2002-2015

L.W. Gustafson[1,2], B.B. Booth[2,3], J. Kahlert[4], G. Ørtoft[5], E. Mejlgaard[6], A.F. Rositch[7], M. Clarke[8], N. Wentzensen[8], A. Hammer[2,9]
[1]Department of Public Health's Programs, Regional Hospital Randers, Randers, [2]Department of Clinical Medicine, Aarhus University, Aarhus, [3]Department of Obstetrics and Gynecology, Randers Regional Hospital, Randers, [4]Department of Clinical Epidemiology, Aarhus University Hospital, Aarhus, [5]Department of Obstetrics and Gynecology, Copenhagen University Hospital, Rigshospitalet, Copenhagen, [6]Department of Pathology, Aarhus University Hospital, Aarhus, Denmark, [7]Department of Epidemiology, Johns Hopkins Bloomberg School of Public Health, Baltimore, [8]Division of Cancer Epidemiology and Genetics, National Cancer Institute, Bethesda, MD, United States, [9]Department of Obstetrics and Gynecology, Aarhus University Hospital, Aarhus, Denmark

Introduction / Background: Corpus uteri cancer is the most common gynaecological malignancy in Denmark. Whereas the incidence of less aggressive histologic subtypes (i.e. endometrioid cancer) has been stable since 2000, the incidence of more aggressive subtypes (i.e. non-endometrioid cancer) has been reported to increase in Denmark. It is important to assess whether this trend may be reflected in an increased mortality rate. Here, we aimed to describe hysterectomy-corrected mortality rates of corpus uteri cancer, overall and stratified by age and histologic subtype.
Methodology: Using data from nationwide registries, we calculated uncorrected and hysterectomy-corrected age-standardized mortality rates of corpus uteri cancer among women aged 35 years and older during 2002-2015. Individual-level hysterectomy status was obtained from the Danish National Patient Registry; hysterectomy-corrected mortality rates were calculated by subtracting post-hysterectomy person-years from the denominator, unless hysterectomy was performed due to corpus uteri cancer.
Results: During 2002-2015, the age-standardized hysterectomy-corrected mortality rate of corpus uteri cancer was 25.5% higher than the corresponding uncorrected rate (12.3/100,000 vs. 9.8/100,000 person-years). Hysterectomy-corrected rates increased with age, reaching 43.2/100,000 person-years in women aged 70 years and older. Among all women, rates decreased from 2002 (14.0/100,000 person-years) until 2009 after which they stabilized at about 11.5/100,000 person-years, This decrease was mainly due to a decline in women aged 70 years and older. Of note, the hysterectomy-corrected mortality rate of endometrioid cancer declined slightly during the study period, from 5.8/100,000 to 4.2/100,000 person-years, whereas the mortality rate of non-endometrioid cancer increased, from 1.4/100,000 to 3.7/100,000 person-years.
Conclusion: Among all women, the mortality rate declined over calendar time and increased with age. While the mortality of less aggressive histologic subtypes declined, although incidence rates were stable in this time period, the mortality of more aggressive histologic subtypes paralleled the incline in the incidence of these subtypes.
Disclosure: The Danish Cancer Society funded this study. All authors have nothing to disclose

P80

Impact of extent of lymphadenectomy on survival in patients with endometrial cancer: a matched cohort

L. Pauly, L. Benoit, M. Koskas
Hôpital Bichat-Claude-Bernard, Paris, France

Introduction / Background: Surgical assessment of lymph nodes for endometrial cancer staging at primary surgery is one of the most varied practices worldwide and controversies remain about the need for and the extent of para-aortic lymph node dissection in all patient. This study aimed to determine whether a pelvic and para-aortic lymphadenectomy improves survivals compared with a pelvic lymphadenectomy alone in patients with endometrial cancer.
Methodology: Data from all women with operated on for endometrial cancer between January 1998 and December 2013 were extracted from the Surveillance, Epidemiology and End Results database. Women treated with pelvic lymphadenectomy were matched with women treated with pelvic and para-aortic lymphadenectomy according to age and risk of recurrence. The primary and secondary endpoints were overall and disease-specific survivals respectively.
Results: One to one matching enabled to compare the outcome of 1015 patients who underwent pelvic lymphadenectomy with 1015 patients who underwent pelvic and para-aortic lymphadenectomy. The 3-years overall survival probabilities for patients at intermediate and high risk of recurrence were similar in the pelvic and para-aortic lymphadenectomy group and the pelvic only group (91.2 and 86.4% respectively, p=0.07). Multivariate analysis of prognostic factors showed that in patients with an intermediate or high risk of recurrence, pelvic and para-aortic lymphadenectomy did not reduce the risk of death compared with pelvic lymphadenectomy (HR 1.08, 95% CI 0.69-1.69; p=0.74).
Conclusion: The present study suggests that for patients with endometrial cancer at intermediate or high risk of recurrence, the extent of lymphadenectomy does not impact overall and disease-free survivals.
Disclosure: Nothing to disclose

P81

Higher detection rate of Lynch syndrome in patients with endometrial cancer by assessment of mismatch repair deficiency

N. Pauly[1], A. du Bois[1], P. Harter[1], S. Prader[1], K. Rhiem[2], B. Schömig-Markiefka[3], S. Heikaus[4], F. Heitz[1], S. Schneider[1], T. Baert[1,5], A. Traut[1], S. Ehmann[1], B. Ataseven[1,6]
[1]Gynaecology and Gynaecologic Oncology, Kliniken Essen Mitte (KEM), Essen, [2]Center for Hereditary Breast and Ovarian Cancer, Center for Integrated Oncology (CIO), [3]Institute of Pathology, University Hospital Cologne, Cologne, [4]Center for Pathology, Kliniken Essen-Mitte, Essen, Germany, [5]Department of Oncology, Laboratory of Tumour Immunology and Immunotherapy, ImmunOvar Research Group, KU Leuven, Leuven, Belgium, [6]Department of Obstetrics and Gynecology, LMU Munich, Munich, Germany

Introduction / Background: Endometrial cancer (EC) is the most common gynaecologic malignancy in Europe. A hereditary background by germline mutations in the DNA mismatch repair (MMR) genes (*MLH1, MSH2, MSH6*, and *PMS2*) as part of Lynch syndrome (LS) is expected in 2-4% of all EC. For women with LS, the lifetime risk of developing EC is up to 60%, which is higher than colorectal cancer risk. EC is considered a sentinel cancer of women with LS, making its proper diagnostic workup key in the detection of LS and prevention of other cancers. Detection of affected women by Amsterdam II, revised Bethesda criteria has low sensitivity. Up to 30% of EC are MMR deficient (MMRd) which is caused in part by germline mutations.
Methodology: We analysed 231 consecutive EC patients treated at Kliniken Essen-Mitte. MMR was either assessed on paraffin-embedded tumour slides by immunohistochemistry (IHC), PCR based microsatellite analysis or both. In case of MLH1 +/- PMS2 deficiency or microsatellite instability additional methylation analysis was performed. Amsterdam II, revised Bethesda criteria and $PREMM_5$ Model were used to detect patients with a familial history at risk for LS.
Results: MMR diagnostic was successfully performed in 213 patients (110-IHC, 69-PCR, and 34-both). MMRd was detected in 45 patients (21.2%). We observed no discordant results between IHC

and PCR. Methylation analysis was performed in 75,5% (n=34) of MMRd EC resulting in the identification of 12 patients at risk for LS. The Amsterdam II and Bethesda criteria identified none of these patients. The $PREMM_5$Model proposed further investigation in seven of these patients. Genetic testing was performed in five patients and revealed two mutation carriers.
Conclusion: General MMRd assessment is a feasible strategy to improve the detection of LS in patients presenting with endometrial cancer as an additional measure to familial history.
Disclosure: NP has nothing to disclose. BA received fees/honoraria (e.g. lectures, advisory boards) from Roche, Amgen, Astra Zeneca, Tesaro, Clovis, Celgene, and non-financial support (e.g. travel grant) from Roche, PharmaMar, Tesaro outside the submitted work . AdB received honoraria for advisory boards outside the submitted work from Roche, Clovis, Astra Zeneca, Tesaro, Pfizer, Pharmar, Biocad and Genmab.PH received: Honoraria: Astra Zeneca, Roche, Sotio, Tesaro, Stryker, ASCO, Zai Lab, MSD Advisory Board: Astra Zeneca, Roche, Tesaro, Lilly, Clovis, Immunogen, MSD/Merck; Research funding (Inst): Astra Zeneca, Roche, GSK, Boehringer Ingelheim, Medac, DFG, European Union, DKH, Tesaro, Genmab.SP has nothing to disclose.KR has nothing to disclose. FH has received Advisory board: Roche, Tesaro, Honoraria: AstraZeneca, Roche, Tesaro, Clovis, travel/accommodation expenses: PharmaMar; Tesaro.TB has reported research grant from Amgen, travel expenses from Amgen and Roche as well as advisory board membership for Tesaro. BSM has nothing to disclose. SS has received fees/honoraria (e.g. lectures, advisory boards) Tesaro, Astra Zeneca, Roche and travel grant from Tesaro outside the submitted work. SH has nothing to disclose.SE has nothing to disclose. AT has nothing to disclose.

P82

Decreased expression of *ABCG2* and *SLC51B* in endometrial cancer

R. Pavlič[1], S. Vidic[1], M. Anko[1], T. Knific[1], T. Büdefeld[1], M. Sinreih[1], S. Frković-Grazio[2], T. Lanišnik Rižner[1]
[1]Institute of Biochemistry, Faculty of Medicine, University of Ljubljana, [2]Department of Gynecological Pathology, University Medical Centre Ljubljana, Ljubljana, Slovenia

Introduction / Background: Endometrial cancer (EC) is an estrogen-dependent disease. Estrogens can be formed locally from estrone-sulfate (E1-S) which is transported into cells by organic anion-transporting polypeptides (OATP), sodium-dependent organic anion transporters (SOAT), organic anion transporters (OAT) or effluxed by ATP-binding cassette transporters (ABC) and organic solute transporter (OST) αβ (Figure1). Currently, 19 transporters are known to transport E1-S but their roles in EC have not yet been determined. In our study, we analyzed the expression of these transporters in model cell lines of EC, Ishikawa and HEC-1-A, and in paired samples of EC and adjacent control tissue.
Methodology: The expression of ´transporter genes´ was evaluated using qPCR and the presence of proteins was analyzed using imunocytochemistry or imunohistochemistry.
Results: In cell lines and tissue samples 15 genes were differentially expressed where *SLC51B, SLC51A, SLC10A6* and *ABCC4* were significantly up-regulated and *SLCO3A1* was significantly down-regulated in both EC cell lines compared to control endometrial cell line HIEEC. The highest difference in expression was seen for *SLCO1B3* (28930.7-fold up-regulation) and *ABCG2* (30.2-fold down-regulation) in HEC-1-A compared to HIEEC. Imunocytochemistry revealed significantly higher levels of OATP1B3 (*SLCO1B3*) (6.3-fold) in HEC-1-A compared to Ishikawa and low concentrations of ABCG2 in both EC cell lines.
In EC tissue significant changes were seen for *ABCG2* and *SLC51B* which were significantly (3.2-fold and 2.1-fold, respectively) down-regulated and *ABCC1* which was significantly (1.6-fold) up-regulated in EC tissue compared to adjacent control tissue. In patients without lymphovascular invasion gene *SLCO1B3* was 15.6-fold up-regulated. Tumor grade had significant effects on expression of *SLC51B*, with lower levels seen in high grade cancers. Immunohistochemistry revealed significantly lower levels of ABCG2, OSTβ (*SLC51B*) and OATP1B3 (*SLCO1B3*) in EC *versus* control tissue.
Conclusion: Our results suggest that OATP, OAT and ABC transporters have important roles in EC pathophysiology. Detailed investigation of E1-S uptake and efflux is thus in progress.

BMJ Publishing Group Limited (BMJ) disclaims all liability and responsibility arising from any reliance placed on this supplemental material which has been supplied by the author(s)

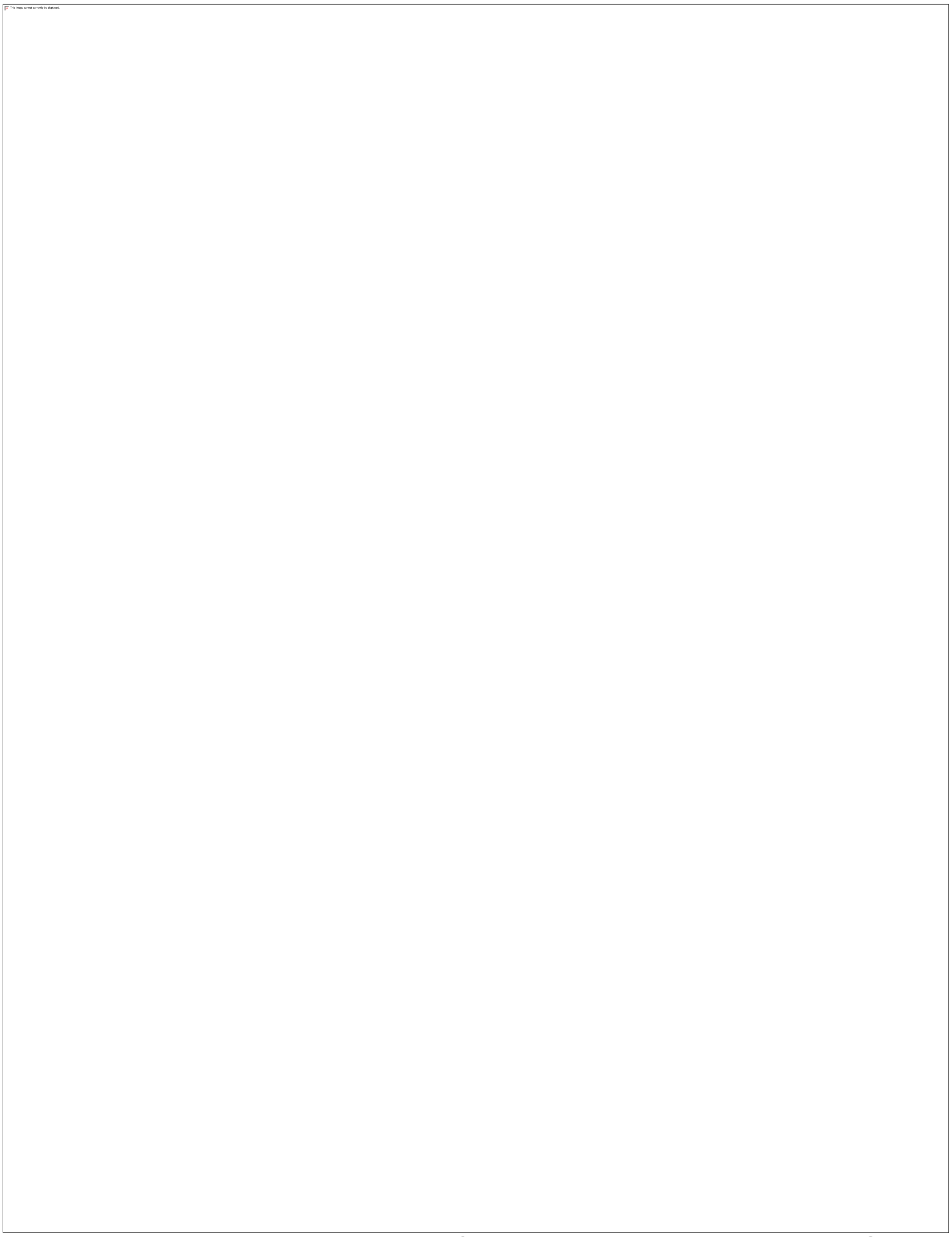

[Figure1: Local estradiol (E2) biosynthesis in EC depends on the uptake and efflux of E1-S by transporters.]

Disclosure: Nothing to disclose

P83

Incorporating artificial intelligence techniques in decision making concerning the optimal management of postmenopausal women with evidence of endometrial pathology

V. Pergialiotis[1], A. Pouliakis[2], V. Damaskou[2], C. Chrelias[1], S. Kalantaridou[1], I. Panayiotides[2]
[1]3rd Department of Obstetrics and Gynecology, [2]2nd Department of Pathology, National & Kapodistrian University of Athens, Athens, Greece

Introduction / Background: The diagnostic accuracy of artificial neural networks (ANNs) and Classification and Regression Trees (CARTs) has been already tested in several studies involving cancer patients. To date, however, its use in the field of gynecologic oncology remains extremely limited. In our study, we aim to investigate the diagnostic accuracy of three different methodologies logistic regression, ANNs and CARTs for the prediction of endometrial cancer in post-menopausal women.
Methodology: We retrospectively searched medical records of all patients that were subjected in endometrial sampling procedures due to post-menopausal vaginal bleeding or ultrasographic evidence of endometrial pathology. Classical regression analysis was performed along with ANN and CART analysis using the IBM SPSS and Matlab statistical packages.
Results: Overall, 178 women were enrolled. Among them, 106 women were diagnosed with carcinoma; whereas the remaining 72 women had normal histology in the final specimen. ANN analysis seems to perform better with a Sensitivity of 86.8%, Specificity of 83.3% and overall accuracy of 85.4%. CART analysis did not perform as well with a Sensitivity of 78.3% a Specificity of 76.4% and overall accuracy of 77.5%. Regression analysis had a poorer predictive accuracy with a sensitivity of 76.4%, a specificity of 66.7% and an overall accuracy of 72.5%.
Conclusion: Artificial intelligence is a powerful mathematical tool that may guide clinicians involved in primary care in decision making when endometrial pathology is suspected. Our study clearly denotes that this can be done with simple indices which may be drawn from the patients` history and a basic anthropometric measurement. However, current data in this field remain in a premature state to support this method as a screening tool as the number of participants remains low to stabilize the diagnostic accuracy of the algorithm; hence, future, multicenter studies are needed to support our findings.
Disclosure: Nothing to disclose

P84

Mismatch repair deficiency as a predictive marker for response to adjuvant radiotherapy in endometrial cancer

C. Reijnen[1,2], H. Küsters-Vandevelde[3], C. Prinsen[3], L. Massuger[1], M. Snijders[2], S. Kommoss[4], S. Brücker[4], J. Kwon[5], J. McAlpine[5], J. Pijnenborg[1]
[1]Obstetrics and Gynaecology, Radboud UMC Nijmegen, [2]Obstetrics and Gynaecology, [3]Pathology, Canisius-Wilhelmina Hospital, Nijmegen, The Netherlands, [4]Women's Health, Tübingen University Hospital, Tübingen, Germany, [5]Division of Gynecologic Oncology, University of British Columbia, Vancouver, BC, Canada

Introduction / Background: Mismatch repair (MMR) deficiency is found in 20 to 40% of endometrial cancers (ECs) and was recently identified as a discerning feature of one of the four prognostic subgroups identified by The Cancer Genome Atlas. There is accumulating evidence that MMR proteins are involved in the DNA repair processes following radiotherapy. We investigated the predictive value of MMR status for response to adjuvant radiotherapy in patients with stage IB/II, grade 3 endometrioid endometrial cancer (EEC).
Methodology: A retrospective multicenter cohort study was performed to compare patients with histopathologically confirmed stage IB/II grade 3 EEC with and without adjuvant radiotherapy. Patients were classified according to the Proactive Molecular Risk Classifier for Endometrial Cancer (ProMisE) identifying ECs as either MMR-deficient, POLE, p53abn or p53wt. Multivariable Cox regression analysis explored associations between adjuvant treatment and outcome.
Results: A total of 128 patients were analyzed, including 57 patients (43.0%) with MMR-deficient EECs. Baseline characteristics were comparable, except a higher proportion of MMR-deficient EECs

were stage II (36.8% vs. 15.5%, p=0.006). Eighty-two patients (64.1%) received adjuvant radiotherapy (external beam [n=55], vaginal brachytherapy [n=27]). In multivariable analysis, adjuvant radiotherapy was associated with improved disease-specific survival in patients with MMR-deficient EECs (hazard ratio 0.19, 95%-CI 0.05 - 0.77), but not in patients with MMR-proficient EECs (hazard ratio 0.92, 95%-CI 0.37 - 2.31).
Conclusion: Adjuvant radiotherapy improved survival in patients with MMR-deficient EECs. MMR status could be used as a predictive biomarker to select patients that benefit most from adjuvant radiotherapy.
Disclosure: Nothing to disclose

P85

Mutational analysis of cervical cytology improves diagnosis of endometrial cancer: a prospective multicenter cohort study

C. Reijnen[1,2], L. van der Putten[1], J. Bulten[3], M. Snijders[2], H. Küsters-Vandevelde[4], S. Sweegers[3], M.C. Vos[5], A. van der Wurff[6], M. Ligtenberg[7], L. Massuger[1], A. Eijkelenboom[3], J. Pijnenborg[1]
[1]Obstetrics and Gynaecology, Radboud UMC Nijmegen, [2]Obstetrics and Gynaecology, Canisius-Wilhelmina Hospital, [3]Pathology, Radboud UMC Nijmegen, [4]Pathology, Canisius-Wilhelmina Hospital, [5]Obstetrics and Gynaecology, [6]Pathology, Elisabeth-TweeSteden Hospital, [7]Human Genetics, Radboud UMC Nijmegen, Nijmegen, The Netherlands

Introduction / Background: Endometrial carcinoma (EC) is traditionally diagnosed by histopathological assessment of an endometrial biopsy, leaving up to 30% of patients undiagnosed due to technical failure of the biopsy procedure or an inadequate amount of tissue. The aim of the current study is to assess whether mutational analyses of cervical cytology including self-samples or pipelle endometrial biopsies can improve the diagnostic accuracy of traditional histopathological diagnosis of EC.
Methodology: A prospective multicentre cohort study was performed in three hospitals in the Netherlands. Patients surgically treated for EC or a benign gynecological condition (control group) were included. A Pap brush sample, a cervicovaginal self-sample, a pipelle endometrial biopsy and the surgical specimen of the endometrial carcinoma (endometrial carcinoma group) or normal endometrium (control group) were obtained. A targeted next-generation sequencing panel was used to analyze these samples for mutations in eight genes.
Diagnostic accuracy was determined by calculating sensitivity, specificity and predictive values.
Results: Fifty-nine EC patients and 31 control patients were included. In these patients traditional histopathological diagnosis by pipelle had a sensitivity of 78.9% and a specificity of 100%. For EC patients, 96.6% of surgical specimens contained at least 1 mutation. Mutational analysis of Pap brush samples, self-samples, and pipelle endometrial biopsies yielded a sensitivity of 78.0%, 67.3% and 96.5% with a specificity of 96.8%, 96.8% and 93.5%, respectively. Combining these three methods individually with histopathological pipelle endometrial biopsy evaluations yielded a sensitivity of 95.8%, 93.0% and 96.5, respectively.
Conclusion: This study has shown that mutational analysis of either cervical cytology, self-samples or pipelle endometrial biopsies improves diagnosis of EC, and is feasible for detection of endometrial pathology. Prospective validation will support implementation in routine clinical practice.
Disclosure: M.L.: advisory board AstraZeneca, Bayer, Bristol-Myers Squibb, Janssen pharmaceuticals, Roche; sponsored research by Astra Zeneca, Bristol-Myers Squibb, Ilumina; royalties by Nimagen. Furthermore, no completing interests were declared.

P86

Diabetes and gynaecological cancers: an umbrella review

A. Semertzidou, I. Kalliala, H. Grout-Smith, M. Kyrgiou
Imperial College London, London, United Kingdom

Introduction / Background: Diabetes mellitus (DM) continues to affect a large proportion of the population and its incidence is on the rise. Many complications associated with it, such as gynaecological cancers, are becoming increasingly apparent. Due to conflicting evidence, it is unknown which outcomes diabetic women are most at risk of. Therefore, the aim of this review is to evaluate the strength and validity of the existing evidence between diabetes and the risk of gynaecological cancers.
Methodology: Design: Umbrella review of systematic reviews and meta-analyses.
Eligibility criteria: Systematic reviews and meta analyses of observational studies investigating the relationship between type I/2 diabetes mellitus and endometrial, ovarian and cervical cancers.
Data analysis: The evidence from the meta-analyses were graded according to a statistical criteria comprised of the random effects estimate of the meta-analyses and of their largest study, the number of cases, 95% prediction intervals, I^2statistic of heterogeneity between studies, excess significance bias, small studies effect and sensitivity analysis using credibility ceilings. From this, meta-analyses were classified as strong, highly suggestive, suggestive or weak evidence.
Results: 60 meta-analyses from 20 eligible papers were assessed, of which 15 looked at the association between type 1/2 diabetes mellitus and gynaecological cancers. A highly suggestive association was found between type 1/2 DM and endometrial cancer incidence. Suggestive associations were found between type 1/2 DM and all-cause mortality in ovarian cancer and overall and recurrence-free survival in cervical cancer.
Conclusion: DM appears to be strongly associated with endometrial cancer incidence, whereas a weaker association was demonstrated with ovarian and cervical cancer survival and mortality.
Disclosure: Nothing to disclose

P87

Mesenchymal splice isoform Fibroblast Growth Factor Receptor (FGFR2c) is an independent prognostic biomarker and further refines risk stratification within endometrial cancer molecular subtypes

A. Sengal[1,2], E. Williams[1], J. McAlpine[3], P. Pollock[1]
[1]School of Biomedical Sciences, Institute of Health and Biomedical Innovation/QUT, [2]Endometrial Cancer, HBI/ Translational Research Institute, Brisbane, QLD, Australia, [3]Department of Gynaecology and Obstetrics, Division of Gynaecologic Oncology, University of British Columbia, Vancouver, BC, Canada

Introduction / Background: Four pragmatic molecular subtypes of endometrial cancer (EC) has been established and these seem promising approach in risk stratification. The mismatch repair deficient (MMRd) and p53 wildtype subtypes are the most common and given their molecular subgroups with intermediate prognosis additional biomarkers to further stratify these groups are needed. Fibroblast growth factor receptor (FGFR2) has two major splice isoforms, FGFR2b and FGFR2c. FGFR2c is an oncogen and has been associated with aggressive tumour behaviour in several cancers. The objective was to investigate the role of FGFR2c in progression and risk stratification within the MMRd and p53 wt subtypes.
Methodology: We have developed, optimized and validated a novel RNA in situ hybridization assay to detect FGFR2c. Expression was determined in screening cohort of ECs (n=78) and the clinically annotated Canadian cohort (n=465). Kaplan Meier curve and Cox regression model analyses were performed to assess the prognostic value of FGFR2c.
Results: There was a significant difference in mRNA FGFR2c expression between endometrioid EC and non-endometrioid EC, 50% versus 23%, $p < 0.0001$. Univariable analysis revealed FGFR2c was significantly associated with shorter Disease Specific Survival ($p < 0.001$) and Progression-free Survival ($p < 0.009$) in endometrioid EC. Multivariable Cox regression analysis showed patients with FGFR2c expression have reduced DSS ($p < 0.022$) and PFS ($p < 0.035$). Specifically, within the MMRd molecular subtype FGFR2c expression was significantly associated with shorter DSS (p <

0.001) and PFS (p < 0.048) and this trend was also evident in the p53 wildtype subtypes.
Conclusion: Conclusion: FGFR2c is an independent prognostic biomarker in EC and its expression further discerns the outcome of EC patients with molecularly classified MMRd and p53 wildtype tumours. Integration of FGFR2c into the new molecular subtyping can further refine risk stratification of endometrioid EC.
Disclosure: Nothing to disclose

P88

Analysis of 93 patients with endometrial cancer using the PROMISE classification and additional genetic analyses for MSI and POLE-EDM

S. Timmerman[1], A.-S. Van Rompuy[2], T. Van Gorp[1], I. Vanden Bempt[3], H. Brems[3], E. Van Nieuwenhuysen[1], S.N. Han[1], P. Neven[1], I. Vergote[1]
[1]Department of Gynaecology and Obstetrics, Division of Gynaecological Oncology, University Hospitals Leuven, Leuven Cancer Institute, [2]Department of Pathology, University Hospitals Leuven, [3]Department of Human Genetics, University Hospitals Leuven, KU Leuven, Leuven, Belgium

Introduction / Background: Recent research has distinguished various molecular classifications for endometrial carcinoma (EC). The Proactive Molecular Risk Classifier for Endometrial Cancer (ProMise, Talhouk, BJC 2015,113:299) classification consists of immunohistochemistry (IHC) (p53 and mismatch repair proteins) and PCR sequencing for Polymerase Epsylon-Extracellular-Domain-Mutations (POLE-EDM).
Methodology: Ninety-three consecutive endometrial cancer patients, diagnosed between March 2017 and November 2018, were subjected to molecular and immunohistochemical analyses, according to ProMise. IHC for p53 and the mismatch repair proteins (MLH1, PMS2, MSH6 and PMS2) was performed. All patients were also tested for POLE-EDM using PCR, followed by Sanger sequencing (exon 9, 11, 13 and 14).
In addition to the ProMise testing, all tumors and corresponding normal tissue of patients with mismatch repair deficient staining on IHC, were tested with PCR using the Microsatellite Instability (MSI) kit (MSI analysis system, Promega). Hypermethylation of MLH1 promotor and the presence of the BRAF p.V600E missense mutation was tested with (methylation specific) multiplex ligation dependent probe amplification.
Results: FIGO classification was stage IA(n= 45), IB(n=17)) II(n=8), III(n=17) and IV(n=6). Classification according to histological type is presented in Table 1. Of the 28 patients with MMR-D (Mismatch Repair-Deficiency) on IHC, 22(79%) showed hypermethylation as the probable cause of MMR-D. The remaining 6 patients were referred for germline analysis of Lynch syndrome. Five patients carried a pathogenic germline mutation in one of the mismatch repair genes: MSH6(n=3), PMS2(n=1), MLH1(n=1). In one patient no germline mutation was identified, hypothesizing a somatic cause. POLE-EDM was identified in 6 (6%) patients. Double positive status (POLE-EDM+MMR-D or POLE-EDM+p53 abnormal) was observed in 4 patients (4%) (Table1).
Conclusion: The ProMise classification proved to be an efficient and easily implementable system. In the group with MMR-D, hypermethylation was the cause in 79%. POLE-EDM was identified in 6% of the patients, but its role needs to be clarified in non-endometrioid tumors.

Histological type	MMR-D on IHC	POLE-EDM	p53 abnormal	p53 wild type	MMR-D + POLE-EDM	POLE-EDM + p53 abnormal
G1-2 Endometrioid EC (n=64)	21	2	3	38	0	0
G3 Endometrioid EC (n=5)	3	0	0	1	1	0
Serous EC (n=11)	1	0	10	0	0	0

Clear cell EC (n=3)	0	0	1	1	1	0
Mixed G3 Endometrioid and serous EC (n=4)	0	0	2	0	1	1
MMMT EC (n=6)	0	0	6	0	0	0
Total (n= 93)	25 (27%)	2 (2%)	22 (24%)	40 (43%)	3 (3%)	1 (1%)

[Table 1: Distribution of molecular subgroups among histopathological types. G: grade; MMMT: Malignant Mixed Müllerian Tumor.]

Disclosure: Nothing to disclose

P89

Implementation of a nationwide guideline to detect of women at risk for Lynch syndrome in endometrial cancer should be improved

A.S. Tjalsma[1], A. Wagner[2], W.N.M. Dinjens[3], P.C. Ewing-Graham[4], H.C. Van Doorn[5]
[1]Gynaecology, Erasmus MC Cancer Clinic, [2]Clinical Genetics, Erasmus Medical Centre, Rotterdam, [3]Pathology, Erasmus Medical Centre, Ro, [4]Pathology, Erasmus Medical Centre, Rotterdam, [5]Gynaecology, Erasmus MC Cancer Clinic, R, The Netherlands

Introduction / Background: Since 2016 routine Lynch syndrome screening (LSS) among women with endometrial cancer < 70 years of age has been incorporated in a nationwide guideline. LSS consists of immunohistochemical (IHC) staining for loss of miss match repair (MMR) protein expression, extended with methylation analysis of the MLH1 promotor in case of MLH1 and PMS2 loss. Test results are discussed by the treating gynaecologist, who refers patients to a genetic counsellor (Figure 1).

Objective: Evaluate the implementation of the Dutch guideline to screen women with endometrial carcinoma under the age of 70 years for Lynch syndrome.
Methodology: From the pathology database women diagnosed with EC < 70 years of age, treated from 1.6.2016 - 1.6.2017, in 14 hospitals, covering about 30% of the Netherlands, were selected. Data collected regarded LS testing and outcome, and follow up. The study was approved by the institutional review boards of participating hospitals.
Results: In 184 out of the 205 tumours (90%) LSS was performed (Table 1). Either on the endometrial biopsy 24%, the hysterectomy (70%), or on both (6%). In 42 cases (22%) MMR protein expression was lost, in 25 cases due to hypermethylation of the MLH1 promotor. LS susceptibility was discussed with 11 of the 17 patients at risk of LS (65%), all were referred for genetic counselling. Three of them declined; eight underwent germline testing for LS after counselling. In one no germline mutation was detected, two were diagnosed with a PMS2 mutation, and five with a MSH6 mutation.
Conclusion: The implementation of the guideline regarding LSS has been successful, though referral for genetic counselling should be improved. Gynaecologist ought to be aware of the benefits and drawbacks of knowing mutational status, and may require training in discussing this with their patients.

Patients with endometrial carcinoma	205	Clear cell	5 (3.5%)	High risk Lynch syndrome	17 (9%)
Patients screened for LS	184 (90%)	undifferentiated	1 (05%)	- results pending	2 (12%)

Immunohistochemistry	179 (97%)	FIGO stage IA	102 (55%)	- known carrier	1 (6%)
Microsatellite instability analysis	3 (2%)	FIGO stage IB	43 (23%)	- result not shared with patient	3 (17%)
Hysterectomy	129 (70%)	FIGO stage II	8 (4%)	- referral for genetic counseling	11 (65%)
Endometrial biopsy	45 (24%)	FIGO stage III	10 (6%)	-- refrained genetic counseling	3 (27%)
Biopsy and Hysterectomy	10 (6%)	FIGO stage IV	2 (1%)	-- germline analysis	8 (73%)
Endometrioid carcinoma	156 (85%)	Unknown FIGO stage	19 (11%)	---no known mutation detected	1 (13%)
Sereus carcinoma	13 (7%)	Loss of MMR-expression	42 (22%)	--- MSH 6 mutation	5 (62%)
carcinosarcoma	7 (4%)	Hypermethylation MLH1	25 (13%)	--- PMS 2 mutation	2 (25%)

[Outcomes]

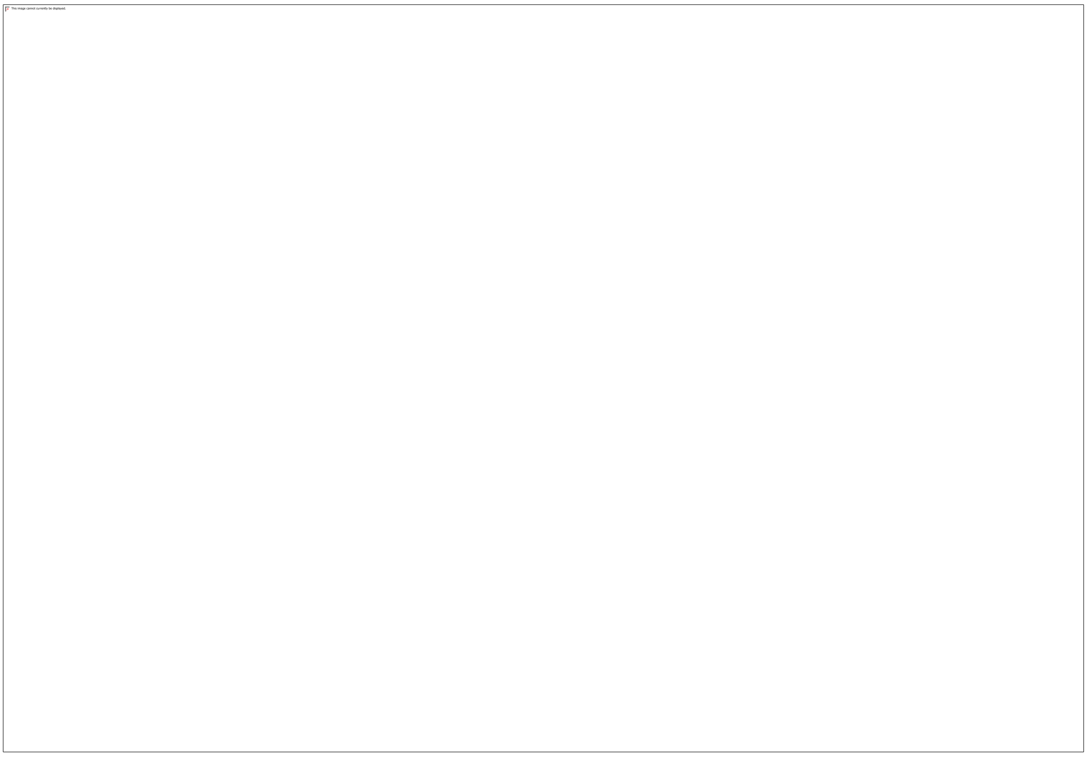

[FIGURE 1 Nationwide Dutch Guideline for detecting Lynch syndrome (LS) in women with endometrial canc]

Disclosure: Nothing to disclose

P90

Complementary role of 18F- FDG PET/CT for sentinel lymph node algorithm in endometrial cancer with high risk factors for lymphatic metastasis

S. Taşkin[1], B. Varli[1], C.C. Ersöz[2], D. Altin[1], Ç. Soydal[3], F. Ortaç[1]
[1]Obstetrics and Gynecology, [2]Pathology, [3]Nuclear Medicine, Ankara University School of Medicine, Ankara, Turkey

Introduction / Background: National Comprehensive Cancer Network (NCCN) sentinel lymph node (SLN) algorithm includes “mandatory steps” for evaluating pelvic lymph nodes but assessment of paraaortic area is left to surgeon's discretion. In this study we aimed to investigate the complementary role of preoperative ^{18}F- FDG PET/CT scan in detecting pelvic and especially paraaortic lymphatic metastasis in endometrial cancer patients with high risk factor(s) according to Mayo Clinic Criteria and underwent SLN algorithm.
Methodology: Patients who underwent preoperative ^{18}F- FDG PET/CT scan, intraoperative SLN algorithm followed by systematic lymphadenectomy and had at least one high risk criterion for lymphatic metastasis were included in this study. ^{18}F- FDG PET/CT and SLN algorithm were compared with final histopathological results of systematic lymphadenectomy.
Results: 38 patients were eligible for the study. Lymphatic metastasis was seen in 10 patients (26.3%). 4 cases had paraaortic lymphatic metastases which were together with pelvic (n:2) or isolated (n:2) metastases. SLN algorithm was able to detect all pelvic lymph node metastases. However isolated paraaortic metastases were diagnosed only by ^{18}F- FDG PET/CT. In 76 hemipelvises, sensitivity and negative predictive value of SLN algorithm for diagnosis of pelvic nodal metastasis were 100%, while sensitivity, specifity, positive predictive value and negative predictive value of ^{18}F- FDG PET/CT were 45.4%, 95.3%, 62.5% and 91.1%, respectively.
Conclusion: Although SLN algorithm has an excellent diagnostic value for pelvic nodal metastasis, paraaortic metastasis might be under-diagnosed. ^{18}F- FDG PET/CT may be a feasible tool to exclude paraaortic lymphatic metastasis in high risk patients for lymphatic metastasis who will undergo SLN algorithm.
Disclosure: Nothing to disclose

P91

Integrative model for prediction of lymph node metastasis in endometrioid endometrial carcinoma

H.F. Berg[1,2], Z. Ju[3], M. Myrvold[4], K.E. Fasmer[5,6], M.K. Halle[1,2], S.N. Westin[7], J. Trovik[1,2], I.S. Haldorsen[5,6], G.B. Mills[8], C. Krakstad[1,2], H.M. Werner[9]
[1]Centre for Cancer Biomarkers, Department of Clinical Science, University of Bergen, [2]Dpt of Obstetrics and Gynaecology, Haukeland University Hospital, Bergen, Norway, [3]Bioinformatics and Computational Biology, UT M.D. Anderson Cancer Center, Houston, TX, United States, [4]Department of Clinical Science, [5]Norway2Section for Radiology, Department of Clinical Medicine, University of Bergen, [6]Dpt of Radiology, Haukeland University Hospital, Bergen, Norway, [7]Dept. of Gynecologic Oncology and Reproductive Medicine, UT M.D. Anderson Cancer Center, Houston, TX, [8]Department of Precision Oncology, Knight Cancer Institute, Oregon Health and Science University, Portland, OR, United States, [9]Obstetrics and Gynaecology, Maastricht University Medical Centre (MUMC), Maastricht, The Netherlands

Introduction / Background: In endometrioid endometrial cancer (EEC), current clinical algorithms do not accurately predict patients with lymph node metastasis, leading to both under- and over-treatment. We aimed to develop an integrative model combining protein data with routinely available clinical information and preoperative imaging to identify EEC patients who may benefit from more aggressive surgery including lymphadenectomy.
Methodology: Using reverse phase protein arrays, expression profiles were generated for 176 proteins in independent training (n=243, Bergen; Norway) and test sets (n=56, Bergen, Norway and

n=100, MDACC, Texas). LIMMA analysis identified significantly differently expressed proteins between cases with and without lymph node metastasis. Generalized linear models were then constructed selecting only the most informative proteins in addition to clinical data. Gene expression data from the same tumours were used for confirmatory testing.
Results: The main model, including fibronectin, cyclin D1 and tumour grade, predicted lymph node metastasis with AUC 0.79 (training); 0.88 (Bergen test set) and 0.83 (RNA expression data). The MRI model, along MRI including fibronectin and grade, resulted in AUC 0.83 (training and Bergen test set). Finally, in grade 1 and 2 EEC, a model was fitted using cyclin D1, SMAD1, fibronectin and beta catenin, with AUC 0.89 (training) and 0.72 (MDACC test set). High levels of fibronectin and cyclin D1 were, associated with metastatic lymph nodes (p< 0.001) and poor survival (p=0.018), and with other markers of tumour aggressiveness; i.e. high FIGO stage, high grade (p< 0.001) and, for cyclin D1, menopausal status (p=0.04), deep myometrial invasion (p< 0.001). Gene set enrichment analysis showed that upregulation of both fibronectin and cyclin D1 was related to cancer invasion and mesenchymal phenotype.
Conclusion: We show that data-driven integrative models, adding protein markers to readily available clinical information, have potential to significantly improve stratification of patients at risk for lymph node metastasis in EEC, including low-risk EEC.
Disclosure: Nothing to disclose

P92

Clinical impact of lymphadenectomy for endometrial cancer in Japan

W. Yamagami, K. Saotome, T. Hirano, Y. Nanki, K. Sakai, T. Makabe, T. Chiyoda, Y. Kobayashi, F. Kataoka, K. Banno, D. Aoki
Department of Obstetrics and Gynecology, Keio University School of Medicine, Tokyo, Japan

Introduction / Background: Hysterectomy,bilateral salpingectomy, and retroperitoneal lymphadenectomy are often performed as standard surgical procedures for endometrial cancer. However, lymph edema and lymphocele, which are adverse effects of lymphadenectomy, degrade a woman's quality of life. The therapeutic significance of lymphadenectomy has not been established. This study aimed to clarify the impact of lymphadenectomy on the therapeutic outcome.
Methodology: The cancer database of Japan Society of Obstetrics and Gynecology provided data on 43,779 patients who started treatment between 2004 and 2011. Among these patients, we retrospectively analyzed the difference in overall survival with and without lymphadenectomy in 36,813 patients who underwent initial surgery and had well registered clinicopathological factors and prognoses. In this study FIGO 1988 staging was used.
Results: The median age was 58 years, and the median follow-up period was 1610 days. Stage I, II, III and IV were 24,296 (66%), 3,313 (9%), 7,362 (20%) and 1,840 (5%) patients respectively. In terms of histologic type, there were 18,406 (50%), 8,098 (22%), 3,681 (10%), and 5,153 (14%) patients with G1, G2, or G3 endometrioid adenocarcinoma, and non-endometrioid adenocarcinoma, respectively. Seventy-three percent of patients underwent lymphadenectomy. Analysis of each histological type showed that prognosis was significantly better in the lymphadenectomy group than in the non-lymphadenectomy group for each histological type. Analysis at each stage showed that prognosis was significantly better in the lymphadenectomy group than in the non-lymphadenectomy group for each stage, except stage Ia. In the low recurrence-risk group (stage Ia-b, G1-2), there was no significant difference in stage Ia, but in stage Ib. A multivariate analysis revealed that the presence or absence of lymphadenectomy was an independent prognostic factor considering age, stage, histological type, and adjuvant therapy.
Conclusion: Although there is a limitation that various biases cannot be eliminated because of retrospective study, lymphadenectomy for endometrial cancer may have a prognostic improvement effect.
Disclosure: Nothing to disclose

P93

Pre-operative levels of circulating cell-free DNA as a risk-assessment tool in endometrial cancer

T. Perri[1,2], I. Zilberman[1,2], A. Douvdevani[3], J. Korach[1,2]
[1]Gynecologic Oncology, Sheba Medical Center, Ramat Gan, [2]Tel-Aviv University, Sackler Faculty of Medicine, Ramat-Aviv, [3]Clinical Biochemistry and Pharmacology, Soroka University Medical Center, School of Medicine, Ben Gurion University of the Negev, Beer Sheva, Israel

Introduction / Background: Different factors were suggested as pre-operative risk grouping predictors in endometrial cancer, to assist in intra-operative decisions including the need for lymph nodes assessment. Circulating cell-free DNA (cfDNA) serum levels were found to correlate with various tumor characteristics, and were suggested to serve as a biomarker of tumor aggressiveness. The level of cfDNA was so far sparsely investigated in endometrial cancer. We aimed to evaluate quantity of serum cfDNA as a pre-operative strategy to determine risk group in endometrial cancer patients.
Methodology: Levels of cfDNA were measured in serum of 80 endometrial cancer patients pre-operatively using SYBR Gold assay (fluorochrome SYBR® Gold) and were correlated with clinical and pathological variables of the patients and tumors.
Results: CfDNA levels were not associated with neither clinical parameter, including age, BMI, parity, diabetes or hypertension, nor with any pathology variable including stage, myometrial invasion, lymphovascular space invasion and lymph nodes metastases. Yet, patients with G1 carcinoma had a lower level of cfDNA compared to G2-3 carcinomas [median 694 (IQR=482-908 vs. 847 (IQR=538-1078 respectively], reaching a borderline statistical significance (p=0.081).
Conclusion: According to our data, cfDNA levels in sera from preoperative endometrial cancer patients correlate weakly with tumor grade. Larger -scale studies are needed in order to assess cfDNA level measurements as a biomarker that might assist clinical decisions in endometrial cancer.
Disclosure: Nothing to disclose

P94

Nature of early stage endometrial cancer recurrence: a cohort study

Z. Zubair[1], S. Masood[2], A. Ali Syed[3]
[1]Skmhc Lahore | Gynaecological Oncology | Surgical Oncology, Shaukat Khanummemorial Hospital and Research Centre Lahore, [2]Student, King Edward Medical University Lahore, [3]Surgical Oncology, Skmch, Lahore, Pakistan

Introduction / Background: Evidence regarding rise of endometrial carcinoma is on uprise which is highlighting the need of appropriate surveillance after the primary treatment. So the study was planned to analyze disease recurrence in cohort of women with early stage endometrial cancer and associated clinico-pathological and sociodemographic predictors of disease recurrence.
Methodology: All the women dignosed with stage 1 endometrial cancer in 2009 __2013 who underwent hysterectomy were included in a hospital based cohort derived from database at SKMCH Lahore. Disease recurrence upto 3 years after the primary diagnosis was assessed and follow up on survival was completed on 31 december 2017. Predictive value of clininco- pathological and socio demographic variables was evaluated .
Results: With in 3 years of primary treatment recurrence identified in 13(5.2%) of the included 250 women. Predictive factors for recurrence included International Federation of Gynaecology and Obstetrics (FIGO) stage(OR 1.89 1B) , Charlson comorbidity index of 3(OR: 1.88) and non endometrial histology(OR 1.77). Overall survival was significantly affected by site of recurrence . 5 year survival was 75 % for women with vaginal recurrence and only 15 % in women with the distant metastasis.
Vaginal recurrence was predicted by FIGO stage only(OR: IB 1.66), while
extra-vaginal recurrence was predicted by FIGO stage(OR: IB 1.88)Charlson comorbidity index of

3(OR 1.77) and non-endometrioid histology(OR 2.31).
Conclusion: Early stage endometrial carcinoma is associated with a low risk of recurrence with in 3 years after hysterectomy and the site of recurrence is highly predictive of overall survival yet future research should seek to understand the underlying mechanism of these predictive factors so as to reduce the morbidity and mortality.
Disclosure: Nothing to disclose

P95

Obstetric and maternal outcome of patients with Hodgkin lymphoma diagnosed during pregnancy: results from the INCIP registry

C. Maggen[1,2], D. Dierickx[2,3], P. Lugtenburg[4], A. Laenen[5], E. Cardonick[6], R.G. Shmakov[7], M. Bellido Casado[8], A. Cabrera Garcia[9], M.M. Gziri[10], M.J. Halaska[11], P.B. Ottevanger[12], K. Van Calsteren[13,14], A. L'Haugklin[6], E. Polushkina[15], L. Van Dam[16], P. Vandenberghe[3], F.J.S.H. Woei-A-Jin[17], F. Amant[18,19,20]
[1]Department of Obstetrics and Gynaecology, UZ Leuven, [2]Department of Oncology, KU Leuven - University of Leuven, [3]Department of Haematology, UZ Leuven, Leuven, Belgium, [4]Department of Haematology, Erasmus MC, Rotterdam, The Netherlands, [5]Leuven Biostatistics and Statistical Bioinformatics Centre, KU Leuven - University of Leuven, Leuven, Belgium, [6]Department of Obstetrics and Gynaecology, Cooper University Health Care, Camden, NJ, United States, [7]Federal State Budget Institution "Research Centre for Obstetrics, Gynaecology and Perinatology", Ministry of Healthcare of the Russian Federation, Moscow, Romania, [8]Department of Haematology, University Medical Centre Groningen, Groningen, The Netherlands, [9]Hospital Regional de Alta Especialidad de Ixtapaluca (HRAEI), Mexico, Mexico, [10]Department of Obstetrics, Cliniques Universitaires St Luc, Sint-Lambrechts-Woluwe, Belgium, [11]Faculty Hospital Kralovske Vinohrady and 3rd Medical, Charles University, 3rd Medical Facculty, Prague, Czech Republic, [12]Department of Medical Oncology, Radboud UMC Nijmegen, Nijmegen, The Netherlands, [13]Department of Obstetrics, UZ Leuven, [14]Department of Development and Regeneration, KU Leuven - University of Leuven, Leuven, Belgium, [15]Federal State Budget Institution 'Research Centre for Obstetrics, Gynaecology and Perinatology', Ministry of Healthcare of the Russian Federation, Moscow, Russian Federation, [16]Department of Oncology, Erasmus MC, Rotterdam, The Netherlands, [17]Department of General Medical Oncology, UZ Leuven, [18]University Hospitals Leuven, Antoni van Leeuwenhoek - Netherlands Cancer Institute, KU Leuven - University of Leuven, [19]Department of Gynaecological Oncology, UZ Leuven, Leuven, Belgium, [20]Centre of Gynaecological Oncology (CGOA), Amsterdam UMC, Location AMC, VUmc, NKI, Amsterdam, The Netherlands

Introduction / Background: The purpose of this study was to assess obstetric and maternal outcome of pregnant patients with diagnosis of Hodgkin lymphoma (HL) to guide physicians in clinical management.
Methodology: Clinical data of pregnant patients diagnosed with HL between 1969 and 2018 were collected from the registry of the International Network on Cancer, Infertility and Pregnancy (INCIP). For survival analysis of classical HL treated with an ABVD-based regimen, non-pregnant controls were selected based on stage and prognostic score at diagnosis.
Results: The median gestational age at diagnosis of 134 eligible patients was 20 weeks (range: 3 - 37). Antenatal chemotherapy was initiated in 53.7% of patients. Ten (7.5%) early pregnancies were terminated. One foetus deceased in the third trimester after three cycles of chemotherapy.
In total, 120 (89.6%) pregnancies ended in a live birth. Preterm delivery was observed in 47 (40.1%) singleton pregnancies. Birth weight percentiles were lower in children prenatally exposed to oncological treatment and 17.9% were small for gestational age at birth (figure 1). Four children (3.5%) had major congenital malformations.
Five-year progression-free survival (PFS) for HL during pregnancy was 82.5% and 90.9% for early (n=62) and advanced stage (n=15). Five-year overall survival (OS) was 97.3% and 100%, respectively. Although not significant, patients with early stage HL appeared to have inferior PFS compared with matched non-pregnant controls (n=62, figure 2), more clearly seen in the subgroup that initiated chemotherapy during pregnancy (n=45). OS was comparable between both groups, supporting the effectiveness of salvage therapy. For advanced stage HL survival was similar to controls, albeit small numbers.

Conclusion: Survival of patients diagnosed with early stage HL during pregnancy appears not statistically different from matched non-pregnant controls, however future prospective research is necessary to investigate the efficacy of chemotherapy during pregnancy. Awareness of complications as preterm delivery and low birth weight is important.

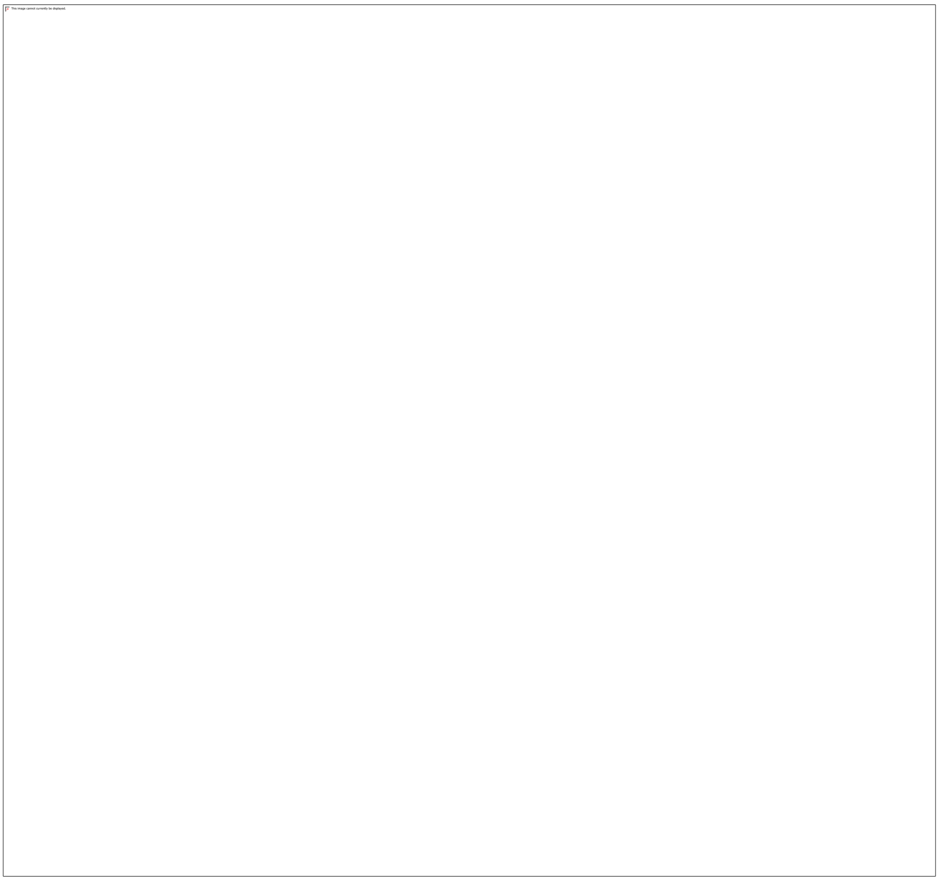

[Figure1: Distribution of birth weight expressed in customised percentile for gestational age (n=117)]

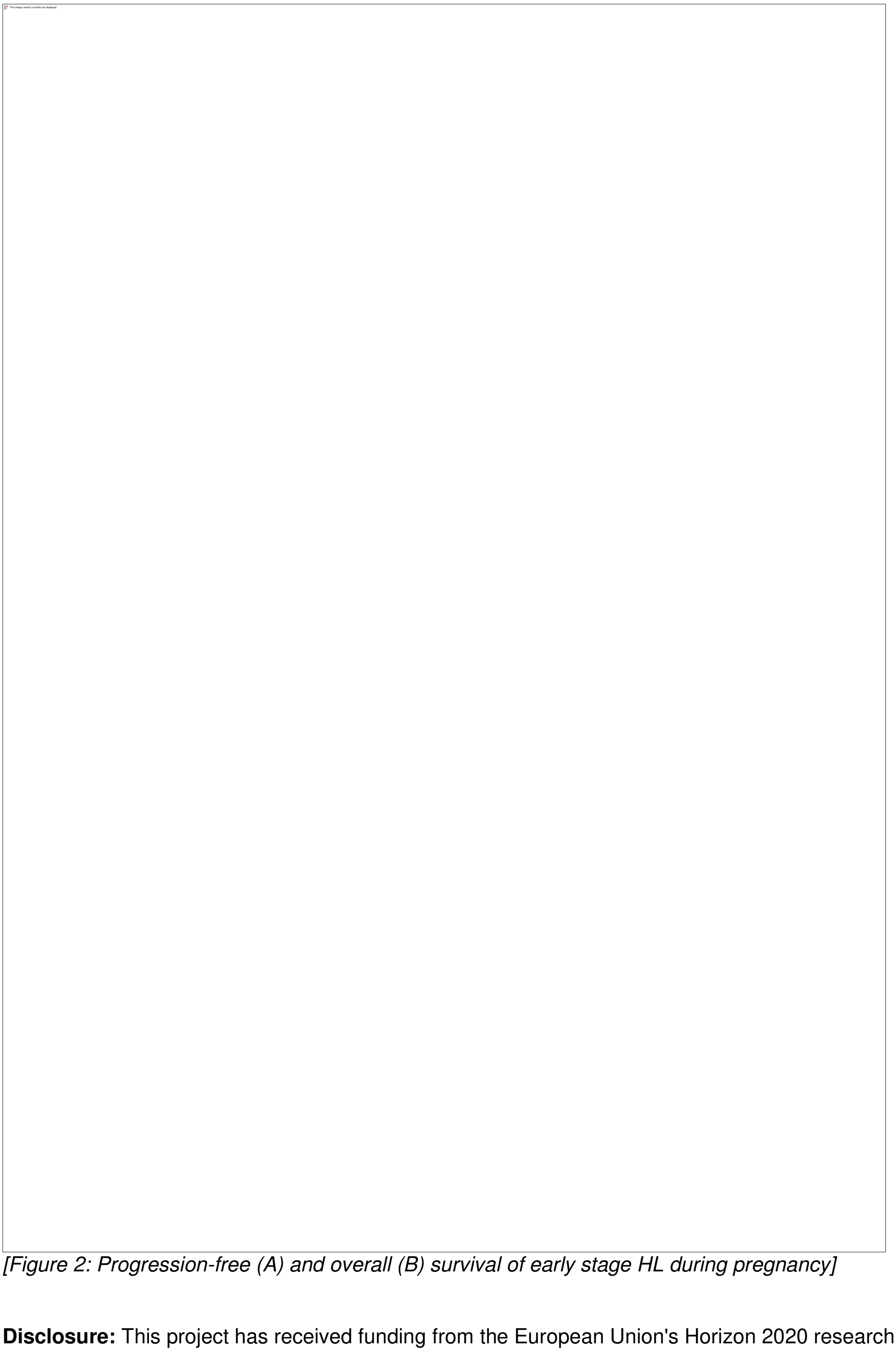

[Figure 2: Progression-free (A) and overall (B) survival of early stage HL during pregnancy]

Disclosure: This project has received funding from the European Union's Horizon 2020 research and innovation program under grant agreement No 647047. We are grateful to the Research Foundation-Flanders (FWO., grant no G070514N) and ESGO (European Society of Gynaecological Oncology) for their support. FA is senior clinical investigator of the F.W.O. MJH was supported by Charles University research project Progres Q28 and Q34 and by grant MH CZ - DRO ("Kralovske Vinohrady University Hospital - FNKV, 00064173"). The funding sources did not influence study design. There are no

conflicting interests to declare.

P96

Fertility outcome after fertility-sparing surgery of malignant ovarian germ cell tumour

J. Bouyou, E. Bentivegna, A. Maulard, P. Pautier, C. Genestie, S. Gouy, P. Morice
Gustave Roussy, Villejuif, France

Introduction / Background: Malignant ovarian germ cell tumours (MOGCT) are the most frequent cancer among women below 20 years old. These rare ovarian tumours have a great sensitivity to platinum-based chemotherapy and nowadays the conservative surgical management is the standard protocol. The aim of this study is to analyse the fertility outcome after conservative treatment of women with malignant ovarian germ cell tumour.
Methodology: We included retrospectively childbearing women with MOGCT who underwent fertility-sparing surgery in our institution between 1995 and 2016
Results: Seventy patients with MOGCT met the inclusion criteria. The median age was 18 years old and 91,4% of the patients were nulliparous. The median follow-up duration was 8,8 years .
Most of patients have a Mixed Tumour (n=27, 38.6%) and FIGO stage was I in in 42 patients (60%), II in 5 patients (7%), III in 17 patients (23%), IV in 5 patients (7%).
Only five (7%) patients have neoadjuvant chemotherapy and the majority of patients have adjuvant chemotherapy (n=59, 84%). We report 14 recurrences for 69 (20%).
We have lost of sight 12 patients after treatment for which we have no data about fertility. Twenty-one patients had no fertility desire after treatment in the study period. Thirty seven patients attempted pregnancy and 30 of them (81%) became pregnant. We report 55 pregnancies with 43 childbirths. The average time to obtain a pregnancy was 4.3 years after the diagnosis of a MOGCT. Three patients went through assisted reproductive technique, two had a successful pregnancy.
Conclusion: Patients treated for a MOGCT can expect to retain a subsequent fertility and become pregnant after fertility sparing surgery. The techniques for preserving fertility even though they are still rarely suggested nowadays in patients with MOGCT, could increase the amount of pregnancies for patients who wish for it.
Disclosure: Nothing to disclose

P97

Assessment of ovarian reserve and reproductive outcomes in BRCA1 or BRCA2 mutation carriers

S. Fernandez-Gonzalez[1], I. Calvo[1], M. Climent[1], J. Peñafiel[2], L. Feliubadaló[3], À. Teulé[3], C. Lázaro[3], J. Brunet[3], B. Candas[4], M. Duran[5], J. Ponce[1]
[1]Gynecology, Hospital Universitari de Bellvitge, [2]Biostatistics, [3]Hereditary Cancer Program, IDIBELL, [4]Clinical Laboratory, Hospital Universitari de Bellvitge, Hospitalet de Llobregat, Barcelona, Spain, [5]The Centre for Reproductive and Genetic Health, London, United Kingdom

Introduction / Background: The clinical impact on fertility in carriers of *BRCA1* and *BRCA2* mutations remains unclear. Our main objective was to assess the ovarian reserve as measured by circulating antimüllerian hormone (AMH) levels in *BRCA1* or *BRCA2* mutation carriers, as well as to investigate the impact of AMH levels on reproductive outcomes.
Methodology: Prospective observational cohort study carried out in Hospital Universitari de Bellvitge. The inclusion criteria were women aged between 18 and 45 years, proven carries of *BRCA1* or *BRCA2* gene mutations. Controls were healthy relatives that tested negative for the family mutation. Exclusion criteria were unilateral or bilateral adnexectomy, history of pelvic radiation therapy, cancer,

chemotherapy, and refusal to participate in the study. AMH measurements were performed in frozen stored samples collected between January 2005 and November 2009. Between March and June 2018, all patients were contacted by telephone to collect data on reproductive outcomes and epidemiological variables.
Results: A total of 135 women divided into three groups: *BRCA* negative (n = 66), *BRCA1* positive (n = 32), and *BRCA2* positive (n = 37). The mean age at AMH serum concentration measurement was 32 years in all groups. Local weighted regression (Loess) analysis showed lower AMH levels in *BRCA2*-positive women (figure 1). Epidemiological variables were assessed at a mean age of 41 years for each group (table 1). Statistically significant differences among groups in parity, infertility, or use of assisted reproductive treatments were not found (table 2). In the multivariable analysis to assess risk factors for infertility (excluding male factor), age at diagnosis of infertility was the only variable independently associated with infertility (OR 1.22, 95% CI 1.11 to 1.36, *P* < 0.001) (figure 2).
Conclusion: *BRCA2* mutation carriers showed decreased AMH levels, although it does not appear to have clinical impact as reproductive outcome was similar among groups.

Variables	BRCA-negative (n = 66)	BRCA1-positive (n = 32)	BRCA2-positive (n = 37)	P value
Age at AMH analysis, years, mean ± SD	32.7 ± 7.3	32.6 ± 6.2	32.1 ± 6.2	0.909
Current age, years, mean ± SD	41.6 ± 7.6	41.4 ± 6.4	41.1 ± 6.4	0.923
Active smokers at AMH analysis	26 (39.4)	12 (37.5)	13 (35.1)	0.912
Body mass index, kg/m2, mean ± SD	22.3 ± 3.02	24 ± 5.1	22.6 ± 5.9	0.201
Serum AMH levels, ng/mL, mean ± SD	2.27 ± 2.03	3 ± 2.27	2.54 ± 2.07	0.280
Ovarian response				0.092
Low	12 (18.2)	6 (18.8)	7 (18.9)	
Normal	20 (30.3)	2 (6.25)	7 (18.9)	
High	34 (51.5)	24 (75)	23 (62.2)	

[Table 1. Age and clinical characteristics of the study groups]

Variables	BRCA-negative (n = 66)	BRCA1-positive (n = 32)	BRCA2-positive (n = 37)	P value
Age at menarche, years, mean ± SD	13.1 ± 1.6	12.1 ± 2.5	12.6 ± 1.7	0.145
Regular menses	50 (75.8)	24 (75)	30 (81.1)	0.787
Childbirths, median (range)	1 (0-4)	1 (0-3)	2 (0-3)	0.854
Spontaneous abortion, median (range)	0 (0-2)	0 (0-2)	0 (0-3)	0.062
Nulliparous	20 (30.3)	7 (21.9)	13 (35.1)	0.478
Age at first birth, mean ± SD	29.0 ± 7.8	30 ± 7	31 ± 7.2	
Age at last birth, mean ± SD	31.4 ± 6.8	32 ± 6.5	32 ± 6.2	
Infertility	9 (18.7)	6 (22.2)	8 (30.8)	0.282
Infertility treatment	7 (14.6)	3 (11.1)	5 (19.2)	

[Table 2. Gynecological and reproductive outcomes according to BRCA status]

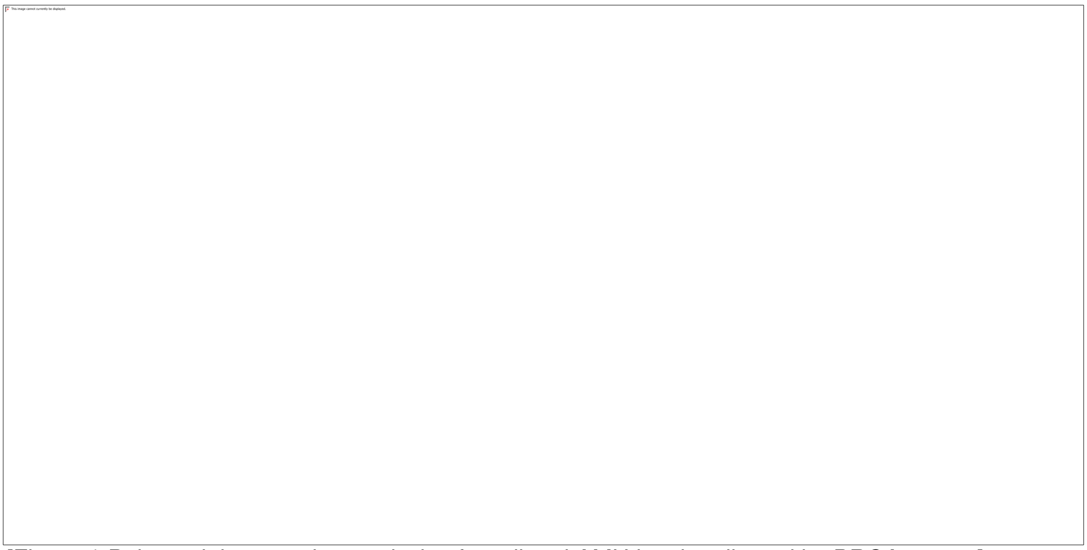

[Figure 1.Polynomial regression analysis of predicted AMH levels adjusted by BRCA status.]

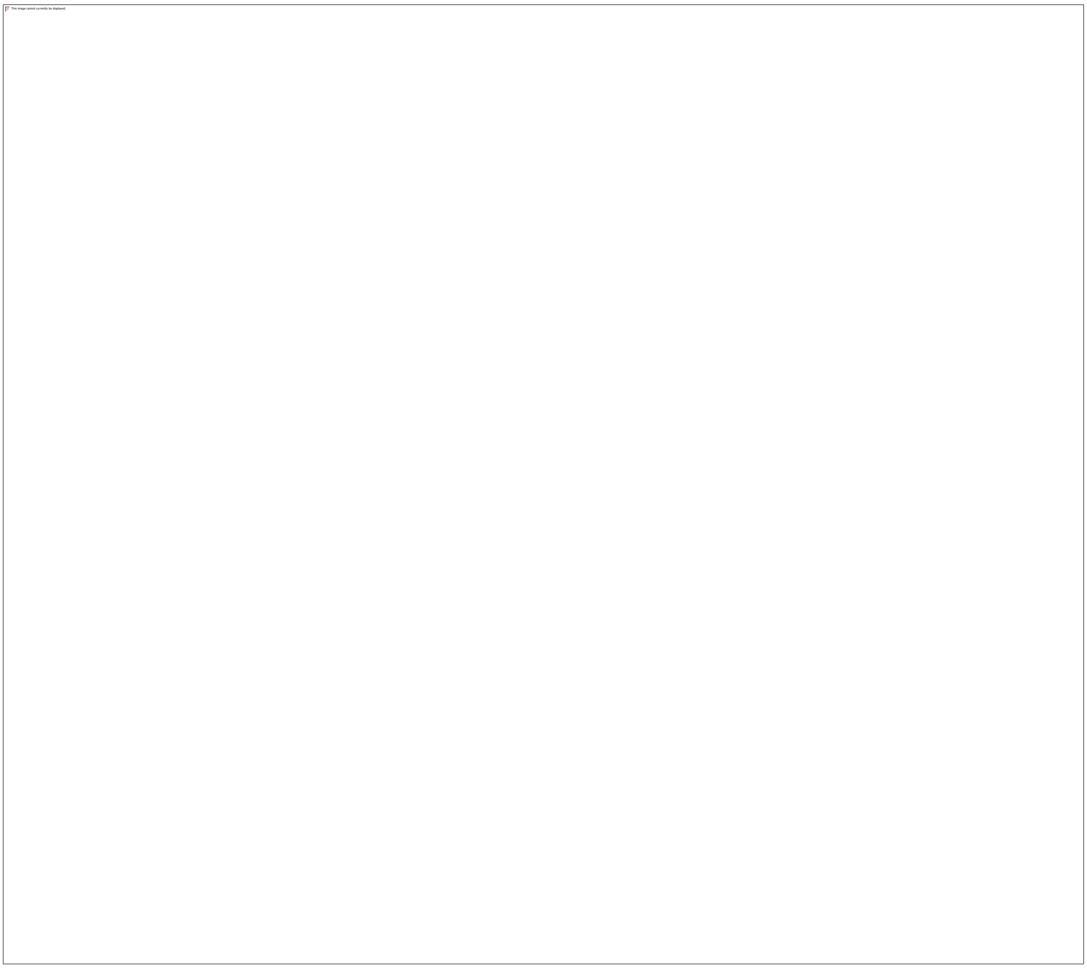

[Figure 2.Clinical characteristics among infertility (male factor excluded).]

Disclosure: Nothing to disclose

P99

Recurrent melanoma after pregnancy and assisted reproductive technology

J. de Haan[1,2], C.J.M. de Groot[1], A. Bouwman[3], M.B. Crijns[4], A.C.J. van Akkooi[5], F. Amant[2,3], C.A.R. Lok[3]

[1]Obstetrics and Gynaecology, Amsterdam University Medical Centers, Amsterdam, The Netherlands, [2]Oncology, KU Leuven, Leuven, Belgium, [3]Department of Gynaecologic Oncology, [4]Department of Dermatology, [5]Department of Surgical Oncology, Center for Gynaecologic Oncology Amsterdam, Location Antoni van Leeuwenhoek, Amsterdam, The Netherlands

Introduction / Background: Melanoma is one of the most common cancers in fertile women. The effect of pregnancy and assisted reproductive technology (ART) on the recurrence of melanoma is still unclear. The aim of this study is to evaluate the incidence of recurrent melanoma in pregnancy after spontaneous conception or after using ART.

Methodology: This study was a single centre questionnaire study among fertile women with a history of invasive melanoma. Questions concerned general health, primary tumour characteristics,

development of recurrent melanoma, use of ART and occurrence of pregnancies.
Results: A total of 354 (49%) questionnaires were available for analyses, 309 from living women and 45 of relatives of diseased women. The majority of women (n=218, 63%) had stage I primary disease. In total, 135 women with a history of melanoma (38%) became pregnant after their melanoma diagnosis. Only 17 women (5%) conceived after using ART. There was no difference in recurrence rate between women who had ever been pregnant (before or after the diagnosis of melanoma) and those who had never been (37% vs 35%, p=0.775). In the small group of women who conceived after ART, recurrency rate was 30% (n= 5).
Conclusion: Pregnancy did not increase the risk of recurrent melanoma. The group of women conceiving after ART was too small to assess a relation. Registration of these women, their oncologic follow-up and possible fertility treatments, will provide more information on the relations between ART and recurrent melanoma in the future which is of importance for preconceptional counselling.
Disclosure: All authors declare that they have no conflict of interest. F. Amant is senior researcher for the Flemish Research Fund (F.W.O.), the other authors did not receive funding for this research project.

P100

Long term obstetric outcomes after less radical fertility sparing treatment in patiens with small volume stage I cervical cancer

L. Rob[1,2], H. Robova[3], M.J. Halaska[3], M. Hruda[3], J. Drozenova[4], T. Pichlik[3], M. Pluta[5]
[1]Gynecology and Obstetrics, Charles University, 3rd Medical Faculty, University Hospital Kralovske Vinohrady, [2]Oncogynecology Center FNKV, Fakultni Hospital Kralovske Vinohrady, [3]Gynecology and Obstetrics, [4]Pathology, 3rd Medical Faculty Charles University and Faculty Hospital Kralovske Vinohrady, [5]Gynecology and Obstetrics, 2nd Medical Faculty, Charles University, FNM, Prague, Czech Republic

Introduction / Background: The purpose of the study is to present our clinical experience, pregnancy management and long term obstetrical outcome after less radical fertility sparing surgery SLNM + laparoscopic lymphadenectomy + simple trachelectomy or large recone.
Methodology: From 1999 - 2018, 91 women with squamous or adenocarcinoma with tumor less than 20 mm in the largest diameter and infiltration less than half of cervical stroma underwent laparoscopic SLNI and select extirpation of afferent parametrial channel and frozen section (FS) of SLN and laparoscopic pelvic lymphadenectomy or only SLN. Second step is simple trachelectomy without cerclage or large recone.
Results: Fertility was definitely spared in 76 women. 3 women (3.3%) not plan pregnancy, 9 currently plan pregnancy in future (9.9%), 64 women (84.2%) wish to be pregnant and 53 was pregnant (82.8%). From 76 fertility spared, 53 was pregnant - pregnancy rate 69.7% , 44 women have 50 baby - delivery rate was 57.9%. 37 was term pregnancy (74%), extreme premature 24-27w - 2 baby (4%), premature (28-32w) - 2 baby (4%), premature (33-37w) - 9 baby (18%). 22 unsuccessful pregnancy (5 artericial abortion, 11 spontaneus abortion in 1 trimestr, 4 spontaneus abortion in 2 trimestr, 2 GEU. Two women after hysterectomy have two baby with donor mother.
Conclusion: SLNM and laparoscopic pelvic lymphadenectomy with simple trachelectomy or large recone in small volume cervical cancer (less than 2cm in the largest diameter and less than half of stromal invasion) have excellent pregnancy result.
This work was supported by Charles University research program PROGRES Q 28 (Oncology).
Disclosure: Nothing to disclose

P1234

Impact of hysterectomy after chemoradiation therapy for locally advanced cervical cancer

L. Ouldamer[1], C. Faussat[2], S. Bendifallah[3], C. Touboul[4], P. Collinet[5], C. Coutant[6], C. Akladios[7], V. Lavoué[8], P.A. Bolze[9], C. Huchon[10], A. Bricou[11], G. Canlorbe[12], E. Raimond[13], E. Darai[14], A. Caille[2], G. Body[15], Francogyn Study Group
[1]CHU Tours | Gyn Oncology, CHU Bretonneau, [2]Université de Tours, Tours, [3]Hôpital de Tenon, Paris, [4]CHIC, Creteil, [5]Chu de Lille, Lille, [6]CAC Dijon, Dijon, [7]Chu de Strasbourg, Strasbourg, [8]CHU de Rennes, Rennes, [9]Chu de Lyon, Lyon, [10]Chu Poissy, Poissy, [11]Chu Jean Verdier, Bobigny, [12]La Pitié Salpétrière Hospital, Paris, [13]Chu de Reims, Reims, [14]Gynecology, Tenon University Hospital, Assistance Publique des Hôpitaux de Paris (AP-HP), University Pierre and Marie Curie, Paris 6, Institut Universitaire de Cancérologie (IUC), Paris, [15]Chu de Tours, Tours, France

Introduction / Background: Standard of care worldwide of locally advanced cancer tend to be non surgical (chemoradiation therapy) with no certainty if this attitude improves survival or not. Therefore, it is important to assess the value of hysterectomy in addition to chemoradiation therapy in this context. The sole randomized controlled trial (Morice 2012) designed to answer a similar question was closed early due to poor accrual and included 61 women, reported no difference in overall and recurrence-free survival between chemoradiation therapy and hysterectomy versus chemoradiotherapy alone
Methodology: Data from 1,963 patients with cervical cancer were collected retrospectively from1 January 2000 to 31 December 2016, from the FRANCOGYN database, pooling data from 12 centres. Survival was determined using the Kaplan-Meier method. Univariate and multivariate analyses were performed to define prognostic factors of survival. Patients included had IB2 to IIB FIGO stage.
Results: 739 were included with a mean age of 53.5 years (±13.5). 634 women had available data of imaging after chemoradiotherapy. Management according to the presence of residue on imaging is presented on figure 1. 376 women had hysterectomy.
There were significant differences for the 5-year Overall Survival (OS) rates between women who underwent hysterectomy and women who did not ($p< 0.0001$). Salvage hysterectomy was an independent negative predictive factor of OS (HR=0.27 (95%CI, 0.16-0.45), $p< 0.0001$)
There were also significant differences for the 5-year Disease-Free Survival (DFS) rates between the two groups ($p< 0.0001$). Salvage hysterectomy was an independent negative predictive factor of DFS (HR=0.45 (95%CI, 0.31-0.66), $p< 0.0001$).
Conclusion: A potential positive effect of hysterectomy after chemoradiation therapy for stage IB2 to IIB cervical cancer should be considered.

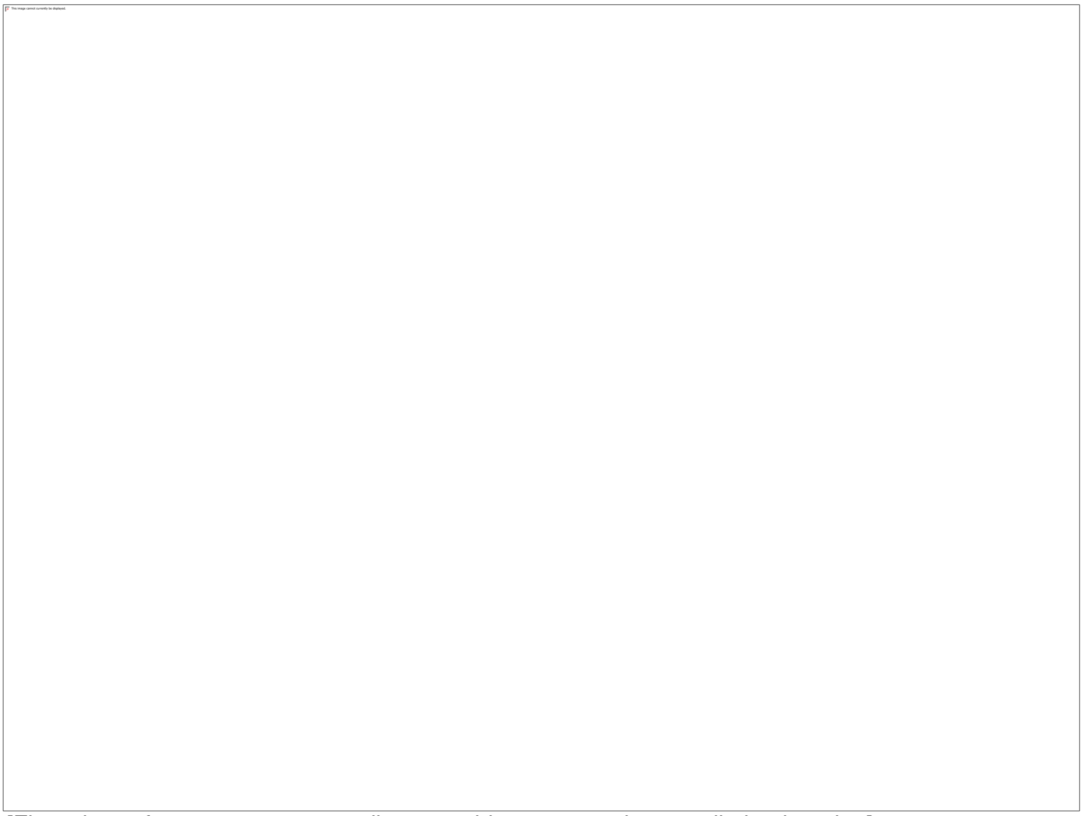

[Flow chart of management according to residue on post chemoradiation imaging]

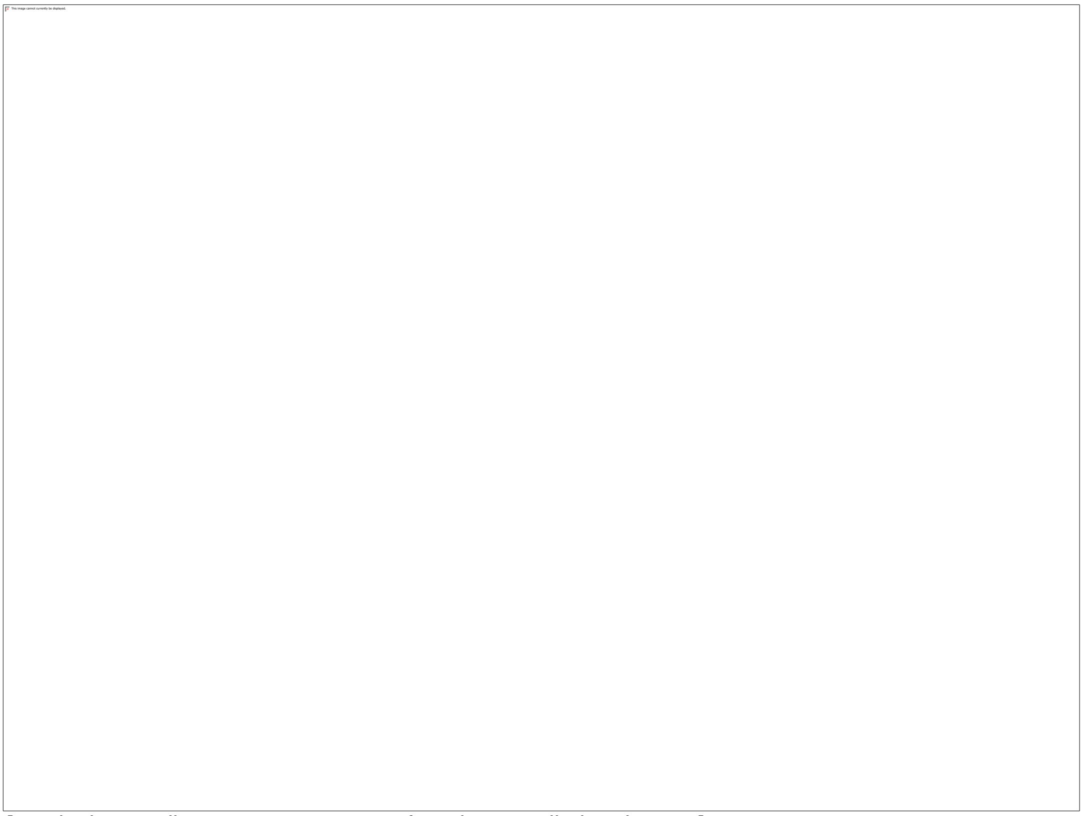

[survival according to surgery or not after chemoradiation therapy]

Disclosure: Nothing to disclose

P1235

Immunologic impact of chemoradiation in cervical cancer and how immune cell infiltration could lead towards personalized treatment

L. Lippens[1], M. Van Bockstal[2], E.A. De Jaeghere[3], P. Tummers[4], A. Makar[4], S. De Geyter[1], K. Van de Vijver[2], A. Hendrix[1], K. Vandecasteele[5], H. Denys[3]
[1]Laboratory of Experimental Cancer Research, Department of Human Structure and Repair, [2]Pathology, Department of Diagnostic Sciences, [3]Medical Oncology, Department of Internal Medicine and Pediatrics, [4]Gynecology, Department of Human Structure and Repair, [5]Radiation Therapy, Department of Human Structure and Repair, Ghent University Hospital, Ghent, Belgium

Introduction / Background: We investigated the potential of tumour-infiltrating immune cells in pre- and post-treatment tissue specimens and their changes during treatment as predictive or prognostic biomarkers for cervical cancer patients.
Methodology: Cervical cancer patients treated with (chemo)radiotherapy and subsequent surgery were included in the current study. Pre- and post-treatment specimens were retrospectively analysed via immunohistochemistry. Immune cell markers for T cells (CD3, CD4, CD8, and FoxP3), macrophages (CD68 and CD163), and B cells (CD20), as well as interleukin-33 (IL33) and (programmed death-ligand 1 (PD-L1), were analysed. Patients were grouped in the low score or high score group based on the amount of positive cells on immunohistochemistry. Correlations to pathological complete response (pCR), cause-specific survival (CSS), and metastasis development during follow-up were evaluated.

Results: In total, 38 patients were included. In analysis of pre-treatment biopsies, significantly more pCR was seen for patients with CD8=CD3, CD8≥CD4, positive IL33 tumour cell (TC) scores, IL33 immune cell (IC)< TC, and PD-L1 TC≥5%. Besides patients with high CD8 scores, also patients with CD8≥CD4, CD163≥CD68, or PD-L1 IC≥5% had better CSS. In analysis of post-treatment specimens, less pCR was observed for patients with high CD8 or CD163 scores. Patients with decreasing CD8 or CD163 scores between pre- and post-treatment samples showed more pCR, whereas those with increasing CD8 or decreasing IL33 IC scores showed a worse CSS. Meanwhile, patients with an increasing CD3 score or stable/increasing PD-L1 IC score showed more metastasis during follow-up (Figure 1).
Conclusion: The intratumoural immune cell landscape is a tool for prediction of outcome and response to (chemo)radiation.

[Figure 1. Response, metastasis development, survival, and scores for each patient.]

Disclosure: This research was supported by a research grant obtained from Roche. E.A.D. received funding from Fonds Wetenschappelijk Onderzoek (FWO) and K.V. received funding from Stichting tegen Kanker.

P1236

Genomic examination of endometrial precancers: serial analysis and diagnostic utility

D. Castrillon[1], H. Zhang[2], M. Aguilar[1], J. Lea[3], D. Miller[3]
[1]Pathology, [2]Population and Data Sciences, [3]Gynecologic Oncology, UT Southwestern, Dallas, TX, United States

Introduction / Background: Endometrial precancer (endometrioid intraepithelial neoplasia/EIN) diagnosis remains contentious, with variable criteria and frequent disagreements among gynecologic pathologists, potentially leading to under/overtreatment. The endometrium is unique in that diagnostic biopsies do not extirpate neoplastic lesions. Our purpose was to 1) characterize serial genomic alterations along the pre-EIN/EIN/cancer continuum in individual women; 2) determine if genomic analysis adds diagnostic value to histopathological review.
Methodology: Inclusion criteria were 1) endometrial cancer diagnosis/hysterectomy 2) preceding serial endometrial biopsies (typically over many years) including for some patients an early biopsy before an EIN diagnosis. A comprehensive panel was designed for genes recurrently mutated in

endometrial cancer, including hereditary cancer loci. Formalin-fixed/paraffin-embedded specimens for each cancer, preceding samples, and matched germline DNA were subjected to barcoded massively-parallel sequencing to identify mutations, hereditary and acquired, and track their origin and allelic frequency progression. 147 samples from 29 women (5.1 samples/patient) were analyzed.
Results: In all but two patients mutations in the cancer were detectable in preceding EIN(s). Notably, ≥1 mutations were detectable in 15/19 patients where a pre-EIN biopsy was available. Serial analysis provided unique insights into the progression of various endometrial cancers. In most cases, the presence of ≥1 mutation could be confirmed by immunohistochemistry, providing unique views of histologic correlates. Class-defining mutations (e.g. POLE) were identified in several cases. Germline mutations were identified in patients with known cancer predisposition syndromes. Mutations were not identified in 15 age-matched control (normal) endometria, arguing that with appropriate thresholds, age-related endometrial mutations should not confound analyses.
Conclusion: Genomic analysis of selected endometrial biopsies has the potential to be a cost-effective and valuable diagnostic adjunct to histology, also replacing in a single assay diverse screening methods to identify cancer predisposition syndromes. Though of considerable promise, further investigations will be required to establish utility and refine diagnostic criteria.

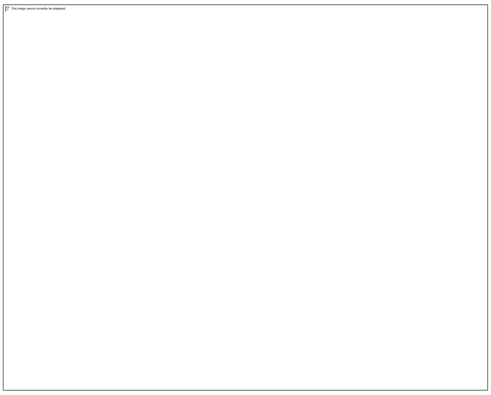

[Allelic frequencies for mutations in biopsies up to 8 years prior to cancer dx (single patient).]

Disclosure: The authors have no conflicts of interest to disclose. Funding to DHC was provided by the National Institutes of Health/National Cancer Institute (USA), the Cancer Prevention Research Institute of Texas (State of Texas) and the UT Southwestern Stembridge Distinguished Professorship.

Poster exhibition Day 2

P101

High-grade and low-grade endometrial stromal sarcoma: descriptive and survival analysis

V. Bebia, Ú. Acosta-Sánchez, S. Monreal-Clua, L. Mañalich-Barrachina, M. Carbonell-Socias, A. Pérez-Benavente, A. Gil-Moreno, S. Cabrera-Díaz
Department of Gynecology - Gynecologic Oncology Unit, Vall d'Hebron University Hospital, Barcelona, Spain

Introduction / Background: Endometrial Stromal Sarcoma (ESS), both Low-Grade (LG-ESS) or High-Grade (HG-ESS), are rare uterine tumours, accounting for less than 1% of all uterine neoplasms. Our aim was to describe a single centre experience and to evaluate which variables affected survival in our cohort.
Methodology: All patients with a histopathological diagnosis of LG-ESS or HG-ESS treated at our centre from January 1995 until February 2018 were included in the study. Data were collected from electronic medical records. Survival analysis was done using Kaplan Meier method and log-rank test

for univariate analysis, and Cox proportional hazards method for multivariate analysis.
Results: A total of 44 patients were included, of which 27 (61.4%) had a diagnosis of LG-ESS and 17 (38.6%) HG-ESS. Median age at diagnosis was 57.5 years (interquartile range, IQR: 47.5-76.5). Most women presented vaginal bleeding as initial symptom (54.8%), were surgically treated (93.2%), and underwent laparotomy (73.2%). Pelvic lymphadenectomy was performed in most patients (56.1%). More than one-half of the patients were diagnosed at initial stages of the disease (Stage I 53.7%), while most patients recurred after surgical treatment (51.2%). HG-ESS patients were older (median age 64.5 vs 51.5 years, p=0.040), presented bigger tumours (Maximum tumour diameter 115 vs 38 mm, p=0.014), and recurred more frequently (81.3% vs 32.0%, p=0.002) than LG-ESS patients.
The median follow-up of the global cohort was 8.15 years (IQR: 1.47-12.85). 5-year overall survival (OS) for the entire cohort was 59.9%, and was significantly better for LG-ESS patients (78.6% vs 31.3%, p=0.001). For LG-ESS patients, only stage remained significantly correlated with survival after multivariant analysis, while for HG-ESS patients, optimal surgery, stage, adjuvant chemotherapy and radiotherapy remained significantly correlated with survival.
Conclusion: HG-ESS patients have worse prognosis than those affected by LG-ESS; proper surgical management and adjuvant therapies are key in order to achieve better survival in this group.

[Overall survival (OS) comparison by groups]

	**General**	**LG-ESS**	**HG-ESS**	**p-value**
**Surgical procedures**				
Pelvic lymphadenectomy	23 (56.1%)	9 (56.3%)	14 (56.0%)	0.987
Paraaortic lymphadenectomy	8 (19.5%)	5 (20.0%)	3 (18.8%)	1.000
Cytoreductive surgery	16 (39.0%)	7 (28.0%)	9 (56.2%)	0.070

**Tumour max diameter (mm) [median, IQR]**	90 (35.5-130)	38 (30-100)	115 (90-160)	0.003
**Stage I**	22 (53.7%)	16 (64.0%)	6 (37.5%)	
**Stage II**	5 (12.2%)	4 (16.0%)	1 (6.3%)	
**Stage III-IV**	10 (24.4%)	4 (16.0%)	6 (37.5%)	0.099
**Recurrence**	21 (51.2%)	8 (32.0%)	13 (81.3%)	0.002

[Tumour and treatment variables]

	**General**	**LG-ESS**	**HG-ESS**	**p-value**
**Age (years) [median, IQR]**	57.5 (47.5-76.5)	51.5 (45.5-64.5)	64.5 (59.5-74.5)	0.040
**Parity**				
Nulliparous	5 (11.9%)	2 (7.7%)	3 (18.8%)	
Primiparous	6 (14.3%)	5 (19.2%)	1 (6.25%)	
Multiparous	30 (71.4%)	19 (73.1%)	11 (68.8%)	0.276
**Diagnosis as a result of previous surgery**	17 (40.5%)	14 (53.9%)	3 (18.8%)	0.024

[Demographic and clinical variables]

Disclosure: Nothing to disclose

P102

Intraoperative cultures in the prediction of surgical site infection in open gynecological surgery

R. Sainz de la Cuesta[1], S. Iacoponi[1], R. Mohedano[2]
[1]Gynecology, Hospital Universitario Quiron Madrid, [2]Faculty of Biomedical Sciences and Health, Universidad Europea de Madrid, Madrid, Spain

Introduction / Background: Intraoperative subcutaneous wound cultures before skin closure may help early recognition and prevention of surgical site infection in open gynecological procedures. The objective was analyze the relationship between intraoperative skin and subcutaneous cultures and the development of surgical site infection (SSI) in women undergoing laparotomy for gynecological surgery.
Methodology: Women who underwent elective laparotomy between January 2012 and December 2017 at our hospital, were included in the study. Skin and subcutaneous samples were taken just prior to skin closure. Patients' demographics, underlying co-morbidities, surgical variables, intraoperative cultures results and 30-days postoperative SSI´s were collected. Univariate and multivariate analyses (logistic regression models) were conducted to explore the association of the studied variables with SSI´s after laparotomy and with positive intraoperative cultures.
Results: A total of 343 patients were included in our study, of which 25 (7.3 %) developed SSI, including 15 (60%) superficial and 10 (40%) organ-space. At univariate analysis, contaminated surgery (P=0.004), length of surgery (P=0.049), transfusions (P=0.006), intra-abdominal drains (P=0.01), days with drainage (P=0.047) and length of hospitalization (P=0.04) were associated with SSI occurrence. Of the 264 women who had skin and subcutaneous cultures obtained, 20 (7.6%) developed SSI. Skin and subcutaneous cultures were positive in 23 (9.2%) and 20 (7.1%) patients, respectively. The most frequently isolated bacteria were intestinal microbiota. SSI occurred in 35% (7/20) of women with

positive subcutaneous cultures versus 4.9% (13/264) with negative culture (*P* < 0.0001); and in 20% (5/25) of those with positive skin cultures versus 5.8% (15/259) with negative culture (*P*< 0.0001). Using multivariate analysis, the only independent risk factor for SSI was a positive subcutaneous culture (OR 6.3; 95% CI 1.6-24.8; *P*=0.009).
Conclusion: Intraoperative identification of potential infectious pathogens with subcutaneous wound cultures before skin closure may help early recognition and prevention of SSI in open gynecological procedures.
Disclosure: Nothing to disclose

P103

Feasibility of post-operative Stereotactic Ablative Radiotherapy (SABR) using cyberknife for positive margins after pelvic exenteration for gynaecological malignancy

G. Imseeh[1,2], D.P.J. Barton[1], M. Llewelyn[1,2], J. Ash[1], A. Fernandes[1], A. Taylor[1,2]
[1]Department of Gynaecological Oncology, The Royal Marsden NHS Foundation Trust, [2]Department of Imaging and Radiotherapy, The Institute of Cancer Research, London, United Kingdom

Introduction / Background: Surgical margin status is a strong prognostic indicator of disease free and overall survival following pelvic exenteration (PE) for recurrent gynaecological malignancy. There have been few attempts at post-operative treatment to improve this and intraoperative radiotherapy was associated with high neurotoxicity. We report our preliminary experience of re-irradiation using stereotactic radiotherapy (SABR) to the positive surgical margin using Cyberknife™.
Methodology: This is a retrospective evaluation of four patients treated at the Royal Marsden Hospital with SABR to the positive margin after pelvic exenteration. Radio-opaque fiducial markers at the positive margin were placed post-operatively in three patients and intra-operatively in one. The Cyberknife system was used for planning and treatment delivery (**figure 1**).
Patients were followed up at 3 monthly intervals and underwent a clinical examination, documentation of acute and late toxicity, and imaging. Patients also completed quality of life questionnaires including ED-5D-3L and Brief Pain Inventory.

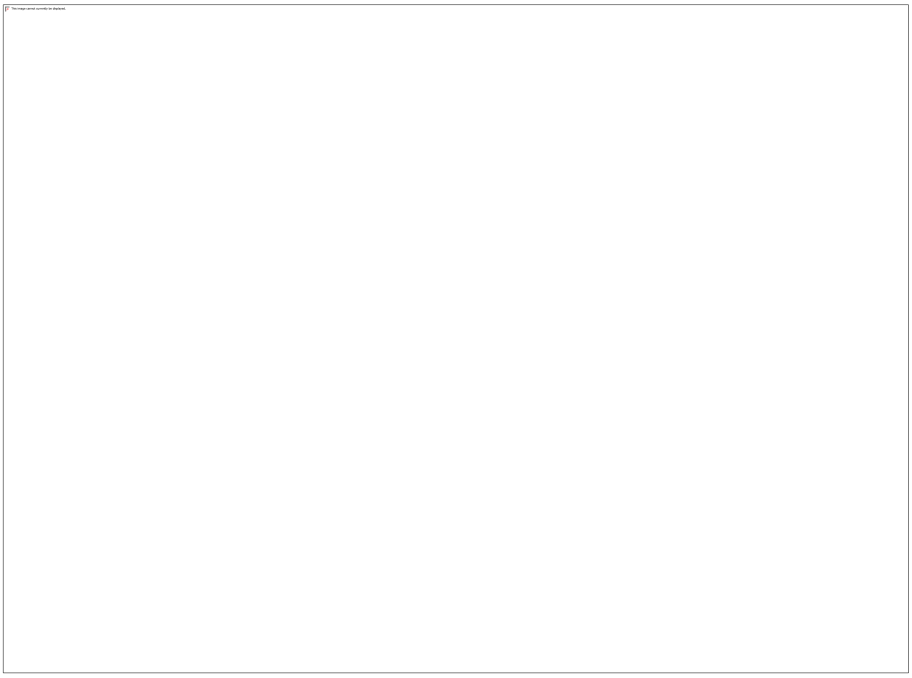

[Figure 1]

Results: Median age was 56 years (30 - 72) and median follow up was 14.3 months. Two patients had an endometrial endometrioid primary, one patient had adenoid cystic carcinoma of the bartholin gland and one had glassy cell carcinoma of the cervix. Three patients had total PE and one had anterior PE. Radiotherapy dose was 30 Gy in 5 Fractions over two weeks prescribed to 70-80% isodose.
There were no Grade 3 or 4 acute or late toxicities related to SABR. One patient died of multifocal recurrence, one is alive with pelvic recurrence and two are alive with no disease. There were no recurrences within the radiotherapy field.
Conclusion: SABR offers an attractive option for complete non-surgical ablation after histopathological assessment of surgical margins. Our early experience shows SABR using Cyberknife to the positive margin after pelvic exenteration is feasible and well tolerated. Work is on-going to determine patient selection criteria and to study efficacy.
Disclosure: Nothing to disclose

P104

Prognostic factors in a large series of patients with stage II and III serous ovarian borderline tumors

S. Maria[1], M. Farron[2], A. Maulard[3], C. Genestie[4], P. Pautier[4], E. Bentivegna[3], A. Leary[5], C. Chargari[2], S. Gouy[2], P. Morice[3]
[1]Gynecologic Surgery, [2]Gustave Roussy, [3]Surgery, [4]Pathology, [5]Medical Oncology, Gustave Roussy, Villejuif, France

Introduction / Background: The aim of this study was to assess prognostic factors in patients with stage II or III serous borderline ovarian tumors (SBOT) after a long term follow up in a large series.
Methodology: Patients with SBOT and peritoneal implants treated or referred to our institution were retrospectively reviewed. All specimens (ovary and peritoneal implants) were reviewed by our expert pathologist.
Results: Between 1971 and 2017, 212 patients were identified. Thirty-three (16%) patients had invasive implants. Sixty-eight patients underwent a conservative (fertility sparing) surgery. After a median follow up of 115 (range 12-512) months, 70 recurrences (33%) were observed (range 4-271 months), 28 (40%) of them under the form of invasive disease. Disease-free survival (DFS) at 5 and 10 years were 73% and 62% respectively. Invasive implants (vs non-invasive) (HR=5.37[1.29-22.26], p=.013), and 3 or more peritoneal sites with implants (vs < 3) (HR=3.56[1.11-11.39], p=.024) were identified as predictors of recurrence in the form of invasive disease.
But invasive implants were not associated with DFS (HR=1.39[0.77-2.51], p=.27) nor with Overall Survival (OS) (HR= 1.76[0.57-5.47], p=.32).
The presence of implants in more than 3 peritoneal sites was the only factor associated with both OS and DFS (p=.049 and p=.045). Conservative surgery was associated with DFS (HR=5.5[3.33-9.08], p< .0001) but not with OS (HR=0.94[0.26-3.41], p=.93). Others factors studied: stage (II vs III), residual disease after surgery, presence of micropapillary patterns, nodal surgery and the use of adjuvant treatment had no impact on OS or PFS.
Conclusion: This study demonstrates that after a long term follow up, the types of peritoneal implants is no longer a prognostic factor on OS.
Presence of implants on at least three peritoneal sites seems to impact significantly the risk of recurrence under the form of invasive disease and OS requiring then a specific follow-up in these patients.
Disclosure: Nothing to disclose

P105

Pelvic exenteration in gynecologic cancer: complications and oncological outcome

T. Marina Martín, V. Lago Leal, P. Padilla Iserte, L. Matute Tobías, M. Gurrea Soteras, T. Viñas Alburquerque, S. Domingo del Pozo
Gynecologic Oncology, University Hospital La Fe, Valencia, Spain

Introduction / Background: Pelvic exenteration (PE) is indicated in cases of unresponsive, recurrent pelvic cancer or for palliative intent. Despite the fact that the surgery is associated with a high rate of morbidity, it is currently the only real option that can effect a cure.
Methodology: Patients who underwent PE between January 2011 and April 2019 in our centre were retrospectively reviewed. Data related to surgery, complications and outcomes were recorded.
Results: Thirty-five patients were included. PE was performed due to recurrent gynaecological cancer, persistence of disease and after first diagnosis in 29 (82,9%), 4 (11,4%) and 2 (5,7%) patients, respectively. Total PE was performed in 23 cases (65,7%), followed by anterior PE in 7 cases (20%) and posterior PE in 5 cases (14,3%). Early grade II, III, IV and V complications occurred in 21 (60%), 9 (25,7%), 4 (11,4%) and 1 (2,9%) patients, respectively. One patient died within the first 30 postoperative days due to surgery-related complications. Medium-late grade II, III, IV and V complications occurred in 20 (57,1%), 16 (45,7%), 5 (14,3%) and 3 cases (8,6%), respectively. Three patients died after > 30-day period from surgery-related complications. The overall survival (OS) and disease-free survival (DFS) at 48 months after PE was 39.8% and 28.7% respectivel.
Conclusion: PE provides about a 40% 4-year survival chance in a selected group of patients. The early-complications rate and 30-day mortality were acceptable. Nevertheless, the medium-late complication grades II-V were 57, 46, 14 and 9%, respectively. We must focus on identifying those patients who could potentially benefit most from P.
Disclosure: Nothing to disclose

P106

Incidence and outcome of occult uterine sarcoma: a multi-centre, retrospective study of 18604 operations performed for presumed uterine leiomyoma

A. Yorganci[1], M.M. Meydanli[2], N. Kadioglu[1], H. Sahin[2], F. Kayikcioglu[3], S. Taskin[4], L. Atasoy[5], M.A. Akgul[3], T. Kinay[3], O.L. Tapisiz[3], N. Haberal[6], O. Evliyaoglu[1], O. Moraloglu Tekin[3], F. Ortac[4], A. Ayhan[5]
[1]Obstetrics and Gynecology, [2]Gynecologic Oncology, Zekai Tahir Burak Women's Health Training and Research Hospital, Faculty of Medicine, University of Health Sciences, [3]Obstetrics and Gynecology, Etlik Zubeyde Hanim Women's Health Training and Research Hospital, Faculty of Medicine, University of Health Sciences, [4]Obstetrics and Gynecology, Ankara University School of Medicine, [5]Obstetrics and Gynecology, [6]Department of Pathology, Baskent University Faculty of Medicine, Ankara, Turkey

Introduction / Background: The objective of this retrospective, multi-centred study was to determine the incidence of occult uterine sarcoma in women operated for presumed uterine leiomyoma.
Methodology: We reviewed all myomectomy and hysterectomy operations performed with a pre-operative diagnosis of uterine leiomyoma at four tertiary centres, from January 2013 until December 2017, in Ankara, Turkey. The medical records of occult uterine sarcoma patients were analyzed to determine prognostic factors for disease-free survival (DFS) and overall survival (OS).
Results: A total 18604 women underwent hysterectomy or myomectomy during the study period. There were 56 cases of occult uterine sarcomas (51 uterine leiomyosarcomas, and five endometrial stromal sarcomas). The incidence of occult uterine sarcoma was 0.30% (56/18604) or approximately 1/332. The median DFS was 31.0 months (95% confidence interval [CI] 9.6-52.3 months). The 3-year and 5-year DFS rates were 44.1% and 34.0%, respectively. The 3-year and 5-year OS rates were 68.8% and 50.0%, respectively. For 5-year DFS, stage of the disease (stage IA vs. more advanced stages; p=0.007), rapidly growing myoma (p=0.01) and complete surgical staging at initial operation (p=0.02) were found to be statistically significant in univariate analysis. However, only stage of the disease (Hazard Ratio: 3.45, 95% Confidence Interval 1.13-10.49; p=0.02) remained as an

independent prognostic factor for decreased DFS in multivariate analysis.
Conclusion: Our results indicated that the incidence of occult uterine sarcoma was 0.3% (1/332) in women operated for presumed uterine leiomyoma. Early and complete surgical resection might offer better DFS for these patients.
Disclosure: Nothing to disclose

P107

Embryonal rhabdomyosarcoma of uterus in adult women: a systematic review and survival analysis of 116 patients

F.-C. Pop[1], C. Stanciu Pop[2], M. Moreau[3], C. Jungels[4], M. Chintinne[5], G.C. Artigas[6], D. Larsimont[5], I. Veys[1]
[1]Service of Surgery, Institut Jules Bordet, Université Libre de Bruxelles, Brussels, [2]Service of Pathology, CHU UCL Namur (Godinne Site), Namur, [3]Data Centre and Statistics Department, [4]Service of Medical Oncology, [5]Service of Pathology, [6]Service of Nuclear Medicine, Institut Jules Bordet, Université Libre de Bruxelles, Brussels, Belgium

Introduction / Background: Uterine embryonal rhabdomyosarcoma (uERMS) in adult women is a malignant entityof mesenchymal origin that very rarely occurs in adult age. Almost all information's about uERMS is coming by case reports, and probably remains an under-recognized neoplasm. The treatment is not codified, and recommended strategies are mostly based on treatments for RMS of paediatric population.
Methodology: A comprehensive database searches were performed for studies ofadult uERMS (> 19 years). Survival probabilities were estimated using Kaplan-Meier Method and potential risk factors for survival were tested using the the Cox model.
Results: 116 patients from 58 articles were included.Mean ages at diagnosis was 36.6 years. Forty-ninewomen were older than 40 years. In half of them, the most common presenting symptoms was vaginal bleeding only. The median tumor size was 5 cm(ranged 0.5-27 cm). Nearly 90% of patients were classified in the IRS Group-I. Surgery was the main therapeutic modality. With a median follow-up time of 24 months, the estimate overall survival (OS) and progression free survival (PFS) at 5 years was 81.4% and respectively 80.6%. In univariate analysis, age >36.6 years, deeply invasive tumors, and IRS Group >IA were associated with worst OS. In bivariate analysis the age higher than 36.6 years and IRSG >IA remained associated with death from uERMS. A trend towards for recurrence was seen in patients with age >36.6 years, deeply invasive tumors, and IRS Group >IA.
Conclusion: Uterine ERMS is a very rare disease among adult women. Multidisciplinary approach is essential taking into account that therapeutic management is still controversial. A relatively good 5-years OS and PFS is possible with a combination of surgery, radiotherapy, and multiagent chemotherapy, but a significantly risk of death exist in women with advanced age, with deep tumor invasion, and with IRS Group >IA.
Disclosure: Nothing to disclose

P108

Local- and distant relapse-free interval and overall survival after morcellation of uterine leiomyosarcomas (LMS)

D. Trojnarska, M. Zygmunt, G. Köhler
Department of Obstetrics and Gynaecology, University Medicine Greifswald, Greifswald, Germany

Introduction / Background: In many cases surgery of LMS is performed under the indication of presumed leiomyoma (LM) resulting frequently in morcellation. This study analysed it's influence on

prognosis.
Methodology: Investigations included: Kaplan-Meier analysis, age, tumour size, mitosis index, estrogen receptor and 5-year-overall survival prediction. In order to exclude the impact of primary extrauterine spread on prognosis, only LMS stage pT1ab were analysed.
Results: From 215 stage pT1ab LMS150 (69.8 %) were operated under the indication of LM. In summary, 89 (41.4 %) tumors were morcellated. In this group local-relapse-free interval (LFI) was significantly shorter than without morcellation with the highest difference between 2 to 7 years, which becomes irrelevant after 8 years. In contrast, patients with morcellation had a significantly longer distant-metastasis-free interval (MFI). The difference in MFI stayed over the whole-time range without becoming similar to the not morcellated tumours. Considering the latter and the results of the other investigated prognosis factors including the overall survival (OS) probability, the morcellated LMS have had a better prognosis primary. A joint analysis of MFI and LFI (fig. 1) showed no differences and the negative effect of morcellation is also after 8 years not relevant. In the same way OS (fig. 2) does not change after the morcellation. In conclusion, the primary better prognosis of morcellated LMS becomes identically with the worse prognosis of not morcellated tumours. Additionally, analysis of time from the first locoregional relapse or metastasis to death shows that women with primary local recurrence have a shorter OS.
Conclusion: Morcellated vs not morcellated LMS show a significant shorter LFI with significantly longer MFI. Combined LFI and MFI like OS are not affected. Due to morcellation the preoperatively better prognosis of morcellated LMS measured by other prognostic factors equals the worse prognosis to tumors without morcellation.

[Joint analysis of MFI and LFI]

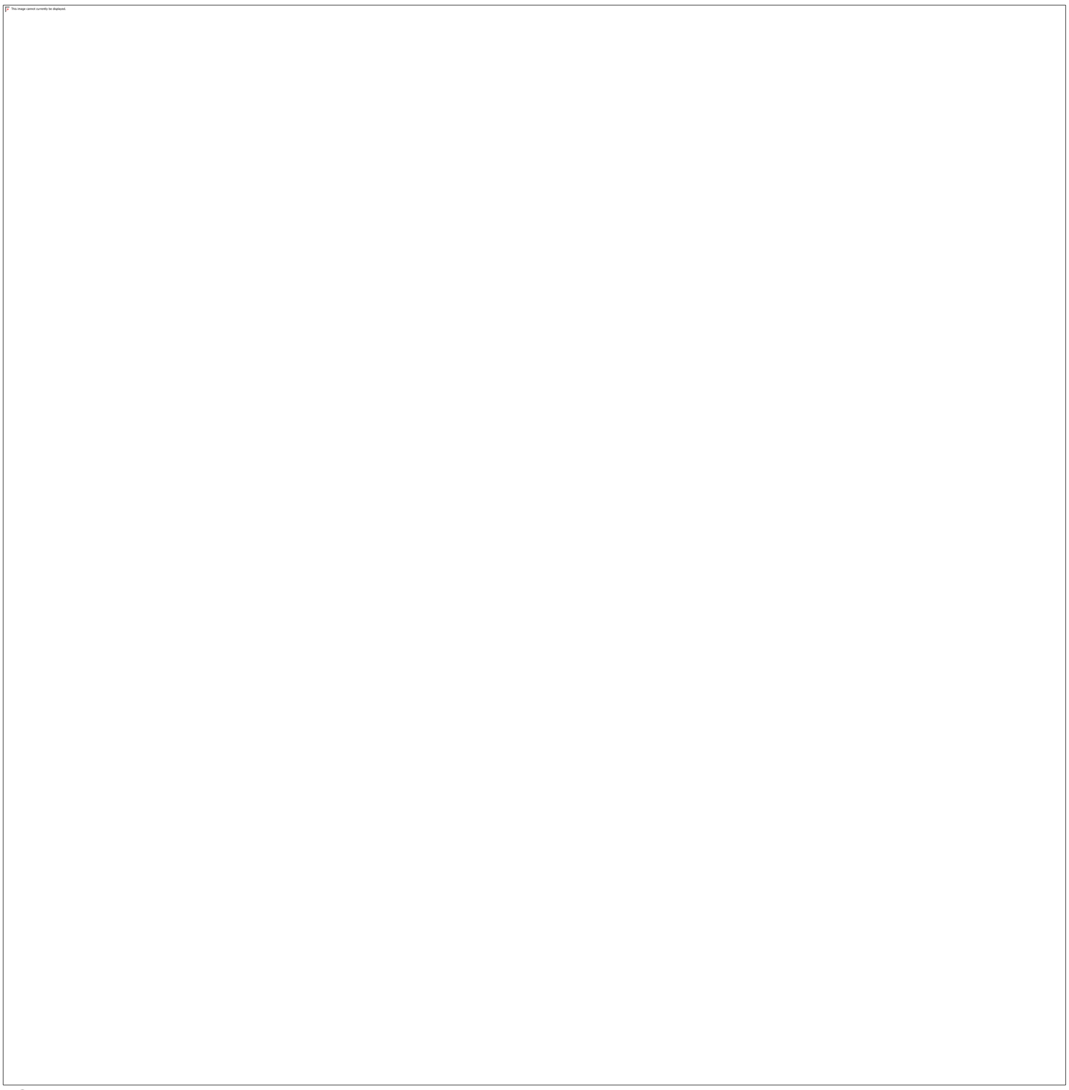

[Overall survival]

Disclosure: Nothing to disclose

P109

Comparison of different staging systems for high-grade uterine leiomyosarcoma

K.Y. Tse[1], H.Y.S. Ngan[1], P.P.C. Ip[2]
[1]Obstetrics and Gynaecology, [2]Pathology, The University of Hong Kong, Pokfulam, Hong Kong

Introduction / Background: The International Federation of Gynecology and Obstetrics (FIGO) had developed a staging system specific to each type of uterine sarcoma in 2009. American Joint

Committee on Cancer (AJCC) also has another staging system for soft tissue sarcoma. However there were limited data to validate these systems due to the rarity of the tumor. Even among uterine leiomyosarcoma (uLMS), the clinical behaviors differ between high-grade and low-grade disease.
Methodology: Patients with uLMS diagnosed in Jun 1993 - Jan 2014 were identified. All specimens were reviewed by gynecologic pathologists using the World Health Organization diagnostic criteria. Survival and recurrence status were recorded from the medical records.
Results: 142 high-grade uLMS patients had enough information for analysis. The median follow-up of the whole cohort was 36 months (inter-quartile range 13 - 77 months). In terms of progression-free survival (PFS) and overall survival (OS), both the 1988 FIGO and AJCC staging systems could classify patients into four distinct groups, while patients with stage 2 in the 2009 FIGO staging system had a worse prognosis compared to stage 3 (Table 1, Figure 1). And neither of the two FIGO staging systems could categorize the patients meaningfully according to their sub-stages (Table 2). In addition, patients are early stage, regardless of the staging systems, had a high recurrence rate up to 50% (Table 1).
Conclusion: None of the staging systems could accurately predict the patients' outcomes according to their stages. Further collaborative studies are needed in order to establish a better staging system to predict the prognosis and guide the adjuvant of the patients.

		stage 1	stage 2	stage 3	stage 4				
1988 FIGO	PFS (months)	108.0	79.5	31.1	20.2				
	OS (months)	132.8	81	47.4	26.8				
	Recurrence rate	51.9%	60.0%	60.0% 53.8%	71.4%				
2009 FIGO	PFS (months)	107.7	19.8	51.9	4.9				
	OS (months)	134.3	30.1	64.8	8.5				
	Recurrence rate	52.9%	50.0%	25.0%	100.0%				
AJCC	PFS (months)		158.8	65.6	27.9				
	OS (months)		160.2	85.5	36.7				
	Recurrence rate		45.0%	55.7%	57.9%				

[Progression-free survival (PFS), overall survival (OS) and recurrence rate according to the two International Federation of Gynecology and Obstetrics]

		1a	1b	1c	2b	3a	3b	3c	4
1988 FIGO	PFS (months)	142.4	19.2	81.4	79.5	12	22.2	67	20.2
	OS (months)	157	30	108.6	81	40.3	28.4	77.5	26.8
	Recurrence rate	20.0%	53.8%	52.0%	60.0%	66.7%	40.0%	50.0%	71.4%
2009 FIGO	PFS (months)	171.3	62.8		19.8	73.5	14.0	33.5	4.9
	OS (months)	172.3	84.4		30.1	78.0	16.0	58.1	8.5
	Recurrence	42.1%	56.5%		50.0%	0.0%	0.0%	50.0%	100.0%

	rate								

[Progression-free survival (PFS), overall survival (OS) and recurrence rate according to the sub-stages of the two International Federation of Gynecolo]

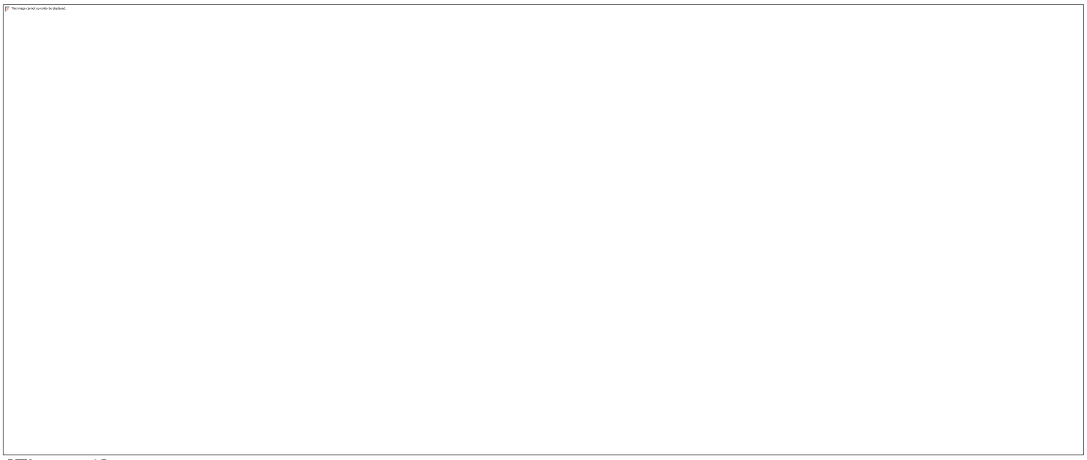

[Figure 1]

Disclosure: Dr. KY Tse had received travel grants from Astra Zeneca, MSD, Transmedic China; lecture honorarium from Zai lab and AstraZeneca; and had a funding project from Pfizer. Prof. HYS Ngan had received travel grant and honorarium from Pfizer, AstraZeneca, Roche, MSD, Zai Lab. Dr. Philip Ip had nothing to disclose.

P110

The role of oral oil administration in displaying the chylous tubes and preventing chylous leakage in laparoscopic para-aortic lymphadenectomy in gynecologic surgeries

G. Zhou, K. Hua, J. Ding
Department of Gynecology, Obstetrics and Gynecology Hospital of Fudan University, Shanghai, China

Introduction / Background: This study is aimed to investigate the possibility of pre-operative oral oil administration in displaying the chylous tubes and preventing chylous leakage in laparoscopic para-aortic lymphadenectomy in gynecologic surgeries.
Methodology: In this retrospective study, 30 patients with gynecological malignancies who had indications for laparoscopic para-aortic lymphadenectomy up to the renal vessels were give pre-operative oral oil (oil administration group) (n=15) or not (control group) (n=15) at our hospital between September 2017 and June 2018. The chylous tubes displaying rate, incidence of chylous leakage and other perioperative datum of the two groups were compared.
Results: uccessful display of chylous tubes was observed in 93.3% (14/15) patients in the oil administration group. The chylous leakage was 0 in the oil administration group, and 33.3% (5/15) in the control group. The postoperative drainage duration (4.1±1.0d vs 7.6±1.4d, P=0.000), somatostatin application time (0d vs 5.9±0.8d), and post-operative hospital stay (6.0±2.3 d vs 9.1±2.1d, P=0.001) were significantly shorter in the oil administration group. The total cost is lower in the oil administration group (4972.52±80.54dollars vs 6260.80±484.47 dollars, P=0.000).
Conclusion: Pre-operative oil administration is a feasible and effective method to display the chylous tubes and to prevent the chylous leakage in para-aortic lymphadenectomy.

Parameter, mean ±SD or n (%)	oil administration group (n=15)	control group (n=15)	p value
Para-aortic lymphadenectomy operating time (min)	73.14±13.22	68.53±11.65	0.327
Chylous tubes diaplaying rate (%)	93.3 (14/15)	0 (0/15)	0.000
Rate of chylous leakage (%)	0 (0/15)	33.3 (5/15)	0.042
Number of lymph nodes excised (n)	10±5	11±5	0.759
Postoperative drainage duration (d)	4.1±1.0	7.6±1.4	0.000
Somatostatin application time (d)	0	5.9±0.8	0.000
Hospital stay postsurgery (d)	6.0±2.3	9.1±2.1	0.001
Operation cost, US $	1303.82±99.54	1363.53±98.57	0.120
Total cost, US $	4972.52±80.54	6260.80±484.47	0.000

[Perioperative datum of the oil administration group and control group]

This image cannot currently be displayed.

[Recruitment and follow-up flow chart.]

This image cannot currently be displayed.

[The left and the right lumbar trunk was displayed. Check the leakage and occlude the lesion.]

Disclosure: Nothing to disclose

P111

Is it the time to move towards early histologic diagnosis before surgical management of advanced ovarian cancers?

W. Akor, R. Oliva, C. Ang
Northern Gynaecological Oncology Centre, Gateshead, United Kingdom

Introduction / Background: Treatment of advanced ovarian cancer is a combination of surgery and chemotherapy. NICE recommends that a confirmed tissue diagnosis is necessary before the commencement of chemotherapy (NICE CG122, 2011). However, for the surgical treatment of advanced ovarian cancer, there is currently no recommendation for pre-treatment tissue diagnosis. The absence of a histologic diagnosis before surgical treatment could lead to less specificity in the selection of patients receiving surgical intervention. This study was designed to explore the frequency of patients who presented and received treatment for suspected ovarian cancer, who did not have gynaecological cancer.
Methodology: A retrospective study was carried out at the Northern Gynaecological Oncology Centre (NGOC), Gateshead the United Kingdom, looking at patient referrals between 2008 - 2011. Extracted data from electronic patient records was analysed to obtain the frequency of non-gynaecological cancer referrals to the study centre, as well as the types of treatment they received on referral.
Results: There were 864 referrals in the study period, of which 764 (88.4%) were confirmed ovarian malignancies. Gastrointestinal malignancies made up 60 (6.9%) of this cohort, breast malignancies 8 (0.9%) and the remaining 32 (3.7%) arising from other sites.
Of 704 (81.5%) patients that had surgical treatment, 48 (6.8%) had primary surgery for non-gynaecological cancers undertaken by a Gynae-Oncologist in a gynaecological cancer specialist referral centre. These 48 patients received treatment by a non-specialist in their disease at the first instance, due to a lack of a definitive diagnosis before surgical treatment.
Conclusion: Findings from this study, suggests that undertaking a pre-treatment histology for all women regardless of the mode of treatment is imperative in ensuring the appropriateness of intervention by the correct specialist and good patient outcomes.
Disclosure: Nothing to disclose

P112

Activation of *PIK3CA* in a model of endometriosis may shed light on the pathogenesis of ovarian clear cell adenocarcinoma

C.F. Barbara[1], C. King[1], M. Jimenez-Linan[2], J. Brenton[3], D.S. Charnock-Jones[1]
[1]University of Cambridge, [2]Addenbrooke's Hospital, [3]Cancer Research UK, Cambridge Institute, Cambridge, United Kingdom

Introduction / Background: Activating *PIK3CA* mutations are described in one third of ovarian clear cell adenocarcinomas (OCCA), an endometriosis-associated cancer, and are likely early events its development. To better characterise *PIK3CA*-mutated OCCA, we designed a xenograft model: we genetically modified human endometrium *ex vivo*, implanted it in an immunocompromised mouse, and studied the consequences of *PIK3CA* activation.
Methodology: We co-transduced healthy human endometrium with luciferase (*in vivo* reporter) lentivirus and either non-targeting control or *PIK3CA* activation lentivirus; this was implanted in Balb/c nu/nu mice. We monitored lesion size and luminescence and examined histology of 9 paired replicates. We performed RNA sequencing of 3 paired matched replicates and used DeSeq2 and PANTHER for differential expression and gene ontology (GO) analysis respectively.
Results: Lesions survived up to 41 days. There was large variation in size and luminescence. Most lesions demonstrated endometrial-type glands in decidualised stroma. Three of 9 PIK3CA lesions, but no controls, showed well-defined hyalinsed stroma with inflammatory infiltration and no endometrial-type glands. Six of 9 PIK3CA lesions, but 1 control lesion, had inflammatory infiltration within the lesion (Fig1). P-value distribution histogram showed PIK3CA activation causes significant differential

expression. 620 genes were differentially regulated (Padj ≤0.05, Fig2). The 2 most significantly enriched GO categories were metalloendopeptidase inhibitor activity (24.37-fold enrichment, Padj=7.06E-04) and metallopeptidase activity (5.15-fold enrichment, Padj=1.27E-04). GO analysis of the genes in the PI3K signalling pathway showed the 2 most significantly enriched transcripts encode the 5-Hydroxytryptamine 2A and 2B receptors (4.6-fold enrichment, Padj=4.95E-08 and 3.3-fold enrichment, Padj=0.00033 respectively).
Conclusion: Constitutive *PIK3CA* activation caused significant differential expression and significant enrichment of metalloendopeptidase inhibitor activity and metallopeptidase activity. 5-hydroxytryptamine 2A and 2B receptor RNAs are also induced. Further research into the role of these molecules in OCCA and other cancers with activating PIK3CA mutations is warranted. Targeted inhibitors may have a role in management of these tumours.

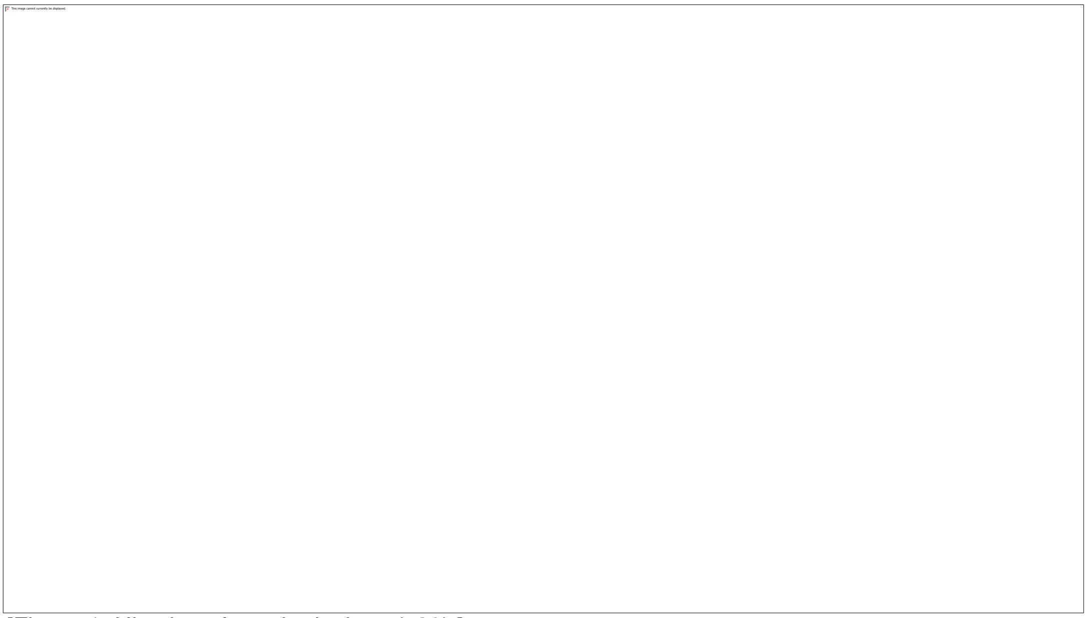

[Figure 1. Histology from the lesions (x20).]

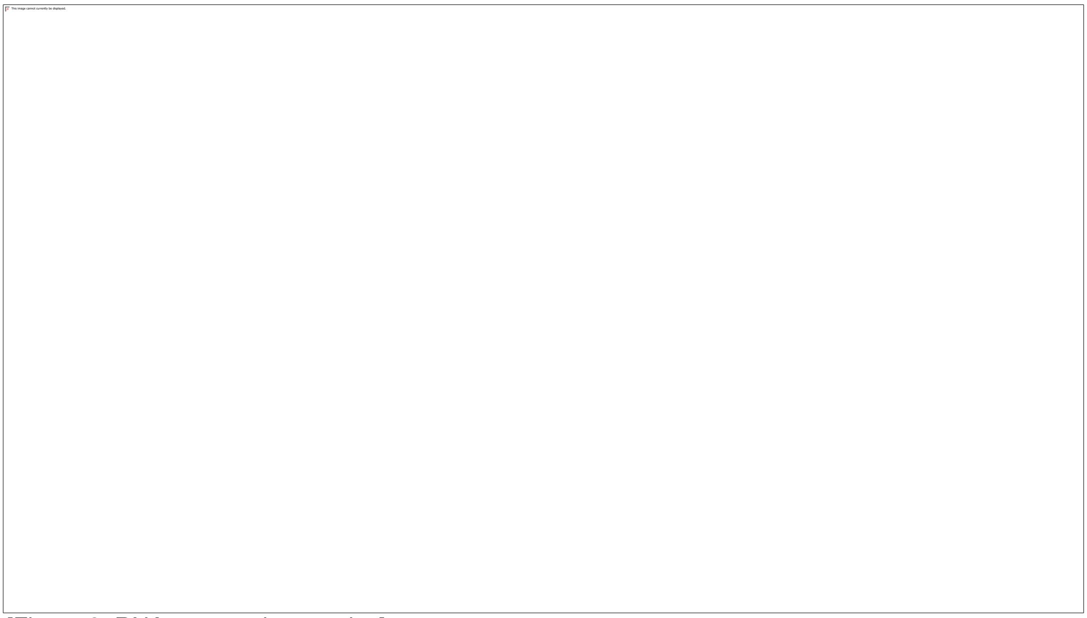

[Figure 2. RNA sequencing results.]

Disclosure: Nothing to disclose

P113

Systemic and surgical treatment of adult granulosa cell tumor of the ovary

E. Bakhidze, A. Belyaeva, I. Berlev
N.N. Petrov Scientific Medical Research Center of Oncology, St. Petersburg, Russian Federation

Introduction / Background: Adult granulosa cell tumor (AGCT) are rare non-epithelial ovarian tumors. Main method of treatment is surgical. Role of systemic treatment due to rarity of AGCT and late recurrence still has not determined.
Methodology: The data of 93 patients in our institution who were diagnosed with AGCT I-IV stage of the disease between 1980 and 2017 were evaluated. The data were obtained from the files of the patients, electronic database of the gynecologic oncology clinic, operation notes, and pathology records.
Results: Stage I disease was established in 51 patients (54.8%), IB - in 6 (6.5%), IC - in 17 (18.3%), II - in 3 (3.2%), III - in 12 (12.9%), IV - in 4 (4.3%). Stage of the disease, the spread of the tumor beyond the capsule and the number of mitoses of more than 10 significantly influenced the OS rates ($p < 0.05$). Relapses were observed in 24.3%. The average time before the onset of the first relapse in IA,IB stage was 134.5 months, in IC stage - 67.4 months, in II-IV stage - 34.3 months. There was no influence of the volume of surgical treatment in stage I disease and adjuvant chemotherapy on DFS and the duration of disease-free period ($p > 0.05$). The duration of the non-progressive period was some, but not significantly more when performing optimal cytoreduction for the recurrence of the disease, and did not depend on the implementation regimen and adjuvant chemotherapy
Conclusion: No significant differences were found in the OS and DFS of patients with AGCT depending on the options of surgical and systemic treatment, which allows to use fertility-preserving operations in patients of reproductive age at IA stage without unfavourable risk factors and only observation after surgical treatment. In case of relapse of the disease intention to perform optimal cytoreduction is justified.
Disclosure: Nothing to disclose

P114

Guidelines and selection criteria for lymphadenectomy in recurrent ovarian cancer patients

V. Gallotta, M. Bruno, C. Conte, M.T. Giudice, F. Davià, C. Lodoli, A. Federico, A. Santoro, F. Moro, C. Moruzzi, A. Fagotti, G. Scambia, G. Ferrandina
Policlinico Universitario Agostino Gemelli I.R.C.C.S. Universita Cattolica Di Roma, Rome, Italy

Introduction / Background: Several retrospective studies suggested that secondary cytoreductive surgery (SCS) could provide improved clinical outcomes in patients with recurrent ovarian cancer (ROC). The goals of this study were 1) to evaluate the prognostic role of clinico-pathological variables conditioning the post-salvage lymphadenectomy progression free survival (PSL-PFS) and 2) to assess which clinical and pathological parameters could drive to a personalized treatment.
Methodology: Retrospective analysis of a single-institutional series of patients with lymph-nodal recurrence (LNR) managed with SCS at Our Institution, from November 2009 to September 2017.
Results:
Seventy two patients underwent SCS (48 laparotomic, 24 with minimally invasive approach). Median age was 58 years (range 37-78), median body mass index was 24.0 kg/m^2 (range: 17-35). The median platinum free interval (PFI) was 14 months (range: 6-175). Preoperative radiological work-up documented the presence of lymph node disease in aortic region (N=27, 37.5%), pelvic (N=21, 29.2%), and both aortic and pelvic (N=17, 23.6%), 5 (6.9%) at the level of hepato-celiac region, and 2 (2.8%) in hepato-celiac and aortic region. Ten patients (13.9%) were found to harbour other sites of disease associated with LNR. Systematic lymphadenectomy was carried out in 48 patients (66.7%), while bulky lymph nodes resection was performed in 24 patients. In all cases optimal residual was achieved. Adjuvant chemotherapy was administered to all patients. As of December 2018, median follow-up after SCS was 33 months (range: 11-111); relapse of disease was documented in 39 (54.0%) cases.
Univariate analysis of variables conditioning PSL-PFS showed that the PFI before LNR >12 months, the negative Ca125 levels, number of metastatic lymph nodes < 3, and documentation of isolate LNR played a statistically significant favorable role.

table 1

Variable	No (%)	HR (95% CI)	Univariate analysis: p value	HR (95% CI)	Multivariate analysis: p value
BRCA status<br>Wild type<br>BRCA mutated<br>n.a.	25 (34.8)<br>23 (32.0)<br>24 (33.2)	0.613 (0,292-1,288)<br>1	0.197		
PFI before LNR<br><12 months<br>>12 months<br>n.a.	31 (43.0)<br>36 (50.1)<br>5 (6.9)	1<br>0,456 (0,237-0,879)	**0.016**	1<br>0,359 (0,183-0,704)	**0.003**
Recurrence site<br>Upper Abdomen<br>Lower abdomen	49 (68.1)<br>23 (31.9)	1<br>0,578 (0,286-1,168)	0.122		
Ca 125 (range)<br>0-35<br>>35	26 (37.0)<br>44(63.0)	0,351 (0,166-0,742)<br>1	**0.004**	0,369 (0,170-0,797)<br>1	**0.011**
Type of surgery<br>Laparotomic	48 (66.7)	1	0.564		

Surgery<br>Minimally Invasive Surgery	24 (33.3)	0,822 (0,422-1,601)			
Type of lymphadenectomy<br>Bulky node resection<br>Sistematic lymphadenectomy	24 (33.3)<br>48 (66.7)	0,883 (0,446-1,748)<br>1	0.719		
Number of metastatic nodes<br>1-3 metastatic nodes<br>>3 metastatic nodes	34 (47.2)<br>38 (52.8)	0,478 (0,246-0,928)<br>1	0.026	0,576 (0,292-1,137)<br>1	0.112
Site of lesion<br>Exclusive LNR<br>Nodal lesion+ other site	61 (84.7)<br>11 (15.3)	0,455 (0,207-1,001)<br>1	**0.045**	0,478 (0,214-1,066)<br>1	0.710

[Univariate and multivariate analysis of prognostic parameters of post relapse disease free-survival]

Conclusion: Our data suggest that particular clinical and pathological features may improve selection criteria for SCS in ROC patients with LNR.
Disclosure: Nothing to disclose

P115

Quality-of-life analysis in the randomized phase II CLIO trial comparing olaparib with standard chemotherapy in platinum-resistant recurrent ovarian cancer

T. Callewaert[1], A. Vanderstichele[1], E. Van Nieuwenhuysen[1], S. Han[1], N. Concin[1], T. Van Gorp[1], P. Berteloot[1], P. Neven[1], P. Busschaert[1], D. Lambrechts[2,3], I. Vergote[1]
[1]BGOG & Department of Gynaecology and Obstetrics, University Hospitals Leuven, Leuven Cancer Institute, Leuven, European Union, [2]Center for Cancer Biology, VIB, [3]Laboratory for Translational Genetics, Department of Oncology, KU Leuven, Leuven, Belgium

Introduction / Background: Patients with relapsed ovarian cancer receive multiple lines of chemotherapy, each negatively affecting their health-related quality of life (HRQOL). The CLIO trial randomized patients with platinum-resistant relapsed ovarian cancer (PROC) to treatment with olaparib versus standard chemotherapy (paclitaxel weekly, or pegylated liposomal doxorubicin, or gemcitabine or topotecan). Olaparib showed a favorable response rate compared with chemotherapy (Vanderstichele A et al., ASCO 2019). We aimed to assess HRQOL and quality-adjusted survival measures including Q-TWIST (quality-adjusted time without symptoms and toxicities of treatment) and QAPFS (quality-adjusted progression free survival).
Methodology: EORTC questionnaires QLQ-C30 and QLQ-OV28 were administered at baseline and every three months until end of treatment. Scoring of HRQOL focused on abdominal/gastrointestinal symptoms. Overall survival was partitioned into three health states to calculate Q-TWIST: time with grade ≥2 nausea, vomiting and fatigue (toxicity), time without toxicities (twist) and relapse. Each health state was adjusted by a utility weight derived from the QLQ-C30 global health status. QAPFS calculation was based on Q-TWIST, but excluded relapse status.
Results: In CLIO, we randomized 100 PROC patients 2:1 towards olaparib (n=67) and chemotherapy (n=33). Baseline compliance rate of questionnaires was 83% (88% olaparib vs. 73% chemotherapy). There was no significant difference in abdominal/gastrointestinal symptoms between both arms (p=0.4). In the olaparib-arm, significant differences were observed between responders and non-responders (p=0.001). Adverse events (AEs) of grade ≥3 occurred in 60% and 52% of patients in the

olaparib and chemotherapy arm respectively (p=0.521). The difference in Q-TWIST was 35.6 days (95%CI: -121.5 - 27.8) in favour of chemotherapy. The difference in QAPFS was 23.8 days (95% CI: -42.2 - 63.5) in favour of olaparib.
Conclusion: In PROC patients, olaparib monotherapy showed similar HRQOL compared to standard chemotherapy. There were no significant differences in abdominal/gastrointestinal symptoms, Q-TWIST and QAPFS between the two treatment arms.
Disclosure: The presenting author, T. Callewaert, has no conflict of interest.

P116

ATHENA (GOG-3020/ENGOT-ov45; EudraCT 2017-004557-17; NCT03522246): a randomised, double-blind, placebo-controlled, Phase 3 study of the Poly(ADP-Ribose) Polymerase (PARP) inhibitor rucaparib + the PD-1 inhibitor nivolumab following frontline platinum-based chemotherapy in ovarian cancer (OC)

A. Christopoulou[1], F. Marmé[2], A. Oaknin[3], D. Lorusso[4], T. Safra[5], G. Lindahl[6], A. Chudecka-Glaz[7], T. Ciuleanu[8], D.C. Collins[9], F. Demirkiran[10], I. Lifrenko[11], T. Van Gorp[12], R.L. Coleman[13], K. Fujiwara[14], A.M. Oza[15], S.N. Westin[13], D.M. O'Malley[16], T.J. Herzog[17], R. Eskander[18], M. Wilson[19], S. Argire[20], N. Grechko[20], I.A. McNeish[21], B.J. Monk[22], R.S. Kristeleit[23]
[1]Medical Oncology Unit, St Andrews General Hospital of Patras, Patras, Greece, [2]Gynäkologische Onkologie, National Center for Tumor Diseases (NCT) Heidelberg, Heidelberg, Germany, [3]Medical Oncology Department, Vall d'Hebron University Hospital, Vall d'Hebron Institute of Oncology (VHIO), Barcelona, Spain, [4]Gynecologic Oncology Unit, Fondazione Policlinico Universitario A. Gemelli IRCCS, Rome, Italy, [5]Sourasky Medical Center, Tel Aviv, Israel, [6]Department of Oncology and Department of Clinical and Experimental Medicine, Linköping University, Linköping, Sweden, [7]Department of Gynecological Surgery and Gynecological Oncology of Adults and Adolescents, Pomeranian Medical University in Szczecin, Szczecin, Poland, [8]Iuliu Haţieganu University of Medicine and Pharmacy and Ion Chiricuta Institute of Oncology, Cluj-Napoca, Romania, [9]Cork University Hospital, Cork, Ireland, [10]Department OB&GYN, Division of Gynecologic Oncology, Cerrahpasa Medical Faculty, Istanbul University-Cerrahpasa, Istanbul, Turkey, [11]Kursk Regional Oncology Dispensary, Kursk, Russian Federation, [12]University of Leuven, Leuven, Belgium, [13]Department of Gynecologic Oncology and Reproductive Medicine, The University of Texas MD Anderson Cancer Center, Houston, TX, United States, [14]Department of Gynecologic Oncology, Saitama Medical University International Medical Center, Hidaka, Saitama, Japan, [15]Division of Medical Oncology and Hematology, Princess Margaret Cancer Centre, University Health Network, Toronto, ON, Canada, [16]Clinical Research Gynecologic Oncology, The Ohio State University, James Cancer Center, Columbus, [17]Department of Ob/Gyn, University of Cincinnati Cancer Institute, University of Cincinnati, Cincinnati, OH, [18]Department of Reproductive Medicine Division of Gynecologic Oncology, Rebecca and John Moores Cancer Center, University of California, La Jolla, CA, United States, [19]Department of Medical Oncology, Auckland City Hospital, Auckland, New Zealand, [20]Clinical Operations, Clovis Oncology UK Ltd., Cambridge, [21]Department of Surgery and Cancer, Imperial College London, London, United Kingdom, [22]Arizona Oncology (US Oncology Network), University of Arizona College of Medicine, Creighton University School of Medicine, Phoenix, AZ, United States, [23]Academic Department of Oncology, University College London Cancer Institute, UCL, London, United Kingdom

Introduction / Background: Rucaparib has clinical activity in patients with recurrent OC with or without homologous recombination (HR) deficiency (HRD; eg, a *BRCA* mutation or high genomic loss of heterozygosity [LOH]) and is hypothesised to provide clinical benefit following frontline treatment. ATHENA is evaluating rucaparib + nivolumab as maintenance treatment following frontline platinum-based chemotherapy in patients with newly diagnosed, high-grade ovarian, fallopian tube, or primary peritoneal cancer. The rationale for this combination includes: tumours with *BRCA* mutations express tumour-specific proteins (neoantigens), which attract PD-L1-expressing, tumour-infiltrating lymphocytes; ovarian tumours with HRD have more neoantigens relative to HR-proficient tumours and may respond to immune checkpoint inhibitors; rucaparib combined with anti-PD-1 demonstrated improved antitumour activity in an OC model; it is hypothesised that PARP inhibitor-induced DNA damage may increase neoantigens, regardless of HRD status.
Methodology: Eligible patients must have completed frontline platinum-doublet chemotherapy and

surgery and achieved an investigator-assessed response without disease progression or rising CA-125 at any time during frontline treatment. Cytoreductive surgery (R0 permitted) could have been completed prior to chemotherapy or following neoadjuvant chemotherapy. Patients will be randomised 4:4:1:1 to receive maintenance treatment in Arm A (oral rucaparib 600 mg BID + intravenous nivolumab 480 mg Q4W), Arm B (oral rucaparib + intravenous placebo), Arm C (oral placebo + intravenous nivolumab), or Arm D (oral placebo + intravenous placebo). Stratification factors include centrally determined tumour HRD status (*BRCA* mutant, non-*BRCA* mutant/LOH high, non-*BRCA* mutant/LOH low, or non-*BRCA* mutant/LOH indeterminate), posttreatment disease status, and timing of surgery. Investigator-assessed progression-free survival (RECIST v1.1; primary endpoint) will be compared between arms. Secondary endpoints include blinded independent central review of progression-free survival, overall survival, objective response rate, and safety.
Results: Patients (n≈1000) will be enrolled at >270 sites worldwide.
Conclusion: ATHENA is evaluating rucaparib + nivolumab as frontline maintenance treatment in patients with OC.
Disclosure: AO: Clovis, AstraZeneca, Genmab/Seattle Genetics, ImmunoGen, PharmaMar, Roche, Tesaro DL: Clovis, AstraZeneca, ImmunoGen, Merck, PharmaMar, Roche, Tesaro DC: AstraZeneca, Genmab, Pfizer, Seattle Genetics, BMS, MSD, Roche RLC: Clovis, AbbVie, AstraZeneca, Esperance, Janssen, Millennium, Merck, OncoMed, Roche/Genentech, Bayer, GamaMabs, Genmab, Gradalis, Pfizer, Tesaro KF: Advanced Oncotherapy, AstraZeneca, Chugai-Roche, Daiichi-Sankyo, Eisai, GSK, ImmunoGen, Kaken-Seiyaku, Lilly, Merck, Ono Pharmaceutical, Pfizer, Shionogi, Taiho, Zeria Pharmaceutical AMO: Clovis, Amgen, Immunovaccine, Verastem, AstraZeneca, WebRx SNW: Clovis, AstraZeneca, Bio Ascend, Genentech, Gynecologic Oncology Group, Medivation/Pfizer, Merck, Ovation, Roche, Takeda, Tesaro, Vermillion, ArQule, Bayer, Cotinga Pharmaceuticals, Novartis DMO'M: Clovis, AstraZeneca, Gynecologic Oncology Group, Janssen, Myriad, Tesaro, Amgen, ImmunoGen, AbbVie, Health Analytics, Ambry, Agenus, Ajinomoto, Array BioPharma, BMS, ERGOMED Clinical Research, Exelixis, Genentech, GSK, INC Research, inVentiv Health Clinical, Ludwig Institute for Cancer Research, Novartis, PRA International, Regeneron, Serono, Stemcentrx, TRACON TJH: Clovis, AstraZeneca, Caris, Johnson & Johnson, Roche, Tesaro RE: Clovis, AstraZeneca, Roche/Genentech, Merck, Pfizer, Tesaro MW: AstraZeneca, MSD, Roche SA, NG: Clovis IAM: Clovis, AstraZeneca, Tesaro BJM: Clovis, AstraZeneca, Janssen/Johnson & Johnson, Myriad, Roche/Genentech, Tesaro, Amgen, Bayer, Biodesix, Cerulean, GSK, Gradalis, ImmunoGen, Insys, Mateon, Merck, NuCana, Perthera, Pfizer, Precision Oncology, Verastem, Vermillion RSK: Clovis, Tesaro, Roche All other authors have nothing to disclose

P117

Centralization and implementation of national guidelines of ovarian cancer improves survival - a population-based nationwide SweGCG study

P. Dahm Kähler[1,2], C. Borgfeldt[3], A. Flöter Rådestad[4], E. Hjerpe[4], J. Marcickiewicz[2], M. Bjurberg[5], B. Tholander[6], K. Hellman[7], P. Kjølhede[8], T. Högberg[9], P. Rosenberg[10], E. Åvall-Lundqvist[11], K. Stålberg[6], SweGCG
[1]Dept Obst & Gynecology, Sahlgrenska University Hospital, [2]Sahlgrenska Academy at University of Gothenburg, Institute of Clinical Sciences, Göteborg, [3]Dept Obst & Gynecology, Skane University Hospital, Lund University, Lund, [4]Dept of Women's and Children's Health, Karolinska University Hospital and Karolinska Institutet, Stockholm, [5]Dept of Clinical Sciences, Skane University Hospital, Lund University, Lund, [6]Dept of Women's and Children's Health, Uppsala University, Uppsala, [7]Dept of Women's and Children's Health, Karolinska University Hospital and Karolinska Institute, Stockholm, [8]Dept Obst & Gynecology, Dept of Clinical and Experimental Medicine, Linköping University, Linköping, [9]Dept of Cancer Epidemiology, Lund University, Lund, [10]Dept of Oncology, University Hospital Linköping, [11]Dept of Oncology and of Clinical and Experimental Medicine, Linköping University, Linköping, Sweden

Introduction / Background: Ovarian cancer is the leading cause of death due to gynecological cancers, the majority are diagnosed in advanced stages. The first Swedish national guidelines for ovarian cancer, implemented in 2012, recommended centralization to tertiary centres and primary debulking surgery (PDS) to no macroscopic residual disease (R0) as gold standard. Neoadjuvant

chemotherapy (NACT) followed by interval debulking surgery (IDS) was recommended according to predefined criteria. The aim of this study was to evaluate overall survival (OS) before and after the implementation of the national guidelines.
Methodology: Women diagnosed with epithelial ovarian/fallopian tube cancers FIGO stages III-IV and registered in the population-based nationwide Swedish Quality Registry for Gynecologic Cancer (SQRGC) between 2008-2016 were eligible excluding those diagnosed in 2012. Comparisons of OS before and after the implementation, 2008-2011vs 2013-2016, were performed in the cohorts of PDS, NACT+IDS and the total cohort. Overall survival (OS) was assessed by the Kaplan-Meier method and log rank test. The validity of SQRGC has previously been reported (1).
Results: In total, 3196 women were included in the study (n=1577 before and n=1619 after the implemented guidelines). PDS was performed in 937 (59%) women before and in 1038 (64%) women after the guidelines were introduced. During the same time periods the proportion of complete cytoreduction to R0 increased from 33% to 58%. In the PDS cohort, 5-year OS rate increased significantly from 29% (95% CI: 26-32%) to 40% (95% CI: 36-44%). In the NACT+IDS cohort, no significant difference in OS was observed. In the total cohort, 5-year OS increased significantly from 25% (95% CI: 23-28%) before to 34% (95% CI: 31-37%) after the introduction of national guidelines.
Conclusion: Centralization and implementation of the first Swedish national guidelines significantly improved 5-year overall survival in the total cohort of stages III-IV epithelial ovarian cancer.
Disclosure: Nothing to disclose

P118

European, non-interventional, phase IV NIMES-ROC trial of trabectedin plus pegylated liposomal doxorubicin in patients with platinum-sensitive recurrent ovarian cancer: an interim analysis

L.M. de Sande González[1], G. Scambia[2], A. Villanucci[3], E. Naglieri[4], M. Arruti Ibarbia[5], F. Brusa[6], H. Bourgeois[7], R. Sorio[8], A. Casado Herraez[9], D. Reichert[10], C. Dopchie[11], S. Pignata[12]
[1]Hospital Universitario de León, León, Spain, [2]Policlinico Universitario Agostino Gemelli I.R.C.C.S. Universita Cattolica Di Roma, Roma, [3]Azienda Ospedaliero-Universitaria Careggi, Firenze, [4]Istituto Oncologico Giovanni Paolo II, Bari, Italy, [5]Hospital de Galdakao, Galdakao, Spain, [6]Ospedale Cardinal Massaia, Asti-Piemonte, Italy, [7]Centre Jean Bernard-Clinique Victor Hugo, Le Mans, France, [8]Centro di Riferimento Oncologico di Aviano (IRCCS), Aviano, Italy, [9]University Hospital San Carlos, Madrid, Spain, [10]Onkologie Westerstede, Medizinische Studiengesellschaft NORD-WEST GmbH, Westerstede, Germany, [11]CHWapi Site IMC, Centre Hospitalier Wallonie Picarde, Tournai, Belgium, [12]Istituto Nazionale Tumori Pascale - I.R.C.C.S. - Fondazione Pascale, Napoli, Italy

Introduction / Background: The non-interventional NIMES-ROC phase IV trial (NCT02825420) evaluated trabectedin (Yondelis®)+pegylated liposomal doxorubicin (PLD) in real-life clinical practice, given in accordance with the marketing authorization to women with platinum-sensitive recurrent ovarian cancer (PSROC) regardless of prior anti-angiogenic treatment.
Methodology: We report the results of an interim analysis of the data from patients with PSROC treated with trabectedin+PLD within the approved schedule (PLD 30 mg/m² followed by 1.1 mg/m² trabectedin as 3-h/every 3 weeks). Eligible were adults with PSROC who have received ≥1 cycle of trabectedin+PLD before inclusion. The primary endpoint was to assess the progression-free survival (PFS) according to investigator criteria.
Results: Overall, 158 adult patients from 50 sites across Spain, Italy, Germany, France and Belgium were evaluated. Median number of trabectedin+PLD cycles received per patient was 6, with 95 patients (50.6%) receiving ≥6 cycles and up 34 cycles. Median treatment duration and cycle duration were 22.2 weeks (range: 3-124) and 25.5 days (range: 21.0-73.0), respectively, with ≥63% of patients treated on an outpatient basis. Twenty-two patients received prior PLD or doxorubicin with no reported safety issues due to retreatment with trabectedin+PLD. With 73 PFS events and 32 deaths recorded, median PFS and overall survival were 11.4 months (95% CI: 10-14) and not reached (16.8-nr), respectively. The objective response rate was 38%, while 28.5% of patients had disease stabilization for a disease control rate of 66.5%. A total of 108 trabectedin-emergent adverse reactions (TEARs) occurred. Most common grade 3/4 TEARs were neutropenia (25%) and asthenia (4%).
Conclusion: Acknowledging the interim results from observational studies need to be interpreted with

caution, our preliminary data confirm that trabectedin+PLD is effective and safe in patients with PSROC. Our data favourably compare with those of the pivotal OVA-301 trial (NCT00113607; i.e. median PFS 9.2 months), even with more pretreated patient population (NIMES-ROC: 39.9% with ≥3 prior chemotherapy lines).

NIMES-ROC*		Total (n=158)
Age at study entry (years)	Median (range)	62.0 (39-86)
ECOG performance status, n (%)	0; 1; 2; Not available*	67 (42.4); 33 (20.9); 5 (3.2); 53 (33.5)
Histopathology, n (%); ≥10% of patients	Papillary/serous	115 (72.8)
Tumor grade at diagnosis, n (%)	High grade; Intermediate grade; Low grade; Not done / Unknown*	111 (70.3); 11 (7.0); 9 (5.7); 27 (17.1)
BRCA 1/2 status tested, n (%)	Positive; Negative; Unknown*; Not done	20 (12.7); 56 (35.4); 15 (9.5); 67 (42.4)
Prior treatments, n (%)	Prior surgery; Prior secondary debulking; Prior radiotherapy; Prior chemotherapy; Prior use of antiangiogenics; Prior bevacizumab; Prior platinum-based therapy; Prior PLD / doxorubicin	144 (91.1); 61 (38.6); 6 (3.8); 156 (98.7); 88 (55.7); 86 (54.4); 156 (98.7); 22 (13.9%)
Platinum sensitivity, n (%)	Partially platinum sensitive; Fully platinum sensitive; Missing*	100 (63.3); 53 (33.5); 5 (3.2)
Number of lines of prior chemotherapy, n (%)	0; 1; 2; 3; 4-7	1 (0.6); 40 (25.3); 54 (34.2); 31 (19.6); 32 (20.3)
*Data not available at cut-off date. CI: Confidence interval; PLD: Pegylated liposomal doxorubicin.		

[Table 1. Demographics]

NIMES-ROC; Interim analysis	Total (n=158)	
Progression-free survival* (PFS); Overall survival** (OS)	months (95% CI)	11.4 (10-14); nr (16.8-nr)
Best response during NIMES-ROC, n (%)	Complete response (CR)	15 (9.5)
	Partial response (PR)	45 (28.5)
	Stable disease (SD)	45 (28.5)
	Progressive disease (PD)	32 (20.3)
	Not evaluable / Missing*	21 (13,3)
	ORR (CR+PR); [95% CI]	60 (38.0); [30.4-46.0]
	DCR (ORR+SD); [95% CI]	105 (66.5); [58.5-73.8]
*Patients who have not died and do not have an assessment of disease progression are censored. **Patients who have not been reported as dead are included as censored. CI: confidence interval; DCR: disease control rate; nr: not reached; ORR: objective response rate.		

[Table 2. Best responses]

Disclosure: Nothing to disclose

P119

An organoid platform for ovarian cancer captures intra- and interpatient heterogeneity

O. Kopper[1,2], C.J. de Witte[2,3], K. Lõhmussaar[1,2], J. Espejo Valle-Inclan[2,3], N. Hami[2,4], L. Kester[1,2], A.V. Balgobind[1,2], J. Korving[1,2], N. Proost[5], H. Begthel[1,2], L.M. van Wijk[6], S.A. Revilla[1,2], R. Theeuwsen[5], M. van de Ven[5], M.J. van Roosmalen[3], B. Ponsioen[2,4], V.W.H. Ho[7], B.G. Neel[7,8], T. Bosse[9], K.N. Gaarenstroom[9], H. Vrieling[6], M.P.G. Vreeswijk[6], P.J. van Diest[10], P.O. Witteveen[11], T. Jonges[10], J.L. Bos[2,4], A. van Oudenaarden[1,2], R.P. Zweemer[12], H.J.G. Snippert[2,4], W.P. Kloosterman[3], H. Clevers[1,2,13]
[1]Hubrecht Institute, Royal Netherlands Academy of Arts and Sciences and UMC Utrecht, [2]Oncode Institute, [3]Center for Molecular Medicine, [4]Molecular Cancer Research, Center for Molecular Medicine, University Medical Center Utrecht, Utrecht University, Utrecht, [5]Preclinical Intervention Unit of the Mouse Clinic for Cancer and Ageing (MCCA), Netherlands Cancer Institute, Amsterdam, [6]Department of Human Genetics, Leiden University Medical Center, Leiden, The Netherlands, [7]Princess Margaret Cancer Center, University Health Network, Toronto, ON, Canada, [8]Perlmutter Cancer Center, NYU Langone Health, New York, NY, United States, [9]Department of Pathology, Leiden University Medical Center, Leiden, [10]Department of Pathology, [11]Department of Medical Oncology, Cancer Center, [12]Department of Gynaecological Oncology, Cancer Center, University Medical Center Utrecht, Utrecht University, [13]The Netherlands Princess Máxima Center for Pediatric Oncology, Utrecht, The Netherlands

Introduction / Background: Ovarian cancer (OC) is a heterogeneous disease usually diagnosed at a late stage. Experimental in vitro models that faithfully capture the hallmarks and tumour heterogeneity of OC are limited and hard to establish.
Methodology: We present a protocol that enables efficient derivation and long-term expansion of OC organoids.
Results: Utilizing this protocol, we have established 56 organoid lines from 32 patients, representing all main subtypes of OC. OC organoids recapitulate histological and genomic features of the pertinent lesion from which they were derived, illustrating intra- and interpatient heterogeneity, and can be genetically modified. We show that OC organoids can be used for drug-screening assays and capture different tumour subtype responses to the gold standard platinum-based chemotherapy, including acquisition of chemoresistance in recurrent disease. Finally, OC organoids can be xenografted, enabling in vivo drug-sensitivity assays.
Conclusion: Taken together, this demonstrates their potential application for research and personalized medicine.

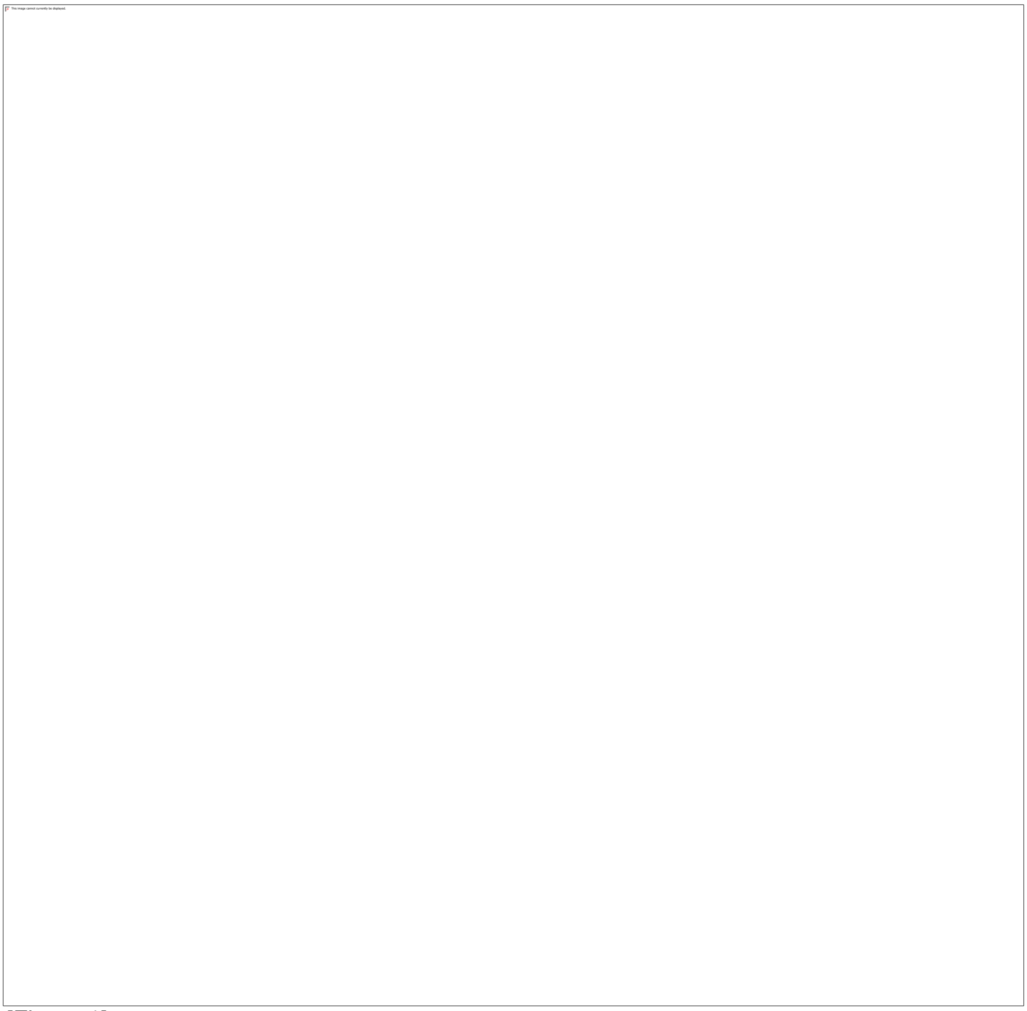

[Figure 1]

Disclosure: No competing interests. Applicable funding sources: EIF | Stand Up To Cancer (SU2C) [Clevers, Kopper, Balgobind, Begthel] KWF Kankerbestrijding (Dutch Cancer Society) - UU2015-7743 [de Witte, van Roosmalen, Witteveen, Zweemer, Kloosterman] Gieskes Strijbis Foundation (1816199) [Clevers, Witteveen, Kloosterman, Zweemer, Lõhmussaar, Espejo Valle-Inclan, Hami, Bos, Snippert] Published in Nature Medicine. April 2019. dx.doi.org/10.1038/s41591-019-0422-6

P120

Intraoperative frozen section could be misleading in the diagnostic evaluation of ovarian granulosa cell tumors

A. Di Cello[1], F. Visconti[2], G. Bitonti[2], K. Cefalì[2], K. Ranieri[2], F. Zullo[3], V. Roberta[2], C. Di Carlo[2]
[1]Magna Graecia University, Lamezia Terme, [2]Magna Graecia University, Catanzaro, [3]Federico II University, Napoli, Italy

Introduction / Background: Intra-operative frozen section (IFS) can provide an instinct guide for treatment of ovarian tumors intra-operatively, though limitations exist. We aimed to determine the value of IFS and US in the diagnosis of ovarian granulosa cell tumors.

Methodology: A retrospective review of granulosa cell tumors (GCTs) surgically treated at our Unit, between June 2006 and March 2019, was done. The diagnostic performance of ultrasound (US) and malignant IFS diagnosis was evaluated. Data of 20 patients were reviewed. Intra-operative FS and US results were compared with the definitive surgical diagnosis. Surgical data were reviewed and analyzed.
Results: A total of 12 cases were correctly identified at the intraoperative FS as ovarian granulosa tumors. There were 8 (32%) false negative diagnoses. According to well defined parameters, all 20 cases were correctly suspected as ovarian GCTs at US. About surgical treatment, in 12 women with positive FS the appropriate surgical staging was performed. In 8 women with negative FS surgical treatment was variable: 4 women underwent laparoscopic salpingo-oophorectomy and the surgical staging was completed after definitive surgical diagnosis; hysterectomy and bilateral salpingo-oophorectomy was performed in 2 patients; the conservative surgery on the healthy ovary was performed in the remaining 2 patients.
Conclusion: This study concluded that FS appears to be an inadequate technique for the histopathological diagnosis of ovarian GCTs, while the US features are really suggestive allowing a right clinical suspicious. Perhaps, when an ovarian GCTs is suspected at the US, intraoperative FS could be misleading and could lead to an inappropriate surgical management.
Disclosure: Nothing to disclose

P121

Surgical secondary cytoreduction in recurrent ovarian cancer patients with liver metastasis

C. Conte[1], V. Gallotta[1], M. d'Indinosante[1], E. Capoluongo[1], A. Minucci[1], A. De Rose[1], F. Ardito[1], F. Giuliante[1], A. Di Giorgio[1], G.F. Zannoni[1], C. Margreiter[2], A. Fagotti[1], G. Scambia[1], G. Ferrandina[1]
[1]Policlinico Universitario Agostino Gemelli I.R.C.C.S. Universita Cattolica di Roma, Rome, Italy,
[2]Medical University of Innsbruck, Innsbruck, Austria

Introduction / Background: To describe accurately the oncological outcomes after hepatic resection (HR) in recurrent ovarian carcinoma (ROC) evaluating clinic-pathological variables and mutational status of BRCA1/2. Although HR is considered a challenging situation in ROC patients, assessment of BRCA1/2 mutational status seems to have a relevant clinical value to guide surgical therapy.
Methodology: Patients who underwent HR for ROC at the Catholic University of Rome, between June 2012 and October 2017 were included. Exclusion criteria were represented by extra-abdominal disease and presence of diffuse peritoneal carcinomatosis requiring more than 2 bowel resections. Details relative to HR were collected and BRCA analysis was performed. Predictive factors for of post-relapse progression free survival (PHR-PFS)were assessed by univariate analyses using Cox-proportional hazard regression models.
Results: Thirty-four patients undewent HR within secondary cytoreductive surgery (SCS). Six patients (17.6%) presented with hepatic relapse only, while the remaining 28 patients (82.4%) had concomitant extra-hepatic disease. In the whole series, the 3-yr PHR-PFS was 49.1% and the 3-yr progression free survival overall survivalwas 72.9%. Univariate analysis of variables conditioning PHR-PFS showed that only BRCA mutational status played a statistically significant favourable role: the 3-yr PHR-PFS rate was 81.0% in BRCA mutated patient compared to15.2% in wild type ones (p value: 0.001).
Conclusion: Our clinical analyses suggest that in ROC patients with liver disease the assessment of BRCA mutational status can help to select patients elegible for SCS.
Disclosure: Nothing to disclose

P122

VATS can be used to effectively triage women with ovarian cancer and moderate to large

pleural effusions to PDS or NACT/IDS

O. Filippova[1], R. Bharathan[2], R. Cowan[1], Y. Lakhman[1], W. Tew[1], R. O'Cearbhaill[1], J. Konner[1], B. Park[1], J. Huang[1], G. Gardner[1], Y. Sonoda[1], K. Long Roche[1], O. Zivanovic[1], D. Chi[1]
[1]Memorial Sloan Kettering Cancer Center, New York, NY, United States, [2]Leicester General Hospital, Leicester, United Kingdom

Introduction / Background: Malignant pleural effusion, denoting stage IV disease, is a commonly cited indication for neoadjuvant chemotherapy (NACT) in women with ovarian cancer. Our objective was to evaluate outcomes of women with moderate to large pleural effusions whose initial management, primary debulking surgery (PDS) vs. NACT, was determined according to our institutional video-assisted thoracic surgery (VATS) algorithm.
Methodology: We conducted a retrospective review of patients with at least a pleural effusion who underwent VATS to guide management between 2001 and 2018. Data collected included VATS indication, findings, subsequent treatment and survival. Appropriate statistical analyses were applied.
Results: 144 women underwent thoracic surgery; 32 were excluded because they had trans-diaphragmatic resection through the abdominal incision and 22 because of no pleural effusion on preoperative imaging or VATS was not performed as part of initial management. The study cohort included 90 women; median age was 59.5 years (range, 38-81). All VATS were performed by a thoracic surgeon; median operative time was 39 minutes (range, 12-100). 50 (56%) patients underwent PDS after VATS demonstrated either no intrathoracic tumor (n=25, 50%) or thoracic disease that was resected to ≤1cm (n=25, 50%). The outcomes of these 50 patients with subsequent abdominal PDS were: complete gross resection, 17 (34%); ≤1cm residual, 25 (50%); and >1cm residual, 8 (16%). In 40 patients (44%), VATS demonstrated chest disease that was not amenable to optimal resection. These patients were treated with NACT and interval debulking surgery (IDS). With a median follow-up time of 36 months, the 3-year survival rate was 79.2% for PDS and 54.4% for NACT/IDS. Median overall survival was 61.6 months and 39.1 months, respectively (p=0.024).
Conclusion: In patients with ovarian cancer and moderate to large pleural effusions, optimal resection in both the chest and abdomen is possible with PDS. VATS can be used to effectively triage patients in this setting.

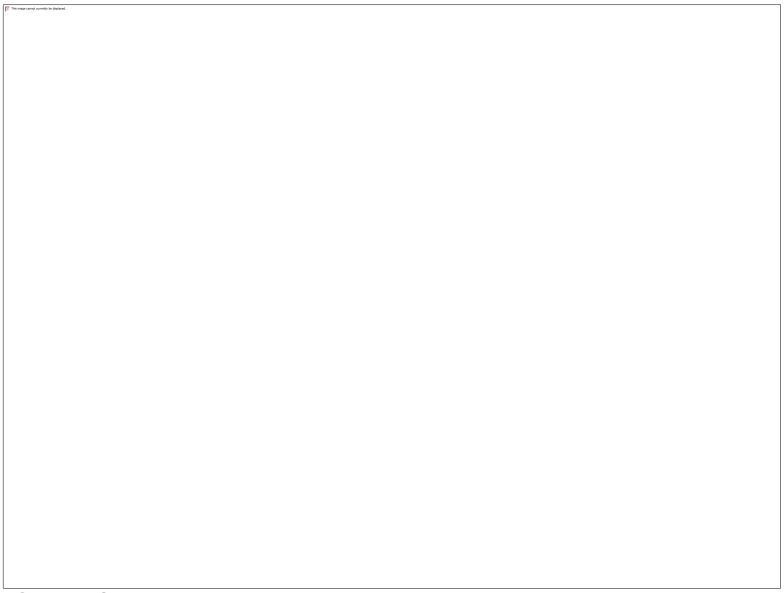

[Overall Survival]

Disclosure: Outside the submitted work, Dr. Chi reports personal fees from Bovie Medical Co., Verthermia Inc., C Surgeries, and Biom ´Up, as well as recent stock ownership from Intuitive Surgical, Inc. and TransEnterix, Inc.

P123

Chemotherapy response score predicts surgical outcome and prognosis in tubo-ovarian cancer

A. Dasgupta, N. Gomes, D. Blake, A. Kucukmetin
Gynaecological Oncology, Northern Gynaecological Oncology Centre, Gateshead, United Kingdom

Introduction / Background: Bohm's 3 tier CRS is a histopathological score for assessing tumour regression in omentectomy specimens after neoadjuvant chemotherapy(NACT).Bohm *et al* has concluded that CRS is more important prognostic factor than completeness of cytoreduction(CC) in IDS, but this has not been proved by other validation studies. There is a conflict in evidence regarding improvement of overall survival (OS) with CRS-3 in available literature(Bohm *et al*, Coghlan *et al,*Lee *et al* no significant difference vs significant difference-Rajkumar *et al*). Evidence of association of CRS with radiological and biochemical(CA-125 decline) response is lacking and conflicting.
Methodology: Patients who underwent IDS between 2010 and 2017 at NGOC were retrospectively analysed. Omental disease was scored by pathologists as per CRS reporting system described by Bohm. Surgical end result and clinico-pathological data was collected and correlated with CRS. Recurrence was assessed radiologically, OS and progression free survival (PFS) calculated in the 3 histopathological sub-groups, and compared with clinical variables using Cox proportional hazard model and log-rank test.
Results: 201 patients who underwent IDS were included in the analysis(table 1). CC was possible in 35 patients with CRS-3(65%) vs 24 patients in CRS-2(37%) and 18 patients in CRS-1(22%)(p< 0.0001), and this is seen after adjusting for stage(stage IV B-2 sided p:0.02, for stage IIIC, p=0.0012). Significant increase in PFS in patients with CRS 3 vs CRS1+2(log rank test for trend;χ^2:41.75,HR:2.8, p< 0.0001) as well as in OS in patients with CRS3 vs CRS1+2(χ^2:28.4, HR:2.6, p< 0.0001) Biochemical response(CA-125 decline after NACT) and radiological response were predictors of chemotherapy response scores.
Conclusion: CRS is an independent prognostic factor and a post NACT pre-IDS omental biopsy may help predict completeness of surgery at IDS and overall good prognosis. Future prospective trials will be useful to identify and understand whether CRS-3 tumours are a different disease entity.

CRS groups	CRS-1,N=82(40.8%)	CRS-2,N=65(32.3%)	CRS-3,N=54(26.9%)
Number of NaCT cycles	3 cycles,n=48(23.9%)	4 cycles,n=136(67.7%)	Greater than 4 cycles n=17(8.4%)
Extent of cytoreduction	Complete n=77(38.3%)	Optimal n=100(49.7%)	Suboptimal n=24(12%)
Radiological response	Good response n=88(43.8%)	Partial response n=82(40.8%)	Poor response n=31(15.4%)
Stages	III C n=113(56%)	IV A n=37(18%)	IV B n=47(23%)
Histology	HGSC n=187(93%)	Other histology n=14(7%)	
Type of NaCT	3 weekly carbo/taxol n=189(94%)	weekly carbo/taxol n=12	carbo/taxol/bevacizumab n=10

[Table-1:Patient characteristics and distribution]

Disclosure: Nothing to disclose

P124

Study of DNA repair gene polymorphisms in ovarian cancer

M. Gomes Ferreira, M. Ovejero-Sanchez, E. Sanchez Tapia, T. Martin-Gómez, R. Vidal, M. Sancho de

Salas, M.J. Doyague Sanchez, R. Gonzalez-Sarmiento
Universidad de Salamanca, Salamanca, Spain

Introduction / Background: Ovarian cancer (OC) is proven to be influenced by alterations in some DNA repair genes (e.g. BRCA1/2). In our work, we analyzed in DNA repair genes polymorphisms (SNPs) by TaqMan genotyping in OC peripheral blood samples.
Methodology: An association study of SNPs rs1799782, rs25487, rs1130409, rs13181, rs11615, rs1799794, rs861539, rs1042522, rs1799977 and rs1800734 of genes XRCC1, APEX1, ERCC2, ERCC1, XRCC3, MLH1 and TP53 was performed in the germinal DNA of 185 patients and 129 healthy controls.
Results: The GA genotype of XRCC1 polymorphism rs1799782, the CC genotype of TP53 polymorphism rs1042522 and the GG genotype of MLH1 polymorphism rs1800734 were associated with increased susceptibility to OC. The T allele of APEX1 polymorphism
rs1130409 was associated with a later onset of the disease and hereditary OC. In relation to XRCC3 gene (rs1799794), the TT genotype was associated with increased OC susceptibility in aging patients and patients with familial OC. In relation to MLH1 gene (rs1800734), the GG genotype was associated with greater OC susceptibility in old patients and in patients with hereditary OC. The SNP rs1799977 of MLH1 gene was
associated with an increased risk of recurrence.
Conclusion: Our study suggests that DNA repair genes different from BRCA1/2 like XRCC1, TP53, MLH1, XRCC3 and APEX1 could modify the risk of developing OC. This project was funded by FIS-FEDER PI16/01920
Disclosure: Nothing to disclose

P125

New horizons in malignant transformation of endometriosis, a retrospective study on all cases in Northern Ireland

M. Graham[1], H. Nagar[1], M. McComiskey[1], S. Dobbs[1], I. Harley[1], D. Hunter[2], G. McCluggage[3], E. Craig[1]
[1]*Gynae Oncology,* [2]*Gynaecology,* [3]*Histopathology, Belfast City Hospital, Belfast, United Kingdom*

Introduction / Background: This study aims to determine the prevalence, risk factors and outcomes of ovarian endometrioid carcinoma (EC) and clear cell carcinoma (CCC) and identify patients at risk of malignant transformation.
Methodology: 161 patients were identified using histopathology records from March 2011 to June 2018. Patient information was collected using an electronic database. All patients had multi-disciplinary team discussion and dedicated histopathological review.
Results: 95 patients had EC (59.%), 55 patients had CCC (34.1%) and 8 patients had synchronous EC and CCC (4.9%).
The mean age of presentation was 57 with the majority of patients (74.5%) presenting over the age of 50. 14 patients had a known clinical history of endometriosis. The mean Ca125 was 514 (Range 9-16042, 95% CI 764.1) with the nearly all patients (81.3%) having a raised Ca125. At time of presentation 129 patients had a pelvic mass ≥10cm. The most common presenting complaint was pressure symptoms/abdominal mass (59- 36.6%) or post-menopausal bleeding (24- 14.9%)
69.5% patients had early disease (Stage 1). 102 patients had endometriosis on histopathology (63.7%). 101 patients had adjutant treatment (64.7%) and 120 (74.5%) patients were still alive as per March 2019 with an Overall Survival of 48 months for both EC and CCC subtypes.
Conclusion: There is an average of 22 cases of ovarian EC or CCC each year in Northern Ireland. The majority of patients present after the age of 50 with pressure symptoms and a pelvic mass ≥10cm. We discuss preoperative risk factors suggestive of malignant transformation and outcomes following treatment. This study has highlighted the need for careful long-term observation of a patient with endometriomata to detect possible malignant change, leading to development of a multi- disciplinary strategy for better prevention, early detection, specific diagnosis and treatment targeting molecular pathogenesis to understand the mechanisms of endometriosis-associated ovarian cancer in Northern Ireland.

Disclosure: Nothing to disclose

P126

Role of tertiary cytoreductive surgery in recurrent epithelial ovarian cancer: systematic review and metanalysis

F. Guida[1,2], A. Fagotti[1,2], A. Grossi[3,4], C. Vascone[1,2], N. Bizzarri[1,2], V. Ghirardi[1,2], S. Boccia[3,4], G. Scambia[1,2]

[1]Università Cattolica del Sacro Cuore, Istituto di Clinica Ostetrica e Ginecologica, [2]Dipartimento di Scienze della Salute della Donna, del Bambino e di Sanità Pubblica, Fondazione Policlinico Universitario A. Gemelli IRCCS, U.O.C. di Ginecologia Oncologica, [3]Università Cattolica del Sacro Cuore, Sezione di Igiene, Istituto di Sanità Pubblica, [4]Fondazione Policlinico Universitario A. Gemelli IRCCS, UOC Igiene Ospedaliera, Roma, Italy

Introduction / Background: Two randomized controlled trials on the role of secondary cytoreductive surgery in platinum-sensitive Recurrent Ovarian Cancer (ROC) patients have shown contrasting results in terms of PFS and OS. Even more debated is the role of surgery in the tertiary setting. This study aims to investigate the current literature on tertiary cytoreductive surgery (TCS) in ROC and to perform a meta-analysis to examine the impact of optimal and sub-optimal TCS on survival.

Methodology: A systematic literature review was performed. PubMed, Scopus and Web of Science were searched to retrieve studies published in English and Italian up to 31st March 2019. Quantitative data were pooled out to compare interventions through a meta-analysis. PRISMA statement was used for reporting.

Results: Literature search led to 706 records. Of 29 full-text articles obtained after deduplication and screening, 17 were excluded as they didn't meet eligibility criteria. The remaining 12 studies were all retrospective. The majority had small size cohorts, and a wide heterogeneity in outcome measures and number and type of covariates was observed. Five studies were eligible for meta-analysis, including a total of 694 patients. Overall pooled HR comparing optimal/complete versus suboptimal/partial cytoreduction was 3.17 (CI95: 1.90-5.29; p< 0.001) with moderate heterogeneity (I^2: 56.2%; p=0.058), suggesting a protective effect of optimal TCS. Pooled HR for studies considering Disease Specific Survival, comparing residual tumor (RT) < 0.5 cm versus >0.5 cm after TCS, was 3.29 (CI95: 1.88-5.73; p< 0.001) with low heterogeneity (I^2: 17.4%; p=0.271). Pooled HR for studies considering Overall Survival, comparing RT < 0. versus >0 after TCS, was 3.29 (CI95: 1.88-5.73; p< 0.001) with very high heterogeneity
(I^2: 80.1%; p=0.025).

Conclusion: Our data suggests surgery might play a major role in improving survival of patients with ROC in tertiary setting. Nevertheless, the topic remains under-investigated and further studies are needed.

Reference	Included Population	Optimal Cytoreduction Criterion (RT, cm)	Optimal Cytoreduction (%)	Complete Cytoreduction (%)	Outcome Measure	Median Survival OC (months)	Median Survival SOC (months)	*P* Value	Median Survival RT=0 (months)
Leitao, 2004	26	≤ 0.5	73	53.8	DSS	36.3	10.6	< 0.00001	36.3
Karam, 2007	47	0	64	-	DSS	24	16	0.03	-
Gultekin, 2008	20	< 2	60	35	OS	15	6	0.12	26
Shih, 2010	77	≤ 0.5	88.3	72.7	DSS	29.7	13.6	< 0.001	60.4
Fotopoulou,	135	0	39.3	-	OS	37.8	19/6.9*	< 0.001	-

2010									
Oksefjell, 2011	28	0	38	-	MST (OS)	84.3	8.9/10.2**	< 0.01	-
OC, Optimal Cytoreduction; SOC, Suboptimal Cytoreduction; RT, Residual Tumor - *RT = 0.5-1/>1; **RT = ≤2/>2									

[Characteristics of the Included Studies - Part 1]

Reference	Included Population	Optimal Cytoreduction Criterion (RT, cm)	Optimal Cytoreduction (%)	Complete Cytoreduction (%)	Outcome Measure	Median Survival OC (months)	Median Survival SOC (months)	*P* Value	Median Survival RT=0 (months)
Hizli, 2012	23	< 1	65.2	NA	OS, TFS	14 (TFS)	0 (TFS)	0.018	NA
Fotopoulou, 2012	406	0	54.4	-	OS, PFS	49 (OS)	12 (OS)	< 0.001	-
Tang, 2013	83	0	70	-	OS	32.9	14.6	0.001	-
Fanfani, 2015	46	< 1	1.9	77.5	PFS, OS	NA	NA	NS	22 (PFS), 99 (OS)
Arvas, 2016	53	< 0.5	49	NA	OS, DSS	40 (DSS)	18 (DSS)	0.003	NA
Falcone, 2017	135	0	68.9	-	OS	43	33	< 0.001	-
OC, Optimal Cytoreduction; SOC, Suboptimal Cytoreduction; RT, Residual Tumor									

[Characteristics of the Included Studies - Part 2]

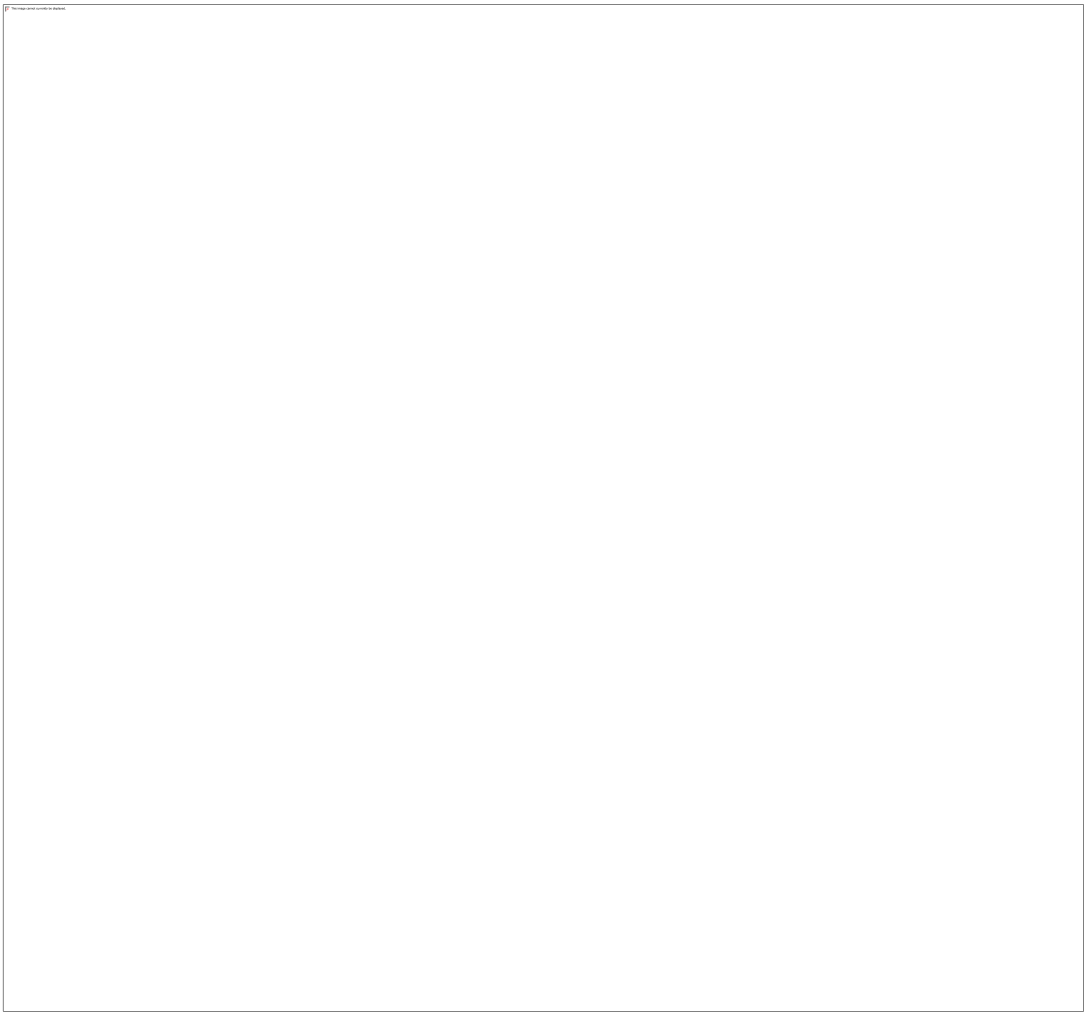

[Overall Analysis (all outcomes included)]

Disclosure: Nothing to disclose

P128

Novel mechanism of acquired platinum resistance for BRCA1-driven malignancies: rapid expansion of pre-existing BRCA1-proficient tumor cells during neoadjuvant therapy for ovarian cancer

E. Imyanitov[1], I. Berlev[1], T. Gorodnova[1], E. Savonevich[2], A. Ivantsov[1], E. Preobrazhenskaya[1], A. Togo[1], A. Sokolenko[1]
[1]N.N. Petrov Institute of Oncology, St. Petersburg, Russian Federation, [2]Grodno Medical University, Grodno, Belarus

Introduction / Background: Somatic loss of the wild-type BRCA1 allele is a key event in pathogenesis of BRCA1-driven cancers rendering their pronounced sensitivity to platinum and PARP inhibitors.
Methodology: We compared the somatic status of BRCA1 and some other genes in serial ovarian

cancer (OC) samples, which were obtained before neoadjuvant chemotherapy (NACT), during surgery and at disease relapse from BRCA1 mutation carriers.
Results: 26/36 (72%) OC had BRCA1 loss of heterozygosity (LOH) before NACT. 15/26 (58%) of the tumors, which had LOH at the beginning of the treatment, showed retention of the normal BRCA1 allele after NACT. The analysis of linked SNPs and FISH assay strongly indicated, that the restoration of BRCA1 function is caused not by the second mutation, but by the selection of pre-existing BRCA1-proficient cells. Tumor relapses were available in 7 patients with BRCA1 LOH in the chemonaive tumor and/or BRCA-proficiency in the residual post-NACT neoplasm; 6 (86%) of these relapses had BRCA1 LOH thus resembling the primary tumor. Secondary open reading frame (ORF) restoring BRCA1 mutation was detected in 1 recurrent OC. Serial samples retained same TP53 mutation during the treatment course. Exome analysis revealed that chemonaive, post-NACT and relapsed tumors had both shared and individual mutations.
Conclusion:
1) BRCA1 LOH is not the first event in the pathogenesis of BRCA1-driven cancer: gain of TP53 mutation probably precedes BRCA1 inactivation;
2) Treatment-naïve BRCA1-driven tumors contain a small fraction of BRCA1-proficient cells, which rapidly repopulate the tumor lump during the first weeks of therapy;
3) Change of BRCA1 status during NACT may call to reconsider the existing approaches to adjuvant therapy;
4) The balance between BRCA1-deficient and BRCA1-proficient cells is a subject of fluctuations, depending whether therapy is applied or not;
5) Clinical trials on BRCA1-driven OCs need to address the issue of intratumoral heterogeneity of BRCA1 status.
Disclosure: This work has been supported by the Russian Science Foundation, grant 19-15-00168

P129

Assessment of cytokine mRNA expression profiles in tumor microenvironment and peripheral blood mononuclear cells of patients with high-grade serous carcinoma of the ovary

P. Israelsson, A. Labani-Motlagh, I. Nagaev, E. Dehlin, O. Nagaeva, E. Lundin, U. Ottander, L. Mincheva-Nilsson
Umeå University, Umeå, Sweden

Introduction / Background: Tumor establishment, metastatic spreading and poor survival in ovarian cancer is strongly associated with progressive derangement of the patient's immune system. Accumulating evidence suggests that immune impairment is influenced by the production and presence of cytokines in the tumor microenvironment.
Methodology: Cytokine mRNA profiles in tumor tissue and peripheral blood mononuclear cells (PBMC) were analyzed in patients with high grade serous carcinoma (HGSC) of the ovary and compared it to patients with benign ovarian conditions and controls with normal ovaries. Cytokine assessment was done by real-time quantitative RT-PCR and specific primers and probes for 12 cytokines-IFN-γ, IL-1β, IL-2, IL-4, IL-6, IL-8, IL-10, IL-15, TNF-α, TNF-β/LTA, TGF-β1, and GM-CSF chosen to distinguish between cytotoxic Th1, humoral Th2, regulatory Th3/Tr1 and inflammatory responses.
Results: The cytokine mRNA response in the HGSC patients was significantly up regulated compared to patients with benign ovarian conditions and normal ovary controls confirming the immunogenicity of HGSC and implying immune recognition and reaction locally in the tumor microenvironment and systemically in the peripheral blood.There was an up-regulation of inflammatory and inhibitory cytokine mRNA promoting tumor progression, T-regulatory cell priming and T-regulatory cell-mediated immune suppression. In contrast, there was an inability to mount the crucially important IFN gamma response needed for upregulation of the cytotoxic anti-tumor response in the local microenvironment. In addition, systemic IL-4- mediated Th2 response prevailed in the peripheral blood deviating the systemic defense towards humoral immunity.
Conclusion: Taken together, these results suggest local and systemic cytokine cooperation promoting tumor survival, progression and immune escape. Our study confirms and extends previous investigations and contributes to the evaluation of potential cytokine candidates for diagnostic cytokine

mRNA profiles and for future therapeutic interventions based on cytokine inhibition.
Disclosure: Nothing to disclose

P130

Doxorubicin-loaded oligonucleotide conjugated gold nanoparticles: a promising drug delivery system for ovarian cancer

S. Jeon, H. Jeon
Soon Chun Hyang University Cheonan Hospital, Cheonan, Republic of Korea

Introduction / Background: Major cause of failure in the treatment of advanced ovarian cancer is chemoresistance. Gold nanoparticles(AuNPs) are promising drug delivery systems to overcome chemoresistance. Doxorubicin(DOX) is one of the representative cancer chemotherapeutic agent and is widely used by many researchers as a chemotherapy agent in the drug delivery system, however there have been limited data on ovarian cancer research. Our aim of study was to compare cytotoxic effect between DOX and Doxorubicin loaded oligonucleotides (ONTs) attached to gold nanoparticles (AuNPs) called DOA on ovarian cancer cell lines
Methodology: We propose Doxorubicin loaded oligonucleotides (ONTs) attached to gold nanoparticles (AuNPs) as a drug delivery system for cancer chemotherapy. We utilized AuNPs as drug delivery vehicle, which were synthesized by chemical reduction to be 13 nm diameter. Oligonucleotide - AuNPs present numerous binding sites for DOX, facilitating the delivery of DOX to cancer cells. Transmission electron microscope(TEM), fluorescence spectrometer and MTT assay were done for characterization and for cell viability assay. **증례**
Results: We characterization of Doxorubicin-loaded oligonucleotide conjugated gold nanoparticles (DOA) using Transmission electron microscope (TEM) image of AuNPs. (Fig 1) And 70% of DOX in solution could be bound to ONTs on AuNPs to became DOX-loaded AuNPs and about 28% of loaded DOX was released from the as-prepared DOA(Fig 2). In MTT assay, significant reduction in the cell viability was detected in DOA treated ovarian cancer cells(SKOV3, A2780, Hey A8) than DOX($p< 0.05$) (Fig. 3).
Conclusion: Our results suggested that ONTs attached AuNP is effective drug delivery system and DOA might be beneficial strategy for chemoresistant patients. We plan to perform in vivo study in the near future for verification from the results from our in vitro study
Disclosure: Nothing to disclose

P131

***Chlamydia trachomatis* and anti-MUC1 antibodies and subsequent risk of high grade serous ovarian cancer: a population-based case-control study in Northern Sweden**

S. Jonsson[1], E. Lundin[2], F. Elgh[3], U. Ottander[1], A. Idahl[4]
[1]Department of Clinical Sciences, [2]Department of Medical Biosciences Pathology, [3]Department of Clinical Microbiology Virology, [4]Umeå University, Umeå, Sweden

Introduction / Background: *Chlamydia trachomatis* (*C. trachomatis)* salpingitis causes inflammatory damage to the fallopian tube, the suggested origin of most high grade serous cancers (HGSC), and could thereby cause initiation and progression of ovarian cancer. Infection with *C. trachomatis* may stimulate production of MUC1 protein and potentially both increase or decrease anti-MUC1 antibody levels. The aim of this study was to examine if serology indicating past infection with *C. trachomatis* and anti-MUC1 antibodies in prospective blood samples were associated with HGSC.
Methodology: In a prospective nested case-control study within the Northern Sweden Health and Disease Study (NSHDS) and the Northern Sweden Maternity Cohort (NSMC), the prevalence of

chlamydial and anti-MUC1 antibodies was analyzed in blood samples drawn more than one year prior to diagnosis from 92 women with HGSC and 363 matched controls. Matching factors were age and date at blood draw. Plasma *C. trachomatis* IgG was analyzed using a MIF-test (Focus Diagnostics), chlamydial HSP60 (cHSP60) and anti-MUC1 IgG were analyzed using ELISA serology (Medac; Thermo-Fisher Scientific). HGSC diagnosis was confirmed by pathology report review.
Data were analyzed using Chi-square test, Fisher's exact test and Mann-Whitney *U* test. Correlation analysis was done by Spearman's correlation test. A two-sided P-value less than 0.05 was considered significant.
Results: The prevalence of *C. trachomatis* IgG and cHSP60 IgG antibodies, as well as the level of anti-MUC1 IgG in women with HGSC compared with controls were similar (16.3% vs. 17.0%, $p = 0.867$; 27.3% vs 28.5%, $p = 0.802$; median 0.24 vs 0.25, $p = 0.700$). A significant correlation was found between anti-MUC1 IgG and cHSP60 IgG (r = 0.169; $p < 0.001$).
Conclusion: There were no significant association between chlamydial or anti-MUC1 IgG antibodies and HGSC in this prospective nested case control study.
Disclosure: This work was supported by grants from The Cancer Research Foundation in Northern Sweden, and The County Council of Västerbotten, Sweden.

P132

Significance of locus-specific TP53 mutations in ovarian cancer

K. Knoll, A. Berger, I. Tsibulak, K. Leitner, C. Degasper, H. Welponer, V. Wieser, C. Marth, H. Fiegl, A.-G. Zeimet
Universitätsklinik für Frauenheilkunde, Medizinische Universität Innsbruck, Innsbruck, Austria

Introduction / Background: The role of *TP53* in tumor initiation and progression is widely studied. Depending on the histological subtype there are mutation rates in ovarian cancer (OC) of up to 95%. Most available studies assess the presence or absence of a *TP53* mutation without taking into consideration the specific localisation and function of the mutation. The influence of exon specific *TP53* mutations on the tumor biology was examined.
Methodology: DNA samples from a total of 189 OC patients that had been diagnosed between the years of 1989 and 2014 were analysed using next-generation sequencing and the TruSight Cancer panel. Correlations between the presence of *TP53* mutation and clinicopathological features were analysed using Chi-quadrat test. Survival analysis were performed with Kaplan-Meier and Cox regression.
Results: Patients in the *TP53* wild-type group demonstrated a statistically significant longer progression free survival (PFS) (p= 0.003) and overall survival (OS) (p= < 0.001) than those in the TP53-mutant group. In HGSOC a statistically significant difference in PFS of patients with tumors harbouring *TP53* mutations in exons 1-5 compared with exons 6-8 (Median: 2.95 vs. 1.31 years) was revealed. A similar effect has been shown for the OS (Median: 3.62 vs. 3.01 years). However, cancers with *TP53* mutations in exons 9-11 exhibited no difference in the PFS (p= 0.87) and OS (p= 0.78) when compared with TP53 wild-type cancers. Tumors with *TP53* mutations with a risk of pathogenicity of >95% had a risk for progression (RR: 1.78; p= 0.025), which was significantly higher in comparison to the wild-type cancers.
Conclusion: This study revealed that different *TP53* mutations have different effects on the biology of OC. Therefore accurate assessment of the locus-specific *TP53* status in OCs is essential to understand their malignant phenotype and differences in chemotherapy responsiveness with the possibility to predict their clinical course.
Disclosure: Nothing to disclose

P133

Low anterior resection syndrome (LARS) in patients with epithelial ovarian cancer after primary debulking surgery

M. Kranawetter[1], C. Grimm[1], B. Ataseven[2], S. Schneider[2], S. Riss[1], P. Alesina[2], S. Prader[2], M. Walz[2], F. Harpain[1], A. Stift[1], F. Heitz[2], A. Reinthaller[1], S. Polterauer[1], P. Harter[2], A. du Bois[2]
[1]Medical University of Vienna, Vienna, Austria, [2]Kliniken Essen Mitte, Essen, Germany

Introduction / Background: The low anterior resection syndrome (LARS) is a long-term complication observed in patients after low anterior resection (LAR). LARS comprises a collection of intestinal dysfunction symptoms, such as diarrhea, fecal incontinence, increased frequencies of defecation, fecal urgency or incomplete emptying.
Diagnosis of LARS is based on the LARS score, a validated questionnaire consisting of five questions and classifies symptoms as “no LARS”, “minor LARS” or “major LARS”. Evidence for LARS is exclusively based on studies for rectal cancer.
The aim of the present study was to evaluate the prevalence of LARS in patients with debulking surgery for epithelial ovarian cancer (EOC) and to identify potential risk factors for development of LARS.
Methodology: We reviewed data on 552 consecutive patients with EOC, who underwent upfront or interval cytoreductive surgery including LAR at two academic institutions. Intestinal dysfunction was assessed by the LARS-questionnaire via telephone call.
Results: In total, 341 patients were eligible and 206 (60.4%) were successfully contacted and provided complete information. Major LARS was observed in 78 (37.9%) patients, minor LARS in 44 (21.4%) patients, and no LARS in 84 (40.8%) patients. The prevalence rate of major LARS was not influenced by time interval between surgery. In multivariate analyses, number of anastomosis was independently associated with an increased risk for presence of major LARS (OR 3.76 [1.95- 7.24]).
Conclusion: LARS is a frequent long-term complication after debulking surgery including LAR in EOC patients. Patients with more than one bowel anastomoses seem to be at an increased risk for major LARS.
Disclosure: MK has nothing to disclose. CG received honoraria from AstraZeneca, Celgene, MSD, PharmaMar, Roche, Tesaro, Vifor Pharma; Amgen, AstraZeneca, MSD, PharmaMar, Roche, Tesaro outside of the submitted work. BA received honoraria from Roche, Amgen, Astra Zeneca, Tesaro, Clovis, Celgene, PharmaMar, outside the submitted work . SS has nothing to disclose. SR received honoraria from Medtronic, Takeda outside of the submitted work. PA has nothing to disclose. SP has nothing to disclose. MW has nothing to disclose. FH has nothing to disclose. AS received honoraria from Takeda, outside the submitted work. FH received honoraria from Roche, AstraZeneca, Clovis, Tesaro, PharmaMar, outside the submitted work. AR received honoraria from AstraZeneca, Celgene, MSD, PharmaMar, Roche, Tesaro, Vifor Pharma; Amgen, AstraZeneca, MSD, PharmaMar, Roche, Tesaro, outside of the submitted work. SP received honoraria from AstraZeneca, Celgene, MSD, PharmaMar, Roche, Tesaro, Vifor Pharma; Amgen, AstraZeneca, MSD, PharmaMar, Roche, Tesaro, outside of the submitted work. PHreceived honoraria from Astra Zeneca, Roche, Tesaro, Sotio, Stryker, ZaiLab, MSD, Lilly, Clovis, Immunogen, outside the submitted work. AdB received honoraria from Roche, Clovis, Astra Zeneca, Tesaro, Pfizer, Pharmar, Biocad, Genmab, outside of the submitted work.

P134

The usability of CA 125 and HE4 tumour markers in the prediction and verification of complete tumour resection and progression free survival in advanced ovarian cancer patients

S. Molnár[1], E. Maka[1], Z. Bagoly[2], R. Lampé[1], R. Póka[1], Z. Hernádi[1], Z. Krasznai[1]
[1]Department of Obstetrics and Gynaecology Gynaecologic Oncology, [2]Department of Laboratory Medicine Division of Clinical Laboratory Sciences, University of Debrecen Faculty of Medicine, Debrecen, Hungary

Introduction / Background: The goal of primary debulking surgery (PDS) of ovarian cancer is complete tumour reduction, since any residual tumour disproves overall survival. In later cases neoadjuvant chemotherapy may be an option. We aimed to investigate the use of CA 125 and HE4

preoperative levels in the prediction of complete tumour reduction during upfront surgery, and the use of postoperative marker levels in the verification of complete tumour reduction and progression-free survival (PFS).
Methodology: 88 advanced ovarian cancer patients (FIGO III-IV), operated between 2010.01.01-2015.12.31 who had attempted curative PDS, had CA 125 and/or HE4 results before and/or after the operation were included. Progression free survival was assessed through Kaplan-Meier analysis, Receiver Operative Characteristics (ROC) curves and area under curve (AUC) was used to evaluate the performance of CA 125 and HE4 markers.
Results: Preoperative HE4 levels differed significantly in cases of successful complete and incomplete reduction (median [IQR]: 192 [80.1-573.7] vs. 1044 [592.8-2912] respectively, p< 0.0001). The ROC analysis gave an AUC of 0.82, and the sensitivity and specificity of complete reduction were 86.67% and 73.33% respectively, at a cut-off level of 496 pmol/l. Preoperative CA 125 proved to be less useful in distinguishing between the two groups. Postoperative HE4 levels differed significantly in the patients who underwent complete reduction from those with residual disease, median [IQR] levels were 54.2 [39.2-82.05] vs. 364.9 [123.3-809.9] respectively (p< 0.0001). HE4 also proved more useful in the prediction of >1 year PFS: AUC 0.786 and at a cut-off level of 138, sensitivity and specificity of >1 year PFS were 75% and 75% respectively (p< 0.001).
Conclusion: HE4 proved to be a more useful preoperative tool in the selection of patients for future successful complete reduction. Postoperative HE4 levels might be used as an independent quality assurance marker for the verification of complete reduction and for prediction of PFS.
Disclosure: Nothing to disclose

P135

Discovery and validation of novel biomarkers for detection of epithelial ovarian cancer

H. Kulbe[1], R. Otto[2], S. Darb-Esfahani[3], H. Lammert[3], S. Abobaker[1], G. Welsch[3], R. Chekerov[1], R. Schäfer[3], D. Dragun[3], M. Hummel[3], U. Leser[2], J. Sehouli[1], E. Braicu[1], Tumorbank Ovarian Cancer Network
[1]Department of Gynecology, European Competence Center for Ovarian Cancer, Campus Virchow Clinic, Charité - Universitätsmedizin Berlin, Corporate Member of Freie Universität Berlin, Humboldt-Universität zu Berlin, Berlin Institute of Health, Department of Gynecology, European Competence Center for Ovarian Cancer, Campus Virchow Clinic, [2]Institute for Computer Sciences, Humboldt-Universität zu Berlin, [3]Charité - Universitätsmedizin Berlin, corporate member of Freie Universität Berlin, Humboldt-Universität zu Berlin, Berlin Institute of Health, Berlin, Germany

Introduction / Background: Early detection of epithelial ovarian cancer (EOC) is poses a critical medical challenge, however, novel biomarkers for early diagnosis remain to be discovered. Here, we investigate both epithelial and stromal compartments, which have been neglected in the past in search for novel candidates.
Methodology: We queried gene expression profiles of microdissected epithelium and adjacent stroma from benign and malignant tumors (GSE29156) and normal epithelium samples (GSE14407). Genes significantly differentially expressed within either the epithelial or specifically the stromal compartments were retrieved. The expression of genes whose products are secreted yet absent in the blood of healthy donors were validated in tissue and blood from patients with pelvic mass by NanoString analysis. Results were confirmed by the independent gene expression database CSIOVDB.
Results: Top 25% of candidate genes were validated, twelve were able to discriminate between benign and malignant tumors on transcript levels (p< 0.05). The stroma-derived candidates versican (VCAN) and syndecan-3 (SDC3) were increased in blood of patients with EOC ovarian cancer compared with benign gynecological conditions. Transcript levels of the nuclear orphan receptor NR2F6, denticleless E3 ubiquitin protein ligase DTL, myelin and lymphocyte protein MAL, aurora kinase A (AURKA), VCAN and SDC3 genes performed significantly better than those of known biomarkers to discern malignant from benign conditions. Furthermore, elevated MAL and AURKA expression levels correlated significantly with poor prognosis.
Conclusion: We identified promising novel candidates and found the stroma of ovarian cancer to be a suitable compartment for early detection biomarker discovery.
Disclosure: Nothing to disclose

P136

Risk factors for anastomotic leakage after colorectal resection in ovarian cancer surgery: a multi-centre study

V. Lago[1], C. Fotopoulou[2], V. Chiantera[3], L. Minig[4], A. Gil-Moreno[5], P.A. Cascales-Campos[6], M. Jurado[7], Á. Tejerizo[8], P. Padilla-Iserte[1], M.E. Malune[2], M.C. Di Dona[9], T. Marina[10], J.L. Sanchez-Iglesias[5], A. Olloqui[8], Á. García-Granero[1], L. Matute[1], V. Fornes[11], S. Domingo[1]
[1]University Hospital La Fe, Valencia, Spain, [2]Imperial College London, London, United Kingdom, [3]University of Palermo, Palermo, Italy, [4]CEU Cardenal Herrera University, Valencia, [5]University Hospital Vall d'Hebron, Barcelona, [6]Virgen de la Arrixaca Clinic and University Hospital, Murcia, [7]University Clinic of Navarra, Navarra, [8]University Hospital 12 de Octubre, Madrid, [9]University of Palermo, Palermo, [10]Valencian Institute of Oncology, [11]Health Research Institute Hospital La Fe, Valencia, Spain

Introduction / Background: In advanced ovarian cancer surgery, there is rather limited published evidence, drawn from a small sample, providing information about risk factors for anastomotic leak.
Methodology: To determine pre-/intraoperative risk factors for anastomotic leak after modified posterior pelvic exenteration (MPE) or colorectal resection in ovarian cancer and to create a practical instrument for predicting anastomotic leak risk. Eight hospitals participated in this retrospective study. Data on 695 patients operated for ovarian cancer with primary anastomosis were included (January 2010-June 2018). Twelve pre-/intraoperative variables were analysed as potential independent risk factors for anastomotic leak. A predictive model was created to stablish the risk of anastomotic leak for a given patient.
Results: he anastomotic leak rate was 6.6% (46/695; range 1.7%-12.5%). A total of 457 patients were included in the final multivariate analysis. The following variables were found to be independently associated with anastomotic leakage: age at surgery (OR 1.046, 95% CI 1.013-1.080, p = 0.005), serum albumin level (OR 0.621, 95% CI 0.407-0.948, p = 0.027), one or more additional small bowel resections (OR 3.544, 95% CI 1.228-10.23, p = 0.019), manual anastomosis (OR 8.356, 95% CI 1.777-39.301, p = 0.007) and distance of the anastomosis from the anal verge (OR 0.839, 95% CI 0.726-0.971, p = 0.018).
Conclusion: Due to the low incidence of AL in ovarian cancer patients, a restrictive stoma policy based on the presence of risk factors should be the actual recommendation. Hand-sewn anastomosis should be avoided.
Disclosure: Nothing to disclose

P137

Management and outcomes of recurrent/progressive ovarian clear cell carcinoma

C.-H. Lai[1,2], H.-J. Huang[1,2], L.-Y. Yang[2,3], H.-J. Tung[1,2], R.-C. Wu[2,4], A. Chao[1,2]
[1]Obstetrics and Gynecology, [2]Gynecologic Cancer Research Center, [3]Biostatistical Unit Clinical Trial Center, [4]Department of Pathology, Chang Gung Memorial Hospital at Linkou, Taoyuan, Taiwan

Introduction / Background: Ovarian clear cell carcinoma (OCCC) with recurrence/progression after treatment has dismal prognosis. We aimed to investigate the management and outcomes of such patients.
Methodology: OCCC patients who received primary surgery between 2000 and 2013 with cancer recurrence or progression after primary treatment were analyzed. Univariate and multivariate analyses were used to identify the independent predictors of survival after recurrence (SAR), progression-free interval (PFI) and cancer-specific survival (CSS).

Results: A total of 64 patients experienced treatment failure (49 with cancer recurrence and 15 with progression without upfront remission). The 5-year CSS rates of recurrent/progressive OCCC patients were 22.9% (progression group: median CSS 5.9 months [range, 0.8-25.2] versus recurrence group 43.6 months [range, 7.1-217.8] ; p < 0.001). Patients with solitary recurrence had significantly better SAR than those with disseminated relapse (median: not reached *vs* 8.9 months, p < 0.001). On multivariate analysis, three models each for SAR and CSS were formulated alternatively including highly correlated variables. Of these, solitary relapse pattern (HR 0.07, p < 0.001), PFI-1 >12 months (HR 0.22-0.40, p = 0.023 to < 0.001), CA125 < 35U/mL at initial recurrence (HR 0.32, p = 0.007), and overall salvage treatment included radiotherapy (HR, 0.19, p = 0.001) were significant predictors of longer SAR, and better CSS (p = 0.034 to < 0.001).
Conclusion: Recurrent OCCC can be treated with curative intent if the relapse is solitary and can be completely resected or encompassed with radiotherapy. Meanwhile, novel therapies are needed for disseminated relapse or progression during primary treatment.
Disclosure: Conflict of interest statement: C.-H. Lai, TTY Biopharm (Research fund to Institution), Roche (fund to Inst), MSD (fund to Inst), AstraZeneca (Advisory board); A. Chao, AstraZeneca (fund to Inst). The other authors have no conflicts of interest to disclose.

P138

Time trends for incidence and survival of epithelial ovarian, fallopian tube, peritoneal and undesignated site cancer in Sweden 1960-2014 - a population-based survey of cancer registry data

P. Leandersson[1], T. Högberg[2], P. Dickman[3], S. Malander[4], C. Borgfeldt[5]
[1]Reproductive Medicine Centre, Skåne University Hospital, Lund University, Malmö, [2]Dept of Cancer Epidemiology, Skåne University Hospital, Lund University, Lund, [3]Dept of Medical Epidemiology and Biostatistics, Karolinska Institutet, Stockholm, [4]Dept of Oncology and Pathology, Skåne University Hospital, [5]Dept of Obstetrics and Gynecology, Skåne University Hospital, Lund University, Lund, Sweden

Introduction / Background: Analysis of survival trends in epithelial ovarian cancer with regard to age, tumour site and morphological subgroup in Sweden 1960 to 2014.
Methodology: Nation-wide population-based study on data from the Swedish Cancer Registry. 46 350 women aged 18 or older with a diagnosis of epithelial ovarian, fallopian tube, peritoneal or undesignated site cancer 1960 to 2014 were included. Analyses of age-standardised incidence and relative survival (RS) were done and time trend graphs modelled according to age, tumour site and morphological subgroup.
Results: Overall incidence of ovarian, tubal, peritoneal and undesignated site cancer declined since 1980. Median age at diagnosis increased. Serous cancer increased in incidence. RS at 1-, 2- and 5-years improved since 1960 although not for the youngest and the oldest patients. 10-year RS did not improve. Best RS was found for fallopian tube cancer and worst RS for undesignated site cancer. Among the histological subgroups endometrioid cancer had the best RS. RS improved most for endometrioid and mucinous cancer.
Conclusion: Survival in epithelial ovarian, tubal, peritoneal and undesignated site cancer in Sweden has improved over the last six decades. Advances in epithelial ovarian cancer treatment have extended life for the first 5 years from diagnosis but not long-term survival.
Disclosure: Nothing to disclose

P139

The role of transcriptomic SMARCA4 expression in ovarian cancer biology

K. Leitner, V. Wieser, I. Tsibulak, C. Degasper, H. Welponer, K. Knoll, D. Reimer, A. Wiedemair, C. Marth, H. Fiegl, A.G. Zeimet
Department of Obstetrics and Gynecology, Medical University of Innsbruck, Innsbruck, Austria

Introduction / Background: The SWI/SNF complex - a chromatin remodelling complex - is recurrently mutated and inactivated in ~20% of human cancers. Mutations of the SMARCA4 gene, which encodes the ATPase of the SWI/SNF complex, can be found in the majority of small cell carcinomas of the ovary, hypercalcaemic type (SCCOHT). EZH2 is the catalytic subunit of the PCR2 complex, which is an opponent of the SWI/SNF complex. EZH2 inhibitors could play a potential role in the therapy of SCCOHT. The aim of this study was to explore the role of SMARCA4 mRNA-expression in ovarian cancer.
Methodology: SMARCA4 mRNA-expression was investigated in 20 non-neoplastic fallopian tubes and 276 ovarian carcinomas (OC) (including 144 high grade serous ovarian carcinomas, HGSOC) in relation to their clinical characteristics, p53- and BRCA1/2- mutational status, BRCA1/2 mRNA-expression and EZH2 mRNA-expression.
Results: The SMARCA4 mRNA-expression in OCs was significantly higher compared to the control group (P< 0,001). There were no significant differences in SMARCA4-expression between the various histological subtypes.
A subgroup-analysis of HGSOC showed higher SMARCA4-expression in patients younger than 65 years (P=0,021), in patients without residual disease after surgical debulking (P=0,008) and in patients with early FIGO stages I-II (P=0,004). High SMARCA4-expression was associated with better progression-free survival (PFS) (P=0,05) and better overall survival (OS) (P=0,002). Patients with p53-mutations showed higher SMARCA4-expression (P=0,004). There was a significant correlation between the expression of SMARCA4 and EZH2 (P=0,000 r=0,377). Multivariate Cox-regression model revealed independency of the prognostic value of SMARCA4-expression for OS in patients under the age of 65 years (P=0,015).
Conclusion: High SMARCA4-expression in patients with HGSOC is associated with younger age at onset of disease, early FIGO stage and with absence of residual disease after primary debulking. In patients younger than 65 years, high SMARCA4-expression is independently associated with better overall survival.
Disclosure: Nothing to disclose

P140
The roles of sex hormone receptors and chemosensitivity in ovarian cancer
H. Lin, H.-C. Fu, Y.-C. Ou, C.-H. Wu, H.-Y. Kang
Kaohsiung Chang Gung Memorial Hospital, Kaohsiung, Taiwan

Introduction / Background: Expression of sex hormonal receptors (SHR) had been reported to influence survival outcomes in patients with epithelial ovarian cancer. In present study, we attempted to investigate the association and mechanism between SHR expression and chemosensitivity in epithelial ovarian cancer (EOC).
Methodology: We reviewed 71 patients with EOC who underwent surgery followed by adjuvant chemotherapy and analyzed sex hormone receptors (SHR) immunohistochemical (IHC) expression of tissue samples obtained from these patients. The disease-free survival (DFS), overall survival (OS), and percentage of chemosensitivity were compared between weak and strong SHR IHC expression. The effects of cisplatin administration on cell proliferation in ovarian cancer OC3-VGH cells with different SHR expression were investigated.
Results: Patients with strong androgen (AR) and progesterone receptors (PR) IHC expression had better 5-year DFS, OS, and chemosensitivity. In cell model data, overexpression of AR and PR OC3-VGH cells caused higher sensitivity to cisplatin treatment. OV3-VGH cisplatin resistant clone expressed lower level of AR and PR than wild-type. Overexpression of AR OC3-VGH cells showed higher DNA damage signals after cisplatin treatment.
Conclusion: Our data indicated that strong AR and PR expression were good prognostic factors via the mechanism of better cisplatin sensitivity. Novel therapeutics against AR and PR might be promising for treatment of EOC patients.

Disclosure: Nothing to disclose

P141

Let's think twice before abandoning secondary cytoreductive surgery in recurrent ovarian cancer

C. Marchetti, A. Rosati, G. Scaletta, A. Pietragalla, M. Arcieri, R. Ergasti, E. Palluzzi, G. Scambia, A. Fagotti

Fondazione Policlinico Universitario Agostino Gemelli IRCCS, Roma, Italy

Introduction / Background: The role of secondary cytoreductive surgery (SCS) in recurrent ovarian cancer (ROC) is controversial after results of randomized trials.

The aim of this study is to evaluate SCS in ROC patients with BRCA ½ (BRCAmut) who received platinum-based chemotherapy followed by olaparib maintenance.

Methodology: This is a case-control study. Patients with recurrent platinum-sensitive high grade serous ovarian cancer admitted to our Gynecologic Oncology Unit between 2014 and 2018 were identified.

Main eligibility criteria were positive BRCA 1/2 germline or somatic mutation status and olaparib maintenance at primary recurrence after response to platinum based chemotherapy.

Cases were those who received SCS followed by medical treatment (SCS-CT-OLA, group 1), controls were those who received medical treatment alone (CT-OLA, group 2).

Results: Overall, 47 patients were identified; 24 (51%) BRCAmut women undergoing SCS followed by platinum based chemotherapy and olaparib maintenance were matched with 23 (49%) BRCAmut women who received medical treatment alone. Groups were well balanced: no statistical differences were found with regard of age, mutational status, outcomes and treatment's approach at diagnosis, timing and patterns of disease presentation at recurrence (Table 1 and 2). Median time to first subsequent therapy (TFST) was significantly longer in the SCS-CT-OLA than in the CT-OLA group (42 months vs 16 months; p=0.05; figure 1). Furthermore, SCS-CT-OLA patients had the best post-recurrence survival (PRS), with a 3-year PRS of 81% in SCS-CT-OLA group versus 43% in CT-OLA group (p = 0.01).

Conclusion: SCS increases TFST and PRS in ROC patients with BRCAmut candidate for olaparib maintenance after platinum-based chemotherapy. Prospective studies are needed. In the era of personalized medicine, indication to SCS should be individualized.

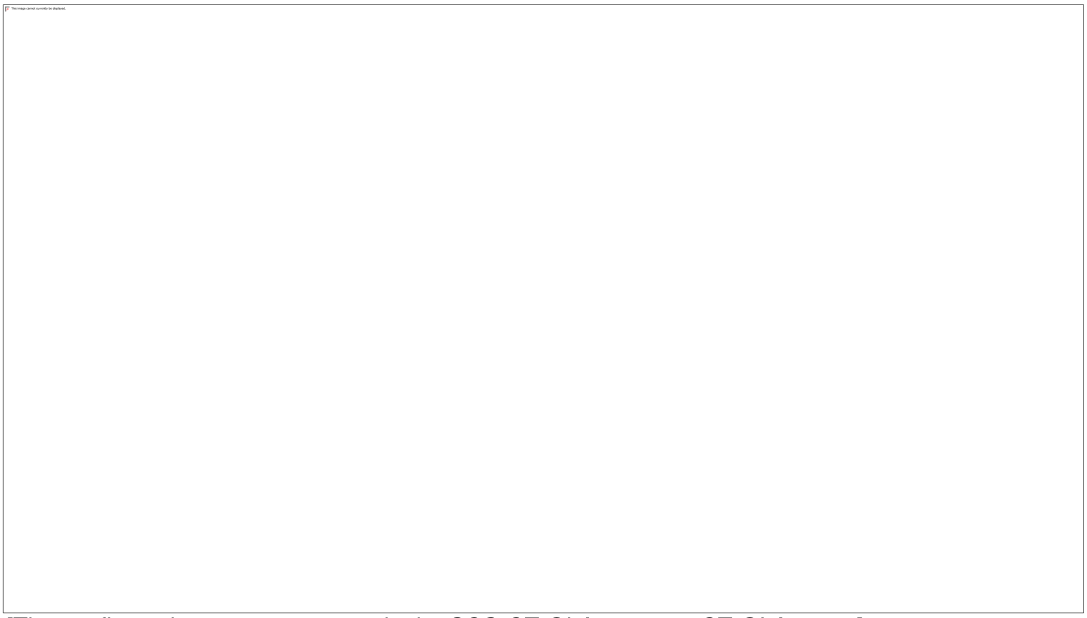

[Time to first subsequent treatment in the SCS-CT-OLA group vs CT-OLA group]

Parameters	All pts	SCS-CT-OLA (group 1)	CT-OLA (group 2)	p value
All cases	47	24 (51%)	23 (49%)	
Age at recurrence (Median and range, years)	54 (36-74)	54 (36-71)	53 (42-74)	0.81
FIGO Stage II	1 (2%)	1 (4%)	0	0.25
FIGO Stage III	45 (96%)	23 (96%)	22 (96%)	
FIGO Stage IV	1 (2%)	0	1 (4%)	
PDS	32 (68%)	17 (71%)	15 (65%)	0.46
NACT	15 (32%)	7 (29%)	8 (35%)	
RT(mm) 0	29 (91%)	15 (88%)	14 (91%)	0.58
RT(mm) 1-10	3 (9%)	2 (12%)	1 (9%)	

[Distribution of patients' clinico-pathological characteristics according with treatment received.]

Parameters	All pts	SCS AND OLA n (%)	CT AND OLA n (%)	p value
PFI (Median and range,months)	22 (8-56)	27 (8-56)	24 (13-54)	0.45
No ascites at recurrence	44 (94%)	23 (96%)	21 (91%)	0.48
Ascites at recurrence	3 (6%)	1 (4%)	2 (9%)	
Lymph nodal recurrence	32 (68%)	16 (67%)	16 (70%)	0.89
Parenchimal (lung,	11 (23%)	7 (29%)	4 (17%)	0.27

liver, spleen) recurrence				
Peritoneal recurrence	34 (72%)	18 (75%)	16 (79%)	0.46
Localized peritoneal disease (up to 3 nodules)	10 (21%)	7 (29%)	3 (13%)	0.39
Diffused peritoneal disease (more than 3 nodules)	24 (51%)	11 (48%)	13 (56%)	

[Patients characteristic and pattern of recurrent disease according with treatment received.]

Disclosure: Nothing to disclose

P142

Results of the 3rd interim analysis of C-Patrol: a non-interventional study on olaparib in German routine clinical practice

F. Marmé[1], F. Hilpert[2], M. Welslau[3], J.P. Grabowski[4], J. Seitz[5], A. El-Balat[6], A.D. Hartkopf[7], R. Glowik[8], J. Sehouli[4]

[1]Department of Gynecology & Obstetrics, University Hospital Mannheim, Medical Faculty Mannheim, University of Heidelberg, Mannheim, [2]Oncology Centre at Jerusalem Hospital Hamburg, Hamburg, [3]Medical office for hemato-oncology, Aschaffenburg, [4]Department of Gynecology and Center for Oncological Surgery, Charité Medical University, Berlin, [5]Department of Obstetrics & Gynecology and National Center for Tumor Diseases (NCT), University Hospital Heidelberg, Heidelberg, [6]Department of Obstetrics and Gynecology, Goethe University Frankfurt, Frankfurt, [7]Center for Women's Health, University Hospital of Tuebingen, Tuebingen, [8]AstraZeneca, Wedel, Germany

Introduction / Background: Olaparib (50 mg hard capsules, HC) was the 1st PARPi approved in the EU in 12/2014 as monotherapy for maintenance treatment of adult patients with platinum-sensitive relapsed *BRCA*-mutated (*BRCA*m) ovarian cancer (PSR-OC) who are in response to platinum-based chemotherapy. In addition, film-coated tablets (FT, 100/150 mg) were approved in 05/2018 regardless of BRCA status. Here, we report for the first time data on real-world olaparib treatment from patients who switched from HC to FT.

Methodology: The German prospective non-interventional study C-PATROL (NCT02503436) collects routine clinical and patient-reported outcome data in *BRCA*m PSR-OC patients treated according to label with olaparib with the recommended total daily dose of 800 mg (HC) or 600 mg (FT). The 3rd interim analysis (cut-off: 01APR2019) for patients treated with olaparib (HC/FT) reflects data on patient characteristics and safety by using descriptive statistics focussing on “switcher” (here patients can be treated with FT since 08/2018).

Results: 252 olaparib-treated patients were analysed; 177 (70.2%) were treated with HC, 47 (18.7%) switched from HC to FT, and 28 (11.1%) were newly initiated with FT.

Median therapy durations for the three subgroups were 9.0 months (HC), 17.4 months (switcher; 13.4 months before [HC] and 4.4 months after [FT] switch) and 9.2 months (FT).

44 switcher (93.6%) had adverse events (AEs); 41 (87.2%) before and 26 (55.3%) after switching. Treatment modifications due to AEs were more frequent prior to switching (i.e. during HC treatment). In total, 30 switcher had therapy modifications; 25 (83.3%) before and 11 (36.7%) after switching.

Conclusion: The 3rd interim analysis indicates that under routine conditions, olaparib is well tolerated in patients who switched from HC to FT. In fact, fewer AEs occurred after switching. The toxicity profile observed so far is in line with the results of the clinical trial program for olaparib in PSR-OC and 1st-line maintenance.

Funding: AstraZeneca

Disclosure: F. Marmé: honoraria received for speaker, consultancy or advisory role, travel, from AstraZeneca, Tesaro, Roche, Novartis, Pfizer, PharmaMar, GenomicHealth, CureVac, Amgen, Eisai,

MSD,Celgene, Clovis. F. Hilpert: membership on advisory boards for AstraZeneca, Roche, Tesaro, Clovis, MSD, PharmaMar, receipt of honoraria from Astra Zeneca, Clovis, Roche, Tesaro, MSD, PharmaMar, Pfizer, Novartis. M. Welslau: No conflict of interest, nothing to disclose. J. P. Grabowski: Advisory, consultancy and speakers activities for AstraZeneca, Tesaro, Clovis, Riemser, honoraria from AstraZeneca, Tesaro, Roche, Clovis, Pfizer, Riemser. J. Seitz: no conflict of interest, nothing to disclose. A. El-Balat: membership on advisory councils, speaker's activities for AstraZeneca, Roche, PharmaMar, Olympus. A. D. Hartkopf has received honoraria (speakers bureau and consulting fees) from AstraZeneca, Eisai, GenomicHealth, Lilly, MSD, Novartis, Pfizer, Roche, Tesaro, Teva. R. Glowik: employee of AstraZeneca. J. Sehouli: Advisory and expert activities for PharmaMar, Clovis, Tesaro, Roche, Roche Diagnostics, Astra Zeneca,Merck, Bayer,Eisei, Johnson and Johnson, MSD, Novocure, Amgen, Lilly; Receipt of professional fees from PharmaMar, Clovis, Tesaro, Roche, Astra Zeneca, receipt of research grant support (institution) from PharmaMar, Clovis, Tesaro, Roche, Astra Zeneca, Medimmune, Bristol Myers, Lilly.

P143

Risk factors for early death among ovarian cancer patients - a nationwide cohort study

B. Mosgaard[1], A. Meaidi[1], C. Hogdall[2], M. Noer[2]
[1]Gynecology, University of Copenhagen - Rigshospitalet, [2]JMC, Rigshospitalet, København Ø, Denmark

Introduction / Background: Denmark has, along with the UK, for many years ranked at the bottom of the statistics regarding ovarian cancer survival. Considering ovarian cancer survival curves, Denmark distinguishes itself by having a steep fall in survival during the first 0-6 months, after which the survival curve is parallel to the curves of the other countries. This high short-term mortality may be the primary explanation for the overall poor prognosis of Danish ovarian cancer patients.
Objective: To characterise ovarian cancer patients who die within six months of diagnosis and to identify prognostic factors for these early deaths.
Methodology: A nationwide cohort study covering women diagnosed with ovarian, fallopian tube, or primary peritoneal cancers in Denmark in 2005-2016.
Results: A total of 5,570 patients were included. Three months after ovarian cancer diagnosis 456 (8.2%) had died and 664 (11.9%) died within six months of diagnosis. Adjusted for age and comorbidity, patients who died early were admitted to hospital significantly more often in a six-month period before the diagnosis (OR 1.61 (1.29-2.00), and OR 1.47 (1.21-1.78)), for patients who died within three and six months respectively). Low educational level, low income and singlehood were factors significantly associated with higher risk of early death.
The discriminative ability of the risk factors family type, employment, income, comorbidity, BMI, admission to hospital due to pain or thromboembolism, tumour stage, performance status, cancer treatment, histology, and residual tumour in identifying early death was assessed by cross-validated area under the receiver operating characteristic curve (AUC). The AUC was found to be 0.91 (0.88-0.93) and 0.90 (0.87-0.92) for death within 3 and 6 months respectively.
Conclusion: Our study found that 90-91 % of deaths within six months from the ovarian cancer diagnosis could be predicted upon admission to the gynecologic department by including known patient- and tumour-related risk factors
Disclosure: Disclosures: None of the authors have conflicts of interest

P144

Use of 3D organotypic models to assess heterogeneity in response to platinum therapy in high grade serous ovarian cancer

K. Nixon, E. Curry, R. Wulandari, C. Morera-Albert, P. Cunnea, C. Fotopoulou
Imperial College London, London, United Kingdom

Introduction / Background: High-grade serous ovarian cancer (HGSOC) is the most common subtype of ovarian cancer, characterised by vast genomic instability and heterogeneity and acquired resistance to platinum-based chemotherapy. A study characterising intra-tumoural heterogeneity (ITH) in HGSOC has identified variations in phenotypic responses to platinum treatment between different metastatic sites. In this study, we aim to investigate the effect of the local microenvironment on metastatic tumour cells' response to treatment through development of 3D organotypic models constructed from omental stromal cells embedded in an extracellular matrix (ECM).
Methodology: 3D models were assembled using normal omental stromal cells embedded in Collagen-I. Metastatic deposits were obtained from defined anatomical regions during upfront radical debulking surgery of advanced stage HGSOC patients. Tumour cells were extracted, cultured short-term, added to 3D models and treated with platinum for 24hrs. Flow cytometry was used to analyse tumour cell apoptosis within the models following treatment. To study variations in ECM composition by anatomical tumour location, ECM proteins were extracted for Mass Spectrometry analysis (4 patients, 5 samples per patient including primary, omentum, diaphragm, spleen, paracolic and rectal deposits).
Results: Tumour cells cultured in 3D showed heterogeneity in response to platinum treatment, data showed a trend towards reduced response to treatment within 3D models compared to 2D (n=8). Changes in patterns of response to treatment between samples from 2D to 3D within the same patient was also demonstrated (n=5). Proteomic analysis displays heterogeneity in ECM composition across patients and different anatomical locations.
Conclusion: Data will be correlated with our ongoing ITH study, including proteomic and copy-number analysis of corresponding disseminated tumour deposits, providing a definitive description of ITH and clonally evolved chemoresistance in HGSOC. Analysis of ECM composition from different anatomical sites will help develop 3D model-matrices further, incorporating more elements of the microenvironment and creating an experimental design better reflecting the *in-vivo* situation.
Disclosure: Professor Christina Fotopoulou, COI: advisory boards and honoraria from Roche, Tesaro, Sequana, Olympus, Astra Zeneca

P145

Preoperative staging of advanced ovarian cancer: comparison between ultrasound, computed tomography (CT) and whole-body MRI with diffusion-weighted sequence (WB-DWI/MRI)

P. Pinto[1,2], R. Kocian[3], F. Fruhauf[3], J. Slama[3], M. Zikan[4], P. Dundr[5], L. Dusek[6], A. Burgetová[7], M. Masek[7], D. Cibula[3], H. Reina[8], D. Fischerová[3]
[1]Obstetrics and Gynecology, Maternidade Dr. Alfredo da Costa - Centro Hospitalar Universitário de Lisboa Central, EPE, Lisbon, Portugal, [2]First Faculty of Medicine and General University Hospital, Charles University, [3]Gynecologic Oncology Center, Department of Obstetrics and Gynecology, First Faculty of Medicine, Charles University in Prague and General University Hospital in Prague, [4]Obstetrics and Gynecology, Bulovka Hospital, First Faculty of Medicine, Charles University, [5]Pathology, First Faculty of Medicine, Charles University in Prague and General University Hospital in Prague, Prague, [6]Institute of Biostatistics and Analysis, Masaryk University, Brno, [7]Radiology, First Faculty of Medicine, Charles University in Prague and General University Hospital in Prague, Prague, Czech Republic, [8]Obstetrics and Gynecology, University Hospital Basel, Basel, Switzerland

Introduction / Background: To compare the diagnostic accuracy of ultrasound (US), CT and WB-DWI/MRI in preoperative staging and assessment of optimal operability in patients with ovarian cancer.
Methodology: Patients planned for ovarian cancer surgery were enrolled. They underwent preoperative work-up with US, CT, and WB-DWI/MRI, following evaluation form. Findings were compared to the reference standard (intraoperative and histopathological evaluation forms).
Results: From 67 patients enrolled, 51 (76%) had advanced and 16 (24%) early stage ovarian cancer. Ultrasound showed the best results in the detection of pelvic carcinomatosis, in contrast with MRI and CT (AUC 0.94, 0.91, and 0.82, respectively), and in the evaluation of the depth of rectosigmoid

infiltration (AUC 0.96, 0.81, and 0.85). In the upper abdomen, all three methods showed comparable results in the detection of liver involvement (AUC 0.78, 0.79 and 0.76 for US, CT and WB-DWI/MRI), while US had the lowest AUC in the assessment of diaphragm (AUC 0.73, 0.84, 0.81). In the middle abdomen, ultrasound reached the highest AUC in the assessment of greater omentum and anterior wall (AUC of 0.87 and 0.76), in comparison with MRI (AUC 0.85 and 0.74), followed by CT (AUC 0.80 and 0.66). Ultrasound, MRI and CT showed comparable results in the assessment of bowel surface involvement (AUC 0.70, 0.69, 0.71), and ultrasound and MRI were better than CT (AUC 0.64, 0.71 and 0.76) in mesenterial involvement detection. In the assessment of retroperitoneal LNs, ultrasound and MRI were comparable (AUC 0.83), followed by CT (AUC of 0.76). All three modalities showed similar AUC in the prediction of optimal operability (AUC of 0.79 for US and CT and 0.80 for MRI).
Conclusion: This is the first prospective study documenting the potential role of ultrasound in ovarian cancer staging, compared to the method of choice (CT) and a novel technique (WB-DWI/MRI).
Disclosure: This work was supported by a grant from the Czech Research Council (AZV NV19- 03-00552).

P146

Patient engagement in rare disease research: a new paradigm

J.F. Roze[1], G.M. Monroe[1], J.W. Groeneweg[1], I. Fransen[2], R.H.M. Verheijen[1], R.P. Zweemer[1]
[1]Department of Gynaecological Oncology, UMC Utrecht Cancer Center, University Medical Center Utrecht, Utrecht University, [2]Member of Dutch and International Granulosa Cell Tumour Patient Organization, Utrecht University, Utrecht, The Netherlands

Introduction / Background: Currently, researchers seem to aim for high impact scientific articles rather than research that is directly beneficial to patients. Patient engagement is therefore an increasingly relevant topic. Grant donors also focus on the translation of a scientific benchside discovery to the patient bedside. However, patients are mostly involved solely at study initiation but ideally should participate throughout the research process. Also, investigators often do not report back the results to the patients. Here, we aim to illustrate successful patient engagement in a Dutch multi-center study on a rare disease: granulosa cell tumours (GCTs) of the ovary.
Methodology: GCTs are neglected and often not included in ovarian cancer research due to their rarity and distinct behaviour. A patient's family initiated a fund to increase awareness and ultimately improve treatment, as it can be challenging to receive regular funding for rare diseases. Her particular experiences helped identifying the challenges that GCT patients face and determined the knowledge gaps. This resulted in patient engagement from the beginning. Additionally, we joined group meetings and informed patients through newsletters, explaining the current state of the research and giving them the possibility to directly ask the research team questions and brainstorm on future directions.
Results: A national, multi-center study was conducted and included 51 patients within a year. This comprises three times the yearly incidence (17-20 cases) of this rare disease in our country. This success is due to successful engagement of patients and their gynaecological-oncologists. Contact with the patient's organization guided future research directions and raised novel research questions. For example, two members who appeared to be distant relatives initiated a project on hereditary factors in these patients.
Conclusion: Patient engagement is crucial in research as it benefits both patients and researchers. Involvement facilitates patient enrollment and study publicity while also focusing on research that directly benefits patients.
Disclosure: No competing interests. Applicable funding sources: Granulosafonds Philine van Esch

P147

Long-term survival in women with high-grade serous ovarian cancer: interplay between RB1 and BRCA1/2

F.A.M. Saner[1], D.W. Garsed[1,2], J.A. Beach[1], A. Pandey[1], S. Fereday[1], K. Alsop[1], M.C.A. Wouters[3], N. Traficante[1], C.L. Pearce[4], M.C. Pike[4], A. de Fazio[5,6,7], S.J. Ramus[8,9], M. Köbel[10], E.L. Goode[11], B.H. Nelson[3], D.D.L. Bowtell[1,2], Australian Ovarian Cancer Study Group, Multidisciplinary Ovarian Cancer Outcomes Group
[1]Peter MacCallum Cancer Centre, [2]Sir Peter MacCallum Department of Oncology, The University of Melbourne, Melbourne, VIC, Australia, [3]Deeley Research Centre, British Columbia Cancer Agency, Victoria, BC, Canada, [4]Department of Epidemiology and Biostatistics, Memorial Sloan Kettering Cancer Center, New York, NY, United States, [5]Centre for Cancer Research, The Westmead Institute for Medical Research, [6]Department of Gynaecological Oncology, Westmead Hospital, Westmead, [7]The Universtiy of Sydney, [8]School of Women's and Children's Health, University of New South Wales, [9]Garvan Institute of Medical Research, Sydney, NSW, Australia, [10]Department of Pathology and Laboratory Medicine, Foothill Medical Center, University of Calgary, Calgary, AB, Canada, [11]Department of Health Science Research, Divison of Epidemiology, Mayo Clinic Hospital, Rochester, MN, United States

Introduction / Background: Only ~15% of patients with high-grade serous ovarian cancer (HGSC) survive >10 years. Those patients with a more typical disease trajectory may benefit from insights gained from long-term survivors (LTS). Here, we investigated genomic and immunologic determinants of exceptional survival *in vivo* and *in vitro* with a focus on the tumour suppressor RB1.
Methodology: Whole-genome sequencing (WGS) was performed on a unique set of primary tumors and germline samples from a cohort of 55 women with >10-year survival following a diagnosis of advanced stage (Stage IIIC/IV) HGSC. Tumors were also characterised by RNA sequencing and immunohistochemistry (IHC). RB1 was depleted in HGSC cell lines through CRISPR-Cas9 knockout (KO). The impact of *RB1* loss was analysed in assays of chemosensitivity, clonogenicity, immunogenicity and transcriptomics.
Results: WGS showed that somatic inactivation of *RB1* was common in LTS, with 33% of tumours showing loss of RB1 protein by IHC compared to 13% of unselected HGSC controls (n=207; P=0.001). IHC revealed that RB1 loss was associated with increased numbers of PD-1+ tumour-infiltrating lymphocytes (P=0.015) and MHC class I on tumor cells (P=0.002). In an independent HGSC cohort (n=847) from the OTTA consortium, co-occurrence of germline *BRCA1/2* mutations and RB1 loss was associated with a significantly longer overall survival than patients with intact *BRCA1/2* and *RB1* (HR: 0.44, P< 0.001). In RB1 negative tumours, top enriched pathways involved E2F targets and interferon response genes. RB1 KO impaired clonogenic survival after cisplatin/paclitaxel combination therapy specifically in the *BRCA1* mutant cell line JHOS2 (P < 0.01). Expression of immune markers MHC Class II and PD-L1 was increased in RB1 KO cells.
Conclusion: In HGSC, concurrent loss of *RB1* and *BRCA1/2* mutation is associated with significantly longer overall survival. RB1 loss appears to result in impaired self-renewal after standard chemotherapy and enhanced host immune response.
Disclosure: F.A.M.S is supported by a Swiss National Foundation Early Postdoc Mobility Fellowship, a Swiss Cancer Research Foundation grant and the Prof. Dr. Max Cloëtta foundation. D.W.G. is supported by U.S. Army Medical Research and Materiel Command grant. AOCS receives funding support from Astra Zeneca and Ovarian Cancer Australia. C.L.P. and M.C.P. acknowledge support from the US Congressionally Directed Ovarian Cancer Research Program administered by the US Army Medical research and Materiel Command, grant number W81XWH-16-2-0010. D.D.L.B. is supported by U.S. Army Medical Research and Materiel Command grant, by the National Health and Medical Research Council of Australia (NHMRC) grants APP1092856 and APP1117044, the US National Cancer Institute U54 program, and receives research funding from Astra Zeneca, Beigene and Genentech-Roche. A.deF. is supported by grants from the U.S. Army Medical Research and Materiel Command, NHMRC, Cancer Institute NSW, Cancer Council New South Wales and AstraZeneca.

P148

Impact of introducing extensive surgical procedures to Cytoreductive surgery (CRS) in

advanced Epithelial Ovarian Cancer (EOC) on rate of complete debulking (CD) and survival

M. Seoud[1], A. El Housheimi[1], I. Jaafar[1], K. Hamed[1], R. Abdallah[1], F. Jamali[2], A. Khalil[1]
[1]*Obstetrics and Gynecology,* [2]*General Surgery, American Universtiy of Beirut Medical Center, Beirut, Lebanon*

Introduction / Background: Following international trends for maximizing CD in EOC, we applied, in 2008, a policy for aggressive pelvic and upper abdominal surgery that included trying to remove any gross visible disease in the abdomen.
Our objective is to asses the impact of extensive procedures (Diaphragmatic resection, large and small bowel resection, complete peritonectomy, complete omentectomy, paraaortic lmph node dissection, splenectomy, resection of all visible disease) in CRS on the rate of CD, progression free (PFS), and overall survival (OS) in patients with advanced EOC.
Methodology: Patients undergoing CRS between 2002-2018 were divided into group#1(2002-2008), and group#2 (2009-2018). Demographic and operative information were retrospectively reviewed .
Results: 204 patient with EOC (IIIC-IV) underwent CRS between 2002-2018. The number of CRS as well as performing extensive procedures (Figure1A) has significantly increased over the whole period (72 in group1 vs 132 in group2: *p*-value< 0.001), and this coincided with a significant increase in the CD rate from 50% to 80% (*p*-value< 0.001) (Figure 1A). In stage IIIC, median PFS was significantly higher in group#2 versus group#1 (22 months, 95%CI 16.80-27.19 and 23 months, 95%CI 19.11-26.80 respectively; HR:0.965, *p*-value=0.036) (Figure 1B). However, OS in stage IIIC was not significantly different. In stage IV, there was no statistically significant difference in PFS or OS between groups.
Conclusion: .Introducing extensive procedures did significantly affect the PFS in stage IIIC but not in IV. OS was not affected in both stages. The increase in number of EOC cases over the years (72 before 2009 versus 132 after 2009), which was associated with more patients with extensive tumor load and low perfomance status being admitted to our service, coupled with their shorter follow up versus patients from before 2009, may have led to the non-significant increase in OS over time

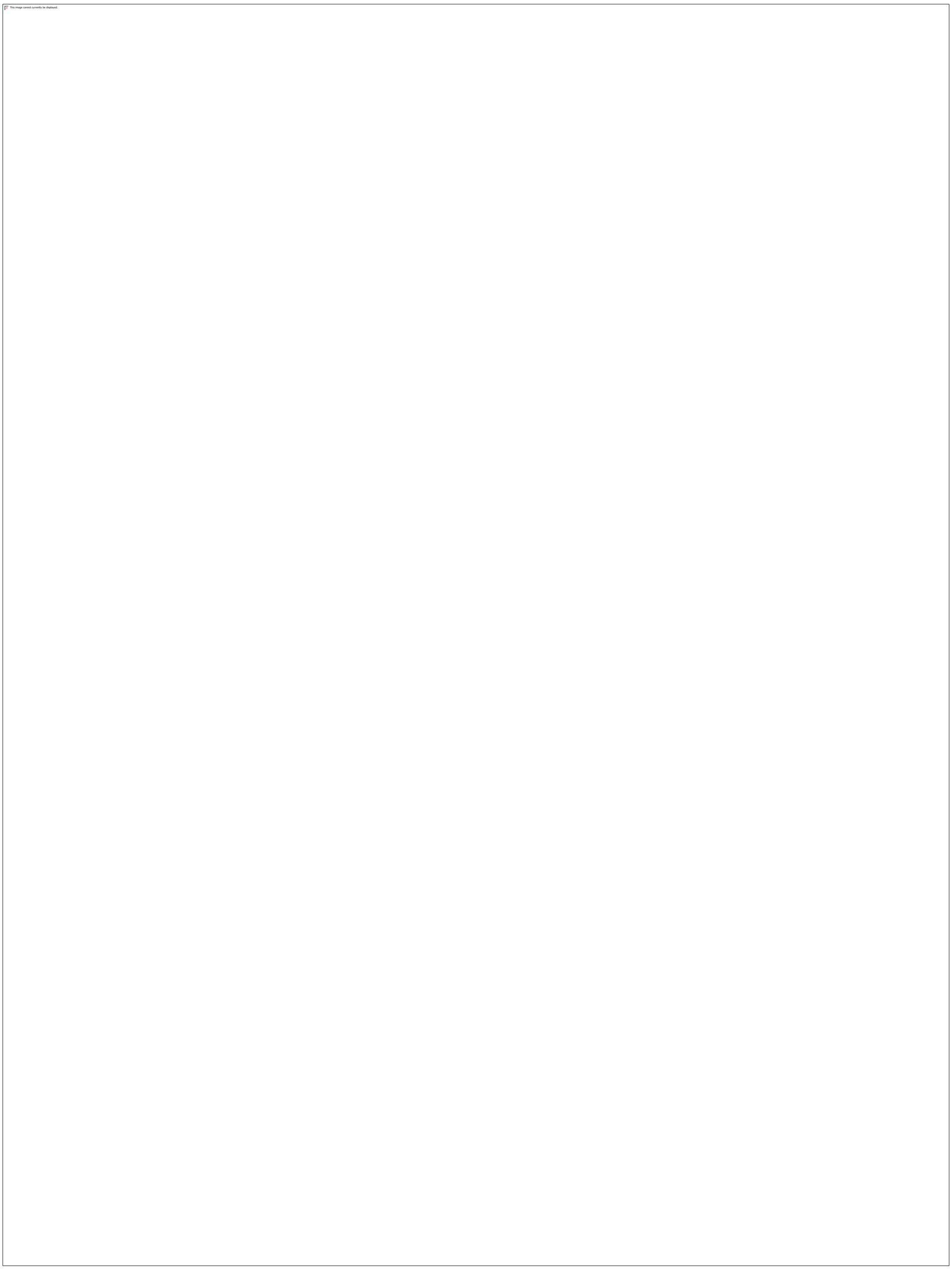

[Figure 1A]

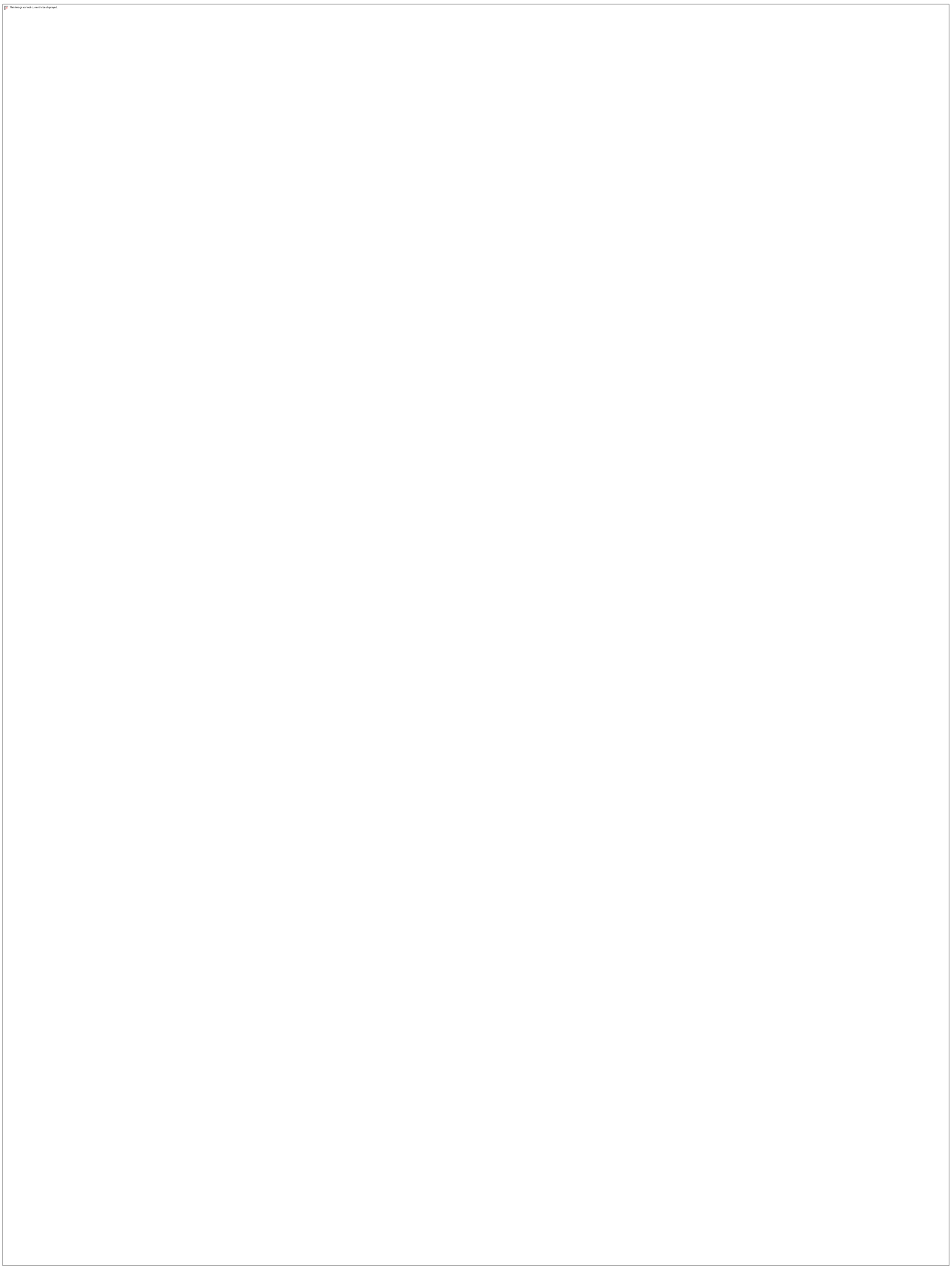

[Figure 1B]

Disclosure: Nothing to disclose

P149

Pregnancy-related risk factors for sex cord-stromal tumors and germ cell tumors in parous women: a registry-based study

C. Sköld[1], T. Bjørge[2,3], A. Ekbom[4], A. Engeland[2,5], M. Gissler[6,7], T. Grotmol[3], L. Madanat-Harjuoja[8,9], A. Gulbech Ording[10], B. Trabert[11], S. Tretli[3], R. Troisi[11], H. Toft Sørensen[10], I. Glimelius[1,4]
[1]Department of Immunology, Genetics and Pathology, Uppsala University, Uppsala, Sweden, [2]Department of Global Public Health and Primary Care, University of Bergen, Bergen, [3]Cancer Registry of Norway, Oslo, Norway, [4]Department of Medicine, Division of Clinical Epidemiology, Karolinska Institutet, Stockholm, Sweden, [5]Department of Chronic Diseases and Ageing, Norwegian Institute of Public Health, Bergen, Norway, [6]National Institute for Health and Welfare (THL), Helsinki, Finland, [7]Department of Neurobiology, Care Sciences and Society, Karolinska Institutet, Stockholm, Sweden, [8]Finnish Cancer Registry, [9]Department of Pediatrics, University of Helsinki and Helsinki University Hospital, Helsinki, Finland, [10]Department of Clinical Epidemiology, Aarhus University Hospital, Aarhus, Denmark, [11]Division of Cancer Epidemiology and Genetics, National Cancer Institute, National Institutes of Health, Department of Health and Human Services, Bethesda, MD, United States

Introduction / Background: Non-epithelial ovarian cancers are rare but affect women of all ages. The etiology is unknown. Non-epithelial ovarian cancers are divided into two major subgroups: sex cord-stromal tumors (SCSTs) and germ cell tumors (GCTs). Whereas parity is protective for epithelial ovarian cancer, its association with non-epithelial ovarian cancer and its subtypes remains less clear, as are associations with other pregnancy-related factors.
Methodology: All parous women with births recorded in the medical birth registries from the Nordic countries with a subsequent diagnosis of SCSTs (n=420) and GCTs (n=345) during 1967-2013 were compared with up to 10 controls (SCSTs n=4041; GCTs n=2942) matched on the cases' birth year and country. We used conditional logistic regression to estimate odds ratios (ORs)with 95% confidence intervals (CI)of associations of pregnancy-related factors.
Results: Number of births was not associated with risk of SCSTs or GCTs. Older age at last birth was inversely associated with the risk of SCSTs (age 30-39 versus < 25 years: OR 0.64, 95% CI 0.45-0.90). The risk decreased gradually with increasing age at birth. Shorter time since first or last birth was also inversely associated with SCSTs. None of the investigated factors were associated with GCTs.

	Sex cord-stromal tumors		Germ cell tumors	
Age at last birth (years)	OR	95% CI	OR	95% CI
<25	1.00	Ref	1.00	Ref
25-29	0.77	0.55-1.08	1.03	0.72-1.46
30-39	0.64	0.45-0.90	1.01	0.68-1.49
≥40	0.48	0.23-0.98	0.82	0.31-2.16
Per year age	0.97	0.94-0.99	1.01	0.98-1.04

[Age at last birth and risk of non-epithelial ovarian cancer by subtype]

	Sex cord-stromal tumors		Germ cell tumors	
Time since last birth (years)	OR	95% CI	OR	95% CI
<10	0.78	0.40-1.53	0.81	0.23-2.78
10-20	0.70	0.39-1.25	0.76	0.24-2.48
20-30	0.97	0.60-1.56	0.61	0.21-1.81

≥30	1.00	Ref	1.00	Ref
Per year	1.04	1.01-1.06	0.99	0.96-1.02

[Time since last birth and risk of non-epithelial ovarian cancer by subtype]

Conclusion: In this large population-based case-control study among parous women, number of births was not protective for risk of SCSTs and GCTs.The inverse associations with greater age at last birth and shorter time since last birth observed for SCSTs, however, suggest that these subtypes may be influenced by the woman's reproductive history.
Disclosure: Ingrid Glimelius have received Honoraria from Janssen for projects unrelated to the current study.

P150

Predictive value of SIRS severity index on survival in elderly women (≥ 70years) with advanced epithelial ovarian cancer

Y.J. Song[1,2], S.L. Todd[2], Y. Xiong[3,4], R. Wenham[2,5], M. Extermann[6], H.S. Chon[2]
[1]Obstetrics & Gynecology, Pusan National University Hospital, Yangsan-si, Republic of Korea, [2]Department of Gynecologic Oncology, [3]Department of Anatomic Pathology, [4]Chemical Biology and Molecular Medicine Program, [5]Department of Oncologic Sciences, [6]Department of Cancer Epidemiology, Senior Adult Oncology, Moffitt Cancer Center, Tampa, FL, United States

Introduction / Background: This study assessed the predictive value of age and Cumulative Illness Rating Scale (SIRS) severity index on the management of advanced stage epithelial ovarian cancer (EOC) patients aged 70 years or older.
Methodology: A retrospective chart review was performed in elderly women (> 70years) with advanced EOC cancer at a Moffitt Cancer Center between January 2007 and April 2016. Exclusion criteria included non-epithelial histology, stage less than IIIC, low grade or incomplete medical records. Clinical data were analyzed according to the following age group categories: (1) group 1: 70 to 74 years versus (2) group 2: 75 to 79 years versus (3) group 3: older than 79 years.
Results: One hundred twenty seven patients were identified (66 in age group 1, 35 in group 2, and 26 in group 3). There was no difference concerning clinicopathologic data between 3 age groups. Optimal debulking rate (77.2% in age group 1, 82.8% in group 2, and 76.9% in group 3, p=0.78) and completeness of adjuvant chemotherapy (80.3% in age group 1, 82.8% in group 2, and 65.3% in group 3, p=0.21) were similar in these groups. The rate of using G-CSF during chemotherapy were not different between age group. Multivariate analyses confirm that only the pretreatment assessment of SIRS severity index was an independent prognostic factor for OS in elderly women (> 70years) with advanced EOC (HR0.52, p=0.02). Older age was not associated with decreased OS in this cohort
Conclusion: High CIRS severity index could be a predictor of poor survival in elderly women (> 70years) with advanced EOC.
Disclosure: Nothing to disclose

P151

Endometriosis and the risk for ovarian cancer

C.N. Trifan[1], S. Pasat[1], D. Hutanu[1], S.D. Tirnovanu[2], C. Mucilenita[1], M.C. Tirnovanu[3]
[1]1st Clinic of Obstetrics and Gynecology, University Women Hospital Cuza Voda Iasi, [2]University of Medicine and Pharmacy 'Gr T Popa' Iasi, [3]Obstetrics and Gynecology, University of Medicine and Pharmacy 'Gr T Popa' Iasi, Iasi, Romania

Introduction / Background: Despite being initially considered a benign disease, the wide opinion nowaday is that ovarian endometriomas are neoplastic condition with the potential to become malignant.
Objective: Our study assess the relationship between endometriosis and clinical features as age, menopausal status, stage for ovarian cancer patients.
Methodology: A retro- and prospective analysis was conducted on 323 patients treated by surgery for ovarian cancer (OC) between 2003-2017 in our clinic. The patients records and pathological results were used. Histological classification of OC was based on WHO system and the stage was according to the current FIGO system. The presence of endometriosis was established on resected specimens.
Results: : The mean age was 49.6 ± 5.2 and 55.2 ± 7.4 years for patients having endometriosis-associated ovarian cancer (EAOC) and OC respectively. From all cases with OC only 25 were associated with lesions of endometriosis: 18 cases with endometrioid epithelial ovarian cancer (from these 5 with synchronous primary endometrioid cancer of endometrium and ovary), 4 with ovarian cancer clear cell, 1 with serous invasive cancer, and 2 patients with mucinous borderline ovarian cancer. From these cases only 6 women had a histology-proven transition from benign endometriosis to atypical endometriosis and cancer. These women were with a long-standing history of endometriosis or with a history of infertility. EAOC was more commenly unilateral (16 cases) and without ascites.
Conclusion: In our study 7.74% cases of epithelial ovarian cancer were associated with endometriosis. There was early onset of ovarian cancer for cases having endometriosis of about 5.6years average. Endometriosis lesions may predispose more freqentlly to endometrioid and clear cell invasive ovarian cancer. Women with EAOC are typically premenopausal and have early stage disease. Screening programs for ovarian cancer are not currently effective, but that may change in the future for premenopausal women with endometriosis.
Disclosure: Nothing to disclose

P152

The effect of T-lymphocytes and tumor-associated macrophages on invasion of peritoneal metastases of epithelial ovarian cancer

J. van Baal[1], C. Lok[1], K. Jordanova[1], W. van Driel[1], F. Amant[1], H. Horlings[1], K. van de Vijver[2]
[1]Netherlands Cancer Institute - Antoni van Leeuwenhoek, Amsterdam, The Netherlands, [2]Ghent University Hospital, Cancer Research Institute Ghent (CRIG), Ghent, Belgium

Introduction / Background: Peritoneal metastases of advanced high-grade serous ovarian cancer (HGSOC) are small-sized tumor depositions with superficial growth towards the peritoneal cavity, rather than invading deeper layers. It is unknown whether depth of peritoneal invasion beyond the peritoneal elastic lamina (PEL) is of prognostic relevance and if the peritoneal tumormicroenvironment (TME) plays a role in this invasion. We explored the integrity of PEL in peritoneal metastases of HGSOC, the composition of TME, and the association with progression-free survival (PFS) and overall survival (OS).
Methodology: .Peritoneal metastases of 69 patients with HGSOC were consecutively collected during primary or interval cytoreductive surgery. Clinical data were collected from medical charts. To evaluate the integrity of PEL with regard to depth of tumor invasion we stained whole slides with histochemistry. To assess the composition of TME, T cell- (CD3, CD8) and M2-macrophage markers (CD163) were analyzed. Intraepithelial and stromal immune cells were scored on whole slide sections using algorithms created in Definiens Tissue studio.
Results: During follow-up, 64 patients (92.8%) had recurrent disease and 54 patients (78.3%; median survival 28.6 months) had died. In 39 patients (56.5%) a disrupted PEL was observed (Figure 1). These patients more often had residual disease after cytoreductive surgery (p=0.050). Integrity of PEL was not correlated with PFS or OS. An intact PEL was associated with higher densities of intraepithelial (ie)CD8+ cells. Abundance of ieCD3+ cells, stromal (s)CD3+ cells and sCD8+ cells was associated with PFS and OS (Table 1). M2-macrophage infiltration was not correlated with PFS or OS.
Conclusion: High density of CD3+ and CD8+ cells in peritoneal metastases of HGSOC is associated with increased PFS and OS, independent of PEL integrity. These results suggest that these immune

cells promote a tumor-suppressive microenvironment and may function as prognostic biomarkers for survival, and perhaps as predictive biomarker for immunotherapy response.

[Figure 1]

[Table 1]

Disclosure: Nothing to disclose

P153

Measuring functional PI3K and NFkB pathway activity to distinguish between short-term and long-term disease-free survivors of high grade serous ovarian cancer

L.A.M. van Lieshout[1,2], A. van de Stolpe[3], R.L.M. Bekkers[2], J.A. de Hullu[2], J.M.J. Piek[1]
[1]Obstetrics and Gynaecology, Catharina Cancer Institute, Catharina Hospital, Eindhoven, [2]Obstetrics and Gynaecology, Radboud Institute for Health Sciences, Radboud University Medical Center, Nijmegen, [3]Precision Diagnostics, Philips Research, Eindhoven, The Netherlands

Introduction / **Background:** The disease-free survival (DFS) of high-grade serous cancer (HGSC) varies greatly, despite comparable clinicopathological features and treatment. It is our hypothesis that the difference in DFS is caused by differences in activity of tumour-driving signal transduction pathways.
Methodology: Using previously described Signal Transduction pathway Activity (STA) assays (table1), signalling pathway activity scores for AR, ER, PI3K-FOXO, HH, NFkB, TGFβ, NOTCH and Wnt were derived from publicly available mRNA expression (Affymetrix) data of primary HGSC, with clinical annotation (GSE9891). K-means cluster analysis was performed on STA-scores of AR, ER, PI3K-FOXO, HH, NFkB, TGFB, NOTCH and Wnt pathway. However, as PI3K and NFkB scores were most discriminating in cluster formation, final clusters were based on these pathways alone.
Results: We selected 81 out of 140 sample data with a DFS < 12 or >24 months. The remaining 59 samples with a DFS of 12-24 months were kept aside to be allocated to identified clusters by discriminant analysis in a later stage. Repeated K-means clustering resulted in two stable clusters; a low PI3K/high FOXO and high NFkB cluster (n=43) with a more favourable prognosis, and a high PI3K/low FOXO and low NFkB cluster (n=38) with a less favourable prognosis (log-rank=0.011, figure 1). The remaining samples (n=59) were then allocated resulting in a low PI3K and high NFkB cluster of 73 samples, and a high PI3K and low NFkB cluster of 67 samples (log-rank=0.036, figure 2).

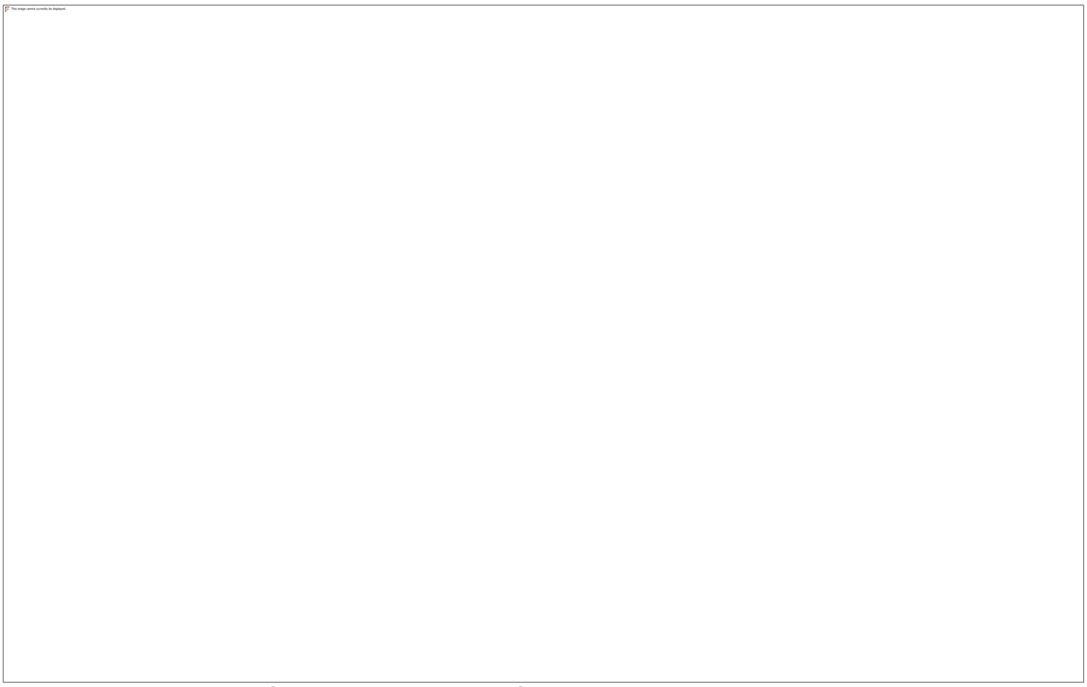

[Figure 1. Kaplan-Meier Curve of Disease-Free Survival for each cluster]

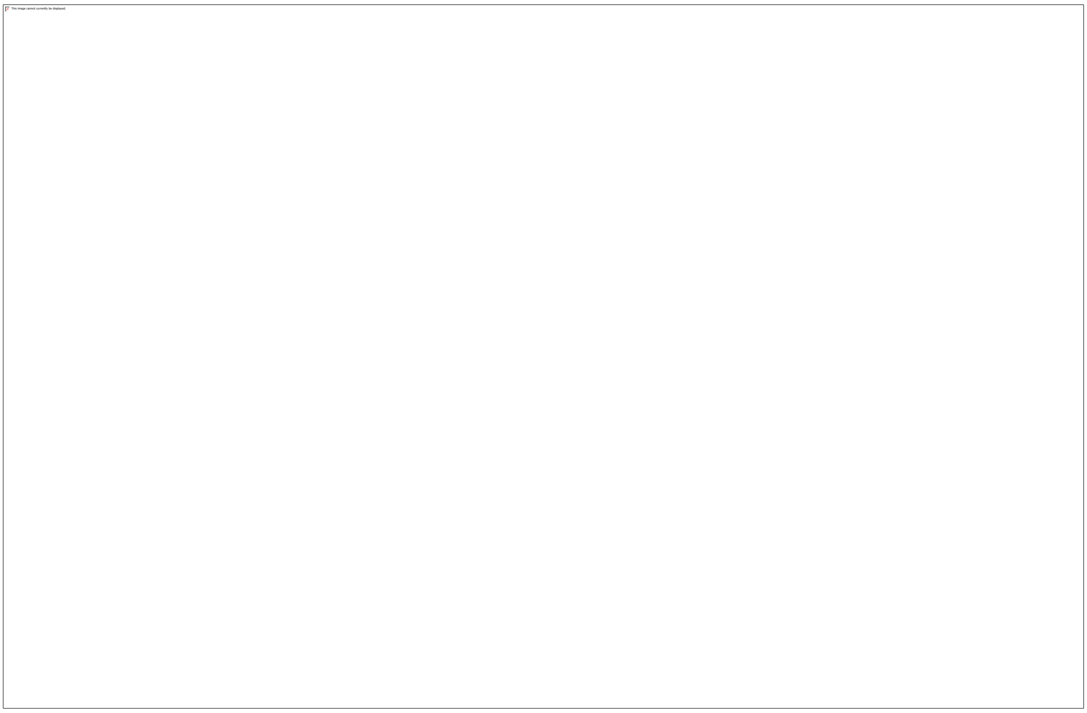

[Figure 2. Kaplan-Meier Curve of Disease-Free Survival, after allocation of remaining samples]

Conclusion: We identified two signalling pathway activity clusters in HGSC with a difference in DFS. The low PI3K/high FOXO and high NFkB activity of the favourable prognosis cluster may indicate apoptosis, while the high PI3K/low FOXO and low NFkB activity of the unfavourable prognosis cluster may indicate high cell division. Elucidating underlying mechanisms of HGSC aids our understanding of the disease and provides possible new leads for targeted therapies.

Authors	Title	Journal	Year
Verhaegh W, Van Ooijen H, Inda MA, Hatzis P, Versteeg R, Smid M, Martens J, Foekens J, Van de Wiel P, Clevers H, Van de Stolpe A	Selection of personalized patient therapy through the use of knowledge-based computational models that identify tumor-driving signal transduction pathways.	Cancer Research	2014
Verhaegh W, Van de Stolpe A.	Knowledge-based computational models.	Oncotarget	2014
van Ooijen H, Hornsveld M, Dam-de Veen C, Velter R, Dou M, Verhaegh W, Burgering B, Van de Stolpe A	Assessment of Functional Phosphatidylinositol 3-Kinase Pathway Activity in Cancer Tissue Using Forkhead Box-O Target Gene Expression in a Knowledge-Based Computational Model.	American Journal of Pathology	2018
Stolpe A van de, Holtzer L, van Ooijen H, de Inda MA, Verhaegh W.	Enabling precision medicine by unravelling disease pathophysiology: quantifying signal transduction pathway activity across cell and	Scientific Reports	2019

	tissue types.		
Van de Stolpe A.	Quantitative Measurement of Functional Activity of the PI3K Signaling Pathway in Cancer.	Cancers	2019

[Publications on STA-analysis]

Disclosure: LvL has nothing to disclose, AvdS is employed by Philips Research, RB has nothing to disclose, JdH has nothing to disclose, JP has nothing to disclose. Funding granted by the Ruby& Rose fund and Catharina research fund.

P154

Tumor Treating Fields (200 kHz) concomitant with weekly paclitaxel for platinum-resistant ovarian cancer: Phase 3 INNOVATE/ENGOT-ov50 study

I. Vergote[1], L. Copeland[2], B. Monk[3], R. Coleman[4], D. Cibula[5], J. Sehouli[6], E.P. Samartzis[7], R. Madry[8], A. González Martin[9], J. Korach[10], V. Salutari[11], P.O. Witteveen[12], D. O'Malley[2], ENGOT-ov50
[1]Universitaire Ziekenhuizen Leuven, Leuven, Belgium, [2]Gynecologic Oncology, Ohio State University Comprehensive Cancer Center, Hilliard, OH, [3]Medical Oncology, University of Arizona Cancer Center, Tuscon, AZ, [4]Gynecologic Oncology and Reproductive Medicine, The M. D. Anderson Cancer Center, Houston, TX, United States, [5]First Faculty of Medicine, Charles University and General University Hospital, Prague, Czech Republic, [6]Gynecology, Universitätsklinik Charité, Campus Virchow-Klinikum, Berlin, Germany, [7]Klinik für Gynäkologie, Universitätsspital Zürich, Zurich, Switzerland, [8]Oddział Ginekologii Onkologicznej Katedry i Kliniki Onkologii, Uniwersytet Medyczny im K. Marcinkowskiego w Poznaniu, Poznań, Poland, [9]Medical Oncology Department, Clinica Universidad de Navarra, Madrid, Spain, [10]Josef Buchmann Gynecology and Maternity Center, Chaim Sheba Medical Center, Ramat Gan, Israel, [11]Gynecology Oncology Unity, Policlico Universitario Agostino Gemelli, Rome, Italy, [12]Medical Oncology, University Medical Center Utrecht Cancer Center, Utrecht, The Netherlands

Introduction / Background: Tumor Treating Fields (TTFields) are a non-invasive, regional antimitotic therapy. The Phase 2 INNOVATE study [NCT02244502] demonstrated safety of TTFields plus weekly paclitaxel in 31 PROC (platinum-resistant ovarian cancer) patients (*Vergote et al Gyn Onc 2018;150:471*). No increase in grade 3-4 TTFields reported; 26 patients (84%) had TTFields-related dermatitis; one patient permanently discontinued TTFields due to dermatitis. Patients received median of 4 prior therapies; 100% prior platinum and 97% prior taxanes. Median PFS was 8.9 months, 25% had partial response and clinical benefit rate was 71%. The median overall survival was not reached: the one-year survival rate was 61%. This phase 3 INNOVATE-3/ENGOT-ov50 study investigates TTFields combined with weekly paclitaxel in PROC patients.
Methodology: Patients (N=540) will have PROC (per RECIST V1.1) within 6 months of last platinum therapy with a maximum of 2-5 prior lines of systemic therapy, ECOG score of 0-1 and no peripheral neuropathy above grade1. Patients with primary refractory disease (progression during first line therapy) will be excluded. Patients will be randomized 1:1 to either weekly paclitaxel alone or weekly paclitaxel plus TTFields (200 kHz). Weekly paclitaxel will be administered at standard starting of dose 80 mg/m^2 weekly for 8 weeks, and then on Days 1, 8, and 15 for subsequent 28-day cycle. TTFields will be delivered for 18 hours/day and continued if no progression in the abdominal or pelvic regions (“in-field region”) per RECIST V1.1. Clinical follow up will be performed q4w, with radiological follow up (CT or MRI scans of the abdomen and chest) q8w. The primary endpoint is overall survival. Main secondary endpoints: progression-free survival, objective response rate, severity and frequency of AEs, and quality of life (EORTC QLQ-C30 with QLQ-OV28). Sample size (n=540) will detect an increase in median overall survival from 12 to 16 months (Hazard ratio 0.75).
Results: N/A TiP
Conclusion: N/A TiP
Disclosure: Ignace Vergote declares:Grants (Amgen and Roche);Consulting and advisory board

(Roche, Genmab,Advaxis, Morphotek, Hoffmann-La Roche, Cerculean Pharma Inc., Novocure GmBh, Astrazeneca, Mateon Therapeutics Inc., Immunogen Inc., Eli Lilly Benelux NV, Amgen Inc., Theradex Europe Limited, Pfizer Inc., Debiopharma International SA, Vifor Pharma België, Novartis Pharma AG, MSD Belgium BVBA, Janssen-Cilag, Bayer Pharma AG, Clovis Oncology, Takeda, Pharma Mar, Oncoinvent, − Contracted Research by Morphotek and travel expenses sponsored by Tesaro, Clovis Oncology, Takeda, Pharma Mar, Roche, Genmab and Oncoinvent.

P155

The microRNA 34 family and its clinical significance in ovarian cancer

H. Welponer[1], I. Tsibulak[1], V. Wieser[1], C. Degasper[1], G. Shivalingaiah[2], S. Wenzel[2], S. Sprung[3], C. Marth[1], H. Fiegl[1], A.G. Zeimet[1]
[1]Gynecology and Obstetrics, [2]Division of Human Genetics, [3]Institute of Pathology, Medical University Innsbruck, Innsbruck, Austria

Introduction / Background: The tumor suppressor miR-34 family is transcriptionally induced by p53. Clinical significance of the various miR-34 family members has not been studied in ovarian cancer.
Methodology: In 228 ovarian cancers and in 19 non-neoplastic fallopian tube samples we analysed miR-34a/b/c expression in relation to clinicopathological characteristics and clinical outcome.
Results: We found significantly lower levels of miR-34a/b/c in ovarian cancers as compared to control-tissues (P=0.002, P< 0.001, P< 0.001, respectively). Expression of miR-34b/c revealed an inverse correlation with *BRCA1/2* mRNA-expression (BRCA1: miR34b/c P=0.002 each; BRCA2: miR-34b/c P< 0.001 each), the same was true for miR-34a and *BRCA2* mRNA-expression (P< 0.001). The miR-34 family expression was found to be significantly lower in type 2 in comparison to type 1 cancers (P< 0.001) and in *TP53*-mutated compared with *TP53*-wild-type ovarian cancers (P< 0.001, P=0.002, P=0.004, respectively). When low grade serous ovarian cancers were compared with high grade serous cancers the respective miR-34a/b/c expression was 2.6-, 40.8- and 32.3-fold higher. The expression of each of the miR-34 family members was revealed to be of independent prognostic relevance regarding progression free survival (miR-34a: HR 0.6, P=0.033; miR-34b: HR 0.2, P=0.001 and miR-34c: HR 0.3, P=0.002, respectively). For overall survival independency of the prognostic value was confined to miR-34b (HR 0.4, P=0.016) and miR-34c (HR 0.6, P=0.049).
Conclusion: Our findings suggest that downregulation of miR-34 family is a crucial part in ovarian cancer development. Low miR-34 levels are linked to a worse overall survival and progression free survival and may indicate a more aggressive disease.
Disclosure: Nothing to disclose

P156

Saliva: Hub of plausible ovarian cancer proteomic markers

S. Yadav[1], M. Tajmul[1], K.B. Chandra[1], A. Saini[1], S.R. Mathur[2], J.B. Sharma[3], S. Kumar[3]
[1]Biophysics, [2]Pathology, [3]Obstetrics and Gynaecology, All India Institute of Medical Sciences, New Delhi, India

Introduction / Background: Ovarian cancer is the most lethal gynecological cancer often termed as a "Silent Killer". It is difficult to diagnose ovarian cancer at early stages as no alarming symptoms are known. A cost effective and non-invasive simple early screening method for ovarian cancer is needed urgently to reduce high mortality rate. Saliva is a clinically informative unique fluid, which is useful for novel approaches to prognosis, clinical diagnosis and monitoring for non-invasive detection of a disease.
Methodology: Differential proteomic expression analysis to identify protein markers of ovarian cancer

in human saliva was performed by fluorescence-based 2D-DIGE and a number of proteins were identified by MALDI/TOF-MS. Expression of selective differentially expressed proteins was also validated by western blotting, ELISA and immunohistochemistry. RT-PCR was performed in an independent cohort of ovarian tumor tissues to check the presence of the proteins at m-RNA level.
Results: A number of differentially expressed proteins were identified and few of them , viz. Multimerin1, Fibroblast Growth Factor 8, Zinc Finger protein 525 and Cystatins SA were validated. Significant over expression of these proteins was observed with fold changes more than 2-3 with $p < 0.01$. Cytoplasmic expression of these upregulated proteins in ovarian cancer tissue were also observed by immunohistochemistry to confirm our proteomics findings. High intensity of these proteins was observed in ovarian cancerous tissues. Furthermore, mRNA expression of these salivary signatures confirms their presence at gene level in both ovarian cancer patients and normal healthy controls. As evident from relative expression analysis of target genes by RT-PCR, mRNA levels of all the targets were found differentially expressed in disease compared to healthy controls.
Conclusion: These differentially expressed proteins can further be explored as plausible markers for screening of ovarian cancer.
Disclosure: Nothing to disclose

P157

Evidence for clinical utility of extended HPV genotyping in persistence tracking and follow-up after abnormal results and colposcopy and test-of-cure

J. Andrews
Medical Affairs, BD, Sparks, MD, United States

Introduction / Background: An analysis of the body of science published recently about the clinical value of HPV genotyping in persistence tracking, follow-up of women with abnormal results, and follow-up after treatment of high-grade cervical intraepithelial neoplasia (CIN).
Methodology: MedLine was searched from 2001 through 2019 for relevant studies. Hand-searching of retrieved article reference lists supplemented the search. Eligible studies included prospective studies of women and retrospective studies of residual specimens from women that were tested using HPV genotyping tests following an abnormal screening result, or colposcopy, or treatment for high-grade CIN. The reference standards were CIN2 or CIN3 or CIN2+ or CIN3+ or invasive cervical cancer. The timeframe for follow-up studies was at least 6-months to determine persistence; periods of 12-months and 24-months were accepted. This systematic review has been registered with PROSPERO. Cochrane risk of bias assessment was performed. GRADE methodology was used to establish quality and strength of evidence.
Results: A PRISMA flow diagram is presented for this systematic review. 32 original research articles met inclusion and exclusion criteria. Reporting genotyping provides profound discrimination of both current and future CIN3+ risks, due to the differential risks of same genotype persistence versus new genotype infection. Within subjects with persistent same genotype, genotype reporting can discriminate risk by more than ten-fold. Genotyping could be utilized as follow-up type-specific persistence versus clearance, to support risk-based clinical decisions. Similar management for similar risk-discrimination is benchmarked.
Conclusion: Based on quality-evaluated studies that met inclusion criteria, there is strong evidence for genotyping to distinguish same-genotype persistence versus clearance, to discriminate risk between same-genotype persistence versus new genotype infection, and support risk-based clinical action steps by the principle of equal management for equal risk. The role of same genotype persistence is critical to test-of-cure assessments. Models for different management paradigms are described.
Disclosure: The speaker is a full time employee and Worldwide Medical Director for Women´s Health & Cancer for BD Biosciences and Diagnostic Systems.

P158

Efficacy of a Coriolus versicolor-based vaginal gel in repairing HPV-dependent cervical lesions (ASCUS/LSIL) in three patients groups according to the risk of HPV strains

L. Serrano[1], A.C. López[2], S. González[1], S. Palacios[3], D. Dexeus[4], C. Centeno[5], P. Coronado[6], J. de la Fuente[7], J.A. López Fernández[8], C. Vanrell[9], J. Combalia[10], J. Cortés[11]
[1]Centro Médico Gabinete Velázquez, Madrid, [2]Hospital Quironsalud, Málaga, [3]Instituto Palacios, Salud y Medicina de la Mujer, Madrid, [4]Women´s Health Institute, [5]Clínica Diatros, Barcelona, [6]Hospital Clínico San Carlos, [7]Hospital Universitario Infanta Leonor, Madrid, [8]Hospital General Universitario, Alicante, [9]Hospital de la Santa Creu i Sant Pau, [10]Procare Health Iberia, Barcelona, [11]Private Practice, Palma de Mallorca, Spain

Introduction / Background: High-risk strains of HPV are well established as the causative agents for cervical dysplasia and cervical cancer. It is also well known that pre-cancerous cervical lesions persist longer and progress quickly in women infected by high-risk HPV than in women with non-oncogenic HPV strains.
The aim of this study was to evaluate the efficacy of Papilocare® -a *Coriolus versicolor*-based vaginal gel- in repairing HPV-dependent cervical lesions (ASCUS/LSIL) in three groups of patients according to the oncogenic potential of HPV strains.
Methodology: Randomized, open-label, parallel-group, controlled clinical trial (Paloma Clinical Trial). HPV+ women aged between 30 and 65 with cytology of ASCUS or LSIL with concordant colposcopy image were randomized into 3 groups. Primary endpoint of the Paloma Clinical trial: percentage of patients with normal cytology and concordant colposcopy image at 6 months, evaluated in total population, high-risk subpopulation (16,18,31,33,35,39,45,51,52,56,58,59,68) subpopulation and the subpopulation infected by any combination of 16, 18 and 31 , is presented. Pap smear evaluation was being centrally-conducted at the IECM laboratory (Lugo, Spain). Papilocare® arms (A+B) were combined for this per-protocol analysis.
Results: A total of 84, 66 and 29 patients corresponding to total population, and high-risk and 16-18-31 subpopulations were evaluated, respectively.
At 6 months, normal cytology and concordant colposcopy image was observed in 85% (45/53), 88% (36/41) and 73% (11/15) of patients treated with Papilocare® vs 65% (20/31), 56% (14/25) and 43% (6 /14) of patients in control group, in the total population, and high-risk and 16-18-31 subpopulations (p=0.0311; p=0.0034; p=0.0959, chi-square test; Fig.1) respectively.
Conclusion: While the spontaneous normalizations of HPV-dependent cervical lesions clearly decreased according to the risk of HPV strains (Total population = 65%, HR-HPV=56%, and HPV-16-18-31=42%), the robust efficacy of Papilocare in normalizing cervical lesions was maintained in the 3 patients groups with statistically significant difference versus control

[Papilocare® on repairing HPV-dependent cervical lesions (ASCUS/LSIL) at 6 months in three groups o]

Disclosure: Nothing to disclose

P159

Human uterine cervix-on-a-chip: establishing the first in vitro model to study the development of cervical carcinoma and human Papiloma virus mechanism of action

D. Khorsandi[1,2], S. Palacios[3], Y. Gaslain[4], C. Emsellem[4], J. Combalia[4], J. Cortés[5], A. Khademhosseini[6]
[1]University of Barcelona, [2]Medical Affairs, Procare Health Iberia, Barcelona, [3]Instituto Palacios, Salud y Medicina de la Mujer, Madrid, [4]Procare Helath Iberia, Barcelona, [5]Private Practice, Palma de Mallorca, Spain, [6]University of California Los Angeles, Los Angeles, CA, United States

Introduction / Background: The cost of drug discovery is steadily increasing owing to the limited predictability of two-dimensional (2D) cell culture and animal models. The objective of this study was to develop a microfluidic 'uterine cervix-on-a-chip', a dynamic 3D platform that let the cultured cells mimic the native human uterine cervix histomorphology *in vitro* aiming to study the transformation zone of cervix during Human Papilloma virus (HPV) infection and cervical cancer development.
Methodology: A microfluidic device prepared by demolding cured polidimetilsiloxano (PDMS). On the chip, we carried out cell culture and co-culture of ectocervical epithelial cells (Ect1/E6E7) and endocervical epithelial cells (End1/E6E7). Endocervix epithelial cells have been marked by AAV-GFP control viruses to provide a convenient way to measure transduction efficiency via fluorescence and to differentiate them from ectocervix epithelial cells. Live/dead assay, prestoBlue cell viability, 2D migration of cells (scratch test) and 3D migration of cells by the use of 3D printers were performed to check the functionality of the chip.
Results: As a preliminary result, we have made a non-toxic, functional, in vitro model for human uterine cervix that allows both types of epithelial cells to grow on both sides of the chip to reach each other in order to make the squamo-columnar junction. This multilayer-cell junction can mimic the

transformation zone of the cervix. The next phase of the project is to mimic the infection via the upper chamber and to test the new treatments on the infected cells in order to evaluate changes in cytokines and immune response cells, as well as HPV mechanism of action.
Conclusion: Uterine cervix-on-a-chip may provide a powerful alternative *in vitro* model for studies on cervix physiology, real-time and high-resolution imaging, and analysis of biological responses in the cervix, as well as drug development.

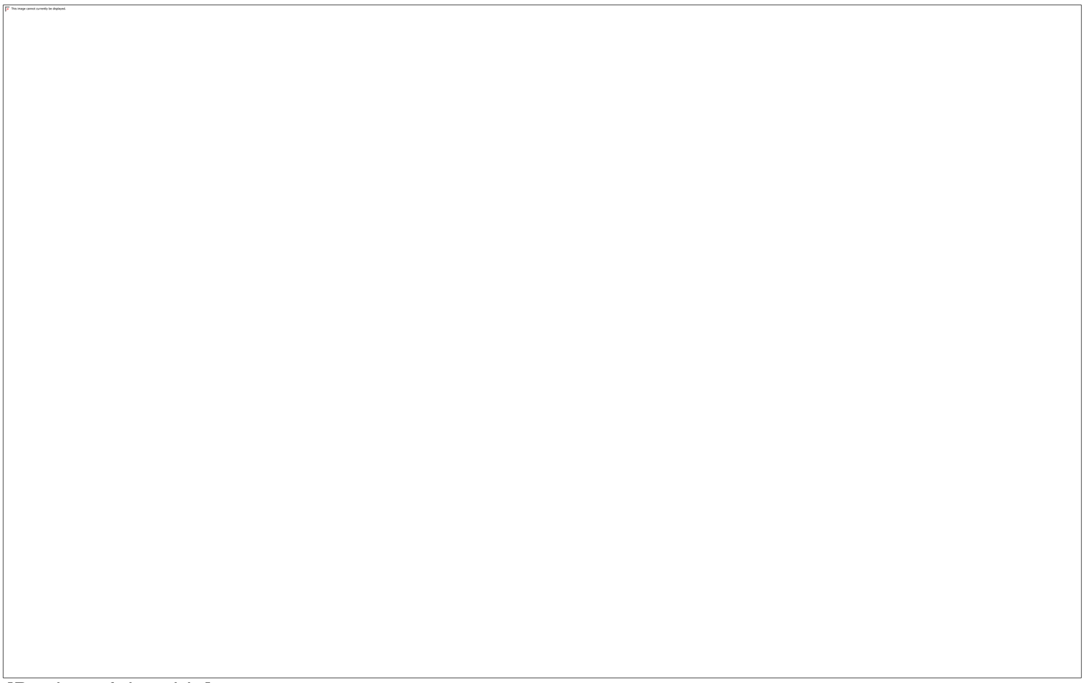

[Design of the chip]

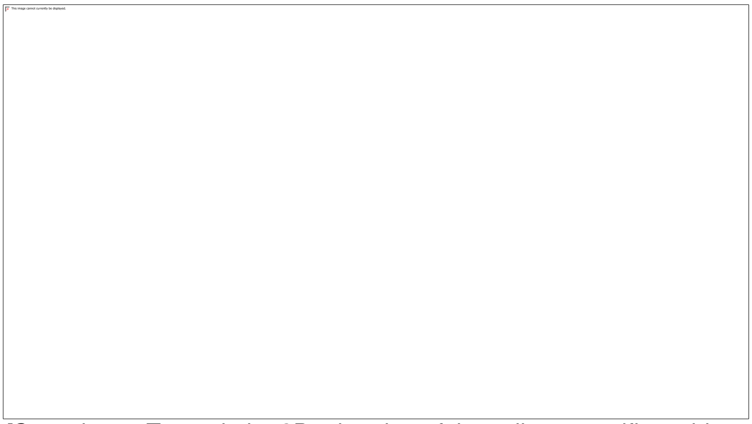

[Scratch test To track the 2D migration of the cells, a specific mold containing 4 cubic holes (5mm]

Disclosure: Nothing to disclose

P160

Utility of HPV genotyping and cytology to predict cervical intraepithelial grade 2 outcome after 2 years of follow-up

A. Salvadó Salvadó, E. Miralpeix Rovira, S. Gayete Lafuente, N. Kajou Auge, M. Castells, J.M. Solé Sedeño, G. Mancebo Moreno
Ginecology, Hospital del Mar, Barcelona, Spain

Introduction / Background: Cervical intraepithelial neoplasia (CIN) commonly occurs in women of reproductive age. Conservative management of CIN1 is widely established, as is excisional treatment for CIN3; however, management of CIN2 remains debatable.

Our objective was to evaluate the utility of human papilloma virus (HPV) genotyping and the previous cytology in the management of patients with CIN2 lesions.

Methodology: Patients newly diagnosed with CIN2 cervical biopsy, older than 16 years old, who agreed to follow up every 6 months for at least 2 years, were prospectively recruited. Unsatisfactory colposcopy and endocervical CIN2 were considered exclusion criteria.

Total regression was defined as two consecutives negative cytologies and no evidence of colposcopic lesions, partial regression if final biopsy showed CIN1, persistence when biopsy showed CIN2, and progression if a CIN3 biopsy was detected during follow-up.

Results: The mean age of 300 patients included was 30 years, 50% were smokers, 76% nulliparous, 9% were vaccinated, 6% were pregnant at moment of diagnosis and 37% affected by HPV16. Overall, the rate of spontaneous total regression at 2 years of follow-up was 59.7%, 20.9% presented partial regression, 13.1% had persistent disease, while 6.3% progressed.

HPV16 infection [OR 4.7 (95% CI 1.6 to 13.7)] and previous HSIL cytology [OR 4.4 (95% CI 1.5 to 12.8)], were statistically associated with progression. Age or smoking were no statistically associated.None of vaccinated patients neither HPV-HR negative lesions progressed in 24 months.

Conclusion: The high regression rate of CIN2 supports clinical observation and conservative management in selected patients.

Patients with CIN2 lesions HPV16 positive and HSIL previous cytology are more likely to progress to CIN3, so tight control is crucial. In contrast, vaccinated patients and HPV-HR negative lesions regress spontaneously.

Study of predictive factors of CIN2 evolution, as HPV16 or previous cytology, would have a great clinical value to reduce unnecessary cone excision and tailored treatment.

Disclosure: The authors declare no conflicts of interest. None received grant support for the study neither for other clinical or research activity, except for the inscription to the current congress, for three of the authors, that was funded by VIFOR Pharma (non related to the study we present)

P161

Hysterectomy with salpingectomy versus hysterectomy alone: a Cochrane review

L.A.M. van Lieshout[1,2], M.P. Steenbeek[1], J.A. de Hullu[1], M.C. Vos[3], S. Houterman[4], J. Wilkinson[5], J.M.J. Piek[2]
[1]Obstetrics and Gynaecology, Radboud Institute for Health Sciences, Radboud University Medical Center, Nijmegen, [2]Obstetrics and Gynaecology, Catharina Cancer Institute, Catharina Hospital, Eindhoven, [3]Obstetrics and Gynaecology, Elisabeth-TweeSteden Hospital, Tilburg, [4]Education and Research, Catharina Hospital, Eindhoven, The Netherlands, [5]Centre for Biostatistics, School of Health Sciences, Faculty of Biology, Medicine and Health, Manchester Academic Health Science Centre (MAHSC), University of Manchester, Manchester, United Kingdom

Introduction / Background: The opportunistic salpingectomy (OS) during hysterectomy for prevention of ovarian cancer is under discussion for the general population. The effect on ovarian cancer risk, surgery-related adverse events and ovarian reserve have not been fully elucidated. We aim to evaluate benefits and potential harms of OS during hysterectomy for benign gynaecological complaints.

Methodology: We searched the Cochrane register of controlled trials, CENTRAL, MEDLINE and Embase from inception to January 2019. Regarding ovarian cancer incidence, both non-randomised trials (NRTs) and randomised controlled trials (RCTs) were eligible. For surgery-related adverse

events and ovarian reserve, only RCTs were included. Two review authors independently screened and selected studies, extracted data and assessed risk of bias.
Results: We screened 3748 trials and identified zero NRTs and seven RCTs with 347 women in total. Five trials were at high risk of bias in at least one domain. No studies were available on ovarian cancer incidence. We found no statistically significant difference in intra-operative or post-operative adverse events or in ovarian reserve, based on post-operative AMH concentration after hysterectomy with or without OS (table 1). Duration of follow-up was limited to a maximum of 6 months.

[Table 1. Summary of main results]

Conclusion: There were no studies available on the effect of OS during hysterectomy on ovarian cancer incidence that met our inclusion criteria. We found no significant difference in surgery-related adverse event or ovarian reserve after hysterectomy with or without OS. To conclude; the OS seems to be a safe procedure both surgically and with regards to ovarian reserve, however the effect on ovarian cancer risk when performed during hysterectomy is not proven. Especially in young women, for whom an effect on ovarian reserve would be more clinically relevant, long term effects should be investigated.
Disclosure: Nothing to disclose

P162

Lynch syndrome followed up in a hereditary gynaecological cancer unit

A. Veiga-Fernández[1], M. Móran[1], M.Á. Oyonarte[1], A. López Picado[2], I. Marquez-Rodas[3], M.S. Luque[1], E.M. Mendizábal-Vicente[1], S. Lizarraga-Bonelli[1]
[1]Obstetrics and Gynaecology, Gregorio Marañón University General Hospital, [2]Research and Clinical Trials Unit, Clinical Hospital San Carlos, [3]Medical Oncology, Gregorio Marañón University General Hospital, Madrid, Spain

Introduction / Background: Women with Lynch syndrome (hereditary nonpolyposis colorectal cancer) have a 40 to 60 percent lifetime risk of endometrial cancer and a 10 to 12 percent lifetime risk of ovarian cancer.
Methodology: Retrospective observational study. Review of patients followed in the inherited cancer unit of the Gregorio Marañón university hospital between 1st January 2012 and 28th February 2019. The statistical analysis was carried out using SPSS 21.0.
Results: During the indicated period, we followed 268 patients with confirmed genetic mutations that predispose to developing gynaecological cancer. Of the 268 patients, 10.8% (29/268) were carriers of genes related to Lynch syndrome. The most frequent mutation was MLH1 (15/29), followed by MSH2 (7/29) and MSH6 (4/29). 24/29 had a first-degree relative affected. Colorectal cancer was the most frequent (20/29).
Within the cohort of patients carrying Lynch syndrome mutations, 12/29 were diagnosed with a first cancer (colorectal cancer in 5/29 and endometrial cancer in 2/29). The mean age at diagnosis of the

primary tumor was 47±13 years. In this subgroup, 4/12 were diagnosed with a second cancer with a mean age at diagnosis of 61±15 years. Characteristics of these patients are summarized in table 1.

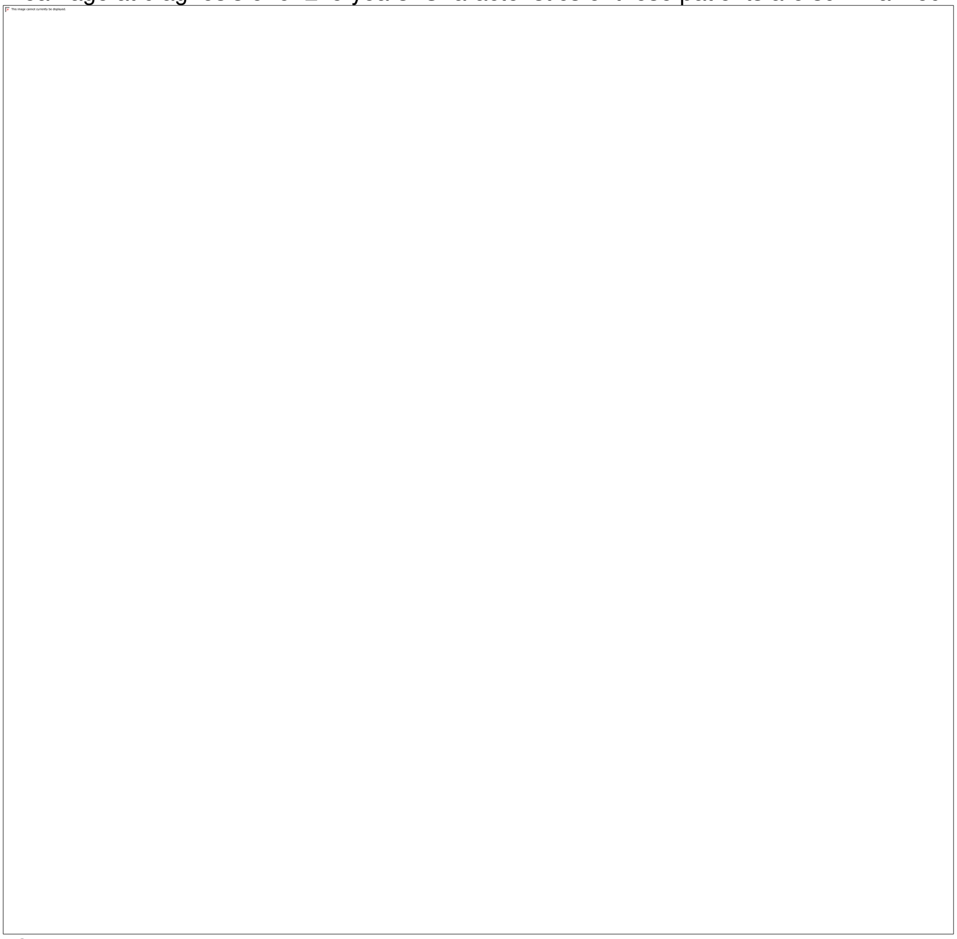

[Characteristics of Lynch syndrome patients.]

The 2 patients with endometrial cancer were diagnosed in early stages and, after oncological surgery, didn´t need adjuvant treatments.
In 7/29 patients, primary prophylactic surgery was performed (total hysterectomy with bilateral salpingo-oophorectomy), with a mean age of 52±15 years. The histological analysis of prophylactic surgeries did not report malignancy.
Conclusion: Patients carrying Lynch syndrome mutations should be followed in specialized hereditary cancer units, in tertiary hospitals.
Prophylactic hysterectomy with bilateral salpingo-oophorectomy is recommended by current guidelines to prevent endometrial and ovarian cancer in these women.
Disclosure: Nothing to disclose

P163

Hormone replacement therapy use after premature surgical menopause and impact on subsequent health services

J.-H. Jang, N. Arora, G. Hanley, J. Kwon
University of British Columbia, Vancouver, BC, Canada

Introduction / Background: It is well-recognized that premature surgical menopause without subsequent hormone replacement therapy (HRT) is associated with increased morbidity and mortality. Our objective was to describe HRT use in a cohort of women from British Columbia (BC), Canada with premature surgical menopause.
Methodology: A population-based retrospective cohort study of women who underwent surgical menopause between ages 19-50, identified from Medical Service Plan Payment Information from BC, and linked to prescription histories from the provincial PharmaNet database from 2004-2014. Women with personal history of cancer were excluded.
Results: A total of 11,864 women were included. Median age of surgical menopause was 43 years. Median follow-up duration was 5.5 years (range: 0-10 years). The majority of women did not have a *BRCA*mutation (99.1%). They underwent bilateral salpingo-oophorectomy (90%) or bilateral oophorectomy (10.4%), and 50.5% had concurrent hysterectomy. There were 196 (1.7%) diagnosed with malignancy. The remainder had benign diagnoses, the most common of which were adnexal masses (17%), endometriosis (17.9%), and abnormal bleeding (14.3%). During the 10-year study period, only 55.4% had ever used HRT. Use of HRT for < 6 months, 6-12 months, 1-5 years, and >5 years after surgery were 38.2%, 8.9%, 32.7%, and 20.2%, respectively. Those who did not use HRT had more physician visits for cardiovascular disease (19% vs. 9%) and osteoporosis (13% vs. 9%) compared to ever-users, respectively (p= 0.002).
Conclusion: In this large population-based study investigating HRT rates following premature surgical menopause, only half of these women ever used HRT, and a greater proportion of those who did not use HRT had physician visits for cardiovascular diseases and osteoporosis. These results underscore a need for education and ongoing follow-up in this patient population.
Disclosure: Nothing to disclose

P164

Reduction of bone mineral density and risk of osteoporosis in gynecologic cancer patients

J.E. Lee, Y.I. Ji
Obstetrics and Gynecology, Inje University, Busan, Republic of Korea

Introduction / Background: The purpose of this study is to evaluate the risk of osteopenia and osteoporosis in patients with Gynecologic cancer without bone metastases and to evaluate the impact of surgical treatment, chemotherapy and radiotherapy on bone mineral density(BMD).
Methodology: From March 2010 to December 2016, this study retrospectively reviewed the medical records of women who were treated at Haeundae Paik Hospital Obstetrics and Gynecology. We compared the lumbar spine and femur neck BMD of 243 women with gynecological cancer and 240 controls. Patients with cervical cancer (n=105), endometrial cancer (n=63) and ovarian cancer (n=75) were treated with radical hysterectomy with bilateral salpingo-oophorectomy, chemotherapy and radiotherapy. As a control group, women with postmenopausal, who did not diagnosed with gynecologic malignancy were selected.
Results: T-scores of cervical cancer patients was significantly lower in L1, L2 and femur neck compared with control before cancer treatment. T-scores of the mean values of lumbar spine and L1-L4 in ovarian cancer patients was significantly lower respectively. On the other hand, T-scores of endometrial cancer patients was not significantly different from the control group.
There was a significant difference in BMD of L3, L4 and femur neck before and after treatment according to treatment method. T-scores change was the smallest in patients who underwent only surgical treatment including bilateral salpingo-oophorectomy, and the T-scores decreased most in patients who underwent postoperative concurrent chemoradiotherapy.
Conclusion: The prevalence of osteoporosis in the gynecologic cancer was significantly higher than in the control. BMD of patients with cervical cancer and ovarian cancer before treatment was significantly lower than control, and BMD after cancer treatment was significantly decreased in endometrial cancer. In patients with gynecologic cancer, the reduction of BMD and the risk of secondary osteoporosis and osteoporotic fracture were identified, and efforts should be made to prevent and early diagnose them.
Disclosure: Nothing to disclose

P165

Comparison of postoperative complications and quality of life between patients undergoing continent versus non-continent urinary diversion after pelvic exenteration

E. Mallet[1], M.A. Angeles[2], B. Cabarrou[3], P. Rouanet[4], P. Méeus[5], E. Lambeaudie[6], J.M. Classe[7], F. Foucher[8], F. Narducci[9], F. Migliorelli[10], C. Martínez-Gómez[11], L. Gladieff[12], A. Martinez[2], G. Ferron[2]
[1]Surgical Oncology, IUCT - Oncopole, Nice, [2]Surgical Oncology, IUCT - Oncopole, [3]Biostatistics Unit, IUCT – Oncopole, Toulouse, [4]Surgical Oncology, Institut du Cancer de Montpellier, Montpellier, [5]Surgical Oncology, Centre Léon Bérard, Lyon, [6]Surgical Oncology, Institut Paoli-Calmettes, Marseille, [7]Surgical Oncology, Institut de Cancérologie de l'Ouest, Nantes, [8]Surgical Oncology, Eugène Marquis, Rennes, [9]Surgical Oncology, Centre Oscar Lambret, Lille, France, [10]Department of Obstetrics and Gynecology, Geneva University Hospitals, Geneva, Switzerland, [11]Surgical Oncology, [12]Department of Medical Oncology, IUCT – Oncopole, Toulouse, France

Introduction / Background: Pelvic exenteration remains as the only curative treatment for patients with recurrent or persistent pelvic disease after prior radiotherapy for gynecologic malignancies. The aim of our study was to compare postoperative complications and quality of life (QoL) of patients undergoing continent versus non-continent urinary diversion after pelvic exenteration.
Methodology: We designed a multicentric French study, including patients from ten centers who underwent an anterior or total pelvic exenteration with urinary reconstruction for persistent or recurrent gynecologic malignancy after prior radiotherapy. From January 2005 to September 2008 we included patients retrospectively, and from September 2008 to May 2009 they were prospectively included, which allowed us to collect QoL data. Demographic, surgical and follow-up data were analyzed. Postoperative complications were classified following Clavien-Dindo Classification. QoL was assessed using the European Organisation for Research and Treatment of Cancer (EORTC) QLQ-C30 (version 3.0) and the EORTC QLQOV28 questionnaires.
Results: A total of 148 patients were included, 92 retrospectively and 56 prospectively. Among these patients, 70 underwent a urinary continent diversion and 78 patients a non-continent diversion. There were no differences between continent and non-continent groups in hospitalization length, postoperative grade III-IV complications, complications needing surgical or radiological intervention and complication type (digestive, urinary, parietal, infectious, hemorrhagic or medical). As well, there were no significant differences in QoL.
Conclusion: Continent and non-continent urinary diversion seems to be equivalent in terms of QoL and postoperative complications. However, global health and QoL in continent group appears to be worse one month after surgery, with a slight long-term improvement when compared to the non-continent group. Body image perception seems to be better in non-continent group one month after surgery with a progressive long-term deterioration, while continent group acquired a better body image perception three months after surgery, which stabilized in the long-term.
Disclosure: Nothing to disclose

P166

Patient-initiated follow-up for low-risk endometrial cancer: an economic evaluation

R. Wickham-Joseph[1], I. Luqman[1], N. Cooper[2], L. Boulter[1], N. Patel[1], E. Moss[2]
[1]University Hospitals of Leicester, [2]University of Leicester, Leicester, United Kingdom

Introduction / Background: Risk stratification has resulted in Patient-initiated follow-up (PIFU) being introduced for low-risk endometrial cancer (LREC) in place of routine hospital follow-up (HFU). The financial benefit to the healthcare economy and the patient of PIFU as compared to HFU as yet to be

explored.
Methodology: Women diagnosed with G1/2 Stage 1A EC enrolled for PIFU were included. Data on the number of appointments and telephone calls actually attended was collected prospectively, and costs calculated at £80/appointment and £43.33/telephone call (assuming £130/hour). This was compared with the cost of the scheduled HFU appointments. Patient travel time to appointments (car and public transport) and mileage were calculated. Travel cost was calculated to be 40p/mile and £2.50 car-parking.
Results: In total 188 women were enrolled between 2014-2018, median follow-up 1.9 years (range 0.1-3.9 years). Median participant age was 61 years and median travelling distance to the hospital was 15.4 miles (maximum 74 miles). There was no public transport option for two patients. The cohort had been scheduled to have attended 1106 appointments with HFU, whereas only 56 appointments were required with PIFU, 94.9% reduction in clinic costs: HFU £88,480 HFU versus PIFU £4,480. The total number of patient miles that would have been travelled with HFU was 20,435 miles (mean 108.7miles/patient) as compared to 888 miles for PIFU (mean 4.7miles/patient). This resulted in an overall mean 94.7% cost saving per patient. Travelling time was also dramatically reduced for PIFU, 13 and 32 minutes/patient versus 293 and 700 minutes/patient for HFU, for travel by car and public transport respectively.
Conclusion: The introduction of patient self-management follow up schemes for LREC can bring considerable financial and time savings to both the patient and the health care economy as compared to HFU.
Disclosure: Nothing to disclose

P167

Development of an EORTC module assessing fertility and patients care needs in young cancer patients (Phase I-III)

R. Schwab[1], A. Kiemen[2], E. Greimel[3], V. Kesic[4], J. Weis[2], A. Hasenburg[1], EORTC QoL Group and ESGO
[1]Frauenklinik, Universitätsmedizin Mainz, Mainz, [2]Tumorzentrum Freiburg-CCCF, Universitätsklinikum Freiburg, Freiburg, Germany, [3]Medizinische Universität Graz, Graz, Austria, [4]University of Belgrade, Belgrade, Serbia

Introduction / Background: Oncological therapy may affect women in terms of future reproductive capabilities. The infertility resulting from the treatment of cancer has a great impact on the quality of life. Patients of reproductive age consider a risk for infertility one of the most difficult components of their illness and treatment.
The aim of the EORTC module is the development of an international questionnaire to assess the individual unmet needs and concerns related to fertility issues in young cancer patients.
Methodology: We conducted interviews with 13 health care professionals and 12 young cancer patients regarding the topic of fertility preservation and related issues (phase I). The relevant issues were assessed by using thematic analysis of the interviews. Additionally, literature research and discussion with the collaborators at the autumn EORTC meeting 2018 helped to identify important missing topics (phase I).
Results: We developed a relevant list of 54 issues in 6 preliminary domains: psychoeducation and counseling; partnership, sexuality and family; financial aspects; quality of life; decision making and coping and global issues (phase I). This list of issues will be translated in national languages. For the next step interviews with young cancer patients and health care professionals during diagnosis and the survivorship continuum will be conducted in eight European countries (phase II and III) for identification of missing issues and to streamline the particular topics with the final goal to develop the international EORTC “fertility module”.
Conclusion: Identification of patients' psychosocial needs is an important part of advanced onco-fertility care including sexual health, intimate relationships and the affordability of fertility preservation measures. This EORTC module will help health care providers to tailor and optimize the individual counseling regarding fertility related issues during cancer diagnosis, treatment and the survivorship continuum and thus to improve the quality of life of young cancer patients.
Disclosure: Roxana Schwab: I have received honoraria and expenses from Roche Pharma AG and

AstraZeneca GmbH and funding by MacoPharma International GmbH, by Max Planck Institute for Polymer Research Mainz and by Geistlich Biomaterials. Annette Hasenburg: I have received honoraria and expenses from AstraZeneca, FBA Frauenärzte BundesAkademie GmbH, KlarigoVerlag, MedConcept, Med public GmbH, Med update GmbH, Medicultus, Pfizer, Promedicis GmbH, Pierre Fabre Pharma GmbH, Softconsult, Roche Pharma AG, Streamedup! GmbH, Tesaro Bio Germany GmbH. I am consultant to PharmaMar, Promedicis GmbH, Pierre Fabre Pharma GmbH, Roche Pharma AG and Tesaro Bio Germany GmbH. I have received funded research from Celgene. Andrea Kiemen: I have no conflict of interest. E. Greimel: I have no conflict of interest. V. Kesic: I have no conflict of interest. Joachim Weis: This study is supported by an EORTC grant.

P168

Influence of first-line treatment for epithelial ovarian cancer on the immune system

T. Baert[1,2], C. Landolfo[3], J. Ceusters[1], G. Thirion[1], A. Van Hoylandt[1], T. Verschuere[4], A.-S. Van Rompuy[5], A. du Bois[2], D. Timmerman[3,6], I. Vergote[1,6,7], A. Coosemans[1,6]

[1]Department of Oncology, Laboratory of Tumour Immunology and Immunotherapy, ImmunOvar Research Group, KU Leuven, Leuven, Belgium, [2]Department of Gynaecology and Gynaecologic Oncology, Kliniken Essen Mitte (KEM), Essen, Germany, [3]Department of Development and Regeneration, Laboratory of Organ Systems, [4]Department of Neuroscience, Laboratory of Experimental Neurosurgery, KU Leuven, [5]Department of Pathology, [6]Department of Gynaecology and Obstetrics, Leuven Cancer Institute, UZ Leuven, [7]Department of Oncology, Laboratory of Gynaecologic Oncology, KU Leuven, Leuven, Belgium

Introduction / Background: The KEYNOTE-021 trial showed a beneficial effect of the combination of chemotherapy and immunotherapy in non-small-cell lung cancer, since then multiple trials have studied combination of chemotherapy and immunotherapy. However, not all combinations yield positive results, as we learned from the JAVELIN Ovarian 200 trial. Therefore, we need to gain insight into the effect of chemotherapy on the immune system *in vivo*. In the current study, we investigated the influence of first-line treatment for ovarian cancer on the immune system.

Methodology: Tumour biopsies and peripheral blood mononuclear cells were collected prospectively in 43 patients with epithelial ovarian cancer. Samples were taken at diagnosis, after cytoreductive surgery, after three courses of (neo-)adjuvant chemotherapy and at the end of their primary treatment. Immunostimulatory cells (T_{helper} cells (Th), $T_{cytotoxic}$ cells (Tc), natural killer cells (NK)) and immunosuppressive cells (regulatory T cells (Treg), mMDSC (monocytic myeloid-derived suppressor cells), gMDSC (granulocytic MDSC)) were measured in blood using fluorescence activated cell sorting. Additionally, we checked for the expression of activation markes and immune checkpoint molecules on immune cells. On tumour biopsies, we performed immunohistochemistry for Tc and Treg.

Results: First-line treatment led to a significant increase in Tc in circulation ($p< 0.05$). During neoadjuvant chemotherapy we observed an increase in Tc in the tumour and in blood ($p< 0.05$ and $p< 0.01$ respectively). In addition, we observed an increased expression of PD-1 on all circulating T cells during neoadjuvant chemotherapy. Chemotherapy after (interval)debulking surgery led to an overall increase in immunosuppressive cells (Treg ($p=0.0001$) and mMDSC ($p< 0.05$)) and a higher expression of PD-1 and CTLA4 on circulating T cells.

Conclusion: We observed a clear change in the immune phenotype of patients during first-line treatment for epithelial ovarian cancer. Chemotherapy led to an increase in the expression of immune checkpoint markers on Th, Tc and Treg in blood.

Disclosure: TB has reported research grant from Amgen, travel expenses from Amgen and Roche as well as advisory board membership for Tesaro. IV has performed contracted research for Oncoinvent AS and Genmab, received research grants from Amgen, Roche, Stichting tegen Kanker, received travel expenses from Takeda Oncology, Pharma Mar, Genmab, Roche, Astrazeneca, Tesaro, Clovis, Immunogen and is a advisory board member for Advaxis, Inc., Eisai Inc., MSD Belgium, Roche NV, Genmab, F. Hoffmann-La Roche Ltd, Pharmamar, Millennium Pharmaceuticals, Clovis Oncology Inc., AstraZeneca NV, Tesaro, Oncoinvent AS, Immunogen Inc and Sotio. CL, JC, GT, AVH, TV, A-SVR, AdB, DT and AC have no disclosures concerning this abstract.

P169

Activated effector T cells co-expressing multiple inhibitory receptors are enriched in the tumor immune microenvironment of HGSOC expressing high and uniform WT-1 antigen

A. Bergamini[1], E. Tassi[2], J. Wignall[2], E. Ruggiero[2], L. Bocciolone[1], M. Candiani[1], A. Potenza[2], F. Manfredi[2], G. Taccagni[3], F. Scalisi[3], C. Doglioni[3], B. Schultes[4], M. McKee[4], G. Mangili[1], C. Bonini[2]
[1]Department of Obstetrics and Gynaecology, IRCCS San Raffaele Hospital, [2]Experimental Hematology Unit, Division of Immunology, Transplantation and Infectious Disease, San Raffaele Scientific Institute, [3]Department of Surgical Pathology, San Raffaele Hospital, Milano, Italy, [4]Intellia Therapeutics, Inc., Cambridge, MA, United States

Introduction / Background: In high grade serous ovarian cancer(HGSOC),the association between tumor-infiltrating lymphocytes(TILs)and survival suggests a role for T cell immune surveillance. Despite this, initial attempts with PD1 checkpoint blockade and adoptive T-cell therapy(ACT) had limited efficacy. This highlights the need to characterize the phenotype of TILs and to find new potential tumor-associated antigens.Wilms tumor-1(WT-1)expression showed a prognostic significance in HGSOC, making it an interesting target.To identify molecules potentially relevant for HGSOC immunotherapy, we evaluated the expression of WT-1 and the pattern of inhibitory receptors(IRs)in TILs.
Methodology: Peripheral blood(PB),tumor and ascites were retrieved from HGSOC patients admitted for primary surgery.WT-1 expression and immune infiltrate were characterized on 34 specimens by immunohistochemistry (IHC) for lineage markers and inhibitory molecules.T cells isolated from tumor, ascites and PB of 14 patients were also characterized by flow cytometry for the expression of lineage and memory markers (CD3, CD4, CD8, CD45RA, CD62L, CD95),inhibitory molecules (PD1, CTLA-4, LAG-3, TIGIT, TIM-3, 2B4, GITR, KLRG1, CD39, CD160)and the activation marker CD137.
Results: WT1 was almost uniformly expressed,with 76% of cases showing high WT1 expression in the majority of tumor cells. 6% of cases were immune desert; all other cases displayed a wide range of immune cell density, dominated by T lymphocytes and macrophages. Intratumoral T cells(ITTCs), mainly CD8, were present in 88.2% of samples. PD-1, LAG-3 and TIM-3 were expressed by 61.9%, 61.7% and 61.7% of ITTCs. PD-L1 was expressed by neoplastic cells and immune cells in 40% and 82.3% of samples.Flow cytometry confirmed the expression of several IRs.Compared with PB and ascites,TILs were enriched in effector memory lymphocytes and in cells coexpressing PD-1, CD39 and CD137.
Conclusion: WT-1 is a potential target for ACT in HGSOC,which is infiltrated by antigen-experienced T lymphocytes displaying features of activation and partial exhaustion.
Disclosure: Nothing to disclose

P171

Development of an *ex-vivo* patient-derived explant model for predicting drug responses in endometrial cancer

A. Collins[1], G. Miles[1], M. MacFarlane[2], C. Pritchard[1], E. Moss[1]
[1]Leicester Cancer Research Centre, [2]MRC Toxicology Unit, University of Leicester, Leicester, United Kingdom

Introduction / Background: Adjuvant chemotherapy confers a modest advantage in progression free and overall survival in endometrial cancer (EC). Response rates to primary chemotherapy regimens are reported as low as 17-36%[1]. Pre-clinical models that better predict patient outcomes to standard of care and novel anti-cancer treatments are urgently required. Patient derived explants (PDEs) retain

histological 3D architecture of the primary tumour, preserve intra-tumour heterogeneity and maintain patient specific stromal cells including tumour associated fibroblasts and immune cells. This project aims to establish optimal culture conditions for EC-PDEs and to evaluate patient-specific responses to SOC chemotherapies.

Methodology: Fresh tumour was obtained post-hysterectomy from patients with EC. Tumour was dissected into 1-3mm^3 explants and placed on PVDF filter inserts in culture media and cultured at the air-liquid interface. Following overnight recovery, explants were moved to fresh media and cultured for 24 hours, optimal media conditions were established and explants were cultured with carboplatin, paclitaxel & pembrolizumab. Multiplexed fluorescent immunohistochemistry for Ki67 (proliferation marker), cleaved PARP (apoptosis marker) and CAM 5.2 (tumour mask) was performed followed by quantitative analysis to evaluate cellular proliferation and apoptosis in tumour and stroma.

Results: Tissue architecture and histology were maintained in PDE culture. Mean tumour cleaved-PARP% at T0 control was 1.4%, increasing to 14.8% following 48 hours (T48) culture in DMEM-F12 media. Autologous serum (AS) supplementation at low concentrations (1%) reduced tumour apoptosis at T48 to 6.7%. Higher concentrations of fetal calf serum/AS resulted in a dose-dependent increase in apoptosis while addition of oestrogen did not confer additional benefit. Explants treated with carboplatin and paclitaxel display increased tumour apoptosis (39.32%) compared to pembrolizumab treatment (11.39%).

Conclusion: PDE viability is preserved following 24h culture with optimal conditions of DMEM-F12 media supplemented with 1% AS. Our results show PDEs are a novel pre-clinical model for predicting drug response in a patient specific setting.

Disclosure: Esther Moss - grants: Hope Against Cancer, CRUK development fund

P172

Preclinical evidences of the therapeutic potential of ABTL0812 in endometrial cancer

A. Gil-Moreno[1], I. Felip[2], C.P. Moiola[3], C. Megino-Luque[4], C. Lopez-Gil[5], S. Cabrera[6], S. Solé-Sánchez[7], P. Muñoz-Guardiola[8], E. Megias-Roda[8], H. Pérez-Montoyo[7], J. Alfon[7], M.Y.-V. Yeste-Velasco[7], M. Santacana[9], X. Dolcet[9], A. Reques[10], A. Oaknin[11], V. Rodríguez-Freixinos[11], J.M.L. Lizcano[12], C. Domènech[7], X. Matias-Guiu[13], E. Colas[14], N. Eritja[9]

[1]Gynecological Oncology Department, CIBERONC., Hospital Universitari Vall d'Hebron, Barcelona, [2]Department of Pathology and Molecular Genetics/Oncologic Pathology Group, Biomedical Research Institute of Lleida (IRBLleida), University of Lleida, CIBERONC, Lleida, [3]Biomedical Research Group in Gynecology, Vall Hebron Research Institute (VHIR), Universitat Autònoma de Barcelona, CIBERONC, Universitat Autònoma de Barcelona, Barcelona, [4]Department of Pathology and Molecular Genetics/Oncologic Pathology Group, Biomedical Research Institute of Lleida (IRBLleida), University of Lleida, CIBERONC, Department of Pathology and Molecular Genetics/Oncologic Pathology Group, Lleida, [5]Biomedical Research Group in Gynecology, Vall Hebron Research Institute (VHIR), Universitat Autònoma de Barcelona, CIBERONC, Universitat Autònoma de Barcelona, Universitat Autònoma de Barcelona, [6]Gynecological Oncology Department, Vall Hebron University Hospital, CIBERONC, Vall Hebron University Hospital, [7]Ability Pharmaceuticals, SL, Cerdanyola Del Vallès, Ability Pharmaceuticals, SL, Cerdanyola Del Vallès, Barcelona, [8]Ability Pharmaceuticals, SL, Cerdanyola Del Vallès, Protein Kinases and Signal Transduction Laboratory, Institut de Neurociències and Departament de Bioquímica i Biologia Molecular, Universitat Autònoma de Barcelona, [9]Department of Pathology and Molecular Genetics/Oncologic Pathology Group, Biomedical Research Institute of Lleida (IRBLleida), University of Lleida, CIBERONC, University of Lleida, [10]Pathology Department, Vall Hebron University Hospital, Vall Hebron University Hospital, [11]Medical Oncology Department, Vall d'Hebron University Hospital, Vall d'Hebron Institute of Oncology (VHIO), Vall d'Hebron University Hospital, [12]Protein Kinases and Signal Transduction Laboratory, Institut de Neurociències and Departament de Bioquímica i Biologia Molecular, Universitat Autònoma de Barcelona, Barcelona, [13]Department of Pathology and Molecular Genetics/Oncologic Pathology Group, Biomedical Research Institute of Lleida (IRBLleida), University of Lleida, CIBERONC, University of Lleida, [14]Biomedical Research Group in Gynecology, Vall Hebron Research Institute (VHIR), Universitat Autònoma de Barcelona, CIBERONC, Universitat Autònoma de Barcelona, Lleida, Spain

Introduction / Background: Endometrial cancer (EC) is the most frequent of the infiltrating tumors of female genital tract. Surgical and adjuvant treatments are the cornerstone treatment for EC patients, however, the response rate to standard therapy is very limited, highlighting the need for novel and efficient treatments. ABTL0812, a small first-in-class molecule, induces cancer cell death by inhibiting the PI3K/AKT/mTOR pathway (frequently overactivated in EC) and inducing endoplasmic reticulum stress (ER-Stress).
Objective: Assess the preclinical efficacy of ABTL0812 in EC.
Methodology: We studied ABTL0812 treatment on viability, autophagy and apoptosis in a wide panel of EC cell lines and in 3D-murine endometrial organoids. ABTL0812 efficacy was evaluated in tumor onset and progression in a tamoxifen-inducible PTEN-KO murine model and cell line- or patient-derived xenograft (PDX) models. TRIB3 mRNA levels were evaluated in blood from EC patients recruited in ongoing Phase 1b/2a clinical trial.
Results: We demonstrated that ABTL0812 treatment in vitro reduced viability, sensitized cancer cells and induced autophagy through ER-stress induction and PI3K/AKT/mTORC1 axis inhibition. In murine PTEN-KO cells, ABTL0812 decreased proliferation and induced nuclear fragmentation/condensation and caspase activation. ABTL0812 in vivo therapeutic benefit was confirmed, leading to a marked reduction in tumor growth ($p < 0.05$), impairing the initial progression of endometrial tumorigenesis in PTEN-KO mouse model, and inhibiting tumor progression in PDX models, potentiating carboplatin-paclitaxel treatment. Finally, ABTL0812 increased TRIB3 mRNA levels after 8h of treatment in blood of human patients.
Conclusion: Our results demonstrated that ABTL0812 treatment promoted autophagy followed by activation of the pro-apoptotic pathway. Collectively, we provided strong preclinical evidences of the therapeutic benefit of ABTL0812 as monotherapy or in combination with current first-line treatment in EC. Our findings present a novel and clinically applicable therapeutic strategy for EC and suggest the potential use of TRIB3 as a pharmacodynamic biomarker to monitor ABTL0812 treatment.
Disclosure: Sonia Solé-Sánchez, Pau Muñoz-Guardiola, Elisabet Megias-Roda, Héctor Pérez-Montoyo, José Alfon, Marc Yeste-Velasco, Carles Domènech are Ability Pharmaceuticals employees. Carles Domènech CD holds shares of the Company. José Miguel Lizcano is member of its Scientific Advisory Board. Antonio Gil-Moreno, Isidre Felip, Cristian Pablo Moiola, Cristina Megino-Luque, Carlos Lopez-Gil, Silvia Cabrera, María Santacana, Xavier Dolcet, Armando Reques, Ana Oaknin, Victor Rodríguez-Freixinos Xavier Matias-Guiu, Eva Colas, Nuria Eritja, declare no conflicts of interest.

P173

Plasma and tumour tissue TP53-gene expression in patients with high grade serous advanced ovarian cancer

J. Martinez-Garcia[1], P. Sanchez Henarejos[1], M.D. Jimenez Lucas[1], A. Puertes Boix[1], A. Anton Garcia[2], F. Perez Sanz[3], E. Rodriguez Braun[4], A. Cano Jimenez[1], D. Collado Martinez[1], A. Lopez Muñoz[1], T. Quiros Figallo[1], D. Sanchez[1], J.L. Alonso Romero[1]
[1]Medical Oncology, Hospital Virgen Arrixaca, [2]Genomic Department, [3]Bioinformatic Department, Murcian Institute Biosanitary Research, Murcia, [4]Medical Oncology, Hospital Santa Lucia, Cartagena, Spain

Introduction / Background: TP53 is a tumour suppressor gene frequently mutated in high grade serous ovarian cancer(HGSOC).The aim of this study is to investigate the expression of TP5-gene in cell-free DNA(cfDNA) in plasma and tumour paraffin-embedded tissue in HGSOC,both platinum-sensitive(PSR)and platinum-resistant relapse(PRR)patients.
Methodology: .This multicentric prospective observational study has been conducted from November/2013 to February/2017 in patients diagnosed of HGSOC.Ampliseq custom panel was designed to amplify TP53-gene.Libraries passing QC were sequenced on the PGM Ion Torrent using a Hi-Q view sequencing kit and 318 chip v.2. The sequence data generated were mapped with the Torrent Suite 5.8.This study was approved by the Central Research Ethics Committee
Results: We analyzed plasma and tumour samples of 30 patients(16 PSR and 14 PRR).The most frequently detected mutations were missense mutations.Mutations in circulating TP53 were found in tumour tissue in only 24% of patients and mutations in tumour tissue were found in circulating TP53 in only 12%.There was no difference in TP53 gene expression in PSR and PRR patients nor with other

clinical variables,neither with circulating DNA nor tumour paraffin-embedded tissue.In 4 tumour tissue were not detected any mutations(13% patients;2 PSR,2 PRR).In 3 circulating TP53 were not any mutations (10% patients;1PSR,2 PRR).We also analysed 5 healthy controls whose we were not detected any mutations in TP53 in plasma.The number of TP53 mutations have not related to the tumour response.The number of TP53 mutations in tumour tissue was related with progression-free survival (p 0,001) and overall survival (p< 0,001), but not with mutations in circulating TP53.
Conclusion: The number of TP53 mutations in tumour tissue have been prognostic factor for progression-free and overall survival in HGSOC.Most of HGSOC had mutation of circulating TP53, both in circulating DNA and tumour paraffin-embedded tissue.We have not found significant difference in the expression of TP53-gene in platinum-sensitive and platinum-resistant HGSOC nor with other clinical variables.
Disclosure: Nothing to disclose

P174

Generation of organoids from ovarian adult granulosa cell tumours for individualized drug screening

G. Monroe[1], J. Roze[1], E. Stelloo[2], C. Stangl[2], F. Sereno[1], J. Groeneweg[1], S. Paijens[3], H. Nijman[3], H. van Meurs[4], L. van Lonkhuijzen[4], J. Piek[5], C. Lok[6], G. Jonges[7], P. Witteveen[8], R. Zweemer[1]
[1]Department of Gynaecological Oncology, UMC Utrecht Cancer Center, University Medical Center Utrecht, Utrecht University, [2]Center for Molecular Medicine, University Medical Center Utrecht, Utrecht University, Utrecht, [3]Department of Obstetrics and Gynaecology, University Medical Center Groningen, University of Groningen, Gronigen, [4]Department of Gynecological Oncology, Centre for Gynaecological Oncology Amsterdam, Amsterdam University Medical Center, Amsterdam, [5]Department of Obstetrics and Gynaecology, Catharina Hospital, Eindhoven, [6]Department of Gynaecological Oncology, Centre for Gynaecological Oncology Amsterdam, The Netherlands Cancer Institute, Antoni van Leeuwenhoek Hospital, Amsterdam, [7]Department of Pathology, University Medical Center Utrecht, Utrecht University, [8]Department of Medical Oncology, UMC Utrecht Cancer Center, University Medical Center Utrecht, Utrecht University, Utrecht, The Netherlands

Introduction / Background: Adult granulosa cell tumours (aGCT) constitute a rare subtype of ovarian cancer, with >90% of tumours characterized by the *FOXL2* c.402C>G mutation. Recurrences occur in nearly 50% of patients and are associated with a poor prognosis. Surgery is the mainstay of treatment, since the effect of adjuvant therapies are limited. Chemotherapy and hormone therapy response is difficult to predict and patient numbers insufficient to conduct informative clinical trials. Patient-specific aGCT organoid and 2D culture establishment could evaluate the effect of novel therapeutic options on a relatively large scale and enable personalized treatment in this neglected patient group.
Methodology: Samples from 46 tumours (4 primary and 42 recurrences) from 18 patients were collected, mechanically homogenized to single cells and small tissue pieces, and seeded in 50% Cultrex Basement Membrane Extract on day of surgery. Basal medium (DMEM/F12) supplemented with various growth factor combinations promoted organoid formation. Medium was refreshed every 3-4 days and cells passaged every 3 weeks. In parallel, 2D monolayer cell cultures were established to perform drug screens for later validation in the more physiologically relevant organoids as they became available. Organoid and 2D tumour origin verification is ongoing by *FOXL2* c.402C>G PCR.
Results: Tumour tissue cultivation resulted in 3D structures that remained viable for 3-4 passages, with an establishment rate of approximately 75% in primary and 26% in recurrences. Morphology of masses ranged from grape-like to cystic, with primary organoids demonstrating a more cystic morphology (Figure 1) that could be robustly passaged. Monolayer cultures in DMEM/F12/FBS were successfully established for future drug screening with carboplatin, paclitaxel, tamoxifen and other first-line treatments.
Conclusion: Patient-derived aGCT organoids can be established. Organoid establishment is more successful for primary tumours than for metastases, and organoid establishment optimization is necessary. Tumour cell culture establishment and drug screens are likely to provide insight into patient-specific treatment response.

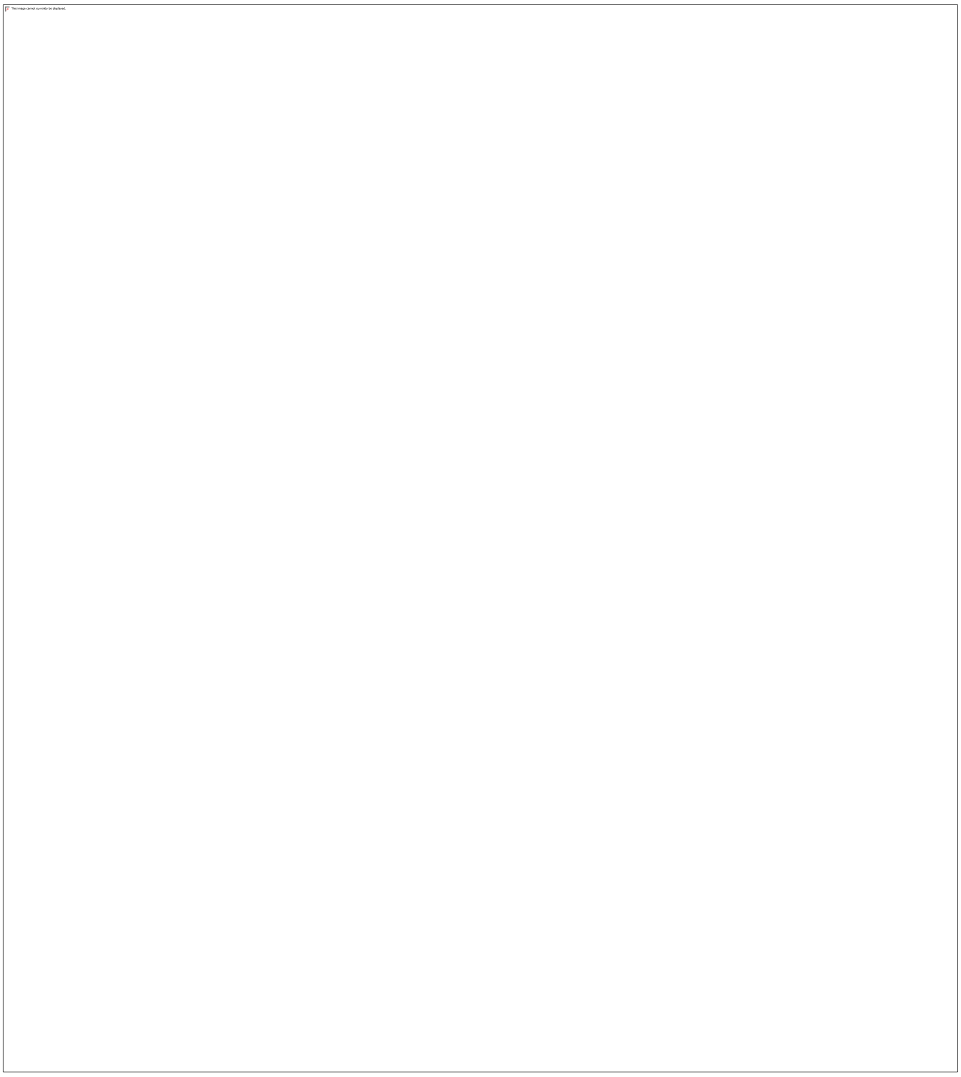

[Figure 1: Organoids (black arrows) derived from primary tumor tissue. Passage III.]

Disclosure: Nothing to disclose

P175

Fetal calf serum alternatives for application in tissue engineering

R. Schwab, T. Helmert, S. Gebhard, K. Peters, A. Hasenburg, M. Heller, W. Brenner
Frauenklinik, Universitätsmedizin Mainz, Mainz, Germany

Introduction / Background: Tissue engineering is an emerging field with growing importance in oncological reconstructive surgery, as it may help to replace damaged or injured body tissue with compatible substitutes. For overcoming the initial loss of the tissue engineered substitutes, pre-vascularized tissue surrogates would be helpful to enable a rapid/fast/quick connection of the blood vessels of the recipient. *In vitro*, culture of tissue engineered body substitutes are performed with fetal calf serum (FCS). Nevertheless, for *in vivo* application in humans, human platelet lysates may

successfully replace FCS.
Methodology: Human umbilical vein endothelial cells (HUVEC) were cultured with FCS and with two commercially available human platelet lysates (MultiPl´30 and MultiPL´100, kindly provided by MacoPharma International GmbH) or one non-commercially available human platelet lysates (hPL) (manufactured by Max Planck Institute for Polymer Research Mainz) in different concentrations. Cell viability, proliferation and tube formation assays were conducted by using platelet lysates and compared with 20 % FCS.
Results: Proliferation and cell viability of HUVEC cultured for 72 hours with the distinct types of human platelet lysates in different concentrations were similar to those of the controls (cells cultured with 20 % FCS). Capillary-like structures of HUVEC cultured in all platelet lysates were more effective than cultured in FCS. The induction of tube formation was most effective by using 5 % MultiPL'30. However, the use of platelet lysates in concentrations between 2.5 % and 5 % were more effective than the cultivation in 20 % FCS.
Conclusion: To prevent tissue rejection or adverse effects like an allergic reaction, autologous cells should be cultivated with human platelet lysates. We showed that human platelet lysates can replace FCS in the *in vitro* cultivation process and, additionally, that these cells are able to form capillary-like structures as prerequisite for pre-vascularized tissue substitutes in humans.
Disclosure: Roxana Schwab: I have received honoraria and expenses from Roche Pharma AG and AstraZeneca GmbH and funding by Geistlich Biomaterials. MultiPl´30 and MultiPL´100 were kindly provided by MacoPharma International GmbH and the human platelet lysates (hPL) were kindly provided by the Max Planck Institute for Polymer Research Mainz. Annette Hasenburg: I have received honoraria and expenses from AstraZeneca, FBA Frauenärzte BundesAkademie GmbH, KlarigoVerlag, MedConcept, Med public GmbH, Med update GmbH, Medicultus, Pfizer, Promedicis GmbH, Pierre Fabre Pharma GmbH, Softconsult, Roche Pharma AG, Streamedup! GmbH, Tesaro Bio Germany GmbH. I am consultant to PharmaMar, Promedicis GmbH, Pierre Fabre Pharma GmbH, Roche Pharma AG and Tesaro Bio Germany GmbH. I have received funded research from Celgene. Tania Helmert: I have no conflict of interest. Susanne Gebhard: I have no conflict of interest. Katharina Peters: I have no conflict of interest. Martin Heller: I have no conflict of interest. Walburgis Brenner: I have no conflict of interest.

P176

Update on UK outcomes in twin pregnancies with complete hydatidiform mole and normal co-twin: a retrospective national cohort study in 153 new cases

J.C. Russell[1], I. Niemann[2,3], N.J. Sebire[3], R.A. Fisher[3], D. Short[3], J. Everard[4], M. Wells[4], P.M. Savage[3], N. Sarwar[3], B.W. Hancock[4], J. Tidy[4], M.J. Seckl[3]
[1]Newnham College, Cambridge University, Cambridge, United Kingdom, [2]Department of Gynaecology and Obstetrics, University Hospital of Aarhus, Aarhus, Denmark, [3]Charing Cross Gestational Trophoblastic Disease Centre, Imperial College NHS Healthcare Trust, London, [4]Gestational Trophoblastic Disease Centre, Weston Park Hospital, Sheffield, United Kingdom

Introduction / Background: Uncertainty remains regarding management of twin pregnancies comprising complete hydatidiform mole (CHM) and healthy co-twin. In 1998, we reported that continuing such pregnancies results in a 38% chance of a healthy baby, no maternal deaths or increased risk of malignancy requiring chemotherapy. However, other studies suggest a higher risk of malignancy and maternal mortality if such pregnancies are not terminated. Here, we re-examine this controversial issue in a new, larger national cohort.
Methodology: The UK hydatidiform mole databases were screened to identify all twin pregnancies with central pathology review confirming CHM and normal co-twin between 1998 and 2018. Data on rates of termination, spontaneous miscarriage, pregnancy complications, successful deliveries and need for chemotherapy were collected.
Results: 153 new patients had a confirmed CHM and healthy co-twin. Fifty electively terminated their pregnancy before 14 weeks. 103 continued beyond 14 weeks but 28 subsequently electively terminated, leaving 75 patients who continued their pregnancy. Their outcomes included: 11 terminations < 24 weeks for complications (4 pre-eclampsia, 1 intrauterine infection and 6 bleeding); 5 stillbirths (27-35 weeks), 2 neonatal deaths and 19 spontaneous abortions/miscarriages. Strikingly,

51% (38/75) delivered a surviving baby at a median gestational age of 30 weeks (range 24-40 weeks). Malignant change requiring chemotherapy was not significantly different whether pregnancies were terminated within 14 weeks (12/56, 21.4%), continued into the second (12/58, 20.7 %) or third trimester (7/39, 17.9%) (p = 0.40). However, malignancy risk is higher in twin (20.3%) than singleton (16%) CHM pregnancies. Importantly, there were no maternal deaths.
Conclusion: Although malignancy risk is slightly greater following CHM and healthy co-twin than in singleton molar pregnancies, the risk does not increase with progression of pregnancy beyond the first trimester. Early termination is not necessary, and the likelihood of a healthy baby appears to be greater than previously reported.
Disclosure: MJS wishes to acknowledge support from the Imperial National Institute for Health Biomedical Research Centre and Cancer Research UK Imperial Experimental Cancer Medicine Centre. NJS is partly supported by an NIHR Senior Investigator award. JCR has nothing to disclose. IN has nothing to disclose. RAF has nothing to disclose. DS has nothing to disclose. JE has nothing to disclose. MW has nothing to disclose. PMS has nothing to disclose. NS has nothing to disclose. BWH has nothing to disclose. JT has nothing to disclose.

P177

What are the real-world outcomes of locally advanced vulval cancer patients who receive neoadjuvant chemotherapy to downstage their disease?

K. Baillie[1], C. Crearie[1], J. Laskey[1], M. Bennie[2,3], A. Sadozye[1], R. Harrand[1], K. Burton[4], N. Siddiqui[4], N. Reed[1], K. Graham[1]
[1]Beatson West of Scotland Cancer Centre, NHS Greater Glasgow and Clyde, [2]Strathclyde Institute for Pharmacy and Biomedical Sciences, University of Strathclyde, Glasgow, [3]Public Health and Intelligence Strategic Business Unit, NHS National Services Scotland, Edinburgh, [4]Glasgow Royal Infirmary, NHS Greater Glasgow and Clyde, Glasgow, United Kingdom

Introduction / Background: Limited studies suggest that locally advanced vulval cancer may be rendered more operable with neoadjuvant chemotherapy (NACT). In the West of Scotland Cancer Network (WoSCAN), NACT is offered to women with sphincter threatening disease. A previous local audit in 2012 reported an operability rate of 35% and median overall survival (OS) of 20 months, following NACT. This study aimed to identify the proportion of patients who proceed to tailored surgery with wide local excision +/- groin node dissection following NACT for vulval cancer in the real-world setting.
Methodology: Data were collected retrospectively from electronic clinical records of WoSCAN patients who commenced NACT between January 2012 and December 2016. Statistical analysis was performed using R®. OS was estimated using Kaplan-Meier method and Cox's proportional-hazards models used to calculate unadjusted hazard ratios (HR).
Results: 57 patients were identified with a median age of 65 years (range 35-88). Cisplatin/ Capecitabine was the most common NACT regimen (80.7%); a median of 3 cycles were administered. 43.9% proceeded to surgery, 35.1% received primary (chemo)radiotherapy and 21.2% did not receive any definitive therapy. Exenteration was avoided in all cases. Data were censored in February 2018 at which time 29 patients (50.9%) remained alive and 35.1% required further treatment for relapse. Median OS was 39.3 months. Survival was shorter in patients receiving NACT alone (HR 3.44 (95% CI 1.33-8.87)) but not significantly different between surgery and radiotherapy groups.
Conclusion: Over 40% of women received surgical resection with sphincter preservation. Our operability rate has increased and OS is longer than our previous audit, reasons for which are currently being explored. Survival with NACT followed by radical (chemo)radiotherapy was comparable to surgery. Local relapse remains an issue and close follow up is advised as salvage therapy may be indicated.
Disclosure: This study was funded by the Scottish Government. The authors declare no competing interests.

P178

Carbon-Ion radiotherapy for malignant gynecological melanoma

A. Barcellini[1], V. Vitolo[1], R. Lazzari[2], F. Consoli[3], A. Ditto[4], A. Facoetti[5], P. Fossati[1], M.R. Fiore[1], B. Vischioni[1], A. Iannalfi[1], C. Laliscia[6], A. Mirandola[7], A. Vai[7], E. Mastella[7], A. Gadducci[8], F. Raspagliesi[4], L. Preda[9,10], R. Orecchia[1,11], F. Valvo[1]
[1]Radiation Oncology, National Center of Oncological Hadrontherapy (CNAO), Pavia, [2]Radiation Oncology, European Institute of Oncology, Milan, [3]Department of Medical and Surgical Specialties, Radiological Sciences and Public Health, Unit of Medical Oncology, University of Brescia, ASST-Spedali Civili, Brescia, [4]Gynecologic Oncology, Fondazione IRCCS Istituto Nazionale Tumori, Milan, [5]Radiobiology, National Center of Oncological Hadrontherapy (CNAO), Pavia, [6]Department of Translational Medicine, Division of Radiation Oncology, University of Pisa, Pisa, [7]Medical Physics, National Center of Oncological Hadrontherapy (CNAO), Pavia, [8]Department of Clinical and Experimental Medicine, Division of Gynecology and Obstetrics, University of Pisa, Pisa, [9]Radiology, National Center of Oncological Hadrontherapy (CNAO), [10]Department of Clinical-Surgical, Diagnostic and Pediatric Sciences, University of Pavia, Pavia, [11]European Institute of Oncology, Milan, Italy

Introduction / Background: We analyzed early clinical outcomes of carbon ion radiotherapy (CIRT) in the first patients with gynecological malignant mucosal melanoma(g-MMM) treated at CNAO
Methodology: Between 2016 and 2018, 9 patients(pts) with g-MMM were treated with CIRT after surgery or in exclusive settings ***(Table 1)***. They had 7 vaginal(VaM),1 cervical(CM) and 1 vulvar(VuM)MMM. One pt with VaM had been previously irradiated with photons;8 pts are considered inoperable and 1 pt underwent adjuvant CIRT on the small pelvic space after radical surgery without lymphadenectomy. Two pts underwent neoadjuvant and sequential anti-PD-1 immunotherapy. Because of the large volume of macroscopic disease,CM and VuM patients were irradiated with up to a total dose of 28 GyRBE(3 fractions) and 68.8 GyRBE(16 fractions), respectively, to the Clinical Target Volume (CTV) defined as the Gross Tumor Volume (GTV)+uterine cervix and corpus for the CM and GTV+vulva for the VuM. For inoperable VaM, small pelvic space including GTV was irradiated with up to a total dose of 38.7-43 GyRBE followed by a GTV boost of up to a total dose of 68.8 GyRBE in 16 fractions***(Fig 1).*** All patients were treated with synchrotron-based scanning carbon ion beams. Early clinical and toxicity profile(according to CTCAE V4.03) were evaluated.
Results: Treatment was well tolerated and no interruption was needed. For the evaluable pts, toxicity profile was favorable and no G≥2 acute/late toxicities were observed. Overall,for pts with a follow-up≥3 months,median LC ranged from 3 to 13 months(< for VuM and CM), median MFS and median OS was 6.5 (range: 3-23) and 9 months (range: 3-29.5) respectively. Data are still ongoing.
Conclusion: Because of the high rate of distant metastasis and unsatisfactory survival benefit, a more conservative treatment approach, instead of extensive surgery, may be warranted. We think that CIRT may serve as a definitive treatment choice for patients with g-MMM.

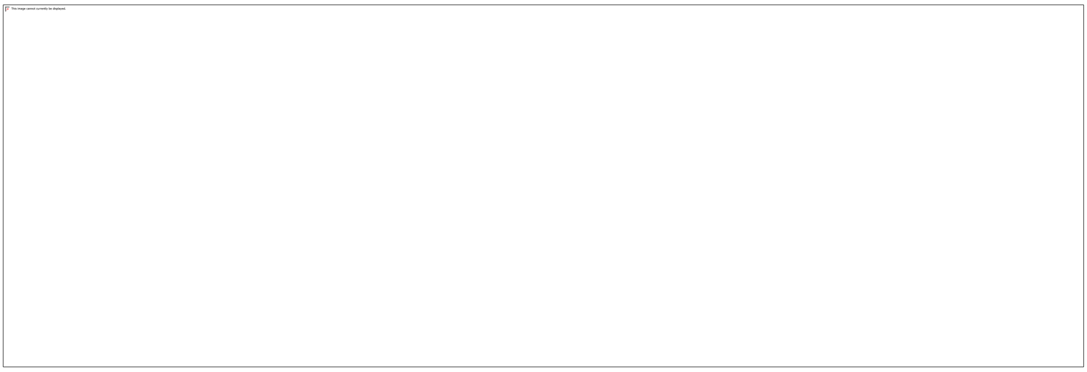

[Fig 1: Case of vaginal MMM received CIRT and dose distribution]

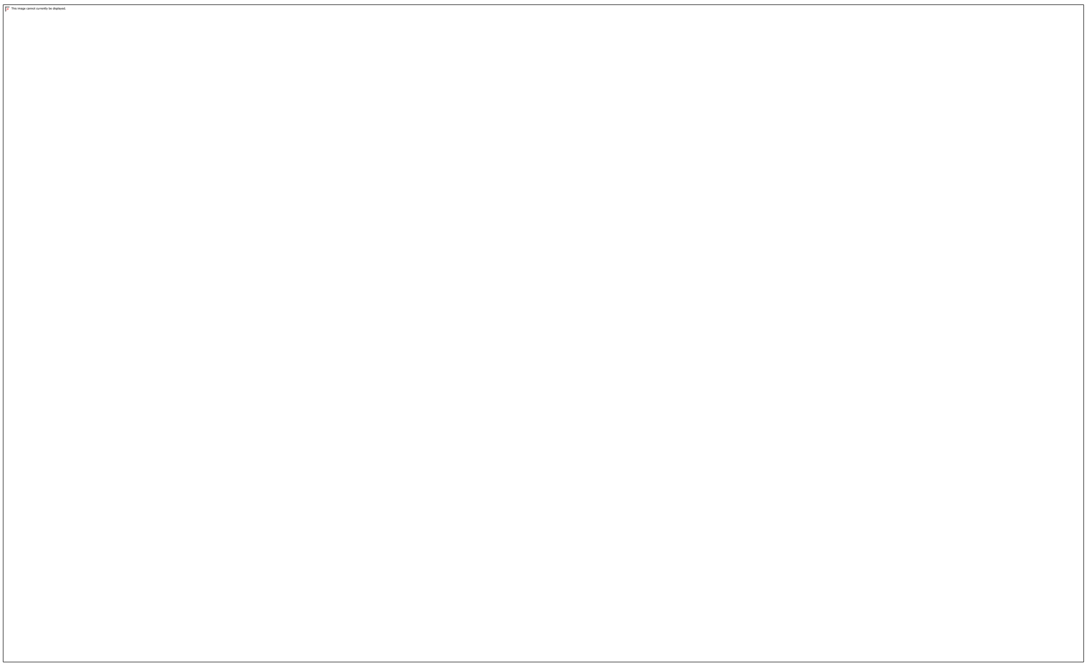

[Table 1]

Disclosure: Nothing to disclose

P179

Treatment and relative survival of vulvar carcinoma in Sweden 2012-16. A population-based cohort (SweGCG study)

K. Hellman[1], M. Bjurberg[2], C. Borgfeldt[2], P. Dahm-Kahler[3], A. Floter Rådestad[4], E. Hjerpe[1], E. Holmberg[5], T. Hogberg[6], J. Marcickiewicz,[7], K. Stalberg[8], P. Rosenberg[9], B. Tholander[10], P. Kjølhede[11], E. Avall-Lundqvist.[6]

[1]Gynecologic Oncology, Karolinska University Hospital, Stockholm, [2]Gynecologic Oncology, Skane University Hospital, Lund University, Lund, [3]Department of Obstetrics and Gynecology, Sahlgrenska University Hospital, Gothenburg, [4]Gynecologic Cancer, Karolinska University Hospital, Stockholm, [5]Regional Cancer Center Western Sweden, Sahlgrenska University Hospital, Institute of Clinical Sciences, Gothenburg, [6]Department of Oncology and Department of Clinical and Experimental Medicine, Linköping University, Linkoping, [7]Department of Obstetrics and Gynecology, Hallands Hospital, Halmstad, [8]Department of Obstetrics and Gynecology, Uppsala University, Uppsala, [9]Department of Clinical Oncology and Department of Clinical and Experimental Medicine, Linkoping University, Linkoping, [10]Department of Gynecologic Oncology, Uppsala University, Uppsala, [11]Children and Women's Health, Department of Clinical and Experimental Medicine Department of Clinical and Experimental Medicine, Linköping University, Linkoping, Sweden

Introduction / Background: Vulvar cancer is a rare disease where treatment guidelines are mostly based on results from retrospective studies with small, heterogeneous materials. There are no data published from the Swedish population.

Methodology: A population-based nationwide study on all women treated for vulvar carcinoma included in the Swedish Quality Registry for Gynecologic Cancer (SQRGC) between 2012-2016. Relative survival (RS) was estimated using the Ederer II method. Simple and multivariable analyses were estimated by Poisson regression models.

Results: 657 women with squamous cell carcinoma were included in the study. The distribution by

FIGO stage for stage IA, IB, II, III, IVA and IVB were18%, 37%, 12%, 19%, 5% and 3% respectively. The distribution by age at diagnosis for age groups 20-59, 60-69, 70-79 and 80-100 years were 20%, 20%, 27%, 33% respectively. 528 patients (80%) had primary surgery, 377 (57%) had surgery alone, 149 (23%) had adjuvant treatment (7% chemoradiation and 16% radiotherapy alone) and 110 (17%) received primary (chemo)radiation (6% chemoradiation and 11% radiotherapy alone). In 84% the treatment intention was curative, but only in 63% in the oldest age group (80-100). The overall 5-year RS was 75% and by stage (I, II, III and IV) it was 90%, 73%, 66% and 50% respectively. The 5-year RS per age groups (20-59, 60-69, 70-79 and 80-100 years) was 90%, 88%, 81% and 61% respectively. In the multivariate analysis there was a significant negative correlation between RS and FIGO stage (p< 0.001). The oldest age group (80-100) had worse RS compared to younger age groups (< 0.001) in stage I-III.
Conclusion: This is the first nation-wide populationbased study reporting on treatment patterns and relative survival in vulvar carcinoma in Sweden with data from SQRGC. The elderly patients more frequently received treatment with palliative intention and had significantly worse relative survival compared to younger patients.
Disclosure: Nothing to disclose

P180

Sentinel node detection in earllystage vulvar cancer: a 14 years experience

J. Klat[1], I. Semeradova[1], S. Bajsova[1], M. Raszykova[1], J. Kummel[1], O. Kraft[2], V. Zidlik[3]
[1]Ob/Gyn, [2]Nuclear Medicine, [3]Pathology, University Hospital Ostrava, Ostrava, Czech Republic

Introduction / Background: Lymph node status is the most important prognostic factor in vulvar cancer. Histologically, sentinel nodes may be representative of the status of the other regional nodes. Identification and histopathologic evaluation of sentinel nodes could then have a significant impact on clinical management and surgery. The aim of the study was to assess the sensitivity, negative prognostic value, accuracy and false negativity of the sentinel lymph node (SLN) procedure during 14 years.
Methodology: From June 2004 to Deceember 2017, 112 patients with vulvar cancer, clinical stages FIGO IA-IB, underwent surgical tretament in onco-gynecologic center of University Hospital Ostrava. Demographic, surgical, and pathologic data on all patients were prospectively entered in a database. Of 86 women with early-stage squamous cell vulvar cancer included in the study, nineteen had lateral lesions and 67 had midline lesions. SLN detection was performed by using a radioactive tracer and blue dye, followed by radical vulvectomy or radical wide excision with uni⁄bilateral inguinofemoral lymphadenectomy. SLNs were subsequently examined with haematoxylin-eosin and immunohistochemistry.
Results: Eighty-six patients underwent the SLN procedure by combined method. The detection rate per patient was 97,7%, and side specific detection rate was 88,1%. For 19 patients with lateral lesion the detection rate per patient and side specific detection rate was 100% and for patients with midline tumor 97,0% a 87,0% respectively. In total, fifty-one positive SLNs were detected in thirty-two of 86 patients (37,2%). From a total 51 positive SLNs, micrometastases were found in ten SLNs. We experienced only one case with a false negativity of SLN. Sensitivity, negative predictive value, accuracy and false negativity of SLN detection were 96,9%, 98,1%, 97,7% and 3.1% respectively.
Conclusion: The SLN biopsy performed by an experienced team during long time period is a accurate method, with high accuracy and low false negativity in patients with early-stage vulvar cancer.
Disclosure: Nothing to disclose

P181

The SARVU study. A novel technique of sentinel lymph nodes detection in vulvar cancer patients

P. Klimczak[1], M. Jedryka[2,3], M. Kryszpin[3], M. Hirowska-Tracz[3], M. Kalus[4], M. Manowiec[3], B. Rossochacka-Rostalska[3]
[1]Gyneacological Oncology, Opole Oncology Center, Opole, [2]Oncological Department, Wroclaw Medical University, [3]Oncological Gynaecology, Lower Silesian Oncology Center, Wroclaw, [4]Oncological Gynaecology, Opole Oncology Center, Opole, Poland

Introduction / Background: Nowadays sentinel lymph nodes (SLN) biopsy is a well accepted technique in vulvar cancer patients with negative groins. The standard for SLN detection uses peritumoral radioisotope injection to locate the groin nodes. A novel non-radioactive method for identifyning SLN uses a superparamagnetic iron oxide tracer (Sienna+®) injected peritumorally and a magnenometer probe (Sentimag®) to find the nodes.
We created the **SARVU** study (Sentinel Lymph Nodes Detection with **S**entimag **a**gainst **R**adiotracer in **Vu**lvar Cancer) to compare and validate the use of ferromagnetic technique of SLN detection with iron oxide tracer (Sienna+®) and magnenometer probe (Sentimag®) vs. standard procedure.
Methodology: We included 20 patients with squamous vulvar tumour less then 4 cm and negative lymph nodes in imaging pre-op work-up. The primary endpoint was the propotion of succesful SLN detection with Siena+® vs. Tc99. The secondary endopoints were: the average of SLN per patient, the proportion of SLN detected (nodal detection rate), the propotion of pathologically positive results (malignancy rate) per patient and per node.
Results: SLN procedure in both studied methods showed equal average distribution (3.3 SLN per patient). SLN detection rate per patient was 100% in both techniques. Nodal detection sensitivity was 98,5% for ferromagnetic and 93,8% for radioactive tracer. Malignancy detection rate per patient was 100% positive with both methods. Malignancy rate for nodes was 21,5% and for patients - 45%.
Conclusion: We consider the new method of SLN detection with the use of ferromagnetic injection in vulvar cancer patients as reliable, safe and non inferior to the standard of care. However these promising data are few thus the SARVU study must be continued to proove the efficacy of a novel technique of Sentimag/Sienna+® use in SLN detection in vulvar carcinoma.
Disclosure: The SARVU study was supported by SYSMEX Europe company in providing the ferromagnetic tracer agent and technical support. No gratification was paid to the study investigators.

P182

The immune landscape is a strong predictive biomarker for clinical outcome in early stage vulvar cancer, irrespective of HPV or p53 status

K.E. Kortekaas[1,2], S.J.A.M. Santegoets[2], Z. Abdulrahman[2], V.J. van Ham[2], M. van der Tol[1], I. Ehsan[3], H.C. van Doorn[4], T. Bosse[5], M.I.E. van Poelgeest[1], S.H. van der Burg[2]
[1]Gynecology, [2]Medical Oncology, Oncode Institute, Leiden University Medical Center, [3]Medical Oncology, Oncode Institute, Leiden University Medical Centre, Leiden, [4]Gynecology, Erasmus MC Cancer Institute, Rotterdam, [5]Pathology, Leiden University Medical Center, Leiden, The Netherlands

Introduction / Background: Vulvar squamous cell carcinoma (VSCC) consists of three subtypes; HPV-related, HPV-negative *TP53* wildtype and HPV-negative mutated *TP53* (HPVposVSCC, HPVnegVSCC/p53wt, and HPVnegVSCC/p53abn, respectively) which are all treated by mutilating radical surgery and/or (chemo)radiotherapy. Despite the fact that the immune system plays a key role in cancer, the knowledge on its effect in VSCC is limited at best. A study, elucidating the clinical impact of tumor-immunity in VSCC was, therefore, performed with the aim to foster the development of immunotherapeutic approaches.
Methodology: Sixty-five patients with early-stage VSCC were categorized based on HPV and p53 status. Archived tissues were analyzed for expression of CD3, CD8, FoxP3, PD-1, and pan-keratin in randomly selected areas using immunofluorescence. Additional phenotyping of T cells was performed *ex-vivo* on VSCC and blood samples by flow cytometry. Healthy vulvar tissue and blood served as controls.
Results: T-cell infiltration of VSCC was highly variable between patients, ranging from completely absent to very high numbers, and differed per VSCC subtype. Approximately 80% of the

HPVposVSCC showed high T-cell infiltration, followed by 60% of the HPVnegVSCC/p53wt, and 40% of the HPVnegVSCC/p53abn. Importantly, high T-cell infiltration was associated with longer recurrence-free period and overall survival, irrespective of the HPV and p53 status. In-depth analysis of tumor-infiltrating T cells with flow cytometry confirmed the tumor-specific presence of activated effector memory T cells in VSCC and revealed that most of the CD4+ and CD8+ T cells expressed PD-1.
Conclusion: This study is the first to show a strong correlation between T-cell infiltration and clinical outcome. Our data suggest the application of two immunotherapeutic strategies depending on immune phenotype. The high expression of PD-1 in T-cell infiltrated tumors alludes to anti-PD1 blockade, whereas VSCC tumors with low numbers of intratumoral T cells should be stimulated with inflammatory reagents to stimulate local immune responses.
Disclosure: Nothing to disclose

P184

Adaptive intestitial brachytherapy of recurrence vulvar cancer based on MR

R. Kwiatkowski, M. Brąclik, D. Nenko
Radiation Oncology, Oncology Centre Katowice, Katowice, Poland

Introduction / Background: Radiotherapy is mainly adjuvant treatment in patients with vulvar cancer after surgery and risk factors of recurrence. Due to high toxicity of adjuvant radiotherapy and risk of developing severe injuries of vulvar and groin skin, brachytherapy as a local treatment of the highest risk area, seems a good option. In asessment of gynaecological cancer MR is recommended.
Methodology: In Radiotherapy Department of KCO in 2017-2019 were admitted 63 patients with vulvar cancer after surgery. 24 patients were recognized recurrence tumour in surgical scars. MR was performed in all of those. 30 patients were treated by interstitial brachytherapy. In patients with tumour after insertion of needles MR and CT were performed. Image fusion was done. 3D brachytherapy treatment plan was prepared using Oncentra Brachy TPS. Parameters V100 and V150 were controlled. Dose range was 18-24Gy/6-8fr delivered twice a day.
Results: All patients after brachytherapy treatment were controlled in our Department. In our observation we performed physical examination and MR or CT in every 3 months. In all patients we observed complete regression of recurrent tumour, but in the bed of tumour radiation necrosis was developed. 4 patients were severe injuries of vulvar tissues due to radiation necrosis and required intensive care, included narcotic drugs. 3 patients were developed another recurrent.
Conclusion: MR based interstitial brachytherapy allow reduced margins of volume of high dose in recurrence tumour of vulva. in the same, it allow reduced volume of tissue necrosis and improve treatment results.
Disclosure: Nothing to disclose

P185

Reduced morbidity by using LigaSure compared to conventional inguinofemoral lymphadenectomy in vulvar cancer patients: a randomized controlled trial

A.W. Pouwer[1], H.J. Arts[2], C.M. Koopmans[3], J. Inthout[4], J.M.A. Pijnenborg[1], J.A. de Hullu[1]
[1]Department of Obstetrics and Gynaecology, Radboud Institute for Health Sciences, Radboud University Medical Center, Nijmegen, [2]University of Groningen, University Medical Center Groningen, Groningen, [3]Medisch Spectrum Twente, Enschede, [4]Department for Health Evidence, Radboud University Medical Center, Nijmegen, The Netherlands

Introduction / Background: The aims of this MAMBO-IC study (Morbidity And Measurement of the

Body) were to study the feasibility of using LigaSure for inguinofemoral lymphadenectomy (IFL) and to assess the differences in the incidence of short-term complications using LigaSure versus conventional IFL. Moreover patients' and surgeons' experience was evaluated.
Methodology: In this multicenter randomized controlled trial (RCT), women diagnosed with squamous cell carcinoma of the vulva planned for bilateral IFL were included. It was randomly assigned for which groin the LigaSure was used; the other groin was treated with conventional IFL (sharp/diathermia). The groin drain was removed when the production of the drain was < 30ml/day with a minimum of three days. Postoperative complications, including lymphocele, wound infection and wound dehiscence, within eight weeks were assessed. We estimated the incidence of ≥ 1 complication(s) per groin using logistic regression and compared this between the two surgical methods, adjusting for possible confounders. Surgeons' experience was evaluated with a questionnaire containing the SURG-TLX workload score and patients' experience at eight weeks postoperatively with a questionnaire regarding pain and restriction of daily activities.
Results: We included 40 groins of 20 patients. The estimated incidence of ≥ 1 complication(s) was 29% after LigaSure versus 70% after conventional IFL (risk difference 41% (95%CI 19-62), p < 0.001). The duration of surgery, number of removed lymph nodes, duration of drainage and volume drained did not notably differ between the two surgical methods. Patients' reported restriction of daily living activities and maximum pain score were equal for both treatment methods. There were no differences in the surgeon reported workload scores.
Conclusion: This RCT shows that LigaSure for IFL is feasible and associated with significantly less short-term surgical complications compared to conventional IFL. Validation of these findings is needed in a large study cohort.
Disclosure: Nothing to disclose

P186

Surgical treatment of vulvar cancer: impact of tumor-free margin distance on recurrence and survival. A multicentre cohort analysis from the francogyn study group

E. Raimond[1], C. Delorme[1], L. Ouldamer[2], X. Carcopino[3], S. Bendifallah[4], C. Touboul[5], E. Daraï[4], M. Ballester[4], O. Graesslin[1]
[1]Department of Obstetrics and Gynaecology, Institute Alix de Champagne, University Hospital REIMS, Reims Cedex, [2]Department of Obstetrics and Gynaecology, Centre Hospitalier Régional Universitaire de Tours, Hôpital Bretonneau, Tours, [3]Department of Obstetrics and Gynaecology, Hopital Nord, APHM, Marseilles, [4]Department of Obstetrics and Gynaecology, Tenon University Hospital, Assistance Publique des Hôpitaux de Paris (AP-HP), University Pierre and Marie Curie, Paris 6, Institut Universitaire de Cancérologie (IUC), Paris, [5]Department of Obstetrics and Gynaecology, Centre Hospitalier Intercommunal, Faculté de Médecine de Créteil UPEC - Paris XII, Creteil, France

Introduction / Background: In vulvar cancer, it is admitted that tumor-free margin distance is one of the most important element for locoregional control. It is currently recommended to surgically remove the tumor with at least an 8 mm tumor-free margin. The aim of this study was to evaluate the impact of tumor-free margin distance on recurrence and survival in vulvar cancer.
Methodology: From 2005 to 2016, 112 patients surgically treated for a vulvar squamous cell cancer were included in a retrospective multicenter study. Overall, disease-free and metastasis-free survivals were analyzed according to tumor-free margin distance.
Results: Patients were divided into three groups : group 1 (margin < 3 mm, n=47) ; group 2 (margin ≥ 3 mm to < 8 mm, n=48) and group 3 (margin ≥ 8 mm, n=17). During the study, 26,8% patients developed recurrence (n=30) after a median of 8 months (1-69). Analysis of 5-year overall survival, as well as disease-free and metastasis-free survivals, did not reveal a difference between groups. We performed a subgroup analysis in patients with a tumor-free margin < 8 mm (group 1 and 2). It showed that histological lesions observed closest to the edge of the specimen were more often invasive or in situ carcinoma lesions in group 1 than in group 2, in which VIN lesions were mainly observed at this location. After re-excision, no patients in group 1 and 50% (n=2) patients in group 2 developed recurrence.
Conclusion: This study did not reveal a significant impact of tumor-free margin distance on recurrence and survival in vulvar cancer. Moreover, the benefit of re-excision seems stronger when

tumor-free margins are positive or very close (< 3 mm), cases in which invasive or in situ lesions are often present closest to the edge of the specimen.
Disclosure: Nothing to disclose

P188

Sentinel lymph node mapping in vulvar cancer - a new approach with indocyanine green and near infrared fluorescence imaging

F. Siegenthaler[1], S. Imboden[1], A. Papadia[2], M.D. Mueller[1]
[1]Department of Obstetrics and Gynecology, University Hospital Bern, Inselspital, University of Bern, Bern, [2]Department of Obstetrics and Gynecology, USI Lugano, Lugano, Switzerland

Introduction / Background: The conventional procedure of sentinel lymph node (SLN) mapping in vulvar cancer includes peritumoral injection of technetium-99m radiocolloid (Tc-99m) the day before surgery and intraoperative injection of methylene blue. However, these techniques have some weaknesses. Near infrared (NIR) fluorescence imaging with indocyanine green (ICG) has recently gained popularity in SLN mapping in different types of cancer. The aim of this study is to evaluate the clinical value of ICG SLN mapping in patients with vulvar cancer.
Methodology: We retrospectively analyzed all patients at our institution with vulvar cancer undergoing SLN mapping using NIR fluorenscence imaging with ICG by applying video telescope operating microscope (VITOM) system technology between April 2013 and May 2019.
Results: 55 groins of 28 patients were analyzed. In 44 groins we used Tc-99m, in four methylene blue and in five both techniques additionally to ICG for SLN detection. ICG was injected peritumorally directly before surgery and no complications due to its administration occurred. 104 SLNs were identified and removed. SLN detection rates were 87.3%, 87.8%, 77.8% and 96.4% for ICG, Tc-99m, methylene blue and the combination of ICG plus Tc-99m, respectively. SLN detection rates of ICG were significantly higher in patients with positive lymph nodes and lymphatic space invasion compared to Tc-99m (p-value 0.035 and 0.005, respectively). We further observed a higher detection rate of ICG in obese patients (94.4% vs. 87.5%); however this observation is non significant (p-value 0.69).
Conclusion: Accurate SLN mapping is a crucial part of vulvar cancer staging and enables avoiding unnecessary inguinofemoral lymphadenectomies. SLN mapping with ICG in vulvar cancer is feasible and safe with a detection rate comparable to Tc-99m, particularly in patients with positive lymph nodes, lymphatic space invasion or obesity. Furthermore, it is easier to perform than mapping with Tc-99m and has less side effects compared to methylene blue.
Disclosure: Nothing to disclose

P189

Host cell DNA methylation markers for cancer risk stratification of vulvar intraepithelial neoplasia

N.B. Thuijs[1], S. Duin[1], A.P. van Splunter[1], D.R.A. Swarts[1], D.A.M. Heideman[1], J. Berkhof[2], M. van Beurden[3], R.D.M. Steenbergen[1], M.C.G. Bleeker[1]
[1]Department of Pathology, Cancer Center Amsterdam, [2]Department of Epidemiology and Biostatistics, Amsterdam UMC, Vrije Universiteit Amsterdam, [3]Department of Gynaecology, Antoni van Leeuwenhoek Hospital, Amsterdam, The Netherlands

Introduction / Background: High-grade vulvar intraepithelial neoplasia (VIN) is the precancerous state of vulvar squamous cell carcinoma (VSCC). Vulvar carcinogenesis is a heterogeneous disease with only a minority of VINs progressing to cancer. Because current clinical and histological classifications are insufficient to predict the cancer risk in women with VIN, affected women are treated

similarly with mutilating interventions. Hence, there is a clinical need for objective biomarkers reflecting the cancer risk. In this study we assessed the potential value of DNA methylation markers for risk stratification of VIN.
Methodology: A series of ~200 vulvar tissues were assessed for methylation status including VSCC, *VIN with VSCC*, *VIN without VSCC* and normal vulvar tissue. *VIN with VSCC* included VIN tissue adjacent to VSCC or VIN tissue of women who developed VSCC during follow-up. *VIN without VSCC* was defined as VIN tissue of women who did not develop VSCC during their follow up. Multiplexed quantitative methylation-specific PCR assays were performed on twelve candidate methylation markers to analyse differential methylation.
Results: Compared to normal vulvar tissue, *VIN with VSCC* showed increased methylation levels. These methylation levels were as high as in VSCC. Interestingly, methylation levels were found to be significantly lower in *VIN without VSCC* compared to *VIN with VSCC* or VSCC.
Conclusion: Our results show that host cell DNA methylation increases with severity of vulvar disease. *VIN with VSCC* demonstrates a 'cancer-like' methylation pattern, reflecting the high cancer risk. Our findings indicate that host cell DNA methylation biomarkers are promising for cancer risk stratification in women with VIN.
Disclosure: RDMS and DAMH have a minority stake in Self-screen BV, The Netherlands.

P190

Vulvar intraepithelial neoplasia: incidence and long term risk of vulvar squamous cell carcinoma

N.B. Thuijs[1], M. van Beurden[2], A.H. Bruggink[3], R.D.M. Steenbergen[1], J. Berkhof[4], M.C.G. Bleeker[1]
[1]Department of Pathology, Cancer Center Amsterdam, Amsterdam UMC, Vrije Universiteit Amsterdam, [2]Department of Gynaecology, Antoni van Leeuwenhoek Hospital, Amsterdam, [3]PALGA Foundation, Houten, [4]Department of Epidemiology and Biostatistics, Amsterdam UMC, Vrije Universiteit Amsterdam, Amsterdam, The Netherlands

Introduction / Background: Vulvar intraepithelial neoplasia's (VIN) comprise a heterogeneous disease entity and have an ambiguous vulvar squamous cancer (VSCC) risk. Human Papillomavirus (HPV) associated VIN accounts for more than 90% of all high-grade VIN, while HPV-induced vulvar squamous cell carcinomas (VSCC) only account for 25% of all VSCCs. This study aimed to investigate the incidence of VIN and the subsequent VSCC risk in relation to age, type of VIN, and presence of lichen sclerosus (LS) and human papillomavirus (HPV), to optimize monitoring and clinical care of women with VIN.
Methodology: The PALGA database, the Dutch Pathology Registry, enabled us to identify a large, historical cohort of women with VIN diagnosed between 1991 and 2011 with long term follow-up, up to 2018. Data on type of VIN, presence of lichen sclerosus (LS), HPV and VSCC development were collected.
Results: In our cohort, 1,148 women were diagnosed with VIN between 1991 and 2011. The incidence of VIN increased with from 2.7 to 4.5 per 100,000 woman-years (67%). Although most women diagnosed with VIN were between 35 and 55 years of age, the incidence rate of VIN peaked in women ≥ 70 years. In women with VIN, the cumulative VSCC risk was 15.7% after 27.4 years. This cumulative cancer risk was increased in women with dVIN (66.7%) compared to women with other types of high grade VIN (15.1%, p< 0.001). Consistently, VSCC risk was higher in elderly women with VIN, in women with LS-related VIN and in women with HPV-negative VIN.
Conclusion: Our findings on a large cohort of women with VIN with long-term follow-up support the different routes in vulvar carcinogenesis. Because of the increased cancer risk in women with VIN diagnosed at a higher age and in women with HPV negative or LS associated VIN, intensified clinical care in these groups is needed.
Disclosure: Nothing to disclose

P191

Paget trial: topical 5% imiquimod for vulvar Paget disease, first results of clinical efficacy

M. van der Linden[1], K. Meeuwis[1], C. van Hees[2], E. van Dorst[3], J. Bulten[1], T. Bosse[4], J. IntHout[5], D. Boll[6], M. van Beurden[7], M. Oonk[8], M. van Poelgeest[4], J. de Hullu[1]
[1]Radboud University Medical Center, Nijmegen, [2]Erasmus Medical Centre, Rotterdam, [3]Universitair Medisch Centrum Utrecht, Utrecht, [4]Leiden University Medical Center, Leiden, [5]Radboud Institute for Health Sciences, Nijmegen, [6]Catharina Hospital, Eindhoven, [7]Antoni van Leeuwenhoek Hospital, Amsterdam, [8]Universitair Medisch Centrum Groningen, Groningen, The Netherlands

Introduction / Background: Vulvar Paget disease (VPD) is a rare skin disorder, which is most common in postmenopausal Caucasian women. They usually present with an erythematous plaque that may show typical "cake icing" scaling and may cause itching, or a burning sensation. Although most cases are noninvasive, VPD may be invasive or associated with an underlying vulvar or distant adenocarcinoma. Treatment of choice is wide local excision with negative margins. Obtaining clear surgical margins is challenging and may lead to extensive and mutilating surgery. Recurrence rates are high, about 35%, which emphasizes the need for new treatment options. A number of reports, have shown promising results using the topical immune response modifier imiquimod. This study aimed to investigate the efficacy, safety, and immunological response in patients with noninvasive VPD using a standardized treatment schedule with 5% imiquimod cream.
Methodology: The Paget Trial (NCT02385188) is a multicenter observational cohort study including seven tertiary referral hospitals in the Netherlands. Twenty-four patients with noninvasive VPD were treated with topical 5% imiquimod cream three times a week for 16 weeks. The primary efficacy outcome was the reduction in lesion size at 12 weeks after end of treatment.
Results: The median age at time of enrollement was 67 (42-84). After treatment with topical 5% imiquimod cream 12 patients (52,2%) had a complete response and 7 patients (30,4%) a partial response. Patients experienced side effects like fatigue, headaches, and almost 80% needed painkillers during treatment. Eight patients adjusted the treatment protocol to two applications a week, and three patients stopped treatment because of the side effects. We found no clear difference in the effect of treatment between patients who used topical 5% imiquimod cream two or three times a week, see table 1.
Conclusion: Topical 5% imiquimod cream seems to be an effective treatment for VPD with a response rate of 82,6%.

Outcome	Treatment according to protocol: 3 times a week	Adjusted treatment schedule: 2 times a week
Complete response	8 (34.8%)	4 (17.4%)
Partial response	4 (17.4%)	3 (13.0%)
No response	3 (13.0%)	1 (4.3%)
Total	15 (65.2%)	8 (34.8%)

[Outcomes in patients with different treatment schedules]

Disclosure: MvdL nothing to declare. KM Consultant and received honoraria from Lilly and Eucerin and advisor for Eucerin, Netherlands. CvH nothing to declare. EvD nothing to declare. JB nothing to declare. TB nothing to declare. JitH nothing to declare. DB nothing to declare. MvB nothing to declare. MvP nothing to declare. JdH nothing to declare.

P192

Nodal debulking or complete inguino femoral lymph node dissection in patients with vulvar

cancer and bulky lymph nodes prior to radiotherapy

J. van der Velden[1], M. Tijong[1], J. Kasius[1], H. Westerveld[2], G. Fons[1]
[1]Gynecologic Oncology, [2]Radiotherapy, Amsterdam UMC, location AMC, Amsterdam, The Netherlands

Introduction / Background: Limited data are available on the preferred policy in patients with vulvar cancer and bulky lymph nodes in the groin. Debulking of the nodes followed by radiotherapy resulted in less morbidity and similar groin control and survival compared with a complete inguino femoral groin dissection (IFL) in studies with limited patient numbers.
The aim of this study was to gather more data and confirm the “safety” of debulking compared to IFL followed by radiotherapy. Historically, patients with bulky lymph nodes were treated by a complete IFL. From 2007 onwards the policy changed into nodal debulking. Groin recurrence-free survival and survival were evaluated in both groups.
Methodology: Between 1982 and 2016, 217 patients were identified with positive groin lymph nodes. Clinically suspicious bulky lymph nodes and histologically confirmed as tumor positive were found in 71 patients. Before 2007, 36 patients with bulky nodes were treated by a complete IFL. After 2007, 35 patients were treated by nodal debulking. All patients received radiotherapy to both groins and pelvis. Clinical (age, tumor diameter) and pathologic (number of positive nodes, extracapsular growth) variables among the two groups were compared. Disease specific survival and groin recurrence-free survival were calculated.
Results: No difference was seen in clinical and pathological parameters between the debulking and IFL groups. Per groin analysis showed no difference in groin recurrence rate (12.3% versus 14.3%, p=0,41). The 2 year groin recurrence free survival for debulking versus IFL was 81,3% versus 73,5%, respectively (p=0.31, fig1). Two years disease specific survival was better for patients after nodal debulking (62.5% versus 31.9%, p=0.04, fig 2).
Conclusion: Because of the limited number of patients analyzed it is difficult to draw firm conclusions, but it is suggested that nodal debulking instead of formal IFL followed by radiotherapy does not compromise groin control and survival.

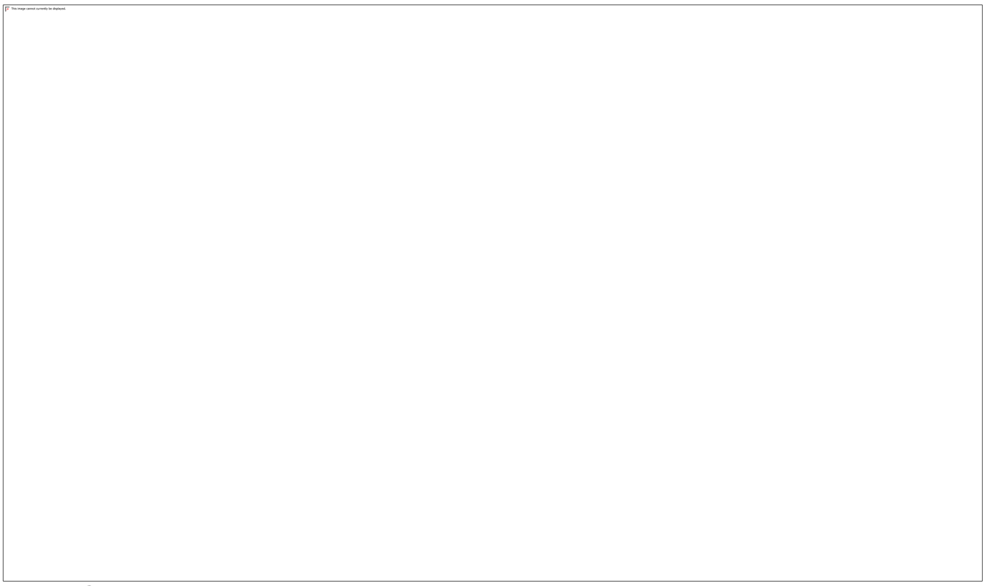

[Fig 1. Groin recurrence free survival for nodal debulking versus complete IFL]

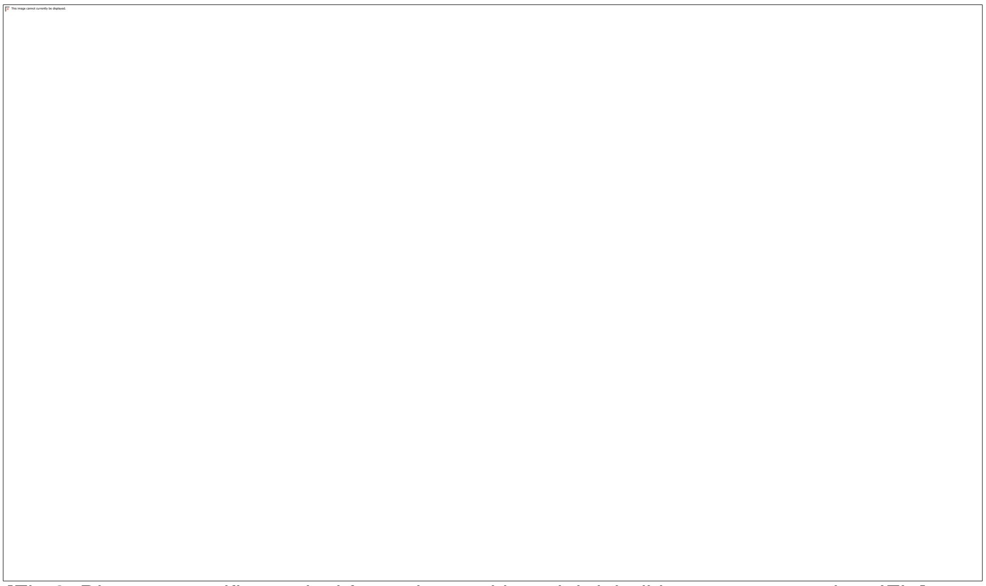

[Fig 2. Disease specific survival for patients with nodal debulking versus complete IFL.]

Disclosure: Nothing to disclose

P193

p53 and p16 expression profiles identify three prognostically subgroups in vulvar cancer: a TMA based study by the AGO-CaRE-translational study group

L. Wölber[1], K. Prieske[1], C. Eulenburg[1], N. de Gregorio[2], R. Klapdor[3], M. Kalder[4], I. Braicu[5], S. Fuerst[6], M. Klar[7], H.-G. Strauss[8], G. Mehlhorn[9], W. Meier[10], A. Ignatov[11], A. Mustea[12], J. Jueckstock[13], G. Schmidt[14], D. Bauerschlag[15], M. Hellriegel[16], U. Canzler[17], A. Luyten[18], S. Kommoss[19], P. Hantschmann[20], M. Heubner[21], S. Mahner[13], E. Burandt[1], AGO Study Group

[1]University Medical Center Hamburg-Eppendorf, Hamburg, [2]University Hospital Ulm, Ulm, [3]University Medical School Hannover, Hannover, [4]University Hospital Marburg, Marburg, [5]Charité University Medicine Berlin Campus Virchow, Berlin, [6]University of Munich (LMU), Munich, [7]University Hospital Freiburg, Freiburg, [8]University of Halle, Halle, [9]Erlangen University Hospital, Erlangen, [10]Evangelisches Krankenhaus Duesseldorf, Duesseldorf, [11]University of Magdeburg, Magdeburg, [12]University Medicine Greifswald, Greifswald, [13]University of Munich, [14]University Hospital Technical University of Munich, Munich, [15]University Medical Center Kiel, Kiel, [16]Georg-August-University Goettingen, Goettingen, [17]Technische Universität Dresden, Dresden, [18]Wolfsburg Hospital, Wolfsburg, [19]University Hospital Tuebingen, Tuebingen, [20]Hospital Altoettingen, Altoettingen, [21]University Hospital Essen, Essen, Germany

Introduction / Background: Today, there are two accepted pathways for tumorigenesis of vulvar squamous cell carcinoma (VSCC): an HPV-dependent with p16 overexpression as a surrogate for HPV-associated transformation and an HPV-independent route linked to lichen sclerosus, characterized by p53 mutation. A possible correlation of HPV dependency with a favourable prognosis has been proposed.
Methodology: The AGO CaRE-1 study is a retrospective survey of pts with primary VSCC FIGO stage ≥1B (UICC-TNM version 6) treated at 29 gynecologic cancer centers in Germany 1998-2008 (n=1,618). For this CaRE-translational sub-study available FFPE tissue was collected centrally (n=648). A tissue micro array (TMA) was constructed; p16 and p53 expression was determined by immunohistochemistry (IHC). HPV status and subtype were analyzed by PCR.
Results: p16 IHC was interpretable in 550 TMA spots and considered positive in 166/550 (30.2%).

HPV DNA was detected in 78.4% of the p16+ tumors, with HPV 16 being the most common subtype (88.3%). p53 IHC was interpretable in 597 spots, 187/597 (31.3%) were considered positive. Pts with p53+ tumors were older at first diagnosis (71 vs. 66 yrs; p=0.001 for p53- tumors) and showed lymph-node involvement more often (43.3% vs. 31.1%; p=0.007). There was a relevant number of tumors with neither p16 nor p53 overexpression (221/535); while co-expression of p53 and p16 was rare (12/535). For survival analyses, three groups were defined: p53+ (n=163), p16+/p53- (n=151) and p16-/p53- (n=221). 2-y-disease-free (DFS) and overall survival (OS) rates were significantly different between the groups: DFS: p53+ 47.0%; p16-/p53- 53% and p16+/p53- 65.5% (p< 0.001); OS: 70.4%, 72.6% and 82.7% (p=0.003), respectively. Adjustment for age and nodal status showed consistent p16 and p53 effects regarding DFS.
Conclusion: p16 overexpression is associated with an improved prognosis in VSCC while p53 positivity is linked to an adverse outcome. Our data provide evidence of a clinically.
Disclosure: The AGO CaRE translational study was supported by medac oncology without restrictions in the study protocol.

P194

Epivin: a phase II double-blind randomised control trial investigating the use of epigallocatechin-3-gallate (Veregen®, Catephen®) vs placebo in the treatment of usual-typed vulval intraepithelial neoplasia

J. Yap[1,2], D. Slade[1], R. Fox[1], B. Kaur[1], A. Hughes[1], R. Ganesan[3], S. Velangi[4], D. Luesley[2]
[1]Institute of Cancer and Genomic Sciences, University of Birmingham, [2]Sandwell and West Birmingham Hospitals NHS Trust, [3]Birmingham Women's and Children's NHS Foundation Trust, [4]University Hospitals Birmingham NHS Foundation Trust, Birmingham, United Kingdom

Introduction / Background: We have shown that Epigallocatechin-3-gallate (EGCG) targets the human papillomavirus (HPV)-encoded E6 and E7 oncoproteins for degradation, resulting in epithelial cell growth inhibition in *in vitro* models. Recent clinical trials indicated that Veregen®, a topical ointment containing 55-72 mg of EGCG, is a safe and effective treatment for vulval warts, benign lesions caused by low-risk HPV strains. Based on our findings, we undertook a phase II randomised control trial (RCT) to evaluate if Veregen® is also effective in the treatment of usual-type vulvar intraepithelial neoplasia (uVIN), a pre-malignant lesion associated with high-risk HPV infection.
Methodology: Eligible patients were randomised to receive either Veregen® or placebo ointment (applied 3 times daily for 16 weeks), and were followed up at 2, 4, 8, 16, 32 and 52 weeks. Outcome measures, recorded at 16 and 32 weeks, were histological (HR) and clinical (CR) response (measured by ≥30% reduction in the sum of the longest diameter of all lesions when compared to baseline) and toxicity. The primary outcome, HR, required a difference in the number of responders ≥3 in favour of Veregen® to detect a difference of 20% between groups (10% for placebo and 30% for Veregen®), as per Jung's design with type I, II errors ≤ 0.15.
Results: 26 patients were randomised, all 13 patients who received Veregen® showed either complete (n=5) or partial (n=8) CR. In placebo group, 3 patients had complete CR, 2 had partial CR and 6 had stable disease. Patients in the Veregen® group showed significant improvement in CR as compared to the placebo group (P=0.0026). There was no evidence of difference in HR and toxicity reported in both groups.
Conclusion: This study indicates that Veregen® application is safe and leads to at least a partial clinical resolution of uVIN lesions, thus warranting a phase III multi-centre RCT.
Disclosure: The study is funded by the National Institute for Health Research via the Research for Patient Benefit programme and Medigene AG supplied Veregen® and placebo.

P195

Effect of a reduced follow-up schedule on patient satisfaction with care among early-stage endometrial cancer patients: one-year preliminary results of the ENSURE randomized controlled trial

N.P.M. Ezendam[1,2], B.H. de Rooij[1,2], C.L. Creutzberg[3], R.F.P.M. Kruitwagen[4], L.R. van Lonkhuijzen[5], M.J.A. Apperloo[6], K. Gerestein[7], A. Baalbergen[8], D. Boll[9], M.C. Vos[10], L.V. van de Poll-Franse[1,11,12], ENSURE Study Group
[1]Netherlands Comprehensive Cancer Organisation (IKNL), Utrecht, [2]Medical and Clinical Psychology, Tilburg University, Tilburg, [3]Leiden University Medical Center, Leiden, [4]Maastricht University Medical Center, Maastricht, The Netherlands, [5]Amsterdam UMC, Location AMC, Amsterdam, New Caledonia, [6]University Medical Center Groningen, Groningen, [7]Meander Medisch Centrum, Amersfoort, [8]Reinier de Graaf Hospital, Delft, [9]Catharina Hospital, Eindhoven, [10]Elisabeth-TweeSteden Hospital, Tilburg, [11]Netherlands Cancer Institute/Antoni van Leeuwenhoek Hospital, Amsterdam, [12]Tilburg University, Tilburg, The Netherlands

Introduction / Background: It has been hypothesized that the frequency of follow-up visits for patients with early-stage endometrial cancer could be decreased. Therefore, the aim of the ENSURE trial was to assess patient satisfaction at 3-years follow-up among patients receiving a reduced follow-up schedule (4 visits/3 years) compared to patients receiving follow-up care according to Dutch guideline (10-13 visits/5 years). In the current analysis 1-year results were evaluated.
Methodology: In this Dutch multicenter non-inferiority trial 320 patients diagnosed with stage 1A/B low-risk endometrial cancer, for whom adjuvant radiotherapy is not indicated, were randomized. Patients allocated to the reduced-follow-up group (n=162) and to the control group (n=158) were asked to complete questionnaires at baseline, after 6 and 12 months. Primary outcome was patient satisfaction with follow-up care as assessed with the Patient Satisfaction Questionnaire III (total scale) at 3-year follow-up. The predefined non-inferiority margin was 6 points (maximum acceptable difference), with a range of 0 to 100. Mixed linear regression and intention-to-treat analyses were used.
Results: At 1-year follow-up, 272 participants had completed the questionnaire (85%)**.** Preliminary results showed that the average satisfaction with care (total scale) score was 83 (SD=13) in the reduced follow-up arm and 82 (SD=13) in the control arm, where a higher score denotes a higher satisfaction (p=0.96). Also, technical competence of the health care provider and interpersonal aspects were scored equally in both groups, 81 (SD=15; p=0.52) and 87 (SD=14; p=0.95), respectively. Finally, access to care was evaluated similarly by patients in the reduced follow-up arm (M=81; SD=15) and the control arm (M=78; SD=15), p=0.39.
Conclusion: These findings indicate no difference in satisfaction with care after 1-year follow-up among early stage endometrial cancer patients who receive a reduced follow-up schedule compared to patients receiving follow-up according to the Dutch guidelines. For final conclusions 3-year results of this trial need to be awaited.
Disclosure: The study was funded by the Dutch Cancer Society (IKZ 2014-6677). The authors report no disclosures. Trial registration: NCT02413606

P196

Preoperative venous thromboembolism screening using enhanced computed tomography based on D-dimer level in patients with gynaecologic malignancy

Y. Habu, A. Mitsuhashi, M. Shozu
Department of Reproductive Medicine, Chiba University, Chiba, Japan

Introduction / Background: Postoperative venous thromboembolism (VTE) is the most important perioperative complication that worsens mortality. Preventive measures for postoperative VTE have been developed recently; however, postoperative pulmonary embolism (PE) still occurs. This might be caused by a preoperative deep venous thrombosis. Therefore, we consider that preoperative VTE screening is important to decrease the incidence of postoperative VTE.
Methodology: We have been performing preoperative VTE screening using enhanced computed tomography (CT) based on D-dimer levels of >1.0 µg/ml for all patients with gynaecologic

malignancies since 2009. We retrospectively examined the diagnostic accuracy of D-dimer level for VTE screening and determined the appropriate cutoff D-dimer level to predict VTE in 1042 patients with gynaecologic malignancies (147 with cervical cancer [CC], 463 with endometrial cancer [EC], and 432 with ovarian cancer [OC]) who underwent surgery at Chiba University Hospital between 2009 and 2017.
Results: VTE was observed in 133 of 432 patients with D-dimer levels of >1.0 µg/ml and in 4 of 610 patients with D-dimer levels ≤1.0 µg/ml (sensitivity, 97.1%; specificity, 66.9%; positive predictive value, 30.1%; negative predictive value, 99.3%). On the basis of the receiver-operating characteristic curve, setting the cutoff D-dimer level to 1.2 µg/ml for EC and 1.5 µg/ml for OC increased the diagnostic accuracy.
Conclusion: We revealed the high incidence of preoperative VTE, including PE in patients with EC and OC. Preoperative VTE screening for patients with gynaecologic malignancies using enhanced CT based on D-dimer level might be a useful strategy for preventing postoperative mortality due to VTE.

	D-dimer >1µl/ml n (%)	VTE n (%)	Positive predictive value	False positive	False negative
CC (51 cases)	5 (10%)	1 (2%)	20%	8%	0%
EC (397 cases)	113 (28%)	44 (11%)	37%	18%	0.5%
OC (359 cases)	245 (69%)	71 (20%)	29%	49%	0.3%

[The D-dimer positivity rate, frequency of VTE, positive predictive value, false positive and false negative according to cancer type]

Disclosure: Nothing to disclose

P1237

Attitudes towards sexual counselling of patients with gynaecological malignancies - preliminary results of the survey examining ENYGO members perspective

J. Kacperczyk-Bartnik[1], K. Nowosielski[2,3], D. Lindquist[4], M. Lanner[5], T. Nikolova[6], D. Vlachos[7], I. Selcuk[8], A. Pletnev[9], K. Zalewski[10,11,12], The European Network of Young Gynaecological Oncologists (ENYGO)
[1]2nd Department of Obstetrics and Gynecology, Medical University of Warsaw, Warsaw, [2]Department of Gynecology and Gynecological Oncology, Teaching Hospital in Czeladz, Czeladz, [3]Department of Physiotherapy, Opole Medical School, Opole, Poland, [4]Department of Radiation Sciences, Oncology, Umeå University, Umeå, Sweden, [5]Division of Gynecology, Medical University Graz, Graz, Austria, [6]University Clinic of Obstetrics and Gynecology, Ss. Cyril and Methodius University, Skopje, The former Yugoslav Republic of Macedomia, [7]Gynecologic Oncology Unit, 1st Department of Obstetrics and Gynecology, Alexandra Hospital, University of Athens, Athens, Greece, [8]Department of Gynecologic Oncology, Zekai Tahir Burak Women's Health, University of Health Sciences, Ankara Training and Research Hospital, Ankara, Turkey, [9]Department of Gynecologic Oncology, N.N. Alexandrov Cancer Center of Belarus, Minsk, Belarus, [10]Department of Gynecologic Oncology, Holycross Cancer Center, Kielce, [11]Department of Obstetrics, Gynecology and Oncology, 2nd Faculty of Medicine, Medical University of Warsaw, [12]Department of Molecular and Translational Oncology, Maria Sklodowska-Curie Memorial Cancer Center, Institute of Oncology, Warsaw, Poland

Introduction / Background: Both the location of primary disease and treatment side effects may have an impact on sexual function in the population of oncogynaecological patients. Sexual health is a major compound of the quality of life, yet due to its intimate nature tends to be underserved in the routine medical care. The aim of the study was to examine prevalence, strategies, barriers and ideas for improvement of sexual counselling among specialists managing patients with gynaecological malignancies.
Methodology: A self-prepared questionnaire concerning sexual counselling in gynaecological

oncology practice (attitudes, behaviours, confronted difficulties and ideas for possible systemic improvements) was used in this cross-sectional study. Paper and online versions were distributed among participants of ESGO-ENYGO-ESO Masterclass. Link to the questionnaire was also shared among ENYGO members via the mailing system, ESGO social media channels, and ENYGO National Representatives. Ongoing data collection was initiated in June 2019. A total number of 99 answers from 39 countries were obtained.
Results: Collecting information concerning sexual function of the majority of managed patients was reported by 36% of respondents, whereas 20% discuss the topic rarely or never. 74% stated that the subject is important or very important. However, 70% are ask
ed by less than half of managed patients about the impact of proposed therapies on sexual function. Most frequently mentioned barriers included time deficiency (74%), insufficient specialist knowledge (53%), patient's embarrassment (46%), and lack of educational materials for patients (35%). According to respondents more widespread sexual counselling in oncological care could be achieved by preparation of educational materials for patients (80%) and healthcare providers (74%) as well as organization of workshops for professionals (64%) and patients (50%).
Conclusion: The patients' needs regarding sexual counselling could be addressed better. Providing access to specialist educational programmes for both patients and healthcare providers may serve as adequate method for achieving this goal.
Disclosure: Nothing to disclose

P1238

Ultra-radical upfront surgery in women with advanced epithelial ovarian cancer confers inferior survival in a population-based setting

S. Salehi[1], U. Joneborg[1], K. Krawiec[2], A. Flöter-Rådestad[1], M. Bottai[3], H. Falconer[1]
[1]Department of Pelvic Cancer | Women's and Children's Health, Karolinska University Hospital/ Karolinska Institutet, Stockholm, [2]Regional Cancer Centre, Stockholm/Gotland, [3]Institute of Environmental Medicin, Division of Biostatistics, Karolinska Institutet, Stockholm, Sweden

Introduction / Background: To assess overall survival after a shift to ultra-radical upfront surgery in women with advanced epithelial ovarian cancer (EOC) in a population-based setting. Furthermore, to assess changes in the distribution of primary treatments after this shift and to investigate to which extent all women with EOC were accounted for.
Methodology: Regional data on women with EOC was extracted from the nation-wide Swedish Quality Registry for Gynecologic Cancer and National Cancer Registry. In addition, women with histologically suspected EOC (without confirmatory biopsy from the adnexa) were included in the analysis. Two 3-year cohorts, based on primary treatment before or after the introduction of a new surgical algorithm to upfront ultra-radical surgery, were selected. The distribution of primary treatment and survival in total and per treatment modality was analysed.
Results: A total of 752 women with advanced EOC were included in the final analysis (*n*= 364 and 388 in cohort 1 and 2 respectively). The shift to upfront ultra-radical surgery increased non-surgically treated women (24 vs 33%, p < 0.001) and decreased neoadjuvant chemotherapy from 34 to 4% (p < 0.001).No improvement of overall survival was observed (HR 1.07 (95% CI, 0.89-1.29); p=0.47)whereas complete resection after the shift to aggressive upfront surgery conferred worse survival (HR 1.76, 95% CI 1.08-2.90; p= 0.02).The majority of patients with suspected EOC (>70%) belong to the spectrum of EOC and make up for 20-35% of women with EOC.
Conclusion: When treating an entire population, without unaccounted selection bias and including the complete spectrum of advanced EOC, an ultra-radical upfront approach confers inferior survival. Accordingly, favourable reports from institutions with unknown selection could not be reproduced, despite careful patient selection that significantly decreased the proportion of women eligible for surgery. Aiming for ultra-radical upfront surgery and using it as a quality indicator in population-based settings may in fact be detrimental.
Disclosure: Nothing to disclose

P1239

Better quality of life in *BRCA* mutation carriers after salpingectomy with delayed oophorectomy compared to salpingo-oophorectomy; first results of the Dutch TUBA study

M.P. Steenbeek[1], M.G. Harmsen[1], N. Hoogerbrugge[2], M. Arts-de Jong[1], A.H.E.M. Maas[3], J.B. Prins[4], J. Bulten[5], S. Teerenstra[6], J.M.J. Piek[7], H.C. van Doorn[8], M. van Beurden[9], M.J.E. Mourits[10], R.P. Zweemer[11], K.N. Gaarenstroom[12], B.F.M. Slangen[13], M.C. Vos[14], L.R.C.W. van Lonkhuijzen[15], N.M. van Mello[16], M.J. Apperloo[17], S.F.P.J. Coppus[18], L.F.A.G. Massuger[1], R.P.M.G. Hermens[19], J.A. de Hullu[1]
[1]Obstetrics and Gynaecology, [2]Human Genetics, [3]Cardiology, [4]Medical Psychology, [5]Pathology, [6]Department for Health Evidence, Radboud University Medical Center, Nijmegen, [7]Catharina Hospital, Eindhoven, [8]Erasmus MC Cancer Clinic, Rotterdam, [9]Netherlands Cancer Institute/Antoni van Leeuwenhoek Hospital, Amsterdam, [10]Gynaecologic Oncology, University Medical Center Groningen, Groningen, [11]UMC Utrecht Cancer Centre, Utrecht, [12]Leiden University Medical Centre, Leiden, [13]Maastricht University Medical Centre, Maastricht, [14]Elisabeth-TweeSteden Hospital, Tilburg, [15]Amsterdam UMC, Location AMC, [16]Amsterdam UMC, Location VUmc, Amsterdam, [17]Medical Center Leeuwarden, Leeuwarden, [18]Maxima Medical Center, Veldhoven, [19]Scientific Institute for Quality of Healthcare, Radboud University Medical Center, Nijmegen, The Netherlands

Introduction / Background: Risk-reducing salpingo-oophorectomy (RRSO) around the age of 40 is recommended for *BRCA1/2* germline mutation carriers. As recent data indicate the Fallopian tube as origin of high grade serous ovarian carcinoma (HGSC), risk-reducing salpingectomy (RRS) with delayed oophorectomy (RRO) is studied as an innovative strategy to delay premature menopause. The TUBA-study (NCT02321228) compares menopause-related quality of life (QOL) between standard RRSO and the innovative RRS with delayed RRO.
Methodology: A multicentre preference trial in 13 Dutch centres started in January 2015. *BRCA1/2* mutation carriers choose between the standard strategy (RRSO at age 35-40 (*BRCA*1) or 40-45 (*BRCA*2)) and the innovative strategy (RRS with delayed RRO until the age of 40-45 (*BRCA*1) or 45-50 years (*BRCA*2)).
Results: August first 2019, 493 participants were included with a mean age of 37 years: 51% carried a *BRCA1* and 49% carried a *BRCA2* mutation. In total, 72% of women chose RRS with delayed RRO. At three months and one year after surgery, data showed a better menopause-related QOL in women after RRS when compared to women after RRSO (P< 0.001), also after adjustment for age, hospital and baseline score. Additionally, a decline in cancer worry was found at three months and one year after surgery in both groups.
Conclusion: The menopause-related QOL is significantly better in *BRCA* mutation carriers after RRS (with delayed RRO) when compared to RRSO. Furthermore, a favourable effect on cancer worry is found among participating women in both arms. At the time of the conference we will have finished recruitment and have one year of follow up in more than 300 women. Based on our results, the Dutch TUBA and American WISP study (NCT02760849) are initializing a combined international TUBA-WISP-II study to assess the safety of RRS with delayed RRO in *BRCA* germline mutation carriers.
Disclosure: Nothing to disclose

P1240

Outcome of different treatment modalities for gestational trophoblastic neoplasia in women at 40 years old or above: a multicenter retrospective study

R. Hemida[1], P. Sauthier[2], E. Toson[3], N. Tsip[4], H. Pradjatmu[5], N. Eladawy[6], N. Anfinan[7], K. Sait[8], H.C. van Doorn[9]
[1]Mansoura University | Obstetrics and Gynecology, Mansoura University, Mansoura, Egypt, [2]Obstetrics and Gynecology Department, University of Montreal, Canada, Montreal, QC, Canada, [3]Clinical Oncology, Mansoura University, Mansoura, Egypt, [4]Gynecologic Oncology Unit, National

Cancer Institute, Kiev, Kiev, Ukraine, [5]Department of Obstetrics and Gynecology, Sardjito Hospital, Faculty of Medicine, Gadjah Mada University, Yogyakarta, Indonesia, [6]Community Medicine, Mansoura University, Mansoura, Egypt, [7]Obstetrics and Gynecology Department, king abdulaziz university, [8]Obstetrics and Gynecology Department, King Abdulaziz University, Jeddah, Saudi Arabia, [9]Gynecologic Oncology Unit, Erasmus MC Cancer Center, Rotterdam, The Netherlands

Introduction / Background: For treatment of GTN patients at old age; there is an ongoing debate about performing upfront hysterectomy to reduce tumor bulk or starting chemotherapy
Methodology: A retrospective analysis of data from 5 referral centers from 5 countries (Egypt, Canada, Ukraine, Saudi Arabia, and Indonesia) during last 5 years. Medical records of 119 women with GTN were retrieved, reviewed and analyzed. Demographic criteria and outcome of different treatment strategies were evaluated.
Results: Mean age was 45.46 years, and median hCG was 1390.5 m.IU/ml. Low-risk GTN represented 81.5% of cases while 18.5% were in high-risk group. Most of the patients were in FIGO stage I (76.2%). Metastases were diagnosed in 28 cases (23.5%), lungs were commonest site. The commonest histopathological type was invasive mole (38.1%). Of 80 patients with low risk non metastatic GTN, 32 women were treated with single chemotherapy. Eighteen of them (56.2%) showed complete response while relapse was reported in one case. In 34 cases a hysterectomy was performed. In four; a wait and see policy was adopted while instant chemotherapy followed in 30, mainly single MTX for 1-12 courses. Only one case 1/34 (3%) failed to respond. Of 14 patients with low risk metastatic GTN, 8 women were treated with single chemotherapy while in 6 patients a hysterectomy was performed and MTX was started immediately in all. Complete response occurred only in 7/14 (50%). Two high risk patients died before treatment could start due to presence of distant metastases. Eleven cases had underwent hysterectomy and chemotherapy as first line; 7/11 showed complete response. Four women used EMA/CO combination chemotherapy alone; two of them (50%) had incomplete response and needed 2nd line EMA/EP combination.
Conclusion: Upfront hysterectomy for treatment low-risk non-metastatic disease and combination chemotherapy for high-risk disease were associated with better prognosis.
Disclosure: Nothing to disclose

P1241

Feasibility of real-time near-infrared fluorescence tracer imaging in sentinel node biopsy for vulva cancer patients

L. Paskeviciute Frøding, V. Zobbe, A. Christensen, J. Mortensen, C. Hoegdall, A. Kjaer, K. Juhl, T. Henrichsen Schnack
Copenhagen University Hospital, Rigshospitalet, Copenhagen, Denmark

Introduction / Background: Sentinel node (SN) biopsy is a safe staging method in patients with vulva cancer (VC). Near-infrared fluorescence (NIRF) imaging using indocyanine green (ICG) has recently been introduced to improve the visual intraoperative SN identification. The purpose of this study was to evaluate the feasibility of fluorescence tracer imaging for SN detection in conjunction with conventional radio-guided technique.
Methodology: Prospective study of patients with primary VC planned for vulvectomy and SN procedure. Bimodal tracer (ICG-99mTc-Nanocoll) was injected peritumorally and followed by lymphoscintigraphy and SPECT/CT to define the SNs. Intraoperatively SNs were detected with a hand-held gamma-probe and NIRF camera. The detection rate was defined as the proportion of patients with at least one SN detected in at least one groin on scintigraphy or perioperatively (using gamma-probe and NIRF camera).
Results: SN procedure was performed in 36 patients (14 uni- and 22 bilaterally), corresponding to 58 groins. At least one SN was detected in 52 of 58 intended groins (90%) on scintigraphy, while perioperatively SN was identified in 54 of 58 groins (93 %). In 14 patients with lateralized tumor and in 22 patients with midline tumor (< 1 cm from midline) the unilateral SN detection rate was 100%. In patients with midline tumors the bilateral detection rate on scintigraphy and perioperatively was 77% and 82% respectively. In one patient with a midline tumor and no-detectable SNs bilaterally on scintigraphy and in three of four patients with midline tumor and no-detectable SNs in one side on

scintigraphy, the SN was detected perioperatively by NIRF only. In 11 of 58 groins (19%) the SN could only be identified by NIRF.
Conclusion: A combined fluorescent and radioactive tracer for SN procedure is feasible, and the additional use of NIRF imaging may improve the accuracy of SN identification in VC patients.
Disclosure: Nothing to disclose

EP889

Improved cytoreduction of ovarian cancer using CD24-targeted fluorescence image-guided surgery in a preclinical murine model

K. Kleinmanns[1], V. Fosse[1,2], B. Davidson[3,4], L. Bjørge[1,5], E. McCormack[1]
[1]Department of Clinical Science, University of Bergen, Bergen, Norway, [2]Department of Radiology, Erasmus University Medical Center, Rotterdam, The Netherlands, [3]Department of Pathology, [4]Faculty of Medicine, Institute of Clinical Medicine, University of Oslo, Oslo, [5]Department of Obstetrics and Gynecology, Haukeland University Hospital, Bergen, Norway

Introduction / Background: Intraoperative tumour-specific fluorescence imaging enables visualisation of cancer lesions[1], and benefits staging and debulking of ovarian cancer[2]. In patients with advanced epithelial ovarian cancer (EOC), the completeness of cytoreduction significantly impacts prognosis[3]. We have demonstrated that EOC cell lines express high levels of the stem cell marker CD24. To improve the degree of cytoreduction in EOC, we aim to develop a near-infrared (NIR) fluorescence image-guided surgery (FIGS) protocol, using CD24-targeting probes in orthotopic xenograft and patient-derived xenograft (PDX) models.
Methodology: $OV90^{luc+}$ and patient-derived EOC cells were injected orthotopically into immunodeficient NSG mice, and tumour growth was monitored by bioluminescence imaging and physical examination until metastatic disease had established. The monoclonal antibody CD24 was conjugated to the NIR fluorophore AlexaFluor750, and injected intravenously into the xenograft and PDX models 48hrs prior to surgery. Survival debulking surgery was performed, using a FIGS camera system for real-time NIR fluorescence-guided resection of tumour tissue. To evaluate the effect of FIGS, mice (n = 16) were operated under fluorescence guidance, and the other half had standard bright light surgery. Resected tumour volume, post-surgical imaging and post-operative survival were measured.
Results: CD24-targeted FIGS allows for improved detection of tumour tissue and metastatic spread with high sensitivity and specificity in $OV90^{luc}$ and PDX models, compared to standard bright light surgery, leading to improved resection rates and improved outcome in these preclinical murine models.
Conclusion: The EOC biomarker CD24 conjugated to AlexaFluor750 demonstrates high sensitivity and specificity of tumour lesion detection in a preclinical murine model. CD24 is a promising agent for clinical implementation of fluorescence image-guided surgery in patients with ovarian cancer.
Disclosure: Nothing to disclose

ePoster

EP197

Nipple skin sparing mastectomy by conventional laparoscopic single port: about four cases

J. Bakenga[1,2]
[1]Gynecologic Oncology, Clinique du Fief de Grimoire, [2]Gynecologic Oncology, Polyclinique de Poitiers, Poitiers, France

Introduction / Background: To assess the feasibility of laparoscopic single port for prophylactic mastectomy before immediate reconstructive surgery

Methodology: Case report: Two patients with proven BRCA1 mutation underwent for nipple skin sparing mastectomy using an anterior axillary line
all descriptive data, a step by step technique is described
exposure
the removal of the gland and at the end , the positioning of trocars
Results: those two cases of bilateral mastectomy are analyzed
the 2, 5 cm incision is done in the anterior axillary line
the first step is done classically with cold scissors until all the outer quadrant of the breast is free
the alexi is positioned
and the mini-Gelpoint of Applied two
insuflation is done to 8mm Hg
we used classical non curve instrument of laparoscopy
no need for energy
removal on the gland is easy
at the end, an implant is set in the cavity easily
Conclusion: This innovative technique of nipple skin sparing mastectomy is feasible without compromising operative time and aesthetics results.
Disclosure: Nothing to disclose

EP198

A prospective pilot study on use of liquid crystal thermography to detect early breast cancer

D. Hodorowicz-Zaniewska[1], A. Kotlarz[1], P. Kasprzak[2], A. Ćwierz-Pieńkowska[3], T.J. Popiela[1], A. Maciejewski[4], R. Giovanazzi[5], S. Zurrida[6], J. Skupień[1], P. Basta[1]
[1]*Jagiellonian University Medical College, Kraków,* [2]*Department of Breast Imaging, Lower Silesia Oncology Center, Wrocław,* [3]*Radiology Department, Ludwik Rydygier Memorial Specialized Hospital, Kraków,* [4]*Department of Medical Rescue, Poznan University of Medical Sciences, Poznań, Poland,* [5]*San Gerardo Hospital, Monza,* [6]*School of Medicine, University of Milan, Milan, Italy*

Introduction / Background: Breast cancer is the most common cancer in women. While mammography is the standard for early detection in women over 50, there is no standard for younger women. The aim of this prospective pilot study was to assess liquid crystal contact thermography, using the Braster device, as a means for the early detection of breast cancer.
Methodology: 274 consecutive women presenting at Polish breast centers for routine breast examination were enrolled to receive thermography; 19 were excluded for errors in thermographic image acquisition. The women were divided according to age (n=135 < 50 years; n=120 ≥50 years). The primary endpoint, stratified by age group, was the C-statistic for discrimination between breast cancer and non-cancer.
Results: In women with abnormal breast ultrasound (n=95 < 50 years; n=87 ≥50 years, respectively) the C-statistic was 0.85 and 0.75, respectively, (p=0.20) for discrimination between breast cancer and non-cancer. Sensitivity did not differ (p=0.79) between the younger (82%) and older women (78%), while specificity was lower in the older women (60% vs. 87%, p=0.025). The false positive rate was similar in women with normal and abnormal breast ultrasound. Breast size and structure did not affect thermography performance. No adverse events were observed.
Conclusion: Thermography performed well in women < 50 years while its specificity in women ≥50 years was inadequate. These promising findings suggest that the Braster device deserves further investigation as a means for the early detection of breast cancer in women under 50 years, particularly since the cost of the device is low.
Disclosure: DH, AK, PK, JS, AC, and PB received compensation from Braster S.A. for consultancy and services provided in relation to this study.

EP199

Nipple areola complex sparing mastectomy on prophylactic and oncologic surgery. Potential impact of its application in a referral centre and literature review

R. Bermejo Martínez, Á. Tejerizo García, S. Aragón Sánchez, M.C. Sanz Ferrández
Hospital Universitario 12 de Octubre, Madrid, Spain

Introduction / Background: Even though breast conserving therapy has become the gold standard surgical treatment for breast cancer, in our institution 34.7% of the patients still undergo mastectomy. Nipple sparing mastectomy(NSM) is becoming widely accepted, particularly in those patients that undergo prophylactic surgery with reconstruction, due to its superior cosmetic results. NSM can also be used in highly selected cancer patients as an effective procedure if it does not compromise oncologic safety.
Methodology: Literature review of the nipple areola complex (NAC) sparing mastectomy on prophylactic and breast cancer management. Additionally we evaluate the impact it would have in our referral centre, selecting patients, among those who underwent a mastectomy between 2015-2018, according to the inclusion criteria of the National Comprehensive Cancer Network (NCCN) 2017 Guidelines.
Results: There are no randomized trials of NSM. Long-term follow-up in clinical series is limited and most of the studies' samples are heterogeneous. The available data shows an overall survival of over 90% and local disease recurrence of 3-4.6%.
2017 NCCN Guidelines suggest the use of NSM in patients with early-stage tumours, biologically favourable carcinomas and infiltrating ductal carcinoma (IDC) or ductal carcinoma in situ (DCIS) that are >2cm from the nipple. Despite total removal of glandular breast tissue and the low rate of recurrence and/or nipple involvement expected, assessment of nipple free margins should be mandatory to reduce the risk of occult NAC involvement.
In our Institution (*H.U.12 de Octubre*, Madrid) 178 mastectomies were performed for breast cancer treatment and 18 for risk reduction between 2015-2018. According to the inclusion criteria of the NCCN Guidelines, 57 patients (29%) could have undergone a NSM (27 patients with IDC, 12 with DCIS and 18 BRCA mutation carriers).
Conclusion: NSM has become a subject of increased attention. It's a safe option of treatment in patients with early-stage tumours and biologically favourable carcinomas.
Disclosure: Prospective trials, with long-term follow up and homogenous samples are needed to establish the inclusion criteria and management of this patients: pathologic factors (tumour stage, molecular subtypes…), preoperative imaging study, safety distance of the tumour to the nipple, surgical technique, intraoperative study of the NAC (frozen section examination), and role of radiotherapy. The authors declare that there is no conflict of interest.

EP200

MammaPrint testing can reduce the costs of treatment in breast cancer patients

B. Crnobrnja, J. Knez, M. Pakiž
Department of Gynaecologic and Breas, University Medical Centre Maribor, Maribor, Slovenia

Introduction / Background: The decision for adjuvant therapy in breast cancer patients is routinely based on clinical and pathological characteristics of the tumour. Recent multi-genetic tests such as MammaPrint can improve patient selection for adjuvant chemotherapy. However, the variation in cost-effectiveness has not been fully evaluated in different health care settings.
Methodology: A single tertiary centre retrospective analysis of breast cancer treatment costs in the time period of 2013 - 2015 in Slovenian health care system. Early breast cancer patients undergoing chemotherapy most likely to benefit from MammaPrint were included. We calculated the costs to our national insurance company for the chemotherapy and chemotherapy-related short-term complications. We also calculated the costs of sick leave compensation due to chemotherapy.
Results: A total of 54 patients were included. The mean cost of chemotherapy and treatment for

chemotherapy-related complications was 2843 EUR (SD 779.7 EUR) In contrast, MammaPrint testing costs 3000 EUR (currently not reimbursed by the insurance company). Considering that MammaPrint may reduce the need for chemotherapy in 46% patients, it is not cost-effective by itself compared to unselected chemotherapy. However, there were 24 women on sick leave (median duration 9 months, range 3-49 months) in our population. When only workers were taken into account and their sick leave costs were included in the cost analysis, MammaPrint was associated with a potential cost-saving of 8,065 EUR (MammaPrint) vs. 12,195 EUR (no MammaPrint). The calculation of cost savings was based on median sick leave duration, average national salary and average costs of chemotherapy.
Conclusion: MammaPrint alone is not cost-effective in our health care system compared to unselected adjuvant chemotherapy. However, when the costs of sick leave reimbursement are considered, MammaPrint is associated with a potential cost savings of approximately 4,130 EUR per patient.
Disclosure: Nothing to disclose

EP201

Free - cell tumor DNA as a biomarker for early breast cancer

B. Crnobrnja[1], J. Knez[1], M. Sobočan[1], N. Čas -Sikošek[1], V. Vogrin[2], K. Repnik[2], E. Heitzer[3], U. Potočnik[2], I. Takač[1]
[1]Department of Gynaecologic and Breast Oncology, University Medical Center Maribor, [2]Faculty of Medicine, University of Maribor, Maribor, Slovenia, [3]Institute of Human Genetics , Medical University Graz, Graz, Austria

Introduction / Background: Currently available imaging techniques are often inadequate for early breast cancer detection, hence the interest for novel non-invasive biologic markers. Liquid biopsies, e.g. the analysis of circulating tumor DNA (ct-DNA), represent an alternative to tissue biopsies and characterize tumor genetic features in a non-invasive manner. ct-DNA is a promising prognostic and predictive biomarker, but in the case of early breast cancer, its role is still unclear.
Methodology: We conducted a prospective analysis of plasma ct-DNA of patients with histologically confirmed breast cancer undergoing surgical treatment. The patients were enrolled between January 2018 and March 2019 at a university based medical center. Prior to surgical treatment patients provided a blood sample and mFAST-SeqS method was used for analysis. The data about tumour size, histological type, lymph node status, hormone receptors, Ki67 were collected and correlation with ct-DNA was analysed. For correlation analysis, non-parametric Spearman´s coefficient was used. Non-parametric tests were used for comparison of continous variables.
Results: We have included 69 patients to the study. The median age of the patients was 64 years (range 31-92 years). The median size of the tumours was 18 mm (range 4-94 mm). In all but one patient, the detected ct-DNA fractions were less then 10% of total cell free DNA (*z*-score < 5). There was no statistically significant correlation between the size of the tumour and ct-DNA (Spearman´s correlation coefficient -0.31, p>0.05). When comparing the tumours < 1 cm in size to the larger tumours, the median z-score was 2.04 vs. 2.59 (p>0.05). Patients with negative lymph nodes had comparable median z-score to patiens with positive lymph nodes (2.34 vs 2.41, p>0.05). There was no statistically significant correlation between ct-DNA z-score and Ki67 expression and tumour marker CA 15-3.
Conclusion:
Our results do not support ct-DNA detection as a feasible biomarker for early breast cancer detection.
Disclosure: Nothing to disclose

EP202

A retrospective case-report study of the DNA methylation patterns of RAR-b & RASSF1A genes from breast FNAC samples in a Greek population

N. Kougioumtsidou[1], E. Vavoulidis[1], M. Nasioutziki[1], E. Mareti[1], G.C. Pratilas[1], A. Liberis[1], S. Petousis[1], E. Chasioti[1], A. Daniilidis[1], A. Papanikolaou[1], L. Zepiridis[2], K. Dinas[1]
[1]2nd Obstetrics & Gynaecology, [2]1rst Obstetrics & Gynaecology, Aristotle University of Thessaloniki, Thessaloniki, Greece

Introduction / Background: Aberrant methylation of CpG islands acquired in tumor cells in promoter regions has been found in many cancers, including breast cancer. DNA hypermethylation of *RAR-b* and *RASSF1A* genes, have been associated with breast cancer in many studies which however focus on the analysis of breast tissue samples. In this study, we determined the frequency of aberrant promoter methylation of the *RAR-b* & *RASSF1A* genes through molecular analysis of cytological material obtained non-operatively with fine needle aspiration (FNAC).
Methodology: Our study retrospectively analyzed 103 patients of our Breast Pathology Clinic. FNAC material was collected in Cytolyt (Hologic). DNA was extracted from the cytological material with Quick-gDNA MiniPrep kit (Zymo) and was subjected to complete bisulfite conversion with EZ DNA Methylation-Gold Kit (Zymo). A methylation specific PCR was carried out with primers specific for either the methylated or unmethylated status for each gene. The final PCR-products were analyzed in 2% agarose gels with electrophoresis.
Results: Aberrant methylation was observed in both *RAR-b* and *RASSF1A* promoters. In fact, 88% and 84% of the C5 cytologically-classified samples (n=50) were methylated for *RASSF1A* and *RAR-b* respectively. Methylated *RASSF1A* and *RAR-b* promoters were also found in 81.2% and 62.5% in the C4 cytologically-classified samples (n=16). In the “grey zone” of the C3 cytologically-classified samples (n=11) and *RASSF1A* and *RAR-b* methylation frequencies were 63.6% and 45.4% respectively. In comparison, over 70% of the C1 and C2 cytologically-classified samples were unmethylated with low methylation frequencies ranging from 0% to 33.3%.
Conclusion: It seems that the higher the cytological diagnosis of the FNA material, the higher the methylation frequency of the RASS1AF and RAR-b promoters. DNA methylation assays during a non-operative FNA procedure could be a valuable pre-operative tool for clinicians in combination with cytology for more precise breast cancer detection and staging.
Disclosure: Nothing to disclose

EP203

Interest and reliability of extemporaneous examination in breast cancer

Z. Edderdabi, A. Touimi Benjelloun, Y. Benchrifi, M. Moutahir, M. Benhessou, M. Ennachit, M. Elkarroumi
Centre Mohamed IV de Chirurgie Oncogynecologique, Hassan II University/Faculté de Medecine et de Pharmacie, Casablanca, Morocco

Introduction / Background: Extemporaneous examination is a pathological examination performed as soon as the specimen is taken, not fixed, during a surgical procedure, in order to rapidly provide the surgeon with a diagnosis likely to modify the course of the surgical procedure.
The objective of our study was to test the reliability of the extemporaneous examination in mammary pathology, to compare our results with those of the literature and to raise the main diagnostic problems with regard to extemporaneous examination (false positives, false negatives).
Methodology: We realised a retrospective study of 80 patients with breast cancer in the Mohammed VI Center for the Treatment of Cancers of the CHN IBN ROCHD of Casablanca during the year 2017.
Results: Reliability of extemporaneous examination in breast cancer was assessed by its performance indices: sensitivity (Ss), specificity (Sp), positive predictive value (VPP) and negative predictive value (VPN).
In our series, the sensitivity was 98% and the VPP was 100%, which matches the results reported in the series of experts, for the specificity and the VPN we could not calculate them since we did not have the "true negative" variant.
Conclusion: In conclusion, EE is very sensitive in breast pathology and it meets its objectives, namely to ensure the diagnosis of malignancy in the breast without false positive.

Disclosure: Nothing to disclose

EP204

Post irradiation primary osteosarcoma of the breast: a case report of an extremely rare entity

M. Elghobashy[1], H. Bartlett[2], R. Rao[2], N. Deshmukh[3], A.M. Shaaban[3]
[1]College of Medical and Dental Sciences, University of Birmingham, Birmingham, [2]Department of Histopathology, County Hospital, Hereford, [3]Department of Histopathology, Queen Elizabeth Hospital Birmingham, Birmingham, United Kingdom

Introduction / Background: Amongst the rarest of breast tumours is the non-epithelial primary mammary osteosarcoma, which has only been reported in ~100 cases in the past 50 years. The differentials include matrix producing metaplastic carcinoma of the breast and malignant phyllodes tumour with osteosarcomatous differentiation.
Methodology: This report describes an extremely rare case of primary osteosarcoma of the breast arising following radiotherapy for primary breast cancer.
Results: A 73-year old female presented with a lump and pulling sensation at the upper outer quadrant of her right breast. She had a history of a 3mm, grade 2, node negative invasive ductal carcinoma of the same breast 10 years prior to presentation, which was treated by wide local excision, axillary node sampling and radiotherapy. On examination, a fixed lump was identified at the lower quadrant of the right breast away from the wide local excision scar but in the field of radiotherapy. No abnormal axillary lymph nodes were found on imaging. A diagnostic excision was fully processed for histological examination and showed the lesion to be markedly calcified. A highly cellular malignant spindle cell lesion merged with an osteoid matrix and foci of calcification and bone formation was found. Immunohistochemistry was negative for a large panel of broad spectrum and basal cytokeratins as well as for S100, Melan A, CD34, SMA, Beta Catenin and Desmin. Due to the lack of epithelial/glandular differentiation, in situ carcinoma or leaf-like pattern, the diagnosis of malignant post irradiation osteosarcoma was made. Subsequent mastectomy revealed no residual disease. The patient remains well 7 months following diagnosis.
Conclusion: Osteosarcoma following radiotherapy should be considered in the differential diagnosis of breast lesions showing malignant osteoid differentiation. Due to the rarity of the lesion, extensive sampling and careful search for epithelial differentiation including basal cytokeratin immunohistochemistry is required to guide management. Complete surgical excision is required.
Disclosure: Nothing to disclose

EP205

Management of subclinical breast cancers: a report of 74 cases

A. Fouad, N. Youssouf, S. Moukhlis, M. Benhessou, S. Ennachit, M. Elkarroumi
Med VI Center for Gyneacologic and Breast Cancer Treatment, UHC Ibn Rochd, Casablanca, Morocco

Introduction / Background: Infraclinical breast cancers represent a particular entity; they occur in an asymptomatic woman without any suspicious abnormalities at clinical examination of the breasts and lymph node areas.The percentage diagnosed cases has increased significantly over the last 30 years thanks to technological advances in radiological detection and biopsy sampling.
Methodology: This work reports the risk factors, the circumstances of diagnosis, the methods of diagnosis, the management and prognosis of infraclinical breast cancer, through a retrospective study of 74 patients treated at the Med VI Center for Gyneacologic and Breast Cancer over a period of 3 years, from January 2015 to December 2017.

Results: Infra-clinical breast cancer accounts for 6.23% of breast cancers treated in our service in the above-mentioned period. The average age of our patients is 43 years.
Regarding risk factors, the average age of onset of menarche is 14 years, 54% of patients used hormonal contraception, 3 patients had a family history of breast cancer (4.05%). 76% of patients never breastfed. 50% of the cases were nulligravida.
In 69% of cases the reason for consultation was mastalgia. For the other 31 cases a breast cancer screening compaign motivated the consultation. Mammography is the only paraclinical examination that has suspected the diagnosis. Microcalcifications were present in 58% .Stellar or linear suspicious opacities were present in 42 % of cases.
In 82.3% the pathological examination revealed infiltrating ductal carcinoma and 17.7% infiltrating lobular carcinoma. As treatment 74% of our patients had a zonectomy, while 26% had a quadrantectomy.
Conclusion: The increasing diagnosis of breast cancer at a subclinical stage suggests the importance of radiological screening rather than clinical examination only, especially in patients with risk factors. This will affect positively the management of the disease, reduce its morbidity and mortality and contribute to the well-being of the patient.
Disclosure: Nothing to disclose

EP206

Serum CA 15.3 and cytokine levels and expression of hormonal receptors in early-stage breast cancer

M. Fuksiewicz[1], B. Kotowicz[1], M. Kowalska[1], M. Rubach[2]
[1]Department of Pathology and Laboratory Diagnostics, [2]Outpatient Chemotherapy Clinic, Maria Sklodowska-Curie Memorial Cancer Centre and Institute of Oncology, Warsaw, Poland

Introduction / Background: Cytokines are known to play a significant role in the regulation of estrogen synthesis in breast cancer tumor cells. The aim of the study was to assess the correlation between serum concentration of the proinflamatory cytokines - Interleukin-6, IL-8 and proangiogenic cytokines - bFGF, VEGF, tumor marker - CA 15.3 in early-stage breast cancer patients and the expression of estrogen and progesterone receptors in tumor cells.
Methodology: We evaluated serum concentration levels of CA15.3 and cytokines in a cohort of 99 untreated early-stage breast cancer patients using either Roche or ELISA system, respectively. The cohort consisted of 44 patients with stage I (T1N0) tumors, 23 stage IIA (T1N1) and 32 stage IIB (T2N1). Immunohistochemical staining was used for estrogen and progesterone receptors status identification.
Results: In the studied group of patients, we observed a significant association between IL-8 and bFGF levels and the state of hormone receptors. The presence of estrogen receptors was associated only with bFGF and its concentrations were significantly higher in the group of patients in IIA (P=0.032) and IIA and IIB (P=0.036) in total. In the entire study group, a relationship between the presence of estrogen receptors and bFGF concentrations was observed, however only on the verge of statistical significance (P = 0.056). However, in patients with stage I (T1N0), significantly higher concentrations of IL-8 (P = 0.028) were associated with absence of the progesterone receptor status in comparison with patients with progesterone-positive tumours.
No association between the progesterone and estrogen receptors status and CA 15.3 and cytokines IL-6 and VEGF was observed.
Conclusion: Our data demonstrates that serum levels of bFGF and IL-8 are associated with predictive-prognostic factors, such as hormonal receptor status in patients with early-stage breast cancer. Determination of these cytokines may be of clinical utility; however, further research is required.
Disclosure: Nothing to disclose

EP207

Evaluation of miRNA-135a and miRNA-9 as prognostic markers in breast cancer patients

M. Gamea
Gharbia Cancer Society, Tanta, Egypt

Introduction / Background: Breast cancer remains one of the top threats to women health. The current lack of tumor markers with desirable sensitivity and specificity arises to be a major obstacle towards future management of breast cancer. Many studies are directed to reveal the diagnostic and prognostic potentials of circulating miRNAs in breast cancer.
Methodology: was used Levels of miRNA-135 and miRNA-9 in sera from 126 participants representing two different groups , 40 healthy group and 86 breast cancer patients
Results: Expression of both miR-135 and miR-9 were evaluated in both groups. Regarding miR-9, its expression was higher in case than in control group, however there were no significant differences in expression between the case and control groups when compared regarding age, menopausal status and BMI. Expression of miR-135 was higher in case group than control group, by comparing the two groups regarding age, menopausal status and BMI, there were significant differences. Higher miR-135 expression was associated with older age (age>35 years), post-menopausal and obese women in case group.
The miR-135levels of 27.59 or lesser provided a sensitivity of 90.70%, a specificity of 90% and a positive predictive value of 95.12%, negative predictive value of 81.82 %. According to these results, miR-135 had a good predictive ability. The miR-9 levels of 27.59 or lesser provided a sensitivity of 75.58%, a specificity of 80.00% and a positive predictive value of 89.04%, negative predictive value of 60.38% ***.***
Conclusion: Our study establishes the role of miRNA 135 and miRNA 9 in breast cancer patients disease progression and prognosis.
Disclosure: Nothing to disclose

EP208

Tumor infiltrating lymphocytes - marker of therapy response in breast cancer

G. Iancu[1], G. Peltecu[1], D. Terzea[2], D. Median[1]
[1]*Filantropia Clinical Hospital, Bucharest,* [2]*Oncoteam Diagnostic, Bucharest, Romania*

Introduction / Background: Tumor infiltrating lymphocytes (TILs) are mononuclear immune cells (mainly CD8+ cytotoxic T lymphocytes), infiltrating tumor and surrounding tissues. High levels of TILs might be associated with better response to chemotherapy and better overall survival and disease free survival in patients with particular subtypes of breast cancer.
Methodology: Our study comprised of an analysis of 45 consecutive patients with HER2 positive or triple negative breast cancer (TNBC), treated in the neoadjuvant setting with chemotherapy (antracycline-based chemotherapy and taxanes) and Trastuzumab (if HER2 positive). TILS were assessed using HE staining on core biopsies (cut-off 5%), in chemo-naive patients. Postoperative, pathological complete response (pCR) was assessed for all specimens.
Results: The median age of our population was 58 years old. 96% of cases were invasive ductal carcinoma NST, 3% - invasive lobular carcinoma and 1% - mixt type. Regarding the molecular subtype, 63.6% were HER2 amplified and 37.4% were TNBC. In our population, 60% of patients had pCR. Of the pCR patients, 81% had TILs positive, while 67% of non-pCR patients had TILs positive. In the HER2 positive subpopulation, 81% of the TILs positive and 40% of the TILs negative had pCR, while in the TNBC subpopulation 55.5% of the TILs positive had pCR. Level of TILs expression did not show a significant difference between pCR and non-pCR groups (39% vs 38%), only the presence or absence of TILs.
Conclusion: In conclusion, increasing pCR in HER2 positive and triple negative breast cancer correlated with the presence, but not with the levels of TILs. TILs presence on biopsy specimen could be used as a marker of therapy response and insertion of a radiopaque marker in tumor bed in

patients with increased likelihood of pCR should be considered, to make breast conserving surgery possible. TILs definite role in breast cancer should be established in future trials.
Disclosure: Nothing to disclose

EP209

miR-190b-5p expression is a highly reliable discriminator between ER-positive and ER-negative breast cancer

A. Iyevleva[1], I. Bizin[1], A. Sokolenko[1], A. Venina[1], E. Anisimova[2], M. Holmatov[1], A. Romanko[1], I. Stepanov[1], A. Togo[1], E. Imyanitov[1]
[1]N.N. Petrov Institute of Oncology, [2]Leningrad Regional Cancer Hospital, St. Petersburg, Russian Federation

Introduction / Background: Various tumor types including breast cancer (BC) are known to have specific microRNA (miR) expression profiles. This work was initially designed to identify microRNAs which are characteristic for hereditary BCs arising in BRCA1, CHEK2, NBS1 or BLM germ-line mutation carriers.
Methodology: Small RNA profiling was performed by the next generation sequencing (NGS). 11 BRCA1-, 9 CHEK2-, 7 NBS1-, 3 BLM-associated and 16 sporadic fresh-frozen BCs were investigated with the use of NEXTFlex Small RNA Seq Kit (Bioo Scientific) on Illumina NextSeq500 platform.
Results: Comparison of different categories of hereditary breast cancer with sporadic tumors did not identify unique miRNAs or specific expression profiles characteristic for BRCA1-, CHEK2-, NBS1- or BLM-dependent BC. However, the analysis of sequencing data revealed that expression of 51, 16, 125, and 168 miRNAs was associated with the ER-status, PR-status, patients' age, and Ki67 proliferation index, respectively. The most significant differences were observed for miR-190b-5p: its expression was extremely low or completely absent in all ER-negative tumors and, conversely, high in ER-positive tumors ($p = 1.43E-59$). This microRNA can be a valuable marker for validation of the tumor ER status. The mechanisms underlying differential expression of miR-190b-5p in hormone-dependent and -independent breast cancer remain unclear.
Conclusion: Hereditary and sporadic breast carcinomas are similar in terms of microRNA expression patterns. A single microRNA, miR-190b-5p, is capable to very reliably discriminate between ER-positive and ER-negative tumors. This finding is of practical importance: ER status determination still remains a manual and somewhat subjective procedure, while microRNA expression measurement can be performed in a semi-automated way and is not prone to inter-observer variations.
Disclosure: This work has been supported by the Russian Science Foundation (grant number 17-15-01384).

EP210

Invasive lobular carcinoma with extracellular mucin production. Presentation of the second case diagnosed within three years in our hospital

S. Kokkali[1], N. Koufopoulos[2], F. Antoniadou[3], E. Pigadioti[3], D. Nasi[1], L. Khaldi[3]
[1]First Medical Oncology, 'Saint Savvas' Anticancer Hospital of Athens, [2]2nd Pathology Department, Attikon University Hospital, Medical School of Athens, [3]Pathology Department, 'Saint Savvas' Anticancer Hospital of Athens, Athens, Greece

Introduction / Background: Invasive lobular carcinoma (ILC) with extracellular mucin production is a recently described new variant of ILC with few reported cases in the English literature. We report another case of this rare entity diagnosed in the pathology department of Saint Savvas hospital.
Methodology: A 65-year-old patient was admitted to the hospital for a palpable lump of the right

breast. Physical examination and imaging studies were consistent with malignancy. Frozen section was positive while sentinel lymph node biopsy was negative for malignancy. A right mastectomy was performed. On gross examination the tumor was soft, pale, grayish blue, gelatin-like and well circumscribed. On microscopic examination the tumor consisted of two foci measuring 13 and 6 mm. It consisted of a non-mucinous ILC component of the classical and solid type. The mucinous area represented about 70% of the tumor. Extracellular mucin production was seen in the form of relatively circumscribed multiple nodular areas. Tumor cells in the mucinous area were arranged in clusters and single cells. Several signet ring cells were identified. Tumor cells showed positive staining for ER and negative for PR, HER-2 and E-Cadherin. Ki-67 stained 25% of tumor nuclei.
Results: The diagnosis of ILC with extracellular mucin production was made. Adjuvant radiotherapy and hormonal therapy was administered.
The patient is alive without evidence of recurrence or metastasis six months after surgery.
Conclusion: Awareness of this new entity will help us avoid confusion with other mucin-producing neoplasms. Differential diagnosis includes mucinous carcinoma, solid papillary carcinoma with extracellular mucin production and composite mucinous and lobular carcinoma. Correct diagnosis is important because both treatment and prognosis are distinct.
Disclosure: Nothing to disclose

EP211

An unusual case of intramedullary spinal cord metastasis from breast cancer

S. Kokkali[1], A. Ntokou[1], M. Drizou[1], E.-S. Tripodaki[1], D. Zylis[1], E. Magou[1], D. Nasi[1], A. Tzovaras[1], N. Koufopoulos[2], A. Ardavanis[1]
[1]First Medical Oncology, Saint-Savvas Anticancer Hospital, [2]2nd Pathology Department, Attikon University Hospital, Medical School of Athens, Athens, Greece

Introduction / Background: Breast cancer (BC), depending on molecular subtype, usually metastasize to the liver, lungs, bone and brain. We report an interesting case of a patient, previously treated for hormone receptor-positive, HER2-negative early BC, who presented 7 years later with HER2-positive metastatic disease. Finally she developed an intramedullary spinal metastasis.
Methodology: A 61 year-old woman underwent left mastectomy in 2010 for BC hormone receptor-positive, HER2-negative, with lymph node involvement. She also received adjuvant chemotherapy, radiation therapy and hormonal treatment until 2017.
In 2017 she received Paclitaxel/Bevacizumab followed by Bevacizumab maintenance for recurrence in the lungs, liver and bones. In February 2018 she underwent rebiopsy in the liver due to progressive disease, which revealed metastasis from hormone receptor-negative, HER2-positive BC. She received Trastuzumab/Pertuzumab/Docetaxel until August 2018. In the meanwhile she receive whole-brain radiation therapy in June 2018 for multiple brain metastases. In August 2018 she started Trastuzumab-Emtansine due to progressive disease.
On January 2019 she presented in our department with paraplegia.
Results: Spine MRI was performed ad revealed an intramedullary lesion on level T12-L1 of 3.6x1.5x1.4 cm with contrast enhancement. Differential diagnosis included metastasis from breast cancer and ependymoma. Blood tests indicated thrombopenia and hepatic disturbances.
After discussion with the neurosurgeons and radiation oncologists we did not offer any treatment to our patient, because of the established paralysis of the lower extremities.
Conclusion: BC can rarely give intramedullary spinal lesions. Their treatment is quite challenging.

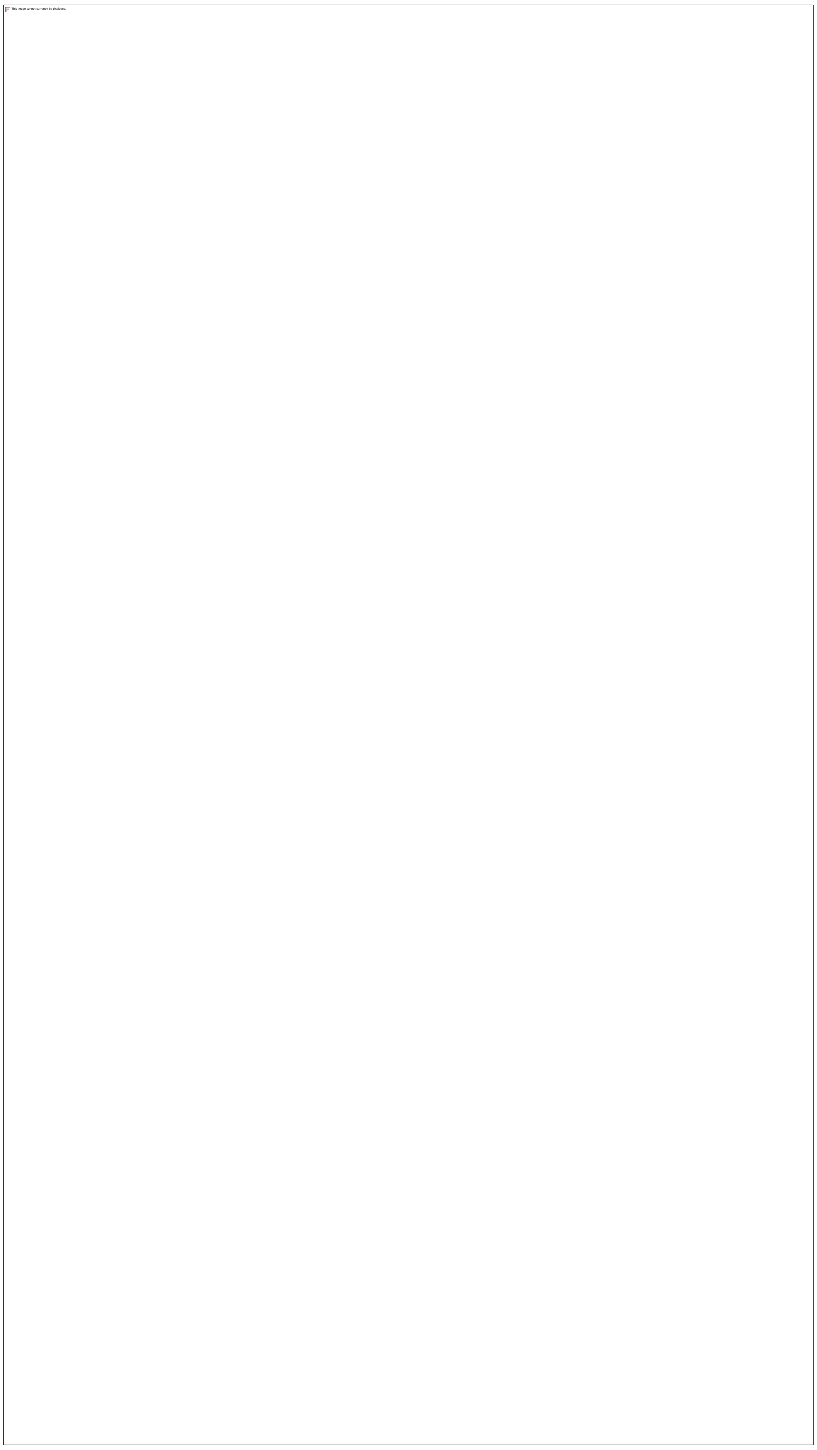

[MRI of lumbosacral spine revealing an intramedullary lesion.]

Disclosure: Nothing to disclose

EP212

Isolated vulvar metastasis of breast cancer of non-special-type after 13 years: a case report

C. Kotanidis, G. Gitas, A. Rody, J.L. Scharf
Gynecological Oncology, University Hospital of Lübeck UKSH, Luebeck, Germany

Introduction / Background: Breast cancer is the most common type of cancer and the most common cancer-related cause of death in women. In general, breast cancer metastasises to regional lymph nodes, lungs, bones, liver and brain, and more rarely to gynecological organs such as the uterus or ovaries. A rarity are metastases of the vulva, especially in isolated form. In the following, an intriguing case of a postmenopausal, 61-year-old female patient with an isolated breast cancer metastasis of the vulva, which occurred 13 years after the initial breast cancer diagnosis, is reported.
Methodology: In 2019, the patient presented herself in our dysplasia unit with pruritus of the vulva with punctum maximum at the right labia majora. After the patient was treated for breast cancer in 2006, she neglected follow-up care recently. The gynecological examination, including vulvoscopy, showed a suspicious area with an ulceration beginning at the right labia majora. A punch biopsy was performed. The staging examination with CT scans of the thorax and abdomen, as well as a bone scintigraphy, revealed no further metastases.
Results: The isolated metastasis of the vulva was excised surgically by resection of the right labia majora. Postoperatively, an adjuvant therapy with a CDK4/6 inhibitor in combination with an aromatase inhibitor was recommended.
Conclusion: This unusual pattern of a metastatic disease highlights the importance of regular gynecological examinations during follow-up in breast cancer survivors and strengthens their unreserved recommendation. Unusual metastasic patterns after long, disease-free intervals can thus be diagnosed and treated at an early stage.
Disclosure: nothing to diclose

EP213

Undifferentiated pleomorphic sarcoma of the breast: presentation of a case

N. Koufopoulos[1,2], S. Kokkali[3], F. Antoniadou[2], D. Nasi[3], E. Pigadioti[2], L. Khaldi[2]
[1]Attikon University Hospital, 2nd Pathology Department, National and Kapodistrian University of Athens, [2]Pathology Department, [3]1st Medical Oncology Department, 'Saint Savvas' Anticancer Hospital of Athens, Athens, Greece

Introduction / Background: Undifferentiated pleomorphic sarcoma (UPS) is a malignant mesenchymal neoplasm presenting very rarely as a primary breast tumor. We present a case of UPS of the breast with aggressive clinical behavior.
Methodology: A 67-year-old patient was admitted to our hospital due to a palpable lump of the right breast. Imaging studies revealed a 7 cm multilobular mass. A lumpectomy was performed. On gross examination, the tumor was solid, white-gray and had a greatest dimension of 7.3 cm.
Results: On microscopic examination, the tumor consisted of spindle cells with alternating cellularity, high-grade atypia, and brisk mitotic activity. Immunohistochemical stains were positive for CD68, CD163, CD10 and negative for AE1/AE3, EMA, CK7, Ck34bE12, p63, SMA, Desmin, S100, CD31, CD34, and CD56. The diagnosis of UPS of the breast was made. The surgical margins were infiltrated. A month later she underwent a modified radical mastectomy. The surgical margins were infiltrated. The patient received first-line systemic therapy. After having received one cycle of systemic therapy, the tumor recurred locally. A third surgical operation was performed to achieve local control. The patient's condition deteriorated very rapidly. A month later she presented with metastatic disease to the lungs. The patient passed away due to respiratory insufficiency.
Conclusion: Primary breast sarcoma diagnosis may occasionally prove very challenging. Before making the diagnosis other more common entities (malignant phyllodes tumor, metaplastic carcinoma and metastatic sarcoma from another primary site) have to be excluded. Extensive tumor sampling, attention to histological detail paired with the use of the appropriate immunohistochemical markers will

usually help in making the diagnosis. Similar to the case we present data found in the literature indicates that breast UPS show an aggressive clinical course and a high incidence of recurrence and metastasis.
Disclosure: Nothing to disclose

EP214

Sebaceous carcinoma of the breast: a rare presentation
I. Marghli[1], S. Kammoun[2], S. Sghaier[1], H. Bouaziz[1], S. Mabrouki[2], R. Ben Romdhan[1], A. Goucha[2], O. Adouni[2], M. Hechich[1], K. Rahal[1]
[1]Surgical Oncology, [2]Immuno-Histo-Cytology, Salah Azaiz, Tunis, Tunisia

Introduction / Background: Sebaceous carcinoma (SC) is an extremely rare malignant epithelial tumor of the breast it is a breast carcinoma with prominent sebaceous differentiation.
Only thirteen examples have been reported.
Thus, the aim of this case is to highlight this rare disease and report its clinical, histological and therapeutic features.
Methodology: We report a case and we reviewed publications using Sciencedirect and PubMed.
Results: A 36-year-old woman presented with a palpable mass in the left breast. She had a family history of breast cancer. Clinically, we noticed a hard and movable mass of 10mm in the lower outer quadrant of the left breast. There were no skin lesions or axillary lymphadenopathies. The imaging described two ill-circumscribed lesions of 11 and 9mm in the lower outer quadrant of the left breast and classified as BI-RADS 4c. Ultrasound-guided microbiopsies demonstrated invasive ductal carinomas grade 3 SBR.
She conducted a left lympectomy and sentinel axillary lymph node. The frozen section showed no lymph node metastasis and a bifocal invasive carcinoma wich were 15 mm apart ; so left mammectomy was performed.
On microscopic examination, the first focus of 10mm was a SC SBR grade III. The second one of 6mm was an invasive ductal carcinoma.
Immunohistochemichal analysis has revealed that tumor cells were positive for AE1/AE3, EMA, HER2, PR, ER and AR. Ki67 was of 70%.
Then, she will receive chemotherapy and radiotherapy after a fertilty preservation consultation.
Conclusion: SC of the breast with positive staining for AR is more aggressive. Thus, a careful follow up is necessary. More cases are warranted to clarify the particularities of this lesion, especially its prognosis.
Disclosure: Nothing to disclose

EP215

Less visible approaches in oncological breast surgery
J.M. Mariconde[1], M. Arrupe[2], J.A. Mariconde[3], C. Biassi[3]
[1]Gynecology Oncology, National University of Cordoba, [2]Breast Cancer, Universidad Nacional De Cordoba, [3]Breast Unit, IMGO, Córdoba, Argentina

Introduction / Background: Breast cancer is a situation that not only affects health but can also affect femininity. taking into account the case, it is feasible to use inconspicuous approaches in conservative surgery for breast cancer
Methodology: We present different approaches for breast conserving surgery: lateral, periareolar, inframmamary fold, axillary, an other situations according to the breat shape, volume and the tumor location and volume.

Results: We present different clinical presentations performing less visible approaches in oncological breast surgery.
the tumor location and approach, were as follow:
lower pole: inframammary fold.
upper pole: peri or para areolar approach.
near external quadrants: lateral, axillary or external approach of the inframmamary fold.
Conclusion: Using this technques, an oncoplastic result was achieve, with the aim of a better wellness in operated patients with breast cancer.
Disclosure: Nothing to disclose

EP216

Breast cancer: tissue expander and implant reconstruction

J. Mariconde[1], C. Biassi[1], M. Arrupe[2]
[1]Breast Unit, IMGO, [2]Breast Unit, Sanatorio La Cañada, Córdoba, Argentina

Introduction / Background: Breast cancer, the most common malignancy in women, comprises 18% of all female cancers. Eventhough there are detection programs and new detection methods, mastectomy is commun intervention nowadays. We present a two stages reconstruction technique using tissue expander and definitive implants in women diagnosed with breast cancer.
Methodology: Tissue expanders and implants are now commonly used in breast reconstruction. The use of tissue expander-implants, is a useful tool en breast cancer, due to the not uncommon association with radiotherapy and chemotherapy. In that cae the management team must consider the timing of each treatment.
Results: ResultsIn a first step, and at the same stage of the oncologyc procedure, the same surgical team performs the retromuscular pocket to locate the expander. Usually anatomical expanders are used with built-in valves and size according to the reconstructive needs of each patient. Usually, and in case of need to perform radiotherapy, it is performed with the expander placed but not fully insufflated. In a second time, the team performs the replacement of the expander by definitive prosthesis. In this stage, defects generated by the previous intervention or by association with radiotherapy can be corrected. The implants used in this stage are those of anatomical profile and height and projection characteristics according to the patient´s case.
Conclusion: The breast reconstruction techniques are a part of the breast treatment, and we strongly consider tha the same surgical team must perform the reconstruction, according to the oncological aspects of each case.
Disclosure: Nothing to disclose

EP217

Frequency of gynaecological symptoms in women treated for up to 5 years with tamoxifen

S. Masood[1], Z. Zubair[2], S. Khattak[3]
[1]King Edward Medical University Lahore, [2]Surgical Oncology, [3]Shaukat Khanummemorial Hospital and Research Centre Lahore, Lahore, Pakistan

Introduction / Background: Increasing evidence regarding the decrease and preventive effect of tamoxifen in Contralateral breast carcinoma has led to its increase use over the span of time. Surveillance for uterine safety becomes important and despite of well documented adverse effect of tamoxifen on endometrium the data is scarce regarding the
endometrial findings with tamoxifen which led to planning of this study to investigate the effects of 5-year's use of tamoxifen in preventive setting on endometrium and gynaecological symptoms.

Methodology: Altogether 750 patients diagnosed with breast carcinoma, who underwent mastectomy and were put on tamoxifen therapy from jan 2012 to 31 may 2013 were included in a hospital based cohort derived from database at Shaukat Khanum Memorial Cancer Hospital & Research Centre , Lahore During the extensive 5 year follow up (ending by 31 May 2018) the patients were inquired about gynaecological symptoms at each routine visit , surveillance of endometrial thickness at 2.5 & 5 yrs via ultrasound and evaluation of endometrium once symptomatic via sampling was done.
Results: The discontinuation rate after the start of tamoxifen was 30 %(n=225) and mean time was 18 months . The median endometrial thickness at 5 years after the use of tamoxifen was 4 mm. these findings were independent of menopausal status. Endometrial sampling was performed in 18 (2.4 %) patients and mean time of referral was 2.5 years. Only 3 patients (0.4 %) underwent hysterectomy and none was histologically proved to have endometrial carcinoma
Conclusion: Although the discontinuation rate was high and endometrial thickness was significantly increased during the tamoxifen treatment yet the serious adverse effects were not significantly high thereby the routine gynaecological surveillance in the preventive setting could not be recommended.
Disclosure: Nothing to disclose

EP218

The primitive breast cancer at the woman of less than 35 years in Oran from 1996 to 2014

H. Messid Bouziane Meflah, F. Bendjelid, F.Z. Besbes, A.B. Derkaoui, N. Nait Bahloul, N. Guetarni, F. Ammour, L. Mokhtari, Z. Ahmed Fouatih
University of Oran 1 Ahmed Benbella, Faculty of Medicine, University Hospital Center of Oran, Oran, Algeria

Introduction / Background: The breast cancer is a disease which is more alarming than it is generally more aggressive. The breast cancer “of the young woman”, is a cancer occurring at a person of less than 35 years. It is more difficult to diagnose, when it occurs before this age.
The objective of this study is to describe the epidemiologic characteristics of the primitive breast cancer in woman of less than 35 years in Oran from 1996 up to 2014.
Methodology: It is a descriptive study of which the data were extracted from the Register from Cancer from Oran which is a register of population going back to 19 years and which is part of the regional network of the Algerian west. The coding of the localizations and morphologies of the tumors is done according to CIMO (CIMO3: International classification of the Diseases for Oncology, 3èmerévision) and CIM 10 (International Classification of the Diseases, version 10). The seizure and the analysis are done by the Edge-reg4 (software bench by the CDC of Atlanta) and Ear-information version 6
Results: Among the 21108 cancers all localizations notified during these 19 years, one finds 623 cases of breast cancer primitive cancer in woman of less than 35 years. The median age is of 30.94 ± 0, 30 years with an extreme 16 years. The most frequent morphology is the canal carcinoma infiltrating followed by the infiltrating ductal Carcinoma and of the Carcinoma lobar SAI.
Conclusion: The breast cancer is the first female cancer: among 21108 of all cancer localizations notified during these 19 years with Oran, one retrouve4148 case (either 19.6%) including 623 case at the young woman (or 15,01%).
Disclosure: Nothing to disclose

EP219

Improvement in quality of life of Indian breast cancer patients by pre and post mastectomy educational counselling

A. Mishra[1,2], M. Gowda[1], S. Deo[1]
[1]Dr Bra Irch, AIIMS, [2]Medeor Hospital, New Delhi, India

Introduction / Background: In India still more than 70% of breast cancer patients are presenting in locally advanced stages, leading them to mastectomies. Being diagnosed as breast cancer patient and further undergoing radical surgery causing them a significat trauma and affecting their quality of life particularly in tems of *body disfigurement, self -esteem, Social & personal relationship etc.* Recognizing these issues, our aim was to assess the impact of educational counselling over quality of life of women who has undergone breast cancer mastectomy.
Methodology: This is a prospective interventional study performed on breast cancer patients who have been treated at IRCH, AIIMS, New Delhi, India. Recruitment of 100 patients and data collection were done between Jan and Dec of 2017. All patients had undergone pre designed questionnaire based assessment of their knowledge regarding breast cancer. Out of 100, 50 patients randomly selected for 20 minutes pre & post mastectomy (POD-1) counselling sessions. After 12 months of mastectomy, all 100 patients again undergone a questionnaire based assessment regarding their quality of life. Statistical assessment done by Stuart Maxwell test & McNamara test.
Results: Mean age of patients was 46.4 years. Almost 60% patients had finished their high school, 94% patients were married while only 15% were working. Out of 50 counselled patients 84.6 % patients completed their treatment well and returned to their previous routine life while only 67.3 % of un-counselled patients. Main issues addressed by patients were disfigurement, hair loss, loss of sensation and pain in operated side upper arm. Prior knowledge and motivtaion for the use of prosthetic bra, wigs, physiotherapy etc help them to return their normal life early.
Conclusion: the short educational counselling sessions have a significant impact in the improvement of quality of life particularly in terms of emotional, physical and social life.
Disclosure: Nothing to disclose

EP220

Survival of Filipino women with metastatic brain disease from breast carcinoma who underwent whole brain radiation therapy in a cancer institute in Cebu city from 2005 to 2016

M.M. Mopon, A.J. Uson
Internal Medicine, Perpetual Succour Hospital, Cebu, Philippines

Introduction / Background: Survival of women with metastatic brain disease from breast cancer who underwent Whole Brain Radiation Therapy vary from institution to institution and influenced by demographic factors, tumor factors such as the immunohistochemical subtype and their treatment. The objective of the study is to determine the overall survival of Filipino women with metastatic brain disease from breast carcinoma who underwent Whole Brain Radiation Therapy in a cancer institutute in Cebu City from January, 2005 to December, 2016.
Methodology: Clinical data of Filipino women with metastatic brain disease from breast cancer who underwent Whole Brain Radiation Therapy were gathered from the cancer institute, medical records, and charts from the clinics of four active oncologists in a tertiary hospital in Cebu. Kaplan-Meier product limit estimate of survival was used to determine the median survival time according to immunohistochemical subtype. The difference on the survival time between variables were tested using Log Rank Test. Cox Regression analysis was done to determine the effects of these variables to the overall survival time.
Results: A total of 1573 patients were diagnosed with breast cancer in the said institute from 2005 to 2016, 106 (6.73%) received Whole Brain Radiation Therapy, 61 patients were eligible for analysis. The median time from breast cancer diagnosis to brain metastasis is 43.9 months. The median time from brain metastasis to death is 2.7 months. Overall median survival time is 2.6 months, with ER(+)PR(+)Her2(-) subtype having the longest survival time among the 3 most common subtypes. Independent factors influencing the overal survival are: initial clinical stage, number of brain metastases, hormonal therapy, and chemotherapy after Whole Brain Radiation Therapy.
Conclusion: Overall survival time of Filipino women with metastatic brain disease from breast cancer who underwent Whole Brain Radiation Therapy in our institution is 2.6 months.
Disclosure: Nothing to disclose

EP221

Ovarian metastases from breast cancer: series over a 20-years period at a Lebanese tertiary care center

R. Eid[1], M. Abdo[1], G. Zgheib[1], T. Zeidan[1], F.G. Haddad[1], A. Khaddage[2], H.R. Kourie[1], M. Moubarak[3], D. Atallah[3]
[1]Oncology, [2]Pathology, [3]Obstetrics and Gynecology, Saint Joseph University/Hôtel Dieu de France University Hospital, Beirut, Lebanon

Introduction / Background: To report the characteristics and outcomes of patients presenting ovarian metastases from a breast.
Methodology: It is a retrospective study reviewing the characteristics of ovarian metastases from a breast cancer diagnosed in 20 years (from 1997 to 2017) at Hôtel-Dieu de France University hospital, a tertiary care center in Lebanon.
Results: We reviewed the data of 1137 patients with ovarian tumors. Ovarian metastases from a breast cancer were found in 13 cases. Mean age was 59 years. 46% of patients received CT-scan and only in 15% of cases a PET-CT scan was performed. The mean interval time between the primary diagnosis of breast cancer and the occurrence of ovarian metastasis was 52 months. The most common histologic type found was invasive lobular carcinoma (60% of cases). Extraovarian metastases were found in 69% of cases (9 out 13 patients). The extraovarian metastases concerned the following organs: uterus (3 cases), bone marrow (5 cases), liver (5 cases), lungs (3 cases), brain (3 cases), stomach (1 case), and adrenal gland (2 cases). All cases were treated surgically and received adjuvant chemotherapy. A cytoreductive surgery was performed in five cases. A unilateral or bilateral adnexectomy was done in one and seven cases respectively. Mean survival was 60 months. Recurrence was noted in 46% of cases (6 out 13 patients). Mean time to recurrence was 38 months.
Conclusion: Ovarian metastases from a breast cancer occur rarely and are associated with worse prognosis. Despite surgical and adjuvant therapy recurrence rate is very high.
Disclosure: Nothing to disclose

EP222

Indocyanine green for sentinel lymph node detection in early breast cancer: prospective evaluation of detection rate and toxicity

C. Ngô[1,2], S. Sharifzadehgan[1], C. Lecurieux-Lafayette[1], H. Belhouari[3], D. Rousseau[4], H. Bonsang-Kitzis[1], L. Crouillebois[3], S. Oudard[5], F. Lécuru[1,2], R.-T. Elaidi[3]
[1]Hôpital Européen Georges Pompidou | Breast Oncological Surgery, APHP, [2]Sorbonne Paris Cité University, [3]ARTIC Association pour la Recherche de Thérapeutiques Innovantes en Cancérologie, [4]Pharmacy, [5]Medical Oncology, APHP Hôpital Européen Georges Pompidou, Paris, France

Introduction / Background: Detection of sentinel lymph node (SLN) in early breast cancer is commonly based on the combination of patent blue dye and a radio isotope (RI) 99m Technetium. Each of these two tracers has advantages and disadvantages leading to the development of the use of Indocyanine Green (ICG) as a new tracer.
Methodology: we conducted a prospective clinical trial to evaluate the detection rate of ICG, the sensitivity and concordance rate as compared to 99mTe. Each patient undergoing a SLN biopsy for an early breast cancer received both ICG and RI. The trial was registered: FLUOBREAST EudraCT N°2015-000698-11, ClinicalTrials.gov Identifier: NCT02875626.
Results: Seventy-seven patients were analysed with a total of 205 nodes. Detection rates were 93% for the RI and 96% for the ICG. Combined detection rate was 99%. Sensitivity of the ICG was 100%.

Overall concordance rate was 91%. Median numbers of excised SLN was 2.3 for both tracers and 2.7 for the combined method (p=0.21). All the macrometastatic nodes were detected by the ICG. Median time between skin incision of the axilla and removal of the last SLN was 14 min. There was no allergy to the ICG. There was no radio-sensitization linked with the use of ICG.
Conclusion: ICG allows high detection rate and high sensitivity for the SLN biopsy in early breast cancer, with short operative time and a usual number of excised SLN. Allergy is extremely rare and there is no toxicity. ICG could be an alternative to RI to provide an accurate staging of the axilla. Its routine use should be approved for SLN biopsy.
Disclosure: Nothing to disclose

EP223

Prognosis factors of breast cancer (about 557 cases)

O. Omran[1], A. Touimi Benjelloun[2], A. Iraqi[1], N. Benchekroun[1], Z. Bouchbika[1], H. Jouhadi[1], N. Tawfiq[1], S. Sahraoui[1], A. Benider[1]
[1]Centre Mohamed IV d'Oncologie, [2]Centre Mohamed IV de Chirurgie Oncogynecologique, Hassan II University / Faculté de Medecine et de Pharmacie, Casablanca, Morocco

Introduction / Background: Breast cancer is a public health problem in Morocco. The objective of this work was to estimate the survival rate and the prognostic factors related to breast cancer in our context.
Methodology: We performed a prognostic study in patients diagnosed with breast cancer in 2008 at the Mohamed VI Oncology Center at the University Hospital Center in Casablanca. Estimation of survival was performed by the Kaplan Meier method, and the comparison between the different classes of a variable was performed by the log rank test. The study of factors associated with survival was performed by the Cox model.
Results: During the study period 557 cases of breast cancer were collected. The percentage of deaths was 18.31%. Overall survival at 5 years was 89.96%. In multivariate analysis, breast cancer survival was statistically associated with a therapeutic delay of more than 3 months ($p < 0.001$), advanced N3 lymphadenopathy ($p = 0.01$), metastasis ($p< 0.001$), stage ($p< 0.001$) PTH was lower for patients not treated with hormone therapy and radiotherapy ($p < 0.001$). The occurrence of recurrence was correlated with a presence of vascular emboli $p< 0.02$), absence of hormone receptors ($p=0.008$), and advanced stage ($p< 0.001$).
Conclusion: An advanced tumor stage and the presence of metastases are factors of poor prognosis of breast cancer, hence the need to strengthen screening programs and early diagnosis.
Disclosure: Nothing to disclose

EP224

Low long-chain polyunsaturated fatty acids levels in breast adipose tissue are associated with multifocality in women with positive hormone receptors tumors

L. Ouldamer[1,2]
[1]Gynoncology, CHU Tours, [2]INSERM UMR 1069, Tours, France

Introduction / Background: In a previous pilot study we found that polyunsaturated n-3 fatty acids of marine origin were associated with Breast cancer multifocality. In the present study, we aimed to confirm these results altogether with investigating clinical and histological factors associated with breast cancer focality in a larger cohort of women with positive hormone-receptors breast cancer.
Methodology: One hundred sixty-one consecutive women presenting with non-metastatic breast cancer with positive hormone-receptors underwent breast-imaging procedures including a Magnetic

Resonance Imaging prior to treatment. Breast adipose tissue specimens were collected during surgery. We established a biochemical profile of adipose tissue fatty acids by gas chromatography. Clinicopathologic characteristics were correlated with multifocality. We assessed whether these factors were predictive of breast cancer focality.
Results: We found that tumor size (OR=1.06 95%CI[1.02 - 1.09], p< 0.001) and decreased levels in breast adipose tissue of long-chain polyunsaturated n-3 fatty acids (OR=0.11 95%CI[0.01 - 0.98], p=0.03) , were independent predictive factors of multifocality.
Conclusion: These differences in lipid content may contribute to mechanisms through which peritumoral adipose tissue fuels breast cancer multifocality.
Disclosure: Nothing to disclose

EP225

characteristics of inflammatory breast cancer within Mohamed VI center for the treatment of cancer, CHU Ibn Rochd, Casablanca

O. Oumaima[1], A. Touimi Benjelloun[2], A. Iraqi[1], N. Benchekroun[1], Z. Bouchbika[1], H. Jouhadi[1], N. Tawfiq[1], S. Sahraoui[1], A. Benider[1]
[1]Centre Mohamed IV d'Oncologie, [2]Centre Mohamed IV de Chirurgie Oncogynecologique, Hassan II University / Faculté de Medecine et de Pharmacie, Casablanca, Morocco

Introduction / Background: Inflammatory Breast Cancer (IBC) is a rare but particularly aggressive form of breast cancer. It represents about 1-5% of all breast cancers. It is characterised by a particular geographical distribution, being described as more common in the region of North Africa, with particular characteristics in comparison to locally advanced breast cancer. The objective of this study is to describe the clinical and progressive characteristics of inflammatory breast cancer within Mohamed VI center for the treatment of cancer, CHU Ibn Rochd, Casablanca
Methodology: In a cross-sectional study, covering the period from January 2010 to December 2015, including all cases of inflammatory breast cancer, clinically diagnosed according to the definition of the AJCC (American Cancer Joint Committee). It excluded the tumors locally advanced.
Results: A total of 57 patients had an IBC, and were taken care of within the Center with an average of 50.2 +/- 11.8 years old with extremes (32-90 years old), 22.8% of cases used oral contraceptives, The histological type the more common was the infiltrating ductal carcinoma in 82.5% of cases. The histological grade SBR 3 was the most common in 47.4 of cases. A positivity of the estrogen receptors was observed in 59.5% of cases and of progesterone receptors in 40.5% of cases. Her2 was overexpressed in 50.0% of cases. After neoadjuvant chemotherapy for localized tumors, 64% of patients had a clinical objective response (COR) and 20% had a histological Complete response (PCR). The survival without relapse (SWR) and overall survival (OG) to five years were respectively 44.5% and 27.4%.
Conclusion: IBC is an aggressive form of breast cancer. It represents 1.7% of cases in our center, with more pejorative criteria in comparison to the noninflammatory Breast Cancer : more youth, more negative ER, more positive HER2, high SBR grade, lymphatic ganglions and The metastatic dissemination,
Disclosure: Nothing to disclose

EP226

The advancing age: breast cancer over 80 years

A.C. Rocha[1,2], M.I. Sá[3], S. Raposo[2], R. Sousa[2], P. Correia[2], P. Aldinhas[2], E. Branco[2], C. Coelho[2], V. Baltar[2], L. Sá[2]
[1]Hospital de Santarém, Santarém, [2]Instituto Português de Oncologia Coimbra, Coimbra, [3]Hospital de

Vila Real - CHTMAD, Vila Real, Portugal

Introduction / Background: Improvements in health care have resulted in a gain in overall life expectancy. Breast cancer is the most common cancer in women, with an incidence that increases with advancing age. However, older women may exhibit a variety of clinical presentations that may influence the treatment and prognosis of this disease.
Methodology: A retrospective cohort study with 130 women aged ≥80 years with breast cancer, referred to the gynaecology appointment of the IPO-Coimbra (2013 and 2018). The information was treated in SPSS DATA editor version 15.0. Statistical significance was set at $p < 0.05$.
Results: The mean age was 83 years. During the study period, 983 women had breast cancer, of which 130 women were aged > 80 years. In terms of the comorbilities, 73.8% presented with HBP and 23.8% DM. The most frequent stage at diagnosis was $T_2N_0M_0$. Regarding the therapy performed, only 9.2% performed neoadjuvant therapy (between hormone therapy (HT) and / or radiotherapy (RT)). In surgical terms, 60.8% underwent surgery and, in most cases (78.5%), no conservative surgery was performed. As regards to adjuvant therapy, 56.7% of the patients performed some type of therapy (RT, HT and / or chemotherapy (QT)). Palliative care was offered to 16.9% of patients as a primary therapy. Concerning the general prognosis, 7.9% of the patients relapsed, but only 10.8% had cancer-related mortality, with a median disease-free time of 16 months. The $\chi 2$ test did not show a statistically significant association between mortality and age when analyzed by age group ($p = 0.32$).
Conclusion: This patients represent an expanding group with specific characteristics that may influence the treatment and prognosis of breast cancer. However, there aren't enough studies to define strategies in this age group. Therefore, it would be important to implement multicenter studies in order to overcome this limitation.
Disclosure: Nothing to disclose

EP227

Ultrasound and MRI evaluation of axillary lymph nodes in breast cancer patients

M.D. Rodriguez Garnica[1], R.M. Alvarez Lopez[1], M. Martin Blanco[1], M.L. Arranz Merino[2], E. Jareño Dorrego[3], A.I. Ballesteros Garcia[4], M.J. Alcaraz Mexia[5]
[1]Department of Gynecology, Santa Cristina University Hospital, [2]Department of Radiology, La Princesa University Hospital, [3]Department of Pathology, Santa Cristina University Hospital, [4]Department of Medical Oncology, La Princesa University Hospital, [5]Department of Radiology, Santa Cristina University Hospital, Madrid, Spain

Introduction / Background: About 30-40% of newly diagnosed breast cancer patients have axillary nodal metastasis. The aim of this study is to analyze the diagnostic performance of MRI and ultrasound for the detection of axillary disease.
Methodology: The medical records of 45 patients diagnosed with breast cancer in the last 6 months have been retrospectively reviewed, including the MRI and ultrasound reports. Sixteen patients were excluded due to having the imaging performed in a different centre.
Results: Twenty-nine patients were finally included. Median age was 58 years old (37-76). Histological type was ductal invasive carcinoma in 22 patients (75.9%) and lobular invasive carcinoma in 7 (24.1%). The molecular subtype was Her2 in 12 patients (41.4%), Luminal A in 9 (31%), Luminal B in 4 (13,8%) and triple negative in 4 (13,8%). Six patients had bulky nodes on clinical examination. Over all patients 12 had suspicious nodes on MRI (44.4%) and 11 on ultrasound (37.9%). A fine-needle aspiration (FNA) of the nodes was performed in 13 patients (44.8%), 9 of them had metastasis of carcinoma (31%) and had neoadjuvant chemotherapy. Twenty patients had upfront surgery, including sentinel lymph node biopsy, in 12 the sentinel node was negative, 2 had micrometastasis and 6 had macrometastasis. At the time of the operation a systematic axillary lymph node dissection was performed in 15 patients (9 with positive FNA and 6 with macrometastasis in sentinel node). The sensitivity in evaluation nodal disease was 78.6% for ultrasound and 91.7% for MRI, the specificity 53.3% for ultrasound and 73.8% for MRI. The negative predictive value was 61.1% for ultrasound and 73.3% for MRI.
Conclusion: The MRI seems to be a more accurate method for detection of nodal disease in breast cancer patients.

Disclosure: Nothing to disclose

EP228

Clear cell carcinoma of the breast. A rare histological variant with a poor prognosis. Case report and literature review

I. Sassi[1], I. Marghli[1], S. Kammoun[2], H. Bouaziz[1], A. Bouida[1], M. Slimen[1], O. Adouni[2], K. Rahal[1]
[1]Surgical Oncology Department, [2]Pathology Department, Institute Salah Azaiez, Tunis, Tunisia

Introduction / Background: Clear cell carcinoma of the breast is a rare histological subtype characterized by carcinoma cells containing an optically clear cytoplasm. It accounts for 1-3% of all breast cancer cases.
Methodology: We report on the prognosis of two female patients treated in our institute in 2007 and 2013.
We reviewed publications since 1981 to date, using PubMed and Sciencedirect.
Results: A 36 year-old women presented with a mass in the right breast. Examination showed irregular lump in the upper outer quadrant of her right breast, measuring 15*10mm. No locoregional nodes were palpable. Breast imaging showed a high density nodule of 20mm with irregular margins classified as BIRADS 4. A Tru-cut needle biopsy was performed. The microscopic examination showed an invasive ductual carcinoma containing clear cytoplasm. Breast-conserving surgery with axillary sentinel lymph node biopsy was initially performed. Surgical margins were positive. A complementary mastectomy was done.The patient was submitted for chemotherapy. Four months after the surgery,examination revealed a permeation nodule. The patient resumed the chemotherapy and radiotherapy. Five months later a CT-scan showed lung and mediastinal node metastases. The patient died of respiratory failure .
A 67 year-old women, presented with a mass in the right breast. Examination revealed a hard and irregular mass of 35mm in the upper outer quadrant of the right breast. Enlarged lymph nodes were palpable in the right axillary. Breast imaging showed a round, high density mass with partially irregular margins measuring 35mm classified as BIRADS 5. Breast-conserving surgery with lymph node dissection was performed. The microscopic examination showed a poorly differentiated carcinoma with abundant clear cytoplasm.Two months after surgery, examination showed a permeation nodule. The patient was sent to chemotherapy. After two cycles of chemotherapy, she developed a rhino-orbito-cerebral mucormycosis and died after a month.
Conclusion: Clear cell carcinoma of the breast is an aggressive subtype with bad prognosis.
Disclosure: Nothing to disclose

EP229

Breast metastasis from a renal cell cancer. Case report and literature review

I. Sassi[1], N. Lamia[1], A. Bouida[1], O. Jaidane[1], H. Bouaziz[1], S. Kammoun[2], M. Slimen[1], O. Adouni[2], M. Hechiche[1], R. Chargui[1], K. Rahal[1]
[1]Surgical Oncology Department, [2]Pathology Department, Institute Salah Azaiez, Tunis, Tunisia

Introduction / Background: Metastases to the breast from extramammary tumours are uncommon representing about 0.2% to 1.3% of malignant tumours in the breast. Metastatic renal cell carcinoma to the breast is extremely rare.
Methodology: We report the case of a female patient treated in our institute in 2019.
We reviewed publications since 1998 to date, using PubMed and Sciencedirect.
Results: A 60 years-old woman with a medical history of renal carcinoma treated by radical nephrectomy. After 7 years of follow-up, CT scan revealed a right breast lump. Clinical examination

found a 2cm hard lump, poorly defined at the union of the upper quadrants of the left breast. A mammography showed a round, relatively circumscribed and intermediate density mass. No calcification was identified. Ultrasonography showed a hypoechogenic, solid vascular mass (1.6*1.4cm) in the left breast and similar intramuscular lesion in the axillary tail of the right breast. No pathological axillary adenopathies were identified. Tumour was classified BIRADS 4.An ultrasound-guided needle biopsy was performed for both left and right lesions. Histopathological examination showed polygonal cells containing clear cytoplasm. Immunohistochemistry revealed that the tumour cells were positive for CD10 and vimentin and negative for oestrogen receptors (ER),progesterone receptors (PR) and cytokeratine CK7. The morphology and immunoprofile were consistent with a metastatic renal clear cell carcinoma.
The case was discussed in the multidisciplinary meeting. Surgery was ruled out and the patient was referred to the medical oncology department to start systemic treatment .
Conclusion: A positive diagnosis of the metastasis from extramammary lesion is crucial to provide proper treatment and avoid unnecessary surgery.
Disclosure: Nothing to disclose

EP230

Solid papillary carcinoma of the breast: about two cases and literature review

S. Sghaier[1], S. Kamoun[2], I. Marghli[1], H. Bouaziz[1], S. Mabrouki[2], S. Nechi[3], R. Ben Romdhane[1], A. Goucha[2], R. Chargui[1], O. Adouni[2], K. Rahal[1]
[1]Surgical Oncology, [2]Immuno-Histo-Cytology, Salah Azaiz, Tunis, [3]Pathology, Tahar Maamouri Hospital, Nabeul, Tunisia

Introduction / Background: Solid papillary carcinoma (SPC) of the breast is a new histological entity described in the last WHO classification (2012). SPC is associated with invasive carcinoma in half of cases. 95% are unilateral.
Methodology: We reported two cases and reviewed publications using PubMed.
Results: Case1: A 73 year-old woman presented a history of adenoid carcinoma of the palate. She presented bilateral breast nodules. Clinically, we found a 25mm nodule and a 10mm condenstaion in the upper of the right and the left breast; respectively. There was no axillary lymph node. Breast imaging demonstrated a heterogeneous nodule of 22x10mm in the right breast, classified as BIRADS 4 and a nodule of 10x5mm in the left, classified as BIRADS 5. Needle biopsies confirmed bilateral invasive ductal carcinoma. She conducted lympectomies. Histologically, it showed a SPC of 9mm in the left breast. In the right side, 2 separate foci was shown; a SPC of 12mm and an intra ductal carcinoma of 22mm.
She refused any complementary treatment.
Case2: A 48 year-old woman presented with a mass of the left breast. Clinically, we noticed a hard and movable nodule of 20mm in the lower outer quadrant of the left breast. There were no lymph nodes. The breast imaging showed an ill-circumscribed mass of 17mm, classified as BI-RADS 5. Needle biopsy concluded to an invasive ductal carcinoma. She undertook a left lympectomy and sentinel lymph node. The frozen examination showed a 17mm invasive ductal carcinoma with negative shaved margins and negative axillary nodes. Histologically, it corresponds to a SPC of 17mm.
She will receive radiotherapy.
Conclusion: Solid papillary carcinoma is a low grade neoplasm occurring in elder women. When solid papillary carcinoma is associated with invasive carcinoma prognosis is poorer. The management of this disease still not well established.
The management still not well established.
Disclosure: Nothing to disclose

EP231

ER, PR and HER-2 status in breast cancer - correlation with clinicopathological characteristics and clinical outcome at a tertiary care centre in South India

D. Shunmugam[1], A. Velappan[2]
[1]Surgical Oncology, [2]Medical Oncology, Tirunelveli Medical College Hospital, Tirunelveli, India

Introduction / Background: Breast cancer has now overtaken cervical cancer to become leading cancer among women in India. The AJCC Staging Manual has included grade and ER, PR and HER-2 status in the Prognostic Stage Grouping thus emphasizing their importance. The main objective of this study was to assess the ER, PR and HER-2 status among breast cancer patients and find out its correlation with age, histological type, grade and stage.
Methodology: In this retrospective study, records of 548 patients in the Medical Oncology Department at Tirunelveli Medical College Hospital were reviewed. Data were analyzed using the Pearson Chi-square test, One-way ANOVA and Kendall tau test. Survival analysis was done using Kaplan- Meier estimator.
Results: The average age at diagnosis was 51.56 years, 70.8% (n=388) belonged to stage III. The most common histological type and grade was Infiltrating Ductal Carcinoma not otherwise specified (94.3%) and Grade II (69.7%, n=382) respectively. The hormone receptor (HR) positive patients constituted about 38.3% (n=210), triple negative breast cancer (TNBC) patients about 27.2% (n=149) and HER-2 positive patients included 34.5% (n=189). Among the HER-2 positive patients, 40.2% (n=76) were also HR positive. The HR-positive patients (53.87) were diagnosed at a later age compared to hormone receptor negative patients (49.46) with a P-value = 0.001. There was no association between histological type and ER, PR, and HER-2 status. Triple-negative breast cancer patients had more of grade III (34.9%) compared to the HR group (17.1%) (P=0.001). The larger number of HR-positive patients presented at stage II (24.8) compared to others (3.8%)(P=0.001). More number of HER-2 positive patients (27.5%) presented in stage IV than others (11.7%) (P=0.001).
Conclusion: TNBC an aggressive subtype was more common in Indians compared to the western population. HR positive status was associated with older age, lesser grade and stage at diagnosis and hence have a better prognosis.
Disclosure: Nothing to disclose

EP232

Clinical prediction factors of prognosis in operable triple negative breast cancer

M. Turk[1], M. Sobočan[1,2], P. Čater[2], N. Čas Sikošek[2], B. Crnobrnja[2], D. Arko[1,2], I. Takač[1,2]
[1]Faculty of Medicine, University of Maribor, [2]Clinic for Gynecology and Perinatology, University Medical Center Maribor, Maribor, Slovenia

Introduction / Background: Triple negative breast cancer (TNBC) is characterized by worse prognosis compared to other breast cancer subtypes. However due to different molecular subtypes within TNBC and thus different disease aggressiveness there is a need to establish clinical markers signalling disease aggressiveness and thus treatment. The aim of this study therefore was to identify clinical markers in TNBC disease prognosis.
Methodology: We identified through a retrospective data analysis at the University Medical Center Maribor 88 patients with operable TNBC between the years 2010 and 2017. By use of multivariate analysis specific factors correlating with poorer prognosis have been identified.
Results: Eighty eight patients with operable TNBC were identified. Node-negative TNBC patients (n=56) were diagnosed at 59.1 years (SD 13.5) and mean tumor size 26.8 mm (SD 17.5). Thirty-two patients (n=32) were diagnosed with node-positive TNBC at mean age 57.4 years (SD 13.1) and mean tumor size 29.8 mm (SD 19.7). In this cohort (n=88), twelve patients (n=12) died and ten (n=10) patients had disease recurrence/metastases. Multifactorial analysis showed, that greater tumor size (p=0.009) and lymph node ratio (LNR) (p=0.011) were correlated with significantly more deaths. Higher LNR was significantly correlated with disease specific death p=0.001 (Mann-Whitney Test;

mean rank 40.3 vs. 63) as well as with greater tumor size p=0.020 (Mann-Whitney Test; mean rank 43.4 vs. 61.8). However, there was no correlation of tumor size (p=0.156) or lymph node ratio (p=0.069) in disease recurrence/metastasis.
Conclusion: Our single-institution analysis shows that LNR and tumor size are predictive factors of overall survival in triple negative breast cancer. Larger studies correlating molecular subtypes of TNBC and patient with high LNR should be explored.
Disclosure: Nothing to disclose

EP233

Prognostic factors in locoregional recurrence of breast cancer

A. Touimi Benjelloun, Z. Edderdabi, M. Moutahir, Y. Benchrifi, M. Benhessou, M. Ennachit, M. Elkarroumi
Centre Mohamed IV de Chirurgie Oncogynecologique, Hassan II University/Faculté de Medecine et de Pharmacie, Casablanca, Morocco

Introduction / Background: The objective was to describe the prognostic factors in locoregional recurrence of breast cancer in our context.
Methodology: We conducted retrospective study in patients diagnosed or treated for breast cancer between January 2016 and December 2017.
To obtain significant results we have deferred to the statistical rules.
Results: During the study period, 703 cases of breast cancer were collected, 47 cases have developed locoregional recurrence. The average time of recurrence was 42 months. The average tumor size was 3.5 The occurrence of recurrence was also correlated with an advanced stage of tumor up to 42.6% in stage II, infiltrating ductal carcinoma was found in (91.4%) with a predominant SBR II grade in (55.3 %), as well as the Presence of vascular emboli (47%), lymph node involvement in (38.3%), Of 34 analyzed tumors, 11% were classified as luminal A, 9% as luminal B 6% as Her-2 type, and 15 % as Basal type. Radical Patey treatment was performed in 24 patients, 17 % of whom received neoadjuvant chemotherapy, 32 %received adjuvant radiotherapy, 17 % had an hormonal treatment and 6.3 % received targeted therapies. The 23 patients were treated by a conservative treatment which (44.6%) patients have benefited radiotherapy and (38.2 %) patients have received adjuvant chemotherapy and 12.7 % had an hormonal treatment.
Conclusion: although enormous progress has been made in understand of the biology of breast cancer and its heterogeneity, there is still much to do to be able to distinguish patients at high risk of recurrence requiring more crucial treatment.
Disclosure: Nothing to disclose

EP234

Breast metastasis from pancreatic neuroendocrine carcinoma misdiagnosed as primary carcinoma. A potential pitfall with significant clinical implications

H. Trihia, G. Stanc, L. Karelis, C. Valavanis
Pathology, 'Metaxas' Cancer Hospital, Piraeus, Greece

Introduction / Background: Pancreatic neuroendocrine tumors (PNETs) are an uncommon malignancy, accounting for a small percentage of all pancreatic malignancies. Although, reports in the literature describe PNET metastasis to the liver, lung and brain, there are very few reports of stage IV disease involving the breast. We report a case of a woman with a history of pancreatic PNET, misdiagnosed as primary breast cancer.
Methodology: A 38-year old woman underwent a wide lumpectomy of her right breast, which was

misdiagnosed as a triple negative, apocrine carcinoma of the breast. Clinical history was not known, at the time.
Results: Following the information of the clinical history of the patient, with metastatic pancreatic PNET in the liver, lung, adrenal gland, lymph nodes and bones, review of the architectural/cytological features of the tumor along with immunohistochemical staining with breast and neuroendocrine markers took place, which showed negative staining for breast markers and positive staining for neuroendocrine markers. Morphology and immunophenotype were compatible with secondary neuroendocrine carcinoma, of pancreatic origin.
Conclusion: Suspicion for metastasis must be high in breast tumors with morphologic unusual features/patterns and in low-grade, non-gland forming tumors that are estrogen receptor, progesterone receptor and HER-2/neu (triple) negative, as a metastasis to the breast can be the presenting sign of a neuroendocrine tumor elsewhere in the body in nearly one-third of the reported cases, or show similar morphologic features with the primary tumors. Increased awareness, the clinical history and review of prior slides are vital for the correct diagnosis, as treatment for metastatic lesion is significantly different from treatment of a primary breast tumor, as radiation to the breast and lymph node sampling may not indicated and systemic therapies will likely be considered if the correct diagnosis can be made.
Disclosure: Nothing to disclose

EP235

The effect of bariatric surgery on breast cancer

D. Vasileiadou[1,2], K. Antiochos[2], M. Litos[1], G. Skrekas[2]
[1]Department of Obstetrics & Gynecology, Konstantopouleio General Hospital of Nea Ionia,
[2]LapEndoSurgery: Advanced Laparoscopy & Bariatric Surgery, Athens, Greece

Introduction / Background: It is well known that obesity is associated with an increased risk of cancer. Considering its high prevalence, obesity is an important risk factor for cancer. Obesity is not only associated with new cancer cases but also with more advanced cancer disease and increased cancer mortality. Hormone - related cancers, such as endometrial and breast cancers, have both been associated with obesity. Bariatric surgery is the most effective treatment for achieving weight loss and sustaining normal weight in patients with obesity.
Methodology: The present study is a review of literature.
Results: Bariatric surgery has been shown to reduce the overall cancer risk in patients with obesity. Regarding breast cancer, several large - scale studies and meta-analyses have shown significant reduction in the risk of breast cancer in women undergoing bariatric surgery, with an odds ratio of 0.25-0.5. Mechanisms that have been proposed as contributors to the increased risk between obesity and breast cancer include estrogen excess through increased aromatase activity in adipose tissue, insulin resistance and increased circulating glucose, that have both shown to be growth factors for cellular proliferation, and obesity-related pro-inflammatory state with higher levels of interleukins, TNF, and adipokines.
Conclusion: Bariatric surgery may protect obese patients from breast cancer. Weight loss and bariatric surgery may be suggested to obese women as an additional preventative modality.
Disclosure: Nothing to disclose

EP236

Breast-conserving surgery (BCS) in the management of Carcinoma Ductal in Situ (DCIS): reoperation rates and safety margins

A. Vazquez Sarandeses, S. Aragon, C. Sanz, I. Muñoz Hernandez, M.L. Arroyo, M. Hernandez Ontoria, B. Garcia Chapinal

Hospital Universitario 12 de Octubre, Madrid, Spain

Introduction / Background: To evaluate the results of BCS for patients with DCIS.
Methodology: We retrospectively analyzed the results of 172 patients diagnosed by vacuum assisted biopsy (VAB) or fine-needle aspiration biopsy (FNAB) of DCIS, between 2011 and 2017. We consider free margin >=2mm. Data was obtained with STATA14.
Results: BCS was performed in 98/172 (57%) patients. In 83.7% of the patients, the diagnosis corresponds to a mammographic finding, whereas, 16.3% had a palpable lesion.
The mean age was 59.2 years (SD± 11.3). After surgery, anatomopathological result showed: 66.3%(65/98) of DCIS, 24.5%(24/98) of infiltrating ductal carcinoma (IDC). 5 benign cases and 4 without residual tumor were found. Therefore, an underestimation in the diagnosis is observed in almost 25% of the cases.
72% had free margins(67/93), 11.8%(11/93)free but < 2mm, 12.9% (12/93)showed positive margins and 5.4%(5/93)focally affected.
19 re-excisions were carried out, obtaining a re-excision rate of 20%. The result was negative for 78.9%(15/19) of them and DCIS was found in the remained 4 cases.
Regarding lymph node involvement, Sentinel Lymph node biopsy (SLNB) was performed in 43/98 patients, of which one was positive presenting micrometastasis.
91 patients received adjuvant radiotherapy. Hormone therapy, Tamoxifen or Letrozole, was also given to 77 patients.
According to the last check, 81.6%(80/98) of the patients are free of recurrence. The remaining 18.4%(18/98) still have a follow-up < 1year.
Conclusion: Re-excision must be individualized according to: life expectancy, DCIS extension, margin' proximity, residual calcifications or cosmetic impact, given the good long-term rates of local control compare to the consequences: increase in surgical complications, stress, cosmetic compromise or greater risk of mastectomy.
We must become more aware of the possible overtreatment of entities with a good prognosis, such as the DCIS, without forgetting the importance of a precise pre-surgical diagnosis.
Disclosure: Nothing to disclose

EP237

Survival outcome and disease free survival of locally advanced cervical cancer with CT-detected para-aortic lymph nodes

J.K. Abrenica, J. Bagadiong
OB-GYN, Jose R. Reyes Memorial Medical Center, Manila, Philippines

Introduction / Background: In the Philippines, cervical cancer ranks second in the list of cancers among female and the country's second leading cause of female cancer deaths. Majority of the patients seek consult with advanced stage of the disease. In the absence of histologically proven lymph node metastasis, imaging may be used to determine patients with para-aortic lymph node metastasis who may benefit with Extended Field Radiation. The aim of this study is to investigate the survival outcome and disease-free survival of advanced cervical cancer with Computed Tomography (CT) Scan detected para-aortic lymph node (PALN) after concurrent chemoradiotherapy using extended field radiotherapy (EFRT) with weekly platinum-based chemotherapy followed by High Dose Brachytherapy.
Methodology: This is a retrospective study which included patients diagnosed with locally advanced cervical carcinoma according to the FIGO (stage IB2 - stage IIIB) who had CT detected PALN metastasis and underwent EFRT with weekly chemotherapy followed by brachytherapy at a single institution from January 2002 - December 2015. The 3 year over-all survival outcome (OS) and disease free survival (DFS) of the patients were documented and analyzed.
Results: There were 51 patients who satisfied the inclusion criteria and were included in the study. The 3-year OS and DFS rates were 81.3% and 58.6%, respectively. The median survival time was 35.5 months and the median disease free time was 25.04 months.
Conclusion: The current recommendation of EFRT with concurrent chemotherapy followed by brachytherapy can be an effective treatment for cervical cancer patients with CT-detected para-aortic

lymph node metastasis with 81.3% 3 year survival outcome. Patients with Non-Squamous Cell Carcinoma have higher risks of recurrence, with central tumor recurrence being the most common site.
Disclosure: Nothing to disclose

EP238

Paraaortic lymph nodes involvement as a prognostic factor in patients with advanced cervical cancer staged by laparoscopy

N. Agusti, B. Diaz-Feijoo, N. Carreras, E. Mension, B. Gil, J. Pahisa, P. Fuste, A. Glickman, M. Del Pino, A. Torne
Hospital Clinic Barcelona, Barcelona, Spain

Introduction / Background: The aim was to evaluate the impact on survival depending on paraaortic (PA) nodes involvement and size of metastasis in locally advanced cervical cancer (LACC) after staging surgery and its safety before applying chemoradiotherapy treatment
Methodology: We conducted a retrospective study in patients with LACC (FIGO stages from IB2 to IVA) from 2000 to 2016. All patients underwent MRI imaging and a laparoscopic staging PA lymphadenectomy (LSPAL). We performed an accurate ultrastaging of positive PA nodes and classified them in two groups (node metastasis >5 mm vs ≤ 5mm) in order to compare the overall survival (OS). Radiation fields were extended to the PA area only in patients with PA nodes involvement. We evaluated the morbidity and mortality of the surgery.
Results: One hundred eighty three patients were diagnosed as a LACC. One hundred fifty patients had squamous carcinoma and thirty three patients adenocarcinoma. We observed 34 (18.5%) patients with PA nodal involvement (8 patients with PA metastasis measuring ≤ 5 mm and 20 measuring >5 mm). Six patients with lymph node metastasis did not have an ultrastaging study and were excluded from the analysis. Hazar Ratio for OS at 15 years were 2.51 (CI 0.90-6.98) for ≤ 5 mm metastasis (p 0.08) and 2.34 (CI 1.27-4.32) for >5 mm metastasis (p 0.007). Regarding surgical complications, 4 intraoperative events were reported (2,19%) although no perioperative mortality occurred. Thirteen (7.14%) early complications during the first month and 7(3.91%) late complications were reported with no mortality events
Conclusion: The size of the paraaortic lymph node metastasis in patients with LACC has prognostic implications. Overall survival in patients with negative nodes or with metastasis ≤ 5 mm is similar. Paraaortic lymph node staging and extended-field radiotherapy represent a survival benefit in cases with positive nodes ≤ 5 mm. In LACC patients LSPAL seems a safe procedure.

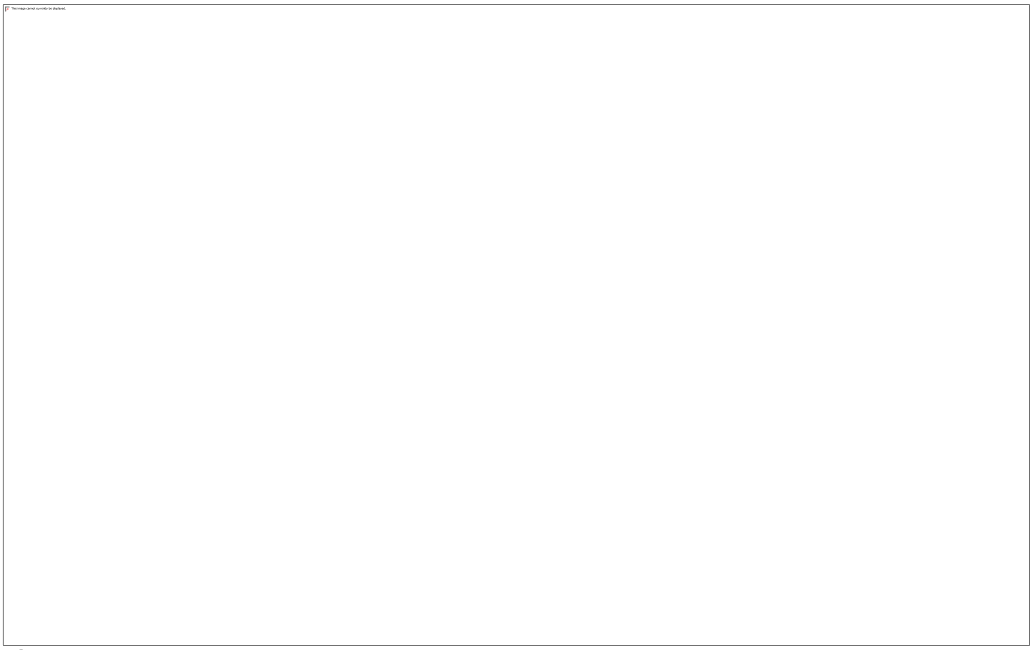

[Size of the paraaortic lymph node metastasis and overall survival]

Disclosure: Nothing to disclose

EP239

Risk factor for the postoperative lymphocele in women with cervical cancer treated with radical hysterectomy and pelvic lymphadenectomy

J.H. Ahn, Y. Jung, K.H. Lee, S.Y. Hur, S.J. Lee
Obstetrics and Gynecology, Seoul St. Mary's Hospital, College of Medicine, The Catholic University of Korea, Seoul, Republic of Korea

Introduction / Background: A lymphocele is a cystic mass that may occur in the retroperitoneum following a pelvic lymphadenectomy. Lymphoceles may be the cause of severe morbidity, or rarely mortality. This retrospective study was aimed to determine risk factor for development of lymphocele after pelvic and/or paraaortic lymphadenectomy in women with cervical cancer.
Methodology: This study was conducted on 547 cervical cancer patients who underwent radical hysterectomy and pelvic and/or paraaortic lymphadenectomy at Seoul St. Mary´s Hospital from 1997 to 2010. We divided them into lymphocele induced and lymphocele free groups, respectively. 6 variables (the number of lymph nodes removed, radiation therapy, operation time, drainage use, BMI, type of energy device) were retrospectively compared between both groups.
Results: Of 547 patients who underwent pelvic lymphadenectomy for cervical cancer, the overall incidence of lymphoceles was 15.5% (85/547) that were detected by computed tomography scan within 8 weeks after operation. To detect the independent risk factor for lymphocele development, we performed multivariate analysis with logistic regression for six variables. Among these variables, we found a significant difference (p=0.029) for only the number of lymph nodes removed. The mean number of lymph nodes removed from the lymphocele group was 55.09 and the mean number of lymph nodes removed from the lymphocele-free group was 51.19. Among 85 lymphocele groups, more than 2 lymphocele were found in 20 patients and the total number of lymphocele was 106. Lymphoceles were predominantly located on the pelvic area. (pelvic wall 54.7%, ext. iliac 26.4%, int. iliac 12.3%, obturator 2.8%, inguinal 2.8%) In only one case, lymphocele was located on the para-

renal area.

	Multivariate-adjusted	
Predictor	OR(95% CI)	*P*
**Number of lymph nodes removed**	1.013 (1.001; 1.025)	**0.029**
Radiation therapy	1.311 (0.728; 2.363)	0.367
Operation time	1.001 (0.993; 1.008)	0.838
Drainage use	1.011 (0.964; 1.059)	0.655
BMI	0.979 (0.909; 1.053)	0.565
Type of energy device	0.651 (0.408; 1.038)	0.071

[Analysis of risk factors for the occurrence of lymphoceles full data set (N=547)]

Conclusion: The number of lymph nodes removed during pelvic and/or paraaortic lymphadenectomy for cervical cancer is a risk factor for the development of lymphocele.
Disclosure: Nothing to disclose

EP240

Predictor factors of Lymph node metastases in cervical cancer

H. Akilli[1], B. Tunc[2], Y.A. Tohma[1], K. Esra[3], A. Ayhan[3]
[1]Gynecology and Obstetrics, [2]Baskent University Faculty of Medicine, [3]Gynecology and Obstetrics, Division of Gynecologic Oncology, Baskent University Faculty of Medicine, Ankara, Turkey

Introduction / Background: The goal of the study was to evaluate risk factors of lymph node metastases ın cervical cancer
Methodology: This retrospective cohort study was done in Baskent University School l of medicine Ankara Turkey.352 cervical cancer patients operated in our clinic was evaluated,all patients underwent radical hysterectomy plus pelvic paraaortic lymhadenectomy
Results: Median age of entire cohort was 50 years (23-81).281 (78,1 %) of cases were SCC ,38 (10,8 %) were adenocancer and and 33 (9,4 %) were adenosquamous cancer. Median tumor size was 3,5 cm (1-10 cm) Of 352 patients 136 (% 37,8) patients had positive lymh node metastases.After univariant analayses ,factors affecting lymh node involvement were subjected to multivariant cox -regression analyses and LVSİ,Parametrial invasion and tumor size were found to be ındependent risk factors .

		95 % CI		P value
Factor	HR	Lower	Upper	
Parametrial involvement	3,07	1,4	6,5	0,003
Tumor size ≥4 cm	1,27	1,01	1,4	0,039
LVSİ	37,7	8,8	160	0,0001

[Multivariant cox-regression analyses of factors affecting lymh node involvement]

.
Conclusion: LVSi ,parametrial invasion and having Tumor size ≥4 cm are the independent factors affecting lymh node metastases in cervical cancer .
Disclosure: Nothing to disclose

EP241

Role of no further treatment after radical hysterectomy in early stage cervical cancer with intermediate risk factors

H. Akilli[1], L. Aksoy Karakaş[1], H. Nihan[2], K. Esra[3], A. Ayhan[3]
[1]Gynecology and Obstetrics, [2]Pathology, [3]Gynecology and Obstetrics Division of Gynecologic Oncology, Baskent University Faculty of Medicine, Ankara, Turkey

Introduction / Background: Radical hysterectomy plus pelvic +- paraaortic lymhadenectomy is the first choice of treatment in early stage cervical cancer and adjuvant treatment is determined according to risk factors.High risk patients (positive surgical margine, positive parametrial invasion, lyph node invoılvement) recieve adjuvant chemoradiotherapy without a doubt .However adjuvant treatment in patients with intermadiate risk factors (tm size ≥ 4 cm ,positive LVSİ and with deep stromal invasion) remains controversial . ESGO, ESTRO and ESP recomend adjuvant radiotheraphy to patients with these risk factors and also offer observation as an option especialy with an experienced team
Methodology: This retrospective cohort study was carried out in Baskent University School of medicine Ankara ,Turkey .All patietns underwent radical hysterectomy plus pelvic ±paraaortic lymhadenectomy ,casese were included with negative lyph node ,negative parametrial invasion and negative surgical margine .Patients with positive LVSİ and Deep stromal İnvasion or LVSİ and tm size ≥ 4 cm or DSİ and tm size ≥ 4 cm or only tm size ≥ 4 cm with minimum 24 months of follow up time were enrolled .
Results: The median age of patients were 50,4 years (23-80 y).66 patients were treated only with surgery and 68 with adjuvant radiotheraphy after surgery. Total recurrence rates were 16,6 % vs 23,5 % respectively p=0,9. Besides each group had similar local recurrence rates .Five year DFS were 80,2 % vs 78,2 % in no adjuvant treatment and radiotheraphy group respectivelly p=0,25.Fİve year OS were 86 % and 88 % p=0,57 . Having tumor size ≥4 cm was found to the only independent factor for recurrence HR 2,426 (1,123 -5,239 with 95,0 Cİ).
Conclusion: No further treatment after radical surgery for early stage cervical carcinoma with intermediate risk factors is a good option with same oncologic outcomes.
Disclosure: Nothing to disclose

EP242

Is the depth of cone related to the endocervical glandular involvement in patients who underwent loop electrosurgical excision procedure or cold knife conization?

S. Akiş[1], Y.E. Purut[1], E. Keleş[1], M. Api[1], C. Kabaca Kocakuşak[1], B. Giray[1], Y. Ceylan[2], M. Güray Uzun[1]
[1]Gynecologic Oncology, Zeynep Kamil Training and Research Hospital, [2]Medicine, Istanbul Medipol University, Istanbul, Turkey

Introduction / Background: The aim of our study was to determine whether endocervical glandular involvement after excisional procedures is related to the depth of the removed conization material.
Methodology: Between April 2016 and December 2019, patients who underwent colposcopy and excisional procedures in the department of gynecologic oncology were investigated retrospectively. Patients with multiple pathology specimen pieces and the patients whose cone depth was not measured were excluded from the study. Age, endocervical curettage (ECC) results during colposcopy, type of excisional procedure (LEEP or CKC), degree of dysplasia in cone material, endocervical glandular involvement and cone depth were evaluated from medical records.
Results: Four hundred thirty patients included in the study. The mean age was 41.3 years. Sixty-one

patients who underwent LEEP and 369 patients who underwent conization were evaluated. Three hundred fifteen (73.2%) patients had no glandular involvement, while 115 patients (26.7%) had glandular involvement. In the analyses, cone depth and endocervical glandular involvement showed statistically significant differences between the patients with LEEP and CKC (p=0,049). Mean cone depth in LEEP group and CKC group were 7.9 mm and 14.5 mm, respectively (p<0,001). The mean cone depth in glandular involvement positive and negative CKC group were 14.94 mm and 14.32 mm, respectively (p=0.54).
Conclusion: The depth of cone was found to be unrelated to determine endocervical glandular involvement. Although LEEP group has lees cone depth and less glandular ınvolvement, the predictive factors for glandular involvement should be determıned in prospective randomized trials.
Disclosure: Nothing to disclose

EP243

Factors that influence surgical margin state in patients undergoing cold knife conization: a single center experience

I. Aluloski[1], M. Tanturovski[1], S. Kostadinova-Kunovska[2], R. Jovanovic[2]
[1]Gynecologic Oncology, University Clinic of Obstetrics and Gynecology, [2]Institute for Pathology, Medical Faculty-Skopje, Skopje, The former Yugoslav Republic of Macedomia

Introduction / Background: To evaluate the factors that influence the surgical margin state in patients undergoing cold knife conization at the University Clinic of Gynecology and Obstetrics in Skopje, North Macedonia.
Methodology: We retrospectively analyzed the medical records of all patients that underwent a cold knife conization at our Clinic in 2015. we cross-referenced the surgical margin state with the histopathological diagnosis (LSIL, HSIL or micro-invasive/invasive cancer), menopausal status of the patients, number of pregnancies, surgeon experience, operating time and cone depth. The data was analyzed with the Chi square test, Fisher's exact test for categorical data and Student's T test for continuous data and univariate and multivariate logistical regressions were performed.
Results: A total of 246 medical records were analyzed. Of those, 29 (11.79%) patients had LSIL, 194 (78.86%) had HSIL and 23 (9.34%) patients had micro-invasive/invasive cervical cancer. The surgical margins were positive in 78 (31.7%) of the patients. The average age of the patients was 41.13 and 35 (14.23%) of the patients were menopausal. The multivariate logistic regression identified preoperative forceps biopsy of micro-invasive SCC, HSIL or higher cone specimen histology and shorter cone depth as independent predictors of surgical margin involvement in patients undergoing cold knife conization.
Conclusion: In the current study, we found no association between the inherent characteristics of the patient and the surgeon and the surgical margin state after a CKC. The most important predictors for positive margins were the severity of the lesion and the cone depth.
Disclosure: Nothing to disclose

EP244

Prevalence and Specific-HPV genotypes distribution among women attending the Ibn Rochd Universitary Hospital in Casablanca: Morocco

B. Amal[1], B. Mustapha[2,3], E.K. Mohamed[2,3], E. Moulay Mustapha[4]
[1]Mohamed VI University of Health Sciences, [2]Gynecology Obstetrics, Faculty of Medicine and Pharmacy, Hassan II University, [3]Oncology, Mohamed VI Oncology Center, Ibn Rochd Hospital, [4]Laboratory of Virology, Microbiology, Quality and Biotechnologies / Biodiversity and Ecotoxicology, Faculty of Sciences and Techniques of Mohammedia, Hassan II University, Casablanca, Morocco

Introduction / Background: HPV infection is strongly associated to cervical cancer. knowledge of the HPV genotypes distribution is requires before vaccination.
Aim: To investigate the HPV prevalence and genotype distribution associated with cervical lesions from women attending the Ibn Rochd Universitary Hospital in Casablanca.
Methodology: A cross-sectional study was conducted and completed with a standardized questionnaire on 121 women aged 21 to 72 years. HPV infection was assessed by nested-PCR using MY09/MY11 and GP5+/GP6+ primers , and direct sequencing. Multiple infections were assessed by type-specific PCR for HPV 6, 11, 16, 18, 31 and 33.
Results: HPV-DNA was detected in 41.1% (95%CI, 35.8-46.5) of the study population. HR-HPV were found in 84.8% of positive samples, of which 15.9% involved multiple-type infections. The five commonest genotypes were HPV16, HPV33, HPV70, HPV18 and HPV31. The prevalence of HPV infection increased with the severity of cervical lesions from 11.7% in N/BRCC to 100% in HSIL/ICC. HPV infection was significant associated with age, number of sexual partners, number of pregnancy, education level and first sexual intercourse age ($p< 0.05$).
Conclusion: Based on the high prevalence of HPV infection found in this study, the necessity of cervical cancer prevention program in Morocco is entirely justified.
Disclosure: Nothing to disclose

EP245

Upstaging of cervical cancer with sentinel lymph node detection: two case reports

J. Amengual Vila[1], A. Torrent Colomer[1], C. Sampol Bas[2], A. Repetto Repetto[2], J. Rioja Merlo[1], O. Córdoba Cardona[1]
[1]Obstetrics and Gynecology, [2]Nuclear Medicine, University Hospital Son Espases, Palma de Mallorca, Spain

Introduction / Background: Lymph node (LN) metastasis is one of the most important prognostic factors of survival among women with cervical cancer (CC). Since October 2018 LN status has become part of the International Federation of Gynaecology and Obstetrics (FIGO) staging.
Benefits of sentinel lymph node (SLN) biopsy include avoidance of morbidity from pelvic lymphadenectomy, and increased detection rate of LN metastases through ultrastaging and removal of SLN in uncommon locations that are not typically included in a standard lymphadenectomy.
Methodology: A retrospective analysis of patients with CC undergoing laparoscopic SLN mapping and frozen section (FS) of SLN biopsy followed by radical hysterectomy or pelvic and/or para-aortic lymphadenectomy, according to the results of the SLN biopsy.
Hybrid technique with indocyanine green ICG-^{99m}Tc nanocoloid was used.
Results: From the introduction of SLN biopsy at our centre in July 2018 to May 2019, 7 patients with clinical FIGO stage IB1/IB2/IIA1 CC underwent SLN detection. Of these, one patient had unilateral SLN detection, while the 6 remaining patients had a bilateral identification of SLN.
Positive SLN was found in 2 patients in the FS analysis. In one case, with negative computed tomography (CT) of pelvic and para-aortic LN involvement, the SLN drained directly into the inframesenteric para-aortic area, and was positive for malignancy. In the other case, the SLN was located by the external left iliac vein.
These 2 patients were upstaged to IIIC1 and IIIC2 and could correctly be triaged to definitive chemoradiotherapy, abandoning radical hysterectomy.
Conclusion: The combination of SLN mapping with hybrid tracer (ICG-^{99m}Tc nanocoloid) and FS seems to have the potential to improve the disease stage with lower morbidity, offering an efficient tool in triaging patients to definitive chemo-radiotherapy. SLN biopsy could upstage patients with presumed early stage cervical cancer and avoid combining radical surgery and chemoradiotherapy due to the high morbidity after combined treatment.

[Figure 1]

[Figure 2]

Disclosure: There are no financial conflicts of interest to disclose in this study.

EP246

Reconsidering the role of ErbB receptors status among young patients with CIN 3 and cervical cancer

G. Michail[1,2], F. Markatos[2], I. Styliara[2], P. Panas[2], D. Koumoundourou[3], P. Ravazoula[3], G. Adonakis[2], G. Androutsopoulos[1,2]
[1]Division of Gynaecological Oncology, Department of Obstetrics and Gynecology, [2]Department of Obstetrics and Gynecology, [3]Department of Pathology, University of Patras, Medical School, Rion, Greece

Introduction / Background: ErbB receptors are trans-membrane glycoproteins with tyrosine kinase activity. Especially in cancer, they are implicated in cell proliferation, transformation, angiogenesis, migration and invasion. The expression of ErbB receptors, has not been studied well in patients with CIN 3 and cervical cancer. The main aim of our study was to investigate the role of ErbB receptors status among young patients with CIN 3 and cervical cancer.
Methodology: We evaluated retrospectively tissue specimens from 75 patients with CIN 3 and cervical cancer, that have been treated in the Division of Gynaecological Oncology of the University of Patras. For ErbB receptors immunostaining, we used: anti-EGFR polyclonal antibody sc-03 (Santa Cruz Biotechnology Inc., Santa Cruz, CA, USA) in a dilution 1:20, anti-ErbB-2 monoclonal antibody CB11 (BioGenex Laboratories Inc., San Ramon, CA, USA) in a dilution 1:100, anti-ErbB-3 polyclonal antibody sc-285 (Santa Cruz Biotechnology Inc., Santa Cruz, CA, USA) in a dilution 1:100 and anti-ErbB-4 polyclonal antibody sc-283 (Santa Cruz Biotechnology Inc., Santa Cruz, CA, USA) in a dilution 1:200.
Results: For ErbB-2 receptor, 8 cases were positive (10.7%) and 67 cases were negative (89.3%).

For ErbB-3 receptor, 24 cases were positive (32%) and 51 cases were negative (68%). For ErbB-4 receptor, 37 cases were positive (49.3%) and 38 cases were negative (50.7%).
Moreover, we found a statistically significant correlation between ErbB-2 expression and invasive cervical cancer.
Conclusion: All these differences in ErbB receptors status among young patients with CIN 3 and cervical cancer could be possibly attributed to well-known alterations in the clinical behavior of both entities. In this perspective, future studies are needed in order to elucidate the clinical role of ErbB receptors status in patients with CIN 3 and cervical cancer.
Disclosure: Nothing to disclose

EP247

Sarcopenia is really prognostic factor of outcome in patients with cervical cancer with concurrent chemoradiotherapy?

Y. Arakaki, Y. Shimoji, T. Nakasone, Y. Taira, T. Nakamoto, T. Ooyama, W. Kudaka, Y. Aoki
Obsterics and Gynecology, Ryukyus University, Nishihara, Japan

Introduction / Background: This study aimed to reveal that pre-treatment sarcopenia is really a prognostic factor of outcome with patients undergoing concurrent chemoradiotherapy (CCRT) in cervical cancer.
Methodology: We retrospectively analyzed pre-treatment CT images of 115 patients with cervical cancer who were treated by same regimen and same irradiation range between 2010 to 2015. All CT images were taken within 30days before radiotherapy. Skeletal muscle index (SMI) was calculated from CT images at the level of third lumbar vertebra, in the range from -29 to +150 Hounsfield Units. We defined sarcopenia as low SMI (less than 38.5cm^2/m^2). Overall survival (OS) and disease free survival (DFS) were analyzed Kaplan-Meier method and Cox hazard regression model.
Results: The median follow-up was 46months(range, 2-104months). 65 patients (56.5%) was defined pre-treatment sarcopenia. The median DFS was 42 months (range, 0-104months). Sarcopenia was not poorer prognostic factor for PFS and OS rates (p=0.4915, and p=0.5811). Multivariate analysis showed same results.
Conclusion: Pre-treatment sarcopenia was not prognostic factor for patients with cervical cancer who treated with CCRT.
Disclosure: Nothing to disclose

EP248

VALHUDES: a protocol for VALidation of HUman papillomavirus assays and collection DEvices for HPV testing on Self-samples and urine samples

M. Arbyn[1], E. Peeters[1], G. Donders[2], P. De Sutter[3], W. Tjalma[4], S. Weyers[5], A. Vorsters[6], S. Van Keer[6], I. Benoy[7], D. Vanden Broeck[7], J.-P. Bogers[7], C. Cocuzza[8], J. Bonde[9], M. Poljak[10], F. Zhao[11], K. Cuschieri[12]
[1]Sciensano, Brussels, [2]Hospital Tienen, Tienen, [3]University Hospital of Brussels, Brussels, [4]University Hospital of Antwerp, Antwerp, [5]University Hospital of Ghent, Ghent, [6]University of Antwerp, [7]Laboratory of Molecular Pathology, Antwerp, Belgium, [8]University of Milano-Bicocca, Monza, Italy, [9]Copenhagen University Hospital, Hvidovre, Denmark, [10]University of Ljubljana, Medical Faculty, Institute of Biochemistry, Ljubljana, Slovenia, [11]Beijing Tongeren Hospital, Beijing, China, [12]Royal Infirmary of Edinburgh, Edinburgh, United Kingdom

Introduction / Background: Systematic reviews have concluded that hrHPV DNA testing using target-amplification tests is as accurate on vaginal self-samples as on clinician-taken specimens for

the detection of cervical precancer. However, insufficient evidence is available for specific HPV assay/self-sample device combinations.
Methodology: The VALHUDES protocol is designed as a diagnostic test accuracy study that aims to compare the clinical sensitivity and specificity of particular hrHPV assay(s) on vaginal self-samples and first-void-urine, collected in agreement with standardized protocols, with hrHPV testing on matched clinician-taken samples. Five hundred enrolled women referred to a colposcopy clinic are invited to collect a first-void urine sample and one or more vaginal self-samples with particular devices before collection of a cervical sample by a clinician. Sample sets are subsequently analysed in a laboratory accredited for HPV testing. Disease verification for all enrolled patients is provided by colposcopy combined with histological assessment of biopsies.
Results: A first VALHUDES study has started in Belgium in December 2017 with enrolment from four colposcopy centres. The following assays are foreseen to be evaluated: RealTi*m*e High Risk HPV assay (Abbott), cobas-4800 and -6800 (Roche), Onclarity (BD), Xpert HPV (Cepheid) and Anyplex II HPV HR (Seegene).
Conclusion: Given empirical evidence that the relative accuracy of HPV-testing on self- vs clinician-samples is robust across clinical settings, the VALHUDES protocol offers a framework for validation of HPV assay/self-sample device combinations that can be translated to a primary screening setting.
Disclosure: A. Vorsters is co-founder and board member of Novosanis NV a spin-off of the University of Antwerp. M. Arbyn, E. Peeters, and M. Poljak are supported by the COHEAHR Network funded by the 7th Framework Programme of DG Research and Innovation, European Commission, Brussels, Belgium (grant No. 603019). M. Arbyn, E. Peeters, K. Cuschieri M. Poljak, and C. Cocuzza are supported by the SME Instrument Grant GA 80551, funded by the Horizon 2020 programme of DG Research and Innovation, European Commission, Brussels, Belgium. I. Benoy, D. Vanden Broeck and J. Bogers are employed by AML, a commercial lab performing cervical cytology and HPV testing.

EP249

Survival in 1B1 cervical cancer treated radical hysterectomy via open or minimal access approaches at a medium sized cancer centre in the United Kingdom

C. Morris, J. Davies, V. Asher, S. Abdul, A. Bali, A. Phillips
Obs and Gyn, University Hospitals of Derby and Burton, Derby, United Kingdom

Introduction / Background: Traditional management of Stage 1B1 cervical cancer has been the radical hysterectomy. Traditionally this was performed via laparotomy (TARH) although increasingly this has been performed via laparoscopy (TLRH). Two trials have demonstrated worse survival in patients undergoing minimal access radical hysterectomies. Our aim was to review the survival outcomes of women who were treated with radical hysterectomies via either open or laparoscopic routes treated at the University Hospitals of Derby and Burton (UHDB).
Methodology: We identified all patients diagnosed with Stage 1B1 Cervical cancer treated with either TARH or TLRH at UHDB with either squamous, adenosquamous and adenocarcinoma histology. A propensity scored analysis was performed to compare 5-year overall survival (OS) and progression-free survival (PFS), and Disease specific survival (DFS) with matching for Grade and Histological Subtype.
Results: 110 patients were identified between 1/11/00-17/8/19. Of these patients 63(57%) underwent TARH and 47(43%) TLRH. OS was 91.2% in those receiving TARH compared to 100% in those receiving TLRH. PFS was 86% in those receiving TARH arm compared to 97.6% in those receiving TLRH. DFS was 94.7% in those receiving TARH arm compared to 100% in those receiving TLRH.
A propensity score was performed to match 47 TLRH patients with 47 TARH patients. Overall survival was greatest for those women who underwent TLRH compared to TARH (100% versus 89% respectively, P= 0.12). PFS was greatest in women who had undergone TLRH (98%) compared to TARH (80%). Women who had undergone TLRH had DFS of 100% compared to 90% of those women who had TARH.
Conclusion: In contrast to two recent studies our data demonstrates a higher 5-year OS and 5 year PFS following TLRH compared to TARH. We wonder if other issues such as laparoscopic surgical technique or differences in tumour biology/patient factors may explain the findings of the LACC trial.
Disclosure: Nothing to disclose

EP250

Hysterectomy of closure after radiotherapy in cancers uterin collar at advance stages (retrospective study)

A. Assal, D. Maazar, M. Serraj, M. Benhessou, M. Ennachit, M. El Karroumi
CHU Ibn Rochd, Casablanca, Morocco

Introduction / Background: The standard treatment of locally advanced cervical cancer is the combination of a concurrent radiochemotherapy brachytherapy. The aime of this study was to evaluate the morbidity, also to determine the post therapeutic prognosis and its factors in disease free survival and overall survival at 2 years.
Methodology: An observational retrospective monocentric cohort concerning 82 patients cared for cervical cancer stage IB2 between 2016-2017 was conducted. All patients included in this study were treated with radiochemotherapy brachytherapy followed by a completion surgery.
Results: In this study, we had patients with epidermoide carcinoma in 93.90% and adenocaricnoma in 4.88%, with advanced stages, IIB (73.17%), IIIB (9.75%), IIA (12.19%), IB2 (1.22%) and IVA (1.22%). All patients recieved radiotherapy with 93.90% of patientes recieving between 41 and 50 Gy. 33 patients (40.24%) recieved a brachytherapy. 74 patientes (90.24%) recieved a chemotherapy (concomitant for 71 patients). The medium period of time between RCC and surgery was 12.5 weeks. The completion surgery was large (ACHE) in 75.61%, with a laparotomy in 63.41%. A lymphadenectomy was done at the same time in 75.61%. Peroperative difficulties were present in 6.10% of cases. Peroperative complications were observed in 16.63% (2.44% vascular lesion, 1.22% visceral lesion, 8.55% bleeding and 2.44% APO), no reanimation, surgical nor radiological treatment was required. None of the patients died in or after the surgery.
Conclusion: Our study found a miner and acceptable rate of per and postoperative complications with no gravity. The analysis of complications risk factors and the prognosis factors suggest that the complete response rate, and higher rate of overall survival and disease free survival could be improved.
Disclosure: Nothing to disclose

EP251

Cervical cancer in HIV-seropositive women

A. Assal[1], S. Moukhliss[1], Y. Benchrifi[1], M. Benhessou[2], M. Ennachit[2], M. El Karroumi[2]
[1]*CHU Ibn Rochd,* [2]*The Division of Gynecologic Oncologic Surgery Mohammed VI Center for Cancer Therapy, CHU Ibn Rochd, Casablanca, Morocco*

Introduction / Background: The aim of our work is to study the epidemiological, clinical, and therapeutic characteristics of cervical cancer in HIV-positive patients under antiretroviral therapy.
Methodology: This is a retrospective study of 13 years extending from 2003 to 2016 in the department of infectious diseases at CHU Ibn Rochd of Casablanca. Were included all patients infected with HIV and presenting a cervical cancer histologically documented. Data were collected from electronic records (Nadis software).
Results: We included 16 cases of invasive cervical cancer. The median age was 41,1 years. The concepts of recurrent STIs and active smoking were found in all patients. Cervical cancer has occurred to the waning of therapeutic followed in the majority of cases. At the time of cancer diagnosis, all patients were immunohierological success. This was squamous cell carcinoma in 7 cases, 4 had stage IIIB and 3 cases in stage II Bd. Five patients were treated with radiotherapy alone. Five patients died by peritoneal carcinomatosis with liver metastasis.

Conclusion: Cervical cancer screening must be systematic for any woman infected with HIV. The rythm of screening should be annual, and the availability and accessibility of screening can have a positive impact on reducing the prevalence and progression of precancerous cervical lesions by providing early access to appropriate care.
Disclosure: Nothing to disclose

EP252

Para-aortic lymph node involvement revisited in the light of the revised 2018 FIGO staging system for cervical cancer: a retrospective, dual-institutional study

A. Ayhan[1], K. Aslan[2], M. Oz[3], Y.A. Tohma[1], E. Kuscu[1], M.M. Meydanli[2]
[1]Obstetrics and Gynecology, Baskent University Faculty of Medicine, [2]Gynecologic Oncology, Zekai Tahir Burak Women's Health Training and Research Hospital, Faculty of Medicine, University of Health Sciences, [3]Gynecological Oncology Unit, Meir Medical Center, Ankara, Turkey

Introduction / Background: This dual-institutional, retrospective study aimed to determine the clinicopathological risk factors for para-aortic lymph node (LN) metastasis among women who underwent radical hysterectomy with systematic pelvic and para-aortic lymphadenectomy for 2009 FIGO stage IB1-IIA2 cervical cancer.
Methodology: Institutional cervical cancer databases of two high-volume gynecologic cancer centers in Ankara, Turkey were retrospectively analyzed. Those featured in the study included women with 2009 FIGO stage IB1-IIA2 disease who underwent radical hysterectomy with pelvic and para-aortic lymphadenectomy between January 2006 and December 2018. We analyzed the patient data and investigated all potential predictive factors for para-aortic LN metastasis.
Results: A total of 522 women met the inclusion criteria. Pelvic LN metastasis was detected in 190 patients (36.4%); para-aortic LN metastasis in 48 patients (9.2%), isolated para-aortic LN metastasis in 4 (0.8 %), and both pelvic and para-aortic LN metastasis in 44 (8.4 %) women, respectively. The independent risk factors identified for para-aortic LN involvement included parametrial invasion (odds ratio [OR]: 3.57, 95% confidence interval [CI]: 1.65-7.72; p=0.001), metastasized pelvic LN size >1 cm (OR: 4.51, 95% CI: 1.75-11.64; p=0.002), multiple pelvic LN metastases (OR: 3.83, 95% CI: 1.46-10.01; p=0.006), and common iliac LN metastasis (OR: 2.97, 95% CI: 1.01-8.68; p=0.04). A total of 196 (37.5%) patients exhibited at least one risk factor for para-aortic nodal disease.
Conclusion: Parametrial invasion, metastasized pelvic LN size>1 cm, multiple pelvic LN metastases, and common iliac LN metastasis seem to be independent predictors of para-aortic LN involvement.
Disclosure: Nothing to disclose

EP253

Is the revised 2018 FIGO staging system for cervical cancer associated with increased discrimination in prognosis for women previously staged as IB disease? A retrospective, dual-institutional study

A. Ayhan[1], K. Aslan[2], A.N. Bulut[1], H. Akilli[1], M. Oz[2], A. Haberal[1], M.M. Meydanli[2]
[1]Obstetrics and Gynecology, Baskent University Faculty of Medicine, [2]Gynecologic Oncology, Zekai Tahir Burak Women's Health Training and Research Hospital, Faculty of Medicine, University of Health Sciences, Ankara, Turkey

Introduction / Background: The purpose of this study was to validate the 2018 International Federation of Gynecology and Obstetrics (FIGO) staging system for cervical cancer.
Methodology: Institutional cervical cancer databases of two high-volume gynecologic cancer centers in Ankara, Turkey were retrospectively analyzed. Only women with 2009 FIGO stage IB1 or IB2

disease that underwent radical hysterectomy with pelvic and para-aortic lymphadenectomy were included. Survival curves were generated using Kaplan-Meier plots, and the log-rank test was used for survival comparisons. The Cox proportional hazards regression model was used to obtain hazard ratios (HRs) and 95% confidence interval (CI).
Results: Data from 425 women were analyzed. Stage migration was observed in 372 (87.5%) patients. According to the 2018 FIGO staging system, there was no significant difference in overall survival rates between stage IB1 (n=53) and IB2 (n=127) disease (95.2% vs. 89.3%, respectively; p=0.23) as well as between stage IB2 (n=127) and IB3 (n=95) disease (89.3% vs. 84.2%, respectively; p=0.12). Similarly, we were not able to demonstrate a significant difference in overall survival rates between stage IIIC1 (n=114) and IIIC2 (n=36) disease (79.0% vs. 67.2%, respectively; p=0.34).
Conclusion: The revised FIGO staging system seemed to reflect OS adequately as there was a clear statistical tendency for poorer OS rates with increasing stage. However, its prognostic discrimination was not statistically significant. The 2018 FIGO staging system seems to suffer from increased number of sub-stages leading to limited number of patients in each sub-stage which results in diminished statistical power.
Disclosure: Nothing to disclose

EP254

The prognostic value of lymph node ratio in stage IIIC cervical cancer patients triaged to primary treatment by radical hysterectomy with systematic pelvic and para-aortic lymphadenectomy: a retrospective, dual-institutional study

K. Aslan[1], M.M. Meydanli[1], M. Oz[1], Y.A. Tohma[2], A. Haberal[1], A. Ayhan[2]
[1]Gynecologic Oncology, Zekai Tahir Burak Women's Health Training and Research Hospital, Faculty of Medicine, University of Health Sciences, [2]Obstetrics and Gynecology, Baskent University Faculty of Medicine, Ankara, Turkey

Introduction / Background: The aim of this study was to determine the prognostic value of lymph node ratio (LNR) in women with 2018 FIGO stage IIIC cervical cancer.
Methodology: In this retrospective dual-institutional study, a total of 185 node-positive cervical cancer patients who had undergone radical hysterectomy with systematic pelvic and para-aortic lymphadenectomy were included. All of the patients received adjuvant chemoradiation after surgery. LNR was defined as the ratio of positive lymph nodes to the total number of lymph nodes removed. The patients were categorized into two groups according to LNR; LNR < 0.05 and LNR≥0.05. The prognostic value of LNR was evaluated with univariate log-rank tests and multivariate Cox regression models.
Results: A total of 139 patients (75.1%) had stage IIIC1 disease and 46 (24.9%) patients had stage IIIC2 disease. With a median follow-up period of 45.5 months (range 3-135 months), the 5-year disease free survival (DFS) rate was 62.5% whereas the 5-year overall survival (OS) rate was 70.4% for the entire study population. The 5-year DFS rates for LNR < 0.05 and LNR ≥0.05 were 78.2%, and 48.4%, respectively (p< 0.001). Additionally, the 5-year OS rates for LNR < 0.05 and LNR ≥0.05 were 80.6 %, and 61.2 %, respectively (p=0.007). On multivariate analysis, LNR ≥0.05 was associated with a worse DFS (HR 2.13, 95% CI 1.21-3.72; p=0.008) and OS (HR 1.95, 95% CI 1.01-3.37; p=0.04) in women with stage IIIC cervical cancer.
Conclusion: LNR ≥0.05 seems to be an independent prognostic factor for decreased DFS and OS in stage IIIC cervical carcinoma.
Disclosure: Nothing to disclose

EP255

Conditional relative survival of cervical cancer: a Korean central cancer registry study

J. Bae
Gynecologic Oncology, Hanayang University, Seoul, Republic of Korea

Introduction / Background: Conditional relative survival (CRS) can complement the conventional 5-year relative survival that does not take into account the time the patient has survived after diagnosis. The purpose of this study was to evaluate the 5-year CRS and associated risk factors among Korean cervical cancer patients.
Methodology: We identified 78,606 cervical cancer cases with a diagnosis between 1996 and 2015 in the Korea Central Cancer Registry. The CRS rates were calculated according to age, history, treatment method and stage at diagnosis.
Results: The relative survival rate for five years was 80.6 %, and for five years the CRS increased for the first two years and remained stable at 94.3 % after five years of survival. Patients under the age of 40 had the highest level (88.9 percent) in five years, while those aged 70 or older had the worst CRS (55.0 percent) in five years. The localized disease had 91.9 % CRS, local transferency had 72.3 % CRS, and remote transitions were 27.0 %.
Conclusion: Patients with cervical cancer showed an increase in CRS rates, which varied with age, histology, and stage at diagnosis. The CRS analysis therefore provides a more detailed perspective on survival over the years after the initial diagnosis or treatment.
Disclosure: Nothing to disclose

EP256

Inhibition of phosphatidylinositol 3-kinase (PI3K) signaling synergistically potentiate antitumor efficiency of paclitaxel and overcome paclitaxel-mediated resistance in cervical cancer

M.W. Baik, Y.J. Choi, S.Y. Hur
Department of Gynecology and Obstetrics, Seoul St. Mary's Hospital, The Catholic, Seoul, Republic of Korea

Introduction / Background: Paclitaxel (PTX) is used as an effective treatment against advanced cervical cancer. However, acquired resistance limits its success and results in cancer progression. We investigated whether the inhibition of phosphatidylinositol 3-kinase (PI3K) signaling overcomes paclitaxel resistance in cervical cancer.
Methodology: Two human cervical cancer cell lines (Me180 and HeLa) and two PI3K inhibitors (BYL-719 and LY294002) were used. We established paclitaxel-resistant cell lines (PTX-R Me180/PTX-R HeLa) and confirmed inhibitory concentrations (IC)50 for each chemicals and combination index (CI) for paclitaxel and PI3K inhibitors by tetrazolium dye (MTT). Flow cytometry with Annexin/PI double or PI single staining were used to detect cell cycle and apoptosis. Migration and invasive abilities were explored by wound healing and transwell assays. Meanwhile, genes related to multiple pathways were assessed by western blot.
Results: PTX-R Me180 and PTX-R HeLa cells were gradually established from parental cell lines by stepwise exposure to paclitaxel over 7 months and with stable resistance at under 20 nM. We found that PI3K pathway was significantly activated in paclitaxel-resistant Hela and Me180 cells compared to its parental cells ($p<0.01$). PTX + PI3K inhibitors） combinatorial therapy potentiated synergistic effect to strengthen paclitaxel induced S and G2M arrest in paclitaxel resistance subline cells by inactivation of cyclin A1(CCNA1), cyclin B1(CCNB1), cyclin E (CCNE), cdc2 expression. Moreover, the combinatory therapy significantly enhanced drug sensitivity and apoptosis through the activation of Bax, cleaved-caspase-9, and cleaved poly ADP ribose polymerase (PARP) compared with paclitaxel alone. In addition, PI3K inhibition also suppressed tumor migration and invasive ability by targeting β-catenin, p-c-raf, matrix metalloproteinase-2(MMP-2) and vascular endothelial cell growth factor (VEGF).
Conclusion: We suggest that a combination of a PI3K inhibitor with paclitaxel may enhance antitumor activity through a cascade of PI3K signaling events.
Disclosure: Nothing to disclose

EP257

Early stage cervical cancer treated by preoperative brachytherapy: prognostic factors ans clinical outcomes

B. Bataille, A. Escande, F. Le Tinier, A. Parent, E. Bogart, P. Collinet, L. Bresson, F. Narducci, E. Leblanc, A.-S. Lemaire, S. Taieb, E. Lartigau, X. Mirabel, A. Cordoba
Centre Oscar Lambret, Lille, France

Introduction / Background: Optimal management of localized cervical cancer is still controversial. The purpose of this study is to report our experience regarding early stage cervical cancer treated with preoperative brachytherapy (BT) and modified hysterectomy (HT).
Methodology: From 2000 to 2013, consecutive patients treated for early stage cervical cancer by preoperative BT and HT were enrolled. They underwent pelvic lymph node assessment by lymphadenectomy prior to BT. Pulse dose rate brachytherapy was performed with either 2D or 3D technique 2D technique. BT aimed to deliver 60 Gray to the clinical target volume, with limited dose to the surrounding tissues, according to GYN-GEC-ESTRO guidelines. Modified HT was performed six to eight weeks after BT.
Results: 80 patients were enrolled. 90% had FIGO IB1 stage. Median clinical tumor size was 25mm (0-40). 61 patients (76%) had a histological complete response. For those with incomplete response, residual tumor was located at the cervix and only one patient had lymphovascular invasion. With a median follow-up of 6.7 years, 16 patients (20%) presented a tumor relapse, with 2 local relapse (3%), 11 regional recurrence, and 9 metastatic relapse. 5-year disease-free survival (DFS) rate and 5-year overall survival (OS) rate were 82% (95% confidence interval [CI]: 71-89) and 88% (95% IC: 78-94) respectively. 24 patients (44%) had grade 2 late toxicity and 10 (13%) had grade 3 late toxicity; with no grade ≥ 4 toxicities. In univariate analysis, poorer DFS was associated with overweight status (≥25 kg/m^2, hazard ratio [HR]: 3.05, 95% CI: 1.20-7.76, p=0.019) and MRI tumor size of >3 cm (HR: 3.05, 95% CI: 1.23-7.51, p=0.016).
Conclusion: Preoperative BT followed by HT may be safe and effective for patients with early stage cervical cancer. Since poorer outcomes were associated with overweight and tumor size >3cm, more agressive treatment should be considered for those patients.
Disclosure: Nothing to disclose

EP258

Long - term outcomes of laparoscopic radical hysterectomy (type III PIVER) for early stage cervical cancer (IA1-IB1 FIGO)

I. Berlev, O. Smirnova, P. Sorokin, S. Sorokina, E. Ulrikh, A. Sidoruk, H. Mkrtchyan, Z. Ibragimov, Y. Trifanov, H. Kotiv, O. Lavrinovich, N. Bondarev, N. Mikaya, A. Urmancheeva
National Medical Research Center of Oncology named after N.N.Petrov, Saint-Petersburg, Russian Federation

Introduction / Background: Minimally invasive surgery was considered as an alternative to laparotomy (open surgery) for radical hysterectomy in patients with early-stage cervical cancer with a similar survival rate. The aim of this study is to evaluate the long-term outcomes of laparoscopic radical hysterectomy (type III Piver) for early stage cervical cancer (IA1-IB1 FIGO).
Methodology: The retrospective study included data of 60 patients with early stage cervical cancer (IA1 - IB1, tumor size ≤ 2cm), who underwent laparoscopic radical hysterectomy (type III by Piver) during the period from 2012 to 2016 at the oncogynaecological department of N.N. Petrov National Medical Research Center of Oncology. The primary endpoint - 4,5 - years disease-free survival.
Results: The median follow-up was 44 months (from 25 to 72 months). A 4.5-years disease-free survival was 94.5%. All patients included in the study were alive at the time of the assessment, 95% (57 patients) without signs of the disease, 5% (3 patients) developed a relapse.

Conclusion: Long-terms outcomes of laparoscopic radical hysterectomy are acceptable for the continuation of such type of surgical intervention. Laparoscopic approach seems to be an effective and safety option for early stage cervical cancer treatment, but the survival rate need to be analyzed in randomized studies.
Disclosure: Nothing to disclose

EP259

Comparison of clinical outcomes of squamous cell carcinoma and adenocarcinoma of the uterine cervix in a Portuguese oncological referral center

C. Reis de Carvalho[1,2], M. Cardoso[2,3], M. Bernardino[2], A.F. Jorge[2]
[1]Centro Hospitalar Universitário de Lisboa Norte, EPE, [2]Instituto Português de Oncologia Franscisco Gentil, Lisboa, [3]Hospital do Divino Espírito Santo de Ponta Delgada, EPE, Ponta Delgada, Portugal

Introduction / Background: Some authors reported that there are clinicopathological differences between squamous cell carcinoma (SCC) and adenocarcinoma (ADC) of the uterine cervix. On the other hand, current studies have failed finding significant differences in survival between the two groups. Still the controversy remains. The purpose of this study was to compare clinical outcomes of squamous cell carcinoma with those of adenocarcinoma of the cervix in our population.
Methodology: We conducted a retrospective analysis of all women with a primary diagnose of squamous cell carcinoma and adenocarcinoma of the uterine cervix, from 2013 to 2014, in a Portuguese oncological referral center. Cases were identified by searching for ICD-9 codes and data was collected through electronic medical records. Our main outcome was overall survival (OS) and recurrence-free survival (RFS). Survival was evaluated using the Kaplan-Meier curves and compared with log-rank test before and after propensity score matching (1:1).
Results: We included a total of 302 cases: 258 (85,4%) of squamous cell carcinoma and 44 (14,6%) of adenocarcinoma. We found differences in patients characteristics between SCC and ADC regarding medium age (61,3 years vs 56,4 years, p=0,02) and initial disease stage (FIGO I-II) at diagnosis (71,2% vs 64,6 %, p=0,034). The 5-year OS and RFS in the SCC group and adenocarcinoma group were respectively 86.2 and 77.4% (p=0.005). After matching, OS and RFS for patients with AC did not significantly differ from those with SCC (p=0,06 and p=0,12).
Conclusion: In our population, both patients with SCC and ADC, with similar baseline characteristics, have no differences in overall and recurrence-free survival. However, it remains controversial if the histologic subtype is an independent prognostic factor. For more conclusive results, more studies focusing adenocarcinoma are needed, in order to understand the differences between these patients and its impact on the natural history of the disease.
Disclosure: Nothing to disclose

EP260

Is there still a role for laparoscopic radical hysterectomy? Personalizing surgical approach in early stage cervical cancer

L. Pedone Anchora[1], N. Bizzarri[1], V.A. Capozzi[2], V. Gallotta[1], V. Chiantera[3], F. Cosentino[4], A. Lombisani[1,5], G. Scambia[1,5], G. Ferrandina[1,5]
[1]Department of Woman and Child Health and Public Health, Woman Health Area, Fondazione Policlinico Universitario A. Gemelli IRCCS, Rome, [2]Department of Gynecology and Obstetrics, University of Parma, Parma, [3]Department of Gynecologic Oncology, University of Palermo, Palermo, [4]Department of Gynecologic Oncology, Giovanni Paolo II Research and Treatment Foundation, Campobasso, [5]Catholic University of the Sacred Heart, Institute of Obstetrics and Gynecology, Rome, Italy

Introduction / Background: To compare survival outcomes of laparoscopic and open surgery for radical hysterectomy (RH) among patients with early-stage cervical cancer (CC) and to identify which subgroups may benefit from one approach rather than the other.
Methodology: 237 and 303 consecutive patients, with clinical FIGO stage from IA1 with lymph vascular space involvement to IB1/IIA1 CC underwent open and laparoscopic RH respectively in 3 Italian Institutions. Differences in terms of progression-free survival (PFS) between the two surgical approaches were tested in the entire population and in different patient's subgroups.
Results: Median follow up was 34 months. Open and laparoscopy procedures had similar 3-yr PFS rate in the entire series (86.4% vs 85.6%, p=0.987). PFS of the two approaches were compared among subgroups stratified by tumor histology, tumor grading, tumor diameter, lymph vascular space status, parametrial invasion, lymph node status and adjuvant treatment.Open and laparoscopic approaches revealed a significantly different 3-yr PFS rate only among patients with >20mm tumor (85.2% vs 69.2% respectively, p=0.026). On the contrary 3-yr PFS in ≤ 2cm tumor did not differ between open and laparoscopic surgery (87.5% vs 93.4% respectively, p=0.151).
Conclusion: Tumor diameter could be considered the most important determinant to choosing to guide the choice of surgical approach in early stage CC. Women with > 20mm disease should undergo open RH. In case of tumor ≤ 20mm, both of the approaches appear to be safe. Therefore, laparoscopic surgery could still be considered an option in this subgroup of patients.
Disclosure: Nothing to disclose

EP261

Cervical cancer tumor size evaluated by preoperative estimation and final pathology report after radical operative procedure and the correlation to survival

S.F. Bjørn[1], C.K. Høgdall[1], T.H. Schnack[2]
[1]Copenhagen University Hospital | Gynecology, [2]Gynecology, Rigshospitalet, Copenhagen, Denmark

Introduction / Background: Tumor-size is an important factor in the managing of early cervical cancer (early-CC). We aimed to evaluate the correlation between the predicted preoperative tumor size and the histologic tumor size and to examine the impact of tumor size on survival.
Methodology: Data on early-CC was retrieved from the Danish Gynecologic Cancer Database (DGCD) and validated against patient files early-CC patients (N=479) treated by radical trachelectomy (n=182) or radical hysterectomy (n=297) at University Hospital Copenhagen 2005-2018. Categorical variables were evaluated by Chi-Square test. Survival estimates were evaluated using in Kaplan Meier or Cox regression analyses.
Results: Among patients treated by radical trachelectomy or hysterectomy 91.4% and 73.4 % of tumors were correctly predicted to ≤2 cm, while 50% and 75.5% of tumors were correctly categorized to > 2 cm preoperatively as compared to the finale pathological examination
(Table 1).
In univariate analysis the cancer specific survival differed significantly according to predicted as well as histologic tumor size p-value=0.03 and p=0.0001, respectively. In adjusted Cox analysis histologic tumor size (size ≤ 2 cm vs. > 2 cm) was associated with a decrease in the cancer specific survival (HR = 5.67; 95% CI:1,78 - 18.09). No significant association was found with preoperative predicted tumor size and cancer specific survival.
Conclusion: In patient with trachelectomy tumor size ≤ 2 cm mainly based on cone biopsies are successfully predicted in 91.4% Whereas the prediction of tumor size in patients referred to radical hysterectomy ≤ 2 cm or > 2 is less precise: 73.4 % and 75.5%, respectively. Increasing histologic tumor size was associated with a decrease in cancer specific survival.
Imaging like MR-scans may improve the preoperative prediction of tumor size in patients referred to radical hysterectomy and thus, the handling and treatment of early-CC.

Histologic tumor size	Radical trachelectomy	Radical hysterectomy	Cancer specifik

							survival
	<= 2 cm n(%)	< 2 cm n(%)	P value	<= 2 cm n(%)	> 2 cm n(%)	P value	HR (95%CI)
<= 2 cm n (%)	159 (91.4)	15 (8.6)	0.014	130 (73.4)	47 (26.6)	0.0001	ref
> 2 cm n(%)	3 (50)	3 (50)		25 (24.5)	77 (75.5)		5.67 (1.78-18.1)
Total	162	18		155	124		

[Table 1 correlation between predicte clinical tumor size and tumor size at the finale pathological examination]

Disclosure: Nothing to disclose

EP262

Primary conization overcomes the risk of local recurrence following laparoscopic radical hysterectomy in early-stage cervical cancer

G. Bogani, A. Ditto, V. Chiappa, F. Raspagliesi
Fondazione IRCCS Istituto Nazionale dei Tumori di Milano, Milano, Italy

Introduction / Background: Accumulating data highlighted that minimally invasive surgery has been associated with higher recurrence rates and worse overall survival than the open approach in women with early-stage cervical cancer. Tumor spread at the time of colpotomy might be advocate as the reason of tumor cells dissemination in the pelvic area. Here, we aimed to investigate if primary conization might overcome the risk of local dissemination in early-stage cervical cancer undergoing laparoscopic radical hysterectomy.
Methodology: Consecutive data of 262 early stage cervical cancer patients were retrieved: 88 women had conization followed by radical hysterectomy. A propensity-matched comparison (1:1) was carried out in order to compare laparoscopy and open surgery, minimizing possible selection biases.
Results: Data of 35 patients´ pair (total 70 patients) were analyzed. No between-group differences in baseline, disease and pathological variables were observed (p>0.20). Patients undergoing laparoscopy experienced a slightly non-significant longer operative time than patients undergoing open surgery (210.8 (±47.7) vs. 187.9 (±24.7) minutes; p=0.089); while laparoscopic approach correlated whit lower blood loss (50 (range, 30-100) vs. 150 (range, 50-500) ml; p< 0.001) and shorter length of stay (3 (±0.8) vs. 5.4 (±1.4) days; p< 0.001) in comparison to open surgery. Morbidity rate was similar between groups (p=1.00). One local recurrence was observed per group (p=1.00). Type of surgical approach did not influence site of recurrence (p=1.00) as well as survival outcomes, in term of 10-year disease-free (p=0.549, log-rank test) and overall survivals (p=0.615, log-rank test).
Conclusion: Our data seems underline that primary conization might overcome the risk of local recurrence following laparoscopic radical hysterectomy in early-stage cervical cancer. Further prospective evidence is needed.
Disclosure: Nothing to disclose

EP263

Laparoscopic surgery improves short- and medium-term outcomes of nerve-sparing radical hysterectomy: a propensity-matched analysis with open abdominal procedures

G. Bogani, V. Chiappa, A. Ditto, F. Raspagliesi
Fondazione IRCCS Istituto Nazionale dei Tumori di Milano, Milano, Italy

Introduction / Background: To investigate the impact of laparoscopic surgery on short- and medium-term outcomes of cervical cancer patients undergoing nerve-sparing radical hysterectomy
Methodology: Data of consecutive patients affected by locally-advaced cervcial cancer who had neoadjuvant chemotherapy followed by laparoscopic nerve-sparing radical hysterectomy were matched 1:1 with an historical cohort of patients undergoing neoadjuvant chemotherapy followed by open radical hysterectomy, using propensity matching algorithm.
Results: Thirty-five patients´ pairs (70 patients: 35 undergoing laparoscopic vs. 35 undergoing open abdominal nerve-sparing radical hysterectomy) were included. Demographic and baseline oncologic characteristics were balanced between groups. Patients undergoing laparoscopic surgery had similar operative time than patients undergoing open abdominal procedures (249 [±91.5] vs. 223 [±65.0] minutes; p=0.066). Laparoscopic approach correlated with lower blood loss (30.5 [±11.0] vs. 190 [90.4] mL; p< 0.001) and shorter hospital stay (3.2 [±1.2] vs. 5.4 [2.0] days; p=0.023). Patients undergoing laparoscopy experienced a lower 30-day pelvic floor dysfunction rate than patients having open surgery (p=0.024). Moreover, they experienced shorter recovery of bladder function than patients having open procedures (median, 7 vs. 9 days; p=0.004, log-rank test). After a median follow-up of 51.7 and 14.7 months for open abdominal and minimally invasive procedures, disease-free (p=0.617) and overall (p=0.814) survivals were similar between groups. Using multivariate model, we observed that the adoption of laparoscopic approach did not impact on disease-free (HR:1.32 (95%CI: 0.58, 3.01);p=0.50) and overall (HR:1.26 (95%CI: 0.41, 3.81);p=0.67) survivals.
Conclusion: Laparoscopic nerve sparing radical hysterectomy resulted in improved short-term outcomes, without impacting on medium-term oncologic outcomes. Further prospective trials are needed to assess long-term outcomes of patients having minimally invasive surgery
Disclosure: Nothing to disclose

EP264

Cervical cancer starring in the love story of brachytherapy planning techniques in an alluring tertiary cancer center

D.-C. Pop[1], T. Kirsch[1], E.-A. Bonci[1,2], D. Portik[1], C. Ordeanu[1], O. Coza[1,2], A. Irimie[1,2], P. Achimaș-Cadariu[1,2], V. Nagy[1,2]
[1]Prof. Dr. Ion Chiricuţă Institute of Oncology, [2]Iuliu Haţieganu University of Medicine and Pharmacy, Cluj-Napoca, Romania

Introduction / Background: After the published guidelines of Group Européen de Curiethérapie and European Society for Radiotherapy and Oncology (GEC-ESTRO) for tridimensional image-guided brachytherapy, many cancer centers have started abandoning the bidimensional conventional brachytherapy (2D-BT). The purpose of this study was to analyze the transition from 2D-BT to tridimensional computed tomography-guided brachytherapy (CT-BT) and its effects on target and organs at risk in cervical cancer patients treated in our institution.
Methodology: We performed a retrospective analysis of 308 patients diagnosed with cervical cancer between 2016 and 2018. All patients were treated with primary chemoradiation, followed by CT-BT. For each patient we used a tandem-ring applicator and retrospectively realized one 2D-BT plan with dose prescribed in International Commission on Radiation Units and Measurements (ICRU) point A and equal dwell times and a second plan with dose prescribed to the high-risk clinical target volume (HR-CTV) and different dwell times.
Results: When prescribed to ICRU reference points, the mean dose to point A was 99.88% of the prescription dose (PD), the mean ICRU bladder and rectum point dose was 102.38% and 100.79% of PD, respectively. When prescribed to HR-CTV, the mean dose to irradiated volume was 96.6% of the PD, mean D2cc for bladder and rectum was 86.76% and 65.58% of PD, respectively. Mean volume of 100% dose was 106.5cc vs 78.25cc when prescribing to point A vs HR-CTV.
Conclusion: The study showed a reduction of the irradiated volume by 23.3% when the prescription of dose was done to HR-CTV compared to point A, the D2cc for bladder and rectum were also lowered. In conclusion, the old and never-ending love story of 2D-BT and CT-BT has to come to an

end in our institution, in order for the tridimensional image-guided brachytherapy techniques to write their own story and for cervical cancer to stop getting the principal role.
Disclosure: Nothing to disclose

EP265

The role of methylation silencing of tumor suppressor genes in cervical HSIL: a prospective cytologic-histologic correlation study of 70 cases

O. Ondic[1,2], J. Bouda[3], J. Nemcova[1,2], I. Kinkorova-Lunackova[2], J. Chytra[4], J. Presl[3], R. Alaghehbandan[5], K. Cerna[2], B. Gomolcakova[2], J. Kostun[4], H. Sidlova[6], P. Vlasak[4], O. Majek[7]
[1]Dept of Pathology, Faculty of Medicine and University Hospital in Pilsen, Charles University in Prague, [2]Bioptical Laboratory, [3]Dept of OBGYN, [4]Faculty of Medicine and University Hospital in Pilsen, Charles University in Prague, Plzen, Czech Republic, [5]Dept of Pathology, University of British Columbia, Royal Columbian Hospital, Vancouver, BC, Canada, [6]Cytopathos, s.r.o., Bratislava, [7]Institute of Biostatistics and Analyses, Masaryk University, Brno, Czech Republic

Introduction / Background: Methylation silencing of tumor suppressor genes has been suggested as one of the epigenetic changes promoting carcinogenesis. The aim of this study was to prospectively evaluate methylation status of CADM 1, MAL, hsa-miR-124 genes in HSIL (high grade squamous intraepithelial lesion) LBC (liquid based cytology) samples with histologic correlation.
Methodology: 70 histologically confirmed cases of HSIL paired with prior screening LBC diagnosis of HSIL within 3 months interval were selected. Histologically, the lesions were reviewed and assessed including 1) number of blocks harboring dysplastic squamous epithelium, 2) number of blocks containing glandular extension of dysplastic epithelium, and 3) the depth of glandular extension (which was assessed semi-quantitatively as graded 1-3). From residual LBC materials, HPV subtyping was performed, using LINEAR ARRAY HPV Genotyping Test and in house PCR targeting HPV E1 gene. Detection of methylation silencing of tumor suppressor genes CADM1, MAL, and hsa-miR-124 was performed by multiplex methylation specific real-time PCR.
Results: Positive methylation status was detected in 41 cases (58.6%). Number of blocks with HSIL varied from 1 to 13. Glandular extension was seen in 44 cases with number of blocks involved ranging from 1 to 10. The depth of HSIL glandular extension varied.
Conclusion: Methylation status in cervical HSIL does not correlate with the size of the lesion (measured by the number of blocks involved) or with HSIL propensity for endocervical glandular extension, nor with HPV type or multi-infection.
Disclosure: The study was partially supported with grant provided by the Ministry of Health of the Czech Republic - Conceptual Development of Research Organization (Faculty Hospital in Pilsen - FNPl, 00669806). There are no any other financial disclosures from the authors.

EP266

Cervical cancer in pregnancy - a clinical case

R. Branco, T. Diniz da Costa, J. Silva Pereira
Ginecology, Hospital Prof. Doutor Fernando Fonseca, Amadora, Portugal

Introduction / Background: Cervical cancer is one of the most common malignancies in pregnancy and frequently diagnosed at an early stage.
Vaginal bleeding is the most frequent symptom, but is dependent upon the clinical stage and lesion size.
Methodology: .
Results: 29-year-old caucasian woman, 13 weeks pregnant (gravida 3 para 1), went to the

emergency department due to vaginal bleeding. Pelvic examination revealed a polypoid cervical tumor, that was excised, sent to histologic review. The patient was referred to colposcopy unit. At 25 weeks, returned to the emergency department with the same symptoms. At this time, pelvic examination unveiled a cervical mass on the posterior lip of the cervix. A pelvic image confirmed a 3cm cervical lesion, without parametrial invasion. Colposcopic evaluation showed a suspicious high-grade disease, and excisional biopsy reported poorly differentiated invasive squamous cell carcinoma, identical to the first biopsy**.** Because the patient explicitly expressed the wish to preserve the pregnancy, an elective cesarean was performed at 34 weeks of gestation. A newborn was delivered with 2120g and Apgar index of 5/6/10.
Five weeks after the delivery, a new colposcopic evaluation showed a suspicious high-grade disease, and the excisional biopsy revealed an high-grade squamous intraepithelial lesion. Laparoscopic radical hysterectomy with ovarian preservation and pelvic lymphadenectomy followed 1 moth later. Histological report disclosed a cervical squamous cell, with glandular differentiation and sarcomatoid carcinoma, margins free of disease, negative parametria and pelvic lymph nodes, compatible with FIGO stage IB1. The patient completed external radiation therapy, and now is under 6 months of follow-up.
Conclusion: The illness trajectory and prognosis of cervical cancer are similar in pregnant and nonpregnant women.
In early-stage cervical cancers, treatment delaying to minimize neonatal risks can be proposed under strict conditions: the patient wish to preserve the pregnancy and obstetrical and oncological favorable prognostic factors.
Disclosure: Nothing to disclose

EP267

Synchronous primary cervical and fallopian tube carcinoma: a case report

C. Cabanag
University of Santo Tomas Hospital, Manila, Philippines

Introduction / Background: Multiple primary malignant tumors (MPMT) are defined as two or more independent primary malignancies from different sites and are of different histologies in the same individual[2]. Incidence of MPMTs ranges from 0.4-21%[7.] Though a primary fallopian tube carcinoma (PFTC) is a rare malignancy with only < 1% of female genital malignancies, a synchronous cervical and fallopian tube carcinoma is a more uncommon clinical entity with only a few documented cases worldwide.
Methodology: This is a case of a 64-year old nulligravid, who presented with post-menopausal bleeding. She underwent fractional curettage. Histopathology of the endometrial scrapings and endocervical tissue showed Squamous Cell Carcinoma, Large Cell Non- Keratinizing. She subsequently underwent exploratory laparotomy for Stage IB cervical carcinoma vs a primary endometrial carcinoma with cervical extension. Contemplated radical hysterectomy was abandoned because of the dense adhesions to the bladder. The fallopian tubes were noted to be bulbous with multiple fleshy papillations, hence bilateral salpingo-oophorectomy, biopsy of the tumor implant, excision of the left external lymph node and infracolic omentectomy was done.
Results: Histopathology showed High-grade Papillary Serous carcinoma of the fallopian tubes with lymphovascular invasion; Metastatic Papillary Carcinoma of bilateral ovaries and external iliac lymph nodes. Final staging according to FIGO system revealed IB1 cervical carcinoma and fallopian tube carcinoma stage IIIB.
Conclusion: She completed 6 cycles of Carboplatin-Paclitaxel regimen. Intracavitary brachytherapy was delivered in four fractions of 6 Gy. Patient has remained in follow-up, and at 8 months, there has been no documented evidence of recurrent disease.

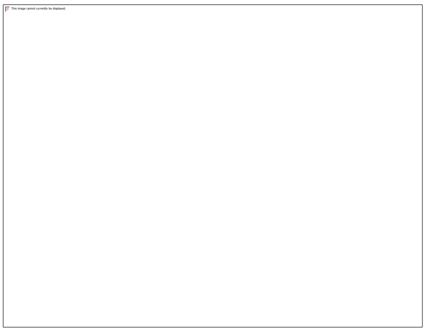

[Microsection from endocervical scrapping showing malignant neoplasm]

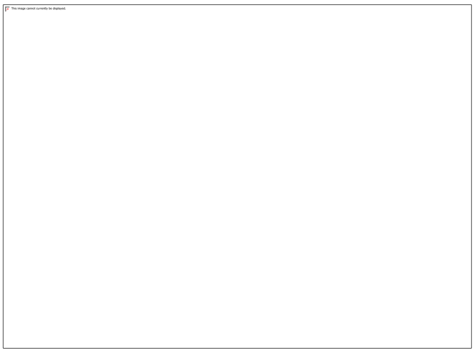

[Left fallopian tube hyperchromatic nuclei, exhibiting hobnailing and with basophilic cytoplasm consistent with Papillary Serous Carcinoma]

Disclosure: Nothing to disclose

EP268

Primary pelvic exenteration for advanced stages of pelvic cancers

M.E. Capilna[1], B. Moldovan[2], A. Fandi[1], M. Gheorghe[1], S.L. Kiss[1], M. Stanca[1], A.L. Cozlea[1]
[1]First Obstetrics and Gynecology Clinic, University of Medicine, Pharmacy, Science and Technology of Targu Mures, Targu Mures, [2]Sf. Constantin Hospital Brasov, Brasov, Romania

Introduction / Background: The main indications for pelvic exenteration are recurrent or advanced pelvic malignancies. This radical surgical treatment that might be performed for some selected advanced gynaecological or urological cancers, without prior radio- or chemotherapy, is still controversial.
Methodology: It is a retrospective study including 18 patients submitted to a primary pelvic exenteration between November 2011 and October 2018, with a mean age of 53.5 years. Regarding the pelvic malignancy, in 11 patients (61.1%), it was a stage IVa cervical cancer; a stage IVa vaginal cancer in 4 patients (22.2%); a stage IVa endometrial cancer in one and a bladder cancer stage IIIb in 2 patients. In all patients, the oncological contraindications were ruled out by MRI.
Results: Out of 18 primary pelvic exenteration, 12 were anterior and 6 were total. Regarding the level of levator ani muscle, 10 exenterations were supralevatorian, 5 infralevatorian and 3 infralevatorian with vulvectomy. The reconstructive phase consisted in a Bricker urinary non-continent ileal or sigmoid conduit. There were no major intraoperative complications. Early complications occurred in 6 patients (33.3 %) including a perioperative death (5.5% %) caused by a pulmonary embolism and 3 bowel fistulas (16.6%), in which a re-operation was needed. Only one patient has experienced a late

complication - a urostomy stenosis which was solved. Among the 18 patients, at this moment, 12 (66.6%) are alive, 5 are dead because of the disease and one is lost to follow-up.
Conclusion: Primary pelvic exenterantion in properly selected advanced pelvic cancers can be associated with long-term survival without high perioperative morbidity or mortality. However, postoperative complications can be lethal.
Disclosure: Nothing to disclose

EP269

Correlation between the level of education and the adequacy of cervical excisional procedures in patients with cervical preinvasive lesions

Y. Ceylan[1], M. Api[2], C. Kabaca Kocakuşak[2], E. Keleş[2], S. Akış[2], Y.E. Purut[2], B. Giray[2], M. Güray Uzun[2]
[1]Medicine, Istanbul Medipol University, [2]Gynecologic Oncology, Zeynep Kamil Training and Research Hospital, Istanbul, Turkey

Introduction / Background: To investigate the factors related to incomplete excision procedure in abnormal cervical cytology among the different levels of surgical education.
Methodology: From the medical records, data were collected from patients with abnormal cervical cytology and/or positive HPV test who have underwent colposcopic biopsy and required to be treated by loop electrosurgical excision procedure (LEEP) or cold knife conization (CKC) by obstetrics and gynecology residents or gynecologic oncology fellows under the supervision of senior attending surgeons from 2009 to 2019 in a university affiliated training and research hospital. Age, menopausal status, parity, body mass index, HPV infection, history of smoking, cervical cytology and characteristics of cone histopathology such as number of specimen, size and glandular involvement were investigated for adequacy of specimen interpretability by the pathologist for margin positivity according to educational level of surgeons.
Results: Total of 1845 cervical excisional procedures, 67% of them LEEP and 33% of them CKC were performed by residents (43%) or fellows (57%) during this period were analyzed. Margin negative complete excision of the LEEP and CKC specimens were found 81% and 86% in fellows and 67% and 80% in residents, respectively (p=0.003 and 0.02, respectively).
Conclusion: Multiple pieces of specimen was the risk factor for incomplete excision of LEEP. Procedures such as cone biopsy should be done only by medical practitioners who have special training, skills, and sufficient experience.
Disclosure: Nothing to disclose

EP270

Adenocarcinoma (ADK) with clear cells of the uterine cervical collar in an young girl in connection with one case

F. Hadjarab[1], C.E. Chekman[2], A. Taibi[1], K. Bouzid[1]
[1]Medical Oncology, Centre Anticancereux Pierre Marie Curie d'Alger, [2]Surgery Pierre Marie Curie, Centre Anticancereux Pierre Marie Curie, Algiers, Algeria

Introduction / Background: ADK of the uterine collar with cells clear is veryrare in the young woman, among the risk factorsone brings back the catch de DISTILBENE, Diéthylstilbestrol at the mom, whereas cancer epidermoïde of the collar the principal disease-causing agent is the papilloma virus HPV, herforecast is pejorative. We report in this poster anobservation illustrating this rare situation withreview of the literature.
Methodology: Young girl S.A 22 years old single person;virgin with ATCD of catch by the mother of

inductor of ovulation.
The patient with presented métrorragies andthe initial gynaecological examination toobjectified a small mass of the collar bleedingin contact with 3cm of diameter, a biopsy ofthe mass to aim diagnosis at summer carried out whose histological results returned in favor of clear ADK with cells of the uterine collar.
Results: Treated by standard RT then surgery CHEL.Cost after 6 months with pulmonary andosseous metastases. having profited from several lines of chemotherapy of which thefirst: Adriamycine/CDDP after 6 curesdevelopment of cerebral localization, irradiated then a second-row forward of chemotherapy (CT):
CPM / Bévacizumab after 7 months of treatment, progression of metastases.
The young patient received two other lines of CT. Deceased after 42 month of the beginning of the treatment following a renal insufficiency; respiratory.
Conclusion: Clear ADK of the uterine cervical collar in the young girl remains very rare; with absence of known factors predisposing with a therapeutic forecast péjoratif. la answer is overall, non satisfactory that it is the radiotherapy or chemotherapy.
Disclosure: no conflict interest

EP271

Primary cervical melanoma: case report and treatment

I. Chelea
Obstetrics and Gynaecology, Hospital Universitario de Torrevieja, Torrevieja, Spain

Introduction / Background: Cervical cancer is one of the most common cancers of the female genitalia in the world. It affects medium-aged and older women above all. Primary malignant melanoma of the uterine cervix is a rare entity characterized by its aggressive development and its poor prognosis. We expose a case of cervical melanoma in an 82 years old woman and its treatment.
Methodology: Case report and treatment.
Results: An 82 years old woman came to our hospital with vaginal bleeding since several months. There were nothing of interest in her clinical history and she had started menopause at 58. The speculum examination revealed a friable, irregular mass in the anterior lip, measuring 3 centimeters of diameter. The biopsy concluded to a cervical melanoma. TC and PET-CT were performed and they suggested that the tumor was not spread. A radical hysterectomy and pelvic pelvic node dissection was performed. The final stage was pT3b, N0, M0, and the pathological analysis confirmed that the lesion was a cervical melanoma BRAF V 600. She did not receive chemotherapy. Five months after the surgery she referred vaginal bleeding and was diagnosed with local recurrence of the tumor, confirmed by biopsy. On PET-CT one nodule over the vaginal dome was seen, consistent with recurrence, also deposit of tracer in rectum to be evaluated with colonoscopy.
Conclusion: Primary cervical melanoma is a rare tumour. The cases are reported in postmenopausal women, with vaginal bleeding. These tumours have a poor prognosis with usual survival of less than 2 years, despite aggressive surgery.
Disclosure: Chelea IG: “I have no conflict of interest” Cánovas Morales E: “I have no conflict of interest” Jara Gálvez R: “I have no conflict of interest” Cazorla Amorós E: “I have no conflict of interest”

EP272

Prognostic value of lymph node characteristics in patients with node-positive or node-negative early-stage cervical cancer

G.O. Chong[1], Y.H. Lee[1], H.J. Lee[2], D.G. Hong[1], Y.S. Lee[1]

[1]Obstetrics and Gynecology, Kyungpook National University/Kyungpook National University Chilgok Hospital, [2]Obstetrics and Gynecology, Kyungpook National University/Kyungpook National University Hospital, Daegu, Republic of Korea

Introduction / Background: This study investigated the prognostic value of lymph node characteristics for prediction of tumor recurrence in patients with early-stage cervical cancer.
Methodology: The medical records of 270 consecutive patients who were performed radical hysterectomy with pelvic and/or para-aortic lymphadenectomy for the treatment of early-stage cervical cancer were reviewed. Lymph node characteristics were included lymph node counts, node-positive counts, lymph node ratio, extent of lymphadenectomy, and node-positive bilaterality.
Results: Among the 270 patients, node-positive were 52 (19%) and node-negative were 218 (81%). In node-negative group, all of lymph node characteristics were not associated with tumor recurrence. In node-positive group, lymph node counts (hazard ratio [HR], 0.476; 95% confidence interval [CI], 0.07-3.17; $p = 0.4423$), node-positive counts (HR, 2.805; 95% CI, 0.80-9.87; $p = 0.1081$), extent of lymphadenectomy (HR, 1.850; 95% CI, 0.58-5.94; $p = 0.3012$), and node-positive bilaterality (HR, 2.196; 95% CI, 0.65-7.39; $p = 0.2043$) did not predict tumor recurrence. Only lymph node ratio was an independent prognostic factor for prediction of tumor recurrence (HR, 6.379; 95% CI, 1.98-20.52; $p = 0.0019$) in the node-positive group.
Conclusion: Lymph node ratio may be a prognostic biomarker for the prediction of disease recurrence in patients with node-positive early-stage cervical cancer.
Disclosure: Nothing to disclose

EP273

A new integrated pre-surgical diagnostic algorithm to define the local extent of disease in women with cervical cancer

G. Sozzi[1,2], C. Cicero[1], M. Ferreri[3], V. Giallombardo[3], R. Berretta[4], V.A. Capozzi[5], S. Finego[6], G. Scambia[7], V. Chiantera[1,2]
[1]Department of Gynecologic Oncology, ARNAS Civico Hospital, [2]University of Palermo, [3]Department of Gynecologic Oncology, University of Palermo, Palermo, [4]Department of Obstetrics and Gynecology, [5]University of Parma, Parma, [6]Department of Woman, Child and General and Special Surgery, University of Campania 'Luigi Vanvitelli', Napoli, [7]Department of Women's and Children's Health, Catholic University of the Sacred Heart, Roma, Italy

Introduction / Background: Survival of patients with cervical cancer is strongly associated with the local extent of the primary disease. For this reason, the new FIGO staging system has given greater importance to instrumental investigations in the pre-surgical evaluation. The objective of this study is to develop an integrated diagnostic algorithm, including ultrasound (US), magnetic resonance imaging (MRI) and clinical examination under anaesthesia (CEUA), to better define the local extent of disease in patients with newly diagnosed cervical cancer, using histology as the referring gold standard.
Methodology: Patients with biopsy proven cervical cancer submitted to primary surgery from January 2013 to December 2018, in four participating centers, were recruited. Data regarding tumor size, parametrial invasion and vaginal involvement, obtained by US, MRI and CEUA were retrieved and compared to final histology. Specificity and sensitivity of the three methods were calculated for each parameter and the methods were compared with each other. An integrated pre-surgical algorithm was constructed considering the accuracy of each diagnostic method for each parameter.
Results: A total of 79 consecutive patients were included in the study. Regarding tumor size, US resulted as the most accurate method, while CEUA was found to be more accurate in prediction of vaginal involvement. About parametrial invasion, CEUA and MRI were found to be superior to US. However, no statistically significant differences were found between these two methods. The use of our algorithm allowed to perform an exact diagnosis in 77.2% of patients, reducing significantly (p: 0.05) the risk of misdiagnosis.
Conclusion: The importance of an accurate pre-surgical staging plays a great role in the management of cervical cancer. Our integrated diagnostic algorithmallows a higher accuracy in the definition of the local extent of disease and can be used as a valid tool to personalize the therapeutic

approach for women with cervical cancer.

Variable	Tumor size	Vaginal involvement	Parametrial invasion
Method to be used	US	CEUA	CEUA or MRI
Score			
0	< 2 cm	NO	NO
1	> 2 cm, < 4 cm	YES	YES
2	> 4 cm	-	-
Total score	-	-	-

[Integrated pre-operative algorythm for the evaluation of the local extension of disease.]

Score	Stage
000	T1a1-T1b1 (<2 cm)
100	T1b1 (> 2cm, < 4cm)
200	T1b2
110	T2a1
010; 210	T2a2
001; 101; 201; 011; 111; 211	T2b

[Correspondence between score and staging of the local extent of disease]

Disclosure: Nothing to disclose

EP274

Effect of a Coriolus versicolor-based vaginal gel in HPV infected women: normalizing HPV-dependent cervical lesions (ASCUS/LSIL) and high-risk HPV clearance

L. Serrano[1], A.C. López[2], S. González[1], S. Palacios[3], D. Dexeus[4], C. Centeno[5], P. Coronado[6], J. de la Fuente[7], J.A. López Fernández[8], C. Vanrell[9], J. Combalia[10], J. Cortés[11]

[1]Centro Médico Gabinete Velázquez, Madrid, [2]Hospital Quironsalud, Málaga, [3]Instituto Palacios Salud y Medicina de la Mujer, Madrid, [4]Women´s Health Institute, [5]Clínica Diatros, Barcelona, [6]Hospital Clínico San Carlos, [7]Hospital Universitario Infanta Leonor, Madrid, [8]Hospital General Universitario, Alicante, [9]Hospital de la Santa Creu i Sant Pau, [10]Medical Affairs, Procare Health Iberia, Barcelona, [11]Private Practice, Palma de Mallorca, Spain

Introduction / Background: To evaluate the efficacy of Papilocare® -a *Coriolus versicolor*-based vaginal gel- in repairing HPV-dependent cervical lesions (ASCUS/LSIL) and in the clearance of high-risk HPV (HR-HPV).

Methodology: Randomized, open-label, parallel-group, controlled clinical trial (Paloma Clinical Trial). HPV+ women aged between 30 and 65 with cytology of ASCUS or LSIL with concordant colposcopy image were randomized into 3 groups: A) Papilocare® 1 cannula/day for 1 month + 1 cannula/alternate days for 5 months; B) Papilocare® 1 cannula/day for 3 months + 1 cannula/alternate days for 3 months; C) Control group: no treatment (usual clinical practice). Main results of the Paloma trial are presented: Percentage of patients with normal cytology and concordant colposcopy image at 6 months (primary endpoint), and percentage of patients with HR-HPV clearance at 6 months (clearance: total and partial clearance with concordant cytological normalization). Pap smear evaluation was being

centrally-conducted at the IECM laboratory (Lugo, Spain). Papilocare® arms (A+B) were combined for this per-protocol analysis.

Results: A total of 84 all HPV genotype patients (total population) of which 66 with High Risk -HPV (HR-HPV subpopulation) were evaluated. In the total population, 85% (45/53) of patients treated with Papilocare® had normal cytology with concordant colposcopy vs. 65% (20/31) in control group (p=0.0311; Chi-square test; Fig.1). In the HR-HPV subpopulation, normal cytology and concordant colposcopy image was observed in 88% (36/41) of patients treated with Papilocare® vs 56% (14/25) of control group patients (p=0.0034; Chi-square test; Fig.2).

HR-HPV clearance was observed in 63% (25/40) vs 40% (10/24) of patients treated with Papilocare® vs. control group (p=0.07; Chi-square), respectively.

Conclusion: Papilocare showed a statistically significant efficacy in normalizing HPV-dependent cervical lesions (ASCUS/LSIL) versus control group. The normalization rate of HPV-dependent lesions was even higher in the HR-HPV subpopulation , in which a positive trend in HR-HPV clearance was also observed at 6 months.

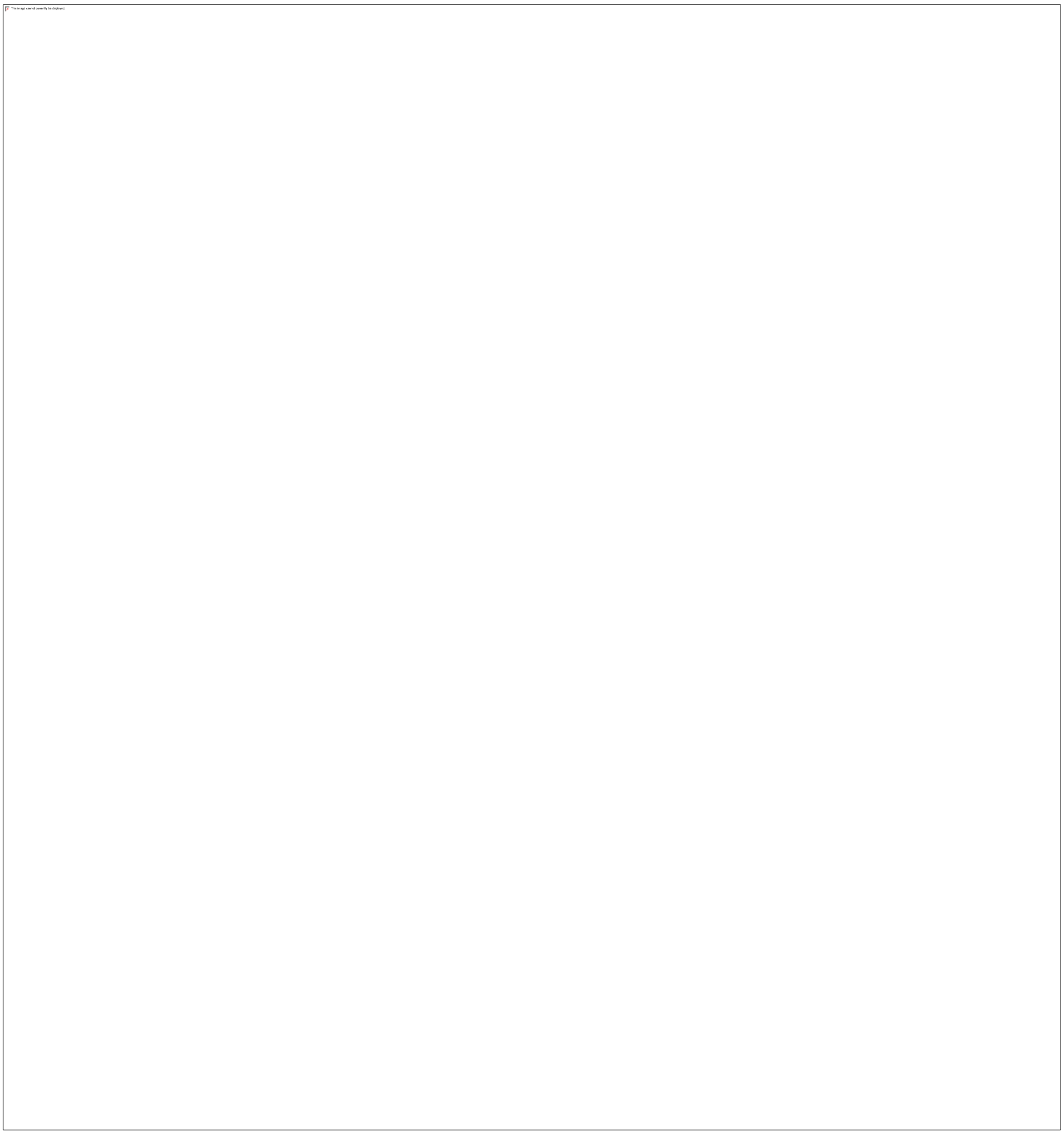

[Papilocare® on repairing cervical esions (ASCUS/LSIL)]

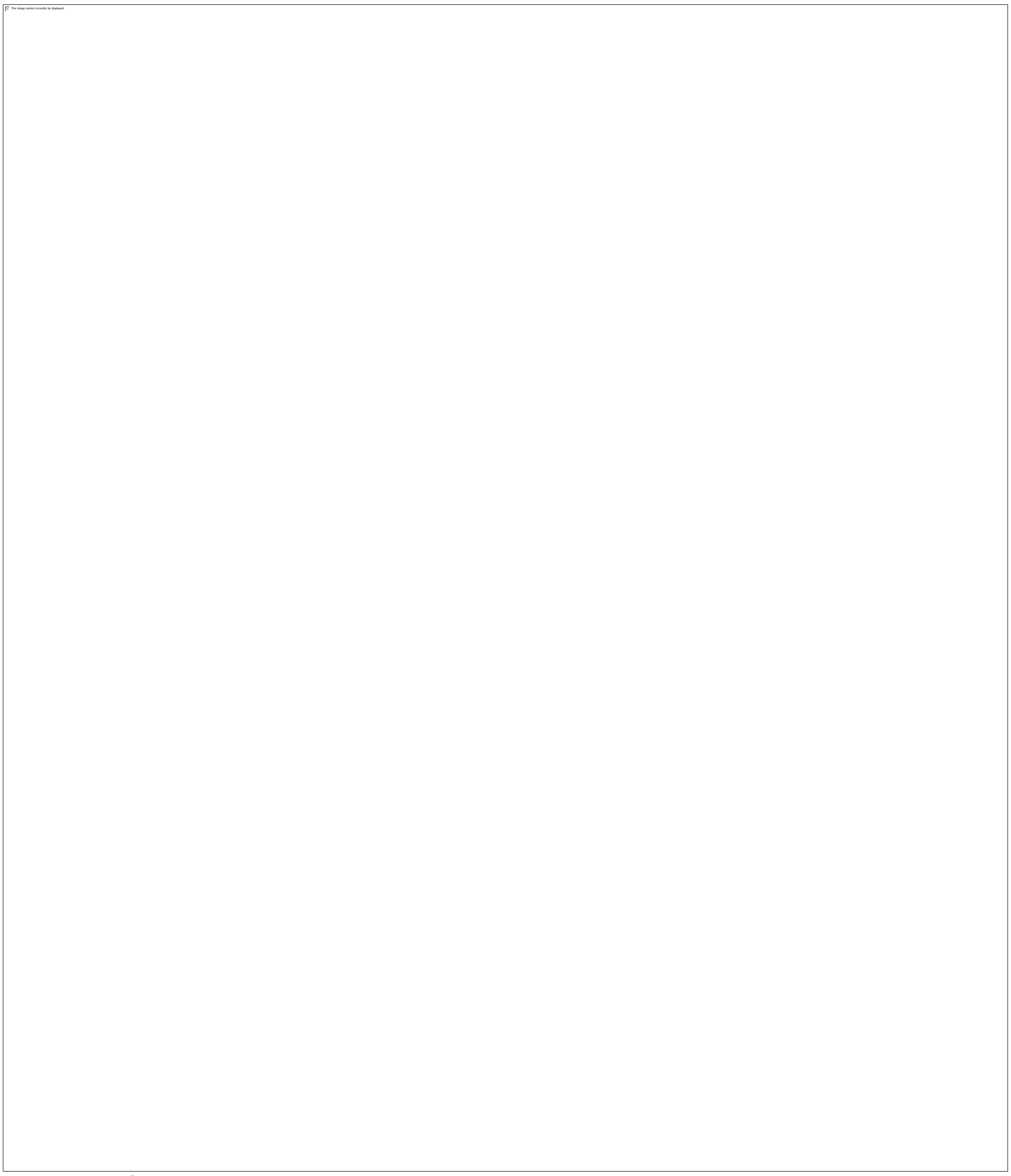

[Papilocare® on high-risk HPV clearance]

Disclosure: Nothing to disclose

EP275

Cervical cancer screening in Africa: a proposal of a different combination of VIA test and PAP smear in Uganda

G. Corrado[1], C. Mazzara[2], C. Certelli[3], J.B. Nsubuga[4], F. Zanetto[5], K. Schurfeld[6], G. Dell'Antonio[7], C. Dagli Orti[8], A. Oryono[9], T. Andriani[10]
[1]Policlinico Universitario Agostino Gemelli I.R.C.C.S. Universita Cattolica Di Roma, Roma, Italy, [2]IPSI-Istituto Pediatrico della Svizzera Italiana, Lugano, Switzerland, [3]Regina Elena National Cancer

Institute, Rome, Italy, [4]St. Kizito Hospital, Matany, Uganda, [5]Fatebenefratelli Hospital, Erba, [6]Policlinico Le Scotte, Siena, [7]San Raffaele Hospital, Milan, [8]INPS Sandrigo (VI), Sandrigo, Italy, [9]St. Joseph Hospital, Kitgum, Uganda, [10]Afron ONLUS, Rome, Italy

Introduction / Background: AFRON Oncology for Africa is an Italian non-profit organization that works for female cancer prevention in the Sub-Saharan area, mainly in Uganda. Although the visual inspection with acetic acid (VIA) is one of the most widespread screening method in African countries, it did not show a high accuracy. The aim of this study is to evaluate the efficacy and usefulness of VIA test combined with the PAP smear in Uganda in order to find possible solutions that may be used in a low-resource setting.
Methodology: Between January 2011 and October 2018, eight screening campaigns were held in Kampala, Kitgum and Karamoja. In the last two campaigns, we decided to try a new approach: we performed the PAP smear before the VIA test and, in case of a positive VIA test, we sent the slide for examination. To compare them, we divided the data collected in two groups: the first five campaigns (2011-2016), in which a standard approach was used, and the last two campaigns (2017-2018), in which the new approach was used.
Results: During the study period, 9,780 women were screened (median age 34), of whom 699 were positive at the VIA test. The VIA test showed 584 (83.5%) false positives. In the first group, the VIA test was positive in 516 women, of whom 483 (93%) were referred for further examinations. In the second group, in which the VIA test was positive in 173 women, the PAP smear was positive in 46 of the 173 slides analysed. Thus, only 46 of the 173 (26.5%) women were referred for further examinations.
Conclusion: Combining the PAP smear, VIA test and slide analysis in positive VIA tests may allow women who need treatment to be selected more effectively, while waiting for other more expensive solutions to become more affordable and suitable for these settings.
Disclosure: Nothing to disclose

EP276

Toxicity evaluation of locally advanced cervical cancer

D. da Silva Dias[1], B. Gosalbez[1], L. Alves[1], P. Luz[1], T. Madureira[1], G. Vieira[2], I. Furtado[1]
[1]Medical Oncology, Centro Hospitalar Universitário do Algarve, [2]Radiooncology, Quadrantes Faro - Unidade de Radioterapia do Algarve, Faro, Portugal

Introduction / Background: Cervical cancer represents 6% of gynecological malignant tumors. The incidence in Portugal is 8,9/100.000, although it is higher in southern region. Concurrent Chemo-radiotherapy (CCRT) is the cornerstone treatment.
Methodology: Retrospective analysis of 109 patients diagnosed with locally advanced cervical cancer in our center between 2010 and 2017. The goal is assessing the efficacy of CCRT overall survival (OS), disease-free survival (DFS) and incidence of toxicity.
Results: 109 patients, with median age 56 years old. Mostly admitted through emergency department (79%), being the most common symptom metrorrhagia (46,8%). 65,1% were Stage IIB. Median time from diagnosis to multidisciplinary committee (MC) was 47 days and from MC to beginning of CCRT was 21 days. CCRT was initiated in 87 (79,8%) patients. DFS was 63% and OS 64% at 5 years. From 522 chemotherapy sessions, only 9 presented severe toxicities, especially haematological grade 3. No relation with OS or DFS was found. 46% (n=40) presented radiotherapy toxicity, especially chronic (n=31). Proctitis (n=24) was the most prevalent. A change was observed in 2015, patients diagnosed before presented an alarming 36,7% of proctitis versus 20% after 2015. A significant difference was observed between radiotherapy toxicity and OS (p=0,018), especially fistulas and proctitis. Median radiation dose was 73Gy. A positive correlation was observed in the incidence of toxicity when exposed to doses ≥75Gy (p=0,007; HR 4,73 [95% CI: 1,52-14,71]), especially diarrhea and proctitis.
Conclusion: The OS and DFS are in concordance with results around European countries.
A high incidence of radiation toxicity was evident, although an improvement was observed since 2015. Our radiation clinic has an intensity-modulated radiation therapy (IMRT) since 2018 and we are eager to evaluate the effect in toxicity in the years to come.
Disclosure: Nothing to disclose

EP277

Special considerations in quality of life of locally advanced cervical cancer patients

D. da Silva Dias[1], B. Gosalbez[1], L. Alves[1], P. Luz[1], T. Madureira[1], G. Vieira[2], I. Furtado[1]
[1]*Medical Oncology, Centro Hospitalar Universitário do Algarve,* [2]*Radiooncology, Quadrantes Faro - Unidade de Radioterapia do Algarve, Faro, Portugal*

Introduction / Background: Cervical cancer (CC) represents 6% of gynecological malignant tumors. The incidence is decreasing in Portugal, especially with the introduction of nationwide screening program in 2008 and Human Papillomavirus vaccination in 2010. Still, special concern should be placed in improving their quality of life (QoL).
Methodology: Retrospective analysis of 109 patients diagnosed with locally advanced cervical cancer in our center between 2010 and 2017. The goal is assessing complications that interfere with the patient´s lifestyle such as venous thromboembolism (VTE), ureter hydronephrosis (UHNF), psychological distress (PD) or Palliative care (PC) referral.
Results: 109 patients, with median age 56 years old. Mostly admitted through the emergency department (79%), being the most common symptom metrorrhagia (46,8%). 65,1% were Stage IIB. Concurrent chemoradiotherapy was applied to 79,8% (n=87). VTE was highly prevalent, present in 12,8% of patients (n=14) and an association with OS (p= 0,014) and tumor staging (p=0,001) was evident. PD including anxiety, depression or uncoping was observed in 25,7% (n=28), especially in younger women (p=0,46; HR 0,96 [95% CI: 0,93-0,99]. Psycho-oncology support was provided to 82% (n=23) of patients. UHNF was present in 21,1% (n=23). Nephrostomy or ureteral stent was performed before the beginning of treatment in 13,8% (n=15). No difference was observed in OS or DFS in this subgroup. PC was presented to 22% (n=24) patients, although 67% of them were only referred to PC in the last month of life.
Conclusion: CC patients have higher incidence of VTE with negative impact in morbimortality and QoL. More studies are necessary to determine the use of prophylactic anticoagulation, especially in patients with concomitant comorbidities. PD may be underdiagnosed and underrepresented in this study. Raised awareness and psycho-oncology support should be provided. PC support should be involved in the patient and families´ lives sooner.
Disclosure: Nothing to disclose

EP278

Atypical presentation of cardiac metastasis in uterine cervical cancer

D. da Silva Dias, B. Gosalbez
Medical Oncology, Centro Hospitalar Universitário do Algarve, Faro, Portugal

Introduction / Background: Uterine Cervical cancer (UCC) represents 6% of gynecological malignant tumors. Cardiac metastasis of UCC are extremely rare as only 4% of them are diagnosed antemortem. The epidemiology and clinical manifestations are not clearly known, only a small number of clinical case reports are published in the literature. The overall survival is expected to be lower than 12 months.
Methodology: Clinical case report with the objective to raise awareness of a rare presentation of cardiac metastasis in UCC.
Results: Woman with 56 years years old is presented, with diagnosis of UCC, stage IIB in May 2014. Proposed to concurrent chemo-radiotherapy (CCRT) which she refused. In February 2015 visited the emergency department with obstructive kidney failure due to bilateral ureter hydronephrosis. Bilateral nephrostomies were applied, and the patient started treatment in May 2015 with CCRT, cisplatin

40mg/m2 and external radiotherapy. Patient had no indication for brachytherapy. In February 2017 presented progressive asthenia and anemia G1 due to radiation proctitis. A computed tomography scan in follow up consultation was performed presenting a lesion of 9cm in the right ventricle with the differential diagnosis of metastasis versus thrombus. A trans-thoracic echocardiogram was performed showing a mass in the right ventricle (RV) without dysfunction of the RV nor left ventricle (LVEF 58%). A magnetic resonance was performed as well to better identify the lesion. After collaboration with a cardiac surgery center no surgical procedure was possible. The patient was discharged and started palliative chemotherapy with Carboplatin and paclitaxel but unfortunately succumbed to disease 4 months later.
Conclusion: The present case report is relevant to raise awareness on a rare, but still possible site of metastasis of uterine cervical cancer.
Disclosure: Nothing to disclose

EP279

A cohort retrospective study of the high-risk HPV recurrence in Greek women after cervical lesion treatment through detection of viral E6/E7 mRNA expression

F. Carcea, A. Daniilidis, E. Vavoulidis, M. Nasioutziki, A. Papanikolaou, K. Dinas
2nd University Clinic of Obstetrics and Gynecology Aristotle University of Thessaloniki Hippokratio General Hospital, Thessaloniki, Greece

Introduction / Background: Long-term persistent infection from high-risk HPV-subtypes is strongly associated with high-grade lesions and cervical cancer due to two overexpressed viral oncoproteins, E6 and E7. Our aim was to detect and evaluate potential alterations in the post-operative status of E6/E7 HPV mRNA in women treated for Cervical Intraepithelial Lesions (CIN) and if so, to evaluate its potential use as a prognostic tool to identify patients with increased risk of treatment failure or recurrent disease.
Methodology: Our study retrospectively analyzed 101 women who visited our Colposcopy Clinic with an abnormal Pap smear, or in some cases with histological reports or molecular analysis, suggesting colposcopy. Thin-prep (Hologic) cytological samples were collected before colposcopy and histology (when necessary). After treatment, all women were scheduled for colposcopy and cytology in six months. The cytological material was analyzed with CLART-2 HPV DNA-test (Genomica) and HPV-PROOFER E6/E7 mRNA-test (Pretect).
Results: No significant correlations were found for smoking, condom use or vaccination status. It seems that the only statistically significant correlation with actual severity comes from mRNA-test after treatment ($\rho=0.261$, $p=0.008$). This probably shows that clinical cases with more severe CIN may have higher chances of unsuccessful treatment. At the first post-op follow-up visit, 83.5% of the HPV-mRNA positive women became negative while only 60.4% of the HPV-DNA positive women became negative. There were 12 HPV-mRNA positive patients both before and after treatment, 3 of were HPV-DNA negative pre- and post-operatively, meaning that if based only on HPV-DNA results, they would have been managed wrongly as successfully-treated patients. Our study shows that E6/E7 mRNA detection has particularly high specificity and positive likelihood ratio for the prediction of treatment failure in comparison with HPV DNA-testing.
Conclusion: E6/E7 mRNA overexpression seems to be a promising candidate as an indicator-biomarker to determine the success of treatment and possibly determine the intensity of post-operative monitoring.
Disclosure: Nothing to disclose

EP280

Role of ganglioside GM1 expression in T lymphocytes membranes in cervical cancer development

D. Danolic[1], M. Heffer[2], J. Wagner[2], I. Skrlec[2], I. Alvir[1], I. Mamic[1], L. Susnjar[1], L. Marcelic[1], T. Becejac[3], M. Puljiz[1]
[1]University Hospital Center Sestre Milosrdnice, University Hospital for Tumours, Zagreb, [2]Faculty of Medicine, University of Osijek, Osijek, [3]University Hospital Center Zagreb, Zagreb, Croatia

Introduction / Background: Cervical cancer is worldwide the second most common cancer in women. Immune response appears to be a key determinant in controlling human papilloma virus (HPV) infection. Cell mediated immunity plays a significiant role in progression or regression of neoplastic cervical lesions. Lipid rafts have essential role in T lymphocite activation. Gangliosides play an important role in the formation and stabilization of specific cell lipid membrane domains. This study aims to determine whether ganglioside GM1 expressions in T cell membrane represent risk factor for cervical cancer.
Methodology: In total, 20 women participated in our study. Peripheral blood samples were obtained from 10 cervical cancer patients and 10 healthy controls at University Hospital for Tumors, Zagreb, Croatia. A 3 mL sample of venous blood was collected from each subject into a test tube containing EDTA as anticoagulant. For T cell subtypes isolation we used Anti CD4 Phycoerythrin antibody and Anti CD8 Peridinin Chlorophyll Protein Complex antibody. Ganglioside GM1 expression in T lymphocytes membranes was analyzed using double labeling antibodies on ganglioside GM1.
Results: We have not found difference in ganglioside GM1 expression in T lymphocytes membranes between the two groups.
Conclusion: The present study did not show association of ganglioside GM1 expression in T lymphocytes membranes with cervical cancer development.
Disclosure: Nothing to disclose

EP281

Observational study of the BGOG on the results of robot-assisted radical hysterectomy for stage I cervical cancer in Belgium

A. de Bruyn[1], K. Smulders[2], F. Peeters[1], K. Traen[3], F. Goffin[4], P. Van Trappen[5], I. Vergote[2]
[1]AZ Klina, Brasschaat, [2]UZ Leuven, Leuven, [3]OLV Ziekenhuis Aalst, Aalst, [4]CHR de la Citadelle, Luik, [5]AZ Sint Jan, Brugge, Belgium

Introduction / Background: The LACC trial (Ramirez P, NEJM 2018; 379:1895-1904) and the Melamed report (Melamed A, NEJM 2018; 379:1905-1914) raise the question whether minimally invasive surgery (conventional and robot-assisted laparoscopy) is inferior to open abdominal surgery in early stage cervical cancer. Notable was the low rate of recurrence and death in the laparotomy group of the LACC trial. The current study investigates the results of patients with FIGO stage I cervical cancer treated with robot-assisted radical hysterectomy in hospitals of the Belgium and Luxembourg Gynaecological Oncology Group (BGOG).
Methodology: In this prospective follow up study combined data of different BGOG hospitals were analysed of patients with stage IA1, IA2 and IB1 cervical cancer with a histologic subtype of squamous-cell carcinoma, adenocarcinoma or adenosquamous carcinoma treated with robot-assisted laparoscopy. Inclusion criteria were the same as in the LACC trial. Outcomes were disease free and overall survival at 3 years.
Results: A total of 177 patients were eligible for analysis. Mean age was 46 years. Most patients had FIGO stage IB1 (84.2%) and squamous cell carcinoma as histological subtype (71,2%). Mean follow up time was 3,5 years, with a range from 0 to 131 months. 22 recurrences and 12 deaths were noted, of which 8 deaths were related to cervical cancer. Disease free survival at 3 years was 85%. Overall survival at 3 years was 96%.
Conclusion: Current results in Belgium show disease free and overall survival rates of patients with early stage cervical cancer after robot-assisted laparoscopy that are consistent with those mentioned by the American and Canadian Cancer Society and earlier reports, except for the LACC trial and the Melamed report.
New randomized trials are needed to investigate whether minimally invasive surgery remains an equal

option in the treatment of early stage cervical cancer in comparison to laparotomy.

Characteristic	
Age at diagnosis (mean)	46 years
BMI (mean)	24.6 kg/m2
FIGO stadium: IA1 - IA2 - IB1	8.5% - 6.2% - 84.2%
Histologic type: Squamous cell carcinoma - Adenocarcinoma - Adenosquamous carcinoma	71.2% - 24.9% - 4.1%
Positive lymph nodes	7.3%

[Patient characteristics]

Disclosure: Nothing to disclose

EP282

Survival analysis of stage IB to IIA2 cervical cancer: a five year single institution review

Z.S. De Leon, B.D. Cuenca
Section of Gynecologic Oncology and Trophoblastic Disease, Department of Obstetrics and Gynecology, Jose R. Reyes Memorial Medical Center, Manila, Philippines

Introduction / Background: This study aims to determine the disease-free survival and overall survival outcome of patients with IB to IIA cervical cancer managed with surgery, chemoradiation, or a combination of both in a tertiary government training hospital.
Methodology: This is a retrospective study of patients diagnosed with stage IB-IIA2 cervical cancer from January 2013 to June 2017. Data were encoded using Microsoft Excel. Statistical analyses were computed using SPSS. Cox regression and Kaplan Meier analyses were used to predict survival outcome.
Results: Out of 135 patients were included in the study, 111 received treatment. 61 had no evidence of disease. Median age is 46 years with stage IB1 disease. Majority of patients underwent surgery followed by adjuvant therapy. Tumor recurrence was highest in the surgery alone group, with median time to recurrence of 19 months. Median follow-up time was 10 months. Overall 5-year survival is 51.4%; 5-year disease-free survival is 54.8%.
Conclusion: Age is a statistically significant factor in survival. Surgery with adjuvant chemotherapy ± radiation had the most favorable survival outcome. Neoadjuvant treatment gave the least number of recurrences. Despite a small sample size, this study provides baseline data into the survival outcome of patients with locally advanced cervical cancer in our institution given the different treatment recommendations.
Disclosure: Nothing to disclose

EP283

Evaluation of quality of life in the patients with infiltrative cervical cancer by improving radical hysterectomy via using nerve-sparing method

T. Dermenzhy[1], V. Svintsitskiy[2], S. Nespryadko[2]
[1]Medical Center, [2]National Cancer Institute, Kyiv, Ukraine

Introduction / Background: The abstract is devoted to the improvement of treatment effectiveness and quality of life (QL) of the patients with infiltrative cervical cancer (ICC) by improving radical hysterectomy via using nerve-sparing method.
Methodology: The study is based on analysis of 120 patients with ICC cured during the last 10 years and prospective data on clinical observation and treatment results of 90 patients with ICC treated in the Department of Oncogynecology of National Cancer Institute in 2012-2017 by nerve-sparing radical hysterectomy (NSRE, the main group, n = 45), and radical hysterectomy III (RHE III, the control group, n = 45).
Results: The conduction of NSRE in the patients with ICC has allowed to decrease significantly the rate of complications of genitourinary system: difficulty with urinary bladder emptying in RHE III group was significantly increased compared with NSRE group (3,93 ± 0,06) and (1,09 ± 0,04) (p < 0,05); dyspareunia - (4,69 ± 0,07) and (2,87 ± 0,07) (p < 0,05).
Conclusion: The data mentioned above evidence on high effectiveness of nerve-sparing technologies for treatment of patients with ICC and their positive influence on the QL.
Disclosure: Nothing to disclose

EP284

Pulsed dose rate brachytherapy in gynecological cancers examples of cervical and endometrial cancer: experience of the radiotherapy service of Martinique

K. Diakité, M.M. San, S. Edelstein-Dit-Edel, S. Bougas, W. Lababidi, E. Papadopoulou, P. Escarmant, C. Cabanel, T. Désiré, V. Vinh-Hung
Service Radiothérapy, CHU Martinique | Radiotherapy | CHU Mohamed VI Marrakech, Fort de France, Martinique

Introduction / Background: Low-dose rate brachytherapy has some radiobiological advantages compared to external beam radiotherapy: subletal damages repair during irradiation leading to a relative protection of healthy tissues; no Tumor cell repopulation, cell cycle redistribution and a low oxygen enhancement ratio.
Methodology: This is a retrospective eight-year study from June 10, 2010 to April 16, 2019. This study was of interest to all Admitted patients after multidisciplinary consultation meeting for gynecological cancer (cervical or endometrial cancer). Ayant bénéficié d'une r curiethérapie à bas de début de dose vaginale ou intra-cavitaire.
Results: We collected 185 patients, with an average age of 45 years. The patients were spread between Martinique and the the other Carribean islands . Cervical cancer was more common. The predominant stage was stage IIA for cervical cancer and stage III for endometrial cancer. The dose of 30Gy was delivered in 3 days for uterovaginal brachytherapy after radiotherapy-chemotherapy combination, 60Gy in exclusive vaginal brachytherapy and 15Gy in barrier brachytherapy.
Conclusion: Brachytherapy retains a key role in the treatment of gynecological cancers. A better knowledge of the dose and its distributions makes it possible to hope for an improvement in the rate of local control and a significant decrease in complications.
Disclosure: Nothing to disclose

EP285

The impact of socioeconomic deprivation on survival in cervical cancer patients: a systematic review

H. Donkers[1], R. Bekkers[2], L. Massuger[2], K. Galaal[3]
[1]Gynae-Oncology, Royal Cornwall Hospital, Truro, United Kingdom, [2]Radboud UMC Nijmegen,

Nijmegen, The Netherlands, [3]Royal Cornwall Hospital Trust, Truro, United Kingdom

Introduction / Background: Factors that increase the risk of cervical cancer are more frequently seen in socioeconomically deprived patients. Furthermore, socioeconomic deprivation is associated with decreased survival in most cancers, mainly due to an advanced stage at diagnosis for more deprived patients. However, in cervical cancer this association is unclear.
Methodology: We performed a systematic search of Medline (1946-2018), Embase (1980 - 2018), Cinahl (1981 - 2018) and the Cochrane Controlled Register of trials to identify studies that reported on the association between socioeconomic deprivation and survival in cervical cancer patients. Included were adult women (age ≥ 18 years) diagnosed with primary cervical cancer. Two reviewers independently selected studies and assessed bias using the Newcastle-Ottawa assessment scale. Data extraction was completed using pre-determined forms and summary tables of evidences from the included studies were created.
Results: Eight studies were included in this review with a total number of at least 38,811 patients. All eight studies showed that socioeconomic deprivation is associated with poorer survival in cervical cancer patients with five studies confirming this association in multivariate analysis.
Conclusion: Socioeconomic deprivation is associated with survival in cervical cancer patients, in which more deprived patients have worse survival. Further research should focus on the interaction between cervical screening attendance, smoking status, stage at diagnosis, socioeconomic deprivation and survival in cervical cancer patients.
Disclosure: Nothing to disclose

EP286

Médecins Sans Frontière's cervical cancer project in Malawi: a collaborative and comprehensive strategy against cervical cancer

P.-F. Dupré[1,2], L. Gadama[3,4], R. Asgary[5], P. Bonongwe[6], M. Iwamoto[7], L. Masamba[8], E. Brown[9], J. Ordi[10,11], P. Poulain[12], C. Rieux[7], C. Mills[13], C. Fotteringham[13], D. Maman[13]
[1]Oncology & Haematology Insitute, Academic Hospital of Brest, [2]INSERM UMR 1078, Brest, France, [3]Obstertic & Gynaecology, Queen Elizabetth Hospital, [4]College of Medicine, Blantyre, Malawi, [5]Internal Medicine, Weil Cornell Medicine, New York City, NY, United States, [6]Obstetric & Gynaecology, Queen Elizabetth Hospital, Blantyre, Malawi, [7]Médecin Sans Frontière, Paris, France, [8]Oncology, Queen Elizabetth Hospital, Blantyre, Malawi, [9]Edinburgh Cancer Center, Edinburgh, United Kingdom, [10]Pathology, Department of Pathology, Hospital Clínic, [11]Centre de Recerca en Salut Internacional de Barcelona (CRESIB), Barcelona, Spain, [12]Douleurs Sans Frontière, [13]Médecins Sans Frontière, Paris, France

Introduction / Background: Malawi has the highest cervical cancer incidence (76/100 000) and mortality rate (50/100 000) in the world representing 3 684 new cases and 2 314 deaths annually.
In response to this need, Médecins Sans Frontière (MSF) in collaboration with the Ministry of Health (MoH) and The College of Medicine (CoM) is implementing a unique comprehensive oncology project from prevention and screening to treatment in the Blantyre and Chiradzulu districts in Malawi.
Methodology: The project aims to encompass all components of gynecologic oncology care through a collaborative work of a multidisciplinary team embedded at the tertiary Queen Elizabeth Central Hospital (QECH) and community health centers.
MSF Set up a dedicated team sustained with a multi-year funding in order to fulfill the following objectives:
· To cover all girls 9 to 14 with HPV vaccines starting 2020.
· To reach 80% screening coverage for women 25 to 49 by 2022 in the targeted areas.
· To strengthen gynecologic oncology treatment and medical education capacity at QECH.
Results: Cervical cancer screening is based on the Visual Inspection with Acetic acid (VIA).
Invasive cancers will be treated according to the National Comprehensive Cancer Network (NCCN) Cervical Cancer Basic Resources Guidelines.
A new Out and In-Patient department have been built as well as a new dedicated Operative theater expected to be active in October 2019.
An Electronical Medical Record and a Cervical Cancer Register are currently being developed in order

to open up opportunities for clinical research.
A multi-step medical education plan is also set up for OBGYN Registrars and Gynecological Oncologist sub-specialists in collaboration with ESGO.
Conclusion: The MoH, the CoM of Malawi and MSF respond to the WHO "global call" towards elimination of Cervical Cancer through a unique comprehensive project.
Transitional support of Gynecol Oncologists is required to ensure its perennial success.
Disclosure: Nothing to disclose

EP287

Iliopsoas muscle metastasis from cervical carcinoma

M. Farazestanian
Fellowship of Gynecology Oncology, Department of Obstetrics and Gynecology, Faculty of Medicine, Mashhad University of Medical Sciences, Mashhad, Islamic Republic of Iran

Introduction / Background: Isolated metastasis of cervical carcinoma to iliopsoas region might be infrequent and its occurrence is a diagnostic challenge. The aim of this study was to report a case of iliopsoas muscle metastasis from cervical carcinoma.
Methodology: Case Presentation: A 52-year-old multiparous females complaining about post menopause bleeding was referred to the oncology department. Gynecological examination revealed cervical mass with involved parameters and biopsy confirming squamous cell carcinoma. In addition, the patient had a large cystic mass on the lateral side of pelvic area and abdomen. Investigations detected solid ovarian mass associated with a rise of tumor marker CA-125 and CEA levels.
Results: Magnetic resonance imaging (MRI) and CT scan reports suggested possible hematoma in iliopsoas muscle. Imaging-guided biopsy detected metastatic squamous cell carcinoma in iliopsoas muscle mass. Based on diagnosis of advance stage cervical cancer, the patient received chemotherapy following adjuvant radiation therapy.
Conclusion: Metastasis of squamous cell carcinoma of the cervix to iliopsoas muscle is considered as a rare event. It is recommended to keep this unusual manifestation in mind.
Disclosure: Nothing to disclose

EP288

Prospective intra/inter-observer evaluation of pre-brachytherapy cervical cancer tumor width measured in TRUS and MR imaging

M. Federico[1], C.R. Hernandez Socorro[2], I. Ribeiro[1], M.D. Rey Baltar Oramas[1], M. Zaiac[1], M. Lloret[3]
[1]*Radiation Oncology,* [2]*Radiology, Hospital Universitario de Gran Canaria Dr. Negrin, Las Palmas,* [3]*Radiation Oncology, Hospital Universitario de Gran Canaria Dr. Negrin, Las Plamas, Spain*

Introduction / Background: US imaging due to its limited cost is under intense investigation as base for dose adaptation in cervical cancer brachytherapy. This work test inter/intra-observer uncertainties between MR and TRUS imaging defining maximum tumor width before first BT application in a prospective cohort of cervical cancer patients undergoing IGABT
Methodology: 110 consecutive cervical cancer patients were included. Before first BT implant underwent MR and TRUS scan with no applicator in place. Images were independently analyzed by three examiners, blinded to the other's results. With clinical information at hand, maximum tumor width was measured on preBT TRUS and MR (fig.1). Quantitative agreement analysis was undertaken. Intra-class correlation coefficient (ICC), Passing-Bablok and Bland Altman plots were used to evaluate the intra/inter-observers agreement
Results: Average difference between $HRCTV_{MR}$ and $HRCTV_{TRUS}$ was 1.3±3.2 mm (p< 0.001); 1.1±4.6

mm (p=0.01) and 0.7±3mm (p=0.01). Error was less than 3 mm in 79%, 82% and 80% of the measurements for the three observers, respectively. Intra-observer ICC was 0.96 (CI95% 0.94-0.97), 0.93 (CI95% 0.9-0.95) and 0.96 (CI95% 0.95-0.98) respectively. Inter-observer ICC for $HRCTV_{MR}$ width measures was 0.92 (CI95% 0.89-0.94) with no difference among FIGO stages. Inter-observer ICC for $HRCTV_{TRUS}$ was 0.86 (CI95% 0.81-0.9). For FIGO stage I/II, ICC $HRCTV_{TRUS}$ values were comparable to respective $HRCTV_{MR}$ values. For larger tumors $HRCTV_{TRUS}$ inter-observer ICC values were lower than respective $HRCTV_{MR}$ although remaining acceptable (tab.1)
Conclusion: TRUS is equivalent to MR in assessing preBT tumor maximum width in cervical cancer FIGO stage I/II. In advanced stages TRUS is slightly inferior to MR.

	Tumor width MR		Tumor width TRUS	
	ICC (intrcalss correlation coefficient)	CI 95%	ICC (intrcalss correlation coefficient)	CI95%
All stages	0.92	[0.89-0.94]	0.86	[0.81-0.9]
FIGO I	0.87	[0.75-0.94]	0.88	[0.76-0.95]
FIGO II	0.81	[0.74-0.87]	0.79	[0.7-0.86]
FIGO III	0.88	[0.75-0.96]	0.69	[0.42-0.87]
FIGO IV	0.93	[0.83-0.98]	0.62	[0.26-0.87]

[Inter-observer agreement analysis of tumor maximum width measurements]

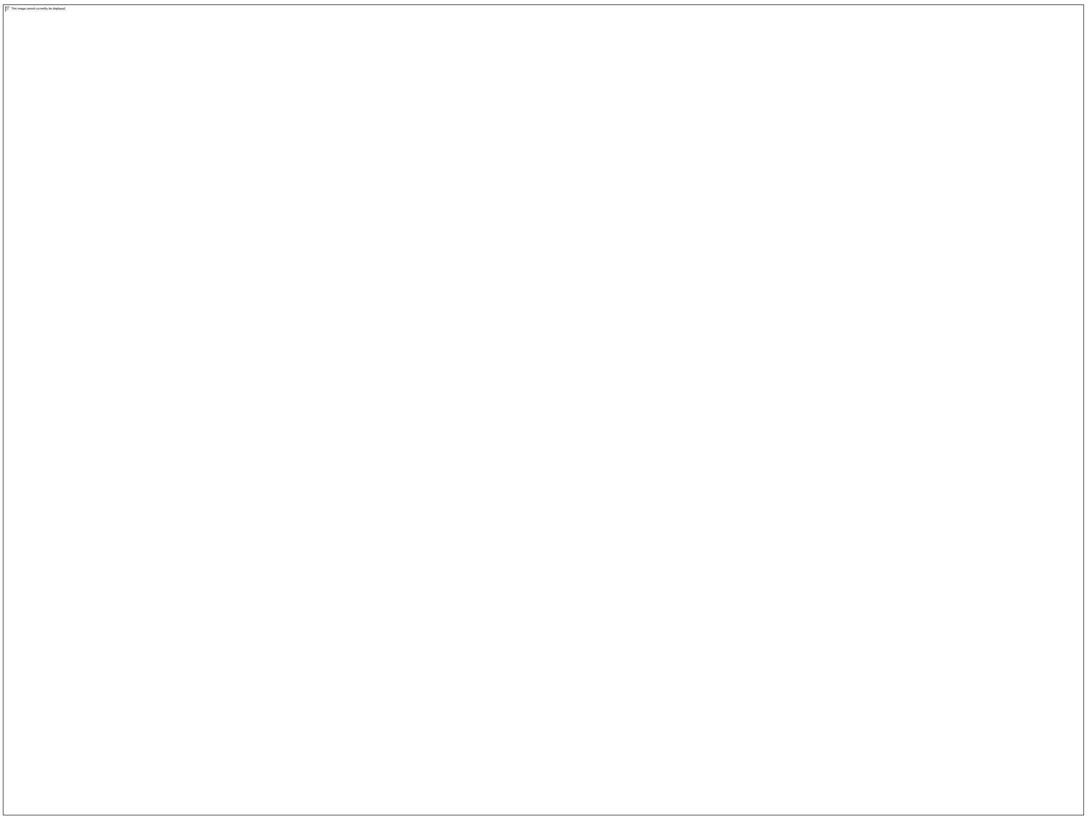

[Figure 1]

Disclosure: Nothing to disclose

EP289

Evolving radiotherapy strategies to treat very advanced (FIGO IIIB/IV) cervical cancers

M. Federico[1], G. Carta[1], V. Benito[2], O. Arencibia[2], M.D. Rey Baltar Oramas[1], A. Ramchandani[3], M. Lloret[1], F. Molano[3]

[1]Radiation Oncology Department, Hospital Universitario de Gran Canaria Dr. Negrin, [2]Gynecology, [3]Medical Oncology, Complejo Hospitalario Universitario Materno Infantil de Canarias, Las Palmas, Spain

Introduction / Background: Treatment of locally advanced cervical cancers with pelvic wall or surrounding organ invasion (FIGO IIIB/IVA) is a challenging situation and the clinical results have been traditionally poor. According the evolving of brachytherapy (BT) and imaging, our treatment aim has progressively become more radical and actually all patients are treated with external beam radiochemotherapy (EBRT) plus MR based Image Guided Adapted Brachytherapy (IGABT) and adjuvant chemotherapy (CT). Aim of this work is to revise this evolving trend over the past 19 years.
Methodology: Since January 2000, 108 cervical cancer patients with FIGO IIIB (because pelvic wall invasion) or IVA were discussed in multidisciplinary tumour board. Patients received radiotherapy treatment or best supportive care (BSC) depending on patient's clinical condition and treatment options available at that time (in our centre 3D-BT was introduced in 2006, IGABT in 2012, adjuvant CT in 2013).
All patients are in prospective follow up in radiation oncology and gynaecology department.
Results: 23 patients receved BSC, 19 EBRT only, 19 EBRT+ standard 3D BT, 28 received EBRT plus IGABT and 19 received EBRT plus IGABT plus adjuvant CT.
with a median follow up time of 53 months, 3 years overall survival was respectively 0%, 16.5%, 26.5%, 48.5% and 77.5%.
Excluding patients who received BSC from further analysis, the 3 years DFS was respectively 11%, 11%, 36% and 58%.
3 year local control was respectively 17.5%, 43%, 91.5% and 100%
3 year systemic control was respectively 53.5%, 62%, 49% and 78.5% (Fig.1)
Conclusion: The evolution the radiotherapy technique is shaping the outcomes of cervical cancer patients, even in the most difficult cases. If tumour control is at hand, chemotherapy strategies to improve systemic control have to be identified.

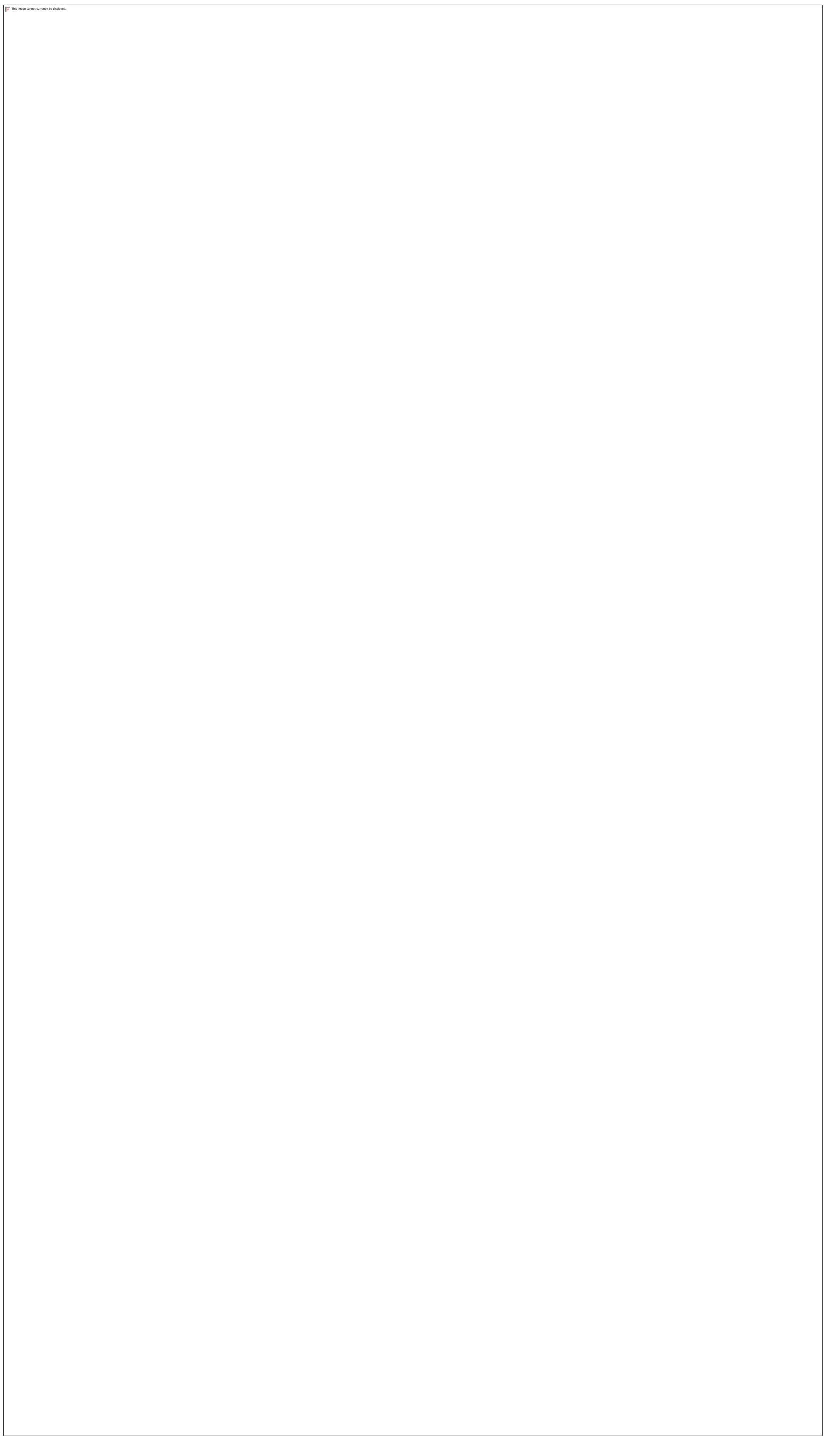

[Cervical cancer FIGO IIIB(pelvic wall invasion) and IVA]

Disclosure: Nothing to disclose

EP290

Epidermal and endothelial growth factors are associated with growth pattern of squamous cell carcinoma of the cervix

E. Frantsiyants, T. Moiseenko, N. Nazaralieva, I. Neskubina, Y. Pogorelova, A. Menshenina
Rostov Research Institute of Oncology, Rostov-on-Don, Russian Federation

Introduction / Background: The disease course, tumor progression and the response to certain types of treatment in patients with similar clinical stages and morphological forms of cancer often vary. The biological aggressiveness of the primary tumor can be assessed by examining its proliferative activity, apoptosis activity and the state of the main regulatory systems. The purpose of the study was a comparative analysis of VEGF-A, VEGF-D and EGF in cervical tissues in exophytic and endophytic growth patterns of cervical squamous cell carcinoma.
Methodology: Cervical tissues were obtained during surgery from 29 patients, mean age 51.5±1.7 years, with histologically verified non-keratinizing squamous cell carcinoma of the cervix T1b-2aN0M0 (endophytic n=9, exophytic n=20). The levels of growth factors (VEGF-A, VEGF-D, EGF) were determined by ELISA using standard test systems (BenderMedSystem, Austria; R&D System, USA).
Results: VEGF-A levels in endophytic tumors were 27.6 times lower than in exophytic tumors, i.e. the latter were characterized with VEGF-A overexpression. The lymphangiogenic growth factor VEGF-D was increased in endophytic tumors by 25.3% ($p< 0.05$). EGF in endophytic tumors was 38% lower than in exophytic tumors.
Conclusion: The expression of EGF and hyperactivation of an angiogenic component VEGF-A were typical of the exophytic growth pattern of cervical squamous cell carcinoma, while the endophytic growth was characterized by the VEGF-D activation accompanied by a moderate stimulation of the angiogenic signaling system (VEGF-A). The study showed fundamental differences in the involvement of growth factors in the formation of the growth pattern of cervical squamous cell carcinoma.
Disclosure: Nothing to disclose

EP291

Surgery for early cervical cancer: a 10-year retrospective outcome comparison between laparoscopic and open radical hysterectomy in an Irish Centre

E. Fullston[1], F. Abu Saadeh[2], N. Gleeson[2], C. Thompson[2]
[1]*Gynaecological Oncology,* [2]*St James's Hospital / Trinity College Dublin, Dublin, Ireland*

Introduction / Background: Following publication of the LACC trial, laparoscopic radical hysterectomy for the treatment of cervical cancer is now debated. We aimed to measure outcomes in our own population.This review aims to compare overall survival and risk of recurrence among women who underwent open versus laparoscopic radical hysterectomy for early cervical cancer at the largest provider of gynaecological cancer care in the Republic of Ireland over 10 years.
Methodology: All cases of cervical cancer diagnosed from January 2005 until January 2016 were reviewed. Patient characteristics, disease stage, histology, node status, method of treatment including surgery, adjuvant radiation or chemotherapy were recorded. Intervals to death and/or recurrence were calculated.
Inclusion criteria were: Stage 1A1, 1A2 or 1B1 adenocarcinoma, squamous cell or adenosquamous carcinoma of the cervix without node involvement; radical hysterectomy (open or laparoscopic).
Results: Of 608 new cases of cervical cancer, 476 were excluded (not treated surgically [n=392]; treated with surgery other than RH [n=18]; histology other than SCC/AC/ASC [n=14]; stage beyond IB1 [n=26]; node positive histology [n=11]; death but no recurrence [n=4]; lost to follow-up [n=11]). For the laparoscopic (n=54) and open (n=78) RH groups respectively, baseline characteristics were similar: mean age at diagnosis was 38.2 vs 43.9 years, histology was SCC in 64.8% vs 66.7%, adenocarcinoma in 7.4% vs 5.1% and adenosquamous in 29.6% vs 28.2%. Death occurred in 13.0% (n=7/54) vs 6.4% (n=5/78) [p=0.19]; recurrence was diagnosed in 13.0% (7/54) vs 9.0% (n=7/78) [P=0.46].

Conclusion: Our findings showed no statistical significance between open versus laparoscopic surgery in terms of recurrence rates and overall survival. However, numerically there were more deaths within the laparoscopic group. A further sub-analysis will be conducted on tumour volume, multimodal treatment with regards to risk of recurrence.
Disclosure: Nothing to disclose

EP292

Difficulties in the diagnosis of premalignant changes in menopausal women
A. Ganovska
Military Medical Academy, Sofia, Bulgaria

Introduction / Background: According to the World Health Organization classification, cervical intraepithelial neoplasia (CIN) is a premalignant condition that subdivides into a low-grade (LSIL) and high-grade squamous intraepithelial lesion (HSIL), depending on the degree of involvement of the multi-layer epithelium of the cervix. HSIL progression in invasive cervical cancer is seen in 10% to 40% of patients, which determines the need for accurate diagnosis and subsequent treatment.
Methodology: We have reviewed the data in the medical literature and summarized the results.
Results: The treatment of premalignant cervical conditions is determined by various factors such as age, parity, lesion size. Histological verification is a gold standard in the diagnosis of cervical dysplasia. There are a number of benign cervical changes such as atrophy and immaturity of the flat epithelium that can mimic cervical intraepithelial dysplasia. This necessitates the detection of additional diagnostic markers for the differentiation of high-grade intraepithelial changes from benign cervical changes.Differential differentiation resulting from age-induced atrophic processes uses immunohistochemical markers such as p16 and Ki67. Immunohistochemical expression of p16 is associated with dysplastic/neoplastic cells and is not detected in normal cervical epithelium. The extent of expression is dependent on the severity of cervical dysplasia. It is characterized by high sensitivity in the diagnosis of CIN 2 and CIN 3, but not of CIN 1.Quantitation of Ki-67 in the cells located in the two-thirds of the epithelium is specific in the detection of CIN. Atrophic changes affecting the uterine cervix Ki-67 only show sporadic staining of the basal/parabasal cells.
Conclusion: Morphological analysis remains the gold standard in the diagnosis and determination of the degree of CIN. Concomitant use of the immunohistochemical marker along with the morphological assessment of biopsy material from the cervix increases the diagnostic value and thus the efficacy of the treatment.
Disclosure: Nothing to disclose

EP293

Advanced squamous cell carcinoma of uterine cervix with metastases- unpredicted long survival obtained after stereotactic radiation directed to the lung metastases
M. Gawkowska, K. Drosik-Rutowicz, M. Steć-Cieślik, M. Stąpór-Fudzińska, R. Tarnawski
M. Sklodowska-Curie Memorial Cancer Branch in Gliwice Centre and Institute of Oncology, Gliwice, Poland

Introduction / Background: Expected median survival for patients with cervical carcinoma stage IV B is 17 - 26 months.
Methodology: This is a case report of 6 years survival for patients with metastatic cervical cancer
Results: 59 years old women was diagnosed with squamous cell cervical cancer in 2012.
MRI- revealed cervical tumor 7 cm involving anterior fornix, vaginal wall, parametrium and metastases to iliac lymph nodes . FDG/PET confirmed disease in pelvis and show metastases to III and X

segment of the right lung.
The patient was treated with palliative radiotherapy directed to pelvis (20 Gy, 4 Gy/fx). Completed response was achieved. After RT 4 cycles of chemotherapy (Carboplatin and Paclitaxel) stabilization was observed but the treatment had to be ceased because of toxicity .
Three months after the chemotherapy, CT show progression of the lung metastases. Stereotactic radiation therapy (SRT) was applied: 40 Gy, 20Gy/fx to the tumors. Complete regression on CT was observed but after 5 months a new lesion in the apex of the right lung was diagnosed. It was treated with SRT (45 Gy, 15Gy/fx) - three months after the treatment a complete response was observed but a new nodules was seen in the right hilum. This one was treated with SRT (30 Gy, 10 Gy/fx). A complete regression was observed.
The patient was well until the February of 2018 when local recurrence was diagnosed. The patient was reirradiated (30 Gy, 10Gy/fx). Near complete regression was seen. After one months a second local progression was observed. The patients underwent palliative chemotherapy, but it was discontinued due to lack of effectiveness.
The treatment described above allowed sixed year survival for patients with metastases to lung
Conclusion: Agressive treatment is warrant in radiosensitive cervical cancer, even in case of multiple metastases
Disclosure: Nothing to disclose

EP294

Comparison of triage management between p16INK4a/Ki67 dual staining and HR-HP test in patients with cervical cytology ASC-US and LSIL

L. Geng, Y. Li, X. Ge, K. You, Y. Guo
Peking University Third Hospital, Beijing, China

Introduction / Background: To compare the triage management between $p16^{INK4a}$/Ki67 dual staining and HR-HPV test detection in patients with cervical cytology ASC-US and LSIL.
Methodology: 143 patients with cervical cytology results as ASC-US and LSIL were enrolled in the gynecological clinic of the Third Hospital of Peking University from June 2016 to July 2018. The average age was 38.83 years. All the participants underwent dual staining test of $p16^{INK4a}$/Ki67, HR-HPV test, colposcopy and biopsy. CINtec PLUS kit (Roche, USA) was used for $p16^{INK4a}$/Ki67 dual staining and Cobas 4800 detection system (Roche, USA) was used for HR-HPV test.
Results: A total of 143 participants were enrolled with 86 ASC-US cases（86/143, 60.1%）and 57 LSIL cases（57/143, 39.9%). Cervical biopsy results showed that 35 cases（35/143, 24.5%）were ≤CIN1, 72 cases (72/143, 50.3%) were CIN2, 29 cases (29/143,20.3%) were CIN3 and 7 cases (7/143, 4.9%) were cervical cancer. In patients with cervical cytology results as ASC-US and LSIL, the sensitivity of $p16^{INK4a}$/Ki67 dual staining and HR-HPV test for detection CIN 2 or above were 85.2% and 75.4% (p=0.078), with the specificity of 54.3% and 22.2% (p< 0.001), and the AUC of ROC curve was 0.697 (95%CI 0.615-0.771), 0.504 (95% CI 0.419-0.588) (P < 0.001), and referral rate of colposcopy was 75.5% and 93.7% (p=0.001).
Conclusion: In patients with cervical cytology ASC-US and LSIL, $p16^{INK4a}$/Ki67 dual staining shows a higher specificity in detecting lesions of CIN2 or above and lower referral rate of colposcopy than that of HR-HPV test, and the sensitivity was also higher in $p16^{INK4a}$/Ki67 dual staining. In patients with cytological ASC-US and LSIL, $p16^{INK4a}$/Ki67 dual staining triage was more effective than HR-HPV test in detecting CIN2 or above lesions.
Disclosure: Nothing to disclose

EP295

Investigating the effect of hypercapnia, hypoxia and pH on cellular proliferation in cervical cancer

L. Gentles[1], S. Sharma Saha[1], M. Case[1], P. Korompelis[2], Y. Drew[1], N. Curtin[1], R.L. O'Donnell[1,2]
[1]Northern Institute for Cancer Research, Newcastle University, [2]Northern Gynaecological Oncology Centre, Newcastle upon Tyne, United Kingdom

Introduction / Background: In the phase III LACC cervical cancer trial, shorter disease-free survival and overall survival was observed in patients treated with minimally invasive radical hysterectomy in comparison to traditional open surgery. One hypothesis for this difference is an adverse effect of the prolonged exposure to the hypoxic, CO_2-rich, acidotic environment created during minimally invasive surgery. Greater cervical tumour hypoxia-inducible factor 1α (HIF-1α) indicating tissue hypoxia has previously been shown to be a strong independent prognostic marker in early stage cervical cancer. This pre-clinical study aimed to evaluate the effect of hypercapnia, hypoxia and pH on cervical cancer cell growth.
Methodology: Three cervical cancer cell lines (Hela, SiHa, C33ca) were incubated in variable CO_2 (5-20%) and O_2 (5% -20%) conditions, in an InvivO_2 incubator with HEPES medium for 3 doubling times, standardised for humidity and temperature. Growth rates were assessed by Sulforhodamine B colorimetric (SRB) assays with measurement of pH in spent media. Matched controls were incubated in routine physiological conditions.
Results: There was a 1.5-fold difference in doubling time of cell lines under physiological conditions with doubling times ranging from 22 to 34 hours. Incubation in a hypercapnic environment persistently resulted in growth inhibition across all three cell lines at all seeding densities, with mean growth inhibition of 45.0% (SD 16.0%) at 20% CO_2. Inhibition of growth was greatest in C33a, the most rapidly dividing cell line with mean reduction in growth of 55.5%. pH remained within physiological range falling by a mean of 0.45 to a minimum of 7.12.
Conclusion: Hypercapnia results in growth inhibition in cervical cancer cell lines, independent of acidosis. It is unlikely that exposure to the CO_2 rich intra-operative environment during minimally invasive surgery is responsible for the poor prognosis reported in the LACC trial.
Disclosure: Nothing to disclose

EP296

Vaginal fertility-sparing surgery and laparoscopic sentinel lymph node detection in early cervical cancer. Oncological outcomes and long-term follow-up

B. Gil-Ibañez, A. Glickman, M. del Pino, D. Boada, P. Fuste, B. Diaz-Feijoo, N. Carreras, N. Agusti, E. Mension, J. Pahisa, A. Torne
Hospital Clinic Barcelona, Barcelona, Spain

Introduction / Background: The aim of this study was to analyze the oncological outcomes and risk factors for recurrence of vaginal fertility-sparing surgery (FSS) and laparoscopic sentinel lymph node biopsy (SLNB) in patients with early cervical cancer (CC).
Methodology: From March 2005 to April 2018, 38 patients diagnosed with early stage CC underwent vaginal FFS at the Hospital Clínic, Barcelona, Spain. Patients with FIGO stage IA1 with lymphovascular space invasion (LVSI) and stage IA2 underwent simple trachelectomy and patients with stage IB1 underwent vaginal radical trachelectomy (VRT). All cases underwent laparoscopic SLNB. In the first cases, laparoscopic bilateral pelvic lymphadenectomy (LBPL) was completed immediately after SLNB. Clinical and oncological follow-up data were prospective collected.
Results: The median age at diagnosis was 33.5 years old (range 22-44). Simple trachelectomy was performed in seven cases (18 .4%) and VRT in 31 (81.6%). Nineteen patients were treated with laparoscopic SLNB exclusively and 19 with SLNB plus LBPL. There were no significant differences between the two groups regarding histology and tumor size. The median follow-up was 73 months (range 1-160 months). There were 4 recurrences (3 patients with IB1 and one in IA2). Two occurred in the SLNB group and 2 in the SLNB plus LBPL group. All recurrences were diagnosed in patients with adenocarcinoma tumors and three without LVSI.
Conclusion: Vaginal FFS with laparoscopic SLNB seems to be a safe oncological procedure in early stage CC patients. Further studies are needed to clarify the role of adenocarcinoma histology as a

prognostic factor of recurrence.
Disclosure: Nothing to disclose

EP297

Different techniques for sentinel lymph node detection in early cervical cancer: one-center experience

A. Glickman[1], B. Gil-Ibañez[1], P. Paredes[2], B. Diaz-Feijoo[1], M. Del Pino[1], P. Fusté[1], N. Carreras[1], E. Mensión[1], N. Agustí[1], A. Perissinotti[2], S. Vidal-Sicart[2], J. Pahisa[1], A. Torné[1]
[1]Gynecological Cancer Unit, [2]Nuclear Medicine, Hospital Clinic Barcelona, Barcelona, Spain

Introduction / Background: In early-stage cervical cancer, nodal involvement remains the most important prognostic factor**.** Imaging evaluation is not sensitive enough to replace histology. Achieving high detection rates is a prerequisite to introduce sentinel lymph node biopsy (SLNB). The aim of this study was to evaluate the evolution of SLNB detection techniques in women with early-stage cervical cancer treated in our institution.
Methodology: From 02/2001 until 01/2019, patients with FIGO 2009 stages IA1-IB1/IIA1 cervical carcinoma underwent SLNB. Those treated until 01/2010 endured SLNB followed by pelvic bilateral lymphadenectomy, thereafter only SLNB was performed. When SLNs were detected unilaterally or not detected, lymphadenectomy of the failed mapped side was performed. SLNB was achieved by laparoscopy after intracervical injection: first with blue dye (BD) plus radiocolloid (Tc) and, since 2014, with indocyanine green (ICG) also. SLN identification was performed with lymphoscintigraphy and SPECT/CT, facilitated by a laparoscopic gamma-probe and a fluorescence camera. Detection rate was calculated as the number of procedures in which at least one SLN was identified intraoperatively, divided by the total number of procedures undertaken.
Results: From 164 patients treated, 129 underwent SLNB with BD+Tc and 35 with BD+Tc+ICG. No differences regarding age at diagnosis, size of the tumor (≤ 2cm vs > 2cm), FIGO stage, histologic subtype and surgical radicality were seen between both groups. In the first group, at least one SLN was identified in 123 cases, for an overall detection rate of 95.4%. The rate of bilateral detection was 61.2%. For the triple-technique group we detected SLNs in 35 cases, prompting a detection rate of 100%. SLNs were bilaterally detected in 34 patients (97.1%).
Conclusion: Both implemented techniques for SLNB in early-stage cervical cancer show a high detection rate. Nevertheless, triple technique with BD+Tc+ICG allows bilateral SLN detection in almost all patients.
Disclosure: Nothing to disclose

EP298

Nerve-sparing versus non-nerve-sparing radical hysterectomy: surgical and long-term oncological outcomes

N.R. Gómez-Hidalgo[1], A. Gil-Moreno[1], M. Carbonell Socias[1], S. Salicrú[2], M. Bradbury[2], A. García[3], R. Vergés[4], O. Puig Puig[1], J.L. Sánchez-Iglesias[1], S. Cabrera-Diaz[1], J. De la Torre[1], A. Pérez Benavente[1], J. Magriña[5], B. Díaz-Feijoo[1]
[1]Department of Gynecology Oncology, [2]Department of Gynecology, [3]Department of Pathology, [4]Radiotherapy Department, Vall d´Hebron University Hospital, Barcelona, Spain, [5]Department of Medical and Surgical Gynecology, Mayo Clinic Hospital, Phoenix, AZ, United States

Introduction / Background: There are controversies regarding the long-term oncological safety of preservation of pelvic innervation during radical hysterectomy (RH). This study aimed to analyze the feasibility and safety of nerve-sparing radical hysterectomy (NSRH) for cervical cancer compared with

non-NSRH following 17 years of experience in a tertiary cancer referral center.
Methodology: Between May 1999 and June 2016, all patients who underwent RH for cervical cancer were followed-up prospectively. Comparison analyses regarding surgical outcomes, complications, overall survival (OS), disease-free survival (DFS), and cancer-specific survival (CSS) were performed between patients treated with NSRH and non-NSRH.
Results: A total of 188 patients were included (113 non-NSRH and 75 NSRH). The median follow-up was 112 months. Estimated blood loss and hospital stay were all significantly lower in the NSRH group. Overall intraoperative complication rate (p=0.02) and need for transfusion (p=0.016) were lower in the NSRH group. There were no differences in the median operation time, OS, DFS, CSS, or recurrence rates between the NSRH and non-NSRH group.
Conclusion: Our study provides a wide perspective on the developments of nerve-sparing procedures for the management of women with early-stage cervical cancer. Our results suggest that NSRH is a feasible and safe procedure, with reduced morbidity outcomes.

This image cannot currently be displayed.

[Nerve Sparing Technique: Key steps]

Disclosure: Nothing to disclose

EP299

The impact of surgical approach on cervical cancer survival rates

M. Gracia Segovia, A. Casajuana Pérez, V. González González, M. Ramírez Mena, M. Bellón del Amo, J. García Santos, P. Coronado Martín, M.Á. Herráiz Martínez
Hospital Clínico San Carlos, Madrid, Spain

Introduction / Background: After LACC trial results, there is controversy about surgical approach in early stage cervical cancer. The aim of this study is to compare morbidity and mortality, as well as disease recurrence using two different techniques at our centre, Hospital Clínico San Carlos.
Methodology: Historical Cohort Study of women who underwent surgery in the initial stage of cervical cancer by total or radical hysterectomy +- lymphadenectomy. 53 patients operated on by laparotomy from 2000-2009 (group A) were compared with 53 patients operated on endoscopically (laparoscopic or robotic surgery) from 2009-2018 (group B).
Results: Both groups were homogeneous. Intra-operative complications were more frequent in Group B (p=0,045) but no differences in post-operative complications were found. Although reinterventions were scarce, they were performed later in laparotomy, while immediate re-operations were more frequent in group B (p = 0.02). Postoperative recovery was faster after an endoscopic approach, leading to shorter hospital stays (p< 0,05). Group A had 16% tumoral recurrence compared to 20% in Group B. There was no significant difference in disease-free survival (DFS) at 5 years, with 87% for laparotomy versus 77% for the endoscopic approach. Mortality in early stage cervical cancer was extremely low in both groups, with no significant difference with regard to surgical approach.
Conclusion: Early stage cervical cancer can be treated by laparoscopy or laparotomy. Although there were no differences in total mortality, DFS appeared to be higher when laparotomy was used. Despite the fact that the endoscopic approach had higher immediate reoperation rates, hospital stays were significantly shorter and subsequent complication rates were lower. Thus, surgical approach should be a surgeon's choice.
Disclosure: Nothing to disclose

EP300

Minimally invasive versus open-way surgery in early stage Cervical Cancer: SENTICOL I and SENTICOL II three years follow up

B. Guani[1], V. Balaya[2,3], L. Magaud[4,5], F. Lecuru[2,3], P. Mathevet[1,6], SENTICOL Group
[1]Department of Gynecology, CHUV - Centre Hospitalier Universitaire Vaudois, Lausanne, Switzerland, [2]Gynecologic and Breast Oncologic Surgery Department, Georges Pompidou European Hospital, [3]Faculty of Medicine, Paris Descartes University, Sorbonne Paris Cité, Paris, [4]Service Recherche et Épidémiologie Cliniques, Hospices Civils de Lyon, Pôle Santé Publique, [5]Univ Lyon, Université Claude Bernard Lyon 1, Université Saint-Étienne, HESPER EA 7425, Lyon, France, [6]University of Lausanne, Lausanne, Switzerland

Introduction / Background: The LACC trial has recently assessed the impact of surgical approach on oncological outcomes in early-stage cervical cancer and found better outcomes in favor of open-way. In this study, we propose to assess this impact through data analysis of 2 French multicenter prospective cohort.
Methodology: We analyzed the data of patients who participated in the prospective study SENTICOL I and SENTICOL II. We analyzed the disease-free survival (DFS) and the overall survival (OS) at 3 years in consideration of surgical access: laparotomy (LPT), laparoscopy (LPS), assisted vaginal laparoscopy (vaginal-assisted LPS) and vaginal. We included only patients with final FIGO 2009 stage IA1 with positive lymph-vascular invasion, IA2 and IB1. We considered age, type of cancer, stage, grade, tumor size, stromal invasion, lymph-vascular invasion, lymph nodal status, preoperative brachytherapy, and adjuvant therapy. The type of surgery was simple or radical hysterectomy and trachelectomy with sentinel lymph node biopsy alone or sentinel lymph node and lymphadenectomy. 3 years follow-up was analyzed with recurrence rate, localization of recurrence and survival rate.

Results: In total, 257 patients were operated by hysterectomy or trachelectomy with a follow up of 3 years. Eighteen patients had recurrences with a global 3-years DFS at 93%.
We found a statistically significant decrease of DFS in patients operated by LPT. In particular, among the 191 radical hysterectomies, patients who underwent surgery by LPT had a significant DFS decrease in comparison with minimally invasive surgical approach, especially with vaginal-assisted LPS (p=0,03). Moreover, only age higher than 70 years influenced the risk of recurrence at 3 years (p<0,01).
Conclusion: Unlike the LACC trial results, our French multicenter prospective studies showed better oncological outcomes in patients operated by minimally invasive surgery. Age is confirmed to be a significant risk factor, as previously suggested in our recent article.
Disclosure: Nothing to disclose

EP301

Free radical processes in the blood of patients with cervical cancer receiving various postoperative treatment modalities

I. Goroshinskaya, N. Popova, A. Menshenina, L. Nemashkalova, A. Shikhlyarova, E. Frantsiyants, I. Neskubina, E. Surikova, O. Kit, M. Gusareva, A. Pustovalova
Rostov Research Institute of Oncology, Rostov-on-Don, Russian Federation

Introduction / Background: The purpose of the study was a comparative analysis of the intensity of lipid peroxidation (LPO) and antioxidant system in the blood of cervical cancer (CC) patients receiving treatment with and without xenon therapy.
Methodology: The study included 20 CC patients (T1B-T2BN_xM0). After extended panhysterectomy, 13 patients received 5 sessions of xenon inhalation therapy, while 7 patients were controls. Blood levels of malonic dialdehyde (MDA) and diene conjugates (DC), the activity of superoxide dismutase (SOD), catalase and glutathione system were measured by standard spectrophotometric methods. The data were analyzed using Statistika 6.0 programs, Student´s t-test and Mann-Whitney U test.
Results: Before treatment, CC patients showed increased levels of MDA in plasma by 101.8%, in erythrocytes by 59.7%, DC in plasma by 4.4 times, in erythrocytes by 6.6 times, compared to non-cancer women (p< 0.01-0.05). LPO intensification was accompanied by a decrease in activity of SOD by 31.4%, catalase by 21.7% (p< 0.001) in erythrocytes on day 2-3 after the surgery. Increased activity of glutathione peroxidase (GPO) by 102.7% before surgery and by 63.4% after surgery (p< 0.001-0.01) probably was compensatory. Xenon therapy led to a decrease in the plasma LPO intensity: DC levels were 2.5 times lower than before treatment, and MDA levels were significantly lower by 25.2% than in patients without xenon therapy. This correlated with partial normalization of antioxidant enzyme activity - SOD activity was 1.9 times higher than in patients without xenon therapy (p=0.0005), catalase activity 13.7% higher, and GPO activity decreased by 30.6% compared to initial levels (p=0.005), while patients without xenon therapy showed increased GPO (81.6% higher than the norm, p=0.003) accompanied by statistically significant decrease in reduced glutathione level.
Conclusion: Xenon therapy decreases LPO, initially increased in CC patients. This is facilitated by partial normalization of the activity of the main antioxidant enzymes.
Disclosure: Nothing to disclose

EP302

Low-dose ionizing radiation activates antioxidative defense system and immunological reactivity in patients with cervical cancer

A. Menshenina, E. Frantsiyants, T. Moiseenko, L. Rozenko, E. Zlatnik, E. Sheiko, M. Gusareva
Rostov Research Institute of Oncology, Rostov-on-Don, Russian Federation

Introduction / Background: Standard doses of ionizing radiation inhibit antioxidant systems (AOS) and immunological reactivity of the body (IRB) in patients with cervical cancer (CC). Our purpose was to analyze AOS and IRB parameters at fractionation of doses of ionizing radiation (FDIR).
Methodology: The study included 69 patients with T3NxMo CC receiving FDIR: single dose of 2Gy daily, 5 fractions per week, intracavitary 60 Co therapy 5Gy twice a week. The total dose at the point A 85±3.6 Hz, point B 59.5±2.2 Hz. AOS and IRB at 10Gy, 20Gy and 40Gy were studied. Comparison group included 45 healthy women.
Results: FDIR 10-20 Gy mobilized endogenous AOS: the content of vitamins A and E and E/A in leukocytes was normal, SOD/TPA decreased by 33.4% and 27%, catalase levels in neutrophils increased by 3 times vs. initial levels ($p \leq 0.05$). Imbalance in enzymatic and nonenzymatic AOC units disappeared. IRB increased: an amount of T lymphocytes with CD4+ and CD8+ markers by 33% and 60%, CIC decreased by 26% ($p \leq 0.05$). Immunoregulatory index decreased by 16.2% ($p \leq 0.05$) and was close to normal levels. The amount of natural killers increased (CD16+) by 34% ($p \leq 0.05$). At a dose of 40 Gy: E and A decreased by 5.6 and 17 times, catalase by 1.9 times ($p \leq 0.05$); SOD and TPA indicators increased by 2.4 times ($p \leq 0.05$). The absolute number of lymphocytes was suppressed due to CD4+ by 3.6 times and CD16+ by 4 times ($p \leq 0.05$). CIC levels were twice higher than initially ($p \leq 0.05$), and immunoregulatory index was lower than the norm and the data at 10 Gy by 27% and 28%, respectively ($p \leq 0.05$).
Conclusion: Doses of 10-20 Gy mobilize endogenous AOS, increasing the content of E and A vitamins in blood cells, normalize the permeability and elasticity of the blood cell membranes, and stimulate the helper-inductor and cytotoxic function of leukocytes.
Disclosure: Nothing to disclose

EP303

Combination treatment of radiation injuries of the rectum in patients with pelvic tumors

O. Rodionova, V. Voshedskiy, S. Vlasov, A. Solntseva, M. Gusareva
Rostov Research Institute of Oncology, Rostov-on-Don, Russian Federation

Introduction / Background: In the overall structure of cancer incidence, pelvic tumors together comprise more than 25%. Currently, radiation therapy is one of the main treatments for malignant tumors of the pelvic organs. According to the literature, the rate of chronic proctitis as a rectal complication varies from 10 to 32%. Our purpose was to evaluate the effectiveness of a multicomponent treatment with pararectal blockade for radiation injuries of the rectum after chemoradiation treatment (CRT) of gynecological cancer.
Methodology: The study included 18 patients with cervical cancer T3NxM0 after CRT; 7-10 months after CRT, all patients were diagnosed with erosive and ulcerative radiation-induced rectitis (1 and 2 degrees by RTOG). Patients were divided into 2 groups: main group - 9 patients, conservative therapy with pararectal blockades; control group - 9 patients, only conservative therapy. Conservative therapy involved hemostatic, anti-inflammatory, antimicrobial therapy, vitamin therapy; drugs improving microcirculation and blood aggregation properties. Pararectal block was performed by administering a set of preparations to the ischemic zones, including: novocaine 0.25%; combilipen; prednisone; ceftriaxone. A course consisted of 10 procedures. The dynamics of clinical symptoms, indicators of clinical blood analysis, RRS data, MRI, ultrasound results were evaluated.
Results: The main group of patients by the end of the first course of therapy showed a decrease in the severity of clinical symptoms, normalization of bowel function, no impurities in feces. The control RRS revealed the relief of exudative inflammation, cleansing and epithelization of ulcers by the end of the therapy course. The control group showed a temporary insignificant improvement followed by the resumption of clinical manifestations with a recurring character.
Conclusion: Pararectal blockades in addition to conservative therapy lead to a more rapid decrease in the severity of clinical symptoms of radiation rektitis and regression of CRT-induced disorders, which improves the quality of life of patients and reduces a rehabilitation period.
Disclosure: Nothing to disclose

EP304

Prevalence of preinvasive lesions in HPV 16/18 positive cases and HPV other positive with cytology negative cases in our region

K. Güzin[1], A. Özer[2], H.N. Şahpaz Kurşun[3], M. Hocaoğlu[4], H. Gökaslan[5]
[1]Kahramanmaraş Sütçüimam University, Faculty of Medicine, Department of Obstetrics and Gynecology, Devision of Gynecologic Oncology, Sütcü Imam University, [2]Kahramanmaraş Sütçüimam University, Faculty of Medicine, Department of Obstetrics and Gynecology, Devision of Gynecologic Oncology, Sütçü Imam University, [3]Kahramanmaraş Sütçüimam University, Faculty of Medicine, Department of Obstetrics and Gynecology, Devision of Gynecologic Oncology, Sutçü İmam University, Kahramanmaraş, [4]İstanbul Medeniyet University Göztepe Education and Resorce, Faculty of Medicine, Department of Obstetrics and Gynecology, Devision of Gynecologic Oncology, İstanbul Medeniyet University Göztepe Education and Resorce, [5]Marmara University Medical Faculty Foundation, Faculty of Medicine, Department of Obstetrics and Gynecology, Devision of Gynecologic Oncology, Marmara University Medical Faculty Foundation, Istanbul, Turkey

Introduction / Background: Cervicalcancer is thefourthmostcommoncancerforwomenworldwidewith a greatproportionprovedto be relatedtohumanpapillomavirus (HPV) infection, especiallytype 16 and 18. Inthisstudy, weevaluatedthefrequency of HPV typedistribution, and colposcopic findings.
Methodology: The results of cytology and colposcopy of 119 HPV positive patients who were referred to the outpatient clinic of Kahramanmaras Cancer Early Diagnosis Screening and Training Center (KETEM) by KSU Gynecologic Oncology outpatient clinic were evaluated. Patients underwent routine colposcopy regardless of their cytology
Results: The mean age of the patients with HPV was 44.2. The most common HPV types are; 16,31,51 (49,19,17 patients). In 31,9% of PPV positive subjects, abnormal cytology was detected. 17,6% of the cases were ASCUS, 5,8% LSIL, 0,84% ASC-H. Was 5.04% HSIL and 2.5% AGC. The number of patients with HPV positivity except than HPV16 and 18 was 60 (50.4%). The number of patients with HPV positivity except HPV16 and 18 was 43 (71.6%) with normal smear results. While the pathological evaluation was normal in 28 (65.1%) of 43 patients who had normal smear biopsy, the pathology result of 8 (18.6%) patients were CIN2 and above.7 (16.2%) patients were reported as CIN 1. HPV 16 and/ or 18 positive patient was 59. Smear results were normal in 33 patients, insufficient in 3 patients and abnormal in 23 patients. As a result of the colposcopic biopsy of 59 patients, 1 (1.6%) patient had anedocarcinoma, 28 (47%) had CIN2 and above, 23 (38.9%) had normal and 7 (11.8%) had CIN 1.
Conclusion: Cytology is negative and in the HPV other type positive group (CIN2 and above in 8 of 43 patients %18,6), high preinvasive lesion is not only the type 16 and / or 18 positive ones in follow-up (especially type 39,31,51). Because of our finding of high preinvasive lesions in other types, review of our guidelines should be considered in the follow-up of patients
Disclosure: Nothing to disclose

EP305

Rhabdomyosarcoma (RMS) of the uterine collar by the way of a case

F. Hadjarab[1], C.E. Chekman[2], A. Taibi[1], K. Bouzid[1]
[1]Medical Oncology, Centre Pierre Marie Curie Alger, [2]Surgery, Centre Anticancereux Pierre Marie Curie, Algiers, Algeria

Introduction / Background: Rhabdomyosarcoma (RMS) of the uterine collar is a rare tumour. It electively occurs atthe young woman. So formerly one preachedan aggressive surgical treatment goinguntil the pelvic exenteration, one currentlyrecommends a preserving treatmentsometimes limited to a conisation, associatedwith L chemotherapy has and whose resultsare encouraging.
Methodology: Miss FN 19 years old, without pathologicalantecedents having regular

menstruationssince the 13 years age followed since 2014 for rhabdomyosarcome of the uterine collar, marked by protrusion through the vagina of anoperated mass has 4 recoveries exérèse typeexteriorized part whose histological study wasin favour of a rhabdomyosarcome embryonicbotriode type keeps II. The therapeutic decision of the multi-fieldcommittee was to make a chemotherapy first has standard 8 IVAO (Ifosfamide, Mesna,Vincristine, Doxorubicine.
Results: Answer is considered to be partial with a tumoral weightsaving, disappearance of necroses as well as the haemorrhage it profited from an ovarian transposition by coelioscopy, then a exérèse of the mass (pédiculée of the uterine collar of 5 cm and been confined by the vagina) by gynaecological way, whose anapath was in favour of cervical Rhabdomyosarcome with healthy zones ofresection the treatment was continued by acuriethérapie (4 fractions) in March 2016. Currently in August 2016, the patient is incomplete remission with a pelvic scannerthoraco abdomino and MRI pelvic of evaluationwithout characteristic and it had a return of themenstruations in May 2016.
Conclusion: The RMS of the uterine collar is a rare tumor which occurs primarily in the young girl. The extension is especially loco régionale. The treatment consists of a surgical gesture with minima associated with an perish-operational chemotherapy.
Disclosure: NO ANY CONFLICT

EP306

Is ultrasound KUB a useful investigation following radical hysterectomy?

A. Haken, A. Sharma, K. Lim, R. Howells
Gynaecology Oncology, University Hospital of Wales, Cardiff, United Kingdom

Introduction / Background: Incidence of iatrogenic ureteric injury during gynaecological surgery occurs in approximately 0.5-1.5% of cases with the majority occurring in benign cases. More than 70% are diagnosed during the post-operative period. However radical hysterectomy for malignancy significantly increases the risk of ureteric injuries (between 5 - 30%) and complications related to it. In view of this, the local guidelines at University Hospital of Wales recommend removing the urinary catheter at day 7 post-operatively, after an ultrasound of the kidneys, ureters and bladder (US KUB) to ensure there are no occult injuries.
Methodology: The aims were to determine: if all patients undergo KUB imaging following radical hysterectomy; incidence of ureteric injury following radical hysterectomy for oncology patients. Retrospective audit of online patient records and clinical notes for all patients undergoing radical hysterectomy between January 2017 and October 2018 at University Hospital of Wales. Data collection and analysis performed using Microsoft Excel Version 16.
Results: A total of 37 radical hysterectomies were performed, 86% (n=32) for cervical cancer. 92% (n=34) patients underwent US KUB (65% of which occurred on day 7[n=22]; 24% [n=8] by day 10 with delay due to Public Holidays in 3 cases; and 12% [n=4] prior to day[AS1] 7, 2 of which were to exclude suspected post-operative complications). Of all US KUB performed 91% (n=31) were normal, and 6% (n=2) showed mild hydronephrosis only and one patient required a CT urogram for clarification. No urinary tract injuries were noted.
Conclusion: The majority of patients had imaging at the appropriate time post-operatively. There were no urinary tract injuries identified with the US KUB however where suspicion was raised further investigation was carried out to exclude injuries. Hence US KUB is a simple yet useful tool, without risk of radiation exposure, to detect injuries in the immediate post-operative period and deal with them.
Disclosure: Nothing to disclose

EP307

The role and optimal regimen of radiation therapy for patients with bone metastasis from

uterine cervical cancer

M. Hata[1], I. Koike[1], M. Asai-Sato[2], H. Kaizu[1], Y. Mukai[1], S. Takano[1], E. Ito[1], M. Sugiura[1], M. Sato[1], R. Taniuchi[1], E. Miyagi[2]
[1]Department of Radiation Oncology, [2]Department of Obstetrics and Gynecology, Yokohama City University Graduate School of Medicine, Yokohama, Japan

Introduction / Background: Patients with bone metastasis from uterine cervical cancer usually have a poor prognosis and are candidates for palliative care. We therefore carried out a review to determine the role of radiation therapy for patients with bone metastasis from uterine cervical cancer and to identify the optimal radiation regimen.
Methodology: A total of 21 patients with bone metastasis from uterine cervical cancer received radiation therapy to the pelvis. All patients received external irradiation and 13 patients also received high-dose-rate intracavitary brachytherapy. The median total dose of 60.4 Gy (range, 32.4-76.2 Gy) was delivered to cervical tumors of all patients and corresponded to 62.1 Gy (range, 31.9-83.3 Gy) in the 2 Gy per fraction-equivalent dose (EQD2). Thirteen patients underwent chemotherapy during and/or following radiation therapy.
Results: In 19 of 21 patients, the primary tumors disappeared or were markedly reduced after radiation therapy. In all but one of 18 patients with pelvic pain and bleeding, these symptoms disappeared or were remarkably relieved. However, three patients had primary tumor progression at 7, 9, and 15 months after irradiation with total doses of 56.8, 58.4, and 68.3 Gy in EQD2, respectively. Two of these patients had relapses of bleeding and pain. The primary progression-free rates in all patients were 73% at 1 year, and 37% at 2 years. The overall survival rates were 38% at 1 year, and 9% at 2 years, with an estimated median survival time of 7 months. The number of metastatic bone sites ($p = 0.016$) and administration of chemotherapy ($p < 0.001$) were significant prognostic factors for survival.
Conclusion: Radiation therapy is very effective for relief of pelvic symptoms in patients with bone metastasis from uterine cervical cancer. For patients who are expected to survive for more than 1 year, almost curative-dose irradiation to primary tumors is recommended.
Disclosure: Nothing to disclose

EP308

Postoperative morbidity of radical hysterectomy and pelvic lymphadenectomy in cervical cancer

M. Hernandez Ontoria, A. Vázquez Sarandeses, G. López González, Á. Tejerizo Garcia
Gynecology and Obstetrics, Hospital 12 Octubre, Madrid, Spain

Introduction / Background: Analysis of postoperative development and complications in cervical cancer treated with radical surgery.
Methodology: Retrospective unicentric study which includes patients with cervical cancerhaving a radical hysterectomy and pelvic lymphadenectomy as a first line of treatment were included between 2009 and 2017 from a Spanish third level hospital.
Results: Forty-one patients met the inclusion criteria. The mean age at the moment of cervical cancer diagnosis was 50,54 (30, 08 - 78,74) years. Depending on stage (FIGO 2016) we found: 17,9% IA, 74,4% IB1, 2,6% IB2 2,6% IIA, 2,6% IIB. Laparoscopy approach were performed in 92,7% of the cases and in 7,3% open approach was performed.
The mean of postoperative length of hospital stay was 5,5 (SD2,31) days. 23 patients were collected data about time to oral tolerance and to removing urinary catheter. All of them started oral feeding the first postoperative day, apart from one of them who started oral tolerance the surgery day. The median of postoperative urinary catheter was 3 (1-15 days).
80,5% (33/41) cases were not register intraoperative adverse events. The urinary and neurologic complications rates were 9,8% and 7,3%. Early complications, defined as complications in the first 30 days after the surgery, were had by 24,4% of the patients. Lower limb lymphedema was the main early complication developed in 7,3% of the cases, other early complications registered in lower rate were: lower limb paresthesia, ureterovaginal fistula, rectovaginal fistula and dehiscence/prolapse of vaginal vault.

Conclusion: Patients with cervical cancerhaving a radical hysterectomy and pelvic lymphadenectomy as a first line of treatment had a low rate of intraoperative and early postoperative complications.
The main intraoperative complications were urologic. In most of the cases early oral tolerance and early removing urinary catheter were possible.
Disclosure: Nothing to disclose

EP309

Survival analysis in advanced cervical cancer

M. Hernandez Ontoria, A. Vázquez Sarandeses, G. López González, Á. Tejerizo Garcia
Gynecology and Obstetrics, Hospital 12 Octubre, Madrid, Spain

Introduction / Background: To assess the survival in advanced cervical cancer treated with external beam radiation (EBRT) concurrent chemotherapy (CT) followed by brachytherapy (BT).
Methodology: Retrospective unicentric study which includes patients with advanced cervical cancer treated with (EBRT) concurrent chemotherapy followed by brachytherapy between 2010 and 2017. Survival analysis was obtained by Kaplan-Meier and logrank test. SPSS 21.
Results: The mean age among the 79 patients studied was 54,75(SD 14,52) years. The mean tumour size was 53,37(SD 17,47) mm. The first line of treatment was EBRT with concurrent CT followed by BT in 83,5% of the cases and EBRT or CT in monotherapy 3,8% and 12,6% respectively. 35,3% of the patients received a second line of treatment. Epidermoid carcinoma (64,6%) was the most frequent histological type. According to the stage (FIGO 2016) these groups were observed: 10,1% IB2, 10,1% IIA, 43,0% IIB, 2,5% IIIA, 12,7% IIIB, 21,5% IV.
After a year, overall survival was 81,0% and at five years was 67,1%. According to histological type, the five-year overall survival rate was: 74,5% epidermoid carcinoma, 66,7% adenocarcinoma, 50% adenosquamous carcinoma, 20% undifferentiated carcinoma. Significant differences were found (p=0,013) in survival curves between histological group of epidermoid/adenocarcinoma vs undifferentiated. Overall survival in five years according the stage was: 75,0% IB2; 87,5% IIA; 76,5% IIB; 50,0% IIIA; 40,0% IIIB y 52,9% IV.
Conclusion: The survival results were similar to others found in the literature, except for the most advances stages what could be explain for the limited sample size.
Histological type was a determining factor in the survival of cervical cancer.
In the light of the results of the study it was found a considerable percentage of patients needed a second line of treatment.
Disclosure: Nothing to disclose

EP310

Incongruous pathological and radiological response in a case of a mucin secreting adenocarcinoma of the cervix

S. Hosni[1], J. Abu[2], R. Oconnor[3], I. Soomro[4], A. Anand[5]
[1]Oncology, University of Nottingham NHS Trust, [2]Gynaecology, [3]Radiology, [4]Histopathology, [5]Oncology, Nottingham University Hospitals NHS Trust, Nottingham, United Kingdom

Introduction / Background: Mucin secreting adenocarcinoma of the cervix is a rare histological subtype of cervical cancer. It is recognised that mucin producing carcinomas have high signal intensity on T2 weighted MRI imaging. This case report describes a patient achieving complete pathological response within the primary tumour following pelvic external beam radiotherapy, correlating to a change in signal on MRI scan but not a reduction in tumour dimension.
Methodology: This is a case report of a female presenting with a large 8 cm biopsy proven mucin

secreting adenocarcinoma of the cervix, staining positive for CEA, CK7 and p16. Initial MRI scan stage was FIGO 3c1 (FIGO 2018) with bilateral pelvic lymph nodes. Distant metastasis was not seen.
Results: Following discussion within the gynaecological multidisciplinary team meeting, the patient underwent definitive treatment with concurrent chemoradiotherapy. Baseline MRI scan (Figure 1) and a repeat MRI scan following external beam radiotherapy (Figure 2) were compared prior to consideration of intracavitary brachytherapy. The primary tumour's dimension remained large. However, there was an increase in signal in both the primary tumour and lymph nodes. Following discussion in a multidisciplinary meeting, the patient had an examination under anaesthetic, where the tumour was deemed operable with no parametrial or vaginal involvement. Rather than proceeding to brachytherapy, the patient went on to have a radical hysterectomy with pelvic and para aortic lymphadenectomy. Final histology revealed presence of acellular mucin indicating complete pathological response within the primary tumour. 1 of 21 lymph nodes contained tumour cells in keeping with metastasis from cervical cancer.
Conclusion: Mucinous adenocarcinoma of the cervix can display an atypical response to radiotherapy with an increase in signal intensity on MRI imaging without a reduction in volume. Further publication of cases are required to help direct future clinical management of these rare histology subtypes.

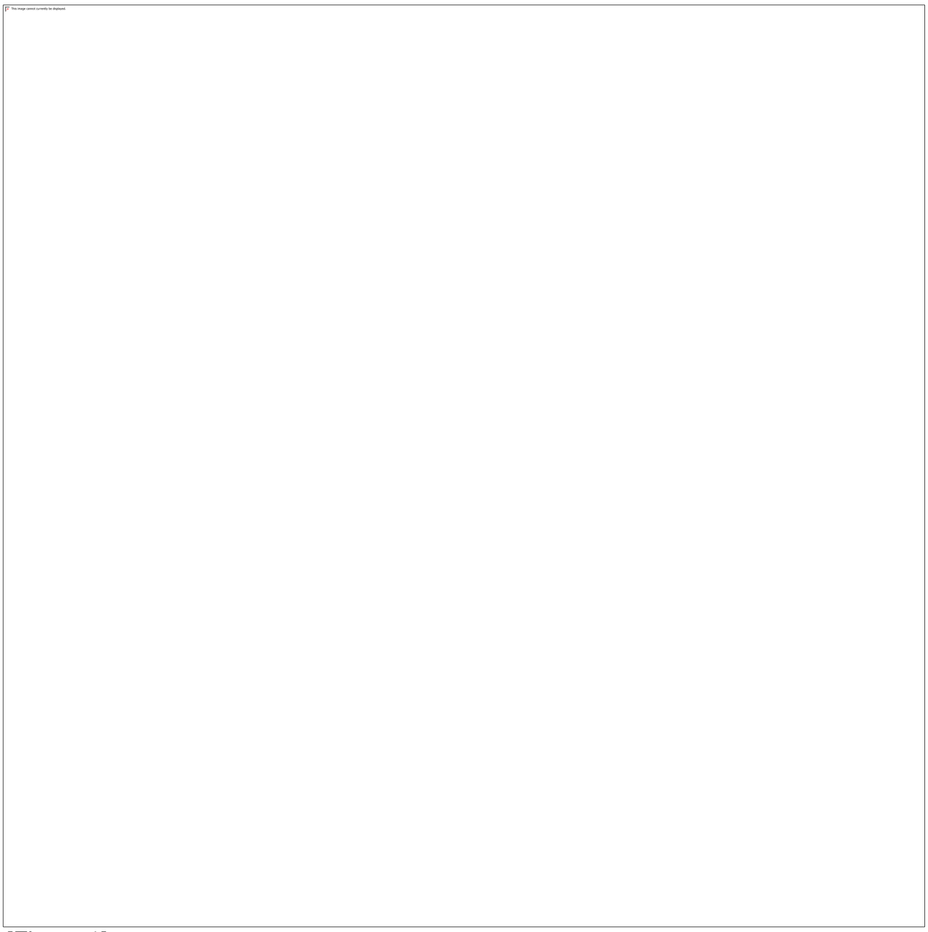

[Figure 1]

BMJ Publishing Group Limited (BMJ) disclaims all liability and responsibility arising from any reliance placed on this supplemental material which has been supplied by the author(s)

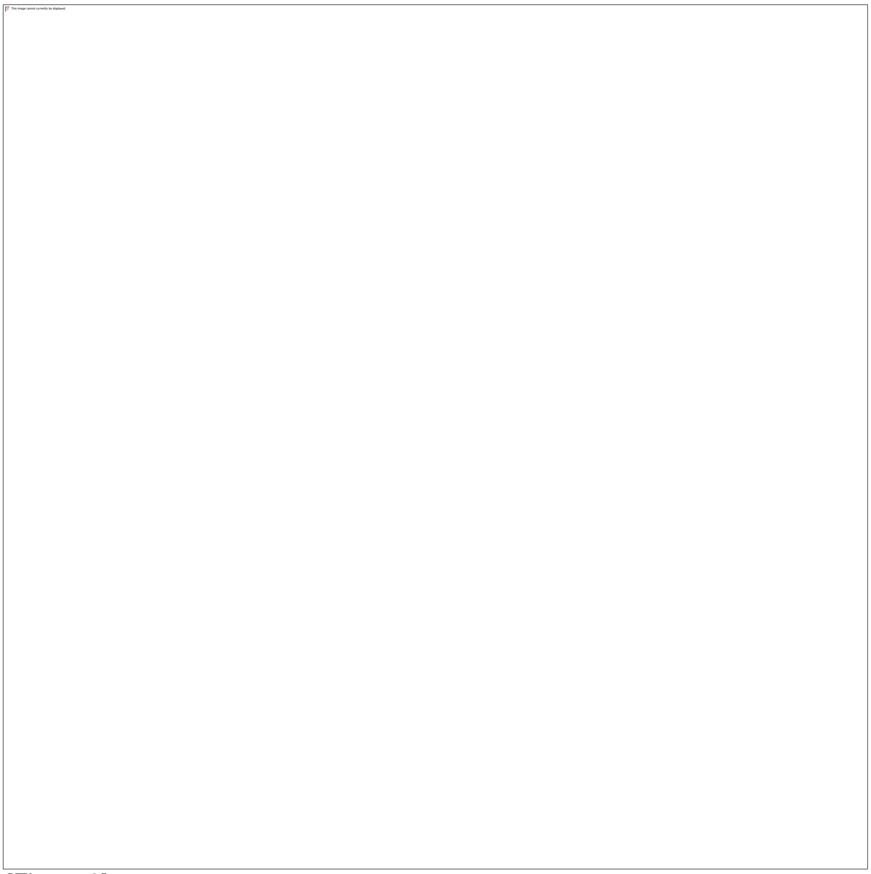

[Figure 2]

Disclosure: Nothing to disclose

EP311

A single centre retrospective review of all patients treated for cervical cancer with high dose rate brachytherapy at Nottingham University Hospital NHS Trust

S. Hosni[1], C. Ellis[1], P. Weber[2], A. Anand[1]
[1]Oncology, [2]Medical Physics, Nottingham University Hospitals NHS Trust, Nottingham, United Kingdom

Introduction / Background: Brachytherapy following external beam radiotherapy is vital in the treatment of cervical cancer. High dose rate (HDR) brachytherapy was introduced in 2011 at Nottingham University Hospitals (NUH), with the introduction of MR guided HDR brachytherapy in 2017. This is a retrospective review of all patients receiving HDR brachytherapy during this period.
Methodology: A database of all patients receiving HDR brachytherapy at NUH identified patients for inclusion. Retrospective review of electronic medical and radiotherapy records enabled data collection and analysis.
Results: 81 patients' records were analysed. 78% of cases had squamous cell histology, 19% adenocarcinoma. Mean age was 54 (range 27-88) years. FIGO staging was as follows: Ib1:6, Ib2:1, IIa:6, IIb:38, IIIa:3, IIIb:7, IIIC1:10, IIIC2:2, IVa:8. 27 patients had a PET CT scan as part of staging, of whom 12 (44%) patients had their cancer upstaged. One case of thyroid cancer and one case of inflammatory bowel disease were diagnosed.

A total of 11 patients received induction chemotherapy, 5 within the Interlace trial and 6 outside of a clinical trial. 22 (27%) patients had a complete response on MRI scan prior to HDR brachytherapy. The most common limiting factor in achieving brachytherapy equivalent dose in 2 Gy per fraction to the high risk clinical target volume of 80 Gy was bowel tolerance.
Median survival has not yet been reached. 57 (70%) patients are still alive. At recurrence, 1 patient out of 6 with local recurrence had operable disease, 1 patient received palliative chemotherapy and the remainder best supportive care. A total of 12 patients received 1st line palliative chemotherapy and 3 patients received second line chemotherapy. Median survival following palliative chemotherapy was 5 months.
Conclusion: This single centre retrospective review reports outcomes for patients treated with HDR brachytherapy for cervical cancer at NUH.
Disclosure: Nothing to disclose

EP312

Laparoscopic radical hysterectomy has higher risk of perioperative urologic complication than abdominal radical hysterectomy: a meta-analysis of 38 studies

J.H. Hwang, B.W. Kim
International St. Mary's Hospital, Catholic Kwandong University College of Medicine, Incheon, Republic of Korea

Introduction / Background: A meta-analysis was performed to assess risks of intraoperative and postoperative urologic complications in laparoscopic radical hysterectomy (LRH) and abdominal radical hysterectomy (ARH).
Methodology: We searched Pubmed, EMBASE, and Cochrane library for studies published up to December, 2018. Manual searches of related articles and relevant bibliographies of published studies were also performed. Two researchers independently performed data extraction. Inclusion criteria of studies were: 1) had information of perioperative complications, and 2) had at least ten patients per group.
Results: A total of 38 eligible clinical trials were collected. Intraoperative and postoperative urologic complications were reported by 34 studies and 35 studies, respectively. When all studies were pooled, odd ratios (OR) of LRH for the risk of intraoperative urologic complications compared to abdominal radical hysterectomy (ARH) was 1.40 [95% confidence interval (CI): 1.05-1.87]. The OR of LRH for postoperative complication risk compared to ARH was 1.35 [95% CI: 1.01-1.80]. However, significant adverse effects of intraoperative urologic complications in LRH were not observed among articles published after 2012 (OR: 1.12, 95% CI: 0.77-1.62) in cumulative meta-analysis or subgroup analysis. The incidence of bladder injury was statistically higher than that of ureter injury (p = 0.001). In subgroup analysis, obesity and laparoscopic type (laparoscopic assisted vaginal radical hysterectomy) were associated with intraoperative urologic complications.
Conclusion: LRH is associated with significantly higher risk of intraoperative and postoperative urologic complications than abdominal radical hysterectomy.
Disclosure: Nothing to disclose

EP313

Additional chemotherapy after six cycles in stable disease of cervical cancer

W.Y. Hwang, J.Y. Kim, D.H. Suh, K.D. Kim, J.H. No, Y.B. Kim
Obstetrics and Gynecology, Seoul National University Bundang Hospital, Seongnam, Republic of Korea

Introduction / Background: Current treatment guidelines don't mention how many cycles of chemotherapy are needed for appropriate tumor remission. This study was performed to evaluate the efficacy of additional cycles of chemotherapy in cervical cancer patients with tumor response of stable disease (SD) after six cycles.
Methodology: A total of 12 women who showed tumor response of SD with tumor≥5mm after six cycles of chemotherapy for the treatment of cervical cancer were reviewed. After excluding two who received radiation therapy, 10 were included in the analysis. Eight received chemotherapy for recurred tumor and two received primary palliative chemotherapy for stage IVB disease.

[Flow chart of patient selection process]

Recurred tumors which were followed up as target lesions during chemotherapy were located at lung, lymph nodes (common iliac, para-aortic, and supraclavicular), bladder, perihepatic, and retrovesical space. Progression-free survival (PFS) was compared according to the cycle number of chemotherapies (6 vs. 9 cycles).

[Summary of patient characteristics and management]

Results: Median age was 50 years (range, 25-78 years). Eight received 6 cycles of chemotherapy. Tumor size on CT scan after six cycles of chemotherapy was 0.8±0.3cm (mean±SD). Among the rest two undergoing 9 cycles of chemotherapy, one received paclitaxel/cisplatin/bevacizumab as primary palliative therapy and the other received paclitaxel/cisplatin for recurrent disease. The regimen used included paclitaxel/cisplatin with (n=3) or without (n=4) bevacizumab and paclitaxel/carboplatin (n=3). There were 6 patients (60%) who showed disease progression. Median PFS was 18 months (range, 10-106 months). Median PFS was similar between the two groups (19 months for 6 cycles vs. 12 months for 9 cycles; p=0.444). Cycle number (6 vs. 9 cycles) was not an independent factor for longer PFS (p=0.859). Neither were bevacizumab containing regimen, old age, and recurrence.

	6 cycles of platinum-based chemotherapy (n=8)	9 cycles of platinum-based chemotherapy (n=2)	p-Value
**Age**	50 (25-78)	50 (47-53)	> 0.999
**FIGO Stage I, II, II**	6 (85.7)	1 (14.3)	> 0.999
**FIGO Stage IV**	2 (66.7)	2 (33.3)	
**Initial tumor size on CT scan**	3.1±1.7	7.0	0.075
**Tumor size after 6 cycles of Chemotherapy**	0.8±0.3	0.6±0.1	0.404
**No Recurrence**	3 (37.5)	1 (50)	>0.999
**Recurrence**	5 (62.5)	1 (50)	
**Progression-free survival (months)**	19 (12-106)	12 (10-14)	0.444

[Characteristics of the patients according to Number of cycles of platinum-based chemotherapy]

Conclusion: Additional chemotherapy does not seem to benefit patients with cervical cancer who showed SD response after six cycles of platinum-based combination chemotherapy in terms of PFS.
Disclosure: Nothing to disclose

EP314

Management of cervical intraepithelial neoplasia 2 in young women

W.Y. Hwang, J.Y. Kim, D.H. Suh, K.D. Kim, J.H. No, Y.B. Kim
Obstetrics and Gynecology, Seoul National University Bundang Hospital, Seongnam, Republic of Korea

Introduction / Background: Cervical intraepithelial neoplasia (CIN) 2 in women younger than 24, observation is preferred in current treatment guidelines. In this study we evaluate the effectiveness of conservative management of CIN2 in Korean young women and find appropriate strategy considering age.
Methodology: A total of 129 women≤30 years diagnosed CIN2 on punch biopsy was retrospectively reviewed. Patient characteristics, initial and follow-up results of cervical examinations including pap smear, human papillomavirus (HPV), and punch biopsy were collected. Treatment was categorized into three: observation (n=30, 23.3%), ablation (n=15, 11.6%), and excision (n=84, 65.1%).

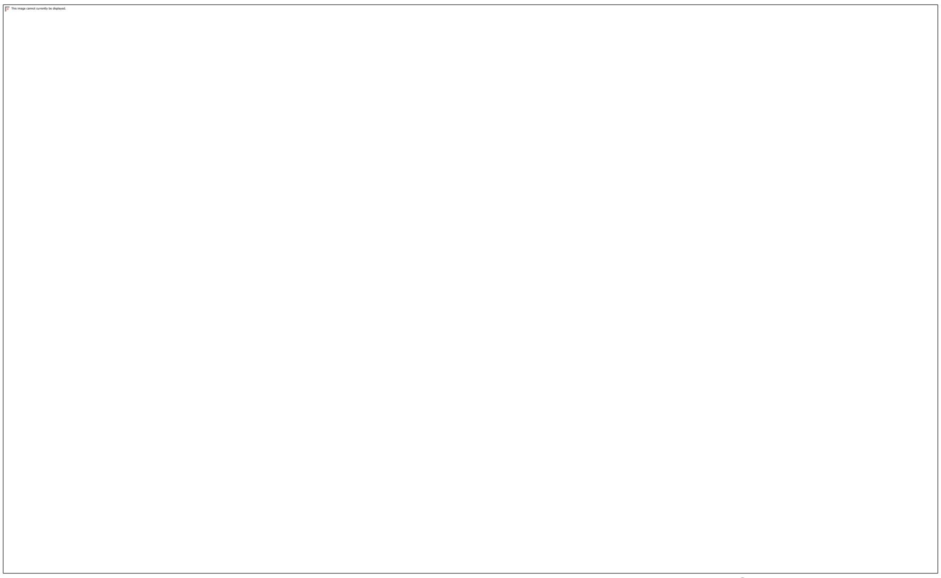

[Recurrence was counted when high-grade lesions on pap smear or CIN1+ in biopsy were detected.]

Recurrence was counted when high-grade lesions on pap smear or CIN1+ in biopsy were detected. Recurrence and recurrence-free survival (RFS) were compared. Survival analysis with Cox proportional hazards regression model was used.
Results: Median age was 27 (range, 14-30). Excision group were older than the other groups (median, 28 vs. 26 years; p< 0.001). Median follow-up was 16 months (range, 0-132 months). There were 14 recurrences (10.9%): 8 CIN1 and 6 CIN2. Observation group had more frequent recurrences (recurrence rate, 23.3% vs. 7.1%; p=0.019) than ablation or excision (treatment group) despite lower rate of HPV-16, 18(+) (10.0% vs. 30.3%; p=0.026).

Variable	Total (n=129)	Observation (n=30, 23.3%)	Ablation (n=15, 11.6%)	Excision (n=84, 65.1%)	p-value
**Age (years)**	25.4±3.2	25.4±3.2	25.0±4.0	27.0±2.9	0.012
**Follow-up (months)**	26.2±28.9	20.4±20.4	26.4±35.1	28.2±30.3	0.447
**HPV-16,-18**	33 (25.6)	3 (10.0)	2 (13.3)	28 (33.3)	0.022
**Recurrence**	14 (10.9)	7 (23.3)	1 (6.7)	6 (7.1)	0.043

[Characteristics according to management (n=129)]

There was no difference in recurrence rate between ablation and excision throughout all age groups (6.7% vs. 7.1%). Observation (HR, 7.2; 95% CI, 2.0-26.0; p=0.003) and HPV 16, 18 (+) (HR, 4.4; 95% CI, 1.3-15.0; p=0.018) were independent factors for early recurrence. Age was not an independent factor. In patients≤24 years, however, HPV 16, 18 (+) was the only independent factors for early recurrence (HR, 23.3; 95% CI, 1.9-285.3; p=0.014). In subgroup analyses of the other age criteria, observation was an independent factor.

Variable	HR	95% CI	p-value
**Observation**	6.1	0.6-62.5	0.129
**HPV-16,-18**	26.8	2.6-278.3	0.006
**Age**	1.1	0.8-1.5	0.542

[Multivariate survival analysis with Cox proportional hazards regression model for recurrence in women ≤ 24 (n=30)]

Conclusion: Observation increase early recurrence in women≤30 years with CIN2. Ablation or careful follow-up could be appropriate in young women≤24 years, which is consistent with guidelines of

western countries.
Disclosure: Nothing to disclose

EP315

Adenocarcinoma versus squamous cell carcinoma of cervix: are we fighting two different beasts with same weapon?

M.R. Islam[1,2], M. Aun[3], S. Kim[2], W.H.E. Park[4], A. Taylor[2]
[1]Southend University Hospital, Southend On Sea, [2]Royal Marsden Hospital, [3]Northwick Park Hospital, [4]Imperial College NHS Healthcare Trust, London, United Kingdom

Introduction / Background: For cervical cancer, squamous cell carcinoma (SCC) accounts for 80% of the histopathological diagnoses and adenocarcinoma (AC) approximately 15%. The literature is varied in terms of outcome data for AC subtypes and few comparative studies exist. At present, the recommendation is to use the same treatment regimens. This study aims to compare rates of regression during radical chemoradiation for squamous cell carcinoma versus adenocarcinoma and assess long-term outcomes.
Methodology: This retrospective study analysed 233 locally advanced cervical cancer patients treated with external beam radiotherapy ±weekly cisplatin chemotherapy (CCRT) combined with brachytherapy from July 2011 to March 2016. 35 patients with AC histology were identified with sequential MRI scans and then chronologically matched with patients with SCC histology. Tumour regression was compared after propensity score matching (1:1). Tumour volumes were measured on the baseline MRI, after 4-5 weeks EBRT. Treatment outcomes were calculated using Mann Whitney test and Log-rank test.
Results: Median follow-up was 38 months (range, 4-83 months). Mean reduction of tumour volume at 4 weeks was less in AC compared to SCC, 70% versus 83%(p=0.0078). Complete radiological response at 3 months post-treatment was also higher in SCC than AC, 80% versus 46% respectively. Although not statistically significant, there was a trend to better 3 years progression free survival at 74% versus 65% (figure 1).
Conclusion: AC of the cervix responds at a slower rate than SCC to radical chemoradiotherapy, and significantly fewer patients have a complete response at 3 months. Longer follow up and a larger cohort is required to assess whether this translates into inferior long-term survival. Future strategies may include earlier assessment for salvage surgery or neo-adjuvant/adjuvant systemic therapies.

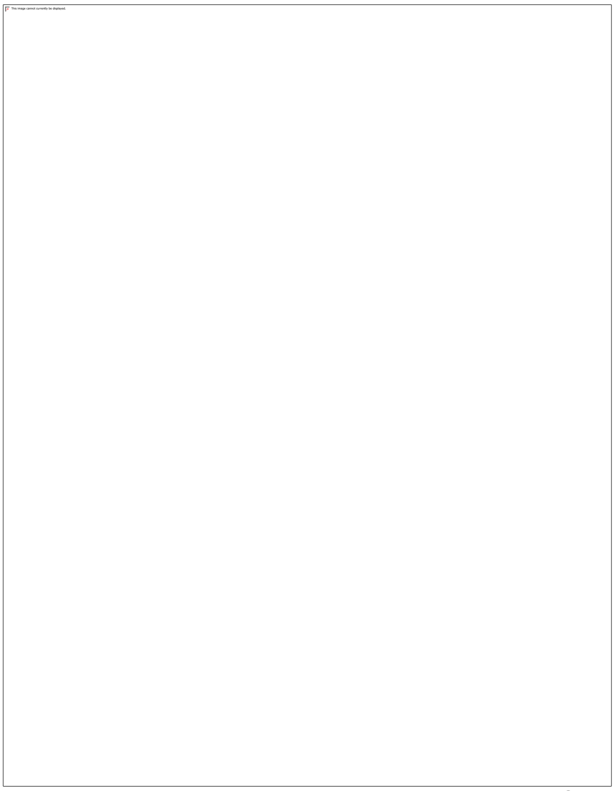

[The percentage reduction (Tumour regression) of AC and SCC after CCRT prior to Brachytherapy]

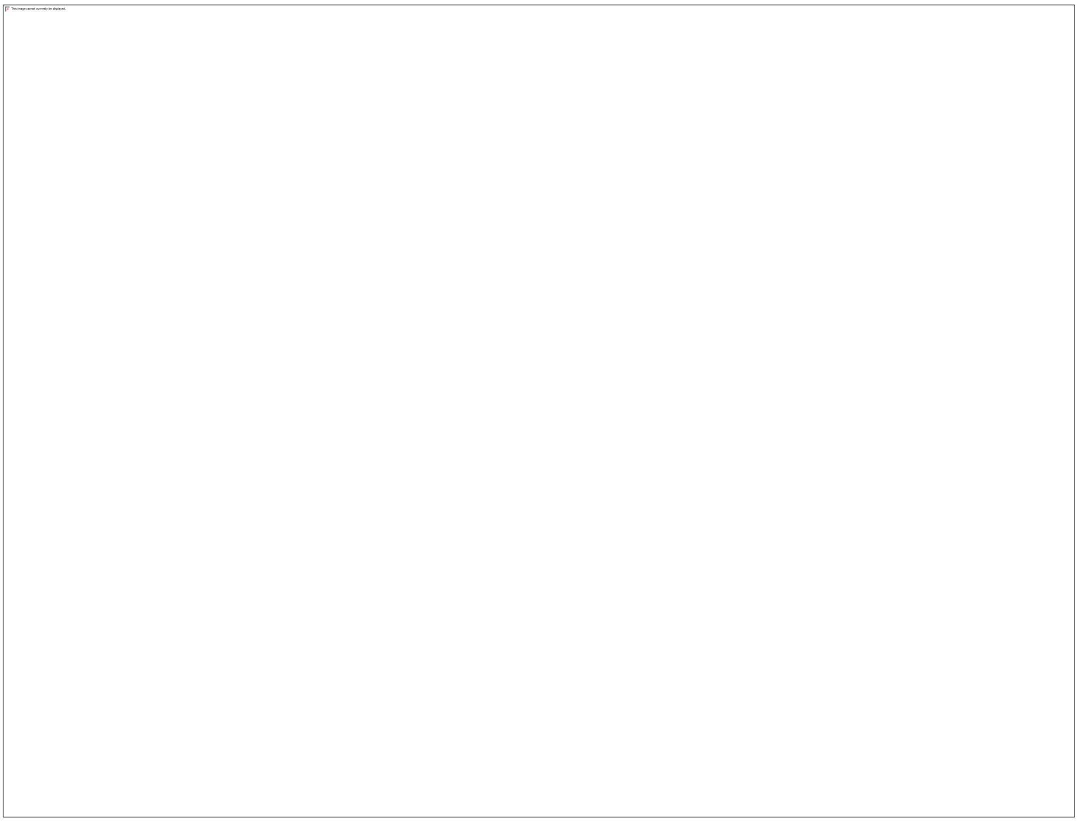

[The Progression Free Survival (PFS) curve of patients with SCC and AC]

Parameters	**AC**	**SCC**
Age(yrs)	52(32-83)	54(26-83)
Grade High Intermediate	22(63%) 12(34%)	22(63%) 11(31%)
FIGO Stage Stage I Stage II Stage III	3(9%) 28(80%) 4(11%)	3(9%) 28(80%) 4(11%)
Para-aortic LN Yes No	2(6%) 33(94%)	4(11%) 31(89%)
Pelvic LN Yes No	17(49%) 18(51%)	23(66%) 12((34%)
Chemo therapy Cisplatin No chemo Carboplatin	31(89%) 3(8%) 1(3%)	29(83%) 6(17%)
Mean % reduction at 4wk	70%	80%
Response at 3 months PR CR PD	19(54%) 16(46%) 0	5(14%) 28(80%) 2(6%)

[Characteristics of cervical cancer patients with SCC and AC]

Disclosure: Nothing to disclose

EP316

Cervical pre-cancer screen and treat program using visual inspection by acetic acid and thermo-coagulation in Malawi

L. Lee[1], A. Guardia[1], S. Sereshki[1], G. Chithyoka[2], D. Mpando[3], T. Phiri[4], J. Kachingwe[5], J. Beijnsberger[1], C. Fotheringham[1], D. Maman[1], M. Iwamoto[1]
[1]Médecins Sans Frontières, [2]Blantyre District Health Office, Ministry of Health, Blantyre, [3]Chiradzulu District Health Office, Ministry of Health, Chiradzulu, [4]Reproductive Health Department, [5]Department of HIV and AIDS, Ministry of Health, Lilongwe, Malawi

Introduction / Background: The scale-up of cervical pre-cancer screen and treat program is feasible in resource-limited contexts in sub-Saharan Africa. HIV-positive women regardless of area (urban or rural) and age were more likely to have pre-cancerous or cancer-suspected lesions, highlighting the importance of integrating cervical cancer screening with routine HIV care.
Methodology: We retrospectively analyzed routinely collected data of women 25-49 years old who were screened for cervical cancer for the first time between March 2018 and February 2019. We used logistic regressions to analyze the association between the presence of a pre-cancer or cancer-suspected lesion and HIV status, adjusting for age and area type (urban or rural).
Results: We retrospectively analyzed routinely collected data of women 25-49 years old who were screened for cervical cancer for the first time between March 2018 and February 2019. We used logistic regressions to analyze the association between the presence of a pre-cancer or cancer-suspected lesion and HIV status, adjusting for age and area type (urban or rural).
Conclusion: The scale-up of cervical pre-cancer screen and treat program is feasible in resource-limited contexts in sub-Saharan Africa. HIV-positive women regardless of area (urban or rural) and age were more likely to have pre-cancerous or cancer-suspected lesions, highlighting the importance of integrating cervical cancer screening with routine HIV care.

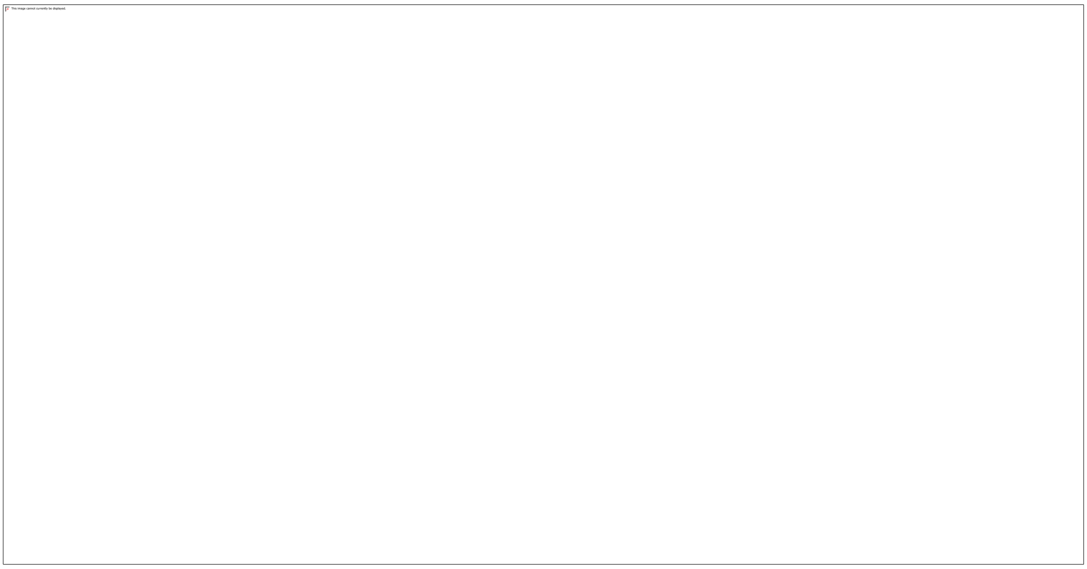

[Association between presence of pre-cancerous lesions and age, HIV status, and area type]

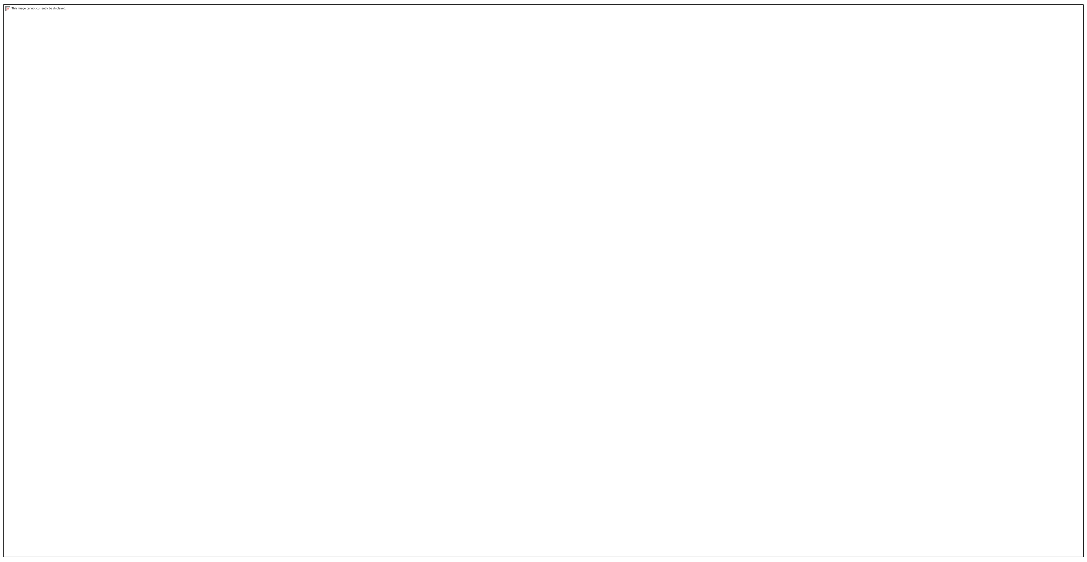

[Association between presence of cancer-suspected lesions and age, HIV status, and area type]

Disclosure: Nothing to disclose

EP317

Efficacy of salvage hysterectomy following radiotherapy for uterine cervical adenocarcinoma

H. Iwase, H. Sato, S. Endo, S. Furukawa, K. Ohnishi, Y. Tazo, T. Takada, M. Arai, O. Yoshino, T. Onda

Department of Obstetrics and Gynecology, Kitasato University School of Medicine, Sagamihara,

Japan

Introduction / Background: Treatment outcome of radiotherapy for uterine cervical adenocarcinoma (CA) is known to be poor compared with squamous cell carcinoma. Especially, the management for residual disease after radiotherapy remains unclear. The aim of this study is to evaluate the efficacy of salvage hysterectomy for residual disease after radiotherapy in patients with CA.
Methodology: Medical records of 28 patients with CA who underwent radiotherapy or concurrent chemoradiotherapy (CCRT) as primary treatment at our hospital between 2004 and 2015 were retrospectively reviewed. Clinicopathological features and prognosis of these patients were analyzed. Fisher's exact test, Kaplan-Meier analysis, and Log-rank test were used for statistical analyses.
Results: Median age was 59 years-old (range 36-87), and stage distribution was 3/10/12/3 in stage I/II/III/IV (FIGO 2008). Histology was adenocarcinoma in 23 and adenosquamous carcinoma in 5. Six patients received radiotherapy alone (RT) and 22 patients received CCRT (one with CDDP and 21 with CDGP as chemotherapy). Eleven patients (39.3%) achieved complete remission (CR) after RT or CCRT (RT-CR group). Five patients with residual disease in uterus and without other extra-uterine lesions achieved CR by means of salvage simple or modified radical hysterectomy following RT or CCRT (OP-CR group). Consequently, 16 patients achieved CR in total. Overall survival (OS) in CR group was significantly favorable compared with non-CR group (74.5% vs 24.2% in 2-year OS, p=0.006). Especially, patients in OP-CR group had excellent prognosis with an 80% 5-year OS, which was comparable with a 61.4% 5-year OS in RT-CR group. There was no significant difference in clinicopathologial features between CR and non-CR groups. Thus, there was no predictive feature for radiation effect.
Conclusion: Salvage hysterectomy should be considered in patients with residual disease after radiotherapy, if they have no extra-uterine disease.
Disclosure: Nothing to disclose

EP318

Laparoscopically assisted vaginal hysterectomy: best surgical approach in patients with endometrial/cervical cancer?

M. Japaridze
Aversi Clinic, Tbilisi, Georgia

Introduction / Background: During TLH (total laparoscopic hysterectomy), uterine perforation may occur while using uterine manipulator. Also, uterus is cut on manipulator without isolating cancer tissue from the pelvic cavity (“open cut”). This later technique often used by surgeons even in specific oncological centers. We think, uterine perforation by vaginal manipulator and the vaginal “open cut” technique may be associated with cancer tissue spillage in pelvic cavity followed by disease progression.
Methodology: Case report.
Results: We had 3 cases of disease fast progression after total laparoscopic hysterectomy using vaginal manipulator: 2 cases after uterine perforation in patients with end/ca and 1 case of fast residual disease after vaginal “open cut” technique on manipulator in patient with cervical cancer. During all three, tumor macroscopic tissue spillage was evident.
After these cases, we avoid using vaginal manipulator in patients with end/ce cancer in order to avoid tumor spillage, associated with uterine perforation or technique of laparoscopic open colpotomy (“open cut”). We use LAVH method and cut uterus by vaginal root, after isolating tumor tissue from entering pelvic cavity and disseminating.
We think, the advantages of LAVH in patients with end/cervical cancer:
• Vaginal colpotomy (vs. intracorp. colpotomy) = No contact of tumor tissue to pelvic cavity!!
• No risks of uterine perforation and disease spread in pelvic cavity.
• Vaginal cuff access technically easy (especially in obese patients);
• No additional time needed for vaginal manipulator installation; no need for 2nd assistant;
• No technical difficulties associated with laparoscopic vaginal cuff cut, (obese patient, unexperienced 2nd assistant).
Conclusion: In patients with endometrial/cervical cancer LAVH technique may prevent tumor spillage

risks associated with vaginal manipulator use.
Disclosure: Nothing to disclose

EP319

Gastric type endocervical adenocarcinoma without delay in diagnosis: uncommon and difficult to diagnosis by screening test

H. Jeon, S. Jeon
Soonchunhyang University Cheonan Hospital, Cheonan, Republic of Korea

Introduction / Background: Gastric type endocervical adenocarcinoma (GAC) is a special variant of cervical adenocarcinoma which is a well-differentiated form of adenocarcinoma. It is uncommon that 1~3% of all cervical adenocarcinoma were GAC. Because of its endophytic growth pattern and its low reproducibility of glandular atypia, it is very difficult to be preoperatively diagnosed by cervical cytology.
Methodology: We present a case of GAC without delay in diagnosis.
Results: A 59-year-old woman who suffered watery vaginal discharge for 3 months visited our hospital. In pelvic examination, the cervix was firm and left parametrium was thickened. Ultrasound showed multiple hyperechogenic cysts. The result of cervical cytology was atypical endocervical cell and the specimen of a punch biopsy was determined as cervicitis. We performed cervical conization and fortunately, she was diagnosed as GAC. The patient underwent diagnostic laparoscopy. There were multiple white nodules scattered over the surface of uterus (Figure A), omentum and peritoneum (Figure B) and both salpinges were edematous (Figure C). Subsequently, laparoscopic bilateral salpingectomy, partial omentectomy and peritoneal biopsy were performed. The results of all specimen were metastatic GAC. She was treated with chemotherapy and pelvic radiation therapy. However the general condition of this patient became worse and she died of disease 15 months after the initial diagnosis in our clinic.
Conclusion: GAC is difficult to diagnosis by cervical cytology. So cervical conization is needed to make an appropriate diagnosis of it preoperatively. In our case, the patient had atypical glandular cell on cervical cytology although punch biopsy didn't reveal GAC. We carried out conization and could diagnosis appropriately before suboptimal or radical surgery so performed proper treatment without delay. GAC is known as unfavorable prognosis and most cases are detected at advanced stages. Because of that, the early detection of GAC is important to avoid a delayed treatment and unnecessary surgical procedure.

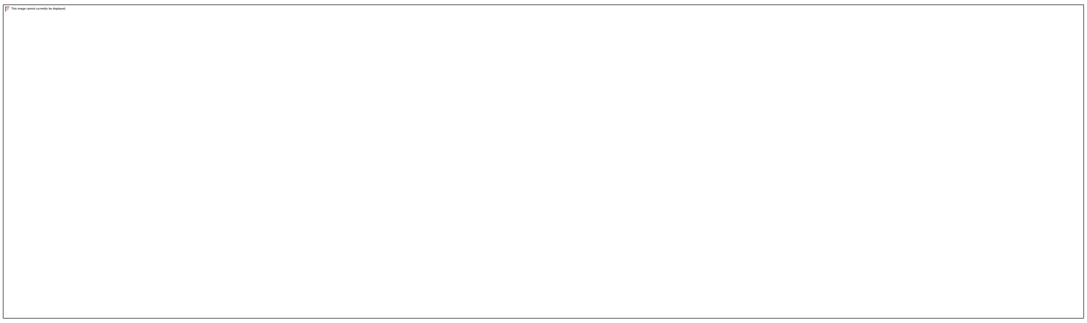

[Intraoperative picture shows multiple white nodules scattered over the intraperitoneal surface and both edematous salpinges.]

Disclosure: No potential conflict of interest relevant to this article was reported and this case presentation was supported by the Soonchunhyang University Research Funds.

EP320

Laparoscopic radical hysterectomy versus open radical hysterectomy in early stage cervical cancer; long term recurrence rates in the South East Wales Gynaecology Oncology Centre

S. Jones, A. Panda, I. Ramzan, R. Jones, R. Howells, K. Lim, A. Sharma
University Hospital of Wales, Cardiff, United Kingdom

Introduction / Background: Radical hysterectomy for cervical cancer may be performed using a minimally invasive approach or and open, abdominal surgical approach. A growing body of evidence provides conflicting evidence regarding survival outcomes between these two surgical approaches. This study contributes a retrospective review of 10 years of data from the South East Wales Gynaecology Oncology Centre.
Methodology: A retrospective review of all cases of radical hysterectomy (open and laparoscopic) carried out between January 2008 and December 2018. Data collected included patient age, BMI, performance status, surgical approach, pre and post op cancer stage, tumour size, length of stay in hospital, complications, recurrence rates and disease free survival. Data were analysed using SPSS software, statistical significance was set at P=< 0.05.
Results: Two hundred and twenty-nine cases of radical hysterectomy were identified. One hundred and forty-one open, abdominal procedures and 88 minimally invasive procedures were carried out. Patient demographics and cancer stage were comparable between the two groups. Mean duration of follow up was 64 months. Nine out of 141 (6.4%) laparotomy cases developed a recurrence and 5/88 (5.7%) patients having had a laparoscopic procedure developed a recurrence.
Conclusion: This retrospective review demonstrates no significant difference in recurrence rates between patients having undergone open, abdominal radical hysterectomy compared to minimally invasive hysterectomy in our centre.
Disclosure: Nothing to disclose

EP321

Potential therapeutic candidate of cold atmospheric plasma for cervical cancer

M.H. Jung[1], Y.I. Ji[2]
[1]*Kyung Hee University, Seoul,* [2]*Inje University, Busan, Republic of Korea*

Introduction / Background: The cold atmospheric plasma has been extensively researched as a new cancer treatment technology. We investigated the selective cytotoxic effects of cold atmospheric plasma in cervical cancer cells.
Methodology: Two human cervical cancer cell lines (HeLa and SiHa) and one human fibroblast (HFB) cell line were treated with cold atmospheric plasma. All cells underwent apoptotic death induced by plasma in a dose-dependent manner. The plasma showed selective inhibition of cell proliferation in cervical cancer cells compared to human fibroblast, control.
Results: To evaluate the cytotoxic effects of non-thermal micro-DBD plasma in both cervical cancer cells and HFBs, we used microscopy to examine the morphological changes of cells and performed MTT assays. The morphology of HFBs, HeLa and SiHa cells did not change significantly after plasma treatment; however, fragmentation of cells was clearly visible after plasma treatment. The cell density decreased in all cases with plasma treatment; however, the decrease was more obvious in SiHa cells. To examine whether apoptosis induced by cold atmospheric plasma is involved in the activation of caspase, cells were incubated with and without plasma treatment for 3 min (E=33 J), and then caspase-3, -6, -8, and -9 activity was evaluated using a commercial caspase assay kit. The flow cytometry shows that cold atmospheric plasma can arrest the cell cycle at subG0 phase in a cell-type-specific manner.
Conclusion: Cold atmospheric plasma showed selective apoptotic effects depending on cell type. Plasma treatment was more effective in cervical cancer cells compared to control. In cervical cancer cells, the effects of plasma on SiHa cells were stronger than its effects on HeLa cells. This evidence of selective apoptosis was in agreement with the observed gene expression alterations induced by

plasma treatment.
Disclosure: Nothing to disclose

EP322

The influence of modulated electrohyperthermia (mEHT) on tumor cell apoptosis in iib−iva cervical cancer patients with deep parametrial invasion

M. Kaskulova, Y. Kreynina, L. Shevchenko, V. Solodky, V. Bozhenko, E. Kudinova
Russian Scientific Center of Roentgenoradiology, Moscow, Russian Federation

Introduction / Background: Incomplete response, associated with unfavorable prognosis after standard chemoradiation with weekly Cisplatinum 40mg/m^2 one observes in more, than 35% of advanced cervical cancer (ACC) patients with deep (≥ half uni- or bilateral) parametrial invasion. mEHT shows to overcome therapy resistance in these pts in pilot study but the mechanism of its input to cell damage effect of radiation and cytotoxic drugs was unclear. mEHT influence on cell apoptosis in ACC pts was evaluated.
Methodology: Tumor samples were taken from 35 pts T2b−T4a ACC, parametrial invasion >1/2 (unilateral − in 14 (40%), bilateral − in 21 (40%) pts.), before the treatment and twice during conformal external beam radiotherapy (3D−CRT, IMRT, Dynamic Arc), in 2Gy, 46−48Gy for pelvic or extended paraaortic fields, Cisplatin 40mgm2 or Carboplatinum AUC2 weekly, with mEHT before irradiation in 18 of them (EHY-2000 device, 60-90' 3 t/week, 90-120Wt, 10−13 fractions per course. 20−gene panel was used (Ki67, STK15, CCNB1,PTEN,CCND1,MYC,P16NK4a,MYBL2,DCL2,BAX,BAG,NDRG1,BIRC5,TERT,CerbB2, GRB7,CTSL2,CD68).
Results: In mEHT group objective response (RECIST 2.0) was reached in all 18 pts: CR − in 13 (73,2%) pts, PR >80% − in 5 (27,8%). In chemoradiation group (17 pts): CR − in 4 (22,9%), PR −1 CR − in 4 (23,5%) pts, PR- in 8 (47%), stable - 4 (23,5%) больных, progression (local+paraaortic LN and liver) - 2(11,7%). Apoptosis induction was associated with complete response in both groups, significantly higher (p= 0,03−0,043) in mEHT group.
Conclusion: mEHT significantly improves the response to chemoradiation in ACC patients with deep parametrial invasion. Gene alteration level at 30Gy has a predictive value.
Disclosure: Nothing to disclose

EP323

Extended nerve-sparing radical hysterectomy

T. Kato, T. Tsukada, Y. Yoneoka, M. Kato, Y. Tanase, M. Uno, M. Ishikawa
Gynecology, National Cancer Center Hospital, Tokyo, Japan

Introduction / Background: Based on our cadaveric studies on the running of autonomic nerves in the female pelvis we modified the Japanese traditional nerve-sparing radical hysterectomy (NSRH) to raise radicality of the cardinal ligament. Since cervical cancer FIGO stage IIB was also applied to the extended NSRH, the oncologic outcome was examined.
Methodology: The extended NSRH was performed using the ureterohypogastric fascia that surrounded the autonomic nerves as an indicator of dissection. The ureterohypogastric fascia were identified during the dissection of the ureter. The study included 108 patients with cervical cancer who underwent the extended NSRH between 2006 and 2017. Their FIGO stages (2008) were 65 IB1, 17IB2, 6IIA1, 1IIA2, and 19IIB. According to pTNM classification, they were divided into 4 groups such as pT1b,2aN0, pT1b,2aN1, pT2bN0, and pT2bN1. None of the pT1b,2aN0 group received adjuvant therapy (radiotherapy, RT; 2006 - 2010, Intensity modulated RT, IMRT; 2011-2014, and Concurrent

chemotherapy with IMRT, CC/IMRT; 2015-2017).
Results: Their pTNM classification was 65 pT1b,2aN0, 11 pT1b,2aN1, 9 pT2bN0, and 23 pT2bN1. The median of number of positive pelvic nodes were three. The para-aortic lymph node metastasis was observed in 22% (5/23) of patients with pT2bN1. After the median follow-up of 74 months (range 8-145), 25% (27/108) had progressed disease, and 13% (14/108) patients died. The 5-year 1ocal recurrence free survival was 89% in the pT1b,2aN0 group, 75% in the pT1b,2aN1, 100% in the pT2bN0, and 77% in the pT2bN1. The 5-year overall survival was 96%, 72%, 100%, and 65%, respectively. Two 2 months after the surgery none needed to perform self-catheterization.
Conclusion: By the procedures of our extended NSRH, patients with negative nodes survived for long time with no evidence of disease. The 5-year OS of node-positive was around 70%, and this improvement could be expected from the effect of adjuvant CC/IMRT.
Disclosure: Nothing to disclose

EP324

MRI-based tumor volume evaluation at the time of brachytherapy as a predictor of survival in cervical cancer patients treated with definitive chemoradiation

C. Khoury[1], D. Atallah[2], J. Naba[3], A. Nassar[3], M. Moubarak[2], F. Azoury[1], T. Felefly[1], J. El Barouky[1], R. Sayah[1], D. Nasr[1], G. Chahine[4], E. Nasr[1]
[1]Department of Radiation Oncology, [2]Department of Gynecology, [3]School of Medicine, [4]Department of Medical Oncology, University of Saint Joseph, Beirut, Lebanon

Introduction / Background: To evaluate the potential impact on survival of residual tumor volume after chemoradiation and prior to brachytherapy initiation determined by magnetic resonance imaging in patients treated for locally advanced cervical cancer.
Methodology: MRI examinations were prospectively performed in patients with advanced cervical cancer (Stages IB2-IVA) after chemoradiation completion and prior to brachytherapy initiation. Region of interest-based 3D volumetry was derived on the treatment planning system by tracing the entire residual tumor region in each T2-weighted MRI slice. All patients received external beam radiation (EBRT) with concomitant weekly Cisplatin followed by high-dose rate endocavitary brachytherapy using volume-based planning. Cumulative EBRT and BT doses were calculated as the total equivalent dose in 2 Gy fractions (EQD2). Potential prognostic factors were selected based on non-parametric tests and then analyzed for survival with a Cox regression model.
Results: Median post-therapy follow-up was 27.4 months (range, 3-57.8). Thirty-seven patients were included. According to the FIGO classification, 8% were stage IB, 75% stage II, 11% stage III, and 6% stage IV. Overall survival was 86.5%. Of the patients evaluated, 29.7% had complete radiological remission and 18.9% were considered having local failure or distant disease. At bivariate analysis, residual volume >2 mL, D90 CTV-HR < 84 Gy and excessive treatment time were all significant predictors of poor overall survival. At multivariate survival analysis, only the residual tumor volume persisted as a significant prognostic factor with survival rates of 95.8% and 69.2% for residual tumor ≤2 mL and >2 mL, respectively (p=0.005).
Conclusion: Our data suggest that residual tumor volume >2 mL at the time of brachytherapy is a predictor of poor survival.

[Overall survival curves according to residual tumor volume ≤2 mL and >2 mL]

Disclosure: Nothing to disclose

EP325

In case of an unusual cervical mass: think to lymphoma

S. Kilzie[1,2], I. Pezzani[3], F. Sartori[4], A. Rossi[1], E. Busato[3]
[1]Gynaecology Department, Conegliano Hospital, Treviso, Italy, [2]Universitat de les Illes Balears, Palma de Mallorca, Spain, [3]Gynaecology Department, [4]Haematology Hepartment, Treviso Hospital Ca' Foncello, Treviso, Italy

Introduction / Background: We present the case of a 46 year-old woman with a rare gynecological presentation of an aggressive lymphoma.
Methodology: Case report.

Results: A 46 year-old woman is referred to the hospital for presenting leucorrea and irregular copious vaginal bleeding; she denies other symptoms. At the physical examination a solid and fixed mass deforming the cervix is observed and the posterior vaginal wall appears thickened . The transvaginal ultrasound shows a solid and vascularized (colour score 4) image of 94x65 mm. The MRI confirms the presence of a pelvic mass at the cervix of 10x9x10 cm with necrotic areas. Other iliac, presacral and inguinal lymphadenopathies are identified by PET scan. Hysteroscopy identifies two typical endocervical polyps and a normal endometrium. Biopsies are realized directly at the surface of the visible lesion through speculum examination. The anatomopathological analysis reveals a diffuse Large B-cell lymphoma so the patient is remitted to the Haematologist to complete the staging and start the chemo-immunotherapy.
Conclusion: Hematologic malignancies rarely present as a primary gynaecologic problem and primary gynaecologic Non-Hodgkin Lymphoma are sporadically reported in the literature. Primary NHL can mimic gynaecological malignancy such as endometrial cancer, cervical cancer, sarcoma or ovarian cancer presenting as a pelvic mass. The primary site of origin for lymphomas is the lymph nodes and other lymphoid tissue but approximately 10 to 35% of patients have a primary extranodal lymphoma at the time of diagnosis. Only less than 0.5% of all extranodal NHLs involve the female genital tract. Most patients lack the classical B symptoms associated with lymphoma: fatigue, fever, night sweats and weight loss. The primary pelvic lymphomas have a five-year survival rate of 80% to 90% if the diagnosis was made early and therapy was adequate so the diagnosis of primary pelvic lymphoma should be considered in the differential diagnosis of gynaecological malignancies.

[MRI: a pelvic mass of the cervix (10x9x10 cm) with necrotic areas.]

Disclosure: Nothing to disclose

EP326

Overexpression of SOCS-1 may inhibit invasion and metastasis of cervical cancer

M.H. Kim[1], S.Y. Ryu[1], B.J. Kim[1], H.J. Jeong[1], J.H. Jeong[2]
[1]Korea Cancer Center Hospital | Ob/Gyn, [2]Research Center, KIRAMS, Seoul, Republic of Korea

Introduction / Background: Cervical cancer is a leading cause of cancer related deaths in women worldwide caused due to infection of high-risk human papillomaviruses. Suppressor of cytokine signaling (SOCS) family is an important negative regulator of cytokine signaling and deregulation of SOCS has been involved in many types of cancer. All cervical cancer cell lines tested showed lower expression of SOCS1, SOCS3, and SOCS5 than normal tissue or cell lines. We published that SOCS genes are silenced by DNA hypermethylation and histone deacetylation and regulate response to radiotherapy in cervical cancer cells (doi:10.1371/journal.pone.0123133).
Methodology: We established stable SOCS-1-overexpressing ME-180 cell line with pLNCX2-mSOCS-1-Myc. Also we established xenograft nude mouse model for tumor growth delay experiment. Immunohistochemistry was employed to elucidate the pattern of expression of VEGF, CD-31, MMP-2, and TUNEL assay for apoptosis in SOCS-1 overexpressing ME-180 cells. Tumor cells were injected to right thigh of mouse to form cancer mass. Lung metastasis assay was performed in day 45 and 60. Metastatic nodules in both lungs were obtained and counted for statistical analysis.
Results: Tumor growth delay after 5 Gy radiation was observed in SOCS-1 overexpressing tumor compared to wild type tumor but the difference did not show the statistical significance ($p>0.05$, Mann-Whitney U test). Immunohistochemistry showed that decreased MMP-2 expression, decreased VEGF expression, decreased CD-31 expression, and significantly elevated index of apoptotic body in SOCS-1 overexpressing tumor. Lung metastasis assay showed statistically significant decrement of metastatic nodule count in SOCS-1 overexpressing tumor ($p< 0.05$, Mann-Whitney U test).
Conclusion: Decrement of radiosensitivity was statistically insignificant in SOCS-1 overexpressing tumor while invasion, angiogenesis, and metastasis were inhibited. Also apoptosis was increased in SOCS-1 overexpressing tumor.
Disclosure: Nothing to disclose

EP327

No difference in overall survival rate between abdominal and minimally invasive radical hysterectomy for early cervical cancer: single institute, retrospective study for more than 10 years

S.I. Kim, J.W. Lee, J.H. Yoon
The Catholic University of Korea/ St. Vincent's Hospital, Suwon, Republic of Korea

Introduction / Background: Recently, results from the Laparoscopic Approach to Cervical Cancer (LACC) trial were published indicating that patients undergoing minimally invasive radical hysterectomy have a lower rate of disease-free survival and overall survival than those who undergo abdominal radical hysterectomy. This is an extremely important trial that has the potential to cause a paradigm shift in how cervical cancer is managed and therefore led us to further evaluate the data from our institution. Our objective was to compare risks of recurrence, and survival in a cohort of women undergoing abdominal radical hysterectomy (ARH) versus minimally invasive radical hysterectomy (MRH) for early stage cervical cancer at a single institution.
Methodology: A retrospective cohort of patients undergoing radical hysterectomy for cervical cancer from 2000 to 2017 was identified. 127 patients with pathologically confirmed 2018 FIGO stage IB1, IB2

and IB3 cervical cancer were included and were grouped by minimally invasive and abdominal radical hysterectomy. Tumor characteristics, recurrence rate, progression-free survival (PFS), and overall survival (OS) were compared between groups.
Results: 127 patients were identified; 44 underwent ARH and 83 underwent MRH. Those who underwent ARH were more likely to have tumors that were ≥ 2 cm, < 4cm (29.5% vs 22.9%, p = 0.41) and ≥ 4 cm (16% vs 8.4%, p = 0.20), but there were no significant differences. There were no significant differences in recurrence rate (12% vs 2.3%, p = 0.06), progression-free survival (PFS) (p = 0.19), or overall survival (OS) (p = 0.24).
Conclusion: Recurrence rate, progression-free survival, and overall survival (OS) of minimally invasive radical hysterectomy (MRH) showed noninferiority compared to abdominal radical hysterectomy (ARH).
Disclosure: Nothing to disclose

EP328

Oncological outcomes and recurrent risk factors following laparoscopic radical trachelectomy with vaginal colpotomy in early stage cervical cancer

Y.-M. Kim[1], J.-H. Kim[2], S.-W. Lee[2], J.-Y. Park[2], D.-Y. Kim[2], D.-S. Suh[2], J.-H. Kim[2], Y.-T. Kim[2], J.-S. Kang[2]
[1]Asan Medical Center, University of Ulsan College of Medicine, Obstetrics and Gynecology, [2]Asan Medical Center, University of Ulsan College of Medicine, Seoul, Republic of Korea

Introduction / Background: We investigated the long-term oncologic outcomes and prognostic factors in CC patients following laparoscopic radical trachelectomy (LRT) with vaginal colpotomy.
Methodology: Between 2004 and 2018, 118 CC patients with FIGO stage IA2-IIA1 were treated with LRT with vaginal colpotomy in Asan medical center. The cases of prior neoadjuvant chemotherapy for CC or intraoperative converted radical hysterectomy were excluded. Total 101 patients were included in this study. Clinicopathological variables of the patients were retrospectively reviewed from electronic medical records.
Results: The median follow-up period was 54.7 months (IQR, 24.7-77.7). The mean age and tumor size were 32.0 (range, 22-43) and 2.03cm (range, 0.1- 7) and Stage IB1 disease was the most common (83.2%), followed by stage IA2 (10%) and IB2 (5.9%). Forty-seven (46.5%) patients had a tumor size greater than 2cm. Twelve patients (11.9%) had lympho-vascular space invasion (LVSI), 26 patients (25.7%) had deep stromal invasion greater than 50%, and 2 patients (2%) had vaginal involvement. Two patients (2.0%) had lymph node metastasis. Twelve (10.9%) had adjuvant chemotherapy after surgery. The 5-year overall survival (OS) rate was 98% and the 5-year disease-free survival (DFS) rate was 91.1%. A deep stromal invasion greater than 50% (P=0.025), tumor grade (P=0.027), vagina involvement (p=0.040), and LVSI (P=0.020) were significant prognostic factors for recurrence. In tumor size below 2cm, the 5-year DFS was 94.4%. Moreover, in tumor size below 2cm with no risk factors, there was no recurrence.
Conclusion: LRT with vaginal colpotomy is feasible and has favorable oncologic outcomes in tumor size below 2cm with no risk factors. And in cases of having above recurrent risk factors, we may consider further adjuvant chemotherapy or warning to the patients about recurrence. However, further study is warranted.
Disclosure: Nothing to disclose

EP329

Real world experience of pembrolizumab in advanced or recurrent cervical cancer

S.H. Kim, J.Y. Park, D.Y. Kim, J.H. Kim, Y.M. Kim

Gynecology Oncology, Asan Medical Center, University of Ulsan College of Medicine, Obstetrics and Gynecology, Seoul, Republic of Korea

Introduction / Background: As several cases of complete response in patients with advanced or recurrent cervical cancer using Pembrolizumab were reported, immunochemotherapy come into the spotlight as one method of treating cervical cancer. The aim of this study was to investigate the real world efficacy of Pembrolizumab in advanced or recurrent cervical cancer.
Methodology: Cervical cancer Patients who treated by Pembrolizumab at Asan Medical Center in Korea between May 2018 and April 2019 were identified, and their medical records were reviewed retrospectively. We assessed Programmed cell death ligand 1(PD-L1) in patients whose cell type was adenocarcinoma(ADCA) or adenosquamous cell carcinoma(ASCC). ADCA or ASCC patients whose PD-L1 was positive, and squamous cell carcinoma(SCC) patients regardless of PD-L1 used Pembrolizumab. Pembrolizumab 200mg was administered intravenously every 3weeks. Best tumour response was assessed using RECIST v1.1 and iRECIST.
Results: Of the 15 patients, 11 (73.3%) patents had SCC. 2 (13.3%), 2 (13.3%) patients had ADCA and ASCC respectively. 4 (26.7%) patients were unable to assess the tumour response, because their condition deteriorated after using Pembrolizumab 1 or 2 times. 2 (13.3%) patients are waiting for follow up computed topography exam after using Pembrolizumab. Therefore, tumour response can be assessed only in 9 (60%) patients. Of these 9 patients, partial response(PR) was showed in just 1 patient. Stable disease(SD) was showed in other 1 patient, and the others were showing progressive disease(PD). Objective response rate was 11.1% and clinical benefit rate was 22.2%. A patient who showed PR had high tumour proportion score(TPS)(90%).
Conclusion: Pembrolizumab can be effective when administered in patients with high TPS. The effects were minimal for patients with PD-L1 negative or low TPS, and PD was often observed. Therefore, adequate patient selection is most crucial before using Pembrolizumab. The necessity of large-scaled multicenter retrospective study was highly required.
Disclosure: Nothing to disclose

EP330

Impact of preexisting bladder outlet obstruction on voiding dysfunction after radical hysterectomy for cervical cancer

T. Kim[1], N.-H. Park[2]
[1]Department of Obstetrics and Gynecol, SMG-SNU Boramae Medical Center, [2]Department of Obstetrics and Gynecol, Seoul National University College of Medicine, Seoul, Republic of Korea

Introduction / Background: The impact Female bladder outflow obstruction (BOO) is commonly detected in the normal population. The aim of this study was to examine of underlying BOO on urinary retention after radical hysterectomy (RH) for treatment of early-stage cervical cancer.
Methodology: A extensive perioperative urologic management protocol was applied to consecutive patients who underwent type III RH performed by a single surgeon between 2006 and 2008. The protocol includes preoperative urologic workup encompassing urodynamic study (UDS), standardized postoperative management, and serial follow-up. If the urologic evaluation at 10 days after RH revealed no spontaneous voiding or a high post void residual (PVR) larger than 100 ml, patients were instructed to perform clean intermittent self-catheterization (CIC) until the PVR decreased to less than 100 ml. Exclusion criteria were preexisting voiding symptom, prior pelvic surgery, and follow-up loss at 3 months after RH. BOO was defined using preoperative UDS findings as detrusor pressure at maximum flow rate (PdetQmax) > 2 × maximum flow rate (Qmax).
Results: Forty-five patients were eligible for the analysis. Seventeen patients (37.8%) met the BOO criteria based on preoperative UDS result. The proportion of high preoperative PVR differs between women with and without BOO (29.4% vs. 7.4%, p=0.089). Fifteen patients (33.0%) were unable to void spontaneously at postoperative 10th day. The rate of preoperative BOO was higher in women without spontaneous voiding than those with spontaneous voiding (60.0% vs. 27.6%, p=0.036). At postoperative 3 months, the proportion of patient with urinary retention requiring CIC was higher in women with preoperative BOO than those without it (52.9% vs. 22.2%, p=0.036).

Conclusion: Preexisting BOO is one of major component determining urinary retention after type III RH for cervical cancer.

Disclosure: Nothing to disclose

EP331

Clinical impact of boost irradiation to pelvic lymph node in uterine cervical cancer treated with definitive chemoradiotherapy

Y.S. Kim[1], K.H. Choi[1], D.S. Lee[1], E.Y. Park[1], J.Y. Kim[1], S.Y. Hur[2]
[1]Department of Radiation Oncology, [2]Department of Gynecology, The Catholic University of Korea, Seoul, Republic of Korea

Introduction / Background: The aim of this study was to analyze tumor control and clinical outcomes of patients with uterine cervical cancer treated by chemoradiotherapy according to pelvic lymph node (PLN) positivity and boost irradiation to PLN and to determine toxicities associated with boost irradiation
Methodology: We retrospectively analyzed clinical characteristics, failure pattern, and survival outcomes of patients with or without PLN metastasis and those with or without boost irradiation were analyzed.
Results: A total of 80 cases were PLN-negative and 46 were PLN-positive. A total of 11 patients underwent PLN boost irradiation. The 2-year and 5-year overall survival (OS) rates showed significant difference between the PLN-positive and PLN-negative groups (P=.010). The 2-year and 5-year progression-free survival (PFS) rates showed significant difference between the 2 groups (P=.032). The 2-year and 5-year OS rates of the no-boost irradiation group were 82.9% and 58.3%, respectively, whereas all patients in the boost irradiation group were alive at the time of analysis (P=.065). There was no recurrence in the boost irradiation group. The difference in PFS was significant between the boost and the no-boost irradiation groups (P=.023). The 2-year and 5-year pelvic recurrence free survival (PRFS) did not show significant difference but the tendency of increased risk of pelvic recurrence in no-boost group (boost vs no-boost; 81.9% and 70.2% vs 100% and 100% in 2-year and 5-year PRFS, respectively, P=.156).
Conclusion: Boost irradiation to PLN could improve locoregional control especially in large pelvic LN (≥1.5 cm). Our results showed that only 1 acute and late toxicity of higher than grade 3 occurred. PLN metastasis was significant prognostic factor in cervix cancer treated by chemoradiotherapy. In the boost irradiation group, there was no recurrence or death with significantly better PFS. Boost irradiation to PLN is expected to improve locoregional control,but further follow-up and assessment are needed.
Disclosure: Nothing to disclose

EP332

Laparoscopic radical hysterectomy in cervical cancer: Is peritoneal contamination with tumor cells an underestimated event?

R. Klapdor, H. Hertel, P. Hillemanns, M. Röttger, P. Soergel, E. Kühnle, M. Jentschke
Department of Gynecology and Obstetrics, Hannover Medical School, Hannover, Germany

Introduction / Background: Peritoneal dissemination of cervical cancer cells during intracorporal colpotomy is discussed to be responsible for reduced disease free survival after laparoscopic radical hysterectomy as shown in the LACC study. However, data on the frequency or mechanisms of peritoneal contamination are missing. Therefore, we aimed to analyze peritoneal contamination of

cervical secretion during intracorporal colpotomy with a novel indocyaningreen (ICG) -based technique.
Methodology: In this prospective proof-of-principle study, we included patients undergoing routine laparoscopic or robot-assisted hysterectomy for benign indications. Routine surgery was performed after application of ICG specifically to the cervical surface (Figure 1). During colpotomy pictures under white and fluorescence light were taken in order to evaluate frequency and extent of contamination.
Results: Peritoneal contamination during intracorporal colpotomy occurred in 9/12 (75%) patients undergoing routine laparoscopic hysterectomy. Laparoscopic instruments were contaminated in 60% of all cases (Figure 1C). Extent of contamination varied between individual patients with a median grade of 2.75 (range 1-4.5; 1 no contamination - 5 strong contamination) at posterior colpotomy. There were no adverse effects during surgery.
Conclusion: Peritoneal contamination with cervical secretion is a frequent event during intracorporal colpotomy. We describe a promising novel tool for feasible and direct visualization of peritoneal contamination during colpotomy. This may be easily implemented in further studies on laparoscopic radical hysterectomy to assess the prognostic role of peritoneal contamination on survival and serve as a quality assessment tool for surgeons and surgical techniques.

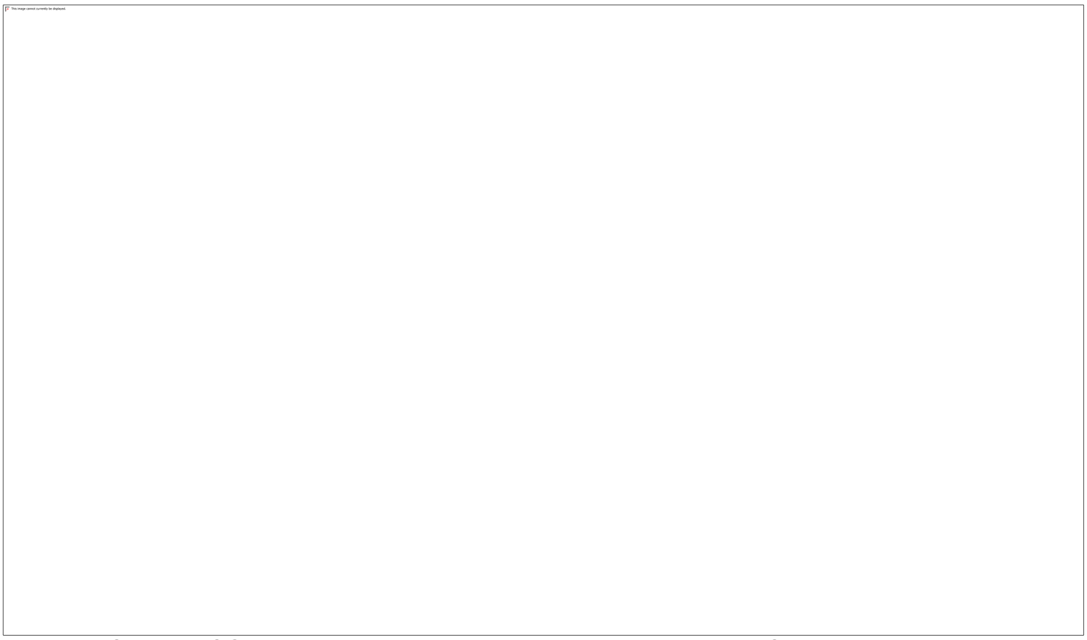

[Figure1: Cervical ICG application, white (A) and fluorescence (B) light. Contamination during anterior(C), posterior(D) colpotomy. U=Uterus, V=Vagina]

Disclosure: The author PH has authored a statement on the LACC study results by the Uterus Comission of the Study Group for Gynecologic Oncologs (AGO) and the and the Study Group for Gynecologic Endoscopy (AGE) of the German Society for Gynecology and Obstetrics (DGGG). All other authors report no conflict of interest.

EP333

Laparoscopic radical hysterectomy compared to laparotomy in patients with early-stage cervical cancer

L. Klasa, S. Sawicki, D. Wydra
Gynecology, Gynecological Oncology and Gynecological Endocrynology, Medical University of Gdansk, Gdansk, Poland

Introduction / Background: The purpose of the study is to compare the two operative techniques in cervical cancer and to determine whether laparoscopy is a safe and effective alternative to conventional abdominal radical hysterectomy (RAH).
Methodology: Fifty-one cases of radical laparoscopic hysterectomy (RLH) and 41 cases of RAH performed in Medical University in Gdansk (Poland) between 2013-2019 were compared and analyzed in a retrospective observational study. Since the publication of prospective LACC trial in 2018 reporting incomparably worse outcomes of minimally invasive techniques in cervical cancer, laparotomy or modified procedure of vaginally assisted laparoscopic radical hysterectomy (VALRH) without uterine manipulator, was carried out in all patients. We included patients with early-stage cervical cancer only: IA1-IB1 according to FIGO 2014 staging.
Results: There were no differences in preoperative clinical FIGO stage in both groups, with the most common Ib1 in 93% and 86% of patients respectively in RAH and RLH group. Although squamous cell cancer was the most frequent, we found a high percentage of adenocarcinoma in RLH (25%) compared to RAH (7%), which can affect the long-term outcomes. We have shown a significantly less blood loss in the minimally invasive technique ($p< 0.001$), a lower rate of postoperative complications ($p=0.003$) and slightly but statistically significant shorter hospital stay ($p=0.04$). RLH required longer operational time ($p< 0.001$) and was associated with a smaller number of lymph nodes removed ($p< 0.001$), with the same rate of intraoperative complication rate ($p>0.05$). There were no differences in recurrences in both groups (5.8% for RLH and 5% for RAH).
Conclusion: As in oncology of the 21st century, we are more and more interested in patients quality of life, we have to focus on the development of minimally invasive techniques. Radical laparoscopic hysterectomy still can be a safe and effective alternative to laparotomy in early-stage cervical cancer.
Disclosure: Nothing to disclose

EP334

SENTIX - Sentinel lymph nodes in patients with cervical cancer: SLN detection and the false negative rate of SLN frozen section (CEEGOG-CX01; ENGOT-CX2; NCT02494063)

R. Kocian[1], C. Köhler[2], J. Klat[3], A. Germanova[1], A. Plaikner[2], S. Bajsova[3], S. Marnitz[2], M. Ostojich[4], I. Zapardiel[5], B. Gil-Ibañez[6], B. Sehnal[7], A. Petiz[8], R. Pilka[9], A. Martin-Martinez[10], J. Presl[11], A. Buda[12], L. van Lonkhuijzen[13], L. Minar[14], M. Barahona[15], D. Wydra[16], P. Blecharz[17], P. Dundr[18], L. Dusek[19], D. Cibula[1], * Central and Eastern European Gynecologic Oncology Group #Belgium and Luxembourg Gynaecological Oncology Group
[1]Gynecologic Oncology Center, Department of Obstetrics and Gynecology, First Faculty of Medicine, Charles University and General University Hospital in Prague, CEEGOG, Prague, Czech Republic, [2]Department of Special Operative and Oncologic Gynaecology, Asklepios-Clinic Hamburg, Hamburg, Germany, [3]Department of Obstetrics and Gynecology, University Hospital Ostrava, CEEGOG*, Ostrava, Czech Republic, [4]Department of Gynecology, Institute of Oncology Angel H Roffo University of Buenos Aires, Ciudad Autónoma de Buenos Aires, Buenos Aires, Argentina, [5]Gynecologic Oncology Unit, La Paz University Hospital, Madrid, [6]Unit of Gynecological Oncology, Institute Clinic of Gynecology, Obstetrics and Neonatology (ICGON), Hospital Clinic of Barcelona, Barcelona, Spain, [7]Department of Obstetrics and Gynecology, Bulovka Hospital, First Faculty of Medicine, Charles University, CEEGOG*, Prague, Czech Republic, [8]Department of Gynecology, Francisco Gentil Portuguese Oncology Institute, Porto, Portugal, [9]Department of Obstetrics and Gynecology, University Hospital Olomouc, Olomouc, Czech Republic, CEEGOG*, Olomouc, Czech Republic, [10]Hospital Universitario Materno Infantil de Canarias, Las Palmas de Gran Canaria, Spain, [11]Department of Gynaecology and Obstetrics, University Hospital Pilsen, Charles University, CEEGOG*, Pilsen, Czech Republic, [12]Clinic of Obstetrics and Gynecology, San Gerardo Hospital, Monza, Italy, [13]Center for Gynecologic Oncology, Academic Medical Centre, Amsterdam, The Netherlands, [14]Department of Gynecology and Obstetrics, Faculty of Medicine, Masaryk University, Brno, Czech Republic, [15]Department of Gynecology, University Hospital of Bellvitge, Biomedical Research Institute of Bellvitge, University of Barcelona, Barcelona, Spain, [16]Department of Gynecology, Gynecologic Oncology and Gynecologic Endocrinology, Medical University of Gdansk, Gdansk, [17]Department of Gynecologic Oncology, Centre of Oncology, M. Sklodowska-Curie Memorial Institute, Cracow Department, Cracow, Poland, [18]Institute of Pathology, First Faculty of Medicine, Charles University*

and General University Hospital in Prague, Prague, [19]Institute of Biostatistics and Analyses, Faculty of Medicine, Masaryk University, Brno, Czech Republic

Introduction / Background: SENTIX is a prospective cohort multicenter international study on sentinel lymph node (SLN) biopsy without pelvic lymph node dissection (PLND) in patients with early-stage cervical cancer. SLN frozen section (FS) and pathological ultrastaging were mandatory. Samples from SLN were reviewed centrally for pathological assessment quality control. Only sites experienced in SLN biopsy technique were eligible.
Methodology: In total, 47 sites from 18 countries participated in the study. Patients with stages T1a1/LVSI+ - T1b2
(< 4 cm or ≤ 2 cm for fertility sparing), with common tumor types and no suspicious lymph nodes on imaging were pre-registered in the study. Patients remained registered after the surgery if SLN were detected on both sides and if SLN were negative on frozen section. Blue dye, radioactive tracer, indocyanine -green (ICG) or their combinations were all eligible tracers for SLN detection. SLN ultrastaging protocol included a complete processing of all SLN tissue in slices of 2 mm thickness, 2 sections in 150 µm from each block until no tissue left, one stained with H&E and second examined immunohistochemically.
Results: Data from 372 patients were analyzed who were pre-registered at the time when the number of cases treated per protocol reached 300. Patient characteristics are shown in Table 1. Bilateral detection rate reached 91%. Table 2 shows factors influencing SLN detection. Frozen section detected macrometastases (MAC) in 22 cases. SLN ultrastaging found additional 7 cases with MAC, 19 with micrometastases (MIC) and 11 with isolated tumor cells (ITC). Sensitivity of FS was 75.9% for the detection of MAC and 37.3% for any type of SLN involvement.
Conclusion: High bilateral detection can be achieved in sites experienced in the SLN biopsy technique. We did not confirm a higher detection rate using ICG. FS failed to detect 26/48 (54%) of cases with MIC or MAC in SLN.

[Table 1: Preoperative characteristics of patients (N = 372)]

This image cannot currently be displayed.

[Table 2: Factors influencing bilateral detection rate of SLN]

Disclosure: This work was supported by a grant from the Czech Research Council (No 16-31643A). Conflict of interest: None of the authors declare a conflict of interest.

EP335

HER3 protein expression is risk factors for postoperative recurrence in patients with adenocarcinoma and adenosquamous carcinoma of the cervix

Y. Kojima[1], K. Yonemori[1], H. Yoshida[2], T. Mizuno[1], Y. Otake[1], T. Seo[1], M. Tanioka[1], K. Sudo[1], A. Simomura[1], E. Noguchi[1], Y. Fujiwara[1], K. Tamura[1]
[1]Breast and Medical Oncology, [2]Department of Pathology and Clinical Laboratories, National Cancer Center Hospital, Tokyo, Japan

Introduction / Background: HER3 (ErbB-3) is a member of the epidermal growth factor receptor family. HER3 overexpression is associated with worse prognosis in ovarian cancer and squamous cell carcinoma of the cervix. Here, the purpose of the current study is to evaluate the expression and risk factors for postoperative recurrence of HER3 in adenocarcinoma and adenosquamous carcinoma of the cervix.
Methodology: This retrospective study included 100 patients diagnosed with primary cervical cancer who underwent primary surgery between 1997 and 2017 was stained immunohistochemically for HER3 expression. Evaluation of HER3 expression was performed by an experienced pathologist in accordance with the HER2 testing guideline for gastroesophageal cancer from CAP, ASCP, and ASCO. A possible influence of HER3 expression on survival was studied by multivariate Cox regression adjusting for established risk factors for postoperative recurrence.
Results: A positive HER3 expression was found in 68.9% (42/61) of squamous cell carcinoma, 85.1% (23/27) of adenocarcinoma, and 58.3% (7/12) of adenosquamous carcinoma. With a median follow-up period for survivors of 63.1 months, the 3-year disease-free survival rates (DFS) in patients with adenocarcinoma and adenosquamous carcinoma of the cervix were 55.9% in HER3-high and 88.9% in HER3-low/negative (p=0.20). In multivariate analysis, HER3 [HR, 6.62; 95% CI, 1.8-37.1; P=0.03], pelvic lymph nodes [HR, 6.72; 95% CI, 2.0-23.5; P< 0.01], vascular invasion [HR, 3.64; 95% CI, 1.1-12.2; P=0.04] were independent predictive factors of DFS.
Conclusion: HER3 overexpression was independent risk factors for postoperative recurrence in

patients with adenocarcinoma and adenosquamous carcinoma of the cervix.
Disclosure: Nothing to disclose

EP336

Glassy cell carcinoma of the uterine cervix. A rare and aggressive entity

N. Koufopoulos[1,2], F. Antoniadou[2], E. Karopoulou[3], A. Karampelas[3], S. Kokkali[4], A. Grivas[5], D. Papatheodorou[3], L. Khaldi[2]
[1]2nd Pathology Department, Attikon University Hospital, National and Kapodistrian University of Athens, [2]Pathology Department, [3]Gynecology Department, [4]1st Medical Oncology Department, [5]2nd Medical Oncology Department, 'Saint Savvas' Anticancer Hospital of Athens, Athens, Greece

Introduction / Background: Glassy cell carcinoma (GCC) of the uterine cervix is a poorly differentiated subtype of adenosquamous carcinoma. It accounts for 1-2% of cervical carcinomas. It has been associated with high-risk human papillomavirus infection (HPV 16, 18 and 32). We present a case of this rare entity.
Methodology: A 46-year-old patient was admitted to the gynecology department. She had a previous diagnosis of poorly differentiated carcinoma on cervical biopsy performed due to an abnormal enlargement of the uterine cervix on routine ultrasound examination. Radical hysterectomy with bilateral pelvic lymph node dissection and omentectomy was performed. On gross examination, the cervix was barrel-shaped, occupied by a bulky, white-gray exophytic mass measuring 60 mm in its greatest dimension.
Results: On microscopic examination, the tumor consisted of solid nests of large, pleiomorphic cells with prominent nucleoli, lacking glandular or squamous differentiation, with ground-glass or finely granular cytoplasm. Several mitoses were identified. Tumor nests were embedded in a heavy inflammatory infiltrate. Immunohistochemically tumor cells showed positive staining for EMA, Vimentin, P16, pCEA and negative for CK7, CK20, P63, ER, PR, and HMB45. The omentum and pelvic lymph nodes were tumor free. Our diagnosis based on morphological and immunohistochemical findings was GCC of the uterine cervix. The patient received adjuvant radiotherapy and chemotherapy. She remains disease-free 9 months post surgery.
Conclusion: GCC is a clinically aggressive subtype of cervical carcinoma with rapid growth, and early metastases. Treatment consists of surgery followed by radio-chemotherapy or neoadjuvant therapy in cases with advanced disease. Multimodal therapy has improved prognosis in patients with FIGO stage I or II but not in higher stage disease.
Disclosure: Nothing to disclose

EP337

The philosophy of setinel lymph node in the management of early stage cervical cancer. Feasible or not?

I. Koutroumpa[1], N. Thomakos[1], M. Sotiropoulou[2], C. Theofanakis[1], V. Theodoulidis[1], A. Pandraklakis[1], D.-E. Vlachos[1], D. Haidopoulos[1], A. Rodolakis[1]
[1]Unit of Gynaecologic Oncology, 1st Department of Obstetrics & Gynaecology, National and Kapodistrian University of Athens, [2]Department of Histopathology, Alexandra Hospital, Athens, Greece

Introduction / Background: A single institution study developing a simple algorithm in sentinel lymph node (SLN) mapping, evaluating the detection rate and diagnostic accuracy between frozen section biopsy (FSB) and final histopathology (FH). We also present an attempt to assess SLN concept for less radical surgery in early stage cervical cancer (CaCx).
Methodology: Prospective study including patients with CaCx, stage IA1- IIA1 (tumour size 0.5-3 cm).

Intracervical injection of methylene blue after induction of anaesthesia, detection of LNs that are dyed and sent for FSB. Bilateral pelvic lymphadenectomy and radical hysterectomy is then performed and correlated with FH.
Results: In our study, 70 patients were eligible. At least one SLN (range 0-6) was identified in 82.4% (58/70), whereas bilateral detection was succeeded in 80% (56/70). SLNs were located at the external (53.8%) or internal iliac region (15.4%), obturator fossa (19.2%), and ventral to the hypogastric vessels (11.6%), whereas 9.1% found in (unexpected) area (parametrium) in cases with tumour size (TS) ≥ 3cm. Frozen section was positive in 4 cases (5.7%) and the procedure was aborted. Sentinel lymph node sensitivity in detection of metastasis was 100% for TS≤ 2cm. False negative SLN and micrometastasis was identified in only two cases (TS>2cm). Parametrial involvement was not detected when SLNs were negative.
Conclusion: Our findings confirm the clinical significance of SLN mapping in minimizing systematic lymphadenectomy and supports less radical surgery of parametrium with greater safety. However, we are still in the process to establish our technique as feasible and equate in early stage cervical cancer.
Disclosure: Nothing to disclose

EP338

Cervical cancer and vaginal flora changes

S. Kovachev
Gynecology, Military Institute of Medicine, Sofia, Bulgaria

Introduction / Background: Microbial changes in vaginal ecosystem may accelerate the process of cervical carcinogenesis. The developed cervical cancer can lead to changes in the vaginal flora. The aim of our study is to determine the vaginal flora changes at women with cervical cancer.
Methodology: We conducted an open, single-site survey in the Department of gynecology of the Military Medical Academy in Sofia, Bulgaria, from 2013 to 2018 year. The study included a total of 32 women aged 38- 55 years with clinical and pathology established cervical cancer (FIGO I stage). The underlying clinical and microbiological studies indicated presence or absence of bacterial vaginosis, other vaginal infections or normal vaginal flora.
Results: Of 32 (100%) women enrolled in our study, 19 (59.4%) was with FIGO IA stage cervical cancer and 13 (40.6%) with IB stage. Disturbances of vaginal flora in we found at 23 (71.9%) of women with cancer cancer included in our study. At the rest of 9 (28.1%) women we found out normal vaginal flora. Bacterial vaginosis was determined clinically and microbiologically in 15 (46.9%) women enrolled in the study. Aerobic vaginitis caused by Group B *Streptococcus* we establish at 4 (12.5%) of women: (*Enterococcus faecalis* -3 /9.4%; *Escherichia coli* - 1/3.1%). *Trichomonas vaginalis* infection have 1 (3.1%) women and *Candida Albicans* the last one 1 (3.1%) from this group with disturbed vaginal microbial balance.
Conclusion: Bacterial dysbiosis, characterized by a predominance of *Gardnerella vaginalis* alone or in complex with other anaerobic bacteria, aerobic vaginitis and other sexually transmitted vaginal pathogens from one side and a concomitant paucity of vaginal *Lactobacillus species* may be an HPV-dependent cofactor for cervical neoplasia development. Only with this single observation it is difficult to confirm that microbiota dysbiosis contributes to HPV infection and carcinogenesis.
Disclosure: Authors declare no conflict of interest. This is an author's own work.

EP339

Urologic complications of cervical cancer radical surgery

S. Kovachev, A. Ganovska
Gynecology, Military Institute of Medicine, Sofia, Bulgaria

Introduction / Background: Ones of the most often serious complications of cervical cancer radical surgery are damages of urinary tract system which healing need multidisciplinary surgical team, prolong hospital stay, are very unpleasant for the patient and expensive as much.
The aim of our study is to establish the urologic complications of radical type C2 hysterectomy.
Methodology: This retrospective study included 52 patients with clinically and pathology establish cervical I and II stages cancer threatened with radical type C2 hysterectomy the period of 5 last years (2013-2018). The urologic complications we included in our study are: intraoperative ureter and urinary bladder lesions and postoperative ureterovaginal and vesicovaginal fistulas.
Results: From 52 (100%) patients included and passed surgery for cervical cancer, urological complications we find out at 8 (15.4%) of women two (3.8%) of them have preoperative radiation therapy. Intraoperative urologic complications are 5 (9.6%) as followed: 3 (5.8%) lesions of ureter and 2 (3.8%) urinary bladder lesions one patient with preoperative radiation therapy. At four of this complications we form multidisciplinary surgical team (urologist included) to restore normal organ anatomy. Postoperative complications are 3 (5.8%): 2 (3.9%) ureterovaginal and 1 (1.9%) with preoperative radiation therapy vesicovaginal fistulas. All they need surgery to be treated and multidisciplinary surgical team involved.
Conclusion: Urology complications are very common after radical surgery (hysterectomy type C2) for cervical cancer. More often they need formation of multidisciplinary surgical team (urologist included) to be treated. Preoperative radiation therapy is a possible predictive factor for common urology complications in our study.
Disclosure: Authors declare no conflict of interest. This is an author's own work.

EP340

Clinicopathologic review of the gastric-type mucinous adenocarcinomas of the cervix in a tertiary referral centre in Singapore

M. Kwek[1], C. Goh[2], Y.C. Yeo[3], Y.K. Lim[4], I. Aggarwal[5]
[1]Obstetrics and Gynaecology, KK Women's and Children's Hospital, Singaporea, [2]Obstetrics and Gynaecology, [3]Pathology and Laboratory Medicine, [4]Gynaecological Oncology, KK Women's and Children's Hospital, Singapore, [5]Gynaecological Oncology, KK Women's and Children's Hospital, Sinagpore, Singapore

Introduction / Background: Endocervical Gastric-type mucinous adenocarcinoma is a rare but recognised subtype of mucinous adenocarcinoma as per the WHO 2014 classification of endocervical adenocarcinomas. Although adenocarcinomas are less common than squamous cell carcinomas, their prevalence is increasing. It is important to distinguish the Gastric type from other endocervical adenocarcinomas as it is has a worse prognosis. Compared to other types of endocervical adenocarcinomas, chemotherapy may be less effective in the gastric-type. Of note, adenocarcinomas are not associated with HPV infection. With increasing uptake worldwide of primary HPV based screening for cervical cancers, diagnosis of non-HPV related cervical cancers might be missed.
Methodology: A retrospective review of clinicopathologic features in patients with endocervical Gastric-type mucinous adenocarcinoma diagnosed between 2015 and 2019 in a single tertiary referral centre in Singapore.
Results: 15 patients were diagnosed with endocervical Gastric-type mucinous adenocarcinoma between 2015 and 2019 with the average age of 56.4 years. 13 patients underwent examination under anaesthesia and clinical staging. Seven (53.8%) had Stage 1 disease,four (30.8%) had Stage 2 disease, one (7.7%) had Stage 3 disease and one (7.7%) had Stage 4 disease. 10 patients underwent primary treatment and follow-up our centre. 9 patients were early stage disease (Stage 1 to 2). Out of the 9 patients with early stage disease, 7 underwent surgery as primary treatment and 2 underwent radiotherapy and/or chemotherapy. 1 patient with Stage 4 disease underwent primary debulking surgery as the tumour was initially thought to be endometrial adenocarcinoma. 2 out of 10 patients have passed away from the disease during follow-up.
Conclusion: The gastric subtype of cervical adenocarcinomas portends a poorer prognosis and good evidence on the optimal modality of treatment is lacking. There is need for multi-centred clinical trials to raise awareness for this rare subtype and formulate treatment options. Newer techniques

incorporating molecular tests may help with the diagnosis.
Disclosure: Nothing to disclose

EP341

Screening capacity and cost-effectiveness of the human papillomavirus test versus cervicography as an adjunctive test to Pap cytology to detect high-grade cervical dysplasia

S.-K. Lee, T. Song, S.J. Seong
OB-GYN, Kyung Hee University, Seoul, Republic of Korea

Introduction / Background: The purpose of this study was to compare the screening capacities and the cost-effectiveness of cervicography and HPV testing as an adjunctive test to Pap cytology to detect high-grade cervical dysplasia in clinical practice in Korea, a country with a high prevalence of cervical cancer.
Methodology: This study was designed by the Korean Cervicography Research Group, established in 1997, and comprises 30 medical university hospital professors who are well trained colposcopists, and who have performed teaching and research activities for cervicography and cervical cancer prevention in Korea. Between January 2015 and December 2016, 33,531 women underwent a cervicography as a screening test for cervical cancer at private clinics and university hospitals in Korea. SPSS 20.0 (SPSS Inc., Chicago, IL, USA) was used for the statistical analysis. Data were presented as mean standard deviation or median (interquartile range) for quantitative variables and frequency (%) for qualitative variables.
Results: In Korea, the cost per patient with CIN2+ is $1474 with the HPV test, and $1135 when cervicography is combined with Pap cytology. Therefore, cervicography co-testing would decrease the cost per patient with CIN2+ lesions by approximately 23% compared to the cost of HPV co-testing.
Conclusion: In conclusion, this study suggests that cervicography as an adjunctive test increases detection of cervical cancer and its precursor lesions through reducing false negative errors (2.5% versus 6.3%), compared to the HPV test.
Disclosure: Nothing to disclose

EP342

Cognitive computing system in cervical cancer IB to IIA: a multicenter retrospective analysis

K.B. Lee[1], S. Lim[1], Y.H. Kim[2], J.-M. Lee[3], C.-H. Choi[4]
[1]Department of Obstetrics and Gynecology, Gachon University Gil Medical Center, Gachon University College of Medicine, Incheon, [2]Department of Obstetrics and Gynecology, Ewha Womans University School of Medicine, [3]Department of Obstetrics and Gynecology, Kyung-Hee University School of Medicine, Kyung-Hee University Hospital at Gangdong, [4]Department of Obstetrics and Gynecology, Samsung Medical Center, Sungkyunkwan University School of Medicine, Seoul, Republic of Korea

Introduction / Background: IBM Watson for Oncology (WFO) has begun to be used in Korea. It provides physicians with evidence-based treatment options. We assessed differences of survival between WFO Rx and the Site Rx for each of the patients with cervical cancer IB to IIA undergoing primary surgical treatment.
Methodology: This retrospective multicenter study collected the database from 4 academic institutes between January 2000 and December 2014. Of the 1612 total, 161 were excluded because they had rare histology, inadequate imaging study, over age-range that covered by WFO or history of neoadjuvant chemotherapy, any of one of which is not applicable to WFO. WFO assign every patient to one of the following subgroups:
A. Unavailable to the site

B. Unknown to WFO
C. Green therapies in WFO: preferred
D. Yellow therapies in WFO: acceptable
E. Red therapies in WFO: unacceptable
Results: The clinical and pathological characteristics of the patients are shown in Table 1. The numbers of each subgroup were as follows: A 0; B 129; C 879; D 80; E 363. The 5-year disease free survival (DFS) were 83.2% in B, 89.0% in C, 93.5% in D, 83.5% in E. The 5-year overall survival (OS) were 93.0% in B, 93.0% in C, 97.4% in D, 92.3% in E. Based on subgroup C, comparison of survival with other subgroups is shown in Table 1.
Conclusion: This retrospective study showed that WFO Rx for the patients with cervical cancer IB to IIA undergoing primary surgical treatment may be effective tools.

subgroup	p-value (log-rank, DFS)	p-value (log-rank, OS)
B	0.04	0.85
D	0.71	0.62
E	0.00	0.92

[Table 1. Comparison of survival with other subgroups based on subgroup C]

Disclosure: Nothing to disclose

EP343

Number of harvested lymph nodes is major risk factor of the postoperative lymphocele after pelvic lymphadenectomy in women with cervical cancer

J.W. Lee, S.I. Kim, J.H. Yoon
The Catholic University of Korea/ St. Vincent's Hospital, Suwon, Republic of Korea

Introduction / Background: A lymphocele is a cystic mass that may occur in the retroperitoneum following a systematic pelvic and/or para-aortic lymphadenectomy. Lymphoceles may be the cause of severe morbidity, or rarely mortality. This study was aimed to determine risk factor for development of lymphocele after pelvic lymphadenectomy in women with cervical cancer, through a retrospective study of patients undergoing sole pelvic or combined pelvic and paraaortic lymphadenectomy for cervical cancer.
Methodology: This study was conducted on 547 cervical cancer patients who underwent radical hysterectomy and pelvic lymphadenectomy at Seoul St. Mary ´s Hospital and St. Vincent's Hospital from 1997 to 2010. We divided them into lymphocele induced and lymphocele free groups, respectively. 7 variables (the type of energy device, the number of lymph nodes removed, radiation therapy, operation time, drainage use, BMI) were retrospectively compared between both group.
Results: Of 547 patients who underwent sole pelvic or combined pelvic and paraaortic lymphadenectomy for cervical cancer. The mean number of lymph nodes removed from the lymphocele group was 55.09 and the mean number of lymph nodes removed from the lymphocele-free group was 51.19. Among 85 lymphocele groups, more than 2 lymphocele were found in 20 patients and the total number of lymphocele was 106. Lymphoceles were predominantly located on the pelvic area. (pelvic wall 54.7%, ext. iliac 26.4%, int. iliac 12.3%, obturator 2.8%, inguinal 2.8%). In only one case, Lymphocele was located on the para-renal area.
Conclusion: The number of lymph nodes removed during pelvic and/or para-aortic lymphadenectomy for cervical cancer is a risk factor for the development of lymphocele.
Disclosure: Nothing to disclose

EP344

Development of nomograms for prediction of disease recurrence according to the surgical approach in patients with stage IB cervical cancer

H.P. Lee[1], S.I. Kim[2], Y.B. Kim[3]
[1]Department of Obstetrics and Gynecology, Soon Chun Hyang University Hospital, [2]Department of Obstetrics and Gynecology, Seoul National University Hospital, Seoul, [3]Department of Obstetrics and Gynecology, Seoul National University Bundang Hospital, Seongnam, Republic of Korea

Introduction / Background: Determination of appropriate surgical approach for each cervical cancer (CC) patient between open radical hysterectomy (RH) and laparoscopic RH without compromising survival outcome is an important issue. Thus, we aimed to develop nomograms predicting disease recurrence according to the surgical approach in early-stage CC.
Methodology: We retrospectively identified 724 patients with FIGO stage IB1-IB2 CC who received either open or laparoscopic Type C RH at two tertiary institutional hospitals between 2000 and 2017. Among them, those who underwent pre-operative MRI were included. Patients' clinicopathologic characteristics and survival outcomes were collected. Considering only the variables that could be obtained before surgery, we constructed predictive models for the 2-year progression-free survival (PFS) rate using logistic regression and a machine learning method called xgBoost.
Results: In total, 572 patients were included; 343 and 229 received open RH and laparoscopic RH, respectively. The median length of observation was 54.7 months during which 79 patients (13.8%) experienced disease recurrence. Similar PFS was observed between the open and laparoscopic RH groups (2-year rate, 90.5% vs. 86.8%; P=0.068). The AUCs of the newly developed nomograms predicting 2-year PFS were up to 0.64 for open RH group and up to 0.65 for laparoscopic RH group.
Conclusion: We preliminarily developed surgical approach-based two independent nomograms predicting disease recurrence in early-stage CC. These nomograms are expected to aid decision-making on the surgical approach of RH in individualized counselling practices. Further studies tailoring the nomograms with large cohorts are warranted.
Disclosure: Nothing to disclose

EP345

Human papillomavirus L1 capsid protein and HPV test as a biomarker for cervical intraepithelial neoplasia 2+ in women with persistent ASCUS /LSIL cervical cytology

H.J. Yoon, M. Jeong, E.Y. Ki, S.J. Lee, Y.S. Lee
The Catholic University of Korea, Seoul, Republic of Korea

Introduction / Background: In spite of the high probability of spontaneous regression of ASCUS and LSIL, persistent minor cervical abnormalities were occasionally observed in women with HPV infection. Until now, efficient predictive biomarker reflecting the prognosis of persistent ASCUS and LSIL was not developed. The aim of this study was to find the best predictive model for diagnosis of CIN2+ using immunocytochemical expression of HPV L1 capsid protein in patients with persistent ASCUS and LSIL with high risk of HPV infection.
Methodology: A total of 285 cervical cytology samples (70 ASCUS and 215 LSIL Pap smear) were analyzed in this study. Immunocytochemical identification of HPV L1 capsid protein in cervical cytology samples was performed using the Cytoactive® HPV L1 screening set. The expression rate of the HPV L1 capsid protein in cervical cytology was calculated, and correlation between HPV L1 expression in cytology and cervical pathologic diagnosis was evaluated. The risk for CIN2+ was calculated using results of immunocytochemistry and HPV DNA test.
Results: Negative results for HPV L1 were more frequently observed in CIN2+, and positive expression of HPV L1 capsid protein was higher in CIN1 or cervicitis (Fisher's exact test, $p < 0.05$). Diagnosis rates for CIN2+ were the highest for the combination of HPV L1 capsid protein immunocytochemistry, cytology and HPV test when compared with other combinations (AIC: 191.7, SC: 206.3, $p < 0.001$).

Conclusion: Negative expression of HPV L1 capsid and HPV type 16 or 18 infection can predict progression to CIN2+ in women showing persistent ASCUS and LSIL. Therefore, HPV L1 immunocytochemistry and the HPV type test can be useful to predict diagnosis of CIN2+ in women with persistent ASCUS or LSIL.
Disclosure: Nothing to disclose

EP346

The prognostic value of hematologic parameters in cervical cancer patients receiving radiation therapy

Y.J. Kim[1], Y.S. Kim[2], K.B. Lee[3], J.W. Shin[3], S.H. Lee[4]
[1]Gachon University College of Medicine, [2]Internal Medicine, [3]Obstetrics and Gynecology, [4]Radiation Oncology, Gachon University College of Medicine, Incheon, Republic of Korea

Introduction / Background: The aim of this study was to determine the prognostic roles of hematologic parameters in patients with cervical cancer received definitive radiation therapy (RT).
Methodology: A total of 128 patients received definitive RT for cervical cancer were enrolled in this study.The clinical data and hematologic parameters were retrospectively reviewed, and analyzed their prognostic roles for survival.Theneutrophil-to-lymphocyte ratio (NLR), platelet-to-lymphocyte ratio (PLR) and lymphocyte-to-monocyte ratio (LMR)) and the changes between pre- and post-treatment in hematologic parameters (ΔNLR, ΔPLR, and ΔLMR) were alsocalculated to analyze the specific roles for survival.
Results: The median follow-up was 36.9 months, with a range of 1-114 months.The 5-year overall survival rate and progression free survival rate was 65.8% and 46.4%, respectively. High pretreatment NLR (NLR>2.84) was a poor prognostic factor for progression-free survival (PFS; HR=2.789; 95%CI 1.497-5.197) and overall survival (OS; HR=4.191, 95%CI 2.002-8.773). ΔNLR> 0 was significantly associated with lower progression-free survival(HR=2.942, 95%CI 1.458-5.938) and overall survival(HR=3.802, 95%CI 1.487-9.725). In addition to NLR and ΔNLR, age (age≤55; PFS: HR=2.538; 95%CI 1.491-4.320; OS: HR=3.475; 95%CI 1.652-7.310) and FIGO stage (Ⅲ-Ⅳ; PFS: HR=3.067; 95%CI 1.790-5.253; OS: HR=4.252; 95%CI 1.974-9.157) were identified as poor predictors of PFS and OS, whereas white blood cell (WBC< 5710; PFS: HR=1.961; 95%CI 1.044-3.682) was significantly predictive of PFS only.
Conclusion: Both pretreatment NLR and ΔNLR were independent prognostic factors for cervical cancer patients treated with RT. Risk scoring system-based prognostic factor analysis could be used to help develop optimized treatment plans for cervical cancer patients receiving RT.
Disclosure: Nothing to disclose

EP347

Preliminary results of primary cancer boost using intensity-modulated radiation therapy for locally advanced cervical cancer

X. Li[1], Q. Song[2]
[1]Renmin Hospital, Wuhan University, [2]Cancer Center, Wuhan University Renmin Hospital, Wuhan, China

Introduction / Background: To evaluate the feasibility of primary cancer boost using intensity-modulated radiation therapy in patients with locally advanced cervical cancer.
Methodology: From January 1st, 2009 to December 31st, 2012, twenty four cervical cancer patients inappropriate or contraindicated for BT were enrolled. The initial plan was designed to deliver 50 Gy in 25 fractions the pelvic cavity and cervix, followed with an IMRT boost of 26-30 Gy with 2 Gy fraction

dose to primary tumor. Most patients were treated with concurrent paclitaxel 50mg/m2 and cisplatin 25mg/m2 weekly.
Results: Of these 24 patients, the median follow-up time was 24 months (ranging from 10 to 44 months). The 3-year overall survival（OS）rate and progression-free survival（PFS）rate was 89.7% and 85%, respectively. Two patients relapsed at 11 and 12 months and died of that, one patient had mediastinal lymph node metastasis at 16 months after definitive diagnosis but is still alive, two patients died of lung metastasis. Acute grade 3 gastrointestinal (GI) occurred in 3 patients (12.5%), and genitourinary (GU) toxicities occurred in 2 patients (8.3%), respectively. Late grade 3 GU toxicity occurred in 1 patient was urinary incontinence. Ten patients had myelosuppression over grade 3.
Conclusion: IMRT may be an effective alternative option for cerevical cancer patients unsuitable for BT or in the regions with limited access to interstitial BT. Larger-scale studies are warranted.
Disclosure: Nothing to disclose

EP348

Fertility-sparing treatment for IB1 cervical cancer with tumors ≥ 2 cm: an updated series with 142 patients

J. Li, X. Li, X. Wu
Fudan University Shanghai Cancer Center, Shanghai, China

Introduction / Background: Although abdominal radical trachelectomy (ART) has become a recommended fertility-sparing procedure by NCCN guideline since 2015, the relative contraindication of a tumor ≥2 cm has been questioned.
Methodology: We conducted a retrospective review of a prospectively maintained database of patients undergoing ART at our institution from 04/2004 to 12/2017. All patients were followed postoperatively.
Results: Of the 343 patients who underwent planned ART, 142 (41.3%) had IB1cervical cancer whose tumors ≥2 cm in size . Among them, 10 had NACT while the other 132 patients underwent upfront ART. In the whole cohort, two hundred and seventy-nine patients had squamous carcinomas (SCC), 53 had pure adenocarcinomas (AC), and 11 had adenosquamous carcinomas (AS). With a median follow up of 65 months,12 patients (3.4%) had recurrence, and five patients (1.5%) died. The cumulative 5-year recurrence-free survival and overall survival rates for the whole cohort were 96.3 and 98.6%, respectively. Among 12 recurrent cases, 8 had tumor larger than 2cm. The recurrence rate in women with tumours ≥2 cm was comparable to that in patients with tumours < 2 cm (P = NS). However, the recurrence rate was significantly higher in patients with AS histology than those with AC and SCC histology (P < 0.05).
Conclusion: These results further supported that upfront ART was a safe option for well-selected patients with stage IB1 cervical cancers ≥2 cm. As for NACT, we need larger sample and longer follow-up to observe the oncological and obstetric results. However, if patients with tumours ≥2 cm have AS histology, they should be advised with great caution when contemplating ART.
Disclosure: Nothing to disclose

EP349

Treatment outcome for primary cervix large B-cell lymphoma: a clinical analysis of 37 cases

X. Liu
Fudan University Shanghai Cancer Center, Shanghai, China

Introduction / Background: Cervical lymphoma is a rare condition and may be difficult to diagnose and treatment. Large B-cell lymphoma is among the most common sub-type of Cervical

Lymphoma.No standard treatments for patients with primary cervix large B-cell lymphoma have been introduced currently. Only case reports published in the literature and indicated the R-CHOP regimens in combination with involved field radiation therapy (RT) are effective. Shanghai Cancer Center is one of the top pathological consultant center in China. We retrospectively analyse the 37 patients with primary cervix large B-cell lymphoma and outcome after R-CHOP in combination with RT.
Methodology: Thirty-seven untreated primary cervix large B-cell lymphoma patients received R-CHOP like regimens. Fourteen of them underwent subsequent RT. Thirteen of them received subsequent salvage chemotherapy.
Results: Of 37 patients underwent R-CHOP chemotherapy, only 3 patients received the radical surgery. An overall response rate (ORR) was 78.3% after completion of chemotherapy.The PFS and OS rate at 5 years were 58% and 69%, respectively. For 14 patients who received RT after R-CHOP, an estimated ORR were 89.2%. For all patients, The PFS reached the platform after 2 years follow up and the OS after 3 years according to the survival curve analysis. The international prognostic index (IPI) score were the only predictor of worse outcome.
Conclusion: R-CHOP regimens plus involved field radiation therapy led high response rate in primary cervix large B-cell lymphoma. Patients with high IPI had a trend of less satisfied with R-CHOP regimens plus IFRT. Future prospective and multicenter studies are needed. By the way, we are conducting a gene expression profile between DLBCL-unspecified and primary cervix large B-cell lymphoma by NGS in order to get the valuable information to improve the treatment outcome even by using some targeted drugs. The data will be show in the next few months.
Disclosure: Nothing to disclose

EP350

Research of 20 years of intraoperative electron radiotherapy for cervical carcinoma stage IIB

Z. Liu, J. Wang, K. Zhang
Department of Radiation Oncology, First Affiliated Hospital of Xi'an Jiaotong University, Xi'an, China

Introduction / Background: Intraoperative radiation therapy (IOERT) is a treatment that combines surgery and radiotherapy. It's been reported in the treatment of gastric cancer, pancreatic cancer and other disease by giving a large dose of radiation to the target area during the operation.
Methodology: 427 patients with cervical carcinoma underwent radical radiotherapy from January 1997 to December 1999 in the First Affiliated Hospital of Xi'an Jiaotong University. Among them , 92 patients with stage IIB (FIGO1985) accepted preoperative radiotherapy $EQD_2$20-30Gy (including external beam radiotherapy with 6MV X-ray 10-20Gy/5-10f to pelvic field and/or brachytherapy with 7-21Gy/1-3f to point A), After 1 week's interval, patients completed total hysterectomy plus double accessory resection(without pelvic lymph node dissection) + IOERT with 12 MeV electron beam 18-20Gy to pelvic field (We have the patent of China for the special applicator used in IEORT.). Patients were discharged from hospital 7 days after surgery and were followed up regularly. Patients didn't receive concurrent chemotherapy. The total treatment time was 4 weeks.
Results: The 20-year survival rate was 85.9%. 3 patients lost follow-up were considered dead. Metastasis was observed in 11 patients (11.9%) (Table 1), 10 patients died of metastasis and 1 patient had vaginal metastasis 13 years after treatment, but achieved a long-term survival after retreatment. Local recurrence was not found. The major complication was hydronephrosis, which occurred in 15 patients (16.3%), including 7 patients with grade 2, whose renal function returned to normal after double J ureteral stents treatment. Other toxicities such as radiation proctitis, radiation cystitis and myelosuppression were shown in Table 2.
Conclusion: The application of total hysterectomy plus double accessory resection combined with IOERT in patients with cervical cancer IIB can obtain good therapeutic effects with acceptable toxicities and shorten the treatment time.

Total patients	92
Patients with metastasis, n(%)	11(11.9%)
Pelvic wall	2(2.2%)

Lung	1(1.1%)
Bone	1(1.1%)
Liver	1(1.1%)
Lymph node	2(2.2%)
Tumor thrombus in lower limb	1(1.1%)
Multiple metastasis	2(2.2%)
Vagina	1(1.1%)

[Treatment outcome]

Hydronephrosis	
Grade1	8(8.6%)
Grade2	7(7.6%)
Radiation proctitis	
Grade1	3(3.2%)
Grade2	1(1.1%)
Myelosuppression	
Grade1	2(2.2%)
Radiation cystitis	0(0.0%)
Radiational vaginal injury	0(0.0%)

[Late toxicities of patients]

Disclosure: No potential conflicts of interest were disclosed.

EP351

Prevalence of genital infection by the human papilloma virus in the female population of Gran Canaria (Spain)

A. Lubrano Rosales[1], A. Torres Afonso[1], B. Vega Guedes[1], M. Andujar Sanchez[2]
[1]Gynecology and Obstetrics, [2]Pathology, Complejo Hospitalario Universitario Materno Infantil de Canarias, Las Palmas, Spain

Introduction / Background: Cervical cancer is the fourth most commun female cancer worldwide after breast, colorectal and lung cancers. In Spain 1942 new cases were diagnosed during 2018, currently occupying the eleventh place among women of all ages. However, this cancer is the fourth in frequency among young women aged 25 to 44 years. Persistent infection with human papillomavirus (HPV) is considered a necessary cause for the development of a cervical carcinoma, and its distribution has been studied in many countries. The objectives of this study were to determine the prevalence of preneoplastic lesions of the cervix and the prevalence of HPV infection through molecular study in the female population of Gran Canaria.
Methodology: A cross-sectional population-based study was conducted among women between 18 and 65 years of age during the years 2004-2007. All the women after the signing of the informed consent were taken samples for cytology and HPV study. The sample size was 2,276 women.
Results: The average age was 41.56 years. 90.4% of cytologies were normal without evidence of intraepithelial lesion , 4.9% were classified as pathological: 3.4% ASCUS; 1.3% LSIL, 0.06% HSIL and 0.06% adenocarcinoma. High-risk HPV was positive in 12.56% (95% CI: 11-14%), 9.8% in negative cytology versus 42.8% in pathological cytology ($P < 0.001$).

Conclusion: The prevalence of HPV in the female population of Gran Canaria was high, similar to previous studies published in Spain. These results support the benefit of routine HPV vaccination to control infection and prevent the burden of HPV-associated disease.
Disclosure: Nothing to disclose

EP352

The clinical utility of MRI and CT-PET following radical treatment for cervical carcinoma

J. Yee Kei To, G. Macdonald, A. Kennedy
Department of Oncology, Aberdeen Royal Infirmary, Aberdeen, United Kingdom

Introduction / Background: Imaging follow up after radical treatment for cervical cancer with chemoradiotherapy and brachytherapy may be warranted if asymptomatic persistent or recurrent disease can be detected such that salvage treatment can be undertaken with curative intent. Since 2013, in Aberdeen Royal Infirmary, follow up MRI at 3 months and/or PET-CT at 9 months post-treatment have been organised for patients in whom salvage pelvic exenteration would be considered in the event of central recurrence.
Methodology: All patients with cervical cancer treated non-surgically with curative intent between 2013 and 2018 at Aberdeen Royal Infirmary were identified from the radiotherapy database. Patients who had undergone a 3 month MRI and/or a 9 month CT-PET scan were identified and the results of these were collated, with the subsequent impact on patient management identified from the electronic patient record.
Results: Twenty-nine and 32 patients underwent a post-treatment MRI and PET-CT scan respectively.
Following MRI, 16 patients had no residual disease (NRD) and have not relapsed to date, 5 had NRD but subsequently relapsed systemically, 5 had residual or new disease unsuitable for salvage, and 3 had potentially salvageable disease (1 undergoing exenteration but subsequently relapsing, 1 progressing prior to exenteration and 1 who did not pursue salvage).
Following PET-CT, 24 patients had NRD and have not relapsed to date, 7 had unsalvageable metastatic or pelvic disease and 1 had persistent pelvic side wall disease that was considered unsalvageable at the time but might now have been considered for salvage ablative radiotherapy.
Conclusion: In our cohort, post treatment MRI at 3 months and/or PET-CT at 9 months have not led to any additional patients being cured of their cancer. The clinical utility of such scans may be limited to providing reassurance to patients with a negative scan, given the high negative predictive value in our series.
Disclosure: Nothing to disclose

EP353

Fertility sparing surgery following neoadjuvant chemotherapy of cervical cancer figo stage IB2 - case report

A. Mandic, M. Davidovic Grigoraki, B. Gutić, N. Prvulović Bunović, N. Šolajić
Oncology Institute of Vojvodina, University of Novi Sad, Medical Faculty, Novi Sad, Serbia

Introduction / Background: During the last three decades, fertility preservation was established as a new treatment modality for young patients with early cervical cancer, less than 2 cm, using different surgical approaches and techniques such as vaginal or abdominal simple or radical trachelectomy, total laparoscopic or robotic trachelectomy even large conisation with laparoscopic lympadenectomy. A further goal for gynecological oncology is to consider a fertility-preserving approach in patients with bulky cervical cancer, without compromising the oncology outcome.

Methodology: The thirty years old nulliparous with uterine sqamous cervix cancer FIGO stage IB2, 3 cm, underwent neoadjuvant chemotherapy, three cycles of Cisplatin and Ifosfamide dose dense regime according to Prague protocol. MRI was performed pre and post NACT treatment
Results: Due to complete pathologic response after three cycles of neoadjuvant chemotherapy conservative surgery was performed. The patient underwent laparoscopic pelvic lymphadenectomy, uterine cervix conisation and the cervical canal curettage.
The histopathology of specimen confirmed the complete response, with H SIL only. There was no need for adjuvant treatment. At the first follow up the length of uterine cervix was sufficient and PAP smear was normal. One year after surgery, patient is without disease. At the last control she was in sixteen weeks of gestation.
Conclusion: Until now, small case control studies concluded that neoadjuvant chemotherapy followed by conservative fertility spare surgery in young patients with bulky cervical cancer who respond well to chemotherapy, is an acceptable approach. Still, more evaluation and data is needed for this group of patients before widespread use.
Disclosure: Nothing to disclose

EP354

Are the cervical cytologic abnormalities, colposcopic and histologic findings reliable in pregnant women?

F. Martinez Hernandez, R. Bermejo Martinez, M. Hernandez Ontoria, A. Villalba Gutierrez, E. Lorenzo Hernando, L. Muñoz Hernando, C. Almansa, V. Bravo Violeta
Gynecology and Obstetrics, Hospital 12 Octubre, Madrid, Spain

Introduction / Background: To determine the correlation between cytologic abnormalities, colposcopic and histologic findings in pregnant women.
Methodology: Retrospective unicentric study of 117 patients with cytologic abnormalities detected during pregnancy at Hospital 12 de Octubre (Madrid, Spain) between 2009-2019.
Results: The mean age at diagnosis was 32,5 years (±5,8), 4,3% were immunocompromised and 19,8 % were smokers. 2,6% of the patients had delivered three times or more. Only 3,6% of the patients had been vaccinated against HPV before diagnosis. Pap test was performed during first trimester in 57.3% of the patients. The most common cervical cytologic abnormality LSIL in 34,2 % of the patients. 85.5% of the study population underwent a colposcopy. Among the patients with lesser cytological abnormalities (ASC-US/LSIL),77,2% of them presented none or minor colposcopic findings. In the group of those with major cytological abnormalities (HSIL/ASC-H), 35,1% of them presented major colposcopic findings. Degree of agreement (Kappa = 0.129).
In 41,9% cases of major colposcopic findings: HSIL in 76,9% and LSIL in 23,1% was found. In 18,8% cases of minor colposcopic findings, a colposcopy-guided biopsy was considered, and the histopathological diagnosis showed 6,3% cases negative, 12,5% of LSIL, 25% others, and 56,3% of HSIL. Degree of agreement (Kappa = 0.198).
Conclusion: Considering minor cytological abnormalities, we found a better agreement between cytology and minor colposcopic findings, however, not so many grade II colposcopic findings were found when patients with major cytological abnormalities underwent colposcopy. Our data suggest a possible overestimate in pregnant women with major cytological abnormalities.
We found a low concordance between colposcopy and anatomopathological results regarding in cases with minor colposcopic changes; that up to 56,3% had a definitive HSIL result in the biopsy. So it is also important that we should take into account the biopsy as a helpful tool to detect malignancy in this challenging population and thus avoiding underdiagnosis.
Disclosure: Nothing to disclose

EP355

A feasibility study of robotic radical hysterectomy in patients with T1b1/2a1, N0 cervical cancer

H. Matsumiya, Y. Todo, R. Yamada, K. Minowa, C. Shimada, T. Tsuruta, S. Minobe
Division of Gynecologic Oncology, National Hospital Organization, Hokkaido Cancer Center, Sapporo, Japan

Introduction / Background: The aim of this study was to evaluate the feasibility of robotic radical hysterectomy (RRH) in patients with early stage cervical cancer.
Methodology: This retrospective study was carried out using data for 166 patients with T1b1/2a1, N0 cervical cancer who underwent radical hysterectomy at Hokkaido Cancer Center from January 2010 to April 2018. Study outcomes including operation time, estimated blood loss (EBL), number of lymph nodes harvested, hospital stay, surgical morbidity, recurrence, and survival were compared between open radical hysterectomy (O group, n=134) and RRH (R group, n=32).
Results: There was no difference in age, body mass index, stage, histology, lymph node metastasis, and tumor diameter between the two group. RRH was significantly associated with longer operation time (268 min vs. 415 min, $P < 00001$), less EBL (492 cc vs. 30cc, $P < 00001$), shorter hospital stay (24 days vs. 10 days, $P < 00001$), and fewer number of lymph nodes harvested (42 vs. 18, $P <$ 00001). RRH was marginally associated with fewer number of severe neurogenic bladder (49% vs, 31%, $P = 0078$). No severe neurogenic bladder was observed in the last eleven cases of RRH. At the time of this report, with a median follow-up of 22 months, only one patient recurred at her vaginal stump.
Conclusion: If RRH was strictly applied to cases of T1b1/2a1, N0 cervical cancer, it could be feasible in early stage cervical cancer. In addition, RRH might decrease occurrence of severe neurogenic bladder compared to open radical hysterectomy.
Disclosure: Nothing to disclose

EP356

Sentinel lymph node vs. lymphadenectomy in patients with early-stage cervical cancer: asingle-center experience

E. Mension, B. Gil-Ibañez, M. Del Pino, N. Agustí, N. Carreras, A. Glickman, F. Pere, B. Diaz-Feijoo, J. Pahisa, A. Torné
Unit of Gynecologic Oncology, Clinical Institute of Gynecology, Obstetrics and Neonatology (ICGON), Hospital Clinic Barcelona, Barcelona, Spain

Introduction / Background: In early FIGO stage patients (IA, IB1 and IIA1) nodal involvement is one of the most important prognostic factors. Pelvic lymphadenectomy (LDN) is performed in order to assess lymph node involvement. Sentinel lymph node (SLN) biopsy has been increasingly used with limited evidence of oncological safety.
Methodology: Patients with FIGO stage IA1-IB1/IIA1 cervical carcinoma treated with radical surgery after lymph node assessment in Hospital Clinic (Barcelona, Spain) from 2001 to 2018 were included. Between 02/2001 and 05/2011 patients underwent SLN biopsy followed by complete pelvic LDN and patients from 06/2011 and 08/2018 underwent SLN dissection alone. Patients in whom SLN were detected unilaterally or not detected underwent a complete LDN of the failed mapped side. Radical surgery after intraoperative negative nodes were laparoscopic assisted vaginal radical hysterectomy or vaginal radical trachelectomy if fertility preservation desire. SLN were postoperative evaluated by pathologic ultrastaging. Intraoperative and postoperative as well as follow up data were prospectively recorded. STATA 15.1 was used for statistical analysis.
Results: In 162 patients lymph node assessment was performed, 87 (53.7%) underwent SLN biopsy followed by pelvic bilateral LDN and 75(46.3%) SLNB only. No differences regarding age at diagnosis, size of the tumor (≤ 2cm vs > 2cm), FIGO stage or histology type were seen between both groups. 151 patients had negative nodes. From these patients, 7 patients (9.1%) recurred in the LDN group and 3 patients (4.3%) in the SLN group. Cox hazard ratios were not statistically significant for lymph

node assessment group.
Conclusion: Sentinel lymph node detection in patients with early cervical cancer does not entail an increased risk of recurrence with respect to the patients in whom a complete pelvic lymphadenectomy is performed.
Disclosure: The authors have no relationships/conditions/circumstances that present a potential conflict of interest

EP357

Transarterial embolisation for the treatment of acute gynecological cancer bleeding

P. Meyer-Wilmes[1], M. Powerski[2], F. Fischbach[2], R. Damm[2], J. Omari[2], M. Pech[2]
[1]Gynecology and Obstetrics, University Hospital of Münster, Münster, [2]Radiology and Nuclear Medicine, University of Magdeburg, Magdeburg, Germany

Introduction / Background: Acute bleeding in patients with gynecological cancer may result in serious life-threatening complications. In these situations, immediate diagnostic and appropriate treatment is a challenge for attending physicians. Accordingly, transarterial embolisation (TAE) has been described as an increasing and effective treatment alternative to surgery and radiotherapy.
Methodology: In the present retrospective study 25 patients were included who underwent a TAE in the period from January 2006 to June 2013 due to acute gynecological cancer bleeding. The objective of this study was to assess the efficiency and outcome of TAE.
Results: Cervical cancer was the most common cause of gynecological bleeding (19/25, 76.0 %). The primary technical success rate was 92.0 % (n = 23). 21 patients were analysed in the 30-day follow-up. The clinical success rate was achieved in 90.5 % (n = 19). A clinical failure due to rebleeding was observed in 9.5 % (n = 2). No associations were revealed between rebleeding and technical/clinical factors. Within 30 days after the last TAE the complication and mortality rates were 0.0 % and 5.0 % (n = 1), respectively.
Conclusion: TAE was found as an effective and safe treatment in the clinical setting of acute gynecological cancer bleeding. An important component of efficient management is an early and interdisciplinary care. Optimal treatment can only be achieved by close cooperation between gynecologists, radiotherapists and interventional radiologists. Further randomised trials are needed to obtain more evidence, especially in predictive factors.
Disclosure: Nothing to disclose

EP358

Rare case of an uterine cervix metastasis from a sigmoid adenocarcinoma

P. Meyer-Wilmes, R. Witteler, L. Kiesel
Department of Gynecology and Obstetrics, University Hospital of Muenster, Muenster, Germany

Introduction / Background: Primary adenocarcinoma of the cervix uteri accounts for 5% to 15% of all malignancies of the cervix uteri. Uterine cervix metastases from carcinomas over the hematogenous or lymphatic pathway are rare. The most frequent non-genital primary sites of adenocarcinomasare stomach and colon.
Methodology: Case report.
Results: A 49-year-old female patient presented with pelvic pain. The gynecological examination showed a well-defined, non-deforming, but ulcerating tumor mass (2.5 cm) on the cervix uteri. The border between squamous and columnar epithelium was not visible on colposcopy, according to T3. Obviously, the lesion had no reference to the transformation zone. By biopsy, an adenocarcinoma was diagnosed. Subsequent immunohistochemical examination revealed a strong nuclear staining of

CDX2 and CK20. This indicated a primary tumor site in the gastrointestinal tract. Colonoscopy detected a stenosing tumor of the sigmoid colon. Due to increased abdominal symptoms, urgent laparotomy was performed. Intraoperative, the primary tumor site in the sigmoid colon was confirmed. Because of multiple metastases (liver, adnexa, lung, omentum), palliative chemotherapy was indicated. Evidence of microsatellite instability could not be demonstrated. At follow-up, 1.5 years later, the patient showed progression of liver and lung lesions. Further therapy included selective internal radiotherapy and oral therapy with Trifluridine/Tipiracil.
Conclusion: The case shows a rare uterine cervix metastasis from a sigmoid adenocarcinoma. Typical character is the recess of the transformation zone. Therefore, the colposcopic image in this case is untypical for primary cervical carcinoma. Management should be adjusted according to the tumor origin.
Disclosure: An image quote about this case was published in the journal "FRAUENARZT" in March 2018.

EP359
Prognostic and therapeutic value of pelvic lymphadenectomy in locally advanced cervical cancer
G. Mezquita, J.C. Muruzabal, S. Aguirre
Complejo Hospitalario de Navarra, Pamplona, Spain

Introduction / Background: The purpose of this study is to assess the safety and feasibility of transperitoneal laparoscopic pelvic lymphadenectomy in patients with locally advanced cervical cancer (LACC), who are going to undergo staging para-aortic lymphadenectomy and to analyze the prognostic value and the impact on survival of this procedure.
Methodology: Data from 67 patients with LACC who underwent transperitonel laparoscopic para-aortic plus pelvic lymphadenectomy and received chemoradiation therapy were analyzed.
Results: Metastatic lymph nodes (LN) were identified in 32 patients (47.7 %), 20 (29.8%) had metastatic LN in the pelvic area and 12 (17,9%) had metastatic LN in the pelvic and para-aortic area. None of them had metastatic LN localized only in the para-aortic area.
After a median follow-up of 54.6 months (5-122.2 months), the 5-year overall survival (OS) for the whole group was 67.3%. Patients with para-aortic nodal metastasis had increased risk of mortality than those with negative nodes (p=0.005), but there were no significant differences in OS between patients with negative nodes and those with only pelvic nodes affected (p=0.380).
The 5-year local control rate for the whole group was 91.1%. The 5-year disease-free survival (DFS) for the whole group was 60.5%. For patients with no metastatic LN the DFS was 86.4%, while for patients with pelvic metastatic LN was 34.2% and 24.2% for patients with pelvic and para-aortic metastatic LN (p< 0.001).
This program allowed a modification of the initial therapeutical plan in the para-aortic area in 13.4% of patients and in the pelvic area in 28.3% of patients. Overall intraoperative morbidity rate was 5.9%.
Conclusion: Transperitoneal pelvic and para-aortic lymphadenectomy provides precise information about nodal state and allows personalized treatment planning in patients with LACC. No differences in OS were observed between patients with negative LN and patients with only positive pelvic LN
Disclosure: Nothing to disclose

EP360
Single-agent amrubicin therapy for recurrent small cell neuroendocrine cervical carcinoma
S. Minobe
Gynecol, Hokkaidou Cancer Center, Sapporo, Japan

Introduction / Background: Small cell neuroendocrine cervical cancer (SCNCC) is a highly aggressive tumor, and there is currently no standard treatment for recurrent SCNCC.
Methodology: We treated three patients with recurrent SCNCC who had received prior etoposide/cisplatin chemotherapy with single-agent amrubicin (35 mg/m^2, days 1-3).
Results: Partial response was achieved in one patient who had no signs of disease progression 14 months after commencing amrubicin. This patient received a total of 10 cycles of amrubicin every 3 weeks, with acceptable adverse effects. Amrubicin treatment was unsuccessful and was discontinued in the other two patients; one received a total of five cycles of amrubicin with acceptable adverse effect, but amrubicin was discontinued because of disease progression, and the other discontinued amrubicin after only two cycles because of grade 4 neutropenia/thrombocytopenia.
Conclusion: This report introduces the possibility that patient with recurrent SCNCC may benefit from the administration of low-dose AMR(35 mg/m^2).
Disclosure: Nothing to disclose

EP361

Evolving and validating tool for capacity building of the existing health services staff for VIA and VILI based cervical cancer screening

G.A. Mishra[1], T. Shylasree[1], S. Noronha[2], C. Pramesh[1], S. Pimple[1], V. Kulkarni[1], H. Shaikh[1]
[1]Tata Memorial Hospital | Preventive Oncology, Tata Memorial Centre, [2]Chemical Engineering, Indian Institute of Technology, Mumbai, India

Introduction / Background: Cervical cancer is the second most common cancer among Indian women. The government is now committed to control common cancers. The objectives are to develop and validate video based training tool for capacity building of existing health services staff in cancer awareness and screening activities, to reduce the expert time involved in the training and to reduce the duration of training.
Methodology: Video based tutorials were prepared in ten modules and three languages by TMH in co-ordination with IIT, Mumbai. The health services staff were invited for the training. The training which was for 12 weeks using standard methods was now planned for two weeks using the new technology. Practical demonstration and micro-teaching of health awareness activities, preparation of dilute acetic acid, lugol's iodine, inserting speculums, performing VIA and VILI, identifying lesions, disinfection and sterilization procedures were combined with video based theory and demonstrations. The tablets pre-loaded with the video based tutorials were retained with the trainees during the training period.
Results: The fifty trainees were evaluated with theory and practical evaluation. Majority scored well in the theory exams. Out of twenty marks allocated for practical evaluation 49 (98%) scored above 10 marks. All trainees found the training to be very informative, easy to understand and felt confident to deliver cancer awareness talk and to perform VIA and VILI based screening.
Conclusion: The outcome of roll out of the cancer control programme will depend on the quality of training imparted to the health services staff. A real-world issue is relieving the health services staff for longer duration for training and the availability of expert time for training. The preparation and validation of these indigenously prepared video based tutorials has opened new avenue by which the vast majority of the health services staff can be trained in relatively shorter duration.
Disclosure: Funding Support for preparation of videos was through TMH-IIT Collaborative projects through Tata Trusts and for testing the videos through TMH Intramural funds.

EP362

Survival rate comparisons in open versus laparoscopic approach in early stage cervical cancer patients treated in Kielce cancer center: a single institution experience

M. Misiek, K. Zalewski, P. Fras, A. Wrona-Cyranowska, B. Gieroń, M. Jedliński, J. Kopczyński, M. Koźmińska, S. Góźdź
Hollycross Cancer Center, Kielce, Poland

Introduction / Background: The purpose of this retrospective study was to assess the 5-year survival outcomes of cervical cancer patients who underwent an open radical hysterectomy (ORH) or laparoscopic radical hysterectomy (LRH) after its introduction in our center for the treatment of this disease.
Methodology: We conducted a retrospective review of cervical cancer patients managed in Kielce Oncology Center, Poland with an ORH or LRH between 2011 and 2016.
Results: A total of 115 women with cervical carcinoma stage IB1 or IIA1 were included in the study. Eighty-eight patients were treated with ORH, 27 were managed via LRH based on surgeon preference. Clinical characteristics did not differ between groups. Duration of total surgery time was longer in patients with ORH compared with that in the LRH group (Pp< 0.001) contrary to blood loss that was higher in the ORH group than in LRH (p < 0.001). Kaplan-Meier survival analysis revealed a progression-free survival at 3 and 5 years (PFS) rate of 95.68% and 90.91% for the LRH group and 95.68% and 85.52% for the ORH group, respectively (p=0,95). Overall survival at 5 years was 89.00% for the ORH group and 100% for the LRH group and 96.6% for the RRH patients (p=0.389).
Conclusion: Results from this retrospective study suggest that introduction of a laparoscopic procedures in the surgical staging and treatment of cervical cancer patients did not have a detrimental effect on surgical or disease outcome, irrespective of operative approach.
Disclosure: Nothing to disclose

EP363

The role of PET-CT in the new FIGO 2018 classification for cervical carcinoma

C. Mom[1], N. van Trommel[2], P. Voormolen[2], E. Vegt[2], J. Adam[3], J. van der Velden[4]
[1]Amsterdam UMC, Location AMC, [2]Antoni van Leeuwenhoek Hospital - The Netherlands Cancer Institute, [3]Radiology, [4]Gynecologic Oncology, Amsterdam UMC, Location AMC, Amsterdam, The Netherlands

Introduction / Background: In the 2018 FIGO classification of carcinoma of the cervix, assessment of lymph nodes by imaging and/or pathological examination is now integrated. Patients with suspicious lymph nodes on imaging are designated as stage IIIC with the annotation 'r'. Compared to the 2009 (clinically based) FIGO classification, we expect that more patients will be assigned a higher stage prior to treatment. Because of the increasingly important role of imaging modalities such as MRI and PET-CT in the staging and treatment of cervical cancer, we analyzed the accuracy of PET-CT in patients with early stage cervical carcinoma, with special emphasis on the positive predictive value (PPV).
Methodology: Patients with early cervical cancer (FIGO 2009 stage IB1/IB2/IIA) that underwent a preoperative PET-CT for standard clinical care between 2010 and 2018 and subsequently a lymph node assessment (histology) were included in this retrospective study. Sensitivity (sens), specificity (spec), negative predictive value (NPV) and PPV of the PET CT scan for the prediction of pelvic lymph node metastases was calculated.
Results: A total of 102 patients was included. Fifty eight percent of this group had positive nodes. Table 1 shows the sens, spec, NPV and PPV. When patients who had a nodal debulking because of bulky suspicious nodes on MRI were excluded (n=37), the remaining 65 patients (with a prevalence of 41% of positive nodes), showed a PPV of 68% (Table 2). This would result in erroneously upstaging to stage IIIC in 32% of the patients with a PET-CT scan and suspicious nodes.
Conclusion: We argue that, depending on the prevalence of positive nodes, PET-CT has a large likelihood to overestimate the incidence of lymph node metastases, theoretically resulting in a non-appropriate treatment. Pathological confirmation of lymph nodes suspicious for metastases on PET-CT scan is essential to guide therapy decisions.

	pelvic nodes pos	pelvic nodes neg	
PET suspicious	49	11	PPV: 60/49=82%
PET non suspicious	10	32	NPV:42/32=76%
	sens:59/49=83%	spec:43/32=74%	

[Table 1. Predictive values of PET CT scan for positive pelvic lymph nodes (prevalence 58%), including bulky nodes]

	pelvic nodes pos	pelvic nodes neg	
PET suspicious	17	8	PPV:25/17=68%
PET non suspicious	10	30	NPV:40/30=75%
	sens:27/17=63%	spec:38/30=79%	

[Table 2. Predictive values of PET CT scan for positive pelvic lymph nodes (prevalence 41%) in 65 patients with pelvic node dissection.]

Disclosure: Nothing to disclose

EP364

Prognostic value of ddPCR HPV in lymph nodes of early cervical cancer

R. Montero Macías[1], D. Veyer[2], M. Mandavit[3], M. Wack[4], B. Rance[4], H. Bonsang-Kitzis[1,5], M. Deloménie[1,5], A.-S. Bats[1,5], C. Ngo[1,5], V. Balaya[1,5], M. Koual[1,5], H.-T. Nguyen-Xuan[1,5], M.-A. Lefrère-Belda[6], V. Taly[7,8], F. Lecuru[1,5], P. Mathevet[9], H. Péré[2,3]
[1]Gynecologic and Breast Oncologic Surgery, [2]Virology Laboratory, [3]INSERM U970, PARCC, [4]Department of Medical Informatics, Biostatistics and Public Health, European Georges Pompidou Hospital, [5]Paris Descartes University, Sorbonne Paris Cité, [6]Anatomo-Pathology Department, European Georges Pompidou Hospital, [7]INSERM UMR-S1147, eDIAG platform, CNRS SNC5014, Paris Descartes University, Sorbonne Paris Cité, [8]National League Against Cancer, Paris, France, [9]Department of Gynaecology, Centre Hospitalier Universitaire Vaudois, Lausanne, Switzerland

Introduction / Background: In early stages (Ia to IIa1) of cervical cancer (CC), nodal status is the cornerstone of treatment algorithm and patients with nodal metastasis (N+) present worse survival. However, 10-15% of patients without nodal metastasis (N0) present the same survival to patients N+. No clinical, imaging or pathological risk-factor is today effective to select these patients for different management.
In HPV-related cancers, like CC, HPV DNA could be assimilated to tumoral DNA and represent an interesting marker of node metastasis.
In this study, we evaluate the presence of tumoral HPV DNA in pelvic lymph nodes (PLN) by new ultrasensitive droplet-based digital polymerase chain reaction (ddPCR) to explore it as a new additional biomarker to classify early stages of CC.
Methodology: This study was conducted on 30 patients from Senticol prospective trials. Formalin-fixed paraffin (FFPE) embedded tumours and associated pelvic lymph nodes samples were available for only 25 patients in our institution.
In HPV16-positive tumour patients (n=18; 3 patients were N+ and 15 N0), we performed the specific HPV16 ddPCR in associated pelvic lymph node samples after a manual DNA extraction. Simultaneously, albumin was detected as internal control.
Results: Unfortunately, 22.2% of PLN were not interpretable (NI) with a very low level of albumin suggesting that no sufficient amount of DNA was extracted for HPV16 tumoral DNA interpretation. It could be explained by DNA destruction over time considering that FFPE biopsies were collected from 2005 to 2011.

Insufficient follow-up data is < 6 months (2 patients).
Conclusion: As expected, HPV16 tumoral DNA was detected in N+ patient PLN.
In the N0 group, 40% (2/5) of patients with HPV16-positive PLN have relapsed regarding 12,5% (1/8) relapsing patients in the group of HPV16-negative PLN.
These interesting preliminary results invite to test this new molecular biomarker in largest cohorts to evaluate its interest in early CC management.

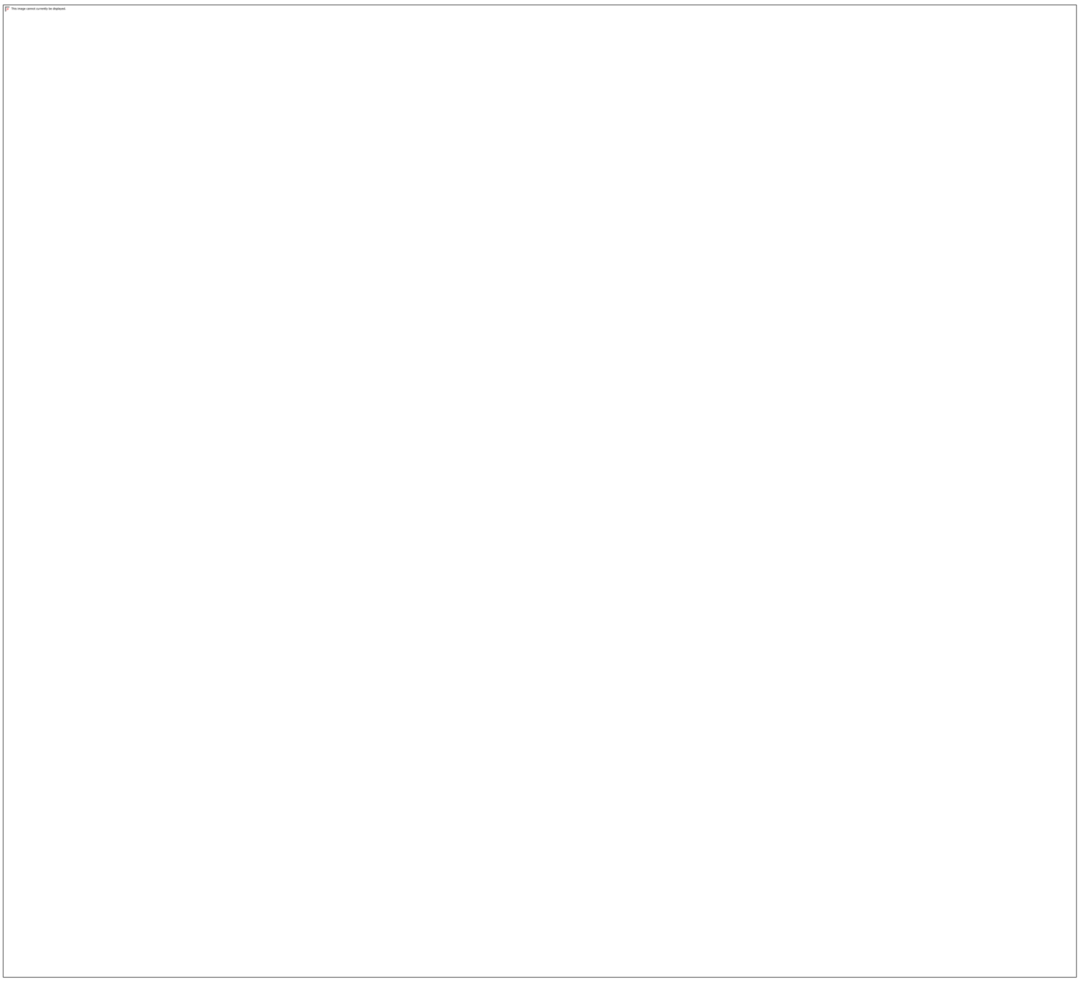

[Figure-1. Association between HPV 16 in PLN, nodes status and recurrences]

Disclosure: Nothing to disclose

EP365

Prognosis factors in patients with early cervical cancer

R. Montero Macías[1,2], H. Bonsang-Kitzis[1,2], V. Balaya[1,2], C. Ngo[1,2], M. Deloménie[1,2], M. Koual[1,2], H.-T. Nguyen-Xuan[1,2], A.-S. Bats[1,2], F. Lecuru[1,2]
[1]Gynecologic and Breast Oncologic Surgery, European Georges Pompidou Hospital, [2]Paris Descartes University, Sorbonne Paris Cité, Paris, France

Introduction / Background: Many factors for recurrence have been described in patients with early

BMJ Publishing Group Limited (BMJ) disclaims all liability and responsibility arising from any reliance placed on this supplemental material which has been supplied by the author(s)

cervical cancer (ECC). Lymph node involvement is considered the main prognostic factor.
Different factors had been associated for risk to involvement lymph pelvic nodes and also with recurrences: FIGO stage, lymphovascular space involvement (LVSI), tumor grade and size of primary tumor.
We studied different risk factors to involvement lymph pelvic node and to recurrence in our population.
Methodology: We retrospectively analysed 85 patients with ECC treated between 2004-2018.
Inclusion criteria: patients with ECC (stage IA1- IIA).
Exclusion criteria: insufficient follow-up (< 6 months), not node stage evaluation and neoadjuvant radiochemotherapy.
No patients with stage FIGO IA had recurrences, so no included in analyse.
Simple logistic regression analyses were performed, expressed as odds ratio (OR) and 95% confidence interval (CI). P values were significance < 0.05. Survival data were analysed using Kaplan-Meier-curves and log-rank-test.
Results: The univariate analysis revealed a relationship between tumor size and risk of recurrences (OR 8,37 ; 95% CI 2,24 - 40,8; p 0,003). No relationship was revealed with the others variables with recurrences and any variables had relationship with the risk of pelvis node metastasis.
There was no statistically significant difference in disease-free survival relative to analyzed prognostic factors in ECC stages, except for tumor size (p< 0,001 Log- Rank).
Conclusion: We don´t found risk factors for pelvic lymph node metastasis.
Only tumor size is a prognosis factor and have impact in progression-free survival. We don´t found relation between other factors described previously like stage, node metastasis, LVSI and recurrences.
Size is a significant and major prognostic factor in cervical cancer, regardless of the type of treatment utilized. The new FIGO staging 2018 system for cervical cancer with the inclusion of size ^{3} 20 mm for Stage IB2 cancers better reflects survival and overall prognosis.

Variable	ORa	IC 95%	p
Age			
≤45 / >45	1 / 1,32	0,41 - 4,66	0,65
B.M.I			
≤25 / >25	1 / 1,01	0,96 - 1,06	0,66
Tumor size			
< 20 mm / ≥ 20 mm	1 / 1,3	0,36 - 4,36	0,67
Lymphovascular space involvement			
Yes / Non	1 / 3,01	0,83 - 12,46	0,103

[Univariate analysis of predictive factors of pelvic nodes mestastasis]

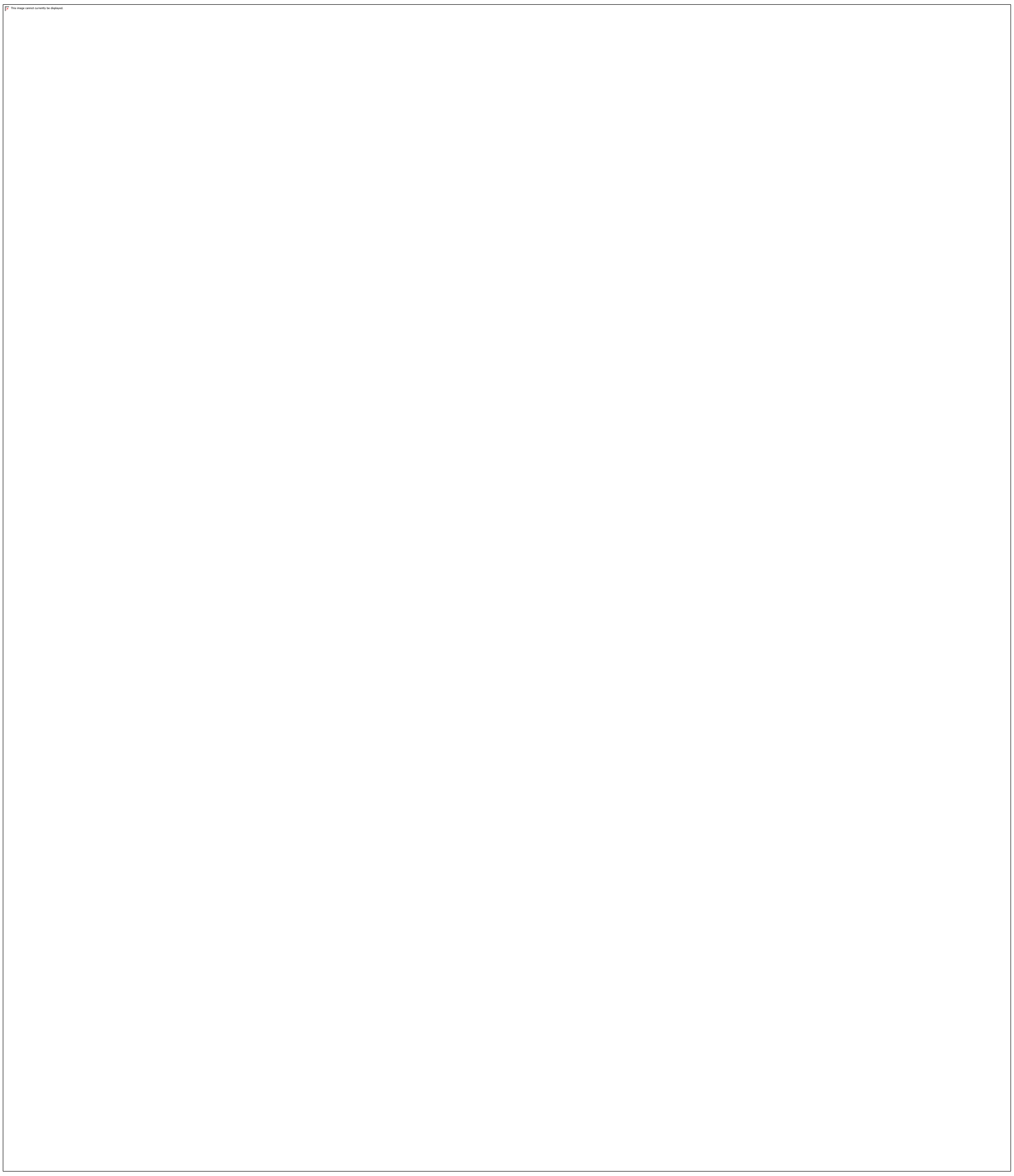

[Univariate analysis of predictive factors of recurrence]

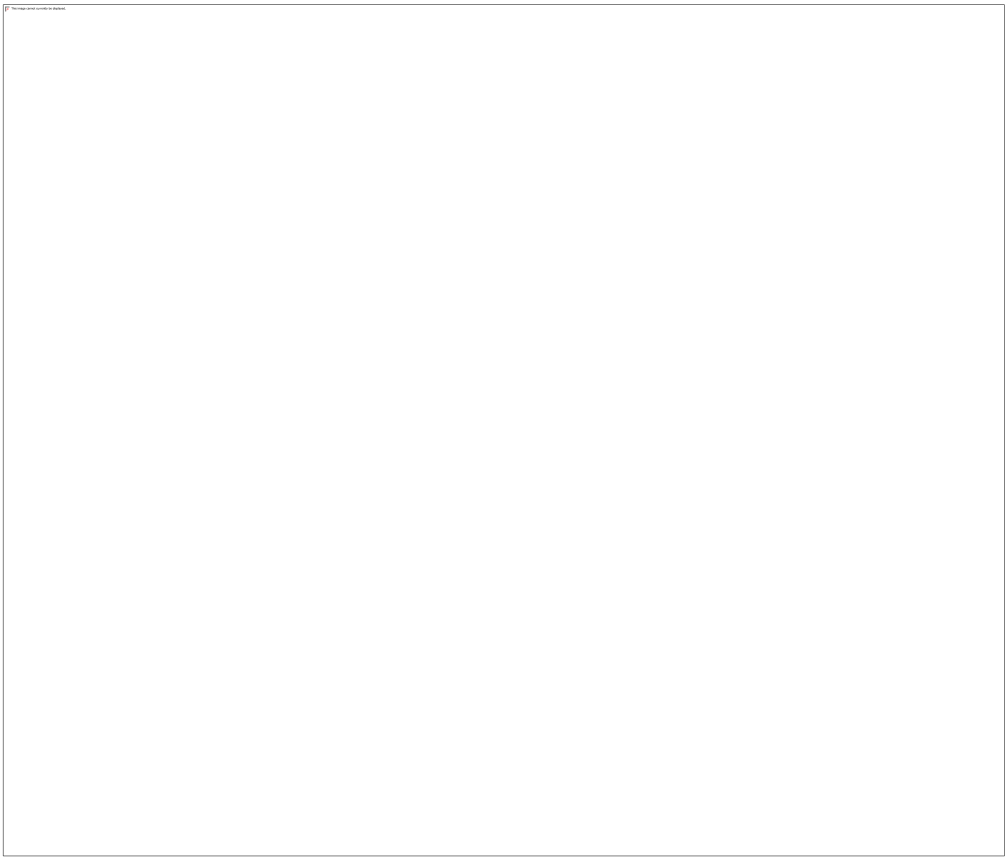

[Survival curves for recurrences, in patients with tumor size < 20 mm and ≥ 20 mm]

Disclosure: Nothing to disclose

EP366

Cervical cancer associated with genital prolapse: a case report

L. Mosca[1,2], A. Cafiero[2], G. Balbi[1,2], L. Cobellis[1,2]
[1]Università degli studi della Campania Luigi Vanvitelli, Napoli, [2]AORN Sant'Anna e San Sebastiano, Caserta, Italy

Introduction / Background: Despite uterine prolapse being a common condition in women, the coexistence of cervical cancer and uterine prolapse is extremely rare. There is no reported incidence, but the available information suggests that the incidence of cervical cancer among those with procidentia is between 0.14 % and 1 %.
Methodology: 86-year-old woman admitted to our Hospital in a condition of a huge, irreducible uterovaginal procidentia with a vegetative and ulcerated lesion on which biopsy is performed; histological examination: squamous carcinoma. Total vaginal hysterectomy with bilateral bilateral salpingo-oophorectomy and total colpectomy were performed.
Results: The histopathology revealed a squamous cell carcinoma of the uterine cervix 11.5 cm in size, stage IIA2 G3.
Conclusion: The coexistence of cervical cancer and pelvic organ prolapse is extremely rare and the best treatment is unclear. A systematic review of the literature suggest that the prognosis following surgery-based treatment of cervical cancer patients with complete uterine prolapse may be favorable,

and thus the recommended treatment option should be based on the surgeon's preference, patient and tumor factors, and consideration of medical comorbidities.
Conclusion: surgical approach needs to be considered in women with cervical cancer complicated by complete uterine prolapse if the patient's condition and medical comorbidity are not limitations to surgery.
Disclosure: Nothing to disclose

EP367

Prevalence of high-risk human papillomavirus by cobas 4800 HPV test and the pathologic findings in women with high risk HPV positive in Mashhad (North-East of Iran)

M. Motaghi
Department of Obstetrics and Gynecology Islamic Azad University of Medical Sciences, Mashhad, Islamic Republic of Iran

Introduction / Background: The prevalence of infection with Human papilloma virus types 16and 18 in south west of Asia was reported by WHO as 4.4 % ,25.1%,46.7% in the normal cytology ,LSIL , and HSIL respectively(1) .In Iran we have 31,715,925 women in the age range of 15-80 years(2) .we have few data about the prevalence of HR HPV in this large papulation . This study was performed to evaluate the prevalence and type specific of high risk HPV in general population and in LSIL and HSIL lesion.
Methodology: A total of 325 women from2015 to 2016 in Arya Hospital(Azad University) that were screened for cervical cancer underwent thin prep and HPV detection by Cobas 4800 in cervical secretions. We determined the prevalence of HR HPV in 24% of 325 and others HPV types in 18.5% ,HPV16 in 9% and HPV 18 in 4% of this group of women .
Results: Also we found HR HPV in 20% of women with normal pap smears. In women with HR HPV positive, colposcopy and biopsy detected normal pathology in 7%, LSIL in 82%, and HSIL in 11%. In LSIL and HSIL group the most HRHPV was others (72%) and 16(50%) respectively.
Conclusion: The present research showed, the prevalence of infection with high risk HPV is as high as other developing regions.While the most common HPV in this region of Iran is Others HPV types but the most sever cellular dysplasia is seen in patients with HPV 16 and 18 positive and Vaccination with bivalent or quadrivalentis effective in prevention of cervical cancer.
Disclosure: Nothing to disclose

EP368

Prognostic value of KI6 biomarker in predict short time prognosis of low grade cervical intraepithelial neoplasia in HPV negative and positive patient

L. Mousavi Seresht
Isfahan University of Medical Science, Isfahan, Islamic Republic of Iran

Introduction / Background: Screening of cervical cancer is the most common gynecologic cancer in developing countries. Despite being preventable, but we have still some problems in the screening of this cancer. Recently, many studies have been done on immunohistochemistry to improve screening of cervical intraepithelial neoplasia (CIN) as precancerous lesion. But, majority of the studies are based on cytological samples.
The objective of this study was to analyze the correlation KI-67 biomarker and HPV infection in predict short time prognosis in CIN as an alternative or auxiliary method to current screening method in a different geographic population.
Methodology: This descriptive cohort prospective study included 40 patients with diagnosis of CIN

based on cervical punch biopsy samples after colposcopy examination. They were referred to the department of gynecology and oncology of an academic hospital, Mashhad University of 2016 to 2017. All samples were investigated for HR- HPV DNA with Cobas test and immunostaining for KI-67 biomarker. Finally, after one year follow up, prognosis for all patients was evaluated. Data were analyzed by SPSS software program version 23.0 and Mann-Whitney U test and Fisher´s exact test. P-value< 0.05 was considered significant.
Results: significant difference was found between HR-HPV positive and negative tests in KI-67 expression (P< 0.001), but no significant difference was observed in reactivity level (P=0.5), also no significant difference was found in KI-67 expression in metaplastic and non-metaplastic epithelium (P=0.88),.
Conclusion: KI-67 biomarker is recommended as complementary screening tests not alternative for differentiating in high risks patients with CIN1. The patients with low KI-67 / HR-HPV positive could be offer for a less aggressive follow-up protocol.
Disclosure: Nothing to disclose

EP369

An evaluation of SMI and SWE utility in the identification of the uterine cervical lesion

T. Nakamura, N. Yoshioka, H. Endo, H. Yamanaka, S. Kuji, I. Deura, T. Ohara, J. Hasegawa, N. Suzuki, Department of Obstetrics and Gynecology
Obstetrics and Gynecology, St. Marianna University School of Medicine, Kawasaki, Japan

Introduction / Background: The purpose of the study is to determine if superb microvascular imaging (SMI) and shear wave elastography (SWE) can be used for the early diagnosis of uterine cervical intraepithelial neoplasia and cancer.
Methodology: A case-control study was carried out in St. Marianna University School of Medicine Hospital between April 2017 and January 2018 on patients with uterine cervical dysplasia and uterine cervical cancer. Seventeen controls with eight subjects were investigated. An evaluation using SMI and SWE was conducted in addition to the conventional ultrasonic tomography and color doppler tests before or after the treatment. The colored races plastic intensity in the SMI was evaluated for three phases, strong, mediate, weak, and the blood flow rate was measured. The SWE was measured at several locations and the mean value was used as signal intensity.
Results: The target breakdown was one patient with uterine cervical cancer and seven patients with high-grade squamous intraepithelial lesions. For the subject and control cases, the intensity of the conventional colored races plastic was 6% vs. 0% (n.s.), and 94% vs. 100% for strong and weak phase, respectively. While for the control the measure, it was 39% vs. 0% and 61% vs. 100% for strong and weak phase, respectively. In addition, one patient with uterine cervical cancer exhibited a strong phase. The SWE evaluation of the uterine cervical hardness resulted in SWE level of 4.4±1.2, 2.2±0.3 for case and control, respectively (p< 0.01).
Conclusion: An evaluation using SMI and SWE utility suggested the identification of histologic changes such as minute vascular hyperplasia or malignant transformation of the uterine cervical lesion.
Disclosure: Nothing to disclose

EP370

The efficacy of hysterectomy for local remnant or recurrent cervical cancer after radical radiotherapy

T. Nakasone, Y. Shimoji, Y. Arakaki, Y. Taira, T. Nakamoto, T. Oyama, W. Kudaka, Y. Aoki
University of Ryukyu, Nishihara, Japan

Introduction / Background: Radiation therapy is an effective treatment for cervical cancer (CC), but remains at a constant rate, and those cases have a poor prognosis. The purpose of this study was to examine the efficacy of adjuvant hysterectomy in local remnant or recurrent (LR) cases of CC after radical radiotherapy (RT).
Methodology: Between May 2007 and September 2018, 21 patients who underwent hysterectomy for LR CC after RT in our department were included in this study. We retrospectively reviewed their medical records for patient background, clinicopathological factors, complications and prognosis. LR CC was determined based on histopathological and imaging examinations 3 months after the end of treatment. We classified surgical specimens according to the treatment effect (effects 0-3) using histological effect criteria. In addition, we used the Kaplan-Meier and log-rank test for statistical analyses.
Results: The median patient age was 55 years, and the FIGO stages were IB2, IIB and IIIB in 11, 8 and 2 cases, respectively. Based on the histological type, there were 14 cases of squamous cell carcinoma and 7 of adenocarcinoma. Tumour markers (TM) were re-elevated in five cases. Perioperative complications included intraoperative bladder injury and postoperative pelvic abscess in two cases. The median observation period was 39 months, and there were 10 recurrences. The median progression-free survival (PFS) was 54 months, and the 3-year overall survival (OS) rate was 61.1%. With regard to prognostic factors, the treatment effect (effect 1 vs. effect 2/3) was significantly associated with PFS ($p = 0.008$) and OS ($p = 0.023$) and the re-elevation of TM was significantly associated with PFS ($p = 0.017$).
Conclusion: Hysterectomy for LR CC after RT might be a tolerable treatment with a long-term prognosis. Histological treatment effect evaluation of surgical specimens might be useful in additional treatment assessment, and TM elevation might be useful in preoperative assessment.
Disclosure: Nothing to disclose

EP371

Non-brachytherapy approach to treatment of locally advanced cervical cancer

N. Salim[1,2], V. Nosov[3], D. Zvereva[1], P. Koposov[2], O. Novikova[3], A. Tedeeva[1], I. Trofimenko[4], E. Moskalets[4], N. Gromova[1]
[1]Radiation Oncology Center, [2]Medical Oncology Department, [3]Clinic of Gynecology and Gynecologic Oncology, [4]Nuclear Medicine and Diagnostic Imaging Department, European Medical Center, Moscow, Russian Federation

Introduction / Background: Concomitant chemoradiation therapy (CRT) that includes both external beam radiotherapy (EBRT) and brachytherapy (BT) is the current standard of care in treatment of locally advanced cervical cancer (LACC). Volumetric Modulated Arc technology provides potential benefits allowing for dose escalation and decreased toxicities. This non-BT approach offers improved accuracy and no geographical miss due to adaptive radiotherapy, but oncologic outcomes still need to be evaluated.
Methodology: Patients with LACC (stages 1B3-IVA) who underwent CRT using EBRT and simultaneous integrated boost at our institution were evaluated prospectively from May 2015 till April 2019. All were initially evaluated by a gynecologic oncologist then with MRI and 18FDG-PET/CT. Histology was confirmed by an expert pathologist. Interval CT were performed during treatment, pelvic exams with cytology every 3 months and PET/CT at 3 and 12 months after completion of treatment. Oncologic outcomes and toxicities were assessed
Results: 21 patients were analyzed: median age was 54 years (30-76), 19 patients had squamous cell histology, 2 had adenocarcinoma. Median follow-up was 26 months (3-44), average dose to the gross tumor volume was 90.2Gy (79.5 - 96.6), 79.8Gy to all PET/MRI positive nodes (63.0 - 89.7) and 56.3Gy (45-67.6) to all elective nodes. No patents received BT, all but three received chemotherapy. 3-year local control was 100%, PFS 90.4% and OS 100%. There were only two recurrences: a skull lesion 18 months following CRT in a patient with mesonephric adenocarcinoma and a transposed ovary metastases 15 months after CRT in the other. No grade III-IV toxicities seen, only one patient (4.7%) had late rectal grade II toxicity.
Conclusion: Non-brachytherapy CRT for LACC is feasible. It allows for a significant dose escalation

thus provides better local control and likely increases PFS and OS at no risk of serious toxicity. Randomised studies comparing this approach to the current standard of care are needed.
Disclosure: Nothing to disclose

EP372

DoVIA and TeleDoVIA as a documentation and consultation media in VIA methods of cervical cancer screening in Indonesia

L. Nuranna[1], G. Purwoto[2], A. Hadisty[2]
[1]University of Indonesia, [2]INASGO, Jakarta, Indonesia

Introduction / Background: In an effort to overcome cervical cancer, Visual Inspections with Acetic Acid (VIA) is very important as a screening methode but there is no an adequate documentation that can be used for consultation or discussion among medical practitioners. For this reason, our team jointly developed a method of documentation VIA (Documentation of Visual Inspection with Acetic Acid (DoVIA)) which uses cellphone's camera to take pictures of the cervix and Portal Telemedicine of DoVIA (TeleDoVIA), which is a model of photography-based consultation that utilizes telecommunication systems through the Whatsapp Messenger feature.
This study aims to assess the use, understanding and satisfaction of the TeleDoVIA Portal as a medium of communication, consultation, and discussion of DoVIA results and the distribution of user
Methodology: Through a descriptive design, datas are analyzed univariate. The research population is medical practitioners who utilize the TeleDoVIA Portal. In the study period, 82 medical practitioners with 177 DoVIA results were in the form of photographs.
Results: Medical practitioners who use the TeleDoVIA Portal are scattered throughout Indonesia starting from the western region (Aceh Province) to the eastern (West Papua Province). 72% work as midwives, and 92.75% work in Puskesmas. About 47.6% stated that they sent DoVIA because they wanted to confirm the diagnosis they had made. They claimed that 100% understand and 100% satisfied with the explanation given by the consultant. The sharpness of cervical photographic images is said to be sharp at 89.27%, only by 10.73% photos that are not sharp enough. A total of 56.5% of diagnoses established by consultants are negative VIA and the suitability diagnosis established by medical practitioners compared to diagnosis by consultants reaches 88.1%.
Conclusion: The TeleDoVIA portal is considered to be quite effective as a medium of communication, consultation, discussion and assistance to medical practitioners who have already attended VIA cervical cancer screening training.
Disclosure: Nothing to disclose

EP373

Humanistic burden associated with cervical cancer: an analysis of patient-reported outcomes in the European Union Five (France, Germany, Italy, Spain, and United Kingdom)

C. Nwankwo[1], N.A. Way[2], V.W. Li[3]
[1]Merck & Co., Inc. | Merck & Co., Inc., Merck & Co., Inc., Kenilworth, NJ, [2]Kantar Health, San Mateo, CA, [3]Kantar Health, New York, NY, United States

Introduction / Background: Although much is known about the prevention, screening, and treatment of cervical cancer (CC), less is known about its humanistic burden. This study compared patient-reported outcomes (PROs) between women diagnosed and treated for CC, women diagnosed and untreated for CC, and women without CC.
Methodology: Data were aggregated from the 2010, 2011, and 2013 EU5 National Health and Wellness Survey, a nationally-representative, self-administered, internet-based survey of adults

(N=177,317). Controlling for baseline patient demographics and characteristics, three matched cohorts were compared in multivariable analysis: treated CC, untreated CC, and no CC. Respondents with other forms of cancer were excluded from analyses. Outcomes included Mental Component Summary (MCS) score, Physical Component Summary (PCS) score, and SF-6D Utility score from the Medical Outcomes Study 12-Item Version 2 Short Form Survey Instrument (SF-12v2). Suicidal ideation and depression were assessed via the Patient-Health Questionnaire 9-Item (PHQ-9).
Results: Adjusted means are reported. Treated CC (N=113), untreated CC (N=646), and no CC (N=759) largely differed by PCS score (44.46 vs. 45.79 vs. 47.28, *p*'s< 0.01) and SF-6D Utility score (0.65 vs. 0.67 vs. 0.69, *p*'s< 0.01), though the difference between treated CC and untreated CC was not significant. MCS score did not differ by cohort (**Figure 1**). Untreated CC reported more depression than no CC, in terms of PHQ-9 score (6.99 vs. 5.82, *p*< 0.05). PHQ-9 score did not differ by any other cohorts (**Figure 2**). Treated CC was more likely to exhibit suicidal ideation than no CC (25.19% vs. 15.15%, *p*< 0.05). Suicidal ideation did not differ by any other cohorts.
Conclusion: Collectively, these results indicate that both treated CC and untreated CC patients experience a greater humanistic burden than women without CC. These differences highlight one aspect of the unmet needs of CC patients in the EU5, namely the humanistic burden they face.

[Figure 1]

[Figure 2]

Disclosure: This study was funded by Merck Sharp & Dohme Corp., a subsidiary of Merck & Co., Inc., Kenilworth, NJ, USA. Chizoba Nwankwo is an employee of Merck Sharp & Dohme Corp., a subsidiary of Merck & Co., Inc., Kenilworth, NJ, USA. Nate Way and Vicky Li are employees of Kantar Health, who received funding from Merck Sharp & Dohme Corp., a subsidiary of Merck & Co., Inc., Kenilworth,

NJ, USA to conduct this study.

EP374

Humanistic and economic burden associated with cervical cancer in Japan

N.A. Way[1], C. Nwankwo[2], V.W. Li[3]
[1]Kantar Health, San Mateo, CA, [2]Merck & Co., Inc., Merck Sharp & Dohme Corp., a Subsidiary of Merck & Co., Inc., Kenilworth, NJ, [3]Kantar Health, New York, NY, United States

Introduction / Background: Although much is known about the prevention, screening, and treatment of cervical cancer (CC), less is known about its humanistic and economic burden. This study compared patient-reported outcomes (PROs) between women diagnosed with CC and women without CC.
Methodology: Data were aggregated from the 2016 and 2017 Japan National Health and Wellness Survey, a nationally-representative, self-administered, internet-based survey of adults (N=69,001). Controlling for baseline demographics and characteristics, two matched cohorts were compared in bivariate analysis (i.e., t-tests): CC and no CC. Respondents with other forms of cancer were excluded from analyses. Humanistic burden was assessed via Mental Component Summary (MCS) score, Physical Component Summary (PCS) score, and SF-6D Utility score from the Medical Outcomes Study 12-Item or 36-item Version 2 Short Form Survey Instrument (SF-12v2 or SF-36v2). Economic burden was assessed via healthcare resource use (HRU) during the past six months: healthcare professional (HCP) visits, emergency room (ER) visits, and hospitalizations. Annualized direct costs were calculated using MEDIAS, Eurostat, and Ministry of Health, Labour, and Welfare data.
Results: Adjusted means are reported. CC (N=197) and no CC (N=197) differed by PCS score (51.70 vs. 53.01, p=0.048), but did not differ by MCS score (47.13 vs. 46.53, *ns*) nor SF-6D Utility score (0.74 vs. 0.75, *ns*) (**Figure 1**). CC and no CC differed by HCP visits (7.93 vs. 4.47, p=.001), but did not differ by ER visits (0.17 vs. 0.08, *ns*) nor hospitalizations (1.19 vs. 0.56, *ns*). CC and no CC differed by HCP direct costs (¥135656 vs. ¥76464, p=.001), but did not differ by ER direct costs (¥10795 vs. ¥5234, *ns*) nor hospitalization direct costs (¥2546173 vs. ¥1191826, *ns*) (**Figure 2**).
Conclusion: In this study, CC was associated with at least one aspect of humanistic burden (physical burden) and at least one aspect of economic burden (HCP visits and associated direct costs).

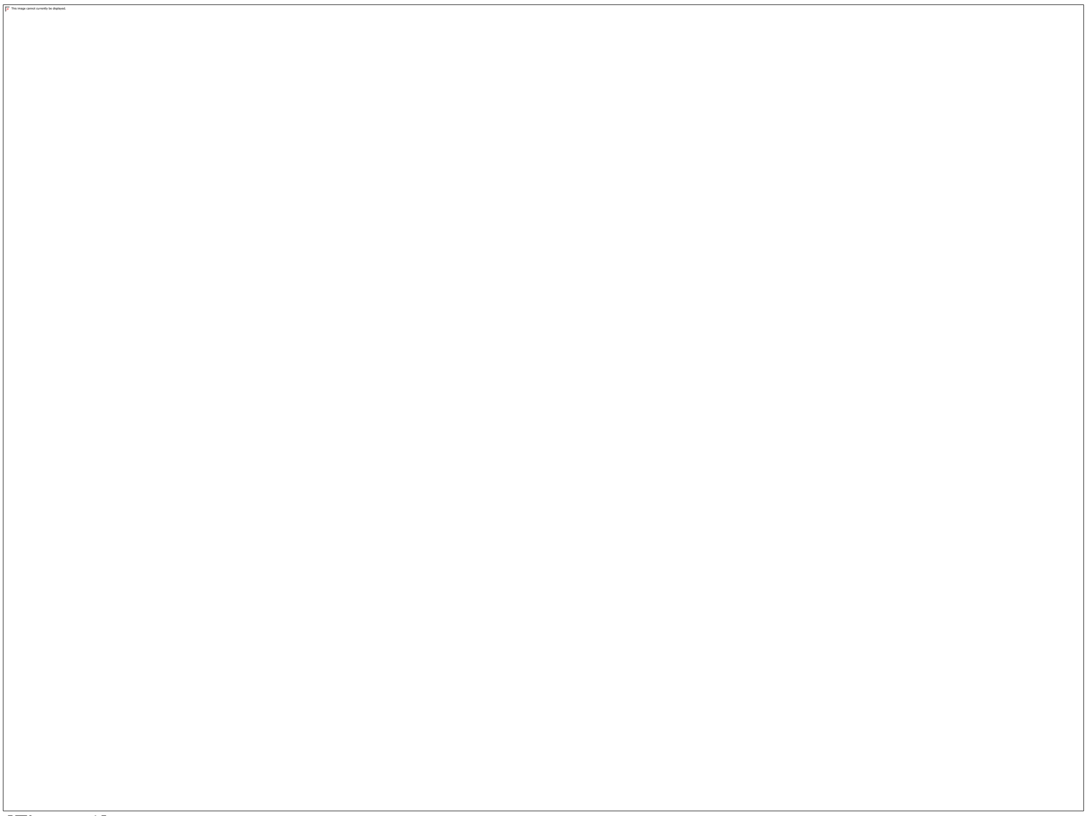
This image cannot currently be displayed.

[Figure 1]

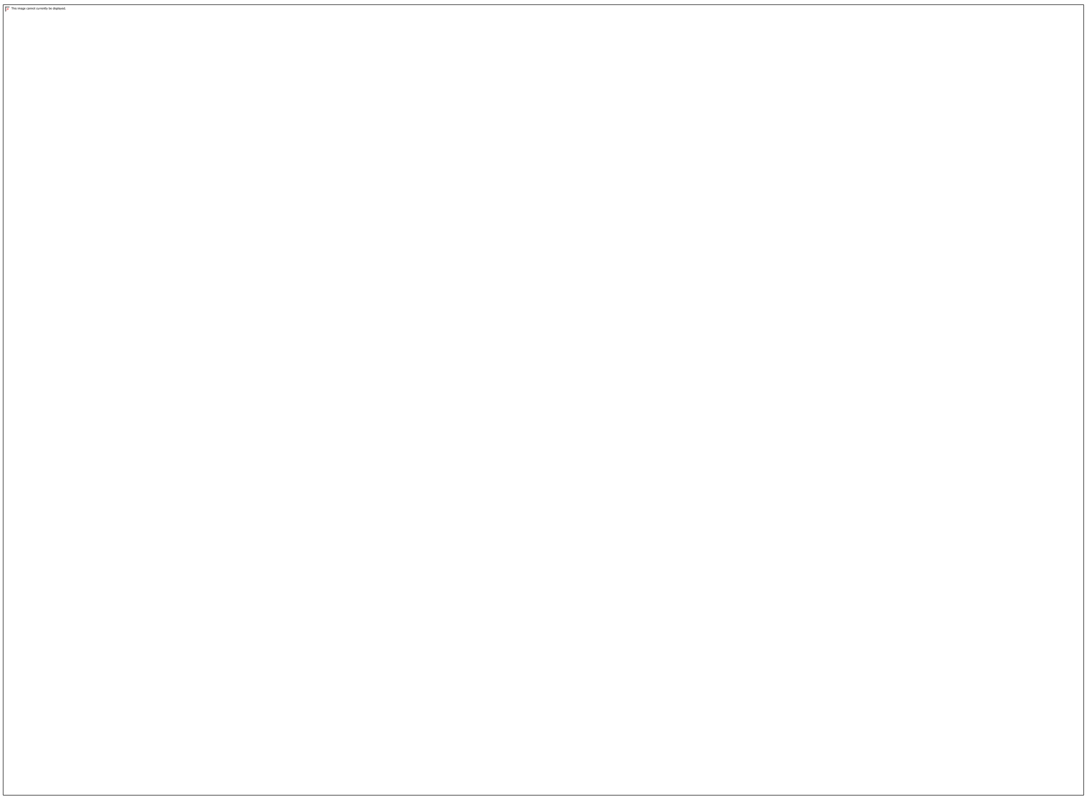
This image cannot currently be displayed.

[Figure 2]

Disclosure: This study was funded by Merck Sharp & Dohme Corp., a subsidiary of Merck & Co., Inc., Kenilworth, NJ, USA. Chizoba Nwankwo is an employee of Merck Sharp & Dohme Corp., a subsidiary of Merck & Co., Inc., Kenilworth, NJ, USA. Nate Way and Vicky Li are employees of Kantar Health, who received funding from Merck Sharp & Dohme Corp., a subsidiary of Merck & Co., Inc., Kenilworth, NJ, USA to conduct this study.

EP375

Abdominal radical histerectomy for cervical cancer: recurrence and disease-free survival in a portuguese oncologic center

C.A. Oliveira, C. Monteiro, P. Serrano
Gynaecology, Hospital de Braga, Braga, Portugal

Introduction / Background: Abdominal radical hysterectomy (RAH) with pelvic lymphadenectomy is the standard treatment for patients with early stage cervical cancer, and in our country this is the only approach possible in most oncologic centres. Studies comparing surgical techniques concluded that minimal invasive surgery (MIS) is associated with better short term outcomes with similar oncologic outcomes, and therefore MIS has been used at several centers. Recently, LACC study concluded that MIS in cervical cancer was associated with higher rate of recurrence and lower disease-free survival (DFS). Some authors argued that this results were due to a high rate of DFS reported in the RAH group.
We evaluated if the DFS in RAH reported in this study was the reality in our oncologic center.
Methodology: We peformed a retrospective study including all cases (n=43) of FIGO stage I-II cervical cancer who underwent RAH in our institution between January 2003 and April 2017. Patient characteristics, post-operative complications, recurrence rate and DFS at 2-years and 5-years follow-up were analyzed.
Results: The mean age of the patients was 51 years. Most patients had clinical stage IB1 disease (74%). Rates of adjuvant radiotherapy was 46%, and was administered in high risk patients. The rate of late post-treatment complications was 30%, most of them associated with dual-modality treatment. 5 patients had a recurrence (11.6%), all occurred in the vaginal vault or pelvis. No carcinomatosis or distant metastases were reported. The rate of DFS at 2 years was 93% and 93% of DFS at 5 years in the patients that had at least this time of follow-up.
Conclusion: Our findings are consistent with the high DFS rates of LACC trial, suggesting that this is not a DFS rate exclusive of referral oncologic centers. Despite the small sample size, our findings confirm the oncologic safety in the open abdominal technique.
Disclosure: Nothing to disclose

EP376

BRCA1 immunohistochemistry expression and survival in cervical cancer

E.S. Paik, J.H. Lee, M. Kang, J.H. Kang, S.Y. Jeong, M.S. Kim, Y.-Y. Lee, T.-J. Kim, J.-W. Lee, B.-G. Kim, D.-S. Bae, J. Seo, C.H. Choi
Samsung Medical Center, Seoul, Republic of Korea

Introduction / Background: BRCA1 expression can be lost by a variety of mechanisms including germline or somatic mutation and promotor hypermethylation. Given the potential importance of BRCA1 loss as a predictive and prognostic biomarker in several cancers, we investigated

immunohistochemistry (IHC) of BRCA1 in cervical cancer and their possible prognostic relevance.
Methodology: Seventy patients with cervical cancer were enrolled in this study. Samples from each tumor were stained for BRCA1 and reviewed independently by gynecologic pathologists blinded to BRCA status. Kaplan-Meier methods were used to estimate overall survival (OS), and the log-rank test was used to assess differences between groups. Gene lists obtained from microarray analysis were analyzed for Gene Ontology enrichment using DAVID tools.
Results: BRCA1 IHC was positive in 62.9% (44/70) of cases. IHC staining confirmed expression of BRCA1 in patients with better OS (p=0.028). Genomic profiling of the tumors showed specific alterations associated to BRCA1 expression in the tumors, affecting relevant cellular processes. Alteration of SNORD8, PGK1, CCR6 and COL1A2 gene signatures was identified to be associated with survival outcomes of patients.
Conclusion: These results support the fact that BRCA1 IHC expression in cervical cancer is associated with survival. In addition, the identification of specific alterations in cervical cancer associated with BRCA1 protein expression would help to provide individualized prediction in these patients.
Disclosure: Nothing to disclose

EP377

Total laparoscopic radical trachelectomy with uterine arteries preservation for stage IB2 cervical cancer

N. Panchbhaya[1], E. Marchand[1], C. Mimoun[1], A.S. Leveau Vallier[2], V. Place[3], M. Mezzadri[1]
[1]Gynécologie-obstétrique, [2]Anatomopathologie, [3]Radiologie, Hôpital Lariboisière, Paris, France

Introduction / Background: Radical trachelectomy is a valuable fertility-sparing treatment option for women with early-stage cervical cancer. Vaginal radical trachelectomy with laparoscopic lymphadenectomy (LVRT) has several limitations (nulliparous patients, previous conization with adverse vaginal anatomy). According to recent studies, 252 patients experienced a total laparoscopic radical trachelectomy (LRT) and there are very few publications about the surgical procedure. In this video, we describe the technique of LRT.
Methodology: A nulliparous 33 years-old woman was diagnosed with a stage IB2 endocervical adenocarcinoma (22mm). To preserve her fertility, we performed a LRT with uterine arteries preservation associated with a laparoscopic cerclage of the uterine isthmus. The pelvic sentinel lymph node biopsy was negative which leaded to bilateral pelvic lymphadenectomy. We opened the para vesical fossa and started the ureteric dissection down to the ureterovesical junction and the dissection of the vesicouterin space. Then, we opened the pararectal fossa and performed a posterior dissection with section of the uterosacral ligaments approximately 20 mm from the uterine insertion and we isolated the ascending branch of the uterine artery. We sectioned the uterine veins. We removed the parametria separately. Trachelectomy was performed after section of the uterine isthmus just below the ascending branches of the uterine arteries and a circumferential colpotomy. Finally, we realized a laparoscopic isthmus cerclage and the uterine isthmus was sutured to the vagina with 2 overlocks, using V-lock thread.
Results: The surgery was performed successfully without per-operative complications and lasted 270 minutes. The intra-operative blood loss was < 100mL. The patient was discharge on day 6 with self-catheterization for urinary retention. The were no early post-operative complications. The resection margins were safe, without lymphovascular space invasion and the parametria were free of disease.
Conclusion: LRT performed by trained surgeon is a feasible option for young women with early-stage cervical cancer who wants to spare their fertility.
Disclosure: Nothing to disclose

EP378

Prognostic importance of lymph node in early cervical cancer

J. Park
Obstetrics & Gynecology, Dong-A University Hospital, Busan, Republic of Korea

Introduction / Background: The purpose of this study was to determine the significance of the positive pelvic lymph node (PLN) metastasis on progression-free survival (PFS) and overall survival (OS) of early cervical cancer patients. The values of other known poor prognostic factors were also analyzed for prediction of the disease prognosis.
Methodology: The stage 1A-IIA 245 cervical cancer patients from 1998 to 2014 who underwent radical surgery were retrospectively reviewed. All of the patients underwent radical hysterectomy. Unilateral or bilateral salpingo-oophorectomy was performed considering their age and the stage. PLN dissection was performed when the nodes were suspicious for malignancy from radiologic evaluation or clinical examination. Adjuvant therapy was decided by the patient's risk factors.
Results: Of the 245 patients, 39 patients had PLN metastasis. 15 patients had one PLN metastasis, and 24 patients had more than 2 PLN metastasis. The multivariate analysis of the prognostic factor, the size (P=0.0198), the number of PLN metastasis (P=0.0005) and the LVSI (P=0.0429) had significant association with PFS. Regarding OS, the number of PLN metastasis (P=0.0044) and stromal invasion (P=0.0427) were significantly associated with OS. Notably the presence of the PLN metastasis was not significant to both PFS and OS, however when specified by the number of PLN metastasis, significant association in both PFS and OS was identified (P=0.0005 and 0.0044 each).
Conclusion: we found that tumor size, existence of LVSI, and number of PLN metastasis independently associated with recurrence of the early cervical cancer who underwent surgical treatment. Particularly the number or the PLN metastasis in early cervical cancer has significant association with both recurrence and survival. This result can help assessing PFS and OS and deciding treatment modality of each patient.
Disclosure: Nothing to disclose

EP379

Prognosis-predicting model based on F-18 PET metabolic parameters in locally advanced cervical cancer patients treated with concurrent chemoradiotherapy: multi-center retrospective study

C.-M. Park[1], Y.H. Lee[2], H.J. Lee[3], D.G. Hong[2], D.H. Lee[4], Y.S. Choi[5], Y.S. Lee[2], G.O. Chong[2]
[1]Obsteterics and Gynecology, Jeju National University/Jeju National University Hospital, [2]Obstetrics and Gynecology, Kyungpook National University/Kyungpook National University Chilgok Hospital, [3]Obstetrics and Gynecology, Kyungpook National University/Kyungpook National University Hospital, [4]Obstetrics and Gynecology, Yeungnam University, [5]Obstetrics and Gynecology, Daegu Catholic University, Daegu, Republic of Korea

Introduction / Background: The aim of this study was to develop a prognosis-predicting model based on F-18-fluorodeoxyglucose positron emission tomography/computed tomography (^{18}F-FDG PET/CT) in locally advanced cervical cancer patients treated with concurrent chemoradiotherapy (CCRT).
Methodology: : The medical records of 270 patients with locally advanced cervical cancer who were treated with CCRT were collected from three institutions and reviewed retrospectively. A nomogram for predicting disease-free survival (DFS) and overall survival (OS) was made using Cox proportional hazards regression. Predictor variables included nodal maximum standardized uptake value (SUVmax), primary tumor SUVmax, age, tumor size, stage, serum squamous cell carcinoma (SCC) antigen level, and human papillomavirus status. Internal validation of the nomogram was performed.
Results: A nomogram for predicting the 2-year DFS and 5-year OS was constructed using 7 parameters. With a focus on 2-year DFS, our model found nodal SUVmax was the highest weighted negative prognostic factor. With a focus on 5-year OS, young age was the highest weighted negative

prognostic factor. The concordance index was 0.75 for the 2-year DFS and 0.78 for the 5-year OS, respectively.
Conclusion: This nomogram is a predictive tool that can be used to counsel patients in predicting survival outcomes. Moreover, our prognosis-predicting model may make it possible to personalize treatment.
Disclosure: Nothing to disclose

EP380

Clinical outcomes and predictive factors for bladder or rectal invasive cervical cancer after definitive radiotherapy

S. Park[1], Y.S. Kim[2]
[1]Department of Radiation Oncology, Korea University Ansan Hospital, Gyeonggi-do, [2]Department of Radiation Oncology, Asan Medical Center, University of Ulsan College of Medicine, Seoul, Republic of Korea

Introduction / Background: This study aims to investigate the clinical outcomes in cervical cancer patients with bladder invasion following definitive concurrent chemoradiotherapy (CCRT) ± brachytherapy.
Methodology: Between January 1999 and December 2015, we reviewed our clinical records of 782 women treated with definitive CCRT for cervical cancer. 57 cervical patients with FIGO stage IVA were identified. We retrospectively analyzed clinical outcomes in 57 patients with bladder or rectal invasion who underwent definitive CCRT ± brachytherapy.
Results: Median follow-up duration was 38.9 months (1-190 months). Five-year cause-specific survival (CSS), disease-free survival (DFS) and local control rates (LCR) were 60.8%, 59.9% and 69.8%, respectively. The median age was 57.0 years (range, 26-81 years). Sixteen (26.7%) had para-aortic nodal involvement at the time of diagnosis. Twenty - seven of the patients (47.4%) received brachytherapy and the remaining patients received external beam radiation therapy (EBRT) boost. In multivariate analysis, para-aortic nodes involvement was associated with poorer CSS and LCR ($p < 0.005$). Especially, brachytherapy significantly increased LCR ($p = 0.003$) on multivariate analysis. After treatment, 6 patients (10.5%) had vesicovaginal fistula (VVF) and 4 patients (7%) had rectovaginal fistula (RVF). Acute hematologic toxicity of grade ≥ 3 was 17.5% and acute genitourinary (GU) / gastrointestinal (GI) toxicity was 1.8%, respectively. Late GU / GI toxicity of grade ≥ 3 was 14.1% and 7.0%, respectively.
Conclusion: A definitive CCRT followed by intracavitary brachytherapy is feasible option for cervix cancer with bladder or rectal invasion. Adding brachytherapy was a significant factor for local tumor control with acceptable toxicity rates.
Disclosure: Nothing to disclose

EP381

The role of diagnostic laparoscopic in locally advanced cervical cancer staging in the bevacizumab era

N. Bizzarri, L. Pedone Anchora, E. Teodorico, A. Lombisani, G. Di Fiore, V. Gallotta, S. Gueli Alletti, V. Ghirardi, C. Conte, A. Fagotti, G. Scambia, G. Ferrandina
Gynecologic Oncology Division, Policlinico Universitario Agostino Gemelli I.R.C.C.S. Universita Cattolica di Roma, Rome, Italy

Introduction / Background: The benefit of surgical staging with laparoscopy remains controversial and the prognostic significance of peritoneal involvement in locally advanced cervical cancer (LACC)

patients has still to be demonstrated. Peritoneal involvement upstages cervical cancer to FIGO stage IVB, which can benefit from addition of bevacizumab to standard chemotherapy, after FDA approval. The aim of this study is to determine the rate of peritoneal involvement in a large series of LACC.
Methodology: Retrospective cohort study approved by the Institutional Review Board. Patients with newly diagnosed LACC (FIGO stage IB2-IVA) and metastatic cervical cancer (FIGO stage IVB), were included. All women underwent diagnostic laparoscopy with peritoneal biopsy at the time of examination under anesthetic (EUA) and cystoscopy between March 2015 and December 2018, during staging of cervical cancer. Clinical and surgical characteristics were extracted from electronic records.
Results: 201 patients underwent EUA in the study period.Of these 9 (4.4%) did not undergo diagnostic laparoscopy. 192 women underwent diagnostic laparoscopy: 171 (89.1%) LACC, 21 (10.9%) IVB (distant/parenchymal metastases). No intra- or post-operative complication was recorded. Peritoneal involvement was present in 30/192 (15.6%) women and it was histologically confirmed in all cases. 18/30 (60%) cases had only pelvic peritoneal involvement, 9/30 (30%) had both pelvic and upper-abdominal involvement, while 3/30 (10%) had only upper-abdominal involvement. PET/CT-scan and MRI-scan detected peritoneal carcinomatosis in 3/30 (10.0%) and 2/30 (6.7%) patients, respectively. In 21/30 (70%) cases, laparoscopy upstaged the tumour to FIGO stage IVB and changed the treatment plan. 9/30 (30%) were staged as IVB for distant/parenchymal metastases (independently from laparoscopic findings). 15/30 (50%) cases underwent chemotherapy+bevacizumab.
Conclusion: Diagnostic laparoscopy as part of cervical cancer staging is an important tool to detect unexpected peritoneal disease and change therapeutic management of LACC patients.
Disclosure: Nothing to disclose

EP382

Regular follow up with cervical/vault cytology is of questionable value in patients treated for microinvasive cervical cancer

S. Hirsi-Farah, S. Kerai, S. Abdul, O. Tamizian, V. Asher, A. Bali, A. Phillips
University Hospitals of Derby and Burton, Derby, United Kingdom

Introduction / Background: Follow up for women treated for microinvasive cervical cancer depends on stage at diagnosis. The BSCCCP states, for completeness, that women treated with fertility preserving surgery for stage 1a1 disease could be offered cervical cytology at 6 and 12 months and yearly for 9 years. Stage 1a1 treated with hysterectomy or 1a2 disease follow up is determined by gynaecological oncologist and local cancer pathways. We aimed to assess the value of various follow up regimes in these patients.
Methodology: A retrospective review of all patients treated for Stage 1a1 and 1a2 cervical cancer from 01/2002-12/2018 at the University Hospitals of Derby and Burton (UHDB) identified from the hospital databases. Only patients with squamous or cervical adenocarcinomas were included.
Results: 101 cases were identified. 84(83.2%) were stage 1a1 and 17(16.8%) were stage 1a2. 75(74.3%) had squamous histology and 26(25.7%) had cervical adenocarcinomas. Median follow up was 64 months.
Only one(1%) case of occult microinvasive cancer was identified post-hysterectomy for presumed benign disease, the other 100(99%) patients were diagnosed/treated with conservative/fertility sparing procedures. A second procedure due to involved/close margins with cancer/persistent precancer was performed in 75(74.25%) cases, of these, 50(66.67%) had conservative/fertility sparing procedures and 25(33.33%) had hysterectomy. Residual cancer was found in 3(4%) of second treatment specimens.
Of patients followed up with vault smears all 45(100%) smears were negative.
For patients treated conservatively (68 1a1 and 7 1a2) 377 smears were performed. 338(89.67%) were negative, 8(2.1%) borderline, 27(7.2%) inadequate, two(0.5%) low grade and two(0.5%) high grade.
Of the two high grade smears only one required a LLETZ that demonstrated CIN3. No cases of recurrent cancer were identified. Only 1/75(1.3%) of patients treated conservatively had recurrent CIN2/3/CGIN.
Conclusion: Microinvasive cervical cancer is effectively treated with conservative surgery and

reducing the intensity of follow of these patients should be considered.
Disclosure: Nothing to disclose

EP383

Serum concentrations of s100-a11 and aif-1 are elevated in cervical cancer patients with lymphonode involvement

R. Pilka[1], D. Stejskal[2,3], G. Krejčí[2], M. Švesták[2], R. Marek[1], J. Hambálek[1], M. Maděrka[1]
[1]*Department of Obstetrics and Gynecology, Medical Faculty Palacký University and Faculty Hospital, Olomouc,* [2]*Department of Laboratory Biochemistry, Central Moravian Hospital Trust, Member of Agel Holding, Prostějov,* [3]*Center of Laboratory Diagnostics, University Hospital Ostrava, Ostrava-Poruba, Czech Republic*

Introduction / Background: The aim of this study was to compare TFF3, AIF-1, S100-A11 and DKK1 serum levels in surgically staged patients with cervical cancer, and in healthy female controls.
Methodology: In total 85 consecutive patients diagnosed with cervical cancer stage undergoing radical hysterectomy or fertility sparing surgery with pelvic lymphadenectomy were included in the study. The control group was comprised of 90 patients who underwent elective total hysterectomy for nonmalignant disorder. In all patients preoperative serum samples were taken, separated and the sera were all stored at -80°C until analysis for TFF3, AIF-1, S100-A11 and DKK1.
Results: According to the final histopathologic examination, 32 (37,6%) out of 85 cervical cancer patients were lymphonode positive. S100-A11 ($p< 0.0001$) and AIF-1 ($p< 0.0001$) were higher in cervical cancer patients than in controls. Furthermore, S100-A11 ($p>0.04$) and AIF-1 ($p>0.01$) were significantly higher in lymphonode positive as compared to lymphonode negative patients. Levels of TFF3 and DKK1 were higher ($p< 0.0001$) in controls than in cervical cancer patients and were not different in groups with or without nodal involvement.
Conclusion: S100-A11 and AIF-1 represent potential biomarkers in patients with cervical cancer. Moreover S100-A11 and AIF-1 levels increase in patients with lymph node involvement.
Disclosure: Nothing to disclose

EP384

Laparoscopic radical hysterectomy with consequent transvaginal closure of vaginal cuff - results of a retrospective analysis of a prospective collected multicentre database

C. Köhler[1], H. Hertel[2], J. Herrmann[3], S. Marnitz[4], P. Mallmann[5], G. Favero[6], A. Plaikner[7], P. Martus[8], M. Gajda[9], A. Schneider[10]
[1]*University of Cologne, Medical Faculty, Department of Gynecology, Cologne,* [2]*Department of Obstetrics and Gynecology, Hannover Medical School, Hannover,* [3]*Department of Obstetrics and Gynecology, Sophien - and Hufeland Clinics, Weimar, Germany, Weimar,* [4]*Department of Radiooncology, University of Cologne, Medical Faculty,* [5]*Department of Gynecology, University of Cologne, Medical Faculty, Cologne, Germany, Cologne,* [6]*Department of Gynecology, Helios-Mariahilf-Clinic,* [7]*Gynecology/Obstetrics | Special Operative and Oncologic Gyne, Asklepios Klinik Altona, Hamburg,* [8]*Institute for Clinical Epidemiology and Biometry, Eberhard-Karls-University, Tuebingen,* [9]*Institute for Pathology, Friedrich-Schiller-University Jena, Jena,* [10]*Center for Dysplasia and Cytology, MVZ Fürstenbergkarree, Berin, Germany*

Introduction / Background: Laparoscopic/robotic radical hysterectomy (RH) has been considered oncologically adequate to open RH for patients with early cervical cancer. However, a recent prospective randomised trial (LACC) demonstrates significant inferiority of minimally-invasive approach. Thereupon we have updated our prospective database of combined laparoscopic-vaginal

RH with respect to oncologic outcome.
Methodology: Between 1994 and 2018 1952 consecutive patients with invasive cervical cancer have been treated using minimally-invasive surgery at the Universities of Jena, Charité Berlin (Campus CCM and CBF) and Cologne and Asklepios Clinic Hamburg. 389 patients with inclusion criteria identical to LACC trial were identified. In contrast to laparoscopic/robotic technique used in LACC trial all patients in our cohort underwent combined transvaginal-laparoscopic approach that does not use any uterine manipulator and comprises creation of a tumor-adapted vaginal cuff by transvaginal approach.
Results: Initial FIGO stage was IA1 LVSI+, IA2 and IB1 in 32 (8%), 43 (11%) and 314 (81%) respectively, and histology was squamous cell cancer in 263 (68%), adenocarcinoma in 117 (30%) and adenosquamous tumor in 9 (2%). LVSI was confirmed in 106 patients (27%). Median number of harvested lymph nodes was 24 (2-86). Lymph nodes were tumor-free in 379 (97%) of patients. Following radical hysterectomy 71 (18%) of patients underwent adjuvant chemoradiation (CRT) or radiation (RT). After a median follow up of 99 (1-288) months 3, 4.5 and 10-ys DFS were 96.8%, 95.8%, 93.1% and 3, 4.5 and 10-year OS were 98.5%, 97.8%, 95.8%, respectively. Recurrences were diagnosed in 50% (n=10) loco-regional. Interestingly, 9/20 recurrences occurred later than 39 months following surgery.
Conclusion: Combined laparoscopic-vaginal technique for RH with consequent avoidance of possible spillage and manipulation of tumor cells provides excellent oncologic outcome for patients with early cervical cancer. Our retrospective data suggest that laparoscopic-vaginal surgery respecting laparoscopic oncologic hygiene is oncologic safe and should be validated in further randomised trials.

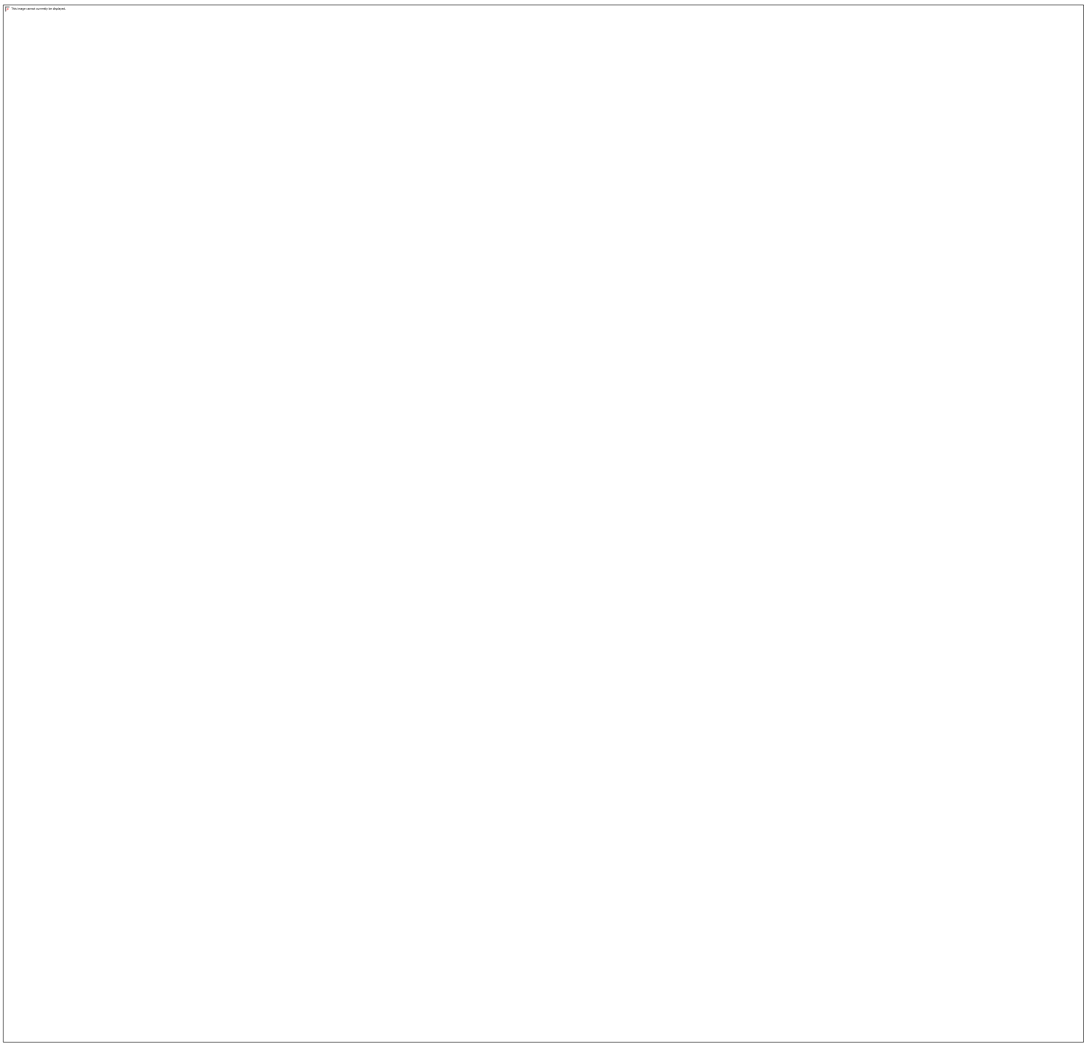

[use of uterine manipulator in cervical cancer]

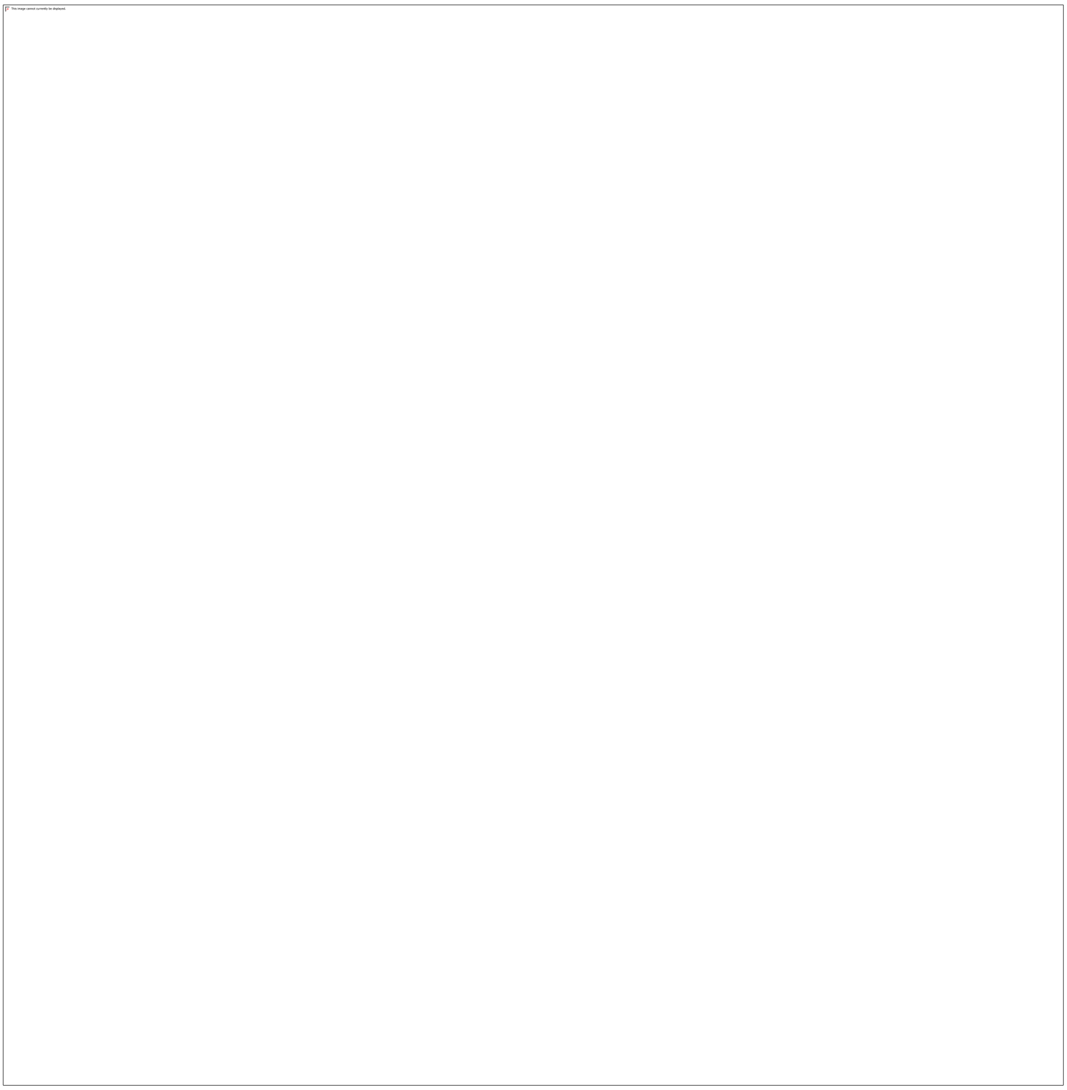

[Incision of vaginal wall using uterine manipulator - tumour spillage may occure]

Disclosure: Nothing to disclose

EP385

A single-center retrospective analysis to evaluate the prognostic role of lymph node density in patients with lymph node positive cervical cancer

A. Polasik, W. Janni, C. Scholz, N. De Gregorio, F. Schochter, T.W.P. Friedl, P. Widschwendter
Department of Gynecology and Obstet., University Hospital Ulm, Ulm, Germany

Introduction / Background: In this analysis we evaluated the prognostic role of lymph node density (LND), i.e. the ratio of positive lymph nodes to the total number of lymph nodes removed during surgery, in nodal-positive cervical cancer (CK) patients.

Methodology: Out of 266 patients with CK that underwent surgery including lymphonodectomy between 2000 and 2017 at our Department for Gynecology and Obstetrics, 86 with positive lymph nodes were included. According to former study results, patients were divided into two groups with LND < 10% vs. ≥ 10%. Univariable and multivariable cox-regression models (adjusted for age, histological subtype, grading, body mass index, R-status, lymphangiosis, histologically confirmed FIGO-status and chemotherapy) were used to evaluate the association between LND and both overall survival (OS) and disease-free survival (DFS).
Results: In the 86 patients, a median of 42 lymph nodes were removed (range 11 - 107), and a median of 2 lymph nodes (range 1 - 25) were found positive. 57 (66.3%) patients had a LND < 10% and 29 (33.7%) patients had a LND ≥ 10%. There was no significant association between LND (≥ 10% vs. < 10%) and OS in both univariable (hazard ratio[HR] 1.49, 95% CI 0.72 - 3.07, p = 0.280) and multivariable survival analysis (HR 1.46, 95% CI 0.64 - 3.32, p = 0.372), respectively. However, LND was significantly associated with DFS in univariable analysis (HR 2.11, 95% CI 1.10 - 4.03, p = 0.024) and was found to be an independent predictor for DFS in adjusted multivariable analysis (HR 2.30, 95% CI 1.08 - 4.91, p = 0.031).
Conclusion: LND ≥ 10% in patients with lymph node-positive CK is associated with a worsened DFS compared to patients with a LND < 10%. Thus, LND may be used as an independent prognostic marker for risk stratification in these patients.
Disclosure: Nothing to disclose

EP386

Cervical cancer screening in PHC organization in Poland

A. Prusaczyk[1], E. Prokurat[1], S. Szafraniec-Burylo[2], M. Bidzinski[3], J. Gronwald[4]
[1]Medical and Diagnostic Centre, Siedlce, [2]Institute of Mother and Child, [3]Oncological Gynaecology Clinic, Maria Skłodowska-Curie Memorial Cancer Center and Institute of Oncology, Warsaw, [4]Department of Genetics and Pathology, Pomeranian Medical University, Szczecin, Poland

Introduction / Background: Incidence and mortality of cervical cancer are high in Poland. The screening for cervical cancer in Poland is based on smear tests examined under optical microscope. Identification of pathological changes in cytology is a key outcome in preventive programmes in primary healthcare (PHC). The aim of the study was to present data regarding to percentage of cervical cancer screening programme execution in a multicentre PHC organization in relation to the voivodships` average.
Methodology: Publicly funded cervical cancer screening programme was available for patients of Polish multicentre PHC organization located in Mazovian and Lublin Voivodships responsible for over 84,000 patients - Medical and Diagnostic Centre (CMD) in Siedlce, Poland. Data were collected by midwifes in respective PHC centres. Tests' results were reported to public database for prophylactic programs and additionally collected locally in MS Excel database.
Results: Participation in prophylactic programme in CMD is relatively high. For Mazovian voivodship 59,5% of eligible CMD patients participated in the programme vs. a mean of 18,0% for entire voivodship, and in Lublin voivodship 65,3% of eligible CMD patients participated in the programme vs. 23,0% for entire region. The percentage of abnormalities found was increasing in these years in which newly screened population was included (no cervical cancer screening for 5 years; new site opened). Among 2310 patients tested, abnormalities were found in 5,24% and 2,25% of all patients required further diagnostics.
Conclusion: Implementation of preventive programmes, especially in new populations is very important. In such populations and among non-compliance patients who do not attend a doctor for many years, the largest number of pathological changes can be found
Disclosure: Nothing to disclose

EP387

The rate of HPV and smear positivity in the follow-up period of patients that underwent cervical excisional procedures

Y.E. Purut[1], E. Keleş[1], S. Akiş[1], M. Api[1], C. Kabaca Kocakuşak[1], Y. Ceylan[2], B. Giray[1], M. Güray Uzun[1]
[1]Gynecologic Oncology, Zeynep Kamil Training and Research Hospital, [2]Medicine, Istanbul Medipol University, Istanbul, Turkey

Introduction / Background: To evaluate the rate of combined Pap smear and human papillomavirus test positivity after cervical excisional procedures.
Methodology: This retrospective study included data of 452 patients who undergone cervical cold knife conization or loop electrosurgical excision procedure (LEEP) and post-cone follow-up co-testing. Age, pre-cone / post-cone: HPV status, cervical cytology and cone margin positivity were recorded. The data were analyzed with the Chi square test, Fisher's exact test for categorical data and Student's T test for continuous data.
Results: A total of 452 women were enrolled and all of them were HPV positive before the procedure. At 6 month follow-up, In margin negative patients, HPV test resulted as positive in 28.1%, while, In margin positive patients, HPV test resulted as positive in 35.4% (p=0.592). In patients with normal precone cytology, 7.9% postcone cytology was positive, whereas, in patients with positive precone cytology, 17.8% postcone cytology was positive (p= 0.006). If the margin is positive, 20.9% of the patients present with positive smears in the follow-up even further excisional procedures are done.
Conclusion: Margin positivity after cone, increases the risk of positive cytology and HPV test. Close monitoring of these groups of patients is recommended.
Disclosure: Nothing to disclose

EP388

Laparoscopic para-aortic lymph node staging in the care of advanced cervical cancer (IB3-IVA): retroperitoneal or transperitoneal?

M. Pécout[1], C. Bourgin[2], H. Azais[3], F. Machuron[4], K. Nyangoh Timoh[5], L. Oulmader[6], G. Canlorbe[3], C. Coutant[7], O. Graesslin[8], C. Touboul[9], A. Bricou[10], C. Huchon[11], E. Daraï[12], M. Ballester[12], J. Levêque[5], V. Lavoué[5], S. Bendifallah[13], E. Raimond[8], Y. Kerbage[1], P. Collinet[1], Francogyn
[1]Jeanne de Flandre Hospital, Lille, [2]René Gauducheau Hospital, Nantes, [3]La Pitié Salpétrière Hospital, Paris, [4]Université de Lille, Lille, [5]Centre Hospitalier de Rennes, Rennes, [6]CHRU Bretonneau, Tours, [7]Centre Hospitalier de Dijon, Dijon, [8]Centre Hospitalier de Reims, Reims, [9]Centre Hospitalier Intercommunal de Créteil, Créteil, [10]Hopital Jean Verdier, Bondy, [11]Centre Hospitalier de Poissy, Poissy, [12]Hopital Tenon, Paris, [13]Hopital Nord, APHM, Marseilles, France

Introduction / Background: Para-aortic lymphadenectomy is currently not systematized in the pre-treatment management of advanced cervical cancer (FIGO IB3-IVA). However, metastatic lymph has prognostic and therapeutic importance. We compared morbidity and mortality of retroperitoneal para-aortic lymphadenectomy with transperitoneal approach, in the pre-therapeutic lymph node staging of cervix cancers at a locally advanced stage (FIGO IB3-IVA).
Methodology: All pre- and postoperative data from 331 patients operated for advanced cervical cancer with a negative Positron Emission Tomography-scanner, managed between 1999 and 2017 in 9 centers in France were listed in the FRANCOGYN database. Morbidity and mortality related to the surgical approach was assessed by studying the rate of per and postoperative complications, as well as the duration of hospitalization. Also analyzed were overall survival as well as survival without recurrence.
Results: 164 patients received a retroperitoneal approach (group 1) and 167 patients received a transperitoneal approach (group 2). Of these 331 patients, 12 had an intraoperative complication (4 of group 1 and 8 of group 2, p = 0.36) and 34 a postoperative complication (12 of group 1 and 22 of group 2, p = 0.044), none of which required surgical revision. Hospital stay was shorter in group 1 than

in group 2 (3.87 ± 2.47 days versus 5.17 ± 3.04 days, $p < 0.001$). There was no significant difference in mortality between the two groups; 25 patients in group 1 died (15.24%), compared with 26 patients in group 2 (15.57%). There was no significant difference in recidivism; 40 patients in group 1 had a recurrence (24.39%), compared with 37 in group 2 (22.16%).
Conclusion: The transperitoneal approach has more complications postoperatively and a longer hospital stay than the retroperitoneal approach. In contrast, there is no significant difference in survival without recurrence or overall survival depending on the approach.
Disclosure: Nothing to disclose

EP389

Does laparoscopic extra-peritoneal para-aortic node dissection aid treatment of women with locally advanced cervical carcinoma? A 9 year review of one institution in Northern Ireland

K. Reilly, E. Craig, S. Dobbs, M. McComiskey, H. Nagar, I. Harley
Gynae-Oncology, Belfast Health and Social Care Trust, Belfast, United Kingdom

Introduction / Background: Cervical cancer is one of the most commonly occurring female cancers with increasing incidence worldwide. The mainstay of treatment for locally advanced cervical cancer is primary chemoradiotherapy. Pre-operative imaging alongside laparoscopic extraperitoneal para-aortic node dissection (LEPAND) has been used in order to best target this treatment. This study looked at all the women in the Belfast Trust over the last 9 years who had this investigation done as part of their pre-treatment workup and their outcomes.
Methodology: Retrospective data was collected for all those who had LEPAND for locally advanced cervical cancer from January 2010 to December 2018. These women all had pre-operative imaging that suggested positive pelvic lymph nodes but negative para-aortic nodes therefore deeming them suitable for the surgery.
Results: Sixty women were identified in this group with an age range from 23-69 and median age of 39. 93.3% had stage 2b cervical cancer, the remainder were 1b2 or 3b. 17% had adenocarcinoma of the cervix and 83% had squamous cell carcinoma.

70% of the women had primary LEPAND surgery before chemoradiotherapy with the remainder having their primary treatment before surgery.
5% (3/60) had positive para-aortic nodes on histopathology although 100% appeared node negative on MRI or PET imaging. None of those with positive nodes had recurrences but 66% died within 2 years.
15% of the study population died with 90% of these in the two years after diagnosis. 67% of these women had primary LEPAND followed by chemoradiotherapy with the others proceeding straight to primary treatment. The median survival for this group is 5 years.
Conclusion: 5% of the group had positive para-aortic lymph nodes on histopathological examination. This was despite imaging stating that they were node negative.LEPAND surgery prevented undertreatment in these women by re-targeting their radiotherapy.
Disclosure: Nothing to disclose

EP390

Open radical hysterectomy for early cancer cervix - 10 years' experience from a tertiary cancer centre

P.N. Rema
Division of Gynaecological Oncology, Department of Surgical Services, Regional Cancer Centre, Thiruvananthapuram, India

Introduction / Background: Carcinoma cervix is the most common cause of cancer related death among women in developing countries. Early cervical cancer is surgically treated by radical hysterectomy and bilateral pelvic lymph node dissection. Recent studies have highlighted the advantage of open radical hysterectomy over minimally invasive surgery in better patient survival. The aim of the study was to assess the survival outcome and post operative morbidity of early cervical cancer patients treated by open radical hysterectomy.
Methodology: Between January 2008 and December 2018, seventy eight cases of early cancer cervix who underwent open radical hysterectomy at our centre, comprised the study group. After obtaining institutional review board approval the case records of these patients were analyzed in detail for clinical and histopathologic details, surgical parameters, post operative morbidity, details of adjuvant treatment and disease status on follow up. All patients were clinically staged according to the International Federation of Gynecology and Obstetrics(FIGO) 2009 staging system. Patients were followed up till February 2019. Survival analysis was done using the Kaplan-Meier curve.
Results: The mean age of presentation was 49.5 years (range 21-72years). The histological type was squamous in 52 patients, adenocarcinoma in 22 and adenosquamous in 4. Most patients belonged to FIGO stage IB1(70.5%) followed by stage IIA1 (11.5%) ,Stage 1B2 (9%)stage IA2(6.4% %) and IA1 with LVSI (2.4%). The most common post-op complication was bladder dysfunction, which was seen in 15.4% followed by urinary tract infection in 9%. 52.6% received adjuvant chemoradiation. 5 patients had locoregional recurrence and three patients had distant metastasis. 5-Year disease free survival was 88.1% and overall 5 year survival was 94.6 %.
Conclusion: This study suggests that open radical hysterectomy has good survival outcome in early cervical carcinoma and with minimal post operative morbidity.
Disclosure: Nothing to disclose

EP391

Rheumatoid arthritis as a risk factor for cervical cancer in Brazil

L. Resende[1], N. Goulart[2]
[1]UniCeub, [2]Clinica Revigore, Brasilia, Brazil

Introduction / Background: Rheumatoid arthritis (RA) is a chronic, polygenic, multifactorial disease that has a systemic inflammatory response.
The prevalence in the world is 1-2% in Western countries. In the United Kingdom, affects about 400,000 people; is more prevalent in women (3:1). In Brazil it is estimated a prevalence of 0.5-1% and age range that ranges between 30-50 years.
Treatment involves the use of anti-rheumatic drug modifying the disease, associated or not with the use of glucocorticoids. Cervical cancer ranks third in prevalence and fourth as cause of death in women worldwide. In Brazil, this disease still represents an important cause of death among women. Persistent High risk HPV (HR-HPV) infection is a necessary condition for the onset of precursor lesions that can progress to cancer.
Methodology: 131 women with HR-HPV positive test, of those 42 diagnosed with rheumatoid arthritis and 89 without autoimmune disease. Oncotic cytology, genotyping for HPV and colposcopy were performed at intervals of 2 years.
Results: The mean age (years) of both groups was 34.7 (RA) and 32.1 (Control). The persistence of HR-HPV infection after 2 years was higher in the study group (61 vs 28%) and the presence of abnormal cytologies was also higher (9.3 vs 2.1%).
Conclusion: Young women have a high rate of negative HPV infection within 2 years. Some studies have evidence that the exposition to long-term immunosuppression increase the risk of pre-invasive cervical lesions than the general population which can be explained by increased persistence of HPV infection, especially HPV 16 and 18. This Brazilian cohort has a small number of women, but has similar statistics to studies in other countries, which also shows an increased risk of those using corticosteroids to develop cervical cancer precursor lesions and, therefore, need an interval tracing. time, associated with colposcopy with biopsies for histological confirmation.
Disclosure: Nothing to disclose

EP392

Abdominal radical trachelectomy as a fertility-sparing treatment for early-stage cervical cancer; a single institution experience

A. Rodolakis[1], N. Thomakos[1], C. Theofanakis[1], D. Haidopoulos[1], M. Sotiropoulou[2], C. Bourgioti[3], L. Moulopoulou[3], D. Loutradis[4]
[1]Unit of Gynaecologic Oncology, 1st Department of Obstetrics & Gynaecology, National and Kapodistrian University of Athens, [2]Department of Histopathology, Alexandra Hospital, [3]Department of Radiology, Aretaieio Hospital, National & Kapodistrian University of Athens, [4]1st Department of Obstetrics & Gynecology, National and Kapodistrian University of Athens, Athens, Greece

Introduction / Background: The recommended surgical treatment for stages IA2 to IB1 cervical cancer is radical hysterectomy with systematic pelvic lymphadenectomy. However, this approach leads to loss of fertility. Data suggest that abdominal radical trachelectomy (ART) is a feasible alternative that preserves the uterus without increasing the risk of recurrence, preserving the possibility for future childbearing. This study was performed in order to assess the safety of this fertility-sparing approach to early-stage cervical cancer. We present the surgical, oncologic and fertility outcomes of patients treated with ART.
Methodology: We conducted a retrospective analysis of patients undergoing fertility-sparing ART at our institution from 2002 to 2016.
Results: A total of 84 patients who underwent ART with bilateral systematic pelvic lymhadenectomy were followed for 2-106 months. Thirty-three patients had undergone conization before surgery. The characteristics of the patients include tutor diameter of 8-34mm (stage IA2=11, stage IB1 [< 3.4mm] =73, average age of 29.5 years (range, 26-43 years), mean operative time of 185 min (range, 142-245 min) and average blood loss of 350 mL (range, 150-750 mL). Uterine arteries were identified at their origin from the internal iliac artery and gently preserved in 80 cases. There were two recurrences in the paraaortic area and one in the remaining part of the cervix. Unfortunately,the last patient developed a kidney metastasis and died of the disease a few weeks later. Forty-eight women attempted to conceive and 35 successful pregnancies were recorded (73%), while 6 of them were preterm (17%).
Conclusion: ART can be successfully performed by gynecologic oncologists, who are experienced in radical surgery. It is a safe and well-established procedure with acceptable oncologic and obstetric outcomes, accompanied by low rates of morbidity and mortality. We strongly believe that is mandatory for young women, diagnosed with early-stage cervical cancer, who desire future fertility, to be offered fertility-sparing procedures.
Disclosure: Nothing to disclose

EP393

Liquid Nitrogen cryotherapy (N_2Cryo) for Cervical Intraepithelial Neoplasia (CIN)

S. Sagae
Gynecologic Oncology, Hokkaido Ohno Memorial Hospital, Sapporo, Japan

Introduction / Background: In general patients with ASC-US/LSIL cytology will be followed periodically with three or six month interval. We performed liquid nitrogen cryotherapy (N_2Cryo) of less than thirty second duration for the purpose of clearance of abnormal cytology and persistent HPV infection.
Methodology: Between April 2015 to May 2019, retrospectively we analyzed patients with abnormal cytology of ASC-US/LSIL into the groups: A group of 37 cases with just follow-up without any

treatment, B group of 39 cases with N_2Cryo according to colposcopic and/or cytological abnormality and HPV infection. Furthermore, 26 patients with HSIL undergone N_2Cryo were also compared to the effectiveness of clearance of abnormal cytology and HPV infection. Patients performed N_2Cryo three times every four weeks with written informed consent.
Results: Among 37 patients with ASC-US/LSIL followed by no treatment, 2 cases recovered spontaneously with normal cytology (NILM) and HPV negative (complete regression: CR), 15 cases with partial regression (PR) (CR+PR Regression Rate (RR): 45.9%) including NILM or HPV negative, 6 cases with persistence, 2 cases with progressed to HSIL. However, 12 cases were missed with no visit during follow-up.
Among 39 patients with ASC-US/LSIL treated by N_2Cryo, 14 cases showed CR, 10 cases showed PR, with RR: 61.5%, including 8 cases with persistence.
Furthermore, 26 cases with HSIL treated by N_2Cryo, 8 cases showed CR, 12 cases showed PR with RR: 76.9%, and 4 cases with persistence.
Conclusion: Liquid nitrogen cryotherapy (N_2Cryo) might be useful for the CIN regression, especially with avoidance of cone biopsy for cases with HSIL.
Disclosure: Nothing to disclose

EP394

The importance of p16Ki-67 in cervical precancerous lesions

B. Sahin[1], D.T. Arioz[1], C. Ozdemir[2], G. Cura[1], M.K. Pektaş[1]
[1]*Obstetric and Gynecology,* [2]*Pathology, Afyon Saglik Bilimleri University, Afyonkarahisar, Turkey*

Introduction / Background: In the presence of high risk HPV, E7 oncogene causes the overexpression of p16 and Ki-67 is an indicator for cell proliferation. This study aims to evaluate HPV positive women in aspect of p16 and Ki-67 presence in tissues.
Methodology: This is a retrospective review of 287 patients who tested positive for HPV DNA.
Results: Colposcopic biopsies revealed normal histopathological findings in 28 patients (9.8%), cervicitis in 48 patients (16.7%), cervical intraepithelial lesion (CIN) 1 in 178 patients (62.0%), CIN2 in 26 patients (9.1%), CIN3 in four patients (1.4%) and carsinoma in situ (CIS) in three patients (1.0%). Cervical tissue positivity for p16 was 2.6% in patients with benign histopathological findings, 16.3% in patients with CIN1, 92.3% in patients with CIN2, 100% in patients with CIN3 and 100% in patients with CIS. Cervical tissue positivity for Ki-67 was 42.1% in patients with benign histopathological findings, 77.5% in patients with CIN1, 96.1% in patients with CIN2, 100% in patients with CIN3 and 100% in patients with CIS. The sensitivity and specificity of p16 positivity for CIN1, CIN2, CIN3 and CIS were 25.1% (95% CI: 19.4%-31.5%) and 97.4% (95% CI: 90.8%-99.7%) respectively. When p16 positivity was combined with Ki-67 positivity, the sensitivity decreased to 20.4% (95% CI: 15.2%-26.5%) and the specificity was 97.4% (95% CI: 90.8%-99.7%). The sensitivity and specificity of p16 positivity for CIN2, CIN3 and CIS were 93.9% (95% CI: 79.8%-99.3%) and 90.6% (95% CI: 86.3%-93.9%) respectively. When p16 positivity was combined with Ki-67 positivity, the sensitivity decreased to 90.9% (95% CI: 75.7%-98.1%) and the specificity increased to 94.1% (95% CI: 90.5%-96.7%).
Conclusion: The presence of p16 and Ki-67 in cervical tissues has a significant role in the detection of precancerous lesions and may help to predict the progression of precancerous lesions into malignancy.

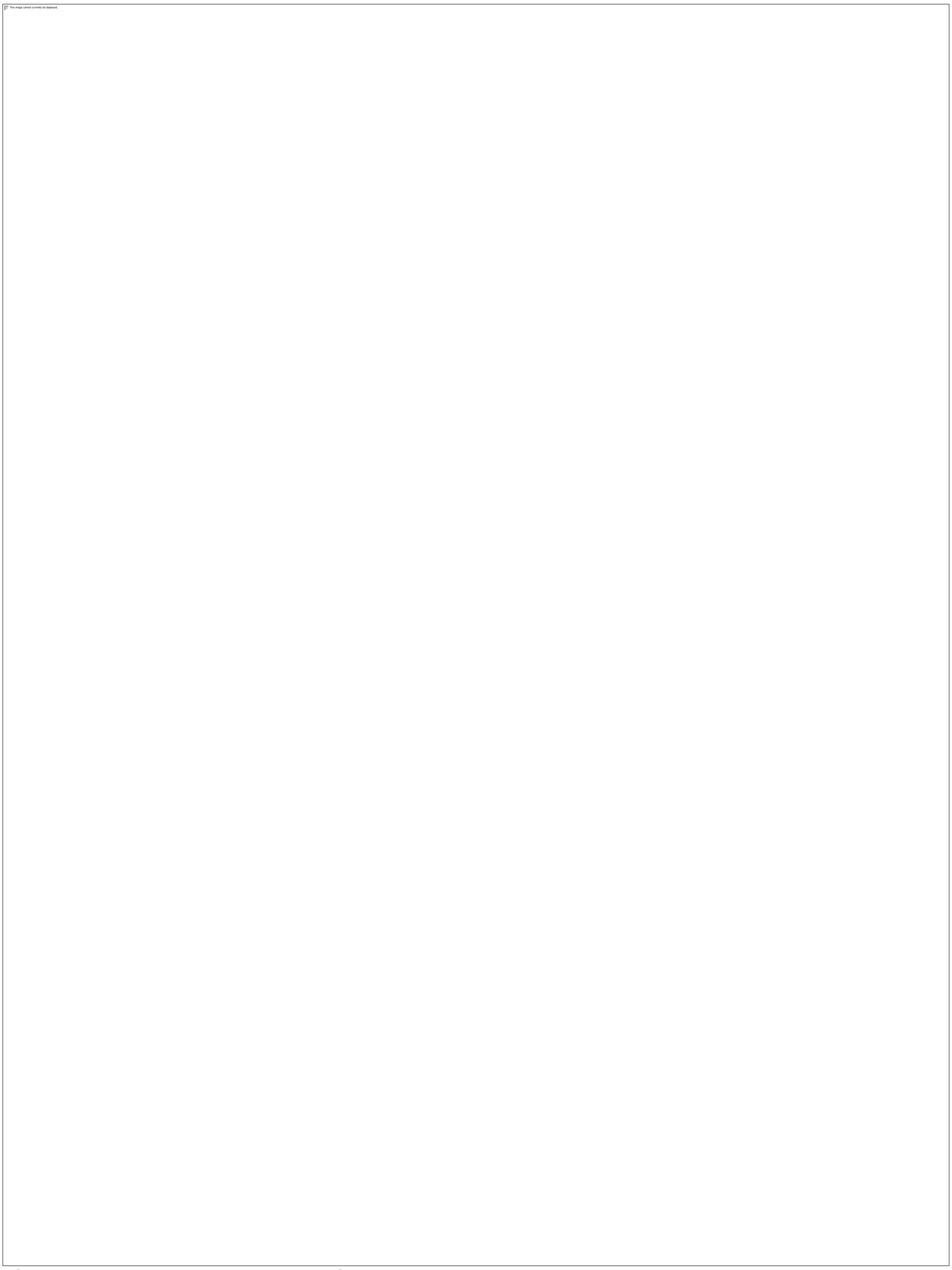

[Sensitivity and specificity of kits in CIN1 and above pathology results]

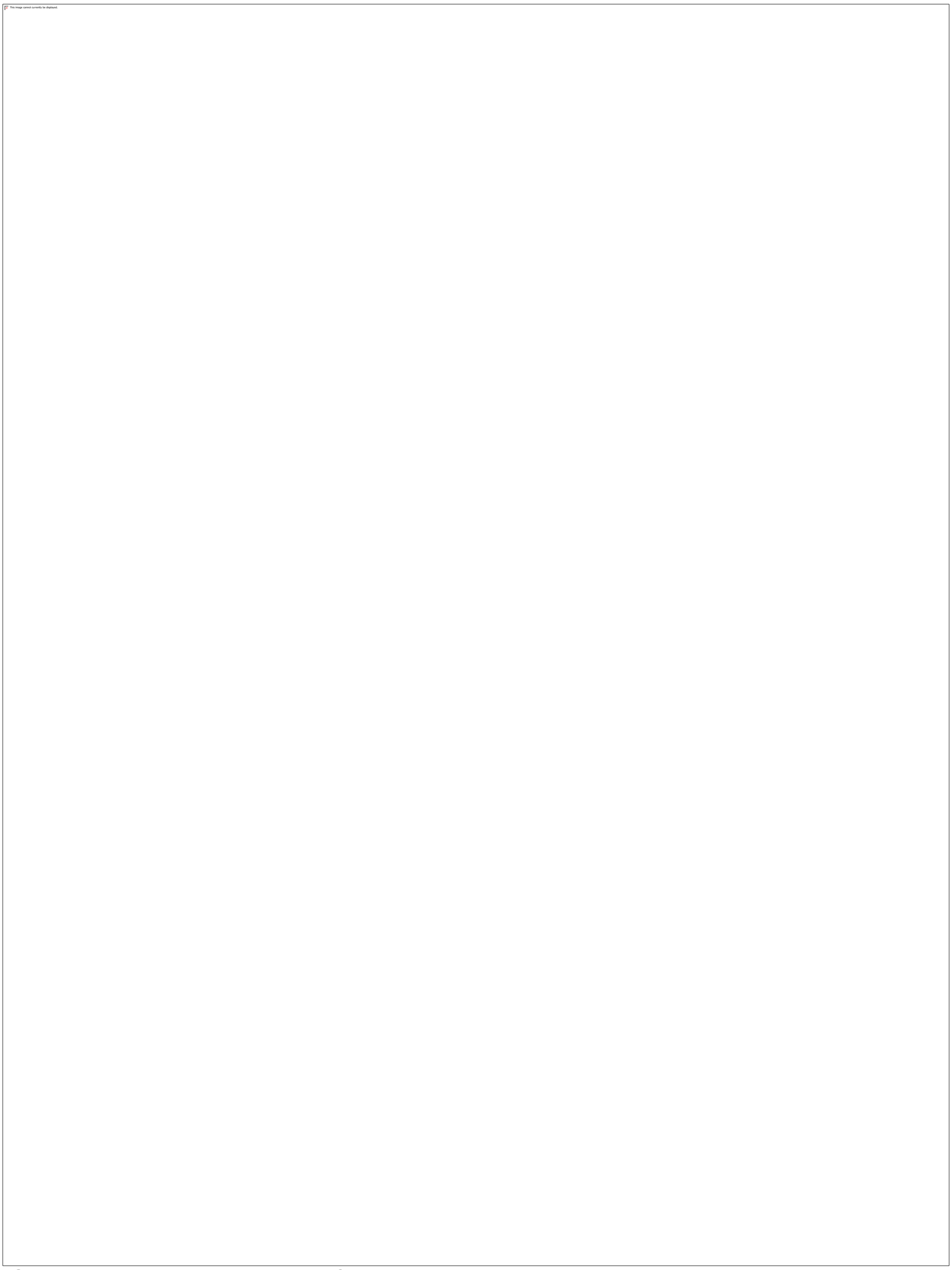

[Sensitivity and specificity of kits in CIN2 and above pathology results]

Disclosure: Nothing to disclose

EP395

The patient had stage IIIB cervical carcinoma with pregnancy has followed up without disease for 22 years : case report

M. Sahin, S.A. Ergen, I. Sahinler
Radiation Oncology, Istanbul University-Cerrahpasa, Cerrahpasa School of Medicine, Istanbul, Turkey

Introduction / Background: Cervical carcinoma is the most common gynecological malignancy during pregnancy. The incidence is expected to increase due to the fact that women delay childbearing. In this case report, we present a patient diagnosed with cervical carcinoma during 6 weeks pregnancy.
Methodology: A 39 years old woman (gravida 6, para 3) presented with the left groin and lower back pain in 1996. She was diagnosed with FIGO Stage IIIB squamous cell cervical carcinoma and also she was six weeks pregnant surprisingly. It was requested that the pregnancy be terminated by curettage, but cervical os couldn't be dilated. She was referred to our clinic for radiotherapy. In the abdomino-pelvic computed tomography, the cervix uteri was enlarged diffusely to 43x70 mm, there was parametrial invasion with surrounding the left ureter and it was diagnosed with grade 2 hydronephrosis. The chest X-Ray was found normal. According to rectovaginal examination, there was endophytic, bleeding mass in cervix uteri and left parametrial involvement up to pelvic side wall. The external beam radiotherapy was applied to pelvis with a total dose of 54 Gy (1.8 Gy fraction dose). In the third weeks, the tumor had regressed more than 50% and the parametrial involvement had regressed almost completely. The uterine cavity was curetted before intracavitary radiotherapy (ICRT). ICRT was applied with tandem and ovoids and the prescribed dose was 24 Gy in 3 fractions to A point.
Results: In gynecological examination after the treatment was completed, the total regression was observed. The patient has been followed up regularly for 22 years without recurrence or metastasis.
Conclusion: The patients with the cervical carcinoma during pregnancy are usually diagnosed at earlier stages. According to our literature review, this is the only patient with long term survival despite having stage IIIB cervical carcinoma.
Disclosure: Nothing to disclose

EP396

Five-year survival and prognostic factors in women treated for cervix cancer at a tertiary care center

K. Sait, N. Anfinan, H. Sait
King Abdulaziz University, Jeddah, Saudi Arabia

Introduction / Background: It is crucial to investigate survival in cervix cancer at the local level to provide an approach on the effectiveness of the overall management, as it reflects the level of care of the patients and the awareness among the population about screening and early diagnosis. Thus, this we conducted this study to provide insight into the medium term prognosis of treated cervix cancer by estimating the 5-year survival rate and investigating the associated clinical, management- and outcome-related factors and predictors.
Methodology: A retrospective study including women with cervix cancer who were treated at the Gynecology Oncology Unit in King Abdulaziz University Hospital, Jeddah, and who achieved at least 60 months (5 years) of follow up. Baseline demographic and clinical data, tumor characteristics, treatment option and outcome including recurrence were collected and analyzed as factors of 5-year survival.
Results: A total 153 patients were included, mean (SD) age was 54.52 (13.47) years. Tumor characteristics showed FIGO stage (I [19.6%], II [50.3%], III [15.7%], IV [14.4%]), (grade I [17.6%], grade II [46.4%], grade III [35.3%]) and the most frequent histological type was squamous cell carcinoma (73.9%). Patients received initial radiotherapy with concurrent chemotherapy (51.6%), systemic CT (3.9%) and palliative care (5.9%). The 5-year survival rate (95%CI) was 51.6% (43.4%,

59.8%), which was independently predicted by tumor grade II (OR=0.08, p=0.009), grade III (OR=0.05, p=0.004) and recurrence (OR=0.09, p< 0.001).
Conclusion: 5-year survival figures are relatively lower than in developed countries but better than in other developing countries. Remarkably, this study evidenced a high percentage of patients diagnosed at advanced-stage, which probably impacts survival and stresses the need of improving early detection.
Disclosure: Nothing to disclose

EP397

Clinical contribution of antecedent ligation of uterine vessels in radical hysterectomy to the prognosis of patients with uterine cervical cancer

F. Saito, M. Katabuchi, K. Takaishi, T. Motohara, I. Sakaguchi, Y. Miyahara, R. Honda, H. Tashiro, T. Ohba, H. Katabuchi
Obstetrics and Gynecology, Faculty of Life Sciences / Kumamoto University, Kumamoto, Japan

Introduction / Background: In Japan, radical hysterectomy (RH) has been widely performed for patients with invasive uterine cervical cancer, resulting in favorable outcome. However, recurrence, which might have been induced by *hematogenous metastasis,* is defined as the development of distant *metastasis in some cases. From 2006, we have performed RH with* antecedent ligation of uterine vessels before pelvic lymphadenectomy t*o prevent hematogenous metastasis. The objective of this study is* to evaluate the clinical contribution of antecedent ligation of uterine vessels in RH to the prognosis of patients with uterine cervical cancer.
Methodology: Two hundred fifty-five patients who underwent RH at Kumamoto University Hospital between 2000 and 2011 were identified. Retrospective analyses were performed between 2 groups according to the surgical approach. The patients who underwent RH with conventional surgical procedures before 2006 were assigned to conventional surgery group (n=98). Another group (antecedent ligation group) consisted of patients who underwent RH with a pelvic lymphadenectomy following antecedent ligation of uterine vessels after 2006 (n=157). The site of initial recurrence within 5 years and histological type were analyzed. Survival estimates used the Kaplan-Meier methods.
Results: At a median follow-up of 88 months, 49 events had been observed and 31 patients had died. The 5-year overall survival rates were similar between groups (83.7% vs 90.4%, p=0.12). Among squamous cell carcinoma (SCC) patients, there were no differences in the 5-year overall survival rates and recurrences between groups. For non-SCC patients, RH with antecedent ligation of uterine vessels improved the 5-year overall survival rates (70.3% vs 88.0%, p=0.06). Furthermore, antecedent ligation of uterine vessels in RH was associated with a lower rate of extrapelvic recurrence than conventional surgery (21.6% vs 6.0%, p=0.03) in patients with non-SCC uterine cervical cancer.
Conclusion: Antecedent ligation of uterine vessels in RH may improve the survival in patients with non-SCC uterine cervical cancer.
Disclosure: Nothing to disclose

EP398

Nerve-sparing radical hysterectomy: a standardized method for precision surgery and local control in cervical cancer

N. Sakuragi[1,2], T. Kato[1], G. Murakami[3,4], C. Shimada[1], Y. Todo[5], M. Kaneuchi[2], T. Mitamura[1], Y. Konno[1], M. Takeda[1], M. Kudo[1], H. Watari[1]
[1]Department of Obstetrics and Gynecology, Graduate School of Medicine, Hokkaido University, Sapporo, [2]Department of Gynecology, Otaru General Hospital, Otaru, [3]Department of Anatomy II, Sapporo Medical University, [4]Division of Internal Medicine, Jikou-kai Clinic of Home Visits,

[5]*Department of Gynecology, Hokkaido Cancer Center, Sapporo, Japan*

Introduction / Background: We aimed to evaluate the efficacy of a systematic nerve-sparing radical hysterectomy (NS-RH) developed by Japanese gynecologists[1-3]in terms of local control of cervical cancer.
Methodology: One-hundred and twenty-one patients with FIGO stage IB1 (n=72)/IB2 (n=13)/IIA (n=9) /IIB (n=27) cervical cancer treated with RH(with or without NS procedure) between 2002 and 2011 were studied. NS procedure was applied when feasible bilaterally or unilaterally. Tumor recurrence included pelvic (local/regional) and distant (extrapelvic) failures. Median follow-up was 106 (range: 6-203) months. The number of loss to follow-up was 4 (3.3%) at 60 months.
Results: Sites of recurrence (some overlapped) were local (n=7), regional (6), and distant (n=11). 5-year overall (OS) and disease-free survival (DFS) for stage IB1 were 97.2%% and 94.1% and for stage IB2 were 92.3% and 84.6%, respectively. For stage IIA (combined IIA1 and IIA2) and stage IIB, 5-year OS and DFS rates were 77.8% and 66.7%; 77.8% and 70.4%, respectively. Overall and local recurrence rates in stage IB1/IB2 were 6/85 (7.1%) and 1/85 (1.2%), respectively; in IIA/IIB was 12/36 (33.3%) and 6/36 (16.7%), respectively. Four (1 stage IB1 and 3 stage IIB) of seven local recurrence occurred in the paracolpium or pararectum area on the same side of NSprocedure and the other three were deemed to have no relationship with NSprocedure. NS-related local recurrence in stage IB1/IB2/IIA and IIB was 1/94 (1.1%) and 3/27 (11.1%), respectively (p=0.034). Both overall recurrence and NS-related local recurrence were better correlated to pT classification (p< 0.001 and p< 0.001) than FIGO stage (p=0.002 and p=0.008).
Conclusion: Japanese method of NS-RH can be safely applied to 2008 FIGO stage IB1/IB2/IIA cervical cancer unless histological vaginal and parametrial invasion is suspected. Modern imaging techniques, which can be used in 2018 FIGO staging, may be useful for personalized application of NS-RH for cervical cancer.

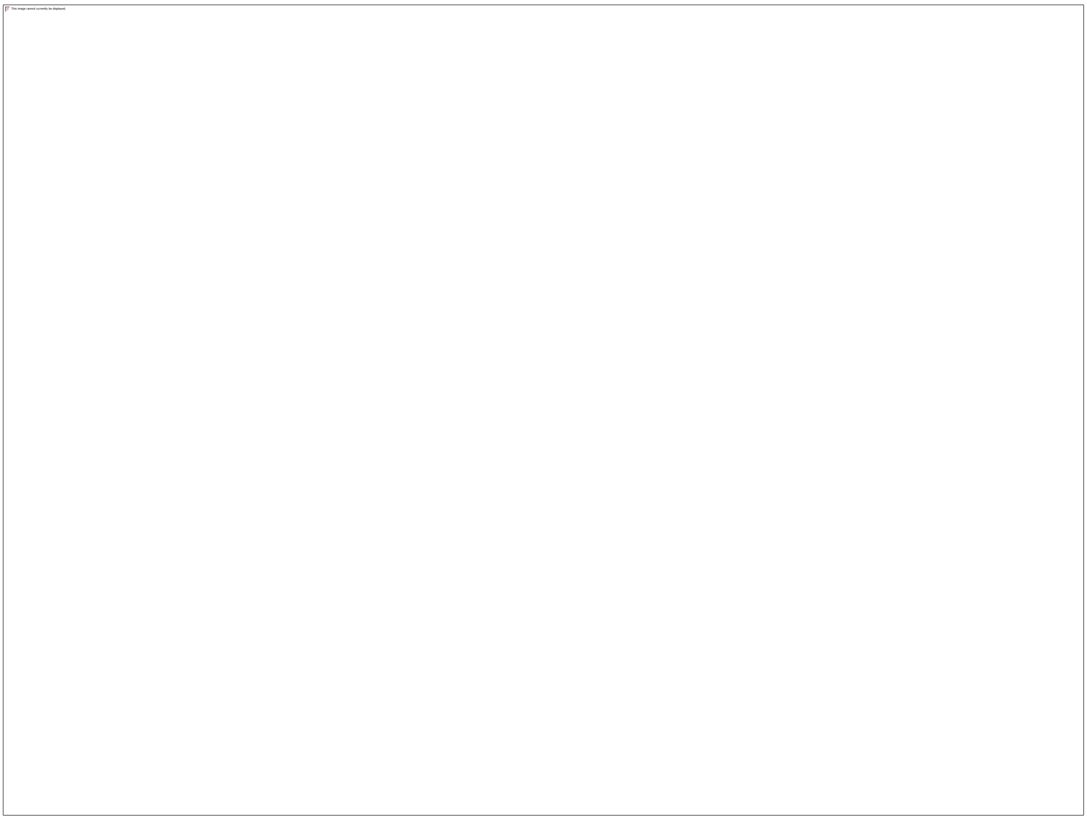

[Various types of RH used in Japan and the corresponding classificatications]

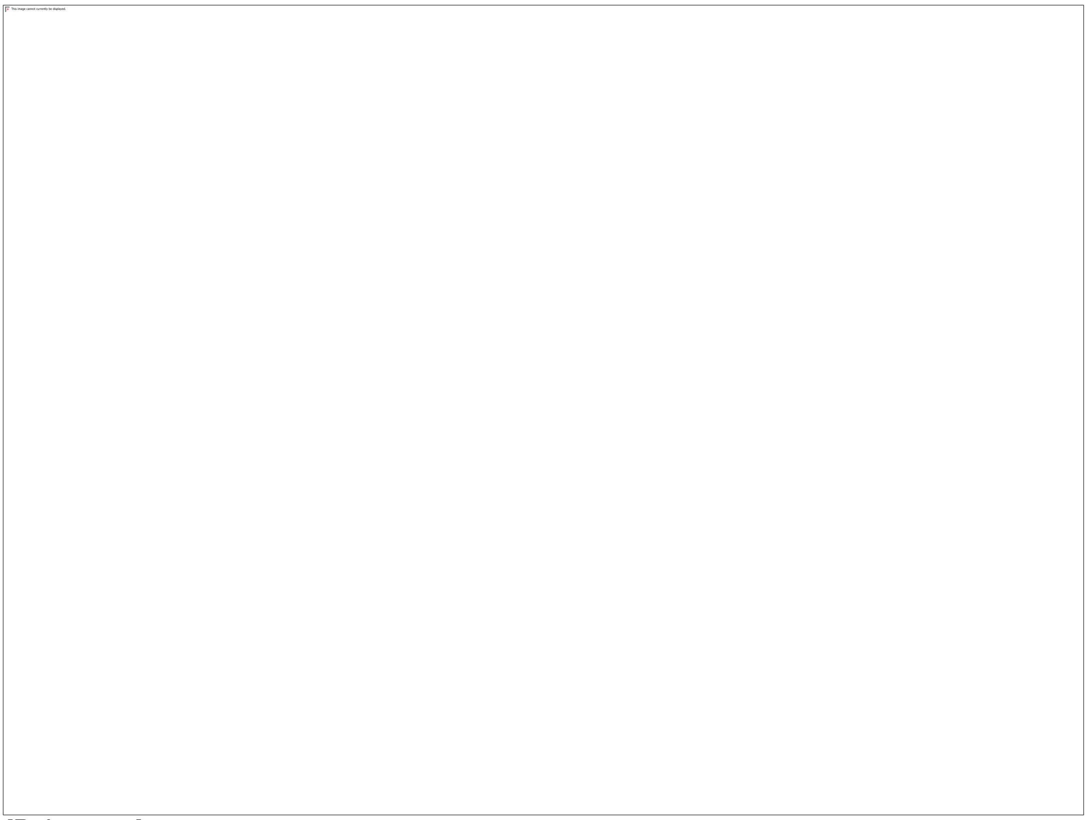

[References]

Disclosure: NS has received chairperson's fees from J & J and Chugai and research grants from Roche and Chugai. HW received research grants or lecture fees from Chugai and Lymphotec. TK, CS, MK, MT and YK declare that they have no conflicts of interest. NS and TK contributed equally to this work.

EP399

Adenoid basal carcinoma combined with invasive squamous cell carcinoma of uterine cervix: a case report of a 37-year-old woman

S. Salman[1], S. Yuksel[1], S. Bektas[2]
[1]Department of Obstetrics and Gynecology, [2]Department of Pathology, GOP Taksim Training and Research Hospital, Istanbul, Turkey

Introduction / Background: Adenoid basal carcinoma (ABC) is an extremely rare neoplasm of the cervix and is seen less than 1% of all cervical malignancies. It can be seen pure or combined with cervical dysplasia or other types of cervical carcinoma. Fewer than 20 cases of ABC with invasive squamous cell carcinoma (mixed carcinoma) have been described in English literature to date.
Methodology: Here we present a 37-year-old female who had complaints of spotting and post-coital bleeding for 6 months. Her last pap-smear, 2 years ago, was negative for dysplasia or malignancy. Vaginal palpation revealed a barrel-shape cervix and endocervical curettage and endometrial biopsy

were performed.
Results: Initial pathology results revealed invasive squamous cell carcinoma. MRI showed an endocervical mass 36x25mm in size with stromal invasion without parametrial invasion (Figure 1). According to the criteria of the International Federation of Gynecology and Obstetrics, our case was staged as IB1.The patient underwent type 3 radical hysterectomy, bilateral salpingectomy, unilateral oophorectomy, pelvic lymphadenectomy and para-aortic lymph node sampling. Macroscopic evaluation of the histological material revealed a tumor surface of 3,5x3,5x1,5 cm in size that dilated the cervix to a barrel-shape. Approximately 50% of non-keratinized less differentiated squamous cell carcinoma with 50% of adenoid basal carcinoma were observed (Figure 2). Focal reaction with CEA,p63, Bcl-2, cytokeratin 8 and a strong reaction with p16 were observed. In our case, 11 types of HPV (6,11,16,18,31,33,42,51,52,56,58) were not detected either in the squamous cell carcinoma component or adenoid basal carcinoma component.
Conclusion: The invasive component of the mixed cervical carcinoma with ABC determines the staging and prognosis. So, we should keep in mind rare mixed cervical carcinoma types when we are doing pathological evaluation of the carcinoma not to overestimate the size of the tumor.

This image cannot currently be displayed.

[MRI image of cervical mass]

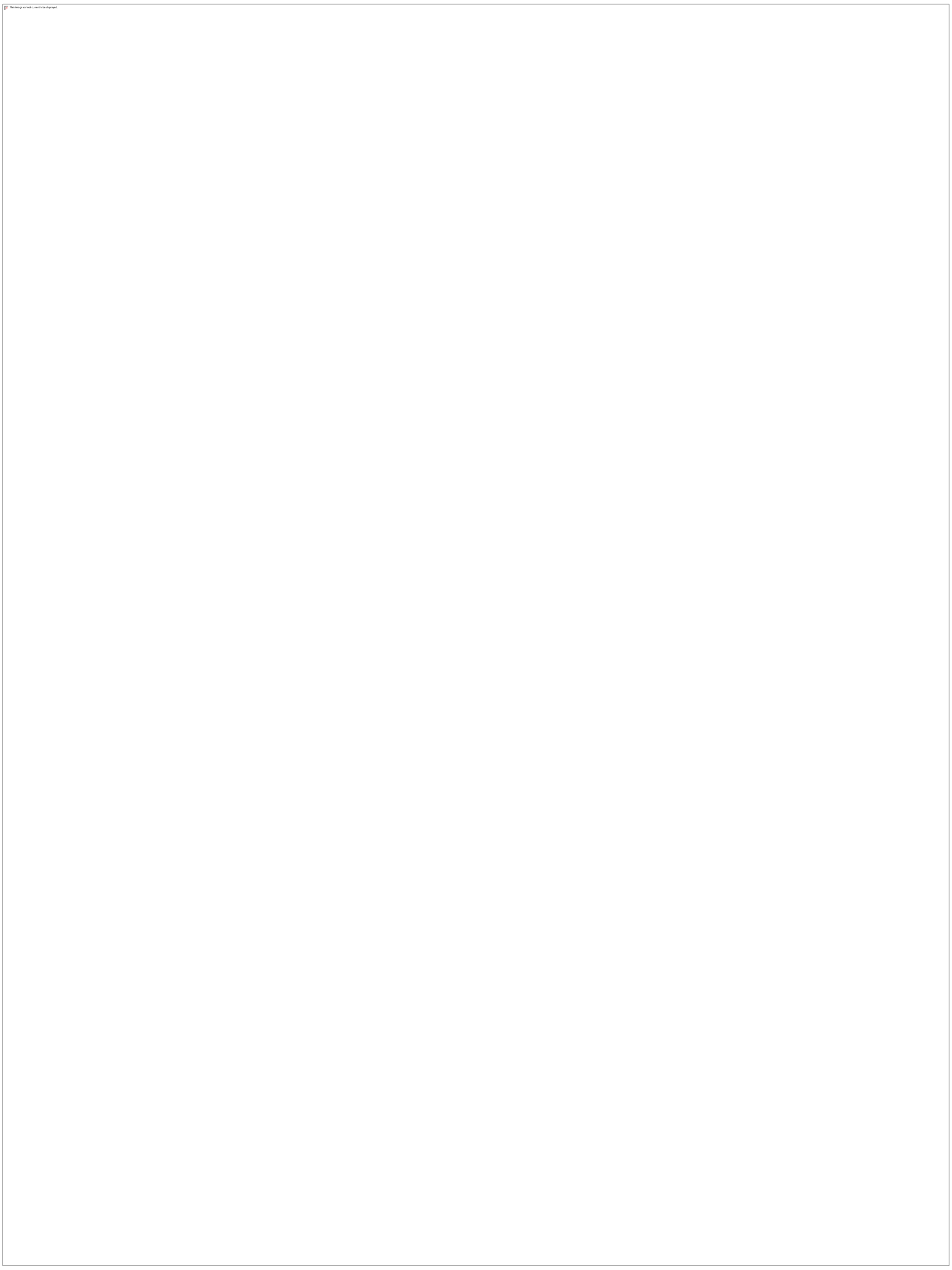

[Microscopic examination of mixed carcinoma]

Disclosure: Nothing to disclose

EP400

Feasibility of FDG-PET-MRI during chemo-radiotherapy for cervical cancer

A. Sarwar, P. Narayanan, I. Kiyani, G. Eminowicz
University College London Hospitals, London, United Kingdom

Introduction / Background: Curative treatment for locally advanced cervical cancer is chemo-radiotherapy and intrauterine brachytherapy. Early identification of patients who are not responding adequately allows treatment adaptation. This has the potential to improve overall survival, spare unnecessary side-effects and reduce healthcare costs associated with ineffective treatment. Diffusion-weighted magnetic resonance imaging (DW-MRI) and fluorodeoxyglucose-positron emission tomography (FDG-PET) may be used to enhance assessment of response during and after chemo-radiotherapy allowing prompt treatment adaptation.
Methodology: A feasibility non randomised cohort study of PET-MRI is currently recruiting cervical cancer patients having definitive chemo-radiotherapy. PET-MRI imaging is added to the standard of care at baseline and week 5 of treatment. A patient experience questionnaire is completed at baseline and week 5. Apparent diffusion coefficient (ADC) for DW-MRI and standardised uptake values (SUV) for PET-MRI were derived, comparing baseline to week 5 and within each scan to investigate the distribution of imaging parameters obtained from areas of responding, progressing and stable disease.
Results: Six patients have been recruited. Two patients did not undergo week 5 imaging. All patients found the imaging attendances acceptable. All patients had a significant reduction in SUVmax in week 5 compared to pre-treatment. All patients had an increase in ADC during treatment which was significant for patient 2 & 3. A significant correlation was seen in reduction of SUVmax and increase in ADC, r= 0.98, p value=0.0172 (95%CI 0.39-0.0.99).
Conclusion: Early data suggests additional imaging to obtain biological data during chemo-radiotherapy is feasible with no detrimental impact on patient experience. Identification of a non-responding 'biological target volume' using ADC and SUVmax parameters could allow personalisation of brachytherapy, delivering a high dose boost to residual disease. This study identifies the feasibility of PET-MRI as an imaging biomarker.
Disclosure: Nothing to disclose

EP401

Ureter delineation and dose reporting in image guided brachytherapy for cervical cancer

A. Sarwar, N. Lalli, G. Eminowicz
University College London Hospitals, London, United Kingdom

Introduction / Background: Ureteric stenosis is a severe complication of pelvic radiotherapy and brachytherapy in patients with locally advanced cervical cancer. Ureter delineation and subsequent dose reporting is not routinely performed. Therefore, the dose-response relationship is not fully understood.
Methodology: A retrospective analysis of 100 cervical cancer patients treated with image guided brachytherapy between January 2014 and March 2019 was performed. All patients received a brachytherapy dose of 21 Gray (Gy)/3 fractions (#) after concurrent chemo-radiotherapy (50.4Gy/28#). Bilateral ureters were delineated with the aid of a consultant radiologist. Maximum dose to 2cc (D2cc) and 0.1cc (D0.1cc) to right and left ureters were recorded for the first brachytherapy treatment and extrapolated for the remaining two fractions. Total ureter dose for the course of treatment was calculated. Ureteric toxicity was recorded and graded according to CTCAE v4.03.
Results: Of the 100 patients treated, 71 had FIGO stage 2B disease, the remainder ranged from stage 1A2 to 4A; 77% were squamous cell carcinomas. Median follow-up was 32 months. Mean (range) right and left ureter D0.1cc were 79.7Gy (56.8-138) and 77.8Gy (52.6-114) respectively. Mean (range) right and left ureter D2cc were 53.3Gy (52.6-60.5) and 53.4Gy (52.6-59.2) respectively. 5 (5%) patients experienced ureteric stenosis. 2(2%), 1(1%), 1(1%) and 1(1%) were grade 1 (G1), G2, G3 and G4 respectively. Further correlation analyses will be performed.

Conclusion: Image guided brachytherapy is the gold standard of treatment for locally advanced cervical cancer. There is limited data on the dose of radiotherapy the ureters are exposed to during a course of radical radiotherapy. Rates of ureteric stenosis were in keeping with published data. Delineation of ureters is possible and can be done with minimal training adding little additional time to the contouring process.
Disclosure: Nothing to disclose

EP402

Low Risk HPV types in CIN 2-3 and in invasive cervical cancer patients

E. Siegler, O. Lavie, L. Mackuli, L. Ostrovsky, N. Kugelman, Y. Segev
Carmel Medical Center, Haifa, Israel

Introduction / Background: 95-99% of patients with invasive cervical cancer are positive to human papilloma virus (HPV). The objective of the current study was to evaluate the incidence of Low Risk (LR-HPV) types among women with Cervical Intra-epithelial Neoplasia 2-3 (CIN 2-3) and cervical cancer (CC).
Methodology: We conducted a nested cohort study of patients diagnosed from May 2008 until October 2017 with CIN 2-3 or cervical cancer.
Results: During the study period we collected clinical data on 608 women of whom 402 were with CIN 2-3 and 206 with diagnosis of cervical cancer (CC). The most common presenting symptom among CIN 2-3 patients was post-coital bleeding (33.3%) compared to post-menopausal bleeding (36.2%) among CC patients. Of patients with CIN 2-3, 90.3% were found positive to at least one type of HR-HPV, compared to 89.3% of CC patients. 4.5% of patients with CIN 2-3 and 3.9% of those with CIN2-3 were positive to only one LR-HPV, or to some of a few LR-HPV types. Among 5.2% with CIN 2-3 and 6.3% of those with CC no HPV types were detected.
Conclusion: The prevalence of the LR-HPV is low but it could not be ignored due to the expected decrease in CC caused by HPV types that are included in the bi-, quadri-, and nano-valent vaccine. The CIN 2-3 and CC patients with LR-HPV types and with negative HPV are challenging the sensitivity of HPV screening that is based on HPV screening of a limited number of HR-HPV types.
Disclosure: Nothing to disclose

EP403

Protein overexpression of c-MET has a significant impact on survival in patients with uterine cervical adenocarcinoma

S. Seo, W.Y. Kim, S.-H. Shim, K.A. So, T.J. Kim, S.J. Lee
Konkuk, Seoul, Republic of Korea

Introduction / Background: The aim of this study was to compare the protein overexpression and gene copy number (GCN) of c-MET in uterine cervical cancer and to assess their prognostic roles.
Methodology: MET protein expression and GCN status were determined using immunohistochemistry (IHC) and silver in situ hybridization (SISH), respectively, in 117 cervical cancers comprising 83 squamous cell carcinomas (SCCs), 23 adenocarcinomas (ACs), 7 adenosquamous cell carcinomas (ASCCs), and 4 other types. All cases had been operated and followed up at a single institute in Seoul, Korea.
Results: MET protein overexpression was observed in 45 of 117 (38.5%) cervical cancers, with IHC 2+ in 40 and IHC 3+ in 5. The overexpression rates of SCCs, ACs, ASCCs, and other types were 31.3%, 73.9%, 14.3%, and 25.0%, respectively. MET protein overexpression with IHC 3+ was only in ACs. MET protein overexpression with IHC 3+ was correlated with worse overall survival (OS) (*P* =

.001) and progression-free survival (PFS) (*P* = .000). High polysomy (HP) of chromosome 7 and gene amplification (GA) were found in 6 (5.1%) and 0 of the 117 cervical cancers, respectively. Three of 6 cases were SCCs and the other 3 cases were ACs. However, GCN was not applicable (NA) to 16 (13.7%) of 117 cases. Cervical cancer cases with negative MET SISH tended to be associated with better prognoses without a statistical significance compared with HP and NA cases (OS, *P* = .302; PFS, *P* = .101). MET protein overexpression with IHC 3+ was significantly correlated with poor OS and PFS.
Conclusion: MET IHC may serve as a biomarker for poor prognosis in patients with cervical cancer.
Disclosure: Nothing to disclose

EP404

Laparoscopic radical trachelectomy with pelvic sentinel node dissection and cervical cerclage of a young woman with cervical cancer wishing to preserve fertility - case report

P. Sklavounos, N. Drandakis, P. Breitbach, I. Juhasz-Böss, E.-F. Solomayer
Department of Gynecology, Obstetrics and Reproductive Medicine, Saarland University Hospital, Homburg, Germany

Introduction / Background: Radical Hysterectomy with pelvic lymph node dissection is to date, the standard Treatment for early-stage cervical cancer (Ia2-Ib1).
However in some cases of women at reproductive age with early-stage disease, a less radical procedure without removing of the corpus, such as a radical trachelectomy can present an oncologic safe approach. This includes patients without risk factors (G1-2, L0, V0 , < 2 cm in diameter tumours).
Methodology: We present a 36-year-old woman gravid 0, stage IA2 cervical cancer, without risk factors, squamous, wishing to preserve fertility. Patient received intraoperatively an intracervical injection of technetium-99m colloidal albumin as well as blue dye to identify the pelvic sentinel nodes. She underwent a laparoscopic radical trachelectomy with bilateral dissection of the pelvic sentinel lymph nodes. Additionally we performed cervical cerclage laparoscopically.
Results: Operation time was 181 minutes, and the estimated blood loss was 100ml. There were no intraoperative or postoperative complications. The final pathology reported a tumour of 7mm with infiltration of 4mm, surgical margins without injury, and 4 pelvic nodes without tumour. After a 12 month follow-up, the patient is having regular periods, but has not yet tried to get pregnant. No evidence of recurrence.
Conclusion: Laparoscopic radical trachelectomy and bilateral pelvic sentinel node dissection is a safe alternative in young patients who wish to preserve fertility with early stage cervical cancer without risk factors and tumor diameter smaller than 2 cm.
Additionally a cervical cerclage should be offered at the same procedure in order to minimise the risks for obstetric complications later.
Disclosure: Nothing to disclose

EP405

Neuroendocrine carcinoma of the cervix (NECC)- case reports

M. Śliwińska, K. Drosik-Rutowicz, M. Cieślak-Steć, K. Raczek-Zwierzycka, M. Gawkowska, R. Tarnawski
III Department of Radiotherapy and Chemotherapy, M. Skłodowska-Curie Memorial Cancer Centre and Institute od Oncology, Branch in Gliwice, Gliwice, Poland

Introduction / Background: Neuroendocrine carcinoma of the cervix (NECC) is a rare variant of cervical cancer. The prognosis of women with NECC is poor and there is no standardized therapy for

this type of malignancy.
Methodology: Three patients with NECC were treated in our department.
Results: The first woman aged 31 was diagnosed with Large cell neuroendocrine carcinoma, Ki67 99%. MR scan showed a tumor of cervix and metastases in pelvic lymph nodes. She received chemotherapy Etoposide and Cisplatin (EP) with complete remission of disease. Subsequently, she underwent surgery and pelvic irradiation. 18 months following the completion of treatment there is no evidence of metastatic or recurrent disease. The second woman aged 47 underwent surgical conization of the cervix, and microscopic examination revealed Carcinoma planoepitheliale praecipue akeratodes, partim keratode, partim neuroendocrinale G3, Ki 67 80%. MRI of pelvis revealed infiltration of cervix. She received EP chemotherapy with complete remission. Afterwards, pelvic irradiation was performed. 6 months following the treatment there is no evidence of metastatic or recurrent disease. The third woman aged 66 was diagnosed with metastatic Small cell neuroendocrine cancer, Ki 67 90%. She received chemotherapy EP. There was partial regression in the assessment of the treatment effect. PET-CT scan revealed a single active localization of cervical cancer. Radical pelvic irradiation was performed. 3,5 months following the completion of treatment she was diagnosed with progressive disease. She received chemotherapy EP and Topotecan. She eventually was qualified to Adriamycin and Cyclophosphamide chemotherapy. So far she received 4 courses and a CT scan showed stable disease.
Conclusion: NECC is a rare variant of cervical cancer with a poor prognosis. Multimodality treatment with radical surgery and chemotherapy with cisplatin and etoposide and radiotherapy is the mainstay of treatment for early stage disease, while chemotherapy with different chemotherapeutics is used for locally advanced or recurrent NECC.
Disclosure: Nothing to disclose

EP406

A year after: lessons learned from Laparoscopic Approach to Cervical Cancer (LACC) trial

M. Śniadecki[1], M. Orłowska-Volk[2], S. Hać[1], M. Liro[1], D. Wydra[1]
[1]Medical University of Gdansk, Gdańsk, Poland, [2]MVZ Clotten, Freiburg im Breisgau, Germany

Introduction / Background: The laparoscopy has been found to be attractive and safe technique in gynaecological oncology. However, some recent retrospective and prospective reports on early-stage cervical cancer have shown unexpected contrary results in terms of overall and disease-free survival. In our opinion, published discussions to date do not fully address the subject and that it is worth considering important issues that were not given enough attention before.
Methodology: We conducted a systematic analysis of current knowledge about laparoscopy in early-stage cervical cancer by reviewing the relevant literature from the fields of basic and clinical research, starting with the results of the Laparoscopic Approach to Cervical Cancer (LACC) study, and including analysis of its appendix and protocol.
Results: The preferred surgical access in cervical cancer cases was the subject of a single prospective randomized clinical trial. Other, similarly designed studies on other pelvic tumours are sparse. The biology of cancer, cancer epidemiology, as well as surgical skills and details of laparoscopic techniques underlie the observed differences in survival rates.
Conclusion: There are some possibilities (such as limiting the manipulation of the uterus, closure of tubes and ligation of the parametria before using the manipulator, closure of the vaginal cuff, applying warmed and humidified CO_2, and limiting intra-abdominal gas pressure) that, if considered and applied, may affect oncological results in selecting laparoscopy as the operative method for early-stage cervical cancer.
Disclosure: Nothing to disclose

EP407

Learning curve could affect the surgical outcome of radical hysterectomy in cervical cancer

S. Kim[1], S. Lee[1], J.Y. Song[1], J.W. Shin[2], J.K. Lee[1], N.W. Lee[1]
[1]Obstetrics and Gynecology, Korea University College of Medicine, Seoul, [2]Gachon University Gil Medical Center, Gachon University College of Medicine, Incheon, Republic of Korea

Introduction / Background: Minimally invasive surgery has become essential technology in field of gynecologic malignancies including cervical cancer. We reviewed our experience and evaluated the results of radical hysterectomy in patients with early stage cervical cancer.
Methodology: This retrospective study included patients with early stage cervical cancer (Ia1 ~ IIa2) who were treated with radical hysterectomy from May 2006 to April 2018. Patients were divided into three groups according to the approach of surgery: open laparotomy (ARH), laparoscopic radical hysterectomy (LRH), and robotic-assisted radical hysterectomy (RRH). The type of hysterectomy included Piver type II and III hysterectomy. Learning curves of each type of surgery were obtained using cumulative sum (CUSUM) method. The survivals were compared using Kaplan-Meier curves.
Results: Learning curves of each groups showed two distinct phases. The minimal cases required to achieve surgical improvement were 16 in ARH, 14 in LRH, and 15 in RRH. Progression-free survival and overall survival were not different between 3 groups (p=0.556 and p=0.273, respectively). But when the groups were stratified by the phases of the learning curves, the patients included in early phase showed poor PFS in RRH (p=0.043).
Conclusion: The learning curve could significantly affect the oncologic outcome in robotic-assisted radical hysterectomy. Enough experience is necessary to improve surgical outcome in RRH. Further, a prospective randomized study regarding sufficient surgical competence is necessary for elaborate analysis of feasibility of minimally invasive radical hysterectomy.
Disclosure: Nothing to disclose

EP408

Usefulness of evaluating lymphatic and venous vessel invasion separately in postoperative cervical adenocarcinoma

Y. Taira, Y. Shimoji, T. Nakasone, Y. Arakaki, T. Nakamoto, T. Ooyama, W. Kudaka, Y. Aoki
Obstetrics and Gynecology, University of the Ryukyus, Nishihara, Japan

Introduction / Background: Surgical treatment of cervical adenocarcinoma is expected to improve prognosis since the cancer is less responsive to radio- and chemotherapy than cervical squamous cell carcinoma. Attempts are made to predict recurrence patterns and prognosis using surgical specimens, including evaluation of vascular invasion. In other types of cancer, such as gastric, colon and breast cancer, it is known that evaluating lymphatic and venous vessel invasion separately helps predict prognosis and recurrence sites, such as lymph node metastasis and distant metastasis. In this study, we aimed to determine the significance of evaluating lymphatic and venous vessel invasion separately in postoperative cervical adenocarcinoma.
Methodology: We retrospectively examined the medical records of 108 patients who were diagnosed with cervical adenocarcinoma or adenosquamous carcinoma and underwent abdominal radical hysterectomy at University of the Ryukyus Hospital from January 1993 to April 2017. The patients were separately evaluated for lymphatic and venous vessel invasion using immunohistochemical staining with D2-40 and haematoxylin and eosin Victoria blue double staining.
Results: Lymphatic and/or venous invasions were observed in 42 (38.9%) patients, including 66 ly(−)/v(−) (61.1%), 7 ly(−)/v(+) (6.5%), 24 ly(+)/v(−) (22.2%) and 11 ly(+)/v(+) (10.2%) patients. In multivariate analysis of risk factors for distant recurrence, stage (IB2 or higher) (odds ratio = 6.21, p = 0.020) and venous invasion (odds ratio = 9.29, p = 0.0047) were considered significant. In multivariate analysis of predictive factors for poor prognosis, stage (IB2 or higher) (hazard ratio = 5.33, p = 0.0315) and pelvic lymph node metastasis (hazard ratio = 7.81, p = 0.0142) were considered significant.
Conclusion: In radical hysterectomy for cervical adenocarcinoma, venous vessel invasion was found

to be a risk factor for distant organ recurrence, but separately evaluating lymphatic and venous vessel invasion is not useful for predicting prognosis.
Disclosure: Nothing to disclose

EP409

Colposcopy findings and biopsy results in HPV tested ASCUS patients

O. Takmaz[1], H.M. Soylu[2], S. Ozaltin[3], Y. Kocyigit[3], M. Celebi[3], S.D. Yozgatli[4], D. Gurleyik[3], A. Catikkas[3], E. Bastu[3], M. Gungor[3]
[1]Department of Obstetrics and Gynecology, Acibadem Mehmet Ali Aydinlar University, [2]Istanbul University, Istanbul Faculty of Medicine, [3]Acibadem Mehmet Ali Aydinlar University, [4]Istanbul University-Cerrahpasa, Cerrahpasa School of Medicine, Istanbul, Turkey

Introduction / Background: To compare the association of high-risk Human Papilloma Virus (HR-HPV) positivity with abnormal colposcopy findings in women with Atypical Squamous Sells of Undetermined Significance (ASCUS) cervical smear result.
Methodology: In this retrospective study, we analyzed the colposcopy findings and the pathology results of the women who had a smear result of ASCUS and HPV test between January 2011 and January 2019 in Acibadem MAA University.
Pap smear was performed using liquid-based cervical cytology (ThinPrep (UHA;Hologic Corp.). HPV tests results were reported as negative or HPV 16 and/or HPV 18 positive or non-16/non-18 positive (positivity of HPV Type 31, 33, 35, 39, 45, 51, 52, 56, 58, 59, 66 and 68).
Colposcopic examination results were grouped into 2 categories: normal and abnormal findings (acetowhite epithelium, mosaic epithelium, leucoplasia and presence of atypical vessels). Biopsies were taken from the abnormal finding areas, histologic evaluations were defined as normal, cervical intraepithelial neoplasia (CIN) 1, CIN2 and CIN3.
The SPSS software version 23.0 (SPSS, Armonk, NY, USA) was used for analyzing data. Pearson Chi-Square tests for contingency tables were used to assess the associations between categorical variables. Statistical significance was set at 0.05 for p value.
Results: Among the 61 patients, HPV test results were 13(21%), 23(38%), 25 (41%) for HPV negative, HPV 16/18 positive and non-16/18 positive, respectively. Colposcopic biopsy results were; 28(46%), 21(35%), 9 (15%) and 3(5%) for normal, CIN1, CIN2 and CIN3, respectively. No significant association was observed between the patterns of abnormal colposcopic findings with 16/18 or non-16/18 HPV positive test result (p>.05). The finding of mosaic pattern during the colposcopy was significantly associated with CIN when compared with asetowhite finding (p< .05). and also, HPV positivity was significantly associated with CIN1-2-3 (p< .05). TABLE 1

	Acetowhite (%)	Mosaic(%)	Normal
CIN1(n=21)	15(53)	4(29)	2(10)
CIN2(n=9)	1(4)	8(14)	0
CIN3(n=3)	1(4)	2(57)	0
Normal Biopsy (n=28)	1(39)	0	17(90)
HPV Negative	4(14)	2(14)	7(37)
HPV 16/18 positive	11(39)	8(57)	4(21)
HPV Non-16/18 positive	13(47)	4(29)	8(42)

[Table-1 Association of abnormal colposcopic findings with biopsy and HPV test results]

Conclusion: Abnormal colposcopic findings does not differ between the high risk HPV subtypes infection.
Disclosure: Nothing to disclose

EP410

Reduced response to controlled ovarian stimulation after radical trachelectomy

S. Tamauchi, H. Kajiyama, S. Osuka, Y. Moriyama, M. Yoshihara, S. Iyoshi, K. Yoshida, Y. Ikeda, N. Yoshikawa, K. Nishino, K. Niimi, S. Suzuki, F. Kikkawa
Obstetrics and Gynecology, Nagoya University Graduate School of Medicine, Nagoya, Japan

Introduction / Background: *In vitro* fertilization is often needed after radical trachelectomy because of difficult spontaneous pregnancy or cervical factor infertility, such as cervical stenosis. At the time of *in vitro* fertilization, patients who have undergone radical trachelectomy usually experience resistance to controlled ovarian stimulation. We aimed to clarify the decrease in response to controlled ovarian stimulation after radical trachelectomy in patients who received *in vitro* fertilization treatment at our hospital.
Methodology: The outcomes of ovarian stimulation were retrospectively evaluated and compared between patients who have undergone radical trachelectomy and control patients who had male factor infertility or unexplained infertility. Patients with ovarian factor infertility, such as those with a history of ovarian endometriotic cyst or ovarian surgery, were excluded. The data obtained were age at ovarian stimulation, body mass index, total dose of gonadotropin, duration of stimulation, peak estradiol concentration, number of retrieved oocytes, and number of fertile ova.
Results: A total of 22 ovarian stimulation cycles in 11 radical trachelectomy patients and 51 cycles in 28 control patients were reviewed. The median age at ovarian stimulation was 35.1 years (range 29.0-41.2 years) in the radical trachelectomy group and 38.2 years (range 22.9-44.8 years) in the control group ($p = 0.034$). Compared with the control group, the radical trachelectomy group had significantly lower mean peak estradiol concentration [1,418 pg/mL (SD 727) vs. 1,845 pg/mL (SD 787), $p = 0.03$] and smaller median number of mature oocytes [5 (range 1-14) vs. 8 (range 1-19), $p = 0.014$], despite the higher use of gonadotropin [3,456 IU (SD 1,406) vs. 2,654 IU (SD 908), $p = 0.005$].
Conclusion: The response to controlled ovarian stimulation decreased after radical trachelectomy. For better reproductive outcome, cryopreservation of eggs before radical trachelectomy should be considered.
Disclosure: Nothing to disclose

EP411

The evaluation of recurrence in vagina after surgery in gynecological cancer after surgery and compare surgery alone to surgery and radiotherapy

E. Telka[1], M. Cieślak-Steć[2], K. Szczepanik[1], P. Chimiczewski[1]
[1]*Department of Radiotherapy, Gliwice Cancer Center Institute, Gliwice,* [2]*The Clinical Center of Woman and Child's Health, Zabrze, Poland*

Introduction / Background: The aim of the study is to evaluate the results of recurrence treatment after the operation of gynecological cancer in patients not previously irradiated and to compare the recurrent operation with IMRT radiotherapy.
Methodology: The subject of the analysis was 121 patients treated surgically for endometrial, cervical, and vulva cancer according to FIGO I-III. The surgical treatment consisted in radical surgery or electro-resection of the vulva with inguinal lymphadenectomy and complementary radiotherapy IMRT 50,4Gy in case of recurrence in the pelvis after surgery. Late effects were assessed according to the EORTC/RTOG scale. The comparison of both treatment techniques was made on the basis of $\chi 2$ and Mann-Whitney analyses.
Results: Local recurrence after surgical treatment in patients not initially irradiated after surgery was

found in 21 patients, which resulted from technical difficulties in performing an effective reoperation with obtaining adequate oncological margins.No patient was diagnosed with local recurrence after surgery and IMRT radiotherapy The surgical technique had an impact on the incidence of postoperative complications.
Conclusion: Radiotherapy is a better therapeutic option in the prevention of local recurrences in pelvis compare to surgery alone.
Radiotherapy treatment with intense modulation of the IMRT radiation beam allows reduce of late 3 and 4-degree radiation reaction.
Disclosure: Nothing to disclose

EP412

Fertility sparing surgery of >2cm cervical cancer following neo-adjuvant chemotherapy: a 12-year experience in a single tertiary care center

F. Tesfai, J. Kroep, R. Nout, K. Gaarenstroom, C. de Kroon, R. van Loenhout, V. Smit, J. Trimbos, M. van Poelgeest, J. Beltman
Leiden University Medical Center, Leiden, The Netherlands

Introduction / Background: To assess feasibility, safety and clinical outcomes (recurrence, obstetric outcomes) in patients with cervical tumors >2 cm treated with neoadjuvant chemotherapy in preparation for fertility sparing surgery (FSS). In addition, to investigate predictive variables in these patients.
Methodology: A retrospective analysis of patients was conducted in patients who were selected to receive upfront chemotherapy before fertility sparing surgery. Surgical and clinical outcomes were examined in relation to radiological and pathological results. In addition, obstetrical outcomes were investigated. We compared our results with other case series in literature.
Results: A total of 20 women were treated with neo-adjuvant chemotherapy for >2 cm cervical tumors at our institution. Mean tumor size was 4,4 cm. Chemotherapy consisted of 6 weekly cycles of cisplatin and paclitaxel. In 15 of 20 cases (75%) fertility was successfully preserved. In 5 of 20 cases FSS failed: 2 of 20 patients had stable disease after 3 cycles and did not meet criteria for surgery and 3 patients had intra- or postoperative indication for adjuvant therapy. Four relapses occurred in total of whom 3 died. Two of these were in the group of the successful ART. Therefore, fertility is currently preserved in 13 patients. Eight spontaneous pregnancies in 3 women occurred with six babies born at term.
Conclusion: Neo-adjuvant chemotherapy followed by FFS is feasible and save option in in selected patients with cervical tumors >2 cm. Unfavorable prognostic factors are defined which can help in patients selection for FSS.
Disclosure: Nothing to disclose

EP413

Definitive radiotherapy with low cumulative doses for uterine cervical cancer: a Japanese prospective study with no midline block

T. Toita[1,2], T. Kusada[2], T. Ariga[2], W. Kudaka[3], H. Maemoto[2], W. Makino[2], K. Ishikawa[2], J. Heianna[2], Y. Nagai[4], Y. Aoki[3], S. Murayama[2]
[1]Radiation Therapy Center, Okinawa Chubu Hospital, [2]Department of Radiology, [3]Department of Obstetrics and Gynecology, University of the Ryukyus, [4]Department of Obstetrics and Gynecology, Okinawa Nanbu Medical Center & Children's Medical Center, Okinawa, Japan

Introduction / Background: A midline block (MB) used in whole pelvic RT (WPRT) hinders accurate

interpretation of the treatment outcome due to uncertainty of the RT dose delivered to cervical tumours.
Methodology: Patients with cervical cancer of FIGO stages IB1-IVA were eligible for this prospective study. The treatment protocol consisted of WPRT (non-IMRT) without a MB (45 Gy/25 fractions) and CT-based high-dose-rate intracavitary brachytherapy (HDR-ICBT; 15 Gy/3 fractions or 20 Gy/4 fractions, prescribed to point A). The total EQD2 to point A was 63 or 69.3 Gy. The HR-CTV was contoured at the time of the present analyses. If optimisation (manual adjustment of the dwell times) was inadequate to meet the organ-at-risk dose constraints, the point A dose was decreased. Graphical optimisation was not performed.
Results: A total of 40 patients were enrolled. The FIGO stage distribution was IB1, 11; IB2, 13; IIA2, 1: IIB, 11; IIIB, 3; IVA, 1. The median pretreatment tumour diameter was 47 mm (14-81 mm). No patients underwent hybrid ICBT. The point A dose was decreased in 19 of 153 ICBT sessions (12%). Twenty-nine patients received concurrent chemoradiotherapy. After a median follow-up of 33 months, the 2-year pelvic progression-free survival (PPFS), and overall survival rates were 83%, and 85%, respectively. A HR-CTV D90 threshold of 70 Gy was correlated with the PPFS: 93% for patients receiving ≥ 70 Gy (n=29) versus 64% for those receiving < 70 Gy (n=11) (P=0.018). Late adverse events (grade ≥ 3) were observed in three patients (bladder in two, rectum in one).
Conclusion: Definitive RT consisting of WPRT without a MB and HDR-ICBT was feasible in our cohort of Japanese patients. A low RT dose might be adequate for patients with non-bulky tumours. Proper 3D-IGBT using graphical optimisation and a suitable hybrid technique, based on appropriate HR-CTV contouring, is recommended to improve outcomes further.
Disclosure: Nothing to disclose

EP414

Completion hysterectomy after radiotherapy in advanced stages of cervical cancers

A. Touimi Benjelloun, Z. Edderdabi, M. Moutahir, M. Benhessou, M. Ennachit, M. Elkarroumi
Centre Mohamed IV de Chirurgie Oncogynecologique, Hassan II University / Faculté de Medecine et de Pharmacie, Casablanca, Morocco

Introduction / Background: The standard treatment of locally advanced cervical cancer is the combination of a concurrent radiochemotherapy (RCC) +/- brachytherapy. Completion hysterectomy remains a therapeutic option, but its place is debated. This surgery is still contraversial because of its morbidity and nonproven benefits. The aime of this study was to evaluate the morbidity, the per and post operative complications and their risk factors, also to determine the post therapeutic prognosis and its factors in disease free survival and overall survival at 2 years.
Methodology: An observational retrospective monocentric cohort concerning 82 patients cared for cervical cancer stage IB2-IV between 2016-2017 was conducted. All patients included in this study were treated with RCC +/- brachytherapy followed by a completion surgery. All surgery complications and morbidity rate were registred. All risk and prognosis factors were studied. We analyzied the post operative prognosis .
Results: In this study, we had patients with epidermoide carcinoma in 93.90% and adenocaricnoma in 4.88%, with advanced stages, IIB (73.17%), IIIB (9.75%), IIA (12.19%), IB2 (1.22%) and IVA (1.22%). All patients recieved radiotherapy with 93.90% of patientes recieving between 41 and 50 Gy. 33 patients (40.24%) recieved a brachytherapy. 74 patientes (90.24%) recieved a chemotherapy (concomitant for 71 patients.
The radicality of the surgery and favoring the laparotomy weren't statistically associated with a higher rate of complications. 70.73% of patients presented a complete response to the treatment. The impact of completion surgery on the quality of life and the sexual and couple's life after the treatment wasn't verified due to a lack of information.
Conclusion: Our study found a miner and acceptable rate of per and postoperative complications with no gravity. Finally, this study has found important benefits of completion surgery on complete response and survival rate with acceptable rate of complications.
Disclosure: Nothing to disclose

EP415

Lymphoepithelial cervical carcinoma

A. Touimi Benjelloun, Z. Edderdabi, Y. Benchrifi, M. Moutahir, M. Benhessou, M. Ennachit, M. Elkarroumi

Centre Mohamed IV de Chirurgie Oncogynecologique, Hassan II University / Faculté de Medecine et de Pharmacie, Casablanca, Morocco

Introduction / Background: Lymphoepithelial like carcinoma occurring in the reproductive organs is a rare variant of squamous cell carcinoma, and this tumor of the uterine cervix accounts for 0, 7% of all primary cervical uterine neoplasms.
Although associations with EBV and HPV have been demonstrated in some studies, the fundamental pathogenesis of LELC is yet to be understood.
Methodology: Here we report the case of a 46 years old woman, gravidity 2 and parity 2. She presented with metrorrhagia of mild abundance induced by sexual intercourses, accompanied by leucorrhea.
Results: The clinical examination as long as the cervical smear a d the MRI was all in favor of a cervical cancer, and neither distal metastasis swelling of lymph was revealed.
The histological study of fragments of biopsy showed an aspect similar to that of LELC. This cervical cancer was stage FIGO IBl and a radical hysterectomy performed for this malignant tumor. The pathological study of the surgical specimen confirmed the diagnosis of LELC.
The patient has been followed up for 12 months since her surgery, and no evidence of recurrence has been detected.
Conclusion: Lymphoepithelial like carcinoma occurring in the reproductive organs is a rare variant of squamous cell carcinoma, and this tumor of the uterine cervix accounts for 0, 7% of all primary cervical uterine neoplasms.The prognosis of LELC is thought to be better than those of other cervical cancer types, but careful follow-up at fixed intervals is recommended.
Disclosure: Nothing to disclose

EP416

Large conization: an effective surgical approach for fertility preservation in young women with early stage cervical cancer

C. Tsaousidis[1,2], F.-A. Taran[1], S. Kommoss[1], A. Hartkopf[1], S.Y. Brucker[1], B. Kraemer[1]

[1]Department of Women's Health, University Hospital of Tuebingen, Tuebingen, Germany, [2]Women's Center Bern, Lindenhofgruppe, Bern, Switzerland

Introduction / Background: The shorter cervical segment after radical trachelectomy (RT) imposes a number of pregnancy-associated risk factors. Large conization (LC) can therefore be an oncologically safe alternative to RT in young women with early stage cervical cancer who want to keep their fertility. Our aim was to evaluate fertility-sparing surgical treatment of early-stage cervical cancer after the introduction of LC. Our objectives were to assess surgical, oncological, fertility, and obstetric outcomes.
Methodology: We followed up the oncological and fertility outcomes of patients who underwent LC in a large oncological single University center between 2009 and 2014. Medical records were reviewed and analyzed for surgical, oncological, fertility, and obstetric outcomes. Postal questionnaires were collected to further evaluate and validate the fertility and obstetric outcomes.
Results: A total of 24 LC's were analyzed. Eight patients had to undergo secondary radical hysterectomy after the LC because of non in sano resection. Nine of 16 women tried to conceive, of which all nine became pregnant. Seven mothers gave birth to nine children; The majority of women conceived spontaneously.
Conclusion: Early-stage cervical cancers treated by LC are associated with acceptable oncological

outcomes. LC appears to be a safe option for eligible women who intend to maintain their wish for a future pregnancy.
Disclosure: Nothing to disclose

EP417

Trends in the incidence of carcinoma in situ and cervical cancer in Ukraine

N. Tsip[1], Y. Mykhaylovich[1], Z. Fedorenko[1], O. Sumkina[1], L. Kuzenko[1], M.A. Khalfalla[2]
[1]National Cancer Institute, [2]Shupyk National Medical Academy of Postgraduate Education, Kyiv, Ukraine

Introduction / Background: To study the dynamics of the incidence of carcinoma in situ of the cervix (code MKH-10 D06), compared with cervical cancer (C53) in Ukraine during the period from 2006 to 2016.
Methodology: The methods of statistical analysis used in descriptive oncoepidemiology were applied. A cohort of patients with cervical cancer included 56579 cases; a cohort of patients with carcinoma in situ - 12387 cases.
Calculation of incidence rates was carried out on the basis of 100 thousand of the population, as well as standardized according to the world standard updated incidence rates of the population for 2006-2016.
The data processing was carried out using the programs of the information system of the National Cancer Registry of Ukraine.
Results: The incidence rate of CC remains virtually unchanged over the past 10 years (about 20 cases per 100,000 female population). However, there was a redistribution of the incidence of CC depicting its increase in women of reproductive age and reduction in women after 50 years (Fig.1). Meanwhile, incidence of cancer in situ has a steady tendency to increase (from 3.5 0/0000 in 2006 to 5.8 0/0000 in 2016).
Despite the increased number of cases of carcinoma in situ, the C53 / D06 ratio remains unsatisfactory: it varies from 0.04 to 0.74 and averages 0.23 in Ukraine.
Conclusion: The problem of prompt diagnosis and treatment of carcinoma in situ and cervical cancer acquires not only medical and biological but also socioeconomic value, since the incidence of this pathology reaches peak values in women of reproductive age, which directly affects the labor and demographic potential of the population in Ukraine.
The way to solve this problem is to strengthen the material and technical base and implement the State Program "Cervical Cancer Screening"

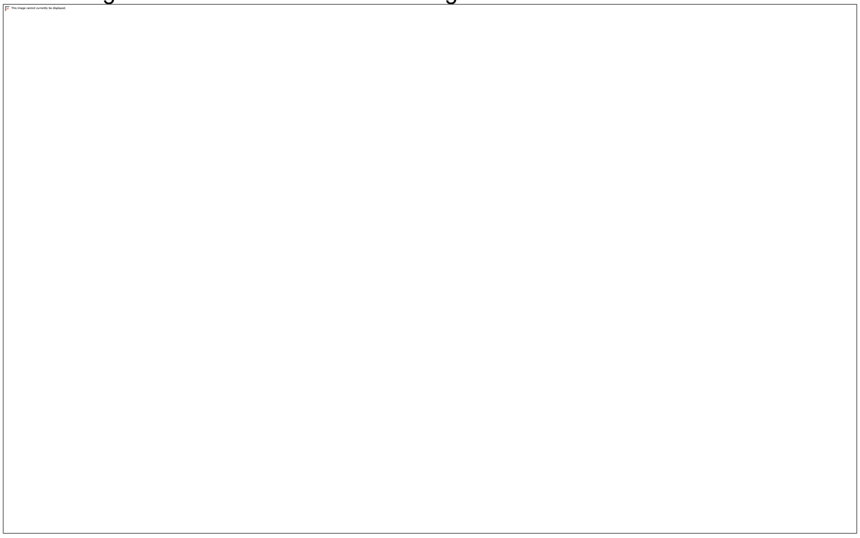

[Age-related rates of the incidence of cervical cancer and carcinoma in situ in Ukraine]

Disclosure: Nothing to disclose

EP418

Immune checkpoint inhibitors in treating stage IV/recurrent cervical cancer and endometrial cancer

H.-J. Tung[1], H.-H. Chu[2], Q.-R. Cheng[1], C.-C. Wang[3], C.-H. Lai[4]
[1]Department of Obstetrics and Gynecology, Chang Gung Memorial Hospital Linkou Branch, [2]Chang Gung University College of Medicine, [3]Department of Radiation Oncology, [4]Department of Obstetrics and Gynecology, Chang Gung Memorial Hospital Linkou Branch and Chang Gung University College of Medicine, Taoyuan, Taiwan

Introduction / Background: Advanced/recurrent gynecologic cancer is a clinical challenge. The ideal treatment was controversial and how to manage recurrent condition was also an unsolved problem. We present four advanced cervical/endometrial cancer cases who were heavily treated before and they achieved complete remission after curative intent including immune checkpoint inhibitors (ICIs).
Methodology: We retrospectively reviewed clinical records of these four such patients.
Results: Case 1.
A 51-year-old nulliparous woman was diagnosed with cervical adenocarcinoma, International Federation of Gynecology and Obstetrics (FIGO) stage IVB, treated by concurrent chemoradiation and maintenance bevacizumab. Bone metastasis treated with radiotherapy focus on metastasis sacrum lesion and pembrolizumab combined with 5-FU and etoposide chemotherapy sequentially. She remains disease free with overall survival (OS) 35.3+ months.
Case 2.
A 34-year-old woman had cervical adenocarcinoma, FIGO stage IVA, treated by chemoirradiation. Central pelvic recurrence at 8 years was treated by total pelvic exenteration with intra-operative radiotherapy (IORT) for involved pelvic side wall margin, and salvage chemotherapy. Re-recurrence at para-aortic lymph node (PALN) found later. She achieved remission with pembrolizumab and radiotherapy.
Case 3.
A 42-year-old woman had cervical squamous cell carcinoma, FIGO IIIC and recurrence once that she received operation and chemoirradiation in China. Second relapse at left para-spinal and paraureter soft tissue noted. Pembrolizumab, cisplatin and topotecan with radiotherapy was applied. She is currently disease free (OS 134+ months).
Case 4.
A 39-year-old woman had endometrial carcinoma, FIGO stage IIIC1. She received primary operation and chemo-radiotherapy. First recurrence at left pelvic lymph node was treated by operation, intra-operative brachytherapy and chemotherapy. 2nd relapse at left PALN was salvaged by proton therapy to PALN, chemotherapy, and pembrolizumab. No disease detected till now (OS 77+ months).
Conclusion: Incorporating ICIs to proton therapy and chemotherapy provide long disease-free survival to advanced/recurrent gynecologic cancer failed traditional chemoirradiation.
Disclosure: Nothing to disclose

EP419

Efficiency of sentinel lymph node detection with indocyanine green in patients with early stage of cervical cancer. Single-institutional experience, n=80

H. Mkrtchyan[1], E. Ulrikh[1,2], N. Mikaya[1], A. Sidoruk[1,2], A. Urmancheeva[1,2], I. Berlev[1,2]
[1]National Medical Research Center of Oncology named after N.N. Petrov, [2]North-Western State

Medical University named after I.I. Mechnikov, Saint Petersburg, Russian Federation

Introduction / Background: Staging lymphadenectomy in cervical cancer (CC) patients is an enforced feasibility, that could be accompanied with long lasting complications impairing the quality of life of the patients. Sentinel lymph node (SLN) mapping with Indocyanine Green (ICG) in patients with early stage of cervical cancer could help to avoid these problems. The safety and efficacy of this procedure was investigated in 80 CC patients treated in National Medical Research Center of Oncology named after N. N. Petrov through the 5-year period.
Methodology: From January 2015 to April 2019 the Data of 80 patients with CC IA1 (with LVSI), IB1, IIA1 stages undergoing minimally invasive surgery and SLN mapping with ICG were analysed in prospective single-center study (National Medical Research Center of Oncology named after N.N.Petrov).
Results: Overall detection rate of SLNs registered in 78/80 patients (97.5%), bilateral - 64/78 (82.0%). SLNs were not detected only in 2/80 patients (2.5%). Lymph node metastasis were diagnosed in 11/80 patients (13.75%), of which 10 were in SLNs (87.5%).
Only in one case metastasis was found in the pelvic lymph node, which was not SLN (1.28%) with IB1 stage CC with clear cell morphological type of the tumor.
The most frequent localisation of SLN was the internal lymphatic group near the bifurcation of common iliac artery - 99/176 (56.2%).
Diagnostic efficiency of the SLN biopsy: sensitivity - 90.90%, specificity -87.34%, accuracy - 86.25%, false-negative results - 1.25%.
Conclusion: SLN mapping with ICG in patients with CC IA1 (LVI) - IB1, IIA1 stages is an effective and safe diagnostic method that allows with high detection rate visualise SLNs, make biopsy, correctly stage the disease, change the surgical plan and individualise treatment for each patient.
Disclosure: Nothing to disclose

EP420

The clinical outcomes of neoadjuvant dose-dense platinum-based chemotherapy in patients with locally-advanced cervical cancer (IB2-IIB FIGO stage)

O. Smirnova, E. Ulrikh, N. Bondarev, A. Petrova, H. Mkrtchyan, O. Lavrinovich, Z. Ibragimov, Y. Trifanov, S. Samus, H. Kotiv, A. Sidoruk, N. Mikaya, A. Urmancheeva, I. Berlev
National Medical Research Center of Oncology named after N.N.Petrov, Saint-Petersburg, Russian Federation

Introduction / Background: Cervical cancer is the most common reproductive cancer in women of childbearing age. The radiation therapy and surgical treatment are currently considered a cornerstone of locally-advanced cervical cancer treatment. For several decades a number of different treatment options aimed to improve the survival of cervical cancer patients. One of these methods studied is the use of neoadjuvant chemotherapy. This study aims to evaluate the clinical outcomes and toxicity of platinum-containing dose-intensive neoadjuvant chemotherapy regimens in patients with locally-advanced FIGO IB2 - IIB cervical cancer.
Methodology: A cohort of 105 consecutive patients with median age of 44 (range 27-68) years was studied. All patients had verified locally-advanced (cT1B2Nx,0M0 - cT2BNxM0) cervical cancer and received 3 dose-intensive intravenous neoadjuvant AP (cisplatin 75 mg/m^2, doxorubicin 35 mg/m^2; n=75) or TP (cisplatin 60 mg/m^2 and paclitaxel 60 mg/m^2; n=30) chemotherapy cycles. The effectiveness of neoadjuvant treatment was determined using magnetic resonance imaging of the pelvic organs (according to the criteria RECIST 1.1.), the operability rate, the pathological response. The toxicity was evaluated according to CTCAE 4.03 criteria.
Results: The objective response rate was 84% (63/75 cases) for AP and 56.7% (17/30 cases) for TP group, accordingly. Also in 66 (88%) patients of AP and 24 (80%) of TP group the surgical intervention was performed. The pathomorphological response rate 89,4% (59/66 cases) for AP and 79,2% (19/24 cases) for TP group. The complete morphological tumor regression (ypCR) was confirmed in 7(10,6%) cases for AP, and 4 (16,7%) cases for TP group. Both dose-intensive regimens were characterized by moderate toxicity, no severe adverse effects during chemotherapy or surgical treatment stages were observed.

Conclusion: The dose-intensive chemotherapy is an effective treatment modality for locally-advanced cervical cancer and may be a feasible alternative for standard treatment approach. It deserves further study in larger patient cohort with evaluation of the long-term results.
Disclosure: Nothing to disclose

EP421

The experience of the N.N. Petrov National Research Center of Oncology in performing radical trachelectomy

A. Sidoruk[1,2], I. Berlev[1,2], A. Urmancheeva[1,3], N. Mikaya[1], N. Bondarev[1,4], E. Ulrikh[1,3], E. Nekrasova[1], T. Gorodnova[1], T. Rogovskaya[1], K. Kotiv[1]
[1]Oncogynecological Department, N.N. Petrov National Research Center of Oncology, [2]Department of Obstetrics and Gynecology, [3]Department of Oncology, North-Western State Medical University named after I.I.Mechnikov, [4]Department of Oncology, Pavlov First State Medical University of St. Petersburg, Saint-Petersburg, Russian Federation

Introduction / Background: One of the most important tasks of modern oncogynecology is the implementation of organ-sparing treatment of young patients and the preservation of reproductive function.
Methodology: From 2008 to 2019 in the oncogynecological department of the N.N. Petrov National Research Center performed 43 radical trachelectomies (IA1-IB1 stages): 27 - abdominal trachelectomies and 13 - Dargent's operations and 3 - laparoscopic trachelectomies. The average age of patients is 30.2 ± 4 years. According to the results of histological examination squamous cell carcinoma was verified in 90% of cases, adenocarcinoma in 10%.
Results: The duration of radical trachelectomy ranged from 150 to 360 minutes, the volume of blood loss ranged from 50 - 1600 ml. The early postoperative period in all patients proceeded without complications. Among the late postoperative complications were recorded: asymptomatic lymphocytes (n = 6), lymphedema (n = 2), cervical stenosis (n = 5). In all patients after surgery, menstrual recovery occurred in the period from 2 to 2.5 months. The uterovaginal anastomosis healed in 2-3 months. The observation period ranged from 3 months to 10 years. Four recurrences of cervical cancer after organ-preserving treatment were established in IB1 stage of the disease after 5 (anastomosis zone), 6 (pelvic wall), 11 (carcinomatosis) and 13 (pelvic wall) months. Three patients died from the progression of the disease. Overall survival was 92% when performing radical trachelectomy. Pregnancy occurred in 8 patients. In five women pregnancies resulted in cesarean section in the third trimester, and three pregnant women had miscarriages in the first trimester. Nineteen patients refused pregnancies due to fear of recurrence of the disease, lack of a partner. Nine women are planning a future pregnancy
Conclusion: The literature data and our experience show that radical trachelectomy is an effective type of organ-preserving treatment for patients with cervical cancer of the initial stages.
Disclosure: Nothing to disclose

EP422

After LACC trial: a single institutional study with our own data

J. Utrilla-Layna, J. Garcia, M. Albi
Hospital Universitario Fundación Jiménez Díaz, Madrid, Spain

Introduction / Background: LACC trial has changed the paradigm regarding the surgical treatment of cervical cancer. This is the first prospective study performed in this field and in which a better prognosis has been observed in patients treated by laparotomy.

Methodology: This is a retrospective observational cohort study without a control group.
Inclusion criteria
Patients with stages Ia1 (with lymphovascular invasion) to IIa1 treated by hysterectomy and trachelectomy including lymphadenectomy between January 2007 and December 2018 at the Jiménez Díaz Foundation Hospital.
Exclusion criteria
Patients under 18 years
Pregnant patients
Older than 80 years
Surgical contraindications
ECOG ≤50% and / or ASA ≥ 4
Parametrial involvement
All patients who met the described inclusion criteria were selected and different clinical parameters were analyzed, including age, menopausal status, physical examination for assessment of parameter involvement. Likewise, anatomopathological characteristics were studied, such as histological type, tumor size, lymphovascular invasion, parametrial infiltration, nuclear grade, stromal infiltration.
The disease-free interval was also studied, as well as the overall survival.
Results: For all the patients recruited, a total of 84 of them were included in the inclusion criteria.
The average age of the patients was 46 years (Minimum 27 and Maximum 70 years).
53 patients studied were in stage IB1 (63%), 14 stage IA1 (16.6%), 12 stage IA2 (14.3%) and 5 stage IIA1 (5.9%).
The disease-free survival studied has been up to> 5 years. There have been a total of 5 recurrences (Minimum 8 and maximum 40 months).
Conclusion: The percentage of relapse and survival is in accordance with the current data.
Laparoscopic surgery presents an acceptable complication rate.
It is important to highlight the number of patients with tumors smaller than 2cm and without other associated risk factors that could have been operated on by type A radical hysterectomy and thus avoid the potential adverse effects of a more extensive surgery.
Disclosure: Nothing to disclose

EP423

Case report of gastric type of invasive adenocarcinoma of the endocervix

V. Uvin, Q. Fontagnes, G. Ena, C. Riera
Gynecology, CHU Charleroi, Charleroi, Belgium

Introduction / Background: Gastric-type endocervical adenocarcinoma (GAS) was recognized in the latest World Health Organisation classification as the second most frequent subtype of adenocarcinoma of the endocervix (EAC) and is HPV-unrelated. GAS is known for its presentation at advanced stage, worse clinical outcome, more metastasis to distant sites and its relative resistance to chemotherapy.
We describe one case of GAS with poor response to chemotherapy and an misunderstanding on imaging.
Methodology: /
Results: A 48 year old Caucasian female patient underwent a Papanicolaou smear during routine gynecological examination. Atypies of Low Grade Squamous Intraepithelial Lesion (LGSIL) were found. Colposcopy and biopsy revealed an invasive adenocarcinoma of the cervix with presence of intestinal cells. HPV PCR was negative.
The conization confirmed a GAS of 15 on 6mm with lymphovascular emboles. The chirurgical margins were non in sano.
Magnetic resonance imaging (MRI) was negative; positron emission tomography (PET) showed a hypermetabolic sigmoidal lesion, but rectosigmoidoscopy was negative. Nevertheless, the clinical examination showed a residual lesion of 30mm and biopsy confirmed persistent disease.
The patient underwent nine courses of neoadjuvant chemotherapy by Carboplatine and Paclitaxel.
Clinical evaluation showed a normal cervix but hard palpation, douglas was negative. Moreover, MRI and PET confirmed the absence of residual disease

Then, Wertheim type II was performed.
The final anatomopathological examination confirmed the diagnosis of a 40mm GAS with tumor-free margins and metastatic invasion of one lymphnode in right parametrium. ypT1B2N1, FIGO: IB2.
Facing this poor result, the patient was offered classical treatment of adjuvant pelvic chemo-radiotherapy (Cisplatin).
Conclusion: This 48 year old female was diagnosed with gastric type of invasive endocervical adenocarcinoma. She underwent neoadjuvant chemotherapy with incomplete response. The persistent disease was not detected by imaging techniques after treatment nor eventually before the treatment.
Disclosure: Nothing to disclose

EP424

Validation of the new FIGO 2018 staging system in clinically early cervical cancer

J. van der Velden[1], C.H. Mom[1], A.M. Spijkerboer[2], L.R.C.W. van Lonkhuijzen[1], J.J. Laan[1], G. Fons[1]
[1]Gynecologic Oncology, [2]Radiology, Amsterdam UMC, Location AMC, Amsterdam, The Netherlands

Introduction / Background: The new 2018 FIGO staging system imaging can be used for staging by analyzing the size of the primary tumor, the presence/absence of parametrial infiltration and the presence of metastatic lymph nodes. It was the aim of this study to compare the outcomes of the “MRI” FIGO 2018 stage (r-stage) with the final pathological stage (p-stage), both in terms of accuracy and survival.
Methodology: A retrospective analysis was performed on patients with early stage cervical cancer who were selected for surgical treatment on the basis of clinical parameters. Patients had a pre-operative MRI (3 x T2TSE, with axial HASTE pelvis and upper abdomen) and partial (lymph node dissection/debulking) or complete (radical hysterectomy and pelvic lymph node dissection) pathological staging. The accuracy of every individual r-stage was defined with the p-stage as “golden standard”. Disease specific survival (DSS) of patients subdivided by r-stage was analyzed.
Results: From 2001 till 2018, 438 surgically assessed/treated patients with FIGO 2009 stage IB1 and IB2 were analyzed. A pelvic lymph node dissection/debulking only or a radical hysterectomy with lymph node dissection was performed in 41 and 397 patients respectively. The accuracy of r-stage to predict p-stage is depicted in Table 1. Cumulative Disease Specific Survival (DSS) according to r-stage is shown in Fig 1.
Conclusion: R-stage IIB overestimates the risk on parametrial invasion (Positive predictive Value (PPV) from 6% to 28%) and therefore results in a better than expected 5 years disease specific survival of 89.7%. Although r-stage IIIC underestimates the risk on lymph node metastases (Negative Predictive Value (NPV) 81%), it correctly distinguishes the group with the poorest survival. The results from this retrospective study suggest that, at this stage, MRI imaging is not reliable enough to accurately predict p-stage IIB.

R-stage	Sensitivity	Specificity	PPV	NPV
IB1	85%	88%	77%	93%
IB2	46%	84%	50%	82%
IB3	43%	93%	44%	93%
IIB/*	31%/50%	83%/83%	6%/28%	97%/95%
IIIC1	42%	98%	88%	81%

[Table 1. Accuracy of r-stage to predict p-stage in clinical early cervical cancer (= accuracy to predict parametrial invasion, irrespective of lymph]*

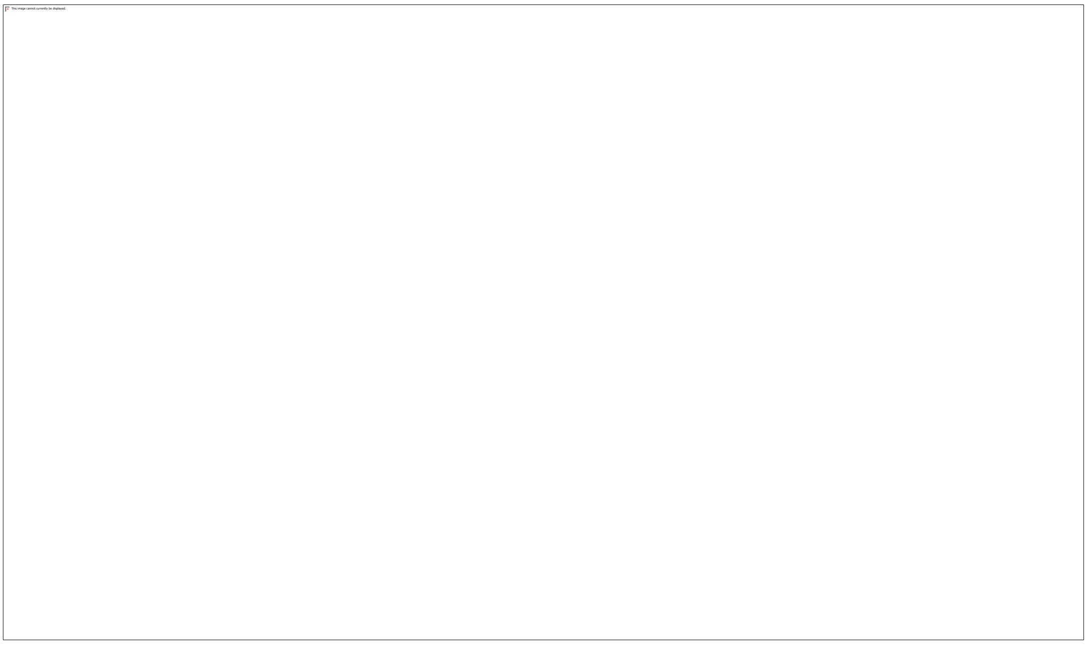

[Fig 1. Five years DSS for R-stage (IB1: 98.4%; IB2: 93.3%; IB3: 77.8%; IIB: 89.7%; IIIC1: 70.6%).]

Disclosure: Nothing to disclose

EP425

Acute and long-term toxicity in patients undergoing induction chemotherapy followed by radiotherapy and hyperthermia for advanced cervical cancer

X.S. Gao[1], I.A. Boere[2], M.J.H.A. Kruip[3], M. Franckena[4], S.T. Heijkoop[4], M.D. Kulawska[5], R. Jonkhoff[6], H.C. van Doorn[1]
[1]Department of Gynaecologic Oncology, [2]Department of Medical Oncology, [3]Department of Haematology, [4]Department of Radiation Oncology, [5]Department of Radiology, ErasmusMC Cancer Institute, University Medical Centre Rotterdam, Rotterdam, The Netherlands, [6]Medical Oncology, Dr. Horacio E. Oduber Hospital, Oranjestad, Aruba

Introduction / Background: To determine survival, acute toxicity (vascular and renal) and chronic renal impairment in high risk cervical cancer patients when treated with platinum-based induction chemotherapy followed by concurrent radiotherapy and hyperthermia.
Methodology: Patients with large primary tumours (>6cm), or para-aortic lymph node (LN) metastases >1 cm, or para-iliac LN >2 cm were treated with weekly platinum-based induction chemotherapy followed by concurrent radiotherapy with hyperthermia. Patient and tumour characteristics, Common Toxicity Criteria (CTC) scores, laboratory tests, and treatment data were retrieved from clinical charts.
Results: From the hyperthermia database 105 patients were included with a median follow-up time of 43 months (95% CI 32-54). Mean age was 47.9 years (range 22-79) and median tumour size 6.0 cm (range 2.6-11.5). Clinical FIGO stage of $\geq$ IIIB was present in 30% and para-aortic LN were present in 47%. Eighty-six patients were scheduled with cisplatin therapy and 20 patients with carboplatin. At baseline a thrombo-embolic event (TEE) was present in 10 and 23 (22%) patients experienced a TEE (18% venous, 4% arterial) during chemotherapy. Because of renal toxicity during chemotherapy in 32 patients (37%), 10 patients stopped with cisplatin and 22 patients switched to carboplatin. At last available follow-up 30 patients (35%) with cisplatin therapy developed a CTCAE grade $\geq$ 2 chronic renal toxicity (GFR < 59 mL/min). Five-year overall survival was 58%. There was no significant

difference between women receiving cisplatin versus carboplatin.
Conclusion: Although platinum-based induction chemotherapy followed by concurrent radiotherapy with hyperthermia is effective as treatment of advanced cervical cancer, there is a high risk of chemotherapy-associated TEE, acute and chronic renal toxicity due to cisplatin. To reduce toxicity, the treatment protocol has been changed to carboplatin based chemotherapy and in an observational study the use of prophylactic LMWH will be implemented.
Disclosure: Nothing to disclose

EP426

Predictive value of the sentinel lymph node biopsy (SLNB) in early-stage cervical cancer: is pelvic lymphadenectomy replaceable?

A. Vazquez Sarandeses, M. Hernandez Ontoria, G. Lopez Garcia, A. Tejerizo Garcia
Hospital Universitario 12 de Octubre, Madrid, Spain

Introduction / Background: We analyzed the predictive value of the Sentinel Lymph Node Biopsy (SLNB) in order to assess its safety and reliability.
Methodology: Retrospective analyses of patients diagnosed with early-stage cervical cancer (ESCC) between June 2010 and June 2017. All patients underwent SLNB followed by pelvic lymphadenectomy(PL). Stage was IA1 to IIA1.
Results: 61(39,8%) met the inclusion criteria. The mean tumor size was 21.1mm preoperatively. A 70% of the patients had a tumor size < = 2cm. We performed 47 (77.0%) SLNB followed by bilateral PL. According to the technique we obtained bilateral detection in 30/47 patients (63,8%). Unilateral detection was observed in 12/47 cases (25,5%) and bilateral failure in 5/47 cases (10.6%). The lymph node status was positive in 4/47 cases (8.5%) in which para-aortic lymphadenectomy was accomplished for staging.
The overall detection rate of the technique was 76.6%. We found a sensitivity of 100%, a specificity of 75,5%, a negative predictive value of 100% and a false negative rate of 0%.
We observed a lymphovascular and neurological morbidity in 6.4%(3/47), and 8.5% (4/47) respectively.
Conclusion: The SLNB would allow a decrease in the morbidity associated with PL.
It seems to be a trend towards a more conservative and less aggressive surgery.
Accurate selection of patients is key point.

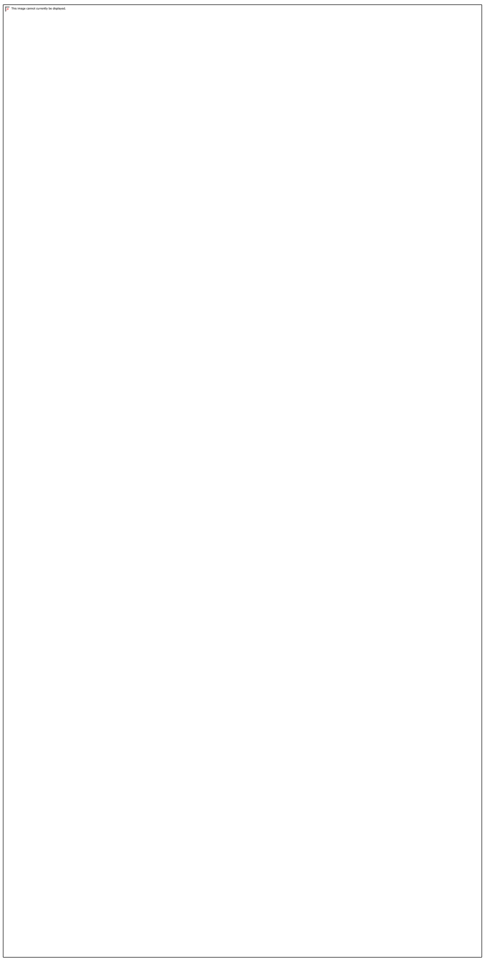

[Patients' and tumor characteristics]

Disclosure: Nothing to disclose

EP427

Comparison of survival curves according to pronostic risk fatcors in patients with early-stage cervical cancer (ESCC)

A. Vazquez Sarandeses, M. Hernandez Ontoria, G. Lopez Garcia, Á. Tejerizo Garcia
Hospital Universitario 12 de Octubre, Madrid, Spain

Introduction / Background: To determine the impact of prognostic risk factors on survival of patients with ESCC.
Methodology: Overall survival was analysed by using Kaplan-Meier analysis and compared by applying log-rank test.
Results: 61 patients with ESCC who underwent surgery as primary treatment were included. We obtained an overall survival of 100% and 97.7% in the first year and up to 5 years of follow-up. Prognostic factors were: TNM and FIGO stage, tumor size, LVSI, depth of cervical stroma invasion, involvement of parametrium, positive surgical margin and lymph node involvement. When comparing the survival curves for each prognostic factor individually, only a statistically significant association

was observed for "surgical margin affected"*(p=0.008).* No other significant association was observed. A trend towards statistical significance is observed if we group several prognostic factors. Hence, the coexistence of ≥3 risk factors reaches almost the level of significance*(p = 0.06).*
Adjuvant radio-chemotherapy was given to 20/57(35%)patients. Among them 65% presented associated morbidity: 30%digestive, 30%cutaneous, 25%haematological and 15%urological. The morbidity was severe in one case, moderate in5/13 and mild in the remained cases(7/13).
Conclusion: Accurate pre-surgical staging would properly select surgical candidates and avoid radio-chemotherapy.
Indications for adjuvant treatment should be reconsidered. LVSI as an independent risk factor is controversial and questionable.

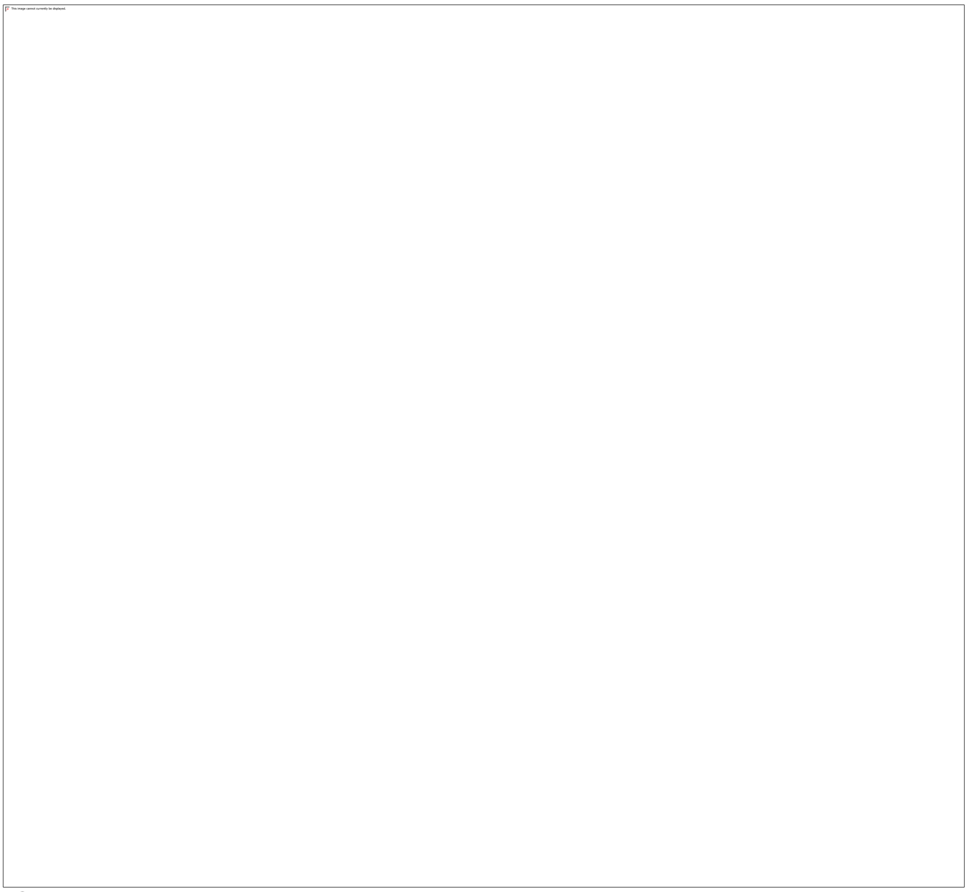

[Overall survival in patients with early-stage cervical cancer]

Disclosure: Nothing to disclose

EP428

Risk of involvement of sentinel lymph nodes in cervical cancer stage IA1 with lymphovascular space invasion and and IA2

M. Vetrovska[1], T. Pichlik[1], L. Rob[1], H. Robova[1], M. Halaska[1], J. Drozenova[2], M. Hruda[1], V. Drochytek[1]
[1]Department of Gynecology and Obstetrics, [2]Department of Pathology, 3rd Faculty of Medicine, Charles University, Faculty Hospital Královské Vinohrady, Prague, Czech Republic

Introduction / Background: To evaluate the risk of involvement of sentinel lymph nodes in cervical cancer stage IA1 with lymphovascular space invasion (LVSI) and IA2 using the detection of sentinel lymph nodes and histopathology ultramicrostaging.
Methodology: The study included women from prospective protocols LAP I and LAP II with cervical cancer squamous or adenocarcinoma stage IA1 with LVSI and stage IA2 from 2002 to 2018 classified according to FIGO 2014 staging, TNM 8. Detection of sentinel lymph nodes throughout this period was performed by using ultra-short protocol with Tc and patent blue and histopathology evaluation by ultramicrostaging histopathological protocol.
Results: In the first group (28 women) with stage IA1 and lymphovascular space invasion diagnosed from cone biopsy (all LVSI cases was confirm by expert oncogynecology pathologist). There were 2 women with positive lymph nodes (7,1%). In the group stage IA2 (34 women) there were 13 women (38,2%) with positive lymphovascular space invasion and 2 women had positive lymph nodes (5,9%). The risk of positive lymph nodes for stage IA1 with lymphovascular space invasion and for stage IA2 is not statistically significant OR = 0.8125 (95% CI: 0.1070 - 6.172).
Conclusion: The detection of sentinel lymph nodes aids to individualize the therapy of early stage cervical cancer and helps to reduce the radicalisation of surgery. The risk of positive lymph nodes in stage IA1 with lymphovascular space invasion and stage IA2 with/without lymphovascular space invasion is the same. The results confirm, that the detection of sentinel lymph nodes in stage IA1 with lymphovascular space invasion is fully indicated. It would be interesting to compare these results with new FIGO classification.
Disclosure: Nothing to disclose

EP429

Laparoscopic management of urinary tract injuries during laparoscopic radical hysterectomy for cervical cancer

S. Baydo[1], A. Vinnytska[2], D. Golub[1]
[1]Surgery, [2]Gynecology, LISOD - Israely Oncological Hospital, Kyiv, Ukraine

Introduction / Background: Urinary tract injury during laparoscopic hysterectomy has become more common. The incidence of ureteral injuries is 0.3-2% for benign diseases and reaches 10% in case of malignancy.
This work represents our experience in laparoscopic management of urinary tract injuries during totally laparoscopic radical hysterectomy (TLRH) for cervical cancer.
Methodology: n 2010-2018 we performed 138 TLRH for cervical cancer. Urinary tract complications occurred in 10 (7.2%) patients. Among them there were 5 (3.6%) ureteral and 5 bladder damages. All ureteral injuries occurred in patients with history of chemoradiotherapy and were diagnosed within 30 days after surgery.
In case of intraoperative bladder damage simple suture and 9 days of catheter were enough. One ureteral damage was treated on 2nd p/o day with relaparoscopy and ureteral suturing on stent. In one case of ureteral necrosis due to the devascularisation of ureter during dissection unsuccessful attempts were made to treat it with stent. After one year uretero-ileo-neocystoplasty was performed. In three cases of ureteral necrosis primary ureterocystoneostomies with psoas hitch were done.
Results: Mean operative time of ureteral reconstruction was 132.3 min. Mean hospital stay was 4.7 days. Cystography showed no urine leak when the ureteral stent was removed at 4-6 weeks after procedure. Ureteral stent was removed after cystography at 4-6 weeks. Ureteroneocystostomy patency was followed up with cystography at 6-8 months.
Conclusion: Urinary tract injury during laparoscopic hesterectomy is rare but in case of ureteral damage very serious complication that can be successfully treated laparoscopically by experienced surgeon.
Disclosure: Nothing to disclose

EP430

Combination of FDG-PET metabolic parameters of cervical tumor and pelvic lymph nodes predict paraaortic lymph node involvement in locally advanced cervix cancer

M. Voglimacci[1], M.A. Angeles[1], A. Lusque[2], H. Leray[1], E. Chantalat[1], A. Ducassou[3], L. Gladieff[4], G. Ferron[1], E. Gabiache[5], A. Martinez[1]
[1]Oncologic Surgery, [2]Biostatistics, [3]Radiation Therapy, [4]Medical Oncology, [5]Nuclear Medicine and Medical Imaging, IUCT Oncopole, Toulouse, France

Introduction / Background: Fluorine^{18} fluorodeoxy glucose positron emission tomography/computed tomography (FDG-PET/CT) is recommended for paraaortic lymph node (PALN) assessment in locally advanced cancer (LACC). Role of surgical staging remains unclear. Aim of our study was to evaluate whether FDG-PET/CT cervical tumor metabolic parameters can predict PALN metastasis.
Methodology: Patients diagnosed with LACC with available pretherapeutic FDG-PET/CT treated between 2006 and 2015 were included. Two senior nuclear physicians experts in gynaecologic oncology reviewed all PET/CT exams, and extracted SUVmax, MTV, TLG (Standardized Uptake Value, Metabolic Tumor Volume, Total Lesion Glycolysis, respectively) for cervical tumor, and for pelvic lymph nodes (PLN) when positive. Patients were divided in three groups following PALN status : PET positive (PET +), PET negative and surgicopathologic positive (PET-/path+), PET and surgicopathologic negative (PET-/path-).
Results: 160 patients were included.
FDG-PET/CT PALN uptake was found in 34 patients (21,3%). 126 (78,8%) patients had laparoscopic paraaortic lymph node retroperitoneal staging :17 patients (13,5%) and 109 patients (86,5%) had negative and positive PALN on surgicopathologic analysis respectively (Figure 1). PLN were positive on FDG-PET/CT in 28 patients (90,3%).

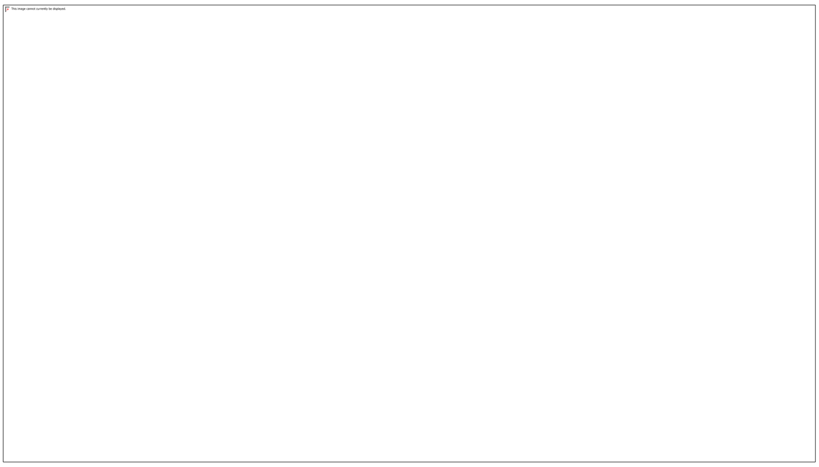

[Figure 1 : flow chart]

Cervical tumor MTV and TLG were significantly different increased in patients with false negative aortic FDG-PET/CT results (table 1). PLN metabolic parameters were also significantly different in the 3 PALN groups (table 2).

	PET+ (n=34)	PET-/path+ (n=17)	PET-/path- (n=109)	p-value
SUVmax: median (range)	14,7 (6,0-23,9)	15,3 (7,4-31,8)	14,5 (2,1-34,4)	0,3866
MTV: median (range)	54 (10,5-299)	73,1 (8,8-120)	34,0 (0,8-162)	0,0004
TLG: median (range)	360,6 (42,2 - 3181,0)	496,8 (43,4-1455,7)	234,0 (1,2-2495,0)	0,0174

[Table 1: Cervical tumor parameters (n=160)]

	PET + (n=28)	PET - / path + (n=10)	PET - /path - (n=30)	p-value
Number of positive PLN : median (range)	4 (1-10)	3 (2-11)	2 (1-5)	0,0008
SUVmax* : median (range)	6,6 (1,8-16,1)	12,6 (4,7-17,1)	4,7 (1,7-16,4)	0,0046
MTV* : median (range)	2,4 (0,2-8,8)	5,1 (1,3-14,3)	1,7 (0,3-22,8)	0,0549
TLG* : median (range)	10,4 (0,4-76,8)	36,2 (3,1-68,4)	5,8 (0,6-152,2)	0,0139

*[Table 2: Pelvic lymph node parameters (n=68). *PLN parameters were studied for the most avid PLN]*

Conclusion: Cervical tumor and pelvic lymph nodes FDG-PET/CT metabolic parameters are predictive biomarkers of paraaortic lymph node involvement in LACC patients.

	PET+ (n=34)	PET-/path+ (n=17)	PET-/path- (n=109)	p-value
SUVmax: median (range)	14,7 (6,0-23,9)	15,3 (7,4-31,8)	14,5 (2,1-34,4)	0,3866
MTV: median (range)	54 (10,5-299)	73,1 (8,8-120)	34,0 (0,8-162)	0,0004
TLG: median (range)	360,6 (42,2 - 3181,0)	496,8 (43,4-1455,7)	234,0 (1,2-2495,0)	0,0174

[Table 1: Cervical tumor parameters (n=160)]

	PET + (n=28)	PET - / path + (n=10)	PET - /path - (n=30)	p-value
Number of positive PLN : median (range)	4 (1-10)	3 (2-11)	2 (1-5)	0,0008
SUVmax* : median (range)	6,6 (1,8-16,1)	12,6 (4,7-17,1)	4,7 (1,7-16,4)	0,0046
MTV* : median (range)	2,4 (0,2-8,8)	5,1 (1,3-14,3)	1,7 (0,3-22,8)	0,0549
TLG* : median (range)	10,4 (0,4-76,8)	36,2 (3,1-68,4)	5,8 (0,6-152,2)	0,0139

*[Table 2: Pelvic lymph node parameters (n=68). *PLN parameters were studied for the most avid PLN]*

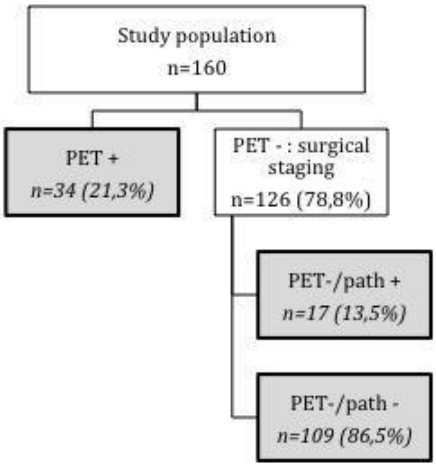

[Figure 1 : flow chart]

Disclosure: Nothing to disclose

EP431

15-year survival and toxicity of locally advanced cervical cancer treatment with hyperthermia in combination with radiochemotherapy

Y. Wang, H. Chen
First Affiliated Hospital of Xi 'an Jiaotong University, Xi'an, China

Introduction / Background: The effect of concurrent chemoradiotherapy (CCRT) for stage IB2 to IVA disease has been proven. However, it has been still unknown whether the additional hyperthermia can render patients survival advantages since the early 1990s. This study was designed to evaluate the effects of hyperthermia (HT) combined with radiotherapy (RT) and / or chemotherapy (CT) in patients with cervical cancer (CC) stage IIB and IIIB.
Methodology: 120 patients with IIB and IIIB CC from the first affiliated hospital of xi ´an jiaotong university were randomized to RT, RCT (radiochemotherapy), RHT(radiotherapy plus hyperthermia), and RCHT (radiochemotherapy plus hyperthermia) groups. There were 7 patients of stage IIB and 23 patients of stage IIIB in each group. All patients were treated with relevant treatments. HT was delivered to the patients in RHT group and RCHT group, and the intracavitary temperature range was 41.5~42.5 ℃. Overall survival time, survival rate, toxicity as well as prognostic factors were evaluated.
Results: The 15-year survival time of RT alone was obviously lower than that of the other groups, 124 months in RT, 136 months in RCT, 147 months in RHT, and 138 months in RCHT, respectively. The 15-year survival analysis showed that combined treatment was associated with better overall survival, although there was not statistically significant(P=0.723). Besides, the combined treatment did not increase the cute or late toxicity. Prognostic analysis showed that the higher pre-treatment hemoglobin (BTHG) and more late toxicity of rectum (LTR) were significantly correlated with better survival(P=0.006, P=0.026).
Conclusion: The therapy of HT combined with RT and CT or RCT for locally advanced CC yielded a longer survival tendency as compared to RT alone, but did not increase acute or late toxicity. we suggest hyperthermia can be an optional or additional treatment for CC that can not respond or tolerate chemotherapy.
Disclosure: The authors declare no conflicts of interest.

EP432

HPV 18 predicts worse prognosis in patients with cervical cancer treated with chemoradiotherapy

Y. Wang, H. Chen
First Affiliated Hospital of Xi 'an Jiaotong University, Xi'an, China

Introduction / Background: Studies have shown that high-risk HPV plays an important role in tumorigenesis. However, the significance of HPV type in the prediction of treatment outcomes of cervical cancer patients is still controversial. This study was designed to evaluate the prognostic value of pre-treatment HPV DNA in correlation to long-term survival.
Methodology: 191 cervical cancer patients from October 2009 to December 2013 at the First Affiliated Hospital of Xi'an Jiaotong University were randomly assigned into radiochemotherapy(RCT) and radiochemotherapy plus hyperthermia (RHCT) therapeutic modalities in this study. The prevalent high risk types of HPV 16, 18, 58 were detected by type specific in situ hybridization (ISH) with paraffin section of 191 cervical cancer patients.
Results: The prevalence of HPV 16, 18 and 58 were 94.2% (95% CI=90.60-97.40), 71.2% (95% CI=64.40-77.50) and 62.30%. (95% CI = 55.50-69.10), respectively. The prevalence of HPV 16/18 double positive, HPV 16/58 double positive and HPV 16/18/58 trible positive were 24.60% (95% CI = 18.80-30.90), 15.20% (95% CI = 10.50-20.40) and 45.00% (95% CI=37.70-52.30), respectively. Only 2.60% (95% CI = 0.50-5.20) of the cervical tissue specimens showe no HPV detection. Univariate Cox regression analysis showed that patients with HPV 16/18 double positive, HPV 16/18/58 three positive showed worse survival than other groups, although the results were not statistically significant(P=0.285). while the patients were divided into HPV18-positive and negative group, we found that HPV18-negative patients were associated with better survival(P=0.034, HR=2.174).
Conclusion: HPV 16 is the most common type in cervical cancer patients in Shaanxi, China. HPV 18 may be a strong independent prognostic factor and associated with relatively poor survival prognosis. However, larger sample sizes and more comprehensive studies are required to verify our findings.
Disclosure: The authors declare no conflicts of interest.

EP433

The efficacy of adjuvant chemotherapy following radical hysterectomy for intermediate risk stage IB-IIA cervical cancer

Y. Wang[1], H. Lin[1], P. Lee[2], C. Huang[2], C. Wu[1]
[1]*Obstetrics and Gynecology,* [2]*Anatomical Pathology, Kaohsiung Chang Gung Memorial Hospital, Kaohsiung, Taiwan*

Introduction / Background: The aim of this study is to evaluate the efficacy of adjuvant chemotherapy following radical hysterectomy (RH) for women with intermediate risk stage IB-IIA cervical cancer.
Methodology: From January 2001 to December 2015, a total of 97 patients of FIGO stage IB-IIA cervical cancer having intermediate risk after radical hysterectomy and pelvic lymphadenectomy were enrolled in this study. Among these patients, 21 patients underwent adjuvant radiotherapy (RH+RT, N=21), 24 patients had cisplatin-based adjuvant chemotherapy (RH+CT, N=24), and 52 patients had surgery only (RH only, N=52). Disease-free survival (DFS) and overall survival (OS) were evaluated with Kaplan-Meier method and the log-rank test. Cox´s proportional-hazards regression model were used to evaluate the impact of prognostic factors in multivariant analysis.
Results: The percentage of histology distribution in 97 patients were 70.1% (68 patients) squamous cell carcinoma and 29.9% (29 patients) non-squamous cell carcinoma. The mean 5-year DFS rate is 88.1% in RH only group, 82.6% in RH+CT group and 70.8% in RH+RT group (P=0.350), respectively. The 5-year OS rate is 95.8% in RH only group , 91.3% in RH+CT group and 85.4% in RH+RT group (P=0.343), respectively. In the univariant analysis, histologic type seemed to be a prognostic factor for recurrence (P=0.014); however, it only showed borderline significance in multivariant analysis

(P=0.053).
Conclusion: Concerning the long-term and irreversible toxicities related to adjuvant radiotherapy following RH, cisplatin-based adjuvant chemotherapy or close surveillance for patients with intermediate-risk cervical cancer after RH showed non-inferior survival impact as compared to adjuvant radiotherapy. Therefore, adjuvant chemotherapy or close surveillance can be an alternative management for patients having intermediate risk of recurrence following radical surgery.
Disclosure: Nothing to disclose

EP434

Survival of patients with early-stage cervical cancer after abdominal or laparoscopic radical hysterectomy in the Netherlands

H. Wenzel[1], R. Smolders[2], J. Beltman[3], S. Lambrechts[4], H. Trum[5], R. Yigit[6], P. Zusterzeel[7], R. Zweemer[8], R. Bekkers[9], M. van der Aa[1]
[1]Department of Research, Netherlands Comprehensive Cancer Organisation (IKNL), Utrecht, [2]Department of Gynaecological Oncology, Erasmus Medical Centre - Cancer Centre, Rotterdam, [3]Department of Obstetrics and Gynaecology, Leiden University Medical Centre, Leiden, [4]Department of Obstetrics and Gynaecology, Maastricht University Medical Centre+, Maastricht, [5]Department of Gynaecologic Oncology, Netherlands Cancer Institute - Antoni van Leeuwenhoek, Amsterdam, [6]Department of Obstetrics and Gynaecology, University Medical Centre Groningen, Groningen, [7]Department of Obstetrics and Gynaecology, Radboud University Medical Centre, Nijmegen, [8]Department of Gynaecological Oncology, University Medical Centre Utrecht - Utrecht Cancer Centre, Utrecht, [9]Department of Obstetrics and Gynaecology, Catharina Hospital, Eindhoven, The Netherlands

Introduction / Background: In the previous decades, laparoscopic radical hysterectomy (LRH) has been introduced as alternative to abdominal radical hysterectomy (ARH) in early-stage cervical cancer. In 2018, results from the first prospective randomised study (the 'LACC' trial) were published, reporting inferior survival in patients treated with LRH. Therefore, we aimed to evaluate survival outcomes for patients treated with ARH and LRH for early-stage cervical cancer, in the Netherlands.
Methodology: In this retrospective study, patients diagnosed between 2010 and 2017 with cervical cancer FIGO (2009) stage IA2 with lymphovascular space invasion, IB1 and IIA1, who underwent ARH or LRH, were identified from the Netherlands Cancer Registry. Weighted multivariable Cox regression with propensity score, based on Inverse Probability Treatment Weighting, was applied to examine the effect of ARH and LRH on overall survival (OS) and disease-free survival (DFS).
Results: Of the 1107 patients, ARH was performed in 738 (67%) and LRH in 369 patients (of which 73% treated by robot surgery). Pathological lymph nodes were more often observed in the ARH group (18% vs. 8%), as were tumours ≥20mm (62% vs. 36%), depth of invasion >10 mm (31% vs. 14%), surgical margin involvement (4% vs. 1%) and recurrences (13% vs. 7%). Patients with ARH also showed higher mortality (9% vs. 5%) and lower unadjusted 5-year OS (85% vs. 92%) and 5-year DFS (83% vs. 91%). However, weighted Cox regression analyses showed similar 5-year OS (95% vs. 95%) and 5-year DFS (89% vs. 89%) in both groups. Subanalyses on clinical tumour size showed similar 5-year OS (98% vs. 99%) and 5-year DFS (93% vs. 96%) for tumours < 20 mm and similar 5-year OS (94% vs. 92%) and 5-year DFS (84% vs. 81%) for tumours ≥20mm.
Conclusion: Our observational data showed no difference in survival between ARH and LRH for early-stage cervical cancer, in the Netherlands.

This image cannot currently be displayed.

[ ]

[ ]

Disclosure: None of the authors received financial support for the research and/or authorship of this article. Hans Wenzel - Nothing to disclose; Ramon Smolders - Nothing to disclose; Jogchum Beltman - Nothing to disclose; Sandrina Lambrechts - Nothing to disclose; Hans Trum - Nothing to disclose; Refika Yigit - Nothing to disclose; Petra Zusterzeel - Nothing to disclose; Ronald Zweemer - Proctor Intuitive Surgical; Ruud Bekkers - Nothing to disclose; Maaike van der Aa - Nothing to disclose;

EP435

Characteristics of cervical cancer in Southwestern China and the clinical significance in young patients

L. Yang, X. Zeng, M. Xi
West China Second University Hospital | Gynecology, Sichuan University, Chengdu, China

Introduction / Background: The incidence of cervical cancer in young women is rising, Several authors have reported that carcinoma of cervix had a poorer prognosis and survival rates in young patients than in elder women while others found that age has no relevance with the prognosis. The aim of this study was to analyze the clinic-pathological characteristics of cervical cancer in southwestern China and discuss the features and prognosis of young patients.
Methodology: A retrospective study was performed, which consisted of 1,543 patients diagnosed with cervical cancer and underwent treatment at West China Second University Hospital between November 2005 and December 2010. Among them, 154 young patients with surgical procedures between November 2005 and December 2008 were selected for a 5-year follow-up and prognostic analysis.
Results: 312 patients (20.2%) were under 35 years old in the last 5 years. The proportion of cervical adenocarcinoma remained high in young patients (13.6%), and young women with adenocarcinoma had a higher rate of LN metastases, comparing with those with squamous cell carcinoma (42.9% vs 15.8%, P = 0.004). Young patients with adenocarcinoma had shorter progression-free survival than those who had squamous cell carcinoma (P = 0.024). Patients aged 35 years or younger with positive postoperative pathological risk factors had shorter progression-free survival, comparing with those with negative factors (P< 0.01).
Conclusion: Regarding to young patients, cervical adenocarcinoma increased the risk of LN metastases and positive postoperative pathological risk factors could apparently worsen the prognosis. Histological type and LN metastases were independent prognostic factors for young patients in southwestern China. We re-emphasize the importance of health education and regular smear screening for elder women, and more attention should be paid to young patients with adenocarcinoma or LN metastases.

BMJ Publishing Group Limited (BMJ) disclaims all liability and responsibility arising from any reliance placed on this supplemental material which has been supplied by the author(s)

This image cannot currently be displayed.

[Fig_1]

This image cannot currently be displayed.

[Fig_2]

Disclosure: Nothing to disclose

EP436

The kinetic profile and clinical implication of SCC-Ag in squamous cervical cancer patients undergoing radical hysterectomy using Simoa assay: a prospective observational study

S. Ye[1], X. Sun[2,3], B. Kang[2,3], F. Wu[2,3], Z. Zheng[1], L. Xiang[1], J. Liang[2,3], H. Yang[1]
[1]Gynecologic Oncology, Fudan University Shanghai Cancer Center, [2]Fudan University Shanghai Cancer Center - Institute Merieux Laboratory, Cancer Institute, [3]bioMerieux (Shanghai) Company Limited, Shanghai, China

Introduction / Background: To study the kinetic profile and clinicopathological implication of squamous cell carcinoma antigen (SCC-Ag) in cervical cancer patients who underwent radical hysterectomy by a self-developed Single Molecule Assay (Simoa) immunoassay.

Methodology: We prospectively enrolled cervical cancer patients who were scheduled for surgery between 2016/04 and 2017/06. Consecutive serum samples were collected at five points: Day 0 (the day before surgery), Day 4 (post-operative day 4), Week 2-4, Month 2-4 and Month 5-7. A total of 92 patients and 352 samples were tested using Simoa, which were compared with commercially available Architect assay. The median SCC-Ag values for each time-point and kinetic profiles were analyzed. We further assessed the correlations between SCC-Ag value/profiles and clinicopathological parameters.

Results: We demonstrated that Simoa was quite comparable to Architect. The median SCC-Ag value for each time-point using Simoa was 2.49ng/mL, 0.66ng/mL, 0.61ng/mL, 0.72ng/mL, 0.71ng/mL, respectively. The kinetic curve showed that the SCC-Ag levels decreased dramatically after surgery, and then stabilized and fluctuated to some extent within 6 months. Patients with some intermediate- or high-risk factors had significantly higher SCC-Ag values than those negative counterparts before surgery and within 3 months after surgery, but reach the same levels after 6 months. Furthermore, although patients with positive lymph node had sustained higher SCC-Ag values compared to those negative counterparts, similar kinetic tendencies were observed in both groups. Different from this finding, in comparison to the patients with surgery only, patients who received post-operative adjuvant treatment had significantly higher SCC-Ag values at the beginning, while no difference existed after completion of treatment.

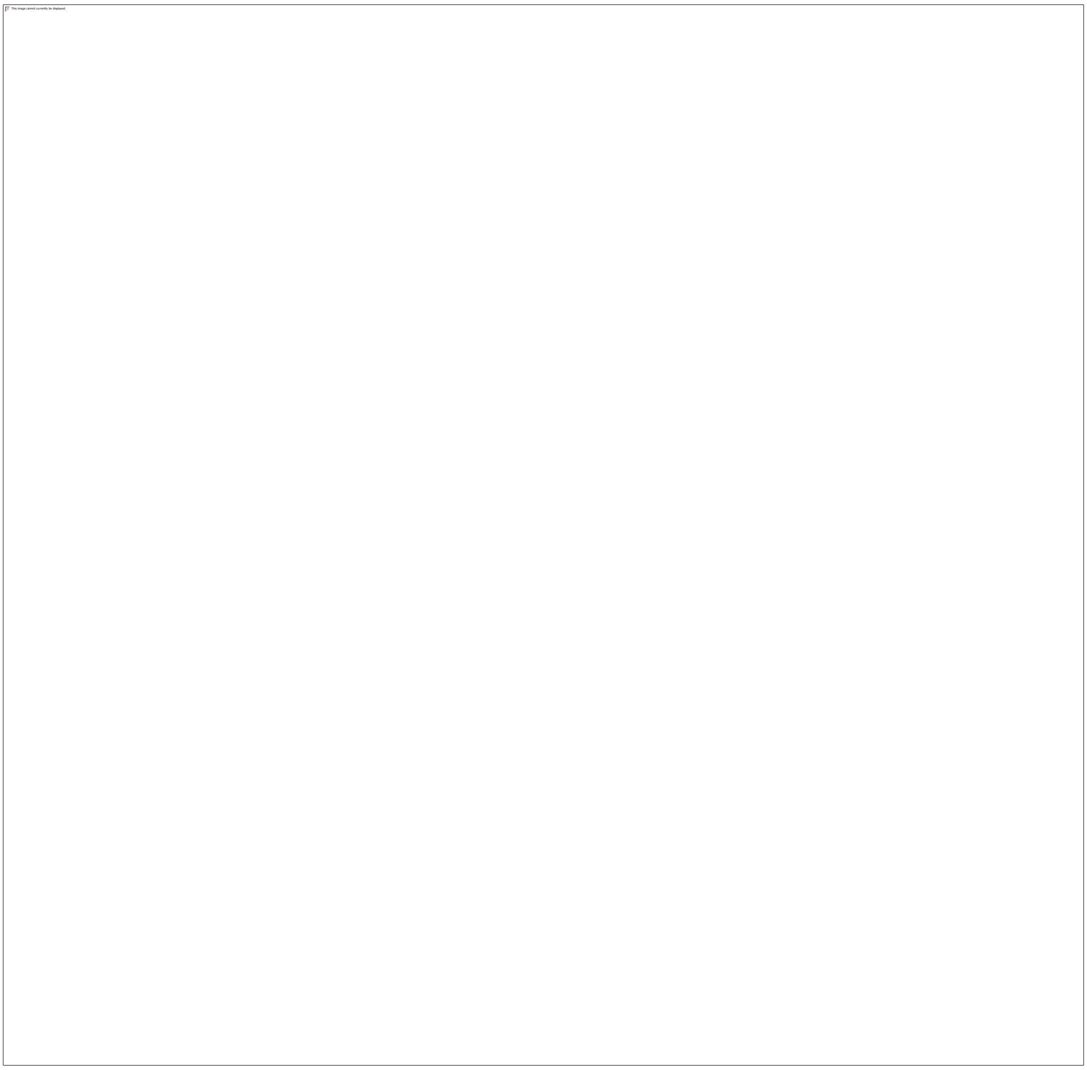

[Figure 1]

Conclusion: The user-friendly Simoa SCC-Ag assay was established for clinical setting. The SCC-Ag levels were higher in intermediate- or high-risk patients, whereas, the kinetic trend of SCC-Ag might be mainly affected by postoperative adjuvant therapy. The prognostic role needs follow-up data, which warranted further investigation.
Disclosure: Nothing to disclose

EP437

Dosimetric analysis of 3D-printed minimally invasive-guided template in the combined intracavitary/interstitial brachytherapy treatment of locally advanced cervical cancer

X. Yuan, Y. Zhang, J. Miao, L. Gao, J. Hu
Radiation Oncology, Hebei Province Cangzhou Hospital of Intergrated Traditional and Western Medicine, Cangzhou, China

Introduction / Background: To compare the dosimetric difference of whether 3D printing minimally invasive guiding template was used in local advanced cervical cancer intracavitary combined with interstitial radiotherapy.
Methodology: A total of 56 patients with locally advanced cervical cancer were performed radical radiation therapy. Intensity-modulated radiation therapy prescription dose of 46.8-50.4Gy/26-28f, 1.8Gy/times, joint intracavitary was inserted between the radiation dose graft 6Gy/times, once per week, a total of 5 times. Fifty-six patients were randomly divided into two groups, among which 25 patients in the template group were assisted with 3d-printed minimally invasive guiding template for intrauterine canal implantation and implant needle, and 31 patients in the free implant group were assisted with intrauterine canal implantation and implant needle implantation by hand. All patients were guided by CT to adjust the position and depth of the insertion needle, and the final CT image was transferred to Oncentra Brachy TPS system to formulate the treatment plan and implement the treatment.
Results: A total of 280 post-radiotherapy plans were made, including 125 in the template group and 155 in the free implant group. Compared with the free implant group, the D_{90} of high-risk clinical target area (CTV) and medium-risk CTV increased ($P< 0.05$) in the template group, and the D_{2cm3} of bladder, rectum and sigmoid colon decreased significantly ($P< 0.05$).Number of CT scans, time of insertion, and number of needles: there were statistically significant differences in CT scan times, implant time and number of needles between the template group and the free implant group ($P< 0.05$).
Conclusion: The application of 3D printing minimally invasive guiding template in the treatment of local advanced cervical cancer has obvious advantages in terms of dosimetry, short operation time and less pain for patients.
Disclosure: Nothing to disclose

EP438

Evaluation of cervical length and optimal timing for pregnancy after cervical conization in patients with cervical intraepithelial neoplasia

X. Zhang[1,2], J. Yang[2]
[1]Sir Run Run Shaw Hospital, School of Medicine, Zhejiang University, [2]Department of Gynecology, Hangzhou, China

Introduction / Background: To evaluate the change of cervical length and the best timng for pregnancy after cervical conization in patients with cervical intraepithelial neoplasia (CIN).
Methodology: This was a prospective study including patients under 40 years with fertility desire treated by cervical conization for CIN. To assess the cervical length, the patients were divided into two groups according to different surgery procedure: loop electrosurgical excision procedure (LEEP) and cold knife conization (CKC). Patients with cervical length < 2.5cm in CKC group were divided into two groups according to whether receiving cervical cerclage.
Results: In LEEP group, the cervical length preoperative was significantly longer than 3 months postoperatively (3.03±0.45 cm VS. 2.84±0.44 cm, $p< 0.001$). The cervical length at 6 and 9 months post-operation (2.97±0.51 cm and 3.01±0.41 cm) present no statistical significance compared to that in pre-operation. In CKC group, the cervical length preoperative was significantly longer than 3 and 6 months postoperatively (2.90±0.41 cm VS. 2.43±0.43 and 2.68±0.41cm, respectively, $p< 0.001$). No statistical significance was seen between the rest of months preoperatively and postoperatively. Cervical length was significantly longer at 12 months and 9 months after cerclage compared to that without cerclage. 18 patients got pregnant in LEEP group, among which one was pregnant at 5 months postoperatively and had premature delivery. There was one inevitable abortion and one preterm birth among 39 pregnant patients from CKC group.
Conclusion: Patients who have fertility desire with CIN were recommended for pregnancy at 6 and 9 months after LEEP and CKC, respectively. Cerclage effectively prolonged cervical length in patents with that less than 2.5cm to prevent cervical incompetence.
Disclosure: no conflict

EP439

Predictive value of preoperative serum squamous cell carcinoma antigen (SCC-Ag) level on tumor recurrence in cervical squamous cell carcinoma patients treated with radical surgery: a single-institution study

Q. Guo, J. Zhu, X. Ju, X. Wu
Department of Gynecologic Oncology, Fudan University Shanghai Cancer Center, Shanghai, China

Introduction / Background: We seek to find the significance of serum squamous cell carcinoma antigen (SCC-Ag) and the optimal cut-off value for predicting tumor recurrence and survival in cervical squamous cell carcinoma receiving radical surgery.
Methodology: A total of 3471 patients with stages IB1 to IIA2 cervical squamous cell carcinoma who underwent radical surgery were enrolled in this study. The cut-off value of serum SCC-Ag for tumor recurrence was calculated using the receiver operating characteristic (ROC) curve. The progression-free survival (PFS) and overall survival (OS) were assessed to estimate the significance of SCC-Ag.
Results: The optimal cut-off value of serum SCC-Ag level for predicting tumor recurrence was calculated and set at 2.75ng/mL. Compared with 1.5 ng/mL used in clinical practice, serum SCC-Ag level > 2.75 ng/mL was closely related to extrapelvic metastatic disease when patients relapse (P = 0.035). Multivariate analysis showed that serum SCC-Ag level > 1.5 ng/mL and serum SCC-Ag level > 2.75 ng/mL were neither independent risk factors for PFS nor OS in all the patients. But in 964 patients with one or more high-risk factors (parametrial invasion, vaginal margin invasion and lymph node metastasis), instead of serum SCC-Ag level > 1.5 ng/mL, serum SCC-Ag level > 2.75 ng/mL could be used as an independent factor affecting PFS (P = 0.018, HR = 1.538).
Conclusion: Preoperative serum SCC-Ag level > 2.75 ng/mL is closely related to extrapelvic recurrence, and is an independent factor for tumor recurrence and survival in cervical squamous cell carcinoma patients with high-risk factors.
Disclosure: Nothing to disclose

EP440

Pembrolizumab efficacy and recurrence pattern of persistent and metastatic cervical cancer

J. Zhu, X. Wu, G. Ke
Fudan University Shanghai Cancer Center, Shanghai, China

Introduction / Background: The aim of this study was to evaluate the efficacy of Pembrolizumab for metastatic, recurrent, or persistent cervical carcinoma.
Methodology: This is a retrospective review of medical records of patients with metastatic, recurrent, or persistent cervical carcinoma. Patients received pembrolizumab 200 mg every 3 weeks for 2 years or until progression, intolerable toxicity, or physician or patient decision. Tumor assessment was performed every 9 weeks. The primary end point was objective response rate (ORR), assessed per Response Evaluation Criteria in Solid Tumors (version 1.1) by independent central radiologic review.
Results: Of the 45 patients, 37 (82.2%), 7 (15.6%) and 1 (2.2%) had squamous-cell carcinoma, adenocarcinoma and small cell neuroendocrine carcinoma, respectively. All the patients had prior platinum exposure and pelvic irradiation. All the patients had previously received one or more lines of chemotherapy for recurrent or metastatic disease. Recurrence included loco-regional (6/45, 13.3 %), nodal (10/45, 22.2 %), and distant site (29/45, 64.4 %). Median follow-up was 8.5 months (range, 0 to 14 months). ORR was 33.3%, with nine complete and six partial responses. Possible Pembrolizumab-related adverse events included hypothyroidism (n=2/45, 4.4%), decreased appetite (n=3/45, 6.7%) and fatigue (n=3/45, 6.7%).
Conclusion: Pembrolizumab monotherapy demonstrated durable antitumor activity and manageable safety in patients with advanced cervical cancer.
Disclosure: Nothing to disclose

EP441

Synchronous small cell neuroendocrine carcinoma and adenocarcinoma in situ of the cervix

G. Zovko, V. Madžarac
Gynecology, University Hospital Merkur, Zagreb, Croatia

Introduction / Background: Small cell neuroendocrine cervical cancer (NECC) is a rare subtype of cervical cancer. This aggressive tumor accounts for less than 1.5% of all cervical cancers. Simultaneous occurrence of small cell NEC and adenocarcinoma of the uterine cervix is considered to be extremely rare.
Methodology: A 35-year-old female patient presented our gynecology department with irregular vaginal bleeding and suspicious lesion of the cervix. Transvaginal ultrasound showed a 3.2×1.9 cm cervical mass with a high blood flow. Diagnosis of neuroendocrine carcinoma was made after biopsy specimen of the tumor was analysed. Human papillomavirus (HPV) polymerase chain reaction (PCR) studies revealed HPV type 18 positivity. PET-CT of the abdomen and pelvis showed an exophytic tumour without lymph node metastasis.
Results: The patient underwent radical hysterectomy with bilateral salpingo-oophorectomy, and pelvic and para-aortic lymphadenectomy. Pathological examination confirmed initial diagnosis of small cell neuroendocrine carcinoma of the cervix, with negative surgical margins. Synchronous adenocarcinoma in situ was also identified in transformation zone. The tumoral tissue stained diffusely positive with CKAE1/AE3, CD56, chromogranin, synaptophysin, thyroid transcription factor-1 (TTF-1). Targeted next-generation gene sequencing revealed it was microsatellite stable (MSS) and mutational burden low (TMB - Low) tumor. The patient received adjuvant treatment according to Hoskins protocol. There was no evidence of disease recurrence during a 14-month follow-up period.
Conclusion: Simultaneous occurrence of small cell NEC and adenocarcinoma of the uterine cervix is a very rare event and there is no consensus with regard to the optimal treatment strategy. Further research in genetic alterations of these tumors will broaden the spectrum and efficacy of targeted therapy and offer better individualized management strategies.
Disclosure: Nothing to disclose

EP442

Laparoscopic nerve sparing radical hysterectomy without uterine manipulator in patients with cervical cancer

D. Zygouris[1], G. Anastasiou[2], N. Chalvatzas[1], A. Gkoutzioulis[1], A. Kavallaris[1,2]
[1]Department of Gynecologic Oncology, St Luke's Hospital, Thessaloniki, Greece, [2]Department of Gynecologic Gynecology, Mother and Child Medical Centre, Nikosia, Cyprus

Introduction / Background: Survival of patients with cervical cancer stage IB - IIA is highly variable and is affected by multiple risk factors, such as tumor size and lymph node metastases. We evaluated the outcome after surgical treatment and survival of patients who underwent laparoscopic radical hysterectomy without uterine manipulator.
Methodology: In time period of 1/2011 to 01/2019 58 patients with cervical cancer stage IB - IIA underwent laparoscopic radical hysterectomy without uterine manipulator at St. Luke's hospital in Thessaloniki, Greece and at Mother and Child Medical Centre in Nikosia, Cyprus. Preoperatively all patients had undergone clinical staging and MRI for the evaluation of lymph node status.
Results: 51 patients with stage IB and 7 patients with stage IIA disease underwent laparoscopic radical nerve sparing radical hysterectomy without uterine manipulator. No patient had any major complication and the mean blood loss was 188 ml (100 - 300 ml). Patients were mobilized the first

postoperative day and the catheter was removed on the third postoperative day with residual urine volume less than 50 ml. The mean follow-up is 53 months (3 - 86). The recurrence rate was 6.9 % (4 pts) and the mortality rate was 1.7 %.
Conclusion: Laparoscopic nerve sparing radical Hysterectomy without uterine manipulator is feasible and safe in patients with cervical cancer St. IB - IIA. It offers oncological safety and the advantages of laparoscopy and nerve sparing technique. Furthermore the patients are able to receive adjuvant therapy directly without losing valuable time in postoperative recuperation.
Disclosure: Nothing to disclose

EP443

Laparoscopic nerve sparing radical hysterectomy for locally advanced cervical cancer, technique and follow - up data

D. Zygouris[1], G. Anastasiou[2], N. Chalvatzas[1], A. Gkoutzioulis[1], A. Kavallaris[1,2]
[1]Department of Gynecologic Oncology, St Luke's Hospital, Thessaloniki, Greece, [2]Department of Gynecologic Gynecology, Mother and Child Medical Centre, Nikosia, Cyprus

Introduction / Background: To evaluate the feasibility and safety of laparoscopic nerve-sparing radical hysterectomy (LNRH) for locally advanced cervical cancer (LACC).
Methodology: 14 patients with stage IB3 and IIA cervical cancer were treated surgically with laparoscopic nerve sparing Radical Hysterectomy without Uterine manipulator at St. Luke's hospital in Thessaloniki, Greece and at Mother and Child Medical Centre in Nikosia, Cyprus. Preoperatively all patients had undergone clinical staging and MRI for the evaluation of lymph node status.
Results: 7 patients with stage IB3 and 7 patients with stage IIA disease underwent laparoscopic radical nerve sparing radical hysterectomy without uterine manipulator. We performed in all patients pelvic lymphadenectomy and in 9 cases we continued with para - aortic lymphadenectomy. No patient had any major complication and the mean blood loss was 265 ml (140 - 300 ml). Patients were mobilized the first postoperative day and the catheter was removed on the third postoperative day with residual urine volume less than 50 ml. All patients underwent adjuvant Chemo - Radio therapy. The mean follow-up is 44 months (6 - 73). The recurrence rate was 7% (1 pt) and the mortality rate was 0% (0pt).
Conclusion: Laparoscopic nerve sparing radical Hysterectomy without uterine manipulator is feasible and safe in patients with advanced cervical cancer. It offers oncological safety and the advantages of laparoscopy and nerve sparing technique. Furthermore the patients are able to receive adjuvant therapy directly without losing valuable time in postoperative recuperation.
Disclosure: Nothing to disclose

EP444

Endogenous intoxication as a symptom of advanced gynecological cancer

I. Neskubina, I. Goroshinskaya, E. Frantsiyants, A. Menshenina, P. Kachesova, L. Nemashkalova, E. Surikova, E. Shalashnaya, M. Adamyan, A. Pustovalova
Rostov Research Institute of Oncology, Rostov-on-Don, Russian Federation

Introduction / Background: Development of endogenous toxicosis in cancer patients is facilitated by intensification of catabolic processes, activation of proteolysis, accumulation of biomolecule oxidation products, together with disrupted detoxification and excretion. The purpose of the study was to evaluate endogenous toxicosis in patients with gynecological cancers of different stages.
Methodology: The blood of 136 patients aged 32-76 years was studied: ovarian cancer, OC (stage I-II) n=10, ascitic OC, AsOC (stage III) n=18, advanced OC, AdOC (stage IV) n=18, non-metastatic

cervical cancer, CC (stage III) n=29, metastatic CC, MCC (stage III-IV) n=27, primary vulvar cancer, VC (stage III) n=34. 20 non-cancer women of comparable age were controls. Medium weight molecules (MWM) at wavelengths of 254 nm and 280 nm were determined, as well as total albumin concentration (TAC), effective albumin concentration (EAC), albumin binding capacity (ABC) - EAC/TAC • 100%, and intoxication coefficient (IC) - MWM254/EAC • 1000.
Results: EAC was decreased in all groups compared to controls, with maximal decrease in AsOC (48.1%) and minimal in OC (I-II st) - 20.3%. In CC and VC, EAC decrease was 33-40.7%. ABC decreased in AsOC by 37.3%, in OC (I-II st) by 14.8%, in OC by 25.5-35.1%, CC and VC - 27.7-36%. The highest concentrations of both MWM fractions (254nm and 280 nm) were noted in AsOC (exceeded the norm by 16.8% and 77.2%) and OC I-II st (exceeded the norm by 25.4% and 74.8%). Elevated MWM_{280} levels were observed in MCC - 26.7%, and AdOC - 49%. IC in AdOC exceeded controls by 2.1 times, in AsOC by 2.3 times. In other groups of patients, IC was 28.5-90.5% higher than in controls (on average by 53.5%).
Conclusion: Patients with advanced cancers showed the greatest abnormalities in the detoxification system associated with the accumulation of endogenous products and dysfunction of natural detoxification and biotransformation systems.
Disclosure: Nothing to disclose

EP445

The ADNEX model to triage adnexal masses: an external validation study and comparison with the IOTA two-step strategy and subjective assessment by an experienced ultrasound operator

E. Viora[1], E. Piovano[2], C. Baima Poma[3], I. Cotrino[3], A. Castiglione[4], C. Cavallero[5], A. Sciarrone[3], S. Bastonero[3], L. Iskra[3], P. Zola[5]
[1]*Obstetrics Gynecological Ultrasound and Prenatal Diagnosis Unit - University of Turin, Turin,* [2]*Obstetrics and Gynecology Unit - Regina Montis Regalis Hospital, Mondovì,* [3]*Department of Obstetrics and Gynecology, Obstetrics-Gynecological Ultrasound and Prenatal Diagnosis Unit - University of Turin,* [4]*Unit of Cancer Epidemiology, CPO Piemonte - University of Turin,* [5]*Surgical Science, University of Turin, Turin, Italy*

Introduction / Background: The ADNEX (Assessment of Different NEoplasias in the adneXa) model was developed using parameters collected by experienced (level III) ultrasound examiners. Our primary aim was to externally validate the ADNEX model, when applied by level II examiners in an academic-women-hospital. The discriminatory performance of ADNEX was compared with the two-step strategy and subjective assessment by an experienced ultrasound operator.
Methodology: From February 2013 to January 2017, all patients who were scheduled for surgery for an adnexal mass at the Sant'Anna Hospital in Turin were enrolled. Preoperative transvaginal sonography was performed, and the two-step strategy was applied. Two ultrasound examiners applied the ADNEX model to all the collected masses based on the ultrasound reports. Finally, an experienced operator assigned the subjective assessment based on recorded ultrasound images. The discrimination and calibration performance of ADNEX were evaluated. The AUC was calculated for the basic discrimination between benign and malignant tumours
Results: A total of 577 patients were included in the analysis: the overall prevalence of malignancy was 25%. With ADNEX, the AUC to differentiate between benign and malignant masses was 0.9111 (95% CI 0. 8788-0.9389). At risk cut-offs of 1%, 10% and 30%, sensitivities were 100%, 89.6% and 79.2%, respectively, and specificities were 2.8%, 76.2% and 89.6%, respectively. Discrimination between benign and stage II-IV tumours was good (AUC 0.935). The model had the most difficulties discriminating between borderline and stage I tumours, and between stages II-IV invasive and secondary metastatic tumours. ADNEX proved to be equally or more accurate than the subjective assessment or the two-step strategy in the discrimination between benign and malignant adnexal masses.
Conclusion: The ADNEX model could probably be successfully applied when a level III examiner is not available and, therefore both a subjective assessment and the two-step strategy cannot be performed.
Disclosure: Nothing to disclose

EP446

When can we suspect gynecological lymphomas? Ultrasound features of ovarian and uterine lymphomas, a single-center experience

S. Boussedra, S. Giunchi, A.M. Perrone, A. De Leo, M. Tesei, P. De Iaco
Sant'Orsola-Malpighi University Hospital, Bologna, Italy

Introduction / Background: Lymphomas involving the gynecologic tract are uncommon and may occur as de novo or secondary as a part of systematic disease. Among gynecological lymphomas, the ovary is the most common anatomic site, accounting for 1.5% of ovarian neoplasm. Involvement of the cervix is more frequent than the uterine body and accounts for less than 1% of extranodal lymphomas. The aim of our study is to improve the use of ultrasound to detect or suspect promptly ovarian and uterine lymphoma through our experience.
Methodology: Patients with histological diagnosis of ovarian and uterine lymphomas, from 2011 to 2018, were retrospectively recruited from the database of our Gynecological Oncology Unit. The sonographic reports and digital images were analyzed, recording specific features.
Results: We recruited seven patients. Five out of seven (71%) of them had ovarian lymphoma, two of them had uterine lymphoma (29%).The echotexture of all the masses had a follicular architecture, reminding the nodular-appearing proliferation in the histology. Median age of the patients was 40 years (range 12-71). All the lymphomas appeared as hypoechoic, without cones of shadows and calcification; lymphomas were discretely solid masses in 5/7 (71 %) (median diameter 71 mm, range 26-103 mm), borders were well defined in all the ovarian lymphoma, and shaded in all uterine lymphoma. Internal cystic lacunae were in 3/7 (43 %). Vascularisation was moderate in 3/7 cases (43 %), and strong in 4/7 (57%), with an evidence of a lead vessel in 5/7 cases (71 %).
Conclusion: Ovarian and uterine solid lesions with heterogeneous nodular echogenicity,follicular architecture, rich in vascularization and presence of lead vessel, should be suspected of being lymphoma.

	Age of diagnosis (years)	Localization	Echogenicity	Cone of shadows	Vascularization (CS)	Lead vessel	Histology	Internal cystic lacunae	Calcifications
Patient 1	43	Ovary	Hypoechoic	No	3	Yes	Diffuse B Non Hodgkin Lymphoma	Yes	No
Patient 2	21	Ovary	Hypoechoic	No	3	Yes	Diffuse B Non Hodgkin Lymphoma	No	No
Patient 3	12	Ovary	Hypoechoic	No	4	Yes	Aggressive linfocitary B lymphoma	No	No
Patient 4	28	Ovary	Hypoechoic	No	4	Yes	Common linfoblastic lymphoma	Yes	No
Patient 5	71	Ovary	Hypoechoic	No	3	Yes	Aggressive linfocitary B lymphoma	Yes	No
Patienr 6	51	Uterus (body)	Hypoechoic	No	4	No	Centrofollicular B lymphoma	No	No
Patient 7	51	Uterus (cervix)	Hypoechoic	No	4	No	Follicular lymphocitic lymphoma	No	No

[Ultrasound features of ovarian and uterine lymphomas]

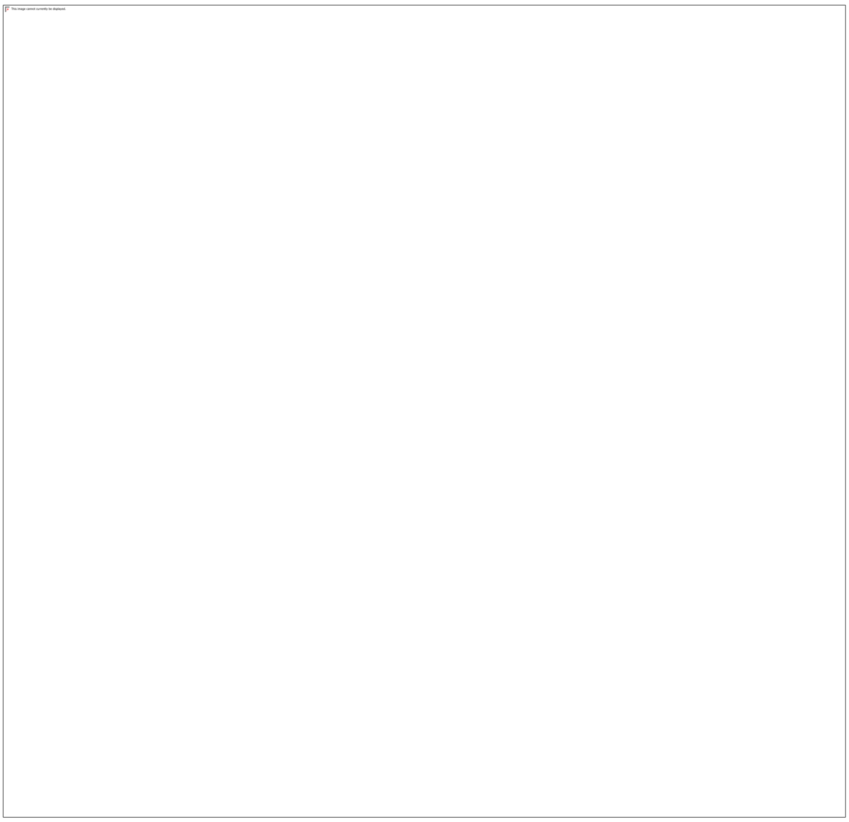

[Gray-scale features in ovarian lymphoma in 12 old patient]

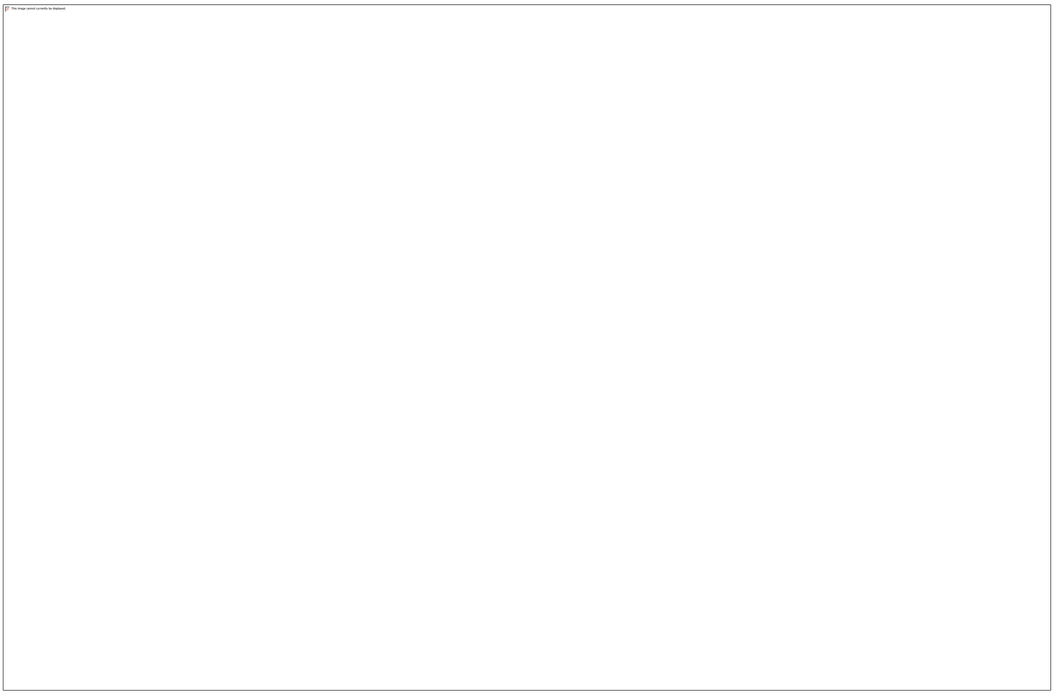

[Vascularization in ovarian lymphoma in 12 old patient]

Disclosure: Nothing to disclose

EP447

Clinical and ultrasound features of extra gastrointestinal stromal tumors (eGIST)

D. Fischerova[1], M. Ambrosio[2], A.C. Testa[3], F. Moro[3], D. Franchi[4], M.C. Scifo[2], N. Rams[5], E. Epstein[6], J.L. Alcazar[7], J.J. Hidalgo[8], C. Van Holsbeke[9], A. Burgetova[10], P. Dundr[11], D. Cibula[1]
[1]Gynecologic Oncology Centre, Department of Obstetrics and Gynecology, First Faculty of Medicine and General University Hospital, Charles University, Prague, Czech Republic, [2]Department of Obstetrics and Gynecology, S. Orsola Malpighi Hospital, University of Bologna, Bologna, [3]Department of Obstetrics and Gynecology, Catholic University of the Sacred Heart, Rome, [4]Division of Gynecology, European Institute of Oncology, Milan, Italy, [5]Hospital Santa Cruz y San Pablo, Barcelona, Spain, [6]Department of Obstetrics and Gynecology, Karolinska University Hospital, Stockholm, Sweden, [7]Department of Obstetrics and Gynecology, Clinica Universidad de Navarra, University of Navarra, Pamplona, [8]Department of Obstetrics and Gynecology, Clinic Hospital Valencia, Valencia, Spain, [9]Ziekenhuis Oost-Limburg, ZOL, Genk, Belgium, [10]Department of Radiology, [11]Department of Pathology, First Faculty of Medicine and General University Hospital, Charles University in Prague, Prague, Czech Republic

Introduction / Background: To describe the clinical and sonographic characteristics of extra-gastrointestinal stromal tumors (eGISTs).
Methodology: This is a retrospective multicentric study. From the database of 9 large European gynecologic oncology centers we identified patients with a histological diagnosis of eGIST, who had undergone preoperative ultrasound. One author from each center reviewed stored images and ultrasound reports, and described the lesions using IOTA and MUSA nomenclatures following ultrasound evaluation form. In addition, clinical information, surgical and pathological reports were recorded.
Results: Thirty-five women with eGISTs were identified, 17 incidental findings and 18 symptomatic

cases. Median age was 57 years (range 21-85). Two patients (6%) had ascites and median CA 125 was 23 U/mL (range 7-403 U/mL). The vast majority of eGISTs were intraperitoneal lesions (33/35, 94%) with few retroperitoneally (2/35, 6%). The most common site was abdomen (23/35, 65.7%), and 12 lesions (34%) were in the pelvis. eGISTs were typically large (median of 79 mm) and solid tumors (31/35, 89%), the remaining eGISTs were multilocular-solid tumors (4/35, 11%). The tumor shape was mainly lobulated (20/35, 57%) or irregular (10/35, 29%). The echogenicity of solid tumors was less often uniform (8/35, 23%) but always hypoechogenic. More frequently solid tumors were non-uniform due to mixed echogenicity (13/27, 40 %) or small irregular cysts (14/27, 37%). eGISTs were typically richly vascularized (color score 3 and 4, 31/35, 89%) with no shadowing (31/35, 89%). Based on pattern recognition, eGISTs were usually correctly classified as malignant lesion (32/35, 91%) and the specific diagnosis of eGIST was the most frequent among the presumed diagnosis (16/35, 46%).
Conclusion: On ultrasound, eGISTs were usually solid non-uniform intraperitoneal tumors with rich vascularization and no shadowing. They may mimic ovarian fibroma or uterine fibroids but the extra-genital origin in the combination with ultrasound characteristics in experienced hands point to the diagnosis of these uncommon tumors.
Disclosure: This work was supported by Charles University in Prague (UNCE 204065 and PROGRES Q28/LF1). None of the authors declare any conflict of interest.

EP448

New diagnositc device for cervical dysplasia using electrical impedence imaging

Y. Gen, S.I. Kim, J.Y. Hong, J. Lee, D.C. Park
Obgyn, St. Vincent Hospital, The Catholic University, Suwon, Republic of Korea

Introduction / Background: To develop a new conceptual diagnostic tool that detects the extent, area, and depth of cervical dysplasia and early cervical cancer using noninvasive electrical property measurement and electrical property distribution imaging method and provides clinical diagnostic information.
Methodology: The electrical properties of biomaterials are measured through the development of electrical property measurement probes and measurement systems using multi-channel electrodes. And then anomaly detection algorithm is made by analyzing the electrical property measurement values after measuring 10 normal cervical tissues and 37 cervical dysplasia tissues (Calculation system).
Results: There was a large difference in the change of the electrical property according to the frequency between the electrophysiological spectrum of the cervical tissue in normal and dysplasia (Figure2).
Conclusion: There was a large difference in the change of the electrical property according to the frequency between the electrophysiological spectrum of the cervical tissue in normal and dysplasia. Using this electrophysiological spectrum may be useful in diagnosis and prediction algorit.
Disclosure: Nothing to disclose

EP449

Magnetic resonance diffusion-weighted imaging in diagnostics of primary fallopian tube carcinoma: preliminary report

L. Grabowska-Derlatka[1], W. Szeszkowski[1], P. Derlatka[2]
[1]2nd Department of Radiology, [2]2nd Department of Obstetrics and Gynecology, Medical University of Warsaw, Warsaw, Poland

Introduction / Background: Primary fallopian tube carcinoma (PFTC) is the rarest form of female

genital malignancy. The imaging applied for suspected adnexal masses includes transvaginal ultrasound (US), computed tomography (CT), and magnetic resonance imaging (MRI), but the vast majority of PFTC is recognised intraoperatively.
Methodology: The study group consisted of seven women with postoperatively histopathological diagnosis of PFTC. To recognise characteristic findings for PFTC, retrospective analysis of preoperative MRI was performed. All patients underwent MRI of the pelvis and abdomen using a 1.5T MR system. Based on the results of the above imaging, suspected adnexal masses were recognised. MRI protocol contained T2-weighted images, fat-suppressed T2-weighted, T2-TIRM, DW EPI, pre- and postcontrast dynamic 3D T1 GRE in transverse orientation, with diffusion weightings of 0, 50, 100, 150, 200, 400, 800, and 1200 s/mm2. Regions of interest were outlined by a radiologist, who documented the character of adnexal masses on diffusion-weighted (DW) images and apparent diffusion coefficient (ADC) maps.
Results: In all seven patients with PFTC unilateral tumour was found. On all DW images (with β values of 0, 50, 100, 150, 200, 400, 800, and 1200 s/mm2) the mean signal intensities of solid parts of tumour were significantly higher than the mean signal intensities of normal ovarian tissue (p = 0.0001). There were no statistically significant differences between eight β values applied for ADC calculations.
Conclusion: Preoperative diagnostics of PFTC is difficult and mainly based on morphological features. Previous research did not show characteristics of PFTC in post-contrast dynamic imaging. In our material a clear increasing of signal intensity in DW imaging occurred independently of the β value.
Disclosure: Nothing to disclose

EP450

The use of Indocyanine Green in gynae-oncology, beyond sentinel pelvic lymph nodes

M. Graham[1], E. Craig[1], I. Harley[1], A. Armstrong[2], S. Dobbs[1], H. Nagar[1]
[1]Gynae Oncology, [2]General Surgery, Belfast City Hospital, Belfast, United Kingdom

Introduction / Background: Indocyanine Green (ICG) is a fluorescent imaging agent which can be given intravenously or subcutaneously and detected using near-infrared light at laparoscopy. It can be injected para-cervically in order to identify pelvic sentinel nodes at laparoscopy. It can also be given intravenously in order to test tissue perfusion.
Methodology: In this study we have reviewed the use of this technology beyond pelvic sentinel nodes. We have identified four interesting cases for discussion.
Case 1 - A total laparoscopic Hudson's, pelvic and para-aortic node dissection, omentectomy with re-anastomosis and loop ileostomy was performed for endometrioid adenocarcinoma detected during prior excision of endometrioma. Following this procedure intravenous ICG was used to test perfusion at the bowel anastomosis.
Case 2 - A total Laparoscopic hysterectomy was performed for Stage 1A endometrial cancer in a patient with a previous renal transplant. Following the procedure intravenous ICG was used to test perfusion to the transplanted kidney.
Case 3 - A Laparoscopic trachelectomy and pelvic node dissection was performed for 1B cervical cancer with incomplete margins. Following this procedure intravenous ICG was used to test uterine perfusion.
Case 4 - Open groin node dissection was performed on a patient with a squamous vulval cancer. Using the technology in the open setting, following subcutaneous injection of ICG around the tumor we were able to identify the sentinel groin node.
Results: We will show edited operative video of all cases mentioned above.
Conclusion: ICG technology has been shown to be a useful adjunct when performing sentinel node sampling. Through this presentation we have discussed a number of other interesting applications in the field of gynae-oncology.
Disclosure: Nothing to disclose

EP451

Significance of CA-125 levels in uterine papillary serous cancer

E. Keleş[1], S. Akiş[1], Y.E. Purut[1], M. Api[1], C. Kabaca Kocakuşak[1], Y. Ceylan[2], B. Giray[1], M. Güray Uzun[1]
[1]Gynecologic Oncology, Zeynep Kamil Training and Research Hospital, [2]Medicine, Istanbul Medipol University, Istanbul, Turkey

Introduction / Background: To investigate CA-125 levels in uterine papillary serous carcinoma (UPSC) patients.
Methodology: From January 2015 to December 2018, we collected data of UPSC and preoperative CA-125 levels of patients. Subsequent parameters were evaluated; age, preoperative endometrial sampling results, tumor size, lymph node involvement, LVSI, depth of myometrial invasion, stage.
Results: For the final analysis, 25 patients were eligible. Mean and %95 confidence interval for CA-125 levels in UPSC patients was 130.56 IU/ml (%95 CI : 5.18 - 255.93). Minimum was 4 and maximum was 1454 IU/ml (median= 19). Mean age was 63.04 + 8.7. In lymph node positive patients mean CA-125 was 114.8 IU/ml and lymph node negative patients 134.5 IU/ml (p=0,9). In patients with LVSI mean CA-125 level was 170.73 IU/ml and patients that didn't present with LVSI 76.77 IU/ml (p=0.77). There was no relationship between the tumor size and CA-125 level (Pearson coefficient 0.069, p=0,745). In patients with more than %50 myometrial invasion, mean CA-125 level was 137.66 IU/ml and was less than 50% 126.56 IU/ml (p=0.932). Mean CA-125 level for tumors that were spread beyond the uterus were 257.08 IU/ml and for the tumors that were confined to the uterus were 13.76 IU/ml (p=0,043).
Conclusion: Our results suggest that, preoperative CA-125 levels is a good predictor for extrauterine involvement and for advanced stage. There was no correlation between CA-125 and LVSI, lymph node positivity, myometrial invasion, age.
Disclosure: Nothing to disclose

EP452

Ultrasound diagnosis of ovarian tumours by pattern recognition

J. Knez, G. Gruškovnjak, M. Pakiž
University Medical Centre Maribor, Maribor, Slovenia

Introduction / Background: Correct diagnosis is crucial for appropriate treatment of ovarian tumours. Ultrasound is the first diagnostic tool used in this process. Several diagnostic models have been developed for classification of adnexal masses as benign or malignant, but pattern recognition by experienced observer remains one of the most reliable approaches. Our aim was to compare the diagnostic accuracy of transvaginal ultrasound in ovarian tumours classification between operators specifically focused on gynaecological oncology and those practicing general gynaecology.
Methodology: We conducted a retrospective analysis of all ovarian tumors treated surgically in the period between January 2016 and December 2017. All patients were seen in two university based units - Department for gynaecologic oncology (GO) and Department for general gynaecology (GG). All women had transvaginal ultrasound examination prior to surgery and all tumours were classified into three categories - benign, malignant or likely malignant by the use of pattern recognition.
Results: Ultimately, 149 women were included in the analysis, 76 were seen by GO and 73 by GG. After histological examination of specimens, 37 women (25%) had various types of malignant tumours, 19 (13%) had borderline tumours and 93 (62%) had benign tumours. Sensitivity of ultrasound analysis for malignant tumour categorisation was 100% when performed by GO and 58% when performed by GG. The specificity was 41% when performed by GO and 87% when performed by GG. This translates to negative predictive value of 100% by GO and 91% by GG.
Conclusion: Ultrasound diagnosis of ovarian tumours by pattern recognition is a reliable diagnostic method that rarely results in false negative diagnosis leading to additional surgical procedures. When performed by operators specifically focused on gynecologic oncology, the false negative rates seem to

be lower.
Disclosure: Nothing to disclose

EP453

Fibrothecomas masquerading as ovarian malignancy; a review of pre-operative imaging

D. Kolomainen, K. Rothnie, S. Liyanage
Southend University Hospital, Essex, United Kingdom

Introduction / Background: Ovarian fibrothecomas are the most common benign solid ovarian tumour, accounting for approximately 4-5% of all ovarian tumours. Imaging can aid diagnosis of fibrothecoma pre-operatively, with its correct interpretation reducing radiological misdiagnosis as an ovarian malignancy. This could decrease physical, psychological, and social costs of placing patients on the cancer pathway unnecessarily. This is a retrospective review correlating radiological diagnosis with final histological diagnosis of fibrothecoma.
Methodology: Over a six-year period from 2013 to 2018, we identified patients with a radiological/histological diagnosis of fibrothecoma from the radiology and histology databases, and multi-disciplinary team (MDT) notes at a Cancer Unit and Centre. Pre-operative imaging, MDT outcomes, operative notes, use of frozen section, and final histology were analysed.
Results: 660 patients with suspicious adnexal masses were discussed at the MDT over the six-year period, of which 31 patients had fibrothecoma. Of this group, the median age was 65(range 29-88), and 27/31(87%) post-menopausal. CA125 was documented in over 90%(28/31), with a risk of malignancy index(RMI) >250 in 42%(13/31). 7 patients(23%) underwent one imaging modality (ultrasound, CT, or MRI), 17(55%) had two modalities, and 7 patients(23%) had all three. In those who underwent ultrasound imaging (19/31(86%)),26% had a documented U-score. Imaging reports suggested fibrothecoma in 24%(9/31), and 56%(5/9) of these underwent surgery. All 31 patients were discussed at the MDT, and 65%(20/31) underwent surgery at the Cancer Centre, all requiring frozen section. The operative morbidity rate was 33%, and median length of hospital stay 3 days(range 1-10).
Conclusion: This study highlights that we can improve the pre-diagnostic pathway for our patients, and avoid unnecessary surgery. We endeavor to achieve this through the implementation of standardized radiological reporting for suspected fibrothecoma.
Disclosure: Nothing to disclose

EP454

Identification of miRNAs associated with endometriosis in Pakistani women

I. Liaqat[1], T. Zia[2]
[1]GC University Lahore, [2]Zoology, GC University Lahore, Lahore, Pakistan

Introduction / Background: Endometriosis is a gynecological condition that affects the female reproductive system and ultimately caused infertility. This disease is present worldwide in all racial groups. Incapacitating signs and symptoms with global prevalence were the reasons of unrevealed pathogenic mechanism of endometriosis. Various risky health phenomenon in relation to endometriosis may triggered by plasma micro RNA (miRNAs) which possibly, could be changed by therapeutic techniques used for endometriosis. For early diagnosis of endometriosis, the miRNAs role as biomarker is crucial.
Methodology: The blood samples of genetically unrelated endometriosis women and controls, 160 each, were used to isolate RNA. This total RNA was used to synthesize cDNA. This cDNA was used to identify miRNAs either down regulated or up regulated for further confirmation of endometriosis by real time PCR.

Results: Twenty microRNAs were differentially expressed in women with endometriosis and their controls. Out of which six microRNAs (miR-15a, miR-15b, miR-16-1, miR-16-2, miR-195 and miR-497) were selected for validation. MiR-15a, miR-15b and miR-195 were significantly down-regulated in women with endometriosis when compared with controls (p= 0.011, 0.002 and 0.002, respectively), yielding an area under the receiver operator characteristics curve of 0.74 (95% CI: 0.58-0.90; 0.79 (95% CI: 0.65-0.93) and 0.85 (95% CI: 0.71-0.98) in endometriosis from controls respectively.
Conclusion: miRNAs appear to be potent regulators of gene expression in endometriosis and its associated reproductive disorders, raising the prospect of using miRNAs as biomarkers and therapeutic tools in endometriosis. Identification of miRNAs lessens the health care cost and improves the work productivity of endometriotic females.
Disclosure: Nothing to disclose

EP455

Diagnostic hysterectomy for clinical suspicion of uterine cancer when histological confirmation is either not possible or inconsistent with clinical findings

S. Makrydima[1], D. Saragli[2], M. Wong[3], R. Wuntakal[4]
[1]Clinical Fellow in Gynaecology Oncology, [2]Specialist Registrar in Obstetrics and Gynaecology, [3]Specialist Trainee in Obstetrics and Gynaecology, [4]Lead Consultant in Gynaecology Oncology Department, Queen's Hospital Romford, London, United Kingdom

Introduction / Background: Traditionally, hysterectomy for endometrial cancer is performed following histological diagnosis. However, there are few cases where histology is impossible, inadequate or inconsistent with clinical findings/ symptoms. Therefore, even though the term “diagnostic hysterectomy” is not used in literature, clinicians occasionally counsel patients regarding hysterectomy in the absence of abnormal histology, but in the presence of clinical suspicion of cancer. Our aim was to evaluate the need of diagnostic hysterectomy when all other diagnostic techniques have been inconclusive, but there is clinical suspicion of uterine cancer.
Methodology: We have analysed all diagnostic hysterectomies (39) performed between 2016-2018 at a UK cancer unit because of clinical suspicion of uterine cancer. All cases were offered surgery following multidisciplinary team discussion.
Results: Four categories of patients were included;
1. symptomatic patients (postmenopausal bleeding/ discharge/ irregular vaginal bleeding) where hysteroscopy was unsuccessful (20) or views were obscured (1),
2. symptomatic patients, where hysteroscopy was suspicious (4), although histology was negative or inadequate,
3. symptomatic patients with atrophic cavity on hysteroscopy or inadequate histology (12) and
4. asymptomatic patients with incidental finding of thickened endometrium and failed hysteroscopy (2).

Final histology revealed 11 (28%) cases of cancer; 6 endometrial cancers (4 endometrioid, 2 carcinosarcomas), 2 uterine sarcomas (1 leiomyosarcoma,1 rhabdomyosarcome), 2 fallopian tube carcinomas (1 mesothelioma, 1high grade serous carcinoma) and 1 sex cord stromal tumour of the ovary. We have to underline that 6 out of 11 cases (50%) were very aggressive with poor prognosis.
Conclusion: Despite the advances of hysteroscopic techniques in the diagnosis of uterine cancer, there is still place for diagnostic hysterectomy when there is clinical suspicion of cancer and no definitive histology. For carefully selected cases, the cancer rate can be up to 30%, which justifies the necessity for intervention.
Disclosure: Nothing to disclose

EP456

Searching HPV genome for methylation sites involved in molecular progression to cervical precancer

C. Kottaridi[1], V. Pergialiotis[2], D. Leventakou[1], A. Pouliakis[1], G. Chrelias[2], E. Patsouri[1], A. Zacharatou[1], E. Panopoulou[1], V. Damaskou[1], V. Sioulas[2], C. Chrelias[2], S. Kalantaridou[2], I. Panayiotides[1]
[1]2nd Department of Pathology, [2]3rd Department of Obstetrics and Gynecology, National & Kapodistrian University of Athens, Athens, Greece

Introduction / Background: Human Papilloma Virus has been considered as the main cause for cervical cancer. In this study we investigated epigenetic changes and especially methylation of specific sites of HPV genome. The main goal was to correlate methylation status with histological grade as well as to determine its accuracy in predicting the disease severity by establishing optimum methylation cutoffs.
Methodology: In total, sections from 145 cases genotyped as HPV16 were obtained from formalin-fixed, paraffin-embedded tissue of cervical biopsies, conization or hysterectomy specimens. Highly accurate pyrosequencing of bisulfite converted DNA, was used to quantify the methylation percentages of *UTR* promoter, enhancer and 5' UTR, *E6* CpGs 494, 502, 506 and *E7* CpGs 765, 780, 790. The samples were separated in different groupings based on the histological outcome. Statistical analysis was performed by SAS 9.4 for Windows and methylation cutoffs were identified by MATLAB programming language.
Results: The most important methylation sites were at the enhancer and especially *UTR* 7535 and 7553 sites. Specifically for CIN3+ (i.e. HSIL or SCC) discrimination, a balanced sensitivity vs. specificity (68.1%, 66.2% respectively) with positive predictive value (PPV) and negative predictive value (NPV) (66.2%, 68.2% respectively) was achieved for *UTR* 7535 methylation of 6.1% cutoff with overall accuracy 67.1%, while for *UTR* 7553 a sensitivity 60.9%, specificity 69.0%, PPV=65.6%, NPV=64.5% and overall accuracy=65.0% at threshold 10.1% was observed.
Conclusion: Viral HPV16 genome was found methylated in NF-1 binding sites of *UTR* in cases with high grade disease. Methylation percentages of *E6* and *E7* CpG sites were elevated at the cancer group.
Disclosure: Nothing to disclose

EP457

Assessment of ovarian tumors with tumor educated platelets (TEPs)

J.M.J. Piek[1], S.G.J.G. In 't Veld[2], M.G. Best[2], B. Tannous[3], A. Supernat[4], C.A.R. Lok[5], C.D. de Kroon[6], T. Wurdinger[2]
[1]Gynaecologic Oncology, Catharina Hospital, Eindhoven, [2]VUmc Cancer Center Amsterdam, Amsterdam, The Netherlands, [3]Massachusetts General Hospital, Massachusetts, MA, United States, [4]Medical University of Gdansk, Gdańsk, Poland, [5]Antoni van Leeuwenhoek Hospital, Amsterdam, [6]Leiden University Medical Center, Leiden, The Netherlands

Introduction / Background: Differentiation of benign and malignant ovarian tumors before surgery is difficult with currently used biomarker based algorithms such as the Risk of Malignancy Index (RMI), and these algorithms are prone to subjective interpretation. This leads to misclassification and incorrect referral to oncologic centers. Therefore, there is an urgent need for more discriminating and objective biomarkers. Recently, “Tumor-Educated Platelets (TEPs)” have shown to be able to distinguish patients with and without a localized malignancy with an accuracy of 96% (Cancer Cell. 2015 Nov 9;28(5):666-676). However, TEPs have never been investigated in clinical early stage ovarian cancer. Therefore, we determined the accuracy of TEPs to discern ovarian malignancies from benign ovarian tumors in patients with an ovarian tumor referred to oncologic centers.
Methodology: The RMI was calculated according to Jacobs e*t al* (Br J Obstet Gynaecol. 1990 Oct;97(10):922-9). The TEP score was calculated according to Best *et al* (Cancer Cell. 2015 Nov 9;28(5):666-676) using a training and evaluation cohort. Pathology results were used for the definitive diagnosis of benign or malignant disease.
Results: The training cohort consisted of 41 women (20 FIGO stage I&II ovarian carcinomas (OC), and 21 healthy controls). The evaluation cohort consisted of 43 women (21 OC, and 20 benign tumors (BT)). When using a RMI cut-off level of 200 , the accuracy of differentiating OC from BT was 44%.

When using TEPs, the accuracy went up to 80% (figure 1 and tables 1&2).
Conclusion: Implementing TEPs as a standard test before referral, is likely to lead to an increase in correct classification of ovarian tumors. Prospective studies will have to confirm whether this results in a decrease of incorrect referrals of patients with an ovarian tumor to an oncologic center.

RMI	Benign	Malignant (stage I-II)
RMI <200 (predicted benign)	5	5
RMI >200 (predicted malignant	15	11

[Risk of Malignancy Index (RMI)]

TEP	Benign	Malignant (stage I-II)
TEP score ≤0.67	16	4
TEP score >0.67	4	17

[Tumor Edicated Platelets (TEPs)]

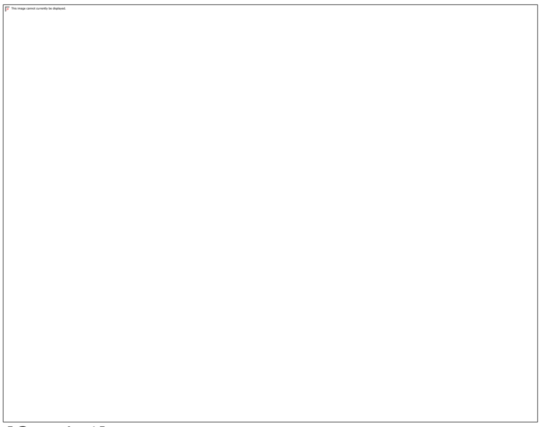

[Graph 1]

Disclosure: Nothing to disclose

EP458

Does HPV type affect the rate of positive surgical margin after cervical cone excision?

Y.E. Purut[1], S. Akiş[1], E. Keleş[1], M. Api[1], C. Kabaca Kocakuşak[1], Y. Ceylan[2], B. Giray[1], M. Güray Uzun[1]
[1]Gynecologic Oncology, Zeynep Kamil Training and Research Hospital, [2]Medicine, Istanbul Medipol University, Istanbul, Turkey

Introduction / Background: Targeted to find the correlation between HPV types and surgical margin positivity in patients exposed the excisional procedure for treatment of precancerous cervical pathologies.
Methodology: We investigated retrospectively 453 patients who recommended Loop Electrosurgical

Excision Procedure (LEEP) or Cold Knife Conization (CKC) between April-2016 and December-2018. The surgical margin was also examined and separately analysed in patients with a surgical margin less than 1-mm. Pre-procedural HPV type and endocervical curettage results, number of surgical specimen pieces, age, and depth of surgical material were recorded. Post-cone sampling of the endocervical canal was performed. HPV types divided into 6 groups as type-16 only, type-18 only, type-16 and type-18 together, other high-risk HPV types (HR-HPV), type-16 and HR-HPV together, type-18 and HR-HPV together.
Results: We performed LEEP in 72 patients and CKC in 376 patients. The mean age was 41.6 years. The surgical margin was positive in 22 patients that underwent LEEP, while in 153 patients that underwent CKC while 1-mm close margin positivity taken into account. Patients (38%) had at least cervical intraepithelial neoplasia (CIN)-1 in the endocervical canal. High-risk types (except 16/18) were presented in 133 patients. Surgical margin positivity was found to be unrelated with the type of HPV in patients either 1-mm close proximity was considered to be positive or negative.
Conclusion: No significant difference was found between LEEP and CKC in terms of surgical margin positivity. In addition, there was no significant difference between HPV groups in terms of surgical margin positivity. Patients' age and the presence of multiple pieces of material had no effect on the surgical margin positivity. There was a relationship between the presence of CIN-1 and higher lesions in the endocervical canal and positive surgical margins. Interestingly, CIN (any grade) in the endocervical canal was more common in the other HR-HPV group excluding type-16/18.
Disclosure: Nothing to disclose

EP459

Clinical management of ovarian masses in pregnancy

F. Mascilini[1], L. Quagliozzi[1], F. Moro[1], A.R. Palumbo[1], I. Peterlunger[1], A.C. Testa[2], G. Scambia[2], A. Fagotti[2]
[1]Fondazione Policlinico Agostino Gemelli, IRCCS, Dipartimento Scienze della Salute della Donna, del Bambino e di Sanità Pubblica, UOC di Ginecologia Oncologica, [2]Fondazione Policlinico Agostino Gemelli, IRCCS, Dipartimento Scienze della Salute della Donna, del Bambino e di Sanità Pubblica, UOC di Ginecologia Oncologica, Catholic University of the Sacred Heart, Rome, Italy

Introduction / Background: The number of women diagnosed with ovarian masses during pregnancy has increased in recent years and the management of these women is controversial.
Methodology: To describe the diagnostic-accuracy of US in the differential diagnosis ovarian masses diagnosed during pregnancy. This is a retrospective single center study, approved by the local IRB. Clinical charts of pregnant women examined at our gynecologic oncology ultrasound center between 2000 and 2019 for a suspicious adnexal mass were retrieved. Ultrasound characteristics of the masses were described by using International Ovarian Tumor Analysis (IOTA) terminology.
Results: 98 patients with an ovarian mass during pregnancy were found. Fifty-five women (56.1%) underwent close US monitoring each four weeks until delivery and one month after delivery. The remaining 43 cases (43.9%) underwent surgery for either high suspicious of malignancy, or high risk of side effects such as torsion, rupture or obstacle to pregnancy. Among them, 14 (32.5%) had a malignant tumor (10 primary ovarian and 4 metastatic tumor to the ovary), 17 (39.5%) had borderline ovarian tumor (4 mucinous and 13 serous histotype), 12 (28.0 %) had a benign ovarian disease. At ultrasound, papillary projections were present in 16 of 31 malignant/borderline tumors (4 primary ovarian cancer and 12 borderline) and in 4 of 12 benign masses (p=0.281). The most frequent US feature was multilocular-solid mass (12/31, 38.7%) followed by unilocular-solid (9/31, 29.1%), solid (7/31, 22.5%) and multilocular (3/31, 9.7%). Accuracy rate of US with respect to final histological diagnosis was 76.7% considering BOT as primary ovarian tumors.
Conclusion: Differential diagnosis of ovarian masses during pregnancy is still challenging and clinical management needs clear US rules.
Disclosure: Nothing to disclose

EP460

Uncommon case of fallopian tubes teratocarcinomas in 36-year-old female

K. Rasoul-Pelińska, M. Czech, M. Bobiński, J. Kotarski, A. Czekierdowski, W. Bednarek
I Department of Gynecological Oncology and Gynecology, Medical University Lublin, Lublin, Poland

Introduction / Background: Teratomas are type of germinal neoplasms derived from multipotential cells, may contain one to all tree germ layers. Usually localised in ovaries and testis due to migration of primary embrionic germ cells in developing embryo, mature teratomas found in other abdominal/pelvic locations are an extremely rare case.
Methodology: A 36-year-old patient reported to the doctor for routine gynecological examination. In transvaginal ultrasound a "small cystic-solid change in the right ovary" was detected. The Ca-125 level was 29.1 U / ml. The patient was sent for a consultative examination to the ultrasound specialist. He found unchanged left ovary, and under it irregular form with mixed echogenicity. In the right adnexa was cystic-solid lesion with a highly vascularized solid field. The patient was referred for minimally invasive surgery. Next obtained results of laboratory tests: Ca-125 20,03U / ml and HE4 50 pmol / l. There were found unchanged uterus and ovaries, intraoperatively but both fallopian tubes were pathologically chanaged. The right fallopian tube was sent for an intraoperative study, giving a result suggesting teratocarcinoma of the fallopian tube.
Results: The histology revealed bilateral teratocarcinomas. Currently, patient stays under clinical surveillance.
Conclusion: In conclusion, teratomas of fallopian tubes are rarely encountred findings still should be considered in the differential diagnosis of extragonadal abdominal masses.
Disclosure: Nothing to disclose

EP461

The role of certain morphological and doppler velocimetric ultrasonographic criteria in the prediction of adnexal mass malignancy

M. Tanturovski, I. Aluloski, S. Stojcevski, V. Jovanovska
Gynecologic Oncology, University Clinic of Obstetrics and Gynecology, Skopje, The former Yugoslav Republic of Macedomia

Introduction / Background: To evaluate the diagnostic performance of certain morphological and Doppler parameters in the preoperative distinction of benign and malignant adnexal masses.
Methodology: We recruited patients that had a newly diagnosed unilateral adnexal mass with no previously recorded gynecologic malignancy and were scheduled for surgery at the Department. We recorded the following morphologic characteristics, in accordance with the terms and measurements described by the IOTA group: tumor morphology (cystic, complex, solid), presence of septa, presence of papillary projections, presence of wall irregularities and presence of ascites. After the detailed gray-scale ultrasound evaluation of the morphology of the mass, we created Doppler flow velocity curves to calculate the PSV, TAMXV, RI and PI, in accordance with the IOTA criteria.
Results: We recruited a total of 216 patients; the pathohistology revealed benign tumors in 77.31% of patients, bordeline tumors in 0.93% and malignant neoplasms in 21.76% of patients. The presence of a unilocular cystic tumor reduced the risk of malignancy by LR+ 0.95. Solid tumor morphology was almost universally associated with malignant neoplasms, increasing the risk of cancer by LR+ 5.04. The presence of septa, wall irregularities and papillary projections were also suggestive of malignancy with LR+ of 8.03, 19.88 and 5.02, respectively. The risk of malignancy was higher in tumors with RI≤0.4 (specificity 98.8%. LR+ 64.76). Most malignant tumors had PI< 1.0 (LR+ 6.63). The criteria based solely on flow velocities performed worse than the indices in our series.
Conclusion: It is very likely that the set of characteristics that we described could be useful for teaching purposes, particularly for clinicians with limited experience in diagnosing ovarian tumors, a process that is automated for experts. The data suggests that these simple morphological traits can be used to characterize the majority of ovarian tumors with a high degree of accuracy.

Disclosure: Nothing to disclose

EP462

Value of tumor markers and pelvic mass diameter in differential diagnosis of peritoneal carcinomatosis

C. Tauste Rubio[1], S. Aguirre Gorospe[2], J. Zabaleta Jurío[2], M. Ruiz García[2], I. Zabaleta Loinaz[2], J.C. Muruzábal Torquemada[2]
[1]Complejo Hospitalario de Navarra | Gynaecologic Oncology, Osasunbidea/ Navarre Health Service,
[2]Complejo Hospitalario de Navarra, Pamplona, Spain

Introduction / Background: Peritoneal Carcinomatosis (PC) is a late stage manifestation of several malignancies like gynecological, gastrointestinal or hepatobiliary cancers, among others. Epithelial ovarian cancer (EOC) is the most common cause in women and has better outcomes in terms of survival compared with other malignanciess like colorectal neoplasms or mesotheliomas. The pathological study is essential to plan the suitable treatment and early intervention in EOC. In this work we analyzed the value of tumor markers CA 125, CEA, ratio CA 125/CEA and diameter of ovarian tumor (DOT) as a pre-surgical predictive tool of ovarian cancer.
Methodology: We recruited patients with suspicious CP studied in the Gynaecological Department of Complejo Hospitalario de Navarra, between 2005 and 2016. CA 125 and CEA were evaluated in preoperative venous blood samples and DOT was estimated by ultrasound or CT scan.
Results: We identified 250 patients with suspicious CP. Median age was 64,5 years and most patients (86.4%) were diagnosed with EOC. Final diagnosis is described in Table 1. CA 125 and ratio CA 125/CEA was higher in those patients with EOC, while CEA was lower with significant differences (p< 0,05). DOT was similar in both groups without signficant differences (Figure 1). Receiver-operating curve (ROC) analysis showed low power diagnostic in EOC patients for CA 125 and CEA (Area under the curve, AUC: 0.698 and 0.642 respectively). ROC analysis of CA 125/CEA ratio improves the results (AUC: 0,747) (Figure 2).
Conclusion: CA 125, CEA and CA 125/CEA ratio showed certain utility to guide the diagnosis of EOC in CP. However the cut-off levels of this study did not show clinical relevance. DOT did not prove any additional information. Other techniques, such as HE 4 or Thermal liquid biopsy could be more specific for EOC

	Number	%
Ovary	216	86.4
pancreas-biliar	5	2
Endometrium	5	2
pseudomixoma	4	1.6
mesothelioma	4	1.6
colon and rectum	3	1.2
Estomach	3	1.2
Lung	2	1
Miscellaneous	8	3

[Final diagnosis of peritoneal carcinomatosis]

	CA 125	CEA	CA 125/CEA	DOT
AUC	0.698	0.642	0.747	0,544

Cut off	>366	<1	>250	non signficant
Specificity (%)	75	75	75	75
Sensibility(%)	57.4	33.5	58,7	non signficant

[Area Under the Curve (AUC), Specificity and Sensitivity of CA 125, CEA, CA 125/CEA ratio and Diameter of Ovarian Tumor (DOT)]

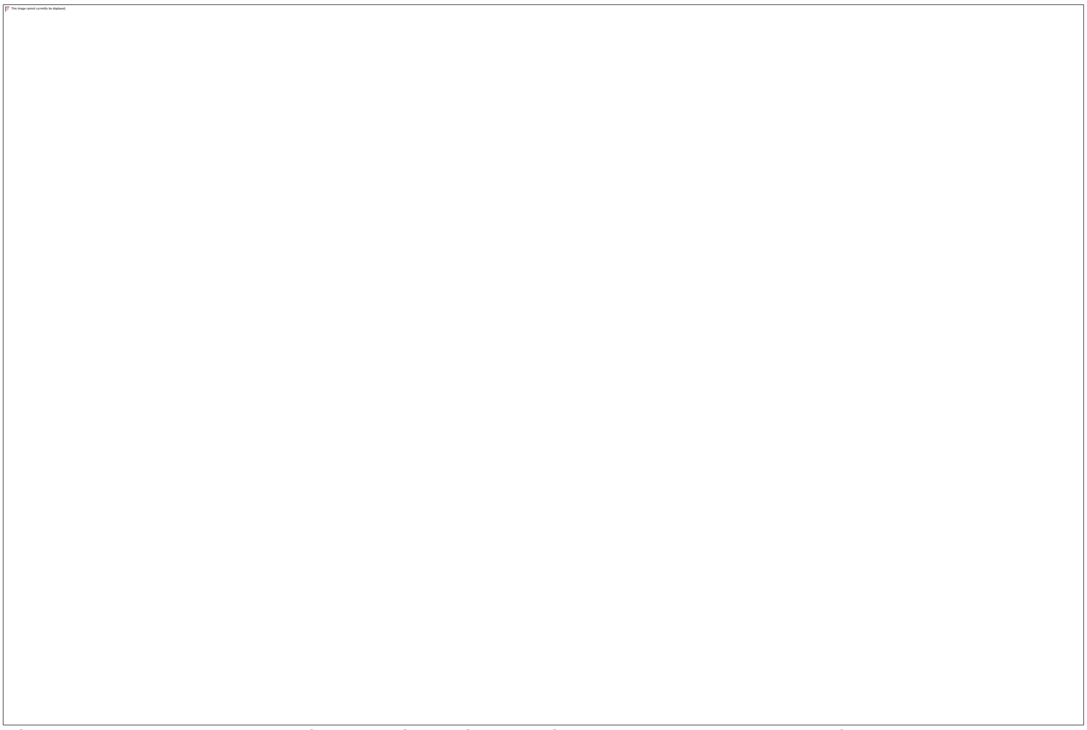

[Stem-and-Leaf Diagram of CA 125, CEA, CA 125/CEA ratio and Diameter of Ovarian Tumor]

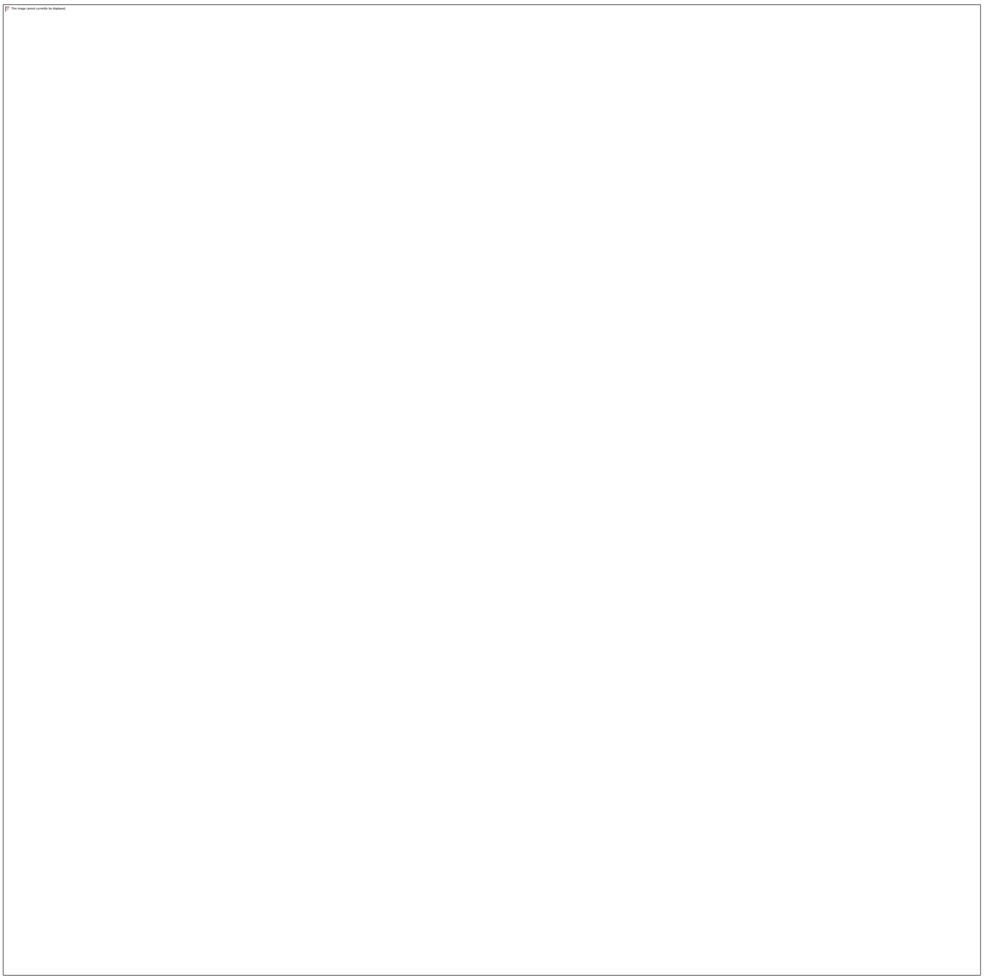

[Receiver Operating Characteristic curve (ROC) of CA 125, CEA. CA 125/CEA ratio, DOT]

Disclosure: Nothing to disclose

EP463

Endometrial cancer recurrence in low risk patients - analysis of potential adverse risk factors in the experience of a single center

A. Abacjew-Chmylko, A.J. Szkop-Dominiak, D. Wydra, J. Kobierski, S. Sawicki, H. Olszewska, E. Tupacz-Mosakowska
Dept. of Gynecology, Gynecological Oncology and Endocrinological Gynecology, Medical Univeristy of Gdansk, Gdansk, Poland

Introduction / Background: Patients with endometrial cancer of low risk (stage IA G1) are recommended for observation or adjuvant therapy (brachytherapy), as recommendations allow, on the basis of the presence of potential adverse risk factors such as: age, positive LVSI, tumor size, lower uterine segment or surface cervical glandular involvement.
The aim of this work was to revise low risk endometrial cancer patients that did not have adjuvant brachytherapy and developed recurrences in the context of their potential adverse risk factors.
Methodology: Among 125 patients of stage IA G1 endometrial cancer, treated surgically in our centre

(total laparoscopic hysterectomy with bilateral salpingoophrectomy and sentinel lymph node dissection or regional lymphadenectomy) with no adjuvant therapy between years 2012 and 2016, recurrences were noted in 2 cases (1,6%), as analyzed below.
Results: In both cases the recurrence occurred in the apex of the vagina.
In the first case the relapse happened after 13 months from primary treatment. Initial surgery was performed in a 62 year-old patient and revealed a G1 endometroid endometrial cancer superficially invading the myometrium, with surface endocervial cancerous implants.
In the second case the recurrence was discovered 16 months after surgery. The primary treatment was performed in a 64 year-old patient and pathologic evaluation described an endometroid G1 endometrial cancer in stage IA involving the lower uterine segment, with positive LVSI.
Both patients after receiving treatment have been now disease-free for 18 months.
The analysis of non-recurrent cases revealed presence of single or multiple potential adverse risk factors.
Conclusion: A combination of potential adverse risk factors must influence the formation of a relapse in low risk endometrial cancer. A calculative score system should be applied to assess potential adverse risk factors and define indications for brachytherapy.
Disclosure: Nothing to disclose

EP464

Relation between Human Epididymis Protein 4 (HE4) and endometrial pathology in patients with postmenopausal bleeding

A. Aboulnasr[1], G. Abdelmoety[1], M. Salem[2], M. Elsharkawy[3], N. Mohamed[1]
[1]Obstetrics & Gynecology, [2]Pathology, [3]Clinical Pathology, Cairo University / Faculty of Medicine, Cairo, Egypt

Introduction / Background: To evaluate the value of human epididymis protein 4 (HE4) in predicting endometrial pathology in patients with postmenopausal bleeding (PMB).
Methodology: 100 patients with PMB were recruited with endometrial thickness (ET) >5mm by transvaginal sonography(TVS).HE4 was measured and hysteroscopic guided fractional curretage (FC) was done. 10 patients were excluded and 90 patients were subjected to abdominal total hysterectomy and bilateral salpingo-oophorectomy with or without pelvic lymphadenectmoy according to pathology .
Results: Level of HE4 in the 90 cases showed a significant difference in various endometrial pathologies with strong positive correlation with severity of the lesion. Mean value ± standard deviation was 38.33±27pmol/L for atrophic endometrium (11 cases), 51.26±28.59 pmol/L for simple endometrial hyperplasia(SEH,51 cases),148.4 ±67.3pmol/L for atypical endometrial hyperplasia (AEH ,16 cases) and 390.9±351.72pmol/L for endometrial carcinoma(EC,12 cases) .Using the cut-off value of 69.5pmol/L for preoperative HE4 yielded a sensitivity of 75% and a specificity of 88.5% in prediction of EC. HE4 was significantly higher (P< 0.001) according to stage (stage I 322.56 ±182.66 and stage II 501.7±423.74) ,grade (grade1 82.16±55.23, grade2 308.89±275.85 and grade3 920.54±166.17) and lymph node involvement (167.84±112.43 in negative and 635.42±426.88 in positive)
Conclusion: : HE4 can predict severity of endometrial pathology in PMB with high specificity and fair sensitivity. HE4 level correlates positively with stage , grade and lymph node involvement in EC .
Disclosure: Nothing to disclose

EP465

Influence of infection in endometrial carcinoma on clinical and morphological characteristics of endometrial cancer

T. Moiseenko, E. Frantsiyants, T. Zykova, E. Nepomnyashchaya, M. Adamyan, A. Menshenina, M.

Vovkochina, E. Verenikina
Rostov Research Institute of Oncology, Rostov-on-Don, Russian Federation

Introduction / Background: Chronic metroendometritis is responsible for 75% of cases of uterine disorders in women of the reproductive age, such as adenomyosis, uterine fibroids or endometrial polyps. Infection is considered a promoter of polyposis and endometrial carcinoma (EC) in postmenopausal patients. Our purpose was to determine the influence of chronic infection on the formation of clinical and morphological variants of EC.
Methodology: Postoperative data on morphological and microbiological examination of EC tissues of 240 patients (mean age 64.5 years, 27% - reproductive age, 73% - peri- and menopausal patients) were studied. After removal of the preparation, the macroscopic state of tumors was evaluated intraoperatively together with the morphologist, and tumor tissues were collected for microbiological examination. The presence of bacterial or viral infections in EC tissues was detected by PCR and ELISA.
Results: EC tissues were morphologically studied with detailed characteristics of the tumor process. 87% - endometrioid EC, 13% - aggressive variants (papillary serous carcinoma and adenosquamous carcinoma). 62% of EC were infected with chlamydia, cytomegalovirus, herpes simplex viruses and HPV. Tumors in patients of reproductive age were infected in 92%. 54% of adenosquamous tumors were infected with HPV 18. Endometrioid tumors showed combined bacterial and viral infections in 83%, monoinfection (bacterial or viral) in 16%; combined viral infections were rare. Patients of reproductive age with monoinfections had the most favorable variants of G1-2 EC in 43% of cases. Older menopausal patients with tumors infected with viral agents demonstrated atrophic endometrium and aggressive G2-3 EC.
Conclusion: The presence of chonic infection in EC is directly associated with the morphological type and aggressiveness of tumors and the patient's age.
Disclosure: Nothing to disclose

EP466

Stage II/III endometrial cancer - how RADICAL should we be? A review of management in the Northern Ireland regional cancer centre

S. Addley, M. McComiskey, S. Dobbs, H. Nagar, E. Craig, I. Harley
Gynae Oncology, Belfast City Hospital, Belfast, United Kingdom

Introduction / Background: ESGO guidance (2016) recommends simple hysterectomy and lymphadenectomy (PLN / PALN) for stage II and III endometrial cancer. BGCS guidance (2017) also recommends simple hysterectomy in both stage II/III disease, but advises reserving lymph node dissection for stage III only.
Methodology: Practice in the Northern Ireland Regional Cancer Centre was compared with current ESGO and BGCS guidance. A retrospective case-note review was performed for patients undergoing surgery for stage II/III endometrial cancer in the NI Regional Cancer Centre between 2013 and 2018.
Results: 53 cases were identified, with mean age 60.5 years and mean BMI 30.6 kg/m2. 62% of the cohort had stage II endometrial cancer on pre-operative MRI; and 38% stage III.
50% of surgeries were performed by Consultants 'buddy-operating'. All patients underwent radical hysterectomy - 84% laparoscopically, with a conversion to open rate of 6.9%. 96% had concomitant BSO; 94% PLND and 23% PALND. PALND was performed in 25% with pre-operative stage III disease. One case of bowel injury occurred intra-operatively.
Histopathology reported parametrial involvement in 19% of pre-operative stage II and 25% of pre-operative stage III cases. Histopathology also identified positive PLN in 19% and 25% of pre-operative stage II and III cases respectively; and positive PALN in 3.8% and 6.3% of these cases.
Post-operatively, 7% of patients required HDU. The mean haemoglobin drop was 15 g/dl. Urinary retention was the most common complication (19%). Mean length of stay was 4.9 days.
Conclusion: In accordance with current guidance and in light of the higher morbidity associated with a radical approach, a change of practice to simple hysterectomy has been recommended within the unit. The role of lymphadenectomy remains controversial, particularly in stage II disease; a population which may particularly benefit from the introduction of sentinel lymph node sampling in the future.

Disclosure: Nothing to disclose

EP467

Endometrial carcinoma in patients with sentinel lymph node biopsy: recurrences

I. Aizpuru, I. Jaunarena, P. Cobas, R. Ruiz, M. Gorostidi, M. Moreno, A. Lekuona
Donostia Hospital, San Sebastian, Spain

Introduction / Background: We describe the cases of recurrence in endometrial carcinoma with sentinel lymph node biopsy (SLNB) study and analyze possible factors associated with recurrence.
Methodology: Retrospective cohort study of patients with recurrent endometrial carcinoma in whom SLNB was performed between 2012-2018. Hysterectomy, double adnexectomy and SLNB were performed by laparoscopy in stages IA G1-G2; while in stages IA G3 and above, we added pelvic and para-aortic lymphadenectomy.
Factors that may associate with recurrence were analyzed: high tumor grade (G3), lymphovascular space invasion (LVSI), myometrial infiltration (MI)> 50% and positive SLNB.
Kaplan-Meier curves, long-rank test and Cox regression were used for statistical analysis.
Results: We followed 159 patients. Recurrence rate was 7.5%. Mean age was 68.4 years (SD = 10.5), BMI 29.4 (SD = 4.9) and tumor size 41.2 mm (SD = 15.2).
Of the 12 recurrences, 6 were serous (50%), 4 endometrioid (33.3%) and 2 clear cell subtype (16.7%). 9 were G3 (75%), in 4 patients (33.3%) MI was > 50% and 5 (41.7%) had LVSI. 3 (25%) and 2 (16.7%) patients had positive pelvic and aortic SLNB respectively. In terms of location of the recurrence, 7 (58.3%) were vaginal and 2 (16.6%) abdominal. The rest were peritoneal, pulmonary and hepatic.
The mean number of months until recurrence was 12.6 (SD = 9.6) and the median was of 9 months [95% CI; 7.3-10.7]. 83% relapsed within the first year of postoperative follow-up (SD = 0.11)and none recurred after 2 years.
No association was found between lower survival and none of the prognostic factors marked.
Conclusion: Recurrence rate of endometrial cancer intervened in SLNB study was 7.5%, being mostly serous adenocarcinoma or G3. 83% recurred within the first year
of postoperative control. No association was found between lower survival and none of the possible factors indicated.
Disclosure: Nothing to disclose

EP468

Tumor size as prognostic factor of lymph node involvement in endometrial cancer

D. Al-Dali[1], M. Pérez de Puig[1], C. López[1], M. Fernández[2], G. Salinas[1], F. Ojeda[1]
[1]*Universitat Internacional de Catalunya, Barcelona,* [2]*Universitat Internacional de Catalunya, Bercelona, Spain*

Introduction / Background: Lymph node involvement in patients with endometrioid adenocarcinoma occurs in 14% of them. There is evidence that lymphadenectomy has no therapeutic value in early stages, but some retrospective studies identify the tumor size greater than 2cm as an independent risk factor for lymph node involvement in patients with early stages (IA and IB).
Our objective is to know the relationship between tumor size and lymph node involvement in patients in early stages of endometrial cancer.
Methodology: An observational analytical study of a retrospective cohort was carried out, whose population was all patients with early stages of endometrial cancer, attended in our center between 2013-2018, who underwent systematic pelvic lymphadenectomy. The early-stage group, as per the

Mayo criteria, was defined by: grade 1 or 2 tumor histology and less than 50% myometrial invasion. We included 58 patients, the study variables were tumor size and lymph node involvement. Fisher´s exact test was used to determine possible associations, differences were considered statistically significant at $p < 0.05$.
Results: According to the tumor size, 50% (29) were < 2cm and 50% (29) >2cm. In the group of patients with tumor size >2cm, the percentage of lymph node affection was 6,89% (n=2) vs. 0% in the group with tumor size < 2cm.
We don't detect statistically significant difference between the two groups (p=0,4912).
Conclusion: No patient with tumor size less than 2cm had lymph node involvement. Although it is not statistically significant, we see a tendency to increase lymph node involvement in tumor sizes larger than 2cm.
Disclosure: Our study supports previously published findings, that patients with and early-stage cancer with a tumor size less than 2cm have a very low risk of lymph node involvement, so routine lymphadenectomy is not recommended. But additional studies with a larger sample would be useful to identify statistical differences in the group of tumor size >2cm.

EP469

Sentınel lymph node biopsy in early stage endometrial cancer: a Turkısh gynecologıc oncology group study (TRSGO-SLN-001)

S. Taşkın[1], D. Altın[2], D. Vatansever[3], N. Tokgözoğlu[4], E. Karabük[5], H. Turan[6], Ö. Takmaz[5], İ. Kahramanoğlu[7], M. Naki[8], Ç. Taşkıran[3], M. Güngör[5], F. Köse[5], F. Ortaç[1], M. Arvas[9], A. Ayhan[10], Turkish Gynecologic Oncology Group
[1]Ankara University School of Medicine, [2]Department of Gynecology and Obstetrics, Ankara University School of Medicine, Ankara, [3]Koc University School of Medicine, [4]Okmeydanı EAH, [5]Acibadem Mehmet Ali Aydinlar University, [6]İstanbul EAH, İstanbul, [7]Diyarbakır EAH, Diyarbakır, [8]Academic Hospital of Brest, [9]Cerrahpasa Faculty of Medicine, İstanbul, [10]Baskent University Faculty of Medicine, Ankara, Turkey

Introduction / Background: This multicentric study aimed to evaluate feasibility of sentinel lymph node (SLN) mapping in clinically uterine-confined endometrial cancer.
Methodology: Indocyanine green (ICG) or blue dye was used as tracer ICG was injected to the uterine cervix and blue dye was injected to uterine cervix or fundus. SLN and/or suspicious lymph nodes were resected. Side specific lymphadenectomy was performed when mapping was unsuccessful.
Results: 357 eligible patients were analysed. The median resected SLN number was 3 per patient. Laparoscopic, open and robotic surgery was performed in 225(63%), 93(26.1%) and 39 (10.9%) of the patients, respectively. ICG was used in 231(64.7%) and blue dye in 126 (35.3%) patients. Dyes were injected into the cervix in 355(99.4%) patients.
In 71(19.9%) patients only SLN biopsy was done whereas 169(47.3%) underwent pelvic and 117(32.8%) underwent pelvic and paraaortic lymphadenectomy.
The overall, unilateral and bilateral SLN detection rates were 91.9%, 20.7%, 71.1%, respectively. These rates were 91.2%, 17.4% and 73.8% by using ICG and 92.2%, 22.5% and 69.7% by using blue dye. There is no statistical significance (p=0.526).
In 29 of 358 (8%) patients there was no lymph node in resected tissue labelled as SLN (empty packets).
There were 40 (11%) patients with lymphatic metastasis. SLN was found metastatic in 32 of 40 patients and 14 of them also had metastasis in non-SLN(s). 4 of 40 cases were diagnosed by side specific lymphadenectomy. However, SLN algorithm were not able to detect 4 of 40 metastatic patients. NPV, sensitivity and false negative rates were 98.4%, 88.8% and 1.57%, respectively.
Conclusion: SLN biopsy has high accuracy in detecting lymphatic metastasis. However nearly half of the cases with metastatic SLN(s) also had non-SLN involvement and the effect of leaving these nodes in situ on survival should be evaluated. To avoid “empty packet” on final pathology, intraoperative frozen section evaluation could be suggested.
Disclosure: Nothing to disclose

EP470

Accuracy of ultrasound evaluation of endometrial cancer for assessing myometrial invasion and cervical stromal involvement

R.M. Alvarez Lopez[1], E. Arevalo Reyes[1], M.D. Rodriguez Garnica[1], M. Martin Blanco[1], M.V. Rodriguez Tabares[1], T. Rivera Garcia[2], E. Jareño Dorrego[2]
[1]Department of Obstetrics and Gynaecology, [2]Department of Pathology, Hospital Universitario Santa Cristina, Madrid, Spain

Introduction / Background: Preoperative assessment of myometrial infiltration in endometrial cancer may help to identify cases that may benefit from extended surgery. The aim of this study is to analyse the diagnostic accuracy of two-dimensional transvaginal ultrasound (2D-TVU) for detecting deep myometrial invasion (MI) and cervical stromal involvement (CSI) of endometrial cancer.
Methodology: All patients with confirmed endometrial cancer underwent a 2D-TVS before surgery. MI and CSI were assessed by subjective impression and recorded prospectively. At the time of the operation the specimens were sent for frozen section and the decision of performing lymph node dissection (LND) was taken intraoperatively. Final histology after hysterectomy served as gold standard.
Results: Twenty-two patients were included. The mean age was 64.3 years old (43-84). The histology was endometrioid in 19 patients (86.4%), serous in 2 (9.1%) and Mixed Müllerian Malignant Tumour in 1 (4.5%). Superficial MI (< 50%) on ultrasound was reported in 15 patients (68.2%) and deep MI (>50%) in 6 (27.3%). Just in one case the cervix was thought to be affected (4.5%). All patients had hysterectomy and bilateral salpingo-oophorectomy, 10 patients underwent bilateral pelvic LND and 4 para-aortic LND, based on MI on frozen section and histological type. Final histology showed pT1A in 14 patients (63.6%), pT1B in 6 (27.3%) and pT2 in 2 (9%). There was one case of no correlation between frozen section and final histology and the patient had a re-operation for LND. Two patients had pelvic and para-aortic lymph node involvement. Accuracy for diagnosing deep MI with 2D-TVU was 86.4%, and for CSI was 95.5%.
Conclusion: Ultrasound evaluation by subjective impression seems to be a good method for assessing MI and CSI in endometrial cancer. The use of preoperative ultrasound in combination with frozen section improve diagnostic performance.
Disclosure: Nothing to disclose

EP471

Molecular analysis of p53 codon 72 polymorphism among women with endometrial cancer in Morocco: a case-control study

B. Amal[1], B. Mustapha[2,3], E.K. Mohammed[2,3], E. Moulay[4]
[1]Gynecology Obstetrics, Mohamed VI University of Health Sciences, [2]Gynecology Obstetrics, Faculty of Medicine and Pharmacy, Hassan II University, [3]Oncology, Mohamed VI Oncology Center, Ibn Rochd Hospital, Casablanca, [4]Laboratory of Virology, Microbiology ,Quality and Biotechnologies / Biodiversity and Ecotoxicology, Faculty of Sciences and Techniques of Mohammedia, Hassan II University, Mohamedia, Morocco

Introduction / Background: This study aims to investigate whether the genotype distribution of p53 codon 72 may be a risk factor for endometrial cancer among Moroccan women.
Methodology: Samples from 20 Moroccan women, 6 diagnosed with endometrial cancer and 14 healthy controls were used. HPV detection was done by nested PCR with MY09/11 and GP5+/6+ primers followed by sequencing for HPV genotyping. p53 codon 72 polymorphism determination was

performed by allele-specific PCR assay. Viral DNA was detected in 16,6% of cases and 14,2% of control. HPV 16 was the most predominant in cancer and controls cases.
Results: The distribution of Arg/Arg, Arg/Pro and Pro/Pro genotypes was 33.2%, 49.8% and 16.6% in the endometrial cancer group and 21.4%, 71,4% and 7,14% in the control group.
Conclusion: No significant association was found between polymorphism of p53 itself as well as in combination with HPV16/18 infection and risk of development of endometrial cancer among Moroccan women. Thus, the polymorphism of p53 codon 72 in exon 4 does not seem to play a role in the development of endometrial cancer among Moroccan women.
Disclosure: Nothing to disclose

EP472

ErbB receptors profile in non-selected patients with endometrial cancer

G. Michail[1,2], I. Styliara[2], P. Panas[2], F. Markatos[2], D. Koumoundourou[3], P. Ravazoula[3], G. Adonakis[2], G. Androutsopoulos[1,2]
[1]Division of Gynaecological Oncology, Department of Obstetrics and Gynecology, [2]Department of Obstetrics and Gynecology, [3]Department of Pathology, University of Patras, Medical School, Rion, Greece

Introduction / Background: ErbB receptors are trans-membrane glycoproteins with tyrosine kinase activity. Especially in cancer, they are implicated in cell proliferation, transformation, angiogenesis, migration and invasion. The expression of ErbB receptors, has not been studied well in endometrial cancer patients. The main aim of our study was to assess ErbB receptors profile in non-selected patients with endometrial cancer.
Methodology: We evaluated retrospectively tissue specimens from 93 patients with endometrial cancer, that have been treated in the Division of Gynaecological Oncology of the University of Patras. For ErbB receptors immunostaining, we used: anti-EGFR polyclonal antibody sc-03 (Santa Cruz Biotechnology Inc., Santa Cruz, CA, USA) in a dilution 1:20, anti-ErbB-2 monoclonal antibody CB11 (BioGenex Laboratories Inc., San Ramon, CA, USA) in a dilution 1:100, anti-ErbB-3 polyclonal antibody sc-285 (Santa Cruz Biotechnology Inc., Santa Cruz, CA, USA) in a dilution 1:100 and anti-ErbB-4 polyclonal antibody sc-283 (Santa Cruz Biotechnology Inc., Santa Cruz, CA, USA) in a dilution 1:200.
Results: For EGFR receptor 53 cases were positive (57%) and 40 were negative (43%), while for ErbB-2 receptor 61 cases were positive (65.6%) and 32 were negative (34.4%). For ErbB-3 receptor 66 cases were positive (71%) and 27 were negative (29%), while for ErbB-4 receptor 72 cases were positive (77.4%) and 21 were negative (22.6%).
There were some differences regarding ErbB receptors profile, especially among different histologic subtypes of endometrial cancer. However, all those differences were not statistically significant mainly because of the small number of cases with papillary serous or clear cell histology.
Conclusion: ErbB receptors profile, should be evaluated independently in patients with type I and type II endometrial cancer. This is mainly based on the fact that those types of endometrial cancer have different pathophysiology and clinical behavior.
Disclosure: Nothing to disclose

EP473

ErbB receptors profiling in selected patients with type II endometrial cancer

G. Michail[1,2], P. Panas[2], F. Markatos[2], I. Styliara[2], D. Koumoundourou[3], P. Ravazoula[3], G. Adonakis[2], G. Androutsopoulos[1,2]
[1]Division of Gynaecological Oncology, Department of Obstetrics and Gynecology, [2]Department of

Obstetrics and Gynecology, [3]Department of Pathology, University of Patras, Medical School, Rion, Greece

Introduction / Background: ErbB receptors are trans-membrane glycoproteins with tyrosine kinase activity. Especially in cancer, they are implicated in cell proliferation, transformation, angiogenesis, migration and invasion. The expression of ErbB receptors, has not been studied well in endometrial cancer patients. The main aim of our study was to investigate ErbB receptors profile in selected patients with type II endometrial cancer.
Methodology: We evaluated retrospectively tissue specimens from 10 patients with type II endometrial cancer, that have been treated in the Division of Gynaecological Oncology of the University of Patras. For ErbB receptors immunostaining, we used: anti-EGFR polyclonal antibody sc-03 (Santa Cruz Biotechnology Inc., Santa Cruz, CA, USA) in a dilution 1:20, anti-ErbB-2 monoclonal antibody CB11 (BioGenex Laboratories Inc., San Ramon, CA, USA) in a dilution 1:100, anti-ErbB-3 polyclonal antibody sc-285 (Santa Cruz Biotechnology Inc., Santa Cruz, CA, USA) in a dilution 1:100 and anti-ErbB-4 polyclonal antibody sc-283 (Santa Cruz Biotechnology Inc., Santa Cruz, CA, USA) in a dilution 1:200.
Results: For EGFR receptor, 5 cases were positive (50%) and 5 cases were negative (50%), while for ErbB-2 receptor 9 cases were positive (90%) and 1 case was negative (10%). For ErbB-3 receptor all cases were positive (100%), while for ErbB-4 receptor 7 cases were positive (70%) and 3 cases were negative (30%).
Overall, we observed high expression levels of all ErbB receptors in our study population and 5 patients were positive (50%) for all ErbB receptors.
Conclusion: The high expression levels of ErbB receptors in patients with type II endometrial cancer possibly indicates a future role of ErbB-targeted therapies in well-defined subgroups of type II endometrial cancer patients with EGFR and ErbB-2 overexpression.
Disclosure: Nothing to disclose

EP474
The prognostic value of preoperative serum CA125 in endometrioid endometrial cancer with cervical stromal and parametrial invasion

A. Bahman, T. Ashraf ganjoie
Gynecology Oncology, Shahid Beheshti University of Medical Sciences, Tehran, Islamic Republic of Iran

Introduction / Background: The correlation between CA125 and epithelial ovarian cancer and its following is already known. However, the correlation of this tumor antigen and endometrial cancer is still under investigation and there is controversy regarding the cut-off value of Cancer Antigen 125 (CA125).
Methodology: A sample of 128 endometrial cancers, surgically staged from 2012 to 2018 in Imam Hossein Hospital were evaluated. According to exclusion criteria, 82 cases were analyzed finally. Receiver Operating Characteristic (ROC) Curve was used to determine the cut-off value of preoperative CA125 for parametrial and cervical stromal involvement.
Results: A high preoperative CA125 level was significantly associated with advanced disease stage, cervical stromal invasion, pelvic lymph node metastases and higher grade (p< 0/05); for parametrial invasion,it was marginally significant(p=.058) .Although the correlation between CA125 and myometrial /lymphovascular invasions were not statistically significant (P= 0/112 & 0/168 respectively). The suitable cut- off for preoperative CA125 in parametrial invasion was 45/5 u/ml (100% sensitivity, 89% specificity, 33/3% Positive Predictive Value, 100% Negative Predictive Value) and for cervical stromal invasion, it was 41/9 u/ml (87/5% sensitivity, 87/8% specificity, 43/75% Positive Predictive Value and 98/48% Negative Predictive Value).
Conclusion: If endometrial cancer invades cervical stroma or parametrium, primary surgical treatment may not be the first treatment approach, neoadjuvant chemoradiotherapy and adjuvant extrafascial hysterectomy could be a better approach with better results and fewer complications. Using preoperative CA125 along with physical examination and imaging modalities would be helpful in predicting cervical stromal and parametrial involvements. More investigations are needed to assess an

agreed cut-off value for preoperative CA125 and endometrial cancer extension.
Disclosure: Nothing to disclose

EP475

Immunohistochemical expression of LHRH receptor in different anatomical compartments of endometrial cancer patients

B. Barczynski[1], K. Fraszczak[1], M. Sobstyl[2], I. Wertel[1], E. Zakrzewska[3], A. Nowicka[4], J. Kotarski[1]
[1]I Dept of Oncological Gynaecology & Gynaecology, [2]Department of Gynaecology and Gynaecological Endocrinology, Medical University in Lublin, [3]Center of Oncology of the Lublin Region, [4]Maria-Skłodowska-Curie University, Lublin, Poland

Introduction / Background: Endometrial cancer is the most common gynaecologic malignancy in developed countries. Despite good overall prognosis, still many patients recur within first 3 years after primary treatment. Recently, new targeted treatment with doxorubicin linked to an LHRH agonist was investigated in phase III trial in patients with locally advanced, recurrent or metastatic endometrial cancer. Unfortunately, this receptor-mediated targeted chemotherapy, despite promising phase II results, showed no benefits comparing to standard treatment. To explain the possible rationale of such unexpected results we decide to assess LHRH receptor (LHRH-R) expression in different compartments of female genital tract in patients with endometrial cancer.
Methodology: Among 100 patients operated due to endometrial cancer in our centre, 92 cases with endometrioid adenocarcinoma were chosen. Immunohistochemical LHRH-R expression in postoperative paraffin-embedded tissue blocks was evaluated in endometrial cancer, ovarian cortex, ovarian medulla, and oviduct tissue. In 10 patients it was also possible to assess LHRH-R expression in areas of endometrial hyperplasia diagnosed concurrently with cancer. The standard semi-quantitive scoring system considering staining intensity and area extent according to Allred was used for expression assessment. The results were presented as negative, moderate and strong expression. Pearson´s chi-square test was used to assess differences in analysed groups.
Results: LHRH-R expression was detected in all compartments studied. Strong expression was found in 77% of endometrial cancer, 61% of ovarian cortex, 28% of ovarian medulla, 85% of fallopian tube, and 7% of endometrial hyperplasia. LHRH-R expression in ovarian medulla was significantly decreased when compared to cancer compartment (p=0,04). Additionally, LHRH-R expression in ovarian cortex was almost significantly decreased when compared to cancer compartment (p=0,06). There was no differences between all other compartments analysed.
Conclusion: Due to observed differences in LHRH-R expression, targeted treatment with chemotherapeutic agents linked to an LHRH agonist may show no benefits in all patients with locally advanced endometrial cancer patients.
Disclosure: Nothing to disclose

EP476

Diagnostic value of office endometrial sampling in postmenopausal bleeding

F. Behnamfar, E. Arshad
Esfahan University of Medical Sciences, Esfahan, Islamic Republic of Iran

Introduction / Background: This study was designed to compare the diagnostic value of Pipelle endometrial sampling with curettage in patients with postmenopausal bleeding and if hysterectomy was done for patients evaluating its pathology with these data´s.
Methodology: Eightyseven patients with postmenopausal bleeding were included in this comparison study. Pipelle sampling endometrial biopsy in office for patients was done at first and maximum 4

weeks later they were transferred to operation room for Dilatation and Curettage. The pathology result of Pipelle sampling was compared with curettage as the gold standard was curettage sampling and if hysterectomy was done for patients this data was compared.
Results: 100% of samples given by curettage while 97.6 % of the samples given by Pipelle were satisfactory for pathological evaluation. Pathological results revealed hyperplasia without atypical in 12 specimens by both methods; while hyperplasia with atypia for 3 patients again in both methods of sampling. Endometrial polyps in 13 for curettage group versus 5 cases in pipelle and malignant endometrium in 13 specimens .25 patients had hysterectomy surgery and pathological result of 17 specimens revealed malignancy. The Pipelle device had 97.7%sensitivity and 97.7 % positive predictive value in diagnosing endometrialmalignancy. Also it had 92.2% sensitivity and 100 % specificity for diagnosing endometrial polyp.
Conclusion: The endometrial sampling with Pipelle is a safe and cost effective in patients with postmenopausal bleeding which avoids general anesthesia and hospitalization .This method has a high sensitivity and specificity for detection of endometrial hyperplasia and malignancy.
Disclosure: Nothing to disclose

EP477

Laparoscopic versus abdominal surgery for 70-years and older women with endometrial cancer

I. Berlev, Y. Trifanov, Z. Ibragimov, O. Smirnova, K. Guseinov, H. Mkrtchyan, N. Mikaya, A. Sidoruk, E. Ulrikh, A. Urmancheeva
Oncogynecology, N.N. Petrov National Medical Research Center of Oncology, Saint-Petersburg, Russian Federation

Introduction / Background: Endometrial cancer (EC) is the most common gynecological malignancy in developed countries. The complexity of treating patients with EC older than 70 years is numerous associated diseases, and high risk of intra- and postoperative complications. Our research compares results of surgical treatment EC performed laparoscopic and abdominal approach.
Methodology: Laparoscopic and abdominal EC operations were collected from 2010-2017. Data included weight, body mass index (BMI), operative time, estimated blood loss, number of removed lymph nodes, hospital stay were collected and compared. Postoperative complications were graded according to the Clavien-Dindo Classification. Survival outcomes within the first 3-years were analyzed using the Kaplan-Meier method.
Results: During the study period, there were 80 elderly women, who underwent surgery with the laparoscopy and 80 women, who underwent traditional abdominal approach. Total hysterectomy with pelvic lymph-node dissection perfomed more than 70% of cases in both groups. Patients undergoing laparoscopy experienced similar operative time ($p>0.05$), lower blood loss ($p< 0.05$), and shorter hospital stay ($p< 0.05$) than patients undergoing open surgery. Laparoscopy is related to a lower rated postoperative complications ($p< 0.05$) and didn't have more than grade II complication. The route of surgical approaches did not influence the 3-year disease-free ($P = 0.84$, log-rank test) and overall ($P = 0.87$, log-rank test) survivals.
Conclusion: Our data suggest laparoscopy is a safe and effective approach in treatment of endometrial cancer in women aged 70 years or older. Laparoscopy improves peri- and postoperative outcomes without any influence to long-term outcomes compare to open abdominal surgery.
Disclosure: Nothing to disclose

EP478

Role of sentinel lymph node mapping with indocyanine green in patients with endometrial

cancer. Single-institution experience, n=100

I. Berlev, H. Mkrtchyan, Z. Ibragimov, K. Guseinov, N. Mikaya, A. Sidoruk, O. Smirnova, Y. Trifanov, E. Ulrikh, A. Urmancheeva
Oncogynecology, N.N. Petrov National Medical Research Center of Oncology, Saint-Petersburg, Russian Federation

Introduction / Background: Sentinel lymph node (SLN) mapping with indocyanine green (ICG) is used for nodal staging in patients with endometrial cancer for last 10 years. Many authors showed the efficiency of using ICG in SLN mapping in endometrial cancer.
As part of surgical management in endometrial cancer, bilateral pelvic lymphadenectomy is considered standard of care. However, pelvic lymphadenectomy is associated with complications, such as lymphedema, the risk of nerve injury, and lymphocyst formation.
Methodology: From January 2016 to March 2018 in single-center study, 100 patients with endometrial cancer IA-IB stages underwent laparoscopic surgery using ICG for SLN mapping.
A unit of 4 cc of this ICG solution was injected into the cervix alone divided into the 3- and 9-o´clock positions, with 1 cc deep into the stroma and 1 cc submucosally on the right and the left of the cervix.
Results: Overall detection rate of SLNs registered in 99/100 patients (99.0%), bilateral - 95/100 (95.0%). Only in one patient SLNs were not detected -1/100(1%).
Lymph node metastatic disease identified in 11/100 patients (11.0%).All metastasis identified in SLNs. The most frequent localization of SLN was in internal lymphatic group 94/220 (46%).
Diagnostic efficiency of the SLN biopsy: sensitivity - 100.0%, specificity -93.0%, accuracy - 93.0%, false-negative results - 0%.
In the high risk group of metastasis, metastasis were detected in 6/100 (6%) patients
In the intermediate group and low risk group - SLN metastasis were identified in 5/100 (5%) patients. No metastatic disease was identified in non SLN.
In the case of absence of metastases in SLNs 88/99 (89%), all non-SLNs were intact.
Conclusion: SLN detection with ICG in patients with endometrial cancer IA-IB stages is an effective diagnostic fluorescence method. Overall and bilateral detection rate of SLN visualization is high, which allows removing the detected SLNs, making frozen-section and individualizing the treatment.
Disclosure: Nothing to disclose

EP479

Agreement between magnetic resonance imaging, transvaginal ultrasound scan and intraoperative biopsy for determining miometrial infiltration in endometrial carcinoma. Tertiary centre results

R. Bermejo Martínez, L. Álvaro Valiente, Á. Tejerizo García, G. López González, M.R. Oliver Pérez, L. Muñoz Hernando
Hospital Universitario 12 de Octubre, Madrid, Spain

Introduction / Background: Miometrial infiltration (MI) is one of the most important prognosis features for endometrial carcinoma (EC). Different methods have been extensively used with the aim of optimize the detection of MI, such us magnetic resonance imaging (MRI), transvaginal ultrasound scan (TUS) and intraoperative frozen section with different results. The aim of our study is to review the diagnostic accuracy of these methods in the preoperative detection of MI in our institution and compare it with the available data.
Methodology: Retrospective unicentric study of 271 patients with EC diagnosed, treated and followed-up at *Hospital Universitario 12 de Octubre* (Madrid, Spain) between 2008 and 2018.
Results: Demographic characteristics of the study population are described in Table 1. Results of sensitivity, specificity, positive predictive value, negative predictive value and validity of the different methods compared with definitive histopathological diagnosis of MI (used as the gold standard) are registered in Table 2.

[Table 1]

[Table 2]

Conclusion: Surgical staging represents the cornerstone in the treatment of EC. Therapeutic role of lymphadenectomy still remains nowadays a controversial issue. Different methods have been used to detect preoperatively those patients with lower risk of lymph node involvement in order to avoid lymphadenectomy. MI is considered as an essential fact when establishing the endometrial carcinoma prognosis. On one side, it determines the modification of the stage during initial staging. On the other side, it has been associated with a higher risk for lymph nodes dissemination, haematogenous spread, higher recurrence rates and lower overall survival rates. In our institution, preoperative imaging studies present an acceptable accuracy for diagnosis of MI according to the available data, but the test with the highest sensitivity and specificity continues to be the histopathological study of intraoperative frozen section. Further studies are needed in order to establish the better approach to determinate preoperative MI.
Disclosure: Nothing to disclose

EP480

The impact of gene-specific germline pathogenic variants on clinical presentation of endometrial cancer in Lynch syndrome

G. Bogani, V. Chiappa, A. Ditto, F. Raspagliesi
Fondazione IRCCS Istituto Nazionale dei Tumori di Milano, Milano, Italy

Introduction / Background: Lynch syndrome (LS) is a well know risk factor for developing endometrial carcinoma (EC). Here, we aimed to investigate the impact of gene-specific germline pathogenic variants on clinical features of EC
Methodology: This is a retrospective case series of consecutive surgically-treated patients with histological diagnosis of EC and with a germline pathogenic variant in mismatch repair genes. Classes of risk are graded per the ESGO-ESGO-ESTRO guidelines.
Results: Overall, 68 patients with EC and LS were evaluated. Ten (14.7%) patients were excluded due to absence of clear information about the gene involved in LS, thus leaving 58 (85.3%) patients available for the final analysis. *MLH1*, *MSH2* and *MSH6* pathogenic variants were observed in 19 (32.7%), 33 (56.9%) and 6 (10.3%) cases, respectively. Mean (SD) age at EC diagnosis was 51 (±6.4), 43.5 (±7.4) and 60.3 (±8.8) years (p=0.0002). Prevalence of non-endometrioid EC were 15.7%, 24.2% and 0% in *MLH1*, *MSH2* and *MSH6* group, respectively (p=0.345). Focusing on classes of risk we observed that patients harboring a *MLH1* or *MSH2* pathogenic variant were at higher risk than patients with a *MSH6* mutation. In fact, according to the ESMO-ESGO-ESTRO classification, low, intermediate, and high risk EC accounted in 47.3%, 10.5% and 42.1% of *MLH1* group, in 57.6%, 3% and 39.4% of *MSH2* group and in 50%, 50% and 0% of *MSH6* group (p=0.009).
Conclusion: Patients with *MLH1* and *MSH2* pathogenic variants are at a higher risk of early onset of EC and are characterized by more aggressive disease than patients with *MSH6* pathogenic variants.
Disclosure: Nothing to disclose

EP481

Sentinel node mapping vs. lymphadenectomy in endometrial cancer: a systematic review and meta-analysis

G. Bogani, A. Ditto, F. Martinelli, V. Chiappa, F. Murgia, U. Leone Roberti Maggiore, F. Raspagliesi
Fondazione IRCCS Istituto Nazionale dei Tumori di Milano, Milano, Italy

Introduction / Background: Sentinel node mapping is increasingly being utilized for endometrial cancer staging. However, only limited evidence supporting the adoption of sentinel node mapping instead of conventional lymphadenectomy is still available. Here, we aimed to review the current evidence comparing sentinel node mapping and lymphadenectomy in endometrial cancer staging.
Methodology: This systematic review was registered in the International Prospective Register of Systematic Reviews. Der-Simonian random-effects models were used to pool log transformed event rates and estimated 95% CI for dichotomous outcomes between the two interventions for each study and we pooled the effect size using the same models
Results: Overall, 3,536 patients were included: 1,249 (35.3%) and 2,287 (64.7%), undergoing sentinel node mapping and lymphadenectomy, respectively. Pooled data suggested that positive pelvic nodes were detected in 184 out of 1,249 (14.7%) patients having sentinel node mapping and 228 out of 2,287 (9.9%) patients having lymphadenectomy (OR: 2.03; (95%CI: 1.30 to 3.18); p=0.002). No difference in detection of positive nodes located in the paraaortic was observed (OR: 93 (95%CI: 0.39 to 2.18); p=0.86). Overall recurrence rate was 4.3% and 7.3% after sentinel node mapping and lymphadenectomy, respectively (OR: 0.90 (95%CI: 0.58 to 1.38); p=0.63). Similarly, nodal recurrences were statistically similar between groups (1.2% vs. 1.7%; OR: 1.51 (95%CI: 0.70 to 3.29); p=0.29).
Conclusion: In conclusion, our meta-analysis underlines that sentinel node mapping is non-inferior to standard lymphadenectomy in term of detection of paraaortic nodal involvement and recurrence rates (any site and nodal recurrence); while, focusing on the ability to detect positive pelvic nodes, sentinel node mapping could be consider superior to lymphadenectomy. Further randomized studies are needed to asses long term effectiveness of sentinel node mapping.
Disclosure: Nothing to disclose

EP482

Associations between thrombocytosis, predictive biomarkers, and survival outcomes in uterine serous carcinoma

P. Bussies[1], S. George[2], A. Pinto[3], M. Huang[2], B. Slomovitz[2], M. Schlumbrecht[2]
[1]University of Miami Miller School of Medicine, [2]Gynecology/Oncology, [3]Pathology, Sylvester Comprehensive Cancer Center, Miami, FL, United States

Introduction / Background: Thrombocytosis is known to be associated with poor outcome in endometrial cancer, though data on uterine serous carcinoma (USC) is sparse. The purpose of this study was to evaluate thrombocytosis and its association with patient survival as well as other predictive biomarkers.
Methodology: We performed a retrospective chart review on 167 patients who presented with USC between 2005 and 2017. Data collected included: age, race, ethnicity, place of birth, overall survival (OS), tumor size, CA125 at diagnosis, CBC count at diagnosis, presence of lymphovascular space invasion (LVSI), and immunohistochemical positivity (>1%) for Estrogen Receptor (ER) and Progesterone Receptor (PR) in the tumor cells. Thrombocytosis was defined as platelets ≥ 450k. Data analysis was performed using Cox proportional hazard models, Spearman rank correlation, and the Kaplan-Meier method, with significance set at p< 0.05.
Results: 18% of women had thrombocytosis at disease presentation. Thrombocytosis prevalence did not vary by race, ethnicity, or place of birth. Thrombocytosis was strongly correlated with rising CA125 levels (Spearman's rho=0.37, p=0.01), tumor size (Spearman's rho=0.36, p=0.04), and presence of LVSI (Spearman's rho=0.26, p=0.04). LVSI was present in 71% of patients without thrombocytosis vs. 100% of those with thrombocytosis (p=0.04). In the hazards model, stage, LVSI, thrombocytosis, and tumor size were associated with worse survival (HR=1.90, 2.28, 2.94, and 1.02, respectively; all p< 0.05). Median OS was negatively affected by thrombocytosis (34.4 vs 16.4 months, p=0.002) (Figure 1). OS was not significantly associated with ER (HR=1.24 [CI 0.65-2.42], p=0.51) or PR (HR=0.95 [CI 0.41-2.20], p=0.90) positivity.
Conclusion: Thrombocytosis is an independent predictor of poor outcome in USC. It additionally correlates with other known poor prognostic factors such as LVSI and rising CA125 levels. Further investigation of these observations is required to understand the biological underpinnings of platelets in tumor progression.

[Figure 1. Overall survival by race, thrombocytosis]

Disclosure: Nothing to disclose

EP483

Biomarker prediction of outcome varies by race in uterine serous carcinoma

P. Bussies[1], S. George[2], A. Pinto[3], M. Huang[2], B. Slomovitz[2], M. Schlumbrecht[2]
[1]Miami Miller School of Medicine, [2]Gynecology/Oncology, [3]Pathology, Sylvester Comprehensive Cancer Center, Miami, FL, United States

Introduction / Background: Minority women are known to have higher risk for uterine serous carcinoma (USC). Limited data exist to establish reliability of prognostic biomarkers by race in these patients. Our objective was to evaluate patterns of clinical and pathologic biomarkers and their effects on outcome between white and non-white patients.
Methodology: A retrospective review was completed on all women diagnosed with USC between 2005 and 2017. Hematologic parameters and CA125 were ascertained at time of diagnosis, and pathology reports assessed for tumor size, lymphovascular space invasion (LVSI), and immunohistochemical (IHC) staining (> 1% tumor cells) for estrogen receptor (ER) and progesterone receptor (PR). Statistics were done using chi-square, Cox proportional hazards, and the Kaplan-Meier method, with significance set at $p< 0.05$.
Results: 59 patients were non-white (35.5%), 57 of whom were black; 107 were white (64.5%). At diagnosis, no differences were seen in mean CA125 levels (745 white vs. 630 non-white, $p=0.83$) or mean platelet levels (320K white vs. 354K non-white). There were no differences in proportion of women with LVSI or positive staining for ER or PR by race (all $p>0.05$). Primary tumors were larger in non-white patients, though this was not significant (55mm vs 43mm, $p=0.06$). Thrombocytosis (platelets ≥450K) was associated with worse overall survival (OS) in whites (HR 4.38 [CI 1.55-12.34], $p=0.005$) but not in non-whites (HR 2.05 [CI 0.73-5.74], $p=0.17$). For both races, tumor size and higher stage were negatively associated with OS ($p< 0.05$), while ER and PR had no effect. Median OS was

worse for non-whites without thrombocytosis (30.1 vs 38.4 months), but was better for non-whites when thrombocytosis was present (22.1 vs 16.4 months) (p=0.01) (Figure 1).
Conclusion: Biomarker variation is minimal by race in USC, while the effect of thrombocytosis on OS varies significantly by race. Further investigation is warranted to elucidate the mechanism behind this observation.

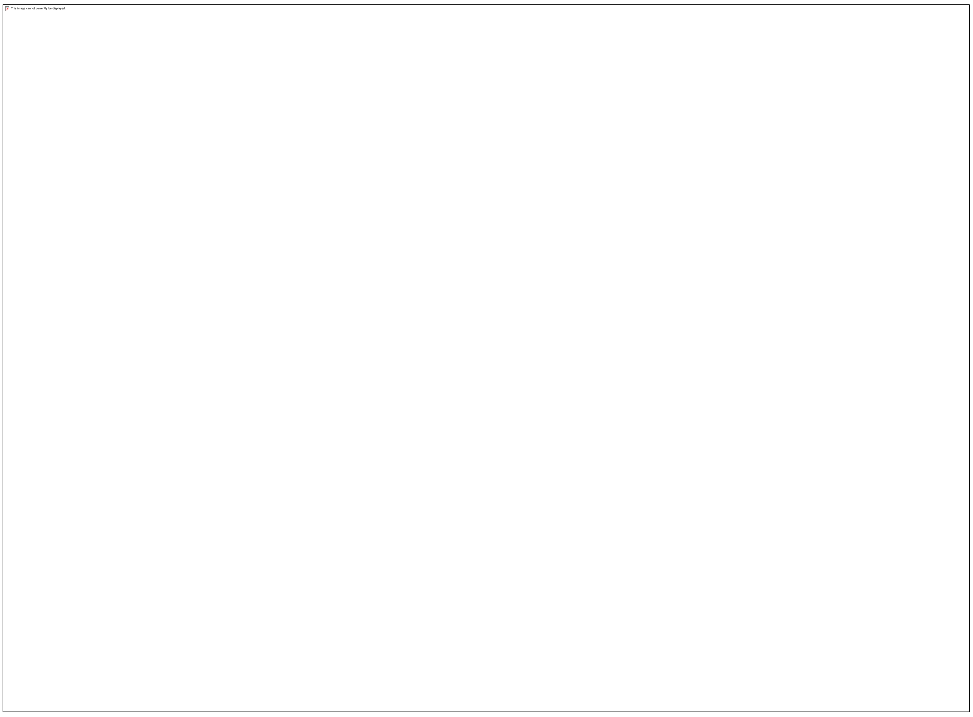

[Figure 1. Overall survival by race, thrombocytosis]

Disclosure: Nothing to disclose

EP484

Warm ischaemia is an important pre-analytical variable affecting the analysis of endometrial biospecimens

L. Button[1], M. Adishesh[2], J. Drury[3], S.B. DeCruze[2], D. Hapangama[1]
[1]Department of Women's & Children's Health, University of Liverpool, [2]Gynaecological Oncology, Liverpool Women's Hospital, [3]University of Liverpool, Liverpool, United Kingdom

Introduction / Background: Biobanks hosting patient biospecimens are central to endometrial research, however pre-analytical variables including specimen handling can lead to misleading, not reproducible and clinically untranslatable results. Warm ischaemia is a putative variable that significantly affects downstream analysis, but its effect on endometrial biopsies is yet to be investigated, and further understanding is vital for generating robust data and the harmonisation of biobanking.
Aims: To examine the effect of warm ischaemia on the analysis of endometrial cancer (EC) and healthy endometrial samples.
Methodology: Paired healthy (n=5) and malignant (n=22) endometrial samples were taken before and after hysterectomy for comparison of expressional analysis. Methods of immunohistochemistry and RT-qPCR were used to examine the expression of HIF-1α and genes of interest *VEGFA*, *CA9* and

PGR.
Results: *VEGFA* (*p*=0.036) and *PGR* (*p*=0.035) genes were upregulated in normal endometrial samples exposed to warm ischaemia, however subsequent analysis confirmed *VEGFA* upregulation to be an artefact due to myometrial contamination. Prognostic gene *PGR* expression in normal post-hysterectomy samples negatively correlated with inter-biopsy time, accounting for warm ischaemia time (R^2=-0.66, *p*=0.0043). Gene expressional analysis in EC samples and immune-staining of HIF-1α was unchanged by warm ischaemia.
Conclusion: Analysis of the markers examined in EC samples is not significantly altered by warm ischaemia. Myometrial contamination may affect gene expressional analysis. Prognostic gene *PGR* is susceptible to changes in expression as a result of warm ischaemia, advocating use of pre-operative biopsies where possible.
Disclosure: Nothing to disclose

EP485

Confounding factors of trans-vaginal ultrasonography accuracy in endometrial cancer

V.A. Capozzi[1], G. Gambino[1], G. Morganelli[1], A. Cianciolo[1], M. Pugliese[1], G. Sozzi[2], G. Armano[1], L. Monfardini[1], M. Gaiano[1], V. Ceni[1], M. Rolla[1], C. Merisio[1], R. Berretta[1]
[1]Department of Gynecology and Obstetrics, University of Parma, Parma, [2]Department of Gynecologic Oncology, ARNAS Civico Hospital of Palermo, Palermo, Italy

Introduction / Background: Endometrial cancer is the most frequently diagnosed gynecological tumor with more than 60.000 new cases each year. Following the ESMO-ESGO-ESTRO consensus conference, patients are classified in different classes of risk for lymph node metastases based on the instrumental suspected stage of the disease. In this scenario, a correct pre-operative workup is essential for a proper planning treatment offered to the patient. Although most of the oncological centers have replaced the MRI with trans-vaginal ultrasound, this, in the international guidelines, is considered only in the fertility sparing and in cases of contraindicated MRI.
The Aim of our study is to evaluate the diagnostic accuracy of transvaginal ultrasonography in relation to confoundable factors that may affect its quality, to a proper patient risk stratification and a consequent better surgical strategy.
Methodology: The study analyzed 290 patients with histological diagnosis of endometrial adenocarcinoma. Each patient was subjected to the anamnestic collection, chest/abdomen/pelvis CT, assessment of Ca-125 and transvaginal ultrasound. The tumor ultrasound description was made according to IETA terminology. According to the FIGO staging system, the depth of myometrial invasion was rated as superficial (M1, < 50%) or deep (M2, >50%).
Results: Of the 272 patients included in the study in 214 (78.7%), there was an agreement between the depth of ultrasound myometrial infiltration and definitive histological examination. The FIGO stage IB (Figure1) was the main statistically significant confounding factor in the univariate analysis about ultrasound myometrial infiltration diagnosis (p = 0.004). Fibromatosis and/or adenomyosis was significantly associated with a discordant ultrasonographic diagnosis in univariate analysis (p=0.04).
Conclusion: The study shows a high concordance between transvaginal ultrasonography and definitive histological examination in predicting myometrial infiltration, with 86,8% sensitivity (CI: 81.1-91), 64.4% specificity. Myometrial infiltration over 50% (Figo stage IB) represents the main confounding factor.

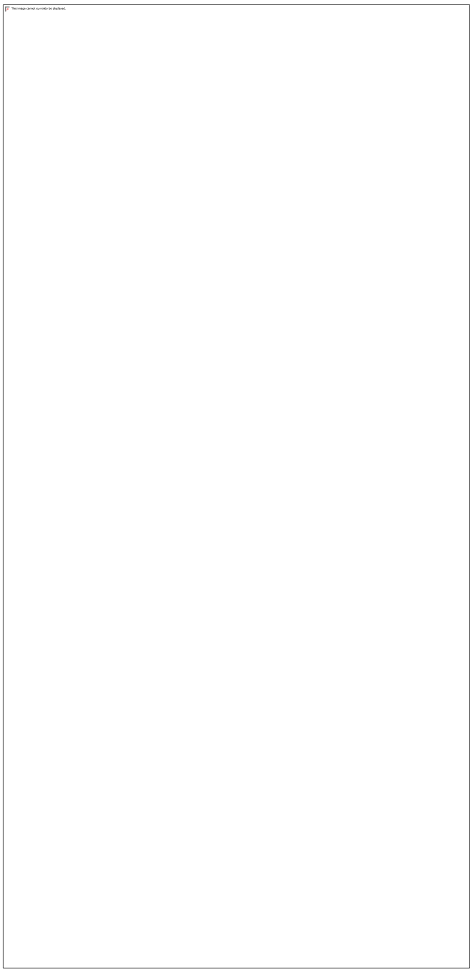

[Figure1: Adenocarcinoma of the endometrium Figo stage IB G2 seen on the intra-operative specimens (A]

Disclosure: Nothing to disclose

EP486

Brain metastases from endometrial cancer: a case report

L. Castro Oliveira[1], A.S. Rolo[1], B. Freitas Lima[1], C. Carvalho[1], I. Faustino[1], C. Fardilha[2], A. Teixeira[1], P. Costa[2], C. Coutinho[1]
[1]Medical Oncology, Hospital da Senhora da Oliveira, Guimarães, [2]Radiation Oncology, Hospital de Braga, Braga, Portugal

Introduction / Background: Endometrial carcinoma (EC) is the most common invasive gynaecologic neoplasm. Brain metastases from EC are rare and they confer a poor prognosis.
Methodology: Clinical case report of a patient with brain metastases from EC.
Results: A 62 year-old woman presented in April 2011 with vaginal bleeding. She was diagnosed with EC and she underwent a total hysterectomy with bilateral salpingo-oophorectomy. Histology showed an endometriod adenocarcinoma grade1, myometrial invasion < 50%, without lymphovascular invasion; FIGO stage IA. In May 2013, she start with abdominal pain and swelling. The CT scan

revealed ascites, peritoneal carcinomatosis and lymph nodes (LN) metastases and she completed six cycles of carboplatin AUC5 and paclitaxel 175 mg/m^2 until December 2013 with partial response. In June 2014, the patient presented with confusion and headache. Brain CT of the brain revealed diffuse brain metastases. She received steroids and whole-brain radiation therapy (WBRT) to a total dose of 30 Gy until July 2014. Thereafter, with no evidence of visceral or LN metastases, she received megestrol 160 mg daily between July and October 2014. In October 2014, peritoneal relapse was diagnosed and she received cisplatin 50 mg/m² and adriamycin 60 mg/m² between October 2014 and February 2015 with partial response. In June 2015, the patient presented headache with new brain lesions and she underwent WBRT to a total dose of 20 Gy until August 2015. In October 2015, peritoneal and LN relapse was diagnosed and she received topotecan 1 mg/m^2 days 1-5 21/21 days between October 2015 and October 2016. The patient died in November 2016, 29 months after brain spread.
Conclusion: Patients with EC that present with neurologic signs and symptoms should be evaluated for brain metastases because early diagnosis and promptly multimodal treatment can improve the prognosis of these patients.
Disclosure: Nothing to disclose

EP487

Panoramic view of endometrioid endometrial cancer in Turkey: a retrospective, multicenter study of Turkish Society of Gynecologic Oncology (TRSGO-END002)

E. Çelik[1], V. Gülseren[2], B. Özge Kayan[3], M. Öz[4], E. Kocaman[5], S. Akgöl[6], S. Alemdoroğlu[7], S. Topuz[1], K. Güngördük[2], F. Demirkiran[3], M.M. Meydanli[4], Ö. Akbayir[6], H. Çelik[7], A. Ayhan[5]
[1]Gynecologic Oncology, Istanbul University, Istanbul Faculty of Medicine, Istanbul, [2]Gynecologic Oncology, Tepecik Education and Research Hospital, İzmir, [3]Gynecologic Oncology, İstanbul University Cerrahpaşa, Istanbul, [4]Gynecologic Oncology, Zekai Tahir Burak Women's Health Training and Research Hospital, Faculty of Medicine, University of Health Sciences, Anakara, [5]Gynecologic Oncology, Baskent University Faculty of Medicine, Ankara, [6]Gynecologic Oncology, Kanuni Sultan Suleyman Teaching and Research Hospital, University of Health Sciences, Faculty of Medicine, Istanbul, [7]Obstetrics and Gynecology, Başkent University Adana Teaching and Research Center, Adana, Turkey

Introduction / Background: The purpose of this study was to investigate prognostic factors associated with overall survival (OS) in endometrioid endometrial cancer (EC).
Methodology: A multicenter, retrospective department database review was performed to identify patients with endometrioid EC at seven high-volume gynecologic oncology centers in Turkey. A total of 4993 women who had undergone primary surgery between 1999 and 2018 were included. Women were staged according to the 2009 FIGO staging system. Kaplan-Meier method was used to generate survival data. Factors predictive of outcome were analyzed using Cox proportional hazards models.
Results: Three thousand two hundred and nineteen women (64.5%) were classified as stage IA, 1034 (20.7%) as IB, 286 (5.7%) as II, 72 (1.4%) as IIIA, 15 (0.3%) as IIIB, 173 (3.55) as IIIC1, 128 (2.6%) as IIIC2, and 66 (1.3%) as stage IV. The median age at surgery was 58 years (range, 21-94 years), and the median duration of follow-up was 47 months (range, 3-227 months). For the entire cohort, the 5-year disease-free survival was 75.1% whereas the 5-year OS was 82.2%. Age ≥60 years (Hazard ratio [HR]: 1.21, 95% confidence interval [CI]:1.06-1.40; p=0.005), myometrial invasion (MMI)≥1/2 (HR: 1.19, 95% CI:1.02-1.41; p=0.02), lymphovascular space invasion (LVSI) (HR: 1.29, 95% CI:1.05-1.58; p=0.01),and baseline serum CA 125≥35 U/ml (HR: 1.29, 95% CI:1.08-1.55; p=0.005) were identified as independent prognostic factors for decreased OS.
Conclusion: Age≥60 years, MMI≥1/2, LVSI, and baseline serum CA 125≥35 U/ml seem to be independent prognostic factors for decreased OS in Turkish endometrioid EC population.

[Clinicopathological characteristics of 4993 endometrioid endometrial cancer patients undergoing surg]

				Multivariate Analyses		
		Events (5-year DFS)	Univariate Analyses p	HR	95% CI	p

**Age, y**	**< 60, ≥ 60**	402/2822 (81.0%), 399/2171 (74.1%)	<0.001	1.24	1.09-1.42	0.001
**Myometrial invasion**	**<1/2, ≥1/2**	461/3493 (81.3%), 340/1500 (70.8%)	<0.001	1.21	1.04-1.41	0.012
**FIGO grade**	**1 or 2, 3**	687/4487 (79.1%), 112/497 (69.1%)	<0.001			
**Primary tumor diameter**	**<3 cm, ≥ 3 cm**	261/2071 (81.9%) 524/2787(74.9%)	<0.001			
**LVSI**	**Absent, Present**	541/3857 (80.5%), 257/1128 (70.1%)	<0.001	1.24	1.03-1.51	0.021
**Baseline serum CA 125**	**<35, ≥35**	553/3357 (77.7%), 143/602 (68.1%)	<0.001	1.25	1.06-1.49	0.009
**Stage**	**I, II, III, IV**	590/4253 (80.6%), 60/286 (74.0%), 120/388 (59.8%), 31/66 (40.0%)	0.003 <0.001 0.006	1.0, 1.30, 2.21, 4.64	1.01-1.68, 1.76-2.78, 3.01-6.97	0.038, <0.001, <0.001
**Abbreviations: DFS: disease-free survival, HR: hazard ratio, CI: confidence interval LVSI: Lymphovascular space invasion**						

[Univariate and multivariate analyses of 4993 women with endometrioid endometrial cancer undergoing surgery with regard to disease-free survival]

				**Multivariate Analyses**		
		**Events (5-year OS)**	**Univariate Analyses p**	**HR**	**95% CI**	**p**
**Age, y**	**< 60, ≥ 60**	326/2822 (84.2%), 303/2171 (79.4%)	<0.001	1.21	1.06-1.40	0.005
**Myometrial invasion**	**<1/2, ≥1/2**	375/3493 (84.5%), 254/1500 (77.1%)	<0.001	1.19	1.02-1.41	0.028
**FIGO grade**	**1 or 2, 3**	539/4487 (83.1%), 90/497 (73.6%)	<0.001			
**Primary tumor diameter**	**<3 cm, ≥ 3 cm**	218/2071 (84.5%), 401/2787 (80.1%)	<0.001			
**LVSI**	**Absent, Present**	444/3857 (83.6%), 185/1128 (77.4%)	0.001	1.29	1.05-1.58	0.012
**Baseline**	**<35, ≥35**	55/3357	<0.001	1.29	1.08-1.55	

**serum CA 125**		(81.2%), 117/602 (73.0%)				
**Stage**	**I, II, III, IV**	476/4253 (84.0%), 48/286 (78.1%), 81/388 (70.9%), 24/66 (44.6%)	0.027, 0.009, 0.002	1.0, 1.27, 1.88, 3.91	0.97-1.67, 1.46-2.41, 2.46-6.22	0.073, <0.001, <0.001
**Abbreviations: OS: overall survival, HR: hazard ratio, CI: confidence interval LVSI: Lymphovascular space invasion**						

[Univariate and multivariate analyses of 4993 women with endometrioid endometrial cancer undergoing surgery with regard to overall survival]

Disclosure: Nothing to disclose

EP488

Is chemotherapy better for early stage high risk histologic types of endometrial cancer

E. Çelik[1], N. Ak[2], H. Sozen[1], P. Saip[2], S. Topuz[1]
[1]Gynecologic Oncology, [2]Medical Oncology, Istanbul University, Istanbul Faculty of Medicine, Istanbul, Turkey

Introduction / Background: The most common cause of death due to endometrial cancer depends on the high risk histologies as we call type 2 cancer. There is no definite recommendation for early stage disease in the international guidelines. We aimed to investigate different adjuvant modalities in high grade endometrioid and non-endometrioid FIGO stage 1 and 2 patients.
Methodology: Retrospective database review was performed to identify endometrial cancer patients which were surgically staged between 2006 and 2016 in Istanbul Faculty of Medicine. Patients were compared regarding their clinical, demographical, surgical and pathological differences using independent two test and chi-square test. Kaplan-Meier method was used to calculate disease progression free survival and overall survival in women with type 2 endometrium cancer followed with two different treatment modalities postoperatively.
Results: A total of 124 patients were included. Mean age and body mass index (BMI) were 63.1 years and 31.8 kg/m^2. Thirty-eight (30.6%) patients were grade 3 endometrioid, twenty-seven (21.8%) patients were serous type endometrial cancer. Pelvic lymphadenectomy were done for all patients and mean lymph node was 13.3. Adjuvant treatments were brachytherapy (12.9%), pelvic radiotherapy (27.4%), chemotherapy+brachytherapy (22.6%), chemotherapy+pelvic radiotherapy (22.6%) and chemotherapy alone (14.5%). Patients were divided into two groups as chemotherapy and radiotherapy. Age, BMI, pelvic and para-aortic lymph node numbers, presence of lympho-vascular space invasion were not statistically significant different between groups. High grade endometrioid cancer and stage 1b disease were statistically significantly higher in radiotherapy group (p< 0.001). In the Kaplan-Meier analysis chemotherapy group had better progression-free survival (log rank p=0.031). But overall-survival was similar between groups (log rank p=0.532).
Conclusion: Chemotherapy seems to be a better option for early stage type 2 endometrial cancer.

**Characteristics**		**mean±SD or number (%)**
**Age (years)**		63.1±9.8
**BMI (kg/m2)**		31.8±6.6
**Histology**	**Clear cell, Endometrioid gr3, Indifferentiated, Mixed, Serous**	6 (4.8), 38 (30.6), 7 (5.6), 21 (16.9), 25 (20.2), 27 (21.8)

**Pelvik lymph node number**		13.3±7.8
**Paraaortic lymph node number**		2.9±4.5
**Omentectomy**	**Not done, Done**	11 (8.9), 113 (91.1)
**Adjuvant therapy**	**Brachytherapy, Pelvic radiotherapy, Chemotherapy+brachytherapy, Chemotherapy+Pelvic Radiotherapy, Chemotherapy**	16 (12.9), 34 (27.4), 28 (22.6), 28 (22.6), 18 (14.5)
**Stage (FIGO 2009)**	**Stage 1a, Stage 1b, Stage 2**	57 (46), 44 (35.5), 23 (18.5)
**Follow-up time (months)**		82.5±44.6

[Clinicopathological characteristics of patients]

**Characteristics**		**Group 1 (radiotherapy) (n=50)**	**Group 2 (chemotherapy) (n=74)**	**p**
**Age (years)**		63.9±9.6	62.5±10.0	0.419
**Histology**	**Clear cell, Endometrioid gr3, Indifferentiated, Carcinosarcoma, Mixed Serous**	0, 31 (62), 3(6.0), 8 (16.0), 4 (8.0), 4 (8.0)	6 (8.1), 7 (9.5), 4 (5.4), 13 (17.6), 21 (28.4), 23 (31.1)	**<0.001**
**Pelvik lymph node number**		13.1±6.5	13.4±8.7	0.807
**Paraaortic lymph node**	**Not done, Done**	28 (56.0), 22 (44.0)	45 (60.8), 29 (39.2)	0.593
**Omentectomy**	**Not done, Done**	4 (8.0), 46 (92.0)	7 (9.5), 67 (90.5)	0.779
**Lymphovascular space invasion**	**Absent, Present**	26 (52.0), 24 (48.0)	35 (47.3), 39 (52.7)	0.390
**Stage (FIGO 2009)**	**1a, 1b, 2**	18 (36.0), 25 (50.0), 7 (14.0)	39 (52.7), 19 (25.7), 16 (21.6)	**0.021**
**Recurrence**	**Absent, Present**	37 (74.0), 13 (26.0)	67 (90.5), 7 (9.5)	**0.014**
**Death**	**Absent, Present**	37 (74.0), 13 (26.0)	59 (79.7), 15 (20.3)	0.454

[Comparision of patients according to adjuvant treatment]

BMJ Publishing Group Limited (BMJ) disclaims all liability and responsibility arising from any reliance placed on this supplemental material which has been supplied by the author(s)

This image cannot currently be displayed.

[Progression-free survival of patient groups]

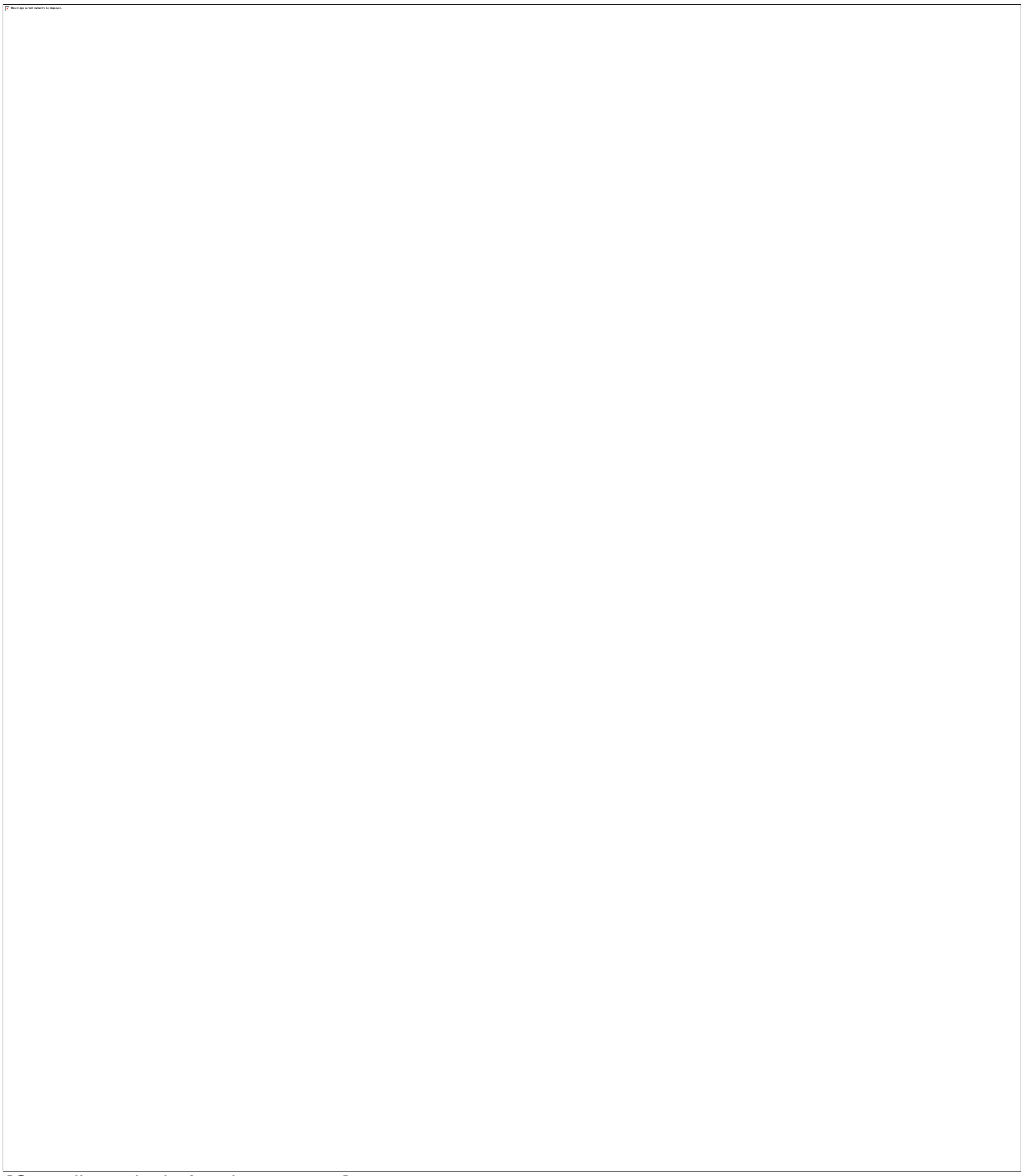

[Overall-survival of patient groups]

Disclosure: Nothing to disclose

EP489

The outcomes of 146 women with surgically staged endometrioid endometrial cancer(EEC): a five years experience from Tata Medical Center , Kolkata

B. Chakraborti, S. Gangopadhyay, J. Bhaumik, A. Mukhopadhyay
Gynaecologial Oncology, Tata Medical Center, Kolkata, India

Introduction / Background: Endometrial cancer is on rise in developing countries with change in socio-economic conditions. Obesity is identified as commonest association along with metabolic co-morbidities. Surgery remains the most important treatment for the endometrial cancer.
Methodology: Clinical, surgicopathologic, recurrence and follow up data of 146 stage I-IV patients with EEC undergoing surgery at Tata Medical Center, Kolkata were collected rerospectively and analyzed in a descriptive manner. Studyperiod was from 2011-2016. Patients operated outside and unstaged patients were excluded. Study analysis: SPSS(Vs 23.0). Multivariate/Univariate, Kaplan Meier test and Cox regression analysis done.
Results: Average age of diagnosis of EEC in the clinic was 58 years. Obesity was common with an average BMI of 30(range 11-48). Seventy six percent of patients had some comorbidity with diabetes being the commonest. Performance status was good. Open surgery were done in 92 patients(63%) and laparoscopy in 54 patients(36%). Seventy one percent of patients were FIGO stage I(IA 39%;IB 32%). FIGO grade 1 accounted for 48% of FIGO stage IA and 19% patients of stage IB. The thirty day post-operative mortality was 0.68% only. Recurrence rate was 8.9%(13 patients). Total 60 patients received vaginal brachytherapy and 12 patients among them also received external beam radiotherapy (EBRT). Another 3 patients received only EBRT and 24 patients received chemotherapy. One hundred and thirty four patients are alive and on follow up, 4 expired and 8 patients defaulted.
Conclusion: Surgical staging and adjuvant therapy resulted in excellent prognosis in EEC patients with 8% and 10% recurrence in early and late stage respectively.

PARAMETERS		NUMBER(%)
**AGE**	< 60yrs	**84(57.5)**
	> 60 yrs	**62(42.%)**
**BMI**	< 30	**87(60)**
	> 30	**59(40)**
**COMORBIDITIES**	Nil	**34(23.3)**
	Multiple	**63(42)**
	Diabetic	**58(39)**
**STAGE**	I	**112(77)**

[Demography]

Disclosure: Nothing to disclose

EP490

A comparative study between laparoscopic and laparotomy approach for surgical staging in early stage endometrial cancer in Rajavithi Hospital, Thailand

S. Chanpanitkitchot[1,2], S. Tuipae[1,2], R. Waesani[2]
[1]College of Medicine, Rangsit University, [2]Obstetric and Gynecology, Rajavithi Hospital, Bangkok, Thailand

Introduction / Background: Endometrial cancer is the third common gynecologic cancer in Thailand after cervical and ovarian cancer. Randomized controlled studies have demonstrated benefit of laparoscopic approach over conventional laparotomy in reduction in operative morbidity and hospital stay. However, laparoscopic approach for surgical staging in developing country, Thailand, is not yet widespread since surgical experience and national health insurance coverage. This study is a retrospective cohort study in single institute to evaluate perioperative outcomes of laparoscopic surgery for surgical staging in early stage endometrial cancer compared with those of the laparotomy approach.

Methodology: Early stage endometrial cancer patients underwent surgical staging from January 2002 to June 2016 at Department of Obstetrics and Gynecology, Rajavithi Hospital, Thailand were extracted for review and matched by age and body mass index (BMI). Demographic data and clinicopathological features including data of perioperative outcomes were analyzed.
Results: A total of 153 patients were included for our study. Eighty-four patients underwent conventional laparotomy and 69 received laparoscopic surgical staging. The demographic data including age, BMI, menopausal status, medical comorbidity and previous pelvic surgery were comparative between both groups. The operative time in laparoscopic surgery had significant longer, 233min ± 55 min vs. 170 ± 50 min (P< 0.001) respectively. However, laparoscopy group had significant less blood loss (164 ± 122 ml vs. 379 ± 234 ml), shorter hospital stays (5 ±1 vs. 6 ±1 days) and needed less postoperative analgesic agents (P< 0.001). The number of harvested pelvic lymph nodes, postoperative adjuvant treatment, perioperative complications of both groups were comparable.
Conclusion: The laparoscopic approach in early stage endometrial cancer is feasible and harmless with better operative outcomes: shorter hospital stays, lesser blood loss and reducing postoperative pain with comparable oncological outcomes.
Disclosure: Nothing to disclose

EP491

Retrospective review of sentinel lymph node mapping in endometrial cancer using indocyanine green and near infra-red fluorescence imaging during minimally invasive surgery- Mater Hospital experience

W. Chaowawanit[1,2], V. Campbell[1], E. Wilson[1], N. Chetty[1], L. Perrin[1], N. Jagasia[1], S. Barry[1]
[1]Gynaecologic Oncology Unit, Mater Hospital, Brisbane, QLD, Australia, [2]Gynecologic Oncology Unit, Department of Obstetrics and Gynecology, Navamindradhiraj University, Bangkok, Thailand

Introduction / Background: Sentinel lymph node (SLN) mapping in endometrial cancer allows us to obtain the lymph node status for prescribing adjuvant therapies and avoids the morbidity of systematic lymphadenectomy. The aim of this study is to determine the feasibility of sentinel lymph node (SLN) mapping in early endometrial cancer.
Methodology: All women with histologically confirmed endometrial cancer, treated with a minimally invasive hysterectomy, bilateral salpingo-oophorectomy and SLN mapping were included. All mapped SLNs were histopathological confirmed.
Results: From January 2017 to April 2019, 138 patients met the inclusion criteria. Eighteen patients were excluded because of unexpected advanced disease, anatomical issues and incomplete medical records. One hundred twenty patients were analysed. A mean age was 62 years old with standard deviation (SD) 11 years and mean Body mass index (BMI) was 33.4 kg/m^2 (SD 7.4). The overall and bilateral SLN detection rates were 85.8% (103/120) and 72.5% (87/120), respectively. BMI < 30 kg/m^2 was a significant predictor of successful detection. 50% of SLNs were detected in the external iliac region, while 15% were found outside the routine lymphadenectomy zone including common iliac, parametrial, infra-mesenteric para-aortic and presacral lymph nodes. Seven patients (5.8%) had positive SLNs.
Conclusion: SLN mapping can reliably detect the SLN in early-stage endometrial cancer. Additionally, some patients having an uncommon site of lymph node metastasis might be missed without SLN mapping.
Disclosure: Nothing to disclose

EP492

Comparison between laparoscopic and robotic surgery, for sentinel lymph node mapping in endometrial cancer using indocyanine green and near infra-red fluorescence imaging

W. Chaowawanit[1,2], V. Campbell[1], E. Wilson[1], N. Chetty[1], L. Perrin[1], N. Jagasia[1], S. Barry[1]
[1]Gynaecologic Oncology Unit, Mater Hospital, Brisbane, QLD, Australia, [2]Gynecologic Oncology Unit, Department of Obstetrics and Gynecology, Navamindradhiraj University, Bangkok, Thailand

Introduction / Background: Indocyanine green (ICG) and near infra-red fluorescence imaging is used in both laparoscopic and robotic surgery to map the sentinel lymph node (SLN). The aim of this study is to compare the outcomes of sentinel lymph node (SLN) mapping between laparoscopic and robotic surgery.
Methodology: One hundred and twenty women with histologically confirmed endometrial cancer, treated with a minimally invasive hysterectomy, bilateral salpingo-oophorectomy and SLN mapping were included. After anaesthetic induction, ICG was superficially injected (2-3 mm) into cervical submucosa and deeply injected into cervical stroma at the 3 and 9 o'clock positions on the cervix (1 ml or 1.25mg per site).
Results: Eleven cases were abandoned after ICG injection (laparoscopic surgery 7 cases and robotic surgery 4 cases) because of obesity, technical difficulty and peritoneal disease. One hundred and nine patients were analyzed. Seventy-six patients (70%) had a laparoscopic procedure and 33 patients (30%) had robotic surgery. The overall and bilateral detection rates were 97% and 83% for laparoscopic surgery and 88% and 73% for robotic surgery. Laparoscopic surgery was superior to robotic surgery in terms of overall detection (p-value 0.046).
There was no significant difference in the intra-operative SLN identification time or SLN dissection time between laparoscopy and robotic surgery.
Conclusion: Further research is required to compared laparoscopy and robotic surgery in terms of SLN detection.
Disclosure: Nothing to disclose

EP493

Does ultra-staging with immunohistochemistry improve the detection of metastasis in sentinel lymph nodes in endometrial cancer?

W. Chaowawanit[1,2], V. Campbell[1], E. Wilson[1], N. Chetty[1], L. Perrin[1], N. Jagasia[1], S. Barry[1]
[1]Gynaecologic Oncology Unit, Mater Hospital, Brisbane, QLD, Australia, [2]Gynecologic Oncology Unit, Department of Obstetrics and Gynecology, Navamindradhiraj University, Bangkok, Thailand

Introduction / Background: Ultra-staging with immunohistochemistry (IHC) increases the detection of sentinel lymph node metastasis in endometrial cancer. The aims of this study were
1. To determine the percentage of positive SLN identified on ultra-staging with IHC which were negative on H&E.
2. To determine if the ultra-staging/IHC result altered the prescription of adjuvant therapy
Methodology: 120 women with histologically confirmed endometrial cancer, treated with a minimally invasive hysterectomy, BSO and SLN mapping were included. All mapped SLNs were histopathological confirmed. If no macroscopic metastasis were identified on H&E staining, ultra-staging with IHC was performed.
Results: SLNs of 103 patients were included in the histopathological analysis. Ninety eight percent (101/103) of all, had ultra-staging performed. Positive SLNs were identified in 7 cases. Two cases had macroscopic metastasis on H&E staining. Using our ultra-staging protocol, 5 patients (5%) had positive SLNs (3 had microscopic metastasis and 2 had isolated tumour cells). According to the Queensland Centre for Gynaecological Cancer (QCGC) protocol for adjuvant therapy, 14 of 101 patients (14%) had their adjuvant treatment altered based on the SLN status after ultra-staging.
Conclusion: Ultra-staging with IHC increased the detection of positive SLNs. Ultra-staging decreased the prescribed adjuvant therapy in 14% of our patients.
Disclosure: Nothing to disclose

EP494

Expression of genes responsible for estrogen metabolism and reception in endometrial cancer depends on tumor grade

A. Menshenina, E. Frantsiyants, T. Moiseenko, D. Vodolazhsky, V. Bandovkina, M. Vovkochina, M. Adamyan, N. Chernikova
Rostov Research Institute of Oncology, Rostov-on-Don, Russian Federation

Introduction / Background: Endogenous or exogenous hyperestrogenism plays the leading role in the pathogenesis of endometrial carcinoma. Estrogens stimulate cell proliferation by inducing the synthesis of growth factors and their receptors, including estrogen receptor alpha (ERα or ESR1). Our purpose was to study the expression of genes responsible for estrogen metabolism and reception in intact and tumor tissues of the uterine body.
Methodology: Relative gene expression was studied in biopsy tissues (tumor and intact) of the uterus from 27 patients aged 38-72 years with histologically verified endometrial cancer (EC). Relative expression of genetic loci responsible for estrogen metabolism and reception (CYP1A1, CYP1A2, CYP1B1, CYP19A, ESR1, ESR2, GPER, STS, SULT1A and SULT1E1) was determined by RT-qPCR.
Results: Patients with G1 tumors showed a significant ($p < 0.005$) increase in the expression of CYP1A1, CYP1A2, CYP1B1, CYP19A, ESR1, ESR2, GPER, STS, SULT1A1 and SULT1E1 genes by 1820%, 845%, 420%, 737%, 198%, 174%, 54%, 746%, 309% and 412% respectively in tumor tissues compared to intact ones. Patients with G2 EC did not have significant changes in the gene expression in tumors compared to normal tissues. Patients with G3 tumors demonstrated a significant ($p < 0.005$) increase in the expression of CYP1A2 (by 90%), CYP19A (by 72%), ESR2 (by 98%), GPER (by 50%), STS (by 130%) and SULT1E1 genes (by 110%).
Conclusion: Only patients with G1 endometrial tumors showed a marked increase in the transcriptional activity of genes responsible for: hydroxylation of estradiol at the C-2 and C-4 positions (CYP1A1 and CYP1B1), formation of estrogens from androgens (CYP19A), regulation of the level of nuclear estrogen receptors (SULT1E1 - sulfotransferase genes), ESR1 and 2 and membrane estrogen receptors (GPER), as well as genes responsible for converting steroids to their active form (steroid sulfatase gene, STS).
Disclosure: Nothing to disclose

EP495

Influence of comorbid estrogen-dependent uterine pathology on morphological characteristics of endometrial carcinoma

T. Moiseenko, N. Chernikova, M. Adamyan, E. Nepomnyashchaya, Y. Poryvaev
Rostov Research Institute of Oncology, Rostov-on-Don, Russian Federation

Introduction / Background: The purpose of the study was to determine special characteristics of endometrial carcinoma (EC) combined with hysteromyoma and/or uterine adenomyosis.
Methodology: We studied clinical, laboratory and morphological data of 345 patients with verified EC with comorbid benign uterine pathology.
Results: Mean age of patients was 62 years. EC with hysteromyoma was noted in patients 5-7 years younger. The combination of all 3 diseases was the most frequent in patients over 62 years. EC with adenomyosis was registered mostly in women of late reproductive age, less often in peri- or deep menopause. All patients were examined and operated on in accordance with the standard of EC treatment. Surgical intervention involved panhysterectomy, pelvic lymphadenectomy. Disease stages were determined using the TNM and FIGO classifications (2009) and were updated according to the results of a postoperative morphological analysis. More than 80% of tumors were endometrioid adenocarcinoma G1-G3. The minimal spread of EC was observed in patients with comorbid hysteromyoma (T1aN0M0), the maximal one (T1-3N0-1M0) in EC+hysteromyoma+adenomyosis.

Macroscopically, EC with hysteromyoma was represented by exophytic or polypoid tumors, EC+hysteromyoma+adenomyosis - by endophytic lesions. Diffuse infiltrative growth of EC was observed in elderly patients with adenomyosis. Aggressive and morphologically rare forms of EC with affected regional pelvic lymph nodes and uterine appendages were recorded in such cases.
Conclusion: More than 50% of EC cases develop in combination with hysteromyoma and/or uterine adenomyosis. Clinically unfavorable disease course was typical of patients older than 62 years with EC in combination with two benign uterine diseases.
Disclosure: Nothing to disclose

EP496

Characteristics of patients with uterine sarcoma depending on morphological tumor structure, methods and results of treatment

E. Verenikina, E. Nepomnyashchaya, N. Chernikova, Y. Poryvaev, O. Zhenilo, P. Kruze, T. Myagkova, M. Adamyan, A. Cherkasova, O. Selezneva
Rostov Research Institute of Oncology, Rostov-on-Don, Russian Federation

Introduction / Background: The purpose of the study was to reveal clinical and morphological characteristics of patients with uterine sarcoma.
Methodology: We studied case histories of 256 patients (24-72 years) with uterine sarcoma. Symptoms, characteristics of the disease stage and histostructure and sarcoma growth, the presence of concomitant diseases and the results of treatment were assessed.
Results: The peak incidence was observed at 50-59 years of age (39.8%); 29.3% patients had stage I, 28.6% stage II, 22.6% stage III, 19.5% stage IV. 45% were diagnosed with leiomyosarcoma (LS), 36% endometrial stromal sarcoma (ESS), 19% carcinosarcoma (CS). Most patients were postmenopausal (71.3%). Most frequent gynecological diseases were hysteromyoma (47.5%) and endometrial polyposis (26.7%). Obesity was observed in 39.8%, hypertension in 32.8%; diabetes in 8.5% of patients. Pain in the lower abdomen was the main complaint reported by 63% of patients, bleeding from the genital tract was the second most common symptom (50.3%); the latter was most frequent in 60% patients with ESS. LS was most often located in the intramural myomatous nodule (50%). ESS showed a polypoidal growth pattern (76.4%); myometrial invasion of CS in 66.6% was >1 cm. Sarcomas were diagnosed by Doppler ultrasonography and dilation and curettage. 22.2% of patients were diagnosed using ultrasound. 5-year survival in patients with surgical treatment only (panhysterectomy) was ≤10% for all stages, in patients with combined treatment (surgery and external radiotherapy) - 49%, in patients with complex treatment (surgery, chemotherapy and external radiotherapy) - 65%.
Conclusion: The disease peak occurred at 50-59 years of age (39.8%); early stages were diagnosed more often (57.9%). Pathognomonic symptoms were not observed. Histologically, LS (45%) and ESS (36%) prevailed. LS was most often located in the intramural myomatous nodule (50%). The best treatment outcomes were obtained in patients receiving complex treatment including surgical treatment, polychemotherapy and external radiotherapy.
Disclosure: Nothing to disclose

EP497

Adjuvant/radical radiotherapy in elderly women with endometrial cancer: is it justified based on survival outcomes?

C. Cunningham, M. Chitoni, A. Kerr, R. Harrand, A. Sadozye, N. Reed, K. Graham
Beatson West of Scotland Cancer Centre, Glasgow, United Kingdom

BMJ Publishing Group Limited (BMJ) disclaims all liability and responsibility arising from any reliance placed on this supplemental material which has been supplied by the author(s)

Introduction / Background: Endometrial cancer is largely a disease of older women. The incidence is increasing and age is a prognostic factor, with subsequent resource implications. Poorer outcomes may be due to biological factors and/or suboptimal treatment. Interestingly, PORTEC-3 indicates that high-risk women >70 years benefit from adjuvant chemoradiotherapy (5-year OS 67%). We investigated real-world outcomes of elderly women in West of Scotland Cancer Network (WoSCAN) who received pelvic external beam radiotherapy (PRT) for endometrial cancer.
Methodology: Data were collected retrospectively from WoSCAN electronic clinical records in women 70 years who commenced PRT for endometrial cancer between January 2011 and December 2015. Statistical analysis was performed using R®. Overall survival (OS) was estimated using Kaplan-Meier method.
Results: 93 patients were identified. The median age was 75 years (range 70-90); 28% >80years. Median number of co-morbidities was 2 (range 0-7). Treatment intent was as follows: adjuvant (60%), primary (inoperable) (21%), salvage (vault/pelvic recurrence) (19%). Only 30% had Stage 1 disease, over 25% had aggressive pathology. 90% completed all radiotherapy as planned (4500cGy/25# +/- brachytherapy), 5% discontinued PRT and 5% completed PRT but defaulted from brachytherapy. 30-day mortality was 5%. Over 50% received chemotherapy. 5-year OS was 50%. Enhanced OS was associated with low-grade pathology, early stage and adjuvant/salvage intent; 5-year OS 55% (adjuvant/salvage) versus 20% (primary). OS was significantly worse in >80 cohort (25%) compared with 70-79 cohort (55%).
Conclusion: Compliance with PRT for endometrial cancer in women >70 years is >90%. OS in adjuvant setting is slightly less favourable than PORTEC-3 but we included very elderly women, often with multiple medical co-morbidities. PRT is justified in patients aged 70-79 years. However, it may be appropriate to consider a more pragmatic approach in women >80 years, especially in the context of inoperable disease, as survival may not be sufficiently long to benefit from aggressive therapy.
Disclosure: Nothing to disclose

EP498

Oncologic and pregnancy outcomes with fertility-sparing management for early endometrial cancer in young women

Y.S. Chung, J.-Y. Lee, E.J. Nam, S. Kim, S.W. Kim, Y.T. Kim
Department of Obstetrics and Gynecology, Yonsei University College of Medicine, Seoul, Republic of Korea

Introduction / Background: This study evaluated the oncologic and pregnancy outcomes of progestin treatment for early endometrial cancer in young women.
Methodology: We retrospectively analyzed the medical records of 82 patients younger than 45 years with early stage endometrial cancer who had received fertility-sparing management between 2006 and 2018. All of the patients were treated with medroxyprogesterone acetate (MPA) or megestrol acetate (MA) or combined MPA/levonorgestrel-intrauterine system (LNG-IUS).
Results: Fifty-nine patients (72.0%) showed complete response (CR) after progestin therapy, and 19 (32.2%) of them experienced recurrence after median follow-up time of 29.2 months. Age ≥ 35 (p=0.011) , body mass index (BMI) ≥ 25 (p=0.015), and myometrial invasion (p=0.002) were significantly negative factors associated with achieving CR. Multivariate analysis showed that myometrial invasion (hazard ratio [HR] = 14.00, 95% confidence interval [CI] 2.45-80.14, p=0.003) was significantly associated with a higher risk of recurrence and maintenance treatment (HR = 0.26, 95% CI 0.08-0.85, p=0.026), and assisted reproductive technology (ART) utilization (HR = 0.20, 95% CI 0.06-0.66, p=0.008) were significantly associated with a lower risk of recurrence. Of the 24 (40.7%) patients who tried to conceive after CR, 21 (87.5%) patients underwent ART, 12 (50.0%) patients experienced pregnancy, 9 patients (75.0%) had live newborn infants.
Conclusion: Fertility-sparing management for early endometrial cancer with progestin is significantly effective. Patients with maintenance treatment, ART utilization, and without myometrial invasion associated with a higher probability of long-term success.
Disclosure: Nothing to disclose

EP499

Volumetric-modulated arc therapy for adjuvant treatment of endometrial cancer

S. Cima, M. Valli, A. Richetti

Radiation Oncology Clinic, Oncology Institute of Southern Switzerland, Bellinzona, Switzerland

Introduction / Background: External beam radiation therapy is prescribed as adjuvant treatment for patients with high intermediate or high risk endometrial cancer (EC). Volumetric modulated arc therapy (VMAT®) is an advanced radiation technique, that achieves highly conformal dose distributions with improved target volume coverage and sparing of normal tissues compared with conventional techniques. The aim of this study was to report clinical outcomes and toxicity of EC patients treated with adjuvant VMAT® in our Clinic.
Methodology: We analyzed 27 women with invasive endometrial cancer, treated with adjuvant radiotherapy after a total abdominal hysterectomy and bilateral salpingo-oophorectomy with or without lymphadenectomy, from 2015 to 2018. Toxicity data were collected according to Common Terminology Criteria for Adverse Events v 4.0. Survival curves were calculated using Kaplan-Meier method.
Results: The FIGO stages were: IB in 33%, II in 15%, IIIA in 11%, IIIB in 4%, IIIC in 37%. The mean age was 67 years. The mean prescribed dose was 49 Gy. Vaginal cuff brachitherapy boost was performed in 16 patients (59%) with a mean prescribed dose of 13 Gy. Fourteen patients (52%) were treated with adjuvant chemotherapy. All patients completed irradiation. No case of acute toxicity G3 or more, was observed. The 1-year and 3-year late grade ≥ 1 GU toxicity free survival was 100% and 64%, respectively. The 1-year and 3-year late grade ≥ 1 GI toxicity free survival was 100% and 64%, respectively. The 1-year and 3-year late grade ≥ 1 GI vaginal toxicity free survival was 68% and 34%. With a mean follow-up of 21 months, local recurrence was observed in 2 patients (7%). The 2-year risk for loco-regional and distant recurrence was 10% and 13%, respectively. The 2-year OS was 92%.
Conclusion: Our experience confirmed the feasibility of this technique with good results in terms of acute and late toxicity.
Disclosure: Nothing to disclose

EP500

The diagnostic and prognostic value of serum L1CAM levels in endometrial cancer

E. Sertel, Ş. Gezer, A. Pulur, A. Çorakçı, İ. Yücesoy

Obstetrics and Gynecology, Kocaeli University Faculty of Medicine, Kocaeli, Turkey

Introduction / Background: Endometrial carcinoma is the most common gynecological malignancy in developed countries. Myometrial invasion and lymph node metastasis are important prognostic factors for endometrial cancer. Although lymphadenectomy is part of the full staging procedure, it is known to be associated with increased complication rates and increased comorbidity with prolonged operation time. For this reason, it is important that biomarkers that can predict preoperative myometrial invasion and lymph node metastasis and which can predict poor prognosis can be used. These biomarkers can help in the adjustment of surgical treatment by identifying patients with advanced disease that will benefit from a wide range of surgical procedures but can also help to prevent over-treatment in low-risk patients. In this context, serum L1CAM examination seems promising in endometrial cancer.
Methodology: Between January and April 2019, 80 patients with endometrial cancer and 37 control patients were included in the study. Blood samples obtained from the patients were diluted and measured by the spectrophotometric microelisa method with HUMAN L1CAM/CD171. Data were evaluated by IBM SPSS Statistics 20.0 statistical data program and p-value < 0.05 was considered statistically significant.
Results: It was found that serum L1CAM values were significantly higher in endometrial cancer patients than in the control group (p: 0.000).Although serum L1CAM levels were higher in patients with

myometrial invasion and lymph node metastasis, statistically significant results could not be obtained.
Conclusion: In this study serum L1CAM measurements were not found to be significantly different in high-risk endometrial cancer patients. We believe that serum L1CAM examination may be effective in the diagnosis of endometrial cancer, but further studies with more patients on low risk and high-risk differentiation should be considered.
Disclosure: Nothing to disclose

EP501

Management of patients with metastatic endometrial cancer at diagnosis: single center experience

A. Cordoba, E. Cerezo, A. Broyelle, A. Parent, M.-C. Le Deley, F. Le Tinier, A. Escande, A. Lesoin, A. Chevalier, A.-S. Lemaire, S. Taieb, D. Hudry, E. Leblanc, F. Narducci
Centre Oscar Lambret, Lille, France

Introduction / Background: Retrospective evaluation of a cohort of patients diagnosed with metastatic endometrial cancer (EC) and to describe the treatment performed as well as the rates of overall survival (OS), progression free survival (PFS) and survival without locoregional recurrence.
Methodology: Between January 2002 and December 2018, all consecutive patients with EC stade IV were included; patients who did not receive any treatment (CT, RT or surgery) at the time of diagnosis were not included. All treatments performed [chemotherapy (CT), surgery and external radiotherapy (RT)] were collected.
Results: 56 patients were identified; the median age of the patients was 64 years (39-83); the median follow-up of patients was 4.4 years 95% CI95% [3.4 - 6]. 53 patients (95%) IVb and 3 patients (5%) Iva. 42 patients (74%) had an endometroid or type 1 histology and 14 patients (26%) had a non-endometroid or type 2 histology. All patients (56) received CT. 37 patients (66%) had surgery, 23 (62%) before CT and 14 (38%) after CT. 25 patients (45%) received adjuvant RT after surgery. 4 patients (7%) presented local-locoregional recurrence, 18 patients (32%) presented metastatic and local-locoregional recurrence and 21 patients (37,5%) isolated metastatic recurrence. The median survival time is 2.9 years 95% CI[1.7 - 4.8]. 5 year-OS is 33%. The median progression-free survival (PFS) rate is 1.5 years 95% CI [0.91 - 1.98]. 5 year-PFS is 19%. Cumulative incidence of local-regional recurrence at 1, 3 and 5 years is 17%, 41% and 45%.
Conclusion: Metastatic EC presents poor prognosis. The use of surgery and adjuvant RT in addition to CT may improve OS, PFS and loco regional recurrence in selected patients.
Disclosure: Nothing to disclose

EP502

Blocking 17b-hydroxysteroid dehydrogenase type 1 in endometrial cancer: a potential novel endocrine therapeutic approach

K.M.C. Cornel[1,2], G. Konings[2,3], S. Xanthoulea[2,3], B. Delvoux[3], M.A. Skowron[4], L. Kooreman[1,5], P. Koskimies[6], C. Krakstad[7,8], H.B. Salvesen[7,8], K. Kuijk van[1,2], Y.J. Schrooders[1,2], M. Vooijs[1,9], A.J. Groot[1,9], M.Y. Bongers[1,2], R.F. Kruitwagen[1,2], A. Romano[1,2], ENITEC (European Network Individualized Treatment Endometrial Cancer)
[1]*GROW - School for Oncology and Developmental Biology, Maastricht University,* [2]*Gynaecology & Obstetrics,* [3]*GROW - School for Oncology and Developmental Biology, Maastricht University Medical Centre (MUMC), Maastricht, The Netherlands,* [4]*Urology, Heinrich Heine University Düsseldorf, Dusseldorf, Germany,* [5]*Pathology, Maastricht University Medical Centre (MUMC), Maastricht, The Netherlands,* [6]*Forendo Pharma Ltd., Turku, Finland,* [7]*Gynaecology & Obstetrics, Haukeland University Hospital,* [8]*Centre for Cancer Biomarkers, Department of Clinical Science, University of Bergen,*

Bergen, Norway, [9]Department of Radiotherapy (MAASTRO), Maastricht University, Maastricht, The Netherlands

Introduction / Background: The enzyme type 1 17β-hydroxysteroid dehydrogenase (HSD17B1), responsible for generating active 17β-estradiol (E2) from low-active estrone (E1), is over-expressed in endometrial cancer (EC) thus implicating an increased intra-tissue generation of E2 in this estrogen-dependent condition.
In this study we explored the possibility of inhibiting HSD17B1 and impairing the generation of E2 from E1 in EC using *in vitro*, *in vivo* and *ex vivo* models.
Methodology: We generated EC cell lines derived from the well-differentiated endometrial adenocarcinoma Ishikawa cell line and expressing levels of HSD17B1 similar to human tissues. In these cells, HPLC analysis showed that HSD17B1 activity could be blocked by a specific HSD17B1 inhibitor. *In vitro*, E1 administration elicited colony formation similar to E2, and this was impaired by HSD17B1 inhibition. *In vivo*, tumours grafted on the chicken chorioallantoic membrane (CAM) demonstrated that E1 upregulated the expression of the estrogen responsive cyclin A similar to E2, which was impaired by HSD17B1 inhibition. Neither *in vitro* nor *in vivo* effects of E1 were observed using HSD17B1 negative cells (negative control).
Results: Using a patient cohort of 52 primary ECs, we demonstrated the presence of HSD17B1 enzyme activity (*ex vivo* in tumour tissues, as measured by HPLC), which was inhibited by over 90% in more than 45% of ECs using the HSDβB1 inhibitor. Since drug treatment is generally indicated for metastatic/recurrent and not primary tumour, we next demonstrated the mRNA expression of the potential drug target, HSD17B1, in metastatic lesions using a second cohort of 37 EC patients.
Conclusion: In conclusion, HSD17B1 inhibition efficiently blocks the generation of E2 from E1 using various EC models. Further preclinical investigations and HSD17B1 inhibitor development to make candidate compounds suitable for the first human studies are awaited.
Disclosure: Nothing to disclose

EP503

Real world outcomes of advanced endometrial cancer treated with neoadjuvant chemotherapy

K. Baillie[1], C. Cunningham[1], C. Crearie[1], J. Laskey[1], M. Bennie[2,3], K. Burton[4], A. Sadozye[1], R. Lindsay[4], R. Harrand[1], N. Siddiqui[4], N. Reed[1], K. Graham[1]
[1]Beatson West of Scotland Cancer Centre, NHS Greater Glasgow and Clyde, [2]Strathclyde Institute for Pharmacy & Biomedical Sciences, University of Stracthclyde, Glasgow, [3]Public Health and Intelligence Strategic Business Unit, NHS National Services Scotland, Edinburgh, [4]Glasgow Royal Infirmary, NHS Greater Glasgow and Clyde, Glasgow, United Kingdom

Introduction / Background: Neoadjuvant chemotherapy (NACT) is increasingly used in locally advanced or selected stage 4B endometrial cancer that is considered unsuitable for primary surgery, despite the lack of randomised evidence. In the West of Scotland Cancer Network (WoSCAN), NACT is offered to women with inoperable locally advanced disease or limited stage 4B disease (omental/peritoneal involvement and/or small volume lung metastases/pleural effusion). This study aimed to identify the proportion of patients proceeding to delayed primary surgery or interval debulking surgery following NACT for endometrial cancer and evaluate survival outcomes.
Methodology: Data were collected retrospectively from electronic clinical records of WoSCAN patients who commenced NACT between January 2012 and December 2016. Statistical analysis was performed using R®. OS was estimated using Kaplan-Meier method and Cox's proportional-hazards models used to calculate unadjusted hazard ratios (HR).
Results: 54 patients were identified; median age was 67 years (range 36-85). All women had Stage III (57.4%) or IV (42.6%) disease. Pathological subtype consisted of serous (42.6%), endometrioid (31.5%), or other (25.9%). NACT regimen was almost exclusively Carboplatin/ Paclitaxel (92.6%); a median of 4 cycles were administered (range 1-6). 66.7% proceeded to surgery, 7.4% received primary (chemo)radiotherapy, and 25.9% received no definitive therapy. Of the 36 patients who underwent surgery, 83.3% received adjuvant radiotherapy. Data were censored in February 2018 at which time 33.3% were alive. Median OS was 31.12 months (95% CI 24.93-58.49); 40.8 months (95% CI 25.6 - Not reached) in the surgical cohort versus 18.6 months (95% CI 8.1 - 60.6) in patients who

received NACT alone (HR 2.28 (95% CI 1.14-4.56)).
Conclusion: 66.7% of women proceeded to surgery following NACT. Median OS is significantly improved with surgery compared to NACT alone. NACT is a valid option in patients with advanced endometrial cancer who are not suitable for primary surgery.
Disclosure: Nothing to disclose

EP504

The role of the CA125 marker as a prognostic factor in endometrial cancer

A. Cymbaluk-Płoska, P. Gargulińska, S. Kwiatkowski, A. Chudecka-Głaz
Pomeranian Medical University, Szczecin, Poland

Introduction / Background: Estimated ASRs per European standard of endometrial cancer, incidence by sex, cancer site and country, in Europe in 2018 was equal to 22.2 per 100,000 person-years (ASR - Age Standardized Rate).
Methodology: The study is based on 199 patients with endometrial cancer and was performed between 2009-2015 at the Pomeranian Medical University. The determinations were made using the ARCHITECT CA 125 II analyzer (Abbott Diagnostics).
Results: Statistically significant differences were found between CA125 levels in patients with endometrial endometrioid adenocarcinoma and non-endometrioid endometrial carcinoma (p=0.044). The mean concentration of the CA125 marker was statistically significantly higher in the group of FIGO III, IV and G3 patients compared to the average CA125 concentrations in the FIGO I, II and G1 patients, respectively (p=0.014 / p=0.039). We have found significantly higher CA125 concentrations in patients with endometrial cancer with invasion of blood vessels (p=0.022) and in patients with lymph node metastases (p=0.016). We did not find statistically significant differences in mean CA125 concentrations by analyzing lymph vessel invasions and depth infiltration myometrium (p=0.056 / p=0.078). In the univariate analysis of the COX regression model, the relationship to disease free time (DSF) shows the cut-off point and the median level CA125 (HR=1.82; p=0.002 and HR=1.76; p=0.033). In the multivariate analysis, the strongest relationship with both the time free from disease and total survival is demonstrated by the CA125 cut-off point (HR=1.45, p=0.026 and HR=1.38, respectively p = 0.037).
Conclusion: CA125 can be a good prognostic factor, isolating the patients with a higher risk of early recurrences and worse prognostications. Determination of the CA125 marker before surgery may allow better treatment individualization and patients follow-up.
Disclosure: Nothing to disclose

EP505

A rare case of squamous carcinoma of the uterine body

C. del Valle Rubido[1], C. Martín Blanco[1], V. Corraliza Galán[1], M.C. Sánchez Martínez[1], E. Díaz González[1], B. Pérez Mies[2], E. Moratalla Bartolomé[1], J. Lázaro- Carrasco[1]
[1]*Obstetrics and Gynecology,* [2]*Pathology, Hospital Universitario Ramón y Cajal, Madrid, Spain*

Introduction / Background: In Europe, endometrial cancer is the fourth most common cause of cancer among women and the most common gynecologic malignancy. Squamous endometrial carcinoma represents an uncommon histology, consisting of 3% of all Type II (non-endometroid) tumors.
Methodology: The following is a rare case of squamous endometrial carcinoma diagnosed and treated at "Ramon y Cajal" University Hospital (Madrid,Spain).
Results: A 57-year-old, gesta2, para2, woman debuted with an HSIL Pap-test and a single episode of

postmenopausal bleeding. Cervical biopsy guided by colposcopy showed a moderately differentiated epidermoid carcinoma. SCC was 3,2. HPV was positive for 16 and 59 genotypes. Preoperative imaging showed no macroscopical tumour.
Radical hysterectomy and pelvic bilateral lymphadenectomy were performed. Hystology showed an infiltrating squamous endometrial carcinoma, FIGO stage IA, and negative lymph nodes. There was overexpression of p63 and p16 and HPV 16 sequencing.
Follow up: controls were negative for four years, after which vaginal Pap-test showed HSIL and HPV 16 persisted. A VAIN3 was located by vaginoscopy at the left vaginal wall. She underwent laser CO2 treatment of the vagina. Controls have been negative, with negative Pap-tests and HPV.
Conclusion: Endometrial carcinoma is traditionally characterized as either endometrioid or nonendometrioid based on histopathologic phenotypes. However, molecular heterogeneity within the two types has become increasingly apparent, and the current classification and treatment paradigms have significant limitations.
Squamous endometrial carcinoma is a rare type II tumour, which etiology could be related to differentiation of pluripotent endometrial precursor cells, heterotopic cervical tissue, or even HPV infection. P16, a tumor suppressor protein that has been associated with HPV, was present in this case, as well as HPV 16. The clinical behaviour of this malignancy, including debut of VAIN3 in the follow-up, supports the similarity to cervical cancer. Therefore, we could consider the treatment and follow-up of squamous endometrial carcinoma as a cervical carcinoma.
Disclosure: Nothing to disclose

EP506

The role of lymphovascular space invasion to define risk groups of endometrial cancer

F. Demirkiran, T. Bese, S. Gokmen, B.O. Kayan, A.S. Acikgoz, I. Kahramanoglu, H. Turan, M. Arvas
Department of Gynecologic Oncology, Istanbul University-Cerrahpasa, Cerrahpasa School of Medicine, Istanbul, Turkey

Introduction / Background: The main objective of this retrospective study was to define risk groups to identify patients at risk of recurrence who may benefit from adjuvant treatment.
Methodology: Patients who had complete staging surgery for endometrial cancer on between 2000 and 2017 in Istanbul University-Cerrahpasa were analyzed. Patients were divided into groups according to grade of the disease and myometrial invasion (MI)
Results: A total of 895 patients were identified. The presence of lymphovascular space invasion (LVSI) increased the risk of nodal involvement and decreased 5-year DFS, significantly in each group (Table 1). Patients with grade 1-2 disease and no LVSI had less than 4% nodal involvement and more than 91% 5-year DFS (Group I and III). However, the presence of LVSI increased the risk of nodal involvement and recurrence significantly in patients with grade 1-2 disease (Group II and IV). Similarly, LVSI increased the risk of nodal involvement and worsen DFS in patients with grade 3 disease (Groups V to VIII).
Conclusion: Along with grade and MI, LVSI is a significant factor for predicting nodal involvement. Patients having grade 1-2 tumor with < 50% MI and no LVSI should be defined as low risk group. Low-intermediate risk group may be consisted of grade 3 tumor with MI of < 50% and no LVSI; or grade 1-2 tumor with MI of < 50% and (+) LVSI; or tumor with any grade, deep MI and no LVSI. Those with LVSI (+) tumors having deep MI and tumor grade of 1-2, or with LVSI (+) tumors with < 50% MI and high grade histology can be classified as high-intermediate risk group. High-risk group should be consisted of patients with deep MI, grade 3 disease and (+) LVSI.

	Group I n=421	Group II n=89	Group III n=77	Group IV n=152	Group V n=38	Group VI n=31	Group VII n=17	Group VIII n=70
Grade	1-2	1-2	1-2	1-2	3	3	3	3
Myometrial invasion	<50%	<50%	≥50%	≥50%	<50%	<50%	≥50%	≥50%

Lymphovascular space invasion	(-)	(+)	(-)	(+)	(-)	(+)	(-)	(+)
Nodal involvement	0.7%	5.6%	3.9%	16.4%	2.6%	9.7%	5.9%	32.9%
5-year disease-free survival	96.0%	93.2%	91.1%	80.0%	79.9%	82.4%	75.6%	74.8%

[Table 1]

Disclosure: Nothing to disclose

EP507

Has the Robot been beneficial to endometrial cancer patients? A prospective quality improvement project at a London Gynae-Oncology tertiary Centre

A.M. Dokmeci[1], M. Abdurrayman[1], S. Drymiotou[2], D. Chandrasekaran[1], N. Rai[3], J. Dilley[1], S. Phadnis[1], R. Manchanda[1], A. Jeyarajah[1], E. Brockbank[1]
[1]Gynaecological Oncology, [2]Obstetrics & Gynaecology, Barts Health NHS Trust, [3]Gynaecological Oncology, The Royal Marsden NHS Foundation Trust, London, United Kingdom

Introduction / Background: Minimally invasive surgery (MIS) is the preferred route for women with endometrial cancer. Although robotic surgery is not yet NHS standard, it was introduced at Royal London Hospital in November 2017. The effect of introducing robotic surgery (RAH) on the length of stay (LOS) and laparotomy rates in women undergoing surgery for endometrial cancer was assessed.
Methodology: Electronic patient records were reviewed for all women undergoing surgery for endometrial cancer in 2016 and 2018 and analysed using Microsoft Excel and IBM SPSS.
Results: A total of 95 and 103 women (same mean age of 66) had surgery in 2016 and 2018 respectively.
In 2016, after examination under anaesthetic (EUA), 53 (56%) proceeded to TAH and 42(44%) to TLH. 2 (0.02%) were converted from TLH to TAH. The median BMI, estimated blood loss (EBL) and LOS for the TAH group were 32kg/m^2, 350ml and 6 days respectively whereas for TLH were 30 kg/m^2, 100ml and 2 days.
In 2018, 37 (35%) had TAH and 67 (65%) MIS hysterectomy. In the MIS group, 21 (31%) had TLH and 45 (69%) RAH. 7 (11%) and 1(1%) were converted from RAH to TAH and vaginal hysterectomy respectively. Of these 8, 7 were draped but not docked. The median BMI, EBL and LOS for the TAH group were 28.6 kg/m^2, 400ml and 5 days, for TLH were 28kg/m^2, 100ml and 1 day and for RAH were 39.3kg/m^2, 100ml and 2 days respectively.
The overall LOS has fallen from 5 days in 2016 to 3 in 2018 ($p< 0.01$). The overall rate of TAH has fallen from 53/95 to 36/103 ($p< 0.01$).
Reasons for conversion included anaesthetic, adhesions, intraoperative complications and uterine perforation.
Conclusion: RAH has enabled complex operating in high-risk cases while significantly reducing LOS and laparotomy rates.
Disclosure: Nothing to disclose

EP508

Could microsatellite instability predict the response to immunotherapy in high-grade endometrial carcinomas?

T. Doulgeraki[1], S. Vagios[1], P. Yiannou[2], E. Giannikaki[3], C. Papadimitriou[4], A. Rodolakis[5], E. Kavoura[2], A. Vlachos[6], K. Pavlakis[1,2]
[1]Pathology Department, National and Kapodistrian University of Athens, [2]Pathology Department, "IASO" Women's Hospital, Athens, [3]Pathology Department, Venizeleio-Pananeio General Hospital, Heraklion, [4]Oncology Unit, 2nd Department of Surgery, Aretaieion Hospital, National and Kapodistrian University of Athens, [5]1st Department of Obstetrics and Gynecology, Alexandra Hospital, National and Kapodistrian University of Athens, [6]Department of Gynecological Oncology, "IASO" Women's Hospital, Athens, Greece

Introduction / Background: The recent food and drug administration (FDA) approval of immunotherapy for all microsatellite unstable carcinomas, has strengthen the need for more detailed studies, in order to highlight the group of endometrial cancers that would probably favor from thisnew therapeutic option. One of the hallmarks of carcinomas with microsatellite instability (MSI), is the increased neo-antigen load which leads to an enhanced immune environment. Our goal was to evaluate by immunohistochemistry, whether mismatch repair (MMR) status was related to CD8 high intratumoral infiltration and PD-L1 expression in a series of high-grade endometrial carcinomas and correlate it with several clinicopathological characteristics and with survival.
Methodology: The cohort comprised 40 high grade endometrioid, 36 serous, 4 clear cell, 5 unclassified and 7 mixed carcinomas as well as 8 Malignant Mixed Mullerian tumors. All patients were fully surgically staged. Postoperative treatment varied according to the tumor's characteristics. Immunohistochemistry for MLH1, MSH2, MSH6, PMS2, CD8 and PD-L1 was performed.
Results: MMR deficient tumors were strongly related to endometrioid type carcinomas (p< 0,001), high CD8 counts (p=0,015), positive PD-L1 tumors (p=0,029) and deep myometrial invasion (p=0,014). Nevertheless, when CD8+ and PD-L1 high tumors were grouped together, they were statistically related to MMR deficiency irrespective of tumor sub-type whereas combined CD8+/PD-L1 low expression was statistically associated with MMR proficient tumors. Both in Kaplan-Mayer univariate and in multivariate analysis, only high CD8+ cells were related to longer progression free survival (p=0,006 and p=0,008 respectively)).
Conclusion: In high grade endometrial carcinomas, both of endometrioid and of non-endometrioid type, a high CD8+ microenvironment represents an independent favorable prognostic factor for progression free survival. The significant statistical relation of MMR deficient tumors with high intratumoral CD8 counts and positive PD-L1 expression might imply that therapy with immune checkpoint inhibitors could be effective in high grade endometrial carcinomas featuring microsatellite instability.
Disclosure: Nothing to disclose

EP509

Case series of uterine sarcomas associated with preoperative treatment with ulipristal acetate for supposed symptomatic myomas

A. Dovnik, M. Pakiž
Department of Gynaecologic Oncology, Maribor University Medical Centre, Maribor, Slovenia

Introduction / Background: Ulipristal acetate is used for preoperative treatment of symptomatic uterine myomas. The diagnosis of uterine myomas is usually clinical and might miss uterine sarcomas.
Methodology: The clinical audit of the regular clinical database of our Department for Gynecologic and Breast Oncology, Maribor, Slovenia, showed in last 6 years a series of three patients with different sarcomas in association with preoperative treatment with ulipristal acetate. All patients were followed, diagnosed and treated outside of our department and referred after the final diagnosis.
Results: The first patient was a 50-years old XY1 who was followed for a symptomatic uterine myoma for years. She was a former infertility patient. Since the persistent bleedings she was put on ulipristal acetate, the tumour grew, laparoscopic myomectomy was performed. The histological report described a low grade endometrial stromal sarcoma.
The second was a 27-years old XY2 who was presented to reproductive medicine gynaecologist for infertility and uterine myomas. MR of the abdomen revealed numerous extrauterine and uterine myomas. She was put on ulipristal acetate for preoperative treatment. Again, the tumours grew. She

was operated, having abdominal myomectomy, and one of the pelvic tumours was histologically STUMP.
The third patient was a 54-years old XY3 who was also followed for a supposed myoma, put on ulipristal acetate, again the tumour grew and laparoscopic hysterectomy followed. The histology revealed leimyomsarcoma.
Conclusion: Our clinical audit showed a series of three different sarcomas associated with preoperative treatment with ulipristal acetate. All three patients were followed for a supposed myomas years before treatment. It is not possible to conclude if the tumours were sarcomas from the beginning or there was a malignant transformation/tumour growth promotion after introducing ulipristal acetate. Our results warrant a research of larger rare tumours databases.
Disclosure: Nothing to disclose

EP510

Robotic surgery: does high body mass index (BMI) matter?

S. Drymiotou[1], A.M. Dokmeci[2], D. Chandrasekaran[2], J. Dilley[2], N. Rai[2], A. Lawrence[2], R. Manchanda[2], S. Phadnis[2], A. Jeyarajah[2], E. Brockbank[2]
[1]Barts Health NHS Trust, [2]Gynaecological Oncology, Barts Health NHS Trust, Royal London Hospital, London, United Kingdom

Introduction / Background: Obesity increases endometrial cancer risk;1.6-fold with every 5kg/m^2 increase in BMI. High BMI is associated with additional surgical and anaesthetic risks and prolonged operating time. Robotic surgery is a promising route in obese women. Short-term data show no statistically significant difference in survival rates between robotic and abdominal (Hinshaw et al. 2016) and laparoscopic surgery for endometrial cancer (Corrado et al. 2018). The aims were to review whether the introduction of robotic surgery has increased minimally invasive surgery (MIS) rate in obese women and to assess the effect of BMI on console time.
Methodology: Data was prospectively collected for all women undergoing surgery for endometrial cancer in a London tertiary unit in 2016 and 2018 (robotic surgery was introduced in 2017) and analysed using Microsoft Excel.
Results: In 2016, 95 women had surgery. Following examination under anaesthetic, 53 (56%) had total abdominal hysterectomy (TAH) and 42 (44%) total laparoscopic hysterectomy (TLH). In 2018, 103 underwent surgery; 37 (35%) TAH and 66 (65%) MIS, of which 21 (31%) had TLH and 45 (69%) robotic hysterectomy (RAH).
The mean BMI for MIS in 2018 was 36.2kg/m^2 vs 32.2 kg/m^2 in 2016 (p=0.023). BMI range was 16-53kg/m^2 in 2016 and 25-62kg/m^2 in 2018.
For BMI>30kg/m^2, 21/42(50%) had MIS in 2016 vs 42/66(64%) in 2018 (p=0.02). In 2016, the median length of stay (LOS) was 5 days for BMI>30 undergoing MIS vs 1 day in 2018 (p=0.04). BMI had no impact on console time (p=0.064).
Conclusion: The introduction of robotic surgery has significantly increased the MIS rate and reduced LOS for obese women with endometrial cancer; with no increase in operative time as BMI rises.
Disclosure: Nothing to disclose

EP511

Consistency between preoperative, intraoperative, and final surgical pathology in endometrial cancer

F.A. Dumlu[1], M.A. Onan[2], O. Erdem[3]
[1]Obstetrics and Gynecology, Ankara Training and Research Hospital, [2]Obstetrics and Gynecology, [3]Pathology, Gazi University, Ankara, Turkey

Introduction / Background: We aimed to evaluate the consistency of the preoperative, intraoperative and postoperative pathology of endometrial cancer.
Methodology: This was a retrospective study on 134 women (mean age 59.2±11.7 years) operated for endometrial cancer.
Results: Preoperative pathologic diagnosis was endometrioid carcinoma in 85.1% of patients. Frozen section evaluation revealed endometrioid adenocarcinoma in 75.7%, and final pathology in 83.6% of patients. Myometrial invasion was 50% or over in 23.5% in frozen section and 27.1% in final pathology. The corpus-cervix border was involved in 25.9% intraoperatively and 18.9% postoperatively. The rate of consistency between preoperative and final assessment was 76.1% for histology. The rate of consistency between frozen section and final pathology was 68.5% for histology, 61.9% for myometrial invasion and 93% for the involvement of corpus-cervix border.
Conclusion: Frozen section pathology results are not consistent with final pathology; thus unreliable in predicting the surgical stage of endometrial cancer.
Disclosure: Nothing to disclose

EP512

Treatment and prognosis factors of endometrial cancer

Z. Edderdabi, A. Touimi Benjelloun, Y. Benchrifi, M. Benhessou, M. Ennachit, M. Elkarroumi
Centre Mohamed IV de Chirurgie Oncogynecologique, Hassan II University / Faculté de Medecine et de Pharmacie, Casablanca, Morocco

Introduction / Background: The endometrial cancer is in the 4th rank among gynecological cancers.
Methodology: Our retrospective study realized between January 2015 and December 2017, included a series of eighteen patients who underwent endometrial cancer surgery.
Results: It is the privilege of menopausal women mainly with an average age of 59 years old and an average parity of 3.88; among the eighteen cases, sixteen patients were menopausal.
The average consultation period was 11 months with extremes ranging …
Two patients were diagnosed at a young age (< 45 years old) with a history of recto sigmoïdian cancer treated surgically in one of the patients.
The diagnosis discovered early the cancer in eleven patients with a predominant symptom of postmenopausal metrorrhagia noticed in 61.11%. The metrorrhagia was associated with pelvic pain in three patients and leucorrhoea in two others.
The presumptive diagnosis reported by the supra-pubic and endovaginal echography revealed a thickening of the endometrium in the half of the patients.
The biopsy curettage of the endometrium was the main diagnostic element, realized in all the patients, it permitted the definitive diagnosis in 100% of the cases, this curettage was realized under hysteroscopy in eight patients.
The endometrioid adenocarcinomas were the most frequent histological type (78%), the other histological types were the carcinosarcoma (2 cases) and the serous papillary carcinomas (2 cases).
At the end of this review, according to the International Federation of Gynecology and Obstetrics, 72.22% were in stage I, 22.22% in a stage II and 5.55% in a stage III.
Conclusion: All the patients underwent surgery, six patients with a total hysterectomy without adnexal preservation, three with pelvic and lumbo-aortic lymph node dissection and twelve patients with enlarged adeno-colpo-hysterectomy. Of the ten patients reviewed, external radiotherapy was performed in four patients with a combination of the vaginal brachytherapy in two patients.
Disclosure: Nothing to disclose

EP513

Intra-operative pelvic sentinel lymph node examination by imprint cytology in endometrial cancer.

A. Elagwany
Alex Uni, Alex, Egypt

Introduction / Background: Intra operative diagnosis of endometrial cancer metastasis in pelvic lymph node is desirable for accurate staging and to avoid second operation for lymphadenectomy or postoperative pelvic radiotherapy especially in endometrial cancer obese patients where lymphadenectomy is difficult with associated intra and postoperative morbidity. Imprint cytology is an easy, popular, simple, rapid and has a good sensitivity
Methodology: thirty patients admitted with endometrial cancer for surgery where methylene blue dye labeling was used for detection of sentinel lymph node .Immediately after obtaining the nodes and prior to adding them in a fixative agent , each specimen was imprinted on several glass slides, fixed immediately and stained with hematoxylin and eosin then, cytological results were reported as suspicious for malignancy ,negative for malignancy and unsatisfactory .Histological examination of the permanent sections was carried several days later.
Results: the imprint cytology resulted in 12 cases with free nodes , 16 cases with malignant ones and 2 unsatisfactory cases while permanent histopathology resulted in 15 cases with no metastasis and 15 cases with malignant ones . The concordance between imprint cytology and paraffin sections was 90% (27 of 30) . the sensitivity and specificity of imprint cytology in detecting malignancy were 88% and 92% respectively .
Conclusion: Imprint cytology is relative simple ,cheap and easy technique that can allow the pathologist to render an intraoperative diagnosis of metastatic sentinel lymph node for selective or systematic lymphadenectomy
Disclosure: Nothing to disclose

EP514

Clinical and survival implications of surgical staging on endometrial cancer in Hospital de la Santa Creu i Sant Pau de Barcelona

P. Español Lloret, C. Soler Moreno, R. Luna Guibourg, R. Rovira Negre
Gynecology Oncology, Hospital de la Santa Creu i Sant Pau de Barcelona, Barcelona, Spain

Introduction / Background: Lymph node (LN) involvement is one of the most important independent prognostic factors in endometrial cancer (EC).
The present study set out to describe the clinical-pathological variables and surgical morbidity of patients undergoing surgical staging in a tertiary referral centre, as well as to compare overall survival (OS), disease-free survival (DFS) and recurrence in relation to LN involvement.
Methodology: The Oncology-Gynaecology Committee evaluated all patients and therapeutic decision was based on the institutional therapeutic protocols for EC.Myometrial infiltration >= 50%, Grade III (FIGO) and EC type II were inclusion criteria; patients with surgical contraindication were excluded. Surgical staging consisted on systematic bilateral pelvic and para-aortic lymphadenectomy. Survival was determined from last treatment (surgical or adjuvant) until July 2018.
Clinical data were retrospectively collected from computerized medical histories, registered on an informatics platform (*Clinapsis*) and exported to a database for statistic evaluation.
Results: Sixty-three patients (2009-2016 period) were analysed. Clinical-pathological, surgical and histopathology results are summarized in Table 1&2.

VARIABLES	VALUES
Age, mean ± SD, years	64,2 ± 11,6
Laparoscopy, n (%)	63 (82,5)
Laparotomy, n (%)	11 (17,5)
Surgical time, mean ± SD, min	223,7 ± 79,8

Blood loss, mean ± SD, cc	366,6 ± 502,9
Intra-surgical complications, n (%)	7 (11,1)
Clavien Dindo Complication >=grade III (<1 month), n (%)	5 (8)
Pelvic LN removed, mean ± SD	12,84 ± 5,32
Para-aortic LN removed, mean ± SD	11,04 ± 5,43

[Clinical and surgical results.]

VARIABLES	RESULTS
Endometrioid, n (%)	3 (53,2)
Serous, n (%)	15 (24,2)
Clear Cell, n (%)	4 (6,5)
Mixed Cell, n (%)	2 (3,2)
Tumour size, mean ± SD, cm.	4,01 ± 1,43
Pelvic lymph node involvement, n (%)	10 (16,4)
Para-aortic lymph node involvement, n (%)	8 (13,1)
Pelvic and/or para-aortic lymph node involvement, n (%)	13 (21,3)
Skip metastasis, n (%)	3 (4,9)

[Histopathology results.]

LN involvement in pelvic (p = 0.049) and para-aortic nodes (p = 0.040) is related to poorer OS. Pelvic node involvement (p=0.006) is related to poorer DFS.
In case of negatives nodes and taking into account the number of nodes removed, it was observed an improvement in DFS in case of more than 10 nodes removed in the pelvic area (0.006)
Conclusion: Lymphadenectomy allows tailoring adjuvant treatment in 21,3% of positive pelvic and/or para-aortic nodes. In our data, 4.9% of skip metastasis was evidenced. There is a potential benefit effect of pelvic lymphadenectomy in those cases of > 10 nodes removed even in negative cases. A better selection of patients who would benefit from LN dissection is mandatory. Sentinel node assessment or molecular characterization should help to avoid morbidity related to surgical staging in those cases with less risk of nodal involvement.
Disclosure: Nothing to disclose

EP515

Fertility-sparing hysteroscopic resection of the endometrium for patients with stage IA endometroid endometrial cancer: Farghaly´s technique

S. Farghaly
Joan and Sanford Weill College of Medicine, Cornell University, New York, NY, United States

Introduction / Background: Surgical treatment of Endometrial cancer is associated with loss of fertility in young women. For young patients who desire future fertility, conservative management of uterine cancer has been advocated as a safe alternative to hysterectomy and bilateral salpingo-oophorectomy.
Methodology: Patients with Endometroid endometrial cancer who have progesterone receptor more than 50% positivity, and serum CA-125 less than 30IU/ml are enrolled. Patients had no evidence of myometrial/cervical invasion, retroperitoneal lymph nodes involvement, ovarian tumors or distant

metastases on transvaginal sonography, abdomen-pelvis magnetic resonance, and chest X-Ray examinations. Female patients who are younger than 40 years and have early stage endometrioid endometrial cancer were selected for the procedure. Interventions: Under general anesthesia; the uterine cervix is dilated to 9/9.5 mm with Hagar's dilators and an 8.5/9 mm resectoscope with a 0° lens is used. The uterine cavity is distended with 1.5% glycine solution with a pressure of 70 mm Hg. A 5-mm loop electrode and 100 W of pure cutting are used to resect the tumor. If the lesion is diffuse, multiple, large, electrode loop biopsy of both the endometrial mucosa and underlying myometrium are performed on the fundus, anterior, posterior, and lateral uterine walls. After one week, patients are prescribed Mega Acetate 120 mg daily for 6 months.
Results: In all patients, intrauterine adhesions are not detected. Relapse of the disease is very small and full-term pregnancies achieved after the end of progestin therapy.
Conclusion: Farghaly´s technique for hysteroscopic resection of the endometrium combined with progestin therapy for stage IA endometrioid endometrial cancer in young patients who wishes to preserve their fertility is safe and has satisfactory oncologic and reproductive outcomes.
Disclosure: Nothing to disclose

EP516

Single port access (SPA) robot assisted sentinel lymph node (SNL) mapping utilizing indocyanine green (ICG) for patients with stage I endometrial cancer: Farghaly's technique

S. Farghaly
WCUMC- NYPH, Cornell University, New York, NY, United States

Introduction / Background: Bilateral pelvic lymphadenectomy in early stage endometrial cancer my result in significant lymphedema. Efforts have been made to reduce the extent of nodal dissection to minimize lymphedema by the application of sentinel lymph node (SNL) mapping with success.
Methodology: A 25 mg single vial of ICG was used at each surgery. ICG was diluted with 20 ml of normal saline. A 4 ml. of the prepared solution was injected into the uterine cervical stroma at 4 and 8 o'clock positions to lessen the staining of the anterior vesical-uterine space. ICG was placed submucosally and in the deep layer of the uterine cervical stroma. Following that A Vcare uterine manipulator is placed vaginally. A veress needle is placed in the umbilicus for peritoneal insufflation. A 2 cm incision over the lower rim of the umbilicus was performed. The patient was placed in Trendelenburg position, and the DaVinci XI robotic surgical system is side-docked parallel to the right side of the patient. The retroperitoneum space is explored. The green stained channels were looked for in the parametrium crossing over the superior vesical artery and presacral spaces and followed to any green stained SLN. The green stained SLN´s are resected and excised. If non green stanning is identified, a complete bilateral lymphadenectomy is performed. In addition, total hysterectomy and bilateral salpingo-oophrectomy are performed in both cases. Abdominal incisions are closed with O vicryl suture.
Results: SLN was identified in 76% of patients. The operating time was maintained at 150 minutes, and console time 100 minutes. Estimated blood loss was 100 ml. Patients discharged home the following day.
Conclusion: Farghaly's technique of robot assisted sentinel lymph node mapping utilizing Indocyanine green identifies more pelvic nodal metastasis than standard lymphadenectomy. It is feasible and has the advantage of decreasing morbidity, reducing risk of dissemination and short hospital stay.
Disclosure: Nothing to disclose

EP517

Cost-effective improvement in endometrial cancer diagnosis by the incorporation of molecular analysis: prospective pilot study

S. Fernandez-Gonzalez[1,2], M. Amoros[2,3], S.M. Arroyuelo[2,3], D. Barazi[2,4], M. Cararach[2,5], J. Fernandez[2,6], L. Fernandez de Castillo[1,2], L. Jofre[2,4], L. Pallares[2,6], M. Palau[2,5], M.J. Rodriguez-Domingo[2,7], L. Santos[2,7], X. Sanz[8], M.E. Fernandez-Montoli[1,2], G. Tena[2,5], J. Ponce[1,2]
[1]Gynecology, Hospital Universitari de Bellvitge, Hospitalet de Llobregat, Barcelona, [2]Gerencia Territorial Metropolitana Sur del Instituto Catalán de la Salud (ICS), Barcelona, [3]Gynecology, ASSIR Gava, Gava, [4]Gynecology, ASSIR Castelldefels, Castelldefels, [5]Gynecology, Hospital de Viladecans, Viladecans, [6]ASSIR Prat de Llobregat, El Prat de Llobregat, [7]Gynecology, ASSIR Viladecans, Viladecans, [8]Health Department, Institut Catala d'Oncologia, Hospitalet de Llobregat, Barcelona, Spain

Introduction / Background: The histopathology remains the gold standard to diagnose endometrial cancer (EC) from endometrial biopsy. Molecular tests have recently emerged as a useful tool to classify EC according to its prognosis. However, there is currently no published protocol that includes molecular diagnosis of postmenopausal women with abnormal uterine bleeding (AUB). We hypothesized that the incorporation of a molecular test in the management of postmenopausal women with AUB improves the cost-efectiveness of the diagnostic process.
Methodology: We present a prospective study performed in postmenopausal women who presented AUB between 2009-2014. Seven centers recruited the patients. Three of them follow the classical diagnosis algorithm (group 1) and four centers follow the one that incorporates a molecular test (Gynecâ-Dx) performed on the remnants of aspirates (group 2). In group 2, when both the endometrial biopsy and the molecular test were negative, the consequent explorations were considered as “out of procotol”. Clinical data, number of biopsies, ultrasounds, hysteroscopies and visits were compared between groups. In addition, the sensitivity (S), specificity (E), positive and negative predictive values (PPV and NPV) of the molecular test were calculated.
Results: 94 patients were recruited. 51 vs 43 women were included in the classical and the molecular algorithm respectively. There were no differences in age, BMI, parity, use of tamoxifen or hormonal treatment. The detailed outcomes of explorations between classical vs molecular group are shown in Table 1. 324 vs 229 explorations were performed respectively (table 2). In the molecular group, 92 explorations were considered “out of protocol”. The test validations showed a 100% S, 92.5% E, 50% PPV and 100% NPV.
Conclusion: According to our results, the incorporation of a molecular test for diagnosing EC in postmenopausal women who complained with AUB reduces the number of explorations. Consequently, the molecular algorithm might be more cost-effective than conventional algorithms.

	Classical group (n=51)	Molecular group (n=43)	p value
Endometrial biopsy, n (%)			0.317
No malignancy	44 (88)	36 (83,9)	
Endometrial cancer	1 (2)	3 (7,14)	
Inadecuate	5 (10)	3 (7)	
Endometrium by US > 4mm, n (%)	22 (43)	26 (61.9)	0.071
Final Outcome			0,143
Discharged	29 (59)	29 (71)	
Hysteroscopy	19 (39)	9 (22)	
Cancer protocol	1 (2)	3 (7)	

[Table 1. Outcomes]

Exploration	Classical group (n=51)	Molecular group (n=43)
	Inside Protocol	Inside Protocol. Out of Protocol
Molecular test	-	43 .... -

Endometrial Biopsy	51	43..................................... 6
Ultrasound	58	41 22
Hysteroscopy	26	2......................................11
Medical visit	189	100.................................. 53
TOTAL	324	229 92

[Table 2. Number of explorations]

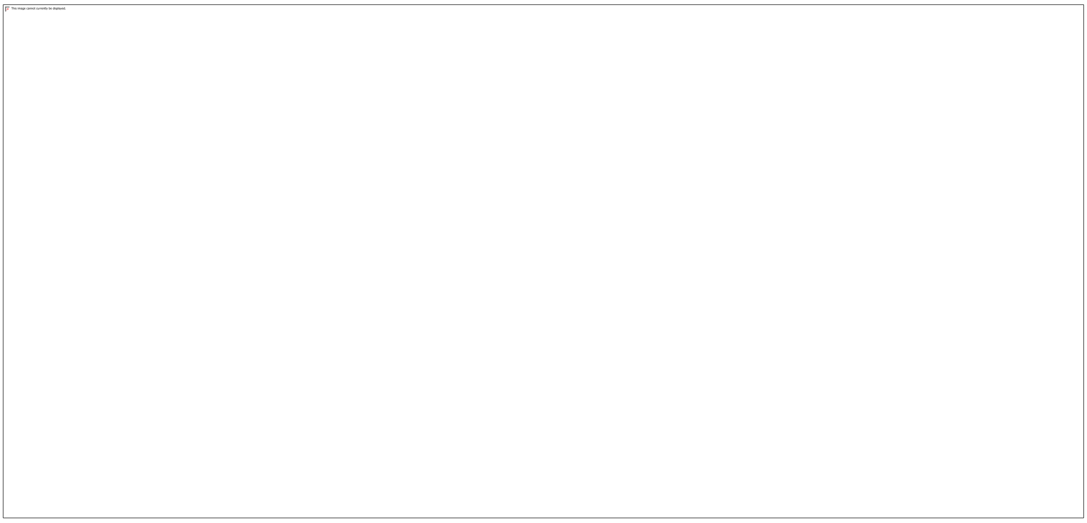

[Figure 2. Outcomes of the molecular test]

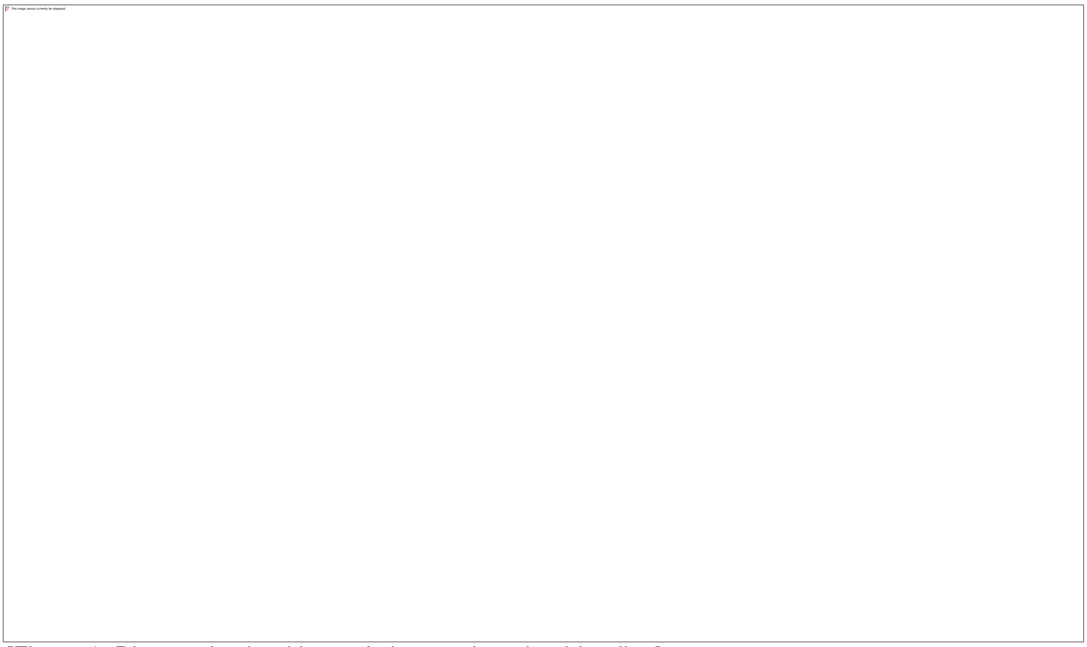

[Figure 1. Diagnostic algorithms of abnormal uterine bleeding]

Disclosure: Nothing to disclose

EP518

'Sandwich' adjuvant chemotherapy and radiotherapy in high risk endometrial cancer: two institution experience

A. Ferrero[1], R. Danese[1], L. Fuso[1], S. Danese[2], S. Gribaudo[3], A. Rossi[4], D. Attianese[1], M. Villa[1], N. Biglia[1]

[1]Gynaecology and Obstetrics, University of Torino / Mauriziano Hospital, [2]Gynaecology and Obstetrics, [3]Radiotherapy, Sant'Anna Hospital, [4]Radiotherapy, Mauriziano Hospital, Torino, Italy

Introduction / Background: High-risk endometrial cancer represents a heterogeneous group of patients with an increased risk of pelvic and distant recurrences and worse outcome. The standard adjuvant treatment has not been established; controversy exists regarding the combination of chemotherapy (CT) and radiotherapy (RT) and their optimal timing. The “sandwich” approach involves adjuvant CT followed by RT and subsequent CT; previous studies investigated different treatment modalities in a limited number of patients. Aim of this study is to assess efficacy and tolerability of a standardized “sandwich” approach in high risk endometrial cancer.
Methodology: A retrospective study was conducted in two Gynaecological Oncology Units (Mauriziano and Sant'Anna Hospitals) in Torino, Italy, from 01/01/2003 until 31/12/2016. High risk patients according to histological type, FIGO stage, grade and lymphovascular invasion, with available clinical data were included. The “sandwich” method consisted of three cycles of platinum based CT, followed by (external and brachy) RT and than three cycles of platinum based CT. Compliance to treatment, CT and RT toxicities, Disease Free Survival (DFS), Cancer Specific Survival (CSS) and Overall Survival (OS) were analyzed.
Results: 98 patients (median age 65 years, 36-77) were selected: 55 hystological type1 and 43 type2; 27 FIGO I-II stages and 71 III-IV. Most of the patients (70.4%) received a Carboplatin-Paclitaxel combination. 558 (94.9%) CT cycles were completed and only 3 required a dose reduction. Grade 2 and 3 hematological toxicity rates were 3.1 and 4.1%. Grade 2 gastrointestinal and grade 2 genitourinary toxicity were reported in 8.2% and 2% of cases. With a median follow-up of 52 months, DFS was 77.6%, CSS 82.7% and OS 69.4%.
Conclusion: In our experience 'sandwich' chemotherapy and radiotherapy combination is an effective adjuvant treatment with a low toxicity in high risk endometrial cancer.
Disclosure: Nothing to disclose

EP519

Cutaneous and muscular metastasis of an endometrial adenocarcinoma: a case report

Y. Fertani, F. Saadallah, O. Jaidane, I. Zemni, M. Hechiche, R. Chargui, K. Rahal

Surgical Oncology, Salah Azaiez, Tunis, Tunisia

Introduction / Background: Endometrial cancer accounts for 6 to 7% of all women's cancer. Most of them are diagnosed with localized early stage disease, explaining a high survival rate.
Endometrial carcinoma rarely metastasis to the skin with a reported prevalence of 0.8% and to the muscle with a prevalence of 2%.
Methodology: We report a case a 72 years old woman treated in our department for a cutaneous and muscular metastasis of an endometrial carcinoma. We did a systematic review of the literature.
Results: We report a case of a 72 years old woman, gravida 1, para 1, postmenopausal for 22 years. she had a hysterectomy for an endometrioid adenocarcinoma grade 1 in February 2014.
She was referred to our department, in December 2015, for an abdominal mass.
Biopsy showed a metastatic localisation of the endometrial adenocarcinoma early treated. She

underwent 3 courses of chemotherapy with carboplatin and taxoter. Then, she had a complete surgical resection.
She presented, 11months later, with a gradually enlarging new abdominal mass which measures 60x50 mm. it was of hard consistency adhering to the skin next to laparotomy scar.
The new CT scan showed a voluminous 158 x 120 mm cutaneous mass invading rectus abdominal muscle with no other metastatic localisation.
She underwent a complete surgical resection. There was no recurrence after surgery.
Conclusion: Although endometrial carcinoma is one of the most diagnosed cancer in women, cutaneous and muscular metastasis is very rare.
This manifestation explains the absence of treatment consensus. This situation can be a challenging for the physician mostly with the poor associated prognosis.
Disclosure: Nothing to disclose

EP520

Comparison of different preoperative biopsy techniques in endometrial cancer

S. Forte[1], G. Arrigoni[1], F. Ferrari[2], M.C. Coppola[1], E. Sartori[1], F. Odicino[1]
[1]Department of Obstetrics and Gynecology, University of Brescia, [2]Department of Obstetrics and Gynecology, Spedali Civili Brescia, Brescia, Italy

Introduction / Background: To assess the agreement for histology and grading, between endometrial biopsy and final diagnosis in patients with endometrial cancer, performed with different biopsy techniques.
Methodology: We performed a retrospective analysis of patients with endometrial cancer and we collected demographic and clinical data, type of endometrial biopsy technique (Dilation & Curettage - D&C, diagnostic hysteroscopy and subsequent D&C, office hysteroscopy and operative hysteroscopy), biopsy and final diagnosis pathological characteristics. We evaluated global agreement for histology and grading using Cohen's Kappa and we performed subgroup analysis based on the biopsy technique and final grading. Agreement was classified in a five stepfashion way ranging from good, moderate, sufficient, fair and poor.
Results: We found 148 patients. Final histology showed 131 (%) endometrioid cancer; 2 patients (1.4%) were finally diagnosed with atypical hyperplasia, 63 (42.6%) with G1, 46 (31%) with G2 and 37 (25%) with G3. Global agreement for histology was fair (K = 0.356; SE 0.081; CI95% 0.08-0.41; p < 0.00), while for grading was moderate (K = 0.458; SE 0.051; CI95% 0.36-0.56; p < 0.00). In well differenciated tumor agreement was fair (k = 0.389; SE 0.074; CI95% -0.24—0.53; p < 0.00), while in G2 and G3 tumors agreement was respectively moderate (k = 0.521; SE 0.074; CI95% -0.38—0.67; p < 0.00) and good (k = 0.710; SE 0.068; CI95% -0.57-0.84; p < 0.00). Subgroup analysis revealed fair agreement for D&C (k = 0.333; SE 0.156; CI95% 0.03-0.63; p < 0.00), as well as for diagnostic hysteroscopy and subsequent D&C (k = 0.334; SE 0.098; CI95% 0.14-0.53; p < 0.00) and for office hysteroscopy (k = 0.313; SE 0.156; CI95% 0.00-0.62; p < 0.03). Operative hysteroscopy showed moderate agreement (k = 0.535; SE 0.084; CI95% 0.37-0.70; p < 0.000).
Conclusion: Operative hysteroscopy is the best technique for endometrial biopsy in case of suspected endometrial cancer.
Disclosure: Nothing to disclose

EP521

Treatment of peritoneal metastases from endometrial cancer with cytoreductive surgery and hyperthermic intraperitoneal chemotherapy, a systematic review of the literature

A. Fotiou[1], N. Blontzos[2], E. Giovannopoulou[3], A. Prodromidou[2], G. Vorgias[1], J. Spiliotis[4], C. Iavazzo[1]

[1]*Gynaecologic Department, Metaxa Cancer Hospital, Piraeus,* [2]*Department of Obstetrics and Gynaecology, Alexandra Hospital, National and Kapodistrian University of Athens, Athens,* [3]*Democritus University of Thrace, Medical School, Alexandroupoli,* [4]*Surgical Oncology and HIPEC Department, Athens Medical Centre, Athens, Greece*

Introduction / Background: Endometrial cancer is the most common cancer of the female reproductive tract in developed countries. Peritoneal metastases from endometrial cancer as advanced stage disease or as a recurrence is not rare. The aim of the present study was to evaluate the efficacy of cytoreductive surgery and hyperthermic intraperitoneal chemotherapy in patients with peritoneal disease from endometrial cancer.
Methodology: A systematic search of the literature for articles published up to February 2019 was performed. All studies that reported outcomes after cytoreduction and hyperthermic intraperitoneal therapy for peritoneal metastases in patients treated for endometrial cancer were independently evaluated by two authors and the discrepancies were resolved by team consensus.
Results: A total of 6 studies were eligible for our review and were included in the present study. 63 patients were recruited by these studies. 11 patients were treated primary with debulking surgery and hyperthermic intraperitoneal chemotherapy while 52 patients underwent this procedure for recurrent disease after their primary treatment. Histologic type, FIGO staging, peritoneal carcinomatosis index, cytoreduction completeness, adjuvant treatment and mean follow up period were documented. Of 63 patients 21(33.3%) were reported to be disease free at the time of study publication.
Conclusion: Cytoreduction and hyperthermic intraperitoneal chemotherapy in selected patients with peritoneal carcinomatosis from endometrial cancer achieves promising outcomes concerning the disease free and overall survival.
Disclosure: Nothing to disclose

EP522

Changes in transcriptional activity of apoptosis-regulating genes associated with age and endometrial cancer grade

E. Frantsiyants, T. Moiseenko, I. Kaplieva, V. Bandovkina, A. Menshenina, M. Adamyan, E. Verenikina, T. Myagkova
Rostov Research Institute of Oncology, Rostov-on-Don, Russian Federation

Introduction / Background: Paradoxically, endometrial tumors demonstrate a significant increase in apoptosis correlating with the disease progression and a poor prognosis. The purpose of the study was to analyze intact and pathological uterine tissues for the relative expression of genetic loci involved in the regulation of signaling pathways of the apoptosis development.
Methodology: Biopsy tissues (tumor and intact) of the uterus from 27 patients aged 38-72 years with endometrial cancer (EC) were studied. Relative expression of 9 genetic loci involved in the regulation of signaling pathways of the apoptosis development (BAX, CASP7, CASP3, CASP8, CASP9, CASP8/FADD, p53, MDM2 and BCL2) was determined by RT-qPCR.
Results: Patients with G1 EC (45-68 years) showed elevated expression of both pro-apoptotic genes CASP3, CASP9, and p53 by 222%, 131% and 98% respectively ($p < 0.05$), and anti-apoptotic genes MDM2 (by 80%) and BCL2 (by 100%) in tumor tissues. Expression of the anti-apoptotic BAX gene was increased by 79% ($p < 0.005$) in patients with G2 EC (38-69 years). In patients with G3 EC (55-63 years), significant ($p < 0.005$) increase in expression of pro-apoptotic genes CASP7, CASP3, CASP8 and p53 by 71%, 90%, 53% and 150% respectively was noted together with decreased expression of the anti-apoptotic c-FLIP gene by 10% in tumor tissues ($p < 0.05$).
Conclusion: The results showed that some characteristics of expression of pro- and anti-apoptotic genetic loci in uterine malignancies were associated with age of patients and tumor grades. G1 EC demonstrated simultaneously elevated expression of pro-apoptotic (CASP3, CASP9, p53) and anti-apoptotic genes (MDM2, BCL2), while in G2 and G3 EC, pro-apoptotic genes (BAX, CASP7, CASP3, CASP8) increased with the inhibition of transcriptional activity of the anti-apoptotic c-FLIP gene neutralizing an external pathway for the apoptosis initiation through the activation of death receptors.
Disclosure: Nothing to disclose

EP523

Preliminary assessing the indication of MPA therapy for early endometrial cancer in the pregnancy point of view

T. Furui, M. Takenaka, K. Terazawa, S. Aikyo, K. Kikuno, S. Yamamoto, T. Shiga, R. Shimaoka, K.-I. Morishige
Obstetrics and Gynecology, Gifu University Hospital, Gifu, Japan

Introduction / Background: High-dose MPA therapy is used as a fertility-sparing treatment for atypical endometrial hyperplasia(AEH) and early stage endometrial cancer(EC). However, this treatment has problems such as uncertain therapeutic effect and high recurrence rate. we studied the indication of "start and continuation of MPA therapy" in the reproductive point of view.
Methodology: Patients: Twelve patients who attempted pregnancy with after treatment with MPA in our hospital from June 2006 to February 2019. Measurement: age, BMI, duration of MPA therapy, the presence of Polycystic ovaries findings in ultrasonography(USPCO) and ovulatory disorder were compared in the pregnancy group and the non-pregnancy group.
Results: In pregnancy group and non-pregnancy, average age was 30.2 ± 2.17, 35.1 ± 3.8 (p = 0.028), BMI was 21.6 ± 3.21, 28.3 ± 6.78 (p = 0.062), MPA treatment period(months) was 6.80 ± 6.06, 28.0 ± 16.7 (p = 0.051), respectively.
Conclusion: In considering the choice and continuation of MPA therapy, it should be more important to sufficiently explain the benefits and risks of this treatment, especially for the elderly, those with high BMI, and those with long-term treatment continuation in the point of pregnancy outcome.
Disclosure: Nothing to disclose

EP524

The significance of positive peritoneal cytology in G1/G2 endometrioid carcinoma of uterine body

S. Furukawa, H. Iwase, S. Endo, K. Ohnishi, Y. Tazo, T. Takada, M. Arai, O. Yoshino, T. Onda
Kitasato University School of Medicine, Sagamihara, Japan

Introduction / Background: Although peritoneal cytology in endometrial carcinoma was excluded from the staging system (FIGO 2008), the management for patients with positive peritoneal cytology remains controversial. The aim of this study is to evaluate the clinical significance of positive peritoneal cytology in G1/G2 endometrioid carcinoma.
Methodology: We retrospectively reviewed medical records of 365 patients with G1/G2 endometrioid carcinoma who were primarily treated at our hospital from 2005 to 2013. We analyzed clinicopathological features and prognosis of patients with positive (P group, N=45) and negative (N group, N=320) peritoneal cytology. Fisher's exact test, Kaplan-Meier analysis, and Log-rank test were used for statistical analyses.
Results: Median age of all patients was 56 years-old (range 27-83), and stage distribution was 290/25/47/3 in stage I/II/III/IV (FIGO 2008). Median follow-up period was 61 months (range 1-130). The frequency of metastasis to adnexa, lymph nodes (LN), and distant organs was significantly high in P group compared with N group (p=0.0002, 0.0034, 0.0018, respectively). However, omental metastasis was not related with the results of cytology. The number of patients in low, intermediate, and high risk for recurrence defined in Japanese guidelines for treatment of uterine body neoplasm was 20/6/19 in P group and 202/62/56 in N group. Forty of 45 patients (89%) in P group received adjuvant treatment, while 110/320 (34%) in N group. Overall survival (OS) in P group was significantly poor compared with N group in patients with high risk for recurrence (75.0% vs 88.4% in 5-year OS,

p=0.0067), although there was no difference in patients with both low and intermediate risks.
Conclusion: As the frequency of lymph node metastasis is significantly high, appropriate evaluation of lymph node metastasis is necessary in P group. Further, more effective adjuvant treatment is desirable for patients with high risk in P group to improve the prognosis.
Disclosure: Nothing to disclose

EP525

The role of LVSI to define the best adjuvant treatment in endometrial cancer (EC)

F. Mirri[1], L. Fuso[2], A. Ferrero[2], M. Ribotta[3], E. Volpi[4], C. Macchi[1], G. Parpinel[1], E. Badellino[1], M.E. Laudani[1], E. Potenza[1], P. Zola[1]
[1]Surgical Science, University of Turin, [2]Gynecology and Obstetrics, A.O. Ordine Mauriziano, [3]Pathological Anatomy, A.O.U Città della Salute e della Scienza, Turin, [4]Gynecology and Obstetrics, Ospedale San Paolo, Savona, Italy

Introduction / Background: Endometrial cancer is the fourth neoplasia in the female population with 88 000 new cases annually in Europe. Recently great efforts have been made to modulate treatments based on the risk of relapse. One of the risk factors included in ESGO guidelines is lymphovascular space invasion (LVSI). In patients LR (IA G1), LVSI determines whether to add EBRT when the lymph node status is unknown. In HR group(IAG3-IBG1-2) LVSI is useful to decide if adding EBRT to the BRT or performing just the follow up.
Methodology: To verify the role of the LVSI in the management of EC, data from 697 patients treated consecutively were collected from hospital databases (01/2011 to 10/2018) in 3 Centres of the Cancer Network of Piemonte and Valle d'Aosta.
Results: LVSI was found in 285 out of 697 tumours collected (40.9%). Stratifying positive LVSI by centre, we found that 12.3% in centre A, 31.7% in centre B, and 5.5% in centre C were positive in LR EC (286 patients G1-2, IA). 43.3% in centre A, 62.5% in centre B, 10.8% in centre C were positive in HR EC (196 patients IA, G3; IB, G1-G2). In order to analyse these differences within centres, we evaluated the determinants of LVSI with mono and multivariate logistic regression analyses. The factors identified related to LVSI are: risk class LR (OR=2.9) vs HR (OR=7.5 p=0.0001); Type I vs II EC (OR=3.3 p=0.001); surgical approach: LPS without manipulation vs LPS with manipulation (OR=2.1) vs LPT (OR=3.1 p=0.001); number of lymph nodes removed during surgery (< 20 vs ≥20 lymph nodes; OR 1.8 p=0.01).
Conclusion: LVSI, a key factor for the decision of adjuvant treatment, depends on different factors that should be considered in quality control studies.
Disclosure: Prof. Paolo Zola has received grants from Astrazeneca, Roche, Pharmamar and Tesaro.

EP526

Inactivating mutations of the *IK* gene inhibit endometrial cancer progression through regulating mitosis and non-homologous end joining DNA repair pathway

C. Gao[1], G. Jin[2], Y. Wang[1], M.E. Forbes[2], Y. Yan[1], W. Tian[1], L.S. Mangala[3], A.K. Sood[3], F. Xue[1], W. Zhang[2]
[1]Gynecology and Obstetrics, Tianjin Medical University General Hospital, Tianjin, China, [2]Cancer Biology, Wake Forest Baptist Comprehensive Cancer Center, Winston-Salem, NC, [3]Gynecologic Oncology and Reproductive Medicine, The University of Texas MD Anderson Cancer Center, Houston, TX, United States

Introduction / Background: IK is a mitotic factor that can promote cell cycle progression. Our recent investigation of the 271 endometrial cancer (EC) samples from The Cancer Genome Atlas (TCGA)

dataset showed that *IK* gene mutations were strikingly enriched in cluster IV which consisted of most of the high grade and high stage patients and this cluster had better overall survival (OS). Therefore, we further characterized the roles of *IK* gene mutations and their coded protein in EC progression.
Methodology: We used bioinformatics methods to analyze the somatic mutational types of *IK* gene and the relationship between *IK* mutation and OS rate of EC patients in the updated TCGA dataset (n = 547). Somatic mutations for 30 DNA repair genes were called to analyze the mutation burdens of EC patients. In vitro and in vivo experiments were used to study whether and how IK influences the EC progression. All statistical tests were two-sided.
Results: The most common somatic mutational type of *IK* was frameshift and this kind of inactivating mutations was unique for alive EC patients. TCGA data analysis showed that *IK/Ku80* mutations caused higher mutation burdens of EC patients and they were associated with significantly better OS. IK attenuation led to enrichment of G2/M phase cells and inactivation of DNA repair signaling by inhibiting the heterodimerization of Ku80 and Ku70. In addition, IK attenuation sensitized EC to cisplatin treatment in vitro and in vivo. IK expressed higher in EC tissue and high IK expression was significantly associated with several clinicopathologic variables, such as age, hypertension, body mass index (BMI) and lymph node metastasis. High IK expression was negatively correlated with OS of EC patients.
Conclusion: Inactivating mutations of the *IK* gene and loss of IK expression are likely to contribute to inhibit EC progression. IK can be considered as an effective therapeutic target against EC.
Disclosure: Nothing to disclose

EP527

Low volume lymph node metastasis discovered by sentinel lymph node evaluation in endometrial cancer: adjuvant and prognostic implication

V. García Pineda, J. Siegrist Ridruejo, I. Zapardiel Gutiérrez, M.D. Diestro Tejeda, A. Hernández Gutiérrez
Gynecologic Oncology, La Paz University Hospital, Madrid, Spain

Introduction / Background: The aim of this study is to evaluate the rate of low volume lymph node metastasis (LVM) diagnosed by sentinel lymph node (SLN) biopsy in order to establish treatment patterns and oncologic outcomes in patients with endometrial cancer.
Methodology: We identified 126 patients who were treated surgically because of diagnosis of endometrial cancer between 2012-2017 in which sentinel lymph node biopsy was performed. We defined micrometastasis as a tumor present in a lymph node measuring >0,2 mm but < 2,0 mm and isolated tumoral cels are those measuring ≤0,2mm. Pathological study is performed by conventional hematosyline and eosin staining and ultrastaging by immunohistochemical analyses and OSNA technique is carried out.
Results: 126 patients with a median age of 63 02±11,051 years. Histology is as follows: endometrioid 80,2%, serous 10,3%, carcinosarcoma 6,3%, clear cell 1,6%.
Concerning stage, 45,3% are low risk, 15,9% intermediate risk, 11,9% high-intermediate risk and 27% high risk. The rate of SLN detection is 100% by dual labeling with Tc99 and ICG.
105 cases (83,3%) of SLN are negative, 6 cases (4,8%) present macrometastasis, 7 cases (5,6%) micrometastasis and finally 3 cases (2,4 %) ITC. 6 patients with micrometastasis receive Radiotherapy and the mayority of ITC patients receive adjuvant treatment. The overall survival (OS) is 93,5%and pathology free survival (PFS) is 87,5% in micro metastasis group, patients with ITC present a PFS of 66,7% with an OS of 100% finally patients with macrometastasis have a PFS of 90% with an OS of 80%.
Conclusion: Patients with LVM frequently receive adjuvant treatment with improve of their oncological outcomes in comparison to those with macrometastasis. More prospective studies are necessary to define optimal adjuvant treatment in patients with LMV.
Disclosure: Nothing to disclose

EP528

Prevalence of pelvic and paraaortic metastasis and low volume lymph node metastasis in endometrial cancer: staging and adjuvant matter

V. García Pineda, I. Zapardiel Gutiérrez, J. Siegrist Ridruejo, M.D. Diestro Tejeda, A. Hernández Gutiérrez
Gynecologic Oncology, La Paz University Hospital, Madrid, Spain

Introduction / Background: We performed a retrospective study in order to define the prevalence and prognostic of lymph node metastasis(LNM) in endometrial cancer, especially low volume lymph node metastasis (LVM).
Methodology: We identified 119 cases of endometrial cancer from January 2012 to December 2016. Pelvic and paraaortic lymph node dissection was carried out in 76 cases (63,8%), only in patients with myometrial invasion ≥50% and G1, G2 or G3 histological grade and myometrial invasion < 50% but high histological grade. We evaluate the rate of LNM and LVM in both areas and establish risk tumor factors as well as the prognosis of LVM taking into account adjuvant treatment.
Results: Of the 119 patients 78 were at risk of which 68 patients (71,1%) underwent systematic lymph node pelvic and paraaortic dissection. A mean of 15±6,4 pelvic and 18,2±7,7 paraaortic nodes were harvested. The prevalence of pelvic and paraaortic LNM was 6,95%(n=5) and 8,9% (n=6) respectively and sentinel lymph node (SLN) was positive in 14 cases (19,7% of cases). Isolated paraaortic LNM with negative pelvic nodes were present in 3 cases (4,8%)
The rate of LVM was 37,5%, 7 cases of total LNM. LVM were present only in SLN.
16,5 % of cases (16/115)presented recurrence, isolated tumoral cels and micrometastasis were present in 13,3 (2 cases) and 1 case, 6,6%. of patients with recurrence respectively.
Most of the patients received adjuvant treatment with radiotherapy and chemotherapy including those who presented LVM.
Conclusion: We evaluated data for the prevalence of LNM in at-risk endometrial cancer patients to guide lymphadenectomy decisions for clinical purposes and the improvement in the diagnosis of LVM thanks to SLN technique.
Disclosure: Nothing to disclose

EP529

Transvaginal ultrasound versus magnetic resonance imaging for preoperative assessment of myometrial invasion in patients with endometrial cancer: prospective study

B. Gastón[1], J.L. Alcázar[2], J.C. Muruzábal[1], A.I. Modroño[1], S. Ostiz[3], R. Guarch[4]
[1]Gynecologyc and Obstetrics Department, Complejo Hospitalario de Navarra, [2]Gynecologyc and Obstetrics Department, Clínica Universidad de Navarra, [3]Radiology Department, [4]Pathology Department, Complejo Hospitalario de Navarra, Pamplona, Spain

Introduction / Background: Endometrial cancer is the most common gynecologic malignancy in developed countries. In 1988, the International Federation of Gynecology and Obstetrics (FIGO) established that endometrial cancer should be surgically staged. However, the therapeutic role of systematic lymph node dissection is still a matter of debate in low risk endometrial cancers. Risk classification is mainly based on tumor histology, tumor grade and myometrial infiltration (MI) depth. Currently, transvaginal ultrasound (TVS) and magnetic resonance imaging (MRI) are the commonest techniques used for assessing preoperatively the depth of MI.
Methodology: Prospective study in the Obstetrics and Gynecology Department of a tertiary hospital. The objective is to compare the diagnostic accuracy of TVS and MRI in for detecting myometrial infiltration in endometrial carcinoma.
Results: Between January 2016 and January 2019, 121 patients with diagnosis of endometrial adenocarcinoma type 1 (63,6%) and 2 (36,4%) have been recruited.85.8% of the patients presented a myometrial infiltration of less than 50% while 14.2% of them presented a myometrial infiltración of more tan 50%. Pooled sensitivity, specificity, LR+ and LR- of TVS for detecting deep myometrial

infiltration were 76.5%, 73.8%, 2.92 and 0.32, respectively.
Pooled sensitivity, specificity, LR+ and LR- of MRI for detecting deep myometrial infiltration were 76.5%, 87.4%, 6.07 and 0.27, respectively.
Pooled sensitivity, specificity, LR+ and LR- of intraoperative anatomopathological study for detecting deep myometrial infiltration were 69.2%, 97.4%, 26.61 and 0.32, respectively.
Conclusion: Data available so far show that TVS has similar diagnostic performance than MRI for detecting deep myometrial invasion in women with endometrial cancer: both of them have the same sensitivity although MRI has a better specificity (no statistically significant differences: McNemar test p>0.05). Therefore, although patients recruitment has not yet been completed, it seems that TVS could be considered as good enough for being used per preoperative diagnosis in these a prioir low-risk cases.
Disclosure: Nothing to disclose

EP530

Indocyanine green in surgical staging of early stage endometrial cancer

I. Gladchuk[1,2], N. Rozhkovska[1,2], V. Marichereda[1], V. Kozhakov[1,2], Y. Petrovskiy[2]
[1]Obstetrics and Gynecology #1, [2]University Clinic, Odessa National Medical University, Odessa, Ukraine

Introduction / Background: Endometrial cancer is one of the most often type of gynecological malignancies all over the world. Lymph nodal status as an important factor of prognosis of the disease and indication to further treatment is still debated. Many investigations shows that sentinel lymphatic node (SLN) dissection using indocyanine green (ICG) is sensitive, safe and effective. In cases of endometrial (submucosal or hysteroscopic applications) or cervical (intracervical administration) cancer, ICG can detect SLN at an accuracy of 95% to 98%. Low toxicity, confinement within the vascular compartment, rapid excretion, and the rarity of allergic reaction are the main advantages of ICG.
Methodology: Study included 32 patients with early endometrial cancer (FIGO stage IA-IB, grade 1-2 endometrioid adenocarcinoma). We made submucosal injection of 1 ml of ICG solution in 4 points at 1,5,7,11 o`clock around cervix. After 15-20 min using near-infrared imaging (D-light P-system, Karl Storz) we visualized pelvic lymph nodes through the peritoneum and performed SLN identification (SLNI) on each side. In cases where we were unable to identify SLN we preferred extended operation.
Results: We had successfully identified SLN in 28 (87.5%) cases. In all cases we had no positive nodes, thus complete pelvic lymph node dissection was unnessesary. Operation time was 81.5 ± 12.2 minutes for TLH with SLN identification with ICG and 175.4 ± 26.5 minutes for SLNI+TLH+PLNE. Patients with only TLH+SLNI had much shorter lymphorrhea duration 2-4 days in post operation period in comparison with patients, who had SLNI+TLH+PLNE (6-7 days). Although time of SLNI+TLH+PLNE is longer then TLH+PLNE without SLNI, overall operation time for selected patient group decreased.
Conclusion: Using ICG in surgical staging of early endometrial cancer may lead to more effective surgical procedures, better detection and removing of cancer cells, reduction of surgical time and postoperative wound-related complications, improved SLN detection rates, avoid radioactivity.
Disclosure: Nothing to disclose

EP531

The presentation, treatment and clinical outcome of patients with dedifferentiated endometrial adenocarcinoma in singapore

C. Goh[1], F.B. Livingston[2], W.Y. Ho[1], S.L. Wong[2], R. Nadarajah[2], C.H.R. Goh[2], S.H. Chew[1], Y.K.T.

Lim[1], T.H. Ho[1,2]
[1]*KK Women's and Children's Hospital,* [2]*Singapore General Hospital, Singapore, Singapore*

Introduction / Background: Dedifferentiated endometroid adenocarcinoma (DEAC) was first described in 2007. However, it has only been recognised as a distinct subtype of endometroid adenocarcinoma in the last 1-2 years. A*s* the diagnosis of DEAC is made based on the presence of any proportion of undifferentiated carcinoma component in coexistence with an endometrioid carcinoma component (usually low grade; i.e grade 1 or 2 endometrioid carcinoma), DEAC can sometimes be misdiagnosed as FIGO grade 2 or 3 endometrioid carcinoma.Distinguishing DEAC from poorly differentiated endometroid adenocarcinoma is important as the former carries a worse prognosis.
Methodology: The study is a retrospective review of all cases of dedifferentiated endometrial cancer diagnosed in two institutions in Singapore between January 2012 and October 2017.
Results: 7 patients were diagnosed with DEAC between April 2013 and October 2017. The mean age was 56.4 years. All patients presented with either abnormal uterine bleeding or post menopausal bleeding. Out of the 7 patients, one was diagnosed with Stage 2 disease, 5 were diagnosed with Stage 3 disease and 1 was diagnosed with Stage 4 disease. The 2-year overall survival (OS) was 31.3%, compared to 82.8% in patients with Grade 3 endometriod adenocarcinoma treated in the same centre. One patient had neoadjuvant chemotherapy, followed by surgery, and completion chemotherapy post surgery. The other 6 patients (87.5%) underwent primary debulking surgery. Out of these 6 patients, 5 patients had adjuvant chemotherapy post surgery and one patient had both adjuvant chemotherapy and radiotherapy. Lymphovascular invasion was found in 71.4% of the cases.
Conclusion: DEAC is a more aggressive histological subtype. Patients with DEAC tend to present with advanced disease compared the other endometroid adenocarcinomas. Better awareness of this condition can lead to proper diagnosis. More research is needed on this rare histology subtype of endometrial cancer in order to tailor better treatment.
Disclosure: Nothing to disclose

EP532

A rare case of uterine body metastasis from cecal adenocarcinoma.

C. Goudeli[1], N. Koufopoulos[2], G. Plataniotis[3], E. Terzakis[1]
[1]*Institouto Gynaikologias,* [2]*2nd Department of Pathology, National and Kapodistrian University of Athens, Athens,* [3]*Anticancer Hospital of Athens 'Saint-Savvas', Ath, Greece*

Introduction / Background: Bowel and uterine carcinomas are often related genetically, through hereditary syndromes or as metastasizing sides. Metastasis to the female genital tract from extragenital malignancies are rare, and the prognosis is extremely poor.
Methodology: We herein report a case of a 62 years old woman with metastatic carcinoma to the uterine body from a colon adenocarcinoma, presenting with vaginal bleeding. 16 months before she had an urgent laparotomy due to obstructive ileus. She underwent right colectomy, right oophorectomy and lymphadenectomy and the histological results reported a colon-cecum adenocarcinoma (pT4aN0 grade2) up to the pericolic fat. The appendix, the right adnexa and the lymph nodes were negative. The imaging showed no metastasis to another organ. She received 9 cycles of chemotherapy with oxaliplatin and capecitabine (Xelox) and was followed up.
The diagnostic curettage that followed revealed a moderate adenocarcinoma of the uterus compatible with the colon adenocarcinoma (CK7 (+), CK20 (+), p16 (+), CDX2(+), ER (-), PR (-)).
Results: Colonoscopy-biopsies were negative for malignancy and PET-CT reported a large uterus with focal disease and suspicious pelvic lymph nodes. The rest body scan was negative. She underwent wide total hysterectomy and left oophorectomy, excision of the mesorectum and parts of peritoneum, omentectomy and pelvic lymphadenectomy. The pathology examination reported the uterine adenocarcinoma infiltrating the serosa and myometrium. The mesorectum and 3/11 left lymph nodes were positive. The endometrium was atrophic and the left adnexa, the omentum and peritoneal parts excluded were negative for disease. The patient received 8 cycles of XELIRI-protocol (Capecitabine plus 3-weekly irinotecan) and is now -14 months after- free of relapse.
Conclusion: According to our findings the tumor was developed of *metastases* in tissue *continuity.* To

choose the best treatment, it is necessary to always diagnose whether a uterine bleeding is due to a primary uterine or a metastatic carcinoma.
Disclosure: Nothing to disclose

EP533

The impact of morbid obesity on overall survival of the endometrial cancer

A.B. Guzel[1], G. Khatib[1], Ü. Küçükgöz Güleç[1], S. Seyfettinoğlu[1], D. Gümürdülü[2], M.A. Vardar[1]
[1]*Gynecologic Oncology,* [2]*Pathology, Çukurova University, Adana, Turkey*

Introduction / Background: World Health Organization had identified morbid obesity as patients with body mass index (BMI) more than 40 kg/m2. Obesity is known as a risk factor for endometrial cancer due to the increase of the deposited estrogen. This study was conducted to evaluate the morbid obesity effect on overall survival of the endometrial cancer.
Methodology: The last 10 years' records and pathological reports of the endometrial cancer cases who were operated and followed up in Çukurova University Gynecologic Oncology Center were reviewed, retrospectively. Data regarding BMI and overall survival was reported in 520 patients of them. These patients were included in this study and stratified into two groups according to their BMI, < 40 and ≥40. Groups' clinic, pathologic features and overall survival rates were compared.
Results: There was 146 cases in the morbid obese group and 374 patients in the non-morbid group (BMI< 40). Mean age of the groups was 58.5 and 56.2, respectively. Mean follow-up time was 51.6 months. There was no difference between groups concerning endometrioid or non-endometrioid histology, grade, stage, lymph node involvement, myometrial invasion, lympho-vascular invasion, postoperative complications, postoperative hospitalization time, adjuvant treatments and recurrence status. Comorbidities were significantly higher in the morbid obese group. Albeit that, groups' clinical and pathological features were homogeneously distributed overall survival rates were significantly different (p=0.054). Five-year survival rate was 85.3% in the morbid obese group and 90.1% in the non-morbid group.
Conclusion: Overall survival of the morbidly obese endometrial cancer patients is significantly lower from those with < 40 kg/m2 BMI. The most acceptable explanation of this result is the most frequent comorbidities in this special group, which could be simply altered by life style changes. Morbidly obese endometrial cancer patients should be encouraged for life style changes and consulted to the dietary and endocrinology specialists.
Disclosure: Nothing to disclose

EP534

Left Parametrial invasion including distal ureter in endometrioid adenocancer

K. Güzin, A. Özer, A. Tok, T. Şahinkanat, M. Kirişçi
Sütçü Imam University, Kahramanmaraş, Turkey

Introduction / Background: It was aimed to present a case of endometrioid adenocancer (EC) which invaded the left parametrium including the distal ureter.
Methodology: A 54-years-old multiparous woman admitted the gynecologic oncology clinic of a tertiary referral hospital with the complaint of abnormal uterine bleeding. Pathologic examination of the endometrial curattege material was reported as grade 3 EC. Abdominopelvic computed tomography revealed left renal pyeliectasia, paraaortic lymphadenopathy, a solid mass of 55-mm in diameter in the left parametrium and a polypoid mass of 43-mm in diameter filling the uterine cavity. During the staging laparatomy, total abdominal hysterectomy, bilateral salphingooopherectomy, omentectomy and pelvic-paraaortic lymphadenectomy was performed.

Results: During hysterectomy procedure, it was seen that the bottom of left parametrium was filled by a solid mass ,extending from the isthmic-cervical portion of the uterus towards the parametrium, which was fixed to the distal ureter resulting in the dilation of the proximal ureter (Figure 1). Ureteroneocystostomy was performed via the bladder mobilisation and tunneling of the ureter in the bladder wall using the Psoas Hitch tecnique. A double J stent was placed and left for 3 weeks. During the pelvic-paraaortic lymphadenectomy, there was a bulky lymph node fixed to the aorta and inferior vena cava at a level just below the left renal artery which was dissected without any complication (Figure 2). A drain was left in the pelvis for 3 days. Postoperative follow up of the patient was uneventful and she was discharged on the 5. Day. A foley catheter was left in the bladder for 14 days. There was no urinary complication developed in the postoperative period. Based on the final pathologic report, the tumor was stage 3

Conclusion: In this case report, parametrial and distal ureteral invasion due to endometriod adenocancer was presented.

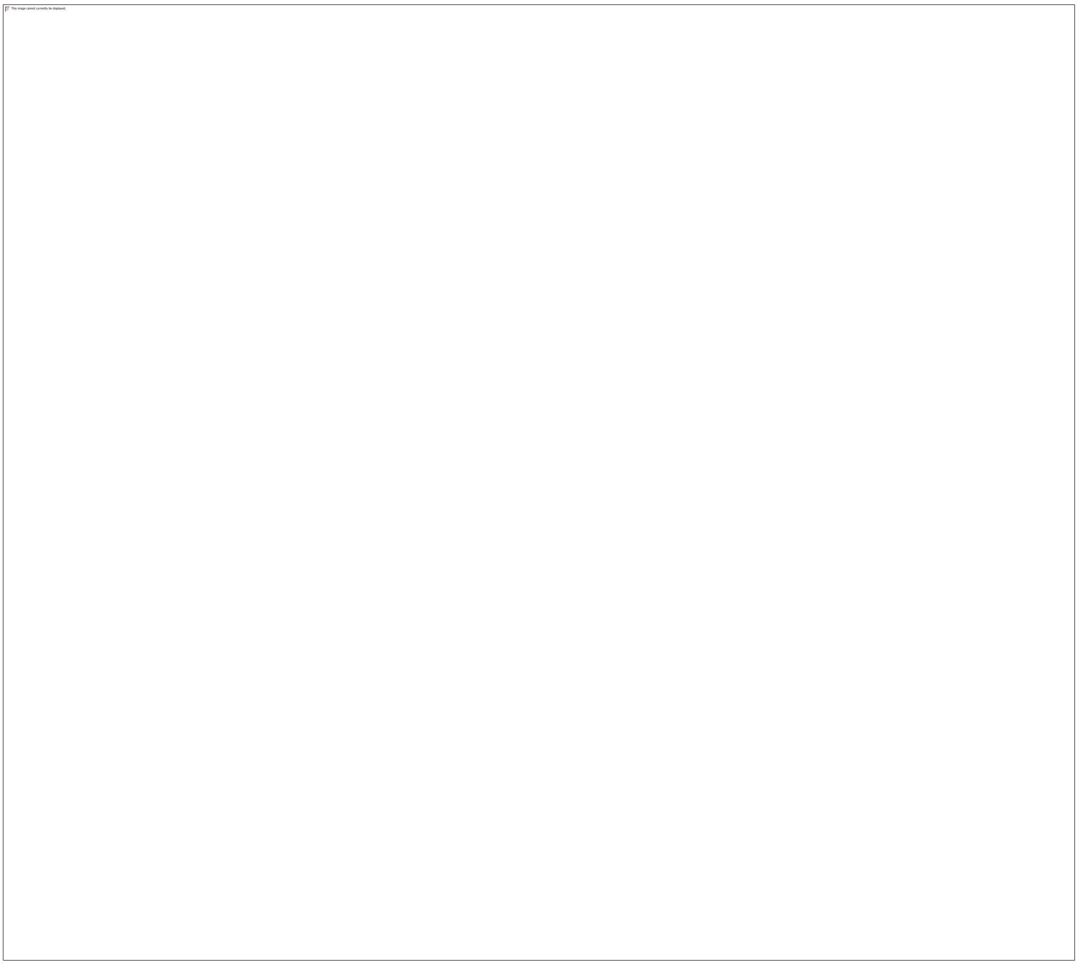

[Figure 1: An intraoperative image showing the bulky lymph node (star) adherent to the aorta (arrow).]

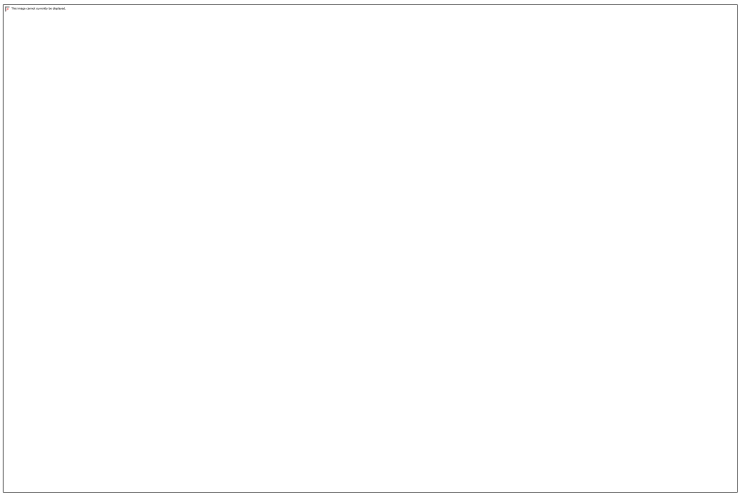

[Figure 2: An intraoperative image of dilated left ureter (arrow) due to distal ureteric obstruction]

Disclosure: Nothing to disclose

EP535

Analysis of endometrial cancer cases seen in a tertiary referral hospital

K. Güzin, A. Özer, A. Tok, Ş. Aydoğdu
Sütçü Imam University, Kahramanmaraş, Turkey

Introduction / Background: It was aimed to present clinicopathologic characteristics of 33 cases diagnosed as endometrium cancer in a tertiary referral hospital.
Methodology: Medical records of 33 patients diagnosed histopathologically as endometrium cancer between June 2017 and March 2019 were analysed retrospectively. For staging, FIGO 2009 staging system was used . Grade 1 and 2 endometrioid adenocarcinomas were classified as type 1 endometrial carcinomas (EC). Grade 3 endometrioid adenocarcinomas and the endometrial cancers with non-endometrioid histologie were classified as type 2 ECs. One patient diagnosed as undifferentiated endometrial stromal sarcoma had not been classified as either Type 1 or 2 EC.
Results: Demographic characteristics were displayed on Table 1. The majority of the cases (90,6%) was postmenopausal and diagnosed as endometrioid adenocancer (81.3%).
90.6 % of the cases was multiparous and 68.8 % of the cases was obese (BMI≥30kg/m^2).
Pelvic and paraaortic lymph node dissection was performed in 88.5% of the endometrioid adenocancers and 75% of the serous papillary cancers.
There was no statistically significant difference between type1 and 2 ECs regarding the mean age (60.2±6.9vs51.8±20.1 years in Type 1 and 2 retrospectively), the rate of obesity, menopausal status and the confinement to the uterus ($p< 0.05$ for all) (Table 2).
Conclusion: Endometrial cancer is the most common gynecologic cancer in women. Type 1 ECs, which consists of endometrioid histology and lower grade, account for 70-80% of the cases and are usually diagnosed at early stages. Type 2 ECs which develop from atrophic endometrium are diagnosed generally in older women and at later stages. However, in the present study the mean age was similar in both groups of type 1 and 2 ECs. In the current study, though statistically insignificant, the number of of the cases confined to the uterus (15/21, 71.4%) in type 1 ECs were higher compared to the type 2 ECs.

BMJ Publishing Group Limited (BMJ) disclaims all liability and responsibility arising from any reliance placed on this supplemental material which has been supplied by the author(s)

This image cannot currently be displayed.

[Table 1: Demographic and clinical characteristics of the cases]

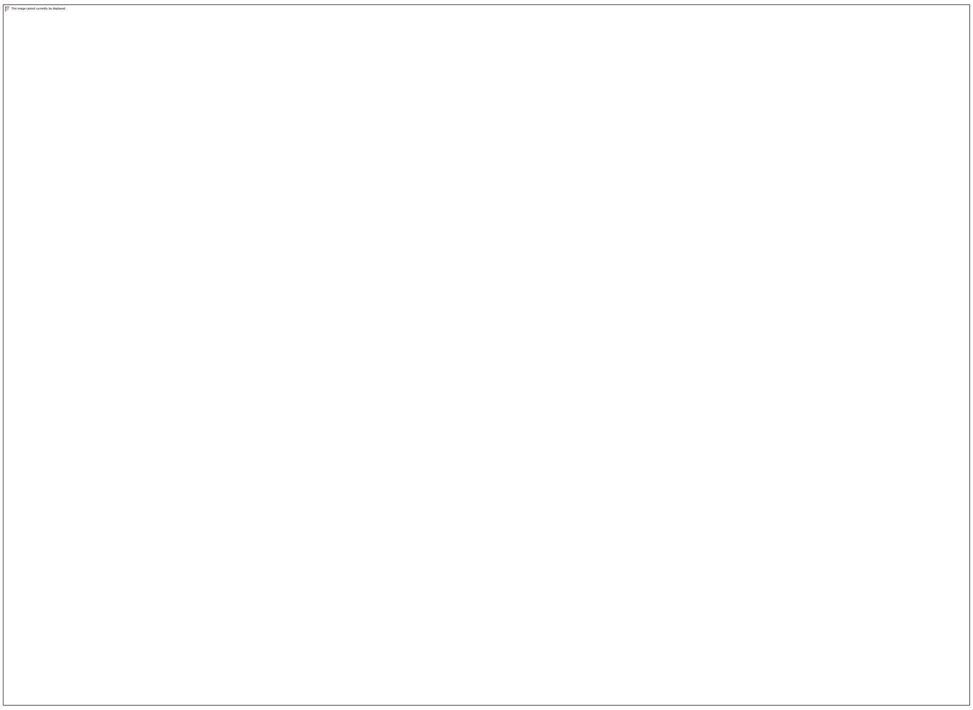

[Table 2: Comparison of Type 1 and 2 endometrium adenocarcinoma]

Disclosure: Nothing to disclose

EP536

Obesity and gynaecological oncology surgery in Wales: rising to the challenge of an expanding population

A. Haken, A. Sharma, K. Lim, R. Howells
Gynaecology Oncology, University Hospital of Wales, Cardiff, United Kingdom

Introduction / Background: Obesity in the UK is a growing problem and is classically associated with increasing comorbidities, longer operative time and more complications. It is increasingly common for gynaecological surgeons to operate laparoscopically which has proven benefits of reducing complications such as wound dehiscence and infection, and shortens recovery time. However there are additional challenges with laparoscopy in these patients including a need for longer instruments, technically difficult access, and restrictions in positioning and insufflation pressures used for surgery.
Methodology: The aims were to determine the number of laparoscopic hysterectomies performed for cancer in University Hospital of Wales over last 5 years, and analyse complication rates in relation to body mass index (BMI).
Retrospective data analysed on laparoscopic gynaecological oncology surgery from the last 5 years. Data was used to assess operation type in association with BMI and complication rates, conversion to open procedure rates, intra-operative blood loss and length of stay.
Results: The Gynae-Oncology service at the University Hospital of Wales has performed 589 laparoscopic hysterectomies since 2015, and over 80% of these in overweight or obese women. This data demonstrates an increasing trend of overweight and obese patients over the last 5 years, and yet no significant increase in complication rates or length of stay. The overall complication rate was 5.1%, and conversion to open procedure was required in 2%.
Conclusion: The findings suggest that surgical skill has developed in line with the additional

challenges of obese patients in gynaecological oncology surgery, enabling a quicker recovery and a smoother return to normal life.
Disclosure: Nothing to disclose

EP537

Dyskerin plays a negative role in endometrial epithelial cell proliferation and is associated with poor survival in endometrial cancer

R. Alnafakh[1], G. Saretzki[2], M. Adishesh[3], S.B. DeCruze[4], A. Midgley[1], J. Drury[1], D. Hapangama[5]
[1]Department of Women's & Children's Health, University of Liverpool, Liverpool, [2]The Ageing Biology Centre and Institute for Cell and Molecular Biosciences, University of Newcastle, Newcastle, [3]Gynaecological Oncology, [4]Liverpool Women's Hospital, [5]University of Liverpool, Liverpool, United Kingdom

Introduction / Background: The dramatic increase of endometrial cancer (EC) incidence with the associated rise in mortality demands the discovery of reliable prognostic and therapeutic biomarkers. High telomerase is an essential feature of most EC, and is needed for telomere maintenance. TERT and TERC components of telomerase holoenzyme is extensively studied yet Dyskerin, the third component of telomerase, in the endometrium is not previously evaluated. The aim of the study is to evaluate the function of dyskerin in EC cells and to examine its prognostic value in EC.
Methodology: The transient over-expression of *DKC1* gene in Ishikawa EC cells line on proliferation was studied in vitro using direct flow cytometry. Immunoblotting and immunoflourescence confirmed the overexpression of dyskerin protein. A human endometrial samples from 173 women were examined with immunohistochemistry to assess prognostic relevance of dyskerin expression in EC.
Results: *DKC1* overexpression down-regulated cell proliferation in ISK cells. Immunoblotting confirmed that dyskerin protein was elevated in transfected cells 24hr after the transfection compared with the negative controls. Exogenous dyskerin localised in cytoplasm and nucleus of transfected cell while endogenous dyskerin was located only in nucleus and has a specific morphological feature. Human endometrium expresses dyskerin (*DKC1*) and dyskerin protein levels are significantly reduced in ECs (p=0.0002) when compared with healthy postmenopausal endometrium and negatively correlated with ki67 proliferative index. Low dyskerin immunoscores were associated with worse outcomes, suggesting a prognostic relevance. *In silico* analysis of the TCGA uterine cancer dataset further confirms changes in *DKC1* gene expression in EC with a prognostic significance.
Conclusion: Our data suggest dyskerin to have a negative role on endometrial epithelial cell proliferation and confirms an aberrant expression and a possible prognostic relevance in EC. Interventions aimed at modulating dyskerin may therefore provide novel therapeutic options in EC.
Disclosure: Nothing to disclose

EP538

Prediction of pelvic lymph node metastasis in endometrioid endometrial carcinoma

A. Jaishuen[1], J. Petsuksiri[2], P. Karavanich[1], V. Achariyapota[1], S. Kuljarusnont[1], P. Inthasorn[1]
[1]Obstetrics-Gynaecology, [2]Radiology, Siriraj Hospital, Mahidol University, Bangkok, Thailand

Introduction / Background: Endometrial carcinoma (EC) is the most common gynaecologic malignancy in developed countries. Approximately eighty percent of cases are endometrioid type which usually presents in early stage and suitable for surgical staging. However, pelvic lymph node (PLN) evaluation mostly depends on surgeon preference. The objective of this study is to explore the associated factors and create the predictive model for PLN in endometrioid EC patients.
Methodology: Medical record of 293 patients with endometrioid EC, who received surgical staging in

single University Hospital during 2009-2016, were reviewed. The relationship between PLN metastasis and these data: demographic factors, biochemical markers, preoperative and intraoperative tumor characteristics were analysed by using a logistic regression model. Consequently, a score was created in order to predict the probability of pelvic lymph node metastasis. The internal validation by bootstrapping 400 times was performed.
Results: From multivariate analysis, associated factors were grade of tumor, platelet count, deep myometrial invasion (deep MI: more than half of myometrial invasion) and size of tumor. Platelet count and size of tumor were re-calculated. Thrombocytosis (platelet count more than or equal to 380,000) and large tumor (tumor size more than or equal to 6 centimetres) were statistically significant for cut-off point. The generated score was: (2 x grade2) + (4 x grade3) + (4 x deep MI) + (2 x large tumor) + (3 x thrombocytosis). The area under curve (AUC) for this score was 0.824. There were 88.9% sensitivity, 57.5% specificity, 40.5% positive predictive value and 94.1% negative predictive value, using score 4 as a cut-off point. The internal validation by bootstrapping 400 times revealed AuROC from 0.820 to 0.807 (optimism 0.013).
Conclusion: Grade of tumor, platelet count, deep MI and size of tumor, were associated with PLN metastasis in endometrioid EC. The generated score yield a promising result to predict PLN metastasis.
Disclosure: Nothing to disclose

EP539

Treatment outcomes of serous carcinoma of endometrium: experience from a tertiary cancer center in Eastern India

D. Jindal[1], J. Bhaumik[1], J. Ghosh[2], D. Midha[3], A. Roy[1], A. Ghosh[1]
[1]Gynecologic Oncology, [2]Medical Oncology, [3]Pathology, Tata Medical Center, Kolkata, India

Introduction / Background: Serous endometrial carcinoma is an aggressive form of endometrial carcinoma accounting for 5% of all endometrial tumours. It is associated with increased mortality and is liable for 40% of the deaths due to endometrial cancers. There is scarcity of outcome data in an Indian setting. Hence, we undertook this study to analyse the clinical characteristics, histopathological features, recurrence and survival outcomes.
Methodology: This is a retrospective observational study of all cases of serous carcinoma endometrium
diagnosed and treated at our institute from June 2011 to March 2018. The data was collected from the electronic hospital database. Survival analysis was done using Kaplan Meir method.
Results: A total of 68 patients were analysed. Median age at diagnosis was 62 years (30-75). The distribution of FIGO stage at diagnosis was: 21 (30.9%) in Stage I, 4 (5.9%) in Stage II, 17 (25%) in Stage III and 26 (38.2%) in Stage IV. Of these 68 patients, 47 underwent surgery, and 13 received palliative chemotherapy in view of stage IV disease, 8 patient received adjuvant treatment after undergoing surgery outside. 32 patients underwent surgery via laparotomy and 15 patients had a laparoscopic surgery performed. Systemic chemotherapy was the commonest form of adjuvant treatment, administered in 24 of the 47 patients who underwent surgery. The median follow up duration was 14 months. There were 34 (50%) recurrences and 23 (33.8%) deaths. The median recurrence free survival (RFS) was 23 months and estimated 5 year overall survival (OS) is 55.5% with recurrence and mortality noted more frequently in advanced stage.
Conclusion: Serous carcinoma is an aggressive form of endometrial carcinoma commonly presenting in
advanced stage. However, patients with early stage disease have better RFS and OS compared to advanced stage disease.

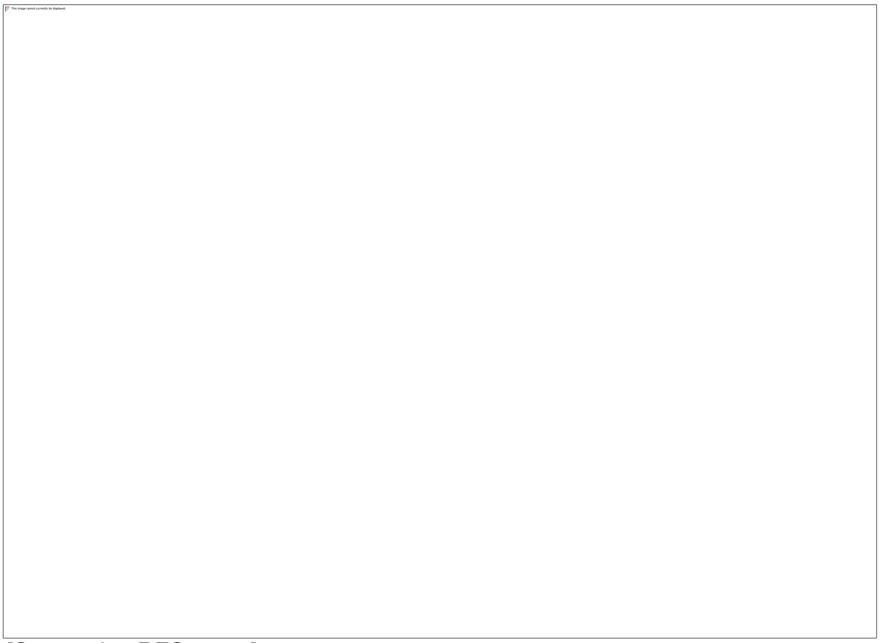

[Stage wise RFS curve]

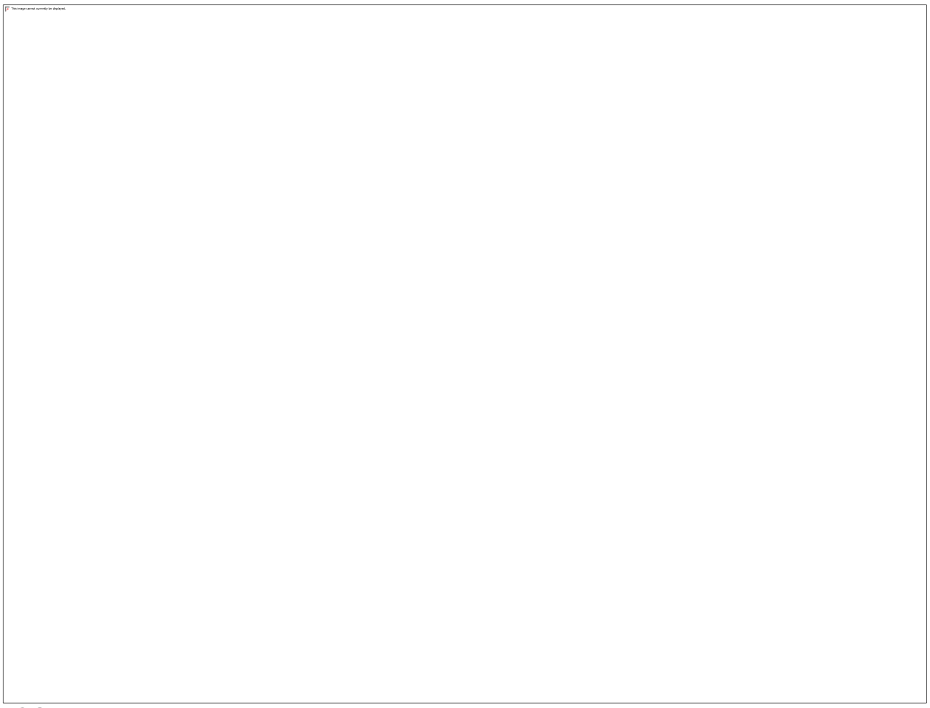

[OS curve]

Disclosure: Nothing to disclose

EP540

Pathological factors associated with adverse outcomes in early stage endometrial carcinoma

D. Jindal[1], J. Bhaumik[1], J. Ghosh[2], D. Midha[3], A. Roy[1], A. Ghosh[1]
[1]Gynecologic Oncology, [2]Medical Oncology, [3]Pathology, Tata Medical Center, Kolkata, India

Introduction / Background: The purpose of this study was to assess the prognostic significance of pathological features in the form of tumour size and lymphovascular space invasion (LVSI) in women with early stage endometrial cancer.
Methodology: This is a retrospective observational study of all cases of early stage endometrial carcinoma (Stage I) diagnosed and treated at Tata Medical Centre, Kolkata- India from June 2011 to December 2018. The Demographic, clinicopathological and survival data was collected from the electronic hospital database and analysed using SPSS version 23. Survival analysis was done using Kaplan Meir method.
Results: Among the women who were diagnosed and treated at Tata Medical Centre, 159 were staged as Stage I endometrial carcinoma. Of these were found to have lymphovascular invasion. Median age at diagnosis was 57 years (53-63). 103 patients (64 %) belonged to Stage IA. Recurrence was noted in 11 patients and mortality in 6 (3.8%). The median tumour size was noted to be 3 cm. Positive LVSI was noted with grade 2 disease more frequently (48%, p=0.47), deeper myometrial invasion (72%, p=0.01), greater tumour size (p=0.35), recurrence (12% v/s 5.9%, p=0.239). The estimated 5 year recurrence free survival (RFS) is 84% in patients with positive LVSI compared to 88% in patients with negative LVSI (p=0.262). However no significant difference was noted in the 5 year overall survival (p= 0.931). Other factor associated with poorer prognosis include age, increased risk of recurrence (p=0.311) but no association with survival. Tumour size was not associated with recurrence or survival.
Conclusion: Lymphovascular invasion was associated with adverse pathological features for early stage endometrial cancer. When compared with patients negative for LVSI, recurrence was more frequently in patients with lymphovascular invasion, though not statistically significant. Tumour size did not contribute to prognosis in early stage endometrial carcinoma.

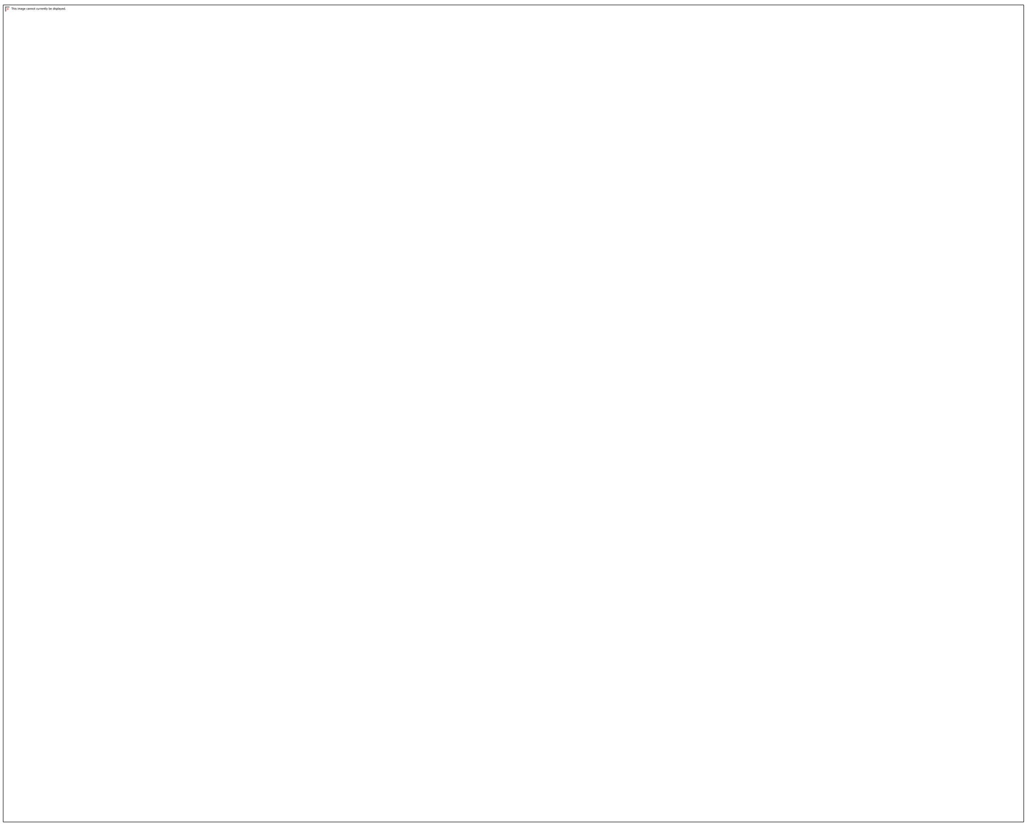

[RFS curve]

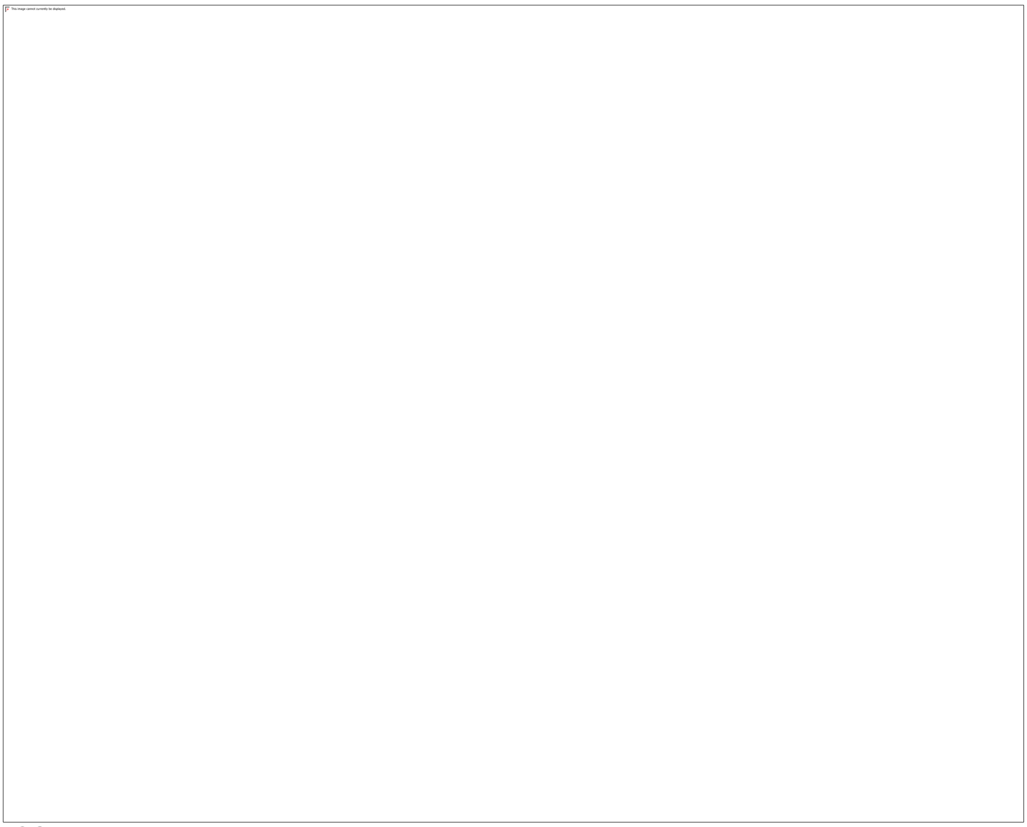

[OS curve]

Disclosure: Nothing to disclose

EP541

Nationwide introduction of robotic minimally invasive surgery was associated with improved survival and decreased odds of severe complications in women with early-stage endometrial cancer: a population-based prospective cohort study

S. Joergensen[1,2,3], O. Mogensen[3,4,5], C.S. Wu[1,3], K. Lund[6,7], M. Iachina[6,7], M. Korsholm[1,2,3], P.T. Jensen[3,4,5]

[1]Research Unit of Gynecology and Obstetrics, [2]OPEN, Odense Patient Data Explorative Network, Odense University Hospital, [3]Faculty of Health Sciences, Clinical Institute, University of Southern Denmark, Odense, [4]Dept. of Gynecology and Obstetrics, Aarhus University Hospital, [5]Faculty of Health, Dept. of Clinical Medicine, Aarhus University, Aarhus, [6]Centre for Clinical Epidemiology, Odense University Hospital, [7]Research Unit of Clinical Epidemiology, Institute of Clinical Research, University of Southern Denmark, Odense, Denmark

Introduction / Background: The oncological safety of minimally invasive techniques versus open access surgery in gynecological cancer is currently debated. Substantial changes in the surgical management of endometrial cancer necessitates evaluation to ensure survival is not compromised

and the risk of severe complications is not increased.
Methodology: A nationwide prospective cohort of all women with early-stage endometrial cancer who underwent surgery between January 1, 2005 and June 30, 2015 were retrieved from the Danish Gynecological Cancer Database. Data were linked with nationwide registers on socioeconomic status, deaths, hospital diagnoses and treatment using each woman´s civil registration number. The cohort was divided according to the time they underwent surgery; group 1 underwent surgery before the introduction of robotic minimally invasive surgery (RMIS) in their respective region, and group 2 underwent surgery after. Women in group 2 were subdivided in total abdominal hysterectomy (TAH), laparoscopic minimally invasive surgery (LMIS), and RMIS. Severe complications encompassed death within 30 days, intraoperative complications, and postoperative complications diagnosed within 90 days. Five-year overall survival was compared by multivariate Cox proportional hazards models stratified by histopathological risk. Odds of severe complications were compared by multivariate logistic regression.
Results: A total of 5654 women were included. Women in group 1 (n=3091) had significantly higher mortality and odds of severe complications compared with women in group 2 (n=2563; hazard ratio [HR], 1.22;95%confidence interval [CI], 1.05-1.42 and odds-ratio [OR], 1.39; 95%CI, 1.11-1.74, respectively). In group 2, TAH was associated with higher mortality and increased odds of complications compared with RMIS in adjusted analyses (HR, 1.70; 95%CI 1.31-2.19 and OR, 3.87; 95%CI, 2.52-5.93, respectively).
Conclusion: The national introduction of RMIS transitioned the surgical approach for early-stage endometrial cancer towards minimally invasive surgery. This change was associated with improved survival and significantly reduced odds of severe complications.
Disclosure: Nothing to disclose

EP542

Computer adaptive testing discriminates early recovery after robotic surgery in women with early-stage endometrial cancer

S.L. Jørgensen[1,2,3], O. Mogensen[3,4,5], M.A. Petersen[6], C.S. Wu[1,3], P.T. Jensen[3,4,5]
[1]Research Unit of Gynecology and Obstetrics, [2]OPEN, Odense Patient Data Explorative Network, Odense University Hospital, [3]Faculty of Health Sciences, Clinical Institute, University of Southern Denmark, Odense, [4]Department of Gynecology and Obstetrics, Aarhus University Hospital, [5]Faculty of Health, Dept. Of Clinical Medicine, Aarhus University, Aarhus, [6]The Research Unit, Department of Palliative Medicine, Bispebjerg and Frederiksberg Hospital University of Copenhagen, Copenhagen, Denmark

Introduction / Background: Knowledge on the individual recovery after robotic surgery for endometrial cancer (EC) is sparse but important for optimisation of pre-operative information and for post-operative supportive care. Generic questionnaires measuring quality-of-life are challenged by the diversity of these women, ranging from women who are part of the active workforce in their thirties to retired women in their nineties. The European Organisation of Research and Treatment of Cancer Computer Adaptive Test Core questionnaire (EORTC CAT Core) provides individualised measurements.
Methodology: 94 consecutive women who underwent robotic minimally invasive surgery for early-stage EC at Odense University Hospital in Denmark were included. The EORTC CAT Core was distributed pre-operatively and every week during the first post-operative month. Repeated measures models were fitted for four domains; physical functioning, role function, fatigue, and pain. Each model was tested for impact of age, ASA score, minor/major surgery, and the individual baseline scores (poorest, intermediate, best). A hypothesis of individual complete recovery of physical health to baseline levels within three post-surgical weeks was evaluated.
Results: Women with the *lowest* physical and role functions, and the highest fatigue and pain levels at baseline recovered within three weeks. Women with the *highest* physical health before surgery did not reach their individual baseline within the first post-operative month but had the most favorable domain-scores three weeks post-operatively.
Conclusion: The individual woman´s baseline score of physical health is predictive for her postoperative recovery following robotic minimally invasive surgery for early-stage endometrial cancer.

Recovery to the individual offset is prolonged in women with the best physical health. The EORTC CAT Core may be a valuable tool for individualised pre-operative information and supportive care during surveillance.
Disclosure: Nothing to disclose

EP543

Safety of ovarian preservation in premenopausal women with endometrioid endometrial cancer clinically confined to the uterus

U.C. Ju, S.M. Kim
Chonnam National University Medical School, Gwangju, Republic of Korea

Introduction / Background: The aims of this study were to determine the frequency of ovarian involvement and to evaluate the impact of ovarian preservation on the recurrence and survival rates in premenopausal women when endometrioid endometrial cancer (EEC) was clinically confined to the uterus.
Methodology: The medical records of premenopausal women under 50 years-old with EEC surgically treated at our institution between 2004 and 2017 were retrospectively reviewed. Patients were excluded if there were extrauterine diseases on imaging (pelvic MRI and PET-CT) or abnormal intraoperative gross findings around adnexa and/or other sites.
Results: A total of 144 patients were found to have EEC clinically confined to the uterus, including 79 patients selectively preserving normal appearing ovaries. Ovarian preservation group was younger ($p < 0.01$) and had significant associations with lower tumor grade ($p = 0.03$) and lower prevalence of lymphadenectomy ($p < 0.01$). Of 65 patients who underwent bilateral salpingo-oophorectomy, microscopic ovarian involvement was detected in only 1 patient, who had grade-3 tumor and deep myometrial invasion. In 79 patients with ovarian preservation, no recurrence at adnexa occurred. Ovarian preservation had no effect on either adnexa or other sites recurrence, and as well overall survival (median follow-up period: 67 months, range 13-133).
Conclusion: Ovarian preservation may be considered in premenopausal women with low-risk endometrioid endometrial cancer clinically confined to the uterus, when there were normal appearing ovaries on preoperative evaluation and intraoperative exploration.
Disclosure: Nothing to disclose

EP544

Saten III - Splitting adjuvant treatment into subgroups of stage III endometrioid endometrial cancers: an international, multicenter study

I. Kahramanoglu[1,2], M.M. Meydanli[3], S. Taranenka[4], A. Ayhan[5], C. Salman[6], M. Sanci[7], F. Demirkiran[1], F. Ortac[8], D. Haidopoulos[9], V. Sukhin[10], D. Kaidarova[11], A. Stepanyan[12], F. Farazaneh[13], S. Aliyev[14], E. Ulrikh[15], D. Kurdiani[16], I. Yalcin[3], S. Mavrichev[4], H. Akilli[5], M.E. Sari[3], A. Pletnev[4], K. Aslan[3], T. Bese[1], M. Kairbayev[17], D. Vlachos[9], M. Gultekin[6], Central Asia and East Europe Trial (CentEAST) Group
[1]Department of Gynecologic Oncology, Istanbul University-Cerrahpasa, School of Medicine, Istanbul, [2]University of Health Sciences, Gazi Yasargil Training and Research Hospital, Diyarbakir, [3]Zekai Tahir Burak Women's Health Training and Research Hospital, Ankara, Turkey, [4]N.N. Alexandrov Cancer Center of Belarus, Minsk, Belarus, [5]Baskent University Faculty of Medicine, [6]Hacettepe University, Ankara, [7]Tepecik Education and Research Hospital, İzmir, [8]Ankara University, Ankara, Turkey, [9]University of Athens, Alexandra Hospital, Athens, Greece, [10]Grigoriev Institute for Medical Radiology, Kharkiv, Ukraine, [11]Kazakh Institute of Oncology and Radiology, Almaty, Kazakhstan, [12]NAIRI Medical Center, Yerevan, Armenia, [13]Shahid Beheshti University of Medical Sciences, Tehran, Islamic

Republic of Iran, [14]National Center of Oncology, Baku, Azerbaijan, [15]N.N. Petrov Research Institute of Oncology, Saint Petersburg, Russian Federation, [16]Tbilisi Cancer Center, Tbilisi, Georgia, [17]Almaty Sema Hospital, Almaty, Kazakhstan

Introduction / Background: The primary aim was to determine whether different adjuvant treatment modalities (CT, RT, and CRT) improve survival outcomes in stage III endometrioid EC patients based on the specific sub-stage of the disease. The secondary aim was to assess the prognostic factors in stage III endometrioid EC.
Methodology: SATEN III was a retrospective study involving 13 centers from 10 countries. Patients who had been operated on between 1998 and 2018 and diagnosed with stage III endometrioid EC were analyzed.
Results: A total of 990 women overall were identified; 317 with stage IIIA, 18 with stage IIIB, and 655 with stage IIIC diseases. The median follow-up was 42 months. The 5-year DFS of patients with stage III EC by adjuvant treatment modality was 68.5% for radiotherapy (RT), 54.6% for chemotherapy (CT), and 69.4% for chemoradiation (CRT) (p=0.11). The 5-year OS for those patients was 75.6% for RT, 75% for CT, and 80.7% for CRT (p=0.48). Negative peritoneal cytology (Hazard ratio [HR]: 0.45, 95% confidence interval [CI]: 0.23-0.86; p=0.02) and performance of lymphadenectomy (HR: 0.33, 95% CI: 0.16-0.77, p=0.001) were independent predictors for improved OS for stage IIIA EC. Independent prognostic factors for better OS for stage IIIC disease were age< 60 (HR: 0.50, 95%CI: 0.36-0.69, p< 0.001), grade 1 or 2 disease (HR: 0.59, 95%CI: 0.37-0.94, p=0.014; and HR: 0.65, 95%CI: 0.46-0.91, p=0.014, respectively), absence of cervical stromal involvement (HR: 063, 95%CI: 0.46-0.86, p=0.004) and performance of para-aortic lymphadenectomy (HR: 0.52, 95%CI: 0.35-0.72, p< 0.001).
Conclusion: Although not statistically significant, CRT seemed to be a better adjuvant treatment option for stage IIIA endometrioid EC. However, we were not able to show the superiority of adjuvant CRT in stage IIIC endometrioid EC.Systematic lymphadenectomy seemed to improve survival outcomes in stage III endometrioid EC.
Disclosure: Nothing to disclose

EP545

Imaging-based definition of lower uterine segment carcinoma to improve the detection sensitivity of probable Lynch syndrome

H. Kato
Hokkaido Cancer Center | Gynecologic Oncology, National Hospital Organization, Sapporo, Japan

Introduction / Background: The aim of this study was to investigate a magnetic resonance imaging (MRI)-based definition of lower uterine segment (LUS) carcinoma.
Methodology: We retrospectively reviewed 587 consecutive patients with endometrial cancer who underwent hysterectomy. LUS carcinoma was determined through pathological examination and MRI assessment. For imaging assessment, the location of the inner lining of the uterus was classified into four equal parts on a sagittal section image. A tumor was defined as LUS carcinoma when its thickest part was located in the second or the third part of the uterine fundus. LUS carcinoma was further divided into LUS in a narrow sense, upon which diagnosis was exclusively based on pathological findings, and LUS in a broad sense. The relationship between LUS carcinoma and probable Lynch Syndrome (LS) was investigated. Patients with loss of MSH2, MSH6, and PMS2 expression or those with tumors with loss of MLH1 and absence of *MLH1* promoter methylation were diagnosed as probable LS.
Results: LUS carcinoma was identified in 59 (10.2%) patients. Twenty-eight (47.5%) patients were categorized as LUS in a narrow sense and 31 (52.5%) as LUS in a broad sense. Among them, probable LS was identified in 12 (20.3%) cases. There was no difference in clinical profiles, including the prevalence of probable LS between the two categories.
Conclusion: An expanded definition of LUS carcinoma might improve the detection of LS-related endometrial cancer.
Disclosure: Nothing to disclose

EP546

Laparoscopy should be the preferable route in the morbidly obese endometrial cancer

G. Khatib[1], A.B. Guzel[1], Ü. Küçükgöz Güleç[1], S. Seyfettinoğlu[1], K. Duran[1], E. Bağır[2], M.A. Vardar[1]
[1]Gynecologic Oncology, [2]Pathology, Çukurova University, Adana, Turkey

Introduction / Background: The risk of endometrial cancer is known to be increased with the increasing body mass index (BMI). Patients with BMI more than 40 kg/m2 are identified as morbid obese according to the World Health Organization. Morbidly obese patients are an enormous problem regarding all of their health procedures including examination, evaluation with imaging methods, surgery, increased per-operative and postoperative morbidity and complications. The laparoscopic approach had been validated for the endometrial cancer surgery in many studies. The present study aimed to investigate the adequacy and oncological safety of laparoscopy compared to laparotomy in the surgery of the morbid obese women with endometrial cancer.
Methodology: Archival records and pathological reports of the endometrial cancer cases who were operated and followed up in Çukurova University Gynecologic Oncology Center between January 2008 and December 2018 were retrospectively reviewed. Patients with BMI ≥40 were divided into laparoscopy and laparotomy groups. Groups were compared concerning cases' clinic, surgical and pathological features. In addition, survival analysis of the groups were performed using Kaplan-Meier and compared with the Log-rank method.
Results: A total of 146 morbidly obese endometrial cancer cases with sufficient data were determined during the study period. Laparoscopic surgery was performed in 65 (44.5%) and laparotomy in 81 (55.5%) of them. Mean age was 58 years for both groups. Mean BMI of the laparoscopy and laparotomy groups was 46.1 kg/m2 and 44.3 kg/m2, respectively. No difference between groups according to the main clinical, surgical and pathological characteristics. Five-year overall survival rates were 86.6% and 83.9% in the laparoscopy and laparotomy groups (p=0.571), respectively. No difference between groups concerning to the disease free survival was noted (p=0.184).
Conclusion: Laparoscopic surgery do not harm the long-term oncologic outcomes of the morbidly obese endometrial cancer patients, while its short-term advantages in this special population are undeniable.
Disclosure: Nothing to disclose

EP547

Clinicopathologic significance of mismatch repair protein expression in endometrial cancer

M.-K. Kim, S.C. Kim
Ewha Womans University School of Medicine, Seoul, Republic of Korea

Introduction / Background: To evaluate the mismatch repair (MMR) protein expression in endometrial cancer and to assess their association with clinico-pathologic outcomes.
Methodology: A retrospective review of the clinico-pathologic data and clinical outcomes was performed on patients who were treated for endometrial cancer at a single center between 2014 and 2018. For the study analysis, patients with results for MMR protein immunohistochemistry were included. MMR-deficient (MMR-d) was defined as absence of expression in any of the 4 MMR proteins (MLH1, MSH2, MSH6, and PMS2). Clinical and pathologic outcomes were compared according to the MMR status.
Results: A total of 146 endometrial cancer patients with available MMR expression data were included. Of these, 41 patients (28.1%) showed absence of MMR protein expression (MMR-d). Compared with MMR-proficient (MMR-p) patients, the MMR-d patients had higher rate of pelvic and/or para-aortic lymph node metastasis and lymphovascular space invasion (p=0.011 and p=0.015, respectively). However, other clinico-pathologic variables, including stage, grade, histologic subtype, and depth of invasion, were not associated with MMR status. During the median follow-up period of

18.2 months (1-39.4), there was no difference in progression-free survival between MMR-p and MMR-d patients (p=0.181).
Conclusion: MMR defects were associated with higher rate of lymphovascular space invasion and lymph node metastasis. However, other prognostic factors and survival outcomes were not different between MMR-p and MMR-d patients.
Disclosure: Nothing to disclose

EP548

Clinicopathologic characteristics and treatment outcomes of Endometrial stromal sarcoma
M.-K. Kim, S.C. Kim
Ewha Womans University School of Medicine, Seoul, Republic of Korea

Introduction / Background: Endometrial stromal tumors are rare uterine sarcomas, comprising low-grade endometrial stromal sarcoma (ESS) and undifferentiated endometrial sarcoma (UES). This study evaluated their clinicopathologic characteristics and clinical outcomes.
Methodology: A retrospective analysis was performed on 18 patients with histologically proven endometrial stromal tumors who were treated and followed-up at a single Korean institution from 2004 to 2015.
Results: Thirteen patients were diagnosed with low-grade ESS and 5 patients had UES. The mean ages at diagnosis of ESS and UES patients were 46.2 and 45.2 years, respectively. Preoperative diagnosis through curettage or hysteroscopic endometrial biopsy was made in 30.8% and 80% in ESS and UES, respectively. Vaginal or laparoscopically-assisted hysterectomy was performed in 6 patients (46.2%) with stage I ESS, of whom there was no recurrence with a median follow-up period of 33.6 months. Lymph node dissection was performed in 61.5% and 60% of ESS and UES patients, and only 1 ESS patient (7.7%) showed microscopic lymph node metastasis. Although the stage distribution was similar between ESS and UES patients, 5-year progression-free survivals were 77.9% and 40% for ESS and UES patients, respectively. The sites of recurrence were loco-regional in ESS patients, whereas UES patients recurred mainly in the distant sites, such as lung. The performance of lymphadenectomy, adnexectomy, or adjuvant treatments was not associated with decreased recurrence rate.
Conclusion: The two endometrial stromal tumors, ESS and UES, show different clinical outcomes despite similar initial presentations. Patients with UES tend to recur at distant sites even in the early stage. On the contrary, ESS show favorable prognosis with longer PFS. In the selected cases of low-grade ESS with small tumor size and without extrauterine involvement, laparoscopic staging also seems to be feasible.
Disclosure: Nothing to disclose

EP549

Clinical implication of preoperative tumor grade results in endometrial cancer with low-risk for lymph node metastasis
M. Kim, S.Y. Won, M.K. Kim, M.-L. Kim, Y.W. Jung, B.S. Yun, S.J. Seong
CHA Gangnam Medical Center, CHA University, Seoul, Republic of Korea

Introduction / Background: To evaluate the clinical implication of preoperative tumor grade results in endometrial cancer with low-risk for lymph node metastasis
Methodology: The medical records of 75 women with endometrial cancer were retrospectively reviewed. Women with (1) non-endometrioid type, (2) deep myometrial invasion on magnetic resonance imaging (MRI), (3) enlarged lymph node on MRI, (4) extension beyond the uterine corpus

on MRI and (5) serum cancer antigen 125 (CA-125) level >35 IU/ml before surgery were excluded. Pattern of pelvic lymph node dissection (PLND) and clinical course by preoperative tumor grade were compared.
Results: Median age was 45 (range, 28-83) years and follow-up period was 35 (range, 1-126) months. Grade 1, grade 2-3 and unknown grade were respectively diagnosed in 50 (66.7%), 11 (14.7%) and 14 (18.6%) women before staging surgery by endometrial biopsy. After surgery, 30% of preoperative grade 1 was confirmed to non-grade 1, and 48.0% of preoperative non-grade 1 was confirmed to grade 1 (p=0.063). PLND was similarly performed between the preoperative grade 1 and non-grade 1 groups (44.0% vs. 36.0%, p=0.507). Lymph node metastasis was diagnosed in 1 women with preoperative grade 1 and 1 women with preoperative non-grade 1 (4.5% vs. 11.1%, p=0.503). There was no recurrence in women with preoperative non-grade 1 during follow-up period (0% vs. 6.0%, p=0.546).
Conclusion: Preoperative tumor grade results occasionally make it difficult to decide PLND omission in low-risk endometrial cancer, however, non-grade 1 tumor seems not to increase the lymph node metastasis and recurrence.
Disclosure: Nothing to disclose

EP550

Prognosis trend of grade 2 endometrioid endometrial carcinoma: toward grade 1 or 3?

U. Kucukgoz Gulec[1], G. Khatib[1], A.B. Guzel[1], E. Bagir[2], B. Bayat[1], M.A. Vardar[1]
[1]*Obstetrics and Gynecology,* [2]*Pathology, Cukurova University Faculty of Medicine, Adana, Turkey*

Introduction / Background: The prognostic significance of grade in the endometrial cancer is well known. However, grade 2 cases were not evaluated separately in most of the previous studies. In this study, we aimed to investigate whether the oncologic outcomes of grade 2 endometrioid endometrial carcinomas is trending to grade 1 or 3 tumors.
Methodology: Patients records and pathological reports were reviewed retrospectively and 776 patients of endometrioid endometrial carcinoma were distributed into 3 groups according to their 1988 FIGO grade. Groups' characteristics and oncologic outcomes were compared. Difference between grades was tested with z-test and adjusted by Bonferroni method. Kaplan-Meier method were performed for the survival analysis.
Results: Mean follow-up time was 52 months. Patients' mean age was 56.29±10.8 years. Five years OS, DFS rates were 95.1%, 83.3% and 91.6%, 79.1% for grade 1 and 2, respectively. These rates were not statistically different (OS: p=0.075, DFS: P=0.939). The statistically difference between grade groups in terms of OS and DFS was mainly noticed between grade 1 and 3 (OS: 75.6%, DFS: 60.3%) Even though, grade 2 endometrioid endometrial carcinomas were different from both grade 1 and 3 in terms of the pathological features, survival analyses demonstrated that their oncologic outcomes were trended to grade 1. Grade was determined as an independent prognostic factor for OS.
Conclusion: The interobserver reproducibility will be improved among pathologists by combining FIGO grade 1 and 2 endometrioid endometrial carcinomas, while prognosis prediction would not likely to be affected.
Disclosure: Nothing to disclose

EP551

A clinicopathologic characteristics and prognosis comparison between uterine carcinosarcomas and uterine high grade endometrial carcinomas

U. Kucukgoz Gulec[1], S. Paydas[2], A.B. Guzel[1], E. Bagir[1], G. Khatib[1], M.A. Vardar[1]
[1]*Cukurova University Faculty of Medicine,* [2]*Medical Oncology, Cukurova University Faculty of*

Medicine, Adana, Turkey

Introduction / Background: Uterine carcinosarcoma (UCS), grade 3 endometrioid adenocarcinomas (G3 EAC) and type two adenocarcinomas (Type2 EAC) are considered as high-grade endometrial adonocarcinomas. The aim of this study was to compare the clinicopathologic features and survival of patients with UCS, G3 EAC, Type2 EAC.
Methodology: A total of 235 patients were included in this study. Of them, 62 cases had uterine HGEAC (group 1), 93 cases had Type2 EAC (group 2), and 80 had UCS (group 3). The groups were compared in terms of clinical and pathological characteristics including age, initial symptom, surgical approach, stage, myometrial invasion (MI), lymphovascular space invasion (LVSI), lymph node invasion, adjuvant therapy, and survival. The Kaplan-Meier analysis was used to compare the survival outcomes.
Results: There were no statistically significant differences among the groups with regard to age, nulliparity, menopausal status, and the main initial symptom. Omentectomy was performed significantly more in group 2, but the rate of optimal cytoreduction was similar among the groups. All groups were similar in terms of stage, depth of myometrial invasion, LVSI and lymph node metastasis. Chemotherapy was the main adjuvant therapy for UCS and Type2 EAC while radiotherapy was the main adjuvant therapy for G3 EAC. There were no differences among the groups in terms of disease-free survival (DFS) (p=0.941) and overall survival (OS) (p=0.504).
Conclusion: Although the survival difference among the groups can not be revealed, these patients have different clinical and pathological features and they should be considered as different groups.
Disclosure: Nothing to disclose

EP552

Comparison of robotic versus laparoscopic surgery for endometrial cancer staging: our institution's experience

M. Kyung[1], Y. Park[2]
[1]Hallym University Dongtan Sacred Heart Hospital, Hwasung, [2]Hallym University Hallym Sacred Heart Hospital, Anyang, Republic of Korea

Introduction / Background: The objective of this study was to evaluate the feasibility and the safety of robot-assisted staging surgery with the DaVinci Xi system in endometrial cancer.
Methodology: From June 2015 to June 2018, we retrospectively analyzed 56 patients who underwent laparoscopic or robotic staging surgery for endometrial cancer in our two hospitals.
Perioperative data including age, parity, body mass index (BMI), previous op. history, preop. CA125 level, operation time, hospital stay, the number of lymph nodes retrieved, postoperative pain score, estimated blood loss and postoperative complications were compared.
Results: Fifty six endometrial cancer patients were admitted for surgical staging of endometrial cancer. Out of these patients, 28 underwent robotic surgery and 28 underwent laparoscopic surgery. There were no differences in age (p=0.523), parity (p=0.432), BMI (p=0.172), op. history (p=0.378), co-morbid medical disease (p=0.130), and CA125 level (p=0.072). There was no difference in stage (p=0.563), extracted pelvic & paraaortic LNs (p=0.076). There were no differences in operative time (p=0.062), blood loss (p=0.056), hospital stay (p=0.112), Hb change (p=0.219), postoperative complications, postoperative pain (NRS score, postop. 6hrs, 24hrs, 48hrs).
Conclusion: Robotic staging surgery with daVinci Xi system is feasible for endometrial cancer. Prospective and randomized studies are needed to assess the benefit of the robotic staging surgery in endometrial cancer.
Disclosure: Nothing to disclose

EP553

An unusual presentation of endometrial cancer with bilateral adrenal metastases at the time of diagnosis

M. Ryan[1], A. Laios[1], D. Pathak[2], R. Hutson[1]
[1]Gynaecologic Oncology, [2]Cellular Pathology, St James's University Hospital, Leeds Teaching Hospitals, Leeds, United Kingdom

Introduction / Background: Endometrial cancer (EC) is the most common gynaecological malignancy with an average of 9,000 new cases diagnosed in the UK each year. If at presentation, the tumour is not amenable to operative management, then the approach must be individualised and chemotherapy or systemic hormonal therapy should be considered. We report an unusual presentation of EC with solitary adrenal metastases at the time of diagnosis.
Methodology: A 68 year old Caucasian woman was referred with post-menopausal bleeding of several weeks' duration. Imaging including urgent ultrasound, pelvic MRI and full body CT revealed a heterogeneous uterine mass and bulky bilateral adrenal masses, which were not in keeping with benign adrenal adenomas. Hysteroscopy and endometrial biopsies diagnosed grade 3 endometrial adenocarcinoma. Central multidisciplinary team meeting (MDT) recommended radiological guided biopsy of the adrenal mass. The histology was consistent with poorly differentiated adenocarcinoma. A PET-CT scan reported the two adrenal metastases being hypermetabolic and suspicious for malignancy. A diagnosis of Stage 4b, Grade 3 EC with adrenal metastases was made.
Results: The patient received neoadjuvant chemotherapy with carboplatin and Paclitaxel. Following six cycles of primary chemotherapy, a CT scan confirmed good response with size reduction of the primary tumour and metastases. The adrenal metastases were no longer PET-avid on repeat PET-CT scan. She received alternating hormonal therapy Megace/Tamoxifen three-weekly. As per adrenal MDT, she underwent a total laparoscopic hysterectomy, bilateral salpingo-oophorectomy followed by a bilateral retroperitoneal laparoscopic adrenalectomy two months later. The patient remains asymptomatic on maintenance hydrocortisone 18 months post diagnosis.
Conclusion: This is the first report of solitary synchronous adrenal metastases in a patient with EC. Central MDT review is key in providing individualised treatment recommendations of such rare entity.
Disclosure: Nothing to disclose

EP554

The effects of *Cimicifuga racemosa* extract on endometrial and ovarian cancer cell lines

K. Marton, T. Lanišnik Rižner
University of Ljubljana, Medical Faculty, Institute of Biochemistry, Ljubljana, Slovenia

Introduction / Background: In postmenopausal women estrogen levels depend solely upon the local formation from steroid precursors, dehydroepiandrosterone-sulfate and estrone-sulfate (E1-S). Decreased estrogen levels are associated with menopausal symptoms, which often occur in peri- and postmenopausal women. Traditionally, hormone replacement therapy (HRT) has been prescribed to ease these symptoms. Due to the reported association with increased risk of breast cancer, stroke and pulmonary embolism the use of HRT has declined dramatically. Women are thus deciding for natural “HRT”, including extracts from *Cimicifuga racemosa* (CE). While CE treatment is considered as safe, little is known about its effects on endometrial or ovarian cancers, hormone-dependent malignancies that arise in this population of women.
Methodology: We aimed to evaluate the effects of CE (BNO 1055, a gift from Bionorica SE) on local formation of estradiol from the steroid precursor E1-S in the endometrial and ovarian cancer cell lines (CLs). We thus exposed poorly differentiated (KLE), and moderately differentiated (RL95-2) endometrial carcinoma, and high-serous ovarian carcinoma (COV362 and Kuramochi) CLs to CE and measured the effect on the expression of 13 E1-S transporter genes and 6 genes encoding estradiol biosynthetic and metabolic enzymes with RT-qPCR.
Results: The E1-S transporter genes were mainly upregulated upon CE treatment, with the highest

differences seen for *SLCO4A1* in KLE and COV362 CL. For genes involved in estradiol biosynthesis and metabolism we saw decreased as well as increased expression in individual CLs. The most significant upregulation was observed for *HSD17B2* in KLE and downregulation for *SULT1E1* in COV362. Interestingly, *STS* was significantly downregulated in all CLs. Genes encoding estrogen receptors *ESR1* and *ESR2* were significantly upregulated only in KLE.
Conclusion: Our research presents an insight of CE effects on endometrial or ovarian cancer CLs at the mRNA level. The effects at the protein level and the level of E1-S metabolism still needs to be evaluated.
Disclosure: Nothing to disclose

EP555

Biomarkers for diagnosis and prognosis of Endometrial Carcinoma: BioEndoCar

T. Knific[1], Š. Smrkolj[2], A. Romano[3], A. Semczuk[4], A. Kaminska[4], A. Adamiak-Godlewska[4], J. Vilo[5,6], D. Fishman[5,6], C. Schröder[7], J. Adamski[8,9,10], T. Lanišnik Rižner[1]
[1]Laboratory for Molecular Basis of Hormone-Dependent Diseases and Biomarkers, Institute of Biochemistry, Faculty of Medicine, University of Ljubljana, [2]Department of Obstetrics and Gynecology, University Medical Centre Ljubljana, Ljubljana, Slovenia, [3]Department of Gynecology, Maastricht University Medical Centre (MUMC), Maastricht, The Netherlands, [4]Department of Gynecology, Lublin Medical University, Lublin, Poland, [5]Institute of Computer Science, University of Tartu, [6]Quretec Ltd., Tartu, Estonia, [7]Sciomics GmbH, Heidelberg, [8]Research Unit Molecular Endocrinology and Metabolism, Genome Analysis Centre, Helmholtz Zentrum München, [9]Lehrstuhl für Experimentelle Genetik, Technische Universität München, [10]German Center for Diabetes Research (DZD), München, Germany

Introduction / Background: Endometrial carcinoma (EC) is the most frequent gynecological malignancy in the developed world. Currently there are no valid non-invasive diagnostic or prognostic methods available and therefore, diagnosis and treatment of EC patients is guided by histopathological and surgical findings. The lack of non-invasive diagnostic and prognostic biomarkers of EC is addressed in the current clinical study titled “**Bio**markers for Diagnosis and Prognosis of **Endo**metrial **Car**cinoma” (BioEndoCar).
Methodology: Prospective observational case - control study. Patient recruitment takes place at three medical centers (University Medical Centre Ljubljana, Slovenia; Maastricht University, The Netherlands; Lublin Medical University, Poland). Plasma samples from women with diagnosed EC and controls will be examined using non-targeted and targeted metabolomics (Helmholtz Zentrum München, Germany) and semi-targeted proteomics approaches (Sciomics GmbH, Heidelberg, Germany). Combined blood metabolome (>850 metabolites), proteome (900 different proteins), clinical and epidemiological data will be analyzed (University of Tartu, Estonia) in order to construct diagnostic/prognostic algorithms for early diagnosis of EC and to identify patients with low/high risk for cancer progression and recurrence.
Results: BioEndoCar project has defined inclusion/exclusion criteria and a strict standard operating procedure for sample collection, processing and sample storage that is followed by in all three medical centers. Since the beginning of the project (April 1st 2018) we recruited nearly 100 patients in total. Great effort was put into informing the lay and expert public about the importance of the translational studies in EC. We have established an official website (https://bioendocar.eu/) and Facebook page where we post all news concerning the project.
Conclusion: We expect to find different metabolic and protein profiles in patients with early stages of EC as compared to controls and in patients with poor prognosis with high risk of disease progression and recurrence as compared to those with favorable prognosis. The BioEndoCar project is currently ongoing.
Disclosure: Nothing to disclose

EP556

Decreased s-Tie2 plasma concentration in patients with endometrioid endometrial cancer

T. Knific[1], L. Roškar[2], Š. Smrkolj[2], T. Lanišnik Rižner[1]
[1]*Laboratory for Molecular Basis of Hormone-Dependent Diseases and Biomarkers, Institute of Biochemistry, Faculty of Medicine, University of Ljubljana,* [2]*Department of Obstetrics and Gynecology, University Medical Centre Ljubljana, Ljubljana, Slovenia*

Introduction / Background: Endometrial cancer (EC) is the most frequent gynecological malignancy in more developed countries. Although the majority of patients are diagnosed at an early stage and cured by hysterectomy, the cancer recurs in about 20% patients with no sign of advanced/metastatic disease with limited response to systemic therapy. Novel biomarkers for EC would be invaluable for preoperative stratification of patients as low/high-risk, detection of primary and recurrent disease and thus enabling personalized therapeutic approaches. Angiogenesis plays an important role in the development and progression of several types of cancer, including EC. This process of is controlled by angiogenic factors (AF). Currently, AF have been studied in some malignancies but their role in EC has not been determined yet. In our study, we analyzed plasma concentration of 20 different AF in patients with the endometrioid type of EC.
Methodology: Prospective case - control study of women who underwent surgical treatment at the University Medical Centre Ljubljana (38 patients with endometrioid EC, 38 control patients with prolapsed uterus or myoma). Plasma samples were measured using commercially available kit that enables simultaneous determination of 20 different AF with the Luminex xMAP multiplexing technology.
Results: Plasma levels of sTie-2 (soluble angiopoietin-2) showed significantly decreased levels in patients with EC compared to the control group ($p < 0.05$). Tenascin C and angiostatin showed decreased levels in patients with EC and present lymphvascular invasion or deep myometrial invasion (DMI), respectively, while osteopontin levels were increased in patients with EC with DMI, but these differences did not reach statistical significance. Based on different grade of endometriod EC neuropilin showed significant difference.
Conclusion: Results of our pilot study indicate that plasma levels of different AF might have a diagnostic and/or prognostic value in endometrioid EC. Further statistical evaluation is currently ongoing.
Disclosure: Nothing to disclose

EP557

Efficacy and survival outcomes of minimally invasive versus open pelvic and para-aortic lymphadenectomy in early stage endometrial cancer

H.S.J. Lee[1], L. Wong[2], S.Y. Yeung[2], S.F. Yim[2]
[1]*Obstetrics and Gynaecology,* [2]*Chinese University of Hong Kong, Hong Kong, Hong Kong*

Introduction / Background: Minimally invasive surgery in endometrial cancer has advantages over open surgery in terms of less morbidity, shorter hospitalization and quicker recovery. Many centers have adopted it as first line management for endometrial cancer. Minimally invasive lymphadenectomy is technically demanding and more evidence is required on whether this approach yields comparable oncological outcomes to open surgery. Our aim is to compare the efficacy and survival outcomes of minimally invasive and open pelvic and para-aortic lymph node dissection in early stage endometrial cancer.
Methodology: Stage I to II endometrial cancer patients with pelvic and/or para-aortic lymph node dissection performed in Prince of Wales Hospital in Hong Kong from January 2007 to April 2014 were included. Operative records, pathology reports and follow up notes were reviewed retrospectively.
Results: A total of 94 endometrial cancer patients with open/ laparoscopic/ robotic pelvic+/-para-aortic lymphadenectomy performed were included. Five year follow up was completed in 89 patients (94%). The two groups were comparable in demographics as shown in Table 1, except the open group had

more stage II disease and larger uterine size. The 5 year disease free survival and 5 year overall survival of the 2 groups were similar. Open surgery was associated with more blood loss (p=0.01) and longer hospital stay (p=0.00), whereas minimally invasive surgery was associated with longer operating time (p=0.00). The number of pelvic/ para-aortic lymph node obtained, surgical complication and lymphedema/lymphocyst formation did not differ significantly. None of the factors such as stage or grade was found to affect the efficacy of minimally invasive pelvic lymph node dissection.
Conclusion: Minimally invasive surgery pelvic/ para-aortic lymphadenectomy in early stage endometrial cancer is as effective as open surgery with similar survival, complication rate and number of lymph nodes obtained. It has advantages of shorter hospital stay and less blood loss.

	Minimally Invasive Surgery (n=43)	Open Surgery (n=51)	P-value
– Age (mean+/-SD) – Parity (mean+/-SD) – Caesarean section (mean+/-SD)	– 55.7 +/-7.31 – 1.62+/-1.31 – 0.3+/-0.62	– 55.37 +/- 8.1 – 2.0 +/-1.76 – 0.31 +/-0.79	– 0.83 – 0.29 – 0.96
Stage	– Stage 1A: 63% (27/43) – Stage IB: 30% (13/43) – Stage II: 7% (3/43)	– Stage 1A: 56.9% (29/51) – Stage 1B: 15.7% (8/51) – Stage II: 27.5% (14/51)	0.02
Grade	– Grade 1: 52.4% (22/42) – Grade 2: 26.2% (11/42) – Grade 3: 21.4% (9/42) – Missing: 1	– Grade 1: 43.8% (21/48) – Grade 2: 35.4% (17/48) – Grade 3: 20.8% (10/48) – Missing: 3	0.18
Histology	– Endometrioid: 95.2% (40/42) – Serous adenocarcinoma: 2.4% (1/42) – Undifferentiated adenocarcinoma: 2.4% (1/42) – Missing: 1	– Endometrioid: 92.2% (47/51) – Serous adenocarcinoma: 2% (1/51) – Undifferentiated adenocarcinoma: 2% (1/51) – Clear cell carcinoma: 3.9% (2/51)	0.48
Lymph node enlarged on MRI	– Pelvic lymph node enlarged: 17.1% (7/41) – Para-aortic lymph node enlarged: 0% (0/40)	– Pelvic lymph node enlarged: 36% (18/50) – Para-aortic lymph node enlarged: 2.3% (1/44)	0.08 0.28
Radical hysterectomy	4.7% (2/43)	11.8% (6/51)	0.39
LVSI present	26.5% (9/34)	28.6% (10/35)	1
– Adjuvant vault radiotherapy – Adjuvant external radiotherapy – Chemotherapy	– 33.3% (14/42) – 9.5% (4/42) – 7.1% (3/42)	– 37.3% (19/51) – 25.5% (13/51) – 9.8% (5/51)	– 0.34 – 0.09 – 0.93
– Recurrence – 5 year disease free survival – 5 year overall survival	– 14% (6/43) – 80% (31/39) – 87% (34/39)	– 5.9% (3/51) – 92% (46/50) – 94% (47/50)	– 0.33 – 0.16 – 0.46

[Table 1. Demographics and Oncological Parameters]

	Minimally Invasive Surgery (n=43)	Open Surgery (n=51)	P-value
Time (mins)	240.59+/-76.97 – Laparoscopy (n=37): 222.81 +/- 64.41 – Robotic (n=6): 350 +/-56.48	182+/-58.76	0.00

Blood loss (ml)	249.71 +/-177.8 – Laparoscopy (n=38): 220.83 +/- 135.06 – Robotic (n=5) : 330 +/- 216.79	416.67+/-367.38	0.01
Complication	2.3% (1/43) – 1 bowel serosal injury	7.8% (4/51) – 2 wound infection – 1 bowel serosal injury – 1 post-operative ileus	0.37
Lymphedema/ Lymphocyst	12.5% (5/40)	2% (1/50)	0.08
Hospital stay (days)	4.52 +/- 2.01 (n=42)	7.62 +/-2.77 (n=50)	0.00
Pelvic lymph nodes number	22.5 +/-10.37 (n=40)	24.06 +/- 12.43 (n=48)	0.53
Para-aortic lymph nodes number	14.8+/-7.11 (n=10)	18.5+/-11.06 (n=14)	0.36
Uterine weight (g)	158.02 +/-184.42	405+/-338.78	0.00
Uterine tumour size (cm)	3.14+/-1.62	3.94+/-2.34	0.07

[Table 2. Intra-operative Parameters and Factors Affecting Efficacy of Minimally Invasive Pelvic Lymph Node Dissection]

Disclosure: Nothing to disclose

EP558

Favorable factors of surgical resection for recurrent uterine leiomyosarcoma

E.J. Lee, H.S. Kim, G.W. Yim, J.W. Kim, N.H. Park
Obstetrics and Gynecology, Seoul National University Hospital, Seoul, Republic of Korea

Introduction / Background: The known prognostic factors of uterine leiomyosarcoma (LMS) are initial stage, tumor size, cellular division (mitotic cell count), tumor grade, etc. In LMS, only radically surgical removal offers a chance of long-term survival. Despite complete surgical resection, about 70% develop recurrence within 8 to 16 months after initial diagnosis (even at stage 1 or 2). Even though there are several researches about prognostic factors of recurrent LMS, no definite conclusions that what the prognostic factors for recurrent LMS is. The objective of this research is to evaluate favorable factors of surgical resection for recurrent LMS.
Methodology: Clinical and pathological data of 38 patients with recurrent LMS who were treated in one center (Seoul National University Hospital) were analyzed retrospectively to identify prognostic factors for their overall survival (OS) and progression-free survival (PFS). Survival curves were generated using the methods of Kaplan and Meier analysis.
Results: The OS was 3.95 years and the median time of first PFS was 17 months and second PFS was 3.4months. Mitotic count in tumor tissues and time to first recurrence were correlated with OS, but recurrent pattern was more important in time to second recurrence. Localized first recurrent site, solitary recurrence, second recurrent site which was limited in intra-pelvic were significantly associated with longer time to second recurrence.
Conclusion: Uterine LMS is a rare disease with extremely poor prognosis. For selected patients who have smaller mitotic count, time to first recurrence, localized and solitary recurrent site, and pelvis-limited second recurrence, secondary cytoreductive surgery can be beneficial and may prolong survival in selected.
Disclosure: Nothing to disclose

EP559

Pelvic vs. para-aortic lymphadenectomy for patients with type I endometrial cancer: a comparison of outcomes

L. Li, Z. Li
West China Second University Hospital, Sichuan University, Chengdu, China

Introduction / Background: To compare surgery outcomes and prognosis for patients with type I endometrial cancer undergoing hysterectomy and bilateral salpingo-oophorectomy (H-BSO) with or without systematic pelvic lymphadenectomy (PLD) or para-aortic lymphadenectomy (PALD).
Methodology: Patients with Type I endometrial cancer in West China Second University Hospital between January 2010 and June 2012 were comprehensively reviewed. Patients with synchronous cancer, synchronous surgery, or neoadjuvant therapy were excluded. Patients were objectively triaged to no-lymphadenectomy, PLD or PALD based on frozen section diagnosis. All patients received telephone follow-up. Surgery outcomes and prognosis were compared among H-BSO, PLD and PALD groups.
Results: Total 333 patients met the inclusion criteria and 291 (87%) patients got effective follow-up data. In all patients, 121 (36%) had PLD, 166 (49%) had PALD and remaining 46 (14%) only had H-BSO. Based on preoperative assessment, there was no significant difference in age, menopause, body mass index among three groups (P>0.05), while H-BSO had higher Charlson Index (P= 0.005). According to surgery and pathology data, PALD had higher laparotomy rate (P=0.00) than other two groups, H-BSO had lower operation time (P=0.001) and blood loss (P< 0.001) while no difference existed between PLD and PALD. There was no significant difference in ascites, blood transfusion rate, FIGO pathology grade, tumor diameter among three groups (P>0.05). Follow-up information showed that three groups had no difference in overall survival, univariate and multivariate Cox regression model survival analyses showed that para-aortic lymphadenectomy was not a risk factor to type I endometrial cancer patients.
Conclusion: Utilization of para-aortic lymphadenectomy for endometrial cancer results in similar surgical and oncologic outcomes and high laparotomy rate to H-BSO combined pelvic lymphadenectomy. The benefit of PALD should be further considered.
Disclosure: Nothing to disclose

EP560

Rare pathology of a malignant uterine myxoid leiomyosarcoma with osteocartilaginous differentiation

Y.H. Lim[1], M.Y. Leong[2], C.L. Ong[3], W.L. Wong[1]
[1]Department of Gynaecological Oncology, KK Women's and Children's Hospital, Singhealth, [2]Department of Pathology, KK Women's and Children's Hospital, Singhealth, [3]Department of Diagnostic Imaging, KK Women's and Children's Hospital, Singhealth, Singapore, Singapore

Introduction / Background: Uterine leiomyosarcomas are a rare form of uterine tumours and are often associated with poor survival prognosis. Heterologous differentiation in leiomyosarcomas is an extremely rare phenomenon with less than 15 cases reported in literature to date. We report a case of primary myxoid leiomyosarcoma of the uterus with osteocartilaginous differentiation.
Methodology: A 44-year old Chinese female first presented to our hospital with complaints of irregular heavy periods and was found to have iron-deficiency anaemia. She underwent a dilatation and curettage which revealed a benign endometrial polyp on a background of late proliferative endometrium. A pelvic ultrasound was performed and she was found to have multiple uterine fibroids with the largest one measuring 4.8cm. She opted for conservative management but repeated follow up scans showed the fibroid enlarging in size to 8.9cm over a period of 2 years. In view of enlarging size of fibroid, she was advised for surgery and underwent a total abdominal hysterectomy and bilateral

salpingo-oophorectomy.
Results: Final histology showed a malignant neoplasm with areas of myxoid leiomyosarcoma and smaller areas of osteosarcomatous and cartilaginous differentation, raising the possible differential diagnosis of malignant mesenchymoma arising from the uterus. She was discussed at the gynae-oncology tumour board meeting and conclusion favoured a myxoid leiomyosarcoma. She was offered complete surgical staging but she declined. Her post-operative CT scan of the chest, abdomen and pelvis was clear with no evidence of lymphadenopathy or distant metastasis. She has remained disease-free for 18 months so far.
Conclusion: Osteocartilaginous differentiation of a malignant uterine myxoid leiomyosarcoma is very rare and very few such cases have been reported in literature. This unique case emphasizes the importance of clinical correlation with radiological and pathological findings in confirming the diagnosis of tumours with unusual histology and presentation.
Disclosure: Nothing to disclose

EP561

The effect of combined progesterone and GnRH agonist on the growth of endometrial cancer cells

S. Lim[1], K.-B. Lee[1], I.-S. Hong[2], S.-H. Lee[1]
[1]Gachon University Gil Medical Center, College of Medicine, [2]Gachon University College of Medicine, Incheon, Republic of Korea

Introduction / Background: Endometrial cancer can occur in young women who need to preserve their fertility. Progestin based hormonal therapy including oral progestin or levonorgestrel-containing intrauterine system can be used in this setting. We performed the present study to evaluate the effect of combined progesterone and GnRH agonist on the growth of endometrial cancer cells.
Methodology: Endometrial cancer cells were obtained from the Korean cell line bank and were cultured in DMEM supplemented with 10% fetal bovine serum (FBS), 100 U/ml penicillin and 100 U/ml streptomycin at 37°C and 5% CO_2. The MTT assay was used to determine the anti-proliferative capacity of GnRH or progesterone, according to the manufacturer's protocol. Cells (1×10^4cells/well) were seeded in 96-well plates. After 24 h of incubation, the cells were treated with GnRH or progesterone for 72 h. The viable cells were measured at 570 nm using a VersaMax microplate reader.
Results: Growth potential was not changed after treatment of progesterone alone at any concentration. GnRH agonist alone and combined progesterone and GnRH agonist showed concentration-dependent growth inhibition of endometrial cells. Growth potential showed 0.8, 0.7, and 0.6 fold change compared control at concentration of GnRH agonist 100nM, 500nM, and 1µM, respectively ($p< 0.05$). Growth potential showed 0.8, 0.7, and 0.5 fold change compared control at concentration of combine progesterone and GnRH agonist 100nM, 500nM, and 1µM, respectively ($p< 0.05$). However, there was no difference of growth potential between GnRH agonist and combined progesterone and GnRH agonist.
Conclusion: GnRH agonist and combined progesterone and GnRH agonist showed a significant inhibited endometrial cancer cell growth by concentration-dependent fashion. Progesterone alone did not show cancer cell growth inhibition.
Disclosure: Nothing to disclose

EP562

Incidence of isolated tumor cells after sentinel lymph node ultrastaging in early stage endometrial cancer

V. Locati[1], E. Corvetto[1], M. Fais[1], M. Russo[1], A. Proto[1], G. Candotti[1], S. Angioni[1], D. Fanni[2], V. Mais[1], M. Peiretti[1]
[1]Department of Obstetrics and Gynecology, [2]Department of Pathology, University of Cagliari, Cagliari, Italy

Introduction / Background: To describe the accuracy and detection rate of sentinel lymph node (SLN) identified by dual cervical indocyanine green (ICG) injection, underlining the incidence of Isolated Tumor Cells (ITC) following SLN ultrastaging.
Methodology: Between December 2015 and May 2019, 40 women with early stage Endometrial Cancer (EC) were scheduled for total laparoscopic or laparotomic hysterectomy, bilateral oophorectomy and SLN detection.
Results: A total of 40 procedures was performed. The patient mean age was 65 years (SD 12.2) and mean body mass index was 26 kg/m2 (SD 6.9). Histological types were endometrioid adenocarcinoma in 29 cases (73%), 7 endometrial hyperplasia (19%), 3 serous adenocarcinoma (5%), 1 mixed histology (3%).
The detection rates were as follow: 97% (39/40) overall for SLNs; unilateral and bilateral mapping of SLNs were achieved in 20% and 72% of subjects respectively (undetermined laterality in 2 cases and unobserved SLN in 1 case). The main lymphatic stations were external iliac (57%) and interiliac (45%). Positive SLNs were 8, identified in 7 out of 40 patients (17%), including 4 patients with ITC (57% - 4/7) (2 in the EI area, 1 in the II area, 1 in the interiliac area). The oncological risk of ITC positive patients was in 1 low, 2 intermediate and 1 high/intermediate risk cases. All patients with ITC were discussed by a multidisciplinary team and none of them received adjuvant therapy. Five months after surgery, 1 patient presented inguinal node and vaginal cuff relapse. She underwent EBRT& BRT with a complete response. At this moment all these patients are free of disease
Conclusion: SLN ultrastaging increases the number of nodes considered positive, especially the identification of ITC. In our population the overall incidence of ITC was 10% and in those who had positive SLN, ITC were detected in 57%. We observed in these patients an excellent prognosis.
Disclosure: Nothing to disclose

EP563

Mismatch repair deficiency glands in normal endometrial tissue as a predictor of endometrial carcinoma in patients with Lynch syndrome

P. Lof[1], C.A.R. Lok[1], P. Snaebjornsson[2], W.G. McCluggage[3], J. Van Dorpe[4], P. Tummers[5], K.K. Van de Vijver[6]
[1]Gynecology, [2]Pathology, Netherlands Cancer Institute - Antoni van Leeuwenhoek, Amsterdam, The Netherlands, [3]Pathology, Belfast Health & Social Care Trust, Belfast, United Kingdom, [4]Pathology, [5]Gynecologic Oncology, Ghent University Hospital, Gent, Belgium, [6]Pathology, Ghent University Hospital, Gent, The Netherlands

Introduction / Background: About 97-100% of Lynch syndrome (LS) associated EC are mismatch repair deficient (dMMR). Previous studies demonstrated the presence of dMMR glands in normal endometrial tissue from LS patients whereas non-LS patients had only MMR proficient (pMMR) glands. We investigated whether there is an association between dMMR glands in normal endometrial tissue and subsequent development of EC in patients with LS.
Methodology: Patients with LS or Lynch-like syndrome in which endometrial tissue was obtained to screen for EC, were retrospectively included. MMR immunohistochemistry was performed and follow-up data was collected regarding development of EC.
Results: We included 54 patients in two centers between March 2004 and April 2019. Mean age at time of biopsy was 46 years (SD 9 years) and was not significantly different between the dMMR and pMMR group. Median follow-up time was 29 months (IQR 11 - 73 months). In the dMMR group (n=10) two patients underwent preventive hysterectomy and three out of remaining eight patients (38%) developed EC. Time between biopsy and developing EC ranged from 10 - 49 months. Follow-up time of the patients who were still being screened ranged from 19 - 40 months.
In the pMMR group (n=44) 12 patients underwent a preventive hysterectomy. Two patients out of remaining 32 (6%) developed dMMR EC at 59 and 119 months after normal biopsy.

Conclusion: We show that dMMR glands can already be detected in endometrial tissue without any (pre)malignant lesion in patients with LS. Although the numbers are small, patients in which dMMR glands were detected appear to have a higher chance of developing EC within four years compared to patients with only pMMR glands. The presence of dMMR glands in normal tissue could therefore be a potential predictive marker for the development of EC in patients with LS. These findings should be validated prospectively in larger cohorts.
Disclosure: Nothing to disclose

EP564

Sentinel lymph node biopsy using indigocyanine green fluorescence in uterine cancer

G.-L. Looser[1], J. Welter[1], W. Sell[2], A. Fleischmann[3], M.K. Fehr[1]
[1]Gynaecology and Obstetrics, Kantonsspital Frauenfeld, Frauenfeld, [2]Gynaecology and Obstetrics, [3]Pathology, Kantonsspital Münsterlingen, Münsterlingen, Switzerland

Introduction / Background: Professional societies recommend sentinel lymph node biopsy using indigocyanine green (ICG-SLNB) as a staging strategy in endometrial cancer. According to a meta-analysis, ICG as a tracer injected into the cervix yields higher detection rates than other tracers or injection sites. Compared to standard systematic lymphonodectomy (LND), ICG-SLNB has lower morbidity, allows detection of lymph nodes (LN) in atypical locations and allows microstaging of LN.
Methodology: This retrospective analysis included 40 uterine cancer patients having ICG-SLNB, using conventional laparoscopy or robotic surgery from 2012-2019. ICG was injected into the cervix (1ml subepithelial, 1ml stromal). Two optical systems were used: Karl Storz Opal-1 NIR/ICG (n=10) and Novadaq Pinpoint (n=30). Pathological ultrastaging was done at 2mm intervals with cytokeratin staining. Technical failures, detected SLN and locations were assessed. Technical failures were defined as no LN staining or only unilateral pelvic staining. We also compared sites of LN metastasis using ICG-SLNB to 16 endometrial cancer patients having metastatic LN assessed by SLNB.
Results: Five technical failures occurred (12.5%). Median number of sentinel LN found using Pinpoint was 10 (range 2-45) and using Opal-1 was 2 (range 1-5), which was significantly less ($p< 0.002$). More technical failures occurred using Opal-1 (30% vs 7%, $p=0.09$). A total of 371 LN were detected (17 metastatic). The most frequent site of SLN was the fossa obturatoria (154, 42%), iliaca externa (107, 29%), iliaca communis (50, 13%), and 12% (44) in the paraaortic region. Nine (2%) SLN were in the parametrium and 7 (2%) in the presacral area, hence, 4% of SLN were found in atypical locations. Metastatic LN location was similar using ICG-SLNB compared to LND.
Conclusion: The imaging device impacts both detection and technical failure rates. 4% of SLN were in atypical locations, however none were metastatic. The pattern of nodal spread was similar in both ICG-SLN and systematic LND.
Disclosure: Nothing to disclose

EP565

Diagnostic performance of transvaginal ultrasonography in the preoperative staging of endometrial cancer

A. Lubrano[1], V. Benito[1], I. Garcia[1], J. Perez[2], M. Andujar[3]
[1]Gynecology and Obstetrics, [2]Radiology, [3]Pathology, Complejo Hospitalario Universitario Materno Infantil de Canarias, Las Palmas, Spain

Introduction / Background: Endometrial cancer is the most common malignant neoplasm of the female genital tract in developed countries, with a prevalence that is increasing. Preoperative assessment of the degree of myometrial invasion is of great importance when choosing the most

appropriate treatment in terms of extension of hysterectomy and sampling of pelvic and / or para-aortic lymph nodes.
Objectives: To determine the value of transvaginal ultrasound to diagnose the degree of myometrial invasion in the preoperative staging of endometrial cancer, comparing the results with the MRI and the anatomical part of pathology, which is the standard gold.
Methodology: Retrospective observational study performed between January 2012 and December 2014. We included women with endometrial cancer diagnosed and treated at the Obstetrics and Gynecology Service of Las Palmas de Gran Canaria, where ultrasound and MRI were performed in the staging preoperative. Patients who underwent surgical treatment due to the need for an anatomical piece were included.
Results: A total of 164 patients were included, with a mean age of 66 years. The endometrioid histological type was present in 82.9% of the patients. 64.2% of the patients had an IA stage at the time of diagnosis and 17.5% had an IB stage. On the other hand, they showed a G1 53.7%, G2 18.3% and G3 27.4%. 12.2% of the patients presented relapse, living 86% of the patients without disease. The percentage of deaths is 6.1%. Transvaginal ultrasound showed a sensitivity of 60% and a specificity of 83.2%, while MRI had a sensitivity of 66% and a specificity of 80%.
Conclusion: Transvaginal ultrasound is a useful, accessible and inexpensive method for the study of myometrial infiltration in Endometrial Cancer, without significant differences with the results obtained with Magnetic Resonance Imaging (MRI). However, the final stage gives us the anatomical piece.
Disclosure: Nothing to disclose

EP566

Location of sentinel lymph nodes in endometrial cancer in a series of 207 patients with dual tracer injection in cervix and uterine fundus

M. Mancisidor, C. Mateos, R. Ruiz, M. Gorostidi, M. Rezola, A. Lekuona
Hospital Universitario Donostia, Donostia, Spain

Introduction / Background: Sentinel lymph node biopsy (SLNB) in endometrial cancer is not standardized yet. Multiple studies have been published using different tracers and injection sites. Most autors prefer cervix injection because of its simplicity, reproducibility and high detection rate, despite detecting few sentinel lymph nodes (GC) in the aortic area. We present our data on location of GCs injecting the green indocyanine tracer (ICG) into both the cervix and uterine fundus in order to increase the detection of GC in the aortic area.
Methodology: We included 207 patients intervened from 2014 to 2018. We injected 2 ml of ICG into the uterine fundus through the cervix and another 4 ml in the cervix, 2 ml at 9 o´clock and 2 ml at 3 o´clock. Then we locate and selectively biopsy the GC, first in the aortic area, after in the pelvic area. We established 10 aortic zones (laterocavo, precavo, interaortocavo, preaortic and lateroaortic, at supramesenteric and inframesenteric zones) and 11 pelvic zones (common iliac, external iliac, interiac, obturator, and pararectal at right and left hemipelvis and also presacral zone). We consider as positive GC macrometastases, micrometastases or isolated tumor cells.
Results: We found 822 GC, 525 pelvic (64%) and 297 aortic (36%). Detection rate was 89.9%, 65.7% and 64.3% for unilateral pelvic, bilateral pelvic and aortic respectively. Of the 60 positive GC, 38 were pelvic and 22 aortic. 7.3 % of GC were positive, similar in both areas (7.24% and 7.4%).
Conclusion: We found less GC in the aortic than pelvic area, even injecting the tracer also in the uterine fundus. However, the percentage of positive GCs in both areas was similar. These data reaffirm the importance of studying the aortic area when performing BGC in endometrial cancer and also injecting the tracer in the uterine fundus to obtain significant detection rates.

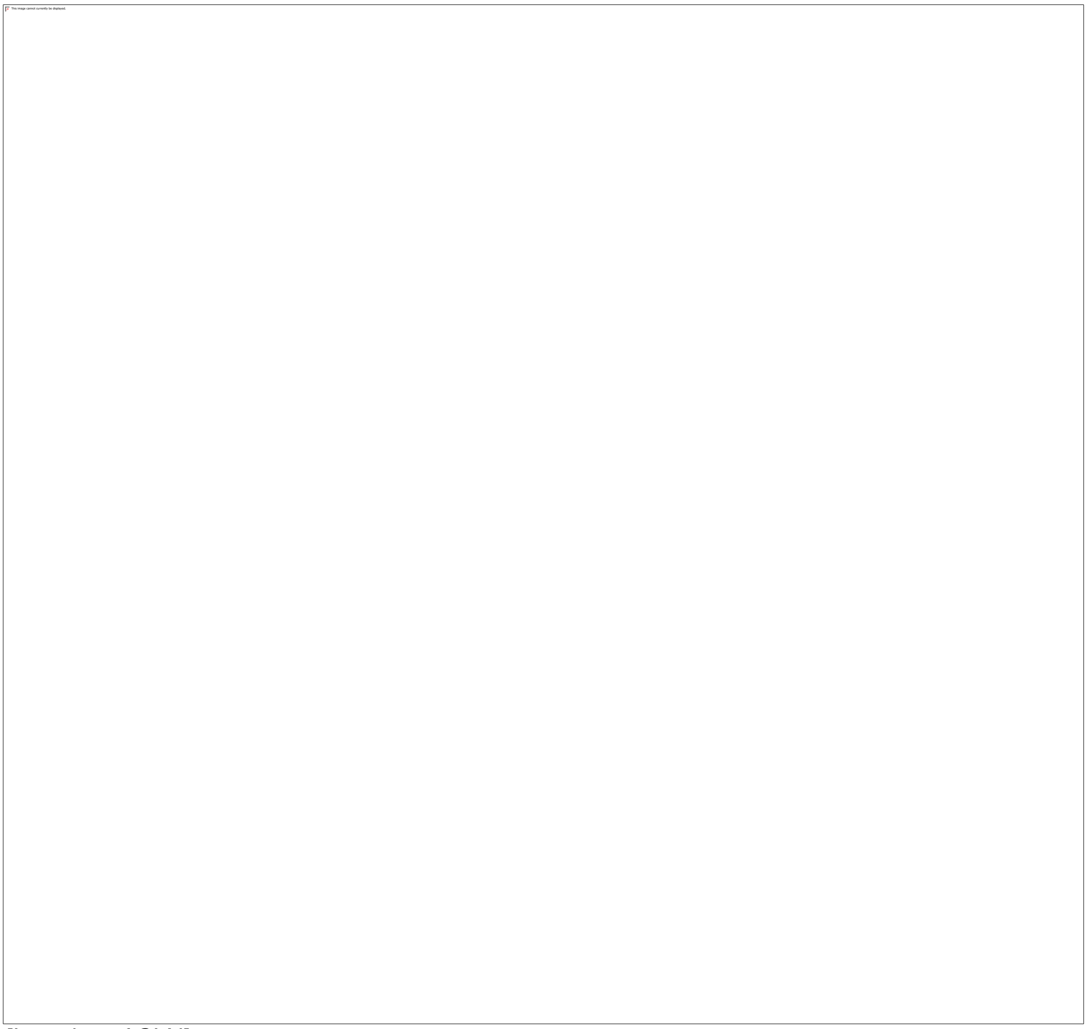

[Location of SLN]

Disclosure: Nothing to disclose

EP567

Low-volume disease, treatment and results in endometrial cancer

M. Mancisidor Iztueta, I. Aizpuru Olalde, M. Gorostidi Pulgar, R. Ruiz Sautua, A. Juaristi, P. Cobas Lozano, A. Lekuona Artola
Donostia Hospital, Donostia, Spain

Introduction / Background: Sentinel node mapping and pathological ultrastaging has increased the detection of low-volume disease (LVD). The prognostic value and appropriate adjuvant treatment of LVD is still controversial in endometrial cancer (EC).
Methodology: 207 consecutive patients undergoing surgery for EC between June 2014 and December 2018 at the Donostia University Hospital in San Sebastian (Spain) were studied. All underwent sentinel lymph node biopsy after a double injection, both in the uterine fundus and cervix, of indocyanine green. Use of adjuvant treatment and overall and recurrence free survival were

analyzed among those showing nodal disease.
Results: Node metastasis was found in 38 patients (18.4%) of which 18 (47.4%) were macrometastasis and 20 (52.6%) were LVD; 8 (21.1%) micrometastasis and 12 (31.6%) isolated tumor cells. Fifteen (75%) of patients with LVD received adjuvant treatment, less than patients with macrometastasis (94.4%).
Overall survival was higher in LVD group compared to patients with macrometastasis (p 0.026). 94.1% and 72.2% of patients with macrometastasis were alive after one and two-year follow-up respectively, while no deaths were reported in patients with LVD in this period.
With a mean follow-up of 41.8 months, progression free survival was also higher (p 0.107) among patients with LVD (90%) compared to those with macrometastasis (66.7%). Both cases with recurrence in LVD group happened within the first year after surgery and showed high risk factors: grade 3 with myometrial invasion and type 2 tumor. Both of them received adjuvant therapy.
No cases of recurrence or death were found among patients with LVD that didn´t receive any adjuvant treatment.
Conclusion: Optimal treatment and prognosis in patients with endometrial cancer showing low volume nodal disease are not well defined. Implementation of a method which would allow clustering these cases for a better study of them would be useful.

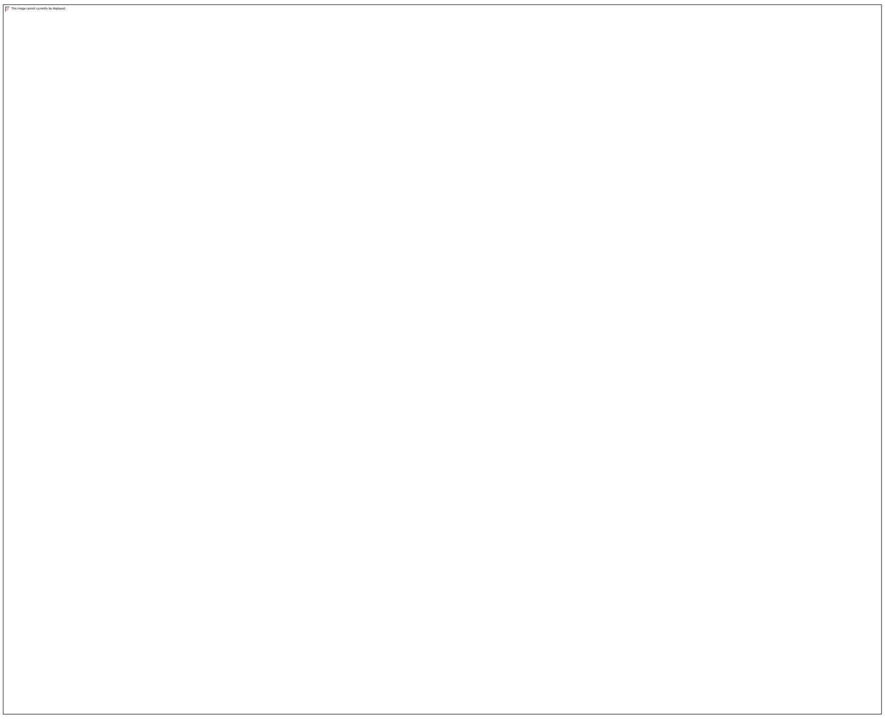

[Recurrence-free survival]

Adjuvant treatment	ITC	Micrometastasis	Macrometastasis	Total
No	3 (23.1)	2 (28.6)	1 (5.6)	6 (15.8)
Yes	10 (76.9)	5 (71.4)	17 (94.4)	32 (84.2)
Total	13	7	18	38

[Adjuvant treatment in node metastasis]

[Overall survival]

Disclosure: Nothing to disclose

EP568

Robotic paraaortic lymphadenectomy in oncogynecology. Double side docking of daVinci S system increases the success rates of high paraaortic lymph node dissection in endometrial cancer

R. Marek[1], V. Jančeková[2], J. Hambálek[2], M. Maděrka[2], P. Dzvinčuk[2], R. Pilka[2]
[1]Gynecology and Obstetrics, [2]Gynecology and Obstetrics Department, Palacky University, Olomouc, Czech Republic

Introduction / Background: To compare two different methods of staging robotic transperitoneal paraaortic lymphadenectomies in patients with early stages of endometrial cancer.
Methodology: In this retrospective study we enrolled 70 patients with early stages of endometrial cancer undergoing staging robotic surgery at the Department of Obstetrics and Gynecology, University Hospital Olomouc from January 2016 to March 2018. Primary systematic pelvic and paraaortic lymphadenectomy was suggested in all patients. In 39 out of 70 patients single docking was used for robotic staging surgery, whereas in 28 patients the procedure was done using double side docking approach. Number of patients with total and infra-renal suprapelvic lymphadenectomy, number of para-aortic lymphonodes retrieved and the rate of lymphadenectomy complications were compared.
Results: Robotic surgery was performed in 67 (96%) out of 70 patients. In three cases (0,4%) laparoscopy was converted to laparotomy. Single side docking was used in 39 cases (58%), whereas in 28 patients (42%) double side docking was used. Paraaortic lymhadenectomy was performed in 45 cases (67%). In 16 patients (24%) the
upper limit of the left renal wein was reached. Upper limit of paraaortic lymphadenectomy was above inferior mesenteric artery but did not reach left renal vein in 19 cases (28%). Inferior mesenteric represented upper limit of paraaortic lymphadenectomy in 10 patients (15%). Number of paraaortic lymphonodes retrived (4,9 ± 3,3 vs 3,7 ± 4,9, p = 0,028) as well as number of paraaortic

lymphadenectomies with upper limit at the left renal vein (p < 0,0001) was higher in double side docking
cases. Complication rates were low in both groups and the differences were not significant.
Conclusion: Number of lymphonodes retrieved as well as the number of paraaortic lymphadenectomy cases with upper limit at the left renal vein was higher in double side docking group.
Disclosure: Nothing to disclose

EP569

Potential role of PAI-1 in endometrial cancer progression and its regulation by epigenetic mechanisms

J. Marí-Alexandre[1], C. Agababyan[2], P. Pascual-Utiel[1], M. Cubertorer-Navarro[1], J. García-Oms[2], A. Estellés[3], J. Gilabert-Estellés[1,2,4]
[1]Laboratorio Investigación Biomarcadores en Reproducción, Ginecología y Obstetricia, Fundación de Investigación Hospital General Universitario de Valencia, [2]Servicio de Obstetricia y Ginecología, Hospital General Universitario de Valencia, [3]Hospital Universitario y Politécnico La Fe, [4]Departamento de Pediatría, Obstetricia y Ginecología, Universidad de Valencia, Valencia, Spain

Introduction / Background: Endometrial cancer (EC) is the most prevalent gyneacological cancer. It presents two subtypes, (Type-1 and Type-2), being the lately associated to worse prognosis and increased epithelial-to-mesenchymal transition (EMT). A growing evidence supports that uPA and PAI-1 play a role in solid tumour progression, though the exact role of PAI-1 and its epigenetic regulation in EC has not been completely established yet.
Methodology: 67 patients with EC and 36 control women were included. 5 miRNAs targeting PAI-1 mRNA (namely miR-99a-5p, miR-143-3p, miR-145-5p, miR-196b-5p y miR-301a-3p) were selected from miRNA expression profiles (Affymetrix, GeneChipmiRNA 2.0) and validated by RT-qPCR. mRNA levels of E-cadherin, Vimentin, Fibronectin, Snail1 and Twist were assessed by RT-qPCR; PAI-1 tissue and plasma levels by ELISA (DuoSet).
Results: At the tissue level, PAI-1 is over-expressed in EC in comparison to control endometria (CE) (88.67±12.65 *vs.* 65.89±9.39 pg/mg), and is significantly increased in Type-2 *vs.* Type-1 EC (124.43±18.31 *vs.* 65.90±13.57 pg/mg; p=0.02). At the plasma level, PAI-1 increases in EC vs. control plasma (3.06±0.34 *vs.* 0.99±0.19 ng/mL, p< 0.001), but is reduced in Type-2 *vs.* Type-1 EC (1.75±0.38 *vs.* 3.49±0.46 ng/mL; p=0.01). The levels of miR-196b-5p were significantly reduced in EC vs. CE (0.42±0.12 *vs.*1.00±0.27; p< 0.05), inversely correlating with PAI-1 levels, especially in Type-2 EC. Nevertheless, miR-301a-3p is significantly increased in EC vs. CE (1.90±0.30 *vs.* 1.00±0.16; p< 0.05), which could act via targeting PTEN. Finally, we observed decreased levels of E-Cadherin (1.00±0.12 vs. 0.57±0.20; p< 0.01) and increased levels of Snail1 and Twist1 in Type-2 *vs.* Type-1 EC (1.00±0.17 vs. 3.49±0.90; p< 0.01; 1.00±0.16 *vs.* 3.56±1.18; p< 0.01; respectively).
Conclusion: PAI-1 plasma and tissue levels are over-expressed in EC *vs.* CE, but only in tissues it is over-expressed in the worst-prognose subtype, which is associated with increased EMT markers. This local action of PAI-1 might be mediated by down-regulation of miR-196b-5p.
Disclosure: All authors have declared no conflicts of interest. This work has been supported by grants from ISCIII (PI17/01945). J. M-A. is supported by a FETH 2016-2018 award and a post-doctoral grant (APOSTD/2019/087).

EP570

Obturator nerve section during laparoscopic pelvic lymphadenectomy, what to do?

C. Martín Blanco[1], C. Del Valle Rubido[1], E. Cabezas López[1], B. Pérez Mies[2], E. Moratalla Bartolomé[1], M.C. Sánchez Martínez[1], J. Lázaro-Carrasco de la Fuente[1]

[1]Gynecology and Obstetrics Department, [2]Anatomical and Pathology Department, Ramon y Cajal Universitary Hospital, Madrid, Spain

Introduction / Background: The injury of the obturator nerve during pelvic oncologic surgery can be a stressful and unexpected complication during otherwise successful surgery. Injuries of obturator nerve can produce numbness or pain of the inner thigh or groin and weakened adduction of the thigh. The immediate repair of a freshly transected nerve can be done successfully, with good postoperative results.
Methodology: We present a case of patient with an endometrial cancer who suffered an obturator nerve section during laparoscopic comprehensive surgical staging surgery.
Results: The patient was a 57 year old women with a serous carcinoma of the endometrium who underwent comprehensive staging surgery. During the dissection of right pelvic lymphadenectomy, the obturator nerve was unintentionally sectioned at the level of the common iliac artery bifurcation. The defect was repaired by end-to-end suture with 5-0 prolene and tissucol sealant was added to reinforce de union. The patient did not developed any functional deficiency and the neurophysiological study performed after showed a complete recovery of the nerve.
Conclusion: Immediate repair of the obturator nerve by laparoscopic suture is a good solution with good postoperative results, in cases of nerve section during laparoscopic pelvic lymphadenectomy.
Disclosure: Nothing to disclose

EP571

Sentinel node mapping in endometrial cancer: analysis of more than 200 cases of hysteroscopic injection of tracers

F. Martinelli, A. Ditto, G. Bogani, U. Leone Roberti Maggiore, V. Chiappa, M. Evangelista, V. Liberale, F. Zanaboni, F. Raspagliesi
Gynecologic Oncology, Fondazione IRCCS Istituto Nazionale dei Tumori di Milano, Milan, Italy

Introduction / Background: To report on performance of hysteroscopic-injection of tracers (ICG and Tc99m) for sentinel-nodes (SLNs) mapping in endometrial cancer.
Methodology: A single center retrospective evaluation of Prospectively collected data of Consecutive patients with endometrial cancer underwent SLNs mapping following hysteroscopic-peritumoral-injection of tracer. Evaluation of Detection Rate (DR) (overall-bilateral-aortic) and diagnostic-accuracy (A) among the entire cohort and comparison between tracers.
Results: 231 procedures were performed. Mean age:60 years (28-84); mean BMI:27.3 Kg/m2 (15-47). In 164 cases (70.9%) hysterectomy and mapping procedure were performed laparoscopically. The overall-DR of the technique was 94.1% (208/221) (10 cases excluded: 9 equipment failure; 1 vagal reaction). Bilateral pelvic mapping was found in 62.5% of cases (130/208) and was more frequent in the ICG group (73.8% vs 53.3%; p:0.001). In 47.6% of cases (99/208) SLNs mapped both to pelvic and aortic nodes, and in 5 cases (2.5%) only in the aortic area. In 8 patients (3.8%) SLNs were found in aberrant (parametrial/pre-sacral) areas. Mean number of detected SLNs was 3.7 (1-8).
26 patients(12.5%) had nodal involvement: 12- (46.2%) macrometastases; 6-(23.1%) micrometastases; 8-(30.7%) ITCs. In 6 cases (23.1%) only aortic nodes were positive; in 6 cases (23.1%) both pelvic and aortic nodes and in 14 cases (53.8%) only pelvic area was involved.
Three false-negative results were found, all in the Tc99m group. All had isolated aortic metastases. Overall-sensitivity was 88.5%(95%CI:71.7-100) and overall-negative-predictive-value (NPV) was 98.4% (95%CI 91.8-100).
No differences in terms of overall-DR, overall-sensitivity and overall-NPV were found between the two tracers.
Conclusion: Hysteroscopic-injection of tracer for SLNs mapping in endometrial cancer is as accurate as cervical injection with a higher DR in the aortic area. ICG improves bilateral DR.
Disclosure: Nothing to disclose

EP572

Analysis of the histological prognostic factors for survival to take into consideration in the management of the endometrial cancer

E. Martínez Lamela[1], J. Molero Vílchez[2], G. Sánchez Rodillo[3], A. Santiago Gómez[3]
[1]Oncologic Gynecology, Hospital Ntra Sra del Rosario, Obstetrics and Gynecology, University Hospital Infanta Leonor, [2]Oncologic Gynecology, Hospital Ntra. Sra. del Rosario, Madrid, Oncologic Gynecology, Clinica TocoGyn, Alcalá de Henares, [3]Institute of Advanced Techniques against Cancer, Madrid, Spain

Introduction / Background: Our purpose is to evaluate the influence of the prognostic factors on the Disease-Free Survival (DFS) and Overall Survival (OS) in endometrial cancer (EC)
Methodology: Retrospective review of 180 patients with EC. Standard surgical staging was performed with lymphadenectomy in 130 cases. Mean age was 63 years (27-94). Pathology most common was endometrioid adenocarcinoma (84.5%). Most frecuent FIGO stage was IA (47.8%). Mean follow-up was 46 months. The factors that impact on survival were compared using univariate and multivariate analysis.
Results: The estimated 5-year DFS and OS comparated rates were respectively according to FIGO local stages (I) 93.9 and 90.2%, vs advanced stages (II-IV) 0 and 53.7% (p=0.00 Hazard ratio 5.58 (CI 95%, 2.74-11.35) and p=0.00 Hazard ratio 4.19 (CI 95%, 2.11-8.34), histological type I 92.3 and 89%, vs type II 28.1 and 58.3%% (p=0.00 Hazard ratio 2.91 (CI 95%, 1.3-6.57) and p=0, 01 Hazard ratio 2.6 (CI 95%, 1.17-5.77), histological low grade (G1) 89.1 and 92%, vs high grade (G2-3) 15.9 and 55.7% (p=0.00 Hazard ratio 5.79 (CI 95%, 2.42-13.85) and p=0.00 Hazard ratio 2.68 (CI 95%, 1.25-5.74); myometrial infiltration <50% 92.5 and 92.9%, vs >50% 63.9 and 66.1 (p=0.00 Hazard ratio 3.7 (CI 95%, 1.73-7.91) and p=0.00 Hazard ratio 4.31 (CI 95%, 1.92-9.7); in the abscence of lymphovascular space invasión (LVSI-) 94.4 and 89.7%, vs LVSI+ 0 and 62.6% (p= 0.00 Hazard ratio 5.88 (CI 95%, 2.92-11.85) and p=0.01 Hazard ratio 2.4 (CI 95%, 1.17-4.89); lack of cervical stromal invasion 94.3 and 91.4%, vs present 26.6 and 87.8% (p=0.00 Hazard ratio 4.33 (CI 95%, 1.54-12.12) and p=0.63) and tumor size <2 cm 88.1 and 89.9%, vs tumor size >2 cm 64.9 and 78.2% (p=0.00 Hazard ratio 4.02 (CI 95%, 1.33-12.15) and p=0.12)
Conclusion: The knowlegde of the histologic parameters is critical to the management of the EC

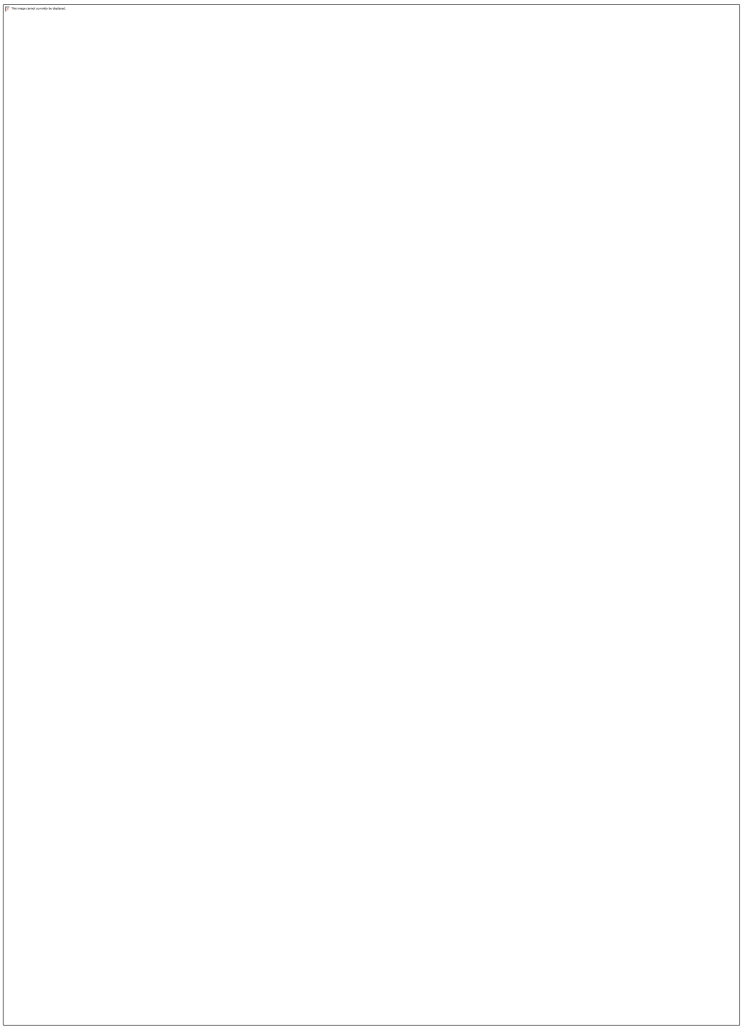

[Disease-Free Survival (DFS) for lymphovascular space invasion (A) and Overall survival (OS) for hist]

[Overall survival (OS) for miometrial invasion (A). Disease-Free Survival (DFS) for cervical stromal]

Disclosure: Nothing to disclose

EP573

The expression of L1CAM and β-catenin in endometrial carcinoma

R. Masadah[1], Y. Manule[1], S. Rauf[2]
[1]Pathology Anatomy, [2]Obstetric and Gynecology, Hasanuddin University, Makassar, Indonesia

Introduction / Background: Endometrial cancer is a fourth most common of female cancer and its prevalence tend to increase in the last decade especially in developing countries. It is important to gain the knowledge of endometrial cancer pathogenesis and metastasis in order to reduce the morbidity and mortality of this disease. L1 cell adhesion molecule (L1CAM) is a glycoprotein transmembrane that plays an important role in neurogenesis, regulation of cell-to-cell interaction, cell adhesion and migration. In cancer, L1CAM has been related to cell motility, invasion and metastasis. β-catenin is a protein coded by CTNNB1 gene in Wnt pathway, and it is important in cell proliferation, differentiation, and metastasis.

Methodology: We analyzed 49 tissue samples of endometrial type endometrial carcinoma by immunostaining using monoclonal antibody of L1CAM and β-catenin. The statistical test was perfomed between L1CAM expression score, β-catenin expression score, miometrial depth of invasion, lymphovascular invasion, and lymphnodes invasion
Results: We found that there was a significant correlation between L1CAM expression, β-catenin expression, and miometrial depth of invasion, lymphovascular invasion, lymphnodes invasion ($p < 0.05$) of endometrioid endometrial carcinoma
Conclusion: This study found that L1CAM and β-catenin are important marker of invasion and metastasis of endometrioid endometrial carcinoma
Disclosure: Nothing to disclose

EP574

Long term results of endometrial cancer stage Ib G1-2 treatment

S. Mavrichev, A. Shushkevich
Oncogynecology, N.N. Alexandrov Cancer Center of Belarus, Minsk, Belarus

Introduction / Background: In retrospective study asses long term results of treatment 548 patients with intermediate risk endometrial cancer in Belarus.
Methodology: In a retrospective study, we include results of treatment of 548 patients with intermediate risk endometrial cancer. We analyzed data from the Belarusian cancer register. Stage and sub-stage aligned with FIGO, 2009.
Results: The overall, cancer-specific and disease-free survival rate was 83.7±1,6; 91,2±1.2; 88.4± 1.4%. Extarfascial simple hysterectomy with bilateral salpingo-oophorectomy combined with radiotherapy such as extra-beam radiation therapy showed the best results of treatment. Adjuvant extra-beam radiotherapy after pelvic lymphnode dissection didn´t affect the survival rate and as the results are impractical. Chemotherapy didn´t improve the treatment results in the studied group. Despite the fact that treatment of EC stage Ib G1-2 In Belarus is standardize, we find the difference in survival rate between different regions. So, 5-year overall survival rate between regions with the highest and the lowest survival rate is 25% and the 5-year disease-free survival rate is 20%.
Conclusion: In regions where the survival rate is the lowest, we should pay attention to the technique of operation, regimen of radiation therapy and equipment for radiation therapy.
Disclosure: Nothing to disclose

EP575

Patterns of relapse in endometrial cancer

G. McCormick, R. Jyothirmayi, K. Nathan, J. Summers, C. Mikropoulos
Kent Oncology Centre, Maidstone, United Kingdom

Introduction / Background: The treatment of patients with high risk endometrial cancer remains controversial, with PORTEC 3 reporting no significant overall survival benefit in patients receiving chemoradiotherapy compared with radiotherapy alone. In this study we reviewed the outcomes of patients who received adjuvant radiotherapy (+/- chemotherapy) to review patterns of relapse.
Methodology: This retrospective study reviewed the clinical notes of 163 patients who were referred for adjuvant radiotherapy in Kent Oncology Centre, England between January 2012 - December 2016. Follow-up clinic appointments and scans were reviewed.
Results:

FIGO Stage	Number of Patients	Histology	% received adjuvant	% received adjuvant

			brachytherapy	chemotherapy
1a	2	100% Carcinosarcoma	0%	100%
1b	5	20% Carcinosarcoma 40% Endometriod 20% Mixed 20% Serous	20%	20%
2	7	86% Endometriod 14% Mixed	72%	14%
3a	2	50% Endometriod 50% Serous	0%	0%
3b	3	33% Carcinosarcoma 67% Serous	67%	67%
3c1	5	20% Carcinosarcoma 20% Clear cell 60% Endometriod	80%	20%
3c2	4	25% Carcinosarcoma 25% Endometriod 25% Missing record 25% Serous	25%	100%
4 (unspecified) and 4a	2	50% Carcinosarcoma 50%Serous	0%	100% (both 4 cycles pre-op; 2 cycles post-op)
4b	1	100% Carcinosarcoma	0%	100%

[Table 1]

163 patients received conformal adjuvant pelvic radiotherapy to a dose of 45Gy in 25 fractions. During the follow-up period 31 patients relapsed. The mean time to relapse was 14.7 months (range 1.3-37.6 months). Table 1 illustrates the staging, histology and treatments received by the patients who relapsed.
28 of the 31 patients with recurrent disease had widespread metastatic disease when diagnosed with recurrent disease, and 3 patients had local recurrence only. The sites of local recurrence, details of the histology and treatment received and site of recurrence in relation to the PTV and isodose level are illustrated in table 2.

Patient	Histology	Stage and grade at diagnosis	Site of recurrence	Did patient have brachytherapy?	Did patient receive adjuvant chemotherapy?	Relation to CTV and isodose
A	Endometriod	2 (Grade 2)	Left common iliac, external iliac and pre-sacral nodes	Yes	No	Within CTV; covered by 95%
B	Endometriod	2 (Grade 2)	Vagina	Yes	No	Inferior to CTV
C	Endometriod	3c1 (Grade 1)	Vaginal vault	Yes	Yes	Within CTV; covered by 95%

[Table 2]

Conclusion: The majority of patients with recurrent endometrial cancer relapsed with systemic disease. No patients with local recurrence only, relapsed within the radiotherapy CTV and all three patients with local relapse had endometriod histology. Although no overall survival benefit with systemic treatment was reported in Portec 3, our results are consistent with the Portec 3 findings that endometrial disease recurrence is usually a systemic event and local recurrence is rare. Our focus for improving outcomes from endometrial cancer should be on systemic therapies.
Disclosure: Nothing to disclose

EP576

A novel multivariable prediction model for lymphatic dissemination in endometrioid endometrial cancer: the lymph node metastasis risk index

M.M. Meydanli[1], K. Aslan[1], M. Oz[1], K.H. Muftuoglu[2], I. Yalcin[1], Y. Engin-Ustun[3]
[1]Gynecologic Oncology, [2]Department of Pathology, [3]Obstetrics and Gynecology, Zekai Tahir Burak Women's Health Training and Research Hospital, Faculty of Medicine, University of Health Sciences, Ankara, Turkey

Introduction / Background: The purpose of this study was to develop a risk assessment index that would determine endometrioid endometrial cancer (EC) patients who would benefit from lymphadenectomy.
Methodology: Final pathology reports of 353 women who underwent complete surgical staging including pelvic and para-aortic lymphadenectomy for endometrioid EC between January 2008 and June 2018 were retrospectively reviewed. Logistic regression was used to investigate clinicopathological factors associated with positive nodal status. Independent risk factors for lymphatic dissemination were used to build a risk model and “lymph node metastasis risk index” was defined as “(tumor grade) x (primary tumor diameter) x (percentage of myometrial invasion) x (pre-operative serum CA 125 level)”. The scores used in the “lymph node metastasis index” were weighted according to the odds ratios assigned for each variable. The lymph node metastasis risk index was calculated for each patient. The diagnostic performance of the model was expressed as sensitivity, specificity, positive predictive value, negative predictive value, positive likelihood ratio and negative likelihood ratio.
Results: The “lymph node metastasis risk index” correctly estimated 35 of 40 lymph node-positive women at a cutoff point of 981.0 (sensitivity 87.5%, specificity 86.3%, negative predictive value 98.2%, positive predictive value 44.9%, positive likelihood ratio 6.37, negative likelihood ratio 0.14). The area under curve of the receiver-operating characteristics was 0.90 (95% Confidence Interval 0.858-0.947) at this cutoff. The clinical accuracy of the model was 86.4%. When a cutoff point of < 981.0 was selected in order to define women at low-risk for lymphatic dissemination, our prediction model classified 275 women (77.9%) as being at low-risk for nodal involvement. Among these 275 women, 5 had actually positive lymph nodes; pointing out a 1.8% of false-negative rate.
Conclusion: After external validation, the “lymph node metastasis risk index” may be a valuable tool in the surgical management of endometrioid EC.
Disclosure: Nothing to disclose

EP577

Is it possible to develop a prediction model for lymphovascular space invasion in endometrioid endometrial cancer?

M.M. Meydanli[1], K. Aslan[1], M. Oz[1], K.H. Muftuoglu[2], I. Yalcin[1], Y. Engin-Ustun[3]
[1]Gynecologic Oncology, [2]Department of Pathology, [3]Obstetrics and Gynecology, Zekai Tahir Burak Women's Health Training and Research Hospital, Faculty of Medicine, University of Health Sciences,

Ankara, Turkey

Introduction / Background: The purpose of this retrospective study was to define a risk index that would serve as a surrogate marker of lymphovascular space invasion (LVSI) in women with endometrioid endometrial cancer (EC).
Methodology: Final pathology reports of 498 women who underwent surgery with curative intent for endometrioid EC between January 2008 and June 2018 were retrospectively reviewed. Logistic regression was used to investigate clinicopathological factors associated with positive LVSI status. Independent risk factors for LVSI were used to build a risk model and "risk of LVSI index" was defined as "tumor grade x primary tumor diameter (PTD) x percentage of myometrium involved". The scores used in the "risk of LVSI index" were weighted according to the odds ratios assigned for each variable. The risk of LVSI index was noted for each patient. The diagnostic performance of the model was expressed as sensitivity, specificity, positive predictive value (PPV), negative predictive value (NPV), positive likelihood ratio (PLR) and negative likelihood ratio (NLR).
Results: According to the "risk of LVSI index", presence of LVSI was correctly estimated in 89 of 104 LVSI-positive women at a cutoff of 161.0 (sensitivity 85.5%, specificity 79.4%, NPV 95.4%, PPV 52.3%, PLR 4.15, NLR 0.18). The area under curve of the receiver-operating characteristics was 0.90 (95% Confidence Interval 0.87-0.93) at this cutoff.
Conclusion: It seems possible to predict the presence of LVSI in women with endometrioid EC when the "risk of LVSI index" is calculated. However, external validation of this model is warranted.
Disclosure: Nothing to disclose

EP578

Detection of lymph vessel invasion and blood vessel invasion using double D2-40 and CD31 immunohistochemistry reveals independent prognostic significance of blood vessel invasion in endometrial cancer

A. Mihaljević Ferari[1], M. Klarić[2], M. Krašević[3], S. Eminović[3], J. Marušić[1], I. Plišić[2], G. Sopta[2], E. Babarović[3]
[1]Clinical Department of Radiotherapy and Oncology, [2]Clinical Department of Obstetrics and Gynecology, Clinical Hospital Center Rijeka, [3]Department Pathology, Faculty of Medicine, University of Rijeka, Rijeka, Croatia

Introduction / Background: Lymph-vascular space invasion (LVSI) is adverse prognostic factor in endometrial cancer (EC). Due to lack in histologic criteria and interobserver variability its role in administering adjuvant treatment and predicting recurrence isn´t well defined. The aim of our study was to utilize sensitive immunohistochemical method for separate detection of lymph vessel invasion (LVI) and blood vessel invasion (BVI) in patients with EC and to investigate their prognostic impact.
Methodology: We retrospectively identified from the institutional database 217 patients with EC and with complete follow-up, between 2002 and 2006. Tumor specimens were retrieved and two-colored double staining immunohistochemistry (D2-40/CD31) was performed to highlight lymphatic and blood vessels in the same slide. Tumors were divided in three groups: LVSI negative, LVI positive and BVI positive.
Results: In the original pathology reports, LVSI was misclassified in 36 (16.6%) tumors. In this study by using immunohistochemistry LVI was detected in 64 (29.5%) and BVI in 32 (14.7%) cases. All tumors with positive BVI also had accompanying LVI. BVI had the strongest impact on the risk for recurrence (OR 9.27 CI 4.47-19.22; $p< 0.0001$) and was, also significantly associated with decreased survival outcomes (overall survival, 59% compared with 96% in LVI group and 98% in LVSI negative group, $p< 0.0001$ and disease-free survival, 5-year rate 42% compared with 96% in both other groups, $p< 0.0001$) in univariate analysis. In multivariate analysis (including: age, tumor type, grade, stage, treatment) BVI remained independent prognostic factor for recurrence (HR 7.1 CI 2.9-16.8), overall survival (HR 8.2 CI 4.1-16.4) and disease-free survival (HR 6.2 CI 2.9-12.8).
Conclusion: Our results suggest that BVI could have greater clinical value in contrast to isolated LVI, and that therapeutic decisions should be based on its presence. We hope that these preliminary results will encourage further application of this inexpensive, readily available technique.
Disclosure: Nothing to disclose

EP579

Our experience with sentinel lymph node mapping in endometrial cancer

M. Miro Matos, C. Millan Lomana, A. Vegas Carrillo de Albornoz, I. López Carrasco, M. Perez Requena, N. Montero Pastor, E. Moratalla Bartolomé
HM Hospitales—Centro Integral Oncológico Hospital de Madrid Clara Campal, Madrid, Spain

Introduction / Background: Endometrial cancer is the most common gynecologic malignancy in Spain. Surgical evaluation of the lymph nodes is important in these patients. Traditionally , lymph node assesment required complete pelvic and paraaortic lymphadenectomy. Nowadays, Sentinel lymph node (SLN) mapping is gaining wide difussion with the goal of reducing the high morbidity associated with complete lymphadenectomy. The detection of nodal metastasis is a good marker of poor prognosis and will influence the decision of giving adyuvant therapy.
Methodology: Patients were surgically staged by two different surgeons in the same institution. The cohort includes patients diagnosed of endometrial cancer at early- stages from 2017-2019 who underwent a SLN mapping. Indocyanine green (ICG) , an methylene Blue in some cases, was injected into the uterine cervix at 3 and 9 o'clock. Fluorescence Imaging was used to visualize ICG tracer.
Results: Twenty patients were enrolled. The median age was 61.35 years. The mean body-mass index(BMI) was 28.84. The overall detection rate(DR) of SLN mapping was 80%(16/20) with a 55%(11/20) bilateral detection. The addition of methylene blue to ICG did not increase the DR. No nodal metastasis were found. Five complete pelvic lymphadenectomies and three para-aortic were perfomed laparoscopically when >50% myometrial infiltration was identified. Lymph node macrometastasis were found in 20%(1/5).
Conclusion: Further studies are needed to standarize the SNL mapping as an alternative to traditional lymphadenectomy. Our results are similar to the ones published in the literature. Nevertheless, much larger sample size is needed to to draw any relevant conclusions.
Disclosure: Nothing to disclose

EP580

Metformin may attenuate the effect of hyperprolactinemia induced by medroxyprogesterone acetate during fertility-sparing treatment for patients with atypical endometrial hyperplasia and endometrial cancer

A. Mitsuhashi, W. Gu, U. Habu, T. Kobayashi, M. Shozu
Reproductive Medicine, Graduate School of Medicine, Chiba University, Chiba, Japan

Introduction / Background: Prolactin (PRL) expression has been reported to stimulate cell proliferation and might be associated with worse survival outcomes in patients with endometrial cancer (EC). We evaluated the effect of PRL during medroxyprogesterone acetate (MPA) plus metformin treatment as fertility-sparing treatment in patients with atypical endometrial hyperplasia (AEH) and EC. Furthermore, we investigated the interaction of PRL and metformin on EC cells.
Methodology: We retrospectively investigated 18 patients with AEH and 41 with EC who were treated with MPA (400 mg/day) plus metformin (750-2250 mg/day) as fertility-sparing treatments. We evaluated serum PRL levels before and during MPA administration. We analyzed the association between serum PRL and relapse-free survival (RFS). The EC-cell lines (ISHIKAWA, HEC-1B, and HEC 265) were treated with recombinant human PRL (500ng/ml) and/or metformin (1mM). The interaction of PRL and metformin on EC cell proliferation was examined with a WST-8 assay and western blot analysis.
Results: Before MPA treatment, the median serum PRL levels were 10.3 (4.5-47.0) ng/ml. During

MPA treatment, the median serum PRL level increased to 34.6 (17.8-78.7) ng/ml. The percent of patients with hyperprolactinemia (>15 ng/ml) increased from 19% to 100% after starting MPA treatment. There was no significant difference in RFS between the patients with serum PRL levels ≥15 ng/ml and < 15 ng/ml before MPA/metformin treatment and between those with serum PRL levels ≥30 ng/ml and < 30 ng/ml during treatment.
PRL promoted the proliferation and significantly increased phospho-ribosomal protein S6 and phospho-extracellular signal-regulated kinase 1/2 in EC cell lines. These effects were attenuated by the combination with metformin.
Conclusion: High-dose MPA using fertility sparing treatment induced hyperprolactinemia that may cause a progressive effect on EC. The addition of metformin may prevent this effect and improve fertility-sparing treatment outcomes for patients with AEH and EC.
Disclosure: Nothing to disclose

EP581

Tissue fibrinolysis and VEGF in tissues of uterine myoma combined with breast cancer

T. Moiseenko, L. Kozlova, E. Frantsiyants, M. Vereskunova, L. Rozenko, N. Chernikova, M. Adamyan, V. Bandovkina, I. Kaplieva, K. Boyko, V. Gurnak, M. Ilchenko, E. Agarkova, D. Spiridonova, M. Ezhova
Rostov Research Institute of Oncology, Rostov-on-Don, Russian Federation

Introduction / Background: The significance of plasminogen regulators and VEGF in cancer has been proven, but is poorly studied in combined uterine and breast pathology in the perimenopausal period. Our purpose was to analyze the fibrinolysis system and VEGF in uterine myoma alone (UM) and combined with breast cancer (BC+UM) in perimenopausal patients.
Methodology: The study included perimenopausal patients (49.3±3.5 years) with UM (n=24) and BC+UM (n=17). Comparison groups: endometrial cancer (EC, n=21, $T_2N_0M_0$) and BC (n=34, $T_2N_0M_0$). Controls: intact breast (IB, n=16) and myometrium in UM resection line (the norm N, n=14). Levels of uPA-Ag, uPA-act, tPA-Ag, tPA-act, plasmin (P), plasminogen (PG) and VEGF were studied by ELISA.
Results: In UM alone, uPA, tPA and VEGF were equal to N; P and PG were 1.8 times higher N. In EC alone, uPA-Ag and uPA-act exceeded N by 3.5 and 1.6 times, tPA-Ag and tPA-act by 1.5 and 2.4, P and PG by 5.0 and 1.5, VEGF by 5.7 times. In tumors of BC alone, uPA-Ag and uPA-act were higher than in IB by 6.5 and 4.5 times; tPA-Ag and tPA-act by 2.1 and 1.5; P and PG by 4.5 and 1.7; VEGF by 84.6 times. In BC+UM, uPA-Ag and uPA-act in BC were higher than in IB by 10.6 and 5.5; tPA-Ag and tPA-act by 3.8 and 2.7; P and PG by 4.1 and 1.2 (p< 0.01); VEGF by 179.0 times. In UM tissues in BC+UM, uPA-Ag and uPA-act were similar to N, tPA-Ag and tPA-act exceeded N by 7.7 and 1.4; P and PG by 6.5 and 1.7; VEGF by 2.0 times.
Conclusion: In BC+UM, cancer affects fibrinolysis and VEGF in UM: tPA, P and VEGF increase compared to N. The direction of changes in tPA, P, PG and VEGF in UM are similar to EC alone.
Disclosure: Nothing to disclose

EP582

Association between changes in relative expression of genetic loci encoding transcription factors and age and endometrial cancer grade

T. Moiseenko, E. Frantsiyants, M. Vovkochina, M. Adamyan, N. Chernikova, D. Vodolazhsky, V. Bandovkina
Rostov Research Institute of Oncology, Rostov-on-Don, Russian Federation

Introduction / Background: Transcription factors encoded by the OCT4 and SOX2 genes are involved in the regulation of human embryonic stem cell function and may play an important role in

tumor progression. Mutant versions of the Myc gene are found in many tumors. Our purpose was to study relative expression of genetic loci encoding transcription factors in intact and pathological tissue of the uterine body.
Methodology: Biopsy tissues of the uterus (tumor - TT and intact - IT) were studied in 27 patients aged 38-72 years with histologically verified endometrial cancer (EC). Relative expression of 3 genetic loci encoding OCT4, SOX2 and c-Myc transcription factors was determined by RT-qPCR. The primer design for RT-qPCR was performed using NCBI GenBank DNA reference sequences. ACTB was used as the reference gene.
Results: Patients with G1 EC, 45-68 years, showed an increased SOX2 expression (by 178%, p< 0.05); G2 EC, 38-69 years - no significant changes in expression of the genetic loci; G3 EC, 55-63 years - expression of SOX2 and c-Myc increased by 63% and 130% (p< 0.05) respectively in TT comparative to IT. Expression of gene pairs OCT4 and SOX2, OCT4 and c-Myc, c-Myc and SOX2 in both IT and TT of different grades demonstrated a strong positive correlation from +0.940 to +0. 998. OCT4, SOX2, and c-Myc expression was 35, 7, and 2 times higher (p< 0.005), respectively, in G3 TT compared to G1 TT; OCT4, SOX2, and c-Myc expression was 32, 12 and 2 times higher in G3 IT compared to G1 IT.
Conclusion: The maximal increase was observed in the OCT4 gene expression, confirming its status as a marker of undifferentiated cells. In tumors of higher grades, changes in OCT4, SOX2, and c-Myc expression affected uterine IT, however with less intensity, especially in later stages of the disease.
Disclosure: Nothing to disclose

EP583

Surgical management of the endometrial cancer and analysis of the surgical prognostic factors for disease recurrence and overall survival

J. Molero Vílchez[1,2], E. Martínez Lamela[1,3], S. Sancho García[4], E. Bancarali Rojnica[5]
[1]Oncologic Gynecology, Hospital Ntra. Sra. del Rosario, Madrid, [2]Oncologic Gynecology, Clinica TocoGyn, Alcalá de Henares, [3]Obstetrics and Gynecology, University Hospital Infanta Leonor, [4]Radiation Oncology, Hospital Ramón y Cajal, [5]Pathology, Jiménez Ayala Institute, Madrid, Spain

Introduction / Background: Our purpose is to evaluate the influence of surgical management and the prognostic factors for Disease-Free Survival (DFS) and Overall Survival (OS) in endometrial cancer (EC).
Methodology: Retrospective review of 180 patients underwent standard surgical staging with pelvic and para-aortic lymphadenectomy in 130 cases. Mean age was 63 years (27-94). Mean BMI 30 kg/m2 (15-51). Mean Ca 1.25 prior to surgery was 61 U/dL (2-4,228). Mean removed nodes was 21 pelvic nodes (9-56, involved nodes 12.3%) and 19 para-aortic nodes (7-51, involved nodes 14.8%). A laparoscopic approach was performed in 65% patients. Mean follow-up was 46 months. The factors that impact on survival were compared using univariate and multivariate analysis.
Results: The estimated 5-year DFS and OS comparated rates were respectively, according to FIGO local stages (I) 93.9 and 90.2%, vs advanced stages (II-IV) 0 and 53.7% (p=0.00 Hazard ratio 5.58 (CI 95%, 2.74-11.35) and p=0.00 Hazard ratio 4.19 (CI 95%, 2.11-8.34); obesity BMI< 30 84 and 85.5%, vs BMI>30 82.7 and 80.8 (p=0.61 and p=0.38); preoperative Ca125 levels < 35 U/dL 86.6 and 88.6%, vs Ca125 level< 35 86.6 and 88.6% (p=0.17 and p=0.02 Hazard ratio 2.67 (CI 95%, 1.09-6.51); laparoscopic surgical approach 86.5 and 88.7%, vs laparotomy 58 and 75.4% (p=0.77 and p=0.27); complete surgical surgery 92 and 92%, vs incomplete 0 and 27.3% (p=0.00 Hazard ratio 3.64 (CI 95%, 1.48-8.39) and p=0.00 Hazard ratio 7.03 (CI 95%, 3.04-16.25); without lymphadenectomy 86.9 and 86.8%, vs performed lymfadenectomy 91.4 and 75.8% (p=0.01 Hazard ratio 2.94 (CI 95%, 1.21-7.16) and p=0.33); negative lymph involvement 81.9 and 85.9%, vs involved nodes 0 and 60% (p=0.00 Hazard ratio 3.17 (1.41-7.11) and p=0.01 Hazard ratio 3.22 (CI 95%, 1.2-8.62).
Conclusion: DFS and OS increased in case of complete debulking, low Ca12.5 leves prior to surgery, the abscence of lymph involvement and patients who underwent lymphadenectomy.

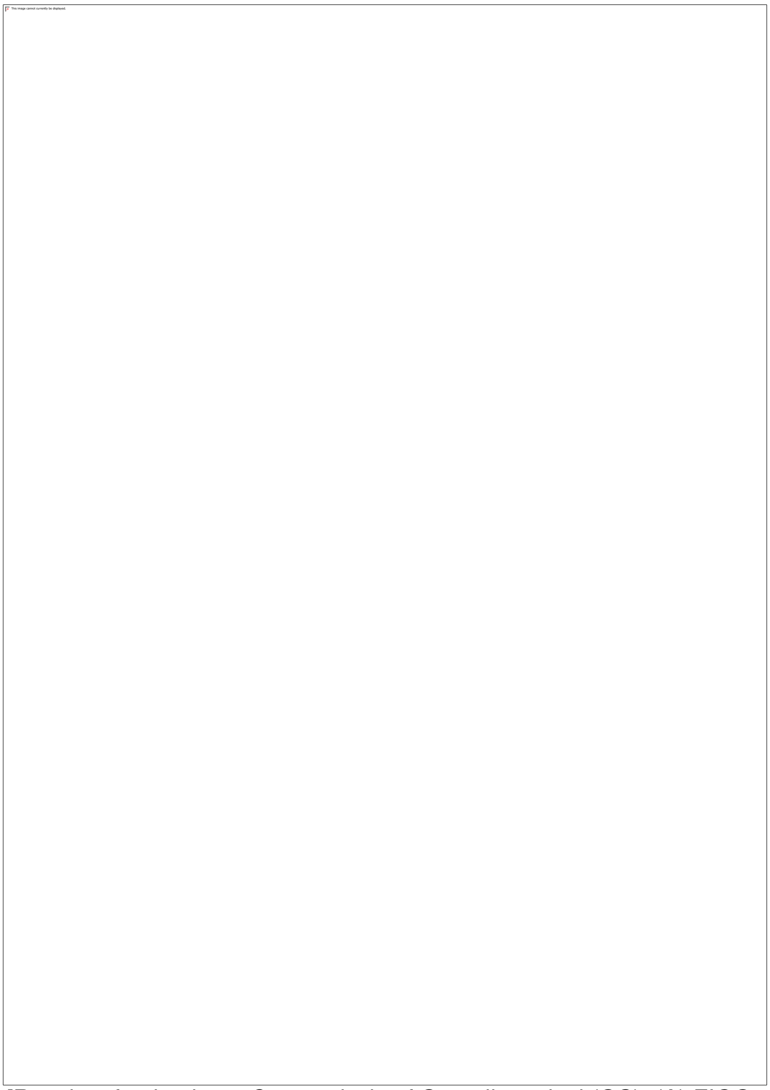

[Results of univariante Cox analysis of Overall survival (OS): (A) FIGO stages. (B) Surgical approach]

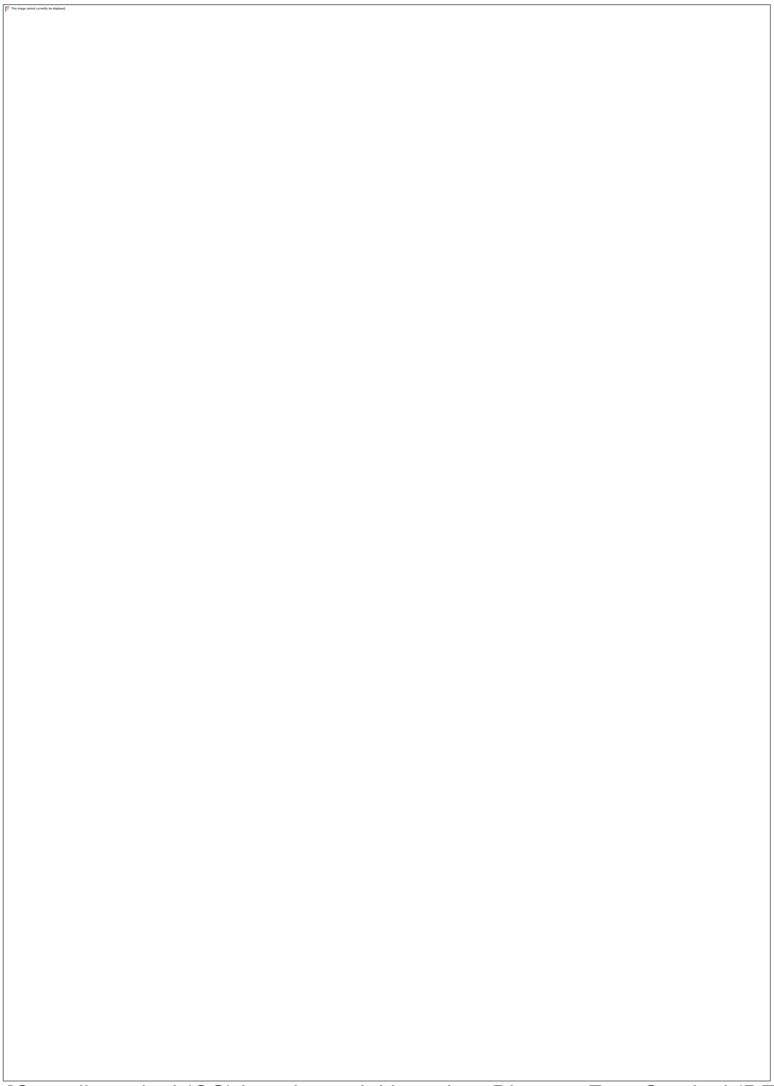

[Overall survival (OS) by miometrial invasion. Disease-Free Survival (DFS) for lymph node involvement]

Disclosure: Nothing to disclose

EP584

Circulating tumour DNA to monitor tumour activity in endometrial cancer

E. Moss[1], D. Gorsia[1], A. Collins[1], A. Whitton[2], D. Guttery[1]
[1]University of Leicester, [2]University Hospitals of Leicester, Leicester, United Kingdom

Introduction / Background: Circulating tumour DNA (ctDNA) has been shown to be a sensitive method for monitoring cancer activity and stratifying patients likely to respond to chemotherapy in numerous solid tumours but its potential in endometrial cancer (EC) has not been established.
Methodology: A prospective cohort study of women with high-risk, advanced and recurrent EC were recruited to have a blood test at every clinic visit until the patient completed follow up or is too unwell

to continue. Clinical examination findings and impression, patient symptoms, imaging results and the site/date of recurrent disease were collected. DNA was extracted from FFPE tumour blocks, 3 ml of matched plasma for cell-free DNA (cfDNA) and buffy coat for germline DNA. DNA was sequenced using the Oncomine Pan-Cancer cfDNA targeted next generation sequencing (tNGS) panel (ThermoFisher) on an Ion S5 GeneStudio and analysed using Torrent Suite v5.6 and Ion Reporter v5.6.
Results: Sixty-two women were recruited. To date 3 patients have recurred, 3 patients with advanced disease have progressed and a further 2 patients have died. Matched mutations were identified within the primary tumour and plasma using tNGS or digital droplet PCR (ddPCR). CtDNA was detected 3-6 months before clinical symptoms. Levels of cfDNA/ctDNA also accurately tracked EC response to chemotherapy and were able to determine chemotherapy response, as well as a lead-time to relapse, before a CT scan performed after Cycle 3.
Conclusion: We have detected ctDNA in patients with high-risk/recurrent endometrial cancer (EC) using a simple, cheap blood test and have shown that it is possible to monitor chemotherapy response to give a real-time assessment of chemosensitivity as compared to waiting for changes on radiological imaging. These results on the utility of ctDNA as a biomarker for EC relapse open the door towards larger scale clinical trials investigating ctDNA for personalising EC treatment and follow-up.
Disclosure: Nothing to disclose

EP585

Attitudes and barriers to window-of-opportunity trials in women who have undergone treatment for endometrial cancer

B. Mandane[1,2], E. Moss[3]
[1]University Hospitals of Leicester, [2]De Montford University, [3]University of Leicester, Leicester, United Kingdom

Introduction / Background: Window-of-opportunity (window) trials are a useful trial design for investigating the biological effect of agents on endometrial cancer (EC). Women from ethnic minority backgrounds are typically underrepresented in clinical trials. However, in light of the differences in EC between different racial groups it is important that these women are recruited to window trials to ensure the results are truly representative.
Methodology: A mixed methods study, comprising of a one-to-one verbal questionnaire and semi-structured interview, was conducted with women who had undergone treatment for EC within the past year. A strategic purposeful sampling was used to obtain opinions of women from diverse/social/ethnic/educational backgrounds. Questions explored the women's background knowledge of clinical research, views of the type of medication they would consider taking as part of a window trial and their perceived barriers to participation.
Results: Over half of women would consider participation although views on optimum time to receive trial information differed; at the time of diagnosis (62.5%), few days after diagnosis (25%). There was greater interest in agents derived from vitamins or food supplements rather than hormone-based drugs (75% compared 0% for 'very likely' to participate, respectively). Potential barriers to participation included the type of substances used and their perceived safety; concerns over potential side-effects; lack of knowledge/information about the trial; emotional/physical burden of the diagnosis whilst preparing for major surgery; perceived waiting time from the patients' perspective from diagnosis to curative surgery on outcome. Positive motivating factors included personal benefit, advancing scientific knowledge within the field, and helping to prevent women developing EC in the future.
Conclusion: This study provides useful insight into women's views on window trials in EC and identifies potential barriers for participation. The results will be used to develop patient information materials addressing women's concerns in order to increase trial participation in the future.
Disclosure: Nothing to disclose

EP586

Robotic surgery and perioperative morbidity in very elderly women (≥75 years old) with type 2 endometrial cancer

E. Myriokefalitaki, B. Winter-Roach, R. Slade, M. Smith
The Christie NHS Foundation Trust, Manchester, United Kingdom

Introduction / Background: To investigate the feasibility, safety, and short-term outcomes for very elderly patients, age ≥75 years undergoing robotic surgery for intermediate and high risk endometrial cancer
Methodology: Retrospective cohort study of all patients ≥75 years old that underwent robotic surgery for the treatment of endometrial cancer in our centre between July 2014 and April 2019
Results: 84 patients identified with a mean age of 79 (75-91) years old. All of them underwent total hysterectomy + bilateral salpingo-oophorectomy and 70 of them underwent also pelvic Lymphadenectomy with a mean count of 17.0. 72% of them had cardiovascular disease and 28% of them chronic obstructive pulmonary disease. 50% of them had previous abdominal surgery and 17% of them had a performance status (ECOG) ≥2. Compared to the younger group of our patients (mean age 63.4, range 32-74 years old), mean length hospital stay was slightly longer 2.7 days vs 2.1 ($p < 0.05$). There were 8.3% grade I-II (Clavien-Dindo) post-operative complications vs 7.4% ($p > 0.05$) and 4.7% grade III-IV vs 3.2% ($p < 0.05$). The difference was observed to complications related to chest infection and postoperative arrhythmia (AF). There were no deaths.
Conclusion: Robotic surgery is feasible and safe in very elderly women with type 2 endometrial cancer. We observed a longer hospital length of stay and greater likelihood of postoperative arrhythmia in comparison to patients ≤75 years old. Patients should not be considered too old for robotic surgery. Pre and perioperative care should take into account their comorbidities and tailored accordingly.
Disclosure: The Christie is an accredited training centre for robotic surgery in Gynaecological oncology.

EP587

Medroxyprogesterone acetate (MPA) maintenance therapy to treat recurrent endometrial cancer: case reports

H. Nagano, Y. Furukawa, Y. Tachibana, M. Akazawa, M. Ueno, M. Ichinoe, K. Hashimoto
Obstetrics and Gynecology, Tokyo Women's Medical University, Tokyo, Japan

Introduction / Background: The Japanese guideline for treatment of endometrial cancer (2018 edition) recommends using hormonal therapy in relapsed cases if hormone receptor status (estrogen receptor/progesterone receptor) is positive.
Methodology: We administered medroxyprogesterone acetate (MPA), 200 mg or 400 mg/day, as maintenance hormone therapy following chemotherapy in two patients with recurrent endometrial cancer.
Results: The first patient with stage IIIC (pT2N1M0) moderately-differentiated endometrioid carcinoma of the uterine corpus underwent initial operation and postoperative chemotherapy (paclitaxel plus carboplatin for six cycles). After 2 years, multiple lung metastases were found. The patient achieved clinical complete response (cCR) after second- (pirarubicin plus carboplatin for six cycles) and third-line (docetaxel plus carboplatin for six cycles) chemotherapies followed by MPA maintenance therapy (200 mg/day) and has been disease-free for over 4 years. The second patient with stage II moderately-differentiated endometrioid carcinoma of the uterine corpus developed multiple lung metastases 29 months after initial operation. Subsequent chemotherapy (paclitaxel plus carboplatin for eight cycles) resulted in cCR. Thereafter, MPA (400 mg/day) was orally administered as maintenance therapy. She has been disease-free for over 2 years. The hormone receptor status in both cases was positive, and no serious adverse events were observed in either patient.
Conclusion: MPA maintenance therapy should be considered after chemotherapy to treat recurrent

endometrial cancer in cases with positive hormone receptor status
Disclosure: Nothing to disclose

EP588

Sentinel lymph node mapping with indocyanine green in laparotomy for endometrial cancer

Z.Y. Ng, K.M.L. Koh, F.H.X. Chin, P.S. Chin, I.M. Aggarwal, W.L. Wong, Y.K. Lim
KK Women's and Children's Hospital, Singapore, Singapore

Introduction / Background: Sentinel lymph node (SLN) mapping with indocyanine green (ICG) is emerging as a feasible alternative to pelvic lymphadenectomy in endometrial cancer staging. Although ICG SLN mapping is well established in robo-laparoscopic surgery, there is little literature validating its use in laparotomy.
Methodology: The technique, effectiveness and feasibility of SLN mapping with ICG during laparotomy for endometrial cancer is discussed. For each patient, intra-cervical ICG injection at the 3 and 9 o'clock positions was performed. Following peritoneal dissection, lymph node chains were inspected in near infrared mode using the Karl Storz Vitom(R) camera system. In each hemi-pelvis, if SLN mapping was not achieved, ipsilateral pelvic lymphadenectomy was performed. Full pelvic lymphadenectomy was also performed if there were suspicious nodes, large tumour size or extensive myometrial invasion.
Results: Between March 2016 and February 2019, 37 patients with endometrial cancer underwent laparotomy. Average operating time was 133 minutes Median blood loss was 200mls. There were no complications related to the use of ICG dye. The detection rate of SLNs was 92%. Bilateral pelvic SLNs mapping was achieved in 81% of patients, whereas 11% of patients had mapping to unilateral pelvic nodes. The median number of SLNs was 3. SLNs were mapped most commonly to the external iliac nodes, followed by the obturator and internal iliac nodes. In 3 patients (8.1%), SLNs were positive for metastases, resulting in a higher surgical stage and influencing the decision for adjuvant therapy. One patient also had mapping to a para-aortic node, which was positive for metastasis. Full pelvic lymphadenectomy was performed in 17 patients (46%). The sensitivity of SLN mapping in these patients was 100%, with no falsely negative SLNs.
Conclusion: SLN mapping with ICG is accurate and feasible in patients with endometrial cancer requiring laparotomy, and has a high detection rate and sensitivity comparable to that of robo-laparoscopic surgery.
Disclosure: Nothing to disclose

EP589

Modified radical hysterectomy reduce the recurrence of the vaginal stump in patients with early-staged endometrial cancer

A. Nishimura, M. Yamaguchi, H. Katabuchi, M. Tominaga
Obstetrics & Gynecology, Kumamoto University, Kumamoto City, Japan

Introduction / Background: The appropriate operative method for patients with early-staged endometrial cancer remains controversial. The aim of the present study was to assess the recurrence rate of the vaginal stump after modified radical hysterectomy for patients with endometrial cancer.
Methodology: Medical records of the patients with FIGO stages Ⅰ, Ⅱ, and Ⅲ endometrial cancer treated with modified radical hysterectomy and pelvic lymph nodes dissection at Kumamoto University Hospital between 2006 and 2014 were analyzed retrospectively. The patients who had been histologically diagnosed with carcinosarcoma or sarcoma were excluded. The recurrence site, FIGO stage, and histological type were analyzed in patients with recurrent disease within 5 years after the

initial treatment.
Results: Two hundred-nine patients with endometrial cancer underwent modified radical hysterectomy during an observational period, and 22 patients (10.5%) had an initial relapse within 5 years. The sites of initial recurrence included the vaginal stump, the urinary bladder, the vaginal inlet, the perineum, the spleen, the adrenal gland, the pelvic and paraaortic lymph nodes, the lung, and the brain in 6 patients with stage Ⅰ and 16 patients with stage 3. Although the recurrence of the vaginal stump was confirmed in 2 patients with stage Ⅲ, there was no recurrence of the vaginal stump in 135 patients with stage Ⅰ and 16 patients with stage Ⅱ. The recurrence rate of the vaginal stump was 0.9% (2/209) in all patients including stages Ⅰ, Ⅱ, and Ⅲ.
Conclusion: Modified radical hysterectomy with sufficient resection margins of the vagina and the parametrium may decrease the postoperative recurrent rate of the vagina for patients with stage Ⅰ and stage Ⅱ endometrial cancer.
Disclosure: Nothing to disclose

EP590

Is tumor free distance an independent prognostic factor for early stage endometrioid endometrial cancer?

T. Oge
Obstetrics and Gynecology, Osmangazi University, School of Medicine, Eskisehir, Turkey

Introduction / Background: Although there are many studies assessing the importance of myometrial invasion (MI) using a cut-off limit as 50% of MI for endometrial cancer, there are limited number of studies evaluating tumor free distance (TFD) to the serosa. The aim of this study is to evaluate the prognostic performance TFD and %MI in patients with Stage IB endometrioid endometrial cancer.
Methodology: We evaluated 133 patients diagnosed and treated as Stage IB endometrioid endometrial cancer. TFD was assessed and recurrence and recurrence free survival were analyzed.
Results: Nine patients had recurrence disease (6.8%). Recurrence free survival was 200 months. Two patients died because of malignancy. In Cox regression model according to TFD, DOI and %MI, it was seen that none of these parameters were significant to predict the recurrence ($p>0.05$).
Conclusion: TFD is not an independent prognostic factor for patients with stage IB endometrioid endometrial cancer.
Disclosure: Nothing to disclose

EP591

Survival and recurrence rates in stage II endometrial cancers in related to uterine-risk stratification after the introduction of lymph node resection and the omission of postoperative radiotherapy. A Danish gynecological cancer study group

G. Ørtoft[1], C. Høgdall[1], E.S. Hansen[2], M. Dueholm[3]
[1]Department of Gynecology, Copenhagen University Hospital, Copenhagen, [2]Department of Pathology, [3]Department of Gynecology, Aarhus University Hospital, Aarhus, Denmark

Introduction / Background: To evaluate the survivals and recurrences in relation to uterine-risk stratification after the introduction of lymph node resection and omission of postoperative radiotherapy in stage II endometrial cancers.
Methodology: This national cohort study includes all 708 cases with cervical stromal invasion of 4380 radically operated endometrial carcinomas (2005-2012). Five-year Kaplan-Meier survival estimates

and actuarial recurrence rates were calculated.
Results: Of 708 cases with cervical stromal invasion 461 were finale stage II. The strongest predictor for survival and recurrences in final stage II cases was myometrial invasion, tumor grade and type e.g uterine risk factors (low-, intermediate- and high-risk) with a very good prognosis for stage II cases with otherwise low-risk (grade 1 or 2 and < 50% myometriel invasion and cervical stromal invasion), almost comparable to that of low-risk stage I, whereas cervical stromal invasion in intermediate and high-risk cases significantly increased the risk of recurrences and decreased cancer-specific survivals compared to corresponding stage I cases (Figure 1). Lymph node resection in 355 cases with cervical stromal invasion diagnosed 27.9% with lymph node metastasis and upstaged 18.1% from stage II to IIIc, resulting in higher survivals and lower recurrences in lymph node resected as compared to non-lymph node resected. Radical as compared to simple hysterectomy did not alter survivals. Stage II (117 cases) treated with external beam radiotherapy had significant lower local recurrence rates, but unchanged survivals compared to non-irradiated.
Conclusion: Uterine risk groups (low-, intermediate- and high-risk) are the strongest predictor for survival and recurrence and should be considered when advising adjuvant therapy for endometrial cancer stage II. Lymph node resected stage II had increased survivals and decreased recurrences. Omitting radiotherapy increased local vaginal recurrences without affecting survivals.

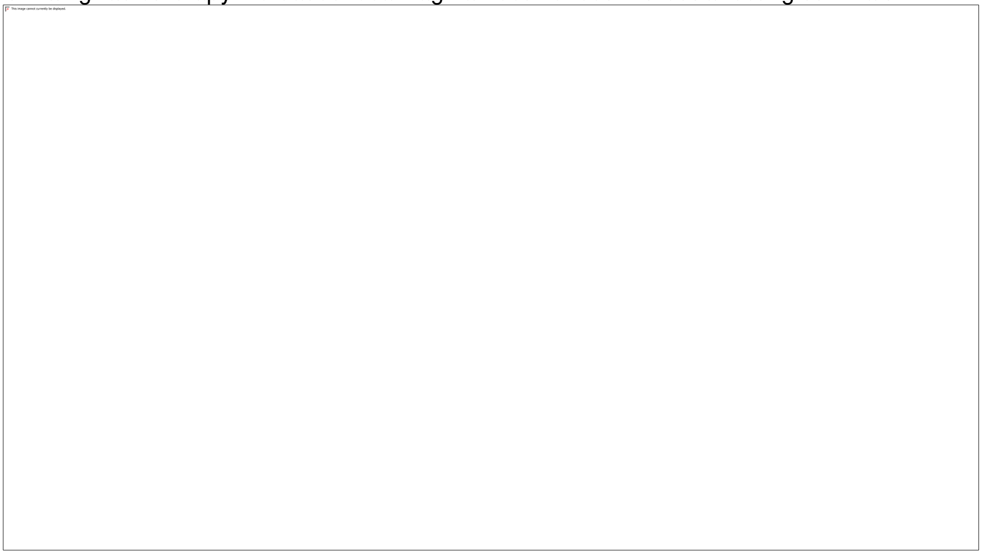

[Figure 1]

Disclosure: Nothing to disclose

EP592

HE4 has a role in identifying high risk prognostic factors in endometrial cancer

S. O'Toole[1], M. Foley[1], S. Rizmee[2], L. Norris[3], W. Kamran[2], N. Ibrahim[2], M. Ward[3], C. Thompson[2], C. Murphy[4], M. Anglim[4], T. D'Arcy[4], N. Farah[4], H. O'Connor[4], J. O'Leary[5], F. AbuSaadeh[2], N. Gleeson[2]
[1]Obstetrics and Gynaecology/Histopathology, [2]Gynaecological Oncology, [3]Obstetrics and Gynaecology, Trinity St. James's Cancer Institute, [4]Coombe Womens and Infants University Hospital, [5]Histopathology, Trinity St. James's Cancer Institute, Dublin, Ireland

Introduction / Background: Endometrial cancer (EC) is the most common gynaecological malignancy. The mainstay of treatment is surgical resection. Currently, there are no sensitive and specific biomarkers for EC. Human Epididymis protein 4 (HE4) shows promise as a diagnostic and prognostic marker. We investigated the sensitivity of preoperative serum HE4 for predicting high-risk EC.

Methodology: 206 patients were recruited from the DISCOVARY bioresource between 2011 and 2016. Demographic and tumour characteristics were recorded. Preoperative serum HE4 and CA125 was measured using Fujirebio Diagnostic ELISA Kits, with the cut-off points of HE4 70 pmol/L and CA125 35 U/ml. ELISA results were correlated with clinicopathological details. Statistical analysis was performed using SPSS.
Results: The cohort comprised of endometrioid adenocarcinomas (n=167) representing over 80% of the malignant cohort, carcinosarcoma (MMMT) (n=14), serous (n=17), mixed (n=6), mucinous (n=1) and clear cell (n=1).
Using a cut-off of 70pmol/L, serum HE4 had a sensitivity of 75.9% for predicting >50% myometrial invasion, more than twice the sensitivity of CA125 for >50% myometrial invasion (27.8%). Specificities were similar at 93.6% for HE4 and 95.1% for CA125.
HE4 was more sensitive for predicting the presence of LVSI in EC (71%), compared to CA125 (34%). HE4 had a sensitivity of 89.6% and specificity of 46.2% for predicting lymphadenopathy, compared to a sensitivity of 46% and a specificity of 90.7% with CA125.
Conclusion: HE4 showed a high sensitivity for predicting the presence of high risk prognostic factors. Clinically, HE4 may have a role in supplementing transvaginal ultrasound in EC diagnosis. Furthermore, preoperative HE4 may supplement imaging studies when stratifying EC patients as high or low risk, enabling personalisation of surgical management, and improved outcomes. Further work is needed on establishing relevant cut-offs for high-risk cohorts.
Disclosure: ELISA kits were supplied by Fujirebio Diagnostics.

EP593

Clinicopathological features of endometrial carcinoma without abnormal uptake of 18F-fluorodeoxyglucose on pretreatment PET/CT

I. Otsuka[1], T. Matsuura[1], Y. Machida[2]
[1]Obstetrics and Gynecology, [2]Radiology, Kameda Medical Center, Kamogawa, Japan

Introduction / Background: PET/CT is an effective imaging modality in evaluating the aggressiveness of a tumour and detecting metastasis. In endometrial carcinoma, degree of 18F-fluorodeoxyglucose (FDG) uptake is associated with tumour grade, myometrial invasion, and extrauterine disease. However, in some types of endometrial carcinoma 18F-FDG uptake is not observed in the uterus whereas biopsy of the endometrium shows a carcinoma. To identify clinical and pathological features of these tumours, we performed a retrospective study.
Methodology: Patients who received pretreatment 18F-FDG PET/CT and underwent staging surgery between 2006 and 2016 at our institution were investigated.
Results: Seventeen cases of endometrial carcinoma without abnormal uptake of 18F-FDG on pretreatment PET/CT were identified. The median age was 62 years (range, 53-73 years). FIGO stage of the disease was IA in 13, IB in 1, II in 2, and IIIC1 in 1 patient. Histology of the tumour was endometrioid carcinoma grade 1 in 6, grade 2 in 2, grade 3 in 2, clear cell carcinoma in 2 and serous carcinoma in 5 patients. Seven patients received PET/CT after dilatation and curettage, and in two of these patients, no residual disease was detected pathologically in the removed uterus. Lymphatic space invasion was observed in three patients, and another patient with serous carcinoma with superficial invasion had pelvic lymph node metastasis and serosal involvement. Tumour recurrence developed in four patients and all of them had a serous carcinoma. The site of recurrence was the abdominal cavity in 2, the lung in 1, and the vaginal stump in 1 patient. Of these, three patients who developed a distant metastasis died of the disease.
Conclusion: Patients with endometrial carcinoma without pretreatment FDG uptake in the uterus usually have a lower stage disease with good prognosis but may have serous carcinoma with poor prognosis.
Disclosure: Nothing to disclose

EP594

Recurrent endometrial adenocarcinoma with pancreatic metastasis

G. Özten[1], E. Uyanık[1], V. Bistika[2], O. Atliya[2], M.C. Salman[1], M. Gültekin[1]
[1]Obstetrics & Gynecology, [2]Hacettepe University, School of Medicine, Ankara, Turkey

Introduction / Background: Endometrial cancer is the most common gynaecological tumour in developed countries. Almost 75-80% of patients present with FIGO (International federation of Gynaecology and Obstetrics) stage 1 disease and a local recurrence rate of 4-8% . An uncommon presentation of metastasis is pancreas.
Methodology: We describe herein the rare case of a pancreas metastasis of grade 2 endometrial adenocarcinoma.
Results: A 58-year-old woman underwent treatment of endometrial adenocarcinoma, staging surgery was performed in 2012. Hysterectomy contained a FIGO grade 2 endometrioid adenocarcinoma featuring depth of invasion more than half of myometrium. Lymh nodes andLymphovascular invasion was negative. The patient underwent adjuvant brachytherapy.
In 2015 resection of umblical mass with diameter of 4x4.5 cm from the rectus sheet was performed. Paclitaxel and carboplatin was administered for 6 cycles. In 2016, the patient was diagnosed with concomitant breast cancer and adjuvant radiotherapy was administered following surgery. After the excision of bilateral inguinal masses in 2017, she was administered 6 cycles of adriamycin and cisplatin. In 2018, during follow up examination CA 125 was detected 86 U / ml, abdominal imaging showed an invasive mass with a diameter of 2 cm in distal anterior pancreas. The cystic mass was removed by distal pancreatectomy. Intraoperatively left hemicolectomy was performed due to the mass detected in distal colon. Final pathology confirmed grade 2 endometrioid endometrial adenocarcinoma metastasis. Postoperatively the patient was discharged without any complication. After 6 cycles of paclitaxel and carboplatin follow up imaging showed no sign of recurrence.
Conclusion: There are limited data in the literature for pancreas metastasis of low grade endometrial cancer. For the last two decades there are 4 cases reported. Although surgical resection appears to be beneficial according to literature, limited number of the cases seem to prevent a consensus on treatment strategies. We believe surgical resection should be offered to these patients.

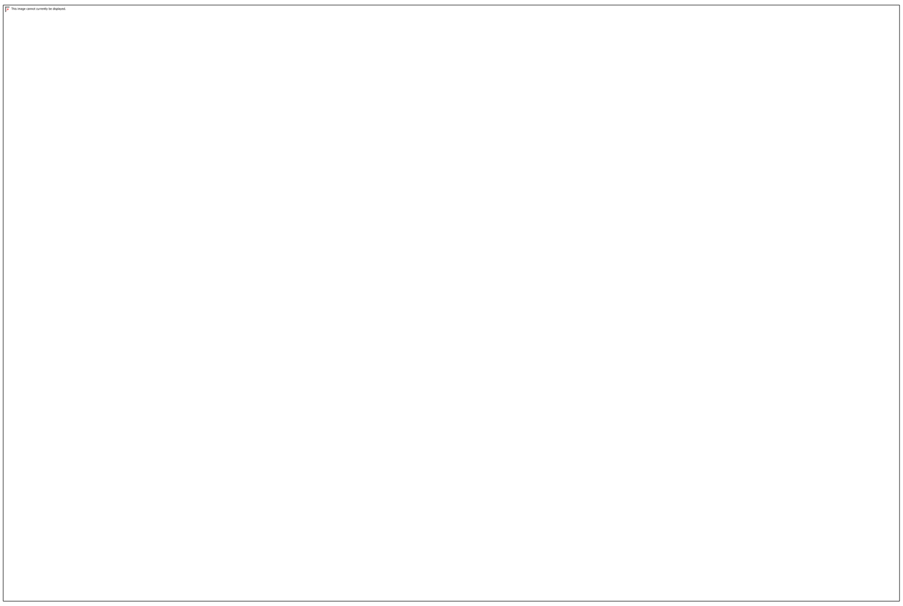

[Abdominal bt image of the mass in the pancreas]

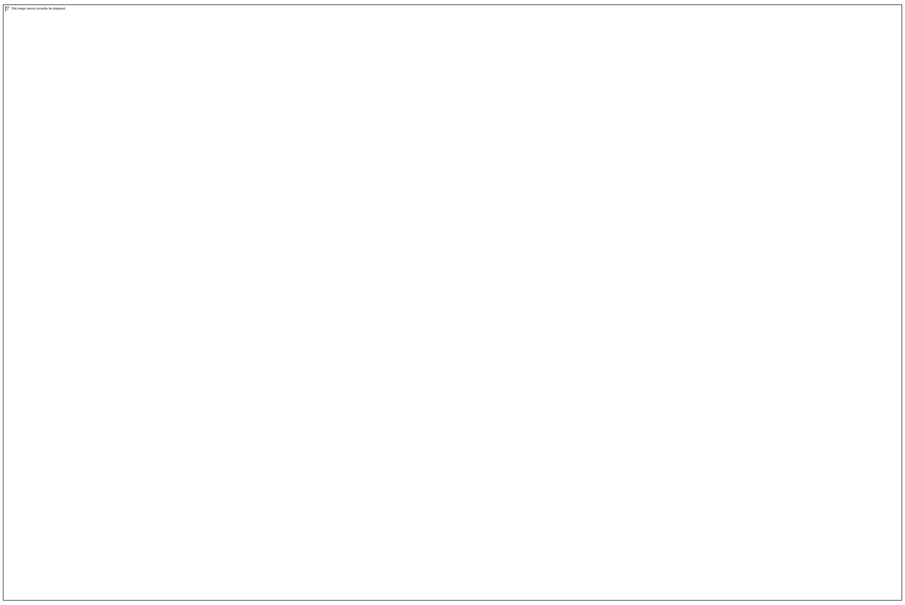

[5.7x 5 cm implant in the distal inferior of the pancreas in abdominal MRG]

Disclosure: Nothing to disclose

EP595

SLN mapping for early endometrial cancer with concurrent two different tracers (radioactive and ICG): a pilot study

A. Cokan, B. Krajnc, M. Pakiž
Department of Gynaecologic Oncology and Oncology of the Breast, UMC Maribor, Maribor, Slovenia

Introduction / Background: The detection of true sentinel node (SN) is crucial to replace the systematic lymphadenectomy. We analyzed whether using two different tracers, applied in slightly different way to the cervix, shows the same SN.
Methodology: A prospective observational study included all consecutive women with apparently early endometrial cancer between 2016-2018 at the Department for Gynecologic and Breast Oncology, Maribor, Slovenia, who signed informed consent. All women had application of radioactive tracer before the surgery to four sites in the cervix and indocyanine green (ICG) to two sites in the cervix in two depths at the beginning of the surgery. First the SN was located with the radioactive tracer and secondly with ICG. Bilateral mapping was demanded. If the SN was not found and the patient had intermediate/high risk disease, one sided pelvic lymphadenectomy was performed.
Results: Forty out of 44 included patients (90.9%) had FIGO I stage and 4 (9.1%) had positive lymph nodes. When using both methods (radioactive and ICG), the detection rate was 70.5%. Bilateral detection rate was very low with radioactive tracer alone (5/44; 11.4%) and moderate with ICG (25/44; 56.8%). The detection rate per hemipelvis with radioactive tracer was 36/88 (40.9%) and with ICG 65/88 (73.9%). At 2 hemipelvises the radioactive labeled node was a different node than ICG labeled node. In one hemipelvis the histology between the nodes differed (ICG node was positive, radioactive node was negative).
Conclusion: We demonstrated different detection rates using different tracers and more importantly when the tracers are applied in different ways to the cervix the sentinel nodes may differ, raising the question which node is true sentinel node. According to our first results we warrant to conduct a prospective multicentric larger study and if possible to use more tracers at the same time.
Disclosure: Nothing to disclose

EP596

Value of fluorodeoxyglucose positron emission tomography /computed tomography (PET/CT) and sentinel lymph node biopsy (SLN) in endometrial cancer patients: a prospective study

I. Palaia, V. Di Donato, A. Musella, L. Muzii, G. Perniola, A. Di Pinto, G. Santangelo, S.M. Boccia, C.M. Sassu, L. Vertechy, L. Musacchio, F. Tomao, A. Giancotti, P. Benedetti Panici
Sapienza University, Roma, Italy

Introduction / Background: This study aimed to define the role of combination of preoperative PET/CT scan and SLN biopsy for detection of nodal metastasis in Endometrial Cancer (EC) patients.
Methodology: All patients affected by EC prospectively collected from January 2014 to August 2016 underwent PET/CT scan and SLN mapping using indocyanine green (ICG) as tracer. Patients with suspicious lymph nodes at FDG-PET/CT underwent selective pelvic lymphadenectomy. In case of undetectable SNL, no further lymphadenectomy was performed if PET/CT scan was negative. Basic descriptive statistics were used to describe outcomes.
Results: A total of 83 patients were enrolled in the study. PET/CT scan was suggestive of nodal involvement in 15 patients. SLN were detected bilaterally in 78% of patients. Detection rate was influenced by patient´ BMI and learning curve. Five patients were node positive: of these all had hyper metabolic nodes at PET/CT scan, in 1 patient SLN was not detected. Ten out of 15 patients, with suspicious nodal at PET/CT scan, was node negative. After a median follow up of 24 months (range 14-45) all patients are alive. Four patients experienced recurrent disease. No nodal relapse was recorded.
Conclusion: Lymphatic mapping with sentinel node biopsy is able to reduce morbidity associated with pelvic lymphadenectomy. Detection rate is strongly improved with learning curve and reduced in morbidly obese patients. PET/CT scan shows high sensitivity for nodal metastasis but moderate specificity and may help to avoid nodal dissection in patients with sentinel node failure.
Disclosure: Nothing to disclose

EP597

The complementary role of serum CA19-9 in maliganat transformation of adenomyosis in endometrial cancer

S.H. Park[1], J.H. Kim[2], K.H. Lee[1], Y.S. Lee[3], T.C. Park[2], C.J. Kim[2]
[1]Department of Obstetrics and Gynecology, The Catholic University of Korea, Seoul, [2]The Catholic University of Korea, Uijeongbu St. Mary's Hospital, Uijeongbu, [3]The Catholic University of Korea, Eunpyeong St. Mary's Hospital, Seoul, Republic of Korea

Introduction / Background: Adenomyosis is usually benign, but it might also be a precursor of malignant disease. As the incidence of adenomyosis malignant transformation is low, and its clinical manifestations are nonspecific. It may only be confirmed by postoperative pathological examination. We report our experience with case of malignant transformation of adenomyosis with endometrial adenocarcinoma.
Methodology: This retrospective study was examined using data at Uijeongbu St. Mary's Hospital from the 2014 to 2018. A total of 27 women has endometrial cancer combined adenomyosis or endometriosis. Ant Only one person has related with elevated CA19-9.
Results: Sixty-two years-old nulliarous woman visited our department at Jan 2019 due to intermittent vaginal bleeding over 1 month. Ultrasound showed hematometra and adenomyosis. The histological examination of the endometrium yielded adenocarcinoma, moderately differentiated. Magnetic resonance imaging (MRI) revealed focal adenomyosis, hematometra and a few small nonspecific LNs

in right external iliac and obturator. Among the pre-operative tumor markers, CA19-9 level was increased (36.80U/ml, normal range < 35U/ml). But CA125 was within normal range (19.90 U/m, normal range < 35U/ml).
Laparoscopic total hysterectomy and bilateral adnexectomy including pelvic lymphadenectomy were performed. The intra-abdominal cytology was negative. In the pathological specimen, poorly differentiated endometrioid adenocarcinoma was present in endometrium with myometrial invasion less then 0.1 cm from endometrial-myometrial junction (T1a, thickness of uninvolved myometrium was 1 cm). Pelvic lymph node metastasis was not found. Very small Foci of adenocarcinoma were observed in adenomyosis (0.1 x 0.1 sized). Although surgical stage was Ia, due to the histologic grade and high risk factors (≥60 yrs), recurrence risk was existed. Adjuvant vaginal brachytherapy was performed after surgery. She is currently undergoing follow-up observations.
Conclusion: From this case, we concluded that if CA19-9 is elevated before surgery, a more detailed histological observation is needed in the adenomyotic foci.
Disclosure: adenomyosis, endometrial carcinoma, CA 19-9, malignancy

EP598

Preliminary results of Immunohistochemical tumor markers evaluation in endometrial cancer patients

M. Fais[1], E. Corvetto[1], M. Russo[1], G. Candotti[1], D. Fanni[2], G. Faa[2], V. Locati[1], A. Proto[1], V. Mais[1], M. Peiretti[1]
[1]Department of Obstetrics and Gynecology, [2]Department of Pathology, University of Cagliari, Cagliari, Italy

Introduction / Background: Endometrial cancer is one the most common female cancers, a better stratification into high- or low-risk is mandatory to identified patients who might benefit of a tailored adjuvant therapy. Recent studies have demonstrated that a new biomarkers panel, assessed immunohistochemically, could be helpful in a better prognosis prediction and thus in patients selection.
Methodology: This is a retrospective analysis of the following Immunohistochemical markers: L1 Cell Adhesion Molecule (L1CAM), p53, β-catenin, Ki67, Estrogen receptor (ER) and Progesterone receptor (PR) in patients with diagnosis of endometrial cancer.
Results: We analyzed twenty-one patients with a median age of 58 years (range, 34-88 years). Final histology was endometrioid in 14 (66,6%) cases, clear cell in 1 (4,8%) case, serous in 5 (23,8%) cases, mixed in 1 (4,8%) case. Seven (33%) patients were L1CAM positive (cut off value 10%). L1CAM expression was associated with advanced age (74 years *vs* 67 years), non-endometrioid morphology and loss of ER and PR expression. An important association was found with p53-mutant tumors often showing diffuse L1CAM expression, mostly in the non-endometrioid subtype. Out of a total of 14 endometrioid endometrial cancer, only one was found to be p53 mutant positive and 13 p53 wild-type (wt) positive. All non-endometrioid EC expressed p53 wt and L1CAM markers and 4/5 (80%) shows a L1CAM positivity > 75%. None association was found between L1CAM and Ki67 expression and depth of myometrial invasion, histologic grade, lymph vascular space invasion and metastatic node. Endometrioid and non-endometrioid subtype demonstrated diffuse β-catenin staining > 50%.
Conclusion: In agreement with the literature, these data confirmed that in high risk endometrial cancer patients L1CAM and p53 expression is more frequent. A high association was found between L1CAM expression and mutant p53 expression in the non-endometrioid subtype endometrial cancer.
Disclosure: Nothing to disclose

EP599

Does intraperitoneal chemotherapy represent a strategy for treatment of malignant ascites in

recurrent endometrial cancer patients? Three cases-report and a literature review

G. Perniola, G. Santangelo, I. Palaia, A. Musella, A. Di Pinto, M. Derme, A. Romito, G. Caruso, V. Di Donato, F. Tomao, L. Muzii, P. Benedetti Panici
Sapienza University, Roma, Italy

Introduction / Background: Endometrial cancer is generally associated with a good prognosis, due to an early diagnosis in stage I and II, 75% and 13% respectively. For advanced-stage disease, the recurrence rate is high and the site of the relapse is heterogeneous with localized or spread peritoneal disease. The treatment strategy is based on site of relapse. For non-localized relapse the chances are limited with a poor prognosis.
Methodology: We reported our experience of three cases with peritoneal spread recurrences, in endometrial cancer patients with advanced stage diagnosis.
Results: The patients had been underwent to multiple lines of chemotherapy including rechallenging with platinum regimens, pegilated liposomal doxorubicin and taxane, with a progression of disease. These patients came at our Department for abdominal distension and breathing difficulty, CA 125 increased and radiology find (CT scan or US scan) of ascites. In these cases, after paracentesis and a single administration of intraperitoneal chemotherapy based on carboplatin (AUC5) were showed quality of life improvement, reduction of fatigue, improved breathing and reduction of anorexia. No complications occurred.
Conclusion: Although only few cases have been reported, the exceptional results and the absence of side effects observed in these cases, strongly warrant future trials to investigate the role that intraperitoneal chemotherapy can have both as palliative treatment of refractory ascites and as salvage therapy in advanced endometrial cancer.
Disclosure: All authors declare that they have no conflict of interest and nothing to disclose.

EP600

Correlation between preoperative imaging biomarkers and histological prognostic factors in endometrial cancer: a prospective study

G. Perniola, M. Derme, I. Palaia, A. Musella, A. Di Pinto, G. Santangelo, V. Di Donato, F. Tomao, L. Muzii, P. Benedetti Panici
Sapienza University, Roma, Italy

Introduction / Background: Endometrial cancer is the most common gynecological malignancy in developed countries.
Transvaginal ultrasound (TVUS) and magnetic resonance imaging (MRI) are widely accepted as imaging tools in preoperative local staging.
In this prospective study, we evaluated the diagnostic accuracy of TVUS and MRI for detecting the depth of myometrial invasion and cervical involvement and for measuring tumor volume. We also correlated tumor volume to negative histological prognostic factors.
Methodology: We enrolled women with a histological diagnosis of endometrial carcinoma. The study was conducted from January 2018 to March 2019. All patients were evaluated by TVUS and pelvic MRI with gadolinium, in the absence of any contraindication. We compared the preoperative imaging results with final histopathology.
Results: The mean age was 63,65 ± 9.79 years. Fifty-four out of the 60 patients resulted suitable for primary surgery. Six patients underwent neoadjuvant chemotherapy for carcinomatosis. Twenty-eight women underwent both TVUS and MRI. The additional 26 patients underwent only TVUS because they were not able to perform MRI.
Both TVUS and MRI showed an accuracy of 85.7% for diagnosing presence or absence of myometrial invasion and an accuracy of 100% and 89.3% respectively, for the assessment of deep myometrial infiltration. The accuracy for the cervical involvement was 92.9% and 96.4% for TVUS and MRI respectively. The coefficient of determination (R^2) of tumor volume measurement was 0.99, 0.95 and 0.98 using 3-Dimensional (D) TVUS, 2-D TVUS and MRI respectively.
A value of tumor volume ≥ 2 ml showed a positive correlation with negative histological prognostic factors, such as high tumor grade, lymphovascular space involvement (LVSI) and deep myometrial invasion ($p < 0.05$).

Conclusion: The accuracy of TVUS and MRI in evaluating deep myometrial invasion, cervical involvement and tumor volume measurement were comparable. A preoperative evaluation of the tumor volume would allow the identification of the patients with a poor prognosis.
Disclosure: Nothing to disclose

EP601

Pelvic and para-aortic versus pelvic lymphadenectomy in intermediate and high risk endometrial cancer: a systematic review and meta-analysis

S. Petousis[1], C. Panagiotis[2], C. Margioula-Siarkou[2], A. Papanikolaou[1], A. Athanasiadis[2], A. Rodolakis[3], I. Vergote[4], I. Kalogiannidis[2]
[1]2nd Department of Obstetrics and Gynaecology, [2]3rd Department of Obstetrics and Gynaecology, Aristotle University of Thessaloniki, Thessaloniki, [3]1st Department of Obstetrics and Gynaecology, Kapodestrian University of Athens, Athens, Greece, [4]Department of Gynaecologic Oncology, KU Leuven, Leuven, Belgium

Introduction / Background: As the extend of lymphadenectomy for endometrial cancer remains controversial, a systematic review and meta-analysis was performed to assess the impact of combined pelvic and para-aortic lymph node dissection (PPALND) compared to only pelvic lymph node dissection (PLND) on survival outcomes of intermediate and/or high-risk patients.
Methodology: Databases were searched up to 20 April 2018. Primary outcomes were overall survival (OS) and disease free survival (DFS). Secondary outcomes were the need for radiotherapy/chemotherapy/combined/adjuvant therapy, pelvic, para-aortic, intra-pelvic and extra-pelvic node recurrence, time to recurrence, pelvic, para-aortic and any node invasion rate at surgery. Pooled risk ratios were calculated using random effects models. Risk of bias of individual studies was assessed using the ROBINS-I tool. PROSPERO's registration number is CRD42017072337.
Results: 13 studies were identified with 7349 patients. PPALND was associated with 46% decreased risk for death (HR:0·54, 95%CI:0·35-0·83) and increased 5-year DFS rate (RR:1·13, 95%CI:1·04-1·23). Furthermore, PPALND was associated with 49% decreased risk for recurrence (HR:0·51, 95%CI:0·28-0·93), decreased need for radiotherapy (RR:0·69, 95%CI:0·52-0·92), while increased risk for chemotherapy (RR:1·71, 95%CI:1·36-2·14) was observed in the present study. There was no significant difference for adjuvant (RR:0·98, 95%CI:0·87-1·10) or combined therapy (RR:1·45, 95%CI:0·75-2·78). Para-aortic recurrence rate was higher for patients with only PLND (9·1% vs 5·2%), however with no significant difference (RR:0·32, 95%CI:0·04-2·55). There was limited evidence for intra-pelvic, extra-pelvic, pelvic node recurrence and time to recurrence. Risk of bias was critical or serious for all studies.
Conclusion: There is low-quality evidence that PPALND contributes to improved survival outcomes in women with intermediate/high-risk endometrial cancer.
Disclosure: Nothing to disclose

EP602

The role of sentinel node assessment and laparoscopic approach in decreasing morbidity of staging surgery for endometrial cancer

R. Poka, I. Baradács, S. Molnár, Z. Krasznai
Obstetrics and Gynaecology, University of Debrecen, Debrecen, Hungary

Introduction / Background: Lymphadenectomy and associated medical disorders of the typical endometrial patient urge gynaecological oncologists to promote the use of laparoscopic approach and sentinel node diagnostics to reduce morbidity.

Methodology: Retrospective chart review was performed to assess the complications of staging surgery of different strategies among endometrial cancer patients. Body mass index (BMI), preoperative (preop) and postoperative (postop) haemoglobin levels (Hb), the duration of hospitalisation, Clavien-Dindo-classified intraoperative and postoperative morbidities were compared between laparoscopic (LSC) and open laparotomy (LAP) procedures, as well as between cases with sentinel-node (SLND) identification and systematic lymphadenectomy (PLND). The Patent-Blau technique was used for sentinel node identification. Significance of differences between continuous and categorical variables was assessed with t-test and χ^2-test or Wilcoxon-test, respectively.
Results: Consecutive cases of 45 PLND and 28 SLND were identified. Preop Hb (135 vs 138 g/100mL) and BMI (34.1 vs 32.8 kg/m^2) showed no difference between PLND and SLND cases. Postop Hb was lower in PLND cases compared to that of SLND cases (120 vs 128 g/100mL, $p < 0.05$). PLND cases required longer hospitalisation (median 9 days vs 5 days, $p < 0.05$). More complications and higher-grade complications occurred in the PLND group then in the SLND group ($p < 0.05$). Subgroup analysis within the LSC group (19 cases with SLND and 27 cases with PLND) revealed similar median duration of hospitalisation (4 days), but there were more complications among PLND cases. Preop and postop Hb did not differ between LSC and LAP cases (134 vs 137 g/100mL, and 120 vs 122 g/100 mL, respectively). Hospitalisation was longer among LAP cases compared to LSC cases (median 9 days vs 4 days, $p < 0.05$). Complication rate was higher in LAP cases than in LSC cases (12 vs 4 cases).
Conclusion: Both the LSC approach and the SLND technique reduces the morbidity of surgical staging of endometrial cancer.
Disclosure: Nothing to disclose

EP603
Three types of sentinel lymph nodes and three routes of lymph drainage for endometrial cancer

O. Postupalenko, K. Kharchenko
Department of Minimally Invasive Surgery, Kyiv City Clinical Oncology Centre, Kyiv, Ukraine

Introduction / Background: Identification of sentinel lymph node (SLN) is an alternative procedure that allows to individualize surgical staging of lymph nodes (LN).
Methodology: Prospective non-randomized trial was performed to investigate the peculiarities of SLN mapping for endometrial cancer with the usage of methylene blue dye.
Results: All cases of SLN mapping (n=89) were analysed according to the spread of contrast by lymphatic pathways and peculiarities of SLN. In all cases at least one lymph-drainage pathway was identified, both-sided pathways were identified in 78 cases (83,9%), right- an left-sided - in 86 (92,5%) and 85 (91,4%) cases respectively. Infundibulo-pelvic pathway was identified in 17 patients (18,3%), upper and lower paracervical pathway in 67 (72%) and 37 (39,4%) cases respectively. It was mentioned that there are three types of SLN. Type I (“classical” SLN) - contrasted afferent lymph vessel and LN. Type II - contrasted afferent lymph vessel that lead to LN which is either not contrasted (contrast breaks off at the confluence) or contain dot blotches of blue dye (“marble” SLN). Type III - any suspicious LN. All of aforementioned lymph nodes require a biopsy. Pelvic LN dissection (referent method) were completed in 41 cases (46%), among them type I SLN was in 24 cases (58,5%), type II - in 3 (7,3%), type III - in 11 (26,8%), SLN was not identified in 3 patients (7,3%). LN metastases were verified in 10 patients (10,8%). Five cases of type I SLN (20,8%) had LN metastases. All three cases of type II SLN were metastatic. Two cases of type III SLN (18,2%) contain LN metastases.
Conclusion: Usage of the concept of three types of SLN and three routs of lymph drainage may be a perspective method for improvement of endometrial cancer treatment results. Further studies with larger number of patients are needed to prove this hypothesis.
Disclosure: Nothing to disclose

EP604

The emerging role of microcystic, elongated, fragmented (MELF) pattern of myometrial invasion in the diagnostic pathway of endometrial cancer: a systematic review of the literature

A. Prodromidou, G. Vorgias, K. Bakogiannis, N. Kalinoglou, C. Iavazzo
Department of Gynecological Oncology, Metaxa Memorial Cancer Hospital, Piraeus, Greece

Introduction / Background: Microcystic, elongated, fragmented (MELF) pattern of myometrial invasion is a recently introduced histological pattern detected in some specimens from patients with endometrial carcinoma (EC) with debatable prognostic and predictive effect. The aim of the present study is to examine the role of MELF in the prognosis and survival of patients with EC.
Methodology: A total of 4 electronic databases **(**Medline, Scopus, Google Scholar, and Clinicaltrials.gov) were searched for articles published up to May 2019, along with the references of all articles. All prospective and retrospective trials which reported outcomes of cases with EC who were examined for the presence of MELF pattern were included in the present systematic review.
Results: Of the 224 records screened, 15 were considered eligible. Fifteen studies which comprised 785 women were finally included in the systematic review. The correlation between MELF pattern and patients' age, tumor grade and stage remained controversial among the included studies. Patients with MELF (+) were detected with larger tumors while lymph node metastasis, lymphovascular space invasion and >50% myometrial invasion were significantly associated with MELF positivity. None of the included studies noted significant difference with regards to disease free survival (DFS) and disease specific survival (DSS) as well as in terms of vaginal recurrence rates. On the other hand, MELF (+) was highlighted as a significant indicator of overall survival.
Conclusion: The present study indicates the potential critical role of MELF pattern of myometrial invasion in the detection of lymphovascular space invasion and lymph node metastasis in patients with EC. Regardless, its implication in survival and recurrences still remains elusive and further studies are needed in the field in order to clarify the exact role of MELF in the prognosis and management of EC patients.
Disclosure: Nothing to disclose

EP605

A single center study of the role of inflammation biomarkers in endometrial cancer

A. Prodromidou, E. Galati, V. Psomiadou, A. Maliaros, F. Lefkopoulos, N. Blontzos, C. Iavazzo, G. Vorgias
Metaxa Cancer Hospital, Piraeus, Greece

Introduction / Background: Endometrial cancer is estimated to be most common female cancer. Among other predictive factors, inflammation biomarkers resulting from white blood cell and platelet counts ratios have been introduced in the assessment of endometrial cancer.
Methodology: Preoperative NMR, LMR, NPR, LPR and MPR ratios in 154 patients of the gynecological department of Metaxa Memorial Cancer Hospital were calculated.
Results: PLR was significantly different between women with negative and women with positive lymph nodes, women with mucinous histology type and those with serous histology type and between patients with stage I and patients with stage III disease. MPR was significantly different between women with stage I and women with stage III too but also between women with stage III and women with stage IV disease as well as patients with positive and patients with negative lymph nodes. LMR was found to be significantly different between patients with negative and patients with positive disease in the upper abdomen, between endometrioid and serous type of histology, grade 1 and grade 3 disease, women with stage I and women with stage IV and between women with stage II and women with stage IV. NPR was significantly different between women with stage I and women with stage III cancer and between women with stage III and women with stage IV cancer and patients with negative and patients with positive lymph nodes. Finally, NLR was significantly different between women with mucinous histology and those with serous histology and between women with stage I and

stage IV.
Conclusion: NPR and MPR were associated with the nodal status while NLR and PLR were correlated with serous or mucinous histology. Upper abdomen metastases and endometrioid histology seem to have an effect on LMR and all of the profiles were found to be affected by the stage and the grade of the tumor.
Disclosure: Nothing to disclose

EP606

Concurrent panniculectomy at the time of gynaecologic surgery in obese women treated for gynaecologic cancer, a single center experience

V. Psomiadou, A. Fotiou, A. Douligeris, M. Drakou, D. Korfias, G. Vorgias, C. Iavazzo
Gynaecologic Department, Metaxa Cancer Hospital, Piraeus, Greece

Introduction / Background: The prevalence of obesity has been rising during the past decades, Greece´s population is not an exception. The treatment of obese female patients with gynaecologic cancer is a challenging task for surgeons. Panniculectomy is a safe procedure that offers better visualization of pelvic anatomy and thus increased lymph node exposure and less intraoperative complications.
Methodology: A retrospective institutional surgical database was used to identify obese women who underwent surgical treatment for gynaecologic cancer with concurrent panniculectomy between January of 2015 and December of 2018. Major intraoperative or postoperative complications as well as wound surgical complications were documented.
Results: Seven obese women were treated with concurrent panniculectomy at the time of gynaecologic surgery for cancer in a single institution. Median age was 55 years old, median blood loss was 228 ml and median operative time was 195 minutes. Three patients had endometrial cancer, two patients complex atypical endometrial hyperplasia, one patient borderline ovarian cancer and one adult granulosa ovarian tumour. One patient suffered from wound infection and was treated with antibiotics and one patient died after wound infection, wound dehiscence and renal failure.
Conclusion: For selected obese patients concurrent panniculectomy with surgical treatment of gynaecological cancer is a safe and efficient procedure.
Disclosure: Nothing to disclose

EP607

Tumor markers and ultrasound findings in endometrial cancer: a single institution 5 year report

V. Psomiadou, E. Galati, A. Prodromidou, I. Mamais, A. Fotiou, A. Douligeris, C. Iavazzo, G. Vorgias
Metaxa Cancer Hospital, Piraeus, Greece

Introduction / Background: Endometrial cancer affects approximately 5% of the female patients. Lately, serum tumor markers such as Ca-125 and HE4 have been suggested as useful prognostic markers of advanced disease. Furthermore ROMA index is used so far to estimate the likelihood of malignancy of an ovarian tumor but its utility in assessing endometrial cancer is under study.
Methodology: 154 endometrial cancer patients of Metaxa Memorial Cancer Hospital were retrospectively studied and the preoperative serum levels of tumor markers HE4 and CA-125 as well as the ROMA index were measured for each case.
Results: Mean age for patients with endometrial cancer was 61,50 years. CA-125 values were significantly different when comparing women with depth of myometrial invasion > 50% and < 50%, the presence of metastases in the upper abdomen, lymph node metastasis and positive or negative cytology.

HE-4 values were significantly different between women with depth of myometrial invasion > 50% and women with depth of myometrial invasion < 50%, women with metastases in the upper abdomen and women with no disease in the upper abdomen. There were also statistically significant differences in HE4 values between groups with positive or negative lymph nodes, and positive or negative cytology . Statistically significant differences were observed when comparing ROMA values for women with depth of myometrial invasion > 50% and women with depth of myometrial invasion < 50%, presence of disease in the upper abdomen or not, positive or negative lymph nodes, and positive or negative cytology.
Conclusion: The present study confirms CA-125 to have a prognostic value in endometrial cancer patients. It also implies a significant association between HE-4 and the cancer stage and grade as well as the metastases in the upper abdomen.By calculating the ROMA score in our patients we combined both marker levels and showed a corresponding difference regarding endometrial pathology.
Disclosure: Nothing to disclose

EP608

Learning curve with ICG SLN mapping in endometrial cancer

A.A. Quesada Lopez-Fe[1,2], J.A. Pérez Álvarez[1], T. De Ganzo Suarez[1], D. Preckler Peña[1], G. Gómez De la Rosa[1]
[1]Hospital Universitario Nuestra Señora de Candelaria, [2]Hospital Nuestra Señora de Candelaria, Santa Cruz de Tenerife, Spain

Introduction / Background: Appropriate extent of lymphadenectomy in clinically, early stage endometrial cancer remains controversial but sentinel lymph node (SLN) mapping has emerged as an alternative staging strategy, until the advent of molecular prognostic markers.
The aim our study was to determine feasibility and mapping rates progression usin indocyanine green (ICG) for sentinel lymph node (SLN) mapping in endometrial cancer
Methodology: we analized the data of our cohort of 46 women.
23 women with les than 50% of myometrial inversion (MI) and 23 with more than 50% of MI.
One vial of 25mg of ICG isd reconstittuted with 10cc of aqueous sterile water. A total of 4cc is used for the intracervical injections at 3 and 9 o´clock deeply into the cervical stroma and superficially.
Results: Our results show global detection rates of 80,5% (37/46) and bilateral detection rate of 45% (17/46). If we analize the results based in the learnig curve. The first 20 women had a detection rate of 80% but only bilateral rate of 30%.
The last 26 women had a detection rate of 81% (21/26) and bilateral rate of 77%.
If we consider only the last 10 patients the detection rate was 100% with a bilateral detection rate of 80%.
There were 2 positive sentynel nodes (5%) both in the group of women with more than 50% MI. The false negative rate of sentynel node was 0%.
There were not further additional complications due to the technique of SNL ICG mapping.
Conclusion: ICG SNL mapping for endometrial cancer is a feasible, safe and cheap technique. During the learning curve we do not have complications and the improvement in the detection rate appears after few cases.
Disclosure: Nothing to disclose

EP609

The prognostic role of LRIG proteins in endometrial cancer

Z. Razumova[1], H. Oda[2], I. Govorov[1], E. Lundin[2], E. Östensson[1], D. Lindquist[3], M. Mints[1,4]
[1]Department of Women's and Children's Health, Karolinska Institutet, Stockholm, [2]Department of

Medical Biosciences, [3]Department of Radiation Sciences, Umeå University, Umeå, [4]4Faculty of Medicine and Health, School of Medical Sciences, Örebro University, Örebro, Sweden

Introduction / Background: Endometrial cancer (EC) is the most common gynaecological malignancy in Sweden. The disease has several prognostic factors. Still, the high amount of EC develops into more aggressive forms of cancer, even though being first considered to be non-aggressive. The LRIG proteins are a family of three integral surface proteins that have a similar domain organisation. The current study evaluated the role of LRIG proteins as prognostic biomarkers in EC.
Methodology: The cohort included 75 women who underwent a hysterectomy and bilateral salpingo-oophorectomydue to EC at the Department of Women´s and Children´s Health, Karolinska University Hospital Solna between 2007 and 2012. The expression of LRIG1, LRIG2, and LRIG3 in paraffin biopsies was analysed by immunohistochemistry (IHC)with applying specific polyclonal antibodies. Evaluation of immunostainings was performed by two senior pathologists without knowledge of the disease outcome.The percentage of positive cells was divided in two groups with median percentage as cut off to have two groups of equal size included in the statistical analysis. Then the groups were assessedin connection with different tumour characteristics and clinical outcomes of EC.
Results: The majority of women in the cohort had >50% LRIG1-, LRIG2-, and LRIG3-positive cells. Among 6047 person-months of follow-up a total, of 14 incident cases of relapsed EC were identified. A statistically significant association between high LRIG3 expression and superior overall survival was observed in the cohort (IRR=2.559, 95 CI=1.054-6.210, p=0.038).LRIG1 and LRIG2 expression did not significantly correlate with survival.
Conclusion: Our results support the hypothesis that LRIG3 expression may have a prognostic role in women with EC. The significance of LRIG1 and LRIG2 expression remains to be clarified.
Disclosure: Nothing to disclose

EP610

Investigation of patient acceptability of ctDNA in endometrial cancer follow-up

A. Relton[1], H. McDermott[1], A. Collins[2], R. Wickham-Joseph[3], D. Guttery[2], D. Gorsia[2], A. Whitton[3], E. Moss[2]
[1]Department of Psychology, National Centre of Sports and Exercise Medicine, Loughborough University, Loughborough, [2]University of Leicester, [3]University Hospitals of Leicester, Leicester, United Kingdom

Introduction / Background: Circulating tumour DNA (ctDNA) is emerging as a potential option to detect disease recurrence in women undergoing follow-up for endometrial cancer (EC). However, ensuring patient acceptability of changing clinical practice and the introduction of new technology is paramount.
Methodology: The aim of this study was to explore women's opinions on the acceptability of ctDNA and their experiences of the ctDNA study. Women undergoing follow-up and participating in a cohort study investigating the utility of ctDNA to detect/monitor recurrent/progressive EC were invited to participate. Semi-structured interviews were conducted by a psychology student (independent from the clinical research team) and analysed through template analysis. Participants were also asked on their views as to the potential role for ctDNA in clinical practice, either in combination with hospital follow-up or as remote monitoring with a patient-initiated follow-up (PIFU) scheme.
Results: Women expressed that their motivations behind participating in the study were primarily due to altruism and a desire to help others, including protecting others from having pelvic examinations. Participants spoke about their dislike of the pelvic examinations, which were deemed unpleasant, and shared their desires to ´find a better way´, and a blood test was viewed as a welcome alternative. The ctDNA blood test was viewed as more physically and psychologically acceptable than the current clinical examination. Participants expressed a wish to know if their ctDNA level was raised, even if a recurrence wasn't detectable on imaging, because it was felt that they would have more certainty in their likelihood of recurrence.
Conclusion: This study provides in-depth insights into patient views on the acceptability of ctDNA for monitoring of EC, with women stating an overwhelming preference for a blood test over pelvic

examination. Participant insights also revealed that PIFU in conjunction with ctDNA, appears to be an acceptable new approach to EC follow-up.
Disclosure: Nothing to disclose

EP611

Microcytic elongated and fragmented (MELF) pattern in early-stage endometrioid adenocarcinoma - is there association with vaginal recurrence? A case report

I. Sá[1,2], A.C. Rocha[1,3], S. Raposo[1], R. Sousa[1], C. Abrantes[1], L. Sá[1]
[1]Instituto Português de Oncologia Franscisco Gentil, Coimbra, [2]Centro Hospitalar de Trás-os-Montes e Alto Douro, Vila Real, [3]Hospital Distrital de Santarém, Santarém, Portugal

Introduction / Background: Prognosis of early-stage endometrial adenocarcinoma is favourable. However, some factors can influence the risk of recurrence: tumour size and grade, depth of myometrial invasion, cervical stromal involvement and lympho-vascular space invasion(LVSI). A microcytic, elongated, and fragmented(MELF) pattern of invasion has also been implicated in poor prognosis. Many studies had describe the association between MELF pattern with non-vaginal recurrences and lymph-node metastases.
Methodology: This case describe an early-stage MELF pattern endometrial adenocarcinoma with no LVSI and multiple vaginal recurrences.
Results: A 74-year-old woman with hypertension and history of cerebral aneurysm was referred to our institution for an endometrial adenocarcinoma G1 diagnosed in a previous biopsy. A total abdominal hysterectomy and bilateral salpingo-oophorectomy was performed (due to her comorbidities, we didn't accomplish bilateral pelvic lymphadenectomy). Histologic diagnosis revealed an endometrioid adenocarcinoma G2, with myometrial invasion depth >50%, negative LVSI, MELF pattern of myometrial invasion and negative peritoneal washing cytology - FIGO stage IB. One month later, the patient referred vaginal bleeding and presented a vaginal vault vegetant lesion measuring 1,5cm. The biopsy showed metastasis of endometrioid adenocarcinoma. She was submitted to a full lesion resection and started adjuvant vaginal brachytherapy (50Gy/5fractions). Four months later, she presented with 2 vaginal vault milimetric lesions whose biopsy revealed metastases of adenocarcinoma. The complete resection of the lesions confirmed the diagnosis. She repeated vaginal brachytherapy treatment (30Gy/5fractions). Pelvic MRI didn't show lymph-node metastases. The patient remains in clinical control in our institution.
Conclusion: The significance of MELF pattern is still unclear. Although many studies describe its association with lymph node metastases and non-vaginal recurrences, this case shows that this histologic pattern can also be associated to vaginal metastases. In the future, MELF pattern could be useful for identifying patients in early-stage endometrial tumours with higher risk of recurrence.
Disclosure: Nothing to disclose

EP612

Repair of external iliac arter with endovascular graft at a case of dedifferentiated endometrial carsinom

S. Şahin Aker[1], F. Ortaç[2]
[1]Gynecologic Oncology Department, Ankara University School of Medicine, [2]Gynecologic Oncology department, Ankara University, Ankara, Turkey

Introduction / Background: Dedifferentiated endometrioid adenocarcinoma (DEAC) is rare and is known to be more aggressive than high-grade endometrioid carcinoma. DEAC may pose a variety of diagnostic challenges. DEAC is defined as a tumor which composed of a mixture of undifferentiated

carcinoma and conventional endometrioid adenocarcinom.
Methodology: This is a case report of DEAC with repair of external iliac arter with endovascular graft which was diagnosed in Ankara University Gynecologic. A 48-year-old perimenopausal woman (BMI: 40 kg/m^2) was referred us endometrioid carcinoma FIGO grade 2 diagnose. She suffered from vaginal bleeding for 2 months.Transvaginal ultrasound revealed abnormally thickened endometrium (5,1 cm) and on examination a gross mass protruding from cervix shown and the entire of endometrial cavity is filled with this mass.Pathology reevoluated the endometrial biopsy and revealed DEAC (endometrioid endometrial carsinom grade 1 (%80) and undifferantiated carsinom(%20). MRI showed a 62*52*42 mm mass at lower uterine segment (Fig. 1c) extending to the cervix. MRI showed a 194*11*95 mm lobulated mass filling right pelvis sidewall.Abdominal pelvic computed tomography (CT) revealed that the mass involving bladder and rectum. TAH+BSO+partial cystectomy+low anterior resection+right external iliac artery repair with endovascular greft was performed. In surgery, pelvic mass was involving the right pelvic sidewall and in order to remove tumor totaly we cutted and removed external iliak artery and there was a 7 cm lenght defect at right external iliac artery. A 12 mm diameter and 7 cm lenght stretch vascular graft (GORE-TEX®) was placed to vascular defect and the graft patency was intact . We achieved complete en bloc tumor resection .
Results: After surgery pathology revealed DEAC (endometrioid endometrial carsinom grade 3 and undifferantiate carsinom). And the patient died 1 week later after the surgery due to tumor lysis syndrome.
Conclusion: Differentiating is important because DEACs have fulminant clinical outcomes and poorer prognosis than high-grade endometrioid carcinoma.

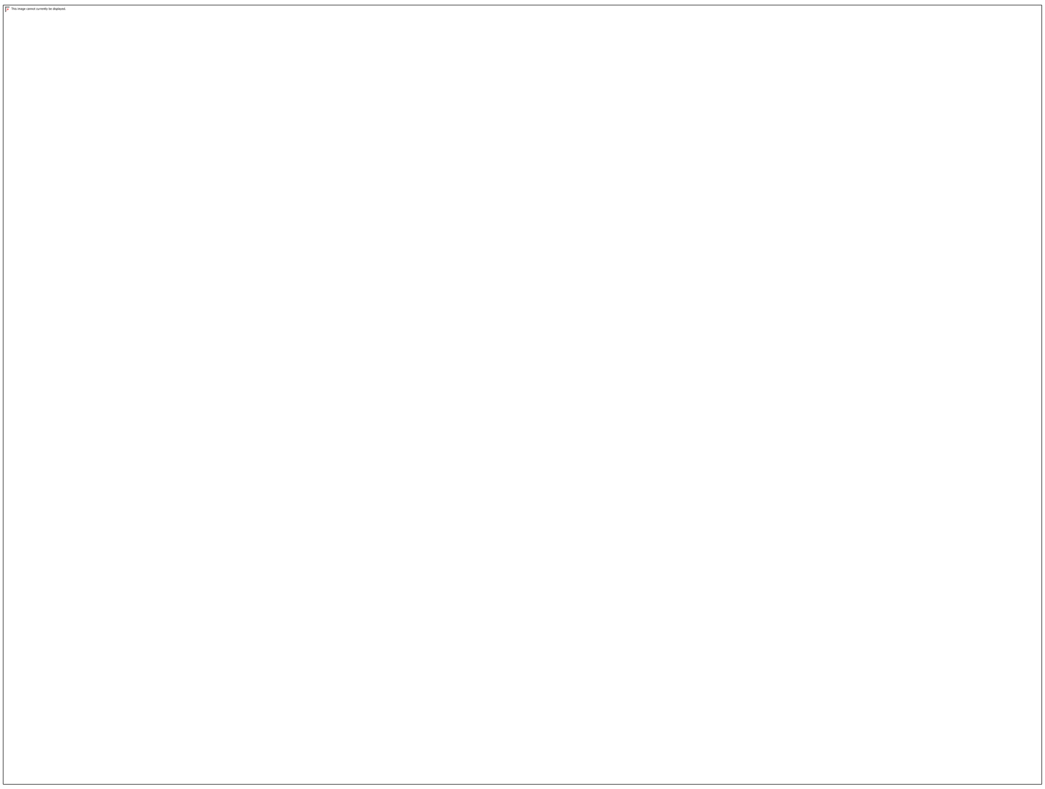

[right external iliac artery defect]

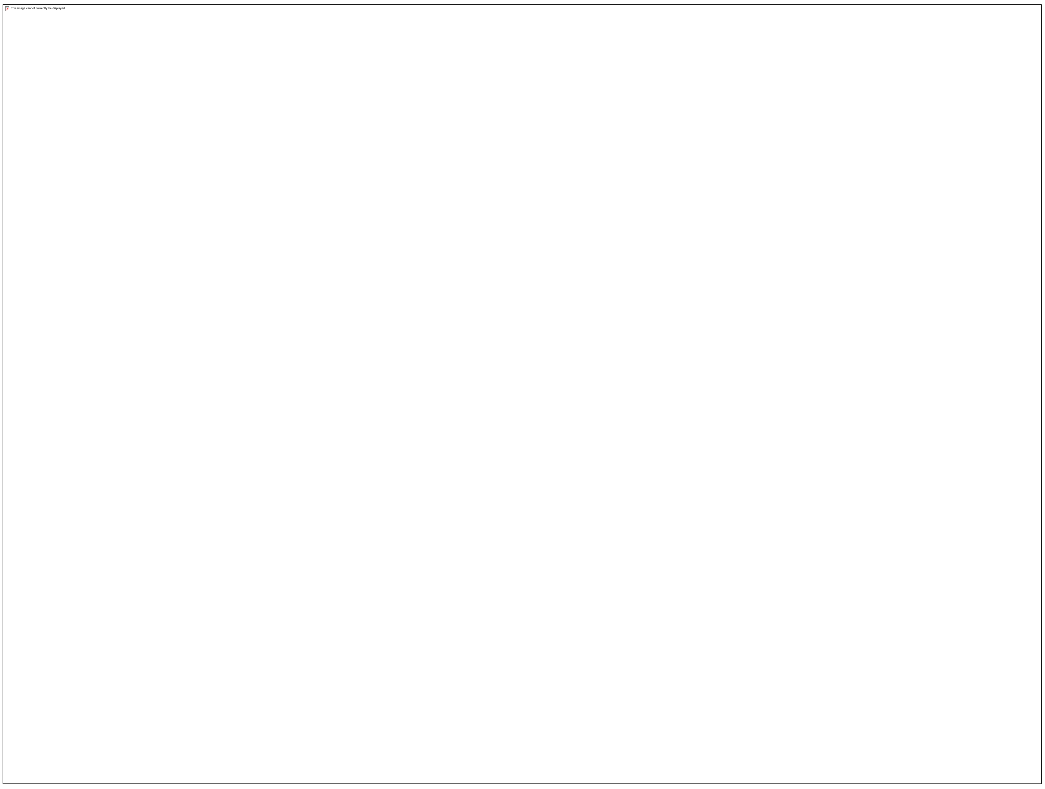

[Repair of external iliac arter with endovascular graft]

Disclosure: Nothing to disclose

EP613

Prospective validation study on feasibility of sentinel lymph node mapping in early endometrial cancer

S. Sambasivan[1], A. G.Nath[2], R. Prabhakaran Nair[1], S. Renjith[1]
[1]Gynecologic Oncology, [2]Regional Cancer Centre, Thiruvananthapuram, India

Introduction / Background: Endometrial cancer is the sixth common female cancer and second common female genital cancer in India. The standard surgical treatment comprise hysterectomy with pelvic and paraortic lymph node dissection.Sentinel lymph node mapping helps to avoid overstaging and associated morbidity.
Methodology: Prospective study from May 2017 to September 2018 . 30 patients diagnosed with endometrial cancer were included in the study. All patients had preoperative MRI as a part of institute protocol.MRI helped to select out patients without nodal enlargement. Informed consent was taken from all patients.Intra cervical injections of 0.2 mL technetium 99 at 3'O clock and 9'O clock position followed by lymphoscintigraphy was done on preoperative day.Intraoperatively Isosulphan blue dye injection 3ml each at 3'O clock and 9'O clock position of cervix.Hot nodes and blue nodes were identified and dissected .All nodes were subjected to frozen examination. Then proceeded with Hysterectomy, bilateral salpingo ophorectomy,bilateral pelvic and paraortic node dissection. Statistical analysis done with SPSS software
Results: Lymphoscitigraphy detected 46.66% (14 out of 30) sentinel node in iliac region.33.3 % cases had 1 hot node and 27.8% cases had 2 hot node on right hemipelvis.50 % cases had hot node at right external iliac region followed by 27.2 % had hot node at right obturator region.50 % cases had

1 hot node and 5.6% had 2 hot node on left hemipelvis.69 % had hot node at left external iliac region and 13.4 % had hot node at obturator region. 38.9 % had blue node on right side and 27.8 % had blue node on left side. 1 case of hot para aortic LN.No blue para aortic LN .None of the cases had frozen positive result.
Conclusion: Detection rate of sentinel node by lymphoscintigraphy is 46.6%.Combined detection rate of SN using both Technitium 99 and iso sulphan blue dye was 73%
Disclosure: Nothing to disclose

EP614

Prognostic implication of primary treatment of uterine low grade endometrial stromal sarcoma: An Institutional experience

S. Sambasivan[1], S. Renjith[2], R. Prabhakaran Nair[2]
[1]Gynecologic Oncology, [2]Regional Cancer Centre, Thiruvananthapuram, India

Introduction / Background: Low grade endometrial stromal sarcoma are a group of indolent tumors with good prognosis. It usually occur in women of 40 to 50 years with a five year survival of approximately 90%. Primary comprehensive surgical management is an independent factor affecting disease free survival and overall survival.
Methodology: We are presenting a series of 4 cases of endometrial stromal sarcoma with diverse pattern of prognosis.
Results: Low grade ESS is usually with good prognosis with long DFS.One patient was presented with immediate post-operative extensive recurrrenc as a result of laparoscopic morcellation. In the second case patient had seven years disease free interval. Then she developed a single site recurrence which was resectable with good prognosis . The third case in the series patient was put on primary hormonal treatment for twenty months as she was not willing for hysterectomy.Later hysterectomy was performed and still patient has got good prognosis with seven years of disease free interval.Fourth case underwent hysterectomy with bilateral salpingoophorectomy and kept under follow up.She alsohas a fairly good disease free interval of 8 years following primary surgery without intraoperative spillage.
Conclusion: Preoperative diagnosis of endometrial sarcoma may not be possible. Hence it is better to avoid open morcellation during laparoscopic myomectomy. Many Gynaecologists are using contained morcellators for tissue extraction . More data is needed regarding safety of the new devices
Disclosure: Nothing to disclose

EP615

Implementation of enhanced recovery after surgery for endometrial carcinoma: a non-randomized controlled trial

A. Sameer[1], E. El-Gindi[2], R.R. El-Khateeb[2], A.N. Abdelrazik[3], A.B. Moussa[1], A.S. Hafiz[4]
[1]Gyn-Oncology, [2]Ob/`Gyn, [3]Anesthesia, Minia University, Minia, [4]Oncology, El-Salam Oncology Center, Cairo, Egypt

Introduction / Background: To study the impact of modified Enhanced Recovery After Surgery (ERAS) program for endometrial carcinoma, on the perioperative outcome.
Methodology: The study included females with physical status I and II according to American Society of Anesthesiologists (ASA) undergoing exploration for endometrial carcinoma. ECOG Performance Status was used to determine patient´s disease progression. The study was done in El-Salam Oncology center; Cairo and Minia Maternity University Hospital, Egypt. The study included two groups of patients diagnosed as endometrial carcinoma prepared for surgical management. First group

included patients prepared for laparoscopy (n=27) and was managed with ERAS protocol. Another group prepared for laparotomy (n=31) and was managed with conventional protocol.
Results: The length of hospital stay (LOS) was reduced in ERAS group when compared with the control group (1.77 ±1.13 days compared with 6.13 ±2.25 days; P < 0.0001; CI 3.4013 to 5.3187). Opioid use was reduced in ERAS group compared to the control group (1.10 ±0.41mg and 9.83 ±3.6 mg; P < 0.0001) and postoperative fluid use was also less in the ERAS group compared to the control group (1362.9 ±234.3 ml compared with 2303.2 ±512 ml; P < 0.0001; CI 725.4990 to 1155.1010). Pain score using VAS on postoperative day 0 was 3.18 ±1.15 vs 4.48 ±1.68 (P = 0.0013) for both active and control groups respectively.
Conclusion: Implementation of ERAS protocols in gynecologic surgery for endometrial carcinoma should be tailored according to the situation of each case. But in general, ERAS was associated with significant reduction in length of hospital stay, reduction in the operative time and blood loss, associated with controlled intravenous fluids utilized and comparable pain control to conventional protocols with improvement in complication rates.
Disclosure: Nothing to disclose

EP616

Risk factors for failure of sentinel lymph node mapping using indocyanine green/near-infrared fluorescent imaging in endometrial cancer

S. Taşkın[1], M.E. Sarı[2], D. Altın[1], C. Ersöz[3], A. Gökçe[1], S. Yüksel[3], D. Kankaya[3], F. Ortaç[1]
[1]Gynecologic Oncology, Ankara University School of Medicine, [2]Gynecologic Oncology, University of Health Sciences, Ankara Training and Research Hospital, [3]Department of Pathology, Ankara University School of Medicine, Ankara, Turkey

Introduction / Background: Side-specific systematic lymphadenectomy is suggested if sentinel lymph node (SLN) mapping failed in early stages endometrial cancer. This study aimed to evaluate the risk factors associated with failed mapping which may lead to modify SLN mapping technique, increase the success of SLN mapping and reduce the necessity of systematic lymphadenectomy.
Methodology: Patients with early stage endometrial cancer were included in this study. All patients underwent SLN mapping with indocyanine green/near-infrared compatible surgical platforms. Indocyanine green was injected intracervical. "Bilateral mapping" and "failed bilateral SLN mapping (unilateral or bilateral failed mapping)" groups were compared for demographic, clinical, surgical, and pathological features.
Results: 101 cases were analyzed. The overall, unilateral, and bilateral SLN detection rates were 94.1%, 19.8%, and 74.3%, respectively. The failed (unilateral or no mapping) bilateral detection rate was 25.7%. Failed bilateral mapping rates were higher in patients with longer cervical and uterine longitudinal lengths, deep myometrial invasion and larger tumor size without statistical significance. Body mass index and operation type were not related with failed mapping. Increasing number of operations or injection of larger volume of indocyanine green (4 mL vs. 2 mL) did not improve mapping rate significantly.
Conclusion: Cervical indocyanine green injection may overcome the negative effect of obesity on bilateral mapping. Although there was a negative correlation trend between the longitudinal cervical and uterine lengths and bilateral mapping, this possible relation needs to be confirmed in further studies.

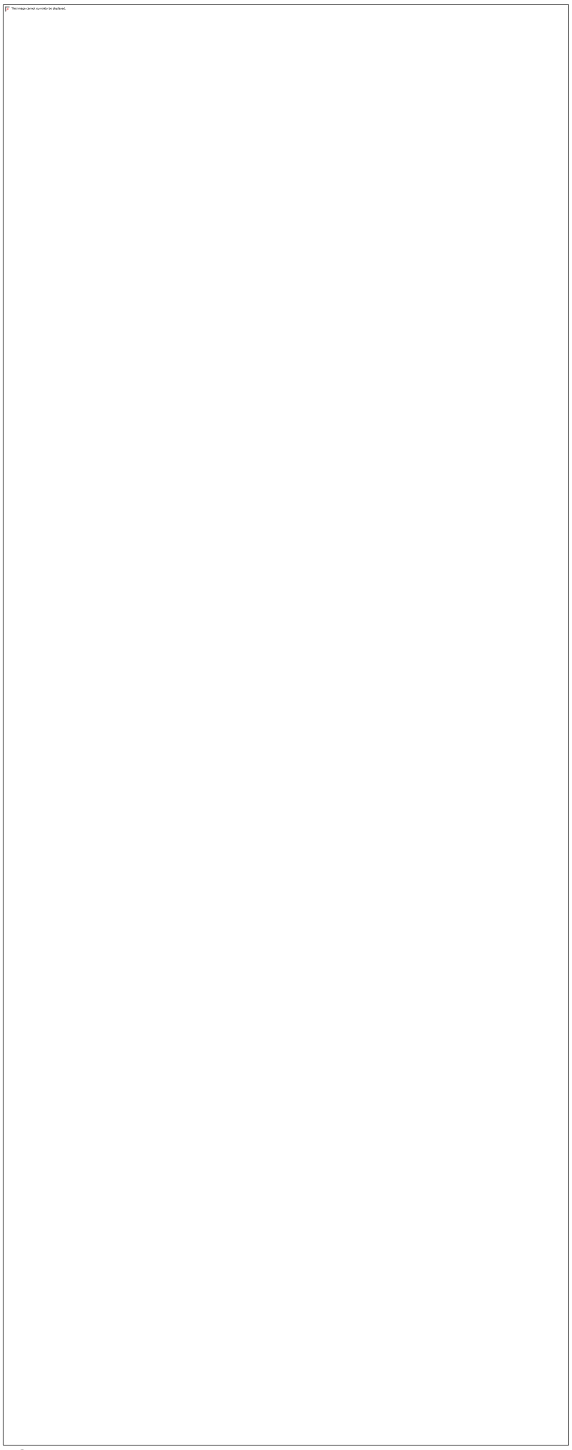

[Comparison of baseline characteristics of patients in terms of SLN detection status]

[Clinicopathological features of patients with no sentinel lymph node detected (n =6)]

Disclosure: Nothing to disclose

EP617

Second line treatment in metastatic uterine pecoma

A. Liapi, R.G. Herrera Gómez, B. Bisig, J.P. Brouland, F. Herrera, P. Mathevet, A. Sarivalasis
CHUV - Centre Hospitalier Universitaire Vaudois, Lausanne, Switzerland

Introduction / Background: Uterine perivascular epithelioid cell tumors (PEComas) are rare neoplasms associated with upregulation of the mTOR pathway, frequently through inactivation of TSC1/TSC2. The treatment of advanced and metastatic PEComas is challenging, especially after progression on mTOR inhibitors. Some reports suggest anti-angiogenic agents as a potent second line treatment. We report the effect of pazopanib for a patient with metastatic uterine PEComa progressing on everolimus.
Methodology: A 53-year-old patient was admitted for a four-month history of vaginal bleeding. Clinical examination and vaginal ultrasound indicated a 6x6 cm uterine mass and PET-CT identified a bone lesion S1. The patient was treated with radical hysterectomy. Both bone biopsy and hysterectomy indicated an oligo-metastatic PEComa with high-grade nuclear features and lymphovascular invasions. The single bone metastasis was treated with radiotherapy, but three months later the patient presented bone, lung and subcutaneous recurrence. Targeted NGS (52-cancer gene hotspot panel) detected no mutation but an heterozygous deletion of 9q34, that contains the *TSC1* gene, prompting a first line everolimus treatment. For the bone metastasis denosumab was initiated. On this treatment, the disease was controlled for two years before symptomatic subcutaneous progression. A second line treatment by pazopanib and denosumab was introduced, based on reports on anti-angiogenic tyrosine kinase inhibitors (TKIs) effect in PEComas.
Results: With the combination of pazopanib and denosumab, there was regression of the subcutaneous disease and stability of lung and bone metastasis for over a year. The treatment toxicities were mild and no dose adaptation was necessary.
Conclusion: Our report provides evidence that anti-angiogenic targeted therapy with pazopanib, a multi TKI, is an effective second line treatment for metastatic malignant PEComas progressing after mTOR inhibition. The toxicity profile of the association was acceptable in line with the previously reported toxicities of the respective drugs.
Disclosure: Nothing to disclose

EP618

The use of preoperative imaging in endometrial cancer: are ontario physicians following GOC guidelines?

K. Schnarr[1], H. Seow[1], L. Elit[1], G. Pond[1], L. Helpman[2], E. O'Leary[1], I. Kong[1]

[1]Department of Oncology, [2]Department of Obstetrics and Gynecology, McMaster University, Hamilton, ON, Canada

Introduction / Background: Joint SOCG-GOC-SCG clinical practice guidelines recommend against preoperative imaging studies (such as CT), except for cases of locally advanced disease or aggressive histologies. This study utilized population-based data to evaluate the use of preoperative imaging in Ontario and factors associated with its use.
Methodology: Ontario women diagnosed with endometrial cancer from 2006-2016 were identified from the Ontario Cancer Registry. Patients with a hysterectomy prior to the date of diagnosis, non-epithelial histology or a prior cancer diagnosis within 5 years were excluded. Age-standardized incidence rate of endometrial cancer and preoperative imaging (CT or MRI) rates were calculated over time. Predictive factors for preoperative imaging use were determined using multi-variable analysis. A subanalysis was performed on low-risk patients, to determine if preoperative imaging use differed in this population.
Results: 21641 cases were included for analysis. From 2006-2016, the number of cases of endometrial cancer increased 48.0%, and age-standardized incidence rate increased 17.6%.
The use of preoperative imaging increased from 19.3% to 32.6%. In a subanalysis of a low-risk population, the rate of preoperative imaging was 16%, compared with 26.8% in the combined population. Factors most predictive of preoperative imaging were: non-endometrioid histology, higher stage, higher grade, comorbidity score and rurality score.
Conclusion: Endometrial cancer incidence and the use of preoperative imaging has increased from 2006 - 2016 in Ontario. Guideline compliance was moderate; while preoperative imaging use was associated with high risk features, inappropriate use was still seen in the low risk population.
Disclosure: Nothing to disclose

EP619

Clinical implications of molecular profiling in patients with primary endometrial cancer

R. Schwameis[1], F. Oberndorfer[2], S. Moling[1], A. Reinthaller[1], S. Polterauer[1], C. Grimm[1], L. Müllauer[2]
[1]Department of General Gynecology and Gynecologic Oncology, [2]Clinical Institute of Pathology, Medical University Vienna, Vienna, Austria

Introduction / Background: In patients with endometrial cancer after surgery adjuvant therapy is frequently administered. In general, indication for adjuvant treatment is established based on clinical pathologic parameters such as patients age, depth of infiltration and pathologic grading. However, although survival has improved, there are still patients over and under treated. Currently, an ongoing prospective trial evaluates risk stratification of patients and treatment modification according to molecular profiling of tumor tissue. This retrospective analysis was performed to investigate, whether molecular profiling of tumor tissue has implication on and is feasible in daily routine.
Methodology: This study included all patients with primary endometrioid endometrial cancer treated at Medical University Vienna between 2017 and 2018. In addition to conventional pathologic analysis, tumor samples were molecular pathologically analyzed. Molecular pathologic evaluation included analysis for micro satellite instability, gen sequencing of 161 different genes, advanced analysis for lymph vascular space invasion and L1CAM analysis. Patients were then stratified into 4 risk groups as previously described.
Results: In total 43 patients were included into this analysis. In 37 cases risk stratification was possible. Microsatellite instability, mutation in the POLE gene locus, and TP53 mutations were described in 5 (11.6%), 3 (7%), und 3 (7%) cases respectively.
In 8 (18.6%) cases molecular profiling would have had an impact on adjuvant treatment. In three cases additional treatment would have been suggested while in five cases molecular profiling would have suggested to restrain from adjuvant treatment.
Conclusion: Molecular profiling is feasible in daily routine, and did not delay therapy. In this small group of patients molecular profiling of tumor tissue would have changed adjuvant treatment in roughly 20% of patients. The current results underline the importance of the PORTEC-4a study that investigates risk stratification of patients according to molecular profiling.
Disclosure: RS Travel expenses: Amgen, Pharma Mar, Roche SP Consultant AstraZeneca, Celgene,

MSD, PharmaMar, Roche,Roche Diagnostics, Meda Pharma, Tesaro, Vifor Pharma, outside the submitted work. CG Consultant: AstraZeneca, Celgene, MSD, PharmaMar, Roche, Tesaro, Vifor Pharma Speaker: Amgen, AstraZeneca, MSD, PharmaMar, Roche, Tesaro Direct research funding: Meda Pharma, Roche Diagnostics AR Research grants: Roche Honoraria as a speaker and for advisory boards: Amgen, Astra Zeneca, MSD, Pharma Mar, Roche, Tesaro, Vifor Pharma Travel expenses: Amgen, Astra Zeneca, Pharma Mar, Roche, Tesaro

EP620

Characterisation of the microbiome along the female genital tract and rectum in endometrial cancer

A. Semertzidou, D. MacIntyre, J. Marchesi, P. Bennett, M. Kyrgiou
Imperial College London, London, United Kingdom

Introduction / Background: Endometrial cancer has a dominant place among gynaecological cancers and is the fourth most common malignancy in women after breast, lung and colorectal disease. Approximately, 2.9 percent of women will be diagnosed with uterine cancer at some point during their lifetime. The intricate relationship between microbes and oncogenesis remains largely obscure. Microorganisms are implicated in 20% of human malignancies and there is currently a wealth of evidence to support this.Functional links between the female genital tract (FGT) microbiome and endometrial cancer have not been established yet, which makes this project conceptually pioneering.
Methodology: Patients with endometrial cancer and benign controls were recruited within Imperial College Healthcare NHS Trust. Eligibility criteria included patients undergoing total hysterectomy with or without bilateral salpingo-oophorectomy either laparoscopically or transabdominally. Microbiome swabs were collected along the female genital tract (lower 2/3 and higher 1/3 of vagina, external cervical os, lower endometrium, fundal endometrium, fallopian tubes and ovaries) and rectum. Bacterial DNA was extracted using QiAmp Mini DNA kit (Qiagen, Venlo, Netherlands). The V1-V2 hypervariable regions of 16S rRNA genes were amplified by PCR and microbial profiling was conducted using a MiSeq platform (Illumina, San Diego, CA, USA). The 16S rRNA gene sequence data were analysed with Mothur software package and OTU taxonomies (from Phylum to Species) were determined.
Results: 65 patients were recruited subdivided into 42 patients with endometrial cancer and 23 with benign conditions.Most common benign indications for hysterectomy were fibroids and dysfunctional uterine bleeding.We observed that bacterial composition and richness differ between women with endometrial cancer and benign controls. In particular, women with endometrial cancer display lower bacterial diversity and richness along their genital tract. Additional evidence will be presented at the conference.
Conclusion: The microbial landscape of women with endometrial cancer differs from that of benign controls.
Disclosure: Nothing to disclose

EP621

Treatment results and patterns of failure of Type II endometrial cancer patients treated at radiotherapy department, national cancer institute, Cairo University, during the period from January 2000 till December 2012

M. Serageldin Abdelaziz[1,2], M.M. Abdelhakim[1], A.S. Fouad[3], M.H. Elnaggar[1]
[1]Radiotherapy, National Cancer Institute, Cairo University, [2]Radiotherapy, Children's Cancer Hospital Egypt (CCHE), [3]Clinical Oncology, Kasr El Ainy Center of Clinical Oncology (NEMROCK), Cairo, Egypt

Introduction / Background: Type II tumors account for 10 to 20 percent of all endometrial carcinomas. These include tumors of non-endometrioid histology: serous, clear cell, squamous, transitional cell,undifferentiated, and recently carcinosarcomas. They are mostly poorly differentiated, with tendency to deep myometrial invasion, distant metastasis, and poor prognosis. Thus, they are considered high-risk. The optimal management is not yet fully settled and still evolving. We sought to identify all clinico-pathological data, different treatment modalities and prognostic factors which affected the locoregional control (LC), disease-free survival (DFS), and overall survival (OS) of Type II endometrial cancer patients presented to our department during a 12-year period.
Methodology: Data records of Type II endometrial carcinoma patients presented to the Radiotherapy department, National Cancer Institute, Cairo University during the period from (2000-2012) were retrospectively reviewed.
Results: The study included 88 patients. 66% were above 60 years of age. Histological subtypes were: Carcinosarcoma 45.5%(n=40), Papillary serous 33%(n=29), Clear cell 12.5%(n=11),Undifferentiated 9%(n=8). Most common stage was FIGO stage IB (33%). 88.6%(n=78) of patients received adjuvant External beam Radiotherapy (EBRT) and 54.5%(n=48) received additional brachytherapy. Only 10.2%(n=9) received adjuvant chemotherapy.
The 5-year LC for the whole group was 65.1%. Age(p=0.028) and brachytherapy boost after EBRT(p=0.05) were significant contributors to local control. The 5-year DFS was 44.6%. Age(p=0.001) and FIGO stage(p=0.004) were significant factors affecting DFS. The 5-year OS was 69.1%. Age(p=0.02) and FIGO stage(p=0.004) significantly affected OS. Locoregional recurrence was noted in 19.3% of patients(n=17), most commonly in the vaginal vault (47.1%) followed by operative bed (23.5%). 31.8% (n=28) developed distant metastasis, commonly in the peritoneum(25%)(n=7).
Conclusion: Type II endometrial carcinomas are a distinct, high-risk group with high tendency for distant failure.Age and FIGO stage were independent significant prognostic factors for OS and DFS. Age and brachytherapy boost after EBRT also significantly affected LC.Larger prospective studies are needed to define the optimum management.
Disclosure: Nothing to disclose

EP622

Use of pelvic lymphadenectomy in the management of high grade & type 2 endometrial cancer: a survey of current practice of gynaecological oncology centres in the United Kingdom

R. Jones, R. Howells, K. Lim, A. Sharma
Department of Gynaecological Oncology, University Hospital of Wales, Cardiff, United Kingdom

Introduction / Background: Previous trials have not shown any therapeutic benefit of pelvic lymphadenectomy (PL) in high grade or type 2 endometrial cancer but doubts have been raised regarding the findings of these trials due to their methodology. The recent ESGO guidelines (2016) have only recommended lymphadenectomy for purpose of comprehensive staging and determining prognosis. Our aim was to assess the current practice of gynaecological oncology centres in the United Kingdom.
Methodology: We surveyed the membership of the British Gynaecological Cancer Society (BGCS) via an online survey platform from 01/11/2017-30/06/2018. A single reminder was sent during the collection period. Information was gathered regarding type of surgery offered, proportion of procedures completed through minimally invasive surgery, whether PL is offered routinely and what adjuvant treatment is offered. Data analysis was with Microsoft Excel.
Results: Responses were received from 17 of the 33 (52%) UK gynaecological oncology centres. All centres reported offering hysterectomy and bilateral salpingo-oophorectomy as the main treatment. Omental biopsy or omentectomy was reported as offered in 4 (24%) centres for high grade and 100% of centres for type 2 disease. 12 (71%) centres reported >75% of cases were completed through a minimally invasive procedure, the remaining 5 (29%) centres reported 50-75% of cases being completed minimally invasively. 15 (88%) centres reported offering PL routinely, 10 of these provided complete information regarding the adjuvant treatment offered, 9 of which reported altering the adjuvant therapy offered dependent on whether PL had been undertaken or not.
Conclusion: Although there is variation in the procedures performed, most of the responding centres

performed surgical assessment of pelvic lymph nodes; the majority of these report using it to guide what adjuvant therapy is offered. Further prospective trials are required to assess the value of PL in management of high grade and Type 2 endometrial cancers.
Disclosure: Nothing to disclose

EP623

Sex hormonal profile during the development from endometrial hyperplastic disease to endometrial cancer

Y. Shi, X. Chen
Gynecology, Obstetrics and Gynecology Hospital of Fudan University, Shanghai, China

Introduction / Background: Type I EC, aka, endometrial endometrioid carcinoma (EEC) is a typical endocrine-related disease, closely associated with both hormonal imbalance and obesity. It has definitive precancerous stages that develops step-by-step from DPE, SH, CH, and EAH to cancer. This progression provides us an excellent model to investigate the mechanisms of sexual hormones and adipocyte involvement in the carcinogenesis of EC.
Methodology: A total of 1874 cases were enrolled, including 764 cases of healthy women, 145 cases diagnosed with disordered proliferative endometrium (DPE), 250 cases with simple hyperplasia (SH), 200 cases with complex hyperplasia (CH), 259 cases with endometrial atypical hyperplasia (EAH), 256 cases of EC. Profiles of BMI and sex hormone levels were analyzed and compared in different groups. Multiple linear regression analysis was done to control for the counfounding factor, age.
Results: Estradiol elevation only took place in pre-cancerous stage. However, progesterone trend is a bit delayed, the significant difference totally vanished until the stage EEC G2, which stage is also a contraindication for conservative treatment with high potency progestins. Nuclear ER-a and membranous GPER also peaked at the phase of EAH and sharply decreased at G1, while continue to further decrease at G2 and G3. No significant difference was found for free estrogen level (FEI) in different groups compared with the control. BMI gradually increases and peaks at EEC (G1, G2), and this group of patients was the only group with both median and mean BMI> 25kg/m2, aka, overweight.
Conclusion: Our hypothesis is cells attain cancerous properties under the stimulation of estrogen, and once cells gain carnogenic mutation, estrogen is no longer the primary engine for cancer progression.
This phenomenon can be explained as “Guider Effect Model” which we made an analogy for E2 as a guider in theater, once the cells seated in the “cancer”, the guider just left immediately.

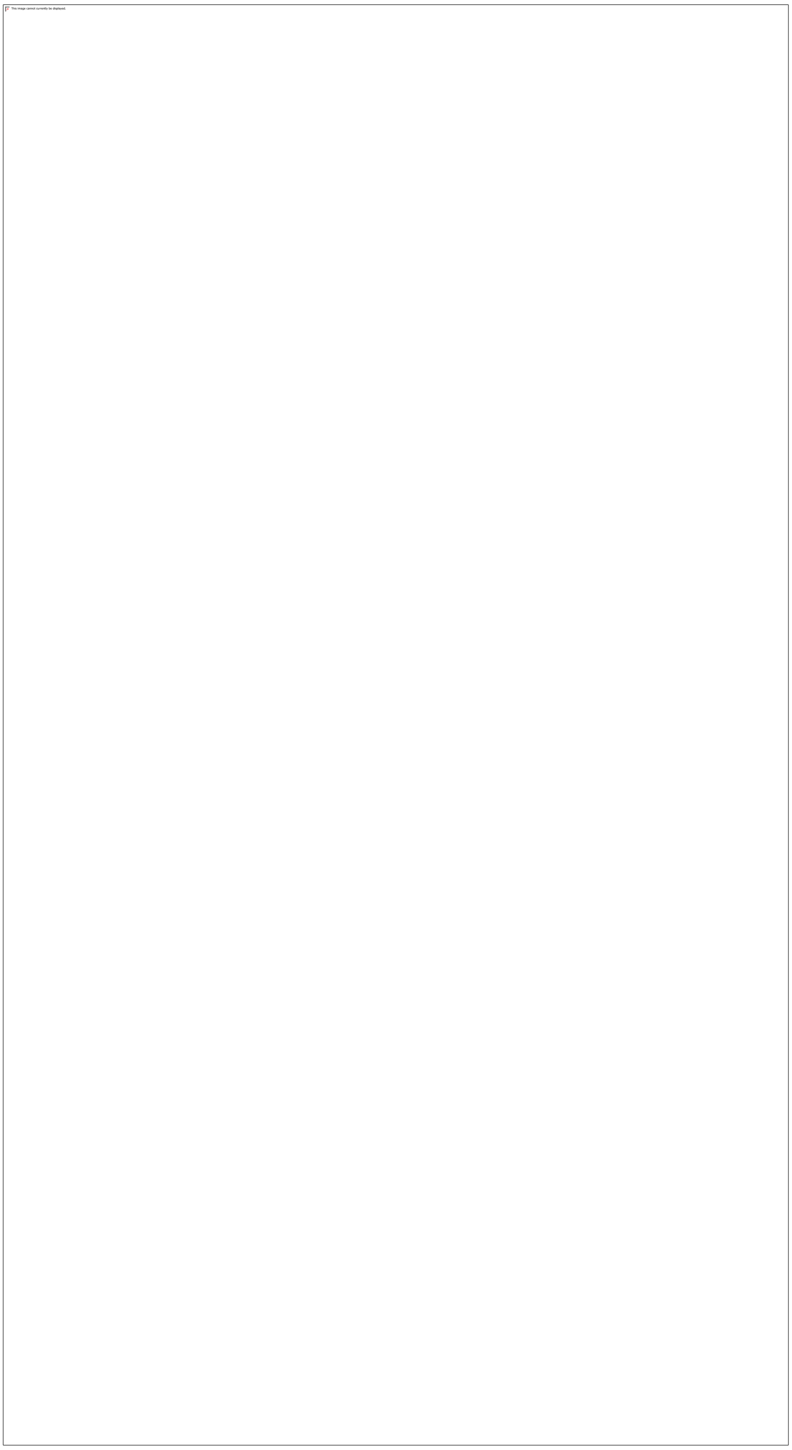

[The characteristics of endocrine profiles at each stage of the disease progression when holding age]

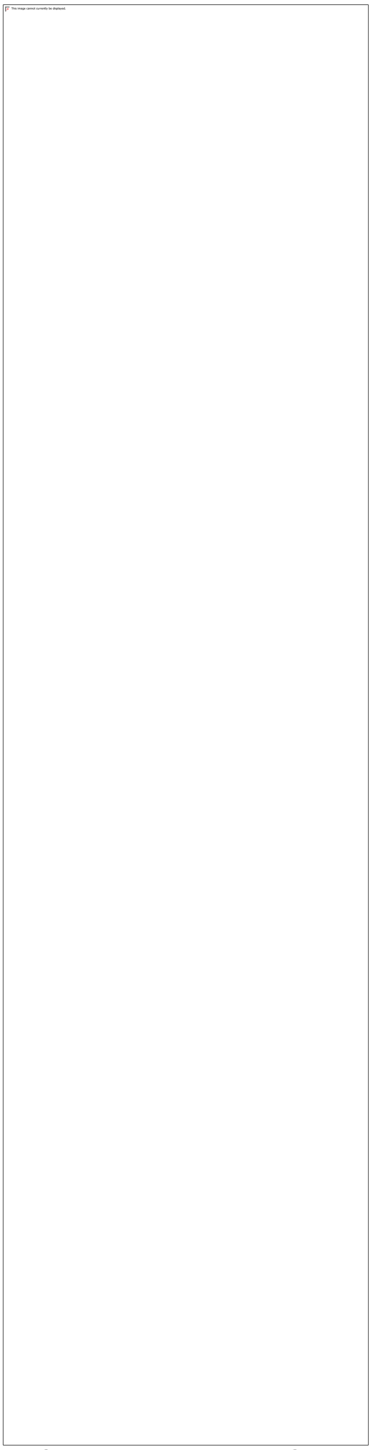

[IHC staining of ER-a and GPER at different stage of the disease]

	Control (n=768)	DPE (n=103)	SH (n=206)	CH (n=158)	EAH (n=131)	EEC G1-2 (n=122)	EEC G3 (n=15)	Type II EC (n=11)
Cases (n)								

Diabetic	49	3	4	3	4	11	1	3
Non-diabetic	715	100	202	155	127	111	14	8
Total	764	103	206	158	131	122	15	11
Diabetes Mellites (%(n))	6.41%	2.91%***	1.94%***	1.90%***	3.05%***	9.01%	6.67%	27.27%***
Age (y)								
	41(10)	42(10)	46(7)*	40(15)*	36(11)***	52(20)***	53(9)***	60(20)***
BMI (kg/m2)								
	22.66(2.42)	23.50(2.22)	23.44(2.51)*	23.28(3.59)*	24.54(4.58)***	24.94(4.81)***	21.78(3.68)	24.97(5.14)

[Cohort characteristics of study population]

Disease progression	β	S.E.	P	Ratio(95% CI)
DPE	0.026	0.035	0.459	1.026（0.991-1.061）
SH	0.089	0.036	0.015	1.093（1.057-1.129）
CH	0.107	0.064	0.046	1.130（1.066-1.194）
EAH	0.103	0.069	0.025	1.110（1.041-1.179）
EC(G1,G2)	-0.129	0.034	0.0001	0.879(0.804,0.939)
EC (G3)	-0.012	0.037	0.756	0.988(0.916,1.061)
Type II EC	-0.025	0.036	0.486	0.975(0.903,1.046)

[Multivariate Linear Regression Analysis of E2 level with disease progression when holding patient age constant]

Disclosure: Nothing to disclose

EP624

Non-endometrioid adenocarcinoma of the uterine corpus: clinicopathological results and treatment outcomes

M. Shouman[1], A. Helal[1], A. Namour[1], E. Hussien[2]
[1]Medical Oncology, [2]Pathology, National Cancer Institute, Cairo, Egypt

Introduction / Background: Uterine corpus cancer is the most common gynecological malignancy in developed countries and the second most common after cancer cervix in developing countries. Non-Endometrioid malignancies have higher risks for recurrence and distant metastasis that may address the need for adjuvant therapy. In this study, we investigated different prognostic factors, clinic-pathological data and treatment outcome to try to find the best treatment modality and its effect on survival.
Methodology: A total of 76 patients with Non-endometrioid adenocarcinoma were treated in NCI,

Cairo University (2009-2013). In which the treatment options, Histological and prognostic factors were correlated to OS and DFS.
Results: The median age at presentation was 62 years. The most frequently encountered pathology was Carcinosarcoma (48.7%) followed by papillary serous carcinoma (36.8%). The 3 years & 5 years OS for the whole group of Non-Endometrioid adenocarcinoma were 35.9 ± 5.6% & 17.5% ± 5.6% respectively and with a median OS of 22.2 +/-3.3 month. Adjuvant radiotherapy (P=0.007), Lymph node negativity (P=0.005) and early stage I/II (P=< 0.0001) had a statistically significant improvement in OS. While the 3 years & 5 years DFS for the whole group of Non-Endometrioid adenocarcinoma were 79.6% ± 7.5% & 30.6% ± 11% respectively and with a median DFS of 46.6 month. When comparing patients with Non-Endometrioid adenocarcinoma who underwent surgery alone VS surgery followed by adjuvant treatment, there was a trend for a better median OS (14.1 ± 1.3 VS 35.1 ± 7.1) and it was statistically significant (p-value= 0.002).
Conclusion: Multivariate analysis revealed that Adjuvant radiotherapy, Lymph node negativity and early stage I/II had a statistically significant impact on OS. On the other hand, there was no significance encountered with the remaining factors. Due to the high incidence of systemic relapse, adjuvant treatment and more in-depth analysis is needed for better management of those high-risk groups of patients.
Disclosure: Nothing to disclose

EP625

Laparoscopic transperitoneal para-aortic lymphadenectomy

P. Sklavounos, N. Drandakis, E.-F. Solomayer
Department of Gynecology, Obstetrics and Reproductive Medicine, Saarland University Hospital, Homburg, Germany

Introduction / Background: Lymphadenectomy in gynaecologic cancer has been traditionally performed by laparotomy. However, in recent decades, the laparoscopic approach has been associated with fewer postoperative complications including less blood loss, minor postoperative adhesion formation, and shorter hospital stay than laparotomy, with an equivalent assessment of lymph node Status.
Methodology: A 47-year-old, otherwise healthy woman, G2 P1 with BMI of 46 kg/m^2, presented with histologically verified endometrioid adenocarcinoma of the endometrium Stage 2 from the International Federation of Gynecology and
Obstetrics (FIGO). The Eastern Cooperative Oncology Group (ECOG) performance was 0.
We present a surgical film focused on the anatomy of para-aortic retroperitoneal spaces and the surgical technique of transperitoneal para-aortic lymphadenectomy.
The standard laparoscopic procedure for surgical staging was carried out. In brief, five trocars were placed at the abdominal wall: one 10-mm trocar at umbilicus and the other 10-mm trocar in at 2 cm above the symphysis pubis . Three 5-mm trocars were punctured right lower abdomen, left lower abdomen and left paraumbillicus.
Results: The operating time was 78 minutes and the estimated blood loss was 110mL We removed 19 paraaortic Lymph nodes; None of them was metastatic. There were no postoperative complications. Patient was dismissed after 4 days
Conclusion: Laparoscopic transperitoneal access to lumboaortic lymph nodes is an effective method of lymphadenectomy, which may bring benefits to a patient and physician. Laparoscopy is the first choice to perform a para-aortic lymphadenectomy. However, an adequate learning curve and an in-depth knowledge of the retroperitoneal anatomy are highly important for this approach
Disclosure: Nothing to disclose

EP626

Lymph vascular space invasion, an independent risk factor of sentinel node mapping failure in endometrial cancer, the sentifail study: a multicentric prospective analysis

G. Sozzi[1,2], F.M. Re[1], R. Berretta[3], G. Lo Balbo[4], V. Giallombardo[4], V.A. Capozzi[5], N. Ceresi[4], G. Scambia[6], V. Chiantera[1,2]
[1]Department of Gynecologic Oncology, ARNAS Civico Hospital, [2]University of Palermo, Palermo, [3]Department of Obstetrics and Gynecology, University of Parma, Parma, [4]Department of Gynecologic Oncology, University of Palermo, [5]Department of Obstetrics and Gynecology, University of Parma, Palermo, [6]Department of Women's and Children's Health, Catholic University of the Sacred Heart, Roma, Italy

Introduction / Background: Sentinel lymph node (SLN) mapping has become the gold standard for surgical staging of early stage endometrial cancer. Side-specific systematic lymphadenectomy is suggested if SLN mapping failed. The objective of this study is to identify clinical and pathological factors associated with failed mapping.
Methodology: We prospectively evaluated a consecutive series of early stage endometrial cancer patients submitted to SLN mapping with indocyanine green (ICG) near-infrared compatible surgical platforms in two oncological referral centers from January 2018 to January 2019. Indocyanine green was injected intracervical. Bilateral mapping and failed bilateral SLN mapping groups were compared for clinical and pathological features.
Results: Over the study period, 78 patients met the inclusion criteria. The overall, unilateral, and bilateral SLN detection rates were 88.5%, 15.4%, and 73.1%, respectively. The failed unilateral or no mapping bilateral detection rate was 26.9%. No association between patient demographic characteristics (age, body mass index, histology, grading, tumor size, myometrial invasion and FIGO stage) and SLN mapping failure was observed. Moreover, uterine manipulator or cervical grasping before ICG injection were not associated to failed mapping, while multivariate analysis identified lymph vascular space involvement (LVSI) as the only independent risk factor of SLN mapping failure [OR 4.09 (1.04 - 1.12), p-value:0.04].
Conclusion: Failed SLN mapping is not a rare event andside-specific systematic lymphadenectomy is required to obtain a comprehensive surgical staging.LVSI resulted as the only independent risk factor of SLN mapping failure, probably due to the occlusion of the lymphatic capillaries and consequent failure of the tracer diffusion.

	Univariate Analysis		Multivariate analysis	
Variables	OR (CI 95%)	p-value	OR (CI 95%)	p-value
Age	1.05 (1 -1.11)	0.05	1.05 (1 -1.11)	0.14
BMI	1.41(0.39 - 5.4)	0.30	-	-
Uterine manipulator	1.35(0.47 - 3.8)	0.57	-	-
Histology	1.23 (0.89 - 1.69)	0.20	-	-
Grading	0.97 (0.48 - 1.93)	0.90	-	-
Tumor size	0.99 (0.97 - 1.01)	0.72	-	-
LVSI	3.5 (1.06 - 11.9)	0.03	4.09(1.04 - 1.12)	0.04
Myometrial invasion	1 (0.04 - 22-7)	1.00	-	-

[Univariate and multivariate analysis of clinic-pathological parameters as risk factor of failed SLN mapping]

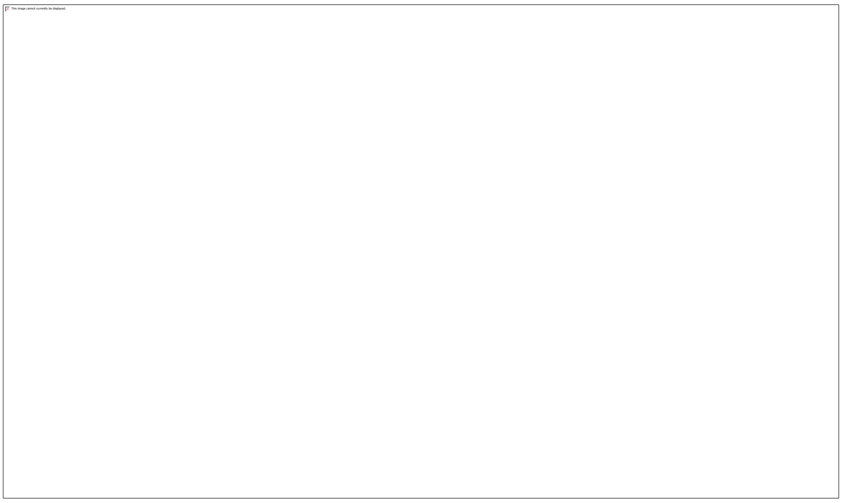

[Upper paracervical lymphatic pathway and obturator sentinel node]

Disclosure: Nothing to disclose

EP627

Clinico-pathological aspects of vanishing endometrial cancer

S. Tangjitgamol, S. Srijaipracharoen
Navamindradhiraj University, Bangkok, Thailand

Introduction / Background: The disappearance of cancer after an initial diagnosis without any definite treatment, particularly without any evidence of disease elsewhere, would be unexpected,so called 'vanishing carcinoma´. This study aimed to assess the prevalence of vanishing endometrial cancer (EMC) in our institution which is a tertiary hospital for cancer care during the 20-year period. The clinico-pathologic features of the patients including pre-operative findings, adjuvant treatment, and clinical outcomes were also studied.
Methodology: Patients who had pre-operative tissue diagnosis of EMC, and had surgical treatment including hysterectomy in the institution between 1995 and 2015 were identified. The patients who had no residual carcinoma in all pathologic specimens including the uterus were included. Pre-and post-operative clinico-pathological data were collected. Clinical outcomes including recurrence and survival of the patients were studied.
Results: Among 422 patients who had tissue diagnosis of endometrial cancer, twenty patients had no residual cancer in the hysterectomy specimens. Three patients who had pre-operative radiation therapy or had evidence of endometrial cancer outside the uterus, so 17 patients (3.8%) were diagnosed as having vanishing cancer. One of the 17 patients, who had pre-operative endometrioid endometrial cancer, had adjuvant chemotherapy for synchronous serous carcinoma of fallopian tube. From a follow-up period of 62.7 months (range, 6.8-184.4 months), no recurrences were found. Only 2 patients were dead of other diseases.
Conclusion: The prevalence of vanishing endometrial cancer was 3.8%. Patients with vanishing endometrial cancer had excellent prognosis. Adjuvant treatment may not be needed. However, periodic surveillance was still recommended because data were derived from only small number of patients.
Disclosure: Nothing to disclose

EP628

Recurrence rate after laparoscopic surgery versus laparotomy for early stage endometrial cancer

R. Sumak, I. Sibli, A. Cokan, M. Pakiz
Maribor University Medical Centre, Maribor, Slovenia

Introduction / Background: To explore long term recurrence rate of patients with early stage endometrial cancer according to the type of surgery (laparoscopy vs. laparotomy).
Methodology: The retrospective analysis included all 144 consecutive early stage patients operated by laparotomy between years 2001 and 2006 and 61 consecutive patients operated by laparoscopy between years 2008-2013, to have at least 5 years of follow up. The general data were compared between patients being operated laparoscopicaly and through laparotomy. Then all patients who had recurrence of the disease were studied in details regarding the location of the recurrence and the risk factors of the primary disease.
Results: Six patients in laparotomy (6/144 [4.2%]) and three patients in the laparoscopy group (3/61 [4.9%]) did not come to follow-up after surgery. Patient characteristics and results are presented in table 1 and table 2.

	Laparotomy (n=138)	Laparoscopy (n=58)	p value
Age (years), mean (range)	63.8 [41-85]	62.6 [23-83]	0.430
Histological subtype			0.247
- Endometrioid	132/138 (95.7%)	58/58 (100%)	
- Non-endometrioid	6/138 (4.3%)	0%	
Risk group model			0.148
- Low risk	67/126 (53.2%)	39/58 (67.2%)	
- Intermediate risk	56/126 (44.4%)	17/58 (29.3%)	
- High risk	3/126 (2.4%)	2/58 (3.4%)	
Concomitant diseases	83/134 (62%)	34/53 (64.2%)	0.599

[Table 1. Patients characteristics]

	Laparotomy (n=138)	Laparoscopy (n=58)	p value
Stage			0.752
- Ia	82/138 (59.4%)	36/58 (62.1%)	
- Ib	56/138 (40.6%)	22/58 (37.9%)	
Pelvic lymph node dissection performed	73/138 (52.9%)	35/58 (60.3%)	0.351
- Pelvic lymph nodes removed, median (range)	23.6 [3-46]	28.6 [4-59]	**0.023**
Postoperative radiation theraphy	66/134 (49.3%)	8/56 (14.3%)	**<0.01**
Time from surgery to recurrence (months), median (range)	74.6 [6-211]	42 [12-72]	0.727
Recurrence rate	9/138 (6.5%)	2/58 (3.4%)	0.512
Distant metastasis	3/9 (33.3%)	1/2 (50%)	1.00

[Table 2. Patients characteristics and outcomes.]

Three (33.3%) patients after laparotomy reccured with not-expected distant metastasis and one (50%) after laparoscopy. All four of them had low/intermediate risk disease. Althought more patients

after laparotomy received adjuvant radiotherapy (change of guidelines) and more lymph nodes were removed through laparoscopy, it did not influence the recurrence rate.
Conclusion: We did not find the difference in recurrence rate or location of recurrence between laparoscopical approach vs. laparotomy in stage I endometrial cancer.
Disclosure: Nothing to disclose

EP629

Two young patients with endometrial cancer who newly developed double cancer in their ovaries after endometrial tumor disappearance through high-dose progesterone therapy and endometrial curettage

N. Susumu, S. Ikeda, E. Saitoh
Mita Hospital, Gynecologic Oncology Center, International University of Health and Welfare, Tokyo, Japan

Introduction / Background: Standard treatment for endometrial cancer (EC) and atypical endometrial hyperplasia (AEH) is total hysterectomy and bilateral salpingo-oophorectomy (BSO), however, young patients with early-stage EC and AEH in reproductive age often hope to preserve their fertility. The oncologic outcomes in long follow-up remain unclear especially regarding the incidences of recurrence or double cancer. We experienced two patients with EC who newly developed double cancer in their ovaries after medication of high-dose medroxyprogesterone acetate (MPA) for fertility-preservation.
Methodology: In principle, we survey the patients after MPA therapy every four months using vaginal ultrasound check, endometrial histological/cytological examinations, measurement of serum CA125, and pelvic MRI once a year.
Results: The 44 y/0 patient had received MPA therapy and cyclic surveillance every 4 to 6 months in the previous hospital, and she was introduced to our hospital with 3 year-recurrence-free interval. However, trans-vaginal (TV) echo showed solid tumor measuring 18 mm in diameter in the right ovary, and the serum CA125 was 32 U/ml. MRI revealed solid tumor with positive Gd-enhancement. Hysterectomy with RSO and LS were performed. Pathological examination revealed endometrioid carcinoma (EMC) G1 in uterus (pT1A), and mucin-producing EMC G1 in the right ovary (pT1A, primary ovarian cancer). Another 38 y/o patient had finished MPA therapy 4 months before. TV echo showed solid tumors measuring 30mm in both ovaries, and the serum CA125 was 112 U/ml. MRI revealed solid tumors in both ovaries with positive Gd-enhancement. Hysterectomy with BSO, retroperitoneal lymphadenectomy was performed. Pathological examination revealed EMC G1 in uterus (pT1A), and mucin-producing EMC G2 in both ovaries (pT1B, bilateral primary ovarian cancers). Both patients have no recurrence after operation without adjuvant chemotherapy for 12 months.
Conclusion: Strictly careful follow-up every 4 months using TV echo and CA125 is needed after fertility-preserving MPA therapy for detecting heterochronous overlapping cancers in ovaries.
Disclosure: Nothing to disclose

EP630

Assumption of responsability of the uterine sarcoma ,experiment of the service of medical oncology center Pierre and Marie Curie. (CPMC) Algiers, Algeria

F. Hadjarab[1], C.E. Chekman[2], A. Taibi[1], K. Bouzid[1]
[1]Medical Oncology, Centre Anticancereux Pierre Marie Curie d'Alger, [2]Surgery Pierre Marie Curie, Centre Anti Cancéreux Pierre Marie Curie d'Alger, Algiers, Algeria

Introduction / Background: The uterine sarcomas account for 1 to 5% of the malignant tumors of the

uterus.
Distribution: - T. epithelial mixed andmesenchymaleusesl (T müllériennes mixed) (50-60%): represented by the carcinosarcome,adenosarcome, - léiomyosarcome. (35%) -sarcoma of the stroma (chorion cytogenous lowrank Bad forecast except SBS 10%) of low rank,and undifferentiated.
Treatment: the improvement of the surgery of theprimary tumour and metastases is essential. RTEplaces auxiliary chemotherapy remains thesensitivity and local control, this, depend, according to the histological type, Place given oftherapeutic targeted, and, to define: the trabectidine drug.
Methodology: The retrospective study of 19 case of uterine sarcoma support to the service of the medical oncology with the CPMC course of the period has 2004 2011.
Results: Median age of the diagnosis: 51 years. The diagnosis made on the histological examination revealed in the operating room. It histological 3varieties: 10 cases of léiomyosarcome, 3 cases ofcarcinosarcome distinguish, 4 cases of sarcomaof stroma (chorion cytogenous of the endometer) stage IV of tumor was found in 50% of the tumors ,then of 25 % III . for the stage is closely related to the phase of tumor ;The forecast treatment is primarily an exclusive radical
surgery in 5 cases, 12 cases have benificiee of atreatment of chemotherapy in the event ofrepetition or metastatic. : Ifosfamide/doxorubicine,DOCÉTAXEL/gemcitabine PXL/CARBO.
The protocols used radiochimio-concomitant was carried out at a patient presenting one To theanswer: RC (7 cases); RP (2case); Ms (4case);failed (4case) carcinosarcome.
to become: 9 patients alive in remission, 4patients died, Sick alive: 4.
Conclusion: The sarcoma of the uterus is the badtumour prognostic one, the surgery of the primary tumor and metastases is essential, chemotherapy is booked in the case of arepetition or metastatic. We pay in this study, the Algerian experiment in favor of uterine sarcomas.
Disclosure: no conflict of interest

EP631
Expression of P-REX2a is associated with poor prognosis in endometrial malignancies
S. Takeshita
Ichinomiya Municipal Hospital, Ichinomiya, Japan

Introduction / Background: Phosphatidylinositol-3,4,5-trisphosphate RAC exchanger 2a (P-REX2a) is a PTEN inhibitor that also activates Rac 1. No associations with P-REX2a and human endometrial cancers have been reported to date.
Methodology: we immunohistochemically analyzed 155 uterine endometrial malignancies for P-REX2a expression.we transduced either P-REX2a expression vector or short hairpin RNAs targeting P-REX2a into 2 uterine endometrioid carcinoma cell lines, OMC-2 and JHUEM-14.we performed expression microarray analysis using these cells, and pathway analysis.
Results: The P-REX2a-positive tumors displayed worse prognosis independent of PTEN expression. Then, Ectopic expression of P-REX2a led to increased cell proliferation only in the PTEN-expressing OMC-2 cells but did not show any change in the PTEN-negative JHUEM-14 cells or the P-REX2a-knockdown cells. Induction of P-REX2a increased and knockdown of P-REX2a decreased cell migration in both cell lines. microarray analysis revealed that the expression of members of the GPCR downstream pathway displayed the most significant changes induced by the knockdown of P-REX2a.
Conclusion: P-REX2a enhanced cell motility via the GPCR downstream pathway independently of PTEN leading to progression of uterine endometrioid malignancies and poor prognosis of the patients.
Disclosure: Nothing to disclose

EP632
Does resection of normal appearing omentum influence survival in patients with type 2

endometrial carcinoma?

O. Tal, O. Elyashiv, E. Ben Shem, O. Peled, T. Levy
Division of Gynecologic Oncology, Department of Obstetrics and Gynecology, Edith Wolfson Medical Center, Holon, Israel

Introduction / Background: Type 2 endometrial cancer includes aggressive histologic subtypes such as serous, clear cell and carcinosarcoma of the endometrium. These tumors have a propensity for intra-abdominal spread, a high recurrence rate, and a poor prognosis. When extra-uterine disease is present, surgical debulking is attempted when possible. The aim of this study was to determine whether resection of normal appearing omentum influences survival.
Methodology: We retrospectively identified cases with type 2 endometrial cancer at our Gynecologic Oncology Division from 1998 to 2017. Medical records were reviewed for the following information: age at diagnosis, preoperative imaging, operative findings, surgical procedure, final histology with definitive International Federation of Gynecology and Obstetrics stage, adjuvant treatment and survival.
Results: A total of 90 patients with type 2 endometrial cancer were identified, of whom 79 had primary operation. All women underwent at least hysterectomy with bilateral salpingo-oophorectomy. Seventeen women (21.5%) were found to have macroscopic extrauterine disease, hence omentectomy was part of the operation and these patients were excluded from evaluation. The final study group included 62 women with no macroscopic evidence of omental metastasis. 36 underwent omentectomy and had no histologic evidence of spread (group 1) compared to 26 women that did not undergo omentectomy (group 2). By multivariate analysis, survival was significantly influenced by stage, deep stromal invasion and lymph-vascular space involvement. However, when only stage I patients (n=32) were compared, the median survival of women who did not have omentectomy was 7.8 ± 0.4 years while the median survival of the women who underwent omentectomy was not yet reached.
Conclusion: It appears that the resection of normal appearing omentum later found to have no metastases improves survival in patients with stage I type 2 aggressive endometrial cancer.
Disclosure: Nothing to disclose

EP634

Comparison of robotic single-site (RSS) and robotic multiport (RM) total hysterectomy (TH) plus sentinel lymph node (SLN) mapping in low risk endometrial cancer (EC): quality of life (QoL) and surgical outcomes analysis

L. Mereu[1], V. Berlanda[1], B. Gardella[2], D. Surico[3], A. Vigone[3], R. Pertile[4], A. Spinillo[2], S. Tateo[1], Robotic Gyne Club RGC
[1]Ob&Gyn, Santa Chiara Hospital, Trento, [2]Ob&Gyn, University of Pavia, Pavia, [3]Ob&Gyn, University of Novara, Novara, [4]Clinical Epidemiology, Azienda Provinciale Servizi Sanitari di Trento, Trento, Italy

Introduction / Background: RSS surgery is an option for EC treatment and its feasibility for lymph node assessment and SLN mapping has been describe.We analyzed RSS and RM approaches to treat low risk EC.
Methodology: Prospective multicenter cohort study**,** setting in Santa Chiara Hospital of Trento and University Hospital of Pavia and Novara**.** Between January 2017 and January 2019, 76 consecutive patients with diagnosis of endometrial atypical hyperplasia or low risk (Grading 1-2, myometrial invasion < 50%) EC undergoing robotic TH and SLN mapping were included. Data on patient's characteristics, surgical outcomes, QoL and cosmesis were prospectively collected and analyzed based on the surgical approach (RSS vs RM). Clinical follow up was performed at 4 weeks, 6 and 12 months after surgery.
Results: 51 patients underwent RM and 25 RSS surgery. Main differences between the two groups were: mean BMI in RM group: 29 Kg/m^2, RSS: 24.8 kg/m^2 (p-value < 0.001), drop haemoglobin in RM: 0.98 mg/mL, RSS: 1.47 mg/mL (p-value 0.013). No differences were found in term of SLN detection: 96.1% in RM (66.7% bilateral, 29.4% monolateral) and 96% in RSS (76% bilateral, 20% monolateral). In one RSS case the removal of right common iliac SLN was not feasible. We found a better physical functioning in RSS (97.1 vs 91.6, p-value 0.006) at 6 and 12 months after surgery and less pain in RM

(98.6 vs 94.4, P-value 0.029) at 6 months. No differences in complications and cosmesis were detected.
Conclusion: In the treatment of low risk EC with SLN mapping, we did not find any substantial advantages between the two approaches but we evidenced technical limitation in the removal SLN in RSS group.
Disclosure: Nothing to disclose

EP635

Minimizing the dose of oral megestrol in combination with the Levonorgestrel-Releasing Intrauterine Device: an equally effective approach for the conservative management of Atypical Hyperplasia and well differentiated endometrial carcinoma

D. Haidopoulos, A. Rountis, V. Pergialiotis, V. Theodoulidis, N. Thomakos, D. Loutradis, A. Rodolakis
1st Department of Obstetrics and Gynaecology, National & Kapodistrian University of Athens, Athens, Greece

Introduction / Background: The purpose of the present study is to evaluate the efficacy of the combination of low dose megestrol acetate (MA) (80mg/day) and the levonorgestrel-releasing intrauterine device (LNG-IUD) for the treatment of complex atypical hyperplasia (CAH) and low-grade endometrial adenocarcinoma (EC) in premenopausal women, 45 years old or younger, who desire to preserve their fertility.
Methodology: A retrospective study that included a consecutive series of patients of reproductive age who were treated conservatively with the combination of per os MA (80mg/day) and the LNG-IUD for CAH and grade I endometrioid EC. The response rate for each of the two conditions was monitored following a 6-month period of treatment.
Results: Twenty-two patients were diagnosed with CAH or grade I endometrioid EC. Eighteen of them were evaluable at the 6-month study point. Among them, 5 patients (27.8%) had an initial diagnosis of CAH and the other 13 (72.2%) were diagnosed with grade I endometrioid EC. Overall, the response rate was 77.7% at the 6-month evaluation exam (80% for the CAH group and 76.9% for the grade I endometrioid EC).
Conclusion: The concomitant use of low-dose MA orally and the LNG-IUD seems to be an efficient option for the conservative treatment of CAH or early- grade EC in young women who wish to preserve their fertility. Future studies should provide head to head comparison with systemic progestin therapy to corroborate our findings.
Disclosure: Nothing to disclose

EP636

Assessing the impact of clinicopathological prognostic variables on survival outcome in patients with clear cell endometrial carcinoma: an institutional analysis

S. Dimopoulou[1], N. Thomakos[1], M. Sotiropoulou[2], K. Ntzeros[1], D.E. Vlachos[1], D. Haidopoulos[1], M. Liontos[3], A. Bamias[3], A. Rodolakis[1]
[1]1st Department of Obstetrics and Gynecology, National and Kapodistrian University of Athens, Medical School, [2]Pathology Department, [3]Department of Clinical Therapeutics, Alexandra Hospital, National and Kapodistrian University of Athens, Athens, Greece

Introduction / Background: Endometrial clear cell carcinomas (ECCC) are rare but aggressive subtype of uterine carcinomas. Several clinicopathological characteristics have been associated with poorer prognosis. The aim of this study is to determine the value of different clinicopathological parameters in predicting prognosis.

Methodology: We retrospectively analyzed files of ECCC patients treated in our institution between 2005 and 2015. Clinicopahtological data and survival outcomes were collected. Our data were analyzed using Cox regression analysis.
Results: Fifty- two patients with ECCC and mean age 65.7 years (range 38-90) were analyzed. Myometrial invasion >50% was present in 59.6% of the cases. Adnexal involvement, lymph node-pelvic and paraortic- metastasis and extra uterine metastasis were present at 21.2%, 7.7%, 17.3 and 32.7%, respectively. Cervical involvement was found in 34.6% of the patients. All patients received adjuvant treatment. The mean follow up period was 6.6 years.13.5% of the cases appeared with recurrent disease. Advanced stage (III and IV), positive peritoneal cytology, paraortic node involvement, extra uterine metastasis and omentum involvement were significantly associated with greater hazard for relapse in univariate Cox regression analysis. Mutlivariate analysis revealed that aortic node metastasis (p=0.013) and omentum involvement (p=0.030) were independent predictors of relapse. 63.4% of the patients were alive at 5 years. Advanced stage, peritoneal cytology, adnexal involvement, aortic node and extra uterine metastasis, omentum and vaginal involvement were associated with worse survival in univariate analysis, while in multivariate analysis peritoneal cytology (p=0.045), adnexal involvement (p=0.036) and aortic node metastasis (p=0.035) were independently associated with survival. Type of adjuvant treatment did not affect prognosis.
Conclusion: Our study suggests that adnexal, omentum and vaginal involvement in addition to aortic node, extra uterine metastases, peritoneal cytology and stage are predictive factors of worse prognosis.
Disclosure: Nothing to disclose

EP637

Are any pathologic variables of lymph node metastases reliable enough to predict survival in high grade endometrial cancer?

K. Ntzeros[1], N. Thomakos[1], M. Sotiropoulou[2], S. Dimopoulou[1], D.E. Vlachos[1], D. Haidopoulos[1], M. Liontos[3], A. Bamias[3], A. Rodolakis[1]
[1]1st Department of Obstetrics and Gynecology, Alexandra General Hospital, National and Kapodistrian University of Athens, Medical School, [2]Pathology Department, [3]Department of Clinical Therapeutics, Alexandra Hospital, National and Kapodistrian University of Athens, Athens, Greece

Introduction / Background: Specific pathologic characteristics of nodal metastases may have prognostic implications on their own. The aim of this study is to identify whether pathologic characteristics of pelvic and para-aortic nodal metastases in high grade endometrial carcinomas affect recurrence and survival.
Methodology: We conducted a retrospective study of patients with stage IIIC1 and IIIC2 high grade endometrial carcinomas (endometrioid, serous and clear cell type) from 2005 to 2012, evaluating the size of lymph node metastases (≤10 mm or >10 mm), number (≥1) and location (pelvic or para-aortic) of lymph nodes involved, duration of follow-up and length of time to recurrence. Cox regression analysis was used.
Results: The study included 68 women with mean age 61.9 years. Thirty were classified as high grade endometrioid, 24 serous and 14 clear cell. Fifty patients had Stage IIIC1 disease and the remaining IIIC2. The mean follow up period was 4.5 years. 52.9% of the patients had a recurrence. Twenty four patients (35.3%) died during the follow up. Advanced local disease as indicated by cervical stroma invasion and adnexa involvement were associated with greater hazard for recurrence (HR=2.83, 95% CI: 1.09 - 7.32, p=0.032 and HR=3.13, 95% CI:1.24 - 7.94, p=0.016 respectively). Cervical Involvement was also associated with worse survival (HR=7.40, 95% CI: 1.57 - 34.84, p=0.011). The pathologic characteristic related with negative prognostic implications was the presence of metastases in >1 lymph nodes. It was associated with greater hazard for recurrence (HR=2.89, 95% CI: 1.10 - 8.01, p=0.025) and worse survival (HR=3.50, 95% CI: 1.41 - 18.72, p=0.014). The other variables were not statistically associated with recurrence or survival.
Conclusion: In patients with high grade endometrial carcinomas where >1 lymph nodes involved with metastases, were associated with poorer prognosis. In contrary, size and location of lymph node metastases did not affect survival or recurrence.
Disclosure: Nothing to disclose

EP638

Lynch syndrome-associated endometrial cancer with BRCA1 mutation

I. Tripac
Institute of Oncology of Moldova, Chisinau, Republic of Moldova

Introduction / Background: Objectives of the study:
1) identify families with accumulation of malignant neoplasms of female reproductive organs (endometrial cancer, ovarian cancer, breast cancer and multiple primary malignant neoplasms);
2) formation of oncology hereditary risk groups based on pedigree analysis, clinical, laboratory, genetic, molecular tests;
3) DNA testing for mutations of gene BRCA1 in patients with endometrial cancer from families with high risk of cancer.
Methodology: In this study we included dates from 361 first- degree relatives of 145 female patients with endometrial cancer. In this study we used methods, as: clinico-genealogical research, DNA testing for germline mutation in BRCA1.
Results: In this study were identified 3 types of mutations in the BRCA1 gene, namely 5382insC (exon 20), 185delAG (exon 5) and 4153delA (exon 11). In patients with endometrial cancer associated with Lynch syndrome 5382insC mutation was the most frequent, its share was 72%.
Conclusion: Prophylactic hysterectomy could be discussed with informed women carrying germline mutatinn BRCA1 from families with high risk of cancer. Additional studies are required to establish future prophylactic surgery guidelines.
Disclosure: Nothing to disclose

EP639

Treatment of elderly patients (≥ 70 years old) with endometrial cancer. Long-term outcome

Y. Gavrish[1], I. Berlev[1,2], A. Urmancheeva[2,3], E. Ulrikh[2,3], A. Artemieva[4], A. Baltrukova[1], Y. Trifanov[2], A. Sidoruk[1,2]
[1]Department of Obstetrics and Gynecology, North-Western State Medical University named after I.I.Mechnikov, [2]Oncogynecological Department, N.N. Petrov National Research Center of Oncology, [3]Department of Oncology, North-Western State Medical University named after I.I.Mechnikov, [4]Department of Pathological Anatomy, N.N. Petrov National Research Center of Oncology, Saint-Petersburg, Russian Federation

Introduction / Background: To evaluate the long-term results of patient treatment with endometrial cancer older than 70 years compared with younger patients.
Methodology: The data of 309 patients with endometrial cancer who received primary surgical treatment in the N.N. Petrov Research Institute of Oncology from 2009 to 2016 were divided into two groups: Group 1-≥70 years (the main group, n=159), Group 2-50-69 years (the control group, n=150).
Results: In the 1st group, a non-endometrioid tumor histotype was more common-17.61% versus 4.67% in the 2nd group (p< 0.001), common (III-IV) stages of the disease-17.61% against 6.67 % respectively (p=0.003). The average observation time in the 1st group was 35.7 ± 30.3 months, in the 2nd group-43.8±31.2 months. Recurrence rate was recorded in 23.36% in the 1st group and 13% in the 2nd group (p< 0.05). The average time of progression in the 1st group was 14.7±16.6 months, in the 2nd group 15.5±13.5 months (p>0.05). In the group of 50-69 years, in most cases, distant metastases were recorded - 46.15% (bones, lungs, supraclavicular lymph nodes, pancreas) versus 18.75% in the group≥70 years (p> 0.05). In the 1st group the leading variant of the progression of the disease was local recurrence (vaginal stump) - 46.88% against 23.08% in the 2nd group, p> 0.05. The

overall survival of patients in the group of ≥70 years was 84% against 98% in the group of 50-69 years (p< 0.01), the relapse-free survival rate was 81% and 89% in the 2nd and 1st groups, respectively (p< 0,05).
Conclusion: Endometrial cancer in the group≥70 years is more aggressive from the point of view of the histological type of tumor, the stage of the disease at the time of diagnosis is characterized by a poor prognosis.
Disclosure: Nothing to disclose

EP640

Retroperitoneal drainage versus no drainage and no peritanization after pelvic lymphadenectomy for the prevention of lymphocyst formation in patiens with endometrial cancer

T. Rogovskaya[1], A. Sidoruk[1,2], A. Urmantcheeva[1,2], E. Ulrikh[1,2], I. Meshkova[1], N. Mikaya[1], I. Berlev[1,2]
[1]N.N. Petrov National Research Center of Oncology, [2]North-Western State Medical University named after I.I.Mechnikov, Saint-Petersburg, Russian Federation

Introduction / Background: Lymphoceles are the most frequent complications after systematic lymphadenectomy in gynaecologic cancers. Drainage that is performed after hysterectomy and pelvic lymph node dissection to prevent postoperative lymphocyst formation and surgical morbidity is controversial.
Objectives: Determine the clinical effect of absence of peritonization and absence of drainage compared with peritonization and drainage after hysterectomy and bilateral pelvic lymphadenectomy for patients with endometrial cancer.
Methodology: 773 endometrial cancer patients underwent hysterectomy and bilateral pelvic lymphadenectomy with laparoscopic and open approach at N.N. Petrov Research Center of Oncology from 2010 to 2018. A total of 430 patients who underwent laparoscopic surgery were compared to 343 patients from open surgery group. According to the type of drainage and peritonization, patients were divided into four subgroups of comparison: A - laparoscopic approach with passive drainage and no peritanization (n=290), B - laparoscopic approach without drainage and peritonization (n=140), C - laparotomic approach with two active drainages and peritanization (n=100), D - laparotomic approach with passive drainage and no peritanization (n=243). Ultrasound examination of the pelvis for all patients after surgery was performed.
Results: The overall incidence of lymphoceles was 337/773 (43.5%): 184/430 (42.8%) after laparoscopy and 196/343 (57,1%) after open surgery (p=0,01). Most of cases were asymptomatic (89.3%).
In the subgroups of laparoscopy: A - overall incidence of lymphocysts formation was 42.4%, incidence of symptomatic lymphocysts was 4.5%; B - 15% and 0% respectively. In subgroups of open surgery: C - overall incidence of lymphocysts formation was 65%, incidence of symptomatic lymphocysts was 17%; D - 52.3% and 8.4% respectively.
Conclusion: The overall incidence of lymphocele after lymphadenectomy for endometrial cancer is high (43.5%). Laparoscopic approach with no peritonization and no drainage in endometrial cancer susgery is associated with a lower occurrence of both asymptomatic and symptomatic lymphoceles compared to open surgery with drainage and peritonization.
Disclosure: Nothing to disclose

EP641

Case report of low grade sarcoma of endometrial stroma with no primitive uterine site

V. Uvin, Q. Fontagnes, C. Riera

Gynecology, CHU Charleroi, Charleroi, Belgium

Introduction / Background: Low grade sarcoma of endometrial stroma with no primitive uterine site is a rare diagnosis.
The diagnosis is challenging. Anatomopathological analysis could be difficult and imaging nonspecific. We describe one case of this pathology.
Methodology: /
Results: A 63 year old Caucasian asymptomatic female patient was referred to the gynecology department for suspicion of ovarian cancer on computed tomography(CT).
Gynecologic examination showed a mass fixed to the left pelvic wall by vaginal examination, without inguinal adenopathies or ascites.
A diagnostic laparoscopy was performed. Multiple intrapelvic adhesions and parauterine solide masses were found and biopsied. Anatomopathological analysis of the biopsies showed a leiomyoma without sign of malignity.
Secondary, a total hysterectomy and bilateral adnexectomy by laparotomy was performed. An enlarged uterus and multiple intrapelvic and intraabdominal masses were present. Peritoneal masses were found on the mesenterium of the ileum and colon, with close adhesion to the bowel. Also a large mass was found in the abdominal left upper quadrant. All the pelvic masses and most of the mesenterial masses were removed. Frozen section pathology peroperatively showed a benign leiomyoma.
Final anatomopathological analysis found a low grade sarcoma of endometrial stroma with no primitive uterine site.
There is no standard treatment protocol available for this pathology. After discussion with different Belgian experts we proposed a third operation with optimal debulking (R0) and adjuvant hormonal therapy. The patient refused the surgery and an aromatase inhibitor was started. CT scan every 3 months is planned for follow up.
Conclusion: This 63-years old female was diagnosed with low grade sarcoma of endometrial stroma with an extra uterine disease only. Most masses intrapelvic and intraabdominal were removed. The patient refused a third operation for maximal debulking. Adjuvant treatment with aromatase inhibitor was started and a 3 monthly CT scan was planned.
Disclosure: Nothing to disclose

EP642

Combined programmed cell death ligand 1 (PD-L1)/CD8 expression in grade 3 endometrial carcinomas

S. Vagios[1], P. Yiannou[2], E. Giannikaki[3], T. Doulgeraki[2], C. Papadimitriou[4], A. Rodolakis[5], A. Nonni[1], A. Vlachos[6], K. Pavlakis[1,2]
[1]*Pathology Department, National and Kapodistrian University of Athens,* [2]*Pathology Department, IASO Women's Hospital, Athens,* [3]*Pathology Department, Venizeleio-Pananeio General Hospital, Heraklion,* [4]*Oncology Unit, Aretaieion Hospital,* [5]*1st Department of Obstetrics and Gynecology, Alexandra Hospital, National and Kapodistrian University of Athens,* [6]*Department of Gynecological Oncology, IASO Women's Hospital, Athens, Greece*

Introduction / Background: The purpose of this study is to evaluate the combined immunohistochemical expression of programmed cell death ligand 1 (PD-L1) and CD8 in high-grade endometrial carcinomas and relate it to several clinicopathological parameters.
Methodology: One hundred and one (101) surgically staged patients with high grade endometrial carcinomas (46.7% endometrioid and 53.5% non-endometrioid) were included in the study. PD-L1 and intraepithelial CD8 expression was evaluated by immunohistochemistry. PD-L1 expression was considered positive when ≥1% of membranous staining was identified. CD8 expression was considered high when the number of CD8+ intraepithelial cells per high-power field (HPF) was equal to or above the median CD8+ cell count (17.6 cells)/HPF. Four groups of tumors were isolated: group 1 tumors were PD-L1 positive/CD8 high, group 2 PD-L1 negative/CD8 low, group 3 PD-L1 positive/CD8 low and group 4 PD-L1 negative/CD8 high.
Results: Combined PD-L1/CD8 expression was significantly associated with tumor histology (p =

0.01). Group 1 tumors were mostly of endometrioid type (71.4%), while group 2 and group 3 tumors were mainly non-endometrioid carcinomas (64.7% and 69.7%, respectively). In group 4, there were nearly equal percentages of endometrioid and non-endometrioid carcinomas (47.8% and 52.2%, respectively). There was no statistical relationship between each group and the clinicopathological features under evaluation (stage, depth of myometrial invasion, lymph node disease and lymph vascular space invasion). Among the four groups there were significant differences in progression-free survival (p = 0.032). Group 4 patients had the longest progression-free survival, whereas group 3 patients the shortest. No statistically significant differences were found regarding overall survival (p=0.336).
Conclusion: Combined PD-L1/CD8 expression in grade 3 endometrial carcinomas, revealed distinct groups with significant differences in progression-free survival.
Disclosure: This research work was supported by the Onassis Foundation - Scholarship ID: G ZO 001-1/2018-2019.

EP643

Sentinel node detection using indocyanine green in intermediate risk of early endometrial carcinoma: initial experiences with the robotic daVinci X system

P. Valha, M. Michal, M. Veleminsky
Oncogynaecology Dept., Hospital Ceske Budejovice a.s., Ceske Budejovice, Czech Republic

Introduction / Background: ICG (Indocyanine Green) and robotic surgery have, in recent years, become an important part in oncoogynaecological surgery. Sentinel node detection (SLN) in endometrial cancer is unfortunetelly still subject of experimental research. Intermediate risk endometrial cancer (IR-EEC - myometrial invasion > 50% or G3 and invasion < 50%) and surgery management with or without systematic lymphadenectomy is also controversal. Combination of fluorometric SLN mapping using ICG and robotic system appears to be an attractive alternative to reliable staging.
Methodology: We have, at our department, enrolled suitable patients for robotic surgery. All cases had diagnosis of IR-EEC established on histology and an oncogynecological ultrasound. Methodology was approved by ethical committe and informed consents were signed. We used of Memorial Sloan Kettering Cancer Center SLN algorithm: 4 ml of ICG (Verdye Diagnostic Green GmbH, Aschheim,Germany) was applied, divided into two injection at 3 and 9 o´clock position of the cervix, with 1 ml in the stroma and 1 ml submucosally. After docking of robotic system daVinci X (Intuitive Surgical Inc., Sunnyvale, CA) we started procedure with opening of retroperitoneal spaces. So called Firefly mode on robotic console - Near Infrared Fluroscence was applied. Detected SLNs were removed and completelly examined with ultra and immunohistochemistry microstaging SLN protocol. Surgery was completed with hysterectomy type B and bilateral salpingo-oophorectomy.
Results: During our starting period, 9 cases were managed. SLN was detected in all patients (9/9), detection rate is100%. In 8 patients (89%) the detection was bilaterally in pelvis. In 2 cases (22%) were SLN visible also in paraaortic region. Alltogether 24 lymph nodes were examined. None of the examined SLNs (0%) contained micrometastases or isolated tumor cells.
Conclusion: Combining the easily applied ICG dye with technical options of robotic system for intraoperative visualization has the potential to replace currently established surgical techniques and can reduce postoperative patients morbidity.
Disclosure: Nothing to disclose

EP644

Trends over time in use of primary and adjuvant hormonal therapy for endometrial cancer: a

population based study

W.J. van Weelden[1], R. Bretveld[2], S. van Erp[1], S. Engels[1], A. Romano[3,4], L. Massuger[5], R. Lalisang[3], J. Pijnenborg[1], M. van der Aa[2]
[1]Radboud Institute for Health Sciences, Radboud University Medical Center, [2]Integraal Kankercentrum Nederland, Nijmegen, [3]Division of Medical Oncology, Department of Internal Medicine, [4]GROW - School of Oncology and Developmental Biology, Maastricht University Medical Center, Maastricht, [5]Obstetrics & Gynaecology, Radboudumc, Nijmegen, The Netherlands

Introduction / Background: Endometrial cancer (EC) is the most common gynaecological cancer in the Western world with rising incidences due to increased life expectancy and obesity. As a result, EC is more frequently diagnosed among young patients and patients with high BMI and comorbidities. Many studies have confirmed the efficacy of hormonal therapy in young women who wish to preserve fertility, as alternative for surgical treatment. Yet, there are less data of hormonal therapy in elderly patients considered unfit for surgery. It is unclear whether the rising incidence of EC has influenced applied hormonal therapy over time. Therefore the aim of this study is to investigate trends in application of primary and adjuvant hormonal therapy in young and elderly patients.
Methodology: Data of all patients diagnosed and treated for EC in the period 1989-2016 was extracted from the Netherlands Cancer Registry (NCR).
Results: A total of 44.209 EC patients were included in the NCR , and analysed in four time periods. Primary hormonal therapy increased from 2.1% (n=184) to 2.7% (n=351), whereas the use of adjuvant hormonal therapy decreased from 2.3% (n=204) to 0.7% (n=91). Primary hormonal therapy among patients ≤ 40 years with FIGO stage I increased from 0% to 29%. The application of primary hormonal therapy also increased among patients > 75 years for both FIGO stage I-II and FIGO stage III-IV disease (***figure 1***). Adjuvant systemic treatment for FIGO III-IV showed a decrease in hormonal therapy in favour of an increase in chemotherapy (***figure 2***).
Conclusion: This study has shown that hormonal therapy is increasingly selected as primary therapy among patients ≤ 40 years and among patients >75 years old. In contrast, the use of adjuvant hormonal treatment has decreased at the cost of chemotherapy although the evidence for both treatments in this setting is limited.
Disclosure: WJ van Weelden, R Bretveld, S van Erp, S Engels, LFAG Massuger, RI Lalisang, JMA Pijnenborg and MA van der Aa declare that there is no potential conflict of interest. A Romano was partly supported for this study by the Dutch Cancer Society (KWF Kankerbestrijding, www.kwf.nl), contract number UM-13-5782.

EP645

Anti-estrogen treatment in endometrial cancer: a systematic review

W.J. van Weelden[1], L.F. Massuger[1], A. Romano[2], J.M. Pijnenborg[1], ENITEC
[1]Radboud University Medical Center, Nijmegen, [2]Maastricht University Medical Center, Maastricht, The Netherlands

Introduction / Background: Hormonal therapy in endometrial cancer (EC) is used for patients who wish to preserve fertility and for patients with advanced or recurrent disease in a palliative setting. Since estrogen plays an important role in the development of most ECs, anti-estrogenic therapy is an alternative treatment to the more frequently used progestin therapy. However, there is limited data on the response rates and toxicity of anti-estrogens in EC. Therefore, we performed a systematic review to investigate response rates and toxicity in anti-estrogen therapy in patients with EC.
Methodology: A systematic search in electronic databases was performed to identify studies on selective estrogen receptor modulators (SERM) and down-regulators (SERD) and aromatase inhibitors that reported on response rates among EC patients. Outcome in estrogen receptor (ER) positive and negative disease was assessed independently.
Results: Fifteen studies were included of which ten investigated anti-estrogen monotherapy and six investigated a combination of anti-estrogenic drugs with either progestin or targeted treatment. Thirteen prospective studies and two case series were included. The median age of the patients in the included studies ranged from 61-71 years and the proportion of low grade tumors ranged from 38-80%. The RR for tamoxifen ranged from 10-53%, for other SERMs and SERDs 9-31%, for aromatase

inhibitors from 8-9%, for combined tamoxifen/progestin treatment 19-58% and for combination of anti-estrogenic treatment with mammalian target of rapamycin (mTOR) inhibitors 14-31%. Toxicity consisted of thrombotic events in 2-6% in all therapies except for the anti-estrogenic/mTOR combination.
Conclusion: Tamoxifen or a combination of tamoxifen and progestin should be the preferred choice when selecting anti-estrogenic treatment because the RRs are similar to progestin treatment and the toxicity is low. The response can be optimized by selecting patients with endometrioid tumors and positive estrogen receptor status, which should be based on a pretreatment biopsy.
Disclosure: Nothing to disclose

EP646

Could the long-term oncological safety of laparoscopic surgery in low risk endometrial cancer be also valid for the high intermediate and high-risk patients? A multi-center Turkish gynecologic oncology group study conducted with 2745 endometrial cancer cases

M.A. Vardar[1], A.B. Guzel[1], S. Taşkın[2], M. Güngör[2], N. Özgül[3], C. Salman[3], Ü. Küçükgöz Güleç[1], G. Khatib[1], İ. Dünder[2], F. Ortaç[2], K. Yuce[3], C. Terek[4], T. Şimşek[5], A. Özsaran[4], Ç. Taşkıran[6], A. Onan[7], G. Çoban[8], S. Topuz[9], F. Demirkıran[10], Ö. Takmaz[11], M.F. Köse[12], A. Göçmen[13], G. Seydaoglu[14], D. Gümürdülü[15], A. Ayhan[16], TRSGO
[1]Gynecologic Oncology, Çukurova University, Adana, [2]Ankara University, [3]Hacettepe University, Ankara, [4]Ege University, İzmir, [5]Akdeniz University, Antalya, [6]Koç University, İstanbul, [7]Gazi University, Ankara, [8]Başkent University, Adana, [9]İstanbul University, [10]İstanbul University Cerrahpaşa, [11]Akdeniz Universitycıbadem University, [12]Medipol University, [13]Umraniye Training and Research Hospital, İstanbul, [14]Biostatistics, [15]Pathology, Çukurova University, Adana, [16]Başkent University, Ankara, Turkey

Introduction / Background: In this study we aimed to compare the long-term oncological outcomes of laparotomy (LT) and laparoscopic (LS) surgeries in endometrial cancer under the light of 2016 ESMO-ESGO-ESTRO risk classification system, focusing on the high intermediate and high-risk categories.
Methodology: Using multicentric database, overall (OS) and disease-free survivals (DFS) of 2745 endometrial cancer cases were compared according the surgery route, laparotomy vs laparoscopy. A sub-analysis was performed considering the 2016 ESMO-ESGO-ESTRO risk classification for endometrial cancer. Thus, high intermediate and high-risk patients were grouped and a comparison was made between the groups.
Results: Among the enrolled patients 1743 (63.5%) were operated by laparotomy and 1002 (36.5%) were operated with laparoscopy. The total number of high intermediate and high-risk endometrial cancer cases was 734 (45%) patients in the LT group and 307 (30.7%) patients in the LS group. OS and DFS were not statistically different when compared between LS and LT groups in terms of low, intermediate, high intermediate and high-risk endometrial cancer.
Conclusion: Regardless of the endometrial cancer risk category, long-term oncologic outcomes of LS were found to be comparable to those treated classically with LT. Our results are encouraging for primarily considering laparoscopic surgery for high intermediate and high-risk endometrial cancer cases in order to minimize surgical morbidity, as adjuvant therapies will mostly be offered to this population. It should be strongly emphasized that this suggestion is limited to gynecological oncology centers with high laparoscopical experience.
Disclosure: Nothing to disclose

EP647

The effect of bariatric surgery on endometrial cancer

D. Vasileiadou[1,2], K. Antiochos[2], M. Litos[1], G. Skrekas[2]
[1]*Department of Obstetrics & Gynecology, Konstantopouleio General Hospital of Nea Ionia,*
[2]*LapEndoSurgery: Advanced Laparoscopy & Bariatric Surgery, Athens, Greece*

Introduction / Background: It is well known that obesity is associated with an increased risk of cancer. Considering its high prevalence, obesity is an important risk factor for cancer. Obesity is not only associated with new cancer cases but also with more advanced cancer disease and increased cancer mortality. Moreover, several cancer types, such as endometrial and pancreatic cancer, are associated with type 2 diabetes, a common co-morbidity related to obesity. Endometrial cancer has the strongest association with obesity compared to all other cancers in women, the risk proportionally increasing as body mass index increases. Bariatric surgery is the most effective treatment for achieving weight loss and sustaining normal weight in patients with obesity.
Methodology: The present study is a review of literature.
Results: Bariatric surgery has been shown to reduce the overall cancer risk in patients with obesity. Regarding endometrial cancer, several large - scale studies and meta-analyses have shown significant reduction in the risk of endometrial cancer in women undergoing bariatric surgery, with an odds ratio of 0.317. The estimated risk reduction has been shown to be as high as 50-70%. Several studies have also reported regression of endometrial hyperplasia after bariatric surgery.
Conclusion: Bariatric surgery may protect obese patients from endometrial cancer and may also benefit patients who are already suffering from its precursors. Weight loss and bariatric surgery may be suggested to patients as an adjunct to their treatment.
Disclosure: Nothing to disclose

EP648

Subungual metastasis of an endometrial carcinoma

A. Vernadou[1], D. Hastir[2], A. Sarivalasis[1]
[1]*Medical Oncology, CHUV- Centre Hospitalier Universitaire Vaudois,* [2]*Pathology, CHUV-Centre Hospitalier Universitaire Vaudois, Lausanne, Switzerland*

Introduction / Background: Metastasis to the nail bed are rare in solid tumor especially endometrial carcinoma. The majority of reported cases are associated with lung, kidney and breast cancers. With only scarce reports on this presentation, subungual metastasis can be misdiagnosed.
Methodology: We present a case of a multi-metastatic progression of a heavily pretreated, endometrioid uterine carcinoma. The occurrence of her subungual metastasis was precursor of a rapid metastatic progression occurring beside an initial partial response to her systemic treatment.
Results: A 52-year-old patient with a 3-year history of metastatic low-grade endometrial uterine cancer presented a multi-metastatic progression to the lymph nodes, bone liver and lung. The oncological history included front line chemotherapy with Carboplatinum and Paclitaxel, oral Medroxyprogesterone acetate, Lurbinectedine and Pembrolizumab-Lenvatinib association. A weekly Carboplatinum and Paclitaxel chemotherapy with concomitant Denosumab was initiated. After 3 months, her imaging concluded to a partial response but she reported a new modification in the shape and the color of her fourth right finger nail. Initially attributed to Paclitaxel, the nail deformation increased and resulted in an ingrown nail treated by surgical excision. The pathological examination confirmed a dedifferentiated adenocarcinoma infiltrating the nail. The radiological assessment excluded an underlying bone metastasis. Soon after the diagnosis, the patient presented multiple symptomatic cerebral metastasis and a rapid progressive disease.
Conclusion: Endometrial carcinoma is rarely associated with subungual metastasis. When observed, subungual metastasis from genitourinary cancers affect mostly lower limbs and terminal phalanges. The metastasis often spreads to the surrounding soft tissues from a primary bone metastasis. Frequent complains include pain and skin erythema. As confirmed by the literature, the occurrence of subungual metastasis is precursor to the rapid fatal progression of the disease. Beside the unexpected topography of the metastasis, the absence of an underlying bone involvement and the mild clinical manifestations were singular features of this progression pattern.
Disclosure: Nothing to disclose

EP649

Pattern of recurrence in patients with endometrial cancer

E. Vizza[1], G. Cutillo[1], V. Bruno[1], I. Sperduti[2], E. Mancini[1], E. Baiocco[1], B. Chiofalo[1], L. Cicchillitti[1], C. Certelli[1], A. Zampa[1], G. Corrado[3]
[1]Department of Experimental Clinical Oncology, Gynecologic Oncology Unit, [2]Scientific Direction, IRCCS 'Regina Elena' National Cancer Institute, [3]Department of Women and Children Health, Gynecologic Oncology Unit, Fondazione Policlinico Universitario A. Gemelli - IRCCS, Rome, Italy

Introduction / Background: Endometrial cancer (EC) is mostly diagnosed at an early stage with a favorable overall survival. 5-year survival decreases from 95% for localized disease to less than 20% for metastatic disease. Survival is related to known prognostic factors. Nevertheless, they are not sufficient to predict either outcome or recurrence rate/site: to decipher the underlying aberrant biomolecular pathways seems to be promising, although it is not yet applicable in a clinical setting. In that purpose, to investigate EC recurrence patterns according to ESMO-ESGO-ESTRO risk classes, could be beneficial for an early recurrences detection and treatment with a survival rate improvement.
Methodology: 758 women diagnosed with EC, and a 5-years follow-up, were enrolled: they were divided into the ESMO-ESGO-ESTRO risk classes (low LR, intermediate IR, intermediate-high I-HR, and high risk HR) and surgically treated as recommended, followed by adjuvant therapy, when appropriated.
Results: Recurrences were detected in 19,5%. Higher recurrence rate (RR) was significantly detected (p< 0,001) in the HR group (40,3%) compared to LR (9,6%), IR (16,7%) and I-HR (17,1%). Recurrences were detected more frequently at distant sites (64%) compared to pelvic (25,3%) and lymph nodes (10,7%) recurrences (p< 0,0001): this trend was evident in all risk classes except for the LR group, where no differences were detected between local and distant recurrences. 5-year distant-free (LR 99%,IR 94%,I-HR 86%,HR 88%) and local-free survivals (LR 99%,IR 100%,I-HR 98%,HR 95%) significantly differ between groups (p< 0,0001 and p=0,003, respectively), even when we stratified according to adjuvant therapy (AT) approach. AT did not modify RRs in all risk classes, except for LR group (p=0,01) (85,71% of recurrences occur if no AT was administered).
Conclusion: These results strengthen the need to identify biological factors to stratify patients at higher risk of relapse, independently or in addition to their risk class prognosis and current surgical and clinical managements.
Disclosure: Nothing to disclose

EP650

Ultrasound screening for endometrial abnormalities in patients on tamoxifen

B. Vranes[1], N. Vulic[1], P. Mitrovic[1], V. Stojanovic[1], N. Filipovic[1], N. Kusljevic[1], D. Tomovic[1], N. Matavulj[1], O. Cegar[2]
[1]Gynecology and Obstetrics, [2]University Clinical Center Zvezdara, Belgrade, Serbia

Introduction / Background: The accuracy of the ultrasound screening for endometrial abnormalities in absence of bleeding is a subject of debate.
We compared pathology reports in 92 postmenopausal patients who underwent D&C based on the ultrasonographic finding of endometrial hyperplasia in the absence of bleeding. The literature review presents data low with low ultrasound accuracy for endometrial abnormalities, at a range of 1:100 or less for asymptomatic patients.
Methodology: Retrospective case-control study was conducted in 2013-2014 and 92 patients were selected. All patients underwent D&C due to endometrial hyperplasia diagnosed with ultrasound only.

Sixty patients were postmenopausal on Tamoxifen treatment for breast cancer and 32 patients were from general population. There was no bleeding registered in any of the patients prior to D&C. The threshold for endometrial thickness was 6 mm for asymptomatic patients from general population, and 4 mm for Tamoxifen receiving patients. No patients were evaluated for endometrial abnormalities before onset of tamoxifen treatment.
Results: Mean endometrial thickness was 15.7 mm in general population and 7.8mm in Tamoxifen group. In general population group endometrial cancer was detected in 1 patient (3.1 %). In 9 patients (28 %) histology revealed simple endometrial hyperplasia, in 7 (22 %) endometrial polyp while 14 (44 %) had normal endometrium or atrophy. One of 60 Tamoxifen patients (1.67%) was found with adenocarcinoma. 1 (1.67%) was found with cystic hyperplasia, 6 (10%) were found with cystic polyps, and 1 (1,67%) with simple polyp. Seventy three percent of patients were found with no endometrial abnormalities.
Conclusion: The accuracy of ultrasound in screening for endometrial abnormalities is low, and it is hard to justify the rate of invasive diagnostic procedures as D&C.
We recommend adopting guidelines as ACOG 336 in identifying high risk patients. Evident uterine bleeding should be invasively investigated.
Disclosure: Nothing to disclose

EP651

The predictive value of preoperative haematological parameters in relation with CA-125 in endometrial cancer

S.W. Vrede[1], C. Reijnen[1], N.C.M. Visser[2], D. Boll[3], P.M. Geomini[4], H. Ngo[5], D. Hamont van[6], B.M. Pijlman[7], M.C. Vos[8], M.P.L.M. Snijders[9], L.F.A.G. Massuger[1], J.M.A. Pijnenborg[1]
[1]Obstetrics and Gynaecology, Radboud University Medical Centre, Nijmegen, [2]Pathology, Amsterdam University Medical Center, Amsterdam, [3]Obstetrics and Gynaecology, Catharina Cancer Institute, Catharina Hospital, Eindhoven, [4]Obstetrics and Gynaecology, Maxima Medical Center, Veldhoven, [5]Obstetrics and Gynaecology, Elkerliek Hospital, Helmond, [6]Obstetrics and Gynaecology, Amphia Hospital, Breda, [7]Obstetrics and Gynaecology, Jeroen Bosch Hospital, 's-Hertogenbosch, [8]Obstetrics and Gynaecology, Elisabeth-TweeSteden Hospital, Tilburg, [9]Obstetrics and Gynaecology, Canisius-Wilhelmina Hospital, Nijmegen, The Netherlands

Introduction / Background: Preoperative risk stratification in endometrial cancer (EC) is mainly based on the histological diagnosis of endometrial biopsies. Yet, easily accessible clinical biomarkers sampled prior to surgery, might contribute to improved risk stratification. Previous studies demonstrated the prognostic value of haematological parameters. Cancer antigen 125 (CA-125) has been widely accepted as tumour marker in EC, yet so far not studied in relation to pre-operative haematological parameters. Our aim is to investigate the prognostic value of preoperative haematological parameters in relation with CA-125.
Methodology: Data collection was performed out of available samples of the prospective multicentre PIPENDO cohort. A total of 285 patients (66%) had complete available serum analysis of at least one. Anaemia (< 12 g/Dl), thrombocytosis (>390x10^9platelets/L), leucocytosis (>10x10^9/L) were related to CA-125 (>35kU/L), patients, histopathological characteristics and outcome.
Results: Preoperative anaemia was present in 9.8% (n=28), leucocytosis in 10.9% (n=31) and thrombocytosis in 7.4% (n=21). Anaemia, thrombocytosis and leucocytosis were associated with BMI, comorbidity of diabetes and hypertension. Age was only associated with anaemia. Histopathological characteristics showed no association between anaemia, thrombocytosis and leucocytosis.
In multivariate Cox regression analysis anaemia and increased CA-125 were associated as risk factor for recurrence with hazard ratio (HR) of 2.50 (95% CI, 1.20-5.24*p*=0.016) and 2.02 (95% CI 1.10-3.71 *p*=0.024). Only increased CA-125 was associated as risk factor for overall survival with HR of 2.12 (95% CI 1.21-3.72 *p*= 0.008).
Conclusion: Preoperative anaemia and increased CA-125 are associated with a two times increased risk of recurrence in women with EC.
Disclosure: Nothing to disclose

EP652

Clinicopathological characterstics and survival analysis of clear cell carcinoma endometrium: a single institution experience of tertiary cancer centre in India

S. Wadhwa[1], A. Ghosh[2], J. Ghosh[3], J. Bhaumik[1]
[1]Gyne Oncology, Tata Medical Center, New Town, [2]Gyne Oncology, [3]Medical Oncology, Tata Medical Center, Kolkata, India

Introduction / Background: Clear cell carcinoma (CCC) comprises a rare yet an aggressive subtype, accounting for less than 5% of all uterine carcinomas. Treatment programs of CCC endometrium at different stages varies yet outcome of this tumor has not improved.
Methodology: This study presents a *single institution experience* regarding the clinicopathological features , treatment strategies outcomes used in CCC endometrium and discuss parameters associated with Recurrence free survival (RFS) and Overall survival (OS).
25 Patients of Endometrial CCC, diagnosed and treated between 2011 and 2018, were reviewed retrospectively. All data related to patient's demographic, clinical, histopathology assessment, adjuvant and survival outcomes was evaluated. RFS,OS were estimated as well as prognostic factors associated with OS were analyzed using Kaplan -Meir's, Cox regression analysis
Results: The median follow- up time was 28 months, and the mean age at diagnosis was 59.8 years. There were 18 (72%) patients with pure clear cell carcinoma and 7(28%) with mixed histology.
The majority were early stage [FIGO IA n= 7 [28%], IB- n= 3[12%], FIGO II n= 4[16%], FIGO III n=6 [24%], FIGO IV n=5[20%].
18 (72%) Patients received adjuvant platinum and taxane based chemotherapy with or without radiotherapy in the form of EBRT and /or vaginal brachytherapy. 5(20%) patients received Palliative chemotherapy only.
6 (24%) patients died during follow up, 6(24%) develop recurrence (local -1, distant-5). The Mean OFS, RFS were 62.98 and 55.83 months respectively.
The OS was significantly shorter in patients with >2 cm size tumour, cervical, adenexal invasion, lymph node metastasis and thus with advanced stage disease.
Conclusion: Patient with early stage UCCC had an excellent outcomes .The use of combined treatment approach is better and it is reasonable to offer the chemotherapy in early stage although its true benefit in stage I are still up for discussion.
Disclosure: Nothing to disclose

EP653

Metabolic surgery for obese patients with early stage endometrial cancer

M. Wilkinson[1,2], T. Walsh[1], R. McVey[1], W. Boyd[1], C. le Roux[3], J. Conneely[4], H. Heneghan[5,6], D. Brennan[7]
[1]Mater Misericordiae Dublin, [2]Conway Institute, [3]Medical Obesity Specialist, University College Dublin, [4]Bariatric Surgery, Mater Misericordiae Dublin, [5]Bariatric Surgery, St. Vincent's University Hospital, [6]University College Dublin, [7]Science Biology Ireland, University College Dublin, Dublin, Ireland

Introduction / Background: Obesity is the greatest risk factor for endometrial cancer. There is a growing need to modify treatment of obesity-related endometrial cancer.
Methodology: We developed a rapid access clinical pathway including a dietician, physician, surgical and psychological review to fast-track metabolic surgery for obese women with early stage endometrial adenocarcinoma (EAC) or complex atypical hyperplasia (CAH). All women have a mirena-IUD in situ and undergo 3 monthly endometrial sampling.
Results: 7 women were referred for assessment, 6 were deemed suitable for surgery, one has been advised to stop smoking. The average age was 45.8 years (range 25-62) and mean BMI was 49.6 kg/m^2(SD 5.5). Three women wished to retain fertility. 5 (71.4%) women had stage 1a EAC and 2

(28.5%) had CAH. All patients had an ASA grade of 3. Thus far 5 women have had a laparoscopic vertical sleeve gastrectomy (VSG) all of which were performed within 12 weeks of referral. All women were discharged on day 3 post op. There was one grade II morbidity. All women who are 3 months post VSG have lost at least 10% of initial body weight. One woman had an uncomplicated TLH/BSO 6 months post VSG following complete resolution of her poorly controlled diabetes and an 8 unit drop in BMI. Her final pathology showed a stage 1B EAC. Endometrial sampling of three patients demonstrated a complete pathological response in 1 and stable disease in 2.
Conclusion: A rapid access clinical pathway for metabolic surgery for obese endometrial cancer patients is feasible and initial results are promising. International collaborative efforts are required to pool data on this intervention and plan appropriate clinical trials.
Disclosure: Nothing to disclose

EP654

Adenosarcoma of the uterus with heterologous elements: early recurrence of a case before first follow-up examination

I. Yalcin[1], M. Yazol[2]
[1]Gynecologic Oncology, [2]Radiology, Şanlıurfa Research and Training Hospital, Şanlıurfa, Turkey

Introduction / Background: Uterine adenosarcoma is a rare and aggressive malignancy. It is defined as a biphasic tumor composed of both benign glandular component and malignant stromal component. Here we report early recurrence of a uterine adenosarcom.
Methodology: A Case Report.
Results: A 46-year-old woman presented with vaginal bleeding and admitted to the outpatient- clinic. Her past medical history was noncontributory. Bimanual examination revealed a normal uterus and bilateral adnexa. Pelvic color ultrasound showed that a 57x38 mm heterogeneous mass occupying the uterine cavity. Fractional endometrial curettage revealed no malignancy. Total abdominal hysterectomy was performed. The diagnosis of the frozen section was uterine adenosarcoma. The patient subsequently received bilateral salpingo-oophorectomy, pelvic and para-aortic lymphadenectomy. Peritoneal cytology revealed nonmalignant cells. Histopathological final diagnosis was adenosarcoma with heterologous elements (cartilage and rhabdomyoblasts). Largest tumor diameter was 6 cm, lymphovascular space invasion was positive, and the tumor was in the inner half of myometrium. There was no metastasis in ovaries and lymph nodes. According to the International Federation of Gynecology and Obstetrics (FIGO) (2009) staging system, the final stage was Ib. She was discharged without postoperative treatment.
A few days before first follow-up visit, the patient was admitted again for abdominal pain. Abdominal ultrasound scans revealed an enlarged pelvic mass with a 17x10 cm diameter (Fig. 1). Contrast-enhanced *Computed Tomography* imaging of the pelvis revealed a large pelvic mass with hypodense areas that indicate cystic componentand peritumoral fat stranding (Fig. 2). The tumor was filling the pelvic cavity (18 width x 15 depth x 12 length cm) and extends into the abdominal cavity. A second surgery was planned but the patient refused to undergo surgery.
Conclusion: Close follow-up of the uterine adenosarcoma patients is recommended because of the high chance of early recurrence.

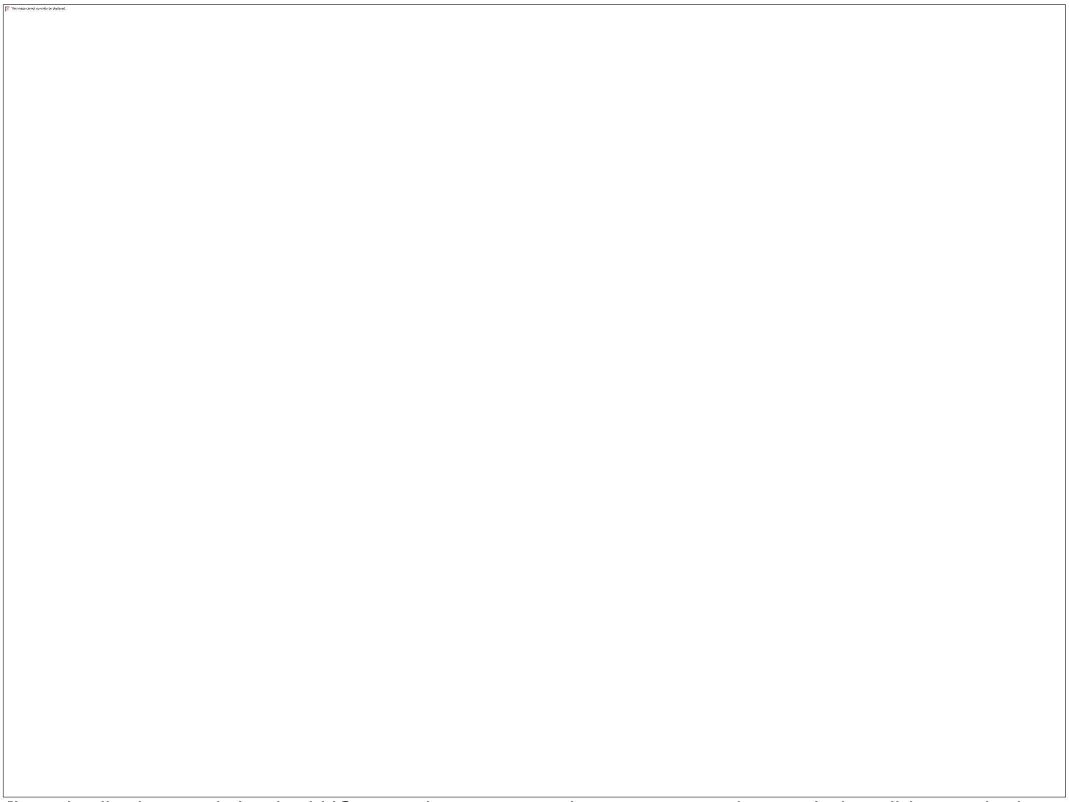

[Longitudinal transabdominal US scan demonstrates heterogeneous hypoechoic solid mass in the pelvis]

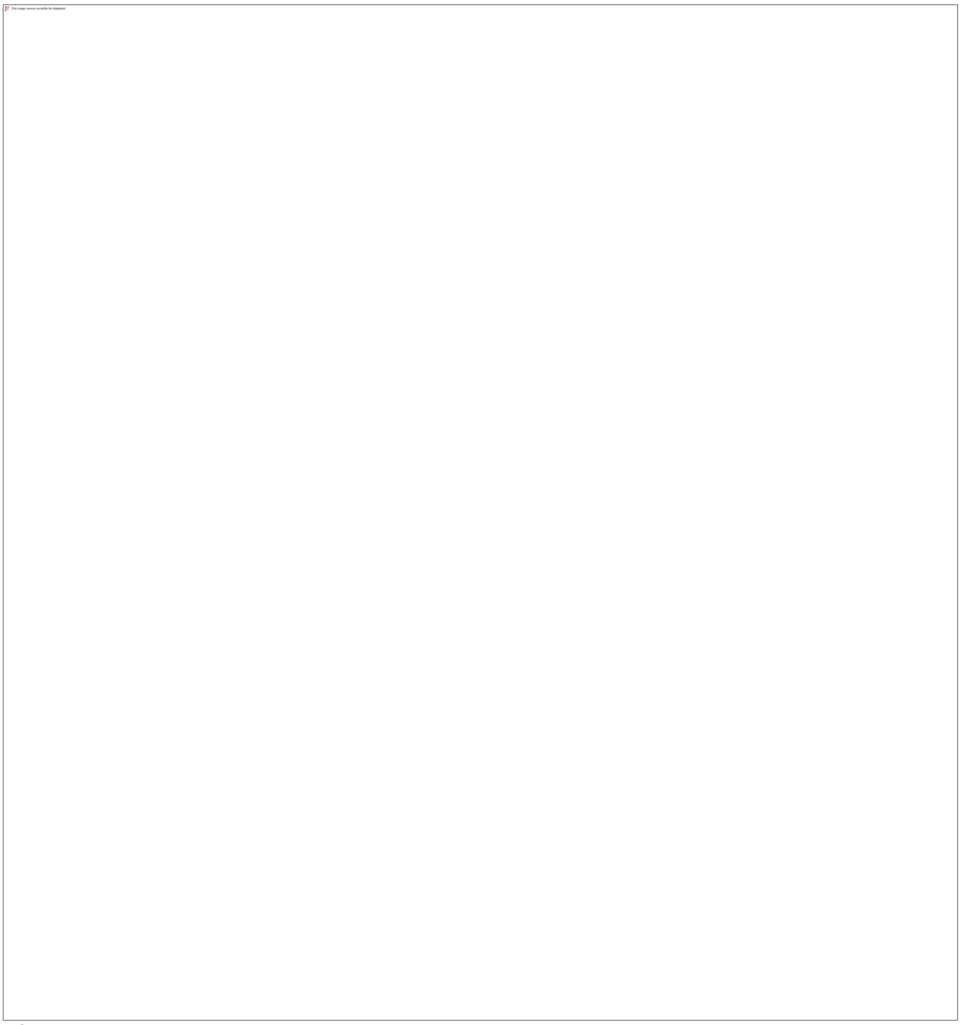

[Computed Tomography imaging of the pelvis revealed a large pelvic mass with hypodense areas]

Disclosure: Nothing to disclose

EP655

A feasibility study of sentinel lymph node surgery for laparoscopic approach in endometrial cancer

R. Yamada, Y. Todo, H. Matsumiya, K. Minowa, C. Shimada, T. Tsuruta, S. Minobe, H. Kato
Hokkaido Cancer Center, Hokkaido, Japan

Introduction / Background: The aim of this study was to verify the feasibility of laparoscopic approach for sentinel lymph node (SLN) surgery in endometrial cancer.
Methodology: This retrospective study was carried out using data for 118 patients with endometrial cancer who had undergone SLN mapping. Technetium colloid (Tc^{99m}) and/or indocyanine green (ICG) was injected into the uterine cervix and a gamma-detecting probe and/or photodynamic eye camera system was used intraoperatively to locate hot spots. SLN detection rate (unilateral or bilateral) and incidence of complications were compared between open approach (O group, n=65) and laparoscopic

approach (L group, n=53).
Results: Patients' median age was 60 years. One hundred and nine (92%) had FIGO Stage I disease. There was no difference in patients' age, BMI, FIGO stage, and histology between the two groups. SLN mapping with a combination with two kinds of tracers (Tc^{99m} and ICG) was performed more frequently in the L group than in the O group (49% vs. 20%, *P*=0.0008). Successful bilateral or unilateral mapping occurred in 91 patients (77%) and 19 (16%), respectively. There was no difference in SLN detection rate between the two groups (*P*=0.73). Perioperative complication rate tended to be lower in the L group (6% vs. 19%, *P*=0.051)
Conclusion: Laparoscopic approach might obtain a SLN detection ability equal to open approach without increasing the number of perioperative complication despite limitation of the present study, i.e. a combination with two kinds of tracers might have given the L group an advantage over the O group.
Disclosure: Nothing to disclose

EP656

Post-transcriptional regulation of prognostic factor PD-L1 expression by 17β-estradiol via PI3K/Akt signaling pathway in endometroid cancer

L. Yang[1,2], X. Zeng[1], M. Xi[1]
[1]West China Second University Hospital | Gynecology, [2]Key Laboratory of Birth Defects and Related Diseases of Women and Children, Sichuan University, Chengdu, China

Introduction / Background: Endometriod cancer (EC) is identified as one of the estrogen-dependent malignancies in women worldwide. Estrogen-stimulated cellular proliferation remains the conceptual underpinning of estrogen receptor(ER) dependent mechanism in EC development and progression. The immune checkpoint PD-1/PD-L1 plays a major role in immune suppression within the tumor microenvironment. The aim of this study is to evaluate the expression and prognostic function of PD-1 and PD-L1 in EC and to assess the effects of 17β-estradiol (E2) on PD-L1 expression in estrogen-dependent cancer.
Methodology: Paraffin sections of EC patients were used for immunohistochemical (IHC) staining using PD-1 and PD-L1 antibodies. All patients were selected for follow-up and prognostic analysis. Real-time quantitative PCR (q-PCR) and Western blot were performed to detect the mRNA and protein levels of PD-L1 after E2 treatment in estrogen-dependent cancer cells. The mechanism was investigated through co-immunoprecipitation, degradation analysis, sucrose gradient centrifugation and polysome analysis.
Results: PD-L1 is highly expressed in epithelium of endometrioid adenocarcinoma, accompanying with more PD-1 positive infiltrating lymphocytes in stroma. Highly expressed PD-L1, associated with advanced stage and high-grade differentiation, shortens the disease-free survival (DFS) and affects prognosis of patients with endometrioid adenocarcinoma. High level and unopposed E2 in tumor microenvironment leads to specific combination of membrane ERα and PI3K p85α regulatory subunit, recruiting PIP3 to phosphorylate No. 473 serine of Akt and forming the active type p-Akt. Activation of Akt can stabilize PD-L1 mRNA and increase PD-L1 translation efficiency. Co-culture results shows that up-regulating PD-L1 expression and activating PD-1/PD-L1 pathway leads to inhibition of T cell activity.
Conclusion: Our results demonstrate a new role of estrogen in regulation of anti-cancer immunity, implying PD-L1 as a potential therapeutic target or an outcome parameter in anti-estrogen treatment of EC and estrogen- dependent cancer.

This image cannot currently be displayed.

[Fig_1]

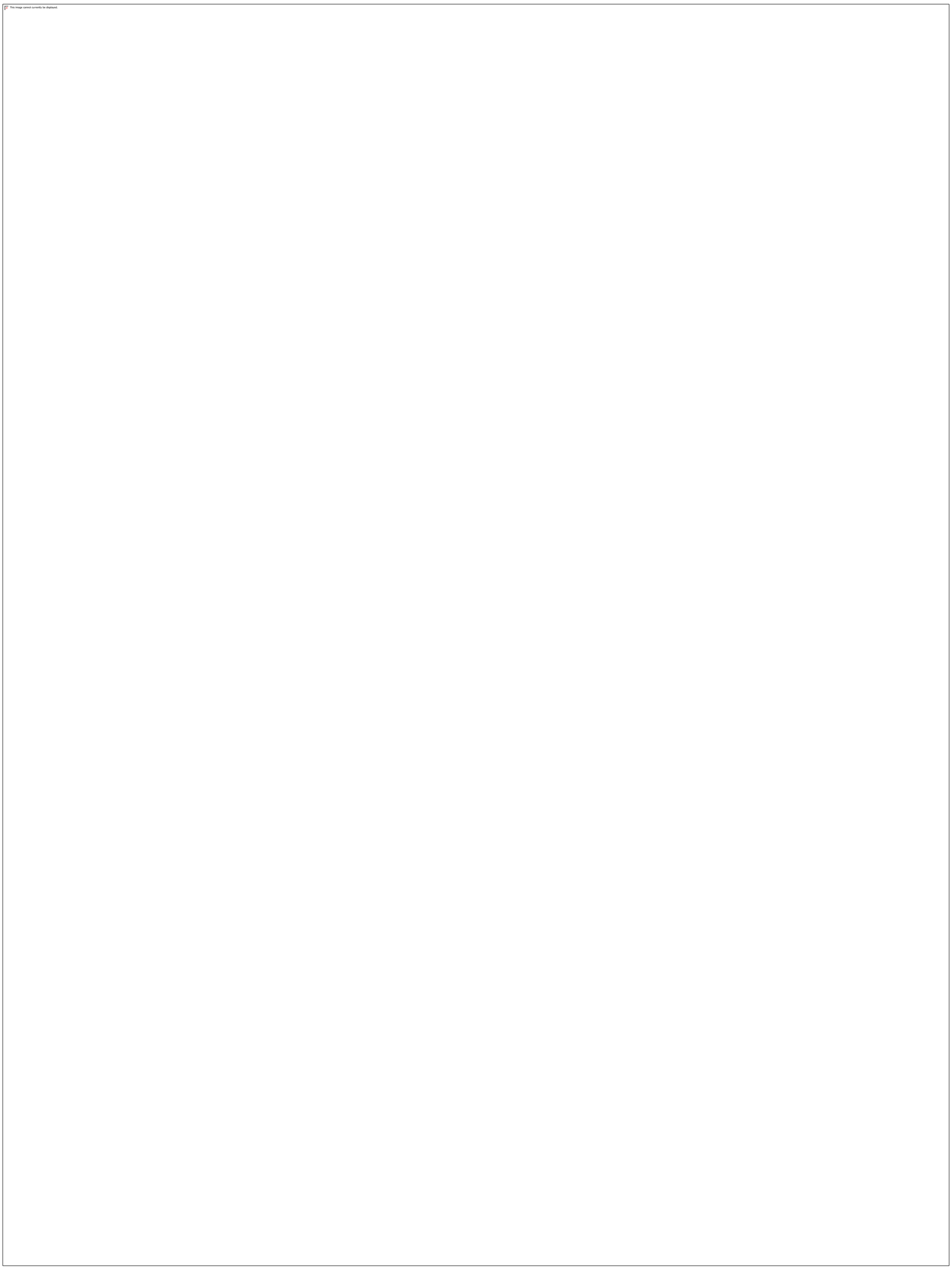

[Fig_2]

Disclosure: Nothing to disclose

EP657

The risk factors and therapeutic regimens analyses in the fertility-sparing treatment of refractory endometrial atypical hyperplasia and endometrial cancer

B. Yang[1], S. Zhou[2], X. Chen[2]
[1]Department of Gynecology, [2]Obstetrics and Gynecology Hospital of Fudan University, Shanghai, China

Introduction / Background: This study investigated the risk factors and therapeutic regimens in refractory endometrial atypical hyperplasia (EAH) and endometrial cancer (EC) patients with fertility-sparing treatment.
Methodology: Patients with EAH (n = 284) or well-differentiated EC (n = 95, FIGO stage IA, without myometrial invasion) were retrospectively included. All patients received progestin therapy combined with hysteroscopy. Therapeutic effects were evaluated by hysteroscopy every three months during the treatment. Profiles of blood glucose levels, sex hormone levels, serum CA-125 and HE4 were tested before progestin treatment, and estorgen receptor (ER)/ progesterone receptor (PR) status of lesions were detailly recorded.
Results: The median age was 32.0 year-old (range, 20-49 year-old). Totally 354 patients (93.4%) achieved complete response (CR) while 6 patients presented with progressed disease (PD), 5 patients remained no response (NR) and then chose hysterectomy, and 14 patients were still in treatment. We defined refractory patients as: (1) presented PD during fertility-sparing treatment; (2) remained no response (NR) after 6 months of treatment; (3) did not achieve CR after 9 months of treatment. 91 patients were refractory. After adjusting for age and type of fertility-sparing therapies, BMI ≥ 30kg/m^2 (OR, 2.880; 95% CI, 1.354-6.126; P=0.006) and testosterone (T) ≥ 0.37ng/ml (OR, 0.448; 95% CI, 0.249-0.805; P=0.007) were significantly correlated with higher risk of refractory disease. After at least 6 months of high-dose progestin treatment, patients who altered to high-dose progestin combined with metformin or Diane-35 combined with metformin may have higher CR rate than those with original regimens (90.9%, 85.7% vs. 61.1%, P=0.132). Diane-35 combined with metformin use up-regulated PR, which in turn may improve treatment efficacy.
Conclusion: BMI ≥ 30kg/m^2 and T ≥ 0.37ng/ml were associated with higher risk of refractory disease. Individualized therapies are needed for refractory patients.
Disclosure: Nothing to disclose

EP658

Adjuvant locoregional chemo-irradiation followed by sequential chemotherapy for high risk endometrial cancer, single centre real-world experience

W.L.W. Yip, N.S.J. Cheng, C.S.F. Wong
Clinical Oncology, Tuen Mun Hospital, New Territories, Hong Kong

Introduction / Background: PORTEC-3 study has illustrated failure-free survival benefit in the subgroup of patients with stage III endometrial cancer. This study reports the toxicities and pattern of relapse for patients with high risk endometrial cancer after adjuvant chemo-irradiation and sequential chemotherapy in Tuen Mun Hospital.
Methodology: Patients with endometrial cancer with concurrent adjuvant chemo-irradiation from 1st January 2015 to 9th May 2019 were reviewed. Those with planned sequential chemotherapy with four cycles of paclitaxel (P)-carboplatin (J) were included.
Results: 25 patients were included. All patients received radiotherapy 45-50.4Gy in 1.8-2Gy per fraction. 76% received concurrent weekly cisplatin 40mg/m2, while after a protocol change in 2018 based on PORTEC-3, 24% of patients received concurrent cisplatin 50mg/m2 every 3 weeks. Vaginal brachytherapy was performed in 76% of the patients. After completion of chemo-irradiation, 72% received at least 1 cycle of 3-weekly P 175mg/m2 and J AUC5, the rest declined further chemotherapy. 72% of those who received PJ had chemotherapy delay, among which 62% was due to treatment related complications (6 patients had at least G2 decrease in white cell count, 1 had non-

neutropenic respiratory infection). There was a statistically significant increase in delay in first cycle PJ in those who received weekly cisplatin compared with PORTEC-3 regime (median 35days versus 28days, p=0.01). Among those on PORTEC-3 regime, 50% received PJ cycle 1 with delay. 56% of all patients completed 4 cycles of PJ. 68% had G3-4 neutropenia and 28% had neuropathy. 20% had residual neuropathy 6months after treatment. After excluding 3 patients with carcinocarcoma and 2 with initial stage IV disease, 15% had a relapse, all occurred within the first year post treatment.
Conclusion: Weekly concurrent cisplatin should be phased out in this setting due to increased marrow toxicity. Further studies comparing chemotherapy first then chemo-irradiation versus PORTEC-3 regime to increase chemotherapy completion rate should be done.

		Tuen Mun Data (n=25)	PORTEC-3 (chemo-irradiation subgroup; n=330)
Median age (year)		55	62
Stage III (%)		88	46
Histology (%)	Endometrioid grade 1-2	40	40
	Endometrioid grade 3	32	27
	Serous	8	16
	Clear cell	0	9
	Mixed	8	5
	Others	12	4
Lymph node dissection > 12 lymph nodes (%)		76	57

[Demographics and baseline treatment details, compared with chemo-irradiation subgroup of PORTEC-3]

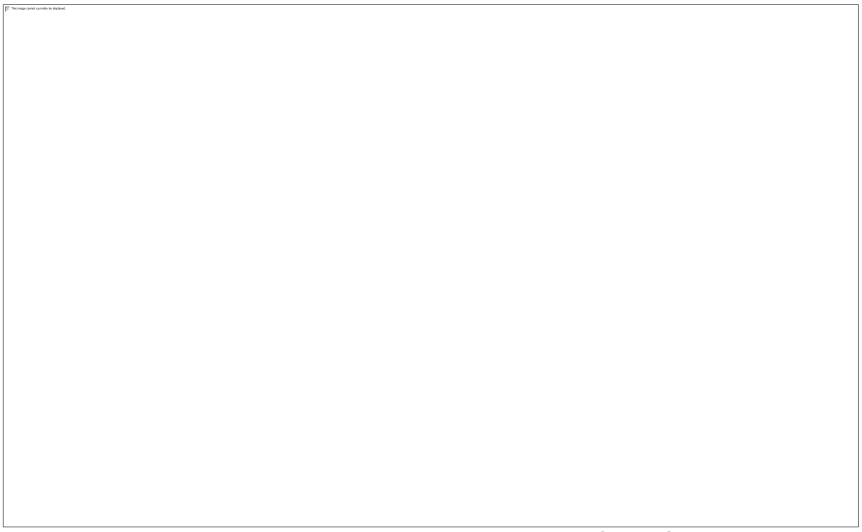

[Toxicities during and after treatment, comparing with PORTEC-3 chemo-irradiation subgroup]

Disclosure: Nothing to disclose

EP659

Role of tumor grade on preoperative endometrial biopsy in predicting recurrence in endometrioid endometrial cancer

J.G. Yoo, J.H. Ahn, Y.Y. Jeong, H.S. Hwang, K.H. Lee
Department of Obstetrics and Gynecology, The Catholic University of Korea, Seoul, Republic of Korea

Introduction / Background: The tumor grade is an important prognostic factor in endometrial cancer. Although most endometrial cancer patients are diagnosed histologically prior to surgery, the results are often inconsistent with the final histological results of hysterectomy.
The aim of this study is to determine which of the two histologic diagnostic methods better reflects the patient's oncological outcome.
Methodology: We reviewed the medical records of endometrial cancer patients who were able to identify both histopathologic results of both endometrial biopsy and hysterectomy specimen between January 2009 and December 2015. The concordance rates for histological types and grades between pre- and post-operative diagnosis were calculated. Recurrence rates of each subgroups were measured, and compared according to the hysterectomy results.
Results: Total 257 patients with endometrial cancer met the basic inclusion criteria (Table 1). The concordance rates were 73.5% for G1, 43.1% for G2, and 62.5% for G3. The overall concordance rate was 61.3%. In patients with endometrioid and serous carcinoma on preoperative endometrial biopsy, 22.0% were up-graded, and 15.2% were down-graded on the final hysterectomy specimen.
133 patients were diagnosed with G1 on the hysterectomy specimen. Among them, 30 patients had been diagnosed with G2 or G3 on their preoperative endometrial biopsy. Interestingly, this group had a significantly higher recurrence rate than the group consistently diagnosed with G1 (20.0% vs 3.1%, OR 8.55, 95% CI 1.98 - 36.85, p=0.002, Table 2B). In the opposite case, 35 patients who were G1 on preoperative biopsy were up-graded to G2 or G3 after hysterectomy. This group showed similar recurrence rate to those consistently diagnosed with G2.
Conclusion: In general, G1 endometrioid endometrial carcinoma has a preferable oncological outcomes, but showed significantly higher recurrence rate for the patients who G2 or G3 features were found on preoperative endometrial biopsy. We suggest this result considered in clinical practice and patient counselling.

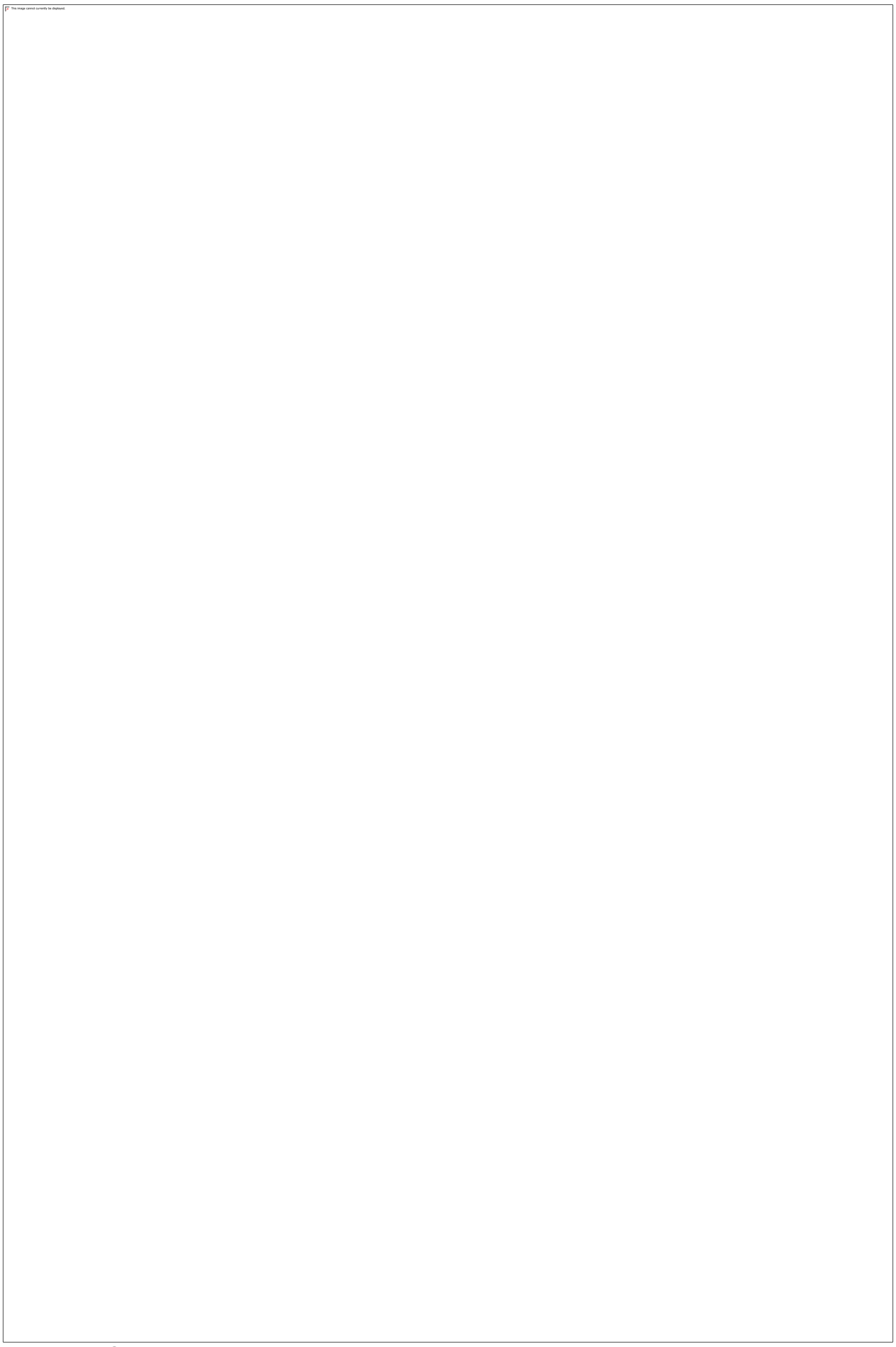

[Table 1. Characteristics of patients]

[Recurrence rates of each subgroups]

Disclosure: Nothing to disclose

EP660

Novel approach to noninvasive assessment of lymph-nodes metastases with one-step isothermal quantification of mRNAs in primary tumor of endometrial cancer

E. Yoshida[1,2,3], K. Usui[3], Y. Ito[1,4], Y. Ueno[5], M. Ozawa[5], S. Nojiri[6], Y. Kogo[4], T. Kato[7], T. Ohtsu[8], H. Kato[5], M. Itoh[4], H. Kawaji[3,4], Y. Terao[1]
[1]Department of Obstetrics and Gynecology, Juntendo University, [2]Diagnostics and Therapeutics of Intractable Diseases, Juntendo University Graduate School of Medicine, Tokyo, [3]Center for Integrative Medical Science, [4]Preventive Medicine and Diagnosis Innovation Program, RIKEN, [5]Department of Gynecology, Kanagawa Cancer Center, Yokohama, [6]Department Center for Lifetime Cancer Education, Juntendo University Graduate School of Medicine, [7]Department of Gynecology, National Cancer Center Hospital, Tokyo, [8]Research Institute, Kanagawa Cancer Center, Yokohama, Japan

Introduction / Background: Lymphadenectomy in endometrial cancer should be considered depending on individual patients owing to its postoperative risks such as lymphedema. Although assessment of lymph node metastasis provides crucial information for appropriate adjuvant management, therapeutic importance of lymphadenectomy for low-intermediate risk patients is a matter of debate. Given that sentinel lymph node biopsy is a beneficial but complex method, noninvasive, simple and high-precision diagnosis method for lymph node metastatic state is highly demanded. In a previous study, we identified SEMA3D and a novel isoform of TACC2 as promising biomarkers to evaluate lymphatic metastasis based on gene expression patterns in the primary lesion. Here, we attempted to optimize sampling method considering tumor heterogeneity and to accelerate gene quantitative analysis for our biomarkers.
Methodology: Endometrial cancer tissues from new several patients were collected in addition to the previous 115 patients. We collected 5-13 pieces of primary tumor from each new patient. We verified gene expressions of each tissue piece by quantitative-PCR to optimize sampling method. Then we assessed whether RT-SmartAmp method, which can detect nucleic acids in one step consisting of a reverse transcription and an isothermal amplification of DNA, could quantify our biomarker RNA rapidly. We measured the speed of amplification and target specificity based on biomarker RNA as the template in one step containing reverse transcription step.

Results: We calculated the average expression levels by bootstrap method It suggested that even when the sampling number is two, each average expression level indicates correct diagnosis with 99%CI. Then we also succeeded to develop a promising candidate of primer set to detect the target mRNA by using SmartAmp. This candidate can detect RNA quantitatively within 30 minutes without non-specific amplification of negative control by using Eprimer as detection fluorescence.
Conclusion: Our findings pave the way for support clinical decisions that minimize irrelevant lymphadenectomy.
Disclosure: I have no COI with regard to our presentation. This work was supported by AMED under Grant Number 18cm0106433h0001 and 19cm0106452h0001, a Grant-in-Aid for Scientific Research (C) from the MEXT (15K10732, 17K11296, 18K09299), a research grant from MEXT through the RIKEN Preventive Medicine and Diagnosis Innovation Program, a research grant from MEXT to RIKEN Omics Science Center and a research grant from MEXT to RIKEN Center for Life Science Technologies (Division of Genomic Technologies).

EP661

Sentinel node mapping with indocyanine green and endoscopic near-infrared fluorescence imaging in endometrial and cervical cancer: a systematic review to assess impact of technical aspects on detection rate and diagnostic performance

K. Zalewski[1], J. Kacperczyk-Bartnik[2], A. Strojna[3], M. Szubert[4], K. Nowosielski[5], P. Lepka[6,7], M. Mardas[8], M. Bałajewicz-Nowak[9], N. Abdalla[10], M. Bobiński[11], Club of Young Gynecologic Oncologists affiliated to the Polish Society of Gynecological Oncology, Poland
[1]Department of Gynecologic Oncology, Holycross Cancer Center, Kielce, [2]Second Department of Obstetrics and Gynecology, Medical University of Warsaw, Warsaw, [3]Department of Gynecological Surgery and Gynecological Oncology of Adults and Adolescents, Pomeranian Medical University, Szczecin, [4]Clinic for Surgical Gynecology and Oncology, I Departament of Gynecology and Obstetrics, Medical University of Lodz, Lodz, [5]Department of Gynecology and Gynecological Oncology, Teaching Hospital in Czeladz, Czeladz, [6]Division of Surgical Oncology, Gynecological Oncology, Chemotherapy and Department of Oncology, Wroclaw Medical University, [7]Gynecologic Oncology Department, Lower Silesian Oncology Center, Wroclaw, [8]Department of Oncology, Poznan University of Medical Sciences, Poznan, [9]Department of Gynecology and Oncology, University Hospital in Krakow, Krakow, [10]Department of Obstetrics, Gynaecology and Oncology, II Faculty of Medicine, Medical University of Warsaw, Warsaw, [11]1st Chair and Department of Gynecological Oncology and Gynecology, Medical University in Lublin, Lublin, Poland

Introduction / Background: Sentinel lymph node (SLN) biopsy has been recently widely investigated in endometrial (EC) and cervical cancer (CC). Near-infrared (NIR) fluorescence imaging is rapidly gaining popularity despite being a relatively new technique associated with lack of uniformity in the application of the method used across centers. The aim of presented systematic review is to gather evidence regarding different technical aspects and their impact on the effectiveness of indocyanine green (ICG) use during SLN mapping.
Methodology: A computer literature search using Medline was conducted for studies in EC and CC patients published in between January 2010 and May 2019. The initial search came up with 207 articles, of which 91 articles used ICG as a tracer.
Results: 7446 patients were included in the final analysis, ranging from 1 to 472 patients per study. The main surgical approach was laparoscopy, followed by robotic surgery, laparotomy, hysteroscopy and natural orifice transluminal endoscopic surgery. The overall SLN detection rate using ICG ranged from 64% to 100% for cervical, from 83% to 100% for fundal and from 33% to 100% for hysteroscopic injections. Sensitivity and negative predictive value varied from 77,8% to 100% and 83% to 100%, respectively. The most common site of injection was the cervix injected submucosally and intrastromally in two quadrants. There is a large variation in the size of the needle, dilution solvent, concentration, dosage and volume of ICG injected as well as the time when the dye is injected and the period between injection and beginning of the dissection.
Conclusion: ICG shows a high overall detection rate as well as sensitivity and specificity in SLN mapping in patients with EC and CC. The differences in results obtained in particular studies suggest that the details of injection procedure might influence the success rate in SLN detection.

Disclosure: Nothing to disclose

EP662

Potential clinical value of Interlikin-31 and Interlukin-33 expressions as diagnostic and predictive factors in endometrial cancer: a case control study

X. Zeng, M.-R. Xi, L.-Y. Yang, G.-D. Liao
Sichuan University, Chengdu, China

Introduction / Background: To evaluate the potential role of interlukin-31 and interlukin-33 in prognosis from endometrial cancer.
Methodology: Tissue and serum samples and clinical data were collected from 260 patients with endometrial cancer and 150 control patients with benign uterine diseases. The expressions of interlukin-31 and interlukin-33 and their receptors were quantified by immunohistochemistry and ELISA testing. After surgery, all patients were followed up for an average of 56.3 months. Surgical effects were evaluated based on the patients' symptoms and signs. A two-sided *P* value < 0.05 was considered significant.
Results: IL-31, IL-33 and their receptors were significantly accumulated with the progression of endometial cancer, in comparison to the controls. Moreover, the expressions were correlated with clinical characteristics, including tumor stage, differentiation and associated with patients' disease-free survival.
Conclusion: Limited data were available between the expressions of IL-31 and IL-33 in patients with endometrial cancer. Our study findings suggested that the expressions of IL-31 and IL-33 might become possible biomarkers for the diagnosis and prediction in endometrial cancer.
Disclosure: This work was supported by the National Natural Science Foundation of China (no. 81572573).

EP663

Correlation between adenomyosis and endometrial cancer: 6-year experience of a single center

D. Zouzoulas[1], D. Tsolakidis[1], I. Efstratiou[2], S. Pervana[2], E. Pazarli[2], G. Grimbizis[1]
[1]1st Department of Obstetrics & Gynecology 'Papageorgiou' Hospital, Aristotele University of Thessaloniki, [2]Anatomical Pathology Laboratory, 'Papageorgiou' Hospital, Thessaloniki, Greece

Introduction / Background: Adenomyosis often co-exist in the pathological reports, after surgery for endometrial cancer. The aim of this study is to describe the clinicopathological and oncological characteristics of these patients and further investigate the malignant transformation of the adenomyotic tissue.
Methodology: We retrospectively reviewed the medical records of all patients that underwent hysterectomy for endometrial cancer (January 2012 - December 2017) in our clinic. The pathological reports were reviewed and when adenomyosis co-existed, the pathological slides were re-examined in order to discover any malignant change in the adenomyotic tissue. The clinicopathological characteristics and oncological results were described.
Results: Out of 229 cases of endometrial cancer, 64 (28%) patients had concurrently endometrial cancer and adenomyosis. Among these 64 patients, 7 (11%) had malignant transformation of adenomyosis. The mean age of patients suffering from both endometrial cancer and adenomyosis was 63.2 years old and 57 (89%) of these patients, had early endometrial cancer (FIGO stage IA-IB). Out of these patients, 22 (34.4%) had grade I, 36 (56.2%) grade II and only 6 (9.4%) had grade III tumors. Moreover, in this special group of patients with concurrent endometrial cancer and adenomyosis, 25 (39%) patients presented in the pathological report fibroids and 2 (3%) endometriosis. Concerning the

patients with malignant transformation of adenomyosis, their mean age was 65 years old with no premenopausal case. There was only 1 case of recurrence and none death due to cancer.
Conclusion: Adenomyosis, as a benign disease, is described in the last decades, but its malignant transformation is not fully researched, due to the low incidence of this entity and the relative diagnostic problems. Further investigation is needed in order to clarify the pathologic progression of adenomyotic lesions to endometrial cancer and the associated prognosis of this disease.
Disclosure: Nothing to disclose

EP664

The effectiveness of the procedure of IVF in patients with uterine myoma

F. Ayupova, D. Mirzaeva, D. Saidjalilova
Obstetric and Gynecology, Tashkent Medical Academy, Tashkent, Uzbekistan

Introduction / Background: Reproductive health in Uzbekistan is one of the priorities way of medicine. Currently, carrying out IVF in patients with uterine myoma is relevant in practical medicine.
Methodology: Studied 86 patients with IVF and uterine myoma. Women were divided into 2 groups: the first was 45 patients with intramural localization of the node, which deformed the uterine cavity with a diameter of not more than 3 cm; the second is 41 patients with a myoma node that does not deform the uterine cavity with a diameter of up to 3 cm.
Results: In patients of 1 group, 6 (13,3%) women had one IVF attempt, only three women (6,7%) had a uterine pregnancy. Further observation showed that two pregnant women had a spontaneous miscarriage, one pregnancy ended in premature labor in the period of 35-36 weeks. Thus, the pregnancy rate for one attempt in patients with an intramural node with a diameter of not more than 3 cm, deforming the uterine cavity, was 13,3%. Of the 41 women with a myoma node with a diameter of up to 3 cm, which do not deform the uterine cavity, pregnancy as a result of IVF occurred in 15 (36,6%) patients. In one case (2,4%), it was ectopic. In two cases (4,9%), spontaneous miscarriages occurred in the first trimester of pregnancy.
Conclusion: The node with a diameter of up to 3 cm, which does not deform the uterine cavity, does not adversely affect the performance of the IVF procedure and was 36,6%. The node with a diameter of up to 3 cm, which deforms the uterine cavity, significantly reduces the effectiveness of IVF by 2,7 times and implies surgical treatment before the IVF program.
Disclosure: Nothing to disclose

EP665

Juvenile granulosa cell tumors of the ovary: a retrospective study of 36 patients

M. Blot-Dupin[1], A. Maulard[1], C. Genestie[2], P. Pautier[3], E. Bentivegna[1], A. Leary[3], C. Chargari[4], P. Morice[1], S. Gouy[1]
[1]Gynecologic Surgery, [2]Pathology, [3]Medical Oncology, [4]Brachytherapy, Gustave Roussy, Villejuif, France

Introduction / Background: Granulosa cell tumors account for approximatively 3% of ovarian tumors. The juvenile histological type (JGCT) represents 5% of them, and remains relatively unknown. Our aim was to describe clinicopathologic characteristics and to evaluate prognostic factors.
Methodology: We retrospectively studied medical records of 40 patients referred to our center for JGCT between November 2010 and 2018.
Results: Thirty-six patients had confirmed JGCT. The mean age was 19,9 years (range 2,5 months - 47,4 years). Abdominal pain (53%), vaginal bleeding (31%), palpable mass (28%), were the most common presenting symptoms. The distribution of FIGO stage was: 16 patients at stage Ia, 17 Ic, 1

IIa, 1 IIIb, 1 IIIc. Thirty-two patients (89%) had conservative surgery. Sixteen (44%) received adjuvant chemotherapy (Ic=13, IIa=1, IIIb=1, IIIc=1), mostly bleomycine/etoposide/cisplatine regimen. The mean follow up was 35 months (range 1 to 204 months). Nine patients recurred (Ia= 1, Ic= 6, IIa=1, IIIc=1) with a mean time of 12,1 months (range 1 -47). Among them, 4 had a cystectomy at the initial surgery: 3 without adjuvant chemotherapy, 2 died of disease; 4 stage Ic did not received adjuvant chemotherapy neither. Three patients died after a mean of 21,2 months (range 9,5 - 32,4). At 36 months: progression free survival was 70,4% [54,9- 90,2], overall survival was 85,6% [71,4-100]. The main prognosis factor of recurrence was advanced FIGO stage *(p < 0,001)*. Age > 15 years *(p= 0,09)*and cytonuclear atypia *(p=0,08)*tended to be associated to relapse too. Intra operative tumor rupture *(p=0,03)* was a significant risk factor of death.We observed 5 pregnancies with term deliveries.
Conclusion: The main prognostic factors are: FIGO stage and intra operative tumor rupture. Fertility sparing surgery is safe for stage Ia, reasonable for stage Ic combined with adjuvant chemotherapy. Patients with cystectomy had poor outcome; unilateral salpingo-oophorectomy must be done sytematically.
Disclosure: Nothing to disclose

EP666

Childbearing desire and pregnancy outcome in patients underwent fertility sparing surgery for Borderline Ovarian Tumours

G. Candotti[1,2], M. Peiretti[2], G. Mangili[1], A. Bergamini[1], R. Cioffi[1], M.L. Fais[2], V. Mais[2], F. Pella[1], A. Proto[2], E. Rabaiotti[1], F. Turba[1], L. Bocciolone[1]
[1]San Raffaele Hospital, Milan, [2]Università degli Studi di Cagliari, Cagliari, Italy

Introduction / Background: Borderline ovarian tumors (BOTs) are characterized by the presence of cellular proliferation and nuclear atypia without stromal invasion. Compared to malignant ovarian tumors, BOTs typically present in younger women and have better prognoses.
Considering the good prognosis of BOT, fertility-sparing surgery can be considered for young women who desire to preserve fertility.
Our study evaluated the pregnancy rate in patients with childbearing desire, the efficacy and risk of recurrence of women affected by BOTs who have undergone fertility-sparing surgery.
Methodology: From June 2000 to December 2017, patients with a diagnosis of BOT have been restrospectively recruited at the San Raffaele Hospital, Vita e Salute University Milan and Policlinico Monserrato, University of Cagliari. Eighty-five patients underwent fertility-sparing surgery for BOT were interviewed about child wishing and pregnancy outcomes. Patients characteristics including age at diagnosis, tumor pathological characteristics, surgical management at primary treatment and relapse, follow up data were collected.
Results: Eithy-five patients were recruited for the study. Median age at diagnosis was 33 years (I.Q.R 29-38). Unilateral salpingo-oophorectomy was performed in 33 patients (38%), unilateral cystectomy in 40 (47%) and 12 underwent both procedures (14%). 40 women (50%) tried to conceive after surgery. We recorded 33 pregnancies, 8 miscarriages and 27 deliveries. 7 patients underwent ART for infertility and 5 of them obtained pregnancy The pregnancy rate was 73% and live birth rate was 67%. Childbearing desire and age at diagnosis were significantly associated with the pregnancy rate (PR).
Conclusion: Conservative surgical treatment seems to be a reasonable therapeutic option for women with BOTs who wish to preserve fertility. Our results suggest that the obstetric outcomes after fertility sparing surgery are promising. Maternal desire and the age of diagnosis are the most important factors affecting PR after surgery. Fertility counselling should be an integral part of the clinical management of women with BOT.
Disclosure: Nothing to disclose

EP667

fertility preservation in breast cancer: the protective role of letrozole

N. Chraiet[1], M. Braham[2], A. Mokrani[1], K. Kacem[2], M. Hamdoun[2], A. Mezlini[1], F. Zhioua[2]
[1]Salah Azaiez Institute, [2]Aziza Othmana Hospital, Tunis, Tunisia

Introduction / Background: Ovarian stimulation with exogenous gonadotropins increases circulating estrogen levels which might stimulate breast cancer sensitive cells. The use of anti aromatase agents such as letrozole could prevent this elevation and protect patients however, it does not reduce the average number of oocytes or embryos collected.
Methodology: It´s a prospective study from January 2015 to October 2018. We have compared the number of mature oocytes obtained in patients with breast cancer stimulated by letrozole (Groupe 1) to those collected in patients with other types of cancer (group 2).
The stimulation is conducted according to an antagonist protocol with random start and GnRH agonist triggering. For patients with breast cancer, the adjunction of letrozole 5mg/day was started the first day of the ovarian stimulation and continued 7 days after oocyte pick up.
Results: We conducted 143 fertility preservation (oocyte/embryo vitrification) with 75 cases in breast cancer.
The average age of patients (year) was respectively 30.3 +/- 3.7 and 26.9 +/- 6.7 with no significant difference (p=0.73). The ovarian reserve was not statistically different: Follicular count AFC (12.3 +/- 6.2 vs 13.9 +/- 6.4; p = 0.7) and Anti Mullerhien Hormone AMH (2.43 +/- 2.3 ng/ml vs 2.8 +/- 2.45 ng/ml, p = 0.5).
The estradiol level on the day of ovulation triggering was significantly lower 479 +/- 323 pg/ml in the breast cancer group versus 1701 +/- 682 pg/ml (p = 0.02)
The number of CCOs obtained in the breast cancer group was 10.76 +/- 8.39 compared with 9.11 +/- 6.81 (p=1.83).
Conclusion: Letrozole as a hormonotherapy in breast cancer, would also allow an efficient ovarian stimulation in women with breast cancer, and safer with a lower estradiol levels
Disclosure: Nothing to disclose

EP668

Cancer in pregnancy: a 15-year descriptive analysis of 86 consecutive patients

E. Di Loreto[1], C. Castellani[1], B. Buonomo[2], G. Polverino[1], E. Grossi[1], L. Buggio[1], M.W. Ossola[1], S. Passera[1], M. Cribiù[1], C. Blundo[1], F. Peccatori[2], G. Scarfone[1]
[1]Fondazione IRCCS Ca' Granda Ospedale Maggiore Policlinico Milano, [2]Istituto Europeo Di Oncologia, Milano, Italy

Introduction / Background: The maternal diagnosis of cancer complicates approximately 0,1% of all pregnancies. The most frequently diagnosed malignancies are breast cancer, cervical cancer, lymphoma, ovarian cancer and melanoma. Although chemotherapy can be administered during pregnancy, its effects on obstetric and neonatal outcome are still largely unknown. The aim of this study is to describe the oncologic management as well as the obstetric and perinatal outcomes in a consecutive series of patients with cancer during pregnancy.
Methodology: We provided descriptive oncologic, obstetric and neonatal data from a cohort of pregnant patients diagnosed with primary invasive cancer between August 2004 and April 2019, retrospectively collected from clinical databases of IRCCS Policlinico di Milano and Istituto Europeo Di Oncologia (Milan, Italy).
Results: We observed 86 consecutive patients with a diagnosis of malignancy during pregnancy: breast cancer was the most common type (70,9%), followed by lymphomas (11,6 %). Patients with ongoing or missing oncological, obstetrical or neonatal data were excluded. Of the 62 eligible patients, 12,9% received surgical treatment, 24% chemotherapy and 54,8% a combination of both. For almost all pregnancies, timing of delivery was previously planned: 38% with labor inductions and 54% with elective cesarean sections, at a mean gestational age of 36 weeks. All pregnancies ended in a

livebirth. Half of deliveries were preterm, mainly iatrogenic due to obstetric or oncologic indications: 3.2% were very low preterm, 6,4% moderate preterm and 45% late preterm. The frequency of small for gestational age (birthweight < 10° percentile) was 11.2%.
Conclusion: This study confirms a high overall frequency of premature birth in pregnant patients with cancer, showing a lower rate of small for gestational age than described in literature. In order to have a better assessment of the toxicity of chemotherapy during pregnancy, we are currently investigating its fetal sequelae through histologic analysis of placental tissue and pediatric follow-up.
Disclosure: Nothing to disclose

EP669

Fertility sparing surgery for borderline ovarian Brenner tumour: a literature review

G. Garofalo, D. Bucella, D. Thomas, F. Buxant
Hôpital d'Ixelles, Université Libre de Bruxelles, Bruxelles, Belgium

Introduction / Background: Borderline ovarian Brenner tumours (BOBTs) represent less than 0,01% of all ovarian cancers with good prognosis. Average age at presentation is 50. We revised the literature to improve our counselling.
Methodology: We reviewed three databases (Medline, LILACS, Central Cochrane Library) and found six case series and ten case reports with a total number of 58 cases.
Results: Four cases are reported under 40 years. Only two cases of fertility sparing surgery (FSS) by unilateral salpingo-oophorectomy were performed, and only one case of spontaneous pregnancy is described (Table 1). In this last case, BOBTs appeared at ultrasound as a four centimetres multilocular anechoic cyst with a papillary projection. Table 2 shows the good prognosis of this tumour: only two cases of recurrence were reported.

Articles	Cases (N.)	Diagnosis ≤ 40 years	Fertility Sparing Surgery	Pregnancy
Hallgrìmsson 1972, Roth 1985, Uzan 2012, Dierickx 2012, Takahama 2004 , Chia 2009, Wang 2010, Aoun 2014, De Cecio 2014, Klasa 2014, Morales-Palacios 2016, Albu 2016	33	0	-	-
Miles 1972	7	1(a)	Yes	NK
Woodruff 1981	10	1	No	-
Cuatrecasas 2009	7	1	No(b)	-
Garofalo 2019	1	1	Yes	Yes
**Total**	**58**	**4**	**2**	**1**
NK: not known; (a): median age 50 (three premenopausal and one at 30 years); (b): FIGO 2A, bilateral				

[Overall BOBTs, Fertility Sparing Surgery and subsequent pregnancy]

Articles	Cases (N.)	Recurrence	Months to recurrence
Hallgrìmsson 1972, Miles 1972, Roth 1985, Cuatrecasas 2009, Chia	33	0(a,b)	-

2009, Aoun 2014, De Cecio 2014, Morales-Palacios 2016, Albu 2016, Garofalo 2019			
Woodruff 1981, Dierickx 2012, Takahama 2004, Wang 2010	14	NK	-
Uzan 2012	10	1(b)	50
Klasa 2014	1	1(b)	24
**Total**	**58**	**2**	**-**
NK: not known; (a) information not for all the cases; (b)data not updated to 2019			

[Overall BOBTs and their recurrences]

Conclusion: Good prognosis of BOBTs supports FSS. However, literature is scarce on BOBTs due to its low prevalence. The prevalence is even lesser in young women which explains the only two cases of FSS. For serous and mucinous borderline tumours, a bilateral cystectomy for bilateral tumours or unilateral salpingo-oophorectomy (or even cystectomy) for unilateral tumours is widely accepted as the treatment to propose to patients willing to conceive. The recurrence rate is higher but the overall survival doesn't change. In these cases, completion surgery is a controversial point. Follow up is commonly intensified in first two years. The same aptitude can be proposed to BOBTs even in the absence of strong studies.
Disclosure: Nothing to disclose

EP670

Oncofertility program in Poland: a single center experience

R. Jach, A. Horbaczewska, W. Pabian, M. Swornik, A. Zmaczyński
Gyneology and Obstetrics, Jagiellonian University Medical College, Krakow, Poland

Introduction / Background: Different types of cancer dominate in different age groups. Oncofertility, a discipline that merges oncology and reproductive medicine, gives adolescent patients a chance to experience the joy of parenthood after the cancer treatment is finished.
Methodology: Purpose is to present the implementation and first outcomes of Oncofertility Programme in the University Clinic of Endocrynological Gynecology and Gynecology, University Hospital in Krakow, Poland.
Patients interested in fertility preservation have been consulted in the University Clinic of Endocrinological Gynecology and Gynecology in Krakow since April 2016. Preliminary qualification to one of the available methods (embryo cryopreservation, oocyte cryopreservation, ovarian tissue cryopreservation) was conducted.
Results: During 24 months, 18 patients were consulted. The youngest consulted patient was 20 years old, the oldest 39.
Time from diagnosis to oncofertility consultation in our Clinic ranged from 2 days to 55 days.
The time from oncofertility consultation to oocyte retrieval varied from 25 to 63 days, to ovarian tissue cryopreservation equaled 7 days.
The time from fertility preservation procedure to onset of the treatment varied from 3 to 6 days.
Two years after the first consultation, telephone survey among consulted patients was carried to verify whether the patients finally underwent oncofertility procedures and ask about their reproductive status.
Four patients ultimately underwent oncofertility procedures: three oocyte cryopreservation and one ovarian tissue cryopreservation and did not use preserved material so far.
Two patients already delivered healthy babies. Both conceived spontaneously during remission of the disease.
Conclusion: The problem of fertility issues being inadequately addressed results in low referral rates to oncofertility programmes.

Attempts to rise awareness of oncofertility possibilities among oncologists should be undertaken, as critically few patients are being referred to oncofertility centres.
Disclosure: Nothing to disclose

EP671

Genital warts in pregnancy-diagnosis and treatement the most common cause of laryngeal paillomatosis in children under 10 years old

I. Jeremic
Policlinic Jeremic, Belgrade, Serbia

Introduction / Background: HPV infection is an epidemic of modern age with the highest number of infected girls between 18 and 30 years of age .Due to the alerted immune status during pregnancy the spreading of HPV infection is progressive .During the labor any retention of the child in the birth canal leads to aspiration of HPV particles witch further represents the most common cause of laryngeal papillomatosis in children.
Methodology: The study involved 60 pregnant women between 18 and 30 years of age diagnosed with genital warts in early and advanced stages that were treated with RF technique which enables the smooth vaginal delivery with no signs of HPV infection on genito-anal region. Radio wave technique involves a special combination of radio wave access evaporisation and radio wave melting . Radio wave access evaporisation causes the evaporation of HPV infected cells and by radio wave melting we get the bloodless removal of condyloma.
Results: With Colposcopic examination we reveal subclinical stages of genital warts on the mucous membrane of the labia and the entrance to the vagina, which provides conditions for their immediate removal. The result of radio wave therapy is a bloodless surgical field with a precise and controlled removal of all forms of genital warts in one act throughout pregnancy. Operation is performed only under local anesthesia with a minimum damage to the surrounding healthy tissue , rapid recovery without accompanying infection, bleeding, recurrence, and a complete protection to the mother and fetus.
Conclusion: Genital warts during pregnancy represent a risk to the fetus during vaginal childbirth regardless of the severity of the clinical picture. Absence of colposcopic diagnosis, avoiding removing warts in the pregnancy, use of the wrong treatment leads to progress of condylomata as for outputting an infection of the fetus, by aspiration of HPV particles in the birth canal.
Disclosure: Nothing to disclose

EP672

The effect of uterine myoma on the course and outcome of pregnancy in women with preeclampsia

M. Kattahodjaeva[1], D. Gaybullaeva[2]
[1]Ophthalmology with a Course of Gynecology, Tashkent State Dental Institute, [2]Obstetric and Gynecology, Tashkent Medical Academy, Tashkent, Uzbekistan

Introduction / Background: The combination of uterine myoma and the terrible complication of pregnancy - preeclampsia (PE) - a common pathology. Probably, these two separate units, judging by the data of numerous authors, have similar facts in the origin of each pathology separately.
Methodology: Surveyed 102 patients. Of these, 72 pregnant women with pre-eclampsia on the background of uterine myoma 27% (20) and without it 72% (52). The comparison group consisted of 30 healthy pregnant patients. Used method of ultrasound scanning (ultrasound), the biophysical profile of the fetus (BPP), Doppler. In the postpartum period the Apgar score was assessed.

Results: The average age of the examined in the group of pregnant women with PE on the background of uterine myoma was 33,34±1,25 years, in the comparison group 26,43±1,76. Premature birth in pregnant women with pre-eclampsia in myoma occurred in 65% (13) and without myoma in 32,6%, detachment of the normally located placenta in 40% opposite 28,8%, operative delivery 45% opposite 34,9%, fetal growth restriction syndrome in 40% opposite to 23%. Doppler readings indicated a maximal change in vascular resistance in gestational terms of 28-31 weeks and 32-35 weeks. BPP in women with uterine myoma and preeclampsia showed changes in breathing and movement parameters - 72% (14) and 68% (35). The state of newborns on the Apgar scale in the first minute in the group with PE combined with uterine myoma and in the group with PE without uterine myoma was 7,031±0,234 and 6,467±0,387 and in the second minute 7,23±0,337 and 7,34±0,46.
Conclusion: The combination of preeclampsia with uterine myoma increases the frequency of complications such as premature labor, placental abruption, and the development of fetoplacental disturbances 2 times.
Disclosure: Nothing to disclose

EP673

Carcinoma sigma and pregnancy in 29 year old women; a case report

V. Krsic[1], J. Krsic[2], B. Jocic Pivac[1], J. Milojevic[3], M. Jovanović[2], L. Srbinović[1]
[1]Obgyn, GAK Nardoni Front, [2]Chirurgico, Millitary Academy of Belgrade, Belgrade, [3]Obgyn, Hospital Lazarevac, Lazarevac, Serbia

Introduction / Background: Carcinoma sigma in young women is a very rare illness, especially in pregnancy. Incidence of colorectal carcinoma in our country is high and it is on third place per incidence and third cause of death of all carcinomas in women.
Methodology: 29 -year old women was admitted in our hospital in 36 weeks gestation because of anemia and general weaknesses. In last month of pregnancy she noticed big mass under right rebated port.We did detail examination. Ultrasound examination showed liver with big multiple focuses of suspected meta changes. Parameter in blood were high like lactic dehidrogenasa , liver enzymes were just little higher than normal and all parameter of red cells were very small. Ultrasound of pregnancy showed one fetus normally developed for gestation of 36 weeks. We were not sure what is primary localization of tumor and what is cause of metasis changes on liver.
Results: We performed Caesarean section and delivered healthy baby boy 2900 grams, Apgar score 8/9.
Detail examination of abdominal cavity showed us big mass inside sigma colon and big multiple metastasis on liver. We performed a colostomy. She left hospital after 15 days and she receives chemotherapy till now.
Conclusion: Carcinomas which are very rare in young women can be diagnosed even in pregnancy.In our case we thought that pregnancy masked the clinical picture so we could not see big liver and it was impossible to diagnosed carcinoma sigma without any specific symptoms.Our country were bombarding with depleted uranium so we have many unusual cases with malignancy in young women ,unfortunately.
Disclosure: Nothing to disclose

EP674

Fertility-sparing surgery for pediatric/adolescent patients with sarcoma involving the uterine cervix

J. Li, X. Wu
Fudan University Shanghai Cancer Center, Shanghai, China

Introduction / Background: Botryoid rhabdomyosarcoma of the uterine cervix and cervical adenosarcoma are uncommon, but usually present with relatively better outcome. In the past, they were often treated with aggressive surgery and there were very few studies focused on fertility-sparing treatment. Here we reported our experience with cervical botryoid rhabdomyosarcoma/adenosarcoma, discussing proper selective criteria and type of fertility-sparing surgery for young patients with these two tumors.
Methodology: We conducted a retrospective review of a prospectively maintained database of patients undergoing fertility-sparing surgery for cervical botryoid rhabdomyosarcoma or cervical adenosarcoma at our institution from 08/2006 to 08/2018
Results: Eleven patients with cervical sarcoma (6 botryoid rhabdomyosarcoma and 5 adenosarcoma) were included in this study. Median age was 16.45 years (range, 11-25). One patient was offered cervical conization while others underwent abdominal radical trachelectomy. They all underwent adjuvant chemotherapy and presented with favorable outcomes at a median follow-up of 73.3 months (range, 30-151months) except for one patient. This young lady with botryoid rhabdomyosarcoma had tumor recurred to her left kidney 5 years after her primary treatment. Although she underwent salvage treatment, tumor metastasized to her lumbar vertebra very soon and she died 6 months after the diagnosis of recurrence. However, there was no evidence of tumor involving her remaining uterus and adnexes.
Conclusion: Although the very patient who died in our study had tumor metastasis to her kidney, we should understand that nearly 70% of treatment failure in patients with botryoid rhabdomyosarcoma and adenosarcoma was due to local recurrence. Our study showed that in properly selected cases of cervical sarcoma, abdominal radical trachelectomy appeared to secure local disease control and may be considered an alternative to hysterectomy.
Disclosure: Nothing to disclose

EP675

Pregnancy after fertility sparing treatment of embrional carcinoma figo III C

D. Ljuca[1], A. Cerovac[2]
[1]Department for Gynecology and Obstetrics, University of Tuzla, Medical Faculty, Tuzla, [2]Department for Gynecology and Obstetrics, Medical Faculty, University of Tuzla, Tešanj, Bosnia and Herzegovina

Introduction / Background: Malignant ovarian germ cell tumors (MOGCTs) account for 2-5% of all ovarian cancers and among them pure embryonal cell cancer is rare condition (1, 2, 3, 4).
Methodology: Aim: To show successful pregnancy after unilateral salpingooopherectomy and chemotherapy in a girl with embryonal carcinoma of ovary (ECO).
Results: Case report: A 11 year old girl presented in January 2008. with pain in abdomen and abdominal wall hardness. CT revealed a 12x10x11 cm heterodense mass. Right sided salpingoophorectomy, omentectomy, biopsy left sided ovary was done. Histopathology examination and immunohistochemistry revealed embryonal cell cancer of ovary. Patient had FIGO stage III c disease. After the surgical removal of the tumour, the patient underwent six cycles of adjuvant chemotherapy with bleomycin, etoposide and cisplatin. Within eight year after the completion of chemotherapy she conceived spontaneously. At 39th gestational week, laparotomy as well as a C-section was done and the patient was managed successfully in giving birth to a healthy female baby (birth weight and lenth 3,430 gm/53cm, Apgar score: 9/9). At the last follow-up visit (May 5, 2018), all the tumor markers were negative, and the control MRI and ultrasound examinations did not reveal tumor recurrence or pathological lymph nodes.
Conclusion: Fertility preservation surgery followed by chemotherapy may be considered as standard care of treatment in embryonal carcinoma of ovary in young girls, who want to retain fertility.
Disclosure: Nothing to disclose

EP676

Outcomes after conservative treatment of stade II and III serous borderline tumors of the ovary

S. Maria[1], A. Maulard[1], C. Genestie[2], P. Pautier[3], E. Bentivegna[1], A. Leary[4], C. Chargari[5], P. Morice[1], S. Gouy[1]
[1]Surgery, [2]Pathology, [3]Gustave Roussy, [4]Medical Oncology, [5]Brachytherapy, Gustave Roussy, Villejuif, France

Introduction / Background: The aim of this study was to assess the outcomes of the largest series of conservative treatment for advanced stages serous borderline ovarian tumor.
Methodology: Between 1973 and 2017, 65 patients were treated conservatively for an advanced-stage serous borderline ovarian tumor. Patient outcomes were reviewed.
Results: 28 patients had undergone a unilateral salpingo-oophorectomy, 12 a unilateral cystectomy and 25 unilateral salpingo-oophorectomy and controlateral cystectomy. Eight patients had invasive implants. The median duration of follow-up was 73 months (range, 12-369). The recurrence rate was high (58%). Eight recurrences were observed under the form of invasive disease: 2 (25%) and 6 (11%) in patients having initially respectively invasive implants and noninvasive implants. Three deaths had occurred, all of them in patients with noninvasive implants and micropapillary patterns (2 of them having a complete resection of peritoneal implants). Twenty-four pregnancies (13 spontaneous) were observed in 29 patients wishing to be pregnant. Seven patients had secondary infertility.
Conclusion: This study demonstrates that the conservative treatment of advanced stage borderline ovarian tumors (with noninvasive implant) can be achieved to preserve the possibility of pregnancy but the recurrence rate is high. Nevertheless, three deaths were observed, all of them concerning patients with noninvasive implants and micropapillary patterns. Initial presence of invasive implant doesn't seem to impact significantly the risk of recurrence under the form of invasive disease. This important data should be shared with such patient when a conservative approach is discussed. A careful follow-up should be done in this subgroup of patients.
Disclosure: Nothing to disclose

EP677

The Asiaglycoprotein receptor expression in placental of HbeAg positive mother could be used as a predictor of hepatitis B virus transmission from mother to child

R. Masadah[1], S. Rauf[2], A. Priyo Wibowo[1], M. Tajuddin Chalid[2], N. Massi[3], R. Sjahril[3], A.D. Bahagia Febriani[4]
[1]Pathology Anatomy, [2]Obstetric and Gynecology, [3]Microbiology, [4]Child Health Science, Hasanuddin University, Makassar, Indonesia

Introduction / Background: The transmision of Hepatitis B virus through placental happened when the virus could passed the placental barrier and infected placental cells before reached the fetus. Asialoglycoprotein recetpot is a transmembrane protein that plays an important role in endocytosis and degradation of lysosomal glycoprotein by binding the terminal end of galactose or N-acetylgalactosamine on the surface of Hepatitis B virus. Asialoglycoprotein receptor could facilitate the entry of Hepatitis B visur into placental cells and inflammation cells surrounding placental villous. Analizing the expression of Asialoglycoprotein receptor would predict the risk of transpacental transmission of the virus from mother to child.
Methodology: We collected 52 placentas from delivered mother who has serum HbsAg positive. The HbsAg positive serum were then examined for HbeAg positive. Immunohsitochemistry staining was performed on block paraffin sections using monoclonal antibody of Asialoglycoprotein receptor. The statistical analysis of Asialoglycoprotein receptor expression was calculated between HBsAg positive-HBeAg positive group and HBsAg positive-HBeAg negative group.
Results: The expression of Asialoglycoprotein receptor of 52 placenta samples found that 37 samples were score I, 5 samples were score II, 2 samples were score III, and 8 samples were score IV. We found that 14 of 52 serum samples were HbeAg positive and 38 negative. Mann Whitney U test by

comparing the expression score of Asialoglycoprotein receptor and HbeAg serum groups resulted of p=0.0001.
Conclusion: There was a significant correlation between Asialoglycoprotein receptor expression on placenta and mother's HbeAg serum positive. The expression of Asialoglycoprotein receptor could increased the risk of Hepatitis B virus transmission. The result of this study could be used as a guidelines for preventing and therapeutic approach of Hepatitis B virus from mother to child.
Disclosure: Nothing to disclose

EP678

Pregnancy and postpartum changes in uterine cervical biopsy abnormalities at our hospital

A. Miura, N. Yoshioka, S. Kuji, I. Deura, T. Ohara, J. Hasegawa, N. Suzuki
St. Marianna University School of Medicine, Kawasaki, Japan

Introduction / Background: In general, abnormal uterine cervical cytology is found in the first trimester, we evaluate the colposcopy, and perform a cervical biopsy if needed likewise in non-pregnancy. As a result, if the case is diagnosed to be cervical intraepithelial neoplasia (CIN), the risk of progression to invasive cancer during pregnancy is low and some cases of regression after delivery have been reported. In this study, we examined the transition of abnormal cervical biopsy from pregnancy to postpartum.
Methodology: We retrospectively examined the transition in histology during pregnancy and postpartum of 44 pregnant women who visited our oncology outpatient clinic from January 2014 to December 2018, including 27 cases of CIN1,6 cases of CIN2, 11 cases CIN3. Their mode of delivery includes 32 cases of vaginal delivery, 9 cases of cesarean section, 3 cases of unknown delivery style.
Results: The diagnosis of cervical biopsy abnormalities was 22 cases before pregnancy and early pregnancy respectively. At the transition change of cervical tissue diagnosis from early pregnancy to postpartum, 19 (73%) cases of CIN1 were improved and 7 cases (27%) were unchanged. There were no cases treated during pregnancy. 5 cases underwent cervical conjunctivectomy after delivery, and a radical hysterectomy was performed on 1 case who had progressed of the medical condition.
Conclusion: We found that the rate of regression of cervical tissue biopsy abnormalities in pregnant women decreased in the order of CIN1, CIN2 and CIN3, however, it is often relieved when giving birth. We assume that accurate diagnosis during early pregnancy is important because cervical cell collection and colposcopy get difficult as the gestational age goes further. In addition, cervical cytology was shown to be an important screen, because the diagnosis was often triggered by cervical cytology in early pregnan.
Disclosure: Nothing to disclose

EP679

Case report: locally advanced rectal cancer in pregnancy

B. Mohelnikova-Duchonova[1], H. Perkova[1], B. Melichar[1], R. Pilka[2], O. Urban[3], D. Klos[4]
[1]Department of Oncology, [2]Department of Obstetrics and Gynecology, [3]Department of Internal Medicine II - Gastroenterology and Geriatric, [4]Department of Surgery I, Palacký University Medical Faculty and Teaching Hospital, Olomouc, Czech Republic

Introduction / Background: Despite recent advances in the treatment of locally advanced rectal cancer and increasing evidence indicating safe administration of chemotherapy in patients in the second and third trimester of pregnancy, the optimal treatment management of locally advanced rectal cancer in pregnancy is largely unknown because of the rarity of the condition.
Methodology: Case report.

Results: We present a case of 29- year-old woman with locally advanced rectal cancer (T3N2M0) diagnosed in 15th week of pregnancy. The patient underwent total neoadjuvant treatment (TNT) consisting of eight cycles of FOLFOX chemotherapy during the second and third trimester of pregnancy and concomitant chemoradiotherapy after a Caesarean section. The Caesarean section was performed in 35th week of pregnancy. The child was in physiological condition, with no signs of growth restriction, malformation, or alterations in blood count, and with normal postpartum adaptation. The patient had a favourable response to systemic and local neoadjuvant treatment and finally underwent a radical surgery.
Conclusion: A concept of TNT of locally advanced rectal cancer starting with induction FOLFOX chemotherapy allows to postpone the delivery of the foetus to healthy childbirth with a good control of mother rectal cancer including downstaging.
Disclosure: Nothing to disclose

EP680

Impact of Pregnancy on the risk of breast and ovarian cancer in *BRCA1* and *BRCA2* mutation carriers

M. Russo[1], E. Corvetto[1], M. Fais[1], G. Candotti[1], V. Locati[1], A. Proto[1], S. Angioni[1], C. Carcassi[2], V. Mais[1], M. Peiretti[1]
[1]Department of Obstetrics and Gynecology, [2]Medical Genetics, University of Cagliari, Cagliari, Italy

Introduction / Background: BRCA 1 and 2 mutated women have a 40-85% lifetime risk of developing breast cancer (BC) and 16-64% risk of ovarian cancer (OC). Literature data evaluating the role of pregnancies on risk of BC and OC in BRCA mutated patients are controversial. The objective of the present study is to evaluate the impact of pregnancy on cancer risk in a cohort of BRCA1/2 mutated patients.
Methodology: This is a retrospective study, conducted on a sample of 106 female patients, 83 (78%) with BRCA 1 mutation and 23 (22%) BRCA 2, involved in a follow-up program of two different divisions of the University of Cagliari. We evaluated the number of BC and OC and the age of cancer diagnosis correlating to the number of pregnancies.
Results: Of 106 patients, 39 (36,8%) patients did´t have diagnosis of cancer and 67 (63,2%) developed cancer: 48 BC, 12 OC and 7 both. Thirthy-nine (36,8%) women didn´t have pregnancy, 21 (19,8%) had one pregnancy, 27 (25,4%) had two pregnancies and 19 (17,9%) more than two pregnancies.
The median age at the cancer diagnosis was 43,3 years (range 19-69 years).
We found a significative difference on the age of cancer diagnosis in patients with two or more pregnancies (p-value < 0,001). Patients with no or one pregnancy developed cancer at a median age of 38,3 years (range 19-59 ys), patients with two or more pregnancies at 49,5 ys (29-69 ys).
Conclusion: Our results seems to suggest a protective effect of pregnancy on the development of cancer in BRCA mutated women. Data show that two or more pregnancies significantly delay the development breast and ovarian cancer.
Disclosure: Nothing to disclose

EP681

Adult granulosa cell tumor associated with peritoneal endometriosis: Is it fair to preserve fertility?

L. Resende[1], M. Genovese[2], G. Cintra[3], A. Junior[2]
[1]Gynecology, UniCeub, [2]Hospital Brasilia, [3]Hospital Sirio Libanês, Brasilia, Brazil

Introduction / Background: The adult granulosa cell tumor (GCT) is derived from ovarian sex-cord stromal cells in the ovaries. It is a rare type of cancer, which accounts for 2-5% of all cases of ovarian carcinoma and have a better prognosis of ovarian epithelial types. The standard treatment is cytoreductive surgery, but in selected cases it is possible to perform conservative surgery with complete staging for patients with a desire to preserve fertility. However, we found a small number of cases described in the literature.
Endometriosis is a common condition present in reproductive-aged women. Some studies have shown endometriosis associated to some types of ovarian cancer, specially endometrioid and clear cells, however, none related to GCT.
Methodology: Case report..
Results: A 37-year-old nulliparous woman presented with abdominal pain and a left ovarian mass. Computed Tomography (CT) confirmed a 5 cm left ovarian mass and underwent a laparoscopic cystectomy. Adult GCT was confirmed stage IA disease. Staging laparotomy including pelvic washed, pelvic lymphadenectomy, infra-colic omentectomy and multiple stripping of the pelvic peritoneum. During procedure peritoneal endometriosis have been identified.
Conclusion: GCTaffects young patients and fertility preservation is thus an important issue. However, the role of fertility-sparing surgery remains unclear.
Recurrences occur 5 years after the first treatment. However, the finding of endometriosis, although peritoneal, should be evaluated with criteria for reducing the chance of spontaneous gestation. It has been advised against use of gonadotrophins to ovarian stimulation and egg collection because of the risk of GCT dissemination. The patient was informed that there is limited evidence in the safety of this approach. Regular follow-up, was proposed including physical examination, tumor markers and CT scan.
Disclosure: Nothing to disclose

EP682

Laparoscopic ovarian transposition in non-gynaecological cancers: more than just oncological outcomes

T. Shylasree[1], P. Singh[1], R. Engineer[2], A. Saklani[3], A. D'souza[3], N. Khanna[2], S. Lasker[2]
[1]Gynaecological Oncology, [2]Radiation Oncology, [3]Colorectal Oncology, Tata Memorial Centre, Mumbai, India

Introduction / Background: Pelvic radiation leads to permanent ovarian failure. Laparoscopic ovarian transposition (LOT) is an established surgical technique to salvage ovaries. The success rate of LOT varies widely and is quoted between 40 to 80% in gynecological cancers In non-Gynecological cancers the available data is limited. We present the data on LOT performed for non-gynecological cancers from a tertiary cancer hospital. **Aims:** Analyze the effectiveness of LOT in non-gynaecological cancers.
Methodology: Forty seven patients who underwent LOT during the years 2013 to 2018 (40 with rectal cancer and 7 with non-rectal cancer mainly pelvic childhood sarcomas and lymphoma) were retrieved from a prospectively maintained data base. Disease characteristics, oncological outcomes and return of menstrual function were analyzed.
Results: Median age of patient was 25 years (range 8 - 35 years). 53% patients were unmarried. Median postoperative stay was 48 hours and postoperative course uneventful in all patients. Of the 40 rectal cancers, 22 had adenocarcinoma and 18 signet ring pathology . 19% patient underwent stoma formation along with ovarian transposition. There was no delay in definitive treatment due to LOT procedure. At the time of analysis a total of 20 patient were excluded. (13 patients had died, 3 defaulted, 2 premenstrual and 2 had progressive disease on CTRT requiring removal of ovaries/uterus). Twenty eight patients were eligible for evaluation, out of which 60.7% were disease free and 53.5% were menstruating.
Conclusion: Laparoscopic ovarian transposition is safe and does not delay primary treatment. Of the the evaluable patients more than half resumed regular menstrual function. Fertility chances and issues will become apparent with long term follow up of the cohort as the uterus also gets irradiated during pelvic radiation.
Disclosure: Nothing to disclose

EP683

Management of pregnancy after fertility-sparing surgery for cervical cancer

J. Slama, P. Simjak, D. Cibula, A. Parizek
Department of Gynecology and Obstetrics, 1st Faculty of Medicine, Charles University and General University Hospital, Prague, Czech Republic

Introduction / Background: Cervical cancer is increasingly diagnosed in women who have not yet completed their reproductive plans. For these patients with stages IA2 and IB1 cervical cancer, trachelectomy is the treatment of choice. However, patients who undergo repeated conization or trachelectomy represent a challenge for obstetricians as they are at increased risk of infertility, mid-trimester abortion, preterm premature rupture of membranes and preterm delivery.
Methodology: So far, the evidence-based guidance on the management of these pregnancies is limited because good-quality studies conducted on this specific population are lacking. Most recommendations originated from underpowered observational studies or are extrapolated from studies conducted in other specific groups of patients, such as patients with cervical incompetence.
Results: Screening and treatment of asymptomatic bacteriuria and screening for cervical incompetence and progressive cervical shortening by transvaginal ultrasonography and proper usage of fetal fibronectin test is desirable in patients after FSS for early stages of cervical cancer. Vaginal progesterone supplementation should be administered to all women after vaginal trachelectomy. It should also be administered to women with asymptomatic short cervix after cervical conization. Women with a prior history of preterm delivery or late miscarriage may benefit from cervical cerclage if they have a short cervix after conization or underwent trachelectomy. The delivery is to be carried out by cesarean section in patients after trachelectomy, even in cases of preterm delivery.
Conclusion: The management of pregnancy in women after FSS is not uniform, which is not surprising given the lack of evidence-based data. Most recommendations so far are derived from personal experience, underpowered observational studies or are extrapolated from studies conducted in other specific groups of patients, such as patients with cervical incompetence.
As pregnancies after FSS become more common, providing guidance for clinicians is worthwhile.
Disclosure: Nothing to disclose

EP684

Ovarian tissue cryopreservation during surgical treatment of patients with gynecological malignancies in Slovenian population

Š. Smrkolj, I. Virant-Klun
Division for Gynecology and Obstetrics, University Medical Centre Ljubljana, Ljubljana, Slovenia

Introduction / Background: As a common side effect of cytotoxic cancer treatments, the probability of young female cancer survivors to conceive after chemotherapy and radiotherapy is reduced. One of the promising and effective methods for fertility preservation of women undergoing these treatments is the autotransplantation of frozen-thawed ovarian tissue. It is still an experimental method, but it resulted in at least 42 healthy babies worldwide without any increased risk for miscarriage or congenital anomalies.
Methodology: We performed a retrospective, cohort study in 24 patients with different advanced gynecological malignancies (predominating ovarian and cervical cancer) treated at the Department of Obstetrics and Gynecology, University Medical Centre Ljubljana between 2001 and 2015. During the same surgery, their ovarian tissue was removed and cryopreserved. We assessed the post-operative outcome in these patients including their natural fertility.

Results: In this study 12 patients with ovarian, 5 with cervical and 3 with uterine cancer, and the rest (secondary cancer) were included. The mean female age at the time of surgery was 29,3 years. In spite of advanced cancer, 18 (75 %) patients survived the cancer, are still alive. At the time of surgery only one patient had a baby after natural conception, while other patients were nullipara; most of them still don´t have a baby. The mean time of ovarian tissue cryostorage is 9,5 years (from 3,4 to 17,9 years). In patients with ovarian cancer the cryopreserved ovarian tissue is the only source of follicles/oocytes; among patients with cervical and uterine cancer, in 9 (50 %) patients the hysterectomy was done, while in the rest the uterus is intact.
Conclusion: Cryopreservation of ovarian tissue retrieved during the cancer surgery is also reasonable in patients with advanced gynecological malignancies but needs to involve the efficient follow-up of these patients and an active communication with gynecologists.
Disclosure: Nothing to disclose

EP685

Management of Acute Lymphoblastic Leukemia in a 26-year-old Primigravida

M. Theodora[1], C. Theofanakis[1], Z. Fasoulakis[1], D. Barbarousi[2], G. Daskalakis[1], A. Rodolakis[1]
[1]1st Department of Obstetrics & Gynecology, National and Kapodistrian University of Athens, [2]Hematolgy Hepartment, Alexandra Hospital, National and Kapodistrian University of Athens, Athens, Greece

Introduction / Background: Acute lymphoblastic leukemia (ALL) during pregnancy represents a challenging disease for both physicians and pregnant women. We present a rare case of a 26-year-old primigravida diagnosed with ALL at 23 weeks of gestation, treated with a modified German multicenter ALL (GMALL) protocol, who gave birth to a healthy baby-girl at 31 weeks of gestation.
Methodology: A 26-year-old primigravida presented at 23 weeks of gestation with persistent leukocytosis with 67,4% B-lymphoblasts. Bone marrow biopsy conducted in 23+2 weeks of gestation revealed precursor B-cell Acute Lymphoblastic Leukemia (ALL).
Continuation of pregnancy was decided after counselling and a personalized treatment protocol was initiated. The patient received 4mg of dexamethasone per day, combined with 300mg of allopurinol. Fetal development was within normal range but during this stage, the patient presented neutropenia and anemia. Treatment continued with 4 doses of 50mg doxorubicin, combined with filgrastim, 2mg of vincristine and 20mg of dexamethasone every 7 days.
Results: At 30 weeks of gestation fetal echocardiography revealed a small pericardial effusion, increased flow velocity, small right ventricular dilatation and a patent ductus arteriosus aneurysm, compatible with cardiac insufficiency either due to chemotherapy-induced cardiotoxicity or due to utero-placental insufficiency. Chemotherapy was discontinuated and a ceasarean section was performed at 31+4 weeks-of-gestation giving birth to a baby girl of 1670gr, with an Apgar score ≥7 at 1 min and ≥8 at 5 min.
Conclusion: ALL requires immediate treatment, regardless of gestational age. Pregnancy termination and risks from chemotherapy should be discussed thoroughly with the patient. More studies are required to determine the prognosis of ALL during pregnancy and the long-term impact of the disease and the CBC on the fetus.
Disclosure: Nothing to disclose

EP686

Breast cancer and pregnancy -our experience and literature data review

C.N. Trifan[1], S. Pasat[1], D. Hutanu[1], V.G. Tirnovanu[2], A. Iov[2], M.C. Tirnovanu[3]
[1]1st Clinic of Obstetrics and Gynecology, University Women Hospital Cuza Voda Iasi, [2]Student,

[3]*Obstetrics and Gynecology, University of Medicine and Pharmacy 'Gr T Popa' Iasi, Iasi, Romania*

Introduction / Background: The pregnancy-associated breast cancer (BC) refers to a breast malignancy diagnosed during pregnancy or in the first postpartum year. This diagnosis is expected to become more frequent since there is an increasing trend for women to delay childbering. Understanding the effect of pregnancy on survival in women with history of BC will help in the counseling of these patients.
Aim: To analize characteristics of pregnancy-associated BC in our clinic. We also evaluated the outcome of pregnancy in women with history of BC.
Methodology: The study evaluated cases from January 2006 to March 2019. We analysed data from the patients record and histological exam results.
Results: We had 6 patients pregnancy-associated cancer: 1 in the first trimester, 3 in the third trimester, and 2 during first year after delivery. The age was between 28 and 38 years. Only one women had familial genetic predisposition - father with BC. The ending of pregnancy was by abortion for the pregnancy at 10 weeks of gestation and delivery at 29, 38 and 37 weeks (all with triple-negative tumors). During the pregnancy the breast tumor had a faster development. Bilateral ovarian micrometastasis were found at patient with 29 weeks of gestation. Radical mastectomy was done as first therapeutic step, excepting one women. We had 3 cases with pregnancy 2 years after BC diagnosis and treatment: one had lung metastasis at 35 weeks of gestation, another one 1 year after delivery (both died), one has 20 weeks pregnancy in evolution (after FIV).
Conclusion: Breast cancer is a rare tumor associated with pregnancy. It's occurring during pregnancy presents a challenging clinical situation since the welfare of both the mother and the fetus must be taken into account in any treatment planning. In our opinion pregnancy after 2 years from BC treatment it seems to reduce survival.
Disclosure: Nothing to disclose

EP687

Cancer during pregnancy: our experience of 16 cases

D. Tsolakidis[1], M. Lioupis[1], M. Lantzanaki[1], E. Bili[1], T. Mikos[1], D. Zouzoulas[1], I. Hatziioannidis[2], G. Mitsiakos[2], G. Grimbizis[1]
[1]*1st Department of Obstetrics & Gynecology 'Papageorgiou' Hospital,* [2]*2nd Department of Neonatology & NICU 'Papageorgiou' Hospital, Aristotele University of Thessaloniki, Thessaloniki, Greece*

Introduction / Background: Diagnosed cancer during pregnancy is a very rare situation related to severe maternal and neonatal morbidity and mortality. Diagnosis, proper management and monitoring of these cases requires specialized knowledge, experience and cooperation of many specialties. The aim of this study is to determine the maternal and neonatal morbidity and mortality in these cases and the impacts caused by a delayed diagnosis or treatment of a malignancy during pregnancy.
Methodology: We retrospectively reviewed the medical records of patients, who were diagnosed with malignancy during pregnancy and gave birth to viable fetuses (>25th week of gestation) from May 2004 to February 2019, in our clinic. Cases with in situ carcinoma were excluded.
Results: Out of 25.672 pregnant women 16 (0.06%) were diagnosed with gynecological malignancy during pregnancy. The average age of patients was 35 years old. 14.2% of the cases were diagnosed in 1st trimester, 64.3% in 2nd and 21.5 % in 3rd trimester pregnancy. Maternal mortality rate raised to 35.7%. The disease was initially diagnosed in 24.1 % of the patients and the recurrence rate of known malignancies in pregnancy was 14.2%. 21,4% of these women were diagnosed with advanced stage cancer. In 57% of the cases there was a delayed diagnosis, mainly caused by underestimation of the symptoms by the doctor. 78.6% were led to premature birth by caesarean section in order to complete the treatment of the malignancy. The mean age of gestation was 31w+3d and the average birth weight of newborns was 1600kg.
Conclusion: In 57% of the cases of cancer during pregnancy there was a delayed diagnosis due to underestimation of the symptoms by the doctor. Only 21.4% of the women were treated for the malignancy continuing their pregnancy in order to prevent premature birth and bad neonatal outcome.
Disclosure: Nothing to disclose

EP688

Perinatal outcomes of Hobnail-like shallow electrosurgical excision prosedure (HEEP) for the treatment of HSIL: prevention of IIOC

J.H. Yooon[1], J.W. Lee[1], B. Lee[2], S.I. Kim[1]
[1]*The Catholic University of Korea/ St. Vincent's Hospital, Suwon,* [2]*Design Team, Medinstru, Young-In, Republic of Korea*

Introduction / Background: We knew that conventional conization can make the incompetent internal os of cervix, which can induce the abortion and preterm delivery. So, we try to minimize the removed volume after conization.
Methods: 34 young patients with HSIL (final diagnosis : CIS or cervical cancer FIGO stage IA1) were enrolled in the Department of Obstetrics and Gynecology of St. Vincent´s Hospital from Jan. 2014 to Aug. 2018. We treated those patients with Hobnail-like shallow Electrosurgical Excision Prosedure(HEEP) of uterine cervix for the treatment HSIL.
Methodology: 34 young patients with HSIL (final diagnosis : CIS or cervical cancer FIGO stage IA1) were enrolled in the Department of Obstetrics and Gynecology of St. Vincent´s Hospital from Jan. 2014 to Aug. 2018. We treated those patients with Hobnail-like shallow Electrosurgical Excision Prosedure(HEEP) of uterine cervix for the treatment HSIL.
Results: 4 women experienced 5 pregnancies. 4 of 5 pregnancies were kept more than 34 gestation weeks. Furthermore, all of 5 pregnancies gave healthy live births.
Conclusion: HEEP could be considered as a treatment of choice for HSIL in young women.
Disclosure: Nothing to disclose

EP689

Cytoreduction and Hyperthermic intraperitoneal chemotherapy followed by Hyperthermic intraperitoneal chemotherapy (CRS+HIPEC) in advanced peritoneal carcinomatous (APC) at the American University of Beirut Medical Center (AUBMC) experience 2007-2018: a retrospective review

R. Abdallah[1], M. Seoud[1], A. El Housheimi[1], A. Shamseddine[2], M. Khalifeh[3], I. Jaafar[1], F. Jamali[1]
[1]*Obstetrics and Gynecology,* [2]*Hematology and Oncology,* [3]*General Surgery, American Universtiy of Beirut Medical Center, Beirut, Lebanon*

Introduction / Background: Peritoneal carcinomatosis (PC) has been traditionally considered a terminal disease with median survivals reported in literature of 6-12 months. Cytoreductive surgery (CRS) & hyperthermic intra-peritoneal chemo-therapy (HIPEC) have gradually gained acceptance as standard of care to manage selected PC cases.
Our objective is to review the outcome of patients with APC who underwent CRS+HIPEC by the HIPEC team at the AUBMC
Methodology: All patients with APC were evaluated by the HIPEC team for eligibility and the majority had either gastrointestinal or gynecological malignancies. We retrospective reviewed data related to their demographics/tumor origin/surgical completeness/adverse events/outcome
Results: Since 2007, 53 patients (45% were females) were had CRS and HIPEC. 20 (37.6%) had Pseudomyxoma peritonei (PMP), 12 (23%) ovarian cancer, 14 (26.4%) colorectal cancer, 4 (7.5%) had gastric cancer, 2 (3.7%) had Mesothelioma and one (1.8%) Desmoplastic round cell tumor. Operative and Postoperative demographics and survivals are shown in tables 1 and 2. Median progression free (PFS) and overall survival (OS) are expressed in months
Conclusion: We report the successful establishment of an active peritoneal surface malignancy

multidisciplinary treatment program with results comparable to other centers. Careful patient selection, a multidisciplinary approach and proper surgical training and technique are essential for the success of such a program

[Table 1]

[Table 2]

Disclosure: Nothing to disclose

EP690

Pelvic exenteration for advanced gynaecological malignancy in South-West Wales

G.O. Abdulrahman Jnr, N. Das, K. Lutchman Singh
Swansea Gynaecological Oncology Centre, Swansea Bay University Health Board, Swansea, United Kingdom

Introduction / Background: Pelvic exenteration is a major surgical procedure for advanced or recurrent gynaecological cancer. It is a potentially curative treatment but it could also be performed with palliative intent.
Methodology: All patients undergoing pelvic exenteration between December 2014 and April 2019 at

the Swansea Gynaecological Oncology Centre were identified through our surgical database. Patient characteristics, the indications for the procedure, pathology results and outcomes were retrospectively analysed. We used frequencies and percentages to describe the sample. Mean values were calculated where appropriate. Statistical comparisons were conducted with chi-squared test and Analysis of Variance where appropriate. A p-value of < 0.05 was considered statistically significant. Statistical analyses were performed with IBM SPSS 25.0 (SPSS Inc, Chicago, IL, USA).
Results: 19 patients were identified who had pelvic exenteration for vulvar, vaginal and cervical cancer during the study period. The mean age of the women was 57.5 years (42 - 82 years). The vulva was the commonest primary site of malignancy. 11 women underwent posterior pelvic exenteration, 6 women underwent total pelvic exenteration and 2 women had anterior pelvic exenteration. 11 women had a form of radiotherapy or chemotherapy or both. 58% of the women were still alive at 1 year and 43% was still living at 2 years. Survival was not associated with margin status (p=0.074), lymphovascular space invasion (p=0.149) or pathological lymph node status (p=0.707). However, there was a strong association between adverse survival and perineural invasion (p=0.009).
Conclusion: Although pelvic exenteration is a major procedure with high morbidity, it is a treatment option with favourable outcomes in some women with advanced gynaecological malignancies even when post-operative pathology reports might appear unfavourable.
Disclosure: Nothing to disclose

EP691

Does apronectomy improve outcomes for the morbidly obese patient undergoing gynae cancer surgery? A review of four cases

S. Addley[1,2], S. Sinclair[3], S. Dobbs[2], M. McComiskey[2], D. Glenn[1]
[1]Gynaecology, Ulster Hospital Dundonald, [2]Gynae Oncology, Belfast City Hospital, [3]Plastic Surgery, Ulster Hospital Dundonald, Belfast, United Kingdom

Introduction / Background: For the morbidly obese patient undergoing laparotomy and pelvic clearance in the context of an oestrogen-driven gynae cancer, concomitant apronectomy confers multiple benefits - optimising intra-operative pelvic access; improving post-operative recovery and general health; as well as also having the potential to reduce future peripheral oestrogen production, with positive implications for cancer recurrence.
Methodology: The aim was to describe the short-term outcomes of performing apronectomy at the time of laparotomy for gynae malignancy in the morbidly obese patient. A retrospective case-note review of morbidly obese patients undergoing apronectomy as a component of gynae cancer surgery between 2017 and 2019 in a district general hospital in Northern Ireland was performed.
Results: Four patients were identified, with mean age 56 years and mean BMI 53kg/m2. Three patients were undergoing laparotomy for endometrial adenocarcinoma, and the fourth for suspected ovarian malignancy.
At each surgery, the operating team consisted of a minimum of three Consultants: one gynae-oncologist, one gynaecologist and one plastic surgeon. No anaesthetic complications occurred. Each patient received intra-operative antibiotic prophylaxis and underwent TAH, BSO, omentectomy/omental biopsy and apronectomy with re-siting of the umbilicus. The average weight of apron excised was 9.6kg. Each patient had two negative-pressure wound drains placed. Skin was closed with monofilament sutures and negative-pressure dressings applied. No intra-operative complications occurred, the maximum blood loss was 300mls and mean operating time 217 minutes. One patient required HDU for 24hours. Immediate post-operative complications included anaemia (50%); wound infection/partial dehiscence (50%); one case of return to theatre for debridement of a non-viable umbilicus; paralytic ileus (25%); and urinary tract infection (25%). The mean length of stay was 16 days. In the 30 day post-operative period, one patient was readmitted with wound infection.
Conclusion: Overall, apronectomy appears to be a safe addition to laparotomy in this challenging patient population.
Disclosure: Nothing to disclose

EP692

FUlvestrant in gynaecological cancers that are potentially hormone sensitive: the FUCHSia study

S. Tuyaerts[1], D. Annibali[1], P. Van Dam[2], L. Lapeire[3], K. Leunen[4], H. Trum[5], J.J. Kasius[6], A.W. Glaudemans[7], C. Deroose[8], F. Amant[1,5,8]
[1]Gynaecologic Oncology, KU Leuven - University of Leuven, Leuven, [2]University Hospital Antwerp, Antwerp, [3]University Hospital Ghent, Ghent, [4]AZ Sint-Maarten, Mechelen, Belgium, [5]Netherlands Cancer Institute/Antoni van Leeuwenhoek Hospital, [6]Academic Medical Center Amsterdam, Amsterdam, [7]University Medical Center Groningen, University of Groningen, Groningen, The Netherlands, [8]University Hospitals Leuven, Leuven, Belgium

Introduction / Background: Many gynaecological malignancies including low-grade uterine sarcomas (LGUS), low-grade endometrial carcinomas (LGEC), sex cord stromal tumours (SCST) and low-grade epithelial serous ovarian cancers (LGSC) express oestrogen receptor (ER). Reports suggest that endocrine therapy can be effective, but due to their rarity, no consensus criteria define which patients would benefit from anti-ER treatment.
Methodology: FUCHSia is an open-label, single-arm, prospective, multi-centre, tandem two-stage, phase II study evaluating Fulvestrant (Faslodex) efficacy in women with recurrent/metastatic ER-positive gynaecological malignancies. Tumour types studied are LGUS (all LG endometrial stromal sarcoma, adenosarcoma without sarcomatous overgrowth and leiomyosarcoma), LGEC, SCST and LGSC.
Fulvestrant is administered (intramuscular injection, 2x 250 mg) once every 2 weeks for the first month, and monthly until progression.
In stage 1, 20 patients will be enrolled per tumour type. When 15 patients per type reach week-24, an interim analysis will be performed. If 1 or more responses are observed per type, 30 additional patients will be recruited in stage 2.
Primary endpoint is response rate, determined by RECIST v1.1 criteria.
Secondary endpoints are progression-free survival, overall survival, time to progression, clinical benefit, safety and tolerability, and quality of life.
Exploratory endpoints are feasibility of sequential ^{18}F-FES PET scans for assessment of ER expression and functional status during treatment, value of ^{18}F-FES PET scans combined with ^{18}F-FDG PET scans in predicting response and patient stratification, and identification of genetic changes in cf-DNA and tumour biopsies potentially predicting response to Fulvestrant.
Results: UZ Leuven is the sponsor and recruitment is ongoing in UZ Ghent, UZ Antwerp and AZ Sint-Maarten. The study will open soon in NKI/AvL Amsterdam. Additional centres in Belgium and The Netherlands will be added later.
Conclusion: In this study, we will assess Fulvestrant activity in low-grade gynaecological cancers, while exploratory endpoints will investigate predictors of response and resistance mechanisms.
Disclosure: The authors declare that they have no competing interests. The study obtained funding from “Kom op tegen Kanker (Stand up to Cancer), the Flemish cancer society” and “FWO - Research Fund Flanders”.

EP693

Disseminated peritoneal leiomiomatosis: report of a case and review of literature

N. Ardila, L.O. Puentes Puentes, M. Pacheco
Bogotá, Fundación Universitaria De Ciencias De La Salud, Bogotá, Colombia

Introduction / Background: Disseminated peritoneal leiomyomatosis (DPL) is a rare disease characterized by the presence of multiple nodules of smooth muscle cells dispersed in the abdominal cavity. To date, only about 150 cases have been reported in the literature. We present a case of

recurrent DPL after laparotomy in a 45-year-old patient treated at San Jose Hospital, in the city of Bogotá, Colombia.

Methodology: We describe a clinical case of a 45-year-old woman with abnormal uterine bleeding, who was taken to total abdominal hysterectomy in November 2010, with a report of pathology with intramural and cervical myomatosis. Subsequently, in 2013, she was taken again to surgical procedure, performing ovarian cystectomy + partial omentectomy + appendectomy, with pathology report leiomyoma, benign stromal tumor and omentum with disseminated peritoneal leiomyomatosis and negative appendix for neoplasia. Due to the persistence of abdominal pain associated with mass, imaging is performed, including a total abdominal ultrasound, Tomography and Nuclear Magnetic Resonance, which reported multiple pelvic masses, compatible with disseminated peritoneal leiomyomatosis, which led to ultraradical surgery in our institution. July 2018, with complete cytoreduction of the lesions and adequate postoperative evolution, who to date has remained without recurrence of the disease.

Results: The differential diagnosis of DPL is a challenge due to its similarity with peritoneal carcinomatosis and other benign abdominal disorders. Malignant transformation is rare, but it can happen, so a close follow-up is necessary. In our literature review we found reports from 1976 to 2017, approximately 150 cases had been reported in the literature.

Conclusion: DPL is a rare but potentially serious disease. In our opinion, larger studies are needed to improve our diagnostic effectiveness and define the best therapeutic strategy. The different studies show that surgical treatment is the first option in women without reproductive desire or postmenopausal women.

Disclosure: Nothing to disclose

EP694

The role of pulmonary metastectomy in gynaecology malignancy

F. Armstrong[1], O. O Brien[2], T. Walsh[2], R. Mc Vey[2], W. Boyd[2], D. Eaton[3], D. Healy[4], K. Redmond[3], D. Brennan[1,5]

[1]Department of Gynaecological Oncology, UCD School of Medicine, Mater Miseraecordiae University Hospital, Dublin, Ireland, Dublin, [2]Department of Gynaecological Oncology, UCD School of Medicine, Mater Miseraecordiae University Hospital, Dublin, Ireland, dublin, [3]Department of Thoracic Surgery, Mater Misericordiae University Hospital, Dublin, [4]Department of Thoracic Surgery, Mater Misericordiae University Hospital, dublin, [5]Systems Biology Ireland, UCD School of Medicine, Belfield, Dublin, Ireland

Introduction / Background: Long term outcomes after pulmonary metastectomy for recurrent gynaecology malignancy are not well documented. We sought to examine the indications and outcomes of patients who had a pulmonary metastectomy for gynaecology malignancy in a tertiary referral centre in Dublin Ireland.

Methodology: A retrospective analysis of medical records for the period 01/01/2014 to 01/01/2019 was performed. Search criteria included history of primary gynaecological malignancy, metastectomy , Video assisted thoracic surgery (VATS), thoracotomy and wedge resection.

Results: A total of 10 patients underwent pulmonary metastectomy for isolated recurrent disease . Median age was 65yrs (range 45-85yrs). Primary diagnosis were ovarian cancer (n=5), uterine sarcoma (n=4) and mucinous adenocarcinoma of the cervix (n=1). The most common procedure was VATS assisted wedge resection (n=8). 37.5% (3/8) also requiring pleurodesis. The other procedures documented are VATS assisted excision of epicaridial lymph node (n=1) and a small anterior thoracotomy (n=1). The time to pulmonary recurrence was 3.6 years (range 1yr to 17yrs). Histology confirmed recurrence in all cases. Two patients were lost to follow up. Of the remaining 8 patients the mean time to disease progression was 8.8 months (range 4-24months) following metastectomy. There were no grade III morbidities in this cohort.

With regards to ovarian carcinoma 40% (2/5) have stable disease following disease progression with 1 patient currently receiving chemotherapy and 1 patient on a PARP inhibitor . Two ovarian cancer patient s died 5 months and 2 years following metastectomy and 1 patient was lost to follow up. Three of the uterine sarcoma patients remain under surveillance, 2 with disease progression.

Conclusion: Pulmonary metastectomy for recurrent gynaecological cancer is an acceptable and safe

form of treatment in carefully selected patients. Patients with isolated pulmonary recurrences should be considered for surgical intervention if their performance status is suitable.
Disclosure: Nothing to disclose

EP695

Uterine arteriovenous malformation after molar pregnancy: a case report and review of literature

N. Babapour
Associate Professor Arya Hospital, Islamic Azad University of Medical Sciences, Mashhad, Islamic Republic of Iran

Introduction / Background: Uterine arteriovenous malformation (AVM) following molar pregnancy is a rare condition. The present study aimed at reporting a case of uterine arteriovenous malformation after molar pregnancy.
Methodology: Case Presentation: A 38-year-old woman with repeated heavy vaginal bleeding was referred to oncology department of Ghaem hospital affiliated to Mashhad University of Medical Sciences in 2016. She had history of evacuation curettage of molar pregnancy in the previous month.
Results: Examinations revealed AVM in fundus of uterus; and the patients was asymptomatic after embolization of the AVM and she is now under serial follow-up.
Conclusion: The diagnosis of AVM should be considered when an unexplained massive hemorrhage occurs after gestational trophoblastic disease. In the case of correct diagnosis, unnecessary surgery is avoided.
Disclosure: Nothing to disclose

EP696

Inoperable pelvic sidewall recurrence of gynecological cancer treated with proton and carbon ion radiotherapy: CNAO preliminary experience

A. Barcellini[1], V. Vitolo[1], R. Lazzari[2], L. Cobianchi[3,4], R. Biffi[5], A. Facoetti[6], M.R. Fiore[1], B. Vischioni[1], A. Iannalfi[1], A. Mirandola[7], A. Vai[7], E. Mastella[7], C. Laliscia[8], A. Gadducci[9], F. Raspagliesi[10], L. Preda[4,11], R. Orecchia[1,12], F. Valvo[1]
[1]Radiation Oncology, National Center of Oncological Hadrontherapy (CNAO), Pavia, [2]Radiation Oncology, European Institute of Oncology, Milan, [3]General Surgery Department, Fondazione IRCCS Policlinico San Matteo, [4]Department of Clinical-Surgical, Diagnostic and Pediatric Sciences, University of Pavia, Pavia, [5]Division of Digestive Tract Surgery, European Institute of Oncology, Milan, [6]Radiobiology, [7]Medical Physics, National Center of Oncological Hadrontherapy (CNAO), Pavia, [8]Department of Translational Medicine, Division of Radiation Oncology, [9]Department of Clinical and Experimental Medicine, Division of Gynecology and Obstetrics, University of Pisa, Pisa, [10]Gynecologic Oncology, Fondazione IRCCS Istituto Nazionale Tumori, Milan, [11]Radiology, National Center of Oncological Hadrontherapy (CNAO), Pavia, [12]European Institute of Oncology, Milan, Italy

Introduction / Background: Re-irradiation of local recurrences of gynaecological cancer pose a difficult challenge to Oncologist. For their biological and physical characteristics particle therapy (PT) could be an interesting treatment.
Methodology: The aim of the study was to evaluate the feasibility and early clinical outcome in patients (pts) with gynaecological pelvic sidewall recurrence (PSWr). Between May 2014 to December 2018, 10 patients (median age 56) with PSWr within or at the edge of the previously irradiated field were treated using PT. They had recurrence of: cervical(5), endometrial(3), uterine(1) and ovarian(1) cancer. Previous radiotherapy prescription dose ranged from 46 to 59.4 Gy and 5 patients underwent

brachytherapy(range: 7-28 Gy).Two patients, with marginal lymph node recurrence, were irradiated with protons with up to a total dose of 25 GyRBE and 51 GyRBE,respectively. The remaining women underwent carbon-ion radiotherapy (median total dose 50.4 GyRBE;range: 36-57) administered in a median number of 12 fractions. Six patients with PSWr received surgical spacer placement by open surgery to keep intestinal tracts apart from the tumour as the distance between tumour and nearest intestinal tracts was not sufficient. No pts received concurrent chemotherapy. Preliminary local control (LC) and toxicity profile(according to CTCAE V4.03 scale) were evaluated
Results: All patients completed the planned treatment and no acute toxicities G>2 were observed. For the evaluable patients, 1 case of intermediate G≥3 toxicity was reported in women received sequential Bevacizumab (BV). For pts with a follow-up≥ 3 months, median LC was 7 months(range: 3-14), median MFS was 4.5 months(range: 3-14,5) and median OS was 7 months(range: 3-14,5). 1 pt experienced local progression and 4 pts died for systemic progression. Data are still ongoing.
Conclusion: For pts with PSWr a PT approach seems to be feasible and our results showed a promising short-term outcome and limited radiation-related side effects. Longer follow-up and large patient accrual are required.
Disclosure: Nothing to disclose

EP697

Establishing the audit and research in gynaecological oncology (ARGO) collaborative in the United Kingdom and Ireland

Y.L. Wan[1,2], R. Bharathan[3], J. Frost[4], M.L. MacKintosh[2], ARGO Collaborative
[1]Gynaecological Oncology Group, Division of Cancer Sciences, University of Manchester, [2]Gynaecological Oncology, St Mary's Hospital, Manchester University Hospitals NHS Foundation Trust, Manchester, [3]Gynaecological Oncology, University Hospitals of Leicester, Leicester, [4]Gynaecological Oncology, Cheltenham General Hospital, Gloucestershire Hospitals NHS Foundation Trust, Cheltenham, United Kingdom

Introduction / Background: Aims: To establish a collaborative to deliver and provide training in research and clinical audit in gynaecological oncology
Background: Trainee-led research collaboratives in general surgery have demonstrated the ability of such groups to rapidly recruit large numbers of patients and deliver high quality multi-centre research. Trainees are ideally placed to deliver research at point of care given the rotational nature of their placements, their established communication networks and the motivation to provide evidence for participation research and audit during their training. In Gynaecological Oncology, where care is centralised to tertiary level units, there is the opportunity to collect nationally impactful data.
Methodology: Members were recruited through the British Gynaecological Cancer Society, National Cancer Research Institute, Royal College of Surgeons and Royal College of Obstetricians and Gynaecologists. A core committee was established to deliver group aims. Webinars and educational events were used to engage with members nationwide. A competitive call for multicentre projects allowed for the selection of our national project.
Results: Between August and November 2018, 52 members, comprising trainees from all fourteen deaneries in the United Kingdom, Northern Ireland and the Republic of Ireland, were recruited. A core committee of six trainees was elected. Two online events and one face to face event have been held. Seven multi-centre project proposals were received. A masked expert panel short-listed four proposals for a ´dragon´s den´ presentation and refinement. Subsequently, the membership selected a project which will establish how frailty in older patients affects outcomes after gynaecological oncology surgery.
Conclusion: There is a desire amongst trainees to engage in research to deliver patient benefit. A collaborative model facilitates efficient implementation of a project and yields nationally actionable information. Our next study will aid clinical decision-making in some of our most vulnerable patients.
Disclosure: Nothing to disclose

EP698

Is chemotherapy induced alopecia an avoidable side effect in routine daily practice?

R. Bracci[1], T. Campanelli[2], G. Casavecchia[2], C. Gasparoli[1], M.L. Iadarola[1], P. Longo[1], R. Mencarelli[1], L. Roberti[1], C. Cappelletti[1], R. Mattioli[1]
[1]Oncology Unit, Fano, [2]Oncology Unit , Fano, Azienda Ospedaliera Marche Nord, Fano, Italy

Introduction / Background: Chemotherapy induced alopecia is one of the most devastating side-effects, , impacting on the patient's psycological well-being , altering body-image, and being also a visible marker of a life-threatening disease.
Means to prevent hair loss with scalp hypothermia, were studied for decades but results were unsatisfactory. New tecnologies as DigniCap, use sensor-controlled scalp-cooling. DigniCap was the first system to receive FDA approval on December 2015.
We wanted to study feasibility and efficacy of DigniCap system in routine practice.
Methodology: From September 2016 to March 2019,136 women underwent chemotherapy treatment with DigniCap. Patients shoud be>18 years old, have a diagnosis of breast or gynecological cancer and candidate for adiuvant/neoadiuvant treatment with alopecia-inducing drugs; 116 women had breast and 20 gynecological cancers. Alopecia was evaluated by WHO grading-system. Grade1:hair loss < 25%; Grade2: between 25-50%, Grade3 between 50-75%, Grade4: > 75%.
Results: A total of 108 (79.4%) women completed all the chemotherapy with DigniCap, 28 stopped (27 breast and 1 gynecological cancer). Main reasons for stopping were: unefficacy, cold intolerance, therapy change.
In the 89 women with breast cancer who completed the treatment, 68 (76.4.%) had a modest hair loss (41 Grade1 and 27 Grade2) , in 21 the treatment was less effective (12 Grade3 and 9 Grade4).
In the 19 women with gynecological cancer who completed the treatment ,15 (78.9%) had modest hair loss (10 Grade1, 5 Grade2), while 1 had Grade 3 and 3 Grade 4.
Conclusion: Around 76-79% of women had satisfactory results.
Coordinating the appointments in order to give the possibility to treat all the women, was one of the main challenges, requiring an optimal organization by the nursing staff. Use of DigniCap is possible and effective in routinary setting
Disclosure: Nothing to disclose

EP699

High incidence with comparable survival of gynecologic sarcomas in israeli population; a comparison to national European and USA reports

Y. Brudner[1], G. Haran[1], A. Blecher[1], B.G. Silverman[2,3], M. Hallak[1], I. Bruchim[1]
[1]Gynecologic Oncology Division, Department of Obstetrics and Gynecology, Hillel Yaffe Medical Center, affiliated with the Technion, Israel Institute of Technology, Hadera, [2]Israel National Cancer Registry, Israel Ministry of Health, Ramat Gan, [3]Tel Aviv University Sackler School of Medicine, Tel Aviv, Israel

Introduction / Background: Survival reports of gynecologic sarcomas are limited and considered poor. The aim of this study is to explore the incidence and outcome of gynecologic sarcomas in different time periods in Israel and to compare to other national reports.
Methodology: Records of gynecologic sarcomas diagnosed between 1980-2014 were extracted from the National Israeli Cancer Registry. Sarcomas were characterized with respect to anatomical site, morphology and demographic variables. Age-standardized incidence rates (ASRs) and 1,3,5 and 10 -year relative survival rates were calculated for 3 time periods (1980-1994, 1995-2004 and 2005-2014) according to age, stage and years of diagnosis.
Results: During this period (1980-2014), 1271 new gynecologic sarcomas were diagnosed in Israel, with incidence slightly increasing between 1980-2004 years up to ASR of 13 per million females. Incidence was highest in women 45-65 years old. The most common histologic diagnosis was leiomyosarcoma (48%) and most common anatomical site was uterus (89%). The ASR for uterine

sarcoma is higher in Israel (10.55 per million females) compared to other national USA and European reports (5.8-9.2/per million). The observed survival rates were comparable to previous reports, with no significant difference through the years of diagnosis and between the Jewish and Non-Jewish populations. The 5 years' overall survival is significantly worse in elderly patients (>70yo) compared to younger patients (P< 0.001) and in patients with leiomyosarcoma compared to endometrial stromal sarcoma (p< 0.001).
Conclusion: Israeli statistics is comparable with global statistics regarding histologic and anatomical variations but the incidence in Israel seems higher than in European population.
Disclosure: Nothing to disclose

EP700

Pelvic exenteration for recurrent gynaecological cancer- outcome of patients referred but not operated on

S. Bryan[1], I. Mahmoodi[1], D. Kolomainen[2], D. Barton[1]
[1]Gynaecology Oncology, The Royal Marsden Hospital, London, [2]Gynaecology Oncology, Southend University Hospital, Essex, United Kingdom

Introduction / Background: The main goal of pelvic exenteration (PEx) for recurrent cancer is to achieve cure. This is most strongly associated with R0 resection, which is also associated with prolonged disease-free interval. Patients typically have been treated with radical surgery, and/or radiotherapy (RT) or chemo-RT. Not all patients referred for PEx will undergo surgery. We set out to establish why PEx was not performed and to determine the outcomes of these patients.
Methodology: We retrospectively collected the data of all patients who were referred for consideration of pelvic exenteration between August 2004 and June 2018. The database was populated from MDT and surgical records. All cases were either recurrent cancer or persistent disease following prior RT/chemo-RT.
Results: There were 90/202 referred during the study period who did not undergo PEx. The mean age at initial diagnosis was 51years. Over half (51%) of the patients had a diagnosis of cervical cancer. The reasons for not undergoing PEx were: 1. R0 would not be achieved (27%), 2. patient declining surgery (21%), 3. Metastatic disease (19%), 4. Extensive disease (14%), 5. Adverse patient co-morbidities (7%), 6. More suitable treatment available (6%), and 7. Procedure abandoned (2%). Of the 90 patients, 64 had died. The mean disease-free interval was 33.7 months, and the mean survival after decision made not for exenteration was 16.4 months. The majority of patients went on to have chemotherapy.
Conclusion: Approximately 50% of patients referred for PEx for recurrent gynaecological cancer are not operated on. The most common reason being that R0 would not likely be achieved, although one fifth declined surgery. Mean survival after decision made not for exenteration was just over a year. Majority of patients went on to have palliative chemotherapy. As expected, those who had no further treatment died sooner than those who underwent other treatments.
Disclosure: Nothing to disclose

EP701

Risk of clinically overt venous thromboembolism in post-operation gynaecological cancer patients: data from a Hong Kong tertiary hospital

Y.K.V. Chai, K.K.L. Chan
Department of Obstetrics and Gynaecology, Queen Mary Hospital, University of Hong Kong, Hong Kong, Hong Kong

Introduction / Background: Venous Thromboembolism (VTE) is known to be a common complication of cancer surgery. In view of this, various international guidelines recommend prophylactic peri-operative anticoagulation for this population. However, there are controversies on whether this should apply to Asian population in whom the VTE risk is perceived to be lower. We retrospectively reviewed the post-op VTE rate in our population whom more than 95% were Chinese or Southeast Asian.
Methodology: Women underwent laparoscopic (conventional and robotic) or laparotomy procedure for malignant or suspected malignant gynaecological pathology during 2012-2016 under gynae-oncology team of Queen Mary Hospital were identified by the Clinical Data Analysis and Reporting System of Hospital Authority. According to our clinical protocol, no routine pharmaceutic thrombo-prophylaxis would be given unless there is any past history of VTE or any medical conditions that require long term anti-thrombotic treatment. There is no routine post-operation biochemical or imaging investigation for non-clinical VTE. Ultrasound Doppler scan of limbs or computed tomography of pulmonary arteries would be arranged if there is clinical suspicion. Amongst these women, those who subsequently diagnosed with clinical venous thromboembolism within 30 days post-operation were identified by the aforementioned electronic system. The medical records of the women with VTE were reviewed for the clinical characteristics.
Results: In the five years, there were 494 laparoscopic and 494 laparotomy procedures performed respectively. Two women who underwent laparoscopic procedure and five women underwent laparotomy procedure were diagnosed to have VTE. The incidences were 0.4% and 1.0% respectively. Table 1 showed a summary of the cases.
Conclusion: The post-operation VTE rate in Chinese women with gynaecological cancer was lower than that of the Western population, which had been reported to range from 4 to 38%.

Patient	Diagnosis	Procedure	Blood Loss	Operation Duration	Intra-op complication	Interval between operation and VTE diagnosis
1	Endometrial cancer	TAHBSO + staging operation	850ml	2 hours 28 minutes	Nil	7 days
2	Ovarian borderline tumour + Pseudomyxoma peritonei	BSO + appendicectomy	100ml	1 hour 58 minutes	Laparoscopy converted to laparotomy due to extensive adhesion	3 days
3	Ovarian cancer	TAHBSO + staging	700ml	3 hours 9 minutes	Nil	1 day
4	Endometrial cancer	TLHBSO + PLND + PALND	100ml	3 hours 49 minutes	Nil	7 days
5	Endometrial cancer	TAHBSO + PLND	300ml	3 hours 12 minutes	Nil	24 days
6	Endometrial cancer	TAHBSO + PLND	1700ml	6 hours 28 minutes	Robotic Laparoscopy converted to laparotomy due to left iliac artery injury	27 days
7	Endometrial cancer	Robotic TLHBSO + PLND + SLNB	1000ml	6 hours 19 minutes	Left iliac vein injury	7 days
Abbreviations: TAH: total abdominal hysterectomy BSO: bilateral salpingooophorectomy PLND: pelvic lymph node dissection PALND: paraaortic lymph node dissection SLNB: sentinel lymph node biopsy						

[Table 1. Summary of the VTE cases]

Disclosure: Nothing to disclose

EP702

Multiple primaries in gynecological cancer-a retrospective observational study from 1990-2017 in a tertiary cancer centre in Eastern India

B. Chakrabarti, N.R. Mondal, T. Chatterjee, R. Roychowdhuri
Gyne-Oncology, SGCC&RI, Kolkata, India

Introduction / Background: Life expectancy in malignancy has increased in recent years.Patients suffering from malignant tumours can expect much longer period of disease-free survival or even cure.Patients may develop second or third primay malignancies.If 2nd.primary is discovered at the same time it isconcurrent,if within 6 months synchronous ,any time after 6 months is metachronous. The term´multiple primary malignant tumour " was first used by Billroth in1889 and the first paper describing multiple primary malignancy was published in1932 by Warren&Gates..
Methodology: Warren&Gate criteria has been followed.a)Histological confirmation of malignancy in both index& 2nd.primary.b)if concurrent there should be at least 2cm. of normal mucosa between the tumours.If the tumours are in the same location they should be separated by time by at least 5 yrs.c)probability of one being metastatic of other must be excluded.Incusion criteria:i)All histologically proven.II)at least one of the sites of malignancy should be female genital tract. Exclusion criteria:i)metastatic disease ii)recurrence III)multifocal /multicentral malignancies.iv)no clear histological confirmation of tumour.iv)hematological tumour.
Results: Synchronous 10 cases(37%)metachronous 17(63%). Of 10 synchronous cases7 were concurrent.cervix&breast-3,cervix&tonsil-1,cervix&endometrium.-1,cervix &ovary,cervix&gestational tumour-1.Rest3 had 2nd primary with in 6 months.cervix followed by ovary-1,cervix followed by kidney-1,cervix followed by breast-1.Of the 17 metachronous-first primary in breast 9,cervix-5,gall bladder-2,ovary-1,2nd primary in ovary-3,endometrium-3,cervix-4,vulva-3,thyroid-1,lungs-1,vagina-1,scalp-1.One patient with primary breast developed 2nd.&3rd.primary in cervix &scalp. Age of1st.primary-28-74 yrs(median51yrs).age of 2nd.primary34-94(median 53yrs).occurance interval in metachronous(11-161)months,median51months.
Conclusion: Approximately 10%of patients develop a 2nd.cancer(Lynch-Seer data 1973-1999)Causal mechanism include genes,environment,iatrogenic(radiation),treatment effects&combination of all.Most common hereditary cancer syndrome related to gynecological cancer include hereditary breast&ovarian syndrome,Lynch syndrome,etc.Treatment schedule for the first maligncy may result in damage of DNA leading to tumouregenesis Microsatelite instability(MSI) is most common in multiple primaries.Peutz-Leghers syndrome(PJS) is an inherited condition that puts people at increased risk for developing hamartomatous polyp in the digestive tract as well as cancer of breast,colon,rectum,pancreas,stomah.testicles,ovaries,lungs,cervix.Multicentric study inTurkey(2012) indicate most common cancer pairs in female are breast-gynecological cancer.(BabacanNA.)
Disclosure: Nothing to disclose

EP703

Comparison of mesonephric adenocarcinoma in the uterine corpus with that in the uterine cervix: a single-institution study of 12 cases

J.-Y. Choi, J.-Y. Park, Y.-J. Lee, S.-W. Lee, D.-S. Suh, D.-Y. Kim, J.-H. Kim, Y.-M. Kim, Y.-T. Kim
Department of Obstetrics and Gynecology, Asan Medical Center, University of Ulsan College of Medicine, Seoul, Republic of Korea

Introduction / Background: Uterine mesonephric adenocarcinoma (MNAC), very rare neoplasm, is most common in the cervix and less common in the uterine corpus. There are limited reports on MNAC regarding its characteristics, depending on the origin site. However, several pathological studies have recently reported that MNAC in the uterine body (UB-MNAC) has a high tendency to metastasise to distant organs. This study compared the clinical characteristics of MNAC cases to help decide adjuvant therapy.
Methodology: We extracted data from the medical records of patients diagnosed with primary uterine MNAC between October 2008 and February 2019 and retrospectively analysed it.
Results: There were seven cases of UB-MNAC and five of MNAC in the uterine cervix (UC-MNAC). Detection rate of distant metastasis/local invasion was higher in the UB-MNAC group (5/7 vs. 1/5). Among advanced UB-MNAC cases, the lungs (n = 2), pelvic peritoneum (n = 2) and ovary (n = 1) were the metastasis sites. Among UC-MNAC cases, only pelvic lymph node metastasis was detected. In the UB-MNAC group, six received adjuvant therapy (Combined chemotherapy - radiation therapy (CCRT): two, chemotherapy: four). In the UC-MNAC group, three received adjuvant therapy (CCRT: two, chemotherapy: one). The follow-up period was 2-78 months (median, 24.5 months). Only one case of UB-MNAC showed progression 7 months after CCRT. There were no significant differences in initial CA-125 levels, clinical symptoms and tumour size
Conclusion: Overall survival (OS) or Progression free survival (PFS) application was limited owing to the overall shortage of cases and duration of follow-up observation. It was difficult to determine post-therapy outcome because there were no treatment guidelines that led to adjuvant therapy administration even in early stages. However, recent studies, including this study, showed that UB-MNAC has more aggressive features compared with UC-MNAC from the point of diagnosis. Therefore, we may consider post-surgery treatment more actively in UB-MNAC cases.
Disclosure: Nothing to disclose

EP704

Is SOFA score reliable in a gynecologic oncology setting or is something missing? A retrospective study on predictors and risk factors for bloodstream infections

G. Corrado, L. Franza, B. Costantini, T. Spanu, M. Covino, V. Ojetti, L. Quagliozzi, A. Biscione, F. Taccari, A. Fagotti, E. Tamburrini, G. Scambia
Policlinico Universitario Agostino Gemelli I.R.C.C.S. Universita Cattolica Di Roma, Roma, Italy

Introduction / Background: Study aim was to determine whether sepsis organ failure assessment (SOFA) score is a useful diagnostic tool in patients with an underlying gynecologic malignancy and if other parameters could be used as bloodstream infection predictors.
Methodology: Between July 2016 and December 2017, 68 patients accessing the Emergency Ward with an underlying gynecologic malignancy were recruited. Variables concerning underlying disease, invasive procedures, laboratory and SOFA score parameters were analyzed. Patients were divided in 3 groups based on their blood and urine specimens. Risk factors for surgical site infections (SSIs), recent (< 30 days) surgery and chemotherapy were studied separately.
Results: Parameters included in the SOFA score and SOFA score itself were not significant predictors of bloodstream infection. Patients who had been treated with chemotherapy were at a higher risk of developing bloodstream infection (p=0.04; OR=7.9). In a rough model, C reactive protein (CRP), bilirubin and oxygen saturation (SO_2) were significantly different between patients with an underlying infection and those who had not. Only CRP maintained its significance in a complex model, with a cutoff of 180mg/L (linear regression, p=0.03; OR=4).
Conclusion: SOFA score does not seem reliable in diagnosing bloodstream infections in patients with an underlying malignancy, but other criteria may help an early diagnosis.
Disclosure: Nothing to disclose

EP705

The uterine mass Magna Graecia risk index in discriminating between benignant and suspicious malignant uterine masses

A. Di Cello[1], M. Borelli[2], M.L. Marra[1], M. Franzon[3], F. Visconti[1], C. Brisinda[1], C. Di Carlo[1], R. Venturella[1], F. Zullo[4]
[1]Magna Graecia University, Catanzaro, [2]Magna Graecia University, [3]Department of Life Sciences, University of Trieste, Trieste, [4]Federico II University, Napoli, Italy

Introduction / Background: Recently, we have proposed a prognostic index named Uterine mass Magna Graecia (U.M.G.) as an algebraic formula combining LDH isoenzymes, for discriminating between benignant and suspicious malignant uterine masses. We decided to prospectively evaluate the prognostic value of this score and then evaluate the role of ultrasound (US) imaging in stratifying the risk of sarcoma for women with uterine mass, when it was integrated to the U.M.G. risk index.
Methodology: From March 2016 to March 2019 we prospectively enrolled all patients with uterine mass referred at the Unit of Obstetrics & Gynecology of Magna Graecia University of Catanzaro (Italy). In all women we measured lactate dehydrogenase (LDH), LDH isoenzymes and U.M.G. risk index. The vascularization pattern of uterine lesions at the ultrasound (US) was recorded. Quantitative relationships between U.M.G., alone and combined to the US vascularization pattern, and the final diagnosis were investigated.
Results: A total of 385 patients with a definitive surgical diagnosis of uterine fibroids and 7 with uterine sarcomas were enrolled. LDH isoenzymes levels significantly differed between patients with benign uterine masses or sarcomas. The accuracy of U.M.G. risk indexin discriminating between benign and suspicious malignant uterine masseswas confirmed with a sensitivity at 100% and specificity at 99.4%, with two false positive over 385 benignant cases and no false negative over 7 sarcomas. The specificity reached 100% when US vascularization pattern was integrated with the U.M.G. score.
Conclusion: Based on preliminary data, U.M.G. risk index was confirmed to be as an inexpensive and accurate prognostic index for discriminating between benignant and suspicious malignant uterine masses. The integration of U.M.G. score and US vascularization pattern could improve the specificity into the uterine masses risk stratification.
Disclosure: Nothing to disclose

EP706

Impact of malignant gynecological disease on occurrence of surgical site infection

A. Stefanovic, I. Likic Ladjevic, S. Kadija, J. Dotlic, Z. Vilendecic, I. Pilic, S. Pantovic, K. Stefanovic, A. Beleslin, N. Topuzovic
Clinic for Obstetrics and Gynecology Clinical Center of Serbia, Medical Faculty University of Belgrade, Belgrade, Serbia

Introduction / Background: Surgical site infections (SSI) in gynaecology are still common. However, it is debated whether malignancy is an independent risk factor for SSI. Therefore, the study aim was to investigate the impact of malignant gynaecological disease on surgical site infection occurrence after abdominal surgical procedure.
Methodology: Study included all patients with SSI in three years period (2016 - 2018). Preoperatively general data as well as detailed medical history were taken from all patients. Surgical procedures were classified as classic or radical. In case of possible SSI signs (red, hot, discharge) swabs were taken from the wound. SSI was diagnosed based on the swab findings and infectious agents causing SSI were registered. Patients were divided according to surgery indications to study group (gynaecological malignancy) and control group (benignant disease excluding inflammatory conditions). Pre and postoperative data were compared and statistically analysed.
Results: Study included 138 patients who in average had 55.71 +/- 14.18 years of age and 27.45 +/- 6.22 Body Mass Index. There were no significant differences between patients with SSI regarding their diagnosis (67 gynaecological malignancies; 71 benignant conditions; $\chi^2=0.116$; p=0.733; carcinoma of

the cervix 14; uterus 25; ovary 28; χ2=4.866; p=0.088). However, in patients with gynaecological malignancies SSI was more often registered in more advanced disease stage (χ^2=17.403; p=0.001) and if radical surgery was performed (χ^2=12.014; p=0.001). In both patients with malignant and benign conditions the most common infectious agents were Staphylococcus (52.9%) and Enterococcus (26.8%) species, while other microbes were rather rare (χ^2=69.594; p=0.001).
Conclusion: Malignancy was not proven as independent risk factor for SSI after abdominal gynaecological surgical procedure. However, SSI is more likely to happen in more advanced stages of gynecological malignancy that require radical surgery.
Disclosure: Nothing to disclose

EP707

Gonadal dysgenesis: a rare congenital hereditary defect

P. Dzvincuk[1], D. Ondrová[1], R. Pilka[1], J. Zapletalová[2], P. Zaoralová[2]
[1]Obstetrics - Gynecology, [2]Pediatrics, Faculty Hospital, Olomouc, Czech Republic

Introduction / Background: Gonadal dysgenesis belongs to the rare congenital abnormalities, affected patients are at increased risk of various types of germline tumors. Symptoms of dysgenetic ovarian tumors are not specific and are usually revealed not earlier then in puberty but it is often already in stage of advanced tumor.
Methodology: A 13 year old girl was admitted to the hospital for suspicious diabetes insipidus, that was not proved. This patient had family history with report of her aunt who at the age of 17 underwent bilateral adnexectomy because of the dysgerminoma and gonadoblastoma of the ovaries followed by chemotherapy and subsequent HRT.
This girl was eunuchoid appearance with primary amenorea, Tanner´s clasification M IV, P IV (sparse), A 0. On the ultrasound there was small uterus with low endometrium and right ovary with oval solid mass 2x1.5 cm. The hormonal profile proved high values of FSH, the karyotype was 46 XY. The findings indicated laparoscopic gonadectomy.
Results: Bilateral ovarectomy was performed and the histopathologic examination proved gonadoblastoma present in both ovaries. There were also signs of tumor transformation into the dysgerminoma in the right ovary. TNM classification was T1a Nx M0, FIGO IA. All findings corresponded to the diagnosis of Swyer´s syndrome. The hormonal replacement treatment and regular check up were suggested.
Conclusion: Gonadal dysgenesis is not a frequent congenital defect and its transfer into the next generations is rather rare. The proper diagnosis is often difficult and it is based mainly on clinical symptoms. The late onset of puberty may be one of the first symptoms of the disease. The early diagnosis may prevent the transformation of the dysgenetic ovaries into the malignant tumors. The girls before the age of 15 should be therefore regulary examined by pediatrician with a careful evaluation of the signs of puberty.
Disclosure: Nothing to disclose

EP708

Multidisciplinary approach to management of fistulae in gynaecological oncology - 5 year experience

A. Farrag[1], D. Chandrasekaran[1], M. Powell[2], E. Brockbank[1], A. Lawrence[1], A. Jeyarajah[1], R. Miller[3], S. Phadnis[1]
[1]Department of Gynaecological Oncology, Barts Health NHS Trust, The Royal London Hospital, [2]Department of Clinical Oncology, [3]Department of Medical Oncology, Barts Health NHS Trust, St Bartholomew's Hospital, London, United Kingdom

Introduction / Background: Fistulas are uncommon complications in gynaecological malignancies; commonest occurring fistulas are vesicovaginal and enterocutaneous fistula. They may develop after primary malignancy, surgery, recurrence or radiation therapy. We report an observational cohort study.
Methodology: Prospective identification and data collection on all cases with fistula related to gynaecological malignancy was undertaken between 2014 and 2018. Patient demographics, co-morbidities, cancer type & stage, investigations, management and outcomes were collated and analysed using descriptive statistics.
Results: A total of 17 patients were identified (1% total n=1485), out of which 12 developed a fistula post operatively, 4 after chemo radiotherapy, and 1 case post recurrence. Among the 17 patients, 5 patients developed Enterocutaneous fistula, 3 uretro-vaginal fistula, 3 vesico-vaginal fistula, 3 rectovaginal fistula and 3 colovaginal fistula. CT scan was the main diagnostic tool in all these case. There were no correlations between developing a fistula and age, BMI, previous abdominal surgery, type or stage of cancer and type of procedure.
All fistulas were managed with multidisciplinary approach from the outset, including medical & clinical oncology, nutrition, interventional radiology, colorectal & urological surgeons, tissue viability, and extensive clinical nurse specialist support. Vesico-vaginal fistulas and post-radiotherapy rectovaginal and colovaginal fistulas were managed surgically. All others were managed conservatively with full resolution.
Conclusion: Fistulas in gynaecological oncology are an uncommon complication. CT scan is the preferred imaging modality for diagnosis. Early multi-disciplinary approach is pivotal to successful management.
Disclosure: AF, DC, MP, EB, AL, ARJ, RM, SP declare no conflict of interest.

EP709

Segmental ureteral resection with ureteroneocystostomy for gynecologic malignances: a case series

A. Federico[1], N. Foschi[2], C. Conte[3], V. Gallotta[3], M.G. Ferrandina[1,3], F. Pinto[2], M. Bruno[1], G. Scambia[1,3], P. Bassi[1,2], F. Zattoni[4], G. Vizzielli[3,4]
[1]Università Cattolica del Sacro Cuore, Istituto di Clinica Ostetrica e Ginecologica, [2]Unit of Urology, Fondazione Policlinico Universitario Agostino Gemelli IRCSS, [3]Unit of Gynecologic Oncology, Fondazione Policlinico Universitario Agostino Gemelli I.R.C.C.S., Rome, [4]Department of Surgery, Oncology and Gastroenterology - Urology, University of Padua, Padua, Italy

Introduction / Background: Primary or recurrent gynecological cancers involving low urinary tract may require demolitive surgery with curative purpose. We aim to assess surgical outcomes in patients undergoing segmental ureteral resection with ureteroneocystostomy as part of surgical procedures in advanced or recurrent gynecological cancers.
Methodology: We retrospectively evaluated post-operative data of all gynecological cancer patients consecutively referred to our Center between March 2012 and July 2018, who underwent unilateral ureteral resection and ureteroneocistostomy with or without psoas-bladder hitch (PBH), in case of macroscopic evidence of ureteral infiltration to achieve oncological radicality.
Results: A total of forty-six patients underwent surgery, including seventeen (37%) locally advanced cervical cancer, nine (19.5%) ovarian cancer recurrence, eight (17.4%) ovarian cancer, twelve (26%) other gynecological malignancies.
Twenty-two patients (47.8%) experienced at least one high-grade post-operative complication and fourteen patients (30.4%) had major urological complications. Four patients (8.7%) died of complications within 90 days from surgery.
Nine (69.2%) of thirteen patients with personal history of pelvic irradiation, had major urological complications, whereas only five (15.2%) among the thirty-three patients with negative history of pelvic irradiation experienced such type of complication.
At multivariate analysis pre-operative radiotherapy (p=0.046) and history of pelvic irradiation (p=0.021) were independently associated with an increased risk of developing severe urinary complications.
Age, peritoneal carcinosis and prolonged operative time did not correlate with an increased rate of such complications.

Type of Complications	N.	Grade*
**Early**		
Uretero-vaginal fistula	2	IIIa
Uroperitoneum	3	IIIa
Ureteral stricture	2	IIIa
**Late**		
Ureteral stricture	2	IIIa
Uretero-enteral fistula	1	IIIa
Uretero-vaginal fistula	3	2 IIIa- 1 IIIb
Renal impairment	1	IIIb

*[Urological complications. *Clavien-Dindo classification]*

Features	p (Univariate analysis)	p (Multivariate analysis)
**Age (< 65 years vs ≥65 years)**	0.634	-
**Neoadjuvant therapy (yes/no)**	**0.053**	n.s.
**Neoadjuvant radiotherapy (yes/no)**	**0.001**	**0.046**
**Pathology (cervical cancer vs others)**	**0.019**	n.s.
**Peritoneal carcinosis (yes vs no)**	**0.053**	n.s.
**Reconstructive technique (PBH vs others)**	0.999	-
**Other severe complications (yes vs no)**	0.226	-
**Previous pelvic radiotherapy (yes vs no)**	**0.000**	**0.021**
**Operative time (<500 min vs ≥500 min)**	0.749	-

[Factors associated with severe urological complications. PBH: psoas-bladder hitch]

Conclusion: In gynecologic oncologic surgery, ureteral resection with ureteroneocystostomy has high rate of related morbidity and mortality. Previous pelvic irradiation is strongly associated with severe urological complications.
Disclosure: Nothing to disclose

EP710

Retinal central vein occlusion with tamoxifen therapy: a case report

Y. Fertani, I. Marghli, L. Naija, O. Jaidane, J. Ben Hassouna, M. Hechiche, R. Chargui, K. Rahal
Surgical Oncology, Salah Azaiez, Tunis, Tunisia

Introduction / Background: Ocular toxicity of tamoxifen can be corneal, neurological or retinal. The risk is low but can occurs in small doses. Indeed, a careful examination is recommended to look for it, specially since continued therapy may decrease reversibility chances.
Methodology: We report a case of a 46 years old woman with retinal central vein occlusion due to

tamoxifen therapy. we did a systematic review of the litterature.

Results: we report a case of 46 years old woman with thyroiditis history, treated for invasive ductal carcinoma of the right breast. She underwent radical right mastectomy with axillary lymph node dissection.

It was a luminal A SBR I Tumour. She underwent hormonotherapy by Tamoxifen and ovarian suppression by radiotherapy.

After a follow-up of 13 months, she presented an ocular redness pain, blurring of vision and loss of eyesight. Funduscopic examination and fluorescein angiography showed a right retinal vein occlusion and an early stage of occlusion of the left one. She received a curative low-molecular-weight heparin. Thrombophilia research was negative, so tamoxifen was incriminated and stopped. Control funduscopic examination was performed and showed a repermeability of both retinal veins. Hormonotherapy was switched to aromatase inhibitor.

However, the patient developed, despite cessation of the treatment, an irreversible right blindness.

Conclusion: ocular side effects of tamoxifen are known since 1978 such as optical neuropathy, vein occlusion and most of all retinal effects. It can occur even for usual doses and can be potentiated by chorio-retinal and thromboembolic diseases.

Treatment and outcome surveillance are non-codified, we must be careful because reversibility of the side effect depends on the earliness of treatment

Disclosure: Nothing to disclose

EP711

Three unexpected cases of gynecological B-cell lymphoma

C.S. Fontana[1], I.L. Domit Empinotti Filho[2], L. I Ching[3], J.A. Gonzaga del Moral[4], A. Barreto Haagsma[3], L.C. Fontana[2]

[1]Gynecology, [2]UFSC - Federal University of Santa Catarina, [3]Anatomo-Pathology, [4]Hematology, UFSC - Federal University of Santa Catarina, Florianopolis, Brazil

Introduction / Background:

Gynecological lymphomas are uncommon and ovary is the most affected site. Their symptoms are similar to non-hematological tumors. Surgical treatments are not suitable for lymphomas, they demand chemotherapy, making etiological differentiation crucial

We report three cases of B-cell lymphoma from gynecological tract: two cases in ovary and another in cervix.

Methodology: Case 1. VS 38-yo, with complaint 1 of menorrhagia. Colposcopy, CT and USG showed an expansive lesion in uterine cervix and with invasion of the posterior bladder wall, pelvic lymphadenopathy. The biopsy showed proliferation of atypical large cells and the diagnosis of Diffuse large B cells lymphoma was concluded.

Case 2. TNM 29-yo, HIV+, with fever and weight loss. Ultrassound scan showed bilateral vascular solid-cystic adnexal masses. She underwent to bilateral salpingo-oophorectomy and biopsy of the peritoneal implants. The pathology reports reveals extensive infiltration by Burkitt's lymphoma

Case 3. CGM 32-yo, history of coughing and weight loss. CT showed a large mediastinal tumor and biopsy reveals High grade B cells lymphoma, double expressor Bcl6(+) and C-myc (+). She underwent to chemotherapy (Hyper-CVAD). Further investigation with PET-CT showed a solid-cystic ovary mass, suspected to be the primary site of the process (confirmed by bilateral salpingo-oophorectomy).

Results: Case 1. VS was submitted to chemotherapy (R-CHOP) and hysterectomy. Remission in 12 months of follow-up

Case 2. TNM underwent chemotherapy (H-CVAD) and radiotherapy. In remission after 9 months of treatment.

Case 3. CGM, after 11 months of diagnosis, she presented CNS infiltration. Chemotherapy, follow by bone marrow transplant is expected.

Conclusion: The cases illustrate the importance of correct diagnosis of primary gynecological lymphomas for proper chemotherapy and surgical treatment.

Disclosure: Nothing to disclose

EP712

Ovarian borderline tumors with invasive peritoneal implants: a long term follow-up study of 30 patients

L. Gaichies[1], A. Maulard[1], C. Genestie[2], P. Pautier[3], E. Bentivegna[1], A. Leary[3], C. Chargari[4], P. Morice[1], S. Gouy[1]
[1]Surgery, [2]Pathology, [3]Medical Oncology, [4]Brachytherapy, Institut Gustave Roussy, Villejuif, France

Introduction / Background: Ovarian serous borderline tumors with invasive peritoneal implants (SBOT-IPI) are associated with serous low grade carcinomas (SLGC) in the 2016 WHO classification of ovarian neoplasms. Even if SBOT-IPI can relapse as SLGC in 30 % of cases, long term survival is not well known. The aim of this study was to describe the frequency and the characteristics of the recurrences of OSBT-IPI.
Methodology: Retrospective study of patients referred to our institution, from 1971 to 2018, for a SBOT-IPI with a minimal follow-up of 12 months.
Results: 30 patients were identified; the median age at diagnosis was 36,5 [19-72] year. FIGO stage was up to IIIA for 26 patients and the average number of peritoneal areas involved was 4,5 [1-12]. The median follow-up was 106,6 [12-366] months. Twelve patients (40%) developed an invasive relapse (4 as SBOT-IPI and 7 as SLGC) with an average time of 53,6 [10,1-175,8] months. In this group, the mean age was 45,3 [23-72] year old at first diagnosis. The initial stage was up to IIIB in 75% of the cases and the average number of peritoneal areas involved was 5,2 [1-12]. Two patients died due to recurrence, one had a recurrence as SBOT-IPI 6 years after and one as SLGC 10 years after.
Conclusion: Relapsing as an invasive disease were more frequent than reported in other series and seems to be associated with the initial FIGO stage and with the number of peritoneal areas involved in the initial diagnosis. However, in this long term follow-up study, overall mortality of SBOT-IPI seems lower than mobility rate of SLGC. This study highlights that SBOT-IPI long term survival is poorly known. Finally, the prognosis of the SBOT-IPI should not be associated with the SLGC.
Disclosure: Nothing to disclose

EP713

Intraoperative repair of obturator nerve injury: experience in a tertiary hospital

B. Gastón[1], C.M. Tauste[1], S. Aguirre[1], J.C. Muruzábal[1], N. Abián[2]
[1]Gynecologyc and Obstetrics Department, Complejo Hospitalario de Navarra, Pamplona,
[2]Gynecologyc and Obstetrics Department, Hospital Reina Sofía, Tudela, Spain

Introduction / Background: Obturator nerve originates from L2-L3 and enters the pelvic cavity after piercing the medial border of the psoas muscle. It is located along the retroperitoneum within the obturator fossa. Finally it leaves the pelvis through the obturator foramen and innervates the medial thigh adductor muscles.
Although rare, obturator nerve injury complicating major pelvic oncologic surgery can occur.
Methodology: Retrospective review of a series of four cases of obturator nerve injury during oncological surgery.
Results: The Oncology Gynecology Department performed 1.226 pelvic lymphadenectomies between January 2000 and December 2018. The incidence of obturator nerve injury in oncological surgery in our Department is 0.32% (4/1.226).
Four cases' most relevant data are summarized in the attached table.
Conclusion: Inmediate laparoscopic repair of a transacted obturator nerve during gynaecologic

surgery is feasible and could prevent or reduce the severity of the postoperative neurological injury.

Tumor (FIGO stage)	Age	Primary treatment	Surgery and approach	Diagnosis	Treatment	Postoperative evolution
Clear cell borderline tumor (IA)	40	Surgery	Laparoscopic debulking surgery	Intraoperative	Intraoperative termino-terminal suture and rehabilitation	Postoperative immediate: monoparesia of the lower right extremity. Conserved sensitivity. Normal ambulation. 1 month postoperative: Right psoas 3/5, hip adductors 3/5.
Mucinous adenocarcinoma of the cervix (IIB)	49	RT-QMT	Laparoscopic radical pelvic and para-aortic lymphadenectomy	Intraoperative	Intraoperative termino-terminal suture and rehabilitation	Immediate postoperative: mild hypoesthesia in the right groin. Right adductors: force 4/5. 3 months postoperatory: normal.
Squamous carcinoma of the cervix (IIB)	59	RT-QMT	Laparoscopic radical pelvic and para-aortic lymphadenectomy	Intraoperative	Intraoperative termino-terminal suture and rehabilitation	Immediate postoperative: cramming of the proximal anterior left thigh. Paresia adduction left lower limb. 4 months postoperatory: normal
Synchronous tumor: endometrial adenocarcinoma and ovarian endometrioid adenocarcinoma (IC)	48	Surgery	Laparotomic radical hysterectomy, double adnexectomy, pelvic and para-aortic lymphadenectomy and omentectomy	Intraoperative	Intraoperative termino-terminal suture and rehabilitation	Fifteen months: pain. Hypoalgesia and hypoesthesia, of the anterior and lateral side of the right lower extremity. Motor difficulty for flexion and extension.

[Cases of obturator nerve injurie during oncology surgery in our Oncology Ginecology Department]

Disclosure: Nothing to disclose

EP714

RaNGO: a study to collect accessible information about patients with Rare Neoplasms of Gynaecological Origin in the UK

M. Hall[1], C. Roberts[2], W.G. McCluggage[3], N. Singh[4], J. Paul[5], R. Glasspoool[6], I. Vazquez[2], I. McNeish[7]

[1]Mount Vernon Cancer Centre, [2]Medical Oncology, Mount Vernon Cancer Centre, Northwood, [3]Dept Pathology, Belfast City Hospital Health and Social Care Trust, Belfast, [4]Dept Pathology, Barts Health NHS Trust, London, [5]CRUK Clinical Trials Unit, [6]Medical Oncology, Beatson West of Scotland Cancer Centre, Glasgow, [7]Dept Surgery and Cancer, Imperial College London, London, United Kingdom

Introduction / Background: According to RARECARE, in Europe rare cancers account for 18% of all female genital tract cancers (Figure 1).[1] Although the quality of UK cancer registry collection has improved significantly recently, national registries do not record treatments or outcomes nor do they obtain patient consent allowing subsequent access for research use of tissue samples. Yet the amalgamation of such clinical information and permissions is essential to facilitate evaluation of existing strategies and improve options/outcomes for patients. RaNGO, a study developed by Gynaecological Clinical Studies Group, demonstrates the feasibility of collecting accessible information about such cancers in the UK. It includes the development of a tissue bank with appropriate patient consents, for suitable ethically approved translational research projects.
Methodology: Patients with a specified rare gynaecological cancer (see Table 1) are offered participation in RaNGO a multicentre, non-randomised study to create an information repository to audit treatments / outcomes and a virtual tissue bank for future translational research. Consent is obtained for collection/retention of anonymised data and the provision of appropriate bloods, bodily fluids and tissue samples. Specific informed consent is sought for the exchange/release of data from the relevant National Cancer Registries (UK) for local audit purposes and for sharing hemi-aggregated RaNGO data internationally in the future.
Results: Of the 44 UK gynaecological cancer centres - 5 are currently fully open to recruitment, 10 are in set-up and 20 patients are recruited but up to date details will be provided in November 2019.
Conclusion: RaNGO demonstrates the safety/feasibility of acquiring blood, tumour samples and fluid from women with rare neoplasms of gynaecological origin from across the UK. The collection of detailed information about such patients will provide a much-needed benchmark of current management strategies. Involving all 44 gynaecological cancer centres will facilitate the development of future trials to improve outcomes for these patients
Disclosure: Nothing to disclose

EP715

Identification and extirpation of local recurrences of gynecological cancer using ROLL technique

S. Iacoponi[1], S. Fuertes[2], R. Sainz de la Cuesta[1]

[1]Gynecologic Oncology Department, [2]Nuclear Medicine Department, Hospital Universitario Quiron Madrid, Madrid, Spain

Introduction / Background: We present 3 cases of gynecological cancer recurrences in which the ROLL technique has been used for its detection and extirpation.
Methodology: The technique consists of the intralesional injection of the CT-guided radiotracer on the day prior to surgery by administering 5 mCi of Tc99m-Macroaggregated albumin. Scintigraphic imaging is performed immediately with SPECT-CT to confirm the correct intralesional injection. In the surgical act, a gamma wireless laparoscopic probe is used to localize the lesions.
Results: The first case was a 25-year-old woman, diagnosed with a breast angiosarcoma treated in 2013 with simple mastectomy and adjuvant chemoradiotherapy.

In 2017 a suspicious lesions were observed in MRI at the level of the right subcostal wall and in the left hemipelvis. The decision was to remove both lesions with ROLL .
The second case was of a 58-year-old woman who underwent primary cytoreduction and adjuvant chemotherapy in 2013 due to borderline ovarian carcinoma.
In 2018 malignancy suspicious implants were visualized by MRI at the paraortic level and at the Douglas sac. They were excised with the ROLL technique.
The third case was a 46-year-old woman diagnosed with high-grade serous ovarian cancer in stage IIIC in 2015, treated with optimal primary cytoreduction and adjuvant chemotherapy. In September 2018, during the control MRI an implant was identified in the right iliac fossa in close contact with the external iliac vessels. It's size was 15mm and it was compatible with recurrence and inguinal node of 9mm suspect. We decided to perform PET-CT for ROLL technique surgery.
In all cases, the implants were successfully removed by laparoscopy with surgical time, morbidity and length of hospital stay reductions.
Conclusion: This technique, already used successfully in other specialties , is increasingly used in oncological gynecology. It allows accurately removing implants that, due to size and location would be difficult to remove otherwise.

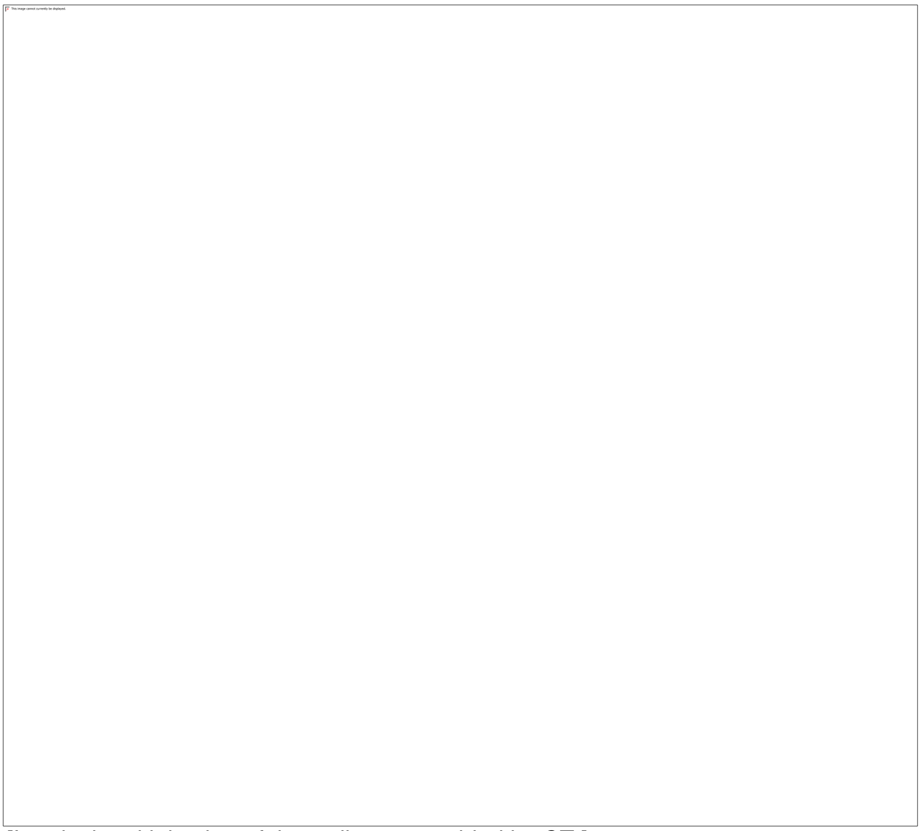

[Intralesional injection of the radiotracer guided by CT]

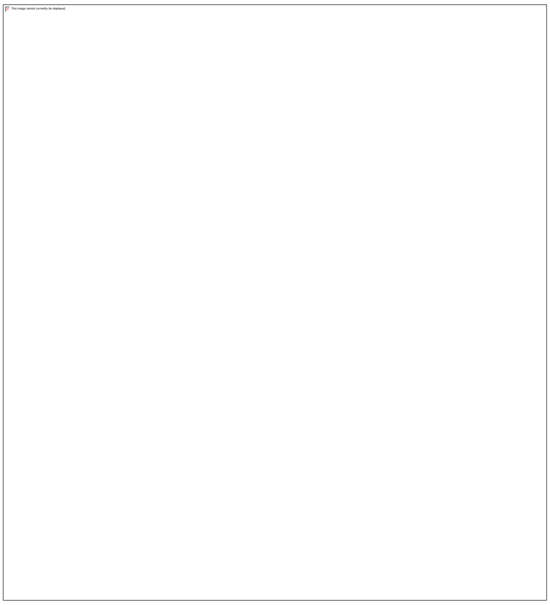

[3D reconstruction of left hemipelvic lesion (case 1)]

Disclosure: Nothing to disclose

EP716

A systematic review and meta-analysis on the effect of extraperitoneal laparoscopic lymph node dissection in patients with gynecological malignancies

A. Prodromidou[1], N. Machairas[2], C. Iavazzo[1]
[1]Department of Gynecological Oncology, Metaxa Memorial Cancer Hospital, Piraeus, [2]Third Department of Surgery, National and Kapodistrian University of Athens, Medical School, Athens, Greece

Introduction / Background: Para-aortic lymphadenectomy is performed for disease staging to tailor the optimal treatment in a plethora of gynecological malignancies such as ın advanced cervical, ovarian and high-risk endometrial cancer. With the advances in minimally invasive procedures, laparoscopic lymphadenectomy approaches have become the gold standard. The present study aimed to evaluate the impact of laparoscopic transperitoneal (TLL) and extraperitoneal (ELL) lymphadenectomy in patients with gynecological malignancies.
Methodology: The Medline, Scopus, Google Scholar, Cochrane CENTRAL Register of Controlled Trials and Clinicaltrials.gov databases were searched for articles published up to April 2019. Prospective and retrospective trials reporting outcomes for women with gynecological malignancies who underwent laparoscopic extraperitoneal or transperitoneal lymphadenectomy were enrolled. Statistical meta-analysis was performed using the RevMan 5.3 software.
Results: Of the 137 records screened, 7 were eligible for meta-analysis. A total of 608 women (329 TLL and 279 ELL) were included in the meta-analysis. Despite the fact that a significantly prolonged lymphadenectomy time was observed in TLL when compared to ELL (284 patients MD 35.18 min 95% CI 5.59 to 64.76 p=0.02) total operative time was not different among the two groups (407 patients MD -10.43 min 95% CI -20.55 to 41.42 p=0.51). No difference was observed with regards to postoperative complications, hospital stay and mean number of resected lymph nodes.
Conclusion: ELL is a safe and feasible. It presents with favorable outcomes in terms of shorter lymphadenectomy times and improved intraoperative outcomes as well as comparable to TLL lymph node yield**.** Further larger-volume studies are warranted to define the optimal approach in patients with

gynecological malignancies.
Disclosure: Nothing to disclose

EP717

Challenging diagnosis: plexiform tumorlet. A rare variant of uterine leiomyoma

E. Karopoulou[1], N. Koufopoulos[2,3], S. Kokkali[4], A. Arapaki[1], K. Strataki[1], S. Polykalas[1], D. Papatheodorou[1]
[1]Department of Gynecology, [2]Department of Pathology, "Agios Savvas" General Anti-Cancer Hospital, [3]2nd Pathology Department, Attikon University Hospital, Medical School of Athens, [4]1st Department of Medical Oncology, "Agios Savvas" General Anti-Cancer Hospital, Athens, Greece

Introduction / Background: Leiomyomas are benign mesenchymal smooth muscle tumors found in 75% of hysterectomy specimens. There are several subtypes, among them plexiform leiomyoma. When they are microscopic findings are referred to as plexiform tumorlets. We report a case of plexiform tumorlet in a patient with history of invasive lobular carcinoma (ILC) and concomitant endometrioid carcinoma.
Methodology: A 64 year-old patient with history of ILC three years ago was admitted to the gynaecology department due to recurrent vaginal bleeding. An endometrial curettage was performed. Histological diagnosis was endometrioid carcinoma grade I arising in a polyp. A hysterectomy and bilateral saplingo-oophorectomy was performed.
On microscopic examination residual endometrioid carcinoma was not present. Six uterine leiomyomas with a maximum diameter ranging from 1.2-4.3cm were found. A small tumor measuring 3 mm was found in the uterine wall. It consisted of small nests, islands, cords and tubules of epithelioid cells surrounded by loose stroma. Mitotic figures, pleomorphism or necrosis were absent. The differential diagnosis included plexiform tumorlet, uterine tumor resembling ovarian sex cord tumor and metastatic ILC.
Immunohistochemical study was positive for SMA, Desmin, CD56 and negative for AE1/AE3, EMA, Inhibin, WT1 and CD99.
Results: The diagnosis of leiomyomatous uterus consisting of six leiomyomas and a plexiform tumorlet was made. The patient is alive without any evidence of recurrence or metastasis fifteen months after surgery
Conclusion: Plexiform tumorlet is a very rare variant of epithelioid leiomyomas. It is usually discovered incidentally, showing a benign clinical course.
Disclosure: Nothing to disclose

EP718

Uterine tumour resembling ovarian sex cord tumour (UTROSCT): a case report

E. Karopoulou[1], E. Arkoumani[2], A. Arapaki[1], K. Strataki[1], S. Polykalas[1], N. Arnogiannaki[2], D. Papatheodorou[1]
[1]Department of Gynecology, [2]Department of Pathology, "Agios Savvas" General Anti-Cancer Hospital, Athens, Greece

Introduction / Background: Uterine tumours with histologic resemblances to ovarian sex cord tumours are very rare neoplasms. The histogenesis of these distinctive lesions is unclear as well as their malignant potential. UTROSCT usually occurs in middle-aged women. Abnormal uterine bleeding and/or abdominal pain are the most common symptoms usually accompanying an enlarged uterus. There are no typical imaging findings and the diagnosis relies exclusively on histopathologic examination.

Methodology: We report a case of a 47 year-old patient who was referred to the gynecologic department due to abnormal uterine bleeding. An endometrial curettage was performed. Histological examination revealed a neoplasm with characteristics of UTROSCT. A total abdominal hysterectomy with bilateral adnexectomy was performed. Under the microscope, the neoplasm found in the uterine cavity consisted of small-medium cells with light cytoplasm and small nuclei arranged in solid nests, trabeculae and ribbons. Mitotic activity was 1 mitosis per 10 HPF and necrosis was absent. On immunohistochemistry, tumor cells were positive for epithelial marker CKAE1/3, smooth muscle markers SMA and desmin and sex cord markers calretinin,CD99 and Melan-A. Staining for h-caldesmon, CD10 WT-1 and inhibin was negative and Ki67 was positive in about 5% of tumor cells.
Results: The morphological and immunohistochemical characteristics of the cells set the diagnosis of UTROSCT. The patient is now without evidence of recurrence disease, 4 months after resection.
Conclusion: Generally, these tumors are considered to be benign and tumors of low malignant potential but can easily relapse after incomplete resection. Hysterectomy with bilateral salpingo-oophorectomy should be performed in cases where fertility spearing is not required.
Disclosure: Nothing to disclose

EP719

Cervical squamous cell carcinoma *in situ* with superficial intraepithelial extension to the endometrium: a rare case

E. Karopoulou[1], F. Antoniadou[2], A. Arapaki[1], K. Strataki[1], S. Polykalas[1], N. Arnogiannaki[2], D. Papatheodorou[1]
[1]Department of Gynecology, [2]Department of Pathology, “Agios Savvas” General Anti-Cancer Hospital, Athens, Greece

Introduction / Background: Squamous cell carcinoma (SCC) of the cervix is the most common type of cervical cancer. It generally invades the endometrial cavity directly with or without parametrium involvement however, in rare cases can develop a superficial spread to the inner surface of the uterus replacing the endometrium. It is an unusual phenomenon for cervical carcinoma in situ to have continuous superficial extension to the endometrium without endometrial involvement.
Methodology: We report a case of a 69 year old female, 19 year post-menopausal, who attended the Gynecologic Department for malignant squamous cells found on her routine cervical smear. The conization biopsy which performed revealed cervical intraepithelial neoplasia III (CIN III) and the patient underwent a hysterectomy with bilateral salpingo-oophorectomy. The final histological examination confirmed the diagnosis of CIN III , non-keratinizing, of small cell type. Cervix and endocervix consisted entirely of CIN III extending up to the endometrial cavity. Both endometrial glands and stroma were completely replaced by CIN III, with involvement of foci of adenomyosis. Immunohistochemical staining for PAS showed that the basement membrane was intact and the cells were positive for p16.
Results: The histology and immunochemistry of the cervical neoplasia were compatible with CIN III with contiguous and superficial intraepithelial extension to the endocervix and the endometrium.
Conclusion: The superficial intraepithelial extension of squamous cell carcinoma in situ of the cervix is very rare and guidelines for the management of these cases are lacking. We treated our patient with simple hysterectomy and did not show any evidence of recurrence 7 months of follow up.
Disclosure: Nothing to disclose

EP720

Endosalpingiosis and papillary serous carcinoma in inguinal lymph nodes with uninvolved adnexae: a case report

V. Kartsiounis, G. Angelopoulos
Department of Gynaecological Oncology, University Hospital Coventry & Warwickshire NHS Trust, Coventry, United Kingdom

Introduction / Background: Endosalpingiosis is the presence of tubal epithelium outside the tube. It is linked to pelvic pain, mass effects, infertility and urinary symptoms. Sometimes concurrent malignancy can exist, low-grade serous carcinoma being the most common. We present a rare case of inguinal node papillary serous carcinoma on the background of endosalpingiosis.
Methodology: This is a 59-year old woman whose tru-cut biopsy of an enlarged inguinal node showed metastatic low-grade papillary serous carcinoma of possible ovarian primary. Staging CT and whole body PET scan confirmed no evidence of metastatic disease.
Results: A laparoscopic bilateral salpingo-oophorectomy and hysteroscopy were performed. The histology showed no evidence of atypia or malignancy. Central MDT review recommended bilateral groin lymphadenectomy. Final histology confirmed 1/6 right node having borderline serous carcinoma and 1/9 left node having invasive low-grade serous carcinoma both arising in lymph-node endosalpingiosis. The patient remains asymptomatic and a CT Thorax-Abdomen-Pelvis performed 6 months after the diagnosis showed no evidence of recurrent or metastatic disease.
Conclusion: There are several theories regarding endosalpingiosis like implantation of tubal epithelium after procedures involving the fallopian tube, direct spread of tubal epithelium cells or metaplasia of the mesothelial cells.
Endosalpingiotic nodal involvement with low-grade serous ovarian carcinoma has been described. However it is the second case report of inguinal node low-grade serous carcinoma arising from endosalpingiosis with no obvious adnexal involvement and the first to histologically exclude adnexal primary.
Disclosure: Nothing to disclose

EP721

Obturator nerve injury in pelvic lymphadenectomy - to stitch or not to stitch?

P. Kaščák, M. Hlavacik
Ob/Gyn, Faculty Hospital Trencin, Trencin, Slovakia

Introduction / Background: In the presented case report we discuss the necessity of immediate treatment of obturator nerve damage in pelvic lymphadenectomy in the light of new surgical procedures in oncogynecology, while considering the abilities and possibilities of the self-repair of damaged peripheral nerves.
Methodology: Case report
Results: Between 2011 and 2018 we performed 482 pelvic lymphadenectomy procedures. 470 cases (97.5%) were laparotomies. In 4 cases obturator nerve was injured (0.8%). In 3 patients, the injury occurred during the laparotomy and the nerve was immediately sutured without any subsequent functional deficiency. We present a case of nerve injury during laparoscopic lymphadenectomy where we did not proceed with the immediate treatment. Despite this, the patient suffered no sensitive or functional impairment, either after surgery or 6 months later. Laparoscopic staging was done due to an early stage ovarian carcinoma in a 67-year-old woman. During the right-sided pelvic lymphadenectomy, the obturator nerve was completely transected. Procedure was completed without further complications however immediate endoscopic treatment of the nerve injury was not possible. After some discussion we forgo the idea of immediate laparotomy. Since the patient did not experience any clinical signs of nerve damage, even after postoperative recovery, we definitely abandoned the idea of treating damaged nerve surgically. We have documented this complication on video.
Conclusion: Immediate treatment and its technique, delayed suture or conservative procedure for obturator nerve injury can all yield to the favourite functional outcome. In our case, we have abandoned the idea of nerve suture after laparoscopic lymphadenectomy and the patient showed no clinical signs of nerve damage.
Disclosure: Nothing to disclose

EP722

A comparative analysis between unidirectional barbed versus conventional vicryl suture for vaginal cuff closure in cases of total laparoscopic hysterectomy

K. Khoiwal, N. Kapoor, A. Gaurav, J. Chaturvedi
AIIMS, Rishikesh, India

Introduction / Background: We compared the use of unidirectional barbed sutures and conventional vicryl sutures for vaginal cuff closure during total laparoscopic hysterectomy. The aim of our study is to evaluate whether the use of unidirectional barbed suture (V-Loc) for vaginal cuff closure is associated with a decrease in vault closure time, operative time, blood loss, and other intraoperative and postoperative complications compared to polyglactin 910 (Vicryl) in patients undergoing total laparoscopic hysterectomy for benign pathology.
Methodology: We performed a prospective comparative study among patients who underwent total laparoscopic hysterectomy between July 2018 and December 2018 at our unit at AIIMS, Rishikesh.
Results: A total of 65 women were studied: 33 were in the vicryl group, and 32 were in the barbed suture group. The mean vault closure time was significantly less with barbed suture (8.84 min) than vicryl group (12.27 min). The mean number of stitches taken was more in barbed suture group (7.46) than vicryl group (7.09), though the difference was not statistically significant. No statistically significant differences were found in operative time, or postoperative outcomes, such as vaginal cuff dehiscence, infectious complications, and the presence of granulation tissue.
Conclusion: The efficacy and safety of unidirectional barbed suture is comparable to the conventional vicryl sutures. Barbed sutures can be safely used to reduce procedure time and surgical difficulty.
Disclosure: Nothing to disclose

EP723

Importance and significance of fertility sparing surgery in borderline ovarian tumors

S. Kim[1], J. Hong[1], J.Y. Song[1], J.W. Shin[2], J.K. Lee[1], N.W. Lee[1]
[1]Obstetrics and Gynecology, Korea University College of Medicine, Seoul, [2]Gachon University Gil Medical Center, Gachon University College of Medicine, Incheon, Republic of Korea

Introduction / Background: This retrospective study aimed to evaluate the safety of ovarian cystectomy in patients with borderline ovarian tumors.
Methodology: Patients with borderline ovarian tumors treated between August 2007 and August 2017 at our institution were divided into two groups according to the type of surgery: cystectomy and oophorectomy with or without salpingectomy. The cumulative disease recurrence was compared using Kaplan-Meier curves. Differences in surgical outcomes were also compared.
Results: Of the 169 patients enrolled, 134 (79.3%) had an oophorectomy with or without salpingectomy and 35 (20.7%) had an ovarian cystectomy. Eight patients (4.7%) developed recurrence of disease: six (4.5%) patients in the oophorectomy group and two (5.7%) in the cystectomy group and one patient died. Median disease-free months were 30 months for oophorectomy patients and 40 months for cystectomy patients, while the two-year disease-free rate was 97.6% and 94.7%, respectively. DFS was not different between the groups (p=0.886). The surgical approach (minimally invasive surgery or laparotomy) was not associated with disease recurrence (p=0.881). None of the parameters increased risk of recurrence on univariate analysis.
Conclusion: In conclusion, ovarian cystectomy can be considered a safe and effective option in young women who desire fertility preservation.
Disclosure: Nothing to disclose

EP724

Morcellation of uterine sarcoma might not be a risk factor for recurrence any more

Y. Kim, D.-Y. Kim, J.-Y. Park, D.-S. Suh, J.-H. Kim, Y.-M. Kim, Y.-T. Kim
Department of Obstetrics and Gynaecology, University of Ulsan College of Medicine, Asan Medical Center, Seoul, Republic of Korea

Introduction / Background: Morcellation has been thought as a risk factor of recurrence or abdominopelvic disease dissemination during surgery for uterine sarcoma. On the strength of advancement in diagnostic technology, capability to predict sarcoma is slightly improved than before. The purpose of this study is to re-evaluate the morcellation in the aspect of risk factor for outcome.
Methodology: From 2007 to 2017, 123 patients who were treated for stage 1-2 uterine sarcoma including leiomyosarcoma, low-grade endometrial stromal sarcoma(ESS), high-grade ESS were analysed retrospectively.
Results: Among 123 patients, 96(78%) had surgery in the presumed benign disease and 53(43%) underwent morcellation to remove uterine mass (morcellation group). Recurrence was detected in 41(33.3%) patients and 37 cases showed abdomino-pelvic recurrence. There was no statistically significant difference in all types of recurrent rate (34.3% vs. 32.1%, p=0.797), abdomino-pelvic recurrent rate (31.9% vs. 30.0%, p=0.827) and survival rate (82.9% vs. 84.9%, p=0.76) between two group (non-morcellation vs. morcellation). Estimated disease free interval were 92.6 months (95% confidence interval(CI), 73.3-112.0 months) for non-morcellation group and 86.0 months (95% CI, 72.3-99.6 months) for morcellation group. Estimated 5-year disease free survival(DFS) rate was 68.5% and 70.2% (p=0.996) in the non-morcellation and morcellation group, respectively. Estimated 5-year overall survival(OS) was not statistically different between two groups (76.7% vs. 83.1% p=0.762) and morcellation was not associated with all types of recurrence (odds ratio(OR), 0.905; 95% CI 0.424-1.934), abdomino-pelvic recurrence (OR, 0.916; 95% CI 0.416-2.015) and OS (OR, 0.991; 95% CI 0.279-3.513; p=0.989).
Conclusion: In the patients with the early stage of uterine sarcoma, morcellation during the first operation is not associated with the recurrence or overall survival. However, this contradiction to current knowledge needs further validation through multi-centre study of large sample size.

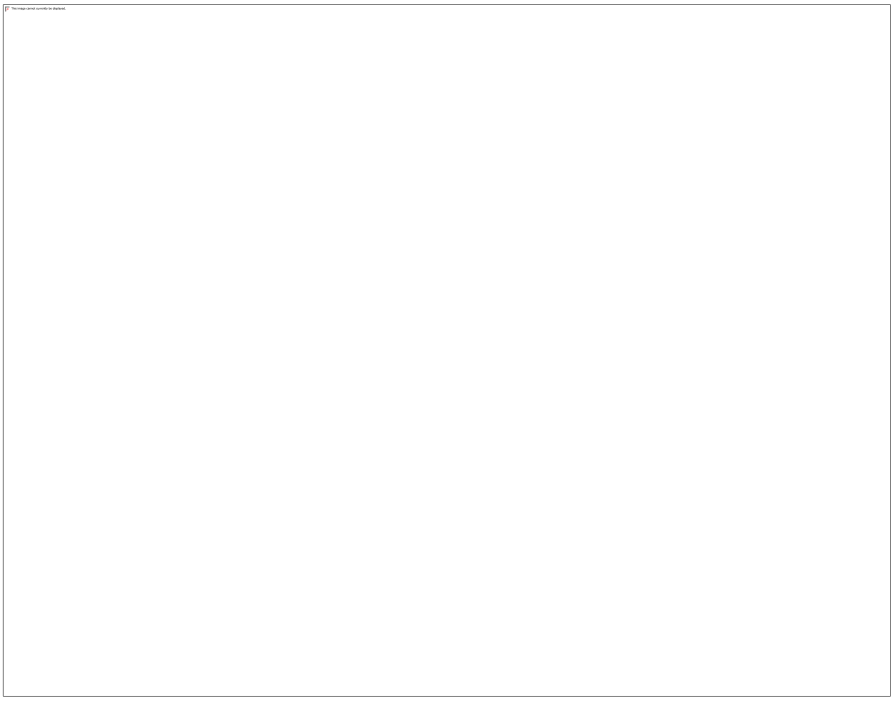

[curnulative overall survival by non-morcellation or morcellation group]

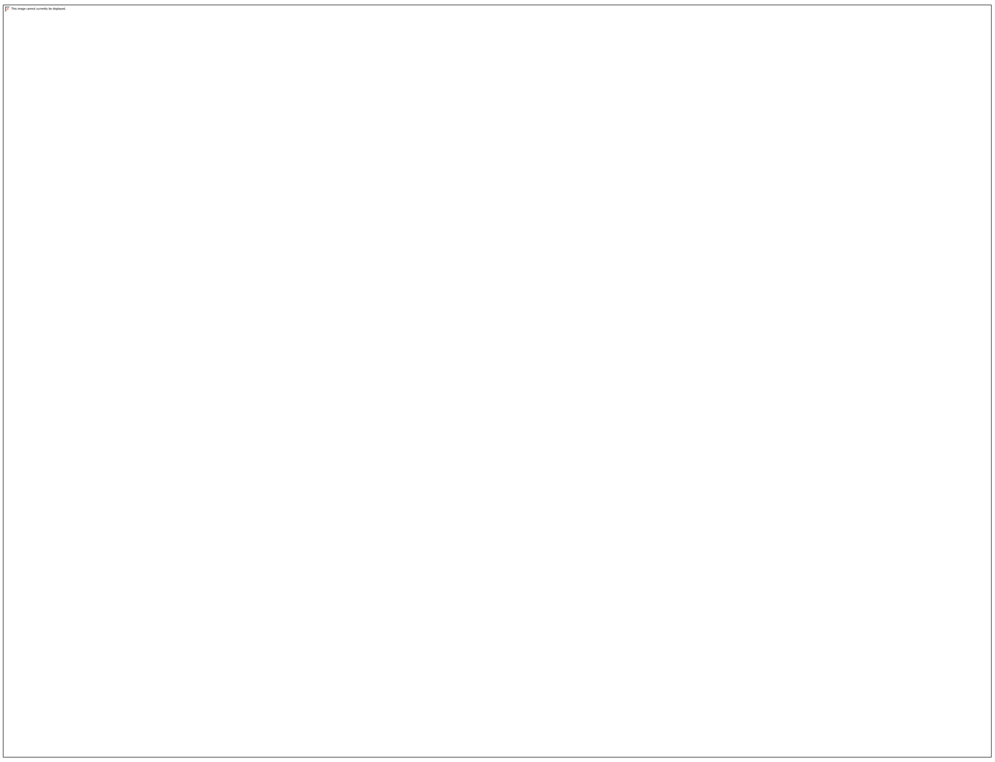

[recurrent rate by non-morcellation or morcellation group]

Disclosure: Nothing to disclose

EP725

Lymphocyte-monocyte ratio predicts uterine sarcoma aggressiveness

H.B. Kim
Hallym University Kangnam Sacred Heart Hospital, Seoul, Republic of Korea

Introduction / Background: The aim of the current study is to assess the prognostic value of the lymphocyte-monocyte ratio (LMR) in patients with uterine sarcoma.
Methodology: We examined the LMR as a prognostic variable in a cohort of 66 patients with uterine sarcoma who underwent surgical resection. Patients were categorized into two groups based on the LMR using cut-off values determined by receiver operating characteristic curve (ROC) analysis. We assessed the effect of the LMR on progression-free survival (PFS) and validated the LMR as independent predictor of survival.
Results: Using data from the whole cohort, the optimized LMR cut-off value selected using the ROC curve was 5.86 for PFS. The LMR-low and LMR-high groups included 45 (68.2%) and 21 (31.8%) patients, respectively. The 5-year PFS rates in the LMR-low and LMR-high groups were 69.0% and 94.4%, respectively (p=0.024). Via multivariate analysis, we identified FIGO, residual tumor after surgery, and LMR as the most valuable prognostic factors affecting PFS (p=0.039, p=0.018 and p=0.043, respectively).
Conclusion: The LMR is an independent prognostic factor affecting the PFS of patients with uterine sarcoma.
Disclosure: Nothing to disclose

EP726

One-Step Nucleic Acid Amplification, are we witnessing a rise of a new method in nodal assessment?

J. Kostun, J. Presl, J. Bouda, P. Vlasák
Department of Gynecology and Obstetrics, University Hospital in Pilsen, Charles University, Pilsen, Czech Republic

Introduction / Background: Lymph node (LN) status is one of the most important prognostic factors in gynecologic cancer. Moreover, in patients with cervical and vulvar cancer, intraoperative LN assessment is important for relevant decision-making regarding therapy. One-Step Nucleic Acid Amplification (OSNA), a rapid molecular analysis (within 16-30 minutes) of the whole LN by quantification of CK19 mRNA expression, should decrease the risk of undertreatment resulting from missing metastatic nodal burden. We performed a review of studies relevant to OSNA with focuse on its perspective in gynecologic oncology.
Methodology: We searched the PubMed for all articles containing keywords “OSNA” or “One-Step Nucleic Acid Amplification” in the title or abstract published until 4/2019. We focused on gynecologic oncologic papers and analysis of their results.
Results: Since 2006, 157 articles were found. OSNA was tested in breast, colorectal, lung, gastric, head-neck, thyroid, endometrial, cervical and prostatic cancer. OSNA achieved certification for use in breast, colorectal and gastric cancer (currently used in more than 300 hospitals worldwide). Five papers were published in the field of gynecologic oncology. Four articles are comparing OSNA and standard histopathology in examining the LNs in endometrial cancer, one in cervical cancer. In the paper Kosťun et al, published 2019, even ultrastaging of sentinel LNs in endometrial cancer was used as the reference method. While ultrastaging detected 8,15% (11/135) of the LNs as metastatically involved, OSNA detected 28 positive LNs 20,69% (28/135), p≤0,0033.

Conclusion: OSNA has already been certified for LN assessment in breast, colorectal and gastric cancer. So far published data indicate high level of sensitivity and specificity even in comparison to ultrastaging, thus providing reassurance in detection of even small metastases. Nevertheless, more statistically significant data is needed for acceptance of OSNA in routine LN analysis in gynecological cancers. Our department is currently working on prospective trials applying OSNA in endometrial and cervical cancer.

Author, year	Cancer	Patients, N	Nodes, N	Sensitivity, %	Specificity, %	Concordance, %	Positive Pred. Value, %	Negative Pred. Value, %
Okamoto et al., 2013	cervical cancer	32	130	50	98.4	96.2	60	97.6
Nagai et al, 2015	endometrial cancer	35	137	82.4	99.2	97.1	93.3	97.5
López-Ruiz et al, 2016	endometrial cancer	34	94	100	87.6	88.3	68.8	100
Fanfani et al, 2018	endometrial cancer	40	110	87.5	100	99	100	99
Kosťun et al, 2019	endometrial cancer	58	135	90.9	85.5	85.9	38	99
	sum/average	199	606	82.2	94.1	93.3	72	98.6

[Table- Results of OSNA related publications in gynecologic oncology until 4/2019, reference method histopathology]

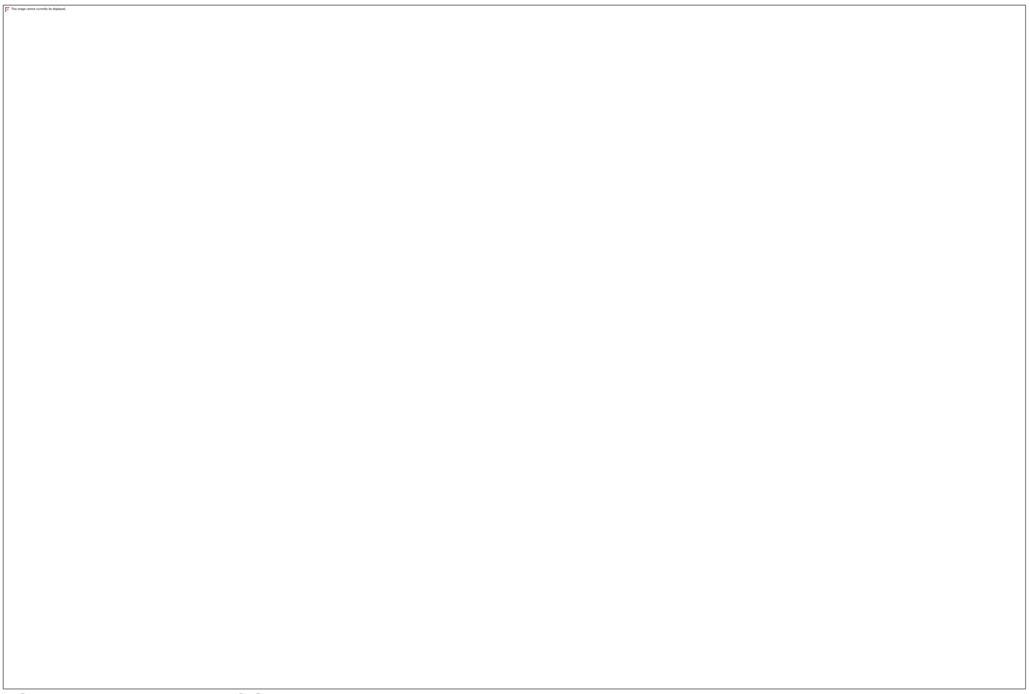

[Graph 1. Number of OSNA related publications per year]

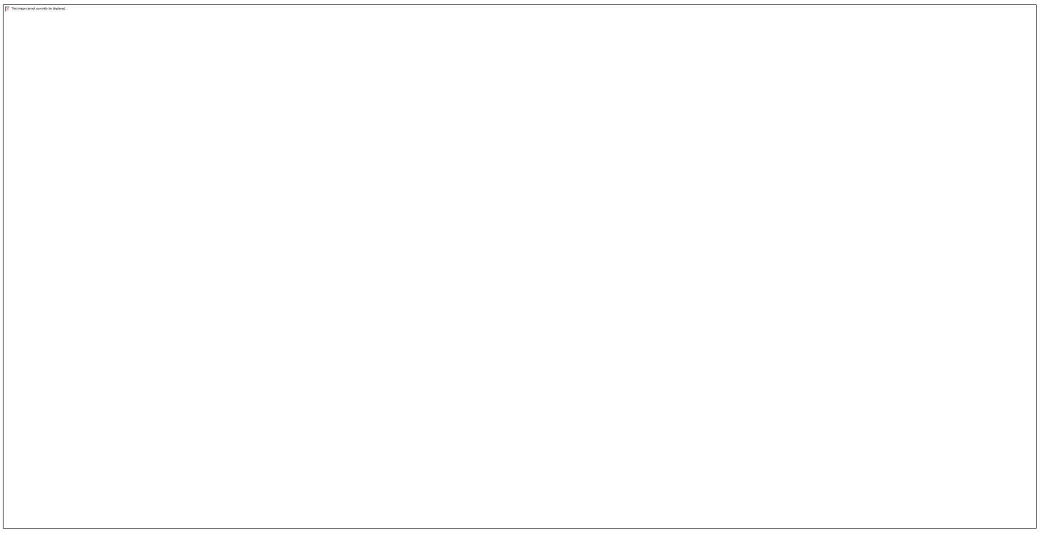

[Graph 2. Number of OSNA related publications by cancer until April 2019]

Disclosure: Nothing to disclose

EP727

Presentation of the 14th case of Leiomyoadenomatoid tumor in the English literature

N. Koufopoulos[1,2], E. Karopoulou[3], A. Arapaki[3], K. Strataki[3], S. Polykalas[3], D. Papatheodorou[3]
[1]Department of Pathology, “Agios Savvas” General Anti-Cancer Hospital, [2]2nd Pathology Department, Attikon University Hospital, Medical School of Athens, [3]Department of Gynecology, “Agios Savvas” General Anti-Cancer Hospital, Athens, Greece

Introduction / Background: Adenomatoid tumor (AT) is an uncommon benign tumor of mesothelial origin. It is most commonly encountered in the male and female genital tracts. Leiomyoadenomatoid tumor (LMAT) is a rare variant of AT with a prominent smooth muscle component. We present a case of LMAT.

Methodology: : A 79 year-old woman without previous history was admitted to the department of gynaecology due to lower abdominal pain. Transvaginal ultrasonography revealed a 7.2 cm fibroid in the anterior wall of the uterine cervix. Hysterectomy and bilateral salpingo-oophorectomy was performed

On gross examination, besides the large leiomyoma of the uterine cervix a second intramural leiomyoma located in the antero-lateral wall of the corpus uteri was observed measuring 2.5 cm.

On microscopic examination, both tumors consisted of smooth muscle bundles arranged in a fascicular pattern. In the smaller one, smooth muscle bundles were separated by slit-like and tubular structures lined by cuboidal epithelioid cells with small, uniform nuclei and scanty, pale, eosinophilic cytoplasm. Mitotic figures were not observed.

Tumor cells were positive for AE-1/AE-3, CK-8/18, CK-7, Calretinin and negative for CD-31 and CD-34. SMA and Desmin highlighted smooth muscle cells. Ki-67 stained less than 1% of tumor nuclei.

Results: The morphological features as well as immunohistochemical results were consistent with LMAT of the uterus. On postoperative evaluation, three months after surgery, the patient shows no evidence of recurrence

Conclusion: LMAT is a rare variant of AT with a prominent smooth muscle component. It was first described in 1992. Our literature review revealed 13 previously reported cases. Awareness of this rare entity is important in order to avoid misdiagnosis as adenocarcinoma.

Disclosure: Nothing to disclose

EP728

Targeted radiotherapy improves progression free survival and delays time to systemic therapy in limited relapse of ovarian cancer

A. Kumar, A. Kapoor, S. Banerjee, A. Taylor, A. George, M. Gore, S. Lalondrelle
Royal Marsden Hospital, London, United Kingdom

Introduction / Background: Treatment for ovarian cancer (OC) usually involves systemic therapy (ST) and surgery. Radiotherapy (RT) is used for palliation, however for limited relapse may provide improved local control (LC) and defer the need for ST. We investigated the benefit of higher doses of RT in localised relapse of OC.
Methodology: We performed a retrospective institutional review of patients with OC who received RT from 07/1998-12/2015. Radical prescription doses >= 30Gy were included, palliative treatments < 30Gy and brain metastases were excluded. Data was collected to determine LC, progression free survival (PFS) and time to next ST (TNT) and correlated with lines of previous treatment, location of disease, histology, RT dose/ fractionation, and platinum sensitivity.
Results: 534 courses of RT for OC were identified. Of these, 65 courses in 59 patients (range 33-84 years)were eligible for inclusion. Histological subtypes were: serous (67.7%), endometrioid (12.3%), clear cell (6.1%), mucinous (6.1%), granulosa cell (3.07%), carcinosarcoma (3.07%) and borderline (1.5%). Table 1 shows the number of lines of previous ST.
Table 2 show sites and modality of RT delivered. The median dose was 45Gy in 25# (range 33-64.8Gy) for stereotactic treatments 30 Gy /3# (range 27-36gy). Table 2 show sites and modality of RT delivered. The median dose was 45Gy in 25# (range 33-64.8Gy) for stereotactic treatments 30 Gy /3# (range 27-36gy).
Conclusion: RT is underutilised and should be considered as an alternative to ST in limited relapse OC. Higher doses of RT can provide high rates of LC, improve PFS and delay time to ST in different histological subtypes and in heavily pre-treated patients.

Lines of previous ST	Number of patients	Platinum sensitive	Platinum resistant	Median PFS (months)
0	4			4
1	21	8	13	15
2	22	2	20	9
3	5	1	4	7
4	7	0	7	10
5	6	0	6	5.5

[Results according to line of previous ST]

Area Treated	Number of patients	RT modality
Axilla	2	conformal(1),IMRT(1)
Neck	2	conformal(2)
Mediastinum	1	conformal(1)
Inguinal lymph nodes	1	conformal(1)
Mesorectum	1	SBRT(1)
PA nodes	8	conformal(8),IMRT (2), SBRT (3)

Pelvic nodes	44	conformal (32),IMRT (9),SBRT(3)
Porta hepatis	1	conformal(1)

[Sites and modality of RT delivered]

Disclosure: Nothing to disclose

EP729

A rare form of ovarian *serous* cystadenoma with monodermal teratoma in a 53 year old patient

G. Boutzios[1], S. Pittokopitou[2], E. Koukoulioti[3], P. Alexandrou[4], K. Giannakopoulos[2], A. Lagkadas[2]
[1]Endocrine Unit, Pathophysiology, Medical School, National and Kapodistrian University of Athens, [2]Gynaecology, Laiko General Hospital, [3]Medical School, National and Kapodistrian University of Athens, [4]Pathology, Medical School, National and Kapodistrian University of Athens, Athens, Greece

Introduction / Background: Struma ovarii is a specialized or monodermal teratoma predominantly composed of mature thyroid tissue. Struma ovarii comprise 1% of all ovarian tumors and 2 to 5% of ovarian teratomas. It is most common between the ages of 40 and 60 years. We present an interesting case of ovarian *serous* cystadenoma with monodermal teratoma, without clinical or biochemical features of hyperthyroidism, as well as the therapeutic approach.
Methodology: A 52 year-old-female, with personal history of polycystic ovary syndrome, multiple micro-nodular goiter and right oophorectomy due to a dermoid cyst of the right ovary, was admitted to the hospital due to continuous low abdominal pain. Blood parameters were within normal range. TSH was 2.5 IU/ml (n,v. 0.27-4.2). An abdominal sonography was performed and was revealed a heterogenous large mass in the left ovary, diameter 8 cm approximately, full of clear liquid. A Computed tomography was performed for the preoperatively assessment. A total hysterectomy with unilateral salpingo-oophorectomy was performed.
Results: The histology of the ovary showed a large *serous* cystadenoma (size 8.5x 5.5cm). Nearby to the tumor was a segment with hyperplastic thyroid follicles and colloid abundance compatible to monodermal teratoma (struma ovarii type) with benign histological features.
Conclusion: Struma ovarii is a rare ovarian tumor mainly with benign histological features. When the cause of hyperthyroidism is not obvious, struma ovarii could be a possible cause.
Disclosure: Nothing to disclose

EP730

Prevalence of germline BRCA mutations among women with carcinoma of the peritoneum or fallopian tube

C. Lee, M.C. Choi, H. Park, S.G. Jung, W.D. Joo, S.H. Song
Comprehensive Gynecologic Cancer Center, CHA University / CHA Bundang Medical Center, Seongnam-si, Republic of Korea

Introduction / Background: The aim of the present study was to assess the frequency of germline mutations
in patients with peritoneal carcinoma (PC) or the fallopian tube carcinoma (FTC), using a multi-gene panel.
Methodology: Twenty-six patients diagnosed with either PC or FTC between January 2013 and December 2016 were recruited consecutively. Germline DNA was sequenced using a 6-gene next generation sequencing (NGS) panel following genetic counseling. Surgico-medical information was

obtained from hospital records. Genetic variations were detected using the panel and were cross-validated by Sanger direct sequencing.
Results: Germline BRCA1/2 mutations were identified in 6 patients (23.1%). Four were detected in patients with PC and 2 were in FTC patients. No mutations were detected in TP53, PTEN, CDH1, or PALB2. We identified 11 variant of uncertain significance (VUS) in 9 patients; 2 in BRCA1, 3 in BRCA2, 2 in TP53, and 4 in CDH1. We also detected a CDH1 c.2164+16->A VUS in 3 patients.
Conclusion: The prevalence of germline BRCA1/2 mutations in patients with PC or FTC is comparable to that of BRCA1/2 mutations in epithelial ovarian cancer patients.
Disclosure: Nothing to disclose

EP731

Comparison of histological outcomes between pre-menopausal and post-menopausal women with report of cervical cytological abnormality

J.E. Lee, Y.I. Ji
Obstetrics and Gynecology, Inje University, Busan, Republic of Korea

Introduction / Background: The aim of this study is to compare and investigate the histological outcomes between premenopausal and post-menopausal women with report of cervical cytological abnormality.
Methodology: From January 2010 to May 2018, patients with abnormal cervical cytology followed by histopathologic examination were collected from Department of Obstetrics and Gynecology of Haeundae Paik Hospital. The patients were divided into two groups according to menopause status and the histologic results. This study included the following cervical cytological abnormalities: atypical squamous cells of undetermined significance (ASC-US), cannot exclude high-grade squamous intraepithelial lesion (ASC-H), low-grade squamous intraepithelial lesion (LSIL), high-grade squamous intraepithelial leision (HSIL).
Results: The results of the cervical cytology among the 438 premenopausal women were ASC-US (n=91), LSIL (n=90), HSIL (n=193) and ASC-H (n=64). The results were ASC-US (n=12), LSIL (n=21), HSIL (n=63) and ASC-H (n=19) in 115 postmenopausal women. According to cytology results, we divide into two groups, ASC-US and LSIL were classified as low risk group, ASC-H and HSIL as high risk group. Within the low risk cytology group, HSIL was 73.5% (133/181) by histologic biopsy in premenopausal women and 42.4% (14/33) in postmenopausal women. In high risk group, HSIL was 88.7% (228/257) in premenopausal group and 70.7% (58/82) in postmenopausal group. In both comparisons, there was a statistically significant difference between premenopausal and postmenopausal women.
Conclusion: In conclusion, our comparative study's finding showed significant difference in correlation of cytology and histology results from premenopausal group and postmenopausal group with cervical cytology abnormality. On the basis of this result, we think that since cytological results in postmenopausal women than premenopausal women have low sensitivity, follow up pap smear with high risk HPV testing would be a more conservative management and appropriate than immediate colposcopic biopsy.
Disclosure: Nothing to disclose

EP732

Bladder endometriosis mimicking invasive pelvic malignancy

M. Loh, C. Goh, Y. Lim
KK Women's and Children's Hospital, Singapore, Singapore

Introduction / Background: Endometriosis, although common, rarely involves of the urinary tract, with bladder being the most commonly involved organ. First described in 1921[1], the pathogenesis, management, impact on fertility, and risk of malignant transformation of bladder endometriosis still remains controversial.
Methodology: We report a case of a patient presenting with an infiltrative pelvic mass with local invasion into the bladder.
Results: The patient, a 49 year old Chinese female with 2 previous cesarean sections and a laparoscopic myomectomy, had no history or symptoms suggestive of endometriosis (dysmenorrhea, dyschezia, dyspareunia, chronic pelvic pain).
An enlarged left ovary was seen on ultrasound pelvis with an irregular papillary focal mucosal lesion in the left lateral wall of the bladder with bladder invasion. A magnetic resonance imaging (MRI) of the pelvis confirmed an invasive bladder lesion (Figure 1). The 4cm ovarian mass was inseparable from the left uterus. There were no enlarged pelvic lymph nodes or ascites. Ovarian tumour markers, renal function and an ultrasound of bilateral kidneys were normal.
She underwent a total abdominal hysterectomy bilateral salpingectomy with bladder tumour excision and bilateral double J stent insertions. Intraoperatively, the solid left ovarian lesion noted on scan was found to be a 5cm uterine fibroid. The 3cm tumour involving the lower uterine segment and bladder extended into the bladder mucosa (Figure 2). The tumour was removed en bloc with the uterus, with frozen section noting endometriosis. Both tubes and ovaries and intraperitoneal survey were normal with no pelvic endometriosis.
Conclusion: Isolated urinary tract endometriosis is rare and may mimic malignancy. It is thus an important differential diagnosis to consider in a woman presenting with an infiltrative bladder mass. Management may be in the form of hormonal therapy or surgery, where complete excision of the bladder mass is ideal to minimize recurrence.
Disclosure: Nothing to disclose

EP733

Incidental finding of vulvar syringoma during the treatment of vulvar intraepithelial neoplasia - a case report

M. Puljiz[1], L. Marcelić[1], D. Danolić[1], I. Alvir[1], I. Mamić[1], L. Šušnjar[1], I. Veliki Dalić[2]
[1]Clinical Department of Gynecologic Oncology, [2]Department of Pathology, University Hospital Center Sestre Milosrdnice, University Hospital for Tumours, Zagreb, Croatia

Introduction / Background: Syringoma is a benign eccrine sweat gland tumor which occurs mostly in females at puberty. The sites of predilection are lower eyelids and malar area. Lesions are usually bilateral and symmetrically distributed and they clinically appear as small, multiple, firm, skin-colored, pink to yellowish papules, 2 to 4 mm in diameter. Syringoma of the vulva is a rare condition with only few cases reported in the literature to date. We report a case of 57-year-old woman who presented to gynecology department with vulvar pruritus.
Methodology: A 57-year-old woman, gravida 3, para 3 was admitted to our department due to abnormal findings of vulvar smear. Patient had been postmenopausal for 6 years. According to the patient, vulvar pruritus had appeared about a year ago. Vulvar smear was taken from indurated area on left labia majora. On left labia majora there were also barely visible confluent nodules on discrete erythematous background. Vulvar smear cytology revealed intermediate vulvar intraepithelial neoplasia and patient underwent wide excision biopsy.
Results: Histological examination showed findings consistent with vulvar intraepithelial neoplasia grade II but also revealed well-circumscribed structure consisting of small solid and ductal structures with comma-like geometric shapes embedded in a fibrotic stroma in the dermis.

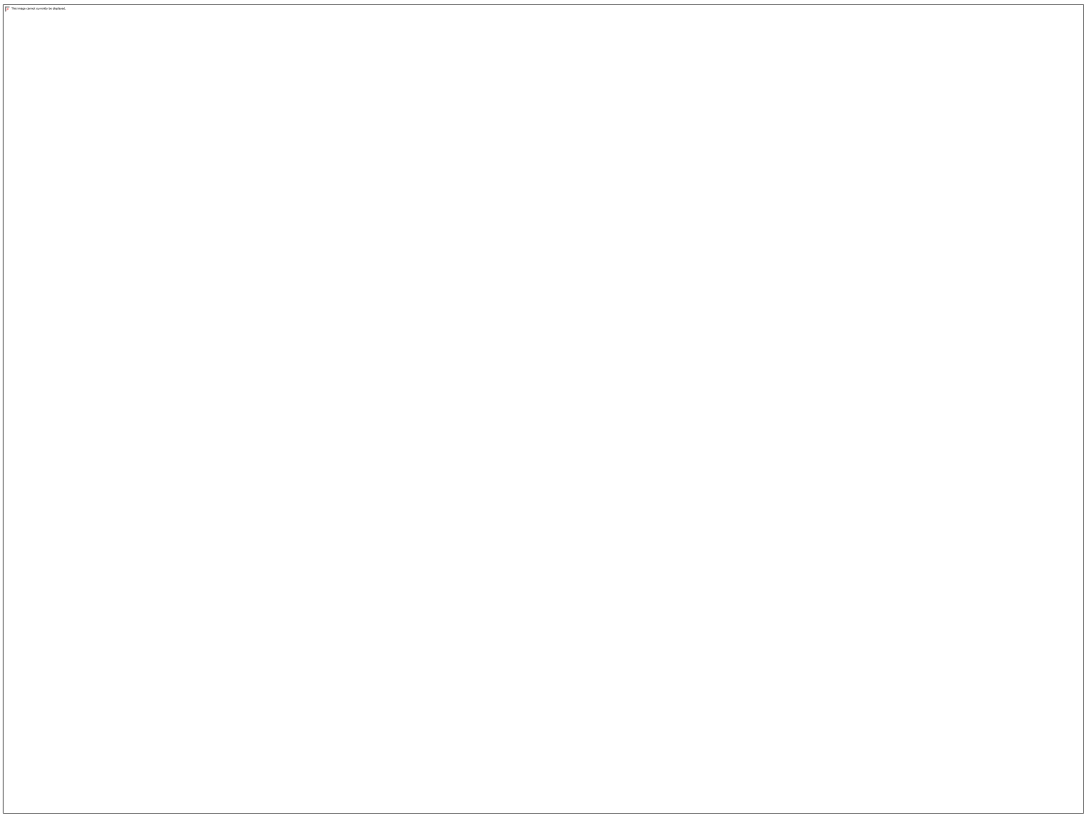

[Figure 1. Tumor is within the dermis, composed of numerous small dilated duct spaces. (HE x50)]

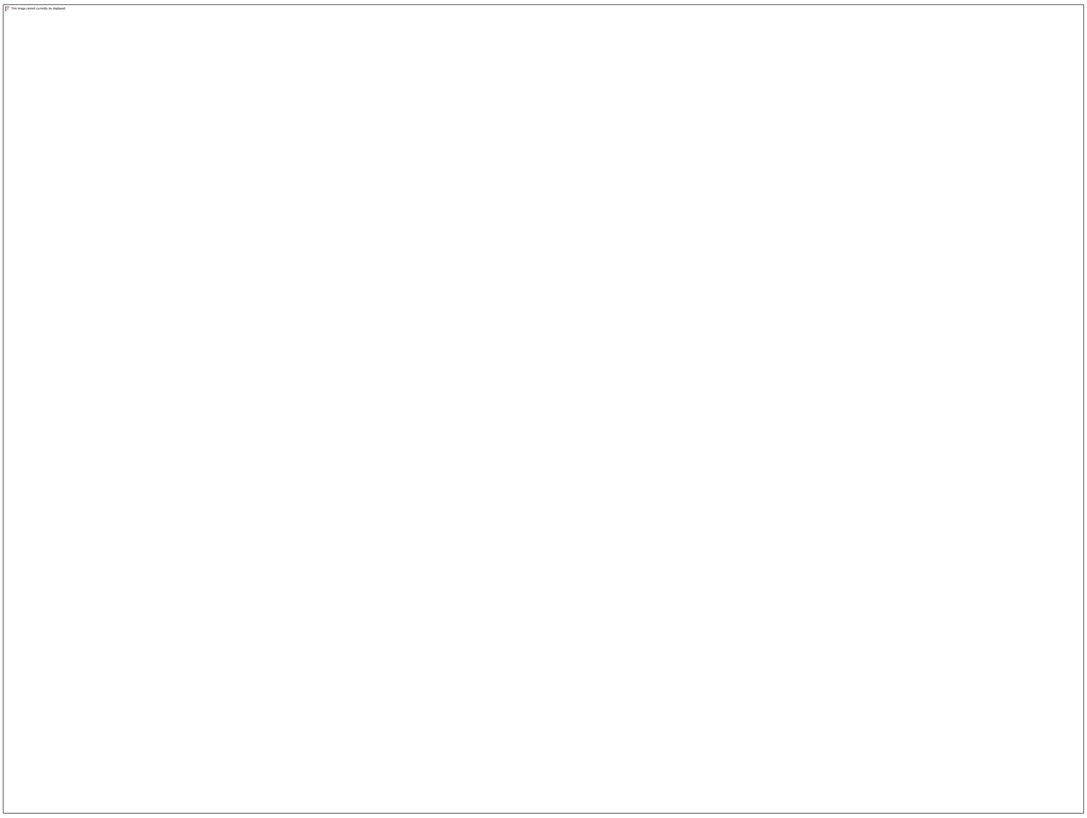

[Figure 2. Tumor is composed of small solid and ductal structures with comma-like geometric shapes.]

The pathological findings confirmed the diagnosis of syringoma.
Conclusion: Despite being a rare diagnosis, vulvar syringoma should be included in differential diagnosis of vulvar pruritus and multiple vulvar papular lesions. Exclusion of malignancy is mandatory in cases of postmenopausal pruritic lesions.
Disclosure: The authors declare no conflict of interest.

EP734

The value of omentectomy for surgical staging of borderline ovarian tumors

S. Maria[1], C. Mimoun[1], E. Marchand[1], M. Mezzadri[1], V. Place[2], F. Cornelis[3], C. Huchon[4], M. Perrin[1], J.-L. Benifla[1]
[1]Gynecologic Surgery, [2]Radiology, [3]Pathology, Lariboisiere Hospital, Paris, [4]Gynecologic Surgery, CHI Poissy/Saint-Germain-en-Laye, Poissy, France

Introduction / Background: Management of borderline ovarian tumors (BOTs) is surgical. The complete surgical staging includes exploration of peritoneal cavity and systematic peritoneal washing, omentectomy and random peritoneal biopsies. The systematic omentectomy is controversial in BOTs. The purpose of the study was to identify risk factors associated with omental involvement.
Methodology: This retrospective bicentric study included 70 patients with BOTs from December 2001 to April 2018. All patients underwent omentectomy (infracolic, infragastric or large biopsy) and pathological analysis of omentectomy was recorded in all cases.
Results: Sixty three patients (90%) were diagnosed at International Federation of Gynecology and Obstetrics (FIGO) stage I. Histologic subtypes were serous (62.9%), mucinous (32.9%) and endometrioid type (4.3%). Macroscopic disease in the omentum was suspected in one case, with a normal histological assessment. Occult omental disease was found in 6 cases (8.6%). In univariate analysis, omental involvement was significatively associated with presumed advanced FIGO stage (> IIB) (p=0.002), Cancer Antigen 125 (>100) (p=0.001), abnormal exploration of peritoneal cavity (p=0.003), peritoneal implants (p=0.00) and microinvasion (p=0.001). In multivariate analysis, omental involvement was significatively associated with presumed advanced FIGO stage (> IIB) and stromal microinvasion (p=0.015 and p=0.012, respectively).
Conclusion: In our study, microinvasion is an independent risk factor for omental involvement. Furthermore, patients with presumed advanced stage BOTs may benefit from systematic omental staging.
Disclosure: Nothing to disclose

EP735

Atypical Leiomyoma (AL) and smooth muscle tumors of uncertain potential (STUMP): a clinicopathological review on 9 cases

C. Martín Blanco[1], C. Del Valle Rubido[1], V. Corraliza Galan[1], B. Pérez Mies[2], E. Moratalla Bartolomé[1], M.C. Sánchez Martínez[1], J. Lázaro-Carrasco de la Fuente[1]
[1]Gynecology and Obstetrics Department, [2]Anatomical and Pathology Department, Ramon y Cajal Universitary Hospital, Madrid, Spain

Introduction / Background: The smooth muscle neoplasms of the myometrium are the most frequent gynecological neoplasms, most of which correspond to benign uterine leiomyoma.Only 1% are malignant tumors o leiomyosarcomas. There are variants of smooth muscle neoplasm in which the neoplasm presents typical histological aspects of malignant neoplasms but not all, consequently they

cannot be definitely classified as benign or malignant tumors and are considered as neoplasms with uncertain clinical behavior.Establishing the malignant potential of this type of tumors constitutes a challenge in clinical practice due to the low incidence of these variants.The aim of these study is to assess the clinicopathological aspects of uterine STUMP and atypical leiomyoma and to correlate with clinical outcomes.
Methodology: We analyzed al cases of AL and STUMP diagnosed between December 1999-February 2019
Results: A total of 9 cases were found, 4 labeled as STUMP and 5 as atypical leiomyoma. Mean age was 48.3 (range 31-78). Surgery was carried out in 88,8%, 4 cases underwent myomectomy and 4 hysterectomy. One patient refused surgical treatment after hysteroscopic diagnosis of atypical leiomyoma, performing uterine artery embolization. This patient developed a leiomyosarcoma 3 years later. The remaining 88.9% of cases continues asymptomatic.
Mean size of tumor was 7cm.100% of AL presented moderate cytologic atypia (60% diffuse and 40% focal), and no mitotic activity was found. STUMP cases showed, moderate atypia associated with low mitotic index (less than 5 per 10 hpf) in 50% and moderate (between 5-9 per 10 hpf). Protein p16 was found positive in all STUMP cases, and p53 in 2 of them.The mean follow-up time was 7 years (range 2 -120 months)
Conclusion: The paucity of data of this type of neoplasms makes it difficult to predict their clinical behavior. Although mostly benign, the ignorance of the probable clinical course, only allows to recommend the narrow follow-up to diagnose relapses
Disclosure: Nothing to disclose

EP736

Postoperative gastric perforation after cytoreductive surgery: an ischemic complication caused by spleen and omental resection

C. Martínez-Gómez, M.A. Angeles, M. Del, M. Voglimacci, E. Chantalat, A. Martinez, G. Ferron
Surgical Oncology, Institut Claudius Regard, IUCT Oncopole, Toulouse, France

Introduction / Background: Cytoreductive surgery (CRS) is the mainstay of some peritoneal malignancies. To obtain complete cytoreduction, extensive surgery with multiple visceral resections may be required. Gastric perforation is an infrequent complication and it has been associated to hyperthermic intraperitoneal chemotherapy (HIPEC), mainly explained by a multifactorial etiology.
Methodology: We designed a retrospective unicentric study identifying all patients who presented a gastric perforation after CRS from March 2007 to December 2018. All relevant clinical and surgical data were collected in a computerized medical database.
Results: Among the 858 cytoreductive procedures performed in our center, 12 gastric perforations (1.3%) were identified. HIPEC was performed in 4 out of these 12 patients. Infragastric omentectomy associated to splenectomy was carried out in all the patients presenting a gastric perforation. Median time to diagnosis was 5 days (range, 2-15). All patients underwent an emergency surgery and perforations were constantly found at the upper part of the greater gastric curvature. In 3 cases a gastropleural fistula was found, with a long and morbid management by thoracic surgeons until closure. Three patients among the 12 with a gastric perforation died during hospitalization at Intensive Care Unit (25%).
Conclusion: Gastric perforation is an infrequent complication after CRS probably due to ischemia at the upper part of gastric curvature caused by the ligature of gastroepiploic and short gastric vessels. This complication may occur independently of the performance of HIPEC and it should be managed with emergency surgery given its high morbimortality. A prophylactic suture of the upper part of the greater curvature in patients undergoing CRS with infragastric omentectomy and splenectomy might prevent this type of complication.
Disclosure: Nothing to disclose

EP737

Intraoperative Brachytherapy (HDR-IOBT) in advanced or recurrence gynecologic cancer: an effective alternative in tumors of poor prognosis

J.C. Muruzábal Torquemada[1], C. Tauste Rubio[2], M. Visus[3], S. Aguirre Gorospe[4], E. Villafranca Iturre[5]
[1]Gynaecologic Oncology, Complejo Hospitalario de Navarra, [2]Complejo Hospitalario de Navarra | Gynaecologic Oncology, Osasunbidea/ Navarre Health Service, [3]Radiotherpay, [4]Complejo Hospitalario de Navarra, [5]Radiotherapy, Complejo Hospitalario de Navarra, Pamplona, Spain

Introduction / Background: The aim of this study is to review our initial results in the treatment with surgery and Intraoperative Brachytherapy (HDR-IOBT) in advanced or recurrence gynecologic cancer.
Methodology: We included two patient groups: Group 1: advanced cervical cancer with poor response to Radiation and chemotherapy; group 2: pelvic recurrence of gynecological tumors of any origin. In all CT and MRI was performed, in recurrences also PET.
The surgical procedure depended on the extent of disease with pelvic exenteration, enlarged cystectomy and/or rectal resection. Surgical specimen was confirmed in all cases with intraoperative biopsy to ensure the absence of macroscopic residual disease. We put clips in the surgical bed. Subsequently the applicator Fleipbrup was placed according to the extension of the bed. After intraoperative planning, a dose of 10 Gy prescribed at 0.5 cm bed depth was administered. Finally, the applicator was removed to complete the surgery.
Results: Between October 2011 and December 2018, 29 patients have been included. In group 1, there were 14 patients with cervical cancer stage IIB in 9 p and IIIB in 6; in group 2 were 15 recurrences.
With a median follow-up of 35 months, the Local DFS 3 years was 59.3% Group 1: 74.5%; Group 2: 41.34%; 3 years Overall Survival 63.3%: Group 1: 70.3%, Group 2: 54%.
The complications were fistula 5p (17.2%): 1 intestinal, 4 bladder , sigmoid stenosis 2p (6.8%), osteitis pubis in 1p (3.4%). Ureteral stenosis 5p (17.1%)
Conclusion: HDR-IOBT associated with pelvic exenteration offers good results of pelvic control and overall survival in patients with gynecologic cancer with a poor prognosis. However the number of complications, especially fistulas has been important, so we must take it into account for prevention. These results are possible thanks to the multidisciplinary approach to these patients.
Disclosure: Nothing to disclose

EP738

Successful management of cervical ectopic pregnancy: a case report

M. Nakhaee
Resident Fellowship of Gynecology Oncology, Faculty of Medicine, Mashhad University of Medical Sciences, Mashhad, Islamic Republic of Iran

Introduction / Background: Cervical ectopic pregnancy is a rare condition with an incidence of less than 0.1% in all ectopic pregnancies. This life-threatening condition is associated with a high morbidity and mortality rates. Recently, the recommended protocol for the treatment of cervical ectopic pregnancy is fertility preservation rather than invasive surgery and hysterectomy. The aim of this report was to introduce a case of successful management of cervical ectopic pregnancy.
Methodology: A 31-year-old woman was presented with her third pregnancy with a history of one cesarean section and spontaneous abortion. She was admitted to an academic hospital with vaginal bleeding following 10 weeks of amenorrhea. Based on transvaginal ultrasound, a live fetus of about nine weeks was reported, located in the cervical canal. The β-subunit of human chorionic gonadotropin (βhCG) titer was reported as 108000 mIU/m.
Results: Cervical pregnancy diagnosis was consistent. In order to preserve fertility based on the patient's hemodynamic status, medical treatment and surgical intervention, including Methotrexate and then intravaginal ligation of cervical branches of uterine arteries, and subsequently cervical tampon was successfully performed.

Conclusion: The early detection and accurate diagnosis of cervical ectopic pregnancy using ultrasound and serial βhCG titer can be a valuable approach. Appropriate and conservative management has decreased the morbidity rate and preserved the ongoing fertility in affected patients.
Disclosure: Nothing to disclose

EP739

Significance of brain metastases in gynaecological cancer patients

A. Ogasawara, E. Katayama, M. Hanaoka, D. Shintani, M. Sato, S. Sato, M. Miwa, A. Yabuno, A. Kurosaki, H. Yoshida, K. Fujiwara, K. Hasegawa
Saitama Medical University International Medical Center, Saitama, Japan

Introduction / Background: Brain metastasis is the most common brain tumors. Brain metastasis is rare in gynaecological cancer, whereas it occurred in 10-30% in other types of cancer. Our aim is to clarify the clinical significance of brain metastasis and assess prognostic factors in those patients.
Methodology: Clinical data of the gynaecological cancer patients who had a brain metastasis were collected from medical records in our center between 2007 and 2019.
Results: We found 18 patients with gynaecological cancer had brain metastases. Nine, five, three and one patients had ovarian, endometrial, cervical cancer and uterine sarcoma, respectively. One, two, nine and six patients were stage I, II, III and IV, respectively. The most common hisotypes were serous and endometriod carcinoma. The median age at diagnosis of brain metastasis was 56.5 years (range 39-83), the median size of the metastasis was 31mm (range 10-70). Fourteen patients (77.8%) had solitary brain metastasis, and six patients (33.3%) had extracranial metastases. The most common part of brain metastasis was frontal part (38.9%) and secondary was cerebellum (27.8%). Three of 18 patients had carcinomatous meningitis. Headache (38.9%) was the most common symptoms, and only one patient appeared without symptom. Seven patients (38.9%) were treated with radiation therapy only and 10 patients (55.6%) were treated with combination therapy. The median time from initial treatment to brain metastasis was 17.5 months (range 0-67). The median overall survival after diagnosis of brain metastasis was 22.0 months (range 2-44).
Conclusion: Gynaecological cancer patients with headache had possibility of brain metastasis.
Disclosure: Nothing to disclose

EP741

100,000 genome project - the future of personalised medicine in gynaecological cancer management?

D. O'Neill, A. El-Ghobashy
Obstetrics and Gynaecology, The Royal Wolverhampton NHS Trust, Wolverhampton, United Kingdom

Introduction / Background: The 100,000 Genomes Project aims to improve cancer services through providing whole genome sequencing (WGS) of normal and tumour tissue in order to determine the genomic mutation responsible. This has the potential to radically change oncology services by offering personalised therapeutics to minimise side effects and optimise response to therapy. This study presents a single-centre experience of results of 'actionable mutations' following WGS and how this may alter subsequent management.
Methodology: Patients diagnosed with ovarian and endometrial malignancies were approached to take part in the project and consent gained. Blood and tissue samples were sent for WGS and the results were discussed within a multi-disciplinary meeting.
Results: Preliminary results from our cohort demonstrate that largely high grade serous ovarian cancers are associated with a somatic TP53 mutation, most frequently of frameshift variant. No

germline mutations were detected within the ovarian malignancies and there were no mutations in BRCA. The majority of endometrial cancers were not associated with a common underlying mutation; one endometrioid endometrial cancer was found to have a germline mutation of MSH2 in keeping with Lynch syndrome whilst one lady with serous endometrial cancer was found to have a somatic frameshift mutation in TP53, similar to those women with high grade serous ovarian malignancy.
Conclusion: Preliminary results suggest that there are no common mutations linking endometrial cancers making it unlikely that personalised medicine will be imminently achievable in the management of endometrial cancer. High grade serous ovarian malignancies are associated with somatic mutation in TP53 but this is yet to be correlated with alternative subtypes of ovarian malignancy and longitudinal studies are required to see how this impacts response to therapy and long-term outcome.
Disclosure: Nothing to disclose

EP742

Cancer genomic and personalized medicine for gynecologic tumors in our hospital

N. Oshima[1], K. Takahashi[1], R. Nakamura[1], Y. Yoshino[1], K. Wakana[1], S. Ikeda[2], N. Miyasaka[1]
[1]Obstetrics and Gynecology, [2]Cancer Center, Tokyo Medical and Dental University, Tokyo, Japan

Introduction / Background: Cancer genomic and personalized medicine has widely used globally and Department of Cancer Genomics was newly opened in our hospital in Tokyo, Japan. The aim of this study was to review the patients who were referred to the department for gynecologic cancers.
Methodology: Medical records of the patients who underwent cancer genome analysis for the gynecologic tumors between September 2017 and December 2018 were retrospectively reviewed.
Results: Ten patients were found including ovarian cancer (n=4), cervical cancer (n=3), peritoneal cancer (n=1), uterine sarcoma (n=1) and ovarian borderline tumor (n=1). All of the cases had completed standard therapy and showed treatment-resistant recurrences. Cancer genome analyses were performed by extracting DNA from the formalin-fixed and paraffin-embedded tumor tissue in 9 cases and from blood sample in one case. Among them, candidate medicines were found in 2 cases. In the other 8 cases, medicines for the targeted gene was under development and clinically unavailable.
Conclusion: This time, treatment was only performed in 2 out of 10 cases (20%). In many cases, the drug could not be reached even after the examination. It would be required to establish medical systems to access to the therapeutic agent without difficulty in the future.
Disclosure: Nothing to disclose

EP743

Abnormally high levels Of Ca19-9 - a mature cystic teratoma case report

G. Özten, E. Uyanık, E. Erçin, M. Gültekin, M.C. Salman
Obstetrics & Gynecology, Hacettepe University, School of Medicine, Ankara, Turkey

Introduction / Background: Mature cystic teratoma (MCT) is one of the most common benign neoplasms, accounting for 10-20% of all ovarian tumors. Several studies showed CA19-9 -associated with gastrointestinal adenocarcinomas, has been suggested as a potential marker in prediction of ovarian MCT. The relationship between CA19-9 elevation and clinical characteristics of MCT has not been well established.
Methodology: To the extent of our database search, we describe a case of MCT with the highest level of the serum Ca19-9 described in literature.
Results: 51 year old women presented with chronic abdominal pain to our clinic, a pelvic examination

and transvaginal ultrasound revealed a 78x59 mm multiloculated complex mass with echogenic areas arising from the left adnexa. The ovary was not visualised seperately from the mass. Abdominal examination was normal. Pre-operative CA-125 level was 23 U/ml (range 0-35 U/ml) and CA 19-9 level was 76.810 (range 0- 35 U/ml) respectively.
Salphingooopherectomy with midline laparotomy was planned. İntraoperatively a 10 cm torsioned adnexial mass was visualied. Oopherectomy specimen was sent to frozen section which revealed a mature cycstic teratoma. Extensive histologic sectioning confirmed the diagnosis. The patient's postoperative period was uncomplicated and Ca19-9 sample retained two days postoperatively was 39.882 U/ml.
Conclusion: Several retrospective studies mentioned CA 19-9 elevation appears to correlate with a larger tumor diameter and higher rate of ovarian torsion which corraletes with our case. Another retrospective study evaluating 215 patients with ovarian MCT concluded that serum CA19-9 levels were correlated with larger tumor size, although they failed to show a positive relationship between bilaterality and CA19-9 elevation.CA19-9 elevation was also correlated with the presence of fat component in the tumor at gross pathology findings. Unfortunately since the specimen was disected in frozen section and the cyst has a large sebum component we were not able to figure fat ratio.

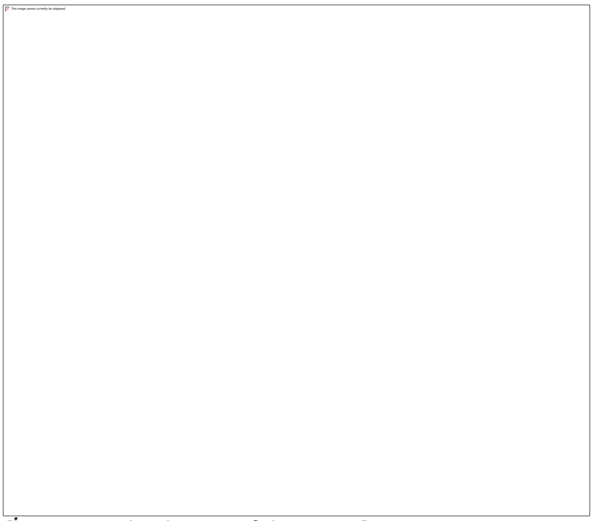

[İntraoperative image of the mass]

Disclosure: Nothing to disclose

EP744

Assessing the impact of clinicopathologic features and recurrence patterns of Uterine Carcinosarcomas: an institutional analysis

I.K. Papapanagiotou[1], N. Thomakos[1], K. Ntzeros[1], D.-E. Vlachos[1], D. Haidopoulos[1], M. Sotiropoulou[2], M. Liontos[3], A. Bamias[3], A. Rodolakis[4]
[1]1st Department of Obstetrics and Gynecology, [2]Pathology Department, [3]Department of Clinical Therapeutics, Oncology Unit, [4]1st Department of Obstetrics and Gynaecology, Alexandra Hospital, National and Kapodistrian University of Athens, Athens, Greece

Introduction / Background: Uterine Carcinosarcomas (UCs) are composed of both malignant epithelial and mesenchymal components, account for < 5% of all gynecological neoplasms and have a highly aggressive clinical course. The aim of this retrospective study was to evaluate the recurrence patterns as well as the clinical and histopathological characteristics of UCs.
Methodology: We retrospectively reviewed the clinical and pathology data from 46 patients who

underwent surgery between 2012 and 2018 and obtained a diagnosis of UCs. Data analysis included histologic subtype, grade, presence of homologous/heterologous elements, depth of myometrial invasion (MI), disease - free interval (DFI), site of recurrent disease, and overall survival (OS). Data was analyzed using Cox-regression and Kaplan - Meier survival analyses.
Results: The median age of our patient sample was 69.9 (range 35-91 years), and median tumor size was 6.4 cm. MI>50% was observed in 68.7%, LVI in 45.6%, and lymph node (LN) metastasis in 19.5%. 60.8% of the patients were diagnosed with stage I disease, 10.8% with stage II, and 28.2% with stage III disease. Serous carcinoma was the predominant carcinomatous component (56.5%), while heterologous elements (43.4%), particularly rhabdomyoblastic differentiation (RMB) (85%) was the most common sarcomatous component. 28% and 65% of patients received adjuvant radiotherapy or chemotherapy, respectively. Median DFI and OS were 10 and 16 months respectively. LN metastasis, tumor size>5cm, MI>50%, LVI, advanced stage and positive cytology correlated with shorter DFI and worse 3 year OS ($p< 0.5$ respectively). Recurrence developed outside the abdomen in 56.5%. Patients with tumors with sarcomatous histology upon recurrence and predominant sarcomatous component in primary tumor (≥50%) had shorter DFI ($p< 0.5\%$).
Conclusion: UCs represent a distinct subtype of uterine malignancy, where, according to our data, LN metastasis, tumor size, MI, stage and sarcomatous (RMB in particular) component have a significant prognostic role.
Disclosure: Nothing to disclose

EP745

Differences in autophagy-associated mRNAs in peritoneal fluid of patients with endometriosis and gynecologic cancers

H.R. Ju[1], S.I. Kim[1], T.S. Lee[2], S.H. Kim[3], S.G. Yeo[3], D.C. Park[1]
[1]Obgyn, St. Vincent Hospital, The Catholic University, Suwon, [2]Obgyn, SMG-SNU Boramae Medical Center, [3]Kyung Hee University, Seoul, Republic of Korea

Introduction / Background: Endometriosis and gynecologic cancer show similar patterns of invasion. Little is known about the roles of autophagy in endometriosis and, to date, the expression of autophagy-associated mRNAs has not been compared in patients with endometriosis and gynecologic cancers. This study therefore compared the levels of expression of autophagy-associated mRNAs in patients with endometriosis and gynecologic cancers.
Methodology: The levels of autophagy mRNAs, including those encoding mTOR, P13KC3, Beclin-1, Bcl-2, LC3 II, FLIP, Rubicon, BIRC2 and BIRC5, were measured by real time polymerase chain reaction in peritoneal fluid of 27 patients with benign masses (control group), 42 patients with endometriosis, and 43 patients with gynecologic (ovarian, uterine, and cervical) cancers. Findings in the three groups were compared.Autophagy mRNAs were present in all samples from patients with endometriosis and gynecologic cancers.
Results: The levels of PI3K, FLIP, and Rubicon mRNAs were significantly higher in the endometriosis than in the control group ($p< 0.05$ each). Compared with the gynecologic cancer group, the levels of LC3II and FLIP mRNAs were significantly lower, and the levels of Beclin-1 and Rubicon mRNAs significantly higher, in the endometriosis group ($p< 0.05$ each). Levels of PI3K and FLIP mRNA were significantly higher in the endometriosis and gynecologic cancer groups than in the control group ($p< 0.05$ each).
Conclusion: PI3K, FLIP, and Rubicon mRNAs are closely associated with the pathogenesis of endometriosis. The similar increases in PI3K and FLIP mRNA expression observed in patients with endometriosis and gynecologic cancer suggest that these conditions have similar autophagic characteristics. The lower levels of Beclin-1 mRNA in the gynecologic cancer than in other two groups suggest that lower Beclin-1 mRNA levels increase the likelihood of developing gynecologic cancer.
Disclosure: Nothing to disclose

EP746

Predictive factors for chemotherapy-induced nausea in patients with gynecological cancer

V.E. Di Mattei[1,2], P. Taranto[2], M. Mazzetti[2], L. Carnelli[2,3], M. Bernardi[4], P.M.V. Rancoita[1], M. Petrone[2], S. De Bastiani[2], A. Bergamini[2], F. Vasta[2], G. Mangili[2], M. Candiani[1,2]
[1]Vita-Salute San Raffaele University, [2]San Raffaele Hospital, [3]University of Milan Bicocca, Milan, [4]University of Parma, Parma, Italy

Introduction / Background: Chemotherapy-Induced Nausea (CIN) is a distressing side effect for patients undergoing chemotherapy, which deeply affects their quality of life and everyday activities. Several treatment-related and patient-related factors may be involved in the expression of CIN. This study aims to identify predictive factors for nausea after the first chemotherapy infusion in a sample of women with gynecological cancer.
Methodology: One hundred and sixty-five patients (mean age = 58.23, *SD* = 13.19) treated for gynecological cancer at the San Raffaele Hospital completed the MASCC Antiemesis Tool (MAT) in order to register the presence of acute (within 24 hours) and delayed (24-72 hours) nausea after the first chemotherapy infusion. Socio-demographic and clinical characteristics that could represent risk factors for nausea were collected with an *ad hoc* questionnaire designed by the research team. Logistic regression analyses were performed for predicting acute or delayed CIN; significance level was set at .05.
Results: Sixty-three (38.7%) patients experienced acute nausea after their first chemotherapy infusion; 46% (n=75) of the sample experienced delayed nausea. Neoadjuvant (vs. adjuvant) chemotherapy represents a protective factor for delayed CIN (OR=0.350; p=.033). Hyperemesis gravidarum and anticipatory nausea both represent risk factors for delayed nausea (OR=2.307, p=.026; OR=4.316; p=.004, respectively). No significant association was found with acute nausea.
Conclusion: Despite advancements in antiemetic therapy, results show that CIN still represents a common chemotherapy side-effect for women with gynecological cancer. Specific patient-related and treatment-related characteristics seem to influence the onset of delayed nausea after the first chemotherapy infusion. The knowledge of risk factors for CIN could help identify patients who are more vulnerable to this symptom and implement specific psychological and medical interventions in order to promote a better adjustment to the disease and its treatment.
Disclosure: Nothing to disclose

EP747

Short-term outcomes of systematic pelvic lymph node dissection in obese patients with gynaecological cancer in the low-volume cancer center

G. Psaras[1], H. Dudarovska[2], S. Voloshin[1], O. Kalinina[1], O. Logutov[1], L. Galla[1], M. Doroshenko[1], Y. Kovalova[1]
[1]Surgical, Mariupol Cancer Center, [2]Surgical, Maria Sklodowska-Curie Institute - Oncology Center, Mariupol, Ukraine

Introduction / Background: Systematic pelvic lymph node dissection (SPLND) is included in the treatment of oncogynecological operative procedure. The aim of this study was to determine short-term outcomes of SPLND in obese patients (pts) with gynaecological cancer in the low-volume cancer center.
Methodology: The analyses were done on a retrospective consecutive series of 109 obese pts with gynaecological cancer treated from January 2015 to December 2018. All pts underwent different kind of hysterectomy with SPLND. Pts were divided into two groups. The first group consisted of 61 pts with a BMI over 30,0, the second group consisted of 48 pts with a BMI less than 30.
Results: Postoperative mortality was not in both groups. Morbidity was 26.2±5.6% in the first group and 8.3±4.0% in the second. The duration of the operation was 201.0±14.1 min in the first group and 218.2±16.7 min in the second. Duration of the operation in pts with Class III obesity was 233.3±56.9 min. The duration of the operation after previously performed gynecological operations was

214.0±25.4 min in the first group and 235.0±34.2 min in the second. The number of lymph nodes removed was 21.5±2.1 in the first group and 23.1±2.9 in the second. In pts with Class III obesity, the number of lymph nodes removed was 19.0±3.6, while in pts with Class I obesity - 22.2±3.0. The duration of hospital stay did not differ in pts of the first and second groups generally, but was significantly higher (by 9 days) in pts with Class III obesity.
Conclusion: Obesity leads to an increase in postoperative morbidity, duration of the operation and an increase in hospital stay in pts underwent different kind of hysterectomy with SPLND.
Disclosure: Nothing to disclose

EP748

Laparoscopically guided TAP block for patients undergoing robotic assisted gynaecologic surgery - a prospective double blind Randomised trial to assess analgesic efficacy

A. Rajanbabu[1,2], N. Puthenveettil[3], A. Appukuttan[3], A. Ashok[3]
[1]Amrita Institute of Medical Sciences, [2]Amrita University, [3]Amrita Institute of Medical Sciences, Amrita University, Kochi, India

Introduction / Background: TAP block has been used regularly as part of multimodal analgesia for cesarean and lower abdominal surgeries. In this study, we are comparing the analgesic efficacy of laparoscopic-guided TAP block with port infiltration in patients undergoing robotic assisted surgery for gynecologic malignancies.
Methodology: This was a single institutional prospective double-blind randomized control trial. Approval was obtained from the hospital ethics committee and Clinical Trial Registry of India. 40 consecutive patients undergoing robotic-assisted surgery for gynecologic malignancy under general anaesthesia were included. Randomisation done by closed envelope technique. First group received bilateral TAP block (Group A) under direct laparoscopic vision with 15 cc of 0.1% ropivacaine on each side. Second group (Group B)received routine port site infiltration with 30 ml of 0.1% ropivacaine. Parameters measured were numeric pain score till 24 hours after surgery , post operative nausea and vomiting, mean arterial pressure (MAP), need for rescue analgesic and time to ambulation, first feed, discharge from recovery and hospital. Chi square test or Fischer's exact test were performed to compare categorical variable. To analyze quantitative data, student t test was used
Results: The pain scores were significantly lower for Group A (Fig 1). The use of rescue Analgesic was also less in group A compared to Group B (15 % vs 90 % respectively)with the difference being significant (p< .001). No complications were noted at the site of injection (Bleeding/haematoma). There was no difference in MAP, Post operative nausea and vomiting, time to ambulation , first feed and also discharge from recovery and hospital
Conclusion: Laparoscopic guided TAP block for patients undergoing robotic assisted surgery for gynecologic malignancies is effective in reducing post operative pain as shown by the reduced pain scores and decreased need for rescue analgesics. There were no increased complications due to the procedure
Disclosure: Nothing to disclose

EP749

A retrospective analysis of factors influencing the survival in patients with uterine Leiomyosarcoma

A. Rajanbabu, V. J Patel
Amrita Institute of Medical Sciences, Amrita University, Kochi, India

Introduction / Background: Leiomyosarcoma is most common subtype among all uterine sarcoma.

Aim of this study was to evaluate prognostic factors affecting oncologic outcome among patients of uterine leiomyosarcoma (uLMS).
Methodology: This was a single institutional retrospective analysis of all patients who underwent treatment for uterine leiomyosarcoma from January 2005 till December 2017. Details of demographics, clinicopathological factors, Intraoperative finding and oncological outcome were obtained with the help of electronic medical records. Stage of disease was assessed as per International Federation of Obstetrics and Gynecologists(FIGO) 2009 classification for sarcoma. Overall survival (OS) and disease free Survival (DFS) was assessed and factors affecting survival were looked into. Chi square test or Fischer's exact test were performed to compare categorical variable. Kaplan-Meier method was used to plot survival curves and to estimate time-to-event analyses
Results: From Jan 2005 to Dec 2017, 34 cases of uLMS were identified. Three cases excluded due to inadequate(< 1 year) follow up details & total 31 cases included in the analysis. Median follow up period was 67.3 months. Median OS & PFS for whole study group was 30.2(0-61) months and 20.3(12-28.5) months respectively. Significant survival advantage were found for stage 1 in comparison with stage 2,3 & 4 (68.5(37.5-99.4) months Vs 16.1(5.6-26.5) months, p=< 0.001). Patients with intact uterus removal experienced an significant better OS of 68.5(31.3-105) month while patients with intraoperative tumor rupture had OS of 12.4(6.1-18.6) months (p=0.02). PFS >15 months was associated with significant better survival [68.5(45-92) Vs 12.4(10.6-14.2), p=< 0.001]. We found more local recurrence in patients with intraoperative tumor capsule breech but was statistically insignificant .
Conclusion: 5 years survival rate for uLMS was 31%. Stage 1, Intact uterus removal intraoperatively and PFS >15 months were favourable prognostic factors for long term survival in uLMS patients.
Disclosure: Nothing to disclose

EP750

Experience of setting up a robotic surgery program for women with gynae cancers at a tertiary level cancer center in Eastern India

A. Roy, S. Wadhwa, J. Bhaumik
Gynaecological Oncology, Tata Medical Center, Kolkata, India

Introduction / Background: The beginning of any robotic program can be challenging as multiple members of the team are learning the technology and their own personal roles on the team.
Methodology: The demographic and perioperative data of the first thirty patients were prospectively recorded. Surgeries done by mentors were excluded. Only surgeries done independently by in house consultants were included. Sentinel lymphnode biopsies were done using ICG dye and near infrared imaging (Firefly).The data was analysed using SPSS software.
Results: 30 women were included with a median age of 60.5 years and median BMI of 26.4. Robotic hysterectomy + BSO + SLNB +/- PLND in 27 women with endometrial cancer. Two women with early cervical cancer underwent Robotic Type B hysterectomy + B/L PLND and one woman had robotic parametrectomy. Docking time ranged from 5 to 100 minutes (median 24.5 minutes). Console time ranged between 70 to 300 minutes (median 140 minutes). SLN mapping was done in 25 patient i.e 50 sites. Unilateral mapping were in 3 women and in another 5 there was no mapping in bilateral pelvis. Hence 37 sites were mapped out of 50 (74%). 18 women (60%) underwent B/L pelvic lymphadenectomy. All women were mobilised and started on diet at four hours from surgery. All women barring one were discharged within 24 hours of surgery. Histopathology revealed one patient with atypical hyperplasia (3.3%), one MMMT (3.3%), three SCC cervix (10%) and rest were endometrial cancer. Median SLN harvest was 2(range 1-9) and pelvic lymphnode yield was 12(range 9 - 45) per patient.
Conclusion: Robotic surgery provides definite benefits of minimal blood loss, excellent vision and dexterity and obviously minimal pain and shorter stay for the patients. It has a short learning curve. Adequate training and team formation are pivotal towards establishing a successful robotic program.
Disclosure: Nothing to disclose

EP751

Cancer in the elderly: do we treat them in the same way?

I. Sá[1,2], R. Ana Carolina[1,3], S. Raposo[1], R. Sousa[1], P. Correia[1], P. Aldinhas[1], E. Castelo Branco[1], C. Coelho[1], V. Baltar[1], L. Sá[1]
[1]Instituto Português de Oncologia Franscisco Gentil, Coimbra, [2]Centro Hospitalar de Trás-os-Montes e Alto Douro, Vila Real, [3]Hospital Distrital de Santarém, Santarém, Portugal

Introduction / Background: The increase of life expectancy is leading to an intensification of number of cancers in older populations. These patients should be managed according to their health status and not only to their age. The aim of this study was to analyse the incidence of cancer in women older than 80yrs and its impact on survival.
Methodology: A retrospective study was conducted, including 177 patients over 80yrs, referred to our department with gynaecological cancer, between 2013-2018, (6,6% of total cases referred in the same period). We collected data about oncological disease, including stage, treatment modalities, disease related survival and mortality.
Results: The average age was 83yrs: 70,0% < 85yrs; 22,1% between 85-90yrs and 7,8% >90yrs. The most frequent cancers were breast cancer (59,9%) and endometrial cancer (17,1%). The other cases were cervical(7,8%), vulvar(7,4%) and ovarian cancer(5,5%), 1 vaginal and 1 tubar cancer. For breast and endometrial cancer, the most frequent stages were stage II and stage IB, respectively. 63,1% of the patients had surgery as a primary treatment; 5,5% had neodjuvant treatment, 51,6% had adjuvant treatment and 26,3% were directed to palliative care. 16 women had recurrent disease. 18,8% died from oncologic disease and 8,9% died from others causes. There were not statistically significant differences in mortality among the age groups (p=0,98).
Conclusion: A high number of cancer cases was observed in older women. Although there is no standard of care for elderly patients, advanced age shouldn't be considered a limitation to treatment. Surgery is an important treatment modality in these age groups, who sometimes haven't conditions for systemic treatment.
Disclosure: Nothing to disclose

EP752

Medical treatment of uterine arteriovenous malformations: a case report

S. Saied
Mashhad University of Medical Sciences, Mashhad, Islamic Republic of Iran

Introduction / Background: Uterine arteriovenous malformation (AVM) one of the complications of cesarean section and uterine curettage which is life-threatening.
The aim of this case report was to assess medical treatment of a symptomatic case of AVM as uncommon cause of vaginal bleeding.
Methodology: A 24-year-old female with an episode of heavy vaginal bleeding after curettage of pregnancy first trimester was referred to Ghaem Hospital of Mashhad University of Medical Sciences in 2014. She had a history of elective caesarian section in first pregnancy. Based on clinical findings and color Doppler ultrasonography, uterine AVM was diagnosed and modality conservative medical management was arranged.
Results: She was successfully treated with GnRH analogs and she had regular and normal menstrual cycles in her 1 year serial follow-up. Now, she has spent three months of pregnancy after 15 months of her disease.
Conclusion: Diagnosis of uterine AVM should be considered in the case of massive uterine vaginal bleeding and a history of prior uterus surgery. The gonadotropin-releasing hormone agonist is useful in this situations.
Disclosure: Nothing to disclose

EP753

Single centre experience with anti-PD1 therapy in gynaecologic cancers

B. Ozdemir[1], C. Dromain[2], A. Sarivalasis[1]
[1]Center of Gynecological Tumors, Department of Oncology, [2]Direction de Radiodiagnostic et Radiologie Interventionnelle, CHUV - Centre Hospitalier Universitaire Vaudois, Lausanne, Switzerland

Introduction / Background: The prognosis of women with gynaecologic malignancies which progress after standard oncological treatment remains poor. While immune checkpoint inhibitors targeting the PD1/PDL1 pathway have provided durable responses in various tumour types, their use in gynaecologic oncology is an area of active research.
Methodology: We retrospectively analysed data from women with advanced or metastatic ovarian carcinoma, cervical cancer or endometrial cancer treated within a compassionate program with nivolumab or pembrolizumab between March 2015 and January 2019 at Lausanne University Hospital. The efficacy endpoint was best overall response rate by RECIST 1.1.
Results: Patients had a mean age of 63 years (range 46-79 years). We included in total 11 patients, with high grade serous ovarian adenocarcinoma (n=4), squamous cell carcinoma of the cervix (n=3), cervical adenocarcinoma (n=1), basal adenoid cervical carcinoma (n=1) endometroid endometrial cancer (n=1) and serous endometrial cancer (n=1). All patients received nivolumab 240 mg every 2 weeks, except one who received pembrolizumab 200mg every 3 weeks and 20 mg lenvatinib daily. Ovarian cancer patients were treated in fourth line or beyond, cervical cancer and endometrial cancer patients in third line. The mean number of therapy cycles was 7 for ovarian cancer, 9 for cervical cancer and 5 for endometrial cancer.
The best overall response was partial response in cervical cancer (n=3) and ovarian cancer (n=1). Stable disease was found in one patient with cervical and ovarian cancer, respectively. Endometrial cancer patients did not respond.
Conclusion: In our small cohort of unselected gynaecologic cancer patients treated with anti-PD1 antibodies after failure of standard of care treatments, response rates were highest among cervical cancer patients. Differences in tumour immunogenicity such as PD-L1 expression, human papilloma virus infection, microsatellite instability and POLE mutations may affect response rates.
Disclosure: Nothing to disclose

EP754

Expression of OCT4, Sox2 / 3 self-renewal genes and gankyrin oncogene in patients with ovarian cancer

N. Sharifi
Department of Mashad Medical University, Mashhad, Islamic Republic of Iran

Introduction / Background: The aim of this study is to investigate the expression of Sox2/3, gankyrin and oct4 biomarkers in tumoral tissues and their comparison with normal and benign cases to clarify the biological role of these biomarkers in emergence and progress of cancer for ovarian carcinoma diagnosis and examination of these biomarkers in different degrees of the disease and different clinicopathological types of this cancer.
Methodology: In this regard, the staining percentage and severity, as well as nuclear, cytoplasmic, epithelial and stromal coloration, were investigated based on clinicopathological and histological grade of tumor in 77 paraffin embedded sampeles from 77 cases of various epithelial ovarian lesions from the pathology archives of Ghaem Hospitalfrom 2015 to 2017 in the form of targetednon random sampling. The results were presented as mean and percentages in the form of tables. Data processing

was done by SPSS-22 software. For statistical analysis, Chi-square or exact Fisher testswere used with a significant level of P-value≤ 0.05.
Results: Overall, concerning gankyrin biomarker, a positive and significant relationship was found between stainability percentage, epithelial, nuclear and cytoplasmic stainability and malignancy (in the study serous adenocarcinomas).
In the case of SOX2-3 biomarker, there was a significant correlation between the stainability percentage and epithelial stainability and ovarian carcinoma.
In the case of OCT-4 biomarkers, there was a significant relationship between stainability percentage and cytoplasmic stainability and ovarian carcinoma.
Conclusion: Regarding the above-mentioned results, gankyrin biomarker is suitable for early diagnosis and prognosis of ovarian carcinoma and should be considered in future studies.
In general, according to the results of this study, more studies are required to select SOX2/3 and OCT-4 biomarkers for early diagnosis and prognosis of ovarian carcinoma, and their stainabilitypercentage is the most appropriate option for this biomarker according to this study.
Disclosure: Nothing to disclose

EP755

Laparoscopically modified laterally extended endopelvic resection for gynecological malignancies involving pelvic side wall: a feasibility analysis

G. Sozzi[1,2], A. Abbate[1], M.C. Di Donna[3], G. Cucinella[3], N. Ceresi[3], G. Lo Balbo[3], N. Buono[3], G. Scambia[4], V. Chiantera[1,2]
[1]Department of Gynecologic Oncology, ARNAS Civico Hospital, [2]University of Palermo, [3]Department of Gynecologic Oncology, University of Palermo, Palermo, [4]Department of Women's and Children's Health, Catholic University of the Sacred Heart, Roma, Italy

Introduction / Background: Lateral pelvic side wall involvement by gynecological malignancies has been considered for a long time an absolute contraindication to curative resection. Recently the surgical paradigm is challenged with the development of the Laterally Extended Endopelvic Resection, LEER. Although the LEER is perfectly standardized in open surgery, just one small experience has been published about its endoscopic feasibility.The objective of this study is to analyze the feasibility of Laparoscopically Modified Laterally Extended Endopelvic Resection, LM-LEER, in patients with gynecological malignancies involving the lateral pelvic side wall.
Methodology: We retrospectively evaluated a consecutive series of women submitted, between July 2014 and November 2018, to LM-LEER for gynecological tumors involving the pelvic side wall. Surgical and post-operative data were analyzed.
Results: Overall, 39 women received LEER due to gynecological cancer infiltrating the pelvic side wall, of them 18 (46.2%) were eligible for laparoscopic approach. No laparotomic conversions were registered. In all patients R0 resection was achieved with negative margins of surgical resection at final histological evaluation. The median operative time was 415 min (285-615 min), estimated blood loss was 285 ml (100-600 ml) and the median length of hospital stay was 10 days (4-22 days). There were three (16.7%) intraoperative complications, all managed laparoscopically. In two (11.1%) cases a second surgical exploration was required, one for hemoperitoneum and another for vaginal cuff dehiscence.
Conclusion: LM-LEER can be safely performed in carefully selected patients with gynecological malignancies involving the lateral pelvic side wall, in particular for those in which a bladder and rectal sparing surgery appears possible. Further larger prospective trials are needed to evaluate the oncological and the long-term functional outcome of patients undergoing LM-LEER.

Variable	Nr
All cases	18

Median operating time (min, range)	415 (285-615)
Intraoperative median blood loss (ml, range)	285 (100-600)
Median number of blood transfusion (range)	0 (0-2)
Intraoperative complications	3 (16.7%)
Median length of Hospital stay (days, range)	10 (4-22)
Median major post-operative complications	6 (33.3%)

[Surgical and perioperative outcomes.]

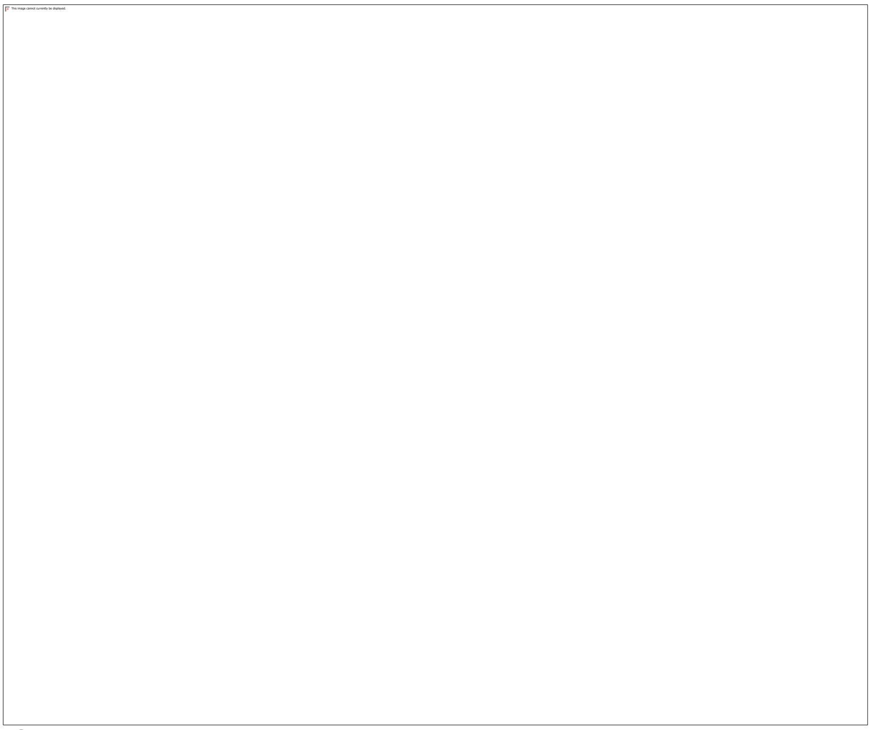

[Surgical specimen of the maximum version of LM-LEER]

Disclosure: Nothing to disclose

EP756

Laterally extended endopelvic resection: a comparative analysis between laparotomic and laparoscopically modified approach

G. Sozzi[1,2], I. Pitruzzella[1], M. Petrillo[3], V. Giallombardo[4], G. Cucinella[4], M.C. Di Donna[4], M. Ferreri[4], N. Buono[4], G. Scambia[5], V. Chiantera[1,2]
[1]Department of Gynecologic Oncology, ARNAS Civico Hospital, [2]University of Palermo, Palermo, [3]Department of Obstetrics and Gynecology, University of Sassari, Sassari, [4]Department of Gynecologic Oncology, University of Palermo, Palermo, [5]Department of Women's and Children's Health, Catholic University of the Sacred Heart, Roma, Italy

Introduction / Background: Laterally Extended Endopelvic Resection (LEER) has been identified as a valid therapeutic option for women with gynecological malignancies involving the pelvic side wall.

The historical approach to LEER is laparotomic, recently we have proposed the so called Laparoscopically Modified Laterally Extended Endopelvic Resection (LM-LEER).The objective of this study is to compare surgical and oncological outcomes between LEER and LM-LEER in a consecutive series of patients with gynecological malignancies infiltrating the pelvic side wall.
Methodology: We retrospectively evaluated women submitted to LEER between October 2012 and January 2019. Inclusion criteria for LM-LEER were tumor size < 10 cm and Body Mass Index < 35. Peri-operative data were analyzed and compared. Recurrence-free survival (RFS) and overall survival (OS) were calculated using the Kaplan- Meier method.
Results: Of the included 39 patients,18 were submitted to LM-LEER and 21 to LEER. Median operative time (425 vs 600 min, p < 0.01) and median blood loss (285 vs 865 ml, p < 0.01) were shorter in the laparoscopic group. Complete tumor resection was achieved in all the patients submitted to LM-LEER and in 90.4% of those submitted to LEER. No laparotomic conversions were observed during LM-LEER. Intra and post-operative complication rate was similar. Median length of hospital stays (10 vs 17 days, p < 0.01), as well as median time to initiation of adjuvant therapies (38 days vs 54, p: 0.20) was shorter in the laparoscopic group.After a median follow-up of 19 months no differences in terms of RFS (p: 0.95) and OS (p: 0.72) were observed in the two groups.
Conclusion: After strict selection, a group of patients with gynecological tumors infiltrating the pelvic side wall may undergo LM-LEER with extremely high rates of complete tumor resection, satisfactory perioperative morbidity, a short interval to adjuvant therapies and encouraging survival outcomes.

Variables	Laparoscopy (N)	Laparotomy (N)	p-value
All cases	18	21	NA
Median operative time (min, range)	415 (285-615)	600(350-900)	<0.01
Median intraoperative blood loss (ml, range)	285 (100-600)	865 (200-2000)	<0.01
Median number of intraoperative blood transfusions (range)	0 (0-2)	2 (0-6)	<0.01
Complete tumor resection	18 (100.0%)	19 (90.4%)	0.18
Intraoperative complications	3 (16.7%)	5 (23.8%)	0.29

[Surgical details]

Variables	Laparoscopy (N)	Laparotomy (N)	p-value
All cases	18	21	NA
Median hospital stays (days, range)	10 (4-22)	17 (5-45)	<0.01
Major post-operative complications (upper than G1)	6 (33.3%)	13 (61.9%)	0.08
Median follow up time (months, range)	16 (2-36)	22 (3-49)	0.18
Median time to initiation of adjuvant therapies (days, range)	38 (26-65)	54 (40-120)	0.20
Recurrence	7 (38.8%)	9 (42.8%)	0.83
Death of disease	6 (33.4%)	10 (47.6%)	0.36
Disease free survival (months, range)	13 (2-31)	20 (3-66)	0.18
Overall survival (months, range)	16 (2-36)	22(3-49)	0.14

[Post-operative and survival details]

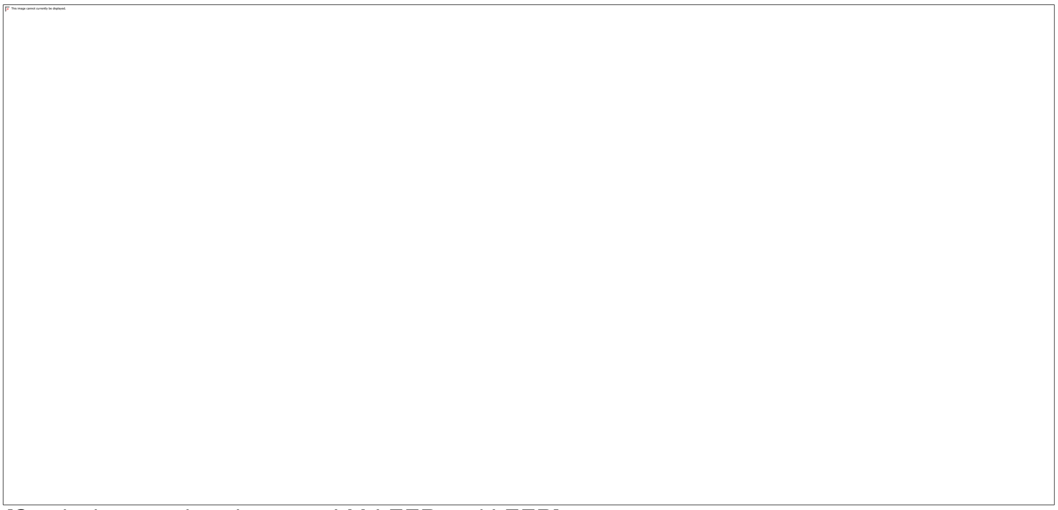

[Survival comparison between LM-LEER and LEER]

Disclosure: Nothing to disclose

EP758

Comparison of outcomes with ileal and colonic incontinent urinary conduits in and irish gynaecological oncology centre

S. Varghese, N. Gleeson, C. Thompson
St James's Hospital / Trinity College Dublin, Dublin, Ireland

Introduction / Background: A comprehensive tertiary gynaecological surgical oncology service includes urinary diversion as part of exenterative surgery and to treat fistulae. Many patients have received pelvic radiotherapy that can adversely and progressively affect the small bowel over a lifetime. As a result there has been a move to colonic conduits in the expectation that there may be less long term adverse sequelae.
Methodology: Retrospective review of all patients undergoing incontinent conduit formation from Jan 2011 - Dec 2017. Major adverse outcomes (MAO) were categorised as immediate (< 4weeks), short term (< 6 months) or long-term (>6 months) following surgery.
Results: Of 27 patients, 18 (66%) had isolated colonic segment and 9 (33%) ileal segment fashioned as urinary conduit.
Immediate MAO: Ureteric anastomotic leak (1 ileal, 0 colonic conduits); Re-laparotomy for adhesional small bowel obstruction (0 ileal, 2 colonic conduits).
Short term MAO: Ureteric obstruction requiring ureteric stenting/nephrostomy (4 ileal, 3 colonic conduits).
Long term MAO: Stomal stenosis (2 ileal, 0 colonic conduits); Recurrent sepsis requiring IR/surgical intervention (3 ileal, 1 colonic conduit); Interval small bowel obstruction (0 ileal,3 colonic conduits); Nephrectomy (1 ileal, 0 colonic conduits).
Conclusion: The absence of anastomotic leaks and stomal stenosis and lower rates of ureteric obstruction even in the short-term (17% vs 44%) with colonic conduits support the view that a colonic segment is less likely to be compromised by prior radiotherapy and is therefore the better choice for urinary diversion in gynaecological cancer care. However, the rate of adhesional small bowel

obstruction was substantial with colonic conduits and this requires further analysis. Retrospective review is compromised by patient selection and operator choice. For now, our centre recommends colonic urinary conduit as standard of care.
Disclosure: Nothing to disclose

EP759

Does bilateral salpingo-oophorectomy influence prognosis of uterine leiomyosarcoma

D. Trojnarska, M. Zygmunt, G. Köhler
Department of Obstetrics and Gynaecology, University Medicine Greifswald, Greifswald, Germany

Introduction / Background: Adequate therapy of uterine leiomyosarcoma (LMS) is total hysterectomy. However, there is little information about the influence of bilateral salpingo-oophorectomy (BSO) on prognosis. This study analysed its influence on survival data.
Methodology: Kaplan-Meier analysis and 5-year-overall survival probability depending on BSO and menopausal status in operated LMS.
Results: From 294 patients with LMS 176 (59,9 %) were postmenopausal. A BSO was performed additionally to total hysterectomy in 167 (56.8%) cases. In all patient's overall survival (OS) with BSO was shorter than in those without BSO but not statistically significant. Irrespectively of this procedure patients with BSO had primary a significantly worse 5-year-OS probability of 40 vs. 47 months (p=< 0.01). In premenopause the OS was identical with and without BSO (fig. 1). In postmenopause the OS was significantly worse with BSO (p=< 0.046. (fig. 2).
But, also irrespective from performed BSO the analysed OS is generally worse in postmenopausal vs. premenopausal patients. Additionally, the primary (!) 5-year-OS probabilitywas shorter than in premenopause (42 vs. 46 month). It also needs to be considered, that in postmenopause BSO was performed in 36 % in stage pT2 and higher vs. 24 % in premenopause. So, the worse OS in postmenopause depends not on BSO. In conclusion the preservation of ovaries in postmenopause does not improve prognosis.
Conclusion: BSO does not influence prognosis in premenopausal patients. So, BSO in premenopause is not necessary if the ovaries are free of disease. In postmenopause the prognosis is generally worse than in premenopause and depends not on BSO.

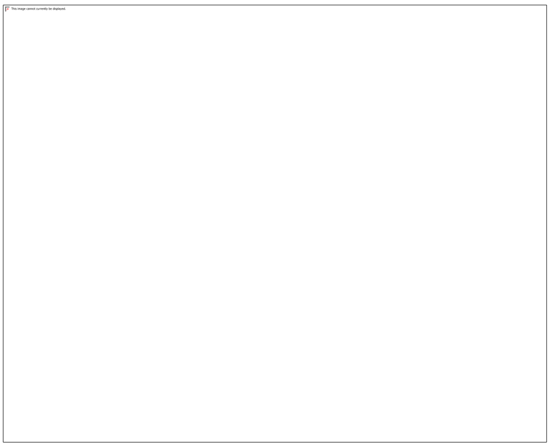

[BSO - Premenopause]

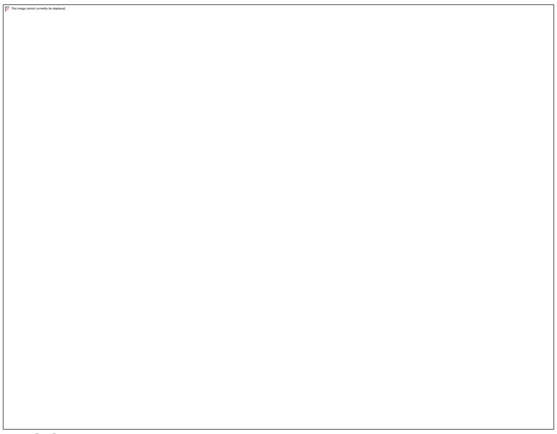

[BSO - Postmenopause]

Disclosure: Nothing to disclose

EP760

Vulvar sarcoma - rare malignancy with favorable prognosis in case of timely diagnosis and treatment

E. Ulrikh[1,2], K. Safronova[1,2], K. Guseynov[1], O. Smirnova[1], A. Sidoruk[1,2], I. Berlev[1,2], N. Mikaya[1], A. Urmancheeva[1,2]
[1]National Medical Research Center of Oncology named after N.N.Petrov, [2]North-Western State Medical University named after I.I.Mechnikov, Saint-Petersburg, Russian Federation

Introduction / Background: Vulvar sarcomas are rare low genital tract malignancies accounting less then 1-3% of all vulvar cancers
The purpose of the study was to assess clinical and morphological features of vulvar sarcomas.
Methodology: All the cases of Vulvar sarcomas treated in the National Medical Research Center of Oncology named after N.N.Petrov were detected during the period 2007-2017.
Results: Through the 10-year period 6 cases of vulvar sarcomas were detected from 251 cases of vulvar malignancies. Five cases were presented by leiomyosarcomas, one case by Dermatofibrosarcoma protuberans. The age of patients with leiomyosarcomas varied over a wide range, 28-85 years, averaging 55 years. The patient with Dermatfibrosarcoma was 63 years old. Four patients with leyomiosarcomas had Stage I disease (the size of the tumor less then 5 cm) only one patient had II stage of disease. No presence of regional node spread was detected. All the patients were surgically treated (vulvectomy/hemivulvectomy), one patient received radiotherapy.
Patient with Dermatofibrosarcoma protuberans stage I was CD34 positive, while EMA, SMA, MSA, Desmin - negative, with Ki67-2%. After surgical treatment in 2014 she is alive without signs of disease. All the patients with Ist stage of leiomyosarcomas were alive through the 5-year period of follow-up. Three of them had no signs of disease through this period of follow-up. One patient had local recurrence surgically treated. One patient with stage II disease experienced repeated local recurrences after initial surgical treatment with secondary surgical treatment. Distant pulmonary recurrence occurred after 21 months after initial treatment successfully treated by local resection and chemotherapy. The patient is stable after 49 months of follow-up after initial treatment.
Conclusion: Vulvar sarcomas are rare malignancies with favourable prognosis in case of timely diagnosis with main management option - surgical treatment.
Disclosure: Nothing to disclose

EP761

Incidentally detected intravenous leiomyomatosis limited to uterus: a case report

E. Uyanık, G. Özten, O.C. Zaim, M. Gültekin, M.C. Salman
Hacettepe University, School of Medicine, Ankara, Turkey

Introduction / Background: Uterin leiomyoma is a benign smooth muscle neoplasm originates from myometrium and the most common pelvic tumor. Among leiomyoma variants, intravenous leiomyomatosis (IVL) is a benign tumor originated from smooth muscle and proliferating within the uterin or extrauterin vessels. Tumor can spread to iliac veins, inferior cava vein, heart or pulmoner arteries via uterin veins. Early diagnosis is difficult and unless cardiac spread occurs, it is asymptomatic. Besides symptoms such as dispnea, pleuretic chest pain, and syncope, it can even lead to cardiac arrest and without treatment it can be fatal.
Methodology: In this case report patient with IVL is presented.
Results: A 54 year old postmenaposal women presented to our clinic with abdominal pain. A mass extending to umblicus is detected with abdominal examination. On ultrasound examination, uterus was larger than usual and filled with myomas. Total abdominal histerectomy and bilateral salpingooferectomy was performed. Pathology reported IVL and thoracal and abdominal tomography was performed to evaluate extrauteine vessels and heart for invasion. In CT, there wasn't leiomyomatosis in venous system and heart and patient discharged.
Conclusion: IVL, even though rare, is a a serious disease that can be fatal. Although disease was diagnosed after pathological evaluation in our case, imaging is essential in diagnosis. Tumor spreading to big vessels, heart or pulmoner arteries can cause severe symptoms like dispnea, syncope and aritmias. Today, surgery is the only effective treatment method and tumor must be completely excised to prevent recurrence. Excision of fibroids only may be recommended in patients with fertility desire, but total excision of pelvic structures is more accepted due to the risk of recurrence. Although originally benign, it should be kept in mind that the disease may cause life threatening complications and therefore patients should be kept under close monitoring.
Disclosure: Nothing to disclose

EP762

Analysis of complications during and after laparoscopic lymphadenectomy by gynecologic malignancies

J. Vasiljeva[1,2], C. Epding[2], M. Mangler[2,3], M. Lanowska[1,2]
[1]KAU; Department of Gynecology and Obstetrics, Vivantes Netzwerk für Gesundheit GmbH, [2]Charité Universitätsmedizin, [3]AVK; Department of Gynecology and Obstetrics, Vivantes Netzwerk für Gesundheit GmbH, Berlin, Germany

Introduction / Background: Lymphadenectomy is an integral part in the management of women with gynecologic malignancies. Pelvic and para-aortic lymphadenectomy is used for staging propose and, in some cases, it is presumed to have therapeutic effect. Surgical technique to perform lymphadenectomy using a laparoscopic approach is still not uniform and keeps being criticized. The purpose of this study was to evaluate our own experience of laparoscopic pelvic and para-aortic lymphadenectomy for gynecologic cancers.
Methodology: We performed retrospective analysis of 144 patients, who were treated in Campus Charité Mitte at the Department of Gynecology between January 2011 and Mai 2015. The pelvic and / or para-aortic lymphadenectomies with or without other operative procedures were performed by five experienced oncology surgeons.
Results: The median age and body mass index (BMI) of the patients were 48 years (range 26-81 years) and 23.9 kg/m2 (range 18-46 kg/m2). All of lymphadenectomies were performed laparoscopically and no conversion to laparotomy was needed. Intraoperative complications occurred in 10 patients (6.9% of cases): five bowel lesions, two urethral injuries, two vascular injuries and one anesthesiologic complication. Early postoperative complications (six weeks after the operation)

occurred in 83 women (57.6% of all patients). There was only one complication - in 46 cases, two in 25 cases, tree in 9 cases and five complications in 3 cases. According to Clavien-Dindo Classification 130 of documented complications (94.2%) were grade I or II and only 8 complications (5.7%) ³grade III. The patients without any complications were 45.3 years old and patients with at least one early complication 50.8 years (p=0.03). BMI, and previous surgeries did not influence on occurrence of intraoperative or early postoperative complication rates.
Conclusion: Laparoscopic lymphadenectomy can still be considered a technically feasible and safe procedure for staging gynecologic malignancies despite BMI, age and previous surgeries as mentioned in earlier studies.
Disclosure: Nothing to disclose

EP763

Primary extraskeletal ewing sarcoma in the retzius space mimmicking adnexial mass: a rare tumour in an uncommon location

C. Yalcinkaya, H.H. Gokaslan, G. Bagran, I. Erbarut Seven, K. Turkoz
Faculty of Medicine, University of Marmara, İstanbul, Turkey

Introduction / Background: Ewing´s sarcoma/primitive neuroectodermal tumor (ES/PNET) family of tumours are rare group of malignant neoplasms. Commonly, they arise from skeletal system, but also they can arise from extraskeletal tissues.
Methodology: A 19-year-old nulliparous female admitted to our outpatient clinic with low abdominal pain. Abdominal ultrasound and MRI findings were revealed an adnexial solid-cyctic mass mimmicking an ovarian neoplasm. After a thorough diagnostic laparatomy; a 14 cm mass arising in the Retzius space with normal internal genitalia were observed. The tumour was firmly adhered to the bladder, urethra and other adjacent tissues. Total resection of the tumour was performed. The frozen section revealed small round blue cell tumour. The patient discharged in a good conditon on the post operative fifth day and she underwent adjuvant chemotheraphy with VAC/IE (vincristine, doxorubicin, cyclophosphamide alternating with ifosfamide, and etoposide). Immunohistochemical analysis revealed negative staining for the CD45, pancytokeratin, chromogranin, CD56, WT1, desmin, myogenin, myoD1, and diffuse positive staining for the CD99. Fluorescence in situ hybridization confirmed EWSR1 gene translocation in the 81.9% of tumour cells. Based on these histopathological, IHC and FISH findings, an extraskeletal Ewing's sarcoma was diagnosed.
Results: Here, we report a case of Ewing sarcoma of the Retzius space treated by surgery and adjuvant chemotheraphy. To the best of our knowledge, this extremely rare clinical condition is the first reported case in English literature.
Conclusion: Extraskeletal Ewing's sarcoma in the Retzius space is extremely rare. It should be included in the differential diagnosis if frozen section reveals small round blue cell tumours. Immunohistochemical analysis and FISH could help to diagnose this rare clinical situation.

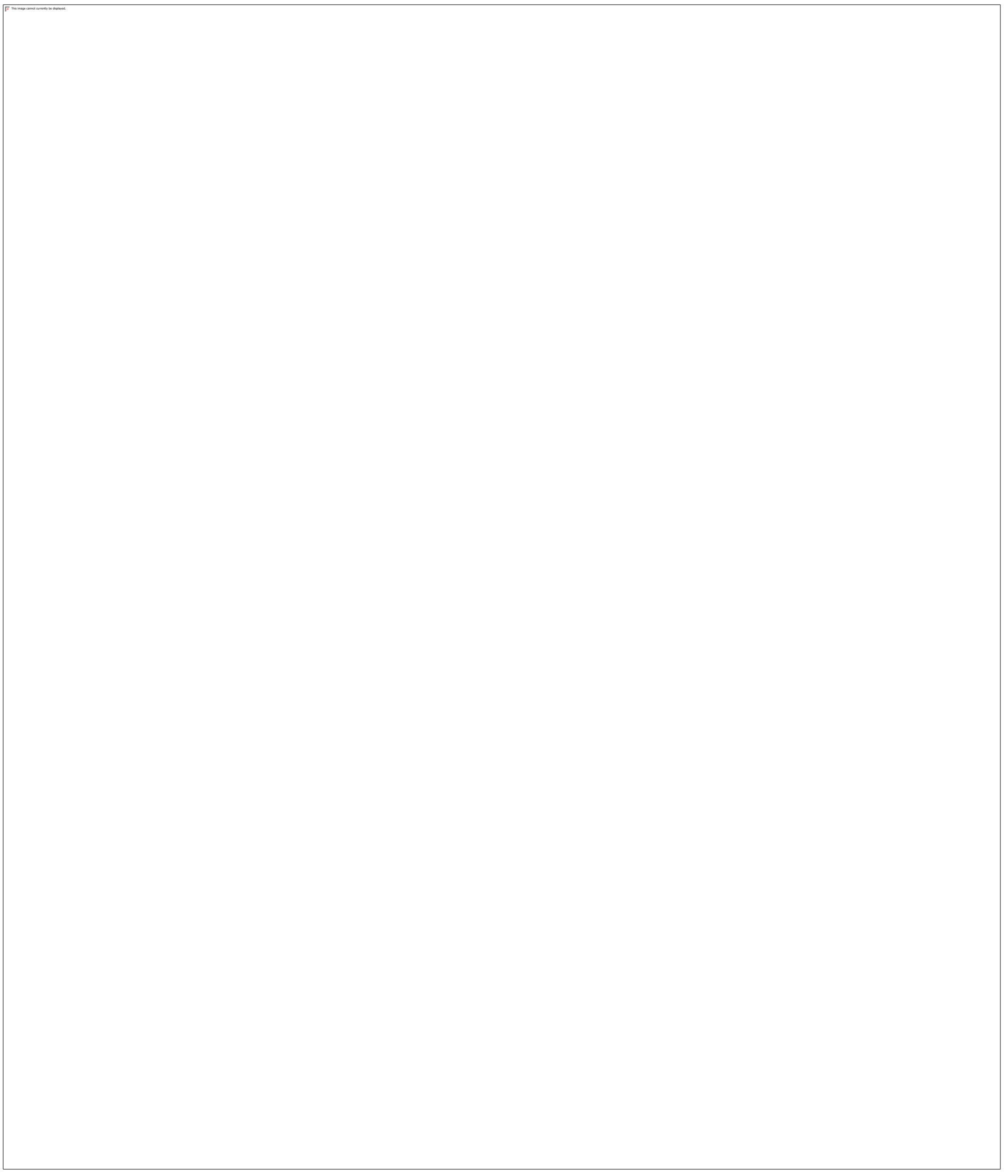

[Ewing MR]

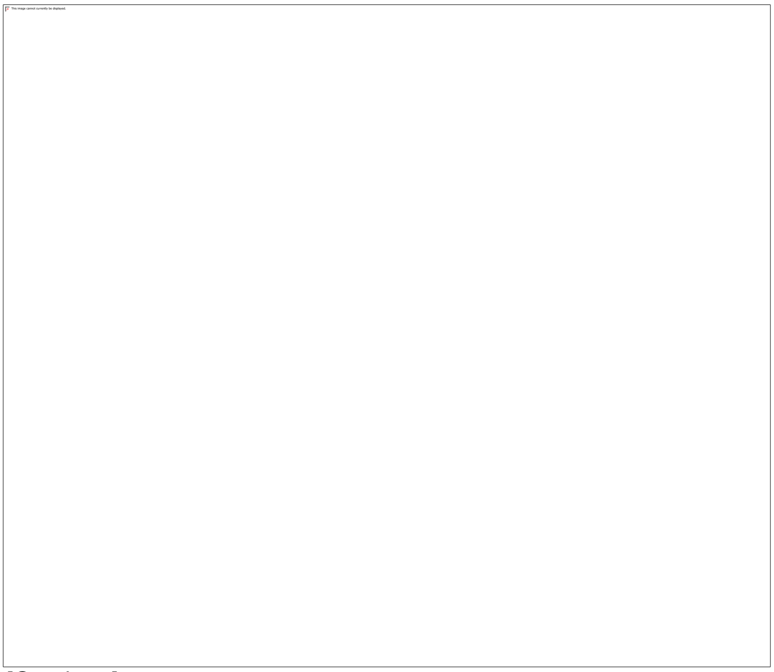

[Op photo]

Disclosure: Nothing to disclose

EP764

A prospective study of closed-incision negative pressure wound therapy following midline laparotomy in gynaecological oncology operations

L. Yin[1], A. Sayasneh[1,2,3]
[1]GKT School of Medical Education, King's College London, [2]Department of Gynaecological Oncology, Guy's and St Thomas' NHS Foundation Trust, [3]Faculty of Life Sciences and Medicine, King's College London, London, United Kingdom

Introduction / Background: Surgical site infections (SSI) have substantial health and economic burdens on society by increasing morbidity, mortality and length of hospital stay. The incidence of SSI after laparotomy are found to be amongst the highest in the surgical field. In gynaecological oncological surgery, this incidence has been reported to be as high as 27%. Thus, there is much interest in continuing to improve practice to minimise SSI. Here, we looked at the effect of using a closed-incision negative pressure wound therapy (ciNPWT) device, PREVENA PLUS™, on the incidence of SSI in gynaecological oncology patients undergoing midline laparotomies.
Methodology: This was a prospective pilot case-control study, with a total of 14 patients who received the PREVENA PLUS™ dressing and 26 patients in the control group whose incisions were dressed with the traditional Tegaderm™ dressing.
Inclusion criteria: Patients undergoing operations under the gynaecological oncology team with a midline laparotomy incision extending above the umbilicus. The vacuum dressing should have been applied directly after primary closure of the laparotomy wound.
All patients were followed up for a period of 30 days after surgery and regular records were kept of the condition of the laparotomy site. The primary outcome was the incidence of SSI after 30 days postoperatively.
Results: The incidence of SSI at 30 days postoperatively was 21% (3/14) in the treatment group and 23% (6/26) in the control group. There was no statistically significant difference between the two

groups (P=0.7). There were 2 cases of deep wound infection with spontaneous dehiscence in the group who received Prevena PLUS™ compared to no deep dehiscence in the control group (p=0.1).
Conclusion: The use of the PREVENA PLUS™ ciNPWT dressing for midline laparotomy wounds does not reduce the incidence of SSI at 30 days postoperatively.
Disclosure: Nothing to disclose

EP765

The effect of Malformina1 on cisplatin-sensitive and cisplatin-resistant ovarian cancer cell lines

N. Abdullah[1], N. Al Balushi[1], S. Al-Bahlani[2], S. Dobretsov[3], I. Hassan[4], T. Sang[5], Y. Tamimi[1], I. Burney[6]
[1]Biochemistry, [2]Allied Health Sciences, [3]Centre of Excellence in Marine Biotechnology, [4]Department of Chemistry, Sultan Qaboos University, Muscat, Oman, [5]Cellular and Molecular Medicine and the Interdisciplinary School of Health Science, University of Ottawa, Ottawa, ON, Canada, [6]Genetics, Sultan Qaboos University, Muscat, Oman

Introduction / Background: High grade epithelial ovarian cancer is often associated with drug resistance and poor outcomes. Previously, we demonstrated significant cytotoxic efficacy of a marine compound MalforminA1 against MCF-7 breast cancer cell line.
Methodology: We tested the anti-cancer potential of MalforminA1on cisplatin-sensitive (A2780s) and cisplatin-resistant (A2780cp) ovarian cancer cell lines using AlamarBlue assay, flow cytometry, western blot and RT-qPCR.
Results: MalforminA1 had significantly higher cytotoxic activity when compared to cisplatin in both A2780s and A2780cp cell lines (IC_{50}0.23µMand 0.34µM compared to 31.4 µM and 76.9 µM p < 0.001respectively).The cytotoxic effect of MalforminA1 was confirmed using flow cytometry where 66% of A2780s and 44% of A2780cp cells underwent apoptosis after48 hours of treatment. Also, morphological changes were seen in the cells upon the exposing to MalforminA1for 24h. To investigate the mechanism of apoptosis induction by malforminA1,we examined the expression of bcl2, p53, caspase3 and caspase9 at the RNA and proteins levels using RT-qPCR and western blot respectively.A significant reduction in m-RNA and protein levels was seen in both bcl2 and p53 in the treated cell lines compared to the non-treated cells(p< 0.001). Similar results were obtained with anti-apoptotic proteins bcl-x and bcl-xl using western blot (p< 0.01). On the other hand, caspases 3/9protein levels were not affected by MalforminA1treatment,suggesting that MalforminA1induces apoptosis by affecting bcl2 signaling pathway.
Conclusion: MalforminA1 showed promising anti-cancer activity by introducing apoptosis, and therefore warrants further investigation.
Disclosure: We are greatly thankful for the support through His Majesty Trust Fund Grant (SR/MED/MEDE/17/01)

EP766

Clinico-pathological features of ovarian granulosa cell tumours

G.O. Abdulrahman Jnr, N. Das, K. Lutchman Singh
Swansea Gynaecological Oncology Centre, Swansea Bay University Health Board, Swansea, United Kingdom

Introduction / Background: Ovarian granulosa cell tumour (GCT) is a rare ovarian malignancy, which is infrequently reported in the literature. However, it generally has good prognosis. We aim to report our experience with GCT in our centre.
Methodology: All patients that were diagnosed with GCT between 2011 and 2018 at the Swansea

Gynaecological Oncology Centre were identified through our cancer database. Patient characteristics, treatment, pathology results and outcomes were retrospectively analysed.
Results: 15 patients were diagnosed with GCT during the study period. This constitutes 2.1% of total ovarian malignancies diagnosed. The mean age of the women was 59.9 years (32 - 81 years). 13 women (86.6%) were diagnosed at FIGO Stage 1, 1 (6.7%) woman was diagnosed at FIGO Stage 3 while 1 was a recurrence after 20 years of initial diagnosis and surgery. Abdominal pain was the commonest presenting complaint. The case of recurrence presented with abdominal pain. Endometrial pathology was detected in 33.3% of patients. 80% were adult-type granulosa cell tumour while 6.7% was juvenile-type granulosa cell tumour. The mean follow-up was 61.7 months with an overall survival of 93.3%. 80% had positive inhibin, 33% had positive calretinin and 46.7% showed evidence of haemorrhage on histology. Only the recurrent tumour stained positive for epithelial membrane antigen (EMA) and CD56 on immunohistochemistry (IHC). 93.3% of the patients had laparotomy with majority having total abdominal hysterectomy, bilateral salpingo-oophorectomy and omentectomy except one patient that had unilateral oophorectomy as she desired fertility. GCTs appear to have a propensity for the left ovary as this accounted for 73% of the diagnosis.
Conclusion: Granulosa cell tumour is a rare ovarian malignancy with favourable prognosis. Abdominal pain presentation with evidence of ovarian cyst on radiological imaging could suggest GCT. Although recurrence is rare, long term follow-up is required. Positive EMA and CD56 on IHC could possibly predict risk of recurrence.
Disclosure: Nothing to disclose

EP767

A survey of germline mutations with epithelial ovarian cancer in Japanese patients

A. Abe, A. Mineda, T. Kagawa, A. Kuwahara, M. Nishimura
Tokushima University, Tokushima, Japan

Introduction / Background: In Japan, the number of BRCA testing is increasing annually. But, till recently, genetic counselling for epithelial ovarian cancer (EOC) has not been generalized. We investigated the awareness survey and the rate of HRD mutation included gBRCA1/2 mutation with EOC, regardless of their family histories.
Methodology: 127 EOC who had treated at our hospital were enrolled. After genetic counselling, DNA was extracted from whole blood, and all coding exons and their flanking intron regions of 11 genes included BRCA1/2, RAD51C/D, ATM, PALB2 and CHEK2 were sequenced with next-generation sequencing in our laboratory.
Results: 127 EOC patients who had treated at our hospital were enrolled. After genetic counselling, DNA was extracted from whole blood, and all coding exons and their flanking intron regions of 11 genes included BRCA1/2, RAD51C/D, ATM, PALB2 and CHEK2 were sequenced with next-generation sequencing in our laboratory.In 121 of 125 patients, consent was obtained, of which 84% wanted to disclose results. Mutations were found in 19 (15.7%) ;7 in gBRCA1 mutation, 10 in gBRCA2 mutation, 1 in gRAD51C mutation, and one in VUS. 12 of pathogenic mutation were high grade serous carcinoma, two; adenocarcinoma, and 4;others.Median age at diagnosis was gBRCA1; 64 years (49-87 years), gBRCA2; 60 years (42-67 years). TC therapy was administered to 10 cases as the first treatment in 19 cases, and all cases PR - CR were obtained. There was no significant difference in OS and PFS with FIGO stage lll of gBRCA mutation (p = 0.3, p = 0.52)
Conclusion: Almost patients with EOC wanted to know genetic risk after genetic counselling. The rate of gBRCA1/2 mutation in tested Japanese EOC patients with an inherited risk, regardless of a family history, was 15.7%. This finding indicates that the cooperation of genetic counselling with other department would be important for patients with ovarian cancers.
Disclosure: Nothing to disclose

EP768

Institutional results of OncoOVARIAN Dx: a novel algorithm for the management of adnexal masses

P. Kubelac[1,2], A.M. Craciun[1], O. Jalba[1], G. Lazar[1,2], F.L. Ignat[1,2], I.C. Lisencu[1,2], A.C. Rancea[1,2], I.C. Vlad[1,2], A. Irimie[1,2], P. Achimas-Cadariu[1,2]
[1]University of Medicine and Pharmacy 'Iuliu Haţieganu', [2]Institute of Oncology 'Prof. Dr. Ion Chiricuţă', Cluj-Napoca, Romania

Introduction / Background: The aim of this study is to evaluate the predictive performance of OncoOVARIAN Dx algorithm, which takes into account tumor markers (beta HCG, CA 19.9, CEA, AFP, CA 125, HE4), general biochemistry and clinical data (age, menopause, comorbidities) in patients scheduled for surgical removal of a suspicious adnexal tumor in comparison with the ROMA model.
Methodology: Consecutive women diagnosed with an adnexal tumor mass and scheduled for surgical intervention at a single tertiary cancer center between October 2018 - April 2019 were enrolled. Preoperative values of tumor markers and general biochemistry (ASAT, ALAT, GGT, total bilirubin, creatinine) were determined. Following surgery, histological type and stage were determined based on FIGO classification.
Results: A total of 44 patients were selected, including 19 benign (BEN), 5 borderline (BOR) and 20 malignant (MAL) epithelial ovarian cancer (EOC) cases on final pathology. BOR tumors comprised 3 serous (S) and 2 mucinous (M) FIGO stage I cases. MAL tumors included 13 high grade S, 4 endometrioid and 3 M histotypes, FIGO stage IA-IIIC. The two models demonstrated very good correlation (Phi 0.78, p< 0.001), however among the 30 cases deemed ROMA low risk there were 5 (1 BEN, 2 BOR/M, 2 MAL/high grade S) cases that had OncoOVARIAN Dx moderate/high risk. Among the 7 BOR/MAL cases labeled OncoOVARIAN Dx low risk 71% were FIGO IA and the remaining two cases were FIGO IB and IIA. The sensibility (Se), specificity (Sp), positive predictive value (PPV), negative predictive value (NPV) of OncoOVARIAN Dx versus ROMA model were 72% vs. 56%, 94.73% vs. 100%, 94.73% vs. 100%, 72% vs. 63.33%, respectively.
Conclusion: OncoOVARIAN Dx model demonstrated higher Se and NPV compared to ROMA and could be a useful marker in the preoperative management of adnexal masses however larger studies are warranted to validate and further refine this algorithm.
Disclosure: This work was supported by a National Grant PN-III-P2-2.1-CI-2018-1509.

EP769

Survival associated with first-line bevacizumab maintenance therapy among older patients with ovarian cancer: findings from the VIERA study

G. Adeboyeje, M. Monberg, S. Iqbal, J. He, K. Desai
Merck & Co., Inc., Kenilworth, NJ, United States

Introduction / Background: Bevacizumab with chemotherapy followed by bevacizumab was approved in 2018 for the first-line (1L) treatment of patients with ovarian cancer (OC). However, little is known about the real-world experience of OC patients treated with bevacizumab.
Methodology: This US SEER-Medicare retrospective cohort study included females ≥ 66 years, with OC (stage II-IV) diagnosis between 2009 and 2015, who continued bevacizumab or observation alone after 4 - 10 cycles of 1L platinum-based chemotherapy with or without bevacizumab. The primary outcome was overall survival (OS), measured from 1L treatment initiation to death from any cause. Safety events of interest included thrombosis, hemorrhage, stroke, and gastrointestinal perforation. Inverse probability of treatment weighting (IPTW) incorporating 32 demographic and prognostic characteristics was used to ensure balance in baseline variables. Cox proportional hazards models with IPTW were used to compare outcomes.
Results: In this cohort (N=2262), 66 (3%) patients received bevacizumab maintenance. Mean age (±SD) was 74.6 (6.2) years, 89% were Stage III or IV, and 26.9% of patients had ≥1 comorbidity.

Patients treated (preapproval) with 1L maintenance bevacizumab increased from 0.3% in 2009 to 4.1% in 2015. Compared to observation alone, median OS in patients treated with bevacizumab maintenance was 43.6 vs 37.9 months (adjusted hazard ratio [HR]=0.83 [95% CI: 0.50 - 1.36]). Among those who received >7 cycles of bevacizumab, OS was longer, though not significantly, compared to observation alone (aHR = 0.62 [95% CI: 0.32-1.18]). Initiation of nonplatinum chemotherapy within 12 months occurred in 36.4% of observation only and 20.9% of bevacizumab maintenance patients. Compared to observation alone, specified safety events were not higher with bevacizumab maintenance, aHR: 0.94 [0.49 - 1.82].
Conclusion: Survival among this small cohort of elderly OC patients in this real-world study is consistent with that observed in trials of 1L bevacizumab maintenance. First-line maintenance bevacizumab therapy appears to provide modest survival benefit.
Disclosure: G Adeboyeje, K Desai, S Iqbal, J He, M Monberg are employees of Merck Sharp & Dohme Corp. This study was funded by Merck Sharp & Dohme Corp., a subsidiary of Merck & Co., Inc., Kenilworth, NJ, USA.

EP770

Real-world treatment experience in patients with ovarian cancer: findings from a retrospective analysis of the SEER-Medicare Database in the United States

G. Adeboyeje, K. Desai, S. Iqbal, J. He, M. Monberg
Merck & Co., Inc., Kenilworth, NJ, United States

Introduction / Background: Despite high response rates to initial treatment, high recurrence rates remain a persistent challenge among patients with ovarian cancer (OC). There is limited data on overall survival (OS) rates and burden of disease with contemporary standard of care. We examined the treatment experience of real-world patients in the United States (U.S).
Methodology: Using a single-arm retrospective cohort study design, we identified OC patients from the SEER-Medicare database, aged >= 66 years and had completed 4 - 10 cycles of platinum-based first-line (1L) chemotherapy from January 2009 to December 2015. The descriptive outcomes were OS rates, rates of hospitalization, mean number of hospitalizations and length of hospital stay (LOS), receipt of bevacizumab, proportion of patients who initiated non-platinum therapies, and transition rates through lines of therapy at 1-, 2-, 3- and 4-year landmarks.
Results: We identified 2,262 patients (mean age was 74.6 ±6.2 years). 29.9 of patients had ≥1 non-cancer comorbidity. 90.2% of patients were white.
At 1 year after initiating chemotherapy, 90.7% of patients were alive, 56.1% were hospitalized at least once with a mean LOS of 16 days; 10.2% had received bevacizumab and 20.5% had initiated a non-platinum-based therapy. At 4 years, 44.1% of patients were alive, 72.6% were hospitalized at least once with a mean LOS of 22 days; 31% had received bevacizumab and 56.2% had initiated a non-platinum-based therapy. The proportion of patients who had died, completed or were receiving 3 or more lines of therapy increased from 10.1% at 1 year to 74.2% at 4 years from initiating chemotherapy. Among 1,538 patients with known treatment or mortality status at 4 years, 251 (16.3%) had not received additional treatment following their initial course of chemotherapy.
Conclusion: The increasing burden of therapy and hospitalizations over time demonstrate the need for more effective front-line therapies to delay progression in ovarian cancer.

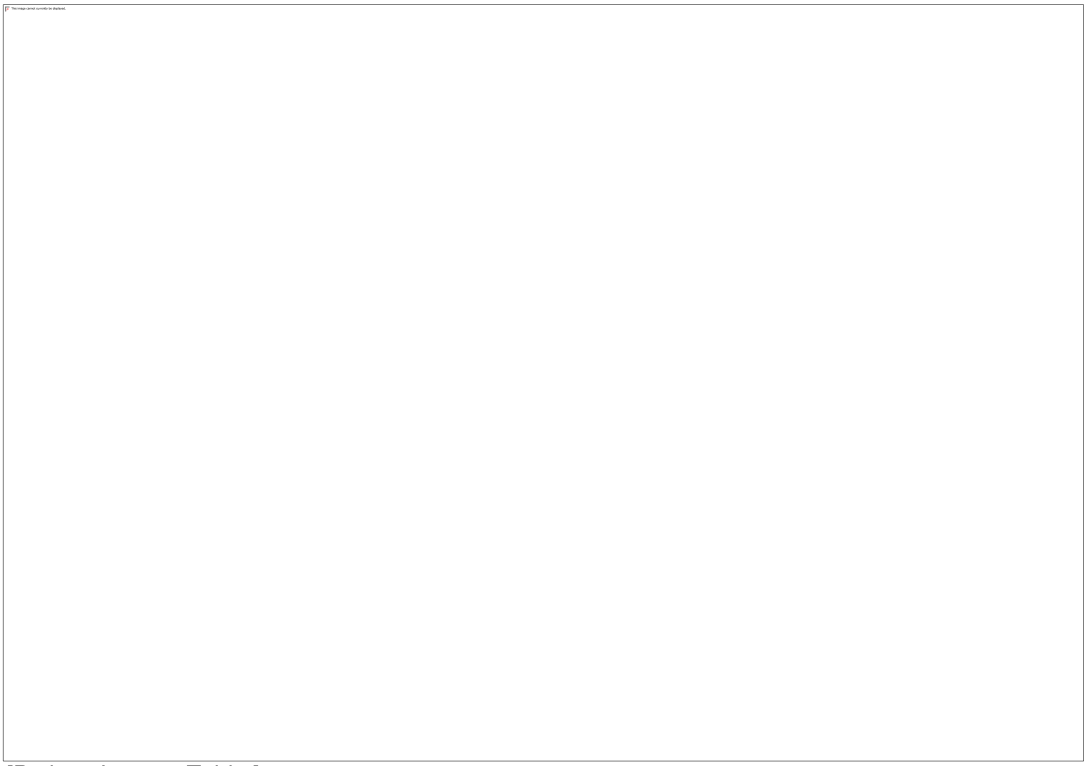

[Patient Journey Table.]

Disclosure: G Adeboyeje, K Desai, S Iqbal, J He, M Monberg are employees of Merck Sharp & Dohme Corp., a subsidiary of Merck & Co., Inc., Kenilworth, NJ, USA. This study was funded by Merck Sharp & Dohme Corp., a subsidiary of Merck & Co., Inc., Kenilworth, NJ, USA

EP771

Could imaging predict lymph node invasion in advanced epithelial ovarian cancer?

C. Adrien[1], C. Mimoun[2], M. Mezzadri[2], E. Marchand[2], C. Touboul[2], V. Place[2], J.L. Benifla[2]
[1]UPMC, [2]Paris Descartes University, Sorbonne Paris Cité, Paris, France

Introduction / Background: Recent french recommandations narrowed indications of lymph node dissection in advanced epithelial ovarian cancer to clinically or radiologically significative adenopathy. The objective was to evaluate the predictive ability of imaging.
Methodology: A prospective tricentric study was conducted in the gynaecologic oncologic departments of Lariboisiere, Creteil and Poissy Saint Germain Hospitals. Between 01st January 2004 and 31 July 2018, we included all women diagnosed with an advanced epithelial ovarian cancer who benefited lymph node resection. Clinical, radiological features of CT and PET scan and pathological reports were reported in our database. Sensitivity (Se), specificity (Spe), positive and negative predictive values (PPV and NPV) and likelihood ratios (pLR and nLR) of each imaging were calculated. We carried out an univariate analysis to find predictors of lymph node invasion based on imaging.
Results: Final population included 84 patients. Mean age was 60 years old. There were 71 serous (85%), 4 endometrioids (5%), 2 mucinous (2%), 1 clear cell (1%), 5 undifferentiated (5%), 1 mixt (1%) adenocarcinomas. 40 patients had positive lymph nodes on the pathological report. 71 patients had a CT, with a sensitivity of 63%, specificity of 92%, pLR of 4,78, PPV of 84%, NPV of 77%. 14 patients

had a PET with a sensitivity of 92%, specificity of 99% pLR of 15, PPV of 99%, NPV of 88%. In univariate analysis for predicting factors of positive lymph node, we did not find any association with the main usually described involvement as ascite, peritoneal carcinosis or metastases.
Conclusion: PET scan is more performing than CT scan to diagnose lymph node invasion. CT and PET scan are probably two relevant imaging exams in the prediction of lymph nodes involvement but further research needs to be conducted.
Disclosure: Nothing to disclose

EP772

Testing for concordance of somatic and germline BRCA1/2 pathogenic mutations in ovarian cancer patients eligible for PARP inhibitors therapy: large single-site institution experience

I. Akaev[1,2], C. Chau[3], M. Uherek[3], S. Rahimi[1,2], F. Gardner[4], C.C. Yeoh[3]
[1]School of Pharmacy and Biomedical Sciences, University of Portsmouth, [2]Histopathology, Pathology Department, [3]Oncology Department, [4]Department of Obstetrics and Gynaecology, Portsmouth Hospitals NHS Trust, Queen Alexandra Hospital, Portsmouth, United Kingdom

Introduction / Background: Incidence of Ovarian Cancer (OC) in Portsmouth is approximately 110 cases per year, standing as the 10th largest city in the UK. Individuals with germline BRCA1/2 (gBRCA1/2) alterations treated with the PARP inhibitors (PARPi) tend to respond better than patients with wild-type BRCA1/2. Additionally, somatic BRCA1/2 (sBRCA1/2) alterations induce the sensitivity to PARPi.
The aim of the study was to investigate the rate of concordance of gBRCA1/2 with sBRCA1/2 pathogenic mutations to increase screening uptake for prescription of the newly NICE approved PARPi tablets available for gBRCA and sBRCA mutation carriers.
Methodology: 70 patients diagnosed with ovarian cancer were screened: 50 with High Grade Serous Carcinoma (HGSC), 2 Low Grade Serous Carcinoma (LGSC), 4 Clear Cell Carcinoma (CCC), 2 Carcinosarcoma, 11 Endometrioid Adenocarcinoma (EdAd) and 1 mucinous carcinoma. Patients were tested for BCRA1/2 germline mutations upfront, followed by testing of tumour specimens for somatic mutations using NGS.
Results: 9 cases had gBRCA1/2 pathogenic mutations: 5 HGSC had gBRCA1, 3 HGSC and 1 EdAd had gBRCA2. 7 cases had sBRCA1/2 mutations: 4 gBRCA1 and 3 gBRCA2 HGSC had sBRCA1 and sBRCA2 respectively. EdAd gBRCA2 had no somatic mutations; 1 HGSC patient with gBRCA1 had no sBRCA1/2 mutations.
1 HGSC wild-type gBRCA showed pathogenic sBRCA1 frameshift mutation. 2 EdAd and 1 CCC wild-type gBRCA showed sBRCA1/2 mutations of unknown clinical significance. LGSC, carcinosaromas and mucinous carcinoma were wild-type gBRCA with no somatic mutations detected.
Conclusion: Detection of both germline and somatic BRCA1/2 mutations is required for effective PARPi treatment. Somatic tests should be offered to increase the number of patients suitable for targeted therapy. The consistency of gBRCA1/2 uptake (13%) was in keeping with published data, whereas the sBRCA1/2 uptake was 11.4%, which is less than the expected 15%. More research into cases with sBRCA1/2 mutations of unknown clinical significance is warranted.
Disclosure: Nothing to disclose

EP773

Splenectomy as primary cytoreductive surgery in advanced stage epithelial ovarian cancer

H. Akilli[1], L. Aksoy Karakaş[1], H. Nihan[2], A. Ayhan[3]
[1]Gynecology and Obstetrics, [2]Pathology, [3]Gynecology and Obstetrics, Division of Gynecologic Oncology, Baskent University Faculty of Medicine, Ankara, Turkey

Introduction / Background: The aim of the study was to evaluate site of splenic metastases and ıts affect on survival in advanced stage epithelial ovarian cancer
Methodology: This retrospective study was done in Baskent University School of Medicine ,Ankara ,Turkey .We evaluated 182 patients who underwent splenectomy as a part of primary cytoreductive surgery between 2008-2018 Besides we invastegated 2,3 and 5 year overall survival rates.
Results: Median age was 61 years (27-81),Median lenght of stay was 13 days (4-87 days). 40 (21,7 %) patients had paranchimal and 144 (78,3 %) had non paranchimal (Hilar or capsular) metastases,Median overal survival was 27 months and 31 months respectively(p=0,6) .

**Site of Metastases**	Number	Median Overall Survival (Months)	2 year OS	3 Year OS	5 year OS	p value
**Paranhimal**	40	27	62%	41%	23,5 %	0,6
**Non-Paranchimal**	142	31	60%	44%	26 %	

[Median Overall Survival rates According to site of Splenic Metastases]

Conclusion: Splenectomy in primary cytoreductive surgery is an effective intervention to achive R0 resection in advanced stage EOC However site of splenic metastases has no impact on short term and long term overall survival .
Disclosure: Nothing to disclose

EP774

Gallic acid exhibits lower cytotoxicity on normal ovarian cells than cisplatin-sensitive and cisplatin-resistant ovarian cancer cells

N. Al Balushi[1], N. Abdulla[2], B. Al Dhahli[3], I. Hasan[4], S. Dobretsov[5], S. Al Bahlani[3], B. Tsang[6], Y. Tamimi[2], I. Burney[1]
[1]Medicine, [2]Biochemistry, [3]Allied Health Sciences, [4]Chemistry, [5]Centre of Excellence in Marine Biotechnology, Sultan Qaboos University, Muscat, Oman, [6]Obstetrics & Gynaecology, Cellular & Molecular Medicine and the Interdisciplinary School of Health Sciences, University of Ottawa, Ottawa, ON, Canada

Introduction / Background: Ovarian cancer is one of the fatal cancers in women, often associated with drug resistance. Therefore, finding effective drugs, including naturally derived compounds is urgently needed. We have previously examined the effect of gallic acid (GA) on breast cancer cell line MCF-7 and found a significant anti-cancer activity. We aimed to test the anti-cancer potential of GA monohydrate and six different derivatives of GA on cisplatin-sensitive (A2780S) and cisplatin-resistant (A2780CP) human epithelial ovarian cancer cell lines and on HOSE6-3 normal epithelial ovarian cell line. The effect of cisplatin and GA in combination was also examined.
Methodology: Cells were exposed to different concentration of GA (0-100µg/ml), and cisplatin (0-24µg/ml) and cell viability was tested via AlamarBlue and CCK08 assays. Apoptosis was determined by Hoechst stain.
Results: GA monohydrate decreased cell viability in a dose- and time-dependent manner in all cell lines. GA monohydrate was demonstrated to have the highest cytotoxicity to both A2780S and A2780CP cell lines compared to all six derivatives. The highest cytotoxicity of GA was observed in A2780S (IC-50= 19.4µg/ml), followed by A2780CP cells (IC-50= 35.6µg/ml), whereas the least cytotoxicity was observed in HOSE6-3 (IC-59= 49.3µg/ml). Similarly, cisplatin displayed a higher cytotoxicity on A2780S (IC-50= 9.4µg/ml) than on A2780CP (IC-50= 23.1µg/ml). However, cisplatin was more toxic to HOSE6-3 than ovarian cancer cells (IC-50= 8.8µg/ml). There was no significant difference in cytotoxicity between cisplatin and GA either combined or used separately. Apoptosis was

estimated to be 49% and 47% in A2780S and A2780CP cell lines respectively at a GA concentration of 25µg/ml.
Conclusion: GA monohydrate exhibited significant cytotoxicity in ovarian cancer cell lines independent of cisplatin, suggesting a cytotoxic effect of GA on ovarian cancer. The growth inhibitory effect of GA, especially on resistant ovarian cancer cells, merits further investigation.
Disclosure: We are greatly thankful for His Majesty Sultan Qaboos Trust Fund Grant (SR/MED/MEDE/17/01). Also, we would like to thank Ms Buthaina Al Dhahli for her endless support and help.

EP775

Differential expression of SOCS2 gene and its putative role in ovarian cancer

F. Al Kindi[1], M. Al Kalbani[2], M. Al Moundhri[2], I. Burney[2], Y. Tamimi[1]
[1]Biochemistry, [2]Medicine and Health Sciences, Sultan Qaboos University, Muscat, Oman

Introduction / Background: Epithelial Ovarian Cancer (EOC) is the most lethal disease among gynecologic cancers. It is usually diagnosed at late stages with a poor outcome due to its asymptomatic nature. In Oman, the incidence rate for 2013 was estimated at 2.9 per 100,000. There is an urgent need to identify biomarkers for an early detection of EOC. we used chromatin immune precipitation (ChIP) to identify downstream genes regulated by the *E2F5* transcription factor (TF) in ovarian cancer cell lines (MCAS and OVSAHO). Results revealed that *SOCS-2* was regulated by the TF *E2F5*. SOCS (1-8) family have a role in JAK/STAT transcription pathway. Any disruption in this pathway may lead to many diseases such as cancers. Some of these SOCS genes found to act as biomarker for some cancers. *SOCS2* was recently shown to act as an early biomarker for prostate and colorectal cancer.
The aim of this study is to explore the role of *SOCS2* gene in EOC by monitoring the expression of the gene in stages variety of EOC tissues and cell lines.
Methodology: We examined the expression of *SOCS2* in tissues and cell lines using the quantitative reverse transcription-polymerase chain reaction (qRT-PCR) to monitor the differential expression of *SOCS2* in cancerous and normal tissues.
Results: Significant down-regulation of *SOCS2* was found in borderline tissues but it was not significant in other cancer stages such as benign and malignant tissues. Although all examined cell lines (MCAS, OVSAHO and 2008OV) showed a significant downregulation of the gene. *SOCS2* methylation was investigated using MSP technique. It was revealed that *SOCS2* is methylated in OVSAHO cell line but not in MCAS cells while cancerous tissues showed partially methylation.
Conclusion: The down regulation of SOCS2 gene in EOC is not caused by methylation; other mechanisms might be involved.
Disclosure: Nothing to disclose

EP776

Müllerian carcinoma in the rectovaginal septum

M. Alonso Espías, V. García Pineda, A. Táboas Álvarez, M. Cabrero Gañán, A. Hernández Gutiérrez, I. Zapardiel Gutiérrez
Hospital Universitario La Paz, Madrid, Spain

Introduction / Background: Endometriosis appears to be associated with some epithelial ovarian cancers (EOC).The types that are specially associated are clear cell carcinoma and endometrioid carcinoma.The risk of malignant transformation of endometriosis has been estimated at 1% for premenopausal women and 1-2.5% or postmenopausal women.Endometriosis-associated EOC

appears to appear in younger women and has a better prognosis than primary EOC.
Methodology: We present the clinical case of a 36-year-old woman,gravida 0,with endometriosis as the only personal history of interest. She came to the emergency room because of rectal bleeding.In the physical examination a pelvic mass of about 9cm in the rectovaginal septum was found.Abdominal sonography, CT-Scan, MRI and PET-CT were performed.They showed a left pararectal heterogeneous mass measuring 90 mm of maximum diameter.Some small locoregional adenopathies were found, predominantly in the left para-aortic area.The levels of tumoral markers were 1694 mUI/ml for Ca125 and 241UI/ml for Ca15.3.The first suspected diagnosis based on the imaging findings was a GIST-tumour.However, an endoscopic ultrasonography and fine needle aspiration showed an adenocarcinoma of müllerian origin.Our tumour board committee formed by oncological gynecologists, medical oncologist, radiotherapists and pathologist decided to perform a cytorreductive surgery.A frozen section confirmed it was a malignant tumour,so a complete excision of the mass with hysterectomy,bilateral adnexectomy and rectal resection was performed.The patient received six cycles of Carboplatin/Paclitaxel as adjuvant treatment.
Results: The pathological report diagnosed a high grade müllerian adenocarinoma with areas of clear cell differentiation.There was no lymphovascular invasion. Rectosigma was infiltrated. Uterus and ovaries were free of tumoural invasion.
After 18 months of follow-up no recurrence of the disease has been detected.
Conclusion: This is a rare case of a carcinoma of Müllerian origin that appeared in the rectovaginal septum in a young woman with endometriosis hystory.In these cases,primary surgery should be the first option of choice as long as complete debulking can be achieved.
Disclosure: Nothing to disclose

EP777

Survival of advanced stage high grade serous ovarian cancer patients in Republic of North Macedonia

I. Aluloski[1], M. Tanturovski[1], S. Kostadinova-Kunovska[2], R. Jovanovic[2], G. Petrusevska[2]
[1]Gynecologic Oncology, University Clinic of Obstetrics and Gynecology, [2]Institute for Pathology, Medical Faculty-Skopje, Skopje, The former Yugoslav Republic of Macedomia

Introduction / Background: The primary objective of the study was to evaluate the overall survival of women with advanced stage (Stage IIIA-IV) high-grade serous ovarian cancer in Republic of North Macedonia.
Methodology: The study was a cross-sectional medical record review of patients diagnosed with advanced stage HGSC. Patients were deemed eligible for inclusion if they were diagnosed with an advanced stage (Stage IIIA-IV) HGSC of the ovary, fallopian tube or peritoneum between 2009 and 2015. The data was analyzed in a descriptive fashion and summary statistics were provided, as appropriate. Survival was calculated using the Kaplan-Meier method.
Results: A total of 81 eligible patients were identified and included in the study. The average overall survival in the studied cohort was 46.59 months (95%CI 39.11-54.06). Patients that were optimally debulked and patients that had a platinum free interval larger than 12 months had significantly longer survival in the current series ($p < 0.001$).
Conclusion: The average overall survival of advanced stage HGSC patients in the studied series was 46.59 months (95%CI 39.11-54.06). Patients aged 65 years or younger had a tendency to live approximately 10 months longer than patients older than 65 years, but this difference was not statistically significant. There was no difference in HGSC survival in the groups of patients with grade 2 and grade 3 disease. However, optimal surgical debulking and platinum sensitivity were associated with significantly better overall survival.
Disclosure: Nothing to disclose

EP778

The role of the E-cadherin/ β-catenin signal transduction pathway in the prognosis of advanced stage high-grade serous ovarian/fallopian tube cancer

I. Aluloski[1], M. Tanturovski[1], S. Kostadinova-Kunovska[2], R. Jovanovic[2], G. Petrusevska[2]
[1]Gynecologic Oncology, University Clinic of Obstetrics and Gynecology, [2]Institute for Pathology, Medical Faculty-Skopje, Skopje, The former Yugoslav Republic of Macedomia

Introduction / Background: The aim of the study was to evaluate the correlation between E-cadherin/ β-catenin expression and the clinical and pathological parameters that influence the overall survival rate in patients with advanced stage high-grade serous ovarian/fallopian tube cancer
Methodology: We performed a retrospective cohort study on patients that underwent primary debulking surgery at the University Clinic of Gynecology and Obstetrics, University “Ss. Cyril and Methodius”, Skopje, Republic of North Macedonia between January 2010 and December 2015.
The following disease characteristics were analyzed: age, stage, nuclear and tumor grade, lymphovascular invasion, the extent of primary debulking, platinum-based chemotherapy resistance and overall survival. Further, we analyzed the expression of E-cadherin/ β-catenin by preforming immunohistochemical staining on deparaffinized and rehydrated tissue specimens with specific monoclonal antibodies.
Results: A total of 84 patients with primary ovarian and 9 patients with primary fallopian tube cancer met the inclusion criteria. The overall survival of patients in the analyzed cohort was 46 months (95%CI 38-53 months). The residual tumor volume after primary debulking surgery was an independent bad prognostic factor with HR=5.32 (p< 0.001). The low frequency of expression as well as the weak staining for E-cadherin were independent bad prognostic factors for the survival of these patients with HR=2.7 (p=0.05) and HR=3.38 (p=0.04), respectively. The univariate analysis also identified that the low frequency of expression and weak staining for β-catenin were bad prognostic factors, but the significance was lost in the multivariate analysis.
Conclusion: In this study, we identified that the residual tumor volume after primary debulking surgery and the low frequency of expression and weak staining for E-cadherin were independent, statistically significant bad prognostic factors for the overall survival of patients with advanced stage high-grade serous ovarian/fallopian tube cancer. Beta catenin expression was not found to be an independent prognostic factor in the multivariate analysis.
Disclosure: Nothing to disclose

EP779

Survival and prognostic factors in women treated for ovarian cancer in western region of Saudi Arabia

N. Anfinan, K. Sait, M. Alam, A.W. Noor Wali, A. Haque, H. Sait
King Abdulaziz University, Jeddah, Saudi Arabia

Introduction / Background: Among gynecological malignancies, ovarian cancer is most common and is currently at number five position in most frequent causes of deaths related to the cancers
Methodology: Clinical data were collected retrospectively for women who underwent primary staging or debulking surgery. Progression free survival (PFS) was defined as the time interval from the date of primary surgery to the date of disease recurrence or death/last follow-up without recurrence. Overall survival (OS) was defined as the time interval from the date of the primary surgery to the date of death or last follow-up.
Results: Total of 144 Epithelial Ovarian Cancer patients were analyzed for progression free survival (PFS) and overall survival (OS). The median PFS and OS were 33 and 43.5 months respectively. Increasing age was found significantly associated with median PFS (p ≤ 0.002) and OS (p ≤ 0.014). A significant association was observed between papillary serous and non serous histopathology in PFS (p≤0.003) as well as in OS (p≤0.044). Patients with non-serous histopathology had better PFS and OS than the patients having serous histopathology. Logistic regression analysis revealed a linear

association between risk of disease progression and death with increasing age. Tumor stage III was significantly associated with the risk of disease progression (OR=8.27, 95% CI, 3.18 - 21.53, p≤0.001) as well risk of death (OR=0.07, 95% CI, 0.02 - 0.19, p≤0.001). Tumor grade 3 was also observed significantly associated with the progression of disease.
Conclusion: There are number of reasonable factors known to be associated with long term survival such as clinical presentation of the disease status i.e., stage, grade, performance status, and burden of the disease.
Disclosure: Nothing to disclose

EP780

Prognostic impact of celiac lymph node involvement in patients after frontline treatment for advanced ovarian cancer

M.A. Angeles[1], G. Ferron[1], G. Balagué[2], B. Cabarrou[3], C. Martínez-Gómez[1], M. Voglimacci[1], L. Gladieff[4], C. Pomel[5], A. Martinez[1]
[1]Surgical Oncology, [2]Radiology, [3]Biostatistics Unit, [4]Oncology, Institut Claudius Regard, IUCT Oncopole, Toulouse, [5]Surgical Oncology, Institut Clermont-Ferrand, Clermont-Ferrand, France

Introduction / Background: Completeness of cytoreduction is the most important prognostic factor in patients with advanced ovarian cancer (OC). Extensive upper abdominal surgery has allowed to increase the rate of complete cytoreduction. The feasibility of resection of celiac lymph nodes (CLN) and porta hepatis disease in these patients has been demonstrated. The aim of our study was to assess the prognostic impact of CLN involvement in patients with primary advanced OC after complete cytoreductive surgery (CRS).
Methodology: We designed a retrospective unicentric study. We reviewed data from patients who underwent CLN resection with or without porta hepatis disease resection, within upfront or interval complete CRS in the frontline treatment of advanced epithelial OC between January 2008 and December 2015. Patients were classified in two groups according to CLN status. Univariate and multivariate analyses were conducted. Survival rates were estimated using Kaplan-Meier method.
Results: Forty-three patients were included and positive CLN were found in 39.5% of them. The median disease-free survival in the group of patients with positive and negative CLN were 11.3 months and 25.8 months, respectively (Figure 1.A). The median overall survival in patients with positive was 31.6 months and in the group with negative CLN it was not reached (Figure 1.B). Table 1 shows the results of univariate analysis. In multivariate analysis, both CLN involvement and high peritoneal cancer index were independently associated with decreased disease-free survival. Relapse within the 6 months after the end of the chemotherapy was significantly associated with CLN involvement, 2/26 (7.7%) in the group with negative CLN vs. 8/17 (47.1%) in the group with positive CLN (p=0.007).
Conclusion: CLN involvement and high preoperative tumor burden are independently associated with decreased survival after complete cytoreduction for OC. CLN involvement is a marker of diffuse disease and an independent risk factor for early recurrent disease.
Disclosure: Nothing to disclose

EP781

Predictive risk factors of acute kidney injury after cytoreductive surgery and cisplatin-based hyperthermic intraperitoneal chemotherapy for ovarian peritoneal carcinomatosis

M.A. Angeles[1], F. Quenet[2], P. Vieille[3], L. Gladieff[4], J. Ruiz[5], M. Picard[5], F. Migliorelli[6], P. Izard[7], L. Chaltiel[8], C. Martínez-Gómez[1], J.J. Torrent Correa[9], S. Carrere[2], O. Sgarbura[2], A. Martinez[1], G. Ferron[1]

[1]Surgical Oncology, Institut Claudius Regard, IUCT Oncopole, Toulouse, [2]Surgical Oncology, Institut du Cancer de Montpellier, Montpellier, [3]Gynecology, CHR, Perpignan, [4]Oncology, [5]Reanimation, Institut Claudius Regard, IUCT Oncopole, Toulouse, France, [6]Obstetrics and Gynecology, Geneva University Hospitals, Geneva, Switzerland, [7]Anesthesiology, [8]Biostatistics Unit, Institut Claudius Regard, IUCT Oncopole, Toulouse, France, [9]Gynecology, Jtorrentinstitute, Hospital El Pilar - Grupo Quirónsalud, Barcelona, Spain

Introduction / Background: Cytoreductive surgery (CRS) followed by hyperthermic intraperitoneal chemotherapy (HIPEC) with cisplatin is a widely used strategy in the treatment of ovarian carcinomatosis. Although it seems to improve the patients' survival, one of its most important adverse effects is postoperative acute kidney injury (AKI). The aim of our study was to assess the incidence and identify the predictive risk factors of AKI after CRS and cisplatin-based HIPEC.
Methodology: This is a retrospective study from two centres evaluating patients with advanced or recurrent ovarian cancer who underwent CRS followed by cisplatin-based HIPEC from January 2007 to December 2013. Patients were classified in two groups according to the occurrence of AKI, defined when glomerular filtration rate at postoperative day-7 was 25% lower than at day-0. We also evaluated AKI following Risk, Injury, Failure, Lost and End-stage kidney function criteria. Univariate and multivariate analyses were conducted in order to assess the association between different variables and AKI occurrence.
Results: Sixty-six patients were included: 29 (44%) underwent first-line treatment while 37 (56%) were treated for recurrent disease. The incidence of postoperative AKI was 48%. Table 1 displays the univariate analysis. After multivariate analysis, hypertension (OR 18.6; 95% CI 1.9-182.3; p=0.012) and low intraoperative diuresis (OR 0.5; 95% CI 0.4-0.8; p=0.001) were associated with AKI occurrence.
Conclusion: The incidence of AKI after CRS and cisplatin-based HIPEC was high. Hypertension and low intraoperative diuresis were independent risk factors for this complication. An adequate perioperative hydration, in order to maintain a correct diuresis, could decrease acute kidney injury occurrence in patients undergoing CRS plus HIPEC. Table 2 shows general recommendations to decrease this complication.
Disclosure: Nothing to disclose

EP782

Transition zones and progenitor cells in ovarian cancer pathogenesis

A. Asaturova[1], M. Sannikova[2], N. Kondrikov[3]
[1]Pathology, [2]Oncogynecology, National Medical Research Center for Obstetrics, Gynecology and Perinatology named after Academician V.I.Kulakov of the Ministry of Healthcare of Russian Federation, [3]Moscow Regional Research Institute of Obstetrics and Gynecology, Moscow, Russian Federation

Introduction / Background: Ovarian cancer remains the most deadly gynecological malignant disease in developed countries. Basically, poor prognosis of this disease can be explained by the ineffectiveness of screening programs due to the lack knowledge of the ovarian cancer pathogenesis. Since pluripotent cells play a key role in the development of neoplastic epithelial changes, verification of the of their localization in transition zones (TZs) will help to determine the possibility of simultaneous existence of several potential sources for ovarian carcinoma development.
Methodology: We investigated 165 tissue samples of the ovary and fallopian tube from patients with benign extraovarian pathology (uterine fibroids and adenomiosis) and 25 samples with high-grade serous ovarian carcinoma (HGSC) [(55 fimbriae, 25 parafimbrial zones (PFZ), 25 paraovarian zones (POZ), 35 samples of ovarian surface epithelium (OSE)]. Transition zones were taken separately for morphological assessment. Immunohistohemiscal investigation involved LGR5, NANOG, LNX9, CD117, CD44 and Oct4 assessment. Statistics was done with Student's test with Boferroni correction.
Results: LGR expressed in 38 fimbriae, 22 PFZ, 21 POZ, 32 OSEs; NANOG expressed in 25 fimbriae, 20 PFZ, 24 POZ, 35 OSEs; LHN9 expressed in 27 fimbriae, 19 PFZ, 19 POZ, 25 OSEs; CD 117 expressed in 30 fimbriae, 10 PFZ, 15 POZ, 11 OSEs. Statistically significant difference in progenitor markers expression were shown between TZs, OSE and fimbriae, as well as between

normal epithelium and HGSC and between TZs and epithelial cells elsewhere (p< 0,05).
Conclusion: We investigated transitional zones of the uterine adnexa with progenitor cell markers and revealed that there is a significantly higher concentration of these cells in the transition zones where two types of the epithelia met. Thus, these zones can be a potential source (hot spot) for neoplasia development due to mutations in progenitor cells and further clonal expansion.
Disclosure: Nothing to disclose

EP783

Epithelial-mesenchymal tranzition and angiogenesis dialog in borderline ovarian tumors complicating pregnancy

A. Asaturova[1], M. Shamarakova[2]
[1]Pathology, National Medical Research Center for Obstetrics, Gynecology and Perinatology named after Academician V.I.Kulakov of the Ministry of Healthcare of Russian Federation, [2]Pathology, Family Planning and Reproduction Center, Moscow, Russian Federation

Introduction / Background: Physiological changes in the mother's body during pregnancy with the primary aim to support the fetus's growth may also suspected to worsen the natural course of various malignancies with even poorer prognosis then in non-pregnant women. Though borderline ovarian tumors generally have an excellent prognosis, the risk of their progression during gestation remain has yet to be investigated. The purpose of this study was to assess the expression of epithelial-mesenchymal markers, angiogenesis and the extent of natural killers in samples of ovarian borderline tumors in pregnant and non-pregnant women.
Methodology: Twenty three samples [13 from pregnant (30,5±4,7 years) and 10 from non-pregnant women (33,6±5,8 years)] were investigated morphologically and immunohistochemically with VEGF, CD31, CD105, CD56, E-cadherin, Vimentin, Mann-Whitney test was used for statistics.
Results: Serous borderline tumors were diagnosed more frequently in pregnant and in non-pregnant women. In addition we diagnosed endometrioid and mucinous borderline tumors with welldiffentiated components in both groups. CD31 expression in pregnant women was significantly higher than that in non-pregnant patients (p< 0.05) with the median number of CD31 positive vessels 28 (range 12 - 68) and 11 (range 4 - 19), respectively. There were no differences in the immunoreactivity of all other markers among both groups (p>0.05).
Conclusion: Borderline ovarian tumors in pregnant women were characterized by an enhanced CD31 expression compared with non-pregnant patients. The increase level of CD31 expression in pregnant women is supposed to be enhanced be pregestrone activity through progestindependent angiogenesis activation. We speculate that the similarity in VEGF, CD-105, Ecadgerin, Vimentin, CD56 expression in pregnant and non-pregnant women's indicates lack of stimulation in these agents-dependent signal pathways on borderline ovarian tumors progression complicating pregnancy
Disclosure: Nothing to disclose

EP784

Innovative diagnostic panel (p16, stathmin1 and laminin-gamma1) for ovarian cancer precursor detection

A. Asaturova[1], N. Kondrikov[2]
[1]Pathology, National Medical Research Center for Obstetrics, Gynecology and Perinatology named after Academician V.I.Kulakov of the Ministry of Healthcare of Russian Federation, [2]Pathology, Moscow Regional Research Institute of Obstetrics and Gynecology, Moscow, Russian Federation

Introduction / Background: Serous tubal intraepithelial carcinoma (STIC) is known to be a putative

precursor for the most common and aggressive ovarian carcinomas, namely ovarian high-grade serous carcinoma. Precise and early diagnose this disease is therefore one of the priorities for tissue diagnosis in gynecologic oncology. At the same time routinely applied algorithm for STIC diagnosis could not cover some grey zones in its diagnostics.
Methodology: The study included 70 patients (125 fallopian tubes), all of the specimens were investigated morphologically and immunohistochemically (p53, Ki-67, p16, stathmin1 and lamininggamma1). Statistics involved exact Fisher's test, χ2-test, Cohen's kappa
Results: Expression of p16, stathmin1 and laminin-gamma1 was revealed in 85%, 77,5% and 90% of STIC consequently. In 4 tubes with STIC, only one of these tree markers was positive but we has no cases with all three negative expression. In STIC cases, expression of p16, stathmin1 and laminin-gamma1 was high both in the presence and in the absence of p53 expression in atypical cells. In most cases, positive expression of at least two of the three studied markers was shown. p16, stathmin1 and laminin-gamma1expressin does not differ between p53positive and p53-negative STICs. Cohen's kappa for STICs with ambiguous p53 expression was moderate (0.55) when we used only p53 and ki-67 expression. P16/lamininggamma1/stathmin1 diagnostic panel let us increased Cohen's kappa to good reproducibility (0.72), ($p < 0.05$).
Conclusion: Lack of grey zones in STIC diagnostics and good reproducibility of diagnoses allows us to recommend p16, stathmin1 and laminin-gamma1 diagnostic panel for STIC diagnostics, especially in case of ambiguous p53 expression and/or borderline KI-67 expression.
Disclosure: Nothing to disclose

EP785

Does progression free survival (PFS), overall survival (OS) or platinum sensitive interval (PSI) differ in patients undergoing intermediate/high complexity surgery for advanced ovarian cancer (AOC) when treated by primary (PDS) or Interval (IDS) debulking surgery?

V. Asher[1], S. Abdul[1], S. Tou[2], M. Persic[3], A. Collins[1], L. Seneviratne[3], D. Gomez[4], O. Glover[1], A. Bali[1], A. Phillips[1]
[1]Obs and Gyn, [2]University Hospitals of Derby and Burton, [3]Dept of Oncology, University Hospitals of Derby and Burton, Derby, [4]Nottingham University Hospitals NHS Trust, Nottingham, United Kingdom

Introduction / Background: Both CHORUS and EORTC 55971 demonstrated no difference in OS/PFS between PDS OR IDS. However, both trials have been criticised due to low surgical quality. Retrospective reviews have suggested differences in OS/PFS/PFI.
Aim: To investigate if PDS or IDS, affects the OS, PFS or PSI in a cohort of AOC patients receiving high quality surgery.
Methodology: All patients with AOC treated between 02/2014-01/2019 obtaining complete cytoreduction with intermediate/high surgical complexity. Recurrence was defined on radiological/CA125 findings. Platinum sensitivity was defined according to international standards.
Results: 53 patients were identified (32 PDS and 21 IDS) with full recurrence data. No difference was seen in Age or Surgical complexity. 25 patients had recurred (15 PDS and 10 IDS). No difference was seen between groups in OS or PFS. Median survival was not yet reached. 56% were alive at 41months and 52% alive at 48 months in PDS and IDS groups respectively. Median PFS was 20.7 months in both groups. PFI did not differ between PDS or IDS patients.
Conclusion: This study supports the findings of previous randomised studies that no difference persists in OS, PFS or PSI in patients undergoing PDS or IDS.
Disclosure: Nothing to disclose

EP786

Telomere length and its implication as prognostic marker in ovarian cancer patients

D. Atallah[1], S. Antoun[2], M. Moubarak[1], N. El Kassis[1], R. Tahtouh[3], G. Chahine[4], G. Hilal[2]
[1]Obstetrics and Gynecology, Saint Joseph University/Hôtel Dieu de France University Hospital, [2]Cancer and Metabolism Laboratory, [3]Saint Joseph University, [4]Oncology, Saint Joseph University/Hôtel Dieu de France University Hospital, Beirut, Lebanon

Introduction / Background: Telomeres are DNA structures protecting the linear ends of eukaryotic chromosomes against degradation and fusion, thereby maintaining genome stability. Telomerase is an enzyme that stabilizes the length of linear chromosomes by de novo synthesizing telomeric repeats during incomplete DNA replication, thus ensuring immortalization. This enzyme is expressed in 80% of cancers, including ovarian carcinoma. The human telomerase reverse transcriptase has been investigated as a detection marker for cancers in early stages, and a prognosis marker in late stages disease. The aim of this study is to investigate telomere length as a marker for survival and recurrence in ovarian carcinoma.
Methodology: 37 ovarian cancer biopsies have been isolated from Lebanese patients. The biopsies were subjected to DNA extraction, and then telomere length was assayed in cancer cells following the method elaborated by R. Cawthon (2002). Clinical data of each patient have been retrieved, including survival, recurrence, lymph node ratio, age, weight.
Results: The results obtained revealed a direct relation between telomere length and survival. In fact, patients with shorter survival rates (less than 1 year) exhibited 30% longer telomeres compared with patients with longer survival rates (3 to 5 years). Moreover, recurrence rates increased with longer telomeres. Higher ratios (35%) were detected in patients with early recurrence (less than 1 year) compared to patients with late or absent recurrence. However, no direct effect between telomere length and the other parameters was spotted.
Conclusion: telomere length could be used as a prognostic marker in patients with ovarian cancer by predicting better survival and lower recurrence in case of shorter telomeres. This will be of a great value when making therapeutic decisions in a heterogeneous and aggressive disease as ovarian cancer.
Disclosure: Nothing to disclose

EP787

Characteristics of ovarian tumors in Lebanon: 20 years of experience in a Lebanese tertiary center

R. Eid[1], E. Lilly[1], F.G. Haddad[1], C. Kesserouani[2], H.R. Kourie[1], A. Khaddage[2], M. Moubarak[3], D. Atallah[3]
[1]Oncology, [2]Pathology, [3]Obstetrics and Gynecology, Saint Joseph University/Hôtel Dieu de France University Hospital, Beirut, Lebanon

Introduction / Background: To report the epidemiological and histological features of ovarian tumors in Lebanon.
Methodology: This is a retrospective study evaluating the characteristics of borderline and malignant ovarian tumors diagnosed in 20 years (from 1997 to 2017) at the Hôtel Dieu de France, University Hospital of Saint Joseph University in Beirut in Lebanon. The data was extracted from the computerized registers of the hospital´s pathology laboratory. Statistical analysis was performed using SPSS 24.0 software.
Results: 1137 ovarian lesions were reported, of which 695 (61.12%) were benign, 50 (4.4%) borderline, 361 (31.75%) malignant and 31 (2.73%) were unspecified. Of the 361 malignant lesions, 54 (4.75%) were metastases from another extra-ovarian primitive. Of the 652 benign neoplastic ovarian tumors, epithelial tumors, stromal and sex cords tumors, germ cell tumors and tumors from the dermoid cyst were 306 (46.93%), 70 (10.73%), 268 (41.1%). %) and 8 (1.24%). The most common benign neoplastic tumor was mature cystic teratoma representing 268 cases (41.1%), followed by 170 (26.07%) serous cystadenomas, and 80 (12.2%) mucinous cystadenomas. Of the 361 malignant ovarian tumors, 246 (68.1%) were malignant surface epithelial tumors. Germ cell malignancies, stromal tumor and sex cords, and metastatic carcinoma were 25 (6.9%), 22 (6.1%), and 54 (15%), respectively. High grade serous cystadenocarcinoma was the most common malignant tumor with 147

cases (40.7%).
Conclusion: The epidemiological characteristics of ovarian tumors in Lebanon are compatible with those published in Western countries and in Asia. This study is the first of its kind in Lebanon and is a database for further research.
Disclosure: Nothing to disclose

EP788

Lebanese experience with cytoreductive surgery in ovarian cancer: a single institution series

D. Atallah[1], M. Moubarak[1], B. Dagher[1], N. El Kassis[1], H. El Hajj[1], G. Chahine[2]
[1]Obstetrics and Gynecology, [2]Oncology, Saint Joseph University/Hôtel Dieu de France University Hospital, Beirut, Lebanon

Introduction / Background: To review the surgical outcomes of cytoreductive surgery for ovarian cancer in a single institution.
Methodology: we reviewed all patients with ovarian cancer who received a cytoreductive surgery between January 2005 and December 2018 at Hôtel-Dieu de France University Hospital, Lebanon.
Results: 161 patients were included. Mean age at surgery was 54 years (range 16 - 83 years). Cytoreductive surgery was primary in 40%of cases. Surgical resection included bowel resection (48%), diaphragmatic peritoneal resection (25%) and splenectomy (15%). 89% of patients received a pelvic and para-aortic lymphadenectomy. Node involvement was noted in 48% of cases. No recurrence was seen in 56% of cases and the mean interval of recurrence was estimated at 21 months. Overall survival was estimated at 40 months (range 2 - 165 months). According to cox regression test, we found that survival is significantly correlated to age, menopausal status, lymph node status, number of positive lymph nodes and lymph node ratio (LNR) ($p = 0.009$, $p = 0.004$, $p = 0.001$, $p = 0.002$ and $p= 0.004$ respectively). Patients with LNR ≤ 0.03 had a survival of 50 months vs. 27 months in patients with LNR > 0.03. Mean survival was estimated at 51 months in patients with only one positive LN vs. 26 months in patients with more than one positive LN. Lymphadenectomy neither increased the postoperative complications nor the transfusion rate and was only associated with longer operative time ($p = 0.7$, $p = 0.85$ and $p = 0.000$ respectively). No impact on survival was detected whether the patient benefited from an upfront surgery or an interval one post neoadjuvant chemotherapy: 36 months vs 30 months respectively, ($p= 0.39$).
Conclusion: Huge efforts including extensive cytoreductive surgeries are being performed at institutions in developing countries in order to improve survival and lower recurrence in ovarian cancer patients.
Disclosure: Nothing to disclose

EP789

Sequential use of Risk Malignancy Index and Ca-125 in evaluating adnexal masses

A. Bahadur[1], K. Khoiwal[2], L. Chawla[2], R. Mundhra[2], A. Gaurav[2], N. Bhattacharya[2], P. Jha[2], A. Yadav[2], S. Kumari[2], S. Prateek[2], J. Chaturvedi[2]
[1]Obstetrics & Gynecology, [2]All India Institute of Medical Sciences, Rishikesh, India

Introduction / Background: Adnexal masses have a wide spectrum with respect to age of presentation, signs & symptoms, imaging findings and histopathology report. Pre-operative identification of malignant potential of adnexal masses is important. Ovarian cancer is a significant cause of mortality in menopausal women, large population-based studies demonstrate that the majority of adnexal masses are benign. Exceptionally an ovarian tumour may be malignant and should be treated as early as possible.

Methodology: This is a prospective study, conducted at All India Institute of Medical Sciences, Rishikesh, Uttarakhand, India. The data was collected from Department of Gynaecology over a period of eighteen months from June 2017 to December 2018. All women who were diagnosed to have an adnexal mass irrespective of age, parity and menopausal status were included in the study. USG abdomen with pelvis was ordered in all patients followed by CECT or MRI in selective patients. Tumor markers such as Ca-125 were measured, and Risk of Malignancy Index (RMI) was calculated for each tumor. The clinical and imaging findings were correlated with intra-operative findings and finally with the histopathology (HPE) report.
Results: A total of 171 women were included in the study who were diagnosed to have adnexal mass. 137 women (80.1%) had benign tumor (Group B) while 34 women (19.9%) were found to have malignant tumor (Group M). Mean age in group B was 37.27$\pm$12.88 and in group M was 46.12$\pm$13.46 (P value=0.001). Ca-125 was significantly higher in group M (1350 U/ml) than group B (175 U/ml) [P value=0.008). The RMI score was also found to significantly increased in group M than group B (P value=0.007).
Conclusion: With respect to adnexal masses, both Ca-125 as well as RMI scoring are important diagnostic tools. RMI scoring has a better overall diagnostic performance than Ca-125 in predicting malignancy.
Disclosure: Nothing to disclose

EP790

What is initial in ovarian cancer, changes in metabolism or in histological structure of the organ?

V. Bandovkina, E. Frantsiyants, T. Moiseenko, I. Kaplieva, E. Surikova, I. Neskubina, N. Chernikova, M. Adamyan, T. Myagkova, V. Nikitina
Rostov Research Institute of Oncology, Rostov-on-Don, Russian Federation

Introduction / Background: The role of sex hormones in ovarian cancer (OC) pathogenesis is indisputable. Serotonin and histamine perform neuroendocrine regulation of reproductive processes. Our aim was to study sex hormones and biogenic amines in tumorous and contralateral ovaries of OC patients.
Methodology: Levels of estradiol (E_2), testosterone (T), progesterone (P_4), histamine (H) and serotonin (5HT) (Hema, Russia) and enzyme activity of MAO-A and DAO were measured in tissues of cystadenocarcinomas and contralateral non-tumorous ovaries of 24 patients (mean age 56.5±2.6 years) with stage III-IV OC using standard ELISA kits. Visually and morphologically unchanged ovaries removed during surgery for uterine fibroids in 21 women of similar age were studied as intact tissues. All patients gave their voluntary informed consent for the study.
Results: Levels of P4 in tumorous ovaries, compared to intact organs, were 1.5 times lower, E2 - 2.1 times higher, 5HT and H - 3.5 and 2 times lower, MAO-A activity - 2.5 times higher, and DAO activity - 3.4 times higher. In histologically unchanged contralateral ovaries, P4 levels were 1.6 times lower than in intact tissues, E2 - 2.3 times higher, 5HT - 2.6 times lower, H - 2 times lower, MAO-A activity - 1.9 times higher, and DAO activity - 4.1 times higher. Levels of T in tumorous and contralateral ovaries were similar and did not differ from the norm. Both tumorous and contralateral ovaries showed hyperestrogenism, hypoprogestinemia, and serotonin and histamine deficiency with an increased activity of amine oxidases.
Conclusion: Contralateral morphologically unchanged ovaries demonstrated similar hormonal and metabolic profile, compared to the organ affected by malignant tumors. The metabolic profile in the ovaries underwent changes similar to the tumor process even before the malignant transformation. The results indicate a systemic reproductive system lesion in OC.
Disclosure: Nothing to disclose

EP791

Dissociation between systemic and local hormonal profiles in young patients with ovarian cancer

V. Bandovkina, E. Frantsiyants, T. Moiseenko, I. Kaplieva, L. Trepitaki, A. Menshenina, E. Verenikina, T. Myagkova, N. Chernikova, N. Cheryarina, I. Goroshinskaya, N. Maksimova
Rostov Research Institute of Oncology, Rostov-on-Don, Russian Federation

Introduction / Background: Blood levels of hormones do not reflect the true picture of hormonal homeostasis due to the local synthesis of sex hormones by tumor tissues. Our purpose was to analyze levels and ratios of sex hormones in the blood and tissues of removed ovaries in ovarian cancer (OC) patients of the reproductive age.
Methodology: Levels of estradiol (E2), testosterone (T) and progesterone (P4) were measured by RIA in the blood and ovarian tissues of patients with unilateral ($T_{1-2}N_0M_0$) and bilateral advanced OC ($T_{3-4}N_{1-2}M_0$), and the E2/P4 ratio was calculated. Mean age of patients 34.7±2.3 years, tumor histotype G2-3 cystadenocarcinoma, menstrual function preserved (luteal phase).
Results: In patients with $T_{3-4}N_{1-2}M_0$, blood levels of E2 exceeded the norm by 1.6 times, T by 1.4 times, and P4 was 7.4 times lower. In unilateral OC $T_{1-2}N_0M_0$, blood levels of E2 were 2.8 times lower than the norm, P4 40 times lower, and T was twice higher. E2/P4 ratios in patients with both $T_{3-4}N_{1-2}M_0$ and $T_{1-2}N_0M_0$ exceeded the norm on average by 13 times. In tumor tissues in $T_{1-2}N_0M_0$, E2 and P4 were 5.5 and 3 times higher, respectively, than in tumor tissues in $T_{3-4}N_{1-2}M_0$; T levels were similar. The E2/P4 ratio in patients with both advanced and unilateral OC exceeded the ratio in intact ovaries on average by 5.5 times.
Conclusion: Relative hyperestrogenism was observed in the blood of patients with $T_{1-2}N_0M_0$ OC, and absolute - in $T_{3-4}N_{1-2}M_0$. Since E2 in tumor tissues of $T_{1-2}N_0M_0$ cancer was higher than in $T_{3-4}N_{1-2}M_0$, we could suppose an activation of extragonadal estrogen synthesis in patients with an advanced process. Changes in the blood and tissue ratios of hormones indicated disorders in the central regulation of the production of sex hormones and the loss of correlative connections typical of normal physiological processes.
Disclosure: Nothing to disclose

EP792

Some biological characteristics of malignant transformation of ovaries preserved after hysterectomy

V. Bandovkina, E. Frantsiyants, T. Moiseenko, I. Neskubina, E. Surikova, I. Kaplieva, L. Trepitaki, N. Chernikova, M. Adamyan, E. Verenikina, I. Goroshinskaya
Rostov Research Institute of Oncology, Rostov-on-Don, Russian Federation

Introduction / Background: Removal of the uterus, the target organ for ovarian hormones, causes disorders in neuroendocrine relationships of the hypothalamic-pituitary-gonadal axis. Long-term effects of hysterectomy (HE) in the subsequent malignant transformation of the ovaries are still poorly studied. Our aim was to study some systemic and local neuroendocrine parameters in women with ovarian cancer (OC) developed after HE.
Methodology: The study included 98 menopausal women with OC, mean age 61.5±3.6: group 1 - OC after HE, group 2 - primary OC; controls - patients with intact ovaries operated on for hysteromyoma. Standard ELISA test systems were used to determine the blood levels of serotonin (5HT), LH, FSH, cortisol (C) and estradiol (E2), and levels of 5HT, 5-HIAA, histamine (H) and activity of MAO-A and DAO in tumor and intact ovarian tissues. All patients gave their voluntary informed consent for the study.
Results: Blood levels of 5HT in group 1 were 2.2 times lower than the norm, FSH and LH - 2.3 and 2.4 times lower, while in group 2 - 2, 1.3 and 1.8 times higher, respectively. Blood levels of E2 in group 1 were similar to the norm, C - 1.6 times higher. In group 2, C in the blood was similar to the norm in 71% cases and exceeded it by 2.2 times in 29%; E2 was 1.6 times lower. In groups 1 and 2,

compared to controls, tissue levels of 5HT were 3.5 and 1.5 times lower, respectively, 5-HIAA 1.6 times lower than the norm, MAO-A activity 2.5 times higher. In both groups, H was lower than in controls, DAO activity in group 1 was decreased by 2.2 times, and in group 2 increased by 3.5 times.
Conclusion: Development of OC in preserved ovaries after HE, unlike primary OC, is not associated with the pituitary stimulation and is more “acquired” than “endogenous”.
Disclosure: Nothing to disclose

EP793

Ovarian teratoma - one case report

D. Barrientos Hernandez[1], M. Barrientos Hernandez[1,2,3]
[1]Departamento de Obstetricia y Ginecología, Hospital 2 de Mayo, [2]San Marcos University, [3]IPSS, Lima, Peru

Introduction / Background: The ovarian cáncer is one of the most common ginaecological tumor after breast and uterus tumor, and the most common are benign (80%) and 20% of malign ovarian tumor. The diagnosis was performed with physical exam. and Abdominal Ultrasound and to complete the study was requested the abdominal. CT Scan and blood tests, It was an Open Surgery with resection of the left ovarian TERATOMA (8cm) with PERITONITIS management during the surgery,drenages was needed.
Methodology: A single woman of 33 y,o, with no historical diseases,no medication, no deliveries, IMC 25%, came to the E.R. with lower abdominal pain, and went to Surgery after Ecography and abdominal CT Scan done,blood test: leucocitosis,with Neutrophylia, fever, and high PCR, HB.: 12.We decided open surgery because of the big size of the tumor (8cm) and we managed the complication of the teratoma (peritonitis).
Results: The Ovarian Teratoma (size 8cm) and a left plastrom was removed by open surgery, and abdominal drenages needed.
After surgery, the patient had SEPSIS (hypotension, tachycardia,hb: 9,PCR:500, and Saturation of Oxygen low: 89%), she went to ICU and required Ventilation, central i.v line,antibiotics, fluids, She was at the intensive Unit for 4 days.
The serum CA 19,9 was 9,000 U/lt, CA 125:; 365and the histopathology study was BENIGN TERATOMA.
Conclusion: The ovarian germinal cells Teratoma (benign) is the most common type of teratoma ,and peritonitis isnt a common presentation, (0,2%), but our patient did.The Laparotomy is the first choice but in our patient the key for Pritonitis complication management was the Open Surgery.
Disclosure: Nothing to disclose

EP794

A meta-analysis of morbidity and mortality in primary cytoreductive surgery compared to neoadjuvant chemotherapy in advanced ovarian malignancy

H.C. Bartels[1], A.C. Rogers[2], D. O'Brien[3], R. McVey[3], W.D. Boyd[3], D.J. Brennan[4,5]
[1]Ireland East Hospital Group Gynaeoncology Group, Mater Misericordiae University, [2]Department of Surgery, Mater Misericordiae Dublin, [3]Ireland East Hospital Group Gynaeoncology Group, St Vincents University Hospital, [4]Ireland East Hospital Group Gynaeoncology Group, Mater Misericordiae Dublin, [5]University College Dublin, Dublin, Ireland

Introduction / Background: The aim of this metanalysis is to compare the morbidity and mortality in patients undergoing Primary Cytoreductive Surgery (PCS) compared to Neoadjuvant Chemotherapy (NACT) and Interval Cytoreductive Surgery (IDS) for advanced ovarian cancer.

Methodology: A literature search using Preferred Reporting Items for Systematic Reviews and Meta-Analyses (PRISMA) guidelines was performed for publications reporting morbidity and mortality in patients undergoing PCS compared to NACT and ICS. A total of 1341 citations were reviewed; 17 studies comprising 3759 patients were selected for the analysis.
Results: Patients in the PCS group were significantly more likely to have a Clavien-Dindo grade ≥ 3 morbidity with an overall rate of 21.2% compared to 8.8% (95%CI 1.9 - 4.0, p < 0.0001). Patients in the PCS group were more likely to die within 30 days of surgery (OR 6.1, 95% CI 2.1 - 17.6, p = 0.0008). Patients who underwent NACT + ICS had significantly shorter procedural times (MD -35 minutes, p = 0.01), lost less blood intraoperatively (MD -382ml, p < 0.001) and had an average admission 5.0 days shorter (MD -5.0 days, 95% CI -8.1 - -1.9 days, p = 0.002) than those undergoing PCS. Patients undergoing NACT were twice as likely to receive optimal and complete cytoreduction (OR 1.9, 95% CI 1.3 - 2.9, p = 0.001, and OR 2.2, 95% CI 1.5 - 3.3, p = 0.0001 respectively).
Conclusion: While NACT and IDCS resulted in significantly less morbidity and mortality with improved complete cytoreduction rates compared to PCS, this did not confer improved survival . Hence NACT is a feasible alternative in particular for patients with co-morbidities or high tumour burden.
Disclosure: Nothing to disclose

EP795

Searching for the best maintenance therapy in platinum-sensitive recurrent ovarian cancer: bevacizumab or PARP-inhibitors? A network meta-analysis

M. Bartoletti[1,2], G. Pelizzari[1,2], L. Bortot[1,2], M. Giavarra[1,3], M.G. Vitale[1,3], V. Andreotti[1,3], A. Parnofiello[1,3], S. Scalone[2], D. Lombardi[2], M. Nicoloso[2], C. Bozza[3], E. Poletto[3], C. Sacco[3], C. Andreetta[3], R. Sorio[2], F. Puglisi[1,2]
[1]Department of Medicine (DAME), University of Udine, Udine, [2]Dipartimento di Oncologia Medica, Centro di Riferimento Oncologico di Aviano (CRO), IRCCS, Aviano, [3]Department of Oncology, ASUI Udine University Hospital, Udine, Italy

Introduction / Background: Patients (pts) with ovarian cancer experiencing a platinum-sensitive (PS) recurrence are generally re-exposed to platinum agents (PCT). The addition of bevacizumab (BEV) or PARP inhibitors (PARPi) as concomitant and/or maintenance therapy has shown to improve progression free survival (PFS). In the absence of direct comparisons coming from randomized trials (RCTs), we have performed a network meta-analysis to evaluate differences in terms of efficacy between BEV and PARPi in pts with PS recurrent ovarian cancer (rOC), according to BRCA status.
Methodology: We searched PubMed, Embase and Medline for RCTs involving pts with PS rOC treated with BEV (n=3, 1563 pts) or PARPi (n=5, 1839 pts). Only trials with PFS as primary endpoint were included. Analyses have been done pooling pts who had received PARPi in three groups, according to the available data on BRCA genes status: all comers (AC), BRCA mutated pts (BRCAm) and BRCA wild-type pts (BRCAwt). A frequentist approach has been used with R statistical software. To rank the effect size of treatments, surface under the cumulative ranking value (SUCRA) has been applied.
Results: In AC pts, PARPi improved PFS compared to BEV (hazard ratio [HR]=0.70, 95% CI 0.54-0.91). In BRCAm pts the gain in PFS for PARPi was even higher compared to BEV (HR=0.46, 95% CI 0.36-0.59). In BRCAwt pts the benefit of PARPi over BEV was not statistically significant (HR=0.87, 95% CI 0.63-1.20) but PARPi had the highest likelihood of being ranked as the best treatment in terms of efficacy according to SUCRA (90% and 60%, respectively for PARPi and BEV).
Conclusion: According to indirect comparisons, PARPi performed the best for the treatment of PS rOC, especially in BRCAm pts who had not previously received PARPi. BEV could be still an option in BRCAwt pts.
Disclosure: Fabio Puglisi: Roche, AstraZeneca (honoraria and research founding). No conflict of interest is to be declared for the remaining authors. The authors receive no financial support for this study.

EP796

Estrogen receptors alpha and beta in prediction of the front-line platinum plus taxane chemotherapy efficacy in III stage serous high-grade ovarian cancer patients

A. Basharina, T. Bogush, A. Tjulandina, E. Bogush, S. Tjulandin
N. N. Blokhin National Medical Research Centre of Oncology, Moscow, Russian Federation

Introduction / Background: Estrogen receptors alpha and beta (ERα and ERβ) are targets not only for antiestrogens, but also for cell proliferation factors. Besides, in most cases, cytostatics efficacy is determined by proliferative activity of malignant cells. Taking these facts into account, we aimed to evaluate the correlation between quantitative indexes of ERα and ERβ expression in tumor tissue and the chemotherapy efficacy in ovarian cancer patients.
Methodology: Patients (n=31) maximally homogenized by clinical parameters (stage III high-grade serous ovarian cancer (HGSOC), suboptimal surgical cytoreduction, six cycles of the front-line platinum plus taxane chemotherapy) were enrolled in the study. Expression of ERα and ERβ was evaluated quantitatively by immunofluorescent method using flow cytometry. The marker level was calculated as the percent of specifically fluorescent cells. The median of the index was used as a cut-off between high and low level marker expression groups. The correlation between ERα and ERβ expression and progression-free survival (PFS) was estimated by both Kaplan-Meier curves and Cox proportional-hazards model (GraphPad Prism 7.0 and SPSS 22.0).
Results: ERα and ERβ were coexpressed in the all studied tumor samples and mean level of ERβ was 1.5 times higher (p=0.001) vs ERα (41.7%±12.4 and 25.9%±11.1 respectively). It was not shown the ERα predicative value for platinum plus taxane chemotherapy efficacy. Opposite, high ERβ tumor expression (in more than 40% of the cells) had positive predictive value for the chemotherapy efficacy. The median of PFS was 2 times greater (18 vs 8 mos in 48 mos patient monitoring, p=0.02) and the risk of recurrence was 2.5 times lower in the group with the high vs low level of ERβ expression (p=0.04).
Conclusion: The level of ERβ and not ERα expression in tumor tissue is a positive predictive marker of the front-line platinum plus taxane chemotherapy in patients with stage III HGSOC and suboptimal surgical cytoreduction.
Disclosure: Nothing to disclose

EP797

20 years of ultra radical surgery for ovarian cancer patients in Perth Western Australia, what have we learnt?

L. Baxter, C. Ayres, P. Cohen, R. Kader Ali Mohan, Y. Leung
King Edward Memorial Hopsital, Subiaco, WA, Australia

Introduction / Background: Primary cytoreductive surgery followed by adjuvant taxane- and platinum-based combination chemotherapy are the standard treatments for advanced ovarian cancer in most patient populations. Much has been debated regarding the competing perspectives of the tumour biology versus the value of aggressive surgical resection in ovarian cancer as major survival determinants.
Many recent studies have showed significant survival advantages for optimally cytoreduced patients. Secondary to this many gynaecological unites, including out own, has adopted a policy of aggressive cytoreduction, often involving bowel resection.
The detraction however from ultra-radical surgery is the associated morbidity of ultra-radical surgery. The aim of this study is to assess radical debulking of ovarian cancer with concomitant rectosigmoid resection, with respect to perioperative complication rates and its impact on survival following surgery in a tertiary unit in Perth Western Australia.
Methodology: A retrospective review was performed of the case notes of 164 consecutive procedures performed between 2000 and 2017 in a regional cancer centre.
Results: 11.4% of patients suffered some form of complication during admission. Anastomotic

complications were rare, occurring in only 1.5% of patients. Perioperative mortality was 2.6%. Overall mean survival was 45 months. Mean survival and progression free survival were significantly longer in the ultra-radical group.
Conclusion: Ultra-radical surgery for ovarian cancer ensure optimal cytoreduction and increased median survival time, with low rates of intra operative and post operative morbidity.
Disclosure: Nothing to disclose

EP798

Diagnostic accuracy of gynecology imaging reporting and data system in evaluation of adnexal lesions

F. Behnamfar, M. Moradi, A. Adibi, H. Khadra
Esfahan University of Medical Sciences, Esfahan, Islamic Republic of Iran

Introduction / Background: Considering the utility of Gynecologic Imaging Reporting and Data System (GI-RADS)in diagnosing malignant adnexal lesions such as ovarian cancer, we aimed to evaluate the diagnostic performance of this reporting system in differentiating between malignant and benign adnexal lesions.
Methodology: In this cross sectional study, women with suspected adnexal lesions were enrolled. For differentiating of malignant adnexal lesions Grade II & III of GIRADs system were classified as low risk for malignancy and grades IV and V as high risk. Results of histopathologic diagnosis were compared with the results of the mentioned GI-RADS system classification and the diagnosed accuracy of the system was determined. Patients who did not have histopathologic diagnosis were followed up.
Results: In this study, 197 women with suspected adnexal lesions were evaluated. Frequency of GI-RADS II,III,IV and V were 34.5%(69 cases),38.0%(76 cases),19.5%(39 cases) and 6.5%(13 cases)respectively. According to the low and high risk classification of GI-RADS, 72.5 % were classified as GIRADs II & III and 26% as GIRADs IV & V respectively. Definitive histopathologic diagnosiswas reported for 158 cases.Histopathologic evaluation indicated that 12(7.6%)of the masses were malignant and 146 (92.6%) were benign.Comparing with the histopathologicdiagnosis, theGI-RADS system sensitivity, specificity, PPV, NPV, (+) LR and (−) LR were 91.6%, 80.82%, 28.2%, 99.1%, 4.77 and 0.10, respectively. The accuracy of the scoring system was 81.64%.
Conclusion: GI-RADS system could be useful for clinicians for performing an appropriate clinical management. However, given that the sensitivity of the system is higher than its specificity, it is recommended to design further studies with additional laboratory tests such as CA-125.
Disclosure: Nothing to disclose

EP799

Intraoperative molecular analysis during interval cytoreduction of high-grade serous tubo-ovarian carcinoma to confirm necessity for visceral resection

J.P. Beirne[1], F. Laura[1], D. McArt[1], I. Harley[2], W.G. McCluggage[3], P. Mullan[1], Ovarian Cancer Research Focus Group
[1]Centre for Cancer Research and Cell Biology, Queens University, Belfast, [2]Northern Ireland Gynaecological Cancer Centre, [3]Institute of Pathology, Belfast Health and Social Care Trust, Belfast, United Kingdom

Introduction / Background: Cytoreductive Surgery (CRS) to zero visible residual disease is the strongest independent predictor of survival in advanced ovarian cancer. High-grade serous(HGSC) is the commonest and most aggressive sub-type. Carcinogenesis begins in the distal fallopian tube (NFT), via serous tubal intraepithelial carcinoma (STIC). Management may be through CRS followed

by Adjuvant Chemotherapy (ACT) or Neoadjuvant Chemotherapy (NACT) followed by Interval CRS. Determining active visceral disease versus chemotherapeutic effect can be challenging during Interval CRS. A proportion of patients may be inappropriately treated and, therefore, potentially have worse survival or increased morbidity. Tumour-specific molecular alterations in surgical margins can predict risk of local recurrence. However, these assays are time-consuming and cannot be used in the intraoperative setting. The objective of this study was to validate tumour-specific methylated DNA assays using that could be used intra-operatively to assess sites of equivocal visceral disease in HGSC patients treated with NACT.
Methodology: Six cases of HGSC were identified through the Northern Ireland Gynaecological Cancer Centre. Formalin-fixed paraffin embedded (FFPE) tissue samples were retrieved via the Northern Ireland Biobank (NIB11:005, NIB13:0094). Matched NFT and HGSC samples were analysed by DNA methylation (DNAme) array profiling. Bioinformatic analyses revealed hypermethylation of multiple loci in HGSC compared to NFT. The top differentially methylated targets were further validated in a second cohort using pyrosequencing and qMSP.
Results: The top differentially methylated DNAme targets were analysed. Four were further validated in a cohort of 48 NFT and 48 HGSC unmatched samples. Pyrosequencing analysis revealed statistically significant differences ($p < 0.0001$) between each cohort with higher methylation in HGSC. A qMSP approach confirmed statistically significant ($p < 0.0001$) hypermethylation in HGSC compared to NFT samples.
Conclusion: Intra-operative tissue biopsy analysis, using qMSP, is technically feasible. This technology may provide a more accurate tool to identify viable, residual HGSC following NACT and therefore assist with more appropriate CRS.
Disclosure: Nothing to disclose

EP800

Outcome and treatment of ovarian cancer: a retrospective study

T. Berg[1], T.J. Nøttrup[1], U.B.S. Peen[2], H. Roed[1]
[1]Department of Oncology, Rigshospitalet, Copenhagen University Hospital, København Ø,
[2]Department of Oncology, Herlev Hospital, Copenhagen University Hospital, Herlev, Denmark

Introduction / Background: Ovarian cancer has the highest mortality rate among gynecological cancer and is the tenth most frequent cancer among women. Eighty percent of patients with advanced stage decease will experience a progression either during or after treatment.
Methodology: A retrospective, observational study of all women referred to adjuvant or neoadjuvant treatment for ovarian cancer between the 1st of June 2013 and 31st of May 2014 at two university hospitals in Denmark.
Results: We included 142 women with diagnosed ovarian cancer. Median overall survival from diagnosis was 48.5 months (95 % CI 36.6-57.9). Median survival after first, second, third, fourth and fifth progression was 19.3 (95 % CI 13.9-27-3), 11.4 (7.7-18.8), 9.5 (6.3-12.7), 8.3 (7.6-11.5), 5.6 (2.9-NA) months, respectively.
Median progression-free survival from diagnosis was 15.6 months (95% CI 14.3-18.4). Median progression-free survival after first, second, third, fourth and fifth progression was 9.2 (95% CI 7.7-10.6), 6.0 (3.5-7.7), 3.3 (2.6-4.6), 4.9 (3.6-8.3), 3.0 (2.4-5.7) months, respectively.
The most frequently used treatment at first progression was carboplatin and liposomal doxorubicin (n=37). The most used non-platinum containing treatment at progression was liposomal doxorubicin (n=26) followed by paclitaxel (n=23).
Conclusion: *:* Ovarian cancer remains a highly aggressive decease with most patients diagnosed in advanced stages. The treatment has not changed a lot in the last 15 years and the same is evident for the overall survival.
Disclosure: Mr. Tobias Berg received an unrestricted scholarship grant from the Danish Cancer Society. The remaining authors have nothing to disclose.

EP801

Malignant ovarian mixed germ cell tumor: a case report of a rare combination

R.M. Betoret Gustems[1], M. Vizcaíno Gómez[1], M. Serrano Jiménez[2], S. Soto García[2], E. Cazorla Amorós[1]
[1]Gynecology, [2]Pathology, Hospital Universitario del Vinalopó, Elche, Spain

Introduction / Background: Ovarian malignant germ cell tumors (OGCT) derive from primordial germ cells of the ovary and arise primarily in young women between 10 and 30 years of age, representing 70% of ovarian neoplasms in this age group. Mixed cell types constitute about 29% of OGCT, following pure dysgerminomas (33%) and immature teratomas (39%), being the most reported combination that of dysgerminoma and endodermal sinus tumor.
Methodology: Hereby we present a rare combination of mixed cell OGCT, comprising both embrionic carcinoma and yolk sac tumor.
Results: A 26-year-old nulliparous woman presented on january 2019, diagnosed by a private gynecologist of left adnexal mass. On ultrasound, an heterogeneus left pelvic mass of 100*65 mm, with peripheric positive Doppler map and minimum ascites was reported. On preoperative work-up a left ovarian tumor without lymphatic or peritoneal extension showed on CT-scan, as well as elevated tumoral markers (BHCG=9402; AFP=5588, Ca 125=109,3; LDH=1279). Medium laparotomy was performed, followed by exploration of abdominal cavity, ascites sampling, left adnexectomy, partial omentectomy and biopsy of a right ovarian, apparently benignant, fibrotic mass.
Pathological findings included ovarian benignant fibroma of the right ovary, negative omentum, positive preoperative cytology and left ovarian mixed cell OGCT, comprising 97% embrionic carcinoma and 3% yolk sac tumor, with focal capsular infiltration, 1C3 surgical stage. Postoperative tumoral markers decrease was monitorized, fertility sparing treatment was rejected and three courses of BEP adjuvant chemotherapy were given. No postoperative disorders were reported, third cycle was delayed due to moderate hepatotoxicity, and follow-up has shortly begun.
Conclusion: OGCT are a group of heterogeneous, sometimes highly malignant neoplasms, so suspiction must always arise in case of young women, presenting with rapidly growing solid pelvic mass. Early sugical intervention is mandatory, fertility sparing must be individually discussed and a thorough pathological examination must be performed, prior to evaluate eventual adjuvant therapies.

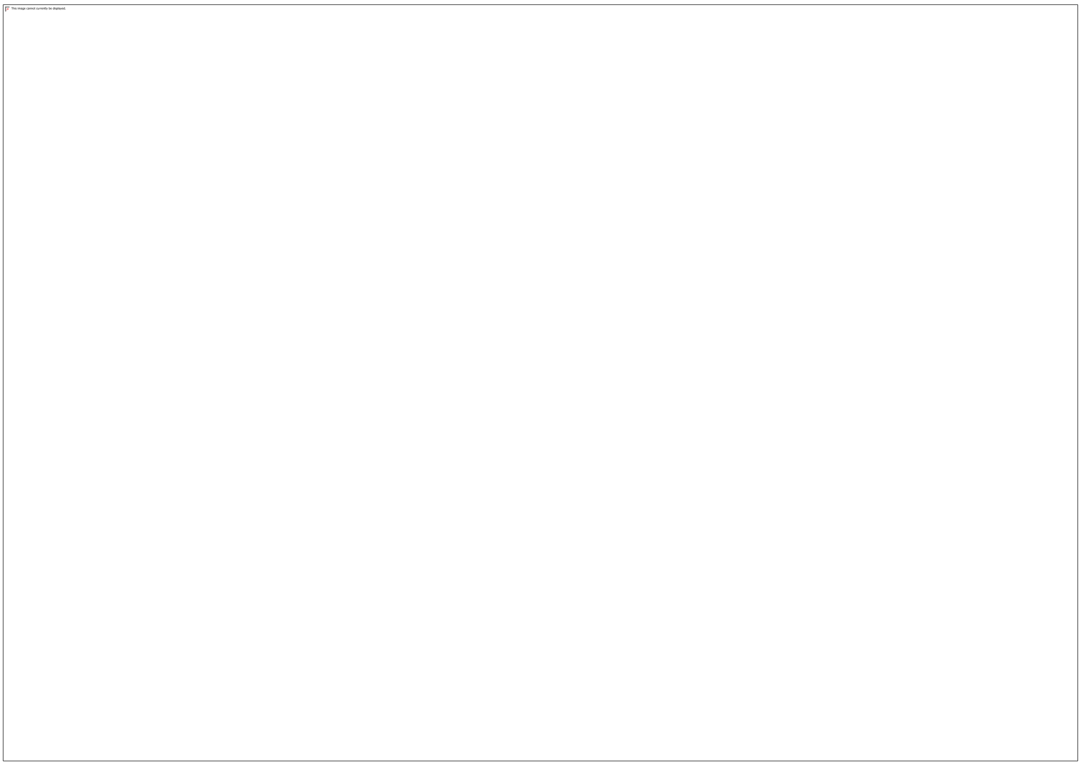

*[Left pelvic mass of 100*65 mm, with peripheric positive Doppler map and minimum ascites.]*

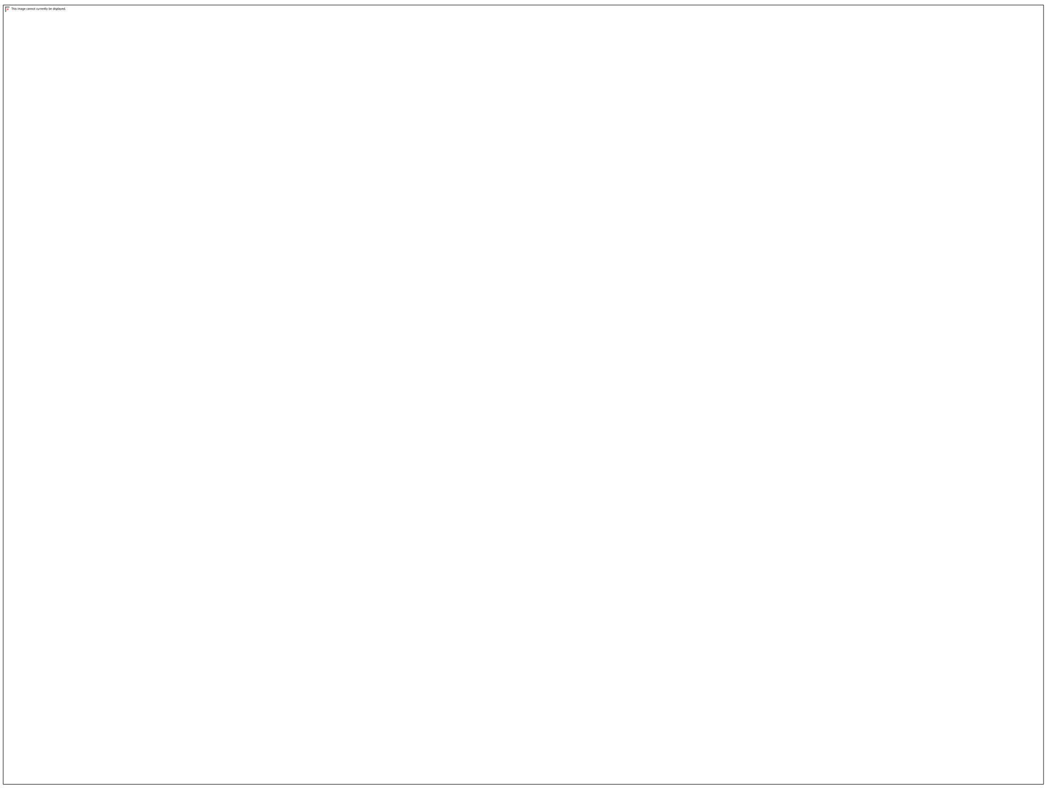

[Embrionic carcinoma on H-E; Alpha1Antitrypsine and AFP+ yolk sac tumor cells; HCG+ cells.]

Disclosure: Nothing to disclose

EP802

Therapeutic role of pelvic and para-aortic lymphadenectomy in apparent early stage epithelial ovarian cancer

N. Bizzarri[1], G. Corrado[1], S. Cianci[1], R. Ergasti[1], M.T. Perri[1], V. Rumolo[1], A. Rosati[1], V. Ghirardi[1], T. Pasciuto[2], G. Scambia[1], A. Fagotti[1]

[1]Gynecologic Oncology Division, [2]Statistics Technology Archiving Research (STAR) Center, Policlinico Universitario Agostino Gemelli I.R.C.C.S. Universita Cattolica di Roma, Rome, Italy

Introduction / Background: The therapeutic role of systematic pelvic and para-aortic lymphadenectomy in surgical staging of apparent early-stage epithelial ovarian cancer (aeEOC) is still unclear. Recently, ESGO-ESMO consensus conference established that re-staging lymphadenectomy is not recommended if patients are due to receive adjuvant chemotherapy for high-risk eEOC. The aim of this study was to define the therapeutic role of systematic lymphadenectomy in patients with eEOC, who received complete adjuvant chemotherapy.

Methodology: Single-center retrospective cohort study with Institutional Review Board approval, comparing women with aeEOC who underwent no (NL) or inadequate lymphadenectomy (IL) versus patients receiving adequate systematic bilateral pelvic and para-aortic lymphadenectomy (AL) (defined as ≥20 lymph-nodes).

Inclusion criteria: epithelial ovarian carcinoma; no bulky (≥10mm short axis) pelvic or para-aortic lymph

nodes at CT-scan; complete intra-peritoneal surgical staging and adjuvant chemotherapy as follow: i) from 3 to 6 cycles of carboplatin/paclitaxel; ii) 6 cycles of carboplatin only.
Results: From January 2006 to December 2016, 570 patients with FIGO stage IA-IIIA1 ovarian cancer were found. Of these 409 were excluded. 161 patients met inclusion criteria. 51 underwent AL, 84 IL and 26 NL. Patients' and surgery's characteristics are showed in Table 1. Patients who underwent AL were younger (p=0.011) and had higher rate of severe post-operative complications (p=0.01). Median follow-up was 45 (41-48) months. 5-year progression-free survival was 78.1% and 66.0% HR(95%CI)=1.47(0.74-2.95) (log rank test p=0.268), and overall-survival was 96.3% and 88.7% HR(95%CI)=2.22(0.64-7.73) (log rank test p=0.194), in IL/NL versus AL, respectively (Figure 1).
Conclusion: Systematic lymphadenectomy in surgical staging of eEOC does not seem to have a therapeutic value and increases the rate of severe post-operative complications. However, results from an ongoing multicentric study, with larger number of cases, will draw definitive conclusions.

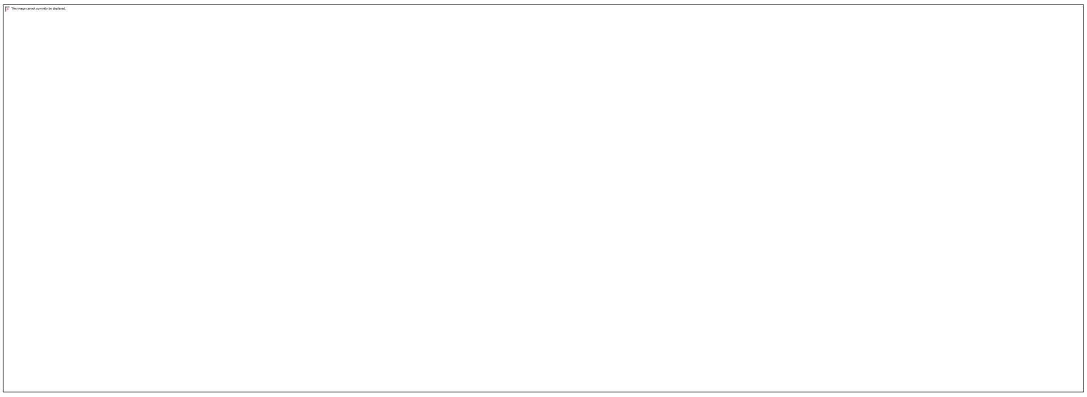

[Figure 1]

This image cannot currently be displayed.

[Table 1]

Disclosure: Nothing to disclose

EP803

The real-world effect of olaparib in BRCA-dependent platinum-sensitive recurrent ovarian cancer patients in Poland

P. Blecharz[1], R. Madry[2]
[1]Center of Oncology, M.Sklodowska-Curie Memorial Institute, Gliwice Branch, Gliwice, [2]Division and Department of Oncology, Poznan University of Medical Sciences, Poznan, Poland

Introduction / Background: Maintenance therapy with PARP inhibitor, olaparib, showed its efficacy in term of prolonged progression-free survival (PFS) and some others endpoints in platinum-sensitive, *BRCA1/2*-dependent, recurrent ovarian cancer (*BRCA1/2*-PSROC) in phase 2 and 3 clinical trials. The aim of the study was to analyze real-world effect of olaparib treatment in *BRCA1/2*-PSROC patients in Poland.
Methodology: Olaparib therapy was funded by Polish Ministry of Health for patients with deleterious *BRCA1*/2 mutations. Inclusion and exclusion criteria consistent with „Study 19” ones were strictly kept and noted in medical records. Follow-up included monthly visits with basic lab tests, Ca125 level and computer tomography (CT) performed three-monthly or if progression was suspected. Treatment was stopped when progression by RECIST criteria was seen, in case of allergy to the drug or long-term grade ≥3 NCI CTC toxicity . No treatment beyond progression was allowed. Date of progression consistent with CT scan date was noted in medical records. Patients were followed until progression or treatment discontinuation. Time to olaparib discontinuation (TTD) as a surrogate of PFS were plotted using Kaplan-Meier survival curves.
Results: Between 19/09/2016 and 30/07/2018, 311 patients with *BRCA1/2*-PSROC started maintenance therapy with olaparib, 400 mg twice daily (capsules). Finally, data on follow-up time and TTD from 301 patients were available for the analysis. The end of therapy in analyzed period was noted in 63(21%) of patients. Median follow up time was 245 days (range 29-668; SD=152,6). Median of TTD was not reached at 22 months.
Conclusion: The effect of olaparib maintenance therapy in platinum-sensitive recurrent ovarian cancer patients in Poland confirms its effectiveness in term of TTD, calculated as a surogat of PFS, when strict inclusion and exclusion criteria are kept.

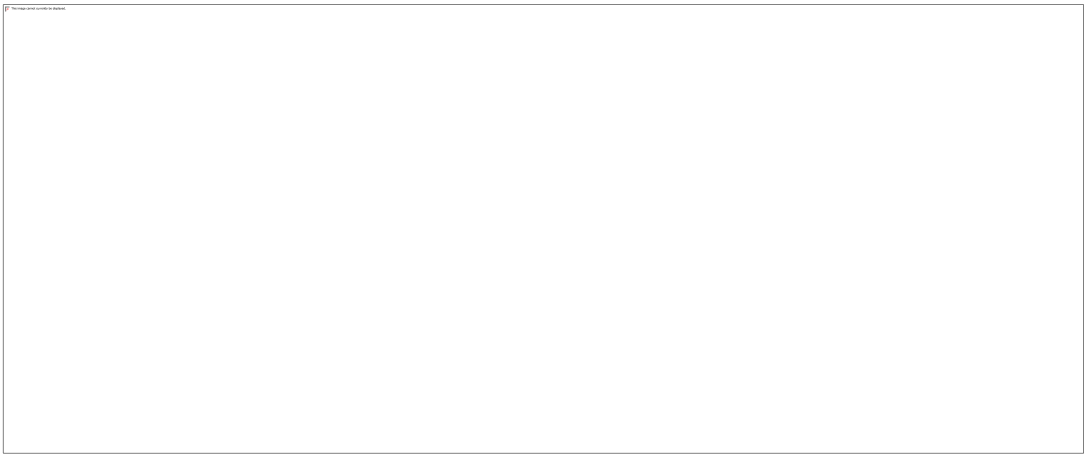

[Time-to-treatment discontinuation curve of Polish patients compared to Study 19 and SOLO2 PFS curves]

Disclosure: Authors received honoraries and travel grants from Astra-Zeneca Polska SA

EP804

Prognostic role of second-line platinum-based therapy in recurrent platinum resistant ovarian cancer patients - data from real-world clinical practice in Poland

L. Bodnar[1], P. Knapp[2], J. Sznurkowski[3], R. Mądry[4], A. Gąsowska-Bodnar[5], P. Blecharz[6], M. Sikorska[7],

A. Timorek[8], A. Ptak-Chmielewska[9], R. Jach[10], Polish Group of Oncological Gynaecology
[1]Department of Oncology, University of Warmia and Mazury in Olsztyn, Olsztyn, [2]Department of Gynecology and Gynecologic Oncology, Medical University of Bialystok, Białystok, [3]Department of Oncological Surgery, Medical University of Gdańsk, Gdańsk, [4]Clinic of Oncology, Poznan University of Medical Sciences, Poznań, [5]Department of Gynecology and Gynecologic Oncology, Military Institute of Medicine, Warsaw, [6]Department of Gynecologic Oncology, Centre of Oncology, Maria Sklodowska-Curie Memorial Institute, Krakow Branch, Kraków, [7]Department of Gynecology and Obstetrics, Faculty of Medical Sciences, University of Warmia and Masuria, Olsztyn, [8]Department of Obstetrics, Gynecology and Oncology, Warsaw Medical University and Brodnowski Hospital, [9]Institute of Statistics and Demography, Warsaw School of Economics, Warsaw, [10]Department of Gynecology and Oncology, Jagiellonian University College, Kraków, Poland

Introduction / Background: Strategy of treatment in patients with recurrent ovarian cancer (OC) dependents on platinum-free interval (PFI). We aimed to compare survival in “platinum resistant” OC patients who received platinum-based or non-platinum chemotherapy at first relapse in real world practice based.
Methodology: Data of OC patients treated with primary debulking surgery and standard chemotherapy (paclitaxel/platinum analogs) between January 1, 2011 and December 31, 2011 were extracted from Polish National Health Fund Database . Further overall survival (OS) was compared between patients who received any type of chemotherapy up to 6 months PFI.
Results: 17% (227/1369) patients classified as “platinum-resistant” by the 6-month threshold of PFI and 29% (66/227) of those group , were treated with Pt-based chemotherapy at second line. Median OS after platinum-based 2-nd line chemotherapy was significantly longer compared to non- platinum chemotherapy group (median OS was 28.1 months, 95%CI: 23.3-42.0 vs. 16.4 months, 95%CI: 13.1-21.0; HR=0.71, 95%CI, 0.51 to 0.99; P=0.049). This effect was seen, among patients with PFI of 3-6 months (median OS was, 28.1 months, 95% CI: 23.1-42.0 vs 14.3 months, 95% CI: 10.4-40.5, respectively; HR=0.61, 95%CI, 0.39- 0.96; P=0.0336). but not in PFI< 3months group (HR=0.88, 95%CI, 0.52 to 1.49, P=0.6342).
Conclusion: Our results from real world data based on nationwide population cohort study shows that six-months PFI as an arbitrary threshold for subsequent Pt-based chemotherapy is questionable. Biomarkers of platinum sensitivity are needed to identify potential responders, especially in group of patients with PFI 3-6 months.
Disclosure: Nothing to disclose

EP805

Primary Malignant lymphoma of cervix misdiagnosed as an ovarian cyst on ultrasonography: a case report

<u>S. Bolandi</u>
Resident Fellowship of Gynecology Oncology, Faculty of Medicine, Mashhad University of Medical Sciences, Mashhad, Islamic Republic of Iran

Introduction / Background: Primary Malignant lymphoma of the uterine cervix can be misdiagnosed due to its rarity and distinct clinical presentation. We introduce a case of PCL which was misdiagnosed as an ovarian cyst on ultrasonography.
Methodology: A 31-year-old woman with chief complain of abnormal uterine bleeding was referred as a suspicious malignant ovarian mass on sonography. The evaluation demonstrated large cervical mass as clinical staging allocated her to stage IB 2 disease of uterine cervix.
Results: After the confirmed diagnosis of non-Hodgkin B-cell lymphoma of cervix, rituximab was added to the systemic CHOP (cyclophosphamide, adriamycin, vincristine, and prednisolone) chemotherapy regimen. Now, the patient is disease-free with no signs of recurrence.
Conclusion: In patients with vaginal bleeding and cervical tumor, it is important to consider the possibility of primary cervical lymphoma in the differential diagnosis of cervical cancer due to different treatment and prognosis.
Disclosure: Nothing to disclose

EP806

First insight of a tailoring Chemotherapy Intensity regimen in a Real life Cohort of Elderly patients with ovarian cancer: The CIRCE study

L. Bortot[1,2], M. Bartoletti[1,2], D. Basile[1,2], L. Gerratana[1,2], C. Corvaja[1,2], C. Lisanti[1,2], G. Pelizzari[1,2], S.K. Garattini[1,2], M. Garutti[2,3], S. Buriolla[1,2], L. Da Ros[2], S. Bolzonello[2], P. Di Nardo[2], S. Spazzapan[2], M.S. Nicoloso[2,4], S. Scalone[2], D. Lombardi[2], G. Giorda[5], R. Sorio[2], F. Puglisi[1,2]
[1]Department of Medicine (DAME), University of Udine, Udine, [2]Department of Medical Oncology, Centro di Riferimento Oncologico di Aviano (CRO), IRCCS, Aviano, [3]U.O.C Oncologia, Fondazione Policlinico Universitario Agostino Gemelli IRCCS, Roma, [4]Department of Translational Research, Division of Molecular Oncology, [5]Department of Gynecologic Oncology, Centro di Riferimento Oncologico di Aviano (CRO), IRCCS, Aviano, Italy

Introduction / Background: Women aged ≥65 represent nearly 50% of ovarian cancer (OC) patients (pts). However, elderly OC pts are less likely to receive optimal treatment. Furthermore, they are significantly under-represented in clinical trials and multidimensional geriatric assessment is still underused. The present study aims to provide an overview of real-life treatment strategies for elderly advanced-OC pts and to investigate clinico-pathological features that could potentially drive choice of first-line treatment.
Methodology: A retrospective analysis was conducted on a consecutive series of 45 OC pts aged ≥69 treated with first-line chemotherapy (1L_CT) from 2011 to 2018 at CRO Aviano National Cancer Institute (Italy). Factors associated with treatment choice and rate of adverse events were investigated through Fisher-exact test; differences in progression free survival (PFS) and overall survival (OS) were tested by log-rank test.
Results: 67% of pts received 1L_CT with a standard carboplatin-paclitaxel combination (CPC). Conversely, 33% received a monotherapy (MT) (31% with carboplatin, 2% with paclitaxel). ECOG PS ≥1 was the only factor significantly associated with choice of MT (P=0.021). No differences were observed between CPC and CPC with dose reductions (CPCdr), neither in terms of PFS (HR=1.29 P=0.59) nor OS (HR=1.40 P=0.56). On the other hand, MT was associated with shorter PFS (HR=4.35 P=0.001) and OS (HR=4.48 P=0.005). No differences in treatment discontinuation rate, neutropenia, thrombocytopenia, neuropathy, constipation, diarrhoea and asthenia in CPC, CPCdr and MT were detected (P>0.05).
Conclusion: The present study confirms CPC as the standard first-line chemotherapy also in advanced OC elderly pts and suggests that CPCdr is preferable to MT regimens. Notwithstanding the limitations due to the small sample size, the evaluated regimens showed a comparable toxicity profile. Notably, clinical decision-making was mainly driven by PS ECOG highlighting the pivotal role of multidimensional geriatric assessment for pts stratification. Further prospective studies are needed to investigate improved tailored strategies.
Disclosure: Nothing to disclose

EP807

The risk of malignancy of unilocular ovarian cysts: a systematic review and meta-analysis

L. Buggio[1], D. Dridi[1], M.P. Frattaruolo[1], M. Reschini[2], E. Di Loreto[1], F. Chiaffarino[3], F. Parazzini[3], P. Vercellini[1]
[1]Gynaecology Unit, Fondazione IRCCS Ca' Granda Ospedale Maggiore Policlinico Milano, Milano, [2]Fondazione IRCCS Ca' Granda Ospedale Maggiore Policlinico Milano, [3]Department of Woman, Newborn and Child, Fondazione IRCCS Ca' Granda Ospedale Maggiore Policlinico Milano, Milan, Italy

Introduction / Background: Unilocular adnexal cysts are a common condition. The oncogenic potential of unilocular adenexal masses has often led to an overtreatment with high health care costs and psychological distress. We conducted a systematic review and meta-analysis to estimate the rate of malignancy in adnexal lesions described as echoic and anechoic unilocular cysts at pre-operative transvaginal ultrasound examination.
Methodology: We performed a Pubmed/MEDLINE search of papers published between January 1990-April 2019, using the terms "unilocular ovarian cysts" or "simple ovarian cysts" combined with "ovarian cancer" or "surgery" or "histology" or "transvaginal ultrasound". Only studies published as full-length in English and reporting original data were included. The present review and meta-analysis were performed according to the PRISMA (Preferred Reporting Items for Systematic reviews and Meta-Analyses) and MOOSE guidelines.
Results: From 35 selected studies, we extracted data on ovarian malignancy in the total series, and separately for premenopausal and postmenopausal women. Flowchart of the study selection process is shown in Figure 1. Of the 2229 surgically removed lesions classified as unilocular cysts at pre-operative transvaginal ultrasound, 24 (1.1%; 95% CI: 0.73-1.60) were malignant (among these 12 had borderline malignancy: 0.5%). The rates were lower for premenopausal women (6/1034, 0.6%;95% CI: 0.27-1.26) than postmenopausal ones (12/377, 3.2%;95% CI: 1.83-5.48). Of the 2296 surgically removed lesions classified as anechoic unilocular ovarian cysts on ultrasound, 20 (0.9%; 95% CI: 0.56-1.34) were malignant (among these 8 had borderline malignancy: 0.3%). The rates were lower for premenopausal women (3/907, 0.3%;95% CI: 0.11-0.97) than postmenopausal ones (13/681, 1.9%;95% CI: 1.56-3.96).
Conclusion: The malignancy rate in surgically removed unilocular adnexal cysts is low. The choice between surgery and expectant management should be individualized, and personalized counseling with the patient is advised to avoid the risk of overtreatment.

This image cannot currently be displayed.

[Flowchart of the study selection process.]

Disclosure: Nothing to disclose

EP808

Evaluating the clinical features of adnexial masses during pregnancy results with radiologic findings

H. Cali[1], H. Sozen[2], Y. Salihoglu[3], S. Topuz[2], V. Dogan[4]
[1]Obstetrics and Gynecology, Bezmialem Faculty of Medicine, Bayrampasa, [2]Obstetrics and Gynecology, Istanbul University, Istanbul Faculty of Medicine, [3]Obstetrics and Gynecology, Istanbul University of Medical Science, [4]Obstetrics and Gynecology, Koc University School of Medicine, İstanbul, Turkey

Introduction / Background: It has been reported that the incidence of ovarian tumours accompanying gestation is between 1-2.%.
Methodology: In our study ,60 pregnant patient were included ,who applied to tertied medical centre of gynecology and obstetrics policlinics in between january 2011- may 2015 , and whose obstetric evaluations complied with the criterion.
Results: During the antenatal track , 33 (%55) pregnannt patients were decided to be followed staying conservative under the control of adnexial masses . for 26 (%43.3) pregnant patients operations in early pregnancy or evaluation of adnexial masses in caesarian , and/or malignity caused staging surgery was planned . 1 (%1.7) pregnant patient was suggested therapotic termination and later chemoteraphy upon clear diagnosis of malignity in early pregnancy .Radiological diagnosis and track of adnexial masses via ultrasound was applied on 11 (18.3) pregnant patients , while magnetic resonance imaging scan wanted from 49 (%81.7)pregnant patients who were strongly doubted to have malignity of ultrasound .and the management /control of adnexial bulk of pregnancy was made through the magnetic resonance imaging symptom and interpretation. Ultrasound and magnetic resonance imaging symptoms were interpreted in the side of strong bening symptoms for 38 (%63) pregnant patients , while it was malign for 22 (%36.7) pregnant patients . under the light of the pathologic results of operated patients , bening cyctic structures were detected on 44 (%73.3) pregnant patients , borderline cyctic structures on 3 (% 5) ang malign cyctic structures on 6 (% 10) of them myoma uteri was detected on 4 (%6.7) of the pregnant patients applied due to adnexial masses.
Conclusion: This facilitates diagnosis as USG and MR are relative cheap and harmless. In this study when radiologic findings in 22 patients who show malignancy symptoms are compared to the post-op pathologic findings, only 8 (%36.4) patients were diagnosed as malignant tumours.
Disclosure: Nothing to disclose

EP809

Survival and prognostic factors in patients with recurrent low-grade epithelial ovarian cancer: an analysis of five prospective phase II/III trials of NOGGO metadata base

E. Canaz[1], J. Grabowski[2], R. Richter[2], E.I. Braicu[2], R. Chekerov[2], J. Sehouli[2]
[1]Gynecology and Obstetrics, Demiroglu Bilim University School of Medicine, Istanbul, Turkey, [2]European Competence Center for Ovarian Cancer (EKZE), Department of Gynecology and Gynecological Oncology, Charite-University Medicine of Berlin, Campus Virchow Klinikum, Berlin, Germany

Introduction / Background: Low-grade epithelial ovarian cancers (EOC) constitute the minority among all epithelial cancers and clinical experience at recurrence is limited. The aim of our study was to investigate the survival in low-grade recurrent EOC and to compare with high-grade disease, as well as in regard to platinum-sensitive and -resistant recurrences.
Methodology: This is an exploratory analysis within the North-Eastern German Society of Gynecological Oncology (NOGGO) metadata base including five randomized phase II/III trials comparing different chemotherapy regimens in recurrent EOC. We conducted survival analyses and cox-proportional regression models.
Results: Out of 1050 patients with recurrent epithelial cancer, 42(4%) patients had low-grade and 1008(96%) patients had high-grade EOC. In the subgroup of platinum-sensitive recurrences, PFS

BMJ Publishing Group Limited (BMJ) disclaims all liability and responsibility arising from any reliance placed on this supplemental material which has been supplied by the author(s)

(*8.7m vs 9.7m, p=0.7*) OS (23.9m vs 24.8m, *p=0.9*) did not differ between low-grade and high-grade EOC. In platinum-resistant recurrences, patients with low-grade ovarian cancer had significantly better PFS (7.6m vs 3.6m, *p=0.03*) and OS (41.9m vs 9.5m *p=0.002*) in comparison to those with high-grade cancer (figure1). In low-grade EOC, there were no significant PFS (8.7m vs 7.6m, p=0.91) and OS (23.9m vs 41.9m, p=0.25) differences between platinum-sensitive and -resistant recurrences. Patients with low-grade non-serous histology had lower PFS (2.8m vs 11.3m, p=0.004) with compared to those with low-grade serous histology (figure2). At cox regression analysis presence of ascites and residual disease after secondary cytoreductive surgery were independent factors associated with poor PFS within low-grade recurrent cancers.
Conclusion: Our study indicates, platinum sensitivity according to platinum-free interval does not have any prognostic significance at recurrent low-grade EOC and non-serous histology is associated with poorer outcome at the recurrence. Secondary surgical cytoreduction to no-gross residual disease and ascites are independently associated with disease progression.

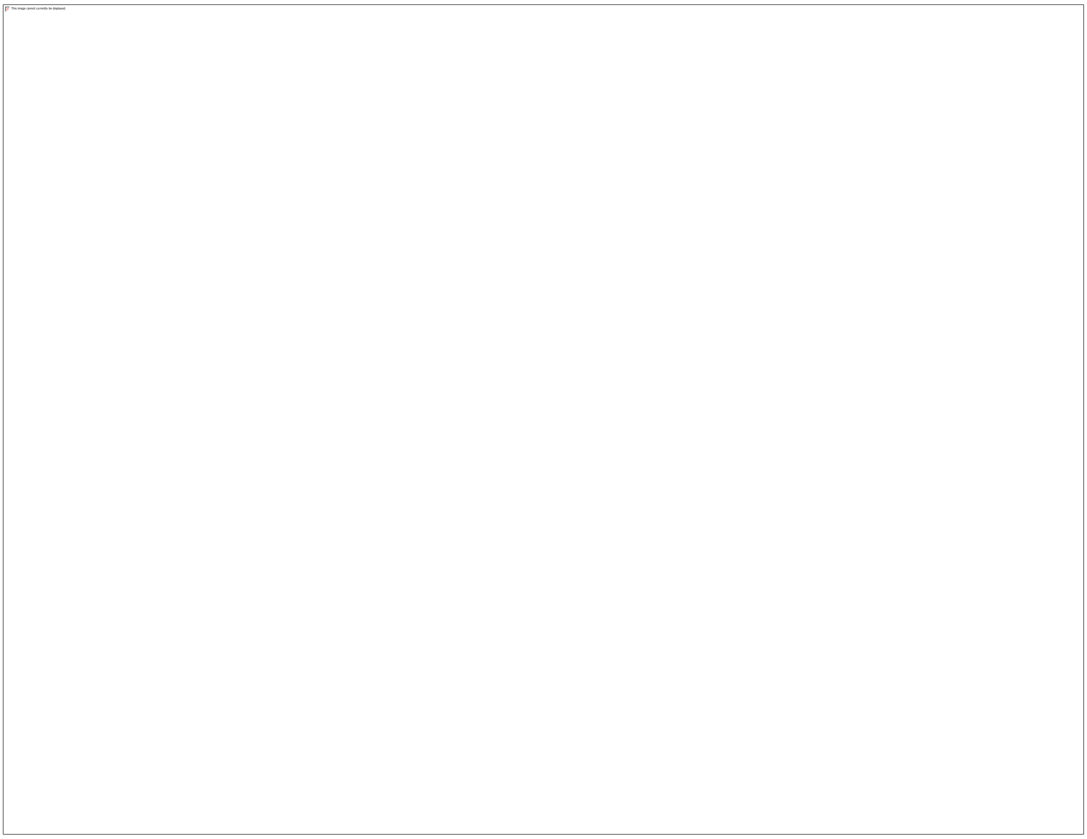

[Figure1. PFS and OS in patients with low-grade and high-grade EOC according to platinum sensitivity]

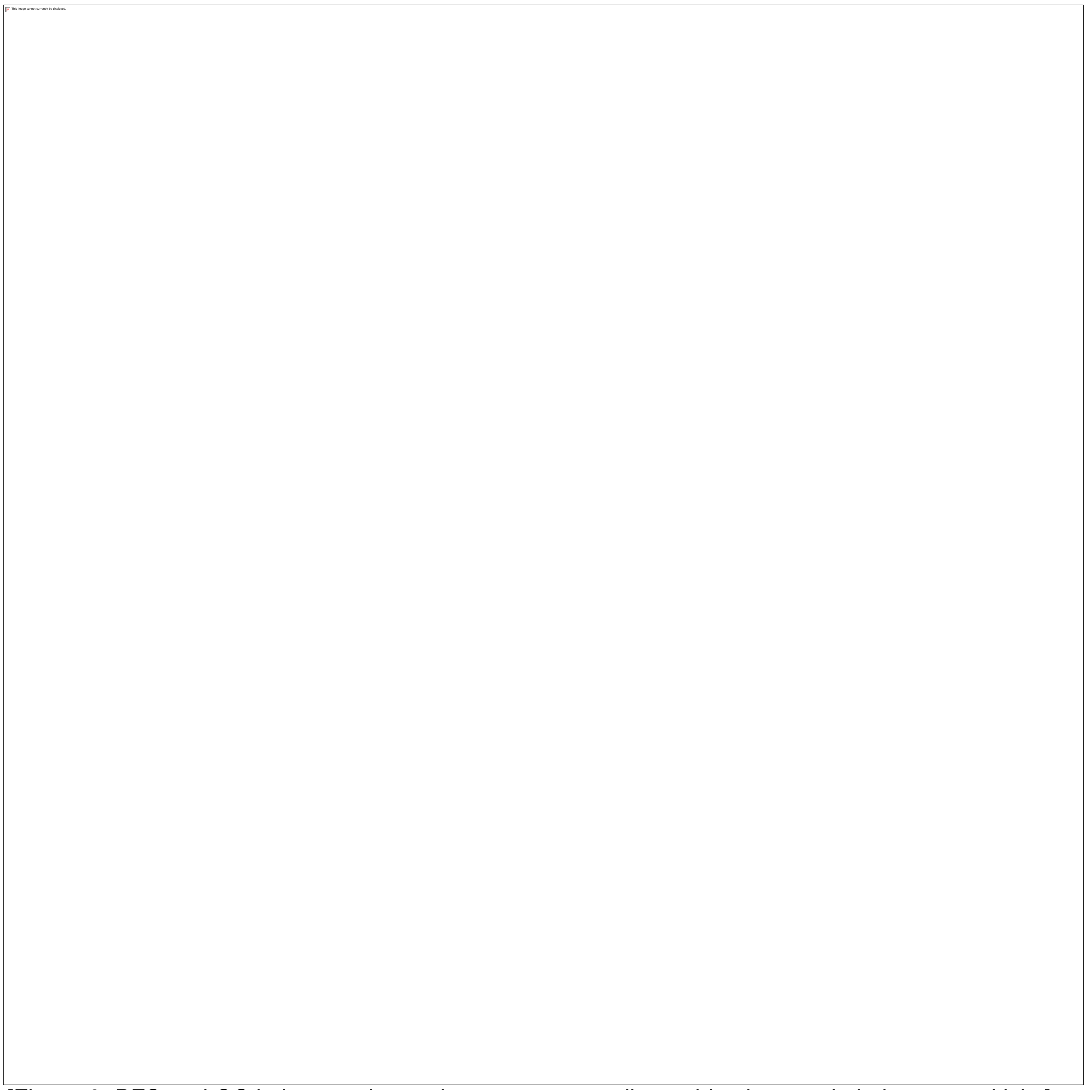

[Figure 2. PFS and OS in low-grade ovarian cancer according to histology and platinum sensitivity]

Disclosure: EIB reports personal fees from Roche Pharma, CLOVIS, Tesaro, AstraZeneca, Incyte, Seattle Genetics, Amgen and grants from Roche Diagnostic outside the submitted work. All remaining authors have declared no conflicts of interest.

EP810

Expression of BRCA1 and prognosis in ovarian cancer: a systematic review and meta-analysis

L.A. Teixeira, F.J. Candido dos Reis
Gynecology and Obstetrics, Ribeirao Preto Medical School - University of Sao Paulo, Ribeirao Preto, Brazil

Introduction / Background: Epithelial ovarian cancer (EOC) is the leading cause of death from gynecological malignancies. Deficiency in BRCA1 function is associated with increased tumor sensitivity to platinum-based chemotherapy. However, the efficacy of immunohistochemistry (IHC) to

identify BRCA1 deficiency is not well established. The aim of this study was to synthesize the evidence on the association of BRCA1 expression by IHC with the prognosis of women with EOC.
Methodology: Using the concepts ovarian neoplasms and IHC testing for BRCA, published titles were systematically searched in four databases (Embase, PubMed, Web of Science, and Scopus). The study was conducted using the PRISMA statement. Meta-analyses for the Hazard Ratios (HR) of overall survival (OS) and progression-free survival (PFS) were calculated with the package "meta" in the R environment.
Results: Fourteen studies comprising 2,071 EOC cases were included. Low IHC expression of BRCA1 was reported in 48.5% of cases. The low expression of BRCA1 was associated with improved OS (HR=0.64; 95% CI 0.53 - 0.76) and with improved PFS (HR=0.75; 95% CI 0.59 - 0.94). The heterogeneity was high (I2=72%) among the studies on OS and moderate (I2=61%) among PFS studies.
Conclusion: Low expression of BRCA1, identified by IHC, was consistently associated with the overall and progression-free survival in EOC. This find may be important in clinical practice, especially with the popularization of the use of PARP inhibition as part of EOC treatment. However, well-designed, prospective studies are still needed to confirm these results.
Disclosure: Nothing to disclose

EP811

A retrospective study regarding the role of cytoreductive surgery (CRS) and hypertermic intraperitoneal chemotherapy (HIPEC) in patients with advanced ovarian, fallopian tube or primary peritoneal cancer

M.E. Capilna[1], B. Moldovan[2], C. Rad[2], A.A. Moldovan[2], A.L. Cozlea[1]
[1]First Obstetrics and Gynecology Clinic, University of Medicine, Pharmacy, Science and Technology, Targu Mures, [2]Sf. Constantin Hospital, Brasov, Romania

Introduction / Background: Emphasizing that complete CRS and HIPEC has potential benefits in patients with ovarian, fallopian tube or primary peritoneal cancer in advanced stages, presenting the morbidity and the oncologic results compared to the traditional surgical debulking combined with conventional intravenous platinum and taxane-based chemotherapy
Methodology: A retrospective study was conducted between June 2013 - April 2019 including 110 patients, with a mean age of 53.94 years old, who benefited from CRS and HIPEC for primary or recurrent peritoneal carcinomatosis.
Results: Upfront CRS and HIPEC was performed in 18 patients (16.36%), while interval surgery and HIPEC was considered in 29 subjects (26.36%). Consolidation CRS and HIPEC was performed in 31 patients (28.18%), while salvage procedure was necessary in 26 patients (23.63%). Six patients (5.45%) required a second CRS and HIPEC. Total peritonectomy following Sugarbaker technique was performed in all patients; large intestine resections were performed in 52 patients (47.27%), small intestine resections in 17 (15.45%), splenectomy in 40 (36.36%), liver resections in 12.72% and diaphragmatic resections in 15.45%. A microscopically cytoreduction (CC0) was achieved in 69 patients (62.73%), macroscopic cytoreduction (CC1) in 36 subjects (32.73%), while gross tumor debulking was performed in 5 (4.54%). Seven patients (6.36%) needed reoperation for postoperative complications. HIPEC was performed using single platinum agents in 30 patients (27.27%), Doxorubicin in 31 (28.18%), while Cisplatin-Doxorubicin association was used in 45 subjects (40.90%), Cisplatin-Mitomicine or Mitomicine alone being applied in smaller percentages. Fifteen patients died from the disease (13.64%), 20 patients are alive with recurrence (18.18%) and 75 are disease free (68.18%), and despite the short follow-up period, survival outcomes are relevant.
Conclusion: Extensive CRS followed by HIPEC is a promising therapeutical option in patients with peritoneal carcinomatosis related to gynecological malignancies.
Disclosure: Nothing to disclose

EP812

Impact of incorporation of extensive surgical procedures on surgical outcomes in advanced ovarian cancer

L. Cárdenas Puiggrós[1], A. Taltavull Pons[1], C. Fina Planas[2], M.P. Barretina Ginesta[2], E. Sala Hernández[1], E. Álvarez Castaño[1]
[1]*Obstetrics-Gynaecology, Gynaecological Oncology, Dr. Josep Trueta University Hospital,* [2]*Medical Oncology, Catalan Institute of Oncology, Girona, Spain*

Introduction / Background: Epithelial ovarian cancer (EOC) is the most lethal gynaecologic malignancy. Most patients present with advanced stage disease. The most important prognostic factor is cytoreduction to no macroscopic residual tumour, which could require the addition of extensive surgical procedures to standard procedures, as diaphragmatic surgery or bowel resection. These procedures improve complete resection rate and subsequent survival of patients with advanced EOC. The aim of this study is to describe the use of extensive surgical procedures in our centre and their impact in terms of results and postoperative complications.
Methodology: A consecutive series of 54 patients with newly diagnosed EOC operated between 2017 and 2018 at Dr. Josep Trueta University Hospital was analysed. Data were retrieved from the departmental database and hospital electronic medical records.
Results: 42 patients (77%) were diagnosed at III/IV FIGO stage and underwent cytoreductive surgery. Of them, 46.5% had primary cytoreductive surgery, while 53.5% underwent surgery following neo-adjuvant chemotherapy.
Additional surgical procedures required to achieve no residual tumour outside of the “standard” procedures are summarized.

	%	N
En-bloc pelvic peritoneum stripping	52.4%	22
Diaphragmatic surgery including peritonectomy and full-thickness resection	21.5%	9
Lesser omentectomy	14.3%	6
Recto-sigmoidectomy	9.5%	4
Small bowel resection	7.1%	3
Large bowel resection beyond recto-sigmoid	4.8%	2
Splenectomy	4.8%	2
Morrison's pouch peritonectomy	4.8%	2
Resection porta hepatis tumour	2.4%	1

[Additional surgical procedures performed]

No macroscopic residual disease was observed in 89.4% of cases, while 2.1% had residual disease ≤ 1 cm and 8.5% >1cm.
Patients were classified in three groups according to the extent of surgery: only standard procedures, standard procedures plus bowel resection and extensive upper abdominal surgery with or without bowel resection. Results in the three groups were compared.

	Standard procedures	Standard procedures + bowel resection	Upper abdominal +/- bowel resection	*p*
Number of patients % (N)	65.2% (30)	8.7% (4)	26.1% (12)	
No complications	63.3%	50%	58.3%	NS

(%)				
Minor complications (Clavien-Dindo I/II) (%)	10%	0%	16.7%	NS
Major complications (Clavien-Dindo III/IV) (%)	26.7%	50%	25%	NS
Median hospital stay (days)	7.03	8.00	11.43	0.049
Median Peritoneal Carcinomatosi Index	2.71	4.00	9.22	0.004
Median operating time (minutes)	258	308	386	<0.001

[Outcomes according to extent of surgery]

Although the median operating time, hospital stay and PCI was significantly higher in upper abdominal group, the rate of major surgical complications was not significantly different. There was no mortality within 30 days of surgery.
Conclusion: The incorporation of extensive surgical procedures is feasible with acceptable morbidity. These procedures could improve the rate of complete cytoreduction and consequently the survival. Dedicated trained teams are needed to achieve the best outcomes in terms of complete cytoreduction and morbidity.
Disclosure: Nothing to disclose

EP813

Different prevalence and pattern of lymph nodes metastases between primary and interval cytoreduction in advanced ovarian cancer

L. Cárdenas Puiggrós[1], A. Taltavull Pons[1], C. Fina Planas[2], M.P. Barretina Ginesta[2], E. Sala Hernández[1], E. Álvarez Castaño[1]
[1]Obstetrics-Gynaecology | Gynaecological Oncology, Dr. Josep Trueta University Hospital, [2]Medical Oncology, Catalan Institute of Oncology, Girona, Spain

Introduction / Background: Complete cytoreduction followed by combination of platinum-taxane chemotherapy remains standard treatment for epithelial ovarian cancer (EOC).
The role of adding systematic lymphadenectomy to cytoreduction procedures in advanced EOC has been a topic of debate. Recently, the LION trial showed that systematic lymphadenectomy after maximal cytoreduction in absence of bulky nodes does not improve survival and may cause additional harm.
The aim of this study is to describe the rate of radiological and histological positive lymph nodes in primary or interval cytoreduction and to estimate the impact that application of the conclusions of LION trial would have in our centre.
Methodology: A consecutive series of 54 patients with newly diagnosed EOC operated between 2017 and 2018 at Dr. Josep Trueta University Hospital was analysed. Data were retrieved from the departmental database and hospital electronic medical records.
Results: 42 patients (77%) were diagnosed at III/IV FIGO stage and underwent cytoreductive surgery. Of them, 46.5% had primary cytoreductive surgery, while 53.5% underwent interval cytoreduction surgery. All patients had a routinely performed preoperative CT-scan and the presence and pattern of bulky nodes was analysed. The most frequent localization of bulky nodes in CT-scan was paraaortic. After neo-adjuvant chemotherapy, bulky nodes responded in almost all cases.

Bulky nodes	Primary citoreduction	Interval cytoreduction	
		Initial CT-scan	Interval CT-scan
Negative	66%	58.3%	95.5%
Only pelvic	8.3%	0%	0%
Only paraaortic	20.8%	18.1%	4.5%
Pelvic and paraaortic	0%	4.5%	0%
Pelvic or paraaortic	29.1%	22.6%	4.5%
Cardiophrenic	4.1%	22.7%	0%

[Pattern of radiological bulky nodes]

Systematic pelvic lymphadenectomy was performed in 80.4% of cases, and paraaortic lymphadenectomy in 78.3%. The pathological involvement of lymph nodes was analysed.

	Primary cytoreduction	Interval cytoreduction
**Pelvic lymphadenectomy**		
Median number of nodes	18.0 (9-37)	19.4 (10-43)
Micrometastasis (≤10mm)	25%	18.1%
Macrometastasis (>10 mm)	8.3%	0%
**Paraaortic lymphadenectomy**		
Median number of nodes	13 (4-29)	12.5 (7-26)
Micrometastasis (≤10mm)	16.7%	13.6%
Macrometastasis (>10 mm)	29.1%	4.5%

[Histological pattern of positive lymph nodes]

Only one case of interval cytoreduction had nodal macrometastasis, which was presurgical identificated on CT-scan.
Conclusion: CT-scan is a good predictor for nodal macrometastasis. In absence of bulky nodes and as a result of applying LION conclusions, near 70% of lymphadenectomies in primary cytoreduction and near 95% in interval cytoreduction could be avoided. Careful selection of patients and discussion of CT-scans in multidisciplinary preoperative tumor conference is crucial to avoid unnecessary procedures and to reduce surgical complicactions.
Disclosure: Nothing to disclose

EP814

TGFβ pathway activation is a predictor of progression free and overall survival in advanced high-grade serous ovarian cancer according to the surgical aggressiveness and rate of cytoreduction of different oncologic centers

L. Ceppi[1], T. Grassi[1], C. Romualdi[2], P. Di Lorenzo[1], S. Palazzi[1], E. Biagioli[3], A. Garbi[4], L. Paracchini[3], F. Landoni[1], G.D. Aletti[4,5], N. Colombo[4,6], R. Fruscio[1], S. Marchini[3], M. D'Incalci[3]
[1]Milano Bicocca University, San Gerardo Hospital, Monza, [2]Department of Biology, University of Padova, Padova, [3]Department of Oncology, Istituto di Ricerche Farmacologiche Mario Negri IRCCS, [4]Division of Gynecologic Oncology, European Institute of Oncology, [5]University of Studies of Milan,

Department of Oncology and Hemato-oncology, [6]Department of Medicine and Surgery, Milano Bicocca University, Milan, Italy

Introduction / Background: The activation of TGFβ pathway in advanced stage high-grade serous ovarian cancer (HGSOC) has been found to be related to short progression free survival (PFS) and overall survival (OS). miRNA181a-5p was found to be a proxy of TGFβ activation. This prognostic role has been identified only in cohorts with low surgical complexity, we evaluated 2 cohorts with different surgical approaches.
Methodology: Series of HGSEOC from two centers (European Institute of Oncology, Milan, *Cohort1*; San Gerardo Hospital, Monza, *Cohort2*) were analyzed for clinical features and miRNA quantification to test their prognostic value using a Cox-proportional hazard regression model.
Results: 118 and 84 patients were included, respectively. *Cohort1* versus *Cohort2* was composed of similar FIGO stages, higher upper abdominal disease spread (UAD)(P< 0.001), higher complexity score (CS) of 6.7 ± 2.4 and 3.5 ± 1.7 (P< 0.001) and lower residual tumor (P< 0.001). *Cohort1* compared to *Cohort2* was associated with a lower number of events of PFS and OS (P< 0.0012).
An optimized threshold of miR181a-5p expression to segregate patients according to their prognosis was identified (miR-low and miR-high): for *Cohort1* no threshold was reached; for *Cohort2* a threshold segregated PFS of 20.9 v. 9.6 months (P:0.0008) and OS of 60.3 v. 23.3 months (P:0.0008), respectively.
In multivariate analysis, no significance was observed for miR-high in *Cohort1.* For *Cohort2*, miR-high was related to worse PFS (P:0.0002), and OS (P:0.007).
Conclusion: miR181a-5p was observed as a prognostic biomarker in HGSEOC in *Cohort2*, -less aggressive surgery-, instead no prognostic role was observed in *Cohort 1* -aggressive surgery-. This question-generating analysis suggests that TGFβ activation, a proxy of tumor aggressiveness, might be withdrawn by an aggressive surgical approach.
A prospective validation of miR181 role is needed, the translational ancillary study of the TRUST trial (NCT # 02828618) may answer this question.
Disclosure: Nothing to disclose

EP815

Ultrasound-based score for predicting debulking surgery outcome in IIB-IV stage epithelial ovarian cancer

E. Chacón[1], J.L. Alcázar[1], J.Á. Mínguez[1], I. Carriles[1], N. Manzour[1], D. Vázquez[2], T. Castellanos[2], A. González-Martín[3], L. Sánchez[3], J. Espinós[3], M. Platero[2], M. Idoate[4], I. Espinosa[5], M. Cambeiro[6], R. Martínez-Monje[7], F. Calvo[6], L. Chiva[2]
[1]Obstetrics and Gynecology, Clinica Universidad de Navarra, Pamplona, [2]Obstetrics and Gynecology, [3]Medical Oncology, Clinica Universidad de Navarra, Madrid, [4]Pathology, Clinica Universidad de Navarra, Pamplona, [5]Pathology, [6]Radiation Therapy Oncology, Clinica Universidad de Navarra, Madrid, [7]Radiation Therapy Oncology, Clinica Universidad de Navarra, Pamplona, Spain

Introduction / Background: To develop an ultrasound-based score for predicting debulking surgery outcome in advanced stage ovarian cancer.
Methodology: Retrospective analysis of data from a prospective cohort study comprising 65 women (mean age: 59 y.o.) with surgically confirmed stage IIB-IV epithelial ovarian cancer and ECOG status performance 0-2. All women had undergone transabdominal and transvaginal ultrasound evaluation for assessing disease spread prior to surgery. 12 anatomical areas were evaluated for the presence/absence of disease (recto-sigma involvement, carcinomatosis of pelvic peritoneum, major omentum, carcinomatosis of upper abdomen peritoneum, root of mesentery, pelvic lymph nodes, aorto-cava lymph nodes, lesser omentum, liver, spleen, hepatic hilum and ascites). All women included underwent surgery at our institution (diagnostic laparoscopy followed by upfront surgery if patient deemed adequate for optimal (R< 1cm) or complete (R0) cytoreduction). Surgical findings and permanent frozen section histologic diagnosis were used as reference standard. The score was developed according to the odd ratio (for optimal or complete cytoreduction) for each anatomical area evaluated and the value of CA-125 serum levels. (Table 1).
Parameter Score

CA-125 >500 1
Bowel 1
Mesogastrium 1
Major omentum 1
Pelvic lymph nodes . 1
Root mesentery . 2
Liver parenchyma . 2
Spleen parenchyma . 2
Hepatic hilium 2
Para-aortic lymph nodes . 2

Results: Cytoreduction was complete or optimal in 51 patients (78.5%), suboptimal in 3 (4.6%) and upfront surgery was discarded in 11 women (16.9%). A score $\geq$ 3 predicted correctly 44 out 51 patients (86%) with complete/optimal cytoreduction in and 8 out of 14 (57%) of those with suboptimal or non-cytoreduction surgery. AUC was 0.76, with correct prediction in 80% of all cases.
Conclusion: Albeit the series is small, the developed ultrasound-based score has a good performance for predicting surgical results in IIB-IV stage epithelial ovarian cancer.
Disclosure: Nothing to disclose

EP816

TRPV1 and PTEN expression is related to poor prognosis in epithelial ovarian cancer

D.B. Chay[1], G.H. Han[2], H. Cho[2], J.-H. Kim[2]
[1]Obstetrics and Gynecology, Yongin Severance Hospital, Yongin, [2]Gangnam Severance Hospital, Yonsei University College of Medicine, Seoul, Republic of Korea

Introduction / Background: Transient receptor potential vanilloid type-1 (TRPV-1) was previously reported to be associated with cancer. This study explored the expression of TRPV-1 and PTEN in epithelial ovarian cancer and evaluated its clinical significance.
Methodology: Immunohistochemical (IHC) staining analyses of TRPV-1 and PTEN were performed using tissue microarray (TMA) constructed from 198 ovarian cancer and 47 borderline ovarian tumor samples. The clinical significance was evaluated by comparing the data with various clinicopathologic characteristics, including survival of patients with epithelial ovarian cancer.
Results: TRPV-1 and PTEN expression was significantly higher in ovarian cancer compared to borderline ovarian tumor and normal human ovarian surface epithelium (HOSE) (both $p < 0.001$). Spearman's rank correlation analysis revealed that TRPV-1 expression shows significant correlation with PTEN expression in ovarian cancer (Spearman's rho = 0.276, $p < 0.001$). Multivariate analysis indicated that advanced FIGO stage (III-IV) (hazard ratio = 2.66 [95% CI, 1.01-6.99], $p = 0.046$), TRPV-1 expression (hazard ratio=3.23 [95% CI, 1.06-9.80], $p = 0.038$), PTEN expression (hazard ratio=2.98 [95% CI, 1.61-5.40], $p < 0.001$) and combined TRPV-1/PTEN(hazard ratio=3.37 [95% CI, 1.85-6.13], $p < 0.001$) were independent prognostic factors for overall survival.
Conclusion: This study reveals the association between TRPV-1 and PTEN expression with epithelial ovarian cancer and shows that these biomarkers predicts poor prognosis. The findings of this study warrants future research evaluating its clinical usefulness.
Disclosure: Nothing to disclose

EP817

The Impact of urological complications during surgical treatment for advanced ovarian cancer

R. Chekerov[1], I. Dr. Braicu[2], C. Fotopoulou[2], G. Dr. Inci[2], Z. Muallem[2], K. Pietzner[2], J. Neymeyer[3], J.P.D.m. Sehouli[1], Ovarian Cancer Study Group of the NOGGO

[1]Gynaecology, [2]Charité – Universitätsmedizin Berlin, corporate member of Freie Universität Berlin, Humboldt-Universität zu Berlin, and Berlin Institute of Health, [3]Urology, Charité – Universitätsmedizin Berlin, corporate member of Freie Universität Berlin, Humboldt-Universität zu Berlin, and Berlin Institute of Health, Berlin, Germany

Introduction / Background: Surgical treatment is the cornerstone in the treatment of ovarian cancer in the primary and in some recurrent situations. Beside the evidence for significant effect on survival, complications caused by the tumor spread or due to techniques of resection are most formidable. There is no clear wich factors may play a predictive role.
Methodology: Between 2010 and 2018 a retrospective analysis of all surgical interventions for primary advanced and recurrent ovarian, Fallopian tube or primary peritoneal cancer were identified. Retrospective analysis of classical patient´s and clinicopathological parameters, type of surgery with surgical techniques, type of complications and their management were evaluated prospectively with special focus on impact of treatment and prognosis.
Results: Of 3486 identified surgeries for primary advanced and recurrent ovarian, Fallopian tube or primary peritoneal cancer we identified 4,1% surgical interventions to the urinary tract with partial resection of the bladder : 39 (17 primary, 22 recurrence), partial resection of the ureter(s): 19 (7 primary, 12 recurrence), cystectomy: 4 (all at recurrence), nephrectomy: 4. the rate of intraoperative injuries of the urinary tract was : 3.9% whit 59 bladder lesions (16 at primary, 20 at recurrence), 15 Ureterallesions (5 at primary, 10 at recurrence). The rate of renal vessel injuries was 0.5% (16 patients and 2 nephrectomies). The rate of post surgical urological complications (30d period) was 1.9% - 8 fistulas, 13 urinomas, 31 ureter obliterations. Furthermore we identified urological complications during the systemic treatment: 24 Fistulas (18 during Bevacizumab treatment).
Conclusion: Urologic surgical interventions and rate of complications was in general low, but in the evaluated cohort they had a significant impact on clinical outcome and prognosis. Further evaluation of possible predictive factors to identify patients at risk for advanced surgical resection on the urinary tract and following complications is needed.
Disclosure: Nothing to disclose

EP818

Primary debulking surgery versus primary neoadjuvant chemotherapy with or without interval debulking surgery for advanced stage ovarian cancer: a single institute study

Y.Y. Chen, Y.C. Ou, H. Lin
Obstetrics and Gynecology, Kaohsiung Chang Gung Memorial Hospital, Kaohsiung, Taiwan

Introduction / Background: Neoadjuvant chemotherapy followed by interval debulking surgery (NACT-IDS) has been recommended as an alternative treatment for selected patients with advanced-stage epithelial ovarian cancer (EOC). The aim of present study was to compare clinical outcomes of primary debulking surgery (PDS) versus NACT with or without IDS in a single institute of women with advanced-stage EOC.
Methodology: Retrospective cohort study of women with stage IIIC(T3cN0-1) and IV EOC diagnosed between 2008 and 2016 treated at Kaohsiung Chang Gung Memorial Hospital. Patients who underwent PDS and NACT were identified. The Kaplan-Meier method was used to compare progression-free (PFS) and overall survival (OS) data.
Results: A total of 143 newly diagnosed stage IIIC (T3cN0-1)and IV EOC patients were included in the final analysis. 92 women (64.3%) underwent PDS, 33 (23.1%) underwent NACT-IDS, and 18 (12.6%) received chemotherapy alone. The patients who received chemotherapy alone had significantly lower PFS of 4 months and OS of 28 months compared to PDS (PFS:13 months; OS:46 months) and NACT-IDS (PFS: 14 months; OS:28 months) groups, respectively.
Conclusion: NACT-IDS did not improve PFS and OS compared to PDS in advanced-stage EOC. Patients received chemotherapy alone had the lowest PFS and OS compared to PDS and NACT-IDS groups.
Disclosure: Nothing to disclose

EP819

Two cases of erythema nodosum in patients on olaparib

M.M.Y. Chu, K.Y. Tse, H.Y.S. Ngan
Department of Obstetrics and Gynaecology, Queen Mary Hospital, University of Hong Kong, Hong Kong, Hong Kong

Introduction / Background: Olaparib has been used both as the maintenance treatment and also monotherapy for patients with ovarian cancer in the past few years.
Methodology: We reported two cases of erythema nodosum associated with the use of olaparib.
Results: Both patients had germline BRCA mutations and were started on maintenance olaparib capsule 400mg twice daily after complete response to platinum-based chemotherapy for recurrent ovarian cancer.
Case 1: A 54-year-old woman complained of bilateral ankle swelling and reddish nodules on her shins 3 months after starting olaparib. Physical examination showed tender, erythematous subcutaneous nodules on her anterior shins compatible with erythema nodosum. Olaparib dose was reduced to 200mg twice daily and the erythema nodosum resolved spontaneously after 2 weeks. Similar skin lesions but of milder degree recurred after she resumed olaparib at 400mg twice daily. Again, the lesions subsided within 2 weeks after dose reduction to 200mg twice daily. The patient was then put on olaparib at 300mg twice daily with no reappearance of the skin lesions. The patient had another recurrence of her ovarian cancer 17 months after olaparib treatment.
Case 2: A 51-year-old woman complained of erythematous skin nodules on her left lower limb one week after starting olaparib, and similar lesions occurred on her right lower limb 2 weeks later. Physical examination showed erythematous subcutaneous nodules compatible with erythematous nodosum. The dose of olaparib was reduced to 300mg twice daily and the skin lesions resolved after 2 weeks. The patient continued with olaparib at 300mg twice daily with no skin reaction but was found to have another recurrence 4 months after olaparib treatment.
Conclusion: We reported two cases of erythema nodosum associated with the use of olaparib. Both of our patients had resolution of the skin lesions within 2 weeks after dose reduction and no other treatment was required.
Disclosure: Dr. KY Tse has received travel grants / lecture honorarium from MSD, ZaiLab and AstraZeneca; and funding for a research project by Pfizer. Prof. HYS Ngan was invited as the expert speaker and received travel grants from Roche, MSD, ZaiLab and AstraZeneca.

EP820

MR and ultrasound fusion imaging to characterize ovarian masses

A. Crestani[1], C. Theodore[2], I. Thomassin[2], J.M. Levaillant[2], C. Touboul[2]
[1]94, [2]Creteil Hospital, Creteil, France

Introduction / Background: The objective was to evaluate the feasibility and the performance of MR and ultrasound fusion imaging (MUFI) for characterizing adnexal masses.
Methodology: A prospective monocentric study was conducted in the gynaecologic oncologic department of Creteil hospital. Between 01st January 2014 and 31 July 2018, we “consecutively” included all women referred for characterization of indeterminate or suspicious pelvic mass who accepted to be enrolled in our study (n=109). We excluded all patients who did not undergo ultrasound or MR imaging (n=3). Feasibility was evaluated on this first population. A subgroup of patients who underwent surgery was identified to evaluate MIFU performance (n=26). Clinical and radiological features were reported in our database. Two independent readers retrospectively and blindly rated ultrasonography according to IOTA simples rules and MR imaging according to ADNEXMR score. Reference standard was final pathology at surgery (n=106). Sensitivity, specificity, positive and negative predictive values and likelihood ratio of each imaging were calculated for predicting malignant

ovarian masses.
Results: Final population included 106 patients (mean age (SD) = 57 (18) years old). Mean time duration between first consultation and MUFI was 15 days (7). The subgroup of patients who underwent surgery consisted in 12 patients with primitive ovarian cancer, 2 with ovarian metastases, 3 patients with borderline and 9 with benign tumours. Sensitivity and specificity were respectively of 94% and 44% for ultrasound, 94% and 89% for MR imaging and 100% and 89% for MUFI for the diagnosis of cancer. In MUFI, high colour score, number of vegetations, carcinosis were the most pertinent malignant features whereas the absence of vascularisation was the most pertinent benign feature.
Conclusion: MUFI is feasible and reproducible. Moreover, MUFI may help improve diagnostic performances of the combination of simple ultrasound and MR imaging. Further research is needed to confirm our results and define proper indications.
Disclosure: Nothing to disclose

EP821

Short-term safety and outcomes of HIPEC in ovarian cancer patients

A. Cruz[1], D. Martins[1], A.R. Monteiro[2], M. Peyroteo[1], B. Pereira[1], F. Coelho[1], A. Martins[1], A. de Sousa[1], D. Pereira[1], F. Faria[1]
[1]Portuguese Institute of Oncology, Porto, [2]Portuguese Institute of Oncology, Coimbra, Portugal

Introduction / Background: Hyperthermic intraperitoneal chemotherapy (HIPEC) for ovarian cancer patients with peritoneal metastasis remains controversial concerning patient selection, safety, efficacy and costs. A randomized phase 2 trial failed to show survival superiority for interval cytoreductive surgery (ICRS) with HIPEC compared to surgery alone. HIPEC group had more anemia and creatinine elevation. Recently, a phase 3 trial showed longer recurrence-free and overall survival for HIPEC group, without increasing toxicity. We aimed to evaluate safety and efficacy of ICRS with HIPEC.
Methodology: Retrospective cohort of patients submitted to ICRS and HIPEC with cisplatin 50mg/m^2, from 2013 to 2018. Data were analyzed using descriptive statistics and survival was estimated by Kaplan-Meier method.
Results: Thirty patients were included, median age was 60 years (IQR, 15) and all had ECOG < 2. Stage was IIIC (FIGO, 2014) in 56.7% (17/30). Median peritoneal cancer index was 11 (IQR, 15). After surgery, 66.7% (20/30) were admitted to the intensive-care unit (ICU), and 33.3% (10/30) to the high-dependency unit (HDU). Mean APACHE II and SAPS II score was 9.7 and 25.5, respectively. Ten percent (3/30) needed invasive mechanical ventilation, despite 23.3% (7/30) developed respiratory failure. None (0/30) had hemodynamic instability or renal failure. Anemia occurred in 73.3% (22/30); mean lower hemoglobin level was 10.5(±1.3)g/dL. Median length of stay in ICU/HDU was 2 days (IQR, 1) and in the hospital was 11 days (IQR, 5). One patient needed reoperation due to gastrointestinal leakage and peritonitis; and one had right femoral nerve neuropraxia. There was no readmission to ICU/HDU or in-hospital mortality. After a median follow-up of 14 months, 40% (12/30) progressed and 33.3% (10/30) died. Median PFS was 8 months (95% CI 4.7-11.3) and OS was 10 months (95% CI 0.7-19.3).
Conclusion: In our cohort HIPEC was a safe technique. Long-term outcomes suggest the benefit of this procedure in the management of well-selected patients.
Disclosure: Nothing to disclose

EP822

Simvastatin enhances the anti-tumoral effect of metronomic cyclophosphamide against cancer-initiating cells (CICs) by reducing ALDH1A1 expression both in vitro and in vivo: an effective combination to overcome resistance and limit the spread of the recurrent disease in

high-grade serous ovarian cancer (HGSOC)

M.A. Cuello[1], M.F. Liberona[1], S. Kato[1], J. Cerda-Infante[2], C. Ibañez[2], V. Montecinos[2]
[1]Gynecology, [2]Hematology and Oncology, Pontificia Universidad Católica de Chile, Santiago, Chile

Introduction / Background: HGSOC is the deadliest gynecologic cancer. Most of cases end up recurring and dying from metastatic/resistant disease. Cancer-initiating cells (CIC) constitute the subpopulation responsible for the acquisition of drug resistance and the progression/recurrence of the disease. ALDH1A1 activity is commonly increased in CICs and correlates with drug resistance (by drug detoxification), metastasis and prognosis. Our group has demonstrated that simvastatin, a lipophilic form of statins, chemo-sensitizes ovarian cancer cells and interferes with cell plasticity of HGSOC-derived CICs reducing metastasis in a mevalonate-dependent manner. Here, we study whether the combination of simvastatin with metronomic cyclophosphamide could be effective in treating recurrent/resistant HGSOC.
Methodology: CICs were isolated from HeyA8 ovarian cancer cell line and from ascites collected from chemo-näive and chemoresistant recurrent advanced HGSOCs using stem cell-selecting culture conditions. CICs were treated with simvastatin (1, 2 and 5 µM), mafosfamide (4,7µM) or its combination (24-hour pulse, 4 days). The induction of apoptosis was measured by detection of PARP cleavage by immunoblotting. ALDH1A1 enzyme levels were determined by q-PCR and immunoblotting. Xenograft tumors were generated by injecting CICs intraperitoneally (IP) in NSG mice. Once established xenografts, mice were treated with placebo, simvastatin (2 mg/kg/day IP), cyclophosphamide (20 mg/kg/day PO) or its combination (n=6 per group). Ascites volume, body weight, distribution of metastatic foci, and ALDH1A immunostaining at the injection site were measured. Survival curves were also made.
Results: Repeated doses of this combination significantly reduced ALDH1A1 expression and induced PARP cleavage compared with each reagent alone *in vitro*. More importantly, simvastatin reduced ALDH1A1 expression, and the combination diminished ascites volume, metastatic disease, and prolonged survival *in vivo*.
Conclusion: Our findings support the effectiveness of simvastatin plus metronomic cyclophosphamide in pre-clinical models of recurrent/resistant HGSOCs.The explanatory mechanism involves a decrease in ALDH1A1 activity (an enzyme regulating the cyclophosphamide detoxification) induced by simvastatin (research support by Fondecyt 1160800)
Disclosure: Nothing to disclose

EP823

Patients with stage IV epithelial ovarian cancer: Understanding the determinants of survival

Y. Dabi[1], C. Huchon[2], L. Ouldamer[3], S. Bendifallah[4], P. Collinet[5], A. Bricou[6], E. Daraï[4], M. Ballester[4], V. Lavoué[7], B. Haddad[1], C. Touboul[1], FRANCOGYN
[1]Centre Hospitalier Intercommunal de Créteil, Créteil, [2]Centre Hospitalier Intercommunal de Poissy, Poissy, [3]Centre Hospitalier Régional Universitaire de Tours, Hôpital Bretonneau, Tours, [4]Hopital Tenon, Paris, [5]Centre Hospitalier Régional Universitaire de Lilles, Lilles, [6]hôpital Jean Verdier Bondy, Bondy, [7]Centre Hospitalier de Rennes, Rennes, France

Introduction / Background: The most appropriate management for patients with stage IV ovarian cancer remains unclear. Our objective was to understand the main determinants associated with survival and to discuss best surgical management in these patients.
Methodology: Data of 1038 patients with confirmed ovarian cancer treated between 1996 and 2016 were extracted from maintained databases of 7 French referral gynecologic oncology institutions. Patients with stage IV diseases were selected for further analysis. The Kaplan Meier method was used to estimate the survival distribution. A Cox proportional hazards model including all the parameters statistically significant in univariable analysis, was used to account for the influence of multiple variables.
Results: Two hundred and eight patients met our inclusion criteria: 65 (31.3%) never underwent debulking surgery, 52 (25%) underwent primary debulking surgery (PDS) and 91 (43.8%) neoadjuvant chemotherapy and interval debulking surgery (NACT-IDS). Patients not operated had a significantly worse overall survival than patients that underwent PDS or NACT - IDS ($p < 0.001$). In multivariable

analysis, three factors were independent predictors of survival: upfront surgery (HR 0.32 95% CI 0.14 - 0.71, p = 0.005), postoperative residual disease = 0 (HR 0.37 95% CI 0.18 - 0.75, p = 0.006) and association of Carboplatin and Paclitaxel regimen (HR 0.45 95% CI 0.25 - 0.80, p = 0.007).
Conclusion: Presence of distant metastases should not refrain surgeons from performing radical procedures, whenever the patient is able to tolerate. Maximal surgical efforts should be done to minimize residual disease as it is the main determinant of survival.
Disclosure: Nothing to disclose

EP824

Ambiguous diagnoses of borderline ovarian tumors at frozen section with a definite diagnosis of invasive carcinoma: consequences for counseling and perioperative treatment

K. De Decker[1], K.H. Jaroch[1], H.G. Ter Brugge[1], J. Bart[1], L.F.S. Kooreman[2], R.F.P.M. Kruitwagen[2], H.W. Nijman[3], A.J. Kruse[1]
[1]Isala Hospital, Zwolle, [2]Maastricht University Medical Center, Maastricht, [3]University Medical Center Groningen, Groningen, The Netherlands

Introduction / Background: Frozen section diagnoses of borderline ovarian tumors (BOTs) are not always straightforward (ambiguous), and sometimes the pathologist may tend towards a diagnosis of invasive carcinoma. It must be decided whether or not additional lymph node sampling (LNS) should be performed to prevent a second procedure in the event of a carcinoma as a definite postoperative diagnosis. The aims of the present study were (i) to compare (un)ambiguous BOT frozen section diagnoses (FSDs) and their definite diagnoses, (ii) to evaluate how often a LNS was performed in these cases and (iii) to discuss managerial implications.
Methodology: All patients who underwent ovarian surgery with perioperative frozen section analysis in the participating hospitals between January 2007 and July 2018 were identified and included in case of a BOT FSD (whether or not tending towards invasive carcinoma) and a definite diagnosis of a BOT or carcinoma.
Results: Of 106 patients with a serous BOT FSD, 92 were unambiguous, of whom two patients were diagnosed with a carcinoma (figure 1). Five of 92 patients underwent LNS (one carcinoma) and 87 patients did not (one carcinoma). Of the remaining 14 patients with an ambiguous serous BOT FSDs, 5 were diagnosed with a carcinoma (35.7%). Two of 14 underwent LNS (two carcinomas) and 12 did not (3 carcinomas). The results with respect to (un)ambiguous mucinous BOT FSDs (n=88) are shown in figure 1 as well.

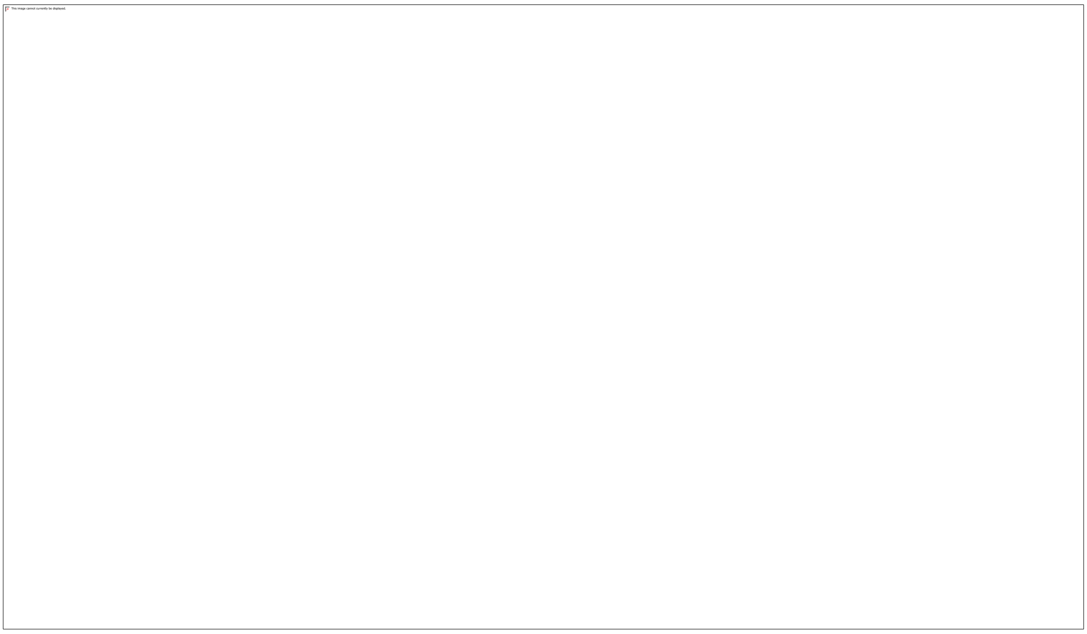

[Figure 1.Overview of lymph node sampling in case of (un)ambiguous BOT frozen section diagnoses.]

Conclusion: The chance of a carcinoma in case of ambiguous serous BOT FSD is 35.7%. Implementation of LNS in case of an ambiguous serous BOT FSD should be discussed with the patient individually. LNS in case of unambiguous BOT FSDs can be omitted, as well as in case of mucinous ambiguous BOT FSDs, since LNS in case of mucinous ovarian carcinomas is not performed frequently because of a low incidence of lymph node metastases.
Disclosure: L.F.S. Kooreman received grant support from Novartis and Roche, intented for financing research into breast cancer.

EP825

The malignant transformation of a dermoid cyst: a case report

M.R. De Guzman, R. Dueñas, L. Reyes
Obstetrics and Gynecology, Far Eastern University Medical Center, Quezon City, Philippines

Introduction / Background: The question of whether patients with squamous cell carcinoma in a mature cystic teratoma should undergo conservative surgery or postoperative adjuvant treatment remains unresolved. Ovarian cancer is the second most common malignancy of the lower part of the female genital tract, occurring less frequently than cancers of the endometrium but more frequently than cancers of the cervix. Germ cell tumors are the second most frequent type of ovarian neoplasm and account for approximately 10-20% of all ovarian tumors. The most frequent germ cell tumor is the Mature Cystic Teratoma; overall, only 2% to 3% of germ cell tumors are malignant**,** and more than 80% of malignant transformations are squamous cell carcinomas arising from the ectoderm. There are currently no reliable procedures for the early detection of ovarian cancer. Available potential screening techniques have included pelvic examination, ultrasound examination, serum CA-125 determination and other tumor markers.
Methodology: Exploratory Laparotomy, Total Abdominal Hysterectomy, Bilateral Salpingooophorectomy was done in this case, with Frozen Section of the affected ovary.
Results: Frozen Section of the affected ovary revealed "Malignant Epithelial Tumor in a Dermoid Cyst" hence, complete surgical staging was done resulting to Intraoperative Stage I-A. It was therefore managed by cytoreductive surgery followed by chemotherapy with Cisplatin and Paclitaxel.

Conclusion: There is no established definitive treatment protocol available due to lack of research on this rare case, thus frozen Section of the affected ovary is imperative in dermoid cysts to ensure that it is benign. In both Gynecologic and General Surgical practice, frozen sections are utilized mainly to aid in the differentiation of benign from malignant disease. Determining the type of tumor and extent of surgery is very important in Gynecologic Oncology.
Disclosure: Nothing to disclose

EP826

Unchanged morbidity despite increased surgical radicality in cytoreductive surgery for an advanced stage epithelial ovarian cancer

A. Kaufmann, A. Laios, T. Broadhead, R. Hutson, D. Nugent, A. Thangavelu, G. Theophilou, D. De Jong
Gynaecology Oncology, St James's Hospital, Leeds, United Kingdom

Introduction / Background: The surgical aim in cytoreduction for an advanced stage of ovarian cancer is to achieve complete cytoreduction (nil macroscopic disease) with minimal peri- and postoperative morbidity.
We assessed the impact of the extent and complexity of surgical cytoreduction on morbidity in patients with an advanced stage of ovarian cancer.
Methodology: A cohort of patients with a diagnosis of advanced stage (FIGO III/IV) high grade epithelial ovarian cancer, undergoing upfront and delayed surgical cytoreduction, from Jan 2016 to Dec 2018 was selected from the ovarian database. We evaluated age, surgical complexity score (SCS) and postoperative morbidity. The SCS was assigned based on the Aletti classification as low, moderate and high. Morbidity was graded based on the Clavien-Dindo score and grouped as 0, I-II, IIIA-IVB and V. The chi square test was used for statistical analysis.
Results: 283 patients were identified. Mean age was 64 $\pm$ 11 yrs. The median SCS was 3 (1-10). The number of patients identified with Clavien-Dindo 0 (no change from normal postoperative course), I-II (any simple deviation from the post-OP course or pharmacological treatment) and IIIa-IVB (intervention or life-threatening complications) were 134, 129 and 20 respectively. None of the patients died within 30-days post-operatively (Clavien-Dindo V).The SCS increased over the years 2016-2018 (p= 0.0035) whilst the Cavien-Dindo remained unchanged (p= 0.764; NS).
Conclusion: We observed that in our cohort of patients with an advanced stage of ovarian cancer, increased surgical complexity does not result in increased surgical morbidity. Therefore radical surgery for an advanced stage of ovarian cancer, to achieve complete cytoreduction, seems justified in our population. Further monitoring and evaluation is ongoing
Disclosure: Nothing to disclose

EP827

Pathologic findings in risk-reducing salpingo-oophorectomy (RRSO) in women with BRCA1 or BRCA2 mutations: a single center experience

A. De Leo[1], C. Ceccarelli[1], D. Turchetti[2], D. Santini[1], A.M. Perrone[3], P. De Iaco[3]
[1]Pathology Unit, S. Orsola Malpighi Hospital, University of Bologna, [2]Medical Genetics, University of Bologna, [3]Gynecologic Oncology Unit, S. Orsola Malpighi Hospital, University of Bologna, Bologna, Italy

Introduction / Background: Bilateral salpingo-oophorectomy offers the greatest risk reduction for ovarian cancer in high-risk women with an identified BRCA germline mutation. The aim of this study was to define the incidence of precursor lesions and cancer after RRSO.

Methodology: We retrospectively reviewed 71 BRCA-mutated patients who underwent RRSO from 2012 to 2017. All cases were examined according to the protocol for Sectioning and Extensively Examining the FIMbriated End (SEE-FIM).
Results: The median age at RRSO was 49.9 years. BRCA 1 mutation was detected in 46/71 (64.8%) patients, BRCA2 mutation in 25/71 (35.2%) patients. 50/71 (70.4%) patients had breast cancer. BRCA1 breast cancer was more frequently high grade (G3) and showed a strong association to triple negative phenotype (p=0.001), high TILs (tumor-infiltrating lymphocytes), and aberrant expression of p53 (p=0.01). Occult invasive gynecological cancer was detected in two patients: a tubal high grade serous carcinoma in a BRCA2+ patient and an ovarian low-grade endometrioid carcinoma in a BRCA1+ patient. 5 (7%) STIC (Serous Tubal Intraepithelial Carcinoma), 5 (7%) STIL (Serous Tubal Intraepithelial Lesion) and 13 (18.3%) SCOUT (Secretory Cell Outgrowth) were detected. 10 patients had endometriosis and benign ovarian lesions were found in 17 cases. The detection rate of STIC/STIL or invasive cancer was 16.9% (12/71). Tubal lesions were more common in BRCA2 mutation carriers (68% vs. 39%).
Conclusion: In our institution, a rigorous surgical protocol with meticulous pathologic review at RRSO yielded an overall detection rate of 9.8% for occult gynecological carcinoma in BRCA mutation carriers.
Disclosure: Nothing to disclose

EP828

Whole genome sequencing and clinical drug response data of 142 ovarian cancer samples

C.J. de Witte[1,2], J. Kutzera[1], J. Espejo Valle-Inclan[1,2], M.J. van Roosmalen[1], S. Boymans[1,2], E. Cuppen[1,2], W.P. Kloosterman[1], R.P. Zweemer[3], P.O. Witteveen[4], Center for Personalized Cancer Treatment, CPCT Consortium
[1]Center for Molecular Medicine, University Medical Center Utrecht, Utrecht University, [2]Oncode Institute, [3]Department of Gynaecological Oncology, Cancer Center, [4]Department of Medical Oncology, Cancer Center, University Medical Center Utrecht, Utrecht University, Utrecht, The Netherlands

Introduction / Background: While most patients with ovarian cancer are initially sensitive to chemotherapy, approximately 75% will develop recurrent disease and become resistant. Predicted response to platinum is considered important in deciding on the best treatment upon recurrence. Often a disease-free interval of 6 months is considered the threshold between predicted resistance and sensitivity. In this study we compare clinical response to predicted response, and aim to identify mechanisms of chemotherapy resistance through whole genome sequencing (WGS) of matched pre-treatment biopsies.
Methodology: Tissue biopsies were obtained in patients with advanced or metastatic ovarian carcinoma. Samples with minimally 30% tumour purity were characterized by WGS to a coverage of ~100X (and 35X for corresponding germline samples). Predicted response to platinum, based on disease-free interval (6-month threshold) was compared to clinical response as measured according to the RECIST criteria on CT imaging.
Results: In total, 137 patients were included and 142 ovarian cancer tissue biopsy samples were obtained, mainly of recurrent disease (111/142, 78%). Up to now, in 49 cases platinum (combination) treatment was given and response was assessed. Predicted response only correlated with measured response in 51% of patients. In the predicted sensitive group 11/29 patients (38%) were sensitive and experienced partial or complete response. In the group with predicted resistance 14/20 patients (70%) had progressive or stable disease. WGS data is being analysed to identify better prognostic criteria. Furthermore, we have started analysing copy number variation profiles of five patients for whom two subsequent biopsies were available. Evolution over time at copy number level did not seem to result from prolonged biopsy intervals or differences in tumour purity, but could be related to the number of previous treatment cycles.
Conclusion: WGS augmented with detailed clinical data can potentially increase knowledge on the development of resistance and aid clinical decision making.
Disclosure: No competing interests. Applicable funding sources: KWF Kankerbestrijding (Dutch Cancer Society) - UU2015-7743 [de Witte, Witteveen, Zweemer, Kloosterman]

EP829

The need for and the development of a decision aid for primary treatment of advanced stage ovarian cancer patients

J.E. den Ouden[1], R. The[2], B.J. Myren[1], D. Boll[3], W.J. van Driel[4], R.I. Lalisang[5,6], R.F.P.M. Kruitwagen[6,7], A.M. van Altena[1]
[1]Obstetrics and Gynaecology, Radboud University Medical Center, Nijmegen, [2]ZorgKeuzeLab, Delft, [3]Obstetrics and Gynaecology, Catharina Hospital, Eindhoven, [4]Gynaecologic Oncology, Antoni van Leeuwenhoek Hospital, Amsterdam, [5]Internal Medicine, Division Medical Oncology, Maastricht University Medical Center, [6]School for Oncology and Developmental Biology, GROW, [7]Obstetrics and Gynaecology, Maastricht University Medical Center, Maastricht, The Netherlands

Introduction / Background: In the light of the growing number of (intense) treatment options for advanced stage ovarian cancer (OC) patients, while survival is still poor, the patient association indicated an increased need for a tool to improve shared-decision-making (SDM) by patients and their clinicians. The Dutch working party on gynaecologic oncology initiated the development of an evidence based online decision aid (DA) to support SDM. The aim of this study was to develop the DA.
Methodology: A four-stage development method was followed: 1: Identifying patients' (N=43) and professionals' (N=22) needs by questionnaires and in-depth patient interviews (N=12). 2: Face-to-face meetings with a multidisciplinary team were organized including representatives of the patient association. 3: Development of the DA using International Patient Decision Aid Standards (IPDAS) and Dutch Guidelines. 4: Usability testing with OC patients and professionals.
Results: Both patients and clinicians supported the need for reliable information that include all available treatment options in the Netherlands. Although the majority of patients were very satisfied about the process of information provision, 90% indicated that no choice in treatment options was offered. Based on study findings and discussions during the meetings, a consultation sheet and a DA were developed. The sheet, presented during first consultations, contains a summary of all treatment options, including intraperitoneal (IP)-chemotherapy and hyperthermic intraperitoneal chemotherapy (HIPEC), and a login code for the online DA. The online part, which can be used at home, consists of information about disease and treatment options, questions about quality of life and treatment preferences, and ends in a summary which patient and clinician can discuss during the next consultation.
Conclusion: A DA was developed in cooperation with patients and professionals to support SDM for advanced stage OC patients and their clinicians. Acceptance was gained among all stakeholders involved. The DA is currently under assessment in a multicenter implementation trial.
Disclosure: Ir. Regina The is the managing director of ZorgKeuzeLab B.V. Delft.

EP830

***DICER1* and *FOXL2* genes mutation in juvenile granulosa cell tumor (A TMRO study)**

C. Genestie[1], P. Baillard[2], F. Descotes[2], E. Rouleau[1], S. Gouy[1], A. Leary[1], I. Ray-Coquard[3], P. Morice[1], M. Devouassoux-Shisheboran[2]
[1]Gustave Roussy, Villejuif, [2]Pathology, Hospices Civils de Lyon, Pierre Bénite, [3]Centre Anticancereux Léon Bérard, Lyon, France

Introduction / Background: Juvenile granulosa cell tumor (JGCT) represent 10% of ovarian tumors in patients less than 20 years of age and 5% of all granulosa cell tumors, most of which are of adult type and bear a mutation in the *FOXL2* gene. Sertoli-Leydig cell tumors show *DICER1* gene mutation

in 60% of cases. However, the exact rate of *FOXL2* and *DICER1* gene somatic mutations in JGCT is not well known.
Methodology: Retrospective study of 48 cases of JGCT referred to the HCL and IGR for second opinion from 1999 to 2018. All cases were reviewed. After DNA extraction, we searched point mutations of *FOXL2* (exon 1 C134W) and *DICER1* (exons 26 and 27 Genbank accession number NM_177438.2). Follow up was available for 40 patients (3 to 324 months, mean 52).
Results: All tumors were unilateral. Patient age ranged from 1 to 70 year-old (median 21). All were stage I (IA : 24 cases, IC: 18 cases), except one case of stage IIA (stage unknown for 5 cases). In 4 cases (Two IA and two IC1) a *DICER1* mutation was seen (8%), and all patients are doing well without recurrence at last follow up (mean 38 months). *FOXL2* mutation was detected in a 31 year-old patient, stage IC1, with a peritoneal recurrence at 12 months but doing well at 58 months. Among cases without any mutation, 17% recurred (1 patient stage IIA, 1 stage IC2, 1 stage IC1 and 1 stage IA) or died (two patients stage IC1).
Conclusion: Pathologists have to be aware that JGCT may show *FOXL2* or *DICER1* somatic mutation in 2% and 8% of cases, respectively. In JGCT, *FOXL2* mutation might be associated with a worse prognosis, while *DICER1* mutation doesn't seem to alter patient's outcome. This need to be confirmed on a larger number of cases.
Disclosure: Nothing to disclose

EP831

Extensive upper abdominal surgery and Hudson hsyterectomy for advanced stage ovarian cancer patients. Our 23 month experience

N.U. Dogan[1], S. Dogan[2], C. Güner[1], A. Hamidova[1]
[1]Gynecology, Akdeniz University, [2]Antalya Resarch and Training Hospital, Antalya, Turkey

Introduction / Background: Optimal and maximal debulking surgery is the gold standard treatment for advanced stage (Stage II and III) epithelial ovarian cancer patients (AOC). In patients with disease confined to pelvis,Hudson hsyterectomy with retroperitoneal retrograde approach is a way to completely excise tumor confined to lower abdominal region. Moreover extensive upper abdominal surgical procedures are obligatory in order to achieve R0 tumor status.
Methodology: Between February 2017 -march 2019, 25 AOC patients operated in Akdeniz University , Department of Gynecology were retrospectively evaluated. All the patients underwent diagnostic laparoscopy and were evaluated for resectability (modified Fagotti score). When resectable, a wide midline incision was carried out. Then the liver was mobilized and upper abdominal procedures was performed. Then for pelvic disease, all retroperitoneal spaced was created, ureters were mobilised and the pelvic tumor was excised en bloc along with total pelvic pertioneum. When required rectosigmoid excision was perfromed. Intraoperative and postoperative parameters and short-term follow-up of 25 advanced stage ovarian cancer patients were analyzed
Results: Seven patients were subjected to neo-adjuvant chemo. Optimal debulking was achieved in 16 patients out of 18 primary debulked patients. In five patients rectosigmoid excision, in seven 7 diaphragma stripping, in two patients celiac truncus lymphadenectomy were performed. Mean operation time was 390 min (300-460 min). Median blood loss was 400 cc (200- 800 cc). Postoperatively 2 patient experienced eviceration and were re-operated. Three patients had wound breakdown. One patient had pulmonary embolus. In four patients post-operative ileus was observed which was handled conservativelyMedian follow-up time was 16 months (3-23 months). Two patients experienced recurrences both of which were treated with second-line chemo.
Conclusion: In patients with AOC extensive upper abdominal surgery and retrograde Hudson hsyterectomy are all feasible surgical options in order to eradicate peritoneal tumor with acceptable morbidty rates.
Disclosure: nothing to disclose

EP832

Mucinous cystic tumor with mural nodules: anaplastic carcinoma and sarcoma like-nodules - case report

K. Drosik-Rutowicz, M. Śliwińska, M. Cieślak-Steć, K. Raczek-Zwierzycka, E. Nowicka, R. Tarnawski
III Department of Radiotherapy and Chemotherapy, M. Skłodowska-Curie Memorial Cancer Centre and Institute od Oncology, Branch in Gliwice, Gliwice, Poland

Introduction / Background: Mucinous cystic tumor with mural nodules: anaplastic carcinoma and sarcoma like-nodules are very rare. Mural nodules are single or multiple nodules arising within the wall of mucinous tumours whether they are benign, borderline or malignant. The prognosis of SLMNs is excellent, their presence does not affect the prognosis of cystic ovarian tumor. In contrast, the foci of anaplastic carcinoma are aggressive components of cystic ovarian tumors. They carry an unfavorable prognosis, and most patients with mural nodules of anaplastic carcinoma have a malignant, frequently rapid course.
Methodology: A woman aged 41 presented abdominal pain. CT scan revealed a cystic mass 110x90x85mm located in retroperitoneal space.
Results: Exploratory laparotomy was performed, and tumor was excised. Grossly the cystic tumor measured 10cm and was partially lined with mucus epithelium exhibiting features of moderate atypia. There was a pleomorphic infiltrate formed in the cyst wall formed of oval spindle cells and giant multinucleated cells. Approximately 50% of the infiltration was necrosis.
The tumor developed within the mucinous cystic tumor of Mullerian epithelium in the form of a mixed wall tumor. She was diagnosed with Mucinous cystic tumor with mural nodules: anaplastic carcinoma and sarcoma like-nodules. Afterwards bilateral salpingo-oophorectomy, appendectomy, pelvic lymphadenectomy and omentectomy were performed. Microscopic examination showed no pathological findings. CT scan performed after laparotomy revealed several suspicious small nodules within both lungs. Subsequentally the patient received chemotherapy consisting of Cisplatin, Adriamycin and Cyclophosphamide. PET-CT performed after completion of chemotherapy showed no evidence of metastatic or recurrent disease. Six months following the completion of therapy the patient is in good condition with no signs of disease.
Conclusion: Careful histologic and immunohistochemical analysis of mural nodules is essential for the determination of treatment and prognosis.
Disclosure: Nothing to disclose

EP833

The prognostic impact of Sphingosine-Kinase 1 expression (*SphK1*) on ovarian cancer

Z. Drosos[1], H. Gevensleben[2], S. Kommoss[3], T. Karn[4], U. Holtrich[4], M. Graeser-Mayer[5], M. Anglesio[6], A. El-Balat[4], A. Rody[1], L. Hanker[1]
[1]Gynecology and Obstetrics, University Lübeck, Lübeck, [2]Institute of Pathology, University Hospital Bonn, Bonn, [3]Department of Woman's Health, Tuebingen University Hospital, Tuebingen, [4]Department of Obstetrics and Gynecology, Goethe University Frankfurt, Frankfurt, [5]Department of Gynecology, Bethesda Hospital, Moenchengladbach, Germany, [6]Department of Molecular Oncology, BCCA Cancer Research Centre, Vancouver, BC, Canada

Introduction / Background: Sphingosine-Kinase 1 (SphK1), encoded by the *SPHK1* gene, is a key enzyme of sphingolipid metabolism and overexpressed in a variety of cancer types. In the present study, we investigated the expression of SphK1 and its prognostic impact on ovarian cancers.
Methodology: We used two large retrospective tissue micro array (TMA) cohorts (in total n=763) for performing immunohistochemical analysis of SphK1. The results were correlated with clinico-pathological characteristics and survival.
Results: We found that SphK1 expression differed between histological ovarian cancer histotypes with most frequent low expression in clear cell carcinoma ($p < 0.001$). In addition, low SphK1 expression was shown to significantly correlate with optimal tumour resection ($p < 0.001$). Kaplan-

Meier analysis further revealed that low SphK1 levels were associated with improved progression-free survival (PFS; 65,23 months [95% confidence interval (CI): 49,95 - 80,50] vs. 51,42 months [95% CI: 27,02 - 75,82], p = 0,052) and overall-survival (OS; 100,01 months [95%CI: 83,64 - 116,38] vs. 75,35 months [95%CI: 59,85 - 90,85], p = 0,049). Subsequently, the prognostic value of SphK1 expression together with clinical factors (i.e. FIGO stage, age, and residual tumour burden after surgery) was substantiated in univariate Cox regression analysis (PFS: hazard ratio (HR) = 0,83 [95% CI: 0,69 - 1,00], p = 0,052; OS: HR = 0,82 [95%CI: 0,68 - 1,00], p = 0,05).
Conclusion: Our results suggest that SphK1 expression might be a prognostic factor in ovarian cancer. Low SphK1 expression seems to be associated with prolonged survival, optimal tumour debulking and clear cell subtype. To corroborate these findings, however, our results need to be validated in an independent patient cohort.
Disclosure: Nothing to disclose

EP834

Surgical management of ovarian epithelial cancer in early stages

Z. Edderdabi, A. Touimi Benjelloun, Y. Benchrifi, M. Benhessou, M. Ennachit, M. Elkarroumi
Centre Mohamed IV de Chirurgie Oncogynecologique, Hassan II University / Faculté de Medecine et de Pharmacie, Casablanca, Morocco

Introduction / Background: Our study has been based on the epidemiologic, diagnostic and therapeutic analysis of patients who have been treated for epithelial ovarian cancer early stage in the Mohammed VI centre-treatment of cancers.
Methodology: Our study has been based on the epidemiologic, diagnostic and therapeutic analysis of 24 patients who have been treated during three years between January 2015 and December 2017.
Results: The mean age was 43 years old
In 75% of the cases, our patients were in the period of genital activity.
The circumstances of discovery were dominated by the pain (83,33%), followed by metrorrhagia (25%)
The abdominal palpation didn't find a tumor mass in 50% of the cases
The echography was done for all the patients, it has suspected the malignancy in all the cases, the mixed echostrucure was dominated (75%), with the presence of vegetations in 79,16%.
The TDM keeps its big interest in case of diagnostic doubt and in the balance assessment of extension of the malignant tumors, it was done for 41,66% of cases. All the patients have received a dosage of tumor markers (CA 125) which was high in 79,16% of cases. the laparoscopic approach was initially performed for 3 patients. unilateral adnexectomy was performed in 3 patients, whereas a total hysterectomy without adnexal preservation was performed in 87.5% of cases
Extemporaneous examination was requested in 41.66% of the cases. It confirmed the malignancy and histological type in all cases, and guided surgical management
The epithelial cancer of the ovary was diagnosed in 91.66% of cases at stage I, with a predominance of stage IA (45.83%).
We noted two histological types serous and mucinous, with a predominance of serous type (70.83%). Of these, five were papillary.
Conclusion: We found a grade I CEO in ten patients, grade II in eight patients and grade III in three patients.
Disclosure: Nothing to disclose

EP835

Value of reducing intraperitoneal cisplatin dose In debulked advanced epithelial ovarian cancer

A. Elagwany

Alex Uni, Alex, Egypt

Introduction / Background: Many centers have switched to using a lower dose of cisplatin IP in an attempt to reduce toxicity and improve patient tolerance.
Methodology: 60 patients were included in this prospective study with 20 cases with primary cytoreductive surgery (PCRS) and 40 cases underwent neoadjuvant chemotherapy followed by IDS (interval debulking surgery) . basal CT and serum ca 125 was done before surgery along with CBC, LFT, RFT . CT and CA 125 was done after surgery as a base line. the IP catheter was introduced during surgery through right upper abdominal quadrant if there is no more than 1 cm residual tumor . half of patients received IP cisplatin 80mg/m2 with IV paclitaxel (group I) and the other half received 50 mg/m2 with IV paclitaxel (group II) . chemotherapy doses was given every 3 weeks for 3 cycles . after completion of the cycles, we measured serum CA l25 , performed CT and assessed the toxicity .
Results: Most patients in our study were with stage III epithelial ovarian cancer. Serum CA l25 decreased significantly after chemotherapy nearly similar in both groups with no statistically significance between groups . toxicity was more in group I and was statistically significant regarding nephrotoxicity , neurotoxicity, nausea and vomiting and loss of appetite . it was not significant regarding neutropenia. Catheter complications were observed in 17.3 % of patients with no significant difference between both groups. The proportion of patients completing all cycles was 47% and 65.2%in group I and II respectively that was not statistically significant .
Conclusion: The use of 50mg/m2 of cisplatin IP was associated with lower toxicity and same response when comparing with the use of 80 mg/m2 of cisplatin IP and completeness of the cycles was better with the lower dose of IP cisplatin.
Disclosure: Nothing to disclose

EP836

Clinical applicability of simplified PCI in advanced ovarian cancer staging and correlation with CT SPCI

A. Elagwany
Alex Uni, Alex, Egypt

Introduction / Background: Optimal cytoreduction surgery in advanced epithelial ovarian cancer is related staging that can be evaluated by CT and laparoscopy . CT PCI evaluate anatomic affection and lesion size in 13 sites while a simplified PCI has been used in colorectal cancer and evaluates the lesion size and 7 anatomic regions . it is used for measuring the amount and distribution of metastatic disease in peritoneal cavity on CT and laparotomy.
Methodology: This was a prospective study performed over 60 patients with stage III epithelial ovarian cancer and excluding those with history of chemo-radiotherapy. the patients were evaluated before surgery within one week for CT SPCI over 7 regions (pelvis , rt lower abdomen , omentum and transverse colon and spleen , rt and lt subdiaphragmatic areas, subhepatic and lesser omental sac , small bowel and mesentery) then correlated with surgical findings (surgical SPCI) regarding accuracy and clinico-surgical applicability .
Results: There was a high level correlation between CT SPCI and surgical SPCI for the all sites with highest correlation for region 2 , 4 , 6 and lowest one for region 3. Surgical SPCI was better evaluated than standard surgical PCI for surgeons in an easy and simple way
Conclusion: Surgical SPCI is highly applicable for surgical staging in advanced ovarian cancer .CT SPCI correlates well with surgical SPCI which is an easier way than complicated standard PCI in advanced ovarian cancer.
Disclosure: Nothing to disclose

EP837

Pelvic schistosomiasis an incidental finding in a suspected gynaecology cancer patient- a case report

A. El-Ghobashy, A. Aggarwal, S. Ijaz
Gynaecological Oncology, The Royal Wolverhampton NHS Trust, Wolverhampton, United Kingdom

Introduction / Background: Schistosomiasis in female reproductive tract is very uncommon. A systematic review estimated that more than 200 million people are infected across the tropical areas, and 800 million at risk of infection. We present case of a patient who underwent surgery for suspected ovarian cancer and was incidentally diagnosed with ectopic schistosomiasis.
Methodology: A 59-year-old lady who was investigated for suspected kidney disease was incidentally diagnosed with on ultrasound to have complex right sided 8 cm ovarian mass. She further had a CT scan which confirmed the same findings. In her personal history she was noted to have type 2 diabetes and hypertension. As per multidisciplinary team advice she proceeded for total abdominal hysterectomy, bilateral oophorectomy and omental biopsy. She had a CA 125 level of 25IU/ml and Risk malignancy index was 150.
Results: She underwent midline laparotomy and findings included an 8x10 cm complex right ovarian cyst with mild omental thickening. Another biopsy was taken from a right sided infundibulopelvic ligament 1 cm white nodule. Histology showed a benign mature cystic teratoma of right ovary and benign endometrial polyp. It also incidentally showed ectopic schistosomiasis in the right infundibulopelvic ligament biopsy. On enquiry she had history of travel to Egypt twice in last 5 years. She underwent HIV and total immunoglobulins serology under infectious disease team. She was treated with two doses Praziquantel.
Conclusion: Schistosomiasis is prevalent in Africa, tropical areas and Asia. It can be acute or chronic and has association with HIV, HPV and STI's. Chronic disease with up to 10 years life span in human body results from the host's immune response to deposited eggs and the granulomatous reaction caused by antigens. It is important to biopsy any suspicious areas during surgery to thoroughly assess for any pathology like in our case to improve diagnosis.
Disclosure: Nothing to disclose

EP838

Current status of oncofertility care for the AYA cancer patients with ovarian germ cell tumors in Japan - a fact-finding survey by Japan agency for medical research and development (AMED)

H. Endo[1], N. Yoshioka[1], H. Yamanaka[1], S. Kuji[1], I. Deura[1], T. Ohara[1], A. Tozawa[1], J. Hasegawa[1], M. Harada[2], Y. Osuga[2], N. Suzuki[1]
[1]Obstetrics and Gynecology, St. Marianna University School of Medicine, Kawasaki, [2]Obstetrics and Gynecology, Graduate School of Medicine, University of Tokyo, Tokyo, Japan

Introduction / Background: The major fact-finding survey on oncofertility care has not been conducted to date in the field of gynecologic oncology. Therefore, we examined AYA (adolescent and adult) cancer patients with ovarian germ-cell tumors (OGT) on fertility preserving therapy and from oncofertility liaisons to elucidate the situation in Japan by AMED survey.
Methodology: AYA (age 15-39 years) cancer patients with OGT who received fertility-preserving therapy at the JSOG (Japan Society of Obstetrics and Gynecology) specialist training facilities nationwide from January 2009 to December 2013 (430 cases) were surveyed with respect to surgery applications, surgical styles, outcomes of pregnancy.
Results: No postoperative recurrence was observed in approximately 90% of 415 cases. The ovarian residual function rate was low at 12.8%. Approximately 80% of the cases were stage I cancer. Of the 415 patients, 82.9% underwent adnexectomy during surgery and 13.5% underwent cyst extraction. Abdominal operation and laparoscopy were performed in approximately half of the patients who underwent cyst extraction. Of the 415 patients who underwent surgery, 97 (23.4%) became pregnant.

In the pregnancy cases, the rate of referal to the REI (reproductive endocrinology and infertility) specialists in the overall and chemotherapy groups remained at approximately 15%. Meanwhile, the reference rate to the REI specialists was low in the non-pregnant cases (0.8%).
Conclusion: AYA patients with OGT in Japan undergo therapies described in JSGO (Japan Society of Gynecologic Oncology) clinical guidelines. However, their pregnancy rate is low, and referral to the reproductive is not sufficiently used. Even though the AYA patients with OGT has normal menstruation, the ovarian reserve should be followed by the specialists. Therefore, referring to the REI specialists early on may improve survivorship for this kind of patients.
Disclosure: Nothing to disclose

EP839

Damage control surgery for the management of a complication of ovarian cytoreductive surgery

S. Espuelas Malón[1], J.M. Solé Sedeño[1], E. Miralpeix Rovira[1], I. Aran Ballesta[1], M. Pera Roman[2], C. Clari Ramos[1], M. Castells Zaragoza[1], G. Mancebo Moreno[1]
[1]Obstetrics and Gynaecology, [2]General Surgery, Hospital del Mar, Barcelona, Spain

Introduction / Background: Damage control surgery (DCS) emerged as a feasible alternative for the management of patients with abdominal infection and sepsis in order to reduce mortality compared with primary definitive surgery.
The phases of DCS are defined by 5-steps: first, identifying injury characteristics and presenting pathophysiology, abbreviated surgery to control bleeding and contamination, dynamic reassessment of patient parameters during the operative course, continued physiological restoration and vital organ support in the intensive care unit (ICU) and finally, delayed definitive surgical repair.
Methodology: We present a case of a 56 years-old woman with history of chronic hypertension and appendicectomy, with suspected ovarian mucinous carcinoma in context of a 25cm right ovarian mass. Complete ovarian cytoreductive surgery was performed successfully including ileocecal resection with mechanical side-to-side anastomosis through midline laparotomy.
Results: Five days after the intervention, the patient presented with septic shock secondary to acute peritonitis due to anastomotic leakage confirmed by urgent surgical evaluation with purulent peritonitis of the four quadrants.
During surgery the patient presented hemodynamic instability with high dose noradrenaline requirements and severe respiratory failure. Given the extreme severity DCS was performed, with excision of the previous ileocecal anastomosis, peritoneal lavage and placement of negative pressure therapy leaving open abdomen.
Subsequently, slow but favourable evolution occurred, performing reintervention at 48h for revision, terminal ileostomy and progressive closure of the abdominal wall according to the Leppäniemi technique until complete closure of the wall (2 more procedures). Finally, the patient was discharged 3 weeks after DCS with good general condition.
Conclusion: There is insufficient evidence to advocate for DCS as a general strategy in patients with secondary peritonitis, but it may be an option in selected significantly physiologically deranged patients with ongoing sepsis.
DCS restores physiological reserve facilitating more definitive surgical treatment resulting in decreased perioperative complications and improved outcomes.
Disclosure: Nothing to disclose

EP840

Hipec at interval debulking surgery in advanced stage ovarian cancer patients: impact of the

OVHIPEC-1 trial in real life experience of a tertiary referral centre

A. Fagotti, C. Ronsini, S. Cianci, B. Costantini, F. Bernardini, G. Corrado, G. Vizzielli, G. Scambia
Fondazione Policlinico A. Gemelli, IRCCS, Roma, Italy

Introduction / Background: The OVHIPEC-1 phase III randomized trial has shown a statistically significant improvement in terms of Progression Free and Overall Survival in patients with advanced ovarian cancer, submitted to neoadjuvant chemotherapy (NACT), and receiving HIPEC at time of Interval Debulking Surgery (IDS).
Methodology: After the publication of the trial, according to our national guidelines, the GYO Hospital Tumor Board decided to include HIPEC as routine practice, in patients achieving complete/optimal cytoreduction up to 2.5 mm at time of IDS. Procedure was performed according to published methods. Cases were collected prospectively to show rate of accrual, feasibility, complications.
Results: From January to April 2019, 40 patients were admitted to our Institution to receive IDS. Among them, 18 (45%) were not eligible due to the presence of strict pre-defined exclusion criteria (age > 70 years old, uncontrolled chronic hyperthension, on-going treatment with ACE inhibitors, autoimmune disease, uncontrolled diabetes, BMI > 35, ASA ≥3, patient included in other clinical trials). 4 women (10%) refused to sign informed consent. Finally, 18 patients had complete cytoreduction and received the planned treatment. Median Operative Time was 399 minutes (including 120 minutes related to HIPEC perfusion) (range 256-587) and median Estimated Blood Loss was 287.5 cc (50-600). 5 patients (29.4%) had grade 3 MSKCC early post-operative complications (3 pleural effusions requiring drainage, 2 wound dehiscence requiring VAC). Neither Acute Kidney Failure nor Grade 4-5 complications were observed.
Conclusion: After adopting strict inclusion criteria, the use of HIPEC in this specific setting of patients, has an accrual rate of 82% (18/22), and a feasibility rate after surgery of 100%. The major early complication rate is 27.8%. A larger number of patients is needed to draw significant conclusions.
Disclosure: Nothing to disclose

EP841

Bilateral ovarian solid tumors in young female with autosomal dominant inherited genetic syndrome

O. Ferreira Rangel Neto[1], L.F. Lessa[1], C.D.C.R. Bertoletto[2], M.A. Pereira[2], S.M. Nicolau[2], M.G.K. Uyeda[2]
[1]Divison of Gynecology Oncology / Departament of Gynecology, Federal University of Sao Paulo, Paulista School of Medicine, Sao Paulo, [2]Divison of Gynecology Oncology / Departament of Gynecology, Federal University of Sao Paulo, Paulista School of Medicine, São Paulo, Brazil

Introduction / Background: Gorlin-Goltz syndrome is an autosomal dominant inherited disorder characterized by the presentantion of tumors in different sites, particularly the skin. Due to the manifestation of recurrent cutaneous tumors, the syndrome is also known as Nevoid Basal Cell Carcinoma Syndrome.
Methodology: This is a case of a woman with Gorlin-Goltz syndrome with bilateral adnexal tumors.
Results: A 28-year-old female from the northeastern region of Brazil, with a history of more than 15 surgical procedures for excision of cutaneous tumors, referred to division of gynecological oncology by bilateral pelvic mass of possible adnexal origin, with antecedent of a son who underwent surgical removal of medulloblastoma at the age of four years. Clinical examination revealed solid, mobile and lobulated mass of 12 cm on right adnexal topography and another 4 cm on the left. In addition, the imaging exam revealed fusion of the fourth and fifth ribs in the right. Negative tumor markers. A laparotomy was performed and revealed solid ovarian masses, with regular limits, 15 cm in the Right and 4 cm in the left, performed bilateral salpingo-oophorectomy. Histopathological analysis revealed multinodular fibroma with dystrophic calcification.
Conclusion: Ovarian Fibroma are benign neoplasm, infrequent, with incidence of 4% in the general population. Patients with ovarian fibroma are usually referred to the oncologic gynecologist for present a solid and expansive mass, that may be associated with ascite and pleural effusion (Meigs Syndrome), being an important differential diagnosis of malignant ovarian tumors.
Women with Gorlin-Goltz Syndrome may be present ovarian fibroma in up to 25% of cases. The main

clinical manifestation of this genetic syndrome is cutaneous basal cell carcinomas. Other findings are skeletal changes in 70% of cases (bifid ribs, fused and widened ribs), odontogenic tumors usually in the first decade of life, neurological changes are rare and include medulloblastoma more frequently.
Disclosure: Nothing to disclose

EP842

Role of thyroid system in malignant transformation of ovaries in women with previously removed uterus

E. Frantsiyants, V. Bandovkina, T. Moiseenko, I. Kaplieva, I. Neskubina, A. Menshenina, M. Adamyan, N. Chernikova, E. Verenikina, T. Myagkova, L. Trepitaki
Rostov Research Institute of Oncology, Rostov-on-Don, Russian Federation

Introduction / Background: Thyroid hormones have a regulatory effect on the synthesis and metabolism of sex hormones by the ovaries. Hysterectomy (HE) in women with functioning ovaries, with benign uterine diseases, leads to homeostasis disorders, disruption of adaptation processes at all levels, exacerbation of menopausal disorders, and contributes to abnormal hypothalamus-pituitary-ovaries-thyroid neuroendocrine relationships. The purpose of the study was to analyze the functional activity of the thyroid gland in women with ovarian cancer (OC) with preserved or removed uterus.
Methodology: In 74 menopausal patients with OC after HE (group 1) and primary OC (group 2), mean age 58.3±1.2, blood levels of thyrotropic hormone (TTH), thyroxine (T4), triiodothyronine (T3) and their free forms - FT4, FT3 (Immunotech, Czech) were determined by RIA. The values in 24 healthy donors were considered normal. All patients gave their voluntary informed consent for the study.
Results: Blood levels of T4 in group 1 were 1.3 times lower, and in group 2 - 1.6 times higher than the norm. FT4 exceeded the norm in all patients - 1.8 times in group 1 and 1.4 times in group 2, while FT3 was decreased on average by 1.7 times. TTH levels in group 1 were decreased by 1.4 times, in group 2 - similar to the norm.
Conclusion: Regardless of the presence or absence of the uterus at the time of the development of a malignant process in the ovaries, the pituitary-thyroid axis imbalance was observed, expressed in violation of direct and inverse relationships between peripheral hormones of the thyroid gland and TSH produced by the pituitary gland. Patients with tumor development in the ovaries preserved after HE showed an initial decrease in the thyroxin formation, not controlled by the pituitary, and secondary hypothyroidism.
Disclosure: Nothing to disclose

EP843

Hyperthermic intraperitoneal chemotherapy (HIPEC) for ovarian cancer: evaluation of side effects in a single institution cohort

M. Friedrich
Academic Teaching Hospital | OB/Gyn, Helios Hospital, Krefeld, Germany

Introduction / Background: Peritoneal carcinomatosis is a sign of advanced disease of ovarian cancer and cancer of the fallopian tubes. If it is possible to achieve a complete tumor free situation by cytoreductive surgery with remaining tumor rest less than 0.25 mm, HIPEC might may improve the prognosis.
Methodology: Patients with ovarian cancer and peritoneal carcinomatosis underwent cytoreductive surgery. In patients with optimal tumor debulking HIPEC was performed. The peri- and postoperative course was observed. Adverse events were recorded after the Clavien-Dindo classification.

Results: 43 patients were treated with cytoreductive surgery and HIPEC. In all 43 patients an optimal cytoreductive situation with remaining tumor rest less than 0.25 mm was achieved. HIPEC was performed with a cisplatin solution (50mg/m2) at 41°C. The mean age of the patients was 57.4 years (range: 32 - 74 years) the median age was 56 years, the mean peritoneal cancer index (PCI) was 11.2 (range: 4 - 21), the median PCI was 13, , the mean operation time was 348.6 minutes (range: 192 - 507 minutes). The mean time to postoperative systemic treatment with chemotherapy was 36.51 days (range 21 - 70), the median time was 29 days. There was no postoperative surgically associated death. No adverse events were recorded in 16 patients of the 43 patients, no grade III or IV adverse events were reported for 33. Grade III adverse events occured in 19; a total of 29 grade III adverse events were reported in these 19 patients. Grade IV adverse events occured in 3 patients. The only adverse event that resulted in a return to the operating room was a fistula of the distal small bowel caused by drainage.
Conclusion: Since we have no marked additional adverse events caused by HIPEC we believe, HIPEC to be an additional additional important treatment option of peritoneal carcinomatosis in ovarian cancer.
Disclosure: Nothing to disclose

EP844

Elaidic acid promotes cell proliferation and migration via LSR in epithelial ovarian cancer

M. Funauchi[1,2], K. Hiramatsu[1], S. Serada[1], M. Fujimoto[1], Y. Ueda[2], T. Kimura[2], T. Naka[1]
[1]Kochi University, Kochi, [2]Osaka University, Osaka, Japan

Introduction / Background: Previously, we identified lipolysis-stimulated lipoprotein receptor (LSR) as a poor prognostic factor of epithelial ovarian cancer (EOC), and we demonstrated anti-cancer effect of our newly developed anti-LSR monoclonal antibody (mAb) against LSR-positive EOC *in vitro*and *in vivo*. We also demonstrated that LSR took up VLDL and promoted cell proliferation, however, lipid metabolic pathway via LSR is still unclear. Thus, we aimed to reveal the function of LSR in lipid metabolism in EOC cells.
Methodology: We administrated fatty acid to LSR-positive EOC cells and investigated the activation of beta-oxidation, ATP production, cell proliferation and cell migration via LSR. We also investigated anti-cancereffect of our anti-LSR mAb against lipid metabolism via LSR.
Results: We administrated fatty acids (Palmitic acid (PA), Myristic acid (MA), Arachidonic acid (AA), Oleic acid (OA) and Elaidic acid (EA)) to LSR-positive and LSR-knockdown EOC cell lines, respectively. OA and EA promoted cell proliferation of LSR-positive cells, however, PA, MA and AA did not. Although OA promoted cell proliferation of LSR-positive cells, EA did not, meaning that EA bind to LSR specifically and promoted cell proliferation via LSR. In LSR-positive EOC cells, EA administration increased intracellular lipid accumulation. Moreover, EA promoted ATP production ($p < 0.05$), suggesting that beta-oxidation, TCA cycle and electron transport system were activated by Elaidic acid in LSR-positive EOC cells. Furthermore, in LSR-positive EOC cells, Elaidic acid administration promoted cell migration ($p < 0.05$). Finally, our anti-LSR mAb inhibited these processes ($p < 0.05$).
Conclusion: Elaidic acid uptake via LSR in lipid metabolic pathway contributed to cell viability and cancer spread, and anti-LSR mAb inhibited these processesin LSR-positive EOC cells. Anti-LSR mAb might be a therapeutic agent against human EOC.
Disclosure: Nothing to disclose

EP845

Serous tubal intraepithelial carcinoma in the opportunistic salpingectomy: a case review at

Mataró Hospital
B. Furriols Raulí, C. Rosado Rubio, P. Carabias Meseguer, À. Altés Planas, A. Danés Barris, M.J. Fantova Cosculluela
Mataró Hospital, Mataró, Spain

Introduction / Background: Ovarian cancer is the leading cause of gynaecologic cancer death. More than 70% of women are diagnosed with metastatic disease where the 5-year survival rates average 25-30%, and. Efforts have been made in order to achieve a screening approach that reduces mortality in these patients but they have been unsuccessful.
In women who carry known pathogenic mutations risk-reducing bilateral salpingo-oophorectomy (rrBSO) is recommended, however they only represent 15-20%, most ovarian cancers (80%) occur in women with no risk factors. The rrBSO has lead to the recognition of a non-invasive precursor lesion, the serous tubal intraepithelial carcinoma (STIC) and it is believed to be the origin of high-grade serous ovarian carcinoma.
Incidental STIC lesions in women without a genetic risk factor are rare (0.1%) and for this reason we reviewed this cases in our hospital and described them.
Methodology: We reviewed all the opportunistic salpingectomies from January 2017 to March 2019 in Mataró Hospital and analysed the 3 pure STIC cases.
Results: Among 254 opportunistic salpingectomies, STIC was found in 3 (1.18%). Patient age ranged from 48 to 72 years (mean 57,6; median 53), one under the age of 50. No patients were known to carry pathogenic mutations. All women were Caucasian and had no familiar or personal history of gynaecologic cancer. Two of them underwent surgery for uterine leyomiomas and the other one for ovarian hypertecosis (BSO). In one case, bilateral oophorectomy was indicated while the other case is being followed annually with ultrasound and CA-125 serum marker.
Conclusion: The role of the fallopian tube in ovarian cancer pathogenesis is a subject of interest and it is starting to change clinical practice. It remains uncertain whether opportunistic salpingectomy will reduce ovarian cancer rates. Further research is needed in this area in order to find evidence and create a screening program for ovarian cancer.
Disclosure: Nothing to disclose

EP846

Parenchymal splenic metastasis from ovarian cancer; a report of two cases and literature review

M. Zuhdy[1], K. Gaballa[1], O. Hamdy[1], R. Alghandour[2], M.G. Elmadawy[1], Y. Laimon[3], G. Saleh[4], A. Hosam[1], M. Saleh[1]
[1]Surgical Oncology, [2]Medical Oncology, [3]Pathology, [4]Diagnostic Radiology, Mansoura University, Oncology Center, Mansoura, Egypt

Introduction / Background: Worldwide, ovarian cancer accounts for 3.4% of newly diagnosed cancer cases and 4.4% of cancer related mortality among females.It spreads mainly through peritoneal dissemination, hematogenous or lymphatic routes, and is optimally managed by cytoreductive surgery.Splenic metastasis from malignant tumors are extremely rare ranging from 2.3 to 7.1 % in autopsy studies , and a reported incidence of 1.3% in splenectomy studies. To the best of our knowledge, approximately 24 cases of isolated parenchymal splenic metastasis from ovarian cancer were reported in English based literature.
Methodology: We here report 2 cases with parenchymal splenic metastasis from ovarian cancer one case was operated in October 2017 and the other was operated October 2018
Results: The 2 cases were managed by doing splenectomy followed by adjuvant chemotherapy and both now are on follow up with no evidence of recurrence or further metastasis
Conclusion: Splenic metastasis from ovarian cancer is relatively rare. Thorough follow up of ovarian cancer cases including careful clinical examination, Computed tomography and tumor markers assessment when clinically indicated can early detect any recurrence events even isolated splenic recurrence. Splenectomy as a secondary cytoreductive surgery is a safe procedure that leads to achieving improved both survival and quality of life.
Disclosure: Nothing to disclose

EP847

CA 125 as a prognostic marker for the stage of patients with ovarian carcinoma

A. Ganovska, S. Kovachev
Military Medical Academy, Sofia, Bulgaria

Introduction / Background: Ovarian carcinomas rank 5th as the cause of death from oncological diseases in women. Because of the lack of specific clinical symptoms, they are most often diagnosed at a later stage.The purpose of the study we conducted was to determine the dependence of the preoperative values of CA 125 and the clinical stage in patients with ovarian carcinomas.
Methodology: The study is retrospective for one year (2017-2018). In the study were included 48 patients aged 39 to 78 years with histologically proven ovarian carcinoma. All patients were operated with subsequent histological examination. Laboratory tests were performed prior to surgical intervention and serum CA 125 levels were assessed. Depending on the CA 125 values, patients were divided into two groups: group 1 with elevated values and group 2 with normal values.
Results: In 38 (79.2%) patients, preoperative CA 125 levels were elevated, and 10 (20.8%) women were found within the reference range. In group 1 the distribution of patients according to the ovarian carcinoma stage is as follows: Stage I - 11 (22.9%) women; Stage II - 3 (6.3%) women, stage III - 15 (31.3%) women, stage IV - 9 (18.8%) women. In group 2 the distribution of patients according to the ovarian carcinoma stage is as follows: I stage - 6 (12.4%) women; Stage II - 1 (2.1%) women, III stage - 1 (2.1%) women, IV stage - 2 (4.1%) women. After the statistical treatment of the results, no relationship was established between the CA 125 values and the ovarian carcinoma stage.
Conclusion: CA 125 is a non-specific marker in the diagnosis of ovarian carcinomas, as it can be elevated in a number of benign diseases.
Disclosure: Nothing to disclose

EP848

Overexpression of DUSP6 enhances chemotherapy-resistance of ovarian epithelial cancer by regulating the ERK signaling pathway

Y. Gao[1], H. Li[2], Q. Han[2], Y. Li[1], T. Wang[1], C. Huang[1], Y. Mao[2], X. Wang[2], Q. Zhang[2], J. Tian[2], D.M. Irwin[3], H. Tan[2], H. Guo[1]
[1]Department of Obstetrics and Gynecology, Peking University Third Hospital, [2]Peking University Health Science Center, Beijing, China, [3]Department of Laboratory Medicine and Pathobiology, University of Toronto, Toronto, ON, Canada

Introduction / Background: DUSP6 is a negative regulator of the ERK signaling pathway. Previously we showed that DUSP6 is overexpressed in ovarian cancer side population(SP) cells that are quiescent and chemotherapy-resistant. Here, we explore the effects of DUSP6 on chemotherapy-resistance by examining its regulation of the ERK signaling pathway and G0/G1 cell cycle arrest.
Methodology: mRNA and protein expression of DUSP6 and G0/G1 cell cycle checkpoint regulating proteins was evaluated among ovarian cancer cell lines and tissue samples. Ovarian cancer cells were transiently transfected to overexpress DUSP6. After treatment with cisplatin, cell viability was measured by the MTS assay at 48 hours and the half maximal inhibitory concentration(IC50) was calculated. Subcellular localization and cell cycle analysis were determined by using immunofluorescence and FACS.
Results: SKOV3 and OVCAR8 SP cells expressed higher levels of DUSP6 and lower levels of CyclinD3 compared with non-SP(NSP) cells($P< 0.001$). Among 39 ovarian cancer tissue samples, expression of DUSP6 in the chemotherapy-resistant group(12 samples) was higher than in the

chemotherapy-sensitive group(27 samples) (P< 0.05). While a lower level of expression of CyclinD3 was seen in the chemotherapy-resistant group, it was not statistically different from the chemotherapy-sensitive group. HO8910 cells where shown to have higher IC50 to cisplatin than SKOV3 or OVCAR8 cells, and this correlated with higher levels of DUSP6 expression. Overexpression of DUSP6 in SKOV3 cells led to an increase in cisplatin IC50 values(P< 0.05), and also markedly reduced the expression levels of phospho-ERK1/2 and CyclinD3 and to the predominance of cells in the G0/G1 phase.
Conclusion: Our findings reveal an enhancement of chemotherapy-resistance and a predominance of cells in G1 cell cycle arrest in DUSP6-overexpressing ovarian cancer cells. This suggests that overexpression of DUSP6 promotes chemotherapy-resistance through negative regulation of ERK signaling pathway, increasing the G0/G1 phase ratio among ovarian cancer cells, and leading to cellular quiescence.
Disclosure: Nothing to disclose

EP849

Evaluating the benefits of neoadjuvant chemotherapy for advanced epithelial ovarian Cancer

Y. Gao, Y. Li, C. Zhang, J. Han, H. Liang, K. Zhang, H. Guo
Department of Obstetrics & Gynecology, Peking University Third Hospital, Beijing, China

Introduction / Background: To compare the chemoresistance and survival in patients with stage IIIC or IV epithelial ovarian cancer (EOC) who were treated with neoadjuvant chemotherapy (NACT) followed by interval debulking surgery (IDS) or primary debulking surgery (PDS). The clinical characteristics of patients who benefited from NACT were further evaluated.
Methodology: We retrospectively analyzed 220 patients who underwent NACT followed by IDS or PDS from January 2002 to January 2017 treated at Peking University Third Hospital. Differences in clinicopathological features, chemoresistance and prognosis were analyzed.
Results: The incidence rate for optimal cytoreduction and chemoresistance in the NACT group was relatively higher than those in PDS group. No differences were observed in progression free survival (PFS) or overall survival (OS). Patients without macroscopic RD in the NACT group (NACT-R0) had a similar prognosis compared to patients in the PDS group who had RD＜1cm, and a relatively better prognosis compared to the PDS group that had RD≥1cm. The survival curve demonstrated that patients in the NACT-R0 group that were chemosensitive seemed to have a better prognosis compared to patients in the PDS group that had RD.
Conclusion: Patients without RD after PDS had the best prognosis, whereas patients with RD after NACT followed by IDS had the worst. However, even if patients achieved no RD, their prognosis varied depending on chemosensitivity. Survival was better in patients who were chemosensitive compared to patients who underwent PDS but had macroscopic RD. Hence evaluating the chemosensitivity and feasibility of complete cytoreduction in advance is crucial.
Disclosure: Nothing to disclose

EP850

Incidence and survival impact of abdominal wall metastases in advanced low-grade serous epithelial ovarian cancer: a single center experience

A. Garbi, F. Multinu, M. Achilarre, I. Betella, A. Aloisi, S. Bogliolo, M. Maruccio, C. Personeni, M. Palumbo, G. Aletti, A. Maggioni, N. Colombo, V. Zanagnolo
IEO, Milan, Italy

Introduction / Background: To estimate the incidence of abdominal wall metastases (AWM) and

their impact on overall survival in patients with advanced low-grade serous ovarian cancer (LGSOC).
Methodology: Patients with advanced LGSOC (FIGO stage III-IV) undergoing primary and/or secondary cytoreductive surgery at our Institution between August 2001 and August 2016 were retrospectively identified. Incidence of AWM was evaluated by review of electronic medical records. Overall survival was estimated using the Kaplan-Meier method.
Results: total of 60 women who met inclusion criteria were identified. Of them, 30 (50%) patients had AWM, including 12 (20%) at primary surgery, 18 (30%) at relapse, or both 8 (13.3%). Among patients with evidence of AWM at primary surgery, 11 (91.7%) had prior surgery (10 laparoscopic, 1 laparotomy). Median follow-up was 87 (IQR 51, 127) years. Among the 60 patients, 24 (40%) died of disease [17/30 (56.7%) vs. 8/30 (26.7%) in the groups with and without AWM, respectively]. The median overall survival in the group of patients with AWM was lower than in the group without AWM (80 months; 95% CI 80-103 vs. 89 months; 95% CI 73-104, respectively, Log rank test, P=0.085), although not statistically significant.
Conclusion: Incidence of AWM in advanced LGSOC is high (50% in the present series) compared to high-grade serous epithelial ovarian cancer (3-5%) (Cormio et al, 2003, Gynecologic Oncology) and is associated with decreased overall survival.
Disclosure: Nothing to disclose

EP851

Treatment outcomes of germ cell tumours of ovary: single institutional study

S. Gargy[1], A. Ghosh[1], J. Bhaumik[1], J. Ghosh[2]
[1]Gynaecological Oncology, [2]Medical Oncology, Tata Medical Center, Kolkata, India

Introduction / Background: Germ cell tumours comprise around 5% of all ovarian cancers. They are highly malignant but chemosensitive affecting mostly affecting young women. Accurate diagnosis and treatment can give best outcomes. This study was conducted with the objective to study the clinical profile and treatment outcomes of germ cell tumours with multimodal therapy at Tata Medical Center, Kolkata, India.
Methodology: Data of histologically confirmed germ cell tumours from August 2011 to May 2018 was collected retrospectively from hospital electronic medical records. Median follow up time was 33 months.
Results: Total number of patients was 29 with Median age being 20.5 years. All had good performance score. 25 underwent upfront surgery whereas 4 received NACT followed by IDS. In upfront surgery group, 80% had fertility preservation surgery which was 75% in IDS group.Most common chemotherapy regimen in NACT and adjuvant therapy group.was BEP (75% in NACT and 80% in adjuvant therapy) with 19.patients receiving 4 cycles, 12 patients received 3 cycles and 2 received 5 cycles respectively. On follow up, 25 patients were alive without disease, 4 had recurrence,1 was lost to follow up and 2 patients did not survive. The estimated 5 year RFS was 84.9% and OS was 87.8%.
Conclusion: Most patients of germ cell tumours were at an early stage of the disease and there was good response to treatment.
Disclosure: Nothing to disclose

EP852

Ovarian cancer recurrence post ultraradical primary debulking. Role of HIPEC

E. Geramani, A. Fotiou, V. Psomiadou, M. Drakou, A. Douligeris, S. Lekka, C. Iavazzo, G. Vorgias
Gynecological Department, Metaxa Cancer Hospital, Pireus, Greece

Introduction / Background: Ovarian epithelial cancer presents in advanced stages (III/IV) and is typically managed by combination of cytoreductive plus adjuvant chemotherapy. Cytoreduction increases the postoperative sensitivity of adjuvant chemotherapy and longer free-disease interval.
Methodology: Our case describes an aggressive surgical primary cytoreductive surgery including total abdominal hysterectomy, bilateral salpingo-oophorectomy ,appendicectomy ,total omentectomy, extended right colectomy, partial pelvic peritonectomy, diaphragm peritonectomy, splenectomy partial pancreatectomy and resection of round ligament of liver (CC=0) followed by 6 cycles of adjuvant chemotherapy carbo/taxol. After 12 months recurrent disease was detected by increased levels of tumor markers and PET scan showing distal metastasis (segment II of liver, enlargement of paraortic lymph nodes).
Results: The patient had a secondary complete cytoreductive surgery followed by HIPEC using cisplatin. After three months of the second debulking operation our patient performed an other PET scan that showed off reccurence of disease in multiple peritoneal sites.
Conclusion: Concluding, HIPEC has promising role in ovarian cancer recurrence. However early relapse can be found even after maximum secondary cytoreduction plus HIPEC.
Disclosure: Nothing to disclose

EP853

The feasibility of robot-assisted laparoscopic staging in early-stage epithelial ovarian cancer

S.J. Karsemeijer, R.P. Zweemer, R.H.M. Verheijen, C.G. Gerestein
Gynecologic Oncology, UMC Utrecht Cancer Center, Utrecht, The Netherlands

Introduction / Background: The aim of this study was to evaluate the feasibility and outcomes of robot-assisted laparoscopic staging in early-stage epithelial ovarian cancer.
Methodology: Women who received robot-assisted laparoscopic staging procedures for early-stage epithelial ovarian cancer at the University Medical Centre Utrecht, Utrecht, The Netherlands, between March 2012 to December 2017, were retrospectively included in the study. All enrolled cases were reviewed for patient and tumour characteristics, surgical outcomes, postoperative parameters and survival.
Results: A total of 27 patients were identified. Median age was 55 years (range 39-74 years). In three (11%) patients, conversion to laparotomy was necessary. Median operating time was 229 min (range 127-312 min) and median blood loss was 50 mL (range 30-200 mL). Median pelvic lymph node yield was 14 (range 5-24), median para-aortic lymph node yield was 5 (range 1-17), and median total lymph node yield was 20 (range 10-39). No perioperative complications were observed. One (4%) patient had a postoperative complication without long-term consequences. There was one (4%) case found to be upstaged. Median postoperative pain score was NRS 4 (range NRS 0-7) and median 24-48 h postoperative pain score was NRS 3 (range NRS 0-6). Median time until full diet resumption was 1.5 day (range 1-3 days), median time until self-mobilizing was 1.5 day (range 1-2 days), and median duration of hospital stay was 2.5 days (range 1-5 days). The median duration of follow up was 13 months (range 0-69 months). The median disease free interval was 40 months (range 12-47 months), the disease free survival was 88%, and the overall survival was 96%.
Conclusion: Robot-assisted laparoscopic staging is feasible and safe for the management of early-stage epithelial ovarian cancer. Further prospective studies, and longer follow-up periods will be necessary to confirm our results in terms of complications, comprehensiveness, and disease recurrence.
Disclosure: Nothing to disclose

EP854

How did to improve the overall survival of patients with advanced serous ovarian cancer?

L.A. Ashrafyan[1], E.V. Gerfanova[1], V.I. Kiselev[1], O.I. Aleshikova[2], I.N. Kuznetsov[1]
[1]Institute of Gynaecological Oncology and Mammology, National Medical Research Center for Obstetrics, Gynecology and Perinatology named after Acad.V.I.Kulakov, [2]Russian Scientific Center of Roentgenoradiology, Moscow, Russian Federation

Introduction / Background: At present, there are numerous confirmed data on multitarget effect of indole-3-carbinol (I3C) and epigallocatechin-3-gallate (EGCG) against tumors of different origin. The aim was to study the effectiveness of combined therapy for patients with ovarian cancer (OC) when I3C and I3C with EGCG are included in its scheme.
Methodology: The study included 284 patients with an ascitic form of advanced serous OC. All of them underwent combined treatment: polychemotherapy (TP, TC, DC) and cytoreductive surgery. At the same time, treatment with neoadjuvant chemotherapy (NACT) was provided in the 1st (n=46), the 2nd (n=76) and the 3rd (n=42) groups and prolonged (60 months) usage of I3C (for the 1st group) and its combination with EGCG (for the 2nd, 3rdgroups). In addition, prolonged polychemotherapy (PPCT) was given in the 3rd group. Two groups of treatment-naive patients (without I3C, EGCG) were taken as control arm: in the 1st group (n=40) treatment included operation+6 courses of adjuvant chemotherapy (ACT); the 2nd group (n=80) was treated by NACT+operation+6 courses of ACT. The observation period was 60 months.
Results: The overall survival (OS) in the 1st and 2nd studies groups were higher than in the control's groups, amounted to 65.2% and 63.2%, against 40% and 36.3%, respectively. OS in 3rd studies group achieved of 71.4%, as was as a longer progression-free survival (PFS) a median was of 48.5 months. In the 1st and 2nd studies group the median of PFS were of 39.5 and 42.5 months, in the control's groups were 24.5 and 22 months.
Conclusion: We result that the usage of drugs with multitarget effects (I3C, EGCG) in addition to standard therapy of OC increased its effectiveness by forming conditions (increasing the efficacy of NACT, maintaining the sensitivity of the tumor to first-line of PCT, reducing the number of ascites relapses) to achieve a longer duration PFS, OS.
Disclosure: Nothing to disclose

EP855

Metachronous brain metastases in ovarian cancer

A. Ghidhaoui[1], A. Staebler[2], J. Schittenhelm[3], S. Brucker[1], C. Bachmann[1]
[1]Department of Obstetrics and Gynecology, [2]Department of Pathology and Neuropathology, University Hospital Tuebingen, [3]Department of Pathology and Neuropathology, Institute of Neuropathology, University of Tuebingen and Comprehensive Cancer Center Tübingen-Stuttgart, Tuebingen, Germany

Introduction / Background: Brain metastasis (BM) from ovarian cancer is a rare disease leading to limited survival. Aim is to evaluate risk factors and survival for BM from epithelial ovarian cancer (EOC).
Methodology: A total of 24 patients diagnosed with metachronous BM after therapy of primary ovarian cancer treated at university Tuebingen from 01/2000 to 3/2019, were retrospectively analyzed.
Results: Age at first diagnosis was 56 years (range 42-71y) in median; 83,3% had advanced FIGO stages (III/ IV); 22 patients had histologic grade 2/3. 46%: node-negative; 46%: node-positive; 8%: Nx. All patients received adjuvant platinum-based chemotherapy +/- bevacizumab according to guidelines. At primary surgery, 58.3% achieved complete cytoreduction (R=0mm), 25.0%: R>0mm-< 1cm, 4.2%: R≥1cm, 12.5%: Rx. 12 patients had recurrence of disease. 22 (91,7%) patients had extracranial disease. At diagnosis of BM all patients received whole-brain radiotherapy; 5 obtained additionally neurosurgical intervention.
Most common distant metastatic site was lymph nodes, followed by liver, lung and bone. Overall, interval from first diagnosis to occurrence of BM was 39 months in median (range 10-98 months). Overall survival from first diagnosis of ovarian cancer was 46 months in median (range 13-108 months) and after diagnosis of BM was 10 months (range 1-84 months). After complete cytoreduction interval to BM was 46.9 months (range 11-98) in median, after R>0mm BM were detected after 30 months in median (range 10-52).

In women without extracranial disease, interval from first diagnosis to BM was 31.5 months in median (range 14-49), in women with extracranial disease this timespan was 39,7 months median (range 10-98). 17 women with extracranial metastases died of disease.
Conclusion: Despite presenting a rare manifestation of ovarian cancer, development of BM should be considered in advanced ovarian cancer. Presence of extracranial disease was associated with poor survival in patients with BM and leads to longer interval to occurrence of BM.
Disclosure: All authors declare that there is no potential conflict of interest.

EP856

Optimal residual disease at primary debulking surgery versus complete tumor resection at interval debulking surgery in advanced epithelial ovarian cancer: a case-control survival analysis

V. Ghirardi, N. Bizzarri, V. Vargiu, M. D'Indinosante, M. Loverro, T. Pasciuto, G. Scambia, A. Fagotti
Gynecologic Oncology Division, Policlinico Universitario Agostino Gemelli I.R.C.C.S. Universita Cattolica Di Roma, Rome, Italy

Introduction / Background: Although the non-inferiority in overall survival (OS) of neoadjuvant chemotherapy (NACT) compared to primary debulking surgery (PDS) has been prospectively demonstrated, complete cytoreduction remains the most powerful determinants of survival among patients with stage III- IV epithelial ovarian carcinoma. In this setting, our study aims to compare survival outcomes between patients with 1-10 mm residual disease at primary debulking surgery (PDS) versus those achieving no gross residual disease (NGR) at interval debulking surgery (IDS).
Methodology: We retrospectively reviewed patients treated in our Institution from 1/2010 to 12/2016. 151 patients were included. Among them, 50 were allocated in PDS group (Cases) and 101 in IDS group (Controls). Patients were matched for clinicopathologic characteristics and appropriate statistical tests were used.
Results: No statistically significant differences were found between the two groups, except for a significant difference in median laparoscopic PIV at time of diagnosis, being lower in the Cases with respect to Controls (p< 0.0001).
After a median follow up of 55.3 months, 130 (86.1%) progression/recurrences and 72 (47.7%) deaths were registered. Median PFS was 16.0 and 18.3 months for PDS and IDS group respectively (p=0.456); median OS was 48.0 and 60.2 months in for PDS and IDS group respectively (p=0.426). Subgroup analysis for PFS and OS showed no statistically significant difference in relation to patient's age, FIGO stage, laparoscopic predictive index value, surgical complexity score.
Conclusion: Our results show that NGR at IDS is associated with the same survival outcomes compared to millimetric residual disease at PDS. In addition, neither older age, nor FIGO stage, and tumour load are correlated with a reduction of survival, irrespectively to the treatment strategy. Research efforts to identify pre-treatment criteria of response to chemotherapy are encouraged to improve patient's treatment selection.

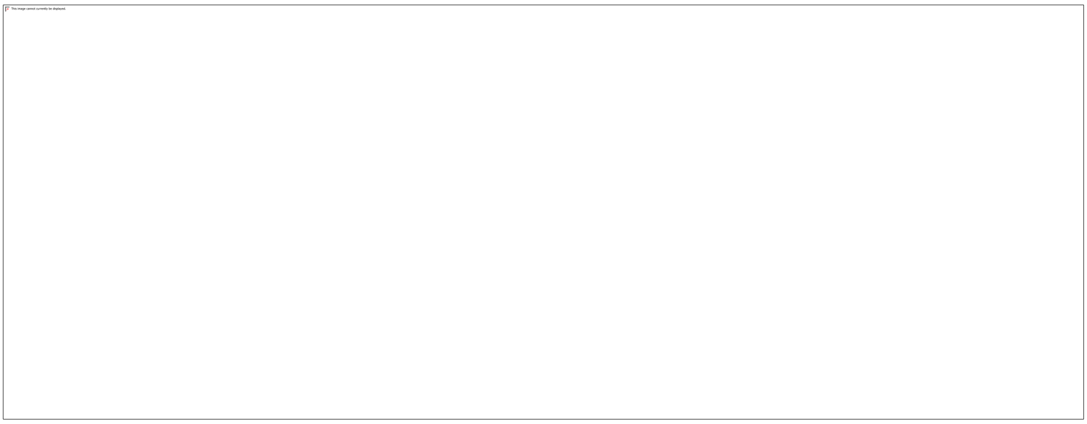

[Figure 1]

Disclosure: Nothing to disclose

EP857

Distinguishing benign vs. cancer states in ovary based on spectrochemical analysis of ascites: a budget omics approach

P. Giamougiannis[1,2], R. Grabowska[1], C.L.M. Morais[1], A. Anagnostopoulos[2], L. Whitham[2], E. Tingi[2], G. Owens[2], P. Hadjiyiannakis[3], N.J. Wood[2], P.L. Martin-Hirsch[2], F.L. Martin[1]
[1]School of Pharmacy and Biomedical Sciences, University of Central Lancashire, [2]Department of Obstetrics and Gynaecology, [3]Department of Clinical Oncology, Lancashire Teaching Hospitals NHS Foundation Trust, Preston, United Kingdom

Introduction / Background: Ovarian cancer is the sixth most common cancer in women in the UK and a leading cause of death from gynaecological cancers, with effective screening and early detection remaining elusive. Vibrational spectroscopy is a rapid, low-cost, non-invasive analytical tool with the potential to classify benign and malignant pathologies according to their biochemical composition. We report on findings of infrared (IR) spectroscopy of ascitic fluid from women with or without ovarian cancer which, to our knowledge, has been conducted for the first time.
Methodology: Attenuated total reflection Fourier-transform IR (ATR-FTIR) spectroscopy was conducted on ascites collected from 20 patients with benign gynaecological pathology, 5 patients with borderline ovarian tumours and 20 ovarian cancer patients. Ten spectra were acquired from each sample. The obtained spectra were pre-processed by cutting the fingerprint region and applying the Savitzky-Golay 2nd derivative and vector normalisation. Classification was conducted by partial least squares discriminant analysis (PLS-DA) with 9 latent variables (94% explained variance) and Support Vector Machines (SVM) algorithm.
Results: Fitting accuracy for discrimination of the 3 classes with PLS-DA was 88% (training set with 70% of samples). When the model was fed with external test samples (30% of samples) the prediction accuracy was 85%. Sensitivity was 0.91, 0.80 and 0.79 for benign, borderline and cancer categories, while specificity was 0.85, 0.96 and 0.93, respectively. However, with SVM, accuracies for fitting and external test samples discrimination were 100%. Additionally, obtained sensitivities and specificities were 100% for the 3 different classes, confirming a better diagnosis response using SVM.
Conclusion: ATR-FTIR spectroscopy demonstrates excellent potential for classifying ovarian cancer and non-cancerous gynaecological conditions in ascitic fluid.
Disclosure: Nothing to disclose

EP858

What you should know about tumor reductive surgery in advanced ovarian cancer - clinical recommendations from a patient perspective

K.R. Gissum[1], S. Drageset[1,2], I. Vistad[3,4], J. Hoel[5], R. Strand[6], L. Bjørge[4,6,7]
[1]Faculty of Health Studies, VID Specialized University, [2]Department of Health and Caring Sciences, Western Norway University of Applied Sciences, Bergen, [3]Sørlandet Hospital, Kristiansand, [4]Department of Clinical Science, University of Bergen, Bergen, [5]GynKreftforeningen, Oslo, [6]Centre for Cancer Biomarkers CCBIO, University of Bergen, [7]Department of Gynecology and Obstetrics, Haukeland University Hospital, Bergen, Norway

Introduction / Background: Little is known about the relationship between tumor reductive surgery

and quality of life in patients diagnosed with epithelial ovarian cancer (EOC). It is therefore unclear whether it is the surgery or the disease itself that is the main factor affecting the patients' quality of life. Engaging the user perspective ensures focus on areas considered relevant and important by patients. The aim of the current study was to examine information requested and perceived about tumor reductive surgery in patients with newly diagnosed EOC.
Methodology: Participants were recruited through the The Norwegian National Gynaecological Patient Organization. They had all received surgical treatment for advanced ovarian cancer at Norwegian hospitals. Focus for the group discussions was to explore the experiences of surgery in women treated for advanced EOC. One focus group was formed and discussions were moderated by members of the research team using a semi-structured interview format. A total of three sessions with this focus group were planned. The focus group discussions were audiotaped and transcribed, and a meaning condensation analysis was performed. Preliminary findings were shared with the participants for validation before further analysis.
Results: Five women were asked and agreed to participate in the focus group discussions. The women had different experiences going through surgery and chemotherapy, but their experiences led to two major themes:
(a) communication gap between the time of diagnosis and surgery, and
(b) insufficient information given about surgical side-effects.
Conclusion: The interviews revealed weaknesses in information given before treatment of advanced ovarian cancer. Guidelines on how to inform the patients should include user-involved research.
Disclosure: Nothing to disclose

EP859

Endocrine-disrupting chemicals present in human follicular fluid disrupt granulosa cell tumors E2 secretion dependent on estrogen metabolism potential

J. Gogola, M. Hoffmann, S. Nimpsz, A. Ptak
Department of Physiology and Toxicology of Reproduction, Jagiellonian University / Institute of Zoology and Biomedical Research, Krakow, Poland

Introduction / Background: Epidemiological studies have shown that the human follicular fluid (FF) include endocrine-disrupting chemicals (EDCs) such as perfluorooctanoate (PFOA) perfluorooctane sulfonate (PFOS), 2,2-dichlorodiphenyldichloroethylene (p,p'-DDE), polychlorinated biphenyl 153 (PCB153) and hexachlorobenzene (HCB) (1,2). Chemicals accumulated in FF may exert their potential effects on granulosa cell tumors (GCTs) that arise from the granulosa cells. Two subtypes of GCTs have been described: the juvenile (JGCT) and the adult (AGCT) form (3). GCTs are hormonally active and produce of 17β-estradiol (E2) (4). Therefore, the aim of this research is to determine whether chemicals with endocrine potential present in FF disrupt E2 secretion by JGCT and AGCT.
Methodology: Two human GCT-derived cell lines: COV434 (ECACC) representing JGCT and KGN (Riken Cell Bank) representing AGCT were cultured in three-dimensional culture. To form spheroids, 6000 cells were seeded in 96-well plates. Spheroids were exposed to single chemicals: PFOA (2ng/ml), PFOS (8ng/ml), DDE (1ng/ml), HCB (50pg/ml) and PCB153 (100pg/ml) or the mixture of these compounds. The CYP19A1 and CYP1A1 expression were determined by Real-Time PCR analysis. The E2, 2-hydroxyestrogen1 (2-OHE1) and 16-hydroxyestrogen1 (16-OHE1) secretion were measured by ELISA.
Results: Firstly, we founded that both basal and T-induced E2 secretion as well as CYP19A1 expression was significantly lower in COV434 than KGN cells. Interestingly, estrogen metabolism potential measured as CYP1A1 expression was contrary correlated. In COV434 cell line, all compounds present in FF and mixtures significantly decreased T-stimulated E2 secretion and simultaneously they increased 2-OHE1/E2 and 16-OHE1/E2 ratio. However, in KGN cells, all compounds and mixtures significantly increased T-stimulated E2 secretion and reduced 2-OHE1/E2 and 16-OHE1/E2 ratio.
Conclusion: These results indicated that EDCs action on E2 secretion depends on estrogen metabolism potential of the target tissue. In JGCT which possess higher estrogen metabolism potential, EDCs accumulated in FF stimulated E2 metabolism. However, in AGCT with lower estrogen metabolism potential, EDCs stimulated E2 secretion.

Disclosure: This study was funded by the National Science Centre (NCN) Poland (grant number 2016/21/B/NZ7/01080). **References:** 1. Petro EM, Leroy JL, Covaci A, et al. (2012); Hum Reprod. 27:1025-33. 2. Petro EM, D'Hollander W, Covaci A, et al. (2014); Sci Total Environ. 496:2828. 3. Pectasides D, Papaxoinis G, Fountzilas G, et al. (2008); Anticancer Res. 28:1421 4. Bulun SE, Simpson ER (2008); Adv Exp Med Biol. 630:112-32.

EP860

Redox status of the blood of patients with ovarian cancer with different prevalence and course of the disease

I. Goroshinskaya, E. Surikova, E. Shalashnaya, G. Nerodo, N. Maksimova, A. Menshenina, M. Sergeeva, P. Kachesova, L. Nemashkalova, A. Chudilova, I. Neskubina, O. Kit
Rostov Research Institute of Oncology, Rostov-on-Don, Russian Federation

Introduction / Background: A comparative analysis of the intensity of oxidative processes and the activity of antioxidant enzymes in the blood of patients with ovarian cancer (OC) was carried out, depending on the prevalence of process and the presence of recurrence.
Methodology: The intensity of lipid peroxidation (LPO), the activity of superoxide dismutase, catalase, glutathione system and ceruloplasmin (main antioxidant protein of blood plasma), levels of proteins of the acute inflammation phase - C-reactive protein (CRP) and haptoglobin were studied in the blood of 114 OC patients. The data were analyzed using Statistika 6.0 programs, Student´s t-test and Mann-Whitney U test.
Results: Ascitic OC was characterized by a high intensity of free radical units of LPO and the maximal increase in CRP and haptoglobin levels (respectively by 17.5 and 2.2 times, compared to stage I-II OC). Patients with advanced OC without ascites showed significantly lower intensity of chemiluminescence (CL) reflecting the formation of free radicals, and higher levels of the molecular product, malondialdehyde (MDA), in the blood plasma compared to patients with ascites ($p< 0.02$). The disease recurrence was accompanied by increased CL by 3 times and MDA level in erythrocytes by 43%, compared to non-cancer women. The key role in the utilization of hydrogen peroxide went from catalase to glutathion peroxidase (GPO), as evidenced by an increase in the GPO/catalase ratio in erythrocytes by 2.6-2.8 times, while in patients with primary OC such changes were not observed. Only patients with stage I-II OC showed all the coefficients reflecting the ratio of activity of antioxidant enzymes similar to the values in healthy women.
Conclusion: The revealed features of the intensity of oxidative processes and balance of activity of antioxidant enzymes in patients with ovarian cancer can contribute to the understanding of disease progression conditions and mechanisms that mediate the effectiveness of antitumor treatment.
Disclosure: Nothing to disclose

EP861

Precision medicine tumor boards: applicability of personalized treatment concepts in ovarian cancer

S. Aust[1], R. Schwameis[1], L. Müllauer[2], G. Prager[3], C. Grech[1], T. Gagic[1], A. Reinthaller[1], H. Kölbl[1], C. Grimm[1], S. Polterauer[1]
[1]*Department of General Gynecology and Gynecologic Oncology,* [2]*Clinical Institute of Pathology,* [3]*Department of Medicine I, Division of Oncology, Medical University Vienna, Vienna, Austria*

Introduction / Background: Treating late-stage cancer according to its genetic mutations is the goal of precision medicine tumor boards (PMTB) that gain importance along with the advances and cost reduction in next generation sequencing (NGS) techniques. Still, their clinical applicability differs

according to tumor origin and has been evaluated in detail for Ovarian Cancer patients within this analysis
Methodology: All ovarian cancer patients whose molecular profile was discussed in a PMTB at the Medical University of Vienna, Austria, from 2015 to 2018 were included in this retrospective analysis (n=33). Not only the NGS results, immunohistochemical (IHC) profiles and respective recommended targeted treatments were determined, but especially the patient characteristics related to actual treatment implementation, duration and outcome.
Results: In 82% at least one mutation, deletion or amplification could be detected. The most frequently altered genes were p53 and PIK3CA, followed by BRCA1, BRCA2, and KRAS. A total of 73% had potentially actionable gene alterations and/or a respective IHC expression profile resulting in a treatment recommendation. Thereof, 7 patients (29%; with 2 to 4 previous lines of standard treatment) received the respective therapy. In 71% the allocated treatment was not administered due to: poor performance status (patients were referred to palliative/hospice care, n=9; or died immediately after the PMTB, n=2); patients received an alternative therapy (n=4); patients were further treated in another clinic (n=2);
Conclusion: Even though a personalized treatment recommendation can be derived frequently, the current treatment implementation in clinical practice remains limited especially due to the poor general condition of ovarian cancer patients after exploitation of standard treatment. However, as each tumor is unique, the obtained molecular genetic data and related clinical characteristics are valuable and will feed the future development of pathway-based drug discovery.
Disclosure: SA: travelling expenses: Roche, Pharma Mar, Amgen, lecture fee: Roche RS: travelling expenses: Roche, Pharma Mar, Amgen GP: speakers fee and/or advisory boards: Bayer, Celgene, Servier, Halozyme, Taiho, Roche, Merck, Sanofi, Amgen, Lilly, BMS, MSD CG, TG none to declare KH: speakers fee and/or advisory boards: Astellas, Pfizer, AMS, Takeda, Johnson & Johnson SP Consultant AstraZeneca, Celgene, MSD, PharmaMar, Roche,Roche Diagnostics, Meda Pharma, Tesaro, Vifor Pharma, outside the submitted work. CG Consultant: AstraZeneca, Celgene, MSD, PharmaMar, Roche, Tesaro, Vifor Pharma Speaker: Amgen, AstraZeneca, MSD, PharmaMar, Roche, Tesaro Direct research funding: Meda Pharma, Roche Diagnostics AR Research grants: Roche Honoraria as a speaker and for advisory boards: Amgen, Astra Zeneca, MSD, Pharma Mar, Roche, Tesaro, Vifor Pharma Travel expenses: Amgen, Astra Zeneca, Pharma Mar, Roche, Tesaro

EP862

Double rare association of malignant ovarian tumors: endocrine and lymphoma ovarian tumors a bout one case

F. Hadjarab[1], C.E. Chekman[2], A. Taibi[1], K. Bouzid[1]
[1]Medical Oncology, Centre Anticancereux Pierre Marie Curie d'Alger, [2]Surgery Pierre Marie Curie, Centre Anticancereux Pierre Marie Curie, Algiers, Algeria

Introduction / Background: The ovarian tumours are rare, often latent andof fortuitous discovery.
The ovarian endocrinetumours, remain in spite of their scarcitypotentially malignant.
The ovarian lymphomas not hodgkiniens areexcrement rare, accounting for 1.5% fromovarian cancers and 0.5% of the lymphomasnot hodgkiniens.
Methodology: We report a rare case of associating anovarian endocrine tumour and an ovarianlymphoma controlatéral at a 61 years oldwoman, revealed approximately a year ago byabdominal pains of the right side. Abdominalechography, it to scan thoraco-abdomino-pelvic and the MRI abdominal assembled ametastatic liver multi nodular, a left ovarianmass and adenopathies retro péritonéales. 4months later, the patient is operated (Colpo hysterectomy, bilateral annexectomy,metastasectomy of segment IV of the liver).
Results: The examination anatomopathologicconcluded with a diffuse lymphoma with largecells from phenotype B (CD20 +) of uterine seat latéro hepatic left andthe localizations and ovarian right-hand side ofa tumour neuro endocrine gooddifferentiated from rank 2.
The assessment of extension (colonoscopy, angio to scan thoraco abdomino pelvic) in particular showed ametastatic liver, a ascite of low abundance,adenopathies retro péritonéales, ganglia interbronchial and hilaires and a pericardialoutpouring.
On the endocrinal level, there are in addition no obviously signs of NEM1 or ovarian hypersecretion.

The patient currently treatedby poly chemotherapy (RCHOP), shows apartial answer estimated according to the imagery at 50%.
Conclusion: The association of these 2 ovarian tumoral forms is exceptional and must be to discuss in RCP(DIALOGUE MULTIDISCIPLINARY OF THEDOCTORS FOR DISCUSSION OF THE FILES OF THE PATIENTS) for an optimal assumption of responsibility the two diseases.
Disclosure: NO ANY CONFILCT OF INTEREST

EP863

Ovarian primitive lymphoma: experience of department of medical oncology Algiers, Algeria

F. Hadjarab[1], C.E. Chekman[2], A. Taibi[1], K. Bouzid[1]
[1]*Medical Oncology, Centre Pierre Marie Curie Alger,* [2]*Surgery, Centre Anticancereux Pierre Marie Curie, Algiers, Algeria*

Introduction / Background: The non-Hodgkin lymphomas primary ovarian malignancies are rare (1.5% of ovarian cancers, 0.5% of NHL) lymphoid tissue that can develop in extranodal sites. whose histogenesis is controversial. Indeed, some authors consider it as an initial manifestation of a generalized lymphomatous disease. The prognosis depends on several factors, including clinical stage, histological type and phenotype, Ovarian primary location is exceptional.
- Criteria for ovarian lymphoma primitive defined by FOX and LANGLEY:
To retain the diagnosis of primary lymphoma of the ovary; Four criteria are required: Location ovary at diagnosis.
Lack of simultaneous extension to another body.
Lack of leukemia.
Monitoring of months to eliminate an extra recurrent genital.
Methodology: We report in this post about 05 comments a retrospective study of 05 cases of ovarian lymphoma supported the medical oncology unit at CPMC, observed 619 cases of ovarian malignancies. This represents 0.80% operated and treated with chemotherapy (CHOP Protocol 4, 1 RCHOP).
TREATMENT: Surgery followed by chemotherapy
- Rests primarily on the standard R-CHOP chemotherapy: - Rituximab (anti CD20 mAb) - Cyclophosphamide - Doxorubicin - Vincristine - Prednisolone.
Results: The therapeutic results were: 03 patients in complete remission; with a decline of 32, 24, and 18 months. Treatment failure in a patient who died after falling 14 months a scheduled these courses of chemotherapy but has not presented patient died as a result of kidney failure two months after surgery.
Conclusion:
- Tumeurs Rare. - Young woman under 50.
- The clinical, biology, imaging are nonspecific making it difficult
Preoperative diagnosis.
- Treatment is primarily based on the medical chemotherapy.
- Key role of extemporaneous analysis before an unusual tumor
young woman to avoid bilateral castration and maintain the possibility of
pregnancy after chemotherapy
FACTORS are ill PROGNOSIS: Medulla injury and neuro-brain - HIV seropositivity - Translocation 14.18 - Failed chemotherapy - but frequently.
Disclosure: no conflict of interest

EP864

BRCA screening and burden of treatment among patients receiving active therapy for ovarian cancer

J. Hall[1], M. Monberg[2], R. Moon[1], K. McLaurin[3], T. Dalvi[3]
[1]Adelphi Real World, Manchester, United Kingdom, [2]Merck Sharp & Dohme Corp., a Subsidiary of Merck & Co., Inc., Kenilworth, NJ, [3]AstraZeneca, Gaithersburg, MD, United States

Introduction / Background: This analysis quantifies the burden among ovarian cancer (OC) patients within 4 years of diagnosis who received chemotherapy (chemo) only as 1L treatment.
Methodology: This chart review and cross-sectional study included advanced OC patients who received chemo at 1L, had a diagnosis within 4 years, and received active treatment at the time of data collection. Between December 2017 and March 2018, 1159 detailed electronic record forms for patients meeting these criteria were provided by 340 oncologists/ gynecologists from France, Germany, Italy, Spain, the UK and the US.
Results: Mean age of all patients was 64 years. Most patients in the study were within 1 year of diagnosis (63%), followed by 1-2 years (22%), 2-3 years (10%) and 3-4 years (5%). BRCA screening rates increased with each line of therapy (42%, 47%, 62%, and 78% for 1L to 4L+). Of those tested, BRCA positivity rates were 20%, 27%, 29%, and 14% for 1L to 4L+. Regardless of the years since diagnosis, the time between lines of therapy decreased with progressive disease. Within the previous year, 33% of all patients had been hospitalized and 13% required a caregiver. Of patients on treatment at 3-4 years after diagnosis, 23% were receiving 3L and 10% were receiving a 4L or later line of therapy. Patients diagnosed 3-4 years ago had received an average of 14.2 months on treatment and a mean 24.1 months treatment-free regardless of line of therapy.
Conclusion: In the real-world setting, a large proportion of advanced OC patients who receive chemo only at 1L are not BRCA tested. Those who receive chemo only as 1L treatment experience multiple lines of therapy within 4 years of diagnosis.

		Years since OC diagnosis			
	All patients	0-1	1-2	2-3	3-4
Patient required a caregiver; N, %	1159, 13	733, 13	254, 11	111, 14	61, 25
Known hospitalizations within prior year, N Hospitalized within prior year; %	703, 33	429, 38	139, 24	83, 22	52, 27
Mean completed duration of 1L treatment; N, Months	386, 4.9	131, 3.9	139, 5.4	73, 5.5	43, 5.4
Mean time from end of 1L to start of 2L; N, Months	389, 9.1	132, 2.1	143, 8.1	71, 16.8	43, 21.4
Mean completed duration of 2L treatment; N, Months	91, 5.0	8, 3.5	30, 4.8	24, 5.3	29, 5.5
Mean time from end of 2L to start of 3L; N, Months	93, 2.3	9, 0.9	30, 1.1	25, 2.6	29, 3.7
Mean time receiving treatment; N, Months	756, 5.6	489, 3.3	150, 8.4	74, 9.7	43, 14.2
Mean time not receiving treatment; N,	387, 9.7	132, 2.1	142, 8.4	70, 17.7	43, 24.1

Months					

[Table]

Disclosure: M.J. Monberg is an employee of Merck Sharp & Dohme Corp., a subsidiary of Merck & Co., Inc., Kenilworth, NJ, USA. K. McLaurin and T. Dalvi are employees of AstraZeneca.

EP865

Forkhead box protein O1 and Paired box gene 3 overexpression is associated with poor prognosis in patients with epithelial ovarian cancer

H. Cho[1], G.H. Han[1], D.B. Chay[2], S. Kim[3], J.-H. Kim[1]
[1]Department of Obstetrics and Gynecology, Gangnam Severance Hospital, Yonsei University College of Medicine, [2]Department of Obstetrics and Gynecology, Yongin Severance Hospital, Yonsei University College of Medicine, [3]Department of Obstetrics and Gynecology, Severance Hospital, Yonsei University College of Medicine, Seoul, Republic of Korea

Introduction / Background: Transcriptional factor FOXO1and PAX3 has been reported to play an imported role in human cancer, but the role in epithelial ovarian cancer (EOC) has not yet been clarified. Here, we evaluated the functional role and expression of FOXO1 with EOC tissues with clinical significance of FOXO1 and PAX3 in EOC.
Methodology: In vitro assessment of cell functions by cell viably assay, cell migration and invasion assay was evaluated using FOXO1 knockdown EOC cell lines. Immuno-histochemical (IHC) staining analyses of FOXO1 and PAX3 were performed using tissue microassay analysis of 141 EOC, 139 borderline ovarian tumors and 48 benign epithelial ovarian tumors and 161 nonadjacent normal epithelial tissues and the data were compared with clinicopathological variables, including the survival of ovarian cancer patients.
Results: In vitro result revealed that knockdown of FOXO1 was associated decreased cell viability (p< 0.001), migration (p< 0.001) and invasion (p< 0.05) supporting the oncogenic role of FOXO1 in EOC. Expressions of FOXO1 and PAX3 were significantly increased in ovarian cancer tissues than in normal epithelium (both $p < 0.001$). Immunoreactivity of FOXO1 significantly correlated with cell type ($p < 0.001$). FOXO1 expression showed strong positive correlation with that of PAX3 (Spearman's rho = 0.430, $p < 0.001$) in cancer patients. Using cox proportional hazards model, we found FOXO1 expression (hazard ratio=4.01 [95% CI, 1.22-13.10], p=0.021) and advanced FIGO stage (hazard ratio=3.89 [95% CI, 1.35-11.19], p=0.012) were independent prognostic factors on overall survival.
Conclusion: This study reveals the association between FOXO1 and PAX3 expression with clinicopathologic variables, including survival of EOC patients. Our results not only suggest the promising potential of FOXO1 as a prognostic and survival marker, but also warrant further studies on a possible link between the biological function of FOXO1 and the pathogenesis of EOC.
Disclosure: Nothing to disclose

EP866

Prognostic and predictive implication of hormonal receptors expression and tumor infiltrating lymphocyte in epithelial ovarian cancer

J.-H. Kim[1], G.H. Han[1], D.B. Chay[2], H. Cho[1], S. Kim[3]
[1]Gangnam Severance Hospital, Yonsei University College of Medicine, Seoul, [2]Yongin Severance Hospital, Kyung-gi do, [3]Severance Hospital, Yonsei University College of Medicine, Seoul, Republic of Korea

Introduction / Background: The effect of systemic treatment depends on the subtypes classified by hormone receptors in breast cancer. However, the effect of hormone receptor status in epithelial ovarian cancer (EOC) is still unclear. Therefore, here we assessed the clinical implication of the hormone receptor status and association with TILs in patients of EOC.
Methodology: Immunohistochemical analysis of estrogen receptor α (ER α), estrogen receptor β (ER β), progesterone receptor (PR), androgen receptor (AR) and glucocorticoid receptor (GR), CD4+ and CD8+ were performed by using tissue microarray analysis of 358 ovarian tumors and the data were compared with clinicopathological variables, including the survival. Cluster analysis was perfored to identify the subgroups by hormone receptor. Receptor expression and correlation with TIL was correlated to uni- and multivariate analysis.
Results: Using cox proportional hazards model, high expression of ER α, ER β and low expression of AR, GR and PR, a combined AR/ ER α expression [HR = 3.25 (95% CI: 1.56-6.98), *p* = 0.003] and a combined GR/ER α expression [HR = 1.93 (95% CI: 1.01-3.68), *p* = 0.046] were revealed to be a poor prognostic subtypes. Also clustering analysis revealed subgroup with hormone receptor ER α positive disease had poor DFS and OS. Finally, in the association between CD4/CD8 and AR-/ER α+ and GR-/ ER α+ were analyzed by cox proportional hazards model. The increased of CD4/CD8 was associated with longer overall survival (OS) in AR -/ER α+ and GR-/ER α+ subtypes [HR = 1.95 (95% CI: 1.02-3.73), *p*=0.041].
Conclusion: In conclusion, increased CD4+ and CD8+ TIL infiltration correlated with improved survival in the whole subtypes as well as in poor prognostic subtypes, AR-/ER α+ and GR-/ ER α+ in EOC. Thus, assessment of these TILs in EOC is essential, and their clinical prognostic implications should be considered according to hormone receptor status.
Disclosure: Nothing to disclose

EP867

Tumor suppression by *FAT4* occurs by acting through retinoblastoma pathway in epithelial ovarian cancer

S. Hanif[1], M. Al Moundhri[2], I. Burney[2], Y. Tamimi[1]
[1]*Biochemistry,* [2]*Medicine and Health Sciences, Sultan Qaboos University, Muscat, Oman*

Introduction / Background: Epithelial ovarian cancer (EOC) accounts for about 70% of all types of ovarian cancer.It is a heterogeneous disease and has variable prognosis. In a previous study, chromatin immunoprecipitation (ChIP) was used to study expression of *E2F5*, a transcription factor, and revealed that *FAT4*, is regulated by *E2F5*. *FAT4*, a tumor suppressor gene, is an upstream regulator of the Hippo pathway and its expression in EOC remains unclear.
Objective: We aimed to investigate the involvement of *FAT4* gene in EOC by quantifying its expression in MCAS, OVSAHO, A2780cp and A2780s ovarian cancer cell lines and compare to HOSE, a normal ovarian cell line.
Methodology: We evaluated the differential expression of *FAT4* and *E2F5* in five ovarian cancer cell lines using qRT-PCR and western blotting. We knocked down *FAT4* and *E2F5* in MCAS and OVSAHO using siRNA and monitored it at the mRNA and protein levels. The effect on viability and invasion following knockdown were assessed by alamar blue and invasion assay. The mechanism linking *FAT4* and *E2F5* was evaluated by assessing proteins involved in Hippo, Retinoblastoma and apoptotic pathways.
Results: MCAS and OVSAHO showed higher *FAT4* expression compared to A2780s and A2780cp. *FAT4* and *E2F5* were knocked down significantly using siRNA in MCAS (FAT4: p< 0.01, E2F5: p< 0.001) and OVSAHO (FAT4: p< 0.001, E2F5: p< 0.00001). Knockdown of *FAT4* promoted cell viability, invasion, upregulation of mesenchymal markers (N-cadherin) and downregulation of epithelial markers (E-cadherin). Also, YAP, an oncogene involved in hippo pathway was upregulated while the pro apoptotic caspase 9 was down-regulated in *FAT4* knocked cell lines. Further, Retinoblastoma and cdk4 was upregulated in *E2F5* knocked compared to *FAT4* knocked cells.
Conclusion: Down regulation of *FAT4* in ovarian cancer cell lines promotes growth and invasion via the modulation of apoptosis, Rb and Hippo pathways.
Disclosure: Nothing to disclose

EP869

Treatment of ovarian cancer in Germany from 2004-2016: results of a nationwide quality assurance program (QS Ovar)

P. Harter[1], J. Pfisterer[2], F. Hilpert[3], J. Sehouli[4], C. Lamparter[5], M. Kerkmann[5], A. du Bois[1]
[1]Ev. Kliniken Essen Mitte, Essen, [2]Zentrum für Gynäkologische Onkologie, Kiel, [3]Krankenhaus Jerusalem, Hamburg, [4]Charité, Berlin, [5]MMF, Dortmund, Germany

Introduction / Background: Treatment of patients with ovarian cancer needs specialized skills and infrastructure. Longitudinal treatment characteristics in Germany are so far unknown at a national level.
Methodology: All German hospitals treating patients with ovarian cancer were asked to document all patients with first diagnosis in the third quarter in 2004, 2008, 2012, and 2016. Details of tumor, treatment and outcome were documented. This analysis is focusing on patients with AOC (advanced ovarian cancer) FIGO III/IV and the endpoint 2Y-PFS (year-progression free survival) (OS data immature for 2016 cohort).
Results: In 2016, QS Ovar included 65% of all patients in Germany treated in 244 hospitals. 2522 of 3512 (71.8%) documented patients have AOC. The rate of complete resection has improved from 33.3% in 2004 to 50.7% in 2016 and the application of platinum + taxane from 83.1 to 89.9%. The additional application of bevacizumab was 54% in 2012 and 67.3% in 2016. The median PFS in patients with complete resection was 26.7 months versus 16.1 months for RD (residual disease) 1-10 mm and 11.1 months for RD> 10 mm (p< 0.001). The 2Y-PFS of all pts was 36.3%. The 2Y-PFS increased in patients with RD > 1 cm from 11.3 to 31.3% and from 27.6% to 40.5% in RD 1-10mm. An increase in 2Y-PFS was also seen in patients with complete resection from 44.8% to 61.8%.
Conclusion: QS Ovar provides representative data of the treatment of patients with ovarian cancer in Germany. Increasing rates of successful surgery and use of standard systemic treatment are associated with an increased 2Y-PFS in all subgroups of patients with advanced OC, but further potentially responsible factors should also be discussed (e.g. centralization). Mature OS data are expected in about 2 years.
Disclosure: QS Ovar was supported by Amgen, Astra Zeneca, Boehringer Ingelheim, Essex, Roche. Harter: Honoraria: Astra Zeneca, Roche, Sotio, Tesaro, Stryker, ASCO, Zai Lab, MSD Advisory Board: Astra Zeneca, Roche, Tesaro, Lilly, Clovis, Immunogen, MSD/Merck; Research funding (Inst): Astra Zeneca, Roche, GSK, Boehringer Ingelheim, Medac, DFG, European Union, DKH, Tesaro, Genmab Pfisterer: Honoraria: Roche, Astra Zeneca, Tesaro, Amgen, Clovis, MSD; Advisory Board: Roche, Astra Zeneca, Tesaro, Clovis, MSD; Travel: Roche, Tesaro; Research funding: Roche, Tesaro Hilpert: Honoraria: Astra Zeneca, Clovis, Roche, Tesaro, MSD, PharmaMar, Pfizer, Novartis; Advisory Board: Astra Zeneca, Roche, Tesaro, Clovis, MSD, PharmaMar Sehouli: Honoraria: PharmaMar, Clovis, Tesaro, Roche, Astra Zeneca, Advisory Board: PharmaMar, Clovis, Tesaro, Roche, Roche Diagnostics, Astra Zeneca, Merck, Bayer, Eisei, Johnson and Johnson, MSD, Novocure, Amgen, Lilly; Research funding (Inst): PharmaMar, Clovis, Tesaro, Roche, Astra Zeneca, Medimmune, Bristol Myers, Lilly; Travel: PharmaMar, Clovis, Tesaro, Roche, Astra Zeneca Lamparter and Kerkmann: nothing to disclose du Bois: Advisory Board: AstraZeneca, Roche, Tesaro, Clovis, BioCad, Genmab, and Pfizer.

EP870

Bevacizumab facilitates surgery in previously inoperable patients with low-grade serous ovarian cancer: a case series

H. Hockings[1,2], G. Wood[1], M. Lockley[1,3]

[1]*Barts Cancer Institute,* [2]*Barts Health NHS Trust,* [3]*University College London Hospitals, London, United Kingdom*

Introduction / Background: Low grade serous ovarian cancer (LGSOC) accounts for 10% of serous epithelial ovarian cancers. In contrast to high grade serous ovarian cancer (HGSOC), it is characterised by an early age of onset, indolent growth rate and relative resistance to chemotherapy. The use of bevacizumab in ovarian cancer has mostly been investigated in HGSOC and is contra-indicated in patients with extensive disease associated with the bowel, due to risks of bowel perforation. Several single-institution studies have reported activity of bevacizumab in LGSOC in the recurrent disease or adjuvant setting. We present two patients with advanced LGSOC and extensive serosal disease involving the bowel that were treated with bevacizumab prior to surgery in the first-line setting.
Methodology: Case notes, pathology results and CT scans were reviewed. Histology slides from initial biopsy and debulking surgery specimens were stained for H&E to assess chemotherapy-response scores.
Results: Both patients had Stage IIIC disease, with extensive disease involving the bowel which prohibited surgery. No response was seen on CT scan or serum Ca125 levels after three cycles of carboplatin and paclitaxel, but following the addition of bevacizumab both patients had a marked reduction in disease volume. Serum Ca125 levels reduced to near-normal values following these three cycles of combined carboplatin, paclitaxel and bevacizumab (438 to 50 and 151 to 54). In both cases surgical debulking with complete macroscopic resection was achieved. There were no adverse events associated with bevacizumab use (18 cycles 7.5mg/kg).
Conclusion: We propose that Bevacizumab may be safe in LGSOC patients with extensive bowel involvement, and can facilitate surgery in those patients that were previously inoperable. Further evaluation of its use in the first-line treatment of LGSOC is warranted as options for neo-adjuvant therapy are severely limited. We have commenced an observational study including all stage III/IV LGSOC patients receiving neo-adjuvant Bevacizumab.
Disclosure: Nothing to disclose

EP871

Impact of surgical restaging on recurrence in patients with borderline ovarian tumors: a meta-analysis

A. Chevrot, C. Huchon
CHI Poissy, Department of Gynecology, University of Versailles, Poissy, France

Introduction / Background: The benefits of restaging surgery for patients with a borderline ovarian tumor (BOT) discovered on initial surgery are debatable. We performed a meta-analysis to evaluate the role of restaging surgery on recurrence in patients with BOTs.
Methodology: We systematically reviewed published studies comparing restaging surgery and incomplete surgery in BOT patients from January 1985 to December 2017. Endpoints were recurrence and mortality rates. Study design features that possibly affected participant selection, reporting of recurrence and death, and manuscript publication were assessed. For pooled estimates of the effect of restaging surgery on recurrence, fixed-effect meta-analytical models were used.
Results: Of the 577 articles initially selected, four retrospective observational studies (Restaging group: 166 patients; Non-Restaging group: 394 patients) met our research criteria. No significant differences in terms of recurrence between the two groups were observed (pooled Peto Odds Ratio [OR]=0.88; 95% confidence interval [CI]: 0.41-1.92). The number of deaths was insufficient for statistical analysis.
Conclusion: This meta-analysis based on retrospective studies, suggests that restaging surgery does not significantly reduce recurrence in patients with BOT.
Disclosure: Nothing to disclose

EP872

Ovarian cancer and HIPEC: Survey for causes, symptoms and treatment multidisciplinary approach - the gynae-oncologists perspective

C. Iavazzo[1], M. Tsiatas[2], A. Christopoulou[3], N. Kopanakis[4], J. Spiliotis[5], P. Sugarbaker[6]
[1]Gynaecological Oncology, Metaxa Cancer Hospital, Piraeus, [2]Medical Oncology Department, Athens Medical Centre, Athens, [3]Medical Oncology Department, Saint Andrews Hospital, Patra, [4]Surgical Oncology, Metaxa Cancer Hospital, Piraeus, [5]Surgical Oncology and HIPEC Department, European Interbalkan Medical Centre, Thessaloniki, Greece, [6]Center for Gastrointestinal Malignancies, Center for Gastrointestinal Malignancies, Washington, WA, United States

Introduction / Background: This research sought to understand Gynaecologists Oncologists knowledge of, experience with, and attitudes toward the use of HIPEC in ovarian cancer patients.
Methodology: A 17-question, self-report survey request was emailed to Gynaecologists Oncologists prior to PSOGI International Symposium on Advanced Ovarian Cancer, Athens April 11th -13th 2019. Doctors submitted responses on the relevant website.
Results: Survey responses were completed by 132 Gynae-Oncologist and reported as a proportion for each query. Sixty percent of the responders were European, 75% male doctors and only 6% were operating on more than 50 patients per year. Ninetyfive percent identified a possible role of pelvic/paraaortic lymphadenectomy during debulking surgery while 48% during fertility sparing operations. Only 25% of the responders check always the BRCA status of their patients however 94% suggest the management can differentiate based on molecular profiling. Sixteen per cent and 9% avoid primary debulking in ovarian cancer patients with mesenteric disease and upper abdominal disease respectively. Fortyseven per cent consider acceptable to administer neoadjuvant chemotherapy based on positive cytology. Fortyeight per cent recognise a possible role of HIPEC during interval debulking, 44.7% the value of surgery in cancer recurrence and 68% the role of HIPEC in recurrent cases. Fortytwo percent are not aware of any evidence regarding the possible role of PIPAC in ovarian cancer. Seventyfive per cent prefer interval debulking for advanced ovarian cancer but only 3% use HIPEC. Tumor resectablility is assessed by 48.5% with upfront laparoscopy. Only 45.5% recognise a survival benefit in patients undergoing extended surgical procedures while the main areas of residual tumor after primary debulking are either mesenteric disease (59.8%) or at porta hepatis (20.5%). Regarding prevention only 18.2% suggest a prophylactic salpingectomy.
Conclusion: We present the findings of our survey which are possibly mirroring the everyday approach of ovarian cancer patients from the Gynae-Oncologists perspective.
Disclosure: Nothing to disclose

EP873

Thinking ouside the box- hyperthecosis in postmenpause

E. Ibrahim
Gynecology, St James's University Hospital, Dublin, Ireland

Introduction / Background:
• Ovarian hyperthecosis is a rare form of tumour. Women present with hyperandrogenism and virilisation. Affecting postmenpause women mainly < 1% and rarely premenopausal women.
• Ovarian hyperthecosis refer to the presence of luteinized thecal cells within the ovarian stroma, separate from the follicles, and is accompanied by a moderate degree of stromal hyperplasia. The luteinized thecal cells may occur as single cells, small nests, or occasionally nodules.
• The ovary produces and releases androgens, including 20% of DHEA, 50% of androstenedione and 25% of circulating testosterone. elevated serum testosterone level provides biochemical evidence of hyperandrogenism. Marked high testosterone levels are an indication to investigate androgen secreting tumor.
Methodology: Case report of 67-year-old referred from the endocrine clinic. Presented with ongoing hirsutism since around the menopause.

Results: All bloods normal
MRI - normal ovaries and adrenal glands.
Thick endometrium 6mm.
Discussion with patient about benefit of oophorectomy in the absence of obvious source of excess androgens.
Pt had laparoscopic bilateral salpingoophrectomy and hysteroscopy D&C .
Procedure was uneventful
Intraop finding- normal anteverted uterus
Endometrium unremarkable
Right ovary slightly enlarged.
Conclusion: The cause of ovarian hyperthecosis is still unknown but there are several factors involved including genetics. Our patient mother had hirsutism which support this theory.
Usually there would be biochemical and radiological evidence pointing to the source of the tumour. In our case though the ovaries looked normal in MRI.the histopathology was positive for bilateral ovarian hyperthecosis. This indicate that negative imaging is not an strong negative predictive test.
Disclosure: Nothing to disclose

EP874

Serologic markers of *Chlamydia trachomatis* and other sexually transmitted infections and subsequent ovarian cancer risk: results from the EPIC cohort

A. Idahl[1], C. Le Cornet[2], S. González Maldonado[2], T. Waterboer[2], N. Bender[2], A. Tjønneland[3], L. Hansen[3], M.-C. Boutron-Ruault[4,5], A. Fournier[4,5], M. Kvaskoff[4,5], H. Boeing[6], A. Trichopoulou[7], E. Valanou[7], E. Peppa[7], D. Palli[8], C. Agnoli[9], A. Mattiello[10], R. Tumino[11], C. Sacerdote[12], C. Onland-Moret[13], I.T. Gram[14], E. Weiderpass[14,15,16], J.R. Quirós[17], E.J. Duell[18], M.-J. Sánchez[19,20], M.-D. Chirlaque[20,21,22], A. Barricarte[20,23,24], L. Gil[24], J. Brändstedt[25], K. Riesbeck[26], E. Lundin[27], K.-T. Khaw[28], A. Perez-Cornago[29], M. Gunter[30], L. Dossus[30], R. Kaaks[2], R. Turzanski Fortner[2]
[1]Department of Clinical Sciences, Obstetrics and Gynecology, Umeå University, Umeå, Sweden, [2]German Cancer Research Center, Heidelberg, Germany, [3]Danish Cancer Society Research Center, Copenhagen, Denmark, [4]Université Paris-Saclay, [5]Gustave Roussy, Villejuif, France, [6]German Institute of Human Nutrition Potsdam-Rehbrücke, Nuthetal, Germany, [7]Hellenic Health Foundation, Athens, Greece, [8]Institute for Cancer Research, Florence, [9]Istituto Nazionale Tumori di Milano Via Venezia, Milan, [10]Federico ii University, Naples, [11]'Civic - M.P. Arezzo' Hospital, Ragusa, [12]Città della Salute e della Scienza, Turin, Italy, [13]UMC Utrecht, YOUth Onderzoek - Universiteit Utrecht, Utrecht, The Netherlands, [14]University of Tromsø, Tromsø, [15]Institute of Population-Based Cancer Research, Oslo, Norway, [16]Karolinska Institutet, Stockholm, Sweden, [17]Conserjeria de Sanidad, Oviedo, [18]Catalan Institute of Oncology, Barcelona, [19]Hospitales Universitarios de Granada/Universidad de Granada, Granada, [20]CIBER in Epidemiology and Public Health, Madrid, [21]Regional Health Council, IMIB-Arrixaca, [22]Universidad de Murcia, Murcia, [23]Navarra Public Health Institute, [24]Navarra Institute for Health Research, Pamplona, Spain, [25]Lund University, Lund, [26]Lund University, Malmö, [27]Umeå University, Umeå, Sweden, [28]University of Cambridge, Cambridge, [29]University of Oxford, Oxford, United Kingdom, [30]International Agency for Research on Cancer, Lyon, France

Introduction / Background: Sexually transmitted infections (STI) and pelvic inflammatory disease may cause damage to the fallopian tube where a substantial proportion of epithelial ovarian cancer (EOC) likely arises. The aim of this study was to determine whether *Chlamydia trachomatis* antibodies are associated with higher EOC risk. As secondary objectives, we investigated *Mycoplasma genitalium,*herpes simplex virus type 2 (HSV-2) and human papillomavirus (HPV) 16, 18 and 45 and EOC risk.
Methodology: In a nested case-control study within the European Prospective Investigation into Cancer and Nutrition (EPIC) cohort,791 cases and 1,669 matched controls with pre-diagnosis blood samples were analyzed. Cases and controls were matched on study center, and at blood collection age, time of day, fasting status, exogenous hormone use, menopausal status, and menstrual cycle phase. Antibodies against *C. trachomatis*, *M. genitalium*, HSV-2, and HPV 16, 18 and 45 (E6, E7, L1) were assessed using multiplex fluorescent bead-based serology. Conditional logistic regression was used to estimate relative risks (RR) and 95% confidence intervals [CI] comparing women with positive

vs. negative serology.
Results: A total of 40% of the study population was seropositive to at least one STI. Positive serology to *C. trachomatis* Pgp3 antibodies was not associated with EOC risk overall, but was associated with higher risk of the mucinous histotype (RR=2.56 [95% CI=1.3-5.05]). Positive serology for chlamydia heat shock protein 60 (cHSP60-1), produced during persistent infection, was associated with higher risk of EOC overall (1.33 [1.09-1.62]) and of the serous subtype (1.42 [1.09-1.84]). None of the other evaluated STIs were associated with EOC risk overall; in analyses by histotype, HSV-2 was associated with higher risk of endometrioid EOC (2.93 [1.50-5.74]).
Conclusion: *C. trachomatis* infection may influence carcinogenesis of serous and mucinous EOC, while HSV-2 might promote endometrioid disease. Mechanisms linking STIs to EOC need to be further elucidated.
Disclosure: Financial support: This work was supported by grants from The Cancer Research Foundation in Northern Sweden and The County Council of Västerbotten, Sweden, and the German Cancer Research Center. The coordination of EPIC is financially supported by the European Commission (DG-SANCO) and the International Agency for Research on Cancer. The national cohorts are supported by grant givers in the separate countries. Detailed information on demand.

EP875

Novel clinical roles for BRCA1/2 germ-line status: 1) Primary surgery is superior to neoadjuvant chemotherapy in sporadic but not in hereditary ovarian cancers; 2) Favorable anatomic pattern of relapses in BRCA1/2 mutation carriers

E. Imyanitov, T. Gorodnova, A. Sokolenko, V. Ni, A. Ivantsov, K. Kotiv, S. Petrik, I. Amelina, I. Berlev
N.N. Petrov Institute of Oncology, St. Petersburg, Russian Federation

Introduction / Background: BRCA1/2-driven ovarian cancers (OCs) compose a distinct group of OC, which is characterized by high sensitivity to platinum-based therapy. This study aimed to compare the disease outcomes in BRCA1/2 mutation carriers vs. non-carriers.
Methodology: The analysis included 283 consecutive OC patients, who underwent optimal cytoreduction (size of residual tumor < 1 cm (n = 156) or complete tumor excision (n = 127)) upon primary debulking surgery (PDS) (n = 168) or after neoadjuvant chemotherapy (NACT) (n = 115). 84 patients carried germ-line mutation in *BRCA1* (n = 78) or *BRCA2* (n = 6) genes, while 199 OCs were classified as sporadic cases.
Results: Patients treated with NACT demonstrated inferior median disease-free interval (DFI) compared to women subjected to PDS followed by adjuvant therapy (7.9 months vs. 19.5 months, $P <$ 0.001). Interestingly, this difference remained clinically meaningful and statistically significant only for sporadic OC (5.1 months vs. 20.7 months, $P < 0.001$), while BRCA1/2-driven carcinomas showed relatively similar DFI irrespectively of the sequence of the treatments (13.1 months vs. 16.7 months, P = 0.11). The disease relapse was observed in 72/84 (86%) *BRCA1/2*-mutated patients and 165/199 (83%) women with sporadic cancer. 35/72 (49%) *BRCA1/2*-associated OCs relapsed by a single tumor lump, which was potentially amenable for radiological or surgical ablation; this estimate was significantly lower in sporadic cancer patients (52/165 (32%); $P = 0.01$).
Conclusion: BRCA1/2 status may contribute to the choice between PDS and NACT, therefore the turnaround time for BRCA1/2 testing may be important for proper treatment planning. BRCA1/2-driven OCs have more favorable pattern of relapses as compared to sporadic cases.
Disclosure: This work has been supported by the Russian Science Foundation, grant 19-15-00168

EP876

Oncologic outcome of cystectomy compared with unilateral salpingo-oophorectomy in the

treatment of stage I borderline ovarian tumors

T.-K. Jang, H. Chung, S.-H. Kwon, S.-J. Shin, S. Lee, C.-H. Cho
Obstetrics and Gynecology, Keimyung University School of Medicine, Dongsan Medical Center, Daegu, Republic of Korea

Introduction / Background: The aim of this study was to compare the oncologic outcomes in stage I borderline ovarian tumors (BOTs) patients treated with unilateral ovarian cystectomy (UOC) versus unilateral salpingo-oophorectomy (USO).
Methodology: This was a retrospective study of 95 patients with stage I BOTs treated with cystectomy (n=22) or unilateral salpingo-oophorectomy (n=73) between 2000 and 2017 at Keimyung University Dongsan Medical Center. The surgical and oncologic outcomes between the two groups (UOC versus USO) were compared.
Results: After a median follow-up of 76 months (range: 10-215), there were 9 patients who had recurrences (UOC group: 4 vs. USO group: 5). The recurrence rate was higher in UOC group (18.2%) than in the USO group (6.8%).
However, there was no statistically significant difference in recurrence rates (p=0.104) and 5-year progression free-survival (p=0.099) between the two groups. All recurred patients except 1 patient (USO group) were successfully treated by secondary operation and were alive with no clinical evidence of disease. Only 1 patient was died due to disease.
Conclusion: Our results support that both surgical methods are an acceptable treatment for women with stage I BOTs. Although USO is an appropriate treatment for BOTs patients, a UOC can be considered for patients who wish to preserve fertility when a unilateral cystectomy is the only surgical method.
Disclosure: Nothing to disclose

EP877

Intestinal (sub)obstruction in ovarian cancer patients: management, complications and survival

J. Jansen, E. Van Nieuwenhuysen, P. Neven, T. Van Gorp, I. Vergote, S.N. Han
Gynaecology and Obstetrics, Division of Gynaecologic Oncology, University Hospitals Leuven, Leuven, Belgium

Introduction / Background: We aimed to describe management, complications and survival of malignant bowel obstruction (MBO) in epithelial tubo-ovarian cancer patients. Our primary objective was overall survival, measured in days. MBO complications and management were considered secondary endpoints.
Methodology: Retrospective monocentric cohort study at the University Hospitals Leuven, Belgium, between January 1st, 2012 until December 31rd, 2016. All primary epithelial tubo-ovarian cancer patients with MBO were identified. The standard conservative approach of MBO in the University Hospitals Leuven is presented in Table 1.
Results: 452 patient files were searched from which 48 patients were included in this study with a total of 84 MBO episodes, (median 1 per patient; range 1-5). Median time interval between two MBO episodes was 59 days (range 2-626). Median hospitalization time was 11 days (range 0-74) per episode. Median time interval between cancer diagnosis and first MBO episode was 342 days (range 0-1155). Further characteristics are noted in Table 2.
Two complications were registered: bowel perforation and bowel ischemia, each in one episode. Conservative treatment was applied in 73 (87%) episodes, including gastrostomy in 3 (4%) episodes. Operative treatment was necessary in 11 (13%) episodes, including exploratory laparoscopy in 2 (2%) episodes and exploratory laparotomy in 9 (11%) episodes. Only one case of postoperative peritonitis was reported. Total parenteral nutrition (TPN) was administered in 15 (31%) patients only. During the study period 35 patients died within a median time interval of 101 days (range 3-917) since the first MBO (Figure 1). There was no significant difference in survival between patient groups regarding TPN use, ascites and obstruction at cancer diagnosis.
Conclusion: Tubo-ovarian cancer patients with MBO have poor prognosis: 73% of our study population died within a relatively short time interval since the first MBO. The majority of patients with

MBO were treated conservatively.

No oral intake of fluid and food
Intravenous fluid therapy 2l/24h
Nasogastric tube when necessary
Antiemetics e.g. Alizapride (Litican®)
Analgesics
Octreotide (Sandostatin®)
Corticosteroids e.g. Methylprednisolone (Solumedrol®)
Abdominal X-ray every two days

[Table 1: Standard conservative approach of MBO in the University Hospitals Leuven.]

Characteristic	Number (n = 48)
Time interval between diagnosis and first MBO episode, days; Median (minimum-maximum)	342 (0-1155)
Episodes of MBO, n (%); 1; 2; 3; ≥4	27 (56,25); 12 (25); 4 (8,33); 5 (10,42)
Episodes of MBO per patient; Median (minimum-maximum)	1 (1-5)
Time interval between MBO episodes, days; Median (minimum-maximum)	58,5 (2-626)
Hospitalization time per MBO episode, days; Median (minimum-maximum)	11 (0-74)
Time to death since the first episode of MBO, days; Median (minimum-maximum)	101 (3-917)

[Table 2: Characteristics of MBO in the study population.]

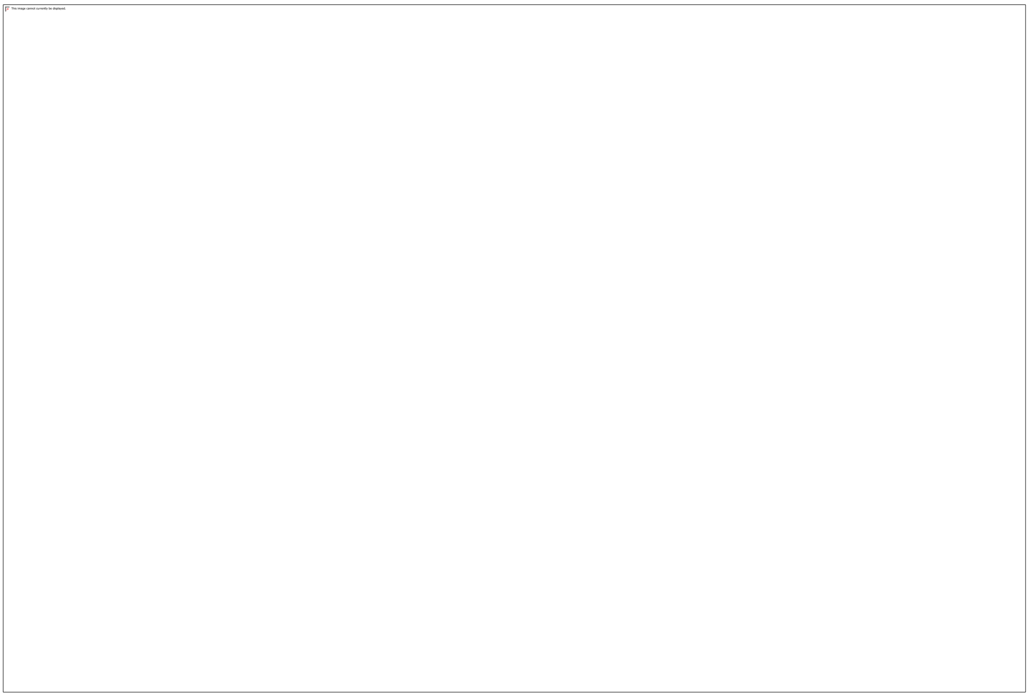

[Figure 1: Kaplan-Meier plot: survival in days from the first episode of malignant (sub)obstruction t]

Disclosure: Nothing to disclose

EP878

Anastomotic bowel leakage after cytoreductive surgery in advanced epithelial ovarian cancer surgery: relation to surgical specialty and complexity score

U. Joneborg[1], S. Salehi[1], H. Falconer[1], P.J. Nilsson[2], A. Flöter Rådestad[1]
[1]Division of Gynecologic Oncology, [2]Division of Colorectal Cancer, Karolinska University Hospital and Karolinska Institutet, Stockholm, Sweden

Introduction / Background: Radical cytoreductive surgery is the most important prognostic factor for survival in women with advanced epithelial ovarian cancer (EOC). Extra-pelvic procedures, including bowel resections, are often necessary to achieve complete tumor resection. Anastomotic leakage (AL) is a severe postoperative complication with risk of perioperative death and delay in time to start of adjuvant chemotherapy. The objective of this study was to evaluate AL rates in relation to surgical specialty and complexity score.
Methodology: Consecutive women with advanced EOC who underwent bowel resection as part of cytoreductive surgery at Karolinska University Hospital between 2004-2011 and 2014-2016 were identified in hospital data bases. Descriptive statistics was performed.
Results: Ninety-three and 101 women were analyzed in the early (2004-2011) and late (2014-2016) cohorts respectively. Demographic data was similar between groups. Bowel surgery performed by colorectal surgeons decreased from 89% to 20% ($p < 0.001$). The rate of upfront surgery increased from 70% to 99% ($p < 0.001$) and surgical complexity score (SCS) from 6.3 to 9.8 ($p < 0.001$) between the cohorts. AL decreased from 6.5 % to 4% ($p=0.52$).
Conclusion: Despite a significant increase of bowel resections performed by gynecologic oncologists, upfront surgery and SCS, there was no increase in AL rate. Bowel surgery can safely be performed by trained gynecological oncologists.
Disclosure: Nothing to disclose

EP879

Peritoneal cancer index (PCI) is a strong predictor of operability in ovarian cancer

B. Jónsdóttir, M. Lomnytska, I. Sundström Poromaa, I. Silins, K. Stålberg
Department of Women's and Children's Health, Uppsala University, Uppsala, Sweden

Introduction / Background: Extent of tumour load is one of the important factors selecting patients with advanced ovarian cancer for cytoreductive surgery (CRS). Peritoneal cancer index (PCI) gives exact information on tumour distribution and is a prognostic factor of survival, but is still not standard in ovarian cancer surgery.
The aim of this study was to find a PCI cut-off value for complete cytoreductive surgery and second aims to identify reasons for open-close, and to correlate tumour burden with surgical complications.
Methodology: One-hundred-sixty-seven women with advanced ovarian cancer planned for CRS were included in the study. Possible predictors of operability were evaluated with receiver operator curves (ROC) and a PCI cut-off was identified. The study population was then divided in groups by cut-off value and differences in peri-or postoperative complications were analysed by one-way ANOVA or Chi-square tests.
Results: The median PCI score for all patients was 22 (range 3-37). Complete CRS was achieved in 148 (88.1%) patients. The median PCI for patients with incomplete surgery was 33 (range 25-37) of whom 14 were open-close. Intraoperative PCI score was an excellent predictor of operability with an AUC of 0.945 (95% CI 0.91 - 0.98) (Fig 1). For patients with PCI > 33, only 28.6% achieved complete CRS (Table 1). In patients with PCI > 24 complete CRS was obtained in 67.2% but with an increased rate of complications (p=0.008). Reasons for all open-close were massive carcinomatosis on the small bowel.
Conclusion: Peritoneal cancer index (PCI) was an excellent predictor of operability and more complications were found with higher tumour burden. Radiologic methods to evaluate preoperative PCI should be further evaluated and PCI used as a tool to compare study populations in surgical studies.

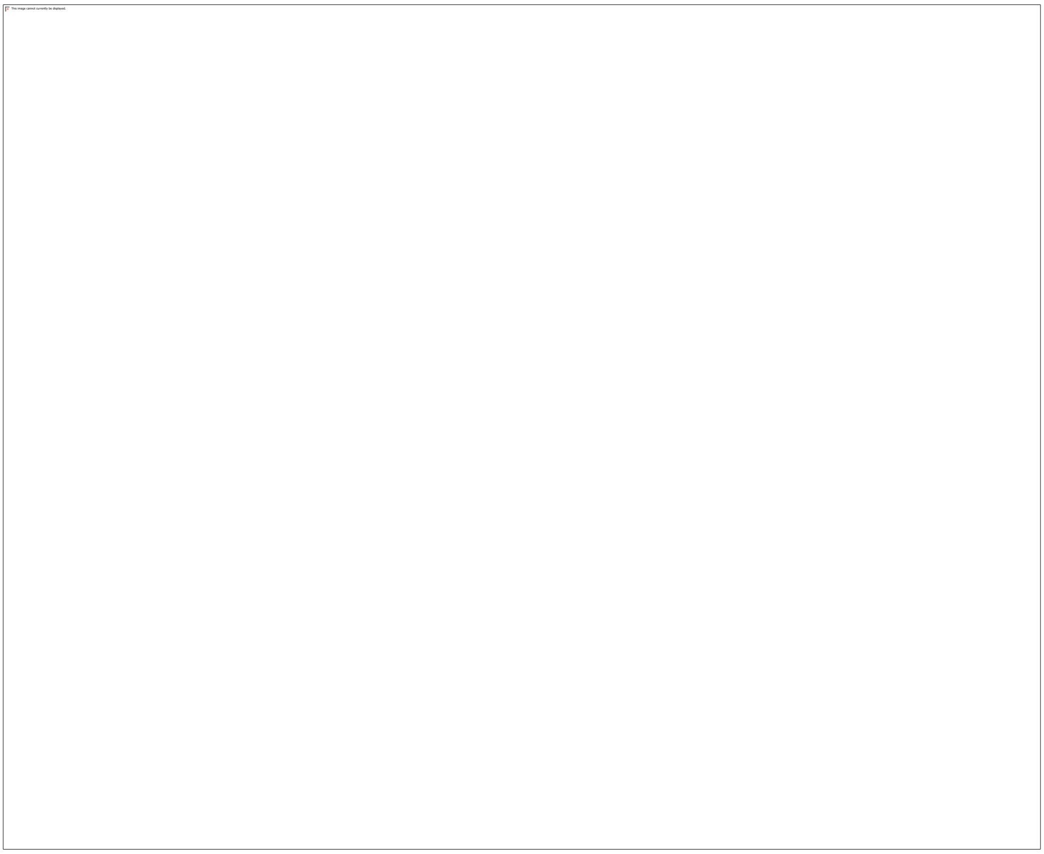

[Figure 1: ROC curve for PCI regarding completeness of cytoreductive surgery]

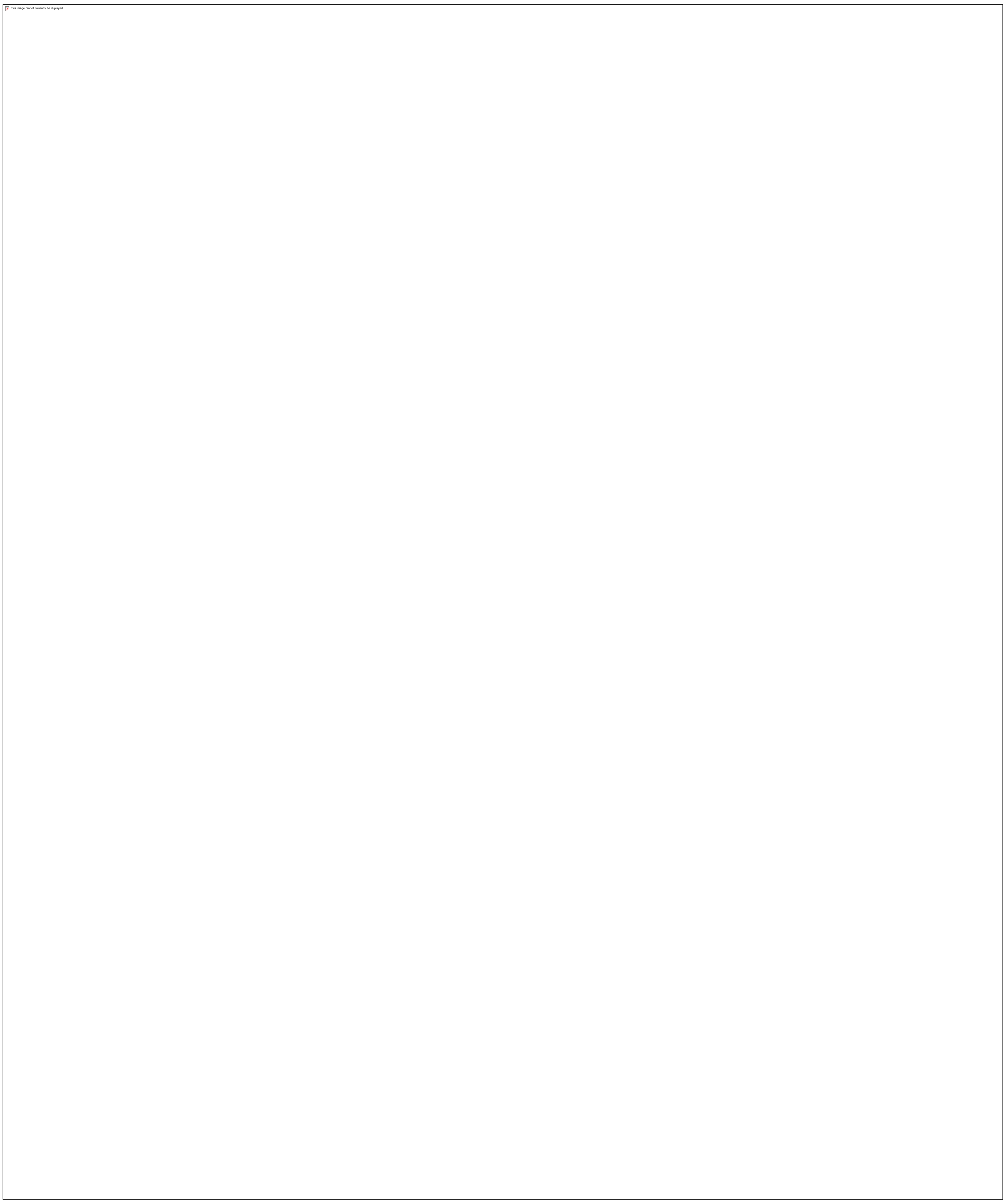

[Surgical data and peri- and postoperative complications analysed by PCI-group]

Disclosure: Nothing to disclose

EP880

Analysis of oncologic and reproductive outcomes after fertility-sparing surgery in young women with malignant ovarian germ cell tumor

U.C. Ju, S.M. Kim
Chonnam National University Medical School, Gwangju, Republic of Korea

Introduction / Background: The aim of this study was to evaluate the oncologic and reproductive outcomes after fertility-sparing surgery (FSS) in young women with malignant ovarian germ cell tumor (MOGCT).
Methodology: The medical records of women aged under 40 years with MOGCT who underwent surgery at our institution between July 2002 and December 2017 were retrospectively reviewed. FSS was defined as the preservation of the uterus and at least one adnexa.
Results: A total of 44 patients who underwent FSS were included. The median age of patients was 23 years (range, 7-39 years). There were 20 patients (45.5%) with immature teratoma, 13 (29.5%) with dysgerminoma, 6 (13.5%) with endodermal sinus tumor, 3 (6.8%) with mixed germ cell tumor, 1 with choriocarcinoma, and 1 with embryonal carcinoma. 29 patients (65.9%) received postoperative chemotherapy with bleomycin, etoposide, and cisplatin (BEP). During a median follow-up period of 62.5 months (range, 7-185), only 4 patients (9.1%) had a recurrence. Notably, all of 2 patients with dysgerminoma had recurrence at para-aortic lymph nodes and all of 2 patients with grade-2 immature teratoma had recurrence at contralateral ovary. Of 14 women desiring pregnancy, 12 achieved term delivery of 12 singleton pregnancies. The pregnancy rate was 85.7% and the live birth rate was 100%.
Conclusion: Fertility-sparing surgery is an acceptable option for young patients with MOGCT who wish to preserve their fertility, provided they are willing to undergo a prolonged follow up. Reproductive outcomes are assuring to them.
Disclosure: Nothing to disclose

EP881

Neoadjuvant chemotherapy for newly diagnosed ovarian cancer: a single center experience

M. Kaparelou[1], R. Zakopoulou[1], A. Tsiara[1], A. Kyriazoglou[1], M. Liontos[1], G. Tsironis[1], K. Koutsoukos[1], F. Zagouri[1], D. Vlachos[2], N. Thomakos[2], D. Haidopoulos[2], A. Rodolakis[2], M.A. Dimopoulos[1], A. Bamias[1]
[1]Oncology Department of Clinical Therapeutics, [2]Department of Obstetrics and Gynecology, Alexandra Hospital, National and Kapodistrian University of Athens, Athens, Greece

Introduction / Background: Ovarian cancer (OC) is one of the most lethal gynecological malignancies. Primary Debulking Surgery (PDS) followed by chemotherapy has been the standard of care. However, two randomized trials have demonstrated that Neoadjuvant Chemotherapy(NACT) followed by Interval Debulking Surgery(IDS) is not inferior to PDS. Therefore, we conducted a retrospective analysis to determine patterns of practice in our institution.
Methodology: Medical records of women with epithelial OC treated at Alexandra Hospital from 2011 to 2016 were retrospectively identified. Clinicopathological data, treatment and survival data were analyzed. Kaplan-Meier Survival curves were generated using IBM SPSS version 20; survival differences were estimated using the long-rank test.
Results: 198 patients were identified. Median age was 60.8 years. 169 patients had serous carcinoma, 10 clear cell, 10 endometrial, 3 mucous and 4 adenocarcinoma. 171 had advanced (stage III/IV) disease. PDS was performed in 128 patients, while 70 received NACT. 48 performed IDS, while 6 had LDS. No surgery was performed in 16 patients. With a median follow-up of 27.3 months, mPFS was 21.8 months and mOS was 58.5 months. Patients treated with NACT-IDS had statistical significant shorter mPFS and mOS than those treated with PDS (PFS: 16.2m vs 25.9m P< 0.001 and OS 44.4m vs Not Reached, P=0.05). NACT-IDS retained its statistical significance as an adverse prognostic factor in multivariate analysis when controlled for stage, grade and histology of the disease (P=0.003). No statistical significant difference in the percentage of patients with platinum resistant disease among PDS and IDS was identified.
Conclusion: NACT followed by IDS is frequently used in the treatment of ovarian cancer patients in a tertiary centre in Greece and was correlated with adverse outcome. A selection bias favoring NACT for patients with high risk for perioperative morbidity may affect the results of this retrospective analysis. Large randomized trials to address this issue are underway.
Disclosure: Nothing to disclose

EP882

Risk of ovarian malignancy algorithm performs better than CA125&HE4 alone in a clinical setting

M. Katsyuba[1,2], R. Khasanov[1], G. Usmanova[2], A. Akhmetzianova[2], G. Muratova[3], A. Saveliev[3], V. Terenteva[1]
[1]Kazan State Medical Academy, [2]Tatarstan Cancer Center, [3]Kazan Federal University, Kazan, Russian Federation

Introduction / Background: Several studies has shown no advantages of Risk of Ovarian Malignancy Algorithm (ROMA) over CA125 and/or HE4 tumor markers alone when ROC-AUC were analyzed. However, the best way of clinical interpretation of CA125/HE4 levels remains unclear. The goal of the study was to comprehensively evaluate several ways of clinical interpretation of CA125/HE4 serum levels in patients with pelvic mass.
Methodology: We analyzed Ca125 and HE4 serum levels in 145 healthy females and in 1019 patients with pelvic mass, scheduled to have surgery.
Results: In healthy women 95th percentile (90% Confidence Interval) of HE4 levels were 38.5(37.5-41.2) and 45.8(41.6-48.1) pmol/L in pre- and post-menopause respectively. When the manufacturer's reference limits were used as a threshold, HE4 showed a sensitivity for epithelial ovarian cancer of 54 % and a specificity of 98.8%. When our local reference population's reference limits were used, the sensitivity and specificity of HE4 were of 94.7 % and of 55.9% respectively. CA125 showed a sensitivity of 96.7% and a specificity of 73.7 %.according to the 35 U/ml threshold. The sensitivity and specificity of ROMA according to its standard cut-off limits were 89.2 and 88% respectively. Even when calculated optimal cut-off levels of CA125 and HE4 and standard cut-off level for ROMA were used, ROMA showed a tendency to a higher performance and a more balanced sensitivity/specificity than CA125/HE4 alone
Conclusion: Reference limits of CA125/HE4 (not proposed by the manufacturer, not determined in a reference population) do not correspond to the optimal cut-off levels and are not of clinical significance in patients with pelvic mass. ROMA performs better in a clinical setting than CA125/HE4 alone even if optimized cut-off levels (instead of reference limits) of these markers are used.
Disclosure: Nothing to disclose

EP883

Repeated use of maintenance therapy olaparib in patients with platinum-sensitive relapse of ovarian cancer

S. Khokhlova
Chemotherapy Department, National Medical Research Center of Gynecology and Obstetrics n.a, V. I. Kulakov, Ministry of Health, Moscow, Russian Federation

Introduction / Background: Experience of repeated use of maintenance therapy olaparib in patients with platinum-sensitive relapse of ovarian cancer in patients with a BRCA1 or BRCA2 (BRCA1/2) mutation
Methodology: In pilot trial evaluated olaparib capsules maintenance retreatment in platinum-sensitive, relapsed ovarian cancer patients with a BRCA1/2 mutation who had received at least four lines of previous platinum -containing chemotherapy and one maintenance line of olaparib therapy. The primary endpoint was progression-free survival the first maintenance therapy of olaparib and then reappointment of olaparib in maintenance therapy.
Results: Between Sept 2014, and May 2019, 21 patients received maintenance olaparib for at least 3 lines of previous platinum-containing chemotherapy lines with a median survival without progression for 17,2 months (6-38 months). Among 21 patients 10 patients have progressed (6-29 months) and they were renewed with platinum-containing chemotherapy (gemcitabine with cisplatin (6pts),

doxorubicin with cisplatin (2pts), paclitaxel with carboplatin (2pts). The complete response was observed in 2 patients and in 8- partial. All 10 patients were restarted olaparib maintenance therapy. Median survival without progression of repeated maintenance treatment was 9,4 months (4-14 months). Two patients reapplication olaparib was longer than at the first use. When repeated administration of olaparib toxicity of 3 or worse severity was not observed. Toxicity of grade 1 or 2 was fatigue or asthenia (8 of 10 pts), anaemia (2 of 10 pts). At the first use of olaparib as a maintenance treatment, grade 3 anemia was observed in 2 from 21 patients, nausea grade 2 in 11 from 21 patients, fatigue and asthenia grade 2 in 17 from 21 patients.
Conclusion: Olaparib maintenance retreatment provided a significant progression-free survival improvement with no detrimental effect on the quality of life in patients with platinum-sensitive, relapsed ovarian cancer and a BRCA1/2 mutation. With repeated use of olaparib, toxicity was less pronounced.
Disclosure: Nothing to disclose

EP884

Synchronous primary carcinoma in left tubal carcinosarcoma and right tubal serous carcinoma: a case report

N.R. Kim[1], S.H. Chae[1], S.-H. Shim[1], K.A. So[1], S.J. Lee[1], S.-B. Kang[2]
[1]Konkuk University Medical Center, [2]Hosan Women's Hospital, Seoul, Republic of Korea

Introduction / Background: Synchronous primary carcinoma of the female genital tract is relative rare cancerous condition. Although the etiology of this cancerous condition is unclear, some postulated that the extended mullerian system comprising the ovarian epithelium, fallopian tube, uterine corpus, and cervix, may respond as a single morphologic unit to produce primary carcinomas in multiple sites. Endometrium and ovary are common sites for coexistence of primary cancers. However, there is no data about adnexa cancers in different pathology. We will introduce synchronous primary carcinoma in different pathology in both adnexa.
Methodology: Case report
Results: Pelvic-abdomen CT announced large multilocular solid mass in left adnexa which should be rule out for malignant epithelial ovarian cancer with peritoneal seeding. Pelvic MRI showed 7cm sized large solid mass in left adnexa which is also diagnosed as malignant epithelia ovarian cancer. On October, 17th, 2017 Cytoreductive operation (Peritoneal washing, hysterectomy, bilateral salpingo-Oophorectomy, pelvic lymph node dissection, para-aortic lymph node dissection, peritoneal mass excision, total omentectomy, appendectomy, diaphragm biopsy) in midline incision from xyphoid process to pubic bone was done. The uterus was atrophied, the right adnexa had small cancerous mass, and the left adnexa had 5cm papillos cancerous mass. The left adnexa done frozen biopsy and serous cancer was diagnosed. The pathology was reported that the patients might be double primary cancer. One is 13x12x2cm sized left carcinosarcoma which is origin from left fimbria or salpinx and the other is 0.3x0.3x0.3cm right serous carcinoma origin from right fimbria.She stated the first chemotherapy on November, 7th, 2017 with paclitaxel (BSAx175) and carboplatin (AUCx5) 3weeks regimen.
Conclusion: Synchronous primary carcinoma is very rare and not much studies are existed. This paper will give some information.
Disclosure: Nothing to disclose

EP885

Oncologic safety of closed suction technique in removal of huge ovarian cyst

N.K. Kim, D.H. Suh, K. Kim, J.H. No, Y.B. Kim

Department of Obstetrics and Gynecology, Seoul National University Bundang Hospital, Seongnam, Republic of Korea

Introduction / Background: To evaluate the effectiveness of closed suction technique (CST) for surgical treatment of huge ovarian cyst in terms of oncologic safety.
Methodology: A total of 211 women who underwent surgery including at least unilateral salpingo-oophorectomy for huge ovarian cyst≥10cm were retrospectively reviewed (Fig 1). Information about tumor characteristics, surgical approach, gross tumor spillage during surgery (no rupture, gross rupture, and CST), pathology, adjuvant chemotherapy and tumor recurrence was collected.
Results: Tumor size was 19.0±6.9cm (mean±SD). Of 211, 129 (61.1%), 34 (16.1%), and 48 (22.8%) were benign, borderline, and cancer, respectively. In table 1 and 2, laparoscopy was used in 132 (62.6%) and open surgery was used in 79 (37.4%). In laparoscopic surgery, vast majority of cysts (73/79, 92.4%) were ruptured and 2.7% (2/73) of the ruptured cysts was cancer. The two received adjuvant chemotherapy, but one had recurrence. In open surgery, 39.4% (52/132) were ruptured and 42.3% (22/52) of them was malignant. CST was used in 34.1% (45/132). Among 46 who were diagnosed with malignancy by open surgery, 15 (34.1%) belonged to stage IC1 because of tumor rupture during surgery. Median follow-up of the 46 patients was 43 months (0-124 months). There were 4 (8.7%) cancer recurrences: 3 in gross rupture group despite adjuvant chemotherapy and 1 in CST group without adjuvant chemotherapy. Recurrence rate was similar between gross rupture and CST groups (13.6%, 3/22 vs. 11.1%, 1/9; p>0.999)(Fig 1). In 12 women who did not receive chemotherapy after open surgery irrespective of stage, recurrent rate was not different between 3 groups. In no rupture group, there was no chemotherapy and no recurrence.
Conclusion: Huge ovarian cyst≥10cm has high likelihood of tumor rupture during surgery even in open surgery. Gross tumor rupture increases recurrence despite adjuvant chemotherapy. CST does not seem to be effective in preventing tumor recurrence.

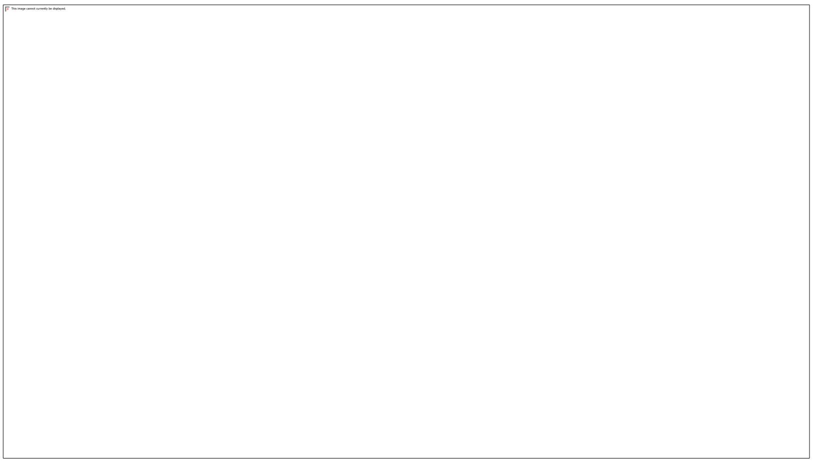

[Fig 1. Rates of malignancy and tumor recurrence according to surgical approach]

Variables		N (%)	Rupture (-)	Closed suction technique	Rupture (+)
Open surgery	Benign	60 (45.5)	11 (18.3)	24 (40.0)	25 (41.7)
	Borderline	26 (19.7)	9 (34.6)	12 (46.2)	5 (19.2)
	Cancer	46 (34.8)	15 (32.6)	9 (19.6)	22 (47.8)
	Total_open surgery	132 (100)	35 (26.5)	45 (34.1)	52 (39.4)
Laparoscopic surgery	Benign	69 (87.3)	6 (8.7)	0	63 (91.3)
	Borderline	8 (10.1)	0	0	8 (100)
	Cancer	2 (2.5)	0	0	2 (100)

	Total_laparoscopic surgery	79 (100)	6 (7.6)	0	73 (92.4)
Total		211 (100)	41 (19.4)	45 (21.3)	125 (59.2)

[Table 1. Tumor rupture and malignancy according to surgical approach]

Variables		Recur (-) (n=43)	Recur (+) (n=5)	P value
Age at surgery (years)		42.98 ± 19.45	44.80 ± 15.72	0.853
Size on preoperative computed tomography (cm)		19.08 ± 7.23	15.60 ± 4.83	0.388
Surgical approach	Open	42 (97.7)	4 (80)	0.199
	Laparoscopy	1 (2.3)	1 (20)	
Presence of gross rupture in open surgery	Rupture (+)	19/42 (45.2)	3/4 (75)	0.338
	Closed suction technique	8/42 (19.0)	1/4 (25)	
	Rupture (-)	15/42 (35.7)	0/4 (0)	
Adjuvant chemotherapy	Yes	30 (73.2)	4 (80)	> 0.999
	No	11 (26.8)	1 (20)	

[Table 2. Characteristics of cancer patients according to tumor recurrence]

Disclosure: Nothing to disclose

EP886

Atypical endometriosis associated with ovarian malignancy

N. Kim[1,2], S.R. Hong[2,3], K.T. Lim[2,4], K.H. Lee[2,5], T.J. Kim[1,2], K.A. So[1,2]
[1]Obstetrics and Gynecology, Konkuk University School of Medicine, [2]Cheil General Hospital & Women's Healthcare Center, Seoul, [3]Pathology, Konyang University College of Medicine, Daejeon, [4]Obstetrics and Gynecology, Hallym University Kangdong Sacred Heart Hospital, [5]Obstetrics and Gynecology, CHA Gangnam Medical Center, CHA University, Seoul, Republic of Korea

Introduction / Background: Atypical endometriosis was first described by Czernobilsky and Morris in 1979. Atypical endometriosis shows dysplastic feature with the cellular atypia that is different from typical endometriosis. It is closely associated with endometriosis-associated ovarian malignancy. This study is to evaluate the clinical outcome of atypical endometriosis and its association with malignant malignancy.
Methodology: A retrospective study included patients who diagnosed with atypical endometriosis between January 2001 and December 2017 at Cheil General Hospital & Women´s Healthcare Center. All patients were surgically treated and histopathology was confirmed to atypical endometriosis. The clinical characteristics, histopathologic results were reviewed.
Results: Among 13,074 patients underwent endomteriosis surgery for the study period, 101 patients (0.8%) were diagnosed with atypical endometriosis. Ninety-eight patients of atypical endometriosis were analyzed and mean age was 34.8 years (range 16~58 years). Ten patients (10.2%) had previously undergone the endomteriosis surgery more than once. Atypical endometriosis associated with malignant ovarian tumor was found in 12 cases (12.2%); 9 cases of carcinomas and 3 cases of borderline tumor. There were classified as follows; five clear cell carcinomas, two endometrioid

adenocarcinomas, one mixed clear cell and endometrioid adenocarcinoma, one seromucinous carcinoma, two mucinous borderline tumors, and one seromucinous borderline tumor. Among atypical endometriosis, the size of ovarian mass was significant difference between atypical endometirosis and ovarian malignancy ($p = 0.025$). However the mean age ($p = 0.452$), serum CA-125 ($p = 0.222$), or CA-19-9 ($p = 0.464$) was not different between two groups.
Conclusion: The overall frequency of malignant transformation of enodmetriosis is 0.3-0.8%. In this study atypical endometriosis associated with malignant ovarian tumor was 12.2%. Atypical endometriosis is considerable precancerous and strongly associated with endometriosis-associated ovarian cancer. Increased awareness of the characteristics of atypical endometriosis will improve early detection of patients with endometriosis who are at risk of endometriotic-associated carcinoma.
Disclosure: Nothing to disclose

EP887

A synergistic anticancer effect of combination treatment with a cell cycle checkpoint kinase 1 inhibitor and PARP inhibitor in BRCA wild type epithelial ovarian cancer

Y.B. Kim[1,2], H.-Y. Cho[3], W.H. Park[1], D.H. Suh[1], J.H. No[1]
[1]Department of Obstetrics and Gynecology, Seoul National University Bundang Hospital, Seongnam, [2]Department of Obstetrics and Gynecology, College of Medicine, Seoul National University, Seoul, [3]Department of Obstetrics and Gynecology, Hallym University Dongtan Sacred Heart Hospital, Dongtan, Republic of Korea

Introduction / Background: Theoretically, PARP inhibitors can function as a DNA damaging agents in BRCA wild type cancer, even if the clinical activity is limited. Most of epithelial ovarian cancer is characterized by TP53 mutation causing dysfunction in the G1/S checkpoint, which makes tumor cells highly dependent on Chk1-mediated G/M phase cell cycle arrest for DNA repair. Therefore, we hypothesized that combination treatment of PARP inhibitors (Rucaparib) and Chk1 inhibitor (LY2606368) would be active in BRCA wild type epithelial ovarian cancer.
Methodology: We investigated the anticancer effects of combination treatment with prexasertib (LY2606368) and rucaparib, in BRCA wild type ovarian cancer cell lines (OVCAR3 and SKOV3) using clinically attainable concentrations.
Results: We found that combination treatment synergistically decrease cell viability in all cell lines and induces greater DNA damage and apoptosis than the control and/or monotherapies. Moreover, we identified that prexasertib can significantly inhibit homologous recombination (HR) mediated DNA repair and thus showed marked synergistic anticancer effect in combination treatment with PARP inhibitor. The synergy between prexasertib and rucaparib was considered to be caused by impaired G2/M checkpoint due to prexasertib treatment, which forced mitotic catastrophe in the presence of rucaparib.
Conclusion: According to our data, prexasertib and rucaparib combination showed synergistic anticancer effects against BRCA wild type epithelial ovarian cancer cells through reduced Rad51 foci formation and greater induction of apoptosis. This might be a novel effective therapeutic strategy for BRCA-wild type epithelial ovarian cancer.
Disclosure: Nothing to disclose

EP888

The impact of levels of biomarkers on overall survival in FIGO stage III-IV ovarian cancer

A. Karlsson[1], E. Lundin[2], P. Lukas[1], G. Lindahl[3], P. Kjølhede[2]
[1]Department of Obstetrics and Gynecology, Linköping University Hospital, [2]Department of Obstetrics and Gynecology, Department of Clinical and Experimental Medicine, Faculty of Medicine and Health

Science, Linköping University, [3]Department of Oncology, Linköping University Hospital, Linköping, Sweden

Introduction / Background: In this study we aim to analyse the impact of five biomarkers on overall survival (OS) in FIGO stage III-IV ovarian-, fallopian tube-, peritoneal- and abdominal cancer, together designated OC, in a cohort of women.
Methodology: All 428 women with FIGO stage III-IV OC who were diagnosed with serous adenocarcinoma (high grade or grade not stated), endometrioid adenocarcinoma or clear-cell carcinoma in the South East Health Region in Sweden in the period January 2008 to November 2017 were included. Only the women who had undergone primary debulking surgery (PDS) or neoadjuvant chemotherapy (NACT) were included. Pre-treatment laboratory, demographic and clinical data were retrospectively retrieved from the patients' charts. The median value for each of the biomarkers (haemoglobin, thrombocytes, albumin, C-reactive protein (CRP) and CA-125) was calculated and the women were dichotomized as having above or below the median. OS was estimated using the Kaplan-Meier method. Comparison of groups was carried out using the Cox Proportional Hazards Regression Analysis. Adjustment was done for age, calendar year of treatment, body mass index and primary treatment modality (PDS/NACT followed by delayed PDS/NACT only).
Results: The women had significantly shorter OS if the albumin level was under the median, or the thrombocytes, CRP or CA-125 levels were over the median. Women with the levels of all three biomarkers thrombocytes, albumin and CRP in adverse direction had significantly shorter OS than the women who had the level of at least one of these biomarkers in the favourable direction.
Conclusion: The levels of thrombocytes, albumin, CRP and CA-125 as well as the combination of thrombocytes, albumin and CRP were independent predictors of OS in FIGO stage III-IV OC even when adjusted for treatment modality. This might imply that biologic factors are the most important determinant for OS in OC.

	Hemoglobin (g/L) n=412	Thrombocytes (x109/L) n=408	Albumin (g/L) n=377	CRP (mg/L) n=333	CA-125 (kIE/L) n=418
Median (range)	126 (78-162)	385 (117-1876)	34 (11-47)	27 (3-330)	607 (11-42365)

[Median concentration of biomarkers in blood.]

Biomarker	Median survival when biomarker level is in adverse direction (months)	Median survival when biomarker level is in favorable direction (months)	5-year-survival when biomarker level is in adverse direction (%)	5-year-survival when biomarker level is in favorable direction (%)
Hemoglobin	30.6	38.3	21.4	31.8
Thrombocytes	30.2	41.2	20.0	34.1
Albumin	28.6	44.2	11.7	38.6
CRP	28.3	43.7	18.2	38.4
CA-125	30.5	37.2	19.8	32.6
Factor	Median survival when all three biomarkers are in adverse direction (months)	Median survival when at least one biomarker is in favorable direction (months)	5-year-survival when all three biomarkers are in adverse direction (%)	5-year-survival when at least one biomarker is in favorable direction (%)
Three biomarkers (thrombocytes, albumin and CRP)	27.2	41.2	13.9	34.2

[Median survival and 5-year-survival in relation to levels of the biomarkers or in relation to the three biomarkers-variable.]

BMJ Publishing Group Limited (BMJ) disclaims all liability and responsibility arising from any reliance placed on this supplemental material which has been supplied by the author(s)

This image cannot currently be displayed.

[Kaplan Meier cumulative proportion surviving in relation to biomarker level.]

This image cannot currently be displayed.

[Kaplan Meier cumulative proportion surviving in relation to biomarker level.]

Disclosure: Nothing to disclose

EP890

Primary ovarian small cell carcinoma of pulmonary type with enlarged paraaortic lymph node masses: a case report and review of the literature

E.G. Klonos[1], D. Tsolakidis[2], A. Papanikolaou[2]
[1]1st. Department of Ob/Gyn, Aristotele University of Thessaloniki, [2]Aristotle University of Thessaloniki, Thessaloniki, Greece

Introduction / Background: Small cell carcinoma of the ovary of pulmonary type, is a rare, aggressive tumor with poor prognosis and its optimal management is unclear.
Methodology: *Case presentation:* A 55 - year - old Caucasian woman presented with abdominal discomfort and left lumbar pain within a three - week period. At exploratory laparotomy, a 8 cm solid cystic mass of the left ovary was found infiltrating the sigmoid colon, and a bulky mass (11x 7 x 4 cm) in the left paraaortic infrarenal region. Histopathological features resembling small cell carcinoma of the lungs and positive immunohistochemical stains provided a define diagnosis of IIIC ovarian small cell carcinoma of pulmonary type. After six cycles chemotherapy with carboplatin and etoposide, the patient is still alive at 75 months (6 years and 3 months) from initial diagnosis.
Results: In this case, the absence of peritoneal involvement and the extensive paraaortic adenopathy is suggestive of a different pattern of spread of this rare tumor.
Conclusion: Optimal treatment seems to be radical primary debulking surgery resulting in no residual disease, maximizing the effect of adjuvant chemotherapy for this biological aggressive tumor.
Disclosure: Nothing to disclose

EP891

Receptor activator of nuclear factor kappa-B ligand expression predicts ovarian cancer outcome

J. Kögl[1], V. Wieser[1], S. Sprung[2], H. Fiegl[1], C. Marth[1], A.G. Zeimet[1]
[1]Department of Obstetrics and Gynaecology, [2]Institute of Pathology, Medical University of Innsbruck, Innsbruck, Austria

Introduction / Background: The receptor activator of nuclear factor-κB (RANK) pathway, activated by RANK ligand (RANKL), critically controls bone metabolism and modulates tumour immune response. Denosumab, a neutralizing RANKL antibody, exerts tumour-suppressive effects in mice and humans. Herein, the relevance of RANK signalling was investigated for the first time in human ovarian cancer (OC).
Methodology: *RANK*, *RANKL* and *OPG* expression of 192 epithelial OCs and 12 non-malignant fallopian tubes was related to clinico-pathological characteristics. Regulation of RANK constituents was studied in four OC cell lines and the impact of RANK ligation or denosumab treatment on cell viability was determined.
Results: In OC, RANK constituents (RANK, RANKL and OPG) were expressed in epithelial and stromal cells. *RANKL* (but not *RANK*) expression was elevated in OC tissue, particularly in *BRCA1/2* mutated tumours and correlated with *BRCA2* expression. *RANKL* expression independently predicted progression free and overall survival. Expression of RANK constituents in OC cells was induced by inflammatory cytokines such as IL-1β and TNFα. However, neither RANK ligation nor denosumab treatment influenced OC cell viability.
Conclusion: This study links RANKL expression independently to patient's outcome in OC and implicates RANK signalling in the pathogenesis and the inflammatory response of OC. Denosumab

may prove efficacious in OC.
Disclosure: Nothing to disclose

EP892

How can collaboration with surgical oncologist change paradigm in the treatment of advanced ovarian cancer: a retrospective cohort study

V. Kopetskyi[1], O. Turckak[2], N. Tsip[2], O. Samokhvalova[2], O. Kolesnik[3]
[1]Hepatopancreatobiliary | Gynecologic Oncology, [2]Gynecologic Oncology, [3]National Cancer Institute, Kyiv, Ukraine

Introduction / Background: The pillar of treatment of advanced ovarian cancer is primary complete or at least optimal cytoreduction with further chemotherapy. Because of complexity of surgical debulking in upper abdomen there is a tendency to perform non-optimal debulking surgery or neoadjuvant chemotherapy that can compromise long-term results. We hypothesized that collaboration with surgical oncologist team treating patients with upper GI tumors can improve both number of primary cytoreductions and completeness of cytoreduction.
Methodology: We have analyzed Prospective database of National cancer institute. In analysis were included patients older than 18 years old with stage III-IV of epithelian ovarian cancer treated in National cancer institute form January 2012 till November 2018. In September 2017 in National cancer institute peritoneal malignancies, treatment program was established. Our primary point was to compare amount of primary cytoreductive surgeries and completeness of cytoreduction during two periods: from January 2012 till august 2017 and from September 2017 till November 2018.
Results: From 551 patient, after primary analysis we excluded 22 where only diagnostic procedures were performed. Among remaining 529 patients primary cytoreduction was performed in 256 cases (48.4%) and interval cytoreduction in 295 (51.6%). In primary cytoreduction group: from January 2012 to august 2017 complete or optimal cytoreduction was performed in 23.6% of cases, from September 2017 to November 2018 in 61.4% ($p< 0.05$). In absolute numbers - 50 cases for the 5 year period vs 27 cases for the 15 month. In group where interval cytoreduction was performed: complete or optimal intervention were in 30% cases in period from 2012 to august 2017 and in 48,2% in period from September 2017 to November 2018. There were no postoperative mortality in both groups.
Conclusion: Collaboration with surgical oncologist can improve both the quality of surgical debulking and amount of primary cytoreductions
Disclosure: Nothing to disclose

EP893

Malignant bowel obstruction in gynaecological oncology: are there alternatives to surgery or palliation following failed conservative management?

P. Korompelis[1], D. Bennett[2], S. Rundle[1], M. Adishesh[1], A. Kucukmetin[1], R. Naik[1], R. O'Donnell[1,3]
[1]QE Hospital, NGOC, Gateshead, [2]Medical School, [3]Northern Institute for Cancer Research, Newcastle University, Newcastle, United Kingdom

Introduction / Background: Bowel obstruction (BO) is an infrequent but challenging complication of gynaecological cancer. Management may be surgical, medical or conservative, but there is a lack evidence-based guidance to inform best practice. This study aimed to review BO management in patients with gynaecological malignancy.
Methodology: In this retrospective study all patients admitted to the NGOC with radiological evidence of BO (2012-2017) were identified. Patient demographics, clinicopathological data and management details were collated. Outcomes were stratified by management

(conservative/Gastrograffin®/stent/surgery).
Results: Of the 82 patients included 62(79%) had ovarian cancer. 67(82%) presented with small BO and 15(18%) with large BO. 13(16%) underwent first-line emergency surgery. All remaining patients were initially managed conservatively with a 32% resolution rate. Following failed conservative management, an algorithm utilising level of obstruction and presence of transition point(s) was used to determine subsequent management.
In the 43 patients for whom conservative management failed, 11 underwent a trial of Gastrograffin®, 5 underwent endoscopic stenting and 26 underwent emergency surgery. The overall rate of resolution using Gastrograffin® was 64%, for stenting was 80% and for surgical diversion was 100%. 5 patients who were not suitable for surgical intervention were palliated.
There were no adverse events from stents or Gastrograffin®. Overall 66(80%) patients were discharged from hospital and 27(40%) received further chemotherapy. In those successfully treated, the median additional survival was 12.4 months (4.2-90.5). Additional survival was greatest in the subgroup with a single distal transition point treated surgically and lowest in the subgroups with multiple or no transition points.
Conclusion: Patients with BO are highly heterogenous and should be stratified into treatment pathways based upon radiological assessment of transition point(s). Non-surgical interventions have high rates of success when used in a highly selected subgroup of patients. Survival following management of BO is highly variable which is likely to be a reflection of disease distribution.
Disclosure: Nothing to disclose

EP894

Evaluation of the clinical usefulness of HE4 and CA125 assays in monitoring treatment response of patients with advanced ovarian cancer

B. Kotowicz[1], M. Fuksiewicz[1], A. Berezowska[1], K. Jalinik[2], M. Bidzinski[2], M. Kowalska[1]
[1]Laboratory of Tumor Markers, [2]Department of Gynecologic Oncology, Maria Sklodowska-Curie Memorial Cancer Center and Institute of Oncology, Warsaw, Poland

Introduction / Background: Evaluation of the utility of HE4 and CA 125 assays in monitoring treatment response before and during treatment, based on the treatment regimen used.
Methodology: CA125 and HE4 levels were determined in 65 ovarian cancer patients FIGO III-IV. 33 patients received surgical treatment before systemic therapy (CHTH) (Group1) and 32 patients received neoadjuvant chemoterapy (NACTH) before surgery (Group2). Determination of markers was performed in serum samples by the Roche: Group1 before treatment, after surgery, after 3 and 6 CHTH; Group2 before treatment, after NACTH, surgery and 6 CHTH. For statistical calculations, the Mann-Whitney and Wilcoxon tests were used.
Results: HE4 concentrations were significantly higher in patients that were assigned to NACTH both before and after treatment, as compared to HE4 concentrations in group I (P=0.003, P=0.0004). In Group1, after surgery HE4 and CA125 median levels decreased respectively by 67% and 66%, depending on the level of treatment. In Group2, after NACTH, the reduction in marker levels was larger (HE4 84%; CA125 94%). In Group1, a similar decrease in HE4 median by 72% and CA125 by 96% was only observed after 6 CHTH cycles. In Group1, concentrations of HE4 (P=0.001) and CA125 (P=0.003) were significantly lower after surgery vs before treatment and CA125 levels were further reduced, sixth vs third CHTH course. Similar differences were observed in Group2: HE4 (P=0.002) and CA125 (P=0.0001) were significantly lower after NACTH vs. pre-treatment and more reduced after six courses of CHTH: HE4 (P=0.009) and CA125 (P=0.02).
Conclusion: At the time of the diagnosis, the median levels of markers HE4 and CA125 are significantly different between patients in the group qualified for NACTH and in patients with advanced ovarian cancer. Observed significant changes in pre-treatment serum concentrations, in particular in the case of HE4 in the schema used, confirm their usefulness in treatment monitoring.
Disclosure: Nothing to disclose

EP895

Primary squamous cell carcinoma of the ovary: review of the literature

N. Koufopoulos[1,2], S. Kokkali[3], F. Antoniadou[2], D. Nasi[3], C. Goudeli[4], E. Terzakis[4], E. Pigadioti[2], L. Khaldi[2]
[1]Attikon University Hospital, 2nd Pathology Department, National and Kapodistrian University of Athens, [2]Pathology Department, [3]1st Medical Oncology Department, 'Saint Savvas' Anticancer Hospital of Athens, [4]Institouto Gynaikologias, Athens, Greece

Introduction / Background: Primary squamous cell carcinoma of the ovary (PSCCO) is a rare entity. Most squamous cell carcinoma cases arise from a cystic teratoma or less frequently are associated with Brenner tumor or endometriosis. We review 36 cases of PSCCO reported in the literature.
Methodology: Data were collected in November 2018. Twenty-three articles from 1964 to 2018 presenting thirty-six cases of PSCCO are reported in the literature. All reviewed cases were analyzed according to diagnosis, surgical approach, adjuvant therapy and outcome.
Results: Patients' age ranged from 27 to 90 years (mean age: 52.9 years). Tumor size ranged from 1.5 to 26 cm in the greatest axis (mean value: 10.3 cm).
The most frequent symptom was abdominal pain. Other symptoms were related to tumor growth, metastasis, local extension or were non specific. Only few patients were asymptomatic.
In 10 cases high grade Cervical Intraepithelial Neoplasia was associated.
Grossly tumors were either cystic or solid. Microscopically several different morphological patterns were present.
All patients were treated surgically, in most cases with hysterectomy and bilateral salpingo-oophorectomy. Adjuvant therapy was administered in twenty-four patients.
Information regarding outcome was available in 31 cases. Follow-up time ranged from 9 days to 90 months. Eleven patients were alive with no evidence of disease, three were alive with metastases and seventeen succumbed to disease.
Conclusion: PSCCO is a rare entity. Treatment includes combination of surgical management with adjuvant therapy when necessary. Its prognosis seems to be poorer compared to other ovarian carcinomas. Further investigations are needed to define optimal treatment and prognosis.
Disclosure: Nothing to disclose

EP896

Ovarian metastasis from colorectal carcinoma: a case report and review of the literature

A. Lagkadas[1], D. Mantas[2], S. Pittokopitou[1], A. Papadimitriou[1], K. Daglas[1], A. Chionis[1], K. Giannakopoulos[1]
[1]Gynaecology, Laiko General Hospital, [2]2nd Department of Propaedeutic Surgery, National and Kapodistrian University of Athens, Medical School, Athens, Greece

Introduction / Background: The most common nongenital malignancies that metastasize to the ovary include gastrointestinal cancers and breast cancer. We report a case of an ovarian tumor metastatic from a primary colon carcinoma and we review the data from the literature.
Methodology: A woman of 79 years old was referred to our department due to an ovarian tumor found at an abdominal ultrasound. From the medical history of the patient there were complaints of bleeding from the intestine. The complete evaluation, including ultrasonography, computed tomography scans, magnetic resonance imaging and colonoscopy with biopsies revealed an adenocarcinoma of the sigmoid colon and reconfirmed the ovarian tumor of diameter 5 cm app.
A laparotomy followed, during which total abdominal hysterectomy with bilateral salpingo-oophorectomy, sigmoidectomy and omentectomy were performed. The ovarian tumor was metastatic from the colonic cancer. The final diagnosis was mucinous adenocarcinoma of the large intestine T3N1aM1c.
Results: The preoperative evaluation of any ovarian tumor suspect for malignancy should include assessment for metastatic disease from another primary cancer. Ovarian metastases (synchronous or

metachronous) are found in 1% up to 14% of patients with colorectal cancer. From all the cases of metastatic neoplasms to the ovary, colon is the most frequent primary site (15-32%). They are more common in premenopausal than postmenopausal women. Other nongenital sites of primary tumors are breast (8-28%), gastric (6-22%) and appendix (2-20%).
The prognosis of the patients with colon cancer and ovarian metastases (especially if bilateral) is poor, but it seems that optimal surgical cytoreduction, compared to suboptimal, is associated to a better overall survival rate.
Conclusion: The evaluation of an ovarian neoplasm should exclude metastasis from another primary carcinoma. Colon is the most frequent primary site of ovarian metastases. Surgical resection seems to have an important role in the management of these patients.
Disclosure: Nothing to disclose

EP897

Clinical application of the sentinel lymph node technique in early ovarian cancer: phase II clinical trial

V. Lago, P. Bello, B. Montero, L. Matute, P. Padilla-Iserte, S. Lopez, M. Agudelo, S. Domingo
University Hospital La Fe, Valencia, Spain

Introduction / Background: There is limited evidence favoring the use of the sentinel lymph node technique in ovarian cancer, and no standardized approach has been studied. The objective of the present pilot study and clinical trial is to determine the feasibility of the sentinel lymph node technique by applying a clinical algorithm and the use of 99mTc and indocyanine green for its detection
Methodology: Patients with confirmed ovarian cancer were included. 99mTc and indocyanine green were injected into the ovarian and infundubulo-pelvic ligament stump.
A gamma probe and near-infrared fluorescence imaging were used for sentinel lymph node detection.
Results: The sentinel lymph node technique was performed in 10 patients in the pilot study and in 20 patients in the clinical trial with a detection rate in the pelvic and/or para-aortic region of 100%.
Conclusion: The sentinel lymph node technique appears to be applicable in early-stage ovarian cancer with excellent detection rate. further studies are needed to establish the negative predictive value of the technique.
Disclosure: Nothing to disclose

EP898

The prognostic role of age, timing of surgery and surgical radicability in patients with advanced stage epithelial ovarian cancer; a single institution experience

A. Laios, A. Kaufmann, M. Otify, T. Broadhead, R. Hutson, D. Nugent, A. Thangavelu, G. Theophilou, D. De Jong
Gynaecologic Oncology, St James's University Hospital, Leeds Teaching Hospitals, Leeds, United Kingdom

Introduction / Background: Our goal in ovarian cancer surgery is to deliver optimal treatment aiming at maximal survival benefits. Quantifying radicality and timing of surgery may help to individualise surgical treatment. We assessed the impact of these parameters on progression free survival (PFS) and overall survival (OS).
Methodology: Patients with a diagnosis of advanced stage (FIGO IIIC/IV) high grade serous ovarian cancer (HGSOC) who underwent surgical cytoreduction from January 2015 to December 2017 was selected from the ovarian database. We evaluated age, timing of surgery (upfront vs delayed cytoreduction), surgical complexity score (SCS), and residual disease (RD). SCS was assigned based

on the Aletti classification as low, intermediate and high. Kaplan Meier curves were constructed for PFS and OS.
Results: Eighty patients were identified. Mean age and BMI were 63.5 ± 11 yrs. and 26.6 ± 4.8 respectively. The mean SCS was 3 ± 1 (1-8). Complete and optimal cytoreduction was achieved in 48/80 (58.7%) and 66/80 (82.6%) patients. Median PFS and OS were 17 and 44 months respectively. The presence of RD had a negative impact on PFS: 20, 16, and 12 months for nil macroscopic, < 1 cm, and > 1 cm, respectively. Although not significant, upfront surgical cytoreduction vs delayed surgical cytoreduction resulted in a better PFS (NS). Patients with a higher surgical complexity score (> 3) showed a favourable trend in PFS compared to those with a low surgical complexity score (< 3): 25 vs. 16 months, p=0.19 albeit not affecting OS (p=0.57). Younger patients (< 75 yrs) are likely to have a better PFS and OS than those > 75 yrs (p=0.21 and p=0.07, respectively).
Conclusion: We observed trends towards favourable prognosis in patients with upfront surgical cytoreduction, more radical surgery and younger age. No significant differences were observed for OS. This cohort is currently expanding to reach significance.
Disclosure: Nothing to disclose

EP899

Diagnostic value of postoperative platelet-to-white blood cell ratio (PC/WCC) after splenectomy in advanced ovarian cancer patients

K. Lathouras[1], G. Panagakis[2], S. Bowden[1], K. Saliaris[3], S. Saso[1], D. Haidopoulos[2], C. Fotopoulou[1]
[1]West London Gynaecological Cancer Centre, Hammersmith Hospital, Imperial College London, London, United Kingdom, [2]Medical School of Athens, Gynaecological Oncology Department of Alexandra University Hospital, Athens Medical School, [3]Medical School of Athens, Athens, Greece

Introduction / Background: Splenectomy is an established procedure in primary and relapsed cytoreduction in order to achieve total macroscopic tumor clearness in ovarian cancer (OC).Reactive, splenectomy-induced thrombocytosis and leucocytosis may obscure the early diagnosis of post-operative infection or sepsis.In trauma patients after splenectomy,a platelet- to white cell count ratio (PC/WCC) of < 20 has been demonstrated to reliably differentiate post-operative sepsis from transient physiological responses to splenectomy.However,this has never been assessed in OC surgery.
Methodology: All consecutive OC-patients who underwent splenectomy as part of their cytoreduction between 01/2013 and 10/2018 in two large European gynaecological cancer centres were retrospectively evaluated.Main outcome measures were WCC,PC and PC/WCC-ratio on post-operative days 1,5&7 and correlated with surgical outcome and morbidity as per the Clavien-Dindo Classification.A binomial logistic regression was applied to assess the predictive value of day five PC/WCC-ratio,WCC and PC for predicting grade III post-operative sepsis.
Results: Ninety-five OC-patients(mean age: 54 years, range:18-75)were identified after primary(73%),interval(5%)and secondary(18%)cytoreductive surgery.Other multivisceral procedures were: bowel resection(74%),diaphragmatic/liver capsule stripping(74%),and distal pancreatectomy(5%).18%(n=17)developed a grade-III post-operative sepsis(infected group)secondary to anastomotic leak(n=2),pneumonia(n=1),left sub-diaphragmatic collection or intra-abdominal collection(n=3)and septicaemia(n=11).
A WCC of more than 15 on day five was significantly predictive of postoperative sepsis(OR=1.32;95%CI 1.12-1.55;p< 0.001). However,PC/WCC-ratio(OR=0.97;95%CI 0.93-1.021; p=0.085)and PC(OR=1.00; 95%CI 0.99-1.00; p=0.409)failed to show any statistical significant predictive value in differentiating between sepsis and reactive,splenectomy induced changes.

Assessing the ability of PC/WCC-ratio on day five to predict post-operative sepsis in ROC curve analysis resulted in an area under the curve of 0.637(95%CI 0.480-0.796).A PC/WCC-ratio cut-off of 26 achieved a sensitivity of 72% and specificity of 53% in predicting grade III post-operative sepsis.
Conclusion: The PC/WCC-ratio after cytoreductive surgery for ovarian cancer with splenectomy does not appear to have the same predictive value in differentiating between sepsis and reactive splenectomy induced changes as observed in trauma splenectomy patients.
Disclosure: Nothing to disclose

EP900

The impact of age on the management of patients with ovarian cancer

M.E. Laudani[1], E. Potenza[1], L. Fuso[2], F. Moro[3], C. Macchi[1], M. Ribotta[4], P. Zola[1]
[1]Surgical Science, University of Turin, [2]Gynecology and Obstetrics, AO Ordine Mauriziano, [3]General Surgery, [4]Pathological Anatomy, Città della Salute e della Scienza, Turin, Italy

Introduction / Background: Incidence of ovarian cancer increases with age and reaches a peak at 70 years.
The aim of this study is to analyse the surgical and pharmacological therapies for patients affected by ovarian cancer identifying the best approach based not on the age but on the performance status.
Methodology: We have conducted a single-center retrospective study. Patients with epithelial and non-epithelial ovarian tumours were included and ranked according to age at diagnosis in group A (≥70 years) and group B(< 70 years). For each patient are considered: co-morbidities, performance status, FIGO stage, grading, histotype and treatment. Surgical treatment was differentiated into primary debulking surgery or interval debulking surgery, reporting extension, residual tumour, complications and days of hospitalization. Chemotherapy was administered for 3 or more cycles and the clinical response was evaluated at the end.
Results: 134 patients were included in the study, 44 (32.8%) in group A and 90 (67.2%) in group B further divided into early and advanced stages. Optimal cytoreduction was achieved in 61.9% of the younger patients and in 36.1% of the older patients (p=0.01), although the surgical extension is not statistically significant different in the two groups. There is good correlation between overall survival and the performance status (p=0.0001), the age at diagnosis (p=0.004), the tumour stage (p=0.03) and the residual tumour (p=0.00001). The residual tumour (OR=2.399; p=0.006) and the performance status (OR=2.50; p=0.0001) resulted as an independent survival prognostic factors according to the multivariate Cox analysis.
Conclusion: Our data suggest that patients aged ≥70 can tolerate radical surgical treatments in the same way as younger patients without a significant increase in morbidity, so an optimal cytoreduction should be considered the gold standard regardless of age.
Therefore, our data underlines the importance of managing these patients within Gynecologic Oncology units equipped with a multidisciplinary team.
Disclosure: Prof. Paolo Zola has received grants from AstraZeneca, Roche, Tesaro and PharmaMar.

EP902

Single port access laparoscopic staging surgery in patient with early stage ovarian cancer

S.-H. Lee[1], J.S. Park[1], Y.T. Kim[2], S.W. Kim[2]
[1]Obstetrics and Gynecology, Gynecologic Oncology, Wonju Severance Christian Hospital, Yonsei University, Wonju, [2]Obstetrics and Gynecology, Gynecologic Oncology, Institute of Women's Life Medical Science, Yonsei University, Seoul, Republic of Korea

Introduction / Background: With an increase in demand for less-invasive surgeries, the role of laparoscopic surgery in the management of gynecological cancers continues to expand. However, the scope of single port laparoscopy is still limited in ovarian cancer. We reported the experience of single port access (SPA) laparoscopic staging of early stage ovarian cancer.
Methodology: We prospectively collected data on women who had SPA laparoscopic staging surgery at Severance Hospital between 2014 and 2017. The surgical outcomes, including intra-operative data, time to interval between the end of SPA laparoscopy and postoperative adjuvant chemotherapy (POAC), and port related complications were measured.

Results: Fifty six patients underwent SPA laparoscopic staging surgery. Mean patient age and BMI were 47.25±13.93 and 23.58 kg/m2 ±3.24, respectively. Mean operation time was 235±93 minutes and median blood loss was 55 ml (range, 10-1100). Median time interval between the end of procedure and the start of POAC was 21 days (range, 14-31). Additional port was needed in 4 cases (7.1%) and the conversion rate to laparotomy was 8.9%. There were no peri-operative moderate to severe complications, including port-site metastasis. The upstaging rate after SPA laparoscopic staging surgery was 30.3% and POAC was performed in 34 cases (60.7%). The overall rate of recurrence with a median follow up period of 26 months was 16% (9 cases) and 4 patients died due to the disease.
Conclusion: The surgical outcomes of SPA laparoscopic staging surgery in patient with early stage ovarian cancer is feasible and could be compatible with conventional laparoscopic approach in terms of operative outcomes and upstaging rate in comparison with previous studies.
Disclosure: Nothing to disclose

EP903

Prognostic factors for BRCA mutation in ovarian cancer patients

N. Lee[1,2], J.H. No[3], Y.B. Kim[3]
[1]Obstetrics & Gynecology, [2]CHA Gangnam Medical Center, CHA University, Seoul, [3]Obstetrics & Gynecology, Seoul National University Bundang Hospital, Seongnam, Republic of Korea

Introduction / Background: Prognostic factors of BRCA1/2 mutation in epithelial ovarian cancer remain controversial. We retrospectively analyzed prognostic factor of patients with ovarian cancer who underwent BRCA testing at our institute.
Methodology: Eighty seven patients who underwent a staging operation for pathologically proven epithelial cancer between February 2006 and August 2017 were enrolled retrospectively. The peripheral germline samples were obtained for BRCA1/2 mutation analysis. Clinico-pathological characteristics were retrospectively reviewed.
Results: Frequency of BRCA1/2 mutation was 18.4% in this study. BRCA 1 and BRCA 2 mutations were founded 13.8% (12 cases) and 4.6% (4 cases) of the ovarian cancer patients, respectively. Mutation carriers were diagnosed at a similar age to non-carrier. There was no difference in FIGO stage, histology, grade, lympho-vascular space invasion, lymph-node metastasis, relapse of tumor between the two group. Only gross residual disease was more frequent in mutation group (50% (n=8) versus 22.5% (n=16); p=0.035).
Conclusion: BRCA1/2 mutation was not associated with any progression factors. There were more residual masses in mutation carriers. It may be more related to PFS or OS due to residual tumor than mutation itself.
Disclosure: The Authors had no conflict of interest in regard to this study.

EP904

Clinical outcomes of post-operative intraperitoneal chemotherapy for patients with early stage high grade serous ovarian cancer treated at British Columbia Cancer Agency

S.(. Lee, A. Albert, A. Kumar, J. Kwon, A. Tinker
University of British Columbia, Vancouver, BC, Canada

Introduction / Background: Intraperitoneal (IP) chemotherapy is recommended for optimally debulked stage III ovarian cancer, and its benefit for prolonging overall and progression free survival for advanced ovarian cancer was previously shown in randomized trials. In British Columbia, we extrapolated from this and have been using IP chemotherapy for stage I high grade serous ovarian

cancer (HGSC) patients for the last 10 years, with IV/IP paclitaxel and IP carboplatin. We conducted a retrospective study to review the clinical outcomes of using IP/IV chemotherapy compared to IV chemotherapy for optimally debulked stage I HGSC cases.
Methodology: A retrospective chart review was performed on women with early stage HGSC, who were optimally debulked at their primary surgery between 2007-2015 and received either IV or IP/IV chemotherapy post-operatively. We analyzed and compared the survival outcomes for women with stage IA-IC HGSC. Kaplan-Meier method was used to correlate chemotherapy delivery method with progression free survival (PFS) and overall survival (OS), using the statistical program R.
Results: We identified 99 patients; 80 (81%) received IV chemotherapy and 19 (19%) received IP/IV chemotherapy. All patients had high grade serous tumors. Among IP/IV cohort, 2/19 (11%) dropped the IP therapy in the middle of their treatment due to abdominal pain at IP port site or hypersensitivity reaction to IV paclitaxel. 5-year PFS was 88.4% (74.5-100%) and 69.7% (58.7-82.7%) among the IP/IV and IV cohorts, respectively (p=0.549). There was a trend for higher 5-year OS for the IP/IV group; however, this did not reach statistical significance (100% vs. 71.4%; p=0.182).
Conclusion: In our study, IP/IV chemotherapy with carboplatin and paclitaxel for optimally debulked stage I HGSC patients was associated with a trend for higher 5-year PFS and OS compared to IV chemotherapy, however this was not statistically significant. Larger prospective study is warranted to evaluate whether early stage HGSC truly benefits from IP/IV chemotherapy.
Disclosure: Nothing to disclose

EP905

Ovarian cancer treatment in the elderly

M. Kaparelou[1], A. Tsiara[1], R. Zakopoulou[1], A. Kyriazoglou[1], A. Cohen[1], M. Liontos[1], G. Tsironis[1], K. Koutsoukos[1], C. Theofanakis[2], N. Thomakos[2], F. Zagouri[1], D. Haidopoulos[2], A. Rodolakis[2], M.-A. Dimopoulos[1], A. Bamias[1]
[1]Dept of Clinical Therapeutics, [2]Dept of Obstetrics and Gynecology, Alexandra Hospital, National and Kapodistrian University of Athens, Athens, Greece

Introduction / Background: Treatment of elderly patients with neoplasia is challenging due to the frailty of the patients, existing comorbidities and increased number of concomitant medications. In addition, elderly patients are usually underrepresented in clinical trials. Age is a known prognostic factor in ovarian cancer but optimal treatment of elderly patients has not been determined. We undertook a retrospective analysis to determine clinical practice in advanced stage ovarian cancer patients older than 75 years of age.
Methodology: Medical records of women with high grade serous and endometrial ovarian cancer, stage III and IV, treated at Alexandra Hospital from 2011 to 2016 were retrospectively identified. Clinicopathological data, treatment and survival data were analyzed. Kaplan-Meier Survival curves were generated using IBM SPSS version 20; survival differences were estimated using the long-rank test.
Results: In total, 158 patients were identified with a median age of 61.1 years. Among them 20 (12.7%) were older than 75 years of age at diagnosis (range 75.03-92.72 years). First line Progression Free Survival (PFS) and Overall Survival (OS) were statistically significant worse in elderly patients in comparison to the younger ones (mPFS 13.4 months vs 21.9 months, P< 0.001 and mOS 25.3 months vs 51,5 months, P< 0.001). However, elderly patients were characterized by worse ECOG-PS, more frequent treatment with Neoadjuvant Chemotherapy followed by Interval Debulking Surgery, more frequently denied debulking surgery, and received monotherapy with platinum as frontline treatment, while were less frequently tested for BRCA mutations. In contrast, there was no statistical significant difference in the outcome of the debullking surgery in comparison to the younger patients. Age over 75 years retained its statistical significance for OS when adjusted for all other reported prognostic factors.
Conclusion: Elderly ovarian cancer patients have worse prognosis independent of treatment. Comprehensive geriatric assessment should be performed for the optimal treatment of these patients.
Disclosure: Nothing to disclose

EP906

Pre-operative prediction of residual disease after interval debulking surgery for advanced stage epithelial ovarian cancer using a multivariable model with HE4

P. Lof[1], R. van de Vrie[1], C.M. Korse[2], W.J. van Driel[1], M.D.J.M. van Gent[3], M.A. Karlsen[4], F. Amant[1,3], C.A.R. Lok[1]
[1]Gynecology, [2]Clinical Chemistry, Netherlands Cancer Institute/Antoni van Leeuwenhoek Hospital, [3]Gynecologic Oncology, Amsterdam UMC, Location Amsterdam Medical Center and University of Amsterdam, Amsterdam, The Netherlands, [4]Gynecology, Copenhagen University Hospital, Copenhagen, Denmark

Introduction / Background: Presence of residual disease (RD) after interval debulking surgery (IDB) is an important negative prognostic factor for patients with advanced stage epithelial ovarian cancer (EOC). Surgery is of limited benefit when the diameter of RD is >1cm. RD is difficult to predict before surgery. The multivariate model Cancer Ovarii Non-invasive Assessment of Treatment Strategy (CONATS) index, based on serum biomarker Human Epididymis 4 (HE4), age and WHO performance status (PS), predicted no visible RD in patients undergoing primary debulking surgery with an AUC of 0.85. The AUC of predicting RD > 1 cm was not reported, although this can be of importance for pre-operative decision making especially in fragile patients. We tested this model for predicting RD > 1 cm in patients undergoing IDB.
Methodology: We retrospectively included patients with advanced EOC who underwent IDB between 2010 and 2017 in two tertiary centers in the Netherlands. HE4 was measured with electrochemiluminescence in pre-operative samples. The CONATS-index was used to predict RD. Areas under the curve (AUC) were calculated to predict RD > 1 cm.
Results: Two-hundred-seventy-three patients were included. Mean age was 64 years (SD 11 years). Following surgery, 232 patients (85%) had RD ≤ 1 cm and 41 patients (15%) had RD > 1 cm. The AUC was 0.80 for predicting RD > 1 cm. In patients ≥ 70 years of age the AUC was 0.82.
Conclusion: The CONATS-index can help to predict surgical outcome after IDB in addition to radiologic parameters and can be useful in counseling patients about the chance whether a meaningful IDB can be achieved. This could be especially helpful in counseling elderly patients in whom surgery has an increased risk of complications.

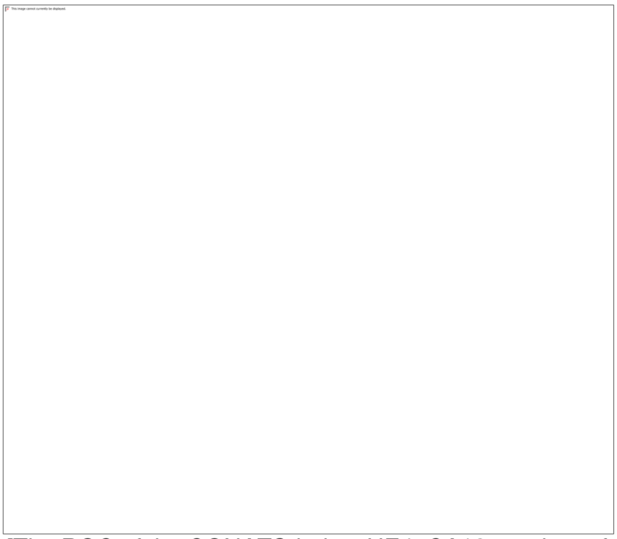

[The ROC of the CONATS-index, HE4, CA125 and age for predicting > 1 cm RD after IDB]

Disclosure: Nothing to disclose

EP907

Hyperthermic intraperithoneal chemotherapy (HIPEC) for ovarian cancer treatment: the feasibility study

M. Lomnytska[1], I. Silins[1], W. Graf[2]
[1]Department of Obstetrics and Gynecology, Academic Hospital, Uppsala University, [2]Department of Surgery, Academic Hospital, Uppsala University, Uppsala, Sweden

Introduction / Background: Survival benefit of the repeated intraperithoneal chemotherapy after surgery was demonstrated in GOG studied for ovarian cancer patients. However, catheter-related complications and accumulated toxicity made the procedure non-feasible. Recent randomized clinical trials OVHIPEC-1 and the Korean trial reported the positive effect on survival of HIPEC after the radical cytoreductive surgery (CRS) for ovarian cancer patients that were not eligible for primary CRS, but for interval CRS only.
Methodology: The feasibility study (NCT03717610) started at October 2018 at the Department of Obstetrics and Gynecology, Academical hospital, Uppsala, Sweden. It comprises 10 patients with ovarian cancer relapse and macroscopically radical surgery outcome. HIPEC with cisplatin 90mg/m2 for 90 min in the hyperthermic phase 41-42°C is performed. Sodium thiosulfate infusion concomitant to the HIPEC treatment protects against the nephrotoxicity. Clinical variables, laboratory tests, EORTC questionnaires are evaluated. The study is performed under the regulations of the local IRB. Study endpoints are toxicity, tolerability, high-grade complications and quality of life after the macroscopically radical ovarian cancer relapse surgery followed by HIPEC.
Results: We observed prolonged surgery time, one case of 3b (reoperation) 30-days morbidity, light to moderate nausea in 3 of 4 cases, no kidney toxicity and no neutropenia. The observations are case-wise related to the clinical parameters from the cohort of ovarian cancer relapse treated in 2009-2018. Table 1 summarizes the clinical and surgical observations.
Conclusion: HIPEC is a feasible procedure, toxicities comprise mild gastrointestinal events, prolonged surgery time corresponds to the HIPEC procedure, no impact on the quality of life is observed.

Surgery Period Patient#	CRS+HIPEC 2018 P1	2018 P2	2019 P3	2019 P4	2019 P5	CRS 2009-2018 n=63 M+/-St dev	
PCI/ Albumin preoperative	4/ 43	16/ 35	3/ 36	30/ 35	5/ 37	15±9,8<br>34,4±4,8	
Surgery time/ Bleeding	300/ 350	506/ 100	585/ 955	575/ 900	310/ 450	320,5±127,4<br>653,2±522,1	
Surgical complexity score	4	12	2	11	6	7,6±3,8	
Time to adjuvant chemotherapy	4	4	6	6	4	4,4±1,2	
Clavien-Dindo>3	0	0	0	3b	0	9 (14,3%)	
Cytoreduction score	0	0	0	1	0	63 (100%)	
Nausea	moderate	light	0	moderate	0	0	
Creatinin elevation	0	0	0	0	0	0	
Neutropenia	0	0	0	0	0	0	

[Table 1. Surgical and clinical parameters of the patients with relapsed ovarian cancer]

Disclosure: Nothing to disclose

EP908

Expression of CD24 in ovarian cancer

J. Lukacs[1], B. Soltesz[2], B. Nagy[2], R. Poka[1]
[1]*Department of Obstetrics and Gynecology, University of Debrecen Hungary,* [2]*Department of Human Genetics, Faculty of Medicine, University of Debrecen, Debrecen, Hungary*

Introduction / Background: Ovarian cancer is really considered to be the fifth most common cause of cancer death among women and the most lethal form of gynecological malignancy. CD24 is a small,cell-surface sialoglycoprotein and an independent marker for poor prognosis in different type of cancers.
Methodology: Our aim was to determine the expression of CD24 in ovarian tissue, plasma, and exosome samples of patients with serous ovarian cancer.
Tissue and blood samples were collected from 21 serous ovarian cancer patients and from 8 healthy controls. RNA was isolated by silica adsorption method, cDNA was synthesized and quantitative real-time PCR was performed. Beta-globin was used as housekeeping gene for the normalisation of the data. Protein-protein and miRNA networking was analysed by using Biogrid and miRTargetLink databases.
Results: Significant difference (p=0.007) was determined in expression of CD24 in ovarian tissue samples between controls (0.16±0.32) and patients (44.97±68.06), while CD24 was not expressed in all plasma and exosome samples. Correlation was observed in expression of CD24 and FIGO grading between controls and patients.
Based on network analysis, the LYN, SELP, FGR and NPM1 proteins showed interaction with CD24.
Conclusion: Higher expression of CD24 was determined in ovarian cancer patients' tissue samples and showed an association with FIGO classification. However, CD24 was expressed in just some cell-free plasma and exosome samples.
Disclosure: Nothing to disclose

EP909

Influence of intraoperative administration of ketorolac on the prognosis of ovarian cancer

M. Luyckx[1,2], C. Verougstraete[1], P. Forget[3], M. Jouret[1,4], M. Waterkeyn[1,5], F. Grandjean[1,5], J.-F. Baurain[6], J.-P. Van Gossum[1,7], J. Squifflet[1]
[1]*Gynaecological Surgery and Oncology - King Albert II Institute, Cliniques Universitaires St Luc,* [2]*De Duve Institute - TIL's Group, UCLouvain University,* [3]*Anesthesiology, UZ Brussel, Brussel,* [4]*Gynaecology and Obstetric, CHWapi Site IMC, Centre Hospitalier Wallonie Picarde, Tournai,* [5]*Gynaecology and Obstetric, Cliniques de l'Europe, Brussel,* [6]*Oncology Departement - King Albert II Institute, Clinique Universitaire Saint-Luc, Bruxelles,* [7]*Gynaecology and Obstetric, Cliniques Saint-Jean, Brussel, Belgium*

Introduction / Background: Several studies suggest that oncological surgery induce an acute inflammatory response, and that it may exacerbate certain mechanisms related to tumor growth and eventual dissemination. We propose to investigates whether the administration of Ketorolac (Taradyl) ®, a nonsteroidal anti-inflammatory drug, given intraoperatively during surgery for ovarian, tubal, or peritoneal cancer, influences disease free survival (DFS) and Overall survival (OS).
Methodology: A Retrospective analysis of all the patients operated at Cliniques Universitaires Saint-Luc; Brussel between 2015 and 2017 for ovarian, fallopian tube or peritoneum cancer. All the patient were included from the UCLouvain Network of Gynaecological Oncology (UNGO), an academic

network in Belgium for centralisation of the surgery for ovarian cancer patient.
Results: Of the 90 patients who met the inclusion criteria, 68% received Ketorolac intraoperatively. Only age was statistically different between the two groups ($p < 0.05$). FIGO stage, Residual disease, rate of primary surgery, neodjuvant chemotherapy was well balanced in the 2 groups. Intra-oprative administration of Ketorolac was statistically significantly associated with an improvement of the DFS (p=0,006). The median DFS in the group receiving ketorolac is 27 months versus 16 months for patients who did not receive ketorolac. There is also a statistically significant impact of peroperative administration of Ketoroloac on the OS ((p = 0.03).
Conclusion: These results suggest that the intraoperative administration of Ketorolac during surgery for ovarian, tubal or peritoneal cancer has a positive effect on the risk of recurrence and median survival
Disclosure: Nothing to disclose

EP910

Non-epithelial ovarian cancer in Tunisia: clinical presentation, treatment and outcome

L. Hmida[1], G. Sahraoui[2], I. Marghli[3], M. Driss[2], R. Doghri[2], A. Mokrani[1], H. Salhi[3], K. Mrad[2], L. Charfi[2]
[1]Medical Oncology, [2]Pathology, [3]Surgical Oncology, Salah Azaiez Institute, Tunis, Tunisia

Introduction / Background: Non-epithelial ovarian tumors represent an heterogeneous group of rare ovarian cancer. The aim of this study was to describe the clinico-pathological features and treatment of those malignancies in a Tunisian single center.
Methodology: Medical records of 134 patients with non-epithelial ovarian tumors treated between 1980 and 2010 were reviewed. Data collected included clinical features, treatment modalities and prognostic indicators.
Results: Overall, 94 were germ cell tumors (70%) and 40 sex cord-stromal tumors (30%). Thirty-six (27%) had immature teratoma, 28 (21%) had dysgerminoma, 13 (10%) had mixed germ cell tumors, 12 (9%) had yolk sac tumor, 5 (4%) had embryonal carcinoma, 37 (27%) had adult granulose cell tumors, 2 (1.5%) had juvenile granulose cell tumors and one (0.5%) had leydig and sertoli cells tumor. Median age at diagnosis was 28 years (range 7-95). Mean age was 27 for immature teratoma, 18 for dysgerminoma, 21 for mixed germ cell tumors, 23 for yolk sac tumor, 13 for embryonal carcinoma, 52 for adult granulose cell tumors and 24 for juvenile granulose cell tumors. Ninety-four (70%) presented with abdominal mass and/or pain. Surgery was the first treatment for 99% (n=133) with fertility sparing management in 79%. FIGO staging was as follows: Stage I in 63% (n=84) (stage IC: 23% of stage I), stage II in 6% (n=8), stage III in 24% (n=32), and stage IV in 7% (n=10). Sixty-four (48%) received adjuvant chemotherapy. Median follow-up period was 45 months. The 5-year odds survival was 78%. Reccurence occurred in 18 % (n=24).The 5-years disease free survival was 75%.
Conclusion: As it is usually unilateral and chemosensitive with high incidence in young women, *fertility-sparing surgery* with optimal staging *should be the standard of care of non-epithelial ovarian cancer.*
Disclosure: Nothing to disclose

EP911

Adjuvant chemotherapy versus surveillance in stage I non epithelial ovarian cancers: a single center experience

L. Hmida[1], G. Sahraoui[2], I. Marghli[3], L. Charfi[2], R. Doghri[2], A. Mokrani[1], H. Salhi[3], M. Driss[2], K. Mrad[2]
[1]Medical Oncology, [2]Pathology, [3]Surgical Oncology, Salah Azaiez Institute, Tunis, Tunisia

Introduction / Background: Non-epithelial ovarian tumors are uncomm malignancies which can be

extremely challenging to treat. The aim of the study was to evaluate the effectiveness of chemotherapy in women with early stage disease.
Methodology: Medical records of 84 patients diagnosed with stage I non epithelial ovarian cancer between 1985-2015 were reviewed. Tumor characteristics and treatment receipt were described.
Results: Median age was 28 years [9-95]. Sixty-five had stage Ia disease at diagnosis while 19 presented with stage Ic disease. Fourteen had dysgerminoma (16.7%), 30 had immature teratoma (35.7%), 5 had yolk sac tumor (6%), 3 had embryonal carcinoma (3.6%), 3 had mixed germ cell tumor (3.6%), 28 had adult granulose cell tumor (33.3%) and one had juvenile granulose cell tumor (2.4%). Most of them (n=55, 65%) underwent fertility sparing surgery. Thirty-one (37%) received adjuvant platinum based chemotherapy, among them 17/65 were stage Ia disease and 14/19 were stage Ic disease. Fifty-two (62%) had surgery followed by active surveillance. Fertility sparing surgery was followed by adjuvant chemotherapy in 25% (n=21). After a median follow up of 45 months, 14 relapsed. Among them 6 received adjuvant chemotherapy and 8 underwent surgery followed by surveillance. The 5-year-disease-free-survival (DFS) and 5 year overall survival (OS) were respectively 79% and 46% in the chemotherapy arm and 80% and 51% in the surveillance arm with no statistically significant difference observed in DFS (p=0.4) and OS (p=0.3).
Conclusion: Fertility sparing surgery with active surveillance is an option which should be discussed within a multi disciplinary team especially in young patients with early stage disease desirous of pregnancy.
Disclosure: Nothing to disclose

EP912

Characterization of the tumour microenvironment in high-grade serous ovarian cancer (HGSOC): prognostic value of the lymphocytic infiltration patterns and immune-related genes

J. Marí-Alexandre[1], F. Herrera-Cañizares[2,3], A. Chipirliu[4], C. Caballero[5], C. Agababyan[4], L. Valdivieso[6], N. Santonja[6], A. Llueca[7,8,9], S. Calabuig[10,11], E. Jantus-Lewintre[10,12], C. Camps[10,13,14], J. Gilabert-Estellés[15,16,17]
[1]Laboratorio Investigación Biomarcadores en Reproducción, Ginecología y Obstetricia, Fundación para la Investigación Hospital General Universitario, [2]Laboratorio de Oncología Molecular, Fundación de Investigación Hospital General Universitario de Valencia, [3]Fundación Carolina-BBVA, [4]Servicio de Obstetricia y Ginecología, [5]Servicio de Oncología Médica, [6]Servicio de Anatomía Patológica, Hospital General Universitario Valencia, Valencia, [7]Servicio de Obstetricia y Ginecología, Hospital General Universitario de Castellón, Castellón, [8]Unidad Multdisciplinar de Cirguía Abdominopélvica, Hospital General Universitario de Castellón, Valencia, [9]Departamento de Medicina, Universidad Jaume I, Castellón, [10]Laboratorio de Oncología Molecular, Fundación de Investigación Hospital General Universitario de Valencia-CIBERONC, [11]Departamento de Patología, Universitat de Valencia, [12]Departamento de Biotecnología, Universidad Politécnica de Valencia, [13]Servicio de Oncología Molecular, Hospital General Universitario de Valencia, [14]Departamento de Medicina, Universidad de Valencia, [15]Laboratorio Investigación Biomarcadores en Reproducción, Ginecología y Obstetricia, Fundación de Investigación Hospital General Universitario de Valencia, [16]Servicio de Obstetricia y Ginecología, Hospital General Universitario de Valencia, [17]Departamento de Pediatría, Obstetricia y Ginecología, Universidad de Valencia, Valencia, Spain

Introduction / Background: Lymphocytic infiltration areas (immunoreactive), frequently found in high-grade serous ovarian cancer (HGSOC), are associated with a better prognosis and increased survival. The cross-talk between tumour cells and lymphocytes conditions the capacity of the immune system (IS) to cope with the tumour in the so-called immune-checkpoints. Therefore, assessing IS-related genes and infiltration patters might provide valuable prognostic biomarkers.
Methodology: This retrospective study includes 57 samples from patients with HGSOC who underwent cytoreductive surgery at Hospital General (Valencia). Clinical variables, the features of the lymphocytic infiltration (pattern, localization and degree) and the expression of immune-related genes (CD4, CD8, FOXP3, ICOSL, ICOS, PD-L2, TGFβ1, CD25, IDO1, IL7R, PD-L1, CTL4, CXCR4, PD1, OX40, LGAL and CD137) were evaluated to assess prognosis.
Results: The median age was 61.5 years, being the majority of patients in advanced FIGO stages (76.3% III-IV *vs.* 23.7%, I-II stages). Patients with ≥65 years and III-IV stages showed a shorter overall

survival (OS, 30.17 *vs.* 99.90 months, p=0.009; 38.73 months *vs.* NR, p=0.005, respectively). Regarding immunoreactive areas, patients with an intratumoural pattern of lymphocyte infiltration had better prognosis compared to those that only had a peritumoural pattern (OS: 44.57 months *vs.* NR, p=0.041). In addition, those with a diffuse infiltration pattern presented a better prognosis compared to those with a focal pattern (OS, 20.20 months *vs.* NR p=0.003). Regarding gene expression, 11 genes (CTLA4, FOXP3, CD25, CSCR4, IDO1, PD-1, PD-L1, PD-L2, OX40L, ICOS, ICOSL, LGAL9 and CD137 were found over-expressed, but only CD137 displayed a significant prognostic value (OS: 50 months vs NR, p=0.020)
Conclusion: HGSOC represents a group of highly immunoreactive tumours. Best prognosis is represented by patients with an intratumoural and diffuse pattern of lymphocytic infiltration and lower CD137 expression, which may be considered as valuable prognostic markers. These interesting findings could open a new window for immunotherapeutic approaches in HGSOC.
Disclosure: All authors have declared no conflicts of interest. This work has been supported by grants from ISCIII (PI17/01945 and CB16/12/00350). J.M-A. is supported by APOSTD/2019/087 post-doctoral grant.

EP913

A comparative study of three different managements after colorectal anastomosis in ovarian cancer: conservative management and observation, diverting ileostomy and ghost ileostomy

V. Lago[1], A. Sanchez-Migallón[2], L. Matute[1], P. Padilla-Iserte[1], T. Marina[1], B. Flor[1], M. Bustamante[1], S. Domingo[1]
[1]University Hospital La Fe, Valencia, [2]Dexeus, Barcelona, Spain

Introduction / Background: Anastomotic leak (AL) remains as the main concern after colorectal anastomosis in ovarian
cancer. Our objective was to compare the use of three different managements after colorectal resection followed by anastomosis in patients with ovarian cancer.
Methodology: Between January 2010 and June 2018, a total of 133 patients FIGO Stage II-IV who underwent colorectal resection and anastomosis were included. According to the approach followed after the colorectal anastomosis, they were classified as: conservative management and observation (W&S) during postoperative period, the performance of a diverting ileostomy (DI) or the use of the ghost ileostomy (GI) technique.
Results: No differences were found regarding the anastomosis leak related factors or the rate of anastomotic leak between the three groups (5,6% vs. 5,3% vs 4,8%; p=0.983). Two patients (50%) died because of the anastomotic leak in the W&S group, and none of them in the DI or GI group. A higher number of patient in the DI group presented complications compared with the GI group (78.9% vs 7,1%; p=00001).
Conclusion: A passive management after colorectal anastomosis based in imaging, blood test and symptoms leads serious consequences in case of AL. The use of DI seems to diminish the consequences of AL but associates specific related complications and it impacts directly in the QoL as well. The use of GI has advantages over routine DI and W&S approaches for ovarian cancer patients undergoing colorectal anastomosis.
Disclosure: Nothing to disclose

EP914

Port site resection after laparoscopy in advance ovarian cancer surgery: time to abandon?

T. Marina Martín, V. Lago Leal, P. Padilla Iserte, L. Matute Tobías, M. Gurrea Soteras, S. Domingo del Pozo

Gynecologic Oncology, University Hospital La Fe, Valencia, Spain

Introduction / Background: The use of laparoscopy in the treatment and management of advanced ovarian cancer is increasing among the gynaecologic oncologists. The development of port site metastases after laparoscopy is a concern and a matter of debate due to theoretical iatrogenic disease spread. Port site resection (PSR) has been proposed as an option to avoid this scenario.
Methodology: One hundred and twenty-three patients with advanced ovarian cancer (FIGO III-IV) and with diagnostic laparoscopy were included and after cytoreductive surgery were classified into two groups: no port site resection (No-PSR) and port site resection (PSR). Based on the pathological results of all port site specimens, PSR was classified as positive port site metastasis (PSM+) and negative port site metastasis (PSM-.
Results: In 82 cases, the laparoscopic port site access was resected in the debulking surgery. At the final specimen examination, 49% presented as PSM+. No statistical differences regarding survival were found, either between the No-PSR and PSR groups (p = 0.28) or between the PSM+ and PSM - groups (p = 0.92). A higher wound complication rate was found in the PSR group (17% vs. 34%; p = 0.047). The RR (Relative Risk) of wound events for PSR was 2.42 (95% CI 1.09-5.35; p = 0.0296).
Conclusion: To date, not only there is no data supporting PSR after laparoscopy in advanced ovarian cancer, but the role of PSM+ in prognosis also remains unclear. In patients in which laparoscopy is performed prior to the debulking procedure, the PSR may not be recommended in those cases of no macroscopic port site metastasis.
Disclosure: Nothing to disclose

EP915

Granulosa cell tumor of ovary: a retrospective study of 16 cases

C. Martín Blanco[1], C. del Valle Rubido[1], E. Cabezas López[1], B. Pérez Mies[2], E. Moratalla Bartolomé[1], M.C. Sánchez Martínez[1], J. Lázaro-Carrasco de la Fuente[1]
[1]Gynecology and Obstetrics Department, [2]Anatomical and Pathology Department, Ramon y Cajal Universitary Hospital, Madrid, Spain

Introduction / Background: Granulosa cell tumors (CGT) are the most common type of potentially malignant ovarian sex cord-stromal being usually diagnosed in the early stages, with 5-year survival rates for completely resected stage I disease close to 90% and tendency for late relapse.
Methodology: All CGT cases diagnosed and treated at the Ramon y Cajal University Hospital in Madrid, Spain from 2000-2019 were retrospectively analyzed.
Results: A total of 16 patient were included.Mean age was 52.4 years (range 14-67) Patients presented mainly with abnormal bleeding (37,5%). 25% of the patients started with abdominal pain and/or abdominal mass, finding 2 cases of acute abdomen requiring urgent surgery.93,75% of the cases were diagnosed in FIGO stage I disease (86,6% stage IA, 13.4% stage IC), and one case of IVC stage with pulmonary involvement. Primary treatment was surgery in all cases, 12 patient underwent total hysterectomy with bilateral salpingoophorectomy. Complete staging surgery was performed in 33,3% of these patients.In 25% of cases, unilateral adnexectomy was performed. Median tumor size was 5.3 cm. 92.3% of the cases were of adult subtype. Mitotic index was measured in 11 patients, being low in 54,5% of cases. Cellular atypia was objected in 5 cases.
Only one patient received adjuvant therapy with tamoxifen due to treatment of a previous breast cancer. Adjuvant chemotherapy was offered to the patient with IVC stage disease, denying treatment. The median follow-up time was 8.4 years. Inhibin determinations in blood were carried out in 5 patients since 2017. Abdominopelvic relapses were reported in 2 patient with FIGO stage I (12.5%).Surgery with optimal resection and platinum based adjuvant chemotherapy were given.The mean relapse free survival was 4.2 years(3-84 months).
Conclusion: GCT tumors are uncommon neoplasm with a late recurrences rate. Therefore, long active follow up is recommended.
Disclosure: Nothing to disclose

EP916

Lymph node involvement pattern and survival differences in a retrospective analysis of individual results prior to change our strategy about de role of lymphadenectomy in management of ovarian cancer

E. Martínez Lamela[1], J. Molero Vílchez[2], A. Santiago Gómez[3], G. Sánchez Rodillo[4]
[1]Oncologic Gynecology, Hospital Ntra Sra del Rosario, Obstetrics and Gynecology, University Hospital Infanta Leonor, [2]Oncologic Gynecology, Hospital Ntra. Sra. del Rosario, Madrid, Oncologic Gynecology, Clinica TocoGyn, Alcalá de Henares, [3]Medical Oncology, [4]Radiation Oncology, Institute of Advanced Techniques Against Cancer, Madrid, Spain

Introduction / Background: Our purpose was to analyse the Overall Survival (OS) and Disease-Free Survival (DFS) comparing subgrous with different tumor spread pattern
Methodology: A total of 118 patients with primary ovarian cancer who received complete cytoreductive surgery were included in current retrospective analysis. Mean age was 57 years (18-90). The histologic type most common was high grade serous carcinoma (48.6%). According to FIGO staging, patients were divided into initial (I-II, 66 cases) and advanced stages (III-IV, 74 cases). The main goal was to compare survival differences in all patients and among groups using standard statistical procedures
Results: In 92 cases lymphadenectomy were performed (lymph node involvement was detected in 14.3%, 20 cases), 78 cases pelvic and para-aortic, 5 cases only para-aortic and 9 cases only pelvic lymphadenctomy. Mean number of removed pelvic lymph nodes was 18 (9-38) (12 cases involved nodes, 8.6%) and 20 (10-51) para-aortic nodes (17 cases involved nodes, 12%). The median follow-up was 41.2 months (SD 33.8).
The estimated 5-year DFS and OS comparated rates respectively were: In all patients if lymphadenctomy was performed were 75 and 79.6%, vs no 72 and 57.6% (p=0.97 and p=0.01 Hazard ratio 2.31 (CI 95%, 1.14-4.68). If lymph node involvement was detected were 33.7 and 65.3%, vs no 76 and 79.3% (p=0.00 Hazard ratio 3.32 (CI 95%, 1.3-8.43) and p=0.17). In initial stages with lymphadenectomy were 88.5 and 88.5%, vs no 82.5 and 90% (p=0.32 and p=0.88). In advanced stages when lymphadenectomy was performed were 27.1 and 56.7%, vs no 65.4 and 46% (p=0.07 and p=0.30). In advanced stages with involvement lymph node were 31.5 and 62.4%, vs no 27.3 and 49.1% (p=0.81 and p=0,69).
Conclusion: In light of our data, OS increased in case of patients who underwent with systematic pelvic and para-aortic lymphadenectomy

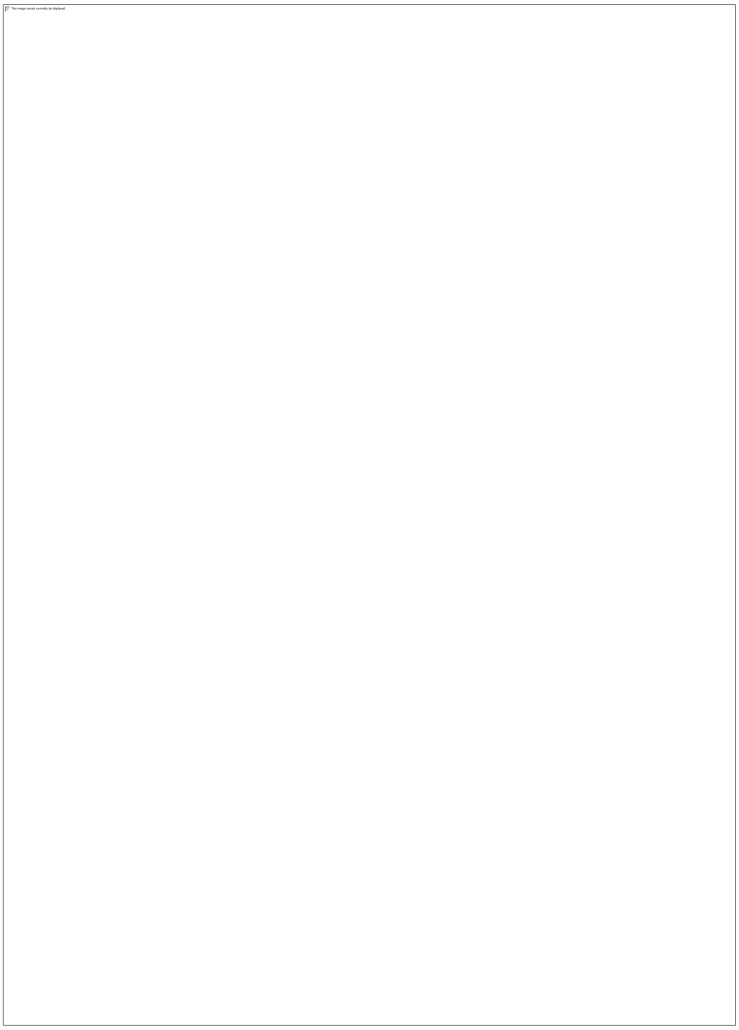

[Results of univariante Cox of Disease-Free Survival (DFS) and Overall survival (OS): (A) Pelvic and]

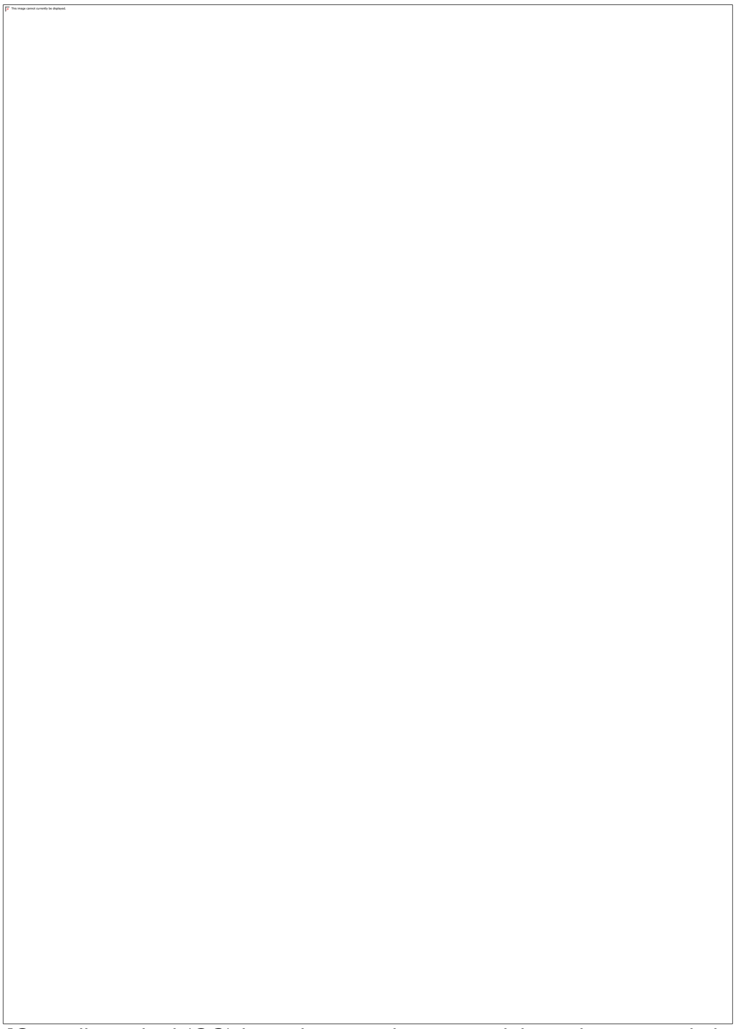

[Overall survival (OS) in patients underwent pelvic and para-aortic lymphadenectomy. (A) Initial stag]

Disclosure: Nothing to disclose

EP917

Retrospective analysis of the prognostic factors for disease recurrence and overall survival in patients with ovarian cancer. A single center study

E. Martínez Lamela[1], J. Molero Vílchez[2], G. Sánchez Rodillo[3], A. Santiago Gómez[4]
[1]Oncologic Gynecology, Hospital Ntra Sra del Rosario, Obstetrics and Gynecology, University Hospital Infanta Leonor, [2]Oncologic Gynecology, Hospital Ntra. Sra. del Rosario, Madrid, Oncologic Gynecology, Clinica TocoGyn, Alcalá de Henares, [3]Radiation Oncology , Institute of Advanced Techniques against Cancer, [4]Medical Oncology, Institute of Advanced Techniques against Cancer, Madrid, Spain

Introduction / Background: Our purpose was to analyse the Overall Survival (OS) and Disease-Free Survival (DFS) comparing the different factors that impact on survival
Methodology: A total of 140 patients with ovarian cancer were included, underwent complete

cytoreductive surgery in 118 cases and chemotherapy in 22 cases. Mean age was 57 years (18-90). Pathology most common was high grade serous carcinoma (48.6%). FIGO initial stages (I-II, 66 cases) and advanced stages (III-IV, 74 cases). Mean Ca 1.25 prior to surgery was 467 U/dL (3-6,790). Exploratory laparoscopy, predictive index value, peritoneal carcinomatosis index, residual tumor and surgical complexity score was reported. We compared differences by survival prognostic factors using univariate and multivariate analysis

Results: In 92 cases pelvic and para-aortic lymphadenectomy were performed. Lymph node involvement was detected in 14.3% cases. 94 patients underwent Primary Debulking Surgery (PDS) (79%), and 24 cases neoadjuvant chemotherapy (NAC) and interval surgery. The median follow-up was 41.2 months.

The estimated 5-year DFS and OS comparated rates respectively were: In initial FIGO stages were 87.3 and 88.7%, vs advanced stages 53.4 and 51.5% (p=0.00 Hazard ratio 4.65 (CI 95%, 1,86-11.62) and p=0.00 Hazard ratio 6.34 (CI 95%, 2.43-16.58). When lymphadenctomy was performed were 75 and 79.6%, vs no 72 and 57.6% (p=0.97 and p=0.01 Hazard ratio 2.31 (CI 95%, 1.14-4.68). If lymph node involvement was detected were 33.7 and 65.3%, vs no 76 and 79.3% (p=0.00 Hazard ratio 3.32 (CI 95%, 1.3-8.43) and p=0.17). In advanced stages, with PDS 44.7 and 59.2%, vs NAC 0 and 24.9% (p=0.05 and p=0.48); microscopical residual 78.8 and 88.1%, vs gross residual 35.2 and 33.4% (p=0.00 Hazard ratio 3.25 /CI 95%, 1.49-7.09) and p=0.00 Hazard ratio 6.89 (CI 95%, 2.98-15.93)

Conclusion: OS increased in case of initial stages, complete debulking with microscopic residual and patients who underwent with lymphadenectomy

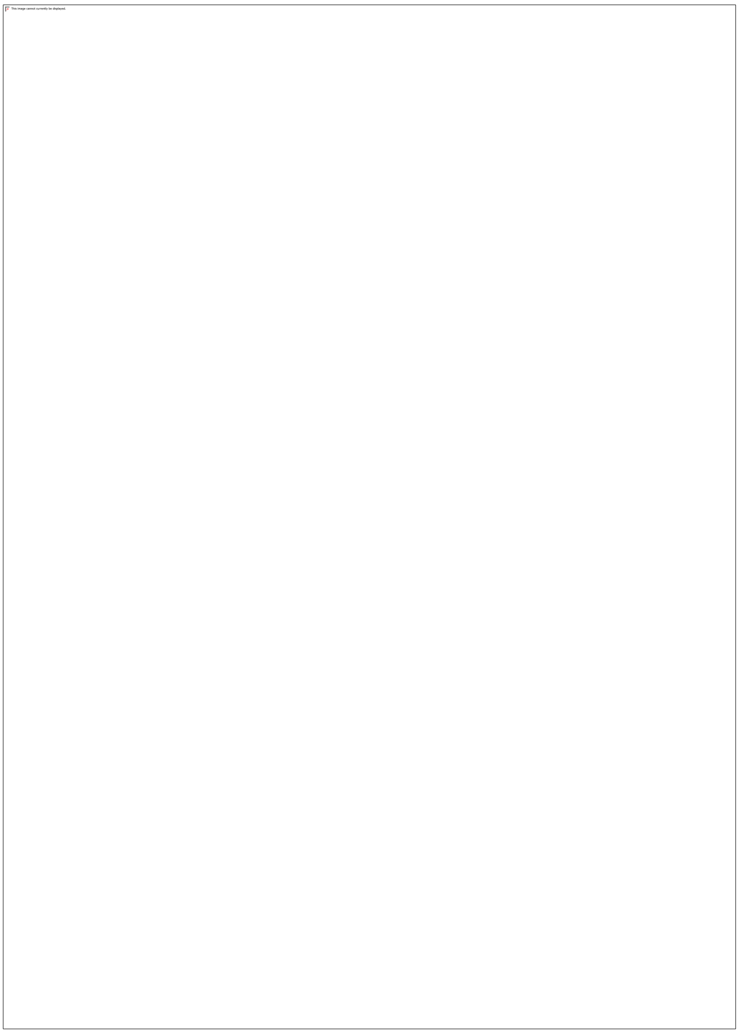

[Results of univariante Cox proportional Hazard ratio analysis of Overall survival (OS): (A) FIGO sta]

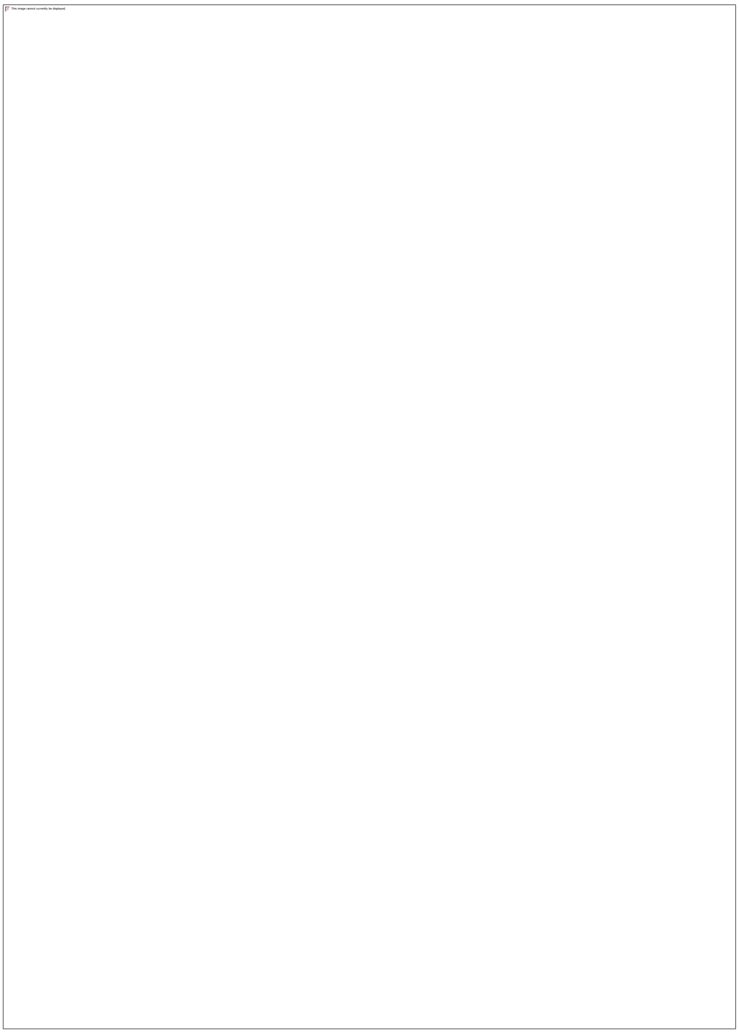

[Disease-Free survival (DFS)and Overall survival (OS) by tumor residual. (A) All FIGO stages. (B) Ad]

Disclosure: Nothing to disclose

EP918

Transvaginal/transabdominal sonography for histological diagnosis in advanced ovarian cancer patients: a single institution prospective study

F. Mascilini[1], L. Quagliozzi[1], M.C. Moruzzi[1], M. Albanese[1], A.C. Testa[2], G. Scambia[2], A. Fagotti[2]
[1]Fondazione Policlinico Agostino Gemelli, IRCCS, Dipartimento Scienze della Salute della Donna, del Bambino e di Sanità Pubblica, UOC di Ginecologia Oncologica, [2]Fondazione Policlinico Agostino Gemelli, IRCCS, Dipartimento Scienze della Salute della Donna, del Bambino e di Sanità Pubblica, UOC di Ginecologia Oncologica, Catholic University of the Sacred Heart, Rome, Italy

Introduction / Background: Ultrasound-guided biopsy is an easy-available technique for obtaining tissue samples, rarely used in suspicious advanced ovarian cancer, in order to plan an early and adequate treatment.

Methodology: Primary endpoints of the study were feasibility, safety and diagnostic accuracy of TA/TV ultrasound guided biopsy with respect to standard approach. Secondary endpoint was patients 'pain assessment during the procedure. This is an interventional prospective single centre study, approved by local IRB. From September 2018, all women admitted in our Institution for suspicious advanced ovarian cancer were consecutively enrolled. They underwent TA/TV ultrasound guided biopsy with an expert ultrasonographer.
Results: Between September 2018 and March 2019, 90 women were eligible in the study but 8 (9.9%) refused to participate. 44 patients were lost before enrolment. The remaining 38 patients underwent a tentative ultrasound transvaginal/transabdominal biopsy, which was not feasible in 12 of them (29%) due to technical difficulties. In the remaining 26 successful procedures, the site of biopsy was ovarian mass in 13 (50%) patients, and peritoneum in the others (50%). Only one patient (3.8%) experienced fever (38°C) 24 hours after the procedure. Five patients (19.2%) experienced mild to moderate pelvic pain during the procedure which was controlled by oral analgesic therapy and lasted for ten minutes (two patients had VAS score 4 and three had VAS score 3). Samples were adequate for histopathological diagnosis in 25/26 (95%) cases: 20 gynaecological cancers, 3 non-gynecological cancers and 2 fibrosis. Sensitivity and accuracy of the procedure, compared to 22 biopsies obtained at time of surgery, were 90.1% and 90.1%, respectively due to 2 FN.
Conclusion: TA/TV ultrasound-guided biopsy is a safe procedure, feasible in 70% of patients with suspicious primary advanced ovarian cancer, with a histological accuracy rate of 90.1%.
Disclosure: Nothing to disclose

EP919

Obesity and inflammation combined are risk factor for early recurrence in High Grade Serous Ovarian Cancer

G. Pizarro[1], M. Cordova[1], M. Cuello[2], S. Kato[2], R. Jensen[2], J. Branes[2], A. Villarroel[3], M. Pinto[1], M. Matias[1], M.L. Bravo[1], C. Ibanez[1]
[1]Facultad de Medicina | Hematologia Oncologia, [2]Facultad de Medicina | Ginecologia y Obstetricia, [3]Facultad de Medicina | Anatomia Patologica, Pontificia Universidad Católica de Chile, Santiago, Chile

Introduction / Background: Ovarian cancer is the deadliest of all gynecological cancers and high-grade serous ovarian cancer (HGSOC) is the most prevalent, aggressive type. Most of the patients are diagnoses at advances stage (III or IV).
Epidemiological studies show that obesity correlates to ovarian cancer incidence, also correlate with a reduced period of overall survivor (OS). Recent studies suggest ovarian tumors are associated to chronic low-grade systemic inflammation. NLR or serum LDH had a significant impact upon OS. Our objective was determine the role of obesity and inflammation as a risk factor in a HGSOC cohort.
Methodology: A retrospective registry was established that includes Chilean patients diagnosed with HGSOC. demographic and clinical data were obtained, after the signing of informed consent
Results: Demographic and clinical information was obtained from 177 patients.
147 (83%) cases were diagnoses in stage III or IV. The median for overall survivor was 49 months. Obesity and systemic inflammation showed a significant reduction in OS, but not affect Progression free survival (PFS). Normoweight patient showed no difference in OS according inflammatory status, but obese patients showed significant difference.
Conclusion: Obesity as inflammation are risk factor for early recurrence in HGSOC. Our results showed a reduction in overall survival according to obesity and inflammatory status. These parameters in combination show that the risk observed by the inflammatory state depends on the BMI of patients with ovarian cancer.
Disclosure: Nothing to disclose

EP920

Ovarian cancer and BRCA mutation typing: a review of cases in Northern Ireland 2018

J. Mcinerney, L. Rainey, G. Popa, M. Robinson, I. Harley
Gynaecology, Oncology, Belfast City Hospital, Belfast, United Kingdom

Introduction / Background: Identification of BRCA mutation is important in the treatment of advanced ovarian cancers. It can improve efficacy of therapies; provide prognostic information and earlier identification in predisposed families.
Aim: To identify the incidence of ovarian cancer in 2018; the variations in histolopathology and the referral rate for genetic testing.
Methodology: Cases were identified through the Cancer Patient Pathway System. Demographic information collected included: age and stage at diagnosis; subtype; family and personal cancer history; referral date for, and date of genetic testing as well as result date and genetic mutation.
Results: There was an incidence of 104 cases. Average 65.7 years (range: 37-93). Cancer stages 1 to 4 had incidence 13.5% : 11.5% : 52% : 23% respectively.
High grade serous carcinoma was the most common histological variant with 76%; endometrioid 10%; clear cell carcinoma 9%; low grade serous carcinoma 5%.
12.5% and 19% of cases had a family history of ovarian or breast cancer respectively, while 66% of cases report a direct family history of cancer.
8 patients reported a breast cancer history and 4 had a history of other cancers.
59% of cases were referred for genetic testing. 41 patients received testing. 10 patients await testing and 10 patients declined or died.
Of the 41 cases tested only 3 patients were identified as BRCA 1 or BRCA 2 mutations.
Conclusion: The majority of cases presenting were advanced stage disease. These represent patients who would benefit from genetic targeted drug therapies or BRCA family identification yet only 59% of cases were referred and only 40% actually received testing by the time of reporting. Interestingly however, 7% of those tested were BRCA positive.
This study highlights the need to further understand our populations' genetic predispositions to ovarian cancer in an effort to improve cancer care for patients and their families.
Disclosure: Nothing to disclose

EP921

Lymphocytic and cytokine microenvironment of ovarian cancer

E. Zlatnik, A. Menshenina, T. Moiseenko, E. Bondarenko, E. Verenikina, D. Yakubova, E. Frantsiyants, I. Nikitin
Rostov Research Institute of Oncology, Rostov-on-Don, Russian Federation

Introduction / Background: Our purpose was to study the content of lymphocytes and cytokines in the ascitic fluid (AF) of patients with ovarian cancer (OC) as probable factors of tumor progression.
Methodology: The study included 20 patients aged 25-73 years with OC with ascites. The percentage content of granulocytes, monocytes, lymphocytes, their populations and subpopulations, as well as naive lymphocytes (Th0) and memory T-cells (Tm) were determined in the blood and AF by flow cytofluorimetry using the BD FACS CantoII analyzer. Levels of IL-1α, IL-1RA and IL-8 cytokines were determined by ELISA.
Results: AF of patients showed elevated, compared to the blood, levels of lymphocytes and monocytes (by 2.8 and 3.3 times, respectively); the content of granulocytes was 3.3 times lower. The percentage content of T lymphocytes and their main subpopulations and the levels of B and NK cells in the blood and AF had no statistically significant differences. However, the amount of Tm in AF was higher than in the blood; the percentage of Th0 in AF was lower. The percentage of Th0 cells in the CD3+CD4+ subpopulation was 18.3±2.6% in the blood and 8.3±1.4% in AF, in CD3+CD8+ 19.7±1.9 and 11.5±1.5% respectively ($p < 0.05$). The results for Tm in CD3+CD4+ were 69.4±2.9 in the blood and 80.6±2.1% in AF, in CD3+CD8+ 40.0±4.3 and 60.0±3.9% respectively ($p < 0.05$). AF showed a

sharp excess of most cytokines over their serum levels, the maximal one for IL-1α (by 273 times) and the minimal one for IL-8 (by 2 times); only levels of IL-1RA in AF were lower than in the blood.
Conclusion: The increase in the content of monocytes and pro- and anti-inflammatory cytokines produced by them and by tumor cells in AF of OC patients supports tumor growth due to inhibition of the activity of memory T-cells, despite their high quantitative level.
Disclosure: Nothing to disclose

EP922

Dependence between the degree of endogenous intoxication and levels of serological markers in ovarian cancer

T. Moiseenko, S. Tumanyan, E. Frantsiyants, A. Menshenina, E. Verenikina, D. Yakubova, V. Nikitina
Rostov Research Institute of Oncology, Rostov-on-Don, Russian Federation

Introduction / Background: The timely use of various detoxification therapy methods during multi-course chemotherapy in patients with ovarian cancer (OC) can reduce the number of side effects and complications. To determine an association between levels of tumor markers and degrees of endogenous intoxication (EI), we studied and compared changes in serum levels of CA125 and HE4 tumor markers in patients with advanced OC with varying EI severity.
Methodology: The study included laboratory data on 44 patients, mean age 58 years, with stage III-IV epithelial OC who received surgical treatment followed by chemotherapy. Integral indices of EI (leukocyte index of intoxication, body reactivity index, neutrophil reactive response, hematological index of intoxication) calculated by the haemogram test results were analyzed before treatment.
Results: 11.5% of patients did not have laboratory signs of EI. 31.8% showed weak EI, 47.8% - medium EI, 8.9% - severe EI. In 75% of patients, EI was accompanied by decreased total body reactivity and uncompensated EI. Patients with CA125=35-120 U/mL and HE4=120-200 pmol/L showed weak EI, patients with CA125=120-600 U/mL and HE4=200-800 pmol/L - moderate EI ($p>0.05$). Patients with severe EI had levels of CA125 and HE4 tumor markers exceeding the 1000 mark ($p>0.05$).
Conclusion: We revealed a stable correlation between CA125 and HE4 and the degree of EI ($r=0.87$) ($p< 0.05$) allowing combination of chemotherapy in OC patients with medical and instrumental methods of EI reduction.
Disclosure: Nothing to disclose

EP923

Smoothened protein expression and promoter methylation status in serous ovarian carcinomas

P. Mioc[1], V. Karin Kujundzic[1,2], R. Matijevic[3,4], A. Skrtic[2,5,6], S. Vranic[7], L. Serman[1,2]
[1]Department of Biology, School of Medicine, University of Zagreb, [2]Centre of Excellence in Reproductive and Regenerative Medicine, School of Medicine, University of Zagreb, [3]Department of Obstetrics and Gynecology, School of Medicine, University of Zagreb, [4]Department of Obstetrics and Gynecology, University Hospital Merkur, [5]Department of Pathology, School of Medicine, University of Zagreb, [6]Department of Pathology, University Hospital Merkur, Zagreb, Croatia, [7]College of Medicine, QU Health, Qatar University, Doha, Qatar

Introduction / Background: Aberrant Hedgehog (Hh) pathway signaling has been implicated in pathogenesis of several human cancers. Recent studies have indicated its active role in serous ovarian carcinomas. Smoothened protein (SMO), a transmembrane co-receptor in Hh pathway signal transduction, is inhibited in non-dividing cells, thus its disinhibition might be a trigger for uncontrolled

cell proliferation and growth. Very few studies have explored the role of SMO in serous ovarian cancers. The aim of the study was to assess the expression of SMO protein and to explore the *Smoothened* gene promoter methylation in a cohort of serous ovarian carcinomas.
Methodology: SMO protein expression was immunohistochemically quantified in 40 high-grade serous carcinomas (HGSC), 12 low-grade serous carcinomas (LGSC), 20 normal ovarian and 9 normal fallopian tube samples (controls). *SMO* gene promoter methylation status was analyzed using methylation-specific polymerase chain reaction (MSP) in randomly selected HGSCs (n=10), LGSCs (n=10), and normal fallopian tube (n=9) samples. Kaplan-Meier survival plots were used to estimate the impact of SMO expression on patients' overall survival (OS).
Results: SMO nuclear expression was significantly higher in HGSCs and LGSCs compared with the fallopian tube samples (p=0.010 and p=0.003, respectively). LGSCs, compared with normal ovarian tissue, exhibited higher total, cytoplasmic/membrane and nuclear expression (p=0.000, p=0.001 and p=0.000, respectively). Comparing HGSCs and LGSCs, significantly higher total and cytoplasmic/membrane expression was found in HGSC (p=0.026 and p=0.030, respectively). *SMO* gene promoter was unmethylated in both LGSCs and HGSCs as well as in fallopian tube. In addition, the SMO protein expression had no significant impact on patients' OS (p=0.07).
Conclusion: Our data indicate the lack of *SMO* gene promoter methylation while a significant overexpression (particularly nuclear) of SMO protein characterized a substantial proportion of serous ovarian carcinomas. Further functional studies should elucidate the clinical relevance of these findings.
Disclosure: Nothing to disclose

EP924

Clinical characteristics predictive of optimal primary cytoreduction in epithelial ovarian malignancy: a five-year retrospective study in a tertiary government hospital

J. Misuari-Alihuddin, J.T. Luna
Section of Gynecologic Oncology, Department of Obstetrics and Gynecol., Philippine General Hospital, Manila, Philippines

Introduction / Background: To determine the clinical characteristics predictive of optimal cytoreduction among women with epithelial ovarian malignancy who underwent cytoreductive surgery in a tertiary government hospital.
Methodology: This retrospective cohort study identified 218 patients with epithelial ovarian cancer who underwent primary cytoreductive surgery in a tertiary government hospital between January 2013 to December 2017. Demographic, imaging, CA-125, surgico-pathologic data were collected. Outcome measures included incidence of optimal cytoreduction and factors affecting its occurrence. Descriptive and inferential analysis were done to estimate odds ratio with 95% confidence interval and p-values. Univariate and multivariate logistic regression analysis were done.
Results: A total of 145 patients had optimal cytoreduction while 73 had non-optimal cytoreduction. A simple logistic regression analysis showed that extent of disease, presence of ascites, CA-125 value, size of tumor, stage and histologic types were independent predictors of optimal cytoreduction. After adjusting the effects of covariates on multiple logistic regression analysis, mid preoperative extent of disease, and low or mid intra-operative extent of disease were associated with more than 900 and 100 times increased odds of optimal cytoreduction (p-values < 0.001).
Conclusion: Pre-operative mid extent of disease and intraoperative low or mid extent of disease were statistically significant predictors of optimal cytoreduction.
Disclosure: Nothing to disclose

EP925

Cytoreductive surgery in Stage IIIC and IV epithelial ovarian cancer, a prospective cohort study

V. Mitsopoulos, A. Innamaa, J. Lippiatt, N. Plevris, I. Biliatis
Gynaecological Oncology, Poole Hospital NHS Trust, Poole, United Kingdom

Introduction / Background: The aim of this study is to estimate the proportion of patients with advanced ovarian cancer requiring radical surgery to achieve complete (no macroscopic residual disease) or optimal cytoreduction (residual diseaze less than 1cm).
Methodology: Data were collected prospectively on consecutive patients undergoing elective cytoreductive surgery for epithelial ovarian cancer (FIGO stage IIIC/IV) at Poole Hospital NHS Trust, a tertiary referral cancer center in the United Kingdom from July 2016 to March 2018.
Results: Over a 33-month period, 84 patients consecutive patients underwent primary (n=45) or cytoreduction after 3 or 4(n=33) or 6 cycles chemotherapy (n=6) [total n=39 in the neoadjuvant chemotherapy (NAC) group]. 32 patients underwent diahragmatic peritonectomy[26 in the primary debulking surgery(PDS) and 8 in the NAC group], 26 underwent bowel resection [22 in PDS, with 3 of them being total colectomies, and 5 in NAC, lymphadectomy (n=20), splenectomy (n=8 in PDS and n=1 in NAC group) and 21 additonal upper abdominal procedures performed, namely cholecystectomy, excision of disease from porta hepatis, lesser omentum, liver´s Glisson´s capsule, pylorous or duodenum.Complete and optimal cytoreduction was achieved in 60.98% and 25.61% respectively (combined rate 86.59%). Of interest, complete cytoreduction was achieved in 68.89% of patients with primary surgery, in contrast to 51.35% of patients in the NAC group. 5 patients (6.10%) underwent a futile laparotomy (3 in primary and 2 in NAC group).Without incorporating radical procedures, the combined rate of complete and optimal cytoreduction would have dropped to only 31.7%.
Conclusion: The majority of our patients required at least one radical surgical procedure to achieve complete cytoreduction emphasizing the importance of advanced surgical skills and effort in management of women with FIGO Stage IIIC and IV ovarian cancer.
Disclosure: Nothing to disclose

EP926

PAD enzymes as a candidate therapeutic target in ovarian cancer

S. O'Toole[1], H. Melarcode[2], S. Elbaruni[3], F. AbuSaadeh[4], M. Ward[5], M. Gallagher[6], L. Norris[1], S. Gray[7], J. O`Leary[6], B. Mohamed[1]
[1]Obstetrics and Gynaecology/Histopathology, Trinity College Dublin,Trinity St. James & Cancer Institute, [2]Obstetrics and Gynaecology/Histopathology, Trinity College Dublin, Dublin, Ireland, [3]University of Tripoli, Tripoli, Libyan Arab Jamahiriya, [4]Gynaecological Oncology, [5]Obstetrics and Gynaecology/Histopathology, Obstetrics and Gynaecology, [6]Histopathology, [7]Thoracic Oncology Research Group, Trinity College Dublin,Trinity St. James & Cancer Institute, Dublin, Ireland

Introduction / Background: Ovarian cancer (OC) is the fifth most common cancer among Irish women with a higher incidence rate than other European countries. OC patients have a good response rate to first line chemotherapy treatment. However, the prognosis for OC following treatment is very poor in the majority of OC patients, especially patients with stage III or IV disease on diagnosis. Patients respond well initially to chemotherapy but go on to develop recurrent chemoresistant disease. Peptidyl arginine deiminase type 4 (PADI4) performs post-translational modification and catalyzes the arginine residue to a citrulline in numerous protein substrates. Citrullination of histones, cytokeratin, antithrombin and fibronectin have been confirmed to be involved in abnormal apoptosis, altered coagulation, and disordered cell proliferation, all of which are main features of primary tumors and metastasis. As PAD enzymes have been shown to be elevated in certain human diseases including autoimmune diseases and cancer, we hypothesized that targeting this enzyme with small molecule inhibitors such as GSK484 and GSK199 could lead to a novel therapeutic strategy. Therefore, we propose to investigate their therapeutic potential in a panel of OC cell lines and *ex-vivo* explants.
Methodology: OC cell lines and ex-vivo explants were exposed to various concentrations of PADI4 inhibitors at different time points. PADI4 expression, cell cycle, viability, caspase 3 and 8 activation were measured using immunoblotting, immunohistochemistry and FACS analysis.
Results: We demonstrated an inhibition of PADI4, changes in cell cycle status, reduction in cell

viability and increased caspase 3,and 8. All these changes were concentration dependent.
Conclusion: Our study observed the role of PADI4 in cell proliferation, apoptosis. These results suggests that PADI4 is involved in cell growth and death. GSK484 and GSK199 presents a promising direction in the search for novel OC treatment strategies.
Disclosure: Nothing to disclose

EP927

Ex-vivo antitumor efficacy of PEGylated-targeted nanodiamonds for docetaxel delivery in ovarian cancer

B. Mohamed[1], S. O'Toole[1], S. Elbaruni[2], L. Norris[1], F. AbuSaadeh[3], S. Gray[4], J. O`Leary[5]
[1]Obstetrics and Gynaecology/Histopathology, Trinity College Dublin,Trinity St. James & Cancer Institute, Dublin, Ireland, [2]University of Tripoli, Tripoli, Libyan Arab Jamahiriya, [3]Gynaecological Oncology, [4]Thoracic Oncology Research Group, [5]Histopathology, Trinity College Dublin,Trinity St. James & Cancer Institute, Dublin, Ireland

Introduction / Background: The use of nanomaterial-based therapeutic systems is rapidly growing and covering several biomedical applications such as detection, diagnosis and treatment. Recently, nanodiamonds (NDs) have been demonstrated to have great potential as a multimodal imaging/therapy platform. NDs are attractive for use in drug delivery because of their rich surface chemistry. NDs enhance the ability of the drug to cross the cell membrane, increase intracellular drug delivery to the cancer cells, improve treatment efficacy, and decrease toxicity to normal cells or tissues. Docetaxel (DTX) “a chemotherapeutic agent” has very low neurotoxic effect to the cancer patients. Therefore, we hypothesized that targeting HER-3 positive ovarian cancer (OC) *ex-vivo* explants by loading NDs with DTX would improve drug targeting and delivery. Thus, we aimed to investigate the efficacy of ND-conjugated DTX on OC *ex-vivo* explants.
Methodology: Bare, uncoated NDs were PEGylated and functionalized with DTX (NDs/DTX) and conjugated with anti- HER-3 antibody. Dynamic Light Scattering (DLS) was used to measure the hydrodynamic diameter of individual NDs dispersed in solutions (NDs,ND/DTX/HER-3). *Ex-vivo* explants from OC patients were exposed to various concentrations of ND/DTX, ND/DTX/HER-3 and DTX for over 24 hour's incubation.
Cytotoxicity was examined by measuring cell viability changes, caspase 3 and caspase 8 activation and the molecular cell stress variation was investigated by examining the activation of transcription factor-2 (ATF-2).
Results: Significant alterations of the examined biological markers were detected in OC *ex-vivo* explants. As expected no nuclear translocation of ATF-2 was observed in the nuclei of untreated explants. Interestingly, the *ex-vivo* explants showed greater responses to NDs/DTX/HER-3 versus ND/DTX and DTX alone.
Conclusion:
Our study demonstrates that NDs loaded with DTX exert significant inhibitory activities on OC explants. Thus, the proposed drug delivery system of ND-conjugated chemotherapy represents a promising, biocompatible strategy for targeting and enhancing chemotherapy efficacy and safety.
Disclosure: Nothing to disclose

EP928

Kallikrein and plasmin in ascitic fluid require exogenous inhibitors in ovarian cancer and tuberculosis of uterine appendages

T. Moiseenko, L. Kozlova, O. Kit, E. Frantsiyants, L. Rozenko, N. Chernikova, M. Adamyan, I. Kaplieva, E. Surikova, V. Bandovkina, I. Neskubina, M. Ilchenko, K. Boyko, V. Gurnak, E. Agarkova

Rostov Research Institute of Oncology, Rostov-on-Don, Russian Federation

Introduction / Background: Ascites is the dominant syndrome in stage III-IV ovarian cancer (OC, cystadenocarcinoma), and the microvasculature is directly exposed to bradykinin. The kallikrein-kinin system (KKS), plasmin (P), and α2macroglobulin (α2M) are involved in the vascular status control. Our aim was to study parameters of KKS in the ascitic fluid (AF) in OC and tuberculosis of uterine appendages (TUA).
Methodology: AF was studied in OC patients (n=26, $T_{3\text{-}4}N_0M_0$), comparison group: AF of TUA patients (n=13); age 57.5±2.6, menopause. AF was considered an exudate due to its high protein content in OC and TUA. Levels of kallikrein (K), prekallikrein (PK), carboxypeptidase N (CPN), P, plasminogen (PG) and α2M were determined by spectrophotometry and ELISA. An exudate comes from vessels damaged by inflammation, so AF was compared with plasma of 32 healthy donors (N).
Results: PK/K balance in N was 7.4±0.5; in AF: in TUA 1.6±0.1, in OC 1.1±0.1. The ratio of kinin formation and degradation rates (K/CPN) in TUA was 195±10.3; in OC 129±6.8, vs. 60.4±3.8 in N. In low PK/K, KKS in AF cannot perform its regulatory and adaptive protective functions.
PG/P ratio was in TUA 2.2±0.1; in OC 0.6±0.1, vs. 2.3±0.2 in N. High P levels are dangerous for all structures, especially since the activity of α2M in OC was similar to N, and in TUA it decreased by 1.4 times, which was not enough in activated proteolysis.
K/ α2M and P/α2M ratios in TUA were 52.1±2.9 and 1.3±0.1, vs. 11.3±0.8 and 0.2±0.01 in N.
Conclusion: Period after the AF removal requires treatment with exogenous protease inhibitors, since the pathological functions of KKS and P in the continuing exudate will ensure the spread of the pathological process.
Disclosure: Nothing to disclose

EP929

Survival impact of different surgical strategies in advanced stages ovarian cancer. Analysis in a single center and self-criticism of our outcomes

J. Molero Vílchez[1,2], E. Martínez Lamela[1,3], S. Sancho García[4], E. Bancarali Rojnica[5]
[1]Oncologic Gynecology, Hospital Ntra. Sra. del Rosario, Madrid, [2]Oncologic Gynecology, Clinica TocoGyn, Alcalá de Henares, [3]Obstetrics and Gynecology, Hospital Universitario Infanta Leonor, [4]Radiation Oncology, Hospital Universitario Ramón y Cajal, [5]Pathology, Jiménez Ayala Institute, Madrid, Spain

Introduction / Background: To evaluate the influence of surgical strategy on survival for patients with stage III or IV epithelial ovarian cancer (EOC).
Methodology: Retrospective data collection in a single and reference cancer center. Currently the strategies in advanced ovarian cancer are changing. Our purpose was to analyse the Overall Survival (OS) and Disease Free Survival (DFS) comparing the ´state of the art´ procedures, Primary Debulking Surgery (PDS) and interval surgery after 3-6 cycles of Neoadjuvant Chemotherapy (NAC). Incidentally, we tested the risk factors for recurrence.
Results: Median age was 59 years (40-80). Exploratory laparoscopy was performed in 52 patients, 28 underwent PDS and 24 NAC. Median Laparoscopic Predictive Score (Fagotti) was 2.2 (0-6) and 5.8 (2-10) (p=0.00), median Peritoneal Carcinomatosis Index was 12.5 (2-18) y 18.3 (10-24) (p=0.00) and mean of Ca125 prior to surgery was 489 y 949 U/dL (p=0.00) respectively. Pelvic and para-aortic lymphadenectomy was performed in 20 patients during PDS (14 cases with involved nodes, 48%) and 19 patients during interval surgery NAC (in 6 cases with involved nodes, 25%)(p=0.11). The median Surgical Complexity Score (Aletti) was 5 and 6.5 (p=0.21), with 80 and 24 points in the Comprehensive Compilation Index (modified from Clavien-Dindo Classification) for complications respectively (p=0.13). There were not significant differences in the complete resection rate (61 vs 63%, p=0.99), DFS (12 vs 15 months, p=0.80), and 5-years OS (59 vs 25%, p=0.32). Complete surgery were significantly associated with a better OS in univariate analysis (Hazard ratio 3.34 (95% CI, 1.19-9.40, p=0.015).
Conclusion: A significative improvement of OS has been observed in relation with complete tumour resection (microscopial residual tumor). In this study, NAC is non-inferior to PDS and is an acceptable standard of treatment of advanced and bulky EOC.

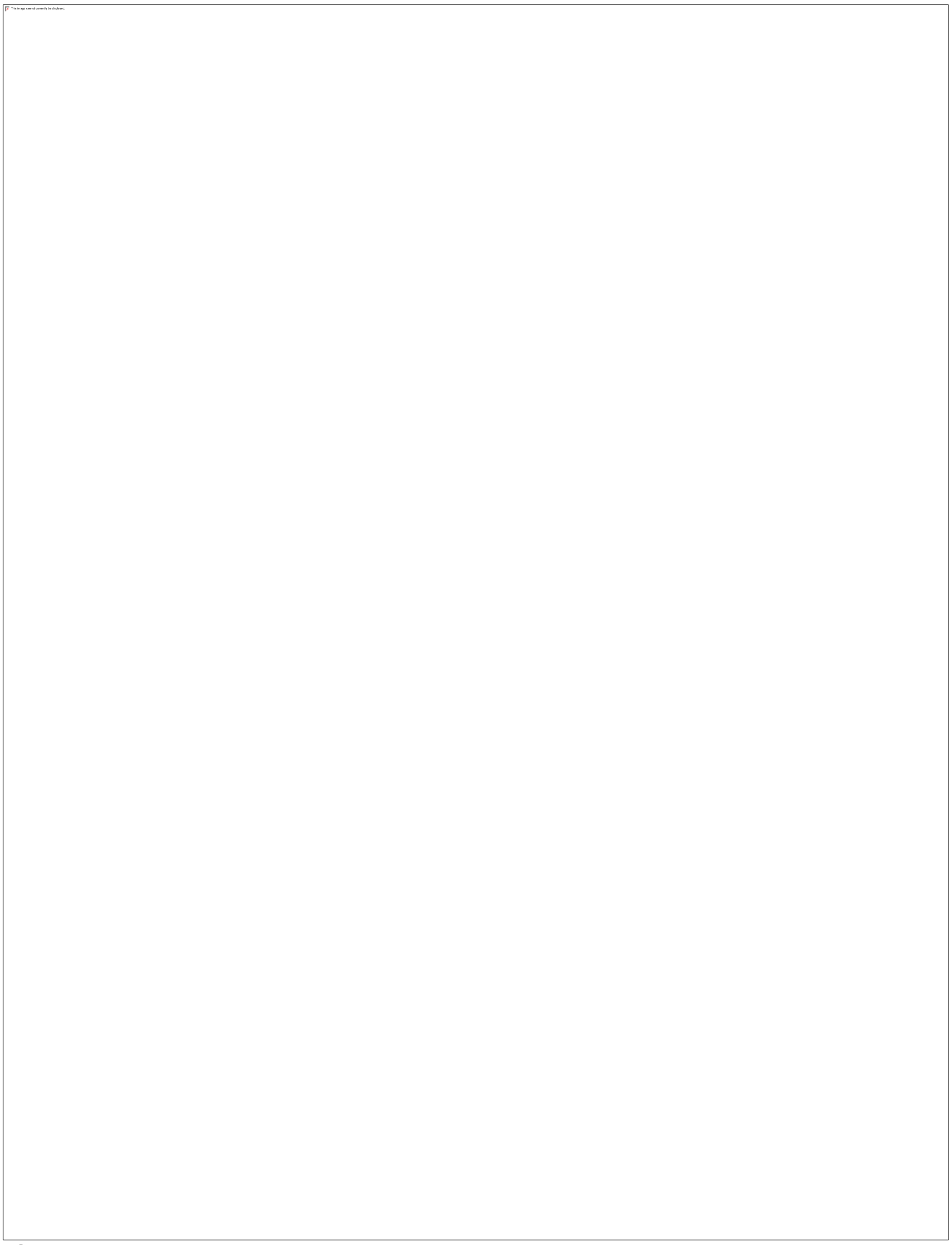

[Survival outcome after extensive cytoreductive surgery and chemotherapy in advanced stages ovarian c]

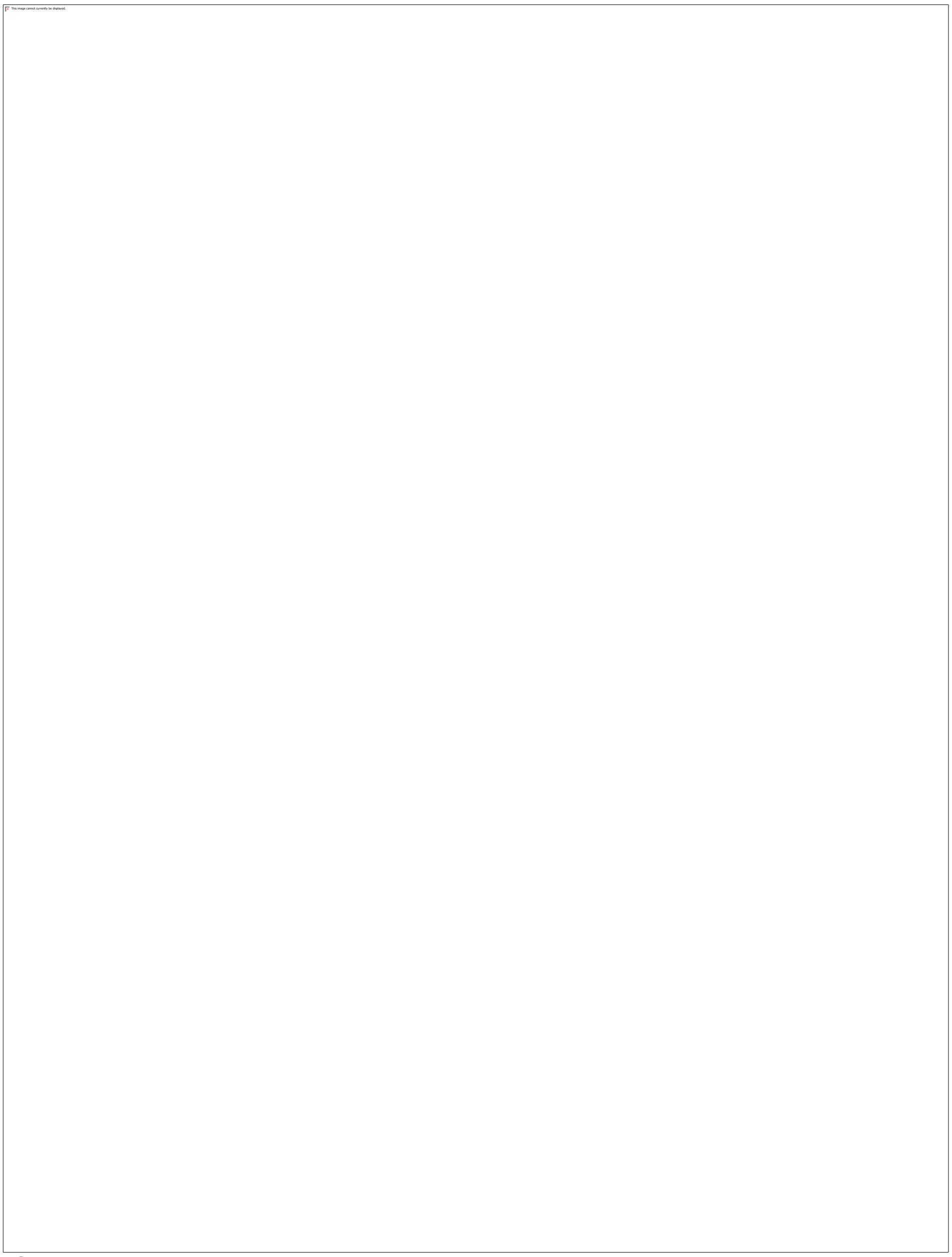

[Survival outcome after extensive cytoreductive surgery and chemotherapy in advanced stages ovarian c]

Disclosure: Nothing to disclose

EP930

The prognostic value of PARPexpression in high grade epithelial ovarian cancer

S. Molnár[1], L. Beke[2], G. Méhes[2], R. Póka[1]
[1]Department of Obstetrics and Gynecology, [2]Department of Pathology, University of Debrecen,

Debrecen, Hungary

Introduction / Background: In an attempt to clarify prognostic relevance of poly-(ADP)-ribose polymerase(PARP) expression we analyzed clinical data of 86 high-grade epithelial ovarian cancer cases in which PARP immunohistochemistry results were available.
Methodology: Immunostaining to highlight PARP protein expression was performed using a Leica Bond MAX Immunostainer (Leica Microsystems, Wetzlar, Germany). We applied the rabbit polyclonal anti-PARP antibody (ab6079 330, Abcam, Cambridge, UK) for the specific reaction. Intensity and distribution of immunostaining were assessed by light microscopy (Leica DM2500 microscope, DFC 420 camera, and Leica Application Suite V3 software; Leica) and evaluated with a four grade (0-3+) system. Median progression-free survival was generated for each semiquantitative group of PARP expression among chemotherapy-naive cases at the time of PARP immunohistochemistry.
Results: Eighty-six cases were chemotherapy-naive at the time of PARP immunohistochemistry, and 41 of them showed no PARP expression. Forty-five cases showed intermediate or high PARP expression. The median progression-free survival among patient without PARP staining was 16 months (Interquartile range; IQR 10,7-35,9 months) and in the PARP positive group was 12 months (IQR 6,1-21,8 months). The difference was significant by Log-Rank test (p=0,01). The median OS was in the PARP negative group 65 months (IQR 43,6-110,8 months) and in the PARP positive group 52 months (IQR 36,9-66,7 months. The difference was significant by Log-Rank test (p=0,28). Multiple comparisons confirmed that PARP expression results in a significant difference in PFS and OS achieved by first-line taxol-carboplatin chemotherapy.
Conclusion: The lack of PARP expression assessed by immunohistochemistry may predict longer progression free survival in ovarian cancer after adjuvant platinum based chemotherapy.
Disclosure: Nothing to disclose

EP931

Variations in BRCA screening and treatment patterns among ovarian cancer patients in Europe and the United States

M. Monberg[1], J. Hall[2], R. Moon[2], K. McLaurin[3], T. Dalvi[3]
[1]Merck Sharp & Dohme Corp., a Subsidiary of Merck & Co., Inc., Kenilworth, NJ, United States, [2]Adelphi Real World, Manchester, United Kingdom, [3]AstraZeneca, Gaithersburg, MD, United States

Introduction / Background: Recent updates to guidelines for the management of ovarian cancer (OC) in Europe and the US include recommendations for BRCA screening and the incorporation of new treatment options. This study sought to quantify regional differences in disease management of OC through analysis of clinical characteristics, treatment patterns and outcomes in Europe and the US.
Methodology: This chart review and cross-sectional study of patients actively receiving treatment for OC, collected data through physician-completed, detailed patient record forms. Data were collected from 2496 patients and 340 oncologists/ gynecologists in France, Germany, Italy, Spain, the UK (EU5), and the US between December 2017 and March 2018.
Results: Stage at initial diagnosis in this sample varied across EU5 countries and the US, with greater proportions of patients diagnosed at Stage III in EU5 (45%) than in the US (30%), and fewer diagnosed at Stage IV (47% in EU5 and 53% in US). The majority of patients were receiving 1L (38% EU5, 45% US) or 2L (54% EU5, 50% US) treatment at the time of data collection. Overall BRCA screening rates were 52% in the EU5 (ranging from 36% in the UK to 64% in Spain) and 46% in the US, with screening rates increasing across line of therapy. Most patients responded to 1L and 2L , although response rates were higher to 1L (76%) than 2L (63%). Bevacizumab use across lines of therapy and PARP inhibitor use in 2L maintenance varied by country.
Conclusion: This study documents regional variances in clinical characteristics at initial diagnosis, BRCA screening rates, and regimens used in frontline and frontline maintenance within the EU5, and when comparing EU5 to the US.

	Global	France	Germany	Italy	Spain	UK	EU5	USA
Total N	2496	407	400	363	361	335	1866	630
Overall BRCA screening rate; %	51%	58%	61%	41%	64%	36%	52%	46%
Received Bevacizumab within completed 1L; %	46%	56%	56%	51%	37%	44%	49%	33%
Initiated 2L; N	1492	253	250	228	225	`92	1148	344
2L+ BRCA screening rate; %	54%	63%	66%	43%	66%	45%	57%	45%
Initiated 2L maintenance; N, %	259, 17%	54, 21%	39, 16%	47, 21%	27, 12%	7, 4%	174, 15%	85, 25%
Of those initiating 2L maintenance, received PARP; N, %	115, 44%	32, 59%	16, 41%	12, 26%	17, 63%	5, 71%	82, 47%	33, 39%
Initiated 3L+; N	177	37	28	35	16	30	146	31
3L+ BRCA screening rate	67%	78%	64%	60%	69%	73%	69%	58%

[Table]

Disclosure: M.J. Monberg is an employee of Merck Sharp & Dohme Corp., a subsidiary of Merck & Co., Inc., Kenilworth, NJ, USA. K. McLaurin and T. Dalvi are employees of AstraZeneca.

EP932

Impact of FDG-PET/CT in initial staging of primary advanced ovarian cancer

B. Morillas[1], A.P. Caresia[2], L. Ribot[1], Y. Garcia[3], C. Codina[4], I. Costa[5], M. Corona[1], J. Antoni[1], Y. Canet[1]
[1]Gynecology, [2]Nuclear Medicine, [3]Medical Oncology, [4]Radiology, [5]Pathology, Parc Taulí Hospital Universitari, Institut d'Investigació i Innovació Parc Taulí I3PT, Univeristat Autònoma de Barcelona, Sabadell, Spain

Introduction / Background: Primary surgical cytoreduction followed by systemic chemotherapy is the preferred initial management for women with stage III or IV ovarian cancer. Patients considered unresectable due to the location and volume of disease involvement or deemed unoperable because of medical comorbidities at the time of diagnosis may be considered for neoadjuvant chemotherapy. The aim of the study is to compare the pre-surgical staging findings in patients with locally advanced ovarian neoplasia by CT and 18F-FDG-PET/CT in our institution.
Methodology: We included 34 locally advanced ovarian cancer patients (mean age 62.6, range 40-88) (IOTA M ultrasound findings) at initial staging. CT and PET / CT were performed and findings are compared. We analyzed changes in FIGO staging. Distant metastasis were pathologically confirmed

when feasible.
Results: PET / CT revealed distant metastasis in 20/28 patients (60.7%), mainly in supradiaphragmatic lymph nodes (18/20 patients), associated with addicional metastasis in pleura, inguinal adenopathies, liver or bone. FIGO stage was modified in 35.3% (12/34 patients) according to PET/CT. All of them were upstaged because of additional peritoneal or distant metastases. In the remaining 22/34 patients (64.7%) CT and PET/CT results were concordant and no change in clinical management was made.
Conclusion: Initial staging of advanced ovarian cancer can be improved using FDG-PET/CT instead of diagnostic CT, mainly due to the detection of unsuspected distant metastasis. Supradiaphragmatic lymph nodes were the most frequent site of metastasis. FDG-PET/CT could make a decisive contribution to final management at least in a third of patients.
Disclosure: Nothing to disclose

EP933

Thromboembolic events post cytoreductive surgery in patients with ovarian, fallopian tube or primary peritoneal cancer

D. Atallah[1], K. Abi Farraj[1], M. Moubarak[1], N. El Kassis[1], G. Chahine[2]
[1]Obstetrics and Gynecology, [2]Oncology, Saint Joseph University/Hôtel Dieu de France University Hospital, Beirut, Lebanon

Introduction / Background: To determine the prevalence of deep vein thrombosis and pulmonary embolism after an extensive cytoreductive surgery for ovarian or fallopian tube cancer, the associated risk factors and to suggest preventive measures pre- and postoperatively to reduce these risks.
Methodology: A retrospective study was conducted at Hôtel-Dieu de France University Hospital and included all patients older than 16 years and receiving a cytoreductive surgery for ovarian, fallopian tube or primary peritoneal cancer between 2004 and 2017.
Results: 123 patients were included. Mean age was 55 years. The prevalence of postoperative thromboembolic events in the studied population was 8.9%. Deep vein thrombosis and pulmonary embolism were found in 6.5% and 4.1% of cases respectively. A correlation was found between the presence of venous catheter and the occurrence of thromboembolic events with a p value = 0.035 (OR = 4, IC [1.019-16.197]).Also, partial colectomy with anastomosis, cholecystectomy and appendectomy were found to be risk factors (0.001, 0.021 and 0.045 respectively). We found a correlation between hospital and intensive care stay and the duration of immobilization as well as a correlation between weight and hospital stay (p value= 0.02). Date of initiation of postoperative thromboprophylaxis was related to the amount of intraoperative bleeding (p = 0.024).
Conclusion: Avoiding the placement of central venous catheter, reducing the patient weight preoperatively, encouraging the early mobilization, reducing the hospital stay as well as stay in intensive care unit, controlling and limiting the intra- and postoperative bleeding are measures that contribute to reduction of risk of thromboembolic events occurrence.
Disclosure: Nothing to disclose

EP934

Predictive factors of postoperative complications after cytoreductive surgery for ovarian cancer

D. Atallah[1], Y. Sarkis[2], F. Richa[2], G. Sleilaty[3], M. Moubarak[1], N. El Kassis[1], G. Chahine[4], V. Chalhoub[2]
[1]Obstetrics and Gynecology, [2]Anesthesiology, [3]Cardiovascular and Thoracic Surgery, [4]Oncology, Saint Joseph University/Hôtel Dieu de France University Hospital, Beirut, Lebanon

Introduction / Background: Ovarian cancer is the leading cause of death from gynecological cancer for women. The standard treatment consists of an extensive cytoreductive surgery followed with adjuvant chemotherapy. This study aims to identify the common postoperative complications as well as to define predictive factors of their occurrence.
Methodology: This study was conducted at Hôtel-Dieu University Hospital in Lebanon between October 2017 and October 2018. All patients older than 18-year-old who underwent cytoredcutive surgery for ovarian cancer were followed up from the postoperative admission in the intensive care unit till discharge from hospital or at least for 30 days. Correlations between perioperative characteristics and complications were searched and analyzed.
Results: 40 patients were included. The mean age was 55 years old. The mean surgical complexity score was 5. Major complications have occurred in 32% of cases. They were associated with neoadjuvant chemotherapy (p = 0.009), elevated surgical complexity (p = 0.037), need for intraoperative transfusion and stay at intensive care unit more than 48 hours (p = 0.05). Complications were infectious, hemodynamic, pulmonary, digestive and surgical. Need for parenteral nutrition was significantly correlated with longer operative time and neoadjuvant chemotherapy. No correlation was found between occurrence of complications and the following parameters: age, stage, APACHE II score, Charlston Comorbidity index and preoperative albuminemia.
Conclusion: Cancer stage, neoadjuvant chemotherapy, high surgical complexity, need for transfusions, delayed extubation and stay at intensive care unit more than 48 hours were predictive factors of higher postoperative morbidity in patients receiving cytoreductive surgery for ovarian cancer.
Disclosure: Nothing to disclose

EP935

The inflammatory response following HIPEC: a novel therapeutic window

K. Mulligan[1], S. Cunningham[2], M. Wilkinson[1], K. Glennon[1], J. Mulsow[3], A. Duffy[4], L. Lynch[2], D. Brennan[1,5]

[1]Department of Gynaecological Oncology, UCD School of Medicine, Mater Miseraecordiae University Hospital, [2]Innate Immunity Group, School of Biochemistry and Immunology, Trinity College Dublin, [3]Peritoneal Malignancy Institute, [4]Department of Medical Oncology, Mater Misericordiae University Hospital, [5]Systems Biology Ireland, UCD School of Medicine, Dublin, Ireland

Introduction / Background: HIPEC improves overall survival in patients with stage III epithelial ovarian cancer after neoadjuvant therapy compared to interval cytoreductive surgery (CRS) alone. The immediate impact of HIPEC on the systemic immune response has not been well characterised. We sought to document the impact of cisplatin-based HIPEC on the systemic immune response.
Methodology: Longitudinal analysis of neutrophil to lymphocyte ratio (NLR) and monocyte to neutrophil ratio (MLR) results in 22 age and stage matched patients with stage IIIc EOC were examined. Flow cytometric analysis (FACs) was performed on intra-operative PBMCs before and after HIPEC in 3 patients. Independent sample t test and one way ANOVA was used to analyse results using SPPS version 24.
Results: 9 patients had primary CRS, 8 had interval CRS+ cisplatin HPEC (50mg/m^2 at 40℃ for 60 min), and 5 had interval CRS with no HIPEC. All patients had aggressive CRS and an CC0 resection. The interval CRS (no HIPEC) group had a significantly lower day 1 NLR compared to interval CRS HIPEC group (p=0.047). The NLR of all groups was similar by day 4. The MLR in the three groups was similar at all time points. FACs of peripheral CD3$^+$ T Cells demonstrated that HIPEC increased circulating CD8$^+$ T Cells and reduced expression of the immune checkpoint protein TIGIT (p< 0.05).
Conclusion: Cisplatin-based HIPEC causes an rapid and short-lived change in the systemic immune response. Our initial findings that HIPEC alters the expression of immune checkpoint proteins on CD8$^+$ T cells suggests that further investigations of the combination of HIPEC and immunotherapy may be warranted.
Disclosure: Nothing to disclose

EP936

A feasibility study of gemcitabine and bevacizumab in women with platinum-resistant recurrent ovarian cancer

S. Nagao[1], A. Kogiku[1,2], M. Ota[1], K. Yamamoto[1], M. Narita[1], K. Shimada[1], H. Nagazawa[1], T. Shibutani[1], K. Yamamoto[1], T. Jimi[1], H. Yano[1], M. Kitai[1], T. Shiozaki[1], K. Matsuoka[1], S. Yamaguchi[1]
[1]Gynecologic Oncology, Hyogo Cancer Center, Akashi, [2]Obstetrics and Gynecology, Nishi Kobe Medical Center, Kobe, Japan

Introduction / Background: In the AURELIA trial, adding of bevacizumab to chemotherapy significantly improved response and survival without problematic toxicity in women with platinum-resistant ovarian cancer. However, information about bevacizumab in combination with gemcitabine is insufficient. We conducted a feasibility study to assess clinical activity and toxicity.
Methodology: Women with platinum-resistant recurrent ovarian cancer who received one to three regimens of platinum-based chemotherapy were registered in this study. They received 15mg/kg of bevacizumab intravenously on day 1 and gemcitabine 1000mg/m^{2}of gemcitabine intravenously on day 1 and 8 every 21 days to disease progression or unacceptable toxicity. The primary endpoint was completion rate of 3 cycles of chemotherapy. The secondary endpoints were clinical response rate, progression free survival,overall survival, and dose-limiting toxicity including bowel perforation, febrile neutropenia, and grade 4 thrombocytopenia. This study is registered with UMIN-CTR, number UMIN000016619.
Results: Between April 1, 2015 and December 31, 2018, 19 patients were enrolled into this study. There were 12 high grade serous, 1 endometrial, 4 clear cell, and 2 other types of carcinoma. Eighteen of 19 (95%) received 3 or more, and 9 (47%) received 6 or more cycles of study treatment. Objective responses were observed in 8 of 19 patients (42% overall; complete response 16%, partial response 26%), with an additional 8 (42%) having stable disease. Hematologic toxicity was neutropenia grade 3/4 in 9 (47%), anemia grade 3/4 in 2 (11%), thrombocytopenia grade 3/4 in 1 (5%). Non-hematologic toxicity included grade 3 hypertension in 1 (5%), grade 3 protein urea in one (5%) and possibly-related grade 3 interstitial pneumonia in 1 (5%). No patient encountered dose-limiting toxicity. Median progression free survival was 5.1 months, and median overall survival was 21.3 months.
Conclusion: Combination chemotherapy with gemcitabine and bevacizumab is feasible in women with platinum-resistant recurrent ovarian cancer.
Disclosure: Nothing to disclose

EP937

Frailty assessment in treatment for elderly patients with ovarian cancer

R. Nakamura, K. Takahashi, N. Oshima, K. Wakana, N. Miyasaka
Perinatal and Women's Medicine, Tokyo Medical and Dental University, Tokyo, Japan

Introduction / Background: We are faced with an increasing proportion of elderly ovarian cancer patients. It is still debating what is adequate treatment for such patients. The aim of this study was to analyze the feasibility of standard surgery and chemotherapy in the elderly ovarian cancer patients.
Methodology: We evaluated 32 patients of 70 years old or older who were treated at our department between 2009 and 2017 retrospectively. Background, type of surgical procedure, complications, schedules of chemotherapy, and clinical outcome were registered.
Results: The average age was 76 years old. Their median performance status (PS) is 1(0-4). The number of patients was 17 in stage I, 4 in stage II, 8 in stage III and 3 in stage IV according to the International Federation of Gynecology and Obstetrics (FIGO). In malignant tumor, the histologic type was serous type (n＝9), clear-cell type (n=4), endometrioid type (n=4), mucinous type (n=3) and other type (n=2). As the primary treatment, twenty-nine patients were performed surgery, and three received chemotherapy. Among the patients who underwent surgery, blood transfusion was needed in 28%. Ileus occurred in 13.7%. Thirteen patients received chemotherapy before or after surgery. The rate of

dose reduction, treatment delay, and discontinuation were 38%, 61%, and 38%, respectively.
Conclusion: Elderly patients are higher rate of advanced cancer and complications during treatment. We need a screening tool, for example CSGA (Cancer Specific Geriatric Assessment), to select adequate therapy.
Disclosure: Nothing to disclose

EP938

Plasmajet In ovarian cancer surgery

P. Natarajan, R. Macdonald, B. Decruze
Gynaecology Oncology, Liverpool Women's Hospital, Liverpool, United Kingdom

Introduction / Background: Ovarian cancer is the second most commonest gynaecological cancer but the leading cause of mortality among the gynaecological cancers. The primary treatment is surgery and adjuvant chemotherapy which is possible in early stage cancers. In advanced stage cancers primary treatment is associated with increased morbidity and mortality. Neoadjuvant chemotherapy with primary chemotherapy and interval debulking surgery as similar outcome to primary surgery with fewer complications.Optimal cytoreduction is associated with improved prognosis however it may be at the expense of stoma, spelenectomy, peritoneal stripping and other extensive surgeries. Plasmajet is an energy device using plasma and reduce the extensive surgery avoding its complications whilst retaining Outcome. There are no studies looking at correlation between clinical findings and Histological findings after treatment with plasmajet. in this study we looked hitological details of the peritonuem after treatment with Plasmajet.
Methodology: This is a prospective conducted in LWH in Liverpool UK where in the recruitment began in January 2019. In women who were diagnosed to have stage 3 or 4 Ovarian cancer are eligible for Plasmajet study. A 2x2 cm involved peritonuem is excised during surgery either at primary or interval debulking surgery. The specimen is sent to the Gynaecological Pathologist . Tumour depth involvement , plasmajet penetration, Viable tumour and damage to other structures are also noted
Results: 10 cases have been recruited until May 2019 . 2 cases did not have tumour and the plasmajet penitration was good and no futher damage was seen. In 2 cases the penteration of Plasmajet and disease on the peritonem was the same and in 2 cases plasmajet depth was more. In 4 cases the depth of disease involvement was more than the plasmajet spread.
Conclusion: Plasmajet may be used in surgical excision whilst ablation may be not effective.
Disclosure: Nothing to disclose

EP939

Neoadjuvant chemotherapy (NAC) for patients with carcinomatosa peritonitis improves prognosis of those patients

M. Nishimura, T. Kagawa, A. Mineda, A. Abe
Obstetrics and Gynecology, Tokushima University, Tokushima, Japan

Introduction / Background: For patients with carcinomatosa peritonitis who are diagnosed to be difficult to completely remove in the first surgery, our hospital is performing tumor reduction surgery after neoadjuvant chemotherapy (NAC).
Methodology: NAC has performed 4-5 courses of dose dense TC, and when it is judged that complete removal is possible, interval debulking surgery (IDS) is performed. Of the 39 cases of advanced ovarian cancer (stage III / IV) whose treatment was started in 2013-2018, 17 cases underwent surgery (PDS) and 22 cases underwent NAC for first treatment. The 22 cases consisted of 2 cases of test laparotomy, 2 cases of partial resection at the first surgery (PDS) and 18 cases of

NAC.
Results: Seventeen of the 18 NAC cases were diagnised by needle biopsy. The response rate for NAC was 94% (17/18). The complete resection rate at IDS was 72% (13/18). Recurrence was observed in 46% (6/13) of the cases achieving complete resection and 80% (4/5) in the remaining cases. The response rate of subsequent chemotherapy was 100% (5/5) in 5 patients with large residual tumors with PDS, and no recurrence was found in 3 patients who could be completely resected with subsequent IDS. The 5-year survival rate was 92% in the PDS group and 48% in the NAC and IDS group. The survival rate of patients with completely resection was 90%, and that of the remaining tumor was 18%. In addition, even in the case of patients with carcinomatosa peritonitis, 5 year survival rate was 85% in cases where there was no residual tumor at IDS.
Conclusion: The prognosis was extremely good if complete resection with NAC and IDS was possible even in cases where complete resection was difficult in the first surgery. There were no surgical complications or deaths. NAC is considered a very effective treatment for carcinomatosa peritonitis cases.
Disclosure: Nothing to disclose

EP940

Chemokine expression in patients with ovarian cancer or benign ovarian tumors

M. Nowak[1], Ł. Janas[1], M. Soja[1], E. Głowacka[2], K. Szyłło[1], M. Misiek[3], M. Klink[4]
[1]Department of Operative Gynecology and Gynecologic Oncology, [2]Laboratory Diagnostics Centre, Polish Mothers' Memorial Hospital - Research Institute, Lodz, [3]Department of Gynecology, Holy Cross Cancer Centre, Kielce, [4]Institute of Medical Biology, Polish Academy of Sciences, Lodz, Poland

Introduction / Background: Chemokines play a crucial role in tumor growth and progression according to proangiogenic and immunosuppressive acting. This study was aimed to investigate the serum levels of selected chemokines in patients with ovarian cancer or benign ovarian tumors to point on their role in tumorigenesis and the potential use in preoperative diagnosing of adnexal mass.
Methodology: The study group consisted of 59 women: 17 epithelial ovarian cancer (EOC) patients (7 in early and 10 in advanced stage) and 42 with benign ovarian tumors (12 endometriotic and 30 other epithelial). We measured in sera obtained preoperatively the level of CA125 and the panel of 5 chemokines: CX3CL1/Fractalkine, CXCL1/GRO-α, CXCL12/SDF-1, CCL20/MIP-3α and IL-17F, using chemiluminescence method with multiplexed bead based immunoassay.
Results: CX3CL1 was significantly elevated in sera of ovarian cancer patients compared to women with benign ovarian tumors (13,3 +/- 7,9 pg/ml vs 6,7 +/- 3,0 pg/ml, p< 0,005). When FIGO stage was taken into consideration we found that only advanced cancer patients showed significantly higher concentrations. The significant elevation of CXCL1, both in early and advanced stage of cancer vs benign tumors was also observed (36,5 +/- 18,4 pg/ml vs 17,6 +/- 8,7 pg/ml, p< 0,01). The similar pattern was present with standard ovarian cancer marker - CA125. In our patients with endometriotic cysts CA125 levels were also significantly higher than in women with other benign tumors whereas both mentioned above chemokines had similar serum titers in patients with endometriotic vs other benign ovarian cysts.
Conclusion: CX3CL1 and CXCL1 are elevated in sera of EOC patients what points on their role in cancer development. Moreover, they might be useful in preoperative differential diagnosis of ovarian tumors, especially as they were not elevated in cases of endometriosis.
Disclosure: Nothing to disclose

EP941

Synchronous ovarian and endometrial cancers; a 10-year retrospective review in a UK tertiary

centre

H. Fairley[1], D. Blake[1,2], S. Rundle[1], A. Fisher[1], R.L. O'Donnell[1,2]
[1]Northern Gynaecological Oncology Centre, Gateshead, UK, [2]Northern Institute for Cancer Research, Newcastle University, UK, Newcastle upon Tyne, United Kingdom

Introduction / Background: The epidemiology of synchronous ovarian (OC) and endometrial cancers (EC) is poorly understood with conflicting hypotheses. Prognosis is uncertain and there is a need to understand if it is advantageous for patients with concordant histology to be treated as two synchronous primaries or a metastatic single primary. There are significant clinical implications with either approach. This study aimed to: 1) describe the incidence and survival outcomes of synchronous primaries, 2) Consider survival outcomes to matched case-control cohorts.
Methodology: All cases of synchronous OC and EC 2007-2017 were identified from the departmental database. Patient demographics alongside clinicopathological data were collated. Survival analysis using 1:1 case-controls for isolated OC and EC, matched for age, diagnosis year, FIGO Stage, histological subtype, grade and surgical outcome was undertaken.
Results: 72 cases of synchronous cancers represented 3.6% of total OC and 2.7% of total EC cases in the study period. 39(54%) presented with presumed/confirmed OC with asymptomatic incidental diagnosis of EC; whilst 46% presented with symptomatic, biopsy proven EC, with incidental diagnoses of tubo-ovarian (88%), or primary peritoneal cancer (12%). Concordant histological subtype was seen in 43(60%) cases, of which 32(74%) were endometrioid. ≥2 antigens in an IHC panel was discordant in 89% of such cases.
Overall survival (OS) was worse in synchronous cases compared to EC case controls (p=0.0419), but no significant difference was observed between synchronous cases and OC controls (p=0.6235, log-rank). In OC cases with concordant EC morphology where endometrial metastases would have resulted in higher stage (n=19), subgroup analysis suggested improved OS in synchronous cases compared with higher stage OC controls (p=0.0282, Wilcoxon).
Conclusion: Synchronous OC and EC cancers occur infrequently with significant challenge in cases with concordant histology. Survival analysis suggests at least equivalent prognosis to matched ovarian cancers. An expanded dataset is needed to establish treatment algorithms.
Disclosure: Nothing to disclose

EP942

Incorporating tumour biology into follow-up strategies in ovarian cancer: can tumour CA125 status dictate utility of serum CA125 in the detection of recurrence?

E. Nevins[1], S. Rundle[1], M. Adishesh[1], P. Korompelis[1], R.L. O'Donnell[1,2]
[1]Northern Gynaecological Oncology Centre, Gateshead, UK, [2]Northern Institute for Cancer Research, Newcastle University, UK, Newcastle upon Tyne, United Kingdom

Introduction / Background: Historically, patients treated for epithelial ovarian cancer (EOC) undergo long-term hospital-based follow-up with serial serum CA125 (sCA125) measurement. It is however unclear whether earlier detection of recurrence through sCA125 monitoring alters survival. This uncertainty is reflected in the variances in follow-up practices.
The aim of this study was to investigate the predictive value of sCA125 at recurrence stratified by tumour CA125 (tCA125) status at diagnosis, determined by immunohistochemistry.
Methodology: All patients diagnosed with EOC at the NGOC 2012-2013 were identified from prospectively maintained data. Demographic and clinico-pathological details, including 5-year survival were collated. Correlation and survival analysis were undertaken stratified by tCA125 status.
Results: 207 patients with available tCA125 underwent treatment for EOC. 176 were FIGO stage IIIC or IV and 184 were high grade serous cancers.
Median sCA125 in the tCA125 positive group was 761 iu/L and in the tCA125 negative group was 151 iu/L (p=0.01).
The median follow-up period was 32 months in which time 181 (87%) developed recurrent disease. sCA125 was elevated (≥twice normal value) at relapse in 131/160 (82%) tCA125-positive group and in 4/21 (19%) tCA125-negative group. Median sCA125 at recurrence was 436 iu/L in the tCA125-positive subgroup in comparison to only 23 iu/L in the tCA125-negative group (p=0.28).

Conclusion: The movement towards precision medicine with treatment pathways dictated by tumour biology is upon us and the utility of tumour biology in the diagnosis of recurrence should not be overlooked. A prospective study powered to include proportional representation of tCA125-negative patients may be justified given the substantial differences in the values demonstrated in this study.
Disclosure: Nothing to disclose

EP943

Prognostic significance of pretreatment serum carcinoembryonic antigen (CEA) level in epithelial ovarian cancer

Y.-C. Ou[1,2], H. Lin[2]
[1]Department of Obstetrics and Gynecology, Chang Gung Memorial Hospital at Chiayi, [2]Kaohsiung Chang Gung Memorial Hospital, Kaohsiung, Taiwan

Introduction / Background: To evaluate the prognostic significance of pretreatment serum CEA level in epithelial ovarian cancer.
Methodology: This retrospective cohort study was carried out in 326 epithelial ovarian cancer patients at Kaohsiung Chang Gung Memorial hospital between 2008-2016. All patients had histopathological confirmed diagnosis and pretreatment measurements of serum CA125 and CEA value. Data acquired from medical records including age, FIGO stage, Histology type, tumor size and debulking status.
Statistical analysis: The difference between proportions was evaluated by chi-square test. Univariate survival curves for progression-free survival (PFS) and overall survival (OS) were estimated using Kaplan-Meier method. Multivariate Cox regression analysis was used to compare independent prognostic factors for PFS and OS. SPSS (version 20) was used for all statistical analyses.
Results: Our analysis showed the elevated pretreatment serum CEA value (cutoff 5 ng/ml) is an independent prognostic factor in epithelial ovarian cancer. These patients has lower PFS and OS compared with patients with normal CEA value.
In multivariate analysis, FIGO stage, pretreatment CEA and CA125 value and debulking status are independent prognostic factors.
The finding of CEA value as an independent prognostic factor was exception of tradition.
CEA may carry prognostic information and this result may indicate that patients showing elevated value of pretreatment CEA could be included in high-risk group for recurrence.
Currently, this study included extended and comprehensive database of relationship between epithelial ovarian cancer and serum CEA value.
Conclusion: Our study included all epithelial ovarian cancer patients with subgroup analysis and the detection of serum CEA value provided the convenience in clinical practice.
Elevated pretreatment serum CEA value is an independent prognostic factor in epithelial ovarian cancer in both univariate and multivariate analysis.
Disclosure: Nothing to disclose

EP944

Isolated lymph node recurrence in epithelial ovarian cancer: a recurrence with better prognosis?

L. Ouldamer[1,2]
[1]Gyn Oncology, CHU Tours, [2]INSERM UMR 1069, Tours, France

Introduction / Background: The aim of this study was tocompare in patients managed for epithelial ovarian cancer; overall survival (OS) between women with isolated lymph node recurrence (ILNR) to those with isolated peritoneal localization of recurrence (ICR).

Methodology: Data from 1,508 patients with ovarian cancer were retrospectively collected, from January 1, 2000 to December 31, 2016, from the FRANCOGYN database, regrouping data from 11 centers specialized in ovary treatment. Median overall survival was determined using the Kaplan-Meier method. .Patients included had a first recurrence defined as ILNR or isolated peritoneal recurrence during their follow up.
Results: 79 patients (5.2%) presented ILNR, and 247 (16.4%) patients recurred with isolated carcinomatosis.
complete lymphadenectomy was more achieved in ILNR group vs ICR group (67.1% vs 53.4%, p=0.004) and the number of pelvic lymph nodes involved was higher (2.4 vs 1.1, p=0.008). The number of involved pelvic LN was an independent predictor of ILNR (OR = 1.231, 95% CI [1.074-1.412], p = 0.0024). The 3-year and 5-year OS in the ILNR were 85.2% and 53.7% respectively, compared to 68.1% and 46.8% in patients with ICR. There was no significant difference in terms of OS after initial diagnosis (p = 0.18). 3- year and 5-year OS after the diagnosis of recurrence were 62.6% and 15.6% in the ILNR group, and 44% and 15.7% in patients with ICR (p = 0.21).
Conclusion: ILNR does not seem to be associated with a better prognosis in terms of OS
Disclosure: Nothing to disclose

EP945

Lymphovascular invasion in ovarian cancer

L. Ouldamer[1,2]
[1]*Gyn Oncology, CHU Tours,* [2]*INSERM UMR 1069, Tours, France*

Introduction / Background: The aim of this study was to evaluate the impact of lymphovascular space invasion (LVSI) on overall survival (OS) and recurrence-free survival (RFS) in patients treated for epithelial ovarian cancer (EOC).
Methodology: Retrospective multicentre study of the research group FRANCOGYN between January 2001 and May 2016. All patients managed for EOC and for whom the presence or absence of LVSI was specified, were included. Patient's characteristics with LVSI (LVSI-1) were compared to those without LVSI (LVSI-0). We evaluated the presence of independent risk factors of LVSI. survival analysis were performed in all population and according to frequent pathological subtypes.
Results: 493 patients were included. Among them, 164 patients had LVSI (33,3%). More LVSI were observed in advanced stage (p< 0,0001), residual disease at the end of surgery (p=0,01), platinum low sensitivity (p=0,03), overall recurrence (p< 0,0001), bilateral ovarian involvement (p=0,003), ovarian capsule involvement (p< 0,0001), positive peritoneal cytology (p< 0,0002), omentum involvement (p< 0,0001), pelvic and para-aortic lymph node involvement (p< 0,0001). Independent risk factors for LVSI were advanced stage (p< 0,001) and ovarian capsule involvement (p< 0,001) in multivariate analysis. LVSI affected OS (p< 0,0002) and RFS (p< 0,0001), OS for early stage (p=0,04) and RFS for advances stage (p=0,007). The related factors to OS were residual disease at the end of surgery (p=0,03), early stage (p=0,0004), presence of LVSI (p=0,04) and pathological type. LVSI and estrogen receptors (ER) affected the RFS of high-grade serous tumors (p=0,0005) with advanced stage (p=0,09) and ER+ (p=0,02) as independent risk factors in multivariate analysis. LVSI affected RFS of endometrioid tumors (p=0,006) and low-grade serous tumors (p=0,018) as well as OS and RFS of mucinous tumors (p< 0,0001).
Conclusion: LVSI in EOC has an impact on OS and RFS. It can be considered as a major prognostic factor to consider in patients with ovarian cancer.
Disclosure: Nothing to disclose

EP946

Differences in correlation of Progression-Free Survival and Overall Survival by clinical variables in epithelial ovarian cancer

E.S. Paik, M. Kang, J.H. Lee, J.H. Kang, S.Y. Jeong, M.S. Kim, Y.-Y. Lee, T.-J. Kim, J.-W. Lee, B.-G. Kim, D.-S. Bae, J. Seo, C.H. Choi
Samsung Medical Center, Seoul, Republic of Korea

Introduction / Background: To investigate significance of PFS as optimal surrogate endpoint for OS, and the differences in correlation of PFS and OS by different clinical variables in epithelial ovarian cancer (EOC) patients.
Methodology: The clinical records of 1134 EOC patients treated at Samsung Medical Center between 2002 and 2015 were retrospectively reviewed. The primary outcome of this study was to evaluate the surrogacy of progression-free survival (PFS) for overall survival (OS) by clinical variables. Correlation analyses with all pair-wise comparison were performed to assess the association of PFS and OS for each clinical variable. After multivariate analysis for PFS and OS, scatter plot with hazard ratio (HR) (with 95% CI) of PFS and OS were drawn for clinical variables.
Results: Of the 1134 EOC patients, 611 (53.9%) experienced relapse and a further 417 (36.8%) died within a median follow-up period of 47 months (range, 3~177 months). For entire cohort, there is a significant linear correlation between PFS and OS (p-value < 0.0001), and the degree of correlation is high (Spearman correlation coefficient = 0.8243). In Z-test using Fisher's transformation, patients with early stage (I, II) (0.9059, p< 0.001), lower grade (0.9019, p< 0.001), and non-serous histology (0.8853, p< 0.001) showed higher correlation coefficient. In patients with no residual disease (0.8661, p< 0.001), no pelvic lymph node metastasis (0.8275, p< 0.001), no paraaortic lymph node metastasis (0.8267, p< 0.001), higher correlation coefficient was shown. In scatter plot for HR of PFS and OS, presence of residual disease, high grade, neo-adjuvant chemotherapy, lymph node metastasis were not located within estimated regression area implicating low correlation coefficient.
Conclusion: The treatment effect on OS is largely predictable according to that on PFS in EOC, especially for patients with early stage, low grade, non-serous, no residual disease, without lymph node metastasis.
Disclosure: Nothing to disclose

EP947

Recurrent ovarian cancer treated by secondary cytoreductive surgery and HIPEC

T. Panoskaltsis[1], C. Tzavara[2], N. Pallas[3], C. Karamveri[3], D. Kiziridis[4], C. Hristakis[4], V. Kyriakopoulos[3], A. Kalakonas[4], D. Vaikos[4], A.-A. Tentes[3]
[1]2nd Academic Department, Aretaieion, Medical School, Gynaecological Oncology Unit, National & Kapodistrian University of Athens, [2]Department of Hygiene, Epidemiology and Medical Statistics, National and Kapodistrian University of Athens, Medical School, [3]Department of Surgical Oncology, Peritoneal Surface Malignancy Program, Metropolitan Hospital, Athens, [4]Department of Surgical Oncology, Peritoneal Surface Malignancy Program, Euromedica Kyanous Stavros, Thessaloniki, Greece

Introduction / Background: Extensive cytoreduction in combination with Hyperthermic IntraPEritoneal Chemotherapy (HIPEC) is emerging as a safe and effective method for the treatment of advanced ovarian cancer. The purpose of this study was the identification of prognostic variables for survival and recurrence in a group of patients with recurrent ovarian cancer undergoing secondary cytoreductive surgery with HIPEC.
Methodology: From 2005-2017, 181 patients with recurrent ovarian cancer underwent treatment with cytoreductive surgery combined with HIPEC. Clinical and pathological variables were correlated to survival and recurrence. Hospital mortality and morbidity were recorded. All patients were treated by a single leading surgeon using a standard surgical technique and HIPEC protocol.
Results: The patients' mean age was 56.6±10.4 years. The mean Peritoneal Cancer Index (PCI) was 14.3. Complete (CC-0) or near complete (CC-1) cytoreduction was possible in 84.5% of the patients. The hospital mortality and morbidity rate were 1.7% and 30.4% respectively. The overall survival for 1, 3, 5, and 10 years was 73%, 48%, 36%, and 32% respectively. The statistically significant favorable prognostic variables of survival were a) limited extent of peritoneal carcinomatosis, b) administration of

adjuvant systemic chemotherapy and c) high histological differentiation. Recurrence was recorded in 55.4% of the patients. The statistically significant favorable prognostic variables of recurrence were
a) limited extent of peritoneal carcinomatosis,
b) limited extent of primary surgical procedures and
c) high histological differentiation.
Conclusion: Complete or near complete cytoreduction is feasible in the vast majority of patients with recurrent ovarian cancer. In our cohort, secondary cytoreductive surgery with HIPEC significantly improved the outcome of those patients. The limited extent of peritoneal carcinomatosis and the high histological degree of differentiation were the most important favorable variables for long-term survival and risk of recurrence.

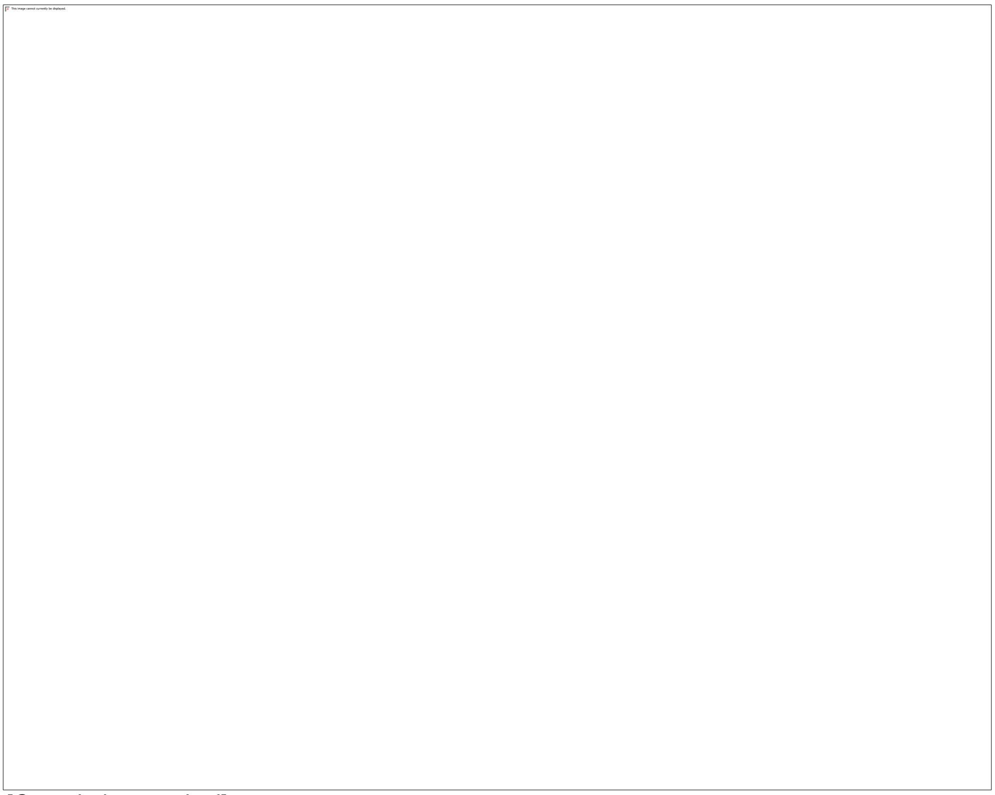

[Cumulative survival]

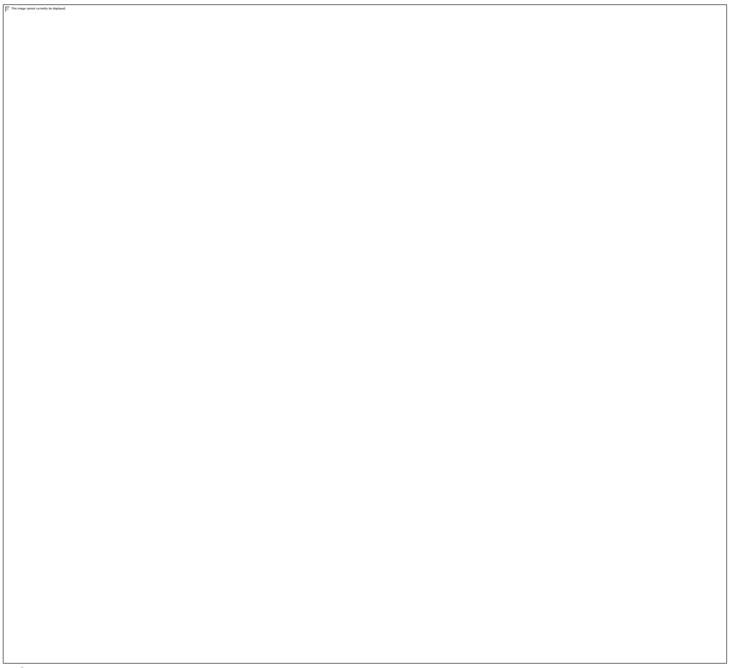

[Cumulative progression-free survival]

Disclosure: Nothing to disclose

EP948

Prognostic value of metastatic lymph nodes in advanced ovarian cancer surgery with HIPEC

T. Panoskaltsis[1], N. Pallas[2], C. Karamveri[2], D. Kiziridis[3], C. Hristakis[3], V. Kyriakopoulos[2], A. Kalakonas[3], D. Vaikos[3], C. Tzavara[4], C. Papadimitriou[5], A.-A. Tentes[2]
[1]Gynaecological Oncology Unit, 2nd Academic Department OB/GYN, Aretaieion Hospital, National and Kapodistrian University of Athens, Medical School, [2]Department of Surgical Oncology, Peritoneal Surface Malignancy Program, Metropolitan Hospital, Athens, [3]Department of Surgical Oncology, Peritoneal Surface Malignancy Program, Euromedica Kyanous Stavros, Thessaloniki, [4]Department of Hygiene, Epidemiology and Medical Statistics, [5]Oncology Unit, 2nd Department of Surgery, Aretaieion Hospital, National and Kapodistrian University of Athens, Medical School, Athens, Greece

Introduction / Background: To identify prognostic factors of survival and recurrence in advanced ovarian cancer patients with peritoneal carcinomatosis, undergoing cytoreductive surgery and Hyperthermic IntraPEritoneal Chemotherapy (HIPEC). In particular, to identify the rate and possible role of metastatic lymph nodes, according to their anatomic distribution.
Methodology: Retrospective analysis of 152 patients with advanced ovarian cancer that underwent complete or near-complete cytoreduction, which included at least one colonic resection and HIPEC. Clinical and histopathological variables were correlated to survival and recurrence. All patients were treated by a single team, with a standard protocol for surgical technique/HIPEC.
Results: Mean age of the patients was 58.8 years and complete cytoreductive surgery was possible in 72.4%. The rates of in-hospital mortality and major morbidity were 2.6% and 15.7%, respectively. Only 122 (80.3%) patients were able to complete adjuvant systemic chemotherapy. Rates of metastatic total lymph nodes, para-aortic and pelvic lymph nodes and large bowel lymph nodes were 58.7%, 58.5%, and 51.3%, respectively. The median, 5- and 10-year survival rates were 39 months, 43%, and 36.2%, respectively. The recurrence rate was 35.5%. On univariate analysis, near-complete cytoreduction, high Peritoneal Cancer Index, in-hospital morbidity, and no adjuvant systemic

chemotherapy were adverse prognostic factors, both, for survival and recurrence. On multivariate analysis, negative prognostic survival indicators were the advanced age of patients, extensive peritoneal dissemination, low total number of resected lymph nodes and no systemic para-aortic/pelvic lymphadenectomy. Metastatic large bowel lymph nodes and segmental resections of the small intestine were associated with a high risk for recurrence.
Conclusion: Retroperitoneal lymph nodes are frequently involved in advanced ovarian cancer. Our data show that extensive lymphadenectomy, including systemic para-aortic/pelvic and large bowel lymphadenectomy, may be important in advanced ovarian cancer surgery, as it may confer to survival improvement and decrease the risk of recurrence.

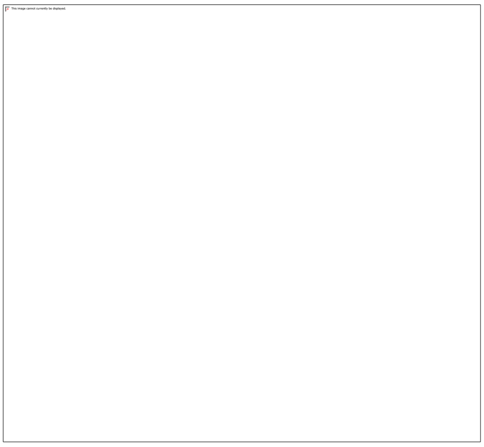

[Recurrence-free survival according to large bowel lymph node invasion]

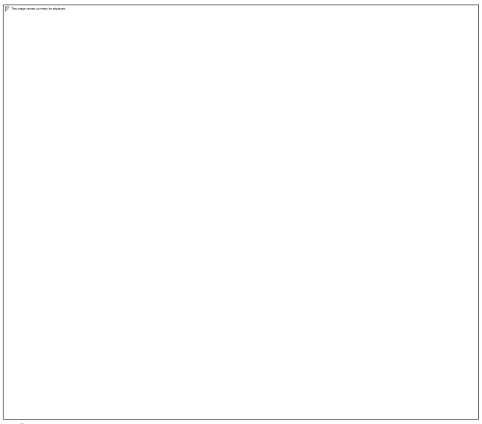

[Cumulative survival according to performing a systemic para-aortic/pelvic lymphadenectomy]

Disclosure: Nothing to disclose

EP949

Cytoreduction plus HIPEC in advanced ovarian cancer: 10-year experience of the Naval and Veterans Hospital of Athens

D. Papageorgiou, K. Papakonstantinou, V. Kalles, M. Zoulamoglou, E. Deskou, N. Stamos, N. Ivros, I. Kyriazanos

Naval Hospital of Athens, Athens, Greece

Introduction / Background: Ovarian cancer is the most common cause of gynecological malignancy mortality in the western world. Hyperthermic intraperitoneal chemotherapy (HIPEC) is a method of locoregional treatment that has recently been applied to selected patients. The present study presents the 10-year experience of our department in the use of cytoreductive surgery in combination with hyperthermic intraperitoneal chemotherapy in patients with advanced stage ovarian cancer.
Methodology: From 2009 to 2018, 30 patients with an average age of 56.6 years (37-78 years) underwent cytoreductive surgery plus HIPEC for ovarian cancer. In 9 cases, interval cytoreductive surgery was performed, and in 21 cases it was performed for tumor recurrence. Outcomes were evaluated by recording the complications and mortality of the method, as well as disease-free survival and overall survival of patients.
Results: The mean intraoperative PCI score was 19 (2-39). The mean operation time was 318 minutes (180 - 490). Optimal cytoreduction (CC-0/1) was achieved in 29 cases. In 1 case CC-2 was conducted. The mean postoperative ICU stay was 1.6 days, and the mean postoperative hospital stay was 21.6 days (9 - 45). Severe (Grade III/IV) complications were documented in 8 cases (26.7%).There was one case of postoperative mortality (3,3%). The mean follow-up period was 28.4 months. The median disease - free survival was 24 months for the interval CRS group, and 17 months for the recurrence group, whereas the median overall survival was 42 months for both groups.
Conclusion: The implementation of CRS and HIPEC for peritoneal carcinomatosis is a safe option, in a certified center for the treatment of peritoneal surface malignancy. Our results indicate a benefit in terms of disease - free and overall survival in patients undergoing CRS and HIPEC, however the small sample and low number of events inhibit an analysis that would provide safe conclusions at present.
Disclosure: Nothing to disclose

EP950
Predictive value of biomarkers during chemotherapy in patients with epithelial malignant ovarian cancer

G. Parpinel[1], E. Potenza[1], C. Macchi[1], A. Carapezzi[1], M.E. Laudani[1], L. Fuso[2], P. Zola[1]
[1]*Gynecologic Oncology, Sant'Anna Hospital,* [2]*Gynecologic Oncology, A.O. Ordine Mauriziano, Turin, Italy*

Introduction / Background: At present there is no predictive value univocally associated with the success of chemotherapy in the treatment of epithelial ovarian cancer. Biomarkers produced by ovarian cancer (HE4 and Ca125) could have a good prognostic significance. The aim of this study is to prove the ability of biomarkers to identify patients with the highest risk of not optimal response during the chemotherapy and to predict which patients will most probably develop recurrence of disease.
Methodology: We analyzed 78 patients with EOC (epithelial ovarian cancer) who underwent surgery in the biennium 2016-2017. All the patients performed chemotherapy after surgery or interval debulking surgery following neoadjuvant therapy. Serum levels of HE4 and Ca125 were measured at the diagnosis and at each cycle of chemotherapy. We established the degree of response to the treatment by CT-scan and the patients were followed up (FU median: 10 months). The parameters PFS (performance free survival) and DFS (disease free survival) were related to serum levels of biomarkers.
Results: The average markers values became both negative at the fourth cycle in the patients with good response to chemotherapy. HE4 rise compared earlier than Ca125. The parameters that best correlated with a long PFS were: negativization of the marker after the third cycle of chemotherapy (HE4: OR 5.5; Ca125: OR 9.1) and biomarker serum levels lower than the mean value in the affected population at the time of diagnosis (HE4: OR 3.4; Ca125: OR 3.7).
Conclusion: Based on the data obtained from this study we can conclude that the monitoring of HE4 and Ca125 during chemotherapy, especially at the third cycle, is recommended, because their variation is a good prognostic factor.
Disclosure: Nothing to disclose

EP951

The predictive value of prognostic nutritional index in diagnosing extent of disease and postoperative course of ovarian cancer patients: preliminary findings from a pilot study

V. Pergialiotis[1], D. Haidopoulos[2], A. Pandraklakis[1], N. Thomakos[1], A. Rodolakis[1]
[1]1st Department of Obstetrics and Gynaecology, National & Kapodistrian University of Athens, [2]1st Department of Obstetrics and Gynaecology, National and Kapodistrian University of Athens, Athens, Greece

Introduction / Background: Prognostic nutritional index (PNI) has been investigated extensively in the field of cancer and seems to be associated both with the extent of disease, perioperative wellbeing of patients as well as with postoperative disease survival. Its value in ovarian cancer patients remains to date unclear. The purpose of the present study is to evaluate the diagnostic accuracy of PNI in detecting metastases in the upper abdominal cavity and correlate its levels to postoperative hospitalization duration.
Methodology: A retrospectively identified consecutive series of patients that were treated in our institution for ovarian cancer was used for analysis. Preoperative PNI was calculated as serum albumin (g/L) + 0.005 × lymphocyte count (per mm^3). Inferential statistics were performed with non-parametric tests and correlation analysis was based in Spearman`s rho.
Results: Overall, 50 patients were included in our analysis with a mean PNI of 41.2±0.7 and a mean age of 62±1.8 years. No differences were observed in PNI levels among patients with ascites (*p=.807*), metastases in the upper abdominal cavity (*p=.658*) as well as among patients at various stages of the disease. Correlation statistics did not reveal significant associations between PNI and postoperative high dependency unit hospitalization (rho=.181, *p=.271*) and total duration of hospitalization (rho=.081, *p=.630*), age (rho=-.239, *p=.143*). The area under the curve (AUC) for detecting metastases in the upper abdominal cavity was poor (AUC=0.456) as well as for cases requiring bowel excision (AUC=0.525).
Conclusion: The findings of our study do not support the use of prognostic nutritional index as a tool for the preoperative assessment of ovarian cancer patients as it does not seem to correlate with the extent of disease and postoperative hospitalization. Larger studies are needed to support our findings and help reach definitive conclusions.
Disclosure: Nothing to disclose

EP952

Novel molecular target selection for tumour-specific imaging of metastases from epithelial ovarian cancer

L. de Muynck[1], K. Gaarenstroom[2], C. Sier[1], M. van Duijvenvoorde[2], T. Bosse[3], C. de Kroon[2], A. Vahrmeijer[1], I. Peters[2]
[1]Surgery, [2]Gynecology, [3]Pathology, Leiden University Medical Center, Leiden, The Netherlands

Introduction / Background: The strongest prognostic factor in epithelial ovarian cancer (EOC) is the completeness of surgery. During both surgical staging in apparent early stage disease and cytoreductive surgery in patients with advanced stage disease, the gynecologic oncologist must rely upon naked eye visual inspection and, if applicable, palpation. As a result, malignant tissue may remain undetected. Tumour-targeted molecular imaging using agents that specifically bind to cell-surface proteins overexpressed on EOC can improve intraoperative tumour detection. Our previous research using a fluorescent folate analogue during cytoreductive surgery has shown a detection of 29% additional metastatic EOC lesions. Yet, some false positive lymph nodes were found. This study aimed to determine which cell-surface proteins are suitable as an additional target for tumour-targeted molecular imaging of EOC metastases.
Methodology: Immunohistochemistry was performed on formalin-fixed paraffin-embedded tissues

from primary ovarian tumours, lymph node, omental and peritoneal metastases from 84 EOC patients who underwent cytoreductive surgery. Tumour-negative tissue specimens were included as a control. The following cell-surface proteins were tested: EGFR (epithelial growth factor receptor), VEGF-A (vascular endothelial growth factor-A), L1CAM (L1 cell adhesion molecule), integrin avb6 and EpCAM (epithelial cell adhesion molecule). FRa (folate receptor alpha) was included as a reference.
Results: EGFR, VEGF-A and L1CAM expression was observed in tumour-negative tissues. In EOC metastases, avb6 showed heterogenous expression patterns. As compared to FRa, EpCAM showed an equally high expression in these metastases. Furthermore, although EpCAM was expressed on tumour-negative epithelial cells lining the intestinal lumen, it was not expressed in lymph nodes that were previously false positive using the fluorescent folate analogue.
Conclusion: In addition to FRa, EpCAM is a suitable target for tumour-targeted molecular imaging of EOC metastases and can contribute to a more reliable detection of EOC lesions intraoperatively. Further research will focus on examining whether serosal metastases can be accurately distinguished from the small bowel.
Disclosure: This work was financially supported by the Zabawas Foundation, which was not involved in any part of the study. The authors have no conflicts of interest to disclose.

EP953

Poor AT and VO2 max recorded during Cardiopulmonary Exercise Testing (CPET) prior to cytoreductive surgery in advanced (Stage 3/4) ovarian cancer (AOC) is associated with suboptimal cytoreduction but does preclude maximum effort cytoreduction

K. Element[1], P. Mehrotra[1], V. Asher[1], A. Bali[1], S. Abdul[1], J. Low[1], R. Curtis[1], S. Tou[1], D. Gomez[2], A. Phillips[1]
[1]University Hospitals of Derby and Burton, Derby, [2]Nottingham University Hospitals NHS Trust, Nottingham, United Kingdom

Introduction / Background: Using CPET pre-operatively to assess functional capacity and help estimate if patients can cope with the physiological stress of major surgery is increasing utilised across many different conditions. No research is currently available on the value CPET can have on predicting outcomes and complications following AOC cytoreductive surgery.
Methodology: A retrospective review of all patients who had CPET prior to AOC cytoreductive surgery with evidence of upper abdominal disease on preoperative imaging at the University Hospitals of Derby and Burton (UHDB) between August 2016 and February 2019. Patients were stratified into two groups, based on their anaerobic threshold (AT) (AT < 11 and AT >=11) and maximum VO2 (VO2 < 15 and VO2 >=15). Cytoreduction (Complete (R0), < 1cm (R1) or >1cm (R2)), surgical complexity, complications within thirty days, length of hospital stay (LOS) and readmissions were assessed.
Results: 36 patients were identified. AT showed no relationship with thirty-day complications or death rates. 100% of patients in the AT>=11 group received R0 (n=18, 90%), or R1 (n=2, 10%) cytoreduction, whereas in the AT< 11 group, only 68.75% underwent macroscopic resection (R0/R1, p=0.01). Surgical complexity was higher in the AT>= 11 group (p=0.003) and the VO2>=15 group (p=0.003). No other correlations were seen between AT or VO2 max and complications, LOS or readmissions.
Conclusion: CPET is not an effective tool for determining appropriateness for surgery. No correlation has been found between performance at testing and complication rates. The majority of patients with AT< 11 achieved R0/R1 resection despite a higher rate of suboptimal surgery. Discounting patients from cytoreductive surgery based on CPET results alone is not supported by our study, however CPET may have a role in conjunction with other investigations
(eg. echocardiogram, PFT´s), or in selected patients.
Disclosure: Nothing to disclose

EP954

Impact of Splenectomy on survival in advanced ovarian cancer (AOC) in a propensity matched cohort

J. Davies[1], V. Asher[1], A. Bali[1], S. Abdul[1], D. Gomez[2], S. Tou[1], M. Persic[1], L. Seneviratne[1], A. Phillips[1]
[1]*University Hospitals of Derby and Burton, Derby,* [2]*Nottingham University Hospitals NHS Trust, Nottingham, United Kingdom*

Introduction / Background: Ultra-radical procedures, including splenectomy, are utilised during cytoreductive surgery in AOC to treat disease that would not be removed with standard procedures, the intention being to increase complete cytoreduction rates. Hypothetically the performance of splenectomy may independently be a marker for worse survival due to detrimental effects on immune function or its complications.
Methodology: A retrospective review of all consecutive patients undergoing cytoreductive surgery for AOC between 16/05/2013-6/02/2019. Survival, complications and surgical parameters were recorded. Propensity scored matching (PSM) was performed, allowing comparison between splenectomy patients with both standard and ultra-radical surgery without splenectomy.
Results: 151 patients were identified within a 71 month time period. Of these 100 underwent standard and 51 underwent ultra-radical surgery. 22 patients received splenectomy (14.5%)
No difference was seen in Overall survival (OS) between all patients (median OS 34 months (95%CI 25.9-41.1) and patients undergoing splenectomy (median OS not yet reached) (p = >0.05).
When comparing UR only patients, neither arm reached median OS; HR = 1.8 (0.64-5.3), p > 0.05.
After PSM for grade, stage, age and cytoreduction, no significant difference in splenectomy versus non-splenectomy patients (3-year survival 54% compared to 56% (hazard ratio - 1.1(95%CI 0.39-3.2). (P>0.05).
When matching only to Ultra radical controls, no significant difference in OS was seen with median OS not reached in either arm (HR - 2.6(0.55-13) p > 0.05).
Splenectomy specific complications were seen in 3 patients; one pancreatic tail injury, one left pleural effusion and one cases of streptococcal pharyngitis during chemotherapy.
No cases of overwhelming post splenectomy infection were identified.
Conclusion: Splenectomy does not negatively impact the complication rates of surgery or survival; and should not be feared by gynaecological oncologist undertaking ultra-radical surgery for AOC.
Disclosure: Nothing to disclose

EP955

Interval debulking surgery for advanced ovarian cancer in elderly patients (≥70y): does the age matter?

C. Pinelli[1], M. Morotti[2], J. Casarin[1], R. Tozzi[2], F. Ghezzi[1], M. Alazzam[2], H. Soleymani Majd[2]
[1]*Obstetrics and Gynecology, University of Insubria, Varese, Italy,* [2]*Gynaecology Oncology, Oxford University Hospital, Oxford, United Kingdom*

Introduction / Background: Elderly ovarian cancer (OC) patients are more likely to be managed suboptimally and experience worse clinical outcomes as a result. Strategies to improve outcomes in this patients are lacking.
Methodology: Retrospective analysis for consecutive patients with advanced stage OC (IIIC-IV) who were managed in Oxford University Hospital between January 2016 and July 2018 were analysed. All patients underwent neoadjuvant chemotherapy (NACT) and interval debulking surgery (IDS) according to our institution protocol. We divided the patients into two groups: an "older group" (age ≥70 years) (Group 1) and a "younger group" (age < 70 years) (Group 2). The primary outcome of the study was the assessment of peri-operative morbidity amongst the two groups.
Results: A total of 153 patients were referred during the study period. 114 patients underwent IDS after NACT (74.5%), of which 46 in Group 1 and 68 in Group 2. Elderly patients were more likely to receive more than three cycles of NACT prior to IDS when compared to younger patients (39% vs.

19%, p=0.03). Elderly patients were more frequently subjected to Cardiopulmonary Exercise Testing (CPET) as part of their pre-operative assessment (63% vs. 27%, p=0.002). For those who underwent IDS; optimal/complete resection was achieved in all patients in Group 1 (100%) and in 97% of patients in Group 2. With the exception of higher postoperative cardiac arrhythmias in Group 1 (11% vs. 1%, p = 0.04), no significant differences in 30-day morbidity were observed. No 90-day death in both groups was registered.
Conclusion: Older age should not preclude clinicians from offering ultra-radical resection, for patients with advanced OC who underwent careful preoperative assessment. In our series, elderly patients received the same treatment with similar outcomes to the younger group. CPET has an important role and clinicians should be encouraged to use it more frequently especially when ultraradical surgery is postponed.
Disclosure: Nothing to disclose

EP956

Cardiophrenic node dissection during interval debulking surgery for stage IV ovarian cancer: a case series

C. Pinelli[1], M. Morotti[2], J. Casarin[1], R. Tozzi[2], F. Ghezzi[1], M. Alazzam[2], H. Soleymani Majd[2]
[1]*Obstetrics and Gynecology, University of Insubria, Varese, Italy,* [2]*Gynaecology Oncology, Oxford University Hospital, Oxford, United Kingdom*

Introduction / Background: Several studies have demonstrated the feasibility and role of cardiophrenic lymph nodes (CPLNs) resection during primary debulking surgery (PDS) for stage IV ovarian cancer (OC). However, no studies, to date, investigated the role of CPLNs removal during interval debulking surgery (IDS) after neoadjuvant chemotherapy (NACT).
Methodology: A retrospective analysis of consecutive stage IV OC patients who underwent NACT followed by IDS with CPLNs resection from July 2017 to June 2018. CPLNs sized >7 mm on the short axis at pre-operative CT-scan were considered for excision if optimal complete resection could be achieved.
Results: A total of 21 ovarian cancer stage IV patients treated with NACT followed by IDS were identified. Seven (33.3 %) patients underwent CPLNs resection. A partial response to NACT by RECIST criteria was observed in 5 of 7 patients (71%) while 2 cases had stable disease (29%). Complete cytoreduction without residual disease was achieved in 5 cases (71%) while in two cases (29%) optimal cytoreduction was performed. All patients underwent full-thickness right diaphragmatic resection and pleurectomy. All excised CPLNs were found in the right side of the lower anterior mediastinum. Intra-operative surgical complications occurred in one patient. One patient (14%) had a major postoperative complication (Clavien-Dindo 3). Two cases of postoperative cardiac arrhythmia were observed. The final histological examination of the CPLNs revealed metastatic disease in 4 (57%) of 7 patients.
Conclusion: CPLNs removal after NACT for stage IV OC is safe and necessary to achieve a complete resection in the context of IDS after NACT.

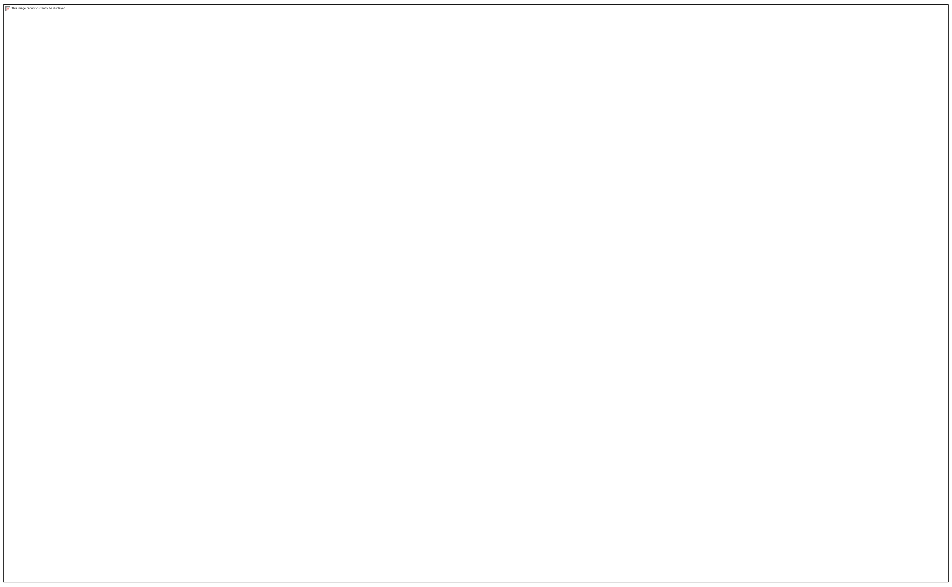

[Demographic characteristics, response to treatment and tumour details of patients who underwent CPLN]

Disclosure: Nothing to disclose

EP957

Inflammation biomarkers and differential diagnosis of pelvic tumors: an observational study

V. Psomiadou[1], A. Prodromidou[1], E. Galati[1], I. Kostakis[2], N. Blontzos[1], F. Lefkopoulos[1], C. Iavazzo[1], G. Vorgias[1]
[1]Metaxa Cancer Hospital, Piraeus, Greece, [2]Guy's and St Thomas' NHS Foundation Trust, London, United Kingdom

Introduction / Background: Carcinogenesis and systemic inflammation have been correlated and the ratios of white blood cell and platelet counts have been suggested to have a prognostic significance in ovarian tumor patients. The purpose of our study was to estimate the prognostic value of inflammation biomarkers in ovarian mass patients.
Methodology: We retrospectively assessed the potential relationship between neutrophil-to-lymphocyte (NLR), platelet-to-lymphocyte (PLR), lymphocyte-to-monocyte (LMR), neutrophils-to-monocyte (NMR), and monocytes-to-platelets (MPR) and malignancy in patients admitted in the Department of Gynaecology of Metaxa Memorial Cancer Hospital due to adnexal mass.
Results: NLR and PLR were found to be significantly higher and LMR significantly lower in patients with malignancy. Platelet and lymphocyte count was also higher in these patients in comparison with those in patients with borderline tumors. On the other hand, white blood cell count , neutrophil-to-monocyte ratio , platelet-to-neutrophil ratio and platelet-to-monocyte ratio did not differ significantly. Cut-off points for identifying malignancy were estimated to be ≥2.4 for NLR, ≥181.1 for PLR and ≤4.55 for LMR.
Conclusion: Differential diagnosis between malignant and benign adnexal masses can be assisted by inflammation biomarkers. Larger prospective studies are needed in order to confirm these findings and strengthen the use of these inexpensive and easy to calculate ratios.
Disclosure: Nothing to disclose

EP958

Effectiveness of diaphragmatic curettage in cytoreductive surgery of epithelial ovarian cancer or primary peritoneal malignancy

R. Puente, E. Schneider
Gynecologic Oncology, Austral University/ Obstetrics and Gynecology, Valdivia, Chile

Introduction / Background: Knowing that diaphragmatic involvement must be resected surgically for optimal cytoreduction in primary peritoneal or ovarian cancer, we performed diaphragmatic curettage instead of resective surgery, with follow-up of the patients to know the specific sites of recurrent disease when it happens.
Methodology: Fifteen patients with primary peritoneal or ovarian carcinoma with diaphragmatic involvement were subjected to diaphragmatic curettage (one or both diaphragms) with a big and sharp curette after liver mobilization as a part of cytoreductive surgery. Eleven (73.3%) patients had suspicious imaging of diaphragmatic involvement preoperative at CT scan. In ten patients with recurrent disease (66.6%), none had suspect of diaphragm involvement at CT scan or intraoperative during secondary cytoreduction.
Results: In all 15 patients the curettage left diaphragms free of disease. In 10 patients with recurrent disease according to the CT scan and/or a secondary cytoreduction, diaphragms were free of disease.
Conclusion: The diaphragmatic curettage is a safe and effective procedure to treat diaphragm involvement during cytoreductive surgery
Disclosure: Nothing to disclose

EP959

Fertility-sparing surgery in patients with early stage clear cell carcinoma of the ovary - is it safe to offer?

M. Qi, I. Aggarwal, Y.K. Lim
Department of Gynecological Oncology, KK Women's and Children's Hospital, Singapore, Singapore

Introduction / Background: Fertility-sparing surgery (FSS) in stage I ovarian clear cell carcinoma (CCC) is not commonly offered due to concern about poorer outcomes despite paucity of evidence. The aim of this study is to review oncological outcomes of CCC patients treated with fertility sparing surgery, to provide evidence to guide practice.
Methodology: Clinical data of all CCC patients treated with FSS and followed-up in a single tertiary hospital were collected and used for analysis.
Results: Over the period from 2005 to 2018, there were in total 180 cases of stage I CCC treated in this hospital, of which five cases underwent FSS. The mean age of FSS patients at diagnosis was 33.6 years. Two cases were stage Ia and the other three Ic. Four cases received adjuvant chemotherapy. During the follow-up (median: 6 years), there was no recurrence. One patient conceived spontaneously and had term live birth, 6 years after completion of treatment. Overall survival of stage I CCC patients treated in this hospital during the same period was 92.8% and disease-free survival 88.3%.
Conclusion: In the present cohort, FSS in selected early stage CCC of ovary did not have a negative impact on oncologic outcome. Further large-scale studies are needed to confirm the safety.
Disclosure: Nothing to disclose

EP960

Total omentectomy versus omental sampling in early ovarian cancer surgery

B. Refky[1], K. Gaballah[2], K. Attallah[2], H. Mohamed[2]
[1]Surgical Oncology, [2]Mansoura University, Oncology Center, Mansoura, Egypt

Introduction / Background: Historically the omentum was found to be a primary site of metastatic spread and invariably involved in all cases of advanced ovarian cancer, omentectomy was included for the staging and treatment of ovarian cancer regardless of whether or not the omentum appeared macroscopically to be involved. However , this dictum is not being routinely applied for the peritoneum , which is in the mainstay a common site for ovarian cancer metastastis and recurrence , only random peritoneal biopsies are obtained in early stage ovarian cancer , rarely total peritonectomy or removal of unaffected peritoneum is done
Methodology: A prospective randomized controlled trial including 150 patients with early ovarian cancer for 5 years period in oncology center, Mansoura University, Egypt randomized in between two groups, Group 1 (control group) underwent conventional total infracolic omentectomy and the other group (Group 2) underwent just omental biopsy in apparently free omentum , just like peritoneal biopsies .Follow up to be done over a period of 5 years , however interim analysis was done after 2 years from the start of the trial.
Results: In this interim analysis 89 patients were included, 46 underwent total omentectomy while 43 underwent omental biopsies only , out of those 43 patients three patients (6.9%) were found to have microscopic omental metastasis alone and thus were upstaged , however 2 years disease free survival was similar in both groups (93 % in group 1 versus 91% in group 2).
Conclusion: Total omentectomy may not be mandatory in the context of comprehensive surgical staging in early stage ovarian cancer , no difference was encountered regarding the disease free survival whether performing total omentectomy versus omental sampling and biopsies in apparently free omentum . We are still waiting for the final results
Disclosure: Nothing to disclose

EP961

Are there opportunities to improve the number of women able to start and tolerate treatment for ovarian cancer? European perspectives from the World Ovarian Cancer Coalition's Every Woman Study

F. Reid[1], A. Oza[2], N. Bhatla[3], S. Blank[4], R. Cohen[5], D. Gardiner[6], A. Jones[7]
[1]The World Ovarian Cancer Coalition, Royston, United Kingdom, [2]University of Toronto, Toronto, ON, Canada, [3]All India Institute of Medical Sciences, New Delhi, India, [4]Icahn School of Medicine at Mount Sinai, New York, NY, [5]Sandy Rollman Ovarian Cancer Foundation, Philadelphia, PA, United States, [6]The World Ovarian Cancer Coalition, Melbourne, VIC, Australia, [7]The World Ovarian Cancer Coalition, London, United Kingdom

Introduction / Background: The aim of the World Ovarian Cancer Coalition Every Woman Study was to address the evidence gap relating to the experiences of women with ovarian cancer globally, with a view to improving survival and quality of life. Reports show 20% of UK women do not receive any treatment, and 15% of women die within 2 months of diagnosis. The chief concerns of European clinicians involved in the study were poor access to specialist surgeons, and delays in diagnosis contributing to poor performance status of women.
Methodology: 1531 women from 44 countries participated in an online survey. Given global 5-year prevalence, results achieve a confidence level of 95% with a confidence interval of +/- 2.5%. Some statistically significant country differences were seen compared to the average for all (p< .01 and *p< .05). 37 clinicians from 15 countries were consulted.
Results: There were wide European variations in a number of metrics relating to the time period leading up to treatment including: women who did not know about ovarian cancer (Hungary 50.9%, Italy 56.3%*, Average 69.1%, Germany 79.3%); proportion of symptomatic women consulting a doctor

(Germany 63.3%, Average 78.3%, UK 87.7%); proportion visiting a doctor within one month (Germany 77.3%, Spain 62.2%, Average 46.3%): diagnosis within one month of visiting doctor (UK 30%, Average 43.2%, Italy 62.3%); total estimated time to diagnosis (21.3 weeks in Germany to 31.5 weeks in Italy: proportion treated by gynaecologic oncologists in high volume centres where established networks and guidelines exist (e.g. Germany 50-60%, UK over 90%).
Conclusion: The study shows wide variations between European countries in the experiences of women leading up to diagnosis and treatment, with no one country without barriers. This suggests there are opportunities to optimise the patient pathway in this critical time period, to ensure as many women as possible are able to start treatment.
Disclosure: None of the authors have any conflicts of interest to disclose in relation to this Study. The study was funded by the World Ovarian Cancer Coalition (WOCC). WOCC received grants and pro bono support to enable translation of the women's survey from the following: Tesaro, Roche, Merck Pfizer Alliance.

EP962

Survival outcome and surgical morbidity analysis of interval cytoreductive surgery performed for advanced epithelial ovarian cancer

P.N. Rema
Division of Gynaecological Oncology, Regional Cancer Centre, Thiruvananthapuram, India

Introduction / Background: In advanced epithelial ovarian cancer (AEOC) patients with unresectable disease in initial presentation neoadjuvant chemotherapy followed by interval cytoreduction is beneficial in improving patient survival. Cytoreductive surgery including extensive upper abdominal surgery although essential in attaining optimal cytoreduction(OCR) leads to increased post operative morbidity. The aim of our study was to assess the survival outcome and post operative morbidity in patients who underwent interval cytoreductive surgery for AEOC.
Methodology: During the time period from January 2014 to September 2018, 415 patients with AEOC were treated in our centre. Patients found to have unresectable disease in initial assessment by CT scan or diagnostic laparoscopy underwent neoadjuvant chemotherapy followed by interval cytoreduction. In this retrospective audit the following parameters were assessed - age at diagnosis, histologic type and grade, medical comorbidities, surgical procedures performed size of residual disease, intra operative complications and post-operative morbidity within 30 days of surgery. Post-operative complications were graded according to the Clavin Dindo grading. Patients were followed up till December 2018. Morbidity profile was assessed using descriptive statistics and Chi-square test for statistical significance. Overall and disease free survival was calculated using Kaplan-Meier method and log-rank test for statistical significance.
Results: Median follow up period was 21 months and median age of patients was 55 years. Study included 273 patients of which 80.58% had in Stage III and 19.42% had stage IV disease. OCR could be achieved in 85% (212 patients). Overall survival was 72.3 % at the end of 3 years and progression free survival (PFR) was 36.6% at 3 years. PFS was significantly higher in patients who attained OCR (P-0.014). Post-operative surgical morbidity was seen in 33 patients (12.08%).
Conclusion: Interval cytoreductive surgeries following neoadjuvant chemotherapy is feasible in AEOC with good rates of optimal cytoreduction and acceptable morbidity.
Disclosure: Nothing to disclose

EP963

Assessment of abdominal resectability with FDG-PET/CT in locally advanced ovarian cancer

L. Ribot[1], P. Caresia[2], B. Morillas[1], Y. Garcia[3], J. del Riego[4], I. Costa[5], J.C. Martin[2], A.A. Rodriguez[2],

M. Corona[1], J. Antoni[1], Y. Canet[1]
[1]*Gynecology Department,* [2]*Nuclear Medicine Department,* [3]*Oncology Department,* [4]*Radiology Department,* [5]*Pathology Department., Parc Taulí Hospital Universitari, Barcelona, Spain*

Introduction / Background: In locally advanced ovarian cancer the primary treatment is debulking surgery followed by chemotherapy. Surgery is recommended in patients who can be debulked upfront to no residual tumour. Therefore, there are some specific criteria against primary surgical approach. In case of inoperable disease, neoadjuvant chemotherapy with interval surgery should be planned. The aim of this study is to evaluate the capability of FDG-PET/CT to assess abdominal resectability in advanced ovarian cancer, before surgery.
Methodology: Unicenter, restrospective, observational study included 35 patients (age 63.11, range 40-82 years) with clinical and radiological suspicion of primary advanced ovarian cancer. All patients underwent contrast enhanced PET/CT before surgery. Based on criteria against surgical approach, patients were classified as resectable or non resectable.
Sensitivity, specificity, NPV, PPV and accuracy for PET/CT to predict complete surgical resectability were calculated.
Results: Thirty-one patients were concordant (18 patients were considered resectables by PET/CT and surgery, 13 patients were considered non resectables by PET/CT and surgery). Only 4 patients were discordant: 1 patient presented inoperable milliary carcinomatosis, not visible by PET/CT. The other 3 patients were finally resectables but it seems not by PET/CT (large and diffuse carcinomatosis of the small bowel or extense involvement of superior abdomen). For detection of abdominal resectability, PET/CT presented a sensitivity of 85.7% (95% CI:64-97), specificity 92.8%(95% CI:66-100), NPV 81.2%(95% CI:54-96), PPV 94.7%(95% CI:74-100) and accuracy of 88.57%(95% CI:73-96). PET/CT also revealed extrabdominal distant metastases in 9/35 patients (29%), mainly represented by supradiaphragmatic lymph nodes (8/9 patients), associated with additional metastases in pleura and/or liver.
Conclusion: In locally advanced ovarian cancer, PET/CT had good results in assessment of surgical abdominal resectability, especially in terms of specificity, PPV and accuracy. PET/CT could be helpful in patients with questionable initial terapeutic decision, due to its ability to detect additional unsuspected extraabdominal disease.
Disclosure: Nothing to disclose

EP964

Targeting AKT and DNA-PK as a therapeutic strategy in platinum resistant high-grade serous ovarian cancer

N. Rinne[1], E. Curry[1], C. Fotopoulou[1], H. Gabra[1,2], P. Cunnea[1]
[1]*Surgery & Cancer, Imperial College London, London,* [2]*Early Clinical Development, IMED Biotech Unit, AstraZeneca, Cambridge, United Kingdom*

Introduction / Background: High-grade serous ovarian cancer (HGSOC) is the most lethal form of gynaecological malignancy. Despite usual initial chemosensitivity, the majority of patients develop resistance to platinum chemotherapy and eventually die. Current treatment options for platinum resistant disease remain limited. The role of the PI3K/AKT/mTOR pathway has been well described in chemoresistant disease, in particular through activation of AKT in response to platinum treatment, by the DNA damage response protein DNA-PK. Our laboratory previously demonstrated that inhibition of AKT or DNA-PK re-sensitises clinically-acquired platinum resistant cell lines to cisplatin. Work to date has been carried out in immortalised cell lines, which inherently develop phenotypic/genotypic alterations over time. The present project aims to confirm our previous findings, using primary HGSOC tumour cells and 3D models, and correlate responses to DNA-PK copy number and gene expression data.
Methodology: Tumour cell cultures established from HGSOC tumour and ascites samples were treated with cisplatin plus/minus AKT or DNA-PK inhibitors, and their apoptotic response and cell viability were measured. RNA and DNA were extracted from tumour cells, and gene expression and copy-number analysis were performed using qPCR.
Results: Following treatment with cisplatin in combination with either AKT or DNA-PK inhibitors, an

enhanced apoptotic response was observed in primary cells from ascites (n=4, p< 0.01, p< 0.05) and tumour (n=4, p< 0.05) compared to cisplatin treatment alone. DNA-PK amplification was detected in a number of HGSOC tumour samples and immortalised chemoresistant cell lines. Strong synergy was observed in a panel of platinum resistant HGSOC cell lines when combining AKT or DNA-PK inhibitors with cisplatin.
Conclusion: In each primary case of HGSOC examined, AKT or DNA-PK inhibition significantly enhanced the apoptotic response of patient derived tumour cells to cisplatin, demonstrating the efficacy of AKT of DNA-PK as potential therapeutic targets for chemoresistant HGSOC.
Disclosure: Professor Christina Fotopoulou, COI: advisory boards and honoraria from Roche, Tesaro, Sequana, Olympus, Astra Zeneca

EP965

The impact of clinico-pathological features and surgery on relapse in women with borderline ovarian tumours: our experience

I. Rizzuto, H.J. Jiang, R. McDonnell, S. Dohr, W. MacNab, B. Rufford
Gynaecological Oncology Department, Ipswich Hospital NHS Trust, Ipswich, United Kingdom

Introduction / Background: The current study was carried out to evaluate the oncological outcomes in patients with borderline ovarian tumours (BOT) according to type of surgery (conservative surgery versus radical surgery), stage at diagnosis and clinicopathological parameters.
Methodology: We enrolled patients with BOT of any FIGO stage, whom were surgically treated from 2005 to 2013 at the Gynecological Oncology Department of the Ipswich hospital, UK. The prognostic effects of stage, clinico-pathological features (micropapillary lesions, invasive implants and bilateral tumors) and surgical variables were evaluated using univariate/multivariate analysis.
Results: A total of 85 eligible patients were enrolled in the analysis. We had 42/85(49%) with serous, 35/85 (41%) with mucinous, 5/85(6%) with mixed mucinous-serous, 3/85 (4%) with endometroid histological subtypes. Among all the cohort of patients, 14/85 (16%) had recurrence within 5 years of follow-up. Multivariate analysis showed a significant association to relapse in the presence of micropapillary lesions and invasive implants at the histological report with RR 1.39 (95%CI 1.39-2.73) *p-value < 0.001* and RR 4.44 (95%CI 2.26-8.69) *p-value 0.002* respectively. FIGO stage RR 0.56 (95%CI 0.23-1.33) *p-value 0.153*, the presence of bilateral tumours RR 2.02 (95% CI 0.95-4.31) *p-value 0.099* and conservative surgery RR 1.25 (95%CI 0.41-3.81) *p-value 0.709* were not significantly associated to higher recurrence rate.
Conclusion: We confirm that BOT patients with micropapillary lesions and invasive implants have high risk of recurrence. These data can support clinicians in tailoring the best surgical strategy in young patients with BOT and in counseling patient about their prognosis after the diagnosis.
Disclosure: Nothing to disclose

EP966

Borderline Ovarian Tumours: a single institute retrospective study

A.C. Rocha[1,2], M.I. Sá[3], C. Abrantes[2], S. Raposo[2], R. Sousa[2], P. Correia[2], P. Aldinhas[2], E. Branco[2], C. Coelho[2], V. Baltar[2], L. Sá[2]
[1]Hospital de Santarém, Santarém, [2]Instituto Português de Oncologia Coimbra, Coimbra, [3]Hospital de Vila Real - CHTMAD, Vila Real, Portugal

Introduction / Background: Borderline ovarian tumours (BOT) are a group of ovarian epithelial tumours (OET) defined by atypical epithelial proliferation without stromal invasion. They represent 10-20% of all OET and have an excellent prognosis. Recurrence and malignant transformation can occur,

however we still cannot predict which women will recur. Our aim is to determine the clinical, epidemiologic and histological features of the BOT.
Methodology: A retrospective *coorte* study involving 79 patients diagnosed with BOT and treated at IPO-Coimbra between 1990-2016. Patients data was obtained from hospital records.
Results: The average age was 50,3 years. 24,1% of the patients were < 40 years. We included 25 mucinous tumors, 46 serous, 2 endometrioid, 3 seromucinous and 1 Brenner tumor. 88,6% of them were in stage-I, 7,6% in stage-II and 3,8% were in stage-III. All patients had surgery as a primary treatment; in 32,7% of the cases was performed a conservative surgery. Three patients were subjected to adjuvant chemotherapy. After a mean follow up of 5,9 years, we reported 4 cases of relapse, 3 with malignant transformation to invasive ovarian cancer. 3 patients died during this period; the overall survival at 5 and 10 years was respectively of 100% and 96,2%. Ca125, FIGO stage and peritoneal implants were associated to disease recurrence. Neither micropapillary growth pattern nor age showed that association.
Conclusion: Our study reveal that the overall prognosis of BOT is good. Relapse cases were mainly related to FIGO stage and the presence of invasive peritoneal implants. Conservative surgery was not related with higher recurrence rate.
Disclosure: Nothing to disclose

EP967

Natural history of patients with BRCA-mutated high grade epithelial ovarian cancer (HGEOC) before the era of PARP inhibitors maintenance in 1st line treatment

C. Romeo[1], P. Meeus[1], M. Rodrigues[2], E. Leblanc[3], A. Floquet[4], P. Pautier[5], F. Marchal[6], M. Provansal[7], L. Campion[8], S. Causeret[9], S. Gourgou[10], I. Ray-Coquard[1], J.-M. Classe[11], C. Pomel[12], T. De La Motte Rouge[13], E. Barranger[14], A.M. Savoye[15], C. Guillemet[16], L. Gladieff[17], T. Petit[18], R. Rouzier[2], C. Labreveux[19], C. Courtinard[19], F. Joly[20]
[1]Centre Léon Bérard, Lyon, [2]Institut Curie, Paris, [3]Centre Oscar Lambret, Lille, [4]Institut Bergonié, Bordeaux, [5]Gustave Roussy, Villejuif, [6]Institut de Cancérologie de Lorraine, Vandœuvre-lès-Nancy, [7]Institut Paoli Calmettes, Marseille, [8]Institut de Cancérologie de l'Ouest (site René Gauducheau), Saint-Herblain, [9]Centre Georges Francois Leclerc, Dijon, [10]Institut du Cancer de Montpellier, Montpellier, [11]Institut de Cancérologie de l'Ouest, Angers, [12]Centre Jean Perrin, Clermont-Ferrand, [13]Centre Eugene Marquis, Rennes, [14]Centre Antoine Lacassagne, Nice, [15]Institut Godinot, Reims, [16]Centre Henri Becquerel, Rouen, [17]Institut Claudius Regaud, IUCT Oncopole, Toulouse, [18]Centre Paul Strass, Strasbourg, [19]Unicancer, Paris, [20]Centre Francois Baclesse, Caen, France

Introduction / Background: In SOLO1 trial, maintenance therapy with olaparib considerably improved progression-free survival (PFS) among women with newly-diagnosed, advanced BRCA-mutated HGEOC. Based on the large real-life ESME ovarian cancer (OC) cohort, we aimed to describe the patient's characteristics and survival outcomes of a selected cohort of BRCA-mutated patients before the era of PARP inhibitors maintenance in first line.
Methodology: ESME OC is a national cohort including all patients (pts) with EOC managed in the 18 French Comprehensive Cancer Centres (NCT03275298). ESME Research program collected retrospective data from patient's medical records. *BRCA1*, *BRCA2*, or both mutated patients with de novo diagnosed advanced (FIGO stage III or IV) high-grade serous or endometrioid EOC were eligible. Primary objective was to describe patient's characteristics, clinical features and treatment patterns.
Results: Of the 4777 patients with OC treated in first line by platinum-based chemotherapy between 2011 and 2016, 266 were included. Median age was 57.0 (33-81). 187 pts (70.3%) harbored a *BRCA1* mutation, and 75 (28.2%) a *BRCA2* mutation. 66.9% of patients had FIGO stage III disease. Almost all patients (95.5%) underwent surgical resection, after neoadjuvant chemotherapy (48%), or primary debulking surgery (44.9%). 119 pts (44.7%) received maintenance therapy and majority with bevacizumab alone (78.9%). After a median follow-up of 51.7months, the median PFS (mPFS) was 28.6months [95 CI: 26.2-32.4]. Patients exhibited higher mPFS in case of FIGO stage III than stage IV (32.4months and 25.4months, respectively), or *BRCA2* mutation than BRCA1 (33.3months and 27.7months, respectively). Although populations were non comparable, mPFS was close between patients treated with or without maintenance (28.3 and 29.5months, respectively). Median PFS2 were

51.4months and 50.4months, respectively. Estimated 5-year OS for the whole population was 69.2% [95 CI: 65.3-73.0].
Conclusion: This large ESME OC cohort described clinical features and real-life survival outcomes among pts with newly diagnosed advanced BRCA-mutated HGEOC treated in specialized centres.
Disclosure: This work was supported by UNICANCER. The ESME OC database is supported by an industrial consortium (Roche, Pfizer, AstraZeneca, MSD, Eisai and Daiichi Sankyo). Data collection, analyses and publications are totally managed by R&D UNICANCER independently of the industrial consortium. All the authors declare that there is no conflict of interest regarding the publication of this article.

EP968
Practice trend of PLD in ovarian cancers: experience from tertiary cancer centre in India
R. Roy[1], S. De[2]
[1]Medical Oncology, [2]Gynaecological Oncology, Saroj Gupta Cancer Centre & Research Institute, Kolkata, India

Introduction / Background: Pegylated liposomal doxorubicin (PLD) remains an active agent in treating epithelial ovarian cancer (EOC). It is mainly preferred in second line after progression on taxanes and platinum. Incidence of alopecia being low, PLD is often preferred over taxanes. This retrospective study looks at the practice trend of PLD in EOC.
Methodology: Retrospective data was collected from 52 patients suffering from advanced epithelial ovarian cancer with a median age of 51 years were administered Pegylated Liposomal Doxorubicin (PLD). All these patients had either undergone a primary debulking surgery or an interval cytoreduction.
Results: 32 (62%) patients received PLD and Carboplatin at an interval of 4 weeks while 20 (38%) patients received PLD as monotherapy (as platinum was considered refractory in these patients). 24(46%) patients received PLD in 2nd line (16 with platinum and 8 as monotherapy) and 24(46%) patients in 3rd line (these patients got rechallenge of Paclitaxel and Carboplatin once disease recurred beyond 6 months in 2nd line). The main reason for choosing PLD in second line was lack of response from Taxane/ platinum rather than toxicity profile of PLD. 4 patients preferred PLD in second line because of lesser alopecia. Average no of cycles of PLD administered were 5 and response rates were 30%. Earlier the introduction of PLD, better was the response. Adverse events noted with PLD were neutropenia 5(10%) patients, alopecia 4(8%) patients, PPE 3(6%) patients. Cardiotoxicity was not detected. There were no treatment discontinuations in the PLD only group. In the PLD and Carboplatin group 2 patients had platinum hypersensitivity, thereby leading to omission of platinum.
Conclusion: Tolerance to PLD amongst patients were high, however paclitaxel and carboplatin still remains as one of the priority regimens in second line if disease recurs > 6 months in this part of world.
Disclosure: Nothing to disclose

EP969
Prognostic value of recurrence patterns in ovarian cancer
J.F. Roze[1], J.P. Hoogendam[1], W.B. Veldhuis[2], R.J.P.M. Scholten[3], R.P. Zweemer[1]
[1]Department of Gynaecological Oncology, UMC Utrecht Cancer Center, [2]Department of Radiology, UMC Utrecht Cancer Center, [3]Julius Center for Health Sciences and Primary Care, University Medical Center Utrecht, Utrecht University, Utrecht, The Netherlands

Introduction / Background: Ovarian cancer has high mortality rates since the majority of patients are

diagnosed with advanced disease and develop a recurrence. Identification of specific recurrence patterns that impact prognosis could be useful to distinguish between patients with a relatively good versus a poor prognosis.
Methodology: 179 patients with epithelial ovarian cancer diagnosed between January 2008 and December 2013 received cytoreductive surgery in the University Medical Center Utrecht. 124 of these patients developed recurrent disease of whom 89 patients met the inclusion criteria and formed the study population. Clinical and pathological data was obtained and >40 anatomical sites were scored on the presence of tumour on Computed Tomography scan (CT-scan). A Cox Proportional Hazard model was used to study the influence of the recurrence pattern on survival.
Results: The median survival of patients with mainly intraperitoneal (n=62), hematogenous (n=13) or lymphatic (n=14) disease at recurrence was 25.8, 27.6 and 52.9 months, respectively. Univariate Cox regression identified the following prognostic factors for overall survival (OS): main type of disease at recurrence (p=0.03) , residual tumour at initial surgery (p=0.001), ascites at primary disease(p< 0.001), BRCA mutation status (p< 0.001) and tumour histology (p=0.04). In the multivariate analysis including these covariates and age, main type of disease retained a prognostic role for OS . Patients with complete debulking had more often mainly lymphatic disease at recurrence (26 vs 7%), as compared to patients with incomplete debulking who had more often mainly peritoneal disease (76 vs 63%, p=0.045).
Conclusion: Patients with mainly lymphatic disease at recurrence had a better OS than patients with mainly peritoneal or hematogenous disease. Classification of the main type of disease at recurrence can help to distinguish between patients with a relatively good versus a poor prognosis.
Disclosure: Nothing to disclose

EP970

PIK3R1 mRNA expression is associated with copy number alteration in ovarian cancer

I.K. Rzepecka, B. Konopka, A. Podgorska, A. Stachurska, R. Lotocka, A. Budzilowska, A. Dansonka-Mieszkowska, P. Leszczynski, M. Lukasik, U. Piekarska, A. Tysarowski, J. Kupryjanczyk
Department of Pathology and Laboratory Diagnostics, Maria Sklodowska-Curie Institute - Oncology Center, Warsaw, Poland

Introduction / Background: The phosphoinositide 3-kinase (PI3K) signaling cascade is one of the most frequently deregulated cascades in human cancers. The possibility of its inhibiting makes the PI3K pathway an attractive target for personalized therapy. We aimed to determine mRNA expression of *PIK3R1*, a gene encoding the p85α regulatory subunit of PI3K.
Methodology: *PIK3R1* mRNA expression analysis was carried out for 144 ovarian cancers and 5 specimens of noncancerous fallopian tube as a control group. Expression was evaluated using the quantitative PCR (qPCR) method and TaqMan Gene Expression Assays. Statistical analyses were done with the Mann-Whitney U test, the Cox's and logistic regression models.
Results: *PIK3R1* mRNA expression was significantly decreased in ovarian cancers compared with control tissues (P = 0.0028). The difference in mean expressions between cancers and normal tissues was 70% (0.1167 ± 0.34 and 0.3981 ± 0.13 in ovarian cancers and a control group, respectively). Reduced expression may be a consequence of allele loss of the *PIK3R1* gene, because there is an association between mRNA level and copy number alteration of the gene (P = 0.0001). Lower level of *PIK3R1* mRNA was observed in non-serous (P = 0.0437), poorly or mostly undifferentiated ovarian cancers (P = 0.0114) diagnosed in patients under 54 years of age (P = 0.077). Reduced expression of *PIK3R1* was associated with the presence of *TP53* mutation (P = 0.0106) but not with *PTEN* loss or *PIK3CA* amplification. Patients overall survival, disease-free survival, complete remission and platinum sensitivity were not associated with *PIK3R1* mRNA expression level.
Conclusion: Ovarian cancers have decreased *PIK3R1* expression at mRNA level and it may be a consequence of the *PIK3R1* gene copy loss.
Grant Support: This study was supported by the grant no. 2016/23/B/NZ5/00572 of the National Science Centre, Poland.
Disclosure: Nothing to disclose

EP971

Survival impact of wide resection of the pelvic peritoneum in patients with epithelial ovarian cancer

A. Sagara[1], T. Motohara[1], Y. Iwagoi[1], F. Saito[1], K. Takaishi[1], Y. Miyahara[1], I. Sakaguchi[1], R. Honda[1], H. Tashiro[2], H. Katabuchi[1]
[1]Department of Obstetrics and Gynecology, Faculty of Life Sciences, [2]Department of Maternal-Newborn Nursing, Kumamoto University, Kumamoto, Japan

Introduction / Background: The present study was designed to evaluate the effect of the peritoneal stripping method termed “wide resection of the pelvic peritoneum (WRPP)” on eliminating disseminated tumors in the pelvic peritoneum and whether there is any survival benefit for patients with epithelial ovarian cancer.
Methodology: From January 2000 to December 2018, 460 patients with epithelial ovarian cancer were treated surgically at Kumamoto University Hospital. The clinical and surgical records of these patients were reviewed retrospectively, and 225 patients with ovarian cancer who underwent standard definitive surgery were included in this study. Eligible patients with ovarian cancer were divided into 3 groups according to surgical approach. Patients in the standard surgery group underwent cytoreduction with standard surgical techniques (n=65). The WRPP group consisted of patients who underwent standard surgery plus WRPP (n=125). The RS group included patients who required standard surgery plus rectosigmoidectomy (RS) (n=35). The primary outcome measure was overall survival. The secondary endpoint was recurrence-free survival.
Results: Overall survival was significantly longer in the WRPP group than in the standard surgery group for patients with stage III-IV disease (P< 0.05). The WRPP group was less prone to recurrence than the standard surgery group (P=0.70). In addition, the intrapelvic recurrence rate differed significantly between the standard surgery and WRPP groups (38.2% vs. 23.8%, P< 0.01).
Conclusion: WRPP is an effective procedure that should be a part of the skill set of any gynecological surgeon performing surgical cytoreduction in patients with advanced ovarian cancer. The addition of this surgical technique has the potential to improve patient outcomes.
Disclosure: Nothing to disclose

EP972

Precision medicine program for epithelial ovarian carcinoma (EOC): first year experience at Clínica Universidad de Navarra (CUN)

L. Sánchez Lorenzo[1], I. Espinosa Mariscal[2], E. Chacón[3], A. Patiño-García[4], J. Espinós[1], J.M. Aramendia[5], T. Castellanos[6], D. Vázquez[6], J.Á. Minguez[3], G. Alkorta-Aramburu[4], M.Á. Idoate[7], J.L. Alcázar[3], L. Chiva[6], A. González-Martín[1]
[1]Medical Oncology, Clínica Universidad de Navarra, [2]Pathology, Clinica Universidad de Navarra, Madrid, [3]Gynaecology, [4]CIMA-LAB Diagnostics, [5]Medical Oncology, Clínica Universidad de Navarra, Pamplona, [6]Gynaecology, Clinica Universidad de Navarra, Madrid, [7]Pathology, Clinica Universidad de Navarra, Pamplona, Spain

Introduction / Background: Next Generation Sequencing (NGS) technology enables for the simultaneous study of multiple gene alterations in the patient's tumor with potential implications for genetic risk assessment, prognosis, and therapy.
Methodology: CUN opened a new hospital in Madrid in January 2018. During the first year, new patients (pts) with EOC at both CUN were offered to perform a NGS-based tumor testing. Local test consisted in Oncomine Comprehensive Assay (161 gene panel, ThermoFisher Scientific) and external test in Foundation Medicine (Foundation Medicine, Cambridge, MA). This is a descriptive analysis of the rate of implementation, the molecular alterations found, and the therapeutic implications derived.
Results: 104 new pts were evaluated from January 1stuntil December 31st. 5 (4.80%) LOGSOC, 81

(77.89%) HGSOC, 11 (10.58%) CCC, 6 (5.77%) Endometrioid, 1 (0.96%) Mucinous. 44 pts with primary disease and 60 pts with recurrent disease. All but two patients were tested with Oncomine with a median turn-around time of 14 days.
Table shows the rate of patients tested according to histology and context of disease.
Subtype N Tested in First Line Tested in recurrence
LGSOC 5 2/2 (100%) 1/3 (33.33%)
HGSOC 81 17/34 (50%) 14/47 (29,78%)
Endometrioid 6 1/2 (50%) 0/4 (0%)
CCC 11 3/6 (50%) 1/5 (20%)
Mucinous 1 0/0 0/1 (0%)
TOTAL 104 23/44 (52.57%) 16/60 (26.67%)
sBRCA mutation was detected in 6/23 primary diagnosis (26.1%) and 2 pts (both gBRCAwt) received olaparib maintenance after SOLO-1 publication. In the recurrent setting, 5/16 pts (31.25%) had at least one actionable mutation with available matched therapy and 3 pts received matched therapy (18.76%). Non-BRCA genes associated to hereditary EOC were detected in 4 pts.
Conclusion: Integration of a precision medicine program with NGS tumor testing was feasible in routine practice in a private university hospital. This is an ongoing program and the rate of tumor testing should improve in subsequent years.
Disclosure: Advisory for Roche and Tesaro

EP973

"Tumour First": an institutional experience of reflex tumour BRCA testing in ovarian epithelial carcinomas

G. Santandrea[1], M.M. de Jonge[2], N. Solleveld-Westerink[2], K.N. Gaarenstroom[3], H.M. Hazelbag[4], M.J. Kagie[5], E.J.M. Ahsmann[6], J.R. Kroep[7], V.T.H.B.M. Smit[2], C.J. van Asperen[8], N. van der Stoep[8], T. van Wezel[2], T. Bosse[2]
[1]Department of Pathology, Sant'Orsola-Malpighi University Hospital, Bologna, Italy, [2]Department of Pathology, [3]Department of Gynecology, Leiden University Medical Center, Leiden, [4]Department of Pathology, [5]Department of Gynecology, Haaglanden Medical Center, The Hague, [6]Department of Pathology, Groene Hart Ziekenhuis, Gouda, [7]Department of Medical Oncology, [8]Department of Clinical Genetics, Leiden University Medical Center, Leiden, The Netherlands

Introduction / Background: Pathology-based "reflex" tumour testing for *BRCA* variants in tubo-ovarian carcinomas (OC) has been proposed as patient-friendly, efficient and cost-effective approach to pre-select women for germline testing and stratify for eligibility of PARP-inhibitor therapy. The aim of this study was to review our first two years' experience of a "tumour first" approach *BRCA* screening in our region, using a previously validated *BRCA* tumour test.
Methodology: *BRCA* reflex testing for OC has been introduced in September 2017. The tumour test consisted of targeted Next Generation Sequencing (NGS) for detection of *BRCA* and (since July 2018) 13 additional HR genes in combination with *BRCA1*-specific copy number variant Multiplex Ligation-dependent Probe Amplification (MLPA). All detected variants and patient characteristics were extracted from the pathology reports. Follow-up data for patients with a likely or known pathogenic variant were collected from the clinical genetics.
Results: 99 reflex BRCA tumour tests were performed on 69 (69.7%) high grade serous carcinomas (HGSC), 12 (12.1%) low grade serous carcinomas, 8 (8%) mucinous carcinomas, 5 (5.1%) endometrioid carcinomas and 5 (5.1%) clear cell carcinomas. Tumour DNA was isolated from 68 (68.7%) resection specimens, 24 (24.2%) biopsies and seven (7.1%) ascites fluid. NGS and MLPA success rate was 100% and 94.6% respectively. 30 (30.3%) OC harboured a variant: 15 (15.2%) pathogenic variants, 6 (6.1%) likely pathogenic variants and 9 (9.1%) variants of uncertain significance. All cases with either a pathogenic or likely pathogenic variant were HGSC. Of the 19/21 (90.4%) patients for which genetic testing was advised, 13 (68.4%) underwent genetic testing and 6/13 cases (46.2%) harboured a germline variant.
Conclusion: Reflex tumour testing confirmed its effectiveness and usefulness as a routine pre-screening tool for patients diagnosed with an EOC. Furthermore all pathogenic or likely pathogenic variants were found in HGSC, which would support a histotype-based tumour testing approach in the

future.
Disclosure: Nothing to disclose

EP974

Review of three cases of high grade serous ovarian cancer associated with cerebellar atrophy and acute inflammatory demyelinating polyneuropathy

A. Liapi[1], R.G. Herrera Gómez[1], E. Sakeliades[2], A. Sarivalasis[1]
[1]CHUV- Centre Hospitalier Universitaire Vaudois, Lausanne, Switzerland, [2]Athens Medical Centre, Athens, Greece

Introduction / Background: Paraneoplastic neurological syndromes (PNS) are rare complications of ovarian cancer. We report three (3) cases of high grade serous ovarian cancer with PNS as presenting symptom.
Methodology: Three previously healthy women aged 61, 70 and 73 years respectively, were referred to Lausanne university hospital (CHUV) for investigation of recent onset neurological symptoms. Two of them suffered from static and kinetic cerebellar ataxia with the latter also experiencing dysarthria. The third patient presented rapidly progressive symmetric ascending limb weakness with areflexia and motor respiratory failure.
Brain and spinal cord MRIs did not reveal any significant pathology. The onco-neuronal serology was positive for anti-Yo antibodies in the patients with ataxia and thus enabled the diagnosis of a PNS. The patient with the paralytic syndrome was tested positive for anti-ganglioside (GD1b, GM1) antibodies and negative for onco-neuronal antibodies. The diagnosis of acute inflammatory demyelinating polyneuropathy was confirmed by elevated cerebrospinal fluid protein and by electromyoneurography. All three patients were eventually diagnosed with a FIGO III high grade serous ovarian cancer.
Results: Following platinum based chemotherapy and complete surgical debulking, two of the patients recovered completely from their neurological symptoms. The third-one, who refused surgery and was only treated with platinum-based chemotherapy, experienced a transient improvement of the cerebellar ataxia and of the dysarthria. All three patients are free of oncological relapse to this day.
Conclusion: Our report indicates that ovarian carcinoma can be associated with both central and peripheral PNS. Optimal oncological management of the underlying malignancy provides the best chances for complete neurological recovery. In our series the occurrence of PNS affected neither the prognosis nor the efficacy of cancer treatment.
Disclosure: Nothing to disclose

EP975

Risk factors for ovarian cancer: an umbrella review

A. Semertzidou, E. Wheelan, I. Kalliala, S. Lever, M. Kyrgiou
Imperial College London, London, United Kingdom

Introduction / Background: Ovarian cancer is one the deadliest gynaecological malignancies and many risk factors for its occurrence have been described. Despite the wealth of data on the subject, the robustness of evidence available has not yet been extensively evaluated. This review aims to fill this gap and assess the strength and validity of evidence regarding the interrelationship between various risk factors and ovarian cancer incidence and mortality.
Methodology: Design: Umbrella review of systematic reviews and meta-analyses.
Data sources: PubMed, Medline, Cochrane Database of Systematic Reviews.
Eligibility criteria: Systematic reviews and meta analyses of observational studies investigating the relationship between various risks factors and ovarian cancer incidence and mortality and all- cause

mortality.
Data analysis: The evidence from the meta-analyses were graded according to a statistical criteria comprised of the random effects estimate of the meta-analyses and of their largest study, the number of cases, 95% prediction intervals, I^2statistic of heterogeneity between studies, excess significance bias, small studies effect and sensitivity analysis using credibility ceilings. From this, meta-analyses were classified as strong, highly suggestive, suggestive or weak evidence.
Results: 205 meta-analyses from 71 eligible papers were assessed and 29 risk factors were identified. We observed strong evidence on the association between BMI as categorical variable (>30 vs. <25) and ovarian cancer incidence: RR 1.27 (1.19-1.36). For BMI as a continuous variable (per 5 unit increase), the summary effect estimate was smaller (1.27 *v* 1.08), and the evidence was judged as only suggestive. Between DM types 1 and 2 together and ovarian cancer incidence and mortality, the evidence was considered weak but suggestive evidence was observed for DM and all- cause ovarian cancer mortality. Additional data will be presented at the conference.
Conclusion: Ovarian cancer incidence appears to be strongly associated with high BMI, while a weaker association was observed with DM.
Disclosure: Nothing to disclose

EP976

The impact of extent of cytoreductive surgery (CRS) on epithelial ovarian cancer (EOC), primary peritoneal carcinomatosis(PPC), and Fallopian tube cancer (FTC) at the American University of Beirut (AUBMC)

M. Seoud[1], A. El Housheimi[1], I. Jaafar[2], K. Hamed[2], F. Jamali[3], R. Abdallah[1], A. Khalil[1]
[1]Obstetrics and Gynecology, [2]American Universtiy of Beirut Medical Center, [3]General Surgery, American Universtiy of Beirut Medical Center, Beirut, Lebanon

Introduction / Background: To study the impact of the extent of CRS [complete Debulking (CD), optimal Debulking (OD) ≤ 1cm, or suboptimal Debulking (SOD) >1cm residual disease] on progression free (PFS) and overall survival (OS) in advanced EOC who underwent primary debulking (PDS) or interval debulking surgery (IDS) between 1998-2018 at AUBMC
Methodology: A retrospective review of the impact of extent of CRS on PFS and OS
Results: of 300 patients with EOC, PPC and FTC, 220 were found to have advanced epithelial cancer (stage IIIB, IIIC and IV). 66.4% had CD, 29.7% OD and 2.6% SOD. Results are shown in tables 1 (Pic 1) and 2 and 3 (Pic 2). Median survival is expressed in months
Conclusion: The extent of the cytoreductive surgery significantly prolonged PFS but not OS in stage IIIC but not IV. However this increase in PFS is only seen in the interval setting. Patients who undergo CD have similar PFS and OS regardless of the timing of CRS.

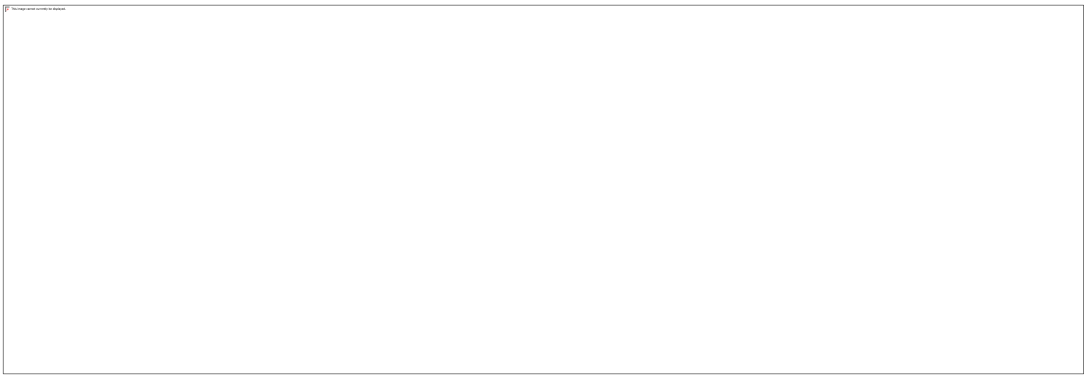

[Table 1]

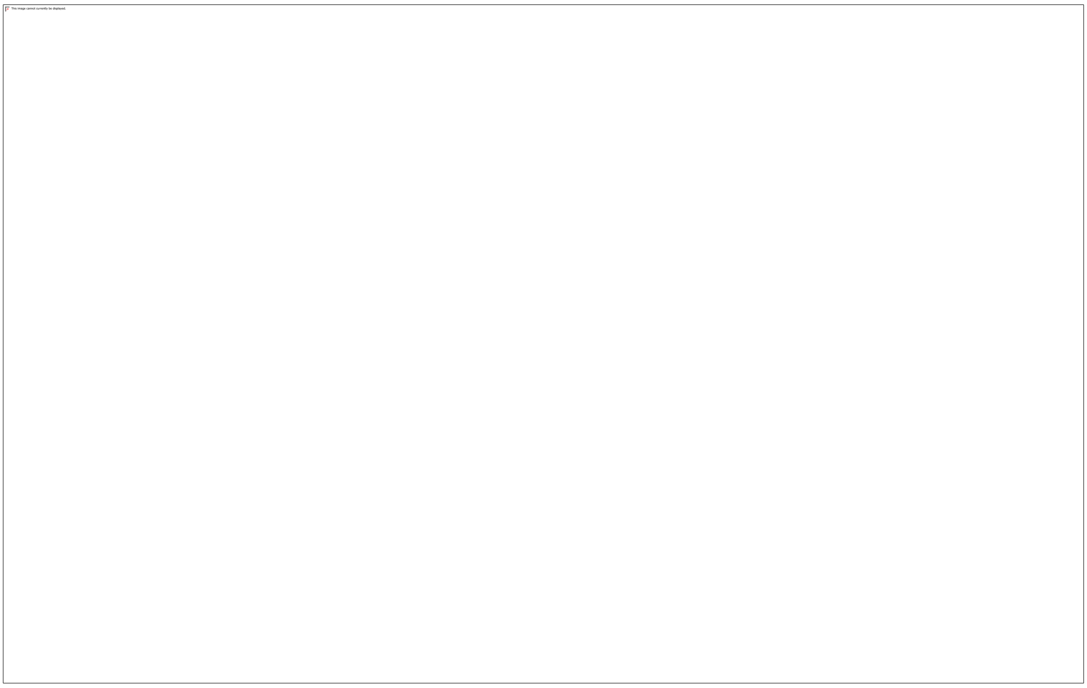

[Table 2 and 3]

Disclosure: Nothing to disclose

EP977

Impact of timing of cytoreductive surgery (CRS) on epithelial ovarian cancer (EOC), primary peritoneal carcinomatosis (PPC), and fallopian tube cancer (FTC) at American University of Beirut Medical Center (AUBMC)

M. Seoud[1], A. El Housheimi[1], I. Jaafar[1], K. Hamed[1], F. Jamali[2], R. Abdallah[1], A. Khalil[1]
[1]Obstetrics and Gynecology, [2]General Surgery, American Universtiy of Beirut Medical Center, Beirut, Lebanon

Introduction / Background: To study the impact of timing of CRS weather done at diagnosis or following neoadjuvant chemotherapy(NACT) on progression free(PFS) and overall survival(OS) of patients with advanced EOC between 1997-2017 at AUBMC. Decision to do primary(PDS) or interval debulking surgery(IDS) is based on factors that help determine the probability to achieve complete debulking (Stage, Tumor markers, tumor burden on imaging, clinical and performance status).
Methodology: A retrospective review of the impact of PDS versus NACT followed by IDS on PFS and OS.
Results: Of 300 patients with EOC, PPC and FTC, 220 were found to have advanced epithelial cancer (stage IIIB, IIIC and IV). 63% had interval debulking surgery (IDS) while 37% had primary debulking (PDS). Results are shown in table #1.
Conclusion: Timing of the CRS (PDS vs. IDS) significantly impacts PFS but not OS in stage IIIC but not IV EOC. This difference in survival is explained by the higher tumor burden, higher morbidity, and worse performance status of patients operated in the interval setting.

[Table#1]

Disclosure: Nothing to disclose

EP978

A radiologic-laparoscopic model to predict suboptimal (or complete and optimal) debulking surgery in advanced ovarian cancer: a pilot study

A. Serra[1,2], K. Delgado[1], K. Maaiochi[1], R. Jativa[1], L. Gomez[1], J. Escrig[1,2], A. Llueca[1,2], MUAPOS working group (Multidisciplinary Unit of Abdominal Pelvic Oncology Surgery)
[1]MUAPOS Working Group (Multidisciplinary Unit of Abdominal Pelvic Oncology Surgery), Hospital General Universitario de Castellon, Castello, [2]Universitat Jaume I, Castelló, Spain

Introduction / Background: Medical models assist clinicians in making diagnostic and prognostic decisions in complex situations. In advanced ovarian cancer, medical models could help prevent unnecessary exploratory surgery. We designed two models to predict suboptimal or complete and optimal cytoreductive surgery in patients with advanced ovarian cancer.
Methodology: We collected clinical, pathological, surgical, and residual tumor data from 110 patients with advanced ovarian cancer. Computed tomographic and laparoscopic data from these patients were used to determine peritoneal cancer index (PCI) and lesion size score. These data were then used to construct two-by-two contingency tables and our two predictive models. Each model included three risk score levels; the R4 model also included operative PCI, while the R3 model did not. Finally, we used the original patient data to validate the models (narrow validation).
Results: Our models predicted suboptimal or complete and optimal cytoreductive surgery with a sensitivity of 83% (R4 model) and 69% (R3 model). Our results also showed that PCI > 20 was a major risk factor for unresectability.
Conclusion: our findings emphasize that the proper application of our R3-R4 models in primary debulking surgery requires maximal surgical effort including UAS techniques and well-prepared multidisciplinary surgical teams. These two models can predict CCS+OCS and SCS, depending on the SCS prevalence of the surgical team. The dynamic nature of the models means that they can be applied in different institutions with different OCS or SCS rates,and they can be changed with changes in the SCS prevalence of the surgical team. The proper integration of the R3 and R4 models with recently-developed molecular and clinical-radiological models, including age, performance status, and comorbidities may help improve the decision-making process in the future.
Disclosure: Nothing to disclose

EP979

Clinicopathological and survival analysis of ovarian malignant germ cell tumors: twenty five years experience of single institution

Y. Sadigova[1], S. Seyfettinoglu[1], M.A. Vardar[1], A.B. Guzel[1], D. Gumurdulu[2], G. Seydaoglu[3], U. Kucukgoz Gulec[1]
[1]Obstetrics and Gynecology, [2]Pathology, Cukurova University Faculty of Medicine, [3]Dep. of Biostatistics, Faculty of Medicine, University of Cukurova, Adana, Turkey

Introduction / Background: Ovarian malignant germ cell tumors (OMGCT's) are derived from primitive germ cells of the embryonic gonad and they are heterogenous tumors. OMGCT should be investigated separately from other tumors for its association to long-term survival, different pathways and biological characteristics. This study was performed to evaluate the clinical and pathological characteristics of patients who were followed up for OMGCT in the last twenty-five years in our clinic. We also aimed to investigate prognostic parameters and the effect of findings on survival rates.
Methodology: One hundred and fifteen patients with OMGCT, treated and followed-up between April 1992 and November 2017 were included to this study. Clinical characteristics, operative information, pathologic findings, treatment and follow-up data of the patients were gathered from medical records. The parameters on survival was investigated. Survival analysis were performed using Kaplan - Meier test. Overall survival (OS) and Disease Free Survival (DFS)'s statistically significance was evaluated with Log-Rank test. Reproductive outcomes were analyzed.
Results: The average age of patients were 27.5±14.3. Mean follow-up time was 71 months. Approximately 61 (53%) of patients was Stage 1. Complete staging surgery (CSS) was performed 60 patients. Fertility-sparing surgery (FSS) was performed 55 patients. Nineteen (34.5%) of the FSS patients had pregnancy and 15 of them had live births. Poor prognostic factors were suboptimal cytoreduction, advanced stage disease, poor differentiation, metastasis, mixed germ cell histological type, co-morbidity of patients. Factors affecting disease-free survival (DFS) in multivariate analyzes are advanced stage, mixed germ cell histology, and suboptimal cytoreduction. The 5-year survival rate of the 115 patients was 74%.
Conclusion: Both DFS and OS period of mixed germ cell ovarian tumors were shown to be significantly lower than other histologic types. The high pregnancy rates after fertility surgery are hopeful for patients.
Disclosure: Nothing to disclose

EP980

Gonadal vein graft after retroaortic left renal vein injury during paraaortic lymphadenectomy: a case report

S. Seyfettinoglu[1], G. Khatib[1], U. Kucukgoz Gulec[1], A.B. Guzel[1], Y. Bayazit[2], M.A. Vardar[1]
[1]Obstetrics and Gynaecology, Gynaecologic Oncology, [2]Urology, Cukurova University Faculty of Medicine, Adana, Turkey

Introduction / Background: Pelvic and paraaortic lymphadenectomy is a major component of the surgical staging procedure for several gynecological malignancies. The removal of these lymph nodes needs expertise as there is a danger of the major vessel injury during the surgery. In this presentation We would like to report a case in which there was an accidental injury to the left renal vein during para-aortic lymphadenectomy.
Methodology: Case: 42 years old patient referred to our clinic with abdominal mass and ascites. After systemic examination, imaging, paracentesis cytology with serous adenocarcinoma and tumor board

decision the patient received three cycles of neo-adjuvant chemotherapy followed by interval debulking surgery. During paraaortic lymphadenectomy nearly half of left renal vein was accidentally ligated and cut with ultrasonic energy device. Left renal vein was retroaortic. After haemostasis we evaluate the renal circulation and was of the opinion that residual patency of the renal vein was not adequate for functioning of the left kidney.The stump of the damaged renal vein was tied, and the venous blood of the left kidney was diverted through an end to side anastomosis of the left gonadal vein with the venacava (ımage 1). On the post operative period the patient was recovered quickly. Two months later her Tc-99 DMSA and other renal examinations was normal. She is presently well.

Results: Retroaortic left renal vein is a normal anatomical variant and its recognition is important in order to avoid complications during retroperitoneal surgery. Management of complications in the literature is various. In our case report, using the gonadal vein as an alternative to the left renal vein saved the patient from nephrectomy.

Conclusion: Gonadal vein graft for maintaining renal circulation can be a good alternative management in renal vein injuries.

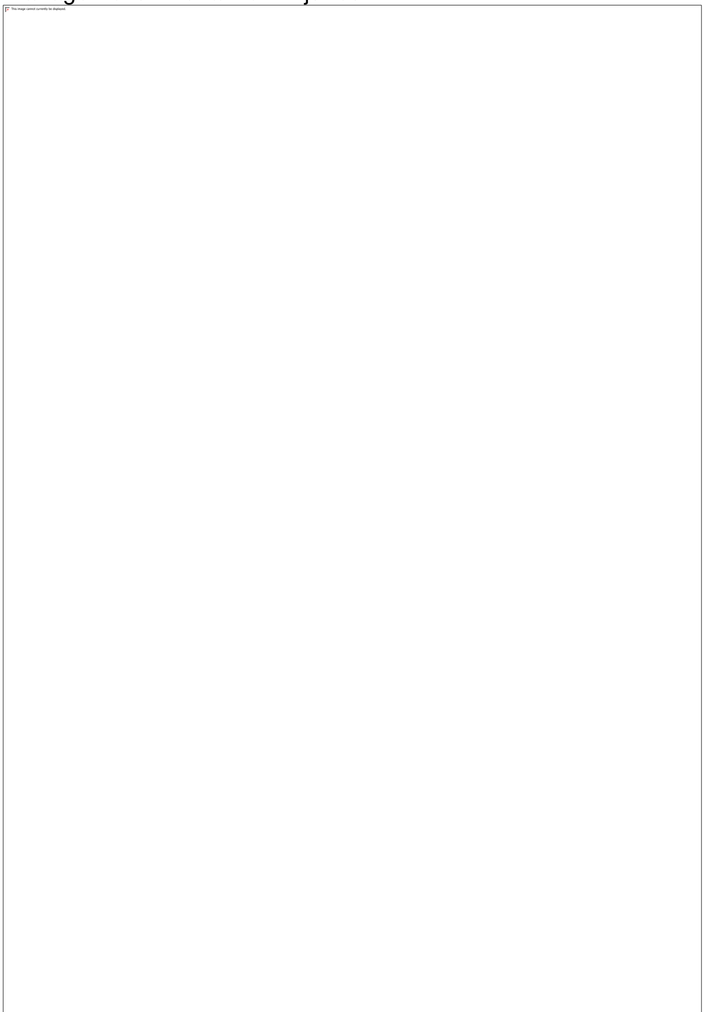

[Anastomosis of left gonadal vein with vena cava]

Disclosure: Nothing to disclose.

EP981

Cytoreductive surgery for tubo-ovarian carcinoma - a study of surgical outcomes with primary surgery

Y. Shahabuddin, R. Daly, C. Thompson, N. Gleeson
Gynaecological Oncology, St James's Hospital / Trinity College Dublin, Dublin, Ireland

Introduction / Background: Primary debulking/cytoreductive surgery (PDS) is considered the optimal approach in management of tubo-ovarian carcinoma (TOC). Not all patients are suitable for primary surgery, and neoadjuvant chemotherapy (NACT) followed by interval cytoreductive surgery (IDS) is considered to be not inferior to PDS. At this Irish tertiary gynaecological oncology centre the treatment approach is determined by the multidisciplinary team (MDT).
Objective: The aim of this study was to analyse the operative outcomes of the MDT decision to assign patients to primary surgery.
Methodology: An observational retrospective study of consecutive patients with TOC presenting during 2018. Borderline, low grade and non-epithelial tumours were excluded.
Results: Eighty-eight patients were assessed by MDT and of 57 eligible, radiological stage was I/II (16, 28%), III (25, 44%), IV (16, 28%). Most were high grade serous histology.
Thirty patients (52.6%) had PDS. Surgery included hysterectomy (29, 96.67%), salpingo-oophorectomy (30, 100%), appendicectomy (27, 90%) , omentectomy (30, 100%) , small bowel resection (2, 6.67%), large bowel resection (11, 36.67%), splenectomy (3,10%), partial gastrectomy (5, 16.67%), diaphragmatic stripping (8, 26.67%), partial resection of diaphragm (7, 23.33%) , pelvic lymphadenectomy (30, 100%), para-aortic lymphadenectomy (30, 100%). Complete cytoreduction was achieved in 27 (90%).
Blood loss average 1361 (50-5100) mls. Duration of surgery average 207 (30-385) mins. Post operative stay average 17.7 (4-84) days . Clavien dindo scores average 2. There were two postoperative deaths. Twenty-four (80%) progressed to chemotherapy.
Conclusion: Most women presenting with TOC had advanced stage disease. Those assigned to primary surgery had multiple procedures but the majority achieved complete cytoreduction. Perioperative morbidity and mortality were substantial. Only 80% of PDS progressed to adjuvant chemotherapy.
Disclosure: Nothing to disclose

EP982

Management of Indeterminate pelvic masses and borderline ovarian tumours: a survey of current practice of gynaecological Oncology Centres in the United Kingdom

R. Jones[1], R. Howells[1], E. Rzyska[2], K. Lim[1], A. Sharma[1]
[1]*Department of Gynaecological Oncology,* [2]*Department of Obstetrics & Gynaecology, University Hospital of Wales, Cardiff, United Kingdom*

Introduction / Background: Borderline ovarian tumours (BOT) represent approximately 15% of ovarian tumours. Currently there is no clear guidance on management or follow up, leading to variation in practice. We aimed to assess current practice in gynaecological oncology centres around the United Kingdom.
Methodology: We surveyed the membership of the British Gynaecological Cancer Society (BGCS) via an online survey platform from 01/11/2017-30/06/2018. A single reminder was sent during the collection period. Information was gathered about use of frozen section for indeterminate mass at laparotomy, surgery offered for those who were post-menopausal or with family completed versus those wanting fertility sparing, offer of completion surgery for those who had initial fertility sparing surgery, whether an agreed protocol for follow up of BOT was in place, length of follow up and whether adjuvant treatment is offered. Data analysis was with Microsoft Excel.
Results: Responses were received from 19 of the 33 (58%) UK gynaecological oncology centres. 13 (72%) centres reported frozen section use. 18 (95%) centres reported offering total abdominal

hysterectomy and bilateral salpingo-oophorectomy for women who were post-menopausal or reported their family was complete. All 19 centres reported offering fertility sparing surgery in those where family not completed. 16 (89%) centres reported offering completion surgery for those who had initial fertility sparing surgery. 16 (89%) centres reported having an agreed protocol for follow up of BOT. Only 5 (28%) centres reported offering adjuvant treatment depending on presence of invasive implants or advanced disease with 12 (67%) not offering any additional treatment.
Conclusion: Based on responses there is variation in the management of indeterminate pelvic masses and borderline ovarian tumours between centres. These findings suggest that there is a need to develop a UK wide management strategy to provide uniformity of care.
Disclosure: Nothing to disclose

EP983

PTEN down regulation induce apoptosis in epithelial ovarian cancer cells

J.W. Shin
Gil Medical Center | Obstetrics and Gynecology, Gachon University College of Medicine, Incheon, Republic of Korea

Introduction / Background: PTEN is known as a tumor suppressor protein that are frequently mutated or down regulated in various human cancers. Down regulation of PTEN, however, may induce anti proliferative effect.
The authors investigate the apoptosis of cancer cells after down regulation of PTEN in epithelial ovarian cancer cells.
Methodology: Epithelial ovarian cancer cells, SK-OV-3 was cultured with 10% FBS in culture medium. The siRNA for PTEN and control were constructed and transfected with Lipofectamine. The experiments were FACS for investigating the proportion of apoptotic cells and Western blot for proteins analysis.
Results: Apoptosis cell proportion was significantly increased (p =0.001) on FACS analysis. Expression of P53 was not detected in both PTEN downregulated cells or not on western blot. Expression of p21was increased and expression of cyclin B1 decreased in PTEN down regulated cells on western blots..
Conclusion: Down regulation of PTEN induce apoptosis in epithelial ovarian cancer cells and could be a new strategy for ovarian cancer treatment.
Disclosure: Nothing to disclose

EP984

Granulosa-cell tumors during ovarian stimulation

L. Shirinzadeh
Fellowship of Gynecologic Oncology, Faculty of Medicine, Mashhad University of Medical Sciences, Mashhad, Islamic Republic of Iran

Introduction / Background: ovarian superovulation and increased follicle-stimulating hormone concentrations in the treatment of infertility may be the risk factors of the developed granulosa-cell tumor. This is a question that the answer is unclear and requires further studies. The aim of this report is to introduce a case of the granulosa-cell tumor which was discovered after ovarian stimulation.
Methodology: A 31- year-old woman with the clinical presentation of massive abdominal distention was referred to oncology department of our academic hospital of Medical Sciences in 2017. She had history of secondary infertility and she was undergoing In Vitro Fertilization protocol and ovarian stimulation) in Aug 2017 but, the cycle was cancelled. She suffered from gradual abdominal distention

one month after the end of IVF procedure despite of failure of pregnancy. After 2-3 months of management ovarian hyper stimulation syndrome investigation revealed large ovarian mass and increased tumor marker of inhibin.
Results: This led to explorative laparotomy that revealed stage III ovarian cancer. Report of final pathology indicated juvenile granulosa cell tumor. So, optimal surgical staging and cytoreductiv surgery without fertility preserving was perfumed. du- to advanced stage of ovarian cancer chemotherapy commended Unfortunately less than six months ,she experienced metastatic diseases in pelvic and abdomen and currently receiving second line chemotherapy.
Conclusion: Persistent ovarian enlargement or ascites during or after infertility treatment should be carefully considered and managed.
Disclosure: Nothing to disclose

EP985

Morbidity outcomes of cytoreductive surgery in advanced low grade serous and sex chord stromal tumor

T. Shylasree[1], B. Dash[1], M. Bhandare[2], R. Bansal[1], J. Ghosh[3], D. Mahajan[1], R. Kerkar[1], A. Maheshwari[1]
[1]Gynaecological Oncology, [2]Surgical Oncology, [3]Medical Oncology, Tata Memorial Centre, Mumbai, India

Introduction / Background: Extensive Cytoreductive surgery (CRS) is the main stay of treatment for advanced Low grade serous ovarian cancer(LGSC) and sex chord tumor (SCST) group, however due to young age at presentation and good performance status , major surgical complications are lower than serous high grade tumors(HGSC).
Aim: To assess Postoperative morbidity of CRS in advanced LGSC/SCST.
Methodology: Nineteen patients with extensive abdominal disease underwent CRS from June 2016-Jan 2019. The median age was 43yrs (22-56). 15 patients had LGSC and 4 patients had SCST confirmed on histology. Ten out of 19 patients had received chemotherapy with no response prior to referral.
Results: Mean PCI was 15(6 - 30). Fifteen patients had complete cytoreduction (CC-0/1). The median surgical time was 9 hrs. (2.5 -10.5). Seventeen patients underwent peritonectomy, 10 patients needed multiple GI resections , 1 patient distal pancreaticosplenectomy, 1 patient had an elective nephrectomy for nonfunctioning kidney. Median blood loss was 2500 ml (250 -11500 ml). Median postoperative ventilation required was 1 day (0-7), Median ICU stay 3 days (1-10) and hospital stay 16 days (9-38).
Seventeen patients had minor postoperative complication. Three had grade 3 -4 complication (aortic graft, wound dehiscence, SIRS with prolonged intubation). There was no postoperative mortality. Median follow up was 14 months (3-30). LGSC patients were put on either letrozole /tamoxifen as maintenance therapy. Nine patients are disease free, 6 have stable disease and 3 have progressive disease, one lost to follow up. Overall median PFS is 14 months. The average cost of treatment was USD 1542 and USD 13878 for subsidized/nonsubsidized category respectively.
Conclusion: CRS in LGSC/SCST has lower morbidity and almost nil mortality compared to published literature in HGSC. This may be attributable to younger age and good performance status of such patients. However the cost of surgery needs to be addressed in mid-income countries.
Disclosure: Nothing to disclose

EP986

Hypoalbuminaemia in Advanced Ovarian Cancer (AOC): primary or interval surgery?

D. Dai[1], J. Balega[2], S. Sundar[2,3], S. Kehoe[2,3], A. Elattar[2], J. Nevin[2], A. Phillips[4], K. Singh[2]
[1]Luton and Dunstable University Hospital NHS Trust, Luton, [2]Pan-Birmingham Gynaecological Cancer Centre, City Hospital, [3]Institute of Cancer and Genomic Sciences, University of Birmingham, Birmingham, [4]Department of Obstetrics and Gynaecology, Royal Derby Hospital, Derby, United Kingdom

Introduction / Background: To investigate the impact of serum albumin (both at diagnosis and pre-operatively) on survival in patients undergoing cytoreductive surgery for AOC and whether any change in albumin achieved following Neoadjuvant chemotherapy (NACT) affects overall survival (OS). Pre-operative albumin is known to be a marker of survival in AOC. However, little is known of the outcomes of hypoalbuminaemic patients after neo-adjuvant chemotherapy (NACT).
Methodology: Outcomes of 441 patients who underwent cytoreduction for AOC between 16/08/2007-03/02/2014) at the Pan-Birmingham Gynaecological Cancer Centre (PBGCC) were reviewed. Albumin was recorded both at diagnosis and immediately pre-operatively. If hypoalbuminaemic at diagnosis, they were further analysed according to whether they received primary debulking (PDS) or interval debulking (IDS) with normalisation of albumin or IDS without normalisation of albumin.
Results: 308 patients had a diagnosis albumin level available for analysis and 400 patients had an immediate pre-operative albumin available for analysis. For patients with a diagnosis albumin ≤35g/L and ≥36g/L median OS was 31.5 (95% CI 23.5 - 39.5) and 50.4 (95% CI 38.9 - 61.9) months respectively (P=0.003). On multivariate analysis (MVA) adjusting for cytoreductive outcome, stage and grade, diagnosis albumin remained statistically significant (p = 0.04, Hazard ratio 1.38, 95% CI 1.01 - 1.89). Preoperative albumin showed a significant difference between low and normal albumin levels (p=0.003) which was not confirmed on MVA.
53% of patients hypoalbuminaemic at diagnosis achieved complete cytoreduction, of which Median OS in those undergoing PDS was 19.7 months (95% CI 11.5 - 27.9) months, those undergoing IDS but remained hypoalbuminaemic 27.9 Months (n = 1) and those undergoing IDS with normalisation of Albumin 42.9 months (95% CI 31.5 - 54.3) (p>0.05).
Conclusion: Hypoalbuminaemia at diagnosis is a poor prognostic factor in AOC. Normalisation of serum albumin after NACT in our exploratory analysis suggests a survival benefit which needs further investigation.
Disclosure: Nothing to disclose

EP987

Prevalence and spectrum of BRCA mutations in patients with platinum-sensitive ovarian cancer from Romania - results of an observational study

D.L. Stanculeanu[1], D.M. Median[2], C.L. Cebotaru[3], G. Teodorescu[4], C. Voinea[4]
[1]Oncology Institute, [2]Filantropia Clinical Hospital, Bucharest, [3]Oncology Institute, Cluj Napoca, [4]AstraZeneca, Bucharest, Romania

Introduction / Background: Knowing BRCA mutation status in ovarian cancer patients is crucial for treatment decisions. Guidelines currently recommend testing of BRCA germline mutations in patients with high-grade serous ovarian cancer (HGSOC), although the implementation in practice is not uniform. The full spectrum of BRCA mutations in ovarian cancer in Romania was not previously characterized.
Methodology: This was an observational, retrospective medical chart review that aimed to describe prevalence of BRCA mutations in HGSOC patients. This study was performed in multiple centers participating in the National BRCA1/2 Testing Program started in 2016. The study collected data from patients included in the Program between 04/2016-04/2017. In total, 233 platinum-sensitive HGSOC patients were included in the full analysis set (FAS). Descriptive statistical methods were applied, using R language version 3.3.1.
Results: Of the 233 patients, 68 (29.2%) patients carried pathogenic BRCA1 mutations (BRCA1m+), 15 (6.4%) pathogenic BRCA2 mutations and 8 (3.4%) variants of unknown significance. The most prevalent pathogenic BRCA1 mutation was c.5266dupC (5382insC) reported for 21/68 patients, followed by c.3607C>T reported in 10/68 patients and two other mutations, c.181T>G and c.4035del A, each found in 7/68 patients. The most prevalent pathogenic BRCA2 mutation was c.9371A>T

reported in 6/15 patients. In FAS, the mean age of patients at the time of HGSOC diagnosis was 53.6 years (50.4 in BRCA1m+ and 54.8 in BRCA2m+ patients) with a mean time from diagnosis to BRCA1/2 testing of 3.1 years (2.8 years in both groups with pathogenic mutations). Most patients had grade 3 advanced disease at diagnosis (~80% in FAS and BRCAm+ groups).
Conclusion: To our knowledge, this is the first comprehensive study of the BRCA1/2 mutation spectrum in HGSOC patients from Romania. These results add to and overlap with data recently provided by a study performed in high-risk breast cancer patients.
Disclosure: Funding: AstraZeneca. Stanculeanu DL - Advisory Board/Consultancy: BMS, Ipsen, Eli Lilly, Merck, Pfizer, Roche, Sanofi; Lecture fees: Novartis, Pfizer, Roche, Teva; Educational grants: BMS, Ipsen; Research support: Amgen, AstraZeneca, Pfizer, Sandoz; Travel grants: Astellas, AstraZeneca, BMS, Ipsen, Merck, Pfizer, Roche, Sanofi. Median DM - Advisory role: Eli Lilly, Novartis, Roche, Pfizer; Lecture fees: AstraZeneca, Roche, Sandoz, Teva; Educational grants: AstraZeneca, Pfizer, Roche; Institutional grants: AstraZeneca, Boehringer Ingelheim, Eli Lilly, Novartis, Roche, Samsung Bioepis, Tesaro; Research grants: Roche; Travel grants: A&D Pharma, Alvogen, Genekor. Cebotaru CL- Advisory Board: Boehringer Ingelheim, Novartis, Pfizer; Lecture fees: Astellas, AstraZeneca, BMS, Bayer, Ipsen, Janssen; Educational grants: AstraZeneca; Travel grants: Alvogen, Boehringer Ingelheim, Merck Serono. Teodorescu G, Voinea C: AstraZeneca employees.

EP988

Hematologic markers of survival outcome in epithelial ovarian, fallopian tube and primary peritoneal cancer patients treated with platinum-based chemotherapy

P. Suprasert, D. Jeerakornpassawat
Department of OB&GYN, Faculty of Medicine, Chiang Mai University, Chiang Mai, Thailand

Introduction / Background: A recent molecular cancer study suggested that inflammatory parameters such as neutrophils and platelet can facilitate tumor initiation, tumor progression, induction of angiogenesis and promote metastatic spreading by inhibiting the natural killer function. Therefore, the elevated absolute neutrophil count (ANC), platelet, neutrophil to lymphocyte ratio (NLR) and platelet to lymphocyte ratio (PLR) in many solid tumors are associated with a worse prognosis. However, only a few studies were conducted to further investigate this association in epithelial ovarian cancer (EOC), fallopian tube cancer (FT) and primary peritoneal cancer (PPA).
Objective: To investigate the association of hematologic parameter and survival outcome in patients with EOC, FT and PPA who treated with platinum-based chemotherapy.
Methodology: 306 medical records of patients with EOC, FT and PPA treated with platinum-based chemotherapy between 2007 and 2017 were reviewed. The association between pretreatment ANC, platelet, NLR and PLR with survival outcome were investigated by using receiver operating characteristic (ROC) curves analysis to determine the optimal cutoff values.
Results: Nearly 80% were diagnosed as EOC. About one-fifth of the patients were received neoadjuvant chemotherapy followed by debulking surgery. Patients with high level of ANC (>5341 cell/cumm^3), Platelet > 325,000 cell/cumm^3 NLR > 3.3 and PLR > 200.3 were significant more common in advanced stage (III&IV) and associated to poor survival outcome with hazard ratio at 1.632,2.072,2.113 and 2.113, respectively.
Conclusion: Pretreatment hematologic parameters were associated to poor survival outcome
Disclosure: Nothing to disclose

EP989

Dependence of blood flow parameters in recurrent ovarian tumors on haptoglobin and ceruloplasmin dynamics in blood

E. Surikova, I. Goroshinskaya, N. Maksimova, P. Kachesova, M. Sergeeva
Rostov Research Institute of Oncology, Rostov-on-Don, Russian Federation

Introduction / Background: Our purpose was to compare haptoglobin concentrations and the oxidase activity of ceruloplasmin in the blood and blood flow parameters in recurrent tumors of patients with a local recurrence of ovarian cancer.
Methodology: The study included 61 patients with stage III-IV ovarian cancer (45-75 years, serous cystadenocarcinoma). All patients underwent hysterectomy and bilateral salpingo-oophorectomy with the greater omentum resection and 6 cycles of adjuvant chemotherapy followed by a complete clinical remission in all patients. Vascularization and blood flow rates were assessed in recurrent tumors in the cavity of the small pelvis found in 50 patients during further monitoring; 11 patients remained in remission. Plasma concentrations of haptoglobin (Hapt) were determined by the immunoturbidimetric method using the COBAS INTEGRA 400 analyzer (Roche), and oxidase activity of ceruloplasmin (OXCP) was determined by the colorimetric method in all patients with ovarian cancer and in 30 healthy women.
Results: Hapt in patients in remission was 50% higher ($p< 0.05$) than in healthy women, but still in the reference interval < 2 g/L. Hapt in patients with avascular recurrence (n=10), patients in recurrence and healthy women were similar, while in patients with hyperintensive blood flow in the tumor (n=20) it was significantly higher than in other patients - on average by 52% ($p< 0.01$). OXCP in the blood of patients in remission did not differ significantly from the levels in healthy women, in patients with avascular tumors increased by 23% compared to patients in remission ($p< 0.05$), and in patients with hyperintensive blood flow in the tumor was similar to patients in remission.
Conclusion: Local recurrent ovarian tumors cause an increase in OXCP blood levels, and Hapt increases significantly together with increased tumor vascularization. This may indicate the involvement of these proteins in angiogenesis at different stages of the formation of recurrent ovarian tumors.
Disclosure: Nothing to disclose

EP990

Activation of *EGFL7* within high grade ovarian serous carcinoma is associated with lower tumor infiltrating CD4+ and CD8+ lymphocytes

J. Sznurkowski, A. Zawrocki, N. Krawczyńska, M. Bieńkowski, B. Wasąg, W. Biernat
Medical University of Gdansk, Gdańsk, Poland

Introduction / Background: It has been demonstrated that EGFL7 promotes tumor cell escape from immunity by downregulating the activation of tumor blood vessels. Therefore, we aimed to analyze its expression with qPCR in cancer cells and adjacent stromal endothelium microdissected from formalin-fixed paraffin-embedded of 59 high-grade ovarian serous carcinoma cases.
Methodology: Subsequently, EGFL7 expression was confronted with the infiltration of intraepithelial lymphocytes (CD4+/CD8+) and ICAM-1 expression (evaluated by immunohistochemistry).
Results: EGFL7 was expressed in cancer cells (9/59, 15.25%), endothelium (8/59, 13.56%), or both cancer cells and adjacent endothelium (4/59, 6.78%). ICAM-1 was expressed on cancer cells (47/59, 79.66%), stromal endothelium (46/59, 77.97%), or both epithelium and endothelium (40 of 59, 67.8%). EGFL7-positivity of cancer cells and endothelium was associated with lower intraepithelial inflow of CD4+ (p=0.0216 and p=0.2892, respectively) and CD8+ lymphocytes (p=0.0039 and p=0.0307, respectively) but impact neither epithelial nor endothelial ICAM-1 expression (p=0.098 and p=0.119, respectively). Patients' median follow-up was 23.83 months (range 1.07-78.07). Lack of prognostic significance of EGFL7-status and ICAM-1 expression was notified.
Conclusion: In summary, EGFL7 is activated in the cancer cells as frequently as in the endothelium of human high-grade ovarian serous carcinoma. Activation of EGFL7 in cancer cells and/or endothelial cells could negatively impact diapedesis regardless to localization.
Disclosure: Nothing to disclose

EP991

Exceptional response with combined anti-hormone and MEK inhibitor therapy in a patient with hormone receptor-positive ovarian cancer and isolated KRAS alteration. Implications for a precision medicine strategy

K. Takahashi[1,2], S. Kato[3], K. Bamel[3], S. Ikeda[1], S. Plaxe[3], B. Parker[3], R. Kurzrock[3]
[1]Cancer Center, [2]Department of Obstetrics and Gynecology, Tokyo Medical and Dental University, Medical Hospital, Tokyo, Japan, [3]Center for Personalized Cancer Therapy and Division of Hematology and Oncology, Department of Medicine, UC San Diego Moores Cancer Center, La Jolla, CA, United States

Introduction / Background: With advances in precision cancer medicine, personalized therapy approaches based on “omic” analyses are being investigated. We present a patient with treatment refractory, metastatic ovarian cancer who achieved dramatic tumor reduction with a combination matched targeted therapy approach.
Methodology: Genomic aberrations were assessed using next-generation sequencing (NGS) from formalin-fixed paraffin embedded surgical tumor tissue (315 genes) (Foundation Medicine). Estrogen receptor (ER) was evaluated by immunohistochemistry (IHC). Patient was consented to our institutional *Profile-Related Evidence Determining Individualized Cancer Therapy study* (NCT02478931).
Results: A-40-year-old with serous papillary ovarian cancer presented after progression on 4 lines of systemic treatment. Evaluation with NGS from her initial surgery revealed only a single alteration, in KRAS G12V. In addition, IHC showed strong positivity for ER. Based on these findings, she was treated with trametinib (MEK inhibitor for KRAS alteration) and tamoxifen (estrogen receptor modulator for positive ER expression.) However, serial images showed gradual progression and the therapy was discontinued after 5 months. The patient was subsequently started on monotherapy with letrozole; however, after 3 months of treatment, the tumor progressed. Letroizole was continued and trametinib was restarted. The patient had a prompt and dramatic response (>50% tumor reduction per RECIST 1.1) with a precipitous decline in CA125 (fall to 243 U/ml from >15,000 U/ml at the time of reintroduction of tarmetenib). Treatment is ongoing 11+ months.
Conclusion: We report a patient with recurrent ovarian cancer with KRAS alteration and positive ER expression who demonstrated a remarkable response to combination therapy with a MEK inhibitor and aromatase inhibitor after failing treatment with the same MEK inhibitor in combination with a SERM. The current case suggests that different hormone modulators may have divergent effects and that selection of specific combination therapies for potentially pharmacologically tractable alterations can be effective.
Disclosure: Shumei Kato serves as a consultant for Foundation Medicine. Razelle Kurzrock receives research funding from Genentech, Incyte, Merck, Serono, Pfizer, Sequenom, Foundation Medicine, Grifols, and Guardant, as well as consultant fees from Loxo, X Biotech, NeoMed, and Actuate Therapeutics, speaker fees from Roche, and an ownership interest in IDby DNA and Curematch Inc. Barbara Parker receives research funding from Novartis, Genentech, Pizer, and Oncoternal Inc. This work was supported in part by Foundation Medicine, the Joan and Irwin Jacobs Fund philanthropic fund; and by National Cancer Institute at the National Institutes of Health [grant P30 CA023100 (Razelle Kurzrock, rkurzrock@ucsd.edu)].

EP992

Iron-dependent cell death "ferroptosis" is a new therapeutic strategy for ovarian cancer

M. Takenaka[1], T. Furui[1], N. Suzuki[1], T. Koike[1], H. Aoki[1], H. Nagasawa[2], K.-I. Morishige[1]
[1]Gifu University Hospital, [2]Gifu Pharmaceutical University, Gifu, Japan

Introduction / Background: Chemo-resistant ovarian cancer is a highly fatal disease and a new

strategy is necessary to improve the prognosis. The oncogenic lethal small molecule erastin triggers a unique iron-dependent form of cell death termed 'ferroptosis'. This new type of programmed cell death (PCD) is expected to induce eradication of chemotherapeutic resistant cancer cells. We demonstrated this new form of cell death in ovarian cancer cells.
Methodology: The epithelial ovarian cancer cell lines were treated with erastin, cisplatin (CDDP) and deferoxamine (DFO). Cell viability, glutathione (GSH) concentrations and whole (cytosolic and lipid) ROS production were assessed. The expression of protein markers for other types of PCD was identified by using western blotting.
Results: TOV-21G cells were the most sensitive to erastin among four ovarian cancer cell lines. The sensitivity to erastin was moderate in SKOV3ip1 cells, and resistant in CaOV3 and KOC-7C cells. DFO rescued growth inhibition induced by erastin. Erastin reduced intracellular GSH level, resulting in increase of whole ROS in TOV-21G cells. The basal GSH level was lower and the basal ROS level was higher in TOV-21G cells than KOC-7C cells. The expression of protein markers for apoptosis, necrosis and autophagy were not changed by erastin. Growth inhibition induced by CDDP was not rescued by DFO. A low-dose erastin combined with CDDP remarkably inhibited cell growth in TOV-21G cells.
Conclusion: Erastin induced ferroptosis in ovarian cancear cells of which intracellular GSH level was low. Ferroptosis had great potential to become a new approach in chemotherapies of ovarian cancer.
Disclosure: Nothing to disclose

EP993

Macrocytosis associated with olaparib

M. Takenaka, T. Furui, Y. Bomoto, S. Murase, Y. Hayasaki, K.-I. Morishige
Gifu University Hospital, Gifu, Japan

Introduction / Background: Anemia is the featured hematological adverse effect associated with olaparib (a poly (ADP-ribose) polymerase (PARP) inhibitor). It was reported that anemia recovered after 18 months of treatment with olaparib, however the reason was unknown. We assessed the feature of anemia during treatment with olaparib.
Methodology: We reviewed 10 patients with advanced ovarian cancer patients underwent maintenance monotherapy with olaparib in Gifu University Hospital between January 1, 2018, and April 30, 2019, retrospectively.
Results: Although the mean hemoglobin levels decreased, it recovered after 16 weeks of treatment with olaparib. The maximum level of hemoglobin was 8.6±2.2 mg/dl at 16 weeks. Mean Corpuscular Volume (MCV) values gradually increased, it remained above the normal range during treatment. It was above 102.0±6.5 (range 102.0-112.2) fl after 12 weeks. Macrocytic anemia occurred during short-term treatment with olaparib and macrocytosis without anemia during long-term treatment.
Conclusion: Olaparib lead to macrocytosis, however alternative mechanism would exists to recover anemia.
Disclosure: Nothing to disclose

EP994

Epigenetic silencing of *FBXW7* through promotor hypermethylation in ovarian cancer

Y. Tamimi[1], M. Al Hinai[2], I. Gupta[3], R. Lakhtakia[4], M. Al Kalbani[5], A. Okamoto[6], M. Al Moundhri[7], I. Burney[7]
[1]Biochemistry, Sultan Qaboos University (SQU), [2]SQU, Muscat, Oman, [3]Qatar University, Doha, Qatar, [4]BenRashed University, Dubai, United Arab Emirates, [5]Gynecology, Sultan Qaboos University (SQU)Hospital (SQUH), Muscat, Oman, [6]Department of Obstetrics and Gynecology, The Jikei

University, Tokyo, Japan, [7]Medicine, Sultan Qaboos University Hospital (SQUH), Muscat, Oman

Introduction / Background: *FBXW7*, a tumor suppressor gene plays a critical role in cell cycle progression under the regulation of the transcription factor E_2F_5. While *FBXW7* is silenced in several cancers; its role in ovarian cancer pathogenesis is still unclear. This study explored *FBXW7* promotor methylation status in ovarian cancer and its functional significance.
Methodology: *FBXW7* expression at mRNA and protein levels were assessed in OVSAHO and MCAS cells using RT-qPCR and western blotting respectively. Expression of *FBXW7* was high in MCAS and low in OVSAHO cells. *FBXW7* gene methylation status was analyzed in cell lines (MCAS & OVSAHO) as well as in 23 EOC patients (5 normal, 8 benign, 4 borderline, 5 high grade tumors) by both methylation-specific PCR and MS-HRM analysis.
Results: In addition to OVSAHO cells, more than 60% of patients analyzed showed methylation. To evaluate its functional importance, *FBXW7* expression was restored in OVSAHO cells by treatment with the demethylation agent, 5'-aza-2'deoxycitidine. Western blotting in *FBXW7*-restored OVSAHO cells and *FBXW7*-positive cell line (MCAS) showed an upregulation in caspases-2, 3, 7, BAX, P53, BAD, Cyclin D1, pNFkB and pGSK3-beta, whereas Bcl-2, Bcl-x, pBad, STAT3, GSK3-beta and NFkB were downregulated; indicating cells to undergo apoptosis *via* STAT3 pathway.
Conclusion: This study described an alternative mechanism for *FBXW7* inactivation, namely promoter specific methylation in ovarian cancer and identified *FBXW7* as a potential target for guiding the development of therapeutic strategies against ovarian cancer.
Disclosure: Nothing to disclose

EP995

Ultrasound patterns compared to histopathological findings in ovarian granulosa cell tumor

C. Prasciolu, C. Del Pezzo, L. Mereu, S. Tateo
Ospedale Santa Chiara, Trento, Italy

Introduction / Background: Granulosa cell tumor (GCT) is the most common sex cord stromal tumor and constitute less than 5% of all ovarian cancers. Pre operative diagnosis is challenging. Sonographic findings are multiple, the mass could present with different patterns.
Methodology: A retrospective study of histopathologically confirmed ovarian granulosa cell tumor that underwent preoperative ultrasound examination according to IOTA classification. Sonographic findings were compared with histopathological findings
Results: A total of 11 patient with confirmed GCT were identified. The median age of patients was 55 years old. Ultrasound patterns were described using IOTA pattern. Almost all the tumors (90%) contained a solid component. Three (30%) were multilocular-solid, six (60%) were solid and one was multilocular. Five of the six solid masses didn't show an omogeneous solid tissue but a large number of small locules or irregular shaped locules with solid tissue around, the only pure solid tumor was seen in the recurrence. Macroscopic appearance was similar to ultrasound description, most of the tumors (60%) were composed by solid and cystic areas in variable proportions. Two (20%) of the tumors were totally composed by cystic lesions. One macroscopic description was not availabile. The subjective assessment of the sonologist correctly suggested the specific diagnosis of GCT in three cases (30%). In three cases (30%) the sonologist impression was non conclusive, in three cases the diagnosis was malignant tumor; two (20%) were described as benign tumor.
Conclusion: A correlation between sonographic findings based on IOTA classification of ovarian masses and pathologic findings was reported, but in 20% of cases the ultrasound scan can underestimate the diagnosis.
Disclosure: Nothing to disclose

EP996

Thermal Liquid Biopsy (TLB) as a new diagnostic tool for patients with ovarian cancer: a pilot study

N. Abian[1], S. Vega[2], S. Hermoso[2], C. Tauste[3], B. Gaston[3], J.C. Muruzabal[3], O. Sanchez[2], O. Abian[2], A. Velazquez-Campoy[2]
[1]Gynecology, Hospital Reina Sofía, Tudela, [2]Aragon Health Science Institute (IACS), Zaragoza, [3]Gynecology, Complejo Hospitalario de Navarra, Pamplona, Spain

Introduction / Background: Thermal Liquid Biopsy (TLB), based on Differential Scanning Calorimetry (DSC, calorimetric thermogram analysis of serum samples), is a new clinical approach for diagnostic assessment for patients with ovarian cancer. TLB is a highly sensitive technique traditionally used to study thermally induced macromolecular transitions. Our main objective is to determine the ability of TLB to differentiate between healthy controls (HC) and patients with Ovarian Cancer (OC).
Methodology: We report in this work the application of a multiparametric analysis of TLB profiles from plasma samples collected from patients with OC before surgery from 2011 to 2018 assisted in a tertiary-level hospital. Plasma samples collected from 120 patients were analyzed and compared with histological result to determine the utility of thermograms for diagnostic and monitoring assessment in ovarian cancer. Data from 100 healthy subjects were used as reference group.
Results: 120 patients diagnosed with OC (women between 29 and 83 years old) with a stage distribution of (I: 18%; II-IV: 82%), histology distribution (69% high-grade serous carcinoma, 18% clear cell carcinoma, 7% endometrioid carcinoma, 3% mucinous carcinoma and 3% others) compared to 100 HC with a homogeneous distribution from a blood bank were included in our study. The parameters included in our multiparametric method showed statistical differences between HC and OC. After developing ROC curves, AUC higher than 0.8 were observed for these parameters, exceeding 0.85 in some of them. The Youden's index was calculated for all of them, finding sensitivity and specificity values higher than 75.0-90.0% (for the best parameter, both indexes were higher than 85.0%).
Conclusion: Results suggest that TLB exhibits high specificity and sensitivity values when comparing to clinical assessment/diagnostic imaging detecting malignancy for ovarian pathology. TLB provides a minimally-invasive, low-risk, low-cost clinical test, allowing personalized patient monitoring for diagnostic assessment of ovarian pathology and facilitating the decision-making process.
Disclosure: Nothing to disclose

EP997

Treatment tailoring in epithelial advanced ovarian cancer: survival results in Navarre

C.M. Tauste Rubio[1], J. Zabaleta Jurío[2], S. Aguirre Gorospe[2], J. Barrenetxea Arrinda[2], B. Gaston Moreno[2], J.C. Muruzábal Torquemada[2]
[1]Complejo Hospitalario de Navarra | Gynaecologic Oncology, Osasunbidea/ Navarre Health Service, [2]Complejo Hospitalario de Navarra, Pamplona, Spain

Introduction / Background: Treatment of advanced epithelial ovarian cancer (EOC) requires an accurate estimation of surgical complexity and residual disease (RD) at primary debulking surgery (PDS). Neoadjuvant chemotherapy (NACT) may be used in non-surgical candidates. However, reaching the interval debulking surgery (IDS) will not be possible in all cases. How do we choose the best option in each case? What are our results?
Methodology: We included stage IIIC/IV EOC patients undergoing PDS from 2005 to 2016. We collected different variables: Age, Performance Status, Stage, CA 125, type of treatment and residual disease (RD). Overall survival (OS) was estimated using the Kaplan-Meier method.
Results: A total of 394 patients were diagnosed with EOC, 216 with IIIC/IV stages were recruited in this study. Average age was 64.7 years and the most common histologic type (88.4%) was serous. Up to 15.3% of the cohort was treated with Palliative Care, 36.7% NACT and 48% with PDS (Figure1). Worst performance status and oldest patients were treated with chemotherapy or palliative care

(Table1). Optimal PDS was achieved in 80.7% while Optimal IDS was possible in 96.3%. Although the length-of-stay was similar, the complexity of the surgery and the later morbidity was higher in PDS compared to IDS.(Table2) Patients with optimal PDS and and Optimal IDS had an OS of 81.1 and 31.4 months respectively, while non optimal PDS obtained an OS of 29 months. (Figure2)
Conclusion: Advanced EOC treatment was adapted according to patients clinical characteristics. There was a clear survival advantage for PDS group . Nevertheless, non optimal PDS increase morbidity without advantages in survival compared to IDS. Further studies are needed to improve the selection criteria for PDS.

	PDS	IDS	Only NACT	Palliative Care	statistically significant difference
Age (years)	59,2	64,6	71,2	77,3	p<0.01
ECOG-PS ≥ 3 (%)	3,1	1,9	12	29,1	p<0.01
FIGO stage IVB (%)	7,1	28,3	28,0	25	Not significant
CA 125 (KU/L)	1145,2	2310,6	1356,8	1004,5	Not significant
Median Overall survival (months)	63,2	31	9,9	3,7	p<0.01

[Table 1. Comparison of initial characteristics among treatment groups]

	PDS N 104	IDS N 79	statistically significant difference
Lenght-of-stay (days)	13,9	12,1	Not significant
Clavien Dindo≥ 3 (%)	14	2,3	p<0,01
Bowel resection (%)	20,7	11,1	p<0,01
Optimal RD results (%)	80,7	96,3	p<0,01
Progression free survival (months	13,9	12,1	Not significant
Overall survival (months)	63,2	31	p<0,01
OS(months)RD 0	98,6	32,8	p<0,01
OS(months)RD ≤1	62,6	29,6	p<0,01
OS(months)RD>1	29	11,236	Not significant

[Primary debulking surgery vs interval debulking surgery. Outcomes]

BMJ Publishing Group Limited (BMJ) disclaims all liability and responsibility arising from any reliance placed on this supplemental material which has been supplied by the author(s)

This image cannot currently be displayed.

[Flow diagram of patients with EOC in Navarre between 2005-2016]

This image cannot currently be displayed.

[Kaplan Meier curves. PDS vs IDS Survival according to residual disease]

Disclosure: Nothing to disclose

EP998

Comparison of laparoscopy and laparotomy in the surgical management of early-stage ovarian cancer

N. Garcia Ginjaume, C. Soler Moreno, N. Teixeira, S. Marin Garrayo, R. Luna Guiborg, R. Rovira Negre
Gynaecology and Obstetrics, Hospital de la Santa Creu i Sant Pau de Barcelona, Barcelona, Spain

Introduction / Background: We aimed to assess the feasibility and survival outcomes of laparoscopic surgical staging for patients with early-stage ovarian cancer compared with laparotomic approach.
Methodology: Retrospective observational study which included forty-six patients who underwent laparoscopy (20 patients) or laparotomy (26 patients) for surgical staging of early-ovarian cancer between 2010 and 2018 were included. We analyzed clinical data and compared surgical and survival outcomes of both approaches.
Results: Mean age was 60±20 years. Nineteen patients were classified as stage IA, 22 as stage IC, 2 as stage IIA and 3 as stage IIB. Histological types included: serous in 16, mucinous in 5, clear cell in 10 and endometrioid in 6 patients, 9 cases were classified as others, including carcinoid, yolk-sac, disgerminoma, granulosa-cell and seromucinous.
The laparoscopy group compared to the laparotomy group presented significantly shorter hospital stay (4,8vs.9,2 days) and lower complication rate (2%vs.22%).
In the laparotomy group 1 patient presented intraoperative vascular injury and another urologic injury. Postoperatively 2 patients presented respiratory infection, 3 urinary infection, 1 haemorrhage, 1 paralytic ileus and 1 asymptomatic lymfocele. In the laparoscopy group no patients presented intraoperative complications. Postoperatively, 1 patient presented symptomatic lymfocele.
Intraoperative blood loss tended to be lower in laparoscopy (197,1 vs 459,1ml), although the difference was not statistically significant(p=0,07).
No significant differences were found in operative time, number of removed lymph nodes, intraoperative tumour rupture rate, postoperative chemotherapy, recurrence or survival rates.
Thirteen patients presented disease recurrence; 10 of which had undergone laparotomy.
Overall survival was 93%, with a median follow-up of 37 months.
Conclusion: Laparoscopic staging performed by a trained gynaecologic-oncologist is a safe and effective alternative for early ovarian cancer. It has the advantage of minimum invasiveness, providing a shorter hospital stay and faster recovery, whilst preserving the requirements of an optimal surgery and the survival outcomes compared to laparotomic approach.
Disclosure: Nothing to disclose

EP999

Bevacizumab-related gastrointestinal perforation in patients with three or more prior chemotherapy regimens: a real-world experience

Y. Todo, H. Matsumiya, S. Minobe, R. Yamada, H. Kato
Gynecologic Oncology, Hokkaido Cancer Center, Sapporo, Japan

Introduction / Background: Associations between the number of prior chemotherapy (CT) regimens and gastrointestinal (GI) perforation in patients receiving bevacizumab treatment has not been fully investigated. The aim of the study was to investigate the impact of ≥ 3 prior
Methodology: We retrospectively investigated the medical records of 133 patients with gynecological cancer who received bevacizumab-containing treatment. Bevacizumab was intravenously administered at a dose of 15 mg/kg every 4 weeks. Incidence of GI perforation was compared between ≤ 2 and ≥ 3 prior CT groups.
Results: Twenty-three (17.3%) patients had a history of ≥ 3 CT; these patients received bevacizumab at 4-week intervals. The percentage of patients with prior surgery was significantly higher in the ≥ 3 prior CT group (70.0% vs, 95.7%, *P*=0.008), while those with prior bowel resection was significantly

higher in the > 3 prior CT group (12.7% vs. 30.4%, *P*=0.034). There was no significant difference in the mean number of bevacizumab cycles between the two groups (8.9 vs. 10.7, *P*=0.19). While GI perforation was observed in three (2.7%) patients in the < 2 prior CT group, no GI perforation was found in the > 3 prior CT group (*P*>0.99).
Conclusion: A history of > 3 prior CT might not increase the risk for GI perforation when bevacizumab is administered at a dose of 15 mg/kg every 4 weeks.
Disclosure: Nothing to disclose

EP1000

Supraclavicular lymph node metastatic spread in a patient with clear cell ovarian carcinoma: an unusual finding

H. Trihia[1], E. Lianos[2]
[1]Pathology, [2]Medical Oncology, 'Metaxas' Cancer Hospital, Piraeus, Greece

Introduction / Background: Tumoral dissemination of ovarian cancer most commonly occurs through the intra-peritoneal route. Nevertheless, although rare, ovarian cancer may also metastasize through lymphatic channels, usually into pelvic and retroperitoneal lymph nodes and even rarely in extra-abdominal lymph nodes. We report a case of a woman diagnosed with clear cell carcinoma of the ovaries, recurring with left supraclavicular lymph node metastases.
Methodology: A 53-year old woman was diagnosed with clear cell carcinoma of the ovaries, stage IC, elsewhere, where she underwent a total hysterectomy with bilateral salpingo-oophorectomy, omentectomy and lymph node pelvic resection.
Results: She was admitted in our hospital, where she underwent 6 cycles of second line chemotherapy with carboplatin and paclitaxel. She remained disease free for the following 21 months, when lymph node disease was revealed in PET-CT examination. Resection and examination of left supraclavicular lymph nodes revealed secondary involvement by ovarian clear cell carcinoma. She received three additional cycles of carboplatin and paclitaxel. The woman remained hospitalized with B symptoms, for five weeks, when after investigation her symptoms were attributed to her disease. She continued with hycamtin every week, as a second line therapy, for seven cycles. Because of anaemia and thrombopenia, she underwent a bone marrow biopsy, where a massive infiltration by carcinoma cells was revealed. She underwent a third-line chemotherapy with cisplatin and bevacizumab for almost a month. After an initial recovery, the fever relapsed. A fourth line chemotherapy was recommended, which the patient refused. She returned after four months with end-stage disease and succumbed one month later.
Conclusion: Reports in the literature on tumoral spread of ovarian cancer to the supraclavicular nodes are rare, however this possible site of metastatic involvement has to be kept in mind by oncologists. Histology remains the only means in identifying specific tumor types and the site of origin.
Disclosure: Nothing to disclose

EP1001

Bilateral neuroendocrine carcinoma of the ovary of the mucinous goblet cell carcinoid type. A very rare tumor entity

H. Trihia[1], E. Bournia[2], A. Douligeris[3], D. Giannoulopoulos[3], N. Novkovic[1], P. Arapantoni-Dadiotis[1]
[1]Pathology, 'Metaxas' Cancer Hospital, Piraeus, [2]Pathology, General Hospital Hospital of Kalamata, Kalamata, [3]Gynaecology, 'Metaxas' Cancer Hospital, Piraeus, Greece

Introduction / Background: Ovarian carcinoids are uncommon tumors. Mucinous carcinoids, particularly if associated with atypical features, may have an aggressive behavior.

Methodology: A 34-year old woman underwent removal of a left ovarian mass which was diagnosed as Krukenberg's tumor, probably of GI tract origin. An upper and lower GI endoscopy and biopsies of the mucosa of the stomach, were unremarkable. One year later the patient experienced a lower abdominal pain which required emergency diagnostic work up and treatment. The patient had an emergency exploratory laparatomy. Peritoneal washings were obtained and a greater omentectomy was performed. The duodenum was mobilized and palpated as well as the small bowel from the ligament of Treitz all the way to the ileocecal region. The head of the pancreas was checked with intraoperative ultrasound which showed no mass in that area. The stomach was palpated carefully and there was no evidence of any lesion. An incidental appendectomy was performed. Dissection of part of the remaining left fallopian tube, ovarian ligament and ovarian vessels was also performed. The pathologic examination showed the same type of lesion in the fallopian tube with lymphatic and vascular invasion. The patient's postoperative course was unremarkable. The diagnosis was transformed to goblet cell carcinoid of the ovary due to positive expression for neuroendocrine markers. Given her age and the desire to have family, the approach was more conservative. Nevertheless, in one year's time, the woman was re-admitted with ascites and underwent exploratory laparatomy with excision of the right ovary, which showed similar histologic features with the left ovary.
Results: The overall findings were of bilateral primary tumor of the goblet cell carcinoid type.
Conclusion: The primary neuroendocrine tumors of the ovary are very rare and their diagnosis needs a detailed work up and long follow up. This case adds to our knowledge for this rare tumor entity
Disclosure: Nothing to disclose

EP1002

Secondary cytoreduction surgery for recurrent ovarian cancer - twelve radiological criteria for case selection: preliminary data

E. Tsahalina[1,2], A. Sohaib[3], N. Mcaddy[3], S. Bryan[4], M. Nobbenhuis[4], T. Ind[4], J. Butler[4], D. Barton[4]
[1]*Gynaecological Oncology|Cancer Division, The Royal Marsden Hospital NHS, London, United Kingdom,* [2]*1st Gynaecological Oncology Department, Hygeia Hospital, Athens, Greece,* [3]*Radiology, The Royal Marsden Hospital,* [4]*Gynaecological Oncology | Cancer Division, The Royal Marsden Hospital NHS, London, United Kingdom*

Introduction / Background: Aim: to identify 12 radiological criteria predictors of complete secondary cytoreductive surgery to assist case selection.
Methodology: This is a retrospective review of all recurrent cases of ovarian, fallopian tube and peritoneal cancer that have undergone secondary cytoreductive surgery (SCS) with the goal of complete cytoreduction from January 2013 till December 2017 in a single institution. Preoperative imaging (CT scans) were available for all patients. Patients with bowel obstruction, Krukenberg tumours and those with imaging older than 2 months prior to surgery were excluded. A consultant Radiologist who have the responsibility for multidisciplinary meetings (Tumour Boards) has reviewed the images and scored the presence or absence of 12 radiological criteria preoperatively for all cases. The Radiologist was blind to surgical outcome. Operation reports of all patients were reviewed.
Results: 83 patients met the above criteria and were included in the study. In 57 complete cytoreduction was achieved (69%). In 26 patients with incomplete cytoreduction the following radiological criteria according to the scoring were present and influenced the outcome: diffuse non-measurable disease (N=15), subcapsular liver lesions (N=12), omentum involvement (N=11) with extension to the lesser sac (superior body of pancreas-inferior stomach antrum) (N=7), other sites of disease (N=22).

Causes for incomplete surgery identified intraoperatively and included: extensive carcinomatosis with wide spread small volume disease, infiltrating plaque-like disease on the upper or lower abdomen with multi-visceral involvement, tumour invading right/left pelvic side wall (including iliac vessels), tumour involving sacral/bladder wall,or small bowel mesentery.
Conclusion: In most cases imaging studies were in accordance with the operating findings. However, further cases are required to validate these 12 radiological criteria, for patients selection, in whom secondary cytoreductive surgery is considered.
Disclosure: Nothing to disclose

EP1003

Meigs' syndrome with elevated serum CA125: case report and review of the literature

C. Tsaousidis[1,2], C.B. Walter[1], J. Braendle[1], C. Oettling[1], D. Wallwiener[1], S.Y. Brucker[1]
[1]Department of Women's Health, University Hospital of Tuebingen, Tuebingen, Germany, [2]Women's Center Bern, Lindenhofgruppe, Bern, Switzerland

Introduction / Background: The ovarian cancer represents the seventh most common cancer in women worldwide. An elevated serum CA 125 level in association with a pelvic mass, pleural effusion, and massive ascites usually signifies a dismal prognosis in a postmenopausal woman. Meigs' syndrome consists of a benign ovarian tumor accompanied by ascites and pleural effusion.
Methodology: Surgery and histopathological examination are required for the correct diagnosis and treatment. The patient underwent a laparotomy with bilateral salpingo-oophorectomy.
All cases of Meigs´ syndrome with elevated CA 125 that have been reported were included at this review.
Results: Meigs' syndrome with marked elevated CA 125 is a rare clinical entity and only 47 cases have been reported. An elevated CA 125 level can be falsely positive for ovarian malignancy because the quantity of ascites might be correlated with a rise in the level of CA 125.
Conclusion: Postmenopausal women with a pelvic mass, pleural effusion, ascites and elevated serum CA 125 levels probably suffer from malignant ovarian tumors. However, it is important to remember that these findings does not always predict a lethal condition, but a benign adnexal lesion as part of Meigs´ syndrome, should be also considered as an unusual but existing diagnostic possibility.
Disclosure: Nothing to disclose

EP1004

Prognostic impact of BRCA2 mRNA expression in ovarian cancer: validation using the TCGA cohort

I. Tsibulak[1], H. Hackl[2], V. Wieser[1], H. Welponer[1], C. Degasper[1], K. Leitner[1], K. Knoll[1], H. Fiegl[1], A.G. Zeimet[1], C. Marth[1]
[1]Obstetrics and Gynecology, [2]Bioinformatics, Medical University of Innsbruck, Innsbruck, Austria

Introduction / Background: Mutations in cancer susceptibility genes *BRCA1/2* are considered to be associated with better survival in ovarian cancer (OC) patients possibly due to a better response to platinum based chemotherapy and PARP inhibitors. However, the impact of the *BRCA1/2* mRNA-expression is not well characterized in OC, but could also influence the platinum response and thus predict the clinical outcome in OC patients. Our previous studies have shown that low BRCA1-expression was associated with favorable overall survival (OS) and low BRCA2-expression with better progression-free survival (PFS) and OS in 201 OC patients. The aim of the present study was to validate the prognostic impact of BRCA expression in OC using the TCGA (The Cancer Genome Atlas) cohort.
Methodology: Clinical and genomic data were obtained from the OC Dataset of The Cancer Genome Atlas (n=581). Overall and progression free survival was estimated using Kaplan-Meier plots and Cox-Regression. Optimal cut off points were defined using Youden index.
Results: Univariate survival analysis showed that low BRCA2 expression was associated with favorable PFS (p=0.029) and OS (p=0.011). Multivariable survival analysis confirmed the prognostic value of BRCA2 expression both for PFS and OS.
Conclusion: Low expression of BRCA2 predicts better survival in patients with OC. Our findings may

reflect higher platinum-sensitivity due to reduced capacity of DNA damage repair in tissues with low BRCA2 expression. In this context, especially in BRCA-wildtype cancers BRCA2 expression could also be potential predictor for PARP sensitivity.
Disclosure: Nothing to disclose

EP1005

Spectrum and frequency of hereditary BRCA 1/2 mutations in ovarian cancer patients in Ukraine

N. Tsip[1], N. Khranovska[1], O. Skachkova[1], M. Inomistova[1], M. Lyzogub[2], O. Kolesnik[1]
[1]National Cancer Institute, [2]National Bogomolets Medical University, Kyiv, Ukraine

Introduction / Background: The purpose of this research is to study the spectrum and frequency of hereditary BRCA1 / 2 mutations in ovarian cancer (OC) patients in Ukraine.
Methodology: The spectrum and frequency of hereditary mutations BRCA1 (5382insC, 185delAG, 4153delA, 300 T>G, 2080delA) and BRCA2 6174delT in 424 OC patients aged 15 to 81 years. In Ukraine, sequencing was not carried out, so the choice of the type of mutations was determined by the type mutations of the neighboring countries where sequencing was carried out.
DNA from peripheral blood samples was extracted using the phenol-chloroform method and the DNA extraction kit “DNA-SORB-B” (AmpliSense, Russian Federation). DNA concentration in all samples was measured by ThermoScientific NanoDrop-1000 (Thermo Fisher Scientific, USA).
Real-time PCR was performed using the 7500 Real-Time PCR System (Applied Biosystems, USA). Each sample was run in duplicates.
Results: Hereditary BRCA1 / 2 mutations were diagnosed in 12% of Ukrainian patients with OC. The average age of mutations carriers is 50 years. The majority of patients have been diagnosed with the BRCA1 5382insC mutation - in 67% of cases. Next in frequency, 300 T> G mutations were diagnosed in 18% of patients, 4153delA in 9%, 185delAG in 4% and 2080delA in 2% of patients.
Conclusion: The spectrum and frequency of hereditary BRCA1 / 2 mutations in patients with OC in different regions of Ukraine differ significantly. However, since the choice of the type of mutations to be determined by PCR was based on the data of sequencing in neighboring countries, the results may not reflect the real situation in the country and require further study.
Such mutation analysis is important not only for precise OC treatment but for screening the relatives of mutation carriers.
Disclosure: Nothing to disclose

EP1006

Subxiphoid approach for the resection of enlarged supradiaphragmatic cardiophrenic lymph nodes during primary cytoreduction for advanced ovarian cancer: a case report

D. Tsolakidis, D. Zouzoulas, P. Pappas, O. Pavlidi, E. Bili, G. Grimbizis
1st Department of Obstetrics & Gynecology 'Papageorgiou' Hospital, Aristotele University of Thessaloniki, Thessaloniki, Greece

Introduction / Background: In ovarian cancer, metastatic cardiophrenic lymph nodes are associated with FIGO stage IV disease. The goal of debulking surgery should be no residual disease and resection of those lymph nodes is mandatory. The aim of this case report is to present the subxiphoid approach, as an alternative for the exploration of both cardiophrenic spaces from one incision, in contrast to the transdiaphragmatic approach.
Methodology: The patient was a 45 years old female. Preoperative work-up revealed an elevated CA-125 of 2521 and a CT-scan showing a pelvic mass, ascites, omental cake, diaphragmatic disease and

enlarged supradiaphragmatic cardiophrenic lymph nodes, without pleural effusion. The patient underwent primary debulking surgery. Cytoreduction included type II radical oophorectomy (en bloc modified radical abdominal hysterectomy, bilateral salpingo-oophorectomy, pan-pelvic peritonectomy, rectosigmoid colectomy), appendicectomy, cholecystectomy, radical omentectomy, paracolic gutters peritonectomy, small - large bowel mesentery electro-coagulation, right diaphragm full-thickness resection and subxiphoid resection of supradiaphragmatic cardiophrenic lymph nodes.
Results: The cardiophrenic incision surgical steps included: subxiphoid peritoneal dissection, cutting of the transverse abdominis muscle, dissection of the retrosternal peritoneum laterally from the midline, digital development of the cardiophrenic space and removal of the enlarged lymph nodes. Total operative time was 450min and estimated blood loss 700ml, with no residual disease. Post-operative intensive care unit (ICU) admission was necessary for three days due to mild hemodynamic instability and the patient was discharged from hospital on the 17th postoperative day. The pathological report showed a grade III serous adenocarcinoma, with metastases to six of seven resected cardiophrenic lymph nodes.
Conclusion: Subxiphoid resection of supradiaphragmatic cardiophrenic lymph nodes is a feasible alternative approach for the exploration of both cardiophrenic spaces with no direct diaphragmatic trauma.
Disclosure: Nothing to disclose

EP1007

Clavien-Dindo classification of postoperative complications in advanced epithelial ovarian cancer treated by primary or interval debunking surgery

D. Tsolakidis[1,2], D. Zouzoulas[1], M. Lantzanaki[1], I. Theodoulidis[1], T. Mikos[1], A. Tolkos[1], G. Grimbizis[1]
[1]1st Department of Obstetrics & Gynecology 'Papageorgiou' Hospital, Aristotele University of Thessaloniki, [2]Center for Gynecologic Surgery, 'Interbalkan' Medical Center, Thessaloniki, Greece

Introduction / Background: Ovarian cancer is usually diagnosed in advanced stage. The goal of debulking surgery should be no residual disease. This may lead to severe postoperative complications, prolonging hospital stay and delaying chemotherapy initiation. The aim of this study is to identify possible predictive factors and minimize the occurrence of postoperative complications.
Methodology: We retrospectively reviewed the medical records of all patients that underwent primary (PDS) or interval (IDS) debulking surgery for advanced epithelial ovarian cancer (January 2018 - December 2018) in two medical clinics. The clinicopathological characteristics, oncological and surgical results were described.
Results: Out of total 79 patients with ovarian cancer, 49 met the eligibility criteria. The mean age was 61 ± 12 years old and the majority of them, 44 (90%), had FIGO stage III serous adenocarcinoma. The median hospitalization was 8 ± 3 days and the postoperative complications ranged from 0 to 100, with one death within 30days. A univariate analysis was conducted: There was a statistically significant difference (p-value = 0.002) between primary and interval debulking surgery: PDS showed increased postoperative complications. Furthermore, Aletti score, prolonged surgery duration (>300min), elevated preoperative CA-125 and postoperative intensive care unit (ICU) admission were statistically significant associated with increased postoperative complications (p-value < 0.05). Last but not least, 17 patients underwent preoperative “Fagotti” diagnostic laparoscopy. The “Fagotti group” had less postoperative complications, but with no statistically significant difference.
Conclusion: Aletti score, prolonged surgery duration, elevated CA-125, postoperative ICU admission and primary debulking surgery were statistically significant associated with more postoperative complications. “Fagotti” diagnostic laparoscopy could be a possible way to decrease postoperative complication but further investigation with more patients is needed.
Disclosure: Nothing to disclose

EP1008

Modified posterior exenteration in advanced ovarian cancer: a single center experience

A. Papanikolaou, D. Tsolakidis, P. Pappas, D. Zouzoulas, S. Pitis, V. Theodoulidis, G. Grimbizis,
Papageorgiou Hospital, 1st Dept Obstetrics & Gynecology, Thessaloniki, Greece
Aristotele University of Thessaloniki, Thessaloniki, Greece

Introduction / Background: Modified posterior exenteration is frequently a necessity in order to accomplish complete cytoreduction in advanced ovarian cancer patients with extensive intra-peritoneal dissemination. The aim of this study is to present the characteristics of patients experiencing bowel resection and to analyse the surgical outcomes and complications after debulking surgery in a tertiary gynecological center for ovarian cancer.
Methodology: Medical records of 79 patients with ovarian cancer who underwent bowel resection between January 2004 and December 2018 retrospectively reviewed. Complications, cytoreductive and oncological outcomes were reported.
Results: 51 (64.5%) patients underwent bowel resection during primary, 17 (21.5%) during interval and 11 (13.9%) during secondary debulking surgery. Intestinal surgeries included: 47 patients underwent rectosigmoid resection, 9 had colectomy and 6 had colectomy plus rectosigmoid resection. From the rest patients 9 underwent small bowel resections and 8 multiple enterectomies. Complete and optimal (< 1cm) debulking was achieved in 44 (55.7%) and 27 (34.1%), respectively. Median hospitalization was 10 days. Median perioperative blood loss was 600cc and postoperative blood transfusion was mandatory in 43 patients. Median resuscitation time was 4 hours and 25 (31.6%) patients required ICU admission after surgery. Postoperative complications such as pelvic abscess formation were observed in 3 patients and fistula in one of them. Median disease-free and overall survival was 25 and 42 months, respectively. Perioperative mortality was 3.9%.
Conclusion: Modified posterior exenteration is the most frequent additional procedure in order to achieve complete or optimal cytoreduction in advanced ovarian cancer. It has acceptable perioperative morbidity, mortality and increased survival rates.
Disclosure: Nothing to disclose

EP1009

Loss of skeletal muscle mass during neo-adjuvant chemotherapy and the relation to survival in patients with ovarian cancer; a prospective analysis of the OVHIPEC-1 cohort

J. Ubachs[1,2,3], S. Koole[4,5,6], L. Bruijs[4], M. Lahaye[7], C. Fabris[8], J. Schagen van Leeuwen[9], H. Schreuder[10], R. Hermans[11], I. de Hingh[12], J. van der Velden[6,13], H. Arts[14], L. Massuger[15], J. Bastings[1], R. Kruitwagen[1,2], S. Lambrechts[1,2], S. Olde Damink[3,16,17], S. Rensen[3,16], T. van Gorp[18], G. Sonke[5,6,19], W. van Driel[4,6,19]
[1]Department of Obstetrics and Gynecology, Maastricht University Medical Centre (MUMC), [2]GROW - School for Oncology and Developmental Biology, [3]Department of Surgery, Maastricht University Medical Centre (MUMC), Maastricht, [4]Department of Gynecology, [5]Department of Medical Oncology, The Netherlands Cancer Institute/Antoni van Leeuwenhoek Hospital, [6]Center for Gynecologic Oncology Amsterdam, [7]Department of Radiology, The Netherlands Cancer Institute/Antoni van Leeuwenhoek Hospital, Amsterdam, The Netherlands, [8]Department of Radiology, University of Verona Hospital, Verona, Italy, [9]Department of Obstetrics & Gynecology, Sint Antonius Hospital, Nieuwegein, [10]Department of Gynecological Oncology, UMC Utrecht Cancer Center, University Medical Center Utrecht, Utrecht University, Utrecht, [11]Department of Gynecology and Obstetrics, [12]Department of Surgery, Catharina Hospital, Eindhoven, [13]Department of Obstetrics and Gynecology, Amsterdam UMC, Location AMC, Amsterdam, [14]Department of Gynecological Oncology, University Medical Center Groningen, Groningen, [15]Department of Gynecological Oncology, Radboud University Medical Center, Nijmegen, [16]NUTRIM, School of Nutrition and Translational Research in Metabolism, Maastricht, The Netherlands, [17]Department of Visceral- and Transplantation Surgery, RWTH Aachen University, Aachen, Germany, [18]Department of Obstetrics and Gynecology, Division of Gynecological Oncology, University Hospitals Leuven, Leuven Cancer Institute, Leuven, Belgium, [19]Dutch

Gynecological Oncology Group, The Netherlands, The Netherlands

Introduction / Background: Skeletal muscle depletion in women with advanced ovarian cancer has been associated with adverse clinical outcome and survival. To validate earlier results in a homogenous population, we analyzed whether a decrease in skeletal muscle index (SMI) during neo-adjuvant chemotherapy (NACT) is associated with worse outcome in patients with stage III epithelial ovarian cancer, who were included in the OVHIPEC trial.
Methodology: Within the phase III OVHIPEC trial, 245 patients with stage III ovarian cancer were randomized after three cycles of NACT with carboplatin and paclitaxel to receive interval cytoreductive surgery (CRS) with or without HIPEC. Randomization was performed after at least stable disease after two cycles of NACT, and when complete or optimal CRS was achieved. CT-scans performed at baseline (timepoint 1), and after two cycles of NACT (timepoint 2) were selected. A slide on the third lumbar level was selected from each CT-scan, and the difference in SMI between both scans (ΔSMI) was calculated using SliceOMatic. Overall and recurrence-free survival of patients with a decrease or increase in ΔSMI were performed using Kaplan-Meier estimates and log-rank tests.
Results: Of the 245 patients randomized in the OVHIPEC trial, SMI and ΔSMI of scans at both timepoints were available for 212 patients (87%). After a median follow-up of 4.7 years, 116 of 212 patients (55%) had died. In survival analysis, 43 of 74 patients (58%) in the group with a decrease in ΔSMI, and 73 of 138 of the patients (53%) in the group with stable/increase in ΔSMI had died. Median overall survival did not differ significantly(p=0.764).
Conclusion: A decreasing skeletal muscle index during neo-adjuvant chemotherapy was not associated with worse outcome in patients with stage III ovarian cancer, who were treated with complete/optimal interval CRS and six cycles of chemotherapy within the OVHIPEC trial.
Disclosure: Nothing to disclose

EP1010

The clinical outcomes of neoadjuvant therapy with cisplatin plus mitomycin in BRCA1-mutated ovarian cancer

K. Kotiv[1], A. Sokolenko[1], E. Ulrikh[1,2], A. Sidoruk[1,2], A. Ivantsov[1], T. Gorodnova[1], A. Urmancheeva[1,2], I. Berlev[1,2], E. Imyanitov[1,2]
[1]National Medical Research Center of Oncology named after N.N.Petrov, [2]North-Western State Medical University named after I.I.Mechnikov, Saint-Petersburg, Russian Federation

Introduction / Background: The aim of the study was to compare the effectiveness of neoadjuvant chemotherapy regimens (NACT): Mitomycin and Cisplatin (MP) vs Paclitaxel and Cisplatin /Carboplatin (TCb/TP) in advanced BRCA1-mutated ovarian cancer (BMOC).
Methodology: In a prospective cohort of 13 patients (38-66 years (mean age 52 years) we analyzed the efficacy of Mitomycin (10 mg/m^2) and Cisplatin (100 mg/m^2) NACT administered intravenously every 3-4 weeks. The control group included 22 patients of retrospective cohort (38-67 years (mean age 51,9 years), who received standard 1-st line NACT (TCb/TP). Clinical efficacy was evaluated according to RECIST 1.1, histopathologic response - according to the Bohm scale (2015).
Results: The objective response was observed in 13/13 (100%) patientsin MP group vs 9/22 (41%) patientsin TCb/TP group (p=0,0006). Complete surgical cytoreduction was achieved in all patients (13/13; 100%) after MP vs 13/22 (59%) cases in TCb/TP group (p=0,01). Complete pathological response was confirmed only in MP group - in 3 out of 13 patients (23%) vs 0/22 patients (0%) in TCb/TP group (p=0,04).
Conclusion: The short-term results of the present study showed that the neoadjuvant chemotherapy with Cisplatin and Mitomycin is an effective option for BRCA1-associated advanced ovarian cancer treatment.
Disclosure: Nothing to disclose

EP1011

CA 125 in borderline ovarian tumors

E. Uyanık[1], G. Özten[2], S. Çetiner[2], M.C. Salman[2], M. Gültekin[2]
[1]Obstetrics and Gynecology, [2]Hacettepe University, School of Medicine, Ankara, Turkey

Introduction / Background: Borderline ovarian tumors (BOT) are defined as atypical epithelial proliferation without histopathological stromal invasion. Tumors with low malignancy potential, tend to be limited to ovary and CA 125 is not elevated in majority of patients. In this study, we aim to investigate CA 125 levels in patients with BOT.
Methodology: 56 patients, final pathologies were reported as BOT were included. Age, menapausal status, histological type and stage of the tumor were evaluated and compared with CA 125 levels, retrospectively.
Results: The mean age of the patients was 42.9 (19-84) and 66.1% of the patients were premenapausal. Serous histology was detected in 67.9% of the patients, the remaining histology was musinous, brenner or mix type. 83.9% of the patients disease was limited to the ovary. 57.1% of the patients Ca 125 levels were above normal range. Mean level of CA 125 was 227.2 U/ml, ranged between 3-5668 U/ml (Table 1). There was positive corelation between CA 125 levels and premenapausal status, serous histology and extraovarian spread (Table 2).
Conclusion: CA 125 is especially used in monitoring the response to treatment in invasive epithelial ovarian cancer. In early stage disease and in premenopausal women its benefits are limited. It is reported that there isn't a significant increase in CA 125 in BOT. However, in our study, elevation in CA 125 level was found in a significant number of patients and it was related to stage and serous histology, and higher levels were detected in the majority of premenopausal patients. This suggests that detection of CA 125 levels in BOT may have prognostic significance in addition to assisting the diagnosis.

Menopausal status	Premenopausal, n (%)	37 (66.1)
	Postmenopausal, n (%)	19 (33.9)
Histological Type	Serous, n (%)	38 (67.9)
	Non-serous, n (%)	18 (32.1)
Stage	1, n (%)	47 (83.9)
	2-4, n (%)	9 (16.1)
Ca 125	Normal	24 (42.9)
	High	32 (57.1)
Ca 125 levels (U/ml)	Min-Max	3 - 5668
	Mean	227.2

[Table 1. Clinical and pathological parameters]

Parameters		Ca 125 Normal	Ca 125 High	p value
Menopausal status	Postmenopausal, n (%)	12 (63.2)	7 (36.8)	
	Postmenopausal, n (%)	12 (63.2)	7 (36.8)	0.028
Histological type	Serous, n (%)	14 (38.8)	24 (63.2)	
	Non-serous, n (%)	10 (55.6)	8 (44.4)	0.045
Extraovarian spread	No, n (%)	24 (51.1)	23 (48.9)	
	Yes, n (%)	0 (0)	9 (100)	0.007

[Table 2. Clinical and pathological parameters which have significant relationship with CA 125 levels]

Disclosure: Nothing to disclose

EP1012

Giant ovarian mucinous cystadenoma: a case report

E. Uyanık, G. Özten, N. Orhan, S. Çetiner, M. Gültekin, M.C. Salman
Obstetrics and Gynecology, Hacettepe University, School of Medicine, Ankara, Turkey

Introduction / Background: Mucinous cystadenoma (MC) is a common benign ovarian neoplasm. The most important feature of a MC is that it can reach much larger sizes than any other adnexial mass.
Methodology: In this case report a MC arises from right ovary with a diameter of 40 cm is presented.
Results: A 38 year old patient presented with recurrent abdominal pain for 2 months. Physical examination revealed severe abdominal distention and laboratory results showed CA 125 was 152 U/ml. Abdominal tomography revealed a massive cystic mass with no solid component and a diameter of 40 cm (Fig 1). Midline laparotomy was performed. In exploration, a smooth surfaced mass derived from the right ovary and extending to the bottom of the liver is observed (Fig 2). Mass was partially adhered to omentum and liver. Salphingoooferectomy was performed. Frozen section was reported as mucinous cystadenoma. Apendectomy was performed. The patient was 72 kilograms before the operation and 57 kilograms in postoperative day 1. Histopathological examination confirmed the diagnosis of benign mucinous cystadenoma.
Conclusion: Diferential diagnosis of adnexial masses is difficult due to its extended spectrum and therefore evaluation and management is challenging. Giant ovarian cystadenomas are rarely observed today because of improved imaging techniques. In most cases, USG examination can reveal a complex adnexal mass, however large ovarian cysts require an evaluation of abdomen. As in our case, we performed a CT scan and preoperative evaluation of tumor markers to exclude malignancy. As a conclusion, benign mucinous tumors can reach excessive diameters when neglected and can be a challenge to surgeon.

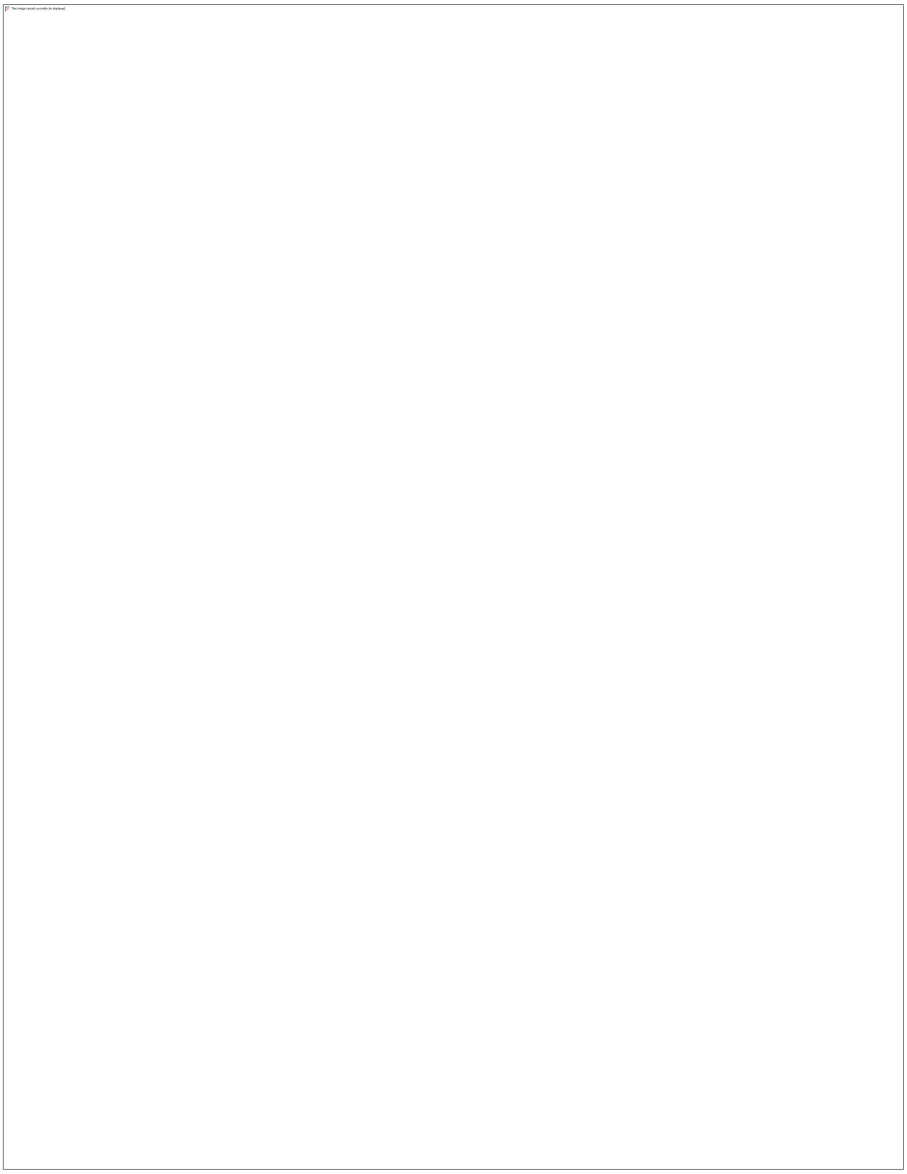

[Figure 1: Abdomen Tomography: the mass fills the entire abdomen]

[Figure 2: The mass during laparotomy]

Disclosure: Nothing to disclose

EP1013

Overview of non-epithelial ovarian tumours: incidence and survival in the Netherlands

O.L. van der Hel[1], M. Timmermans[1,2,3], A.M. van Altena[4], R.F.P.M. Kruitwagen[2,3], B.F.M. Slangen[2,3], G.S. Sonke[5], K.K. van de Vijver[6], M.A. van der Aa[1]
[1]Department of Research and Development, Netherlands Comprehensive Cancer Organisation (IKNL), Utrecht, [2]Department of Obstetrics and Gynaecology, Maastricht University Medical Center, [3]School for Oncology and Developmental Biology, GROW, Maastricht, [4]Department of Obstetrics and Gynecology, Radboud University Medical Center, Nijmegen, [5]Department of Medical Oncology, Netherlands Cancer Institute, Amsterdam, The Netherlands, [6]Department of Pathology, Ghent University Hospital, Cancer Research Institute Ghent (CRIG), Ghent, Belgium

Introduction / Background: About 5% of ovarian tumours have a non-epithelial histology, including germ cell tumours (GCTs), sex cord-stromal tumours (SCSTs), and sarcomas. Since the rarity of these non-epithelial ovarian tumours and the scarcity of population-based studies, the aim of this population-based study is to describe trends in incidence, treatment, and survival of these women in the Netherlands.
Methodology: All Dutch women diagnosed with non-epithelial ovarian malignant tumours between 1989 and 2015 were identified from the Netherlands Cancer Registry. Demographics, tumour characteristics, and initial treatment were collected and overall survival was analysed.
Results: A total of 1258 women were diagnosed with non-epithelial ovarian tumours in the Netherlands, including 752 GCTs (60%), 341 SCSTs (27%), and 165 sarcomas (13%). The European age standardized incidence rate (ESR) was 0.4 per 100,000 persons per year for GCTs, 0.2 for

SCSTs and 0.1 for sarcomas. Approximately 97% of patients underwent surgical resection for the primary tumour, whereas 31% received systemic treatment and 3% radiotherapy. Five-year overall survival improved for all histologic subtypes between the late 1980's until 2015: GCTs from 73% to 88% (p=0.03) SCSTs from 64% to 81% (p=0.57) and sarcomas from 20% to 29% (p=0.14).
Conclusion: Malignant GCTs and SCSTs are rare and its incidence did not significantly change during the last decades. They have a good prognosis which also improved slightly. Women with primary sarcomas of the ovary are extremely rare and still have a poor prognosis.
Disclosure: Nothing to disclose

EP1014

Signal transduction pathway activity in normal Fallopian tube epithelium and high-grade serous carcinoma

P. van der Ploeg[1], K. Bucks[1], R.L.M. Bekkers[1], J. de Hullu[2], A. van de Stolpe[3], A. van der Wurff[4], M.C. Vos[4], J.M.J. Piek[1]
[1]Obstetrics & Gynecology, Catharina Hospital, Eindhoven, [2]Obstetrics & Gynecology, Radboud University Medical Center, Nijmegen, [3]Molecular Diagnostics, Philips Research, Eindhoven, [4]Obstetrics & Gynecology, Elisabeth-TweeSteden Hospital, Tilburg, The Netherlands

Introduction / Background: High-grade serous carcinoma (HGSC) is the most lethal gynecological malignancy due to late detection and high recurrence rates. New treatment options focus on targeted therapy based on signal transduction pathways (STPs). Tumour growth is induced by aberrant STP activation leading to disturbed cell proliferation and differentiation. However, knowledge about STP activity in healthy tissue is needed to discriminate STP activity in HGSC. As the Fallopian tube epithelium (FTE) is considered the tissue of origin of most HGSC, the aim of our study was to determine STP activity in normal FTE during the menstrual cycle compared to HGSC.
Methodology: We included 10 lasermicrodissected FTE samples and 17 HGSC*. This study used Philips OncoSignal PCR, a diagnostic approach based on a computational model which infers quantitative STP activity from mRNA levels of pathway-specific target genes.
Results: Preliminary data suggest that the follicular phase is characterized by high phosphoinositide 3-kinase (PI3K) pathway activity and the luteal phase by high androgen receptor (AR) pathway activity. Remarkably, no significant differences in estrogen receptor (ER) pathway activity were seen in the FTE during the menstrual cycle. In addition, ER activity was low in HGSC compared to normal FTE. High PI3K activity in HGSC was comparable with activity in the follicular phase. As proliferation is a hallmark of the follicular phase, it is suggested that up-regulation of the PI3K pathway in HGSC could be promoting proliferation and therefore be an interesting target for new therapeutic options.
Conclusion: During the menstrual cycle, PI3K and AR pathway activity are activated in a strictly controlled manner. High PI3K activity in HGSC could indicate aberrant activation and may therefore be an attractive target. Future investigations should focus on post-menopausal FTE in order to further discriminate STP activity in HGSC.
*Sample size will be expanded as the study is still ongoing.
Disclosure: Nothing to disclose

EP1015

Is endometriosis always benign?

D. Vasileiadou, M. Litos, V. Triantafyllidi, A. Gerolymatos
Department of Obstetrics & Gynecology, Konstantopouleio General Hospital of Nea Ionia, Athens, Greece

Introduction / Background: The aim of this study is to present a case of an ovarian endometrioma with atypia.
Methodology: The present study is a case report and literature review.
Results: A 43-year-old patient underwent laparoscopy for the excision of a cystic mass of the left ovary. Intraoperatively the mass was recognized as an endometrioma and other findings included multiple adhesions and a rectovaginal septum nodule. The endometrioma was excised and adhesiolysis was performed. The histology report was diagnostic of atypical endometriosis of the ovary, due to the presence of epithelial micropapillary tufts and extensive nuclear atypia.
Atypical endometriosis is characterized histologically as a lesion with features that fall between benign and malignant, such as nuclear enlargement and hyperchromatism, a high nuclear to cytoplasmic ratio, cellular crowding and stratification or tufting and the presence of atypical glands. It is observed in less than 1-3% of endometriomas and is considered to have a malignant potential to endometriosis - associated ovarian cancer, such as the endometrioid and clear cell subtypes. Literature is limited and inadequate in order to safely estimate the risk of malignant transition, the natural course or the best clinical management of atypical endometriosis.
Conclusion: Atypical endometriosis may be considered as a precancerous lesion and requires close follow - up or prophylactic treatment.
Disclosure: Nothing to disclose

EP1016

Evaluation of the antitumor effect of dioxade on the change in the content of free DNA in the blood

I. Vasilyeva[1], V. Bespalov[1], A. Semenov[1], G. Tochilnikov[1], A. Stukov[1], K. Senchik[2], G. Gafton[2]
[1]Research Laboratory of Cancer Prevention and Oncopharmacology, [2]Department of General Oncology and Urology, N.N. Petrov Medical Research Center of Oncology, Saint-Petersburg, Russian Federation

Introduction / Background: Tumor growth is characterized by a modified ratio of apoptotic and mitotic indexes. Purpose of research was to assess the cfDNA levels change during tumor growth and the effect of antitumor drug from group of acetylated cytostatic dioxadet in a model of transplanted ascites ovarian cancer (OC) in female rats.
Methodology: Tumor was transplanted intraperitoneally to 12-week-old Wistar rats (n=20) by introducing 1×10^7 tumor cells in 0.5ml of saline. 48 h after OC inoculation dioxadet at the maximum tolerated dose of 1.5 mg/kg b.w. was injected intraperitoneally to rats (n=10) and saline was injected intraperitoneally to control rats (n=10). The results were evaluated by the increase in median survival (MS) of rats and by the content of cfDNA. Blood from rats was taken from the peripheral vein at intervals of 0, 2, 4, 6 and 8 days after inoculation and EDTA-plasma was separated. The cfDNA content was measured by ELISA assay.
Results: The blood plasma cfDNA level of rats initially, before OC transplantation, was 1.6 ±0.33 ng/µl, without changing within 2 days, was significantly higher after 6 days (9.84±3.96 ng/µl, p< 0.01) and remained elevated after 8 days. 2 days after the dioxadet administration the cfDNA level in the experimental group was lower than in the control group (0.78±0.33 vs 2.33±1.17 ng/µl, p< 0.025), but 6 and 8 days after OC transplantation the cfDNA level did not differ significantly from the baseline. MS of rats treated with dioxadet was 46.0 days, control - 29.5 days (p=0.0049).
Conclusion: A significant (56%) increase in rat's life expectancy caused by dioxadet is accompanied by cfDNA level decrease in the 2 days after injection of the drug. Thus, the measurement of cfDNA can be used to assess the effectiveness of the cytostatic effect in the clinic.
Disclosure: Nothing to disclose

EP1017

Prognostic and therapeutic factors for low-grade serous ovarian carcinoma: a multicentric, retrospective study of the Turkish society of gynaecologic oncology

D. Vatansever[1], C. Taskiran[1], M.M. Meydanlı[2], K. Gungorduk[3], O. Akbayır[4], I. Yalcin[2], F. Demirkiran[5], H. Sozen[6], N. Ozgul[7], H. Celik[8], M.A. Onan[9], S. Taskin[10], T. Oge[11], T. Simsek[12], S. Abboud[13], A. Ayhan[14]
[1]Obstetrics and Gynaecology, Division of Gynaecologic Oncology, Koc University School of Medicine, Istanbul, [2]Obstetrics and Gynaecology, Division of Gynaecologic Oncology, Zekai Tahir Burak Women's Health Training and Research Hospital, Faculty of Medicine, University of Health Sciences, Ankara, [3]Obstetrics and Gynaecology, Division of Gynaecologic Oncology, Tepecik Education and Research Hospital, Izmir, [4]Obstetrics and Gynaecology, Division of Gynaecologic Oncology, Kanuni Sultan Suleyman Training and Research Hospital, University of Health Sciences, Faculty of Medicine, [5]Obstetrics and Gynaecology, Division of Gynaecologic Oncology, Cerrahpasa Faculty of Medicine, [6]Obstetrics and Gynaecology, Division of Gynaecologic Oncology, Istanbul University, Istanbul Faculty of Medicine, Istanbul, [7]Obstetrics and Gynaecology, Division of Gynaecologic Oncology, Hacettepe University, School of Medicine, Ankara, [8]Obstetrics and Gynaecology, Division of Gynaecologic Oncology, Baskent University Faculty of Medicine, Adana, [9]Obstetrics and Gynaecology, Division of Gynaecologic Oncology, Gazi University, School of Medicine, [10]Obstetrics and Gynaecology, Division of Gynaecologic Oncology, Ankara University School of Medicine, Ankara, [11]Obstetrics and Gynaecology, Division of Gynaecologic Oncology, Osmangazi University, School of Medicine, Eskisehir, [12]Obstetrics and Gynaecology, Division of Gynaecologic Oncology, Akdeniz University, School of Medicine, Antalya, Turkey, [13]Obstetrics and Gynecology, Saint Joseph University/Hôtel Dieu de France University Hospital, Beirut, Lebanon, [14]Obstetrics and Gynaecology, Division of Gynaecologic Oncology, Baskent University Faculty of Medicine, Ankara, Turkey

Introduction / Background: Low-grade serous ovarian carcinoma (LGSOC) is a rare subtype of epithelial ovarian cancers (EOC), and is molecularly and clinically distinct from its high-grade counterpart. The aim of this study was to describe the clinico-pathological features of patients diagnosed with LGSOC and to analyse the factors affecting recurrence free survival (RFS) and overall survival (OS) rates.
Methodology: Databases from 13 participating centres in Turkey were searched retrospectively for women who had been diagnosed with stage I to IV LGSOC between 1997 and 2018.
Results: Overall 191 eligible women were analysed. Median age at diagnosis was 49 years (range, 21-84 years), and median Ca-125 value was 164 U/mL (range, 7-8685 U/ml). One hundred seventy-five (92%) patients underwent primary cytoreductive surgery and 65 patients (34%) were found to have FIGO stage I disease. Complete and optimal cytoreduction was achieved in 148 (77.5%) and 33 (17.3%) patients, respectively. Lymph node dissection was performed in 155 patients (81.2%), and 158 (83%) patients received adjuvant chemotherapy. Median follow-up period was 44 months (range, 2-208 months). Multivariate analysis showed that presence of endometriosis (p=0.012), LVSI (p=0.022), any residual disease (p=0.023) and FIGO stage II-IV (p=0.045) were negatively correlated with RFS, while only presence of residual disease (p=0.002) and FIGO stage II-IV (p=0.003) significantly shortened OS. Age, neoadjuvant or adjuvant chemotherapy, and lymph node metastasis did not affect survival outcomes.
Conclusion: Patients with LGSOC diagnosed at an early stage have the best survival outcomes. The results of the current study suggest that maximal effort is warranted for complete cytoreduction since LGSOC appears to be relatively chemo-resistant in all settings. The prognostic role of both LVSI and endometriosis should be evaluated by further studies since both significantly affected RFS in this population.
Disclosure: Nothing to disclose

EP1018

Abnormal antioxidant status of lymphocytes in tumor and blood in ovarian cancer as the cause

of their functional failure

E. Verenikina, E. Frantsiyants, T. Moiseenko, T. Myagkova, V. Bandovkina, N. Maksimova
Rostov Research Institute of Oncology, Rostov-on-Don, Russian Federation

Introduction / Background: Maintaining the metabolic processes in lymphocytes (LF) is one of the conditions for their effective functioning. The purpose of the study was to analyze the antioxidant defense in tumor-infiltrating LF and blood LF of patients with ovarian cancer (OC).
Methodology: Levels of A and E vitamins and activity of catalase (C), SOD and glutathione peroxidase (GP) were determined by ELISA in LF isolated from malignant ovarian tissues and blood of patients with OC $T_{3-4}N_xMx$.
Results: The amount of LF in tumors was 1.5 times higher than in intact ovarian tissues, while protein amount in them was 1.8 times lower. E and A vitamins in tumor LF were 3.1 times lower than in intact ovaries, and their ratio did not change. C activity in tumor LF was 2.1 times lower, SOD and GP were similar to control values, but a natural enzyme cascade characterized by their ratio was 1.9 times higher than in intact organs.
The amount of LF in the blood of OC patients was similar to the norm, while protein amount in them was 1.8 times higher. Levels of E and A vitamins in blood LF in patients were 2.4 and 4.1 times lower, and their ratio was 1.8 times higher than in healthy donors. SOD activity was 2 times lower than in donors, and GP was 6 times higher. The SOD/GP ratio was 3 times higher than in the blood of healthy people.
Conclusion: Tumor-infiltrating LF in ovarian cancer demonstrated disrupted natural cascade of antioxidant enzymes with the suppression of C activity. This may result in hyperproduction of superoxide anion radical in cells leading to self-poisoning of lymphocytes by active oxygen species. Low levels of E and A vitamins playing an important role in the maturation of immunocompetent cells can be the main cause of lymphocyte functional failure.
Disclosure: Nothing to disclose

EP1019

Antioxidant status of tumor and blood neutrophils in ovarian cancer patients as an indicator of immune homeostasis

E. Verenikina, E. Frantsiyants, T. Moiseenko, T. Myagkova, I. Kaplieva, E. Surikova, V. Bandovkina
Rostov Research Institute of Oncology, Rostov-on-Don, Russian Federation

Introduction / Background: Animportant role of neutrophils in antitumor immunity does not cause doubt. Assessment of the functional status determined by the state of cell metabolism is considered one of methods to control immune homeostasis. Our purpose was to study the antioxidant defense in tumor-infiltrating neutrophils and blood neutrophils of patients with ovarian cancer (OC).
Methodology: Levels of A and E vitamins and activity of catalase, SOD and glutathione peroxidase (GP) were determined by ELISA in neutrophils isolated from malignant ovarian tissues and blood of 45 patients with OC $T_{3-4}N_xMx$. Intact ovaries and blood of healthy donors were used as a control.
Results: The amount of neutrophils in tumors was 2.9 times higher than in intact ovaries, while protein amount in them was 2.9 times lower. E and A vitamins in tumor neutrophils were decreased by 2.5 and 1.9 times compared to control values, and their ratio did not change. SOD and GP activity did not differ significantly from the values in intact tissues, but their ratio was 12.5 times lower. Catalase activity in neutrophils of tumor and intact tissues was similar.
The amount of neutrophils in the blood of patients was 1.5 times higher than in healthy donors, while protein content in the cells did not differ significantly. Levels of E and A vitamins and their ratio in blood neutrophils of OC patients were 8.3, 4.7 and 1.8 times lower, respectively, than in healthy people. Activity of SOD and GP was similar to the control values, but their ratio was 2 times lower than in donors. Catalase activity in patients was 2.5 times lower.
Conclusion: Tumor-infiltrating neutrophils had abnormalities in both units of the antioxidant defense which may cause a decrease in their functional activity impeding an adequate antitumor response, but their normal ability to chemotaxis was maintained.
Disclosure: Nothing to disclose

EP1020

Luminescent characteristics of peripheral blood lymphocytes reflect efficiency of treatment in patients with ovarian cancer

E. Verenikina, E. Frantsiyants, T. Moiseenko, E. Sheiko, T. Myagkova, L. Rozenko
Rostov Research Institute of Oncology, Rostov-on-Don, Russian Federation

Introduction / Background: The alpha parameter characterizes the ratio of single-stranded and double-stranded nucleic acids in the lymphocyte. The values of this parameter can be used as a signal test containing generalized information about the complex of changes in the body of a cancer patient. The purpose of the study was to perform a luminescent-spectral analysis of peripheral blood lymphocytes in patients with ovarian cancer (OC) during antitumor treatment.
Methodology: Peripheral blood lymphocytes of 20 patients with T3NxMo OC were studied. Patients received endolymphatic polychemotherapy (thiotepa 200 mg, metatrexate 100 mg, 5-fluorouracil 200 mg) twice with a 1-week interval. Lymphocytes isolated during the treatment were stained by the Karnaukhov-Rigler method and studied using a microspectrofluorimeter. Fluorescent spectral analysis was performed.
Results: The spectra of OC patients before treatment had only one emission band at 530 nm, with low α parameter values (0.098-0.1 SU). After the beginning of the treatment, a second band appeared on the spectrum at 640 nm, however the band at 530 nm prevailed. During the antitumor treatment and achievement of clinical effect, the intensity of the band at 530 nm significantly decreased on the spectrum in favor of a wavelength of 640 nm. After the end of antitumor therapy, both peaks of approximately the same intensity were present on the spectrum. With the normalization of patients' state, α parameter values increased from 0.44 SU to 2.12 SU ($p \leq 0.05$). The values were low (0.22-0.048SU) when the state of patients worsened, without clinical effect.
Conclusion: The shape of the luminescence spectrum and values of the α parameter vary during the treatment and depend on its results. Luminescent characteristics of peripheral blood lymphocytes allow monitoring the treatment effectiveness and can help to objectively assess the condition of ovarian cancer patients.
Disclosure: Nothing to disclose

EP1021

Laparoscopic primary cytoreduction with multi visceral resection in advanced ovarian cancer: the first experience of 5 procedures

S. Baydo[1], A. Vinnytska[2], D. Golub[1]
[1]Surgery, [2]Gynecology, LISOD - Israely Oncological Hospital, Kyiv, Ukraine

Introduction / Background: Standard approach for the initial treatment of advanced ovarian cancer (AOC) is surgical removal of all visible implants of the disease through a large midline laparotomy. Although laparoscopy has been shown to be safe and effective in early stage EOC, few surgeons have adopted this approach for patients with advanced disease. We want to present our fist experience of laparoscopic complete primary cytoreduction in AOC.
Methodology: Having an extensive experience in performing laparoscopic radical operations for early OC and interval debulking for AOC, since 2018 we'd started to perform primary cytoreduction (PC) in AOC. Till now 5 PC were done. Average age 50,8 (44-54) years. Average PCI 14 (4-29) and Fagotty score 3(2-8). In all cases total hysterectomy with omentectomy, peritonectomy, pelvic and paraaortal lymphadenectomy were done. Other procedures were: diaphragmatic stripping (2), colorectal resection with primary anastomosis (4), resection of small bowel (1), splenectomy (1), hepatic

resection (2).
Results: Complete cytoreduction (CC0) with no residual tumor was achieved in all cases. Average operative time was 285 (150-370) minutes. Estimated blood loss - 175 (50-470) ml. Mean length of hospital stay - 14,4 (4-46) days. Mean number of lymph nodes harvested 23,2 (13-31). Postoperative complications grade IIIB-IV occurred in 1 patient: intraoperative heart failure and anastomotic leak with relaparoscopy. No patients died.
Conclusion: Laparoscopic primary cytoreduction with multivisceral resection in AOC is technically feasible in experienced hands and can be offered to carefully selected patients with AOC. Potential postoperative advantages of laparoscopic approach are faster recovery and relatively low morbidity.
Disclosure: Nothing to disclose

EP1022

Primary vs interval debulking surgery for advanced epithelial ovarian cancer: survival outcomes from a tertiary Greek centre

D.-E. Vlachos, D. Haidopoulos, N. Thomakos, E. Kalogeris, A. Rodolakis
First Department of Obstetics and Gynecology, National & Kapodistrian University of Athens, Athens, Greece

Introduction / Background: The standard treatment for advanced stage epithelial ovarian cancer consists of surgery and chemotherapy. Multiple studies have shown that complete removal of the disease is associated with better survival. However, primary debulking surgery (PDS) is not always possible, either due to the disease's extent or the patient's poor physical condition. In such cases, neoadjuvant chemotherapy (NACT) and interval debulking surgery (IDS) is preferred. The aim of the present study is to present the results from a Greek tertiary referral center as to which of the two methods has the best survival outcome.
Methodology: We retrospectively analyzed all advanced stage epithelial ovarian cancer patients that were operated in our department from 1/1/2007 till 31/12/2017. We recorded the debulking type, residual disease and follow-up of the patients. Overall and progression free survival were the primary outcomes of the study. Kaplan - Meier estimator and Cox regression analysis was conducted in order to find survival rates and associated factors.
Results: A total of 409 patients were recorded (214 in the PDS and 92 in the IDS group). Complete cytoreduction was achieved in 61/132 patients (46,2%) in the IDS group and 95/277 (34,3%) in the PDS group (p=0.019). The recurrence rate was 73.6% and 74% for the PDS and IDS groups respectively. The mean follow-up period was 6 years with a median equal of 5.1 years. The cumulative 5-year survival rate was 56% for the PDS and 40% for the IDS group, respectively. Kaplan Meier analysis revealed worse survival rates for the IDS group while no significant difference was found regarding the recurrence rates.
Conclusion: Our findings support that PDS with adjuvant chemotherapy is the optimal treatment for advanced stage epithelial ovarian cancer patients. Cytoreduction rate remains the most import prognostic factor.
Disclosure: Nothing to disclose

EP1023

The effect of surgery on patients with stage III ovarian cancer and enlarged supradiaphragmatic lymph nodes

O. Weitzner, Y. Yagur, Y. Kadan, A. Fishman, R. Zissin, E. Ben Ezry, M. Beiner
Gynecological Oncology Unit, Meir Medical Center, Kfar Saba, Israel

Introduction / Background: Enlarged supradiaphragmatic lymph nodes (ESDLN) in patients with advanced epithelial ovarian cancer (EOC) are associated with poor prognosis. This study assessed whether the prognosis is also affected by the treatment approach and surgical outcomes among these patients.
Methodology: A retrospective cohort study comparing patients with Stage 3 EOC and ESDLN to those without SDLN, who underwent surgery at a single center from 2006 to 2016. CT scans were retrospectively reviewed by the same radiologist for disease extent and ESDLN. Extent of disease at diagnosis (defined by carcinomatosis score), results of surgery, and progression-free survival (PFS) were recorded.
Results: Based on imaging, among 71 women who met the inclusion criteria, 47 (66%) had ESDLN and 24 (34%) did not. There was no significant difference between groups in baseline characteristics except for elevated CA125 at diagnosis in the ESDLN group (1,797 U/ml vs. 708 U/ml, p=0.04). There was a trend toward longer PFS in the negative SDLN group (19.6 months vs. 12.6 months, p=0.09). Although not significant, among patients who underwent complete cytoreduction, those without ESDLN had longer PFS compared to patients with ESDLN (21.9 vs. 13.6 months, p=0.29). Similarly, a trend toward better prognosis was observed in the negative SDLN group when comparing treatment modalities (21.5 vs. 15.1 months for primary surgery, and 16.5 vs. 9.7 months after neoadjuvant chemotherapy and interval debulking, p=0.16).
Conclusion: ESDLN in patients with advanced EOC are associated with poor prognosis. In seems that even when optimal intra-abdominal cytoreduction is achieved, whether at primary surgery or after neoadjuvant chemotherapy, PFS is compromised in patients with ESDLN.
Disclosure: Nothing to disclose

EP1024

Assessment of the clinicopathological relevance of interleukin 17 in ovarian cancer patients

I. Wertel, M. Bilska, A. Pawłowska, D. Suszczyk, R. Tarkowski, B. Barczyński, J. Kotarski
Department of Oncological Gynecology and Gynecology, Medical University of Lublin, Lublin, Poland

Introduction / Background: The pathogenesis of ovarian cancer (OC) is still unknown.The particular immune system factors may actively promote tumor growth by maintaining inflammation, promoting immunosuppression and creating new blood vessels. The role of interleukin 17 (IL-17) in OC immunology is controversial. On the one hand, IL-17 seems to stimulate tumor growth by inducing tumor vascularization or enhancing inflammation. On the other hand, IL-17 can reduce invasiveness and induce specific immunity to cancer cells.
The aim of the study was evaluation of IL-17A concentration in plasma and peritoneal fluid (PF) of patients with ovarian cancer (n=71) in relation to their clinicopathological characteristics. The studies were also conducted in benign ovarian tumor patients (n=35) and healthy donors (n=10).
Methodology: The concentration of IL-17A in plasma and PF were determined by enzyme linked immunosorbent assay (ELISA) according to manufacturer's instruction.
Results: The highest concentration of IL-17A was detected in plasma of OC patients and it was higher (p< 0.0001) than in the both, benign tumors and control group. The peritoneal fluid IL-17A level in OC patients was higher (p< 0.0001) than in women with benign ovarian tumors. There were no significant differences in IL-17 level in OC patients depending on FIGO stage, histological grade, cancer type according to Kurman and Shih classification. The concentration of IL-17A in the PF was higher (p=0.04) in OC patients before menopause than in patients after menopause.The patients survival with higher level of IL-17A in the PF was longer than patients with lower Il-17A level.
Conclusion: The concentration of IL-17A in the PF of OC patients is significantly higher than in patients with benign tumors.
There are differences in the concentration of IL-17 in OC patients depending on microenvironment. Higher IL-17A level in the PF correlate with longer survival of ovarian cancer patients.
Disclosure: Nothing to disclose

EP1025

Serum calretinin as liquid biopsy marker for identifying ovarian cancer patients with poor prognosis

P. Wimberger[1,2], T. Link[1,2], S. Passek[1,2], Y. Vassileva[1,2], M. Kramer[2,3], J.D. Kuhlmann[1,2]
[1]Department of Gynecology and Obstetrics, TU Dresden, [2]NCT Dresden, [3]Medical Clinic I, TU Dresden, Dresden, Germany

Introduction / Background: Calretinin (CRT) is a calcium-binding protein, controlling intracellular calcium signaling. Besides its prominent expression in neurons, CRT has diagnostic implications in cancer, particularly in mesothelioma. In a recent liquid biopsy approach, plasma CRT level has been suggested for pre-diagnostic detection of mesothelioma. CRT is also expressed in serous ovarian cancer in about 23% of cases; however, clinical relevance of serum CRT is completely unknown and shall therefore be analyzed in the present study.
Methodology: Serum calretinin (sCRT) was determined by CRT enzyme-linked immunoabsorbent assay (DLD-Diagnostika GmbH, Hamburg, Germany) in a total of 380 serum samples from 134 ovarian cancer patients (thereof n=115 (86%) with FIGO III or IV), including samples at primary diagnosis and at four follow-up reading points in the course of adjuvant treatment.
Results: sCRT levels were significantly increased in ovarian cancer patients compared to healthy controls (ED=0.3ng/ml, p< 0.001) and enabled an accurate discrimination between cancer and controls (AUC=0.85). High sCRT levels at primary diagnosis correlated with platinum resistance (p=0.002), predicted suboptimal debulking surgery without achieving macroscopically complete tumor resection (p< 0.001) and were associated with advanced FIGO-stage (p< 0.001) and high volume of ascites (p< 0.001). Increased sCRT levels at primary diagnosis were an independent predictor of poor PFS (HR=1.99, p=0.018) and indicated poor OS (HR=2.49, p=0.008). Higher sCRT levels before and after platinum-based chemotherapy were independent predictors of poor OS (HR=15.4, p=0.01; HR=5.59, p=0.026). Moreover, in the course of debulking surgery and adjuvant treatment, individual progression of sCRT levels provided independent prognostic information (OS: HR=7.38, p=0.034; PFS: HR=2.90, p=0.035) and additionally identified patients with poor prognosis.
Conclusion: This is the first study, suggesting sCRT as an innovative liquid biopsy marker for the identification of ovarian cancer patients with primary platinum resistance and poor prognosis.
Disclosure: Nothing to disclose

EP1026

CD4+IL-21+ and IL-22+ and CD4+FOXP3 in a algorithm ROMA relationship in patients affected by ovarian tumours

I. Winkler[1,2], J. Woś[3], A. Karczmarczyk[4], P. Miotła[2], M. Gogacz[2], K. Skorupska[2], T. Rechberger[2], J. Tabarkiewicz[5]
[1]II Department of Gynecology, St'Johns Center Oncology, [2]II Department of Gynecology, [3]Department of Clinical Immunology, [4]Department of Experimental Hematooncology, Medical University in Lublin, Lublin, [5]Centre for Innovative Research in Medical and Natural Sciences, Medical University in Rzeszów, Rzeszów, Poland

Introduction / Background: The aim of the study was to estimate the relationship of Treg (CD4+ FOXP3+) and Th17 cells producing IL21 and IL-22 in the peripheral blood and the tissue of the epithelial ovarian tumor, to blood serum levels of markers HE4 and CA125 and to assess the application of the risk of ovarian malignancy algorithm (ROMA).
Methodology: Mononuclear cells (PBMCs) obtained from the peripheral blood and the ovarian tissue of patients suffering from ovarian pathology were isolated by density gradient centrifugation. The control group consisted of patients who had undergone surgery for unexplained infertility. The percentage of Treg and Th17 producing IL-21+ or IL-22+ lymphocytes in peripheral blood and tissue was assessed using the flow cytometry method according to the manufacturer´s instructions (Figure.1).The ROMA index was calculated by way of levels of HE4 and CA125 in serum.

Results: A larger concentration of marker HE4 and value of ROMA was shown in patients with ovarian cancer, when compared with women without ovarian pathology and cystadenomas. A negative correlation was also found in the percentage of CD4+/IL-21+ in the peripheral blood and the amount of Treg infiltrating normal ovarian tissue. Moreover, we observed a relationship between the ROMA percentage in the serum and Treg in the peripheral blood of women suffering from benign ovarian tumors (Figure.2).
Conclusion: In patients with benign tumors, we found for the first time, significant negative correlation between percentages of circulating Treg cells in the peripheral blood and with ROMA assessment in the serum. This result could be explained by the negative influence of Treg on inflammation and secondary on malignancy induced by chronic inflammation. Furthermore, the imbalance in Treg percentage in normal ovarian tissue of patients suffering from unexplained infertility, could induced immunotolerance, and hence, infertility.

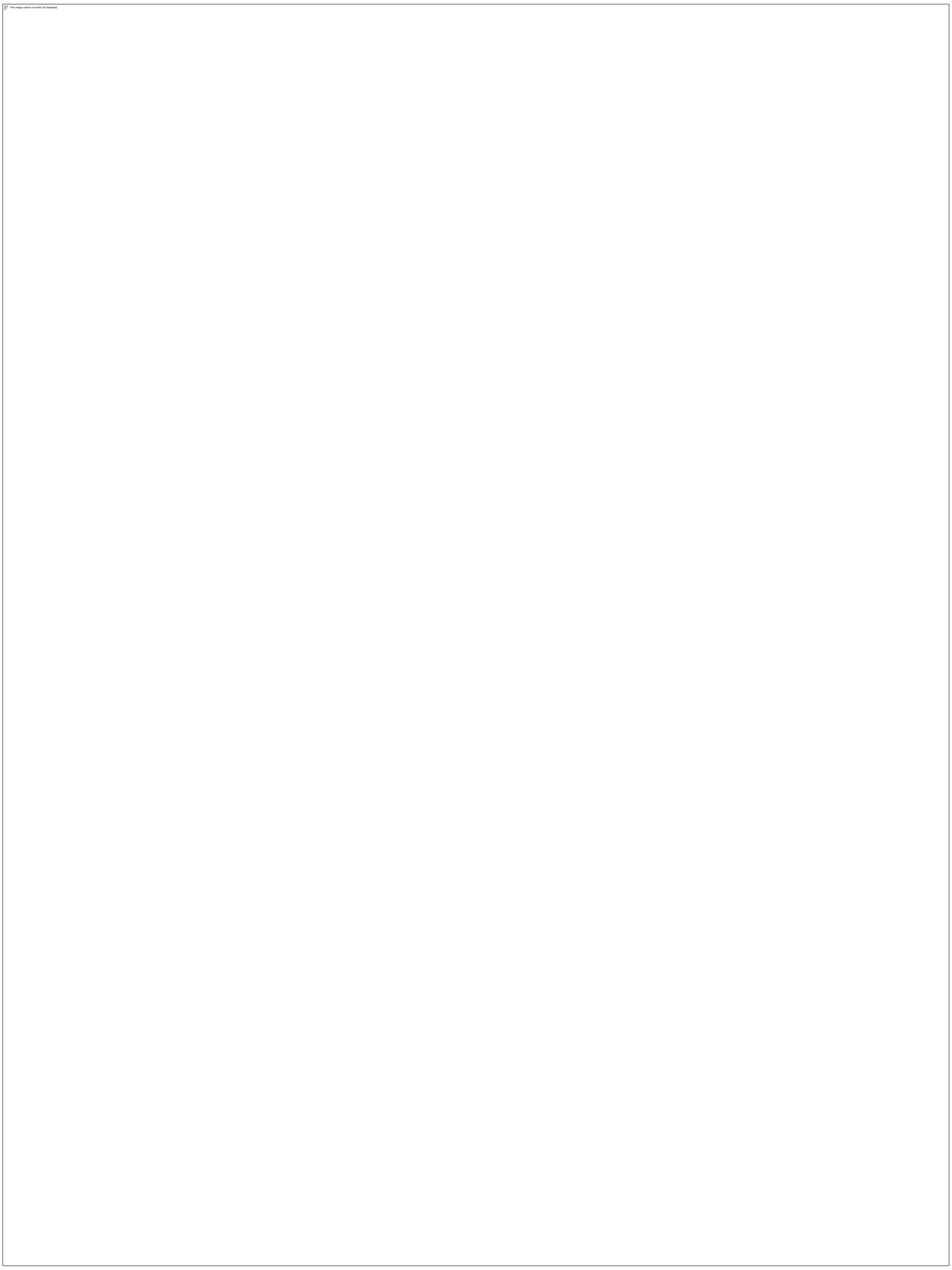

[Figure 1. Representative dot plots illustrating the analysis method for the identification of Treg c]

[Figure 2. The relationship between serum ROMA percentage and Treg in the peripheral blood of women s]

Disclosure: Nothing to disclose

EP1027

Ovarian cancer with and without other primary cancers - are these different entities?

L. Wong[1], T.H. Lao[2], H.S. Lee[1], S.F. Yim[1], S.Y. Yeung[1]
[1]Prince of Wales Hospital, [2]Chinese University of Hong Kong, Hong Kong, Hong Kong

Introduction / Background: Multiple primary cancers (MPC) refer to more than one cancer in the same or different organs being diagnosed in the same patient, which are uncommon. But as the population ages, the growing number of cases of multiple primary cancer becomes an increasingly important medical issue. The purpose of this study was to evaluate the clinic-pathological characteristics of ovarian cancer patients who had multiple primary cancers.
Methodology: In a retrospective study, we reviewed 534 patients presenting with primary ovarian cancer between January 1, 2006, and December 31, 2017. Patients were divided into those with primary ovarian cancer only and those who had in addition other primary cancers for comparison of patient characteristics and presentation, including the staging and histological types, and short term (two-years) mortality from ovarian cancer.
Results: 17.0% (91/534) of patients with ovarian cancer had multiple primary cancers. The most common other malignancies were endometrial cancer (57.1%), followed by breast cancer (27.5%) (Table 1). Patients with multiple primary cancers presented at earlier stages for the ovarian cancer at FIGO stage 1 or 2 (OR 1.217, 95% CI 1.034-1.432), they had more epithelial than other histological types of ovarian cancer (OR 1.076, 95% CI 1.039-1.115), with more being nulliparous (OR 1.336, 95% CI 1.034-1.727) and having family history of malignancies (OR 1.866, 95% CI 1.221-2.853). There was no significant difference in the disease-free survival up to 24 months (OR 1.098, 95% CI 0.966-1.248) (Table 2).
Conclusion: The finding of this study suggested that patients with multiple primary cancers have different characteristics which may represent different entities, but without apparent prognostic implications.

Sites of other primary cancers	Number (%)
Endometrial cancer	52/91 (57.1%)
Breast cancer	25/91 (27.5%)
Cervical cancer	6/91 (6.6%)
Colorectal cancer	3/91 (3.3%)
Thyroid cancer	3/91 (3.3%)
Nasopharyngeal cancer	2/91 (2.2%)
Hepatocellular cancer	2/91 (2.2%)
Others	4/91 (4.4%)

[Frequency of other primary cancers in patients diagnosed with ovarian cancer and multiple primary cancers]

	Ovarian cancer with MPC (n=91)	Ovarian cancer only (n=443)	P value	Relative Risk (95% CI)
Nulliparity (%)	46.2	34.5	0.036	1.336 (1.034-1.727)
Family history of cancer (%))	25.3	13.5	0.005	1.866 (1.221-2.853)
FIGO stage I/II at presentation (%)	68.1	56.0	0.032	1.217 (1.034-1.432)
Epithelial type ovarian cancer (5)	98.9	91.9	0.016	1.076 (1.039-1.115)
Disease-free survival up to 24	76.9	70.1	0.188	1.098 (0.966-1.248)

months (%)				

[Patient characteristics between those with ovarian cancer with and without multiple primary cancers]

Disclosure: Nothing to disclose

EP1028

Metastatic patterns do not provide additional prognostic information in patients with FIGO stage IV high-grade serous ovarian cancer

Z. Feng, H. Wen, X. Ju, R. Bi, X. Chen, W. Yang, X. Wu
Fudan University Shanghai Cancer Center, Shanghai, China

Introduction / Background: The aim of our study was to investigate whether metastatic patterns were associated with prognosis in patients with FIGO stage IV high-grade serous ovarian cancer (HGSC).
Methodology: We retrospectively investigated FIGO stage IV HGSC patients who underwent primary staging or debulking surgery between April 2005 and June 2013 at our institution. None of these patients received neoadjuvant chemotherapy. Metastatic patterns were defined as pleural effusion (stage IVA), parenchymal metastases (stage IVB), and extra-abdominal lymph node metastases (stage IVB). Clinical characters and metastatic patterns were compared with chi-squared or Fisher's exact tests as appropriate. Overall survival was analyzed using Kaplan-Meier method and Cox proportional hazards model.
Results: A total of 83 consecutive stage IV HGSC patients were identified. 42 (50.6%) patients were sub-classified into stage IVA with pleural effusion. Among the rest stage IVB patients, 19 (22.9%) patients had parenchymal metastases and 22 (26.5%) had extra-abdominal lymph node metastases. Five-year overall survival of stage IVA and IVB patients was 39.7% and 39.4%, respectively (p=0.711). Besides, there were no survival differences among three metastatic patterns (p=0.701).
Conclusion: Our study demonstrated that neither the sub-classification into FIGO IVA and IVB nor metastatic patterns of FIGO stage IV provided additional prognostic information.
Disclosure: Nothing to disclose

EP1029

A preliminary study of genetic susceptibility and treatment related gene profiling in Chinese ovarian cancer patients

Z. Feng, H. Wen, X. Ju, X. Chen, X. Wu
Fudan University Shanghai Cancer Center, Shanghai, China

Introduction / Background: The purpose of our study was to presentgenetic susceptibility and treatment related gene profiling in Chinese ovarian cancer patients.
Methodology: We have applied next-generation sequencing in consecutive patients who underwent primary surgery for ovarian cancer since 2016 at our institution. 241 patients have been enrolled for blood tests so far. Paired tumor samples from 62 patients have been tested for somatic deleterious mutations.
Results: The median (range) age of patients was 54 (35-83) years old. 27.4% (66/241) patients had germline deleterious mutations. The most frequently reported genes were as follows: 29 (12.8%) BRCA1 mutations, 19 (7.9%) BRCA2 mutations, 6 (2.5%) RAD51C mutations, and 6 (2.5%) RAD51D mutations. Other mutated genes investigated included BRIP1 (2), CHEK2 (2), RAD50 (2), MRE11A (2), PMS1 (1) and MLH1 (1). 4 patients had dual gene mutation, that is BRCA1/PMS1,

BRCA1/RAD50, BRCA2/BRIP1, and BRCA2/RAD51D. For the paired tumor samples, 19 (30.6%) deleterious TP53 mutations were identified, 4 patients had somatic BRCA1 mutations and 1 patient had somatic BRCA2 mutation.
Conclusion: Our results suggest a comprehensive profiling for Chinese ovarian cancer patients. Somatic genetic test could help better stratify patients for individualized treatment.
Disclosure: Nothing to disclose

EP1030

Apatinib monotherapy in patients with advanced ovarian cancer after platinum resistance: a prospective, exploratory study

X. Xia, W. Zhao
Anhui Province Cancer Hospital, Hefei, China

Introduction / Background: Apatinib is a novel tyrosine kinase inhibitor targeting vascular endothelial growth factor receptor 2. This prospective, exploratory, single-arm, multicenter clinical study to observe and evaluate the efficacy and safety of apatinib in patients with advanced ovarian cancer(OC) who failed standard treatment.
Methodology: Fourty-five female patients (age ≥18 yrs,≤70 yrs) with pathologically diagnosed OC after standard treatment failure (which advanced OC with platinum resistantce) met the inclusion criteria andwere treated with apatinib (250/500 mg/day). Clinical information on the patients was collected. Clinical efficacy (complete response,CR;partial response,PR; stable disease,SD; progressive disease,PD; objective response rate,ORR;duration of response,DRR) based on mRECIST criteria, progression-free survival(PFS) andadverse events(AEs) were evaluated.
Results: From June 1, 2018 to April 26, 2019. 45 patients were included in the analysis. The age of the patients was 53 yrs. Patients with Eastern Cooperative Oncology Group (ECOG) score 1 accounted for 86.7%, 24(55.8%) patients received less than third-line treatment, and 19 (44.2%) patients received more than third-line treatment. Total 71.1% patients received apatinib at the dose of 500mg. There were 28 patients eligible for efficacy evaluation. Among them, 6 achieved PR, 21 achieved SD, and 1 achieved PD, resulting in an ORR of 21.4% and a DCR of 96.4%. The mPFS and OS data was still in follow-up.
This study showed the incidence of AEs was 85.7% and the grade 3/4 treatment-related AEs was 11.4%. The most common treatment-related AEs were hypertension (37.5%), hand-foot syndrome (28.6%), debilitation (17.2%), proteinuria (5.7%), anorexia (2.9%), and diarrhea (5.7%).
Conclusion: In this exploratory study, apatinib monotherapy exhibited potential efficacy and manageable toxicity for OC patients who failed standard treatment. Although PFS and OS have not been achieved, it could be predicted that PFS will exceed 6 months and OS will exceed 10 months.
Disclosure: Nothing to disclose

EP1031

Ovarian high-grade sarcoma: case report and literature review

S. Yazdani, A. Hosseini, M. Abedisamakoush, M. Naeimi Rad
Babol University of Medical Sciences, Babol, Islamic Republic of Iran

Introduction / Background: These biologic lesions are invasive and most patients were observed with metastases. Therefore, this article introduces a 42-year-old female case with a metastasis aromatic sarcoma admitted to Ayatollah Rouhani Hospital in Babol in 2017.
Methodology: A 42-year-old female patient with abdominal pain and abnormal vaginal hemorrhage which had a large pelvic mass showed in CT scan with the size of 186 × 196 × 146 mm, with a

compressive effect on the rectum and bladder, bilateral hydronephrosis and the involvement of brain, liver and the inguinal and pelvis lymph nodes (Fig. 1-2).. In the initial tests, the hemoglobin level was 9 mg and creatinine 10 mg / dL, so she was dialyzed due to the impossibility of nephrostomy and the patient´s creatinine was decreased.
Results: In the normal bimanual examination of cervix, there was a large mass in the umbilical cord with a clear compression effect on the posterior Choledosac area. The patient was underwent laparotomy operation 5 days after hospitalization, in which a large tumoral mass was observed with severe adhesion to the uterus; so part of the sigmoid colon was removed with the uterus and ovaries and colostomy was performed. There were liver metastasis and tumor invasion into the retroperitoneal area, and the Cytoreduction was performed as much as possible. The patient was admitted to the ICU after surgery and had seizure and electrolyte disturbances due to the fever resistant to drug therapy expired. In the pathology of ovarian sarcoma with IHC, Vimentine Ki67, positive CD10, IHC, AE1-AE3, negative SMA and S100 were reported.
Conclusion: Ovarian sarcoma tumor is rare but highly invasive. In order to improve the survival of patients, it is necessary to find early diagnostic methods in these patients and to conduct a faster surgical operation with Cytoreduction Optimal.
Disclosure: Nothing to disclose

EP1032

Late vaginal recurrence of ovarian serocyst adenocarcinoma

S. Yazdani[1], M. Abedisamakoush[2], F. Mohammadian Amiri[1], H. Ghorbani[1]
[1]Babol University of Medical Siences, Babol, [2]Mazandaran University of Medical Sciences, Sari, Islamic Republic of Iran

Introduction / Background: Most patients with epithelial ovarian cancer are diagnosed at high stages of the disease. Therefore, the recurrence of ovarian cancer, which is usually diagnosed with clinical symptoms, tumor markers or imaging techniques, is commonly found in these patients. This report is an interesting case of late recurrence of epithelial ovarian cancer in the vaginal cuff area, diagnosed with a clinical examination.
Methodology: A 68-year-old woman who had undergone hysterectomy, ovarian and ovarian cancer staging surgeries 10 years ago due to ovarian mass was under 8-period chemotherapy based on a pathology report of serocyst adenocarcinoma.
Lately, she referred with a complaint of weight loss and vaginal bleeding, and a large mass which covered the vagina and extended to the vaginal cuff was diagnosed in the examination. Abdominal and pelvic sonography was normal. During surgery, the entire mass with a size of 4*8*10 was removed from the vaginal cuff area and sent to the laboratory for pathology. A recent pathology report showed the recurrence of serocyst-adenocarcinoma (Fig 1). The patient was referred for chemoradiotherapy
Results: -
Conclusion: Although, in a study, the average duration of recurrence of ovarian cancer was 17 months and the most common sites of recurrence were in peritoneum, patients with a history of epithelial ovarian cancer might experience late metastasis. Hence, the symptoms of patients should be paid more attention and vaginal examinations in addition to other examinations should be performed during each visit to diagnose an unusual recurrence like this.
Disclosure: Nothing to disclose

EP1033

^{18}F-FDG PET/CT-based metabolic metrics in recurrent tumors of ovarian clear cell carcinoma

and their prognostic implications

S. Ye, S. Liu, L. Xiang, X. Wu, H. Yang
Fudan University Shanghai Cancer Center, Shanghai, China

Introduction / Background: Glucose metabolism has been suggested as a therapeutic target in ovarian clear cell carcinoma (CCC). We attempted to clarify ^{18}F-FDG PET/CT-based metabolic metrics in the recurrent ovarian CCC patients and their prognostic values.
Methodology: Quantitative metabolic parameters included maximum standardized uptake value (SUVmax), metabolic tumor volume (MTV) and total lesion glycolysis (TLG). Two different methods were employed for defining the threshold SUV to delineate MTV: 1) SUV of 2.5 (designated as MTV); 2) a fixed ratio including 40% (MTV40), 50% (MTV50) and 60% (MTV60) of SUVmax. The Kaplan-Meier model and Cox regression were used in survival analysis.
Results: Among the 35 patients, platinum-resistant recurrence accounted for 34.3% and the median progression-free survival was 13 months (range, 2-135). Fifteen (42.9%) patients presented with single tumor recurrence, while 51 recurrent lesions were identified, with the most common sites in pelvis (29.4%), followed by lymph node metastases (19.6%) and peritoneal carcinomatosis (15.7%). Except four patients with FDG-inavid tumor, the median SUVmax of the 31 patients with high glucose metabolic activity was 7.10 (range, 3.00-20.60). After a median follow-up of 36.5 months (range, 7-155), 22 patients (64.7%) were dead from disease. The median post-relapse survival (PRS) was 17 months (range, 4-126). High TLG60 level was associated with shorter overall survival (OS) (*P*=0.044) and PRS (*P*=0.031) on univariate analysis, while retained significance on multivariate analysis for OS (P=0.024).
Conclusion: PET/CT-based metabolic volumetric parameters might be predicators for survival in recurrent ovarian CCC patients. More patients should be included in further study.

[Figure 1. Representative PET/CT images and Kaplan-Meier survival curves]

Disclosure: The study was supported by grants from National Natural Science Foundation of China (81702558) and Fudan University Shanghai Cancer Center (YJ201603). The funding bodies didn´t participate in the design of the study and collection, analysis, and interpretation of data and in writing the manuscript. Shuang Ye declares that she has no competing interest. Shuai Liu Shuang Ye declares that she has no competing interest. Libing Xiang declares that he has no competing interest. Xiaohua Wu declares that he has no competing interest. Huijuan Yang declares that she has no competing interest.

EP1034

Comparing the efficiency of interval debulking surgery after neoadjuvant chemotherapy with primary debulking surgery in patients with stage 3 and 4 ovarian carcinoma: multicenter real world data

M. Yılmaz[1], M. Başak[2], V. Sunar[3], F. Gürler[4], D. Tataroğlu Özyükseler[2], B. Kurt Inci[4], K. Bir Yücel[4], O. Yazıcı[5], M.E. Yıldırım[2], G. Tahtacı[4], M.M. Meydanlı[6], M.A. Onan[7], A. özet[5], M. Gültekin[8]
[1]Medical Oncology, Cumhuriyet University, Sivas, [2]Medical Oncology, Kartal Training and Research Hospital, Istanbul, [3]Medical Oncology, Dr. Zekai Tahir BURAK Medical Oncology Women Health Education and Research Hospital, [4]Medical Oncology, Gazi University, [5]Medical Oncology, Gazi University, School of Medicine, [6]Gynecology and Obstetrics, Zekai Tahir Burak Women's Health Training and Research Hospital, [7]Gynecology and Obstetrics, Gazi University, School of Medicine, [8]Gynecological Oncology Department, Hacettepe University Medical Faculty Hospital, Ankara, Turkey

Introduction / Background: General survival (GS) and clinical characteristics of the patients (NACT) undergoing interval debulking after neoadjuvant chemotherapy and the patients undergoing adjuvant chemotherapy after primary debulking surgery (PDS) were compared retrospectively among the patients with FIGO stage 3 and 4 ovarian carcinoma.
Methodology: In this study, 141 patients who were diagnosed with FIGO stage 3 or 4 epithelial ovarian or fallopian tube carcinoma between 2009 and 2017 and had completed file data were included.
Results: 73 (51.8%) patients and 68 (48.2%) patients were included in the in the NACT and PDS groups, respectively. 56 patients (81.2%) in the NACT group and 67 patients (98.5%) in the PDS group had an ECOG 0-1 performance score (p= 0.001). Average age was 60.4 ± 10 and 52.2 ± 10 years in the NACT and PDS groups, respectively (p< 0.01). In the diagnosis, median CA 125 level was significantly higher in the NACT group than the PDS group (1068 vs 343 U/mL, p=0.01). When NACT and PDS groups were compared in the diagnosis in terms of the radiological stage, NACT group was more advanced stages (p< 0.01). Complete resection (R0) rate in the PDC group was higher than the NACT group (80.9% (n=55) and 55% (n=33) , p= 0.015, respectively). Among the patients younger than seventy years of age, median GS was statistically significantly higher in the PDS group than the NACT group (50 months vs 38 months, respectively), p< 0.001].
Conclusion: NACT group in this study , these patients were older, they had lower ECOG performance scores, and their CA 125 levels were higher. Unlike the randomized trials, this study is important in order to show the characteristics of the patient group sent to neoadjuvant therapy in ovarian cancer as well as the GS results in real life clinical practices.
Disclosure: Nothing to disclose

EP1035

Comprehensive miRNA sequencing of squamous cell carcinoma arising from ovarian mature teratoma

K. Yoshida, A. Yokoi, S. Tamauchi, Y. Ikeda, N. Yoshikawa, K. Nishino, K. Niimi, S. Suzuki, H. Kajiyama, F. Kikkawa
Obstetrics and Gynecology, Nagoya University Graduate School of Medicine, Nagoya, Japan

Introduction / Background: Owing to its rarity, the carcinogenesis and molecular biological characteristics of squamous cell carcinoma arising from mature teratoma (SCC-MT) remain unclear. This study aims to elucidate the molecular background of SCC-MT from the aspects of microRNA (miRNA) profiling.
Methodology: Between 2003 and 2016, all the seven patients with SCC-MT were identified, and their formalin-fixed paraffin-embedded (FFPE) cancer and contralateral normal ovarian tissues were used. Moreover, 20 mature teratoma (MT) samples were also used as control. Total RNA was extracted from FFPE tissues, and small RNA libraries were prepared. Then comprehensive miRNA sequencing was performed, and data was analysed using Rstudio. Moreover, the results were validated in patient-derived xenograft (PDX) models. We extracted total RNA from fresh frozen tissues and plasma of PDX models and analysed as described above. Finally, we performed target prediction and functional annotation analysis using miRSystem.
Results: Heatmap and principal component analysis revealed markedly different miRNA profiling in cancer, normal ovarian and MT tissues. While normal ovarian tissues exhibited almost similar profiles, SCC-MT tissues differed from each other. Then we narrowed down cancer-related miRNAs. Comparing paired-cancer and normal ovaries, 15 dysregulated miRNAs were identified. Moreover, we compared SCC-MT and MT statistically. As a result, two markedly upregulated miRNAs (miR-151a-3p and miR-378a-3p) and two markedly downregulated miRNAs (miR-26a-5p and miR-99a-5p) were identified. In PDX model, the four cancer-related miRNAs were also dysregulated in cancer tissue. In addition, the two upregulated miRNAs were elevated in the murine plasma when tumour tissues were enlarged. Finally, *in silico* analysis indicated that targets genes of these miRNAs markedly correlated with cancer-related pathways, including 'pathway in cancer' and 'cell cycle.'
Conclusion: This is the first study on miRNA sequencing for SCC-MT. The study identified four cancer-related miRNAs that were related to the feature of SCC-MT.
Disclosure: This study was supported by a Japan Society for the Promotion of Science Grant-in-Aid for Scientific Research: Grant Number 17H04338, 16K15704, and 16H02616.

EP1036

Association of TNF-308 G>A polymorphism located in tumor necrosis factor a with the risk of developing cervical cancer and results of pap smear.

Z. Yousefi
Department of Obstetrics and Gynecology, Mashhad, Islamic Republic of Iran

Introduction / Background: Tumor necrosis factor a (TNFa) is an inflammatory cytokine that plays a crucial role in the immune response and the progression of cervical lesions. There is a growing body of data evaluating the value of a genetic variant in the TNFa gene with the risk of developing cervical cancer. The aim of this study was to explore the association of a variant, TNF-308 G>A, residing in the TNFa gene with cervical cancer.
Methodology: A total of 91 women with cervical cancer and 161 women as the control group were recruited. DNA was extracted, and Taqman®-probes-based assay was used for genotyping. Our results showed that the minor allele frequency was 0.3 in total population, and the frequency of minor allele A was more in the case group compared with the control.
Results: The regression models in different genetic models also revealed that the allele A is a potential risk factor for the development of cervical cancer. In particular, in the dominant model, patients with AG and AA genotypes had a higher risk of developing cervical cancer with odds ratio (OR) of 2.75 (95% confidence interval [CI]: 1.57-4.83, < 0.001) and OR of 7.27 (95%CI: 2.5-20.8, < 0.001), compared with the GG genotype.
Conclusion: Moreover, a similar outcome was obtained for smear test results. Our study demonstrated that TNF-308 G>A located on TNF-a was associated with the risk of cervical cancer, supporting further studies in a larger population and multicenter setting to show the value of emerging markers as risk stratification biomarkers in cervical cancer.

Disclosure: Nothing to disclose

EP1037

Predictive accuracy of the risk of malignancy index and clinicopathological results of patients with probably malignant pure adnexal mass (without acidity and implantation)

H. Yumru Celiksoy, H. Sozen, M. Baktiroglu, E. Çelik, Y. Minareci, Ö. Tosun, Y. Salihoglu, S. Topuz
Istanbul Faculty of Medicine | Gynecologic Oncology, Istanbul University, Istanbul, Turkey

Introduction / Background: Adnexal mass is one of the leading causes of admission to the gynecology clinics. Distinction and early diagnosis of malignancy are critical points that the surgical procedure (laparotomy/ laparoscopy, incision type, debulking) and experience/ competence of the surgeon play a major role in the prognosis of the patient who has malignancy. Some of existing indexes are too complex to be used in daily practice, and some contain markers that cannot be done anywhere.Risk of malignancy index(RMI) is practical and simple.
Methodology: Between January 2010 and December 2018, 264 patients who had purely adnexal mass and underwent surgery due to presumed malignancy were analyzed. Since 56 patients had data loss, 208 patients were included in the study. RMI-3 and RMI-4 scores were calculated retrospectively.
Results: According to the final pathology reports, 52 (25%) of 208 patients were benign, 46 (22%) were borderline and 110 (53%) were malignant. Age and menopausal status were significantly different only in borderline group(tab_1). Of malignant tumors, 3 were uterine sarcomas, 8 were metastatic ovarian tumors (all gastrointestinal origin), 99 were primary ovarian cancers. 76 of primary ovarian cancers were epithelial and 23 were nonepithelial. The most common type were respectively serous and endometrioid adenocarcinoma. 76% of primary ovarian cancers were at early stage (stage1-2) and 24% were at advanced stage (stage 3-4). RMI-3 and RMI-4 were both have low specificity, sensitivity and predictive values(tab_2). The most unexpected benign tumors were serous cystadenofibroma(%24).
Conclusion: For patients with adnexal masses who are not easily accessible to gynecological oncologists, the ability to differentiate benign/ malignant in less specialized hospitals by simple methods will prevent unnecessary referrals and inadequate surgical procedures. This index and our approach are not sufficient.New techniques need to be developed.

	Benign (n:52)	Borderline (n:46)	Malignant (n:110)	x2	p
Age(y)	51,5 (13,7)	41,6 (13,4)	51,9 (16,0)	F=0,832*	<0,05
CA125,U/ml	55,5 (20-204)	63 (23-213)	98 (28-261)	1,969	0,37
Postmenopausal,n(%)	30(57,7)	14(30,4)	61(55,5)	9,565	<0,05
Tumor diameter, cm	11 (7-15)	10 (6-14)	10,5 (8-17)	2,208	0,33
Bilaterality, n(%)	9 (17,3)	14 (30,4)	16 (14,5)	5,470	0,07
USG Findings,n(%) a)Solid	25 (17,3)	26 (8,70)	66 (11,8)	3,132	0,54
b)Solid+multiloculated	18 (34,6)	16 (34,8)	31 (28,2)		
RMI-3	195 (48,5-790,5)	189 (70-447)	271,5 (81-876)	1,476	0,48
RMI-4	520 (100-2160)	424 (128-1192)	700 (168-2288)	1,971	0,37

[Demographic Datas and Prognostic Factors]

	Specificity	Sensitivity	PPV	NPV

RMI-3	53,1	56,4	57,4	52,0
RMI-4	48,0	55,5	54,5	49,0
CA-125	59,2	46,4	56,0	49,6

[Specificity, Sensitivity, Positive Predictive Value (PPV), Negative Predictive Value(NPV) of RMI-3, RMI-4 ad CA-125]

Disclosure: Nothing to disclose

EP1038

Ovarian malignancy risk stratification using a third-generation multivariate index assay

Z. Zhang[1], R.G. Bullock[2], H. Fritsche[3]
[1]Johns Hopkins University School of Medicine, Baltimore, MD, [2]Vermillion Inc., [3]Aspira Laboratory, Austin, TX, United States

Introduction / Background: To further extend and improve the utility of two FDA-cleared multivariate index assays for preoperative malignancy risk assessment of adnexal masses, we developed a third generation multivariate index assay MIA3G that stratifies patients with an adnexal mass into three risk groups. A small high-risk (HR) rule-in group captures a majority of the total cancer cases with a clinically actionable positive predictive value (PPV). A low-risk rule-out group includes a significant portion of the remaining test population with a very high negative predictive value (NPV).
Methodology: Seven serum protein analytes: apolipoprotein A-1, beta-2 microglobulin, CA125, follicle stimulating hormone, HE4, transferrin (TRF), and transthyretin (TT) were analyzed on samples from four IRB-approved independent multicenter study cohorts (Table 1). The OVA1 Study set was used for training. Two algorithms and corresponding cutoffs were derived for pre- and post-menopausal patients separately. They were then independently validated on the remaining three study sample sets. In addition to sensitivity/specificity, PPVs/NPVs were estimated after adjusting the pre-test cancer prevalence of the validation sets to 5% for premenopausal and 10% for postmenopausal patients.
Results: The results are summarized in Table 2 and Figure 1. They were relatively consistent across the 3 validation sets. As an example, for the premenopausal patients in the validation sets, with an assumed prevalence of 5%, the HR group identified < 10% of the test population yet captured 52% - 81% of the cancer cases, resulting in a PPV>33%. On the other hand, >64% of the premenopausal samples were assigned to the LR group at an NPV of 99%. MIA3G performed well for stage I/II invasive cancer and multiple histologic subtypes (data not shown).
Conclusion: With further validation in a prospective studies, MIA3G could be potentially used in patients with suspicious adnexal masses as part of the decision of surgery.

	Training:		Validation						
Sample Sets	OVA1		OVA500		FHCRC#7788		OVA1-PS1-CO4		Validation Sets Total
Menopausal Status	Pre-	Post-	Pre-	Post-	Pre-	Post-	Pre-	Post-	
Benign	230	177	255	160	47	77	576	166	1281
LMP	11	18	5	12	9	8	6	3	43
Stage I/II Ovarian Cancer	22	32	11	26	3	17	5	5	67
Stage III/IV	18	66	16	26	11	46	12	16	127

Ovarian Cancer									
Total	281	293	287	224	70	148	599	190	1518

[Table 1. Training and Validation Sample Sets.]

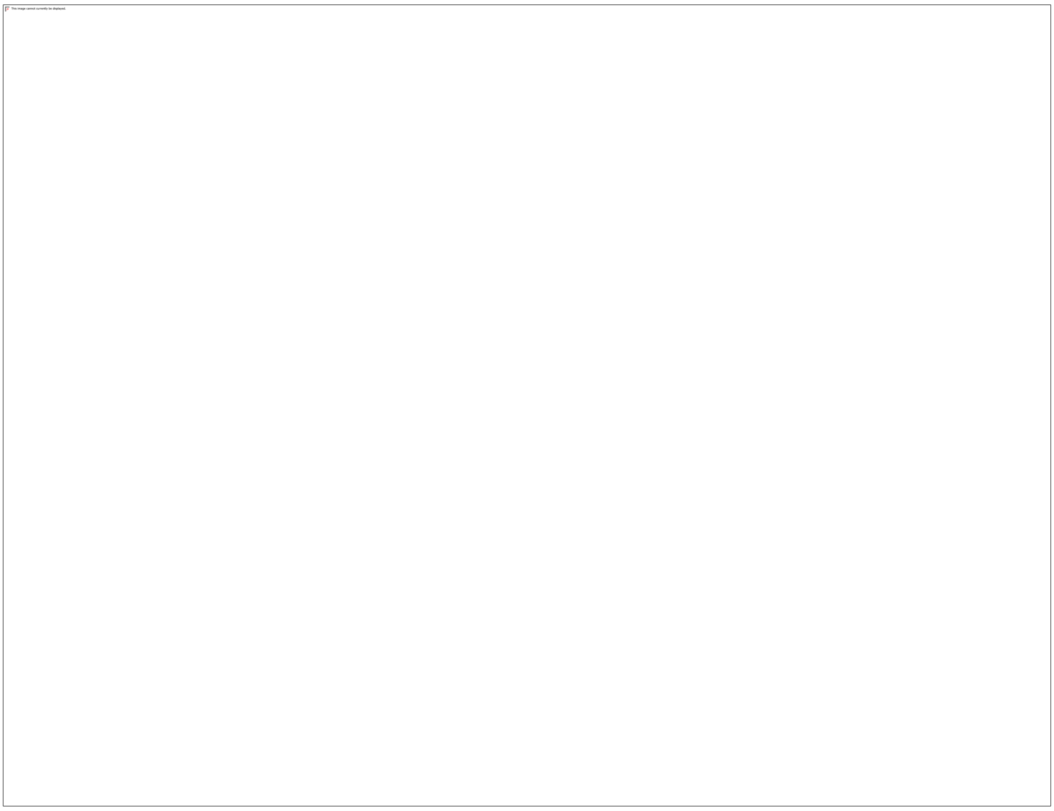

[Table 2. Estimated performances at fixed rule-in and rule-out cutoffs with assumed prevalence]

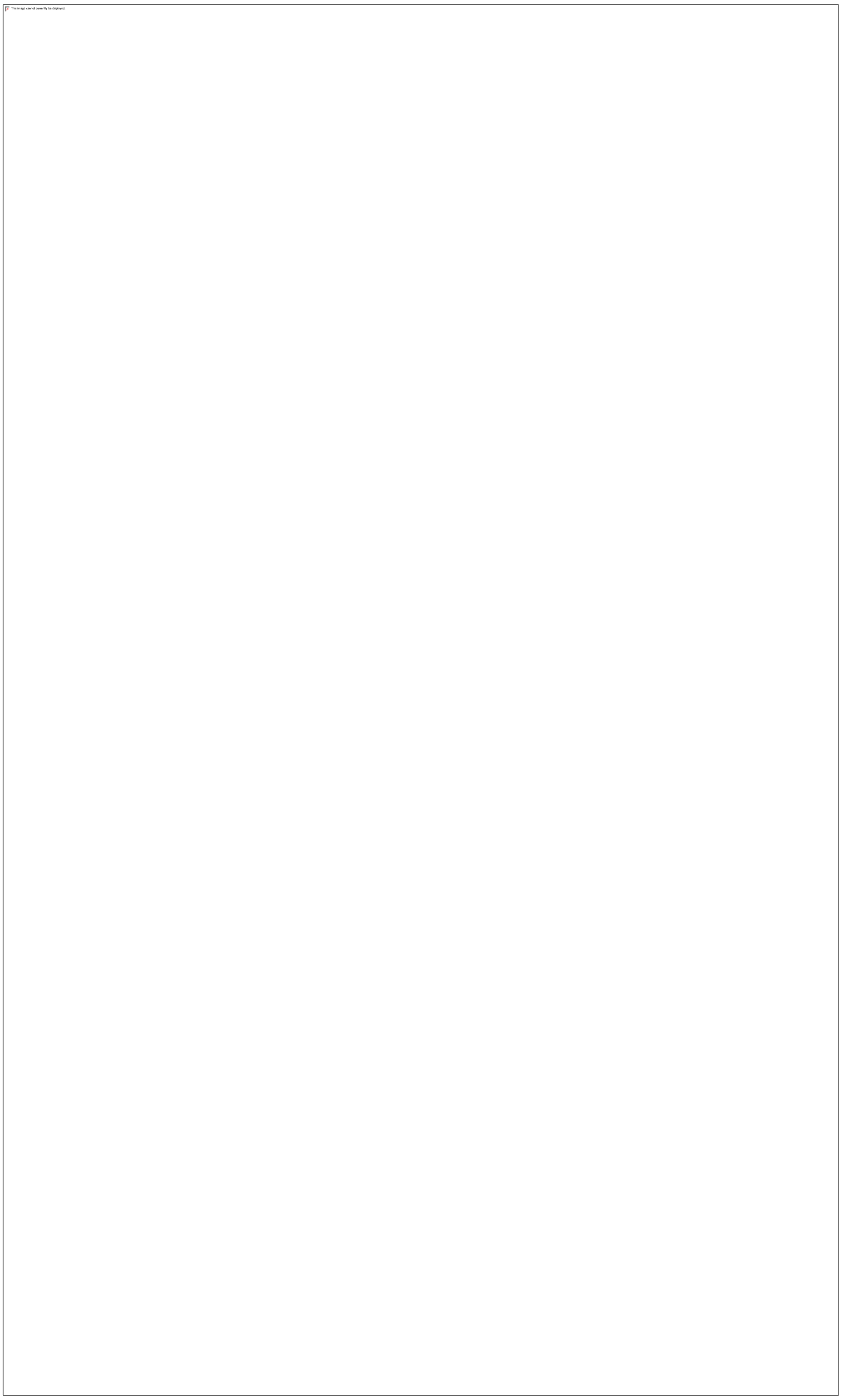

[Figure 1. Projected distribution of benign, LMP, early/late stage ovarian cancer in risk groups]

Disclosure: RG Bullock and HA Fritsche are employees of Vermillion Inc. which funded this work. Z Zhang is a consultant for Vermillion Inc. and contributed to this work independent of his affiliation with Johns Hopkins University.

EP1039

Resibufogenin inhibits ovarian clear cell carcinoma (OCCC) growth *in vivo*, and migration of OCCC cells *in vitro*, by down-regulating the PI3K/AKT and actin cytoskeleton signaling pathways

G. Zhou, Z. Zhu, J. Ding
Department of Gynecology, Obstetrics and Gynecology Hospital of Fudan University, Shanghai, China

Introduction / Background: Ovarian clear cell carcinoma is a rare ovarian cancer which presents poor prognosis owing to its chemotherapeutic resistance. There is an urgent need to identify new therapeutic options for these patients.
Methodology: Drug screening among 172 traditional Chinese medicines to find out the impact of resibufogenin on ovarian clear cell carcinoma. CCK-8 assay was used to detect cell viability. Transwell assay was used to examine cell migration and invasion. Flow cytometry assay was applied to evaluate cell apoptosis. Xenograft mouse models were constructed to clarify the treatment response in vivo. Then mRNA sequencing assay, qPCR assay, immunohistochemistry assay and western blotting assay were used to detect and verify the targeted pathways.
Results: Resibufogenin inhibited proliferation, migration, and invasion of OCCC cells, and induced apoptosis in them. Resibufogenin also suppressed the growth of xenograft tumors, which consequently showed lower Ki-67 and higher terminal deoxynucleotidyl transferase dUTP nick end labeling (TUNEL) expression. We observed down-regulation of (a) PI3K and AKT in the PI3K/AKT signaling pathway, and (b) MDM2 and myosin in the actin cytoskeleton pathway upon resibufogenin treatment. Thus, resibufogenin inhibits growth and migration of OCCC cells *in vitro* and suppresses OCCC growth *in vivo* through the PI3K/AKT and actin cytoskeleton signaling pathways.
Conclusion: Resibufogenin inhibits growth and migration of OCCC cells *in vitro* and suppresses OCCC growth *in vivo* through the PI3K/AKT and actin cytoskeleton signaling pathways.

This image cannot currently be displayed.

[Resibufogenin inhibits ovarian clear cell carcinoma growth and migration in vivo and in vitro]

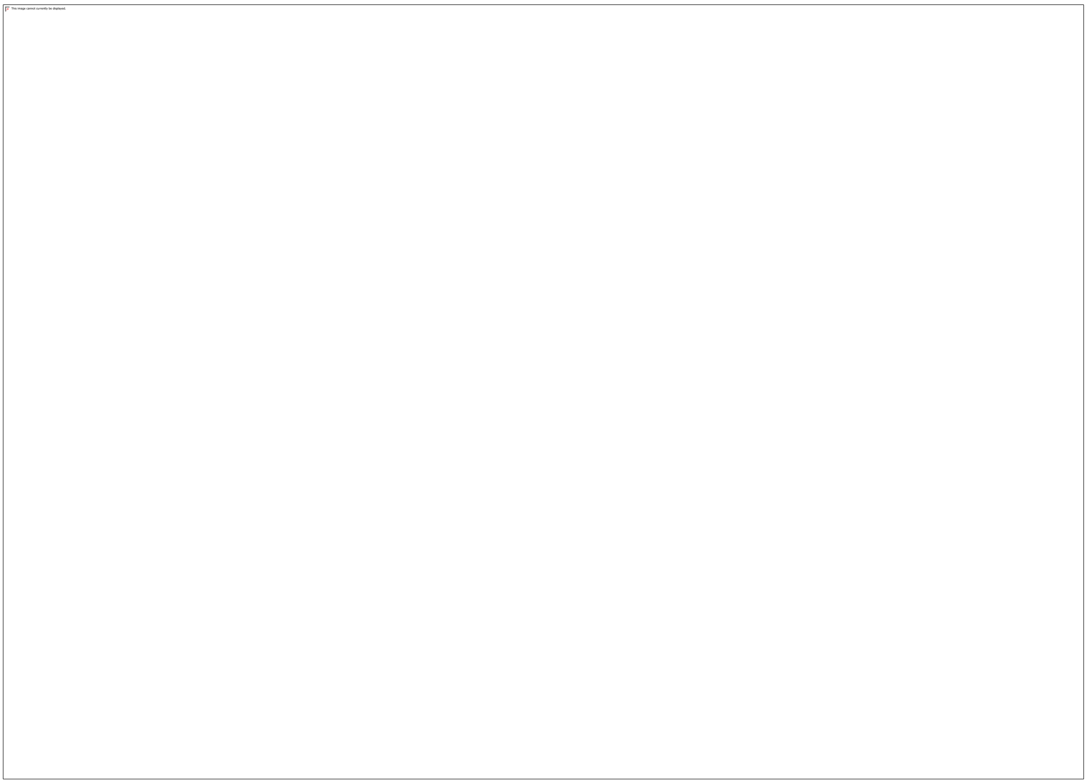

[The mechanism of resibufogenin inhibits ovarian clear cell carcinoma growth and migration]

Disclosure: Nothing to disclose

EP1040

Engineered bovine-milk-exosome-mediated delivery of resibufogenin and its enhanced suppression effect in platinum-resistant ovarian cancer cells

G. Zhou, J. Ding
Department of Gynecology, Obstetrics and Gynecology Hospital of Fudan University, Shanghai, China

Introduction / Background: Platinum-resistant ovarian carcinoma presents poor prognosis owing to its chemotherapeutic resistance. Numerous studies reported that exosomes are naturally occurring membrane particles that mediate intercellular communication by delivering molecular information between cells. Herein, we investigated whether bovine-milk-derived exosomes as carriers of resibufogenin (RBG) could inhibit platinum-resistant ovarian carcinoma and what is the specific mechanism.
Methodology: First, exosomes and exosome-encapsulated resibufogenin were isolated using differential centrifugation and were characterized by electron microscopy, nanoparticle tracking analysis, and western blot. Then, the loading efficiency was measured by Ultra Performance Liquid Chromatography-tandem mass spectrometry (UPLC-MS) method. Then, the anti-cancer efficacy of exosome-mediated drug was assessed in vitro and in vivo. Meanwhile, the distribution of Milk-exosome-RBG in cells was inspected by confocal microscopy. High-throughput RNA sequencing was used to detect and verify the targeted pathways and GO analysis and western blot were applied to clarify the function.
Results: Bovine-milk-derived exosomes, which expressed CD63 and TSG101 and ranged from 35 to 201 nm, loaded resibufogenin with certain loading efficacy(A and B). Bovine-milk-derived exosomes

delivered resibufogenin into platinum-resistant ovarian carcinoma(E,F), the dose-depended process enhanced the cytotoxicity of resibufogenin(C). In vivo experiment demonstrated that intragastric administration of milk-exosome-RBG suppressed the growth of xenograft tumors(D), the effect of which was superior to that of intraperitoneal injection of resibufogenin, and had lower ki-67 expression and higher tunel expression. The mRNA sequencing assay(G), qPCR assay, immunohistochemistry assay and western blotting assay showed that the PI3K/AKT and Regulation of actin cytoskeleton signaling pathways accounted for the results above.
Conclusion: Bovine-milk-exosome may be used as effective carriers in delivering resibufogenin into platinum-resistant ovarian carcinoma via clathrin-independent endocytosis and macropinocytosis. Bovine-milk-exosome-RBG enhancing resibufogenin's cytotoxicity in vitro and in vivo through PI3K/AKT and Regulation of actin cytoskeleton signaling pathway. This study supports the potential use of bovine-milk-exosome as a drug carrier for the treatment of platinum-resistant ovarian carcinoma.

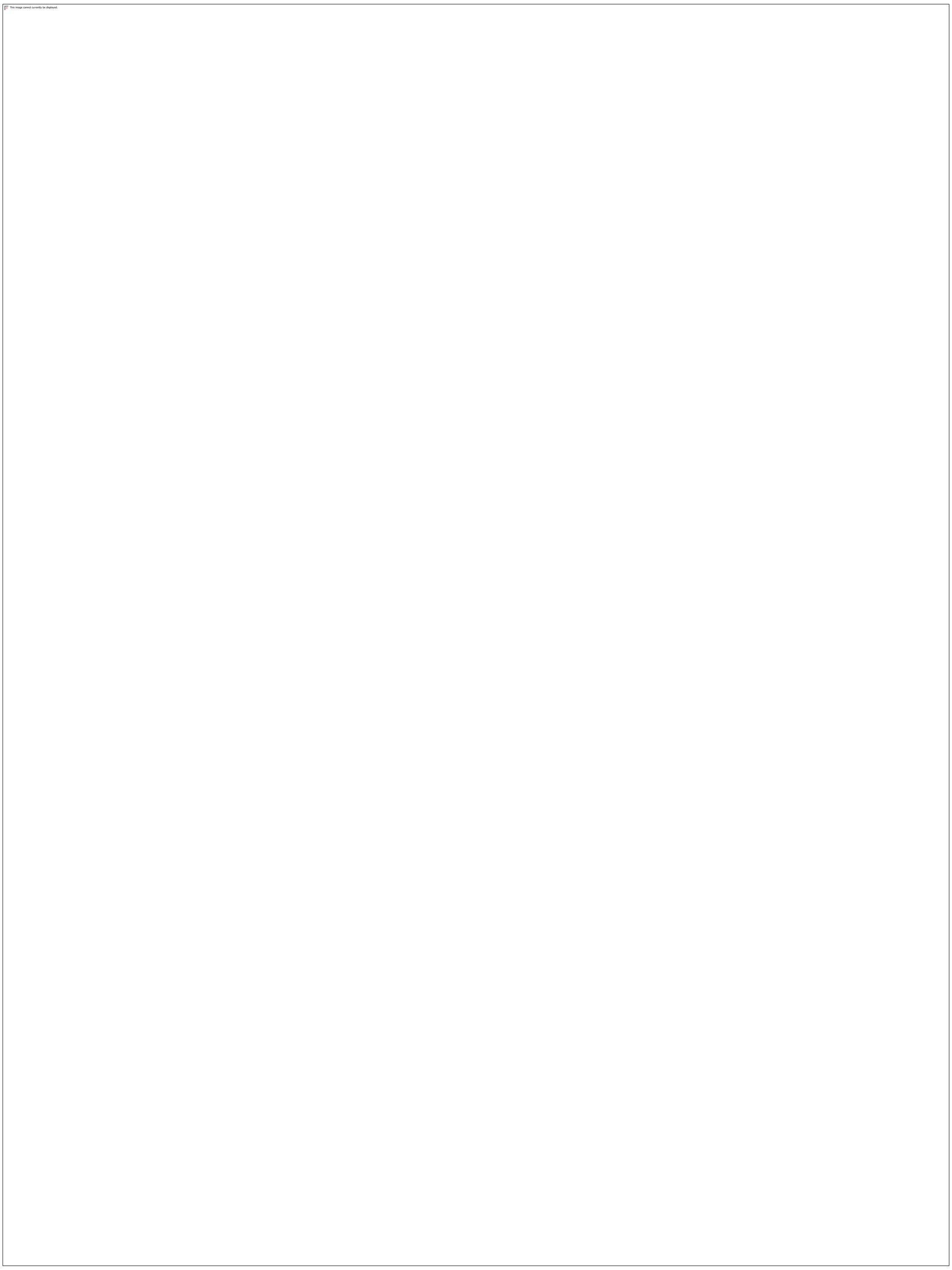

[Milk-exosome-resibufogenin inhibits platinum-resistant ovarian cancer in vitro and in vivo]

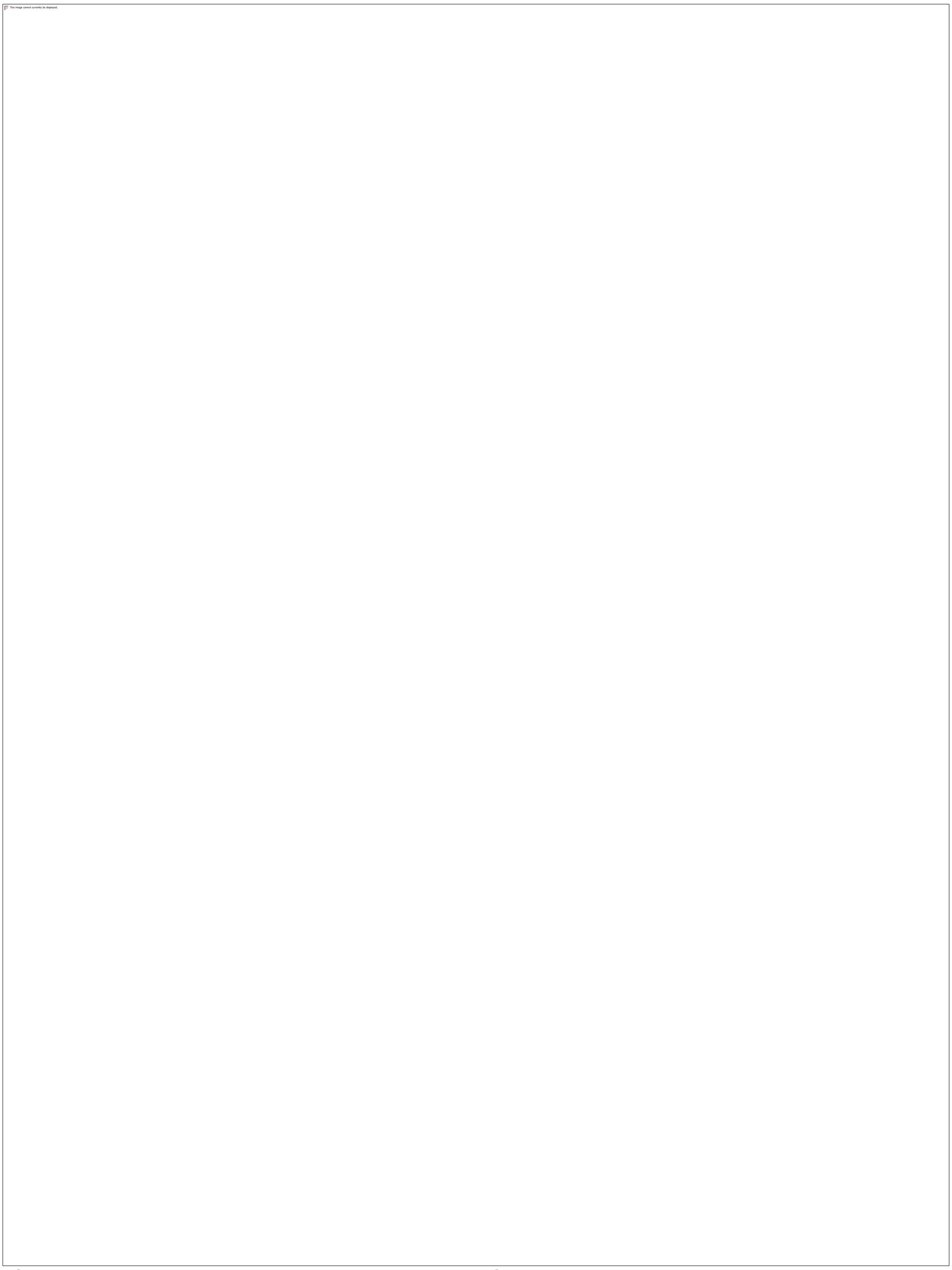

[Schematic representation showing the milk-exo-RBG inhibits platinum-resistant ovarian cancer cells]

Sample/Method	Size by NTA (nm)	Loading Capacity (%)
milk-exosome	98±5.4	n/a

milk-exosome-resibufogenin	128±6.3	16.1%
empty milk-exosome loading	105±6.1	n/a

[Characterization of milk-exosome-resibufogenin formulation]

Disclosure: Nothing to disclose

EP1041

Is there a place for platinum reinduction in BRCA positive platinum-resistant ovarian cancer?

M. Zikan, O. Dubova, B. Sehnal
Dept. of Gynecology and Obstetric, Na Bulovce Hospital, Charles University - 1st Faculty of Medicine, Prague, Czech Republic

Introduction / Background: Increasinf data supports fact that biological behavior of ovarian cancer in BRCA positive individuals differs from others. It is also generally assumed that germline BRCA mutations are found exclusively in patients with platinum sensitive disease.
The aim of this retrospective study was 1) to examine the prevalence of germline BRCA mutations in unselected ovarian cancer patients population, 2) to analyze whether biological behavior differs in BRCA+ and negative patients, and 3) to analyze possibility of platinum reinduction in platinum-resistant BRCA+ patients.
Methodology: BRCA1/2 mutational analysis in peripheral blood samples from biobank by NGS and MLPA. Retrospective analysis of clinical data.
Results: 364 unselected ovarian cancer patients in stages IIC-IV were included into study. Median follow-up of these patients was 37 months.
1) 44 of them harboured BRCA1 or BRCA2 mutation (12.1%). 29 of mutation carriers developed platinum sensitive recurrent disease (defined as PFS >6 months from each and last platinum-based chemotherapy), 15 of them developed platinum resistant disease.
2) there was no difference in PFS comparing BRCA positive patients to BRCA negative; there was a difference in PFS comparing BRCA negative vs. BRCA positive platinum sensitive vs. BRCA positive platinum resistant patients.
3) 7 of 15 BRCA positive platinum resistant patients were reinduced with platinum after one non-platinum chemotherapy line and they achived PFS median of 8 months (compared to others in this group with median PFS 4 months).
Conclusion: 1) germline BRCA mutations are not exclusive for platinum sensitive ovarian cancer patients
2) we did not find difference in PFS for platinum sensitive BRCA positive and negative patients
3) platinum reinduction may be considered for BRCA positive platinum resistant ovarian cancer patients to prolong PFS
Even these data describes only small population, it opens several clinically important questions.
Supported by Ministry of Health of the Czech Republic, grant nr. 17-32030.
Disclosure: Nothing to disclose

EP1042

Evaluation of PCTAIRE2 kinase expression in ovarian cancer and ovaries and fallopian tubes with BRCA1 mutation

P. Ziober-Malinowska, M. Bobinski, K. Barszczewska, K. Fraszczak, J. Kotarski, R. Tarkowski
Ist Chair and Department of Oncological Gynecology and Gynecology, Medical University Lublin,

Lublin, Poland

Introduction / Background: Ovarian cancer in about 10% is an inherited cancer. The BRCA1 gene and/or the BRCA2 gene play a role not only in breast cancer but also in ovarian cancer. Cyclin-dependent kinases regulate the cell cycle by activating the phosphorylation (activation) reaction. They combine with proteins in complexes regulating the passage of cells into individual phases of the cycle. An interesting group of kinases is the PCTAIRE family of kinases, consisting of PCTAIRE1 (CDK16 / PCTK1 / PCT1), PCTAIRE2 (CDK / PCTK2 / PCT2) and PCTAIRE3 (CDK18 / PCTK3 / PCT3). Their common feature is the protection of the amino acid sequence of PCTAIRE in the helical alpha-C region of kinase binding to related cyclins.
Methodology: The study included 88 patients operated on in the 1st Department of Oncological Gynecology and Gynecology of the Medical University in Lublin (41 patients with ovarian cancer, 13 ovaries prophylactically removed in patients with confirmed the BRCA1 gene mutation and 21 normal ovaries of patients operated on due to other non-neoplastic diseases. Immunohistochemistry was performed using a PCTAIRE2 IgG polyclonal rabbit antibody from Bethyl Laboratories, USA. Paraffin sections after H+E staining were evaluated under a microscope and evaluated for the intensity of the abscess (0 - no expression, 1 - weak, 2 - medium, 3 - strong expression). The final result was the average of the three readings obtained.
Results: Higher PCTAIRE2 expression was obtained in patients with a higher degree of disease (FIGO III and IV). A shorter survival time has been demonstrated in patients with elevated PCTAIRE2 expression.
Conclusion: Increased expression of the PCTAIRE2 nuclear kinase may be responsible for adverse disease course and worse prognosis of patients with ovarian cancer.
Disclosure: Nothing to disclose

EP1043
Comparison between "Fagotti" diagnostic laparoscopy and "Suidan" criteria for the avoidance of suboptimal laparotomies in advanced ovarian cancer

D. Zouzoulas, D. Tsolakidis, T. Theodoridis, L. Zepiridis, M. Lioupis, G. Pados, G. Grimbizis
1st Department of Obstetrics & Gynecology 'Papageorgiou' Hospital, Aristotele University of Thessaloniki, Thessaloniki, Greece

Introduction / Background: Ovarian cancer is usually diagnosed in advanced stage. The goal of primary debulking is to achieve no residual disease (RD). Proper selection of candidates for complete debulking is crucial for the avoidance of suboptimal laparotomies. The aim of this study is to compare “Fagotti” diagnostic laparoscopy and preoperative “Suidan” criteria (age, ASA score, CA125, CT results) in the selection of patients for primary debulking.
Methodology: We retrospectively reviewed the medical records of patients with advanced ovarian cancer that underwent “Fagotti” diagnostic laparoscopy (January 2017 - January 2019) in our clinic.
Results: 25 patients with advanced ovarian cancer underwent “Fagotti” diagnostic laparoscopy, in order to decide their treatment plan. 7 patients were excluded from the study, because they did not meet the inclusion criteria. According to the preoperative predictive score, half of the patients (n=9) were assigned for primary debulking (PDS) (Group A) and the other half (n=9) for neoadjuvant chemotherapy (NACT) followed by interval debulking (IDS) (Group B). After “Fagotti” diagnostic laparoscopy, the treatment plan changed in a total of 9 patients: 4 from Group A (from PDS to NACT+IDS) and 5 from Group B (from NACT+IDS to PDS), but with no statistically significant difference. From all patients (n=16) that underwent PDS or IDS, no case of suboptimal cytoreduction was documented (13 RD=0 and 3 RD< 1). On the other hand, if the treatment plan was based only in the preoperative predictive score, 4 patients would have been offered an unnecessary suboptimal laparotomy and 5 patients would have been offered NACT, when they could have been offered a successful primary debulking.
Conclusion: “Fagotti” diagnostic laparoscopy continues to be the method of choice for the proper selection of patients for primary debulking, despite being an invasive technique.
Disclosure: Nothing to disclose

EP1044

Preoperative computed tomography predict unresectability in primary cytoreductive surgery for advanced ovarian cancer

J. Zuo[1], L. Wu[1], J. Dai[2]
[1]Department of Gynecologic Oncology, [2]Department of Radiology, National Cancer Center/National Clinical Research Center for Cancer/Cancer Hospital, Chinese Academy of Medical Sciences and Peking Union Medical College, Beijing, China

Introduction / Background: The volume of residual tumor is a independent prognosis factor for advanced ovarian cancer. computed tomography (CT) is a widely adopted non-invasive methods for access the tumor dissemination and respectability. With neoadjuvant chemotherapy gain popularity, the extent of multivisceral techniques require further evaluation by CT imagings. The objective of this study was to estimate the value of preoperative CT in predicting suboptimal cytoreductive surgery in advanced ovarian serous cancer.
Methodology: From January 2011 to December 2018, patients histologic diagnosed of stage IIIC-IV ovarian cancer enrolled in this retrospective study, underwent primary cytoreductive surgery in national cancer center china, Abdomen/pelvis CT scans were obtained 2 weeks before primary surgery, an experienced radiologist was trained by 50 cases and then proposed 13 radiologic criteria measured by 2-4 quantitative scores, all CT imagings were retrospectively analyzed by 13 radiologic criteria which were blinded to the surgical exploration results.
Results: A total of 302 patients were enrolled in this study; optimal cytoreduction was achieved in 72.8%. The overall sensitivity of CT in predicting suboptimal primary Cytoreduction was 91%, the specificity was 84% and the positive predictive value was 69% and the negative predictive value was 96%. all patients were set up on condition that no more than two muiltivisceral procedures were applied. On multivariate analysis, five radiologic criteria were significantly associated with suboptimal cytoreductive surgery: perisplenic and diaphragm lesion>1cm (P< 0.0001); Liver metastasis or hilar lesion>1cm (P< 0.0001); suprarenal retroperitoneal lymph nodes >1cm (P =0.0011), Spleen metastasis or spleen hilum lesion>1cm (P=0.0213), colon and mesentery more than one invasion (P=0.006).
Conclusion: Preoperative CT analyze shows high specificity and sensitivity in detecting high tumor dissemination in advanced ovarian cancer. 5 criteria were identified associated with suboptimal cytoreduction when applied no more than two muiltivisceral procedures.A predictive model was developed for initial treatment strategy.
Disclosure: Nothing to disclose

EP1045

Differences of tumour behaviour among various types of epithelial ovarian cancer

昱. 陳, H. Lin
Kaohsiung Chang Gung Memorial Hospital, Kaohsiung City, Taiwan

Introduction / Background: Epithelial ovarian cancer (EOC) consisted of several histologic types, including serous, mucinous (MC), clear cell, endometrioid carcinoma (EC), etc. Few studies evaluated the rare histologic types, such as seromucinous carcinoma (SMC) and mixed-type carcinoma (MTC). The aims of present study were to investigate the clinical characteristics and outcomes of this uncommon histology and to compare with other types of EOC.
Methodology: A total of 414 EOC patients of various histologic types between Jan. 2008 and Dec. 2016 were included for analysis. The clinical and pathological characteristics of SMC and MTC were

compared with other common histologic types. Chi-square test was used for analysis of distribution of category variables. Kaplan-Meier method and Cox regression were used for analysis of survival outcomes.
Results: SMC and MTC comprised 1.2% and 4.6% of all epithelial cell types in our series, respectively. The mean age of diagnosis of both was 52.8 years old which was older than other EOC (51.2 years old). Most SMC was diagnosed at early stage (I, II, 80%), while the stage of MTC was equally distributed. MTC (9.6cm) and SMC (8.9cm) had bigger mean size than high-grade serous carcinoma (HGSC, 7.1cm), but much smaller than MC (14.9cm). MTC predominantly occurred unilaterally (70.6%). SMC and MTC were associated with endometriosis, especially SMC (60%). With a median follow up of 43 months, 5-year progression free/ overall survival rate of SMC and MYC were 60.0%/60.0% and 64.7%/81.3%, respectively. The survival rate of SMC and MTC were worse than EC, similar to MC, but better than HGSC.
Conclusion: SMC and MTC possess relatively special clinicopathologic features, including age, size, bilaterality and component of endometriosis. But there are no significant differences in survival outcomes among SMC and MTC, and EC.
Disclosure: Nothing to disclose

EP1046

Living life in the face of loss: parenteral nutrition in ovarian cancer patients in bowel obstruction

A.M. Sowerbutts[1], S. Lal[2], J. Sremanakova[1], A. Clamp[3], G.C. Jayson[3], A. Teubner[2], C. Todd[1], A.-M. Raftery[3], E. Sutton[4], S. Burden[1]
[1]University of Manchester, Manchester, [2]Salford Royal NHS Foundation Trust, Salford, [3]The Christie NHS Foundation Trust, Manchester, [4]University of Bristol, Bristol, United Kingdom

Introduction / Background: Malignant bowel obstruction (MBO) is a complication of advanced ovarian cancer and these patients may receive home parenteral nutrition (HPN). This study investigates patients' and relatives' experiences of HPN, alongside patients' nutritional status and survival outcomes.
Methodology: A mixed methods study involving 38 patients with ovarian cancer in MBO admitted to a tertiary referral hospital over 15 months. Data on nutritional status, body composition, and patient characteristics were collected from patients' notes and computerised tomography scans.
In addition, seventy-five longitudinal in-depth interviews were conducted with 20 patients considered for HPN, their relatives and healthcare professionals. Interviews were transcribed verbatim and analysed thematically influenced by the techniques of Van Manen
Results: Of the 38 women, 32 received parenteral nutrition in hospital and 17 were discharged on HPN. Nutritional status was poor with 31 of 33 women who had a CT scan having low muscle mass, although 10 were obese. Median overall survival from admission with MBO for all 38 women was 70 days (range 8 - 506) and for those 17 on HPN was 156 days (range 46 - 506).
Patients experienced HPN as one facet of their illness, but viewed it as a “lifeline” that allowed them to live outside hospital. Nevertheless, HPN came with losses including erosion of normality through an impact on activities of daily living and dealing with the bureaucracy surrounding the process. Relatives coped but were often left in an emotionally vulnerable state.
Conclusion: Patients and relatives recognised HPN as a “lifeline” and were grateful for it. However this benefit came with losses and healthcare professionals need to be realistic about these and aware of relatives' needs. Nevertheless, patients are prepared to suffer losses in order to continue living.
Disclosure: Nothing to disclose

EP1047

The prognostic nutritional index is useful for predicting the prognosis of patients with terminal-stage gynaecologic cancer

T. Hirayama, Y. Terao, A. Itakura
Obstetrics and Gynaecology, Juntendo University, Tokyo, Japan

Introduction / Background: An accurate, objective prognostic index based on disease pathology has not been established for gynaecologic cancer. Therefore, many cases have been treated aimlessly and highly invasively, rapidly aggravating patients' general condition and eventually causing death. Recently, the Prognostic Nutritional Index (PNI), an indicator of nutritional status, has shown to be related to cancer prognosis. In this study, we aimed to verify if the PNI is an useful prognostic predictor in terminal-stage gynaecologic cancer.
Methodology: The study cohort consisted of 104 patients who died of gynaecologic cancer at related facilities, including our hospital, between January 2013 and September 2018. We retrospectively analyzed the correlation between prognosis and trends in PNI in cases of cervical, endometrial, and ovarian cancers by using laboratory data obtained during the initial examination and during the last 6 months before death. We investigated whether initiating chemotherapy when the patient's PNI was lower than the cutoff value could improve prognosis.
Results: For all cancer types, PNI decreased significantly 2 months before death. The PNI values that indicated 2-month survival were 36.9, 30.2, and 33.6 for cervical, endometrial, and ovarian cancers, respectively. There was no difference in the prognosis between chemotherapy group and best supportive care group after when the PNI became lower than the each cutoff value.
Conclusion: For terminal-stage gyneacologic cancer patients, the PNI was useful for predicting prognosis. Since initiating chemotherapy when the PNI was below the cutoff value did not improve prognosis, the cutoff PNI could instead be used as the basis for a switch to palliative care.
Disclosure: Nothing to disclose

EP1048

Imaging-guided percutaneous radiofrequency ablation of retroperitoneal metastatic disease in patients with MMMT: clinical experience with two patients

M. Jeong, S. Lee, K. Lee, C. Kim, Y. Lee
The Catholic University of Korea / Department of Obstetrics and Gynecology, Seoul, Republic of Korea

Introduction / Background: Uterine carcinosarcoma is a rare uterine tumor with an extremely poor prognosis and a high recurrence rate. 36% of carcinosarcoma patients with disease clinically confined to the uterus have nodal metastasis at the time of diagnosis and the majority of these patients will die of metastatic disease within 2 years of the diagnosis. There are no clearly defined treatment guidelines and no large controlled clinical trials have been conducted on metastatic carcinosarcoma. So, we tried to radiofrequency ablation procedure for retroperitoneal metastatic region in two cases.
Methodology: The rational of RFA is based on the induction of coagulation necrosis in tissues secondary to hyperthermia. After the electrode was advanced into the LN and positioned against its deepest margin with US-CT fusion image guidance, ablation was performed with an impedance-control algorithm. Radiofrequency energy was applied with a mean power of 100 W (range, 80-120 W) for 5-10 minutes per radiofrequency application.
Results:
Case1: A 55-year-old woman underwent total hysterectomy , bilateral salpingo-oophorectomy due to abnormal uterine bleeding. The final pathological report was carcinosarcoma. Six months after chemotherapy, she developed distant metastasis to obturator lymph node and paravesical area. RFA was underwent for 10 minuites. 4 months after procedure, CT showed a remarkable reduction in the retroperitoneal metastatic mass.
Case2: A 62-year-old female underwent optimal surgery for MMMT. The final pathological report was carcinosarcoma, stage IV. Almost 5 months after adjuvant chemotherapy, a new retroperitoneal metastatic lesion was detected by CT scan. The lesion was subsequently treated with radiofrequency ablation.

Conclusion: We can found the reduced RF ablated mass without local tumor progression in follow-up CT. Image-guided radiofrequency ablation (RFA) could be an alternative curative option for patients of uterine carcinosarcoma with retroperitoneal metastasis.
Disclosure: Nothing to disclose

EP1049

Perceived stress, self-esteem and self-rated health in women with breast cancer receiving chemotherapy treatment

A. Koumarianou, K. Kampoli, A. Ntavatzikos, E. Rizos, A. Papanastasiou, N. Arkadopoulos, C. Tsionou
Attikon University Hospital, Hematology Oncology Unit, Fourth Department of Internal Medicine, National and Kapodistrian University of Athens, Athens, Greece

Introduction / Background: Stress, self-esteem and self-rated health are important parameters which have been extensively studied in breast cancer research. However, the correlations between these variables and the proportion of patients experiencing different stress and self-esteem levels are foggy. The aim of this study was to search for correlations between perceived stress, self-esteem and Self-Rated Health (SRH), as well as for their relationship with sociodemographic parameters in breast cancer patients treated with chemotherapy.
Methodology: The data were collected from 100 patients with breast cancer. Sociodemographic variables, the Perceived Stress Scale (PSS), a 1-5 Lickert scale measuring SRH and the Rosenberg Self-esteem Scale (RSES) were administrated. Pearson's correlation, Independent Sample's t-test and ANOVA were utilized. Differences in RSES and PSS levels were presented using proportions based on indicated cut-offs.
Results: The majority of patients were found to have normal self-esteem (72%), while a high proportion experienced low (38.4%) and mild perceived stress (34.3%). Severe stress was experienced only by 8.1% of the participants. The PSS and RSES scores were inversely correlated (P=0.005), while income satisfaction was positively correlated with SRH (P=0.000) and RSES (P=0.006), and inversely to PSS scores (P=0.033). There were also significant inverse associations between BMI and RSES (P=0.016) and between the number of children and PSS (P= 0.002). Higher PSS (P=0.032) and SRH (P=0.008) scores were recorded for those cohabitating. Finally, there was a significant association between smoking and SRH (P=0.021).
Conclusion: Only a minority of the participants experienced severe stress, while most of them had normal self-esteem levels. Stress was inversely associated with self-esteem, having more children and a higher income. No relationship between SRH and stress was observed. Future research on the identification of related parameters and the development of interventional programs to address stress should be intensified.
Disclosure: Nothing to disclose

EP1050

Effects of an 8-week stress management program in women with breast cancer: a randomized controlled trial

A. Koumarianou, T. Seliniotaki, F. Bacopoulou, K. Kampoli, G. Chrousos, A. Ntavatzikos, C. Tsionou, C. Darviri
Attikon University Hospital, Hematology Oncology Unit, Fourth Department of Internal Medicine, National and Kapodistrian University of Athens, Athens, Greece

Introduction / Background: Stress management programs have demonstrated benefits for patients

with breast cancer, but their adoption in clinical practice is limited mainly due to the absence of necessary resources. The aim of this study was to investigate the effect of an 8-week stress management program carried out by one psychologist, in women treated for breast cancer.
Methodology: In this randomized controlled trial patients were allocated to two groups (control and intervention groups) that received standard care; women in the intervention group also participated in an 8-week stress management program. Intervention included stress and diet related psychoeducation, diaphragmatic breathing, guided imagery, progressive muscle relaxation and cognitive reconstruction. Anthropometric and psychological measurements were carried out in both groups, pre- and post-intervention, using a battery of questionnaires.
Results: A total of 53 patients participated in the study (27 in the intervention group). Parametric analysis revealed statistically significant differences post-intervention in body mass index (p=0.040) and quality of life including global health status (0.019), emotional functioning (p=0.024), cognitive functioning (p=0.041) and diarrhea (p=0.012). There was a statistically significant effect between role functioning and type of surgery (partial or total mastectomy) (p=0.030) with major benefits identified in the subgroup of patients that had undergone mastectomy with immediate reconstruction.
Conclusion: This stress management program, carried out by a single health professional, significantly improved psychosomatic health parameters of patients with breast cancer. Further implementation of similar supportive approaches in cancer units are warranted.
Disclosure: Nothing to disclose

EP1051

Is treating malignant obstructive uropathy in advanced cervical cancer as futile as we think?

D. Mahajan[1], T. Shylasree[1], U. Mahantshetty[2], N. Shetty[3], S. Chopra[2], R. Kerkar[1], A. Maheshwari[1]
[1]Gynaecological Oncology, [2]Radiation Oncology, [3]Intervention Radiology, Tata Memorial Centre, Mumbai, India

Introduction / Background: Advanced cancer cervix with obstructive uropathy has poor prognosis and these patients are rarely eligible for curative treatment. Overall survival of advanced stage III/IV cancer cervix with obstructive uropathy is less than 15-20%. However, performing percutaneous nephrostomy (PCN) might prolong life to some extent in order to bid good bye needs to be balanced against the complications and side effects of the procedure itself. Also it is thought that death from uremia is kinder in this situation. **Aim:** To assess the feasibility of treatment interventions in prolonging life of patients diagnosed with advanced cancer cervix with obstructive uropathy.
Methodology: Retrospective analysis of 15 patients with a diagnosis of advanced cancer cervix and impaired renal function underwent PCN & ureteral catheterization during a 12 month period (2016) at a tertiary cancer center.
Results: Out of 15 patients , 9 patients had stage III B and 6 stage IV disease. Following PCN, 3 patients received radical treatment with curative intent and 12 treated with palliative intent (Radiotherapy/chemotherapy). The time period from PCN to starting treatment was 48 days (20 - 140), and total duration of treatment was 114 days(28 - 311). All patients died within 12 months of initial diagnosis of malignant obstructive uropathy.
Conclusion: Treatment of uropathy in advanced Cancer cervix does help to tide over the imminent uremic state. This will make them eligible for some form of palliative treatment to prolong life in months when uropathy is treated instead of weeks if left untreated. The goals of intervention in such patients needs to be redefined and should be discussed on one on one basis. Patient's wishes for prolongation of life for various reasons and QoL issues needs to be factored in during consultations.
Disclosure: Nothing to disclose

EP1052

First experience in palliative care for patients with cancer type pain at the Mohammed VI center for the treatment of cancer

O. Omran, S. Ridai, A. Iraqi, N. Benchekroun, Z. Bouchbika, H. Jouhadi, N. Tawfiq, S. Sahraoui, A. Benider
Centre Mohamed IV d'Oncologie, Hassan II University/Faculté de Medecine et de Pharmacie, Casablanca, Morocco

Introduction / Background: Hospice palliative care is defined as acute care delivered in a comprehensive approach to the person with a progressive or terminal illness. Pain is one of the most common symptoms experienced by patients with palliative care needs.
Methodology: For this purpose, we carried out a descriptive, observational retrospective study of 107 cases, the data collection was made on the basis of a standardized data sheet after analysis of the literature, and in order to evaluate the knowledge and practices Clinics we sent an anonymous questionnaire to all the healthcare staff of the oncology center of the CHU Ibn Rochd of Casablanca.
Results: During our study year 107 patients were cared for at the level of palliative care whose pain was the reason for consultation in 95%. All types of tumor localizations were included, with a prevalence of lung cancer in 34%. Integration of palliative care is at an advanced stage of cancer in 73.8%. The pain was intense in 51.4%, this allowed the use of analgesics of the pallium III of the WHO in 66.3%. Analysis of the overall dimensions of painful cancer patients in palliative care found that 77.5% of patients with AEG, and 34% had psychological disorders, 30% of whom could receive psychological support. As part of palliative care training, 56% of physicians are trained in the service, with 19% continuing education; on the other hand, ¾ of the nurses who did not have a basic training in the management of cancer pain.
Conclusion: Finally, proposals for recommendations for the development of palliative care and clinical practice are proposed to improve the management of end-of-life patients.
Disclosure: Nothing to disclose

EP1053

Palliative urinary diversion in patients with malignant ureteric obstruction due to gynecologic cancer

T. Perri[1,2], E. Meller[2], G. Ben-Baruch[1,2], Y. Inbar[2,3], S. Apter[2,3], L. Heyman[2], Z. Dotan[2,4], J. Korach[1,2]
[1]Gynecologic Oncology, Sheba Medical Center, Ramat Gan, [2]Sackler Faculty of Medicine, Ramat-Aviv, [3]Diagnostic Imaging, [4]Urology, Sheba Medical Center, Ramat Gan, Israel

Introduction / Background: We aimed to identify factors aiding selection gynecologic cancer patients with malignant urinary obstruction that are least likely to benefit from palliative urinary diversion (UD), and to create a risk-stratification model for decision-making.
Methodology: This historic cohort study comprised 74 consecutive patients with urinary obstruction resulting from gynecologic malignancies. All underwent palliative UD by percutaneous nephrostomy (PCN). Using the Cox proportional-hazards regression model and Kaplan-Meier curves with the log-rank test, we developed a prognostic score identifying candidates least likely to benefit from the intervention.
Results: Median follow-up was 4.72 (range 0−5.71) years. Hydronephrosis was diagnosed in most patients upon recurrent or persistent disease (81%). It was bilateral in 37.8%. Intervention-related complications included urinary sepsis (8%), catheter dislodgment requiring replacement (17%), and gross hematuria necessitating blood transfusions (13%). After PCN, conversion to an internal ureteral stent was feasible in 46%. Median survival was 11.13 (range 0-67) months. Two patients died within a month of UD. Multivariate analysis identified diabetes mellitus (DM), poor ECOG performance status >1, and ascites as significant negative survival factors. A prognostic index based on those factors identified the short-term and long-term survivors. Risk-factor-based mortality hazard ratios were 11.37 (95% CI, 4.12−31.37) with one factor, 26.57 (95% CI, 9.14−77.26) with two, and 67.25 (95% CI, 15.6−289.63) with three (all with $p< 0.0001$).
Conclusion: Our proposed prognostic index, based on ascites, ECOG performance status and DM, might help select gynecologic cancer patients least likely to benefit from palliative UD.

Disclosure: Nothing to disclose

EP1054

Symptom burden and operational metrics in gynecological cancer patients cared by a palliative care team, in Greece

A. Tserkezoglou, H.A. Mullali, A. Tsiatsouli, H. Avgetidou, J. Liakopoulos, S. Amoiridou, S. Tsotakos, E. Patiraki
'Galilee' Palliative Care Unit, Spata, Greece

Introduction / Background: The integration of palliative care into standard gynecologic oncology care, in developed countries, is considered to be associated with cost-savings, longer survival, lower symptom burden, and better quality of life for patients and caregivers. “GALILEE” is the first comprehensive Palliative Care Unit in Greece.
The aim of the study is to characterize symptom prevalence and identify operational metrics in patients with gynecologic cancer, cared at home by an interdisciplinary palliative care team
Methodology: Retrospective analysis of all women, with gynecologic cancers, cared from 01.03.2015-31.12.2018, by “GALILEE” was performed. Electronic patient records were reviewed for demographics, disease characteristics and end of life discussions. Performance status was measured by the Palliative Performance Scale (PPS) and symptom burden by the Edmonton Symptom Assessment System (ESAS).
Results: Out of 520 patients cared in the same time period, only 42 (8)%) were identified with gynecologic cancers. Their median age was 68,5 years (range, 44-94). The majority had ovarian cancer (47,6%). Half of the patients were admitted during first line treatment. Only two patients (4,7%) were referred by their gynecologic oncologist. Median time in care was 97,5 (5-2151) days.
At study entry, most patients (61%) had a good performance status (PPS>50%), while the most common disease-related symptoms were pain (78,6%), fatigue (54,8%), nausea (52,4%), anxiety (45,2%) and depression (31%). 43,9% of the patients had no emergency admission to the hospital , 42,8% of patients died of their disease and 50% were supported to die at home. In the last 2 weeks of life 7,1% of patients were receiving chemotherapy and 31% had a hospital admission.
Upon admission, only 23,8% of patients were aware of the diagnosis and 11,9% of the prognosis.
Conclusion: Gynecologic oncology outpatients have a high symptom burden and information needs and could thus profit by concurrent provision of palliative care, early on their disease trajectory.
Disclosure: Nothing to disclose

EP1055

Interprofessional simulation training to improve gynecologic oncology palliative care knowledge, interdisciplinary education perceptions and teamwork attitudes in health professional students

F. Uslu Sahan[1], F. Terzioglu[2]
[1]Faculty of Nursing, Hacettepe University, [2]Faculty of Health Sciences, Atılım University, Ankara, Turkey

Introduction / Background: In order to improve the palliative care for gynecologic oncology and provide qualified care for the patients and their families, the palliative care team should develop a common approach on their disciplinary knowledge and skills and work in interprofessional cooperation. Interprofessional simulation training may contribute the healthcare professionals to work in cooperation by providing them to know and understand each other as from their undergraduate education. This training program may provide to gain experience to manage the process optimally as a

professional palliative care team.The aim of the study was to determine the effect of a interprofessional simulation training on palliative care knowledge to gynecologic oncology, interdisciplinary education perceptions and teamwork attitudes of the health professional students.
Methodology: A randomized controlled trial was conducted with a pre-test and two post-tests. Eighty four health professional students (nursing, medicine, nutrition-dietician, social worker) were stratified by their profession and randomized by four blocks to the high fidelity simulation (HFS), hybrid simulations (HS), high fidelity simulation and hybrid simulations (HFS+HS) and control group. Data were collected semi-structured questionnaire form, gynecologic oncology palliative care knowledge test, interdisciplinary education perception scale, teamwork attitudes questionnaire.
Results: The HFS+HS and HS groups made more significant improvement in gynecologic oncology palliative care knowledge, ınterdisciplinary education perception and teamwork attitudes from pre-test to first and second post-test than the control group.
Conclusion: The introduction of interprofessional HFS+HS or HS based simulation training in undergraduate education could enhance students´ gynecologic oncology palliative care knowledge, interdisciplinary education perception and teamwork attitudes.Interprofessional simulation training programs which are used together with HFS and HS applications in interdisiplinary training should be integrated into the undergraduate education curriculums of the future cooperating health professions.
Disclosure: This study (Project No: 117S900) was supported by 1002-Short Term R&D Funding Program, the Scientific and Technological Research Council of Turkey (TUBITAK).

EP1056

Quality of life assessment before and after concentrated ascites reinfusion therapy in patients with advanced or recurrent gynecologic cancer: a prospective cohort study

K. Yamamoto, S. Nagao, K. Shimada, M. Narita, K. Suzuki, H. Nakazawa, T. Shibutani, T. Jimi, H. Yano, M. Kitai, T. Shiozaki, K. Matsuoka, S. Yamaguchi
Gynecology, Hyogo Cancer Center, Akashi, Japan

Introduction / Background: Concentrated ascites reinfusion therapy (CART) is currently applied widely to relieve symptoms in patients with malignant ascites. However, only few studies have investigated the ability of CART to improve or maintain quality of life (QOL). Therefore, we performed a prospective cohort study to evaluate the efficacy and safety of CART.
Methodology: From April 2018 to March 2019, 22 patients with gynecological malignancies received CART. We performed QOL assessment using the Japanese version of the M.D. Anderson Symptom Inventory (MDASI-J) at 24 h before and after CART.
Results: The 22 patients received a total of 41 CART sessions. This included 16 patients with epithelial ovarian cancer, 5 with peritoneal cancer, and 1 with fallopian tube cancer. CART was performed 24 times before or during neoadjuvant chemotherapy, twice before primary debulking surgery, and 15 times during chemotherapy to prevent recurrence. The mean amount of ascites collected was 2.9 L (0.75-6 L). The concentrations of total protein and albumin were maintained before and after CART (2.5 ± 0.58 g/dL and 2.8 ± 1.72 g/dL, respectively). The most common adverse event was fever [63.6% (14/22 patients) and 39.0% (16/41 sessions)], which is easily controllable by NSAIDs. No patient experienced severe adverse events. CART improved the symptom and interference scores of the MDASI-J significantly within 24 h of the procedure. Various symptoms associated with malignant ascites, especially appetite loss and fatigue, improved within the 24-h period following CART. The mean symptom scores of the MDASI-J decreased from 3.3 to 2.5, and the interference scores of the MDASI-J decreased from 5.2 to 4.
Conclusion: CART is an effective and relatively safe procedure for malignant ascites in patients with gynecologic cancer. In particular, QOL scores were improved by CART.
Disclosure: Nothing to disclose

EP1057

Utilization and timing of Advance Statements on Life-Sustaining Treatment decisions in patients with recurrent gynecologic cancer

G.W. Yim, E.J. Lee, M. Lee, H.S. Kim, H.H. Chung, J.-W. Kim, N.H. Park, Y.-S. Song
Department of Obstetrics and Gynecology, Seoul National University Hospital, Seoul, Republic of Korea

Introduction / Background: A new legislation on Hospice, Palliative Care and Life-sustaining Treatment Decision (LSTD) has been enforced in Korea since early 2018. This study is to investigate the incidence and timing of LSTD registration among recurrent gynecologic cancer patients.
Methodology: Retrospective observational study was performed of patients that were treated for recurrent gynecologic cancer at a tertiary hospital from February 2018 to January 2019. The number, type and the main person to sign the LSTD form, previous number of treatments, time from last treatment to end-of-life (EOL) care decision and time from decision to death were analyzed.
Results: Among 1,162 patients admitted during the study period, 309 patients had recurrent disease. Among them, 51 patients (16.5%) made EOL care decisions by themselves (n=44) or by the family (n=7). In detail, the number of LSTD registration was made in 30 (58.8%) patients during terminal phase and in 19 (37.3%) patients during dying phase and the number of previous advance directives had been documented in only 2 patients (3.9%). Only seven out of 49 patients (14.2%) stated previous knowledge of the new LSTD Act. The majority (92.2%) of patients were in disease progression state, and the median number of chemotherapy regimen was 4 (range 1-9). Among those who have stopped treatment, the median time from the last palliative treatment to LSTD was 42 days (range 7-468). Time from LSTD registration to death was median 10 days (range 0-232). EOL decisions that were made within 3 days of death occurred in 10 patients (19.2%) and most (n=8) of them were surrogate decision-making by the family.
Conclusion: The median time from LSTD to death was 10 days. Completion rate of advance directives by the patient was very low. These findings suggest that early patient-family-doctor discussions on LSTD is needed to help patients make advance EOL care decisions.
Disclosure: Nothing to disclose

EP1058

Efficacy of the endocervical curettage vs endocervical brushing for the detection of high grade endocervical dysplasia

C. Almansa González, M.R. Oliver Pérez, M. Hernandez Ontoria, A. Coves Segarra, C. Bellido Bel, V. Bravo Violeta
Hospital Universitario 12 de Octubre, Madrid, Spain

Introduction / Background: The principal aim is to compare the efficacy of the detection rate of high and low grade endocervical dysplasia between endocervical curettage (EC) vs endocervical brushing (EB). Also complicacion rates, pain and inadequate specimens.
Methodology: Randomized clinical trial
Estimation of sample size for sensibility of 84% for EC and 96% for EB in acordance to previous literature, and alpha error of 5% and beta error of 80%: 54 patients. Simple aleatorization was used. After informed consent was signatured we include 100 patients who undergo office conization. Exlusion criteria were macroscopic tumor, pregnancy or prior conization. We performed either EC o EB prior to conization and compare it result with endocervical margin of the cone specimen.
Results: 100 patienst were aletaorized. None of them had exlusion criteria. Demographic caracteristics were comparable between both gropus. Sensibility for EC was 91.27% and specificity 34.26%.
Sensibility for EB was 75% and specificity was 24.65%. Pain, complications or inadequate specimens were similar in both groups.
Conclusion: Endocervical curetagge has a higher rate of detection of high grade endocervical

dysplasia versus endocervical brushing without more pain or complications. No diferences in inadequate specimens were found.
Disclosure: Nothing to disclose

EP1059

Effects of oral probiotics administration in the human papillomavirus infection and its intraepithelial cervical lesions

M.C. Alonzo Martinez[1], E. Canovas Morales[1], V. Navarro-Lopez[2,3], E. Cazorla Amoros[1]
[1]Gynecology and Obstetrics, Hospital Universitario de Torrevieja, Torrevieja, [2]Hospital Universitario del Vinalopó, Elche, [3]Grupo MiBioPath, Universidad Católica de Murcia, Guadalupe, Spain

Introduction / Background: The human papillomavirus (HPV) infects 75% of sexually active women. Most of these infections are transitory and resolve spontaneously in less than two years. The persistence of high grade HPV genotypes is the main risk factor for cervical cancer and its precursory lesions. Recently, new evidence suggests that the state of vaginal microbiota plays an important role in the persistence and elimination of HPV. The main objective of our study is to observe the changes on the HPV infection and its intraepithelial cervical lesions after a long term administration of an specific oral probiotic formulation.
Methodology: The study is a randomize double-blinded clinical intervention trial. The sample will be taken from patients that attend the Gynecological consult of Hospital Universitario de Torrevieja, Spain, from 2018-2020. They must comply with the inclusion and exclusion criteria. The study has been divided in two parts. In Part I, we will analyze the vaginal samples taken after the oral administration of the probiotic to a large sample of patients for 28 days. In this part, we will evaluate the presence of the probiotic strain in vaginal microbiota. In Part II, the treatment with probiotic vs placebo, will be administered to HPV-positive women and observe its effect on the infection status and its cervical lesions.
Results: Preliminary results in Part I (ended on April 30th,2019) demonstrated, using massive sequencing techniques, that the administration of the oral probiotic allows for its vaginal colonization (Results details will be shown on the poster).
Conclusion: Through this study we attempt to demonstrate that an oral probiotic can colonize the vagina and consequently change its microbiota. In Part II of the study, we will study if an oral probiotic facilitates the elimination of HPV. In consequence, we would achieved to decrease the persistence of the intraepithelial cervical lesions, and therefore, cervical cancer.
Disclosure: Nothing to disclose

EP1060

Optimal triage of HPV-positive test results

J. Andrews
Medical Affairs, BD, Sparks, MD, United States

Introduction / Background: An analysis of the body of science published recently about the clinical value of triage options after HPV-positive test results.
Methodology: MedLine was searched from 2001 through 2019 for relevant studies. Hand-searching of retrieved article reference lists supplemented the search. Eligible studies included prospective studies of women and retrospective studies of residual specimens from women that were tested using HPV genotyping tests. The reference standards were CIN2 or CIN3 or CIN2+ or CIN3+ or invasive cervical cancer. The comparator was cytology as a triage for HPV-positive results.
Results: Reporting genotyping provides profound discrimination of both current and future CIN3+

risks, due to the differential risks. Genotyping combined with cytology improves risk discrimination for Bethesda categories NILM, ASC-US, LSIL. Improvements in referral rates may be achieved. Similar management for similar risk-discrimination is benchmarked. Immunohistochemical staining with p16 and Ki67 has good evidence for triage of HPV-positive screening results. Methylation of viral HPV and host markers has fair evidence for triage of HPV-positive screening results, and may be applicable to self-collected samples.
Conclusion: Based on quality-evaluated studies that met inclusion criteria, genotyping combined with cytology discriminates risk and supports risk-based clinical action steps by the principle of equal management for equal risk. P16/Ki67 staining could be used as an adjunct to cytology or to replace cytology. Methylation tests could be developed as a screening test, an adjunct triage test with cytology, or to replace cytology as triage. Future research is needed to determine if genotyping could have clinical use without cytology. Inclusion of vaginal swab and/or urine self-collection as initial screening samples will stimulate development of triage tests that do not require direct sampling of the transformation zone. Models for different management paradigms are described.
Disclosure: Speaker is a full-time employee of BD, as Worldwide Medical Director of Women´s Health & Cancer.

EP1061

Risk-reducing surgery in patients with high risk of ovarian and endometrial carcinoma: single institution experience over 10 years using a standardized surgical-pathological protocol

A. van der Leden[1,2], R. Arora[1]
[1]*University College London Hospitals, London, United Kingdom,* [2]*Maastricht University Medical Centre (MUMC), Maastricht, The Netherlands*

Introduction / Background: Risk-reducing bilateral salpingo-oophorectomy (RRBSO) is currently the most effective method for reducing ovarian cancer risk in patients with strong family history or BRCA mutation. Lynch syndrome patients are offered hysterectomy with bilateral salpingo-oophorectomy due to a 40-60% lifetime risk for endometrial cancer and 10-12% lifetime risk for ovarian carcinoma.
Methodology: For this retrospective study at our institute, the pathology database was searched for patients that underwent RRBSO with or without hysterectomy between 2009 and 2019, with no clinical suspicion of malignancy. The ovaries and fallopian tubes were examined by sectioning and extensively examining the fimbrial end (SEE-FIM protocol). The endometrium of the hysterectomy specimens was examined in its entirety.
Results: 92 patients fit the study criteria and had an age range from 35 to 77 years. 24 patients had BRCA 1 mutation, 32 had BRCA 2 mutations,1 had both BRCA1 and 2 mutations, 7 had Lynch syndrome (LS) and 28 had strong family history. We found carcinoma in 2 patients (2.2%): 1 high grade serous carcinoma and 1 endometrial endometrioid carcinoma.
There were 5 serous tubal intraepithelial carcinomas (STIC), 3 showed p53 signature and 1 non-atypical endometrial hyperplasia. Benign findings were seen in 22 patients.
Conclusion: In our study, 8 patients (8.7%) had (pre)malignancy. Our data is consistent with the literature that reports an incidence of 2-10% (pre)malignancy in risk reducing surgeries. 35-45 years is deemed an appropriate age for women to be considered for risk reducing surgery but our study had a wider age range.
With lack of effective screening modality for high risk ovarian and endometrial carcinoma, our study highlights the importance of risk reducing surgery.
The SEE-FIM protocol is effective and should be routinely used. We also want to raise awareness of importance of embedding the entire endometrium in patients with increased risk of endometrial carcinoma.
Disclosure: Nothing to disclose

EP1062

Hyperplastic processes in hormone-dependent organs in women after IVF

F. Ayupova, D. Mirzaeva, D. Saidjalilova
Obstetric and Gynecology, Tashkent Medical Academy, Tashkent, Uzbekistan

Introduction / Background: The main complications of IVF are ovarian hyperstimulation syndrome, which increases the risk of hyperplastic processes in the female reproductive system. However, the protocol for the management of women with IVF does not take into account the dynamic control of possible hyperplastic processes of the female organs. Therefore, the frequency of the risk of ovarian cancer may increase against the background of stimulation of superovulation.
Methodology: Examined 116 patients after IVF. Depending on the outcome of IVF, women were divided into 2 groups: the first was 52 patients with successful IVF; the second - 64 patients with an unsuccessful outcome of IVF. Depending on reference, women are divided into 2 subgroups: A subgroup consisted of women who were traditionally led; B subgroup consisted of women using anti-proliferative progestogens.
Results: In group 1, women of subgroup A, when stimulating ovulation in the IVF program, increased the frequency of formation of hyperplastic processes: uterine myoma were detected in 10%, endometrial hyperplasia in 11%, yellow body cyst in 21% of women. Whereas, in subgroup B uterine myoma was detected in 5% of women, endometrial hyperplasia in 4%, yellow cyst in 19%. In group 2 in women of subgroup A, hyperplastic processes were detected after 3 months in 19% of women, 6 months in 24% of women. Whereas in the subgroup B hyperplastic processes were observed after 3 months only 3% of women; 6 months - at 8%.
Conclusion: After stimulation of ovulation with IVF after 1, 3, 6, 12 months, it is necessary to conduct an examination of the reproductive organs in women to identify hyperplastic processes. If pregnancy has not occurred during IVF, then over the next month, recommend the use of contraceptives with an anti-proliferative gestagen to prevent hypergonadotropic state.
Disclosure: Nothing to disclose

EP1063

Analysis of expression of microRNA a new method for the diagnosis and prognosis of preinvasive cervical carcinoma

E. Bakhidze, P. Arkhangelskaya, I. Berlev
N.N. Petrov Scientific Medical Research Center of Oncology, St. Petersburg, Russian Federation

Introduction / Background: Cervical cancer remains the leading cause of death among women of reproductive age, even in countries with existing cytology screening program. In recent years it was shown that infection with HPV, development and progression of dysplasia of cervical epithelium are accompanied by changes of microRNA expression profile of epithelial cells. Evaluation of these changes may have diagnostic value in doubtful cytological data (ASCUS) and prognostic value in identifying of LSIL and HSIL that was the aim of this study aim of this study.
Methodology: Study enrolled patients with cervical cancer (n = 33), intraepithelial neoplasia of high (HSIL, n = 28) and low grade (LSIL, n = 34) and healthy women (n = 20). RNA was isolated by original method from material of cytological smears, dried on glasses. miRNA expression analysis was performed by semi-quantitative RT-PCR. Also in all cases it was carried out research of fifteen HPV types of high oncogenic risk, viral load and genotyping.
Results: It was found that progression of the neoplastic process was accompanied by increased expression of miR-20a and decreased expression of miR-375. It was developed the classifier that allows by assessing expression of the three microRNAs to confidently differentiate cervical cancer from dysplasia (AUC = 0,876), or divide norm from group with neoplastic changes of the squamous epithelium (cervical cancer, HSIL) that implies further thorough examination (AUC = 0,94). The observed changes in microRNA expression correlated with presence of HPV, but not correlated with the viral load. Samples of the LSIL group were characterized by the most heterogeneous profiles of

microRNA expression, which may indicate the heterogeneity of this category of patients in regard to the risk of cervical cancer development.
Conclusion: Search for specific changes of microRNAs opens possibility of developing new methods of screening and early diagnosis of cervical cancer.
Disclosure: Nothing to disclose

EP1064

Clinical and pathologic findings of risk-reducing surgery in high-risk breast/ovarian cancer women

M. Cardoso[1,2], C. Reis de Carvalho[2,3], J. Aquino[2,4], L. Correia[2], B. López[2], M. Bernardino[2], A.F. Jorge[2]
[1]Hospital do Divino Espírito Santo de Ponta Delgada, EPE, Ponta Delgada, [2]Instituto Português de Oncologia Franscisco Gentil, [3]Centro Hospitalar Universitário de Lisboa Norte, EPE, [4]Maternidade Dr. Alfredo da Costa - Centro Hospitalar Universitário de Lisboa Central, EPE, Lisboa, Portugal

Introduction / Background: Inherited germline mutations in BRCA1/BRCA2 increase significantly the risk of developing breast and ovarian cancers. For women in whom a BRCA pathogenic variant is identified, risk-reducing bilateral salpingo-oophorectomy (rrBSO) is recommend by age 35 to 40 years or when childbearing is complete. This procedure is associated with reduction of ovarian cancer risk, reduction in breast cancer, cancer-related mortality and overall mortality. The incidence of unsuspected neoplasia in these women varies in the literature.
Methodology: We performed a retrospective review of all women undergoing risk-reducing surgery between January 2013 and December 2018, in an oncological referral center - Instituto Português de Oncologia de Lisboa Francisco Gentil, Lisbon, Portugal.
The purpose of this study was to analyze the results of anatomo-histopathologic examinations and to identify the rate of occult cancer diagnosis in a high-risk population.
Results: Overall, 83 high-risk women (75 known BRCA1/2 carriers; 8 with unknown mutation status) underwent risk-reducing surgery. The median age at the surgery was 48,76 years and the median body mass index was 25,65 kg/m^2. 42,2% (n=35) of these women had personal history of breast cancer. Preoperative CA-125 levels were considered normal in 97,6%(n=81).
We performed rrBSO on 22,89%(n=19) of cases and concomitant hysterectomy on 77,1%(n=64). Laparoscopic approach was used in 77,1%(n=64) of procedures. The surgical complication rate was 3.6%(n=3).
The anatomopathological analysis of specimens revealed: 5 ovarian serous cystadenoma, 1 adenomatoid tumor of the fallopian tube, 1 ovarian cystic teratoma and 1 ovarian endometrioma. The presence of occult tumor cells, including epithelial ovarian cancer and serous tubal intraepithelial carcinoma, was not reported.
Conclusion: Risk-reducing surgery is a safe and effective strategy to manage BRCA mutation carriers. Even though our median age at surgery is high, the incidence of unsuspected neoplasia identified in this study was unexpectedly low compared with the current literature.
Disclosure: Nothing to disclose

EP1065

Comparison of two self-sampler devices for cervical screening in a South African population

A. Breidenthal[1], F.H. van der Merwe[1], G. Dreyer[2], L. Snyman[2], C. Visser[2], M.H. Botha[1]
[1]Department of Obstetrics and Gynaecology, University of Stellenbosch, Cape Town, [2]Department of Obstetrics and Gynaecology and Gynaecological Oncology Unit, University of Pretoria, Pretoria, South Africa

Introduction / Background: Self-sampling is an attractive addition to cervical screening programmes. It can remove barriers to testing as some women fear a gynaecological examination, and offers potential task shifting and resource savings for overburdened health care systems often in low resource settings.
Methodology: We performed a large cervical cancer screening study with ultimate objective to determine optimal screening algorithms for South African women. This interim analysis describes the experiences of a subset of women who were asked to fill in a questionnaire about the self-samplers. The aim was to compare user experiences of two different self-samplers in a South African public sector setting.
253 participants received a short education by the Health Care Worker (HCW), and were given either the Digene HC2 DNA Collection Device (Qiagen)or the Evalyn® Brush (Rovers Medical). Participant and HCW filled in a questionnaire afterwards.
Results: Analysis was done for 253 women. Mean age in group was 41.33 years (range [24 - 63], median 41 years). 82 women were in the Digene group, and 171 in the Evalyn group.
HCWs reported more bleeding with the Digene vs. Evalyn users (37.9% vs. 20.4%).
Both groups felt it was very or somewhat easy to use (98.8% vs. 94.2%). Women had little fear of using the devices, (61.0% vs 71.3%), and felt sure of how to use them. There was more bleeding reported by Digene vs Evalyn users (9.8% vs. 4.1%), and lastly, there was little pain experienced by users of either brush, 91.5% vs 93.6%.
Conclusion: In this cohort, both the self-samplers were well tolerated; there was more bleeding associated with use of the Digene device, both seen by participants and HCW, which will be analysed further once results are available. Self-sampling is feasible and acceptable in this public setting.

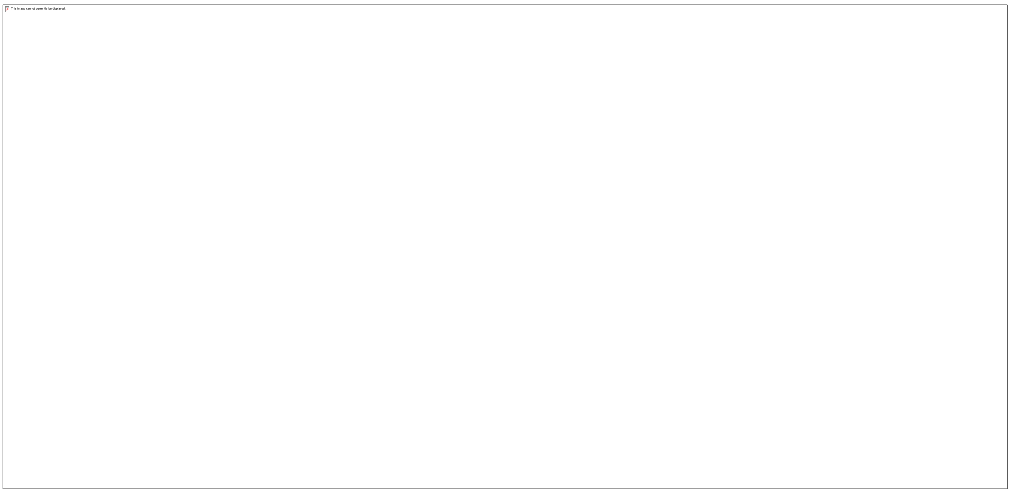

[Proportion of responders % : Digene]

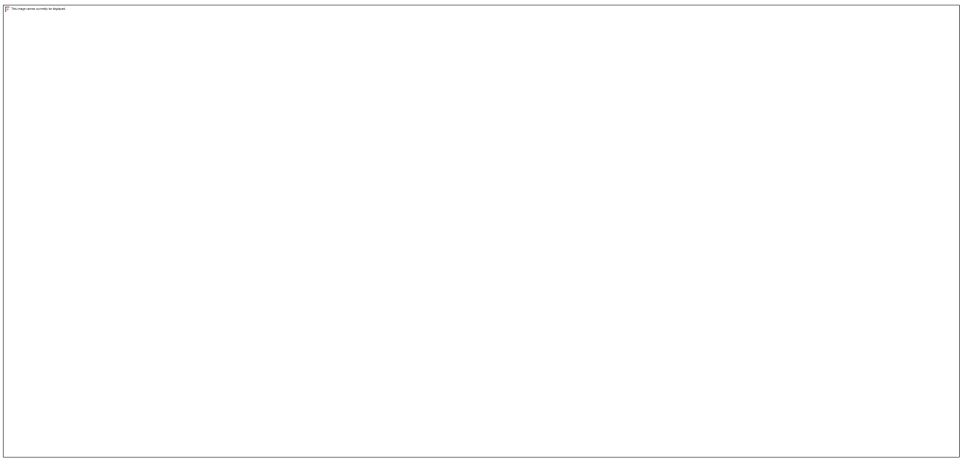

[Proportion of responders % : Evalyn]

Disclosure: Rover BD donated Evalyn brushes for the study, and Qiagen provided digene collection devices, but neither were involved in preparing papers.

EP1066

Opportunistic salpingectomy as a risk reducing strategy for high-grade serous carcinoma: a Northern Ireland opinion

H.J. Agnew, A.G. Brown, I.J.G. Harley
Gynaecology Department, Belfast City Hospital, Belfast, United Kingdom

Introduction / Background: Serous tubal intraepithelial carcinoma (STIC) is the precursor of high-grade serous carcinoma (HGSC), and its site of origin is well recognised as the fallopian tube. With the lack of effective screening and often advanced stage at presentation, opportunistic salpingectomy has been proposed as an intervention to decrease the incidence of HGSC in the general population. The logical step would be to introduce this into our routine obstetric and gynaecological surgeries. We assessed the opinions of consultant and trainee obstetrician/gynaecologists in Northern Ireland to this intervention, given that the benefit is currently unknown.
Methodology: An anonymous online survey was sent to consultants and trainees to determine their understanding of STIC, barriers to counselling patients and performing opportunistic salpingectomy, in 3 specific scenarios: caesarean section sterilisation, vaginal hysterectomy and sterilisation requests.
Results: As stage of training increased so did knowledge regarding STIC. Barriers to counselling included lack of knowledge and evidence, and no accurate risk/benefit ratio. Trainees were more willing to consider opportunistic salpingectomy than consultants; with 90% versus 68% for caesarean section sterilisation, 76% versus 67% for vaginal hysterectomy and 98% versus 77% for sterilisation. Consultant concerns included fertility, lack of evidence and increased complication risk; the latter shared by trainees. The other factor that prevents trainees considering opportunistic salpingectomy is the consultant involved.
Conclusion: Overall trainees are much more enthusiastic in their willingness to consider opportunistic salpingectomy. If we can target knowledge gaps, consultant concerns and instigate surgical/procedural teaching for trainees at an early stage of their career, we could improve the uptake of this procedure. This could then potentially have an impact as a risk reducing strategy in those without a defined genetic risk for HGSC.
Acknowledgements: The Ulster Obstetrical&Gynaecological Society, Dr P. Birkett, Dr J. Breen, Dr K. Devlin, Dr R.Farr, Dr A. McNally and Dr A. Wilson, for encouraging responses.
Disclosure: Nothing to disclose

EP1067

Malignant transformation of extra-ovarian endometriosis: a PRISMA-compliant systematic review

C. Cheng, Z. Pan
Womens Hospital School of Medicine Zhejiang University, Hangzhou, China

Introduction / Background: Malignant transformation of extra-ovarian endometriosis is a rare and aggressive disease. The purpose of this study is to provide a clinical overview, focusing on risk factors affecting prognosis.
Methodology: We performed a Preferred Reporting Items for Systematic Reviews and Meta-Analyses-compliant systematic review based on prior reviews and case reports regarding the

phenomenon published in English, from 1954 to 2019. Descriptive data were collected and analyzed.
Results: Of 316 patients (mean age was 51.2) included, the common primary clinical presentation was mass and /or abdominal pain (72.3% and 46.2%, respectively).The frequent sites of extra-gonadal involvement are the intestine (26.3%) and the abdominal wall (16.9%). Endometrioid carcinoma was the most prevalent histology (40.1%), followed by clear-cell carcinoma (21.3%). Extensive Surgery was the major part of the therapeutic approach in patients, with/without adjuvant treatment. The median survival time was 132 months since diagnosis. According to our data analysis, patients who younger than 45-years-old presented a trend of survival lower than elder ones (p=0.024). Tumor diameter ≥8cm showed a tendency toward worse prognosis relative to both overall survival (P=0.0081) and disease free survival(P=0.024).Clear-cell histology has a higher recurrence rate versus endometrioid carcinoma (P=0.0098) and stroma (P=0.0045).
Conclusion: Malignant transformation of extra-ovarian endometriosis is a rare entity with poor prognosis. Patiens who are menopause, who have long term history of endometriosis and with growing mass should pay special attention. Large size of tumor and clear-cell histology tends to poor prognosis. Complete tumor resection is the primary treatment, but it remains unclear whether adjuvant chemotherapy is suitable. Further investigation need to be conducted.
Disclosure: Nothing to disclose

EP1068

CO2 Laser ablation for cervical intraepitelial neoplasia:retrospective study at "Ramón y Cajal" University Hospital

C. del Valle Rubido[1], C. Martín Blanco[1], M. Salvador Osuna[1], E. Díaz González[1], M.C. Sánchez Martínez[1], E. Cabezas López[1], C. Miguel Bayo[2], B. Pérez Mies[3], E. Moratalla Bartolomé[1], J. Lázaro-Carrasco[1]
[1]Obstetrics and Gynecology, [2]Hospital Universitario Ramón y Cajal, [3]Pathology, Hospital Universitario Ramón y Cajal, Madrid, Spain

Introduction / Background: CO2 ablation of cervical lesions is used for treatment of persitent CIN1 or CIN2/3 with adequate colposcopy. In our Department, laser ablation is performed under colposcopic guidance by specially trained clinicians.
Methodology: We have performed a retrospective study of CIN that has been treated by laser ablative therapy at "Ramón y Cajal" University Hospital from January 2018 to April 2019.
Results: 51patients have undergone laser ablative therapy in the past 1,3years.
Mean age is 36,9years-old and parity is 0.54children per woman in the study group. The subject´s nationality is 62%Spanish, 23% from South America and 13% from Eastern Europe. 33%use condoms as contraceptive method, 29%oral contraceptives, 23,5%do not use any method, 3.9%IUD and only 1.9%vasectomy or vaginal ring. None of the patients are inmunodepressed. 37.2%are active smokers vs 49%non-smokers. Only 27%have received HVP vaccination. HPV infection is present in all but one patient: 55.9% high-risk HPV(not16 or18), 17%HPV16, 13.7% HPV16 and another high-risk HPV, 3.9%HPV18 and 1.9%HPV18 and another type.
Colpo-histologic study previous to ablation shows:
- Pap-smear: 47% LSIL, 19%ASCUS, 15.6%negative, 7.8%ASC-H and 7.8%HSIL.
- Colposcopy: 17.6%negative, 3.9%erithroplakia, G1 changes(19.6% mosaicism, 19.6%,19.6%acetowhite epithelium, 11.7%punctation), G2 changes (3.9%acetowhite epithelium,1.9%mosaicism, 1.9%punctation), 3.9%condyloma, 13.7%negative Schiller test and 1.9%insatisfactory colposcopy.
- Histology: 64.7%CIN1, 7.8%CIN2/3, 7.8%chronic cervicitis and 3.9%negative.

Indications for cervical ablation are: 58%persistent CIN 1, 11.7% CIN2/3, 3.9% postcoital bleeding and 1case persistent ASCUS.
At the moment, 25patients have undergone cervical ablation with laser CO2, 22 are pending and 3 have been lost. No complications have taken place.
14 patients have reached the 6-month follow-up visit, all of whom had negative results except 1ASCUS Pap-smear and 3LSIL (all arising in previous CIN2/3-HSIL). HPV has become negative in 6patients. Only one patient has required surgery because of persistent CIN2.
Conclusion: Ablative treatment for cervical intraepitelial neoplasia appears to be effective, with low

rates of disease persistence.
Disclosure: Nothing to disclose

EP1069

Vulvar and vaginal intraepithelial neoplasia: CO2laser ablative therapy at Ramón y Cajal University Hospital

C. del Valle Rubido[1], C. Martín Blanco[1], E. Díaz González[1], M. Salvador Osuna[1], M.C. Sánchez Martínez[1], E. Moratalla Bartolomé[1], C. Miguel Bayo[2], B. Pérez Mies[3], J. Lázaro- Carrasco[1]
[1]Obstetrics and Gynecology, [2]Hospital Universitario Ramón y Cajal, [3]Pathology, Hospital Universitario Ramón y Cajal, Madrid, Spain

Introduction / Background: Laser vaporization is an alternative to excisional therapy in women with VIN and VAIN, especially with multifocal and extensive lesions.
Methodology: Retrospective study of 12 cases of VIN or VAIN, treated with CO2 laser ablation at RamónYCajal University Hospital.
Results: Nine patients with VAIN and 4with VIN have been selected for ablative therapy with CO2laser.
Mean age of the VAIN group is 41.9years and parity 0.66births per woman. 55%are Spanish, 22%South American and 22%from Eastern Europe. Half of the patients do not use any contraceptive method, 2use condoms, 1implant and 1DMPA. One third are active smokers, and two of them have inmunodepressant conditions:HIV and chronic lymphocitic leukemia. Only one patient has received HPV vaccination, 3have undergone cervical conization, 1vaginal ablation, 1exeresis of condylomas and one histerectomy.
Vaginoscopy and histology study has shown the following results:
- Positive HPV in all but one patient(the one with leukemia):66%HPV16 and 22%high-risk HPV(not16 or18).
- Pap-smear:55%LSIL, 33%HSIL, 11%ASCUS and 11%ASC-H.
- Vaginoscopy: condylomas in 4patients, negative Schiller test in 3, colpitis in 2 and acetowhite epithelium in another two.
- Vaginal biopsy:55% VAIN2/3 and 33%VAIN1.

At this moment,vaginal vaporization has been performed in 6cases,3 are pending and one has been lost.Complications have taken place in two cases:vulvar edema and vulvitis. Two patients have reached the six-months follow-up, with negative Pap-smears and vaginoscopy, but persistent HPV in one of them.
Mean age of the VIN group is 53.7 years-old and parity 0.5. 100%are Spanish. Two use condoms and the other 2 are postmenopausal. All have inmunodepressant conditions: 3 HIV and 1 CLL(with concomitant VAIN). HPV is positive in 50%. Vulvoscopic - histologic findings are:condyloma-VIN3, vulvar and vaginal warts-VAIN1, G1acetowhite epithelium-VIN3 and clitoris erythema-VIN3.
The patient that has reached follow-up has persistent VIN1 and re-vaporization is pending.
Conclusion: VAIN and VIN lesions can be effectively treated with CO2 laser ablation.
Disclosure: Nothing to disclose

EP1070

Risk-reducing salpingo-oophorectomy in Brca mutation carriers. a prospective multicentric study

V. Del Vecchio[1], V. Loizzi[1], E. Cicinelli[1], X. Deromemaj[1], A. Kardhashi[2], A. Paradiso[3], F. Legge[4], M.I. Natalicchio[5], L. Resta[6], N. Resta[7], G. Cormio[1,2]
[1]Obstetrics and Gynecology Unit, Department of Biomedical Sciences and Human Oncology,

University of Bari, [2]2. Gynecology Unit, IRCCS Istituto Tumori "Giovanni Paolo II", [3]3. Institutional BioBank, Experimental Oncology and Biobank Management Unit, IRCCS Istituto Tumori 'Giovanni Paolo II', Bari, [4]Obstetrics and Gynecology Unit, Regional General Hospital "F. Miulli", Acquaviva delle Fonti, [5]Molecular Oncology and Pharmacogenomics Laboratory, University of Foggia, Foggia, [6]Department of Pathology, University of Bari, [7]Medical Genetics Unit, Department of Biomedical Sciences and Human Oncology, University of Bari, Bari, Italy

Introduction / Background: Ovarian carcinoma is the leading cause of death among gynaecological malignancies, and there is a current lack of effective screening procedure. More than one-fifth of ovarian tumours have genetic predisposition and, in about 65-85% of these cases, the genetic abnormality is related to a germline mutation in Breast Related-Cancer Genes (*BRCA)* 1 and 2 which are tumour suppressor-autosomal dominant inherited genes encoding proteins involved in homologous recombination. Different studies evaluated the origin of early High Grade Serous Ovarian Cancer (HGSOC) in the distant portion of the fallopian tube and this discovery led to develop a risk-reducing strategy through a prophylactic bilateral salpingo-oophorectomy (BSO) in women with BRCA1/2 genes mutation.
Methodology: Risk-reducing surgery (RRS) was performed in 74 patients carrying BRCA1 (aged between 34-73 years, median age was 50 years) and BRCA 2 mutation (aged between 41-69 years, median age was 52 years). 39 patients had previous history of breast cancer.
Results: Between the 74 patients, 58 women (78,4 %) underwent risk-reducing salpingo-oophorectomy (RRSO) through a laparoscopic minimally invasive approach, 5 (6,7%) underwent laparoscopic RRSO and contextual hysterectomy, 1 woman (1,4%) underwent RRSO through a laparotomic approach and 10 women (13,5%) laparotomic RRSO and hysterectomy. During 2 (2,7%) laparoscopic RRSO, prophylactic bilateral mastectomy was also performed. Early and late complication occurred in 3 patients (4%). 2 patients (2,7%) were found to have occult Serous Tubal Intraepithelial Carcinoma (STIC) and 3 patients (4%) occult cancer.
Conclusion: RRSO is safe and feasible in BRCA mutation carriers. The procedure is effective for genetic prevention of ovarian cancer.
Disclosure: Nothing to disclose

EP1071

Genotype distribution of HPV in patients with histologically proven findings for cervical intraepithelial neoplasia or invasive cervical cancer in Macedonia

G. Dimitrov[1], E. Dzikova[1], G. Dimitrov[2], G. Tofoski[3], A. Daneva Markova[3], M. Joksimovik[3], I. Kjajova[3]
[1]Gynecologic Oncology, University Clinic for Gynecology and Obstetrics, [2]Private General Hospital Re-medika, [3]University Clinic for Gynecology and Obstetrics, Skopje, The former Yugoslav Republic of Macedomia

Introduction / Background: The aim of this study was to provide basic data on genotype distribution of HPV in patients from Macedonia with histologically proven findings for squamous intraepithelial lesions or invasive cervical cancer, in order to better plan the vaccination program and screening for cervical cancer and to plan and with hope to introduce a National cervical cancer screening programme with HPV genotyping as a primary screening test.
Methodology: We analyzed the histological findings positive for cervical intraepithelial neoplasia or invasive cervical cancer and genotype HPV distribution results in 564 patients treated at the University Clinic for Gynecology and Obstetrics in Skopje, Macedonia, during 2017.
Results: 78% of the total of 564 subjects appeared to be positive for HPV. The genotype distribution of HPV in cervical squamous intraepithelial lesions or invasive squamous cell cancer and adenocarcinoma of the uterine cervix was 69.2%, 87.2%, 97.6% and 71.4%, respectively. The distribution of HR HPV types was for 21% higher in HSIL lesions than in LSIL. The most frequent isolated HPV types were: 16, 18, 35, 31, 33, 58, 6, 11 and 40. The most common genotype was HPV 16 with the highest rate in HSIL (48.4%), a little bit lower in invasive squamous cancer (30.1%) and the lowest in LSIL (19.6%). On the other hand, the prevalence of HPV 18 was the highest in patients with invasive adenocarcinoma of the uterine cervix, 30.1%.
Conclusion: In Macedonia, the most predominant genotypes of HPV in squamous intraepithelial

lesions and invasive cervical cancers are the human papillomavirus types 16, 18, 35, 31, 33, 58, 6, 11 and 40 which is very important to know in order to plan the vaccination program and to create an expedient National cancer screening programme that will be more effective strategy against invasive cervical cancer.
Disclosure: Nothing to disclose

EP1072

Diagnostic performance of p16/Ki67 immunostaining in postmenopausal women with ASC-US and LSIL cytology

A. Dovnik[1], A. Repše Fokter[2]
[1]Maribor University Medical Centre, Maribor, [2]General Hospital Celje, Celje, Slovenia

Introduction / Background: Interpretation of postmenopausal smears can often be time consuming and difficult. With our study we aimed to evaluate the diagnostic performance of p16/Ki67 immunostaining for detection of high-grade squamous intraepithelial lesion (HSIL) in postmenopausal women with low grade cytology.
Methodology: All consecutive ASC-US and LSIL smears in a one-year period were collected and p16/Ki-67 immunostaining was performed retrospectively. All patients were followed-up for the period of six years. Results were compared with histology results or long-term cytology follow-up in cases with no biopsy.
Results: 270 postmenopausal patients were included in the study. 241 patients had their Pap smear interpreted as ASC-US (89.3%) and 29 patients had LSIL (10.7%). 20 patients (7.4%) had histologically confirmed HSIL (CIN2+). The sensitivity of p16/Ki-67 immunostaining for the detection of CIN 2+ was 57.0% and for the detection of CIN 3+ 85.0%. The specificity for the detection of CIN 2+ and CIN 3+ was 94.3% and 94.6%, respectively.
Conclusion: With high specificity levels for the detection of CIN 2+ and CIN 3+, p16/Ki67 immunostaining is useful for postmenopausal women with low grade cytology and could be used as a triage method for colposcopy.
Disclosure: Nothing to disclose

EP1073

The prevalence of squamous intraepithelial lesions according to ethnic affiliation in Macedonia

E. Dzikova[1], G. Dimitrov[1], G. Dimitrov[2], G. Tofoski[3], A. Daneva Markova[3], E. Djikova[4], M. Avramovska[5]
[1]Gynecologic Oncology, University Clinic for Gynecology and Obstetrics, [2]Private General Hospital Re-medika, [3]University Clinic for Gynecology and Obstetrics, [4]Private Health Institution:'Dr. Emilija Trajkovska', Skopje, [5]Clinical Hospital 'Dr. Trifun Panovski', Bitola, The former Yugoslav Republic of Macedomia

Introduction / Background: To determine the prevalence of abnormal Pap smears in different ethnic population in women from Macedonia. In our country, Pap smear test is included in the prevention program against cervical cancer to be performed every year in women aged 18 to 65.
Methodology: We analyzed 1145 female patients, 129 with abnormal Pap smear and 1016 controls. The study was performed in 2015 in women aged 18-65, at the University Clinic for Gynecology and Obstetrics, Private General Hospital Re-Medika, Clinical Hospital "Dr. Trifun Panovski" and in the Primary Health Institution “Dr. Emilija Trajkovska”.
Results: From 1145 analyzed female patients and 129 (11.3%) abnormal Pap smears, we found 9 patients (6.97%) with cell changes from viral infection, 50 (38, 76%) HPV positive patients on cytology

test, CIN 1 in 34 patients (26.35%), CIN 2 in 9 patients (6.97%), ASC in 19 patients (14.72%), ASC-H in 6 patients (4.65%), and CIS in 2 patients (1.55%). Furthermore, from all Pap test positive patients, 15 patients had previous cervical conisation and following re-infection.
The distribution of ethnicity among abnormal Pap smear women was as following: 107 (82.94%) were Macedonians, 12 Serbians (9.30%) with all types of cervical intraepithelial lesions, 5 Roma ethnicity (3.87%), 3 (2.32%) Albanians, 1(0.77%) Bosnian and 1(0.77%) Turkish ethnicity all with CIN1 lesion.
Conclusion: Our study showed that the Macedonian population as the predominant population in Republic of Macedonia, was the most prevalent ethnicity with cervical intraepithelial lesions.
Disclosure: Nothing to disclose

EP1074

Prevalence of high risk human papillomavirus infection among Iranian women using COBAS HPV DNA testing

F. Farzaneh[1], F. Jamdar[1], F. Navidpour[2], S. Yunesi[3], P. Balvayeh[4], M. Hosseini[1], R. Robabeh Ghodssi-Ghasemabadi[5]
[1]Preventative Gynecology Research Center, Shahid Beheshti University of Medical Sciences, Tehran, [2]Manager of Post Analytical Quality Control Department in Nilou Lab, Theran, [3]Technical Manager of Nilou Laboratory, [4]Anatomical and Clinical Pathologist, Nilou Laboratory, [5]Department of Biostatistics, Faculty of Medical Sciences, Tarbiat-Modares University, Tehran, Islamic Republic of Iran

Introduction / Background: Persistent infection with High Risk Human Papillomavirus (HR HPV) types plays a major role in the development of cervical cancer. Therefore, the detection of HR HPV types is an essential part of cervical cancer screening. The aim of this study was to estimate the prevalence of HR HPV infection among healthy women undergoing routine cervical cancer screening in Iran.
Methodology: In this cross-sectional study, 2453 normal Iranian women who were referred for routine cervical cancer screening from September 2015 to March 2017 were recruited. Participants were screened using COBAS assay for HPV DNA typing and liquid based cytology. The results of HPV DNA typing and Cytology were analyzed.
Results: A total of 2453 healthy sexually active women were included in this study. The mean age was 35.1±8. 08 years. The overall prevalence of HR HPV infection was 10.3%. HPV16 was found in 73 (3%) women. The prevalence of HPV18 and other HR HPV typeswere 16(0.7%) and166 (8.2%),respectively. Approximately, 5% of the study population had an abnormal cervical cytology (ASCUS or worse), of whom 34% were infected by HR HPV.
Conclusion: The prevalence of HR HPV infection among Iranian women has increased in the recent years which indicates the need for public education and health planning to prevent this cancer through vaccination and early diagnosis using screening tests.HPV DNA typing, diagnosis and the distribution of prevalent genotypes should be considered in the development of comprehensive cervical cancer prevention programs in Iran
Disclosure: Nothing to disclose

EP1075

Immunohistochemistry improves specificity in the diagnosis of preneoplastic fallopian tube lesions of BRCA mutation carriers

I. Cipullo[1], P. Modaffari[1], R. Ponzone[2], F. Maggiorotto[2], P. Campisi[3], G. De Rosa[3], D. Balmativola[4], E. Maldi[4], L. Fuso[1], A. Ferrero[1], N. Biglia[1]
[1]Gynaecology and Obstetrics, University of Torino / Mauriziano Hospital, Torino, [2]Gynaecological Oncology, Candiolo Cancer Institute, Candiolo, [3]Pathology Unit, Mauriziano Hospital, Torino,

[4]*Pathology Unit, Candiolo Cancer Institute, Candiolo, Italy*

Introduction / Background: Serous Tubal Intraepithelial Carcinoma (STIC) is considered the main precursor of high-grade serous carcinoma (HGSC). There are no standardized diagnostic criteria; an algorithm combining histological and immunohistochemical (IHC) analysis has been proposed by Kurman to improve the standardization. In women undergoing Risk Reducing Salpingo Oophorechtomy (RRSO), the diagnosis of STIC is essential for the assessment of the risk of recurrence in this group of women.
Methodology: A retrospective study was conducted in two Gynaecological Oncology Units (Mauriziano Hospital Torino and IRCC Candiolo, Italy). Pathological reports of the fallopian tubes of 128 BRCA mutation carriers/high risk women (who underwent RRSO or surgery for ovarian cancer) and 222 women at low risk for the development of breast and ovarian cancer (who underwent surgery for ovarian cancer or benign diseases) were analyzed searching hyperplasia, atypia, dysplasia, STIC and tubal carcinoma and classifying these lesions as not suspicious/suspicious/unequivocally STIC. Subsequently, in high risk patients with histological alterations, IHC stains (p53 and Ki67 index) were performed, to apply Kurman's algorithm.
Results: Prevalence of pathological tubal lesions was higher in BRCA/ high risk patients compared to low-risk (14,8% vs. 4% p = 0.0004; OR 4,2 (IC 1,8-9,4)). After IHC analysis 78.6% of "not suspicious for STIC" alterations, were defined as normal/reactive epithelium. The remaining lesions (21.4%) were classified as Serous Tubal Intraepithelial Lesions (STIL). The Cohen concordance index K between histological diagnosis alone and its association with IHC was 0,019.
Conclusion: The routinely association of IHC and histological analysis may help pathologists in producing more congruous and reproducible diagnoses, improving the assessment of atypical intermediate tubal lesions. Since the prognosis and the correct management of women undergoing RRSO diagnosed with STIC is still debated, this new type of combined technique of analysis may influence clinical attitudes when STIL or STIC are found.
Disclosure: Nothing to disclose

EP1076

Dependence between arterial hypertension and endometrial hyperplasia in pre- and postmenopausal women

A. Ganovska, S. Kovachev
Military Medical Academy, Sofia, Bulgaria

Introduction / Background: Endometrial hyperplasia is a condition of the endometrium characterized by hyperplasia of the endometrial glands. According to histological morphology, the presence of atypical cells is subdivided into typical and atypical hyperplasia. Atypical hyperplasia is a premalignant condition that can progress to endometrial adenocarcinoma.
Methodology: The study is one-central, retrospective over a two-year period (2017-2018), conducted at a clinic of general and oncology gynecology. The study included 308 patients with symptomatic abnormal uterine bleeding. All women were given a history of gynecological examination followed by a test sample of abrasion under short-term venous anesthesia. For arterial hypertension are taken blood pressure values exceeding 140/80 mmHg for a period of six months prior to surgery.The aim of the study is to determine the relationship between arterial hypertension and the risk of endometrial hyperplasia in pre- and postmenopausal women.
Results: The average age of patients in the study was 51.1 years (24 to 87 years). In 6 (1.9%) women, arterial hypertension and atypical endometrial hyperplasia were detected. In 21 (6.8%) patients, arterial hypertension and typical endometrial hyperplasia were found, and in 7 (2.3%) endometrial carcinoma and arterial hypertension. After the statistical treatment of the results, we established a relationship between arterial hypertension and the incidence of both typical and atypical endometrial hyperplasia. We have not found a similar relationship between arterial hypertension and enometric carcinoma.
Conclusion: In patients with arterial hypertension and uterine bleeding, the risk of endometrial hyperplasia is higher. Timely diagnosis of this type of pathology is necessary, which allows for timely treatment and prophylaxis for progression in endometrial cancer.

Disclosure: Nothing to disclose

EP1077

Argon-assisted loop electrosurgical excision procedure, "Argon-Leep": a feasible and cheap technique to reduce the smoke and thermal damage during cervical conization

C. Goudeli[1], N. Koufopoulos[2], E. Laini[3], E. Terzakis[1]
[1]Institouto Gynaikologias, [2]2nd Department of Pathology, National and Kapodistrian University of Athens, [3]Department of Cytology, Alexandra Hospital, National and Kapodistrian University of Athens, Athens, Greece

Introduction / Background: After evaluating the Argon insufflation (99, 9%) effectiveness on improving the method of cervical tissue excision by loop electrosurgery (leep) in bovine cervical specimens we use it safely in our everyday office practice in many leep cases. *(the experiment mentioned was presented in ESGO congress2015).*
Methodology: Argon is a chemical element in group 18 of the periodic table and is a noble gas. It is colorless, odorless, nonflammable and nontoxic as a gas. Argon is chemically inert under most conditions and forms no confirmed stable compounds at room temperature. Argon constitutes a cheap material. It has low thermal conductivity and is sometimes used even for extinguishing fires. Based on the argon characteristics above and our previous experience we herein use the simultaneous argon insufflation in the area before cervix so as there is no oxygen to be burned and provoke smoke or tissue damage during leep.
Results: We apply a small plastic tube that isufflates argon connected to a 5-litre bottle of argon gas on the diathermy pen. The diminution of the thermal injury of the cone while using this patent is macroscopically obvious and microscopically significant. Especially, the zone of carbonization and coagulation of the entrance, middle and exit margins of the loop is decreased.
Conclusion: Although argon in Gynecology is mostly known in its liquid form via Cryosurgery procedures or in argon plasma beam electrosurgery the argon gas can also be used as a safe and cheap material. The use of lower watts electrosurgery allows us to excise high quality histological specimens inexpensively by altering the air medium of loop electrosurgical procedure to Argon or cooled-Argon.
Disclosure: Nothing to disclose

EP1078

HPV self-sampling as an alternative to pap smear collection in outpatient office - women´s considerations

J. Kacperczyk-Bartnik[1], P. Bartnik[1], N. Zawrotna[2], J. Wabiński[3], J. Sozańska[4], E. Rola[5], E. Romejko-Wolniewicz[1], K. Czajkowski[1], K. Zalewski[6]
[1]2nd Department of Obstetrics and Gynecology, Medical University of Warsaw, [2]University of Warsaw, [3]Military University of Technology, Warsaw, [4]Jagiellonian University, Cracow, [5]Warsaw University of Technology, Warsaw, [6]Department of Gynaecologic Oncology, Holycross Cancer Center, Kielce, Poland

Introduction / Background: Despite the development of a national population-based cervical cancer screening program in 2006 by the Polish Ministry of Health, the National Health Fund and the Polish Gynecological Society, following World Health Organization and International Agency for Research on Cancer (WHO/IARC) guidelines, percentage of women performing the test in Poland is still unsatisfactorily low.
The aim of this study was to evaluate women's interest in participation in cervical cancer screening

program based on HPV testing of self-sampled specimen.
Methodology: It was a cross-sectional study with data collected via online distribution of surveys among women aged 17-68 years old. Designed questions regarded prophylactic behavior, interest in new method of screening, women's fears and considerations of proposed self-sampling method.
Results: Altogether 1158 answers were obtained. Main reported reasons for undersceening included: no regular gynecologic control (32.7%), feeling ashamed of visiting gynecologic office (26.9%), anxiety associated with gynecological examination (22.8%). Sixty-seven percent of women with no history of cervical screening expressed positive interest in participation in self-sampling program. More than 40% of ladies in this group admitted decrease in anxiety and 70% reported decrease in feeling ashamed of the examination due to different specimen collection method. Over one third of women regularly visiting gynecologist's office was also willing to substitute collection of specimen by a healthcare professional with self-sampling as in case of 90% of respondents this would be more convenient.
Conclusion: Introduction of population-based screening cervical cancer program using self-sampling of specimen could be an effective way of increasing cervical screening coverage among women in Poland as reported patients' interest is encouraging.
Disclosure: Nothing to disclose

EP1079

Clearance of HPV in women received conization related with mycoplasma and Ureaplasma

C.J. Kim[1], S.J. Lee[1], K.H. Lee[2], Y.S. Lee[2], J.S. Park[3], T.C. Park[4]
[1]Department of Obstetrics and Gynecology, Uijongbu St. Mary's Hospital The Catholic University of Korea College of Medicine, Uijongbu-si, [2]Department of Obstetrics and Gynecology, [3]Catholic University of Korea, Medical College, Seoul, [4]Uijongbu St. Mary's Hospital The Catholic University of Korea College of Medicine, Uijongbu-si, Republic of Korea

Introduction / Background: Little is known about risk factors for persistent high-risk infection after cervical conization. We compared the duration of high risk HPV among women with cervical conization by exposure to sexually transmitted infections(STIs) using a real time PCR methods.
Methodology: This study was conducted on 28 patients who received conization due to persistent CIN-1, CIN-2,3 and CIS at the St Paul's Hospital of the Catholic University of Korea from Jul, 2014 until Feb, 2018. They all were positive for high risk HPV at initial visit. A specimens were tested using realtime based assay, Anyplex II STI-7 (Seegene, Korea). tients were followed in 4-6 month interval with Pap smear and Anyplex TM Real time PCR HPV 28 detection method which detect 19 high-risk HPV genotype , 9 low-risk genotypes
Results: The mean age was 39.3 years (Min-Max : 26-50) and mean follow-up month was 20.2 month(Min-Max :3-41 month). ASCUS, LSIL,ASCH and HSIL were 8,6,3 and 11 women. Single and multiple HPV detection was 14 and 14 (50%).
Of the women received conization, median clearance time of those multiple type HPV was 26 month and that was slightly longer than single type HPV(24.5 month, P>0.05) . Mycoplasma, Chlamydia and Ureaplasma positive cases were 10(35.7%),1(3.6%) and 18(64.3%).The median clearance of women infected with Mycoplasma and Ureaplasma were 22.4 and 29.8 months. Ureaplasma positive women had longer clearance time compared with negative women(29.8 month *vs* 24.8 month p>0.05).
HPV types were related with clearance time. Mean clearance of alpha-9 group HPV(HPV-16,31,33,35,52,58) was shorter than alpha-7(HPV-18,39,45,59,68,70) and alpha-6(HPV-53,56,66). (20.9 month vs 28.8 month p< 0.05).
Conclusion: In a follow-up after conization, women with Ureaplasma showed relatively long time persistence. Management of this STI could reduces risk for high risk HPV persistence.
Disclosure: Nothing to disclose

EP1080

Oooops...very low incidence of HPV infection in patients with CIN2+ in Serbia! Why?

V. Krsic[1], I. Vujicic[2], B. Jocic Pivac[3], V. Jovanovic[4], J. Milojevic[5]
[1]Obgyn, GAK Nardoni Front, Beograd, [2]Institut of Epidemiology of University Belgrade, Belgrade, [3]Obgyn, GAK Nardoni Front, Novi Beograd, [4]Higher Education School of Professional Health Studiea in Belgrade, Beograd, [5]General Hospital Lazarevac, Lazarevac, Serbia

Introduction / Background: Serbia has incidence of cervical carcinoma 20,3/100000 , what means twice than west Europe countries.Aim of this study was to evaluate the incidence of HPV infection and to identify specific types of human papiloma virus in cervical intraepithelial lesions CIN 2+ before conisatio and in healthy women without CIN2+.
Methodology: The study was performed on a cohort of 53 patients with CIN 2+ and 84 patients without CIN+2, patients who during primary examination had already normal PAP smear test. Human Pappiloma Virus (HPV) genotyping was analyzed by multiple Polymerase Chain Reaction (PCR) methods.
Results: The mean age of enrolled women was 37 (minimum of 20 and maximum 67 years. Among the patients with CIN2+ , the presence of HPV by using PCR was detected in 24,58 % (13patients) and among control group was 22,37% .The incidence of HPV was highest (66,1%) in women aged 30-40 years old and it decreased with age and was lowest (6,5%) among patients older than 50 years. The prevalence of oncogenic types of the virus was highest then presence low risk type in both group. Results of HPV typing showed that HPV 16,58 and 31 were the most common types detected among the patients with CIN2+and some of them have mulptiple infection.HR HPV type 16 was the most common type in control group and 31.LR HPV type 6 is most common type in both group. Surprisingly, in our population with CIN 2+ and control group we did not find any patients with type 18 and 11.
Conclusion: Our study showed very low incidence of HPV infection in CIN2+ group,almost the same as in control healthy patients.We did not find any patients with type HRHPV18 and LRHPV11.Other factors could be reasons for developing CIN2+in our country because we were bombarding with depleted uranium and maybe it can has influence.
Disclosure: Nothing to disclose

EP1081

Human papillomavirus genotype and E6 oncoprotein molecular test among cervical cancer patients at Center Mohammed VI for cancer treatment

Z. Lakehayli[1], K. Bendahou[2], I.K. Ahmadaye[2], A. Kaufmann[3], J. Schweizer[4], J. Saidi[3], J. Sehouli[3], A. Benider[1]
[1]Radiotherapy, Centre Mohammed VI pour le Traitement des Cancers - CHU Ibn Rochd, [2]Cancer Registry of Casablanca, Casablanca, Morocco, [3]Charite-University Medicine of Berlin, Campus Virchow Klinikum, Berlin, Germany, [4]Arbor Vita Foundation, California, CA, United States

Introduction / Background: Human Papillomavirus (HPV) DNA detection is a sensitive technique for cervical cancer screening but its specificity remains limited.
New molecular tests are available, easy to use. In our study we used The *OncoE6 test* to detect E6 Oncoprotein from cervical specimens.
The aim is to establish the HPV genotype profil in cervical cancer patients in Casablanca, and compare the results of genotyping with *the OncoE6 test* results. So we can determin the feasibility of a screening with the new HPV generation test in Morocco.
Methodology: It's cross-sectional study that was conducted at Mohammed VI Center for Cancer treatment. The study included patients newly diagnosed with cervical cancer. Patients who received previous treatment (surgery, chemotherapy, radiotherapy) were excluded.
Two samples were taken from the cervix for each patient, One sent to Berlin for multiplex Polymerase Chain Reaction (PCR) genotyping, second samples were analyzed in Casablanca by a different investigator, using the OncoE6 test.

Results: Thirty patients were included in our study. The average age was 58,65±11,83. The histopathological examination showed Squamous Cell Carcinoma in 80% of the cases and Adenocarcinoma in 20%.
The OncoE6 test was positive in the two thirds of cases. 42% of the cases tested positive to the Oncoprotein E6 HPV 16 type, and 23% were positive to the OncoE6 HPV 18 type.
Genotyping revealed the presence of 12 serotypes of High Risk HPV, the most frequent was HPV 16. In 7 cases there was more than one serotype.
For the patients who tested negative to the OncoE6 test, the genotyping identified 8 different high risk HPV apart from HPV 16 and 18.
Conclusion: High risk HPV serotypes found in our patients apart from 16 and 18 suggest that the test must be adapted to our context before using it for screening.
Disclosure: Nothing to disclose

EP1082

Study on the knowledge of the human papilloma virus: survey of 538 adult women

A. Lubrano, L. Gutierrez, M.A. Nieto, C. Molo, E. Cortes
Gynecology and Obstetrics, Complejo Hospitalario Universitario Materno Infantil de Canarias, Las Palmas, Spain

Introduction / Background: The human papillomavirus (HPV) is one of the most important sexually transmitted infections in the world due to its health implications. Systematic vaccination against HPV is currently considered the most effective intervention for the control of infection and prevention of the disease burden associated with HPV. The aims of this study were to estimate the knowledge about HPV infection and the prevalence of adult women who received a vaccine to prevent HPV in our population.
Methodology: A transversal descriptive design was carried out in adult women between 18 and 60 years old from the island of Gran Canaria (Spain) through a questionnaire with 10 questions about HPV and vaccines. 538 women participated anonymously and voluntarily.
Results: The average age was 40 years. The 89.9% had heard about HPV. The 10.1% say they do not know anything about HPV. The 92.6% mentioned that the main route of transmission is through sexual contact. The 76.2% think that HPV has a cure. 10.5% reported having had HPV infection at some point in their lives. The main means of information are through a health professional (31.4%), through friendships or family (29.1%) and through television (18.2%). 68.4% were aware of the existence of HPV vaccines, however only 9.5% reported having been vaccinated.
Conclusion: Despite the dissemination of information, there is still part of the population that has not heard about HPV or that lack knowledge about its form of infection. Knowledge is a key point to increase vaccination rates, and health care providers play an important role in providing information on HPV prevention.
Disclosure: Nothing to disclose

EP1083

DNA methylation of L1 region of HPV 16, 18 as an approach for hrHPV-positive pregnant women triage

I. Muryzina[1], V. Lazurenko[2], N. Pasieshvili[3], O. Gradil[2]
[1]Obstetrics and Gynaecology N1, [2]Obstetrics and Gynaecology N2, Kharkov National Medical University, [3]Kharkov Regional Perinatal Center, Kharkov, Ukraine

Introduction / Background: Recognition of high-grade severe cervical intraepithelial neoplasia

(CIN2+) during pregnancy is a matter of concern in areas where pregnant women are covered with well-arranged screening, but generally cervical screening is rather opportunistic due to low awareness among population about its vital significance (like in Ukraine). According to guidelines during pregnancy CIN2+ are left untreated with next check-up when puerperal period comes to the end, unless invasive cancer is suspected. But if CIN2+ was misconstrued as low-grade lesion and a patient was not properly alerted, she may fail to come for check-up. Reliable tools for recognition CIN2+, especially those destined to progress are very important for cervical triage during pregnancy
Methodology: Study comprised 85 HPV16,18-positive pregnant women, all of them underwent colposcopy. Every cervical sample was tested for expression of p16INK4A, Ki-67, HPV L1, DNA methylation of L1 region of HPV 16 and 18. Results were collated with colposcopy conclusion verified by biopsy.
Results: Colposcopy revealed 47,1% patients with occult LSIL and 39,4% with occult HSIL (normal PAP-smear). Hypermethylation of L1 region was detected in all cases of CIN2+, being much more significant in CIN3. L1 gene methylation matches colposcopy in capacity to recognize CIN2+ (positive PV >95%) but less affected by human mistake. L1 gene methylation proved to be much more informative for distinguishing LSIL from mimics than p16INK4A. Alarming expression of p16INK4A, Ki-67 was spotted sporadically and its sensitivity for CIN2+ fell behind sensitivity of L1 gene methylation.
Conclusion: DNA methylation of L1 region of HPV 16 and 18 can improve workflow for hrHPV-positive pregnant women triage because of its high sensitivity and specificity for CIN2+. It is plausible, that CIN3 lesions destined to progress were consistent with higher level of methylation comparatively to those of indolent entity, but further study is necessary in order to confirm that assumption.
Disclosure: Nothing to disclose

EP1084

The change of opinions on HPV vaccination in Japan: a 5-year follow-up survey of obstetricians and gynecologists regarding their opinions about the HPV vaccine

Y. Nagase, S. Nakagawa, Y. Ueda, A. Yagi, M. Sawada, S. Matsuzaki, E. Kobayashi, T. Kimura
Osaka University | Obstetrics and Gynecology, Osaka University, Suita, Osaka, Japan

Introduction / Background: In Japan, the governmental recommendation for the HPV (Human papillomavirus) vaccination has been suspended since June 2013 because of Japanese media reports of so-called adverse events.
As a result, the HPV vaccination rate in Japan was almost 70% before suspension of recommendation, but has decreased to less than 0.5% in recent years.
We conducted surveys on the opinions about the HPV vaccine of obstetricians and gynecologists three times between 2014 and 2019. Here, we report the change of opinions in a 5-year follow-up survey.
Methodology: We posted our questionnaire on HPV vaccine in August 2014, January 2017, and June 2019 to 500-600 obstetricians and gynecologists in Osaka, Japan. We used the same structured format questionnaire, including questions about their personal opinions about HPV vaccination, cervical cancer screening, and the HPV vaccination status of their own daughters.
We compared results of three surveys and investigated their thoughts regarding whether the government should restart its recommendation for the vaccine and their intentions to recommend HPV vaccination to adolescence.
Results: The rate of responders with thoughts that the Japanese government should restart its HPV vaccine recommendation was increased significantly, from 61.0% in survey of 2014 to 73.6% in 2017 ($p=0.0027$). The rate of doctors intending to recommend the HPV vaccine for teenagers also increased, from 65.2% in survey of 2014 to 70.1% in 2017 ($p=0.26$). The same tendency was observed in survey of 2019.
Conclusion: Our three surveys indicated that opinions about HPV vaccination of obstetricians and gynecologists in Japan have been changed positively toward recommendation of HPV vaccination during 5 years. We must make an effort to give a correct understanding to not only obstetricians and gynecologists but all of women and healthcare providers.
Disclosure: YU and AY received a lecture fee, and YU received a research grant (J550703673), from Merck Sharp & Dohme (MSD). TK received a research fund (VT#55166) from MSD.

EP1085

Surgical margins of large loop excision of the transformation zone: histological comparison with patient characteristics and colposcopists' experience

K. Razvi, K. Rothnie, T. Speed, S. Clark
Southend University Hospital, Essex, United Kingdom

Introduction / Background: Large loop excision of the transformation zone (LLETZ) is a standard procedure for the treatment of cervical intra-epithelial neoplasia (CIN). This audit aims to assess compliance with the NHS Cervical Screening Programme (NHSCSP) at Southend University Hospital, regarding recommended minimum excision depth of 7mm and specimen removal as a single loop. The results were correlated to the experience of the colposcopist.
Methodology: Retrospective audit identifying 224 patients who underwent LLETZ in 2015 from a colposcopy database. Patient age, colposcopist experience, margin involvement, depth excised, fragmentation, and recurrence at one year analysed. Patients were allocated to groups based on years of colposcopist experience - Group A (< 5 years), Group B (5-10 years), Group C (>10 years). Results were compared to the NHSCSP guidelines.
Results: Of the 224 patients, 51.79% (n=116) aged < 35, and 19.64% (n=44) smokers. 26.34% (n=59) samples were from Group A, 51.34% (n=115) from Group B, and 22.32% (n=50) from Group C. The minimum depth of 7mm achieved in 69.33% (n=156), with the highest rate in Group C (82.00%, n=41) - this is below the NHSCSP target of 95%. NHSCSP recommends an 80% target of specimens removed as a single loop. We achieved 72.77% (n=163), with the lowest proportion in Group A (66.10%, n=39). Group C met the target at 82.00%. Recurrence rate from the test of cure cytology was 15.11% (n=34).
Conclusion: Our results are below NHSCSP standards. However, women in the younger age group may have less radical approaches to depth of excision in view of future fertility. The audit suggests years of experience affects the adequacy of LLETZ samples, with a higher rate of positive margins and specimens not removed as a single loop in Group A. The ability to obtain adequate LLETZ samples appears to be dependent on colposcopic experience.
Disclosure: Nothing to disclose

EP1086

Diagnostic accuracy of conventional cervical cytology (Papanicolau smear), liquid based cytology and visual inspection with acetic acid in detecting premalignant and malignant cervical lesions among Filipino women in a tertiary hospital

R. Rivera, J. Madera
Obstetrics and Gynecology | Section of Gynecologic Oncology, Jose R. Reyes Memorial Medical Center, Manila, Philippines

Introduction / Background: Carcinoma of the cervix remains to be the second most common malignancy and is the most common cause of cancer-related mortality among Filipino women despite being a preventable disease. Screening can reduce both the incidence and the mortality rate. However, after more than a decade of strong efforts from the national government and different medical organizations in promoting cervical cancer awareness, prevention and screening, we still see a steady rise in the number of newly diagnosed cases every year. Most of these newly diagnosed cases are in advanced stage. This study compared the diagnostic accuracy of the screening methods used in the country.
Methodology: This was a prospective cross-sectional study done in a tertiary hospital. 249 patients

were included in this study. Diagnostic accuracy of Papanicolau smear (Papsmear), liquid based cytology (LBC) and visual inspection with acetic acid (VIA) was compared, and results were confirmed by biopsy.
Results: Among the three screening methods, LBC (65%) had the highest probability to detect a premalignant or malignant cervical lesion, followed by VIA (60.5%). Papsmear (10%) which is commonly used by majority had the lowest probability in detecting premalignant or malignant lesions.
Conclusion: This study has shown that LBC is the best screening method for cervical cancer compared to VIA and papsmear. VIA is an adequate and acceptable screening method for cervical cancer. Furthermore, in low-resource areas, VIA is a better screening method than papsmear for its ease of use and lower cost. In areas where LBC is not available, it is highly recommended to do papsmear in conjunction with VIA.
Disclosure: Nothing to disclose

EP1087

HPV infection and histological abnormality of uterine cervix in relation to sexual behavior in Japanese young women

M. Sekine[1], M. Yamaguchi[1], R. Kudo[1], S. Adachi[1], Y. Ueda[2], E. Miyagi[3], M. Hara[4], J.B.H. Sharon[5], T. Enomoto[1]
[1]Obstetrics and Gynecology, Niigata University, Niigata, [2]Obstetrics and Gynecology, Osaka University, Osaka, [3]Obstetrics and Gynecology, Yokohama City University, Yokohama, [4]Preventive Medicine, Saga University, Saga, [5]Women's Health Medicine, Hokkaido University, Sapporo, Japan

Introduction / Background: In Japan, the incidence of cervical cancer and high grade cervical intraepithelial neoplasia (CIN) has been markedly increasing in young women. However, Japan has several problems about cervical cancer prevention, such as suspension of proactive recommendation of human papillomavirus (HPV) vaccination, low cervical cancer screening coverage and insufficient sex education. In this study, we aimed to investigate the correlation between sexual behavior and HPV infection and histological abnormalities of cervix in young women for a comprehensive approach to cervical cancer prevention in Japan.
Methodology: We recruited women aged 20-30, 35-36, 40-41 years attending for public cervical cancer screening at Niigata city from April 2014 to March 2016 under informed consent. Residual Pap smear specimens were collected for HPV testing. The histological diagnosis for cervical abnormalities was examined using the database of the Niigata city health center. Abnormal histology was defined as CIN2 or worse. Sexual behavior was gathered from a questionnaire.
Results: The study enrolled a total of 3968 participants and included 3231 in the analysis. Seventy-two percent of women become sexually active as a teenager. There was a correlation between lower age at first intercourse and multiple numbers of sex partners ($p< 0.01$). In the multivariate analysis, HPV infections were associated with number of sex partners. Especially, women who had 6 or more sex partners were extremely high risk of HPV16/18 infection (aOR = 74.9), however, logistic regression analysis revealed that number of sex partners was no longer a significant factor for abnormal histology. There were strong correlations between high-risk HPV infection (HPV16/18: aOR = 113.8, HPV16/18/31/33/45/52/58: aOR = 93.6) and abnormal histology.
Conclusion: We recognized that sexual activity was closely correlate with HPV infection, and the highest-risk HPV types of abnormal histology were preventable by vaccination regardless of the sexual activity.

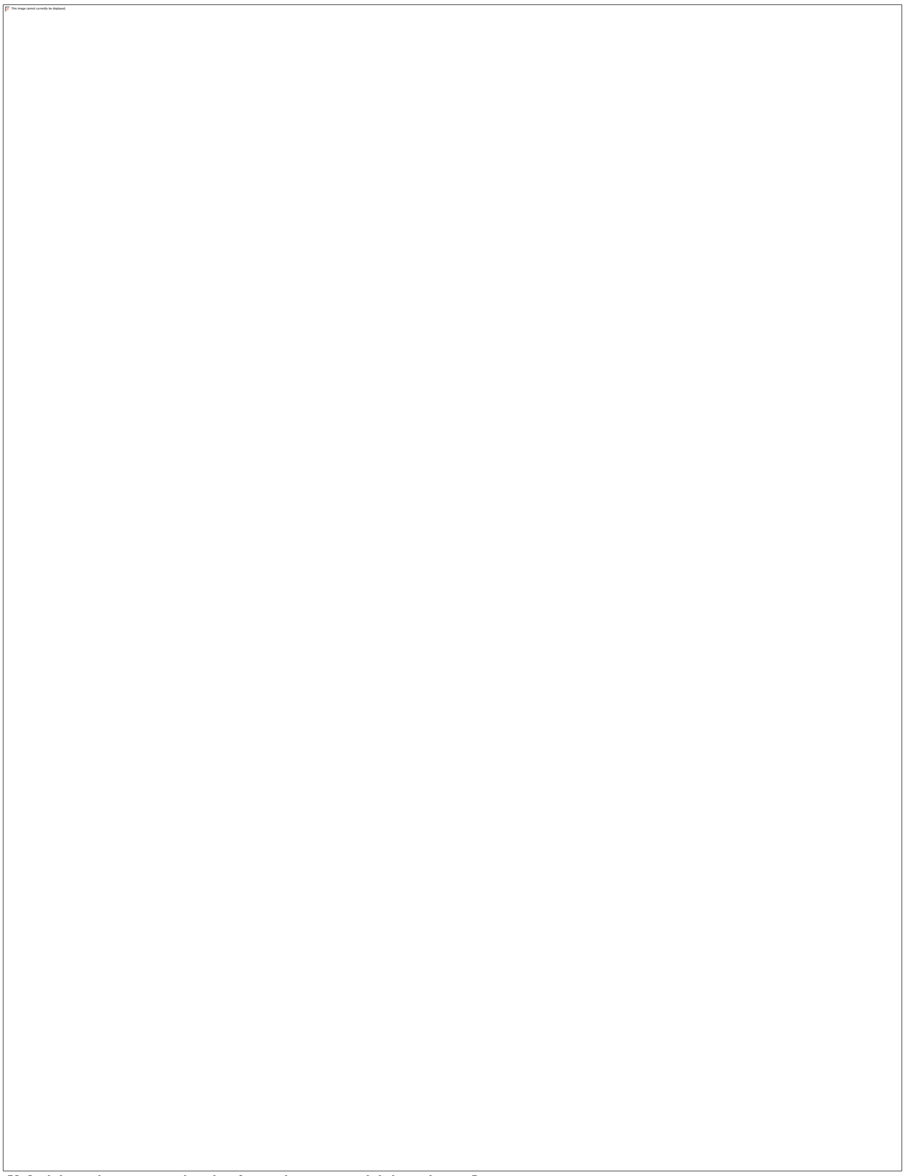

[Multivariate analysis for abnormal histology]

Disclosure: Nothing to disclose

EP1088

Pelvic leiomyomatosis (about a case and literature review)

M. Serraj, A. Assal, O. Baroud, M. Benhessou, M. Ennachit, M. El Karroumi
CHU Ibn Rochd, Casablanca, Morocco

Introduction / Background: The Pelvic Leiomyomatosis a benign disease, rare, characterized by the proliferation of various nodules in smooth muscle cells in the peritoneal cavity, which usually affects young women of childbearing age.

Methodology: We report a case of disseminated peritoneal leiomyomatosis in a woman aged of 40 years old, nulligeste and nulliparous, who had never used oral contraceptives, suffering from chronic pelvic pain with abdominal-pelvic mass, she had undergone a subtotal hysterectomy because of myomatous uterus by laparotomy way, the various radiological studies have found a parietal mass in the right lower quadrant with tissular density, a large mass in retro and sus vesicle, some peritoneal pelvic nodules and a
nodule under the skin in front of the pubic symphysis. The patient had an exploratory laparotomy with resection of the parietal mass, the histological examination showed a much limited cellular leiomyomas.
Results: The evolution was characterized four months later by a parietal recurrence and development of multiple nodules in the peritoneal cavity, the patient had a second laparotomy with resection of the graft nodules on the paries, the peritoneum and the digestive structures, histological examination showed a leiomyomatosis without malignancy.
The patient used analogues GnRH during 1 year with poor supervision of the treatment, and had been lost to sight despite repeated attempts to contact her.
Conclusion: The definite diagnosis is histological. The evolution is generally favorable. The malignant transformation in a leiomyosarcoma is rare but its prognosis is catastrophic.
When a hormonal factor is recognized, the stop of the estrogen exposures allows the regression of the disseminated peritoneal leiomyomatosis in the majority of the cases. Some cases will require more expensive management. The extreme gravity of the prognosis in the cases of sarcomatous change requires the utmost vigilance in the supervision of the patients.
Disclosure: Nothing to disclose

EP1089

The screening and deasise histories of women with invasive cervical cancer

V. Terenteva[1], R. Khasanov[1], M. Katsyuba[1], A. Akhmetzianova[2], M. Nukhnin[1], G. Fakhrutdinova[2], G. Khasanova[2]
[1]Kazan State Medical Academy, [2]Tatarstan Cancer Center, Kazan, Russian Federation

Introduction / Background: Presently, a few organizational patterns of screening against cervical cancer (CC) coexist in Russia. These include organized screening under a federal governmental program, opportunistic screening on visiting a gynecologist, obligatory annual medical checkup of working class people with inclusion of Pap-test for women and some regional programs.The objectives of this observational study were: (1) to evaluate shortcomings and faults of the screening chain (attendance to a primary screening, screening test performance, management of abnormal results of cervical cytology, follow up after treatment etc.) and a gynecologic care which lead to invasive CC development or late diagnosis in Tatarstan (Russian Federation); (2) to evaluate the effectiveness and limitations of different organizational patterns of CC screening.
Methodology: Ninety consecutive patients with newly diagnosed invasive CC in the Tatarstan Cancer Center (Kazan, Russia) were carefully interviewed on their anamnesis morbi, previous screening history, history of attendance to governmental or private gynecologic care, history of their Pap-smears and other details. Medical records from primary hospitals and Pap-smear results for five years prior to the CC diagnosis were evaluated, where possible. Histology and stage of diseases were also analyzed.
Results: Within 3 years before CC diagnosis only 14 (15.5%) patients had not visit no gynecologist no medical check-up with inclusion of gynecologic examination.
Conclusion: Non-attendance to CC screening is not a main cause of cervical cancer occurrence in Russia. Faults in the management of abnormal results of Pap-test are the common causes of CC development and late diagnosis. Organized screening offered by the federal governmental program is a best way of CC screening organization in Russia. A well organized service (within the healthcare system) aimed on managing patients with abnormal results of Pap-smear, their subsequent treatment and follow up is needed.
Disclosure: Nothing to disclose

EP1090

Use of HPV-vaccine in prevention of recurrent HSIL after LEEP in women of reproductive age

A. Vinnytska[1,2]
[1]LISOD - Israeli Oncological Hospital, Kyiv, [2]Kharkiv Medical Academy of Postgraduate Education, Kharkiv, Ukraine

Introduction / Background: Women who have been treated for high-grade squamous intraepithelial neoplasia (HSIL) face an elevated risk of recurrence and invasive cervical cancer. The likelihood of recurrence depends on the grade of dysplasia, the treatment used, persistence of high-risk human papillomavirus (HR-HPV) and the woman's age. We represent our experience in performing HPV-vaccination in previously unvaccinated women of reproductive age at the time of treatment cervical HSIL in order to prevent recurrent disease.
Methodology: In 2010-2018 we performed 106 loop electrosurgical excision procedures (LEEP) in patients of reproductive age with histological confirmation of HSIL. There are two groups - vaccination group (76), who were vaccinated with LEEP with bivalent / quadrivalent HPV-vaccine at the time of treatment, and non-vaccination group (37), which were performed LEEP and followed without vaccination. The vaccination group received the first dose at 2 month before LEEP and the remaining two doses at the same day of LEEP and four months later. Women had post-operative surveillance with cytology and colposcopy at 3, 6, 9, 12, 18 and 24 months, and yearly thereafter. Also HPV DNA tests were performed in order to determine if the persistence of HR-HPV infection is associated with the development of recurrent HSIL.
Results: In the vaccination group (76 patients), 7 patients (9.2%) developed recurrence, whereas 6 patients (16.7%) in the non-vaccination group (37 patients) developed recurrence. In patients infected with HPV of 16 and/or 18 type, 2 patients (2.6%) in the vaccination group (76 patients) and 3 patients (8.1%) in the non-vaccination group (37 patients) developed recurrent disease related to vaccine HPV types after LEEP.
Conclusion: HPV-vaccine can be used in prevention of recurrent HSIL at the time of treatment by LEEP. The persistence of HR-HPV infection is associated with the development of recurrent HSIL and can be reduced by combination of HPV-vaccination and LEEP.
Disclosure: Nothing to disclose

EP1091

Possible influencing the quality of life of patients with endometrial cancer and cervical cancer after radical treatment

M. Adamyan, T. Moiseenko, A. Menshenina, S. Tumanyan, E. Verenikina, N. Popova
Rostov Research Institute of Oncology, Rostov-on-Don, Russian Federation

Introduction / Background: Modern therapeutic possibilities allow achieving high rates of survival in patients with some oncogynecological diseases. In this regard, the problem of medical and social rehabilitation of patients who have completed radical treatment is particularly acute. The purpose of the study was to assess prospects of menopausal hormone therapy (MHT) in patients of the reproductive age with cervical (CC) and endometrial (EC) cancer.
Methodology: Clinical and laboratory data and survey findings of 736 patients of the reproductive age with CC Ib-IIb and EC I-IIa (FIGO system), STRAW scale + 10: (-4) - (-3a).
In accordance with the clinic-based algorithm of managing postovariectomy syndrome, rehabilitation of patients began 48 hours before the surgery by the appointment of deposited androgens. 3-4 weeks after surgery, combined MHT (estrogen+progesterone) or phytoestrogen therapy was prescribed upon the consent of patients: 501 patients preferred MHT, 197 patients - phytoestrogen therapy, 48 patients - symptomatic treatment. The quality of life of patients was assessed by a questionnaire survey during 5 years of the follow-up monitoring.

Results: None of the observed 736 patients had recurrences or metastases of the primary disease. MHT ensured a good quality of life in all patients. Phytoestrogens did not completely compensate for the hormonal imbalance. Symptomatic therapy did not allow control of the postovariectomy syndrome and was accompanied by the development of somatic and psycho-emotional disorders.
Conclusion: Combined MHT in CC and EC patients of reproductive age, the STRAW scale + 10: (-4) - (-3a), after radical treatment does not affect the disease prognosis and maintains the quality of life at a comfortable level.
Disclosure: Nothing to disclose

EP1092

Predictors of gas passage time interval at postoperative period in gynecologic oncologic surgery: is drainage necessary?

M. Api[1], S. Akiş[1], Y.E. Purut[1], E. Keleş[1], Y. Ceylan[2], C. Kabaca Kocakuşak[1], M. Güray Uzun[1], B. Giray[1]
[1]Gynecologic Oncology, Zeynep Kamil Training and Research Hospital, [2]Medicine, Istanbul Medipol University, Istanbul, Turkey

Introduction / Background: Drain placement remains controversial and there has been nearly no prospective studies evaluating the effectiveness on the gynecologic oncology cases. While some surgeons put on drains most of the cases after surgery, the literature search on general surgery and gynecologic cases suggest that the benefits are questionable. The aim is to assess the effectiveness of drain placement in patients who have underwent gynecologic oncology surgery for benign or malign diseases.
Methodology: Quasi randomized 144 patients were divided into two groups; 75 of them were placed drainage (D) and 89 of them without drainage (WD).The subsequent parameters; operation time, BMI and age were recorded and mobilization time, oral intake and gas discharge after surgery were evaluated.
Results: Mean ± SD operation time (136.2 ± 50.84 vs 123.64 ± 45.48, minutes p= 0.126), age (45.56 ± 12.82 vs 45.91 ± 14.28 years, p= 0.876), BMI (27.23 ± 6.37 vs 27.98 ± 4.93 kg/m2, p= 0.436) and oral intake after the operation (9.88 ± 12.03 vs 7.17 ± 1.96 hours, p= 0.058), was not statistically significant between D group and WD group respectively. Mean ± SD gas discharge time after the operation was 20.92 ± 14.74 and 16.86 ± 8.62 hours in D group and WD group respectively (p= 0.048). Mobilization time (7.08 ± 1.56 vs 6.09 ± 1.54 hour, p<0.001) was statistically significant and differed between D and WD groups respectively. There was no drain related complications or re-operations reported.
Conclusion: There was obvious advantage of not using drain in gynecologic oncology patients in terms of gas discharge time and mobilization time without compromising morbidity.
Disclosure: Nothing to disclose

EP1093

Comparison of the effects of vaginal honey jelly with vaginal lubricant gel on atrophic vaginitis symptoms and quality of life in post-menopausal women with endometrial cancer

H. Ayatollahi, Z. Yekta, S. Mirzaie
Urmia University of Medical Sciences, Uromieh, Islamic Republic of Iran

Introduction / Background: General health and welfare of middle-aged women has become a major public health concern around the world. More than 80% of women experience physical or mental disorder symptoms during their menopausal years, which decrease the quality of their life.

To compare to the effect of vaginal honey jelly with vaginal lubricant Gel as an alternative treatment for vaginal atrophy and increasing the quality of life in post-menopausal women with endometrial cancer who have limitation for estrogen use.
Methodology: This is a clinical trial study on 62 women with endometrial cancer referred to the Kosar Hospital in orumiyeh, Iran due to post-menopausal symptoms. The data were collected using a Vulvovaginal Symptom and a Quality of Life Questionnaire (VSQ) and MENQOL questionnaire. At the beginning of the study, patients were taught how to use vaginal lubricant and honey gel. The intervention period was 2 months and the collected data were analyzed using SPSS23 software.
Results: The mean age in the study group was 55.09 ± 2.25 years and 59.67 ± 1.59 years in the control group. The mean of atrophic vaginitis score before the intervention was 4.58 ± 0.83 in the study group and 2.16 ± 0.55 in the control group. (P = 0.02), that after using the medication changed to 3.45 ± 0.62 in study group and 1.96 ± 0.47 in the control group (P = 0.07). Mean score of quality of life before intervention in the study group and control group was 29.64 ± 3.93 and 21.29 ± 3.83 respectively (P = 0.24), which decreased to 26.35 ± 3.71 and 17.09 ± 3.43.
Conclusion: The present study showed that vaginal honey jelly can be an appropriate alternative for vaginal atrophy, sexual problems and improving the quality of life in postmenopausal women with endometrial cancer who cannot use estrogen and other hormonal medications.
Disclosure: Nothing to disclose

EP1094

Quality of life assessment in gynae-onco survivors

A. Bahadur, N. Vdovichenko, R. Mundhra, L. Chawla, A. Yadav, P. Kumari, S. Kumari, N. Bhattacharya, J. Kashibhatla, D. Das, S. Prateek, J. Chaturvedi
All India Institute of Medical Sciences Rishikesh, Rishikesh, India

Introduction / Background: Quality of life (QOL) is of vital concern to cancer survivors, their families and care providers. Patients' go through emotional distress, altered life priorities, & fear of unknown. Primary objective of this study was to compare quality of life of disease-free patients after therapy for gynecologic malignancies. We assessed difference between emotional, physical, spiritual, social/family, functional well-being, financial burden, treatment satisfaction with QOL score according to FACIT.
Methodology: This cross sectional study was conducted in Gynaeoncology OPD at AIIMS, Rishikesh over a period of ten months from March 2018 to December 2018. Participants completed self-report assessments of psychosocial, spiritual, and physical well-being, including social support, optimism, hopelessness, depressive and anxious symptoms. Patients were asked to consider how they felt in the previous 7 days.
Results: 60 patients with a mean age of 44.3 years (32-69) were evaluated. Among malignant we found of 52.3 % had endometrial cancer, 14.2 % each had ovarian, cervical and vulval cancer and 5 % breast cancer. 70% patients did not go to hospital despite being symptomatic. 45% were not aware that these symptoms needed evaluation, 25% stated hospital was far away, 20% had financial constraints, 10% Scared of cancer diagnosis. The mean FACIT score in malignant group was 39.5 (32.5-58.6), lowest score observed in cervical cancer. Higher levels of optimism were significantly associated with fewer anxious and depressive symptoms, less hopelessness, and better QOL. Higher levels of perceived social support were also significantly associated with better QOL
Conclusion: Optimism and social support serve as protective factors against distress in gynae malignancy survivors. QOL of cancer survivors requires comprehensive care & is best described from patients' perspective. Future clinical research must focus on interventions that attend to patients´ expectations for positive experiences and in expansion of social support.

Disclosure: Nothing to disclose

EP1095

Timing of urinary catheter removal after radical hysterectomy. A single institution study

J. Billod
Baguio General Hospital and Medical Center, Baguio City, Philippines

Introduction / Background: Radical hysterectomy remains to be the primary surgery for selected early stage cervical and endometrial cancer. Functional disorders of the lower urinary tract are the most common complications following radical surgery necessitating catheterization. This study was undertaken to evaluate the average day of removal of urinary catheter after radical hysterectomy and assess the practicability of earlier removal of catheter without compromising the bladder function.
Methodology: A descriptive retrospective study of patients who underwent radical hysterectomy for cervical or endometrial cancer. Medical , intraoperative and anesthesia records and results of the histopathologic diagnosis of each patient were reviewed. Demographic, clinical and histopathologic data needed in this review were recorded.
Results: Between January 2016 and July 2018, a total of 26 patients underwent radical hysterectomy (25 patients for cervical cancer and 1 for endometrial carcinoma), with a median age of 51. Among patients with cervical cancer, 1 patient is stage IA2, 16 patients are stage IB1, 3 patients with stage IIA1, and 5 patients had central tumor recurrence after primary concurrent radiation plus brachytherapy. The average operative time is 2.48 hours and the average blood loss is 500 ml. No morbidities reported related to the surgery. The average size of cervix, length of vagina and lateral width of parametria were 3.0 cm , 2.2 cm and 4.2 cm, respectively. There was no parametrial metastasis. One patient had pelvic node metastasis. Catheters were removed between 3rdand 10thpostoperative day (median 4 days). All patients had adequate (more than 100 ml) spontaneous void within 6 hours after removal.
Conclusion: The primary outcome supports the hypothesis that an earlier removal of catheter seems to be a safe and practical option compared to long-term catheterization for patients undergoing radical hysterectomy without causing morbidities thus providing better quality of life after a radical surgery.
Disclosure: Nothing to disclose

EP1096

Implementing enhanced recovery after surgery (ERAS) pathways in major gynecologic oncology operations in Greece (The pre-eliminary results of our department)

M. Bourazani[1], E. Karopoulou[2], N. Fyrfiris[1], S. Poulopoulou[1], G. Fasoi[3], M. Kelesi[3], D. Papatheodorou[2]
[1]Department of Anesthesiology, [2]Department of Gynecology, 'Agios Savvas' General Anti-Cancer Hospital, [3]Department of Nursing, University of West Attica, Athens, Greece

Introduction / Background: The Enhanced Recovery After Surgery (ERAS) program includes preoperation counseling, avoidance of fasting, nonopioid analgesia, fluid balance, normothermia and early mobilization. ERAS pathways were developed to reduce hospital length of stay, reduce costs and decrease perioperative opioid requirements.
Methodology: Patients were randomly allocated in two groups: An Eras pathway group including preoperative counseling, early feeding/mobilization, and opioid-sparing multimodal analgesia; and a classic model group of post operative recovery as control.
Results: Sixty-one patients were recruited in our study who underwent gynecologic oncolgy surgery (35 in the ERAS group and 26 in the control group) with mean age 52.9 years (SD=11.7). Hospital stay was significantly longer in the control group as compared to the ERAS group (5.1±2.4 days vs. 3.1±1.3 days, p=0.001). Pain levels according to Numeric Rating Scale (NRS) and Present Pain Intensity (PPI) scale were higher in the control group at all-time points except from baseline. Pain according to NRS at rest was significantly decreased from 24h to 48h and from 48h to 72h (p< 0.001) in the control group; while in the ERAS group there was a continual and significant reduction from 6h

to 72h (p< 0.05). Additionally, pain according to (NRS) at movement was found to be significantly increased from 45min to 2h (p=0.045) in the control group and afterwards remained stable until 24h where a significant decrease was recorded continuously until 72h. NRS at movement in the ERAS group showed a significant and continual decrease from 6h to 72h (p< 0.001). Pain according to PPI scale was significantly decreased from 24h to 48h (p< 0.001) and from 48h to 72h (p=0.011) in the control group.
Conclusion: Implementation of ERAS pathways was associated with reduction in length of stay, lower pain-scores levels according to NRS and PPI scale (both at rest and at movement), and generally better surgical.
Disclosure: Nothing to disclose

EP1097

Quality of life and sexuality in breast and gynecological cancers, a pilot study

R. Bracci[1], T. Campanelli[1], F. Labianca[2], E. Prospero[3], R. Mattioli[1]
[1]Oncology Unit , Fano, Azienda Ospedaliera Marche Nord, Fano, [2]Scienze Infermieristiche, Università Politecnica delle Marche, [3]Igiene, Università Politecnica dell Marche, Ancona, Italy

Introduction / Background: Quality of life women undergoing therapies for breast and gynecological cancers can be decreased in many dimensions. Sexuality is one othe issues, since alterations of body image, symptoms due to chemo-radiotherapy, psychological and emotional problems with partners. It is very difficul for medical and nursing staff to investigate sexual problems and give support.
The aim of this study was to explore sexual problems and quality of life (QOL) in a sample of women with breast and gynecological cancer soon after or during treatment.
Methodology: From February 2018 to March 2019, we enrolled 132 patients, 105 accepted and 27 refused. We administered to patients QOL questionnaires EORTC-QLQ-C30 with the appendix BR23 for breast, CX24 for cervical; OV28 for ovarian cancer, together with FSFI (Female Sex Function test) , a validated questionnaire that investigates sexual disfunction. Women were given detailed explanation and reassurance about their privacy. We excluded patients >69 years old. Mean age was of 56.4.
Results: *Breast cancer:* 68 women completed the questionnaires 37 (54%) had no or minimal sexual disfunction, in 31(46%) was serious or severe. In the last month 37% had sexual activity and 39% reported it positively. Sexual activity correlated with QOL global score.
Cervical cancer: 22 women completed the questionnaires 9(41%) had no or minimal sexual disfunction, in 13(59%) was serious or severe. 78% had sexual activity but 52% reported sexual experience as unsatisfactory. QOL improvement was correlated to improvement in sexual activity, athough not so strictly as in breast cancer.
Ovarian cancer: 15 completed the questtionnaires, (33%) had no or minimal sexual disfunction, in 10 (66%) was serious or severe. 55% had sexual activity. QOL was non so strictly correlated to improvement in sexual activity.
Conclusion: Most women have serious/severe sexual disfunction during therapy although mantainig interest for sex. Ovarian cancer patients had more problems
Disclosure: Nothing to disclose

EP1098

Sexual health in women with vaginal atrophy. Results of a non-hormonal vaginal gel in women with severe dyspareunia

J.L. Delgado Marín[1], J. Estévez Tesouro[2], J. Suárez Almarza[3], C. Nieto Magro[3]
[1]Hospital Virgen Arrixaca, Murcia, [2]Hospital de Marqués de Valdecilla, Santander, [3]Italfarmaco, Alcobendas, Spain

Introduction / Background: Sexual dysfunction is prevalent among cancer survivors as a result of surgery, radiation, and chemotherapy. Severe dyspareunia due to the atrophy of vaginal tissues can cause a negative impact in women's sexual activity, and hence their quality of life. Vaginal moisturizers used two or three times a week are considered first line treatment to reduce symptoms of vulvovaginal atrophy, help to moisturize the vaginal lining and facilitate the flexibility of the vulvar and vaginal tissues. The effect of a mucoadhesive vaginal gel in women with severe dyspareunia was evaluated.
Methodology: 29 women with severe dyspareunia received 1g of a mucoadhesive moisturizing vaginal gel daily for 3 weeks (3w) and twice weekly up to 12 weeks (12w). Change in the symptom intensity (graded as 0=absent, 1=mild, 2=moderate, 3=severe) was evaluated at w3 and w12.
Results: After 3w of treatment, 28% of women improved from severe to moderate intensity, 24% to mild intensity and 17% did not present dyspareunia anymore (41% improved the symptom to mild or absent). After 12w, 21% of women presented moderate intensity, 24% had mild intensity and dyspareunia was absent in 35% of patients (59% improved the symptom to mild or absent). Improvement vs baseline was significant both at w3 and w12 *(p< 0.001, Mc Nemar test).*
Conclusion: These results confirm that vaginal moisturizers should be considered in the management of vaginal symptoms, even when these symptoms are severe. An early effect has been demonstrated after only 3 weeks of daily use and continued ameliorating until the end of treatment with a twice weekly regime. The use of vaginal moisturizers is a therapeutic alternative for women who prefer not to use hormonal products. Physicians should consider women´s preferences regarding their treatment by asking about sexual health issues in the clinical practice routine.
Disclosure: These clinical trials were funded by Italfarmaco S.A. Dr Estévez and Dr Delgado participated as investigators in the clinical trials carried out with this product (EudraCT Numbers: 2007-003399-20 and 2014-004517-84). J. Suárez and Dr Nieto are full time employees of Italfarmaco S.A.

EP1099

Intravesical sodium hyaluronate for treatment of cystitis & bladder pain in women with pelvic malignancies

A. DeMaio, N. Gleeson
Department of Gynaecology Oncology, St James's Hospital, Dublin, Ireland

Introduction / Background: Radiotherapy is commonly indicated in the treatment of gynaecological malignancies. Pelvic radiation can lead to a condition known as radiation cystitis in at least 5% of women cancer survivors. Treatment of gynaecological malignancies with radiotherapy delivers high-energy radiation to the pelvis, which can include the bladder epithelium and/or vasculature, and the detrusor muscle. This in turn can have long-term sequelae of incontinence, obstruction, pain, haematuria, ulceration, and fistulation.
Sodium hyaluronate (Cystistat®) bladder instillations have been established as a treatment option for bladder pain syndrome and recurrent urinary tract infections. There has been preliminary data to suggest its usefulness in radiation cystitis in men treated for prostate cancer. However, data is lacking in the cohort of women with radiation cystitis following treatment for gynaecological malignancy.
Methodology: This was a retrospective review of patients attending a tertiary referral centre for gynaecology oncology over a 5-year period. Following cystoscopic assessment of bladder, patients received a standard regimen of sodium hyaluronate bladder instillations of weekly for 6 weeks, followed by 2 treatments at 6-weekly intervals.
Results: Seventeen (17) patients were reviewed. Eleven patients were labelled as radiation cystitis, two patients had bladder pain syndrome following surgery alone for gynaecological malignancy, and four patients had bladder pain syndrome but no history of malignancy.
Of the patients with pelvic malignancy, 84% (11/13) reported improvement in symptoms following treatment and 76% (10/13) reported significant improvement. Of the patients treated with pelvic radiotherapy, 81% (9/11) reported significant improvement. All patients without history of malignancy (4/4) reported some improvement, and of these 75% (3/4) reported significant change.
Conclusion: Bladder instillation of sodium hyaluronate was effective in reducing the symptoms of radiation cystitis in women treated for gynaecological malignancies.

Disclosure: Nothing to disclose

EP1100

Body Mass Index and sexual function in women with gynaecological cancer

H. Donkers[1], A. Smits[1], A. Eleuteri[2], R. Bekkers[3], L. Massuger[3], K. Galaal[1]
[1]Royal Cornwall Hospital Trust, Truro, [2]Royal Liverpool and Broadgreen University Hospitals, Liverpool, United Kingdom, [3]Radboud UMC Nijmegen, Nijmegen, The Netherlands

Introduction / Background: Obesity negatively impacts the quality of life of gynaecological cancer patients. Sexual function is an important part of woman's quality of life, however the association between Body Mass Index (BMI) and sexual functioning in gynaecological cancer patients is unclear. In addition the relationship between socioeconomic deprivation, which is linked to obesity, and sexual function is unclear.
Methodology: This is a prospective cohort study on women undergoing surgery for suspected or proven gynaecological cancer between September 2014 and February 2016 in the Royal Cornwall Hospital Trust. Patients were invited to participate by completing the Female Sexual Function Index (FSFI) at three time points: pre-operative, three months post-operative and one-year post-operative. A semi-parametric model of the FSFI score was used to establish the association between BMI and sexual functioning.
Results: A total of 257 patients were approached of which 166 patients were included. 52 patients (33.8%) were overweight (BMI 25-29.9 kg/m^2), 44 (28.6%) were obese (BMI 30-39.9 kg/m^2) and a further 20 (13.0%) morbidly obese (BMI ≥ 40 kg/m^2). Overweight and obese women reported improved sexual functioning compared to normal-weight women in endometrial, ovarian and vulvar cancer. Among cervical cancer, worse sexual functioning was seen in women with an increased BMI, however this was not significant. Younger age was associated with improved sexual function and sexual functioning was better postoperatively for all patients compared to preoperatively. There was no evidence of relationship between deprivation and sexual functioning in gynaecological cancer patients.
Conclusion: Higher BMI is associated with improved sexual functioning in endometrial, ovarian and vulvar cancer**,** however this was not seen in cervical cancer patients. There is no evidence of correlation between deprivation and sexual functioning.
Disclosure: Nothing to disclose

EP1101

Efffect of a non-hormonal mucoadhesive polycarbophilic vaginal gel on prevalent vaginal symptoms in women who develop vaginal atrophy

J. Estevez Tesouro[1], J.L. Delgado Marin[2], M. Palma Santisteban[3], C. Nieto Magro[3]
[1]O&G, Hospital de Marqués de Valdecilla, Santander, [2]O&G, Hospital Virgen Arrixaca, Murcia, [3]Med Dept Italfarmaco, Madrid, Spain

Introduction / Background: Gynecological and breast cancer treatments can cause early ovarian failure with vulvovaginal atrophy (VVA) in the medium-long term. Also, external radiotherapy or brachytherapy for cervix, endometrium or digestive cancer has a potential toxicity risk to develop VVA, loss of elasticity and vaginal stenosis. These changes alter vaginal and sexual health in up to 90% patients. Vaginal moisturizers are recommended periodically in the prevention and treatment of these disorders. Efficacy of a non-hormonal mucoadhesive vaginal gel on symptoms of VVA is evaluated.
Methodology: 62 women presenting bothering symptoms of VVA (98% with moderate or severe symptoms) received 1g of a mucoadhesive moisturizing vaginal gel daily for 3 weeks (w3) and twice weekly up to 12 weeks (w12). Vaginal dryness (VD), dyspareunia and pruritus were registered at

baseline (B), w3 and w12 and symptom intensity was individually scored as 0=absent, 1=mild, 2=moderate, 3=severe. Symptom variation was evaluated as response at w3 and w12 vs B. Patients who improved (change in symptom intensity) and who turned symptom-absent were evaluated.
Results: All symptoms improved significantly at w3 and w12. VD improved in 71% and 69% of women at w3 *(p=0.01)* and w12 *(p=0.02)* respectively. Dyspareunia improved in 62% (w3, *p< 0.001*) and 67% (w12, *p< 0.001*) of women presenting this symptom, and pruritus improved in 34% (w3, *p=0.03*) and 42% (w12, *p=0.008*). VD, dyspareunia and pruritus had disappeared in 13%, 22% and 29% of women at w3, and in 21%, 38% and 34% at w12, respectively.
Conclusion: Discussing vaginal health concerns and treatment options should be considered in the oncologist's regular clinical practice. Management of vulvovaginal symptoms can impact significantly on cancer survivors' quality of life as these symptoms are very prevalent and disturbing. The regular use of this mucoadhesive vaginal gel has proved to be efficacious on this purpose.
Disclosure: These clinical trials were funded by Italfarmaco S.A. Dr Estévez and Dr Delgado participated as investigators in the clinical trials carried out with this product (EudraCT Numbers: 2007-003399-20 and 2014-004517-84). M.Palma and Dr Nieto are full time employees of Italfarmaco S.A.

EP1102

Post-therapeutic impact of gynecologic cancer on urinary symptoms, psychosexual health and quality of life

S. Gayete Lafuente, L. Pérez, E. Miralpeix Rovira, A. Salvadó Salvadó, M. Farré, M. González Comadran, M. Castells, J.M. Sole-Sedeno, G. Mancebo Moreno
Hospital del Mar, Barcelona, Spain

Introduction / Background: The survival rates improvement of oncogynecologic surgeries and adjuvant therapies has led to focus on their long-term effects. Lower tract urinary symptoms (LTUS), sexual dysfunction (SD) and impaired quality of life (QoL) have been described as common complications in survivors. Disruption of paracervical autonomic fibers during hysterectomy and radiation cystitis could be main LTUS causes; while psycho-affective deterioration may play great role on SD.
Our aim was to assess changes on these aspects after oncogynecological radical treatment.
Methodology: This prospective cohort study included oncogynecological patients (endometrial, cervical and ovarian) undergoing radical-intent treatment in our unit, March2017-June2018, classified according the received therapy. ERAS protocol (Enhanced Recovery after Surgery) was performed. Baseline features as age, surgical-obstetric history, comorbidities, emotional traits and relationship stability were considered. All patients were surveyed before and 6 months after treatment, using validated questionnaires: ICIQ-UI-SF (International-Consultation-Incontinence-Questionnaire) evaluating LTUS impact on QoL, CACV (“Cuestionario-de-Autoevaluación-de-Control-Vesical”) for detrusor dysfunction, ISFS (Index-of-Feminine-Sexual-Function) measuring SD risk, and SF-12 providing QoL profile.
Results: 67 patients were finally included, without finding differences in baseline characteristics among the 46 ones completing both surveys and analysed. Pre-treatment, 50.7% patients reported urinary incontinence (UI), 74.6% no sexual activity and all high QoL scores. In the follow-up, an UI worsening tendency with advanced age was found independently of treatment modality, not reaching statistical significance. Mental QoL score in SF-12 reduced ~2.5 points if undergoing radiotherapy. SD did not differ from pre-treatment status.
Conclusion: Urinary and sexual function, as global QoL, remained stable 6 months after oncogynecological treatment. Although LTUS is difficult to assess being common in gynecological patients baseline, special attention is recommended in older women to avoid UI worsening. Those receiving radiotherapy may benefit from mental QoL support.
Further research with larger populations is needed to accurately assess the impact of radical oncogynecological treatments; probably leading to develop patient-targeted recovery programs.
Disclosure: The authors declare no conflicts of interest. None received grant support for this study neither for other clinical or research activity, except for the inscription to the current congress for 3 authors that was funded by VIFOR Pharma (non related to the study we present).

EP1103

Can fibrin sealant agents applied after lymph node dissection in patients with gynecological malignancies prevent lymphocele formation? A systematic review and meta-analysis of randomized controlled trials

A. Prodromidou, C. Iavazzo, A. Fotiou, V. Psomiadou, M. Drakou, G. Vorgias, N. Kalinoglou
Department of Gynecological Oncology, Metaxa Memorial Cancer Hospital, Piraeus, Greece

Introduction / Background: Pelvic and para-aortic lymphadenectomy has been considered a standard procedure in encountering and treating patients with various gynecological malignancies. On the other hand, formation of lymphoceles are considered the most common early or late lymphadenectomy-related complications. The present study aimed to evaluate the safety and efficacy of application of fibrin-collagen sealants on lymphadenectomy areas in the formation of lymphocele after lymphadenectomy in patients with gynecological malignancies.
Methodology: A systematic search of 6 electronic databases for articles published up to April 2019, along with the reference lists of articles which were retrieved in full text, was performed. All randomised controlled clinical trials (RCTs) which evaluated the effect of fibrin sealant patches in the prevention of lymphocele, were considered eligible for inclusion in the present meta-analysis. Statistical meta-analysis was performed using the RevMan 5.3 software.
Results: Six RCTs with a total of 481 patients were finally recruited. Among them, 178 patients were treated with fibrin sealant while the remaining 192 women underwent lymphadenectomy without application of fibrin sealant. A significant decline was observed either in the total amount of fluid which was postoperatively drained or in mean duration of drainage in fibrin sealant group, compared to control, (187 women MD -86.40 ml 95% CI -100.2 to -72.60 $p < 0.00001$ and 113 patients MD -1.00 days 95% CI -1.13 to -0,87 $p < 0.00001$, respectively). On the other hand, the incidence of lymphoceles, overall and symptomatic ones, did not differ among the two groups ($p = 0.08$, and $p = 0.22$, respectively).
Conclusion: According to the findings of the present meta-analysis, the application of fibrin sealants in pelvic and/or para-aortic areas after lymphadenectomy due to gynecologic cancer is safe and efficient. However, its benefit still remains elusive. In that setting, despite associated with significant decrease in the duration and volume of drainage, it was not proved efficient in minimising the incidence of lymphocele.
Disclosure: Nothing to disclose

EP1104

Lower extremity lymphedema in gynecologic cancer patients: propensity score matching analysis of external beam radiation versus brachytherapy

H.-C. Kang
Radiation Oncology, Seoul National University Hospital, Seoul, Republic of Korea

Introduction / Background: To compare the risk of lower extremity lymphedema (LEL) between pelvic external beam radiation therapy (EBRT) and brachytherapy and to identify risk factors for LEL in gynecologic cancer patients treated with adjuvant radiation therapy (RT).
Methodology: International Federation of Gynecology and Obstetrics (FIGO) stage I-III gynecologic cancer patients who underwent adjuvant RT between January 2013 and December 2017 were retrospectively reviewed. Patients were diagnosed as LEL when the difference in leg circumference was 2 cm or more. One-to-one case-matched analysis was conducted with propensity scores generated from patient, tumor and treatment characteristics. Using the risk factors found in this study,

high- and low-risk groups for LEL were identified.
Results: With a median follow-up of 30.6 months, 34 (13.5%) patients developed LEL. In the multivariate analysis, laparoscopic surgery (hazard ratio [HR], 2.548; p=0.025), harvesting more than 30 pelvic lymph nodes (HR, 2.167; p=0.037), and para-aortic lymph node dissection (PALND, HR, 2.537; p=0.009) were identified as independent risk factors for LEL. After propensity score matching, the EBRT group showed a significantly higher LEL rate than the brachytherapy group (3-year LEL rate: 30.3% vs 14.3%, p=0.034). The three-year LEL rate was 3.5 fold higher in the high-risk patient group (34.3% vs 9.8%).
Conclusion: Laparoscopic surgery, harvesting more than 30 pelvic lymph nodes, and PALND were risk factors for LEL in gynecologic cancer patients. Compared with brachytherapy, adjuvant pelvic EBRT was also associated with an increased risk of LEL. High-risk group patients with these risk factors require a closer observation for LEL.

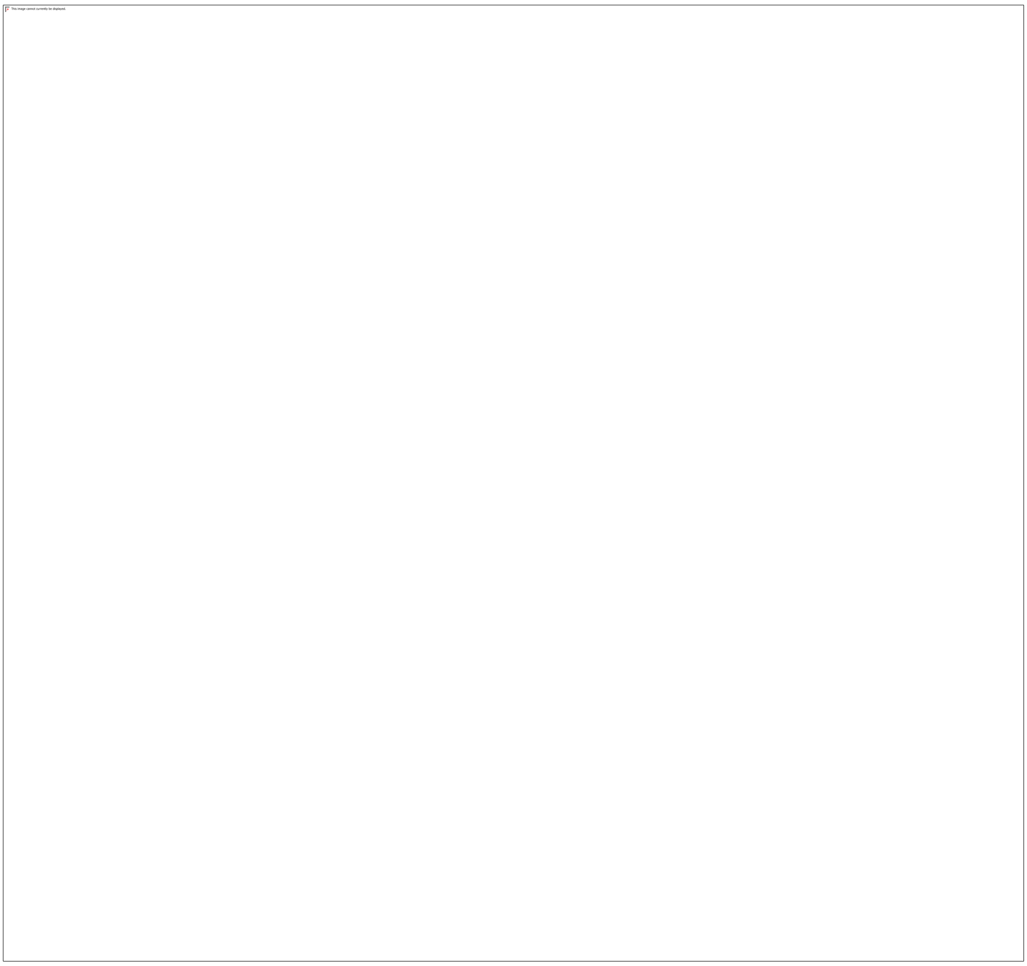

[Cumulative incidence of lower extremity lymphedema (LEL) in the matched cohort according to modality]

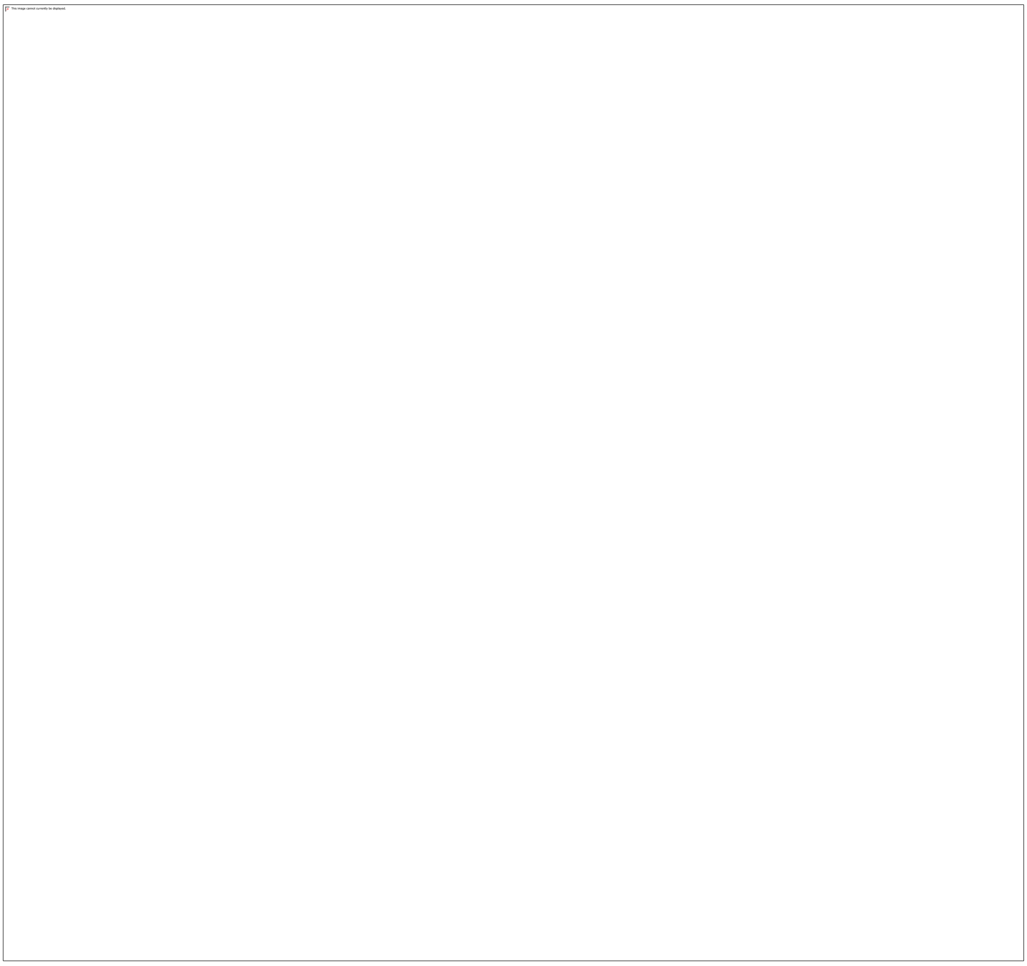

[Cumulative incidence of lower extremity lymphedema (LEL) according to risk groups]

Disclosure: Nothing to disclose

EP1105

The role of stress, age and adjuvant therapy in cognition of patients with breast cancer: a systematic review

A. Koumarianou, A. Papanastasiou, K. Kampoli, E. Rizos, A. Ntavatzikos, T. Seliniotaki, N. Arkadopoulos, C. Tsionou
Attikon University Hospital, Hematology Oncology Unit, Fourth Department of Internal Medicine, National and Kapodistrian University of Athens, Athens, Greece

Introduction / Background: Based largely on data from retrospective studies it has been postulated that chemotherapy has an aggravating effect on the cognitive function of breast cancer patients. Potential individual factors related to the effect of chemotherapy on cognitive function have been indicated, such as age-related cognitive dysfunction and stress. Elderly patients differ from non-elderly for higher cognitive related comorbidities, such as dementia, as well as for lower stress levels, indicating that chemobrain may affect differentially these two age groups. The aim of this review is to investigate the effect of stress and chemotherapy on the cognitive dysfunction and identify any

potential age-related differences in breast cancer patients treated with adjuvant chemotherapy.
Methodology: A systematic review of the literature was carried out on Medline, Scopus and Web of Science. The inclusion criteria were original articles published in peer-reviewed journals, elderly and non-elderly patients with breast cancer, reporting on stress and at least one cognitive parameter pre- and/or post-treatment.
Results: Eight studies met the preset criteria and were further analyzed. 1253 women participated, of whom 800 breast cancer patients treated with surgery only, systemic treatment only, or both. Although all studies included a non-elderly breast cancer patient subpopulation, just two of the studies included patients over 65 years of age. All studies indicated a statistically significant association of stress with various domains of cognitive dysfunction in patients compared to controls, as shown by either self-completed questionnaires, neuropsychological testing or both. Age over 60 was linked to fewer cognitive difficulties mediated by lower levels of stress.
Conclusion: The evidence supports the association of stress with cognitive deficits in breast cancer patients, regardless of the type of cancer-related treatment. Therefore, stress should be addressed appropriately. Further research is needed to investigate the correlation of stress with cognitive function in elderly patients with breast cancer.
Disclosure: Nothing to disclose

EP1106

Evaluation of a late gastrointestinal effects of radiotherapy clinical nurse specialist attending a gynaecological oncology follow-up clinic

N. Malik[1], L. Hanna[2], L. Harris[2], E. Hudson[2], S. Burton[2], M. Maurice[2], J. Green[3], J. Turner[3], H. Ludlow[3]
[1]Cardiff University, [2]Gynaeoncology, Velindre Cancer Centre, [3]Gastroenterology, Cardiff and Vale UHB, Cardiff, United Kingdom

Introduction / Background: Patients who receive pelvic radiotherapy may develop gastrointestinal (GI) symptoms which impact on their quality of life (QoL). A GI Specialist Nurse (CNS) has attended our gynaecological follow up clinic since January 2018. This is a new service in addition to a Late GI Effects Clinic based in the local Gastroenterology Department.
Methodology: From January 2018 to March 2019, 185 patients had received pelvic radiotherapy. 35% (65) of patients with GI symptoms were identified using the ALERT-B (Taylor et al 2016), a quick and simple screening tool. Those who answered yes to any question were seen by the GI CNS and investigated using the “Guidance: The Practical Management of Gastrointestinal Symptoms of Pelvic Radiation Disease” (Andreyev et al 2014).
Results: Some of the 65 patients had also previously been investigated in the main Late Effects Clinic. The most common findings are shown in Table 1 below. Many patients reported more than one symptom.
Table 1

**Symptom**	**Prevalence**
Diarrhoea	45% (29)
Abdominal pain	34% (22),
Urgency	31% (20),
Faecal incontinence	29% (19)

Table 2 below shows the most common abnormal investigation results.

**Investigation**	**Number of abnormal findings**
Colonoscopy/CT	38% (25) proctopathy, colitis, diverticular
Blood tests	Haematology 63% (41) low Vit B12, FeDef anaemia

Biochemistry: 66% (43) low Vit D
Fructose breath test
8% (5)
SeHCAT scan
9% (6) Bile acid malabsorption
It was also identified that 22% (14) of patients required specialist dietary advice including certain carbohydrate avoidance, low fat and/or a low FODMAP diet.
Conclusion: Late GI effects are not uncommon following pelvic radiotherapy and can have a significant impact on the patients QoL. A GI Late Effects CNS in a gynaeoncology follow-up clinic quickly identifies, investigates and treats affected patients thus avoiding further clinic referral and speeding up the patients recovery.
Disclosure: Nothing to disclose

EP1107

Implementation and feasibility of a digitized self-management intervention targeting cancer patients

M.L. Olesen[1], J. Uffelmann[2], V. Zoffmann[3]
[1]Gynecological Department & Research Unit Women's and Children's Health 7821, Copenhagen University Hospital, Rigshospitalet, København Ø, [2]The Public Danish eHealth Portal, Sundhed.dk, [3]Copenhagen University Hospital, Rigshospitalet & Copenhagen University, Copenhagen, Denmark

Introduction / Background: In Denmark, evidence-based individualized follow-up is recommended after gynecological cancer (1) in addition to medical follow-up. The recommendation includes psychosocial and self-management supportive interventions. We have developed a nurse-led self-management intervention called Guided Self-Determination Gynecological Cancer (GSD-GYN-C) (2) comprising preparatory paper reflection sheets completed by patients before an individual number of conversations with a certified nurse. In a randomized trial, we observed that GSD-GYN-C improved gynecological cancer survivors' physical quality of life after 9 months (3). A digitized version of GSD-GYN-C was sought by the women due to long transportation times. In 2016/17, we therefore collaborated with The Public Danish eHealth Portal, Sundhed.dk, to develop a digitally assisted version. When moving from a paper reflection tool and face-to-face conversations into a virtual world with a digitized reflection tool and sometimes video conversations, it is important to address how interactions may change in a real-life clinical setting and document the maturity of the digital GSD in a feasibility study.
Methodology: Action research (AR) (4) was applied. From the autumn of 2018, patients have been invited to use the digital version in three departments treating oncology patients. Data has been continuously collected in terms of summary of meetings, diaries, questionnaires and interviews with patients, nurses and leaders.
Results: The data collection is ongoing, six patients have received digitally assisted GSD-courses and 12 interviews have been conducted with participants. Preliminary findings include that the digitized intervention was easy and intuitive to use despite some technical barriers. The patients receive a support they otherwise would not have had. Some patients with cognitive challenges like memory loss experienced difficulties. Delivering the intervention through video conversation has been challenging. Further analysis of data will be presented at the conference.
Conclusion: Our preliminary findings suggest the digitized version of GSD-GYN-C appears to be feasible in clinical practice.
Disclosure: Nothing to disclose

EP1108

Creatsas modified vaginoplasty as stenosis vaginal treatment after pelvic radiotherapy

P. Padilla-Iserte, V. Lago, T. Marina, L. Matute, M. Gurrea, S. Domingo
Department of Gynecologic Oncology, Hospital Universitario La Fe, Valencia, Spain

Introduction / Background: Female sexuality is a complex phenomenon with important repercussions on their overall quality of life. In the case of women who have undergone gynecological interventions, their quality of sexual life may be diminished.
Methodology: Women survivors of gynecological cancers usually have important sequelae derived from the treatment. The radicality of the surgery and / or adjuvant radiotherapy treatments are sometimes unavoidable and they involve reconstructive surgery to restore the anatomical defect (vaginal stenosis) and, in this way, the woman can recover her sexual life and body self-perception as before.
Results: The role of reconstructive surgery for the anatomo-functional sequelae and body self-perception of the onco-gynecological patient has already been evaluated in previous studies (Hawighorst-Knapstein 2004, Chen 2015) with promising results since women who underwent reconstructive techniques such as Vaginoplasty with autologous peritoneal tissue (Chen 2015) improved sexual functioning and quality of life. Similar results were obtained in non-oncological patients with vaginal aplasia using the technique of vaginoplasty modified by Creatsas in which up to 95.5% of women reported feeling satisfied in their sexual life (Creatsas 2007). The surgical video is presented. With the goal of visualization this technique in the treatment of vaginal stenosis after pelvic radiotherapy for gynecological cancer.
Conclusion: Reconstructive surgery such as vaginoplasty modified by Creatsas, can help to restore the anatomical defect and, ultimately, improve the patient´s body perception and quality of sexual life.
Disclosure: Nothing to disclose

EP1109

Hormone replacement therapy for BRCA-mutation carriers: attitudes of Israeli health-care providers before and after a brief educational intervention

T. Perri[1,2], Y. Farhadian[1,2], E. Friedman[2,3], J. Korach[1,2]
[1]Gynecologic Oncology, Sheba Medical Center, Ramat Gan, [2]Tel-Aviv University, Sackler Faculty of Medicine, Ramat-Aviv, [3]Oncogenetics Unit, Institute of Human Genetics, and Meirav High Risk Clinic, Chaim Sheba Medical Center, Ramat Gan, Israel

Introduction / Background: *BRCA*-mutation carriers are offered risk-reducing bilateral salpingo-oophorectomy (RRBSO) at age 35−40 years, leading to major life-quality and health-related issues associated with early menopause. Hormone replacement therapy (HRT) may significantly alleviate menopausal symptoms without increasing breast or ovarian cancer risk in those patients. We investigated attitudes of Israeli health-care providers to HRT post-RRBSO in *BRCA*-mutation carriers, before and after a brief educational intervention.
Methodology: In this pre−post survey of gynecologic departments in Israel, health-care providers were given questionnaires (based on scores of 1−4) assessing attitudes to prescribing HRT in different clinical scenarios, before and after an educational intervention on current knowledge about HRT in *BRCA*-mutation carriers. Higher scores indicated higher tendency to prescribe HRT. Mean score was calculated for each scenario and the association between scores and various health-care providers´ characteristics was assessed. The change in attitude pre- vs. post-intervention was evaluated.
Results: Of the 200 health care providers who were offered participation, 162 responded, 25.3% were obstetricians, 13.6% gynecologists, 5.55% gynecologic-oncologists, 8% oncologists, 38.9% Obstetrics-gynecology residents and 8.6% nurses. Median age was 44 (IQR 36−58); 42.6% were males. Higher score correlated weakly with older age, but did not correlate with gender or personal HRT/menopause experience. Significantly higher mean pre-intervention scores were obtained by gynecologists (3.33±0.93) and gynecologic-oncologists (3.6±0.78) than by oncologists, obstetricians, residents or nurses (2.59±1.06, 2.7±1.09, 2.48±0.99, and 2.2±0.92, respectively). Overall scores were higher post-intervention ($p < 0.001$). The change in scores post intervention was most prominent among younger precipitants and nurses.

Conclusion: In Israel, it is acceptable to offer HRT post-RRBSO to healthy *BRCA*-mutation carriers. Younger health care workers and nurses tend to be more hesitant, yet they are more likely to adopt a pro- HRT attitude after an educational intervention. Such intervention is likely to improve overall care for *BRCA*-mutation carriers.
Disclosure: Nothing to disclose

EP1110

Sexual dysfunction impairment in gynaecological cancer survivors: systematic review

S. Petousis, G. Mavromatidis, A. Sotiriadis, A. Daniilidis, A. Papanikolaou, K. Dinas
2nd Department of Obstetrics and Gynaecology, Aristotle University of Thessaloniki, Thessaloniki, Greece

Introduction / Background: Gynecological cancer treatment often leads in subsequent sexual function impairment both in short-term and long-term outcomes.Main objective was to perform a systematic review of relative publications concerning sexual dysfunction parameters in gynaecological cancer patients, namely endometrial cancer, ovarian cancer, cervical cancer and vulvar cancer.
Methodology: A systematic review was performed in Pubmed and Scopus databased regarding the period 2016-2018. Searching strategy used for the systematic review included the words “sexual[tiab] AND endometrial cancer/carcinoma[tiab] OR cervical cancer/carcinoma [tiab] OR ovarian cancer/carcinoma [tiab] OR vulvar cancer/carcinoma” regarding the period 2016-2018. Primary and secondary outcomes of studies were comprehensively reviewed and summarized for each kind of cancer.
Results: There were overall 211 abstract retrieved, of which 22 studies were finally included in the systematic review. Main outcome of studies reporting on endometrial cancer (N=5) was that post-operative radiation significantly affects the majority of scored sexual function parameters, while laparoscopic approach was associated with favourable outcome. Studies reporting on ovarian cancer (N=3) concluded that salpingooophorectomy significantly affected sexual desire, while hormonal replacement therapy mitigated the detrimental effect. Regarding cervical cancer (N=8), the detrimental effect of radiotherapy on vaginal atrophy and dyspareunia was outlined, while minimally invasive surgery was indicated by one study to be associated with reduced pain intercourse and sexual function impairment. Finally, BMI, physical activity, smoking and coexisting pathology were also indicated to affect measured scale scores regarding sexual dysfunction parameters.
Conclusion: The majority of gynaecological cancer patients suffers from severe sexual function impairment, therefore indicating the need to implement strategies mitigating and improving post-operative and post-radiation status of these patients. Minimally invasive surgery, hormonal replacement therapy and improvement of co-morbidities may significantly improve outcomes.
Disclosure: Nothing to disclose

EP1111

Effectiveness of drainage following laparoscopic pelvic lymph node dissection

K. Sakai[1,2], M. Nakamura[1], W. Yamagami[1], T. Chiyoda[1], Y. Kobayashi[1], H. Nishio[1], S. Hayashi[1], H. Nomura[1], F. Kataoka[1], E. Tominaga[1], K. Banno[1], D. Aoki[1]
[1]Obstetrics and Gynecology, Keio University School of Medicine, Tokyo, [2]Obstetrics and Gynecology, National Hospital Organization Saitama National Hosipital, Saitama, Japan

Introduction / Background: Drainage, following pelvic lymph node (PLN) dissection for gynecological oncologic surgery to prevent postoperative lymphocyst formation and postoperative infection in pelvis, is controversial. The aim of this study is to elucidate the efficacy of drainage following PLN dissection

especially in laparoscopic surgery.
Methodology: We evaluated 374 patients who underwent systemic PLN dissection at our institution between January 2012 and October 2018. The drainage tubes were placed in the retroperitoneal fossa in all patients. All tubes were removed a day after laparoscopic surgery. For those who underwent laparotomy, tubes were removed four days after operation until March 2016, and a day after operation in March 2016 or later. Clinical information such as drainage volume, lymphedema and lymphocysts were collected from medical records and analyzed retrospectively.
Results: 80 patients were treated by laparoscopy (Group A), while 294 patients were treated by laparotomy (Group B). No patients were re-operated due to postoperative hemorrhage.
The median drainage volume a day after operation is 176.5ml in Group A and 172ml in Group B ($P>0.05$). Laparoscopy had significantly lower rates of lymphocysts (5% vs 16.7%, $P< 0.05$), however incidence of lymphedema (5% vs 3%) and symptomatic lymphocysts (1.3% vs 3.4%) were similar between two groups.
In laparotomy group, tubes were removed a day after operation (Group B1) in 172 patients, while tubes were removed four days after post operation (Group B2) in 122 patients. Between these two groups, there was no significant difference in incidence of lymphedema (3.5% vs 2.5%), lymphocysts (16.3% vs 17.2%) and symptomatic lymphocysts (1.3% vs 3.4%).
Conclusion: Our data suggests drainage has low importance both as an informative means and as an preventative means. Moreover, as incidence of lymphocyst was lower in laparoscopic group, drainage may not be necessary especially in laparoscopic surgery.
Disclosure: Nothing to disclose

EP1112

The impact of chemo radio therapy: a qualitative study

N.J. Schuur, H.A. van der Steege, M. Folsche, H.J. van Beekhuizen
Gynaecological Oncology, Erasmus MC, Rotterdam, The Netherlands

Introduction / Background: Although chemoradiotherapy for uterine cervical carcinoma is an effective treatment there is less knowledge about the impact on daily life for these women. What limitations do they have to face and what is the impact in social and economic functioning? There is a need to know what is the best way to support these women.
Methodology: A qualitative study was performed using semi structured interviews among nine women with cervical cancer. All nine women filled in the EORTC-QLQ-C30 and the EORTC-QLQ-Cx24 questionnaires to see if these questionnaires can be used to identify potential health issues and supportive care needs.
Results: Even years after chemoradiotherapy therapy women are struggling with physical and mental limitations like fatigue, bowel problems, anxiety, and decreased sexual and cognitive functioning. Limitations which have a negative effect on social-economical functioning. Women are in need to receive individual supportive care and practical help returning to work. The impact on quality of life can be monitored using the EORTC-QLQ-C30 and the EORTC-QLQ-Cx24 questionnaires.
Conclusion: Supportive care for women after chemo radiation therapy needs more attention. Women need a dedicated healthcare professional who helps her to deal with physical, mental and sexual limitations. Women are capable to return to work when they get support. The EORTC-QLQ-C30 and the EORTC-Cx24 can be used to identify potential healthcare problems and supportive care needs in the follow up period.
Disclosure: Nothing to disclose

EP1113

How a gynecologic oncology unit became "the happiest place on earth"

S. Pils[1], J. Ott[2], R. Schwameis[1], A. Reinthaller[1], E. Steiner[3], S. Springer[2], R. Ristl[4]
[1]Department of General Gynecology and Gynecologic Oncology, [2]Department of Obstetrics and Gynecology, Medical University Vienna, [3]Department of Obstetrics and Gynecology, General Hospital of Vienna, [4]Section for Medical Statistics, Center for Medical Statistics, Informatics, and Intelligent Systems, Medical University of Vienna, Vienna, Austria

Introduction / Background: Beside treatment efficacy, evaluation of side effects and quality of life (QoL) assessments have become more and more relevant in oncology. In this study, the influence of Disney movies during chemotherapy on emotional and social functioning as well as fatigue status was evaluated.
Methodology: 50 patients with gynecologic cancers were either shown Disney movies or not during six cycles of chemotherapy. Before and after every cycle, patients filled out standardised questionnaires from The European Organisation for Research and Treatment of Cancer. Changes in QoL were evaluated.
Results: In the course of six cycles of chemotherapy, patients in the Disney group felt less tensed and worried less, than patients in the control group according to the questions about emotional functioning (p=0.0217). Furthermore, watching Disney movies seemed to be associated with less encroachment in patients´ family life and social activities evaluated by the social functioning questions (p=0.0143). Moreover, this intervention led to less fatigue symptoms (p=0.0092). Perceived global health status was not affected by watching Disney movies (p=0.1576).
When comparing the results to the normative data for the EORTC QLQ-C30 in non-oncologic women aged 50 to 59 years, the control and the Disney group before intervention rated their emotional and social functioning as well as their global health status below the published data, while only the Disney group after the sixth chemotherapy/Disney movie valued them above.
Conclusion: Watching Disney movies during chemotherapy leads to improvements in emotional functioning, social functioning and fatigue status in gynecologic oncology patients.
Disclosure: SP: lecturing fee: MSD (not realted to submitted work) AR:Research grant: Roche lecturing and advisory boards fee: Amgen, Astra Zeneca, MSD, Pharma Mar, Roche, Tesaro, Vifor Pharma Travel expenses: Amgen, Astra Zeneca, Pharma Mar, Roche, Tesaro (not realted to submitted work) RS: Travel expenses: Amgen, Pharma Mar, Roche (not realted to submitted work) JO: lecturing fee: lenus pharma (not realted to submitted work) ES, SS, RR: non to declare

EP1114

Assessment of quality of life after diagnosis and treatment of gynecologic-oncology patients: review of the literature

M. Tanturovski, I. Aluloski, V. Jovanovska, S. Stojcevski, D. Tanturovski
Gynecologic Oncology, University Clinic of Obstetrics and Gynecology, Skopje, The former Yugoslav Republic of Macedomia

Introduction / Background: To sum up a part of the published results from studies focused on quality of life in populations of patients treated for gynecological cancer.
Methodology: The PubMed index was searched to identify relevant studies and abstracts published in English in the past 16 years (2000-2016). The following keywords were used: gynecologic cancer, long term effects, survivor-ship, quality of life, psycho-social adjustment.
Results: Of the initially identified 117 studies, only 24 publications met the inclusion criteria. There is a higher incidence of depression and anxiety in the population of patients undergoing treatment for gynecologic malignancies. This higher incidence is probably a result of the associated toxicity of the treatment as well as the poor prognosis in some of these patients. Factors influence the quality of life most negatively in the period from diagnosis until the end of treatment. Quality of life worsens significantly more in patients during treatment for gynecologic cancer compared to treatment for other non-gynecologic malignancies, but usually starts to improve in the first 6-12 months and stabilizes at base level approximately 2 years after treatment. The quality of life in patients with no relapses is comparable to that of the general population of healthy women.
Conclusion: Further prospective studies focused on the effects of possible psychosocial treatment on

the quality of life and prognostic factors in these patients should be conducted. The results of such studies could help these patients cope with the challenges posed by the diagnosis itself, as well as the associated treatment toxicity.
Disclosure: Nothing to disclose

EP1115

Parametrium tumor (lymphocyst) after adnextomy on the same side: case report

A.Z. Tolkos[1], T. Theodoridis[1], E.G. Klonos[1], L. Zepeiridis[2], G.F. Grimbizis[2]
[1]Aristotele University of Thessaloniki, [2]Aristotle University of Thessaloniki, Thessaloniki, Greece

Introduction / Background: Lymphocyst is rare disease,and mainly reported in some case reports.Occurrence of lymphocyst has been described in relation to surgery in a wide variety of other areas including the mediastinum, axila, neck, aorta and peripheral vasculature.The pathophysiology is based in insufficient lymphatic stasis with gross postoperative lymph leakage which cannot be absorbed in the peritoneum.
Methodology: A 46-year-old woman, was admitted to the emergency department, with abdominal pain. The pain was located to the abdomen.Her vital signs were non-reassuring.A mass was palpated arising from the pelvis on the left side.Her medical history included aleft oophorectomy due to a cyst form about 13 centimeters. The tumor makers were within normal range. Moreover, an abdominal MRI scan was carried out, that described the presence of a cyst measured 10 x 8 cm in the left parametrium.She was scheduled for surgery a few days later. The cyst was above the division of iliac vessels cling to left ureter and left uterine artery. The operation was uncomplicated and her post-operative period was uneventful. Histology showed **lymphocyst** , without any signs of malignancy Fifteen days later after a routine check, an entrenched retention of fluid was found in the pouch of Douglas 5 cm which was aspirated by needle under guidance of Computed Tomografy. The cytological examination showed lymphpocytes as an inflammatory response.
Results: In 5 to 10% of patients, the lymphocyst become symptomatic, manifesting fever, pain, constipation, prolonged ileus, urinary frequency, obstructive uropathy, leg edema, erythema of overlying skin or deep vein thrombosis.
Conclusion: Most lymphocysts are asymptomatic and are only diagnosed accidentally. A symptomatic lymphocyst may compress adjacent structures, for example, ureter, the urinary bladder, rectum or the iliac vein. Pelvic lymphocysts may be treated conservatively or surgically, by laparoscopy or laparotomy as well as using the methods of intervention radiology.
Disclosure: Nothing to disclose

EP1116

Integrated pathway analysis identifies a 3-gene signature predicting platinum response and outcome of high grade serous ovarian carcinoma patients

E. Bignotti[1,2], G. Benvenuto[3], L. Paracchini[4], L. Zanotti[2], C. Romani[2,5], G. Tognon[1], E. Calura[3], D. Vicini[6], M. Adorni[6], M.C. Paderno[6], T. Bianchi[6], F. Odicino[7], E. Sartori[7], A. Ravaggi[2], M. D'Incalci[4], S. Marchini[4], P. Todeschini[2], C. Romualdi[3]
[1]Obstetrics and Gynecology, ASST Spedali Civili di Brescia, [2]Angelo Nocivelli' Institute of Molecular Medicine, University of Brescia and ASST-Spedali Civili of Brescia, Brescia, [3]Department of Biology, University of Padova, Padova, [4]Department of Oncology, IRCCS - 'Mario Negri' Institute for Pharmacological Research, Milan, [5]Department of Molecular and Translational Medicine, University of Brescia, Brescia, [6]Clinic of Obstetrics and Gynecology, University of Milano Bicocca, San Gerardo Hospital, Monza, [7]Department of Clinical and Experimental Sciences, Division of Obstetrics and Gynecology University of Brescia, Brescia, Italy

Introduction / Background: High-grade serous ovarian carcinoma (HGSOC), the most common and deadly epithelial ovarian cancer histotype is characterized by a heterogeneous genomic landscape. After surgery, all patients are treated with adjuvant platinum (Pt)-based chemotherapy to which the response is heterogeneous, ranging from cases sensitive (Platinum-sensitive, Pt-s cases), to intrinsically resistant patients, who relapse within 6 months from the end of therapy (Pt-resistant, Pt-r). The aim of the study was to investigate the mechanisms characterizing the biology of primary resistance to upfront Pt-based chemotherapy through an integrated pathway analysis.
Methodology: Gene and miRNA microarray experiments were carried out on 36 Pt-s and 41 Pt-r tumor samples. Gene and miRNA expression have been integrated using Micrographite algorithm. Expression levels of selected predictive and prognostic genes were validated on a total of 242 HGSOC samples by qRT-PCR and on the Curated Ovarian Database (including 838 HGSOC).
Results: Expression profiles of 131 mRNAs and five miRNAs, belonging to five different and functionally-related molecular pathways, discriminate Pt-s and Pt-r cases. We selected 23 elements of the networks for orthogonal validation and 19 coding genes confirmed as differentially expressed between Pt-r and Pt-s cases. Among them, 16 elements also showed independent prognostic value in terms of PFS and OS in multivariate analysis. The prognostic impact of this signature was in silico validated on the Curated Ovarian Database, resulting in a 3-gene signature as independent prognostic biomarker of survival for HGSOC.
Conclusion: In the current study, we used an integrated pathway analysis on miRNA and gene expression signatures to capture the key pathways shaping the different biology of Pt-r and Pt-s tumors, validating our results using two independent cohorts of HGSOC biopsies. Finally, we investigated the association of the validated signature with survival across multiple HGSOC databases. This strategy identified a three-gene signature with predictive and prognostic impact in HGSOC.
Disclosure: Nothing to disclose

EP1117

Unravelling BRCA1-dependent molecular mechanisms in ovarian cancer by multi-level proteomics

M. Bradbury[1,2,3], E. Borràs[2,3], A. Pérez-Benavente[1,4], J. De la Torre[4], A. Gil-Moreno[1,4], E. Sabidó*[2,3], A. Santamaria*[5]
[1]Biomedical Research in Gynaecology, Vall d'Hebron Research Institute, [2]Proteomics Unit, Centre for Genomic Regulation, [3]University Pompeu Fabra, [4]Gynaecological Oncology Unit, Vall d'Hebron University Hospital, [5]Biomedical Research in Urology, Vall d'Hebron Research Institute, Barcelona, Spain

Introduction / Background: Our understanding of the molecular events associated to the development of high-grade serous ovarian cancer (HGSOC) in BRCA1 mutated patients and the differences observed in treatment response remain to be elucidated. Recent advances in large scale genomic analysis have shed a light on the importance of homologous recombination DNA repair and the beneficial use of PARP-inhibitor therapy. Nevertheless, additional cellular functions of BRCA1 in ovarian cancer are poorly understood.
Methodology: We analysed thirty-two patient-derived fresh frozen tissues from healthy ovaries and chemotherapy-naïve advanced high-grade serous tumours by mass-spectometry based proteomics. BRCA1 mutation status was known for all patients ($BRCA1_{mut}$ and $BRCA1_{wt}$). We quantitatively evaluated the protein changes and post-translational modifications focusing in the phosphorylation and ubiquitylation events. Comprehensive network analysis was performed comparing both healthy and cancer tissues based on their BRCA1 mutation status.
Results: We identified a variety of differentially expressed proteins discriminating between cancer from healthy tissues predominantly related to cell cycle control, extracellular matrix organisation, mitochondrial translation and immunological response. Cancer tissues harvesting $BRCA1_{mut}$ showed specific changes related to DNA damage repair and activated mTOR and insulin signalling pathways. In addition, downregulation of active mRNA processing and RNA splicing proteins were also observed in $BRCA1_{mut}$ tumours.

Conclusion: We demonstrate that direct patient tissue samples represent a valuable source for molecular investigation and provide new insights into the mechanisms of BRCA1 in ovarian cancer. We also highlight potential druggable target pathways in this group of patients. Finally, a deeper understanding of the RNA splicing events related to BRCA1 could unveil new clinically relevant cellular processes in ovarian cancer.
Disclosure: Nothing to disclose

EP1118

Patient-derived ovarian cancer organoids resemble clinical response to chemotherapeutics and provide insights into response mechanisms

C.J. de Witte[1,2], E. Stelloo[1,2], N. Hami[2,3], J. Espejo Valle-Inclan[1,2], K. Lõhmussaar[2,4], O. Kopper[2,4], C. Vreuls[5], T. Jonges[5], P.J. van Diest[5], H. Clevers[2,4,6], H.J.G. Snippert[2,3], W.P. Kloosterman[1], R.P. Zweemer[7], P.O. Witteveen[8]
[1]Center for Molecular Medicine, University Medical Center Utrecht, Utrecht University, [2]Oncode Institute, [3]Molecular Cancer Research, Center for Molecular Medicine, University Medical Center Utrecht, Utrecht University, [4]Hubrecht Institute, Royal Netherlands Academy of Arts and Sciences and UMC Utrecht, [5]Department of Pathology, University Medical Center Utrecht, Utrecht University, [6]The Netherlands Princess Máxima Center for Pediatric Oncology, [7]Department of Gynaecological Oncology, Cancer Center, [8]Department of Medical Oncology, Cancer Center, University Medical Center Utrecht, Utrecht University, Utrecht, The Netherlands

Introduction / Background: Epithelial ovarian cancer is characterized by intratumour genetic heterogeneity, chemotherapy resistance and poor survival. The advent of ovarian cancer organoids, that recapitulate histological and genetic features of the original lesions, has providedopportunities for personalized assessment of drug response. Here, we assessed if the ovarian cancer organoid system can predict patients' clinical response to chemotherapeutics. In addition, we evaluated the link between intratumour genetic heterogeneity and drug response using multiple organoids derived from individual patients.
Methodology: In total, we included 34 organoid lines derived from 22 patients with ovarian cancer at primary surgery or interval debulking. Organoid drug responses to chemotherapeutics as well as targeted drugs were determined. The IC50 and area under the curve values were computed from the drug response curves. Patients' recurrence-free and overall survival were compared to the organoid drug response. For patients who received carboplatin and paclitaxel chemotherapy prior to interval debulking (N=7), we additionally compared organoid drug response to biochemical (CA125), pathological, radiological outcomes. The organoids and corresponding tumour tissues were subjected to whole genome sequencing for genomic analysis.
Results: Organoid drug response correlated to clinical response as defined by histopathological tumour assessment (chemotherapy response score (CRS)). Organoids of patients with no or minimal pathological response (CRS=1) were less sensitive to carboplatin and paclitaxelcompared to organoids of the patients with appreciable pathological response (CRS=2). The presumed additive effect of paclitaxel on carboplatin was only observed in a subset of organoids. Organoid exposure to eight additional drugs revealed that multiple tumour locations of the same patients exhibited varying responses to specific drugs. Genomic analyses are being performed to identify underlying mechanisms of differential drug responses.
Conclusion: Ovarian cancer organoids provide a valuable preclinical system with the potential to guide treatment choice through prediction of drug response, avoid unnecessary overtreatment and provide insights in response mechanisms.
Disclosure: No competing interests. Applicable funding sources: EIF | Stand Up To Cancer (SU2C) [Clevers, Kopper] KWF Kankerbestrijding (Dutch Cancer Society) - UU2015-7743 [de Witte, Witteveen, Zweemer, Kloosterman] Gieskes Strijbis Foundation (1816199) [Clevers, Witteveen, Kloosterman, Zweemer, Lõhmussaar, Espejo Valle-Inclan, Hami, Snippert]

EP1119

Fischer rate 344 with syngeneic epithelial ovarian cancer (EOC) cells NuTu 19: key points for building a reliable ovarian carcinomatosis animal model

P.-F. Dupré[1,2], M. Muller[3], T. Le Gall[2], S. Loisel[4], T. Montier[2,5]
[1]UF Chirurgie Oncologique Gynécologique et Mammaire, CHU Brest, [2]INSERM UMR 1078, UBO, [3]Gynecologie & Obstétrique, CHU Brest, [4]Service Général des Plateformes Technologiques de l'UBO, UBO, [5]Laboratoire de Génétique Moléculaire et Histocompaptibilité, CHU Brest, Brest, France

Introduction / Background: Animal models are required for better understanding of EOC natural history and therapy assay.
We set up an experimental peritoneal carcinomatosis in an immunocompetent female rat model Fischer F 344 using a syngeneic epithelial ovarian cancer cell line NuTu 19.
Methodology: Twenty height female Fischer rats' F 344 were used in keeping with ethical in animal testing.
NuTu19 were transfected using the lipophosphoramid KLN-47 reagent carrying the luciferase-encoding plasmid pTG11033.
NuTu 19 Luc+ cells were injected into the peritoneum in the left iliac fossa with various concentration and dilution.
Bioluminescence monitoring was performed day 4, 10, 17, 25, 31, 38 and 49.
Surgery was performed under general anesthesia and pain management at day 17, 31, 49, 77.
Two Peritoneal Carcinomatosis Index (PCI) were used.
Results: Significant luciferase-activity was observed 24 hours following the injection, increased gradually and stabilising from day 10 (table 1).
Site-injection and sub-diaphragmatic areas were the two most common bioluminescent locations whatever cell concentration.
Macroscopic tumour graft was found delayed at day 21 and Iterative surgery show increasing PCI over time.
Medium PCI 1 was 22.5 [17-41] and PCI 2 22 [13-29].
Pathological exam (PE) was performed in 21 rates and results did not correlate with PCI and bioluminescence.
Lymphoid hyperplasia without carcinomatosis was found for low PCI (< 6) in four rates, however high PCI (>12) were associated with tumour graft at PE.
No carcinomatosis was found at PE before day 10 and bioluminescence's positive predictive value was 62.5% from day 35.
Conclusion: Developing a non-short term -life-threatening microscopic homogeneous carcinomatosis remains a challenge for in-vivo therapy assay.
The choice of a suitable animal model for surgery requires high quality experimental plan and systematic sampling for pathological exam to avoid incorrect assessment.

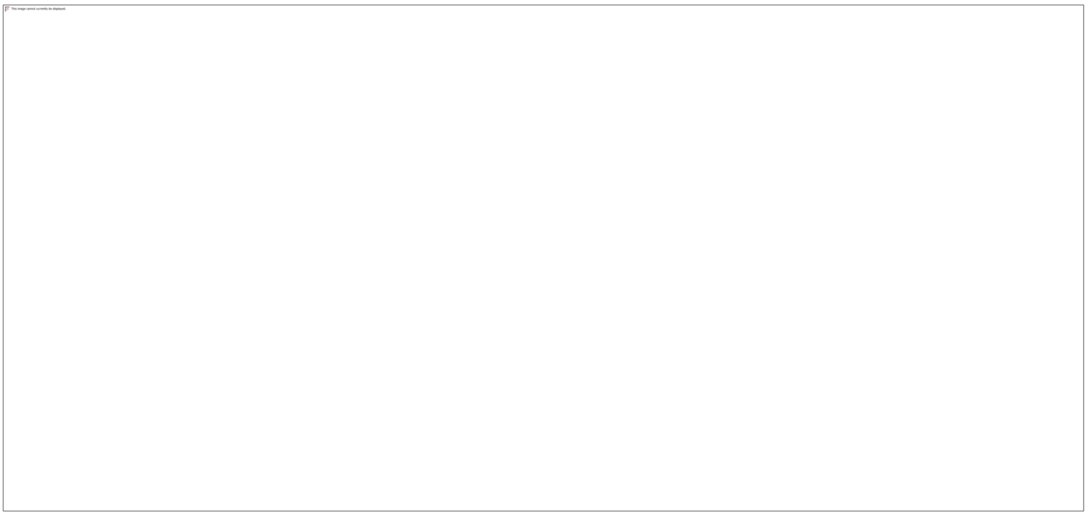

[Bioluminescent Imaging]

Disclosure: Nothing to disclose

EP1120

Application of the Proactive Molecular Risk Classifier for Endometrial Cancer (ProMisE) to patients conservatively treated: outcomes from an Institutional series

F. Falcone[1], N. Normanno[2], N. Losito[3], G. Scognamiglio[3], R. Esposito Abate[2], N. Chicchinelli[2], C. Scaffa[1], S. Greggi[1]
[1]Gynecologic Oncology Surgery, [2]Cell Biology and Biotherapy Unit, [3]Pathology Unit, Istituto Nazionale Tumori, IRCSS, “Fondazione G. Pascale”, Naples, Italy

Introduction / Background: To test the Proactive Molecular Risk Classifier for Endometrial Cancer (ProMisE) and determine the frequency of specific/prognostic molecular alterations within a cohort of endometrial cancer (EC) women conservatively treated by combined resectoscopy and progestin therapy.
Methodology: We used blocks of formalin-fixed paraffin-embedded tissue from the primary tumors of patients enrolled into the ECCo trial (EudraCT 2010-018581-23) between 2007 and 2016. In order to assign EC resectoscopic specimens to one of four ProMisE subgroups, testing involved sequential assessment of i) immunohistochemistry (IHC) for mismatch repair (MMR) proteins MLH1, MSH2, MSH6 and PMS2; ii) sequencing for POLE/POLD1
exonuclease domain mutations (EDMs); iii) p53 IHC.
Results: Molecular analysis methods were used in 25 patients (stage IA, G1-2 endometrioid EC), of whom 15 (60%) represented fully evaluable cases. Seven cases (46.7%) had abnormal MMR IHC, POLE/POLD1 EDMs were found in 3 cases (20%), and abnormal p53 IHC in 1 case (6.6%). Three patients (20%) had more than one molecular feature. Among 10 (40%) 'unclassifiable' patients, six failures in achieving complete molecular categorization were due to the low tumor volume. Molecular classification of the 15 fully evaluable cases yielded the following ProMisE subtypes: 7 (46.7%) MMR IHC abnormal, 1 (6.6%) POLE EDM, 0 (0%) p53 IHC abnormal, 7 (46.7%) p53 IHC wild-type.
Conclusion: Although larger series are needed to further assess the feasibility of a molecular categorization in a fertility-sparing setting, data presented are promising. In women with early stage low-volume disease, resectoscopy could be advantageous to provide samples allowing complete genetic risk assessment.
Disclosure: Nothing to disclose

EP1121

The clinical potential of *FOXL2* c.402C>G mutation detection in circulating tumour DNA of patients with granulosa cell tumours

J.W. Groeneweg[1], J.F. Roze[1], G.M. Monroe[1], S.T. Paijens[2], H.W. Nijman[2], H.S. van Meurs[3], L.R.C.W. van Lonkhuijzen[3], J.M.J. Piek[4], C.A.R. Lok[5], G.N. Jonges[6], R.P. Zweemer[1]
[1]Gynaecological Oncology | UMC Utrecht Cancer Center, University Medical Center Utrecht, Utrecht, [2]Obstetrics and Gynaecology, University Medical Center Groningen, Groningen, [3]Gynaecological Oncology, Amsterdam UMC, Location AMC, Amsterdam, [4]Obstetrics and Gynaecology, Catharina Hospital, Eindhoven, [5]Gynaecological Oncology, Antoni van Leeuwenhoek Hospital - The Netherlands Cancer Institute, Amsterdam, [6]Pathology, University Medical Center Utrecht, Utrecht, The Netherlands

Introduction / Background: Adult granulosa cell tumours (aGCT) are rare ovarian malignancies, molecularly characterized by a single somatic c.402C>G mutation in *FOXL2*. Recurrent disease after initial surgical treatment occurs in 30-50% of patients. Serum markers are used for diagnosis and

follow-up, but are not always accurate for GCT monitoring. The use of circulating tumour DNA (ctDNA) to monitor disease has been suggested in many cancer types. We aimed to assess the presence of *FOXL2* mutant ctDNA in aGCT patients and perform an initial evaluation of its potential as a marker for disease activity.
Methodology: In a national, multicenter GCT study, plasma samples (n=27) were prospectively collected from 7 patients with primary (n=1) or recurrent (n=6) aGCT harbouring the *FOXL2* c.402C>G mutation. Circulating cell-free DNA was extracted and assessed for the *FOXL2* mutation using droplet digital PCR. Identification of *FOXL2* mutation positive ctDNA, defined as ≥ 3 mutant droplets, was correlated with clinical parameters.
Results: The *FOXL2* c.402C>G mutation was found in the plasma of 5 out of 7 aGCT patients (71%), with mutant ctDNA fractions ranging between 0.4% and 47.8%. The tumour load in two patients without *FOXL2* mutant ctDNA was limited, while all aGCT patients with *FOXL2* mutant ctDNA had multifocal recurrent disease. In all ctDNA positive patients, increasing *FOXL2* mutant ctDNA levels were associated with disease progression or relapse defined by a rise in serum markers and/or CT scan findings. In 3 out of 4 ctDNA positive patients with plasma taken before and after treatment, the observed clinical response to therapy was accompanied by a marked decrease in *FOXL2* mutant ctDNA fractions.
Conclusion: *FOXL2* c.402C>G mutant ctDNA can be detected in patients with aGCT. Our results suggest that this test may have clinical value in disease monitoring, particularly when standard biomarkers inaccurately reflect tumour burden. Further research in a larger cohort is currently ongoing.

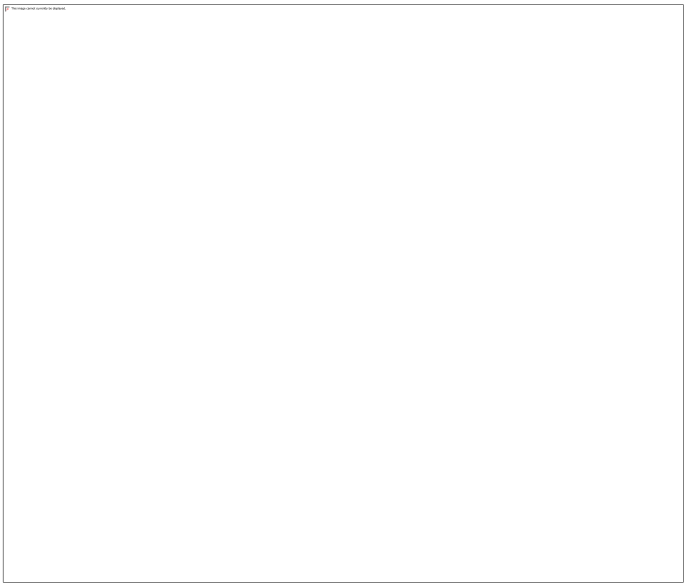

[FOXL2 ctDNA of recurrent aGCT patient, before (A) and after (B) complete cytoreductive surgery.]

Disclosure: Nothing to disclose

EP1122

Detection of non-haematopoetic circulating cancer-related cells in patients with rare gynaecological cancers

J. Jeyaneethi[1], M. Hall[1,2], E. Karteris[1]
[1]Life Sciences, Brunel University London, Uxbridge, [2]Medical Oncology, Mount Vernon Cancer Centre,

Northwood, United Kingdom

Introduction / Background: .Over the past decade, the concept of 'liquid biopsies' has been introduced as an alternative to conventional tissue examination for cancer. Advantages of this approach include potentially earlier warning of treatment resistance or new mutational changes as cancer progresses and the significantly greater acceptability of repeated testing for patients. In this study, we report detection of non-haematopoietic circulating cells (CCs) from patients participating in the **Ra**re **N**eoplasias of **G**ynaecological **O**rigin (RaNGO) study. This is a UK study designed to demonstrate the feasibility of collecting accessible information about specified rare gynaecological cancers, to facilitate in-depth studies of their clinical and biological behaviours leading to a greater understanding of mechanisms of disease progression and response to treatment, possibly also applicable to common tumours. It includes the development of a tissue bank, with appropriate patient consents for suitable ethically approved translational research projects. Patients with advanced stage rare gynaecological tumours are also eligible to donate blood for the evaluation of potential circulating biomarkers
Methodology: Venous blood was collected prospectively from 3 patients diagnosed with either FIGO stage IIIc squamous cell carcinoma (SCC) arising from a dermoid, adult granulosa cell tumour (GCT), or small cell cancer of the ovary cancer (SCOC). All patients were on active (palliative) treatments for their inoperable malignancies. CCs were evaluated using ImageStream Technology^{TM} and specific antibodies to differentiate epithelial from haematopoetic cells.
Results: Significant numbers of CCs determined by being $CK^{+}/CD45^{-}$ were detected in all 3 patients. Numerically, 195, 750 and 60 per millilitre of blood for SCC, GCT and SCOC respectively.
Conclusion: This is the first study to demonstrate that liquid biopsies might have a role in a number of different rare gynaecological malignancies. Future studies will concentrate on CC characterisation using multiple antibodies and enumeration from sequential samples in relation to treatment.
Disclosure: Nothing to disclose

EP1123

Apoptotic effect of herbal product, Gyejibokryunghwan on uterine sarcoma cells

M.H. Jung[1], Y.I. Ji[2]
[1]Kyung Hee University, Seoul, [2]Inje University, Busan, Republic of Korea

Introduction / Background: Diagnosis of uterine sarcomais is a challenging task for clinicians because its position is not easily accessible by current conventional techniques. In addition, standardized treatment for uterine sarcoma has not yet been established due to its rarity and heterogeneity.
We investigated the apoptotic cell death of uterine sarcoma cells (SK-UT-1B) induced by Gyejibokryunghwan (GBH).
GBH, an herbal medicine, has been widely used for gynecological diseases in oriental medicine.
Methodology: SK-UT-1B cells were treated with GBH of varying concentrations from 0 to 500 µg/ml. The mechanism of cell death was investigated through multiple analysis methods, including flow cytometry, cell cycle, and western blotting.
Results: Flow cytometric analysis revealed that the number of apoptotic cells increased in a GBH dose-dependent manner. The cell populations of sub-G1 and G0/G1 phases were increased by GBH treatment, indicating apoptosis and cell arrest, while the population of S and G2/M phases decreased. With GBH, the expression levels of cleaved caspase-3, -6, and -9 were upregulated, while the expression levels of pro-caspase-3, -6, and -9 were downregulated in SK-UT-1B cells.
Conclusion: These results are the first observation of uterine sarcoma cell death induced by GBH and confirmation of the mechanism of cell death, which occurred through the intrinsic apoptotic pathway. Clinically, uterine sarcoma has a poor prognosis with no appropriate treatment. GBH may become a new treatment modality for uterine sarcoma.
Disclosure: Nothing to disclose

EP1124

CKD-602, a topoisomerase I inhibitor, induces apoptosis and cell-cycle arrest and inhibits invasion in murine cervical cancer

Y. Jung, S.Y. Hur, Y.J. Choi
Department of Obstetrics and Gynecology, Seoul St. Mary's Hospital, College of Medicine, The Catholic University of Korea, Seoul, Republic of Korea

Introduction / Background: .Cervical cancer is the third most common gynecological malignancy. Conventional treatment options are known to be ineffective for the majority of patients with advanced or recurrent cervical cancer. Therefore, novel therapeutic agents for cervical cancer are necessary. In this study, the effects of CKD-602 in cervical cancer were investigated
Methodology: Three established human, immortalized, cervical cancer cell lines (CaSki, HeLa and SiHa) were used in this study. Following treatment with CKD-602, apoptosis was quantified using fluorescein isothiocyanate Annexin V-FITC and propidium iodide (PI) detection kit and cell cycle analysis was analyzed using fluorescence activated cell sorting (FACS). Transwell chambers were used for invasion assays. Western blot assay was performed to analyze proteomics. CaSki cells were subcutaneously injected into BALB/c-nude mice and cervical cancer xenograft model was established to elucidate the antitumor effect of CKD-602 *in vivo*.
Results: Treatment with CKD-602 induced apoptosis and increased expression of the enzyme PARP, cleaved PARP, and BAX. In addition, expression of phosphorylated p53 increased. Cell cycle arrest at G2/M phase and inhibition of invasion were detected after treatment with CKD-602. A significant decrease in cervical cancer tumor volume was observed in this *in vivo* model, following treatment with CKD-602.
Conclusion: This is the first report of CKD-602 having an antitumor effect in cervical cancer in both an *in vitro* and *in vivo* models. The results of this study indicate that CKD-602 may be a novel potential drug, targeting cervical cancer, providing new opportunities in the development of new therapeutic strategies.
Disclosure: This work was supported by Chong Kun Dang Corporation and Bio & Medical Technology Development Program of the National Research Foundation (NRF) funded by the Ministry of Science & ICT (2018M3A9E8021512).

EP1125

Chemotherapy reduces par glycohydrolase (PARG) expression in high-grade serous ovarian cancer patients

V. Lopez-Ozuna[1], I.Y. Hachim[2], L. Kogan[1], W. Gotlieb[1], A. Yasmeen[1]
[1]*Gynecology Oncology, Jewish General Hospital, McGill University,* [2]*Medicine, McGill University, Montreal, QC, Canada*

Introduction / Background: PAR glycohydrolase (PARG) enzyme was found to play an essential role in the synthesis and hydrolysis of poly (ADP-ribose) (PAR), which play an important role in maintaining genomic integrity. Recently, PARG inhibitors were developed, however the effects of PARG in ovarian cancer treated-patients has not been evaluated yet. Here, we evaluate the effects of chemotherapy in PARG of high-grade serous ovarian cancer patients (HGSOC).
Methodology: Two HGSOC cohorts were evaluated by immunohistochemistry: 54 chemo-naïve HGSOC patients (45 HGSOC, 9 borderline, 4 normal tissue) and 53 HGSOC chemo-treated patients (44 HGSOC, 9 borderline, 7 normal tissue). In addition, we used in silico analysis to evaluate the effect of PARG mRNA expression in ovarian cancer and its relation with patient outcome.
Results: Our results showed that chemo-naïve patients have significant higher levels of PARG expression compared to borderline and normal (62.2%, 44.4% and 0% respectively). Interestingly, these levels were reduced significantly in HGSOC patient samples that have received chemotherapy

(45.44%, 44%, 0%, respectively, p< 0.03). Indeed, this demonstrated that chemotherapy induces a reduction in PARG expression to levels equal to the borderline tumors. Furthermore, we found a dramatic re-localization of PARG protein to the cytoplasm in chemo-treated patients (100%) compared with chemo-naïve HGSOC samples that were localized in the nucleus (80%, p< 0.05). In silico analysis of 1500 ovarian cancer patients revealed that PARG is up-regulated in ovarian cancer in comparison with normal tissue and highly express in advance-metastatic HGSOC. Moreover, its expression in advance disease was associated with shorter overall survival.
Conclusion: Our results showed that chemotherapy decreased PARG expression and re-localized it into the cytoplasm. Moreover, our findings highlights the possible use of PARG inhibitors as an adjuvant therapy to treat recurrent ovarian cancer together with chemotherapy and other new-targeted drugs such as PARP inhibitors.
Disclosure: Nothing to disclose

EP1126

Single-cell RNA-sequencing of 7 HGSOC cases reveals multiple prognostic cell subtype

S. Olbrecht[1,2], P. Busschaert[2], J. Qian[3,4], A. Vanderstichele[1,2], D. Lambrechts[3,4], I. Vergote[1,2]
[1]Department of Gynaecology and Obstetrics, University Hospitals Leuven, [2]Division of Gynaecological Oncology, [3]Laboratory for Translational Genetics, Department of Oncology, Catholic University of Leuven, [4]Center for Cancer Biology (VIB), Leuven, Belgium

Introduction / Background: High grade serous ovarian cancer (HGSOC) is generally detected in an advanced stage with poor long-term survival. This therapeutic resistance is attributed to its extensive inter- and intratumoural heterogeneity. Several non-tumoural cells within the tumour specimen — such as immune cells, fibroblasts and endothelial cells — are increasingly recognized as important contributors to tumoural development as they shape the microenvironment and the tumour's ability to metastasize.
Methodology: In this project, we employed ´3 single-cell RNA-sequencing (scRNA-seq) on randomly dissociated cells from tumour biopsies of seven HGSOC patients before first-line treatment.
Results: Based on gene expression and principal component analysis, we clustered 18,403 cells into 11 tumoural and 35 non-tumoural cell subtypes (Figure 1). Analysis of differential gene expression enabled us to correlate known and new biological functions to most subtypes. Furthermore, we selected specific markers genes (MG) for each subtype by differential gene expression analysis within and across cell subtypes. As a downstream analysis, we evaluated these subtype-specific MG in publically available datasets containing bulk expression and curated clinical annotation of 1467 HGSOC patients. We performed a meta-analysis using multivariate Cox proportional hazards regression, adjusting for age, residual disease and FIGO stage. This allowed us to discover a prognostic effect for 7 cell subtypes across cohorts, of which 3 were associated with improved overall survival (OS) (1 type of dendritic cell, b-cell and t-cell) and 4 were associated with worsened OS (2 types of fibroblasts, 1 type of tumour cell and endothelial cell).
Conclusion: ScRNA-seq enabled us to disentangle the heterogeneous microenvironment of HGSOC and detect several relevant tumoural and non-tumoural cells that influence OS. These subtypes can act as novel biomarkers for treatment stratification. Quantification of these novel cell types by specific MG in bulk RNA data can make clinical implementation feasible and pave the way to a more personalized therapy.

This image cannot currently be displayed.

[Figure 1]

Disclosure: Nothing to disclose

EP1127

Compared the expression of endoplasmic reticulum stress and their adaptive molecule mRNA in endometriosis and gynecologic cancer

H.R. Ju[1], Y. Gen[1], S.I. Kim[1], M.K. Baek[1], T.S. Lee[2], S.G. Yeo[3], D.C. Park[1]
[1]Obgyn, St. Vincent Hospital, The Catholic University, Suwon, [2]SMG-SNU Boramae Medical Center, [3]Kyung Hee University, Seoul, Republic of Korea

Introduction / Background: Endometriosis is most serious disease which combined chronic inflammatory process and invasion and those are very similar characteristics with invasive cancer. However the precise pathogenesis is not still clear. We compared the expression pattern of ER stress and their adaptive molecule mRNA in Endometriosis and gynecologic cancer to investigate the inflammatory mechanism.
Methodology: Specimens were obtained peritoneal fluid from 33 patients with benign tumor without endometriosis, 44 patients of endometriosis, and 40 patients of gynecologic cancer. The levels of expression of various ER stress transcription factor were assessed quantitatively using real-time RT-PCR.
Results: The mRNA expression of sXBP1 and ATF6 were significantly higher in gynecologic cancer group than control or endometriosis group ($p< 0.05$). And the expression of CHOP and BIP were significantly lower in gynecologic cancer group ($p < 0.05$). On the contrary, in endometrial group, only ATF6 was increased than control group ($p< 0.05$). Also, ATF6 is associated with severity of endometriosis or CA125 elevation.
Conclusion: ATF6 is likely to be involved in inflammatory reaction of both endometriosis and gynecologic cancers. Also, ATF6 is associated with severity of endometriosis or CA125 elevation. Therefore, it is suggested that the ER stress mRNA affects inflammatory reaction and the pathophysiology of endometriosis.
Disclosure: No potential conflict of interest relevant to this article was reported.

EP1128

Differential DNA methylation in High Grade Serous Ovarian Cancer (HGSOC) is associated with tumor behaviour

H.D. Reyes[1], E. Devor[1], A. Warrier[1], A. Newtson[1], J. Mattson[1], V. Wagner[1], G. Duncan[2], D. Ikoma[1], K. Leslie[1], J. Gonzalez- Bosquet[1]
[1]Obstetrics and Gynecology, [2]Health and Human Physiology, University of Iowa, Iowa City, IA, United States

Introduction / Background: The epigenome represents and adds another facet of complexity to cancer that needs to be understood to categorize patients into those at risk of disease, recurrence, or treatment failure. Our goal is to compare DNA methylation status between controls vs HGSOC, and relate it with clinical outcomes.
Methodology: A retrospective review of our institution's advanced/ recurrent HGSOC patients yielded 97 cases with good quality DNA and RNA. 12 normal fallopian tubes were also collected and used as controls. Infinium Illumina methylationEPIC was used to characterize DNA methylation status and RNA seq to evaluate gene expression. T- tests were used to compare methylation patterns between controls vs HGSOC, primary vs recurrent, and optimal vs suboptimal surgical outcomes. Spearman's rank correlation was used to evaluate association between degree of methylation and expression of identified genes. Validation of our findings used the cancer genome atlas (TCGA) database followed by C-statistics to assess degree of agreement.
Results: Out of 66,069 methylation probes that interrogate known genes, 5,852 probes were significantly different between normal and HGSOC. This includes genes that are enriched in several pathways such as the RAS signaling pathway. A similar analysis of the TCGA database showed 2,075 differentially methylated probes. 1,891 probes were significant in both datasets giving a 70.1% degree of agreement.

When comparing differential DNA methylation between primary and recurrent disease, there were 57 probes which represent 17 genes that were significantly different. When comparing optimal vs suboptimal surgical outcomes, 99 probes were significantly different wherein 29 genes show expected inverse correlation between methylation status and gene expression.
Conclusion: There are significant differences in methylation patterns between HGSOC and fallopian tubes, primary vs recurrent disease, and optimal vs sub-optimal surgical outcomes. Cataloging these phenomena in a well characterized clinical population will aid in determining groupings for precision medicine treatment cohorts in the future.
Disclosure: Nothing to disclose

EP1129

The association among cervical, anal and oral HPV infections in women with and without high-grade cervical lesions

B. Sehnal[1], M. Nipcova[1], M. Zikan[1], J. Slama[2]
[1]Department of Gynaecology and Obstetrics, Hospital Na Bulovce and 1st School of Medicine, Charles University, [2]Department of Gynaecology and Obstetrics, General University Hospital and 1st School of Medicine, Prague, Czech Republic

Introduction / Background: The human papillomavirus (HPV) can cause premalignant and malignant tumors in the anogenital and oropharyngeal regions.
Methodology: A total of 718 immunocompetent women were enrolled in the study. The high-risk (HR) group consisted of 473 patients with biopsy-confirmed high-grade cervical lesion while the low-risk (LR) group consisted of other 245 women. All participants completed an anonymous self-administered questionnaire and were subjected to cervical, anal, and oral HPV genotyping using the Linear array HPV test.
Results: A total of 81.4 % women were infected in the cervix, 43.3 % in the anus, and 2.7 % in the oral cavity in the HR group in comparison with only 26.9 %, 24.5 %, and 1.4 % in the low-risk LR group, respectively. The cervical and anal HPV infections were much more frequent in the HR patients ($p < 0.001$); the difference in the oral HPV prevalence was not significant ($p = 0.511$) between groups. Concurrent cervical-anal infection was observed in 39.3 % of HR women and in 8.3 % of the LR patients ($p< 0.001$) and it significantly increased with the grade of cervical lesion ($p^{trend}< 0.001$). The higher prevalence of concurrent cervical-oral, anal-oral, and cervical-anal-oral infections in HR women was statistically not significant according to the generally small oral HPV prevalence.
Conclusion: All HPV infections occurred more often in HR than in LR women but not all results were statistically significant. The genotype HPV 16 was found in approximately half of all infections at all sites.
Disclosure: This study has been supported by Charles University in Prague (UNCE 204065) and by Ministry of Health of the Czech Republic, grant nr. 17-32030A.

EP1130

Clinical significance of autophagy related molecules in liquid biopsy of cervical cancer

A. Sharma[1], R. Khan[1], N. Gupta[1], M. Sharma[2]
[1]Department of Biochemistry, All India Institute of Medical Sciences, [2]Department of Radiation Oncology, Maulana Azad Medical College, New Delhi, India

Introduction / Background: Cervical cancer (CaCx) is the second most common malignancy in women in India. Autophagy is one of the significant hallmarks of cancer in which high mobility group box 1 (HMGB1) plays a crucial role. Aberrant expression of HMGB1 is associated with tumor

development, progression and poor prognosis. There are no reports available studying HMGB1, autophagy related molecule in context to clinical significance in cancer cervix. Thus, we aim to investigate the association between HMGB1 and its associated molecules (RAGE, p53 & p62) in CaCx. We have also evaluated the clinical significance of serum HMGB1 in CaCx diagnosis.
Methodology: 50 subjects including 20 CaCx patients, 20 healthy women (controls) and 10 controls having gynecological disorder other than malignancy were recruited. Circulatory levels of HMGB1 were measured by ELISA. mRNA and protein levels of HMGB1 and its associated molecules were quantitated using Q-PCR and western blotting, respectively in tissues of patients and controls. HeLa cells were used as positive control for HMGB1. Data was statistically analyzed.
Results: Circulatory levels of HMGB1 were significantly higher in patients as compared to controls. mRNA and protein expression of HMGB1 were significantly higher in tumor tissues in comparison to controls. The levels of RAGE, p53 and p62 were also significantly elevated than their expression in controls at mRNA as well as at protein levels. ROC curve analysis showed better sensitivity and specificity for HMGB1 for non-invasive diagnosis of CaCx in liquid biopsy.
Conclusion: HMGB1 level could be a useful and specific marker for evaluating the disease and diagnosis in non-invasive liquid biopsy. Autophagy mediated HMGB1/RAGE pathway might play a significant role in the pathogenesis of CaCx. Validation in larger patient cohort might exploit HMGB1 as a novel non-invasive diagnostic marker for CaCx in liquid biopsy in future.
Disclosure: Nothing to disclose

EP1131

The iKnife and its application for the treatment of cervical abnormalities

M. Tzafetas, A. Mitra, I. Kalliala, S. Lever, Z. Bodai, F. Rosini, A. Savage, J. McKenzie, D. MacIntyre, S. Ghaem-Maghami, Z. Takats, M. Kyrgiou
Imperial College London, London, United Kingdom

Introduction / Background: Surgical management of presumed early stage cervical cancer presents challenges as clearance of surgical margins avoids adjuvant chemoradiotherapy that can substantially increase morbidity and may allow fertility preservation in young women. Improved intra-operative diagnostic tools have the potential to improve outcomes. In this study, we explore whether the Rapid Evaporative Ionization Mass Spectrometry (REIMS), also known as the iKnife (intelligent Knife), can accurately discriminate between healthy, pre-invasive and invasive cervical tissue ex vivo.
Methodology: We prospectively collected fresh frozen cervical tissue samples in normal controls and women with Human Papilloma Virus (HPV) +/- cervical intraepithelial neoplasia (CIN) and cervical cancer (Cancer) between 2006 and 2018. A handheld diathermy device was used to burn the biopsies; the surgical aerosol produced was subsequently transferred into a mass-spectrometer. The tissue was then stained for histopathological validation. We used principal component and linear discriminant analysis to calculate the accuracy of the iKnife in discriminating between the 3 groups. Cancerous and normal tissues were then processed with REIMS tandem MS (REI-MS/MS) to further delineate the most significant peaks allowing separation of the two classes and identify their lipid profile.
Results: The iKnife had an overall 97.8% accuracy in discriminating accurately normal (100%) vs HPV±CIN (100%) versus cancer (83.3%) when compared to histology as the gold standard. REI-MS/MS and LipidMAPS revealed twelve significant MS peaks in the cancer to normal separation.
Conclusion: This study shows that REIMS iKnife has the potential to accurately discriminate healthy, from precancerous and invasive lesions on the cervix. The overexperessed lipids in cancer may be used as markers of disease. If these finding are replicated in larger cohorts, the iKnife may allow real-time intra-operative diagnosis of the presence of disease in surgical margins and lymph nodes.
Disclosure: Nothing to disclose

EP1132

A comperative molecular analysis of DNA damage response and apoptosis of malignant granulosa cells after exposure to gemcitabine and cisplatin

D. Vatansever[1], G. Bildik[2], C. Taskiran[1], O. Oktem[3]
[1]*Obstetrics and Gynaecology, Division of Gynaecologic Oncology, Koc University School of Medicine,* [2]*Koc University, Graduate School of Health Sciences,* [3]*Obstetrics and Gynaecology, Division of Reproductive Endocrinology and Infertility, Koc University School of Medicine, Istanbul, Turkey*

Introduction / Background: We aimed to compare gemcitabine vs. cisplatin in terms of DNA damage response, viability/apoptosis of malignant granulosa cells.
Methodology: Malignant granulosa tumour cell lines (COV434 and KGN) were used for the experiments. Cell viability, proliferation, DNA damage response and apoptosis were investigated using immunofluorescence staining and immunoblotting. Cell cycle analysis was carried out using flow cytometry. In vitro oestradiol and AMH productions were analysed by ECLIA method. Gemcitabine and cisplatin were used at four different concentrations corresponding to their therapeutic blood levels.
Results: Gemcitabine treatment caused DNA damage, cellular stress, inhibited proliferation and activated cell cycle check-point sensors and induced apoptosis as shown by increased expression of g-histone H2AX, p-JNK, Chk-1/Chk-2, cleaved forms of PARP and caspase-3 in the asynchronous cells in a dose dependent manner.
As a result, the proliferation and in vitro AMH and oestrogen production of the cells were decreased at post-exposure 24h. In the cells synchronized at S phase gemcitabine significantly inhibited DNA synthesis and blocked their proliferation. Similar effects were also observed after cisplatin treatment. Exposure of the cells to gemcitabine at G2/M transition abolished the progression of mitosis, caused mitotic arrest and failure to exit mitosis as evidenced by the inhibition of Cyclin B degradation and absence of de-phosphorylation of cdc-2 at Tyr 15 residue. However, such an effect was not observed in the cells synchronized and treated with cisplatin at G2/M.
Conclusion: These results may suggest that anti-metabolite chemotherapy drug gemcitabine might have anti-neoplastic actions on granulosa cell tumour.
Disclosure: Nothing to disclose

EP1133

Duration of chemotherapy for normalization of BHCG and treatment outcomes of gestational trophoblastic neoplasia at a tertiary cancer centre in Saudi Arabia

F. Azam, W. Al-Selwi, W. Taha AbdelGhafar, A. Zahralliyali, A. AlWbari, N. Bukhari, F. Ibnshamsah
Adult Oncology, King Fahad Specialist Hospital, Dammam, Saudi Arabia

Introduction / Background: Gestational trophoblastic neoplasia (GTN) constitutes rare group of benign and malignant neoplasia. BHCG (Beta human chorionic gonadotropins) is serum tumor marker that indicates a treatment response. This study is aimed to evaluate treatment outcomes, epidemiologic profiles and duration of chemotherapy for normalization of BHCG for (GTN) patients.
Methodology: This retrospective study included GTN patients treated with chemotherapy at King Fahad specialist Hospital during the period January 2016-December 2018. Clinical data were collected patients electronic and paper files
Results: Total of 22 patients with GTN received chemotherapy during the study period. Median age was 32 (18-53) years. 17 cases (77%) were diagnosed as GTN following molar pregnancy, and 5 (23%) cases were identified after abortion. GTN Stages of I, II, III and IV on diagnosis were noted in 12, 1, 7 and 2 patients respectively. According to WHO prognostic scoring system, 16 (72%) patients were classified as low risk, and 6(28%) as high risk. Intramuscular Methotrexate was the most frequently used first-line single-agent chemotherapy (n=16, 72%) for low risk disease, and EMACO regimen was the most frequently used combination chemotherapy (n=6, 28%) for high risk disease. The median number of chemotherapy cycles were 6 (1-16) and median duration of chemotherapy resulting in normalization of BHCG was 3 (1-8) months. Only 3 patients developed resistance to single

agent chemotherapy and were switched to second line combination chemotherapy with median duration for normalization of BHCG of 6 months after chemotherapy switch over. 19 patients achieved complete response with first line and only 3 (14%) patients needed 2ndline EMACO chemotherapy. All patients achieved complete response and currently on follow up.
Conclusion: All patients achieved complete response with chemotherapy. Median duration of chemotherapy for normalization of BHCG was 3 months and 6 cycles. Only 3 patients required 2ndline chemotherapy after single agent chemotherapy in 1stline.
Disclosure: Nothing to disclose

EP1134

Gestational trophoblastic disease (GTD): a 23-years single institution experience

S. Baptista de Almeida[1], F. Ferreira da Silva[2], F. Gomes[1]
[1]Oncology Department, Hospital Prof. Doutor Fernando Fonseca, Amadora, [2]Oncology Department, Hospital da Luz, Lisboa, Portugal

Introduction / Background: Gestational trophoblastic neoplasia (GTN) is a rare potential curable malignancy that consists of abnormal proliferation of trophoblastic tissue following a hydatiform mole or a nonmolar pregnancy. Both a stage and a risk score are determined at baseline to help decide the best treatment. We aimed to review all cases of histologically diagnosed GTD, focusing on GTN.
Methodology: Retrospective analysis of all GTD cases diagnosed at our institution since 1996.
Results: A total of 101 GTD cases were identified, divided by histological subtypes as follows: 46 complete hydatiform moles, 34 partial hydatiform moles, 12 invasive moles (IM) and 9 choriocarcinomas (CC). Median age at diagnosis was 31 years. Regarding GTN (CC and IM, 21 cases), 4 cases were treated at another hospital and so excluded from this analysis. Of the remaining 17 cases, 12 were stratified as low risk and 5 as high risk according to modified WHO prognostic scoring system. Most (9 cases) had stage III by FIGO staging. All low risk cases (12) were treated with single agent methotrexate (MTX) (median of 6.5 cycles) and 4 non-responders had a second line chemotherapy (ChT) with dactinomycin with complete response (CR). None of these low risk patients had disease relapse. Regarding high risk cases (5), 3 were treated with first line MTX (4, 8 and 10 ChT cycles) followed by second line dactinomycin (10, 5, 8 ChT cycles respectively), 2 with CR and 1 progressed to third-line EMA/CO (ongoing). The remaining 2 high risk cases were treated with EMA/CO (3 and 8 ChT cycles), one of them retreated after 7 years. All patients are alive.
Conclusion: As expected GTN is a rarer condition than non-invasive moles. Despite its invasive nature shows a very good prognosis after treatment with ChT. Sequential single-agent ChT should be studied as an option even in high risk patients.
Disclosure: Nothing to disclose

EP1135

A series of missing primaries: pulmonary metastasis in gestational trophoblastic neoplasia in the absence of uterine tumors

G.P. Gonzalez[1,2], A. Estrella[3]
[1]Obstetrics and Gynecology, Philippine General Hospital, Manila, [2]Obstetrics and Gynecology, The Medical City South Luzon, Santa Rosa, [3]Philippine General Hospital, Manila, Philippines

Introduction / Background: Gestational trophoblastic neoplasias (GTNs) are extremely aggressive tumors derived from placental trophoblasts. These tumors are always the sequalae of a pregnancy. Choriocarcinoma is the most common of these, in which early extra-pelvic hematogenous spread is typical. As progression of illness is rapid, timely diagnosis and treatment will favor improved chances

for cure, whereas late commencement of therapy will make resolution difficult. The diagnosis of GTN is straightforward, because of elevated beta human chorionic gonadotropin (bhCG) and distinct sonographic features of the tumors inside the uterus. However, very rarely, this disease may occur in the absence of uterine tumors.
Methodology: This presents a series of three patients with choriocarcinoma who consulted for evaluation of abnormal clinical and radiologic chest findings.
Results: In all cases, the patients presented with a radiographically documented pulmonary mass with accompanying lower respiratory symptoms. Pulmonary carcinoma was ruled out. All patients underwent chemotherapy with 2/3 achieving a favorable outcome
Conclusion: It is imperative for any clinician to have a high index of suspicion, backed by a thorough analysis of the clinical findings, in order to appropriately diagnose and treat patients with choriocarcinoma. Ample recognition of the various manifestations of metastatic choriocarcinoma and greater use of assays for HCG should result in prompt diagnosis and an improved long-term prognosis in these patients. Varied forms of treatment are available. Based on sound judgment, the clinician must decide which of these will best attain the treatment goals set the patient and the physician.
Disclosure: Nothing to disclose

EP1136
Outcome of gestational trophoblastic disease in Lower Egypt
R. Hemida[1], A. Zakaria[2], W. Elrefaie[3], E. Refaie[2]
[1]Mansoura University | Obstetrics and Gynecology, [2]Mansoura University, Mansoura, [3]Port Said University, Port Said, Egypt

Introduction / Background: Gestational trophoblastic disease (GTD) defines a spectrum of proliferative disorders of trophoblastic epithelium of the placenta. Incidence, risk factors, and outcome may differ from a country to another.
Methodology: An observational prospective study was conducted at the GTD Clinic of Mansoura University. The patients were recruited for 12 months from September 2015 to August 2016. The patients' characteristics, management, and outcome were reported.
Results: We reported 71 clinically diagnosed GTD cases, 62 of them were histologically confirmed, 58 molar (33 CM and 25 PM) in addition to 4 initially presented GTN cases. Mean age of the studied cases was 26.22 years ± 9.30SD. Mean pre-evacuation hCG was 136170 m.i.u/ml ±175880 SD. Most of the cases diagnosed accidentally after abnormal sonographic findings (53.2%). Rate of progression of CM and PM to GTN was 24.2% and 8%, respectively.
Conclusion: The incidence of molar pregnancy and GTN in our locality was estimated to be 13.1 and 3.2 per 1000 live births respectively. We found no significance between CM and PM regarding hCG level, time to hCG normalization, and progression rate to GTN.
Disclosure: Nothing to disclose

EP1137
Primary gestational choriocarcinoma of the uterine cervix and fallopian tube: the diagnosis of ectopic pregnancy is not always a benign condition
E. Karaman[1], A. Kolusarı[1], I. Alkış[1], O. Karaaslan[2], H.G. Şahin[2], A. Gül[2]
[1]Gynecologic Oncology, [2]Obstetric and Gynecology, Yuzuncu Yil University, Van, Turkey

Introduction / Background: Choriocarcinoma is the most aggressive, malignant form of gestational trophoblastic disease. The most common localization is uterus. However, in some cases, it can be found in cervix, tuba uterina or ovaries. Here, we present two different clinical cases of which were

misdiagnosed as ectopic pregnancy.
Methodology: A 36 year-old patient admitted to our department for investigation of irregular vaginal bleeding. On ultrasound examination, the endometrial line was thin and bilateral adnexial structures were normal. B-hcg level was 2815 mIU/L, progesteron was 0.2. On B-hcg follow-up, the levels were 2764 and 2877 at two days intervals. The patient was given methotrexate for the diagnosis of ectopic pregnancy. One week later, B-hcg level increased to the 5373 mIU/lt. The MRI showed a 25x22 mm cervical mass with parametrial invasion with rectal serosal involvement (Figure 1). Biopsy confirmed the cervical choriocarcinoma. The patient underwent chemotherapy (EMA-CO).
Results: The second case was a 31 year-old woman (gravida 2 para 2) was operated due to the ruptured ectopic with B-hcg of 29.251. On exploration, a ruptured and actively bleeding ectopic mass with size of 4x3 cm was noted on the ampulla of left fallopian tube as well as abundant haemoperitoneum with 600 cc of blood. A complete left salpingectomy was performed (Fig-2,3). The final diagnosis was primary tubal choriocarcinoma of 2 cm in size which involved the whole layer of tubal wall and ruptured into pelvic cavity at the ampulla region. Computed tomography scans were negative for metastasis. Patient underwent monochemotherapy.
Conclusion: Knowledge of the characteristics of the choriocarcinoma is very important for accurate diagnosis and treatment, especially when the tumor is localized on the rare locations and where a high level of serum B-hcg is absent. We aimed to implicate that when diagnosing the ectopic pregnancy, even very rare, the gestational choriocarcinoma should be kept in mind.

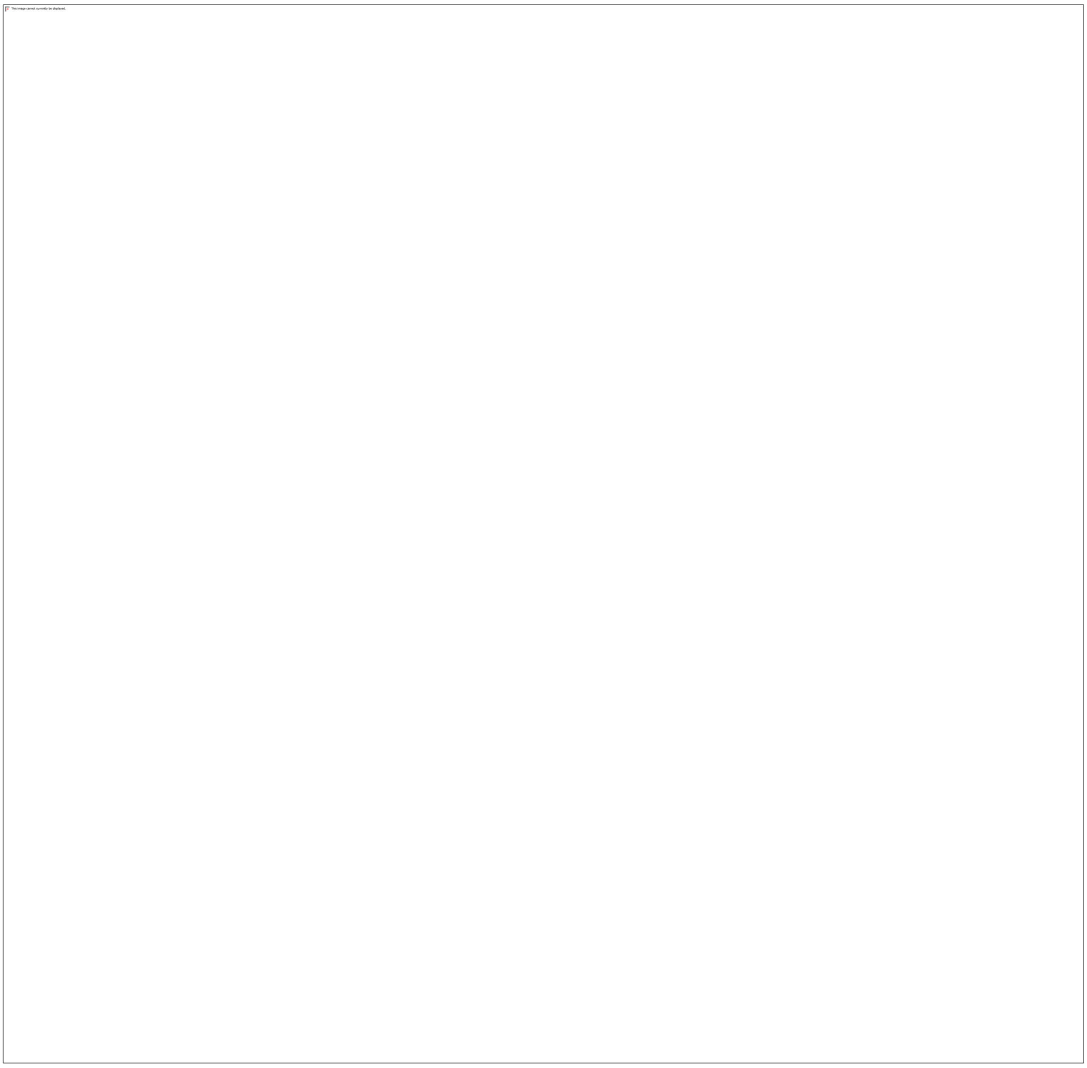

[Figure 1]

[Figure 2]

Disclosure: Nothing to disclose

EP1138

Ovarian vein thrombosis in a gestational trophoblastic neoplasia patient

I.H. Kim, I.Y. Kim, S.H. Kim
Department of Obstetrics and Gynecology, Eulji University Hospital, University of Eulji School of Medicine, Daejeon, Republic of Korea

Introduction / Background: Ovarian vein thrombosis (OVT) is a rare medical condition most often seen in the immediate postpartum period. OVT has been reported in approximately 0.05-0.18% of vaginal births and in 2% of births by Caesarean section. Anticoagulation is the main therapy used to alleviate symptoms
Methodology: A 23-year-old female (gravida 0, para 0) presented to obstetrics and gynecology department with symptoms of amenorrhea for 15week 6days and intermittent abdominal pain. One month before admission, she was tested positive for urine HCG.
Results: Laboratory data revealed a b-HCG was elevated (over 200,000 mIU/ml).
A transvaginal ultrasound scan revealed a bulky uterus with honeycomb pattern in the endometrial cavity (Fig 1). She subsequently underwent a dilatation and curettage. Following this, b-HCG level fell to 43,996.2 mIU/ml. 3 months later, Her b-HCG level again started rising, reaching a level of 138,103.3 mIU/ml. A transvaginal ultrasound scan revealed the right adnexa showed a 9.1x5.9cm multilobular and mixed echogenicity (Fig 2a-b). A CT with intravenous contrast revealed multilobulating & tortuous tubular hypodense mass with strong enhencing septae & peripheral wall at right adnexa and extent of

the right ovarian vein thrombus which did not reveal other abnormlity (Fig 2c).
she was started on methotrexate chemotherapy combined with oral anticoagulation with rivaroxaban immediately. 2 weeks later, the b-HCG level showed a fall to 12,364.1 mIU/ml. After several cycles of chemotherapy, the b-HCG level reached a normal level of 2.6 mIU/ml.
Conclusion: OVT is a rare disorder commonly associated to the peripartum period. But, there was no report of OVT in GTN patients. OVT with only acute abdominal pain is unlikely to be suspected in GTN patients, and thus is found incidentally on CT for metastatic evaluation. Therefore, it is important to have a CT scan if GTN patients have symptoms such as abdominal pain or dyspnea.

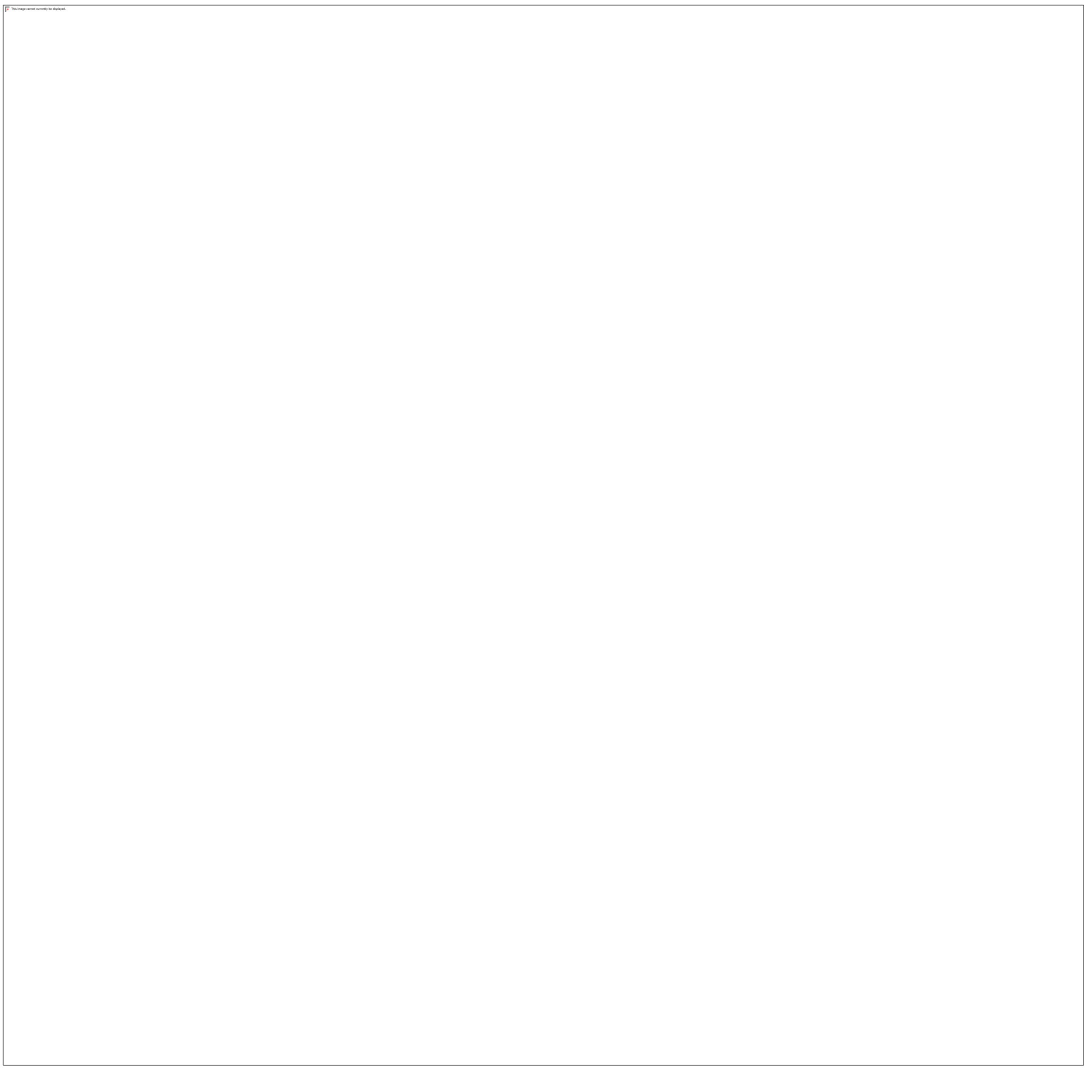

[Fig 1. Transvaginal ultrasound a-b) uterine, c) Right anexa, d) Left adnexa]

This image cannot currently be displayed.

[Fig 2. Transvaginal ultrasound a) Endometrium. b) Right adnexa , c) CT image (OVT)]

Disclosure: Nothing to disclose

EP1139

Successful treatment of choriocarcinoma with brainmetastasis with cisplatin etoposid: a case report

F. Metke, L. Kiesel, R. Witteler
Department of Obstretics and Gynecology, University Hospital Münster, Münster, Germany

Introduction / Background: Ultra high risk gestational trophoblastic neoplasia with brain metastasis have high mortality due to early death during chemotherapy. The recommended treatment is a therapy induction with cisplatin and etoposid followed by systemic polychemotherapy with etoposide, methotrexate, actinomycin D, cyclophosphamide, and vincristine (EMA/CO) with intrathecal

methothrexat application. In our reported case complications prohibited standard treatment.
Methodology: Case report.
Results: A 26-year-old patient, after being initially treated with methotrexate for suspected extrauterine pregnancy, presented at our hospital with gestational choriocarcinoma with pulmonary, brain and intraabdominal metastases. During induction therapy with cisplatin and etoposide the patient had intracerebral hemorrhage. After the implantation of a Rickham-reservoir she developed intracranial parenchymatous lesions which led to the explantation of the reservoir. Due to these events we could not apply EMA/CO chemotherapy. Since etoposid has a good brain penetration we chose to continue the etoposide cisplatin regimen. After six cycles she had complete remission according to human gonadotropin levels and imaging studies. We continued up to eleven cycles. The patient is now in complete remission with a follow-up of six months.
Conclusion: In the case presented we successfully applied etoposide cisplatin treatment in a patient with CCA with brain metastasis, when standard chemotherapy regimen could not be given due to complications.
Disclosure: Nothing to disclose

EP1140

Evaluation of the relation between treatment results and predictive factors in metastatic and high risk gestational trophoblastic neoplasia

M. Hasanzadeh Mofrad[1], L. Mousavi Seresht[2]
[1]Mashhad University of Medical Sciences, Mashhad, [2]Isfahan University of Medical Science, Isfahan, Islamic Republic of Iran

Introduction / Background: High risk gestational trophoblastic neoplasia is considered a treatable malignancy due to recent advancements in chemotherapy. This report describes treatment outcomes as a predictor of prognosis in one institute.
Methodology: We performed a retrospective analysis of the treatment results from 41 patients diagnosed with high risk and metastatic gestational trophoblastic neoplasia who received treatment at Mashhad University of Medical Sciences, Mashhad, Iran from January, 2013to May, 2017.
Results: Patients had a mean age of 31.31 years. Average treatment time was 3.5 months. Within the participant; 19 patients with World Health Organization scores over 7; received methotrexate at the first line of treatment. 11 cases (26.8%) of the 19 patients with single agent chemotherapy showed resistance. The patients who were resistant to treatment received a combination chemotherapy as the second line of treatment. The response rate of the Etoposide, Methotrexate, Actinomycin D, Cyclophosphamide and Oncovin chemotherapy regimen as the first line of treatment was 93.7%, which decreased to approximately 83.3% when administered as the second line of treatment. There were 76.4% of cases in remission at the one year follow-up and a successful pregnancy rate of 17.5%. A statistically significant relation existed between chemotherapy response rate with disease stage, score, site, and number of metastases ($P< 0.05$).
Conclusion: The World Health Organization/International Federation of Gynecology and Obstetrics staging-scoring system is appropriate for gestational trophoblastic neoplasia management. The Etoposide, Methotrexate, Actinomycin D, Cyclophosphamide, and Oncovin regimen showed superior efficacy. The importance of accurate patient selection for adjuvant surgery in high risk gestational trophoblastic neoplasia cannot be under emphasized.
Disclosure: Nothing to disclose

EP1141

Reproductive outcomes after treatment of patients with gestational trophoblastic neoplasia

S. Srijaipracharoen, S. Tangjitgamol
Obstetrics and Gynecology, Faculty of Medicine Vajira Hospital, Navamindradhiraj University, Bangkok, Thailand

Introduction / Background: To assess reproductive outcomes including menstrual and fertility function of gestational trophoblastic neoplasia (GTN) patients after treatment.
Methodology: Patients with GTN who had clinical data in our institution present were identified. Data collected were age at GTN diagnosis, menstrual and obstetrical history, characteristics of GTN, treatment and reproductive outcomes including menstrual and fertility function after treatment.
Results: From 115 GTN patients, 73 patients had available data and were included in the study. Median age at GTN diagnosis was 32.8 years (range, 17-52 years), with 75.7% aged ≤ 40 years and 97.3% ≤ 50 years. Clinico-pathological data of the patients were presented elsewhere. In brief, 74.3% had FIGO stage I disease and 85.1% were classified as low risk by WHO scoring system. Total hysterectomy was performed in 20.3% (or 21.6% including one patient who had hysterectomy prior to GTN diagnosis): prior to or after few cycles of chemotherapy were performed in 12.2% and 8.1% respectively. Median age of the patients who had or did not have hysterectomy were 41.2 years (range, 27-52 years) vs 31.4 years (range, 17-50 years) respectively (p< 0.001). Among 59 patients who did not undergo hysterectomy, all except one had chemotherapy. Methotrexate and actinomycin-D were the most common first-and second-line chemotherapy, 74.1% and 80.0% respectively. Menstrual history was uncertain in almost all patients. Total of 27.0% of all patients or 34.5% of patients who had preserved uterus achieved pregnancy at least once with the median interval time of 44.0 months (range, 2.7-97.3 months) after complete treatment.
Conclusion: Approximately 76% of GTN patients aged 40 years or younger. Majority were in early stage and low risk. Nearly 35% of the patients who did not have hysterectomy could have at least 1 pregnancy after treatment with chemotherapy.
Disclosure: Nothing to disclose

EP1142

Gestational trophoblastic neoplasia: clinical presentation, treatment and outcomes

S. Tangjitgamol, S. Srijaipracharoen
Obstetrics and Gynecology, Faculty of Medicine Vajira Hospital, Navamindradhiraj University, Bangkok, Thailand

Introduction / Background: To assess clinical and pathologic features of patients with gestational trophoblastic neoplasia (GTN), treatment and oncologic outcomes.
Methodology: Patients with GTN who had clinical data in our institution were identified. Data collected were age at GTN diagnosis, preceding pregnancy and interval, FIGO stage and WHO score, treatment and oncologic outcomes after treatment.
Results: From 115 patients, 73 with available data were included in the study. Median age at GTN diagnosis was 32.8 years (range, 17-52 years). The most common antecedent pregnancy was molar pregnancy (85.1%). Stage I disease was most common (74.3%) followed by stage III (23.0%) and stage IV (4.0%). According to the WHO score, 85.1% were classified as low risk, 10.8% as high risk, and 4.1% as ultra-high risk. All patients with metastasis had pulmonary lesions. Other metastatic sites were brain, abdominal wall, vulva, and colon. Surgery was performed in 27.0% as resection of lesions, primary or secondary hysterectomy prior to or after few cycles of chemotherapy respectively. Chemotherapy was given in 94.6% of patients, with methotrexate as the most common first-line chemotherapy (75.0%). Response rate was 86.3%. The patients who had persistence or rising of serum hCG (13.7%) as well as those who experienced side-effects from first-line chemotherapy (4.1%) or recurrence (6.8%) had second-line chemotherapy. After a median follow-up of 22.7 months (range, 0.37-210.9 months), only 1 patient was dead from progressive diseases in brain and lung at 15.1 months after GTN after diagnosis. Two patients with ultra-high risk or recurrence diseases were still in the course of chemotherapy treatment. The others were alive and doing well without any evidences of diseases.
Conclusion: Majority of GTN had antecedent molar pregnancy, stage I and low risk disease. Surgery had certain role in selected patients. Chemotherapy was the mainstay of treatment yielding good

oncologic outcomes.
Disclosure: Nothing to disclose

EP1143

Psychological portrait of women after hydatidiform mole evacuation

N. Tsip[1], M. Lyzogub[2], V. Zaviryukha[3]
[1]Oncogynaecological, National Cancer Institute, [2]National Bogomolets Medical University, [3]Psychological, Kyiv National Economic University, Kyiv, Ukraine

Introduction / Background: The aim of this study was to explore the psychological symptoms of patients after hydatidiform mole evacuation.
Methodology: 30 patients with morphologically verified diagnosis of hydatidiform mole aged from 21 to 35 years were examined. The personality dispositions were studied by using the clinical interview. SF-36, WPPF and test-assessment of stress-strain to global changes were used to define the attitude of patients to their "I", "Health". To study the level of personal and situational anxiety, as well as the structure of each patient's anxiety Spielberg-Khanin test was used.
Results: According to the study in patients were identified high level of personal (76,3 ± 6,53)% and the situational anxiety (78,7 ± 6,4)%, resentment (77 ± 5,72) %, fear (49,2 ± 7,9)% and anger (47,5 ± 4,56)%. The analysis revealed the presence of psychosomatic disorders in (95,43 ± 8,2)% of patients. The most frequent manifestations of such violations: the need for empathy - at (84,3 ± 9,03)%, depreciation of themselves as women - at (69,7 ± 5,2)%, the rejection of sexuality - at (52,1 ± 6,1)% , a tendency to blame themselves - at (37,2 ± 4,3)% and isolation - at (43,1 ± 4,7)%.
Most of the surveyed allocate altruistic, family life and existential categories of meanings.
Particular attention is drawn to the fact that the vast majority of patients (79,5 ± 5,4)% observed low degree of resistance to stress.
Conclusion: These results testify the importance of medical and psychological support of patients after hydatidiform mole evacuation based on positive psychotherapy and self-control techniques.
Disclosure: Nothing to disclose

EP1144

Stage IV choriocarcinoma: case report and review of literature

A. Veiga-Fernández[1], C. García-Díaz[1], J.Á. Arranz[2], E.M. Mendizábal-Vicente[1], P. Rincón-Olbes[1], S. Lizarraga-Bonelli[1]
[1]Obstetrics and Gynaecology, [2]Medical Oncology, Gregorio Marañón University General Hospital, Madrid, Spain

Introduction / Background: Choriocarcinoma is the most aggressive histologic type of gestational trophoblastic neoplasia (GTN). It consists of invasive, highly vascular and anaplastic trophoblastic tissue made up of cytotrophoblasts and syncytiotrophoblasts without villi, and it is characterized by early vascular invasion and widespread metastases.
Methodology: We present a clinical case and a review of literature.
Results: A 33-years-old woman with 2 previous caesarean deliveries, last one in July 2018, was referred to our hospital in December 2018 after a seizure secondary to a brain tumour. Human chorionic gonadotropin (b-hCG) was raised with a normal gynaecological ultrasound except for a suspicious sonographic image of ectopic pregnancy. She was treated with one dose of Methotrexate, without decrease of b-hCG levels. Finally, the patient underwent brain surgery and the tumour was completely resected. After that, b-hCG decreased quickly.
Histological study showed a choriocarcinoma. No primary tumour was found in complementary

imaging tests.
Choriocarcinoma occurs in about 1 in 20,000 to 40,000 pregnancies in the United States and three to nine per 40,000 pregnancies in Southeast Asia and Japan. About 50% of all choriocarcinomas arise from a complete molar gestation, 25% following a normal pregnancy, and 25% after a spontaneous miscarriage or ectopic pregnancy.
Diagnosis is often delayed following a nonmolar pregnancy and thus, metastases are more common than after a molar pregnancy.
Clinical presentation of choriocarcinoma depends on the extent of disease and location of metastases. The most frequent sites of GTN metastases are: lung (80%), vagina (30%), central nervous system (10%) and liver (10%).
Conclusion: Choriocarcinoma is a rare disease. Patients should be managed by a gynaecologist oncologist and/or medical oncologist experienced with GTN. Treatment includes chemotherapy combined with uterine surgery.
According to the International Federation of Gynecology and Obstetrics, the majority of cases of choriocarcinoma are chemotherapy sensitive, with a survival rate of nearly 80-100%.
Disclosure: Nothing to disclose

EP1145

Choriocarcinoma after term delivery: a difficult diagnosis

M. Vizcaíno Gómez, R. Betoret Gustems, E. Cazorla Amorós
Obstetrics and Gynecology, University Hospital of Vinalopo, Elche, Spain

Introduction / Background: Choriocarcinoma following a term pregnancy is an uncommon disease with an estimated occurrence of 1 per 160,000 pregnancies. The interval between delivery and treatment is an important prognosis factor. We present a case of a placental choriocarcinoma with no metastases in a full-term intrauterine pregnancy. A first curettage because of abnormal persistent bleeding did not reveal the true diagnosis of choriocarcinoma.
Methodology: A 34-year-old patient, gravity 3, miscarriage 1, parity 2 presented an episode of abnormal vaginal bleeding 6 weeks after term delivery. First curettage was performed without abnormal histopatholgical findings. Due to the persistence of bleeding second curettage was done 4 weeks after the first one. Pathologic report was Choriocarcinoma and it was possible to review the original material that confirmed the diagnosis. Ultrasound revealed an intracavitary uterine tumoral mass with strong Doppler signal(Fig1). Stage 1 was confirmed by MNR (Fig2), pretreatment human chorionic gonadotropin (HCG) level was 92.770 mUI / ml and FIGO risk factor score was 6.
The treatment was Methotrexate (MTX)50 mg by intramuscular injection and Calcium folinate after each injection. During chemotherapy, the HCG measured once per week was decreasing to become negative after 6 weeks. After a new increase of HCG levels in the third month of negativity, an early uterine relapse was suspected and confirmed by CTscan and PET-CT.
Results: Hysterectomy was performed with double salpinguectomy and right oophorectomy due to the presence of ovarian cyst. HCG levels became negative three weeks after. Patient is asymptomatic without any abnormality in image or laboratory tests 3 years after the diagnosis.
Conclusion: Although posterm delivery choriocarcinoma incidence is very low, postpartum placental histopathological examination and HCG monitoring in case of abnormal bleeding are necessary for early diagnosis an d improvement in prognosis.

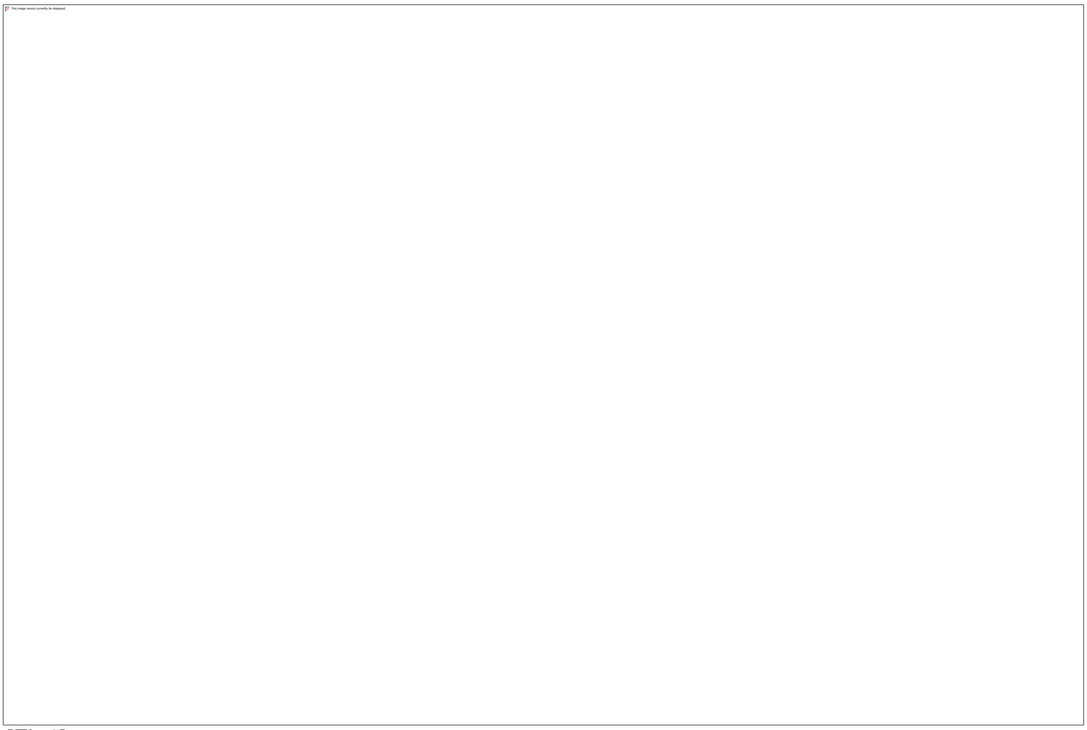

[Fig1]

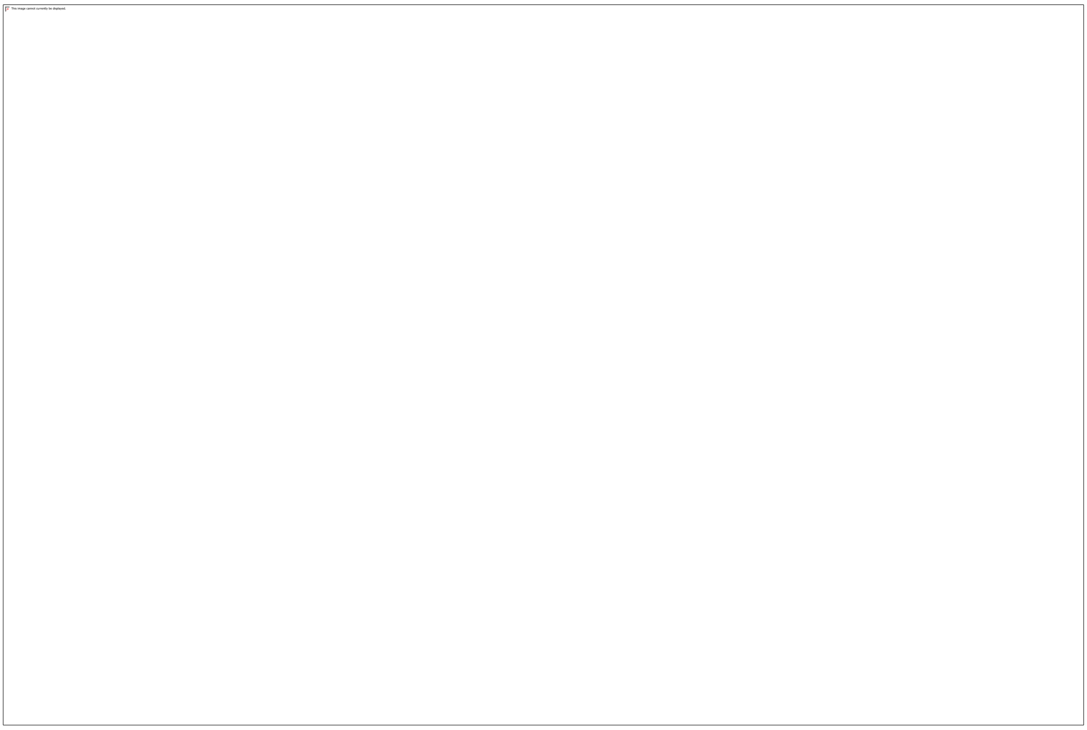

[Fig2]

Disclosure: Nothing to disclose

EP1146

Clinical response to a second uterine curettage in patients with low-risk gestational trophoblastic disease: a pilot study

F. Yarandi, E. Shir Ali
Teharn University of Medical Scienceis, Tehran, Islamic Republic of Iran

Introduction / Background: To determine the curative effect of a repeat uterine evacuation in patients with low-risk gestational trophoblastic neoplasia.
Methodology: Patients with low-risk gestational trophoblastic neoplasia (GTN)(N=12), diagnosed according to the International Federation of Gynecology and Obstetrics 2002 guidelines, were enrolled in a prospective cohort study. Primary outcomes were need for chemotherapy after second uterine evacuation and number of chemotherapy courses needed to achieve complete remission.
Results: Ten patients (83%) did not require chemotherapy and were cured bya second curettage. Two patients failed to respond to the second curettage and received single-agent chemotherapy with actinomycin-D (1.25 mg/m2 biweekly, slow intravenous administration). Both patients responded to chemotherapy as second-line therapy. A 100% remission rate was achieved, with no recurrence at the 1-year follow-up. One patient (8%) had a uterine perforation.
Conclusion: Second curettage has a favorable response rate. It seems reasonable to perform a second curettage in patients with low-risk GTN in settings where serum beta-hCG assay follow-up is highly reliable and available. However, its potential complications and inconvenience must be discussed critically with each patient.
Disclosure: Nothing to disclose

EP1147

Limb preserving surgery for necrotic vulvar cancer groin recurrence infiltrating the femoral artery and pubic bone

A. Abdelbadee[1], A. Elnaggar[2], M. Morsy[3], D. Badary[4], G. Seifeldein[5], S. Abdalla[6], H. Abou-Taleb[1]
[1]Obstetrics & Gynecology, [2]Vascular Surgery, [3]Orthopedic Surgery, [4]Pathology, [5]Diagnostic Radiology, Faculty of Medicine/Assiut University, [6]Diagnostic Radiology, South Egypt Cancer Institute/Assiut University, Assiut, Egypt

Introduction / Background: Management of recurrent vulvar cancer involving the femoral artery after primary surgery and irradiation is challenging. Few case reports exist describing surgical management of advanced recurrent vulvar cancer in the groin region in the context of limb salvage.
Methodology: A 60 years old female who underwent a left hemivulvectomy at another institution 1 year earlier for vulvar squamous cell carcinoma. The patient was diabetic, hypertensive and had left hemiplegia. Two months later, local recurrence occurred in the left inguinal lymph nodes and was treated by bilateral inguinal lymphadenectomy and adjuvant radiotherapy. Amidst her radiotherapy course, she refused to continue treatment. The following year, she presented to our institution with a large infected necrotic fungating recurrence in the left groin region. Evaluation by a multidisciplinary team revealed a 15x7 cm left inguinal mass infiltrating the ipsilateral superior pubic ramus, inguinal ligament and encasing the external iliac and femoral vessels. The family was counselled about urgent palliative surgery for possibility of femoral vessels blow out.
Results: Surgery was performed by a team of gynecologic oncologists, vascular, orthopedic, general and reconstructive surgeons. It entailed enbloc radical excision of the tumor with safety margins including excision of the superior pubic ramus. The femoral vessels were dissected from the lateral border of the tumor. Partial reconstruction of the soft tissue defect was done by a right rectus

abdominus muscle flap to cover the exposed major vessels. Estimated blood loss was 1600 ml and operative time was 10 hours. The patient survived postoperatively and was stable but at 3 weeks she deteriorated quickly and died of septic shock.
Conclusion: Tumor resection for patients with recurrence of vulvar cancer in the groin region involving the femoral artery after primary surgery and pelvic irradiation is a complex yet feasible palliative surgical option, thus avoiding classic hemipelvectomy.

Author (year)	Age (years)	Medical comorbidities	Primary tumor therapy	FIGO stage (initial presentation)	Management of Groin recurrence with limb preserving procedures	Postoperative outcomes
Deppe et al (1984)	63	---	Radical vulvectomy and bilateral inguinal lymphadenectomy.	II	Radiation therapy and femoral blow-out was managed by external iliac-femoral bypass utilizing 6-mm expanded polytetrafluoroethylene vascular graft (PTFE).	Persistence of right leg lymphedema 5 months after surgery
Chao et al (2001)	51	Diabetes mellitus, hypertension	Radical vulvectomy and bilateral inguinal lymphadenectomy	III	Radical enbloc excision of tumor along with the involved femoral artery and vein followed by Gore-Tex vascular graft and rectus abdominis myocutaneous flap reconstruction.	Occlusion of the grafted vessels occurred 21 months following surgery. Patient survived more than 48 months
Sevin et al (2001)	66	---	Excision and postoperative external beam radiation	I	Wide excision of the recurrence including resection of the pubic bone and adjacent muscles with extra-anatomic axillo-popliteal bypass. Wound closure by a myocutaneous flap from the contralateral rectus abdominis.	Death 7 months later due to rapidly progressing pulmonary recurrence.
Pararajasingam et al (2001)	55	---	Vulvectomy and bilateral inguinal lymphadenectomy	I	Common femoral artery blow-out was managed by a bypass from the left external iliac artery to the superficial femoral artery using a 6-mm expanded polytetrafluoroethylene vascular graft (PTFE) after external iliac artery ligation.	No early postoperative complications.
Kim et al (2008)	52	---	Right radical vulvectomy and bilateral inguinal lymphadenectomy	I	First groin recurrence was managed by excision, radiation and chemotherapy. Second recurrence was managed by resection and reconstruction with a right gracilis muscle	Death from widely metastatic disease 3 months later.

					flap. Third recurrence and femoral blow-out was managed by embolization coils and percutaneous endovascular stent-graft placement from the distal left external iliac artery to the left superficial femoral artery.	
Trompetas et al (2010)	50	---	Radical vulvectomy and bilateral inguinal lymphadenectomy with adjuvant radiotherapy	III	Palliative chemotherapy (Carboplatin and Gemcitabine). Femoral blow-out was managed by a combined open surgical and endovascular intervention with 2 stents deployed across the bifurcation of the common femoral artery into the profunda femoris artery.	Death from progressive disease without further bleeding 3 months later.
Horta et al (2011)	68	Diabetes mellitus	Radical vulvectomy with bilateral inguinal lymphadenectomy	II	Radical tumor excision, ligation and excision of the femoral vein over a length of 7 cm. Reconstruction of the defect with a contralateral vertical rectus abdominis musculocutaneous (VRAM) flap based on the inferior epigastric pedicle.	No early postoperative complications.
Nassiri et al (2015)	50	---	Excision	---	Bland endovascular embolization of the tumor as well as 3 vessel covered stent revascularization in the common femoral, superficial femoral and profunda femoral arteries.	No early postoperative complications.
Abdelbadee et al (2019)	60	Diabetes mellitus, hypertension, cerebrovascular stroke	Left hemivulvectomy	I	Recurrence initially managed by bilateral inguinal lymphadenectomy and postoperative radiation. Subsequent management by radical enbloc tumor excision including left superior pubic ramus and dissection of femoral vessels. Reconstruction was done by a right rectus abdominus muscle	Death 3 weeks later with septic shock.

					flap to cover the exposed major neurovascular bundle.	

[Table 1: Summary of case reports involving limb preserving procedures for vulvar cancer groin recurrence]

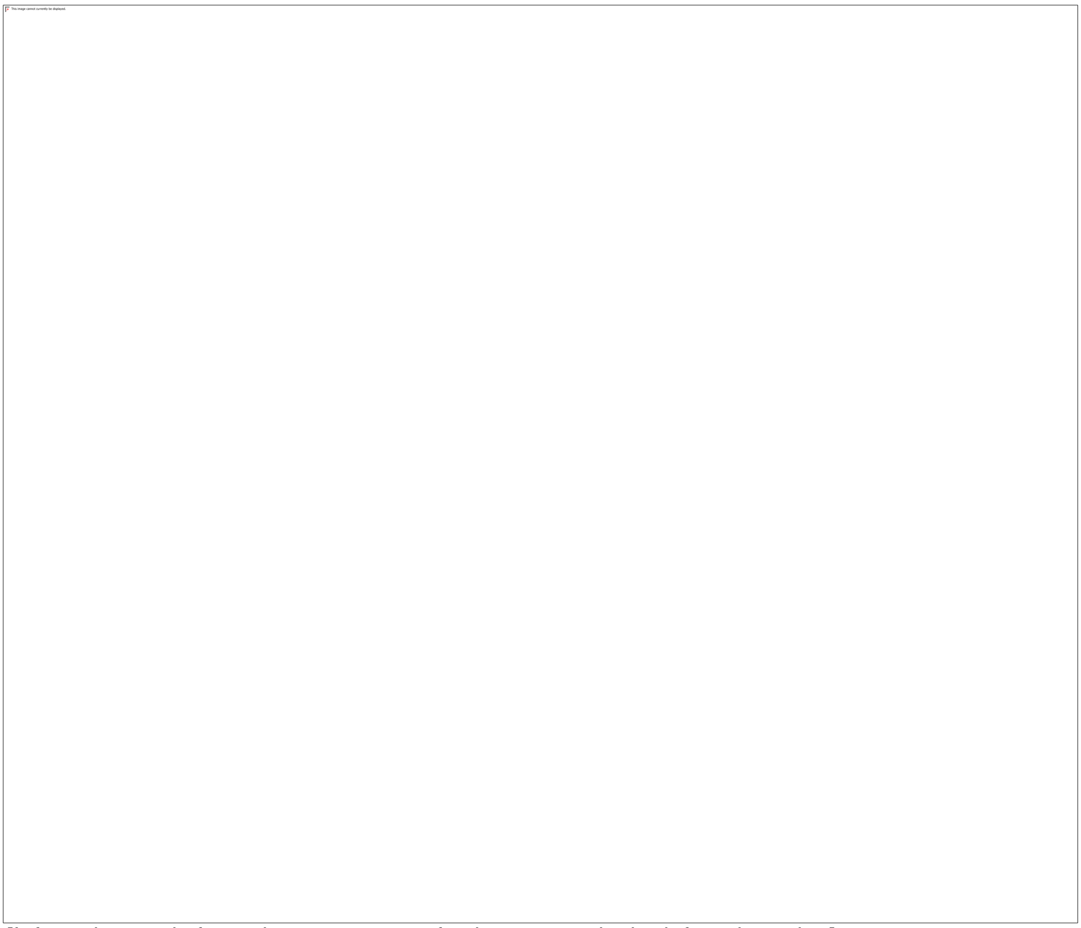

[Infected necrotic fungating recurrence of vulvar cancer in the left groin region]

[3D reconstruction of the pelvic CT showing the groin recurrence encasing the left femoral vessels]

Disclosure: Nothing to disclose

EP1148

Treatment outcome of patients with carcinoma of vulva - NCI - Cairo experience

A. Abdelhamid[1], M. El Naggar[1], E. El-Kest[1], E. El Nakoury[2]
[1]Radiation Oncology Department, National Cancer Institute, Cairo University, [2]Kasr El Ainy Center of Clinical Oncology (NEMROCK), Cairo, Egypt

Introduction / Background: Treatment of patients with vulvar carcinoma is challenging for oncologists, owning to the low incidence of the disease and the poor level of evidence for different treatment modalities.
Methodology: This is a retrospective study involving patients with pathologically proven vulvar carcinoma, who presented to the National Cancer Institute, Cairo University during the period from January 2001 to December 2016.
Data was retrieved from the patients' medical records. The collected data included the patients' names, age, menopausal status, comorbidities, obstetric history, performance status, pathological subtype, FIGO stage, grade, date and type of surgery, details of adjuvant treatment and response to treatment, side effects and
complications of treatment, follow-up details and patterns of failure, salvage
treatment and patient status at last follow up.
Results: This study highlights and confirms the data stating that lymph node status is the most important predictor for outcome of patients with carcinoma of vulva and also confirms that < 2 lymph nodes have a better OS and DFS than patients ≥ 2 positive lymph nodes.
Postoperative radiotherapy significantly improved survival for patients who underwent surgical

resection which was statistically significant.
It shows that tumor size at a cut-off value of ≤ 5 cm had better OS than patients with tumors >5 cm which was statistically significant.
Old age had significantly worse survival at a cut-off value of ≤ 69 years.
Conclusion: patients with < 2 positive lymph nodes had better OS than patients ≥ 2 positive lymph nodes, lack of prognostic significance of 2009 FIGO staging system should be studied carefully in larger studies in the future, also this study calls considering to include tumor size in staging system for cancer vulva, and downstage the patients with one positive lymph node.
Disclosure: Nothing to disclose

EP1149

A rare vaginal lesion - the potential for malignancy

W. Abdelrahman Ramadan[1,2], N. Halder[3], H. Samir[4], C. Jones[3], I. Abdelrahman[3]
[1]University of Birmingham, Birmingham, [2]Hywel Dda University Health Board, [3]Obstetrics and Gynaecology, [4]Radiology, Hywel Dda University Health Board, Carmarthen, United Kingdom

Introduction / Background: Epithelioid leiomyomas have been well studied in the gastrointestinal tract however, its presence in the female genital tract is extremely rare. Epithelioid leiomyomas include leiomyoblastoma, clear cell and plexiform leiomyoma. Consensus is lacking on their differentiating features and malignant potential.
Methodology: A 48-year-old P2 lady with BMI 50 presented to Emergency Department with 3-week history of vaginal bleeding and passing grape-like products vaginally. Clinical examination elicited a cystic lesion measuring 2cm in diameter on the anterior vaginal wall that was also noted on pelvic TAS. Histology of the tissues passed vaginally suggested microscopic features of malignancy suggestive of Embryonal rhabdomyosarcoma. Her case was discussed at the regional Sarcoma MDT. MRI showed a 2.3cm hypo-intense vaginal mass with no evidence of fat-stranding or lymphadenopathy and CT scan ruled out metastatic disease. Although the morphological features mimicked sarcoma with strong expression of muscle-related antigens however immunophenotyping did not support malignancy and confirmed a cellular epithelioid leiomyoma. Due to the diagnostic complexity, excisional biopsy and hysteroscopy was decided for. The entire tumour was excised through transvaginal route with hydro-dissection of its planes and enucleation of its capsule leaving clear margins around. Hysteroscopic finding was normal. Tumour biopsy showed lack of necrosis or mitotic activity thereby excluding neoplastic process and confirmed the diagnosis of an epithelioid vaginal wall leiomyoma. MDT recommended close post-operative surveillance.
Results: Wherever possible TVS is superior to TAS for vaginal lesions. MRI is excellent in tissue characterisation of the lesion and essential to evaluate relation / invasion to adjacent structures. Histological confirmation remains the gold standard for diagnosis. Tumour recurrence or rapid enlargement alongwith microscopic features of >5 mitoses/10 HPF with atypia suggests sarcomatous change and removal *en-bloc* is recommended.
Conclusion: Sarcomatous transformation and recurrence after 11 months and 10 years have been reported hence close surveillance is essential.
Disclosure: Nothing to disclose

EP1150

Pelvic exenteration for locally advanced vulvar cancer in South-West Wales

G.O. Abdulrahman Jnr, N. Das, K. Lutchman Singh
Swansea Gynaecological Oncology Centre, Swansea Bay University Health Board, Swansea, United Kingdom

Introduction / Background: Pelvic exenteration is a major surgical procedure for advanced or recurrent gynaecological cancer. It is a potentially curative treatment but it could also be performed with palliative intent. Locally advanced vulvar cancer is rare and extremely challenging to manage. We aim to report our experience with pelvic exenteration for locally advanced vulvar cancer in our centre.
Methodology: All patients undergoing pelvic exenteration between December 2014 and April 2019 at the Swansea Gynaecological Oncology Centre were identified through our surgical database. Patient characteristics, the indications for the procedure, pathology results and outcomes were retrospectively analysed. We used frequencies and percentages to describe the sample. Mean values were calculated where appropriate. Statistical analyses were performed with IBM SPSS 25.0 (SPSS Inc, Chicago, IL, USA).
Results: 6 women were identified who had pelvic exenteration for locally advanced vulvar cancer during the study period. All were squamous cell carcinoma. The mean age of the women was 54.2 years (42 - 64 years). 5 women underwent posterior pelvic exenteration while 1 women underwent total pelvic exenteration. 3 patients required VRAM flap. 2 women died during the study period but they had pelvic exenteration with palliative intent and both had positive margins on histology, with an average survival of 8.5 months. The remaining 4 women had negative margins on histology and are still alive despite 3 of them having positive lymph nodes. 4 women had a form of radiotherapy or chemotherapy or both.
Conclusion: Pelvic exenteration is a major procedure with high morbidity but it is a reasonable treatment option for women with locally advanced vulvar cancer for both curative and palliative intent. We continue to closely follow up all our patients.
Disclosure: Nothing to disclose

EP1151

A case report on primary cutaneous mucoepidermoid carcinoma of the vulva and its distinct clinico-pathologic identity

J.K. Abrenica, L.M. Cole, J. Bagadiong
OB-GYN, Jose R. Reyes Memorial Medical Center, Manila, Philippines

Introduction / Background: Mucoepidermoid Carcinoma (MEC) is an epithelial malignant tumor that was first described as a salivary gland malignancy. Though common in salivary gland, it is extremely rare in the vulva with only 2 cases reported in the English language literature and none yet in the Philippines. The objectives of this case report are to present an extremely rare case of MEC in the vulva and describe its clinico-pathologic characteristics, explain how MEC may occur in this unusual location and discuss its management and other treatment options.
Methodology: This is a case of a 68 year-old woman with a history of vulvar pruritus and vulvar mass at the left labia majora. Punch biopsy and review of slides revealed Invasive Squamous Cell Carcinoma, Non-Keratinizing type. She underwent Radical Vulvectomy and Bilateral Lymph Groin Dissection; Wide Excision of Perineal Area; Protective Transverse Loop Colostomy; Gracilis Myocutaneous Flap with Identification of Right and Left Median Circumflex Artery with a final histopathology report of Primary Cutaneous MEC of the vulva with lymph node metastasis.
Results: Due to its low incidence, prognosis and definitive management is still unclear. The patient was started on adjuvant chemotherapy, followed by Intensity Modulated Radiation Therapy, as in other histopathologic diagnosis of advanced stage vulvar carcinoma.
Conclusion: This is a case of a primary cutaneous MEC of the vulva in a 68 year-old woman who presented with a history of intermittent vulvar pruritus and vulvar mass. To the best of our knowledge, this is the first reported case of vulvar carcinoma having this rare pathology in our local setting. Due to limited studies available, tailored management should still be investigated.
Disclosure: Nothing to disclose

EP1152

Prevalence of occult cancer in biopsy proven VIN3 and dVIN in lesions suspicious and not suspicious of malignancy

A. Shaban[1], S. Abdul[1], V. Asher[2], A. Bali[1], A. Phillips[1]
[1]Obs and Gyn, University Hospitals of Derby and Burton, [2]Obs and Gyn, University Hospital of Derby and Burton, Derby, United Kingdom

Introduction / Background: The prevalence of occult invasive malignancy in biopsy proven VIN3/dVIN has been suggest to be between 9-22%. Traditionally precancerous vulval lesions have been treated with a wide local excision which can significantly alter the vulval anatomy
Aim To determine the prevalence of invasive cancer in biopsy proven VIN3/dVIN. To determine whether clinical suspicion (or not) of invasion in lesions where the biopsy shows precancerous lesion changes can help tailor management allowing less radical treatment.

Methodology: Retrospective study of all patients at the Royal Derby Hospital from 2009-2018 who received wide local excision or skinning vulvectomy (WLE/SV) for a biopsy proven VIN3 or dVIN lesion within 6 months from initial diagnosis. Patient, surgeon and pathological data was recorded.

Results: 86 patients were identified. 71 patients were diagnosed with VIN3 at the initial biopsy of which 7(9.8%) showed invasive cancer after WLE/SV. When subgrouped by clinical suspicion (if recorded) 4/11 (36%) suspicious VIN3+ve lesions showed cancer compared to 2/46 (4.3%) that did not. Overall the PPV and NPV for clinical assessment in biopsy proven VIN3 was 36% and 96% respectively.
15 patients were diagnosed with dVIN, of which 6 (40%) demonstrated cancer after WLE/SV. Clinical suspicious was recorded in five cases of which four (80%) ultimately had a cancer after WLE/SV. The PPV and NPV for clinical suspicion of invasion in dVIN was therefore 80% and 78% respectively

Conclusion: Prevalence of occult carcinoma in biopsy proven VIN3 and dVIN is 9.8% and 40% respectively in this cohort. Clinical suspicion at the time of biopsy alters the subsequent risk of malignancy and may allow less radical surgery in selected women with VIN3 with no clinical suspicion of invasion

Disclosure: Nothing to disclose

EP1153

Skinning Vulvectomy and Conization together in the treatment of Vulvar Intraepithelial Neoplasia (VIN) 3 of the vulva and High Squamous Intraepithelial Neoplasia (HSIL) of the uterine cervix: oral presentation

M.C. Ateş[1], S. Akar[1], A.H. Güler[1], E. Cintesun[2], C. Ugurluoglu[3], Ç. Çelik[1]
[1]Gynaecological Oncology Surgery, [2]Gynaecology and Obstetrics, [3]Pathology, Selcuk University Medical Faculty, Konya, Turkey

Introduction / Background: Vulvar intraepithelial neoplasia (VIN) is termed premalignant situation of the vulva (1). VIN incidence is about 1.4/100.000. Risk factors for VIN are Human Papilloma Virus (HPV) infection, HIV(Human İmmunodeficiency Virus) infection, other sexual transmitted diseases, smoking and other neoplasms(2).In 2015, İnternational Society for the Study of Vulvovaginal Disease(ISSVD) released that describes VIN; Low-grade squamous intraepithelial lesion(LSIL) of the vulva(HPV effect), High grade squamous intraepithelial lesion(HSIL) of the vulva (VIN usual type) and VIN differentiated type(dVIN) (3). The increase of VIN usual type among younger patients is generally due to the infection of high risk HPV (4). The dVIN is seemed commonly in older women with a median age of 67(5), and is usually associated with skin disases like lichen sclerosus.Suspicious for malignancy or-premalignant lesions of the vulva may be excised in a conservative fashion, protecting as much of the vulvar architecture as possible(6).
Cervical intraepithelial neoplasia is termed premalignant situation of the uterine cervix (7). HPV infection is considered the most important factor for the development of CIN and cervical cancer. High grade lesions have significant risk of disease progression to the cervical cancer and should be

managed with conization. This will be therapeutic as well as diagnostic.
Methodology: Our patient was 42-years old and multiparous patient with main complaint of vulvar pruritus and genital condyloma.Her outer hospital vulvar and cervical-biopsy result was squamous carcinoma in situ for vulva and HSIL for cervix.We re-evaluated the pathology in our hospital. The biopsy revealed for cervix VIN 3. Then she underwent conization and vulvectomy with a 2 cm disease free-margin.
Results: After the excision procedure patient´s skinning vulvectomy pathology was VIN3, psoriasis, lichen sclerosis and conization pathology was LGSIL.Both surgical margins were clear. After the surgery there wasn´t any wound dehiscence, infection or abscess.
Conclusion: In VIN3 lesions skinning vulvectomy appears to be a favourable and safe treatment however the recurrence of VIN is frequent (8). It should-be applied carefully evaluated patients.
Disclosure: Thanks for your concern. References 1- Reyes MC,Cooper K. Anupdateonvulvarintraepithelialneoplasia:terminologyand a practicalapproachtodiagnosis. J Clin Pathol 2014; 67:290. 2- Iavazzo C,Pitsouni E,Athanasiou S,Falagas ME.Imiquimod for treatment of vulvar and vaginal intraepithelial neoplasia. Int J Gynecol Obstet 2008; 101:3-10. 3- CrumCP,HerringtonCS,McCluggageWG,et al. Tumours of the vulva; epithelial tumours. In: WHO Classification of Tumours of Female Reproductive Organs, 4th ed, Kurman RJ, CarcangiuML,HerringtonCS,et al (Eds), IARC Press, Lyon 2014. 4- TanJ,ChettyN,Kondalsamy-ChennakesavanS,CrandonA,GarrettA,LandR,et al. Validation of the FİGO 2009 staging system for carcinoma of the vulva. Int J Gynecol Cancer.2012;22(3):498-502. 5- JouraEA,GarlandSM,PaavonenJ,FerrisDG, Perez G, Ault KA, et al. Effect of the human papilloma virüs (HPV) quadrivalent vaccine in a subgroup of women with cervical and vulvar disease: retrospective pooled analysis of trial data. BMJ. 2012;344:e1401. 6- von GruenigenVE,GibbonsHE,GibbinsK,et al. Surgical treatments for vulvar and vaginal dysplasia: a randomized controlled trial. Obstet Gynecol 2007; 109:942. 7- MontzFJ.Management of high-grade cervical intraepithelial neoplasia and low-grade squamous intraepithelial lesion and potential complications. Clin Obstet Gynecol 2000;43:394. 8- WallbillichJJ,RhodesHE,MilbourneAM,et al.Vulvar intraepithelial neoplasia (VIN 2/3): comparing clinical outcomes and evaluating risk factors for recurrence. Gynecol Oncol 2012;127:312.

EP1154

Vulvar midline tumors: is it really necessary bilateral inguinofemoral groin evaluation, in an era of de-escalation surgery?

J. Bernardeco, L. Correia, B. López, M. Bernardino, I. Santana, A.F. Jorge
Instituto Português de Oncologia Franscisco Gentil, Lisboa, Portugal

Introduction / Background: Sentinel lymph node (SLN) biopsy is the recommended staging method for single vulvar tumors, <4 cm, without suspected groin metastasis.
The vulva has an ample lymphatic system and especially for midline tumors (tumors that cross midline), its drainage can be made to both groins. According to international guidelines, bilateral detection of SLN is recomended for midline tumors and, in case of unilateral detection, inguinofemoral lymphadenectomy should be performed on the opposite groin.
In an era of de-escalation surgery, should bilateral groin evaluation be mandatory for midline vulvar tumors?
Methodology: Retrospective observational study (2005-2018) of midline squamous-cell vulvar tumors, fulfilling criteria for SLN biopsy. In our oncology center, we detect SLN by performing lymphocintigraphy and using dual-mapping technique (radioactive tracer and blue-dye). In case of unilateral detection we do not execute contralateral lymphadenectomy.
The aim of the study is to evaluate recurrence on the contralateral groin when SLN was negative. To accomplish this, the study-population was divided into two groups, according to SLN biopsy: 1) unilateral; 2) bilateral.
Results: We included 34 cases with a median follow-up time of 37,5 (1-111) months. During surgery, the detection rate of the SLN was 100% and always in accordance with the previous lymphocintigraphy. Group 1 included 16 cases (47%) and SLN biopsy was negative in 13 cases (81,3%). There were 2 cases of groin recurrence, one of which on the opposite side of the previous

SLN. Group 2 included 18 cases (53%) and SLN biopsy was negative in 13 cases (72,2%). There wasn´t any case of groin recurrence.
Conclusion: SLN biopsy is a feasible staging technique and should be directed by previous lymphocintigraphy. Because vulvar cancer is rare, few cases were described, but our approach does not seem to increase the groin recurrence rate on the non-evaluated side. More studies are needed.
Disclosure: Nothing to disclose

EP1155

Endoscopic near infrared and indocyanine green to verify the viability of the subcutaneous flap for vulvar cancer

V.A. Capozzi[1], M. Pugliese[1], A. Cianciolo[1], G. Gambino[1], G. Sozzi[2], G. Armano[1], L. Monfardini[1], M. Gaiano[1], V. Ceni[1], M. Rolla[1], C. Merisio[1], R. Berretta[1]
[1]Departement of Gynecology and Obstetrics of Parma, University of Parma, Parma, [2]Department of Gynecologic Oncology, ARNAS Civico Hospital of Palermo, Palermo, Italy

Introduction / Background: Vulvar cancer often requires radical vulvectomy with subsequent vulvar flap due to tumor aggressiveness and a high percentage of relapses. Approximately in 20-60% of cases, there are post-operative complications ranging from infection to wound dehiscence, lymphocele and flap necrosis that often require reoperation. Several methods have been described to verify the vitality of the flap, especially in plastic surgery, but these are often expensive and require specific machinery that is not generally present in a gynecological clinic. We present a viability verification of V-Y fasciocutaneous advancement flap for vulvar reconstruction by Endoscopic Near-Infrared and Indocyanine Green.
Methodology: The patient was a 67-year-old woman with FIGO IB ≤ 4 cm squamous cell vulvar cancer with the absence of inguinal lymphadenopathy. The lesion appeared about 35 mm from the lateral margin of the large left lip and extended to the left inguinocrural fold (**Figure 1**).
The patient underwent left inguinal lymphadenectomy and left radical hemivulvectomy with a left fasciocutaneous medial-thigh advancement flap.
For the evaluation of the flap, we endovenous administered 50 mg of Indocyanine Green diluted in 10 ml of saline solution. After 10 minutes we visualized the flap margin with a near-infrared laparoscopic view and we noticed a tracer uptake on the whole edge of the flap, the perforating branches were also evident. The evaluation was repeated at the end of the surgical procedure and we confirmed the good vascularization of the flap (**Figure2**).
Results: No early or late post-operative complications were obtained. There was no wound dehiscence, marginal necrosis or surgical site infection. The patient stated satisfaction of the aesthetic level obtained.
Conclusion: Verifying the viability of the vulvar flap using near-infrared laparoscopic optics was easy to use, reproducible and extremely economical technique. This could be an extremely useful and reproducible alternative to other more expensive techniques.

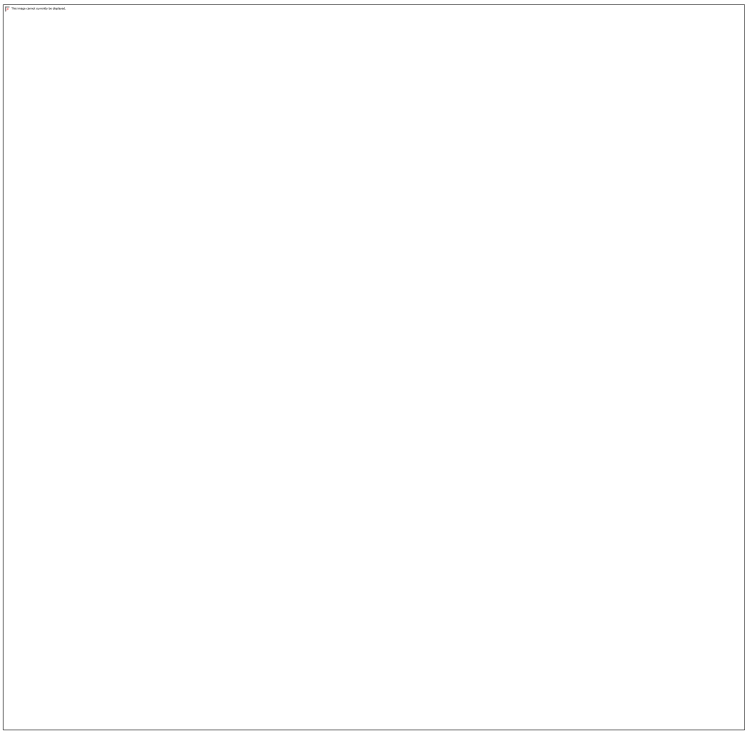

[Figure1]

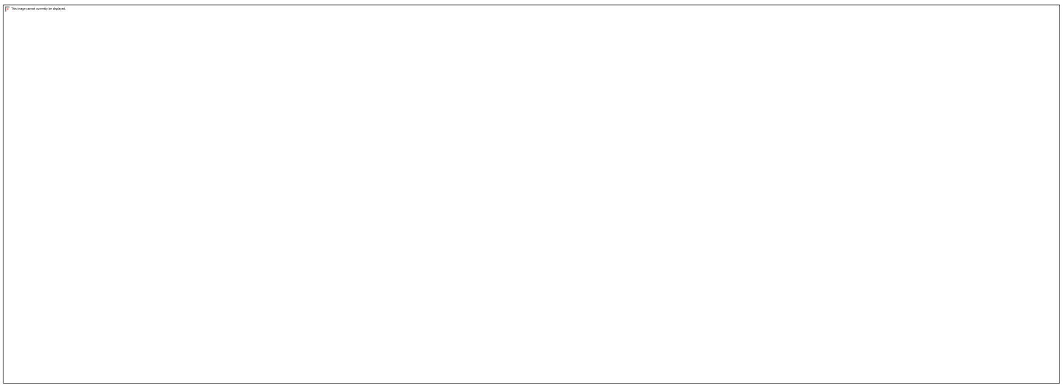

[Figure2]

Disclosure: Nothing to disclose

EP1156

Invasive extramammary paget of the vulva: a case report

I. Chelea
Obstetrics and Gynaecology, Hospital Universitario de Torrevieja, Torrevieja, Spain

Introduction / Background: : Extramammary Paget diseases includes a set of rare entities. Among

those, invasive Paget´s disease of the vulva is a rare disease, accounting for < 1% of all vulvar malignancies. It can be classified into primary and secondary, according to immunohistochemistry. There are three entities into the primary group: intraepithelial Paget´s disease, invasive Paget and Paget´s disease as a manifestation of an underlying adenocarcinoma of a skin appendage or a vulvar gland. In this communication we describe a case of invasive Paget of the vulva.
Methodology: A 73 years old woman came to our hospital because of an asymptomatic irregular lump in the vulva that had appeared several years ago. The result of the biopsy was Paget's disease with no invasion. The TC scan informed about a tumor of about 6 cm in diameter with no lymphatic nodes affected. We performed a radical hemivulvectomy with sentinel lymph node evaluation with technetium.
Results: The intraoperative study of the sentinel node was negative. The patient could be discharged 3 days after surgery. The final results of the pathological examination of the hemivulvectomy informed about Paget disease with invasion lesser than 1 mm with tumor free edges, and the final pathological report of the sentinel node was isolated malignant cells. FIGO stage IIIA.
Conclusion: Paget's disease of the vulva is a rare entity than sometimes can be confused with squamous cell cancer because of its clinical presentation, but we have to suspect it and dismiss it order to be able to bring the best treatment to our patients.
Disclosure: Nothing to disclose

EP1157

A preliminary analysis of adverse prognostic factors in vulval cancer patients treated with radical electro-resection surgery and groin lymphadenectomy

M. Cieslak-Stec[1], E. Telka[1], K. Drosik-Rutowicz[1], M. Sliwinska[1], P.M. Gawkowska[1], P. Chimiczewski[2], K. Raczek-Zwierzycka[1], R. Tarnawski[1]
[1]*Oncology Centre (MSCI), Gliwice MDe Branch,* [2]*iOncology Centre (MSCI), Gliwice MDe Branch, Gliwice, Poland*

Introduction / Background: Vulval cancer is a rare malignancy, accounting for 3-5% of all cancers of female genital organs, with approximately 500 new cases diagnosed in Poland yearly. The study has been aimed at the analysis of the frequency of adverse prognostic factors in patients undergoing radical electro-surgery and groin lymphadenectomy.
Methodology: A total of 28 cases have been analysed. All patients covered by the study had FIGO stage IB-III vulval cancer and were treated with radical electro-surgery and groin lymphadenectomy between March 2016 and December 2018 The patients' age ranged from 46 to 82 years old, and their eligibility for treatment was decided by a designated panel of specialists made up of gynaecologic oncologists, radiotherapy specialists and clinical oncologists.. Surgical treatment consisted in radical electro-resection and groin lymphadenectomy.
Results: Early recurrence was found in 6 patients (22%). 4 out of 6 patients had local vulvar recurrence, which was treated with a wide excision with a surgical margin = 1 cm. In all cases of early recurrence, the primary tumour had involved the clitoris.
The remaining 2 patients suffered from groin recurrence. In all these cases, a narrow original surgical margin ≤ 0.3 cm was determined, and the patients had post-surgically received adjuvant intensity modulated radiation therapy (IMRT) in a dose of 50 Gy/25 fr.
Conclusion: 1.Adverse prognostic factors in vulval cancer patients treated with radical electro-resection surgery and groin lymphadenectomy include:
-clitoral involvement,
-narrow surgical margin ≤ 0.3 cm, the latter correlating with a higher incidence of groin recurrence.
Disclosure: Nothing to disclose

EP1158

Case report: Leiomyosarcoma of the Vulva (STUMP) an incidental finding at marsupialization of Bartholin´s cyst

T. Davidson, G. Angelopoulos
Gynae-Oncology, Women and Children's Department, University Hospital Coventry & Warwickshire NHS Trust, Coventry, United Kingdom

Introduction / Background: Leiomyosarcoma of the Vulva is very rare. It accounts for 1-2% of gynaecological cancers. [1,2,3] Leiomyosarcoma originates from smooth muscles, blood vessels, round ligaments, and erector-pili muscles.[2,3] On the Bartholin's area it may show similarities to cysts therefore causing delayed diagnosis. [3]
Methodology: We report the case of a 40-year-old woman who was referred to our tertiary hospital following a routine marsupialization for Bartholin's cyst. The histology confirmed incomplete excision of leiomyosarcoma - smooth muscle tumour of uncertain potential (STUMP). The management of her case was referred to the central Gynaecological Oncology Multidisciplinary Team (MDT). She underwent staging CT and MRI investigations, followed by radical wide local re-excision. Post-surgery adjuvant radiotherapy was considered. However, the patient refused due to insufficient literature support. Instead, close clinical and radiological surveillance was commenced.
Results: Pre-surgery CT and MRI investigations did not reveal metastatic disease. Re-excision performed confirmed clear margins with no residual tumour. Eighteen months later she remains tumour free.
Conclusion: Atypical presentation of Vulval lesions including at the area of the Bartholin's gland should raise suspicion. Appropriate investigations and referral to a central cancer MDT ought to be considered. Radical wide local excision is the recognized treatment. Adjuvant radiotherapy and/or chemotherapy needs to be considered but individualized to the patient. Intense long term surveillance is required due to possibility of delayed recurrence.
References:
1. Nielsen GP et al: Smooth muscle tumors of the vulva: A clinicopathological study of 25 cases and review of the literature. Am J Surg Pathol 1996;20:779-793
2. Losch A et al: Leiomyosarcoma of the vulval A case report; J Repro Med 2001;46(6):209-612
3. Chokoeva AA et al: Vulvar sarcomas: Short guideline for histopathological recognition and clinical management. International J Immuno Pharm 2015;28(2):168-177.
Disclosure: Nothing to disclose

EP1159

Staging system comparison in vulvar squamous-cell cancer

F. Ferrari[1], S. Forte[2], E. Bonetti[2], E. Sartori[2], F. Odicino[2]
[1]Department of Obstetrics and Gynecology, Spedali Civili Brescia, [2]Department of Obstetrics and Gynecology, University of Brescia, Brescia, Italy

Introduction / Background: Currently, there is no agreement regarding the most important prognostic factors of vulvar cancer used to build staging system. The aim of this study is to test the homogeneity, monotonicity and survival classification of FIGO 1988 and 2009.
Methodology: We evaluated cases of vulvar squamous-cell carcinoma treated with primary surgery at Spedali Civili of Brescia (Italy) from January 2005 to December 2015. We compared stage distribution of the following poor outcomes of interest between FIGO 1988 and 2009 staging system: local recurrence (LR), nodal recurrence (NR), distant recurrence (DR), multiple recurrence (MR) and disease-specific death (DSD). We used McNemar test to calculate homogeneicity and monotonicy and Kaplan-meier curves with log-rank test to calculate survival differences.
Results: We found a total of 123 patients. FIGO 2009 shows a worse homogeneity for poor outcomes compared to FIGO 1988 respectively for stage I with 54 (44%) and 18 (15) events (p = 0.0027) and for stage II with 8 (7%) and 44 (36%) events (p = 0.00). FIGO 2009 has a better monotonicity compared

to FIGO 1988 for stage III with 52 (42%) versus 36 (29%) events (p = 0.05), while it has a worse monotonicty in stage IV with 12 (5%) versus 22 (18%) with p = 0.02. Survival rates for every stage were compared between FIGO 1988 and 2009 and are as follows: stage I 89.5% versus 89.4% (p = 0.89), stage II 83.9% versus 50% (p = 0.04), stage III 52.9% versus 38.1% (p = 0.07) and stage IV 28.6% versus 0% (p = 0.03).
Conclusion: FIGO 2009 generally fails to classify poor events when compared to FIGO 1988, however it has a better survival classification. Further prognostic factors should be implemented in future revision of the staging system.
Disclosure: Nothing to disclose

EP1160

Prognostic factor in primary vulvar squamous-cell cancer in the context of FIGO 1988 and 2009 and Brigham and women's vulvar tumor classification system

F. Ferrari[1], S. Forte[2], E. Bonetti[2], E. Sartori[2], F. Odicino[2]
[1]Department of Obstetrics and Gynecology, Spedali Civili Brescia, [2]Department of Obstetrics and Gynecology, University of Brescia, Brescia, Italy

Introduction / Background: The aim of the study is to identify prognostic factors associated with poor outcomes in vulvar squamous-cell cancer and to compare the prognostic discrimination of FIGO 1988 and FIGO 2009 staging systems with the new Brigham and Women's Vulvar Tumor Classification system (BWVTC).
Methodology: We retrospective evaluated patients with vulvar squamous-cell carcinoma treated with primary surgery at Spedali Civili of Brescia (Italy) from January 2005 to December 2015. We collected dmeographic data and relevant prognostic factors such as age, tumor size, length of free surgical margin in millimeters, depth of invasion (DOI) in millimiters, presence of perineural invasion (PNI), lymph vascular space invasion (LVSI) and lymph nodal status. We used logistic bivariate regression models to investigate survival outcome. We developed multivariate regression Cox models for survival in FIGO 1988, 2009 and BWVTC using significant risk factors.
Results: We found a total of 123 patients. We identified five relevant risk factors as follows: DOI $\geq$ 9 mm (HR 2.9 - CI95% 1.10-8.35; p = 0.04), PNI (HR 15 - CI95% 4.34-51.82; p = 0.00), LVSI (HR 5.3 - CI95% 1.74-15.82; p = 0.00), free surgical margin < 8 mm (HR 4.9 - CI95% 1.02-23.31; p = 0.04) and positive lymph nodes (HR 6.3 - 1.81-22.11; p = 0.00). Multivariate regression Cox models were not significant for FIGO 1988 (p = 0.20) and 2009 (p = 0.24); in FIGO 2009, positive lymph node status was excluded from analysis as factor since its direct correlation with stage. In BWVTC two factors resulted significant: PNI (HR 4.17; CI95% 1.19-14.65; p = 0.02) and positive lymph node (HR 4.24; CI95% 1.23-14.63).
Conclusion: PNI and positive lymph nodes remain the most important prognostic factors in vulvar cancer, regardless staging system.
Disclosure: Nothing to disclose

EP1161

Exome analysis of vulvar and vaginal tumors reveals an antigen processing and presentation deficiency

L. Bengrine[1], J.-D. Fumet[1], L. Favier[1], C. Richard[2], L. Arnould[3], R. Boidot[4]
[1]Medical Oncology, [2]Statistics, [3]Biopathology, [4]Research Platform in Biological Oncology, Centre Georges Francois Leclerc, Dijon, France

Introduction / Background: The rare vaginal and vulvar cancers are considered to be histologically

similar to cervical squamous cell carcinoma. However, mutational landscape differences between them are unclear. In the time of precision medicine and immunotherapy, a personalized therapy approach based on a detailed biological characterization of each tumor entity should be considered. This study aimed to identify differences and specific genetic alterations in vaginal and vulvar compared to cervical squamous cell carcinoma.
Methodology: Using next generation sequencing (NGS), both somatic and germline exome sequencing of vulvar (n=11), vaginal (n=') and cervical cancer (n=11) were analyzed. To determine a potential sensitivity to immunotherapy, we studied tumor mutation burden, ratio indels, TCR clonality, neopeptides, Alexandrov's signatures, and copy number variation. Additionally a signaling pathway analysis was performed applying 2 different approaches (NCI and KEGG).
Results: No significant differences were seen in the respective immunologic predictive biomarkers comparing the three tumor entities. The signaling pathway analysis revealed an alteration of antigen processing and presentation pathways in vulvar and vaginal cancer compared to cervical cancer (77 genes). Moreover, an activation of Notch signaling was found in vulvar and vaginal tumors. Vulvar and vaginal cancer reveal a comparable mutational landscape in the analyzed genes.
Conclusion: NGS determines a potential immune evasion strategy of vulvar and vaginal tumors related to an alteration of antigen presentation. Our findings suggest that immunotherapy might be less efficient compared to cervical cancer. Targeting Notch and mTOR pathway may be a promising therapy approach in these rare tumors.
Disclosure: Nothing to disclose

EP1162

Adenoid Cystic Carcinoma of Bartholin´s gland as a challenging clinical and surgical problem. Reporting two new cases and their outcome

C. Goudeli[1], N. Koufopoulos[2], E. Laini[3], D. Vassilakos[4], E. Terzakis[1]
[1]Institouto Gynaikologias, [2]2nd Department of Pathology, National and Kapodistrian University of Athens, [3]Department of Cytology, Alexandra Hospital, National and Kapodistrian University of Athens, [4]Mikroskopisi, Athens, Greece

Introduction / Background: Adenoid cystic carcinoma (ACC) is an aggressive slow-growing tumor occurring in women with mean age 49 years. The neoplasm is similar to those of salivary gland, evolving from chromosomal translocation (NFIB) and having no HPV-correlation. ACC accounts for 15% of Bartholin´s gland (BG) malignancies and for 2-7% for vulvar carcinomas, with less than 90 cases described. Treatment remains individualistic as there is no level I evidence available, making the new cases crucial to be reported.
Methodology: A 46-year-old female, presented after a positive biopsy for ACC-BG. She reported a small swelling in her left posterior labia minor during the last 6 months. She had the BG cyst removed and the pathology showed ACC 3x3cm encapsulated inside the cyst. Another 45-year-old female, presented after an excision of a vulvar mass 3,5x2,5x2,5cm of the left labia minor after swelling for the last 2 years which resulted histologically in ACC-BG with positive margins and neural and muscular invasion.
Results: Supplementary surgery was decided and the first patient underwent radical left hemivulvectomy with ipsilateral inguinofemoral lymph node excision. There was no residual disease and no adjuvant therapy was needed. In the second case we performed imaging control and radical vulvectomy with bilateral inguinofemoral lymph node dissection. The pathology-report described a middle line tumor 2x1,25x1,2cm infiltrating the outer third of vagina plus one left lymph node, having negative margins (FIGO stIIIa); she then underwent adjuvant radiotherapy. Both women are disease-free after 16 and 20 months respectively.
Conclusion: Radical excision with negative margins is beneficial, while adjuvant radiotherapy should be considered for patients with risk factors (tumor size, lymph node status, surgical margins). Lung is the most common site of distant metastasis and several chemotherapeutic agents have been used such as cyclophosphamide, adriomycin, 5-FU, cisplatin and tamoxifen. ACC should always be considered in patients older than 40 years with BG lesions.
Disclosure: Nothing to disclose

EP1163

2 cases of vaginal melanoma treated by robot assisted total vaginectomy

S. Hwang
Obstetrics and Gynecology, Inha University Hospital, Incheon, Republic of Korea

Introduction / Background: Vaginal malignant melaona is rare non-cutaneous melanoma. It account less than 5% of vaginal cancers and 0.5% of all malignant melanomas. I experienced 2 cases of primary malignant melanoma.
Methodology:
Case 1: 58 years old patient was referred due to vaginal black discoloration. She was noticed black discoloration 3 months ago at local OBGY clinical.
3 months later, local clinical doctor said black discoloration increased and recommended to our hospital. Punch biopsy was done on several black colored vaginal surface.
Case 2: 62 years old patients visited my office due to vaginal discharge. There was black discolortaion spreading whole vaginal surface including uterine cervix. Punch biopsy was done on vagina and uterine cervix.
Results: I did Robot assisted radical hysterectomy with pelvic lymph node dissection. I did upper 1/3 vaginal dissection via intraperitoneal approach and via vaginal approach I did total vaginectomy. Plastic surgeon did vulvovaginal reconstruction surgery.
Conclusion: 1st case there was pelvic lymph node positive and 2nd case was lymph node negative. I did adjuvant immounotherapy on 1st case patient and did not done any adjuvant therapy on 2nd patient.
Disclosure: Nothing to disclose

EP1164

Modified oblique versus classical 'lazy S' incision for inguino-femoral lymph node dissection; complications, node count and hospital stay

S. Jones, J. Davies, R. Jones, K. Lim, R. Howells, A. Sharma
University Hospital of Wales, Cardiff, United Kingdom

Introduction / Background: Inguino-femoral lymph node dissection plays a crucial role in the management of vulval cancer. The procedure is associated with high complication rates including infection, lymphocysts and wound dehiscence. Several skin incision techniques exist and practice amongst gynaecology oncologists is variable. Little evidence exists to guide surgeons regarding optimal surgical approach. This study aimed to determine the difference in complication rates, node harvest and length of stay between patients undergoing the modified oblique and classical 'lazy S' skin incision.
Methodology: A retrospective review of 4 years of data between 2014 and 2018 was carried out in the UHW, Cardiff. Data collected included age, BMI, incision, length of stay, complications, cancer stage, suture material, node count and recurrence of disease. Data were analysed using SPSS software and clinical significant was set as p=< 0.05.
Results: Thirty-five cases of classical 'lazy S' and 14 cases of modified oblique. More serious, grade 3/4 complications were significantly more common following classical 'Lazy S' versus modified oblique (20/35, 57.1% vs. 2/14, 14.3%). Mean number of nodes harvested was significantly higher in the classical 'Lazy S' group compared to the modified oblique (11.1 vs. 7 nodes). Mean hospital stay was significantly higher in patients undergoing classical 'Lazy S' vs. Modified oblique (10.7 vs. 4.5 days). One case of groin node recurrence occurred and this patient was in the classical 'lazy S' arm.

Conclusion: This study demonstrates significantly lower rates of overall and serious complications following modified oblique skin incision compared to classical 'Lazy S'. However, this would appear to be at the compromise of a reduced node count. This study was not designed to determine the clinical significance of node count and recurrence but this is also a subject upon which the literature is scarce. More research is required to determine optimal surgical incision in this challenging procedure.
Disclosure: Nothing to disclose

EP1165

Induction of chemokine receptor and suppression of inhibitory receptor of CD8 T cell effectively controlled HPV-associated tumor

S. Lee, Y. Jung
Seoul St. Mary's Hospital, OBGY, The Catholic University of Korea, Seoul, Republic of Korea

Introduction / Background: Activation of exhausted CD8 T cell and migration of immune cells into tumor site is indispensable for overcoming resistance to cancer therapy. We evaluated the role of suppression of inhibitory receptor and chemokine axis in HPV-associated tumor bearing mouse.
Methodology: C57BL/6 mice were categorized into four groups according to treatment modality. Mice were challenged with 1×10^5 TC-1 cells on vulva. HPV DNA therapeutic vaccine was injected intramuscularly and Imiquimod cream was applied on vulva cancer. The mice were harvested on day 21 and immune cells were investigated by flow cytometry. We checked the expression of inhibitory receptors of CD8 T cells, including PD1, TIM3 and LAG3. Chemokine axis such as CXCL9, CXCL10, and CXCR3 were evaluated to demonstrate migration mechanism.
Results: Combination of HPV DNA vaccine and Imiquimod resulted in a significantly lowest percentage of inhibitory receptor of CD8+ T cells including PD1, TIM3 and LAG3 in spleen and tumor ($p< 0.05$). They significantly induced accumulation of tumor specific CD8 T cell in tumor site and increased expression of CXCR3 on tumor infiltration CD8 T cell ($p< 0.05$). Chemokine such as CXCL9, CXCL10 was overexpressed in tumor site after combination therapy ($p< 0.05$). Finally, mice treated with combination therapy survived significantly longer than other groups ($p< 0.05$).
Conclusion: In conclusion, we overcame T cell exhaustion and identified chemokine axis during migration of CD8 T cell into TC-1 tumor site using HPV DNA vaccine and Imiquimod. This mechanism can be ideal target for future immunotherapy.
Disclosure: Nothing to disclose

EP1166

A huge vulva tumor treatment

K. Kim[1], A. Moshi[2], H.S. Kang[3], J.T. Kim[4], S.L. Nam[5]
[1]Obsterics and Gynecology, Presbyterian Medical Center, Jeonju, Republic of Korea, [2]Obsterics and Gynecology, Muhimbili National Hospital-Mloganzila, Dar es Saalaam, United Republic of Tanzania, [3]Nursing, Kongju National University, Kongju, [4]Plastic and Reconstructive Surgery, Hanyang University Hospital, Seoul, Republic of Korea, [5]Obsterics and Gynecology, Muhimbili National Hospital-Mloganzila, Dar es Salaam, United Republic of Tanzania

Introduction / Background: Angiofibroblastoma is a mesenchymal tumor that affects mostly reproductive aged woman although very rare case have been reported in male. It is generally asymptomatic mass that primaily arises in the vulva region The treatment require s simple local excision. We present a case which managed with total vulvectomy , wet dressing and negative pressure wound dressing and reconstructive surgery with full thickness skin graft from both thigh.
Methodology: A 29 -year- old female , nullioarous patient presented with five year s hystory of

progressive growing of both labia. The mass was 3x10x3cm of Rt side and 5x12x9cm of Lt vulva that were rubbery firm abd replace both labia major(Fig!)
Results: The patient underwent total vulvectomy with partial closure was done, She had everyday wet dressing and colostomy due to risk of wound contamination, She was started negative pressure dressing to help wound healing. Vulva reconstruction surgery and colostomy closure after 2months care.
Conclusion: After total vulvectomy, huge operative wound can be controlled negative pressure dressing and skin graft reconstructive surgery .A gynecologist and plastic surgern cowork could lead to good result for patients.
Disclosure: Nothing to disclose

EP1167

Vaginal leiomyoma: a case report

N.W. Lee, Y.Z. Kim, K.-J. Min, J.-K. Lee, J.Y. Song
Department of Obstetrics and Gynecology, Korea University Ansan Hospital, Ansan, Republic of Korea

Introduction / Background: Different from Uterine leiomyomas which are common benign tumor in woman, Vaginal leiomyomas are very rare, having only 350 cases reported in the medical literature. However, leiomyomas are the most common mesenchymal tumors of the vagina in adult women, and can easily be misdiagnosed due to its low incidence and diverse, nonspecific clinical features.
Methodology: A 43-year-old woman, para 2, visited our OPD department with chief complaint of about 4cm sized prolapsing soft mass through vagina. Sonographic findings and physical examinations suggested vaginal cystic mass and surgery was recommended but the patient did not visited our center since she had so symptom other than intermittent prolapse. After 4 years later, the patient visited our center again with symptom of vaginal bleeding and discharge. About 6.0cm by 5.0cm semisolid vaginal mass near the urethra and bladder was found by sonography, and 8.3 by 5.6 cm sized mass lesion in the vagina was shown in abdomen CT finding, suggesting leiomyoma, most likely.
Results: The patient received vaginal mass excision and anterior repair. About 8cm protruding solid mass was found inside the anterior vaginal wall. Excision and anterior repair was done without any significant complications, and her hospital stay was 24 hours. Clinical and sonographic examination at a follow-up consultation, after 1 month, showed healed vaginal wall without any mass like lesion.
Conclusion: Occurrence of leiomyoma in vagina is very rare, and its sarcomatous transformation is even rare but some case has been reported. Preoperative diagnosis is somewhat difficult and the gold standard of diagnosis is histopathological confirmation. Surgical removal with urethral protection should be done and histopathologic diagnosis should be made to rule out any possibility of malignancy.
Disclosure: Nothing to disclose

EP1168

The influence of obesity on tumor recurrence in vulvar cancer patients

R. Klapdor[1], P. Hillemanns[1], L. Wölber[2], J. Jückstock[3], F. Hilpert[4], N. de Gregorio[5], A. Hasenburg[6], J. Sehouli[7], S.T. Fürst[3], H.G. Strauß[8], K. Baumann[9], F. Thiel[10], A. Mustea[11], W. Meier[12], P. Harter[13], P. Wimberger[14], L. Hanker[15], B. Schmalfeldt[2], S. Mahner[3]
[1]Department of Gynecology and Obstetrics, Hannover Medical School, Hannover, [2]Department of Gynecology, University Medical Center Hamburg-Eppendorf, Hamburg, [3]Department of Obstetrics and Gynecology, Ludwig Maximilian University of Munich, Munich, [4]Jerusalem Hospital, Hamburg,

[5]*Department of Gynecology, University of Ulm, Ulm,* [6]*Department of Gynecology and Obstetrics, Johannes Gutenberg University, Mainz,* [7]*Department of Gynecology, University Medicine Berlin, Berlin,* [8]*Department of Gynecology, University of Halle, Halle,* [9]*Department of Gynecology, Klinikum Ludwigshafen, Ludwigshafen,* [10]*Department of Gynecology, Alb Fils Kliniken, Klinik am Eichert, Erlangen University Hospital, Goeppingen,* [11]*Department of Gynecology, University Medicine Greifswald, Greifswald,* [12]*Department of Gynecology, Evangelisches Krankenhaus Duesseldorf, Duesseldorf University Hospital, Düsseldorf,* [13]*Department of Gynecology & Gynecologic Oncology, Kliniken Essen Mitte, Essen,* [14]*Department of Gynecology and Obstetrics, Carl-Gustav-Carus University Dresden, TU Dresden, Dresden,* [15]*Department of Gynecology, UKSH Campus Lübeck, Lübeck, Germany*

Introduction / Background: Obesity is associated with decrased survival in several cancer entities. The influence of obesity on prognosis of vulvar cancer patients is not clear. However, knowledge about this may have consequences on treatment and follow-up.
Methodology: This is an analysis of the large AGO-CaRE-1 study which included vulvar cancer patients (UICC stage IB and higher), treated in 29 cancer centers between 1998 and 2008, in order to analyze treatment patterns and prognostic factors.
Results: In total, 849 eligible patients were divided into two groups depending on their body mass index (BMI, < 30 vs. ≥30 kg/m²). We did not detect differences in the baseline variables (age, tumor diameter, depth of infiltration, tumor stage, nodal invasion, tumor grade) and treatment variables (R0 resection, chemotherapy, radiotherapy and continuation of adjuvant therapy) between both groups (p>0.05). Radical vulvectomies were performed more frequently in patients having a BMI ≥30 kg/m² (61.1 % vs. 51.8%, p=0.042). Interestingly, we detected a higher recurrence rate in the group having a BMI ≥30 kg/m² (43.4%, vs. 28.3%, p< 0.01) due to an increased rate of local recurrences (33.3% vs. 18.5%, p< 0.01). This led to significantly shorter disease free survival of obese patients in univariate analysis (HR 1.362, 95%CI 1.093-1.696, p=0.006) and multivariate Cox-regression analysis (BMI < 30 kg/m² vs. ≥30 kg/m², HR 1.811, 95%CI 1.005-3.262, p=0.048).
Noteworthy, this association was not linear: in an additional subgroup analysis on five BMI groups we detected a longer time to recurrence in women with a slightly increased BMI 25-30kg/m² compared to those with a normal BMI between 19 and 25kg/m² (OR 0.55, 95%CI 0.33-0.91, p=0.02).
Conclusion: In this first large study about the association between obesity and prognosis of vulvar cancer patients, we observed that a BMI ≥30kg/m² was associated with shorter DFS, mainly attributed to a higher risk for local recurrence.
Disclosure: Conflicts of Interest and Source of Funding: All authors declare that there are no conflicts of interest involved with the presented data. The CaRE-1 study was supported by medac oncology without restriction in protocol or analysis

EP1169

Laparoscopic pelvic lymphadenectomy in vulva cancer vs postoperative radiation in pelvic area

E.G. Klonos[1], M.Z. Muallem[1], J. Muallem[1], I. Hatoum[1], A.Z. Tolkos[2], J. Sehouli[1]
[1]*Charité – Universitätsmedizin Berlin, Corporate Member of Freie Universität Berlin, Humboldt-Universität zu Berlin, and Berlin Institute of Health, Department of Gynecology, European Competence Center for Ovarian Cancer, Campus Virchow Clinic, Berlin, Germany,* [2]*Aristotle University of Thessaloniki, Thessaloniki, Greece*

Introduction / Background: Vulval Cancer constitutes worldwide 5.1% of all gynaecologic malignancies.The treatment is mainly based on the surgical approach. Recently, due to the modern surgical treatment, pelvic lymphadenectomy is crucial in the treatment of vulva cancer. Reviewing the literature about this topic the patients are divided into two subgroups: 1) those receiving post-operative radiotherapy in the inguinal and pelvic area 2)and in those with the pelvic lymphadenectomy after having undergone radical vulvectomy and inguinal lymphadenectomy without any postoperative radiotherapy of inguinal area although the positive groin nodes.The results showed a superiority of radiotherapy compared to surgical treatment.
Methodology: From January 2015 to December 2017, after vulvectomy pelvic lymph nodes were

removed in seven patients , five of whom were laparoscopic performed and in two with laparotomy..Four patients were stage T1b,two T2p and one patient T1a. The age of the patients ranges from 44 to 81 with an average age of 63.3. All operations were performed at the Charite Campus Wirchow Berlin in Gynecology Oncology Department by the same surgeon.The including crtiteria for undergoing pelvic lymphadenectomy were : i) ≥ 2 positive inguinal Lymphnodes, ii)1 inguinal Lympnode with size ≥ 10mm or iii)1 Lymphnode with extracapsular spread.
Results: In our study we present a number of patients,who were radical vulvectomy as well as inguinal and pelvic lymphadenectomy operated. In the case of positive inguinal and negative pelvic lymphnodes , postoperative radiation was limited locally only in the inguinal area and not in the pelvic, since only pelvic lymphnodes excision was sufficient and the patients showed an excellent follow-up.
Conclusion: Laparoscopic Lymphadenectomy seems to be a safe method to avoid postoperative radiation in the pelvic area regarding the excellent follow up. But due the low number of the patients, it is necessary to be more researched to find out the proper treatment in vulva cancer.
Disclosure: Nothing to disclose

EP1170

Development of a risk model for survival and recurrence in patients with vulvar squamous cell carcinoma

K.E. Kortekaas[1], E. Bastiaannet[1], H.C. van Doorn[2], I.F. Goddijn[1], H.J. Rogaar[1], P.J. de Vos van Steenwijk[3], P.C. Ewing[2], C.L. Creutzberg[1], K. Akdeniz[2], L.S. Nooij[1], S.H. van der Burg[1], T. Bosse[1], M.I.E. van Poelgeest[1]
[1]Leiden University Medical Center, Leiden, [2]Erasmus MC Cancer Institute, Rotterdam, [3]Maastricht University Medical Center, Maastricht, The Netherlands

Introduction / Background: Increasing evidence points to better prognosis for human papillomavirus (HPV)-positive vulvar squamous cell carcinoma (HPVposVSCC) compared to HPV-negative VSCC (HPVnegVSCC). The aims of this study were to evaluate the clinical significance of HPV and other prognostic factors in a large cohort with VSCC patients and to develop risk models for survival and recurrence.
Methodology: Patients with primary VSCC, surgically treated with curative intent between 2000 and 2015 (n=421) were included. HPV was determined by HPV-PCR and p16 immunohistochemistry. Overall survival (OS), relative survival (RS), and recurrence-free period (RFP) were determined in patients with HPVposVSCC and HPVnegVSCC. A risk model based on prognostic factors with significant impact on OS and RFP was designed to predict risk of death and shorter RFP. Data were internally validated using a bootstrap resampling procedure.
Results: Patients with HPVnegVSCC had worse 5-year OS, RS, and RFP than those with HPVposVSCC (52%, 64%, and 46% *vs* 82%; p=0.001, 90%; p=0.010, and 82%; p< 0.001, respectively. Independent prognostic factors for unfavorable OS were FIGO stage III, age ≥70 and tumor-positive resection margins, and for shorter RFP HPV status and age ≥70. Risk models for OS and RFP were developed in which patients were classified into low, intermediate and high-risk categories for death or recurrence (AUC were 0.77 and 0.73 for OS and RFP, respectively). According to this model, 5-year OS was 89%, for the low, 65% for the intermediate, and 29% for the high-risk groups. In HPVposVSCC, 70% and 100% of patients were classified as low-risk for death and recurrence, respectively, compared to 22% and 21% of HPVnegVSCC patients.
Conclusion: The developed risk models contribute to better prediction of survival and recurrence in surgically-treated VSCC patients. They may aid trial design and encourage the selection of high-risk patients potentially benefitting from new therapies or intensified follow-up.
Disclosure: Nothing to disclose

EP1171

A rare cause of postmenopausal bleeding - vaginal melanoma- case report

L. Kovac[1], B. Cvjetićanin[1], M. Jakimovska[1], M. Velikonja[2], M. Barbič[1]
[1]Department of Gynaecology, [2]Department of Pathology, University Medical Centre Ljubljana, Ljubljana, Slovenia

Introduction / Background: Primary vaginal melanoma is a rare carcinoma, representing less than 3% of vaginal carcinomas. It normally presents in postmenopausal women with vaginal bleeding and discharge. No standard of treatment has been established in literature, with poor prognosis after diagnosis, with overall survival rate less then 12 months.
Methodology: 62 years old female was referred to our center due to postmenopausal bleeding. Hysteroscopy of uterine cavity was unremarkable, however pigmented lesions in the vagina were revealed. Biopsy confirmed a diagnosis of primary vaginal melanoma. Imaging studies with CT and MRI scan of pelvis; abdomen and thorax have not shown spread of the disease. Results were evaluated by a multidisciplinary team, which recommended surgical therapy. A combined laparotomy and vaginal en bloc resection with radical hysterectomy, bilateral salpingo-oophorectomy and total colpectomy was preformed. Additionally omentectomy, pelvic lymphadenectomy and biopsy of sentinel inguinal lymph nodes were performed.
Results: Histopathological examination revealed primary vaginal melanoma in situ with no signs of distal spread, maximum invasion at 1,1mm, with 10 mm negative margins. No additional therapy was indicated. 6 month after procedure the patient is recovering well, with no sign of relapse. Abdominal ultrasound at check up was unremarkable.
Conclusion: Primary vaginal melanoma is a rare carcinoma that can present as postmenopausal bleeding. Our case report highlights that vagina should be evaluated in case of unremarkable examination of uterine cavity. As no standard of treatment has been established, good preoperative imaging and multidisciplinary approach should be considered. Radical excision is the best approach if no signs of distal spread are present.
Disclosure: Nothing to disclose

EP1172

Skinning wide local excision with delayed healing for the surgical treatment of vulval intraepithelial neoplasia

P. Owegie, A. Laios, R. Hutson
Gynaecologic Oncology, St James's University Hospital, Leeds Teaching Hospitals, Leeds, United Kingdom

Introduction / Background: In the management of vulval intraepithelial neoplasia (VIN), surgical excision such as wide local excision (WLE) with primary closure or superficial skinning vulvectomy has been the mainstay of treatment. WLE or SV with delayed wound healing without primary closure can be an alternative, particularly when perianal or periclitoral areas are involved with less flexibility of surrounding tissues offering less perioperative complications and better cosmesis. We aimed to assess the safety and efficacy of surgical excision with delayed wound healing in the surgical management of VIN.
Methodology: We retrospectively reviewed all the cases with a biopsy proven diagnosis of (high grade) VIN, which presented to our centre between January 2010 and December 2015. All cases were followed up until June 2017. Patient clinicopatholgical characteristics were reported when available. Treatment responses, positive margins, recurrence rates, disease free intervals (DFI) were the primary endpoints. Direct surgical morbidity according to the Clavien-Dindo classification and surgical outcomes including operative time, blood loss, length of stay, hospital readmission and long-term pain were the secondary endpoints. All patients had at least one postoperative follow-up.
Results: Twenty-four women underwent the treatment. Final histology confirmed the preoperative diagnosis in all cases. The margins were positive in 15/25 cases. The procedures were uncomplicated

with minimal bleeding. None of the patients were readmitted for complications of surgery. Only 6/24 relapsed with a mean time to relapse 44+-26 months. All relapsed cases were marked by re-emergence of vulval lesions. All multifocal lesions relapsed. There were no cases of occult cancer during the follow-up period. One third of women responded with a letter of satisfaction at subsequent follow-up.
Conclusion: Surgical excision with delayed healing is feasible and efficient for the surgical treatment of VIN. This technique should be offered irrespective of age considering the nature of the disease, the clinician´s experience, the patient characteristics and preference.
Disclosure: Nothing to disclose

EP1173

Vulvar Cancer in association with Muir-Torre syndrome

S. Lekka, M. Drakou, R. Geramani, G. Stanc, C. Valavanis, A. Douligeris, G. Vorgias, C. Iavazzo
Metaxa Cancer Hospital, Piraeus, Greece

Introduction / Background: Muir-Torre Syndrome (MTS) is a rare autosomal dominant inherited disease that appears to be a variation of non polyposis colorectal carcinoma-HNPCC , known as Lynch syndrome. Current criteria for its diagnose include at least one sebaceous malignancy and a visceral cancer, most commonly colonic and genitourinary.
Methodology: We report a very interesting case of a 73-year old European woman that presented with four different types of neoplasms. These neoplasms encompass two types of sebaceous tumors, as well as vulvar and urothelial cancer.
Results: As far as we know, concerning vulvar cancer while coexisting with MTS, this case is the first to be described in literature.
Conclusion: Although MTS is very rare , we should pay considerable attention when dealing with more than one malignancy, including sebaceous tumor. Then, we should take a detailed family history as well as advise the family members to undergo genetic counseling about the risk of possible occurrence.
Disclosure: Nothing to disclose

EP1174

Female genital tract melanoma: a 12-year case series with expert pathological review

J. Lewin[1], P. Ellery[2], N. Wilkinson[2], I. Kotsopoulos[1], A. Olaitan[1]
[1]Gynaeoncology, [2]Pathology, University College London Hospitals, London, United Kingdom

Introduction / Background: Malignant melanomas of the female genital tract (FGT) are rare and carry poor prognosis.They have a poorly characterised natural history & lack consensus on optimal management. We reviewed the 12 year experience of a tertiary centre treating this disease.
Methodology: A text search of the pathology reporting system was used to identify all FGT malignant melanoma cases from 2007 to present. Electronic patient records were used to record clinical information. Histopathology was reviewed by a gynaecological and dermatological pathology specialist. Data were analysed using Chi square, Mann-Whitney-U, or Student's t-test.
Results: We identified 30 cases of FGT melanoma; Mean age at presentation was 64 years (range:32 - 87). Median Breslow thickness was 4.55mm. 17 patients were treated by wide local excision, 12 by radical surgery and 3 received no surgical treatment. Median follow-up time was 67 months. Survival rates at 1, 3 and 5 years were 87%, 70% and 67% respectively.
19 presented with lower (vulvar) and 11 presented with upper FGT (vaginal or cervical melanoma). Upper FGT lesions were significantly associated with higher mortality compared to vulval lesions

(p=0.002).5 patients were diagnosed at in-situ stage, all of these were lower FGT. Even after removing these from analysis, upper genital tract melanomas were still significantly associated with mortality (p=0.01).
There was no association between Breslow thickness and site (p=0.156), or mortality (p=0.857). No association was found between mortality and microscopic ulceration, lymphovascular invasion, pigmentation, or completeness of primary excision.
Conclusion: This case series, one of the largest available, helps to further demonstrate the natural history of this rare disease. Patients presented at advanced stage, indicated by high Breslow thickness. Upper FGT melanomas had significantly poorer prognosis than lower FGT melanomas, which was not fully explained by a difference in stage.
Disclosure: Nothing to disclose

EP1175

Small cell neuroendocrine tumour of the vagina

N.K. Mendoza[1], D.G. Mata-Carrillo[2]
[1]Northern Mindanao Medical Center, [2]Obstetrics and Gynecology, Northern Mindanao Medical Center, Cagayan de Oro City, Philippines

Introduction / Background: Primary small cell neuroendocrine tumor of the vagina was first reported by Sculty et al in 1984. This is a rare neoplasm with only a few cases reported in literature. This type of tumor exhibits aggressive clinical behavior and has a poor prognosis. Since the disease is so rare, there is still no consensus regarding the optimal therapy. This is a case report of a malignant polypoid mass in a postmenopausal woman and was managed by surgical approach followed by radiation therapy.
Methodology: Treatment modalities were surgical excision of the mass with ligation of bleeders followed by radiation therapy.
Results: Standard therapy has not been established because of the very small number of cases. Surgical resection with partial or total vaginovulvectomy with bilateral or ipsilateral lymphadenectomy followed by radiotherapy and chemotherapy may provide increased local control. Radical hysterectomy, partial vaginectomy and pelvic lymphadenectomy can be done to small lesions involving the upper third of the vagina. Surgery was proven to be the main therapeutic approach but surgery alone has a high recurrence rate of 70-75%.
Conclusion: Small cell neuroendocrine tumor of the vagina is a rare tumor with aggressive behavior and poor prognosis commonly seen in postmenopausal women as in this case. Its clinical behavior is usually early with widespread dissemination. Most patient, like in this case, presents with an introital mass associated with vaginal bleeding. Mortality is secondary to distant metastasis regardless of the extent of the tumor upon diagnosis. Standard therapy has not been established for the disease, which needs further cases and discussions.
Disclosure: Nothing to disclose

EP1176

Treatment of early stage vulvar carcinoma in a pregnant woman: a case report

F. Metke[1], E. Bösken[2], M. Pixberg[3], A. Bräuer[3], P. Kies[3], L. Kiesel[1], R. Lellé[1], R. Witteler[1]
[1]Department of Obstretics and Gynecology, University Hospital Münster, Münster, [2]Private Practice, Soest, [3]Department of Nuclear Medicine, University Hospital Münster, Münster, Germany

Introduction / Background: The incidence of invasive squamous cell carcinoma of the vulva is increasing in young women in the last decades. Early stages T1b are treated by partial or total

vulvectomy and inguinal lymphadenectomy, which has a high morbidity rate due to seromas. Occurrence during pregnancy greatly increases the complexity of the surgical management. Only few cases have been reported.
Methodology: Case report.
Results: A 39 year old pregnant woman presented at our clinic with newly diagnosed early stage squamous cell carcinoma of the vulva, stage T1b, at 32 weeks of gestation. Surgery was performed at 34 weeks of gestation. In order to minimize morbidities associated with inguinal lymphadenectomy we performed bilateral sentinel lymph node dissection. Risk and dosage were calculated and the patient was counseled accordingly. After delivery of a healthy child the patient received successful reexcision due to insufficient resection margins of the first operation. Now after 6 months she has had no recurrence of the disease.
Conclusion: Sentinel lymph node dissection is a viable option for treatment of stage T1b vulvar carcinoma even during pregnancy and could be considered when postponing the treatment until after delivery imposes too great a risks for the patient.
Disclosure: Nothing to disclose

EP1177

New surgical approaches in management of pre-invasive diseases of vulva. Preliminary experience with Plasmajet surgery in management of vulvar intraepithelial neoplasma and Morbus Paget

M. Michal, P. Valha, M. Veleminsky
Obstetrics and Gynaecology Department, Ceske Budejovice Hospital, Ceske Budejovice, Czech Republic

Introduction / Background: The Plasmajet surgery system (Plasma surgical, Atlanta, GA, USA) was developed for atraumatic surgery using kinetic disection, microlayer vaporisation and surface sealing. Allows enhanced visualization, opening, and dissection of tissue planes. Thermal effect is limited, less than 0.3 mm depth and 0,5mm lateral. That allows to perform safe surgery close to key structures such as bowel, veins or nerves. Exept abdominal surgery, Plasmajet can be also used in lower genital region in management of pre-invasie diseases of vulva.
Methodology: Pacients were selected by results of histology and expertise colposcopical examination. Plasmajet skinning hemi/vulvectomy were performed at pacients with multifocal HG vulvar intraepithelial neoplasia. Pacients with multifocal Morbus Paget of vulva were treated by deeper excizion including dermis. Moist wound healing was performed using Nu-Gel (SWML, Gargrave NY, U.K.) and Mepilex Border (Molnlycke Healt Care AB, Goteborg, SE).
Results: So far we had 5 pacients with multifocal HG vulvar intraepithelial neoplasia treated by Plasmajet skinning vulvectomy. In all cases final histology was without invasion. Clear margins were obtained in four cases. Healing was without infalmatory complications in all cases, median healing time was 8 weeks. Two pacients with multifocal Morbus Paget of vulva were treated by deeper Plasmajet excision, clear margins were obtained and final histology was without invasion. Healing was without inflamatory complications, median healing time was 11,5 weeks.
Conclusion: The Plasmajet surgery is suitable technique for management of pre-invasive diseases of vulva allowing precise dissection of tissue. In larger leasions, where size of defect doesnt allow performing suture, can decrease time of healing due to more gentle surgery performed. In management of Morbus Paget of vulva, where laser surgery doesnt allow required radicality, Plasmajet can be optimal technique allowing precise atraumatic surgery with required radicality.
Disclosure: Nothing to disclose

EP1178

Diagnosis of the vagina´s melanoma by vaginoscopy

E. Cabezas López, C. Martín-Blanco, E. Moratalla Bartolomé, M.J. Pablos Antona, C. Sánchez Martínez, J. Lázaro De La Fuente
Ramon y Cajal Universitary Hospital, Madrid, Spain

Introduction / Background: The incidence of vagina´s melanoma is about 3/10.000.000 women/year. It is typically diagnosed in elderly women at an advanced stage and characterized by early recurrence and a poor prognosis.
Methodology: We present a case report.
Results: An 86 years-old woman came to our hospital reporting vaginal bleeding. Vaginal bleeding was the common presenting symptom. Other symptoms were vaginal discharge and vaginal mass. It was typically located in the distal third of the vagina. In our patient vaginal examination was not possible because she had vaginal narrowing, but we could see a black solid mass of 4 cm in the lower third of the vagina, although it was impossible to see the origin.
Immunohistochemical analysis of the biopsy of the mass indicated the present of S100 and HMB-45 and other molecular finding as Melan A and SOX10.
We perform a vaginoscopy to find out the origin of the mass. We could see two lesions in the distal third of the vagina, in the anterior wall and in the left lateral wall; which otherwise would have been impossible to observe without a hysteroscopy because the patient had a vaginal narrowing.
In our case, an expectant treatment was performed given the patient´s advanced age and general status.
Conclusion: Vagina´s melanoma is a rare type of cancer. Diagnosis of the vagina´s melanoma is made by histological and immunological examination, using special immunostains. If you can´t see the tumor, you can do a vaginoscopy to see them and to do biopsies. To date, there is no consensus regarding the optimal treatment. Surgery is the mainstay of treatment for localized disease while systemic chemotherapy and radiotherapy are appropriate for disseminated or recurrent primary melanoma. Immunotherapy with interferon alpha has been used in some patients and may be useful adjunct therapy, but evidence for this efficacy is lacking.
Disclosure: Nothing to disclose

EP1179

Keystone flap application in vulvo-perineal reconstructive surgery: a case series of patients with vulvar cancer

E. Corvetto[1], A. Proto[1], M. Fais[1], V. Locati[1], M. Russo[1], A. Figus[2], G. Candotti[1], V. Mais[1], M. Peiretti[1]
[1]Institute of Obstetrics and Gynecology, Department of Surgical Sciences, [2]Plastic Surgery Division, University of Cagliari, Cagliari, Italy

Introduction / Background: This study aimed to evaluate the application of Keystone perforator island flaps (KPIF) technique, complications and long-term results in vulvovaginal reconstruction after wide vulvar surgery.
Methodology: This is the first case series describing the technique of Keystone perforator island flaps to close a wide defect after radical vulvectomy. We prospectively collected patient demographics, operative times, intraoperative, early and late complications, pathologic results, and length of stay on all patients. The benefits, cosmetic results and satisfaction of patients were analyze in the follow up.
Results: Between December 2016 and July 2018, 5 patients were selected for the study: four patients underwent radical vulvectomy for squamous cell vulvar cancer and one underwent a vulvar wide excision for Paget disease, in all cases a reconstruction with KPIF technique followed the demolitive surgery. The defects were successfully covered by the key stone flap technique in all patients. There were no major intraoperative complications and early postoperative complications such as suture dehiscence occurred in only one patient, suffered a partial dehiscence of the wound. None skin flaps necrosis were observed. All patients were functionally and aesthetically satisfied in the long term.
Conclusion: Keystone flaps seems to be easy to design, elevate, and offer rapid fasciocutaneous closure in wide vulvo-perineal defect, with few intraoperative, early and late complications and excellent long-term results.

Disclosure: Nothing to disclose

EP1180

Incidence and prognostic factors for inguinofemoral lymph node metastasis in patients with a first local recurrence of vulvar squamous cell carcinoma

N. Pleunis[1], A.-F. Pouwer[1], A. van Heertum[1], N. te Grootenhuis[2], L. Massuger[1], M. Tjiong[3], M. Oonk[2], H. van Doorn[4], J. de Hullu[1]
[1]*Obstetrics and Gynecology, Radboud University Medical Center, Nijmegen,* [2]*Obstetrics and Gynecology, University Medical Center Groningen, Groningen,* [3]*Obstetrics and Gynecology, Amsterdam UMC, Location AMC, Amsterdam,* [4]*Obstetrics and Gynecology, ErasmusMC Cancer Institute, University Medical Centre Rotterdam, Rotterdam, The Netherlands*

Introduction / Background: Local recurrences of vulvar squamous cell carcinoma (VSCC) are reported in up to 40% of patients. Knowledge of the risk of inguinofemoral lymph node metastases in local recurrences is necessary to optimally counsel patients with respect to treatment of the groins, and to explore alternative treatment regimens. The aim of our study is to determine the incidence and prognostic factors of lymph node metastases in women with a first local recurrence of VSCC.
Methodology: A nation-wide multicenter retrospective study was performed, including all patients with a first local recurrence of VSCC after primary surgical treatment with curative intent between 2000-2015. Interim analysis was conducted on the results of four out of eight participating gynecologic oncology centers (Academic Medical Center, Amsterdam; Erasmus Medical Center, Rotterdam; University Medical Center Groningen, Groningen and Radboud University Medical Center, Nijmegen).
Results: We retrieved 292 patients with a macro-invasive local recurrence of VSCC. The median follow-up after local recurrence was 61 months (range 2-208). The median interval time between the primary VSCC and the first local recurrence was 27 months (range 1-201) with a median age of 74 years (range 28-99). Positive inguinofemoral lymph nodes were present in 15.8% of the patients with a local recurrent VSCC. Significant differences for tumor size (median 25mm vs 14mm, $p< 0.01$) and depth of invasion (4.8mm vs 3.1mm, $p=0.01$) were found in relation to the lymph node status.
Conclusion: Our study demonstrates an incidence rate of 15.8% of inguinofemoral lymph node metastases in women with a first local recurrence of VSCC. A larger tumor size and depth of invasion were significant prognostic factors for the presence of positive lymph nodes at the time of recurrent disease. More research is needed to accurately identify patients at high risk for lymph node metastases, in order to individualize treatment and follow-up.
Disclosure: Nothing to disclose

EP1181

Clonal relationship between lichen sclerosus, differentiated vulvar intra-epithelial neoplasia and non human papillomavirus (HPV) related vulvar squamous cell carcinoma

A.W. Pouwer[1], L.C.G. van den Einden[2], M. van der Linden[3], J.Y. Hehir-Kwa[4,5], J. Yu[5], K.M. Hendriks[4], E.J. Kamping[4], A. Eijkelenboom[6], L.F.A.G. Massuger[2], J. Bulten[6], A.A.G. van Tilborg[6], R.P. Kuiper[4,5], J.A. de Hullu[3]
[1]*Radboud Institute for Health Sciences, Radboud University Medical Center,* [2]*Department of Obstetrics and Gynaecology, Radboud University Medical Center,* [3]*Department of Obstetrics and Gynaecology, Radboud Institute for Health Sciences, Radboud University Medical Center,* [4]*Department of Human Genetics, Radboud University Medical Center, Nijmegen,* [5]*Princess Máxima Center for Pediatric Oncology, Utrecht,* [6]*Department of Pathology, Radboud University Medical Center, Nijmegen, The Netherlands*

Introduction / Background: Differentiated vulvar intraepithelial neoplasia (dVIN) is mostly found in a background of lichen sclerosus (LS) and can give rise to vulvar squamous cell carcinoma (VSCC), but genetic evidence is currently still limited. We hypothesise that LS and dVIN are lesions showing early neoplastic alterations which are also present in VSCC together with other accumulating events. We aimed to determine genetic abnormalities in VSCC and backtrack these abnormalities in the dVIN and LS lesions.
Methodology: We included twelve patients, surgically treated non HPV-related VSCC with a history of LS and dVIN in the surgical specimen. DNA was isolated from the VSCC and the patient-matched dVIN and LS samples. High-resolution genome-wide copy number analysis was performed for six VSCC samples and three paired dVIN samples. Subsequently, we sequenced in eight VSCC samples *TP53*, a recurrently mutated driver in VSCC.
Results: Copy number alterations were identified in all VSCC samples. One dVIN lesion presented with three copy number alterations that were preserved in the paired VSCC sample, including a 11q23 high-level amplification. Targeted sequencing of *TP53* identified mutations in five VSCCs. Targeted deep-sequencing in the available precursor lesions revealed that all five mutations were traced back in the dVIN (n=5) or the LS (n=1) with frequencies ranging from 3 to 19%, suggesting that mosaic subpopulations of cells in the LS and dVIN lesions gave rise to the VSCC.
Conclusion: Our data provide genetic evidence for a clonal relationship between VSCC and dVIN or LS.
Disclosure: Nothing to disclose

EP1182

Signal transduction pathways in vulvar squamous cell carcinoma: a pilot study

A.W. Pouwer[1], A. Bhadai[2], E.M.G. Aussems-Custers[3], J. Bulten[4], A. van de Stolpe[3], J.M.A. Pijnenborg[1], J.A. de Hullu[1]
[1]Department of Obstetrics and Gynaecology, Radboud Institute for Health Sciences, Radboud University Medical Center, [2]Department of Obstetrics and Gynaecology, Radboud University Medical Center, Nijmegen, [3]Philips Research, Eindhoven, [4]Department of Pathology, Radboud University Medical Center, Nijmegen, The Netherlands

Introduction / Background: Vulvar squamous cell carcinoma (SCC) has two etiologic mechanisms , non human papilloma virus (HPV) related often in a background of lichen sclerosis (LS), and HPV-related. A novel approach to determine tumor signaling pathway activity was developed by Philips, using knowledge-based Bayesian computational models which infer pathway activity from mRNA expression levels of target genes.
The primary aim is to explore differences in signal transduction pathway activity between HPV-related and non HPV-related vulvar SCC. The secondary aim is to assess the correlation between hormone receptor pathway activity and immunohistochemical expression.
Methodology: Pathway analyses was performed on mRNA (after microdissection) of 25 formalin fixed paraffin embedded (FFPE) samples: vulvar SCC without lymph node metastases (N=5 HPV associated, N=10 non-HPV associated) and with lymph node metastases (N=3), lichen sclerosus (N=5) and normal vulvar tissue (N=2). Activity of the oestrogen receptor (ER), androgen receptor (AR), transforming growth factor beta (TGFβ), hedgehog (HH) and Wnt pathways was measured. Immunohistochemical analysis of ER, PR, AR and Ki67 was performed interpreted by an expert gynecopathologist.
Results: HH and PI3K pathway activity were increased in vulvar SCC (n.s.). ER signal transduction pathway activity was low but significantly higher (p = 0.027) in non HPV-related compared to HPV-related vulvar SCC. Increased PI3K pathway in HPV-related compared to non HPV-related vulvar SCC did not reach significance. There was no significant difference in AR, and TGFβ pathway activity between HPV-related and non HPV-related vulvar SCC. ER, PR and AR immunohistochemical expression was not detected in vulvar SCC., A higher AR expression was observed in LS compared to normal vulvar tissue. No correlation between pathway activity and immunohistochemical expression was observed.
Conclusion: We showed significantly higher *functional* ER pathway activity in non-HPV-related compared to HPV-related vulvar SCC, and this was not correlated with immunohistochemical ER

expression. These findings are currently validated in larger series.
Disclosure: Nothing to disclose

EP1183

Sebaceous carcinoma of the vulva: an unexpected diagnosis and review of the literature

A. Rocha[1,2], M.I. Sá[3], C. Abrantes[2], S. Raposo[2], R. Sousa[2], P. Correia[2], P. Aldinhas[2], L. Sá[2]
[1]Hospital de Santarém, Santarém, [2]Instituto Português de Oncologia Coimbra, Coimbra, [3]Hospital de Vila Real - CHTMAD, Vila Real, Portugal

Introduction / Background: Sebaceous carcinoma(SC) of the vulva is a rare malignancy with potentially aggressive behavior of the sebaceous glands that is most commonly found in the periocular area. Nonetheless, there are sebaceous glands in the vulva and this diagnosis is especially rare with only nine previous cases described in the literature.
Methodology: Case Report.
Results: We report a case of a seventy-eight-year-old female patient who presented with vulvar pruritus, previously treated with topic steroids and antifungal, without improvement. The vulvar exam presented with a visible yellow papule which was biopsied and the microscopic examination revealed a sebaceous intra-epithelial carcinoma of the vulva in situ. We perform an excisional biopsy, uneventfully, with a posterior margin widening. One year after the diagnosis, she recurred with a lesion that was larger and in a multidisciplinary meeting it was decided that the patient should be submitted to a partial right vulvectomy with homolateral inguino-femoral sentinel node research (one node with was negative). Two weeks after the surgery, she presented a wound dehiscence in the groin area, re-sutured. The patient is without evidence of recurrence three months' post-surgery.
Conclusion: In summary, SC of the vulva is rare variant of SC, that appears to have many clinicopathologic differences as compared to the classic disease. Our case reports the first one with an intra-epithelial component. It is important to recognize this tumor, with a microscopic evaluation and diagnosis, to report in detail any future cases towards a better understanding of the natural history of this disease.
Disclosure: Nothing to disclose

EP1184

Tissue engineered autologous oral mucosa as an innovative procedure for reconstruction surgery in patients with vaginal or vulvar malignancies

R. Schwab, C.M. Pfeifer, M. Heller, A. Hasenburg, W. Brenner
Frauenklinik, Universitätsmedizin Mainz, Mainz, Germany

Introduction / Background: Premalignant or malignant disease of the vulva or vagina and require appropriate surgical oncological treatment. In these patients, wound breakdown occurs in 20-40% and, additionally, some patients might not be eligible for standard procedures of reconstructive surgery. In these cases, tissue engineered buccal mucosa might promote wound healing, reduce hospital days and improve satisfaction and long-term outcomes of patients. In this project we tested the use of collagen matrices for generation of engineered mucosa replacements.
Methodology: Primary buccal epithelial cells and fibroblasts were seeded on three commercially available (BioGide®, TissueFoil E®, Sugsisis ES®) and one non-commercially available (BioGide-Pro®) biodegradable membranes. After 21 days cultivation the tissue engineered oral mucosa (TEOM) equivalents were evaluated.
Results: Each examined TEOM displayed multi-layered epithelial cells on the bottom side of the membrane and mono-layered fibroblasts on the underside of each membrane. Immunohistochemical

staining showed collagen IV as a marker of the basement membrane, detected in all TEOMs. Additionally, the BioGide® membrane enabled deep invasion of fibroblasts, whereas cells seeded on TissueFoil E® were not able to penetrate the membrane. Suboptimal invasion of fibroblasts was displayed by Sugsisis ES® and BioGide-Pro®. The BioGide® membrane showed a clear shrinkage during TEOM formation.
Conclusion: In summary, the BioGide® scaffold showed the best results regarding formation of TEOMs. We showed that tissue engineered oral mucosa equivalents can be engineered under *in vitro* conditions. This could enable surgeons to apply the engineered tissue for reconstructive purposes in patients with benign or malignant vulvar or vaginal conditions, who are not eligible for standard surgical approach but who may benefit from innovative procedures.
Disclosure: Roxana Schwab: I have received honoraria and expenses from Roche Pharma AG and AstraZeneca GmbH and funding by MacoPharma International GmbH and by Max Planck Institute for Polymer Research Mainz. BioGide-Pro® was kindly provided by Geistlich Biomaterials. Annette Hasenburg: I have received honoraria and expenses from AstraZeneca, FBA Frauenärzte BundesAkademie GmbH, KlarigoVerlag, MedConcept, Med public GmbH, Med update GmbH, Medicultus, Pfizer, Promedicis GmbH, Pierre Fabre Pharma GmbH, Softconsult, Roche Pharma AG, Streamedup! GmbH, Tesaro Bio Germany GmbH. I am consultant to PharmaMar, Promedicis GmbH, Pierre Fabre Pharma GmbH, Roche Pharma AG and Tesaro Bio Germany GmbH. I have received funded research from Celgene. Celine Pfeifer: I have no conflict of interest. Martin Heller: I have no conflict of interest. Walburgis Brenner: I have no conflict of interest.

EP1185

Uncommon direct lymph drainage pathway to deep pelvic nodes in patient with cancer localized in lower third of the vagina

I. Semeradova[1,2], J. Kümmel[1], S. Bajsová[1,2], J. Klat[1,2]
[1]*Obstetrics and Gynecology, University Hospital Ostrava,* [2]*Dept Surg Studies, Faculty of Medicine, University Ostrava, Ostrava, Czech Republic*

Introduction / Background: Lymphatic drainage pathways in vaginal cancer depend on the localization of the tumor. The upper two thirds of the vagina usually drain primarily to deep pelvic nodes, the lower one-third drains into the inguinal and femoral nodes. However, some studies documented large variance of the lymph drainage. The recomended surgical approach for distal vaginal lesions involved radical resection of infiltrated vagina to achieve adequate negative margins and inguinal lymph node dissection.
Methodology: A case report.
Results: 72 year-old woman has been referred to University hospital of Ostrava with histologically confirmed adenocarcinoma of the vagina. Gynaecological examination revealed the exophytic tumor size of 3x1.5x1.5 centimeters involving lower posterior wall of the vagina. The distance from the vaginal entrance was 1 centimeter . Ultrasounography detected vascularised exophytic tumor of distal part of vagina with no spread to the rectal wall or lymfadenopathy in the groins and in the pelvis. Surgical treatment has been recommended. The radiolabeled technetium-99 m colloid has been injected in four quadrants around the lesion directly into the junction of tumor and normal vaginal mucosa two hours before surgery. Following lymphoscintigraphy showed no activity in the groins but detected sentinel lymph nodes in pelvis. In operating room the isosulfan blue has been injected in the same manner. Pelvic sentinel nodes were detected by gamma counter and blue dye and removed by laparoscopic approach. After systematic pelvic lymphadenectomy the radical excision of vaginal tumor with clear margin was performed. Histological findings confirmed adenocarcinoma of vagina, clear margins of excision and no involvement of lymph nodes.
Conclusion: The lymph drainage of the vagina has a large variance. Preoperative lymphoscintigraphy is able to identify the nodes most at risk for metastatic disease (the sentinel nodes) and helps to choose optimal surgical approach in treatment of vaginal cancer patients.
Disclosure: Nothing to disclose

EP1186

Basal cell carcinoma of the vulva: a case report

A. Seneris, J.T. Luna
Obstetrics and Gynecology | Gynecologic Oncology, Philippine General Hospital, Manila, Philippines

Introduction / Background: Basal cell carcinoma (BCC) is a rare tumor of the vulva. It is frequently seen in skin sites exposed to sun. Vulvar basal cell accounts for < 1% of all BCC and 2-5% of all vulvar carcinoma. The standard of care for treatment is surgical excision with good outcomes.
Methodology: We report a case of an 83-year old Gravida 3 Para 3, widow for 20 years who presented with vulvar nodule associated with pruritus and bleeding and later underwent concurrent chemoradiation therapy with Cisplatin. Tumor persistence was noted after chemoradiation and she subsequently underwent radical vulvectomy upon documentation of persistence of tumor. Patient had no evidence of disease at 8 months of follow up.
Results: To the author's knowledge, this was the only well-documented case of vulvar BCC in the Philippines that was initially managed by chemoradiation followed by radical vulvectomy.
Conclusion: Vulvar BCC is a rare type of vulvar carcinoma. Awareness of its presentation in the genitalia is important as early detection is critical to allow complete surgical resection. Health care providers must maintain a high index of suspicion for such lesions in the vulva and a low threshold to biopsy any abnormality on the vulva. Wide local excision is the preferred treatment for primary tumors and local recurrences.
Disclosure: Nothing to disclose

EP1187

Malignant melanoma of the vulva: a rare presentation

S. Sghaier, I. Ben Safta, I. Zemni, I. Marghli, H. Mansouri, R. Chargui, K. Rahal
Surgical Oncology, Salah Azaiz, Tunis, Tunisia

Introduction / Background: Melanoma of the vulva (MV) is rare; accounting for 10% of vulvar malignancies. Although there is not a standard management for MV, the surgical resection remains the mainstay of the treatment.
Methodology: We report a case of MV diagnosed and treated at Salah Azaiz institute in 2019.
Results: A 76-year-old woman presented with a nodule of the vulva with urinary burning. Clinically, we noticed a hard and movable reddish nodule of 30mm on the clitoris with irregular borders. This lesion seemed to be invading the urethra and the lower third of the vagina. There were no associated inguinal lymph nodes. A biopsy of this lesion concluded to the diagnosis of MV. Pelvic MRI showed a heterogeneous mass of 29*16mm of the clitoris extended to the labia minora. It invaded the terminal part of the urethra and reached the lower edge of the anterior vaginal wall without extension into it. It also demonstrated millimetric bilateral inguinal lymph nodes. No secondary lesions were found in the CT scan.The patient underwent anterior pelvic exenteration and radical vulvectomy with bilateral inguinal lymph node biopsy. Histological examinations showed free margins and no lymph node metastasis. The patient is alive, with no recurrence, after three months of follow up.
Conclusion: The MV represents an uncommon tumor; occurring mostly in older women. Because of its poor prognosis, early diagnosis and staging of this cancer is mandatory.
Disclosure: Nothing to disclose

EP1188

Topic treatment of vulvar lichen sclerosus: symptoms control and risk of progression to malignancy

F.P. Sina, P. Blangiardo, M. Lamanna, S. Magni, F. Vecchione, S. Chiari, L. Gnecchi, F. Landoni, R. Fruscio
Milano Bicocca University, Ospedale San Gerardo, Monza, Italy

Introduction / Background: vulvar lichen sclerosus (VLS) is a chronic inflammatory dermatosis usually associated with symptoms that cause significant physical discomfort and emotional distress. The therapy of VLS is controversial, and factors that can influence the progression to neoplastic lesions are not clear.
Methodology: this is a retrospective analysis of women with a diagnosis of VLS made between 2005-2010 at San Gerardo Hospital, Monza. Patients were treated with mometasone furoate 0.1% ointment for a 30-days period in association with emollient creams followed by a maintenance phase with emollient creams only at least twice a day. The efficacy of therapy was evaluated in women with at least one follow-up visit; the development of preneoplastic and neoplastic lesions was evaluated only in patients with a follow-up of at least 1 year. The association between clinical data (including demographics and response to therapy) and progression to cancer was evaluated using univariate and multivariate analysis.
Results: a total of 251 women were included in this retrospective analysis. Median age at diagnosis was 63 years. The efficacy of therapy was evaluated in 231 patients. Symptoms remission was obtained in 65% of patients, while 16% required topical steroids in addition to emollient creams in the maintenance phase. Therapy was not effective in 19% of patients. Neoplastic progression was evaluated in 144 patients. 28 women developed a vulvar carcinoma (24) or VIN (4) with a median time from VLS diagnosis of 51 months. Patients without symptoms control were significantly more likely to develop a neoplastic lesion during follow up at a multivariate analysis (p: 0.004).
Conclusion: symptoms remission can be obtained in the majority of patients with VLS using emollient creams only in the maintenance phase. Patients who do not respond to therapy should be carefully monitored, as they have a higher risk of developing neoplastic lesions.
Disclosure: Nothing to disclose

EP1189

Intensity of oxidative processes in blood of patients with vulvar cancer is associated with remission duration

E. Surikova, I. Goroshinskaya, G. Nerodo, P. Kachesova, E. Shalashnaya, E. Nerodo, L. Nemashkalova, A. Leonova
Rostov Research Institute of Oncology, Rostov-on-Don, Russian Federation

Introduction / Background: The purpose of the study was to reveal the intensity of oxidative processes and the state of the antioxidant system in the blood of patients with vulvar cancer depending on the remission duration.
Methodology: The study included 46 patients (64.2±1.7 years) with squamous cell vulvar carcinoma, stage I-III, G1-G2. All patients received a timely combination treatment according to the disease stage at Rostov Research Institute of Oncology. Remission periods were up to 3 years in 22 women (group 1) and 3-23 years in 24 women (group 2). The control group included 30 healthy women (58.4±2.7 years). We determined plasma intensity of peroxide-induced luminol-dependent chemiluminescence (CL), catalase activity (CAT), ceruloplasmin concentration (CP), malondialdehyde (MDA), carbonyl derivatives of amino acid residues of proteins (CDP). Statistical processing of results was performed using the Student and Mann-Whitney criteria.
Results: In group 1, CL was increased by 2.9 times, MDA by 2.3 times and CDP by 1.5 times compared to healthy women ($p < 0.05$-$p < 0.001$); CAT was similar, and CP was increased by 1.5 times ($p < 0.01$). In group 2, CL, MDA and CAT were 1.5 times higher than in healthy women

(p< 0.01-p< 0.001), while CDP and CP were similar.
Conclusion: Patients with a short remission period were characterized with significant activation of oxidative processes in the blood, manifested by an increased generation of free radicals, oxidation of lipids and proteins, lower CAT and increased CP, which may indicate the activation of copper and iron metabolism in this group of patients. Apparently, this created conditions for the development of disease recurrence. In patients with long-term remission, a lower activity of oxidative processes in the blood was noted, according to some indicators similar to that in healthy women, due to reduced intensity of generation of free radicals and the CAT increase.
Disclosure: Nothing to disclose

EP1190

MAGE-A4 expression depends on p16 status of squamous vulvar cancer

K. Sznurkowska, A. Zawrocki, J.J. Sznurkowski
Medical University of Gdansk, Gdańsk, Poland

Introduction / Background: Objective: To explain the difference in immune cell infiltration of vulvar squamous cell carcinomas (vSCCs) with different p-16 status we aimed to analyse the correlation between vSCC expression of cancer testis antigens (CTA): MAGE-A1, MAGE-A4, immunosuppressive enzyme indoleamine-2,3-dioxygenase (IDO), as well as the number of tumor infiltrating lymphocytes (TILs) and tumor associated macrophages (TAMs) within p16-positive and negative cancer nests.
Methodology: Expression of MAGE-A1, MAGE-A4 and IDO, the numbers of TIL and TAM, as well the data on of $p16^{Ink4a}$ status were retrieved from our previous studies on cohort of 85 vSCC patients. Numbers of intraepithelial (IE) CD8+, CD4+, FOXP3+, CD56+, CD68+ and GrB+ cells were compared between tumors presenting different expression of MAGE-A1, MAGE-A4 and IDO. CTA and IDO expressions were compared between tumors with different $p16^{INK4a}$ status.
Results: Index of MAGE-A4 expression was higher within p16-negative vSCCs (p=0.0005) while indices of MAGE-A1 and IDO expression were similar in both p16 negative and positive tumors (p=0.23 and p=0.069 respectively). MAGE-A4 expression was correlated with higher infiltration of (IE)GrB+ cells (p=0.02). MAGE-A1 expression was correlated with higher infiltrates of (IE)CD68+ immunocytes (p=0.01). IDO expression did not correlate with immune cell infiltrates.
Conclusion: Expression of CTAs could depend on $p16^{INK4a}$ status of vSCC. The combined cytotoxicity of innate and adaptive immune effectors infiltrating cancer nests (IE GrB+) correlates with MAGE-A4 expression. Correlation between MAGE-A1 and innate immune effector (TAM) needs to be verified.
Disclosure: Nothing to disclose

EP1191

Recurrent vaginal mucosal melanoma treated with salvage hypofractionated external beam radiotherapy and interstitial brachytherapy

H. Vaghefi[1], H. Fornalik[2], J. Wheeler[3], A. Moore[3], L. John[3], I. Sparks[3]
[1]*Goshen Hospital, Department of Radiation Oncology,* [2]*Gynecologic Oncology/Surgical Oncology,* [3]*Radiation Oncology, Goshen Center for Cancer Care, Goshen, IN, United States*

Introduction / Background: Malignant melanoma of the vagina is rare and portends a poor prognosis. Primary treatment for vaginal melanoma is oncologic resection (1). Systemic therapy has been utilized in the case of inoperable tumors (2). Postoperative adjuvant radiation therapy with or without chemotherapy has been utilized; however definitive radiation therapy for vaginal mucosal melanoma is rarely used (3-5).
In this presentation, we discuss utilization of combination of external beam radiation therapy and

interstitial brachytherapy as a salvage modality for recurrent vaginal mucosal melanoma.
Methodology: 62 year old treated with partial vaginectomy in Sept 2016 for vaginal cancer. Pathology showed a malignant melanoma with 6 mm thickness with positive deep margin. She had a completion radical vaginectomy and inguinal and pelvic node dissection in December 2016. PDL-1, BRAF V600, c-kit and N-ras were all negative. In Nov 2016, PET/CT was negative. In January 2017, MRI showed a 6.6x3.8x4.3 cm left vaginal mass and left inguinal lymphadenopathy.
On March 9, 2017, she completed salvage radiation to the pelvic nodes, vaginal mass to a total of 62.1 gray with Simultaneous integrated boost, followed by interstitial brachytherapy in 4 fractions of 6.4 gray per fraction to a total of 26 Gy on April 24, 2017.
Results: She developed pulmonary metastasis. However, she refuses all systemic therapy. She enjoys excellent local control in the vaginal and inguinal nodes for 2 years. She complains of fatigue; she remains sexually active. She reports occasional pelvic discomfort.
Conclusion: This presentation demonstrates that hypo-fractionated radiation therapy plus interstitial brachytherapy can offer excellent local control. Based on radiobiological principles, the use of high-dose-per treatment regimens for such relatively radioresistant tumor is understandable (6). We propose that radiation therapy should be considered for patients with unresectable melanoma.

References: 1. Schmidt M, Honig A, Schwab M, Adam P, Dietl J. Primary vaginal melanoma: a case report and literature review. Eur J Gynaecol Oncol. 2008;29:285-288. 2. Baloglu A, Bezircioglu I, Cetinkaya B, Yavuzcan A. Primary malignant melanoma of the vagina. Arch Gynecol Obstet. 2009;280:819-228. 3. Lin LT, Liu CB, Chen SN, Chiang AJ, Liou WS, Yu KJ. Primary malignant melanoma of the vagina with repeated local recurrences and brain metastasis. J Chin Med Assoc. 2011;74:376-379. 4. Androutsopoulos G, Terzakis E, Ioannidou G, Tsamandas A, Decavalas G. Vaginal primary malignant melanoma: a rare and aggressive tumor. Case Rep Obstet Gynecol. 2013;2013:137908. 5. Kirschner AN, Kidd EA, Dewees T, Perkins SM. Treatment approach and outcomes of vaginal melanoma. Int J Gynecol Cancer. 2013;23:1484-1489. 6. Rofstad EK. Radiation Biology of Malignant Melanoma. Acta Radiol Oncol 1986 Jan -Feb ; 25(1):1-10.

[References]

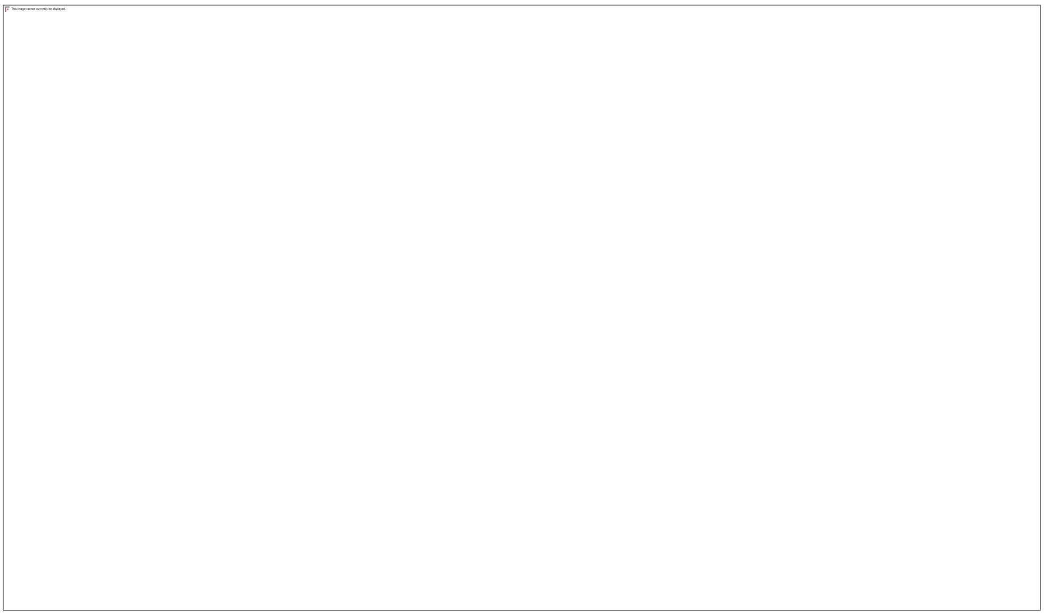

[Radiographic Images and Radiation Therapy plans (External Beam and Brachytherapy)]

Disclosure: Nothing to disclose

EP1192

Surgical treatment of the vulva in the absence of a conclusive diagnosis of squamous cell carcinoma, data from a 10 year cohort

L.W. Jonker[1], S. Dasgupta[2], P.C. Ewing - Graham[2], H.C. van Doorn[1]
[1]Department of Gynaecologic Oncology, [2]Department of Pathology, ErasmusMC Cancer Institute, University Medical Centre Rotterdam, Rotterdam, The Netherlands

Introduction / Background: Treatment decision in a vulvar lesion is guided by the histopathological diagnosis of the lesional biopsy. For vulvar intraepithelial neoplasia (VIN), a local excision can be performed, whereas for vulvar squamous cell carcinoma (SCC), more radical procedures are mandatory. In certain cases, despite the lack of a conclusive biopsy diagnosis of SCC, the surgeon opts for a radical treatment, to avoid repeated surgery. This exploratory retrospective study was conducted on a cohort of patients who underwent vulvar excision, in the absence of a conclusive biopsy diagnosis of SCC. We aimed to identify the predictive factors for the presence of a SCC, and describe the treatment chosen.
Methodology: All patients who underwent vulvar excisions (2005 - 2016) at Erasmus MC, without a definitive diagnosis of vulvar SCC on the preoperative biopsy were included. Patient characteristics and treatment details were retrieved. The surgical treatment was categorized as definitive, under- or over-treatment, based on the histopathology of the excision specimen. Logistic regression analysis was performed. Approval was obtained of the Erasmus MC Ethical Board.
Results: Of 1245 patients who underwent vulvar excisions, 113 met the inclusion criteria. In 57% (64/113), SCC was diagnosed in the excision specimen. [Figure 1] A higher age (p = 0.03), and a suspicion of SCC on pre-operative biopsy (p < 0.01) were found to be predictive of a final diagnosis of SCC on univariate analysis. On multivariable analysis, suspicion of SCC on biopsy was the only predictor (p < 0.01); with a positive predictive value of 82%. The surgical procedure was definitive for 72%, under-treatment for 25%, and over-treatment for 3%.
Conclusion: Suspicion of SCC on biopsy is strongly predictive of a final diagnosis of carcinoma. In these cases more radical (definitive) treatment can be chosen to avoid second surgical procedures, at the cost of minimal overtreatment.

	Final pathology SCC	Final pathology no invasive carcinoma	p value (univariate)
Age (in years) Mean (95% CI)	67.9 (64.8-71.2)	61.2 (57.1 - 66.4)	0.03
Tumor diameter< 40 mm	48 (75%)	40 (82%)	0.53
Preoperative histopathology suspicion of invasive SCC	45 (70%)	10 (20%)	<0.0001
Surgical procedure Local treatment only	40 (63%)	45 (92%)	<0.001
Surgical procedure; local treatment and groin surgery	24 (37%)	4 (8%)	
treatment categorization: Definitive	36 (56%)	45 (92%)	<0.01
treatment categorization: under-treatment	28 (44%)	0	
treatment categorization: Over-treatment	0	4 (8%)	

[Characteristics of the patients with and without SCC at final pathology]

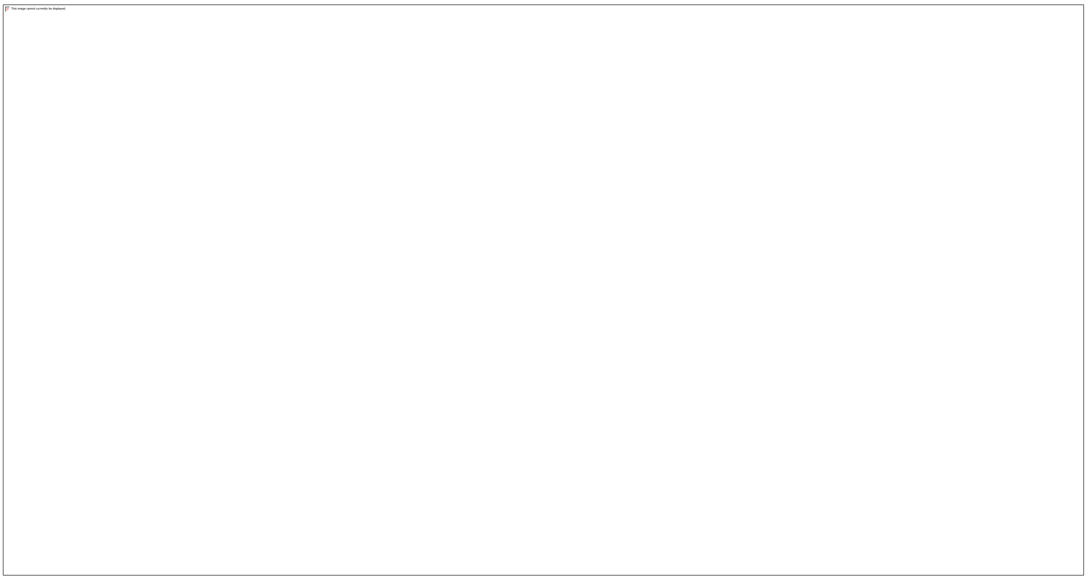

[Distribution of patients according to their pre-operative biopsy findings, and treatments received.]

Disclosure: Nothing to disclose

EP1193

Value of radiotherapy in the management of Paget's disease of the vulva: a retrospective study of 31 patients

C. Nawara, M. Christiaens, E. Van Limbergen, A.-S. Van Rompuy, E. Van Nieuwenhuysen, T. Van Gorp, S. Han, P. Neven, I. Vergote
Universitaire Ziekenhuizen Leuven, Leuven, Belgium

Introduction / Background: Surgical treatment is mutilating and resection often results in involved margins. As a consequence, local recurrences are frequent after surgery.
Methodology: Retrospective study of patients with vulvar Paget's disease treated in our hospital between 1993 and 2018.
Results: Thirty-one consecutive patients with vulvar Paget's disease were included, whereof twenty patients were irradiated in the primary or recurrent setting. Radiotherapy on the vulva and perineum was performed at a median dose of 60 Gy in fractions of 2 Gy. The median follow-up after radiotherapy was 75 months. Eight (26%) patients underwent as primary treatment radiotherapy only. Only one (13%) had a recurrence (after four months) and was operated. Fifteen (48%) patients had surgery as only primary treatment; five (33%) of them had a recurrence. Four of these had, after 3 or 4 surgical interventions, radiotherapy for recurrence. Only one of these 4, had a relapse after 85 months. Eight (26%) patients had as primary treatment surgery followed by adjuvant radiotherapy (due to positive margins), one (13%) of these recurred and died of disease.

Treatment	N	Recurrence after primary treatment N (%)	Surgical treatment for relapse N (number of re-interventions per patient)
Surgery only	15	5 (33)	4 (2-3-2-3)

Radiotherapy only	8	1 (13)	1 (1)
Surgery + radiotherapy	8	1 (13)	0

[Treatment and recurrences]

Conclusion: Surgery is the most commonly used therapy for vulvar Paget's disease, even though it is difficult to excise the disease adequately and often multiple surgeries are necessary. According to our experience, radiotherapy is a good adjuvant treatment option in patients with vulvar Paget's disease with positive resection margins. Moreover, in our experience, primary radiotherapy seemed to be a safe alternative to surgery, that can prevent patients with Paget's disease of the vulva to undergo mutilating and often repeated surgery.
Disclosure: Nothing to disclose

EP1194

Primary vaginal leiomyosarcoma: a case report with complete morphological, immunohistochemical and ultrastructural study

E. Vizza[1], V. Petrozza[2], P. Natale[2], C. Certelli[1], E. Battaglione[3], G. Corrado[4], G. Familiari[3], R. Heyn[3]
[1]Regina Elena National Cancer Institute, Rome, [2]Sapienza University, Latina, [3]Sapienza University, [4]Policlinico Universitario Agostino Gemelli I.R.C.C.S. Universita Cattolica Di Roma, Rome, Italy

Introduction / Background: Primary vaginal leiomyosarcomas (LMS) are rare, recurrent tumours with an unknown etiology; the prognosis is poor and there is no consensus guideline on their management. A nodular, 25 x 23 x 28 mm-mass, infiltrating the urethra but not the rectovaginal septum, was found in a 58-year-old previously hysterectomized woman. A biopsy showed a LMS of the vagina and an anterior pelvic exenteration was performed.
Methodology: The sample was fixed and prepared for light microscopy, transmission and scanning electron microscopy. An immunohistochemical analysis was performed.
Results: The results confirmed a LMS of the vagina that was positive for vimentin, alpha-smooth muscle actin, caldesmon, desmin, p16 and p53. Light microscopy revealed that the mass contained a storiform pattern of spindle-shaped cells with blunt-ended nuclei. Cells were arranged in interwoven fascicles within a dense and richly vascularised stroma, suggesting an active neoangiogenesis. The histopathological analysis revealed a coagulative focal necrosis and low to moderate mitotic indexes, about 1-4/10 high power fields (HPF). Scanning Electron Microscopy (SEM) evidenced a dense collagenous stroma with numerous small blood vessels. Transmission Electron Microscopy (TEM) showed invasive neoplastic and pleomorphic cells with complex labyrinthic cytoplasm projections. Tumoral cells contained paranuclear crowds of dilated mitochondria, free ribosomes and a well-developed rough endoplasmic reticulum. There were atypical mitotic figures. Blood vessels were usually lined by a high and reactive endothelium.
Conclusion: The histopathological and ultrastructural analyses confirmed the malignancy of this tumor. Best outcomes occur when the tumour is small, localized, and can be removed surgically with wide, clear margins, as in this case. As there are different kinds of malignant mesenchymal tumours, biopsy followed by immunohistochemistry and electron microscopy still represents a good diagnostic choice.
Disclosure: Nothing to disclose

EP1195

Circulating microRNAs in differential diagnosis of vulvar intraepithelial lesions and vulvar

squamous cell carcinoma

K. Zalewski[1,2,3], M. Szczyrek[1], A. Kowalik[4], A. Długosz[5], M. Misiek[2], M. Bidziński[6], K. Goryca[7], M. Kowalewska[1,5]
[1]Department of Molecular and Translational Oncology, Maria Sklodowska-Curie Institute - Oncology Center, Warsaw, [2]Department of Gynecologic Oncology, Holycross Cancer Center, Kielce, [3]Chair and Department of Obstetrics, Gynecology and Oncology, 2nd Faculty of Medicine, Warsaw Medical University, Warsaw, [4]Department of Molecular Diagnostics, Holycross Cancer Center, Kielce, [5]Department of Immunology, Biochemistry and Nutrition, Medical University of Warsaw, [6]Gynecologic Oncology Department, [7]Department of Genetics, Maria Sklodowska-Curie Institute - Oncology Center, Warsaw, Poland

Introduction / Background: Vulvar squamous cell carcinoma (VSCC) develops from vulvar squamous intraepithelial lesions (VIN). VIN is subdivided into two types, high grade-squamous intraepithelial lesion (HSIL) and differentiated vulvar intraepithelial neoplasia (dVIN), which differ by pathogenesis and clinical course. No molecular markers allowing precise and non-invasive diagnosis and/or differentiation of malignant and premalignant lesions of the vulva have been developed to date. This study aimed to identify circulating microRNAs as markers for differential diagnostics of VIN and VSCC.
Methodology: Expression levels of 20 microRNAs were analyzed by quantitative RT-PCR in the plasma samples of 19 patients with VIN (HSIL and dVIN), 41 patients with VSCC and 15 healthy female donors (control group). The microRNA levels were normalized to hsa-miR-93-5p and hsa-miR-425-5p.
Results: The levels of three microRNA molecules, namely miR-7-5p, miR-630 and miR-23b-3p were found to differentiate plasma samples of obtained from healthy donors, VIN and VSCC patients. The levels of miR-7-5p and miR-630 were decreased in blood of patients with VSCC as compared to VIN and controls . In contrary, the levels of miR-23b-3p were decreased in the blood of VSCC patients as compared to VIN and healthy donor samples. These differences were statistically significant.
Conclusion: Our data reveal high diagnostic potential of the assessment of levels of circulating miR-7-5p, miR-23b-3p and miR-630 in VIN and VSCC patients.
Disclosure: Nothing to disclose

EP1196

Radioguided detection of sentinel lymph node in vulvar cancer. Achievements and limitations

J. Zekan[1], J. Juras[2], A. Mutvar[2], D. Karelovic[1], I. Pitner[2]
[1]School of Medicine, University of Split, Split, [2]School of Medicine, University of Zagreb, Zagreb, Croatia

Introduction / Background: To evaluate the effectiveness and limitations of Technetium-99m for lymphoscintigraphic mapping and sentinel lymph nodes (SLNs) localization in early stage vulvar cancer patients.
Methodology: In prospective observational case series study 34 consecutive patients with histologically proven vulvar squamous cell cancer ≤ 4 cm in size were enrolled. Technetium-99m colloid albumin was injected intradermally around the tumor at least 3 h before surgery for lymphoscintigraphic mapping and intraoperative mobile gamma ray probe detection of sentinel nodes (SLN). SLNs were identified and removed before a complete inguinofemoral lymphnode dissection was performed, regardless of the SLN status. If no metastases were found in hematoxylin-eosin slides, immunohistochemical staining was performed for the definitive pathohistological diagnosis.
Results: The detection of SLN was successful in all patients. There was one patient with metastatic non-sentinel lymph node despite the negative sentinel node in which the previously performed diagnostic complete excision of tumor. The sensitivity, specificity and negative predictive value of sentinel node assay with immunohistochemistry included were 90,0% (95% CI: 55.5 to 99.7%), 100,0% (95% CI: 86.3 to 100.0%), and 96.1% (95% CI: 79.6 to 99.4%), respectively.
Conclusion: Lymphoscintigraphy and SLN biopsy under gamma-detecting probe guidance proved to be a reliable method for the detection of sentinel node in early vulvar cancer. The SLN biopsy is highly

accurate in predicting the status of the remaining inguinofemoral lymph nodes and represents a true advance in the selection of patients for less radical surgery.
Protocol of radioguided detection of SLN includes the injection of the technetium-99m-nanocolloid at least 3 h before surgery which involves two invasive procedures, transport of patient from the Nuclear medicine unit into the Operating suite. The local colloid application is almost always painful procedure for patients. All this points to the need to change the application protocol or tracer.
Disclosure: Nothing to disclose

EP1197

Types and viral load of human papillomavirus in vaginal intraepithelial neoplasia: a cross-section study

F. Zhou, J. Ding
Obstetrics and Gynecology Hospital of Fudan University, Shanghai, China

Introduction / Background: It is widely acknowledged that infection with human papillomavirus (HPV) is a significant risk factor for vaginal lesions. Previous studies regarding HPV types in vaginal intraepithelial neoplasia (VAIN) and vaginal cancer indicated inconsistent conclusions, and did not explore the correlation of HPV viral load and vaginal lesions.
Methodology: A total of 176 women who underwent examining for Lower genital tract lesions were enrolled in this cross-sectional study with definite pathological diagnosis, among which, 99 patients with vaginal lesions and 77 patients without vaginal lesions were included. HPV types and viral load were detected by BioPerfectus Multiplex Real-Time PCR assay, and 109 cases were HPV positive, which included separate vaginal lesions (n=44), negative for intraepithelial lesion or malignancy (NILM, n=31), separate cervical lesions (n=16) and vaginal lesions concomitant with cervical lesions (n=18). The relevance was analyzed between viral load and vaginal lesions.
Results: In patients with vaginal lesions, the HPV detection rate was 89.7% (87/99). Women with VAIN2/3 (n=12) or vaginal cancer (n=2) were all HPV positive and 57.14% (8/14) were HPV16 positive. Top five leading HPV types of vaginal lesions were HPV16 (24.2%), HPV52 (24.2%), HPV53 (16.1%), HPV58 (14.5%) and HPV66 (14.5%). Compared with histopathologically normal cases, separate vaginal lesions had a higher rate of HPV multiple infection (47.7% vs 29.0%). Besides, the viral load of HPV type 16, 52, 58 appeared higher for women with separate vaginal lesions than those with NILM (P=0.026, 0.002, 0.013 respectively). For women with cervical lesions, the viral load of HPV showed little difference whether there was coexistence of vaginal lesions or not.
Conclusion: Our findings suggest that HPV16, 52, 53, 58, 66 are more prevalent in vaginal lesions, and infection with HPV16 may have a higher risk of VAIN2/3 or vaginal cancer. Some type-specific HPV viral load may provide indication for vaginal lesions.
Disclosure: Nothing to disclose

EP1198

Interobserver variability of Eastern Cooperative Oncology Group (ECOG) performance status in gynecologic oncology patients

M. Api[1], E. Keleş[1], S. Akiş[1], Y.E. Purut[1], Y. Ceylan[2]
[1]Gynecologic Oncology, Zeynep Kamil Training and Research Hospital, [2]Medicine, Istanbul Medipol University, Istanbul, Turkey

Introduction / Background: Eastern Cooperative Oncology Group performance status (ECOG-PS) is the most widely used measurement of patient's ability to perform daily activities. Although the prognosis and effectiveness on therapies based on the PS are assigned to patients, the reliability is

still questionable and manifests great variability among the doctors and patients. The objective is to conduct a prospective randomized triple blinded study on 127 gynecologic oncology patients in which, three gynecologic oncology fellows (observers) grade them one by one without having knowledge of each other´s score results.
Methodology: The patients were questioned by the observers in a randomized sequential manner. Linguistic validation was also conducted by our group for Turkish version of ECOG performance scale.
Results: Kendall's W coefficient was found out to be 0.043. Overall agreement was 74.42% in terms of ECOG PS between the three observers and the average agreement according to ECOG scores; 0, 1, 2 and 3 were 76.16%, 68.3%, 61.6% and 91.63% respectively. There were no patients that were scored as 4. The agreement was the least variable for the score 3.
Conclusion: Our results showed that ECOC performance scale in gynecologic oncology patients having good interobserver variability and this questionnaire can be reliably used in gynecologic oncology patients in the assessment of physical health status.
Disclosure: Nothing to disclose

EP1199

Validation of Turkish version of Eastern Cooperative Oncology Group Performance Status (ECOG-PS) for gynecologic oncology patients

M. Api[1], Y.E. Purut[1], S. Akiş[1], E. Keleş[1], Y. Ceylan[2], C. Kabaca Kocakuşak[1], B. Giray[1], M. Güray Uzun[1]
[1]Gynecologic Oncology, Zeynep Kamil Training and Research Hospital, [2]Medicine, Istanbul Medipol University, Istanbul, Turkey

Introduction / Background: Although studies on performance in cancer patients are increasing, there has not been enough efforts to validate the Eastern Cooperative Oncology Group Performance Status (ECOG-PS). Therefore, we aimed to translate and validate the ECOG into Turkish.
Methodology: This was done by drawing on the original version and by employing more rigorous translation methods to identify and resolve the translation problems. An expert panel was also employed to adjudicate on final translation and item selection issues. The ECOG questionnaire was validated between January and April 2019. Two bilinguals independently translated the English version of the ECOG questionnaire into Turkish, and then reconciled the forward translation of the Turkish version. The original ECOG was back-translated into English, then assessed for equivalence to the original. Gynecologic fellows interviews with 15 patients to test the interpretation of the translation were made, then modified and distributed to 114 patients for re-evaluation. A statistical analysis of inter-scale correlation, and test re-test consistency was performed with the Cronbach´s alpha coefficient. The changes from patient interviews were reflected in the final version.
Results: In an intra-class correlation, the Cronbach´s alpha was high in all of the questions (0.81, $P < 0.001$). Test re-test Cronbach´s alpha analysis of reproducibility was higher than 0.75 for all of the 5 questions ($P < 0.001$).
Conclusion: Translation and linguistic validation of Turkish version of the ECOG questionnaire was completed by a proper process, with high reliability and validity.
Disclosure: Nothing to disclose

EP1200

Impact of socio-demographic characteristics on the quality of care in endometrial cancer: an analysis of a national database in the United States

L. Benoit[1], L. Pauly[2], J. Phelipeau[2], M. Koskas[2]
[1]Bichat | Gynecology Obstetrics, [2]Bichat Hospital, Paris, France

Introduction / Background: Endometrial cancer (EC) is the most frequent gynecological malignancy and studies have shown that demographic attributes could influence quality of care. We aim to review their impact on compliance to standard treatment guidelines in EC with already published quality metrics.
Methodology: Using the Surveillance Epidemiology and End Results (SEER) database 1988-2013 we identified 151,752 patients treated for EC in the United States. The quality indicators (QI) were extracted from a Belgian study on quality of care in EC. Ten QI were selected because of their relevance and applicability to the SEER. These QI either evaluated the surgical management, the adjuvant treatment decision, or the outcome in EC. We examined the association between socio-demographic characteristics (race, age, insurance status, income, and marital status) and quality of care using a logistic regression model.
Results: Concerning the six surgical QI, one was accurately met (>80% population received the recommended treatment), three were moderately met (50-79%) and two were poorly met (< 50%). For example, 91% of the patients with a type 1/2, FIGO stage I EC underwent a total hysterectomy with bilateral salpingo-oophorectomy. Concerning the three QIs reporting outcome, one was well met (99.3% alive one month after surgery) and two were moderately met (71% alive at 5 years and 52% of the patients deceased, died from EC). The adjuvant QI was not well met (30% FIGO I/II EC who did not undergo surgery, received radiotherapy). All socio-demographic characteristics studied were associated with low adherence to the QI. Age over 75 years old, black ethnicity, lower income group, without partner and un-insured, had a negative impact on the compliance to standard treatment.
Conclusion: The 10 Belgian QI used to assess quality of care in the United States showed an overall correct compliance to standard treatment. Demographic discrepancies persist in the management of EC, impacting evidence-based care.

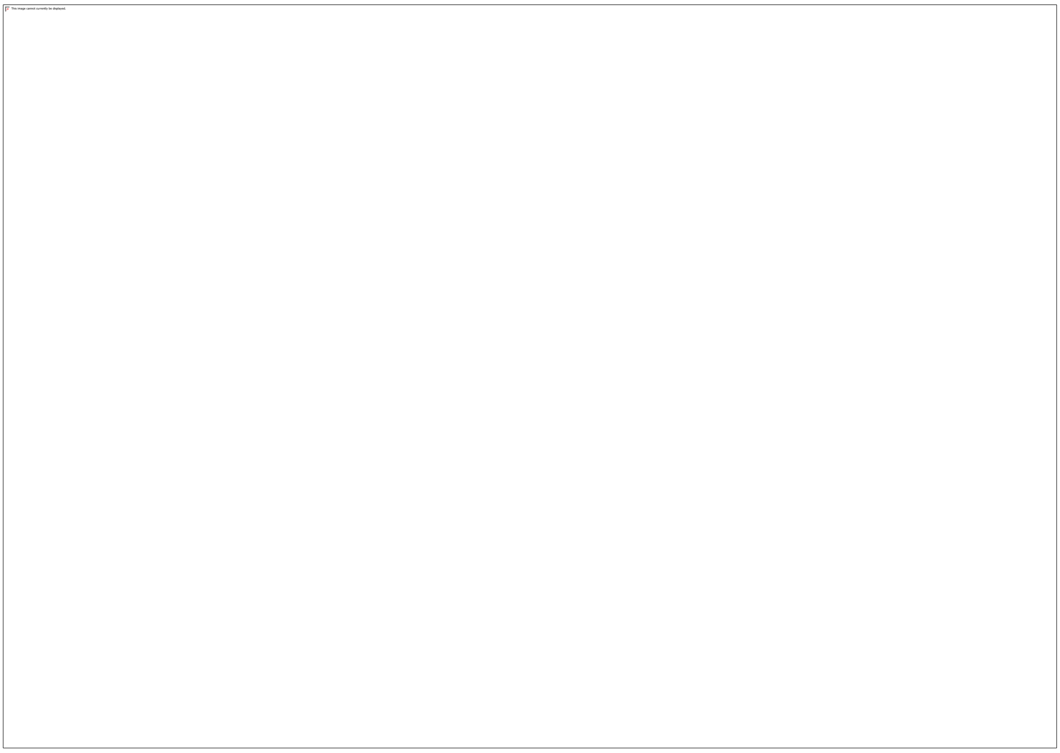

[Impact of the socio-demographic factors on the adherence to the quality indicators in endometrial ca]

Disclosure: Nothing to disclose

EP1201

Laparoscopy versus laparotomy for surgical treatment of obese women with endometrial cancer: a cost-benefit comparative analysis

V.A. Capozzi[1], A. Cianciolo[1], M. Pugliese[1], G. Gambino[1], G. Sozzi[2], G. Armano[1], L. Monfardini[1], M. Gaiano[1], V. Ceni[1], C. Merisio[1], R. Berretta[1]
[1]Department of Gynecology and Obstetrics, University of Parma, Parma, [2]Department of Gynecologic Oncology, ARNAS Civico Hospital of Palermo, Palermo, Italy

Introduction / Background: Obesity represents a major health problem. Several studies reported that morbid obesity is associated with an 81% greater health care expenditure per capita compared with normal weight adults in the US. Although many articles have investigated costs of laparoscopic versus open approach for endometrial cancer, very few data are available in selected population of patients, such as obese women. The aim of this study has been to evaluate pre-, intra- and post-operative costs in obese women affected by endometrial cancer comparing laparoscopic versus open abdominal surgery.
Methodology: Economic expenditure in pre-operative, intra-operative and post-operative phases of the selected patients was evaluated. Costs were analyzed for all blood tests, instrumental examinations, consultations, operating materials, drugs, gynecological examinations, hospital stay, intensive care hospitalization and management of operative complications.
Results: The average length of stay was longer for patients operated by laparotomy with an almost double median hospitalization cost in the open abdominal group compared to the laparoscopic group (€ 4805.37 vs. € 2589.25; $p < 0.0001$). Evaluation by another specialist (cardiologist, diabetologist, internist) was necessary in 30.9% of laparotomies versus 10.4% of laparoscopies ($p = 0.003$). Antibiotic and pain-relief therapies resulted in a significantly higher cost for the open abdominal than for the minimally-invasive approach ($p = 0.027$). Considering all the pre-, intra- and post-operative course, the expenses for an obese patient operated by laparoscopy was € 4412.41 vs. € 7323.17 by open surgery.
Conclusion: This study shows that in obese patients with endometrial cancer, minimally invasive surgery is more advantageous both in terms of costs and post-operative complications. Laparoscopic surgery in obese patients allows an economic saving of about 60% less than open surgery.
Disclosure: Nothing to disclose

EP1202

The SORT and ACS-NSQIP scores in assessing the surgical outcome of gynaecological oncology patients; performance comparison

A. El-Ghobashy, A. Aggarwal, S. Ijaz
Gynaecological Oncology, The Royal Wolverhampton NHS Trust, Wolverhampton, United Kingdom

Introduction / Background: Surgical specialities are using different preoperative patient scoring systems to predict postoperative outcomes. The Surgical outcome Risk Tool (SORT) score and the National Surgical Quality Improvement Programme (ASC-NSQIP) are two such risk prediction models.However, the use of these risk prediction models across surgical specialties is quite variable, mainly due to lack of local validation. We compared these two risk prediction tools to determine the best model suited to our patients.
Methodology: We carried out a retrospective comparative study including 84 patients at New cross Hospital in 2018 having elective Gynaecology oncology operations. Both scores were calculated preoperatively. We noted intraoperative time, intraoperative complications, estimated blood loss, Postoperative inpatient stay and complications. We analysed the data using Microsoft Excel and SPSS software. Correlation between the respective risk scores and postoperative variables was calculated using Pearson correlation coefficient.
Results: 73 patients had major surgery while 11 had minor procedure. 46 patients had open and 38 had minimal access surgery, including 14 robotic procedures. The average post-op inpatient stay for

open, laparoscopic and robotic surgery was 5, 2.8 and 1.4 days, respectively. The correlation coefficient for SORT score and post op stay was weak 0.167 (p value 0.13) as compared to ACS-NSQIP r=0.554 (p< 0.01). In comparison to SORT,NSQIP was better predictor of post-op complication. {r_1=0.356 (p< 0.01) vs r_2=0.492 (p< 0.01}. The correlation between duration of surgery and post-op stay was strong at 0.538 (p< 0.01).
Conclusion: In our experience ACS-NSQIP was a better predictor of postoperative outcomes as compared to SORT score. However, our study group is small and there are other factors that affect outcomes. It should be noted ACS-NSQIP requires more data input and comparatively less user friendly. Further studies are needed to standardise the perioperative prediction tool in Gynaecological cancer patients to improve surgical outcomes and provide safer patient care.
Disclosure: Nothing to disclose

EP1203

Cytoreductive surgery for tubo-ovarian carcinoma - a study of MDT decision-making for primary vs NACT-interval surgery

R. Daly, Y. Shahabuddin, C. Thompson, N. Gleeson
St James's Hospital, Dublin, Ireland

Introduction / Background: Primary debulking/cytoreductive surgery (PDS) is considered the optimal approach in the management of tubo-ovarian carcinoma (TOC). Neoadjuvant chemotherapy (NACT) with interval cytoreductive surgery (IDS) is not considered inferior to PDS. The aim of this study was to analyse the multidisciplinary team (MDT) decision-making process in assigning patients to PDS or NACT at this Irish tertiary gynaecological oncology centre.
Methodology: This is an observational retrospective study on consecutive patients with invasive serous and non-serous TOC, presenting from 1st January to 31st December 2018. Borderline, non-epithelial and low grade tumours were excluded. Patient demographics, staging, histology and pre-treatment biochemistry and tumour markers were reviewed. Treatment modalities (PDS, NACT-IDS, chemotherapy alone) were documented.
Results: Of 88 patients assessed by MDT, 31 were excluded. 41 patients had radiological stage III (61%, n=25) or IV disease (39%, n=16). 90% (n=37) were high-grade serous. 30 patients (52.6%) underwent PDS. 44.6% (n=14) were radiological stage III 14 (46.6%) and 3.33% Stage IV (n=1). Median age was 55.6 years and median Ca125 was 929. Complete cytoreduction was achieved in 27 patients (90%).
24 patients underwent primary chemotherapy. 45.9% (n=11) were radiological stage III and 54.1% (n=13) were Stage IV. Median age was 64 years and median Ca125 was 1902. 13 patients did not progress to IDS due to suboptimal chemotherapy response (n=5) and poor surgical candidacy due to comorbidities (n=4).
Conclusion: High grade serous was the most common histology. Achieving complete cytoreduction in the majority of the PDS group suggests MDT decision was correct. Patients selected for chemotherapy were more likely to have stage IV disease, higher age and Ca125. Half of patients receiving primary chemotherapy never progressed to surgery. Their poor baseline performance status may also be as important as chemo-resistance in that outcome. Robust comparisons of outcomes for PDS vs NACT-IDS need to pay close attention to patient stratification.
Disclosure: Nothing to disclose

EP1204

Knowledge, attitudes, and practices of resident trainees regarding screening for sexually transmitted diseases in cervical cancer patients

G.P. Gonzalez, S.L. Bravo
Obstetrics and Gynecology, Philippine General Hospital, Manila, Philippines

Introduction / Background: Cervical cancer is one of the leading malignancies affecting Filipino women. Its necessary cause is Human Papilloma Virus (HPV) infection. Because of the sexual nature of garnering HPV infections, other Sexually Transmitted Diseases (STDs) may also be present. This study aims to determine the knowledge attitudes and practices (KAP) of Obstetrics and Gynecology residents regarding screening for STDs in cervical cancer patients.
Methodology: The study design is cross-sectional and was approved by the Ethics Board. The population consists of Obstetrics and Gynecology residents who were made to answer a database questionnaire which was adopted from Roelens et. Al., and Barnett et. Al. Descriptive statistics provided an overview of the study population. Logistic regression analysis was used to determine the dependent variables such as the KAP. The mode was extracted.
Results: This study has described the KAP, of resident trainees regarding the screening of STDs in cervical cancer patients. Based on our findings, the respondents perceived that STDs in cervical cancer are common, hence their attitudes and practices emanated from this unsupported premise.
Conclusion: There is a gap of knowledge between the perception of respondents and the actual prevalence of STDs.
Disclosure: Nothing to disclose

EP1205

Enhanced recovery after surgery program in older patients undergoing gynaecologic oncological surgery is feasible and safe

C. Jauffret[1], A. de Nonneville[2], C. Braticevic[2], M. Faucher[3], G. Houvenaeghel[1], E. Lambaudie[1]
[1]Surgical Oncology, [2]Medical Oncology, [3]Anesthesiology, Institut Paoli Calmettes, Marseille, France

Introduction / Background: Enhanced Recovery After Surgery Programs (ERP) include multimodal approaches of perioperative patient´s clinical pathways designed to achieve early recovery after surgery and a decreased length of hospital stay (LOS). By allowing patients to return rapidly to their everyday surroundings, older patients are those who could take the greatest benefit from ERP. This is the first study to date to assess feasibility and safety of ERP on older patients undergoing gynaecologic oncological surgery.
Methodology: Data were prospectively collected between December 2015 and September 2017 at the Institut Paoli-Calmettes, a French comprehensive cancer centre. All the patients included in the study were referred for hysterectomy and/or pelvic or para-aortic lymphadenectomy for gynaecological cancer. The primary objective was to achieve similar LOS in patients ≥70 years old compared to younger patients without increasing the proportion of complications and readmission rates. A binary (LOS < or ≥ 2 days) logistic regression was built, including age, Charlson score, BMI, ASA score, oncological indication, surgical procedures and surgical approaches.
Results: Of a total of 329 patients, 75 were ≥70 years old and 254 were < 70. There were no differences in patient´s characteristics and surgical procedures. Age ≥ 70 years was associated with a longer LOS (means, 3.88 vs. 3.11 days; $p = 0.024$) only in univariate analysis. Considering the logistic regression, age was no longer associated with LOS. Total hysterectomy with pelvic lymphadenectomy and ASA score ≥ 3 were independently associated with longer LOS while mini-invasive techniques were associated with a shorter LOS. Morbidities and readmissions occurred respectively in 23% and 8% of the total population without any difference between the two groups. In the ≥70 years old population.
Conclusion: Our study shows that ERP for patients over 70 years of age undergoing gynaecologic oncological surgery is as safe and feasible as on younger patients.
Disclosure: Nothing to disclose

EP1206

Timely access to genetic counseling for women with epithelial ovarian cancer in Nova Scotia

A. Warias[1], M. Ferguson[2], E. Chamberlain[3], L. Currie[3], N. Snow[3], K. Matheson[4], L. Penney[3], K. Kieser[1]
[1]Obstetrics and Gynecology, Dalhouisie University, Halifax, NS, Canada, [2]MyGene, Miami, FL, United States, [3]Medical Genetics, IWK Health Centre, [4]Research Methods Unit, NSHA, Halifax, NS, Canada

Introduction / Background: Prompt access to genetic counseling and testing is increasingly important for women with epithelial ovarian cancer (EOC) and their families. We evaluated the efficacy of a new collaborative model of care and preferences on timing for genetic counseling in Nova Scotia between Divisions of Medical Genetics and Gynecologic Oncology at Dalhousie University.
Methodology: A retrospective cohort of all women diagnosed with EOC from 2012 to 2017 (n=386) was identified. Two cohorts were identified, pre and post implementation of a genetic counsellor attending weekly tumor board rounds. Wait-times to different nodes of the clinical pathway assessed. A questionnaire was sent to identify preference for timing of genetic counselling, and factors that may contribute to the decision to pursue genetic testing (n=103 living). Chi-square and Wilcoxon rank sum tests were used to compare demographic and clinical variables pre and post implementation of new model.
Results: There were 386 women with EOC and 196 women were referred for genetic counseling with 89.8% seen. Most women chose testing after counseling, 93.1%. Following the implementation of a new care model in May 2016, there was a 48.2% (95%CI 39.4 to 56.7) increase in referrals for genetic counseling from 40% to 88%. The wait time from the referral to the first genetic counseling appointment decreased by 67.8 days, p< 0.001. Seventy percent of survey respondents expressed that they wished to be referred for genetic counseling at the time of diagnosis or with initiation of chemotherapy. Women seen for genetic counseling, expressed that the most important reason for pursing genetic testing was for information for their family, 62%.
Conclusion: This novel model of care for EOC in Nova Scotia was successful in increasing referral rates for genetic counseling, and genetic testing. Women favored referral to genetic services at the time of diagnosis, highlighting the importance for timely access.
Disclosure: Disclosures: This project was funded by the Atlee Foundation and Astrazeneca

EP1207

Adolescents education about sexuall responsible behavior and preventing HPV infections-health education in Croatia

D. Lepusic
Gyn/Ob, KBC/Aktiva, Zagreb, Croatia

Introduction / Background: HPV is one of the most frequent sexually transmitted diseasees in the world, and according to some authors can be found in over 75% of sexually active women. Sexually active adolescents face serious health risks associated with unprotected sexual intercourse including HPV and other sexually transmited diseases as well as unwanted pregnancy. Behaviours particularly relevant to HPV transmission are: early age of sexuall debut, poor contraceptiv/condom use, multiple sexual partners, certain sexual practices and the use of substances such as alcohol and drugs. Adolescents may not have sufficient cognitive skills to foresee risks in sexual relationships and this may be compounded by unrealistic perceptions of themselves as relatively invurnerable.
Methodology: We want to do something for that young population for education of Human Papillomavirus and sexuall responsible behaviour, so we organized multemedia presentations Name of the project „Knowledge is pleasure“. Adolescents joind project active, by making their own web sites, scene performances,poems,posters,lectures.... all with sexuall responsible behaviour themas.
Results: By organizing multimedia presentations the interest of that population to attend would be greater. Questions they made after the lecture were those usual for that age. They asked about the way of contracting HPV and other STD-s, medical treatment of partners and use of contraceptives and

also about vaccina.
Conclusion: The most effective prevention of sexually transmitted diseases is a stable, harmonious relationship, with a faithful partner. A necessary condition prior to the onset of sexual intercourse is a physical and mental maturity of both partners. For systematic prevention of sexually transmitted infections diseases in this moment it is necessary: introduce an effective sexual education in schools starting from primary schools.
Disclosure: Nothing to disclose

EP1208

Enhanced recovery after surgery (ERAS) implementation and outcomes in a UK tertiary gynaecological oncology centre

E. Myriokefalitaki, M. Smith, B. Winter-Roach, R. Slade
The Christie NHS Foundation Trust, Manchester, United Kingdom

Introduction / Background: Enhanced Recovery Programmes have been proven to successfully prepare patients undergoing major abdominal surgery for an earlier and safe discharge.
Methodology: We are presenting The Christie Gynaecological oncology ERAS programme (pre-operative assessment and rehabilitation to peri-operative and inpatient care to post operative discharge support)
Results: Substantial reduction of length of hospital stay without compromising in morbidity, re-admissions or return to theatre and excellent patients´ feedback on satisfaction surveys.
Conclusion: ERAS programme is feasible and safe in Gynaecological oncology surgery. It order to succeed, requires a robust programme and collaboration with a vast multidisciplinary team.
Disclosure: Nothing to disclose

EP1209

Patient evaluation of group counselling after fast track surgery for gynaecologic cancer

D. Reventlow, T. Bjerre Kanstrup, C. Stroemberg, L. Sigaard
Copenhagen University Hospital, Copenhagen, Denmark

Introduction / Background: Previously, patients had individual follow-up in the outpatient clinic 5 years after fast track surgery.
Today patients are offered one follow-up visit after 4 weeks and a four-hour multidisciplinary group counselling session with: Physician, psychologist, nurse and sexologist 6 weeks after surgery. No individual consultations are planned afterwards.
The purpose of the new follow-up program was to enable patients to live with various physiological and psychological challenges, and how to be aware of a possibly relapse. The purpose of this study was to evaluate group counselling session as a method of achieving self-care.
Methodology: Five group counselling sessions with a total of 42 patients all previous diagnosed with either cervical or endometrial cancer radically operated with no need for adjuvant oncology treatment. In the beginning of each counselling, patients were asked to put their expectations into words. Finally, all patients completed an open-ended questionnaire focused on outcomes. The evaluation included the following phases:
1) Transcription
2) Content analysis
3) Thematizing.
Results: Overall expectations were to achieve knowledge about: Lymphedema, fatigue, infertility, fear of recurrence, a changed body, emotional- and sexological challenges.

Theme 1: Community
Patients emphasized the advantages of being in a smaller group, reflecting on and listening to other patients´ problems.
Theme 2: Counselling
Patients highlighted the benefits of counselling based on specific problems rather than education in general topics.
Theme 3: Coherence
Patients experienced a united and appreciatively multidisciplinary team supporting each other´s guidance.
Conclusion: The patients obtained an immediately gain from group counselling. They benefitted in meeting other patients in the same situation. In addition, the patients had a sense of being acknowledged. No one expressed the feeling of being insecure with no outpatient visits subsequently. The patients´ needs following the counselling session are unknown. We will conduct a follow-up study investigating how patients handle various challenges forward.
Disclosure: Nothing to disclose

EP1210

Follow-up in gynecologic malignancies, correspondence with ESGO Guidelines- preliminary result of ENYGO survey

I. Selcuk[1], M. Lanner[2], A. Pletnev[3], D. Vlachos[4], T. Nikolova[5], D. Lindquist[6], K. Zalewski[7], European Network of Young Gynae-Oncologists National Representatives 2017-2019
[1]Zekai Tahir Burak Women's Health Training and Research Hospital, Faculty of Medicine, University of Health Sciences, Ankara, Turkey, [2]Gynecology and Obstetrics, University Hospital Graz, Graz, Austria, [3]Gynecologic Oncology, Alexandrov National Cancer Center of Belarus, Minsk, Belarus, [4]Obstetrics and Gynecology, University of Athens, Athens, Greece, [5]Obstetrics and Gynecology, University Clinic of Obstetrics and Gynecology, Skopje, The former Yugoslav Republic of Macedomia, [6]Medical Oncology, Umeå University Hospital, Umea, Sweden, [7]Gynecologic Oncology, Holycross Cancer Center, Kielce, Poland

Introduction / Background: To identify the follow-up strategies of gynecologic malignancies across Europe and compare the correspondence with ESGO Guidelines.
Methodology: In 2019, a web-based survey was sent to the National Representatives of European Network of Young Gynae-Oncologists (2017-2019). The questionnaire consists of the questions related with vulvar cancer, cervical cancer, endometrial cancer and ovarian cancer.
Results: A total of 15/31 (48.3%) National Representatives responded the survey. Despite having a national guideline (>50%), the topic of follow-up was missing in some countries. The follow-up was mainly led by clinicians and performed by gynecologic oncology team (Table 1). For each malignancy, gynecologic examination is performed at every visit. Responders stated that Pap-test is routinely performed during the follow-up of cervical cancer 8/8 (100.0%), additionally in endometrial cancer 4/7 (57.1%) of responders perform routine Pap-test. Serum Ca-125 level is never routinely preferred during the follow-up of endometrial cancer, however serum Ca-125 is mostly preferred routinely for ovarian cancer. Complete blood count-biochemical tests and chest X-ray are not routine tests to consider at every visit. Routine imaging is mostly preferred for ovarian cancer and computed tomography is the most common imaging technique in case of a suspicion of recurrence except vulvar cancer in which magnetic resonance imaging is preferred (Table 2).
Conclusion: This preliminary results indicated that follow-up periods are similar with ESGO Guidelines. One of the major differences is that cytology is not recommended after radical/simple hysterectomy or chemoradiation in cervical cancer and the other one is serum Ca-125 is not a reliable marker in non-High grade serous carcinoma of the ovary. ESGO Guidelines generally don't recommend routine imaging unless a clinically indicated situation. Finally, these preliminary results showed that majority of the responses are similar with ESGO Guidelines.

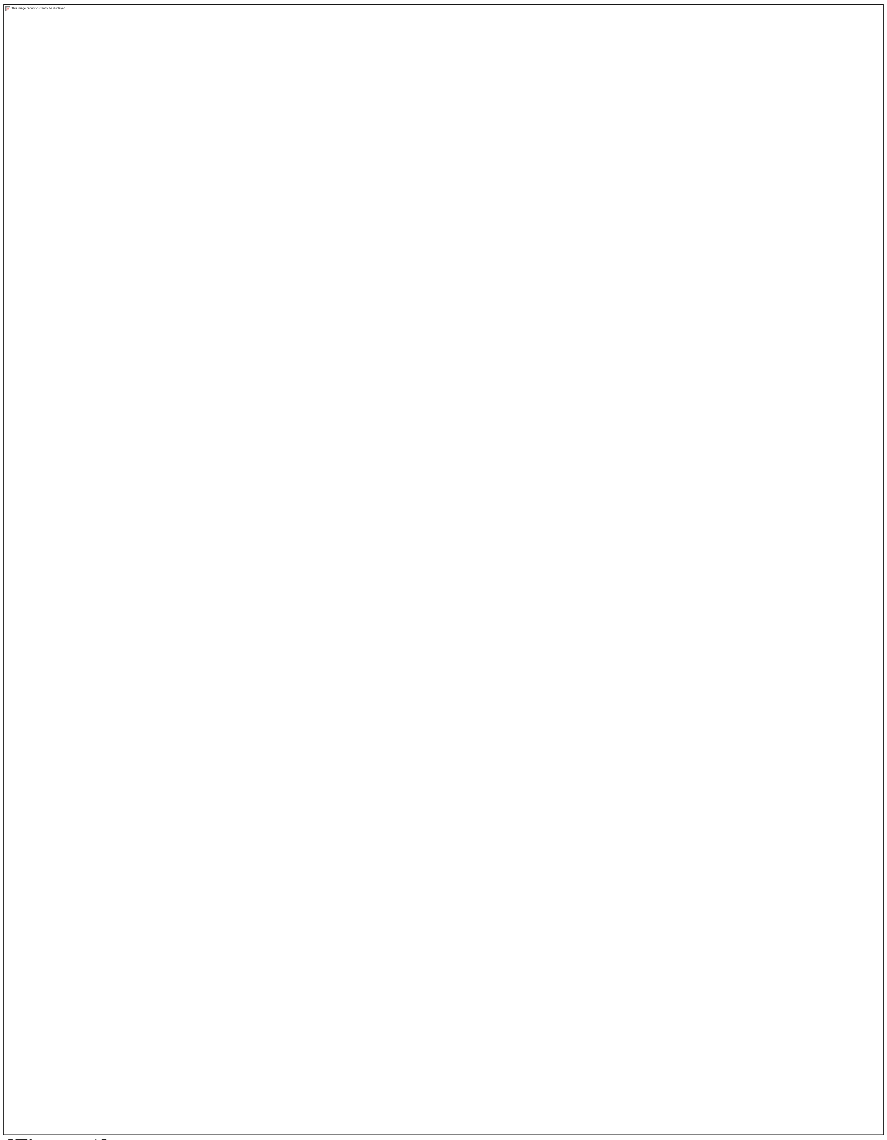

[Figure 1]

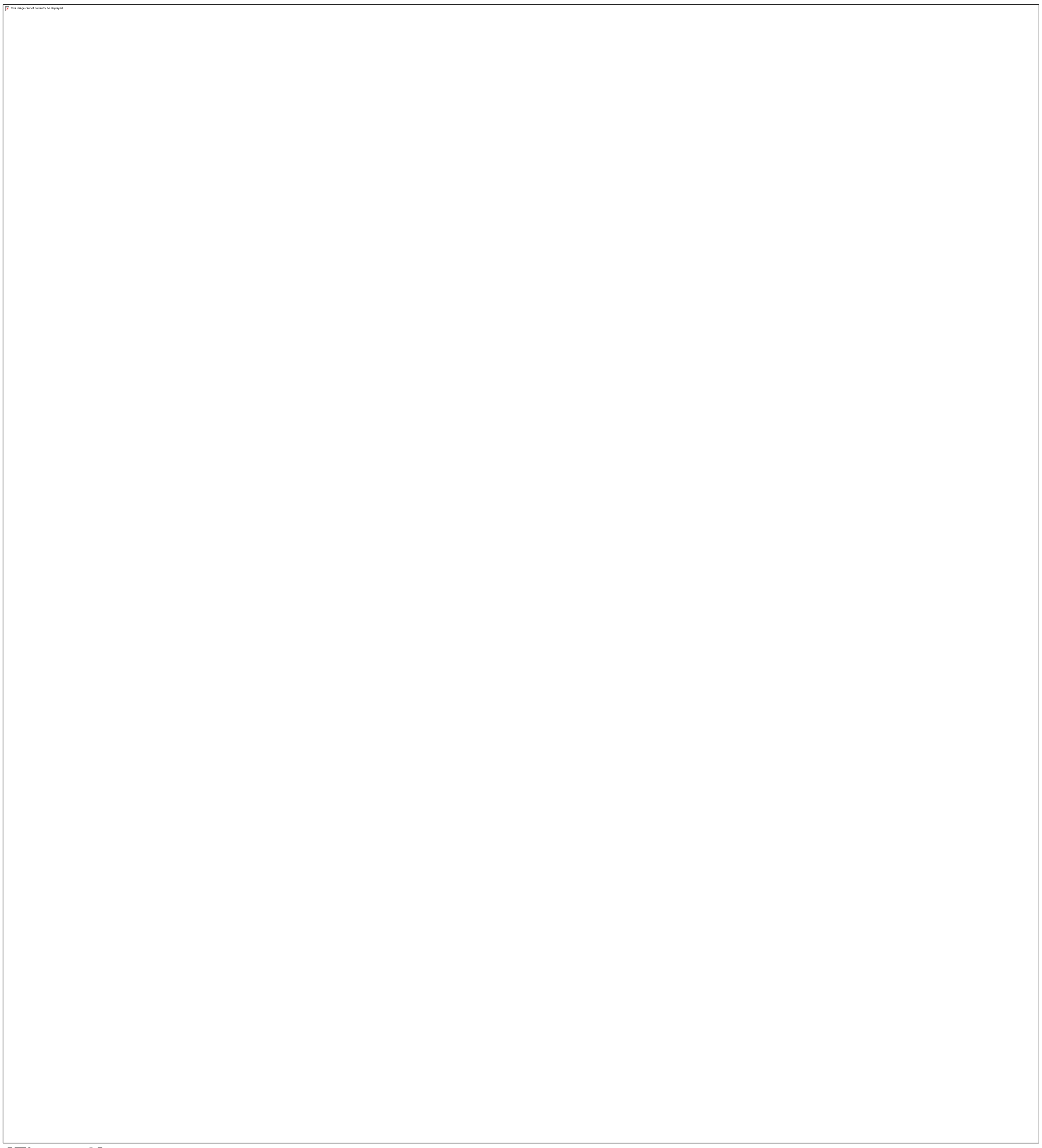

[Figure 2]

Disclosure: Nothing to disclose

EP1211

Observance of hormonal therapy in breast cancer centre Mohammed Vi for cancer treatment

H. Zaine, A. Iraqi, Z. Lakehayli, N. Benchakroun, A. Benider
Radiotherapy, CHU Ibn Rochd, Casablanca, Morocco

Introduction / Background: Hormonal adjuvant therapy in breast cancer with positive hormone receptors have reliably reduced the risk of recurrence and death.

As with any oral treatment, the simplicity of taking seems to lead patients to underestimate the value of hormonal therapy.
The objective of our study is to assess the degree of compliance with hormone therapy, and to discuss the different risk factors for poor compliance.
Methodology: This is a retrospective study based on observations collected during the first 6 months of 2014, about patients with breast cancer who have received hormonal therapy. With patient follow-up until January 2019.
Results: In study, poor compliance with hormonal therapy was objective, with up to three-quarters of the patients in our sample discontinuing their treatment.
Several factors were identified in our study, including drug toxicity and the precarious situation of patients. Depression, although it reached only a small percentage of our sample, nevertheless had a significant impact on poor treatment compliance. The lack of awareness and misinformation of patients about the benefit reported by hormonal therapy was also an important factor in poor compliance. This category of patients feels that they have been informed in a standardized way without personalization.
Conclusion: Our results have shown that good compliance with treatment does not depend solely on prescription; many additional factors must be taken into account.
Disclosure: Nothing to disclose

EP1242

Palbociclib in the treatment of metastatic breast cancer. Institutional experience

A. Mainella, S. Vigo, F. Marmissolle, P. Price, M. Sansano, M.L. Luján, M.P. Dominguez, G. Zapata Caamaño, E. Menna, N. Giacomi
Centro Oncológico Integral - COI, La Plata. Buenos Aires, Argentina

Introduction / Background: In Argentina, 18.000 new cases of breast cancer are diagnosed each year and it is the commonest cause of cancer death in women reaching 5600 deaths per year.
About 70% of all breast cancers are estrogen receptor-(ER) positive.
In postmenopausal women with advanced or metastatic, ER-positive, Her2-negative breast cancer the combination of Palbociclib (P) + Letrozol (L) or Fulvestrant (F) is a good option of treatment.
The objetive was to assess clinical benefit, evolution and safety with P associated with L or F in the context of daily practice.
Methodology: We performed an observational, descriptive and retrospective study.
All patients who started treatment with P in combination with L or F between April 2016 and August 2019 were included.
Results: We included 48 patients (pts), with median age 62 years (r37-85). 10 premenopausal women.
26 pts (54%) performed P + L and 22 pts (46%) P + F.
5 pts (10%) had metastatic onset. 22 pts (46%) visceral disease and 21 pts (44%) exclusive bone disease.
Clinical benefit: 8 pts (17%) stable disease, 16 pts (34%) partial response and 3 pts (6,25%) complete response.
In 23 pts (48%) an extension of 7 to 14 days of rest between cycle and cycle was necessary. Dose reduction in 12,5% (6 pts).
The most frequent toxicity were neutropenia in 25 pts (52%), G3: 60%, G2: 40%, asthenia (33,3%) and fatigue (23%). No cases of febrile neutropenia were reported.
Only 2 pts discontinued P because of adverse events.
26 pts (54%) still under treatment.
Conclusion: In our experience, the response rates observed were similar with reported data. Despite the high incidence of hematological toxicity (52% neutropenia), adherence to treatment with acceptable toxicity profile was maintained.
A clinical benefit was observed in 57% of our pts with an adequate tolerance.
Disclosure: Nothing to disclose

EP1243

Anthropometric marker of breast cancer

A. Rawat, N. Ganesh
Department of Research, Jawaharlal Nehru Cancer Hospital & Research Centre Affiliated to Barkatullah University, Bhopal, India

Introduction / Background: Breast cancer is the most prevalent cancer in females and accounts for nearly 23% of all cancers worldwide. There is a need to look for novel, cost-effective and noninvasive tumor marker for its early and effective diagnosis. In this regard, the present research work aims to generate an anthropometric marker for the diagnosis of Breast Cancer and screening females having high risk of developing Breast Cancer by exploring the Dermatoglyphics tool of 'atd' angle measurement in the palms of females screened
Methodology: The study consists of dermatoglyphics tool of 'atd' angle measurement in the palms of breast cancer females and their healthy counterpart for which informed consent were taken from each individual. "Angle of triradius" (atd) angle of around 100 Breast Cancer patients (age group 18-70 years) along with age matched 100 Healthy Control females were taken (Harold Cummins, 1960) and analyzed.
Results: In the Anthropometric Analysis using dermatoglyphic tool of 'atd' angle, Breast Cancer patients were found to be having wider atd angles (mean 'atd' angle of 48°) than their healthy counterparts (mean 'atd' angle of 39°) which was statistically significant by Student's t test with a p value of < 0.001.
Conclusion: This simple technique has a huge potential in screening females at high risk for developing breast cancer as an adjunct to the existing screening techniques like mammography so that the females with wider 'atd' angle can be sent for further investigations for early detection and treatment of breast cancer. It is not only cost effective but also non-invasive which makes them more accessible especially for screening females who are otherwise extremely anxious to go for mammographic screening for Breast Cancer.
Disclosure: Nothing to disclose

EP1244

Populational trends and outcomes of postoperative radiotherapy for high-risk early-stage cervical cancer with lymph node metastasis: concurrent chemo-radiotherapy *versus* radiotherapy alone

M. Klar[1], D.J. Nusbaum[2], H. Machida[3], Y. Huang[4], V. Khetan[2], B.H. Grubbs[2], L.D. Roman[2], J.D. Wright[4], K. Matsuo[2]
[1]Department of Obstetrics and Gynaecology, University Clinics of Freiburg, Freiburg, Germany, [2]Department of Obstetrics and Gynecology, University of Southern California, Los Angeles, CA, United States, [3]Department of Obstetrics and Gynecology, Tokai University School of Medicine, Kanagawa, Japan, [4]Department of Obstetrics and Gynecology, Columbia University College of Physicians and Surgeons, New York, NY, United States

Introduction / Background: Pelvic lymph node metastasis carries the highest impact on decreased survival among surgical-pathological risk factors for early-stage cervical cancer. The goal of this study was to examine the association between the use of concurrent chemotherapy and survival in women with early-stage cervical cancer and nodal metastasis receiving adjuvant radiotherapy.
Methodology: This is a population-based cohort study using the Surveillance, Epidemiology, and End Results Program from 1988-2016. Women with stage T1-2 cervical cancer with pelvic lymph node metastasis who underwent hysterectomy and received postoperative radiotherapy were examined. Trends, characteristics, and overall survival (OS) were compared between women who received postoperative radiotherapy alone (*n*=729) or in combination with CCRT (*n*=1,809). Propensity score-

based inverse probability of treatment weighting (PS-IPTW) was used to account for the effect of measured covariates on treatment selection.

Results: Among 2,538 women, there was a marked increase in the use of concurrent chemotherapy from 1997-2000 (20.7% to 78.5%, *P*=0.052) followed by a more gradual rise through 2016 (88.3%, *P*< 0.001). In a multivariable model, women with non-squamous cell carcinomas and those diagnosed more recently were more likely to receive CCRT, while older women were less likely to receive CCRT (all, *P*< 0.05). At the population level, the 5-year OS rates remained unchanged (annual percent change for 1997-2012 -0.1, 95% confidence interval [CI] -1.2 to 1.0, *P*=0.776). In a propensity score weighted cohort, women who received CCRT had a 5-year OS rate similar to those treated with radiotherapy alone (73.1% *versus* 73.6%, hazard ratio 1.004, 95%CI 0.887-1.136, *P*=0.955). Significant differences were also not seen in older women, non-squamous types, stage T2 disease, and multiple node metastases (all, *P*>0.05).

Conclusion: Despite the marked increase in the use of CCRT for women with early-stage cervical cancer and nodal metastases, there was no association between use of concurrent chemotherapy during postoperative radiotherapy and improved survival.

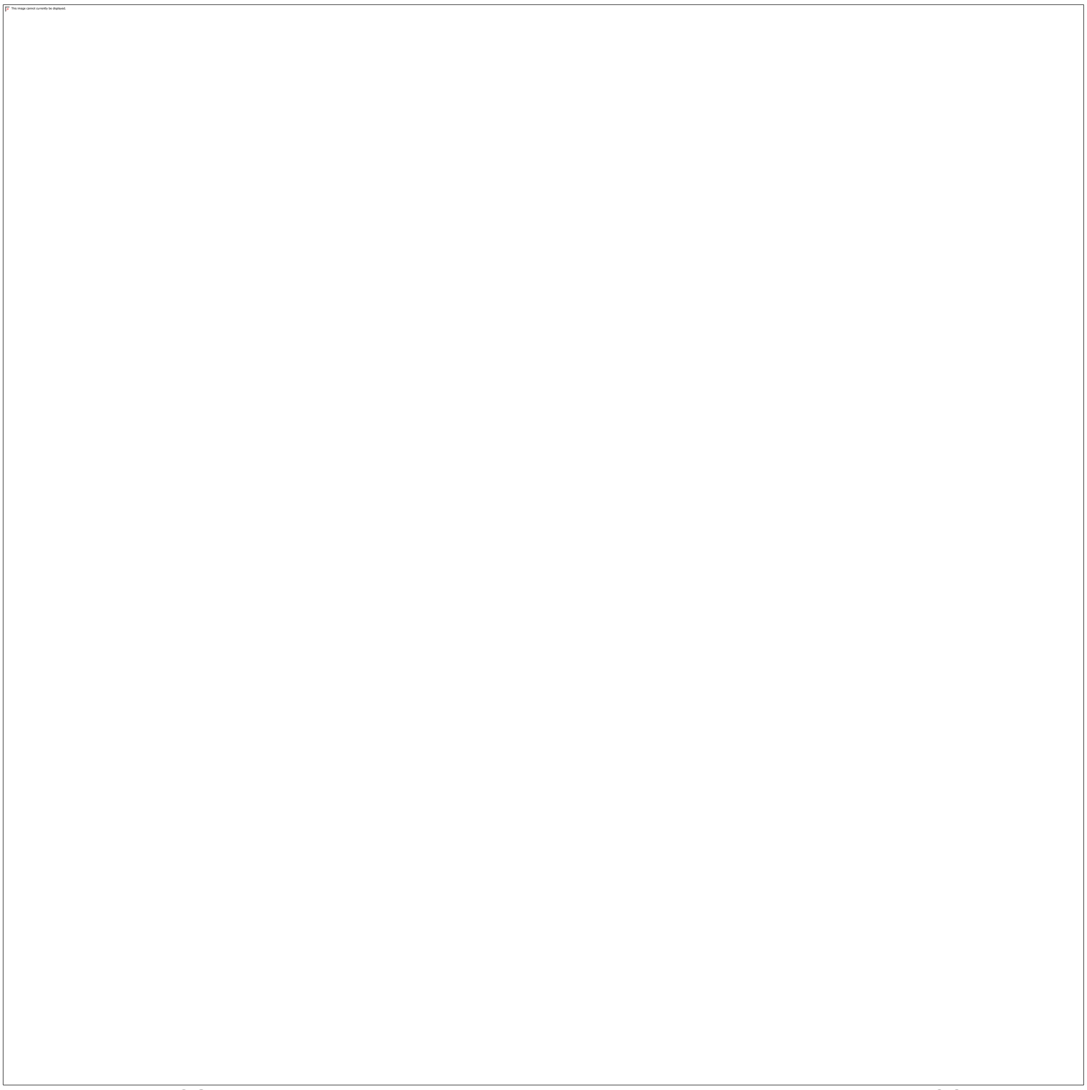

[Post-operative OS rates stage T1-2, N1 cervical cancer pts undergoing RT (n=729) or CCRT (n=1,809)]

Disclosure: Consultant, Tesaro and Clovis Oncology, research funding Merck (J.D.W.); consultant, Quantgene (L.D.R.); honorarium, Chugai, book editorial expense, Springer, and investigator meeting attendance expense, VBL therapeutics (K.M.); none for others.

EP1245

Topical Imiquimod treatment of high-grade cervical intraepithelial neoplasia (TOPIC-3) study: a multicentre, non-randomized controlled study

N. Hendriks[1], M. Koeneman[1], M. van de Sande[2], L. Kooreman[1], S. van Kuijk[1], L. Hoosemans[1], S. Sep[1], T. van Gorp[1], B. Slangen[1], J. Piek[3], H. van Beekhuizen[2], H. Nijman[4], R. Kruitwagen[1], A. Kruse[1]
[1]Maastricht University, Maastricht, [2]Erasmus MC, Rotterdam, [3]Catharina Hospital, Eindhoven, [4]UMC Groningen, Groningen, The Netherlands

Introduction / Background: High-grade cervical intraepithelial neoplasia (CIN) is the premalignant condition of cervical cancer. Treatment is usually performed by large loop excision of the transformation zone (LLETZ), but is associated with an increased risk of premature birth in subsequent pregnancies. Topical imiquimod cream may be an alternative treatment modality. This study aims to provide evidence on treatment efficacy and side-effects of topical imiquimod cream compared to standard LLETZ treatment.
Methodology: A multi-centre, non-randomized controlled trial was performed among women with a histological diagnosis of CIN 2/3. Women who opted for standard treatment, underwent LLETZ treatment. Successful treatment was defined as no need for second LLETZ after 6 months follow-up. Women who opted for imiquimod treatment, administered 6.25mg imiquimod 5% cream vaginally up to three times per week during 16 weeks. Treatment efficacy was evaluated with colposcopy at 20 weeks follow-up and defined as CIN 1 or less.
Results: 123 patients were included. 62 women underwent LLETZ treatment, which was successful in 58 women (95%). 37% of women reported severe side-effects (Visual analogue scale [VAS] ≥ 8).
61 women underwent topical imiquimod treatment. Twelve women discontinued treatment due to side effects. Treatment was successful in 27 (59%) of remaining women. Side effects were reported by all women who underwent imiquimod treatment and consisted most frequently of headache and fatigue. 69.0% of women reported severe side-effects (VAS ≥ 8). All reported side-effects can be found in tables 1 and 2.
Conclusion: Topical imiquimod is a feasible alternative treatment for patients with high-grade CIN, although efficacy is considerably lower than LLETZ treatment. It could be an alternative treatment, especially for women with a future pregnancy desire. Predictive biomarkers are necessary to better select those patients who will benefit from imiquimod treatment.

	Any reported side effect		Side effect VAS ≥8	
	No. of patients (% of total)	Mean no. of days (if recorded)	No. of patients (% of total)	Mean no. of days (if recorded)
Abdominal pain	35 (77.8%)	4.8	4 (8.9%)	1.5
Vaginal discharge	45 (100%)	8.9	14 (31.1%)	3.8
Vaginal blood loss	45 (100%)	9.0	23 (51.1%)	2.2
Vaginal pruritus/pain	7 (15.6%)	4.6	4 (8.9%)	3.0
Fever	2 (4.8%)	1.0	0 (0.0%)	0.0
Hospital admission due to hemorrhage	7 (11.2%)	-	-	-

[Table 1. Side-effects of LLETZ treatment, reported during 2 weeks]

N=42	Any reported side effect		Side effect VAS ≥8	
	No. of patients (% of total)	Mean no. days per patient (if recorded)	No. of patients (% of total)	Mean no. days per patient (if recorded)
Headache	39 (92.9%)	13.9	22 (52.4%)	3.3
Fever	29 (69.0%)	4.5	13 (31.0%)	2.4
Fatigue	36 (85.7%)	11.4	16 (38.1%)	6.4
Myalgia	34 (81.0%)	8.9	11 (26.2%)	4.5
Vaginal blood loss	27 (64.3%)	6.0	5 (11.9%)	5.2
Vaginal pruritus/pain	34 (81.0%)	9.5	16 (38.1%)	3.1
Vaginal discharge	29 (69.0%)	10.4	6 (13.3%)	3.5
Vaginal redness	18 (42.9%)	2.4	6 (14.3%)	4.7

[Table 2. Side-effects of imiquimod treatment, reported first 8 weeks]

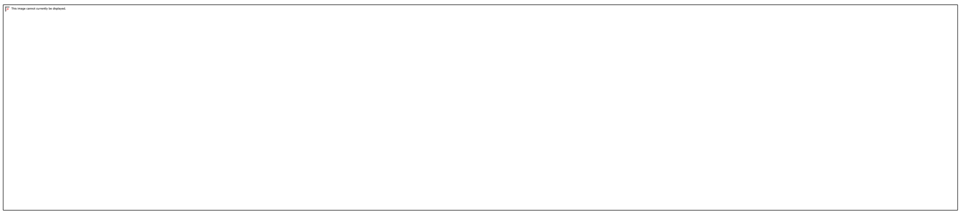

[Figure 1: imiquimod treatment regime]

Disclosure: The authors declare that they have no competing interests. This study and manuscript preparation were funded by the Academic Hospital of Maastricht (Academic Fund) and MedaPharma. Both funding bodies were not involved in the study design and will not be involved in the collection, analysis, and interpretation of data, in the writing of the manuscript and in the decision to submit the manuscript for publication.

EP1246

Role of transforming growth factor beta 1 (TGF-β1) polymorphisms in susceptibility to cervical cancer: a case-control study

M.N.H. Apu
Clinical Pharmacy and Pharmacology, University of Dhaka, Dhaka, Bangladesh

Introduction / Background: TGF-β signaling pathway is one of the most commonly altered cellular signaling pathways in human cancers. Till now limited pharmacogenetic study of TGF- β1gene C509T and T869C polymorphisms has been reported for cervical cancer. So, the aim of the study is to determine whether there is an elevated risk of cervical cancer development with TGF- β1 C509T and T869C polymorphisms in Bangladeshi women.
Methodology: A case-control study was conducted with 100 cervical cancer patients and 100 age-sex matched healthy controls using polymerase chain reaction-restriction fragment length polymorphism (PCR-RFLP) method.
Results: In case of TGF- β1 C509T polymorphism, the frequency distribution of genotypes in controls was 43% CC, 45% CT, 12% TT; in cervical cancer it was found to be 41% CC, 44% CT and 15% TT. No statistically significant differences were observed among heterozygous (OR= 1.083, 95% CI= 0.589-1.993, p>0.05) and mutant homozygous (OR= 1.27, 95% CI= 0.524-3.075, p>0.05) variants compared to normal homozygous variant. In case of T869C polymorphism, the frequency distribution showed the similar trend with 49% TT, 40% TC, 11% CC in controls and 39% TT, 49% TC, 12% CC in

patients indicating no association with cervical cancer risk (heterozygous: OR= 1.38, 95% CI= 0.753-2.538, p>0.05; mutant homozygous: OD= 1.46, 95% CI= 0.573-3.730, p>0.05). Moreover, no clinicopathological characteristics i.e. age, dwelling status, menstrual status, parity, contraception of patients was found to be significantly associated with cervical cancer risk. On the contrary, patients with a first-degree relative cancer patient possesses 5.51 folds more risk (p=0.031) of carrying a T allele in C509T variant of the TGF- β1 gene compared to those patients who does not have such relative.
Conclusion: This is the first study to identify that Bangladeshi women with C509T and T869C polymorphisms in the TGF- β1 gene are not in risk of developing cervical cancer.
Disclosure: Nothing to disclose

EP1247

Early-stage cervical cancer outcomes after open, minimally invasive and vaginal surgery. Single centre experience

S. Fernandez-Gonzalez, I. Calvo, M. Barahona, L. Marti, J. Ponce
Gynecology, Hospital Universitari de Bellvitge, Hospitalet de Llobregat, Barcelona, Spain

Introduction / Background: The management of surgical stages in cervical cancer need to be redesign based on the recent literature. However, it is not less important to know about the own experience in that field in order to reconsider further changes in daily practice. We aimed to study our outcomes in women affected by early-stage cervical cancer.
Methodology: This retrospective study analyzed data for all patients with early stage (IA1, IA2, IB1) cervical cancer treated by surgery between July 2000 and April 2017 at our institution. Clinical and pathological features, recurrences, disease free-survival (DFS) and disease specific survival rates were compared between patients who received Open Surgery (laparotomy), Minimally Invasive Surgery (MIS: laparoscopy or robotic) and Vaginal Surgery (Schauta assisted by laparoscopy for lymphadenectomy).
Results: A total of 107 patients were included, of whom 24 (22.4%), 65 (60.7%) and 18 (16.8%) received open, MIS or vaginal surgery respectively. The three groups were similar with respect to age, histologic subtype, FIGO stage and ECOG. Higher rates of lymphovascular invasion and tumor grade 3 were observed in vaginal and open group respectively, and lower rates of none adjuvant treatment in open surgery were observed (Table 1). After a median follow-up of 99, 50 and 117 months, the DFS was 82.6%, 93.8% and 86.7% in Open, MIS and Vaginal respectively and Disease specific survival rate was 100%, 98.4% and 93.8% respectively (Table 2).

	Open Surgery (n= 24)	Minimally Invasive Surgery (n= 65)	Vaginal Surgery (n= 18)	p value
Age (median, years)	44 (28-79)	47 (27-79)	44 (31-66)	0.897
Squamous-cell carcinoma (%)	16 (66.7)	45 (69.2)	11 (61.1)	0.814
Satge of disease IB1(%)	23 (95.8)	59 (90.8)	15 (83.3)	0.228
Lymphovascular space infiltration (%)	7/23 (30.4)	22/62 (35.5)	9/17 (52.9)	0.166
Tumoral Grade 3 (%)	5/12 (41.7)	15/57 (26.3)	2/8 (25)	0.283
ECOG performance-status 0 score	18/19 (94.7)	61/61 (100)	1/12 (83.3)	0.503
Adjuvant treatment-None (%)	14/20 (70)	36/65 (55.4)	6/14 (42.9)	0.219
Chemotherapy +	4/20 (20)	15/65 (23.1)	5/14 (35.7)	

Radiotherapy				
Radiotherapy	2/20 (10)	14/65 (21.6)	3 (21.3)	

[Table 1. Baseline patient characteristics]

	Open Surgery (n= 24)	Minimally Invasive Surgery (n= 65)	Vaginal Surgery (n=18)	p value
Hospital stay (median, days)	5 (4-15)	2 (1-18)	5 (3-12)	0.001
Recurrences (%)- None	19/23 (82.6)	60 (93.8)	13 (86.7)	0.6
Vaginal	2/23 (8.7)	2/64 (3.1)	1/15 (6.7)	
Pelvic	2/23 (8.7)	1/64 (1.6)	1/15 (6.7)	
Abdominal	0/23	1/64 (1.6)	0/15	
Disease specific survival (%)	23/23 (100)	63/64 (98.4)	15/16(93.8)	0.397
Overall Survival (%)	21/23 (91.3)	63/64 (98.4)	14/16 (87.5)	0.825

[Table 2. Outcomes]

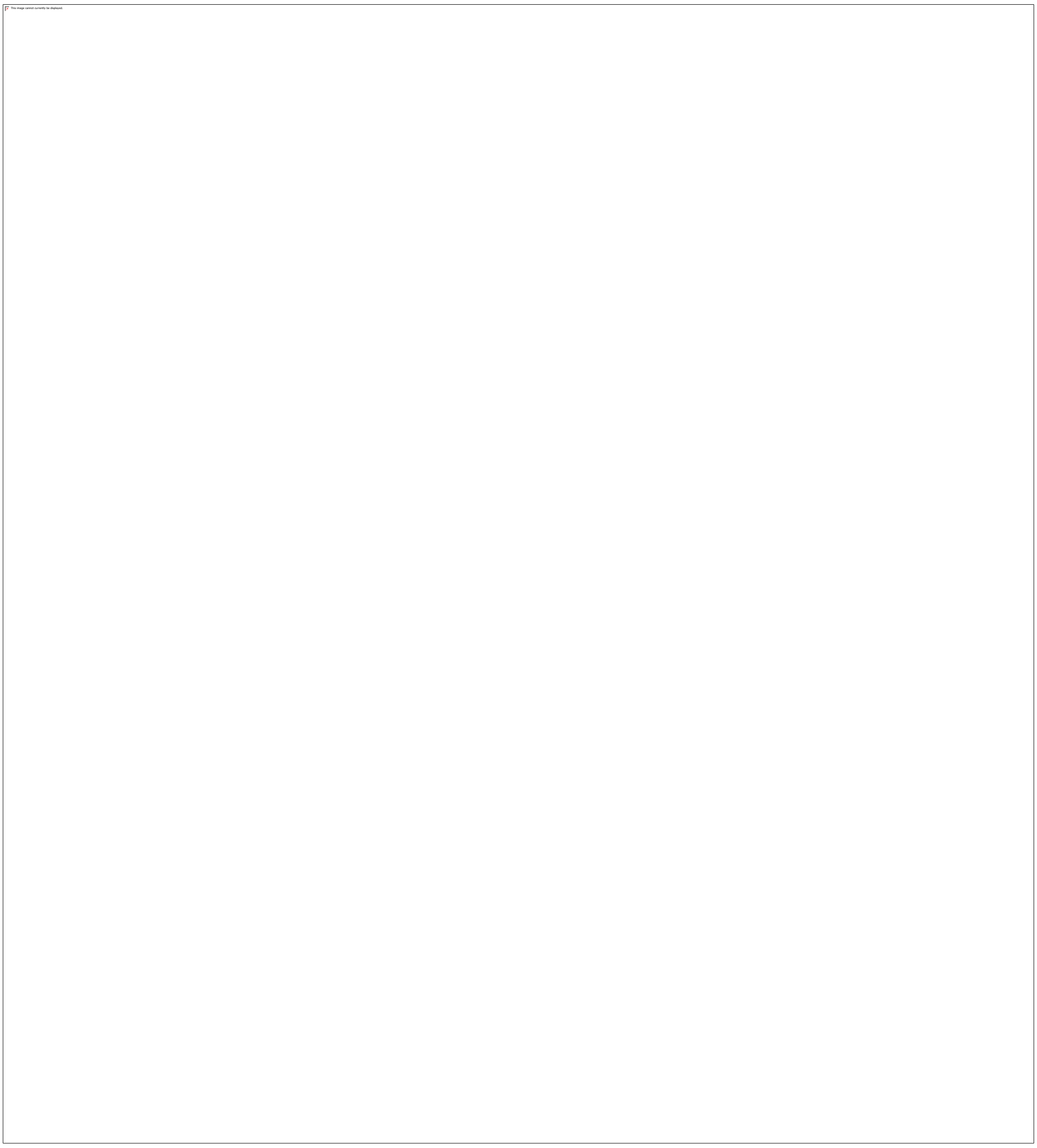

[Figure 1. Disease-free survival rate]

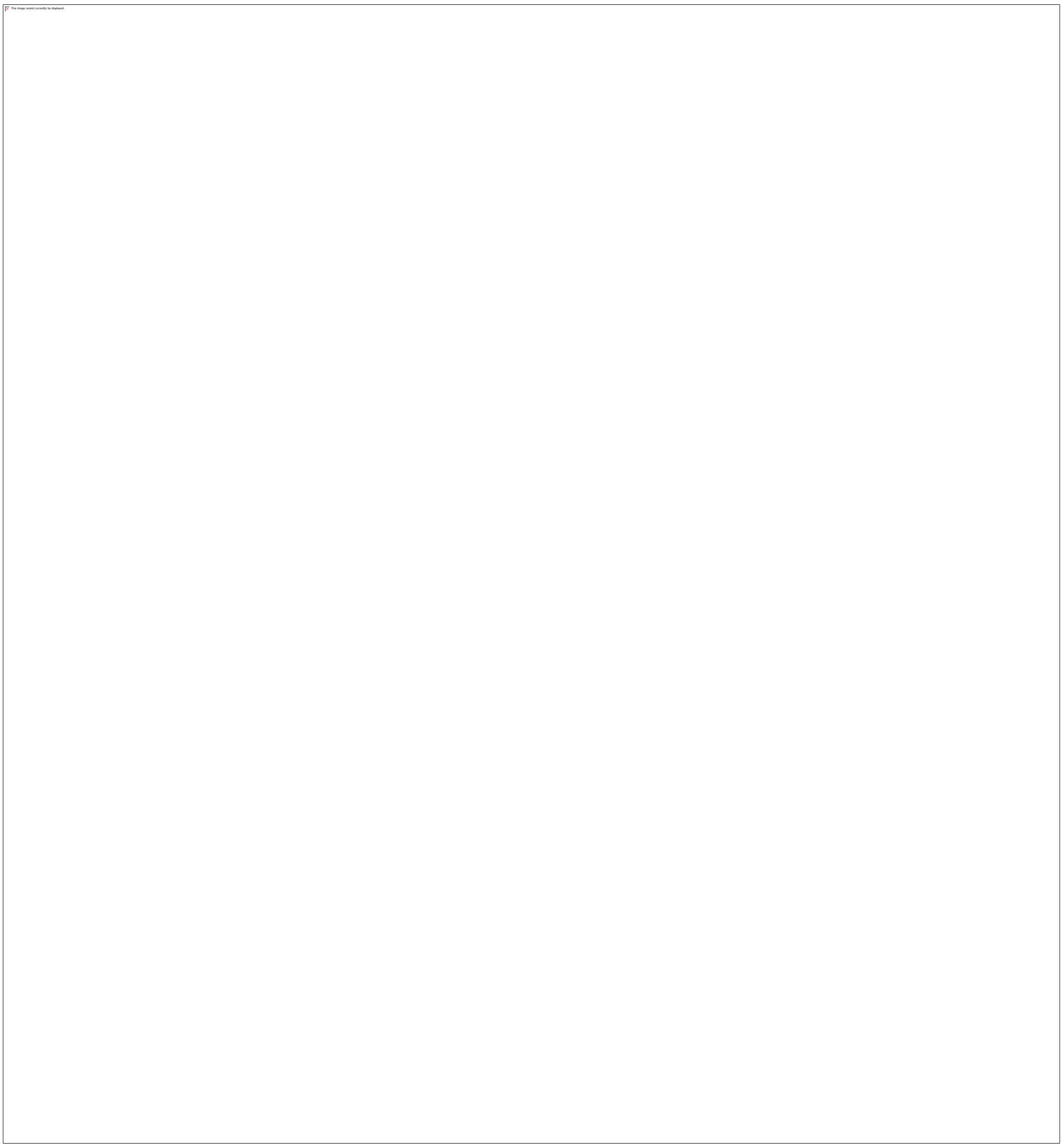

[Figure 2. Disease specific survival rate]

Conclusion: In our experience, minimally invasive radical hysterectomy was associated with higher rates of DFS and similar disease specific survival rate than Open and Vaginal radical hysterectomy among women with early-stage cervical cancer.
Disclosure: Nothing to disclose

EP1248

Laparoscopic radical hysterectomy for early stage cervical cancer

N. Doan Trong[1,2], T. Nguyen Van[1], L. Ta Thanh[1], T. Nguyen Duy[1]
[1]Oncology Hospital at Ho Chi Minh City, [2]Oncology, University of Medicine and Pharmacy at Ho Chi Minh City, Ho Chi Minh, Viet Nam

Introduction / Background: Although phase 3 LACC trials showed that minimally invasive radical hysterectomy in early stage cervical cancer was associated with lower rates of disease-free survival as well as overall survival than conventional laparotomic hysterectomy, this trial did not have enough power to be generalize to low risk patients. Therefore, laparoscopic radical hysterectomy is still be considered in that context.
Methodology: This was a retrospective trial. We review data of early stage cervical cancer who were underwent laparoscopic total hysterectomy at Oncology Hospital at Ho Chi Minh city from June 2016 to January 2019.
Results: Seventy-five patients were enrolled in this study. Most of them (89.3%) were stage IB1 disease. Overall, the average of tumors diameter was 17 mm, the median time of the surgery was 190 minutes and the median of blood lost was 150 ml. The rate of surgical complications was 14.6% (11/75 patients). Among of them, 2 patients had vesicovaginal fistulas, 7 patients had ureteral perforations and 2 patients had rectal perforations. The median follow-up time was 12 months. The rate of pelvic recurrences was 2.6%.
Conclusion: Laparoscopic total hysterectomy in early stage cervical cancer is a safe approach in women with lowest-risk disease. It reduces the operative morbidity as well as increases the quality of life. However, the limit of our study was that the follow-up time was still short. Therefore, we will continue this study to explore the disease-free and overall survival rates.
Disclosure: Nothing to disclose

EP1249

Correlation between cytological and histological characteristics of abnormal cervical smears

S. Argyridis
Archbishop Makarios III Hospital | Obstetrics and Gynaecology Clinic, University of Nicosia, Nicosia, Cyprus

Introduction / Background: Cytological classification and management of abnormal smears remains a controversial issue, as various classification systems have been proposed over the years. Management of most abnormal smears is by colposcopic guided biopsies, while management of cervical intraepithelial lesions varies according to the degree. Correlation between cytology and colposcopy is very important for assessment of sampling technique, sensitivity and specificity of methods used for screening and diagnosis.
Methodology: 431 abnormal smears were included that were managed at the cervical pathology department between 2013-2017. The Bethesda and the CIN classification systems were used to classify the results.
Results: ASC-US with positive HPV DNA testing was 37.04%, LSIL 32.44%, ASC-H 17.67% and HSIL 12.83%. The ASC-US smears were 27.45% normal, 29.41% HPV infected, 27.45% CIN 1, 13.07% CIN 2, 01.96% CIN 3 and 0.65% cancer, while the LSIL smears were 20.14% normal, 08.95% HPV infected, 45.52% CIN 1, 20.89% CIN 2, 04.47% CIN 3. The ASC-H smears were normal in 15.06%, HPV infected 10.95%, CIN 1 26.02%, CIN 2 17.80%, CIN 3 28.76% and cancer 01.36%, while the HSIL smears were normal 09.43%, HPV infected 01.88%, CIN 1 15.09%, CIN 2 16.98%, CIN 3 50.94% and cancer 05.66%.
Conclusion: ASC-US and LSIL have a low risk of moderate-severe intraepithelial neoplasia or cancer (15.68% vs 25.39% respectively), therefore monitoring with repeat smear or using surrogate markers (HPV DNA methylation) may be of use in clinical management. ASC-H and HSIL have a high risk of moderate-severe intraepithelial neoplasia or cancer (47.97% vs 73.60% respectively), which indicates that excisional procedures (cone biopsy) are usually required.
Disclosure: Nothing to disclose

EP1250

Distribution of stages of cervical cancer in a University Hospital of Dhaka, Bangladesh

S. Khatun
Bangabandhu Sheikh Mujib Medical University, Bangabandhu Sheikh Mujib Medical University, Dhaka, Bangladesh

Introduction / Background: Cervical cancer is the second most cancer in women of Bangladesh. Clinical stage is the single most important prognostic factor. Acording to GOG report ,the errors in FIGO clinical staging ranges from 24% in stage IB to 67% in stage IIA.MRI is used for diagnosis of the stromal ,parametrial, bladder and vaginal invasion. In Bangladesh, MRI is not easily available,costly and interpretation is often not correct.We have to depend on Examination Under Anesthesia (EUA) which is more objective and informative.
Methodology: Aim of the study was to know the distribution of stages of cervical cancer in BSMMU Hospital. Objective was to do clinical staging together with cystoscopy and proctoscopy.A retrospective study done at Gynaecologic Oncology Department of BSMMU on 735 patients from December 2011 to June 2019.Results are described in two groups, 08-12-11 - to 01-08-17 (group I) and 02-08-17 to 30-06-19 (group II).
Results: Statistical analysis done in two groups.Parameters were age, size of tumor ,cystoscopy, biopsy and final diagnosis of the disease. No difference found regarding age distribution,size of tumor and rate of biopsy taking. Differences found in rate of performance of cystoscopy which was lower in second group of patients (27.3% vs 2.73%).Another difference found in stage of the diasease. Most common stage in first group was stage II B (28.26%). In second group also Stage II B.(50.78%)
Conclusion: Rate of cystoscopy decreased in 2017 and 2018. Stage II B cases has been increased in 2017 and 2018. This difference may be due to change in operators performing the procedure and due to observer variation.
Disclosure: Nothing to disclose

EP1251

The robotic surgical management of upper paraaortic lymph node recurrancy of uterine cervical cancer in lateral decubitus position - *Video presentation*

F. Demirkiran, T. Bese, B.O. Kayan, S. Acikgoz, S. Gokmen
Division of Gynecologic Oncology, Istanbul University-Cerrahpasa, Istanbul, Turkey

Introduction / Background: Uterine cervical cancer (UCC) is the third-most common malignancy among gynecological cancers . Although metastasis at the time of initial diagnosis of UCC is rare, 15-61% of patients eventually develop metastases within 28 years. Recurrence rates for patients with Stages IB, IIA, IIB, III, and IV have been 10%, 17%, 23%, 42%, and 74%, respectively. The 5-year overall survival (OS) has been < 5%. In this case we have present the robotic surgery which is done in lateral decubitus position in a patient who have paraoartic lymph node recurrence.
Methodology: The 45 year old patient had a primary radiochemotherapy treatment for stage 2b cervix cancer at the year of 2011. The patient applied to our clinic, 2 years later of the initial treatment, for the isolated lymph node(6-4 cms) recurrency in the paraaortic area between inferior mesenteric artery and left renal artery. The patient had been done robotic lymph node dissection surgery in lateral decubitus position so that the clinical and radiologic assessment revealed that there is no metastatis in other areas. Visualization of inferior part of left renal vessels can be achieved with lateral decubitus position. Operation time was 130 minutes after docking procedure.
Results: In this video, we are presenting robotic surgery for bulky metastatic upper paraaortic lymph nodes.
Conclusion: Advantages of the lateral decubitus position for this surgery include easier trocar setup, better visualization and instrument access for certain procedures.
Disclosure: Nothing to disclose

EP1252

Magnetic resonance fingerprinting of cervical carcinoma- a feasibility study

E.Y.P. Lee, J.A. Perucho, D. Cui, E.S.K. Hui
Department of Diagnostic Radiology, University of Hong Kong, Hong Kong, Hong Kong

Introduction / Background: Magnetic resonance imaging (MR) offers high soft tissue contrast to allow accurate assessment of local disease extent in cervical carcinoma. Magnetic resonance fingerprinting (MRF) is a new technique that allows simultaneous generation of multiple contrast with T1 and T2 quantitative maps. It has been shown to be useful in the grading of prostate carcinoma and detection of breast carcinoma. The aims of this study were to evaluate the feasibility of applying MRF in cervical carcinoma and quantifying the T1 and T2 values of tumour tissue.
Methodology: Consecutive patients with newly diagnosed cervical carcinoma and MR examinations before treatment were prospectively recruited for MRF. MR examinations were performed on a 3T system (Philips Achieva TX). Multi-slice MRF was acquired using an inversion recovery steady-state free precession sequence with spiral-in-spiral-out trajectory, pseudorandomized flip angles and repetition times over 1000 dynamics. T1 and T2 maps were obtained using the MRF dictionary matching algorithm. Values were expressed as mean ± standard deviation.
Results: Eleven patients (54.9 ± 16.8 years) with FIGO stage IB2 to IVA (IB2 1, IIA1 2, IIB 4, IIIC1 3, IVA 1) were recruited. All patients had squamous cell carcinoma, except for one with adenocarcinoma. The T1 value of tumour was 1534 ± 115 msec and the T2 value was 67 ± 12 msec. It was noted that the adenocarcinoma had higher T1 and T2 values (1721 msec and 93 msec) than squamous cell carcinoma (Figures 1 and 2).
Conclusion: Magnetic resonance fingerprinting was feasible in evaluation of cervical carcinoma and permitted T1 and T2 quantification of the tumour thereof with one acquisition.

[Figure 1. Patient with adenocarcinoma (A) standard T2W, (B) T1 map, and (C) T2 map]

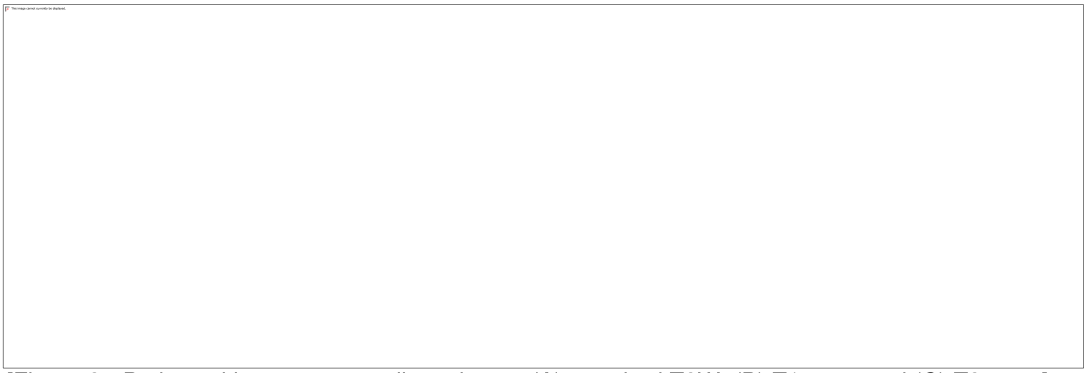

[Figure 2 - Patient with squamous cell carcinoma (A) standard T2W, (B) T1 map, and (C) T2 map]

Disclosure: Nothing to disclose

EP1253

A novel model to predict cancer-specific survival in patients with early-stage uterine papillary serous carcinoma (UPSC)

C. Lihua, C. Xi
Fudan University Shanghai Cancer Center, Shanghai, China

Introduction / Background: Uterine papillary serous carcinoma (UPSC) has aggressively biological behavior and leads to poor prognosis. However, clinicopathologic risk factors to predict cancer-specific survival of patients with stage I-II UPSC were still unclear. This study was undertaken to develop a prediction model of survival in patients with early-stage UPSC.
Methodology: Using SEER database, we identified 964 patients with FIGO stage I-II UPSC who underwent at least hysterectomy between 2004-2015. Using proportional subdistribution hazards regression to compare cancer-specific death (CSD) for all patients. Based on the results of univariate and multivariate analysis, the variables were selected to construct a predictive model; and the prediction results of the model were visualized by nomogram to predict the cancer-specific survival and the response to adjuvant therapy of stage I-II UPSC patients.
Results: The median age of the cohort is 67 years old. 17.1% (n=165) of the patients died of UPSC, while 8.6% of the patients died from other causes. On multivariate analysis, age (HR=1.45, P=0.021), tumor size (HR=1.81, P=0.014) and number of regional nodes removed (HR=0.52, P=0.003) were significantly associated with cumulative incidence of CSD. In the age≥67 years cohort, FIGO stage I-II was a risk factor for CSD (HR=1.83, P=0.036), and ≥ 10 lymph nodes removed was a protective factor (HR=0.50, P=0.01). Both combination of adjuvant chemotherapy and radiotherapy and adjuvant chemotherapy alone decreased the CSD of patients with stage I-II UPSC older than 67 years (HR=0.47, P=0.022; HR=0.52, P=0.024, respectively). The prediction model had great risk stratification ability as high-risk group had higher cumulative incidence of CSD than low-risk group (P< 0.001). In the high-risk group, patients with adjuvant chemoradiotherapy had improved CSD compared with patients who did not receive any adjuvant therapy (P=0.037).
Conclusion: Our prediction model has a good performance in predicting cancer-specific survival of early-stage UPSC patients and contributes to guide clinical treatment decision.
Disclosure: Nothing to disclose

EP1254

Pericardial effusion by neoplastic infiltration as one of first clinical manifestation of metastatic uterine carcinosarcoma

O. Ferreira Rangel Neto, L.F. Lessa, C. de Carvalho Ramos Bortoletto, M.A. Pereira, S.M. Nicolau, M.G.B.K. Uyeda
Oncological Gynecology Division / Departament of Gynecology, Federal University of Sao Paulo, Paulista School of Medicine, São Paulo, Brazil

Introduction / Background: Uterine Carcinosarcoma (UCS) accounts 40 to 50% of all uterine sarcomas. It was known as a malignant mixed Mullerian tumor (MMMT) and recently has been categorized as high-grade endometrial cancer. UCS are aggressive tumors, presents with extrauterine disease in 60% of cases in initial presentation.
Methodology: This is a case of a woman who presented with a pericardial effusion by neoplastic infiltration as a clinical manifestation.
Results: A 60-year-old woman was admitted to the emergency room with dyspnea, chest pain, hypotension, distant heart sounds, elevated jugular venous pressure. Echocardiogram identified a pericardial collection and a drainage was performed, with an output of 268cc of bloody serous fluid. Liquid cytology was positive for neoplastic cells. Pericardial sampling revealed infiltration by adenocarcinoma. Immunohistochemical (IHC) suggested serous adenocarcinoma of uterine origin. Patient with a history of postmenopausal bleeding 6 months ago. Magnetic Resonance Images (MRI) were obtained, which demonstrate a 12x9cm heterogeneous intrauterine mass, with invasion of more than 50% of the myometrium, presence of bladder wall implants, multiples pelvic, paraortic and mediastinal lymph nodes. Endometrial sampling revealed high grade carcinoma and immunohistochemical conclusion was serous endometrial adenocarcinoma.
The woman was initially treated with six cycles of Paclitaxel and Carboplatin and during chemotherapy experienced moderate vaginal bleeding. She was submitted to five cycles of hemostatic radiotherapy (2000cGy). Subsequently, was performed total hysterectomy with bilateral salpingooforectomy. Final histopathological evaluation revealed endometrial carcinosarcoma with heterologous rhabdomyosarcomatous component with differentiation of peripheral nerve sheath. Eighteen months after the beginning of the systemic treatment (5 months after the surgery) she presents with peritoneal recurrence (ascites and intestinal subocclusion).
Conclusion: UCS are relatively rare but very aggressive, treatment include surgery and combination with chemo and radiotherapy. Recurrence rates are high despite adequate treatment even in early stages. UCS accounts for all 15% of all deaths caused by uterine corpus malignancy.
Disclosure: Nothing to disclose

EP1255

The effect of lymph vessel invasion on the survival rate in low-risk endometrial cancer

S. Taranenka, A. Shushkevich, S. Mavrichev
N.N. Alexandrov Cancer Center of Belarus, Minsk, Belarus

Introduction / Background: We evaluate the effect of lymph-vascular space invasion on survival rate in stage I endometrial cancer (EC)
Methodology: 625 patients with low-risk stage I EC were included in a prospective randomized study who underwent treatment at N.N. Alexandrov national cancer center of Belarus. After risk group determining, all patients were randomized by means of a computer program. All patients have performed hysterectomy with bilateral salpingo-oophorectomy. Groups were differed by presence or absence radiation therapy (postoperative brachytherapy).
Results: In the group without lymph-vascular space invasion, the 5-year overall survival rate (OS) was 95.6% (95% CI 93.5-97.2%), cancer-specific survival rate (CSS) was 97.7% (95% CI 96.0-98.8%) disease free survival rate (DFS) was 95.9% (95% CI 93.9-97.5%). In the group with lymph-vascular space invasion, the 5-year OS was 90.2% (95% CI 83.0-95.3%), CSS - 92.0% (95% CI 85.4-96.6%),

DFS 90.7 % (95% CI 83.0-95.3%). Statistically significant differences in OS (p=0.019) and absence of differences in CSS (p=0.102) and DFS (p=0.144) for lymph vascular space invasion were obtained between the groups. OS and DFS were higher in the sub-group without lymph vascular space invasion on stage Ia G1 with myometrial invasion (p(OS)=0.005 and p(DFS)=0.030). There were not statistically significant differences in OS, CSS, DFS (p=0.929;; p=0.838;; p=0.802) in stage Ia G2 with myometrial invasion
Conclusion: Low-risk EC represents a non-homogeneous group. The survival rate in the study group is determined by the degree of tumor differentiation and depth of myometrial invasion. The effect of lymph-vascular space invasion needs to be clarified in further studies, because of the significant effect on survival rate in stage Ia G1 EC with myometrial invasion.
Disclosure: Nothing to disclose

EP1256

Long-term results of high-risk stage I endometrial cancer depending on the morphological tumor type

S. Taranenka, A. Shushkevich, S. Mavrichev
N.N. Alexandrov Cancer Center of Belarus, Minsk, Belarus

Introduction / Background: We evaluated the long-term results of treatment and effect of adjuvant chemotherapy on the survival rate of patients with high-risk EC stage I.
Methodology: In the retrospective study were include 225 patients who underwent treatment in Belarussian cancer clinics between 2006-2010. EC Ib G1 stage and nonendometrioid stage I cancer (serous, clear cell, undifferentiated carcinoma, carcinosarcoma) were included in the study. We received all the data from the Belarusian cancer registry.
Results: The overall (OS), cancer-specific (CSS) and disease-free (DSF) 5-year survival rate in high-risk EC was 66.7±3.2, 77.4±2.9%, 77.0±2.9%. 10-year old ¬ 53.7±4.5, 69.7±4.2 and 69.3±4.2% respectively. There were no statistically significant differences in the survival depending on morphological tumor type, but the endometrioid carcinoma of the Ib G3 stage is defined as a most favourable form of the high-risk EC, and the undifferentiated carcinoma and carcinosarcoma are the most unfavourable forms. The were no statistically significant differences in OS, CSS and DFS between the endometrioid carcinoma of the Ib G3 stage EC and nonendometrioid forms of the EC stage I. Adjuvant chemotherapy did not improve the long-term outcome of treatment in general, as well as separately in the Ib G3 stage of the endometrioid carcinosarcoma and the nonendometrioid EC stage I.
Conclusion: International collaborative research may define the role of chemotherapy in patients treatment with high-risk EC
Disclosure: Nothing to disclose

EP1257

High grade endometrial stromal carcinoma: rare but challenging diagnosis in nulliaprous women requesting conservative surgery, a case report

A.K. Tellisi[1], A. Hamcho[2], J. Ramawat[2], R. Belkhir[2], D. Kumar[2], E. Amir[2]
[1]*Ob/Gyn,* [2]*Hamad Medical Corporation, Doha, Qatar*

Introduction / Background: Case report: we present a case of 48 yr old single lady presenting with weakness, severe anemia and abnormal uterine bleeding and large pelvic mass of 30 week size. Ultrasound abdomen and pelvis : showed Mild right hydronephrosis; large heterogeneous lesion in pelvis likely to suggest a ?fibroid. MRI showed large posterior wall fibroid presenting as pelvi

abdominal mass with cystic and hyaline degeneration for excision and pathological confirmation to rule out sarcomatous change.. As patient was single she requested conservative surgery. She was counseled about the risk of hemorrhage and need for hysterectomy and underwent total abdominal hysterectomy with removal of tubes and ovaries.
Histopathological examination showed undifferentiated endometrial sarcoma (high grade) in background of atrophic endometrium and leiomyomata uteri
Post surgery she was investigated with oncology and multidisciplinary team and CT scan of abdomen and pelvis showed lung metastasis.
Patient was counseled but she was in denial and did not attend for follow up and palliative surgery.
Methodology: Case report
Results: Histology slides and MRI slides will be uploaded in the poster
Conclusion: High grade endometrial stromal sarcoma is rare but serious diagnosis. High Degree of suspicion pre operatively specially in large fibroids and patient requesting conservative surgeries is required.
We present this case due to its rarity and difficulty in managing the patient pre and post operatively as she was requesting conservative surgery pre operatively and due to denial failed to continue post operative follow up and therapy .. Also this case again emphasized that most of this cases present as common perimenopausal complaints like abnormal menstrual bleeding and pelvic masses and diagnosed in late stage of disease which carries poor prognosis. Therefore high degree of suspicision is required for early diagnosis, counseling and management of such cases.
Disclosure: Nothing to disclose

EP1258

Predictors of node metastasis in endometrial cancer at an early stage

E. Lopez Gonzalez, F. Montero Venegas, M. Peña Salas, J.A. Rojas Luna
Juan Ramon Jimenez Hospital, Huelva, Spain

Introduction / Background: Objective: To determine predictor factors of node metastasis in patients suffering from endometrium cancer at an early stage.
Methodology: Retrospective research of 170 patients with endometrium cancer between 2013 and 2017 at the Juan Ramon Jimenez Hospital. 56 patients with complete surgical staging susceptible to the node involvement analysis.
Variables such us age, histological type, degree of tumour differentation, lympho-vascular space infiltration (LVSI), miometrial invasion (MI) were taken into account among the groups. Statistical analysis was done using SPSS 23v program.Once the analysis of Kolmorov-Smirnov concluded in an unusual result; the Chi-Squared test was used in order to obtain parametric categorical data.
Results: Among the patients with complete surgical staging, pelvic lymph node metastasis was found as well as para-aortic lymph node metastasis in ten of them (18%). In the univariate analysis it was concluded that the degree of tumour differentation (p=0,015), miometrial invasion < 50% (p= 0,042) and LVSI (p< 0,01) were significantly related to the lymph node matastais although with a mild correlation level; underlining that the node affection is related to the lymphovascular infiltration.
The existence of LVSI was assessed in all the patients. LVSI was negative in 141 cases and one of these cases had lymph node metastasis. 86 % of the patients with node metastasis had positive LVSI (6 out of 7).
Conclusion: A high percentage of the patients with node metastasis have positive LVSI and if the LVSI is negative, there is also a risk of the 0.7 % of finding node metastasis. Although prospective multi-center studies would be convenient, this preliminary study shows the relevance and neccesity of including the infiltration of the lymphovascular space in the staging of the endometrium cancer and this could also be used when beginning with the adjuvant treatment in patients with the illness in an early stage.
Disclosure: Nothing to disclose

EP1259

Predictors of the endometrium cancer recurrence

E. Lopez Gonzalez, F. Montero Venegas, M. Escribano Cobalea, C. Daza Manzano
Juan Ramon Jimenez Hospital, Huelva, Spain

Introduction / Background: Objetive to find out the predictors in the recurrence of the illness in patients suffering from endometrium cancer at the differet stages of it.
Methodology: Retrospective study of 170 patients who were diagnosed with endometrium cancer between 2013-2017 at “Juan Ramon Jimenez” Hospital in Huelva.
Variables such us age, degree of tumour differentation, miometrial invasion (MI), post surgical staging and lympho-vascular space infiltration (LVSI) were analysed in all those patients that had recurrences during the period of the study.
The study was sone by using SPSS 23rd V.
Once the analysis of Kolmorov-Smirnov concluded in an unusual result; the Chi-Squared test was used in order to obtain parametric categorical data; whereas the U (by Mann Whitney) test was used for the non-parametric data. P< 0,01 was accepted as significant from a statistic point of view
Results: During the study, 9 patients were diagnosed with tumoral affects recurrence. This means 5.3 % out of the whole sample.
The statistic analysis concluded that there is no relation of dependence among the recurrence varible and the Miometrial Invasion variable (X2=4780,p=.092) recurrence and Tumor grade (X207765,p=.051) and recurrence and post surgical (x2=10200,p=.070). On the contrary, it is stated that there is a relation of dependence among lymph-node damage variable and LSVI+(x209954,Cc=.235,p< .01). The existence of LSVI was evaluated in all the patients. lVSI was negative in 141 cases and 4 of these cases had recurrence of the illness.55.5 % of the patients in which the illness recurred had positive LVSI (5 out of 9).
Conclusion: Our results proved that more than a half of the patients with recurrence of the illness have Lympho-vascular infiltration and also; if the LVSI is negative, there is a risk of 2.8% of having recurrence.
Disclosure: Nothing to disclose

EP1260

Endometrium cancer and its features in our enviroment

E. Lopez Gonzalez, F. Montero Venegas, P. Barbero Duran, L. Fernandez Villarrenaga
Juan Ramon Jimenez Hospital, Huelva, Spain

Introduction / Background: Objetive to describe the different features of the endometrial cancer in our environment in the course of five years.
Methodology: Descriptive study of 170 patients who were diagnosed with endometrium cancer between 2013 and 2017. A study of different variables have been carried out; these variables include: age, presentation, diagnosis, histology, type II, LSVI; pre and post surgical, treatment given and adjuvancy, tumor grade, miometrial invasion (MI),lymphadenectomy,node involvement, reappearence and the existence or absence of surgical complications.
In the analysis of the data, the 23rd version of SPSS has been used.
Results: The main reason why 85.5% of the patients attended surgery was metrorrhagia, being 50.6% diagnosed through hysteroscopy and 44.7% through endometrial aspirate. The most common found histology has been ca. Endometroid in 84.1% of the cases and lymphovascular invasion in 14.1%. Looking at the staging of the illness, it has been concluded that 54.1% of the cases are in AI stage, 22.1% in IB stage; according to this, it has been noticed that HT+DA is the most frequent procedure , being used in 61.2% of the cases.
On the other hand, it has been detected that 53.5% of the cases presented miometrial invasion >50% and 55% of the cases were in G1. Laparoscopic surgery has been the most common choice of approach, doing lymphadenectomy in 32.9% of the cases and finding pelvic metastasis in 2.4% and

para-aortic in 3.5% of the patients.
Finally, it is neccessary to remark that adjuvant treatment has not been given to 54.1% of the patients and 5.3% of recurrences in our patients was detected.
Conclusion: The most usual is the endometrioid in early stages and highlighting almost a 20% of lympho-vascular space infiltration, even in those patients with an early stage in which no adjuvant treatmnent was applied.
Disclosure: Nothing to disclose

EP1261

Malignization of abdominal wall endometriom case report

A. Córdoba, O. Puentes, E. Mora, Woman and Neoplasia
Department of Gynecology Oncology, University Foundation of Health Sciences, FUCS, Bogotá, Colombia

Introduction / Background: Endometriosis can occur in extra pelvic locations. Abdominal wall endometriomas (AWE) usually occur as a secondary process to gynecological surgeries, most often caesarean, with an incidence between 0.03 to 0.45% and only 1% can be malignant.
Methodology: 34-year-old patient who consulted due to dysmenorrhea, antecedent of two caesarean, to physical examination with nodular lesion in abdominal wall in relation to surgical scar, Ca 125: 696.60, pelvic ultrasound normal, in Abdominal and pelvic resonance of thickening and distortion of the straight muscles in the suprapubic region of the abdominal wall, conglomerate 84 * 68 * 39 mm. The diagnosis of AWE is made, she´s carried out to resection where a 10 x 15 cm lesion is evidenced, pathology report a moderately differentiated adenocarcinoma of endometrioid appearance.
The rarity presentation of a primary endometrioid adenocarcinoma in the abdominal wall, possible endometrial or ovarian origin are studied. She´s led to gynecological curettage reported as negative for hyperplasia / malignancy, CT scan with adnexal cystic lesion, primary ovarian is suspected, is decided to cytoreduction. Pathology report: adenomyotic uterus, parametria with endometriosis, 72 lymph node free of tumor. She´s assessed by clinical oncology and before non-standard management, decided clinical observation without evidence of tumor after 8 months of follow-up.
Results: It was considered a rare case of a primary endometrioid adenocarcinoma of the abdominal wall and the most common histological type is clear cell adenocarcinoma (62%). The histopathological criteria of Sampson-Scott, such as the coexistence of benign and malignant endometrial tissue in the tumor, histological appearance consistent with endometrial origin and excluding another primary tumor site allow to confirm the diagnosis.
Conclusion: The malignization of the AWE is a rare clinical entity and the histological type endometrioid is even more rare, in terms of management wide local excision with free margins is the key point to prevent recurrence.

This image cannot currently be displayed.

[Abdominal and pelvic resonance]

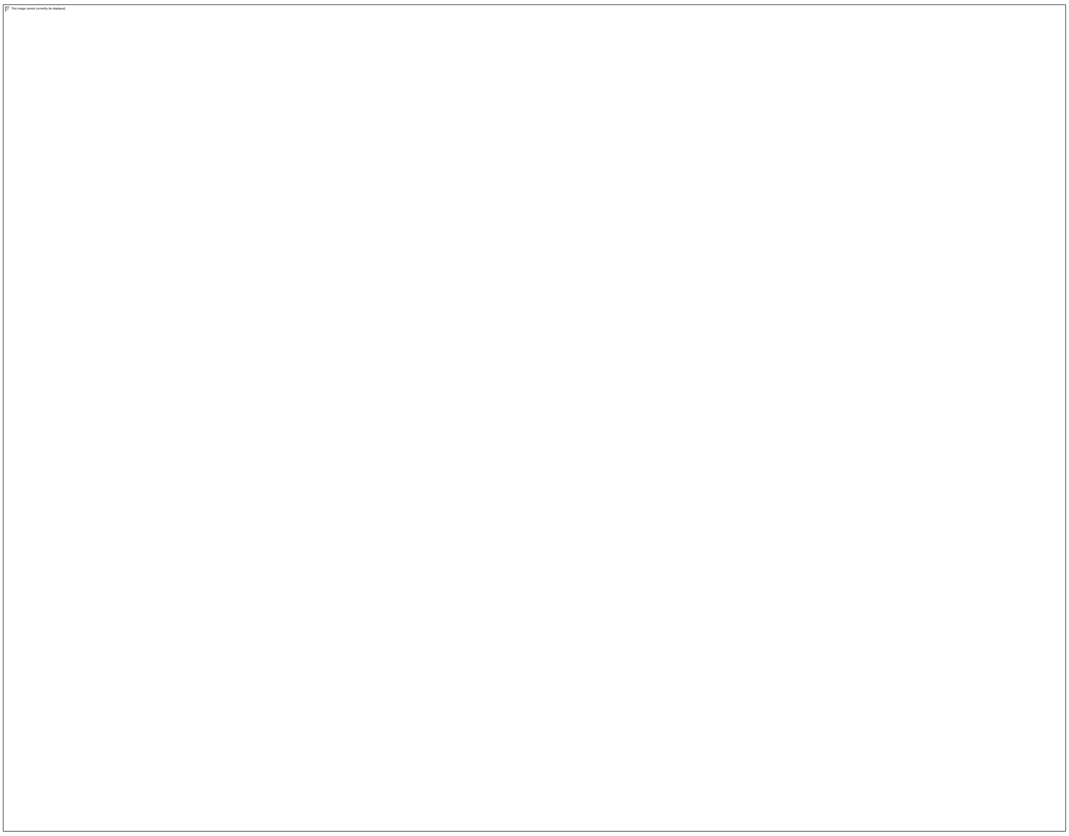

[Immunohistochemistry]

Disclosure: Nothing to disclose

EP1262

Cervical cancer during pregnancy and diagnostic problems - a case report

A. Słabuszewska-Jóźwiak[1], M. Janicka[2], K. Zaręba[1], J. Szymański[1], G. Jakiel[1]
[1]The First Department of Obstetrics and Gynecology, Medical Centre of Postgraduate Education, Warsaw, [2]Żelazna Medical Center, Warszawa, Poland

Introduction / Background: An invasive cervical cancer discovered during pregnancy is a rare clinical feature. However, this is one of the most common malignancies occurring during pregnancy. The prevalence according to population ranges is estimated between 1 and 10 for 10,000 pregnancies. Diagnostics in pregnant women are similar to those in non-pregnant women, including colposcopy and Pap smear. We report a case of pregnant patient with advance cervical cancer treated by radical surgery and chemoradiotherapy.
Methodology: Case report
Results: 32 -years-old pregnant woman, with no significant medical history, with no history of vaginal blooding and a low grade squamous intraepithelial lesion in Pap smear (LSIL) was admitted to the First Department of Obstetrics and Gynaecology at 38 weeks' gestation. A colposcopy and a cervical biopsy during pregnancy confirmed a cervical intraepithelial neoplasia grade 1 (CIN1). At the admission, a physical examination performed a lesion located in proximal part of cervix that suggested leyomyoma. An ultrasonographic examination showed the presence of hipervasculr and limited cervical tumour. Both lateral parametria were not involved. Because of uncertain diagnosis at 39

weeks' gestation a caesarean section was performed. A tumour biopsy revealed a low differentiated squamous cell carcinoma. Abdominal radical hysterectomy and pelvic lymphadenectomy with adnexal transposition was performed. The final pathological result showed presence of a low differentiated cervical carcinoma and macro metastasis in 2 pelvic lymph nodes. An adjuvant chemoradiotherapy was proposed
Conclusion: Physiological pregnancy changes make Pap smear and colposcopy difficult to interpret therefore such patients should be followed in specialized centres. However, expert ultrasound examination should be consider as routine examination.
Disclosure: Nothing to disclose

EP1263

Adnexial mass at pregnancy: case report series

E. Keleş Peker, M. Api
Gynecologic Oncology, Zeynep Kamil Women's and Children's Disease Training and Research Hospital, Istanbul, Turkey

Introduction / Background: To evaluate adnexial masses at pregnancy whom were referred to gynecologic oncology department.
Methodology: Referred cases their different stages of pregnancy were investigated according to sonographic appearence , magnetic resonance images, blood tumor markers and final pathology.
Results: 15 cases referred from perinatology department examined and sonographic mass sizes were max 234- min 43 mm. Average CA125 levels were 47,8 and 40% of them were above 35 mIU/ ml. Diagnosis were made 60% at first trimester , 20% at second trimester , 20% third trimester . 40% of the cases managed conservatively and 60% were laparatomy of them 55% at cesarean delivery.None of the patients have malignant neoplasm but 4 out of 15 cases were borderline ovarian tumors.
Conclusion: Most of the cases in pregnancy diagnosed in the first trimester and confirmed to be benign, consequently adnexial masses at pregnancy should be evaluated gynecologic oncology department and managed accordingly.
Keywords: Adnexial mass, Pregnancy,Teratoma
Disclosure: Nothing to disclose

EP1264

Simultaneous integrated intensity modulated radiotherapy boost for gynecological cancer when brachytherapy is not an option: presentation of nine cases

E. Kucuktulu[1], A.F. Yurekli[2]
[1]University of Health Sciences, Trabzon Training and Research Hospital, Trabzon, [2]University of Health Sciences, Gülhane Training and Research Hospital, Ankara, Turkey

Introduction / Background: Brachytherapy (BT) lowers the risk of recurrence and improves the survival rates when used after external beam radiotherapy (EBRT), in cervical cancers. In endometrial cancers vaginal cuff BT lowers the risk of vaginal recurrences. New treatment methods have been investigated since BT is not an option for every patient because of the difficulties in placement of BT applicators. Simultaneous integrated boost (SIB) has recently been investigated as an alternative modality in patients BT is not suitable after EBRT.
Methodology: Nine patients were treated with the SIB IMRT for cervical and endometrial cancers (Table1)In SIB volume 74 Gy in 2,54 Gy/fraction, in common iliac external-internal iliac and obturator lymph nodes and presacral lymphatic lymph node volumes 50,4 Gy in 1,74 Gy/fractions were administered.(Figure1) In patients with endometrial cancer vagina, paravaginal-parametrial regions

received 60 Gy in in 2,3 Gy/fraction with 10 mm margin. 45 Gy in 1,73/fraction was administered to common iliac, external-internal iliac and obturator lymph nodes.

CASE	I	II	III	IV	V	VI	VII	VIII	IX
Age	55	54	56	88	66	72	55	55	56
Primary Cancer	Cervical Cancer	Cervical Cancer	Cervical Cancer	Cervical Cancer	Cervical Cancer	Endometrial Cancer	Endometrial Cancer	Endometrial Cancer	Endometrial Cancer
Surgery	Ø	Ø	Ø	Ø	Ø	TAH + BSO	TAH + BSO	TAH + BSO	TAH + BSO
Histology	SCC	SCC	SCC	SCC	SCC	Serous + Clear Cell	Serous + Clear Cell	Adenocancer	Adenocancer
Stage	IIB	IVA	IIIB	IIB	IIB	IB	IIIA	IB	IB
Tumor Size (cm)	4	7,5	6	6,5	6	Ø	Ø	Ø	Ø
Chemotherapy	Cisplatin	Cisplatin	Cisplatin	Ø	Cisplatin	Ø	Taxol + Carboplatin	Ø	Ø
Dose (Gy)	74	74	74	74	74	60	60	60	60
Fractions (Gy)	2,54	2,54	2,54	2,54	2,54	2,30	2,30	2,30	2,30

[Patients characteristics and SIB dose.]

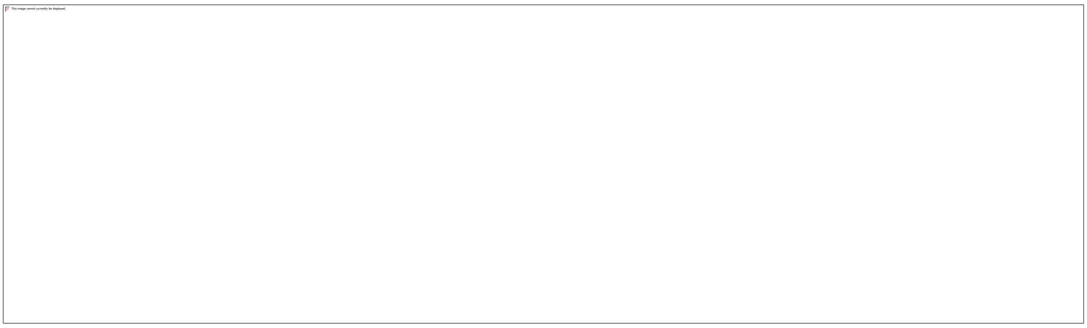

[Radiotherapy plan]

Results: All patients with the diagnosis of inoperable cervix carcinoma were in complete remission (CR) The median follow-up was 45 months (18-78 months) with local control (LC) rate of 100%. Acute side effects were seen in 2 patients with cervical carcinoma and in 2 patients with endometrial carcinoma. Late side effects were observed in 1 patient both in cervical and endometrial carcinoma patients (Table2)

CASE	I	II	III	IV	V	VI	VII	VIII	IX
Follow up (Months)	77	24	78	27	30	18	25	60	68
Local Control (%)	100	100	100	100	100	100	100	100	100
Acute side effect	Ø	Proctit (grade 3)	Ø	Vaginitis (grade 2)	Ø	Ø	Disuri (grade 1)	Disuri (grade 2)	Ø
Late side effect	Ø	Rectal bleeding (grade 3)	Ø	Ø	Ø	Ø	Ø	Ureteral stenosis (grade 2)	Ø

[Outcome of nine cases]

Conclusion: SIB-IMRT is an alternative treatment option for patients with cervix and endometrial

carcinoma. This pioneering study, although contains limited number of patients, presents long term favorable follow up results.
Disclosure: Nothing to disclose

EP1265

Advanced bipolar seal and cut technology device (Caiman®) versus traditional bipolar electrosurgery in laparoscopic hysterectomies

D. Danolić[1], L. Marcelić[1], I. Alvir[1], I. Mamić[1], L. Šušnjar[1], M. Banović[2], M. Puljiz[3], A. Bezić[4], M. Puljiz[1]
[1]University Hospital for Tumours, Clinical Department of Gynecologic Oncology, University Hospital Center Sestre Milosrdnice, [2]School of Medicine, University of Zagreb, Zagreb, [3]Institute of Emergency Medicine Split-Dalmatia County, Split, [4]B. Braun Adria, Zagreb, Croatia

Introduction / Background: Advanced bipolar technology devices are now routinely used for sealing and transecting blood vessels in many surgical specialties including gynecology. The main advantage is enhanced sealing capability, especially in blood vessels larger than 2 mm in diameter. These technologies are particularly efficient for the sealing of large vessels of up to 5-7 mm in diameter through compression and efficient energy delivery to the tissue. The aim of our study was comparison between the reusable traditional bipolar device and the disposable Caiman® 5 instrument, regarding the operating time in patients undergoing laparoscopic hysterectomies for various gynecological conditions.
Methodology: We retrospectively recruited 51 patients who underwent laparoscopic hysterectomies in 2017 and compared them to 62 patients who underwent the same procedures in 2018. They were further divided in two groups regarding the type of hysterectomy performed, subtotal laparoscopic hysterectomy (SLH) or total laparoscopic hysterectomy (TLH). First group in our study underwent procedures in 2017. using traditional bipolar forceps jaws, while in second group laparoscopy was performed with Caiman® 5. The difference in operating time was investigated.
Results: Results revealed considerable shorter operating time since introduction of avanced laparoscopic technology in our department (56.5 minutes ± 6.8 for SLH in 2017. using traditional bipolar device vs 42.1 minutes ± 5.8 using Caiman® 5 in 2018., $p< 0.0001$) (Figure 1.)
This beneficial effect appears more noticeable in more difficult procedures (110.7 minutes ± 8.5 for TLH in 2017. using traditional bipolar device vs 76.5 minutes ± 11.4 using Caiman® 5 in 2018., $p< 0.0001$). (Figure 2.)

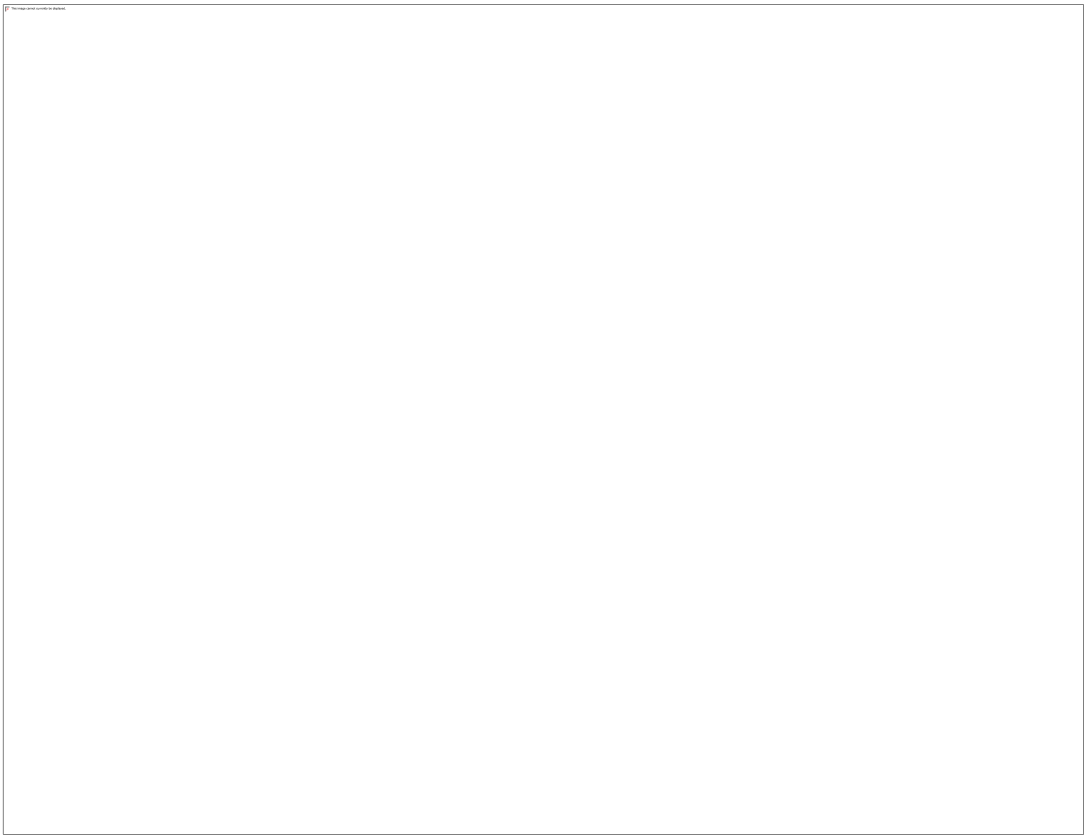

[Figure 1. Box plot diagram of of all measured durations of subtotal hysterectomies.]

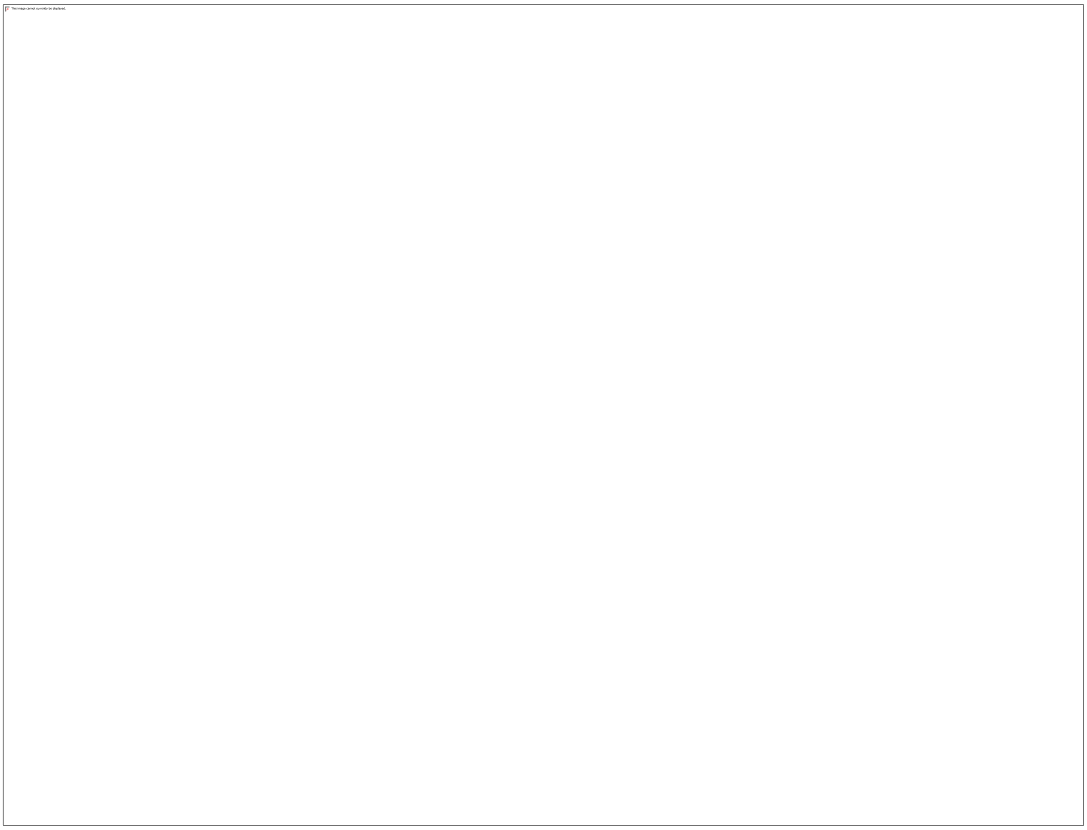

[Figure 2. Box plot diagram of of all measured durations of total hysterectomies.]

Conclusion: In conclusion, we observed a significantly shorter operating time using Caiman® 5 than traditional bipolar device in both SLH and TLH. This difference was more pronounced in more complex procedure such as TLH. However, the main issue with this instruments is that they are disposable and represent significant financial burden.
Disclosure: Nothing to disclose

EP1266

Assessment of effectiveness of post-operative triptorelin administration in alleviating gastrointestinal symptoms in patients with deep infiltrating endometriosis in a multicentre, non-interventional study

X. Zhang[1], V. Perrot[2], H. Li[3]
[1]Women's Hospital School of Medicine, Zhejiang University, Hangzhou, China, [2]Ipsen Pharma, Boulogne-Billancourt, France, [3]Ipsen (Beijing) Pharmaceutical Science and Technology Development Co., Ltd, Beijing, China

Introduction / Background: Adjuvant use of gonadotropin-releasing hormone agonists (e.g. triptorelin, Ipsen Pharma) can improve gastrointestinal (GI) symptoms in patients with deep infiltrating endometriosis (DIE) following conservative surgery. From a multicentre, non-interventional, observational study assessing the effectiveness of triptorelin in premenopausal Chinese patients with DIE (NCT01942369), this subgroup analysis evaluated the effectiveness of triptorelin in alleviating post-operative GI symptoms.
Methodology: The study included premenopausal patients with DIE aged ≥18 years who underwent

surgery 1 month before the study inclusion and received triptorelin intramuscular injections (3.75 mg every 28 days for up to 24 weeks). We assessed the intensity of GI symptoms using visual analogue scale (VAS) at pre-surgery and at 3, 6, 9, 12, 18- and 24-months post-surgery. The patients were categorized based on the number of triptorelin injections received (< 3, 3-5 or >5 injections). Logistic regression was used to compare the improvement in GI symptoms across subgroups, and a *P*< 0.05 was considered statistically significant.
Results: Of 384 study patients, 119 (31%) women had GI symptoms, with 52, 32 and 35 patients reporting the intensity of GI symptoms as mild, moderate and severe, respectively, at the baseline. At 3 months, the proportion of women with GI symptoms decreased to 3% and remained low until the last follow-up (3% at 24 months) (Table 1). At 24 months, the improvement in the intensity (a decrease of at least 3 points) was higher in patients receiving >5 injections (81%) or 3 to 5 injections (68%) in comparison with those receiving < 3 injections (33%; with *P* values 0.006 and 0.060, respectively). Similar results were obtained at all-time points.
Conclusion: This study confirmed the effectiveness of post-operative triptorelin therapy in alleviating GI symptoms in patients with DIE. The post-operative medical treatments for optimal outcomes need to be further addressed.

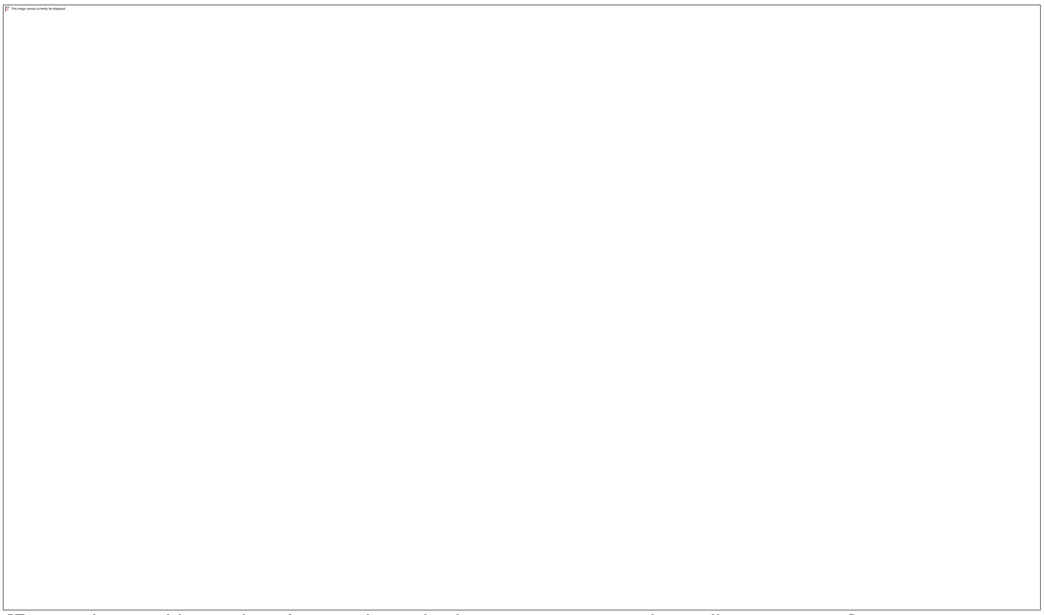

[Proportion and intensity of gastrointestinal symptoms post triptorelin treatment.]

Disclosure: This study was sponsored by Ipsen. The authors declare the following potential conflicts of interest with respects to the research, authorship and/or publication of this article: Valerie Perrot and Hongbo Li are employees of Ipsen. Xinmei Zhang has no conflict of interest to declare.

EP1267

Assessment of efficacy of post-operative triptorelin in improving pain symptoms from deep infiltrating endometriosis: a 24-month multicentre, prospective, non-interventional study in highest pain intensity patient population

X. Zhang[1], V. Perrot[2], H. Li[3]
[1]Zhejiang University, Hangzhou, China, [2]Ipsen Pharma, Boulogne-Billancourt, France, [3]Ipsen (Beijing) Pharmaceutical Science and Technology Development Co., Ltd., Beijing, China

Introduction / Background: Symptom recurrence in patients with deep infiltrating endometriosis

(DIE) undergoing conservative surgery is common. Adjuvant use of gonadotropin-releasing hormone agonists (e.g. triptorelin, IPSEN Pharma) can improve postoperative pain caused by DIE and prolong the asymptomatic period. This analysis from a multicentre, non-interventional, observational study (NCT01942369) assessed the effect of triptorelin administration on the most severe intensity symptoms reported by the patient.
Methodology: This study included patients with DIE aged ≥18 years who underwent conservative surgical treatment within 1 month before inclusion and were prescribed triptorelin (3.75 mg) as part of their treatment for up to 24 weeks. The intensity of symptoms was assessed using a 10-cm visual analogue scale (VAS) or numerical scale ranging from 0 to 10 at baseline and 3, 6, 9, 12, 18 and 24 months. At baseline (pre-surgery assessment), the most severe intensity for all the symptoms reported by the patient was identified, and changes in selected symptoms' intensity were assessed. Paired t-test was used to assess the change in intensity.
Results: In total, 384 patients (mean ± standard deviation [SD] age: 33.4±6.2 years) were included. Among the symptoms identified with the most severe intensity at baseline, dysmenorrhea (69.5%), pelvic pain (28.9%) and dyspareunia (13.8%) were most frequently reported. At baseline, the mean (SD) VAS scores for pelvic pain (n=111), dysmenorrhea (n=267) and dyspareunia (n=53) were 5.25 (3.11), 6.32 (3.06) and 3.68 (3.09) cm, respectively, which significantly decreased at all-time points (mean VAS decrease < 1 cm, $P < 0.0001$ for all). Irrespective of the number of injections, the mean VAS score of these three symptoms significantly decreased from baseline ($P < 0.05$).
Conclusion: This observational study confirmed that postoperative triptorelin administration was beneficial in women with DIE suffering from the highest pain intensity. However, individualised treatment for optimal pain relief needs to be further addressed.
Disclosure: This study was sponsored by Ipsen. The authors declare the following potential conflicts of interest with respect to the research, authorship and/or publication of this article: Valerie Perrot and Hongbo Li are employees of Ipsen.

EP1268

Burden of female genital tract malignancies at a tertiary care teaching hospital

P. Bharti
DMCH, Darbhanga, India

Introduction / Background: Genital tract malignancies are associated with high mortality, morbidity and shortening of lifespan in women.The tumors of female genital tract have different patterns of distribution worldwide.Cervical cancer is the most common genital tract malignancy among women world wide.
The aim of this study was to determine the prevalence of female genital tract malignancy in a tertiary care teaching hospital of Bihar,India
Methodology: It is a retrospective study.The data of female genital tract malignancy patients at DMCH hospital in Darbhanga,Bihar were collected.All the data were retrieved from OPD registers
Results: There were 4,830 gynaecological cases seen during the study period.Among them 118 cases of genital tract malignancies were recorded.The percentage of different gynaecological malignancies recorded were-Cancer cervix 48.3%, ovarian cancer 33%,endometrial cancer 13.5%,choriocarcinoma 2.5%, vulval cancer 0.8%,vaginal cancer 0.8%,fallopian tube malignancies 0.8 %
Conclusion: High frequency of malignancy was detected in our study.Commonest genital tract malignancy detected was carcinoma of the cervix.
Disclosure: Nothing to disclose

EP1269

Quality of life after surgery of varying surgical complexity in advanced ovarian cancer: results from the international, prospective, multicenter cohort SOCQER2 study

S. Sundar[1], C. Cummins[2], S. Kumar[2], J. Long[3], V. Arora[4], J. Balega[3], T. Broadhead[5], T. Duncan[6], R. Edmondson[7], C. Fotopoulou[8], R. Glasspool[9], D. Kolomainen[10], S. Leeson[11], R. Manchanda[12], O. McNally[13], J. Morrison[14], A. Mukhopadhyay[15], J. Paul[16], J. Tidy[17], N. Wood[18]

[1]Institute of Cancer and Genomic Sciences University of Birmingham, [2]University of Birmingham Institute of Applied Health Research, [3]Institute of Applied Health Research, University of Birmingham, Birmingham, United Kingdom, [4]Bondi Women's Health, Sydney, NSW, Australia, [5]Leeds Teaching Hospitals NHS Trust, Leeds, [6]Norfolk & Norwich University Hospital, Norwich, [7]University of Manchester, Manchester, [8]Imperial College London, London, [9]Institute of Clinical Sciences, University of Glasgow, Glasgow, [10]Southend University Hospital NHS Foundation Trust, Westcliff-on-Sea, [11]Betsi Cadwaladr University Health Board, Bangor, [12]Barts Cancer Institute, London, United Kingdom, [13]Peter MacCallum Cancer Centre, Melbourne, VIC, Australia, [14]Taunton and Somerset NHS Foundation Trust, Taunton, United Kingdom, [15]Tata Medical Center, Kolkata, India, [16]University of Glasgow, Glasgow, [17]University of Sheffield, Sheffield, [18]Lancashire Teaching Hospitals NHS Foundation Trust, Preston, United Kingdom

Introduction / Background: Extensive surgery has been shown in retrospective studies to improve survival from advanced ovarian cancer. UK uptake of extensive surgery has been variable, owing to concerns regarding the impact on quality of life (QoL) and organizational factors. We investigated QoL after surgery in parallel, multicentre, international, prospective cohorts, here reporting UK and Kolkata, India results.

Methodology: Patients completed EORTC-QLQ-C30 and other Patient Reported Outcome Measures pre-operation and at 6 weeks, 6, 12, 18 and 24 month postoperatively. Surgical complexity scores (SCS), intraoperative disease load and survival up to two years were collected and Kaplan Meier survival analyses and forward stepwise Cox proportional hazard models of overall survival conducted. The association of SCS with QLQ-C30 global scores at 6 weeks, 6 months and 12 months post-surgery was evaluated using general linear repeated measures models (SPSS 24).

Results: In 247 patients, those undergoing high (64) or intermediate (70) SCS procedures were younger, showed better performance status, higher disease load, more upper abdominal disease and greater cytoreduction rates although all SCS groups included patients with a higher burden of disease. (Table 1). Progression free and overall survival was poorer with residual disease in all SCS groups (log rank p=< .001) (Figure 1). Age Adjusted Charlson Comorbidity Index, pre-operative albumin and Peritoneal Carcinomatosis Index were independent predictors of survival (Table 2). In a complete case analysis, post operation QLQ-C30 improved in all SCS groups between 6 weeks and 12 months (p< .001) (Figure 2) with no statistically significant or large differences between SCS groups over that time.

Conclusion: In this observational study, QoL of patients improved postoperatively, and was no worse in patients undergoing complex surgery during that period. Extensive surgery to achieve complete cytoreduction for high disease load was associated with improved survival and no deleterious impact on QoL in the post operative year.

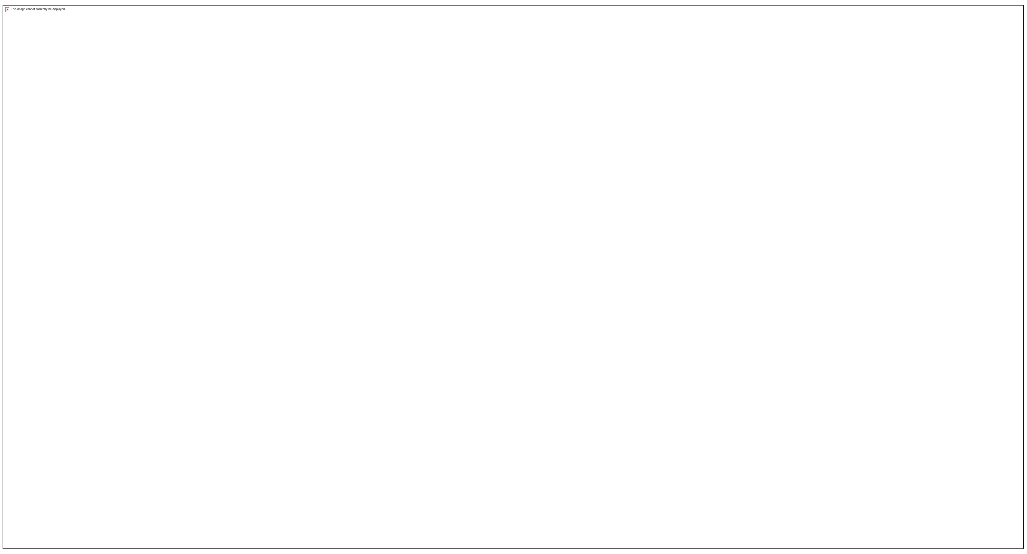

[Figure 1 Survival by Surgical Complexity Score and by presence of residual disease]

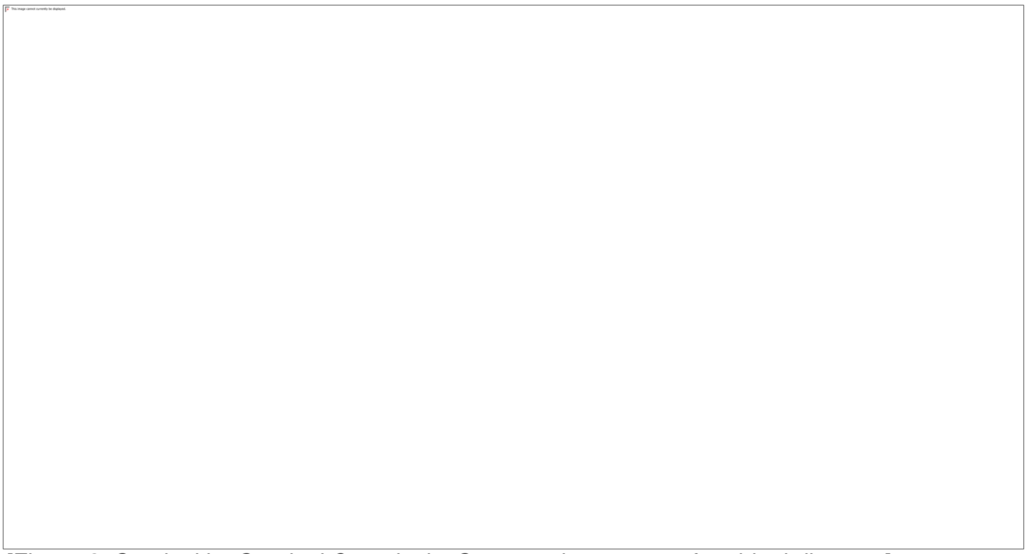

[Figure 2: Survival by Surgical Complexity Score and presence of residual disease]

Patient characteristics	Low SCS N=113		Intermediate SCS N=70		High SCS N=64		p value
Age >65 years	62	54.9%	26	37.1%	16	25.0	<0.001
Age adjusted Charlson Co-morbidity Index >=3	51	45.1%	21	30.0%	18	28.1	0.033
Neoadjuvant chemotherapy	103	91.2%	44	62.9%	25	39.1%	<0.001
Peritoneal Carcinomatosis Index <=6	65	57.5%	18	25.7%	2	3.1%	<.001
7 to 12	21	18.6%	29	41.4%	6	9.4%	
>12	27	23.9%	23	32.9%	56	87.5%	
No visible residual disease	63	55.8%	50	71.4%	40	62.5%	0.007
<1cm residual disease	29	25.7%	17	24.3%	21	32.8%	
>=1cm residual disease	21	18.6%	3	4.3%	3	4.7%	

[Table 1: Patient characteristics by Surgical Complexity Score (SCS)]

	Hazard ratio	95% Confidence Interval		p
Intermediate SCS (reference)				0.034*

Low SCS	2.63	1.23	5.64	0.13
High SCS	1.60	0.68	3.79	0.282
ACCI>2	2.08	1.21	3.59	0.008
Pre-operative albumin <35g/l	2.00	1.14	3.50	0.016
Pre-operative PCI <5 (reference)				0.006*
Pre-operative PCI 6-14	2.18	0.99	4.79	0.052
Pre-operative PCI <=15	3.80	1.67	8.64	0.001
*for variable contribution				

[Table 2: Overall survival adjusted hazard ratios (Cox proportional hazards)]

Disclosure: Nothing to disclose

EP1270

Risk of ovarian cancer in women with histological proven endometriosis. The ENOCA a population-based cohort study

M. Hermens[1,2], A. van Altena[2], B. Nieboer[2], D. Schoot[1,3], H. van Vliet[1], B. Siebers[4,5], R. Bekkers[1,6]
[1]Obstetrics and Gynaecology, Catharina Hospital, Eindhoven, [2]Obstetrics and Gynaecology, Radboudumc, Nijmegen, The Netherlands, [3]Obstetrics and Gynaecology, University Hospital Ghent, Ghent, Belgium, [4]Pathology, Radboudumc, Nijmegen, [5]PALGA, the Nationwide Network and Registry of Histo- and Cytopathology in the Netherlands, Houten, [6]GROW School for Oncology and Developmental Biology, Maastricht University Medical Center, Maastricht, The Netherlands

Introduction / Background: Several studies have suggested that endometriosis is associated with an increased risk of ovarian cancer. However they lack sufficient power, follow-up data, or diagnostic certainty. We used a large nationwide pathology database to assess whether there is an association between endometriosis and ovarian cancer.
Methodology: We identified all women with a histological diagnosis of endometriosis between 1990 and 2015 from the Dutch nationwide registry of histopathology and cytopathology (PALGA), and matched with women with a benign dermal nevus. Histology results for cancer of the ovaries, fallopian tubes, and peritoneum between January 1990 and July 2017 were retrieved. Incidence rate ratios (IRR) were estimated for ovarian cancer.
Results: The crude IRR for ovarian cancer in endometriosis patients was 4.80 (95%CI 4.34 to 5.32), and the age-adjusted IRR was 7.22 (95%CI 6.20 to 8.41) (table 1). Median age at ovarian cancer diagnosis was significantly different, 56 years (IQR 49 to 63) and 60 years (IQR 53 to 67) in the endometriosis and nevus group respectively. After excluding cases with less than a half-year of follow-up, the age-adjusted IRR was 1.09 (95%CI 0.88 to 1.35). In both analyses endometrioid and clear cell cancers were significantly more associated with endometriosis (figure 1).
Conclusion: These findings show a five to seven times higher prevalence of ovarian cancer in endometriosis patients. To our knowledge, this is the largest study to investigate the risk of ovarian cancer in women with endometriosis and the first study to only include patients with histological proven endometriose. Women with endometriosis are on average four years younger at ovarian cancer diagnosis. We found a large proportion of women with synchronous endometriosis and ovarian cancer after the average menopausal age, this suggests that the risk of cancer remains, even when clinical symptoms of endometriosis are no longer present.

	Endometriosis group		Nevus group			
	N	Incidence rate per 100,000 person years	N	Incidence rate per 100,000 person years	Crude Incidence Rate Ratio (95% CI)	Age-adjusted Incidence Rate Ratio (95% CI)
By ovarian cancer subtype:						
Clear cell (n=381)	347	18.90 (17.01-20.99)	34	1.67 (1.20-2.34)	11.50 (8.08-16.88)	41.02 (24.15-69.69)
Endometrioid (n= 594)	546	29.74 (27.34-32.34)	48	2.37 (1.78-3.14)	12.82 (9.54-17.60)	39.83 (23.22-68.34)
Serous (n=980)	703	38.29 (35.56-41.22)	277	13.65 (12.13-15.35)	2.86 (2.49-3.30)	6.06 (4.48-8.21)
Mucinous (n= 285)	238	12.96 (11.42-14.72)	47	2.32 (1.74-3.08)	5.70 (4.16-7.98)	3.14 (0.88-11.23)
Adenocarcinoma NOS (n=277)	212	11.55 (10.09-13.21)	65	3.20 (2.51-4.08)	3.68 (2.77-4.93)	4.01 (1.00-16.06)
Overall (n=2,517)	2,046	111.43 (106.70-116.36)	471	23.21 (21.20-25.40)	4.80 (4.34-5.32)	7.22 (6.20-8.41)

[Incidence rate per 100,000 person-years, crude incidence rate ratios and age-adjusted incidence rate ratios of ovarian cancer]

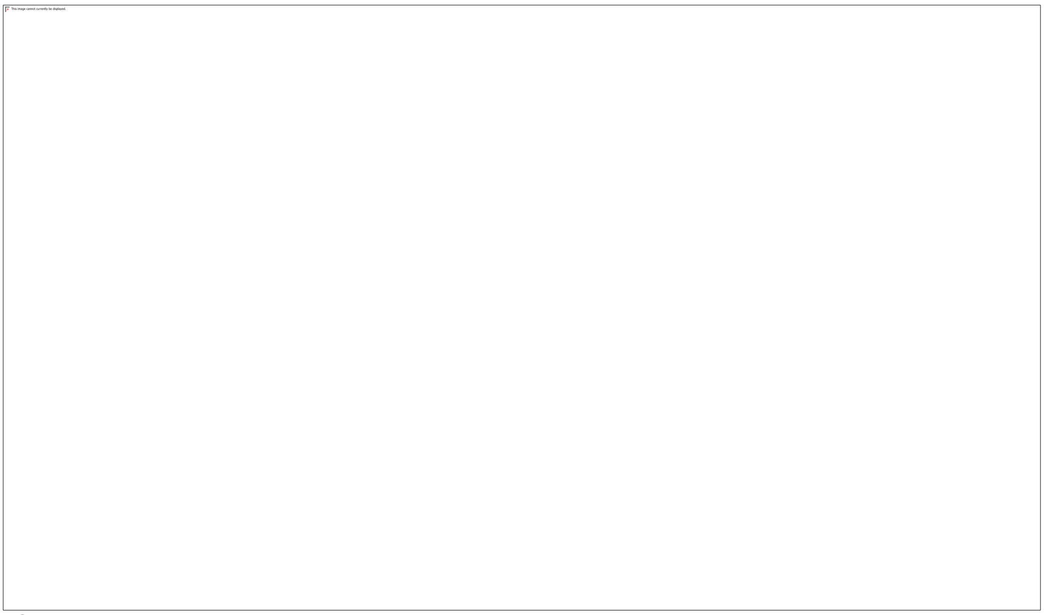

[Ovarian cancer distribution per histological subtype in the endometriosis group and nevus group]

Disclosure: Nothing to disclose

EP1271

Prognostic implications of histological tumor regression (Böhm´s score) in patients receiving neoadjuvant chemotherapy for high grade serous tubal & ovarian carcinoma

S. Rajkumar[1], A. Polson[2], R. Nath[1], G. Lane[1], A. Sayasneh[1], A. Tranoulis[1], A. Jakes[1], S. Begum[3], G. Mehra[1]
[1]Department of Gynaecological Oncology, [2]Department of Pathology, Guy's and St Thomas' NHS Foundation Trust, [3]Department of Women and Children's Health,, School of Life Course Sciences, King's College London & King's Health Partners, St Thomas' Hospital,, London, United Kingdom

Introduction / Background: Primary objective of this study was to determine prognostic significance of Bohm´s histopathological regression score in patients who received neoadjuvant chemotherapy (NACT) for treatment of high grade serous (HGS) tubal & ovarian carcinoma.
Methodology: This was a retrospective cohort study of patients who received NACT between 2010 and 2015. The 3 point histopathological regression score of Böhm was used to classify chemotherapy response. Survival outcomes between the 3 different subgroups was analysed and compared with standard clinico-pathological variables using the Cox proportional hazards model and log-rank test.
Results: Study cohort comprised 111 patients. Chemotherapy response score (CRS) 3 was observed in 47 (42.4%) and CRS 1and CRS 2 in 22 (19.8%) and 42 (37.8%) women respectively. Women with CRS score of 1 and 2 combined showed a three-fold increased risk of progression on both univariate and multivariate assessment (HR 3.54; C.I 2.19-5.72, $p < 0.001$). The median overall survival for patients with CRS 1 was 34 months, CRS 2 was 30 months and 47 months for CRS 3. CRS 1 and 2 combined was the only variable that held significance in prediction of reduced overall survival on multivariate assessment (HR 3.26, C.I 1.91-5.54, p 0.0006). CRS 1 and 2 were also associated with 5.15-fold increased risk of relapse within 6 months of completion of chemotherapy (Odds ratio OR 5.15, C.I 0.07-0.47, p - 0.002).
Conclusion: CRS is an independent prognosticator of survival and reliable predictor of relapse within 6 months in advanced high grade serous tubal and ovarian carcinoma patients receiving NACT.
Disclosure: Nothing to disclose

EP1272

Loss of fragile histidine triad protein expression is associated with homologous recombination deficiency in high-grade serous ovarian cancer

N. Sarvilinna[1], A. Virtanen[2,3], M. Tumiati[1], L. Kauppi[1]
[1]Biomedicum Helsinki 1, Systems Oncology Research Program/Genome Stability Group, Helsinki University, Finland, [2]Department of Pathology, Helsinki University Hospital, [3]Helsinki University Hospital, HUSLAB, Helsinki, Finland

Introduction / Background: We previously showed that low homologous recombination (HR) scores correlate with platinum sensitivity and better outcome in high-grade serous ovarian cancer (HGSOC) (Tumiati et al. 2018). Previous studies have reported loss of the tumor suppressor gene Fragile Histidine Triad (*FHIT*) in many cancers including HGSOC. *FHIT* contains FRA3B, a common fragile site, which is prone to aberrations under replication stress. Fhit protein protects cells from DNA damage accumulation through modulation of DNA damage checkpoint proteins, and some previous publications have reported a link between *FHIT* and HR. We analyzed Fhit protein expression in HGSOC and tested for correlation between Fhit expression and HR status.
Methodology: 25 FFPE samples from 13 HGSOC patients were studied by immunofluorescence against Fhit. Only vital tumor cells were analyzed and scored. Percentage of positive tumor cells and staining intensity were analyzed by a pathologist and a FHIT score was calculated (intensity multiplied by percentage of positive cells). FHIT score was plotted against HR score and their association was calculated by linear regression.
Results: Fhit staining intensity varied from negative to strong (Figure 1). 16% of the samples were negative (n=4) and 25% were weakly positive (n=6) for Fhit, while 60% were moderately or strongly

positive (n=15). Both cytoplasmic and nuclear staining was seen (Figure 1). Most of the samples were HR proficient (n=15; 65,2%), two were HR deficient (8,7%), and the rest were HR low (n=6; 26%). FHIT score values ranged from 0 to 285. There was a positive correlation between Fhit expression and HR score: both HR deficient samples were negative for Fhit and the higher the HR score, the higher the Fhit expression (Figure 2, Pearson´s correlation coefficient 0,27).
Conclusion: Loss of Fhit is associated with HR deficiency in HGSOC. Fhit expression positively correlates with HR score suggesting low FHIT expression indicates platinum sensitivity.

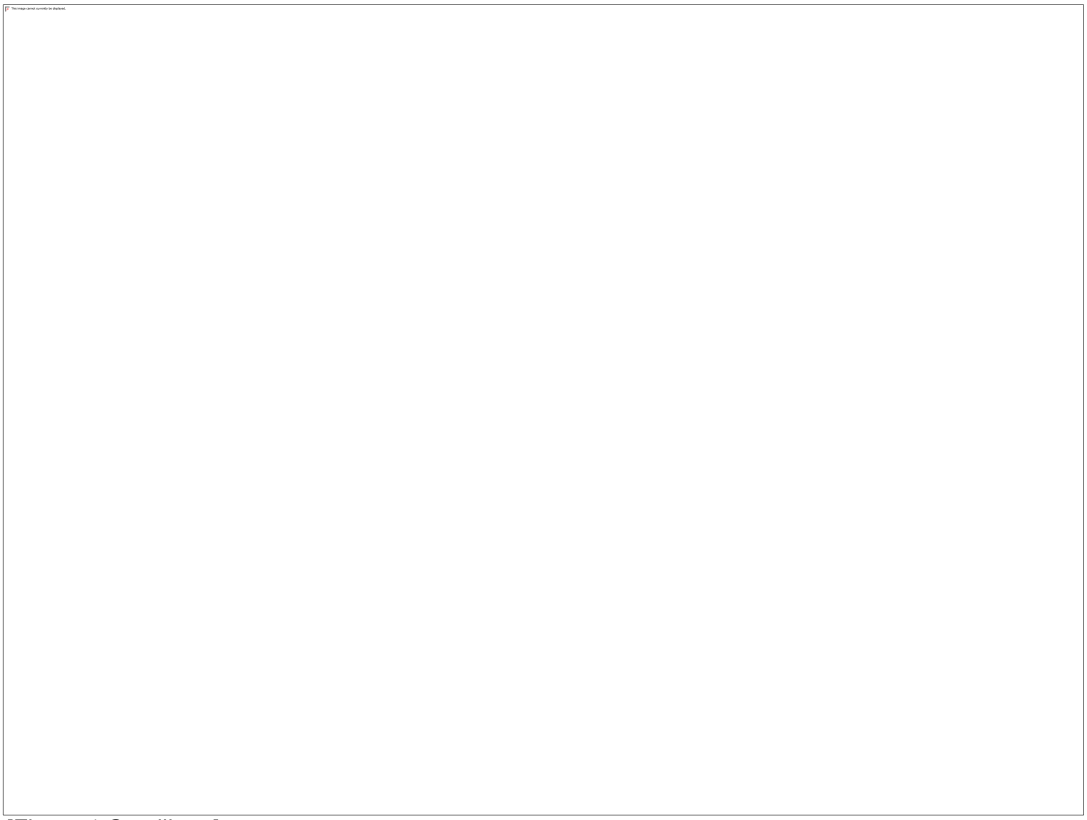

[Figure 1 Sarvilinna]

[Figure 2 Sarvilinna]

Disclosure: Sources of funding: the Finnish Medical Foundation (N.S.), the Maud Kuistila Memorial Foundation (N.S.), University of Helsinki (A.V. and M.T.), Helsinki University Hospital (A.V.), the Academy of Finland (M.T. and L.K.), the Sigrid Juselius Foundation (L.K.), and the Finnish Cancer Foundation (M.T. and L.K). The authors have no conflicts of interest to disclose.

EP1273

Fertility preservation in malignant ovarian tumors: experience of the French ESGO labeled - ovarian tumours center and pregnancy associated cancer network (CALG) national expert center at Tenon University Hospital

A. Lier, S. Geoffron, M. Prades, N. Sermondade, K. Kolanska, J. Varinot, E. Wafo, C. Uzan, E. Daraï, S. Bendifallah, N. Chabbert-Buffet
Université Pierre et Marie Curie, Paris 6, Paris, France

Introduction / Background: Progress in early diagnosis, treatment efficacy have allowed increased life expectancy in cancer patients (1). Therefore, approximately 6% childbearing age individuals are cancer survivors (2). In this specific setting, 20% of malignant epithelial ovarian tumours (MEOT) are diagnosed before the age of 44, with a 91.2 % five years survival rate for stage IA and IB (3). Similarly rare malignant ovarian tumors (RMOT) and borderline ovarian tumours (BOT) more frequently occur in women of childbearing age (4). Personalized counseling on post treatment ovarian function and possibilities of fertility preservation (FP) are part of oncological patients' care (5). However the safety and efficiency evidence level is currently too limited to allow specific recommendations. Therefore, we report the experience in FP for patients with malignant ovarian tumours, at a French ESGO labeled national expert center and pregnancy-associated cancer network (CALG).

Methodology: Data from 43 patients with malignant ovarian tumors who underwent a FP between February 2013 and July 2019 were analysed from the prospective unicentric database.
Results: Pathological diagnosis was 5 (11.6 %) MEOT, 14 (32.6 %) RMOT, 24 (55.8 %) BOT, mostly unilateral (76.5 %) and stage I (87.9 %). Mean age at diagnosis and tumor size was 26.8 and 97.4 mm respectively. Before fertility preservation, mean antral follicle count and AMH levels were 9 [0-20], and 2.26 ng/ml [0.1-10]. Mean age at fertility preservation was 29.2 [17.4-37.1]. Six ovarian tissue freezing procedures were performed out of 14 proposed. 23 oocyte freezing procedures were performed after tumor surgical treatment out of 26 proposed, with a median delay of 188 days. The mean mature oocytes collected number was 12.9. No FP was proposed for 5 patients.
Conclusion: Oocyte and ovarian tissue cryopreservation can be offered to patients with malignant ovarian tumor. More data are needed to confirm ovarian stimulation and ovarian tissue grafting safety.

Histology [n]	Maligant epithelial ovarian tumor [n=5]	Rare malignant ovarian tumor [n=14]	Borderline ovarian tumor [n=24]	All tumors [n=43]
Age at diagnosis (mean, years)	29.4 (24-36) [n=5]	24.9 (9-35) [n=14]	27.4 (17-39) [n=24]	26.8 (9-39)[n=43]
BMI (mean, kg/m²)	23.5 (19-29.7) [n=4]	21.8 (18.1-32) [n=13]	24.7 (17.8-43.4) [n=22]	24.2 (17.8-43.4) [n=38]
Parity (%)	25 [n=4]	15.4 [n=13]	4.2 [n=24]	9.8 [n=41]
Smoking (%)	0 [n=4]	18.2 [n=11]	42.9 [n=21]	30.6 [n=36]
Fertility preservation consultation preoperative (%)	20 [n=5]	23.1 [n=13]	29.2 [n=24]	26.2 [n=42]
AFC before multidisciplinary committee (mean)	10.5 (8-13) [n=2]	9.25 (2-20)[n=12]	12.7 (3-30) [n=12]	11 (2-30) [n=26]
AMH before multidisciplinary committee (mean, ng/ml)	1.3 (0.7-2) [n=2]	1.8 (0.03-4.26) [n=9]	3.4 (0-23) [n=16]	2.7 (0-23) [n=27]
Bilateral lesions (%)	66.7 [n=3]	22 [n=9]	18.2 [n=22]	23.6 [n=34]
Ovarian tumor size to imaging (mean, mm)	68.9 (20-155) [n=5]	84.1 (7-260 [n=17]	91.8 (18-240) [n=26]	96.8 (7-260) [n=48]

[Table1: Population caracteristics]

Histology [n]	Malignant epithelial ovarian tumor [n=5]	Rare malignant ovarian tumor [n=14]	Borderline ovarian tumor [n=24]	All tumors [n=43]
Ovarian tissu freezing realised/proposed	0/1	2/4	4/9	6/14
Fragment number	NA	11 [n=1]	27.4 (16-30) [n=3]	17 (1-30) [n=5]
Fragments follicular density (mean, primordial follicles/mm²)	NA	0.08 (0-0.16) [n=2]	0.045 (0.02-0.07) [n=2]	0.06 (0-0.16) [n=4]
Mature oocytes freezing realised/proposed	3/3	6/8	9/16	18/27
Average time after surgery (mean,	429 (34-1446) [n=4]	2232 (61-8348) [n=6]	298 (46-643) [n=13]	824 (34-8348) [n=23]

days)				
Total gonadotrophine dose (mean, IU)	2450 (1200-4050) [n=3]	4083 (2750-5850) [n=6]	3521 (950-6300) [n=13]	3528 (950-6300) [n=22]
Stimulation duration (mean, days)	8 (6-11) [n=3]	11.6 (8-15) [n=6]	11.1 (10-14) [n=13]	10.8 (6-15) [n=22]
Expected oocytes number (mean)	10 (3-17) [n=2]	11.8 (0-33) [n=6]	14.2 (6-29) [n=13]	13.1 (0-33) [n=21]
Mature oocytes number (mean)	7 (1-18) [n=3]	14.8 (6-29) [n=5]	13.6 (3-58) [n=13]	13 (3-58) [n=21]

[Table 2: Fertility preservation results]

Disclosure: Nothing to disclose

EP1274

Distribution of histopathology types in stage 1A ovarian cancer

F. Kokka[1], R. Xiong[1], N. Singh[2], N. Kamel[3], D. Oram[4], A. Olaitan[3], A. Nordin[1]
[1]Queen Elizabeth the Queen Mother Hospital, East Kent Gynaecological Oncology Centre, Margate, [2]Cellular Pathology, Barts Health NHS Trust, [3]Gynaecologic Oncology, University College Hospitals NHS Foundation Trust, [4]Gynaecologic Oncology, Barts Health NHS Trust, London, United Kingdom

Introduction / Background: Epithelial ovarian cancer (EOC) is the 6th most common cancer among women in the UK (2014). It has the highest mortality of all gynaecological cancers. More females with a known stage are diagnosed at a late stage (55-58% are diagnosed at stage III or IV), than an early stage (42-45% are diagnosed at stage I or II).It is increasingly recognized that the distribution of histopathology types of EOC differs between early and late stages.
The objective of this study was to identify the distribution of the histopathology types of stage 1A EOC cases in three cancer centers in the UK and compare the results with the available published data.
Methodology: We identified retrospectively over a 7-year period stage 1A ovarian cancer patients who underwent primary surgical treatment in two cancer centers. We then compared the results with similar data from another cancer center over a 10-year period.
Results: 105 patients in total with surgical stage 1A EOC were treated at three different gynaecological cancer centers over a period of 7-10 years. In each of these centers, the majority were mucinous ovarian cancers (MOC) (54.28%, 46.15%, 36.36%), followed by clear cell/endometrioid types.
Conclusion: In our study the commonest type of stage 1A EOC was MOC. This may partly be due to different thresholds in diagnosing MOC, but the fact that three Centers showed a similar proportion suggests this is a true incidence. Our findings support the hypothesis that there are biological differences in the behavior of the tumor types between early and advanced EOC. MOC accounts for approximately 3% of all ovarian cancers with recent genomic studies suggesting it is a true gynaecological cancer and not a distant metastasis of a cancer that started elsewhere. Awareness of the histopathological distribution of EOC between different stages may guide screening research and aid management guidelines.
Disclosure: Nothing to disclose

EP1275

Concordance of peritoneal cancer index using a two-step surgical protocol to select patients for cytoreductive surgery in ovarian cancer

M. Del[1], M. Angeles[1], F. Migliorelli[2], C. Martínez-Gómez[1], M. Voglimacci[1], A. Provendier[1], S. Bétrian[3], E. Gabiache[4], G. Balagué[5], S. Leclerc[6], E. Mery[7], L. Gladieff[3], G. Ferron[1], A. Martinez[1]
[1]Surgical Oncology, Institut Claudius Regaud, IUCT Oncopole, Toulouse, France, [2]Obstetrics and Gynecology, Geneva University Hospitals, Geneva, Switzerland, [3]Medical Oncology, [4]Nuclear Medicine, [5]Radiology, [6]Anesthesiology, [7]Anatomo-Pathology, Institut Claudius Regaud, IUCT Oncopole, Toulouse, France

Introduction / Background: The aim of our study was to assess concordance of the staging laparoscopy and cytoreductive surgery peritoneal cancer index (PCI) when applying a two-step surgical protocol. We also aimed to evaluate the accuracy of diagnostic laparoscopy to triage patients for complete cytoreduction, and to define optimal time between staging laparoscopy and cytoreductive surgery.
Methodology: We designed a retrospective review of prospectively collected data from patients with advanced ovarian cancer who underwent a diagnostic laparoscopy followed by a cytoreductive surgery a few weeks later (two-step surgical protocol), from January 2010 to April 2019. Only patients selected for complete cytoreduction, and with available PCI score evaluation during laparoscopy and laparotomy were included.
Results: During the study period 543 patients underwent a laparoscopic staging for ovarian carcinomatosis. Among them, 43 patients fulfilled the inclusion criteria. The ICC between laparoscopic and laparotomic PCI was 0.54. After applying the linear regression equation: laparoscopic PCI + 0.2x[Days between surgeries] + 2, ICC increased to 0.79. Completeness cytoreduction score and laparoscopic PCI were significantly associated (OR 1.27). The AUC of laparoscopic PCI to predict a complete cytoreduction was 0.90.
Conclusion: The concordance between laparoscopic PCI assessment and PCI score at the end of cytoreductive surgery is fair within a two-step surgical management. Laparoscopic assessment underestimates final PCI score by two points, and this difference increases with the delay between both surgeries. Diagnostic laparoscopy can adequately select patients for cytoreductive surgery, and the optimal time to perform it is no more than ten days after laparoscopy.
Disclosure: Nothing to disclose

EP1276

Microinvasive tubal carcinomas: cases of poor outcome despite 'very early' diagnosis. A systematic review of the literature

S. Piedimonte[1], C. Frank[2], C. Laprise[3], A. Quaiattini[4], W.H. Gotlieb[5]
[1]Obstetrics and Gynecology, Jewish General Hospital, McGill University, [2]Obstetrics and Gynecology, [3]Epidemiology and Biostatistics, [4]Centre for Medical Education, Faculty of Medicine, McGill University, [5]Gynecologic Oncology, Jewish General Hospital, McGill University, Montreal, QC, Canada

Introduction / Background: Fortuitous cases of invasive tubal carcinoma in BRCA patients have been reported at the time of risk reducing salpingo-oophorectomy (RRSO). We performed a systematic review of the literature to determine the incidence of microinvasive tubal carcinoma in BRCA or high-risk patients undergoing RRSO, and evaluated treatment outcomes, recurrence and overall survival.
Methodology: The systematic review of the literature was done using OVID, EMBASE, Medline and PubMed with keywords selected by an expert medical librarian. Two independent reviewers performed study selection, data extraction, data analysis and quality assessment. Data was extracted,recorded in a database, and synthesized using tables. Quality was assessed using an index for non-randomized studies (MINORS). Statistical analysis was done with SAS.
Results: Among 2314 studies, 35 met inclusion criteria and were retained for qualitative analysis; 27 were used for quantitative analysis. A total of 6425 patients underwent RRSO between 1990 and 2019; 2963 were BRCA1 and 1605 BRCA2. Among these, 86 patients had occult tubal carcinoma. The pooled incidence of occult tubal carcinoma after RRSO in BRCA mutated or high-risk women was

1.34%, occurring at a median age of 54 years (range 42.4-67). After a median follow-up of 47.5 months, 13 recurrences and 27 cases of post-RRSO peritoneal cancer were reported. BRCA1, older age, previous breast cancer and extensive sampling of the fimbriated end were more often associated with occult malignancy.
Additionally, we report a case of a 57 year old BRCA2+ patient with microinvasive tubal carcinoma found at prophylactic surgery. Despite chemotherapy and complete interval debulking, she recurred after 33 months with diffuse carcinomatosis, treated with second line calyx/carboplatin and olaparib maintenance. After 16 months, she developed acute myeloid leukaemia and died from this secondary malignancy.
Conclusion: Despite early diagnosis and aggressive chemotherapy, microinvasive tubal carcinoma found at the time of RRSO can recur and result in poor outcomes
Disclosure: Nothing to disclose

EP1277

Clinical evaluation of miR-34a in ovarian carcinoma

A. Arapaki[1], A. Karabelas[1], K. Panoutsopoulou[2], G. Papachristopoulou[3], M. Avgeris[2], A. Scorilas[2], D. Papatheodorou[1]
[1]Department of Gynecology, 'Saint Savvas' Cancer Hospital of Athens, Athens, Greece, [2]Department of Biochemistry and Molecular Biology, Faculty of Biology, National and Kapodistrian University of Athens, Athens, Greece, [3]Department of Pathology, 'Saint Savvas' Cancer Hospital of Athens, Athens, Greece, Athens, Greece

Introduction / Background: Ovarian cancer (OC) is the most common cause of gynecological cancer-related death in developed countries. The absence of evident early symptoms and effective asymptomatic population screening leads to diagnosis in advanced stages for the majority of patients and, thus, to poor prognosis. Consequently, the identification of predictive markers of patients´ treatment outcome could ameliorate disease management. microRNA-34a (miR-34a) is highlighted to exert a tumor suppressive role in several malignancies and is downregulated in numerous cancers. According to TarBase v.8, highthroughput studies have reported the negative correlation of miR-34a with kallikrein-related peptidase 6 (KLK6) expression levels. In the present study, we aimed to investigate the expression profile of miR-34a in ovarian carcinomas and to explore its clinical value in OC patients.
Methodology: Ovarian cancer specimens and matched adjacent tissues were obtained from St. Savvas Regional Anticancer Oncology Hospital of Athens. Following homogenization, total RNA was extracted using TRI reagent. Thereafter, the RNA was polyadenylated in the 3'-end and first-strand cDNA synthesis was performed by MMLV using an oligo (dT) adapter. A SYBR-Green fluorescent-based qPCR assay was developed to quantify miR-34a, using snoRNA RNU48 as endogenous reference control gene for normalization purposes. Extensive statistical analysis has been conducted to explore the clinical utility of miR-34a levels.
Results: The expression analysis demonstrated that miR-34a levels are downregulated in ovarian tumors compared to normal epithelium. With regard to clinicopathological characteristics, patients with tumor grade 3 (G3) were highlighted to underexpress miR-34a compared to OC patients with tumor grade 1 (G1) or 2 (G2). Ultimately, no association was observed between FIGO stages and miR-34a expression levels.
Conclusion: The tumor-suppressor miR-34a is downregulated in ovarian malignant epithelium and correlated with higher tumor grading, supporting its further evaluation as a potential molecular marker in OC prognosis.
Disclosure: Nothing to disclose

EP1278

Sentinel lymph node technique in early stage ovarian cancer (SENTOV): a phase II clinical trial

V. Lago, P. Bello, B. Montero, L. Matute, S. Lopez, T. Marina, M. Agudelo, S. Domingo
University Hospital La Fe, Valencia, Spain

Introduction / Background: There is limited evidence that favours the use of the sentinel lymph node (SLN) technique in ovarian cancer. A standardised approach has not been studied. The objective of the present study was to determine the safety and feasibility of the SLN technique by applying a clinical algorithm.
Methodology: Twenty patients with histologically confirmed ovarian cancer were prospectively included. ^{99m}Tc and indocyanine green were injected into the ovarian and infundibulopelvic ligament stump. An intraoperative mobile gamma camera, a gamma probe and near-infrared fluorescence imaging were used for SLN detection.
Results: The SLN technique was performed in 20 patients. The SLN detection rate at both the pelvic and para-aortic region was 19/20 (95%); this value represented a 100% SLN detection rate at the pelvic and/or para-aortic region. The SLN migration and tracer distribution rates of SLN were 100% for ^{99m}Tc + ICG, 100% for ^{99m}Tc alone and 95% for ICG alone. The mean number of harvested SLNs was 2.2 ± 1.5 (range 0-5) nodes in the pelvic field and 3.3 ± 1.8 (range 1-7) nodes in the para-aortic region. There were no adverse events reported during surgery or the 30-day post-surgery.
Conclusion: Detection of SLN in early-stage ovarian cancer appears to be a safe and feasible technique (NCT03452982).
Disclosure: Nothing to disclose

EP1279

The role of HE4 for prediction and prognosis of recurrence for different histologic subtypes of epithelial ovarian cancer- results from single institution

A. Strojna[1], A. Chudecka-Głaz[1], K. Safranow[2]
[1]*Department of Gynecological Surgery and Gynecological Oncology of Adults and Adolescents, Pomeranian Medical University, Szczecin, Poland, Pomeranian Medical University, Szczecin, Poland,*
[2]*Department of Biochemistry, Pomeranian Medical University in Szczecin, Szczecin, Poland, Pomeranian Medical University in Szczecin, Szczecin, Poland, Szczecin, Poland*

Introduction / Background: Ovarian cancer (OC) is one of the leading causes of cancer-related death in women worldwide. The majority of OC patients will relapse following primary treatment. HE4 has been approved by the FDA in monitoring the follow-up of OC patients. Expression of the HE4 in OC depends on the histological subtype. HE4 is expressed almost in all serous and endometrioid type. In contrast, mucinous OC rarely show protein expression. The aim of this study was to compare the role of HE4 in predicting recurrence for different histologic subtypes. We also evaluate the role of HE4 in predicting relapse after second-line of treatment.
Methodology: We retrospectively analysed 188 patients with OC (10% FIGO I, 4%- II, 81%- III, 5%- IV) treated between November 2005 and September 2017. The patients were followed up during the first and second line treatment using tumour marker (CA 125, HE4) together with rectovaginal examination, or CT if necessary.
Results: We identified 188 patients with OC. Of 188 patients evaluated, 160 had serous, 10 endometrioid, 9 mucinous and 9 clear cell carcinoma. We evaluated median PFS in all histopatological subtypes. Patients were divided into groups: group A: HE4 (< 70pmol/l) after first line treatment and group B: HE4 (>70 pmol/l). The median PFS for mucinous histotyp for group A and B was 28,7 and 34 months respectively and for clear cell carcinoma was 30 and 16 months respectively. 25% of patients with serous OC without normalization of HE4 after I line treatment developed reccurency within the first year of follow-up. CA125 and HE4 value after the first line treatment, and during follow-up were found statistically significant and included into a multivariate logistic regression model to determine the risk of recurrency.

Conclusion: Our study showed that HE4 marker is a useful tool for early detection of recurrency excluding mucinous type OC.
Disclosure: Nothing to disclose

EP1280

Thrombocytosis in ovarian tumours: a single institute experience

A. Slabuszewska-Jozwiak[1], E. Dmoch-Gajzlerska[2], B. Kozakiewicz[3], G. Jakiel[1]
[1]The First Department of Obstetrics and Gynecology, Medical Centre of Postgraduate Education, [2]Department of Obstetrics and Gynecology Didactics, [3]Department of Oncological Prophylaxis, Medical University of Warsaw, Warsaw, Poland

Introduction / Background: To evaluate the platelet counts as a prognostic parameter for ovarian tumours.
Methodology: Between 2000 and 2005, 349 patients with ovarian tumours (aged 12 - 88-years-old) underwent primary surgical treatment at First Department of Obstetrics and Gynecology. 97 patients were diagnosed with ovarian malignancies and borderline tumours: adenocarcinoma (64,9%), carcinoma endometrioides (13,4%), carcinoma clarocellulare (10,3%), carcinoma endometrioides and adenocarcinoma serosus(1,0 %) cystadenocarcinoma papillarae of borderline malignancy (5,1 %), cystadenofibroma papillarae of borderline malignancy (2,0%) cystadenoma mucinosum of borderline malignancy (1,0%), Sertoli Leyding tumour (2,0%).
Thrombocytopaenia was assumed with a platelet count below 150G/L and thrombocythaemia at 350G/L and higher. The patients were retrospectively evaluated and compared.
Results: Thrombocytosis often coincides with ascites and the cytoreduction decreases platelet count. There is a positive correlation between platelet count and tumour grading. Thrombocytosis was more frequently found in high grade tumours. There is also a positive correlation between platelet count and tumour stage according the International Federation of Gynaecology and Obstetrics (FIGO). Thrombocytosis was more frequently found in stage III and IV cancers. Patients with co-occurring thrombocytosis were found to have shorter survival periods and shorter time free from disease.
Conclusion: The present study suggested that the platelet count should be included in the panel of prognostic factors for patients with ovarian tumours
Disclosure: Nothing to disclose

EP1281

Total parietal peritonectomy: what morbidity? and which sites of recurrence?

D. Hudry[1,2], A. Berthet[1], R. Saadeh[1], M.-C. Le Deley[1], F. Narducci[1,2], E. Leblanc[1,3]
[1]Centre Oscar Lambret, [2]Inserm U-1192, [3]Université de Lille, Lille, France

Introduction / Background: To evaluate the morbidity of perioperative total parietal peritonectomy (TPP) during cytoreduction surgery (CRS), and its impact on the site of recurrence of different peritoneal surface malignancies (PSM).
Methodology: We led a retrospective study in a French tertiary cancer institution (Centre Oscar Lambret - Lille) experienced in treating PSM over a 6-year period from 2012 to 2018. All patients underwent a total parietal peritonectomy during a debulking surgery for PSM including ovarian cancer, appendiceal pseudomyxoma peritonei or peritoneal mesothelioma.
Results: Sixty-one patients were included in this study with 79% of them having ovarian cancer. The rate of optimal surgery reached 87% with almost 70% of surgeries being highly complicated. 74.2% were transfused during the surgical procedure. The median hospitalization stay was 10 days including 7 days in Intensive Care Unit (ICU). 30% had early postoperative complications with no grade 4 and

42% Grade 3 complications. After a 30-months median follow-up, 84% of ovarian cancer group had no recurrence of the disease the first year and a 3-year survival of 77%. The main site of first and second recurrence was peritoneal (51% and 56.3%). In this series, there have been no cases of metastatic evolution of the abdominal wall.
Conclusion: TPP is a feasible surgical procedure to treat peritoneal surface malignancies and their recurrences with a low rate of Grade 3-4 morbidity. Even though, TPP doesn't prevent peritoneal recurrence, it allow optimal surgery.
Disclosure: Nothing to disclose

EP1282

Evaluation of factors predicting recurrence in borderline ovarian tumors

V. Jain
Gynaecologic Oncology, Rajiv Gandhi Cancer Institute & Research Centre, Delhi, India

Introduction / Background: The objective of our present study was to identify the Clinicopathologic factors that are associated with increased risk of recurrence for borderline ovarian tumors.
Methodology: We performed a retrospective review of all cases of BOT treated at our institute from January 2005 to December 2017. Disease free survival (DFS) was calculated from the date of surgery to the date of recurrence or last follow up. Overall survival (OS) was calculated from the date of surgery to date of death or last follow up. The effect of various variables on recurrence was evaluated by univariate and multivariate Cox-proportional hazard method.
Results: A total of 75 patients were identified. The median age of patients was 42 years. Abdominal pain was the most common symptom (54.6%). Complete staging was done in 47 patients (62.7%). 64 patients had stage I, 5 had stage II and 6 had stage III disease. 27 patients underwent fertility sparing surgery. Implants were seen in 11 patients, with invasive implants in 6 patients. 7 patients received adjuvant chemotherapy. The median follow up was 53 months (range, 6- 165 months). 7 patients developed borderline recurrence and 5 developed invasive recurrence. All the patients with borderline relapses were successfully managed by second surgery. 3 patients with invasive recurrence died of disease. On univariate analysis, the factors significantly associated with worse DFS were Nulliparity (p= 0.015), advanced stage (stage Ic and above) (p= 0.003), incomplete staging (p= 0.006), fertility sparing surgery (p= 0.0007) and presence of implants (p= 0.026). On multivariate analysis, only incomplete staging (p= 0.003) and advanced stage (p= 0.02) held significance.
Conclusion: Advanced stage and incomplete staging are associated with increased risk of recurrence in borderline ovarian tumors. Borderline relapses are usually managed successfully by second surgery and have good prognosis while invasive relapses are often associated with fatal course.
Disclosure: Nothing to disclose

EP1283

Preliminary experience in the use of J-Plasma device in gynecological malignancies

S. Gueli Alletti[1], A. Rosati[2], V.A. Capozzi[3], B. Costantini[1], G. Vizzielli[1], A. Fagotti[1,3], F. Fanfani[1,3], C. Fedele[3], S. Cianci[1], G. Scambia[1,3]
[1]Obstetrics and Gynecology | Gynecologic Oncology, Fondazione Policlinico Universitario A. Gemelli IRCCS, [2]Obstetrics and Gynecology | Gynecologic Oncology, Università Cattolica del Sacro Cuore, Rome, Roma, [3]Obstetrics and Gynecology | Gynecologic Oncology, Università Cattolica del Sacro Cuore, Istituto di Clinica Ostetrica e Ginecologica, Rome, Italy

Introduction / Background: The present study investigated the role of this novel plasma energy device in gynecological malignancies in the context of a modern personalized approach delivered by a

tertiary care referral center. The indication was the resection/ablation of peritoneal and/or serosal carcinomatosis, either in laparotomy or in laparoscopy.
Methodology: From January 2019 to April 2019 six patients, referring to the Division of Gynecologic Oncology, Fondazione Policlinico Universitario A. Gemelli IRCCS in Rome, were selected for a feasibility study on the use of J-Plasma in advanced gynecological cancer conditions. The setting of the device used was Coagulation 45, Cut 45, Bipolar Macro 1, J-Plasma Power 40%, Gas Flow 4.0 l/min, Pulsing 80.
Results: All patients showed peritoneal/serosal carcinomatosis with an indication of regional peritonectomy and/or ablation respectively. Three patients underwent laparoscopic cytoreduction (Group 1= for interval debulking surgery (n=2) and stage IV endometrial cancer (n=1). Three patients underwent laparotomic primary debulking surgery for advanced ovarian cancer (Group 2). All patients achieved optimal residual tumor at the end of surgery. The device was used for parietal, diaphragmatic, and mesenteric peritonectomy and to ablate nodules on the bowel serosa.
The median OT and median EBL were 195 min and 100 ml for Group 1, and 420 min and 500 ml for Group 2. The median hospital stay was 4 days for Group 1 and 13 days Group 2 respectively. No intra- and post-operative complications were registered within 60 days after surgery.
Conclusion: The use of J-PLASMA in the laparoscopic or laparotomic cytoreductive surgery could enhance the removal of peritoneal and serosal carcinomatosis. This kind of energy seems to be safe in performing peritoneal stripping and serosal ablation without increasing the morbidity. Further experience and a longer follow-up period are required to establish the use of the J-PLASMA in cytoreductive surgery for gynecological cancer.

	LPS	LPT
**Number of patients (N)**	3	3
**Median Age (years)**	67	58
**Median BMI (N)**	24	30
**Indication for surgery**		
**PDS in AEOC**	-	3
**IDS in AEOC**	2	-
**Stage IV endometrial cancer**	1	-

[Patient characteristics]

	LPS	LPT
**Median operative time (min)**	195	420
**Median EBL (ml)**	100	500
**Residual tumor (mm)**	0	0
**Median hospital stay**	4	13
**Surgical procedures with J-Plasma**		
**Parietal/Pelvic Peritonectomy**	3	2
**Diaphragmatic Peritonectomy**	1	3
**Mesenteric Peritonectomy**	1	1
**Bowel serosal ablation**	-	1

[Perioperative variables]

Disclosure: Nothing to disclose

EP1284

Does co-administration of Resveratrol (RSV) and Cisplatin (CP) protects against Cisplatin - induced ovarian and uterine toxicities in female Westar rats?

I. Albahlol
Obstetric & Gynecology, Jouf University KSA,Mansoura University Egypt, Mansoura, Egypt

Introduction / Background: The multiple systemic adverse effects of CP including ovarian and uterine toxicities restrict its use. Resveratrol supplementation has significantly increased uterine artery velocity and fetal weight in mice. Due to its role in reducing the oxidative stress damage in the ovary, Resveratrol has been suggested to be an effective option in protecting ovarian tissue
Methodology: Cisplatin and female Westar rats were obtained. The animals were divided into four groups of eight rats each and were treated as follows. Group I (control): received normal saline orally for 28 days. Group II (CP): received a single dose of CP (7 mg/kg of body weight intraperitoneal: IP) on day 21. Group III (RSV): received RSV (10 mg/kg body weight through oral gavage daily) for 28 days. Group IV (CP+RSV): received 10 mg/kg body weight dose of RSV once a day for 28 days along with single dose of CP (7 mg/kg body weight IP) on day 21. At the end of the treatment, animals were sacrificed. Blood samples were collected for Follicle stimulating hormone (FSH) and Estradiol analyses. One of the ovaries and a part of uterus were immediately washed with ice-cold physiological saline, blotted dry and then stored at−20 °C for biochemical analysis. The other ovary and the remaining part of the uterus were transferred into 10% buffered formaldehyde for histopathological and immunohistochemical examinations
Results: Treatment with Cisplatin resulted in significant increase in the oxidative stress marker, Malondialdehyde (MDA), compared to control group ($p<0.05$). This increase was dramatically ameliorated with co-treatment with RSV. Cisplatin administration significantly increased serum FSH, this effect was ameliorated by concomitant administration of RVS.
Conclusion: Co-administration of Resveratrol and Cisplatin protects against ovarian and uterine toxicity. This open the way for further human studies to prove these results and gain maximum benefits in clinical practice
Disclosure: There was no conflict of interest and no fund

EP1285

Pregnancy and epithelial ovarian cancer: case report

O. Puentes[1], T. Quijano[2], E. Mora[1], C. Rojas[1]
[1]Fundación Universitaria de Ciencias de la Salud, [2]FUCS, Fundación Universitaria de Ciencias de la Salud, Colombia, Colombia

Introduction / Background: Epithelial ovarian cancer in pregnant patients, an uncommon problem, delay maternity increases frequency. Pregnancy patient 18 5/7 weeks and a complex adnexal mass of 13 centimeters and elevated tumor markers is found. In surgery on 20 weeks, abdominal-pelvic mass is found diagnosing epithelial ovarian cancer Our aim was to present the case of a pregnant patient with and epithelial ovarian cancer and review the literature concerned.
Methodology: We present the case of a patient with a pregnancy of 18 weeks of gestation and mucinous epithelial cáncer.
Results: The diagnosis is difficult, pregnancy characteristic symptoms create distractions. The tumor markers have limited value. Human epididymal protein 4 may have utility in diagnosis and management Ultrasound is the preferred tool, because of its high sensitivity and specificity. Magnetic resonance imaging is performed on masses suggestive of malignancy, the use of contrast and computerized axial tomography is discouraged. It is recommended to treat the pregnant patient as well as the non-pregnant patient. Adjuvant chemotherapy is administered with greater security during the second and third trimester, suspended three weeks before delivery, to avoid fetal myelosuppression The oncological outcome is not conclusive. This increases fetal losses, prematurity and fetal growth restriction. The patient pregnancy is interrupted by cesarean, on week 32, a male newborn of 1645 g

is received with apgar 8-9-9 and the oncological surgery is completed.
Conclusion: Epithelial ovarian cancer in pregnancy is rare, there is a clear lack of information. The diagnosis, surgical and adjuvant treatment should be performed in the same way as a non-pregnant patient. The surgical approach and chemotherapy are recommended in the second and third trimesters. Oncological results are like non-pregnant patients. Fetal results are encouraging.
Disclosure: Nothing to disclose

EP1286

Single institution experience with neoadjuvant chemotherapy compared to primary debulking surgery in patients over 75 with high grade ovarian cancer

S. Piedimonte[1], R. Kessous[2], I. Laskov[2], J. Abitbol[2], L. Kogan[2], A. Yasmeen[2], S. Lau[2], S. Salvador[2], W.H. Gotlieb[2]
[1]Obstetrics and Gynecology, [2]Gynecologic Oncology, Jewish General Hospital, McGill University, Montreal, QC, Canada

Introduction / Background: Patients over 75 with high grade ovarian cancer have poorer prognosis compared to younger patients due to decreased physiologic reserves, delayed diagnosis, and undertreatment due to fear of toxicity. We performed a hypothesis generating evaluation of outcomes following neoadjuvant chemotherapy compared to primary debulking surgery in patients over 75 with high grade ovarian cancer.
Methodology: This is a retrospective cohort study of consecutive patients 75 years and above, with high grade stage III-IV ovarian cancer. Patients with inadequate follow-up were excluded. Descriptive statistics were performed to characterize groups based on primary treatment modality. Kaplan Meier survival curves were used to estimate overall and progression free survival with log rank tests for significance. Cox proportional hazards was performed for analysis of confounders.
Results: Among 429 patients with stages III-IV high grade ovarian cancer (endometrioid and serous), 55 were over 75 years old and met inclusion criteria; 42 were treated with neoadjuvant chemotherapy followed by interval debulking compared to 13 patients undergoing primary debulking. There was no significant difference in demographic parameters. More patients were optimally debulked following neoadjuvant chemotherapy (50% vs 23%, p=0.003) and had a shorter length of stay (5 vs 7 days, p=0.018). Overall survival (mean 67.3 vs 59.7 months, p=0.765), and progression free survival (31.1 vs 47.1 months, p=0.143) were similar in patients treated with neoadjuvant chemotherapy compared to primary debulking .
Conclusion: In this limited number of older patients with Stage III-IV high grade ovarian cancers, there was no significant difference in survival between those treated with neoadjuvant chemotherapy and primary debulking surgery.
Disclosure: Nothing to disclose

EP1287

A unique case report: postoperative palmar digital thrombophlebitis in a young primer Sjögren's syndrome patient with high grade serous ovarian cancer

E. Keles[1], C. Kabaca Kocakuşak[2], Z. Çelik[3]
[1]Department of Gynecologic Oncology, Zeynep Kamil Women's and Children's Disease Training and Research Hospital, [2]Department of Gynecologic Oncology, [3]Department of Obstetrics and Gynecology, Zeynep Kamil Training and Research Hospital, Istanbul, Turkey

Introduction / Background: Sjögren´s Syndrome (SjS) is a chronic autoimmune inflammatory disease of unknown etiopathology, characterized by focal lymphocytic infiltration of the exocrine

glands (predominantly lacrimal and salivary glands) , mainly affects middle-aged women. SjS have an increased risk of developing malignant neoplasm, particularly non-Hodgkin lymphoma.SjS carries an elevated risk of deep vein thrombosis (DVT) and pulmonary embolism (PE).
Methodology: While (SjS) mostly affects patients in between the fourth and sixth decade of life, a young SjS patient is a rare event. Additionally the patient diagnosed with high grade ovarian cancer is an uncommon phenomenon, judging by scarcity of published data.
Results: What makes our case unique is ; there is a paucity of data on postoperatively palmar digital thrombophlebitis which seen infrequently.We present a 35 year SjS patient with high grade serous ovarian cancer; postoperatively developed pneumothorax in right lung and atelectasıa with effusion ın left lung concomitant with thrombophlebitis in the 5th finger.
Conclusion: Herein, we report a unique case report: Postoperative palmar digital thrombophlebitis in a young primer Sjögren's Syndrome patient with high grade serous ovarian cancer.
Keywords: Sjögren´s syndrome, thrombophlebitis , ovarian cancer
Disclosure: Nothing to disclose

EP1288

The role of angiogenesis in early detection of epithelial ovarian carcinoma (EOC) - VEGF D (vascular endothelial growth factor D) as a potential novel biomarker

M. Nalezinska
Masaryk University, Brno, Czech Republic

Introduction / Background: Present pilot study discusses the role of pathologic angiogenetic pathways in ovarian tumours. The basement for this study is the knowledge of physiologic angiogenesis in corpus luteum as the most vascularised part of the ovarian tissue. Surprisingly bening and malignant leasions of ovary have similar symtoms due to high vascular permeability. In present study we have examined the role of VEGF D as a potent biomarker for early EOC detection.
Methodology:
Enzyme linked immunoassay kits detecting the level of VEGF D in blood serum specimens form biobank. Statistic analysis: Mann-Whitney test with the significance level set at p< 0,05.
Total number of samples: 75 (n=100%), benign 39 (52,7%), malignant 35 (47,3%)
Results: Median score of VEGF D in the group of benign tumours was 319,1ng/l. Median score of the VEGF D in the EOC group was 423,8 ng/l.
Conclusion: We have obtained promissing data that may lead to identify a novel presymtomatic biomarker in the secondary screening of EOC.
Disclosure: Nothing to disclose

EP1289

When ovarian cancer surgery cannot be achieved: an interview study of patient perspectives

L. Seibæk, H. Vind Thaysen, K. Lomborg
Aarhus University Hospital, Aarhus, Denmark

Introduction / Background: Previous research has documented that patients undergoing specialized, surgical treatment for peritoneal carcinomatosis of ovarian and colorectal origin perceived the cytoreductive surgery (CRS) as their best treatment option. Rather than discussing the alternatives, it was therefore vital for them to be candidates for surgery. However, CRS cannot be completed in all cases. We aimed to identify in which areas patients wish to be involved when CRS was not completed.
Methodology: We applied a qualitative approach with individual interviews, generated in two rounds, a first during 2016-2017, and a second that finished August 2019. Participants were undergoing (CRS)

and hyperterm intraperitoneal chemotherapy (HIPEC) for peritoneal carcinomatosis of ovarian and colorectal origin, and purposively included to ensure variation regarding age, sex, and socio-demographics. The interviews followed a semi-structured guide, were digitally recorded, and transcribed verbatim. Subsequently, thematically structured content analyses were made (Kvale and Brinkmann).
Results: The data material consisted in 14 interviews (with 12 patients and five relatives). The first warning of a changed treatment strategy was experienced when waking up earlier than intended or when noting that there was no ostomy. In this situation most patients wished to be informed as quickly as possible together with their relatives. First and foremost they needed: A plan for future treatment; to maintain a realistic hope and a meaningful everyday life; to rebuild their functional level; counseling concerning symptom management, sick leave and finances; support for relatives; and insight into and influence on decision-making processes.
Conclusion: The transition from intentional curative to palliative care constituted a life-changing process containing elements of reorientation, restitution and new treatment cycles. However, this phase was by patients experienced as integrated part of the overall treatment course, and there was a great and often unmet need to regain physical strength and psychosocial well-being.
Disclosure: Nothing to disclose

EP1290

The possible role of cell polarity for cancer disease - the implication of cancer regression by human body axes interaction

M.C. Ou[1], D. Ou[2], C.C. Pang[3]
[1]Taipei City Hospital | Obstetrics & Gynecology, Zhong-Xiao Branch, Taipei City, Taiwan, [2]Carnegie Mellon University, Pittsburgh, PA, United States, [3]National Taiwan University, Taipei City, Taiwan

Introduction / Background: The human body anatomical axes (HBAAs) interaction shows an anti-inflammatory effect (Ou MC decrescendo phenomenon, OuDP, Am J Emerg Med. 2012) which indicates a normalization of dysfunctional cells and has shown to induce tumor regression (TJOG, 2017; Cancer arch, 2019).
Methodology: OuDPt is performed by putting the contralateral hand over the lesion along human body anatomical axis of left-right, dorsoventral or vertical axis by the patient.
Results: Ten patients with cancer diseases including gynecological cancers as uterine leiomyosarcoma (1/10), endometrial cancer (2/10), ovarian cancer (1/10) and breast cancer (1/10) showed initial cancer suppression or regression. However, long term follow up showed tumor regrowth in endometrial cancer (2/10) and the ovarian cancer.
Conclusion: The signaling system of embryonic axes has shown to impart polarization of individual cells in Drosophila. Normal cell polarity is essential for normal cell function. The effect of OuDP is rapid, which indicates a direct mending effect on cell function (ICST, 2015).Normalizing the tumor cells and microenvironment function may make tumor cells conform to the regulations with apoptosis, metastasis suppression, preventing uninhibited proliferation, minimizing angiogenesis and supervision by host immunological systems. Long term follow up of advanced cancer diseases treated with OuDP showed tumor regrowth after initial regression, which may be due to poor accessibility, insufficient efficacy or escape phenomenon. However, OuDP may be availed for an active prevention for cancer.

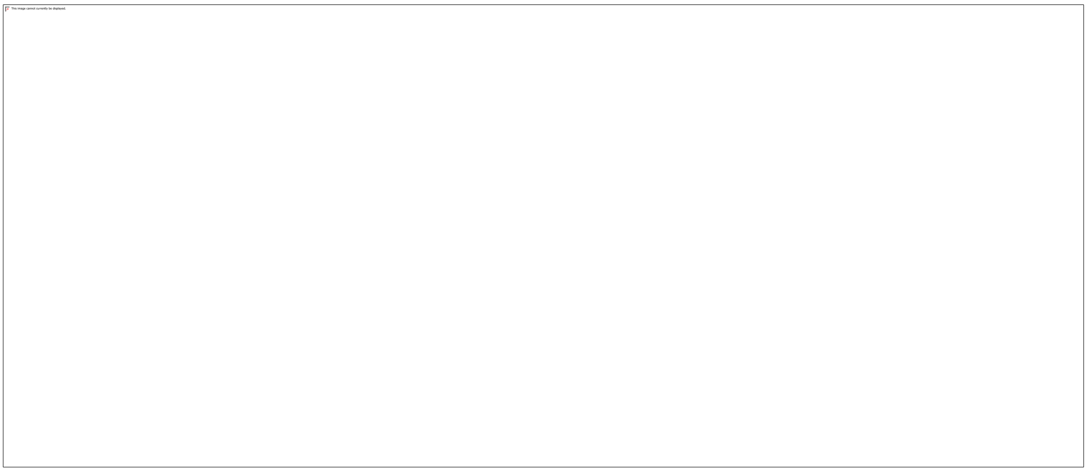

[Figure 1. The relation between OuDPt and stage II uterine endometrioid adenocarcinoma]

Disclosure: Nothing to disclose

EP1291

What factors affect quality of life in patients with gynaecoloc cancers? A prospective cohort study at the University Hospital of the West Indies

N. Medley, A. Robinson, I. Bambury, C. Rattray
Gynaecologic Oncology | Obstetrics and Gynaecology, University of the West Indies, Kingston, Jamaica

Introduction / Background: The impact of cancer and cancer treatment on quality of life (QoL) plays an increasingly important role in management decisions in gynaecologic oncology. This is the first study in our institution looking at determinants of QOL. The aim of this study is to identify factors impacting QoL in patients newly diagnosed with endometrial, ovarian and cervical cancer and to determine the effect of treatment in these patients.
Methodology: All patients newly diagnosed with ovarian, endometrial and cervical cancer were recruited from the gynaecologic oncology out-patient department between November 2018 and May 2019. Participants completed the FACT-G questionnaire at baseline, 6-weeks and 6-months after treatment. The domains assessed were physical well- being (PWB), emotional well- being (EWB) social well-being (SWB) and functional well-being (FWB).
Results: A total of 112 participants were enrolled: 40 persons with cervical cancer, 39 with endometrial cancer and 33 with ovarian cancer representing 36%, 35% and 29% respectively. The mean age of the cohort is 53.7 (±15.5) years. Fifty- eight per cent of participants presented at stage one (n=65, 58.0%) and fifty-five per cent reported only mild restriction in physical activity at diagnosis. Participants had good FACT- G scores across all domains of QoL. The mean scores for PWB and EWB improved from baseline to six- weeks after treatment and then returned to basleine by six months after treatment (p=0.03). There was no statistically significant change in the SWB and FWB scores at six weeks or six months after treatment.
Conclusion: Patients with cervical, endometrial and ovarian cancer have a good quality of life across all domains of physical, emotional, social and functional well-being. This is attributed to younger age, good functional status and early stage disease at diagnosis. Treatment did not significantly affect the quality of life in these women.
Disclosure: Nothing to disclose

EP1292

Minimally invasive palliative treatment of vulval cancer: is there a role for electrochemotherapy?

A. Tranoulis[1], D. Georgiou[2], G. Mehra[1], S. Rajkumar[1], C. Founta[1], A. Sayasneh[1], R. Nath[1]
[1]Gynaecological Oncology, Guy's and St Thomas' NHS Foundation Trust, [2]Gynaecological Oncology, Chelsea and Westminster NHS Trust, London, United Kingdom

Introduction / Background: Electrochemotherapy (ECT) represents a locally enhanced chemotherapy that combines the administration of chemotherapeutic agents with cell membrane electroporation via electric pulses. ECT has gained popularity as minimally invasive treatment in vulval cancer. The aim of this meta-analysis was to ascertain the efficacy and safety of ECT amongst vulval cancer patients treated in the context of palliative care.
Methodology: A systematic literature review was conducted to identify relevant studies that reported response to ECT amongst women with vulval cancer treated with palliative intent. We performed a single-proportion meta-analysis for the outcomes of overall response, complete response and partial response rate respectively. Sensitivity analysis was performed to address heterogeneity.
Results: Four studies were included. The studies were of moderate quality. Pooled results from four studies rendered a summary proportion of 78.81% (95% CI 70.39 - 86.15) for the outcome of overall response. The pooled proportion for the outcomes of complete and partial response rate was 48.74% (95% CI 30.74 - 61.49) and 30.18% (95% CI 21.69 - 39.40) respectively. The outcome of the leave-one-out analysis demonstrated that no single study was found to significantly affect the overall outcome. By omitting one study at a time, overall and complete response ranged from 73.21 to 80.89 and from 45.19% to 53.31% respectively. The follow up ranged from one to 51 months. Significant improvement in quality-of-life was reported. The treatment was generally well-tolerated. The length of stay in the hospital ranged from one to eight days. The safety profile of ECT is favourable. No severe adverse effects were reported.
Conclusion: Our study showed that palliative ECT is associated with high response rate, significant improvement in quality-of-life and limited side-effects amongst palliative vulval cancer population. Further studies with longer follow-up and quality-of-life data will be required to further assess its efficacy in this setting.
Disclosure: Nothing to disclose

EP1293

Medical treatment of vaginal intraepithelial neoplasia (VaIN): evaluating the efficacy and safety of 5-flouorouracil (5-FU)

A. Tranoulis[1], D. Georgiou[2], C. Founta[1], A. Sayasneh[1], R. Nath[1]
[1]Gynaecological Oncology, Guy's and St Thomas' NHS Foundation Trust, [2]Gynaecological Oncology, Chelsea and Westminster NHS Trust, London, United Kingdom

Introduction / Background: Medical treatment has gained popularity as an alternative treatment option in vaginal intraepithelial neoplasia (VaIN). The aim of this meta-analysis was to ascertain the efficacy and safety of 5-flouorouracil (5-FU) in this context.
Methodology: A literature search was conducted throughout the PubMed, EMBASE, SCOPUS, ClinicalTrials.gov, and Cochrane Databases for relevant studies from inception until September 2019. We computed the summary proportions of women treated for VaIN with 5-FU for the outcomes of complete response and recurrence by random-effects meta-analysis. Sub-group analyses were performed to address heterogeneity.
Results: 14 observational studies were included. The studies were of moderate quality. Pooled results from 14 studies enrolling 358 VaIN women rendered a summary proportion of 82.18% (95% CI 69.80% - 88.82%) for the outcome of complete response. Pooled results from six studies enrolling 184

high-grade VaIN women rendered a summary proportion of 77.53% (95% CI, 59.90% - 91.15%) for the outcome of complete response. Pooled results from five studies enrolling 26 VaIN women rendered a summary proportion of 53.92% (95% CI 34.62% - 72.61%) for the outcome of complete response amongst persistent lesions. Pooled results from six studies enrolling 98 VaIN women rendered a summary proportion of 16.42% (95% CI, 7.39% - 28.14%) for the outcome of recurrence. The agent was well tolerated by most women and only few studies reported discontinuation of the treatment owing to severe adverse effects.
Conclusion: 5-FU is seemingly an effective and safe treatment modality in the treatment of VaIN. It can be considered as an alternative conservative treatment option, leading to less aggressive and morbid interventions, especially amongst young women with multifocal lesions. Further studies with longer follow-up will be warranted to further assess its efficacy and safety in this setting.
Disclosure: Nothing to disclose

EP1294

Vulvar melanoma: case report

T. Quijano, E. Mora, O. Puentes, C. Rojas, C. Quintero
Fundación Universitaria de Ciencias de la Salud, Bogota, Colombia

Introduction / Background: Melanoma, neoplasm originating in melanocytes 90% of them in skin and 1% originating in mucous membranes, including the ano-vagino-vulvar mucosa. Melanomas of primary gynecological origin are extremely rare tumors with reported incidences between 0.48 and 1.4 per 100,000; 70% of cases occur in vulva, followed by vagina and more rarely in cervix. With a 5-year overall survival that ranges from 30% to 50%.A peak incidence in the fifth or sixth decade of life, 75% of patients are diagnosed after 60 years. They have been described associated with mutations in a third of cases such as cKIT, NRAS and BRAF.
A 45-year-old patient who consulted due to edema and erythema in the left hemivulva, a Bartholin gland abscess was diagnosed and marsupialized. It consults 3 days later finding persistence of inflammatory process and evidence of mass. A biopsy is taken which reports a poorly differentiated malignant tumor of large cells of poorly defined cytoplasms and large nuclei.
Methodology: We present a case report of a patient with vulvar melanoma.
Results: Clinical oncology indicated a BRAF mutation study to begining immunotherapy (Nivolumab vs Vemurafenib); 10 sessions of radiotherapy are indicated like palliative intent, with marked decrease in tumor size.Functional status of the patient progresses to deterioration
Conclusion: The behavior of a large simulator presented by melanomas, a homologated management is proposed between cutaneous melanomas and other types of melanoma in mucous membranes. Evidence is enough to support neoadjuvant management. The role of chemotherapy and immunotherapy is conditioned to immunohistochemical. Unresectable cases or recurrent disease or with nodal involvement, the use of radiotherapy is favored.
Disclosure: Nothing to disclose

EP1295

Primary vaginal Non-Hodgkin´s lymphoma: a case report of a rare entity

O. Ferreira Rangel Neto, L.M. Martins, M.G.F. Sartori, M.G.B.K. Uyeda, M.J.B.C. Girão
Gynecology Department, Federal University of Sao Paulo, Paulista School of Medicine, São Paulo, Brazil

Introduction / Background: The female genital tract may be affected by Non-Hodgkin lymphoma in about 30%, most due to secondary systemic involvement. Primary lymphoma of gynaecological tract is

an extremely rare entity, comprising 0.2-1.1% of all cases of extra nodal lymphoma. Primary vaginal Non-Hodgkin lymphoma (PVNHL) accounts for less than 0.1%. PVNHL is most often presented as solid consistency mass of defined limits. It may also infiltrate subcutaneous tissue and infiltrate deep fat tissue. Patients may also present abnormal vaginal bleeding, dyspareunia, vaginal discharge, and irritative urinary symptoms.
Methodology: This is a case report of a young woman whose diagnosis of PVNHL came after symptoms of genital discomfort and the presentation of vulvar mass of progressive enlargement.
Results: Woman, 32 years old, that initially presented with urinary symptoms. After a few weeks a large vaginal mass appeared, and a sampling of tissue was performed. The histological diagnosis of a Non-Hodgkin's lymphoma of the vagina (diffuse large B-cell lymphoma) was made. After 6 cycles of R-CHOP chemotherapy (Rituximab, Endoxan, Doxorrubicina, Predinisone) there were full regression of symptoms and involution of vulvar mass. Patient were under follow up without evidence of relapse.
Conclusion: The diagnosis of PVNHL is very difficult for several reasons: First because of this rarity, after the similarity with day-to-day gynecological symptoms and other more commons affections that involves vaginal discomfort and pain. The PVNHL management is challenging and remains unclear due to the lack of specific guideline protocols. Therefore, a multimodal treatment is usually the approach which involves association of surgery and chemo and radiotherapy.
Disclosure: Nothing to disclose

Video Session 1: Lymphadenectomy. Step-by-step procedures and new approaches

The technique of sentinel lymph node and afferent lymphatic channel detection in radicality reducing early stage cervical cancer surgery

M. Hruda, H. Robová, M.J. Halaska, T. Pichlík, L. Rob
Kralovske Vinohrady University Hospital, Prague, Czech Republic

Introduction / Background: In our video we would like to demonstrate the technique of identification of sentinel lymph nodes and afferent lymphatic channels in paracervix using the combination of Tc radiocolloid and Patent Blue in patients with early stage cervical cancer.
Methodology: The patients included in this study were diagnosed with early stage spinocellular or adenocarcinoma of uterine cervix stage IA and IB1 up to 2cm maximal diameter with maximal depth of invasion of less than half of the stroma. We use an ultrashort protocol consisting of application of 2ml Tc (20 MBq) and 2ml of Patent Blue diluted in 2ml of saline. Tc is applicated in surgical theatre at the beginning of anaesthesia using 4 quadrant technique followed by the application of 1 ml Patent Blue into each quadrant. The surgical extirpation was performed in interval of 12 to 15 minutes after the application.
Results: During the period 2009-2018 we included 123 women. In this group we detected positive sentinel lymph nodes in 6 cases. The detection rate (DR) of sentinel lymph nodes was 122/123 - 99,2%, specific side detection rate (SSDR) 232/244 - 95,1% Our aim was to detect 246 channels in 123 women. No channels were detected in 6 women (4.9%), unilateral channel was detected in 25 women (20,3%) and bilateral channels were detected in 93 cases (75,6%).
Conclusion: Our video illustrates the safe technique of identifying and extirpation of sentinel nodes and afferent channels in the paracervix. In early stages of cervical cancers this technique can achieve very good detection rate of sentinel nodes on both sides of the pelvis.

Work was supported Charles University research program PROGRES Q 28 (Oncology).
Disclosure: Nothing to disclose

SENTOV (SENtinel lymph node technique in OVarian cancer): video technique

V. Lago, P. Bello, P. Padilla-Iserte, L. Matute, T. Marina, M. Gurrea, S. Domingo
University Hospital La Fe, Valencia, Spain

Introduction / Background: There is limited evidence favoring the use of the sentinel lymph node technique in ovarian cancer, and no standardized approach has been studied. The objective of the video is to show the standardized sentinel lymph node technique in early ovarian cancer
Methodology: In the case of malignancy, the sentinel lymph node technique was performed. We subperitoneally injected 0.2 mL of saline solution containing 37 mBq of 99mTc nanocolloid (Albu-res, Pharmaceutical Nycomed Amersham, Braunschweig, Germany). At
the same time, 0.5 mL of indocyanine green (concentration 1.25 mg/mL) was injected. We used a 27 G needle at each injection point
Results: The sentinel lymph nodes were checked with an intra-operative mobile gamma camera (Sentinellatm, Oncovision) for descriptive purposes only. Thirty minutes after the injection, the SLN procedure was started regardless of probe migration with the IMGC.
Guided by the acoustic signal of a gamma probe (Wprobe wireless gamma probe STD and LAP, Oncovision), we performed a minimum dissection looking for the hottest sentinel lymph node(s) in the pelvic/para-aortic region. We simultaneously used the Imagen1
HUB-OPAL1 (NIR/ICG system) (Karl Storz Endoscopy, GmbH, Mittelstrasse, Tuttlingen, Germany) to detect the sentinel lymph node(s) dyed with indocyanine green. Any lymph node with a remarkably higher count than the background was considered a sentinel lymph
node and was harvested separately.
Conclusion: In light of the findings of SENTOV clinical trial, the sentinel lymph node technique appears to be applicable in early-stage ovarian cancer
Disclosure: Nothing to disclose

Robotic assisted ingunofemoral lymphadenectomy for vulval carcinoma

Y. Kulkarni
Gynecologic Oncology, Centre for Cancer, Kokilaben Dhirubhai Ambani Hospital and Research Centre, Mumbai, India

Introduction / Background: Vulval carcinoma accounts for 3-5% of all gynaecological cancers. The primary treatment of vulval carcinoma is Local excision +/-inguinal lymphadenectomy. Inguinal node status is an important prognostic indicator in predicting outcomes. It makes lymph node assessment Mandatory for all case of vulval carcinoma except the superficially invasive cancers. Sentinel node mapping is a useful technique, still around 50% of women require inguinal lymphadenectomy. Here we demonstrate our technique of Robotic Assisted Inguinal Lymphadenectomy.
Methodology: Various techniques have been tried to reduce the morbidity associated with inguinal lymphadenectomy both short term and long term. It includes separate incisions, sentinel node mapping, saphenous sparing and video endoscopic approach. From June 2014 to December 2018, 15 patients of carcinoma vulva underwent 21 (9 unilateral, 6 bilateral) Robotic assisted inguinal lymphadenectomy at our institute. This video demonstrates the port placement and technique of the procedure.
Results: 15 patients of vulval carcinoma underwent 21 robotic assisted inguinal lymphadenectomy (9 unilateral / 6 bilateral) underwent the procedure. Mean age of patients was 59yrs (32-73). Mean operative time was 69 min and mean blood loss was 40 ml. The mean number of nodes harvested were 13 (8-23). There were no conversions. No intraoperative complications were observed. Postoperative superficial wound infection was seen in two of the twelve cases and prolonged serum aspiration was required in four cases. Final histopathology showed metastasis in 4 of 21 inguinal node dissection procedures.
Conclusion: Robotic assisted inguinal lymphadenectomy is safe and feasible with less wound related morbidity that conventional procedure. Need multi-institutional studios to evaluate long term complications, safety and survival data.

Disclosure: Nothing to disclose

Video Session 2: Surgical approach in bulky disease in gyneacological tumors

Laparoscopic - laterally extended pelvic resection

M.C. Di Donna[1], C. Cicero[2], G. Sozzi[1,2], V. Chiantera[1,2]
[1]University of Palermo, [2]Department of Gynecologic Oncology, ARNAS Civico Hospital, Palermo, Italy

Introduction / Background: Lateral isolated gynecological cancer infiltrating the pelvic side wall (PSW) has been considered as a not operable surgery with poor oncological outcome. The development of the laterally extended endopelvic resection (LEER) and surgical progress for disease overcoming the endopelvic fascia infiltrating muscles and nerves, have achieved the possibility to treat those patients with a curative intent. We describe for the first time the feasibility of laparoscopic laterally extended pelvic resection (L-LEPR), with complete removal of disease.
Methodology: LEPR was defined as an en bloc lateral resection of a pelvic tumor involving sidewall muscle, and/or bone, and/or major nerve, and/or major vascular structure. We performed the first L-LEPR and a subsequent vascular by-pass for an ovarian cancer recurrence.
Results: A 71 year-old patient, with significant swollen right leg, affected by isolated ovarian cancer recurrence with involvement, on the PET/CT, of the right external iliac vessels and ileo-psoas muscle. L-LEPR was performed with external iliac artery decompression and right obturator, genito-femoral nerve and external iliac vein resection. A vascular bypass with bovine patch reconstruction was performed. A complete removal of recurrence was obtained (R0). Estimated blood loss was 700 mL and operative time was 385 min. No intraoperative complications were observed and the hospital stay was 11 days. Histology confirmed the isolated nodal relapse with infiltration of the surrounding tissue and in the sano margins. After 30 days, an improvement in the swelling of the leg was observed despite a deficiency in adduction and an obturator neuropathy. A re-evaluation at 6 months showed an absence of recurrence.
Conclusion: We describe for the first time L-LEPR with a vascular bypass. Never before this technique was performed by laparoscopy. Our single experience shows that in highly selected cases and in referral centre, L-LEPR can be safely offered with excellent results in terms of morbidity and oncological outcome.
Disclosure: Nothing to disclose

Anterior infra levator exenteration plus colpectomy and intraoperative radiation for recurrent cervical cancer

D. Vázquez Vicente[1], Á. Cabello[2], M. Cambeiro[3], T. Castellanos[1], E. Chacón[1], I. Espinosa[4], J.Á. Minguez[1], J.L. Alcázar[1], L. Chiva[5]
[1]Gynecology, [2]Plastic Surgery, Clínica Universidad de Navarra, [3]Radiotherapy, Clinica Universidad de Navarra, [4]Pathologist, Clínica Universidad de Navarra, [5]Gynecology, Clinica Universidad de Navarra, Madrid, Spain

Introduction / Background: In this educational video, we try to show and step by step procedure of anterior infra levator exenteration with colpectomy plus intraoperative radiation for recurrent /persistent cervical cancer in a patient a solitary kidney. She underwent a urinary diversion and a neovagina reconstruction.
Methodology: A 31 yo woman with a past medical history of systemic lupus erythematosus and congenital absence of the left kidney was diagnosed of a 4 cm-IIA squamous cervical carcinoma in the context of immunosuppressive therapy. Beyond the proximal vaginal involvement, the work up failed to

show any extra cervical disease.
She received standard concomitant chemoradiotherapy showing a questionable partial response.
Four months later after the chemoradiation, an pelvic MRI showed a 4.5 cm persistent cervical mass along with mild right hydronephrosis and vaginal involvement.
Again, a new PET-TAC confirmed the metabolic activity of the cervical tumor without demonstrating distant metastases.
Results: In this video the following procedures are shown, highlighting the tips for the most complex steps:
- Anterior exenteration plus colpectomy (“en bloc” resection of uterus (Type D Radical Hysterectomy), bladder and vagina)
- Intraoperative radiation with electron therapy over the right pelvic side wall (infiltration of the right parametria)
- Neovagina reconstruction from a rectus abdominis musculocutaneus flap
- The urinary diversion was accomplished by means of an ileal urinary conduit (Bricker procedure) using the only right available renal unit.

The final pathological reported was 3 cm epidermoid cervical tumor with involvement of the superior vagina and right parametria.
Follow up: ten months after the surgery the patient has no shown any sign of recurrence.
Conclusion: Pelvic exenteration after radiation is a challenging surgical procedure that may prolong survival in selected patients with local and persistent disease. Intraoperative Radiation allow us in provide an extra amount of radiation in the context a a previous radiated field.
Disclosure: Nothing to disclose

Minimally-invasive cytoreduction in recurrent endometrial cancer: laparoscopic and uniportal video-assisted thoracoscopic surgery (VATS) combined approach

N. Bizzarri[1], E. Meacci[2], F. Giuliante[3], V. Ghirardi[1], G. Scambia[1], A. Fagotti[1]
[1]Gynecologic Oncology Division, [2]Thoracic Surgery, [3]Hepatobiliary Surgery, Policlinico Universitario Agostino Gemelli I.R.C.C.S. Universita Cattolica Di Roma, Rome, Italy

Introduction / Background: Cytoreductive surgery for recurrent abdominal endometrial cancer is typically performed with laparotomy approach. Furthermore, thoracic procedures are usually performed with a thoracotomy approach. Recently, the uni-portal video-assisted thoracoscopic surgery (VATS) has been proposed as the new evolution of VATS, with even less morbidity and faster recovery.
Methodology: We present the video of a third abdominal and thoracic recurrence of endometrial cancer, managed with a combined laparoscopic and uni-portal VATS approach.
Results: The patient is a 58-year-old woman, who was initially diagnosed with FIGO IB grade-2 endometrioid endometrial adenocarcinoma. 19-months after initial surgery she developed a pelvic recurrence, so underwent robotic colpectomy and removal of left pelvic peritoneal nodules, followed by adjuvant chemotherapy. 17-months later, she developed a single splenic recurrence, for which she underwent robotic splenectomy and subsequent chemotherapy. After 18-months, a PET/CT-scan showed avid uptake in the liver capsule/right diaphragm and a 1cm non-avid right pulmonary nodule. Diagnostic laparoscopy revealed a right diaphragmatic plaque of disease as the only intra-abdominal site of recurrence. She underwent laparoscopic full-thickness right diaphragm resection (5x3cm) followed by uni-portal VATS with removal of right pleural nodules, diaphragm defect suturing and an atypical resection of right middle lobe. Operation time was 400 minutes and estimated intra-operative blood loss was 80mls. No macroscopic residual tumour was present at the end of the surgery. No intra-operative complication was recorded. Post-operatively she developed a small right apical pneumothorax, which did not require any intervention. She was discharged on day 6. Histology confirmed recurrent endometrial cancer in all specimens but in the pulmonary nodule (atypical hyperplasia). Adjuvant chemotherapy was started 18-days after the surgery. She is alive and disease-free at 6 months from the surgery.
Conclusion: Minimally-invasive debulking abdominal and thoracic surgery to no residual disease for recurrent endometrial cancer, is feasible and ensure a rapid post-operative recovery to start medical

treatment.
Disclosure: Nothing to disclose

Cinema Viewing

Video endoscopic inguinal lymphadenectomy using 3D scope with sparing saphenous vein

J.S. Park, S.-H. Lee
Obstetrics and Gynecology, Wonju Yonsei College of Medicine / Wonju Severance Christian Hospital, Wonju, Republic of Korea

Introduction / Background: Inguinal lymphadenectomy is necessary when FIGO stage IB or higher. However, conventional inguinal lymphadenectomy has a high morbidity. Video endoscopic inguinal lymphadenectomy(VEIL) is emerging as a minimally invasive treatment to reduce postoperative complications. We report the case of the first VEIL using 3D scope performed in a patient with early stage vulvar carcinoma.
Methodology: 62 years-old woman visited for right vulvar area pain. The excisional biopsy was done and pathologic diagnosis was squamous cell carcinoma. Preop imaging study revealed Ill-defined hypermetabolic activity in right inguinal area on PET-CT and a 1.2cm hypointense lymph node in right inguinal area on MRI(T2WI).
Modified right radical hemivulvectomy, coverage with V-Y advancement flap, right inguinal lymphadenectomy, Video-endoscopic guided left inguinal lymphadenectomy was planned. VEIL was not available on right side due to flap surgery. Surface marking for femoral triangle was made on the skin. Three ports were used. Balloon trocar was used for avoid leakage of carbon dioxide gas. The main landmarks of dissection were medially the adductor longus muscle, laterally the sartorius muscle, superiorly the inguinal ligament and the inferior margin was the apex of the femoral triangle. After identified the saphenous vein, we preserved it to reduce the risk of lymphedema. Several lymph nodes were obtained. At the end of the surgery, a drain was inserted through the port.
Results: The total operation time was 335 minutes and left VEIL took 54 minutes. The total number of nodes removed was 8. The pathologic diagnosis was invasive squamous cell carcinoma. moderately differentiated(stage IB). There is no metastasis on any lymph node. Drain was removed on the 15th postoperative day. Hospital stay after operation was 18 days. No postoperative complication was noted.
Conclusion: Use of 3D scope in VEIL may provide a less invasive approach and aid surgeons by enhancing vision enabling saphenous vein-sparing technique.
Disclosure: Nothing to disclose

Creation of an Y-shaped ileal neobladder in 10 logical steps

C. Martínez Gómez[1], M.A. Angeles[1], M. Del[1], B. Malavaud[2], A. Martinez[3], G. Ferron[1]
[1]*Surgical Oncology,* [2]*Urology,* [3]*Institut Claudius Regard, IUCT Oncopole, Toulouse, France*

Introduction / Background: Anterior pelvic exenteration is a salvage procedure carried out in patients with recurrent or persistent gynecological malignancies involving the bladder. The type of urinary reconstruction is chosen according to the patient preferences after preoperative counselling. Bricker´s ileal conduit is a non-continent urinary diversion, and it is the most frequently employed among gynecological oncologists. However, continent diversions seem to improve patient´s quality of life in patients. Unfortunately, these techniques are not well-known among surgeons and have a low acceptance. In this video, we aimed to describe and standardize orthotopical ileal neobladder creation.
Methodology: This video shows the creation of an orthotopical Y-shaped neobladder in ten

consecutive steps.
The surgery was performed on a 34-year-old woman treated by extended chemoradiotherapy for a stage IIIC2 cervical cancer. At the end of the treatment, a symptomatic vesicovaginal fistula was evidenced at physical examination and at magnetic resonance imaging. In this situation, a closure surgery was proposed to the patient. Taking into consideration that the urethra and the neck of the bladder were competent and not involved by the tumor or the fistula, an orthotopical ileal neobladder was performed following patient's choice.
Results: This video displays the creation of a Y-shaped orthotopic ileal neobladder in the 10 following steps:
1: Patient selection
2: Omental j-flap
3: Selection of the ileal loop
4: Stapled side-to-side ileoileal anastomosis
5: Y-shaped detubularization of the ileal loop
6: Ureter spatulation and ureteral stents insertion
7: Ileoureteral anastomosis
8: Posterior Ileovesical anastomosis
9: Anterior Ileovesical anastomosis
10: Blue dye testing
Conclusion: Orthotopic ileal neobladder reconstruction may be an option after anterior pelvic exenteration in selected patients fulfilling a restrictive selection criteria. As previously published, standardization of surgical procedures in 10 steps enhances learning of complexes techniques.
Disclosure: Nothing to disclose

Total infralevator pelvic exenteration
I. Kahramanoglu
University of Health Sciences, Gazi Yasargil Training and Research Hospital, Diyarbakir, Turkey

Introduction / Background: Pelvic exenteration is a treatment option for select patients with recurrent cervical cancer.
Methodology: Total infralevator pelvic exenteration was performed in a 55-year old patient, who had undergone simple hysterectomy for cervical carcinoma and received adjuvant chemoradiotherapy 9 months ago. Even though the development of pelvic spaces was not succeeded as planned, total exenteration was performed by a gynecologic oncology team. Ileal conduit was preferred for urinary diversion. Surgical details (exenterative and reconstructive phases, in particular) were shown in this video.
Results: Pathologically free margins were obtained. No adjuvant treatment was given. Follow-up at 11th month showed no evidence of recurrence.
Conclusion: Pelvic exenteration should be considered in patients with central recurrence of cervical cancer.
Disclosure: Nothing to disclose

Preliminary outcomes of tumor-sealing laparoscopic radical hysterectomy using the BUMI (Bora's Uterine Manipulation Instrument) for early cervical cancer
B. Lee[1], J.H. Yoon[2]
[1]*Medinstru Ltd., Young-In,* [2]*The Catholic University of Korea/ St. Vincent's Hospital, Suwon, Republic*

of Korea

Introduction / Background: Minimally invasive surgery including laparoscopy and robotic surgery have been increasingly employed in cervical cancer. However, there are concerns that the use of uterine manipulator and Intracorporeal colpotomy may cause tumor injuries and tend to spread tumors. This study introduces a novel tumor-sealing procedure using BUMI (Bora's Uterine Manipulation Instrument, Sejong Medical, Korea) manipulator in early stage cervical cancer patients.
Methodology: The 5 patients presented with FIGO stage IB1 cervical squamous cell cancer. After the outer wall of the vagina was incised circumferentially at the level of the junction between the upper and the middle thirds, the vaginal cuff was folded over the cervix to cover it by continuous suture (Tumor-sealing).
Results: Laparoscopic type C1 radical hysterectomy with pelvic lymph node dissection was successfully performed without tumor exposure or manipulation. The average time of operation was 240 minutes; the average blood loss was 400 ml. The most of patients were discharged at postoperative 9 days without any complication.
Conclusion: Tumor-sealing procedure before colpotomy provides a safe method to prevent potential harmful tumor spread and the BUMI system can help to perform radical surgery effectively without unnecessary tumor manipulation during minimally invasive surgery in patients with early stage cervical cancer.
Disclosure: Nothing to disclose

Modified Martius flap: an easy fistula repair technique (video)

S. Domingo del Pozo, T. Marina Martín, V. Lago Leal, P. Padilla Iserte, L. Matute Tobías, M. Gurrea Soteras, T. Viñas Alburquerque
Gynecologic Oncology, University Hospital La Fe, Valencia, Spain

Introduction / Background: Rectovaginal and vesico-urethrovaginal fistulas after gynecologic surgical procedures are a major concern and represent a challenge for surgeons.
Some decades ago H Martius described an interpositional tissue flap using the bulbocavernosus muscle with fibroadipose tissue from the labia majora for urethrovaginal fistula repair. Subsequently, the technique has been modified developing a less invasive technique.
Methodology: For a proper technique, the patient is placed in a modified lithotomy position and a temporary transurethral urinary catheter is placed during the surgery. The procedure starts with direct primary anatomical repair: after the identification of the fistulous tract, a Foley catheter is placed through it, this maneuver allows a gentle traction of the fistula. Then, a circumferential incision around the fistula is made in order to separate and mobilize bladder and vaginal walls. Anterior and posterior vaginal wall flaps are developed in healthy tissue using sharp dissection to expose the perivesical fascia. Once the bladder wall is mobilized the defect is closed by suturing.
Results: A lateral incision on the labia majora to expose the fatty pad is performed. There is a natural tissue plane around the pad for the dissection, it´s important to ensure that the blood supply is maintained. Once posterior dissection is performed, vascularized labial fatty flap is mobilized and transposed into the vagina by a clamp under the vaginal wall, for covering the fistula repair site without tension. Care should be taken to stay lateral to the bulbocavernosus and ischiocavernosus muscles in order to prevent significant bleeding or scar deformity. Flap must be fixed with absorbable suture. Finally, the skin and vaginal incisions are closed with an absorbable synthetic suture.
Conclusion: In general, the Martius technique and its modifications are used for the repair of complex fistulas in the perineal region, either urogynecological or rectovaginal including recurrent, radiation induced or large fistulas
Disclosure: Nothing to disclose

Cisterna Chyli: an important landmark in laparoscopic paraaortic lymphadenectomy

N.U. Dogan[1], S. Dogan[2], M. Erol[3], B. Tas Uzun[1]
[1]Ob &Gyn, Akdeniz University, [2]Antalya Resarch and Training Hospital, [3]Akdeniz University, Antalya, Turkey

Introduction / Background: Cisterna chyli is a pearl shaped elongated lymphatic structure generally located at the level of L1-L2 vertebra just beneath the aorta. It recieves lymphatic drainage of intestines and lower body structures. Size, shape and location are all highly variable and in some autopsy series only in 50 % of cases CC was identified. During the laparoscopic paraaortic lymphadenectomy, inadvertant injury of otherwise unidentified CC could lead to refractorry chlous ascites.
Methodology: video presentation of two cases
Results: Cases: The first case is a 51 year old woman with grade III endometrioid adenocarcinoma of uterus who was subjected to laparoscopic staging (laparoscopic hysterectomy + BSO + pelvic and paraaortic lymphadenectomy). Intraoperatively in paraaortic lymphadenectomy an incidental injury to cisterna chyli occurred which was sealed and repaired immediately. The second case is a woman with stage IIB clear cell cervical cancer who had been laparoscopically staged (pelvic and paraaortic lymphadenectomy). In this case cisterna chyli located just under the renal vein could be recognized and preserved. Having identified the cisterna chyli laparoscopic paraaortic lymphadeenctomy was completed without any complications.
Conclusion: Conclusion: Anatomical variations and malformations of lymphatic system could be encountered in paraaortic region similar to vascular malformations. During the laparoscopic paraaortic lymphadenectomy presence of Cisterna chyli below the renal vein should always be kept in mind and this structure should be preserved. Any iatrogenic injury of cisterna chyli could lead to refractory chlylous ascites
Disclosure: Nothing to disclose

Minimally-invasive radical hysterectomy for early-stage cervical cancer: a no-touch, no-spill approach

J.P. Beirne[1,2,3], N. Chetty[4]
[1]Queensland Centre for Gynaecological Cancer, Brisbane, QLD, Australia, [2]Centre for Cancer Research and Cell Biology, Queens University, [3]Northern Ireland Gynaecological Cancer Centre, Belfast Health and Social Care Trust, Belfast, United Kingdom, [4]Mater Misericordiae Hospital, Queensland Centre for Gynaecological Cancer, Brisbane, QLD, Australia

Introduction / Background: Several high profile studies, particularly Laparoscopic Approach to Cervical Cancer (LACC) trial, have reported MIRH was associated with worse prognosis than its open counterpart for the management of early-stage cervical cancer. Additional studies are needed to better understand the mechanisms explaining the worse survival associated with minimally-invasive surgery. However, it has been postulated that use of a uterine manipulator and an open colpotomy are responsible. We hypothesise that a no touch, no spill approach to MIRH will reverse this finding. This video presentation outlines a novel surgical technique that ensures cervical tumour does not encounter the surgical field during Minimally-Invasive Radical Hysterectomy (MIRH).
Methodology: A case of a 38 year old female with FIGO IB1 Squamous Cell Carcinoma of the Cervix. Pre-operative staging MRI revealed a tumour size of 2cm. A type III, nerve-sparing radical hysterectomy, bilateral salpingoophorectomy and pelvic node dissection (PND) was performed. A minimally-invasive approach was employed with assistance of a suprapubic Alexis GelPort© (Applied Medical). No uterine manipulator was used. An Endoloop© (Ethicon) suture to the adnexae aided manipulation. Routine PND was completed bilaterally. Routine ureteric tunnel dissection and bladder reflection was performed. Development of the recto-vaginal space and vaginal cuff was completed

with hand-assistance. Infundibulopelvic and uterosacral ligaments were transected as standard. The GelPort was opened to allow cross-clamping of the vagina and excision of the specimen. Vaginal vault and all port sites were closed as standard.
Results: The use of the Alexis GelPort© system allowed hand-assisted uterine manipulation, vaginal cuff development and an enclosed colpotomy technique.
Conclusion: This novel approach to MIRH reduces the risk of intra-operative tumour spillage. Further refinement of the technique is in progress. However, further investigation of oncologic outcomes in larger prospective studies would be necessary to confirm our hypothesis.
Disclosure: Nothing to disclose

Surgical video presentation: surgical tecnique in retroperitoneal paraaortic lymphadenectomy
N. Teixeira, C. Soler Moreno, M.P. Español Lloret, R. Luna Guiborg, R. Rovira Negre
Gynaecology and Obstetrics, Hospital de la Santa Creu i Sant Pau de Barcelona, Barcelona, Spain

Introduction / Background: Cervical cancer is the 4th most common malignancy in women. It is essential to stage the disease. International Guidelines recommend para-aortic lymphadenectomy as staging procedure in patients diagnosed as Locally Advanced Cervical Cancer (defined as Bulky tumor > 4 cm and/or parametrial involvement) and determining level of radiotherapy.
We aim to describe surgical approach and technical landmarks in retroperitoneal laparoscopic para-aortic lymphadenectomy.
Methodology: We present a case report of a 51-year-old woman presenting 12-month history of lumbar pain and irregular menses. Physical examination revealed a 4cm cervical tumour with bilateral parametrial involvement. Biopsy informed squamous carcinoma. MRI and PET-CT described a 30x36x47mm tumor with bilateral parametrial involvement and bilateral infracentimetric hyper-metabolic iliac lymph nodes.
Results: The patient was classified as FIGO stage IIIC1r (T2bN1M0). After discussion in multidisciplinary committee, retroperitoneal lymphadenectomy was indicated.
Surgical strategy consisted in creating retroperitoneal space, identifying left ureter and gonadal vessels and remaining adherent to the peritoneum anteriorly. Left common iliac artery, aorta and inferior mesenteric artery (IMA) were identified. Left ovarian vein was followed until left renal vein. Lymphadenectomy started at the level of left iliac bifurcation, respecting the ureter, IMA and left sympathetic chain, continuing along the aorta. Upper limit of the lymphadenectomy was the left renal vein. Infra- and supra-mesenteric left para-aortic nodes were dissected from the level of the iliac bifurcation until IMA and from IMA to left renal vein, respectively. Pre-caval and para-caval lymph nodes were removed. In visual screening of pelvis an enlarged external right iliac node was identified and removed.
Postoperative course was uneventful. The patient was discharged on the 2nd-day. Histopathological analysis revealed 52 para-aortic and one iliac lymph node free of malignancy.
Conclusion: Retroperitoneal laparoscopic para-aortic lymphadenectomy is a safe reproducible technique in referral centres.
Disclosure: Nothing to disclose

Sentinel node byopsy in cervical cancer
O. Rosato[1], J.M. Mariconde[2]
[1]Gynecology Oncology, Uiniversidad Nacional de Cordoba, [2]Gynecology Oncology, IMGO, Córdoba, Argentina

Introduction / Background: Sentinel node biopsy is a mini-invasive technique used in the staging of cervical and endometrial carcinoma.
Methodology: The laparoscopic approach allows a better visualization, thus achieving punctual dissections. Sentinel node detection methods include vital dyes such as patent blue and fluorescent dyes such as indigocyanine green (ICG).
Patent blue is a low cost and high availability method.
The use of ICG requires specific and high-cost equipment. This marker has shown a higher rate of detection and bilaterality than blue and TC99 in early stages of cervical and endometrial cancer.
Results: In the present video, the use of both methods of detection in each patient with carcinoma of the cervix and initial endometrium is shown.
Conclusion: We perform rutinelly the blue dye detection, eventhoug ICG is a better method. The reason are cost effective results.
Disclosure: Nothing to disclose

Surgical video: laparoscopic complete resection of bulky precaval nodes by extraperitoneal approach in a patient with advanced endometrial cancer
I. Jaunarena, R. Ruiz, M. Gorostidi, P. Cobas, D. Del Valle, A. Lekuona
Gynecologic Oncology, Donostia Hospital, San Sebastian, Spain

Introduction / Background: 10-15% of endometrial cancers are advanced but are responsible for more than half of the recurrences and deaths.The benefit in survival of cytoreduction surgery imitating advanced ovarian cancer is suggested and complete resection and lymphadenectomy up to the left renal vein is mandatory (Havrilesky et al. 2015). In stages IIIC the size of the lymph nodes technically conditions its possibility of resection and this challenging surgical effort often is performed by laparotomy.
We suggest benefits and feasibility of conventional laparoscopic approach due to its high definition and technical accuracy and this is the aim of this surgical video which describes the entire removal of precaval bulky nodes by conventional laparoscopic extraperitoneal approach during standard aortic lymphadenectomy.
Methodology: A 49-year old woman was referred for incidental endometrial cancer after hysterectomy; 3.5x2.5 cm sized endometrioid carcinoma invading more than 50% of the myometrium with ILV positive and suspicious evidence of metastasis in pelvic and paraaortic lymph nodes in TC scan.Thus, following ESGO guidelines, surgical staging and debulking was planned through minimal invasive technique.
Results: After extraperitoneal paraaortic space exposure, enlarged pelvic, left aortic and precaval lymph nodes were confirmed. A primary complete laparoscopic lymphadenectomy was performed up to the left renal vein using an harmonic sealing device. Right ureter was firmly attached to infra mesenteric precaval 2-3cm bulky nodes. Ureter was released, and bulky nodes resection was accomplished by careful rome dissection.
The surgery also comprised other transperitoneal procedures, which are not included in this video (Pelvic lymphadenectomy and ooforectomy). The surgery was carried out in Donostia Hospital (Spain) by a senior oncological surgeon and a fellow. The final biopsy confirmed metastasis in pelvic and paraortic nodes (FIGO IIIC2) and extensive LVI.
Conclusion: Laparoscopic complete resection of bulky precaval nodes in advanced endometrial cancer is feasible and provides desirable accuracy in trained surgeons hands.
Disclosure: Nothing to disclose

Tips of laparoscopic para-aortic lymphadenectomy for patients with endometrial cancer
N. Takahashi, M. Sugiyama, A. Mochizuki, Y. Kasamatsu, N. Kado, M. Abe, M. Takekuma, Y. Hirashima
Shizuoka Cancer Center, Nagaizumicho, Japan

Introduction / Background: Laparoscopic para-aortic lymphadenectomy (L-PALA) for patients with endometrial cancer has been performed in some hospital (lees than 30) in Japan. To perform the surgery safely, it is important to avoid injuries against organs such as ureter, inferior mesenteric artery, duodenum, left renal vein.
Methodology: The indication of L-PALA in our hospital was endometrioid carcinoma (EM) of grade 3 and serous, clear cell carcinoma, carcinosarcoma or more than half myometrial invasion from an intraoperative frozen section diagnosis and a tumor clinically confined to the uterus (clinical stage I) based on a pre-operative evaluation.
Lymphatic tissues were resected from the anterior, lateral, and medial aspects of the aorta and vena cava to the level of the renal veins after total hysterectomy (TH), bilateral salpingo-oophorectomy (BSO) and pelvic lymphadenectomy (PLA).
There are three tips to avoid injuries in operating field of L-PALA.
Tip1: Dissection bilateral ureter the cranial level of aorta during performing PLA.
Tip2: Confirmation of fellow's vein in front of vena cava.
Tip3: Lifting up duodenum sufficiently with wide instrument.
We show these tips with this video.
Results: We have performed the surgery for 11 cases since 2018. The post-operative stage of them was 1A: 8, 1B: 2, 3C2: 1. Histopathologically, they had EM G1:1, EM G2:3, EM G3:1, and others:6. The median age was 60 y.o. (52-70), Body mass index (BMI) 22 (18-27), operative time 443min. (393-501), blood loss 75ml (0-421).
The median number of resected pelvic and para-aorta lymph nodes was 58 (34-86) and 31 (13-53). There was no blood transfusion, organ injuries, surgical site infection, pelvic peritonitis, and ileus.
Conclusion: The rate of peri- and post-operative complications was very low.
Our laparoscopic surgery with para-aortic lymphadenectomy for early-stage endometrial cancer is safe and minimally invasive in our opinion.
Disclosure: Nothing to disclose

Robotic resection of pelvic recurrence of endometrial cancer. Fighting the odds in minimally invasive surgery era
H. Fornalik[1,2], N. Fornalik[1]
[1]*Surgical Oncology, Goshen Center for Cancer Care, Goshen,* [2]*Obstetrics and Gynecology, Indiana University, Indianapolis, IN, United States*

Introduction / Background: Isolated pelvic recurrences of endometrial cancer are associated with only 40% complete response to salvage radiation therapy. We discuss treatment options for bulky pelvic recurrences of endometrial cancer. We demonstrate our robotic surgical technique for resection of tumor from the pelvic sidewall without compromise of vascular structures. We discuss potential advantages of minimally invasive approach to resection of the tumor wrapping around iliac vessels.
Methodology: Surgical video and literature discussion. Dissection technique is demonstrated. Adjustments of surgical technique to peculiarities of surrounding anatomy are discussed. Presentation is enhanced with diagrams.
Results: Successful and uneventful robotic resection of 8 cm tumor with recovery typical for minimally invasive approach.
Conclusion: Combination of surgery and radiation may offer better chances for local tumor control. Robotic surgery may allow for hemostatic, organ sparing, anatomical resection of recurrent pelvic

tumors. Minimally invasive approach may facilitate faster recovery resulting in earlier initiation and better tolerance of adjuvant treatment.
Disclosure: Nothing to disclose

Laparoscopic management of major vascular injury during paraaortic lymphadenectomy
H.A. Tuncer
Gynecologic Oncology, Akdeniz University, Antalya, Turkey

Introduction / Background: Laparoscopy is increasingly being used for comprehensive surgical staging in women with endometrial cancer. However if there is a major vascular complication during lymphadenectomy, vascular bleeding is associated with significantly high rate of perioperative mortality and conversion to laparotomy is necessary for control of bleeding in many cases.
Methodology: A 68-year-old gravida 7, para 5 woman with body mass index of 41 was admitted to our clinic for postmenopausal bleeding. The result of an endometrial biopsy was endometrioid adenocarcinoma with grade 3. Laparoscopic hysterectomy, bilateral salpingoooferectomy, infracolic omentectomy, pelvic and paraaortic lymphadenectomy was performed.
Results: In this video, we report an immediate repair of an incompletely lacerated inferior vena cava during laparoscopic paraaortic lymphadenectomy in a patient with endometrial cancer. After injury, lacerated edges of inferior vena cava were held by two graspers and approximated wit 5/0 polypropylene suture.Beside this, we aim to present following errors during management of vascular bleeding i; improper use of irrigator aspirator system during control of minor vascular bleeding by electrothermal bipolar tissue sealing system ii; how improper intraabdominal cleaning of telescope leads to waste of time and massive bleeding iii; how careless suppression of an irrigator aspirator instrument above the level of cauterized edge of inferior vena cava leads to laceration of inferior vena cava.
Conclusion: Laparoscopic surgery of endometrial cancer requiring lymphadenectomy should only be managed by experienced and skillful laparoscopic surgeons with extensive knowledge about vascular surgery.

[Suturing inferior vena cava injury]

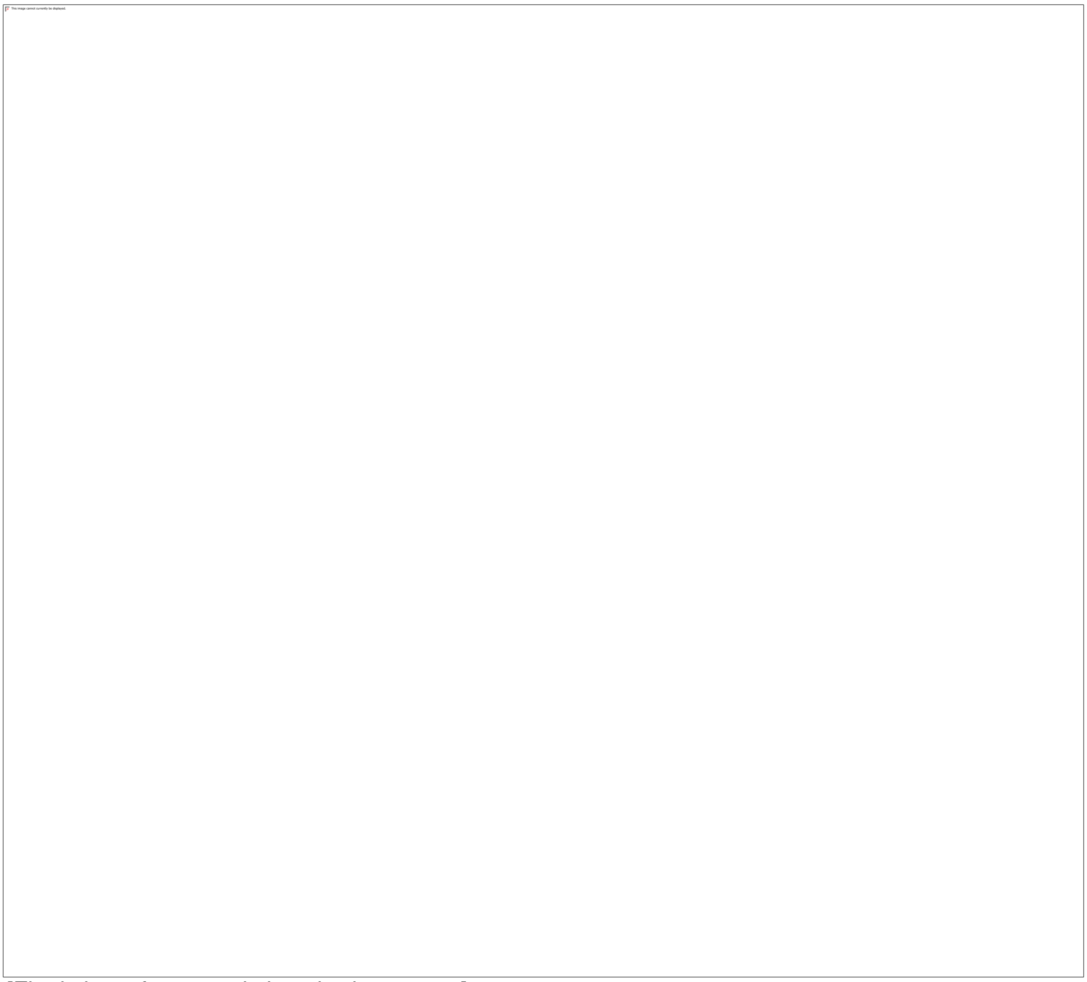

[Final view of paraaortic lymphadenectomy]

Disclosure: Nothing to disclose

Laterally extended endopelvic resection with iliac vessels resection and preoperative femorofemoral crossover bypass grafts after aorta uni-iliac endovascular graft in recurrent endometrial cancer extended to and fixed to the right pelvic side wall

J. Molero Vílchez[1,2], E. Martínez Lamela[3,4], S. Sanchez Coll[5], F. Ruiz Grande[5]
[1]Oncologic Gynecology, Hospital Ntra. Sra. del Rosario, Madrid, [2]Oncologic Gynecology, Clinica TocoGyn, Alcalá de Henares, [3]Oncologic Gynecology, Hospital Ntra Sra del Rosario, [4]Obstetrics and Gynecology, Hospital Infanta Leonor, [5]Vascular Surgery, Hospital Ntra Sra del Rosario, Madrid, Spain

Introduction / Background: To present in video format the most relevant performed procedures, step by step.
Methodology: Current case was analysed in a restrospective analysis of 19 patients underwent PE for recurrent and bulky gynecologic cancer during last ten years. Pelvic exenteration is an ultraradical surgery and it is imperative a preoperative assessment to evaluate the indication for surgery.
A 60-year-old woman operated on 2014 by laparotomy presented endometrioid adenocarcinoma, IIIc1

FIGO stage (5/19 involved pelvic nodes, 0/45 paraa-ortic nodes). Adjuvant treatment was indicated with chemotherapy, concomitant with external radiation therapy and brachytherapy, with complete response. Two years later, in a CT control, a mass of 3,5 cm appears in the right pelvic side and another mass of the 1,3 cm in right kidney (renal cell carcinoma G1). Chemotherapy and radiation therapy directed at the area was performed.
Results: In RMI control, six months later, the tumor had grown (size 5 cm) and extended to and fixed to the right pelvic side wall. PET-CT was applied, and it excluded extrapelvic disease so the patient was proposed to LEER in the right pelvic side and right nephrectomy. Preoperative femorofemoral crossover bypass grafts after aorta uni-iliac endovascular graft and anticoagulant therapy was performed by vascular surgeons. Three weeks later, LEER was performed. Even a correct proper performance, presence of severe arterial and venous thrombosis during postoperative period was reported with critical ischaemia of the right lower limb with distal necrosis. It was proposed the amputation of his right leg from mid-thigh level. The patient, admitted to Intensive Care Unit, had a shock, multiorgan failure, and death.
Conclusion: LEER is a potential salvage treatment in selected patients with recurrent gynecologic malignancies, including those with pelvic side wall disease, traditionally not considered for surgical therapy. LEER could be the last possible treatment, but cancer morbility begins in operating room.
Disclosure: Nothing to disclose

Inframesenteric retrocaval bulky lymph node excision
I. Selcuk, Z. Firat Cuylan, C. Kose, B. Ozdal, H. Yalcin
Gynecologic Oncology, Zekai Tahir Burak Women's Health Training and Research Hospital, Faculty of Medicine, University of Health Sciences, Ankara, Turkey

Introduction / Background: During excision of bulky lymph nodes, there is an increased risk of operative vessel injury.
Methodology: 59 years old woman with a preoperative endometrial biopsy result of Grade 3 endometrioid endometrial cancer admitted to the Gynecologic Oncology clinic with the abdominal computed tomography result which indicates bulky lymph nodes at the paraaortic retrocaval region.
Results: Entering the retroperitoneal paraaortic area by cutting the peritoneum with an incision beginning below the cecum and extending along the base of small bowel mesentery up to the level of ligament of Treitz (suspensory muscle of duodenum).
Small bowels are kept outside the surgical field; right lateral traction of ascending colon and right ureter, cranial traction of duodenum and left lateral traction of mesentery of sigmoid colon.
At the level of L3-L4 vertebra; inferior vena cava is isolated and retracted over the bulky lymph node. Tape traction of inferior vena cava from the superior and inferior part of bulky lymph node facilitates the surgery and provides a hemostatic control.
A cleavage is developed on the right border of inferior vena cava and the bulky lymph node is excised from the retrocaval and inter aortocaval space.
Conclusion: Meticulous dissection with proper surgical and anatomic landmarks is extremely important in resection of bulky lymph nodes especially if they are located on the retrocaval or retroaortic region.
Disclosure: Nothing to disclose

Sentinel lymph node biopsy in endometrial cancer: dual injection, dual tracer. Could this increase the detection rate of para-aortic sentinel lymph nodes?

A. Torrent[1], J. Amengual[1], C. Sampol[2], J. Rioja[1], O. Córdoba[3]
[1]Gynaecological Oncology, [2]Nuclear Medicine, [3]Head of Department, Hospital Universitari Son Espases, Palma, Spain

Introduction / Background: Sentinel lymph node (SLN) mapping has recently been introduced into many Gynaecological Oncology Consensus Recommendations in an effort to secure information concerning lymph node status for treatment planning and in order to have information about prognosis, yet minimise collateral damage if complete lymphadenectomy can be avoided.
The aim of this video is to present ara-aortic and pelvic SLN detection using a **Dual Tracer:** radiolabeled colloid Technetium 99 (Tc99) and Indocyanine green (ICG), and **Dual Injection** (cervical and fundal).
Methodology: In this video we present one of our case-series (61 year-old woman diagnosed with initial stage IAG3 endometrial adenocarcinoma). The day before the intervention a dual Tc 99 injection was performed:
1) **cervical** injection: 2 ml at 3 o-clock and another 2 ml at 9 o'clock at only 0.5cm intracervical depth;
2) Intramiometrial **fundal** injection: 4 ml (1 cm deep), under ultrasonographic vision. Intraoperative dual injection: first, the fundal ICG injection (2ml at 1 cm depth) was performed under laparoscopic vision; and then the cervical injection
(1 ml at 3 o'clock and another 1 ml at 9 o´clock (0.5 cm deep)
Results: In this case detection is seen in 2 para-aortic SLN and 2 bilateral pelvic SLN nodes. The most interesting thing about this video is to observe that ICG uptake correlates perfectly with the sound capture of the laparoscopic nuclear probe.
Conclusion: This video shows para-aortic and pelvic dual uptake and the concordance using two tracers. A dual tracer could help in the ICG learning curve and ensure that the right SLN is removed. Dual injection could increase para-aortic SLN detection using a simple, safe, reproducible technique.

This image cannot currently be displayed.

[Figure 1]

[Figure 2]

Disclosure: Nothing to disclose

How to develop and appropriate extraperitoneal space before a laparoscopic para-aortic lymphadenectomy

I. Zapardiel, F. Boria, M. Cabanes, A. Hernandez
Gynecologic Oncology Unit, La Paz University Hospital, Madrid, Spain

Introduction / Background: The development of the extraperitoneal space uses to be the most challenging step to perform an extraperitoneal para-aortic lymphadenectomy. Few papers describe the technique, but none explain step by step how to develop this virtual space effectively. The success on developing it will influence the rest of the surgical procedure.
Methodology: In the video a step by step technique is carefully explained, adding tips a tricks on haw to avoid peritoneal rupture of development of wrong spaces that may difficult the dissection. Additionally graphs and pictures have been added to the video to illustrate the technique.
Results: The crucial steps include: diagnostic laparoscopy to assess spread of the disease, development of the extraperitoneal layer under direct vision, first trocar placement and insulation of the extrapneumoperitoneum, accessory tracers placement and dissection of the para-aortic space.
Conclusion: A systematic technique can improve the success rate of exztraperitoneal para-aortic lymphadenectomy.
Disclosure: Nothing to disclose

Laparoscopic Lymphadenectomy (Pelvic and Para - aortic) step by step technique and follow up data

D. Zygouris[1], G. Anastasiou[2], N. Chalvatzas[1], A. Gkoutzioulis[1], A. Kavallaris[1,2]
[1]Department of Gynecologic Oncology, St Luke's Hospital, Thessaloniki, Greece, [2]Department of Gynecologic Gynecology, Mother and Child Medical Centre, Nikosia, Cyprus

Introduction / Background: The aim of this study is to illustrate the effectiveness and feasibility of a standardized surgical procedure. We present our technique for laparoscopic pelvic and para - aortic lymphadenectomy step by step and our follow up data.
Methodology: Our study includes cancer treated patients between January 2011 and January 2019. 140 women underwent laparoscopic pelvic lymphadenectomy for primary gynaecologic pelvic malignancy, of which 58 patients were diagnosed having cervical cancer and 82 patients were diagnosed having endometrial cancer, all of them were retrospectively analysed.
Moreover we performed Laparoscopic Para - aortic Lymphadenectomy in 20 cases of patients with cervical cancer and 14 cases of patients with endometrial cancer.
Results: The median number of yielded pelvic lymph nodes was 26 (range 13 - 58) and the median number of para - aortic lymph nodes was 16 (range 10-29). Pelvic right and left sided lymphadenectomy mean time was 27 minutes and 33 minutes respectively. Para - aortic lymphadenectomy mean time was 45 minutes.The number of the yielded pelvic lymph nodes and the duration of pelvic lymphadenectomy were independent from the body mass index on the other side the duration of the paraaortic lymphadenectomy was dependent of the BMI of the patient. The overall intraoperative complications rate was 4%. No Laparoconversion was necessary. No major postoperative complications were encountered during the hospital stay for 3.8 days (range 2-7).
Conclusion: Laparoscopic pelvic and para -aortic lymphadenectomy is a save and possible procedure. An adequate number of lymph nodes can be removed in an adequate time. The complication rate is low and can be minimized by standardization of the procedure.
Disclosure: Nothing to disclose

How to get an excellent anatomical landmarks exposure in trasperitoneal paraaortic lymph node dissection. Systematic lymph node dissection in five steps

V. García Pineda, I. Zapardiel Gutiérrez, J. Siegrist Ridruejo, M.D. Diestro Tejeda, A. Hernández Gutiérrez
La Paz University Hospital, Madrid, Spain

Introduction / Background: The aim of this surgical video is to demonstrate step by step how to do a systematic transperitoneal paraaortic lymph node dissection in 5 steps.
Methodology: In order to be useful for the surgeon we are going to explain tips and tricks to achieve a good exposure of anatomical landmarks using determined laparoscopics devices as well as how to avoid surgical risks. Finally we take into account different techniques and laparoscopic sealers to avoid symptomatic lymphoceles and chylous ascites.
Results: The exposition of systematic surgical technique.
Conclusion: For physician is very important to get an excellent exposition of anatomical landmarks in order to be comfortable even in obese patients.
Disclosure: Nothing to disclose

Sentinel node identification with the Sentimag/Sienna+ system

T. Panoskaltsis[1], J. Kontis[2], A. Zouridis[1], P. Theodorou[1], F. Anyfantaki[1], A. Nonni[3], N. Vlahos[1]
[1]Medical School | Gynaecological Oncology Unit | 2nd Academic Department, Aretaieion, [2]Academic Department of General Surgery, Aretaieion Hospital, [3]1st Department of Histopathology, Medical School, National & Kapodistrian University of Athens, Athens, Greece

Introduction / **Background:** The identifcation of sentinel node in the surgical management of endometrial cancer has become a standard procedure in many countries worldwide. However, different media and techniques have been used with various sensitivity and specificity rates. We present a method, which has not been extensively studied so far.
Methodology: We have started a pilot feasibility study on the use of Sentimag/Sienna+ system. With a handheld non-radioactive detector we sought to magnetically mark and locate sentinel nodes in patients operated for endometrial cancer. A solution of superparamagnetic iron oxide particles (60nm) was injected at 3 and 9 o'clock of the uterine cervix and the probe aimed to detect the magnetic signature generated by these particles in the pelvic lymph nodes. So far, the method has been evaluated mostly in breast cancer patients and there is limited experience with prostate cancer and only one report on 12 endometrial cancer patients.
Results: So far, we have evaluated the method in two patients with a grade2 endometrioid adenocarcinoma , where Magnetic Resonance Imaging (MRI) showed <50% myometrial invasion, and one patient with a grade3 endometrioid adenocarcinoma, with >50% myometrial invasion on MRI. In none of these cases, enlarged lymph nodes were identified. The tracer injection occurred just before the operation in two and one hour before in another case. Sentinel nodes were easily identified in all cases, except for one pelvic side in one patient. Visibly, pigmented nodes were observed with an earlier injection. Identification was easy with standard histopathology.
Conclusion: We believe that the technique deserves further evaluation as it appears to easily identify sentinel nodes, with no major costs, in endometrial cancer patients. A laparoscopic probe would help to establish its role in the majority of cases performed with endoscopic surgery.

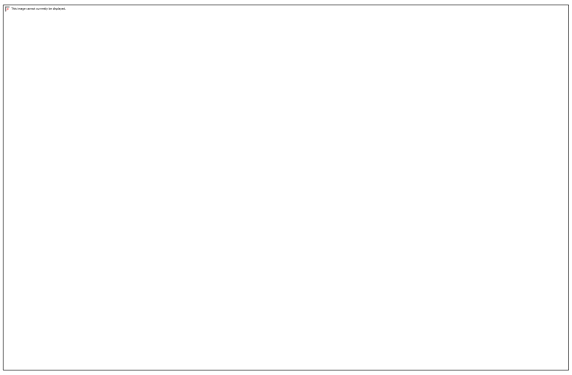

[H+E Lymph node with brownish pigmentation within the histiocytes (X200)]

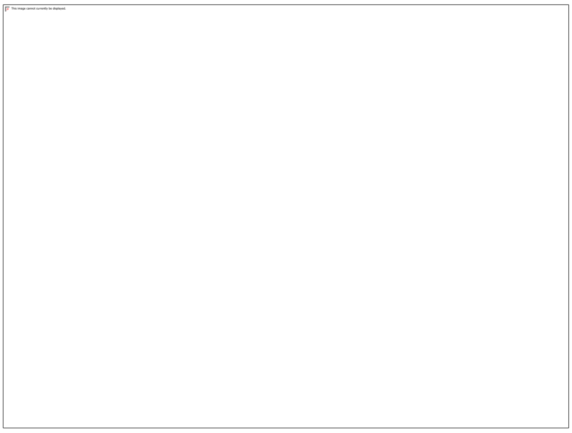

[Histochemistry, the granules are Pearl's positive (X200)]

Disclosure: Nothing to disclose

Identification of upper and lower lymphatic pathways during mapping of sentinel pelvic lymph nodes in a patient with endometrial cancer

I. Biliatis, V. Mitsopoulos
Gynaecological Oncology, Poole Hospital NHS Trust, Poole, United Kingdom

Introduction / Background: it is well known that the lymphatic drainage of the uterus is mainly performed through 2 distinct pathways. The first is called upper pathway and drains the lymph from the parametrium and through the external iliac artery, while the second is called lower pathway and uses a system through the internal iliac artery. These 2 pathways most of the times are not connected. The lower pathway usually drains at the presacral lympatic basin.
Methodology: With ICG injection at the cervix and using the Xi Robotic system we managed to clearly identify these 2 distinctive pathways
Results: We believe that this is important as identifying a pre-sacral lymph node through the identification of the lower lymphatic pathway could potentially improve the sensitivity of the sentinel node technique in patients with endometrial cancer
Conclusion: Identification of both lymphatic pathways using ICG and Robotic technology is feasible and might improve the sensitivity and detection rate of sentinel node in endometrial cancer patients
Disclosure: Nothing to disclose

Successful laparoscopic drainage of symptomatic lymphocele araising after laparoscopic paraaortic lymphadenectomy

N.U. Dogan[1], S. Dogan[2], M. Erol[1], A. Uren[1]
[1]Gynecology, Akdeniz University, [2]Antalya Resarch and Training Hospital, Antalya, Turkey

Introduction / Background: Asymptomatic lymphocele formation after lymphadenectomy is quite common but in some symptomatic cases surgical intervention is essential to relieve the symptoms.

Methodology: Video presentation of case
Results: 51 year old woman with postmenopausal bleeding was subjected to laparoscopic staging (laparoscopic hysterectomy + BSO + pelvic and paraaortic lymphadenectomy) who had grade III endometrioid adenocarcinoma of uterus in her endometrial biopsy. Intraoperatively in paraaortic lymphadenectomy an incidental injury to cisterna chyli occurred which was sealed and repaired immediately. She was discharged in post-op day 2. Three day after her discharge she referred to our clinic once again with complaints of right lower back-pain. Ultrasonographic evaluation revealed grade 2-3 hydroureteronephrosis (HUN) . In CT scan a 5 cm lymphocele located in paraaortic area was observed which causes ureteral obstruction at that level. Because of the sympyoms and HUN re-laparoscopy was planned. Laparoscopically lymphocele located laterally in right low-paraaortic region was detected and aspirated. A window was created in the lymphocel sac in order to prevent recureence. In postoperative period the patient developed ileus which was handled conservatively. In P.O. day 4 she was discharged without any symptoms. One week after re-laparoscopy complete resolution of HUN was confirmed and she was referred to brachytherapy unit.
Conclusion: Laparoscopy is a useful and safe tool in the management of symptomatic lymphocele. With minimally invasive techniques recovery is fast and there is no delay in the planned adjuvant therapy.
Disclosure: Nothing to disclose

New device for ICG cervical injection and different NIR/ICG light modality to detect Sentinel Lymph Node Endometrial Cancer
M. Peiretti, G. Candotti, V. Mais
Department of Obstetrics and Gynecology, University of Cagliari, Cagliari, Italy

Introduction / Background: The aim of the present video is to show the feasibility and the utility of a new device used for the ICG cervical injection and to evaluate the accuracy rate in the detection of sentinel lymph node with different NIR/ICG light modality.
Methodology: The video demonstrates the feasibility of ICG cervical injection using 85 mm - 21 G needle generally adopted for local anesthesia. Furthermore we showed the new color system OPAL1 NIR/ICG visualization (Karl Stortz), i.e. blue or green or different Furthermore, S-Technologies can be used under white light or in combination with the NIR/ICG mode.
Results: 20 procedures were performed using both systems. None technical problems were observed. The overall detection rate was 97%; unilateral and bilateral mapping of SLNs were achieved in 20% and 77% of subjects respectively.
Conclusion: The use of this device will provide an augmentation of the precision especially in narrow vagina. The new color system NIR/ICG provides more intense fluorescence or better background lighting with a high SLN detection rate.
Disclosure: Nothing to disclose

The importance of an appropriate identification of structures in ginecologyc oncology
J. Utrilla Layna, J. Garcia, M. Albi
Hospital Fundacion Jimenez Diaz, Universidad Autonoma de Madrid, Madrid, Spain

Introduction / Background: Realización de video de cirugia laparoscopica en que se muestra la importancia de una adecuada identificación de estructuras en la linfadenectomia aortica y pelvica.
Methodology: Video production of laparoscopic surgery showing the importance of an adequate

identification of structures in aortic and pelvic lymphadenectomy. In this systematic way it is possible to save unexpected structures or anatomical malformations.
Results: This video shows retroaortic renal vein, double obturator nerve, left ovarian vein towards vena cava, high obturator vein, crown of death vein, lateral inferior mesenteric artery...
Conclusion: the importance of reviewing images available preoperatively is essential, as well as performing systematic surgery step by step
Disclosure: Nothing to disclose

Double-barreled wet colostomy after total pelvic exenteration (video)

T. Marina Martín, V. Lago Leal, P. Padilla Iserte, L. Matute Tobías, M. Gurrea Soteras, S. Domingo del Pozo
Gynecologic Oncology, University Hospital La Fe, Valencia, Spain

Introduction / Background: Double-barreled wet colostomy (DBWC) is an alternative method to classical reservoirs using two stomas for patients who underwent total pelvic exenteration for advanced pelvic malignancies. It was firstly described and performed by MF Carter in the late eighties.
Methodology: The procedure starts with the mobilization of the left colon beyond the splenic flexure. A sufficient length of the colon is necessary to perform a well everted loop colostomy at a previously marked site. Afterwards, a double-barreled colostomy is created in which the distal part of the colon is sectioned and sutured at about 15 cm to the stoma. Both ureters are mobilized towards the colostomy and the healthier ends of them are re-implanted separately along the anti-mesenteric tenia of the distal segment. Subsequently, a pig tail ureteral stent is placed bilaterally which runs from a curl in the renal pelvic to the skin through the stoma. It must be removed 4-6 weeks after surgery. After the intervention, the urine reservoir, which has been created distal to the stoma, empties out freely without fecal contact.
Results: Surgically the double-barreled wet colostomy has many advantages when compared with other incontinent cutaneous diversion: is technically easier, requires only one stoma, avoids the use of small bowel and thus reduces surgical time and length of stay in hospital.
Furthermore, its avoids the use of over-irradiated small bowel for urinary diversion in patients who underwent previous radio-chemotherapy. The use of small bowel in this patients may predispose to leaks.
Several studies have demonstrated there is no statistically significant difference in the rate of postoperative complications. Furthermore, with an adequate stoma care, peristomal dermatitis and other local skin complications can be avoided or easily controlled.
Conclusion: Finally, one of the most important advantages is the improvement in the quality of life and self-perception of these women.
Disclosure: Nothing to disclose

Control of operating field during difficult robotic vascular dissections

N. Fornalik[1], H. Fornalik[1,2]
[1]*Surgical Oncology, Goshen Center for Cancer Care, Goshen,* [2]*Obstetrics and Gynecology, Indiana University, Indianapolis, IN, United States*

Introduction / Background: Bulky tumors surrounding large pelvic vessels typically create vast collateral circulation and pose risk for direct injury. Such cases present significant surgical challenges, especially in elderly and medically compromised patients. Debulking technique via laparotomy is

thought to be quicker in controlling catastrophic bleeding but may be less precise in dissection and isolation of large vessels and collaterals. Typically, minimally invasive approach (MIS) to difficult pelvic surgery is associated with lower blood loss (EBL). Rate of locoregional control with use of radiation corelates with size of residual tumor.
Methodology: Surgical video. We demonstrate our technique for preparation for difficult dissections in cases at high-risk of large blood loss and vessels injuries. Robotic adaptation of vascular technique including unobscured exposure, identification of landmarks, identification and control of collateral blood supply, proximal and distal control of large vessels are discussed and demonstrated. Repair of 8 mm external iliac vein injury with utilization of vessel loops and 5-0 Prolene suture is demonstrated.
Results: Resection of 8 cm recurrent endometrial cancer wrapping around iliac vessels with minimal blood loss.
Conclusion: Robotic approach may facilitate precise dissection of pelvic vasculature. Control of blood supply to the dissection field and control of large vessels, before engaging in tumor debulking, may result in minimal blood loss. Large vessels injuries can be repaired quickly if prior proximal and distal control is assured. Robotic debulking surgeries require meticulous preparation, time and experienced staff. MIS approach may result in faster recovery and easier tolerance of adjuvant treatment.
Disclosure: Nothing to disclose

Management of vascular complications in laparoscopic lymphadenectomy: four brief case presentations
S. Dogan[1], N.U. Doğan[2], E. Uysal[3], A. Uren[3]
[1]Antalya Resarch and Training Hospital, [2]Gynecology, [3]Akdeniz Üniversitesi, Antalya, Turkey

Introduction / Background: Vascular malformations and variations are commonly recognized in pelvic and paraaortic area which makes retroperitoneal surgery challenging. Laparoscopic approach is gaining popularity in every aspect of gynecologic oncology and vascular complications could rarely occur in these regions. Laparoscopic surgeon should be aware of and prepared for vascular complications which carry a high morbidity rate.
Methodology: Video presentation of four cases
Results: In this video in the first two cases the management of bleeding due to injury to branhces of hypogastric artery (vesical and obturatory) during laparoscopic pelvic lymphadenectomy is presented. The third case is about management of plexus bleeding just above the anteriror vertebral ligament in interaorticocaval region during laparoscopic paraaortic lymphadenectomy. Fourth case presents an aberrant ovarian artery located just under IMA which was recognized and preserved without any complications in paraaaortic lymphadenectomy.
Conclusion: There is a small but significant risk of vascular injury and related morbidities in laparoscopic pelvic and paraaortic lymphadenectomy due to vascular anomalies ,variations, suboptimal visiulisation of surgical field and patient parameters such as obesity and previous surgeries. Laparoscopic surgeon should be familiar with vascular complications and should be able to manage the complicaitons to mimimize the morbidity related to vasuclar injury.
Disclosure: Nothing to disclose

Laparoscopic debulking surgery in pacient with nodal involvement
J. Utrilla Layna, J. Garcia, M. Albi
Hospital Universitario Fundación Jiménez Díaz, Madrid, Spain

Introduction / Background: Importance of adequate hemostasis and precision in vascular and nervous ganglionar surgery.
Methodology: We present a case of a patient with adenopathies larger than 2 cm on the superior mesenteric artery, interaortocaval and adhered to the obturator nerve, which are completely resected.
Results: The entire disease has been removed by practicing debulking surgery.
Conclusion: Knowledge of the anatomy to perform better surgery.
Disclosure: Nothing to disclose

Fascia closure technique using BERCI© instrument for preventing trocar-site hernia after robot-assisted gynecologic surgery

E.J. Lee, H.S. Kim, G.W. Yim, J.W. Kim, N.H. Park
Obstetrics and Gynecology, Seoul National University Hospital, Seoul, Republic of Korea

Introduction / Background: We introduce fascia closure technique using BERCI© instrument after robot-assisted gynecologic surgery, which is necessary for management and prevention of trocar site hernia. We design a step-by-step explanation of the procedure using a video. Totally 4 trocars were inserted, including 12mm sized umbilical trocar, bilateral 8mm sized robot arm trocar, and 5mm sized extra-trocar for assistant.
Methodology: This video presents a systemic approach to fascia closure clearly devided into 5 steps after the main operation: (1) Right lateral trocar site fascia was closed first with BERCI© Instrument. Then, Umbilicus, left lateral, assistant port were remained. (2) Vicryl suture was inserted with BERCI© Instrument. (3) Hanging Vicryl suture on the left lateral trocar site, camera was moved from umbilical trocar to left lateral trocar site without trocar. (4) Watching the umbilical trocar site via camera, fascia was closed in the same way with BERCI© Instrument. (5) Finally, removing the camera, Vicryl suture was pulled out and tied on left lateral trocar site. All trocar site fascia was closed. At every step, it is necessary that kelly was inserted in each fascia to check Vicryl suture both sides of fascia.
Results: The procedure was performed without any complications. The patient had an uneventful postoperative course and was discharged home after routine postoperative care. Since then, she showed no evidence of trocar site hernia and doing well.
Conclusion: Fascia closure technique using BERCI© Instrument after robot-assisted gynecologic surgery is very safe and certain way because fascia was sutured watching with laparoscopic camera. The patient presented by us is an example showing that BERCI© Instrument can be used in the suture of trocar site. The key point in this procedure is to completely closure of fascia of trocar insertion site compared with conventional technique which is blind closure technique, so certain way to prevent trocar site hernia.
Disclosure: Nothing to disclose

Secondary debulking surgery for isolated para-aortic nodal recurrence in ovarian cancer requiring partial resection of the inferior vena cava

K. Kato
Department of Obstetrics and Gynecology, Tokyo Medical University, Tokyo, Japan

Introduction / Background: Complete secondary debulking surgery for recurrent ovarian cancer presenting as isolated lymph node metastases has an acceptable morbidity, and is associated with a favorable long-term survival outcome. However, the resection of enlarged lymph nodes can result in severe intraoperative complications, including the laceration of major retroperitoneal vessels and

increased blood loss. Here, we report the details of a surgical technique for the resection of metastatic para-aortic lymph nodes with involvement of the inferior vena cava (IVC) requiring partial resection of the IVC.
Methodology: A 55-year-old woman was initially managed for FIGO stage IIIA1 (pT1c pN1; metastasis to right pelvic lymph node, M0) ovarian cancer at another hospital. At a follow-up, computed tomography examination revealed para-aortic adenopathy in the lateroaortic and retrocaval regions. No other lesions were observed. The patient subsequently underwent a secondary debulking surgery. During surgery, enlarged lymph nodes that had involved the IVC were identified. Therefore, an *en bloc* resection of the lymph nodes with the involved portion of the IVC was performed with the clamping of the IVC.
Results: In this case, a partial resection of the IVC was performed to enable a complete cytoreduction without encroaching on the tumor planes. Consequently, the suprarenal and retrorenal para-aortic lymph nodes were safely removed and tumor involvement of the lymph nodes was histopathologically confirmed in all the patients. Serious intra or early postoperative complications were not noted.
Conclusion: An *en bloc* resection of the para-aortic lymph nodes with the involved segment of the IVC can be adopted for the management of metastatic para-aortic lymph nodes in selected patients. In this Surgical Video Session, I am presenting the nuts and bolts of this surgical technique.
Disclosure: Nothing to disclose

Follow the yellow brick road - Supra-renal para-aortic lymphadenectomy for metastatic ovarian cancer

M.T. Daia, D. Chandrasekaran, A. Lawrence, E. Brockbank, R. Manchanda, A. Jeyarajah, S. Phadnis
Gynaecological Oncology, Barts Health NHS Trust, London, United Kingdom

Introduction / Background: Complete cytoreduction along with platinum sensitivity are the most important prognostic factors in advanced ovarian cancer. Cytoreductive surgery should include excision of enlarged lymph nodes in order to achieve R0 resection. Although pelvic, inguinal and infra-renal para-aortic lymphadenectomy (PALND) is commonplace in gynae-oncological surgery, there is limited literature on supra-renal PALND. We aim to present in this video the advantages, challenges and superior exposure of the upper abdominal aorta when approaching from the left side, as presented in this video.
Methodology: Real-time video of PALND from above the renal vessels to the bifurcation of the aorta was recorded. This was performed at the time of primary cytoreductive surgery for clinical stage 3c ovarian cancer with involvement of para-aortic nodes above the renal vessels. Descending colon was mobilised up to the splenic flexure in order to expose the left kidney and the aorta. The duodenum was reflected off the left kidney. From the pelvis, the left ovarian vessels were identified and lateralizing along with the ureter, enabling identification and exposure of the renal vessels with ease. Enbloc excision of enlarged nodal tissue undertaken with harmonic focus.
Results: This video demonstrates the left side surgical approach which enables superior exposure of high Para aortic nodes. Following the ovarian vessels enables easy and safe identification and exposure of renal vessels.
Conclusion: Supra renal PALND should not be an obstacle in achieving complete cytoreduction in women with ovarian cancer and the left side approach enables better exposure compared to the right-sided classic approach. Moreover, this video adds valuable teaching resource to the current limited reports of this surgical approach in ovarian cancer.
Disclosure: Nothing to disclose

Laparoscopic tertiary cytoreduction in ovarian cancer relapse

I. Zapardiel, V. García-Pineda, A. Hernandez
Gynecologic Oncology Unit, La Paz University Hospital, Madrid, Spain

Introduction / Background: The use of laparoscopy for the treatment of ovarian cancer recurrences is very controversial. However, based on the published evidence, it can be very useful for tumor debulking in selected cases. We present a case of hepatic second relapse completely resected by laparoscopy.
Methodology: A 37-year-old patient presented her second relapse of granulosa cell tumor in segment VI of liver, cranially to Morrison pouch (Figure 1). The area was previously operated 18 months before due to carcinomatosis (first relapse) at that point. AT the time of surgery it measured 25mm in largest diameter. CT scan and PET-CT did not show any other intra-abdominal or extra-abdominal disease. A laparoscopic resection was planned to perform the tertiary cytorreduction.
Results: One umbilical 10mm-trocar was placed at the umbilicus, and 3 accessory 5mm-trocars as shown in Figure 2. Full inspection of abdominal cavity and assessment of no other disease was performed. Careful dissection of Morrison pouch was carried out that was completely covered in adhesions. After that, we followed the right renal surface to reach the most cranial aspect of the segment VI of liver. The relapse was identified and removed without incidences.
Conclusion: Laparoscopic approach to perform tertiary cytoreduction is feasible in very selected cases, and it should be considered when single relapse is evidenced and it is accesible to laparoscopy.

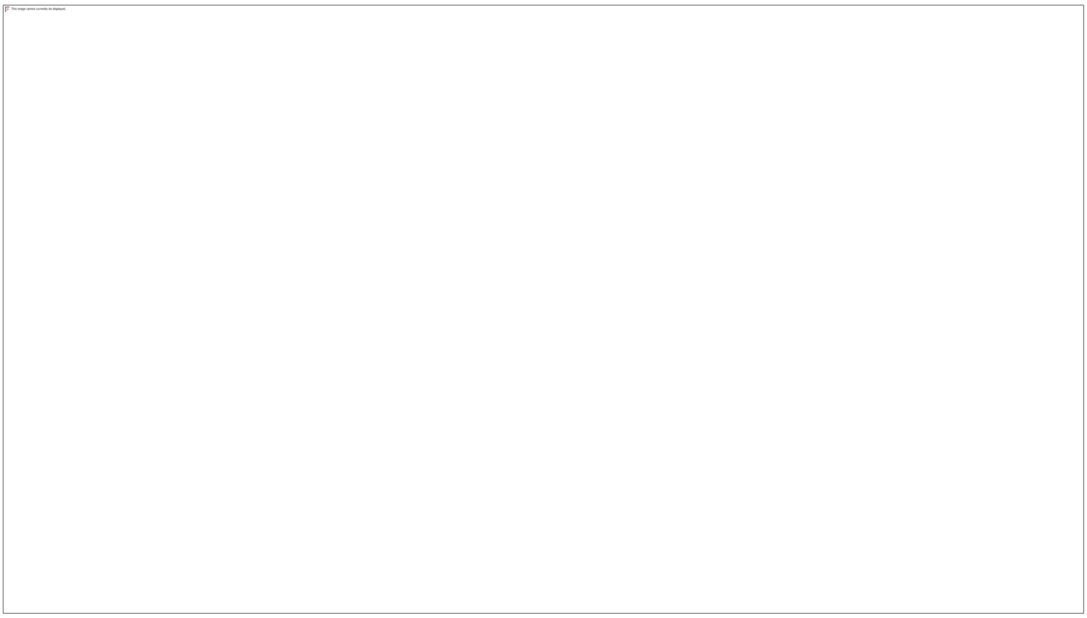

[Figure 1: Hepatic second relapse in granulosa cell tumor]

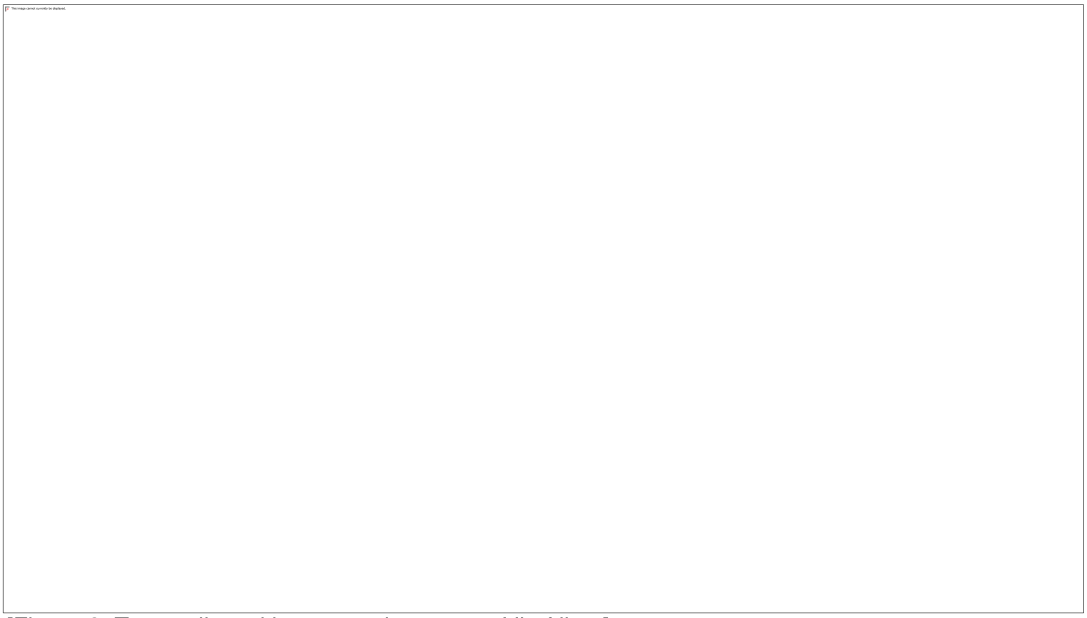

[Figure 2: Trocar disposition to reach segment VI of liver]

Disclosure: Nothing to disclose

Total pelvic peritonectomy for ovarian cancer with extensive peritoneal carcinomatosis in pelvic cavity

L. Xiang, S. Ye, H. Yang
Gynecologic Oncology, Fudan University Shanghai Cancer Center, Shanghai, China

Introduction / Background: *En bloc* pelvic resection including hysterectomy, bilateral salpingo-oophorectomy and rectosigmoid colon resection and anastomosis is often performed to achieve optimal cytoreduction in ovarian cancer patients with extensive pelvic carcinomatosis. We presented specific surgical details of total pelvic peritonectomy avoiding rectosigmoid colon resection in a well-selected patient.
Methodology: A 41 year-old women with good performance status (ECOG 0) presented with disseminated disease in abdominal and pelvic cavity. The patient underwent primary debulking surgery including total pelvic peritonectomy. The procedure is initiated by accessing the retroperitoneum by incision of the anterior and lateral peritoneum. After retroperitoneum exposure, the round ligament, infundibulo-pelvic ligament, medial umbilical ligament, and umbilical artery are dissected and ligated. The bladder is mobilized caudally and the vesico-vaginal space is exposed after completely dissecting off the prevesical peritoneum. In the next step, the ureter is isolated and mobilized laterally. Then, uterine vessels and parametria are divided and ligated, which is followed by colpotomy to access the recto-vaginal septum. By retracting the total specimen cranially, the Douglas pouch is dissected from the anterior side of rectum. The mesorectum is further divided and dissected in the extraperitoneal space. The specimen is removed *en bloc* with the uterus, adnexa, pelvic peritoneum, and tumor nodules, leaving a tumor-invisible pelvis (Figure 1). The sigmoid is also deperitonized.
Results: Complete removal of the disseminated tumors in two layers of peritoneum (parietal and visceral) was achieved by the method introduced in our video.
Conclusion: Total pelvic peritonectomy is effective for complete cytoreduction in selected ovarian cancer patients with extensive peritoneal carcinomatosis sparing rectosigmoid resection.

[Figure 1]

Disclosure: Nothing to disclose

Total laparoscopic Hudson's procedure, pelvic and para-aortic node dissection, omentectomy with primary re-anastomosis and loop ileostomy for endometrioid adenocarcinoma in association with endometriosis

M. Graham[1], I. Harley[1], A. Armstrong[2], E. Craig[1], C. Wilson[1]
[1]Gynae Oncology, [2]General Surgery, Belfast City Hospital, Belfast, United Kingdom

Introduction / Background: Video presentation of procedure.
Methodology: This video will outline the procedure above.
Results: This patient previously underwent a right ovarian cystectomy for endometrioma with final histopathology confirming an endometrioid adenocarcinoma in association with endometriosis - at least FIGO 1C1. Pre-operative MRI and CT suggested endometriosis/disease in recto-uterine pouch, with no evidence of disease outside the pelvis. Completion surgery with a Hudson's and comprehensive surgical staging was planned as a laparoscopic procedure. On initial inspection Intravenous Indocyanine Green (ICG) was used to facilitate the identification of endometriosis/disease in the pelvis. Following surgical staging, including en-bloc dissection of the uterus, tubes, ovaries and rectosigmoid, the specimen was removed via the vagina with the aid of an Alexis retractor. For re-anastamosis the distal descending colon was delivered through the vagina, the anvil applied, and the anastomosis completed laparoscopically. This anastomosis was then de-functioned via a loop ileostomy. ICG was used to confirm perfusion of the anastomosis.
Conclusion: In this video we will show the steps of this laparoscopic procedure where bulky disease was resected en-bloc laparoscopically.
Disclosure: nil

Complete tumor resection from porta hepatis in a patient with recurrent epithelial ovarian cancer

D. Vatansever[1], S. Misirlioglu[1], B. Giray[2], M. Erkan[3], E. Balik[3], M. Arvas[4], C. Taskiran[1]
[1]Obstetrics and Gynecology, Koc University School of Medicine, [2]Obstetrics and Gynecology, Zeynep Kamil Training and Research Hospital, [3]General Surgery, Koc University School of Medicine, [4]Obstetrics and Gynecology, Cerrahpasa Faculty of Medicine, Istanbul, Turkey

Introduction / Background: Ovarian cancer is the leading cause of death among gynecological malignancies. Even if a complete cytoreduction is achieved at the time of first surgery, about 60-70% of advanced stage patients develop a recurrence. Secondary cytoreductive surgery is associated with improved overall survival in patients with recurrent ovarian cancer. The aim of this video is to present complete tumor resection from porta hepatis in a patient with recurrent epithelial ovarian cancer.
Methodology: A 50 years-old woman referred to our clinic with a diagnosis of recurrent epithelial ovarian cancer. She has undergone a primary maximal debulking surgery two years ago. The magnetic resonance imaging revealed multiple metastasis in liver. Total colectomy, ileostomy, liver metastasectomy, cholecystectomy, splenectomy, peritonectomy, lymphadenectomy, unilateral diaphragmatic stripping, and bilateral ureteroneocystostomy were performed as a part of maximal secondary cytoreduction.
Results: She stayed at the intensive care unit for two day and discharged on post-operative day 6 without any grade 3 or 4 adverse event in post-operative period.
Conclusion: Secondary cytoreductive surgery is associated with improved results in patients with recurrent ovarian cancer, and maximal cytoreduction is necessary in selected cases.
Disclosure: Nothing to disclose

A primary extended cytoreductive surgery for ovarian cancer: total abdominal hysterectomy and bilateral salpingo-oophorectomy, total omentectomy, total peritonectomy, bilateral diaphragm stripping, cholecystectomy, total colectomy, splenectomy, bilateral pelvic-paraaortic lymphadenectomy, dissection of porta hepatis, liver metastasectomy, and bilateral cardiophrenic lymphadenectomy

C. Taskiran[1], D. Vatansever[1], A. Eraslan[1], B. Giray[2], S. Misirlioglu[1], E. Balik[3], T. Kumcular[4], M. Arvas[5]
[1]Obstetrics and Gynecology, Koc University School of Medicine, [2]Obstetrics and Gynecology, Zeynep Kamil Training and Research Hospital, [3]General Surgery, Koc University School of Medicine, [4]Women's Health Center, American Hospital, [5]Obstetrics and Gynecology, Cerrahpasa Faculty of Medicine, Istanbul, Turkey

Introduction / Background: Ovarian cancer is the leading cause of death among gynecological malignancies. Primary cytoreduction for ovarian cancer is associated with significantly improved survival. We aimed to present a primary extended cytoreduction performed in this video.
Methodology: A 37 years-old woman was admitted with abdominal swelling and pelvic pain. Pelvic examination revealed out ascites and bilateral adnexal masses. MRI showed 11-cm right adnexal mass, 7-cm left adnexal mass, omental cakes, disseminated peritoneal implants, liver metastases, and enlarged lymph nodes in the right obturator fossa. Total abdominal hysterectomy, bilateral salpingo-oophorectomy, total omentectomy, total peritonectomy, bilateral diaphragmatic stripping, total colectomy, splenectomy, bilateral pelvic-paraaortic lymphadenectomy, cholecystectomy, dissection of the porta hepatis, liver metastasectomy, and transabdominal cardiophrenic lymph node dissection were performed as a part of maximal primarily cytoreduction.
Results: We did not encounter any grade 3 or 4 adverse event in post-operative period.
Conclusion: Primary cytoreduction for ovarian cancer with no residual disease is a major impact on survival. The management of this condition should be performed with expert multidisciplinary teams in gynecological oncology.
Disclosure: Nothing to disclose

Para-aortic lymphadenectomy step-by-step landmarks, boundaries and avoiding complications
F. Alkhan[1], E. Karabuk[2], M.M. Naki[2], M. Gungor[2], M.F. Kose[2]
[1]Salmaniya Medical Complex Hospital, Manama, Bahrain, [2]Acibadem Mehmet Ali Aydinlar University, Istanbul, Turkey

Introduction / Background: The goal of this video to demonstrate step-by-step paraoartic lymphadenectomy
Methodology: It is important to recognize the landmarks to open the spaces and mobilize the bowel. It is important to open the space laterally until the ureters and superiorly until left renal vein. In addition, the procedure should be performed systemically to accomplish a complete lymphadenectomy
Results: In the video a successful para-aortic lymphadenectomy performed for stage IIIC ovarian cancer
Conclusion: Para-aortic lymphadenectomy needs an expert surgeon with major blood vessels to achieve a successful procedure with minimal or no complications
Disclosure: Nothing to disclose

Resection of enlarged retrocaval lymph nodes in a patient undergoing surgery for recurrent ovarian cancer
C. Liakou, J. Nieto, N. Burbos
Obstetrics and Gynaecology, Norfolk & Norwich University Hopsital, Norwich, United Kingdom

Introduction / Background: To demonstrate the technique of resection of enlarged retrocaval lymph nodes in a patient undergoing cytoreductive surgery for recurrent ovarian cancer.
Methodology: A 70-year-old patient undergoing follow up for previously treated stage IIIA1 serous carcinoma of the ovary was found to have enlarged retrocaval lymph nodes extending from the level of right common iliac vein to the level of the right renal artery. A PET-CT scan showed no evidence of peritoneal or extra-abdominal disease.
Results: The patient underwent a midline laparotomy. Exploration of the abdomen showed no evidence of peritoneal disease. Ascending colon and mesentery of the small bowel was mobilised. The second and third part of the duodenum was mobilised and inferior vena cava (IVC) was exposed. Renal veins were identified and exposed bilaterally. Right renal artery and ureter were identified and mobilised. IVC was mobilised from the anterior surface of the tumour from the level of the right common iliac artery to the level of the right renal artery and the aorta. Lumbar veins were identified and ligated. Tumour was mobilised from the anterior aspect of the vertebral bodies and removed using bipolar diathermy. Complete tumour resection was achieved.
Postoperative recovery was uncomplicated and the patient was discharged home after 5 days. Histological examination of the retrocaval lymph nodes showed metastatic high grade serous carcinoma.
Conclusion: Resection of enlarged retrocaval lymph nodes is feasible. Careful consideration of the relevant surgical anatomy is required and steps should be in place to deal with complications if necessary.
Disclosure: Nothing to disclose

Excision of paraaortic bulky disease in a patient with advanced ovarian cancer after treatment with 3 cycles of neoadjuvant chemotherapy
V. Mitsopoulos, I. Biliatis

Gynaecological Oncology, Poole Hospital NHS Trust, Poole, United Kingdom

Introduction / Background: A 55 year old patient presented with advanced ovarian cancer with disease at the mediastinal lymph nodes, the right diaphragm, the omentum and a 7 cm lymph node block at the paraaortic area. The MDT decided to commence with neoadjuvant chemotherapy taking into consideration the mediastinal lymoh nodes. She had 3 cycles of carbo/taxol. On post chemo CT scan she had complete response at the chest and partial response intraperitoneally. Thus, We decided to offer interval debulking surgery.
Methodology: In this video monopolar diathermy is uded to excise bulky disease from the paraaortic area after mobilising the duodenum
Results: She underwent liver mobilisation and right diaphragmatic excision, en-block resection of the spleen and omentum, excision of disease from the paraaortic area and stripping of pelvic peritoneum
Conclusion: Excision of disease from the paraaortic area after chemotherapy is feasible and should be offered in patients with advanced ovarian cancer in the setting of interval debulking surgery
Disclosure: Nothing to disclose

Video assisted thoracoscopic surgery - VATS in advanced ovarian cancer

A. Conde Adán, S. Alonso Salvador, M. Fernandez Chereguini, C. Yelo Docio, R. Moreno Valsalobre, J. de Santiago Garcia
MD Anderson Cancer Center, Madrid, Spain

Introduction / Background: Complete citorreduction continue to be the aim in ovarian cancer treatment. Pleural disease is in some cases a limitation for a surgical approach, malignant pleural effusion is the most common site of stage IV ovarian cancer but the accuracy rate of pleural cytology is low.
Methodology: We show through 3 cases the impact of VATS in the management of ovarian cancer with suspicious pleural disease
Results: Patient 1, 49 years old, showed in PET TAC a pelvic mass, adenopathies, peritoneal carcinomatosis, pleural effusion and pleural carcinomatosis; an omento biopsy was taken by laparoscopy, aspiration of pleural effusion and biopies were performed by VATS, all of them positive for malignancy, so stage IV ovarian cancer was diagnosed and neoadyuvant chemoterapy started.
Patient 2, 49 years old, showed in PET TAC suspicious right ovary, right pleural effusion, peritoneal carcinomatosis, supra and infradiaphragmatic adenopathies, positive for malignancy pleural fluid citology and inguinal adenopathy, neoadyuvant chemotherapy was started in other centre, histerectomy, doble anexectomy, omentectomy were performed, She came to our center with progressive elevation of CA 125 marker; in diffusion MRI pleural effusion was detected. Diagnostic laparoscopy and VATS showed miliar carcinomatosis in pleura, a biopsy was taken; peritoneal carcinomatosis, a 3 cm nodule in caecum, a 4 cm nodule in diaphfram and multiple adenopathies were resected, complete secondary citorreduction was achieved.
Patient 3, 47 years old, diagnosed with granulosa cell tumor after left anexectomy, 7 years later right ovary tumour and carcinomatosis were seen in TAC, the patient refuse treatment; one year later pleural effusion and multiple milimeter nodules in diaphfram were detected by MRI, negative biopsies for malignancy were obtained by VATS and complete citorreduction was performed.
Conclusion: VATS procedure is feasible and can alter the stage and the therapeutic management in advanced ovarian cancer choosing between a surgical or chemotherapy approach

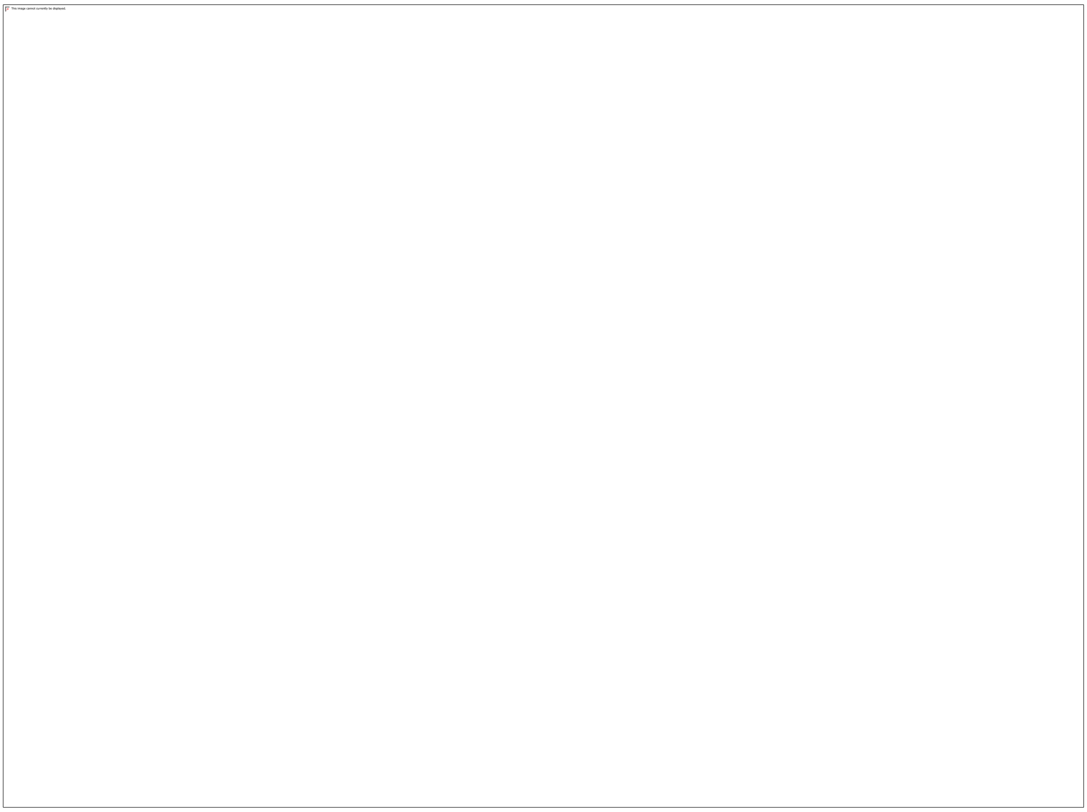

[pleural carcinomatosis]

Disclosure: Nothing to disclose

Ovarian cancer with metastatic inguinal lymphadenopathy: an abdominal approach to inguinal nodes dissection

F. Gaba[1,2], A. Farrag[1], A. Lawrence[1], E. Brockbank[1], A. Jeyarajah[1], R. Manchanda[1,2], S. Phadnis[1]
[1]Barts Health NHS Trust, [2]Barts Cancer Institute, London, United Kingdom

Introduction / Background: Autopsy studies report inguinal metastasis in up to 3% of patients with advanced ovarian cancer. Metastasis occurs via a minor lymphatic drainage pathway running through the round ligament of the uterus toward the inguinal region. Postoperative complications after inguinal surgery performed through an incision crossing over Scarpa´s triangle extending to the anterior superior iliac spine, are common (seroma/lymphocele 6-40%, dehiscence 17-65%, infection 6-20%, lymphoedema 22-80%), debilitating and harbour considerable socioeconomic costs. To reduce surgical morbidity, an alternative approach per abdomen has been proposed in patients with ovarian cancer with metastatic inguinal lymphadenopathy.

Methodology: A fifty-four year old woman underwent primary cytoreductive surgery for histologically proven metastatic high grade serous carcinoma of tubo-ovarian origin. CT/MRI had revealed bilateral inguinal lymphadenopathy with a 16mm right external iliac lymph node (FIGO stage IVB). Surgery involved a midline-laparotomy, total omentectomy, enbloc resection of appendix/recto-sigmoid/uterus/cervix/both tubes and ovaries/pelvic peritoneum and excision of enlarged right external iliac lymph node and enlarged bilateral inguinal nodes. R0 resection achieved.

Using an abdominal approach, Scarpa´s fascia was identified and the nodal bundle dissected from

inferior surface of Scarpa. External oblique fascia identified and nodal tissue dissected, preserving the long saphenous vein. Nodal tissue removed within femoral triangle medial to femoral artery and vein with inguinal ligament superiorly.
Results: Operative time was 12 minutes per side. Drain anterior to rectus sheath was removed on the seventh postoperative day. No postoperative complications were observed. Histology confirmed bilateral tumour infiltration in the inguinal nodes which measured 35 x 15 x 12 mm on the left and 55 x 30 x 20 mm on the right.
Conclusion: An abdominal approach to inguinal node dissection in metastatic ovarian cancer has the potential to reduce surgical morbidity and reduce operating time when compared to the standard approach without compromising complete cytoreduction.
Disclosure: FG, AF, AL, EB, ARJ, SP declare no conflict of interest. RM declares research funding from The Eve Appeal and Cancer Research UK into population testing and from Barts & the London Charity and Rose Trees Trust outside this work, an honorarium for grant review from Israel National Institute for Health Policy Research and honorarium for advisory board membership from Astrazeneca/MSD. RM is supported by an NHS Innovation Accelerator (NIA) Fellowship for population testing.

Ovarian cancer presenting with bulky lymph node situated in paraaortic area with anatomical variations

P. Español Lloret, C. Soler Moreno, R. Luna Guibourg, R. Rovira Negre
Gynecology Oncology, Hospital de la Santa Creu i Sant Pau de Barcelona, Barcelona, Spain

Introduction / Background: To describe surgical approach and strategy in the excision of enlarged paraaortic node by laparoscopy.
Video presentation offers a thorough review of CT images and its correspondence with anatomical findings in surgery, permitting a complete view of retroperitoneal anatomy.
Methodology: Case report of 86-year-old woman presenting 2 month history of abdominal pain which leads to detection by imaging tests of multilocular solid cyst in left ovary and a suspicious node situated above left renal vein without peritoneal carcinomatosis signs.
CT shown two vascular anomalies in the paraaortic area consisting on a retroaortic left renal vein and a polar artery, in between of which, bulky node was described.
After Oncology-Gynaecology Committee evaluation, a laparoscopic approach was performed to undertake adequate staging and complete cytoreduction.
Results: Abdominal cavity exploration was performed confirming enlarged left ovarian and no peritoneal carcinomatosis signs.
An extraperitoneal laparoscopic approach was chosen to get access to the bulky node; permitting more secure access to left paraaortic zone and less bowel interference. After retroperitoneal space creation, establishment of anatomical landmarks was determined as first step. Dissection of anterior aspect of the aorta allowed identification of the anatomical variations described on CT and the enlarged node, which was carefully dissected. Due to anatomopathological study confirmation of ovarian cancer metastasis; hysterectomy, bilateral adnexectomy, omentectomy and systematic paraaortic and pelvic lymphadenectomy was completed.
Postoperative course was uneventful and the patient is currently undergoing chemotherapy treatment. Definitive histological findings confirmed high-grade serous carcinoma of 5cm located in left ovarian and a macrometastasis of 4,5cm in the paraaortic node. Paraaortic and pelvic lymphadenectomy study were 1/10 and 0/18 respectively.
Uterus, right adnexa and omentum were negative for malignancy. FIGO Stage: IIIA.
Conclusion:
- Carefully review of preoperative imaging studies before the surgery is a mandatory step.
- Extraperitoneal laparoscopic approach is a feasible technique to remove paraaortic bulky nodes.

Disclosure: Nothing to disclose

Fertility-sparing surgical technique in clinically early stage epithelial ovarian carcinoma: a video surgical skills

S. Monreal Clua[1], N. Rodriguez Gómez-Hidalgo[2], S. Franco Camps[2], M.A. Pérez Benavente[2], A. Gil Moreno[2]
[1]Gynecology Oncology, Vall d'Hebron University Hospital, [2]Gynecology Oncology, Vall D´hebron University Hospital, Barcelona, Spain

Introduction / Background: Fertility sparing surgery is becoming an important technique in the surgical management of young women with early stage ovarian carcinoma (OC). The aim of this video is to show the surgical skills to reach a minimally invasive approach in giant cyst mass.
Methodology: A description of the minimally invasive fertility sparing surgical technique in a young woman diagnosed with a giant cystic mass.
Results: A nulliparous twenty-six years old female, was incidentally diagnosed with left ovarian mass. The vaginal ultrasound described an unilocular, hypervascular cyst of 280 x140 mm in size with a solid portion, well-delimited.
The Ca 125 tumor marker raised to 42.9 U/mL, HE4 155.1 pmol/L and ROMA index 56 %. The magnetic resonance (MRI) described a 23 x 12 x 30 cm cystic mass with hematic and papillary proyections.
The patient underwent primary conservative left oophorectomy, intra-operatory biopsy was positive for mucinous carcinoma; the omentectomy, bilateral pelvic and para-aortic lymphadenectomy and ascites fluid cytology was complete. The final histopathological conclusion was mucinous carcinoma, grade I, expand type.
The key steps of fertility sparing surgery technique consist in an incision and dissection pfannenstiel type, peritoneal dissection and abdominal cavity aperture, placement of intra-abdominal alexis to create the surgical field, plastic bag fix to the ovarian cyst with surgical glue and two stitches, a control-guide drainage of the cyst, expose the left ovary and fallopian tube out of abdominal cavity with previous cyst close by an endoloop to perform the unilateral salpingo-oophorectomy.
The laparoscopic staging was performed in a second time after closing the abdominal incision.
Conclusion: The laparoscopic equipment and surgical skills play an important role in the fertility sparing surgical in young women with early stage OC.
Disclosure: Nothing to disclose

Pelvic lymphadenectomy: step-by-step surgical education video

I. Selcuk[1], B. Uzuner[2], E. Boduc[2], Y. Baykus[3], B. Akar[4], T. Gungor[5]
[1]Zekai Tahir Burak Women's Health Training and Research Hospital, Faculty of Medicine, University of Health Sciences, Ankara, [2]Anatomy, [3]Obstetrics and Gynecology, Kafkas University Faculty of Medicine, Kars, [4]Obstetrics and Gynecology, Istinye University Faculty of Medicine, Istanbul, [5]Gynecologic Oncology, Zekai Tahir Burak Women's Health Training and Research Hospital, Faculty of Medicine, University of Health Sciences, Ankara, Turkey

Introduction / Background: Pelvic lymphadenectomy is a supplementary part of staging and treatment in gynecologic oncology.
Methodology: This video demonstrates a right side systematic pelvic lymphadenectomy in a cadaveric model with proper anatomic landmarks.
Results: The borders of pelvic lymph nodes are genitofemoral nerve laterally, bifurcation of common iliac artery cranially, deep circumflex iliac vein caudally, obturator nerve inferiorly and obliterated umbilical artery medially
The fibroadipose lymphatic tissue over the external iliac artery is gently elevated and mobilized

medially, a tiny dissection is applied to separate the lymphatic tissue from the fibrous sheath. While dissection is carried out longitudinally over the external iliac artery; at the mid-level a cleavage is opened to clear the lymphatic tissue over the external iliac vein until the level of deep circumflex iliac vein whichis the caudal border. Therefore, internal iliaclymph nodes are also removed over the anterior part of internal iliac artery. Afterwards, lymph node dissection turns around the superior pubic ramus of pubic bone which forms a part of obturator foramen and pubic vein the connection between the external iliac and obturator vein (corona mortis) is identified. By the way, dissection of obturator lymph nodes starts from this point after retraction of external iliac vessels laterally to the psoas muscle and maintaining a medial retraction on the paravesical space (that retracts the obliterated umbilical artery medially). All the lymphatic tissue over the obturator nerve medial to the obliterated umbilical artery is stripped from the attachments and finally the lymphatic tissue is removed.
Conclusion: Learning the proper technique of pelvic lymphadenectomy with anatomical landmarks will improve the surgical skills and confidence.
Disclosure: Nothing to disclose

Paraaortic lymphadenectomy: step by step surgical education video
I. Selcuk[1], M. Oz[1], A. Toyran[1], I. Tatar[2], Y. Engin-Ustun[1], D. Demiryurek[2]
[1]*Gynecologic Oncology, Zekai Tahir Burak Women's Health Training and Research Hospital, Faculty of Medicine, University of Health Sciences,* [2]*Anatomy, Hacettepe University, School of Medicine, Ankara, Turkey*

Introduction / Background: Paraaortic lymphadenectomy is a part of surgical staging in gynecologic oncology practice.
Methodology: This cadaveric educational video shows the technique of paraaortic lymph node dissection in a step by step manner.
Results: Boundaries of paraaortic lymphadenectomy; right-right psoas major muscle, ascending colon, right ureter; left-mesentery of descending colon and sigmoid colon, inferior mesenteric vein; caudal-mid common iliac level, below the aortic bifurcation; cranial-left renal vein; posterior-anterior longitudinal ligament.
Small intestines are packed cranio-laterally and mesentery of sigmoid colon is retracted caudo-laterally. Posterior parietal peritoneum is cut from the level of ileocolic junction to the level of ligament of Treitz. Paracaval space is developed; right ovarian vessels and right ureter is identified.
Horizontal part of duodenum is mobilized and retracted superiorly. Left renal vein is identified.
The areolar tissue between the left common iliac artery and mesentery of sigmoid colon is opened, by the way the left ureter and inferior mesenteric artery is identified.
The paraaortic lymph node dissection begins over the right common iliac artery from the mid-level caudad to cephalad direction. Precaval and preaortic lymph nodes are dissected to the level of left renal vein.
Lateral aortic lymph nodes are dissected from the infra-mesenteric region (low paraaortic), below the level of inferior mesenteric artery. Lateral aortic lymph nodes superior to the level of inferior mesenteric artery (high paraaortic) are identified by sharp and blunt dissection from the mesentery of descending and sigmoid colon and underlying left Gerota's fascia. Lateral aortic lymph nodes are dissected from the supra-mesenteric region, between the inferior mesenteric artery and left renal vein.
Conclusion: Aortic bifurcation, common iliac artery, common iliac vein, abdominal ureter, inferior mesenteric artery, ovarian artery, ovarian vein, left renal vein, lumbar vein, lumbar artery, inferior vena cava and aorta are the basic anatomic landmarks for a proper paraaortic lymphadenectomy.
Disclosure: Nothing to disclose

Laparoscopic para-aortic lymph node dissection

F. Alkhan[1], E. Karabuk[2], M.M. Naki[2], M. Gungor[2], M.F. Kose[2]
[1]Salmaniya Medical Complex Hospital, Manama, Bahrain, [2]Acibadem Mehmet Ali Aydinlar University, Istanbul, Turkey

Introduction / Background: The goal of this video to demonstrate Laparoscopic Para-aortic Lymph Node Dissection
Methodology: It is important to recognize the landmarks to open the spaces and mobilize the bowel. It is important to open the space laterally until the ureters and superiorly until left renal vein. In addition, the procedure should be performed systemically to accomplish a complete lymphadenectomy.
Results: Patient was discharged 2nd post-operative day
Conclusion: Laparoscopic para-aortic lymphadenectomy needs an expert surgeon with laparoscopy to achieve a successful procedure with minimal or no complications
Disclosure: Nothing to disclose

Reconstruction of the vulva and vagina with a lotus flap after resection of cancer of the Bartholin gland

K. Hatch
Gynecology, University of Arizona, Tucson, AZ, United States

Introduction / Background: The majority of flaps used in reconstruction of the vulva are local flaps with a random blood supply and consist of the skin and subcutaneous tissue. They include the V-Y advancement flap, the transposition flap and the rhomboid flap. The lotus flap is a transpositional flap with the blood supply from the perineal branches of the internal pudendal artery. It has an axial blood supply at the base and a random blood supply at the tip. It can be 4 times longer than its width and can be rotated more than 90 degrees. The type of flap chosen is based on the size and location of the defect. The V-Y is best for lateral defects, the rhomboid is best for perineal defects and the lotus can be used for anterior lateral or posterior defects.
Methodology: The video will show the defect in the vulva and lower vagina that needs to be covered. It will show the harvesting of the flap, rotating it under the vulva tunnel, preparing the recipient graft site and sewing the graft into place.
Results: The patient healed primarily. She received 4400 cGy of radiation postop. She did not have stricture or skin breakdown. Photos of the result are shown. She is alive without disease at 2 years.
Conclusion: The lotus flap is an excellent flap for reconstruction of the vulva or the lower and mid vagina.
Disclosure: Nothing to disclose

Conservative management of a clear cell vaginal adenocarcinoma with laparoscopic staging, ovarian transposition, and brachytherapy

A. Protopapas[1], D.-E. Vlachos[1], G. Vlachos[1], M. Sotiropoulou[2], K. Sarris[3], M. Terzi[4], D. Loutradis[1]
[1]First Department of Obstetics and Gynecology, National & Kapodistrian University of Athens, [2]Pathology Department, [3]Radiotherapy Department, 'Alexandra' General Hospital of Athens,

[4]*Pathology Department, 'Hygeia' General Hospital of Athens, Athens, Greece*

Introduction / Background: Clear cell adenocarcinomas (CCA) of the lower genital tract associated or not to previous in utero diethylstilbestrol exposure, are very rare tumors. Such a diagnosis in a young and nulliparous woman poses difficult dilemmas regarding treatment. Conservative management with preservation of fertility may be an option in carefully selected patients offering good results in terms of survival rates, giving a chance to achieve a pregnancy.
Methodology: A 24 years old nulliparous patient presented with irregular vaginal bleeding from a solitary 2cm upper third exophytic vaginal lesion. Histology of colposcopically directed biopsies revealed the presence of a grade 2 CCA. Subsequent hysteroscopic examination showed a normal uterine cavity with open tubal ostia. Endometrial biopsy was negative and the preoperative evaluation was unremarkable for metastatic disease. The patient was managed with laparoscopic staging including systematic pelvic lymphadenectomy plus infracolic omentectomy, and bilateral ovarian transposition with tubal conservation, followed by vaginal brachytherapy.
Results: The procedure was carried out without any intraoperative complications. Ovaries were transposed outside the pelvis, and tubes were suspended separately with non-absorbable sutures for future reconstruction. Twenty pelvic lymph nodes were excised. All specimens were negative for metastatic disease. The patient made an uneventful postoperative recovery, and concluded brachytherapy with complete disappearance of the vaginal lesion. Six years after the initial treatment the patient is without evidence of disease.
Conclusion: Vaginal CCA remains a rare malignancy of the urogenital tract, occurring mainly in young women with in utero exposure to diethylstilbestrol or history of vaginal endometriosis. Our experience agrees with the international literature, that conservative treatment of early stage disease is oncologically safe and the fertility of these young patients can be spared.
Disclosure: Nothing to disclose

Inguinofemoral lymphadenectomy for the treatment of vulvar cancer
J. Laufer, S. Scasso, C. Olmedo
Department of Gynecology, Universidad de la Republica, Montevideo, Uruguay

Introduction / Background: Management of vulvar cancer requires a tailored approach with the objective of reducing morbidity associated with radical procedures. The compromise of regional lymph nodes remains the most important prognostic factor. The introduction of sentinel lymph node biopsy instead of total lymphadenectomy has drastically reduced the morbidity associated with the surgical procedure without compromising oncological safety. However, for tumours ≥ 4 cm and/or in case of multifocal invasive disease, inguinofemoral lymphadenectomy (IFL) by separate incisions is recommended. Superficial and deep femoral nodes should be removed. The main objective of this video is to review the surgical anatomical landmarks of the inguinofemoral region as well as useful tips of the procedure performed in a university hospital.
Methodology: We hereby demonstrate the surgical technique of IFL while identifying the major anatomical landmarks in live tissue as well as in cadaveric dissection models. Surgical steps for the dissection of superficial and deep femoral nodes are shown. Special emphasis is placed on guiding the surgeon using still photographs and videos of live tissue and cadaveric dissection to optimize the understanding of the anatomy involved in this procedure. Key components of the surgery, including identification of Scarpa triangle components, vascular structures like the great saphenous vein, and recognition of the fossa ovalis, are shown.
Results: The material shown in this video allows the identification of the anatomical landmarks and the step by step systematization of the IFL procedure.
Conclusion: Through the use of still photographs and video, we hereby demonstrate the surgical technique as well as useful tips of IFL for the treatment of vulvar cancer.
Disclosure: This procedure was performed at Pereira Rossell Hospital, a tertiary referral and educational center in Montevideo, Uruguay. The patient gave consent for the use of the surgery footage for educational purposes.

Inguinofemoral lymphadenectomy technique

D. Altin, S. Taskin, F. Ortac
Department of Gynecology and Obstetrics, Ankara University School of Medicine, Ankara, Turkey

Introduction / Background: For all vulvar cancer patients with tumour diameter greater than 2 cm and more than 1 mm of stromal invasion, inguinofemoral lymphadenectomy is indicated. In this video we present inguinofemoral lymphadenectomy technique with a patient with vulvar squamous cell cancer.
Methodology: A linear incision made 1 cm above and parallel to the groin crease starting 3 cm distal and medial to the anterior superior iliac spine and ending below the superficial inguinal ring. The borders of the operation are sartorius muscle laterally, adductor longus muscle medially and inguinal ligament superiorly. The inguinofemoral lymph nodes are divided into superficial and deep. The superficial lymph nodes are above the fascia lata and the deep nodes lye below the fascia lata and within the femoral sheath. To remove the superficial nodes, incision is carried through the subcutaneous tissues to the Camper's fascia. The dissection should not be too close to the skin flaps as flap necrosis may develop. The fascia is incised and grasped with forceps for traction, and the fatty tissue between it and the fascia lata is removed. Usually the dissection is carried from lateral to medial and superior to inferior. Care should be taken to identify and preserve the great saphenous vein, which is superficial to the fascia of the medial thigh. To access the deep nodes, an incision is made through the cribriform fascia. Lymph nodes are always situated medial to the femoral vein in the opening of fossa ovalis. The proximal deep femoral node (node of Cloquet) is removed from the femoral triangle. The incision is closed in layers.
Results: not applicable
Conclusion: not applicable
Disclosure: Nothing to disclose

Inguinofemoral lymphadenectomy and femoral dissection: cadaveric educational video

I. Selcuk[1], H. Akdemir Aktas[2], B. Ersak[3], I. Tatar[2], M. Sargon[2], T. Gungor[3]
[1]Gynecologic Oncology, Zekai Tahir Burak Women's Health Training and Research Hospital, Faculty of Medicine, University of Health Sciences, [2]Anatomy, Hacettepe University, [3]Zekai Tahir Burak Women's Health Training and Research Hospital, Faculty of Medicine, University of Health Sciences, Ankara, Turkey

Introduction / Background: Vulvar cancer is rarely seen
Methodology: This step by step cadaveric educational video was recorded on the left groin.
Results: Between the anterior superior iliac spine and pubic tubercle the skin incision is performed, 8cm in length, and 2cm below and parallel to the inguinal ligament. After the incision, dissection deepens to identify the Camper's fascia over the Scarpa's fascia. Identification and preservation of the Camper's fascia is critical in securing the skin flap and preventing skin necrosis. To dissect the fibrofatty tissue containing the superficial inguinal lymph nodes between the Camper's fascia and fascia lata; firstly, the fibrofatty tissue is mobilized under the Scarpa's fascia and dissected 2cm cephalad to the inguinal ligament where the aponeurosis of external iliac muscle is seen. Afterwards, excision of the lymphatic and fibrofatty tissue is performed from lateral (superficial circumflex iliac vein) to medial (superficial external pudendal vein) and superior to inferior (inferomedial end of inguinal ligament where it intersects with the adductor longus muscle). The fossa ovalis is encountered after

resection of superficial inguinal lymph nodes. Medial to the falciform edge of fossa ovalis, dissection of the cribriform fascia will lead to the deep inguinal lymph nodes which are located medial to the femoral vein. Great saphenous vein enters the femoral vein from the opening of fossa ovalis, so a careful dissection is needed while removing the cribriform fascia. Lymphadenectomy is performed towards the apex of femoral triangle, and there is no need to dissect the femoral sheath to excise the deep inguinal nodes completely. Finally, Cloquet's lymph node which is the most superior of deep inguinal nodes could be resected from the femoral canal under the level of inguinal ligament.
Conclusion: Proper anatomical knowledge of inguinofemoral anatomy, the superficial fascia structures and deep vessel structures facilitates the inguinofemoral lymphadenectomy procedure.
Disclosure: Nothing to disclose

Sentinel node in vulvar cancer: anatomical landmarks and technique
J.M. Mariconde[1], E. Castellani[2]
[1]Gynecology Oncology, University of Cordoba, [2]Cuniversity of Cordoba, Córdoba, Argentina

Introduction / Background: To describe and to analyze the technique steps and anatomical landmarks for the sentinel node biopsy in vulvar cancer.
Methodology: Surgery remains the cornerstone in the treatment of vulvar cancer. Radical vulvectomy with inguinofemoral lymphadenectomy has been replaced by radical local excision with sentinel node procedure for early disease.
Results: We perform the sentinel node biopsy using a double method: Tc99 and blue dye. However, the fluorescence substances is a feasible technique. First of all, the injection of Tc 99 is performed the day before the surgery, and during the surgery we inject the blue dye. A 3 cm insicion is performed at the Douglas arcade. A delicate dissection is performed trying to localize tha blue collector or a hot-spot area. At the time that the node is located, it is taken out and correlated with the gamma probe.
Conclusion: Patients with unifocal disease, tumor size less than 4 cm, and clinically negative groins are candidates to sentinel node procedure. The learning curve of this technique can be improved with a correct knowledge of surgical anatomy of Scarpa´s triangle. The goal of this technique is the less index of morbidity, specially due to lymphedema
Disclosure: Nothing to disclose

Patient Seminar

Awarness campaign (TV, cinema, social media) about gynaecological cancer
C. Marjollet, B. Massicault, G. Hamon, IMAGYN
IMAGYN, Paris, France

Introduction / Background: In France in 2017*, 15 300 women are affected by gynaecological cancers.
There are a lot of media campaigns about breast cancer. What about gynaecological cancers ?
The risks of gynaecological cancers are not known. The level of information is too low to promote reduction of diagnosed cases.
How to break the taboo and encourage women to have a gynaecological follow-up once a year to avoid the risks of gynaecological cancers?
That is why we did this campaign to raise awareness of the existence of gynaecological cancers and improve the diagnosis and to detect the disease earlier.
*National Cancer Institute (INCa) 2017
Methodology: To break the taboo, spread a message with a humorous and connivent tone.

To promote identification, carry the message by women well known to the general public.
To talk to all women: 2 TV spots with 40 women from different backgrounds (for example Chantal Thomas, designer, Helene Darroze, chef and famous french actresses and TV presentors).
In the first, «the small words», they had volunteered to named the female sex with their own words.
In the second, «once a year», they invited the audience to have a gynaecological consultation every year.
You can find the spots here: https://www.imagyn.org/qui-somme-nous/on-parle-de-nous/
Results: For the first time, we spoke about gynaecological cancers in french television
453 spots on national television channels in May 2018 70 press articles Television, Radios, Magazines, National Press, Regional Press
More than 2 million views on social networks
Conclusion: To continue this awareness campaign, we decided to do a poster campaign for the waiting room of gynecologists associated with a postcard showing the symptoms of gynaecological cancers and the importance of the vaccination against HPV for girls and boys at twelve.
Disclosure: Nothing to disclose

Deciding together

A. van der Kolk, K. Hulscher, L. Snoep
Stichting Olijf, Utrecht, The Netherlands

Introduction / Background: Nowadays much is said about the patients voice when it comes to deciding on the best suitable treatment: What are the options from a medical perspective? What counts from the patients perspective? Must one seek every possible treatment or are other factors just as important?
Methodology: Stichting Olijf cannot support every patient. Our patient advocates are limited in their possibilities due to health issues, energy required for family and work. Despite this we found ways to voice the patients perspective, that reach us through our media channels (internet, fb, email, phone, presentations).
Results: In this triptych (poster-series of 3) we would like to illustrate our practice and its results of patient participation fron three perspectives:

1. Participation in medical research: from the phase of application and during the project - our patient advocates are involved in every step.
2. Contributing to the revision of Dutch gynaecological oncological guidelines and adjusting the patientinformation to the patients' needs.
3. Involvement in ppecial projects such as:

- Participation of our patient advocates in the development of two 'choice tools' for advanced ovarian cancer including quality of life issues: 1) the issue of 'chemo or debulking operation first' in a combination therapy; 2) the aftercare regime: how to choose for a periodical or a health-related aftercare regime.
- Hearing at the Dutch Health Council: Olijf pleaded for HPV vaccination for all children, not only girls.
- Testimonials of patient advocates at the Dutch Care Institute on their experiences on the HIPEC procedure and the risks they encounter when having to travel to other countries in order to get their desired HIPEC therapy.

Conclusion: The patients voice is more and more included and we are heard in the development of research designs, (medical) guidelines and patient information.
Disclosure: Nothing to disclose

Evaluation of doctor-patient communication in a gynaecological oncology outpatient setting

K. Mulligan[1,2], S. Egan[1], D. Brennan[1,2], Y. O'Meara[1,3], S. O'Toole[1,4], Irish Society of Gynaecological Oncology Public and Patient Involvement Group Members
[1]Irish Society of Gynaecological Oncology Public and Patient Involvement Group, [2]University College Dublin, [3]Our Lady's Hospice and Care Services, Harolds Cross, [4]Obstetrics and Gynaecology/Histopathology, Trinity College Dublin, Dublin, Ireland

Introduction / Background: The Irish Society of Gynaecologic Oncology Public and Patient Involvement Group (ISGOPPI) was established "to achieve a true partnership between public/patients and the society which will promote the quantity and quality of health research and ultimately improve health services and outcomes." At the groups first workshop, a dominant theme emerged from the patient group centred around doctor-patient communication, in particular the doctor's body language. The aim of this study was to evaluate doctor patient communication within gynaecological oncology services in Ireland.
Methodology: An anonymous and confidential 20 question survey was designed by the patient advocacy group ISGOPPI and distributed in three gynaecological oncology outpatient clinics in tertiary referral centres.
Results: A total of 84 patients completed the survey. Doctors surveyed ranged from senior house officer to consultant level. Overall women were very satisfied with the communication they had received from their doctor. 85% felt that they the doctor listened to them and took their opinion into account. 84% of patients felt that the doctor's body language was appropriate throughout the consultation. One of the main issues for women surveyed was waiting times. 33% of women waited over an hour to see their doctor and over 30% of women did not receive contact details of the clinical nurse specialist.
Conclusion: Overall our study shows that patients in gynae-oncology clinics are satisfied with the communication from their doctors. The main issues for patients were waiting times and contact details for follow up questions.
Disclosure: Nothing to disclose

Patient experiences on long term side effects of gynecological cancer treatment in Finland

M. Sorsa, E.-M. Strömsholm, E. Toivonen, M. Tran Minh
Association of Cancer Patients in Finland, Helsinki, Finland

Introduction / Background: The Association of Cancer Patients in Finland coordinates a network for gynecological cancer patients with approximately 500 members. The network's members brought forward the need to raise awareness about gynecological cancers and the long term side effects of treatments. Patients expressed frustration over not much media coverage on gynecological cancers and concern over the lack of attention to long term side effects within the health care system.
Methodology: Volunteers from the network and staff at the association agreed a campaign targeting the media and the general public would be planned to create more discussion around mentioned issues.
The volunteers and staff arranged meetings to plan and discuss the aims of the campaign. Once aims were agreed upon, tasks were defined and divided between volunteers and staff according to wishes and skill sets.
The association launched a survey on experiences in long term side effects among gynecological cancer patients which was distributed in the closed Facebook group for the network. The survey received 133 responses.
Materials were written by the campaign team and distributed to target groups in order to gain visibility

for the topic. A campaign launch event was also organised in order to get the media interested.
Results: As the campaign is still taking place (1st-31st May 2019), it is not possible to give results. However, it can be stated that there is already one published article on a popular news website (reach ~1 million readers) and an opinion piece was published in the main newspaper (reach ~800k). More coverage is expected within the coming weeks.
Conclusion: The campaign was born from patients' initiative and contributions. It appears it has been received well and will serve as a great starting point for generating discussion around the issue of long term side effects.
Disclosure: In 2019 Association of Cancer Patients in Finland has received financial support from AstraZeneca in total 2900 euros. Association of Cancer Patients in Finland received 85 % of its funding from the Funding Centre for Social Welfare and Health Organisations (STEA), which operates under the Ministry for Social Affairs and Health.

Ricerca In-Acto: a multicentre, perspective survey about knowledge, attitudes and experience on randomized controlled trials among women with ovarian cancer

N. Cerana[1], P. Mosconi[2], Acto - Alleanza Contro il Tumore Ovarico
[1]Acto Alleanza Contro il Tumore Ovarico, [2]Laboratorio di Ricerca Coinvolgimento Cittadini in Sanità, Istituto di Ricerche Farmacologiche Mario Negri, Milano, Italy

Introduction / Background: Although there are several initiatives by research groups, regulatory authorities, scientific associations to engage citizens/patients on clinical research, obstacles still prevent people from participating. Among the main discouraging aspects, the incomplete understanding of the concepts related to the proposed study, and the scarce, sometimes confused, explanations received.
This study aims to investigate knowledge and understanding, trust in clinical research, obstacles and motivation in participation, satisfaction with the information received and involvement of ovarian cancer patients on clinical trials.
Methodology: Ricerca In-Acto is a prospective, multicentre study using self-administered questionnaires. After the signature of inform consent, the first questionnaire is delivered by the physician of the participating centre to eligible ovarian cancer women during the first follow-up visit or during the first therapy session. A follow-up questionnaire is mailed after 3 months.
The questionnaire has been developed starting from a research of literature, and through a validation process that involved researchers, clinicians and ACTO regional groups (Bari, Campania Lombardia, Piemonte, Roma).
The study will be carried out in 39 centres of the MaNGO (Mario Negri Gynecologic Oncology) and MITO (Multicenter Italians Trials in Ovarian Cancer) groups, and coordinated by Mario Negri Institute. A recruitment period of 6 months is expected allowing to involve about 400-500 women.
Results: During 2018 the protocol and the questionnaires have been finalised, as well the submission to the competent ethics committees. Currently eight centres started the recruitment after approval from ethics committee.
Conclusion: A large number of clinical centres participate in this research showing the great interested in this issue. Results of this study will contribute to figure out knowledge, and attitude of women with a history of ovarian cancer in relation to clinical studies suggesting initiatives aimed at improving the culture of clinical studies and compliance as well new ways of communication between doctor and patient.
Disclosure: This research is funded by ACTO Onlus - Alleanza contro il tumore ovarico Both authors Nicoletta Cerana and Paola Mosconi have no conflict of interest to declare.

Role of effective communication and international advocacy to improve cancer management in Georgia

E. Sanikidze[1,2]
[1]David Tvildiani Medical University, [2]Georgian Patients' Union, Tbilisi, Georgia

Introduction / Background: Cancer management is one of the priorities of the country´s health system. However, National Strategy for Cancer Control(NSCC) is not approved yet, many issues are not solved and funded from public insurance.
Methodology: Official data, results of the questionnaires(149) and modeling were elaborated via correlative and regressive analysis method, using key indicators system.
Results: Objectives were: 1. Assessment of the official data, including informational-educational campaign(IEC)/behavior-changing(BCC) campaign; 2. Interview of the target groups 3. Assessment of HE-modeling 4. Assessment of the "triangle partnership"(TP=Public+private+civil -PPC) model for international advocacy (IA).
1. Cancer cases among women still high 56-57%(total-234.3/100,000/2017), only 39.5% is diagnosed on the I-II stage. 24-27 % of all cases is registered in reproductive age. Although screening is implemented, IEC is permanent, all communication channels are used, 7270 USD is spent, beneficiary involvement in the screening still low-12%. No social discussion and relevant IEC/BCC was done in frames of the new HPV vaccination program for 9-years old girls (WHO) and as a result, only 2% of Tbilisi cohort was involved.
2. Results of interview of target groups- patients, general population and medical personnel (p value 0.05) show low knowledge/information, lack of HE and PE. This process was conducted only in 16.7%. In 19.2 % complications were linked with low awareness.
3. Small experiment-modeling of IEC/BCC show positive changing of knowledge by 7%, behavior - 11%. 4. Active advocacy via TP model show excellent results-inclusion Tbilisi in the "Cancer Challenge City" project and signing memorandum between the Ministry of Health, Georgian Patient Union and the Union International for Cancer control -UICC.
Conclusion: 1. Role of relevant IEC/BCC is important for timely detection and treatment success. National HE and PE strategies for general population, patients and medical personnel should be developed and implemented
Disclosure: Efficiency of TP model (PPC instead of PPP) is clear and should be implemented. Active advocacy (including IA) for fundraising, approval of the NSCC, providing management of whole chain of cancer control, is effective method to influence on the cancer policy in Georgia.

Some Cha Cha moves of battling with cancer

C. Coskun
Dance with Cancer Association / Kanserle Dans Dernegi, Istanbul, Turkey

Introduction / Background: Dance with Cancer Association is the one of the important advocacy and patient organization dealing with awareness of all types of cancer especially focusing on woman in the country often faced.Our main vision is aiming for early diagnosis and prevention, to make the future cancer treatment patient-centered not forgetting either you, me or beloved one danced with cancer, and will go on.Over the last year, we have created awareness on colon cancer, lung cancer, gynecologic and breast cancer for woman with some special events.
Methodology: Dance with Cancer Association worked with ESGO Engage in translation of ovarian, cervical cancer leaflets into Turkish. We also worked on psycho oncology and palliative care and nutrition leaflets that will be printed in 2019. Immunotherapy leaflets were prepared for lung cancer and a YouTube video was taken with sign language and subtitle by a well-known physician in Turkey.In the three largest cities in Turkey, Ankara,İstanbul,İzmir, Booths are opened and brochures were distributed for giving information.Webinars were held on reaching 20.000 people.With the

collaboration of a hospital in Ankara, yoga has been done in every week.A seminar was given at 3 locales of Cankaya Municipality and at 2 dormitory for girls in the breast cancer awareness event to around 120 guests.Self-examination was described with silicone breast model by a well-known physician and a physician radiologist.In Ankara, Kugulu Park, leaflets were given and again self-examination was described for 4 days and last two days concerts took place with the participation of approximately 1000 people. In 2018, our colon model has visited 18 cities including Ankara, Istanbul, Izmir and Bursa and thousands of women have been reached.Since March was the month of colon cancer awareness, many seminars were held in cooperation with the hospitals.
Results: Awareness
Conclusion: More awareness, fewer cancer for women.
Disclosure: Nothing to disclose

The Venus Project by BRACHA - living with a high risk of hereditary cancer

L. Cohen
BRACHA - Living with a High Risk of Hereditary Cancer, Kfar Saba, Israel

Introduction / Background: BRACHA is the only organisation in Israel focused on raising awareness about HBOC - Hereditary Breast and Ovarian Cancers. The aim of BRACHA is to provide current knowledge and education about genetic testing, ways to reduce the risk of HBOC and information on research and medical innovations. The BRACHA community is diverse: Healthy mutation carriers, Previvors - women who have undergone risk reducing surgery, women and men with breast cancer, women with ovarian cancer as well as couples undergoing PGD - pre-implantation genetic diagnosis. Hereditary cancers are present in the Jewish, Muslim, Christian and Druze communities. Knowing you have a high risk for breast and ovarian cancers gives you the opportunity to undergo risk-reducing surgeries, thereby reducing your risk by 90%.
Methodology: BRACHA brought together British artist David Scheinmann, curators Tamar Arnon and Eli Zagury and producer Osnat Arnon Kashtan to create a touring exhibition featuring photography, video art, a book and a documentary to raise awareness of hereditary cancers. This empowering art project features ten individuals affected by hereditary cancer.
Results: This powerful and educational project exhibited at The Design Gallery, Tel Aviv in 2017 and The Rosenfeld Porcini Gallery, London in 2018. The exhibition reached thousands of people who visited the art exhibition, read the numerous media articles or saw the news items on TV. The prestigious British Medical Journal gave a double spread report on the importance of the exhibition. Awareness was raised, genetic testing increased and thus more cancers were prevented.
Conclusion: Awareness of hereditary cancer is low. If there was comprehensive genetic testing for all, cancer rates would be hugely reduced which would lead to increased cancer prevention. The BRACHA travelling art exhibition increases awareness and reduces global cancer rates. The exhibiton can be translated to all languages and appear globally.Please join us in raising awareness!

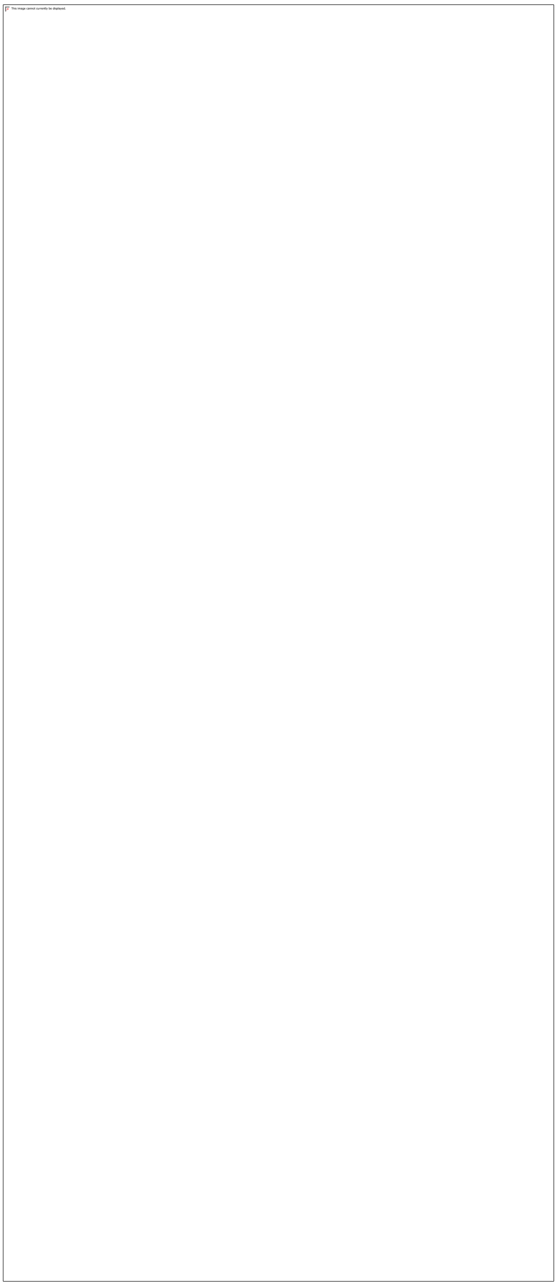

[BRACHA Venus Project]

Disclosure: Nothing to disclose
